# A novel antibiotic class targeting the lipopolysaccharide transporter

**DOI:** 10.1038/s41586-023-06873-0

**Published:** 2024-01-03

**Authors:** Claudia Zampaloni, Patrizio Mattei, Konrad Bleicher, Lotte Winther, Claudia Thäte, Christian Bucher, Jean-Michel Adam, Alexander Alanine, Kurt E. Amrein, Vadim Baidin, Christoph Bieniossek, Caterina Bissantz, Franziska Boess, Carina Cantrill, Thomas Clairfeuille, Fabian Dey, Patrick Di Giorgio, Pauline du Castel, David Dylus, Pawel Dzygiel, Antonio Felici, Fernando García-Alcalde, Andreas Haldimann, Matthew Leipner, Semen Leyn, Séverine Louvel, Pauline Misson, Andrei Osterman, Karanbir Pahil, Sébastien Rigo, Adrian Schäublin, Sebastian Scharf, Petra Schmitz, Theodor Stoll, Andrej Trauner, Sannah Zoffmann, Daniel Kahne, John A. T. Young, Michael A. Lobritz, Kenneth A. Bradley

**Affiliations:** 1 Roche Pharma Research and Early Development, Immunology, Infectious Disease and Ophthalmology, Roche Innovation Center Basel, F. Hoffmann-La Roche, Basel, Switzerland; 2 Roche Pharma Research and Early Development, Therapeutic Modalities, Roche Innovation Center Basel, F. Hoffmann-La Roche, Basel, Switzerland; 3 SixPeaks Bio, Basel, Switzerland; 4 Roche Pharma Research and Early Development, Pharmaceutical Sciences, Roche Innovation Center Basel, F. Hoffmann-La Roche, Basel, Switzerland; 5 Preclinical Sciences and Translational Safety, Janssen Pharmaceutica, Beerse, Belgium; 6 AutoChem R&D, Mettler-Toledo International, Greifensee, Switzerland; 7 Independent consultant, Cambridge, Great Britain; 8 Department of Chemistry and Chemical Biology, Harvard University, Cambridge, MA USA; 9 Discovery Microbiology, Aptuit (Verona) Srl, an Evotec Company, Verona, Italy; 10 Infectious and Inflammatory Disease Center, Sanford Burnham Prebys Medical Discovery Institute, La Jolla, CA USA; 11 Roche Pharma Research and Early Development, Informatics, Roche Innovation Center Basel, F. Hoffmann-La Roche, Basel, Switzerland; 12 Therapeutics Discovery, Janssen Pharmaceutica, Beerse, Belgium

**Keywords:** Antibiotics, Pharmaceutics, Target identification

## Abstract

Carbapenem-resistant *Acinetobacter baumannii* (CRAB) has emerged as a major global pathogen with limited treatment options^1^. No new antibiotic chemical class with activity against *A. baumannii* has reached patients in over 50 years^1^. Here we report the identification and optimization of tethered macrocyclic peptide (MCP) antibiotics with potent antibacterial activity against CRAB. The mechanism of action of this molecule class involves blocking the transport of bacterial lipopolysaccharide from the inner membrane to its destination on the outer membrane, through inhibition of the LptB_2_FGC complex. A clinical candidate derived from the MCP class, zosurabalpin (RG6006), effectively treats highly drug-resistant contemporary isolates of CRAB both in vitro and in mouse models of infection, overcoming existing antibiotic resistance mechanisms. This chemical class represents a promising treatment paradigm for patients with invasive infections due to CRAB, for whom current treatment options are inadequate, and additionally identifies LptB_2_FGC as a tractable target for antimicrobial drug development.

## Abstract (web summary)

A tethered macrocyclic peptide antibiotic class described here—which shows potent antibacterial activity against carbapenem-resistant *Acinetobacter baumannii*—blocks the transport of bacterial lipopolysaccharide from the inner membrane to its destination on the outer membrane through inhibition of the LptB_2_FGC complex.

## Main

Antibiotic-resistant bacterial infections are an urgent global threat to public health^2^. The effective treatment of bacterial infections is a foundation of modern health care, enabling medical technologies such as transplantation, cancer chemotherapy and surgery. The rise of antibiotic-resistant bacteria represents a silent pandemic and is eroding the safety of these basic medical interventions and is an increasing cause of mortality globally^1,3^. Antibiotic resistance has disproportionately accumulated among specific Gram-negative pathogens. To better align global efforts, the World Health Organization (WHO) and US Centers for Disease Control (CDC) have categorized antimicrobial-resistant pathogens for which new antibiotics are urgently needed and which pose the greatest threat to human health^4^. Antibiotic-resistant *A. baumannii* emerged as a priority 1: critical WHO pathogen and a CDC urgent threat^1^.

*A. baumannii* is the most frequently encountered member of the *A. baumannii–calcoaceticus* complex (ABC) of opportunistic bacterial pathogens that causes invasive infections in hospitalized patients and patients with critical illness, most commonly nosocomial pneumonia and bloodstream infections^5,6^. The rapid accumulation of resistance mechanisms to multiple antibiotic classes and the global spread of CRAB has rendered this preferred class of antibiotics obsolete^7^. Increasingly, the emergence of pan-drug-resistant *A. baumannii* has been documented^8,9^. Recent approvals of the siderophore-conjugated β-lactam cefiderocol and β-lactamase inhibitor durlobactam in combination with sulbactam offer new treatment options for infections caused by ABC^10,11^. However, older or repurposed agents (such as the polymyxin class) with unfavourable safety and efficacy profiles continue to define the standard of care^12–14^. Mortality estimates for invasive CRAB infections range from 40 to 60%, in part due to the lack of effective treatment options^15–17^. In the absence of any viable antibiotic treatment options, patients have also been treated with experimental cocktails of bacteriophages^18,19^.

Here we report the identification and optimization of a structurally novel antibiotic class, tethered MCPs, culminating in the selection of a clinical candidate, zosurabalpin. We further identify the lipopolysaccharide (LPS) transport machinery as an unprecedented antibiotic target for MCPs in *Acinetobacter*^20–22^. The in vitro antibacterial and pharmacokinetic properties of zosurabalpin translated into potent in vivo efficacy in animal models of infection, including infections caused by pan-drug resistant strains of *A. baumannii*. Collectively, non-clinical data supported the selection of zosurabalpin as a suitable clinical development candidate that has the potential to address the urgent threat of invasive, drug-resistant *Acinetobacter* infections.

## MCPs are active against *A. baumannii*

**Fig. 1:**
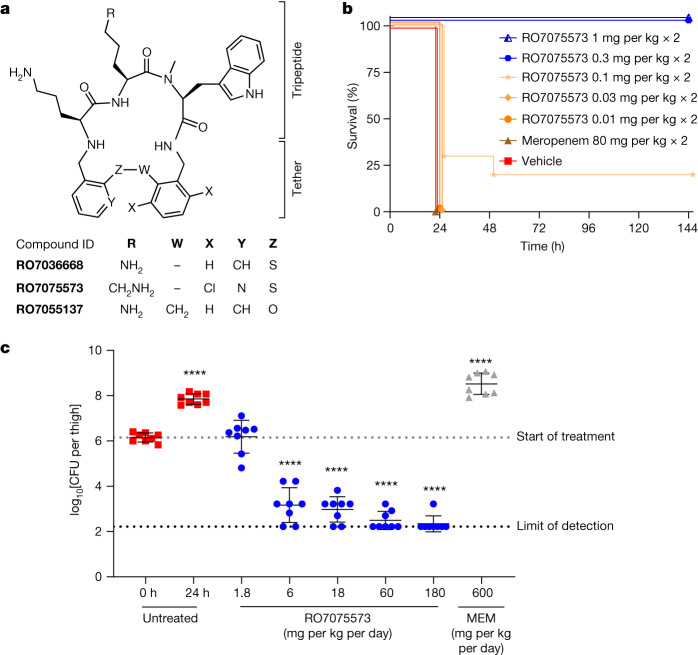
First-generation lead RO7075573 protects mice from *A. baumannii* infections. **a**, The chemical structure of tethered macrocyclic peptides, from the screening hit RO7036668 to the first-generation lead RO7075573. RO7055137 is an inactive control compound (MIC > 64 mg l^−1^) (Fig. 3c). **b**,**c**, The in vivo efficacy of RO7075573 in a mouse model of infection induced by *A. baumannii* ACC00535 (RO7075573 MIC = 0.12 mg l^−1^ in CAMHB with 20% human serum). **b**, Sepsis was induced by intraperitoneal bacterial inoculation in immunocompetent mice. Doses (mg per kg) were administered subcutaneously at 1 and 5 h after infection. The Kaplan–Meier survival curve shows the percentage of mouse survival for each group treated with vehicle, meropenem (80 mg per kg) or varying doses of RO7075573 (*n* = 10 per group) over 6 days. **c**, Thigh infection was induced by bacterial intramuscular inoculation in immunocompromised mice. Starting 2 h after infection (0 h), mice were given s.c. administration of RO7075573 or meropenem (MEM) (*n* = 4 mice per treatment group or vehicle) every 4 h over 24 h. The dose–response curve of RO7075573 total daily doses (mg per kg per day) is shown, measured as the bacterial burden reduction (CFU) in infected thigh (8 thighs, 8 read-outs for bacterial counts). Results are presented as mean ± s.d. The statistical significance of the difference in bacterial counts between control and treated mice was calculated using one-factor analysis of variance (ANOVA) followed by Dunnett’s multiple-comparison test (*P* < 0.05 was considered to be significant versus *T* = 0 h); ****P < 0.0001. Source Data

A novel class of small-molecule antibiotics was identified through whole-cell phenotypic screening of 44,985 MCPs from Tranzyme Pharma^23,24^ against a collection of type strains, including Gram-negative and Gram-positive human pathogens. A cluster of compounds with antibacterial activity featured a tripeptide subunit and a diphenylsulfide tether to close the ring. RO7036668, which possessed an l-Orn-l-Orn-l-*N*-Me-Trp subunit (Fig. 1a), was found to have a minimum inhibitory concentration (MIC) of 4 mg l^−1^ against *A. baumannii* ATCC 19606. RO7036668 was inactive against other Gram-negative bacteria (MIC > 64 mg l^−1^), had limited activity against Gram-positive bacteria and yeast (32–64 mg l^−1^) (Table 1), and was non-cytotoxic (Extended Data Table 1 and Supplementary Table 1). Replacement of the central l-Orn by l-Lys, dichloro substitution at the southeastern benzene ring and replacement of the southwestern benzene ring by pyridine produced the lead compound RO7075573 (Fig. 1a).

**Table 1:** Spectrum of activity of MCPs against selected Gram-positive and Gram-negative bacterial species and *C. albicans*

Microorganism	MIC (mg l^−1^)				
	RO7036668	RO7075573	RO7202110	ZAB	MEM
*E. coli* ATCC 25922	>64	>64	>64	>64	≤0.06
*K. pneumoniae* ATCC 700603	>64	>64	>64	>64	≤0.06
*P. aeruginosa* ATCC 27853	>64	>64	>64	>64	0.5
*S. aureus* ATCC 29213	32	>64	>64	>64	0.12
*C. albicans* ATCC 90028	64	>64	>64	>64	>64
*A. baumannii* ATCC 17978	NA	≤0.06	≤0.06	≤0.06	0.5
*A. baumannii* ATCC 19606	4	0.12	≤0.06	0.25	1
*A. baumannii* (10 MDR isolates)	16 (1–16)	0.5 (≤0.06–0.5)	0.12 (≤0.06 − 0.12)	0.25 (0.12–1)	64 (1–64)

MIC was determined in CAMHB. MIC values represent the mode of at least three replicates, with the following exceptions: for ATCC 17978, the MIC value of one replicate is reported for all compounds; for the compound RO7036668, the MIC value of one replicate is reported for all isolates. RO compound and zosurabalpin (ZAB) end-point MIC values versus *A. baumannii* were read at 80% of growth inhibition due to the observed trailing effect. The MIC_90_ (range) is reported for the ten MDR isolates (bottom row). MIC values shown here are from the screening campaign. MEM, meropenem; *C. albicans*, *Candida albicans*; NA, not available; *S. aureus*, *Staphylococcus aureus*. Source data are in Supplementary Data 1.

**Extended Data Fig. 1:**
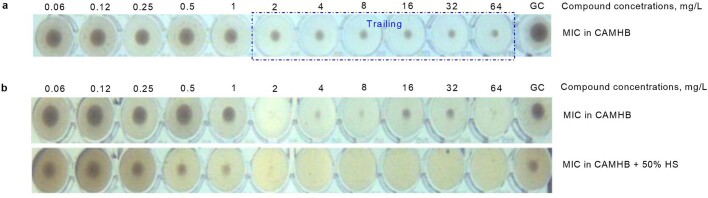
MIC of RO7075573 in CAMHB and in CAMHB supplemented with 50% human serum. **a**, MIC conducted in CAMHB with reading endpoints at 80% and 100% growth inhibition corresponding to 2 mg/L and > 64 mg/L respectively. **b**, MIC conducted in CAMHB with and without 50% human serum showing that the MIC read at 80% growth inhibition in CAMHB is correlating to MIC read at 100% growth inhibition in CAMHB + 50% human serum. GC, growth control.

**Extended Data Fig. 2:**
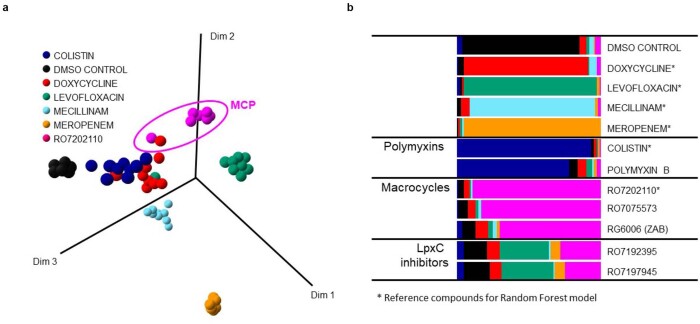
Compound induced phenotypic profile similarity derived with random forest analysis. For the random forest model generation, analysis was applied to the reference compound set Doxycycline (Red), Levofloxacin (Green), Mecillinam (Pale blue), Meropenem (Orange), Colistin (Dark blue), RO7202110 (Magenta) as well as samples with no compound (DMSO, black) as previously described in Zoffmann et al with data from three independent experiments run in triplicates. **a**, In the left panel is a similarity-based projection of the correlating random forest distance matrix into 3 dimensions showing clear separation between data points belonging to different compounds and close proximity of those belonging to the same compounds, forming clusters. For the random forest analysis data proximity analysis from 4x and 8x LOED are pooled and the number of datapoints are reduced by down sampling to be equal for all reference conditions the maximal projection of the analysis. **b**, In the right panel an out-of-bag validation of the random forest classification model is indicated for the reference compounds. For the test compounds (MCPs RG7075573 and zosurabalpin (RG6006, ZAB), polymyxin B and the two LpxC inhibitors) a similarity score was derived from the model. The degree of frequency of matching prediction correspond to the length of the individual bar segment, where a longer segment represents a higher similarity. Thus there is a high similarity for polymyxin with >70% similarity score towards colistin fitting with the known similar MOA, while the LpxC inhibitors as expected does not have a strong correlation with any of the 6 reference compounds all known to have different MOA. For the macrocycles RG7075573 and zosurabalpin (RG6006, ZAB) > 70% similarity was seen towards the reference compound RO7202110 supporting a similar MOA for the three. Source Data

RO7075573 was found to be 4- to 64-fold more potent than RO7036668 against a panel of *A. baumannii*, and displayed improved selectivity against *Acinetobacter* (MIC > 64 mg l^−1^ against all of the other tested species) (Table 1). The MIC readings for MCPs were impacted by a trailing effect, which was previously reported for treatment of *A. baumannii* with other standard of care antibiotics^25^, that was alleviated by inclusion of serum in the testing medium (Extended Data Fig. 1). RO7075573 was inactive against wild-type and efflux-impaired and porin-deficient *Escherichia coli*, *Klebsiella pneumoniae* and *Pseudomonas aeruginosa*, indicating that access to the target was not the primary determinant of pathogen selectivity of MCPs (Extended Data Table 2). RO7075573 antibacterial activity was similar for antibiotic-susceptible type strains and for multidrug-resistant (MDR) *A. baumannii* strains (Extended Data Table 3 and Supplementary Tables 2 and 3), with MICs ranging from ≤0.06 to 0.5 mg l^−1^ (Table 1). This suggested that MCPs may interact with a new target compared with current clinical standard-of-care antibiotics. To investigate this possibility, we applied bacterial phenotypic fingerprint profiling^26^. The approach combines multiparametric high-content screening with random-forest-based machine learning analysis and can identify similarities in compound-induced phenotypes, indicating a similar mode of action. MCPs displayed a highly similar phenotypic profile across several tested compounds, while clearly differentiating from other known antibiotic classes (Extended Data Fig. 2). Taken together, these data support the hypothesis that antibacterial activity is mediated through a new target.

To assess the potential of MCPs to treat bacterial infections in vivo, RO7075573 was tested in two mouse models of infection induced by the MDR and CRAB strain ACC00535. Treatment with RO7075573 at subcutaneous (s.c.) doses of between 0.1 and 0.3 mg per kg given at 1 h and 5 h after inoculation provided complete protection in a lethal sepsis model in immunocompetent mice (Fig. 1b). Furthermore, treatment of neutropaenic mice with RO7075573 every 4 h for 24 h at s.c. doses between 0.3–30 mg per kg resulted in dose-dependent reductions in thigh bacterial burden, achieving a >4 log decrease in colony-forming units (CFU), while treatment with either vehicle or meropenem resulted in bacterial outgrowth, as expected (Fig. 1c). Thus, the in vitro activity of MCPs translated into a robust antibacterial effect in vivo, including the treatment of infections caused by CRAB.

## Zwitterions improve tolerability

Despite the promising properties of RO7075573, including favourable absorption, distribution, metabolism and elimination properties and in vitro safety profiles (Extended Data Table 1), intravenous administration of 6 mg per kg per day of RO7075573 (1.2 mg ml^−1^) in rats revealed a substantial tolerability issue, including mortality and moribund animals (Extended Data Table 4). A rapid decrease (>40%) in lipid parameters (cholesterol, triglycerides and high-density lipoprotein) was observed after intravenous drug administration to rats, which correlated with in vitro plasma incompatibility (Extended Data Table 1). Specifically, RO7075573 caused formation of aggregated low-density lipoprotein/high-density lipoprotein vesicles through an unknown mechanism.

**Fig. 2:**
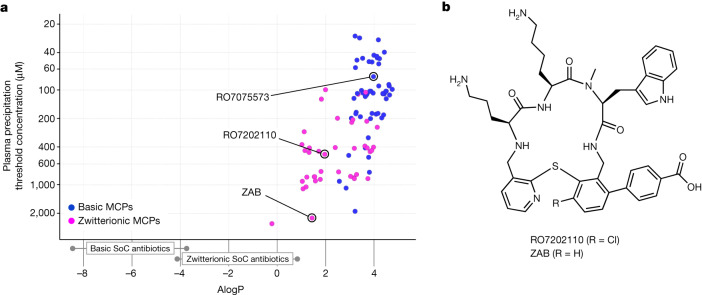
The second-generation lead zosurabalpin demonstrates low lipid plasma precipitation. **a**, The drug lipophilicity (AlogP) of basic MCPs and zwitterionic MCPs in correlation with plasma precipitation. The standard of care (SoC) antibiotics and their AlogP lipophilicities are described in Extended Data Table 5. **b**, The chemical structure of the second-generation tethered macrocyclic peptides: zwitterions RO7202110 and zosurabalpin (ZAB).

Poor plasma compatibility precludes intravenous drug candidates from clinical development and, to address this liability, a customized precipitation assay in rat plasma was used. This assay identified minimum concentrations of MCPs causing precipitation, and was used to guide the optimization of second-generation MCPs. The threshold concentration for RO7075573 causing precipitation was 52 μM (0.038 mg ml^−1^), which was far below the concentration of the formulated drug used in the intravenous tolerability study. A correlation between lipophilicity (calculated partition coefficient (AlogP)) and the minimum concentration causing plasma precipitation was identified (Fig. 2a). Comparison of the lipophilicity of MCPs with that of standard-of-care antibiotics^27^ revealed that basic (positively charged) standard-of-care antibiotics (such as polymyxins and aminoglycosides; AlogP < −3.5; Extended Data Table 5) are far more hydrophilic than the basic tethered MCPs. This information was used to inform the design of zwitterionic second-generation MCPs.

Zwitterionic tethered MCPs showed reduced plasma precipitation compared with basic compounds (Extended Data Table 1). Zwitterionic standard of care antibiotics (such as fluoroquinolones and β-lactams; AlogP between –4.1 and +0.8; Extended Data Table 5) are close to the lipophilicity of the zwitterionic tethered MCPs RO7202110 and zosurabalpin (Fig. 2 and Extended Data Table 1). On the basis of this analysis, the zwitterionic benzoic acid derivative zosurabalpin, which displayed potent in vitro activity against MDR *A. baumannii* (Table 1 and Extended Data Table 3) and greatly reduced plasma precipitation (threshold concentration of 1.76 mg ml^−1^), was selected for in-depth profiling and was found to be better tolerated when infused intravenously into rats (Extended Data Table 4).

## Zosurabalpin inhibits LPS transport

To identify the potential molecular target of MCPs, resistance development to zosurabalpin was assessed using a standard spontaneous mutation approach with eight *A. baumannii* isolates (Supplementary Table 3). Moreover, a dynamic culture model (morbidostat) was used, performing eight complete cycles of experimental evolution in four *A. baumannii* strains and two distinct growth media (cation-adjusted Mueller–Hinton broth (CAMHB) with or without 20% human serum) under conditions of gradually increasing zosurabalpin concentrations^28^. Whole-genome sequencing (WGS) of single colonies with elevated MIC identified 43 distinct mutations: 24 unique mutations arising only in the morbidostat analysis, 11 unique mutations were identified only in spontaneous resistance studies and eight mutations were identified using both methodologies. Mutations primarily arose in genes encoding the LPS transport and biosynthesis machinery (Extended Data Table 6 and Supplementary Tables 4–6). A total of 28 different mutations was identified in the gene encoding LptF, and two unique mutations were identified in LptG. These proteins are components of the LptB_2_FGC complex in Gram-negative bacteria, which is part of the LPS-transport system^29^. Specifically, LptF(Glu249), LptF(Ile317), LptF(Lys320), LptF(Arg322) and LptF(Ile323) were implicated by several amino acid substitutions that emerged independently in several independent experiments spanning multiple strains (Extended Data Table 6 and Supplementary Tables 4–6). These altered residues are predicted to colocalize to the lumenal region of LptF (ref. ^30^), suggesting that the compounds may affect LPS transport in *A. baumannii*.

**Fig. 3:**
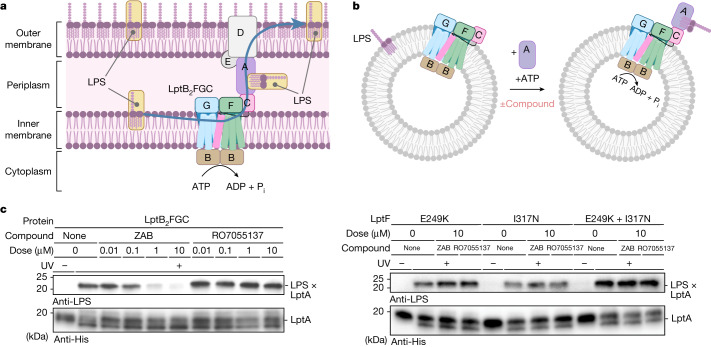
Zosurabalpin kills cells by inhibiting LptB_2_FGC function. **a**, Schematic of the trans-envelope lipopolysaccharide transporter. The inner-membrane complex LptB_2_FGC is an ATP-binding cassette that uses ATP hydrolysis to extract LPS from the inner membrane and transport it to the cell surface. P_i_, inorganic phosphate. **b**, In vitro assay monitoring the release of LPS from proteoliposomes containing LptB_2_FGC complexes to LptA^I36*p*BPA^–His7 by ultraviolet irradiation cross-linking and detection of LPS–LptA^I36*p*BPA^–His7 adducts by LPS immunoblotting (Methods). The diagrams in **a** and **b** were created using BioRender. **c**, Zosurabalpin (ZAB) inhibits LPS transport in vitro by wild-type LptB_2_FGC to LptA, whereas the structurally related inactive control compound RO7055137 (Fig. 1a) displays no LPS transport inhibition at comparable doses. Two amino acid substitutions, LptF(E249K) and LptF(I317N), that decreased the susceptibility of *Acinetobacter* to zosurabalpin were tested both individually and together. All three variants were resistant to compound treatment (Extended Data Table 6). Activity assays were conducted in biological triplicate, and representative blots are shown. UV, ultraviolet irradiation.

A biochemical assay was next used to directly assess the potential of zosurabalpin to affect the function of the LptB_2_FGC complex. The protein complex from the experimentally tractable and zosurabalpin-susceptible species *Acinetobacter baylyi*^30^ was reconstituted in proteoliposomes and monitored for ATP-dependent LPS extraction from the membrane to the periplasmic transport component LptA. Zosurabalpin blocked LPS extraction at concentrations comparable to growth inhibitory concentrations (Fig. 3c). By contrast, the compound had no effect on LPS extraction when *E. coli* LptB_2_FGC proteins were used (Supplementary Fig. 1), consistent with the observation that MCPs are lethal only to *Acinetobacter* strains. To confirm target specificity, five *A. baylyi* strains were engineered, each containing one of the *lptF* mutations found to decrease susceptibility to *A. baumannii* (E249K, I317N, K320T, R322C and I323R; Extended Data Table 6). Four out of the five mutations decreased the susceptibility of *A. baylyi* to zosurabalpin by at least ninefold (Extended Data Table 6). Protein complexes, in which the two least susceptible LptF variants E249K and I317N (>100× MIC shift) were incorporated, were tested for their ability to rescue LPS extraction in the presence of zosurabalpin. Zosurabalpin did not inhibit LPS transport using complexes containing either mutation (Fig. 3c). Taken together, the convergent and colocalized resistance mutations in *lptF* along with biochemical data provide strong evidence that zosurabalpin targets the inner-membrane LptB_2_FGC complex to block LPS transport.

## Characterization of resistance

Spontaneous mutation frequency to zosurabalpin ranged from 10^−7^ to <10^−9^ at 4× to 16× MIC (Table 2), that is, within a range comparable to current clinical standard-of-care antibiotics in *Acinetobacter*^31–33^. No colonies were recovered for 6 out of 8 strains tested at 8× MIC, and no colonies from any parental strain were recovered at 16× MIC. Increases in MIC values for zosurabalpin ranged from 2× to >256× in derivative colonies relative to the parental strains, and were generally stable after passage in drug-free medium (Extended Data Table 6 and Supplementary Table 4). Importantly, MIC values for colistin and meropenem were not affected in strains with elevated zosurabalpin MICs (Supplementary Table 4).

**Table 2:** Spontaneous mutation frequencies to zosurabalpin of eight *A. baumannii* MDR isolates

*A. baumannii* isolates	Agar MIC (mg l^−1^) ZAB		Single-step spontaneous mutation frequencies against ZAB			
			2× MIC	4× MIC	8× MIC	16× MIC
ACC00535		0.12	2 × 10^−7^	2 × 10^−7^	9 × 10^−8^	<3 × 10^−9^
ACC00445		0.25	2 × 10^−7^	7 × 10^−8^	3 × 10^−9^	<3 × 10^−9^
ACC01073		1	1 × 10^−8^	7 × 10^−9^	<4 × 10^−9^	<4 × 10^−9^
ACC01077		0.25	4 × 10^−9^	<4 × 10^−9^	<4 × 10^−9^	<4 × 10^−9^
ACC01085/AR0307		0.25	1 × 10^−6^	<2 × 10^−9^	NA	NA
ROB08706		1	4 × 10^−8^	6 × 10^−8^	<2 × 10^−8^	<2 × 10^−8^
ROB08708		0.12	2 × 10^−7^	1 × 10^−7^	<4 × 10^−9^	<4 × 10^−9^
ATCC BAA-747		0.12	2 × 10^−7^	<1 × 10^−8^	<1 × 10^−8^	<1 × 10^−8^

MIC determinations were performed by agar dilution according to CLSI guidelines (CLSI M07-A11 2018), using Mueller–Hinton agar supplemented with 20% human serum. AR0307 is a CDC isolate. ZAB, zosurabalpin; NA, not assessed. Source data are in Supplementary Data 2.

Three main groups of mutations were identified in the colonies sequenced from spontaneous mutation studies: (1) target-based mutations (for example, in *lptF* and *lptG*); (2) mutation of the gene encoding the final enzyme of lipid A synthesis (*lpxM*); and (3) genes encoding regulators of efflux (*adeS* and *adeR*). Single-nucleotide polymorphisms were recovered in genes encoding LptF and LptG when selected at 2× and 4× MIC, but not when selected at 8× or 16× MIC. Mutations in *lptF* were associated with MICs from 8 to >64 mg l^−1^, and mutant strains retained partial to full virulence in a mouse septicaemia model (Extended Data Table 7). Mutations in *lpxM* led to a complete loss of virulence in mice (Extended Data Table 7), consistent with previous report of an in vivo fitness cost in *Galleria mellonella*^34^. Finally, mutations in *adeRS* typically resulted in lower fold changes in MIC, with MICs ranging from 1–8 mg l^−1^ and, in some cases, were not stable when mutant isolates were passaged in the absence of drug pressure. *adeRS* mutants retained virulence in mice but, consistent with lower MIC fold increases, also retained susceptibility to zosurabalpin treatment in vivo on the basis of the survival of mice in the treated groups (Extended Data Table 7).

## Zosurabalpin is efficacious in vivo

**Fig. 4:**
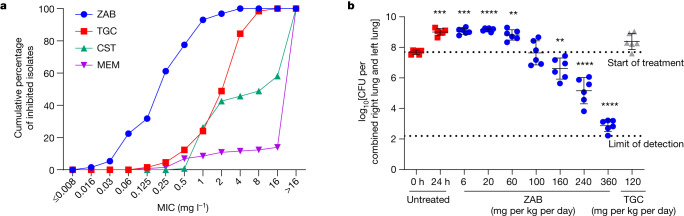
In vitro activity and in vivo efficacy of zosurabalpin against clinical *A. baumannii* isolates. **a**, In vitro MIC activity of zosurabalpin against 129 *A. baumannii* clinical isolates shown as the cumulative percentage (MIC_90_: zosurabalpin (ZAB) = 1 mg l^−1^; tigecycline (TGC) = 8 mg l^−1^; colistin (CST) > 16 mg l^−1^; meropenem (MEM) > 16 mg l^−1^). Line listing of the data is provided in Supplementary Table 7. **b**, The in vivo efficacy of zosurabalpin in a mouse model of infection induced by pan-drug-resistant *A. baumannii* ACC01073 (zosurabalpin MIC = 2 mg l^−1^ in CAMHB with 20% human serum). Lung infection was induced by bacterial intratracheal inoculation in immunocompromised mice. Treatment, starting 2 h after infection (0 h), was administered subcutaneously (*n* = 6 mice per treatment group or vehicle) every 6 h over 24 h for zosurabalpin and every 12 h for tigecycline. Dose–response curve of zosurabalpin total daily doses (mg per kg per day) measured as the bacterial burden reduction (CFU) in infected lungs. Results are presented as mean ± s.d. Statistical significance of the difference in bacterial counts between the control and treated mice was calculated using the one-factor ANOVA followed by Dunnett’s multiple-comparison test (*P* < 0.05 was considered to be significant versus *T* = 0 h); **P < 0.01, ***P < 0.001. Source Data

To assess the potential of zosurabalpin for the treatment of severe invasive CRAB infections, its in vitro activity was evaluated against 129 human clinical isolates of *A. baumannii* derived from a range of infection sites. This panel was enriched for difficult-to-treat isolates (78%)^35^ and MDR (80%) isolates. The MIC required to inhibit growth of 90% of these isolates was 1 mg l^−1^ (MIC_90_; range, ≤0.016–4 mg l^−1^) (Fig. 4a and Supplementary Table 7).

**Extended Data Fig. 3:**
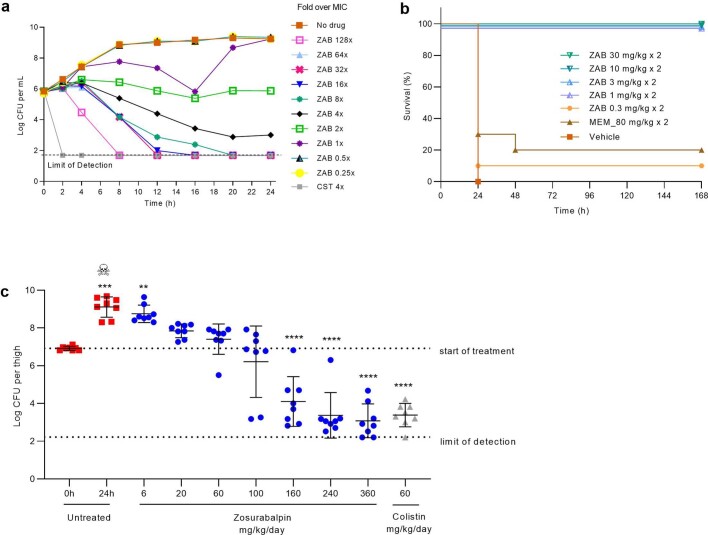
In vitro and in vivo bactericidal activity of zosurabalpin against MDR resistant *A. baumannii* isolates. **a**, An exponential culture of ACC01073 was challenged with sub MIC, MIC (2 mg/L in CAMHB with 20% HS) and multiple MIC of zosurabalpin (ZAB) or colistin (CST) at 4x MIC. Kinetic of killing determined by sampling, plating and CFU counting at different time points over 24 h. Data from one representative replicate of 3 independent experiments displayed. **b**, **c**, In vivo efficacy of zosurabalpin in mouse model of infections induced by MDR *A. baumannii*. **b**, Sepsis induced by intraperitoneal bacterial inoculation of ACC00445 (zosurabalpin MIC = 0.25 mg/L in CAMHB with 20% HS) in immunocompetent mice. Treatment administered subcutaneously (s.c.) at 1 and 5 h post-infection. Kaplan-Meier survival curve shows percentage of mice survival for each group treated with vehicle, Meropenem (MEM, 80 mg/kg) or varying doses of zosurabalpin (n = 10/group) over 7 days. **c**, Thigh infection induced by bacterial intramuscular inoculation of ACC01085 (zosurabalpin MIC = 0.5 mg/L in CAMHB with 20% HS) in immunocompromised mice. Treatment, starting 2 h post-infection (0 h), administered s.c. (n = 4 mice/ treatment group or vehicle) each 6 h over 24 h for zosurabalpin and each 8 h for colistin. Dose response curve of zosurabalpin total daily doses (mg/kg/day) measured as bacterial burden reduction (colony forming units, CFU) in infected thigh (8 thighs, 8 read outs for bacterial counts). Data are expressed as scatterplot distribution. The centre bars represent the mean ± SD (error bars). Statistical significance of the difference in bacterial count between control and treated mice was calculated by using the one-factor ANOVA followed by Dunnett’s Multiple Comparison Test (p < 0.05 significant), **p < 0.01, ***p < 0.001 and ****p < 0.0001 vs T0h. The ‘skull and crossbones’ symbol indicates mortality prior to 24 h endpoint (n = 1). Source Data

The pharmacokinetic properties of zosurabalpin were examined both in single-dose and multiple-dose pharmacokinetic studies in mice, revealing acceptable plasma exposures after s.c. administration with high clearance (51 ml min^−1^ kg^−1^), a low volume of distribution (0.7 l kg^−1^), a short terminal half-life (0.3 h) and moderate protein binding (fraction unbound, 37%) (Extended Data Table 1). Zosurabalpin lacked off-target activities in a 50-receptor panel assay, and biotransformation studies found no substantial interactions with the cytochrome P450 system, neither as inhibitor nor inducer (Extended Data Table 1 and Supplementary Table 1). The physicochemical properties were found to be consistent with those required for clinical antibiotics, for which gram-scale doses are common, including low lipophilicity (distribution coefficient logD_7.4_ = –2.46) and high aqueous solubility (>100 mg ml^−1^ at pH 1–9)^36^. Finally, zosurabalpin was tested for efficacy in a neutropaenic mouse model of pneumonia using a pan-drug resistant contemporary clinical isolate (*A. baumannii* ACC01073). zosurabalpin treatment of mice resulted in a dose-dependent decrease in bacterial burden, achieving a >5 log reduction in CFUs at the upper total daily dose of 360 mg per kg per day (Fig. 4b). Efficacy in vivo was further confirmed in a neutropaenic mouse thigh infection model and an immunocompetent mouse intraperitoneal induced sepsis model (Extended Data Fig. 3b,c). In time–kill kinetic studies, zosurabalpin tested against the same pan-drug-resistant isolate used in the pneumonia model confirmed a bactericidal effect above 4× MIC (Extended Data Fig. 3a).

## Discussion

Here we report MCPs as a class of antibiotic with potent activity against CRAB and pan-drug-resistant *Acinetobacter*. Optimization of physico-chemical properties, supported in part by a bespoke serum precipitation assay, led to the identification of the clinical development candidate zosurabalpin. This compound has favourable non-clinical pharmacokinetic and safety profiles and demonstrated in vivo efficacy in multiple mouse infection models, including sepsis and thigh and lung infection induced by CRAB strains. These data collectively demonstrate the potential of zosurabalpin as an antibiotic, and human clinical trials have been initiated to further develop this compound with the goal of providing a treatment option for invasive infections caused by CRAB.

New classes of antibiotics inhibiting previously undrugged targets are needed to overcome pre-existing resistance mechanisms. The tethered tripeptide structure of MCPs is not expected to be susceptible to existing mechanisms of resistance. Indeed, these compounds are able to kill *Acinetobacter* clinical isolates displaying a wide range of resistance mechanisms. Moreover, MCPs inhibit the LPS-transport machinery LptB_2_FGC, which represents an antibiotic target in *Acinetobacter*. Antibiotics inhibiting a single target may have resistance liabilities^36^ and, indeed, point mutations in LptF were identified at a frequency of ≤1 × 10^−^^8^ at exposures ≤4× MIC, resulting in a high fold change in MIC. This information can be used alongside human pharmacokinetic data to guide assessment of resistance potential under clinically relevant conditions. Resistance may be further mitigated by current guidance to use at least two active agents in treating infections caused by CRAB. Additional insights into the target and mechanism of action for MCPs is reported in the companion paper^30^. The combination of a unique chemical scaffold and a new target could lead to MCPs’ becoming a new class of antibiotic against *Acinetobacter*.

## Methods

### MIC determination

MIC determinations were performed using the broth microdilution method and in line with CLSI guidelines M07 and M100 (refs. ^37,38^). Bacterial inocula were prepared by diluting a 0.5 McFarland suspension in CAMHB or lysogeny broth (LB) specifically for *A. baylyi* isolates. Then, 96-well microtitre plates containing serial twofold dilution solutions of MCPs or standard of care antibiotics were inoculated with an appropriate volume of bacterial cells to give a final inoculum of around 5 × 10^5^ CFU per ml and the desired test concentrations of antibacterial agents. The test plates were incubated for 20 to 24 h (for all *Acinetobacter* isolates) or 16 to 18 h (for non-*Acinetobacter* isolates) before visual inspection or reading the optical density at 600 nm (OD_600_) in the case of *A. baylyi* ATCC 33305 and the respective constructed mutants. MIC values of all of the tested antibiotics were read as the lowest compound concentration inhibiting bacterial growth by naked eye or beyond which the OD_600_ ceased to decrease. When testing MCP compounds using standard CAMHB non-supplemented medium, a trailing phenomenon was observed for part of the isolates rendering the MIC end-point reading ambiguous. Thus, for MCP tested in non-supplemented medium, the lowest concentration that demonstrated at least an 80% reduction in growth (MIC 80%) in comparison to the growth control was recorded, in addition to the MIC read as full growth inhibition (MIC 100%). Alternatively, the MIC testing was performed in CAMHB supplemented with 20–50% human serum, a condition that ameliorates trailing, and was read only at the end point of 100% growth inhibition.

### Bacterial phenotypic fingerprint profiling

Sample preparation and analysis was conducted as previously described^26^ for *A. baumannii*, with the generated and analysed dataset consisting of three independent experiments, *n* = 3, as the only modification to the protocol.

### Cell-viability assay

Compounds were prepared in serial dilutions (3.125–100 μM in 1% DMSO) and transferred to 384-well plates together with a positive (Staurosporine) and a negative (no compound) control. In total, 25 μl of HEK293 cells (ATCC; verified by short tandem repeat PCR and mycoplasma negative) (50,000 cells per ml) were added in either low-serum-containing (0.5% fetal bovine serum (FBS)) or high-serum-containing (12.5% FBS, 1% BSA) medium (Dulbecco’s modified Eagle medium (DMEM), glucose, l-glutamine). The plates were incubated for 24 h at 37 °C and a mixture of CellTiterGlo and DMEM was added to the plates. After incubation at room temperature, the luminescence signal was read using a BioTeK reader and a calculated IC_50_ was derived from the data.

### Off-target activity screening

Off-target activity screening was conducted at Eurofins CEREP SA using the customized panel of 50 off-targets previously described^39^ and was run as single point measurements in duplicates in the presence of 10 μM of the test compound and reported as percentage inhibition of the radioligand signal or control enzymatic activity.

### Rat plasma precipitation assay

Whole blood was obtained from WISTAR rats and collected in 1.2 ml heparin tubes for the preparation of heparin plasma. The tubes were then centrifuged for 5 min at 5,200*g* at room temperature to isolate the plasma supernatant. Test compounds were received as powder and solubilized at various concentrations in 0.9% aqueous sodium chloride solution and adjusted for pH with phosphate-buffered saline (PBS). The assay was conducted in 384-well plates. Rat plasma (10 µl) was added to 10 µl of the various compound solutions. A total of 10 µl of vehicle (0.9% aqueous sodium chloride solution, PBS) added to the plasma was used as a negative control. The absorption was measured at 362 nm. Raw data were obtained as absorbance data. The difference between the absorbance of the sample and the mean absorbance of the vehicle was calculated. The minimum effect concentration was defined as the lowest concentration giving an absorbance difference of OD_362_ ≥ 0.05.

### Human plasma haemolysis and precipitation assay

For the in vitro haemolysis test, blood was provided by Roche medical services through an anonymous blood donation for research program, approved by the Ethics Committee Northwestern Switzerland and Central Switzerland (EKNZ) and collected with informed consent. Blood was collected by venipuncture in ethylenediaminetetraacetic acid (EDTA) and Li-heparin-coated tubes. Plasma was prepared from anticoagulated blood by centrifugation (2,500*g*; 10 min; at room temperature) and the haematocrit was measured to ensure that it was within normal reference values. Each formulation of compound in saline was added at a concentration of 8 mg ml^−1^ into test tubes containing exactly 0.5 ml of heparinated blood to give a final assay volume of 1 ml and a concentration of 4 mg ml^−1^ or less (following further dilutions). After incubation in a water bath for 10 min at 37 °C, the tubes were centrifuged (10 min, 1,811*g* at room temperature) and 100 μl of the supernatant was subsequently transferred into test tubes containing 5 ml Drabkins solution (RANDOX Laboratories) and 1 ml phosphate buffer. Haemoglobin was photometrically determined at 540 nm according to the RANDOX test-kit instructions. The results were expressed as percentage haemolysis extrapolated from an internal standard curve with 0%, 50% and 100% haemolysed blood samples.

For the plasma precipitation assay, test item formulations of compounds were diluted as follows with the vehicle (0.9% aqueous sodium chloride solution): undiluted, 1:2, 1:4, 1:8 and 1:16. These serial dilutions were then added into test tubes containing 0.5 ml of plasma to give a final assay volume of 1 ml with a final concentration of 4 mg ml^−1^ assay volume or less. After centrifugation of the samples (1,811*g*; 10 min; at room temperature), plasma precipitation was visually determined by scoring the resulting pellet (score: 0, none; 1, mild; 2, moderate; 3, marked).

### Kinetic of killing determination

Time–kill studies were performed according to the CLSI standard procedure M26-A^40^. Zosurabalpin was tested at concentrations ranging from above, at and below MIC (twofold dilutions from 128 down to 0.25× MIC) and the control compound colistin was used at 4× MIC. *A. baumannii* colonies from agar plates were inoculated into CAMHB supplemented with 20% human serum and incubated overnight at 35 ± 2 °C in ambient air under shaking. The overnight culture was diluted 1:10,000 and further incubated for 2 h. A total of 5 ml of the bacterial log-phase suspension was transferred into six-well plates. A total of 50 μl of 100× antibiotic serial twofold dilution solutions was added according to the established multiple of MIC testing range. The six-well plates were incubated at 35 ± 2 °C in ambient air under shaking (100 rpm) for 24 h, 150 μl was withdrawn at the selected timepoints (0, 2, 4, 8, 12, 16, 20 and 24 h), diluted, plated on a Mueller–Hinton agar plate and incubated overnight for CFU determination. GraphPad Prism v.8 was used for graphical presentation of the data.

### Resistance studies

For the single-step spontaneous mutation studies, four concentrations of zosurabalpin corresponding to multiples of agar MIC (2×, 4×, 8× and 16× MIC) were tested for each strain. Molten Mueller–Hinton agar (19 ml) supplemented with 20% human serum was added to 1 ml of compound at 20× the final concentration, poured immediately into Petri dishes (100 mm diameter) and gently mixed. Then, 2–3 colonies from agar plates were inoculated into CAMHB and incubated overnight under shaking (150 rpm). A total of 100 μl of the bacterial suspension (inoculum of around 10^8^ CFU) was spread onto agar plates containing zosurabalpin and growth control plates without antibiotics. The agar plates were incubated aerobically at 35 ± 2 °C. After 24 h, the colonies grown on plates were counted and the spontaneous mutation frequencies were determined as the number of colonies counted on compound-plates divided by the inoculum size. Up to 8 colonies per condition including different morphology types were picked, tested for MIC determination and whole-genome sequenced.

#### Genomic characterization of mutants

Genomic DNA was extracted using the MagNA Pure Pathogen Universal Protocol 200 (MagNAPure 96 system, Roche) and used as the input for library preparation. For short-read sequencing, libraries were prepared using the Illumina Nextera XT library preparation kit (Illumina). The libraries were multiplexed, clustered and sequenced on the Illumina NextSeq system using a paired-end 150 bp cycles protocol at DDL Diagnostik Laboratory. Reads containing adapters and/or bacteriophage PhiX control sequences were removed and trimmed using Trimmomatic (v.0.36)^41^. Trimmed reads of parent strains were used to generate draft genomes by performing de novo assembly using SPAdes (v.3.12)^42^ with MismatchCorrector activated (--careful parameter) and annotation with Prokka (v.1.14.0)^43^ using the NCBI *A. baumannii* assembly (ASM975968v1; GCA_009759685.1) as the reference. Single-nucleotide polymorphism detection in derivative mutants was performed by mapping trimmed Illumina reads from derived colonies to the draft genome of the corresponding parent with the Genomic Short-read Nucleotide Alignment Program (GSNAP v2016-08-24)^44^ using the default parameters. Duplicate reads were removed using samtools^45^, awk scripts and Picard tools (Broad Institute). Variant calling was performed using Freebayes (v.1.1.0)^46^ followed by filtering using bcftools^47^ to remove variants present in the corresponding parent strain and requiring a read depth >5 and a variant frequency >0.8.

For long-read sequencing of genomic DNA, libraries were prepared using the amplification-free SQK-LSK109 library preparation protocol (Oxford Nanopore Technologies (ONT)). Libraries were multiplexed using either the EXP-NBD104 or EXP-NBD196 protocols (ONT), and sequenced on the ONT GridION Sequencer using an R9.4.1 flow cell over 72 h. Raw sequencing data were base-called and demultiplexed live during sequencing using Guppy (either v.3.2.8 or v.3.2.10) and a high-accuracy base-calling model (dna_r9.4.1_450bps_hac.cfg; ONT). Hybrid assemblies were generated first from raw ONT reads by CANU (v.2.0)^48^ followed by realigning ONT reads to the draft assembly by Minimap2 (v.2.17-r941)^49^. The alignment was used for assembly polishing first with ONT reads with Racon (v.1.4.16)^50^ and then by ten rounds of polishing using trimmed, unmapped Illumina reads by Pilon (v.1.23)^51^. Protein and gene annotations for polished hybrid assemblies were performed using Prokka (v.1.14.5)^43^ as described above. Hybrid assemblies were used to identify large insertions in derived mutants compared to parent strains using a sliding-window approach. Insertion elements were annotated using ISfinder (database from 10 November 2020)^52^.

#### Morbidostat-based experimental evolution and genomic profiling of zosurabalpin resistance

The experimental evolution approach using a custom-engineered continuous culturing device, morbidostat, was based on the principles previous introduced^53^. Implementation of morbidostat as well as the entire experimental and computational workflow were established and validated in model studies with triclosan in *E. coli*^54^ and ciprofloxacin in three Gram-negative species, including *A. baumannii* ATCC 17978 (ref. ^28^). In brief, the morbidostat-based workflow included: (1) competitive outgrowth of *A. baumannii* in 6 parallel reactors with regular computer-controlled medium dilutions leading to a gradual increase in drug concentration; (2) sequencing (with ~700–1,000× genomic coverage) of total genomic DNA from bacterial population samples taken as time series; (3) identification and quantitation of sequence variants (mutations, small insertion–deletion mutations, insertion sequences insertions, genomic rearrangements) to deduce evolutionary dynamics and resistance mechanisms; and (4) confirming the impact of major mutational variants by sequencing and MIC determination for selected clones. *A. baumannii* strains ATCC17978 and ATCC19606 were from ATCC, and two clinical isolates, ROB08705 and ROB08706 were from Roche collection. Starter cultures and growth media were as follows. Aliquots of glycerol stocks (from 6 colonies per strain) were grown to an OD_600_ of around 0.3 in CAMHB (TEKNOVA) at 37 °C and 2 ml of each culture was used to inoculate morbidostat reactors with 20 ml of the same medium with or without 20% human serum (Sigma-Aldrich, H4522). Drug dosing was as follows. A 10 mM stock solution of zosurabalpin compound in DMSO was used for preparing drug-containing medium and later for MIC measurements by serial dilutions in MHB medium with 2% DMSO in microtiter plates. Experimental evolution runs in morbidostat included two phases, starting with 2 μM zosurabalpin in drug medium until the intermediate resistance plateau was reached (typically within 48 h) followed by a tenfold increase in zosurabalpin up to 20 μM (over the next 24–36 h). Samples (10 ml) of evolving bacterial populations were typically taken once per day and were used to (1) isolate total genomic DNA for sequencing and (2) prepare glycerol stocks for further clonal analysis.

#### Genome sequencing and assembly for parental strains

All six starter cultures of each strain (A1–A6) were analysed using high-coverage Illumina sequencing (see below), assembly and RAST-based annotation as described for ATCC17978 strain^28^. The obtained genomic assemblies (provided in the Supplementary Information genome assembly file), including the identified pre-existing sequence variants, were used as a framework for the identification and analysis of sequence variants in evolved samples.

#### WGS of evolved population and isolate clones

DNA was extracted using the GenElute Bacterial Genomic DNA Kit (Sigma-Aldrich), analysed by Qbit and used for library preparation using one of the two protocols (kits): (1) the NEBNext Ultra II FS DNA Library Prep Kit for Illumina (New England BioLabs) using TruSeq DNA UD Indexes 20022370 (IDT) without PCR amplification; or (2) the PlexWell PW384 kit with included adapters (seqWell). After quantification using quantitative PCR and quality-control analysis (2100 Bioanalyzer), the libraries were sequenced by Novogene (2 × 150 paired-end) with an average 500–1,000× genomic coverage for populations and 100–200× for isolated clones. To verify IS insertions, some of the clones were additionally analysed by Nanopore (MinION with FLO-MIN106 flow cell) sequencing using the Nanopore Rapid Barcoding kit SQK-RBK004 (Oxford Nanopore Technologies).

#### WGS data processing, variant calling and ranking

The computational pipeline for the initial variant calling was performed as described previously^28^ and is available for download online (https://docs.conda.io/projects/conda/en/latest/index.html). Potentially relevant non-pre-existing and non-synonymous mutational variants were ranked by (1) maximal relative abundance (*A*_max_, %). All genes implicated by at least one event with *A*_max_ ≥ 10% distinct events with *A*_max_ ≥ 2% were selected for further ranking by (2) the number of independent occurrences (*N*) of the mutational events per gene (*N* ≥ 2).

### SDS–PAGE and immunoblotting

Homemade Tris-HCl 4–20% polyacrylamide gradient gels or 4–20% Mini-PROTEAN TGX precast protein gels (Bio-Rad) were used with Tris-glycine running buffer. The 2× SDS sample loading buffer refers to a mixture containing 125 mM Tris (pH 6.8), 4% (w/v) SDS, 30% (v/v) glycerol, 0.005% bromophenol blue, and 5% (v/v) β-mercaptoethanol. SDS–PAGE gels were run for 45 to 60 min at 200 V. Protein complexes purified were analysed by SDS–PAGE followed by staining with Coomassie blue (Alfa Aesar) and imaging using the Gel feature of an Azure Biosystems C400 imager. For western blotting, proteins were transferred onto Immun-Blot PVDF membranes (Bio-Rad). Membranes were then blocked using sterile-filtered Casein blocking buffer (Sigma-Aldrich) for 1 h, and then incubated with the appropriate antibodies. Mouse monoclonal antiserum against the LPS core (Hycult Biotechnology), sheep anti-mouse horseradish peroxidase (HRP) conjugate secondary antibody (GE Amersham) and mouse anti-His tag HRP conjugate antibody (BioLegend) were used for the immunoblots. Bands were visualized using the ECL Prime Western blotting detection reagent (GE Amersham) and the Azure c400 imaging system. Uncropped immunoblots are provided in Supplementary Fig. 1.

### Plasmids, strains and oligonucleotides

Genes encoding LptB, LptC and LptFG were amplified by PCR from *A. baylyi* ADP1 (ATCC 33305) genomic DNA. *lptB* and *lptFG* PCR products were inserted into pCDFduet by Gibson assembly (New England Biolabs) to generate plasmids analogous to those used for other LptB_2_FG homologues^55^. *lptC* PCR products were inserted into pET22/42 with a C-terminal thrombin cleavage site and a His7 tag. Oligonucleotide primers were purchased from Eton Biosciences or Genewiz. Plasmids and strains used in this study are reported in Supplementary Tables 8 and 9 with plasmid sequences provided.

### Construction and use of mutant *A. baylyi* strains

Culture, genetic manipulation and MIC measurements of *A. baylyi* ADP1 were conducted according to previously reported procedures^56,57^. Point mutants were constructed in a two-step procedure as described previously^58^ with the introduction and excision of the integration cassette at codon 66 of *pepA*, wherein the excising fragment of otherwise wild-type chromosomal DNA sequence from codon 406 of *pepA* to codon 193 of *lptG* bore the desired mutation, and the resulting clones were screened by amplicon sequencing from codon 81 of *holC* to codon 501 of *gpmI*. After amplicon confirmation, three validated isolates of each constructed mutant were tested for susceptibility to a panel of antibiotics with known mechanisms of action as a further validation step to ensure congruence of phenotypes across replicates, which was confirmed in all cases, and one of the validated replicates was later used for MIC measurements reported here.

### Purification of LptB_2_FGC complexes for biochemical reconstitution

LptB_2_FGC complexes were purified as previously described for LptB_2_FG with slight modifications^59^. Overnight cultures of *E. coli* C43(λDE3) containing pCDFduet-LptB-LptFG and pET22/42-LptC-thrombin-His7 were diluted 1:100 into LB containing 50 mg l^−1^ spectinomycin and 50 mg l^−1^ carbenicillin. Cells were grown at 37 °C to an OD_600_ of around 0.8. Then, 200 μM isopropyl β-d-1-thiogalactopyranoside (IPTG) and 0.2% glucose were added and cells were allowed to grow for another 2–3 h. Cells were collected by centrifugation (4,200*g*, 20 min, 4 °C). Cell pellets were flash-frozen using liquid nitrogen and stored at −80 °C. All of the subsequent steps were performed at 4 °C unless otherwise noted.

Thawed cell pellets were resuspended in lysis buffer (50 mM Tris (pH 7.4), 300 mM NaCl, 1 mM phenylmethylsulfonyl fluoride (PMSF), 100 μg ml^−1^ lysozyme, 50 μg ml^−1^ DNase I, 1 cOmplete Protease Inhibitor Cocktail tablet per 40 ml) homogenized, and subjected to passage using an EmulsiFlex-C3 high-pressure cell disruptor three times. The cell lysate was centrifuged (10,000*g*, 10 min), and the supernatant was further centrifuged (100,000*g*, 1 h). The resulting pellets were resuspended and solubilized in solubilization buffer (20 mM Tris (pH 7.4), 300 mM NaCl, 15% glycerol, 5 mM MgCl_2_, 1% (w/v) DDM (Anatrace Maumee), 100 μM PMSF, 2 mM ATP) and rocked at 4 °C for 2 h. The mixture was centrifuged (100,000*g*, 30 min), and the supernatant was spiked with imidazole to a final concentration of 15 mM and then rocked with Ni-NTA Superflow resin (Qiagen) for 1 h. The resin was then washed with 2 × 10 column volumes affinity buffer (300 mM NaCl, 20 mM Tris (pH 7.4), 10% glycerol, 0.015% (w/v) DDM) containing 20 mM imidazole, followed by 2 × 15 column volumes affinity buffer containing 35 mM imidazole. Protein was eluted with 2 × 2 column volumes affinity buffer containing 200 mM imidazole, concentrated using a 100 kDa molecular mass cut-off Amicon Ultra centrifugal filter (Millipore) and purified by size-exclusion chromatography on a Superdex 200 increase column in SEC buffer (300 mM NaCl, 20 mM Tris (pH 7.4), 5% glycerol, 0.05% DDM, 0.5 mM tris(hydroxypropyl)phosphine). Fractions collected after size-exclusion chromatography were incubated overnight with restriction-grade thrombin (Sigma-Aldrich) to cleave the His tag. The solution was spiked with 8 mM imidazole, and the uncleaved protein was removed by passage through Ni-NTA resin and benzamidine Sepharose. The fractions were pooled, and concentrated to 7–8 mg ml^−1^ using a 100 kDa molecular mass cut-off Amicon Ultra centrifugal filter. Protein was then prepared in liposomes as described below.

### Purification of LptA^I36*p*BPA^

LptA^I36*p*BPA^ was purified as described previously^59^. In brief, *E. coli* BL21 (λDE3) cells containing pSup-BpaRS-6TRN and pET22b-LptA(I36Am) were grown to an OD_600_ of around 0.6 at 37 °C in LB medium containing 50 μg ml^−1^ carbenicillin, 30 μg ml^−1^ chloramphenicol and 0.8 mM ultraviolet-irradiation-cross-linkable amino acid *p*-benzoyl phenylalanine (pBPA) (BaChem). Cells were then induced with 50 μM isopropyl IPTG; allowed to grow for 2 h; collected; resuspended in a mixture containing 50 mM Tris-HCl (pH 7.4), 250 mM sucrose and 3 mM EDTA; incubated on ice for 30 min; and pelleted (6,000*g*, 10 min). The supernatant was supplemented with 1 mM PMSF and 10 mM imidazole and pelleted (100,000*g*, 30 min). The supernatant was incubated with Ni-NTA resin, which was then washed twice (20 column volumes of 20 mM Tris-HCl (pH 8.0), 150 mM NaCl, 10% (v/v) glycerol and 20 mM imidazole). LptA was eluted twice (2.5 column volumes of wash buffer supplemented with an additional 180 mM imidazole), concentrated using a 10-kDa-cut-off Amicon centrifugal concentrator (Millipore), flash-frozen and stored at −80 °C until use.

### Preparation of LptB_2_FGC liposomes

Proteoliposomes were prepared as described previously^59^. Aqueous *E. coli* polar lipid extract (Avanti Polar Lipids) (30 mg ml^−1^) and aqueous LPS from *E. coli* EH100 (Ra mutant; Sigma-Aldrich) (2 mg ml^−1^) were sonicated briefly for homogenization. A mixture of 20 mM Tris-HCl (pH 8.0), 150 mM NaCl, 7.5 mg ml^−1^ *E. coli* polar lipids, 0.5 mg ml^−1^ LPS and 0.25% DDM was prepared and kept on ice for 10 min. Purified LptB_2_FGC was added to a final concentration of 0.86 μM, and the mixture was left on ice for 20 min. The mixture was diluted 100-fold with cold 20 mM Tris-HCl (pH 8.0) and 150 mM NaCl and kept on ice for 20 min. The proteoliposomes were pelleted (300,000*g*, 2 h, 4 °C), resuspended in 20 mM Tris-HCl (pH 8.0) and 150 mM NaCl, diluted 100× and centrifuged (300,000*g*, 2 h, 4 °C). The pellets were resuspended in a mixture of 20 mM Tris-HCl (pH 8.0), 150 mM NaCl and 10% glycerol (250 μl per 100 μl of the original predilution solution), homogenized by sonication, flash-frozen and stored at −80 °C until use.

### LPS-release assay

The levels of release of LPS from proteoliposomes to LptA were measured as previously described^55^. Assays used 60% proteoliposomes (by volume) in a solution containing 50 mM Tris-HCl (pH 8.0), 500 mM NaCl, 10% glycerol and 2 µM LptA^I36*p*BPA^. Reaction mixtures were incubated with drug for 10 min at room temperature, as applicable. Reactions were then initiated by the addition of ATP and MgCl_2_ (final concentrations of 5 mM and 2 mM, respectively) and proceeded at 30 °C. Aliquots (25 µl) were removed from the reaction mixtures and irradiated with ultraviolet light (365 nm) on ice for 10 min using a B-100AP lamp (Thermo Fisher Scientific). After ultraviolet irradiation, 25 µl 2× SDS–PAGE sample loading buffer was added, the samples were boiled for 10 min and proteins were separated using Tris-HCl 4–20% polyacrylamide gradient gels with Tris-glycine running buffer. Immunoblotting was conducted as described above.

### Animal experiments ethical statement

Mouse pharmacokinetic studies and rat safety studies were conducted at Roche and all of the procedures were performed in accordance with the respective Swiss regulations and approved by the Cantonal Ethical Committee for Animal Research and conducted in a facility accredited by the Association for Assessment and Accreditation of Laboratory Animal Care International (AAALAC) (animal research permit, 2395). The pharmacodynamics studies assessing the efficacy of the compounds were performed at Aptuit Verona, an Evotec company, and were subject to both the European directive 2010/63/UE governing animal welfare and protection, which is acknowledged by the Italian Legislative Decree no. 26/2014 and the company policy on the care and use of laboratory animals. All animals studies were revised by the Animal Welfare Body and approved by Italian Ministry of Health (51/2014-PR) and conducted in a facility accredited by the Association for Assessment and Accreditation of Laboratory Animal Care International (AAALAC) (accredited unit, 001090). CD-1 mice were 6 weeks old at arrival (minimum acclimatization 5 days). Wistar Han IGS Crl:WI(Han) rats were 8 weeks old at the start of dosing. Mice were randomly allocated to treatment groups on arrival. Rats were randomly assigned to group/cage based on body weight.

### Mouse pharmacokinetics study

Three male CD1 mice were administered with compound formulation (0.5 mg ml^−1^ in 0.9% aqueous sodium chloride solution) as an intravenous bolus dose of 1 mg per kg. Blood was sampled at 0.08, 0.25, 0.5, 1, 2, 4, 7 and 24 h after administration and the blood collecting tubes were centrifuged for 5 min at 5,200*g* at room temperature to isolate the plasma supernatant. The concentrations of compound in the plasma were analysed using a liquid chromatography–mass spectrometry method with a calibration range of 5–10,000 ng ml^−1^. The pharmacokinetic parameters were derived from the individual concentration data and were estimated by non-compartmental analysis.

### Five-day repeat dose tolerability study

Four male Wistar rats per group were administered 0 (vehicle control), 0.6 or 6.0 mg per kg per day of RO7075573 or zosurabalpin as a slow intravenous infusion for five days (0, 0.12 or 1.2 mg ml^−1^ in 0.9% aqueous sodium chloride solution). Assessment of tolerability was based on mortality, in-life observations, body weight, food consumption and clinical pathology during the in-life phase. Moreover, gross pathology and histopathology were performed at unscheduled or scheduled euthanasia on day 6.

### Immunocompetent mouse septicaemia infection model

Septicaemia was induced in CD-1 immunocompetent male mice by an intraperitoneal inoculation of a bacterial suspension of the tested *A. baumannii* isolate at a challenge of approximately 1–2 log[CFU] above the determined median lethal dose (resulting in 10^5^ to 10^7^ CFU per mouse). Doses of RO7075573 (ranging from 0.01 mg per kg to 1 mg per kg) or zosurabalpin (ranging from 0.3 to 30 mg per kg), of control standard-of-care antibiotic (meropenem tested at a single dose) and vehicle (sterile saline solution) were administered subcutaneously 1 and 5 h after infection. Mouse survival was followed over 6–7 days. GraphPad Prism 8 was used for graphical presentation of the data. Septicaemia studies with mutant derivative isolates obtained in resistance studies were performed as described above, using a bacterial challenge as determined for the parental isolate. Zosurabalpin was administered subcutaneously at a dose of 30 mg per kg twice.

### Neutropaenic mouse thigh and lung infection model

Neutropenia was induced in male CD-1 mice by administration of two successive intraperitoneal injections on day −4 and day −1 of cyclophosphamide monohydrate (CPM) before the start of treatment with MCPs (RO7075573 or ZAB) or control standard of care antibiotics (colistin, meropenem or tigecycline). An intramuscular inoculation of a bacterial suspension of approximately 10^6^ CFU per thigh was used to induce the infection. Treatment started 2 h after infection. Total doses of RO7075573, administered subcutaneously every 4 h, ranged from 1.8 to 180 mg per kg per day. Total doses of zosurabalpin, administered subcutaneously every 6 h, ranged from 6 to 360 mg per kg per day. Thigh bacterial burden was determined after 24 h of treatment. In the pneumonia model, an intratracheal inoculation of approximately 10^7^ CFU per lung was used to induce the infection. Treatment started 2 h after infection. Total doses of zosurabalpin, administered subcutaneously every 6 h, ranged from 6 to 360 mg per kg per day. Lung bacterial burden was determined after 24 h treatment. Standard of care antibiotic was tested at a single dose in both infection models. GraphPad Prism 8 was used for graphical presentation and to analyse data.

### Reporting summary

Further information on research design is available in the Nature Portfolio Reporting Summary linked to this article.

## Online content

Any methods, additional references, Nature Portfolio reporting summaries, source data, extended data, supplementary information, acknowledgements, peer review information; details of author contributions and competing interests; and statements of data and code availability are available at 10.1038/s41586-023-06873-0.

### Supplementary information

Supplementary Information Supplementary Tables 1–4 and 7–9, the sequences of the plasmids used in this study, analytical data of the target compounds, synthetic chemistry of all of the compounds described in this paper and genome assemblies of the four *A. baumannii* isolates used in morbidostat studies.

Reporting Summary

Supplementary Fig. 1 Biochemical assay uncropped immunoblots. Uncropped gels and blots for data shown in Fig. 3. For each figure, the cropped regions are denoted by boxes.

Supplementary Table 5 WGS data: highly ranked variants and graphs. Summary of all of the results obtained in the run of four *A. baumannii* strains with zosurabalpin (RO7223280/RG6006) in MHB medium with and without HS.

Supplementary Table 6 Combined sequence and MIC measurement data for a collection of 84 non-redundant clones selected from eight evolutionary runs of *A. baumannii* (4 strains × 2 media) with zosurabalpin (RO7223280/RG6006).

Supplementary Data 1 Source data for Table 1.

Supplementary Data 2 Source data for Table 2.

Supplementary Data 3 Source data for Extended Data Table 7.

### Source data

Source Data Fig. 1

Source Data Fig. 4

Source Data Extended Data Fig. 2

Source Data Extended Data Fig. 3

## Figures and Tables

**Extended Data Table 1:**
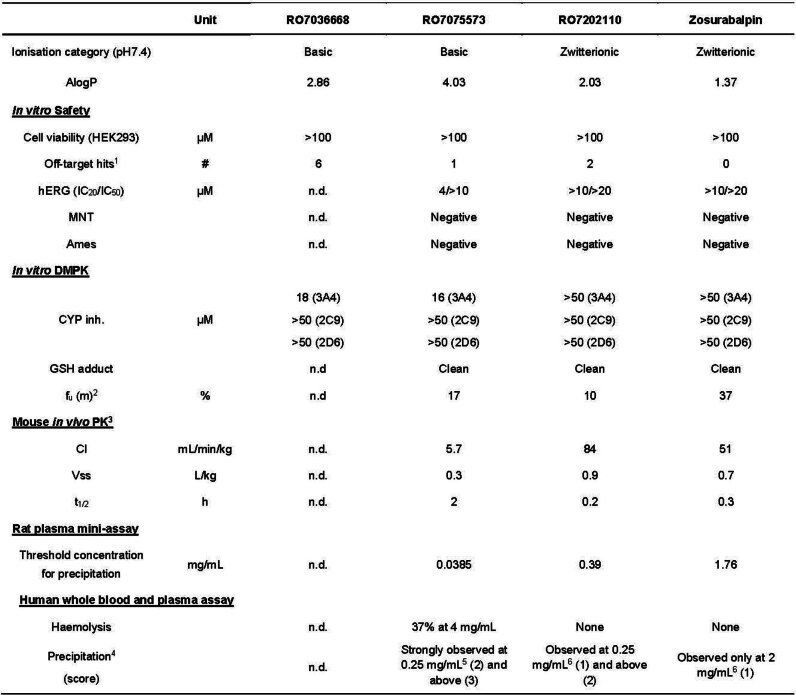
Summary of DMPK & in vitro safety profile of RO7036668, RO7075573, RO7202110, zosurabalpin

^1^ > 30% inhibition, see Supplementary Table 1 for details; ^2^Fraction unbound in mouse plasma; ^3^1 mg/kg IV; ^4^Scores from 0 (no precipitation) to 3 (strong precipitation), ^5^Concentrations of formulated compound from 0.06–4 mg/mL tested; ^6^Concentrations of formulated compound from 0.125–2 mg/mL tested. MNT, micronucleus test; Cl, Clearance; Vss, volume of distribution at steady state; t_1/2_, terminal plasma half-life; n.d., not determined.

**Extended Data Table 2:**
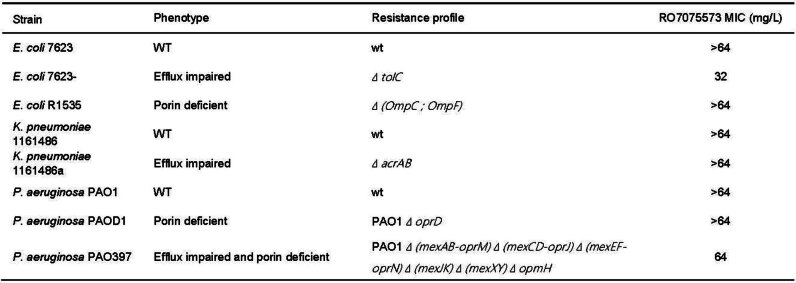
MIC of RO7075573 versus efflux impaired and porin deficient strains of *E. coli, K. pneumoniae* and *P. aeruginosa*

MIC broth microdilution conducted in CAMHB, read at 100% bacterial growth inhibition. *acrA/B*, acriflavine resistance protein A/B; *mexA/B/C/D/E/F/J/K*, multidrug efflux protein A/B/C/D/E/F/J/K; *ompC/F*, outer membrane porin protein C/F; *opmH*, outer membrane protein M family protein H; *oprD/J/M/N*, outer membrane porin protein D/J/M/N; *tolC*, colicin tolerance protein; WT/wt, wild-type.

**Extended Data Table 3:**
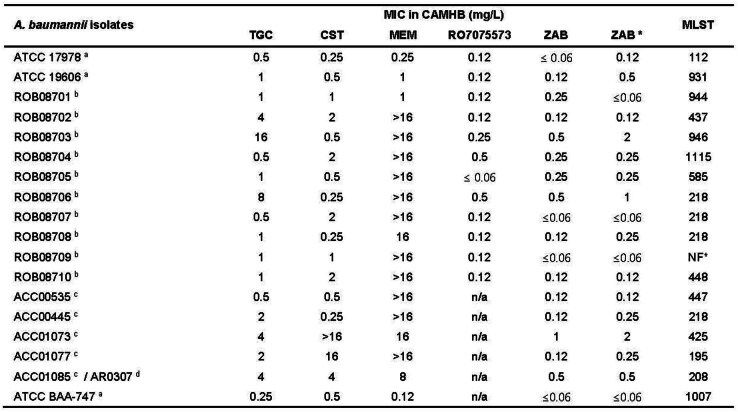
*A. baumannii* isolates, including their phenotypic and MLST profile, selected for use in in vitro and in vivo studies

MIC broth microdilution conducted in CAMHB for tigecycline (TGC), colistin (CST), meropenem (MEM), RO7075573, and zosurabalpin (ZAB), and in CAMHB with 20% human serum (HS) for ZAB*. RO7075573 and ZAB endpoint MIC value in CAMHB read at 80% of bacterial growth inhibition due to observed trailing effect. MIC values represent the mode of ≥3 replicates, with the following exceptions: for ATCC 17978, the MIC value of 1 replicate is reported for RO7075573; for isolates ROB08701, ROB08702, ROB08704, ROB08707, ROB08709, ROB08710, the MIC value of 1 replicate is reported for TGC, CST, MEM and ZAB. MLST, multilocus sequence typing; n/a, not assessed; NF, Not Found (MLST could not be determined because one housekeeping gene necessary for MLST classification was not identified in this isolate). ^a^ from ATCC; ^b^ from LGC; ^c^ from Aptuit; ^d^ from CDC.

**Extended Data Table 4:**
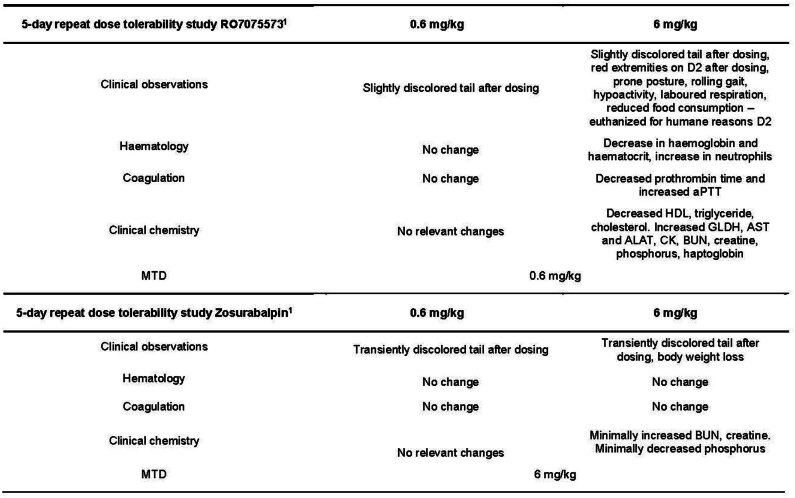
Summary of safety findings of RO7075573 and zosurabalpin in rats

^1^Slow IV bolus administration once daily for 5 days, n = 4 males per group; MTD, Maximum Tolerated Dose; aPTT, activated partial thromboplastin time; HDL, high density lipoprotein; GLDH, glutamate dehydrogenase; AST, aspartate aminotransferase; ALT (or ALAT), alanine aminotransferase; CK, creatine kinase; BUN, blood urea nitrogen.

**Extended Data Table 5:**
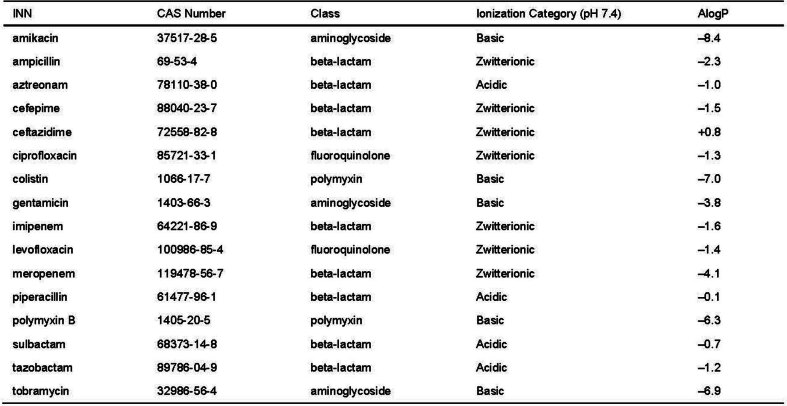
Standard of care antibiotics for hospital-acquired and ventilator-associated pneumonia caused by gram-negative bacteria

Ionization and AlogP values for standard of care antibiotics^27^.

**Extended Data Table 6:**
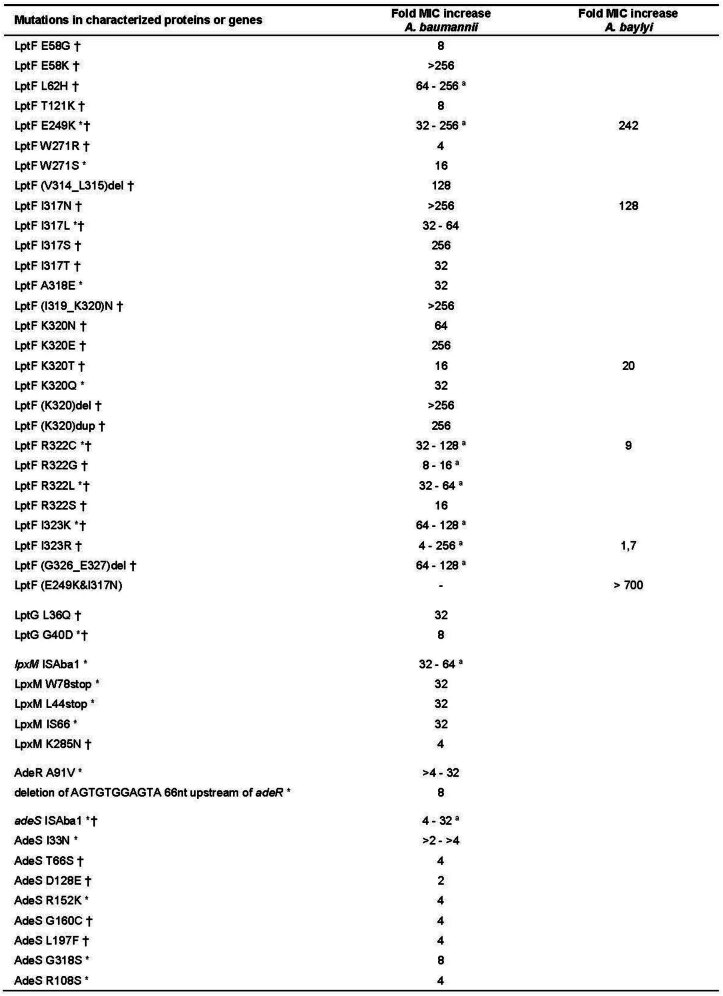
Fold MIC of zosurabalpin versus *A. baumannii* resistant mutants selected in spontaneous mutation and morbidostat studies and *A. baylyi* constructed mutants

Fold MIC increase of resistant mutants compared to parental strains. MIC conducted as broth microdilution in CAMHB with 20% HS for *A. baumannii* and read by naked eye as complete growth inhibition. *A. baylyi* MICs were measured similarly to the *A. baumannii*, except that the cells were grown in LB instead of CAMHB, and OD600 nm was recorded using a plate reader. MIC values corresponded to the lowest compound concentration inhibiting bacterial growth beyond which OD600 nm ceased to decrease.

* *A. baumannii* resistant mutants selected in spontaneous mutation studies; † *A. baumannii* resistant mutants selected in morbidostat studies; ^a^ fold MIC ranges provided when several mutants were characterized and showing different MIC values; ISAba1, gene interrupted by insertion sequence *Aba*1.

**Extended Data Table 7:**
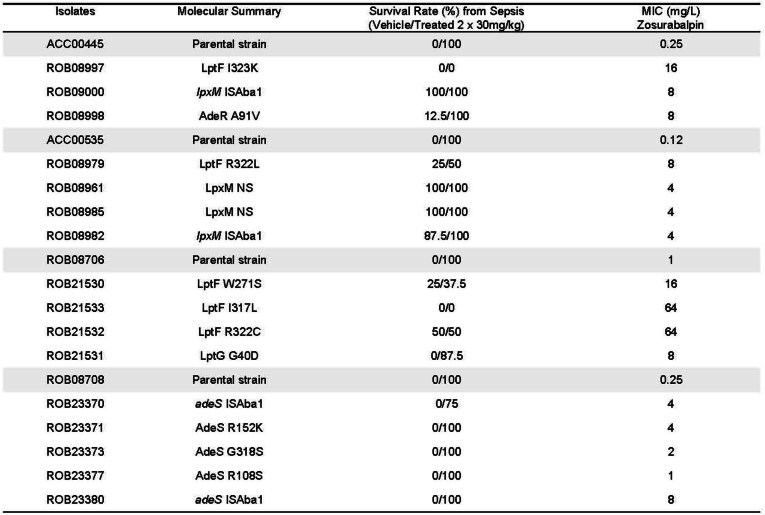
Summary of immunocompetent mouse septicemia infection induced by selected *Acinetobacter baumannii* mutants with and without zosurabalpin treatment

MIC broth microdilution conducted in CAMHB with 20% HS. ISAba1, Insertion sequence Aba1; NS, Nonsense mutation (gain of stop codon).

## Data Availability

All data supporting the finding of this study are available within the Article and its Supplementary Information or have been deposited to the indicated databases. Sequencing reads are deposited in the NCBI Sequence Read Archive (SRA) under accession code PRJNA1026547 (spontaneous mutant profiling) and PRJNA1016345 (morbidostat). Source data are provided with this paper.
