# Author Correction: A novel antibiotic class targeting the lipopolysaccharide transporter

**DOI:** 10.1038/s41586-024-07641-4

**Published:** 2024-07-11

**Authors:** Claudia Zampaloni, Patrizio Mattei, Konrad Bleicher, Lotte Winther, Claudia Thäte, Christian Bucher, Jean-Michel Adam, Alexander Alanine, Kurt E. Amrein, Vadim Baidin, Christoph Bieniossek, Caterina Bissantz, Franziska Boess, Carina Cantrill, Thomas Clairfeuille, Fabian Dey, Patrick Di Giorgio, Pauline du Castel, David Dylus, Pawel Dzygiel, Antonio Felici, Fernando García-Alcalde, Andreas Haldimann, Matthew Leipner, Semen Leyn, Séverine Louvel, Pauline Misson, Andrei Osterman, Karanbir Pahil, Sébastien Rigo, Adrian Schäublin, Sebastian Scharf, Petra Schmitz, Theodor Stoll, Andrej Trauner, Sannah Zoffmann, Daniel Kahne, John A. T. Young, Michael A. Lobritz, Kenneth A. Bradley

**Affiliations:** 1 Roche Pharma Research and Early Development, Immunology, Infectious Disease and Ophthalmology, Roche Innovation Center Basel, F. Hoffmann-La Roche, Basel, Switzerland; 2 Roche Pharma Research and Early Development, Therapeutic Modalities, Roche Innovation Center Basel, F. Hoffmann-La Roche, Basel, Switzerland; 3 SixPeaks Bio, Basel, Switzerland; 4 Roche Pharma Research and Early Development, Pharmaceutical Sciences, Roche Innovation Center Basel, F. Hoffmann-La Roche, Basel, Switzerland; 5 Preclinical Sciences and Translational Safety, Janssen Pharmaceutica, Beerse, Belgium; 6 AutoChem R&D, Mettler-Toledo International, Greifensee, Switzerland; 7 Independent consultant, Cambridge, Great Britain; 8 Department of Chemistry and Chemical Biology, Harvard University, Cambridge, MA USA; 9 Discovery Microbiology, Aptuit (Verona) Srl, an Evotec Company, Verona, Italy; 10 Infectious and Inflammatory Disease Center, Sanford Burnham Prebys Medical Discovery Institute, La Jolla, CA USA; 11 Roche Pharma Research and Early Development, Informatics, Roche Innovation Center Basel, F. Hoffmann-La Roche, Basel, Switzerland; 12 Therapeutics Discovery, Janssen Pharmaceutica, Beerse, Belgium

**Keywords:** Antibiotics, Pharmaceutics, Target identification

Correction to: *Nature* 10.1038/s41586-023-06873-0 Published online 3 January 2024

In the version of the article initially published, three references were missing and have now been added as refs. 20–22: Schuster, M. et al. Peptidomimetic antibiotics disrupt the lipopolysaccharide transport bridge of drug-resistant Enterobacteriaceae. *Sci. Adv*. **9**, eadg3683 (2023); Martin-Loeches, I., Dale, G. E. & Torres, A. Murepavadin: a new antibiotic class in the pipeline. *Expert Rev. Anti-infect. Ther*. **16**, 259–268 (2018) and Li, D. & Schneider-Futschik, E. K. Current and emerging inhaled antibiotics for chronic pulmonary *Pseudomonas aeruginosa* and *Staphylococcus aureus* infections in cystic fibrosis. *Antibiotics (Basel)* **12**, 484–505 (2023). These have been added to the HTML and PDF versions of the article.

