# Supplementary material for: A novel antibiotic class targeting the lipopolysaccharide transporter
Source: Nature. 2024 Jan 3;625(7995):566–71. doi: 10.1038/s41586-023-06873-0 (PMC10794144; doi:10.1038/s41586-023-06873-0)
Supplement: Supplementary file 1 — Supplementary Tables 1–4 and 7–9, the sequences of the plasmids used in this study, analytical data of the target compounds, synthetic chemistry of all of the compounds described in this paper and genome assemblies of the four A. baumannii isolates used in morbidostat studies. [file 41586_2023_6873_MOESM1_ESM.pdf]

nature portfolio

https://doi.org/10.1038/s41586-023-06873-0

Supplementary information

A novel antibiotic class targeting the lipopolysaccharide transporter

In the format provided by the authors and unedited

Supplementary Table 1 │ Off-target activity screening for MCP compounds

Assay (species)	RO7036668	RO7075573	RO7202110	zosurabalpin
5-HT transporter (human)	13	16	13	2
5HT1A (human)	33	11	13	0
5HT2A (human)	-2	-17	4	3
5HT2B (human)	27	1	14	-1
5HT3 (human)	6	8	6	3
Abl kinase (human)	-340	-85	-24	-18
Acetylcholinesterase (human)	9	4	40	13
Adenosine A1 respetor (human)	1	-10	1	-7
Adenosine A3 respetor (human)	32	11	9	2
Alpha1A-adrenoreceptor (human)	15	22	7	7
Alpha2A-adrenoreceptor (human)	-2	-5	-2	4
Androgen receptor (human)	6	-4	3	-5
Angiotensin converting enzyme (human)	-7	-7	3	-16
Angiotensin II receptor 1 (human)	14	-7	14	6
Beta1-adrenoreceptor (human)	12	-2	-2	10
Beta2-adrenoreceptor (human)	3	-7	-11	6
Ca2+ channel (diltiazem site) (rat)	12	-1	2	-11
Cannabinoid receptor CB1 (human)	-26	-16	-24	-20
CDK2 (human)	9	-5	17	9
Cholecystokinin 1 receptor (human)	22	32	0	-1
Cyclo oxygenase 2 (human)	4	-46	12	-7
Dopamine D1 receptor (human)	27	8	-7	-3
Dopamine D2 receptor (human)	74	28	28	14
Estrogen receptor alpha (human)	24	-3	-7	7
GABA Cl- channel receptor (rat)	10	-9	-1	1
GABA-A (benzodiazapine binding site) (rat)	6	-20	14	-1
Glucocorticoid receptor (human)	3	9	9	-1
Glycine receptor, strychnine insensitive (rat)	-27	-29	-25	15
GSK-3A (human)	-12	-1	22	-1
GSK-3A (human)	-2	10	-6	-3
Histamine H1 receptor (human)	16	4	-3	-4
Histamine H2 receptor (human)	12	11	3	-2
Histamine H3 receptor (human)	12	-4	5	5
HIV-1 protease (HIV-1)	19	-4	12	16
Kappa opioid receptor (human)	69	26	32	9
Matrix metalloprotease-9 (human)	6	-31	13	10
Monoamine oxidase-A (human)	-2	3	11	2
Mu opioid receptor (human)	63	21	26	4
Muscarinic receptor M1 (human)	26	20	-6	-3
Muscarinic receptor M2 (human)	14	11	-1	17
Nicotinic receptor, muscle (human)	18	5	11	-16
Nicotinic receptor, neuronal (human)	-2	-7	4	-11
Norepinephrine transporter (human)	43	1	14	12
PCP receptor (rat)	-12	-10	9	-24
Phosphodiesterase 3B (human)	17	-3	2	2
Phosphodiesterase 4D2 (human)	n.d.	0	-3	12
PPAR gamma (human)	-1	-16	-8	-4
Prostaglandin F receptor (human)	2	-4	-3	5
Xanthine oxidase (bovine)	-16	6	0	5
ZAP70 kinase (human)	2	-47	-39	1

The assays given in the table above were conducted at Eurofins CEREP SA (Celle-Lévescault, France) and were run as single point measurements in duplicates in the presence of 10 µM of the test compound and reported as percent inhibition of the radioligand signal or control enzymatic activity. n.d., not determined.

Supplementary Table 2 │Standard-of-care antibiotics antibiogram of *A. baumannii* isolates used in *in vitro* and *in vivo* studies

**Bacterial species**	**VITEK data**											
	**GM**	**AMC**	**AMI**	**CTX**	**IPM**	**TZP**	**CIP**	**CAZ**	**TGC**	**SXT**	**FEP**	**MEM**
A. baumannii ATCC 17978	≤ 1 S	4 R		8 S	≤ 0.25 S	≤ 4 S	≤ 0.25 S	4 S	≤ 0.5 S	160 R		≤ 0.25 S
A. baumannii ATCC 19606	8 I	≥ 32 R		32 I	≤ 0.25 S	8 S	1 S	16 I	≤ 0.5 S	≥ 320 R		1 S
A. baumannii ROB08701	8 I			≥ 64 R	0.5 S	≥ 128 R	≥ 4 R	≥ 64 R	≤ 0.5 S	≥ 320 R	32 R	0.5 S
A. baumannii ROB08702	8 I			≥ 64 R	≥ 16 R	≥ 128 R	≥ 4 R	≥ 64 R	2 S	≥ 320 R	≥ 64 R	≥ 16 R
A. baumannii ROB08703	≥ 16 R			≥ 64 R	≥ 16 R	≥ 128 R	≥ 4 R	≥ 64 R	≥ 8 R	≥ 320 R	≥ 64 R	≥ 16 R
A. baumannii ROB08704	≥ 16 R			≥ 64 R	≥ 16 R	≥ 128 R	≥ 4 R	≥ 64 R	4 I	≥ 320 R	≥ 64 R	≥ 16 R
A. baumannii ROB08705	≤ 1 S			≥ 64 R	≥ 16 R	≥ 128 R	≥ 4 R	16 R	≤ 0.5 S	≤ 20 S	≥ 64 R	≥ 16 R
A. baumannii ROB08706	≥ 16 R			≥ 64 R	≥ 16 R	≥ 128 R	≥ 4 R	≥ 64 R	≥ 8 R	≥ 320 R	≥ 64 R	≥ 16 R
A. baumannii ROB08707	≥ 16 R			≥ 64 R	≥ 16 R	≥ 128 R	≥ 4 R	≥ 64 R	≤ 0.5 S	160 R	≥ 64 R	≥ 16 R
A. baumannii ROB08708	≥ 16 R			≥ 64 R	≥ 16 R	≥ 128 R	≥ 4 R	≥ 64 R	≤ 0.5 S	160 R	32 R	≥ 16 R
A. baumannii ROB08709	≥ 16 R			≥ 64 R	≥ 16 R	≥ 128 R	≥ 4 R	≥ 64 R	2 S	≤ 20 S	≥ 64 R	≥ 16 R
A. baumannii ROB08710	≥ 16 R			≥ 64 R	≥ 16 R	≥ 128 R	≥ 4 R	≥ 64 R	≤ 0.5 S	≤ 20 S	≥ 64 R	≥ 16 R
A. baumannii ACC00535	≥ 16 R	≥ 32 R		≥ 64 R	8 I	≥ 128 R	≥ 4 R	≥ 64 R	≤ 0.5 S	≥ 320 R		8 I
A. baumannii ACC00445	≥ 16 R	≥ 32 R		≥ 64 R	≥ 16 R	≥ 128 R	≥ 4 R	≥ 64 R	2 S	≥ 320 R		≥ 16 R
A. baumannii ACC01073	≥ 16 R		8 S		≥ 16 R	≥ 128 R	≥ 4 R			≤ 20 S		≥ 16 R
A. baumannii ACC01077	≥ 16 R		≥ 64 R		≥ 16 R	≥ 128	≥ 4 R		1	≥ 320 R		≥ 16 R
A. baumannii ACC01085 / AR0307	≥ 16 R		8 S		2 S	≥ 128	≥ 4 R		4	≥ 320 R		8 I
A. baumannii ATCC BAA-747	≤ 1 S	4 R		8 S	≤ 0.25 S	≤ 4 S	≤ 0.25 S	4 S	≤ 0.5 S	≤ 20 S	2 S	≤ 0.25 S

Amoxicillin/Acid clavulanic (AMC), Amikacin (AMI), Ceftazidime (CAZ), Cefotaxime (CTX), Ciprofloxacin (CIP), Cefepime (FEP), Gentamicin (GM), Imipenem (IPM), Meropenem (MEM), Trimethoprim/Sulfamethoxazole (SXT), Tigecycline (TGC), Piperacillin/Tazobactam (TZP).

Supplementary table 3 │*A. baumannii* isolates, including their genotypic profile, selected for use in *in vitro* and *in vivo* studies

***A. baumannii* isolates**	**Resistance profile**
ATCC 17978 [a]	ant(3'')-IIa, OXA-259, ADC-26, sul2
ATCC 19606 [a]	ADC-158, ant(3'')-IIa, OXA-98, sul2
ROB08701 [b]	OXA-90, ADC-152, floR, aac(6')-Ian, sul2, aph(3')-Ia, ant(2'')-Ia, ant(3'')-IIa
ROB08702 [b]	OXA-66, sul1, qacEdelta1, OXA-20, aac(6')-Ib3, aph(3')-VIa, ADC-175, OXA-58, ant(3'')-IIa
ROB08703 [b]	ant(3'')-IIa, catA1, TEM, aac(3)-I, aadA1, qacE, tet(A), ADC-11, OXA-23, OXA-69, sul1
ROB08704 [b]	aph(3')-VIa, OXA-51, ant(3'')-IIa, sul2, ant(2'')-Ia, ADC, OXA-23
ROB08705 [b]	ADC-76, OXA-68, ant(3'')-IIa, aph(3')-VIb, OXA-23
ROB08706 [b]	tet(B), aph(6)-Id, aph(3'')-Ib, sul1, qacEdelta1, aadA1, catB8, aac(6')-Ib', mph(E), msr(E), armA, ADC-30, OXA-66, OXA-23, aph(3')-Ia, ant(3'')-IIa
ROB08707 [b]	OXA-66, aac(6')-Ib', msr(E), mph(E), catB8, aadA1, qacEdelta1, sul1, armA, ant(3'')-IIa, aph(3'')-Ib, aph(6)-Id, tet(B), ADC-30, OXA-23, aph(3')-Ia
ROB08708 [b]	ant(3'')-IIa, aph(6)-Id, aph(3'')-Ib, tet(B), aac(6')-Ib', msr(E), mph(E), catB8, aadA1, qacEdelta1, sul1, armA, OXA-66, ADC-30, OXA-23, aph(3')-Ia
ROB08709 [b]	ADC-25, ant(3'')-IIa, aph(6)-Id, aph(3'')-Ib, tet(B), OXA-66, PER-1, aac(3)-I, aadA1, qacEdelta1, sul1, aph(3')-VIb, OXA-58
ROB08710 [b]	aph(3'')-Ib, aph(6)-Id, tet(B), OXA-66, ADC-30, aac(3)-I, aadA1, qacEdelta1, sul1, ant(3'')-IIa, OXA-23, TEM-1, aph(3')-Ia
ACC00535 [c]	ADC-type, ant(3'')-IIa, OXA-68, tet(B), aph(6)-Id, aph(3'')-Ib, OXA-366, aac(3)-IIa, aac(6')-Ian, TEM-1, sul2
ACC00445 [c]	aph(3'')-Ib, aph(6)-Id, tet(B), ant(3'')-IIa, OXA-66, aac(6')-Ib', msr(E), mph(E), catB8, aadA1, qacEdelta1, sul1, armA, ADC-30, OXA-23, aph(3')-Ia
ACC01073 [c]	ADC-73, aph(6)-Id, aph(3'')-Ib, tet(B), ant(3'')-IIa, armA, OXA-66, mph(E), msr(E), OXA-23, TEM-1, aph(3')-Ia
ACC01077 [c]	sul1, qacEdelta1, OXA-23, OXA-66, aph(3')-VIa, aph(3'')-Ib, aph(6)-Id, tet(B), ADC-73, mph(E), msr(E), armA, ant(3'')-IIa, catA1, aadA1, aac(3)-I
ACC01085 [c] / AR0307 [d]	ADC-30, sul2, aph(3')-Ia, OXA-237, OXA-66, aph(6)-Id, aph(3'')-Ib, ant(3'')-IIa, aac(6')-Ib', msr(E), mph(E), catB8, aadA1, qacEdelta1, sul1, armA, tet(B)
ATCC BAA-747 [a]	ADC-type, OXA-383

ADC, Acinetobacter-derived cephalosporinases; OXA, Oxacillinase; PER, Pseudomonas extended resistant extended-spectrum beta-lactamase; TEM, Temoniera beta-lactamase.
[a] from ATCC; [b] from LGC; [c] from Aptuit; [d] from CDC.

Associated resistance: Beta-lactams: ADC-type, OXA-type, TEM-1, PER-1; Aminoglycosides: aac-type, aadA1, ant-type, aph-type, armA; Sulphonamide: sul1, sul2; Chloramphenicol: catA1, catB8, floR; Tetracycline: tet(A), tet(B); Macrolide, Lincosamide and Streptogramin B: mph(E), msr(E). Quaternary ammonium compounds: qacE, qacEdelta1.

Supplementary Table 4. Line listing of all mutants obtained in spontaneous mutation.

Roche ID	parental strain	Drug pressure	Mutations in characterized genes	MIC in CAMHB (mg/L)			
				ZAB (p1)	ZAB (p10)	CST	MEM
parental strain	ACC00445 [FoR: 2×10^{-7}]			0.25		0.25	>16
ROB08997	ACC00445	2x MIC	LptF I323K	16	16	0.25	16
ROB08998	ACC00445	2x MIC	AdeR A91V	8	8	0.25	16
ROB08996	ACC00445	2x MIC	*lpxM* 412::IS*Aba*1	16	8	0.25	16
ROB08999	ACC00445	2x MIC	*lpxM* 856::IS*Aba*1	8	8	0.25	8
parental strain	ACC00535 [FoR: 2×10^{-7}]			0.125		0.5	>16
ROB08979	ACC00535	2x MIC	LptF R322L	8	8	0.25	16
ROB08980	ACC00535	2x MIC	*lpxM* 90::IS*Aba*1	4	2	0.25	16
ROB08981	ACC00535	2x MIC	LpxM W78stop	4	4	0.5	16
ROB08982	ACC00535	2x MIC	*lpxM* 412::IS*Aba*1	4	4	0.5	16
ROB08983	ACC00535	2x MIC	LpxM L44stop	4	4	1	16
ROB08984	ACC00535	2x MIC	*lpxM* 781::IS66	4	4	0.25	16
parental strain	ACC01073 [FoR: 1×10^{-8}]			2		>16	>16
ROB21502	ACC01073	2x MIC	LptF K320Q	64	64	>16	16
ROB21503	ACC01073	2x MIC	LptF K320Q	64	32	>16	16
parental strain	ACC01077 [FoR: 4×10^{-9}]			0.25		16	>16
ROB21506	ACC01077	2x MIC	LptF E249K	8	4	16	>16
parental strain	ACC01085 [a] [FoR: 1×10^{-6}]			0.5		8	8
ROB21523	ACC01085	2x MIC	deletion of AGTGTGGAGTA 66nt upstream of *adeR*	4	4	2	8
ROB21524	ACC01085	2x MIC	deletion of AGTGTGGAGTA 66nt upstream of *adeR*	4	4	2	16
ROB21525	ACC01085	2x MIC	deletion of AGTGTGGAGTA 66nt upstream of *adeR*	4	4	2	8
ROB21526	ACC01085	2x MIC	deletion of AGTGTGGAGTA 66nt upstream of *adeR*	4	4	8	8
ROB21527	ACC01085	2x MIC	deletion of AGTGTGGAGTA 66nt upstream of *adeR*	4	4	2	8
ROB21528	ACC01085	2x MIC	deletion of AGTGTGGAGTA 66nt upstream of *adeR*	4	4	4	8
ROB21529	ACC01085	2x MIC	deletion of AGTGTGGAGTA 66nt upstream of *adeR*	4	4	2	8
parental strain	ATCC BAA-747 [FoR: 2×10^{-7}]			≤0.06		0.5	0.5
ROB23898	ATCC BAA-747	2x MIC	AdeS I33N	0.5	0.25	0.25	0.5
ROB23899	ATCC BAA-747	2x MIC	AdeS I33N	0.5	0.5	0.5	0.5
ROB23900	ATCC BAA-747	2x MIC	AdeR A91V	0.5	0.5	0.5	0.5
ROB23901	ATCC BAA-747	2x MIC	AdeS I33N	0.5	0.25	0.5	1
ROB23902	ATCC BAA-747	2x MIC	AdeS I33N	0.5	0.25	0.25	1
ROB23903	ATCC BAA-747	2x MIC	AdeS I33N	0.25	0.25	0.5	1
parental strain	ROB08706 [FoR: 4×10^{-8}]			1		0.5	>16
ROB21531	ROB08706	2x MIC	LptG G40D	8	8	0.25	>16
ROB21530	ROB08706	2x MIC	LptF W271S	16	8	0.25	>16
parental strain	ROB08708 [FoR: 2×10^{-7}]			0.25		0.25	16
ROB23371	ROB08708	2x MIC	AdeS R152K	4	2	0.25	16
ROB23370	ROB08708	2x MIC	*adeS* 369::IS*Aba*1	4	2	0.25	16
ROB23372	ROB08708	2x MIC	*adeS* 369::IS*Aba*1	4	0.5	0.5	16
ROB23373	ROB08708	2x MIC	AdeS G318S	2	4	0.25	16
ROB23374	ROB08708	2x MIC	*adeS* 372::IS*Aba*1	4	4	0.25	16
ROB23375	ROB08708	2x MIC	*adeS* 369::IS*Aba*1	4	0.25	0.5	16
ROB23376	ROB08708	2x MIC	*adeS* 369::IS*Aba*1	4	0.25	0.5	16
ROB23377	ROB08708	2x MIC	AdeS R108S	1	2	0.5	16
parental strain	ACC00445 [FoR: 7×10^{-8}]			0.25		0.25	>16
ROB09000	ACC00445	4x MIC	*lpxM* 412::IS*Aba*1	8	8	0.25	8
ROB21501	ACC00445	4x MIC	*lpxM* 129::IS*Aba*1	8	8	0.25	16
parental strain	ACC00535 [FoR: 2×10^{-7}]			0.125		0.5	>16
ROB08985	ACC00535	4x MIC	LpxM L44stop	4	4	0.25	16
ROB08986	ACC00535	4x MIC	LpxM L44stop	4	4	0.25	16
ROB08987	ACC00535	4x MIC	*lpxM* 828::IS*Aba*1	4	4	1	16
ROB08988	ACC00535	4x MIC	*lpxM* 524::IS66	4	4	0.25	16

Roche ID	parental strain	Drug pressure	Mutations in characterized genes	MIC in CAMHB (mg/L)			
				ZAB (p1)	ZAB (p10)	CST	MEM
ROB08989	ACC00535	4x MIC	*lpxM* 760::IS66	4	4	0.25	16
ROB08990	ACC00535	4x MIC	*lpxM* 110::IS*Aba*1	4	4	0.5	>16
ROB08991	ACC00535	4x MIC	*lpxM* 412::IS*Aba*1	4	4	0.25	>16
ROB08992	ACC00535	4x MIC	*lpxM* 811::IS66	4	4	0.25	16
parental strain	ACC01073 [FoR: 7 x 10^{-9}]			2		>16	>16
ROB21504	ACC01073	4x MIC	LptF A318E	64	64	>16	16
ROB21505	ACC01073	4x MIC	LptF R322C	>64	>64	>16	16
parental strain	ROB08706 [FoR: 6 x 10^{-8}]			1		0.5	>16
ROB21532	ROB08706	4x MIC	LptF R322C	64	64	0.25	>16
ROB21533	ROB08706	4x MIC	LptF I317L	64	64	0.25	>16
parental strain	ROB08708 [FoR: 4 x 10^{-7}]			0.25		0.25	16
ROB23378	ROB08708	4x MIC	*adeS* 369::IS*Aba*1	4	0.25	0.5	16
ROB23379	ROB08708	4x MIC	*adeS* 369::IS*Aba*1	4	0.25	0.5	16
ROB23380	ROB08708	4x MIC	*adeS* 370::IS*Aba*1	8	0.5	0.25	16
ROB23382	ROB08708	4x MIC	*adeS* 369::IS*Aba*1	4	0.5	0.5	16
ROB23383	ROB08708	4x MIC	*adeS* 369::IS*Aba*1	4	0.5	0.5	16
ROB23384	ROB08708	4x MIC	*adeS* 369::IS*Aba*1	4	0.25	0.5	16
ROB23385	ROB08708	4x MIC	*adeS* 370::IS*Aba*1	4	0.25	1	16
ROB23381	ROB08708	4x MIC	LptF R322C	8	8	0.25	16
parental strain	ACC00535 [FoR: 3 x 10^{-9}]			0.125		0.5	>16
ROB08993	ACC00535	8x MIC	LpxM L44stop	4	4	0.25	16
ROB08994	ACC00535	8x MIC	*lpxM* 582::IS*Aba*1	4	4	0.25	16
ROB08995	ACC00535	8x MIC	*lpxM* 412::IS*Aba*1	4	4	0.5	16

MIC broth microdilution conducted in cation-adjusted Mueller-Hinton broth (CAMHB) for colistin (CST) and meropenem (MEM), and in CAMHB with 20% human serum (HS) for zosurabalpin (ZAB).

[a] CDC isolate AR0307;

p1, passage 1; p10, passage 10; passages were performed on drug-free Tryptic Soy Agar (TSA) plates;

IS*Aba*1, gene interrupted by insertion sequence *Aba*1, IS66, gene interrupted by insertion sequence 66.

Supplementary Table 7. Line listing of zosurabalpin, TGC, CST and MEM MIC against the 129 clinical isolates

Bacterial isolates	Year of isolation	Country of isolation	MIC in CAMHB (mg/L)			
			ZAB	TGC	CST	MEM
A. baumannii 1072184	2014	United States	0.25	2	2	32
A. baumannii 1098735	2014	Spain	1	4	32	32
A. baumannii 1104061	2014	United States	0.25	2	2	32
A. baumannii 1104062	2014	United States	0.25	4	2	32
A. baumannii 1104059	2014	United States	1	4	32	32
A. baumannii 1089975	2014	Hungary	0.125	2	2	32
A. baumannii 1089062	2014	Greece	0.125	4	2	32
A. baumannii 1065391	2014	Italy	0.25	2	32	32
A. baumannii 1140100	2014	Jordan	0.06	1	2	8
A. baumannii 1093170	2014	Greece	0.25	4	32	32
A. baumannii 1065279	2014	Venezuela	0.25	2	1	16
A. baumannii 1154091	2014	Emirates	1	4	16	32
A. baumannii 1061067	2014	Mexico	0.25	4	4	32
A. baumannii 1061145	2014	Mexico	0.125	4	1	32
A. baumannii 1065549	2014	Mexico	0.06	1	1	2
A. baumannii 1126813	2014	United Kingdom	0.25	2	1	2
A. baumannii 1171976	2014	Argentina	0.06	1	2	32
A. baumannii 1172109	2014	Argentina	0.016	0.25	2	0.125
A. baumannii 1172126	2014	Argentina	0.06	0.5	1	32
A. baumannii 1161864	2014	Thailand	0.016	0.25	2	0.25
A. baumannii 1115481	2014	Panama	0.06	0.25	1	0.5
A. baumannii 1115495	2014	Panama	0.5	0.5	32	0.5
A. baumannii 1115510	2014	Panama	0.5	0.5	32	0.5
A. baumannii 1115514	2014	Panama	0.25	0.5	32	0.5
A. baumannii 1069554	2014	Panama	0.06	0.5	1	0.5
A. baumannii ROB08701	n/a	Italy	0.06	1	1	1
A. baumannii ROB08702	n/a	Italy	0.125	4	2	32
A. baumannii ROB08703	n/a	Italy	0.5	8	1	32
A. baumannii ROB08704	2008	Turkey	0.25	0.5	2	32
A. baumannii ROB08705	2008	n/a	0.06	0.5	1	32
A. baumannii ROB08706	n/a	Italy	0.125	8	2	32
A. baumannii ROB08707	n/a	Italy	0.03	0.5	2	32
A. baumannii ROB08708	n/a	Italy	0.125	1	4	32
A. baumannii ROB08709	2008	Turkey	0.03	1	1	32
A. baumannii ROB08710	2008	Turkey	0.125	1	2	32
A. baumannii 1630178	2017	Spain	0.25	0.5	32	32
A. baumannii 1630190	2017	Spain	1	1	32	32
A. baumannii 1630410	2017	Spain	0.25	0.25	32	0.5
A. baumannii 1631625	2017	France	0.06	1	1	4
A. baumannii 1631735	2017	France	0.5	4	1	32
A. baumannii 1631846	2017	United States	0.125	2	1	32
A. baumannii 1631847	2017	United States	2	4	32	32
A. baumannii 1631848	2017	United States	4	2	32	32
A. baumannii 1631849	2017	United States	0.06	2	1	32
A. baumannii 1631949	2017	Italy	0.5	4	32	32
A. baumannii 1631953	2017	Italy	0.5	4	8	32
A. baumannii 1631954	2017	Italy	0.25	4	2	32
A. baumannii 1631955	2017	Italy	0.06	2	4	32
A. baumannii 1632073	2017	United Kingdom	0.06	2	1	32
A. baumannii 1632166	2017	United States	0.25	2	32	2
A. baumannii 1632273	2017	United States	0.25	0.125	8	16
A. baumannii 1633081	2017	Italy	0.25	4	16	32

Bacterial isolates	Year of isolation	Country of isolation	MIC in CAMHB (mg/L)			
			ZAB	TGC	CST	MEM
A. baumannii 1633082	2017	Italy	0.5	4	16	32
A. baumannii 1633098	2017	Italy	0.25	2	16	32
A. baumannii 1633115	2017	Italy	0.25	4	32	32
A. baumannii 1633117	2017	Italy	1	4	8	32
A. baumannii 1633121	2017	Italy	0.5	4	32	32
A. baumannii 1633124	2017	Italy	1	4	32	32
A. baumannii 1633269	2017	Italy	2	4	32	32
A. baumannii 1633271	2017	Italy	1	4	32	32
A. baumannii 1633272	2017	Italy	1	8	32	32
A. baumannii 1728772	2017	Greece	0.25	4	16	32
A. baumannii 1728773	2017	Greece	0.5	8	32	32
A. baumannii 1728774	2017	Greece	0.25	8	16	32
A. baumannii 1728884	2017	Greece	0.5	8	32	32
A. baumannii 1729042	2017	Greece	2	4	32	32
A. baumannii 1729043	2017	Greece	1	4	32	32
A. baumannii 1729044	2017	Greece	1	8	32	32
A. baumannii 1729049	2017	Greece	2	8	32	32
A. baumannii 1729050	2017	Greece	2	8	32	32
A. baumannii 1729052	2017	Greece	1	8	32	32
A. baumannii 1729102	2017	Greece	4	8	32	32
A. baumannii 1729123	2017	Greece	0.5	4	32	32
A. baumannii 1729351	2017	Greece	0.5	4	32	32
A. baumannii 1729352	2017	Greece	0.25	4	32	32
A. baumannii 1729353	2017	Greece	0.25	4	32	32
A. baumannii 1729358	2017	Greece	0.25	4	32	32
A. baumannii 1729362	2017	Greece	1	8	32	32
A. baumannii 1729379	2017	Greece	1	4	32	32
A. baumannii 1864609	2018	United States	0.06	2	16	32
A. baumannii 1864833	2018	France	0.5	1	4	1
A. baumannii 1865057	2018	Germany	0.25	4	1	32
A. baumannii 1865083	2018	Italy	0.5	2	32	32
A. baumannii 1865084	2018	Italy	0.5	1	32	32
A. baumannii 1865085	2018	Italy	0.5	4	1	32
A. baumannii 1865086	2018	Italy	0.5	4	32	32
A. baumannii 1865266	2018	Italy	1	1	32	32
A. baumannii 1865269	2018	Italy	0.25	2	2	32
A. baumannii 1865273	2018	Italy	0.06	2	1	32
A. baumannii 1865274	2018	Italy	0.25	2	32	32
A. baumannii 1865386	2018	Spain	0.125	2	2	32
A. baumannii 1865387	2018	Spain	0.125	2	2	32
A. baumannii 1865494	2018	Spain	0.06	4	1	32
A. baumannii 1865714	2018	Spain	0.25	4	1	32
A. baumannii 1866281	2018	United States	0.125	2	16	32
A. baumannii 1866282	2018	United States	0.06	2	1	32
A. baumannii 1866283	2018	United States	0.03	2	1	32
A. baumannii 1866370	2018	United States	0.25	2	2	32
A. baumannii 1866376	2018	United States	0.25	8	1	32
A. baumannii 1868315	2018	United States	0.5	8	2	32
A. baumannii 1869068	2018	United States	1	8	0.5	32
A. baumannii 1869084	2018	Greece	4	16	32	32
A. baumannii 1869085	2018	Greece	1	8	32	32
A. baumannii 1869086	2018	Greece	1	4	32	32
A. baumannii 1869087	2018	Greece	1	4	32	32
A. baumannii 1869088	2018	Greece	0.5	4	32	32
A. baumannii 1869089	2018	Greece	0.25	4	32	32
A. baumannii 1869090	2018	Greece	0.25	4	32	32

Bacterial isolates	Year of isolation	Country of isolation	MIC in CAMHB (mg/L)			
			ZAB	TGC	CST	MEM
A. baumannii 1869091	2018	Greece	0.25	4	32	32
A. baumannii 1869093	2018	Greece	0.25	4	32	32
A. baumannii 1869173	2018	Greece	0.25	2	16	32
A. baumannii 1870044	2018	United States	0.03	0.125	2	0.5
A. baumannii 1870131	2018	United States	1	2	16	32
A. baumannii 1870152	2018	United States	0.25	2	8	32
A. baumannii 1870171	2018	United States	0.5	0.5	16	32
A. baumannii 1871948	2018	Turkey	1	8	32	32
A. baumannii 1872001	2018	Turkey	0.25	4	32	32
A. baumannii 1872360	2018	United States	4	16	1	32
A. baumannii 1887389	2018	Romania	0.06	1	1	32
A. baumannii 1887397	2018	Romania	0.06	2	1	32
A. baumannii 1887497	2018	United Kingdom	0.06	1	1	32
A. baumannii 1887498	2018	United Kingdom	0.06	8	1	32
A. baumannii 1897312	2018	Turkey	0.03	1	1	32
A. baumannii 1897314	2018	Turkey	0.25	4	16	32
A. baumannii 1897317	2018	Turkey	0.25	2	32	32
A. baumannii 1914727	2018	Turkey	0.5	2	32	32
A. baumannii 1914733	2018	Turkey	0.06	4	1	32
A. baumannii 1930837	2018	United States	0.06	2	1	32
A. baumannii 1930840	2018	United States	0.125	2	1	32

Minimum inhibitory concentration (MIC) broth microdilution conducted in cation-adjusted Mueller-Hinton broth (CAMHB) for tigecycline (TGC), colistin (CST) and meropenem (MEM), and in CAMHB with 20% human serum (HS) for zosurabalpin (ZAB).
n/a, not available.

Supplementary Table 8. Bacterial strains used in this work

Strains	Genotype	Source
NovaBlue	*endA1 hsd*R17 (r_K^-, m_K^+) *sup*E44 *thi*-1 *rec*A1 *gyr*A96 *rel*A1 *lac* F′[*pro*A$^+$ B$^+$ *lac*I^q ZΔM15::Tn*10*]	Novagen
BL21(DE3)	*fhuA2 [lon] ompT gal (λ DE3) [dcm] ΔhsdS*	Novagen
C43(DE3)	*F – ompT hsdSB (rB- mB-) gal dcm (DE3)*	Lucigen

Supplementary Table 9. Bacterial plasmids used in this work

Plasmid	Description	Source
pET22/42-LptC-thrombin-His$_7$	pET22/42 encoding full-length *Acinetobacter baylyi* LptC with a C-terminal cleavable His$_7$ tag	This paper
pCDFduet-LptB-LptFG	pCDFduet encoding full-length *Acinetobacter baylyi* LptB at the first site and *Acinetobacter baylyi* LptFG at the second site.	This paper
pCDFduet-LptB-LptF^{E249K}G	pCDFduet-LptB-LptFG with LptF(E249K)	This paper
pCDFduet-LptB-LptF^{I317N}G	pCDFduet-LptB-LptFG with LptF(I317N)	This paper
pCDFduet-LptB-LptF^{E249K,I317N}G	pCDFduet-LptB-LptFG with LptF(E249K, I317N)	This paper
pET22b-LptA(I36Am)	pET22b encoding LptA(I36Am) with a His tag.	Okuda, S. et al. 2012

Sequence of the plasmid pET22/42-LptC-thrombin-His7

CTAACAGCGCGATTTGCTGGTGACCCAATGCGACCAGATGCTCCACGCCCAGTCGCG
TACCGTCTTCATGGGAGAAAATAATACTGTTGATGGGTGTCTGGTCAGAGACATCAA
GAAATAACGCCGGAACATTAGTGCAGGCAGCTTCCACAGCAATGGCATCCTGGTCA
TCCAGCGGATAGTTAATGATCAGCCCACTGACGCGTTGCGCGAGAAGATTGTGCACC
GCCGCTTTACAGGCTTCGACGCCGCTTCGTTCTACCATCGACACCACCACGCTGGCA
CCCAGTTGATCGGCGCGAGATTTAATCGCCGCGACAATTTGCGACGGCGCGTGCAG
GGCCAGACTGGAGGTGGCAACGCCAATCAGCAACGACTGTTTGCCCGCCAGTTGTT
GTGCCACGCGGTTGGGAATGTAATTCAGCTCCGCCATCGCCGCTTCCACTTTTTCCC
GCGTTTTCGCAGAAACGTGGCTGGCCTGGTTCACCACGCGGGAAACGGTCTGATAA

GAGACACCGGCATACTCTGCGACATCGTATAACGTTACTGGTTTCACATTCACCACC
CTGAATTGACTCTCTTCCGGGCGCTATCATGCCATACCGCGAAAGGTTTTGCGCCAT
TCGATGGTGTCCGGGATCTCGACGCTCTCCCTTATGCGACTCCTGCATTAGGAAGCA
GCCCAGTAGTAGGTTGAGGCCGTTGAGCACCGCCGCCGCAAGGAATGGTGCATGCA
AGGAGATGGCGCCCAACAGTCCCCCGGCCACGGGGCCTGCCACCATACCCACGCCG
AAACAAGCGCTCATGAGCCCGAAGTGGCGAGCCCGATCTTCCCCATCGGTGATGTC
GGCGATATAGGCGCCAGCAACCGCACCTGTGGCGCCGGTGATGCCGGCCACGATGC
GTCCGGCGTAGAGGATCGAGATCTCGATCCCGCGAAATTAATACGACTCACTATAG
GGGAATTGTGAGCGGATAACAATTCCCCTCTAGAAAGAATTTTGTTTAACTTTAAGA
AGGAGATATACATATGGATACCAGAGCTCTTTATATTGCTGCTGTTGTAATCGCAAG
TATTAGTGGCGGTTACTATTACTATAGTGGTAAGGCAAAAAAACTTGATGTCGACTC
GGCTCGAAATATGACTTATTCGGCCGAGGGAGTAAATCTTACACAAACGGATGATC
AAGGGCATTTGTATGTACGTGCAACAGTAGATCGTCTAGAGCAAAATATGCAAAAT
CAGACATCTAAGTTGGAAAATCTGAATGCATCCACATATAAAGCTGGCAATGTCGAT
TCAACATTTTTTTCTAAATATGCCTATGGTTACAATGACAATGAAAAGGTTGTCCTTT
CAGATCAAGTTGTTGCAACTAAACTCTCTGATCATGGAAAAATGCAATTTCAAACTG
ATGAGTTAACCACCTATCCTCCGAAAAAAATGATTGAGACGACTCATCAAGTCAATG
TGGATTCTCCACAGTCATCTTTTATCAGTCAGGGATTAAAAGCAGATTTAAACACTG
GTCAATACGAGTTTTTTAATATTCGAGGAAAGTATGCACCGAATTCTCTGGTACCGC
GCGGCAGCCACCACCACCACCACCACTAATTGATTAATACCTAGGCTGCTAAAC
AAAGCCCGAAAGGAAGCTGAGTTGGCTGCTGCCACCGCTGAGCAATAACTAGCATA
ACCCCTTGGGGCCTCTAAACGGGTCTTGAGGGGTTTTTTGCTGAAAGGAGGAACTAT
ATCCGGATTGGCGAATGGGACGCGCCCTGTAGCGGCGCATTAAGCGCGGCGGGTGT
GGTGGTTACGCGCAGCGTGACCGCTACACTTGCCAGCGCCCTAGCGCCCGCTCCTTT
CGCTTTCTTCCCTTCCTTTCTCGCCACGTTCGCCGGCTTTCCCCGTCAAGCTCTAAAT
CGGGGGCTCCCTTTAGGGTTCCGATTTAGTGCTTTACGGCACCTCGACCCCAAAAAA
CTTGATTAGGGTGATGGTTCACGTAGTGGGCCATCGCCCTGATAGACGGTTTTTCGC
CCTTTGACGTTGGAGTCCACGTTCTTTAATAGTGGACTCTTGTTCCAAACTGGAACA
ACACTCAACCCTATCTCGGTCTATTCTTTTGATTTATAAGGGATTTTGCCGATTTCGG
CCTATTGGTTAAAAAATGAGCTGATTTAACAAAAATTTAACGCGAATTTTAACAAAA
TATTAACGCTTACAATTTAGGTGGCACTTTTCGGGGAAATGTGCGCGGAACCCCTAT
TTGTTTATTTTTCTAAATACATTCAAATATGTATCCGCTCATGAGACAATAACCCTGA
TAAATGCTTCAATAATATTGAAAAAGGAAGAGTATGAGTATTCAACATTTCCGTGTC
GCCCTTATTCCCTTTTTTGCGGCATTTTGCCTTCCTGTTTTTGCTCACCCAGAAACGCT
GGTGAAAGTAAAAGATGCTGAAGATCAGTTGGGTGCACGAGTGGGTTACATCGAAC
TGGATCTCAACAGCGGTAAGATCCTTGAGAGTTTTCGCCCCGAAGAACGTTTTCCAA
TGATGAGCACTTTTAAAGTTCTGCTATGTGGCGCGGTATTATCCCGTATTGACGCCG
GGCAAGAGCAACTCGGTCGCCGCATACACTATTCTCAGAATGACTTGGTTGAGTACT
CACCAGTCACAGAAAAGCATCTTACGGATGGCATGACAGTAAGAGAATTATGCAGT
GCTGCCATAACCATGAGTGATAACACTGCGGCCAACTTACTTCTGACAACGATCGGA

GGACCGAAGGAGCTAACCGCTTTTTTGCACAACATGGGGGATCATGTAACTCGCCTT
GATCGTTGGGAACCGGAGCTGAATGAAGCCATACCAAACGACGAGCGTGACACCAC
GATGCCTGCAGCAATGGCAACAACGTTGCGCAAACTATTAACTGGCGAACTACTTAC
TCTAGCTTCCCGGCAACAATTAATAGACTGGATGGAGGCGGATAAAGTTGCAGGAC
CACTTCTGCGCTCGGCCCTTCCGGCTGGCTGGTTTATTGCTGATAAATCTGGAGCCG
GTGAGCGTGGGTCTCGCGGTATCATTGCAGCACTGGGGCCAGATGGTAAGCCCTCCC
GTATCGTAGTTATCTACACGACGGGGAGTCAGGCAACTATGGATGAACGAAATAGA
CAGATCGCTGAGATAGGTGCCTCACTGATTAAGCATTGGTAACTGTCAGACCAAGTT
TACTCATATATACTTTAGATTGATTTAAAACTTCATTTTTAATTTAAAAGGATCTAGG
TGAAGATCCTTTTTGATAATCTCATGACCAAAATCCCTTAACGTGAGTTTTCGTTCCA
CTGAGCGTCAGACCCCGTAGAAAAGATCAAAGGATCTTCTTGAGATCCTTTTTTTCTG
CGCGTAATCTGCTGCTTGCAAACAAAAAAACCACCGCTACCAGCGGTGGTTTGTTTG
CCGGATCAAGAGCTACCAACTCTTTTTCCGAAGGTAACTGGCTTCAGCAGAGCGCAG
ATACCAAATACTGTCCTTCTAGTGTAGCCGTAGTTAGGCCACCACTTCAAGAACTCT
GTAGCACCGCCTACATACCTCGCTCTGCTAATCCTGTTACCAGTGGCTGCTGCCAGT
GGCGATAAGTCGTGTCTTACCGGGTTGGACTCAAGACGATAGTTACCGGATAAGGC
GCAGCGGTCGGGCTGAACGGGGGGTTCGTGCACACAGCCCAGCTTGGAGCGAACGA
CCTACACCGAACTGAGATACCTACAGCGTGAGCTATGAGAAAGCGCCACGCTTCCC
GAAGGGAGAAAGGCGGACAGGTATCCGGTAAGCGGCAGGGTCGGAACAGGAGAGC
GCACGAGGGAGCTTCCAGGGGGAAACGCCTGGTATCTTTATAGTCCTGTCGGGTTTC
GCCACCTCTGACTTGAGCGTCGATTTTTGTGATGCTCGTCAGGGGGGCGGAGCCTAT
GGAAAAACGCCAGCAACGCGGCCTTTTTACGGTTCCTGGCCTTTTGCTGGCCTTTTG
CTCACATGTTCTTTCCTGCGTTATCCCCTGATTCTGTGGATAACCGTATTACCGCCTT
TGAGTGAGCTGATACCGCTCGCCGCAGCCGAACGACCGAGCGCAGCGAGTCAGTGA
GCGAGGAAGCGGAAGAGCGCCTGATGCGGTATTTTCTCCTTACGCATCTGTGCGGTA
TTTCACACCGCAATGGTGCACTCTCAGTACAATCTGCTCTGATGCCGCATAGTTAAG
CCAGTATACACTCCGCTATCGCTACGTGACTGGGTCATGGCTGCGCCCCGACACCCG
CCAACACCCGCTGACGCGCCCTGACGGGCTTGTCTGCTCCCGGCATCCGCTTACAGA
CAAGCTGTGACCGTCTCCGGGAGCTGCATGTGTCAGAGGTTTTCACCGTCATCACCG
AAACGCGCGAGGCAGCTGCGGTAAAGCTCATCAGCGTGGTCGTGAAGCGATTCACA
GATGTCTGCCTGTTCATCCGCGTCCAGCTCGTTGAGTTTCTCCAGAAGCGTTAATGTC
TGGCTTCTGATAAAGCGGGCCATGTTAAGGGCGGTTTTTTCCTGTTTGGTCACTGATG
CCTCCGTGTAAGGGGGATTTCTGTTCATGGGGGTAATGATACCGATGAAACGAGAG
AGGATGCTCACGATACGGGTTACTGATGATGAACATGCCCGGTTACTGGAACGTTGT
GAGGGTAAACAACTGGCGGTATGGATGCGGCGGGACCAGAGAAAAATCACTCAGG
GTCAATGCCAGCGCTTCGTTAATACAGATGTAGGTGTTCCACAGGGTAGCCAGCAGC
ATCCTGCGATGCAGATCCGGAACATAATGGTGCAGGGCGCTGACTTCCGCGTTTCCA
GACTTTACGAAACACGGAAACCGAAGACCATTCATGTTGTTGCTCAGGTCGCAGAC
GTTTTGCAGCAGCAGTCGCTTCACGTTCGCTCGCGTATCGGTGATTCATTCTGCTAAC
CAGTAAGGCAACCCCGCCAGCCTAGCCGGGTCCTCAACGACAGGAGCACGATCATG

CGCACCCGTGGGGCCGCCATGCCGGCGATAATGGCCTGCTTCTCGCCGAAACGTTTG
GTGGCGGGACCAGTGACGAAGGCTTGAGCGAGGGCGTGCAAGATTCCGAATACCGC
AAGCGACAGGCCGATCATCGTCGCGCTCCAGCGAAAGCGGTCCTCGCCGAAAATGA
CCCAGAGCGCTGCCGGCACCTGTCCTACGAGTTGCATGATAAAGAAGACAGTCATA
AGTGCGGCGACGATAGTCATGCCCCGCGCCCACCGGAAGGAGCTGACTGGGTTGAA
GGCTCTCAAGGGCATCGGTCGAGATCCCGGTGCCTAATGAGTGAGCTAACTTACATT
AATTGCGTTGCGCTCACTGCCCGCTTTCCAGTCGGGAAACCTGTCGTGCCAGCTGCA
TTAATGAATCGGCCAACGCGCGGGGAGAGGCGGTTTGCGTATTGGGCGCCAGGGTG
GTTTTTCTTTTCACCAGTGAGACGGGCAACAGCTGATTGCCCTTCACCGCCTGGCCCT
GAGAGAGTTGCAGCAAGCGGTCCACGCTGGTTTGCCCCAGCAGGCGAAAATCCTGT
TTGATGGTGGTTAACGGCGGGATATAACATGAGCTGTCTTCGGTATCGTCGTATCCC
ACTACCGAGATATCCGCACCAACGCGCAGCCCGGACTCGGTAATGGCGCGCATTGC
GCCCAGCGCCATCTGATCGTTGGCAACCAGCATCGCAGTGGGAACGATGCCCTCATT
CAGCATTTGCATGGTTTGTTGAAAACCGGACATGGCACTCCAGTCGCCTTCCCGTTC
CGCTATCGGCTGAATTTGATTGCGAGTGAGATATTTATGCCAGCCAGCCAGACGCAG
ACGCGCCGAGACAGAACTTAATGGGCCCG

Sequence of the plasmid pCDFduet-LptB-LptFG

TCGAACAGAAAGTAATCGTATTGTACACGGCCGCATAATCGAAATTAATACGACTC
ACTATAGGGGAATTGTGAGCGGATAACAATTCCCCATCTTAGTATATTAGTTAAGTA
TAAGAAGGAGATATACATATGATTATTCGACGTTATCTTGTCAAACAAGTGGTATCG
ACGTCCTTGGTAGTGATTGCCTTATTGACCTTAATCATGATGGGTGGTCGTTTAATCA
AGTATTTTGGCGTGGCTGCTCAGGGTCGTTTGGATGCTGGCGTACTGTTTAGCATCAT
TGGCTATCGTATGCCTGAATTTTTAACCCTGATCTTACCGTTAGGATTCTTTATTGGT
TTGATGCTGGTGTTTGGTCGACTTTACGTTGATCATGAAATGGCTGTACTCAATGGC
AGTGGCATCAGTCGAATTCGACTTGGACAGCTATTGATTCCACTGGCTTTGGTCTTCT
TGGTGATACAAGGCATTTTGATGCTATGGATGACGCCTTGGGGGCTACGCCAGTTTG
ATCAACTCTCCAGTAGTCAGGCTGTTCGTACAGGCTTTGATTTAGTCAGACCCAAAG
AATTTATATCATCGGGGCCTTATACCATCTATGCAGGGGATTTATCCGAAGACCGTA
AAAACCTGAAAGATATTTTCTTCTACCAGCGAGCACAAAAAGAAGGCAAACCCGAT
GTCATGATTCTGGCCAAAGAAGCCACGCGTGTGGTCATGGAAAATGAAACGGCCAA
TGTAGTGGACTTGATTCAGGGACGTCGCTACGAAATTTATCCAGGAAAGGCAAAAT
ATTCACAGGCCGAATTCCAGCGCTATCGTTTACGTCTAGAAAATGATAAGTCGGCAA
CCTTTGAAACTGACAAAGTTGAGGCATTGCCAAGTTCAAAACTCTGGAACAAATGG
AACGATCCAGTCATTGCCAGTGAAATGGGCTGGCGTGTATTTGGCCCTTTTACCATT
GTCATTGCCTTGATGATGGCTGTTGCACTGTGTGAGGTAAGTCCGCGTCAGGGACGT
TATTATCGACTTATTCCTGCGATTTTTATCTTTGCGAGTTTAATTGTATTGTTAATCGC
AATTCGTACACGTATTAGTCGTGATGAACTAGGTGTATGGGCTTATCCAGCAGCCTT
GGCTGTGTATGGTATTGCTGCGGCGTTATTCTCACGCAAGCAAAAGCTGGCGCCTAA
AATTAAGAAACAGATCAAACGAGTGAGAGCATAATGTTAGCACGTCGAATCGTCGC

AAAGCATGTGACCAAAACCACGGCGCTAGCAATGCTAGGAACCACGATTGTTTTGG
TGATTTTGCAGGTTTTATTTACCTATTTAGGTGAGCTGTCCAATCTTAAAGCAGACTA
TAGCGCCTGGCAGGCATTTTTATATGTTTTATGGGGAGCACCGCGCTATCTCTATGA
AATTCTGCCTATCTCGGCGTTGATTGGTGCAATTTTAGGATTGGGTACACTGGCATCC
AACAGTGAATTGATTGTGATGCGCTCGGTGGGAATCAGTTTATGGCGGATTGTCGGT
TGGGTCATTCGTTCGGCATTAGTACTGGTTTTACTCTCGTTTGCATTGAGTGAGTGGG
TCGTGCCGTATACCAATGAGCGAGCAAATAGCGTGAAGAGCCATCAAAGTGTTGCG
GCACTGGGCGAGGTACGAGGTTACTGGTCACGTGAAGGACAGCGCTTTATTTATGTG
GATTATGCCAACTCACAAGGTCAGCTTAAACGGATTCAGGTGGTCGATTTTGATGAC
AACTATCGTTTAAAGTCGGTAACCAATGCCGAGCAAGGACAGTTTGTCAAAGATGG
TCAATGGTTGTTAAATCATTCGCAGCAGATGGCGATTCAGGGACAAGGCGATGCTGT
ATTGGCAAATGCAGCTAAACAGCCATTTTCATTGGCATTGCAGCCTAAGTACGTGCA
TATGGTGACGATTGATCCAGAAGATTTATCCTTTAGCCAACTGGTCAGTTTTATGAA
CTATATGCGTGAATACAGCCAAGTGCCGAAAACCTATCAGTTGGCATTCTGGAAGA
AAGTGGCTTCACCTTTTGCATTGATTACGTTGGTATTGGTTGCCTGTTCTTTTATTTTT
GGGCCACTACGCCAGCAATCGATGGGTTTTCGCTTGGTGATCGCGCTGTTTATTGGA
CTAGGTTTTTACTATTTACAAGATTTTCTTGGATATGCAAGTTTGGTTTATAACCCAT
CACCCGCATGGTTTGTACTTGGGCCAATTGTACTCATGTTTGTTGCGGGGAGTTACTT
GTTATATCGGGCAAGATAAGGTACCCTCGAGTCTGGTAAAGAAACCGCTGCTGCGA
AATTTGAACGCCAGCACATGGACTCGTCTACTAGCGCAGCTTAATTAACCTAGGCTG
CTGCCACCGCTGAGCAATAACTAGCATAACCCCTTGGGGCCTCTAAACGGGTCTTGA
GGGGTTTTTTGCTGAAACCTCAGGCATTTGAGAAGCACACGGTCACACTGCTTCCGG
TAGTCAATAAACCGGTAAACCAGCAATAGACATAAGCGGCTATTTAACGACCCTGC
CCTGAACCGACGACCGGGTCATCGTGGCCGGATCTTGCGGCCCCTCGGCTTGAACGA
ATTGTTAGACATTATTTGCCGACTACCTTGGTGATCTCGCCTTTCACGTAGTGGACAA
ATTCTTCCAACTGATCTGCGCGCGAGGCCAAGCGATCTTCTTCTTGTCCAAGATAAG
CCTGTCTAGCTTCAAGTATGACGGGCTGATACTGGGCCGGCAGGCGCTCCATTGCCC
AGTCGGCAGCGACATCCTTCGGCGCGATTTTGCCGGTTACTGCGCTGTACCAAATGC
GGGACAACGTAAGCACTACATTTCGCTCATCGCCAGCCCAGTCGGGCGGCGAGTTC
CATAGCGTTAAGGTTTCATTTAGCGCCTCAAATAGATCCTGTTCAGGAACCGGATCA
AAGAGTTCCTCCGCCGCTGGACCTACCAAGGCAACGCTATGTTCTCTTGCTTTTGTCA
GCAAGATAGCCAGATCAATGTCGATCGTGGCTGGCTCGAAGATACCTGCAAGAATG
TCATTGCGCTGCCATTCTCCAAATTGCAGTTCGCGCTTAGCTGGATAACGCCACGGA
ATGATGTCGTCGTGCACAACAATGGTGACTTCTACAGCGCGGAGAATCTCGCTCTCT
CCAGGGGAAGCCGAAGTTTCCAAAAGGTCGTTGATCAAAGCTCGCCGCGTTGTTTCA
TCAAGCCTTACGGTCACCGTAACCAGCAAATCAATATCACTGTGTGGCTTCAGGCCG
CCATCCACTGCGGAGCCGTACAAATGTACGGCCAGCAACGTCGGTTCGAGATGGCG
CTCGATGACGCCAACTACCTCTGATAGTTGAGTCGATACTTCGGCGATCACCGCTTC
CCTCATACTCTTCCTTTTTCAATATTATTGAAGCATTTATCAGGGTTATTGTCTCATG
AGCGGATACATATTTGAATGTATTTAGAAAAATAAACAAATAGCTAGCTCACTCGGT

CGCTACGCTCCGGGCGTGAGACTGCGGCGGGCGCTGCGGACACATACAAAGTTACC
CACAGATTCCGTGGATAAGCAGGGGACTAACATGTGAGGCAAAACAGCAGGGCCGC
GCCGGTGGCGTTTTTCCATAGGCTCCGCCCTCCTGCCAGAGTTCACATAAACAGACG
CTTTTCCGGTGCATCTGTGGGAGCCGTGAGGCTCAACCATGAATCTGACAGTACGGG
CGAAACCCGACAGGACTTAAAGATCCCCACCGTTTCCGGCGGGTCGCTCCCTCTTGC
GCTCTCCTGTTCCGACCCTGCCGTTTACCGGATACCTGTTCCGCCTTTCTCCCTTACG
GGAAGTGTGGCGCTTTCTCATAGCTCACACACTGGTATCTCGGCTCGGTGTAGGTCG
TTCGCTCCAAGCTGGGCTGTAAGCAAGAACTCCCCGTTCAGCCCGACTGCTGCGCCT
TATCCGGTAACTGTTCACTTGAGTCCAACCCGGAAAAGCACGGTAAAACGCCACTG
GCAGCAGCCATTGGTAACTGGGAGTTCGCAGAGGATTTGTTTAGCTAAACACGCGGT
TGCTCTTGAAGTGTGCGCCAAAGTCCGGCTACACTGGAAGGACAGATTTGGTTGCTG
TGCTCTGCGAAAGCCAGTTACCACGGTTAAGCAGTTCCCCAACTGACTTAACCTTCG
ATCAAACCACCTCCCCAGGTGGTTTTTTCGTTTACAGGGCAAAAGATTACGCGCAGA
AAAAAAGGATCTCAAGAAGATCCTTTGATCTTTTCTACTGAACCGCTCTAGATTTCA
GTGCAATTTATCTCTTCAAATGTAGCACCTGAAGTCAGCCCCATACGATATAAGTTG
TAATTCTCATGTTAGTCATGCCCCGCGCCCACCGGAAGGAGCTGACTGGGTTGAAGG
CTCTCAAGGGCATCGGTCGAGATCCCGGTGCCTAATGAGTGAGCTAACTTACATTAA
TTGCGTTGCGCTCACTGCCCGCTTTCCAGTCGGGAAACCTGTCGTGCCAGCTGCATT
AATGAATCGGCCAACGCGCGGGGAGAGGCGGTTTGCGTATTGGGCGCCAGGGTGGT
TTTTCTTTTCACCAGTGAGACGGGCAACAGCTGATTGCCCTTCACCGCCTGGCCCTG
AGAGAGTTGCAGCAAGCGGTCCACGCTGGTTTGCCCCAGCAGGCGAAAATCCTGTTT
GATGGTGGTTAACGGCGGGATATAACATGAGCTGTCTTCGGTATCGTCGTATCCCAC
TACCGAGATGTCCGCACCAACGCGCAGCCCGGACTCGGTAATGGCGCGCATTGCGC
CCAGCGCCATCTGATCGTTGGCAACCAGCATCGCAGTGGGAACGATGCCCTCATTCA
GCATTTGCATGGTTTGTTGAAAACCGGACATGGCACTCCAGTCGCCTTCCCGTTCCG
CTATCGGCTGAATTTGATTGCGAGTGAGATATTTATGCCAGCCAGCCAGACGCAGAC
GCGCCGAGACAGAACTTAATGGGCCCGCTAACAGCGCGATTTGCTGGTGACCCAAT
GCGACCAGATGCTCCACGCCCAGTCGCGTACCGTCTTCATGGGAGAAAATAATACT
GTTGATGGGTGTCTGGTCAGAGACATCAAGAAATAACGCCGGAACATTAGTGCAGG
CAGCTTCCACAGCAATGGCATCCTGGTCATCCAGCGGATAGTTAATGATCAGCCCAC
TGACGCGTTGCGCGAGAAGATTGTGCACCGCCGCTTTACAGGCTTCGACGCCGCTTC
GTTCTACCATCGACACCACCACGCTGGCACCCAGTTGATCGGCGCGAGATTTAATCG
CCGCGACAATTTGCGACGGCGCGTGCAGGGCCAGACTGGAGGTGGCAACGCCAATC
GGCAACGACTGTTTGCCCGCCAGTTGTTGTGCCACGCGGTTGGGAATGTAATTCAGC
TCCGCCATCGCCGCTTCCACTTTTTCCCGCGTTTTCGCAGAAACGTGGCTGGCCTGGT
TCACCACGCGGGAAACGGTCTGATAAGAGACACCGGCATACTCTGCGACATCGTAT
AACGTTACTGGTTTCACATTCACCACCCTGAATTGACTCTCTTCCGGGCGCTATCATG
CCATACCGCGAAAGGTTTTGCGCCATTCGATGGTGTCCGGGATCTCGACGCTCTCCC
TTATGCGACTCCTGCATTAGGAAATTAATACGACTCACTATAGGGGAATTGTGAGCG
GATAACAATTCCCCTGTAGAAATAATTTTGTTTAACTTTAATAAGGAGATATACCAT

GGAGCAAATCGCGCAACAACAACCTCAGACTTTATGCATTAAGCATCTTGCAAAGA
ATTACAGCAAACGTTGGGTGGTAAAAGACGTATCGTTTGAGATGCAAAGTGGACAA
ATTGTTGGTTTGCTTGGGCCGAATGGTGCTGGTAAAACAACCAGTTTCTATATGGTT
GTCGGGTTGGTGCGCATGGATAAAGGTGAAATTCATCTTGATAATCTTGATTTGTCT
GATCTAGCTATGCACGAACGCGCGAGAAAGGGAATTGGTTATTTGCCTCAAGAAGC
TTCTATTTTCAGAAAACTTACGATTGCTGAAAATATTATGGCTATTCTTGAGACTCGA
AAAGACCTGAATAAGCAACAGCGTCAGCAACGTCTCCAAGAGTTATTGAATGATTTT
AAAATCACGCATATTAAAGATTCTTTAGGGATGAGTGTATCTGGTGGTGAGCGACGA
CGTGCTGAAATTGCGCGTGCATTGGCCGCAGACCCAAAGTTTATGCTGCTTGATGAA
CCTTTTGCGGGGGTGGATCCGATTTCGGTCGGTGATATTAAAGACATTATTCGTAAT
TTAAAAGATCGCGGTATTGGCGTACTCATTACCGATCATAATGTGCGTGAAACTTTG
GCTATCTGTGAGCATGCTTACATTGTAAGTGAAGGCGCTGTAATTGCAGAGGGTTCT
CCGCAGGATATTTTAGAAAATGAACAGGTACGTAAGGTATATCTAGGAGATGATTTT
ACAGTCTAAGAATTCGAGCTCGGCGCGCCTGCAGGTCGACAAGCTTGCGGCCGCAT
AATGCTTAAG

Sequence of the plasmid pCDFduet-LptB-LptF(E249K)G

TCGAACAGAAAGTAATCGTATTGTACACGGCCGCATAATCGAAATTAATACGACTC
ACTATAGGGGAATTGTGAGCGGATAACAATTCCCCATCTTAGTATATTAGTTAAGTA
TAAGAAGGAGATATACATATGATTATTCGACGTTATCTTGTCAAACAAGTGGTATCG
ACGTCCTTGGTAGTGATTGCCTTATTGACCTTAATCATGATGGGTGGTCGTTTAATCA
AGTATTTTGGCGTGGCTGCTCAGGGTCGTTTGGATGCTGGCGTACTGTTTAGCATCAT
TGGCTATCGTATGCCTGAATTTTTAACCCTGATCTTACCGTTAGGATTCTTTATTGGT
TTGATGCTGGTGTTTGGTCGACTTTACGTTGATCATGAAATGGCTGTACTCAATGGC
AGTGGCATCAGTCGAATTCGACTTGGACAGCTATTGATTCCACTGGCTTTGGTCTTCT
TGGTGATACAAGGCATTTTGATGCTATGGATGACGCCTTGGGGGCTACGCCAGTTTG
ATCAACTCTCCAGTAGTCAGGCTGTTCGTACAGGCTTTGATTTAGTCAGACCCAAAG
AATTTATATCATCGGGGCCTTATACCATCTATGCAGGGGATTTATCCGAAGACCGTA
AAAACCTGAAAGATATTTTCTTCTACCAGCGAGCACAAAAAGAAGGCAAACCCGAT
GTCATGATTCTGGCCAAAGAAGCCACGCGTGTGGTCATGGAAAATGAAACGGCCAA
TGTAGTGGACTTGATTCAGGGACGTCGCTACGAAATTTATCCAGGAAAGGCAAAAT
ATTCACAGGCCGAATTCCAGCGCTATCGTTTACGTCTAGAAAATGATAAGTCGGCAA
CCTTTGAAACTGACAAAGTTAAGGCATTGCCAAGTTCAAAACTCTGGAACAAATGG
AACGATCCAGTCATTGCCAGTGAAATGGGCTGGCGTGTATTTGGCCCTTTTACCATT
GTCATTGCCTTGATGATGGCTGTTGCACTGTGTGAGGTAAGTCCGCGTCAGGGACGT
TATTATCGACTTATTCCTGCGATTTTTATCTTTGCGAGTTTAATTGTATTGTTAATCGC
AATTCGTACACGTATTAGTCGTGATGAACTAGGTGTATGGGCTTATCCAGCAGCCTT
GGCTGTGTATGGTATTGCTGCGGCGTTATTCTCACGCAAGCAAAAGCTGGCGCCTAA
AATTAAGAAACAGATCAAACGAGTGAGAGCATAATGTTAGCACGTCGAATCGTCGC
AAAGCATGTGACCAAAACCACGGCGCTAGCAATGCTAGGAACCACGATTGTTTTGG

TGATTTTGCAGGTTTTATTTACCTATTTAGGTGAGCTGTCCAATCTTAAAGCAGACTA
TAGCGCCTGGCAGGCATTTTTATATGTTTTATGGGGAGCACCGCGCTATCTCTATGA
AATTCTGCCTATCTCGGCGTTGATTGGTGCAATTTTAGGATTGGGTACACTGGCATCC
AACAGTGAATTGATTGTGATGCGCTCGGTGGAATCAGTTTATGGCGGATTGTCGGT
TGGGTCATTCGTTCGGCATTAGTACTGGTTTTACTCTCGTTTGCATTGAGTGAGTGGG
TCGTGCCGTATACCAATGAGCGAGCAAATAGCGTGAAGAGCCATCAAAGTGTTGCG
GCACTGGGCGAGGTACGAGGTTACTGGTCACGTGAAGGACAGCGCTTTATTTATGTG
GATTATGCCAACTCACAAGGTCAGCTTAAACGGATTCAGGTGGTCGATTTTGATGAC
AACTATCGTTTAAAGTCGGTAACCAATGCCGAGCAAGGACAGTTTGTCAAAGATGG
TCAATGGTTGTTAAATCATTCGCAGCAGATGGCGATTCAGGGACAAGGCGATGCTGT
ATTGGCAAATGCAGCTAAACAGCCATTTTCATTGGCATTGCAGCCTAAGTACGTGCA
TATGGTGACGATTGATCCAGAAGATTTATCCTTTAGCCAACTGGTCAGTTTTATGAA
CTATATGCGTGAATACAGCCAAGTGCCGAAAACCTATCAGTTGGCATTCTGGAAGA
AAGTGGCTTCACCTTTTGCATTGATTACGTTGGTATTGGTTGCCTGTTCTTTTATTTTT
GGGCCACTACGCCAGCAATCGATGGGTTTTCGCTTGGTGATCGCGCTGTTTATTGGA
CTAGGTTTTTACTATTTACAAGATTTTCTTGGATATGCAAGTTTGGTTTATAACCCAT
CACCCGCATGGTTTGTACTTGGGCCAATTGTACTCATGTTTGTTGCGGGGAGTTACTT
GTTATATCGGGCAAGATAAGGTACCCTCGAGTCTGGTAAAGAAACCGCTGCTGCGA
AATTTGAACGCCAGCACATGGACTCGTCTACTAGCGCAGCTTAATTAACCTAGGCTG
CTGCCACCGCTGAGCAATAACTAGCATAACCCCTTGGGGCCTCTAAACGGGTCTTGA
GGGGTTTTTTGCTGAAACCTCAGGCATTTGAGAAGCACACGGTCACACTGCTTCCGG
TAGTCAATAAACCGGTAAACCAGCAATAGACATAAGCGGCTATTTAACGACCCTGC
CCTGAACCGACGACCGGGTCATCGTGGCCGGATCTTGCGGCCCCTCGGCTTGAACGA
ATTGTTAGACATTATTTGCCGACTACCTTGGTGATCTCGCCTTTCACGTAGTGGACAA
ATTCTTCCAACTGATCTGCGCGCGAGGCCAAGCGATCTTCTTCTTGTCCAAGATAAG
CCTGTCTAGCTTCAAGTATGACGGGCTGATACTGGGCCGGCAGGCGCTCCATTGCCC
AGTCGGCAGCGACATCCTTCGGCGCGATTTTGCCGGTTACTGCGCTGTACCAAATGC
GGGACAACGTAAGCACTACATTTCGCTCATCGCCAGCCCAGTCGGGCGGCGAGTTC
CATAGCGTTAAGGTTTCATTTAGCGCCTCAAATAGATCCTGTTCAGGAACCGGATCA
AAGAGTTCCTCCGCCGCTGGACCTACCAAGGCAACGCTATGTTCTCTTGCTTTTGTCA
GCAAGATAGCCAGATCAATGTCGATCGTGGCTGGCTCGAAGATACCTGCAAGAATG
TCATTGCGCTGCCATTCTCCAAATTGCAGTTCGCGCTTAGCTGGATAACGCCACGGA
ATGATGTCGTCGTGCACAACAATGGTGACTTCTACAGCGCGGAGAATCTCGCTCTCT
CCAGGGGAAGCCGAAGTTTCCAAAAGGTCGTTGATCAAAGCTCGCCGCGTTGTTTCA
TCAAGCCTTACGGTCACCGTAACCAGCAAATCAATATCACTGTGTGGCTTCAGGCCG
CCATCCACTGCGGAGCCGTACAAATGTACGGCCAGCAACGTCGGTTCGAGATGGCG
CTCGATGACGCCAACTACCTCTGATAGTTGAGTCGATACTTCGGCGATCACCGCTTC
CCTCATACTCTTCCTTTTTCAATATTATTGAAGCATTTATCAGGGTTATTGTCTCATG
AGCGGATACATATTTGAATGTATTTAGAAAAATAAACAAATAGCTAGCTCACTCGGT
CGCTACGCTCCGGGCGTGAGACTGCGGCGGGCGCTGCGGACACATACAAAGTTACC

CACAGATTCCGTGGATAAGCAGGGGACTAACATGTGAGGCAAAACAGCAGGGCCGC
GCCGGTGGCGTTTTTCCATAGGCTCCGCCCTCCTGCCAGAGTTCACATAAACAGACG
CTTTTCCGGTGCATCTGTGGGAGCCGTGAGGCTCAACCATGAATCTGACAGTACGGG
CGAAACCCGACAGGACTTAAAGATCCCCACCGTTTCCGGCGGGTCGCTCCCTCTTGC
GCTCTCCTGTTCCGACCCTGCCGTTTACCGGATACCTGTTCCGCCTTTCTCCCTTACG
GGAAGTGTGGCGCTTTCTCATAGCTCACACACTGGTATCTCGGCTCGGTGTAGGTCG
TTCGCTCCAAGCTGGGCTGTAAGCAAGAACTCCCCGTTCAGCCCGACTGCTGCGCCT
TATCCGGTAACTGTTCACTTGAGTCCAACCCGGAAAAGCACGGTAAAACGCCACTG
GCAGCAGCCATTGGTAACTGGGAGTTCGCAGAGGATTTGTTTAGCTAAACACGCGGT
TGCTCTTGAAGTGTGCGCCAAAGTCCGGCTACACTGGAAGGACAGATTTGGTTGCTG
TGCTCTGCGAAAGCCAGTTACCACGGTTAAGCAGTTCCCCAACTGACTTAACCTTCG
ATCAAACCACCTCCCCAGGTGGTTTTTTCGTTTACAGGGCAAAAGATTACGCGCAGA
AAAAAAGGATCTCAAGAAGATCCTTTGATCTTTTCTACTGAACCGCTCTAGATTTCA
GTGCAATTTATCTCTTCAAATGTAGCACCTGAAGTCAGCCCCATACGATATAAGTTG
TAATTCTCATGTTAGTCATGCCCCGCGCCCACCGGAAGGAGCTGACTGGGTTGAAGG
CTCTCAAGGGCATCGGTCGAGATCCCGGTGCCTAATGAGTGAGCTAACTTACATTAA
TTGCGTTGCGCTCACTGCCCGCTTTCCAGTCGGGAAACCTGTCGTGCCAGCTGCATT
AATGAATCGGCCAACGCGCGGGGAGAGGCGGTTTGCGTATTGGGCGCCAGGGTGGT
TTTTCTTTTCACCAGTGAGACGGGCAACAGCTGATTGCCCTTCACCGCCTGGCCCTG
AGAGAGTTGCAGCAAGCGGTCCACGCTGGTTTGCCCCAGCAGGCGAAAATCCTGTTT
GATGGTGGTTAACGGCGGGATATAACATGAGCTGTCTTCGGTATCGTCGTATCCCAC
TACCGAGATGTCCGCACCAACGCGCAGCCCGGACTCGGTAATGGCGCGCATTGCGC
CCAGCGCCATCTGATCGTTGGCAACCAGCATCGCAGTGGGAACGATGCCCTCATTCA
GCATTTGCATGGTTTGTTGAAAACCGGACATGGCACTCCAGTCGCCTTCCCGTTCCG
CTATCGGCTGAATTTGATTGCGAGTGAGATATTTATGCCAGCCAGCCAGACGCAGAC
GCGCCGAGACAGAACTTAATGGGCCCGCTAACAGCGCGATTTGCTGGTGACCCAAT
GCGACCAGATGCTCCACGCCCAGTCGCGTACCGTCTTCATGGGAGAAAATAATACT
GTTGATGGGTGTCTGGTCAGAGACATCAAGAAATAACGCCGGAACATTAGTGCAGG
CAGCTTCCACAGCAATGGCATCCTGGTCATCCAGCGGATAGTTAATGATCAGCCCAC
TGACGCGTTGCGCGAGAAGATTGTGCACCGCCGCTTTACAGGCTTCGACGCCGCTTC
GTTCTACCATCGACACCACCACGCTGGCACCCAGTTGATCGGCGCGAGATTTAATCG
CCGCGACAATTTGCGACGGCGCGTGCAGGGCCAGACTGGAGGTGGCAACGCCAATC
GGCAACGACTGTTTGCCCGCCAGTTGTTGTGCCACGCGGTTGGGAATGTAATTCAGC
TCCGCCATCGCCGCTTCCACTTTTTCCCGCGTTTTCGCAGAAACGTGGCTGGCCTGGT
TCACCACGCGGGAAACGGTCTGATAAGAGACACCGGCATACTCTGCGACATCGTAT
AACGTTACTGGTTTCACATTCACCACCCTGAATTGACTCTCTTCCGGGCGCTATCATG
CCATACCGCGAAAGGTTTTGCGCCATTCGATGGTGTCCGGGATCTCGACGCTCTCCC
TTATGCGACTCCTGCATTAGGAAATTAATACGACTCACTATAGGGGAATTGTGAGCG
GATAACAATTCCCCTGTAGAAATAATTTTGTTTAACTTTAATAAGGAGATATACCAT
GGAGCAAATCGCGCAACAACAACCTCAGACTTTATGCATTAAGCATCTTGCAAAGA

ATTACAGCAAACGTTGGGTGGTAAAAGACGTATCGTTTGAGATGCAAAGTGGACAA
ATTGTTGGTTTGCTTGGGCCGAATGGTGCTGGTAAAACAACCAGTTTCTATATGGTT
GTCGGGTTGGTGCGCATGGATAAAGGTGAAATTCATCTTGATAATCTTGATTTGTCT
GATCTAGCTATGCACGAACGCGCGAGAAAGGGAATTGGTTATTTGCCTCAAGAAGC
TTCTATTTTCAGAAAACTTACGATTGCTGAAAATATTATGGCTATTCTTGAGACTCGA
AAAGACCTGAATAAGCAACAGCGTCAGCAACGTCTCCAAGAGTTATTGAATGATTTT
AAAATCACGCATATTAAAGATTCTTTAGGGATGAGTGTATCTGGTGGTGAGCGACGA
CGTGCTGAAATTGCGCGTGCATTGGCCGCAGACCCAAAGTTTATGCTGCTTGATGAA
CCTTTTGCGGGGGTGGATCCGATTTCGGTCGGTGATATTAAAGACATTATTCGTAAT
TTAAAAGATCGCGGTATTGGCGTACTCATTACCGATCATAATGTGCGTGAAACTTTG
GCTATCTGTGAGCATGCTTACATTGTAAGTGAAGGCGCTGTAATTGCAGAGGGTTCT
CCGCAGGATATTTTAGAAAATGAACAGGTACGTAAGGTATATCTAGGAGATGATTTT
ACAGTCTAAGAATTCGAGCTCGGCGCGCCTGCAGGTCGACAAGCTTGCGGCCGCAT
AATGCTTAAG

Sequence of the plasmid pCDFduet-LptB-LptF(I317N)G

TCGAACAGAAAGTAATCGTATTGTACACGGCCGCATAATCGAAATTAATACGACTC
ACTATAGGGGAATTGTGAGCGGATAACAATTCCCCATCTTAGTATATTAGTTAAGTA
TAAGAAGGAGATATACATATGATTATTCGACGTTATCTTGTCAAACAAGTGGTATCG
ACGTCCTTGGTAGTGATTGCCTTATTGACCTTAATCATGATGGGTGGTCGTTTAATCA
AGTATTTTGGCGTGGCTGCTCAGGGTCGTTTGGATGCTGGCGTACTGTTTAGCATCAT
TGGCTATCGTATGCCTGAATTTTTAACCCTGATCTTACCGTTAGGATTCTTTATTGGT
TTGATGCTGGTGTTTGGTCGACTTTACGTTGATCATGAAATGGCTGTACTCAATGGC
AGTGGCATCAGTCGAATTCGACTTGGACAGCTATTGATTCCACTGGCTTTGGTCTTCT
TGGTGATACAAGGCATTTTGATGCTATGGATGACGCCTTGGGGGCTACGCCAGTTTG
ATCAACTCTCCAGTAGTCAGGCTGTTCGTACAGGCTTTGATTTAGTCAGACCCAAAG
AATTTATATCATCGGGGCCTTATACCATCTATGCAGGGGATTTATCCGAAGACCGTA
AAAACCTGAAAGATATTTTCTTCTACCAGCGAGCACAAAAAGAAGGCAAACCCGAT
GTCATGATTCTGGCCAAAGAAGCCACGCGTGTGGTCATGGAAAATGAAACGGCCAA
TGTAGTGGACTTGATTCAGGGACGTCGCTACGAAATTTATCCAGGAAAGGCAAAAT
ATTCACAGGCCGAATTCCAGCGCTATCGTTTACGTCTAGAAAATGATAAGTCGGCAA
CCTTTGAAACTGACAAAGTTGAGGCATTGCCAAGTTCAAAACTCTGGAACAAATGG
AACGATCCAGTCATTGCCAGTGAAATGGGCTGGCGTGTATTTGGCCCTTTTACCATT
GTCATTGCCTTGATGATGGCTGTTGCACTGTGTGAGGTAAGTCCGCGTCAGGGACGT
TATTATCGACTTATTCCTGCGATTTTTATCTTTGCGAGTTTAATTGTATTGTTAAACGC
AATTCGTACACGTATTAGTCGTGATGAACTAGGTGTATGGGCTTATCCAGCAGCCTT
GGCTGTGTATGGTATTGCTGCGGCGTTATTCTCACGCAAGCAAAAGCTGGCGCCTAA
AATTAAGAAACAGATCAAACGAGTGAGAGCATAATGTTAGCACGTCGAATCGTCGC
AAAGCATGTGACCAAAACCACGGCGCTAGCAATGCTAGGAACCACGATTGTTTTGG
TGATTTTGCAGGTTTTATTTACCTATTTAGGTGAGCTGTCCAATCTTAAAGCAGACTA

TAGCGCCTGGCAGGCATTTTTATATGTTTTATGGGGAGCACCGCGCTATCTCTATGA
AATTCTGCCTATCTCGGCGTTGATTGGTGCAATTTTAGGATTGGGTACACTGGCATCC
AACAGTGAATTGATTGTGATGCGCTCGGTGGGAATCAGTTTATGGCGGATTGTCGGT
TGGGTCATTCGTTCGGCATTAGTACTGGTTTTACTCTCGTTTGCATTGAGTGAGTGGG
TCGTGCCGTATACCAATGAGCGAGCAAATAGCGTGAAGAGCCATCAAAGTGTTGCG
GCACTGGGCGAGGTACGAGGTTACTGGTCACGTGAAGGACAGCGCTTTATTTATGTG
GATTATGCCAACTCACAAGGTCAGCTTAAACGGATTCAGGTGGTCGATTTTGATGAC
AACTATCGTTTAAAGTCGGTAACCAATGCCGAGCAAGGACAGTTTGTCAAAGATGG
TCAATGGTTGTTAAATCATTCGCAGCAGATGGCGATTCAGGGACAAGGCGATGCTGT
ATTGGCAAATGCAGCTAAACAGCCATTTTCATTGGCATTGCAGCCTAAGTACGTGCA
TATGGTGACGATTGATCCAGAAGATTTATCCTTTAGCCAACTGGTCAGTTTTATGAA
CTATATGCGTGAATACAGCCAAGTGCCGAAAACCTATCAGTTGGCATTCTGGAAGA
AAGTGGCTTCACCTTTTGCATTGATTACGTTGGTATTGGTTGCCTGTTCTTTTATTTTT
GGGCCACTACGCCAGCAATCGATGGGTTTTCGCTTGGTGATCGCGCTGTTTATTGGA
CTAGGTTTTTACTATTTACAAGATTTTCTTGGATATGCAAGTTTGGTTTATAACCCAT
CACCCGCATGGTTTGTACTTGGGCCAATTGTACTCATGTTTGTTGCGGGGAGTTACTT
GTTATATCGGGCAAGATAAGGTACCCTCGAGTCTGGTAAAGAAACCGCTGCTGCGA
AATTTGAACGCCAGCACATGGACTCGTCTACTAGCGCAGCTTAATTAACCTAGGCTG
CTGCCACCGCTGAGCAATAACTAGCATAACCCCTTGGGGCCTCTAAACGGGTCTTGA
GGGGTTTTTTGCTGAAACCTCAGGCATTTGAGAAGCACACGGTCACACTGCTTCCGG
TAGTCAATAAACCGGTAAACCAGCAATAGACATAAGCGGCTATTTAACGACCCTGC
CCTGAACCGACGACCGGGTCATCGTGGCCGGATCTTGCGGCCCCTCGGCTTGAACGA
ATTGTTAGACATTATTTGCCGACTACCTTGGTGATCTCGCCTTTCACGTAGTGGACAA
ATTCTTCCAACTGATCTGCGCGCGAGGCCAAGCGATCTTCTTCTTGTCCAAGATAAG
CCTGTCTAGCTTCAAGTATGACGGGCTGATACTGGGCCGGCAGGCGCTCCATTGCCC
AGTCGGCAGCGACATCCTTCGGCGCGATTTTGCCGGTTACTGCGCTGTACCAAATGC
GGGACAACGTAAGCACTACATTTCGCTCATCGCCAGCCCAGTCGGGCGGCGAGTTC
CATAGCGTTAAGGTTTCATTTAGCGCCTCAAATAGATCCTGTTCAGGAACCGGATCA
AAGAGTTCCTCCGCCGCTGGACCTACCAAGGCAACGCTATGTTCTCTTGCTTTTGTCA
GCAAGATAGCCAGATCAATGTCGATCGTGGCTGGCTCGAAGATACCTGCAAGAATG
TCATTGCGCTGCCATTCTCCAAATTGCAGTTCGCGCTTAGCTGGATAACGCCACGGA
ATGATGTCGTCGTGCACAACAATGGTGACTTCTACAGCGCGGAGAATCTCGCTCTCT
CCAGGGGAAGCCGAAGTTTCCAAAAGGTCGTTGATCAAAGCTCGCCGCGTTGTTTCA
TCAAGCCTTACGGTCACCGTAACCAGCAAATCAATATCACTGTGTGGCTTCAGGCCG
CCATCCACTGCGGAGCCGTACAAATGTACGGCCAGCAACGTCGGTTCGAGATGGCG
CTCGATGACGCCAACTACCTCTGATAGTTGAGTCGATACTTCGGCGATCACCGCTTC
CCTCATACTCTTCCTTTTTCAATATTATTGAAGCATTTATCAGGGTTATTGTCTCATG
AGCGGATACATATTTGAATGTATTTAGAAAAATAAACAAATAGCTAGCTCACTCGGT
CGCTACGCTCCGGGCGTGAGACTGCGGCGGGCGCTGCGGACACATACAAAGTTACC
CACAGATTCCGTGGATAAGCAGGGGACTAACATGTGAGGCAAAACAGCAGGGCCGC

GCCGGTGGCGTTTTTCCATAGGCTCCGCCCTCCTGCCAGAGTTCACATAAACAGACG
CTTTTCCGGTGCATCTGTGGGAGCCGTGAGGCTCAACCATGAATCTGACAGTACGGG
CGAAACCCGACAGGACTTAAAGATCCCCACCGTTTCCGGCGGGTCGCTCCCTCTTGC
GCTCTCCTGTTCCGACCCTGCCGTTTACCGGATACCTGTTCCGCCTTTCTCCCTTACG
GGAAGTGTGGCGCTTTCTCATAGCTCACACACTGGTATCTCGGCTCGGTGTAGGTCG
TTCGCTCCAAGCTGGGCTGTAAGCAAGAACTCCCCGTTCAGCCCGACTGCTGCGCCT
TATCCGGTAACTGTTCACTTGAGTCCAACCCGGAAAAGCACGGTAAAACGCCACTG
GCAGCAGCCATTGGTAACTGGGAGTTCGCAGAGGATTTGTTTAGCTAAACACGCGGT
TGCTCTTGAAGTGTGCGCCAAAGTCCGGCTACACTGGAAGGACAGATTTGGTTGCTG
TGCTCTGCGAAAGCCAGTTACCACGGTTAAGCAGTTCCCCAACTGACTTAACCTTCG
ATCAAACCACCTCCCCAGGTGGTTTTTTCGTTTACAGGGCAAAAGATTACGCGCAGA
AAAAAAGGATCTCAAGAAGATCCTTTGATCTTTTCTACTGAACCGCTCTAGATTTCA
GTGCAATTTATCTCTTCAAATGTAGCACCTGAAGTCAGCCCCATACGATATAAGTTG
TAATTCTCATGTTAGTCATGCCCCGCGCCCACCGGAAGGAGCTGACTGGGTTGAAGG
CTCTCAAGGGCATCGGTCGAGATCCCGGTGCCTAATGAGTGAGCTAACTTACATTAA
TTGCGTTGCGCTCACTGCCCGCTTTCCAGTCGGGAAACCTGTCGTGCCAGCTGCATT
AATGAATCGGCCAACGCGCGGGGAGAGGCGGTTTGCGTATTGGGCGCCAGGGTGGT
TTTTCTTTTCACCAGTGAGACGGGCAACAGCTGATTGCCCTTCACCGCCTGGCCCTG
AGAGAGTTGCAGCAAGCGGTCCACGCTGGTTTGCCCCAGCAGGCGAAAATCCTGTTT
GATGGTGGTTAACGGCGGGATATAACATGAGCTGTCTTCGGTATCGTCGTATCCCAC
TACCGAGATGTCCGCACCAACGCGCAGCCCGGACTCGGTAATGGCGCGCATTGCGC
CCAGCGCCATCTGATCGTTGGCAACCAGCATCGCAGTGGGAACGATGCCCTCATTCA
GCATTTGCATGGTTTGTTGAAAACCGGACATGGCACTCCAGTCGCCTTCCCGTTCCG
CTATCGGCTGAATTTGATTGCGAGTGAGATATTTATGCCAGCCAGCCAGACGCAGAC
GCGCCGAGACAGAACTTAATGGGCCCGCTAACAGCGCGATTTGCTGGTGACCCAAT
GCGACCAGATGCTCCACGCCCAGTCGCGTACCGTCTTCATGGGAGAAAATAATACT
GTTGATGGGTGTCTGGTCAGAGACATCAAGAAATAACGCCGGAACATTAGTGCAGG
CAGCTTCCACAGCAATGGCATCCTGGTCATCCAGCGGATAGTTAATGATCAGCCCAC
TGACGCGTTGCGCGAGAAGATTGTGCACCGCCGCTTTACAGGCTTCGACGCCGCTTC
GTTCTACCATCGACACCACCACGCTGGCACCCAGTTGATCGGCGCGAGATTTAATCG
CCGCGACAATTTGCGACGGCGCGTGCAGGGCCAGACTGGAGGTGGCAACGCCAATC
GGCAACGACTGTTTGCCCGCCAGTTGTTGTGCCACGCGGTTGGGAATGTAATTCAGC
TCCGCCATCGCCGCTTCCACTTTTTCCCGCGTTTTCGCAGAAACGTGGCTGGCCTGGT
TCACCACGCGGGAAACGGTCTGATAAGAGACACCGGCATACTCTGCGACATCGTAT
AACGTTACTGGTTTCACATTCACCACCCTGAATTGACTCTCTTCCGGGCGCTATCATG
CCATACCGCGAAAGGTTTTGCGCCATTCGATGGTGTCCGGGATCTCGACGCTCTCCC
TTATGCGACTCCTGCATTAGGAAATTAATACGACTCACTATAGGGGAATTGTGAGCG
GATAACAATTCCCCTGTAGAAATAATTTTGTTTAACTTTAATAAGGAGATATACCAT
GGAGCAAATCGCGCAACAACAACCTCAGACTTTATGCATTAAGCATCTTGCAAAGA
ATTACAGCAAACGTTGGGTGGTAAAAGACGTATCGTTTGAGATGCAAAGTGGACAA

ATTGTTGGTTTGCTTGGGCCGAATGGTGCTGGTAAAACAACCAGTTTCTATATGGTT
GTCGGGTTGGTGCGCATGGATAAAGGTGAAATTCATCTTGATAATCTTGATTTGTCT
GATCTAGCTATGCACGAACGCGCGAGAAAGGGAATTGGTTATTTGCCTCAAGAAGC
TTCTATTTTCAGAAAACTTACGATTGCTGAAAATATTATGGCTATTCTTGAGACTCGA
AAAGACCTGAATAAGCAACAGCGTCAGCAACGTCTCCAAGAGTTATTGAATGATTTT
AAAATCACGCATATTAAAGATTCTTTAGGGATGAGTGTATCTGGTGGTGAGCGACGA
CGTGCTGAAATTGCGCGTGCATTGGCCGCAGACCCAAAGTTTATGCTGCTTGATGAA
CCTTTTGCGGGGGTGGATCCGATTTCGGTCGGTGATATTAAAGACATTATTCGTAAT
TTAAAAGATCGCGGTATTGGCGTACTCATTACCGATCATAATGTGCGTGAAACTTTG
GCTATCTGTGAGCATGCTTACATTGTAAGTGAAGGCGCTGTAATTGCAGAGGGTTCT
CCGCAGGATATTTTAGAAAATGAACAGGTACGTAAGGTATATCTAGGAGATGATTTT
ACAGTCTAAGAATTCGAGCTCGGCGCGCCTGCAGGTCGACAAGCTTGCGGCCGCAT
AATGCTTAAG

Sequence of the plasmid pCDFduet-LptB-LptF(E249K I317N)G

TCGAACAGAAAGTAATCGTATTGTACACGGCCGCATAATCGAAATTAATACGACTC
ACTATAGGGGAATTGTGAGCGGATAACAATTCCCCATCTTAGTATATTAGTTAAGTA
TAAGAAGGAGATATACATATGATTATTCGACGTTATCTTGTCAAACAAGTGGTATCG
ACGTCCTTGGTAGTGATTGCCTTATTGACCTTAATCATGATGGGTGGTCGTTTAATCA
AGTATTTTGGCGTGGCTGCTCAGGGTCGTTTGGATGCTGGCGTACTGTTTAGCATCAT
TGGCTATCGTATGCCTGAATTTTTAACCCTGATCTTACCGTTAGGATTCTTTATTGGT
TTGATGCTGGTGTTTGGTCGACTTTACGTTGATCATGAAATGGCTGTACTCAATGGC
AGTGGCATCAGTCGAATTCGACTTGGACAGCTATTGATTCCACTGGCTTTGGTCTTCT
TGGTGATACAAGGCATTTTGATGCTATGGATGACGCCTTGGGGGCTACGCCAGTTTG
ATCAACTCTCCAGTAGTCAGGCTGTTCGTACAGGCTTTGATTTAGTCAGACCCAAAG
AATTTATATCATCGGGGCCTTATACCATCTATGCAGGGGATTTATCCGAAGACCGTA
AAAACCTGAAAGATATTTTCTTCTACCAGCGAGCACAAAAAGAAGGCAAACCCGAT
GTCATGATTCTGGCCAAAGAAGCCACGCGTGTGGTCATGGAAAATGAAACGGCCAA
TGTAGTGGACTTGATTCAGGGACGTCGCTACGAAATTTATCCAGGAAAGGCAAAAT
ATTCACAGGCCGAATTCCAGCGCTATCGTTTACGTCTAGAAAATGATAAGTCGGCAA
CCTTTGAAACTGACAAAGTTAAGGCATTGCCAAGTTCAAAACTCTGGAACAAATGG
AACGATCCAGTCATTGCCAGTGAAATGGGCTGGCGTGTATTTGGCCCTTTTACCATT
GTCATTGCCTTGATGATGGCTGTTGCACTGTGTGAGGTAAGTCCGCGTCAGGGACGT
TATTATCGACTTATTCCTGCGATTTTTATCTTTGCGAGTTTAATTGTATTGTTAAACGC
AATTCGTACACGTATTAGTCGTGATGAACTAGGTGTATGGGCTTATCCAGCAGCCTT
GGCTGTGTATGGTATTGCTGCGGCGTTATTCTCACGCAAGCAAAAGCTGGCGCCTAA
AATTAAGAAACAGATCAAACGAGTGAGAGCATAATGTTAGCACGTCGAATCGTCGC
AAAGCATGTGACCAAAACCACGGCGCTAGCAATGCTAGGAACCACGATTGTTTTGG
TGATTTTGCAGGTTTTATTTACCTATTTAGGTGAGCTGTCCAATCTTAAAGCAGACTA
TAGCGCCTGGCAGGCATTTTTATATGTTTTATGGGGAGCACCGCGCTATCTCTATGA

AATTCTGCCTATCTCGGCGTTGATTGGTGCAATTTTAGGATTGGGTACACTGGCATCC
AACAGTGAATTGATTGTGATGCGCTCGGTGGGAATCAGTTTATGGCGGATTGTCGGT
TGGGTCATTCGTTCGGCATTAGTACTGGTTTTACTCTCGTTTGCATTGAGTGAGTGGG
TCGTGCCGTATACCAATGAGCGAGCAAATAGCGTGAAGAGCCATCAAAGTGTTGCG
GCACTGGGCGAGGTACGAGGTTACTGGTCACGTGAAGGACAGCGCTTTATTTATGTG
GATTATGCCAACTCACAAGGTCAGCTTAAACGGATTCAGGTGGTCGATTTTGATGAC
AACTATCGTTTAAAGTCGGTAACCAATGCCGAGCAAGGACAGTTTGTCAAAGATGG
TCAATGGTTGTTAAATCATTCGCAGCAGATGGCGATTCAGGGACAAGGCGATGCTGT
ATTGGCAAATGCAGCTAAACAGCCATTTTCATTGGCATTGCAGCCTAAGTACGTGCA
TATGGTGACGATTGATCCAGAAGATTTATCCTTTAGCCAACTGGTCAGTTTTATGAA
CTATATGCGTGAATACAGCCAAGTGCCGAAAACCTATCAGTTGGCATTCTGGAAGA
AAGTGGCTTCACCTTTTGCATTGATTACGTTGGTATTGGTTGCCTGTTCTTTTATTTTT
GGGCCACTACGCCAGCAATCGATGGGTTTTCGCTTGGTGATCGCGCTGTTTATTGGA
CTAGGTTTTTACTATTTACAAGATTTTCTTGGATATGCAAGTTTGGTTTATAACCCAT
CACCCGCATGGTTTGTACTTGGGCCAATTGTACTCATGTTTGTTGCGGGGAGTTACTT
GTTATATCGGGCAAGATAAGGTACCCTCGAGTCTGGTAAAGAAACCGCTGCTGCGA
AATTTGAACGCCAGCACATGGACTCGTCTACTAGCGCAGCTTAATTAACCTAGGCTG
CTGCCACCGCTGAGCAATAACTAGCATAACCCCTTGGGGCCTCTAAACGGGTCTTGA
GGGGTTTTTTGCTGAAACCTCAGGCATTTGAGAAGCACACGGTCACACTGCTTCCGG
TAGTCAATAAACCGGTAAACCAGCAATAGACATAAGCGGCTATTTAACGACCCTGC
CCTGAACCGACGACCGGGTCATCGTGGCCGGATCTTGCGGCCCCTCGGCTTGAACGA
ATTGTTAGACATTATTTGCCGACTACCTTGGTGATCTCGCCTTTCACGTAGTGGACAA
ATTCTTCCAACTGATCTGCGCGCGAGGCCAAGCGATCTTCTTCTTGTCCAAGATAAG
CCTGTCTAGCTTCAAGTATGACGGGCTGATACTGGGCCGGCAGGCGCTCCATTGCCC
AGTCGGCAGCGACATCCTTCGGCGCGATTTTGCCGGTTACTGCGCTGTACCAAATGC
GGGACAACGTAAGCACTACATTTCGCTCATCGCCAGCCCAGTCGGGCGGCGAGTTC
CATAGCGTTAAGGTTTCATTTAGCGCCTCAAATAGATCCTGTTCAGGAACCGGATCA
AAGAGTTCCTCCGCCGCTGGACCTACCAAGGCAACGCTATGTTCTCTTGCTTTTGTCA
GCAAGATAGCCAGATCAATGTCGATCGTGGCTGGCTCGAAGATACCTGCAAGAATG
TCATTGCGCTGCCATTCTCCAAATTGCAGTTCGCGCTTAGCTGGATAACGCCACGGA
ATGATGTCGTCGTGCACAACAATGGTGACTTCTACAGCGCGGAGAATCTCGCTCTCT
CCAGGGGAAGCCGAAGTTTCCAAAAGGTCGTTGATCAAAGCTCGCCGCGTTGTTTCA
TCAAGCCTTACGGTCACCGTAACCAGCAAATCAATATCACTGTGTGGCTTCAGGCCG
CCATCCACTGCGGAGCCGTACAAATGTACGGCCAGCAACGTCGGTTCGAGATGGCG
CTCGATGACGCCAACTACCTCTGATAGTTGAGTCGATACTTCGGCGATCACCGCTTC
CCTCATACTCTTCCTTTTTCAATATTATTGAAGCATTTATCAGGGTTATTGTCTCATG
AGCGGATACATATTTGAATGTATTTAGAAAAATAAACAAATAGCTAGCTCACTCGGT
CGCTACGCTCCGGGCGTGAGACTGCGGCGGGCGCTGCGGACACATACAAAGTTACC
CACAGATTCCGTGGATAAGCAGGGGACTAACATGTGAGGCAAAACAGCAGGGCCGC
GCCGGTGGCGTTTTTCCATAGGCTCCGCCCTCCTGCCAGAGTTCACATAAACAGACG

CTTTTCCGGTGCATCTGTGGGAGCCGTGAGGCTCAACCATGAATCTGACAGTACGGG
CGAAACCCGACAGGACTTAAAGATCCCCACCGTTTCCGGCGGGTCGCTCCCTCTTGC
GCTCTCCTGTTCCGACCCTGCCGTTTACCGGATACCTGTTCCGCCTTTCTCCCTTACG
GGAAGTGTGGCGCTTTCTCATAGCTCACACACTGGTATCTCGGCTCGGTGTAGGTCG
TTCGCTCCAAGCTGGGCTGTAAGCAAGAACTCCCCGTTCAGCCCGACTGCTGCGCCT
TATCCGGTAACTGTTCACTTGAGTCCAACCCGGAAAAGCACGGTAAAACGCCACTG
GCAGCAGCCATTGGTAACTGGGAGTTCGCAGAGGATTTGTTTAGCTAAACACGCGGT
TGCTCTTGAAGTGTGCGCCAAAGTCCGGCTACACTGGAAGGACAGATTTGGTTGCTG
TGCTCTGCGAAAGCCAGTTACCACGGTTAAGCAGTTCCCCAACTGACTTAACCTTCG
ATCAAACCACCTCCCCAGGTGGTTTTTTCGTTTACAGGGCAAAAGATTACGCGCAGA
AAAAAAGGATCTCAAGAAGATCCTTTGATCTTTTCTACTGAACCGCTCTAGATTTCA
GTGCAATTTATCTCTTCAAATGTAGCACCTGAAGTCAGCCCCATACGATATAAGTTG
TAATTCTCATGTTAGTCATGCCCCGCGCCCACCGGAAGGAGCTGACTGGGTTGAAGG
CTCTCAAGGGCATCGGTCGAGATCCCGGTGCCTAATGAGTGAGCTAACTTACATTAA
TTGCGTTGCGCTCACTGCCCGCTTTCCAGTCGGGAAACCTGTCGTGCCAGCTGCATT
AATGAATCGGCCAACGCGCGGGGAGAGGCGGTTTGCGTATTGGGCGCCAGGGTGGT
TTTTCTTTTCACCAGTGAGACGGGCAACAGCTGATTGCCCTTCACCGCCTGGCCCTG
AGAGAGTTGCAGCAAGCGGTCCACGCTGGTTTGCCCCAGCAGGCGAAAATCCTGTTT
GATGGTGGTTAACGGCGGGATATAACATGAGCTGTCTTCGGTATCGTCGTATCCCAC
TACCGAGATGTCCGCACCAACGCGCAGCCCGGACTCGGTAATGGCGCGCATTGCGC
CCAGCGCCATCTGATCGTTGGCAACCAGCATCGCAGTGGGAACGATGCCCTCATTCA
GCATTTGCATGGTTTGTTGAAAACCGGACATGGCACTCCAGTCGCCTTCCCGTTCCG
CTATCGGCTGAATTTGATTGCGAGTGAGATATTTATGCCAGCCAGCCAGACGCAGAC
GCGCCGAGACAGAACTTAATGGGCCCGCTAACAGCGCGATTTGCTGGTGACCCAAT
GCGACCAGATGCTCCACGCCCAGTCGCGTACCGTCTTCATGGGAGAAAATAATACT
GTTGATGGGTGTCTGGTCAGAGACATCAAGAAATAACGCCGGAACATTAGTGCAGG
CAGCTTCCACAGCAATGGCATCCTGGTCATCCAGCGGATAGTTAATGATCAGCCCAC
TGACGCGTTGCGCGAGAAGATTGTGCACCGCCGCTTTACAGGCTTCGACGCCGCTTC
GTTCTACCATCGACACCACCACGCTGGCACCCAGTTGATCGGCGCGAGATTTAATCG
CCGCGACAATTTGCGACGGCGCGTGCAGGGCCAGACTGGAGGTGGCAACGCCAATC
GGCAACGACTGTTTGCCCGCCAGTTGTTGTGCCACGCGGTTGGGAATGTAATTCAGC
TCCGCCATCGCCGCTTCCACTTTTTCCCGCGTTTTCGCAGAAACGTGGCTGGCCTGGT
TCACCACGCGGGAAACGGTCTGATAAGAGACACCGGCATACTCTGCGACATCGTAT
AACGTTACTGGTTTCACATTCACCACCCTGAATTGACTCTCTTCCGGGCGCTATCATG
CCATACCGCGAAAGGTTTTGCGCCATTCGATGGTGTCCGGGATCTCGACGCTCTCCC
TTATGCGACTCCTGCATTAGGAAATTAATACGACTCACTATAGGGGAATTGTGAGCG
GATAACAATTCCCCTGTAGAAATAATTTTGTTTAACTTTAATAAGGAGATATACCAT
GGAGCAAATCGCGCAACAACAACCTCAGACTTTATGCATTAAGCATCTTGCAAAGA
ATTACAGCAAACGTTGGGTGGTAAAAGACGTATCGTTTGAGATGCAAAGTGGACAA
ATTGTTGGTTTGCTTGGGCCGAATGGTGCTGGTAAAACAACCAGTTTCTATATGGTT

GTCGGGTTGGTGCGCATGGATAAAGGTGAAATTCATCTTGATAATCTTGATTTGTCT
GATCTAGCTATGCACGAACGCGCGAGAAAGGGAATTGGTTATTTGCCTCAAGAAGC
TTCTATTTTCAGAAAACTTACGATTGCTGAAAATATTATGGCTATTCTTGAGACTCGA
AAAGACCTGAATAAGCAACAGCGTCAGCAACGTCTCCAAGAGTTATTGAATGATTTT
AAAATCACGCATATTAAAGATTCTTTAGGGATGAGTGTATCTGGTGGTGAGCGACGA
CGTGCTGAAATTGCGCGTGCATTGGCCGCAGACCCAAAGTTTATGCTGCTTGATGAA
CCTTTTGCGGGGGTGGATCCGATTTCGGTCGGTGATATTAAAGACATTATTCGTAAT
TTAAAAGATCGCGGTATTGGCGTACTCATTACCGATCATAATGTGCGTGAAACTTTG
GCTATCTGTGAGCATGCTTACATTGTAAGTGAAGGCGCTGTAATTGCAGAGGGTTCT
CCGCAGGATATTTTAGAAAATGAACAGGTACGTAAGGTATATCTAGGAGATGATTTT
ACAGTCTAAGAATTCGAGCTCGGCGCGCCTGCAGGTCGACAAGCTTGCGGCCGCAT
AATGCTTAAG

Analytical data of the target compounds

LC-MS high resolution spectra were recorded with an Agilent LC-system consisting of Agilent 1290 high pressure gradient system and an Agilent 6545B QTOF. The separation was achieved on a Zorbax Eclipse Plus C_{18} 1.8 µm 2.1*50 mm column at 55°C; A=0.02% formic acid in water; B= acetonitrile with 0.01% formic acid at flow 0.8 mL/min. gradient: 0 min 5%B, 0.3 min 5%B, 4.5 min 99%B 5 min 99%B. The injection volume was 2 µL. Ionization was performed in Agilent's Multimode source. The mass spectrometer was run in “2 GHz extended dynamic range” mode, resulting in a resolution of about 20,000 at m/z=922. Mass accuracy was ensured by internal drift correction.

**Structure**	**Compound ID**	**EF**	**calculated $[M+H]^+$**	**HRMS found (*m/z*)**	**Difference (mDa)**	**HPLC Purity (%)**
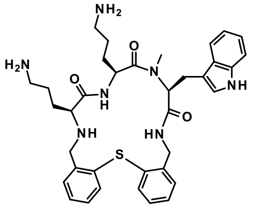	RO7036668	$C_{36}H_{45}N_7O_3S$	655.3305	655.3302	–0.3	98.3
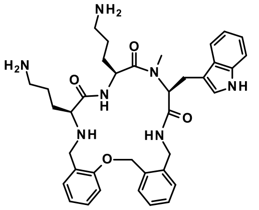	RO7055137	$C_{37}H_{47}N_7O_4$	653.3690	653.3693	–0.3	99.8
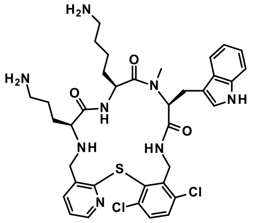	RO7075573	$C_{36}H_{44}Cl_2N_8O_3S$	738.2634	738.2630	–0.4	99.1
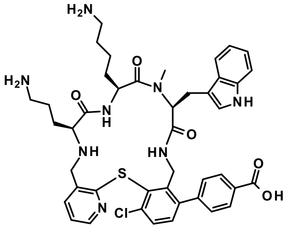	RO7202110	$C_{43}H_{49}ClN_8O_5S$	824.3235	824.3232	–0.3	99.7
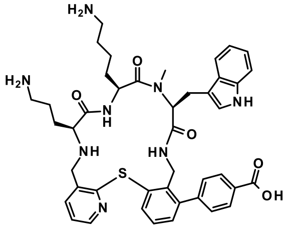	zosurabalpin RG6006	$C_{43}H_{50}N_8O_5S$	791.3698	791.3693	–0.5	100

Synthetic Chemistry

4-[(11S,14S,17S)-14-(4-Aminobutyl)-11-(3-aminopropyl)-17-(1H-indol-3-ylmethyl)-16-methyl-12,15,18-trioxo-2-thia-4,10,13,16,19-pentazatricyclo[19.4.0.0^{3,8}]pentacosa-1(25),3(8),4,6,21,23-hexaen-22-yl]benzoic acid (zosurabalpin; RG6006)

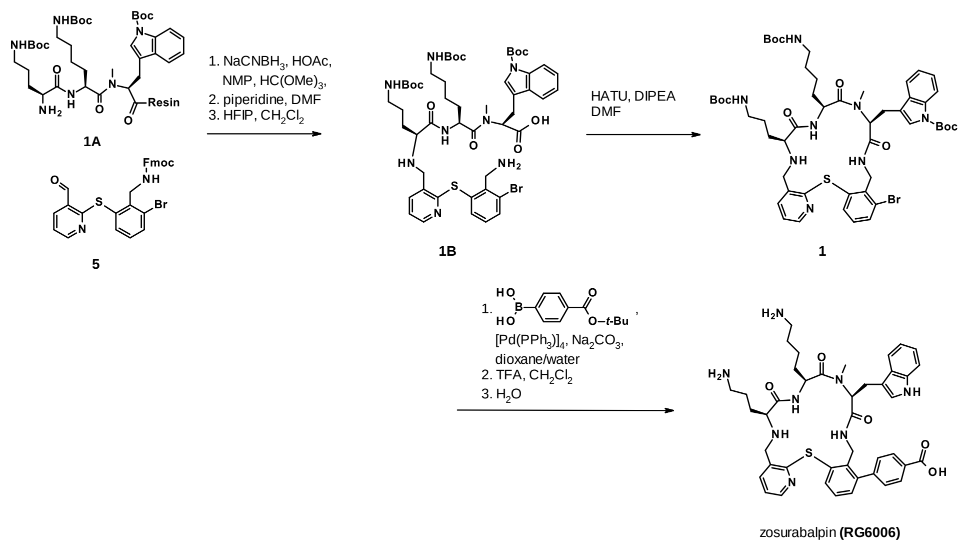

*a) (2S)-2-[[(2S)-2-[[(2S)-2-[[2-[2-(Aminomethyl)-3-bromo-phenyl]sulfanyl-3-pyridyl]-methylamino]-5-(tert-butoxycarbonylamino)pentanoyl]amino]-6-(tert-butoxycarbonylamino)-hexanoyl]-methyl-amino]-3-(1-tert-butoxycarbonylindol-3-yl)propanoic acid (**1B**)*

A mixture of Orn(Boc)-Lys(Boc)-N-Me-Trp(Boc)-O-(2-chlorotrityl resin) (**1A**; 5.0 g, 3.0 mmol), 9H-fluoren-9-ylmethyl N-[[2-bromo-6-[(3-formyl-2-pyridyl)sulfanyl]-phenyl]methyl]-carbamate (intermediate **5**; 2.29 g, 4.2 mmol), trimethyl orthoformate (50 mL), 1-methyl-2-pyrrolidone (50 mL), acetic acid (0.6 mL) were shaken for 8 h at room temperature, then treated with sodium cyanoborohydride (1.89 g, 30 mmol), and the reaction mixture was shaken at room temperature overnight. Finally, the resin was washed with N,N-dimethylformamide, dichloromethane, methanol/dichloromethane (1:1) and N,N-dimethylformamide. The 9-fluorenylmethoxycarbonyl-group on the tether was cleaved with piperidine/N,N-dimethylformamide 1:4 (60 mL) for 15 min at room temperature, followed by washings with N,N-dimethylformamide and dichloromethane. A cleavage-cocktail of hexafluoroisopropanol/dichloromethane 1:4 (60 mL) was added to the resin and the mixture was stirred for 2 h at room temperature. The resin was filtered off and the filtrate was evaporated to dryness. The residue was dissolved in

water/acetonitrile and lyophilized to produce the title compound (3.2 g), which was directly used in the next step. Light yellow oil, MS: 1069.5 $[M+H]^+$.

*b) tert-Butyl 3-{[(11S,14S,17S)-22-bromo-14-(4-{[(tert-butoxy)carbonyl]amino}butyl)-11-(3-{[(tert-butoxy)carbonyl]amino}propyl)-16-methyl-12,15,18-trioxo-2-thia-4,10,13,16,19-pentaazatricyclo[19.4.0.0^{3,8}]pentacosa-1(25),3(8),4,6,21,23-hexaen-17-yl]methyl}-1H-indole-1-carboxylate (**1**)*

O-(7-Azabenzotriazol-1-yl)-N,N,N′,N′-tetramethyluronium-hexafluorophosphate (1.37 g, 3.6 mmol) was added to a solution of (2S)-2-[[(2S)-2-[[(2S)-2-[[2-[2-(aminomethyl)-3-bromo-phenyl]sulfanyl-3-pyridyl]methylamino]-5-(tert-butoxycarbonylamino)pentanoyl]amino]-6-(tert-butoxycarbonylamino)hexanoyl]-methyl-amino]-3-(1-tert-butoxycarbonylindol-3-yl)propanoic acid (intermediate **1B**; 3.2 g) and diisopropylethylamine (0.77 g, 6.0 mmol) in N,N-dimethyl-formamide (250 mL) at room temperature, then after 2 h the reaction mixture was diluted with ethyl acetate, washed with water and brine, dried over magnesium sulfate, filtered, and evaporated. The residue was chromatographed with silica gel using a heptane–ethyl acetate gradient as the eluent yielding the crude product. The crude product was purified by reversed phase high-performance liquid chromatography (RP-HPLC) using a Phenomenex Gemini-NX 5u 110A column (100 x 30 mm) as the stationary phase and a gradient from water (+0.05% trifluoroacetic acid) to acetonitrile as the eluent to afford the title compound (673 mg, 32%).

White solid, MS: 1051.7 $[M+H]^+$. ^1H NMR (600 MHz, DMSO-d_6): δ 8.59 p.p.m. (br s, 1H), 8.24 (br d, J=3.3 Hz, 1H), 8.02 (br d, J=8.2 Hz, 1H), 7.81-7.75 (m, 1H), 7.71 (d, J=7.9 Hz, 1H), 7.69-7.64 (m, 1H), 7.50 (s, 1H), 7.38-7.32 (m, 1H), 7.29-7.25 (m, 1H), 7.26 (br s, 1H), 7.22 (d, J=7.9 Hz, 1H), 7.20-7.14 (m, 1H), 5.05 (br s, 1H), 4.87 (br dd, J=14.2, 4.6 Hz, 1H), 3.82 - 4.00 (m, 1H), 3.93 (br s, 1H), 3.68 (br s, 2H), 3.15-2.99 (m, 2H), 2.78 (br d, J=5.8 Hz, 3H), 2.66 (br s, 2H), 2.77-2.59 (m, 1H), 1.62 (s, 9H).

c) 4-[(11S,14S,17S)-14-(4-Aminobutyl)-11-(3-aminopropyl)-17-(1H-indol-3-ylmethyl)-16-methyl-12,15,18-trioxo-2-thia-4,10,13,16,19-pentazatricyclo[19.4.0.0^{3,8}]pentacosa-1(25),3(8),-4,6,21,23-hexaen-22-yl]benzoic acid (zosurabalpin; ***RG6006****)*

tert-Butyl 3-{[(11S,14S,17S)-22-bromo-14-(4-{[(tert-butoxy)carbonyl]amino}butyl)-11-(3-{[(tert-butoxy)carbonyl]amino}propyl)-16-methyl-12,15,18-trioxo-2-thia-4,10,13,16,19-penta-azatricyclo[19.4.0.0^{3,8}]pentacosa-1(25),3(8),4,6,21,23-hexaen-17-yl]methyl}-1H-indole-1-carboxylate (intermediate **1**; 2.15 g, 1.97 mmol) 4-(tert-butoxycarbonyl)phenylboronic acid (CAS-RN 850568-54-6; 675 mg, 2.95 mmol) and sodium carbonate (521 mg, 4.91 mmol) were dissolved in dioxane / water 6:1 (22 mL). The reaction mixture was sparged with argon for 2 min while sonicating the vessel in an ultrasonic bath. Then tetrakis(triphenylphosphine)–palladium(0) (459 mg, 393 µmol) was added, degassing continued for another 2 min, then the tube was sealed and heated at 120°C under microwave irradiation for 30 min. After cooling the reaction mixture was partitioned between ethyl acetate and 1 M aqueous sodium carbonate solution. The combined organic layers were washed with brine, dried over magnesium sulfate, filtered and evaporated to afford the crude product (1.95 g, light orange foam). The material was purified by MPLC using a RediSep Rf Gold® C18 Reversed Phase (100 g column, 20–40 µM particle size) as the stationary phase and a gradient from water (+0.05% trifluoroacetic acid) to acetonitrile as the eluent. After lyophilisation 2.21 g of a light yellow product were obtained. This material was dissolved in dichloromethane (22 mL) and treated at room temperature with trifluoroacetic acid (22 mL, 295 mmol), then after 90 min the reaction mixture was evaporated in vacuo. The residue was dissolved in water (22 mL), stirred at room temperature for 2 h, and then lyophilised. The residue was dissolved in water/acetonitrile 10:1 and purified by preparative HPLC using a C18 Reverse Phase column (Phenomenex Gemini-NX 5u 110A, 100 x 30 mm), and a gradient from water (+0.05% trifluoroacetic acid) to acetonitrile as the eluent. Lyophilisation of the pure fractions produced the title compound as the trifluoroacetate salt (1.65 g). This material was transferred into a smaller flask using 1 M aqueous hydrochloric acid solution (50 mL) and acetonitrile (50 mL) and freeze dried again. This procedure was repeated two more times to afford the title compound as the hydrochloride salt (1.30 g, 69%).

White lyophilised powder, HRMS (*m/z*): $[M+H]^+$ calcd for $C_{43}H_{50}N_8O_5S$, 791.3690; found, 791.3693. ^{1}H NMR (600 MHz, DMSO-d_6): δ 11.02-10.82 p.p.m. (m, 1H), 10.70-10.31 (m, 1H), 9.21 (br s, 1H), 9.15-8.86 (m, 1H), 8.52 (t, J=4.5 Hz, 1H), 8.44 (dd, J=4.8, 1.8 Hz, 1H), 8.20-

8.12 (m, 1H), 8.16 (dd, *J*=7.7, 1.6 Hz, 1H), 8.08-7.90 (m, 3H), 7.90 (br d, *J*=8.3 Hz, 1H), 7.70 (d, *J*=7.9 Hz, 1H), 7.59-7.54 (m, 2H), 7.45 - 7.49 (m, 1H), 7.42 - 7.45 (m, 1H), 7.41 (d, *J*=8.1 Hz, 1H), 7.35 (dd, *J*=7.4, 1.5 Hz, 1H), 7.34 (br s, 1H), 7.30 (dd, *J*=7.6, 4.9 Hz, 1H), 7.19 (d, *J*=2.1 Hz, 1H), 7.13-7.08 (m, 1H), 7.06-7.01 (m, 1H), 4.80 (br dd, *J*=10.6, 4.5 Hz, 2H), 4.37 (br d, *J*=13.1 Hz, 2H), 4.32 (br d, *J*=3.5 Hz, 1H), 4.31-4.20 (m, 2H), 4.17 (br s, 1H), 4.05 (br d, *J*=13.1 Hz, 1H), 3.25-3.16 (m, 1H), 3.15-3.09 (m, 1H), 2.89 (s, 3H), 2.87 (br d, *J*=3.9 Hz, 2H), 2.64-2.54 (m, 2H), 2.02-1.94 (m, 1H), 1.93-1.84 (m, 1H), 1.87-1.73 (m, 2H), 1.35-1.23 (m, 2H), 1.19-1.08 (m, 1H), 1.11-1.01 (m, 1H), 0.84 (br d, *J*=11.6 Hz, 1H), 0.39 (br t, *J*=12.2 Hz, 1H). ^{13}C-NMR (150.9 MHz, DMSO-d_6): δ 171.8, 169.5, 167.4, 167.3, 157.6, 150.6, 145.3, 143.6, 140.3, 137.3, 136.5, 135.0, 133.3, 130.4, 130.3, 129.7, 129.5, 128.6, 127.4, 127.0, 124.4, 121.4, 121.3, 118.8, 118.6, 112.0, 110.1, 60.0, 59.1, 50.9, 46.5, 40.6, 38.5, 38.3, 29.4, 29.2, 27.4, 26.8, 25.6, 22.6, 22.2.

4-[(11S,14S,17S)-14-(4-Aminobutyl)-11-(3-aminopropyl)-25-chloro-17-(1H-indol-3-ylmethyl)-16-methyl-12,15,18-trioxo-2-thia-4,10,13,16,19-pentazatricyclo[19.4.0.0^{3,8}]-pentacosa-1(25),3(8),4,6,21,23-hexaen-22-yl]benzoic acid (RO7202110)

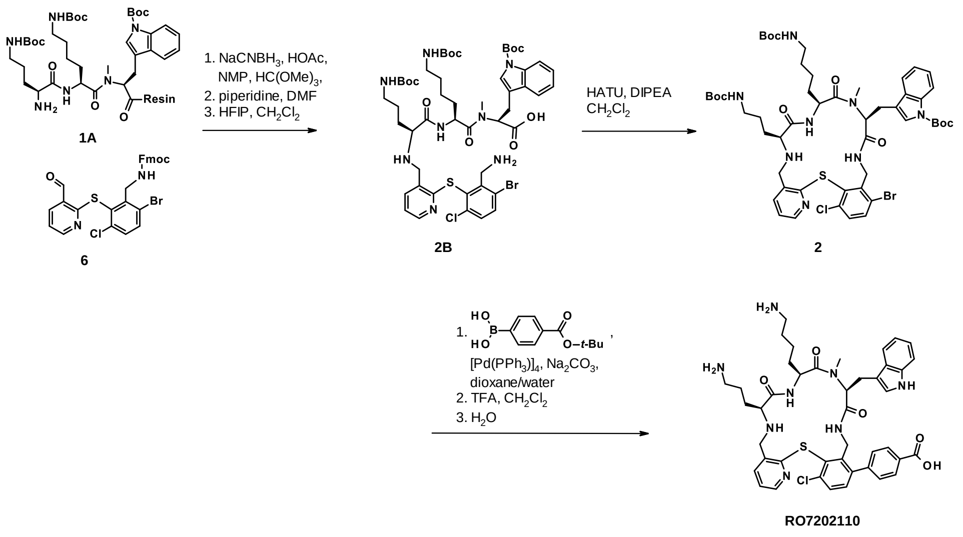

*__a) (2S)-2-[[(2S)-2-[[(2S)-2-[[2-[2-(Aminomethyl)-3-bromo-6-chloro-phenyl]sulfanyl-3-pyridyl]methylamino]-5-(tert-butoxycarbonylamino)pentanoyl]amino]-6-(tert-butoxycarbonyl-amino)hexanoyl]-methyl-amino]-3-(1-tert-butoxycarbonylindol-3-yl)propanoic acid (**2B**)__*

A mixture of Orn(Boc)-Lys(Boc)-N-Me-Trp(Boc)-O-(2-chlorotrityl resin) (intermediate **1A**; 10.0 g, 6.0 mmol), 9H-fluoren-9-ylmethyl N-[[6-bromo-3-chloro-2-[(3-formyl-2-pyridyl)sulfanyl]phenyl]methyl]carbamate (intermediate **6**; 4.06 g, 7.0 mmol), trimethyl orthoformate (50 mL), 1-methyl-2-pyrrolidone (50 mL), acetic acid (1 mL) were shaken for 8 h at room temperature, then treated with sodium cyanoborohydride (3.14 g, 50 mmol), and the reaction mixture was shaken at room temperature overnight. Finally, the resin was washed with N,N-dimethylformamide, dichloromethane, methanol/dichloromethane (1:1) and N,N-dimethylformamide. The 9-fluorenylmethoxycarbonyl-group on the tether was cleaved with a piperidine/N,N-dimethylformamide 1:4 (100 mL) for 15 min at room temperature followed by washings with N,N-dimethylformamide and dichloromethane. A cleavage-cocktail of hexafluoroisopropanol/dichloromethane 1:4 (100 mL) was added to the resin and the mixture was stirred for 2 h at room temperature. The resin was filtered off and the filtrate was evaporated to dryness. The residue was dissolved in water/acetonitrile and lyophilized to produce the title compound (8 g), which was directly used in the next step. Yellow oil, MS: 1103.4 $[M+H]^+$.

*b) tert-Butyl 3-[[(11S,14S,17S)-22-bromo-25-chloro-16-methyl-14-[4-[(2-methylpropan-2-yl)oxycarbonylamino]butyl]-11-[3-[(2-methylpropan-2-yl)oxycarbonylamino]propyl]-12,15,18-trioxo-2-thia-4,10,13,16,19-pentazatricyclo[19.4.0.0^{3,8}]pentacosa-1(21),3,5,7,22,24-hexaen-17-yl]methyl]indole-1-carboxylate (**2**)*

O-(7-Azabenzotriazol-1-yl)-N,N,N′,N′-tetramethyluronium-hexafluorophosphate (2.76 g, 7.3 mmol) was added to a solution of (2S)-2-[[(2S)-2-[[(2S)-2-[[2-[2-(Aminomethyl)-3-bromo-6-chloro-phenyl]sulfanyl-3-pyridyl]methylamino]-5-(tert-butoxycarbonylamino)pentanoyl]amino]-6-(tert-butoxycarbonylamino)hexanoyl]-methyl-amino]-3-(1-tert-butoxycarbonylindol-3-yl)propanoic acid (intermediate **2B**; 8 g) and diisopropylethylamine (1.88 g, 14.5 mmol) in dichloromethane (250 mL) at room temperature, then after 2 h the reaction mixture was diluted with ethyl acetate, washed with water and brine, dried over magnesium sulfate, filtered, and evaporated. The residue was chromatographed with silica gel using a heptane–ethyl acetate gradient as the eluent yielding the crude product. The crude product was purified by reversed phase high-performance liquid chromatography (RP-HPLC) using a Phenomenex Gemini-NX 5u 110A column (100 x 30 mm) as the stationary phase and a gradient from water (+0.05% trifluoroacetic acid) to acetonitrile as the eluent to afford the title compound (1.74 g, 27%). White solid, MS: 1085.7 $[M+H]^+$.

*c) 4-[(11S,14S,17S)-14-(4-Aminobutyl)-11-(3-aminopropyl)-25-chloro-17-(1H-indol-3-ylmethyl)-16-methyl-12,15,18-trioxo-2-thia-4,10,13,16,19-pentazatricyclo[19.4.0.03,8]penta-cosa-1(25),3(8),4,6,21,23-hexaen-22-yl]benzoic acid (**RO7202110**)*

tert-Butyl 3-[[(11S,14S,17S)-22-bromo-25-chloro-16-methyl-14-[4-[(2-methylpropan-2-yl)oxycarbonylamino]butyl]-11-[3-[(2-methylpropan-2-yl)oxycarbonylamino]propyl]-12,15,18-trioxo-2-thia-4,10,13,16,19-pentazatricyclo[19.4.0.03,8]pentacosa-1(21),3,5,7,22,24-hexaen-17-yl]methyl]indole-1-carboxylate (**2**; 2..00 g, 1.84 mmol) 4-(tert-butoxycarbonyl)phenylboronic acid (CAS-RN 850568-54-6; 626 mg, 2.77 mmol) and sodium carbonate (586 mg, 5.53 mmol) were dissolved in dioxane / water 6:1 (80 mL). The reaction mixture was sparged with argon for 2 min while sonicating the vessel in an ultrasonic bath. Then tetrakis(triphenylphosphine)–palladium(0) (215 mg, 184 µmol) was added, degassing continued for another 2 min, then the tube was sealed and heated at 100°C for 4 h. After cooling the reaction mixture was partitioned between ethyl acetate and 1 M aqueous sodium carbonate solution. The combined organic layers

were washed with brine, dried over magnesium sulfate, filtered and evaporated to afford the crude product. The material was purified by MPLC using a RediSep Rf Gold® C18 Reversed Phase (100 g column, 20–40 µM particle size) as the stationary phase and a gradient from water (+0.05% trifluoroacetic acid) to acetonitrile as the eluent. After lyophilisation 2.23 g of a light yellow product were obtained. This material was dissolved in dichloromethane (60 mL) and treated at room temperature with trifluoroacetic acid (17 mL, 226 mmol), then after 90 min the reaction mixture was evaporated in vacuo. The residue was dissolved in water/acetonitrile 9:1 (50 mL), stirred at room temperature for 16 h, and then lyophilised. The residue was dissolved in water/acetonitrile 10:1 and purified by preparative HPLC using a C18 Reverse Phase column (Phenomenex Gemini-NX 5u 110A, 100 x 30 mm), and a gradient from water (+0.05% trifluoroacetic acid) to acetonitrile as the eluent. Lyophilisation of the pure fractions produced the title compound as the trifluoroacetate salt. This material was transferred into a smaller flask using 1 M aqueous hydrochloric acid solution (50 mL) and acetonitrile (50 mL) and freeze dried again. This procedure was repeated two more times to afford the title compound as the hydrochloride salt (1.37 g, 77%).

White lyophilised powder, HRMS (*m/z*): $[M+H]^+$ calcd for $C_{43}H_{49}ClN_8O_5S$, 824.3235; found, 824.3232. ^{1}H NMR (600 MHz, DMSO-d_6): δ 13.84-12.18 p.p.m. (m, 1H), 11.13-10.94 (m, 1H), 10.94-10.58 (m, 1H), 9.31-9.07 (m, 1H), 8.32, (br s, 1H), 8.14-8.03 (m, 2H), 8.09-7.99 (m, 6H), 7.98 (br s, 2H), 7.85-7.72 (m, 1H), 7.74 (br d, *J*=7.7 Hz, 1H), 7.60-7.51 (m, 2H), 7.53-7.47 (m, 1H), 7.39 (br d, *J*=8.0 Hz, 1H), 7.23 (br s, 1H), 7.27-7.15 (m, 1H), 7.10 (br t, *J*=7.4 Hz, 1H), 7.06-6.99 (m, 1H), 4.88 (br s, 1H), 4.58 (br s, 2H), 4.50 (br s, 5H), 4.22 (br s, 2H), 4.32-4.10 (m, 2H), 3.77 (br s, 1H), 3.35-3.11 (m, 2H), 2.94 (br s, 2H), 2.84 (br s, 3H), 1.93 (br d, *J*=5.8 Hz, 3H), 1.88-1.66 (m, 2H), 1.33 (br s, 3H), 1.19 (br s, 1H), 0.69 (br s, 1H). ^{13}C NMR (150.9 MHz, DMSO-d_6): 171.85, 169.60, 167.50, 150.29, 144.64, 142.40, 140.72, 140.05, 136.67, 132.94, 130.64, 129.91, 129.68, 127.57, 124.49, 121.51, 120.70, 118.91, 118.67, 112.11, 110.22, 60.23, 59.31, 46.62, 42.10, 38.60, 38.37, 29.48, 29.30, 27.57, 26.90, 22.59, 22.29.

(11S,14S,17S)-14-(4-Aminobutyl)-11-(3-aminopropyl)-22,25-dichloro-17-(1H-indol-3-ylmethyl)-16-methyl-2-thia-4,10,13,16,19-pentazatricyclo[19.4.0.03,8]pentacosa-1(25),3(8),4,6,21,23-hexaene-12,15,18-trione (RO7075573)

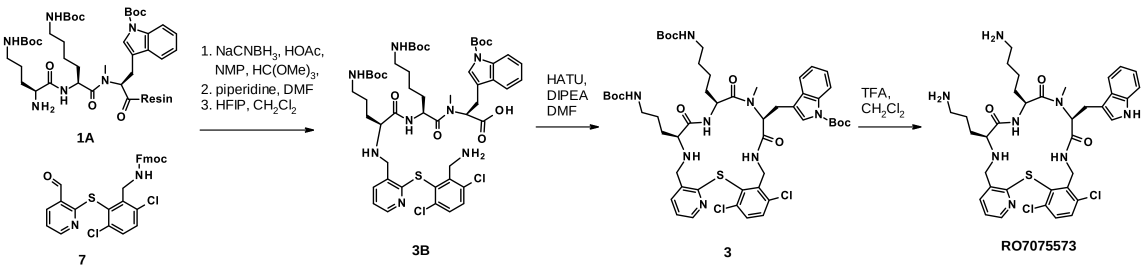

*a) (2S)-2-[[(2S)-2-[[(2S)-2-[[2-[2-(Aminomethyl)-3,6-dichloro-phenyl]sulfanyl-3-pyridyl]-methylamino]-5-(tert-butoxycarbonylamino)pentanoyl]amino]-6-(tert-butoxycarbonyl-amino)hexanoyl]-methyl-amino]-3-(1-tert-butoxycarbonylindol-3-yl)propanoic acid (**3B**)*

A mixture of Orn(Boc)-Lys(Boc)-N-Me-Trp(Boc)-O-(2-chlorotrityl resin) (intermediate **1A**; 5.9 g, 3.4 mmol), 9H-fluoren-9-ylmethyl N-[[3,6-dichloro-2-[(3-formyl-2-pyridyl)sulfanyl]-phenyl]–methyl] carbamate (intermediate **7**; 2.03 g, 3.8 mmol), trimethyl orthoformate (30 mL), 1-methyl-2-pyrrolidone (30 mL), acetic acid (0.6 mL) were shaken for 8 h at room temperature, then treated with sodium cyanoborohydride (2.14 g, 34 mmol), and the reaction mixture was shaken at room temperature overnight. Finally, the resin was washed with N,N-dimethyl-formamide, dichloromethane, methanol/dichloromethane (1:1) and N,N-dimethylformamide. The 9-fluorenylmethoxycarbonyl-group on the tether was cleaved with a mixture of piperidine/N,N-dimethylformamide for 15 min at room temperature, followed by washings with N,N-dimethylformamide and dichloromethane. A cleavage-cocktail of hexafluoroisopropanol/di-chloromethane 1:4 (60 mL) was added to the resin and the mixture was stirred for 2 h at room temperature. The resin was filtered off and the solution was evaporated to dryness. The residue was dissolved in water/acetonitrile and lyophilized to produce the title compound (2.4 g), which was directly used in the next step. Orange foam, MS: 1057.4 $[M+H]^+$.

*b) tert-Butyl 3-[[(11S,14S,17S)-22,25-dichloro-16-methyl-14-[4-[(2-methylpropan-2-yl)oxycarbonylamino]butyl]-11-[3-[(2-methylpropan-2-yl)oxycarbonylamino]propyl]-12,15,18-trioxo-2-thia-4,10,13,16,19-pentazatricyclo[19.4.0.0^{3,8}]pentacosa-1(25),3(8),4,6,21,23-hexaen-17-yl]methyl]indole-1-carboxylate (**3**)*

O-(7-Azabenzotriazol-1-yl)-N,N,N′,N′-tetramethyluronium-hexafluorophosphate (1.56 g, 4.1 mmol) was added to a solution of (2S)-2-[[(2S)-2-[[(2S)-2-[[2-[2-(aminomethyl)-3,6-dichloro-phenyl]sulfanyl-3-pyridyl]methylamino]-5-(tert-butoxycarbonylamino)pentanoyl]-amino]-6-(tert-butoxycarbonylamino)hexanoyl]-methyl-amino]-3-(1-tert-butoxycarbonylindol-3-yl)propanoic acid (**3B**, 3.2 g) and diisopropylethylamine (2.2 g, 17 mmol) in N,N-dimethylformamide (40 mL) at room temperature, then after 45 min the reaction mixture was diluted with ethyl acetate, washed with water and brine, dried over magnesium sulfate, filtered, and evaporated. The residue was chromatographed with silica gel using a heptane–ethyl acetate gradient as the eluent yielding the title compound (683 mg). MS: 1039.4 $[M+H]^+$.

*c) (11S,14S,17S)-14-(4-Aminobutyl)-11-(3-aminopropyl)-22,25-dichloro-17-(1H-indol-3-ylmethyl)-16-methyl-2-thia-4,10,13,16,19-pentazatricyclo[19.4.0.03,8]pentacosa-1(25),3(8),-4,6,21,23-hexaene-12,15,18-trione (**RO7075573**)*

tert-Butyl 3-[[(11S,14S,17S)-22,25-dichloro-16-methyl-14-[4-[(2-methylpropan-2-yl)-oxycarbonylamino]butyl]-11-[3-[(2-methylpropan-2-yl)oxycarbonylamino]propyl]-12,15,18-trioxo-2-thia-4,10,13,16,19-pentazatricyclo[19.4.0.0^{3,8}]pentacosa-1(25),3(8),4,6,21,23-hexaen-17-yl]methyl]indole-1-carboxylate (**3**; 683 mg) was dissolved in dichloromethane and treated with trifluoroacetic acid at room temperature, then after 2 h the reaction mixture as concentrated under vacuum and the residue was lyophilised to produce the title compound as the trifluoroacetate salt (716 mg). This material was transferred into a smaller flask using 1 M aqueous hydrochloric acid solution (20 mL) and acetonitrile (20 mL) and freeze dried again. This procedure was repeated two more times to afford the title compound as the hydrochloride salt (490 mg, 25%).

Off-white solid, HRMS (*m/z*): $[M+H]^+$ calcd for $C_{36}H_{44}Cl_2N_8O_3S$, 738.2634; found, 738.2630. ^{1}H NMR (600 MHz, DMSO-d_6): δ 11.03 p.p.m. (s, 2H), 9.55-8.63 (m, 2H), 8.54-8.20 (m, 1H), 8.16-7.90 (m, 7H), 7.72-7.61 (m, 2H), 7.39 (br d, J=8.1 Hz, 1H), 7.25-7.08 (m, 1H), 7.10-7.03 (m, 1H), 6.99 (br s, 1H), 4.91 (br s, 1H), 4.43 (br s, 1H), 3.22-3.03 (m, 2H), 2.84 (td,

J=13.1, 6.9 Hz, 4H), 2.62-2.54 (m, 2H), 2.08-1.62 (m, 4H), 1.37-0.80 (m, 4H). ^{13}C NMR (150.9 MHz, DMSO-d_6): 171.61, 169.80, 167.91, 150.31, 143.29, 140.30, 136.61, 134.22, 132.58, 130.59, 127.63, 124.47, 121.43, 120.74, 118.93, 118.49, 112.11, 110.19, 60.98, 59.13, 50.88, 46.58, 46.57, 41.71, 38.57, 38.39, 29.35, 29.12, 27.56, 26.86, 24.58, 24.57, 22.56, 22.24.

(12S,15S,18S)-15,18-bis(3-Aminopropyl)-12-(1H-indol-3-ylmethyl)-13-methyl-2-thia-10,13,16,19-tetrazatricyclo[19.4.0.0^{3,8}]pentacosa-1(21),3,5,7,22,24-hexaene-11,14,17-trione (RO7036668)

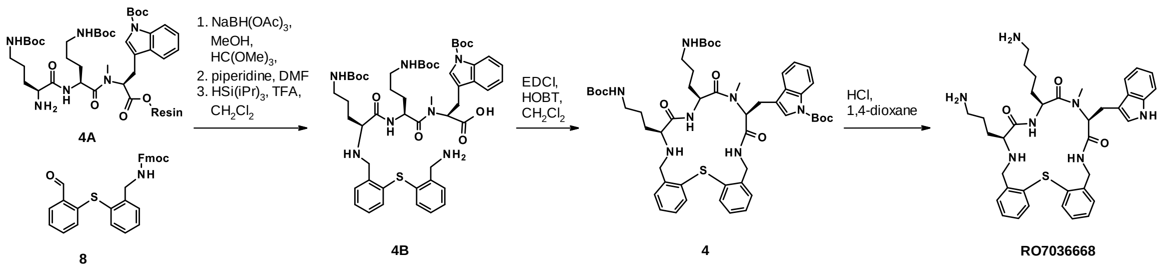

*a) (2S)-2-[[(2S)-2-[[(2S)-2-[[2-[2-(Aminomethyl)phenyl]sulfanylphenyl]methylamino]-5-(tert-butoxycarbonylamino)pentanoyl]amino]-5-(tert-butoxycarbonylamino)pentanoyl]-methyl-amino]-3-(1-tert-butoxycarbonylindol-3-yl)propanoic acid (**4B**)*

A mixture of Orn(Boc)-Orn(Boc)-N-Me-Trp(Boc)-O-(2-chlorotrityl resin) (**4A**; 1.2 g, 0.8 mmol), 9H-fluoren-9-ylmethyl N-[[2-(2-formylphenyl)sulfanylphenyl]methyl]carbamate (**8**; 521 mg, 1.12 mmol), trimethyl orthoformate (5 mL) and methanol (15 mL) was shaken for 1 h at room temperature, then treated with sodium triacetoxyborohydride (848 mg, 4 mmol), and the reaction mixture was shaken at room temperature overnight. Finally, the resin was washed with N,N-dimethylformamide, dichloromethane, methanol/dichloromethane (1:1) and N,N-dimethylformamide. The 9-fluorenylmethoxycarbonyl-group on the tether was cleaved with a mixture of piperidine/N,N-dimethylformamide for 15 min at room temperature, followed by washings with N,N-dimethylformamide and dichloromethane. A cleavage-cocktail of dichloromethane/trifluoroacetic acid/triisopropylsilane 94:1:5 (10 mL) was added to the resin and the mixture was stirred for 1 h at room temperature. The resin was filtered off and the solution was evaporated to dryness. The residue was dissolved in water/acetonitrile and lyophilized to produce the title compound (69 mg, 7%), which was directly used in the next step. Light yellow solid, MS: 973.5 $[M+H]^+$.

*b) tert-butyl 3-[[(12S,15S,18S)-15,18-bis[3-(tert-butoxycarbonylamino)propyl]-13-methyl-11,14,17-trioxo-2-thia-10,13,16,19-tetrazatricyclo[19.4.0.0^{3,8}]pentacosa-1(21),3(8),4,6,22,24-hexaen-12-yl]methyl]indole-1-carboxylate (**4**)*

To a solution of 1-(3-dimethylaminopropyl)-3-ethylcarbodiimide hydrochloride (**4B**, 79 mg, 0.41 mmol) and 1-hydroxybenzotriazole hydrate (63 mg, 0.41 mmol) in dichloromethane (15 mL) to give a colorless solution at 0°C for 5 min, which was added a suspension of (2S)-2-[[(2S)-2-[[(2S)-2-[[2-[2-(aminomethyl)phenyl]sulfanylphenyl]methylamino]-5-(tert-butoxycarbonyl-amino)pentanoyl]amino]-5-(tert-butoxycarbonylamino)pentanoyl]-methyl-amino]-3-(1-tert-butoxycarbonylindol-3-yl)propanoic acid (160 mg, 0.16 mmol) in dichloromethane (5 mL) under ice-water bath. After addition, the suspension was stirred at 25°C for 1 h to give a light yellow solution, then concentrated under vacuum. Chromatography (petroleum ether/ethyl acetate 1:3) produced the title compound (80 mg, 50%). Light yellow solid, MS: 956.4 $[M+H]^+$.

*c) (12S,15S,18S)-15,18-bis(3-Aminopropyl)-12-(1H-indol-3-ylmethyl)-13-methyl-2-thia-10,13,16,19-tetrazatricyclo[19.4.0.0^{3,8}]pentacosa-1(21),3,5,7,22,24-hexaene-11,14,17-trione (**RO7036668**)*

A solution of tert-butyl 3-[[(12S,15S,18S)-15,18-bis[3-(tert-butoxycarbonylamino)propyl]-13-methyl-11,14,17-trioxo-2-thia-10,13,16,19-tetrazatricyclo[19.4.0.0^{3,8}]pentacosa-1(21),3(8),-4,6,22,24-hexaen-12-yl]methyl]indole-1-carboxylate (**4**, 80 mg, 0.08 mmol) in 1,4-dioxane (1 mL) was added hydrogen chloride solution (4 M in 1,4-dioxane, 4 mL, 16 mmol). After addition, the mixture was stirred at 25 °C for 2 hours, during which a white solid precipitated. The solid was collected by filtration and washed with acetonitrile. The filter cake was dissolved in water (20 mL) and lyophilised to produce the title compound as the hydrochloride salt (38 mg, 61%).

White solid, HRMS (*m/z*): $[M+H]^+$ calcd for $C_{36}H_{45}N_7O_3S$, 655.3305; found, 655.3302. ^1H NMR (400 MHz, D_2O): δ 8.86-8.77 p.p.m. (m, 1H), 7.77 (d, J=7.75 Hz, 1H), 7.58-7.53 (m, 1H), 7.52-7.48 (m, 1H), 7.47-7.39 (m, 3H), 7.34-7.28 (m, 2H), 7.25-7.16 (m, 2H), 7.07 (s, 1H), 7.03-6.97 (m, 1H), 6.48-6.39 (m, 1H), 4.62-4.58 (m, 3H), 4.22-4.14 (m, 1H), 4.12-4.04 (m, 1H), 4.02-3.95 (m, 1H), 3.32-3.21 (m, 1H), 3.17-3.03 (m, 1H), 2.96-2.88 (m, 2H), 2.80 (s, 3H), 2.76-2.58 (m, 1H), 2.20-2.05 (m, 2H), 1.94-1.84 (m, 1H), 1.80-1.69 (m, 1H), 1.64-1.51 (m, 2H), 1.11-0.92 (m, 1H), 0.60-0.42 (m, 1H), 0.15-0.06 (m, 1H), (0.62)-(0.79) (m, 1H). ^{13}C NMR (150.9 MHz, DMSO-d_6): 171.05, 169.72, 139.15, 136.58, 135.55, 133.89, 132.73, 132.03, 130.97, 130.66, 129.53, 128.23, 128.01, 127.64, 124.55, 121.51, 119.03, 118.61, 111.89, 110.36, 61.45, 58.89, 49.75, 48.09, 42.17, 42.15, 38.60, 38.58, 28.95, 26.71, 23.93, 23.58, 22.70.

(13S,16S,19S)-16,19-bis-(3-Amino-propyl)-13-(1H-indol-3-ylmethyl)-14-methyl-2-oxa-11,14,17,20-tetraaza-tricyclo[20.4.0.0^{4,9}]hexacosa-1(22),4,6,8,23,25-hexaene-12,15,18-trione (RO7055137)

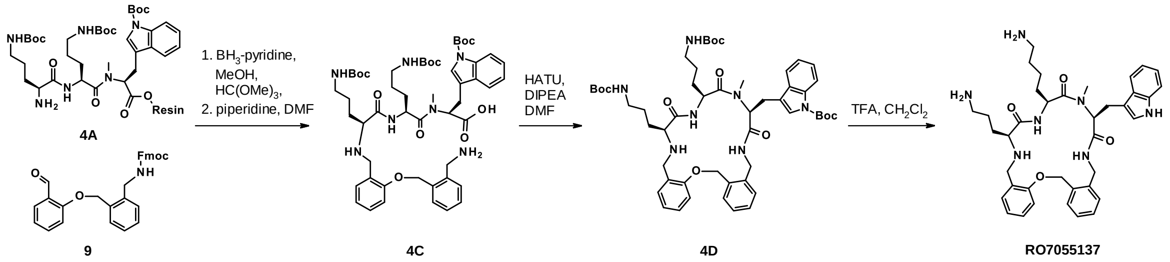

*<u>a) 3-{(S)-2-[((S)-2-{(S)-2-[2-(2-aminomethyl-benzyloxy)-benzylamino]-5-tert-butoxycarbonylamino-pentanoylamino}-5-tert-butoxycarbonylamino-pentanoyl)-methyl-amino]-2-carboxy-ethyl}-indole-1-carboxylic acid tert-butyl ester (**4C**)</u>*

Orn(Boc)-Orn(Boc)-N-Me-Trp(Boc)-O-(2-chlorotrityl resin) (**4A**; 1 equivalent, 0.16 mmol) was first swelled in dichloromethane (1.8 mL) and methanol/trimethyl orthoformate (3:1, 1.8 mL)for 30 min in a sealed tube, then 2-(2-formyl-phenoxymethyl)-benzyl]-carbamic acid 9H-fluoren-9-ylmethyl ester (**9**; 78 mg, 0.24 mmol) was added and shaken for 1 h at room temperature followed by the addition of borane pyridine complex (0.04 mL, 0.42 mmol), then after 15 min pressure was released by opening cap slowly, re-closed and allowed to shake at room temperature for 16 h. The resin was filtered off and the filtrate was evaporated to get the crude product. The residue was treated with 20% piperidine in N,N-dimethylformamide (4 mL) at room temperature for 1 h, then evaporated. Purification by preparative HPLC (Column name: YMC-Triart C18 (250 x 20 mm, 5µm). Flow rate = 20.0 mL/min. Gradient Profile: acetonitrile / 10 mM aqueous ammonium acetate solution) produced the title compound (50 mg).

*<u>b) tert-Butyl 3-[[(13S,16S,19S)-14-methyl-16,19-bis[3-[(2-methylpropan-2-yl)oxycarbonyl-amino]propyl]-12,15,18-trioxo-2-oxa-11,14,17,20-tetrazatricyclo[20.4.0.0^{4,9}]hexacosa-1(22),4,6,8,23,25-hexaen-13-yl]methyl]indole-1-carboxylate (**4D**)</u>*

To a stirred solution 3-{(S)-2-[((S)-2-{(S)-2-[2-(2-aminomethyl-benzyloxy)-benzylamino]-5-tert-butoxycarbonylamino-pentanoylamino}-5-tert-butoxycarbonylamino-pentanoyl)-methyl-amino]-2-carboxy-ethyl}-indole-1-carboxylic acid tert-butyl ester (**4C**; 50 mg, 0.051 mmol) in N,N-dimethylformamide (1 mL), 1-[bis(dimethylamino)methylene]-1H-1,2,3-triazolo[4,5-b]pyridinium 3-oxide hexafluorophosphate (20 mg, 0.051 mmol), diisopropylethylamine (0.012 mL, 0.062 mmol) was added and the resulting reaction mixture was stirred at room temperature for 2 h. The crude reaction mixture was purified by preparative HPLC (Column name: YMC-

Triart C18 (100 x 30 mm, 5µm). Flow rate = 30.0 mL/min. Gradient Profile: acetonitrile/ 20 mM ammonium hydrogen carbonate in water) to the title compound (25 mg) as off-white solid.

c) (13S,16S,19S)-16,19-bis-(3-Amino-propyl)-13-(1H-indol-3-ylmethyl)-14-methyl-2-oxa-11,14,17,20-tetraaza-tricyclo[20.4.0.0^{4,9}]hexacosa-1(22),4,6,8,23,25-hexaene-12,15,18-trione ***(RO7055137)***

A solution of tert-butyl 3-[[(13S,16S,19S)-14-methyl-16,19-bis[3-[(2-methylpropan-2-yl)oxycarbonylamino]propyl]-12,15,18-trioxo-2-oxa-11,14,17,20-tetrazatricyclo[20.4.0.0^{4,9}]hexa-cosa-1(22),4,6,8,23,25-hexaen-13-yl]methyl]indole-1-carboxylate (**4D**; 70 mg, 0.07 mmol) in hydrogen chloride solution (4 M in 1,4-dioxane, 3.5 mL, 14 mmol) was stirred at 40°C for 2 h, then the solid was collected by filtration and washed with acetonitrile (3 mL). The crude product was dissolved in water (20 mL) and freeze dried to produce the title compound (32 mg, 59%) as the hydrochloride salt.

White solid, HRMS (*m/z*): $[M+H]^+$ calcd for $C_{37}H_{47}N_7O_4$, 653.3690; found, 653.3693. ^1H NMR (400 MHz, D_2O): δ 8.11-8.06 p.p.m. (m, 2H), 8.01-7.99 (m, 1H), 7.96-7.94 (m, 2H), 7.89-7.87 (m, 2H), 7.74-7.72 (m, 2H), 7.67-7.64 (m, 2H), 7.60 (s, 1H), 7.40-7.37 (br t, *J*=7.2 Hz, 1H), 5.84 (d, *J*=11.6 Hz, 1H), 5.72 (d, *J*=12.4 Hz, 1H), 5.08-4.96 (m, 3H), 4.85 (br d, *J*=13.6 Hz, 1H), 4.39 (br d, *J*=13.6 Hz, 1H), 4.24-4.19 (m, 1H), 3.94 (br d, *J*=16.0 Hz, 1H), 3.75 (br d, *J*=10.0 Hz, 1H), 3.71-3.62 (m, 1H), 3.31 (s, 3H), 3.26-3.18 (m, 2H), 2.68-2.57 (m, 2H), 2.12-2.03 (m, 1H), 1.87-1.78 (m, 1H), 1.69-1.59 (m, 2H), 1.50-1.40 (m, 1H), 1.24-1.11 (m, 1H), 0.78-0.65 (m, 1H), 0.07-(0.05) (m, 1H). ^{13}C NMR (150.9 MHz, DMSO-d_6): 171.32, 170.03, 157.68, 138.69, 136.65, 134.89, 132.07, 131.17, 130.61, 130.13, 129.29, 127.85, 127.58, 124.59, 121.54, 121.49, 119.09, 118.57, 113.74, 111.96, 110.37, 69.46, 61.50, 58.16, 49.93, 44.61, 38.55, 29.27, 26.86, 24.28, 24.18, 23.89, 23.73, 22.58, 22.56.

Intermediates

*<u>Orn(Boc)-Lys(Boc)-N-Me-Trp(Boc)-O-(2-chlorotrityl resin) (**1A**)</u>*

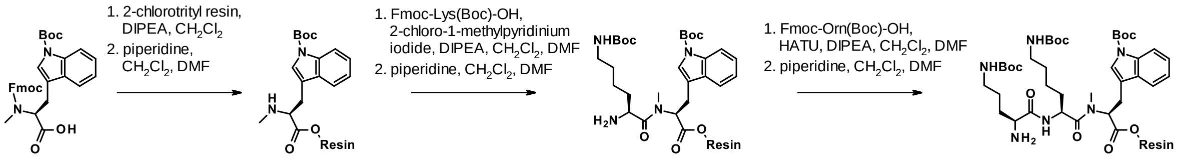

1A-1 1A-2 1A

*<u>a) N-Me-(Boc)Trp-O-(2-chlorotrityl resin) (**1A-1**)</u>*

2-Chloro-tritylchloride resin [CAS-RN 934816-82-7]; 1.6 meq/g, 100-200 mesh) was loaded with 1-[(1,1-dimethylethoxy)carbonyl]-N-[(9H-fluoren-9-ylmethoxy)carbonyl]-N-methyl-L-tryptophan [CAS-RN 197632-75-0] (0.6 equivalents) and diisopropylethylamine (8 equivalents) in dry dichloromethane overnight at room temperature. After extensive washing with N,N-dimethylformamide and dichloromethane, the 9-fluorenylmethoxycarbonyl protecting group was cleaved off with a mixture of 50% piperidine in dichloromethane/N,N-dimethylformamide (1:1) in N,N-dimethylformamide for 30 min at room temperature. After washing with N,N-dimethylformamide, dichloromethane and methanol the resin was dried under vacuum at room temperature overnight. The resin loading was determined via weight increase.

*<u>b) Lys(Boc)-N-Me-Trp(Boc)-O-(2-chlorotrityl resin) (**1A-2**)</u>*

N^6-[(1,1-Dimethylethoxy)carbonyl]-N^2-[(9H-fluoren-9-ylmethoxy)carbonyl]-L-lysine [CAS-RN 71989-26-9] (4 equivalents) was coupled to N-Me-(Boc)Trp-O-(2-chlorotrityl resin) (**1A-1**) with 4 equivalents of 2-chloro-1-methylpyridinium iodide as coupling reagent, 6 equivalents of N,N-diisopropylethylamine in dichloromethane/N,N-dimethylformamide (1:1) overnight at room temperature. The resin was extensively washed with N,N-dimethylformamide and dichloromethane and the coupling rate was controlled by a test-cleavage.

The 9-fluorenylmethoxycarbonyl-group from the dipeptide was cleaved with a mixture of piperidine / dichloromethane / N,N-dimethylformamide (2:1:1) for 5 min followed by washings with N,N-dimethylformamide and dichloromethane. The cleavage rates were again controlled by test-cleavage.

*<u>c) Orn(Boc)-Lys(Boc)-N-Me-Trp(Boc)-O-(2-chlorotrityl resin) (**1A**)</u>*

N^2-[(9H-fluoren-9-ylmethoxy)carbonyl]-L-ornithine [CAS-RN 109425-55-0] (4 equivalents) was coupled to Lys(Boc)-N-Me-Trp(Boc)-O-(2-chlorotrityl resin) (**1A-2**) using 4 equivalents of O-(7-azabenzotriazol-1-yl)-N,N,N′,N′-tetramethyluronium-hexafluorophosphate as coupling reagent and 6 equivalents diisopropylethylamine. Complete couplings were accomplished at room temperature for 2-4 hours with the coupling rate again controlled by a test-cleavage.

The 9-fluorenylmethoxycarbonyl-group from the tripeptide was cleaved with a mixture of 20% piperidine in N,N-dimethylformamide for 15 min at room temperature followed by washings with N,N-dimethylformamide and dichloromethane to produce the title compound.

*Orn(Boc)-Orn(Boc)-N-Me-Trp(Boc)-O-(2-chlorotrityl resin) (**4A**)*

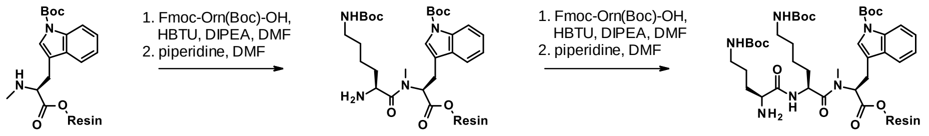

1A-1 **4A-2** **4A**

*a) Orn(Boc)-N-Me-Trp(Boc)-O-(2-chlorotrityl resin) (**4A-2**)*

N-Me-(Boc)Trp-O-(2-chlorotrityl resin) (**1A-1**) was coupled to N^6-[(1,1-dimethylethoxy)carbonyl]-N^2-[(9H-fluoren-9-ylmethoxy)carbonyl]-L-ornithine [CAS-RN 109425-55-0] (4 equivalents) using 4 equivalents of (2-(1H-benzotriazol-1-yl)-1,1,3,3-tetramethyluronium hexafluorophosphate as coupling reagent, 6 equivalents of N,N-diisopropylethylamine in dichloromethane/N,N-dimethylformamide (1:1) overnight at room temperature. The resin was extensively washed with N,N-dimethylformamide and dichloromethane and the coupling rate was controlled by a test-cleavage.

The 9-fluorenylmethoxycarbonyl-group from the dipeptide was cleaved with a mixture of piperidine / N,N-dimethylformamide (2:1) for maximally 5 min followed by washings with N,N-dimethylformamide and dichloromethane. The cleavage rates were again controlled by test-cleavage.

*b) Orn(Boc)-Orn(Boc)-N-Me-Trp(Boc)-O-(2-chlorotrityl resin) (**4A**)*

Orn(Boc)-N-Me-Trp(Boc)-O-(2-chlorotrityl resin) (**4A-2**) was coupled to N^2-[(9H-fluoren-9-ylmethoxy)carbonyl]-L-ornithine [CAS-RN 109425-55-0] (4 equivalents) using (2-(1H-benzotriazol-1-yl)-1,1,3,3-tetramethyluronium hexafluorophosphate as coupling reagent as coupling reagent and 6 equivalents diisopropylethylamine. Complete couplings were accomplished at room temperature for 2-4 hours with the coupling rate again controlled by a test-cleavage.

The 9-fluorenylmethoxycarbonyl-group from the tripeptide was cleaved with a mixture of 20% piperidine in N,N-dimethylformamide for 2 x 15-20 min at room temperature followed by washings with N,N-dimethylformamide and dichloromethane (test-cleavage).

*9H-Fluoren-9-ylmethyl N-[[2-bromo-6-[(3-formyl-2-pyridyl)sulfanyl]phenyl]methyl]–carbamate **(5)***

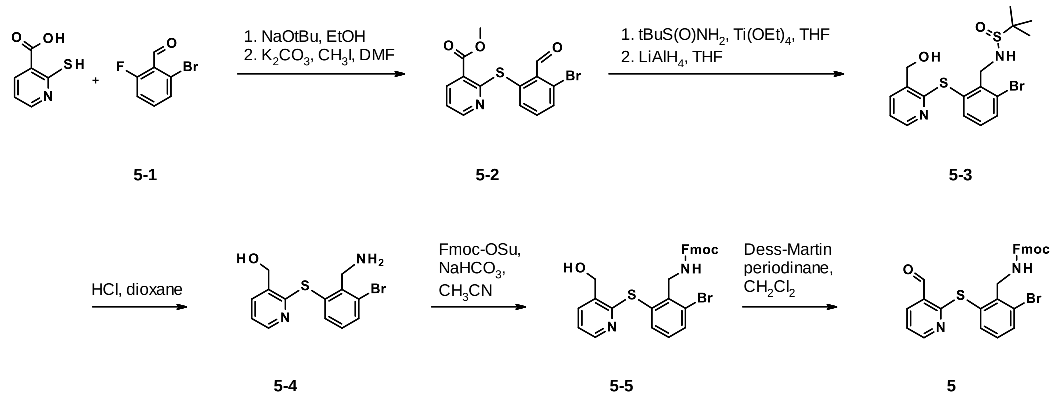

*a) Methyl 2-(3-bromo-2-formyl-phenyl)sulfanylpyridine-3-carboxylate (**5-2**)*

To a stirred solution of 2-bromo-6-fluorobenzaldehyde (**5-1**; 37.0 g, 182 mmol) in N,N-dimethylformamide (370 mL) was added potassium tert-butylate (40.9 g, 365 mmol) and the reaction mass was stirred at 25°C for 30 min. Then, 2-mercaptonicotinic acid (CAS-RN 38521-46-9; 31.1 g, 200 mmol) was added and reaction mixture was stirred at 80°C for 4 h. After cooling to room temperature potassium carbonate (75.5 g, 547 mmol) was added followed by addition of iodomethane (77.6 g, 547 mmol), then after 16 h the reaction mixture was partitioned between ethyl acetate and water. The separated organic layer was washed with brine, dried over sodium sulfate, filtered, and evaporated under reduced pressure. Chromatography (silica gel; hexane/dichloromethane 85:15) produced the title compound (27.5 g, 43%).

Yellow solid, MS: 351.9 $[M+H]^+$. ^1H NMR (600 MHz, $CDCl_3$): δ 10.30-10.14 p.p.m. (m, 1H), 8.38-8.33 (m, 1H), 8.27-8.22 (m, 1H), 7.76-7.72 (m, 1H), 7.74 (dd, *J*=8.1, 1.2 Hz, 1H), 7.60-7.56 (m, 1H), 7.58 (dd, *J*=7.7, 1.1 Hz, 1H), 7.41-7.35 (m, 1H), 7.13-7.06 (m, 1H), 4.09-3.92 (m, 3H).

*b) N-[[2-Bromo-6-[[3-(hydroxymethyl)-2-pyridyl]sulfanyl]phenyl]methyl]-2-methyl-propane-2-sulfinamide (**5-3**)*

To a stirred solution of methyl 2-(3-bromo-2-formyl-phenyl)sulfanylpyridine-3-carboxylate (**5-2**; 27.0 g, 76.7 mmol) in tetrahydrofuran (270 mL) was added 2-methyl-2-propanesulfinamide (CAS-RN 146374-27-8; 9.29 g, 76.7 mmol), titanium(IV) ethoxide (73.9 g, 384 mmol), and the

reaction mixture was heated at 70°C for 4 h. After cooling the reaction mixture was treated with brine, then insoluble material was removed by filtration through diatomaceous earth. The filtrate was extracted with ethyl acetate, the separated organic layer was washed with water, dried over sodium sulfate and evaporated under reduced pressure. The residue was dissolved in tetrahydro-furan (280 mL) and treated with lithium aluminum hydride solution (2.5 M in tetrahydrofuran, 48.4 mL, 121 mmol) at -40°C and the reaction mixture was stirred at -40°C for 2 h, then excess reagent was destroyed by addition of saturated aqueous sodium sulfate solution. The reaction mixture was extracted with ethyl acetate, the organic layer was washed with brine, dried over sodium sulfate, filtered, and evaporated to produce the title compound (22.6 g, 69%).

Yellow semisolid, MS: 431.0 $[M+H]^+$. ^1H NMR (600 MHz, DMSO-d_6): δ 8.20 p.p.m. (dd, J=4.7, 1.8 Hz, 1H), 7.83-7.78 (m, 1H), 7.71 (dd, J=8.0, 1.3 Hz, 1H), 7.48-7.44 (m, 1H), 7.27-7.23 (m, 1H), 7.24-7.19 (m, 1H), 5.55-5.49 (m, 1H), 5.17 (dd, J=6.4, 4.9 Hz, 1H), 4.62-4.57 (m, 2H), 4.47 (dd, J=12.7, 4.8 Hz, 1H), 4.36 (dd, J=12.7, 6.6 Hz, 1H), 1.06 (s, 8H).

*c) [2-[2-(Aminomethyl)-3-bromo-phenyl]sulfanyl-3-pyridyl]methanol hydrochloride (**5-4**)*

To a stirred solution of N-[[2-bromo-6-[[3-(hydroxymethyl)-2-pyridyl]sulfanyl]phenyl]-methyl]-2-methyl-propane-2-sulfinamide (**5-3**; 58.0 g, 135 mmol) in tetrahydrofuran (550 mL) and methanol (10 mL) was added hydrogen chloride solution (4 M in 1,4-dioxane, 67.5 mL, 270 mmol) at room temperature, then after 2 h the reaction mixture was concentrated to produce the title compound (46.1 g, 94%).

Off-white solid, MS: 327.0 $[M+H]^+$. ^1H NMR (600 MHz, DMSO-d_6): δ 8.44 p.p.m. (br s, 3H), 8.20 (dd, J=4.7, 1.8 Hz, 1H), 7.85-7.82 (m, 2H), 7.62 (dd, J=7.8, 1.2 Hz, 1H), 7.40 (t, J=7.9 Hz, 1H), 7.25 (dd, J=7.6, 4.8 Hz, 1H), 4.64 (s, 2H), 4.27 (q, J=5.7 Hz, 2H).

*d) 9H-Fluoren-9-ylmethyl N-[[2-bromo-6-[[3-(hydroxymethyl)-2-pyridyl]sulfanyl]phenyl]-methyl]carbamate (**5-5**)*

To a stirred suspension of [2-[2-(aminomethyl)-3-bromo-phenyl]sulfanyl-3-pyridyl]-methanol hydrochloride (**5-4**; 46 g, 127 mmol) in 5% aqueous sodium hydrogen carbonate solution (150 mL) was added (fluorenylmethoxycarbonyl)hydroxysuccinimide ester (CAS-RN 82911-69-1; 51.5 g, 153 mmol) in 1,4-dioxane (25 mL) at room temperature, then after 15 h the

reaction mixture was partitioned between water and dichloromethane/methanol 9:1. The organic layer was washed with brine, dried over sodium sulfate, filtered, and evaporated. Chromatography (silica gel; dichloromethane/ethyl acetate 19:1) produced the title compound (34.3 g, 49%).

Off-white solid, MS: 547.1 $[M+H]^+$. ^{1}H NMR (600 MHz, DMSO-d_6): δ 8.15 p.p.m. (br d, *J*=3.6 Hz, 1H), 7.88 (d, *J*=7.5 Hz, 1H), 7.81-7.78 (m, 1H), 7.73 (d, *J*=7.9 Hz, 1H), 7.68 (d, *J*=7.5 Hz, 1H), 7.49 (d, *J*=7.7 Hz, 1H), 7.45 (br t, *J*=4.4 Hz, 1H), 7.40 (t, *J*=7.5 Hz, 1H), 7.32-7.29 (m, 1H), 7.29-7.26 (m, 1H), 7.26-7.22 (m, 1H), 7.19 (dd, *J*=7.5, 4.8 Hz, 1H), 5.50 (br d, *J*=0.7 Hz, 1H), 4.58 (s, 1H), 4.47 (d, *J*=4.4 Hz, 1H), 4.24 (d, *J*=7.2 Hz, 1H), 4.18 (br d, *J*=6.9 Hz, 1H).

*e) 9H-Fluoren-9-ylmethyl N-[[2-bromo-6-[(3-formyl-2-pyridyl)sulfanyl]phenyl]–methyl]carbamate (**5**)*

To a solution of 9H-fluoren-9-ylmethyl N-[[2-bromo-6-[[3-(hydroxymethyl)-2-pyridyl]sulfanyl]phenyl]methyl]carbamate (**5-5**; 16.0 g, 29.2 mmol) in dichloromethane (160 mL) was added 1,1,1-tris(acetyloxy)-1,1-dihydro-1,2-benziodoxol-3(1H)-one (CAS-RN 87413-09-0; 18.6 g, 43.8 mmol) at 0°C, then the reaction mixture was allowed to reach room temperature over 1 h. The reaction mixture was partitioned between saturated aqueous sodium hydrogen carbonate and dichloromethane. The organic layer was washed with water and brine, dried over sodium sulfate, filtered, and concentrated. Chromatography (silica gel; dichloromethane/ethyl acetate 19:1) produced the title compound (11.9 g, 68%).

Off-white solid, MS: 545.2 $[M+H]^+$. ^{1}H NMR (400 MHz, DMSO-d_6): δ 10.28 p.p.m. (s, 1H), 8.44 (dd, *J*=4.7, 1.8 Hz, 1H), 8.23 (dd, *J*=7.6, 1.8 Hz, 1H), 7.82 (dt, *J*=7.5, 1.0 Hz, 2H), 7.73 (dd, *J*=8.0, 1.3 Hz, 1H), 7.61 (dq, *J*=7.5, 1.0 Hz, 2H), 7.55 (dd, *J*=7.7, 1.2 Hz, 1H), 7.39 (tt, *J*=7.5, 1.0 Hz, 2H), 7.36-7.25 (m, 4H), 6.65 (br s, 1H), 4.53 (d, *J*=4.9 Hz, 2H), 4.33-4.22 (m, 2H), 4.21-4.11 (m, 1H).

*9H-Fluoren-9-ylmethyl N-({6-bromo-3-chloro-2-[(3-formylpyridin-2-yl)sulfanyl] phenyl} methyl)carbamate (**6**)*

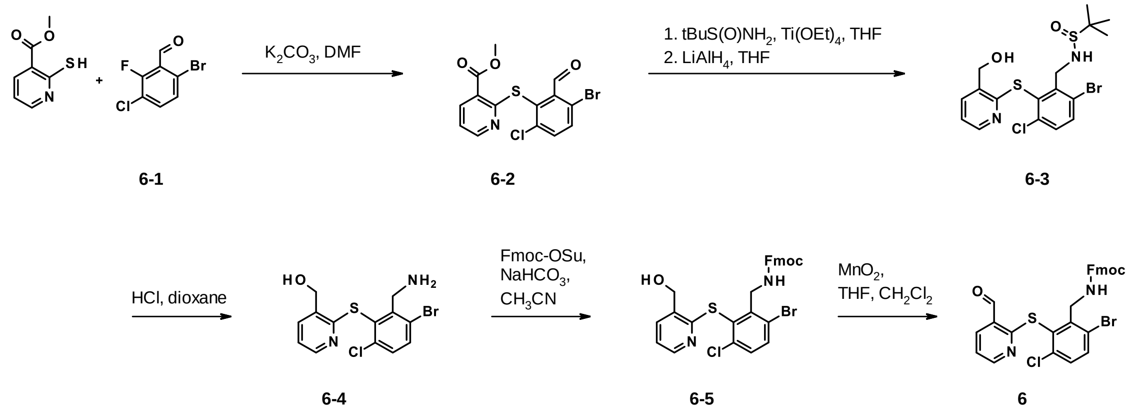

*a) Methyl 2-[(3-bromo-6-chloro-2-formylphenyl)sulfanyl]pyridine-3-carboxylate (**6-2**)*

To a stirred solution of methyl 2-sulfanylpyridine-3-carboxylate (CAS-RN 74470-32-9; 20.0 g, 118 mmol) in N,N-dimethylformamide (100 mL) was added potassium carbonate (32.7 g, 236 mmol) at 0°C in portions and stirred for 15 min. Then 6-bromo-3-chloro-2-fluoro-benzaldehyde (**6-1**; 22.4 g, 94.6 mmol) in N,N-dimethylformamide (100 mL) was added slowly at the same temperature and slowly allowed to reach 25°C and stirred for 2 h, then cooled to 0°C, quenched with water (500 mL) and allowed to stir at 25°C for 30 min. The yellow solid thus precipitated out, was collected by filtration, washed with water (100 mL), followed by n-hexane and then dried well under vacuum to produce the title compound (35 g, 77%).

Green solid, MS: 387.9 $[M+H]^+$. ^{1}H NMR (400 MHz, DMSO-d_6): δ 10.11 p.p.m. (s 1H), 8.46 (dd, J=4.7, 1.6 Hz, 1H), 8.33 (dd, J=6.2, 1.6 Hz, 1H), 7.90 (d, J=8.6 Hz), 7.33 (dd, J= 6.2, 4.7 Hz, 1H), 3.93 (s, 3H).

*b) N-[(6-Bromo-3-chloro-2-{[3-(hydroxymethyl)pyridin-2-yl]sulfanyl}phenyl)methyl]-2-methylpropane-2-sulfinamide (**6-3**)*

To a stirred solution of methyl 2-(3-bromo-6-chloro-2-formyl-phenyl)sulfanylpyridine-3-carboxylate (**6-2**; 35 g, 91 mmol) in tetrahydrofuran (400 mL) were added 2-methyl-2-propanesulfinamide (12.1 g, 99.8 mmol) and titanium(IV) ethoxide (68.9 mL, 272 mmol) and heated at 70°C for 2 h, then the reaction mixture was quenched with brine (250 mL) and diluted with ethyl acetate (300 mL). Insoluble material was removed by filtration and the filtrate was extracted with ethyl acetate (2x150 mL). The combined organic extracts were dried over anhydrous sodium sulfate and concentrated under vacuum. The residue (40 g) was dissolved in

tetrahydrofuran (400 mL) and slowly treated with lithium aluminum hydride solution (2.5 M in tetrahydrofuran, 63 mL, 159 mmol) at -40°C. The reaction mixture was then slowly allowed to reach -5°C over 30 min, then quenched with saturated aqueous sodium sulfate solution (500 mL) and diluted with ethyl acetate (1 L). Insoluble material was removed by filtrated, and the organic layer of the filtrate was separated, dried over anhydrous sodium sulfate and concentrated under vacuum to afford the title compound (32 g), which was used directly in the next step.

Yellow solid, MS: 464.9 $[M+H]^+$. ^1H NMR (400 MHz, DMSO-d_6): δ 8.15 p.p.m. (dd, *J*=4.8, 1.5 Hz, 1H), 7.80 (br d, *J*=7.6 Hz, 1H), 7.77 (d, *J*=8.7 Hz, 1H), 7.51 (d, *J*=8.7 Hz, 1H), 7.20 (dd, *J*=7.6, 4.8 Hz, 1H), 5.56 (t, *J*=5.4 Hz, 1H), 5.25 (t, *J*=5.0 Hz, 1H), 4.65 (d, *J*=5.4 Hz, 2H), 4.60 (dd, *J*=12.6, 4.6 Hz, 1H), 4.45 (dd, *J*=12.6, 6.8 Hz, 1H), 1.05 (s, 9H).

*c) (2-{[2-(Aminomethyl)-3-bromo-6-chlorophenyl]sulfanyl}pyridin-3-yl)methanol hydrochloride (**6-4**)*

To a stirred solution of N-[[6-bromo-3-chloro-2-[[3-(hydroxymethyl)-2-pyridyl]sulfanyl]phenyl]methyl]-2-methyl-propane-2-sulfinamide (**6-3**, 32 g, 69 mmol) in tetrahydrofuran (400 mL) was added hydrogen chloride solution (4 M in 1,4-dioxane, 50 mL) at 0°C. The reaction mixture was allowed to reach 25°C and stirred for 1 h. then concentrated under vacuum to afford the title compound (26 g), which was used directly in the next step.

Off-white solid, MS: 362 $[M+H]^+$. ^1H NMR (400 MHz, DMSO-d_6): δ ppm 8.55 (br s, 3H), 8.16 (d, *J*=4.8 1.5 Hz, 1H), 7.88 (d, *J*=8.6 Hz, 1H), 7.83 (br d, *J*=7.1 Hz, 1H), 7.65 (d, *J*=8.6 Hz, 1H), 7.24 (dd, *J*=7.5, 4.8 Hz, 1H), 4.89 (br s, 1 h), 4.67 (s, 2H), 4..37 (br d, *J*=5.6 Hz, 2 H).

*d) 9H-Fluoren-9-ylmethyl N-[(6-bromo-3-chloro-2-{[3-(hydroxymethyl)pyridin-2-yl]sulfanyl}phenyl)methyl]carbamate (**6-5**)*

To a stirred suspension of [2-[2-(aminomethyl)-3-bromo-6-chloro-phenyl]sulfanyl-3-pyridyl]methanol hydrochloride (**6-4**, 25 g, 69.6 mmol) in 5% aqueous sodium hydrogen carbonate solution (300 mL) was added N-(9-fluorenylmethoxycarbonyloxy)succinimide (16 g, 48.7 mmol) in acetonitrile (200 mL) and stirred at 25°C, then after 16 h the product was collected by filtration to produce the title compound (27 g), which was used directly in the next step.

Off-white solid, MS: 583 $[M+H]^+$. ^1H NMR (400 MHz, DMSO-d_6): δ 8.15-8.10 p.p.m. (m, 1H), 7.90-7.70 (m, 4H), 7.66 (d, *J*=7.4 Hz, 1H), 7.55-7.30 (m, 5H), 7.20-7.15 (m, 5H), 5.56 (br s, 1H), 4.63 (s, 2H), 4.57 (br d, *J*=4.0 Hz, 2H), 4.15-4.05 (m, 3H).

*e) 9H-Fluoren-9-ylmethyl N-({6-bromo-3-chloro-2-[(3-formylpyridin-2-yl)sulfanyl]phenyl}methyl)carbamate (**6**)*

To a stirred solution of 9H-fluoren-9-ylmethyl N-[[6-bromo-3-chloro-2-[[3-(hydroxy-methyl)-2-pyridyl]sulfanyl]phenyl]methyl]carbamate (**6-5**, 25 g, 43.1 mmol) in tetrahydrofuran (150 mL) and dichloromethane (150 mL) was added manganese(IV) oxide (74.8 g, 861 mmol) at 25°C, then after 1 h insoluble material was removed by filtration and the filtrate was concentrated under vacuum. The residue was triturated in ethyl acetate/heptane 1:1 to produce the title compound (16.5 g, 64%).

White solid, MS: 581 $[M+H]^+$. ^{1}H NMR (400 MHz, DMSO-d_6): 10.19 (s, 1H), 8.44 (dd, *J*=4.8, 1.8 Hz, 1H), 8.37 (d, *J*=7.5 Hz, 1H), 7.88 (d, *J*=7.6 Hz, 2H), 7.81 (d, *J*=8.6 Hz, 1H), 7.67 (d, *J*=7.4 Hz, 2H), 7.56 (d., *J*=8.6 Hz, 1H), 7.55-7.50 (m, 1H), 7.45-7.35 (m, 3H), 7.31, t, *J*=7.5 Hz, 2H), 4.46 (d, *J*=3.9 Hz, 2H), 4.25-4.15 (m, 3H).

*9H-Fluoren-9-ylmethyl N-[[3,6-dichloro-2-[(3-formyl-2-pyridyl)sulfanyl]phenyl]–methyl] carbamate (**7**)*

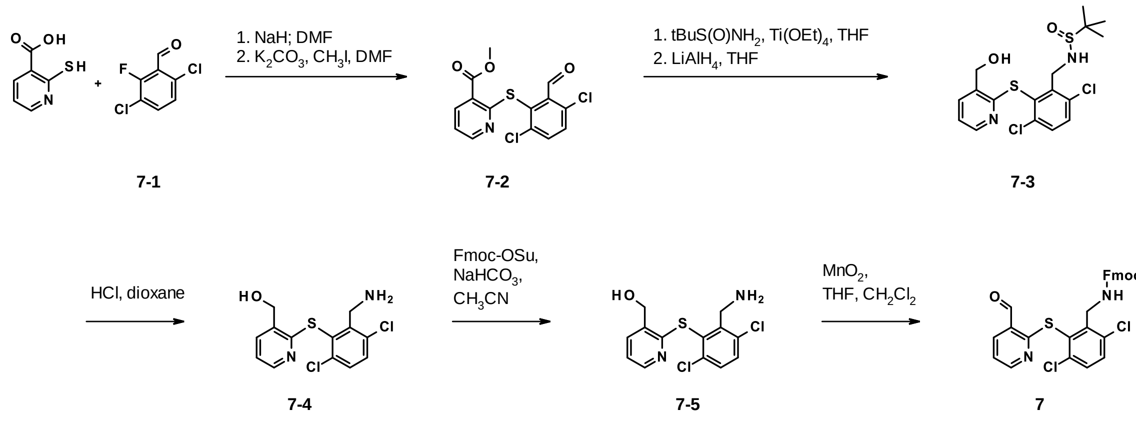

*a) Methyl 2-(3,6-dichloro-2-formyl-phenyl)sulfanylpyridine-3-carboxylate (**7-2**)*

To an ice-cooled suspension of sodium hydride (18.7 g, 777 mmol) in N,N-dimethyl-formamide (200 mL) was added 2-sulfanylpyridine-3-carboxylic acid (40.2 g, 259 mmol) and stirred was continued for 15 min at 0°C. Then the resulting reaction mixture was stirred at room temperature for 30 min, then the reaction mixture was again cooled to 0°C and a solution of 3,6-dichloro-2-fluoro-benzaldehyde (**7-1;** 50 g, 259 mmol) in N,N-dimethylformamide (300 mL) was added dropwise. The reaction mixture was stirred at 25°C for 16 h, then cooled to 0°C and to it was added potassium carbonate (71.6 g, 518 mmol), followed by methyl iodide (48.4 mL, 777 mmol) in a dropwise manner. Stirring was continued for 16 h at 25°C. Then the reaction mixture was quenched with saturated aqueous ammonium chloride solution (100 mL) and poured onto crushed ice (2 kg). The precipitate thus obtained was filtered and washed with water (2000 mL) followed by n-hexane (100 mL) to produce the title compound (70 g, 79%).

Pink solid, MS: 342.0 $[M+H]^+$. ^1H NMR (400 MHz, $CDCl_3$): δ 10.27 p.p.m. (s 1H), 8.33 (dd, *J*=4.7, 1.7 Hz, 1H), 8.27 (dd, *J*=6.2, 1.7 Hz, 1H), 7.59 (d, *J*=8.6 Hz), 7.10 (dd, *J*= 6.2, 4.7 Hz, 1H), 3.99 (s, 3H).

*b) N-[[3,6-dichloro-2-[[3-(hydroxymethyl)-2-pyridyl]sulfanyl]phenyl]methyl]-2-methyl-propane-2-sulfinamide (**7-3**)*

To a stirred solution of methyl 2-[(3,6-dichloro-2-formylphenyl)sulfanyl]pyridine-3-carboxylate (**7-2**; 50 g, 146 mmol) in tetrahydrofuran (500 mL) was added titanium(IV) ethoxide (61.23 mL, 292 mmol) followed by tert-butyl sulfinamide (35.4 g, 292 mmol) and the reaction mixture was heated at 60°C for 2 h. Then the reaction mixture was diluted with water (300 mL),

passed through diatomaceous earth, and the filter pad was washed with ethyl acetate (4000 mL). The combined organic layer was washed with brine (300 mL x 3), dried over anhydrous sodium sulfate and solvent was distilled off under reduced pressure. The residue (60 g) was dissolved in tetrahydrofuran (200 mL) and added dropwise to an ice-cooled suspension of lithium aluminum hydride (9.96 g, 269 mmol) in tetrahydrofuran (300 mL), and the resulting reaction mixture was stirred at 25°C for 2 h. Then the reaction mixture was quenched with saturated aqueous ammonium chloride solution (100 mL) and passed through diatomaceous earth, and the filter pad was washed with ethyl acetate (3 x 300 mL). The combined organic extract was washed with brine (300 mL x 3), dried over anhydrous sodium sulfate and solvent was distilled off under reduced pressure to produce the title compound (45 g, 73%).

Pale yellow solid, MS: 444.9 $[M+H]^+$. ^1H NMR (400 MHz, DMSO-d_6): δ 8.14 p.p.m. (dd, *J*=4.8, 1.7 Hz, 1H), 7.79 (dd, *J*=7.6, 1.7 Hz, 1H), 7.63 (d, *J*=8.6 Hz, 1H), 7.59 (d, *J*=8.6 Hz, 1H), 7.20 (dd, *J*=7.6, 4.8 Hz, 1H), 5.59 (t, *J*=5.3 Hz, 1H), 5.35 (t, *J*=5.9 Hz, 1H), 4.64 (d, *J*=5.2 Hz, 2H), 4.55 (dd, *J*=12.6, 4.9 Hz, 1H), 4.43 (dd, *J*=12.6, 6.6 Hz, 1H), 1.04 (s, 9H).

*c) {2-[(3,6-dichloro-2-ethylphenyl)sulfanyl]pyridin-3-yl}methanol hydrochloride (**7-4**)*

To an ice-cooled solution of N-[(3,6-dichloro-2-{[3-(hydroxymethyl)pyridin-2-yl]sulfanyl}phenyl)methyl]-2-methylpropane-2-sulfinamide (**7-3**, 67 g, 160 mmol) in methanol (450 mL) and 1,4-dioxane (150 mL) was added hydrogen chloride solution (4 M in 1,4-dioxane, 130 mL) dropwise and the reaction mixture was stirred at 25°C for 1 h. Volatiles were removed under reduced pressure, and the residue was triturated in ethyl acetate to produce the title compound (56 g), which was used without further purification.

Orange solid, MS: 314.9 $[M+H]^+$. ^1H NMR (400 MHz, DMSO-d_6): δ 8.52 p.p.m. (br s, 3H), 8.17 (d, *J*=4.8 1.7 Hz, 1H), 7.83 (dd, *J*=7.6, 1.7 Hz, 1H), 7.75 (s, 2H), 7.24 (dd, *J*=7.6, 4.8 Hz, 1H), 6.30 (br s, 1 h), 4.67 (s, 2H), 4.40-4.30 (m, 2H).

*e) 9H-fluoren-9-ylmethyl-N-[(3,6-dichloro-2-{[3-(hydroxymethyl)pyridin-2-yl]sulfanyl}phenyl)methyl]carbamate (**7-5**)*

To an ice-cooled solution of {2-[(3,6-dichloro-2-ethylphenyl)sulfanyl]pyridin-3-yl}methanol hydrochloride (**7-4**; 56 g, crude) in acetonitrile (200 mL) was added 5% aqueous sodium hydrogen carbonate solution (640 mL) followed by the N-(9-fluorenylmethoxy-carbonyloxy)succinimide (43.1 g, 128 mmol) in acetonitrile (300 mL) dropwise and the reaction mixture was stirred at 25°C for 3 h. Then the reaction mixture was diluted with cold water (500 mL) and the precipitate thus obtained was filtered. The precipitate was washed with water (2 x

400 mL) followed by n-hexane (400 mL) to produce the title compound (70 g, 82% over two steps).

Off-white solid, MS: 537 $[M+H]^+$. ^1H NMR (400 MHz, DMSO-d_6): δ 8.10 p.p.m. (br d, *J*=3.5 Hz, 1H), 7.88 (d, *J*=7.4 Hz, 2 H, 7.78 (br d, *J*=7.5 Hz, 1 H, 7.55-7.70 (m, 5H), 7.41 (t, *J*=7.4 Hz, 2H), 7.30 (t, *J*=7.4 Hz, 2H), 7.17 (dd, *J*=7.5, 4.8, 1H), 5.56 (t, *J*=5.2 Hz, 1H), 4.62 (d, *J*=5.4 Hz, 2H), 4.54 (d, *J*=4.5 Hz, 2H), 4.24 (d, *J*=7.0, 2H), 4.20-4.15 (m, 1H).

*f) 9H-Fluoren-9-ylmethyl N-[[3,6-dichloro-2-[(3-formyl-2-pyridyl)sulfanyl]phenyl]–methyl] carbamate (**7**)*

To an ice-water solution of 9H-fluoren-9-ylmethyl N-[(3,6-dichloro-2-{[3-(hydroxy-methyl)pyridin-2-yl]sulfanyl}phenyl)methyl]carbamate (**7-5**; 30 g, 55.8 mmol) in tetrahydrofuran/dichloromethane (600 mL, 1:1) was added manganese(IV) oxide (72.2 g, 837 mmol) portionwise and the resulting reaction mixture was stirred at 25°C for 4 h. The reaction mixture was filtered through diatomaceous earth and the pad washed with dichloromethane/methanol 9:1 (3 x 500 mL). The filtrate was concentrated under reduced pressure to produce the title compound (25.8 g, 86%).

White solid, MS: 535 $[M+H]^+$. ^1H-NMR: (400 MHz, DMSO-d_6): 10.19 p.p.m. (s, 1H), 8.43 (dd, *J*= 4.8, 1.8 Hz, 1H), 8.36 (dd, *J*=7.5, 1.5 Hz, 1H), 7.88 (d, *J*=7.5 Hz, 2H), 7.70-7.60 (m, 4H) 7.55 (t, *J*=4.4 Hz, 1H), 7.40-7.35 (m, 3H) 7.31 (t, *J*=7.3 Hz, 2H), 4.45 (d, *J*=4.4 Hz, 2H), 4.20-4.15 (m, 3H).

*9H-Fluoren-9-ylmethyl N-[[2-(2-formylphenyl)sulfanylphenyl]methyl]carbamate (**8**)*

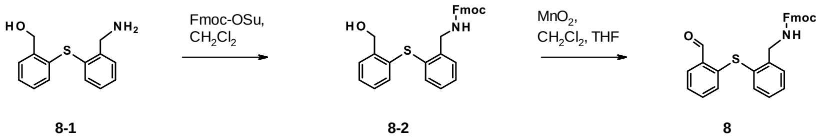

*a) 9H-Fluoren-9-ylmethyl N-[[2-[2(hydroxymethyl)phenyl]sulfanylphenyl]methyl]-carbamate (**8-2**)*

To the stirred solution of 2-[2-(aminomethyl)phenylthio]benzyl alcohol (**8-1**, CAS-RN 79467-22-4; 10.0 g, 40.8 mmol) in dichloromethane (10 mL) and tetrahydrofuran (10 mL) N-(9-fluorenylmethoxycarbonyloxy)succinimide (20.6 g, 61.1 mmol) was added and stirred at room temperature for 1 h, then the reaction mixture was diluted with water and insoluble material was removed by filtration. The filtrate was azeotroped with toluene, the obtained solution was washed with saturated aqueous sodium hydrogen carbonate solution, dried over sodium sulfate and concentrated to produce the title compound (16.0 g, 76%). MS: 450.3 $[M+H–H_2O]^+$.

*b) 9H-Fluoren-9-ylmethyl N-[[2-(2-formylphenyl)sulfanylphenyl]methyl]carbamate (**8**)*

To a stirred solution of 9H-fluoren-9-ylmethyl N-[[2-[2-(hydroxymethyl)phenyl]-sulfanylphenyl]methyl]carbamate (**8-2**; 5.0 g, 10.7 mmol) in dichloromethane (10 mL) and tetrahydrofuran (10 mL) was added manganese(IV) oxide (15.0 g, 35.4 mmol) then after 1 h the reaction mixture was filtered over diatomaceous earth. The filtrate was concentrated under vacuum and the residue was triturated in heptane/ethyl acetate 4:1 to produce the title compound (4.6 g, 83%).

Orange solid, MS: 466.1 $[M+H]^+$. ^1H NMR (600 MHz, $CDCl_3$): δ 10.40-10.10 p.p.m. (m, 1H), 7.86 (d, *J*= 7.6 Hz, 1H), 7.76 (d, *J*= 7.6 Hz, 2H), 7.60-7.25 (m, 12H), 7.25-7.15 (m, 1H), 6.77 (d, *J*= 7.8 Hz, 1H), 5.20-5.10 (m, 1H), 4.48 (d, *J*= 6.3 Hz, 2H), 4.38 (d, *J*= 6.9 Hz, 2H), 4.20-4.15 (m, 1H).

2-(2-Formyl-phenoxymethyl)-benzyl]-carbamic acid 9H-fluoren-9-ylmethyl ester (**9**)

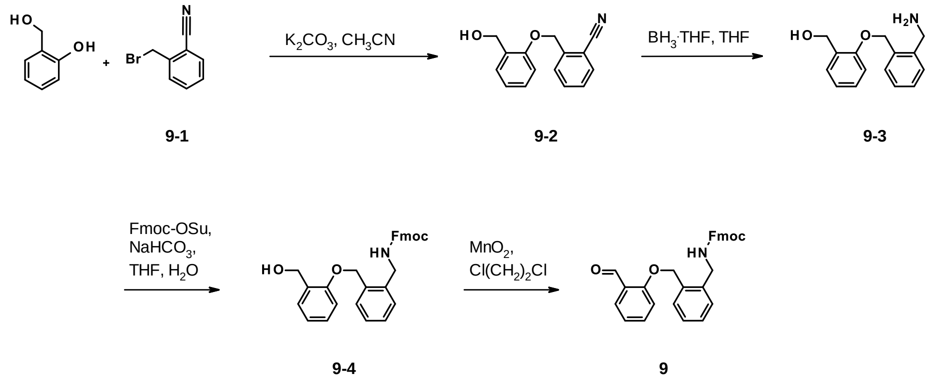

(9H-2-((2-(Hydroxymethyl)phenoxy)methyl)benzonitrile (**9-2**)

To a solution of 2-hydroxybenzyl alcohol (2.3 g, 18.5 mmol) in acetonitrile (30 mL) was added potassium carbonate (7.68 g, 55.6 mmol) and 2-(bromomethyl)benzonitrile (**9-1**; 2.34 mL, 18.5 mmol. After addition, the yellow suspension was stirred at 80°C for 16 h, then partitioned between water (40 mL) and ethyl acetate (2 ×30 mL). Combined organic extracts were washed with brine (60 mL) and dried over sodium sulfate. The solid was filtered off and the filtrate was concentrated under vacuum to give a residue, which was purified by flash chromatography (SiO_2; ethyl acetate / petroleum ether 2:3) to produce the title compound (3.75 g, 85%).

Light yellow solid, MS: 222 $[M-H_2O+H]^+$. ^1H NMR (400 MHz, $CDCl_3$): δ 7.76 p.p.m. (d, *J*= 7.8 Hz, 1H), 7.71-7.63 (m, 2H), 7.53-7.44 (m, 1H), 7.42-7.36 (m, 1H), 7.35-7.29 (m, 1H), 7.04 (t, *J*= 7.5 Hz, 1H), 6.99 (d, *J*= 8.2 Hz, 1H), 5.34 (s, 2H), 4.80 (s, 2H).

(2-((2-(Aminomethyl)benzyl)oxy)phenyl)methanol (**9-3**)

To a solution of 2-[[2-(hydroxymethyl)phenoxy]methyl]benzonitrile (**9-2**; 400 mg, 1.67 mmol) in tetrahydrofuran (1 mL) was added borane tetrahydrofuran complex (1 M in tetrahydrofuran, 8.36 mL, 8.36 mmol), and the solution was stirred at 60°C for 3 h, then methanol (10 mL) was added dropwise and the solution was stirred at 65°C for another 0.5 h. The solution was then concentrated produce the title compound (400 mg, 98%).

Colourless oil, MS: 2244.0 $[M+H]^+$. ^1H NMR (400 MHz, DMSO-d_6): δ 7.50-7.38 p.p.m. (m, 3H), 7.33 (br t, *J*= 7.4 Hz, 1H), 7.29-7.19 (m, 2H), 7.09 (d, *J*= 8.1 Hz, 1H), 7.00-6.93 (m, 1H), 5.18 (s, 2H), 4.53 (s, 2H), 3.81 (s, 2H).

*(9H-Fluoren-9-yl)methyl (2-((2-(hydroxymethyl)phenoxy)methyl)benzyl)carbamate (**9-4**)*

To a solution of [2-[[2-(aminomethyl)phenyl]methoxy]phenyl]methanol (400 mg, 1.64 mmol) in saturated aqueous sodium hydrogen carbonate solution (4.0 mL, 0.94 mmol) was added a solution of N-(9-fluorenylmethoxycarbonyloxy)succinimide (0.33 g, 0.98 mmol) in tetrahydrofuran (4 mL). After addition, the mixture was stirred at 20°C for 16 h, the partitioned between ethyl acetate (20 mL) and water (10 mL). The organic extract was washed with brine (30 mL) and dried over sodium sulfate and concentrated under vacuum. The residue was purified by flash chromatography (SiO_2, petroleum ether – ethyl acetate gradient) to produce the title compound (440 mg, 58%).

White solid, MS: 448.2 $[M–H_2O+H]^+$. ^1H NMR (400 MHz, DMSO-d_6): δ 7.89 p.p.m. (d, *J*= 7.6 Hz, 2H), 7.70 (d, *J*= 7.5 Hz, 2H), 7.48 (br d, *J*= 7.0 Hz, 1H), 7.45-7.39 (m, 3H), 7.35-7.28 (m, 4H), 7.23-7.19 (m, 1H), 7.10-7.05 (m, 2H), 7.02-6.92 (m, 2H), 5.17 (s, 2H), 5.04-4.97 (m, 1H), 4.55 (d, *J*= 5.5 Hz, 2H), 4.38-4.28 (m, 4H), 4.26-4.18 (m, 1H).

*(9H-Fluoren-9-yl)methyl (2-((2-formylphenoxy)methyl)benzyl)carbamate (**9**)*

To a solution of 9H-fluoren-9-ylmethyl N-[[2-[[2-(hydroxymethyl)phenoxy]methyl]phenyl] methyl]carbamate (**9-4**; 340 mg, 0.73 mmol) in 1,2-dichloroethane (7 mL) was added manganese(IV) oxide (635 mg, 7.3 mmol), then the black suspension was stirred at 60°C for 3 h. The solid was filtered off and the solid was washed with 20 mL of 1,2-dichloroethane, then the filtrate was concentrated to give a residue, which was purified by flash chromatography (SiO_2, petroleum ether – ethyl acetate gradient) to produce the title compound (260 mg, 77%).

White solid, MS: 464.2 $[M+H]^+$. ^1H NMR (400 MHz, DMSO-d_6): δ 10.40 p.p.m. (s, 1H), 7.93-7.82 (m, 3H), 7.76-7.63 (m, 4H), 7.55 (br d, *J*= 7.1 Hz, 1H), 7.45-7.36 (m, 4H), 7.34-7.27 (m, 4H), 7.11 (t, *J*= 7.5 Hz, 1H), 5.34 (s, 2H), 4.43-4.26 (m, 4H), 4.25-4.17 (m, 1H).

LOCUS NODE_2_length_148137_cov_371.33_ID_22131 148137 bp DNA linear UNK
DEFINITION Acinetobacter baumannii ATCC 17978
ACCESSION NODE_2_length_148137_cov_371.33_ID_22131
KEYWORDS .
SOURCE Acinetobacter baumannii ATCC 17978.
ORGANISM Acinetobacter baumannii ATCC 17978
Bacteria; Proteobacteria; Gammaproteobacteria; Pseudomonadales;
Moraxellaceae; Acinetobacter; Acinetobacter
calcoaceticus/baumannii complex; Acinetobacter baumannii.
FEATURES Location/Qualifiers
source 1..148137
/mol_type="genomic DNA"
/db_xref="taxon:400667"
/genome_md5=""
/project="sleyn_400667"
/genome_id="400667.82"
/organism="Acinetobacter baumannii ATCC 17978"
CDS complement(472..810)
/locus_tag="AUO97b_00001" /db_xref="SEED:fig|400667.82.peg.3653"
/translation="MMKEFNLDGYSSIELENLIKAASLEKGKREIEEKVAAFGQLEEI
AKSVGLTLDQLIEFGKTHNKKKKTKTVEPRYRSKADPENTWTGRGQMPRWLKSEMEES KLKLSEFLIK"
/product="putative DNA binding protein"
/transl_table=11
CDS complement(852..2576)
/locus_tag="AUO97b_00002" /db_xref="SEED:fig|400667.82.peg.3654"
/translation="MNVQSQELTSSDAESLSPADLGYSQANSLSQGHPNLASFKYETE
FKLPVSLIHVTHECSLVEHKIWLCLLNHACEILENFQDNGSHPIVNFKIPFDLLRFKT
NSQHRLKEINKSLSNLRDAEIQIDIFNAQGLKSSSSQFSFIQDFAIYNKNMMLNREEV
EADNKPDYQSLEIVYSLPPFFSSIITDSISIEKVKRLFSWTQIQSLSTSTGRNGKIEL
LIYKLLEGTDEPITLAYETFKQYLGYGDVEENRYFLRDTLKPALKNFNANELLEYEVE
TDFIRGKRQKIESIKLFKKYKFVDNEQAAHAWQSLLDSPPENTTNLRIRIKQLVPENY
DIDSLTDDSIYTILSRLKQWINGLKKKGQTINFVGACRSAFKDKSWIDSKNGKGQSSL
ILGEEVDDVVVKASRTNYESAVGKGDIPKTSQESDYNNPVNKIAKSVATLISTLAIKG
SKTTFFENISKENFDELTNNNLKYFNYFSENIDSFKNDYVIDMVKRIFFVGNENALNS
LLNQSNQSKVLYPYLFSVNFAMQNYTQIISDPIRSGAYSTNLANNELAVNIVVGDLIK YFKSQLSI"
/product="hypothetical protein"
/transl_table=11
CDS complement(2732..3397)
/locus_tag="AUO97b_00003" /db_xref="SEED:fig|400667.82.peg.3655"
/translation="MTNLASMPYAHSLYLNEPAEWKKQSSVQLSLLDDVQEPSVVERS
LPALPLKRFSKLFSFLDFDFDTKSYEYMVGSDRFTIETKIDKADQAFVCLINEVLMAY
KQKNILLTSFDSKLTHTINASEISKVVGISLDEFEKRMKSLASCSLSAHQHRLLRHTK
TGRESFRFVHMFSTKLVSEYVSFDKDGQIFYSFNYAPFFETMFKENLHKKNQSQPYLD QVV"
/product="hypothetical protein"
/transl_table=11
CDS 4176..5552 /locus_tag="AUO97b_00004" /db_xref="SEED:fig|
400667.82.peg.3656"
/translation="MIKNNDLDNLRNLNRNIIQNKPNSAPATPQRLFVYLDGIAQNND
GHDYAYGTNVLTNEKVKVRLTTIDERIDDLVDSGRAKNRQQVASQVTNQYQNSANPRQ
SLKARLNSNVVVLSFDQSFEIKSEDNYRSYRSHWAESVTSNPSSQLITAPVHIQFVNN
QRPFGRVEILEENFKIDIGSSKENIKDAFLKSLVHQDEFSAERSGSLNYILRYKDTNE
PIYQGSLQTFLQQVSVGPDRSIRIPADINTTLNNLMRKPLTTQDFTPDDSMMFNHDLN
RILVNRFLGVEFSQKLETSNPQLQDNLRLISGKLKKGEIYLDAYSTQNIFFGRDTLKT
YQAKANRNSSPLKIYTVTDMHNEQPVYQKGYIHSLVGIDRHSDTLAPFVTFSSTTDPM

AVSKKIQDLNINEENLKRTIYLKEEFQKHPLPNSAAPVVDKERANINRIREEMQNQSN NSINFNNI"
/product="hypothetical protein"
/transl_table=11
CDS 5567..11527
/locus_tag="AUO97b_00005" /db_xref="SEED:fig|400667.82.peg.3657"
/translation="MSVLDKKGFIWVNLESFGYLTRPSRRKENNQIGIEIVDLNNQLD
IDKLRSFNVHVEKVNKSSVLDVYWSSISIKDFSNFIGVDNNNIPFQSITLTQLNAEFN
NSVRSLAQQRVDYAIRHATLIGQNLNKQYVYATQYSRFYLDQPLTEIAKNGTISIDPA
QFEARLPQEDYWKILKVGNQPKFKRANMAHCAKSFASSIVEGTTNIHDSDVELFLKKI
LNDNHDRSYLNEIKPTIKQFFYEQLECCFNSLSNQFNVHNFNSFEKIIEIHNKQPVKP
IIGISELQLQQFSTPITISAICQKLLFNPETLDTGKTLLEPTIGNGSLVAHFIKKPQL
KIVGVEIDSNRVKNTQLFLDANIEHSNLRVIEGDYSKIKLKQLNNNELFDFTIANPPF
GKIDKTTLTLNQAGSLERLNFSTQRLDHKILLETLSLRKDKGRSVFIIGSDSFYEAGV
VKGGSKNLLNYLYDNYNVEAAVELDGSLYKKQGTRVNVRVLVIGDLLENNRTYEVPHE
LPIINNVQDLWRWSENVLQKRLQPELSYDEPSPSINQSESVISEANLDQNLDVVDDAD
FLTSALDSYLNDQIVPDESGNVGDLNKPDDDDFFGNEEEDTAVKPLSTGDEHQNNEIL
DLEIVEDESQAQEIIFESKSLQVAYVPVSQLSLPETMLPADQAILIKQAQDNQIKKIL
LLAEVNHELQKSIDLANNNPFDGFLLYKLQYSDLSSLSKAFSAEQVDAVTSAILSIEN
DRSYILGDQTGIGKGRSLAGMLRYSLINGFRPVFFTRNAQLFNDLWRDIVDTGTDKFI
SNPFIFNSDGFITHKDKVVYSPMKTSDIYNLDRIGNQFDIIFSTYSQFSGRSGKKENL
FLSSIDKNTLLFLDESHVAASGESNISRFLSVVKERTGPTYHSSATDRKKANNLLFYS
DIFPASFKGQLKSLIENKPSNELLEAISMALVKDGVFLRREHDFSKLEFRTYHASTQY
QTASRELSDQLAQVLSKMAYLSGDVEEKVAKMNSEIKHNLEHMKEGSWQENRMQVNSM
NFASRMHNISRQILLSVTTPLIIDQALESLKENRKPVIGLENTGESLLKMLLLKDSKA
FELLDQINELKDSSDYDRALSDLNSGKMNQNEFDQEPIIVKLKKLEQDLAQFKQEDTI
LAKPPTIADLLENMVHKLDIIQVRDRYGQVRSETIDDEIYSDFKQDILDNIRKLPDIP
LCPLDLIKAELESRGYKVGEISGREYQLKKIDSNQGSVFKVEVRQENNPTAKTKACNE
FQSGILDAMIITRAGSTGLSLHAIPVNDGSPSQSDHLRQREFLTGQSPQAIDEFLQMI
GRVDRKGQVSAPIISQFDTGLPIQRKFLMMHNAKLAELSANVTSNRENVNKQVTDVDL
LNSYGDDVALEYLVQNRSVAQMLVINLPKENDKNGAEGMVKKIFNRLNFVSIYQQEKI
IDDLTFAYNEKIQKLNEMGINPFVVQVCDWKAQTISKQELQSGLGIMSHVSDSAFFEP
AYFQKVSYKSETKPFHGDDVISLIDKSRQLTRQQLFKHLCNFDDNFASTMEGKDQELS
LSSLIKFYNDAQKQILERELRESIKYSASQSFFKSKIDKYEFTEIENFILNKPNLDEI
KEFLKISAENNNFLHIYQGLNKALTVVEFVEKLQILEEHQSFNINFAISMKESLWDQH
YHRETNENALIRVMFPKLGASSLLASNYEIRYVYPSSDFVHSSKLKYLIEDAALTPTL
LRNVGTHLFIDGNKKEEEFSFMKTLNEIEYLKTNSPLLLSFDKYEPTAIERTATLLTG
NIINALNAASVAKLSSQMINYTDASGNRQRAVMLYDDCELEEVLDKVKGLSTIQEAIT
YFERVHDAIEMNEKPTLPDFYIVRSNSNGNPTIELNLKPLNEETYNFVITGSRKHIKQ
VAEMDSIFVQAGSGNRSDSLNLVLKGDLSQGTRSSQTVQVPIEKVTKLLTNLSTEFST
VKINNLDPDVLDQLKPNTSNKLTQNLMY"
/product="Probably methylase/helicase"
/transl_table=11
CDS 11541..13592 /locus_tag="AUO97b_00006" /db_xref="SEED:fig|
400667.82.peg.3658"
/translation="MDNKEEMKAAPQALNEIDWKDEIVFDELLSKVGPITPPDTTLKS
SDLEAYGYQSQVMLPVSEARAKELYEKIELYRLYEDGTEALVEDLADLNSHAQKDGLF
GVQENDWRNYQLKIEKSNDSENSNDLENFQYFSYPALAQSIIKREGDQFFANYELSRS
LGSDEDITILNKEFYKEHKADLDILLNELTISEQYEHQGYAVLYATKSNLSALNSDEE
IEAEANEIKLFLEGNNDDPKYAYVYDTLYELSAENVFNEFKALETRIERDMDLEEGNF
DLPFQHAVEETMETFDNNAPISNLPFDSLKEPEGFMEDIEQYSYAEDQNFGSSLPINN
EEMIQKSEVKEEKEKIDVNDVGVPEQLIINGKEFNFEQIDPNDIEKHEQLRAALSQLL
GNTNASTFQSPSQINPVTSTPLIPPSMPDFNENSAQPSTVKNYSLFPSFANLKKATPD
NALSLLQAEQLLQTSQNIQAPASENSGAHMNTQNVQHMLSSHLASSKYNDLVNSHKNY
SDSINEFWKFDKFEDFRGTLIERGQKENKDLKTLINEMNTDPNSPYRKEFQDLIVNVG

STEKGKALLSKLNNSHQDFLSRSDSMFEVIQKSELSPDDKTKFENKLSEVVENQAELS
STLPNYINDDGVRISLADEMKKYAESLKKALQVVVDFVKSKVGLSVDGPTEQPSSSPS P"
/product="hypothetical protein"
/transl_table=11
CDS 14087..14659
/locus_tag="AUO97b_00007" /db_xref="SEED:fig|400667.82.peg.3659"
/translation="MESLVERSAKQDLDTFLRFICTYALNDTYVSKDLITESLKDLPQ
SVVTFVYLLEFSPNDQISSITSISDSVLKTLSELIHNFRNERINSNGYSIIFDFFSAN
PFSYFIYFLRNVITANIDELPLVVNLNITDFFQFLLDRKSSVFHSRIFYFIRAWQTVS
VYNEANISELKETMLYASQNKVFPNISVSS"
/product="hypothetical protein"
/transl_table=11
CDS 14677..15723
/locus_tag="AUO97b_00008" /db_xref="SEED:fig|400667.82.peg.3660"
/db_xref="GO:0009986"
/translation="MHKRTSALNKNPQKLNSVEKKHSFKPKLGFTTGIVLGVSSAIAL
STTASVGDGLSTIQSSLNQTSNTSTNITNSYTNTTTSTIPTLSASPFAASSNATAAQS
AIEASSVGCGDASQQGSIAWTQQEVIKNRQILNTAPVDIDALFNGSKGSSSGSCFSDI
TKIIDLSVAIPDWGGMRAAAIAALRQYAQRKVCTMVNEATKKALEPLNNAIDIINKNG
TIDLNGAVQGAITGGLGKLDPDLANIYQTQAPQGETVNIFDSNQTTFNTDVAKVQDQV
NGAINQIGTIGTNTGSTGSTNPTGTSGTTWSNNTSTQSIPNLSANTSNQTQQKAQPQE ASRSSIFDALFGGS"
/product="Predicted cell-wall-anchored protein SasA (LPXTG motif)"
/transl_table=11
CDS 15731..16492
/locus_tag="AUO97b_00009" /db_xref="SEED:fig|400667.82.peg.3661"
/translation="MSKLVKVALSTAITMSLIGSSYAQIAHTTYNQNTRDLTSAIGVL
TKQEGLSAQQQAESAMISSKALATAIGAINMSDRVLRFIVDDAGRLGSTQTTTCTVFA
EQQAEKIKILQSSINAANMVSNYMKVMMSNSVESEKDKILRHENYCSIAEAAMGTCTL
LPNGLQAADSNYALVFSKSKLQDDTFNAATDYIANITDSRFSAQDICSNTQCMTKQID
FLNKTSGNSLAAFVLSNQVSDRLSYSLNANSSGAN" /product="hypothetical protein"
/transl_table=11
CDS 16493..17620
/locus_tag="AUO97b_00010" /db_xref="SEED:fig|400667.82.peg.3662"
/db_xref="GO:0005515"
/db_xref="GO:0005524"
/db_xref="GO:0005694"
/db_xref="GO:0051276"
/translation="MKYKYLCVVAALPFLGLSFKAWAAADVLYGSVAPTFHQNVQTLN
SKMVSEFASTQANLLNAIQENTRQLQSALSVSIAQSSAGTTQVIDARKRAGQALATAY
QAIEMQDKVIDTVLNYGSTGQGYNTCKVLVSNATLSKGKEVSRQTAAKMVNETSTGTG
RVVHDLNQVQKETVQKHRELFCTQAEASLGLCTVSKLAGGDTNAALLFQPAKPNSLEA
EARRTLRGNILGSPDVALNAVNGMTPRGQQYLFALNRKTALQAFPAYSLAAIDAANLQ
TIKNEAGELRSPNQMIDETIGRYYGTDEAKKFQATMMNQSARGLLVELARQRGAAIWL
LNEDYERGLRLEGNLASLLLISAEESKSKMRDSYRTMAGRS" /product="Dehydrogenases with
different specificities (related to short-chain alcohol dehydrogenases)"
/transl_table=11
CDS 17623..18756
/locus_tag="AUO97b_00011" /db_xref="SEED:fig|400667.82.peg.3663"
/translation="MKKKTTFLLLSVFSTSVIAAYCPPFSNPLILRDFISGFQQLNAT
FTTGLNTLDQSVSAALTTERQLLLNAIALKTTQEAMNASSIAHADQEIAKATANAIGA
VKQSERLKETAIDFGAAGQGYNPCKVLNERRTITETQERDKENRPDLIFKEISAGTGK
YANPVVAQEQTLKLHHDYFCTSEQVSAGLCMSVGEMSGANLQASTLFEYSSANDKYAI
AKTSFINNMVGLPDAPLSANAAKTPRGKEYMRQKVENDAVISPAINSLMAIKSEFTEN
PNGGEFGQPVKPLRQQYREQVDRYLGHGEEYKNWNRTLVAAPERGVLVEILKEGALEL

AIMGNKHAQNERIEANLASLVALENKKANSLNKSNIAGSAKAK" /product="hypothetical protein"
/transl_table=11
CDS 18880..22614
/locus_tag="AUO97b_00012" /db_xref="SEED:fig|400667.82.peg.3664"
/translation="MKWLNLSEFGLHLGFRKATSFRDKVAVLTVNDLNTLSRFIHLND
FSEKLQAVGFNRVNSDDGNAKFVKADNSINPKQLASILPITNKHLQDMPENDARAKFH
VDKQAWIDLTAKLAQQGSVWKLDENYYNEAGIHWKNKISENYLDLSTKQDDRYINLAF
ESPLPVNMLETYKYDSRALGVLTTNKEILIRKGLPEEAFTEVQLNFMLPLLSLEDEVH
IVLNDIREVPEILELHPDIWRQSINSFESFLTRSKLLEKINNLLINDELLEKSNEIVN
DIKVFYETYNNCLLSKEEFSNDDDLTNHRIQLLKNQIIKLDKFGVINENLLPKLDNIY
DSSIEDTVLEPEKENKSLNERIEDYGEYVFGARKHFYQKSKNALFEELNNSKDLSLDV
LIDKFQKRNLWSFKNETMSQIEDNNVIFFLAQSVYNMIPSKISFSKDERKDSEECIDK
LKSYMYSVNHFKEMVHTAIELDKNERMPIANFVNQMVNYRNVLRGYAQDPQQFLFDEL
DEDRLLADRIYKVVKGTTLRGSPLFQIMNNLDTVKTQYCKMMFNEVYPETKEVSYDQF
QDYVESGGHIYDGSAIKQFIDAANGKLDYTYLSRQFERLEDSLGQELFLNMIVTGNKP
FSDALFKNVEKILESDKSIKTPLDFEKDDSKLNYLLSLYRFKFDSKYSIPDIYIKDEN
QHTQVITDLSSTDKFDGLVRKILKDQEPNRLDLRYPSIVSGLAHETSKNFRHGENVNP
EQFSNVFGFRAIQFGNWVTQQERQHLLNITFDGLSELRDALGFDSKNISLNGTLAIAF
GARGQAGRNAASAHYEYASKIFNLTKQNGAGCLAHEWFHGLDAYMAQLVRKDVEKLIY
KSGNFESVENNYFLTHLMSDVDYKTCKTVIDNNPLLKSVYELQLKLNGKMHRDEIFSE
NGKELIVNRIHELVDSNLIKVNVLKDAISKSLEKQGLSRIASLLDGVTTNHVDNVKKL
KNHELTKNIKNYLVDNDLHIKRDQMYSAVKTIFKELQNNNAPVGKYTIANPITREFNS
LVRGSQVNDDLAKSEKEILKHLRSHKIIKEMEKLLHESVFYLVQRQTLPLFIDQVNSI
GGNKISILKGVALDHYQSYMEKSFQTGKGIANKLDLDLVFNNNNFASQSKLLDKDKKQ
YFSLVAERFARCGEALVTDLLAEKSVQVDYLVTCMEDPQTVGQRAGITPSGNDRTEIL
KAFKSVIEACRSYAFNLDKDIKEENEVNQKLSTTLSFGAN" /product="hypothetical protein"
/transl_table=11
CDS 22820..23014
/locus_tag="AUO97b_00013" /db_xref="SEED:fig|400667.82.peg.3665"
/translation="MYSKNTTGSIFQPYKVEPTSLLSTISFSLISPFFFFWMKNLIEN
YASQLELMYIHFPDSFIFKN"
/product="hypothetical protein"
/transl_table=11
CDS 23068..23721
/locus_tag="AUO97b_00014" /db_xref="SEED:fig|400667.82.peg.3666"
/translation="MNASQTIQTVQFGLDQYSKLVKDLKVLSGIDAFYTAKCLIESFP
NVVNDEFYGYDFYADEKILDISSYIHIDENKWFNLNCLIDLEEKIVSINLASEMENGE
IFLFRTDKNYQVAVNYNYSFENFFSLQSNFEYSLFDLVLSSMGLSLLPKKIFNNFELK
DIFLFSMSIVTGRYKNYIPDQLFFNSIHKNIVLDLKSDELELPSRLINYRDEIDLKW"
/product="hypothetical protein"
/transl_table=11
CDS 24364..24855
/locus_tag="AUO97b_00015" /db_xref="SEED:fig|400667.82.peg.3667"
/translation="MKKSMSKADFERIRAHDFVVLKGVNNKLIVGQVSVDKSCIFLSL
TNNELSRLAMIPELILENLGSIVELMKKYKEVFSRLNDVIEKSNLPELNEYKNDSLFK
IKALYALRELLDSAEVNEDVTTDTLEIAVKKALEECVYIQDVQFTLSYYILNRPKQNT QMM"
/product="hypothetical protein"
/transl_table=11
CDS 24886..26379
/locus_tag="AUO97b_00016" /db_xref="SEED:fig|400667.82.peg.3668"
/translation="MRIFKYELEDALQSLDNSVSYAGFINKLFPKFISKDVDNINTIT
INIILKKIQDLLSSGYYSVFINYKNYMTNGERVWGLHHFCMCIYLHENFEPFGTYSWD
ASSHPQMILDFWSLTSSNRYELHEVGLTYNQVNNSDHTERMNIDIEMAWEELVILNPT
FNFPFFRNFFYLSCLSIFNISDYQKFGKLLSLNYSIDHLLDQLRHNLFNFLDLLDDYM

LNIDLSESTYSFADGTSFTMFAEVSEQDLLNYDFSFVSDSSSSEDSTAQQSQTFSTSD
QHSVPSRVDNSYLSFLSKQDIHEEITSNYVRELIKQFFYNYSKVSFSIHNTSIPCGFV
ADFLDYLNRSLEQIFIKVESLKVNYLKLQFIYQTSHTLSSMYFYKYIVDNYTSIFINI
SVPESLKKEVLVKVRSLSIFIKNKINKLNDVLVHDPLFINVYSLIFFYLDSFSQSLNY
IRCSGGELDKDRLDLIYNNELEYIDALDFNFFQYLARRLPRQVYRLP" /product="hypothetical
protein"
/transl_table=11
CDS complement(26383..26880)
/locus_tag="AUO97b_00017" /db_xref="SEED:fig|400667.82.peg.3669"
/translation="MKSENSIECSKIIPLTGILNQSPLLNTLHKSVKLEIPEINNQRR
ALSNIFNRLGIDNIQQVIISEPKLNKEVLSELANNFFTSSFPQKAEELIRKMSMPEYG
FTDPKQPSPSTLAMNTVTQPQSDIQKQIIEEIKQRSAESMQEFNQQKNESNNKTNEID LNNFF"
/product="hypothetical protein"
/transl_table=11
CDS complement(26922..27491)
/locus_tag="AUO97b_00018" /db_xref="SEED:fig|400667.82.peg.3670"
/translation="MYSSTKSFSNLYASIVHMQEHQSLEELQANQIINHAMKNGPDHK
LNRLLEDQILNGVKGFNIENMGMKHEIFNDKVLMNLKRERATLQDINSIIIDTALQFS
RNPDQIELNEEKLKSSLNTIKNLHCERSLKIDLIRNMKEGVLTFVPEEQRNDIERVII
SKQREIERTATQSNKVVLESKIPNSFNPR"
/product="hypothetical protein"
/transl_table=11
CDS 27825..30479
/locus_tag="AUO97b_00019" /db_xref="SEED:fig|400667.82.peg.3671"
/translation="MSIFCPSQTDSTCQNDAAIDLLGKIFGDGFIQAFVLGKSAAATA
PATGELTPVLFGALGHLALVFAAFLFLFLVFTSVLHTARDGEVFGAGKKKHWVILRIL
MSGVMILPTTSLYSVSSVVLMMVVLASNGETNKAYREFLNTVVMNPSPSFASVDGLDP
YGFRAYAIQKLRQYTCVRVLNQNFAAEGVSVGEKKYSDGQTYYVSNIGGERQFNSSLV
DRNNVLDQGYFGAICGTSSYIVANADSVDSNFKYLTSENPDLESYKDFKKRLLLNIQN
LKFQGIVRLNEAVLAYVNRLPSEAISPEEYARNFNQAFNNEEFDKIITDEIRRTDASI
NSHLQSNQNSYINKTIQYFAERGWTQVALTKKKLGEFQRDIYEARAKTIYEGTNPDLQ
RLNREGVASEKERMIYNVLVTQMDSTVNNYLKTPQVVTTLSTNDIQNLIPEDITNSDV
SFKQISKDVEAANTTLVSNTMRIFIETFTGTGQDDGVDAVTRMQNTGNVLHIALTAVD
TTLTGVRTGLSAAGATGAVFSDNIGTAAEIVIGLVDYYSEKLGKIMVMGKLVAYYLAV
IIPTLPFVFFFFAVIGWIIHILQTMVGLILWSVMHMIPEQSFVGSQTQGYLTVVALFF
RPMLSLVGFFLAFIISDPLLTFTTDGFFAIMGAVQFTNNGFIAALAQFFWLMGWVILY
CLILLPIIYLIFGLPSSLADSVLAWLGTNLSRSLGETNLGQNVGRAANPAAGYYNTSG
GGRGGSGGGSGGNGGGGSPAGGGGAGTRGDGSVLMNKGQPNEQSSGAQTHLPNATPSS
SNSNWQSAAAAGAREGGIGGAIASSVSFAGRRGLQNEASAGTSVLSDVNAGANQTGYI
PTEYRIVPSSASGANSVAPDPVDKKPKN"
/product="hypothetical protein"
/transl_table=11
CDS complement(30531..30890)
/locus_tag="AUO97b_00020" /db_xref="SEED:fig|400667.82.peg.3672"
/translation="MSNMRYSNDGIDNGLFAPHVLDRNARDFDQYMKDHTPAAVARIR
NFTRNINTPVHQNAYSQEYVNRLLNYISQLENSNSNYETRLGNAFKSINNANATIDRL DDLVTDLKKRLSKYENV"
/product="hypothetical protein"
/transl_table=11
CDS 31163..32998
/locus_tag="AUO97b_00021" /db_xref="SEED:fig|400667.82.peg.3673"
/translation="MMSAAVIADLHASATNFEYAKPSGQTGKPSNLDAGDFPLTGNLL
VTSRFGITRFRSDGSPRSHSGLDIINSNGNHSLYAIADGVVIANGWMTGGGNYIRVKR
KENNDVYQYLHLATRSPLNIGTEVKKGQYLGTMGNTGISFGTHLHFDYAIPQKEGSRA
RNAWLGSPKGSANANPYQNIQTIGLSKNGIEGYYVTDPTPYLKDDIKITDSTYKNYLG

STIRQQFNILYGANLPVGAGATQPKFAGVQFKLPQGYGATDEQLAKVNISVIEARQKA
LENGEITEAQAYDPNITYREVEDIFKDSGQVGSSEETKLDIGDDGSVKGHIENLAFKR
FASAGWAQDLVKASNRSLWVEFLNIEIAKNHLKTQLLKQNERIEALFAAYAIAKARVL
QGKIEELRQAIENGRNIALISRLELEDLPPIASDTEVMNATVTKRNGSYGLTADKKAA
IIAVAQAIGANPNDLAAVISFETAGSFSPSIRNPKGSATGLIQFMQYTDGTGNDKTPR
DQWDYWGMTRSQFGSLSFTEQMKYVEKYFKDRGLRESKPVSLATLYGLVMGVPKGGYT
IARHSAVFRDNPAWDVNGDGVITAAEAVNGSKFKAHIRVYFPDFP" /product="Peptidase, M23/M37 family"
/transl_table=11
CDS 33013..33354
/locus_tag="AUO97b_00022" /db_xref="SEED:fig|400667.82.peg.3674"
/translation="MLTKNILLAITLAGSIGTSYAADLNDQFYQVMDEADANPITLNT
KEDDKNFISRYTKLNQKLASINQQKNAKVYSQYKARIEQNCSKLKTNGTESSSSAYCK YILNYSIFNML"
/product="hypothetical protein"
/transl_table=11
CDS 33368..33763
/locus_tag="AUO97b_00023" /db_xref="SEED:fig|400667.82.peg.3675"
/translation="MKKLLLISLILGASVTASANSKFQNQFKQVEQMTDSCGEANYGD
SLTCYNEVIAKHAQLAETIKGHSPEKSKIPAWNKAVSDLSSINNLCNQIGQIQTGMQG
AASLGTGDYCRAKIMGLTSYKAAQIFGSK"
/product="hypothetical protein"
/transl_table=11
CDS 33774..34034
/locus_tag="AUO97b_00024" /db_xref="SEED:fig|400667.82.peg.3676"
/translation="MNNLNPIERSALTSVSESLIAIRVIIRRAQGNGGLSVKDIQNIE
SLADACHNIPSVIANNESEQLKFLLESGTREAKMILAQGAVF" /product="hypothetical protein"
/transl_table=11
CDS 34058..35716
/locus_tag="AUO97b_00025" /db_xref="SEED:fig|400667.82.peg.3677"
/translation="MDELSNTRIEDAGEKFEGARKDAASPIFNQPFKLEKNDSKITKK
KIWPSPNFREDVHNGIKTKEHALLFMLVYEGLCNKPLNGGWFNISGQSFEKAYEDTLN
FLKEAYEKKSYENRDVLVQDFNDFMKAKYSGNELYAAGRRKGRVVQHPLYFNLHSRIR
FKNLLNIGWPHKLILDSDCFGAYEFSDNDSKNLFWYAIEGIKERRIRSLDNWAKHDHY
EAALQVAKSAFRSIIQKRKEEQPENNSKVRSKPPKRPTFNRPFVRVGETYRQSNISTE
EFLSTFKFRGIEWGNYVTQSERQGFLDATYDSFIDLTRLCGLPPSFASLGGKLGIAFG
SRGRGYDGAAAHFELDQWLIHLTKTKGVGALAHEFGHALDAYLALKSKTKRDFLTESF
ITKYNNHSLSLNTNSLKVSESESLRCFKVLLENMIFTSDKNIIDEDQVRINSTFAKNA
CLLDQGRKEAYWTDPTELFARVFEVWVSDKLKASGYINEFLVYGPDEPPSSWHTKVNT
YPEDQERIRIVHYLDRWLSELVTTWKFEKKRKLTISRIMSKNSQ" /product="hypothetical protein"
/transl_table=11
CDS 35998..36501
/locus_tag="AUO97b_00026" /db_xref="SEED:fig|400667.82.peg.3678"
/translation="MNRVINSELEHAKLLLGLGQVCTDVLSMHEVVGSSIKEIPFVSE
RISAGFPSPAQDYVDKLMDMNEHLIRNEAATFIVKVCSLSMINAGIDINDELVVDRSL
EAKNNDIVVALIENEFTVKRLKIEGSKIWLKAENPDYQDIYFNEGQKLIVWGVVTFIL KNVRKRS"
/product="Error-prone repair protein UmuD"
/transl_table=11
CDS 36498..37793
/locus_tag="AUO97b_00027" /db_xref="SEED:fig|400667.82.peg.3679"
/translation="MNQSKKIFFLVDVNNMYTSVETAFDPSLTGRSVIVLSSNDGNVV
ARSPAAKKLGIKMGEPLFQILDLVMRNDVIVLSSNYIIYGEMSRRFHKILAEYVSPEN
HEIYSVDEAFLELTSYRGIYNLNQCAQDIKEKLLKLLSLPVCVGIGRSKTEAKIMNYI
AKTYPHLNGICNVFDLAEVKDSIFRNTDVGEVWGVGSQQKKKLRLMNINTVYDLMTAS
PSHIQSVFSVVLKRTVLELNGISCIDIEHTPPTKKQIVSSRTFGQRITDIHDLKEAVI

KRTQEAFTRARNEQVLVGCIIVFAYSNPFDKTKPFYKKEQSSSFAVPTDDLRLLVQTA
TRLMDQVYKSGIEFKKAGVILTCLEPKHTYTYDLLTDHQDLEKTEQLMKAIEEVQQIF
GKNKIGFGGSMFKNRIWNLTANYQTRNYFSFDGMIKIKN" /product="Error-prone, lesion bypass DNA polymerase V (UmuC)" /transl_table=11 CDS 38001..38996
/locus_tag="AUO97b_00028" /db_xref="SEED:fig|400667.82.peg.3680"
/translation="MSDFISTPRLSTYQNILKLTDPNQILRAYYWNIALAGAIYPLMQ
TLEITLRNAIDVSVKNNHQPKSANGSNFVSYKNNDLWFEQLVTAVQDRKITKMRPHQA
LKWVRGGKRIKFSTTESHVKKARDDASTVKSWVKGEDILSRLPFGFWTTLLSKDYEDV
TNKHLLWPNLLHHVFPNAPSHIKRKDIEDHFNLIREFRNRLSHHEPIWKFYYRNPANN
ALDYTTPIFGLNASLNLLNNQYDDMLMLLQWMSASAYDNFNYSRIGNEFKKLCSIDGF
YAYVDREKVANCYPASRAKREFFKLAETLQNVNVVYMKTNGKRGYILGLNEPSLP" /product="Tiorf34 protein"
/transl_table=11
CDS complement(39052..39933)
/locus_tag="AUO97b_00029" /db_xref="SEED:fig|400667.82.peg.3681"
/translation="MSSLLDLVLDGDESKANSAKQTEIELHLIDLDPENPRKTFIEAK
EKDLEESIRESGVRIPVILIKHPHDDNRFIVKDGNRRVKNSLRAGLQKIPAVVVESFS
ELDQLTANTMNEDMTIYDISRAIVRLKAKGYSQRQIGKTLAYGDAKVSKIVAITDGLD
EEILETLEDMCNRNLLNDFSAMYEIAVLYRDHKVDVRAWVEKQSATAKKIGIAEVQKF
KKTFETDLEKDKPKSKEVSLNETVIEDILSKAKESLPLVSKLHKGEIKDPEEREEVVN SLGELIKDLTLILKQYK"
/product="Chromosome (plasmid) partitioning protein ParB" /transl_table=11
CDS complement(39937..40821)
/locus_tag="AUO97b_00030" /db_xref="SEED:fig|400667.82.peg.3682"
/translation="MNDKTSQIKDAGILTIGNGKGGIGKSTFTVHSAYFFSQHGYKVI
VVDLDNQGNSSGHLLKLNSNTVLPEYVTRPSTPVHNLGDAVSLFHKDNQNLKCDLQNG
SIGVFRPTKALASINTSSDKELVAIFQENINKLLKYKTVIIFDTPPTLSNLMLLPLSV
SNVVIIPTMLSQYALDGIRDYISFTNNIKKALNTKLLLGGIVPNRVNMKSSIQKEELA
KLLAEGESNSIFYKDKNNIAYITDKNVIEEAVNYGCACWHINKTSSRDVKRTFFNIFK
QVGVQLKLAAFNSSSKGE" /product="Chromosome (plasmid) partitioning protein ParA"
/transl_table=11
CDS complement(40955..41602)
/locus_tag="AUO97b_00031" /db_xref="SEED:fig|400667.82.peg.3683"
/translation="MQLSLELLERVFRSNQNDPNKFMSRLKQSPNPKHVLDVVAASEM
LQKANLLIHDLMSNGQKQSHIFKEKKEYESKDQIFTLRSDDDPNKFYVYVFHLIPENS
LTVKFSYCLGTLNAPNVFGPESALKLKGVLLNIFELHLKQEFIFPSWCNVFYINNLTS
RAMPNIALSSIEDQLSNPLDFFPDAIETLKMYSINIKNIDLDIMDMFVSSDQMSN"
/product="hypothetical protein"
/transl_table=11
CDS complement(41619..42227)
/locus_tag="AUO97b_00032" /db_xref="SEED:fig|400667.82.peg.3684"
/translation="MAKKLPPHIVDALEALEENEIESASLNALRLRAWLYFIRFLSSF
QTNYETKNPYSSIQFTHHQASLFAFGIDDKNPENFLLFAQENDFWWLSEIGIEYFTFI
EKFLIIKFLPLNYVNLNLSSENKIRIPSLTISIPLSDMYYDHLQQYIPAFKNAYIGKI
GPSPAGINKWNISEFSNLPIPVRPQLTPINRQHALSNKTSFL" /product="hypothetical protein"
/transl_table=11
CDS complement(42220..43188)
/locus_tag="AUO97b_00033" /db_xref="SEED:fig|400667.82.peg.3685"
/translation="MESKQRLNFSLDEYYSAITHLIASKPKKEASDFSILENGFFTDD
KELSLKITRGEKSNLHNPLSSSNKQYPLFNDNQLADLINFFDTAIWTVERRLPVQSYC
RAISQNFDLIVDLKAEFVIALSILNNLEFSKIQSQFHIDGTSHLIDKTRQLSRLICDI
NDPKVLQDIVAQCNKRIDDLDTAPYIERMVVTGHDHIINSLQLYSKVKPYANMVQDGN
SDFSIGKTLDIRKCDVKRIRKMSNIEGLLNTDNRSARSRALKTDIEKILDTPSYSWVL
EQSQTHGISEHQALKKIIKEHKKFTLKSNLDSSDEDEIENTGEVNG" /product="hypothetical protein"

/transl_table=11
CDS complement(43198..44055) /locus_tag="AUO97b_00034" /db_xref="SEED:fig|400667.82.peg.3686"
/translation="MKLFNKKVSLPDTTHLPSVTSNVHEKEDKSFLTIQVHTDDNCTI
HNRAPKLVSIQLPELPSFNYALDFDQSTLNADPVHTSEEVPYKIVKFKAVLEISPSFK
NETDETKTYEIADIDFKVYPNDYDSKADKYFFNTPDEIKEYLNGQWEDAFNSIISTYN
LNNRTFGNQNNGGGYTPITPSGHASVSTVRQPRTFSIFGYEFSLLQLFLAAIVLLLLA
FISLSLWGHYVNSKNGYANSSQNPYSSSSIDQQVQIAEDVVSRVQGKLGVPATAKSND LGCLQDSLN"
/product="hypothetical protein"
/transl_table=11
CDS complement(44077..45021)
/locus_tag="AUO97b_00035" /db_xref="SEED:fig|400667.82.peg.3687"
/translation="MKTKSLGNSNKKSSFKFPHVALAALLGFGAAGGWFYYESHNVPT
GVTSNSTNLYSENLFAQMSKLGQVSNVQPMAGELLAWTMLINGQPQVVFTTKDEKVVI
TGNAFDTATMDSVSLPILQKAQQGGAPANSTGVAAPTGGNVAVDFAEIVGKWDKEVPP
SISLLDKLKGAKEGNASAVDTLYIVFDPRCSNCHDAYRATRKYVEKGFSIKWIPTTLL
GKHEEGAKLAAAVLRDPKQLERAFSKDPNIVVNPTPEEKKALEENVQFLTEATVQSTG
SQTIAVPTAFYLNKKTGEPKMTTGLSDESILEMIFGKI" /product="Thiol:disulfide interchange protein DsbG precursor" /transl_table=11
CDS complement(45103..46473)
/locus_tag="AUO97b_00036" /db_xref="SEED:fig|400667.82.peg.3688"
/translation="MSNNHNASHGSTDDTLILIGVLCVGIPLLVFGGWKIGKVAIITI
TRNVYGLIELPFYYLQLFIQKITGSNAGYLDFTTNAITKLCYPDPNHAMNPLMHCTAN
LKVVPFSFYSENCIFPTILIGALLVGWIWYKHAINLKYLPDTRYVQEHDLESLIGELG
SYQPHLRYIQRFIQSNYATDKGFYRLLLSPREFIKIHNLVYGFRKYEIENAEDFDDFK
GSNSPKDDAYPLLDQSKFEKLMMHQLGQMLDKPSNLSDGEIILYALFLPMACATDERM
SDKEFKKIKAQKDMLLHYFWDKAIEHLTSEKAFINPKNYEPREFKYFERKRLQKIVER
YWNHPVAKEVFSKHAYVRTALIALIYKTRTLGVLAPCELRWLRNYDRTLWALVQNVGR
PSVYYVEQLAAFSHYTSEAYYHRRKISPDFSYGWNGLLESLKQYRYADPHVYLPAPPKA NEITVN"
/product="IcmP protein"
/transl_table=11
CDS complement(46492..47241)
/locus_tag="AUO97b_00037" /db_xref="SEED:fig|400667.82.peg.3689"
/translation="MPQKHRLRISVSLAKLPKRQRDFLDKFKNIKEIDVHSFYAHLIS
YFFFHPTKSLQLIGAVGKNESKIALNFHHEALVKTLTGKTQYQRELYCLTALYEMSNM
LAYKLFMYYTYVTNRNFNELAALENQFFELGISAFLDDFIELTNETKFFQNIQIMASS
FNTESTRAFALLESYVHSETMITATSHPDPLQKPILDDLTTNALSPTPEYTLASPKQE
TIDQIQNPVDQESFDEQSSSTIEDWSQIQVG"
/product="hypothetical protein"
/transl_table=11
CDS complement(47255..48313)
/locus_tag="AUO97b_00038" /db_xref="SEED:fig|400667.82.peg.3690"
/translation="MKGYKTLTVGVDDGHDGIKIYCGEVGKSFRLPSRVANGRTIIGD
TDEVNKQIIHVNGKYFTVDEFTKEHIDTRTEDYPLSDANVALVHHALHQAFDGQYRKF
KIATGLPLNRYYGGKDKAKNEKLIADKTQNLLINKDFNNPTVYNLYEHDKKNDPLQIL
NHIVLSEGQCAYFDALMDDNGKRSSMYEDLWEGGCAIIDIGGRTTDIAMINPRGGTMQ
ASRCDTLDVGIITLKNKVSQNLKEFFGLSSNITDWRLSKALKTGIYNHGGKDHDISKI
LNAAKVEITDQIENSIKVNVQDGQDLGAVLLVGGGSITLGDELLKRFNYDNWHLVKQP
EFANARGMYKCAKYISKL"
/product="StbA protein"
/transl_table=11
CDS complement(48598..49887)
/locus_tag="AUO97b_00039" /db_xref="SEED:fig|400667.82.peg.3691"
/db_xref="GO:0005524"

/db_xref="GO:0005622"
/db_xref="GO:0006810"
/translation="MNVYARDTVAEEPKAEHPYQMLRHETIDLDNISINSEEDVRKIL
TVANAFNASDVLINSGRPILIRRYNKLYALTLKNLKDVELNMFLTIINGNEALSSIKK
GKGLNFAYNCSAFQDRNSKSGKGYITTQNDESWLHVNAVEKEKIIFRCNVSACNDRTG
NSSFQIVQRIIPSDPPTYDQIGLTEEFVLKAIPRLGITYVVGETGHGKSTTLASMVRY
VYEEDTHIQGNIITLEEPIEFRYDGIKSKHSIIVQSQIPEHFMTFGLAVREAMRRKPA
LLLVAELRDQESFSAAIELSKTGHPVMSTIHSNDVANVLPRIMDLVPPDQQHRKLTET
ISTAHAFIAQRLVETPEGKLIGVREHLFFSKSIRNDLLDLAIQAGGYGLVIQYIRKMM
NDNPQGTENSSPNFENQAKDLLNKGLISKKGFDALIA"
/product="Twitching motility protein PilT"
/transl_table=11
CDS complement(49900..50718)
/locus_tag="AUO97b_00040" /db_xref="SEED:fig|400667.82.peg.3692"
/translation="MADLPNYQYYLQQSPIIISQDDSAKQAESSARLESIKTLVHEAA
TRNALRARLTEIDKVVYNPNNVRKLDSIYDFQRLMINTVVVPPVIVQANNISDVQNPR
TLSVLGVRYEIKENARFSHTPPNWRDFLQFNVKSYEIGVDALPNELKPKNSAEERAFK
EAMNKGIEDGVREADAIFKHQLNRLNQTYKGMIAFHRFVMDGKITMPAIARRDIALTG
NGNTLMLNQTNFEITSLPQFVNNTKLWNSRILSIKERSVVKDPVETPVAIETQQ" /product="DotC
protein"
/transl_table=11
CDS complement(50760..51230)
/locus_tag="AUO97b_00041" /db_xref="SEED:fig|400667.82.peg.3693"
/translation="MKIVPSIRRSLTVLCISVATLTGCQSYYYVPSPKFDVESPLGIL
SQAAQDAVAEQQTYALDKKAFNEDLALRQRSFRTDVIRVDYVGEPQTLLQSAAYHFGY
RFIEAGNIHQLPIVNFSKRKLTGFDLIKDVGLQLGNSATVDVDENDKTITLIHR"
/product="hypothetical protein"
/transl_table=11
CDS 52168..54072
/locus_tag="AUO97b_00042" /db_xref="SEED:fig|400667.82.peg.3694"
/translation="MKAGSELEFNAKKYPKSIKGLETRVFDYSEKTIIRYTNVWKKLL
KQYGLEDQVLVNPEYTLETVVNNLISEYGNNELRNSTFRHYRSALIHRMGVILSEQKR
DGEKLVALPKLARLFESLKAVEATDDKKQPNRSSSSKLKYFPKDLYEFVLGSSFNLNR
SNLGLMLKLFIEANTMIGLRPVEWQSLRLACDVEERCLSLVVDNAKNTQGRANGDIRI
LNMIEPDSIKLKNQLRAILGFKRLLDLELEARARKFLTERSRETNETYKIYIASKEPD
SFEFEFIHWKGSQTVNCIEAVNCIKPVNGTGETVNWISTENELEQNIEDRTNGALPSI
NGPDTVNRISSGHGQDTTDDNKAGGGEFNYERVIEDGAHSSVNGINTVDRIKTVDGIN
KVDGSDHGALPSANGPDTVNGIKAIDGNNTVDGINAVAGIKGGGTNQAISTASTAPAP
ELTDFGFNSVAGEIQQTGRNTDHGKMAALLGAYNAVVIESYVDRYGTIQYALCQSVMD
SLQRKLKDMSNEFYEKKKFGLPEELASNKANATIYSTRHQAVANRKKYKWKEEEVAGW
FGHASVQTASRHYGVAGKGWGDKPMFRTSKFSLEKVRMREQQLNQTKQLTEEDVLNSV LSDKDLDDLL"
/product="Phage integrase"
/transl_table=11 CDS 54303..54563
/locus_tag="AUO97b_00043" /db_xref="SEED:fig|400667.82.peg.3695"
/translation="MKDIHKKEISDKAYFRLSSEYGFVKASHAGDEMRSKIIKALSDG
IIDGNEYVSITGEEPDYDLSTDAEKATYKEAKQKFIDEFKPS" /product="hypothetical protein"
/transl_table=11
CDS 54701..54904
/locus_tag="AUO97b_00044" /db_xref="SEED:fig|400667.82.peg.3696"
/translation="MYEHVKNSKAGDELEIRLHTALDDGIITQAEYTYLTDSELPSMT
VQASDKEYKEAKLKILKEFKKVS"
/product="hypothetical protein"
/transl_table=11
CDS 55028..55330

/locus_tag="AUO97b_00045" /db_xref="SEED:fig|400667.82.peg.3697"
/translation="MKTDDVIEHIEKQNVTAVELLDEFDPNLQFKFAKAMTALKQVID
DVRILYPDASYYIQDDAFYLMLGDSHSGDHKQNANTNLIALTDNHKLAGCISGGGF"
/product="hypothetical protein"
/transl_table=11
CDS 55508..55657
/locus_tag="AUO97b_00046" /db_xref="SEED:fig|400667.82.peg.3698"
/translation="MTSEEIHQLSEAFMEALFSRVFMLVIFIFFGGFALLNLIKLIQL FDSGK"
/product="hypothetical protein"
/transl_table=11
CDS 56321..56614
/locus_tag="AUO97b_00047" /db_xref="SEED:fig|400667.82.peg.3699"
/translation="MKESQQQDIELSSSSDEYSTWGNPRNSLNVDVRTKEFYSNLLTR
IINFKIIVLVALTLVVLAQVAYIAVKRNFEQLVFDDGSSTPCIYNPLTKTFRQ" /product="hypothetical
protein"
/transl_table=11
CDS 56632..57348
/locus_tag="AUO97b_00048" /db_xref="SEED:fig|400667.82.peg.3700"
/translation="MSEQKQNQNPKTKPRFPTTPEQVNSGLVEMAKKNDELEDKIKSQ
RRQLLGCIIGIGVLGTAVAVLGGIIGLMPKVEYFQTVNNEVICKINPNDNPAFTDVNV
AEFASEGLLNAYSLDYQNADADINNTLRRYFTDRGRESFITTLRQSGLIDQIRNNFLV
LRTSKLQTPEVAKNNGIDSRGNRFWIVMQPVRMDYFNGKSQPADTRQYMAEIRVEVTP
RDVYNPKGLGIASIVLRAKN"
/product="IncI1 plasmid conjugative transfer protein TraM" /transl_table=11
CDS 57366..58496
/locus_tag="AUO97b_00049" /db_xref="SEED:fig|400667.82.peg.3701"
/translation="MQLKFKNLALSMALVVAGSSYAEPSQQQSGQQQQATAQQTVPSS
QPQGNNQSQGNNQQQGQQQANNQQAQAEAPSYIGTPPPNTINLGNVQTFGNSFPPPVR
VAKTKDEIVAEILSMELTPQQVKNIKDLLLRQERDASTPYTTTARPIVRSVPIDLSPA
AQPPVLRLSMGMLTSIVFSDLSGNPWEIEKVTFNKELFADDRPEQGQGQGEKSDISSK
NTNIFSIEPLTATAYGNVTVRLKGLSTPVIFILATGQNEVDMRIDARLAGRSPTSVNS
SLRANMNATGVMSSIDSQTLLFLDGTPPVEAERIRTSDTSVEAWNFNGQYIVRSNDTV
LYPSYKSTARSTGGTSIYVYPDPINQVTISRNGQPTTVYFNN" /product="IncI1 plasmid
conjugative transfer protein TraN" /transl_table=11
CDS 58505..60136
/locus_tag="AUO97b_00050" /db_xref="SEED:fig|400667.82.peg.3702"
/translation="MSAENNTNKPDDIQSSQEQFTGQNEATHSTHNSDPERRDPLTPE
YETFEENQTATWIPRNDADNRINMKFGDKFKLYWRENGVFRFIILTVGAVILLILFFL
MKFIFRQDAPEQINPANIKMDAPQSNTKQAEYVSQEQYTAIRKQEEILARQAAAKGES
YTPRNLVIAPTDAQGQVVLPSTSNAQYGAMNPQQAGLMNSTGQLTYANPAVAGQQGQM
QQQGMQQGQPQPQYIPVQTQDYVSDVAANYEQNNNQFANWFDEESQRQITANKEQEQT
INSFVLKQIGQITGAEFDANGNMIPKSKDKKTKAGSYAQKTYYTAPASADNSGNGSNT
GNGANNAQDASPFSANGNNPVNGKKSGTGKVLIPAGTRSIARLLGEVNTDDGNQVFAQ
VTNGPLKGRKVFGTVSKSNDNIQFKIDRVLSDGKNKEFSIDAVGQTLQGSYGMATYIK
RHTLQRYGALIASGIAQGYGQAYSQAYGQQTYTSGATVIETKDPSGERIAGNIIGQVG
QNASQEIQQIGRKQVTYITPVGTVFYIFFNQDVTE"
/product="IcmE protein"
/transl_table=11
CDS 60161..61015
/locus_tag="AUO97b_00051" /db_xref="SEED:fig|400667.82.peg.3703"
/translation="MSNEPQQPNYDDDFDYQTQTQQPVDSTDRRNKFILIGAVIFAIC
VLTFMFISTKMKKAPPKDQTIEAPAPDQTAYQPPPQPVQMQPQQPVGYSQNSMVAVPE
TMPKSTDPVNGTVMVKDMTGKDVDAYSVDGQNAIIQFNNANGLQPIPLNMPAPVAPNI
NNQANNFNEQRVITLEQENSQLKAQNNSLRTLAQDQQLKIGQYQALIKNARANNGANI

VQLRETKKGVALNKALPRGQEVVATVGNRVWLSDGKQYNSYGVGQRLPTGYRVLDVNE MAGEASFK"
/product="hypothetical protein" /transl_table=11
CDS 61054..61632
/locus_tag="AUO97b_00052" /db_xref="SEED:fig|400667.82.peg.3704"
/translation="METIDILQNMGFFFVAATELLRYFFVVSGFYIFLHALVDFVQIS
QPALAGRFMTQIRRPSAFGASMKLIIAAFLCTLGLRLQLLDIVGSLISDTTPAGVTYY
DYTQYTGSSYTDELNLAVLVIIGVLQFFGLLAIGRGLWIWRDNADGIAKLSVGQGTMF
MIGGMLAYKVEVVHAFLASWFGLDFFKIIGLT"
/product="hypothetical protein"
/transl_table=11
CDS 61737..62156
/locus_tag="AUO97b_00053" /db_xref="SEED:fig|400667.82.peg.3705"
/translation="MSKINNLQKVKNVGQDIYAQLGVAFTMGSLGITNAIAAPDTTNL
TGGNRQGQATGGDSVGDIRDRGINAMDAFMSFLQYAGMLVGFIVFVMGVIDFLNIGKP
GKDATAGKAGLKLLIGAVLAGAGYFYWSAANSASGNV" /product="hypothetical protein"
/transl_table=11 CDS 62427..62993
/locus_tag="AUO97b_00054" /db_xref="SEED:fig|400667.82.peg.3706"
/translation="MPACTLCHRAHHLAWVLTDRSGLLGICHELDQIRINHLQRFVLV
LANHPDPKLKAFFIKGGMFQTVLDNIAMEYYRPDVDEKTIRESIDSEFPQATIDLKES
LFTDRINAIYVRQSLSSNDGLERLIYAINEVKQSNKTIAPNAFFPFVLLYNQNIFSKE
QIDYYLSLDEFNPEKWTQLFEEQLLKAG"
/product="hypothetical protein"
/transl_table=11
CDS 63005..64012
/locus_tag="AUO97b_00055" /db_xref="SEED:fig|400667.82.peg.3707"
/translation="MIESNLNPFLFRALPNIEHACIGNAYDLMANQFEQAPINVQEWF
HLHTGFIDTGLELTDDVETAFVTILSNQVVRLLEQTRLPLFSTPDPELEPFIADSMDD
ELCLVSISLENLTAQQIKIRTLLGFGPQLVILKSNSDFFDMHGFELVVTDSFPCTSFR
LFIESSKRNLLSTFNKTFMLQQNTIANELLNGPAIYSDLFKQALRQQICSKLCAEDDL
EISVHDKELCDDAGNLYFHIGFNQTFKHQFFHLVSKNILISPFHIERLIGEGNELLET
PAAAQSNLESLVTFMLVYMAIYEKEALDSINSTNINDDDVLNMIMSKLEQHSNNYPSF S"
/product="hypothetical protein"
/transl_table=11
CDS 64029..67301
/locus_tag="AUO97b_00056" /db_xref="SEED:fig|400667.82.peg.3708"
/translation="MVSTATPKRADTRSTANNSKANTKPTKVKRRSSLVSSLELILVS
LAKMFKASANDYCEIETVDGRFNLVMQDGTMVTILKYEGIRSTIDYHQTFMGEIDRLN
NYLKNNLNAVNSHKIMVLFRRDDAVNSELNRIVDIQKNTARKVNIRLDSFIDEMRDVY
APYIYDEEIYFALMTNPKILDKSEIAGDYRRTADIVKKYNIPPMSDAQNLLHGLSSLK
SIHDTFVSGVISELEHQDFKGVVDKLDVSAAITATGRMMNPRYFDPNFKAFLPNSGMP
IPLRWKNRTDPQDLSHVLYPRISDQLMTQTITPLNSKDKAGDITVPKSAVQCGDMVYV
PIPLKTPPSPSFSGTSSVMPTFIDFFDRMNQQTTETSDGSSRAIPYAVSFMLSGDGMA
SIGIKRAVTAVVGWQGAANQQIKAAGDSLQRYKDYGGCVVGLQVSLMTWDEATPRGIE
RLQIRRSKLWRAAQNWGHAVPLERTGDAVKAYLTHFPLSTMHHAPLCPAPLPDALHLL
PLDRPASPFKKGTMVQRTPCGKLIRIEHFSEELAYNYTIISGTMGSGKSMRMNDELFE
ACLAAGLERLPYIFISDVGVSSSGLVYGIQNALDPSRKHWCVYKRISKSAKDAINPME
TAVGLQYPLSNEFDQIRAFLVTLVTPAENEHPEPEMSAVISLIIREAFKFFATDNDQS
QPKIYQTGQSHELDLLLRELRISLSETLPLHDLALLCHQLAEQIVSNEEIQQVRKNKL
LRARNLAHRYAMPILSDLITVAKKEEIQRIYGDYKTSHGENILDAVTRSLSQAANDYP
NFSTFTQFDISDARIASVDLQDVIVKNNRKNNSLFFQIVRMFYKKRIAYSEEDLTRGE
IPKDYLDYYSNLVAELKIDKKYMVFDEMHNIKSDNFMIAELERDSREIRKWGIAYYLS
SQYISDFGILTKAASRFVVCSQGSPEERKDIVKYLQLTPAQEKRLTSIVGLRSSGMTY
LSKVINERNDQYIQFFTSMIGPKRLWLLTTNPEDQAVRSIMRKKHDLETCIDILATVH
PRGVKTIVNTLKEQRALEITSSAALSLEEEENIKLGIYTELVDEAIKTYNFIQSQKLR VA"

/product="hypothetical protein"
/transl_table=11
CDS 67365..67775
/locus_tag="AUO97b_00057" /db_xref="SEED:fig|400667.82.peg.3709"
/translation="MNFHGNSLSQIQDKIQILIFESDKEESFLGETIHFYQFWIELLF
FLNQKKEVEIDMSKIQSSEDLQDLAELAYGHLYTSFMEKYGNDYYQTVKPLREYLDSL
PAFNPEDPNSYESTATKEQHAKIICKFSNTGLKQ"
/product="hypothetical protein"
/transl_table=11
CDS complement(67883..67996)
/locus_tag="AUO97b_00058" /db_xref="SEED:fig|400667.82.peg.3710"
/translation="MINIAIGVAIMSNKGKKKLTKTTIVIVMAKYIDPSDV" /product="hypothetical protein"
/transl_table=11
CDS 68064..70520
/locus_tag="AUO97b_00059" /db_xref="SEED:fig|400667.82.peg.3711"
/translation="MPDGSFDGVARKVNLRKNPEKAYGQGVYYYGIDRQNHQQIWKSD
SDERVHDLLIGTTGAGKTETIKMVLTNPLLQNSGFILSDAKGTTELVAELMYLLTRFM
RQDDYLVTNFITAGRDLLVRQEDKMTNTFNLMSYTSAAMLSEQLVYLLADSEGSGGDM
WKDRTITYLTATTHLLTFLRDRGLLLLEPKTYVEYQELNQLIRAAYGEDFDDNYKNFG
EIYITAVSPLRAYLKSLAGFDEGKLYKQPEKCVEQHGYVIMQFTRAISDLSFNFGHIY
GTKSGEIDMFDVVLNRRCLINVLPALQNAPPTIRMLGKLFVGSIKQMMAGALGNRIDG
MVRNVIEAKITASATCFKIVLDELAYQIVKGLSVIPAQARGLTFSLTFSAQELKDIFV
ADEAEGEAIVANTGTKTFGRINSGTEGLSMQRLRSLVDQVTQSVVNGYNIASSAMGIN
YQSSTNVNIEKQDRISYQDIASQQEGQFTIVVAKKIDGGKEGGVFAIHAQTMFVQPPK
LAKIRLNDFVSIEKELTYFDSKKYDNELKNFAYQLAENKILQSCPNYKKHAIGALNQV
DTHLESISKMAKLYETDVKSGFFAIMRTMQNMLDSVQISGFSAPFYEVVDSTFANEID
GALDSIFTEIKLDTEEVDVVLNKTIDHIQDERSQDLLTKTSETIIDEIVDQYLNLDSS
EIETIEAVNAYIEALPNVYDDQLYSEEFATTVREHLKQHDIYIQSLDIPSFEDLLELD
QCAAEAQKSIFSDDFEEDDLFSDAQERYNYVTDFIKCKNLVFEEKEKEIESEDQEILS
KLEKYHEIVYRMISAKLKDK"
/product="hypothetical protein"
/transl_table=11
CDS complement(71844..72008)
/locus_tag="AUO97b_00060" /db_xref="SEED:fig|400667.82.peg.3712"
/translation="MNRRSHEYQAQPIVSNKAYRYLSGKTSDLEFHEIFDGSFKVYGV KAYQPTTPKI"
/product="hypothetical protein"
/transl_table=11
CDS complement(72134..72763)
/locus_tag="AUO97b_00061" /db_xref="SEED:fig|400667.82.peg.3713"
/translation="MNNIVADIVNSYEVKSFEQVKEFIAIEKLKYSFYNKCFKVVSLL
NLIGWPPFIVCVLVGLAAFCFSKAELVFFSTSLVYFLLLYCLIVTALWMYTASKADKE
SDLLIKKNFPNYFNVYGHHDLLDEESLKYLLSTEVTLDEYKIIKSLAELSPEFNIKLK
EIMKFRNGVFTIYDYRVLNCKRLLSISEKENNRSKEQAEIDEFKKEIIK" /product="hypothetical protein"
/transl_table=11
CDS complement(72804..73265)
/locus_tag="AUO97b_00062" /db_xref="SEED:fig|400667.82.peg.3714"
/translation="MKNIFVLSLALLGAPVAYADFQGKVIKVADGDTLTVLVNGNTQE
RVRLENIDAPESSQAYGQQARNFLNSQVYGKFVYVKSNKMDLYKRHIGTIYLGNYNIN
QSIVQNGYAWAFREYLTDPNYIKYEQYARQNRLGLWKDNNPIYPSNWRRSK" /product="Nuclease (SNase-like) precursor"
/transl_table=11
CDS complement(73294..73560)

/locus_tag="AUO97b_00063" /db_xref="SEED:fig|400667.82.peg.3715"
/translation="MPEPSKRNGGKLVKTPSEKVVDYRNRRKQRIDSGKPDQWDCKDC
AHILATDYLREKLGLLAWLRIAELNGYKFPPKKTNQNEINNPKG" /product="hypothetical protein"
/transl_table=11
CDS complement(73553..73993)
/locus_tag="AUO97b_00064" /db_xref="SEED:fig|400667.82.peg.3716"
/translation="MKAITVNFVQNQAVLETHVIHLTEASYELIKAEILPIINQSSSI
LSFLKKLNNAKNIIQISSAFRLKKQLHTLLSATGQIKDQNDLASLQHFVLILGKVFDR
LVNQYHEPNLYIGAYVLSVTTLPVLNLALDTLEVDPDSLGALHA" /product="hypothetical protein"
/transl_table=11
CDS complement(74858..76372)
/locus_tag="AUO97b_00065" /db_xref="SEED:fig|400667.82.peg.3717"
/db_xref="GO:0003676"
/db_xref="GO:0003677"
/db_xref="GO:0003896"
/db_xref="GO:0006260"
/db_xref="GO:0006304" /db_xref="GO:0008270"
/translation="MRRNHRFQRTLQRIAKTWSRIEYNRAQPVQSIHEHPYLKNKAAF
PEGYNLDLVRIIKKQPLFAKSLHIAISRVVKKAEAKGLNIDYIKPKYFQHAWFQNHHI
RPTLINAYYDVDLKIQNCQLISALQDRETGKLKFFKGNLTHGRKDGVFCPIHRLPAQD
EKIFALTEGFATGKSFARICKEKPNTIAAFDKESLISVAIAIRNKWPDTTIYIAADND
LNEQLNGKTKTNGGLIKAYEATQAVSGYVLIPPIKKDELTLSDWNDLELKYGTQHCRE
IIKQQLIDIAEFNQSRNMSNFKMPAMASANIHKPQINIQKTTAALDPAIFTSWLIHLM
ENQKEKDVDLSMAKDIVQAVSSNEQKQVLPMLKVICQKNGWHDLDQALNLMSDKVEIR
NNNEKEALNRVNMTLQAIFYASNNTTIDASKKEKAEEMLKNTIEFIGYLPISRAEKVN
IYTNIKDNIFEATDEAEPRWSKTLLKLCKETISEMENKQVNQFENQKSLSYERT" /product="DNA
primase, phage associated"
/transl_table=11
CDS complement(76829..77254)
/locus_tag="AUO97b_00066" /db_xref="SEED:fig|400667.82.peg.3718"
/translation="MGEKRKISISSEVSNYIESLARTSKENDQDQISWSMHINKMLCD
YISIVQSNLPESLSPQMADALVFICQNYIDDLYKINYRDDLKHYLYNFYFNLEKESQI
PPYIKSEFLDHFMEMELTHIEALSILHFLKKTLAKHHRN" /product="hypothetical protein"
/transl_table=11
CDS 78172..78903
/locus_tag="AUO97b_00067" /db_xref="SEED:fig|400667.82.peg.3719"
/translation="MLKKIGKAFVYLYVTLPLKGIAGNLGYKHLKNDYEFNKQQYKSL
KKIHCPACFKSIVQTSQIKLTDNEIVEHKAPAKAVKVTLKCANPECSFENSFISGPSK
KEISEKTNNAILAVGRESAKRIFEVIPVGQVNNSIKEHYEMAIMLRNISIFCVIGCFA
FTFYQIFFKDNSLFFTALSAFSWLVIGLGIFLNALKYSHKSWQAQTLKVFEQQSQFYF
WFKNHAWFSKPKYWRISGEGKNATK"
/product="hypothetical protein"
/transl_table=11
CDS 78890..79717
/locus_tag="AUO97b_00068" /db_xref="SEED:fig|400667.82.peg.3720"
/translation="MLQSKRLFYFLGCMVCVSGITNAKVPQMSSQQLGLYANQLNGSP
RANFVNSGTTSNHSINPKTANTSVVLPQRRTGNYTTISYTNSPAPEAKQLEIVQQSQT
SNTNWVQEYAFRGNVAAPEVLNNYSYQKPKVNINYVRGREANLTCVIAAARSEGVPLY
VLLGIQSKERGRNGEVNATNDMGHFQVNKQHFRNGGMFNPQDMERARTDGCYNAVLAA
KILRDRLSTKVASVDFWTRAAAYHSWTPSVNARYRPDLIRYSMQWKQWLTQNGINPN"
/product="hypothetical protein"
/transl_table=11
CDS 79698..80027
/locus_tag="AUO97b_00069" /db_xref="SEED:fig|400667.82.peg.3721"

/translation="MELIRTEGYEDKMVQKHISQNYIFKKLVHLTNLNDDIETLITLF
KEKDIVVDSALIESWLSEEGQSNFKEISENNLFSFFEILFSVKKEALSKDINLFDLSY IFEDIRN"
/product="hypothetical protein"
/transl_table=11
CDS 80152..80286 /locus_tag="AUO97b_00070" /db_xref="SEED:fig|
400667.82.peg.3722"
/translation="MDFSHAISLQITIKKDGSKLYQPFGISSKKGTPITTIVYENDIV "
/product="hypothetical protein"
/transl_table=11
CDS 80327..80662
/locus_tag="AUO97b_00071" /db_xref="SEED:fig|400667.82.peg.3723"
/translation="MSKKLKFNFKNIINLKPFDENEDNSRDDFTGSKTRSFFIYLAFF
CSIAGISAVFIYPMTKAPIILISLGCASSAILSGIEWHLGYKSKALNKLFEATVLLVL LILVRLFTW"
/product="hypothetical protein"
/transl_table=11
CDS complement(80694..81605)
/locus_tag="AUO97b_00072" /db_xref="SEED:fig|400667.82.peg.3724"
/translation="MLFNNNFGIDPLVGYHKPRIIACLNIKKFSQVESHIPSILHFCE
QHLQKMYGECIATVLVATPEEISIPDDDNLILNISLYNIFNLQNSHHYNEKQIGIEIQ
HCIKNFTPAHTDLSASSRDLINEQLSKMLVIANAVQKTVEEKELFKDGEQFIALDLKG
NPIVFNSTNAINNRISLGYGIVDAHDHLLTTFSGHLKTENIVRFLECLAQSQSNIENY
YNNFLSNRILSFLDGTLNFLDCSDPEFALEKCRKLYEESKSALLDIIDEICISDLNKF
LFNQFEKSINYVCLDFKDNLQDYCRLR"
/product="hypothetical protein"
/transl_table=11
CDS complement(81921..83024)
/locus_tag="AUO97b_00073" /db_xref="SEED:fig|400667.82.peg.3725"
/translation="MNVKPLDYLATIEHIFEHVKRTFYQTVADTKSINKGRLNAPITE
YKWHPCVFLPTNLWQEVLRPTIMAKLYEQVQINKTKDPFVIDAYFANFVAAFTWRYTK
SIYRFDEYTFECLIKHSAGKYLDHQLLTNLPEGCIYIDLATPLNIADHKVYGVYAILD
LSSNSIDTLKNTLPDQLIITLDTHNPFEENNFALLKPLPTIVFDIKRSGKVSQQIKFK
WGINLESPEELNEHIAPVISLISSICTDEIAIDNFTKNSTRQNVTKKPSFKDLEAAYW
QKGEYRVNAPANHKMWRIGVKYGVDYRAFVARQQKYESKTEYRITGVHWTAYIQDYEI
RLKFMPPRMNSPVEIDEQINNFFDELNASNSLK"
/product="hypothetical protein"
/transl_table=11
CDS 83198..85459
/locus_tag="AUO97b_00074" /db_xref="SEED:fig|400667.82.peg.3726"
/db_xref="GO:0003917"
/translation="MTDLVIVESSNKVDSIQGYLDSLFGRGKFKVSFSKGHIRDLPKD
SLGLTAEYTPNYVFNDSGASRVRELKALVEKSQKVYLATDPDREGEAIAWHLKTVLRL
NENYGRVTFQEITKTAVAQAMGNERKLNMQVVQAQETRRILDRIIGYIGTPAFTLICG
EKQIAGRVQSSIVWYLVDLENKIKQFSSVLHFGVTFNFGSVDESWEAKWDTSNFITKL
DPFVIDRKLAEEVSKLREFTVETFEEKPEKSSPPAPFTTPTYLRAAHVQLKLKTKRAM
EIAQKLFEDGHITYIRTDSPNLSDDAIQAIREFALKNKLPIPATPRKFKSKALAQEGH
EAIRPTDFNQLKVSDDSTIQAVYQMIWLRSVACQLDDATFTARIVRMKGRQLVQGREV
FVKARGRKLIDQGWLRLTAKLNFSEDENEDIPEDAAQELNNPVPLLQKAGNLVALKSD
LNEKRTEPPRRHSEASLIKFMEDVGIGRPSTFVSIVSKIIDHGYVHLDSKNKIHATDL
AQRLVGKISNQFEFCNLKYTAELEGKLDLIAHGENIGKRLLKETHLKIETQAKESIKN
LDQGGPKQYCSKCSEQLVNMTSANGKPYFKCTNLKCGHMMMNQDGIAIDRESLTSEFK
CIVCESGLISRSGQNGEFFGCSGFFKKRNKCTASYQKKPDGTPDYQAFLAKRELKESK
QEQGEKPVPKNTEKKEKEFNCPECKALLQQRTGKSKTTNKKWEIWSCSSPNCKNEYWG ERNKPIFDKKIVK"
/product="IncQ plasmid conjugative transfer protein TraB"
/EC_number="5.99.1.2"

/transl_table=11
CDS 85657..85863
/locus_tag="AUO97b_00075" /db_xref="SEED:fig|400667.82.peg.3727"
/translation="MMQVVAVMANKTSHIKYLLLGSYFFLFCTTFYLDNFFYGSTAGF
FVRFLCIYSILQGCFIIWLYEKNK"
/product="hypothetical protein" /transl_table=11
CDS complement(85912..86316)
/locus_tag="AUO97b_00076" /db_xref="SEED:fig|400667.82.peg.3728"
/db_xref="GO:0004375"
/translation="MDYQILDLQTIYAIKDRCKETLNIEENDYEEEKSFSKRFSYKLK
DNIKLDTKIFNECIDMDLKKLPLSQENLDILMNNEDYLKFKDNKSVRELLNKAKRDSF
ISIYEGLEIKDALYTEHLKFKDNNYKKNIKSL"
/product="Glycine dehydrogenase [decarboxylating] (glycine cleavage system P protein) (EC 1.4.4.2)" /EC_number="1.4.4.2"
/transl_table=11
CDS 86526..88313
/locus_tag="AUO97b_00077" /db_xref="SEED:fig|400667.82.peg.3729"
/translation="MPVNNDKITTIDCFPNDNISRVVYRYGGLVRNEDNDSTNPFVEV
LLIEIRKSDQWLFLDKCSTFLVPVLDLDAVQHGSIWDGNVLTNYTYRFSGKLITKRFS
FDFSNNKPRNIKLSDKIPDTSEYYIPLKNYFLPNKVDYVLQGYPFTKYTNDSYRKVNH
CLMHSNDGTQVITSSIHILHSLFVNRKDIRGLLLSTSGQSIINRFLESYTTEVENDNI
EYKIKIRKPYEDIGETAIIFLANLALNPYVQTIVDKIQRSMEVTEFHHLQHGTGIRYP
IVYPPHPTKLFLEAEGIWLDDNKTRFFITRVKKFDPINDHMIDVNKDLTNTVSKTDEK
NPRPREKSQNKNEHINTQKPPSRTSGEYRKRSDVETGNTQNILKYSFNEPANDPIEVG
TSNHPYSDKSEDVETSSDEPYGNKNTKTKKSETTDNPPSRDERFDIQYIIQSLNHLTL
DTTSPLERVYSIDILGNEIHGFNLLQIKKLVPQPKHPSWIDAEFGRKLLFLKLELKDQ
HDQYYLIDIHKNKSHEAFCAFIIVSPHKLTREQIKNICIELENAKGIKKWTLRCESFT
KKIISIKHSSATPDEWKNRFKNLFSTLIN"
/product="hypothetical protein"
/transl_table=11
CDS 88654..88887
/locus_tag="AUO97b_00078" /db_xref="SEED:fig|400667.82.peg.3730"
/translation="MYLSDLNAQQRAIVTKVQASLILSNRLETLGFIPQAEVRVITKG
IFGGDPILVQIGFTRFALRKSEAKCIYVEGLNS"
/product="Ferrous iron transporter-associated protein FeoA" /transl_table=11
CDS 88884..90731
/locus_tag="AUO97b_00079" /db_xref="SEED:fig|400667.82.peg.3731"
/db_xref="GO:0001882"
/db_xref="GO:0005525"
/db_xref="GO:0005622"
/db_xref="GO:0015093"
/db_xref="GO:0015684"
/db_xref="GO:0016021"
/translation="MTTDLRIALVGNPNCGKTSLFNHLTGTRQKVANYAGVTVEKKVG
HFTLPSSNKVRILDLPGTYSLSATSPDEAITRDVCNGLTEEAQQDAFICVVDATNLKL
HLGLVLEVLALGRPVLVVLNMMDEARRRGIQINIEKLSQHLGVPVVETVAVKSSGLTQ
LIDALDKEVFHTPHTDFTAKIDSPQKHIEYVLATTITDQGKVDTRTEFLDKILLHPIA
GLLILAITMFIVFQAVFAWANPFIEMLENLFSFIGEFLGSIITQPLLNSLVVDGIIGG
VGSIIVYLPQILILFFFIFVLEESGYLPRASFLLDKLMVSAGLSGRAFIPLLSSFACA
IPGIMATRTITDRTDRIATIMVAPLMTCSARLPVYALLIAAFIPNQTVAGIFNLQGVV
LFGLYIAGIVSALVVSLVMKLFRKNKENSLFLMELPAYRMPNWRSVFLGVYERAWIFI
RRVGGIIFALTILLWFLCTFPTPPENATLPAIDYSFAGMLGHLLQPIFAPIGFNWQIT
IALIPGLAAREVVVAALGTVYALSGSEDAQAETLVNLITADGTGWSLATGLSLLVWFI
YAPNCLATLATIRRETGSMRTVTIMVVYMFALAYLMAFFTYQLASYYWG" /product="Ferrous iron

transporter FeoB"
/transl_table=11
CDS 90912..91283
/locus_tag="AUO97b_00080" /db_xref="SEED:fig|400667.82.peg.3732"
/db_xref="GO:0003700"
/db_xref="GO:0005622"
/translation="MLEKNALSAFAALSQETRLRIVRLLVVAGAEGMASGQIAEALDG
AAPSRISFHLSHLENAGLIYSRREGRQIIYCAIWSTLADLVAFLMHDCCQGHCEYCDK
AIELFAKCTGEPTEGYIKIGE" /product="Transcriptional regulator, ArsR family"
/transl_table=11
CDS 91321..92283
/locus_tag="AUO97b_00081" /db_xref="SEED:fig|400667.82.peg.3733"
/db_xref="GO:0006814"
/db_xref="GO:0008508"
/db_xref="GO:0016020"
/translation="MENLRSLLEDHQVKIYFITIFIGTIFGLTISGVSSLESAINPAL
ALMLFATFLQVPIAEIGKAFKNLKFISALLITNFIALPAITAGLIQFLPENPLIRLAV
LFVLLAPCIDYVITFTHVGKGNARLLLAMTPILLLIQMILLPVYLGTLLGDEVSQLVE
MEPFLHAFIWFIIVPLTLAGIVQFISNKSESLATLSAPLNLLPVPATALVLFIVLASV
TPQIGLAQSAALQALPIYIAFAIIAPFIGWAIASLFKLESSSARAVSFSASYRNSLVI
LPLALVVPGSMPIIPAVILTQTIVELCFLPIYTRLIPRMFPSKD" /product="Putative sodium-
dependent transporter" /transl_table=11
CDS complement(92529..93443) /locus_tag="AUO97b_00082" /db_xref="SEED:fig|
400667.82.peg.3734"
/translation="MIAAGCADLCADCYWHKSLWNKFDQNQNAFEFTYLKQQYESYID
WLEKKVGSHKATLYINKHTHFFLKTEVNWNQSVPTPMQLLAVLRSSGLRKFELVMQWL
EEMHGIQVDTDNKKGCSERDQMEKLVQSILQPSLAYDVVFGYKNKLEEKIKRGDTSIR
SARLAVKPAVALMLSIGSESTQLPNLEHIKAYLADYSGQAAALTGFINFLNENYGTSI
DYLKLKKSDFLRIKQKQKLEKAIVALAQTDLSNREELIGWVRNGLRYFHQLSYIDSLK
VKVEMITEVNNGFAVRFNEQSYWLPNNQ"
/product="FIGfam110555"
/transl_table=11
CDS 93750..94067
/locus_tag="AUO97b_00083" /db_xref="SEED:fig|400667.82.peg.3735"
/translation="MTKLKYTPEIRERAVQLVIESEKDYPSNWAAVSAIAPKIGCTPE
TLRVWYQKYLDQQNPIKVQQFSDQERIKQLERENKELQRANEILRKAAAFFAQAELDR PYK"
/product="Insertion element IS401 (Burkholderia multivorans) transposase"
/transl_table=11
CDS 94067..94969
/locus_tag="AUO97b_00084" /db_xref="SEED:fig|400667.82.peg.3736"
/translation="MVDFIHNNKALYGVDAICRILPIAASTYYRTLDLVNNPEFRAKR
ALHDLHHAEQIKRIWKESLGRYGIRKVWQQLRREGYVIARCTVVRLMRKLNIQGVWRG
KNKQTTRSRDDQKRADDLVKRNFKADHPNHLWVADFTYIQTNSGWVYTAFIIDVFSRA
IVGWKVSTRMNTDMVLDALEQALHDRGMPKNVIHHSDRGVQYLSIRYTNRLEAANLRA
SVGTTGDSYDNALAETVNGLYKTEVIEYLKADWQGLADVQLATLNWVDWFNKKRIHSA
LGYVSPFDFEAMYYDKINPLGQVA"
/product="Transposase OrfAB, subunit B"
/transl_table=11
CDS complement(94966..95286)
/locus_tag="AUO97b_00085" /db_xref="SEED:fig|400667.82.peg.3737"
/translation="MELGLLTENVTEHDCHGCNQAVSFIKKRYKGKKYCSTCYARIFK
RRICSGCGDFARLPRDDEQAICNECIKKQPCIRCNQSNKPIGKLTEYGVEPYRVCRRL FYLS"
/product="FIGfam110555"
/transl_table=11

CDS complement(95264..96274)
/locus_tag="AUO97b_00086" /db_xref="SEED:fig|400667.82.peg.3738"
/translation="MVLVEALWGKWRALTIDLDKGVDAEKFDALLSHSIETRQNIQQS
MLSSFVSEIQPNYDPKQTIPWVLSLGTRNRSNTSGRQVCVECLKSRENPPYFRLMWRM
GWHCSCDRHQVSLIDHCPECGVTLQAFKTDLEHGCLAICTACGFDLRHCKQSQNINLN
SLNFQNKADQVLKQKVGFYNQSIITSQEWFEIARVWLSEIRFLINTSNKNVIQLFESF
DVNLHLSHPVTPLAFEYLNTQERIVLLSILDQIMDIPCDLLVQRSNEYGISHANFWDK
RKKLPVQLQQMKDLMIKPTRRYPVSRAAITVTKPKSKASVQRQWLNLLRRSNNSGARL ID"
/product="Mll9366 protein"
/transl_table=11 CDS complement(96406..97128)
/locus_tag="AUO97b_00087" /db_xref="SEED:fig|400667.82.peg.3739"
/translation="MGKTTIIHEFARQYYTKTVSDADMDLLSVTKPVLPILAPAKANV
KELYINILNHFFVPFRASDPEAKLRNQAVHLMRKYETKMLIIDEIHNCLTGSAKLLPE
VMNTLKNLSNELSLNVIGVGTREAITILHTDSQYASRFDVVNLPKWELNQDFLRLLMS
YVRLLPLKKQSNLASKEIATLIFEVSGGNLGDLNRLLVECAKEAILQGEEEITYDIVH
KFKWLKPTEGLRNIRNINLSVL"
/product="TniB NTP-binding protein"
/transl_table=11
CDS 97261..97437
/locus_tag="AUO97b_00088" /db_xref="SEED:fig|400667.82.peg.3740"
/translation="MPLSPRNCGLFFVCVWHDLATGGSAKHRLSMALKLVLWFGDAMI DADTMLRSFVCSWY"
/product="hypothetical protein"
/transl_table=11
CDS 97428..98729 /locus_tag="AUO97b_00089" /db_xref="SEED:fig|
400667.82.peg.3741"
/translation="MVLIFYKLLFRKKKYDKFEIFSAWYRRRRRTRYMLTDTKLRNLK
PRDKLYKVNDREGLYVAVTPAGSISFRYNYSINGRQETITFGRYGVGGITLAEARELL
GDAKKMVAAGKSPAKEKARDKARVKDAETFGAWAEKWLRGYQMADSTRDMRRSVYERE
LKPKFSNQKLVEITHEDLRALADAIVERGAPATAVHVREIVLQVFRWAIERGQKVENP
AELVRPTSIARFEPRDRALTPEEIGLMYQYMERVGTSPTNRAAAKLLLLTMVRKSELT
NATWSEINFSEALWTIPKERMKRRNPHLVFLSQQALDIFIAMKTFAGGSDFVLPSRYD
SDAPMSAATLNQVLTLTYKAAQKDGKSLTKFGPHDLRRTASTLLHEAGYNTDWIEKCL
AHEQKGVRAVYNKAEYREQRAAMLQDWADMIDEWTSGGSKG" /product="Integrase"
/transl_table=11
CDS 99103..99930
/locus_tag="AUO97b_00090" /db_xref="SEED:fig|400667.82.peg.3742"
/translation="MALDLMAAFTELPPPIDYVLPNMVAGTVGALVSPGGAGKSMLAL
QLAAQIAGGPDLLEIGEFPTGQVVYLPAEDPPAAIHHRLHALGAHLSAAERQAVADGL
LIEPLIGKCPNIMAASWFDALKRAAEGRRLMILDTLRRFHIEEENASGPMAQVVGHME
AIAADTGCSIVFLHHASKSAAMMGSGDQQQASRGSSVLVDNIRWQSYLSGMTQGEAEI
LGVDDCQRGYFVRFGVSKANYGAPFQELWFRRHDGGVLKPAVLERQCKVKRRQREEA"
/product="Replication protein RepA"
/transl_table=11
CDS 99917..100798
/locus_tag="AUO97b_00091" /db_xref="SEED:fig|400667.82.peg.3743"
/translation="MKKPKHDLTHVRHDPAHCLAPGLFRSLKRGDRKRCKLDVTYTFG
EDESMRFVGFEPLGADDMRLLQGIVALGGPNGILLTPEPTSETGRQLRLFLEPRFEAI
EQDGLVVRESLTKLLSETGMTDSGDNIKALKASLLRMSNVTILVTKGRRQAAFHLMSH
AFDETDGRLWVALNPRIAEAILGHRPYARIDMAEVRVLQTDPARLMHQRLCGWIDPGK
SGRVELDTLCGYVWPDEANAEAMKKRRQTARKALAELAAVGWVVNEYAKGKWEIKRPG PTATAPVYRRNVPLLPS"
/product="Replication protein RepC"
/transl_table=11
CDS 101493..101624
/locus_tag="AUO97b_00092" /db_xref="SEED:fig|400667.82.peg.3744"

/translation="MSSDFEATMSIAGRFGDDYGYVAKNRCLFDDKTLYVRYALDFQ"
/product="hypothetical protein"
/transl_table=11
CDS 101831..102019
/locus_tag="AUO97b_00093" /db_xref="SEED:fig|400667.82.peg.3745"
/translation="MANVLIRNLPAEVHRALKVRAALNDRSTDAEIREILTAAVQPLE
LEEIGREVGLSESDKKTE"
/product="IS, phage, Tn; Transposon-related functions" /transl_table=11
CDS 102088..102939
/locus_tag="AUO97b_00094" /db_xref="SEED:fig|400667.82.peg.3746"
/translation="MKRCREQAQRLVKAMPSFHRRSTTMKSKILAAKKTALAVALATG
FITTTTAPVQAGIPVIDGGNLAQNIMTAIESVAQTLKQIEQYQTQLQQYENQLQNTMA
PAAYIWDQAQTTINRLIAAQNTLAYYENQLGSLDRYLAKFQDVAYYRSSPCFNGSGGC
TPAEKAAMEENRRLASESQKKANDALFQTVADQQKALKDDARTLERLQGAAQGATGQL
QAIGYANQLASQQANQLLQIRTMLTAQHNAEAARIAAELDAEARGDARAEQMRTWTFR PSPADNY"
/product="Conjugative transfer protein TrbJ" /transl_table=11
CDS 103073..104626
/locus_tag="AUO97b_00095" /db_xref="SEED:fig|400667.82.peg.3747"
/translation="MKCAAKRTERPLPRNAEHSRRPSRCVSGSSSPALKMIIEVVAMR
KKIALGGLMMVATMVLAEPAMAQELSSSGVMNDVLKRFHDAAATWGPAIESAASRLFW
TLVVISMVWTFGMMALRKADIGEFFAEFVRFTIFTGFFWWLLTNANQGMNIAGTIVQS
LQTLGAQAGGLSNSNLGPSSILDLGFELYNRTVQATSELGWRQMATALVMELMALAVL
FVLALIAVNLLLLLASAWILLYAGVFFLGFGGSRWTSDMAINYYKTVLGLAAQLMAMV
LLVAIGKEFINHYYTQISENMASQELAVMLVISVILLFLVNKVPPMISGLVSGGGIGA
AGGIGNFGAGAAVGAAVTAASMATGGAALAGKAVMGAAAGAAGGASALQAAFQKASAS
METGGDMSSMGSVVSSGGNGGGEAGTAGSSPFAQAAGFGDSGSSSSGGGFAKAAKLAT
GTASELAKGVGSQVKQGFQERVSETTGGKLAASIRESMEPKEASQSGQFEGNSLGADS GPDSNEVRS"
/product="Conjugative transfer protein TrbL" /transl_table=11
CDS 104943..105167
/locus_tag="AUO97b_00096" /db_xref="SEED:fig|400667.82.peg.3748"
/translation="MTTQHIIEPGQAVHQAAAILSSLEYINQAEARSLGPLAEAVANA
FMVVYYQAETGRATQADFQEAMNALRQACS"
/product="hypothetical protein"
/transl_table=11 CDS complement(105522..106589)
/locus_tag="AUO97b_00097" /db_xref="SEED:fig|400667.82.peg.3749"
/db_xref="GO:0006814"
/db_xref="GO:0008508"
/db_xref="GO:0016020"
/translation="MSAGAQAIAAKPRQAPMSGFERYLTLWVALCIVAGVALGQGLPD
VFQAIGRMEVAQVNLPVGLLIWVMIVPMLVKIDFGALHQVKEHWRGIGVTLFVNWAVK
PFSMALLGWLFIRQVFAPFLPADQLDSYIAGLILLAAAPCTAMVFVWSRLTNGDPLFT
LSQVALNDAIMIVAFAPIVGLLLGMSSISVPWDTLLISVVLYIIVPVILAQLLRRHLL
KQGQAAFERAMQKIGPWSMAALLLTLVLLFAFQGEAIIRQPLVIAMLAVPILIQVFFN
SGLAYWLNKRAGEKHSVACPSALIGASNFFELAVAAAISLFGLHSGAALATVVGVLVE
VPVMLLVVRVVNRSKSWYERG"
/product="Arsenical-resistance protein ACR3" /transl_table=11
CDS complement(106586..107092)
/locus_tag="AUO97b_00098" /db_xref="SEED:fig|400667.82.peg.3750"
/db_xref="GO:0008794"
/translation="MTERIYNVLILCTGNSARSIMAEALINTMGQGRFRAYSAGSHPT
GKVNPFAVEKVESVNYPTENLRSKSWDEYATPDAPKMDFIITVCDNAAGEMCPVWPGQ
PISAHWGFEDPAAVEGTDAEKRRAFEQTFRHMMNRVRLFVNLPLKMLDQTAIKRELAN IGKTEQEA"
/product="Arsenate reductase (EC 1.20.4.4) thioredoxin-coupled, LMWP family"
/EC_number="1.20.4.1"

/transl_table=11
CDS complement(107089..107856)
/locus_tag="AUO97b_00099" /db_xref="SEED:fig|400667.82.peg.3751"
/translation="MRLRHLSDPDSLPALDKSFAIERPALGLAPDAPPVRILLLYGSL
RARSFSRLAVEEAARLLQFFGAETRIFDPSDLPLPDQVQSDDHPAVKELRALSEWSEG
QVWCSPERHGQITSVMKAQIDHLPLEMAGIRPTQGRTLAVMQVSGGSQSFNAVNTLRL
LGRWMRMFTIPNQSSIAKAFQEFDAAGRMKPSPYYDRIADVMEELVRFTALVRPHREA
LTDRYSERKAAGHVIDEATDLSSIAIAPQPLPESETS"
/product="Arsenic resistance protein ArsH"
/transl_table=11
CDS 108333..109070
/locus_tag="AUO97b_00100" /db_xref="SEED:fig|400667.82.peg.3752"
/translation="MRRTKPVAAPMVARVYLRVSTDAQDLERQEAITTAAKAAGYYVA
GIYREKASGARADRPELLRMIGDLQPGEVVIAEKIDRISRLPLPEAERLVASIQAKGA
RLAVPGVVDLSDLAAEAQGVAKIVLEAVQIMLFRLALQMARDDYEDRRERQRQGIELA
RQAGRYKGRRADPKRRAQVVALRKSGYSINKTAELAGYSAAQVKRIWAEVSQAEAKQH
GAFVEDALTEADALAAVGQDERQEERA"
/product="plasmid replication and transfer functions" /transl_table=11
CDS 109502..110995
/locus_tag="AUO97b_00101" /db_xref="SEED:fig|400667.82.peg.3753"
/translation="MPHVAARTASRDRDTGRYQSHRPEQTLLYQIVDEYYPAFAALMA
EQGKELPGYVQREFEEFLQCGRLEHGFLRVRCESCHAEHLVAFSCKRRGFCPSCGARR
MAESAALLVDEVLPEQPMRQWVLSFPFQLRFLFASRPEIMGWVLGIVYRVIATHLVKK
AGHTHQVAKTGAVTLIQRFGSALNLNVHFHMLFLDGVYVEQSHGSARFRWVKAPTSPE
LTQLTHTIAHRVGRYLERQGLLERDVENSYLASDAVDDDPMTPLLGHSITYRIAVGSQ
AGRKVFTLQTLPTSGDPFGDGIGKVAGSSLHAGVAARADERKKLERLCRYISRPAVSE
KRLSLTRGGNVRYQLKTPYRDGTTHVIFEPLDFIARLAALVPKPRVNLTRFHGVFAPN
SRHRALVTPAKRGRGNKVRVADEPATPAQRRASMTWAQRLKRVFNIDIETCSGCGGAM
KVIACIEDPIVIKQILDHLKHKAETSGTRALPESRAPPAELLLGLFD" /product="Mobile element protein"
/transl_table=11
CDS complement(111560..112426)
/locus_tag="AUO97b_00102" /db_xref="SEED:fig|400667.82.peg.3754"
/db_xref="GO:0004156"
/translation="MSYLFLSCTEIIIIEAPMNKSLIIFGIVNITSDSFSDGGRYLAP
DAAIAQARKLMAEGADVIDLGPASSNPDAAPVSSDTEIARIAPVLDALKADGIPVSLD
SYQPATQAYALSRGVAYLNDIRGFPDAAFYPQLAKSSAKLVVMHSVQDGQADRREAPA
GDIMDHIAAFFDARIAALTGAGIKRNRLVLDPGMGFFLGAAPETSLSVLARFDELRLR
FDLPVLLSVSRKSFLRALTGRGPGDVGAATLAAELAAAAGGADFIRTHEPRPLRDGLA VLAALKETARIR"
/product="Dihydropteroate synthase type-2 (EC 2.5.1.15) @ Sulfonamide resistance protein" /EC_number="2.5.1.15"
/transl_table=11
CDS 112552..113037
/locus_tag="AUO97b_00103" /db_xref="SEED:fig|400667.82.peg.3755"
/translation="MLVIILKQTCNLSSASKALPIKCLPQSFYRRMQRFFAGQYFDYR
QISQLIFNMFSFDQVQLTLDRTNWKWGKRNINILMLAIVYRGIAIPILWTLLNKRGNS
DTKERIALIQRFIAIFGKDRIVNVFADREFIGEQWFTWLIEQDINFCIRVKKTSLSPI I"
/product="Transposase"
/transl_table=11
CDS 113124..113558
/locus_tag="AUO97b_00104" /db_xref="SEED:fig|400667.82.peg.3756"
/translation="MKLYISALQLENGELLLVVSPQFNANAIQDYALRWEIETLFSCL
KGRGFNLENTRLTDPRRVKKLIAVLAISFCWCYLTGEWQHDQKKAIKIKKHGRLSMSL
FRYGLDYVQMAIQRLIGFGKKEEFKEILAILRRQNPDRIRVL" /product="hypothetical protein"

/transl_table=11
CDS complement(113805..113975)
/locus_tag="AUO97b_00105" /db_xref="SEED:fig|400667.82.peg.3757"
/translation="MEKYEYLAEHVLEIMQLSDSERIETLFTDRWIGYKKAHSIVNKT FLSFIFNGLFSC"
/product="TniB NTP-binding protein"
/transl_table=11
CDS complement(113980..115890) /locus_tag="AUO97b_00106" /db_xref="SEED:fig|
400667.82.peg.3758"
/translation="MIESNAVSIDRSRITLKPNVVVGYEGNPYKIVNVLNANDIVISS
LDSVRSLQVNSKSLTVFEGENTLTENLNKGDKDISNEAWQIALQRYEIIKPLIEYSTT
ELVENRANEYDVNRSTLWKWLRDYRENNSLMTLIPKKRGWTTEKSRLSPQVSNIIKQA
INDEYLNAKKPSISKTIEIVKAECSRLQLEAPHENSIRRRIEALNNYQVTKARLGSKA
AIDKFKAAAGSFPNADYPLAYVQIDHTPLDIEIVDDEFREAIGKPHLTLAIDVFSRMI
VGYYLSLEAPSTTSVAMCIASCILSKKRKLIELNIDAEWQVEGIMDSVHTDNGPDFRT
NHISKACLKYGIHWEYRPIGGARFGGHIERMLGIVNLEMHVLDGTTFSNVQQRGTYDS
AKQACMTLKELEYYIVYWITKVYHQKKHSALGTSPIVKWEEGVWGTKTTAGTGLKERV
SDEDTLFIDFLPEFEATIQRTGVQKDNLFYFADCLRQWVNSIDPDDNNRKRKRKFLFK
RDPRDISQIWFYEPFSNTYFKVPTAKREIPPISLFEYRQVQNYLKNGRQDVQNQDEIY
KAILHLREQLNQARSLTRKQRRSNQRKKENEKAITQLSEQNQSKKAVVSESLQTSDDL WSTPLTAFDDLR"
/product="TniA putative transposase"
/transl_table=11
CDS complement(115891..116601)
/locus_tag="AUO97b_00107" /db_xref="SEED:fig|400667.82.peg.3759"
/translation="MKKLSDEYLPVRKAQTVYGSISGNYAFRGEKTIWFESTLERDFI
LKQEFNNNVIDVIGQPVVIPYITELGNQSNYTPDFLVQFSSSNCDDFEDFPVPMLIEI
KPKKKLAEDWDKLKPKFRAAHRFAADKGWKFKIISETRLYDQYWENINFLKKFRRSHI
DAVDQQLLLDTLQKLGQSTINQLPAHLFKDKTNVLKGIHQVWALVAKRMIACDLYCPL
TAETVIWVNQNDAFVRNI"
/product="FIGfam050825"
/transl_table=11
CDS complement(116913..117182)
/locus_tag="AUO97b_00108" /db_xref="SEED:fig|400667.82.peg.3760"
/translation="MASTAAERKAKQRQEMLKKGFTRKDLWLSKESLEVIEKFKSEHK
LSSNDEAINLLLKTIVVIEKFKSEHKFNTIDEVINLFLKSLNECK" /product="hypothetical
protein"
/transl_table=11
CDS complement(117236..117592)
/locus_tag="AUO97b_00109" /db_xref="SEED:fig|400667.82.peg.3761"
/translation="MKNSVSNSYIVQANGVKVVKADVNSIVTTNKRIHVGDDTPAFAF
YVCGFWDSIDQFERYGMTPHVTLYKTESEVKEYCKGKVVSYREYYANSEKEVDDLIAT NRMSHENHARSLAELF"
/product="hypothetical protein"
/transl_table=11
CDS complement(117638..117955)
/locus_tag="AUO97b_00110" /db_xref="SEED:fig|400667.82.peg.3762"
/translation="MNEVKAYKLTVENVELFEEIEHEVLFQTTIKTKKSILNYLTPVS
TLATLLQRHNIILPKGIFLGSIIEIYDEEVEPRVIIGHEGCDFGNVKTVRQLEIYWEE VAF"
/product="hypothetical protein"
/transl_table=11
CDS complement(118162..119094) /locus_tag="AUO97b_00111" /db_xref="SEED:fig|
400667.82.peg.3763"
/translation="MLDREILLKKWLDEYLLANKSNETIYTLKNSLNLFFEYTNLALE
EIEASDIRTFIAYRAQNGVGVSTLKKNISAIRTFFHYLTKEKLINFNPAEDIKIKKSS
KVLPKFHEVTVINEVLDSNKNLEFERPSSTVIFKRDLALIEIAYSCGLRLEEIHSLQI
ENVEVKRKQVRVTGKGNKTRIIPLGSKAIEAYLEWLPLREEIMNKDTNQKHNYVFVTT

TGAQLSRVQINKRIKNAFKLAGYPIQSNPHMLRHSFATHLINNSVGIREIQEMLGHSN
LNTTQIYTDLDHTSMTNVYMDTHPRAVKNTEGEN" /product="Site-specific tyrosine
recombinase XerC" /transl_table=11
CDS 119195..119422
/locus_tag="AUO97b_00112" /db_xref="SEED:fig|400667.82.peg.3764"
/translation="MNSKFSVDQSNKISLKAVLEDLGDQMFEANKCPDTHPVYDQLRE
RLRKDRQLLDALDAQDENANTEERSRSLDIN"
/product="hypothetical protein" /transl_table=11
CDS 119423..120925
/locus_tag="AUO97b_00113" /db_xref="SEED:fig|400667.82.peg.3765"
/translation="MTQKNELDTKYAVIVAGGNGAGKSTLIDNVIIPKFNSLNLDINF
INADVWQLQHFGHFDNTNPTHAREAQKWAEAERQKHLDEGRSFIAETVFSHPSKVDLI
KEAKSKGFYVVLYHIHLENSDIALDRIKDRVRKGGHDVDEDKVKARYERVLPLVAEAT
QYADLTFVLDNSVRDRPHQQVFKLEYGKITAIDNNVPDWAIKAYEKQLTDYLGLDQAT
KISNTNEDLTMSNTKEKLGFEYNARLLANDIQPQHRETVVANYRKNFVEHVANSAAVA
GNSHKLDDVASLINGTTVGGHSVKDQRQVLNLIDASNELVNMVRNGNFALNKSTYLKV
NGIVADRESIEAGVLRGEGVEQSFTPDVGIRHFRTHRPMKTEEDAPALNKFMQEGFDH
INSLKNPLEKGIATFLYGSLNQFTFEGEKRTAHLMMNGVLMSAGLDAISIPADKAIDF
RQKMVRFYESKNADEVMEFLADLHPALNNTKKLDAEKEKKSENDFGRSFQ" /product="Predicted
kinase"
/transl_table=11
CDS 121010..122173 /locus_tag="AUO97b_00114" /db_xref="SEED:fig|
400667.82.peg.3766"
/translation="MASLNSEFQIPLKVVTTRLNHMKINLPQLVKKIEKRAIENLKRR
KDKKEQIDLLITWDDWIYAALDADHERIHGSADPHPLPPTFKNNMVSGVYEKALKNVM
GHFGVKATVPAWAANIKVGEHIPKELLDRLPVTTGELVTLGNWMLTKNIYRFEDIVIN
ELIKTGFNGVIPNHILNLPDLCVYIQTDNAKGLTFENRQVVGVLFCVTELCGDRLLVS
TMYLDDGMPRTIAIMLNEDQDIEASLTNFVDQFQQDYDPETMASDLKERLKIQKKLIN
LVLWFSQSKPEVTPLTPDTNKPVQFVEIKKEKRLFEAGKYKTFKIGSETARKLTKLYE
EIEVAKAEGKASGREPHLRKSHWHLYWYGKKGRYERYDFLLIPVTIVGGVSKD" /product="FinQ"
/transl_table=11
CDS 122194..122775
/locus_tag="AUO97b_00115" /db_xref="SEED:fig|400667.82.peg.3767"
/translation="MDNSESKILAEYEKAKKWDKYEKVFSILGVLSLFIFMVMIIISA
ITLSLNDTSPTKIFNSYCWAAILFLAVLFTSLRNIAEQISFKKSMEILIDSKDAEDEK
DQLAVVRRQHTIICITAFCALIVADFILFYFIAFNLTLPLNILIHKPLFIIMLACLIF
FTINYLIYKYKFNKLNNEAMVAFGKHLDNKRKK"
/product="hypothetical protein"
/transl_table=11
CDS 123062..124858
/locus_tag="AUO97b_00116" /db_xref="SEED:fig|400667.82.peg.3768"
/translation="MPVNNDKITTIDCFPNDNISRVVYRYGGLVRNEDNDSTNPFVEV
LLIEIRKSDQWLFLDKCSTFLVPVLDLDAVQHGSIWDGNVLTDQSYRFSGKLITKRFS
FDFTNNKPKNIKLTDRIPNTSDYYIPLKNYFLPNKVDYVLQGYPFTKYTNDSYRKVNH
CLMHSNDGTQVITSSIHVLHSLFVNRKDIRGLLLGTSSQSIINRFLESYTTEIVDDNV
EYKIKIRKPYEDIGETAIIFLANLALNPYVQTIVDKIQRSMEITEFNHLQQGTGKRYP
IVFPPHPTKLFLEAEGIWLDDNKTRFFITRVKKFDPINDHMIDVNKDLTNTISKPDEK
NPRPREKSQNKNEHINTQKPPSRTSGEYRKRSEVETGNTQGILKYSFNEPTDDPIEVG
ASNQPYSDKSEEVETSSDEPYGNQNTKIKKSETTDTPPSRDERFDIQYIIQSLQELAS
EMGSPLEHLSAINEHGEMIASINLLQIKKLVPEPKHPSWIDYDKGRKLLFLKLDLKDQ
NGFSYLIDIHKNKNHEAFCAFLIFTQNKLTSEQIKKICIELENAKGIKKWALYCDSFI
HKMIAIKHLYATPDEWKNRFKNLFISLQKDKS"
/product="hypothetical protein"
/transl_table=11

CDS 125153..126640
/locus_tag="AUO97b_00117" /db_xref="SEED:fig|400667.82.peg.3769"
/db_xref="GO:0005215"
/db_xref="GO:0006810"
/db_xref="GO:0006813"
/db_xref="GO:0008324"
/db_xref="GO:0015137"
/db_xref="GO:0015746"
/db_xref="GO:0016021"
/translation="MLSTIREQWFSNIRGDVLAGIVVALALIPEAIAFSIIAGVDPKV
GLYASFCIAVVIAFVGGRPGMISAATGAMALLMVTLVKEHGLQYLLAATILTGFIQIL
AGYLKLGALMRFVSKSVVIGFVNALAILIFMAQLPELTNVTWHVYAMTIGGLAVIYLF
PYIPVIGKLLPSPLICIVLLTLFALFIGLDVRTVGDMGQLPDTLPIFLLPDIPLNLET
LAIILPYSLGLAAVGLLESMMTATIVDDLTDTTSDKNRECKGQGVANVASGFLGGMAG
CAMIGQSIINVKSGGRTRLSSFSAGVFLLIMVVFISDWLKAIPMAALVAVMIMVSIGT
FNWDSLRNIRQYPLSSNIVMIVTVVVVVATHNLAYGVLVGVLLSALFFANKIERYMAI
QSVFDESESTRTYTVTGQVFFSSADKFTSAFDFKEALSKVVIDVSQAHFWDVSAVAAV
DKVVIKFRREGTEVELIGMNEASKTLVDKFGIHDKPDALDRLNSH" /product="Sulfate permease"
/transl_table=11
CDS 126653..127504
/locus_tag="AUO97b_00118" /db_xref="SEED:fig|400667.82.peg.3770"
/translation="MSKIIACIDGSLVTNTVCDYAAWFSDKLNSPIKLLHVIDKPKAK
APQDLSGAIGLGSRETLLKELVELEERKGKIELEHGQILLREAKNYLLEKFSIDAQSF
QRHGSVLETIMGMEDDIRVLVMGKHGNETEHDSSKIGTHIENVVRALHKPVLITSAPF
SPPKSFLIAFDGSQTARKCVERIASSPLLKGLAVHLVYVGNPNSEMQNQLSWAKEQLE
SQGFNITSNTLDGEVDKAIINYAEQHQIDLIVVGAYGHSKIRQFFIGSTTTKVITSAN KPVLLLR"
/product="UspA-related nucleotide-binding protein" /transl_table=11
CDS 127944..128315
/locus_tag="AUO97b_00119" /db_xref="SEED:fig|400667.82.peg.3771"
/translation="MGQAKQRGTLEQRVKQAQEQKDDFYGTERSVAEVLAELGLPKES
TVKGYVIHLPEKDEFVAAINDNNISFSVAYARTPELAMIYDEPQKAISDAKKISKHKL
LVCVLFETSNQYMINDIWANY"
/product="hypothetical protein"
/transl_table=11
CDS 128424..128543
/locus_tag="AUO97b_00120" /db_xref="SEED:fig|400667.82.peg.3772"
/translation="MIFIFMSLKLYCVNTAFKASLGDFLGGGDNLINTWKSTL" /product="hypothetical
protein" /transl_table=11
CDS complement(128690..130120)
/locus_tag="AUO97b_00121" /db_xref="SEED:fig|400667.82.peg.3773"
/translation="MTENVIEHDCHGCNQSVSFIKKRYKGKKYCSTCYARIFKKRLCP
SCGDFARLPRDDEQAICNECIKKQPCIRCNQTNKPIGKLTEYGVVCNSCSVYFRPIEP
CERCGTPSQKLTRISPFNDDLRVCPKCATRDYETCPSCQKHRLLESDVSGQRTCKKCR
DKPQKSCKACHCMIAAGCADLCDDCYWHQNLWNKFDQNQKVFESSDLKQQYENYIGWL
EKKVGSHKAALYINKHTHFFIKTEIDWNQSVPTPKQLLVRLRSSGLRKFELVMQWLEE
VHDIRIDMDNKKSCSERDQMEKLVQRILQPSLAYDVVLEYKNKLEEKIKRGETSIRSA
RLAVKPAVALMLSMEGESAQLPNLEHVKAYLAEYSGQAAALTGFINFLNENYGASIDY
LKLKKSDFLKTKQKKKLEMELIALTQTDLNDSELILSWVRNGLRYFHQLPYIDALKIK
TEMITEIEDGFTVVLNGQYYWLPKTQ"
/product="FIGfam110555"
/transl_table=11
CDS complement(130113..131228) /locus_tag="AUO97b_00122" /db_xref="SEED:fig|
400667.82.peg.3774"
/translation="MSQPEQCWYIRTPIQQGEIFSSWLIRSALDVGCSPMVLIEALWG

KWRALTIDLDKGVDAERFDALLSHSMESKQKIQQSMLSSVVSQIQPNYDSNQNIPWVL
SLGTRNRSNTSGRQVCVECLKSHENPPYLRLMWRIGWHCSCVEHQLSLIDHCPECGVT
IQPFKADMEHGCLAICTTCGFDLRRCEESKNINLNALNFQNKAEQVLKQKIGFYNQSP
VTTQVWFEIARSWLSEIRFLVNTPNKNVIQLFESFDVNLHLSHPVTPLAFEYLSTQER
IVLLSMLDQIMDIPCDLLVQRSKEYGVSRANFWDKRKKLPVQLQQMKDLMIKPTRRYP
VSRAAITVTKPKSKATVQRQWLNLLRRSNNSGAMHID"
/product="Mll9366 protein"
/transl_table=11
CDS complement(131258..132178)
/locus_tag="AUO97b_00123" /db_xref="SEED:fig|400667.82.peg.3775"
/translation="MEKYEHLAEHVLEIMQLSDSERIETLFTDRWIGYKKAITIMNTL
TDILNRPRKLRPECLLIVGDSNMGKTTIIHEFARQYYTKTVSDADMDLLSVTKPVLPI
LAPAKANVKELYINILNHFFVPFRASDPEAKLRNQAVHLMRKYETKMLIIDEIHNCLT
GSAKLLPEVMNTLKNLSNELSLNIVGVGTREAITILHTDPQYASRFDVVNLPKWELNQ
DFLRLLVSYVRLLPLKKQSNLASKEIATLIFEVSGGNLGDLNRLLVECAKEAILQGDE
EITYDIVHKFKWLKPTEGLRNIRNINLSLS"
/product="TniB NTP-binding protein"
/transl_table=11
CDS complement(132183..134093)
/locus_tag="AUO97b_00124" /db_xref="SEED:fig|400667.82.peg.3776"
/translation="MIESNAVSIDRSRITLKPNVVVGYEGNPYKIVNVLNANDIVISS
LDSVRSLQVNSKSLTVFEGENTLTENLNKGDKDISNEAWQIALQRYEIIKPLIEYSTT
ELVENRANEYDVNRSTLWKWLRDYRENNSLMTLIPKKRGWTTEKSRLSPQVSNIIKQA
INDEYLNAKKPSISKTIEIVKAECSRLQLEAPHENSIRRRIEALNDYQVTKARLGSKA
AIDKFKAAAGSFPNADYPLAYVQIDHTPLDIEIVDDEFREAIGKPHLTLAIDVFSRMI
VGYYLSLEAPSTTSVAMCIASCILSKKRKLIELDIDAEWQVEGIMDSVHTDNGPDFRT
NHISKACLKYGIHWEYRPIGGARFGGHIERMLGIVNLEMHVLDGTTFSNVQQRGTYDS
AKQACMTLKELEYYIVYWITKVYHQKKHSALGTSPIVKWEEGVWGTKTTAGTGLKERV
SDEDTLFIDFLPEFEATIQRTGVQKDNLFYFADCLRQWVNSIDPEDNNRKRKRKFLFK
RDPRDISQIWFYEPYSNTYFKVPTAKREIPPISLFEYKQVQNYLKSERQDVQNQDEIY
KAILHLRDQLNQARSLTRKQRRSNQRKKENAKAITQLSEQNQSKKAVVSESLQTSDDL WSTPLTAFDDLR"
/product="TniA putative transposase"
/transl_table=11
CDS 134505..135467
/locus_tag="AUO97b_00125" /db_xref="SEED:fig|400667.82.peg.3777"
/translation="MTEPNSISCTQLAETYNISHDSVNRFLEREDYTPHDLYQEAIQH
IDNNKLIVSIDDTVLDKPYSQHMDLVSYFWSGKHHRSVKGINLITLYATDQNGQNIPI
NFRIYDKSESKTKNDYFMDMLSEVLSWGAKIQFITGDSWYSSTGNLKTIRKYGIRFMF
GIDCNRKVSPEKGQLFQLRLLPDFHQGQVVWLKDFGFVQLFKTQLKEQQRFYIVHQDE
DDLLSFEGFHELHSSHWKIEQYHRVIKQVCHIEKFQVRRSKLILNHIFSALMAYVEIQ
KNQFEGIFENVYRWQKKLFRPIIKDFIDDFILDKNHLLPQRIYK" /product="hypothetical protein"
/transl_table=11
CDS complement(135458..135910)
/locus_tag="AUO97b_00126" /db_xref="SEED:fig|400667.82.peg.3778"
/translation="MKKLSDEYLPVRKAQTVYGSISGNYAFRGEKTIWFESTLERDFI
LKQEFNNNVIDVIGQPVVIPYITELGNQSTYTPDFLVQFSSSNCDDFEDFPVPMLIEI
KPKKKLVEDWDKLKPKFRAAHRFAADKGWKFKIISETRLYVGLTHYHL" /product="FIGfam050825"
/transl_table=11
CDS complement(136100..136438)
/locus_tag="AUO97b_00127" /db_xref="SEED:fig|400667.82.peg.3779"
/translation="MKKQVDKIILVLFGIQEINMLIPKKRGKGYLKQPLGHYDCPLAA
LSRDIGFDFNGLDGYLEIQTGYLTDKDKVDLTQRVVVPISNFYDYKWQEVDRNTFFET LKGNIARVDK"
/product="hypothetical protein"
/transl_table=11

CDS complement(136442..138556)
/locus_tag="AUO97b_00128" /db_xref="SEED:fig|400667.82.peg.3780"
/translation="MTSTTKKFKLHPKFIVNAIEQQANGLPKAIVELIMNSIDANATQ
INIKLWLNAKKHLQFEIIDDGKGFTKKSIDEYFAMFGAPHEEGDATYGKFRIGRGQVF
NYAITTWTSNNYQLKVDIYKPLREKSDEELGFTITRLAKKFNGCKISGYIYTNITDSM
GSITEIIEEIQSIIKYVQIPVFINKNQINKNISDCLDSECKIIEDDYAYYILDPNSDS
LKVYNIGVWVPYARFKNAIFKGSIISKSQFKITSSRNEIIEDRCELFKNIEGKIRDVV
DPTFIKLTTINEKKNKKLSSKLALGMLANILKNPLKDCRYDKEAILMMLNTPLDVFTD
YKGYKQSLIDISKSKAIMMLIRSDYDVLVVEKIEKRTNCLILEETYDISHLQGYLRTR
LIQEYIDKNEQPDNENLYDYDYMLMKLLSEFKDIVVAYTNYEMFTDIPPVTLLNYKDE
KEKVCFDRKIIDQKDLNNEQTGFLEFLKKLNTSFCDAYPDFINDCYGKREIFLGKSEL
DNAWTNMTSNIVFDIRTISFYQQNSYLRLLLILIHEYAHLLDDEEGHNFEFYERFHDF
VIRNDFIPLLESLSSSNNSIHSNSSSTAPDETELKLLAMIDQPTPQNARELYNILHDL
NYSFKAVEEGKKNKELFGLSYDTFNAWLADPKSTRYRNPKLQTWQSFIYETIAKKRGF
NDFNAMVLSNVFNCGTTTKEKV"
/product="hypothetical protein"
/transl_table=11
CDS complement(138718..139512)
/locus_tag="AUO97b_00129" /db_xref="SEED:fig|400667.82.peg.3781"
/translation="MINKALSVILISSVLSGCMTTIQNSASGLSRKQFLLIPSQDYYI
EANKGYVVLMNNYKNSGALDRKPELTKRVVNIAKKITAQVQDIKPETNQWTWELHVIN
TTTINAFCVGGQGKMAIFEGMIKTLNLNDDEIAAIIGHEIAHALLEHGREKASRNLVSD
LALGQIGGNAQILAYFGTKLGLTLPHSRSQESEADLLGLEIAAKAGFNPDAAITLWKK
VGDQQKGQNNKLSQMLSTHPMPQKRMEDLALAAPKFRPFYLATLHK" /product="Zn-dependent
protease with chaperone function PA4632" /transl_table=11
CDS 139971..140528
/locus_tag="AUO97b_00130" /db_xref="SEED:fig|400667.82.peg.3782"
/translation="MTKVISKRKTILDTALSLFKQYSFKFVGVDRIINESQVAKMTFY
KHFPSKTLLIQACLCEEQKTIEESILNELSLLSEAGNIARLKALLNWHVAYINQQNFN
GCLFQKAVYENEVSEEVLSVIQAHKQWKFKLVSDLMEVPECCTAFVSSSMVYSMLEGM
LLPANINPCVDHETAIKNLIQTFEA"
/product="Transcriptional regulator, AcrR family" /transl_table=11
CDS 140552..140878
/locus_tag="AUO97b_00131" /db_xref="SEED:fig|400667.82.peg.3783"
/translation="MNNLFDTIYSDMFVMIVASAFIAVMITSLTSILVKVNLSGYAIP
LTSFIWFLFLYGPIPAPAQQALKKDLVFLKNNNVPTNAMINTIILSCSDALSAVCTGL CTTIKI"
/product="hypothetical protein"
/transl_table=11
CDS 141027..141923
/locus_tag="AUO97b_00132" /db_xref="SEED:fig|400667.82.peg.3784"
/translation="MIDTLLSPFSVKHELYSKLKKHQENRINIVDKDSIYLFDIFSFE
SFKIKSKSPYSHLAYFSCSYTDDVTELELCFNPIFQDYDIPKLTIDISQLCGFGHSKS
DLFKYKSIDEFVENECPSYIETISQTQLRLMLSHNGIQIVNSPDSTFDNFYMYGWQPK
VFLSNEDGSHHLASAQYIALRLNQPVPLTARLIYKVIRGEQLANFHDRYAAFITPLHE
LDDYFKWFDNSNIKFIRLDDSLHEYAVYFFKRTSENERIIELFSSKFSSLNQILWKRL
SDQTNNMLLKALVPLNSGCWSY"
/product="hypothetical protein"
/transl_table=11
CDS 142045..142410
/locus_tag="AUO97b_00133" /db_xref="SEED:fig|400667.82.peg.3785"
/translation="MSKDDALFPCHKTVGTARTNLNEALELLEDELAFTGLSQNLTAR
KELEEKYQIDNLQEALLKDMKSEKVCAGWLILGKKEQIINNNFPLRLAQMQGLLQLND
LTREDEIYDSIEQAIEDHS"
/product="hypothetical protein"

/transl_table=11
CDS 142753..142869
/locus_tag="AUO97b_00134" /db_xref="SEED:fig|400667.82.peg.3786"
/translation="MRNKIFIKDPLRFEDDLLITKASTPHIYFDPKTLKVKG" /product="hypothetical protein"
/transl_table=11
CDS 142922..143491
/locus_tag="AUO97b_00135" /db_xref="SEED:fig|400667.82.peg.3787"
/translation="MTNKASQPRTFTLSDEKRNHILTTALNLFDQFGFQHISIMRIIT
EAKIAKQSFYMAFPSKDSFIIECLDMEINRLKQSLSNLLGQCGKDDHTSILKSFYQWH
VDLAYREGYNGTLLTKAVIEYWNLPDIKMKVEDFNKWKYEIFAEYLAEEINNARLRII
VSAMNGILLPASTYLPTWEDIESLYGEQF" /product="Transcriptional regulator, AcrR family" /transl_table=11
CDS 144221..145012
/locus_tag="AUO97b_00136" /db_xref="SEED:fig|400667.82.peg.3788"
/translation="MSNEKKFFLNSFKRNAKLLNTSFDVVSNTLNTPFKAPTYTQCLE
ISARALGFKTFKGLTLSDLTEVNEIRYLSDFDKTKLIESYLDVNRKKITTMPLKIDSS
LDLNSTIDAVCTLAYHIQTFPLLRDFYLPKRYLSKHLDFDKGPFTINDTLSKICAHWC
ISLLIEKPKGYEFYKGVYENNNDILYDVPMSFFTKKKINDWYKSRDFLHDFTDLFQHC
DFIRVQSGWTDFDNMSNSKINIFIPPALHERITELVNPLKGGPAN" /product="hypothetical protein"
/transl_table=11
CDS complement(145077..146459)
/locus_tag="AUO97b_00137" /db_xref="SEED:fig|400667.82.peg.3789"
/translation="MNQNNTEAEVNLFRFTFHFITQNTNSFFVSVEGPDENETLINFV
NDMYRVLYASNVKQDLDPAHPNQIKASALQRMTPSLRTQVNAMLSDYTKLKPSIHKAI
SKLNSIEDDEYFFVFPYEFTEKMQAQMLLQTFLRAKNSLNSDEEVNQYMQTHMEGFNS
LFDDLLEQYNIAVFGETERKTVIGQKPTKEHGVCRFCKSPVDENTTFRKVAHTISEAL
GNKSIVTADECDTCNEKFGRDFEPHLIQYLDILRVYYGTRGKKGIPHLKFKNGQVYQH
KLEGSEEHSIVIASQDIVETENGLIVSLLAHQKVNKLKIFKSLCKYAISVLPAEELPA
FESTINWLFLDPELGEEEQQFHIAKMYGQSYTEHPELTLYTRISDTDPEWPHVVAEFK
LGTLHLIFILPFSKKDSSIFNLETDFPKLCQLFRHYQKFEDQIIFENLSNSEDRECDY KVRFSTDKIN"
/product="hypothetical protein"
/transl_table=11
CDS complement(146532..146945)
/locus_tag="AUO97b_00138" /db_xref="SEED:fig|400667.82.peg.3790"
/translation="MKIMSSKATVQGGASILLFHCVYNGSDSITHFMQDIDPEIYNEL
PNLYSVCVADNSTGNKKITATFFIKTTRHHNDPDFLDSLLSIMALSPDLLAHWKEKTS
LIPAHHLVNGPPLSENEYVHFSQKLYMKHNIDGRA" /product="hypothetical protein"
/transl_table=11
CDS complement(147175..147288)
/locus_tag="AUO97b_00139" /db_xref="SEED:fig|400667.82.peg.3791"
/translation="MKKTGINVSSHIGFGASHQWAIQLLDELHKALVLEFS" /product="hypothetical protein"
/transl_table=11
CDS 147860..148051
/locus_tag="AUO97b_00140" /db_xref="SEED:fig|400667.82.peg.3792"
/translation="MKKFCLVLIPALIAGCSSPATEAEIRAAVQECEAVMHDIENQIQ
PALLHKPICKGFFSDIIFK"
/product="Mobile element protein"
/transl_table=11
mobile_element 93700..95008
/mobile_element_type="insertion sequence:ISAba18" mobile_element 112397..113576 /mobile_element_type="insertion sequence:ISAba1"

mobile_element 134383..135483
/mobile_element_type="insertion sequence:ISAba11" mobile_element
147994..148137
/mobile_element_type="insertion sequence:ISAba12"
BASE COUNT 46504 a 26858 c 28624 g 46151 t
ORIGIN
1 cccacttacc caagttacgc cttaaatttg gctcaattag gggcgctttg catttcaaat
61 ctttgtgcaa caaagccaat gaacgctaat cattatattc aacttgctag agaatttcgc
121 aagtctaatt cggaaccaat tttagctagt cagaaaattg agcatttagc acgatatata
181 aaattgcatc ctaagattgg tgcatacgaa gattatattc gtaagctacc gcttggcagc
241 cgcttaccac gtggtaagta ttttaaaggt cgtaaagctg gtgacaatat aagagttatg
301 gatgaatact ggagtatgtt caatcaactg aggaacgaaa gtttaattcc aaaccaaaac
361 gatgcattga gcaactaatg aaaaatcata atgaatatca tctttaatat gaatccatta
421 ttttagatag aaaaaagcct agtatatgaa ctaggctttt tttatacatc attatttaat 481
caggaattca ctcagcttta acttgctttc ttccatttca cttttcaacc atcttggcat
541 ctgtcctcgt cctgtccaag tgttctccgg atcagcctta cttctataac gtggctcaac
601 agtctttgtt tttttctttt tattatgggt tttcccaaat tcaatgagct ggtctaaagt
661 cagtccaaca cttttggcaa tttcctctaa ttggccaaag gctgccactt tctcttcaat
721 ttcccttttt cctttttcaa gacttgcagc cttgatcaaa ttctctagtt ctattgatga
781 ataaccatct aaattaaatt ctttcatcat taatactcta tatgttttaa agtaaatatt
841 ttaatggaat gttaaattga taattgagat ttaaaatatt taattaaatc tccaactaca
901 atattaaccg caagttcgtt atttgctaag tttgtactat aggctccaga tcggataggg
961 tcactaataa tctgagtata gttttgcatc gcgaaattta cactaaataa ataaggatag 1021
agtactttac tctgattact ttgattcagt aaagaattta atgcattttc atttccaaca 1081
aagaatattc gcttgaccat gtcaataaca taatcatttt tgaatgaatc aatattttcg 1141
gaaaaataat taaagtactt caaattatta ttggtcaatt cgtcaaaatt ctctttagat 1201
atattttcaa agaacgttgt tttagatcct ttgatagcta aagtagaaat caaagtcgca 1261
acagatttag caattttatt cactggatta ttgtaatcag actcttgtga agttttggga 1321
atatcgcctt tacccacggc actttcgtag tttgttctac tagccttcac aacaacatca 1381
tcgacttctt cgcctaaaat taatgatgat tgccctttac catttttaga atcaatccag 1441
ctcttatctt taaatgctga acggcatgcc ccgacaaagt taattgtctg gcccttttc 1501
ttcaatccat ttatccattg tttaagacgc gatagaatcg tatatataga atcatctgtt 1561
aaactatcga tatcataatt ttccggcact aactgcttaa tacggattcg taaatttgtt 1621
gtattctcag gtggactatc taagagtgat tgccatgcat gagcagcttg ttcattatca 1681
acaaacttat attttttgaa tagttttatc gactctattt tttgacgctt acctcttata 1741
aagtctgttt caacttcata ttcaagcaat tcgtttgcat taaaattttt taaagctggt 1801
ttaagagtat cacgcaagaa ataacgattt tcttctacat caccatagcc tagatactgt 1861
ttgaatgttt cgtatgccaa agtaattggc tcatcagttc cttccagtaa tttataaatt 1921
aataattcaa tttttccatt tcttcctgtt gatgtactta aagattgtat ttgtgtccaa 1981
ctaaataaac gttttacttt ttcaatcgaa atagaatcag taataattga actaaaaaaa 2041
ggaggtaatg aatatacaat ttcaagactt tggtagtctg gtttgttatc agcttccacc 2101
tcttctcgat taagcatcat atttttatta taaatcgcaa aatcttgaat aaagctgaat 2161
tgtgaggaag atgactttaa tccttgtgca ttgaagatat cgatttgtat ttcagcatct 2221
cttaaattac ttagtgattt attaatctcc ttaagtctgt gttgactatt tgttttgaat 2281
cttaatagat caaacggtat tttaaaatta acaataggat gagaaccatt atcttgaaaa 2341
ttttcaagaa tttcacaagc atgattcaag agacacaacc aaatcttatg ttcaactaat 2401
gaacattcat gagtcacatg aattaatgaa actggcagtt taaactctgt ctcatactta 2461
aaagaggcta aatttgggtg cccttgacta agactattag cttgactata gcccagatca 2521
gcaggagaca agctctctgc gtcagaacta gtcaattctt gtgattgtac gttcatttga 2581
tctaataatg acgtttgtta gtaatgacaa agttatatca taaagaatgt gtgagttaaa 2641
tagaaatacc tcacatttag aacactcaat gtgtgagaaa agtaaaaaaa cctcacataa 2701
aaaaaagatc ccatatttgg gatcttctta tttacacaac ttgatctaaa taaggttgtg 2761
attgattttt cttatgtagg ttttctttaa acatcgtttc aaagaaaggt gcataattaa 2821
agctgtagaa aatctgccca tctttatcaa aagaaacgta ttcactaacg agtttggttg 2881

aaaacatatg tacaaatcta aaagattcgc ggccggtctt tgtatgacga agtaagcggt 2941
gttgatgagc actcaaagaa cagcttgcta agcttttcat tctttttca aactcatcta 3001
atgaaatacc aacaactttg gaaatttcac ttgcattgat agtatgagtc agtttgctat 3061
caaatgaagt aagcaaaata tttttttgct tataagccat taaaacttca ttaattaaac 3121
acacaaaagc ttgatcggct ttatcaattt tagtttcaat agtaaatcga tctgatccga 3181
ccatatattc atatgactta gtatcaaaat cgaaatctaa aaatgaaaac aatttagaaa 3241
atctttttaa tggtaaagca ggtaaagaac gttcaactac agaaggctct tgtacatcat 3301
ccaataatga taattgaaca gatgactgtt ttttccattc tgccggctca ttcaaatata 3361
atgagtgagc atagggcata gatgctaaat tagtcatatc ctcactcctt ttaaattgaa 3421
aataatttgc catattaatt tgaatataaa tatatccata ccaaatttta gcataggcac 3481
atagcgaata ctattatgtt tacaaaaaat gcaaagttaa ctctttgaat agttttacaa 3541
aaacatagtt ttttttaact taattgaaat aaaaaagccg aaccaaaaat gttttccaga 3601
tattgagcac attggaaatg aaacctcaca tatatataag acttatccac aggctttgaa 3661
atgaaacctc acacattgga aatgaaacct cacatatatt ttttaataat tatatttcaa 3721
tactttattg tagatgccta aattgaaatg aaacctcaca catcggaaat gaaacctcac 3781
atttgttgaa atgaaacctc acacaaaatt ttataattta atattttaag acttaagtta 3841
ttgaaaataa attaaataaa tgaaacctca cattagttga aatgaaacct cacatatcgg 3901
aaatgaaacc tcacattgca agataaaaat taataaaatc aatgaaatac aattacccc 3961
tatataaata taaatttaaa tttatattta tataaattta tatatatagg aaggactttc 4021
ttaggttttt tgaatgtttt aatctttaaa atacggatat atcttttttt taaaatcatt 4081
tccattttcc tttttttgtt ttaaaatcat atttcgctat ccactattct ttttgacacc 4141
agctcataca acagagcaat atagtaggta ttaacatgat taaaaacaat gacttggata 4201
atctaagaaa tttgaataga aatattattc aaaataaacc taattctgct ccagctactc 4261
ctcaaaggct tttcgtttat cttgatggta tcgcacaaaa caacgatggc catgattatg 4321
cttatggaac aaacgtccta accaatgaaa aagtgaaagt tagactaaca acaattgatg 4381
aaagaattga tgacttagtt gactctggta gagcaaagaa cagacaacaa gttgcctctc 4441
aagtaacaaa tcagtatcaa aactctgcca atcctcgtca atctttaaaa gcgcgactga 4501
acagcaatgt agttgtactt agttttgatc aatcttttga aataaagagt gaagataatt 4561
accggtcata tcgtagccat tgggcagaat cagtaacaag caatcctagt tcacaactaa 4621
ttacagctcc cgttcatatc caatttgtaa ataatcaaag acctttcgga cgtgtcgaaa 4681
ttttagaaga gaatttcaaa atagatatag gatcctcaaa ggaaaatata aaagatgcat 4741
ttttaaaatc tcttgtacat caagatgagt tcagcgctga aagatccgga tctttaaatt 4801
atatactgcg ttataaagat actaatgaac caatctatca aggttctttg caaacttttt 4861
tacaacaagt atctgtaggt cctgaccgat ctatccggat accagcggat attaatacta 4921
ctctcaataa tttaatgagg aagcctttaa ccacgcagga tttcacacct gacgatagta 4981
tgatgtttaa tcatgattta aatcgaatat tggtaaatag atttcttggt gttgaatttt 5041
ctcaaaaatt agaaacttct aatccacaat tacaagataa cctacgtctt atttctggaa 5101
agttgaaaaa gggtgaaatt tatcttgatg cttattcaac tcaaaatatc tttttggta 5161
gagatacact taagacatat caagcaaaag caaatagaaa tagcagtcca ttaaaaatct 5221
atactgttac tgatatgcac aatgaacaac cagtttatca aaaaggttat atacattctt 5281
tagttggcat tgatagacat tcagacactt tagctccttt tgtcacattt agttctacta 5341
ctgatccaat ggcagtatca aagaaaattc aagatttaaa tattaatgag gaaaatttaa 5401
agcgaaccat ttatctaaaa gaagaattcc aaaagcatcc attacctaat agtgctgctc 5461
cagttgtgga taaagaaaga gcaaatatca atagaattcg ggaagaaatg caaaatcaat 5521
ctaataactc aataaatttt aataatattt aaaaggatta gatgatatga gcgttttaga 5581
taaaaaaggt tttatttggg ttaatttaga atcttttgga tatttaacta gaccatcacg 5641
acgtaaagaa aacaaccaaa tcggtattga aatagttgat ctcaataatc aactagatat 5701
cgataaatta agatccttta atgttcatgt tgaaaaagtg aataaatcaa gtgttcttga 5761
tgtttactgg tcaagcattt caattaagga cttttctaat tttattggtg ttgataacaa 5821
caatattccc tttcaatcca ttacattgac tcagcttaat gctgagttca ataactctgt 5881
tcgttctctt gcccaacaaa gagttgacta tgccattaga catgcgacat taattggtca 5941
aaacctgaat aaacaatatg tttatgctac acaatattca cgattttatt tggatcagcc 6001
tttaactgaa attgctaaga atggaacaat ctcaattgat cccgcgcaat ttgaagcacg 6061
attacctcaa gaagattatt ggaaaatact taaagttggc aatcagccaa agtttaagcg 6121

agctaatatg gcacattgtg caaaaagctt tgctagtagc atagttgaag gaacaacaaa 6181
cattcatgat tcagatgttg agttgttttt aaaaaaaata ttaaacgaca accatgatag 6241
atcttatttg aatgaaataa agcctacaat taagcaattt ttctatgaac agcttgaatg 6301
ttgttttaat agtttaagta atcaatttaa cgtacataat ttcaattctt ttgaaaaaat 6361
tattgaaatc cataacaaac aaccagttaa accgatcatt ggtatttctg agctgcaact 6421
gcaacaattt tcaactccta taacaatttc agcaatatgt cagaaattat tattcaatcc 6481
tgaaactcta gatactggaa aaactctact tgaacctact attggtaatg gatccttagt 6541
tgctcatttc ataaaaaaac cgcaacttaa aatcgttggt gttgaaattg attcgaatcg 6601
ggtgaaaaat acccagctct ttctcgatgc aaacattgaa cattcaaatc taagagtaat 6661
tgagggagat tattcaaaga ttaaattgaa gcaattaaac aataatgaat tatttgattt 6721
tactatcgca aatccacctt ttggcaaaat cgataaaact acacttactt tgaatcaagc 6781
gggatcatta gaacgtttaa atttttctac tcaaaggtta gatcataaaa ttttattaga 6841
gacactttct ttgagaaaag acaaaggaag aagtgttttt attattggtt cagacagctt 6901
ttatgaagct ggcgtagtca agggtggaag taaaaattta ttaaattatc tctatgataa 6961
ttacaatgtt gaagcggccg tagagctcga cgggagttta tataagaaac aagggactcg 7021
cgtcaatgtt cgtgttcttg tcattggtga tcttctggaa aataaccgta cttatgaagt 7081
tcctcatgag ttgcccatca taaataatgt tcaagattta tggagatggt cggaaaatgt 7141
cctacagaaa agattacaac ctgagttaag ttacgatgag ccctcccctt ctattaatca 7201
aagtgaatca gtcatatccg aagctaattt agaccagaat ttagatgtgg tagacgatgc 7261
tgatttctta acaagtgcat tggattcata tttaaatgat caaatagttc cggatgagtc 7321
cggtaatgtt ggggacttga ataaacctga tgatgacgac ttctttggta atgaggaaga 7381
agatacggct gttaaacctc tttctactgg agatgaacat cagaacaatg agattcttga 7441
tctagaaata gtagaggatg aatctcaagc tcaggaaatt atttttgaat ctaaatcatt 7501
gcaagttgca tatgtgccag tttcacaact aagcttgcca gaaacaatgt tgccagcaga 7561
tcaagcaata cttattaaac aagcgcaaga taatcagatt aagaagatcc tgcttttagc 7621
tgaagtaaat catgaattgc aaaaatctat cgatttagcc aataacaatc ctttcgacgg 7681
ctttctactt tacaagctac agtattcaga tctttcatct ctttctaaag ctttcagtgc 7741
tgaacaggtt gatgctgtta cttccgcaat attaagcatt gaaaatgacc gctcttatat 7801
tcttggtgat caaacaggta ttggtaaagg tcgctcttta gctggcatgt tgcgttattc 7861
tttaatcaat gggtttagac cagtattttt cacaaggaat gcacaattat ttaatgattt 7921
atggcgggat atcgtagata caggaacaga taaatttatt tctaatcctt ttatctttaa 7981
tagtgatgga ttcattacgc ataaagacaa ggttgtttat tctcctatga aaaccagtga 8041
tatctataat ctagatagaa ttggtaatca gtttgatatt atctttagta catacagtca 8101
attttctggt agaagcggta agaaagaaaa tctttttcta tcaagtattg ataagaatac 8161
cctattattt ttagatgaat cgcatgtggc tgcaagtggc gaatcgaata ttagccggtt 8221
cctttcagtc gttaaagagc gtacaggtcc tacttatcat tcttcagcaa cagatagaaa 8281
gaaagcaaac aatctacttt tctattctga tattttccct gcatctttta aagggcaatt 8341
gaaaagctta atcgaaaata aaccatctaa tgaattacta gaagctatct ctatggcttt 8401
ggtcaaagac ggtgttttct taagacgtga acatgatttt tctaaacttg aattccggac 8461
atatcacgca tcgactcagt atcaaactgc ttcacgtgag ttatccgatc aattagctca 8521
ggtactttca aaaatggcat acctttctgg tgatgtagaa gagaaagttg ccaaaatgaa 8581
tagtgagatt aaacataatc ttgagcacat gaaggaggga agttggcaag agaatcgtat 8641
gcaagtgaat tcgatgaact ttgcttcaag aatgcacaat ataagccgtc aaatccttct 8701
ctcagttact accccttttga ttattgatca agcacttgag tctctcaaag aaaatcggaa 8761
gccggttatt ggtttggaaa ataccggtga atcgttgctt aaaatgttgc ttctaaaaga 8821
ttcaaaagca tttgagttgc tggaccagat taatgaactc aaggattctt cagattatga 8881
tcgagcactg agtgacctaa actcagggaa aatgaatcaa aatgaatttg atcaagagcc 8941
aattattgta aaactaaaaa aattagaaca ggatctagct cagttcaaac aagaagatac 9001
tattcttgca aaacctccga cgatagctga cttacttgag aatatggttc ataagttaga 9061
tattattcaa gttcgtgacc gatatggcca agtacgaagt gaaaccattg atgacgaaat 9121
ctattctgat ttcaaacaag acatcttgga taatattcgg aaattaccag acattccttt 9181
atgtccttta gatctgataa aagctgagct ggagtctcgt gggtataaag tcggtgagat 9241
ttctggtaga gagtatcagc tcaaaaaaat tgattctaat caaggttcgg tttttaaagt 9301
tgaagtacgt caagaaaata atccgacagc aaagacgaaa gcttgtaatg aatttcagtc 9361

tggcattctt gatgcaatga ttattacgag agctggtagt actggccttt cattacatgc 9421
tattcccgtt aatgatggta gtcctagcca atcagatcat ttgaggcaac gtgagttcct 9481
tacaggccag tcacctcaag caattgatga attcctacaa atgattggac gtgttgatag 9541
aaaaggccaa gttagtgcgc cgattatttc ccaatttgat acggggcttc caattcaacg 9601
caaatttcta atgatgcata atgctaaact tgccgaatta tcagcgaatg tcacaagtaa 9661
ccgtgaaaat gttaataagc aggtaactga cgtcgattta cttaatagtt atggtgatga 9721
tgttgctctg gaatatctag tccagaatcg ttctgtagct caaatgcttg tcataaattt 9781
acctaaagaa aatgataaaa atggcgctga aggtatggtt aagaaaatct ttaaccgtct 9841
taattttgtt tccatttacc agcaagagaa gattatcgat gatttaactt ttgcttataa 9901
cgaaaagatt cagaaactta atgaaatggg aattaatcca ttcgtagttc aagtatgtga 9961
ttggaaagct caaactatct ctaaacaaga gttacaaagt ggcttgggca ttatgtcgca 10021
tgtttctgat agtgcatttt tcgaaccggc ttatttccaa aaagttagct ataaatctga 10081
aacaaaacct tttcatggcg atgatgttat ctctttaatt gataagtcga gacagcttac 10141
acgccagcaa ttgttcaagc atttatgtaa ttttgatgat aattttgctt ctactatgga 10201
aggaaaggat caggagctat ctttatctag tttaattaag ttctacaatg atgctcaaaa 10261
acagattctc gaaagagaat taagagagtc aattaaatac tcagcttcgc aatctttttt 10321
caaatctaaa attgataaat atgaatttac cgagattgaa aactttattc tgaataaacc 10381
caatcttgat gaaatcaaag aattcttgaa aatatctgct gaaaataata attttcttca 10441
tatttatcaa gggttaaata aagctttaac tgtggtggaa tttgtagaaa agcttcagat 10501
cttagaagaa catcagagct ttaatataaa tttcgcaata agcatgaagg aatcactttg 10561
ggatcagcat taccatcgtg aaacaaatga aaatgctttg atacgagtta tgttcccgaa 10621
attaggcgct tctagtcttt tagcgagtaa ttatgaaatt cgttacgttt atccaagttc 10681
agatttgta cacagtagta agctcaaata tttaatagaa gatgctgctt taactcctac 10741
tctattacgg aatgttggta ctcatttatt tattgatgga aataaaaagg aagaagagtt 10801
ttcatttatg aaaactttaa atgaaataga gtacttaaaa actaatagtc ccttttgct 10861
ttctttcgat aagtacgaac caaccgctat tgagcgaact gcaacccttt taactggcaa 10921
tattatcaat gcccttaatg ctgcctcagt agctaaatta tcttctcaaa tgattaatta 10981
caccgatgct agtggaaata gacaacgagc agtcatgcta tatgacgact gtgagttgga 11041
agaagtttta gacaaagtta aaggtctttc aacaatacaa gaggcgatta cttattttga 11101
acgtgtacat gatgctatag agatgaatga aaaacctact cttccggatt tctatattgt 11161
tcgttcaaat tcaaatggga acccaactat tgaactcaat ctaaaacctc taaatgaaga 11221
aacatataac tttgttatta ctggtagcag aaaacatata aaacaagttg ctgagatgga 11281
ttctatattt gtacaagctg gctcaggaaa tagatcagat agtttgaatt tagtccttaa 11341
gggagatctt tcacaaggaa ctcgttctag ccagacagtt caggtcccta ttgaaaaagt 11401
aactaaactg ctaaccaacc tttccactga attttctact gtgaaaatta ataatcttga 11461
tccagacgtc ttagatcaac ttaaaccgaa tacgtcgaat aaacttactc agaatctcat 11521
gtattagaga ggaaaatatc atggacaata aagaagaaat gaaagcagct ccacaagctt 11581
taaacgaaat agactggaaa gatgaaatcg tctttgatga gctactatct aaagtaggtc 11641
ccataacacc accagacacc actttaaaat cgagtgattt ggaagcttac ggttatcaaa 11701
gccaagtgat gctaccagtt agcgaagcaa gagccaaaga gctatatgaa aaaattgaat 11761
tatatcgatt atatgaagat ggaacagaag cgttagtaga agatcttgca gaccttaata 11821
gtcatgcaca aaaagatgga ctatttggtg ttcaggaaaa cgattggaga aattatcaat 11881
taaaaataga aaaaagcaat gattccgaaa atagtaatga tcttgaaaac tttcagtatt 11941
tttcgtatcc agctttagct caatctataa ttaagcgtga aggtgaccaa ttttttgcaa 12001
attatgaact atctcgaagc ttgggttctg atgaagatat aacgattttg aataaagagt 12061
tttacaaaga acacaaagca gacttagaca ttttgttaaa cgaattaact atatctgagc 12121
aatatgaaca tcaaggctat gctgttttat atgcaaccaa gagtaattta tctgccctaa 12181
atagtgatga agaaatagaa gcagaagcaa atgagattaa attattttta gaaggaaata 12241
atgatgatcc aaaatatgct tatgtatacg atactcttta tgaattgagc gcagaaaacg 12301
tgttcaatga atttaaggca cttgaaacta gaattgaaag agacatggat ttagaagaag 12361
gcaattttga tttaccattc cagcatgctg tagaagagac tatggaaact tttgataata 12421
acgcccctat ttctaacctt ccctttgata gtttaaaaga gcccgaagga tttatggaag 12481
atatagagca gtactcttat gcagaggatc aaaattttgg ttcttcatta cctatcaata 12541
acgaagaaat gatccaaaaa tctgaagtaa aggaagaaaa agaaaaaatt gatgttaacg 12601

atgtgggggt gccagaacaa ctcataatca atggtaaaga gttcaacttt gaacaaatag 12661
atcctaatga tatagaaaag catgagcaat taagagcagc tttgtcgcag ttattgggta 12721
ataccaatgc ttcaactttt caaagtccaa gccaaataaa tccagttact tccactcctt 12781
tgattcctcc atcaatgcca gattttaacg aaaattcggc acagccttct actgttaaaa 12841
attattcgct ttttcctagt tttgcgaatc ttaaaaaggc gactccggat aatgcattat 12901
cgttattaca agctgaacaa cttctacaaa catcacaaaa tatacaagca ccagcttcag 12961
aaaattctgg tgctcatatg aatacacaaa atgttcaaca tatgctttca agtcatttag 13021
caagctcaaa atataatgat ttggtaaatt cacataaaaa ttattcagac tcaataaatg 13081
agttctggaa atttgacaaa tttgaggatt ttcgtggaac cctaattgaa cgtggacaaa 13141
aagaaaataa ggatttgaaa acattgatta atgaaatgaa tacagatcct aattcaccat 13201
atcgcaaaga gtttcaagat cttattgtta atgttggtag tactgagaaa ggcaaagctt 13261
tattgagtaa attgaataat tcacatcaag actttttgtc gcgctcagat tcaatgtttg 13321
aagttattca aaaatcagag ctttcacctg atgacaagac aaaatttgaa aataagctaa 13381
gtgaggttgt tgaaaaccaa gctgaattat cctctaccct acctaattat attaatgatg 13441
acggtgtacg tattagtctt gcggatgaaa tgaagaagta tgcagaaagt ctcaagaagg 13501
ctctacaagt tgttgtggac tttgttaaat ctaaagtggg tttatctgta gatggcccta 13561
cagaacagcc ttctagctca ccgtcacctt aacaatgtat tgatatagag gtttttatga 13621
acatgagcac tctcccctac cctaacatat atgaagatgg ttgttttgct ggggcagcag 13681
aaatgaactg gctaaaacca atttttgaaa aacagatata tgttaaattt aaaaaaaatt 13741
ccttttctcc tttttttctt gataaggtta aaagtgatta ttactgtgaa gcagacattt 13801
ctgaaattga gaaagatgaa gtttatattg tctatataaa ttatggagaa acaaagcttt 13861
atgagaagga gaatcaatat agccttatat taaaagtaaa aatcattaat ggacagtata 13921
gggaatatgt agaaggttgc tcactcaggg gtgaacagac gccgcttgaa tctataaatt 13981
atagcttgag ggcaattttc gactttaatt gaatatagtc gaaaaacact ctaaaattaa 14041
gcctactgat aatgcaaata cccctgcggg gatttgggat aataaaatgg aaagtttagt 14101
tgaaagaagt gccaaacaag atttggatac tttcttacgt tttatatgca catatgcctt 14161
aaatgataca tatgtgtcta aagatcttat aacggaaagt cttaaagact taccgcaatc 14221
cgttgtgacg tttgtatact tacttgagtt ttctccaaac gatcagatca gttctatcac 14281
ttctatctca gatagtgttt taaaaacatt gtcagagctt attcataatt ttagaaatga 14341
aagaattaat tcaaacggct actcaataat ttttgatttt ttttccgcaa atcctttcag 14401
ttatttcatt tattttttac gtaatgtgat tacagcaaat attgatgagt taccacttgt 14461
tgttaatttg aatatcacag atttcttcca gtttttacta gatcgtaaaa gttcagtctt 14521
tcactcaaga atttttttatt ttatacgggc ttggcaaact gtttctgtat ataacgaagc 14581
aaatatcagc gagctaaaag aaacaatgct gtatgccagt caaaataaag tctttccaaa 14641
catttcagtt tcatcttgat caggtggtgg tgggttatgc ataaacgtac atcagcttta 14701
aataaaaatc cgcagaaatt aaactctgta gaaaagaaac attcgtttaa accaaaatta 14761
ggttttacaa cgggtattgt tcttggtgtg tcctcagcaa ttgcattgtc gaccactgca 14821
agtgtcggag atggtttatc aactattcaa tcctcattga accaaacttc taatacctct 14881
acaaacatta cgaatagtta tacgaataca acaacttcta ctattccgac cttatcagct 14941
tctccatttg ctgctagtag taatgctact gctgcccaat ctgcaattga agcttctagc 15001
gtgggttgtg gtgatgcttc acaacaaggt tcaattgctt ggactcaaca agaagtaatt 15061
aaaaatcgtc agattttaaa tacggcacca gttgacatcg atgcattgtt caacggatct 15121
aagggttctt cttctggaag ctgtttttca gatatcacga agattattga cttgagtgta 15181
gcaattcccg attggggtgg tatgcgagct gctgctattg ctgctttacg tcaatatgct 15241
caacgtaaag tatgtactat ggttaatgaa gcaaccaaaa aagcgttgga gccactcaat 15301
aatgctattg atattattaa taagaatgga accattgatt tgaatggagc tgtacaaggg 15361
gctataactg gtggtttggg taagttagat ccagatttag caaatatata tcaaacacaa 15421
gcacctcaag gcgaaactgt taacattttt gattctaatc agactacttt taatactgat 15481
gttgctaaag ttcaagatca agttaatggt gctattaatc aaattggcac aatcggaacg 15541
aataccggta gtactggttc aacgaatcct actggaactt cgggtactac atggtctaat 15601
aatacatcta cgcaatctat accaaatttg agcgcaaaca cttctaatca aacacaacag 15661
aaggctcaac cacaagaagc ttctcgttct tcaatttttg atgcgctttt tggcggtagt 15721
tgaggtggca atgagtaagc ttgttaaagt tgccttgagt actgcgataa caatgagttt 15781
aattggttct agttatgctc agattgctca tacaacgtac aaccaaaata cgagagattt 15841

aacatctgcc ataggtgttt tgactaagca agaaggtctg agcgcccagc aacaggcaga 15901
atccgcaatg atctcttcta aagcactagc tactgcaatt ggtgcgataa atatgtctga 15961
tcgtgtatta cgctttattg ttgacgatgc tggaagactc ggtagtacgc aaaccactac 16021
ttgtactgtt tttgctgaac agcaagcgga aaaaataaag attcttcagt catctattaa 16081
tgcggccaat atggttagta attatatgaa agttatgatg tctaattcag ttgagtctga 16141
aaaggacaaa atacttcgtc atgaaaatta ctgttctatt gccgaagcag caatgggtac 16201
atgcacactt ctaccaaatg gtttacaagc ggctgactca aattatgcat tagttttctc 16261
aaaatctaag cttcaagatg acacttttaa tgcggctaca gattatatag cgaatattac 16321
tgattcgcgt ttttctgctc aagatatctg ttcaaacact cagtgtatga ctaaacaaat 16381
cgatttttta aacaagactt caggtaatag tcttgcagcg tttgtattga gtaatcaagt 16441
tagtgatcga ctttcttatt cattgaatgc taattcatct ggagctaact aaatgaaata 16501
taaatattta tgtgtggttg ctgccctccc cttcttgggt cttagtttta aggcatgggc 16561
tgcggcagat gtattgtatg gaagtgttgc acctaccttc catcaaaacg ttcaaacact 16621
taatagtaag atggtttcag aattcgcttc tactcaagca aatttgttga atgcaattca 16681
agaaaatact cgtcaattgc agagtgcact tagtgtttct attgctcaat caagtgctgg 16741
aacaacacaa gtaattgatg cacgtaaacg agctggacaa gctctagcaa cggcttatca 16801
agcaattgaa atgcaagata aagtgattga tacggtattg aactatggat ctactggaca 16861
aggttacaat acatgtaaag tactagttag taacgcaaca ctgagtaaag gtaaagaagt 16921
ttcgagacaa actgctgcca aaatggtgaa tgaaactagt actggtactg gtcgtgtagt 16981
acatgattta aaccaagttc aaaaagaaac ggttcaaaag caccgtgagt tattttgtac 17041
ccaagctgag gcaagtctag gattatgtac ggtaagtaag ctggctggtg gagatactaa 17101
tgcggcttta cttttccaac ctgctaagcc taattcatta gaggctgaag cgagacgtac 17161
attaagagga aatatcttag gttcaccaga tgtcgcttta aatgctgtta atggtatgac 17221
acctagaggt caacaatatt tatttgcttt aaaccgtaaa acagctttac aagccttccc 17281
tgcttattca ttggctgcta ttgacgcagc aaacttacaa acaatcaaga atgaagctgg 17341
tgaactgcga tcacctaatc agatgattga tgagactatt ggccgttatt acggtactga 17401
cgaagccaaa aagtttcagg caacaatgat gaaccagtca gctcgtggat tgctagttga 17461
gttggcaaga cagcgtggtg cggctatttg gttacttaat gaggactatg aacgtggttt 17521
acgtctagaa ggtaatttag cctcacttct cttaattagt gctgaagaat caaagtcgaa 17581
aatgcgtgat tcttatcgca caatggctgg ccggagctaa ttatgaaaaa gaaaacgacg 17641
tttttactat tatcagtttt tagtacttca gtaattgctg cttactgtcc tcctttttca 17701
aatccattga tactcagaga ttttataagc ggttttcagc aattaaatgc gacattcacg 17761
actggactta atactttgga tcaaagtgtt agtgcagctt taactacaga acgccaatta 17821
cttttaaatg caattgcttt gaagacaaca caagaagcaa tgaatgcttc ctcaattgct 17881
catgcggacc aagaaattgc aaaggcgact gctaatgcta ttggcgctgt taaacaatca 17941
gagcgtttaa aagaaacagc tattgatttt ggagctgctg gacaaggcta taacccatgt 18001
aaggttctaa atgaacgtag aacaattact gaaactcaag aaagggataa agaaaataga 18061
ccagatttaa tttttaagga aatttcagcg ggaacgggaa aatatgcaaa tcctgtagtt 18121
gctcaagaac aaacacttaa gcttcatcac gattattttt gtacatcaga acaagtatca 18181
gccggcttat gtatgagcgt aggtgaaatg tctggtgcaa atttgcaagc ctctacttta 18241
tttgagtatt catcagcgaa tgataagtat gctattgcaa aaactagctt cattaacaat 18301
atggttggtt tgccggatgc acccttatct gcaaatgcag ccaaaacgcc tcgtggtaaa 18361
gaatacatga gacagaaagt tgaaaatgat gcagttattt caccagctat taatagttta 18421
atggcaatca aatctgaatt tactgaaaac cctaatggtg gtgagtttgg tcaacctgtt 18481
aaaccattgc gacaacaata tcgggaacaa gtagaccgtt atcttggtca tggtgaagaa 18541
tataagaatt ggaatagaac attagttgct gcacctgagc gtggggtact tgtagagatt 18601
cttaaagaag gtgcattaga gcttgcaatc atgggaaata aacatgctca aaacgaacgt 18661
attgaagcaa atttagcttc tttagttgca ttagaaaata aaaaggcaaa ctcgcttaat 18721
aaatctaata tagctggttc tgctaaagct aaataataca tagttttcat aaatgaaaac 18781
ttttagtttg aactattctt gctagaagtt ttcataaatg aaaacttttt tattgggtaa 18841
gttttcataa atgaaaacta atctaaaagt ggtgatgata tgaaatggtt gaatttatca 18901
gaattcggac tgcatctagg gtttcgaaaa gcaacaagct ttagagataa agttgccgta 18961
ctgactgtca atgatttaaa tactttatca cggttcattc atttaaatga ttttagtgaa 19021
aaactacaag cggtaggatt taaccgtgtc aattcagatg acgggaatgc aaaatttgta 19081

aaagctgata attcaatcaa tccaaaacaa ttggcttcaa tattgccaat cacaaataaa 19141
catttacaag atatgccgga gaatgatgct agagcgaaat ttcatgtaga taagcaagca 19201
tggatagatt tgactgcaaa actagctcaa caaggttcag tttggaaact agatgaaaat 19261
tattacaatg aagcaggtat acattggaaa aataaaatta gtgaaaacta cctagatcta 19321
tctacaaagc aagatgatag atacataaat ttagcttttg aaagcccttt accagtaaat 19381
atgttagaaa cttataaata tgattcaaga gcattgggtg tactaactac taataaggaa 19441
atactgataa gaaaaggctt gccagaagag gcttttacag aagtacaatt aaactttatg 19501
ctgccattat tatcattgga agatgaagta catatagttc taaatgatat ccgtgaagta 19561
ccagaaatat tagagttgca tccagatata tggcgtcaat cgatcaatag ttttgaatcc 19621
tttttaacaa gatcaaaatt actagaaaaa ataaataacc ttttgataaa tgatgaactc 19681
ttggaaaaat ctaatgaaat tgtaaatgat ataaaagtgt tttacgaaac gtataacaat 19741
tgcttactaa gtaaagaaga attttcaaat gatgatgatt taacaaatca ccgtattcag 19801
ttgttgaaaa accaaattat aaagctcgat aaatttggtg tgattaatga aaatttatta 19861
ccaaagcttg ataacatcta tgactcttca attgaagata ctgtactaga acctgaaaag 19921
gaaaataagt cccttaatga aagaattgag gactatggag aatatgtttt cggagctaga 19981
aaacattttt atcagaaatc taaaaatgca cttttcgaag aattgaataa ttcaaaagac 20041
ctatctttag atgttctcat agataaattc caaaaacgaa atttatggtc attcaaaaat 20101
gaaaccatga gccagattga agacaacaat gtgatctttt tcttagctca atcagtctac 20161
aacatgattc cttctaaaat ctctttctcc aaagatgaga gaaaagactc tgaagaatgc 20221
attgataagt taaaaagcta tatgtattca gttaaccatt ttaaagaaat ggtccatacg 20281
gctatagaat tagacaaaaa tgaaagaatg cctatagcta attttgtcaa tcaaatggtt 20341
aactatagaa acgtcttaag aggttatgct caggatcctc agcaattctt atttgatgaa 20401
ttagatgagg accgtttatt agcagatagg atatataagg ttgtaaaagg tacgactctt 20461
agaggaagtc ctttatttca aattatgaat aatttggata ctgtcaaaac acagtattgc 20521
aaaatgatgt ttaatgaagt ctatccagaa actaaagaag tctcatatga tcaatttcaa 20581
gactatgttg agtccggcgg tcacatatat gatggtagcg caataaaaca atttatagac 20641
gcagctaatg gtaagttaga ttacacatat ctttcgcgcc aatttgagcg tttagaagat 20701
agcttgggtc aggaattatt cctaaacatg attgttacag gaaataagcc attttcagat 20761
gctttattta aaaatgttga aaagatttta gaaagcgata aaagcattaa aacaccttta 20821
gatttcgaaa aagatgattc taaacttaat tatctacttt ctttatatcg ttttaaattc 20881
gattcaaagt attcaattcc tgatatctat atcaaagatg aaaaccaaca tactcaagtt 20941
atcactgact tgagttcaac tgataaattt gatggcttag ttagaaagat attaaaagac 21001
caagagccta atcgacttga tttacgctat ccatcaattg tatctggtct tgcacatgag 21061
acatcaaaaa attttcggca tggtgaaaat gtaaatccag aacaatttag taatgtattt 21121
ggttttcgag caatacagtt tggaaactgg gtgacacaac aagaaagaca gcatttattg 21181
aatatcacat ttgatggtct aagtgagcta agagacgctc tcggatttga ttcaaagaat 21241
atttctttaa atggaacttt agctatcgct ttcggtgcgc gggggcaagc tggacgaaat 21301
gcagcatcag ctcactatga atatgctagc aaaattttta acctaaccaa acaaaatgga 21361
gcaggttgtt tagcacatga gtggttccac gggcttgatg catatatggc acaactagta 21421
agaaaagatg tagagaaatt gatttataaa agtggaaatt ttgaatctgt agaaaacaac 21481
tattttttaa cacatttaat gtcagacgtt gactataaaa catgtaaaac agttatagat 21541
aacaatccat tattgaaaag tgtttatgag cttcaattaa aactgaatgg aaagatgcat 21601
agagatgaaa tttttagtga aaatggaaaa gagttaatag taaatcgtat acatgagcta 21661
gttgatagca atttaataaa agtaaatgta ttaaaagatg caatttcaaa gtctttggaa 21721
aagcaaggtc ttagtagaat agcatcattg ttagatggag tgacaacaaa ccatgttgac 21781
aatgtaaaaa aattaaaaaa tcacgaatta acaaagaata ttaagaatta cttggtagat 21841
aatgatctgc atataaaaag agaccaaatg tatagtgcgg tgaaaacaat ctttaaagaa 21901
ttgcaaaata acaatgcgcc agttggaaaa tatacaattg cgaatccaat tacccgtgaa 21961
ttcaattcat tggttcgtgg ttcacaagtc aatgatgatc ttgcgaaatc agagaaggaa 22021
atattgaaac atctaagatc acataagatt attaaagaaa tggaaaagtt actgcatgaa 22081
tcagtattct atttagtaca aagacaaaca ttacctttgt ttatagatca agtgaattct 22141
ataggtggaa ataaaataag cattctaaaa ggggttgcgc tagatcacta ccagtcttat 22201
atggaaaaat catttcagac agggaagggg atagcaaata aacttgattt agaccttgtc 22261
tttaataata acaattttgc aagccagtca aaattactgg ataaagacaa gaagcaatat 22321

ttttcattag ttgcagaaag gttcgcacga tgtggagaag cgctggtaac agatttactt 22381
gctgaaaaaa gcgtacaagt agattactta gtaacttgca tggaggatcc acaaacagta 22441
gggcaacgag ctggtatcac tccatctggt aatgaccgaa ctgaaatttt aaaagctttt 22501
aaatcagtga ttgaagcttg ccgtagttat gcttttaatc tggataaaga tatcaaagag 22561
gaaaatgaag ttaatcaaaa attgtctact acactgtcat ttggagctaa ttgatgaaac 22621
ttaacaatac ctttgtacaa ttaaaaatat gcttatattc attttgcaaa aaaacgattt 22681
ggattttaag tatatgaaca cgagtctaga tcaccagact gctgctgagt atatctatcc 22741
gttaatttgt cacttgtgtg agtctgagtt tatttattat ttccatagag aaaagctgga 22801
ggatttagaa gcccttttta tgtatagtaa gaatactaca gggtcaattt ttcaacctta 22861
taaggtagaa cctacaagtt tactttcaac catttcattt agccttattt ctcctttttt 22921
cttcttttgg atgaagaact taattgaaaa ttatgcgtca caacttgagc taatgtatat 22981
acattttcca gactcattta tttttaaaaa ttgacagttt aaaattttta aaatacgatg 23041
aattatagct tttgcgagag ttgtaccatg aatgcatcac aaactatcca gactgtacaa 23101
tttggtttgg atcaatactc taaattagta aaagatctca aagtcttatc cggcattgat 23161
gcattctata cagctaaatg cttgatagaa tcatttccca atgtagttaa tgatgaattt 23221
tatggctatg atttttatgc tgatgaaaag atcttagata tttcaagcta catacatatt 23281
gatgaaaata agtggtttaa tttaaattgt ctaattgatt tagaagaaaa gattgtctca 23341
ataaatttag cttcagaaat ggaaaatggt gagatttttc tctttcgaac agataaaaat 23401
tatcaggtgg ctgtaaatta taattattca ttcgaaaatt tcttctcact ccaaagtaac 23461
ttcgaatata gtctatttga tttagttctt tcatcgatgg gtttatcttt attaccgaaa 23521
aaaatattta acaactttga gctgaaggat atattcttat tctcaatgtc gatagttact 23581
ggcagatata aaaactatat tccagatcag ctattttta actcaataca caaaaatatt 23641
gttctagatt taaaaagtga tgaattagaa ttaccatcac gattaattaa ttacagagat 23701
gagatagatc ttaaatggta atcagtgatg aaaaaacttt tttaaattat ctaaatttaa 23761
atcaaagttc gggtttgtca tcaaatgaat ataaacagct tcttgaaatt aaatcagtaa 23821
ttgataagaa aaatgataaa ataaactatg tttatacttc catttgaaa gacaatttcg 23881
ttaaaatttt aacccctta aatgataata tatttcagga tgtaaaaaag cagaaaagtg 23941
tgaaaaaaca tattagtctc ttttttaatt ctgaggtgat gaacacatta tttatcaata 24001
accttataga tgagttgtta aaatttaaat ttgtagatct gttatcaatg actaatacat 24061
ttatgagtaa agtattagat tctacaaata cgtccggaat aagattaaag atcgaattta 24121
gttatgaggg ggaagagtat aatttttacc ttataacaat attacaaaca cttaattacc 24181
tcatcgaaga ccagaaagat gtttcttctc ttaagaaaat gattcttcta cctgaattca 24241
agctcataac atattaatat ttagattaat tacaaatttt ctctaatttt tttttgctcc 24301
tatattatct tctataggct atattgtaga attagcagat aagctaaaga tttggaattt 24361
atgatgaaaa aatcaatgag taaggctgat tttgaaagaa ttagagctca tgactttgta 24421
gttctaaagg gagtcaacaa taagcttatt gttggtcaag tttcggttga taaaagttgt 24481
atattcttat ctctaacaaa taacgaactt agtcgtttag ctatgattcc tgaattaatt 24541
ttagagaatt taggtagtat cgttgaactc atgaagaaat acaaagaagt tttttctcgg 24601
ttaaatgatg ttattgaaaa gtccaatttg cctgaattaa atgagtataa gaatgattca 24661
ctatttaaaa ttaaagcttt atatgctttg agagaattat tggattctgc tgaagtgaac 24721
gaagatgtaa caactgatac attagaaata gcggtcaaaa aggcattaga agaatgtgtt 24781
tatattcaag atgttcagtt tacactgtct tactatattt taaatcgacc taagcaaaat 24841
acacaaatga tgtagttttc aatataagcg agtagggtgg caagaatgcg tatattcaaa 24901
tatgagctcg aagatgctct acaatcgctt gataatagtg ttagttatgc aggttttatt 24961
aataaattat ttccaaagtt tatatctaaa gatgttgata acatcaatac cattaccata 25021
aacattattc tgaagaaaat tcaagattta ttatcttctg gttattactc cgtatttatt 25081
aattataaaa actatatgac caatggcgag agggtatggg gtttacatca tttttgtatg 25141
tgtatttact tacatgaaaa ttttgaacca tttggtactt attcatggga tgcatcttca 25201
catcctcaaa tgatattaga tttttggtcc ttaacatctt caaatagata cgaattacat 25261
gaggtgggtt taacttataa tcaagttaac aattcagatc atacagaaag gatgaacatc 25321
gatattgaaa tggcttggga agaattagtt atattaaatc caacttttaa ttttcctttc 25381
tttaggaact ttttttactt aagttgttta tcaattttta atatttcgga ttaccaaaag 25441
tttggcaagt tgctttcatt aaattatagc attgatcatt tacttgacca gttgagacat 25501
aatttattta atttttaga cttacttgat gactatatgc ttaatataga tttaagtgag 25561

tcaacatact cttttgctga tggcactagt tttacaatgt ttgctgaagt aagtgaacag 25621
gacttactca attatgactt ctcatttgtt tctgacagtt caagttctga agattctaca 25681
gctcaacaga gccaaacgtt ttccacatcc gatcaacatt cggttccttc acgtgtagac 25741
aactcttatc tatcttttct ttcaaagcag gatatccacg aagaaatcac ttctaactat 25801
gttcgtgaac ttataaaaca gttcttttat aattattcta aagtcagctt ttctattcat 25861
aatacatcga ttccgtgtgg attcgtggct gattttcttg attacttaaa ccgatcacta 25921
gagcaaatat ttattaaagt tgaaagttta aaagtcaatt atttgaagct acaatttatt 25981
taccagactt ctcatacttt atcgagtatg tatttttata aatatatagt tgataactat 26041
acttctattt ttatcaatat ttctgtaccg gaaagcctaa aaaaagaagt tttagttaag 26101
gtgagatctt tatcaatttt tattaaaaat aagattaata aattaaatga tgttctcgtt 26161
catgatccac ttttcataaa tgtttactct ttaatctttt tttacctaga tagtttttca 26221
caatctttaa actatattcg atgttctgga ggggagctag acaaagatag attagattta 26281
atatataata atgaattgga gtatatcgat gctttggatt ttaacttttt tcaatacttg 26341
gcgcgaagat taccacgcca agtatataga ttgccttaat atttaaaaga aattatttaa 26401
atcaatttca ttcgttttgt tgttactttc atttttctgc tggttaaact cctgcataga 26461
ttctgccgat ctttgtttga tttcctcaat gatctgcttt tgaatgtcac tctgaggttg 26521
ggtaactgta ttcatggcca gtgtagaagg ggaaggttgt tttggatctg taaatccata 26581
ctcaggcata gacatcttgc gaataagttc ttcagctttt tgaggaaaac tgctagtaaa 26641
gaagttattt gctagttcac taagtacttc tttatttaat ttcggttcac tgataattac 26701
ttgctgaata ttatctattc caagtctatt aaaaatattg cttaaagctc ttcgttggtt 26761
attaatttca ggaatttcaa gctttacact tttgtgtaat gtatttaata aaggactttg 26821
atttaatatt cccgttaagg gaatgatctt tgaacactca attgaatttt cacttttcat 26881
ggcgatcctc atttttttat catgctgtcc agcagtcatg attatcttgg attgaagctg 26941
ttaggaatct ttgattctaa tactacttta tttgattgag tagctgtacg ttcaatttca 27001
cgttgcttac taattattac tcgttcaata tcatttcttt gttcttctgg aacaaacgta 27061
agtacacctt ctttcatgtt ccggattaga tcaattttta gcgaacgttc gcaatgcaga 27121
tttttgattg tatttagaga gctctttaat ttttcttcat tcaattcgat ttgatccgga 27181
ttgcgcgaaa attgtaatgc tgtatcaatg atgatgctgt taatgtcttg gagagtggca 27241
cgttctcttt taagattcat tagaactttg tcattaaaaa tttcgtgctt cattcccata 27301
ttttcaatat tgaacccttt aacaccatta agtatttggt cttctagaag acgattcagt 27361
ttgtgatcag gaccattctt cattgcatgg ttaatgattt gattcgcttg taattcttca 27421
agagattggt gctcttgcat atgaacaata gaagcataca aattgctaaa cgattttgtg 27481
ctactataca tacatacctc gaaaacagcg atatatctat caaaatcaaa ggaaaaagtt 27541
aacgttaact ttttacatta aacaccataa ttaatgtgct aacaatgcat tctaccttta 27601
ttttttttgc gaatcttaat ttattgttat atgtgtccga atgatagtca attgcatttt 27661
gtttttttaaa aattgtctat ttccattttt taaattaaaa ttaattgtgc tttgaaaact 27721
aatattttga cagcattccg aataatttaa gacaagatag ttgagctact tgtttaaaaa 27781
cctaacagac ctgtggtcct tgttagaaat aaaggataag agtaatgagc atattttgcc 27841
cttcgcagac tgacagtaca tgtcaaaacg atgcagcaat agacctgctg ggaaaaattt 27901
ttggtgatgg gtttattcaa gcattcgtgc tgggtaagtc cgctgctgca actgcacctg 27961
ctacgggtga attaacgcct gtactctttg gtgcattagg tcatttagcc ttagtgtttg 28021
ccgcttttct ttttctcttt ctagttttta cttccgtatt acacactgca cgagatggtg 28081
aagtctttgg tgctggaaag aaaaaacatt gggtaatact tcgtatcttg atgtctggtg 28141
tcatgattct acccaccaca tcattatatt cagtttccag tgtggtactt atgatggttg 28201
tattagcctc gaatggtgaa actaacaagg catacagaga atttttaaat actgttgtta 28261
tgaacccatc gccatccttt gcttcagtag acggtttaga tccgtatggc tttcgcgcat 28321
acgcaatcca aaagcttcgg caatacacat gtgtacgtgt tttgaatcag aattttgcgg 28381
ctgaaggtgt ttctgtcggt gaaaaaaaat attcggatgg ccaaacttat tatgtttcta 28441
atattggagg agaaagacaa tttaacagct ctctagttga cagaaataat gtcttagatc 28501
agggatattt cggagcaata tgtggtacat cctcttacat tgtagctaat gctgactctg 28561
ttgattcgaa ttttaaatat ttaacatctg aaaatccgga tttagaatca tataaagatt 28621
tcaaaaaacg tttattactc aatatacaaa atcttaaatt tcagggtatt gttcgattaa 28681
atgaagcagt acttgcatat gtaaatagat tgccatctga agcgattagt cctgaagagt 28741
atgcacgtaa ttttaatcaa gcttttaata atgaagaatt tgataaaatt attactgatg 28801

aaattagacg aactgatgcg tctattaact cccacttaca gtcaaatcaa aatagttata 28861
ttaataaaac aattcaatat tttgctgaac gtggttggac acaagtagct ctcactaaga 28921
agaaacttgg tgaattccag cgtgatattt atgaggctcg cgcaaaaacc atttatgaag 28981
gtacgaatcc agacttacaa cgtcttaaca gagaaggcgt tgccagtgaa aaagaacgta 29041
tgatttataa cgttctagtc acacagatgg actctactgt aaacaattac ctcaagacac 29101
ctcaagtagt aacaacccta tcaactaacg atattcagaa tttaattcct gaagatataa 29161
ctaattcaga tgtttcattt aagcagatct ctaaagatgt tgaagctgca aataccacac 29221
tagtttcaaa taccatgaga atctttattg agacttttac tggcactgga caagacgatg 29281
gcgtagacgc tgtaactcgt atgcagaaca cgggtaacgt acttcacatt gctttaacgg 29341
ctgtagatac tactctaact ggcgtgagaa ctggtctatc tgctgctggt gcaactggtg 29401
ctgttttctc agataatatc ggaactgctg ctgaaattgt cattggtctt gttgactatt 29461
attctgaaaa gctaggcaaa attatggtga tgggtaaact cgttgcctat tatcttgctg 29521
taattatccc gactttaccg tttgtattct tcttcttcgc tgttattggc tggattattc 29581
atatcctgca aactatggtt ggcttaatcc tttggtcagt aatgcatatg atccctgaac 29641
aaagttttgt tggtagccag acacaaggtt acttaactgt agttgcctta ttctttaggc 29701
cgatgctatc gctagtcgga ttcttcttgg cgtttattat ttccgatcct ttattaacat 29761
ttactactga tggtttcttc gcaatcatgg gagctgtaca gttcactaat aatgggttta 29821
ttgctgcttt agctcagttc ttctggctaa tggggtgggt aattttatat tgcttaatcc 29881
ttttaccaat tatttattta atttttggat tacctagttc gcttgcggat agtgtattag 29941
cttggttggg tactaattta tctcgttcac ttggcgagac aaatttaggt cagaatgttg 30001
gtcgtgctgc aaatccggct gctggttact ataatacctc tggtggtgga cgtggtggct 30061
ctggcggtgg ttccggtggt aacggtgggg gtggtagtcc tgctggtggc ggtggagccg 30121
gtacacgtgg tgatggatca gtattgatga ataaagggca gcctaatgaa caatcatcgg 30181
gagctcaaac tcacttacct aatgctaccc caagctcaag taattctaat tggcagtctg 30241
cggcggctgc tggagctaga gagggtggta taggtggtgc tattgcttcg agtgttagct 30301
tcgcgggtag acggggtctt cagaatgaag cttcggccgg aacgagcgtc ctttcagatg 30361
taaatgctgg agctaatcaa acgggttata ttccaactga atatcgtatt gtcccatctt 30421
ctgcttctgg agccaacagt gttgctcctg atccagtgga taagaagcca aaaaactgat 30481
agtaaaaagg ccggtacata accggccttc tttttttggt ttatttaaaa ttagacattt 30541
tcatatttag ataaccgttt tttcaaatca gttaccaaat cgtctagtct gtctatagtc 30601
gcgtttgcat tgttgatgct tttgaatgca ttacctaatc gagtctcata attagaattt 30661
gagttttcta attgacttat gtaattcaaa agacgattta catattcttg agaataagca 30721
ttttgatgaa caggtgtatt aatatttctt gtgaaattac gaatacgtgc aacggcagcc 30781
ggagtatgat ctttcatata ttgatcaaaa tcacgggcat ttctgtctaa aacatgtgga 30841
gcaaataagc cattatcaat gccgtcattt gaatagcgca tatttgacat actcaaactc 30901
ctttaacatt cttatatata tcttgattaa attataaatt agcattcaca aggatcaagc 30961
tattttatgt aaggaatttt taaaaaactg atatattcaa aaaataaaaa tcagaatcaa 31021
caaacttgca taattttaag gattagaaga aaattagagg tagaaaattc agatggttta 31081
ccactgaaaa aaataaccta cctttagggc aaacggtaaa atgataacac ttggttcaac 31141
aaaactaagg cttcttgtct tgatgatgtc tgctgctgtt attgcagatt tacatgcttc 31201
ggccaccaac tttgaatatg ccaaaccgtc tggtcaaact ggtaaaccat caaatttaga 31261
tgctggtgat tttcctttaa ctggtaatct tctcgttaca tcacgttttg gtattactcg 31321
atttcgatcg gatggatcac cgcggtcaca ttccggttta gatatcatca actcaaatgg 31381
taatcatagt ctgtacgcaa ttgcggatgg tgtcgttatt gcgaatggat ggatgaccgg 31441
tggtggtaat tatattcgcg ttaaaaggaa agaaaataat gacgtatatc agtatcttca 31501
tttagctacg cgctcccctt taaatatcgg tactgaagta aagaaggggc aatatttagg 31561
gacaatgggt aataccggta tatcgttcgg tacacattta cattttgatt atgcaattcc 31621
acaaaaagaa ggatcccgtg ccagaaatgc atggcttggt agtccaaagg gaagcgctaa 31681
tgcgaatcca tatcagaata ttcagacaat aggtctaagt aagaatggaa tcgaaggata 31741
ctacgtaact gatccgacac catatttaaa agatgatata aaaataacgg atagtaccta 31801
taaaaactat ctcggttcta ctattagaca acagtttaat atcctctatg gagctaattt 31861
gccagtagga gctggtgcaa cacagccaaa atttgctggg gttcagttta agttacctca 31921
aggatatgga gctactgatg agcaactagc aaaagtaaat atttctgtca ttgaggctag 31981
acaaaaagct cttgaaaatg gtgagatcac tgaagcacaa gcttacgatc caaacattac 32041

ttatcgagaa gtagaagata tttttaaaga ttcagggcag gtggggagtt ctgaagaaac 32101
taaacttgat attggtgatg atggatcagt aaaaggccac attgaaaatt tagctttcaa 32161
acgtttcgct tctgctggct gggctcaaga tctggtaaaa gcttcaaatc gatctttatg 32221
ggtcgaattt ttaaatattg aaattgctaa aaaccatctt aaaacacaac ttttaaagca 32281
aaatgaacgc attgaagcac tttttgcagc ttacgcaatt gcaaaggcac gagtcctaca 32341
aggtaagatt gaagagttaa gacaggctat tgaaaatggc cgtaatattg ctttaatcag 32401
tcgcttagag cttgaagatc ttccaccgat tgcttcagat acagaagtaa tgaatgcaac 32461
agttacaaaa cgtaatggaa gttatggttt aactgctgat aagaaagctg caattattgc 32521
agtcgctcag gcaattggtg caaaccctaa tgacttagct gctgttatta gttttgaaac 32581
ggctggttca tttagtccaa gcatacgcaa tccaaaaggg tctgcaaccg gtttgattca 32641
atttatgcag tatacggacg gaactggaaa tgataagacg ccgcgagatc aatgggatta 32701
ttggggcatg acacgttcac aatttggatc attatcattt acagaacaaa tgaagtatgt 32761
tgaaaagtac tttaaagatc gtggattacg tgaatctaaa ccagttagtt tagcgacact 32821
ttatggttta gttatgggcg tgccgaaggg aggatatacg attgcaagac attcggctgt 32881
cttcagagat aacccagcat gggatgtgaa tggagacggt gttatcacgg ctgctgaagc 32941
tgtaaatgga tcgaaattta aagctcatat tcgagtttat ttccctgatt tcccataata 33001
aggagtaaga aagtgttaac aaaaaatatt ttattagcca ttactttggc cggttcaatt 33061
ggtacaagtt acgcagccga tttgaatgat caattctatc aagtaatgga tgaagcagat 33121
gcaaatccta tcacgttaaa cactaaggaa gacgataaaa actttatttc gcgttataca 33181
aaattgaatc aaaagctggc ttcaattaac cagcaaaaga acgcaaaagt ttatagccaa 33241
tataaggcta gaattgagca aaattgttca aagcttaaaa ctaatgggac tgagtcatcg 33301
agttcagcct attgtaaata tattttgaac tattcgattt ttaacatgct gtgattggga 33361
gccagaaatg aaaaaattac tcttgatatc tttaattcta ggtgcttctg taactgctag 33421
tgcgaactcc aagtttcaaa accagttcaa gcaagtcgag caaatgacag atagttgcgg 33481
tgaagcaaac tatggggatt cgcttacttg ttataacgaa gttatagcta agcatgccca 33541
acttgctgaa acaattaagg gtcattcacc ggaaaaatcc aaaattcctg catggaataa 33601
agccgtttcg gatctatcat cgattaacaa tttatgcaat cagattggcc aaattcagac 33661
cggtatgcaa ggtgcagcaa gtttaggcac cggtgattac tgtagagcta aaataatggg 33721
cttaacttca tataaggcag ctcaaatttt tggcagtaaa taaggcgcat atattgaata 33781
acctgaatcc aatagagcga agtgcgctga cgtctgtatc tgaaagttta atcgcgatca 33841
gggtaattat aagaagagct caaggcaatg gtgggttatc agttaaagat attcaaaata 33901
ttgaatcatt agctgatgct tgccataaca taccttcagt tattgccaat aatgagtcgg 33961
aacagctcaa gtttttgctt gagtcgggta caagagaggc gaagatgatt cttgctcaag 34021
gagctgtatt ttaatctgat ctgaatggcg ggcttaaatg gatgaattaa gtaatacaag 34081
aattgaagat gctggagaaa aattcgaagg tgcgcgtaaa gatgcggctt ctccgatctt 34141
caatcaacca tttaagctag aaaaaaatga cagcaaaata acaaaaaaaa agatatggcc 34201
ttcaccgaat tttagagaag acgttcataa cggaattaaa actaaagagc atgcattact 34261
ttttatgctt gtgtatgaag gtttatgcaa taagccttta aacggggggt ggttcaatat 34321
tagtggccaa tcatttgaaa aagcgtatga agatacattg aatttttga aagaagctta 34381
tgaaaaaaaa tcttatgaaa atagagatgt attagttcaa gacttcaacg attttatgaa 34441
agcgaaatat tctggtaatg agttgtatgc tgctggtaga agaaaggggc gggttgtcca 34501
gcatccatta tatttcaatt tacattctag aattcgtttt aaaaatctac ttaatattgg 34561
ctggccacat aagttaattc tggattcaga ttgttttgga gcttatgagt tttcggataa 34621
tgattcaaaa aacctatttt ggtatgcaat agaaggcatc aaggagcgga ggatccggag 34681
cttagataat tgggccaagc atgatcatta tgaggctgca ttacaagtgg ccaaatctgc 34741
attcagaagt attattcaaa aaaggaaaga agagcagcca gaaaataact caaaagtgcg 34801
ttctaagcct ccaaaacgcc ctacatttaa tcgaccattt gtacgggtag gggaaacata 34861
tcgtcaatca aatatttcaa ctgaggagtt tttgagtacc tttaaattta gagggattga 34921
atggggaaat tatgtaactc aatctgaaag acaaggtttc ctagacgcta cttatgattc 34981
ctttatagat cttacaaggc tttgtggttt accaccttct tttgcctcac taggtggtaa 35041
attgggtatt gcttttggca gcagagggcg agggtatgat ggggcagcag cacattttga 35101
gcttgatcaa tggctaattc atttgacaaa gacaaaaggt gtgggagcac ttgctcatga 35161
atttggccat gcgttagatg cttatttagc tttaaagagt aaaacaaaaa gagattttct 35221
aactgaatct tttattacta aatacaataa tcactcgctt agccttaata caaatagttt 35281

aaaggtgagt gaatccgaaa gcctgagatg ttttaaagtt cttcttgaaa atatgatttt 35341
tacttcagat aagaacataa ttgatgaaga tcaagtaagg attaattcta catttgcaaa 35401
gaatgcgtgt ttattagatc aagggagaaa agaagcatat tggactgatc caactgagct 35461
ttttgctaga gtctttgaag tgtgggttag tgataagcta aaagcttcag gatatatcaa 35521
tgagtttctt gtttatggtc cagatgaacc accttcaagc tggcacacca aagttaatac 35581
ataccctgaa gatcaggaaa gaataagaat agtccattat ctagatcgat ggttatctga 35641
acttgtaact acatggaagt ttgaaaaaaa gagaaaactc actatatcta gaattatgtc 35701
taaaaactcc caatgatagc tacaaccacg ttttaatttt tgtcagcaaa aaatgacttt 35761
tacaaacaat gtttgtttga agacatcgag tacggtcttt actgaatgaa gctataagta 35821
aaattgttta aaaactgttc tataagcggt agaggcccgc ataaaaaaat taaatattcc 35881
tttatccgtt gatcattttt tctagatcaa tttgtttatc cacaactttt aaatttgaaa 35941
ttaagctgtg ctctacttta ttattttgat tacgttacgt tttcaagagg ttttaatatg 36001
aatagagtaa ttaattcaga attggagcat gccaagttgt tgctcggtct cggtcaggtt 36061
tgtacggatg tcctttccat gcatgaggta gttggatctt caataaaaga aatacctttc 36121
gtaagtgaaa ggatatcagc aggatttcca tcaccggcac aagattatgt tgataaattg 36181
atggatatga atgagcattt aatccggaac gaagctgcga catttattgt gaaagtgtgc 36241
tcattatcaa tgataaatgc tggaatcgat atcaacgatg aattggttgt tgatcgaagt 36301
ttagaagcga agaataacga tattgttgta gcgcttatag agaatgaatt cactgttaaa 36361
cgtttaaaga ttgaaggttc gaagatttgg ttaaaggctg aaaatccaga ttaccaagat 36421
atatatttta atgaaggtca aaaactaatc gtatggggag tcgtcacgtt catactaaaa 36481
aatgttagaa aaagatcatg aatcaaagca agaaaatttt tttccttgtt gatgtaaaca 36541
acatgtatac ctcggtagaa acagctttcg atccctctct aacgggccgc agtgttatcg 36601
ttttatcctc gaatgatggc aatgtcgtag cccgtagtcc tgctgcaaaa aaacttggta 36661
ttaagatggg ggagccctta tttcaaatcc tagatttagt gatgaggaat gatgtaattg 36721
tactttcaag taactatatt atttatggag aaatgagccg acgttttcat aaaatactag 36781
cggaatacgt atctccagaa aaccatgaaa tatactctgt tgatgaagca tttttggagc 36841
ttacttcata cagaggtata tataatctga atcaatgcgc tcaagacatt aaagaaaaat 36901
tattgaaact tttgtctttg cctgtctgtg ttggtatagg tcgttccaag actgaagcta 36961
agataatgaa ttatatagca aaaacctatc cgcacttgaa tggtatttgt aatgtatttg 37021
atttagctga agtaaaagat tctatttttc gtaacactga tgtaggagaa gtttgggggg 37081
taggcagtca acaaaagaag aagcttcgac tcatgaatat taatactgtc tacgacctaa 37141
tgacggcctc accttctcat atacaaagtg ttttttcagt tgtactaaaa cgaactgtct 37201
tagagttaaa tggcatatca tgtattgata tcgaacatac gcctccgaca aaaaagcaaa 37261
ttgtatcgtc acgaactttt ggtcagagaa taacagatat tcatgacttg aaagaagcag 37321
taattaaaag aacacaagag gcgtttacaa gagcgagaaa tgaacaagtt cttgtggggt 37381
gcattattgt gtttgcttat tcaaatcctt ttgataaaac taaaccattt tataaaaagg 37441
aacaaagttc atcttttgca gtaccgactg atgatttaag gttactggtt caaacagcaa 37501
caagattaat ggatcaagtt tataagtccg gaatcgaatt taaaaaagcc ggagtgattt 37561
tgacttgtct cgaaccaaaa catacgtaca cctatgatct gttaactgac catcaagatc 37621
tggagaaaac agagcaactt atgaaggcta ttgaggaagt gcaacagatc tttggcaaaa 37681
acaaaattgg atttgggggga agtatgttta aaaaccggat ttggaacctt acagcaaatt 37741
atcaaactag aaattatttt agttttgatg gaatgataaa aattaagaat taaaaaaatg 37801
ataagaaaca aaactataga tagagaaaaa ccacctttcg gtggtttttc caaattgcgt 37861
acattatgtt tatagaattg ggtttttgca attctaggga acgcataacg tgtctatggt 37921
tgttataata cactcatttt tcgggtagta caactaaccc atactttaga tcttaaaaac 37981
aaggattact tcttcaaaaa atgagcgatt ttatttctac ccccagacta agcacatatc 38041
aaaatatttt gaagctaaca gatccgaatc agatactaag ggcatattat tggaacattg 38101
ctttagctgg agcaatttat ccgttaatgc agactttgga aattacttta agaaatgcta 38161
tagatgtctc agtaaaaaac aatcatcaac ctaaaagtgc aaatggatct aactttgttt 38221
cttataaaaa taatgatcta tggtttgaac aactggttac agcagtacaa gatagaaaga 38281
taactaaaat gcgaccgcac caagctttga aatgggtgag aggtggaaaa cgtataaaat 38341
tcagtaccac ggaaagtcat gtgaaaaaag cacgtgatga tgcatcaact gtaaaaagct 38401
gggttaaagg ggaggatata ctaagccggt tacctttgg attctggaca actttgctta 38461
gcaaggatta tgaagatgtt acgaacaaac atttactatg gccaaatctt ttgcatcacg 38521

tttttccaaa cgcaccttca catattaaga ggaaagacat tgaagaccat tttaacttaa 38581
ttagagaatt caggaaccga ttatcccacc atgaacctat ctggaagttc tattatagaa 38641
atcctgcgaa taatgcatta gattatacaa caccaatatt cggcttgaac gcttctttaa 38701
atctattaaa taatcaatat gatgatatgt tgatgcttct tcagtggatg tcagcaagtg 38761
cttacgataa ttttaattat tcacgtatag gcaatgagtt caagaaatta tgttctattg 38821
atggcttcta cgcttatgtg gacagggaaa aagttgccaa ttgttatcca gcttcaagag 38881
ccaagagaga gttctttaaa ctcgcagaaa cattacagaa tgtgaatgtt gtttatatga 38941
aaactaacgg aaaacgtggg tatattctag gattaaatga accatcttta ccctaacaaa 39001
tctaataaaa aagagatcta taaagatctc tttttttatta ctgatgaacg cttatttata 39061
ttgcttcaga attaaagtta gatccttaat cagttcacct aaagaattta caacttcttc 39121
acgttcttcc ggatctttga tttcgccctt atgtagtttc gatactaaag gcaagctttc 39181
tttagctttc gatagaatat cctcaattac agtttcattg agtgaaactt cttttgattt 39241
aggtttatct ttttctagat ctgtttcaaa tgttttttttg aatttctgta cttccgcaat 39301
accaattttt ttagctgtgg cagattgttt ctcaacccaa gcacgtacat ctactttatg 39361
atcacgatat aaaactgcaa tctcatacat tgctgagaag tcatttaata gattgcgatt 39421
acacatatct tccagagttt cgagaatttc ctcatccaac ccatcggtga tagctacaat 39481
ctttgaaact tttgcatcac cgtaagccag cgttttaccg atttgacgtt ggctgtatcc 39541
tttagccttt aagcgaacaa tagcacgact aatatcataa atagtcatgt cttcattcat 39601
ggtattagct gttaattggt caagttctga aaaactctcg accactacag cagggatctt 39661
ttgaaggcca gctcgcagac tgttcttaac ccgtctattg ccgtctttga caataaaccg 39721
gttatcatca tgaggatgct taattaggat tactggaatg cggacaccgc tttcacgaat 39781
actttcttct aaatcctttt ctttagcttc aatgaatgtt ttacgtgggt tttctggatc 39841
aagatcaata agatgtagtt cgatttctgt ttgtttagca ctattagctt ttgattcatc 39901
accgtcaaga actaaatcta ataaactgct catggattat tctcctttgc tcgatgaatt 39961
gaatgcagct aatttaagtt gtacacccac ttgtttgaag atattgaaaa aggtacgttt 40021
tacatcacgc gaagaagttt tattaatatg ccagcatgca catccgtaat taacggcctc 40081
ttcaatgacg ttcttatctg taatataagc aatgttgttt ttgtctttat agaaaatgct 40141
gttactttca ccttcagcta aaagcttcgc taactcttcc ttttgaattg aagatttcat 40201
attcacacga tttggaacaa taccaccaag aagtaattta gtatttaatg ctttcttaat 40261
attgttagta aaagaaatgt agtcacgtat accgtctaaa gcatattggc taagcattgt 40321
tgggataata actacatttg atactgataa aggtaaaagc atcaaattac ttagagtagg 40381
gggagtgtca aaaataataa ctgttttata tttcaataac ttattaatat tttcttggaa 40441
tatagcaacc aattctttgt cactacttgt attaatgctt gcaagtgctt tagttgggcg 40501
aaatacgcct attgaaccat tttgtaaatc acacttaagg ttttgattat ccttgtgaaa 40561
aagtgaaacg gcatcaccta gattgtgaac aggagtagaa ggacgggtta catattcagg 40621
caatacggta tttgaattaa gttttaataa atgtccggag ctattacctt gattatcaag 40681
gtcaactacg atcactttgt agccgtgttg agaaaagaaa tatgcagaat gtacggtaaa 40741
tgtactttta ccaataccac cttttccatt accaatagtt aagataccgg catctttaat 40801
ttgtgaagtc ttgtcgttca tgtgcttatc ctatttataa aaatcagggc gaactcttgt 40861
agaaatcact acccgtccac cgcatgatta atagaatagc agaatattaa atttatgaaa 40921
gtatttttcc aaaaaaattt tcatttatga aaacttaatt agacatttga tccgaactaa 40981
caaacatatc cataatatct agatcaatgt tttttatatt tattgaatac atttttaatg 41041
tttctatcgc atcaggaaaa aaatccaatg ggttacttaa ctgatcttca atagaagaga 41101
gggcaatatt tggcattgct cttgaggtta agttgttgat ataaaacaca ttacaccaag 41161
atgggaaaat aaattcttgc ttgaggtgaa gctcaaaaat atttagtaaa acacctttta 41221
gttttaaggc agactcagga ccaaaaacat ttggtgcatt tagcgtacct aagcagtagg 41281
agaactttac agtaagagag ttttcaggaa ttaagtggaa aacgtataca taaaatttat 41341
ttggatcgtc atcagatctt aaagtgaaga tttgatcctt actctcatat tcctttttt 41401
ctttaaaaat atgtgattgc ttttgaccat tcgacattaa atcatgtatc agcaagttag 41461
ctttctgcaa catttctgag gctgccacaa catctaaaac atgtttcgga ttaggtgatt 41521
gcttgagtct actcataaat ttattaggat cattctgatt tgaacggaat actcgttcaa 41581
gtaattctag agaaagctgc attcggcacc tttttatctt aaaggaatga agttttgttg 41641
gataaagcgt gttgacgatt aattggagtc aattgtggtc gaacaggtat aggaagatta 41701
ctgaactcag aaatattcca tttattaata ccagccggag aaggtccaat cttaccgata 41761

tatgcatttt tgaatgctgg aatatattgc tgaagatgat catagtacat gtcgcttaac 41821
ggaatactaa tagttaagct aggaatccgg atcttattct cactggataa atttaaattg 41881
acataattta gaggtaaaaa cttgataatc aagaattttt caataaacgt aaaatactca 41941
ataccaattt cacttaacca ccaaaagtca ttttcttgag cgaataacaa gaaattttca 42001
ggattcttgt catctattcc aaatgcaaac aaacttgctt ggtgatgtgt aaattgaata 42061
ctagagtaag ggtttttagt ttcatagttt gtttgaaaag aacttaagaa tcggatgaaa 42121
tacaaccatg ctcttaatcg aagtgcattt aaagatgcac tttcaatttc attctcttct 42181
aacgcttcaa gagcgtcaac gatatgtggg ggtagctttt tagccattta cttcaccagt 42241
gttttcaatc tcatcttcat ctgaagaatc tagattcgat ttcaaagtga actttttatg 42301
ttctttaata atcttcttaa gagcttggtg ttcactgatc ccgtgcgtct gtgattgctc 42361
taaaacccaa ctatatgatg gagtatcaag gatcttctca atatcagttt taagtgcacg 42421
acttcttgct gaacgattat ctgtattcaa tagcccttca atattagaca tctttcggat 42481
gcgctttaca tcacatttac ggatatcaag agttttaccg atcgaaaaat cactattgcc 42541
gtcttgaacc atgttcgcat acggctttac tttgctgtaa agctgaagac tattaataat 42601
gtggtcatgg ccagttacaa ccattcgttc aatatatggc gcagtatcca agtcatcgat 42661
acgcttatta cattgagcaa cgatatcctg aagaactttt ggatcattga tatcgcaaat 42721
caaccgtgaa agttggcgcg ttttatcaat aagatgagaa gtaccatcaa tatggaactg 42781
actttgaatt ttagagaatt cgagattgtt taggattgat aaagcaatca caaattcagc 42841
ttttaggtca acaataaggt caaagttctg tgaaatcgct cgacaataac tctgtactgg 42901
taaacgacgt tcaacagtcc aaatagcagt atcaaagaaa ttaatgagat cggctaattg 42961
attgtcattg aaaaggggat attgcttatt tgaagatgaa agagggttat gcaagttcga 43021
cttttcacca cgagttattt ttaaactcag ttctttgtca tcagtaaaaa aaccgttttc 43081
taagatggaa aagtccgaag cctctttttt aggtttagag gcgatcagat gtgtaatagc 43141
cgaatagtac tcatctagtg agaaatttaa acgttgttta ctttccataa taacctttta 43201
atttaaacta tcttgtaagc aacctagatc attgcttttg gcggtagctg gaacccctag 43261
cttgccctga acacgcgaaa ccacatcttc tgcaatttga acttgttgat caattgaaga 43321
tgaagaatac gggttttgtg aagaatttgc atagccattt tttgaattaa cataatggcc 43381
ccataaagat agtgagataa atgcaagtag aagtaaaaca atagcggcta ggaagagctg 43441
taaaagtgaa aactcatagc caaatattga aaaggttctt ggctgtctta cagttgatac 43501
agatgcatga cctgaaggtg tgatagggt atatccacca ccgttattct ggttgccaaa 43561
agttctatta tttaagttat aagttgagat gatactatta aatgcatcct cccactggcc 43621
atttaaatac tctttaattt catcaggggt gttaaagaaa tatttatctg ctttagaatc 43681
ataatcgttt ggatagactt tgaagtcgat atcagcaatt tcatatgtct tagtttcatc 43741
agtttcattc ttaaaggaag gtgatatttc tagaactgct ttgaatttga caattttata 43801
aggtacttcc tctgaagtat gcaccggatc tgcattcaat gtactttggt caaagtctaa 43861
agcgtaatta aatgatggta gttctggaag ttgaattgat actaatttgg gagcacgatt 43921
gtgaattgtg caattatcat cagtatgtac ctgaattgtc agaaaacttt tatcttcctt 43981
ttcgtgaaca ttgctagtca ctgaaggtaa atgagtcgta tcaggtaatg acactttttt 44041
attaaacagt ttcataggtt atgccttcgc caattcttag atttttccga aaatcatttc 44101
taaaatgctt tcatctgata agccagtagt catttttggc tcacctgttt ttttgtttaa 44161
gtaaaatgct gtaggaacag caattgtttg actacctgta ctttgtacgg ttgcttcagt 44221
aaggaattgc acgttttctt caagggcctt cttctcttca ggagtaggat tgaccactat 44281
atttggatct tttgagaacg ctctttcgag ttgctttgga tcacgaagaa cagcagccgc 44341
caatttagcg ccttcttcat gtttaccaag tagcgtggtt ggaatccatt tgatcgaaaa 44401
gcctttttct acatatttac gtgttgcgcg gtaggcatca tgacaatttg aacaacgagg 44461
atcaaaaacg atatacagtg tatcaactgc tgaagcatta ccttcttttg caccttttaa 44521
tttatctagt aaagagatag aaggaggaac ttctttatcc catttcccta cgatttcggc 44581
aaaatcaaca gctacgttac cgccagtcgg tgcagcaact ccggttgaat ttgctggagc 44641
accaccttgt tgtgcttttt gcaaaatagg aaggcttaca ctatccattg ttgctgtatc 44701
aaaagcattt ccagtaatga ccactttttc atctttagtt gtaaaaacaa cttgtggctg 44761
gccattaatt agcatggtcc aagcaagtag ttcgccagcc ataggttgaa cgttagaaac 44821
ttggcccagc ttactcattt gagcaaaaag attttcacta taaaggtttg tgctgtttga 44881
agttacccct gtaggaacat tatgtgattc gtaataaaac catcctccag ccgcaccaaa 44941
gccaagtaga gctgccaaag caacatgggg aaatttaaag cttgattttt tattgctgtt 45001

accaagtgat ttggttttca taaaattcac cttgaaggaa ttattttata gaataatacc 45061
tgattttaaa aaaaacatat atgcaaaaaa taaatatttt tgctagttga cggtgatctc 45121
attagctttc ggaggcgctg gtaaataaac atgaggatcg gcgtagcgat attgtttaag 45181
gctttctaaa agcccattcc atccatagct gaaatctgga gaaatctttc tccggtggta 45241
ataagcttct gaggtgtagt gtgaaaatgc cgctagttgt tctacataaa cactaggtct 45301
accaacattc tgaacaagag cccaaagcgt gcggtcataa tttctcaacc agcgaagctc 45361
acatggagca agtaccccta acgttctggt tttatagatc agtgcaataa gggcagtacg 45421
tacatatgca tgctttgaaa agacttcttt agctacaggg tgattccagt atctctcgac 45481
aattttctgt aatctttttc tttcaaaata cttaaattca cgtggctcat aatttttagg 45541
attaataaaa gctttttctg aagttaagtg ctcaatggct ttgtcccaaa agtagtgaag 45601
taacatatct ttttgagctt taattttttt gaactcttta tcagacatgc gttcatctgt 45661
agcacaagcc attggtaaaa aaagtgcata aagaataatt tcgccatcgc ttagattgga 45721
tggtttatct agcatttgac caagttgatg catcatcaat ttttcaaatt tgctttggtc 45781
tagcagtgga taagcatcat cttttggaga atttgagcct ttgaaatcat caaaatcttc 45841
ggcattttca atttcgtatt ttctgaatcc ataaacaaga ttatgtattt tgataaattc 45901
tcttggcgat aaaagaagtc tataaaaccc tttgtcggtc gcatagttgg attgaataaa 45961
acgttgtata tagcgtaaat gtggctgata agatcccagc tctccaatta atgattcaag 46021
atcatgttct tgaacatagc gtgtgtccgg taagtatttc aaattaattg catgtttata 46081
ccagatccag ccgactaaga gagctccaat aagaatcgtt ggaaaaaatgc agttttcaga 46141
ataaaaggaa aaaggaacta cttttaaatt cgctgtacaa tgcattaacg gattcattgc 46201
gtgatttgga tccggataac atagctttgt aatagcatta gttgtgaaat ccagataacc 46261
agcgttagaa ccagttatct tttgaataaa taattgaagg taataaaatg gaagttcaat 46321
taacccgtac acgtttcgag taattgtgat aattgccact ttaccaattt tccatccccc 46381
aaaaactaat agagggatgc ctacacataa aacaccaatc aaaatgagtg tgtcatcagt 46441
agatccatga cttgcattat gattatttga catatacggc ctatattaag cttatccgac 46501
ctgaatctga ctccagtctt caatagttga acttgattgt tcatcgaagg actcttgatc 46561
aacgggattt tggatttgat caatcgtttc ttgttttggt gaagctaaag tgtattcagg 46621
ggtgggtgaa agagcattgg tagttagatc gtctaagata ggtttctgta gaggatctgg 46681
atgactagtt gctgtaatca tagtttcgct atgaacataa ctttctaaaa gtgcaaaggc 46741
tctggtgctt tcagtattga agctggatgc catgatttga atattctgga aaaacttcgt 46801
ttcattcgta agctcaataa aatcatcaag gaatgcagag attcccaatt caaaaaattg 46861
attctcaaga gctgccagtt cgttgaaatt cctattagta acatacgtgt aatacataaa 46921
aagtttgtat gccaacatat tggacatttc atataaagcg gttaggcaat aaagctctct 46981
ttgatattga gttttaccag taagtgtttt aacaagcgcc tcatgatgaa agttaagggc 47041
aattttgctc tcattttttc caacggctcc aattaactga agtgatttag taggatgaaa 47101
aaagaaatat gaaatgagat gtgcatagaa gctatgcaca tcaatctctt ttatattctt 47161
aaacttgtcc aaaaaatcac gttggcgctt aggtagcttg gctaaactaa cagagattct 47221
taaccggtgt ttttgaggca taagtcacct caaatcaaag cttcgaaata tactttgcac 47281
atttatacat gccacgtgca ttcgcaaatt caggctgttt tacaaggtgc cagttatcat 47341
agttgaatcg tttaagtagc tcgtcaccaa gcgtgattga acctccacca acgagcaaca 47401
ctgctcctag atcttgaccg tcttgcacat tcactttgat agagttttca atctgatcag 47461
tgatttcaac ctttgcagca tttaagattt ttgaaatatc gtgatctttc ccgccatgat 47521
tataaatgcc ggtcttcaag gctttagaga gtcgccaatc tgtaatattt gaagaaagcc 47581
cgaaaaactc cttaagattt tgacttacct tattttttaa agtaattatc cctacatcta 47641
gagtgtcgca gcgagaagct tgcatggtgc cgccacgggg attgatcatt gcaatatcgg 47701
tagtgcgacc accaatatcg ataattgcac aaccaccttc ccataaatct tcatacatac 47761
ttgagcgttt gccattatca tccatgagag catcaaaata agcacattga ccttcagaaa 47821
gaacgatatg gtttaaaatt tgcaaaggat cattttttttt gtcgtgctca taaagattgt 47881
aaacagtagg gttgttaaaa tccttattaa tcaatagatt ttgagttttg tcagcaatca 47941
acttctcatt tttagctttg tctttaccac cgtagtaccg atttaatggt aagccagttg 48001
caattttaaa tttacggtat tggccatcaa aagcttggtg aagagcatgg tgcacaagtg 48061
cgacattcgc atcacttagc ggataatctt cagtacgtgt atcaatatgc tcttttgtaa 48121
attcatcgac agtaaaatac ttgccattaa cgtgaatgat ttgtttgttt acttcatcgg 48181
tatcaccaat aatggtgcgt ccattggcta ctcgacttgg tagacgaaat gatttaccca 48241

cttcaccaca ataaatcttg attccgtcat ggccatcatc gacgccaact gtcaaagttt 48301
tataaccttt catatctatt tcctttaatt atttgggaga tttaattttt ccgaagaaaa 48361
agccgataat gagctggaaa aaatacttgt atggcatttt taaatagttc tcgattagcc 48421
agcatatcgg gacagcaaag ataccgacga ttatcggaat ttttgaaata ttccaaaaca 48481
aacatccgaa agcgataaga aatagggcta tagggttaat gccttttaaa ctagggttat 48541
ttgaagcttt accccaatct atgaaattat tgttgtcttc catatttaac tccaaaacta 48601
ggctataagg gcatcaaagc ctttttttgct gatgagtccc ttattcaata aatctttagc 48661
ctgattttca aaatttggtg atgagttttc tgtaccttgt ggattgtcat tcatcatctt 48721
tcgaatgtat tggataacaa gtccataccc accggcctga atagccagat ccaaaaggtc 48781
attacgaatg gatttagaga aaaagaggtg ttctcgtaca cctattaatt tcccctctgg 48841
cgtctcaacg aggcgctgag caataaatgc atgtgctgta gagatagttt cggttaattt 48901
acgatgttgt tggtctggtg gaaccaaatc catgatacga ggtagtacgt ttgcaacatc 48961
gttactatgg attgttgaca taacggggtg tcccgtcttt gagagctcaa ttgcagcaga 49021
gaaagattct tggtcacgta attcagcaac tagtagtagt gctggtttac gtctcattgc 49081
ttctcttaca gctaggccaa aagtcatgaa atgttcaggt atctgacttt gaacaatgat 49141
tgaatgctta cttttaatgc cgtcgtatct gaattcgata ggttcttcaa gagtaataat 49201
attgccctga atatgtgtat cttcttcata tacgtaacga accattgaag ctaatgttgt 49261
tgattttccg tgaccagttt caccaacaac ataagtgatg cctagtctcg gaatggcttt 49321
tagaacgaac tcttcagtta ggccgatttg atcataggta ggtggatcag atggaataat 49381
acgttgaaca atttgaaagg aagagttgcc agttctatca ttacaggcac tcacattaca 49441
gcggaaaatg attttctctt tttccactgc attcacatgt aaccagcttt catcattctg 49501
tgtagttata taccctttac cacttttcga attgcgatct tgaaatgcgc tacagttata 49561
cgcgaaattt aaacctttcc ctttttttaat tgagcttagt gcttcattac cattaataat 49621
ggttaaaaac atattaagct caacatcttt aaggttcttc agagttaatg cataaagctt 49681
attgtaacga cgaatgagta ttggtctacc ggagttgatc aatacgtcac tcgcgttaaa 49741
agcattagca acagttaaga ttttacgcac atcttcttca gaatttatgc tgatgttatc 49801
caaatcaatt gtttcatgtc gcaacatttg ataggggtgt tcagctttgg gctcttcagc 49861
gacggtatct ctagcataaa cattcataag ctgtttcttt tattgttgag tttcaatcgc 49921
aacaggtgtt tcaaccggat ccttaacgac tgaacgttct ttgatgctta aaattcggct 49981
attccacaat ttagtgttgt tcacaaattg aggtaaagaa gtaatctcaa agttggtctg 50041
gttcaacata agtgtattgc cgttaccagt aagggcgata tcccgacgag caattgctgg 50101
catcgtaatt ttcccgtcca tgacaaaacg atggaatgca atcattcctt tatatgtttg 50161
gttgagacgg tttaattgat gcttgaaaat agcgtctgct tcacgaacac catcttcaat 50221
tcctttattc attgcttctt taaaagcacg ttcttcggca ctatttttag gttttaactc 50281
gttaggtaag gcgtccacac cgatttcata gctcttcaca ttaaactgta aaaagtcacg 50341
ccagtttggt ggggtatgtg agaaacgagc attttcttta atttcataac ggacaccaag 50401
cactgaaagt gtacgagggt tttgaacatc actaatattg tttgcctgaa caatgacagg 50461
gggaacaaca acagtattaa tcattaaacg ctgaaagtcg taaatagaat caagcttgcg 50521
tacattgttt gggttgtaaa cgactttatc aatttcagtc agtcgagcac gtaatgcatt 50581
tcgtgtagct gcttcatgta ctagtgtttt gattgattca agtcgggctg agctttcagc 50641
ttgttttgca gagtcatctt gagaaatgat gattggtgat tgctgcaagt aatattggta 50701
attaggtaaa tctgccatag tcttaatcct tatctttatt taaatgacgg ttcatttgat 50761
taacgatgga tcaatgtgat tgttttatca ttttcatcta catcaacagt tgcagagtta 50821
ccaagttgca agccaacatc tttaatgaga tcaaatccag ttaatttgcg tttgctgaag 50881
tttacgattg ggagctgatg aatgttacca gcttcaataa agcgatatcc gaagtgataa 50941
gctgcacttt gaaggagggt ctgaggttcc ccgacatagt caacacgaat aacgtcagtt 51001
ctaaagctac gttggcgtaa agctaaatct tcgttaaagg ctttttttatc tagggcataa 51061
gtttgttgtt cggctacagc atcttgagca gcttgactca aaatacctaa aggcgattca 51121
acatcaaatt tagggcttgg aacgtagtag tagctttggc atccagtcaa agtggcaaca 51181
gagatacaaa gaacagtgag tgaacgacga attgaaggta ctattttcat aattacacct 51241
agtgtttgct attgttagta gaagtgctca aggcagcttc ggcttgtagc aacaagtata 51301
aattctaaat tttaaaaaat atatagtatt aaaaaataaa aaagaagaat aagcggtctt 51361
aagttcatat aagttgcgaa ccaaaaacgt caaatcaaaa gttattgcta gttgagctgg 51421
taaggttaat ttgtgaacag tttctaagac gagaaaagac gaaaattctt taaaaaatta 51481

aataactagt taatttcatc taaaaaatat gaaatgtaat gaaataaaag aatttaattc 51541
caaataaatt tggaaacttg taattaaatt taaagaaatt cacaaattag taaaacagtt 51601
aaaatatttg gacaaaggtg gacaaggcta gtctctaatt aaaatcaaaa ttgaaagtta 51661
tataatgtaa agcgtggtat ataacatggt gaattttata aaattatttg aattatataa 51721
tattatttta tttttataat gcaaattaat tatgtatatg agacaatttt tatcgaataa 51781
tataagaatg aataattttc tatataatta ttaaaaatgt atagttttaa aaaataaaat 51841
ttcaaagatt aattatttaa aatttaaaga tcaataaatc tttttatac gtttcgataa 51901
atttaaaaaa aataaattac atgttgcatc acgtgttagg ttgcgagtga tatctgctta 51961
caggataatg aacgtactta gtgatagctc aaaagtggaa tttaataaag ttaaggatgg 52021
gtaacgagca aggagtgttg caaacggtaa gtttttaagc aaaactgcaa tctgatgaga 52081
catggtctag aacgaagtga aagcagttga gcaacactcc gtatctgata gatgtaataa 52141
attaaaagtt atcgtggtgt ttgaattatg aaagctggct cggaattaga attcaatgca 52201
aaaaaatatc caaaaagtat taaaggcttg gagacacgtg tatttgatta ttctgagaaa 52261
acaataatca gatataccaa tgtttggaaa aaactattaa aacaatatgg gttagaagat 52321
caagttttgg ttaatcccga atatacgttg gaaacagtag taaataatct tatttctgaa 52381
tatggaaata atgaattgcg aaactctact tttagacact atcgatctgc acttattcat 52441
cgtatgggag tgatcttgag tgaacaaaag agggatggtg agaagcttgt agctttgcct 52501
aagctggcaa ggctttttga aagtttgaaa gctgttgagg cgacagatga taagaaacag 52561
ccgaaccgtt ctagtagttc gaagctcaag tactttccaa aagatcttta tgaatttgtg 52621
ttaggcagca gttttaattt gaacaggtct aatttgggat tgatgttaaa gctgtttata 52681
gaagcaaata ctatgattgg attgaggcca gtggagtggc aaagcttaag gttggcatgt 52741
gatgttgaag agagatgctt atctcttgtg gtcgataatg caaaaaatac tcagggtaga 52801
gcgaacggcg acataaggat tcttaatatg attgagcctg attcaatcaa gcttaagaat 52861
caattaaggg caatacttgg gtttaagaga ttgctcgatt tagaactgga ggcaagagct 52921
agaaagttct taactgaaag aagtagagag actaatgaaa cttataaaat ctacatcgca 52981
agtaaagagc cagacagttt tgaatttgag ttcattcatt ggaaaggttc ccaaaccgtt 53041
aactgtatag aagccgtaaa ctgtataaaa cctgtgaatg gaactggaga aaccgtaaac 53101
tggatttcta ctgaaaacga acttgagcaa aatattgaag atagaacaaa cggcgcatta 53161
ccttccataa acggaccaga taccgtaaac agaatatcgt ccggacatgg ccaggatacg 53221
actgacgaca ataaagctgg aggtggtgaa tttaattatg agcgagtgat tgaagacggc 53281
gctcattctt ccgtaaacgg gataaatacc gtagacagaa taaagaccgt agacggaata 53341
aataaagttg atggaagtga tcacggcgca ttaccttccg cgaacggacc agataccgta 53401
aacggaataa aggccataga cggaaataat acagttgatg gaattaacgc cgttgccgga 53461
ataaaggggg gtgggacaaa tcaagccatt tctactgctt ctactgcgcc agctccagag 53521
ttgaccgact tcggatttaa ttcagttgcc ggtgaaatac agcaaacggg tagaaatacc 53581
gatcacggaa aaatggctgc cttattgggt gcttataacg ccgtggtaat tgagagttat 53641
gttgatcgtt atggcacgat tcaatatgca ctatgtcaaa gtgtgatgga ttcattgcaa 53701
cgtaaattaa aagatatgag caatgaattt tatgaaaaga aaaagtttgg tttacctgaa 53761
gagttagctt caaataaagc aaatgctacg atttacagta cacgccatca agcggtagcc 53821
aatcggaaaa aatataagtg gaaagaagaa gaggtcgcag gttggtttgg gcatgcaagt 53881
gttcaaacgg cgtcaagaca ctatggtgtg gctggtaagg gttggggtga taaacctatg 53941
tttagaactt caaaatttag tctggaaaaa gtcagaatga gagagcaaca acttaatcaa 54001
accaaacagc tcactgaaga agatgtatta aattcggttt tatcagataa agatttagat 54061
gatctgttgt aattaaaaat gtattccggt aatttttaaa aaatagatat atcttttttt 54121
aaaaaatcgg tggtaatatg aaagcgagac ttttgctggc ggctatcata attattgtga 54181
ttggtgggcc actctcattt ttttattatt tatacggcgg ttgagcagca atgaagatac 54241
ccctaatcat aattgtggtt ttatgtactt tgtatttttt aaatctttca ttgaaatcaa 54301
atatgaaaga cattcacaaa aaagagatca gcgataaagc ttattttcgt ctgagtagcg 54361
aatatggctt tgttaaggca agtcatgctg gagatgagat gcggtcaaaa atcatcaaag 54421
cattatctga tggcataata gatggcaatg aatatgtatc tataaccgga gaagagcctg 54481
actatgatct tagtactgat gctgaaaaag caacatacaa agaggcaaaa caaaaattta 54541
ttgatgaatt taaaccttca taaatagagg ccaattaatg aaaaaactat ttataggct 54601
cgtaataata gtaattgcac tgatcatcgt tactcagtac ttcaaagcta ggagttacaa 54661
agttgaggtt gatgataagg gttactacat actcaatgaa atgtatgagc atgttaaaaa 54721

tagtaaggct ggagatgagc ttgaaatacg gcttcacacg gcgttggatg atgggattat 54781
cactcaggct gaatatacat acctcacgga ctctgaattg ccatcgatga cagttcaagc 54841
cagtgataaa gaatataaag aagccaagct aaaaatttta aaagagttta aaaaagtttc 54901
ataaatgaaa gtttgtattt atagcgcggt ttagttccgt tctcggacta acatacagta 54961
aacggtatta ccgccgtgga cggataaagg ctctccccaa tagccctaat ttatataggt 55021
ggcttaaatg aaaacagatg atgttattga acacatcgaa aaacaaaatg tgacagcagt 55081
agaattactc gatgagtttg atccgaactt gcaattcaaa ttcgctaaag caatgacggc 55141
gttaaaacag gttattgatg atgttcggat tctttatcca gatgcctctt actacattca 55201
agatgatgct ttttatctaa tgctaggaga tagccattct ggtgatcata aacagaatgc 55261
aaatacaaat ttaattgcat tgactgataa tcataaattg gcagggtgta tctctggtgg 55321
aggattctaa atgaatatgt caaacgaata taaaccaatg cttatcaagc acaataagtc 55381
acagccacgt attaaaagta tcgaggacag ggtatttcaa gtctttggca tttttgcgct 55441
aattgctttt gggttaacaa cgtgggacct ctttgcctct ggggagctct cagttctaag 55501
ttttaatatg acttctgagg aaatacatca gttatctgaa gcttttatgg aagcgctctt 55561
ttcccgtgta tttatgttgg tcatttttat tttcttcggt ggttttgccc tacttaattt 55621
aatcaaactt attcaattgt ttgattctgg caaataaatt acataaataa cttaatagga 55681
ctttattatg aaatcagtaa tgcaattagg caaaccgaaa gctgaaaaaa gtatttttct 55741
atcccagcta ctttgtgaat atgaaaatga tcccaccaaa tttaagatta tatcagctcc 55801
agtagatacc tctcagctaa accaagttta tagaagtaca tgcaacgtcg aggttaagtg 55861
tattaagaaa tcaaagtagg ataagccgtt ctcattagga acattacata gacacggcgt 55921
accaattccg gctacggaaa aatacagtaa acgggcttgg gtccggacac ggcgaatatt 55981
aagttttcag ttatgaaaga tgaaaggact aattccggaa gcggaggaat acaggaaacg 56041
gaaataggtc cgtacacgga aacacagctt catacgccgt gataatttaa aaatagacca 56101
gctttaaagc tggttttttt gtgcaagcta tttataatta gaaactataa attttttaaa 56161
actaaaaagt tttctgaaat atttttattt tcttaggtaa ttttaaaaaa aagatattgc 56221
agatttttaa aaatactaag acaatagcca ctataagcaa ctgacactag atgtgcagtt 56281
taattcaagc tcgtgcctta ataatgacga gatgagaact atgaaagagt cacaacaaca 56341
agacattgaa ctgtcttctt cttcagatga atacagtacg tgggggaatc ctcgcaattc 56401
tctgaatgta gatgttagaa ctaaagagtt ctattcaaat ctgcttacgc gcattatcaa 56461
cttcaagatt attgttcttg ttgctttgac attagttgta cttgcacaag ttgcctatat 56521
cgctgttaaa agaaattttg aacagcttgt ttttgatgat ggttcaagta ctccttgtat 56581
ttataaccct ctaactaaaa cctttcggca ataataagca aggaataggt aatgtctgaa 56641
caaaagcaga atcagaaccc gaaaactaaa cctcgttttc caactacacc agaacaggtt 56701
aatagtgggt tggtcgaaat ggccaaaaag aatgatgaac ttgaagacaa gattaaaagt 56761
caacgtcgtc aattattagg ctgcattatc ggtattggtg tgttgggtac agcggttgca 56821
gtacttggtg gcattattgg attgatgcca aaagtcgaat actttcagac cgtcaataat 56881
gaagtaattt gtaaaatcaa tccgaatgac aatcctgcat ttactgacgt aaatgttgct 56941
gaatttgctt cggaaggttt gctaaatgcc tattcacttg attatcagaa tgccgacgct 57001
gatatcaata acactttacg ccgttacttc acagatcgtg ggcgtgaaag cttcattact 57061
acgctacgtc aatcaggttt gattgaccag atccgtaaca atttcttagt ccttagaact 57121
tctaagctgc aaacacctga agtcgctaaa aataatggca ttgatagtcg tggcaatcga 57181
ttctggattg ttatgcaacc agttcgcatg gactacttca acggtaaatc tcaaccggct 57241
gatactcgac aatatatggc tgaaattcgt gttgaagtta cgccacgtga tgtctataac 57301
ccgaaaggtt taggtatcgc cagtatcgtt ttacgtgcaa aaaattaatt tagggaagtc 57361
tcaagatgca gctaaagttt aaaaatcttg cactgagtat ggcgctagtc gttgctggta 57421
gttcttatgc tgaaccaagc caacaacaat caggccagca acagcaagct acggcgcagc 57481
aaactgtgcc ttcttctcaa ccacagggta ataaccaatc tcaagggaat aaccagcaac 57541
aaggtcaaca gcaagccaat aatcaacagg ctcaagctga agcgccttcg tatattggaa 57601
cacctccacc aaatacgatt aatttaggta atgttcaaac ttttggtaat tcgttcccac 57661
caccagtacg tgtagcgaaa actaaagatg agattgttgc tgaaatttta agtatggaac 57721
ttaccccgca acaggttaaa aacatcaagg atctgctctt acgtcaagag cgggatgctt 57781
ctacaccata tacgacaacg gcgcgaccaa ttgtacgctc tgttccaatt gatttgagcc 57841
cagcagctca accgccagtc ttacggcttt ctatgggaat gttaaccagt attgttttta 57901
gcgatctgag tggtaatccg tgggaaattg aaaaagttac gttcaacaaa gaactgtttg 57961

cagacgacag accagaacaa gggcagggtc aaggtgaaaa atctgatatt tcatctaaaa 58021
acacgaacat attttcaatt gaacctctta ccgctaccgc ttacggaaac gtgacagtac 58081
gtttaaaagg tttatcaaca ccagtaattt tcattttagc cactggtcaa aatgaagtgg 58141
atatgcgtat tgatgcgcga ctagctgggc gtagtccaac atctgtcaac tcttcgttac 58201
gcgcaaatat gaatgccact ggcgttatgt cgagtattga tagtcagaca ctactatttc 58261
tagatggtac gcctccagta gaagcagaac gcattagaac ttcagataca agtgttgaag 58321
cttggaattt taacggtcaa tacattgttc gtagtaacga tacagtgctt tacccaagtt 58381
ataaatcaac agcacgttca acaggcggca caagtattta tgtctatccg gatcctatta 58441
atcaggtaac gattagtaga aacggacaac cgactacagt gtattttaat aattaaggtg 58501
tgctatgagt gctgaaaata atacaaataa accagatgac atccagtctt cgcaagagca 58561
attcactggt caaaatgaag cgactcactc gactcataat tccgatcctg aacgcaggga 58621
tcctttgacg cctgaatatg agacgtttga agaaaatcag acagcaactt ggataccaag 58681
aaatgatgca gataatcgca tcaatatgaa atttggtgat aagttcaagc tctactggcg 58741
tgaaaacggt gtgttccgtt ttatcatctt aactgttggt gcagtaattc ttcttatcct 58801
atttttcctc atgaagttca tcttcagaca ggatgcgccg gaacaaatta atccggcaaa 58861
tattaagatg gatgctccgc aaagcaatac taagcaagcg gaatatgtat cacaagagca 58921
atatacagct atacgtaaac aagaagaaat tctagcaaga caggcggcgg ctaaaggcga 58981
aagctataca ccacgtaatc ttgttattgc tccaacagat gctcaaggtc aagtggtatt 59041
accttcaaca tctaatgctc aatacggcgc tatgaacccg caacaagctg gtttaatgaa 59101
tagtactggc caattaactt atgcaaatcc agcagtggca ggacaacaag gtcagatgca 59161
acagcaggga atgcagcaag gccaaccgca acctcaatac ataccagttc aaacacaaga 59221
ttacgtaagt gatgttgctg cgaattatga gcaaaataac aatcaatttg cgaactggtt 59281
tgatgaagaa agtcaacgcc aaatcactgc aaataaagag caagaacaaa caattaacag 59341
ttttgttctt aaacagattg gtcagatcac gggtgctgag tttgacgcta acggaaatat 59401
gattccgaag agcaaagaca aaaaaactaa agccggttca tacgcacaaa aaacctatta 59461
cacggcgcca gcaagcgcag acaattccgg aaacggaagt aatacaggta acggtgcaaa 59521
caatgctcag gatgcttcgc cgttttccgc taacggaaat aatccagtaa acggtaaaaa 59581
gtccggaacc ggaaaagtgt tgattccagc cggaactcgt tcaattgctc gtttattggg 59641
tgaagtgaat acagatgacg gcaaccaagt ctttgcacaa gttaccaatg gcccattaaa 59701
aggccgcaaa gtgtttggaa cagtttctaa gtcaaatgac aatattcaat tcaaaattga 59761
tcgtgtcttg agtgatggta agaacaaaga attttctatc gacgccgtgg gtcaaacgtt 59821
gcaaggctca tatggaatgg cgacatatat caaacgtcat actttacagc gttatggtgc 59881
attaatcgca agtggtattg cacaaggtta cggtcaggca tatagccaag cttatggcca 59941
acaaacttat acaagtggtg caacagtgat tgagactaag gatccatctg gtgagcgtat 60001
tgccggaaac atcattggtc aagttggcca aaatgcaagt caggaaattc agcaaatcgg 60061
gcgtaaacaa gtgacatata tcactccagt cggcaccgtt ttctacattt tctttaacca 60121
agatgtgact gaataacaat cgaatttgga gaaattaaaa atgtcaaacg aaccacaaca 60181
acctaattat gatgacgatt ttgactatca gacacagaca caacaaccag ttgattcgac 60241
tgatagacgt aataagttca ttttaattgg cgcagttatc ttcgctattt gtgttcttac 60301
gttcatgttt atttcaacaa aaatgaaaaa agcaccaccg aaagatcaaa ctattgaagc 60361
tccagctcca gatcaaacgg catatcaacc accgccacaa ccagtgcaaa tgcagccaca 60421
acaaccggtt ggatattcgc aaaattcaat ggtggccgtg ccagaaacga tgccaaagtc 60481
tactgatcca gtgaacggta cagtgatggt taaagatatg actggtaaag acgttgatgc 60541
ttattccgtg gacggacaaa acgccattat tcaattcaac aatgctaatg gtttacagcc 60601
gatcccattg aatatgccag ctccagtagc gccaaatatc aataatcagg cgaataattt 60661
taatgagcaa cgtgtgatta cgttagagca agaaaattca cagctcaagg ctcaaaacaa 60721
ttcactacgt acattggccc aagatcagca acttaaaatt gggcagtatc aagcattgat 60781
taaaaatgca cgcgcaaaca acggcgcaaa tattgtgcaa ttacgtgaaa ctaaaaaagg 60841
tgttgctttg aataaagcat taccacgtgg acaagaagta gtagcgactg taggcaatcg 60901
tgtttggtta tctgacggta agcaatataa ctcatatgga gtaggccaac gtttaccaac 60961
gggttatcgc gttcttgatg taaatgaaat ggctggcgaa gcttcattta agtaacaaag 61021
ctacaaacta gaccaagatt atagggggcg cgcatggaaa ctatagatat tttgcaaaat 61081
atgggtttct tcttcgtagc agctaccgaa ttgcttcgat acttctttgt agtgtctggt 61141
ttttacatat ttcttcatgc gctggttgat tttgttcaaa tttcacaacc agctttagca 61201

ggacgattta tgactcaaat acgccgacca agcgcatttg gggcatcgat gaaacttatt 61261
atcgcagcgt ttctttgtac gttgggttta agacttcagt tgctagacat cgttgggtca 61321
ctcattagtg acacaacacc agcaggggta acttattacg attacacaca gtacacaggc 61381
agctcttata cagacgagct caacttagca gtattggtaa tcatcggggt tttacaattt 61441
tttggattat tagccattgg tcgtggtcta tggatctggc gcgataatgc tgacggcatt 61501
gccaaattgt ctgtaggtca aggaacaatg ttcatgatag gaggaatgtt agcttataaa 61561
gttgaagttg tgcacgcatt tttagcgtcg tggtttggct tagacttttt caaaatcata 61621
ggcttaacct agaaatgaat attaattttt ttaacatctg agctggtaga tccactcagt 61681
ccaaaaccaa ctcctagcca atttcggcgg aaaaaggaaa gaaggaaaaa ttttttatga 61741
gcaaaatcaa taacttacag aaagtaaaaa acgttggtca agatatctat gcacaacttg 61801
gcgttgcttt cactatgggt tcattaggta ttactaatgc gattgcggct ccagatacta 61861
ccaatctaac tggtggtaat cgtcagggac aagcaacagg tggtgactct gtgggtgaca 61921
tccgtgatcg tggtattaac gcaatggatg cattcatgtc attccttcaa tatgcgggaa 61981
tgttagttgg tttcatcgtt ttcgtaatgg gtgttatcga cttcttaaat atcggtaagc 62041
cgggtaaaga tgccactgct ggtaaagctg gattgaagct cttgattggt gctgttttag 62101
cgggtgctgg ttacttctac tggtctgctg cgaactctgc ctctggcaac gtctaagcgc 62161
gtttaagagg tattgtaggg agagctccgg ctttccctac agcttaattt aaatatgcga 62221
ttaaggggat tatcatgata aacaaaagca ttcaactcga taccataggt gatactgaga 62281
agtttttaca tgctttaaaa attgattctt cttccttttt caaaaagcat gaccatcgtt 62341
gctattttttg tgattttcgg gatcttcgtt ttcttgaggt ccaccatcta gatggaaatc 62401
acaacaataa taatgagtcg aatcttgtgc cagcttgtac tttatgtcat cgagcgcatc 62461
atttagcttg ggtgctaaca gatcgttcgg ggttgttagg tatatgtcat gagttggacc 62521
agatcagaat taatcatcta caacgttttg ttctggtttt agctaaccat ccggatccta 62581
agctgaaggc attttttatc aagggtggga tgttccagac cgtacttgat aatattgcaa 62641
tggagtatta ccgtccggat gttgatgaga agacgattcg tgaaagtatc gattctgaat 62701
tccctcaagc aacgattgat ttaaaagaat cactctttac tgaccgtatt aatgcaattt 62761
atgtgagaca gtctctatca tcaaatgatg ggttagaacg cctcatttat gcaattaatg 62821
aggttaagca gagtaataag acgatagcgc cgaatgcgtt tttcccgttt gttctgttat 62881
ataaccaaaa tatcttttct aaagagcaaa ttgattatta cctttcatta gatgagttta 62941
atccggagaa gtggactcag ctttttgaag aacaacttct taaagccggt taatggaggt 63001
cattatgatt gaaagtaacc taaatccatt tctgtttaga gctctgccaa atatagagca 63061
tgcttgtatt ggtaacgctt acgatttaat ggccaatcaa ttcgaacaag cgcctataaa 63121
tgttcaagaa tggtttcact tgcataccgg ctttatagat accggcttag agcttactga 63181
cgatgttgag acggcttttg taacgatact atcgaaccaa gtagtacgtt tactggagca 63241
aacacgattg cctctattca gtactccgga tcctgaatta gaaccattta ttgccgatag 63301
tatggatgat gagctctgtc tggtatctat ttcactagaa aatctgacgg cgcaacaaat 63361
caagattcgt actttattag gttttggtcc tcaactcgtt atattgaagt caaatagtga 63421
ttttttttgac atgcatgggt tcgaactggt tgttactgat tctttccctt gtacttcatt 63481
tcgattgttt attgagtcaa gtaagcggaa tttattatct acgttcaaca aaacttttat 63541
gcttcagcaa aacactattg caaatgaact tttaaatggt ccagcaatct atagtgattt 63601
atttaagcaa gcattacgtc aacaaatctg ttcaaagctg tgtgctgaag atgatttgga 63661
aatatcagta catgacaagg aactttgtga tgatgctgga aatttatatt ttcatattgg 63721
ctttaatcaa acttttaagc atcagttttt tcatttggta tctaaaaata ttcttatttc 63781
tccttttcat atcgaacgtc taattggcga aggaaacgaa cttcttgaaa caccggctgc 63841
ggctcaatcg aatttagaaa gtttagttac gttcatgttg gtgtacatgg ccatatatga 63901
aaaagaggct ctagattcaa tcaattcaac aaatattaat gatgatgatg ttctaaatat 63961
gattatgtct aaattggaac aacattctaa taattaccca agttttagtt aaaaaggtga 64021
gatcagctat ggtaagcact gcgactccaa aaagagcaga tactagaagt actgcgaata 64081
attctaaagc gaacaccaag ccaactaaag tgaaaagacg ctcttcttta gtgtcttcac 64141
ttgagctgat actcgtatcg cttgcgaaaa tgtttaaagc ttcagcaaat gactattgtg 64201
aaatagaaac ggttgatggt cgtttcaatt tggtcatgca agacggtacg atggttacga 64261
tcttgaagta tgaaggtatt cgttcaacaa ttgattatca ccaaacattt atgggtgaaa 64321
ttgaccgctt aaataactat ctgaagaata atttaaatgc agtaaacagc cataagatta 64381
tggtgttatt tagacgtgat gatgcagtaa attcagaatt aaatcgtatt gttgatatcc 64441

agaaaaatac agcacgtaaa gtcaatattc gtctggattc tttcattgat gaaatgcgtg 64501
atgtttatgc accgtatatt tacgatgaag agatctattt tgctttaatg accaatccga 64561
aaattttgga taaatccgaa attgccggtg attatagacg aactgctgat attgttaaga 64621
aatacaatat tccaccgatg tcagatgcac agaacctttt acatggcttg tcttcgttaa 64681
aatcaattca tgatacattc gtatctggtg tgattagtga acttgaacat caagacttta 64741
aaggggttgt agataagcta gatgtaagtg ctgctattac ggcaactggc cgtatgatga 64801
atccacgcta ctttgatcca aactttaaag cattttttgcc gaatagtgga atgcctattc 64861
ctttacgttg gaaaaaccgt actgatcctc aagatctgtc tcacgtttta tatcctcgta 64921
tttctgatca attaatgact cagacgatta ctccattaaa cagtaaagat aaagccggag 64981
acatcactgt tccaaaaagt gctgttcaat gcggtgatat ggtctatgtt ccaattcctt 65041
taaaaacacc tccatcacca agtttctcag gcacatcttc tgtaatgcct acatttattg 65101
atttcttcga ccgaatgaat caacaaacaa ctgaaacatc agatggttca agtcgtgcaa 65161
ttccttatgc cgtctcattt atgttatctg gcgatgggat ggcatctatc ggtatcaaac 65221
gagccgtaac ggcggtagta ggctggcaag gagctgcaaa ccagcaaatt aaggccgctg 65281
gtgattcatt gcagcgttat aaagattatg gtggttgtgt agtcggttta caggtttcgc 65341
tcatgacttg ggatgaagct actccaagag gtattgagcg tttacagatt cgccgatcta 65401
aactttggcg tgctgctcaa aactggggac atgccgttcc actagagcgt acaggagatg 65461
ctgttaaagc ttatttaact cattttcctt tatctactat gcatcatgcg ccactttgcc 65521
cagcaccttt acctgatgca ttgcatttac ttcctcttga ccggccagct tccccattta 65581
aaaagggtac aatggtacaa cgtacacctt gcggaaaatt aattcgtatc gagcacttct 65641
ctgaagagct ggcatataac tacaccatta tttccggtac gatgggttcc ggtaaatcga 65701
tgcgtatgaa cgatgagctg tttgaagctt gtttggcggc cggtctagaa cgtttacctt 65761
atatctttat ttcagacgtt ggtgtgtcat cttcaggtct ggtttatggc atccagaatg 65821
ctttagatcc ttcacgcaaa cattggtgcg tgtataagcg aatctcgaag agtgctaagg 65881
acgcaattaa cccgatggaa actgcggttg gtcttcagta tccattatca aacgagtttg 65941
atcagatccg cgcattcctt gtaacgcttg ttactccagc ggaaaacgaa catcctgaac 66001
ctgagatgag cgcggtaatc tctttgatta ttcgtgaagc atttaaattc tttgctactg 66061
ataatgatca aagtcagcca aagatctatc aaacaggtca gtcacatgaa ttagatctac 66121
ttttacgtga attaagaatt tcattatctg aaacacttcc gttgcatgat ctggctttat 66181
tgtgtcatca acttgctgaa caaattgttt ctaatgaaga aatacaacaa gttagaaaga 66241
ataagctttt acgtgcacgt aatttggcac accgttatgc aatgccaatt ttgagtgact 66301
taattacggt tgccaagaaa gaagaaattc agcgtattta tggtgattat aaaacctcac 66361
acggcgaaaa tattcttgat gcagtaaccc gttctctttc tcaagcagca aacgattacc 66421
ctaactttag tacgtttact caatttgata tttctgacgc gcgtattgct tctgtggatc 66481
tgcaagacgt aattgttaaa aacaaccgta aaaataactc attattcttc cagatcgtgc 66541
gtatgttcta taaaaaacgt atcgcataca gtgaggaaga cttaacccgt ggtgagatac 66601
ctaaagatta tcttgactat tacagcaatt tagttgcaga gctgaaaatc gataaaaaat 66661
atatggtttt cgatgagatg cacaacatta aatctgacaa ctttatgatt gctgaacttg 66721
agcgtgacag tcgtgagatc cgtaaatggg gtattgctta ctacttaagt tcacaatata 66781
tcagtgactt tggcattctt accaaagctg ccagtcgttt tgttgtatgt tctcaaggtt 66841
cacctgaaga acgtaaagac attgtgaaat acttacaact tactccagct caagaaaaac 66901
gtttaacttc cattgtgggt ttacgttcat ctggtatgac gtacctttct aaagtgatta 66961
atgagcgtaa tgatcaatat attcaattct tcacctctat gattgggcct aagcgccttt 67021
ggttattaac gacgaatccg gaagatcagg ccgttcgaag catcatgcgt aaaaaacatg 67081
atctggagac atgtattgat atcttggcta cagtgcatcc acgtggtgtg aaaaccattg 67141
taaataccct gaaagagcaa cgtgctcttg aaatcacttc ttcagcagct ttaagtctgg 67201
aagaggaaga gaatattaag ctgggtattt ataccgaact ggtggatgag gcgattaaaa 67261
cttataactt cattcaaagc caaaaactca gagtcgcata attaaggtta aggaaaaagc 67321
tttcatttat gaaacctttt tccttttttt cagaaatttg agtcatgaat tttcacggta 67381
attcactaag tcagattcaa gataagatcc agatcttaat tttgaaagt gataaagaag 67441
agtccttttt aggtgaaacg attcattttt atcagttctg gattgaactc ttgtttttttc 67501
tgaatcaaaa gaaagaggtt gagattgata tgagcaaaat acaatcatca gaagaccttc 67561
aggatttggc agaattggcc tacggtcact tatatacaag cttcatggaa aagtatggca 67621
atgattacta ccagacagta aagccgctgc gtgaatatct tgattcgctg ccagctttta 67681

atccggagga tcctaattct tatgaatcta ctgccactaa agaacaacat gcaaagatca 67741
tttgtaaatt ttcaaatacc ggtttaaagc aatagggata aatgatggct acaccacgtt 67801
ttaaaggtgc atcggataga tttaaaccga aggaccagcg tgttgataca cgggatatca 67861
tcactaaaat acacgtagct cttcaaacgt cagaagggtc gatgtatttt gccataacga 67921
tcacaattgt agtttttgta agtttctttt ttcctttatt actcataatt gcaacgccaa 67981
ttgcaatatt gatcacacgt aaatatttaa taccccagct tcgctatatc aatatgcctt 68041
atcgggtgcc gaagcatgca aatatgccag atggatcatt cgatggtgta gcgcggaaag 68101
taaatttaag gaaaaaccct gaaaaagctt atgggcaggg tgtttattat tacggtattg 68161
accgtcaaaa tcatcagcaa atctggaaaa gtgatagtga tgaacgtgta catgacttgc 68221
taattggtac gactggtgct ggtaaaactg aaaccattaa gatggttttg acgaatcctt 68281
tacttcaaaa ttctggtttt atcttatcgg atgcgaaagg tacgactgaa cttgtagccg 68341
aacttatgta tttactgacg cgattcatgc gtcaagatga ttatctagtc acaaacttca 68401
ttactgctgg ccgtgatttg ctggtacgcc aagaagataa aatgacaaac acatttaatc 68461
ttatgtcgta cacttctgct gcaatgttga gtgaacaact cgtctacctt ctcgcggact 68521
ctgagggttc tggcggtgat atgtggaagg accgtaccat tacctattta acggccacaa 68581
cacacttatt aacttttttta agggatagag gattattatt acttgagcca aaaacatatg 68641
ttgaatacca agagctcaat caattaatac gagcagctta cggcgaagat tttgatgata 68701
attataaaaa ctttggtgag atttatatta cggccgtttc tcctttacgt gcttatctta 68761
aatccctagc aggcttcgat gaagggaaac tttataaaca acccgaaaaa tgtgttgaac 68821
agcatggtta tgtaatcatg caatttacac gtgcaatttc ggatctaagt tttaacttcg 68881
gccatatcta tggaaccaaa tctggcgaaa ttgatatgtt cgacgtagtt ctaaaccgtc 68941
gttgcttgat caatgtttta ccggcattgc agaatgcgcc ccctacgatc cggatgcttg 69001
gtaaactttt tgtaggctct attaagcaga tgatggctgg agcgctgggg aaccgtatcg 69061
atggcatggt acgtaatgtc attgaagcga aaattacggc ttcagcgaca tgttttaaga 69121
ttgttctaga tgagctggca tatcaaatcg taaaaggcct gtctgtgatc ccagctcaag 69181
cacgtggttt gacattcagc ttaacgtttt cagcgcaaga gcttaaagat atcttcgttg 69241
ccgatgaagc tgaaggtgaa gcgattgtag ctaacaccgg tactaaaact tttggacgta 69301
tcaactccgg tacggaaggt ttatcgatgc agcgtttacg tagtctggtg gaccaagtga 69361
cacagtcagt agtaaacggc tacaacattg cttcttcagc aatgggaata aattatcaat 69421
cttctacaaa cgttaatatc gaaaagcagg accgaattag ttatcaggat attgcatcac 69481
aacaagaagg tcagttcacg atcgtagttg ctaaaaagat cgatggtggt aaagagggcg 69541
gtgtattcgc tattcatgct caaacaatgt ttgtacaacc acctaaacta gcaaagatcc 69601
ggttaaatga ttttgtttcg attgaaaaag agctgaccta ttttgatagt aagaaatatg 69661
ataatgagct taaaaacttt gcttatcagt tagcagagaa caagatcctt caaagttgtc 69721
caaattataa aaaacatgcc attggtgctc taaatcaggt cgatactcac cttgaaagta 69781
tcagtaaaat ggcgaaatta tacgagactg atgttaagtc cggtttcttc gcaattatgc 69841
gtacaatgca aaatatgctg gattcagttc aaatatctgg ctttagtgct ccttttttatg 69901
aagttgttga ttcaactttt gcaaatgaga tcgatggagc tctagattca atatttactg 69961
aaattaaact tgatactgaa gaagtcgatg ttgttttaaa taaaacaatt gatcacattc 70021
aggatgaacg ttctcaggat ctattaacaa aaactagcga aacaattatc gatgagatcg 70081
tcgatcaata cctaaattta gattcaagcg aaatagagac aatagaagct gttaatgcat 70141
atatcgaagc acttccaaac gtttacgatg accaacttta tagcgaagag tttgcaacaa 70201
cggttagaga gcatttaaag cagcatgata tctatattca atcgttagac attccatcat 70261
tcgaagactt attagaactt gatcaatgtg cggccgaagc tcaaaagagt atcttcagtg 70321
atgattttga agaagatgat ttattttcag atgctcaaga gcgatacaac tatgtaaccg 70381
acttcattaa atgcaaaaac ttagtttttg aagaaaagga aaaggaaatc gaatctgaag 70441
atcaggagat cttgagcaag ctggagaagt atcacgaaat tgtatatcgc atgattagtg 70501
cgaagcttaa agataaataa accatttttt gattctatta catcctctaa aaaaggccct 70561
aaaattaatt tagggccttt ttaaaaaatt atgctaccgt gctatagaga aatctaaaaa 70621
tgagcttatg cgtgaaatga gcgcaaaata gggcataaag attaagttag cttaagtggg 70681
ttaaggtttt ttaattgtta tgatatcaag cacacaataa ctgagtatag ccaatccgct 70741
gaaaagacca ccagcaaatg cagatataac aataatggac gcattcagac ttctggttaa 70801
gggactgaat atagccaaat ttataagtat agctattgaa aaaagtagaa ttaaagatat 70861
tcgcaagacc tttctaatgt atttattaag tttatcaata tcattgttca taaatttacc 70921

cttattttaa ttacaaatat ataaatacta aagatgatat ataaacaaaa tgaaaatgga 70981
agaactaaga caaatataac caaaatttta ggaagtagat tgaaactagg attctctaga 71041
attacatgca ttttatagac aataaatata atacccataa ggggtaagat tgatacaaat 71101
gatgcaaaat atagagggag atttttttata ttcatattga tcaactcctt aatccttttt 71161
attccataaa accttaaaaa acttcagaga tattcttact gctagtaaaa aaaggattga 71221
gagaaatact aatacggcgt atttaattac aaagtgacgt gagtcatcca ttgcaacaag 71281
ataaaattta tatcccatgt agggaaaact cgaaattaat gaaagtaaaa caacgcttgc 71341
ggcaatctta tttgttattt cttcaaaagt catatgaata cctttaataa gtatctaagt 71401
atggaattaa taatcccatc tactatttac aactctaaag tatatgcttt tcttgaaacc 71461
catctttctt tctacggctg atatccatag ttgtactgaa gatttatcga tacctgaata 71521
aagtaaaggt ttaaagtttg gtactttaaa gtactttctt gattttgctt taggtacaca 71581
gtctaatatt tcatctgcaa tttttttcatc atcacactca agaataaaat caaaaccttt 71641
aatttttttct tttgttttaa tgagtgttcc tattgaattt acagcaatat aaaacctgta 71701
tatttgagaa atatcgacag tatatttccc cttgtaattt gcataacatt cttctttttc 71761
tacttcgtat tccggaagat ctaaactcag ttgattactt ctattcatgg tctagtgcct 71821
ctaaaatatc aaaacaaaat ttgttagatc tttggtgtgg ttggttgata agctttaaca 71881
ccataaacct taaaacttcc gtcaaaaatt tcatgaaact ctagatctga agttttacca 71941
gataaataac gataagcttt attacttaca attggttgag cctgatattc atgtgatcga 72001
cgattcattt catcaatcca gttagttcgg tgtatagggc atttaaaagc aaatatagat 72061
gcttcacctt ttactgtgga attaagatct ttgcttgtta aaagagcgta gaaatatcgt 72121
gttggctttt tagtcattta attatttcct ttttaaattc atctatttca gcttgttctt 72181
tagaacggtt gttttctttc tcagatatac ttagaagtcg tttgcagtta agtacccgat 72241
agtcgtaaat tgtaaaaact ccatttctaa acttcataat ttctttaagc tttatattga 72301
attctggact aagttcagcc agtgatttta taattttata ttcatctaaa gtaacttctg 72361
ttgaaagcag atattttaat gactcttcat ccagcaaatc atgatgtccg tatacattaa 72421
aatagttcgg gaaattcttc ttaattaata gatccgattc tttatctgct tttgatgcgg 72481
tatacatcca taaagcagta acaataaggc aatagagaag aagaaaataa actaatgatg 72541
tagagaagaa tactaattct gctttagaaa aacaaaaagc tgctaaacca actaatacgc 72601
aaacaataaa agggggccag ccgataaggt ttagtaagga aaccacctta aaacatttgt 72661
tatagaagct atattttaat ttttcgatag caatgaattc tttaacctgt tcaaaagatt 72721
tgacttcata actgttaact atgtcagcaa cgatattatt catgtgttat tcctttttaa 72781
aaaaataagt aaactttttga agtctactta gatcttctcc aattgctagg atatatagga 72841
ttgttgtctt tccataaacc taatctgttt tgtctagcgt attgctcata cttaatataa 72901
ttaggatccg ttaaatattc acgaaatgcc catgcataac cattttgaac aattgactgg 72961
ttaatgttgt aatttccaag atatatagtc ccgatatggc gtttatataa atccatttta 73021
ttagacttaa catatacgaa ctttccataa acctgactat ttaagaaatt tcgtgcttgc 73081
tggccgtatg cttgtgaaga ctcaggagca tctatattct ccagacgaac tcgttcttgt 73141
gtatttccgt ttactagaac ggtaagagta tctccatcag cgactttaat aactttgcct 73201
tgaaaatctg catatgcaac gggtgcccct aaaagagcta gtgataaaac aaaaatattc 73261
ttcataagtg tgtaccaagt gtccaaaggg ggattagccc tttgggttgt taatttcatt 73321
ctgattggtt tttttaggtg ggaacttata gccatttaat tcagctattc tcaaccatgc 73381
taataaaccc aacttttctc ttaggtaatc agtcgcaagt atgtgagcac aatctttgca 73441
atcccattga tccggtttac ccgaatcaat tctttgctta cgtcgatttc tataatccac 73501
aactttttct gaaggggttt taaccagctt gcccccattt cttttacttg gttcaggcat 73561
gtaatgctcc tagagaatcg ggatcaacct ctagagtatc taaagctagg tttagaacag 73621
gtaaagttgt aactgagagt acataagctc caatgtataa atttggctca tggtactggt 73681
ttactaagcg atcaaaaact ttgcctaaga tcaaaacaaa atgctgaagc gaagctaaat 73741
cgttttggtc cttaatttgt ccggttgcag acaacaaagt atgaagctgc ttcttaagtc 73801
gaaatgcaga gctaatttga ataatatttt tggcgttgtt gagcttttta agaaagctca 73861
aaatacttga gctttgatta atgataggga gtatttcagc tttgataagc tcatagctcg 73921
cttcagttaa atgaataaca tgagtctcca gtacagcttg gttttgtaca aaatttactg 73981
taattgcttt cataagtgtc ttcctcatca gttcatcaag aatctgactg ttttgttaat 74041
tataagacct taagtctatg ttgtagatag agaacaacct acaatttcat tgtagatgaa 74101
ttttatgtga agtcaatata aattgtacaa agtacaataa atatataaat tatattgtaa 74161

tgctttcttt atataaatat aatggttaga tgcttataag atatttggtg taagaagtga 74221
aaaaatataa tgtaaatata tttgatggta ataatataat tttaaataaa attgtaagag 74281
ctagatctct ggatgctgca tattattgtg aagatattat taatgatctt aaaaaggagg 74341
taggtgtttc tgtattaaat ttaacttaca gtactattta tgaatttcaa ggaagtgcag 74401
aattatggct ttcaaatact aaaacaggta gaaaaatgtt taggttggaa gctataatag 74461
tttcgtaaaa tataaaccct tttaaggtat caactatgaa tataaatttt aagtttgtta 74521
cttggcatta tatcattccg attatttatc taagtgtttc ttttttctca tgttattgta 74581
ttttttggct atatggcaat gagattaaca atagtatact aaccattatt ggtactgttt 74641
tctttttgga aggaaatgat ctaaatagat ctatttctta tctcaattgg tctactgttg 74701
atggtgctta ctcgttagtt ttatttatgc ttgcgaatgc tatagttttg agcattaaat 74761
ttattatttg gggataccct ttttgtaaac taggaattaa tattctagag aagcagcaaa 74821
tgaaagttaa aactgctcac taagagcagt tttttttatta agttctctca taacttagtg 74881
atttttgatt ttcaaactgg ttaacttgtt tgttttccat ttcgctaatt gtttctttac 74941
aaagcttgag taatgttttg ctccatcgtg gttccgcttc atctgttgct tcaaatatat 75001
tatctttaat atttgtgtag atgtttactt tttcggctct actaatgggt agatatccaa 75061
tgaactcaat ggtgttttta agcatttctt ctgctttttc ctttttagaa gcatctatag 75121
tggtgttgtt agaagcatag aagattgctt gtaaagtcat atttacccga tttagagctt 75181
ctttttcatt gttatttctt atctcaactt tatcgctcat caggtttaat gcttgatcca 75241
gatcgtgcca tccatttttc tgacaaatta ctttaagcat cggtaggact tgcttttgtt 75301
cattgcttga aacagcttga acaatatctt tggccatact taaatcgaca tccttttcct 75361
tctgattctc catgaggtgt attaaccacg aagtaaagat tgccggatct agagctgctg 75421
tcgttttctg aatattgatt tgaggtttat gaatgtttgc ggatgccatt gctggcattt 75481
taaaatttga catattacgg ctttggttga attctgcgat atcgataagc tgttgtttaa 75541
tgatctctcg gcaatgctgt gtaccgtact tcagttcaag atcattccaa tcagagagcg 75601
ttaactcatc ctttttgata ggaggtatta gtacgtatcc ggatacagct tgagttgctt 75661
catatgcttt tattaaccct ccatttgtct tggttttgcc atttaattgt tcatttaaat 75721
cattatcagc ggcaatatat atggtggtat ctggccattt gtttcgtatt gctattgcta 75781
ctgagattaa tgactcttta tcaaaagctg cgatagtatt aggtttttct ttacagatcc 75841
ttgcaaaact ttttccagtg gcaaaacctt cagtgagtgc aaatattttt tcatcttgcg 75901
ctggcaatct gtgaattggg caaaaaactc catctttccg gccatgagtt aaattccctt 75961
taaagaattt tagtttaccg gtctctctgt cttgtagtgc tgagataagc tggcaattct 76021
ggatttttaa atcaacgtcg taatatgcgt taatcaaggt aggtctaatg tgatgatttt 76081
ggaaccatgc gtgctgaaaa tatttcggct tgatatagtc aatatttaaa cctttcgctt 76141
cagctttttt tactactcgg ctaatagcaa tatggagtga tttagcaaac aatggttgct 76201
ttttaataat gcgaacaagg tctaagttgt atccttcagg aaatgcggct ttatttttaa 76261
gataagggtg ttcatgtatg gactgaactg gctgggctct gttgtattca atgcgcgacc 76321
atgtcttagc aatgcgttgt aaggttctct gaaagcgatg atttctacgc attcgaattt 76381
cgtattgttt cttttccctt tccaattttc tgagttcttg aagtgataga ttggactctt 76441
tctttaacaa tttgggatct aggtgccata cctctttatt gccggtatta taattgcccc 76501
aaattacact aggcacgtca aaagcgaaat ttaatctgta ccacccagac tttgagtgct 76561
tattcttttg cgatcgcttt aagcatttaa cgtaatggcg ggtgccatca acttttagtt 76621
gatccgctgt gatgtctaat tcagccgttt cccttaaata gttggcaaaa atttgacgtg 76681
catcatattc ggaaatattt aatctcattc ccatacctca attgttgtgt tcctagcatc 76741
aaggtgtaga tgtctaggtg atttatataa aaagggtatt agttatgcga ttttaacagg 76801
aatatgaaat gtttgctttt gtttaatgtt aatttcgatg atgttttgcg agtgtctttt 76861
ttaaaaagtg aagaatagag agggcttcaa tgtgggtaag ttccatttcc ataaaatgat 76921
ctaaaaactc actttttatg tatggcggta tttgactttc tttttctaaa ttaaaataaa 76981
agttatataa ataatgcttt agatcatccc tataattaat tttatagagg tcatcaatat 77041
aattttggca tataaaaact agtgcatcgg ccatttgggg tgatagtgac tctggaaggt 77101
tgctctgaac aatagaaata taatcacaaa gcattttatt gatatgcata gaccaagaaa 77161
tttgatcttg gtcattttct ttgcttgtac gagccaaact ttctatgtag ttcgaaactt 77221
ctgatgaaat ggaaatcttt cttttttcgc ccatgattaa gcaagcttga ttaattaaga 77281
tctatagtaa tatttttttag taaatatagc caaaaaatca ttacttacaa gctttaaaaa 77341
agtgcagttt tgagattaaa gtcattaatg attaattgag gttgtacagt cttaggatct 77401

gattgagtgc tgctccaatt ctgctgataa gataggtaaa agagcccaac attcttcaac 77461
tcatgatcat acgatttaga ggaagatgta aagaagctgt gctttttgc aagagatacg 77521
gtgtataaat tagaaaaatc caaaataagt ttttcgttct tttggggtga gattttacta 77581
ttccctctta agtttttgta atcaatatag aggtcaacac aatagtcctt gctggttaga 77641
gttgtgttga cttcattgtt gttagtcaca actccaatgt aggggcggtc acatttgtaa 77701
aaaccagcat gtactggaat gaagaatata tctcgtatag cacaaatcca aaaagtcaaa 77761
acgactaagg ttataaagat tattaagcat atgatcttta agatcttaac taacagggct 77821
gtattcatac gttgaccttt agatatattt ttctattaac agttcttgaa agtaatttat 77881
aaatccagat aaccctaaga tcttataaag caaaatctga agagaaatta gaagaatgta 77941
acgaaagaaa aaaccatgaa aaatggatgg gccattaggg aaaaaccttt tgattttaaa 78001
aggaaagtaa attaaaaatg cttgagctat caaagccaat attccgtaaa gtagaaacat 78061
ggaaaagcct aatcaaaata ttttaaaaaa attatagtgc ataaatttaa aaataacatt 78121
aaaatatttt aaaaaattga tactgcaatt ttttaaaaaa gggcgttcat tatgctgaag 78181
aaaattggta aggcttttgt ctatttgtat gtgacgttgc cattgaaagg tatagctgga 78241
aatttaggtt ataaacacct gaaaaatgac tatgaattca ataaacaaca gtataaaagt 78301
ctaaagaaaa tacattgtcc tgcatgcttc aaaagcattg ttcaaacatc gcaaatcaag 78361
ctaactgata atgaaattgt tgagcacaaa gcaccagcaa aagcggttaa agtcacgtta 78421
aaatgcgcta atcctgaatg ttcttttgag aattcattca tttcgggacc tagtaaaaaa 78481
gaaattagtg aaaagactaa taatgcaatt ttggcagttg gccgcgaaag tgctaaacgg 78541
atctttgaag taattccggt tggtcaagtc aataattcaa ttaaagaaca ttatgaaatg 78601
gcgattatgt taagaaatat aagtattttt tgcgtaatcg gttgttttgc atttactttt 78661
tatcagattt ttttcaagga taactcatta tttttcactg ccttatctgc cttctcgtgg 78721
ctggttatag ggttaggtat atttcttaat gcccttaagt attcacataa gtcatggcaa 78781
gcacagactt taaaggtttt tgaacagcag tctcagtttt atttttggtt taaaaatcat 78841
gcatggtttt caaaacctaa atactggcgt atcagtggag aagggaaaaa tgctacaaag 78901
taaaagactt ttctactttc ttggttgtat ggtgtgtgtg tctggcataa cgaatgctaa 78961
agttccacaa atgagctccc agcaacttgg tttatatgct aatcagctta atggttctcc 79021
tagagccaat tttgttaata gcggtactac tagtaatcat tcaattaatc cgaaaactgc 79081
aaacacttct gtagttttac cacaaagaag aactggtaat tatacaacta taagttatac 79141
caattctcca gctccagaag ctaagcagct cgaaattgta caacaaagcc aaacatcgaa 79201
tacaaattgg gttcaagaat acgcatttag aggtaatgtc gctgcgcctg aagtactaaa 79261
taattacagt tatcaaaaac caaaagttaa tatcaattac gttcgtggac gtgaagctaa 79321
cttaacatgt gtaatcgctg cggctcgatc agaaggtgtg cctctttatg tgctattggg 79381
aattcaaagt aaagaaagag gtagaaatgg tgaagttaac gcgacaaatg atatgggtca 79441
tttccaagta aataaacagc attttagaaa tggaggaatg tttaacccgc aagacatgga 79501
gagagctcgt acagatgggt gttataacgc tgtattggcc gctaaaatat tacgtgatcg 79561
gctgagtact aaagttgcat ctgtagattt ctggactcgc gccgcagctt atcattcatg 79621
gactccttcc gtgaacgcaa gatatcgacc agatctaata agatattcta tgcaatggaa 79681
acagtggttg acacaaaatg gaattaatcc gaactgaagg ttatgaagat aaaatggttc 79741
aaaagcatat ttctcaaaat tacatcttta agaaacttgt tcacttaaca aacctaaatg 79801
atgatattga aactttgata acactgttta aagagaaaga tatcgtggtt gattcagctt 79861
tgattgaatc gtggttaagc gaagaaggtc aatctaactt taaagaaatt tcagaaaata 79921
atttattttc ctttttttgag attcttttta gcgttaaaaa ggaagctcta tctaaagata 79981
taaatttatt tgatttaagt tatattttttg aagatatcag aaactaatag atgcgctttg 80041
acaatcacga ggcagagttt aaaagtaaaa atgagtgact atatgtttaa aaaattgatt 80101
agattcttta taggaaaaaa gactattgta ccaaaggcta tttttgatcc tatggacttc 80161
tcccatgcta tcagcttgca aattacaata aagaaagatg gctcgaaatt gtatcaacca 80221
tttggtattt caagtaaaaa gggcacacct attacaacaa tagtttatga aaatgatatt 80281
gtttaatatt tgaaatactt aatttcatac tgtttgggca tcttatatga gtaaaaaatt 80341
gaaatttaat ttcaaaaata ttattaactt aaaacctttt gatgaaaatg aagataatag 80401
tcgggatgat tttacgggaa gtaaaactag atctttcttt atttatttgg cctttttttg 80461
cagtatagct ggaatttctg cggtatttat ctatcctatg actaaagcac caataatttt 80521
aatatcatta ggttgtgctt cttcagctat tttaagtggt atcgagtggc atttaggata 80581
taagtcaaaa gcattgaata agttatttga agcaacagtt cttttagtgc ttttgattct 80641

tgtaaggtta tttacttggt aataaaaaag cccttttttaa gggcttttta tctttatctt 80701
aagcggcaat aatcctgaag gttatcttta aaatctagac atacataatt tatagatttt 80761
tcaaactggt taaaaagaaa cttatttaaa tcagaaatac atatttcatc aattatgtct 80821
aataaagccg atttagactc ttcataaagt ttacgacatt tctctaatgc aaactccgga 80881
tctgaacaat ctaagaagtt tagcgtgcca tccaagaagg ataaaattct gtttgataag 80941
aagttgttgt aatagttttc aatattgctt tgtgattgtg ctaagcactc caaaaaacga 81001
actatatttt cagtttttaa atgaccactg aaggtagtta aaagatgatc atgtgcatca 81061
acaattccat aacctaaact aatacgatta ttaattgcat ttgtagaatt gaacactata 81121
ggattgcctt ttagatccag tgcaataaat tgctcaccat ctttaaacag ttccttttct 81181
tcaacagttt tttgtacggc attggcaatc actaacattt ttgacagttg ctcgttaata 81241
agatctcttg aagatgcaga aaggtctgta tgcgccggag tgaaattttt tatacagtgc 81301
tgaatttcaa tgccaatttg tttctcatta tagtgatgtg aattctgtag attgaagatg 81361
ttatacaacg agatatttaa gattaaattg tcatcatcag gaatcgaaat ctcttctgga 81421
gtagcgacta aaacggttgc tatacactca ccatacatct tttgtagatg ttgttcgcag 81481
aagtgaagaa tagaagggat gtgagactca acctgagaaa actttttat gttcaagcaa 81541
gcaatgattc gcggcttatg gtaaccaact aaaggatcta ttccgaaatt gttattaaac 81601
agcatagaat tgctcggcat atattcaggc taagtttgtt tctttaacta ccaaatgagc 81661
aaacttgttg actcacgaag taaataagtt caaatcataa tgattataaa ctaaccaaag 81721
tttaaaattt tgaaaaatgt ttcaaaattg tatgtttgtt aacaaatcgt acagaacgat 81781
ccgcttttgt gtaattttac cgaaaaatag aggttatgag aacgaaattt gtatcaaaat 81841
taaaaattag ataaattcaa ttatctgatt ttgtaaagta aatataagaa aggccaaatt 81901
tagtattggc ctttttcctt ttattttaat gaattagagg cattgagctc atcaaagaaa 81961
ttattaattt gttcgtcgat ttcgaccggt gagttcatgc gtgggggcat aaatttaagg 82021
cgaatttcat aatcttgaat ataagctgtc cagtgaacac ctgttattct atattcagtt 82081
ttggattcat atttttgctg acgtgctacg aaagctcgat aatctactcc gtatttgacg 82141
ccaattcgcc acattttatg attagctggt gcatttactc tatattcacc tttctgccaa 82201
taagcggcct ctagatcctt aaatgagggt tttttagtta cattctgtcg agtagagttc 82261
ttagtaaaat tatcaatagc tatttcatct gtacatattg aagaaattag cgaaattact 82321
ggagcaatat gttcattgag ttcttctgga ctttccagat taataccccca tttgaacttg 82381
atttgttgac tcaccttgcc agaacgtttt atgtcaaaaa cgatagtagg gagaggcttg 82441
agcagagcaa aattattttc ttcaaatggg ttatgagtgt ctaatgtaat aatgagttgg 82501
tcaggtagag tattctttaa agtatctatg ctgttcgaag atagatcgag gattgcatac 82561
acaccataaa ccttatgatc tgcaatattt aaaggtgttg ctagatcaat gtagatacaa 82621
ccttctggta aattagtcaa aagttgatga tcaagatatt ttcctgctga atgtttaatc 82681
aaacattcaa acgtgtattc atcaaaacga taaatacttt tagtgtatct ccatgtaaat 82741
gctgcaacaa aatttgcgaa atatgcatcg attacaaatg gatctttagt tttatttatt 82801
tgcacttgtt catatagttt ggccatgatg gttggtctta agacttcttg ccataagttt 82861
gttggtaaaa atacacatgg atgccactta tattctgtta ttggtgcatt caggcggcct 82921
ttatttattg attttgtgtc tgcaactgtt tgataaaacg ttcgtttaac atgctcaaat 82981
atatgttcaa ttgttgctaa ataatcgagt ggtttaacat tcataattta ttcaggccct 83041
tcatgtcctt agacatatta agtttgtctc gattctatca tttaaaaatt gacactttaa 83101
aaaaataaat tgaaaaaata cataggtaac ttatcatcta tacttgaata cacaagttca 83161
gatcgctggc gactgaatga taagaaggaa tgtagaaatg actgatttag ttatcgtaga 83221
gtcatctaat aaggttgact ctatacaggg ctatctagat agccttttttg gtcgaggtaa 83281
atttaaagtt tcatttagta agggccatat acgtgactta cccaaagact cactaggatt 83341
aactgccgaa tatacaccta actatgtgtt taatgattct ggtgcaagtc gtgtaagaga 83401
gctaaaagca ttagttgaga aatcacaaaa agtatatctt gctacggatc cggatcgaga 83461
aggtgaggct attgcatggc atcttaaaac ggttctccgg cttaacgaaa attatggtcg 83521
agttacattt caggagatta caaagacagc agtagcacaa gctatgggta acgagcgtaa 83581
gttaaatatg caagttgttc aagcacaaga aacacgaaga attctagacc gaattattgg 83641
atacattgga acaccggctt ttactttaat atgtggtgaa aaacagattg ctggtcgagt 83701
tcaaagtagt atcgtatggt atctagtcga tcttgaaaat aagatcaaac aattctcgtc 83761
agttttgcat tttggtgtaa ctttcaattt tggttctgta gatgagtctt gggaagcaaa 83821
atgggataca tcaaatttca taactaagtt agatcctttt gtaattgatc gaaagcttgc 83881

tgaagaagtt tcaaaattac gtgaattcac tgtagaaact tttgaagaaa aaccagagaa 83941
aagctctcca ccggctcctt ttacaactcc tacttatcta cgggccgcac acgttcaatt 84001
aaaacttaaa acgaaaagag caatggaaat tgctcaaaaa ttatttgaag atggccatat 84061
aacatacatt cgtactgaca gcccaaattt aagtgatgat gccattcaag ctattcgtga 84121
atttgcactg aagaacaaat tacctatacc ggcaacacca cgaaaattta aatctaaagc 84181
tttggctcaa gaaggccatg aagctatcag accgactgat tttaatcaat taaaagtatc 84241
agatgattca accattcagg cggtatatca aatgatctgg ttgcgttcag tggcttgtca 84301
gcttgatgat gcgacattca cagcgcgtat tgtccgtatg aaaggtcgcc agttagtgca 84361
aggtcgagaa gtctttgtta aagctcgtgg acgtaaacta attgatcaag gctggttacg 84421
acttacagca aagctaaact tttctgaaga cgagaatgaa gatattccgg aagacgctgc 84481
acaagagttg aataatccag tgccgttatt acaaaaggca gggaatttag ttgcattaaa 84541
atcagatctg aatgagaaaa gaaccgaacc cccacgtagg cattctgaag ctagtctcat 84601
taagttcatg gaagatgtgg gtataggtcg cccatcaact ttcgtaagca tcgtttctaa 84661
aattattgat catggttatg tacacttaga cagtaaaaat aaaatacatg ctactgatct 84721
ggctcaaaga ttggttggga aaatttcaaa ccaatttgaa ttttgcaatc tcaaatatac 84781
agcagagtta gaaggaaagt tggatctcat tgcacatggt gaaaacattg gtaagaggct 84841
attaaaagag acacatctca aaattgaaac ccaagcaaaa gagtctatta aaaatttaga 84901
tcaaggtgga cctaaacaat attgttctaa gtgctcagaa caattggtga atatgacaag 84961
tgcgaacggt aagccttatt tcaaatgtac aaatttgaag tgtggtcaca tgatgatgaa 85021
tcaagatggg atagcaattg atagagaatc gcttacatca gaatttaagt gcattgtgtg 85081
cgagagtggc ctaatttcgc gttcaggcca aaatggtgag ttctttggat gctcaggttt 85141
cttcaagaag agaaataaat gtacagccag ttatcaaaag aaaccagacg gtacacctga 85201
ctatcaagca ttccttgcca aaagggagct gaaggaatct aagcaagaac aaggtgaaaa 85261
acctgttcct aaaaatacag agaaaaagga aaaagaattt aactgtccgg agtgtaaagc 85321
tctactccaa caacgtactg gtaaatcaaa aacaacaaac aagaagtggg agatttggtc 85381
gtgttcttcc cctaattgta aaaatgaata ttggggtgag cgaaacaaac caattttcga 85441
taaaaagatc gttaaatgat cagaatttga ggtgaaatcg tggatatagt aaatacattg 85501
aaagcatact cattgagtga gaaacaatat tttcagatca atagtattct actgctggtt 85561
ctcgtaatta tattaggtgt tcttgctaaa ggctcctaca atggtgtgct gacaagcttg 85621
aaggtatctc tgtatataca atgttttatt tgtattatga tgcaagtcgt agcagtgatg 85681
gcaaataaaa cttcacatat aaaatattta ttacttggta gttatttttt ccttttctgt 85741
acaacatttt atttagataa cttcttttat gggagtactg ccggattttt tgtgagattc 85801
ttatgtatct acagtatttt acaaggctgt tttattatct ggctttatga aaaaaataag 85861
tgaatatatt tatacagtct tatactatgt ataagactgt aaataaatct cttataaaga 85921
tttgatattt tttttgtaat tattgtcctt gaattttaag tgctcagtat ataatgcatc 85981
ttttatttca agaccttcat aaattgaaat gaaactgtcc cttttagcct tatttaaaag 86041
ttcgcgcacg gacttgttgt ctttaaattt taagtaatct tcattattca ttaaaatatc 86101
aagattttct tgagataatg gtaacttctt taaatccata tcaatgcatt cattgaatat 86161
ttttgtatcg agtttaatat tatctttaag tttatatgaa aagcgttttg aaaatgactt 86221
ttcctcttca taatcatttt cttcaatatt taatgtttcc ttacatctat ctttaattgc 86281
atatattgtc tgaaggtcaa gtatttgata atccaaattc tcatcattaa aggtgtttac 86341
cgattcaaag ctattcttaa aggatgcgtt gagatgttga tatagcagtt tttcagagat 86401
ttgtgattta gctacaagat attcttgtca gttccacaaa taaatcagag tttacacaaa 86461
taaactagag caagaataaa aagtatatta tctataagct ttaatttttc attttgtaaa 86521
aatatatgcc tgttaataat gataaaataa cgactattga ttgtttccca aatgataata 86581
ttagtcgagt ggtttatcga tatggtggac tcgttcgaaa cgaagataac gactcaacta 86641
atccttttgt tgaagtgtta ttaattgaga ttagaaaaag tgatcaatgg ttatttttag 86701
ataagtgctc aacattcctt gttcccgtac ttgatcttga tgctgttcaa catggcagta 86761
tttgggacgg gaatgttctg accaattaca cttatagatt ttcaggaaaa ctcatcacaa 86821
aacgattttc tttcgacttc agcaataata aaccgaggaa cataaaactt tcagacaaaa 86881
ttccggatac gtcagagtac tacattccat taaaaaacta ttttcttccc aataaagttg 86941
attatgttct acaaggctat ccattcacta aatacaccaa tgattcatat cggaaagtga 87001
atcactgctt aatgcattct aatgatggaa ctcaagtcat cacttcctca atccatattt 87061
tgcatagctt attcgtcaat cggaaggata ttcggggact cttattaagc acttcaggcc 87121

aatctatcat taaccgcttt ttagaatctt atacaacaga agttgagaat gataatattg 87181
aatataaaat aaaaatcaga aagccatatg aagatattgg cgaaacagca attatatttc 87241
tggctaatct agccttaaat ccatatgtgc agacgatagt cgataaaatt caacgaagta 87301
tggaagttac tgagtttcat catcttcagc atggcacagg cataagatac cccattgtat 87361
atccgccaca tcctacaaag ctttttcttg aagcagaagg aatttggcta gatgacaata 87421
agacacgttt ttttattact agagtgaaaa aatttgatcc tattaacgat cacatgattg 87481
atgtcaataa agaccttacc aatactgttt caaaaactga tgaaaaaaac cctcgtcctc 87541
gtgaaaaaag tcaaaataaa aatgagcata tcaatacaca aaaaccacca tcaagaacaa 87601
gcggtgaata ccgtaagcgt tctgatgtgg aaactggaaa tacacaaaat attttgaagt 87661
attcttttaa tgaaccagcc aatgacccaa ttgaagttgg cacatccaat catccatatt 87721
cagataaatc agaggatgtt gaaacatcat ctgatgaacc atatggcaat aaaaatacta 87781
aaactaaaaa gtctgaaaca actgataatc cgcctagcag agatgaacgc tttgatattc 87841
aatatattat tcaatcactt aaccatttaa cactagatac aacctctcca ttagaacgtg 87901
tgtattcaat tgatatattg gggaatgaga tacacggatt caacttactt caaataaaga 87961
aactcgttcc tcaaccaaag catccatctt ggattgatgc agaatttgga aggaaattgt 88021
tatttttaaa actggaattg aaagatcagc atgatcaata ttatttaatt gatatacata 88081
aaaataaaag ccatgaagca ttctgtgctt ttattatcgt gtcaccacat aagttaaccc 88141
gtgagcaaat aaaaaatata tgtatagagc ttgagaatgc aaaaggaatt aaaaaatgga 88201
cgctacgttg tgaaagtttt accaaaaaaa ttatttcgat taaacattca tctgctactc 88261
ccgatgagtg gaaaaataga tttaaaaatt tattttcaac attaattaat tgatttttat 88321
taaattacaa aaaactgtcg tactaaaaat aagaaaggta cttcataaag gagttgcaag 88381
aatcctaacc tttttttaca attcaatagt tgttgaaata ttgaaatatt atttctaaag 88441
taaccgtgca tcggaagtta atatttctag tattgtttcc tgtcataagc atcaaggtct 88501
tagctaataa tataaattag ctcatttgat acttttcaaa cttaatctcc gtcgatataa 88561
atcaattaat atcaatatct tgaagatagg aaatgcataa tcagtgttcg tttcaagaat 88621
cattagcatt attatttaag gtttagctat cctatgtact tatctgattt aaacgcacaa 88681
caaagggcta tcgtaacgaa agttcaagct tctttaatct tatcaaatcg tctagaaaca 88741
ttaggtttta ttccacaagc agaagttcgt gtcattacta aaggaatctt tggtggtgac 88801
cctattctcg ttcaaattgg atttactcgt tttgctttaa gaaagtcaga agctaaatgt 88861
atttatgttg aaggacttaa ttcatgacaa ccgacctgag aattgcctta gttggtaatc 88921
caaattgtgg taaaacttca ttatttaacc atttaactgg tacacgtcaa aaagttgcaa 88981
attatgctgg tgtaacagtt gagaaaaaag ttgggcattt tacacttccc tcatccaata 89041
aagtacgaat acttgatctt cctggtacat atagtttatc tgcaacaagc ccagatgaag 89101
caattactcg tgatgtttgc aacggtctta cagaagaggc acaacaagat gctttcattt 89161
gtgtcgtaga tgctacaaat ctaaaacttc atttaggatt agttttagaa gtactggctc 89221
taggccgtcc cgtattagtt gttttaaaca tgatggatga agctcgtcgt cgaggcattc 89281
agattaatat cgaaaaatta tctcagcatt tgggtgttcc tgtggttgaa acagttgctg 89341
tcaaaagctc aggtttaacc caattgatag atgccctaga taaggaagtc tttcacaccc 89401
ctcatactga tttcactgcc aagattgatt ccccacaaaa acatattgaa tatgtcctag 89461
caaccacgat tacagatcaa ggtaaagttg atacccgaac agagtttcta gataagattt 89521
tacttcatcc tattgctgga cttcttattc tagcaatcac tatgtttatc gtattccaag 89581
ccgtatttgc ttgggcaaat ccattcattg agatgcttga gaatctattt agttttattg 89641
gtgaatttct cggaagtatc atcacccaac ccccttttaaa tagtcttgtc gttgatggca 89701
ttatcggagg agtgggaagt attattgttt atttacccca gattctgatt ttgttcttct 89761
ttatctttgt actagaagag tcaggctatc ttccccgtgc ctcctttttа ttagataagc 89821
ttatggtaag tgcaggttta agtggtcgag catttattcc tcttctttcc agttttgcct 89881
gtgcaatccc tggcattatg gcaacaagaa ctatcactga tcgtaccgac cgtattgcga 89941
cgatcatggt tgctcccctc atgacgtgct cagcacgact acctgtttat gccttattga 90001
tcgcagcgtt catcccaaat caaactgttg caggtatttt taacttgcag ggagttgtac 90061
tatttggact ctatattgca ggaattgtca gtgcattggt tgtgtcactg gtgatgaaac 90121
tgtttagaaa gaataaggaa aatagcttat tcctaatgga actacctgct tatcgaatgc 90181
ctaactggcg tagtgttttc cttggggttt atgaacgtgc ctggatattc atcagacgtg 90241
tgggcggaat catctttgca ctaaccattc ttctttggtt tctgtgtaca tttccaacac 90301
ctccagaaaa cgccacgtta ccagcgatag attatagttt tgcaggcatg cttggacatc 90361

ttctacaacc aatattcgca ccaatcggtt tcaactggca aattactatt gcgctgatac 90421
ctggacttgc tgcccgtgaa gtggttgttg ctgccctagg tacagtttat gcgctctctg 90481
gcagtgaaga tgctcaggca gaaacactgg tcaacttgat cacagccgat ggtacaggct 90541
ggtcactagc gacaggctta tctttactgg tgtggtttat ctatgcaccc aactgtttgg 90601
caactttagc caccatacga agagaaactg gctctatgcg aacggtgaca attatggtgg 90661
tctatatgtt tgcccttgca tacttaatgg cctttttcac ttatcaacta gcttcctatt 90721
actggggcta agatggagaa agaaagccag ctttctcctg attaattgaa tcattttgac 90781
tcatagttta cgaacagtgg atcattatat agaagatata atagacgctt gttagcccgt 90841
atgttgagta aacttgcagc aatgcttgtg acgtcctaca atctttttaaa gacgagaata 90901
ctggaaattc aatgttagaa aaaaacgcct taagtgcctt tgcggcttta tctcaagaaa 90961
cacgcttacg aattgtgcgt ttacttgtgg tcgctggtgc tgaaggcatg gcttcaggac 91021
aaattgctga ggcactagat ggcgcagcac catcaagaat ttcttttcat ttaagtcatt 91081
tggaaaatgc agggctgatc tacagtcgcc gagagggtcg gcaaattatc tattgtgcaa 91141
tttggtctac tctagcagat ctcgttgcat tcctgatgca tgactgttgc caagggcatt 91201
gtgaatattg cgacaaagca attgagttgt ttgctaaatg tacaggtgaa ccgaccgagg 91261
gttatatcaa aataggagaa tagttttctg tcctctccat atacctgagc aaacacacct 91321
atggaaaatc tacgcagctt acttgaagac catcaagtca agatctactt tattacgata 91381
tttattggaa ccatttttgg gctaactata agtggggtaa gtagtttaga aagtgcaatt 91441
aatccagcat tggcattaat gttatttgcc acttttttac aggtacccat tgctgaaata 91501
ggtaaagcat ttaagaacct aaaattcatt tctgctttat taattaccaa ctttattgcc 91561
ttacctgcta tcaccgcagg cctaatccag tttctacctg aaaatccatt gattcggctg 91621
gctgttttat ttgtactctt agctccttgc attgattatg tgattacctt tacacatgtg 91681
ggaaaaggta atgcgaggtt actgcttgca atgacaccaa tattattact tattcaaatg 91741
attctgcttc cagtctacct tggaacatta ttaggtgatg aggtatcaca gcttgtcgaa 91801
atggaaccat ttttacatgc tttcatttgg tttattattg taccccctgac acttgcaggc 91861
attgttcagt ttattagcaa taaaagtgaa tcattggcta cgctttccgc acctttaaat 91921
ttactgcctg tacccgctac agcacttgtt ctgtttattg tgcttgcatc agtgacgcct 91981
caaattggac tcgctcagag tgctgctctt caggctttac caatttacat cgcctttgcc 92041
attattgctc cctttatagg ctgggctatc gcatccttgt tcaaactgga gtctagttct 92101
gcaagagctg tatctttttc agcatcatat cgaaattctt tggttatcct accattagct 92161
ctagtagtac ctggaagtat gcctattatc cctgctgtaa ttctaacgca aacaattgtt 92221
gaactttgtt ttctaccaat ttatacacga ttgattccca gaatgtttcc atctaaagat 92281
tagtaatcac tttccattta tacaacttgg tgatcaatta acaacgacca ctgaagtagg 92341
taactggctg ggcgacccag aaggtttaac tgctcctgca cttatggagc aaatgcaagc 92401
ccatgctgaa cgctttagat accgatcaat tgagcaatta aaatacacgt ttaactgttt 92461
gaaatttaca taaaatttgt ccagcttatt aaataggctg ggcttctctt ttaattcttt 92521
tcaatcaatt attggttgtt tggcagccaa tatgattgct cattaaatct tacagcaaaa 92581
ccattattta cctctgtaat catttcaact ttaactttta aagaatcaat gtaagaaagt 92641
tgatggaaat acctaagacc atttctaacc catccgatca attcttcacg gttacttaga 92701
tcagtttgag ctaatgcaac aatcgccttt tctaattttt gtttctgctt aatccttaaa 92761
aaatcagact ttttcaactt taaataatca atggaagtgc cataattctc atttagaaaa 92821
ttaatgaatc ccgtaagcgc agcagcttgt cccgaatagt cagccaaata cgccttgata 92881
tgttccaaat ttggtaattg ggtactttct gacccgattg acagcatcaa cgcaacagca 92941
ggctttactg ccaaccgagc tgaacgtatt gaggtatcgc ctcgtttgat tttttcttct 93001
aatttattct tgtatccaaa tacaacatca tacgccaaag atggttgtaa aatactttgc 93061
acaagctttt ccatctgatc tcgttctgaa caaccttttt tgttatccgt atctacttga 93121
atgccatgca tctcttcaag ccattgcatc accaattcaa attttctaag cccactgctt 93181
cgtaatactg ctaacaactg cattggtgta ggtacggatt ggttccaatt cacttctgtt 93241
ttcaaaaaga aatgggtatg tttatttata tataacgtag ctttatgcga tccgactttc 93301
ttttctaacc agtctatata gctctcatac tgctgtttta agtaagtaaa ctcaaatgca 93361
ttctgatttt gatcaaattt attccataag cttttatgcc aatagcaatc agcacagaga 93421
tcagcacaac ctgctgcaat catacgatga catgacatac aagatttctg aggattatct 93481
ctacattttt tacaggcttt ttgtcctgat gcatcagatt ccagtaaacg atgtttctga 93541
cacaatggac aagtttcgta atctcttgtc gaacatttgg gacatacccg tagatcgtca 93601

ttaaatcgtg aaattcgagt caatttttgt gatggtgtac cacatcgctc acaaggctca 93661
ataggtctaa aatacacgga acaactatta cagaccacat gaaccgtacc gggtttgtcg 93721
gagagtcaat attctgagag actattccga tgacaaaatt aaaatatacc cctgaaatca 93781
gagaaagagc ggttcaactt gtgattgaat ccgaaaaaga ttatccatcg aactgggcag 93841
cagtttccgc aattgctcct aaaattggtt gtactcctga aacgctgcgt gtttggtatc 93901
aaaaatattt agatcaacaa aacccaatca aagtacaaca attttcagat caagagcgta 93961
ttaaacaact cgaacgtgaa aataaagaac tgcaacgcgc taacgaaatt ctacgcaaag 94021
cagccgcttt tttcgcccag gcggagctcg accgcccata caaataatgg tggatttcat 94081
tcataacaat aaagcgttat acggtgttga tgcgatttgt agaatattac ccatcgcagc 94141
atcaacctat taccggactt tagatctcgt taataatcca gaatttcgag cgaaacgtgc 94201
tctgcatgat ttacatcatg cagagcaaat taaacgtatt tggaaggaaa gtttaggccg 94261
atatggcata cgtaaagtat ggcaacaact gagacgtgaa ggctatgtga tcgcacgttg 94321
cacagtagtt cgcttgatgc gaaagctaaa catacaaggt gtttggcgtg gtaagaataa 94381
acaaaccacc cgtagccgag atgatcaaaa acgagcagat gatttagtga aacgtaattt 94441
taaagctgat caccctaatc acttgtgggt tgctgacttc acctatattc aaaccaattc 94501
aggctgggtg tataccgcat ttattattga tgtgttctca cgagcaattg ttgggtggaa 94561
ggtatcgaca cgcatgaata cggatatggt gctcgatgca ttggagcaag cattgcatga 94621
tcgaggtatg ccaaagaatg ttattcatca ttctgacaga ggggttcaat atctctccat 94681
tcgctatact aatcgtttag aagcagcaaa tttaagagca tcagtcggta caactggtga 94741
ttcttacgat aatgctttgg ctgaaacggt gaatggctta tacaaaactg aggtgattga 94801
atatttaaaa gcagattggc agggtttagc tgatgtacaa cttgccacat tgaattgggt 94861
ggattggttc aataaaaagc gtatacacag tgcacttggt tatgtatcac cttttgattt 94921
tgaagcaatg tactatgata agattaaccc gttaggtcag gtggcctaac ttaaataaaa 94981
aagtctccga caaactcggt acggttcaac accatactcg gtcagcttac caatcggctt 95041
attactctga ttacatcgaa tacagggctg tttcttaata cattcattac aaatagcttg 95101
ttcatcatcc ctagggagcc gagcaaagtc accacagcct gaacatatgc gtcttttaaa 95161
gatacgagca taacaagtcg aacaatattt cttgccttta tatcttttttt ttatgaatga 95221
aacagcctga ttgcatccat gacagtcatg ctcagtaaca ttttcagtca ataagcctag 95281
ctccactatt attgcttctc cttaataaat ttaaccattg acgttgtaca ctggctttgg 95341
atttcggctt ggtaacagtt attgctgcac gagagacggg ataacgtctt gtaggcttaa 95401
tcattaaatc tttcatctgt tgaagctgaa caggcaactt cttacgctta tcccaaaagt 95461
ttgcatgact gattccgtac tcatttgaac gctgtaccaa taagtcgcat ggaatatcca 95521
taatctgatc caatattgaa agcaaaacta ttcgctcctg agtattcaga tattcaaatg 95581
ctaatggggt aactggatgt gaaagatgta aatttacatc aaaactttca aacaattgaa 95641
tcacattttt attggatgta ttgatcaaaa atctaatctc actgagccat acacgagcaa 95701
tttcaaacca ttcttgagag gtaataattg attgattata aaagccaact ttttgtttta 95761
aaacctgatc cgctttattt tggaagttta aagagtttaa attgatattt tggctttgct 95821
tacaatgcct taagtcaaaa ccacatgctg tacagatcgc caagcaacca tgttccaaat 95881
cagttttaaa tgcttggagc gtcacaccgc attcagggca atgatcgatt aaacttacct 95941
gatgccgatc acaactacaa tgccatccca tgcgccacat taaacgaaaa taaggcggat 96001
tttcacgact ttttaagcat tcaacacaca cttgccgacc tgaggtattt gaacggtttc 96061
gtgtgcccaa ggacaaaacc caagggatag tttgctttgg atcataattt ggctgtatct 96121
cagaaacaaa gctactcaac atcgattgct gtatattctg ccttgtctca atactatgag 96181
ataacagagc atcaaacttt tctgcatcta ctcctttgtc taaatcaatc gttaaagctc 96241
tccatttccc ccataaggcc tcaacgagaa ccataggtga gcaacctaca tctaaggcgg 96301
atcgaatcaa ccacgatgag aatatttcgt cctgttgaat cggagttctg atataccacc 96361
gtggttcaga ttgactcatc ttcccccccc ataaacaatt taaagctaaa gtacgctgag 96421
atttatattt ctaatatttc ttaaaccttc agtcggttta agccatttaa acttatggac 96481
aatgtcatat gtaatttctt cttcaccttg taaaatggct tctttggcac actcgaccag 96541
taatcggttt aaatccccca aatttccgcc tgatacttca aaaatcaacg tggcaatctc 96601
tttagatgca agattagact gttttttaa cggcaatagt cgaacatagc tcattaataa 96661
tcttaaaaaa tcttggttga gttcccattt tggtaaattc accacatcaa aacgactggc 96721
atattgcgaa tcagtatgca gtatggtgat tgcttcacga gtaccaacac caatgacatt 96781
cagactcaat tcattgctga gattcttgag cgtattcatg acttcaggaa gcaatttggc 96841

tgaacctgtt aaacaattat gaatttcatc aataatcagc atttttgtct catacttacg 96901
catcaagtgt actgcttgat ttcgtaattt tgcttcagga tcacttgccc gaaatggcac 96961
aaagaagtga tttaaaatat taatatagag ttctttaaca ttagcttttg caggagcaag 97021
tattggcaaa acaggcttgg tcacggacaa taaatccatg tcggcatcac tcacggtttt 97081
tgtataatac tgtctagcaa actcatgaat gatggttgtt ttgcccatat ttgagtcacc 97141
aactatcaac aagcattcgg gtcttaattt cctagggcga ttcaaaatat cggtcaatgt 97201
attcatgatg gtaatggaat gattccgcgt ctggcacagc caggcacctg atggcatgaa 97261
atgccgctaa gcccgcgtaa ttgcgggctt tttttgtttt gtgtttggca cgatctggca 97321
acgggaggaa gcgccaagca ccgtttgagc atggcattaa agctggtatt atggtttggc 97381
gatgccatga ttgatgccga taccatgctg cgttcttttg tgtgttcatg gtattaatat 97441
tctataagtt actgtttcgt aagaaaaaat acgataaatt cgagattttt tcagcatggt 97501
atcggagacg aagaaggaca aggtacatgc tgaccgatac caagctgcgc aatctcaagc 97561
ccagggacaa actctacaaa gtgaatgacc gggaaggtct ctatgtggca gtgactccag 97621
ccggctccat ctcgttccgt tacaactact caatcaacgg tcggcaggag accatcacct 97681
ttgggcgtta tggtgtcggt ggcatcaccc tggccgaagc ccgcgagctg ttgggtgacg 97741
ccaagaagat ggttgcggcg ggcaagtcgc cggccaagga gaaagcccga gacaaggcgc 97801
gggtgaaaga tgcagagacg ttcggtgcct gggcggagaa gtggctgcgt ggctaccaga 97861
tggccgactc cacccgcgat atgcgccgct cggtttatga gcgtgagctg aagccgaaat 97921
tcagcaatca gaagctggtg gagatcaccc acgaagactt gcgggcgctg gctgatgcca 97981
ttgttgagcg aggcgcaccg gccaccgccg ttcatgtgcg tgaaatcgtg ttgcaggtat 98041
ttcgctgggc catcgagcgt gggcagaagg tcgaaaaccc ggcagaactg gtgcgcccaa 98101
caagcatcgc tcgattcgag ccacgtgacc gagcgttgac gccagaagaa attgggctga 98161
tgtaccagta catggagcga gtgggcacaa gcccaacaaa ccgtgcggcg gccaagctgt 98221
tattgctgac gatggtgcgc aagagcgagc tgaccaatgc gacctggagc gagatcaatt 98281
tcagcgaagc gttgtggacg attccaaagg agcggatgaa acgccgtaac ccgcacctgg 98341
tatttctgtc ccagcaggct ctggatattt tcattgccat gaaaaccttt gccggtggtt 98401
ccgacttcgt tttgccatca cggtacgact cggatgcgcc gatgagcgct gccacactta 98461
accaggtgct gacgctgact tacaaggcgg cgcagaaaga tgggaagtca cttaccaagt 98521
tcggaccgca tgatttgcgg cgcacagcca gcacgctgtt gcatgaggcc ggctacaaca 98581
ccgactggat cgagaagtgc ctggcgcacg agcagaaggg cgtgagggct gtctacaaca 98641
aggctgagta ccgcgagcag cgggcggcga tgttgcagga ttgggccgat atgattgatg 98701
agtggacttc gggaggcagt aagggttgat gtttttgcta tctttgatag cattgaagtt 98761
gatgccgtga atttataaat aaggccgcca ttggcatcgg gcaggtcaat taccgattag 98821
ctgcttcgcg cagctactcg atgaatagca actattcatt tgagaggtat tgacccatgc 98881
aaaatataaa gaccctcatc aacaggaaga agctcctgga gatgatcccg ctttcgacac 98941
gaacaattta taacctggag cagcgagggg attttccacg ccgtatcgcg ttaaccagta 99001
gaaatgtcgc ctgggatttg tcagaggtcg aagagtggat tgaggcacgt aaatcatcgg 99061
gtgatcaagc tgcgcgacct ggccctatag agggctagtt atatggctct tgatcttatg 99121
gcggctttca cggaattgcc gccacccatt gattatgttc tgcctaatat ggttgctggc 99181
actgttggtg ctcttgtgtc gcctggtggg gctggtaaat ccatgttggc ccttcaattg 99241
gctgcacaga ttgcaggcgg gcctgattta cttgaaatag gcgagtttcc caccgggcaa 99301
gtggtctatc tgcctgctga agatccaccg gccgctattc accatcgtct gcacgccctt 99361
ggggcacacc tcagcgcagc ggaacggcaa gccgtggctg atggtttgct cattgaaccc 99421
ttgatcggta aatgcccaaa catcatggct gctagctggt tcgatgctct caaacgagcc 99481
gctgaaggtc gtcgcttgat gatcttggac actttgcggc gcttccacat tgaggaggag 99541
aacgctagcg ggccgatggc gcaggttgtt gggcacatgg aggccatagc cgctgataca 99601
ggctgctcca ttgtgtttct gcaccacgcg agtaaaagcg cagccatgat ggggtcgggc 99661
gatcaacagc aggccagtcg tggatcgtcc gtactggttg ataacatccg ttggcaatcg 99721
tacctatccg gcatgacgca aggcgaggcc gagatactgg gggtcgatga ttgtcagcgt 99781
gggtattttg tccgctttgg cgtcagcaag gccaactatg gcgcaccttt tcaagaactc 99841
tggtttagac ggcacgacgg cggcgtgttg aagcctgctg tactggagcg gcagtgcaag 99901
gtgaaaagga gacagcgtga agaagcctaa gcatgacctg acccacgtcc gacatgatcc 99961
cgcgcactgt ttggcacctg gcctgttccg cagcctcaag cgtggcgatc gcaaacgctg 100021
caagctggac gtgacctaca cctttggcga ggacgaatcc atgcgtttcg tcggattcga 100081

acctctcggg gccgatgata tgcgtctttt gcaaggcatc gtggcccttg gcggcccgaa 100141
cggcatcttg ctaaccccgg aaccgaccag tgagacgggg cgacagctac ggctattcct 100201
tgaaccccgt ttcgaagcca ttgagcaaga cggcttggtg gttcgtgaga gcctgaccaa 100261
actgctctca gaaacgggca tgacggatag cggcgacaac atcaaggcgc tcaaagccag 100321
cctgctgcgc atgtcgaacg tcaccatcct tgtgacgaag ggacggcggc aagccgcgtt 100381
ccacctgatg agtcatgctt ttgacgagac ggacggcagg ctatgggttg ccctgaatcc 100441
gcgtattgcc gaagcgatcc tggggcatcg tccatatgcc cgtatcgaca tggcggaagt 100501
gcgggtgcta cagactgatc cggcacggct gatgcaccaa cggctatgcg gctggatcga 100561
ccccggcaaa tccgggcgcg tggaactgga cacgctttgc ggctatgtct ggccagatga 100621
agccaatgcc gaagctatga aaaaacgccg tcagactgcc cggaaggcac tggccgaact 100681
tgccgccgtg ggttgggtag tgaacgaata cgccaaggga aaatgggaga tcaagaggcc 100741
tggccccacg gcaactgcac ccgtttaccg tcgtaacgtt cccttgttac cgtcgtaacg 100801
ctccccttct gccgtcgtaa cgttccccgt cccaatttgg aaaacccaga cggtgcgctg 100861
ctttgcgggc agtttgggaa atccatccaa gattatctaa agataatcca tcatgcgcgg 100921
tgcagctcgc gccttgaggg cgcgttctga ccttgccaat aaagtcctgg cgggctttat 100981
caatcggcca taggccgatg gtttaacgcc aagaagagaa ggcggcttca gtggccgctg 101041
aaacgccttg tgagagggga aaacaacccg gagtccctta ttccatgagc tgaagcagaa 101101
tctgcgtcta acggtgctgg agggccgctg tgtgcacttg tagttatctg tactgaaacc 101161
ttggctcgaa acgtgaaatg gctaagcagt gcccattcgt tcggtaagaa taataagaag 101221
attattattc ttattattac caagcaggaa tgagccgtgt gtgttggacg cccataacca 101281
gtaacctgcg tgaaggtgtc aacatcagga aaggttgccc ctcaagtatc aatacgtcgc 101341
cttagtggta tgacgggagg ggaggcaaat ttcttgtgtt cattgacact tgaggggcgg 101401
gttatgccct tctttttgg cgtccaactc gccccatcgg ggcgaatggt ttgtgtcgat 101461
tgttggcatt ttaggttccg atttaaagcc gattgagcag cgattttgaa gcgactatgt 101521
cgattgcagg ccgatttggt gacgattacg gttatgttgc caagaatcgg tgtttgtttg 101581
acgataaaac gctttacgtt cgatatgcgc tagattttca atagtgggtg aaatgcgggc 101641
aggatgggtg aagttagcta accggcaagc cggttatcta tcttcactgt cccttattcg 101701
cgccgggggc actcaacggg aatcctgccc tgcggggctg atcggctacc gccggttgca 101761
agagtatagg tcttgctatt tttaatatct ttgatagcat ggttgtcggg tacaccaaaa 101821
gggtattgat atggcaaatg tgcttatcag gaatttaccc gccgaggtgc accgtgcgtt 101881
gaaagttcga gcagcgctca acgataggag caccgacgcg gaaatacgcg aaatactgac 101941
ggcggcagta cagccgctag agcttgaaga gattggccgg gaagttggct tgtccgaaag 102001
cgataaaaaa acagagtagt tttacgtttg taagtccggg cctcagcgcc aagggcaata 102061
ccgatcagct agtggcgtag ctcttcgatg aaacgttgcc gagaacaagc acaacgcttg 102121
gtcaaggcaa tgccttcttt tcatcgacgg agtacgacca tgaagtccaa aattttagcg 102181
gctaaaaaaa ccgctctagc cgttgcactc gcaaccggct ttatcaccac taccaccgcc 102241
cctgtgcaag ctggtatccc cgtcatcgac ggcggcaacc tggcccagaa catcatgacg 102301
gccatcgagt cggtggcgca gacgctcaag cagattgagc agtaccagac ccagttgcag 102361
cagtacgaaa atcagctcca gaacacgatg gccccagccg cgtatatctg ggatcaggcg 102421
cagactacga tcaaccggct gattgccgcg caaaacacgc tggcctatta cgagaaccag 102481
ctaggcagcc tggatcgtta cctggccaag tttcaggacg tggcctatta ccgcagctcg 102541
ccatgcttca acggcagcgg cggatgcacg ccggccgaaa aggcagcgat ggaagagaac 102601
cgccgcctgg cgtcagagtc gcaaaagaag gccaacgatg ccctgttcca gaccgttgcc 102661
gaccagcaaa aggctttgaa ggatgacgcc cgtaccctgg agcggctgca aggcgcggcc 102721
cagggtgcaa ctggccagct acaggccatc ggctacgcca accagctcgc cagccagcag 102781
gccaatcagc tcttgcagat tcgcaccatg ttgaccgccc aacacaacgc ggaggcagcc 102841
cggattgcag cagaactcga tgccgaggcg cgaggtgatg ccagggccga gcaaatgcgt 102901
acctggacgt ttcgcccgag tccggcagac aactactagg gaggcggtga gatgaaaaaa 102961
atcttttttta ctaccgcggc tacgttgctg ttgttggctg gatgtgaaca ggaaaggatg 103021
cccgaaccaa atgcggcgac gtgtgccccc gatgcgttcc aggcagcact gaatgaaatg 103081
cgcagcgaag cgaaccgaga ggcctttacc gaggaatgca gagcattcca gaaggccaag 103141
cagatgcgtc agtgggagtt caagcccagc cctaaagatg attattgagg tggtcgccat 103201
gagaaagaaa attgctctag gtggcttgat gatggtcgcc acaatggtgc tggctgaacc 103261
tgcaatggcg caggagctaa gtagcagcgg tgttatgaat gatgtgctca agcgtttcca 103321

tgatgctgcc gcaacatggg ggccagctat tgagtccgct gcatcgcgtt tgttctggac 103381
gctggttgtg atttcgatgg tctggacatt cggcatgatg gctttgcgca aggccgacat 103441
tggcgagttc ttcgcggagt tcgttcgctt cacgatcttc accggctttt tctggtggtt 103501
gctgacgaat gcgaaccagg gcatgaatat cgccggtacg attgttcagt cattgcaaac 103561
tctgggcgcg caggcggggg gactttccaa tagcaatctt gggccctcca gcatccttga 103621
tcttggtttc gagttataca accgcacagt acaggccacc tcggagctgg gatggcggca 103681
gatggcaact gctctggtca tggagctaat ggccctggct gttttgtttg tcctggcgtt 103741
gattgcggtc aacctgttgc tgttgctcgc atcagcctgg attctgttgt atgccggtgt 103801
gttcttcctt ggctttggtg gaagtcgttg gacttccgac atggcgatca attactacaa 103861
gaccgtgctg ggcctggccg cacagcttat ggcaatggtg cttctggttg cgattggcaa 103921
agagttcatc aatcactact acacgcaaat cagcgagaac atggcatccc aggaactggc 103981
cgtgatgttg gttatatcgg tcatcttgct tttcttggtc aacaaagttc cgccgatgat 104041
ttctggtctt gtgtctggtg gtggtattgg cgcagccggt ggaattggaa acttcggtgc 104101
gggtgcggca gttggggcgg cggtgacggc ggccagtatg gcaactggcg gcgctgccct 104161
ggcaggtaaa gcggttatgg gtgccgcagc cggtgcagcc ggaggtgcaa gtgcactcca 104221
agcggctttt cagaaagcat cagcgagtat ggaaaccggc ggtgacatgt ccagcatggg 104281
gtcagttgtc agcagtggcg gaaacggtgg tggtgaagcg ggtactgctg gcagtagccc 104341
attcgcccaa gcggctggct ttggtgacag cggcagtagc tcaagcggtg gcggctttgc 104401
caaggccgcg aagctggcca caggcacggc ctccgagtta gccaagggtg tcggctctca 104461
agtgaagcag ggattccagg agcgagtgag cgaaaccaca ggcggaaaac tggctgcttc 104521
gatacgcgaa agcatggagc cgaaagaagc aagccaatct ggccagttcg agggcaatag 104581
cttgggcgcc gattctggcc cagatagtaa cgaagtcagg agttagcacg atgacgacat 104641
caacatacat ggccgcattg gcggccctgg atgaagcgcc aagggaagaa gtcagccagg 104701
tatttatttc gccgctgggt gatcaatcga ccgaggtcga tgcagagaca gaaacagccg 104761
cttttgtatc acggccagac cctatgagag agagcgaaac taagccgata agtattgagg 104821
ttaatggcgg caggaatgag cggtcagcat tcgcggaggc ggcggggctg tacctgtagg 104881
cgcaccgggc tggccacaac cagcccggca ctttcaatca acacctactg gagaggtgaa 104941
ttatgacaac acaacatatt atcgaaccag ggcaagcagt gcatcaagca gcggctattc 105001
tttcttcttt ggagtacatc aaccaagcgg aagcgcggag ccttgggcca ttggccgaag 105061
ccgtcgctaa tgcttttatg gtggtgtact accaagctga aacgggccgg gcgacacagg 105121
ctgattttca agaagcaatg aacgccttgc gccaagcgtg cagctaatga cgaaagggcg 105181
gccactcgaa ctggccgcct ttttcaatct tcactgtttg gccattcccg acccacagac 105241
ggcgcatcca tccattcgcg ttcctccggc ggcagcgggt aagccccgga tgcttccgcg 105301
tcggccagta gttcggccag tgtgtagcgc acccgaccag tggaaacggc gcgggccggg 105361
tcggccttct catgcagcct gttctctagg ttctttgggt ctgtcatcgg gcgacctcag 105421
caagcatgga tactaggtgt tccaggtcag tgatcgaatg cgccaggaag cggccggcgc 105481
ggcaatctgc aatcatttcc tcagtttttt cctactcatg ttcaacctcg ttcgtaccag 105541
ctcttcgagc gattcaccac gcgcacgacc agcagcatca ccggcacttc gaccaggacg 105601
ccgaccacgg ttgccaatgc agcgccggag tgcagaccga acaagctgat ggcggccgcc 105661
acggccagct cgaagaagtt gctggcaccg atcagggccg aaggacaggc gacagagtgc 105721
ttttccccg cccgcttgtt cagccagtag gctaacccgg aattgaagaa cacttgaatc 105781
aagatcggca cggctagcat ggcgatcact aacggctgcc ggatgatggc ctcgccctgg 105841
aaggcgaaca gtagaaccaa cgtcagcagc agcgcggcca tcgaccacgg cccgatcttc 105901
tgcatggccc gttcaaaggc ggcctgtcct tgcttgagca gatgccgccg caagagctgc 105961
gccaggatca ccggcacgat gatgtagagc acgaccgaaa tcagcagcgt gtcccaaggc 106021
accgagatcg aggacatgcc cagcagcagg ccgacgatgg gcgcgaaggc cacgatcatg 106081
atggcgtcgt tcagagccac ttgcgagagc gtgaaaagcg ggtcgccgtt ggtaaggcgg 106141
ctccacacga acaccattgc cgtgcagggc gcggcggcca gcaagatcaa gcccgcgatg 106201
tagctgtcca gttgatcggc cggcaggaac ggagcgaaca cttgtcggat gaacagccac 106261
cccagcaggg ccatcgagaa tggcttgacg gcccaattca cgaacagcgt gacgccgatg 106321
ccgcgccaat gctctttgac ctggtgcagc gccccgaagt cgatcttgac cagcatcggg 106381
acgatcatta cccaaatcag caggcccacc ggcaaattga cctgggccac ttccatgcgg 106441
cctatggcct ggaacacgtc aggaaggccc tggccgagcg caacaccggc gacgatgcac 106501
agggccaccc acagcgtcag gtaacgctcg aagccgctca tcggggcctg gcgcggcttc 106561

gcggcgatgg cctgtgcgcc ggcgctcatg cttcctgctc ggtcttgccg atgttcgcca 106621
gctcgcgctt gatggccgtc tggtcaagca ttttgagagg cagattcacg aacaggcgga 106681
cgcggttcat catgtgccgg aaggtttgtt cgaaggcccg gcgcttctcg gcgtcagtgc 106741
cttcgacggc ggccgggtct tcaaatcccc aatgcgccga gatcggctga ccaggccaca 106801
ccggacacat ttcgccggcg gcgttgtcgc agacggtgat gatgaagtcc atcttcggtg 106861
cgtcgggcgt ggcgtactcg tcccagctct tgctgcgcag attctcggtc gggtaattca 106921
ccgactccac cttttcgacg gcgaagggat tgaccttgcc ggttgggtga ctgccggcgc 106981
tgtaggcgcg gaagcgccct tgccccatcg tgttgatgag agcttcggcc atgatgctgc 107041
gcgccgaatt gccggtgcag aggatgagga cgttatagat tctttcggtc atgatgtttc 107101
gctttctggt agtggctggg gagcaatggc gatggatgaa agatcggtgg cctcgtcgat 107161
gacgtggccg gccgctttcc gttcggaata gcggtctgtc agtgcttcac ggtgcggccg 107221
taccagcgcc gtgaagcgca ccagttcttc catcacatcg gctattcggt cgtagtacgg 107281
cgagggcttc atgcggcccg ccgcgtcgaa ctcttggaac gctttggcga tactcgactg 107341
gttcggaatg gtgaacattc gcatccagcg gccgagcaga cgcaaggtgt tcacggcgtt 107401
gaagctctgc gagccgccgg atacctgcat cacggccagg gtgcggcctt gggtcggccg 107461
gatgccggcc atttcaagcg gcagatggtc aatctgcgcc ttcatgacac tggtaatctg 107521
accgtggcgt tccgggctgc accagacttg tccctctgac cactcggaca gggcgcgcag 107581
ctccttgacg gccgggtgat cgtcgctttg cacttgatcg ggcaacggca aatcggacgg 107641
gtcgaagatg cgtgtttctg cgccaaagaa ttgcagcagc cgggccgctt cctcgacggc 107701
cagccgtgag aacgagcggg cgcgcagcga gccatacagc agcaggatac gcaccggcgg 107761
ggcgtcgggt gccagcccga gcgccgggcg ctcgatggca aaggatttgt ccaaagcggg 107821
caaagaatcg gggtcggaaa gatggcgaag tctcatgcca acaccttccc ggcttccgtg 107881
gtgcaagaca ggttgcacac ctggccgccg cagcagtttt ccgtgagaaa ggccaccagg 107941
ttagtggccg tcgaaaagtt cgcctcgtag atcacaaatc ggccttcttg ccgcgacgtg 108001
accatgccgg cgtgtgccag ctctttcagg tggaaggata gcgaagacgg cgggatgccg 108061
gccgcttcgc tgatttttcc ggcggccatg ccggcggggc cggcctggac gagaagccgg 108121
aacaccgcga ggcgcgattc ttgagcgatg gccgccaggg cggctacagc atttatcgtt 108181
tccatgtttc aataatagtc gaaatatgga gtgtgctcaa tagctcattt aagctgtaaa 108241
aatgagctag gcgcatttag atcgatatgt gataaattgg cctgccagtt taagatgggt 108301
gcatttgagt atgcccaaag gagcccgcaa gtatgcgcag gacgaagcca gtagccgcgc 108361
cgatggtggc gcgggtctat ctgcgcgtca gcaccgacgc gcaggacttg gaacgccaag 108421
aggcgatcac tacggccgcg aaggccgccg gctactacgt cgccggcatc taccgtgaga 108481
aggcatccgg cgcacgcgcc gaccggcctg agctgctgcg catgatcggc gacctacagc 108541
ccggcgaggt ggtcattgcc gagaagatcg accgcatcag ccgcctacct ttgcccgagg 108601
ccgagcgcct ggtggcctcg atacaggcca aaggcgcacg cctggccgtc cctggcgtgg 108661
tcgatctatc cgacctggcg gccgaggccc agggcgtcgc caagatcgtg ctggaagccg 108721
tgcagatcat gctttttcgc ctggccttgc agatggcccg cgacgactac gaggacaggc 108781
gcgaacgcca gcgccaaggc attgagttgg cccgccaggc cgggcggtac aagggccgcc 108841
gtgctgatcc gaagcgccgc gcccaagttg tcgcgctgcg caagtccggc tacagcatca 108901
acaagaccgc cgagctggcc gggtacagtg cggcccaggt gaaacggata tgggccgagg 108961
tcagccaggc cgaagcgaag cagcacggcg cgttcgtgga ggacgcattg acggaagccg 109021
atgccctggc cgctgtcggc caggatgagc gccaggagga aagggcatga agaagccgaa 109081
ccaagacgac gagccgtttt tcatcaccga ggagattgcg gccgaaatga tcgccggcgg 109141
ctatgagttc gagctgccgc ccattccttg caccatccgc ctacgcgacg tgctggagcg 109201
catgaccgat gctgagctag cattgcagcc gggcgagatc gccgaccagg agcgtgaacg 109261
ctgccggcgc aagccgtgtt caacctcatg atctggtcat ggtatttttc atggcactga 109321
gcctgatagt tcttgcaaat tgttgtcact aaagggtttt gtgtgcttgt ttacaatcga 109381
gtgggagtga cgggcactgg ctggcaatgt ctagcaacgg caggcatttc ggctgagggt 109441
aaaagaactt tccgctaagc gatagactgt atgtaaacac agtattgcaa ggacgcggaa 109501
catgcctcat gtggcggcca ggacggccag ccgggatcgg gatactggtc gttaccagag 109561
ccaccgaccc gagcaaaccc ttctctatca gatcgttgac gagtattacc cggcattcgc 109621
tgcgcttatg gcagagcagg gaaaggaatt gccgggctat gtgcaacggg aatttgaaga 109681
atttctccaa tgcgggcggc tggagcatgg ctttctacgg gttcgctgcg agtcttgcca 109741
cgccgagcac ctggtcgctt tcagctgtaa gcgtcgcggt ttctgcccga gctgtggggc 109801

gcggcggatg gccgaaagtg ccgccttgct ggttgatgaa gtactgcctg aacaacccat 109861
gcgtcagtgg gtgttgagct tcccgtttca gctgcgtttc ctgtttgcca gccggcccga 109921
gatcatgggg tgggtgctgg gcatcgttta ccgcgtcatt gccacgcacc tggtcaagaa 109981
agcgggccat acccaccaag tggccaagac gggcgcggtc accctgatcc agcgttttgg 110041
atcggcgctc aatctgaatg ttcacttcca catgctgttt ctcgacggtg tgtatgtcga 110101
gcaatcccac ggctcagcgc gtttccgctg ggtcaaggcg ccgaccagcc cagagctcac 110161
ccagctgacg cacaccatcg cccaccgggt gggtcgctat ctggaacggc aaggcctgct 110221
ggaacgggat gtcgaaaaca gctatctggc ctcggatgcg gtggatgacg acccgatgac 110281
acccctgctg gggcactcga tcacttaccg tatcgctgtc ggttcacagg cggggcgaaa 110341
ggtgttcact ttgcaaactc tgccgaccag tggtgatccg ttcggtgacg ggattggcaa 110401
ggtagccggg tccagcctgc acgccggcgt ggcggccagg gccgatgaac gcaagaagct 110461
cgaacggctg tgccggtaca tcagccgccc ggcggtatcc gagaagcggc tgtcgttaac 110521
acgaggcggc aacgtgcgct accagctcaa gacgccgtac cgggacggca ccacgcacgt 110581
cattttcgaa ccattggatt tcattgcaag gctggccgcc ctggtaccga agcccagagt 110641
caacctaacc cgcttccacg gggtgttcgc acccaacagt cggcaccggg cgttggtcac 110701
gccggcaaaa cggggcaggg gcaacaaggt cagggtggct gatgaaccgg caacaccagc 110761
acaacggcga gcgtcgatga catgggcgca acggctcaag cgtgttttca atatcgacat 110821
cgagacctgc agcggctgcg gcggcgccat gaaagtcatc gcctgcattg aagaccctat 110881
agtgatcaag cagatccttg atcacctgaa gcacaaagcc gaaaccagcg ggaccagggc 110941
gttacccgaa agccggcgcc caccggctga gctgctcctg ggtctgtttg actgacgagc 111001
ctgaaggcca acgataccaa tcaaaatgct gcgttcacag cgccgcggca gggatccgcc 111061
gtgctggttg tcggaaaagg agccgctagt gggaaagagg agggtaaatt ttcagcgttg 111121
ctggctcccc gtcagccgga ttgggttgca tcgcaggggt gtcgaaagag tcaactgcgg 111181
tccaaagctg ttggacttgg gtgaaaaggg cgtttattct tcctatacgt tgtcggcagc 111241
gggccaaaaa ggaatacgtc catgcccatc gaggtgaaac cggctgtgag cgcgggttca 111301
agcatatagc ccgacaggcg cgtatccttg ccgatcacga cacgatggcg gtggtcaccg 111361
cgacgaaaga cacggccagc cgccatgccg acgcgcaagg cggtttccgc cgtcatcgcg 111421
ccttcgttgg ctttgccacg aataccgtct gtgccgaaat atttgcgcac cataaggtcg 111481
attatcctgt cgtcggtcg ccctcaaagg ggacatgcct gctgaaccgc gaatatagag 111541
aaatatcccg aatgtgcagt taacgaattc ttgcggtttc tttcagcgcc gccaataccg 111601
ccagcccgtc gcgcaagggg cgcggctcgt gtgtgcggat gaagtcagct ccacctgcgg 111661
cggcggcaag ctctgcagcg agtgtcgcgg ccccgacatc ccccggacca cggcctgtga 111721
gcgcgcgcag aaaggatttg cgcgaaacag acagaagcac cggcaaatcg aagcgcagcc 111781
gcaattcatc gaaccgcgcc agcaccgaga gcgaggtttc gggagcagcc cccagaaaaa 111841
accccatgcc gggatcaagg acaaggcggt tgcgtttgat accggcaccc gtcagcgccg 111901
cgatgcgcgc gtcaaagaac gccgcaatgt gatccatgat gtcgccagcg ggtgcctcgc 111961
gccgatctgc ctgcccgtct tgcaccgaat gcataacgac gagtttggca gatgatttcg 112021
ccaattgcgg atagaacgca gcgtctggaa aaccgcgaat atcattgaga taggccacac 112081
cacgcgacaa ggcataggct tgcgtcgcgg gttgataact gtcgagcgag acgggaatgc 112141
catctgcctt gagcgcgtcc agcaccggcg cgatacgcgc gatttctgtg tcggacgaaa 112201
caggcgcggc gtcgggattg ctggatgccg gaccgaggtc gatcacatct gccccctcgg 112261
ccatcagctt acgcgcctgc gcaatggctg cgtctggcgc cagataccgg cctccatcgg 112321
agaaactgtc cgaggttatg ttgacgatgc cgaaaatgat gagcgattta ttcatggggg 112381
cttctataat aataatctct gtacacgata aaaatagata actcattgaa ataatgtcat 112441
aataattgtt ttctaacgac gaatactatg acacatctca atgagttata tcttatctta 112501
aacaaatatc taaaatggaa caagtcacat ttaaagtgct ttgcgctcat catgcttgtg 112561
attattttaa agcaaacatg taatctttct tctgcatcta aagccttgcc catcaagtgc 112621
ttaccacaat cattttatcg acgtatgcag cgcttctttg caggtcagta ttttgattat 112681
cgtcaaattt ctcagttgat tttcaatatg ttttcattcg accaagtgca actgacttta 112741
gatagaacca attggaaatg gggaaaacga aatattaata tcctgatgct cgcaatcgtt 112801
tatcgtggaa tagcgatacc tatcctttgg acattgctta ataaacgtgg aaattcagat 112861
acgaaagagc gtattgcttt gattcaacgc tttatagcca tttttggtaa agaccgtatt 112921
gtgaatgtgt tcgcagacag agagtttatc ggtgagcagt ggtttacatg gttaattgaa 112981
caagacatca acttctgcat tcgtgttaaa aaaacttcat tgtcaccaat catttaggaa 113041

agaatcataa aattagtgat ttatttcgcc atcttaaagt tggtcaaatt gaatgtcgta 113101
aacgacggat tttggttggt cgggtgaaac tatatataag tgcactacag ttagaaaatg 113161
gagagctttt actcgtcgtt tctcctcagt ttaatgccaa tgctattcag gattatgcat 113221
tacgctggga aattgaaacc ttattcagtt gtctcaaagg acgcgggttt aatcttgaaa 113281
atacgcgctt gacagaccct agacgagtga aaaaattgat tgcggtgtta gctataagct 113341
tctgttggtg ttacttaacg ggtgaatggc aacatgatca aaaaaaagcg ataaaaataa 113401
agaagcatgg acgactctca atgagtttat ttcgctatgg tttagactat gttcaaatgg 113461
cgattcagcg tttaattggt tttgggaaaa aagaagagtt taaggaaatt ttggcaattt 113521
taagaaggca gaaccctgat aggataaggg ttctgtgaaa tttgtcgtgt acagagaata 113581
ataataatcg agcatgagtc tcatacggat gctcgggtcg aaagggaatc cccaggcgag 113641
taacctgttt gcggtgatcc attagctgca ggagcagaag agcatacatc tggaagcaaa 113701
gccaggaaag cggcctatgg agctgtgcgg cagcgctcag taggcaattt ttcaaaatat 113761
tgttaagcct tttctgagca tggtattttt catggtatta ccaattagca ggaaaataag 113821
ccattgaata taaaagataa aaatgtcttg tttacaatag agtgggcttt cttatagcca 113881
atccaccgat cagtgaataa tgtctcaatc cgttcactat cgcttaattg catgatttca 113941
agtacatgct ctgcaagata ttcatatttt tccatcacat caccttaaat catcaaatgc 114001
agttaaagga gtactccaca aatcatcact tgtctgaagg ctttcagaaa ctacagcctt 114061
tttagactga ttctgttcgg atagctgagt aatggctttt tcattttcct tttttctttg 114121
gtttgagcga cgttgctttc gagtcaagct tcgagcctga ttaagctgct cacgtagatg 114181
taaaatcgcc ttgtaaatct catcctgatt ctgaacatcc tgacgcccat tcttgagata 114241
gttctgtacc tgtctatatt caaataagct aatcggtggt atctctcgct ttgcagtggg 114301
tactttaaaa taggtattag aaaatggctc ataaaaccaa atctgtgaaa tatccctcgg 114361
atcacgctta aataagaatt ttcttttgcg ctttcgatta ttgtcgtctg gatcaataga 114421
attaacccat tgccttaaac agtctgcaaa atagaataag ttatctttct gcacccctgt 114481
acgctgaatg gtcgcttcaa attcaggtag aaaatcaata aacaaggtat cttcatctga 114541
tacacgttct ttcaaacctg ttcctgctgt cgtcttcgtt ccccaaacac cttcttccca 114601
tttaacaata ggtgatgttc ctaaagctga atgcttcttc tgatgataaa ctttagtaat 114661
ccaataaacg atatagtatt caagttcctt taatgtcata caggcttgtt tagcggaatc 114721
ataagttcct cgctgctgca catttgaaaa agttgtacca tccagcacat gcatttcaag 114781
attgacaata ccaagcatac gttcaatatg accgccaaat ctcgccccac caattggtct 114841
atattcccaa tgaatcccat attttaggca agctttactg atgtgattgg ttctaaaatc 114901
gggtccatta tctgtatgca ccgagtccat aataccttcg acctgccatt cagcatcaat 114961
atttagttcg atcaacttac gttttttga caaaatacaa gatgctatac acatcgccac 115021
tgaagtcgta cttggtgctt ctagtgatag gtaatatcca acaatcatcc gactgaaaac 115081
atcgattgcc aaggttaagt gaggttttcc aatcgcttct ctgaactcgt catccacgat 115141
ttcaatatct agaggtgtat gatcaatctg cacatatgcc aaagggtaat cagcatttgg 115201
gaatgaaccc gcagcagcct taaatttatc tattgcagct ttagatccaa gacgtgcttt 115261
cgttacttgg taattattta aagcctcgat tcttctacga atagagtttt catgtggtgc 115321
ttcaagctga agccgtgaac actctgcttt tacaatttca atcgttttgg aaatactagg 115381
tttcttcgca ttcaaatatt catcattgat agcttgttta atgatattgc tcacttgagg 115441
tgacaacctt gatttttcag ttgtccaacc tcgttttttg ggaatcaacg tcattagaga 115501
gttattttca cgataatctc taagccactt ccacaatgta gaacgattta catcatattc 115561
attcgctcta ttttcaacca gttcagtggt tgaatactca atcaaaggct taataatctc 115621
ataacgttgt aaagcaatct gccaagcttc attgctgata tccttatcac ctttgtttag 115681
gttttcagtt agcgtatttt ctccctcaaa aacggtcaaa cttttagaat tgacctgtaa 115741
actacgtacc gaatcaagac ttgatataac aatgtcatta gcatttaaaa cattcacaat 115801
tttataaggg tttccctcat aaccaacaac cacattgggt ttaagtgtga tacgactacg 115861
atcaatactg acagcattag attcaatcat ttagatattc ctcacaaacg catcattttg 115921
gttaacccat atcacagttt cagccgttaa agggcaatac aagtcacaag caatcattcg 115981
ctttgccact agtgcccaaa cttggtgaat cccttttaga acattggttt tatctttaaa 116041
aagatgagca ggtaattgat taattgttga ttgacctaac ttttgtaggg tatcgagtaa 116101
taattgttga tcaacggcat ctatatgaga gcgtctaaat ttttttaaga aattaatatt 116161
ttcccaatac tgatcatata atcttgtttc actgataatt ttgaacttcc atcctttatc 116221
agcagcaaaa cgatgagctg ctctaaattt tggttttaac ttatcccagt cctcagcaag 116281

cttttcttg ggcttaattt caataagcat aggtacaggg aaatcctcaa aatcatcaca 116341
attcgagctt gaaaactgca ccaaaaagtc aggggtgtag tttgattgat tacccaattc 116401
agtgatgtag gggataacta caggttgacc gataacatca atcacattgt tattaaattc 116461
ttgcttaagt atgaaatcac gctcaagggt cgattcaaac catatggttt tttcacctct 116521
aaaagcataa tttcctgaaa tactcccata aacagtttga gcttttctaa caggtaagta 116581
ttcgtcactc aattttttca taaaaaattc accattcagg cactcgtgct tattaatgta 116641
attttgtaag ccactcttat ctatttgtat aaataatctg atttaattgt gtgaaattag 116701
agtgacaacc tattgattat ctataatgcc actctattct attgctgtaa atgacaattc 116761
tgattcggag gtgcctgagt caaaacaagc agtaatgaaa agacttgtgg agataagaat 116821
acataacttt gaatatttca tagtttagtc ctagatatga caattaaatt agagcatgtt 116881
cttttattta gaatcatgca tttattaaaa aattatttgc actcattaag agattttaag 116941
aaaagattaa ttacttcatc tatagtgtta aatttatgct ccgatttgaa tttttctatt 117001
accacaattg tttttagtaa aagattaatt gcttcatcat ttgagctcaa tttatgctct 117061
gatttaaatt tttctattac ctctaaactt tcttttgata accaaagatc cttacgcgtg 117121
aacccttttt ttaacatttc ttggcgttgt tttgctttac gctctgctgc tgtgcttgcc 117181
ataatctacc tgaaataatg ttaataactt tggccaaaag gatgtggagt agcgatcaga 117241
acaattcagc taatgaacgt gcgtgatttt catgtgacat tctattagtt gcaattagat 117301
catcaacttc tttttccgaa tttgcgtaat actcgcgata agacaccact ttgcctttgc 117361
agtattcctt cacctcagac tcggttttat acaatgttac atgcggtgtc ataccataac 117421
gttcaaattg gtcaatactg tcccaaaaac cacacacata aaaagcgaaa gcgggagtgt 117481
catcacctac atgaattctt ttattcgtgg ttactataga atttacatct gccttaacaa 117541
cttttacgcc gttagcttga acaatataac tattactaac tgaatttttc attaatctaa 117601
gtctcctaat gatattaagt ttttatttat tccggaatta aaatgccact tcttcccagt 117661
agatctctaa ctggcgaact gtcttaacat ttccaaagtc acaaccttca tgtccgataa 117721
tgacacgagg ctcaacttcc tcatcataga tttcaattat gcttcccaaa aaaataccttt 117781
tcggaagtat gatgttatgt ctctgaagca atgtggccaa tgttgaaaca ggcgtcaaat 117841
aattcaaaat tgatttttta gttttaatag ttgtttgaaa aagtacctca tgctcaattt 117901
cttcaaacaa ttcaacgttc tcaacagtta atttatatgc tttaacttca ttcattttta 117961
acctcagctc ataggcatta ttgtacatat tgttattata aattttatat tgtgaccgct 118021
cacaaagcaa gaactatttt taatcaatgt taagtatcta gtctgcttta agttgtttca 118081
aactaagaaa tctactccat ttaacataat tatgaaactt tatccttatc tagttcttac 118141
ttattgtgaa aatttaactt tttagttctc accttcagta ttttttacgg ctcttggatg 118201
tgtatccata taaacatttg tcattgaagt atggtccaga tctgtataga tctgagtagt 118261
attcaaatta ctatggccca gcatttcctg aatttcacga atacctacag aattatttat 118321
tagatgagtt gcaaatgaat ggcgtaacat gtgtgggttg gattgaattg gataaccagc 118381
aagtttaaaa gcatttttaa ttcttttatt tatttggact cttgatagtt gagctccagt 118441
tgtggtgaca aaaacataat tatgtttttg attggtatct ttattcataa tttcttctct 118501
taaaggcaac cattctagat acgcttcaat agcttttgaa cccaaaggta taatacgagt 118561
tttatttcct tttcctgtaa ctcttacttg tttgcgtttt acctctacat tttctatttg 118621
taatgagtgt atttcttcaa gtcttaaacc acatgagtat gctatttcaa ttagtgctaa 118681
gtctctttta aaaataactg tgcttgatgg tcgttcaaat tctaaattct tatttgaatc 118741
taatacttca tttattactg tgacttcatg aaatttaggt aaaactttac tacttttttt 118801
aattttaata tcttctgctg gattgaaatt aattaatttt tctttagtta aataatggaa 118861
aaatgttctt atagcagaga tatttttttt tagtgtagaa actcctactc cattttgagc 118921
tctataagct ataaatgttc taatatctga tgcttcgatt tcctctaatg ctaaattggt 118981
atattcaaag aatagattta aactattttt aagcgtgtat attgtttcat tagatttatt 119041
tgctaataaa tattcatcta accatttttt tagaagtatt tctctgtcta gcattaataa 119101
atcctattaa caaactgtaa tgatattata tacgtttaaa caaaataagt ggtaaaatat 119161
agctatcgca agacctgtcc ttatggtggt gcttatgaat agcaaatttt ctgttgatca 119221
atcgaacaag atttctctta aagcagtgct tgaagatctt ggtgatcaaa tgtttgaagc 119281
aaataaatgt cctgatactc atcctgtcta tgatcagttg cgtgagcgtt tacgtaaaga 119341
tcgtcaactt ctagatgcct tagatgctca agatgagaac gcaaatactg aagaacgttc 119401
taggagtttg gatataaatt aaatgacaca aaaaaatgaa ctagacacca agtatgcagt 119461
gatagttgct ggtggtaatg gagctggtaa atccactctg atagacaatg tgataattcc 119521

aaaatttaat agtttaaatc ttgatataaa ttttattaat gctgatgttt ggcaattaca 119581
acactttggt cattttgata ataccaatcc tacccatgca cgcgaagctc agaaatgggc 119641
tgaagctgaa cgtcaaaaac atttagatga aggacgttct tttattgctg aaacagtttt 119701
ttcgcatcct tctaaagttg atctcatcaa ggaagctaag agtaagggat tttacgtagt 119761
cctgtatcac atccatttag aaaactcaga tatcgcactt gatcgtatta aagatcgagt 119821
tcgtaaaggt ggtcatgatg ttgatgagga taaggtaaaa gctagatatg agcgtgtact 119881
acccttagtt gctgaagcta ctcaatatgc agatttgact tttgtacttg ataacagcgt 119941
gagagatagg cctcatcagc aagtatttaa attagagtac gggaaaatta ctgcgattga 120001
caacaatgtt ccggattggg ccattaaagc ttatgagaag cagctcactg attatcttgg 120061
attggatcaa gcaaccaaaa tttcaaatac taatgaagat ttaactatgt caaatactaa 120121
agagaaactt ggattcgagt ataacgctag actgctggct aatgatattc aacctcaaca 120181
tcgagaaaca gttgttgcta attatcgaaa gaattttgtt gaacatgtag cgaactctgc 120241
tgcggtagct ggtaattcac ataagcttga tgatgtagca tctttaataa atggcaccac 120301
tgtaggtgga cacagtgtta aggaccagcg acaagtatta aatttaattg atgcgtcaaa 120361
tgaactggta aatatggttc gtaatggaaa ttttgcatta aataaatcta catatttaaa 120421
agtcaatgga attgttgcag atcgtgaatc gattgaagct ggtgttctac gtggcgaagg 120481
agttgaacaa tcatttactc cggatgtagg aattagacac tttagaaccc atcgaccgat 120541
gaaaactgaa gaagatgctc cggcgttaaa taaattcatg caagaagggt ttgatcatat 120601
caactcactc aagaacccac tagaaaaagg tattgctaca ttcctttatg gaagcctcaa 120661
ccaattcaca tttgaaggtg agaaaagaac agctcacctt atgatgaatg gagtattgat 120721
gagtgctggt ttagatgcta ttagcatacc ggctgataag gcaattgact tccgtcaaaa 120781
aatggttcgt ttctatgaat caaaaaatgc ggatgaagtg atggagtttt tagctgactt 120841
gcatccggct ctcaataata cgaaaaaatt agatgcagaa aaggaaaaga aatcagaaaa 120901
tgactttggc cgcagctttc agtaattttc gtttatcctt ttaatatcaa tacgagagac 120961
actataatta tagtgtctct tttttaattt aaagtatttt gtattattta tggccagcct 121021
taattctgag ttccagatcc ctttaaaagt ggttacaacg cgcttaaatc acatgaaaat 121081
taatttgccg caattggtaa agaaaattga aaagagagcg attgaaaact taaaaagacg 121141
taaagataag aaagaacaaa ttgatctact gataacttgg gatgattgga tttatgccgc 121201
gcttgatgct gatcatgaac gtatacatgg aagcgcggat cctcacccac ttccaccaac 121261
attcaaaaat aatatggtta gtggtgtata tgaaaaagct ttaaaaaatg tcatggggca 121321
tttcggagtt aaagctactg ttccagcatg ggcggcaaat ataaaggttg gtgagcatat 121381
tccaaaggaa ttgcttgatc gtctgcctgt gaccactgga gagcttgtaa ctctaggaaa 121441
ttggatgctc acaaaaaata tataccggtt cgaagacata gtaattaatg agttaattaa 121501
gaccggcttt aatggggtta taccaaacca tattttgaac cttcccgatt tgtgtgtata 121561
cattcagact gataatgcta agggtcttac attcgaaaat agacaagttg tgggtgttct 121621
attctgtgta actgagcttt gtggcgaccg ccttttagtt tcaacaatgt accttgatga 121681
tggtatgcca cgcacaattg caattatgtt gaatgaggac caagatattg aagcaagcct 121741
cacaaatttt gtggaccaat ttcaacaaga ctatgatcca gaaacaatgg cttcagatct 121801
gaaagaaaga cttaaaatac agaaaaaatt aatcaatctt gttttatggt ttagtcaatc 121861
aaaacctgaa gtgactcctt taactccaga taccaataag ccggtacaat ttgttgagat 121921
taagaaggaa aagcgtttat ttgaagctgg caaatataag acttttaaaa ttggatctga 121981
aacagctagg aaattaacta aattatacga agaaattgaa gtggccaaag ctgaaggaaa 122041
agcatctggt agagagccac atttaagaaa atcacattgg cacctttact ggtatggtaa 122101
gaaaggtcgt tatgaacgtt atgatttttt attaattcct gtcacaattg ttggcggcgt 122161
ttcaaaagat taaactttat aagtagagaa aaaatggata attcagaatc taaaatttta 122221
gctgaatacg agaaagcgaa gaagtgggat aaatacgaaa aagtattttc tattcttgga 122281
gtactttctt tgtttatatt tatggttatg atcataattt cagcaattac tttgagttta 122341
aatgatactt caccaactaa aatatttaat tcttattgtt gggctgcaat attattttg 122401
gcagttttat tcacctcact aagaaatatt gctgagcaga tttcttttaa gaaatctatg 122461
gaaatcttaa tagactcaaa agatgctgaa gatgaaaaag atcaattggc agtagtaaga 122521
aggcagcata caattatttg cataactgca ttttgtgcat taatagttgc ggattttatt 122581
ttattctact ttattgcttt taatctcacc cttcccctaa atatactgat tcataaaccc 122641
ttatttatta ttatgctggc ttgtttaata ttttttacta ttaattattt gatttataaa 122701
tataagttta ataaattaaa caacgaagca atggttgcat tcggtaagca ccttgataat 122761

aaaagaaaga aataatataa gaaaaggctc attatgatga gccttttttc taatctttag 122821
ttttattaac gatgccattg cagcatttac atgtatagat atattcagta ggatccaaaa 122881
gaattttaat aaaaatacta tttaaaaagt tatagattat gaacctaaca attccgagac 122941
acgccaaaaa gctagaacta ttgtcagttc cacaaataaa tcagagttta cacaaataaa 123001
ctagagcaag aataaaaagt atattatcta tatgctttaa tttttcattt tgtaaaaata 123061
tatgcctgtt aataatgaca aaataacgac tattgattgc ttcccaaacg ataatattag 123121
tcgagtggtt tatagatatg gtggacttgt tagaaacgaa gataacgatt caactaatcc 123181
ttttgttgaa gtgttattaa ttgagattag aaaaagtgat caatggttat ttttagataa 123241
gtgctcaaca ttccttgttc ccgtacttga tcttgatgct gttcaacatg gcagtatttg 123301
ggatggaaat gttttaaccg atcaatccta tcggttttca ggaaaactca ttacaaaacg 123361
attttctttc gacttcacca ataataaacc taagaatata aaacttacgg acagaatccc 123421
caatacttct gactactaca ttcctttaaa aaactatttc ctcccaaata aagttgatta 123481
tgttcttcaa ggctatccat ttaccaaata taccaatgat tcatatcgca aagtgaatca 123541
ttgcctaatg cattccaatg atggaactca agtcatcaca tcatcaattc atgtattgca 123601
tagcttattc gtcaatcgga aggatattag gggactttta ttaggaactt cgagtcaatc 123661
catcattaat cgttttttag aatcttatac aacagaaatt gtggacgata atgttgaata 123721
taaaattaaa atcagaaagc catatgaaga tataggcgag actgcaatta tttttcttgc 123781
taatcttgcc ttaaacccat atgtgcaaac gatagttgat aaaattcaac gaagtatgga 123841
aattaccgag tttaatcatc ttcagcaggg tacaggcaaa aggtatccca ttgtgtttcc 123901
accacatccc acaaagcttt tccttgaagc agaaggaatt tggctagatg acaataagac 123961
tcgttttttt atcactagag tgaaaaaatt tgatcctatt aacgatcaca tgattgatgt 124021
caacaaagac ctgaccaata caatttcaaa gcctgatgag aaaaatccta gacctcgtga 124081
aaaaagtcaa aataagaatg agcacatcaa tactcaaaaa ccaccatcaa gaacaagcgg 124141
tgaatatcgt aagcgttctg aggtggaaac tggaaataca caaggtattc tgaagtattc 124201
ttttaatgaa ccaaccgatg atccaattga agtcggagca tctaatcaac cttattcaga 124261
taaatcagaa gaggttgaaa catcttctga tgaaccctat ggtaatcaaa atactaagat 124321
caaaaaatct gaaacgacag atacaccacc tagcagagat gaacgttttg acattcaata 124381
cattatccaa tctctccaag aacttgcttc tgaaatgggt tctcctctgg aacatctgtc 124441
tgcaattaat gaacatggtg aaatgatagc gagtattaat ttgcttcaaa tcaaaaaact 124501
tgtgcctgaa cccaagcatc cctcatggat tgattatgat aaagggcgaa aattattgtt 124561
cttaaaatta gacttaaaag atcaaaatgg attttcctat ctcatcgata tacataaaaa 124621
taaaaatcat gaagcatttt gtgcctttct tatttttact cagaataagt taacaagcga 124681
acaaattaaa aaaatctgta ttgaactaga aaatgccaag ggaataaaaa aatgggcact 124741
ctactgtgat agtttcattc ataaaatgat tgcaataaaa catttatacg caacaccaga 124801
tgaatggaaa aatcgcttta aaaatttatt cataagctta cagaaggaca aatcctaatt 124861
aaagaatatt ttttatacaa tcatatgttc atgaaattac ttggtatttc atgttttcta 124921
taaatttgcg aaataaaaaa gagtgaaatt ttcgttcagt tcgcctatac taacagttca 124981
atttaaattt ctattcgtat tcacacactt atgcttttga ctccacccta gtttatttac 125041
ccatcttttt gtaaataact catcactgtc tagtgatgtg gtgtagtcgc ttgtgtgttg 125101
tatatatcac atcactagcc tttcctaaaa tttaaaaaaa taggctattt tcatgttatc 125161
cactattcga gaacaatggt tctccaatat tcgcggggat gttcttgcgg gtattgttgt 125221
tgctttggct ctaattcctg aagcaattgc tttctccatt attgcaggcg ttgatcctaa 125281
agtggggcta tatgcttcat tctgtattgc tgttgtgatt gcttttgtgg gcggtcgtcc 125341
tggtatgatt tctgctgcaa ctggtgctat ggctttatta atggtcacac tggttaaaga 125401
gcatggtctg caatatttat tagcagccac aattttgaca ggatttattc aaatcctagc 125461
aggctatcta aaattaggag ccttgatgcg ttttgtatca aagtcagtcg ttattggctt 125521
tgtaaatgcc ttggctattc taatttttat ggctcaattg cctgaactta ccaacgtcac 125581
ttggcatgtt tatgcgatga caatcggtgg tttagcggtt atttatctat ttccctatat 125641
cccagtgata gggaaacttt taccctctcc cctcatctgt attgtcctac ttacgctctt 125701
tgccttattc attggtttag atgtaagaac tgtcggtgat atggggcaat tgccggatac 125761
gcttccaatt ttcttgcttc ctgatattcc tttaaatctt gaaactttag ctattatttt 125821
accctattca ctaggtttgg cagccgtggg tttacttgag tccatgatga ccgcaacaat 125881
tgtcgacgat ttaacagata ccaccagtga taaaaaccga gaatgtaaag ggcaaggtgt 125941
tgccaatgtc gcttcaggct tcttaggcgg aatggcaggc tgtgccatga ttggtcaatc 126001

cattattaat gtgaaatctg gtggacgtac ccgtttatca tcatttagtg caggggtctt 126061
tctgttaatt atggtggtgt ttatcagtga ctggcttaaa gccattccaa tggctgcact 126121
ggttgctgtc atgattatgg tatcaattgg aacatttaac tgggattctc tacgcaatat 126181
ccgccaatat cctctctcta gcaatatcgt gatgatcgtt acagtcgtcg tagttgttgc 126241
cacacataac ctggcttatg gtgtattagt cggtgttctt ctttctgcat tattttttgc 126301
caataaaatt gaacgctata tggcgattca atctgtattt gatgaatcag aaagcacacg 126361
tacttatacg gtaacaggtc aagtgttctt cagttcagca gataaattta cttctgcatt 126421
tgatttcaaa gaggcacttt caaaagtggt gattgatgtc agtcaggcac acttctggga 126481
tgtttctgcg gttgctgccg tagataaagt ggtcattaag tttagacgtg aaggaacaga 126541
agtcgaactt attggaatga atgaagctag taaaacatta gtcgataagt ttggtatcca 126601
tgataagcca gatgctttag atcgcttaaa tagccactaa gagggattag ttatgtcaaa 126661
aattattgca tgtattgatg gatcattagt aacaaatact gtgtgtgatt atgcagcttg 126721
gtttagcgat aaacttaatt cacctattaa actattgcat gtcattgata agccaaaagc 126781
aaaagcacca caagatttaa gtggtgcaat tggtctagga agtcgagaaa ccttgctcaa 126841
agaattggtt gaactcgaag aacgtaaggg aaaaatcgag ctagagcatg gccagattct 126901
tcttcgggaa gcaaagaact atttattgga gaagttctct attgatgctc aaagttttca 126961
acgccatggc agtgttttag aaacaatcat gggaatggaa gacgacatta gagttcttgt 127021
tatgggtaag catgggaatg aaaccgagca tgattccagt aaaattggaa cccatattga 127081
aaatgttgtt cgtgcccttc ataaacccgt attaataacg tctgcaccat ttagtccacc 127141
taagtctttt ttaattgctt ttgatggaag tcagactgca agaaaatgtg ttgaacgcat 127201
tgcgtcaagt ccattactta aaggtcttgc agtacatttg gtttatgtcg gaaatccgaa 127261
tagtgaaatg caaaatcagc tctcttgggc aaaagagcaa cttgaatcac aaggttttaa 127321
tatcacctcg aatacgttgg atggtgaagt tgataaggca attatcaatt acgctgaaca 127381
gcatcaaatc gatttgattg tggttggggc ttatggtcat tcaaagatac gtcaattttt 127441
tattggcagc accaccacaa aagtgatcac aagcgctaat aaaccagtcc tgttactacg 127501
ataatttcat gaaggggtag aattttgatc tatcccttta tttttaataa gttataaaag 127561
gaatcataaa tatgaatacg atgttaataa gcccaactct aaatgtttct tgttccgaaa 127621
atgtgctgtt aaaaatatcc tcatactttg gtgtatctca tgaacaagct tatcgaaaag 127681
gcgtagattc tcttttaaaa ttaacagaga tggttatagc aaataatgaa cattctgaaa 127741
ttattaaact tttttgtgt gattgtcatt taagaacatc tatcaattta gatgatatat 127801
ccgatatgcc tactcaattg ataggcgatt gccttaatgt gattgttcta cgttatgcat 127861
ttgtagtttg acaatcaggt cgtaaactag acattaatgt tctaaactta aacaatatat 127921
ttgtaaacca aaaggttgaa taaatgggac aagctaaaca aaggggtaca cttgagcaaa 127981
gagtaaagca agctcaagaa caaaaagatg atttttatgg aactgaaaga agtgtagcag 128041
aagttctagc tgaactaggc ttaccaaaag aaagtacagt aaaaggttat gttatccatc 128101
tacctgaaaa agatgaattt gtagctgcaa ttaatgacaa taatatttct ttttcagttg 128161
cttatgctcg gacacctgaa ttagcgatga tatatgatga accacaaaaa gcgatttcag 128221
acgctaaaaa gatttcaaaa cataagctat tggtttgtgt tttatttgaa acttcaaacc 128281
aatacatgat taatgatatt tgggctaatt attagtctga gtttaaatct attatgtcaa 128341
aaattgcata tagttctaaa cttatgtttg attgagttta gaactataat gtaaatctat 128401
tcaattgttt ttgttataag ttattgattt tcatctttat gagtttaaaa ctttattgtg 128461
taaacacagc atttaaagct tcattaggtg actttttagg tggtggtgat aacttaatta 128521
acacatggaa atctactcta taaaggtctc acatactact atctcagaat tttcagcgcg 128581
tcttttaata tttcattaat gatcggtgtg acaattacta aaataatctg ctgaaatctc 128641
aataataatg atcagctaac tcgctgatca ttattttaag tgcttcaact tattgtgttt 128701
ttggcaacca ataatattgc ccattaagca caacagtaaa accatcctca atctcagtaa 128761
tcatttccgt tttaatcttt agagcatcta tgtaaggaag ttgatgaaaa tacctaagcc 128821
catttctgac ccaacttaag ataagttcac tgtcatttaa atcagtttgg gtaagtgcaa 128881
taagttccat ttctagtttt ttcttttgtt tagtctttaa aaaatcagat ttttttaatt 128941
ttaaataatc aatggaagca ccgtaattct catttaaaaa gttaatgaat ccagtgagcg 129001
ccgcagcttg accagaatat tcagcaagat acgctttgac atgctccaaa tttggtaatt 129061
gagcactttc tccctccatt gacagcatca acgccacagc aggctttaca gccaacctcg 129121
ctgaacgtat tgaagtctca cctcgtttga tttttcttc taatttattt ttgtattcca 129181
gtacaacatc ataagccaaa gatggttgca aaatacgttg cacaagcttt tccatctgat 129241

ctcgttctga acaactttt ttgttatcca tatctattcg aatgtcatgc acctcttcaa 129301
gccattgcat caccaattca aattttctaa gcccactact tcgtaacctt actaataact 129361
gctttggagt aggcacagat tgattccaat ctatttcggt tttaataaag aaatgggtat 129421
gtttatttat atataatgca gctttatgtg atccaacttt cttttctaac cagcctatat 129481
agttttcata ctgctgtttt aagtcactag actcaaatac tttctgattt tgatcaaatt 129541
tattccataa attttgatgc caataacaat catcgcataa atcagcacaa cctgctgcaa 129601
tcatacaatg acatgcttta caggatttct gaggcttatc tctacatttt ttacacgttc 129661
tttgtcctga tacatcagac tccaataagc gatgtttctg gcatgatggg catgtctcat 129721
aatctcgtgt cgcacatttt ggacataccc gtagatcatc attaaatggt gaaattcggg 129781
tcaatttttg tgatggcgta ccacatcgtt cacaaggctc aatgggtcta aaatacacgg 129841
aacaactatt acacaccaca ccatattcgg tcagcttacc tattggttta tttgtctgat 129901
tacatcgaat acagggttgt ttctttatac attcattgca aatggcttgt tcatcatctc 129961
tagggagtcg ggcaaagtca ccacaacttg ggcataatcg ctttttaaag atacgggcat 130021
aacaagtcga acaatatttc ttgcctttat atcgtttttt tataaaagaa acagactggt 130081
tgcatccatg acagtcatgt tcaataacat tttcagtcaa tatgcatcgc tccactatta 130141
ttgcttcttc ttaataaatt taaccattga cgttgtacag tcgctttaga ttttggctta 130201
gtaacagtta ttgctgcacg agagacagga taacgtcttg taggcttaat cattaaatct 130261
ttcatctgtt gaagctgaac aggcaacttc ttgcgcttgt cccaaaaatt tgcacgactt 130321
actccatatt cctttgatcg ttgtaccaat aagtcacatg gaatatccat aatctgatcc 130381
aacattgaaa gcaaaactat tcgttcctga gtactcagat attcaaatgc taatggggta 130441
actggatgtg aaagatgtaa atttacgtca aaactttcaa ataattgaat cacattttta 130501
ttgggcgtat tgaccaaaaa tctaatctca ctgagccatg aacgagcaat ttcaaaccat 130561
acttgagtag tcactgggga ttgattataa aaaccaattt tttgttttaa gacttgctct 130621
gctttatttt ggaagtttaa agcatttaaa ttgatgtttt tgctttcctc acaacgcctt 130681
aaatcaaagc cacatgtcgt acagatcgct aaacaaccat gttccatatc ggctttaaat 130741
ggttggatcg tcacaccaca ttcagggcaa tggtcaatta aacttaactg atgctcaaca 130801
caactacaat gccacccaat acgccacatt aagcgcaaat aaggcggatt ttcatgactc 130861
ttcaggcatt caacacatac ttgccgacct gatgtatttg agcggtttcg tgtgcccaag 130921
gataaaaccc aagggatatt ttgatttgaa tcataatttg gctgtatctg agaaacgacg 130981
ctactcaaca tcgattgctg tatcttctgt tttgactcca tactatgaga taacagagca 131041
tcgaatcttt cggcatctac tcctttgtct aaatcaatcg ttaaagctct ccatttcccc 131101
cataaagcct caatgagaac cataggtgag caacctacat ctaaggctga acggatcaac 131161
caagatgaaa atatttcccc ttgctgaatg ggagttctga tataccaaca ttgttcaggt 131221
tgactcatct ctacctccct ataatgattt cgcaaaatta actcaggctg agatttatat 131281
ttctaatatt tcttaaaccc tccgtaggtt taagccattt aaacttatgt acgatgtcat 131341
aggtaatttc ttcatcacct tgtaaaatgg cttctttggc acactccacc agtaatcgat 131401
ttaaatcccc taaatttcca cctgacactt caaaaatcaa cgtagcaatc tctttggatg 131461
caagattaga ctgttttttt aacggcaata gtcgaacata gctgactaat aatcttaaaa 131521
aatcttgatt gagttcccat ttaggtaaat tcaccacatc aaagcgactg gcatattgcg 131581
gatcagtatg cagtatggtg attgcctcac gagtaccaac acctacgata ttcagactca 131641
attcattgct gaggttcttg agcgtattca tgacttcggg aagcaatttg gctgaacctg 131701
tcagacaatt atgaatttca tcgataatca acatttttgt ttcatactta cgcatcaagt 131761
gtacggcttg atttcgtaat ttggcttcag gatcacttgc ccgaaatggc acaaagaagt 131821
gatttaaaat attaatatag agttctttaa cattggcttt tgctggagca agtatcggta 131881
aaactggctt agttacggac aataaatcca tgtcagcatc acttacggtt tttgtataat 131941
actgtctagc aaactcatga atgatagttg ttttgcccat attggagtca ccaacaatca 132001
acaagcattc aggtcttaat ttcctagggc gattcaaaat atcggtcaat gtgttcatga 132061
tggtaatggc tttcttatag ccaatccatc tatcagtaaa taatgtctca atccgttcac 132121
tatcgcttaa ttgcatgatt tcaagtacat gctctgcaag atgttcatat ttttccattg 132181
catcacctta aatcgtcaaa tgcagttaaa ggagtactcc acaaatcatc acttgtctga 132241
agactttcag aaactacagc ctttttagac tgattctgtt cggaaagctg agtaatggct 132301
tttgcatttt cctttttctt ttggtttgag cgacgttgct ttcgagtcaa gcttctagcc 132361
tgattaagtt gatcacgtag atgcaaaatc gccttgtaaa tctcatcctg attctgaaca 132421
tcctgacgtt cacttttgag atagttctga acctgcttat attcaaataa gctaatcggt 132481

ggtatctctc gctttgcggt aggtacttta aaataggtat tagaatatgg ctcataaaac 132541
caaatctgtg aaatatccct cggatcacgt ttaaacaaga attttctttt gcgctttcga 132601
ttattgtcct ctggatcaat agaattaacc cattgtctta aacagtcggc aaaatagaat 132661
aagttatctt tttgcacccc tgtacgctga atagtcgctt caaattcagg tagaaaatca 132721
ataaacaagg tatcttcatc tgatacacgt tctttcaaac ctgtacctgc tgtcgtcttc 132781
gtcccccaaa caccttcttc ccatttaacg attggggatg ttcctaaagc ggaatgtttc 132841
ttctggtgat aaactttagt aatccaatac acgatatagt attcaagctc ttttaatgtc 132901
atacaggctt gtttagcgga atcataagtc ccacgctgct gtacatttga aaaagttgta 132961
ccatccaaga catgcatttc aagattgacg ataccaagca tacgctcgat atgaccgcca 133021
aatcttgccc caccaattgg tcggtattcc caatgaatcc catactttag acaagcttta 133081
ctgatgtgat tggttctaaa atcggggcca ttatctgtat gcaccgagtc cataatccct 133141
tcgacctgcc attcagcatc aatatcgagt tcgatcaact tacgtttttt tgacaaaata 133201
caagatgcga tacacatcgc cactgaagtc gtacttggcg cttctagtga taggtaatac 133261
cctacgatca tccgactgaa aacatcgatt gccaaggtta aatgaggttt tccaatcgct 133321
tctctaaact cgtcatccac gatttcaata tctagaggtg tatgatcaat ctgcacatat 133381
gccaaagggt aatcagcatt tggaaatgaa ccagcagcag ccttaaattt atctattgca 133441
gctttagatc caagacgtgc tttcgttact tggtaatcat ttaaagcctc gattcttcta 133501
cgaatagagt tttcatgcgg tgcttcaagc tgaagccgag aacactctgc ttttacaatt 133561
tcaatcgttt tggaaatact gggtttcttc gcattcaaat attcatcatt gatagcttgt 133621
ttaatgatat tgctcacttg aggtgacaac cttgattttt cagttgtcca acctcgtttt 133681
ttgggaatca acgtcattag agagttattt tcacgataat ctctaagcca cttccacaat 133741
gtagaacgat ttacatcata ttcattcgct ctattttcaa ccagttcagt ggttgaatac 133801
tcaatcaaag gcttaataat ctcataacgt tgtaaagcaa tctgccaagc ttcattgctg 133861
atatccttat cacctttgtt taggttttca gttagcgtat tttctccctc aaaaacggtc 133921
aaacttttag aattgacctg taaactacgt accgaatcaa gacttgatat aacaatgtca 133981
ttagcattta aaacattcac aattttataa gggtttccct cataaccaac aaccacattg 134041
ggtttaagtg tgatacgact acgatcaata ctgacagcat tagattcaat catttagata 134101
ttcctcacaa acgcatcatt ttggttaacc catatcacag tttcagccgt taaagggcaa 134161
tacaagtcac aagcaatcat tcgctttgcc actagtgccc aaacttggtg aatccctttt 134221
agaacattgg ttttatcttt aaaaatatgg gcaggtaatt gattaattgt tgattgacct 134281
aacttttgta gggtatcgag taatagttgt tgatcaacgg cacctacatg agagcgtcta 134341
aatttttta agaaattaat attttcccaa tactgatcat attaggactt acgcattgac 134401
ggattcaaaa aagtattaaa atactttata aagtatctgt gatcagacga attaaaaatg 134461
cttctacagc aacttgtact ctacccattt atatgggctt tctcatgaca gaaccgaact 134521
ctattagctg cacacaactt gccgagactt ataatatctc gcatgatagt gtaaatcgct 134581
ttctagagcg tgaagactac acacctcacg acctatatca agaagcaatt caacatattg 134641
ataataataa acttatagtc agtattgatg atactgtttt agataaacca tatagtcaac 134701
atatggactt agttagctat ttttggtcag gcaaacacca ccgatccgtc aagggtatta 134761
atctcattac cttgtatgcg acagatcaaa atggtcaaaa tattccaatt aatttccgaa 134821
tttatgacaa atctgaaagt aaaaccaaga atgattactt tatggatatg ttaagtgaag 134881
tactcagttg gggtgcaaag attcaattta ttacaggtga tagttggtat tcatcgactg 134941
gaaatctaaa aaccataaga aaatatggta ttcgatttat gtttggtatc gactgtaacc 135001
gtaaggtttc cccagaaaaa ggacaattgt ttcaactgcg tttattgcca gatttccatc 135061
agggtcaagt ggtctggctc aaagattttg gctttgtaca attatttaag actcagttaa 135121
aagaacagca gaggttttat attgtgcatc aagatgaaga tgatttattg tcctttgagg 135181
gttttcatga attacattca agtcattgga aaatagaaca atatcaccga gtgattaaac 135241
aggtttgtca tattgaaaag tttcaagtaa gacgatctaa actgattttg aatcatattt 135301
tttcagcctt gatggcctac gtcgagatac aaaagaacca gtttgagggg atctttgaaa 135361
atgtatatcg ttggcagaag aaattattta gaccaatcat caaagacttc attgatgact 135421
ttattctcga taaaaatcat ctgctaccac agagaatcta taaatgatag tgcgtaagtc 135481
ctacatataa tcttgtttca ctgataattt tgaacttcca tcctttatca gcagcaaaac 135541
gatgggctgc tctgaatttt ggttttaact tatcccagtc ctcaacaagc tttttcttag 135601
gcttaatttc gatgagcatt ggtacaggaa aatcctcaaa atcatcacaa ttcgagcttg 135661
aaaactgcac taaaaagtca ggagtgtagg ttgattggtt acccaattca gtgatgtagg 135721

ggataactac aggttgaccg ataacatcaa tcacattgtt attaaattct tgcttaagta 135781
tgaaatcacg ctcaagggtc gattcaaacc aaatggtttt ctcacctcta aaggcataat 135841
ttcccgaaat actcccataa acagtttgag cttttctaac aggtaagtat tcgtcactca 135901
atttttcat aaaaaagtca ccattcaggc actcgtgcct attaatgtaa ttttgcaagt 135961
cactcttatc tatttatgta aataatctaa tttaattgtg tgaaattaga gtgacaacct 136021
attgattatc tataatgcca ctctattcta ttgctgtaaa tgacaataat tttgccaatt 136081
ttgtaattaa ttggattatt cacttatcca ccctagctat attccctttg agagtttcaa 136141
aaaaagtatt tctatcaact tcttgccatt tataatcata gaagttactt attggtacaa 136201
ccacgcgttg agtgagatct actttatcct tatctgttag gtatccagtt tgaatttcta 136261
agtagccatc caaaccatta aaatcaaaac caatatctct acttaaggca gcaagtggac 136321
aatcataatg gcctagaggc tgttttaaat acccttttcc tctcttttg ggaattagca 136381
tattgatttc ttgaatccca aagagtacta aaatgatctt atcaacttgt tttttcatct 136441
ttcaaacctt ctctttagta gttgttccac aattgaagac attactgagt accattgcat 136501
tgaaatcatt aaagcctctt ttctttgcta tagtttcata aatgaaactt tgccaagttt 136561
gcagctttgg atttctgtag cgtgtagact taggatcagc cagccaagca ttaaaggtgt 136621
catagcttaa tccgaataac tctttattct ttttcccttc ctcaactgct ttgaatgaat 136681
aatttaagtc atgcagtatg ttatagagtt ctcgcgcatt ttgaggcgtt ggttgatcga 136741
tcattgctaa aagttttaat tcagtttcat ccggagcggt tgagctcgaa ttactatgta 136801
tggagttatt agatgaactt aggctctcca gaagtggaat aaagtcattg cgaataacaa 136861
aatcatggaa tcgttcatag aattcaaaat tatggccttc ttcatcgtcg agaagatgtg 136921
catactcatg gattaaaatt aatagtaatc ttaaatatga attctgctgg taaaagctga 136981
ttgtccttat atcgaagaca atattcgatg tcatgttggt ccatgcatta tctagctctg 137041
atttgcctag aaaaatctct cgcttaccat aacaatcatt tataaaatct ggataggcat 137101
cacaaaaact agtattcaat ttttttagaa attcaagaaa gccggtttgc tcgttattta 137161
gatcttttg gtcaattatt ttccgatcaa aacatacctt ttcctttca tccttataat 137221
ttaaaagggt tactggaggt atgtctgtga acatttcata gttggtatag gctacaacta 137281
tatctttaaa ctcagataac agcttcataa gcatataatc ataatcatat aagttttcat 137341
tatccggctg ttcattctta tcgatatact cttgaattag gcgtgtgcgg agatagcctt 137401
gcaaatgcga aatatcatag gtttcctcca agatgagaca gtttgttctc ttttcgattt 137461
tttcaactac gagcacgtcg taatctgacc taattagcat catgatagct ttagattttg 137521
aaatatcgat aaggctttgt ttataacctt tatagtcggt aaagacatcg agaggtgtat 137581
tgagcatcat caatattgcc tctttatcat accggcaatc tttcagtgga tttttagaa 137641
tattagctag catacctaaa gctaatttgc tcgatagctt cttatttttt ttctcattga 137701
ttgtggtgag ttttataaag gttggatcta cgacatctcg gatcttccct tctatgtttt 137761
taaatagctc acaacgatcc tcaataattt cattacgtga agatgtaatc ttgaattgag 137821
atttagaaat aatactacct ttaaaaatag catttttaaa acgggcataa ggaacccaaa 137881
cacctatatt atagacttta agggaatcag aattcggatc tagaatataa tatgcataat 137941
catcttcaat gattttgcat tcagaatcta aacaatctga tatattttta ttaatttgat 138001
ttttatttat gaatactgga atttgaacat atttaataat tgattggatt tcttctatta 138061
tttcagtaat agaacccatc gaatctgtaa tgtttgtata aatataaccg gatattttgc 138121
atccattgaa ctttttggcg agtctagtaa tagtaaaccc aagttcttcg tctgactttt 138181
ctcttagcgg cttatagata tctactttga gttggtagtt attacttgtc catgtggtta 138241
tagcgtaatt aaagacctgt ccgcgaccaa tacgaaattt tccgtatgta gcatcaccct 138301
cttcatgagg tgcaccaaac attgcaaagt attcatcaat agattttttg gtaaaacctt 138361
ttccatcatc aataatttca aactgaagat gtttttggc atttaaccat aattttatat 138421
ttatttgcgt ggcatttgca tcaatagagt tcattatcaa ctctactatc gctttaggta 138481
aaccgttggc ttgctgctca atggcattca cgatgaattt gggatgcagc ttaaattttt 138541
ttgttgtaga ggtcataatt atatccggtt aaggtaatat aattattata ttcaatgtac 138601
aaagtacaat caataaaaat taataataaa ttgtacaaag aacaataaaa ttaattataa 138661
ttttaaatgt actttgtaca ataataacaa gttttatgcg tgatgtacaa agtacattta 138721
tttgtgcaat gttgcaaggt agaaaggtct aaatttaggt gctgccaatg ccaagtcttc 138781
catacgtttt tgtggcatag gatgagtgct tagcatttgc gagagtttat tgttctgccc 138841
tttctgttgg tccccaacct tcttccataa ggtaatggct gcatctggat taaaaccggc 138901
tttggctgct atttctaatc ccaatagatc cgcttcactc tcctgacttc ggctatgcgg 138961

taatgttaat cctaactttg taccgaagta cgccaagatc tgagcattgc ctccaatttg 139021
gcctaacgct aaatccgata cgagatttct tgaagctttt tctcggccat gctcaagtag 139081
tgcatgagca atttcatggc cgatgattgc tgcaatctca tcgtcattta aattaagagt 139141
tttgatcata ccttcaaaaa ttgccatttt gccttgacct acacaaaagg cattaattgt 139201
agtggtatta attacatgca gctcccaagt ccattgattc gtttcaggtt taatgtcttg 139261
aacttgcgcc gtaattttct tggctatatt gactactctt tttgttaatt caggttttct 139321
atccaaagcg ccagaatttt tataattgtt cattaaaaca acataacctt tgtttgcttc 139381
gatgtaatag tcttgagacg ggattaagag aaattgtttt cttgagagtc cagatgctga 139441
attttgaatt gtagtcatgc aaccagataa gacgcttgaa attagaatca cactcaaagc 139501
tttatttatc ataaacttaa ccctgtatta tattgtctaa ttttatatct ctaaaaagag 139561
tataccaaaa aaaacaagat ttttaaaatt tagatatatg caaaaaataa aaatcttgct 139621
acgattattt taatacagtt ccgacctaaa agtcgaagga gccgcttcat gcgttcacca 139681
caagaacata tgcaacttga tgttcatata gttataaaaa tacgccttct cgaacttgaa 139741
tataagctgc acttaagaac cgctatctgt tttctattgg gtgctgtttt aagcttaagc 139801
ttcgccttcc cttttgattt tggcttttct ttatccacac tgtttttaat agcctcgttt 139861
gcatctggtg cagttgcttt gtactggtta aactcggccc aagaatttaa agcagcccac 139921
cgaagcttat gtgatgagta ttcacggtac tgagtatgga gtttgaaatc atgactaaag 139981
ttatttcaaa aagaaaaacc attttggata ccgctttatc actctttaag cagtacagct 140041
ttaagtttgt cggtgtagat cggatcatca atgagtcaca agttgcgaaa atgacttttt 140101
ataaacactt tccgtctaag actttgttga ttcaggcttg cttatgtgaa gaacaaaaga 140161
caattgaaga gtctatttta aatgagctgt ctttactttc tgaagcggga aacattgccc 140221
gtttaaaagc attgttaaat tggcatgttg cgtatatcaa tcagcaaaac tttaatggct 140281
gtctttttca aaaagccgtg tatgaaaatg aggtgagtga ggaagtttta tcagttattc 140341
aagcccataa acaatggaaa ttcaaattag tttcagactt aatggaagtt ccagaatgct 140401
gtactgcctt tgtctcttct tcgatggtgt atagcatgtt agaaggcatg ctattgcctg 140461
caaatataaa tccatgtgtg gaccacgaaa cggcaataaa aaacctaatt cagacatttg 140521
aggcttaaag cctcaaataa ggaaaataaa tgtgaataat ttatttgata cgatttattc 140581
agatatgttt gtaatgattg ttgcaagtgc ttttattgca gtgatgatca cttctttaac 140641
ctcaatatta gtgaaagtaa atttaagtgg ttacgccata ccattgacat cttttatttg 140701
gtttcttttt ttatacggtc caattccagc tccggcccaa caagctttaa agaaagatct 140761
agttttttta aaaaataata atgttccaac gaatgcaatg ataaatacaa ttattctttc 140821
atgcagtgat gctttaagtg ctgtttgtac cggattatgc acgaccatta aaatttaatg 140881
tctctccaga tgggaataca atatttttta tctatacgaa ttgtaagatg caatttaaaa 140941
aaattatcgt ggatattgaa cgtgtacaga tcatgagcaa ttcaaaattt gcaattaaac 141001
acggattcta taaaaaagga taagaaatga ttgatacctt attgtctcct ttttcagtga 141061
aacacgagct ctatagtaaa ctgaagaagc atcaagaaaa cagaatcaat attgtcgata 141121
aagactcaat ttatctattt gatattttta gttttgaatc tttcaaaata aagagtaaaa 141181
gcccttattc ccatcttgct tatttttctt gttcctatac agatgatgtc actgagctgg 141241
agctttgctt taatccaatt tttcaggatt acgatatccc taaattgaca attgatattt 141301
cacagctatg tggttttggc cactctaaat ctgacctgtt taagtataaa tctatagacg 141361
aatttgttga aaacgagtgc ccttcttata ttgagacaat tagtcagaca cagttaagat 141421
taatgctctc tcataatggg atccagattg taaactcgcc agattcaact tttgacaatt 141481
tctatatgta tggctggcag cctaaagtat ttttgagcaa tgaagatggt tcacatcatc 141541
ttgcttcagc tcaatacata gctttaagac ttaatcaacc agtgccattg actgcacgac 141601
ttatttataa agtcattaga ggtgagcaac ttgctaattt tcatgatcga tatgcagcgt 141661
tcataacgcc tctccatgaa ttagatgact actttaagtg gtttgataat tccaatatta 141721
aatttatccg gttagatgat tctttgcatg aatatgctgt gtatttcttt aaacgtacaa 141781
gtgaaaacga gcggatcata gagctgttta gttctaagtt ttcttcatta aaccaaatat 141841
tatggaaaag actgtcagat caaacaaata atatgctgtt aaaagcgctt gtaccgctta 141901
attcaggctg ttggagctat tagtatggct accccccaat cttgcaataa atttgcccta 141961
aaagcctcct gtaaggattg tccgtttaga aaagatagcg gaggttatct acatccggaa 142021
agggtacggg aaatagttaa ttacatgagt aaagatgatg ctcttttccc ttgtcataaa 142081
actgtgggta cagctcggac taatttaaac gaagctttag agttgctaga agatgaactg 142141
gcgtttaccg gactctcaca aaatcttacg gcacgaaaag aacttgaaga gaagtaccag 142201

attgacaatc ttcaggaagc actacttaaa gatatgaagt cagaaaaggt ttgtgctggc 142261
tggttaatat tagggaaaaa agagcaaatc attaacaata atttcccatt gagattagct 142321
caaatgcaag gactcttaca gctaaatgac ttaacaaggg aggatgagat atatgattct 142381
attgagcaag caattgaaga tcattcttga gcagctccaa tattgtattt ataggaatta 142441
aactgtttat ctatcctttt ttaattttaa aatgtacata ggcttgtctt cacaagccgt 142501
attataacca attttgaggg ttctttaatg attacattcc ttaacgagca ctggtatttc 142561
gtttatatca ttgcaataac gattggtttg ctgtctcttt ttatccgtct ttatgtatta 142621
cctaaaatac gatcttcgga gggttcacag agtgatattt ttttcgctaa gaatttaata 142681
gaaatattaa attccgatca taacttagtg ttgtatgtga ataactctga tggtaaatct 142741
gtggattcca atatgcgtaa taagatattt attaaagatc ctttacgttt tgaagatgat 142801
ctattgatta ctaaagcatc cacgccacat atttactttg atccaaaaac attaaaagtg 142861
aaaggttaag ttaagtccaa aatcaaagta gaaatttata gatacacctg agaaatcact 142921
gatgacaaat aaagcttcac agcctagaac atttacatta tctgatgaga agaggaacca 142981
tatcttaacg acagctctaa acttatttga tcagtttggt tttcagcata taagcattat 143041
gcgtattatc actgaagcaa aaattgctaa acaaagtttc tacatggctt tcccttccaa 143101
agattctttc atcattgaat gtttagatat ggaaatcaat agactgaagc aatcactaag 143161
caacctcttg ggccagtgtg gtaaagatga ccatacaagt attttaaaat ccttctatca 143221
gtggcacgtg gacctagctt atagagaagg atataacggt acgctgttaa caaaagctgt 143281
gatcgaatat tggaatttac cagatataaa aatgaaggtt gaagacttca acaaatggaa 143341
atatgaaatt tttgcagagt atctggcgga agaaatcaac aatgctcgac taagaattat 143401
agtaagcgca atgaatggga ttttattgcc ggctagtact tacttgccta catgggaaga 143461
tattgaaagt ttatatggtg agcaattttg aaaaaaaata gtttgaatcg aatcatcttc 143521
aatgaaggac taaatacatt aaaaatttta gtttttgtag tatttgtagt ctatattcta 143581
aagtacttct taaatgtata tttaggtgtc gtattaagtg gagaagtagc cctcttcttt 143641
ggcattttta tgacactatt aattgttatc tatgattata tctcaggaat tattgataaa 143701
ttatctaata gacttcatga agaggctgat cgaaaagaag ccattaaaat acttgaagca 143761
aatcaattaa aagtaggttt aatcttatca gaattacctt tggatttaaa taatttagaa 143821
aagcgtgaat gtgtggaagc ttatataact ttaaagcatc aaaattcaca attggtcctc 143881
gttaattcag aaaacgaagt aaatgcacga ttgtactttt taggaaaata tgcggataaa 143941
gatcacaata agtacaagct tatacttaag agaaatttag attaagccta tttccctttt 144001
gctattcttt taattgatac cattttaatt tgcatcaaat cgcccttgca atgcaccgtt 144061
aatatgataa cattgacttg ctaaatgcgg ctctggatgc agcttacttg tgtgaaagta 144121
aaagctatct ttttaaatct tatatcgcaa tatttaaacg aaccttgttt gtctatttgt 144181
ttaatttcac accagataga attgtattca aggaatcgtt atgtctaatg aaaagaagtt 144241
cttcttaaac tcttttaaac gcaatgcaaa attgcttaat acttcttttg atgttgtttc 144301
aaatacactt aacacaccat ttaaagctcc cacttacacc caatgcttag aaatttctgc 144361
acgagcgctc ggctttaaaa cttttaaagg tttgacttta agtgacctta ctgaagtaaa 144421
cgagatacgt tatttaagtg attttgataa aactaaatta atagaaagtt acttagatgt 144481
taatcgaaaa aaaattacta cgatgccgct taaaatagat agctctttag atttaaattc 144541
aacaatcgat gctgtatgta ctctcgcgta tcatatacaa acttttccat tgcttcgcga 144601
tttctatcta ccaaaacgat atctcagtaa acatctagat tttgataaag gtcccttcac 144661
aattaatgat acgttatcaa aaatatgcgc acattggtgt atatcgctat tgattgaaaa 144721
accgaagggc tatgaatttt ataaaggtgt ttatgagaac aacaatgaca ttctttatga 144781
tgtaccaatg agtttcttca caaagaaaaa aatcaatgat tggtataagt cacgtgactt 144841
tttgcacgac ttcactgatt tgtttcagca ctgtgacttt atacgtgtac aaagtggttg 144901
gaccgatttt gacaacatga gtaattcgaa aatcaatata tttattcctc cggctttgca 144961
tgaacggatt acagaattag taaatccatt aaaaggtgga cctgcaaact aaagttgata 145021
gtacaaagta caatatcaaa taaaaaggag ttcatagaac tcctttttt tcactgttaa 145081
ttaattttat ctgtgctaaa ccttacttta taatcacatt ctctatcttc agagttcgat 145141
aaattttcaa aaatgatttg atcttcaaat ttttggtaat gcctgaagag ctgacacaat 145201
tttgggaagt ctgtttctaa gttgaatatg gaagaatctt ttttagaaaa aggaagaatg 145261
aagattaaat gcaacgtacc taacttgaat tctgctacca cgtgcggcca ctccggatcc 145321
gtatcactga ttcgtgtata tagtgttaac tccggatgtt ccgtataact ttggccatac 145381
attttggcta tatggaattg ctgttcttct tcaccaagtt ccggatctag gaacaaccaa 145441

```
ttaattgtcg attcaaatgc cggtagttct tcggccggca atactgagat agcgtatttg 145501
cataatgact taaagatttt tagtttgtta actttttgat gggctaacag gctgacaatt 145561
aaaccgtttt ctgtttcaac aatatcctgc gaagcaatca caatcgagtg ttcttcagat 145621
ccttctagct tgtgctggta cacttggcca ttcttaaact tcagatgggg aatgcccttc 145681
tttcctcttg ttccataata tacacgtagg atatcaaggt actgtatgag atgaggttca 145741
aaatcacggc caaacttctc attacatgta tcgcattcat ctgccgtcac gattgattta 145801
ttgcccagcg cttcagagat cgtatgagcc acttttcgga aggttgtgtt ttcatcaacc 145861
ggtgatttac aaaacctaca aacgccgtgt tcttttgttg gtttttggcc tataacagtt 145921
ttacgttctg tttcaccaaa cacagctatg ttgtattgct ctaataaatc atcaaataag 145981
ctattaaacc cctccatatg agtttgcatg tattgattta cttcttcatc gctattcaat 146041
gaatttttgg ctctaagaaa agtttgcaaa agcatttgtg cttgcatctt ttctgtaaac 146101
tcatagggaa atacaaagaa gtattcatca tcttcaatcg aattgagctt actaatcgct 146161
ttatgaatac taggcttgag tttagtgtaa tcgcttaaca tagcattaac ttgtgtccgt 146221
aaacttggtg tcatgcgttg taaagcgctg gctttgattt gattgggatg tgctggatcg 146281
agatcctgtt tgacattgct ggcgtataaa actctgtaca tatcattgac gaagtttata 146341
agagtttcat tctcatctgg cccttctacg ctaacaaaaa aactattcgt attttgagta 146401
ataaagtgga aagtaaatcg aaatagattt acttcagctt cagtattgtt ttggttcata 146461
tcgatgtcac cccaaattta ttaaagtggc caacatgttt ggccacagaa ttgaaaatgc 146521
cataaggcta attatgctcg tccatcaata ttgtgtttca tatataactt ctgagaaaaa 146581
tgaacatatt cattttctga gagcggtgga ccattaacca aatgatgtgc tggtattaag 146641
gatgttttct ctttccagtg agccagcaaa tccggactta aagccatgat gcttagtagt 146701
gaatctagga aatctggatc gttgtgatgg cgtgttgttt taataaagaa agttgccgtg 146761
atctttttat taccggttga attgtctgct acgcaaacag aatataaatt aggtagttcg 146821
ttatagatct ctggatctat atcttgcata aagtgtgtga tcgagtcact accgttataa 146881
acacaatgaa atagtaaaat actagcacca ccttgaacag tggccttaga agacataatt 146941
ttcattttgg aatcctgttt gaaaattaaa tggaactcat caaaagctta taattttgag 147001
tttaaagcta tctaaactag atactagtgc atctacaccc tatcttcaaa tgcccacctt 147061
aaacgcgctc tacggcgctg ctataacgaa gaatttttta attctcgccg tgaaaaaaag 147121
atttacgcaa atccggagac ggttttactg taaatttgat ctggtttatc tagatcaact 147181
aaattcaagt accaaagctt tatgtaattc atctagtagc tgaatagccc attgatgtga 147241
cgcgccaaaa ccaatatgag agctcacatt gataccggtt tttttcattt gcggccgtaa 147301
atgatcttaa ccgataacta taagaaatgt atgcagaatt ataccgttga catgatatta 147361
taaaataaga cttaagctca ctaaccagct taagaatata taaattgtac gccaaaatag 147421
cctaaggaga ttttatcgaa ccccataaaa ggactcacgg cgtccccttt ttacacttta 147481
caaaaatcgc ttcttccgtt ggcggtcttt ctagtccggt tacggtattt ataattccgc 147541
ttacggtttt atctattccg gatacggaaa tatctaccac aaaaaaacca tactaaattt 147601
ttattttttg catatattaa atttttaaaa attagtttat tctattatta acgcagatca 147661
gccccccttat tccggagccg gaataatcca taaacggtat taaaaccatg aacggaccta 147721
taccagaaat ggaaaaggtc catagacgga aagtccggca atggaagagt cacttaaata 147781
aggtactcaa aggtagagcg aaatcggcta actataaagc aatagacctg cttaggctat 147841
tttataatag gcgtttaaca tgaaaaaatt ttgtttggta cttataccag cacttatcgc 147901
tggttgttcc tccccagcaa ctgaagctga aattcgtgcc gccgtgcaag aatgtgaagc 147961
tgttatgcat gatattgaaa atcagattca accggctttg ttgcacaaac ctatctgtaa 148021
aggcttttttc agcgatataa ttttcaaatg aagaagccta cacacaaaat ctaccgcaca 148081
accaattggc ccgcatataa ccgagcactc atgagtcgtg gaaatattgc catttgg
//
LOCUS NODE_1_length_3909467_cov_533.478_ID_22129 3909467 bp DNA linear UNK
DEFINITION Acinetobacter baumannii ATCC 17978
ACCESSION NODE_1_length_3909467_cov_533.478_ID_22129
KEYWORDS .
SOURCE Acinetobacter baumannii ATCC 17978.
ORGANISM Acinetobacter baumannii ATCC 17978
Bacteria; Proteobacteria; Gammaproteobacteria; Pseudomonadales;
Moraxellaceae; Acinetobacter; Acinetobacter
```

calcoaceticus/baumannii complex; Acinetobacter baumannii.
FEATURES Location/Qualifiers
source 1..3909467
/mol_type="genomic DNA"
/db_xref="taxon:400667"
/genome_md5="" /project="sleyn_400667"
/genome_id="400667.82"
/organism="Acinetobacter baumannii ATCC 17978"
CDS complement(34..336)
/locus_tag="AUO97b_00141" /db_xref="SEED:fig|400667.82.peg.1"
/translation="MQLSFNKRTIFPSVYRGENKKTGEPTCYLSATVFSPVKYNLKPA
AGMMPIEQIQAILEECADNGQEVEIEFTEQQTKFGAEMQIFSVKPLPKKNPMESKA"
/product="hypothetical protein"
/transl_table=11
CDS complement(352..1578)
/locus_tag="AUO97b_00142" /db_xref="SEED:fig|400667.82.peg.2"
/translation="MLDFMRLAIPIIPTYVRSLDNHHWFNGDIRDYGIPAATRHVSKT
DDGQTITGELYHPYESLPSDYTDMAVKFYTNTMNTPPYVEIKASPLKLLQGHNVYGFE
SIELGSDHMLGMLLEAFPQLAPILDLANTEVLHLDTTYLFRLPHQNMVQPTLDYMANL
ASGHRKAREVKYDNYITWGNDSASVRPKAYGKFEEVKSQLNKIQKKADKGCMRSKSLV
IAMNGALQFANAVLRLEARICKTYLTKNGYPSNLFQLIKLQHEQPELLLRLWHVAFDP
ILKTMEGEYMNFASDDELEALLKSKLVTYTKKGNPSYTKAYNAFDFYRSLRIDGYKKV
KSRHLESRFYKRERELISCGISRSHLQNLHKNPNGKVIPFVRLFELKMADQLPPDYVQ PVSQYSPKNQGLHLVA"
/product="phage replication protein Cri"
/transl_table=11
CDS 1724..2098
/locus_tag="AUO97b_00143" /db_xref="SEED:fig|400667.82.peg.3"
/translation="MRNNCYKIDESEKGHEMNSIGERIEKKCKELNISIPELANIAGV
NYKTLKRNMTAEDPNPTLQHLKRLSIALGMSIDNLAFGEEGSTDEEIAIILNDLKNIE
KEDKKRILYMVRMMIAESKNRK"
/product="helix-turn-helix family protein"
/transl_table=11
CDS 2218..2475
/locus_tag="AUO97b_00144" /db_xref="SEED:fig|400667.82.peg.4"
/translation="MIGIEKIKKDHDEKTFKKYMNELKKCGITEKYIREEHEKERFKR
KSQKVEYVELVFDLNNQVPEGYIPPEGQYNIEEIIGKKVKI" /product="hypothetical protein"
/transl_table=11
CDS complement(3236..3493)
/locus_tag="AUO97b_00145" /db_xref="SEED:fig|400667.82.peg.5"
/translation="MMSLADISALHNLVIHIFVAGAILGFILSGFFKTLLNMWAYRFE
RPKRIKTDTGFLYFWRGKYYPLEQRNKFIEEHRKKIRASFS" /product="hypothetical protein"
/transl_table=11
CDS complement(3506..4720)
/locus_tag="AUO97b_00146" /db_xref="SEED:fig|400667.82.peg.6"
/translation="MAIKLITAQPGSYKTAMMMEIASKMASENRPIYLCNIRGLKPEI
PFPYQVLDHFKDWIDTPETSVIFIDEVQEFTRDVPTNCKTEDLPKWLTLLEKHRHEGK
DIFIVTQHPMFIHTHVRRLTSEHVHLVRNGNVPFAAKRTWGFVESDPDDFQKATVKNG
CTTSIYRPNKDVFNWYESTVLDTHKFKFPTKLIKTVSLLAALVGFSVYIGYPVFNKYF
GSKEQEVSATDTSPQPNNSNMTLAEKAKLDAAMAGLTPEQYADLMHPEKRNAELQAVN
DVRMETIAVKYNPNRPYDMDTSQIQYEVTAKPVFSGCMKKNGKYVAYTQQGTILHDVS
QSDCRKLMEDGDRPFNYFQVQNNRPAQVNNAMPQVQMQPNYSSYQANNYVQPNLQRSS VDGANSQSSFSF"
/product="Zona occludens toxin-like phage protein" /transl_table=11
CDS complement(4724..5065)
/locus_tag="AUO97b_00147" /db_xref="SEED:fig|400667.82.peg.7"

/translation="MPLFIGAIVAALLKVLFRYAVFKIFAKLILGTATAGIIYLFLTS
TIKPFIDEMQQKIVDKAAELSTIGGTAAEVIQYFDFIQCVNIILSASAACFSLKLMSV AIRAFGINTGG"
/product="hypothetical protein"
/transl_table=11
CDS complement(5065..6561)
/locus_tag="AUO97b_00148" /db_xref="SEED:fig|400667.82.peg.8"
/translation="MVGSGNACYLSGSLYASKVSETYICPPIDYPAYYYFDAGSPIPI
QECYPTGDKFCVFKAKPNSIILNHANNRQSTVLYNTSPTPVASCTSLKSGQCDKNDPY
GDCYQPPNDGCTRLADGSITCPDGAAPPSPTGTCSGATYCNRPPTGCGTGYVSGSFNG
QALCVKSSNSGTGSGTGTGSGDGGSGTGTGSGDGGSGTGTGSGDGGSGTGTGSGDGGG
GSGSGTPIDTGTGSTNINNSGSGSGSSTGGSGGGTTSTSFTIDLSPVVRAISALSDKL
TWVKSELVNAVSRVEDKLTQTNSKLDTTNSKLDSVKSSVDQTTAAVNANATTVKTAVE
ANTAATNNVKSAVDANTNSTANKLNEVVNAINNKPVGGGGGGTTDVKPVVDAIEKQTT
DFKDMMKTDSSDFDTSQYEKIGDASDDPRYLNAQSEATNALQNLSNKLTFSNTACVQD
FTVDFPYFGSFVVPISRWCELLALIKILIHLSTLILAFRMLDSTVRAI" /product="hypothetical
protein"
/transl_table=11
CDS complement(6822..7073)
/locus_tag="AUO97b_00149" /db_xref="SEED:fig|400667.82.peg.9"
/translation="MENQIVVQEKRGVVSLRNASRYGLGAVLSAGILSSASAATLVDE
QAAQFKTDGIAMVTAIGVAMISVAVVAVLIKWAKATFFS" /product="hypothetical protein"
/transl_table=11
CDS complement(7570..7872)
/locus_tag="AUO97b_00150" /db_xref="SEED:fig|400667.82.peg.10"
/translation="MQLSFNKRTIFPSVYRGENKKTGEPTCYLSATVFSPVKYNLKPA
AGMMPIEQIQAILEECADNGQEVEIEFTEQQTKFGAEMQIFSVKPLPKKNPMESKA"
/product="hypothetical protein"
/transl_table=11
CDS complement(7888..9114)
/locus_tag="AUO97b_00151" /db_xref="SEED:fig|400667.82.peg.11"
/translation="MLDFMRLAIPIIPTYVRSLDNHHWFNGDIRDYGIPAATRHVSKT
DDGQTITGELYHPYESLPSDYTDMAVKFYTNTMNTPPYVEIKASPLKLLQGHNVYGFE
SIELGSDHMLGMLLEAFPQLAPILDLANTEVLHLDTTYLFRLPHQNMVQPTLDYMANL
ASGHRKAREVKYDNYITWGNDSASVRPKAYGKFEEVKSQLNKIQKKADKGCMRSKSLV
IAMNGALQFANAVLRLEARICKTYLTKNGYPSNLFQLIKLQHEQPELLLRLWHVAFDP
ILKTMEGEYMNFASDDELEALLKSKLVTYTKKGNPSYTKAYNAFDFYRSLRIDGYKKV
KSRHLESRFYKRERELISCGISRSHLQNLHKNPNGKVIPFVRLFELKMADQLPPDYVQ PVSQYSPKNQGLHLVA"
/product="phage replication protein Cri"
/transl_table=11
CDS 9260..9634
/locus_tag="AUO97b_00152" /db_xref="SEED:fig|400667.82.peg.12"
/translation="MRNNCYKIDESEKGHEMNSIGERIEKKCKELNISIPELANIAGV
NYKTLKRNMTAEDPNPTLQHLKRLSIALGMSIDNLAFGEEGSTDEEIAIILNDLKNIE
KEDKKRILYMVRMMIAESKNRK"
/product="helix-turn-helix family protein"
/transl_table=11
CDS 9754..10011
/locus_tag="AUO97b_00153" /db_xref="SEED:fig|400667.82.peg.13"
/translation="MIGIEKIKKDHDEKTFKKYMNELKKCGITEKYIREEHEKERFKR
KSQKVEYVELVFDLNNQVPEGYIPPEGQYNIEEIIGKKVKI" /product="hypothetical protein"
/transl_table=11
CDS 11996..12514
/locus_tag="AUO97b_00154" /db_xref="SEED:fig|400667.82.peg.14"
/translation="MIAALSVVGIANAQAADGTITINGLVTDKTCDIVTPAGKDFTVT

LPTVSKQTLAVAGDVAGRTPFQIKLANCSEGKVATYFEPGANVDFNTGRLLNKDATGA
KNVNVQLLGSNNNFIPVLAAGANGAQANSQWVDVAAAGSADLNYYAEYYATGASTAGK VNTSVQYTIIYQ"
/product="Fimbrial protein"
/transl_table=11
CDS 12601..13335
/locus_tag="AUO97b_00155" /db_xref="SEED:fig|400667.82.peg.15"
/translation="MKLNSYNFLLGLAFASAVVAPATQAEIVIHGTRVIYPSDAREVT
LQVSNNGSKPALVQAWLDEGDSKSTPDQSKVPFMIAPPISRVEATKSQTLRITALPTA
SQLNQKQETVLWLNVLDIPPRPTSKGENATPDNFLQLAIRSRIKFFYRPASIKEDANL
AADKLQWVKSGQNLKVKNPTPFHITMTSVYQKVGDKKVDLLPQGLMIKPFSEESVQLK
NGNLQDMSFMNVNDYGGRVEHQIKLQ"
/product="P pilus assembly protein, chaperone PapD" /transl_table=11
CDS 13394..15952
/locus_tag="AUO97b_00156" /db_xref="SEED:fig|400667.82.peg.16"
/translation="MPKKRQESYKLLKRHICYYLASGVVTMTMPVFVHAEETTANAPV
EAEFDSAFLIGDAQKVDISRFKYGNPVLPGEYNVDVYVNGQWFGKRRMFFKALDPNKN
AVTCFTGNNLLEYGVKQEVLSQHTTLQKENSSCYKIEEWVENAFYEFDTSRLRVDISI
PQVALQKNAQGYVDPSVWDRGINAGFLSYNGSAYKTFNQSGNIRETSNAFMGVTAGLN
LAGWQLRHNGQWQWQDTPAENQSKSSYEETSTYLQRAFPKYRGVLTLGDSFTNGEIFD
SYGYRGIDFSSDDRMLPNSMVGYAPRIRGNAKTNAKVEVRQQGQLIYQTTVAPGNFEI
NDLYPTGFGGEIEVSVIEANGEIQKFAVPYASVVQMLRPGMNRYALTIGQFRDSDIDI
DPWVVQGKYQQGINNYLTGYTGIQATENYAAILLGAAFATPIGAVALDITHSEADFEK
QSSQSGQSFRLSYSKLITPTNTNLTLAAYRYSTENFYKLRDALQIRDFEEKGINTYSA
GRQRSEFQITLNQGLPEGWGNFYVVGSWVDYWNRSESTKQYQVGYSNNFHGLTYGLFA
INRKVEYGSTNQTHDTEYLMTLSFPIDFKKNSVNVNVTASEDSRTVGASGMVGDRFSY
GASMSHQDYSNPSFNVNGRYRTNYTTVGGSYSVADSYQQAMVSLTGSVVAHSEGVLFG
PEQGQTMVLVHAPEAAGAKVNNAVGLSVNKAGYAVVPYVTPYRLNDITLDPQEMSSQV
ELEETSQRIAPFAGAIAKVDFATKTGYAVYITSKTADGNSLPFAAQVFNQKDEAVGIV
AQGSMIYLRTPLAQDRLYVKWGDESNERCSVEYNISNQLQNKQQSMVMTEAVCK" /product="outer membrane usher protein precursor" /transl_table=11
CDS 15949..16098
/locus_tag="AUO97b_00157" /db_xref="SEED:fig|400667.82.peg.17"
/translation="MKRILLTGALFTAGLGVYGEANAYCTLGNKKGGSFSSVDISMAV GRVVV"
/product="Fimbrial adhesin"
/transl_table=11
CDS 16117..16977
/locus_tag="AUO97b_00158" /db_xref="SEED:fig|400667.82.peg.18"
/translation="MVLRKATFPISDNGSTAICTSYSDTITAALTQGYPLSSLGNNIY
STNIPGIGIRLYREAENATNFSGYYPYTRNLTPGVQYFLASGYFVVEIVKTAAQTGSG
ALVPGLYSRYYVSGYMNRPFLTSTVNGDAITIASSSCEIQGNINKVVQLPTVTKAGFK
GVGSTQGEQTFDMNILCNGGINPTGYEEKNVISLSYDFTQDGTNSQVLANTAAASEKA
NGVGVQLLWNYQNKNQVIKKGDKLALGTLSSNQTLQYNVPMTARYYQTATNVTAGKVR AMATVTIQYD"
/product="fimbrial adhesin precursor"
/transl_table=11
CDS 17262..18044
/locus_tag="AUO97b_00159" /db_xref="SEED:fig|400667.82.peg.19"
/translation="MAQTTSLNYSVPVCQYNFDATQEDFDVLNAQDPDRPPMKIFPLI
NGQKFGFKVEPVTEDNYGYLDYNAKSKAKINSPSYEGDFLLPNKGIVAFEMELKVPTL
SSSSSSYSADISFNGVTNNNYTIRSNYHFDIGAHDFEFGENPPRLYYSVSSEMGDYEF
FDNYFKNKQMTDNTNEYQRLGVYINQDTNQVGFISNGVDEGYQFKLPGALQKIAFSMN
GNINILSTNLFGQELSNELITDRTALQFNYPQGTTDICGNAI" /product="hypothetical protein"
/transl_table=11
CDS complement(18102..19211)
/locus_tag="AUO97b_00160" /db_xref="SEED:fig|400667.82.peg.20"

/translation="MKSRAAVAFAPGKPLEIVEVDVAPPKAGEVLIKITHTGVCHTDA
FTLSGDDPEGVFPAILGHEGAGVVVEVGEGVTSVQPGDHVIPLYTAECKECLFCKSGK
TNLCVAVRATQGKGVMPDGTTRFSYNGEPIYHYMGCSTFSEYTVVAEVSLAKINPEAN
HEQVCLLGCGVTTGIGAVHNTAKVQEGDSVAVFGLGGIGLAVVQGARQAKAGRIIVVD
TNPDKFELAKQFGATDFLNPKDYDQPIQQVIVEMTGWGVDHSFECIGNVNVMRSALEC
AHRGWGQSVIIGVAGAGQEISTRPFQLVTGRKWLGTAFGGVKGRSQLPKMVEDAMKGE
IQLEPFVTHTMPLQDINTAFDLMHEGKSIRTVIHF" /product="S-(hydroxymethyl)glutathione
dehydrogenase (EC 1.1.1.284)" /EC_number="1.1.1.284"
/transl_table=11
CDS complement(19441..21312)
/locus_tag="AUO97b_00161" /db_xref="SEED:fig|400667.82.peg.21"
/translation="MITNRQNVQNNTNTSPHPITQNTLIDRFSPIYWRIDGPQTMSFA
ITNYGNGFEASFRSRMTNDLVGISWDSYDTKDHKLLAYETKYSYAGVVWDFDIELSAS
MPVLNNPSLTPTLTVYYHDNGVDKIAYIVLFNYANNPSSRTAHIHINWDTVKAGFSAT
DSFPVTNIQRISFSGFTSHYNGQSATPLSQPEDGYIRITNSVVTGTNAKLNLSRVVVP
QHNYGICTSYDDHYDLNPQRLVNNMVALGYQGLVNHYCGMSHYPEMTWKSDINKWQIP
DTLVTGEAVVNACTRKWHEKYAQALHNANMQPIFGVSFEMYSLGANEFWAQRDWNSNL
GKTGYQPPSYFFSLSDQNALAYLHKVFIEFADTMVAGGCNVNMQIGEPWWWYNTDTNL
PCVYDYPTKLAFNADTGLYAPDLGTIYEAMNKTGTPYDEFKTWLRNKLGQTCQNIRAA
IKAKYANAQVCPLIFFPSIRSPIQTLATYINYPSQHYSYPNFDYIMTEAYDWLLEAKL
DLAHQAVSQIPTQDLGYPANKVAYLSGFVPDASIAYIYGFDKNKPYRTPIWQRIFGDM
KNNVSLGLMKQFIWAYPQVMFDSITIDTTQAPNGFFVENTLYSPISDNTPYPPDIYL" /product="Phage
capsid and scaffold"
/transl_table=11
CDS 21495..22658
/locus_tag="AUO97b_00162" /db_xref="SEED:fig|400667.82.peg.22"
/db_xref="GO:0004096"
/translation="MSKLIQPISFKALFIFTPLLFVSIGCSEALYSQTIPSVKSSSEN
IDYPEIDTALGEKLQPNEEVIAHQITQVIEKSIREQYTAGNALRDAHPKAHGCVRAEF
HVFKNIPAQLAKGIFIPDKNYEAWIRFSNASNDASRADIEKDARGKAIKILGVSDQKI
LENEKEATTQDFIMINHPVFFVNDGQRYLSFMNDVNSHNMIRKLHIPFALGFKGTMNA
LGARNSKIANPLYARYWSMVPYQLGLGVDRQAVKYSVRNCSTVSATLPDHPSHNFLRD
ALKDTLQKQYVCMEFLIQPRTSTKMLVEDAMTEWKENEAPFYQVATIHIPKQSFDTPE
QNQFCENLSFTPWHALPEHKPLGTINRMRKIIYENISRVRHDINSAPRQEPKN" /product="Catalase-
like heme-binding protein" /EC_number="1.11.1.6"
/transl_table=11
CDS complement(22736..23101)
/locus_tag="AUO97b_00163" /db_xref="SEED:fig|400667.82.peg.23"
/translation="MPKYLLLAEKISKNIKQDSSNNYLKNLNNLIIEIKKAITGTELK
FLYNFLDFGGSLLNLQDEVLLKFDSSLIPSYKNEEEFILWLADFIDRITIDTSKGDYP
IKKNLANLSSIAEKLMYRH"
/product="FIG00350165: hypothetical protein" /transl_table=11
CDS complement(23914..24381)
/locus_tag="AUO97b_00164" /db_xref="SEED:fig|400667.82.peg.24"
/translation="MPQYLMIAEKVYKKIKEDNLFSDTPTEHLNNLIGVIRKEIKGTK
FKLKYNFIDFDEYLTKPLDECTVKIDISLMPSHKNKDEYILWLAGFIERMTTGGETHL
PPTSKFIPPDFKFTCEFDNAKTPAKSSHTDDEGELIINYFKSEAFMKIINEEK" /product="hypothetical
protein"
/transl_table=11
CDS 24846..26372
/locus_tag="AUO97b_00165" /db_xref="SEED:fig|400667.82.peg.25"
/db_xref="GO:0003824"
/db_xref="GO:0008152"
/translation="MALSAWGFTDLPSYTDKYDPNEKDIVIKTKNWLNDEAAEEKTQQ

GLTAFVALDDAFMSIASRIYLIRNAKETIDLQYYIWTNDFVGNLILHELLKAADRGIK
IRLLIDDQNGIKLDGILRSLLQHTNFEIRLFNPYKFRYLRVFDYLFRFKKVNHRMHNK
LIIADASIAVTGGRNISSEYFEASSKFQFTDMDILFYGHAVRHAEAVFTDFWESTLSV
NATDIIGTCAEHHLKALREHYEQLHHEDHSLTEDKLYDAQSYLKEILEHNPIQWSKAH
FVADSPKKILGTATEHEMLYGQVLSIMGKPKQHLELASAYFVPTQQGTYYLKQLRVEG
IKVRALTNSFAANDVAIVHAFYSQYRVEMLKNGIELYEFKPVLERRRRTWYEIVTGSV
IPAKGKNKSSLHAKFFDVDGKVFIGSFNFDPRSTYLNTEVGLVIESSQLQTQISVMLD
QHLPQVAYQLKLNSQGQITWLDYQANGQVIEYDKDPGTSRFQRTMIKAVSYLPIEWMM "
/product="Cardiolipin synthase (EC 2.7.8.-) phosphatidylethanolamine-utilizing, bacterial type ClsC" /transl_table=11
CDS complement(26381..26968)
/locus_tag="AUO97b_00166" /db_xref="SEED:fig|400667.82.peg.26"
/translation="MIIEIETHPLQPFLPENARLLMLGSFPPPRNKWKMDFYYPNFQN
DMWRIFGLIFFQDKTYFLSENQKSFNEEAIRSFLIETGIAIFDTAYQIKRLKGNASDK
FLEIVTPTDLAVLLKQIPQCHTIMTTGDKATDTLISVLPENTEKPLIGTSTSTYFADR
DINLYRLPSSSRAYPLALEKKAEAYQKFFKEIGFL" /product="G:T/U mismatch-specific uracil/thymine DNA-glycosylase (EC 3.2.2.27)" /transl_table=11
CDS complement(27135..27371)
/locus_tag="AUO97b_00167" /db_xref="SEED:fig|400667.82.peg.27"
/translation="MSKRHILKEVNAKSMCGMEIVVEQIFENTLEKNLASAEIEQNWL
PLSKIVISDKVINLDQDNTFAHPRTGKVFKVLNS"
/product="FIG00350577: hypothetical protein" /transl_table=11
CDS complement(27625..27801)
/locus_tag="AUO97b_00168" /db_xref="SEED:fig|400667.82.peg.28"
/translation="MLKSILVLGTGIAIGMCMYKKKQKNSKSFSTDSDTDSLISKDNN KSKDDNSLDNAVQV"
/product="FIG00351524: hypothetical protein" /transl_table=11
CDS complement(28384..29436)
/locus_tag="AUO97b_00169" /db_xref="SEED:fig|400667.82.peg.29"
/db_xref="GO:0004237"
/translation="MNHGTFPVFDGHNDVLTRLWLSDHNNPAQAFIQEGLAGHLDLKR
CQQAGFVGGMFAIFLPPFHYVKQHHPNKLFDQDASDFTQQQIEHICLAQLDLAKQLAE
YSNDIQICTTVQDIQHCLTQQKLAIVLHMEGAEALQHNPDLLDVFYDAGLRSIGPLWN
RPSRFGHGLNAKFPHSPDTGAGLTNEGKAFIKRCADKKMVIDVSHMNERAFWDTANIL
QQPIVATHSNVHALCPQARNLTDNQLKAIKDSKGIVGLNFDVAFLRKDGQRDANTSIE
VLLEHLEYLIDQMGENHVGFGSDFDGALISHEIEDVRGLHLLIEAMQKRHYSNEIIEK ICFSNWLTVLNRILGK"
/product="Microsomal dipeptidase (EC 3.4.13.19)" /EC_number="3.4.13.19"
/transl_table=11
CDS complement(29453..30607)
/locus_tag="AUO97b_00170" /db_xref="SEED:fig|400667.82.peg.30"
/translation="MTEGVGLPLWLLAELTYRCPLQCPYCSNPLDYAQHKNELTTQEW
FDVFDQARQMGAVQLGFSGGEPLVRQDLEQLVAHAHQLGFYTNLITSGMGLTEQRISH
LKQAGLDHIQISFQASDPVVNDALAGSKHAFEQKYEMCRLVKKYDYPMVLNFVIHRHN
IDQIDKIIELCLELNADTVELAICQFYGWAFLNRQGLLPTQEQLIRAERITNEYREKL
KAQNHPCKLIFVVPDYYEERPKACMNGWGKIFFTVAPDGMALPCHAARQLPISFPNVR
EQSLSRIWYESTGFNHFRGDAWMPEGCRSCPDKDRDFGGCRCQAYMLTGDASNADPVC
GKSPYHQLIEQARAESEIDSSLEKLVFRNSRNSKQFTVQQNIPVQNIVDD" /product="Coenzyme PQQ synthesis protein E" /transl_table=11
CDS complement(30604..30888)
/locus_tag="AUO97b_00171" /db_xref="SEED:fig|400667.82.peg.31"
/translation="MNKEQFDVNLVPTWRQGYRFQFEPAQNGFVILYPEGMIKLNESA
GTIGQYIDGTNNVSAIIAQLKQQFGDIAEIDNDVIDYMLVAQQQHWIDLV" /product="Coenzyme PQQ synthesis protein D" /transl_table=11 CDS complement(30885..31643)
/locus_tag="AUO97b_00172" /db_xref="SEED:fig|400667.82.peg.32"
/translation="MTQTPEALTTEQFKQAIIDKGQYYHIYHPFHVMMYEGKATQQQI

QAWVANRYYYQINIPLKDAAIMANCPDQRVRQEWIQRMIDQDGEYPDGGGREAWLRLA
EAVGLSREQVISEELVLPGVRFAVDAYVNFARRASWREAASSSLTELFAPQIHQSRLE
SWPQHYPWIDDKGYEYFRSRLSQARRDVEHGLTITLDSFTTHEQQQRMLEILQFKLDI
LWSILDALTLAYVHNEAPYHSVTQERVWHKGLFK" /product="Pyrroloquinoline-quinone
synthase (EC 1.3.3.11)" /transl_table=11
CDS complement(31652..32563)
/locus_tag="AUO97b_00173" /db_xref="SEED:fig|400667.82.peg.33"
/translation="MHIYILGSAAGGGFPQWNCNCPNCHGVRTGTINAKVRTQSSIAI
SENGVDWILLNASPDIRQQLFDFKAAQPARKLRDTGITNVILMDSQLDHTTGLLTLRE
GCPMNVWCTEMVYQDLTTGFPVFNMLKHWNGGLQYHQIDPKQAFKIDGFENLEFLPLI
IQSAAPPYSPHRHDPHEGDNIALIIKDHKTQKQLFYAPGLGKIDDQIMQIMQDSDCVM
IDGTLWTDDEMQQTGVGKKTGREMGHLYISGEGGSLSYLNQLSTPKKVLIHINNTNPI
LNEDSTQFAELKANGVEVAFDGMQIEL"
/product="Coenzyme PQQ synthesis protein B" /transl_table=11
CDS complement(32639..32770)
/locus_tag="AUO97b_00174" /db_xref="SEED:fig|400667.82.peg.34"
/translation="MRFNQVIHFTRLILTGVKFMQWTKPAFTDLRIGFEVTMYFEAR" /product="Coenzyme
PQQ synthesis protein A" /transl_table=11
CDS complement(32937..35012)
/locus_tag="AUO97b_00175" /db_xref="SEED:fig|400667.82.peg.35"
/db_xref="GO:0008976"
/translation="MTSLPTLASSNQHNLETYINRELSLLEFHKRVLAQAKDIEHPLL
ERLNFLIIFSRNLDEFFEIRVASLIQKISLMSQATGPEGLPLVEVLKQISKNAHEAVE
EQYKILNYTLLPQLQGYGVHFIQHGDILEKHKEWIKEYFFKEVQPVVTPISLDPAHPF
PRLVNKSLNFIVTLEGKDAFGRQIDLAIVPAPRSLPRLVKIPEHISGGAEHYILLSAI
IHQHISDLFPGMKATGCHQFRVTRNADLTVSEDVEDLAAALKGELSSRRFGQAVRLEV
AKKCPEEIANYLLEQFDLDAEHLYYVDGPVNLARFISNFDLPELRYKPYQQVLPQLLY
TKKPIFDVLKAGDVLLHHPFDSFAPVIKFLREAAQDPNVLAIKQTLYRSGANSEIVQL
LAEAARNGKEVTAVIELRARFDEESNIEVANVLQEAGAVVVYGIVGYKTHAKMILVVR
REQDKIKRYVHLGTGNYHAVNAKIYTDFGLLTNDAEICEDVHKIFQELTGMGKMAKLK
KLFHAPFTLHSQLLELIEQEVKNSLAGKKAHIIIKVNALTEPQLISALYKASQAGVKI
DLIIRSICCLRPQVAGLSENIRVRSIVGRYLEHTRVYYFYNDGDTKVYCASADWMERN
LFSRIETCFPIENKKLKKQVIEDGLNNYLKDNRQAWELQADGTWVQCHPKPEEELYIA QQHLMNLAG"
/product="Polyphosphate kinase (EC 2.7.4.1)" /EC_number="2.7.4.1"
/transl_table=11
CDS complement(35296..35415)
/locus_tag="AUO97b_00176" /db_xref="SEED:fig|400667.82.peg.36"
/translation="MLESLSSLSLQPTNKTIARHKDVVLNSMFKVVIFYQLMN" /product="hypothetical
protein"
/transl_table=11
CDS 35366..37099
/locus_tag="AUO97b_00177" /db_xref="SEED:fig|400667.82.peg.37"
/db_xref="GO:0008253"
/translation="MVLLVGCNDNDDNDSSTPAPRSNQTVNILAFNDFHGNLEPPKRY
VEAANPNDASQTVRIPVGGVSYFADAIKKLKAQNPNNAVVSAGDLISASPLTSSLFLD
EPTIEVMNDIQIDFNAVGNHEFDRGTDELRRLQNGGCQQYTSTAPCQINKNFSGAKFN
FLAANVAMKNDTSKTIFPAYKVKTYGGIPVAFIGLTLKTTPSIVSAAGIKDVEFRDEA
DTVNALIPELQKQGIEAIVVVVHEGAAPSTKLNQKTCDGLSGPILGILDRLNPAVDIV
VSGHTHQSYICDYATKNPAKPFLLTSAGQYGTLITDIKVELDGKTGDIIKKDAKQIPV
QSEAYTSGTTTVSLTDLYQKFSKTPSIEAILDKYRQAVTTISGRVVGTSTAVVSRTQV
ESGESPLGDMIADAQQAAALQASNQGSDFTLMNPGGVRADLLINSSNQITFGDIFAVQ
PFGNSIVTLSLTGKQIRDVLEQQWSGANANSPRILQPSKELSYQYAANTSVSPRASNI
MVAGSPLVDTKVYRVTVNSFLADGGDNFTVLKEGQNRVGGGQDIDALESYVAKNSPLI IPATTRIKVIK"
/product="5'-nucleotidase (EC 3.1.3.5)" /EC_number="3.1.3.5"

/transl_table=11
CDS complement(37167..37568)
/locus_tag="AUO97b_00178" /db_xref="SEED:fig|400667.82.peg.38"
/translation="MTTISKSLLNGLPIKKSRVQVFEILNQNPQGLSAVSIYAQIKNE
HKITLGTIYRILSEFENRKIIKRVFLGKSKSIYKLRKDKMSCNLISLKTSEALNFENE
KVKELLHEVTDIIFANTGVDISSVELNLYTE"
/product="FIG00351880: hypothetical protein" /transl_table=11
CDS complement(38095..38736) /locus_tag="AUO97b_00179" /db_xref="SEED:fig|
400667.82.peg.39" /translation="MSIPKIASYSMPQAHEFTPNKTNWPLHTSRAVLLVHDMQQYFLD
FYDLTQEPIPELIQNTKALIDAARQSNIPVVYTAQPGNQSPEYRQLLTDFWGPGLKDE
PSITQIFPKISPQKNDTVLTKWRYSVFKFSPLEQLMRDSGRDQLIICGVYAHIGCLMS
AAEAFMLNIQPFLCGDALADFSREEHDMALKYASTRCAQVMTTQQVIQAWIQE"
/product="Isochorismatase (EC 3.3.2.1) of siderophore biosynthesis"
/EC_number="3.3.2.1"
/transl_table=11
CDS complement(38809..39579)
/locus_tag="AUO97b_00180" /db_xref="SEED:fig|400667.82.peg.40"
/translation="MRSTIVVTGAARGIGAAIAKQLLDQGYHVIGIDCQKNPEQWDIS
KTMTSDQSSQWQGISQDITHQQETQTLISELLEKYEITGLVNAAGVLIMRSMLEAKTE
DWETLFAVNVMAPIAISQQVAKHFCGKKQGSIVTISSNSARMPRMQLGMYATTKAALS
HFCRNLALEIAPYQVRLNIVSPGSTLTQMQQQLWTDNTPPPAVIDGDLSQYRTGIPLR
KLAQPDDIANTVSFLLSDRAAQITMQEIVVDGGAALGV" /product="2,3-dihydro-2,3-
dihydroxybenzoate dehydrogenase (EC 1.3.1.28)" /EC_number="1.3.1.28"
/transl_table=11
CDS complement(39939..40529)
/locus_tag="AUO97b_00181" /db_xref="SEED:fig|400667.82.peg.41"
/translation="MGQFHIEPSFQSDAAVIGLFGPSGSGKTSILHAIAGLNTPRSGL
IKIQDQTWFDSNQKINVSTQKRHVGLVFQDAQLFPHKTVKQNLLFGLKRLSQQERHFE
ADYIIELLKLGHLLQRMPIKLSGGEKQRVALGRALLYSPKLLLLDEPLSALDANHKAE
IIPFFQKVKEEIKIPMIYVSHDIQEIKQLTETMWIL"
/product="Molybdenum ABC transporter ATP-binding protein ModC"
/transl_table=11
CDS complement(40564..41184)
/locus_tag="AUO97b_00182" /db_xref="SEED:fig|400667.82.peg.42"
/translation="MVSIIPAICVGWFLARKDFWGKSFIETLVFLPLVLPPVVPGYLL
LQVFGSRGIIGQYLAPLGIDFVFNWKGAVLASAVMGFPLLVQSIRLAIELVDQRLEMA
AKTLGASSLGAFFHVTLPLASSGILIGAILCFCRSLGEFGATITFVGNISNETRTLPL
AMYSLMQQPDTEAAIQRLIVLSLSVAFLALWFAQWFHRYQKKRLGR" /product="Molybdenum ABC
transporter permease protein ModB" /transl_table=11
CDS complement(41253..42038)
/locus_tag="AUO97b_00183" /db_xref="SEED:fig|400667.82.peg.43"
/translation="MVSDTKIKKLALACSVLSIGLVFNISAKAESVTVYAAASLTNAI
NDLEKIYEKQNKVEVKTSYAGSSTLAKQVEAGAPADIFISADTQWMDYLQNKKRVAAN
DRINLLGNRLVLITPKGQSLIIKLDKTTDPNKVFTGKICTGDTKSVPVGKYAKQAFTN
LGWWSRIEPKLVETEDVRAALNFVARGECQVGIVYATDAAISKDVKVAGVFPENTHSP
IIYPLGLIKKNPSSTKFYQFLQSNQAKAVFKKYGFSVLAPVKP" /product="Molybdenum ABC
transporter, substrate-binding protein ModA" /transl_table=11
CDS complement(42227..42577)
/locus_tag="AUO97b_00184" /db_xref="SEED:fig|400667.82.peg.44"
/translation="MKEQNLKLQIRILLEQNIAFGPGKADLLEAIERTGSISQAAKSM
NMSYRRAWQLVDTMNQCFETALVETQTGGTHGGGAAVTALGQKVLQHYRQMENNARQA LEHDYQIMSSYLKG"
/product="DNA-binding domain of ModE"
/transl_table=11
CDS 42800..44215

/locus_tag="AUO97b_00185" /db_xref="SEED:fig|400667.82.peg.45"
/db_xref="GO:0018623"
/translation="MTSSNLKQWNDFIDGCIDFRPNDGVFRIARDMFTEPELFDLEME
FIFEKVWIYACHESEIPNNHDFLTVQIGRQPIIVSRDGKGELHAMVNACEHRGATLTR
VAKGNQSTFTCPFHAWCYKSDGRLVKVKAPSEYCEDFDKSSRGLKQGRIASYRGFVFV
SLDTQATDSLEDFLGDAKLFLDLMVNQSPTGELEVLQGKSSYTFAGNWKLQNENGLDG
YHVSTVHYNYVSTVQHRQQVNASKGAELDTLDYSKLGAGDSETDDGWFSFKNGHSVLF
SDMPNPTVRPGYSTVMPYMVEKYGEKYAEWAMHRLRNLNLYPSLFFMDQISSQLRIVR
PVAWNKTEVISQCIGVKGESTEARRNRIRQFEDFFNVSGLGTPDDLVEFREQQKGFQA
RLERWSDISRGCQSWEYGATKNSQDLGIQPVITGREFTHEGLYVNQHGHWQRLMLDGL
NKKALKMQDITFENNAVMDEV"
/product="Benzoate 1,2-dioxygenase alpha subunit (EC 1.14.12.10)"
/EC_number="1.14.12.10"
/transl_table=11
CDS 44218..44709
/locus_tag="AUO97b_00186" /db_xref="SEED:fig|400667.82.peg.46"
/translation="MSQELHFAVSQFLYKKAELCDAYDWDAYLELYDEDSEYHIPQWI
DDHNYVQDPNQGLSYIYYADRTGLEDRVFRIRTGKAASATPLPRTLHSINNIRVKTLE
DGLIEAKVAWQTLYNRQGLEGCFYGHATYLLRQTADGFRIRRQHSILLNDKIDSVLDF YHV"
/product="Anthranilate dioxygenase small subunit" /transl_table=11
CDS 44721..45749
/locus_tag="AUO97b_00187" /db_xref="SEED:fig|400667.82.peg.47"
/translation="MGHSVALNFADGKTFFISVNKDELLLDAAVRQGINLPLDCREGV
CGTCQGQCETGIYEQEYVDEDALSERDLAERKMLACQTRVKSDAAFYFDHDSAICNAG
DTLKIETKVTAVELVSETTAILHLDASSHAEQLQFLPGQYARLQIPDTEDWRSYSFAN
RPNATNQLQFLIRLLPDGVMSNYLRDRCQVGQRLLIEAPLGSFYLREVERPLVFVAGG
TGLSAFLGMLDNLVDQPNSPAVQLYYGVNSETDLCEQQRLQAYTEQLPNFSYHPIVTK
ATETWQGKAGYIHEHLNKDQLAEQAFDMYLCGPPPMIEAVKNWLDEQSLQNYRIYSEK FLQSNTSR"
/product="benzoate dioxygenase, ferredoxin reductase component; Anthranilate dioxygenase reductase" /transl_table=11
CDS 46199..47257
/locus_tag="AUO97b_00188" /db_xref="SEED:fig|400667.82.peg.48"
/translation="MNQANSHELNGRHFQNESIFTDHNLVFDHHDLSETSRNVGQIFK
PHDLKISHQKRDFSATMHHVKTGALSISRLEYGADVIIEPDHLDNFYLIQIPTQGYAE
IEFGSQKFISYSQVASLISPQQSLRMCWHANSPQLILKVSKDDFTYHCRQHIADSENN
LLIFDPKLDFSTQGGAYFLQLVRTLMDALACEQHPLHHPLAFKQFESNLFNALIYGQP
NNALHKLDHYKEKTVSPYFVKRTEAYIKEHLHEPLNVEILAEHAGVSVRTLFTGFKNY
LGTTPMSYLKELRFEQAHLELMHNENLSVTDVAFKWGFTHLGRFSQEYKRRYGELPSS
TRRSGQSEGSGLITSIFS"
/product="benABC operon transcriptional activator BenR" /transl_table=11
CDS complement(47314..48261)
/locus_tag="AUO97b_00189" /db_xref="SEED:fig|400667.82.peg.49"
/translation="MKSTLIKHGFLAGTLLGLPWVTHAYDLPTVNLGMTSFLDGGLPA
GPGWYAQTYFQNYDSNKLVDANGQKLGLPKTDLNYHVLVEQISYLSNVRVKDKASLGM
NFLIPFVSKMDMDDGLNNAAIKAQSGFGDIMIGPFIQFDPVMGAQGPKFVHRFEFQVN
LPTGEYDQQKDINPGNNTVSFDPYWAATYWFNPKWTVSTRIHYLYNFKNDDPSYAFAG
ANDMQAGQAIHANFATDYAITEQFRLGLNDYWLKQITDTEVDGEKVKGRREQVWAIGP
GAMYSFSKNDHLVANAYFEQDAENRPEGTRLQVRYVHHF"
/product="Protein involved in meta-pathway of phenol degradation"
/transl_table=11
CDS complement(48315..48845)
/locus_tag="AUO97b_00190" /db_xref="SEED:fig|400667.82.peg.50"
/translation="MDTIDTLALQAINAENPREIRNLLGQFATGVTVITTRGKDGRKI
GMTANSFSSLSLDPPLILWSLSKTAPSLPDFVEAEYFAIHMLAQEHHQLSGHFARGAE

DKFAGIAHQQCNAGVPILTDALATLVCRNVNQYEGGDHLIFIGQIEQYQQRQGEPLVF HAGKYRIAAEHPELAH"
/product="Nitrilotriacetate monooxygenase component B (EC 1.14.13.-)"
/transl_table=11 CDS complement(48874..50112)
/locus_tag="AUO97b_00191" /db_xref="SEED:fig|400667.82.peg.51"
/translation="MRRIAIVGAGQSGLQLGLGLLDTGYDVTMITNRTADEIRQGKVM
SSQCMFHTALQTERDLGLNFWEEQCPAVEGIGLALVNPENGSKAFEWSARLERYAQSV
DQRVKMPYWMEEFERRGGRLVIQDVGIEELEQLANDYELVLLAAGKGEVVKQFERDDI
RSVFDKPQRALALTYVKNMKPISPYSRVTFNIIPEVGEYFSFPALTINGPCDIMVFEG
IPNGPMDCWQDVKTPEQHLQCSLDLLKKFVPWEYERCANVELTDAGGYLTGRFPPTVR
KPVLTLPSGKKIFGMADALVVNDPITGQGSNNAAKCSKIYFDAILANDNQSFSEQWMV
QTFERYWAYAEKVVAWTNSLLLPPEPHVVELLAAASQNQSIASIMANNFDDPRAFAPW
WFDANQAQVFLASKNTAKVA"
/product="putative oxygenase subunit"
/transl_table=11
CDS complement(50156..50941)
/locus_tag="AUO97b_00192" /db_xref="SEED:fig|400667.82.peg.52"
/translation="MNIDLKGKVAVVSGGATLIGKAVVQALVSAGAHVAILDIDAKGK
AIAESFNHDVMFIQTDLTSDAAIQQAVADIHQHLGEVSYLVNLACTYLDDGFKSSRQD
WLQALDINLVSTVELSRALYNDLKKQQGSIVNFTSISAKVAQTGRWLYPVSKAAIRQL
TQSMAMDFAADGIRVNSVSPGWTWSRVIAEVSGNNREKADSVAADYHLLGRLGHPEEV
ANVVLFLLSSAASFVTGADYAVDGGYSVMGPEGSQPAIPRLAE" /product="Oxidoreductase, short-chain dehydrogenase/reductase family" /transl_table=11
CDS complement(50954..52204)
/locus_tag="AUO97b_00193" /db_xref="SEED:fig|400667.82.peg.53"
/translation="MAGQTIQIKTASGKQFSAYLATPETGKGPGVVLCQEIFGVNAAM
REKAEFLAEEGYTVLVPDLFWRVAPNIELGYTPEDFQKAFELYQQYDENLGVEDIQDS
LAFLKTRPECDTSTGLGVVGYCLGGKLAYLAGCRLSEVACAVGYYGVGIEKALDELAN
VKGRLVLHIAELDQFTPPDARQAIVDAANQYSNVQAYVYEGVGHAFARPKSNHYHKPS
ARFAHERTVTALHETIGPKYDLVALWEEHIRHEFDTRDVPATMATMVAEPYVNHIPTL
TGGVGHSQLARFYQYHFVHQNPKDMKITSISRTVGSTQVVDEFIMSFTHDAEIDWLLP
GVKPTGKYVEIPMLGVIQFRGSKLCHEHIYWDQASVLVQIGLLDPTGLPVAGVETARK
LLDEDLPSNTLMPSWSSSEGKPVN"
/product="Dienelactone hydrolase family protein" /transl_table=11
CDS 52534..53877
/locus_tag="AUO97b_00194" /db_xref="SEED:fig|400667.82.peg.54"
/translation="MKTIDANTIINREKLSPLQWVVFILGFFVFFCDGLDTGIIGFVA
PALLDDWGITKPQLAPVLSAALVGMSIGAIISGPLADKFGRKGIIVFTSLLFSIFTIL
CGFANSTQDLMIYRFITGVGLGAAMPNISTIVSEYMPVKRKAFLTGLAGCGFMLGISC
GGLLSAYLLESYGWAKVIIIGGSIPLILVVALLLKLPESTQYLITRHQQEKAQRILEN
IQGHSFQEPVKLVLTQAETASDENPVKVVLGKYLWGSSMLWLCCFTSLLVFYLLTSWM
PTILKTAGFSTQQFSLIAAIFPFGGVIGATIMGWYMDKLNPTTVIKYSYLIAFVLFIV
AGLVSSNIFLLGLTIFLIGALLAGAQSSLLPLAAMFYPAVCRAVGVSWMHGIGRIGAI
LGAFFGSLIFTFSLSLSGIFFILAIPTFISFIALSLKVIYEKSKHKQVLKLEESL" /product="4-hydroxybenzoate transporter"
/transl_table=11
CDS complement(53945..54910)
/locus_tag="AUO97b_00195" /db_xref="SEED:fig|400667.82.peg.55"
/translation="MELNHYSRKAEFAQNLMSTICGEHRLDTIYKDNLDFHYDGMRLP
NKKMAIGTISYGANVAININISNLKAYSISLPIQGRQALNIRGAHYQSDENRGLIVSNND
QQDLIIDKDCRKFQVVIPERSMQIVLADLLNKPIETPIIFNPEMHLDSEQLIGAWWKN
IQNFLQLKSQYSNFYGLQMLSEDYENFVIKALLLSQENNYSEALKSLSHQDEPAYIRK
VRNFIIEHAHEEIYAEDLQRLAGVSKSKLYDEFQQYYGTSPMSYLKKYRLQQIYKILS
TTGADKKVSISKLAYDWGFNHLSRFSQEYREEFGENPSETKGKIF" /product="Transcriptional regulator"

/transl_table=11
CDS complement(55262..56047) /locus_tag="AUO97b_00196" /db_xref="SEED:fig|400667.82.peg.56"
/db_xref="GO:0003858"
/translation="MTKLLDGKVAFITGSASGIGLEIAKKFAQEGAKVVISDMNAEKC
QETANSLKEQGFDALSAPCDVTDEDAYKQAIELTQKTFGTVDILINNAGFQHVAPIEE
FPTAVFQKLVQVMLTGAFIGIKHVLPIMKAQKYGRIINMASINGLIGFAGKAGYNSAK
HGVIGLTKVAALECARDGITVNALCPGYVDTPLVRGQIADLAKTRNVSLDSALEDVIL
AMVPQKRLLSVEEIADYAIFLASSKAGGVTGQAVVMDGGYTAQ" /product="3-hydroxybutyrate dehydrogenase (EC 1.1.1.30)" /EC_number="1.1.1.30"
/transl_table=11
CDS complement(56081..57502)
/locus_tag="AUO97b_00197" /db_xref="SEED:fig|400667.82.peg.57"
/db_xref="GO:0015137"
/db_xref="GO:0015746"
/db_xref="GO:0016021"
/translation="MWELFVILLSLGLLMYTAYKGFSVILMAPICALLAVLLINPANV
LPFYSGVFMPKMVNFIKDYFLVFLLGAIFGKVVEMSGIAESIARTIVRWIGAKKAILT
VVLLGAILTYSGVSLFVAVFAIYPFANQLFRQANIPKRLIPGTIALGAFTFTMDALPG
TPQIQNVIPTTFFGTNIYAAPFLGIIGAIFVLCMGLAYLEWRRRAAAKAGEGFSGFGV
DNTSSQEIEADMNLQNNGSTARQFLAFVPLILVAVMNKYLSKAIKEWYPNGFDFAAVG
LADYTVDVAKTGAIWAVGLALIVGIITAILFDYQRVVTNFKEGINASIGGSLLAVMNT
ASEYGFGAIIASLPGFAMISHAMSSTFTNPLVNGAVTTTVLAGITGSASGGMSIALSA
MAEQYNAAILAAGIPPEVMHRVVAMASGGMDTLPHNGAVITLLAVTGLTHKQSYKDIF
AVTVIKTLAVFVVITVFTVTGIV"
/product="D-beta-hydroxybutyrate permease"
/transl_table=11
CDS complement(57969..58385)
/locus_tag="AUO97b_00198" /db_xref="SEED:fig|400667.82.peg.58"
/translation="MKKNIFLALVLGLVGCSTAPVVPNTAKPIPNERVYNQNYLKKQT
PEQAKVTFSRDKGFLGSGCTHDMYVNNEKVFSIRSNEIATIYLEPKYYIFRLETGGGM
CPNIATSQETEVKPGAEIEYRILLPSDFNLRLTRMK" /product="hypothetical protein"
/transl_table=11
CDS complement(58487..59101)
/locus_tag="AUO97b_00199" /db_xref="SEED:fig|400667.82.peg.59"
/translation="MISESTKTNQIDNKNRLIIQTVGDLFLEYGYSRVSINLIISKIG
GSKRDLYAQFGDKEGLFRSVIADVCQQVLDPLKALPVEGGSIEQALTSFGRIFLSVLL
SSRVIALQKLVLSEATRYPEFAKTFVQLGPVSAYNLAAELLMKRAEVGEIKVSKPKVM
GALFCDMLISDLQFKIISGEIVTDSQIDERVEMAVLTFLNGIKS" /product="Transcriptional regulator, AcrR family" /transl_table=11
CDS 59211..59465
/locus_tag="AUO97b_00200" /db_xref="SEED:fig|400667.82.peg.60"
/translation="MASLIHNTLSSNYTLVIGRIVLSSFFLIAGIFGVFNFDTVVNEM
IGANLPLPQLFAFATIAIQLIGSILLISNFAGLTWLVQEY" /product="FIG00351389: hypothetical protein" /transl_table=11
CDS 59444..59611
/locus_tag="AUO97b_00201" /db_xref="SEED:fig|400667.82.peg.61"
/translation="MVGAGILAIFTLLCIPIGHPFWKLAEPQRTQDFQIVLEHIALSG GLLLAAIATKK"
/product="FIG00351389: hypothetical protein" /transl_table=11
CDS complement(59814..60641)
/locus_tag="AUO97b_00202" /db_xref="SEED:fig|400667.82.peg.62"
/translation="MNKTILGLMVVPLVLSGCIKKAEEKPTEKPEKTSTIKATEQPPK
EENIIPAMLVRNFLVKFQQPKICAPNPDVDGIVCTSKEAIALKSNIEWIDQVIDKEIR
KDYADALTPPSEKQKQLNQTQAEGFNNWSQSVVYRFNGQFGQYVQISKVSSEYSGGAH

GIATFEDYIFDLKSKKRIELKDIVINGKMNALKEELWKQYGYWCQEKDMQPYISKEDF
KVSENFYINQSGGITFDFQLYELAPYAAGPVSLELYDTDQLIKSDFLPPMPTLKTEM"
/product="Chromosome segregation ATPases"
/transl_table=11
CDS 60983..61228
/locus_tag="AUO97b_00203" /db_xref="SEED:fig|400667.82.peg.63"
/translation="MQILIGYKIPHLAPKYKFVVSATYVILFTFLLAIYSTTVLLEGL
KVAPQAIWLGYLFFHVIGVFIYFSNNRNEFCWDNLLK" /product="hypothetical protein"
/transl_table=11
CDS 61376..62257
/locus_tag="AUO97b_00204" /db_xref="SEED:fig|400667.82.peg.64"
/translation="MTLKQLKAFLALARTLNYANASLELHLSQSALSLTIKSLEEELG
GKLFNRNTRRVELTQEGKSLIPYAKKLLANWDEMENDVKQRFKLNRGTLSIASMPFVT
HAILPEVIHQFLAVHPNLNFSIHDIPNETIIEKVQDGIFELGICFEPDLTEGLEFKPL
FEEDFLALLPKSHPLAQSSSITWHELCSNPFVTLQKPSIVRYLVEQNCLRNNITLDLK
VECHQISSLSSFVAYGIGVSAIPRHFAKHIDKEHNVLIDLKDEAIHKTVGIVYKKNFE ISNISAQFIEALVQYKF"
/product="LysR family transcriptional regulator near succinyl-CoA:3-ketoacid-coenzyme A transferase" /transl_table=11
CDS 62503..63207
/locus_tag="AUO97b_00205" /db_xref="SEED:fig|400667.82.peg.65"
/translation="MNKVYANAEAALKDVIADNQTLAVGGFGLCGIPEKLIVAVKQSG
VTGLTCISNNAGVDDFGLGILLQTKQIKKMISSYVGENKEFERQYLNGELEVELTPQG
TLAEKLRAGGAGIPAFYTQTGVGTLIAEGKEERIFNGKNYILEESLTADVALVKAYKA
DKAGNLVFRKTAQNFNPECAMAGKITIAEVEQVVEIGELDPDEIHLPGIYVNRIVLNA SPEKRIEHKTLSTEAV"
/product="Succinyl-CoA:3-ketoacid-coenzyme A transferase subunit A (EC 2.8.3.5)" /EC_number="2.8.3.8"
/transl_table=11
CDS 63209..63850 /locus_tag="AUO97b_00206" /db_xref="SEED:fig|400667.82.peg.66" /translation="MAWSRNEMAQRAAKELEDGFYVNLGIGLPTLVANYIPENINVWL
QSENGLLGIGEFPTEETVDADLINAGKQTVTARPGAAFFSSSESFAMIRGGHVNIAIL
GAMEVSEKGDLANWMIPGKKVKGMGGAMDLVVGVQKVVVLMEHCAKDGTPKIVKNCSL
PLTGKGVVHRVITDLGVMDITPQGVELIELAKDVTLEQIQAATVVEFYKTLVA" /product="Acyl-CoA:acetate CoA-transferase, beta subunit (EC 2.8.3.8)" /EC_number="2.8.3.8"
/transl_table=11
CDS 63967..65376
/locus_tag="AUO97b_00207" /db_xref="SEED:fig|400667.82.peg.67"
/db_xref="GO:0015635"
/db_xref="GO:0015912"
/db_xref="GO:0016020"
/translation="MEKQQGIFERFALRISDWSEKWFPDSYIFALLGVIIVSVAALGI
GAPIHDVATSFGNGFWSLIPFTLQMTMLIIVGYVVSVSKPVKFLIQKMARIPSSGRGA
IVLVATVSLLISLVNWAISTILTALLVIALAKRKELNMDYRAAAAAAIIGMGATWALG
ISSSAAQLQANKTSLPESIYNLTGVIPFTETIFLWQSIAMTIILVIVSIAIAYWSAPK
GNSVKTIDSFDVQFEEEKTNEAKSTRPGDWLENSPILTIIVVVLGLIWMFFEFSKSNP
IIAISSLNTYNFVFLMLGLALHGTPRNFLNAVAKAVPAVSGILIQFPLYGSIAFIMTQ
ALNSQDLSLSHYIAEFFVSIASKETFAIVMGIYSAVLGFFVPSGGGKWIIEAPYVMQA
ANDLKVHLGWSVQIYNAAEALPNLINPFFMLPMLGILKLKAKDVIGFTVTQLVVHFPL
VLFLLWFFGRTLSYTPPTF"
/product="Short-chain fatty acids transporter" /transl_table=11
CDS 65442..66614
/locus_tag="AUO97b_00208" /db_xref="SEED:fig|400667.82.peg.68"
/db_xref="GO:0003988"
/db_xref="GO:0003985"
/translation="MSSIVIVSGSRTAMGGFQGSLSSLTAPELGAAVIRESIQRAGVK

PEQVDEVIFGCVLSAGIGQGPARQAMRKAGVPDHVGAVTINKLCGSAMKAVLMASDTL
KAGSANIIVAGGMESMTNAPYILPKARGGYRMGHGEIKDHMFLDGLEDAETGRLMGSF
AQDMANTKGFTREQMDDFAISSLKKAQTAITEGYFKEEIVPVEVKTRKGVEVIDQDEQ
PLKANLEKIPTLRPAFSKDGTITAANSSSISDGAAALVLTTEEYAQSHGLNTLAKIVA
TSTHSQHPSEFTIAPISAIQKVLERAGWSVDEVDAWEINEAFAMVAMAPIHELGIDEA
KVNVHGGACALGHPIGATGSRIILSLIYALKRLGKKKGVAALCIGGGEATAVAIEI" /product="3-ketoacyl-CoA thiolase (EC 2.3.1.16) @ Acetyl-CoA acetyltransferase (EC 2.3.1.9)" /EC_number="2.3.1.9"
/EC_number="2.3.1.16"
/transl_table=11
CDS complement(66675..67505)
/locus_tag="AUO97b_00209" /db_xref="SEED:fig|400667.82.peg.69"
/translation="MAADAIGLTQPSVSNGLNKIRQHFNDPLFIRVGNEMIPTELAKQ
IFPLISEVIDKVESINNFNVNFDPLTSDQLFTIAMTDVSHLVLLPQLTNYLKEKAPLI
RLNIRPITPETSYQMANGEIDLAIGFLPQLEEGFYQQKFFRQHYVVISSKSHPRLDPN
NFTLEDYMRESHIDIDAGIGHYHIKNELQNKGLDRNILIRLPSYLGVGLVVQETDAIA
TVPYYLSQVLLVRENLQILPAPLDFPTYDVKQHWHMSCHHKSSHKWFRQTCYELFKTA "
/product="Transcriptional regulator, LysR family" /transl_table=11
CDS complement(67809..68705)
/locus_tag="AUO97b_00210" /db_xref="SEED:fig|400667.82.peg.70"
/translation="MNLEVRWVEDLLTLERERSISKAAEKRFISQSAFTRRIQQIEEM
IGAEVIIRNNKNNIEFTDIGRILLVMSKNIEKQVEETAKLIKNIKNENESTIRFCVVH
SLASGFLTTFLKKFPTLIRDLKIEIVATNSGEGLSLLKEGACDFMICYADKSNIRKVG
DDILSGIKIAETVIVPVSATEPDHSPKHTIQSNFALLAYSKHAYLRKLVDQLIEGKLI
YKTLYETDNATNLKELVLQGLGVAWIPKINVEEDLAAGKLVILDQQEYCLAQDIYIFK
NNLSDNKSVQKIWNGFKGQQDL" /product="Transcriptional regulator, LysR family"
/transl_table=11
CDS complement(69483..69710)
/locus_tag="AUO97b_00211" /db_xref="SEED:fig|400667.82.peg.71"
/translation="MRMKFIAYDPNSGEKLSLKEHIHHMDKYTTYVFFSDEKDNMVFN
PQKKQIFEISQYTSNMMVEISRKLYYLIGEY"
/product="FIG00352393: hypothetical protein" /transl_table=11
CDS complement(69796..69981)
/locus_tag="AUO97b_00212" /db_xref="SEED:fig|400667.82.peg.72"
/translation="MLKVTAALLILGSFACYLLWGNEEKAETKSSHMLKEAEQSIKNA ELSIQKAKQNIPTASYQ"
/product="hypothetical protein"
/transl_table=11
CDS 70395..71813
/locus_tag="AUO97b_00213" /db_xref="SEED:fig|400667.82.peg.73"
/db_xref="GO:0008797"
/translation="MMNADITTRIEKDLIGHREINNDHYYGVQTLRALENFNLTNSKV
GNFPNLIKALAMVKLACAEANYNLKNLDQTKFEAIEYSCNQLIRGNFHDQFPIDMMQG
GAGTSTNMNANEVLANVALEYMGHSKGQYQYLHPNNDINMSQSTNDVYPTAIRLGLLL
SLDELNLPFNNLILSFLNKSQEFAHILKMGRTQLQDAVPMTLGQEFGAFAVTLQKDLE
QINTLIPNVLAYMNLGGTAIGTGITTEYSYRELAIQALGKISGKKIKSAPDLIEATSD
MGDFVLLSGLLKRTATKLSKIANDLRLLSSGPRTGINEINLEARQPGSSIMPGKVNPV
IPEAMNLACFQIIANDLAVTLAAEAGQLQLNAMEPLIAFKLFESMDLLGKAMAMFQTK
CIDTITANPEHCKALVENSIGIVTALNPYLGYETTTRIAKTANETGQSVLALVQNEGL
LSAQVLDDILSINNMVKPKMSA"
/product="Aspartate ammonia-lyase (EC 4.3.1.1)" /EC_number="4.3.1.1"
/transl_table=11
CDS complement(71870..74080)
/locus_tag="AUO97b_00214" /db_xref="SEED:fig|400667.82.peg.74"
/db_xref="GO:0004872"

/db_xref="GO:0005215"
/db_xref="GO:0006810"
/db_xref="GO:0016020"
/translation="MHGMAGSYSMERRSLNLHFMTCSKTALASSIALITSVVYANEAE
VSQLPTITVNATQNSDALYTSKKVSLSGFQTGDVKKVPASITTITSERIADQHAKILT
DVIKNDSSLGDGYAAIGYYPNFVARGFALDLGSSYLINGHTIRGEQNVALENKEQVEI
LKGISAIQSGMSTPGGVVNYVTKRPADVKNITVDANSEGGYTLATDVGGFVNDSFGYR
INLAHESIHPNVHHANGKREFGSVALDWKINERSKLLFDIEAQHQSQRSVPGYQLLDG
QEVPTNVDQDRLLGYQSWSKPVVNNSLNASLKYQYAFNDQWNGSLSASQSRVVIDDYI
VFPYGSYYSSGNELAVFDKDGNYDIYDYRSPDDARITNQFAAGLNGQFNTGNIQHQIA
FELQHTYKTLKHHTPLNGFIGTGNIYENTVDYNPSLESPGDRYKALQSDQTAFTVSDR
VQFNDHWSTLLGGKLIHLDEQAYNSDGQQSRDTDLNKFLPQLALMYSPTATTNLYASY
AKGLSDGGEAPWYTNNIKNNIGPSVLSPRSSEQYEIGIKQQIRNFLVTAAIFDLKQDN
QYSKVNAEGTFDFVSQGEQHNQGIELGLTGALTDKLDISSGITYTKSRLVDIDTDSYK
GHQTQNVPKVRATAQLSYKVPSVEGLRLLSGMQYSSSKYANKEGAAKVGGYSVFNIGA
AYKTNFAGHDTTFRFNIDNLFNKKYWRDVGAFMGDDYLFLGNPRTAQFSTTFSF"
/product="Ferrichrome-iron receptor"
/transl_table=11
CDS 74599..75885
/locus_tag="AUO97b_00215" /db_xref="SEED:fig|400667.82.peg.75"
/translation="MTYTQEEKRSRIRGIVGAASGNLVEWFDFYIYAVFAAYFTQALT
APDMDSTTKSIYVWGVFAASFFMRPIGSWLFGRIADRHGRKKSMIISICLMALSSFLF
AALPTYDQVGLFAPFLLLLVRLLQGLSVGGEYGAVATYMSELGLKGQRGFYSSFQYVT
LSGGQLLASLLGVILLAFLSEQQLHDGGWRIPFVIGGIAAILSLLARSRLEETLSHQD
SERKESGSLRELFKKHWKTFLLVVGYTSAGSLTFYVETVYSKTYLTNLGMDGKTVGYI
MTFALFVYMCAQPVFGAISDRIGRRSSMLAFSLLSAIFIYPVMVIGMRAYIDSPIIIT
LLLIFMMMLLSFYTSISGLVKAEMFPPHVRALGVGFSYAVGNALFGGSAPSVALQFKA
AGIENTFFIYVIIMLVICFLCSLALPKKPDYLEHDH" /product="dicarboxylic acid transporter
PcaT" /transl_table=11
CDS complement(75925..76206)
/locus_tag="AUO97b_00216" /db_xref="SEED:fig|400667.82.peg.76"
/translation="MQIRPDIRVDTINKQVHFIWTDGSSICLSHQSLREICPCGFCRA
KRIKQIKIEYQNVEVTAMFDQGYGAQICFSDGHDKGIFPWAFLKEFAKS" /product="FIG028220:
hypothetical protein co-occurring with HEAT repeat protein" /transl_table=11
CDS complement(76190..77188)
/locus_tag="AUO97b_00217" /db_xref="SEED:fig|400667.82.peg.77"
/translation="MSFYYQALDDIDEYNQEFLAQLNHADEKIRFVALMNIGDEENPD
LLPWLHTALQHDSSALVREEAAKNLESWEDPTSLQVLAQALFDVDLNVRQAASQSLSE
VKNAASAQILAPYLKHTDTFALSAILRALKPLRPQQLFEEISALVHHEDAFVRREAVS
ALSWLQQDQAITILAKIAETDTDDEVRRIATGGLAYSQNISVEVIQALQHSLTSESWQ
LRVEAALTIGKLRIVQLEAPLIHALSDLYWQVRIAAVRSLGLIKSHLAVTHLTENFKH
EISNLRKEVALALGEIGTSEAQTLLEQHQNDPDPEVRKAIRIGLNQIGEVKYANQT" /product="HEAT
repeat-containing protein"
/transl_table=11
CDS complement(77185..78024)
/locus_tag="AUO97b_00218" /db_xref="SEED:fig|400667.82.peg.78"
/translation="MSITQGHVRIQNLSLHLGQAKNRFQALDRVNFEIQPGEFICLLG
PSGCGKSTLLGALAGHLPISSGSLTVDDESIFEPHPDRGLVFQQHTLFPWKSVLENIA
FGLKMKGIAKQKRIEQAQKMIDLVGLKGFEHKFPAELSGGMQQRVEIARVLINQPRIL
LMDEPFGALDAQTRLKMQMLLLEIWQEIQTSVLFITHDIDEALFLADRVLIMSHRPGR
IIEEISLKFERPRDVELVTSSEFTAIKKHCIQTLKEATQQEELSRLNPLGLGKKSQIK EYE"
/product="Organosulfonate ABC transporter ATP-binding protein"
/transl_table=11
CDS complement(78026..78703)

/locus_tag="AUO97b_00219" /db_xref="SEED:fig|400667.82.peg.79"
/translation="MNFSNVPTPLDVIHALWAFFQLSTAFEHVESSVFRVVVGFVLAA
LIGVGIGLLAGRYQKLAAFLMPPLEVLRPIPAVAWIPLAILIFPSSEASMIFITFIGA
LFPILLNTVHGVGAVDPRLVASAKSLGASDLAIIYEVIIPGALPNIITGLSIGMGTCW
FCLVTAEMISGQHGIGYFTWESFTLQNYSNIVVGMVLIGALGMFSSTLVRVIGNRFTP WYQLRKS"
/product="Organosulfonate ABC transporter permease protein" /transl_table=11
CDS complement(78915..80345)
/locus_tag="AUO97b_00220" /db_xref="SEED:fig|400667.82.peg.80"
/translation="MRQNKLINKKLLLSSLIGAIVLGTLGCGSKTETVRVAIGTQDTT
INCATGGLLIRELNLLPKYLPKDGKYKHVKYDIQWKNFTSGAPLTSEMVAGRLDLGAM
ADFPAVNNLAAFKKAGKESIFLAPLSGSVNGSGNAIVVPIDSKVTSLKELKGQTISVP
FASTAHGLLLRAIQAEGWQLDKDIKVIAQAPEVAGPALKSHKIAAHADFVPFGELFAY
QGFAKKIYDGSQAKSPTFHGSLASKDYAQQHPEVIKAYLQATIEANRLIREQPEKYSE
LIAEKTGIPAEVVYLFHGPLGLQTRDLTWKPEYRKATQIAIDTLKVLGKNDGTLDVNK
FIDDQYIKDAFQASGLNYSQQLADYAKSPLVANDALTGQPIKTFDRVTQIWVKGEEKV
RSYETPEHAFSDLKKIQADGKTVRVVYSQDHQSDIKLLANLAWYATDKAGQIQAFLLK
DDAEKWAKQQGGKVYDFKAIQLVTQS"
/product="Organosulfonate ABC transporter substrate-binding protein"
/transl_table=11
CDS complement(80365..80610)
/locus_tag="AUO97b_00221" /db_xref="SEED:fig|400667.82.peg.81"
/translation="MAFIFKEVQHRTVAPVIIDEDKCIADKGCTVCVDVCPMDLLAID
PTTQKAYMQFDECWYCMPCEKDCPTNAVKVNIPYLLK" /product="Adenylylsulfate reductase
beta-subunit (EC 1.8.99.2)" /EC_number="1.8.99.2"
/transl_table=11
CDS complement(80631..82364)
/locus_tag="AUO97b_00222" /db_xref="SEED:fig|400667.82.peg.82"
/translation="MENKYLEFDIVVIGGGTAGPMAAIKAKQENPNLKVLLIEKANVK
RSGAISMGMDGLNNAVIPGYATPEQYTKEITIANDGVVNQTTVYAYAKHSFTTIQQLD
QWGIKFEKDETGEFAVKKVHHMGAYVLPMPEGHDVKKVLYRQLKRAQVKINNRIVTTK
LLKNEQGAINGVLGFDCRSGDFYVIKTKAAILCCGAAGRLGLPASGYLMGTYENPTNA
GDGYAMAYHAGAELANLECFQINPLIKDYNGPACAYVTGPLGGYTANSKGERFIECDY
WSGQMMWEFYQELESGNGPVYLKVDHLAEETIQTIEEILHTNERPSRGRFHEGRGTNY
RQDMVEMHISEIGFCSGHSASGVWVNEKAETSVKGLYSAGDMAAVPHNYMLGAFTYGW
FAGVNAAQYVNAVEDSELNQSEIEAEKARIFAPLNCSEGLPPEQVEYKLRRFVNDYLQ
PPKTRQKMEIGLKRFEEIKQDIKRISARNPHELMRAAEVSFIRDCAEMAARASLFREE
SRWGLYHYRADFPQKNNSDWFCHAHLKKDEHGNMVSFKKPIEPYVVPINQDEAVSYDH LRIQKDVALAD"
/product="Adenylylsulfate reductase alpha-subunit (EC 1.8.99.2)"
/EC_number="1.8.99.2"
/transl_table=11
CDS complement(82393..83136)
/locus_tag="AUO97b_00223" /db_xref="SEED:fig|400667.82.peg.83"
/db_xref="GO:0003677"
/db_xref="GO:0003700"
/db_xref="GO:0005622"
/db_xref="GO:0006813"
/db_xref="GO:0008324"
/db_xref="GO:0009058"
/db_xref="GO:0030528"
/db_xref="GO:0045449"
/translation="MSQLPQDPKNHKPLYDQLRELILNKIVNGEYAVLSQIPSENEFA
EQFGVSRITVRQALNQLQLEGYIFKVPGKGTFVSKPKTFQNISSLQGFAEAMSSAGHE
ILNRVISAELKQIPMHVVPKLKLPVKANVYEIQRVRLLNRQPVSYELTYLPEHIGLKL
KEKAIDLRTTDIFKALEQECDIPLGHADLSIDATVADEELAALLQVEIGTPVLRVERL

THDANGQPIDYEYLYFSGDTFQYRLRIHR"
/product="Putative alkanesulfonate metabolism utilization regulator"
/transl_table=11
CDS 83378..84727
/locus_tag="AUO97b_00224" /db_xref="SEED:fig|400667.82.peg.84"
/translation="MQQKQLNTPDDSVEQVTFWQAFLFWLKLGFISFGGPAGQIAVMH
QELVEQKRWISEKRFLHALNYCMLLPGPEAQQLATYIGWLMHRTVGGLVAGILFVLPS
LFILIGLSWVYIKFGDVPVIAGIFYGIKPAVTAIVFHATYRIGSRSLKNKFLWVVAIA
AFFAIFVLKLPFPLIVLSAGIAGYLASKKYPELFSSAGGHKASQKDYGPAYIDDDTPT
PEHAKFHWSRLARLIFVAIALWLLPILALGAYFGWHHSYTQMAWFFTKAALLTFGGAY
AVLPYVYQGAVNHFGWLSPTQMIDGLALGETTPGPLIMVVAFVGFLGGVNHSLLGSEH
LFFAGALGAVIVTWFTFLFSFVFIFAGAPIIESTHNEIKFTAPLTAITAAVVGVILNL
ALFFSYHVLWPQGFSGHFDIVAALITVAAFIALFRFNINVLYVIFASALVGLAVTFF" /product="Chromate transport protein ChrA"
/transl_table=11
CDS complement(84811..85419)
/locus_tag="AUO97b_00225" /db_xref="SEED:fig|400667.82.peg.85"
/db_xref="GO:0006118"
/translation="MKLLQIDTSILGEQSVSRQLTSSVIRQLSTAYPDAEIIHHDFAL
EPIPHLSDAEFLAWQGVEPNNEAAQQRVARNTQYLDEFLSSDIVVIGAPMYNFSFPSQ
LKAWLDRLSVAGKTFRYTENGPQGLVEGKRVIIASSRGGVYSEGSPAEALDYQETYIK
AFFNFIGVTDITFVRAEGIAFGPEARQAALDNAAAQIAQLAG" /product="FMN-dependent NADH-azoreductase (EC 1.7.1.6)" /transl_table=11
CDS 85683..85910 /locus_tag="AUO97b_00226" /db_xref="SEED:fig|400667.82.peg.86" /translation="MLTLCLRGLERDGLVKRTVYPVVPPHVEYELTPLGHSLTEPVIA
LGQWAQQHIADIDAARAAFDAAQEKPITLDT"
/product="Transcriptional regulator, HxlR family" /transl_table=11
CDS complement(85907..86761)
/locus_tag="AUO97b_00227" /db_xref="SEED:fig|400667.82.peg.87"
/translation="MAYQTLEQLQQSKAKLHETVVLDENMQLAFWSNQQDRVSVCSDH
HTLSLYIQDGYESYQKTPAGWKNGGGPGRFCLLPEHQESTWDIRGGLKFVHLYYTDQH
LRDVAEKIWDKEPNQIELNEVVFNDDERIRALYQFFLLDCDWQDSSNHLQMSTTASLL
LNHLIRKYSSVQWQQPTIKGGLAPHKLKYIQEWIEDHLDQALTLSDLANVVNLSEYHF
AHMFKQSMNMAPHQYVMQRRLTKAKGLIQSSQLSLQDIALVCGFSSASHLSHRFKQFY GMSPSQLR"
/product="Transcriptional regulator, AraC family" /transl_table=11
CDS 86928..87845
/locus_tag="AUO97b_00228" /db_xref="SEED:fig|400667.82.peg.88"
/db_xref="GO:0016020"
/translation="MNILLYVAVVLIWGTTWIGIAVQSHYATPVVAILWRFAISALLL
WVVLLFLGKLQRIALKDHLFCMLQGLCVFGLNFICFYTAVKYVNSGLESVIFSMAVFF
NAINALIFFKQKPSPNFAPAALLGLSGIVLLFWHDLINSQFNAQLLMGIGLCAIGTYG
FSLGNMISVRHQKRGLNIFSTNCYAMTYGALVMLILALFTGQDLMPPMNFPFLSSVLY
LAVFGTVIGFSAYFSLVGRIGAAKAAYSTLLFPLVALTVSTFFEGYEWTLNAVFGILL
ILLGNYLMFSKFEIGKKLFSNQKNCEKQA"
/product="Permease of the drug/metabolite transporter (DMT) superfamily"
/transl_table=11
CDS complement(87878..88882)
/locus_tag="AUO97b_00229" /db_xref="SEED:fig|400667.82.peg.89"
/db_xref="GO:0004412"
/translation="MKRINVAICGFGRIGQQIAELLLNRSTYYKQKYQIDARLVGVCN
SSSGLIDQEGLQASKWLDKTQYQAGLTEQKFLEQVQADVIIETGPSDYVTGGKGLFYL
NYALTHNMNAIAVSKGALVVEGKKIISLAHQHNKKLFFSGATAAALPTVDLFEYNLAG
CQILEIEGVFTGTTNFILNDMLQHECAFADSLEKAQAKGIAEPNSSFDVDGWDTAAKI
TILANTVLGADVKIQDIPRQGISHVTADHIRDWKKENVIPRLVGFIHVKNQQIQTGVE

LRLVPANHPFAHLQGSNKCIRVLTQEMGEFVVSGGASAPLATAAAALKDFELMLKTGC "
/product="Homoserine dehydrogenase (EC 1.1.1.3)" /EC_number="1.1.1.3"
/transl_table=11
CDS complement(88992..89783)
/locus_tag="AUO97b_00230" /db_xref="SEED:fig|400667.82.peg.90"
/translation="MLLLVVEGIVIGFLLGLTGAGGGILAVPALMTSQGWSVAQAAPI
GLLAVALSTLIGTIEGLFKKIVRYRAAIWIALIGAPMAHIGILIANTISPIWLMLMFS
LVMFTVGYRLISNRVSDFHNPPCQVNPSTGRFIWNFKTGSVLASIGIIAGLLTGMLGV
GGGFVIVPALRKVTNLDMHSIVATSLMIIFLISGMSIVMHIAEGFHYPVAITSAFALA
CAFGMLLGRRAIRFIPSAIVQRVFALMVFAVAIYMVIKIVNLTNI" /product="putative membrane protein"
/transl_table=11
CDS complement(89959..91614)
/locus_tag="AUO97b_00231" /db_xref="SEED:fig|400667.82.peg.91"
/translation="MELKRVNQDFYVAGQITANDIVKIADQGIKTLICNRPDGEGADQ
PNVIEIEEAAQQYGLNVIYQPVISGKITDQQVTEFKQLYQNAQKPVLAYCRSGMRAIS
LWALAEVAPQDAALLVESGNKLGFNLKGLVPRILKRDHEPATIPCYSVVIVGAGAAGI
SVASSLLCREPHLDIVIIDPADTHYYQPGWTMVGGGIFKPQVTARSMVSVIPSKVKWM
KAAVAGFDPEHNQVILEGCQPIQYKALVVCPGLKLNWHGIEGLVETLGKNGVTSNYRY
DLAPYTWELVQQLNSGTAIFTQPPMPIKCAGAPQKAMYLSADYWLKQGKLKDISIHFY
NTGAVLFGVKEYVPALMQYVEKYGTELHFNHQLVKVDGSAKKAWFKVVNDENAALVET
DFDMLHVVPPQQAPDFIRASTLTDEASWVSVNPQTLQHTQHANIFALGDVMNAPNAKT
AAAARAQAPIVAVNVIAQLKGEQNFCEYNGYGSCPLTVERGKIVLAEFGYGGKLLPSF
PKWVIDGQKPSRLAWLLKEQILPPIYWQGMLKGREWMVKPERG" /product="Sulfide:quinone oxidoreductase, Type II" /transl_table=11
CDS complement(91624..92319)
/locus_tag="AUO97b_00232" /db_xref="SEED:fig|400667.82.peg.92"
/translation="MFFKQFFEKESSTYTYMLGCEETRGAVLIDPVASDIEIYAKELE
QHQFTLIYTLDTHVHADHITAADLLRERFHCKSVLHRNSEVSCGDILITDGCMLKVGN
LSIEARYTPGHTNACTSYLVGNMIFTGDALLIDGCGRTDFQQGNAGTLYDSIHKQLFS
LPDNTILYPGHDYKGRLSSTIGYERLNNSRLGQNRSREDFIELMNNLNLPYPKQIDKA LPANQACGSISQL"
/product="MBL-fold metallo-hydrolase superfamily" /transl_table=11
CDS 92512..93783
/locus_tag="AUO97b_00233" /db_xref="SEED:fig|400667.82.peg.93"
/translation="MAKLNFELEQDFYILFEQLKALFPNASLFLADTTTQAIHYKFNT
DDISVENIAALTQDHHNQYLEINTPKMRKRFIYQEHDIYIAEHRIIGKLHILQPVSIE
NDIVDEVELTEQLQDLAKVFNSCSPEMTRMFSIIQRVAITEFPVLVRGESGSGKELVA
KAIHDYSQRKNKIFVAINCAALNSNILESELFGHVKGAFTGAIRDHKGVFERAAGGTL
FLDEIAEIPLELQAKLLRVLETGEFTPVGGEKPIQANVRIITATHKALREEAKAGRFR
HDLLYRLRVIPIFIPPLRERKVDIPLIVDQILKESSTQLDTMPRHVNQKAMQVLLNYD
WPGNVRELKNTLLYALTMANGKDEIEVEDLPNELLDADEYVKLPENNAELTPENVQAA
LEKYNHNLNMVANIFGISRTTLWRYRQKHKL"
/product="Transcriptional activator of acetoin dehydrogenase operon AcoR"
/transl_table=11
CDS 93853..94764
/locus_tag="AUO97b_00234" /db_xref="SEED:fig|400667.82.peg.94"
/translation="MIVRDKSNVFKLLFAWKGTILPKVLPALSFVVLISACVVWLSYY
HWIQIPTVPAIGFTIFGVILSICTGFRNNACYDRWWEGRKLWGALIANARHIVRDSHV
LSNEKREHLIHQVLIFSNLLRDRLRQQTVEPTKFLEHAYLNNSSLNYLNEHINAPQFV
LENIQKDLVKALKDGEISDIIYSTLNRHIIELGNIQAGCDRIAGTPLPYSYSVLLHRA
VYCFCFILPFSLEAALGIWTPLIVGLIAYMFLGLDALSAQIEEPFGLQENDLPLDSIV
RLIERESLSSLGQPLPPIIEAQNNNLL"
/product="probable membrane protein STY1534" /transl_table=11
CDS complement(94812..95870)

/locus_tag="AUO97b_00235" /db_xref="SEED:fig|400667.82.peg.95"
/translation="MKAARFYDRGDIRIEDIPEPEVTPGTVGIKVAWCGICGTDLHEF
MEGPIFIPPCGHPHPISGESAPITMGHEFSGVVYAVGEGVDDIEIGQHVVVEPYIVAD
DVPTGPGDNYHLSKNMNFIGLGGRGGGLSEKIAVKRRWVHPISNKIPLDQAALIEPLS
VGHHAFVRSGAKAGDIALVGGAGPIGLLLSAILKAKGLKVIITELSAKRKEKAKESGV
ADYILDPSEVDVVSEVMKITNGDGVDVAFECSSVNKVLDTLVAAVKPTGVIVIVSIWS
HPASINVHSVVMKELDVRGTIAYVNDHQETIKLVEEGKINLEPFITQRIQLDDLISQG
FETLIHNNESAVKIIVHP"
/product="2,3-butanediol dehydrogenase, R-alcohol forming, (R)- and (S)-acetoin-specific (EC 1.1.1.4)" /EC_number="1.1.1.4"
/transl_table=11
CDS complement(95900..96685)
/locus_tag="AUO97b_00236" /db_xref="SEED:fig|400667.82.peg.96"
/translation="MVKGLKNKVVLVTGAAQGIGRGIALRLAQEGAHIALVDMKQDRL
NDVQQEIQALGVNASTFIADVSDREQVYAAINHAEQALGGFDVMINNAGIAQVQAIAD
ITPDEFQKIVDINIGGVLWGIQAAAAKFKQREHKGKIINACSIAGHDGFALLGIYSAT
KFAVRALTQAAAKEYASHGITVNGYCPGIVGTDMWVEIDKRFAEITGAAIGETYKKYV
EGIALGRAQTPDDVAALVAFLASEDSDYITGQSILTDGGIVYR" /product="2,3-butanediol dehydrogenase, S-alcohol forming, (S)-acetoin-specific (EC 1.1.1.76)"
/EC_number="1.1.1.76"
/transl_table=11
CDS complement(96712..98115)
/locus_tag="AUO97b_00237" /db_xref="SEED:fig|400667.82.peg.97"
/db_xref="GO:0005737"
/db_xref="GO:0006118"
/db_xref="GO:0015036"
/db_xref="GO:0016491"
/translation="MSEAQFDLIVIGAGPGGYVAAIRAAQLGLKTAIVEERHLGGICL
NWGCIPTKALLAGAELATQFKHAGQFGFQVSQLDFDIQKLVQHSRQVSAQLVQGIDYL
LKKNQVTVFSGRAQLTAKEKIEVTDAQGNRQALSAPHIILATGAKARHVPQLPVDGTY
VWSYKEALVPEQLPKSLLVVGSGAIGSEFASLYQDLGCQVTLIDLAKQILPTEDVEVA
QFVRKQFEQKGMKVLTDAVVQSIQIENEQVHCVVETANDVQTLVFDRVLSAIGVQPNT
TGLGLERLGVELNPQGFVAIDDYCKTNVAGLYAIGDVAGAPCLAHKASHEAMICVEKI
AGVKNVHSLDRSQIPGCIFTHPQVASIGLTENAAKAKNLPIRIGKFSLTANGKALAIG
DASGFVKTVVHAETGELLGAHMVGHEVTEHIQGFAIAKYLEATDESLAQVIFPHPTLS EAMHESILASMQRAIHM"
/product="Dihydrolipoamide dehydrogenase of acetoin dehydrogenase (EC 1.8.1.4)" /EC_number="1.8.1.4"
/transl_table=11
CDS complement(98126..99616)
/locus_tag="AUO97b_00238" /db_xref="SEED:fig|400667.82.peg.98"
/db_xref="GO:0005515"
/db_xref="GO:0008152"
/db_xref="GO:0008415"
/translation="MEEGTIAQWLIKEGDSFNKGDEICEIETTKIVNVLEAPFAGTLR
KILAKDGDTLPVGGLIAVCADSEVSDAEIEKFIASLGGSAAQAPEAPSEQSKAETSAP
VAEKTEQPQTVAASASAPAKVAKEDYAVPESLQGYQTSNELFATPHALKLAEKHNVNL
AKVTGSGREGRISVQDIQKAVQAAGGQWPDVKQQTQAKVVKSTADDSQVLATPVARRL
AKQWGINLNDCRVSGTRGRVCKEDVEAVYYRNNPTSVNEQPVQCAAQPQSTVTTVAMN
GMRKAIASRLQAAKRNAPHFRLVVDLNVEALQKLRKQINETVPQVKLSINDMLIKAAA
AALIKVPEVNVQFDEATQSILQFSQADISVAVAIPNGLITPIVKAANQKSLAQISDDM
RDLATRAKTGKLQPDEFQGGSFSISNLGMLGIKQFDAIINPPQGAIMALGASESRAVV
ENGNVVVREIVTATLSCDHRVIDGAVGAKFLASFKQFVENPALILV" /product="Dihydrolipoamide acetyltransferase component (E2) of acetoin dehydrogenase complex (EC 2.3.1.12)" /transl_table=11

CDS complement(99663..100682)
/locus_tag="AUO97b_00239" /db_xref="SEED:fig|400667.82.peg.99"
/translation="MPNKSFRNAIKEAIESEMRRDPTVFVVGEDVRGGHGGKNTEENQ
LEGFGGVLGVTKGLWTEFGSERVIDTPITESAIIGMAAGAAATGLRPVADLMFMDFYG
VCHDMLYNQAAKFRYMFGGKAKAPMVVRGMIGAGFSAAAQHSQSPYNVFAAVPGLKVV
VPSSPYDVKGLLIQAIRDDDPVVFCEHKMLYDIKGEVPDDAYTIPFGVANYTREGTDV
TIIALGLMVHRANEVADKLAKDGISVEVVDPRTISPLDEEGILESVASTGRVVIVDES
AARCGFGHDVAALIAQKGFHYLKAPVELVTPPHTPVPFSPVLEKEWIPSVERIEQAVR KTLEA"
/product="Acetoin dehydrogenase E1 component beta-subunit (EC 2.3.1.190)"
/transl_table=11
CDS complement(100696..101658)
/locus_tag="AUO97b_00240" /db_xref="SEED:fig|400667.82.peg.100"
/translation="MQLTEEQLLAAYKRMRDIREFEDRLHEENTNGDIPGFIHLYSGE
EAVAVGICENLTDKDYITSTHRGHGHCIAKGCDIHAMMLEIFGKDDGLCRGKGGSMHI
ADLDKGMLGANGIVGGGPPLAIGAALTAKTLKTGGVGLSFTGDGGSNQGLTFEAMNMA
VVLKLPVIFVFENNGFGEGTGHDYAVGSKDIAGRAAGFGLPAVKVDGTDFFAVYEAAQ
TAIERARRGEGPSVIETITNRFYGHFEGDPGLIRSKEEVEFIKENKDPLKIFREKVKG
KIDEAKLDEIDAASKANVDDAVAKARAAAYPKPEQLLTDVYVSY" /product="Acetoin dehydrogenase
E1 component alpha-subunit (EC 2.3.1.190)" /transl_table=11
CDS 102023..103009
/locus_tag="AUO97b_00241" /db_xref="SEED:fig|400667.82.peg.101"
/db_xref="GO:0003824"
/db_xref="GO:0005506"
/translation="MDLKRQIETGIKLRGIDKVSRIPIKILPTEELPKKPEWIRAKIT
DFEEIQRIKNLLRQQKLHSVCEEAACPNLPECFGGGTATFMIMGDICTRRCPFCDVAH
GRPMPLDENEPKHLAETVANLNLKYVVITSVDRDDLHDGGAAHFVKCIEEIRNRCPDT
LIEILVPDFRGRLETALSTLSLSPPDVFNHNIETVPRLYKAMRPGSDYQHSLNLLKRF
KKYCPDIKTKCGLMVGLGEIEAEVIALLNDLKDHDVDLVTIGQYLQPSKSHAPIHRFV
NLQEFERYTQHGQRLGFKNIWSAPMVRSSYFADRQYFGESAPKPFSREDVLS" /product="Lipoyl
synthase (EC 2.8.1.8)"
/transl_table=11
CDS 103167..104306
/locus_tag="AUO97b_00242" /db_xref="SEED:fig|400667.82.peg.102"
/translation="MDMSKQLLLPSTDNLPSFFRPETISETPCLGKKPSLDLDAAPIF
DLDHEWQHSLFGRSIQECHRNLMHIAEDADMAIGVTDPHGTLLWTWSSTPMRSSAEQV
HFVEGGQWSTQAVGQNAIGLALNTHSASCVYSHENQMNSVRDWVCYAAPITDANTGQF
YGIMNLSTKYQKHNSLGILAVERCADIIKQAIQLHQKNILYIKALGTPKVQYNQHGLT
LTQRQIEILCILALCPEGINLEELHYALYGDRPVSTTTLKAELSQLRNLIPDVIESRP
YRLNCEIQCDFLMAEQALNLGFTATTLTLYRGSFLAKSESPFLCAWRDCFDARLSHVI
YQIDDVDQLLRVVSRVPDRVDAVERLLELLPEETPYRAKLLKLLE" /product="Transcriptional
regulator, ArsR family" /transl_table=11
CDS complement(104353..105105)
/locus_tag="AUO97b_00243" /db_xref="SEED:fig|400667.82.peg.103"
/translation="MTALNQYGMSLEITPHNGWSQRFWEDLLPVKERVSKHPFFTEMA
NGGLSLESFRYALLNFYPLVAHFPSYMAGALAKATAFAEPGVTETRDWLIQNIKVEER
HLQWYRDWARGFGLTVEQLDSVRPPASMNAVNHFLWNVSHRGNLAECLAATNLAIEWA
TGDWSIQVYKGIHAYTDHPEVTIDKRSLAWLRAHAHYDDLHPYEAMELIKRLCNERPD
WQQKAFHAAEEGLRYYELALDDCYRVQLQASA" /product="Pyrroloquinoline quinone
(Coenzyme PQQ) biosynthesis protein C" /transl_table=11
CDS 105426..106655
/locus_tag="AUO97b_00244" /db_xref="SEED:fig|400667.82.peg.104"
/translation="MKLQGSNILGQEQIDLLTTRGLNFVWFPKQLETIYRFQYQNGAA
YEFRYRAPIILILYLFLSFGIYQVLPTEQVLSWLSYYSWVGIIVLIAWILSFIKKLNQ
WFDYYVGIGSSAAVAITFILINVLENGQDNVLFHAAMMYAIVIIYGAVGMRFYTAIIA

GWVGGLIGILVSTYLNGDIDWTFLNRTYTFSSFLGMTLAYATDRQHRENYLQNCMIEL
NRIELMQQAQQLSLLSQQDALTGLANRRYLDETLDNEWRRALRHETPLTIMMVDIDFF
KPYNDSLGHLKGDQCLKDIATAISSIAARSGDLVARYGGEEFLLLFPMTNAQQAKIQA
ERLMNAIKKIAIVHPCSSVSPYVTISVGVATTIPRLNDSISAFVSRADHALYQAKTNG RNQYQIALNEEQIVDLT"
/product="Phytochrome-like protein; Cph2"
/transl_table=11
CDS complement(106752..107843)
/locus_tag="AUO97b_00245" /db_xref="SEED:fig|400667.82.peg.105"
/db_xref="GO:0004107"
/translation="MAGNSIGQLFRVTTCGESHGVGLMAIVDGVPPGLALTEEDLQKD
LDRRKPGTSKFATQRKEPDQVEIISGVFEGKTTGTPIGLLIRNTDQKSKDYGNIAQTF
RPGHADYTYTQKYGFRDYRGGGRSSARETAMRVAAGAIAKKYLAEKFGVLIRGHVTQI
GNEVAEKLDWNEVPNNPFFCGDVDAVPRFEALVTSLREQGTSCGAKLEILAEKVPVGW
GEPVFDRLDADIAHAMMSINAVKGVEIGDGFAVAGQFGHETRDELTSHGFLANHAGGI
LGGISSGQTIRVAIALKPTASITTPGKTINLNREDTDVLTKGRHDPCVGVRATPIAEA
MLAIVLMDHFLRHRAQNADVVPPFAPIEP"
/product="Chorismate synthase (EC 4.2.3.5)" /EC_number="4.2.3.5"
/transl_table=11
CDS complement(107856..108860)
/locus_tag="AUO97b_00246" /db_xref="SEED:fig|400667.82.peg.106"
/translation="MERPTISPEHLQEAAENLSTIRDFIRFGVSALRQHDAHLGQGTE
DYFAESSALVLQTLSLEWSADSEILDAKLLPSEKAEFLSLLERRINERIPTSYLLNLA
YFFNKPFYVDERVLIPRSPIAELIEQRFAPYCLDENGQMREALNNLPENTKPKTPQRI
LDMCTGSGCIAVALAYAYPDAEVDATDISKEALEVASINVEHHNKQYQVALLESDLFE
KIPAENQYDLIVSNPPYVDAEDMADLPEEFLHEPELALAAGQDGLDLVRKMLAQAADY
LTEDGLIVIEVGNSEWAMRQNFNTVDFNWLTFQKGGSGIFALTAEQCRRYRKLFEEQV "
/product="Ribosomal protein L3 N(5)-glutamine methyltransferase (EC
2.1.1.298)" /transl_table=11 CDS complement(109097..110302)
/locus_tag="AUO97b_00247" /db_xref="SEED:fig|400667.82.peg.107"
/db_xref="GO:0004834"
/translation="MQHQINGIALPNEQGFFGEYGGQFIPPHLKEAMDEINVAYEEIR
HTPEFQNELADLFANYVGRPSPLFYAKRLSEQLGGAQIYLKREDLNHTGAHKINHCLG
EALLAKYMGKKKIIAETGAGQHGVALATACALVGIPCEIHMGQVDIEKEHPNVVKMKI
LGAHLVSVTRGTATLKDAVDSAFEEYLKDPKNFIYAIGSVVGPHPFPKMVRDFQSIIG
KEIKEQTHQRFGKNPDYIVACVGGGSNAIGAFTAFLNDSDVKLVGVEPAGHGLDTNMH
SATLTLGKPSQIHGMACYVLENEAGEPLPVHSIASGLDYPGVGPQHSFLKDLGRVEYS
TATDQECLDAFMTLSRVEGIVPALESSHAVAWAIREAPKLPKETMIVVNLSGRGDKDS DYVAEKLKL"
/product="Tryptophan synthase (indole-salvaging) (EC 4.2.1.122)"
/EC_number="4.2.1.20"
/transl_table=11
CDS complement(110339..111526) /locus_tag="AUO97b_00248" /db_xref="SEED:fig|
400667.82.peg.108" /translation="MFVRLFYTLRKYGVPVTTRELIDLNRAVSEGLVFADQEDFYQLA
KTILVKDERFFDKFDRAMKDYFDGIETFDIDELLKQVHKLPKDWFDLELLEKHLTPEQ
RAELQKAGSLEELMKMLEERLREQHKKHQGGNRMVGTGGTSPFGAFGDHPEGVRIGGP
GRKRSAVKVWEQRKYQNLDDDQVLGTRQMQIALRRLRKFARQGAAEELDVDGTIRETA
KQGILDVQMVPERRNRIKVLMLFDVGGSMDAHIAQCEKLFSAAKTEFKTLEYFYFHNC
LYDYVWKDNYRRTATRMNTWDLFHTYGRDYRVIVVGDASMAPYELKSVGGSVEYMNDE
AGEVWLRRLRQHFDKTAWLNPETEGYWHYTQTIGWIKEIFENHMYPMTLKGIEDLTRY LSR"
/product="hypothetical protein"
/transl_table=11
CDS complement(111542..112393)
/locus_tag="AUO97b_00249" /db_xref="SEED:fig|400667.82.peg.109"
/translation="MSADTQHFTGTDQYIATDSLKLAVKAARALQKPLLVKGEPGTGK
TLLAEQVAESLDLKLITWHIKSTTKAQQGLYEYDAVSRLRDSQLGDDRVYDIKNYIKP

GKLWEAFTSEERCVLLIDEIDKADIEFPNDLLHELDKMSFYVYETGETITATQRPIVI
ITSNNEKELPDAFLRRCFFHYIEFPDEATMREIISVHFPNISATLVNEALQVFFKLRE
IPNLKKPPSTSELIDWLSLLMADDMPEDVLRNRDTSKAIPPLYGALIKNEQDVQLLER LAFMSRR"
/product="ATPase associated with various cellular activities, AAA_5"
/transl_table=11
CDS complement(112423..112809)
/locus_tag="AUO97b_00250" /db_xref="SEED:fig|400667.82.peg.110"
/translation="MREDQAGILNQLLSSLDAIPEEIIALTAYGVGAIIALWCWWRLM
RRLPTTLGAISWLVVFAILVTPTVSEGPNASVAPAIFGLLFGVLTKDSPLIWSNLSLI
LFVIGLGLVIGYCWSKYITNKSMRSI"
/product="putative membrane protein"
/transl_table=11
CDS complement(112802..113107) /locus_tag="AUO97b_00251" /db_xref="SEED:fig|
400667.82.peg.111" /translation="MHCDIYRSSKKDEMYIYIARPNYPDETEQADPFEKVPEAVLQAF
GRATFVMHLELAPTRKLARVNVLHVLDSLQTKGFFIQMPPEGLINPNAVEPEGLRGA" /product="Protein
YcgL"
/transl_table=11
CDS 113550..113714
/locus_tag="AUO97b_00252" /db_xref="SEED:fig|400667.82.peg.112"
/translation="MSRGDKSSYTAKQKRQAKHIVESEVDRGHSQEEAERIAWSTVNK QDGGGKKKSH"
/product="hypothetical protein"
/transl_table=11
CDS complement(113785..114120)
/locus_tag="AUO97b_00253" /db_xref="SEED:fig|400667.82.peg.113"
/translation="MNFQTILSSFKNQSTGTDAFKNLKSACEHHLKHSSDLNEKAVIY
LIYGFARSYVILYEDEAVTTEFAQASKEMLVNYMNCLNEALRTQDDHIILNTLNQVSN DYMQGSRIF"
/product="FIG00353503: hypothetical protein" /transl_table=11
CDS complement(114257..114523)
/locus_tag="AUO97b_00254" /db_xref="SEED:fig|400667.82.peg.114"
/translation="MTNNFNAEQARKNAKDVKIDQALLLEKILSGTEEISKEGKRRAV
FQFPIDALHPEHITQVEEELKTRGFNVSTNIEQSGTTVNIEVSF" /product="hypothetical protein"
/transl_table=11
CDS complement(115026..116150)
/locus_tag="AUO97b_00255" /db_xref="SEED:fig|400667.82.peg.115"
/db_xref="GO:0004525"
/translation="MFQFIQQQTELVDVLQQMDQCSTYGLDTEFIKVDTLWPKLGVCQ
VNVNGNVYLLDGVSLDLSEFWKKIFLAQQNIFHACGEDIDLIYHYADEQNLLNVFDTQ
VGLSFLGHGLQVSYQGALKLCLEIDIEKDQTRSDWLARPLSPQQLCYAANDVLYLMQL
ANHIKDQLKQKGLYEYVLEDCSSLTKEIISETPTPLLYTDVGNYRHSRRQLMQLQNLS
EWREEVVRATNQPRSFILRNSTMIDLVEKNPRNSFQLSQVKDIRPNVVREYGKIILDL
LKDLPVEGHWPAKIAKPFKITSKETLNKMDVLIAKAIEQTSIPKEVLLRKKWLNAIHQ
HVLTKGDEQDLPDYLLGWRYELLTKPLIQLFKEEKASLLF" /product="Ribonuclease D (EC
3.1.26.3)" /EC_number="3.1.26.3"
/transl_table=11
CDS complement(116456..117052)
/locus_tag="AUO97b_00256" /db_xref="SEED:fig|400667.82.peg.116"
/db_xref="GO:0003676"
/db_xref="GO:0006281"
/db_xref="GO:0006304"
/db_xref="GO:0006310"
/translation="MFSDRFEQLVQALRILPSVGPKSAQRMALHLLMKNREGAFALAH
ALHEASSYIHECSVCHSLTEHEICDICASTERDDQLLCVVESPADVMAIEQSGSFRGK
YHVLGGHLSPLDGIGPEEIGIPYLIQRLSQGTIEEVILATNATVEGQATAHYLVEATK
HLPIHMTRIAQGVPQGGELEYVDSHTLSQAVHNRMRMK" /product="Recombination protein RecR"

/transl_table=11
CDS complement(117062..117391)
/locus_tag="AUO97b_00257" /db_xref="SEED:fig|400667.82.peg.117"
/translation="MNINMLMQQAQRMQKDMESNIKKAKEELAQTEVHAEAGGGLVKV
TMTGRYIVKRIEINPELLQDEPDMIEDLIAAAVNDAVRQAEVVSEEKMQKANSGMGLP PGLAGMF"
/product="Nucleoid-associated protein YaaK" /transl_table=11
CDS complement(117657..117893)
/locus_tag="AUO97b_00258" /db_xref="SEED:fig|400667.82.peg.118"
/translation="MLKPIIILVAIGAIGLVGYKVFAQKDEPTQINETVLLINDANKS
VEEADHTIEKANASFQKIPLNEYKPDNSTLQASQ"
/product="hypothetical protein"
/transl_table=11 CDS complement(118071..119282)
/locus_tag="AUO97b_00259" /db_xref="SEED:fig|400667.82.peg.119"
/db_xref="GO:0003961"
/translation="MTSKGEHLMNQSDNLEYQLDTLAIRTGHTRSFEGEHGEPIFLTS
SFVYENAAEAAAKFSGQVQGNIYSRFTNPTVSMFEKRLAALDGAERAVATSSGMGAIL
SIAMAYLKAGDHVICSRAVFGSIVALFEKYIAKFGVDITFVDLRDLDAWKNAVRPETK
LLFVESPSNPLAEIADIQALADIAHANGALLAVDNSFCTPVLQQPLKLGADLVIYSAT
KYLDGQGRALGGAVVGNHQLLEEVFSVVRTLGPSMSPFNAWIFLKGLETLRLRMKAHC
ENAQKLAEWLSTHEKVEKVYYGGLPTHEGHELAAKQQNGFGGIVSFVVKGGREGAWKV
IDNTKFISITGNLGDVKSTITHPATTTHGKLSAEAKQAAGIQEGLIRVSVGLEDINDI IGDISRGLDLI"
/product="O-acetylhomoserine sulfhydrylase (EC 2.5.1.49) @ O-succinylhomoserine sulfhydrylase (EC 2.5.1.48)" /EC_number="2.5.1.48"
/EC_number="2.5.1.49" /transl_table=11
CDS 119392..120477
/locus_tag="AUO97b_00260" /db_xref="SEED:fig|400667.82.peg.120"
/db_xref="GO:0008415"
/db_xref="GO:0042619"
/translation="MKRLKSLVSEQSQIKHLSTRLFRPQTLVLSQSTPFEVIGEFNQT
RVRYYAATEKSFREPLIFVAPLAINMAIYDLYPYRSLIKYFQNAGFDVYLVDWGRLGF
KDRHLNFLSFIEDFIPKAIEFVRTHSGSDQISLHGWSMAGIFVTLYTAHNHPNYVKNL
IVLGSPIDSYASGYIGKLYRTINKTIARNKKLQERIYSGLPKRLIHTPGILNSLGFKI
LDPKGWFDGHIQLLKNLNDLQFVQEHATLSSFLNNMIDYPGGINQDMLFNVWLQNPLR
QGSIQLKDKKIELKNIDCSLLVGAGRSDQLVTADAAQPLSQLTSSQDVTFTLIPGGHL
GLMSSQASAQEFWPKLATWLSERSTKI"
/product="Polyhydroxyalkanoic acid synthase" /transl_table=11
CDS complement(120558..121325)
/locus_tag="AUO97b_00261" /db_xref="SEED:fig|400667.82.peg.121"
/translation="MLNQHEINLKQYQNKSLSYLNSTAHSEGVEFDKFIKEIDEYPNA
VVLDLGCGGGHVAYNVARHADLVFAYDLSHEMLDTVSKAANARKIKNIFVQQGIAEDM
PFTDEQFDVIISRYSAHHWQHVPTAMKEINRVLKPNGTVIFVDIISSSFPILDTFLQT
IEVIRDPSHVRNYSIKDWVHFIEDAGFELTTLEKQTLKLDFDSWVQRMKTPEDQIKTL
KYLQENAADVVKKYFNIQKDGSFESKVGYFVFKKLSF" /product="SAM-dependent methyltransferase YafE (UbiE paralog)" /transl_table=11
CDS complement(121500..121874)
/locus_tag="AUO97b_00262" /db_xref="SEED:fig|400667.82.peg.122"
/translation="MKNVIILTSILLLSGCTINSKLLSPAKSDDDKIIVEAEKYKQQY
QKKIFKIWNVPPSSVGMSASVKVFLSDTGEIEQIIFLDKEDQKFKSSIEKAIWRASPF
VLPSNPEVRKQVRKFNIKFTAK"
/product="hypothetical protein"
/transl_table=11 CDS complement(122053..122277)
/locus_tag="AUO97b_00263" /db_xref="SEED:fig|400667.82.peg.123"
/translation="MKRLLLVLATGFGLDGCASLTQKADENYITKSPVTPVNTAFNAT
KVLEHGIFTEQDIADMPEHSSINSRYKISF"

/product="hypothetical protein"
/transl_table=11
CDS complement(122777..122977)
/locus_tag="AUO97b_00264" /db_xref="SEED:fig|400667.82.peg.124"
/translation="MIDEEKPLNFEDDDEPLDFEDEEFIDDKKEDEMYNSITKDGSSV
DPADDGTRHIRPEDGDPIEIDE"
/product="hypothetical protein"
/transl_table=11
CDS complement(123108..123533)
/locus_tag="AUO97b_00265" /db_xref="SEED:fig|400667.82.peg.125"
/translation="MSNIGSCLCHKVKFSVPCEIKTVYHCHCSLCRKQTGTGANAATL
VKENQFNWLMGKEYVATYKKETGFTSSFCNRCGSPVPNKINGTNYMWIPLGLLEQPFK
PDLKLNFCIESKHDWVVLGDAYKNFAHLPELEEFLKYFE" /product="FIG00350723: hypothetical
protein" /transl_table=11
CDS 123798..124067
/locus_tag="AUO97b_00266" /db_xref="SEED:fig|400667.82.peg.126"
/translation="MNKLMVAVFAGMVTLAGFSAQAATPEQECQKLQDDYNLIYASKG
FCFKDQDAKEKYGNENCHTTKPKFSDKEQQRLDAIKERQKELKCK" /product="hypothetical
protein"
/transl_table=11
CDS 124086..124499
/locus_tag="AUO97b_00267" /db_xref="SEED:fig|400667.82.peg.127"
/db_xref="GO:0004081"
/translation="MAYDDQNIFAKILRGELPAIKLYEDDQVLAFMDIMPQADGHALV
IPKTPAVTLLDLPPEAAAYTIQIVQKIAKAMETALNLDGIVLMQLSGAAAGQTVPHVH
FHLIPTNVHQLGKHAAQLGDQDKIKALAEKIKAAL" /product="Bis(5'-nucleosyl)-
tetraphosphatase (asymmetrical) (EC 3.6.1.17)" /EC_number="3.6.1.17"
/transl_table=11
CDS 124754..125860
/locus_tag="AUO97b_00268" /db_xref="SEED:fig|400667.82.peg.128"
/translation="MKKLLLAAAVATLSVNAVQAAPTLYGKLNVSMNQVDNKNFDGKS
DVTEVNSNSSRIGVKGEEKLTDKLSAVYLAEWAISTDGSGSDTDLSARNRFIGLKTEG
VGTLKVGKYDSYFKTSAGSNQDIFNDDTRLDITNIMYGENRLDNVVGFELDPKLLAGL
TFNIMAQTGESTSDSKKGETGKDSKNDSFDSVSTSLGYENKDLGLAIAAAGDFGIKGK
YAAYGLKDVYTDAYRVTGSYDIAKSGFVVGALWQHAEPTDDLTAYGQTYKSDGSIDKA
GKAYRGLEEEAYAVTAAYKIPNTKIKVKAEYASAETQVSGQADRKIDLYGLGLDYQIN
KQARFYGIVAQQKRDWLNDDDKQTVVGTGIEYNF"
/product="outer membrane porin, putative"
/transl_table=11 CDS complement(125969..126559)
/locus_tag="AUO97b_00269" /db_xref="SEED:fig|400667.82.peg.129"
/translation="MTNLSNIVEVLAKQALGGNQQASGQSGLGGILGSVLGQMGGNTS
SGAQGGLGGVLGSVLGQVTGNNNTPQAGGGVQSLLIAVVPLILGWVQQQGGLQAALEK
LKGAGLGSQVQSWVDPNQSNSEVPTQQLQSLFNPADIEQVAQQAQAPKEQVYGAIASV
LPQVIDSLTPQGESTDHQEANQDIQNVMNLVSGFLK" /product="hypothetical protein"
/transl_table=11
CDS complement(126659..127120)
/locus_tag="AUO97b_00270" /db_xref="SEED:fig|400667.82.peg.130"
/translation="MAEQNVITSMLDDLSNEQPIHTALCIGQKIDQNNAIQWHYFTVT
ELLSLPFTQRYDLGFVLFDTDEMQDISDVQKSQLLVKLRDLLAKRIVVVSKRSDEQLL
RSLGFTQLIDKTSHDSDFALWQFNILTYKHVPDWFNSKFWANPENWNKFRW" /product="FIG00350803:
hypothetical protein" /transl_table=11
CDS 127187..128029
/locus_tag="AUO97b_00271" /db_xref="SEED:fig|400667.82.peg.131"
/translation="MHAGLKDASVTDQQTTKANVEYKFVLDRSEIDLPFNLGQELEIE

WTGNIYCTSCGAKTPKSYSQGHCFKCLKTKAECDLCIMKPETCHYHLGTCREDDFAHS
VCFQPHIVYLANSSALKVGITRVSHMPGRWLDQGATQALPILKVGSRRLSGQLETMFG
SLIADKTDWRKLLKGEAEPLNLIEQRDQLIEEFAPKIQTIREEFSQNLEFNETVELLE
NELPREFVYPVEQYPEKIKSLNLDKTPKIRGVLQGIKGQYLIFDIGVINIRKYTGYEL IVRA"
/product="hypothetical protein"
/transl_table=11
CDS complement(128079..129197)
/locus_tag="AUO97b_00272" /db_xref="SEED:fig|400667.82.peg.132"
/translation="MWQQLITWLKQLSFLVKKEFLTIFSDPANRAILFVPALMQALLF
GYAATYDVNNVDYAILDQSNGQVSHELISKLDGSGIFKRVATLEYTEQIKQVIDNRQA
LLVITIPNDFESKLNNNQSSPIQIIVDGRNSSTAMVAGSYLSKIIGQFNQQKFNSALP
ISLETRTWYNPNQESRWSLMPALIAALSMMQTLLLSALSVAREREQGTFDQLLVTPYT
PLQIMIGKALPPIFVGLMQSTIILLIILFWFKIPMNGSIGLLYFGLFSFNVAVVGVGL
SISALSLNMQQAMLFTFLLIMPLMLLSGLLTPVENMPKALQVATYANPLRFGINLVQR
VYLEGASFAQVKLNFLPMIVLGIVTLPLAAWLFRNRLS"
/product="ABC-type efflux pump permease component YbhR" /transl_table=11
CDS complement(129202..130326)
/locus_tag="AUO97b_00273" /db_xref="SEED:fig|400667.82.peg.133"
/translation="MSFAFWSRLKALVRKETKQLLRDKSSLGIGLVLPVILILLFGYG
LSFDLNQARVGVVVDQSSPQVNQVLAGLNGSRYLTSLEFHNLPEAEQAIRNGKIDAIL
HLPSDFANQAQQGNAKVQLLLNGRSTTIATALEGYVAGALATAPSIQIDRSPILASAS
AVKIEQRIWFNESGNSTWFLVPGLMVLILTLIGAFLMGLLIARERERGTLEALFVTPV
RPFEIVLAKLIPYVVVGMIDIVICIVAAHFIFEVPMRGSLFSILFASFLYLIVSLLLG
LTISGFAQSQFQASQIALLASFMPALMLSGFVFDTRNLPLVVQIISQLLPATHFMVLI
KTLFMGGDDWKLWLKECGILLGYIVVLICAVNFSLKKRLR" /product="ABC-type efflux pump
permease component YbhS" /transl_table=11
CDS complement(130337..132094)
/locus_tag="AUO97b_00274" /db_xref="SEED:fig|400667.82.peg.134"
/translation="MIDSKAVVQAHNLFMTFKAENKNSADISAIVDLNMQIKKGELTA
LVGPDGAGKTTLLRLIAGLYKASSGSLHVLGFDIEKYPQVVQDRISYMPQRFGLYEDL
SVQENLNLYADLHGVPKNVRNQRFKRLLEITDLTQFTQRLAGQLSGGMKQKLGLACTL
VRSPELLLLDEPSVGVDPLSRRDLWIIIEQLVQEENLSVIISTAYMDEAEKCAYVYIM
HEGKILRQGSPEQLKALVHEQTWQIKPSEQIKTRIVQAQLLGNSGEIIDAVPRGEQVN
FISRQKELSTNILPLGLVANRRPPELEDAFMMLLQQNQKQQISISQQTFRSEQNNNSQ
SDQAVIVVKDLVRTFGDFTAVANTSFTVQRGEIFGLLGPNGAGKTTTFRMLCGLLSAS
SGYLEVAGKNLRTARAEARAKVGYVSQKFALYSNLTVLENLKFFGGAYGLSGKKLDQQ
IDKALQQYDLEPQIKSGDLPGGYKQRLSMAAALLHEPEILFLDEPTSGIDPLARRSFW
YSIGKLANQGITIIITTHFMEEAEYCDRIAIQDAGKLLALGSPQQVRELASKDKHIVD
MNEAFIAIVEQARALRHAG"
/product="ABC-type efflux pump, duplicated ATPase component YbhF"
/transl_table=11
CDS complement(132087..133109)
/locus_tag="AUO97b_00275" /db_xref="SEED:fig|400667.82.peg.135"
/translation="MNKKVIAGILVVIAVVAIGFWVWGYNHKKQLDNDVLTLYGNVDI
RQVSLAFEQSGRIQKLLVQEGDKVQAGQVLATLNTNALQIQAKQAQAQLKAQQEAIIK
QEVGARPEEITQAKAQLASAQAELDKTNKNLQRLQILVSSTDGRAISQQELDYAKSNQ
HSAEAAVRERQANLELLIKGARKEDREATRAQYEVTKANLDLIKYNLTQAELRSPVNA
VVRARLQEVGDMTTAQKAVYTLALTNPKWIRVYVNEQDLSSIKMGGTAQVIRDAYPNQ
PINGKIGYISSVAEFTPKTVQTEEIRTTLVYEVRVYVNDPNDQLKMGQPVTVKVPLAF SEKAHD"
/product="ABC-type efflux pump membrane fusion component YbhG"
/transl_table=11
CDS 133258..133875
/locus_tag="AUO97b_00276" /db_xref="SEED:fig|400667.82.peg.136"
/translation="MTKTKIIEAAGPLIAQYGFAKTANKTIAKVANVDLAAINYHFDG

RDGLYQAVLMEAHAHYLDEQYLLELVESTYSPEEKLSLLLETLLHKLTEKDVWHGKVF
IRELFSPSEHLLSFIELTGMRKFFLIRKLISQVANLDENDPAVLPCILSVMTPCMMLI
IAGPNAQAPEPLKNIAQMPLHDLVEHFKKFSLAGLKAISQSNLKN" /product="Transcriptional regulator YbiH, TetR family" /transl_table=11
CDS complement(133872..135047)
/locus_tag="AUO97b_00277" /db_xref="SEED:fig|400667.82.peg.137"
/db_xref="GO:0008915"
/translation="MANRKLKIGIVVGEVSGDTLGVKLMRSFREQGIDAEFEGIGGPQ
MIAEGFNSYYPMETLSVMGIVEVLKDLKKLFAVRDGLINQWTQHPVDIFIGIDAPDFN
LRLSKSIKEKNLPIKTVQYVSPSVWAWRQGRVHGIKQSIDLVLCLFPFEKVFYEQYEV
PAAFVGHPLAKQLPLENPIQIAKQELGVDENQKHIALLPGSRKGEVERLLPMLLGAAN
ILHTKYPDIQFLIPAINDARKQQIEQGVEQLAPQLKAKIHILENTDSESKIGRMVMNA
SDIIALASGTATLEAMLMHRPMVTFYKLHWLTYLIAKFLVKIPYYSLPNIIAGKKVIE
ELIQADATPENLAAEIEKLMNVETAQIQVMQHLTMHKQLISGNTEDPVQAILQCLNS" /product="Lipid-A-disaccharide synthase (EC 2.4.1.182)" /EC_number="2.4.1.182"
/transl_table=11
CDS 135269..137404
/locus_tag="AUO97b_00278" /db_xref="SEED:fig|400667.82.peg.138"
/translation="MNIPPRPFKLSVIACAICYANLTYAQDAQVQALQTIQVKASDAE
QSSEQTKAYNVKNSSSATKLNIEAKETPQTINVVTRQQIEDFGLTSTRDVLRNTPGVT
VSNQETERTTYMARGFEISNILTDGVGFPLSGYNYNNTNPDTYFYDRVEVVKGADSLT
NAFGDPSATINNIRKRPTQEFQASDGVSYGSWDTQRYEADVSGSILPSGKVRGRIMGY
EQTGDSYLDRYSAEKNGFAGIVEADLTDSTLLTAGYSQEQNKPNANNWGALPLLDANG
KQISYDRSYNPNPDWAHWDNETQNAFVELKQKLNDQWNAKLTYNYLDTKHNSRLLYYY
GYPKADGSGVSLTPWGGQEHQEKHAVDFNLEGTYKLFNREHEATLGYSYVRNHQQDKQ
STGTINDSNVIKSTTTDWASWTPQSITWSAFTEAANYKQNINSIYAATRLHLNEDLKL
LLGVNYVQAESKGESYSSPMSYSESKVSPYVGLTYNFTPEYTGYMSYTSIFRPQTGID
KDTNQALKPIEGKSYEMGVKSSWLDDRLTGTLSVFKTEQNNYPLRNSDGNPLNRKVPT
SDLESQGVEVGLSGQITDNVNLSLGYAQFSIKDTKNGGEARIYNPNQTLNLLTTYTPP
VLPKLKVGAGLQWQDGIKLYDSNVNGTIKQDAYALVNLMASYEVNDHITLQANGNNIF
DKKYLNSFPDGQAFYGAPANYTVAVKFKY" /product="Outer membrane receptor for ferric coprogen and ferric-rhodotorulic acid" /transl_table=11
CDS 137539..137766
/locus_tag="AUO97b_00279" /db_xref="SEED:fig|400667.82.peg.139"
/translation="MGYACMAYLSLGLTEIFHLNLDKPEAIYLAAFISILFYVFFVIV
GFCIQSLLRLTILSIALFALFFIVTRTVGYA"
/product="FIG00351117: hypothetical protein" /transl_table=11
CDS 137783..139315
/locus_tag="AUO97b_00280" /db_xref="SEED:fig|400667.82.peg.140"
/translation="MAWLHSWTGLIFGWLVFAIFLMGSLSYYRHEINLWMQPPLAQFE
IKQDVAIKTAYQYLQEHASDAKSWYLTVATSESPVNTMYWEKPDGSYGNATLDANTGQ
ELKLSATEGGDFFYRFHYQLFGVPILIGRLVVSLAAFIMLIALISGIITHKKIFTDFF
TLRTFKSQRSWLDFHNVSSVIALPFFLTVTFTGLAIFFYLYLPWGMQKLYPENPYQYF
NDIRTKTVTESTTPHPAQNLPVEKLLAQLKQRWGNQTLATISVKNPNTNQAQITFIQQ
EDHSITRNQAQITLDATNAKVLGDTRNHSPIATLYAGVYGLHMATFAQPLLRLGLFFS
GILGCVMIASGLLLWSLKRQLQNKNSQFHFGHYLVDRLNVATFVGLPCATLAYLYANR
LFTVTATTVNYEIYSFFFVWLMSFIIALITKKQYLWQTQLGIFILLCIALPILNISYL
LKNNYIQNFSGYWDFARIDIFFWLFAALAFFIFLKIKPIQHKASDKIQKKLSKLNIEV NS"
/product="FIG138928: iron-regulated membrane protein" /transl_table=11
CDS 139312..139614
/locus_tag="AUO97b_00281" /db_xref="SEED:fig|400667.82.peg.141"
/translation="MMFFVFILTLLGMYLLFISTEKYRSPKSTGHFKPLAQKYYSLLR
ILAFLLFALSGFILIKKYGFSIGFVSWWIFATPLTFLLILWLNPLKPSKTSSKSSI"
/product="hypothetical protein"

/transl_table=11
CDS 139747..140634
/locus_tag="AUO97b_00282" /db_xref="SEED:fig|400667.82.peg.142"
/translation="MSGAFEFILAGMLVGFCVGITGVGGGSLMTPILIGLFRIEPHIA
IGTDLLYAAISKFCGSMVHAKKLNIVWPIVLWLAVGSIPASFGTAWVLEHYLSQSTHY
KAVLTMVLGFMLTLTGVSIIFRTRIEKFFNKFRNKENTQTENEQLAVQNKRTYIVIMG
IILGVFVTLSSVGAGAFGIMALVIMFPNLPMIRIIGSDVVHAVLLTLVAGLGHMSAGN
VDFVLLMWLLVGSIPAIIIGTLISSRMPERLIRKILGITLFALGVNFMVHPVKAKPKA
AVVQQQAVVAEKTGNSTER"
/product="Putative sulfate permease"
/transl_table=11
CDS 140731..141840
/locus_tag="AUO97b_00283" /db_xref="SEED:fig|400667.82.peg.143"
/translation="MIEPVTAMTTHSNSVSKPDIDDVNIKSIQTLVTPAELKSELPLS
DVASQTVLKGRETIRNILDGSDKRLFVVIGPCSIHDPKAAHEYADRLKALSEKVKDTL
YLVMRVYFEKPRTTIGWKGLINDPDMNDSFNIEKGLRIGRKLLLELNEKGLPCATEAL
DPNSPQYYQDLISWSAIGARTTESQTHREMSSGLSSPVGFKNGTDGGLTVATNAMQSV
KYGHSFLGLNEDGQVSIINTKGNPYAHVVLRGGNGKPNYDAGSVAEAENALAKAKVSN
KIMIDASHANSNKDPYLQPLVLKNITEQILDGNKSIVGIMVESHLKGGRQDIPDNLCD
LEYGKSVTDGCIDWETTEKTLLEMHEALKEVLPNR" /product="2-keto-3-deoxy-D-arabino-heptulosonate-7-phosphate synthase I alpha (EC 2.5.1.54)"
/EC_number="2.5.1.54"
/transl_table=11
CDS complement(141917..142660)
/locus_tag="AUO97b_00284" /db_xref="SEED:fig|400667.82.peg.144"
/translation="MTPFWIALQFLTVLPIELKTIPTAQQNGRAILFYPLVGLIIGGI
LFLVTCIFVKLPALLLAAIVLALWIWLTGGLHLDGLADTADAWVGGFGDKLRTLQIMK
DPSCGPIGVLSLVIICLLKFALVYVLIEQHQSLFLICIPILGRVVPSILFLTTPYVRE
KGLGRSLTDHLPKTASWIITGFVLLLPLYWGWQGLIAIIGFLISLVYLRHVFIKRIGG
ITGDTVGAAIEIGETVLMFTFVVSYFYLV" /product="Cobalamin synthase (EC 2.7.8.26)"
/EC_number="2.7.8.26"
/transl_table=11
CDS complement(142767..144335)
/locus_tag="AUO97b_00285" /db_xref="SEED:fig|400667.82.peg.145"
/translation="MNILFKTTVLAASLLLAACNNNDDQEAQTAPSTNPSKYYQTKTP
YQPQQDLKSYEQAPTGFQPVFTELVARHGSRGLSSLKYDLALYNLWKQAKAENALTPL
GEQLGADLEAMMKANILLGYGVEGIRQYGYGNETMTGILEHRGIADRLLQRLPTLLNT
QASILVQSSGVDRAVDSAKFFTAELIKQQPQLKDKIVPLSYTNLSSENVPSVEDGGVD
RFKLYFHSLNADEDLAQPLSVSQQKIYDASQAYQDFEENNKDLAQKLDELTKNTQAEK
TAQLVLSPIFKADFIKKLGTTGYSFSNTGSFTVTSPKGEQITEKGKGKNTIASAVDAA
AYIYELYSISGGMKDELKGIDFDKYMPLEAAKFYAEFNDANDFYEKGPSFTESNKVTS
EIAQGLKQDLFQQVDAVVNKAQPYKAVLRFAHAEIIIPLATSLDLHNMMQPLPLRQTY
NYSTSAWRGEVVSPMAANVQWDIYQNSQGNTLVKMLYNEKETLFKSACNYARYTPTSF YYDYIKLKQCYQMQ"
/product="FIG00351429: hypothetical protein" /transl_table=11
CDS complement(144460..145038)
/locus_tag="AUO97b_00286" /db_xref="SEED:fig|400667.82.peg.146"
/translation="MLRHGESQYSHTLRGHLDDELTAKGWQQMQSTIEQVTNQTWDVI
VSSSLKRCACFAEQLAKTAKLPLLVNHDLKEMYFGEWEGVSTQQIYETSPELLANFWQ
KPSQYCPPRAETLDQFQTRVLKGFQDLLEHMQKQNLQHALVVTHGGVIKLLACLARQQ
PLDDLLKMPAELGKLYSLEFSETDGQLTFKLR" /product="Alpha-ribazole-5'-phosphate phosphatase (EC 3.1.3.73)" /EC_number="3.1.3.73"
/transl_table=11
CDS complement(145052..146107)
/locus_tag="AUO97b_00287" /db_xref="SEED:fig|400667.82.peg.147"

/db_xref="GO:0008939"
/translation="MNWWLESVQQPNLDAKQQAEQHQLQLTKPTGALGDLEQIAITLA
SLQSNAHPQVSHPWITIFAGDHGVVEENISAYPQAVTRQMLQNFTTGGAAISVIAKYH
QAHLQVIDCGTVGEAYEYAGVERHCIRAGTANFAKQAAMNADECRAALELGKKSVDTA
KANGADIYIAGEMGIGNTCSASALACLLLNDTAEQLTGVGTGIGADQLRHKIEVIEKA
IELHHKHVIGDVFKTLCAVGGLEIAAIVGAYIRCAQAGLPIIVDGFISSVAALCAGRM
NPQVRDWMLFGHQSAEYGHRRILQELNADPILKMNLRLGEGSGAGAALALVKMACVLH NQMATFAQAAVSGNKIG"
/product="Nicotinate-nucleotide--dimethylbenzimidazole
phosphoribosyltransferase (EC 2.4.2.21)" /EC_number="2.4.2.21"
/transl_table=11
CDS complement(146109..146627)
/locus_tag="AUO97b_00288" /db_xref="SEED:fig|400667.82.peg.148"
/translation="MLQLILGGARSGKSRLAEQTAISMQLAVTYVATAQALDPEMQSR
IVHHQNQRPAHWSLVEEPLFLAKTLQEIDRPNQIILVDCLTLWLTNLLLLEDQNIQQF
ECEQLLRVLPTLESEIILVSNETGLGVVPLGEISRRFVDEAGRVHQALGQIADKVVFC VAGFPMILKGDK"
/product="Adenosylcobinamide kinase (EC 2.7.1.156) / Adenosylcobinamide-
phosphate guanylyltransferase (EC 2.7.7.62)" /EC_number="2.7.7.62"
/transl_table=11
CDS complement(146615..147241)
/locus_tag="AUO97b_00289" /db_xref="SEED:fig|400667.82.peg.149"
/translation="MVPTPIRDYPEWHLGRQNYALWYLEINDQKLVHYLDQLRAHFSE
YLVEPNHRQYHVTLFICGFLTHETKVHCDDFSFTEFEQHKKVLIQENFAPFQLKIGSV
DSFSSALFVEVQDTENNLSLIRQKLGIVSNEIAALDYCPHITLGLYKKDYSSNLILQK
ISQLPVQYTQAEFDLKVEHLSFGYYQAQILQGQLYPYQHLFLGDSCCN" /product="FIG00350035:
hypothetical protein" /transl_table=11
CDS 147240..147368 /locus_tag="AUO97b_00290" /db_xref="SEED:fig|
400667.82.peg.150" /translation="MSVESFNSTVLIQNYSNFVLKHVCLSIVEVNSISKLKHIDLM"
/product="hypothetical protein"
/transl_table=11
CDS complement(147346..149448)
/locus_tag="AUO97b_00291" /db_xref="SEED:fig|400667.82.peg.151"
/db_xref="GO:0008926"
/translation="MDGTMFDTERLRFQTLQQASQELIGQEFSHEYLMQCLGLSATTA
EKLAQRLYGVDVPYKEIRKRADEMELEHIRKHGVPIKKGLVQVLERLRKSGLRMAVAT
SSRRAIAEEYLINANVYKFFDVITCGDEVEQGKPHPEIFLKAASQLHLDANQCLMFED
SENGLTSAHTSKGLTILLKDIKEPNDEMLEKAHFYYEQMYDFLTDLDQFIPVMDTPEI
QEPFPQSLNQLTVGIHGFGAIGGGYIAQILSHWDGYTKPKRIIASTRNSLFREAVNAF
GTYSIRYGQFSYDERIENMTIVDSDNEQQMLEMYTHSSLIALCLPEQAIEPESKIIAK
GLYARFNSPLETCIEPLTFLIILNKVGAKYLVMKHLKEALLELTNDEDVTEHILKEHY
FCDTVVNRMVSKLSNQNLYRQLRIKHNFLEQHLEDVEQEDQIEIEDCNKLTPDQLNQA
SIYVDNMRRNFQPGHILQSMDLILFHSETDMPIYVEKGSPLLEKLRQVVLVDQITDIQ
LIKNRLWNGVHAMLAWYASLMGYESIGVAMGDHLVKAFAENLIAEVKQGLAIVLPNYA
KDLDRMSQSFLDSCEYAFKDPCQRVARDPLRKLNHNERVMASIAVNIRHDLPYKNLLK
GAALGYAYAIQFLEIEETKAVEHLQQQIQNLDLSTTQRRQFEAELVQLIQYLFSEQGK
QPLDIKLNNTKTTSNQYV"
/product="CbbY family protein"
/EC_number="1.1.1.17"
/transl_table=11
CDS complement(149824..150444)
/locus_tag="AUO97b_00292" /db_xref="SEED:fig|400667.82.peg.152"
/translation="MLTLSQKLPDYFEYIENDVSYSLRQVQYPQDIPLLHRWMHEPHV
IPQWQLNKSEIELKVYFEKMLADDHQRLMIVGINGQDVGYAEIYEGKRDRLARYYKGE
DNDLGWHLLFGEKSAFGQGFLRPTIRLLNFYIFENSPTNRIVGEPDHTVKPYAAVVED
MCYEEQGLIPMPEKTAMLYYCFREKFYDRFFELYQASQQKLQQKAS" /product="Siderophore

synthetase small component, acetyltransferase" /transl_table=11
CDS 150704..150838
/locus_tag="AUO97b_00293" /db_xref="SEED:fig|400667.82.peg.153"
/translation="MAKNHDKTPTELPSKEQFKNNELSKNNMVALYKTRLDVLVSQLY "
/product="hypothetical protein"
/transl_table=11
CDS complement(150915..152438)
/locus_tag="AUO97b_00294" /db_xref="SEED:fig|400667.82.peg.154"
/translation="MNFRQIQAILHSWFGIIVLWVIFFIFFTGSIAYFWTEINVWAQP
EAISHIQTIPSAQHSAQTAFNYLNQHAPNAKRWRVTVANERMPVNLLQWQDKKGKHQE
LQNPNTGELLGPVRKTLGGDFFFKLHYTLYPLPSKFGSLVVAVVALILLISLITGVIT
HKKIIKEFFTFRAFKGQRSLLDLHHITGVITFPFYLVMAFTGLLILFYLVLPWGLSEQ
YGKAGIPKFYNEMQFTEVAKPREPSLTEAMQPFNQFMAQMPKRTESGAILDKFEVQKP
NTADGMITFDYNYKNIITLNTPQYIFATNTAERLNQPRNLSAIAQLGSSSYGIHLGYF
ASIWMRFILACLGIIGCVMLVAGALLWQKKRIKEQKKFEYRLVQHLNFFTFLGLPFAS
AFYLMVGRIIPASFEPRELYEVSAFYIAWLLSLLISFSCSIRKGIIIMLYITAAVLLL
IPVISVVLVPEASLLNSLKSVHWSLAGVDLALILLGLFYLIVLRFYQTKFITLGEAK" /product="putative iron-regulated membrane protein" /transl_table=11
CDS complement(152439..152630)
/locus_tag="AUO97b_00295" /db_xref="SEED:fig|400667.82.peg.155"
/translation="MAKWLPFAKAENVYFSVFISFIFFIFYIVFTSSILSKLWFWMIN
SLGVVLLVSYWLLAKWGVA"
/product="hypothetical protein"
/transl_table=11
CDS complement(152817..155135) /locus_tag="AUO97b_00296" /db_xref="SEED:fig|400667.82.peg.156" /translation="MEKMNVNNKIGVKSRNHIRSKQVLFKMTPCCLAISAIFAQQAYA
ETVTQTAEVSENATQKPVAQLQKIVVTATRTPKNIAEIAGTVQSIDQKQIIQQATAGR
KVADILAQLVPSLASSSGTTSNYGQTMRGRNVLVMIDGVSQTGSRDVSRQLNSISPGM
IERIEVISGATSIYGSGATGGIINIITKRADTSKPLSFETKVGITSSDTFRSDGLAYE
VGQSVSFNKGNIDGFLGANFTSRGSQFDGNGDRISLSPWQGSTMDTDTIDVNGRLNFN
LNDTQTLSFGAQYYKDKQDTDYGPDYSYLPTTSKSNDATTPTYKAIKGLKLSNPLFTE
RYAVNSQYQNQDFLGQILNVEAYYRNEKSRFFPYGLSNKSVTSVNQSQSEIEVAGLRS
TMQTDLNIANRDMKITYGLDYDWEKDKQFVDILATQYPYLVYTPTGQRKGYGPNTEIQ
NIGAFVQSDYAVTDKLNLQAGIRYQYIQADTDAYIPSRETTMVPAGSTHDDKPLFNLG
AVYKLTDAQQVYANFSQGFSFPDVQRMLRDVSTYTVSTANLQPITVNSYELGWRLNQD
DGLNLGLTGFYNTSDKTVQFNNRAAKVVDTDQRVYGAEATISYPFMENYKVGGTLGYT
RGQYKDVANKWHELNSFTVAPVKGTLFAEWDNNEGYGVRVQMQAIKGTNKAYKDDREL
AAFATTQDEAFQNAVKNDANSAAQIKGYTTMDVLAHFPAWKGRVDFGVYNVWNRQYRT
VFAQQAAVSNANPLLAIPAEGRTYGLSYTFNY"
/product="Outer membrane receptor proteins, likely involved in siderophore uptake @ TonB-dependent siderophore receptor" /transl_table=11
CDS complement(155221..155838)
/locus_tag="AUO97b_00297" /db_xref="SEED:fig|400667.82.peg.157"
/translation="MNQIDTSKALVTAYKEIATSTIGHVLDQGYIPQVFPLRPEDKVV
GIVRTVRIESINASGLRQVLMSCQPNEVLVIDARYDLQRACWGEQRSVAAIHCGLAGV
VVLGAITDRQALLKLKLPIFAHTTSCLTTRNEGESLVEIDAKIHINHTIVQTGDLIVG
DADGIFIIKMDVAQQYLKEFQRLEQNEQNKKNEFFLKNKIDDYYF" /product="Putative siderophore biosynthesis protein, related to 2-demethylmenaquinone methyltransferase"
/transl_table=11 CDS complement(155825..156454)
/locus_tag="AUO97b_00298" /db_xref="SEED:fig|400667.82.peg.158"
/translation="MLQDFATRIHAKNLTCATSLFVKYWCTSWILPFLYCHVAVLPFV
KWDSSALVIDLLEQWYWDRTLQLNQTSFYSFQIIHLQEFNDLIEQLNVLFKQLAKIGR
VPYVLLWENVAVRVVQFYHSFTNQNLNPDIQSRLEQQKQFFKSKAAESFYLTENPFVR
LWNGWHPEFNTFMRQKCCFYFQLEEAEQTLCRNCPLRLKEIGKFKDESN" /product="Atr protein"

/transl_table=11
CDS complement(156608..158518)
/locus_tag="AUO97b_00299" /db_xref="SEED:fig|400667.82.peg.159"
/translation="MNALQPVYQVMNQGILQDFINSLLVETIIPEQFIFDFATAQTAL
NQHGKNLQEVFKNTSEQFSFVLLHEENRQGLVMFAVKKGITQAWKFANETEVFLLERT
NDHVQINVIGIIELFEFIQAIGLFNHCSADKVQAFYEQLQKCLKQYHLLQQHRVNAHD
LLNQSSAHRFRILEQYAGYRDRPYHPLAKLKEGLSQQEYMQYCPEFAQELSIHWVAVH
KDKMMFGEGVENIFKQQPSEIFIPRAERYQLKQEMFQRGLNETHIAMPIHPWQFEHLL
PKFYADDIAEGICHPLNFMSKGMYASASMRSLLSKNVLEESLKLPIGIKALGSLRFLP
IVKMINGEKNQKLLQQAKAKDAVLKQKLWLCEETQWWSYLPEKQNDRTADNEWLFVEK
PTHLAAQRRHIPAELLQEPYQLIPMASLGHTITGQPAIFDYILQLQHKDINSKQILIE
FEKLCTCFFDVNLRLFRLGLMGEIHGQNICLVLKNGEFDGLMFRDHDSLRIYLPWVEQ
SGLKDPNYLSPHDFRNTLYHESIEALLFYIQTLGIQVNLGCIVDNLASHYQIEVKDLW
SVLAHALQQVIQNLNFQPEILAQLQHLLFEVPEWPYKQLLRPLLEQDTRIGSMPSGIG KTRNPLWYALNC"
/product="Uncharacterized siderophore S biosynthesis AcsA-like protein @ Siderophore synthetase superfamily, group B" /transl_table=11
CDS complement(158528..160369)
/locus_tag="AUO97b_00300" /db_xref="SEED:fig|400667.82.peg.160"
/translation="MGQLHPVLSNTHVIQNTLQAWQHPNSDQAKYVEQRVIKQLLQAL
IFEDIIHSEYDGKNFIIEVQNSQGQTIRYVAAGQRQYSYKLVRLARNQDVFRQDENGH
YQIATLNLVIDEILRTITDAAKVEDFIFELKRTFIHDLQSQACFDHYALPAIQYPYDI
LESYLMDGHPYHPCYKSRVGFSLQDNVRYGVEFAQPIALVWLAVHQDIVAKKHSEDIE
PDSFFKEQLNSQDQELFLQHLSDRDLKVDEYIWIPVHPWQWENHLISIFAEEILNGKI
VYLGQSQDRYLAQQSLRTMTNLQHPEKPYIKLSMSLTNTSSSRVLAKHTVMNGPIITD
WLQRLIKQSKTAQELDFAVLREVYGLSVDFTKLPKSHAQQAYGTIGCLWRESVHQYLR
EGEDAIPLNGVSHIQKDGQALIAPWLQQYGVESWTRQLLKVVITPLIHLLFAEGIATE
SHGQNIILVHKQGWPTRVLLKDFHDGVRYSPAHLAHPELAPELDQLPPEHAKTNSMSF
ILTDDLNGIRDFSCACLFFVALTDIAIFLNQDFDLPEKNFWQWAAEVIQNYQQQHPEH
ASRYQLFDVFAEKLRIESLTKRRLFGDRSIQIKFVDNPLAPFKLQVK" /product="Uncharacterized siderophore S biosynthesis protein, AcsC-like @ Siderophore synthetase superfamily, group C @ Siderophore synthetase component, ligase"
/transl_table=11 CDS complement(160381..161820)
/locus_tag="AUO97b_00301" /db_xref="SEED:fig|400667.82.peg.161"
/translation="MERSSVSAAQPHEQSLQRYVLLTVMLGTGTVSLNNSSFNPAIPH
LMSYFQVGEVWASWVVVAFLLAMSISLPLAGFLSQRFGKRSIYLIALLGFALASTAGG
LFNQFESVLIARALQGFCSGLMIPLSLGLIFSVTPSEQRGSTTGLWGAMIMLTLAVGP
MLGALVLVWLNWKALFFINLPVACLALILGYVFLPKEQGDNKQEFDWAGFFFLGSSIV
LLLGTLSQIHQIQDLFQPLYGALLVLSVLLFIRFIFLQKNKSMPLIEPALFATKGFRY
SLVICVAQTVGLFIGMLLIPLWIQHLLKLSPLWTGFALMSSAVVTGICSQPAGKYLDH
YGAAKIMSLGLMITVASFLLLAWAPIQNVWFIVFCMILHGLGMGLSYMPSTTAGLNSL
RQQQQHLVTQAAALNNLFRRIFAAVAVVIAALYLQLRQQSLPLNTQAIFTSFHTMQEI
FVCCAILILCTLPFAWRFPEQYRIDQLKK"
/product="Permease of the major facilitator superfamily in uncharacterized siderophore S biosynthesis operon" /transl_table=11 CDS complement
(161841..163274)
/locus_tag="AUO97b_00302" /db_xref="SEED:fig|400667.82.peg.162"
/translation="MLDFIGIGLGPFNLSLASLLHDKSGLQYRFFDQREKFDWHAGMQ
LPNAMLQVPFMADLVSMVEPTSPFSFLNYLKHHQRLYKFYFRENLYIPRQEYNHYCQW
VVEQLNHIEFSSHVLDIKPIKGGFDVEVVCNGHHQHYRTRQLVLGVGTTPHLPESLQQ
IYSKSGTCLHSSEYLQYSSQKLSGNVVLIGSGQSAAEIFLDLFDRQYMNGRTTPDFHL
YWLTRSAGFFPMENTPFGLEHFSPDYMHYFYHLPSGLKAELPAQQANLYKGISSKTIR
DIYERLYHRSIGGMPTDVTLAGHHQLQYAELLENQKTKLCFEHRQQNKNYILEADCVI
AATGYRYPNPKFLQNLQSLIIRDQADHWQVGSNFKVNYQGEGSIFIQNMEVYHHGVGT
PDLGLGAYRAATIANQLVGEELFKLDHSQSFQQFGVNASAQEEVSVRNIAASKITEFS

RTLNKSGPMHHSALGAYARSKLSEHIE"
/product="Probable Lysine n(6)-hydroxylase associated with siderophore S biosynthesis (EC 1.14.13.59) @ Siderophore biosynthesis protein, monooxygenase" /EC_number="1.14.13.59"
/transl_table=11
CDS complement(163267..165051)
/locus_tag="AUO97b_00303" /db_xref="SEED:fig|400667.82.peg.163"
/translation="MQELANRLAIQNFVNAYMQETGKGYLLSFDQQSSTQQAFSSGLT
LLTLPLPSIQAECSVPLSYVSRVGRHRLAALPKMCIDGQWQKFSAGTIVSLLLEELVI
ESQFKLDAASLLEKWIQSRDALLQFLKQRHNDFDDLVKAGQNFIESEQALILGHSMHP
APKSRNGFVHEDWLKFSPEHAGKTQLHYWLVHQNYIAEGCATEQPISDQVKDAIRWYL
SESDLNLLKTHVEFKLLPLHPWQARYLQGKPWFEQLKQTGQLIDIGLRGWQFSPTTSI
RTLASFNAPWMVKTSLSVMITNSIRVNLAKECHRGEISYRLWHSDLGKKILKQCPTLK
AVNDPAWIALQIDGEIINETICIFRDQPFAVQQQVTCIASLCQDHPNKELNRFNALFD
QIAQKNQQTNFKEIALDWFDHFLKIGLAPLMYVYHKYGMAFESHQQNVLLELEDGLPK
NLWLRDNQGFYYIEEFATEIVEALPDLLEKAHAVGPKDFVDERFSYYFFGNTLFGLIN
AIGPTGYISEDELLIHLQQNLLQLLEQYPDSTLLQGLLFNDSLPYKGNLLTRLHELDE
LIAPLEHQSVYVQLPNPLYVEQKDVSYA"
/product="Uncharacterized siderophore S biosynthesis protein, AcsD-like @ Siderophore synthetase superfamily, group A @ Siderophore synthetase large component, acetyltransferase" /transl_table=11
CDS complement(165425..165889)
/locus_tag="AUO97b_00304" /db_xref="SEED:fig|400667.82.peg.164"
/translation="MTPACRLLKSNKIEFSIHEYEHDANAKSFGLEAAEKLNLNVNEV
FKTLLVTDEKNYFVAVIPVNHQLNLKKVATAVGCKKLQMANPKDAERLTGYLVGGISP
LGQKKRLKTVIDASAQELSKIYVSGGKRGLDIGVNPKDLAQILTAQFVDVLD" /product="Cys-tRNA(Pro) deacylase YbaK"
/transl_table=11
CDS 166104..166382
/locus_tag="AUO97b_00305" /db_xref="SEED:fig|400667.82.peg.165"
/translation="MAMRHRFWDETSDHVLNEKQFLQQTLIEQGIFNNATFDDVKYFF
YTLPSIVIVKAHALGFMHDSVKQMVIQHIQANRMHLMQKAELKIQFKM" /product="hypothetical protein"
/transl_table=11
CDS 166619..167992
/locus_tag="AUO97b_00306" /db_xref="SEED:fig|400667.82.peg.166"
/translation="MSNQNDKLKSLKTSSMDRRLSIAKASLLAGTRWATASASSLFSS
EEEKEKKRKKAMSEQANYLVSEIGKLKGSIVKIGQMMALYGEHFLPEEITQALNTLNN
QTVALDWPAIKPHLQEQLGSKLNDLTIDHEPIGTASLAQVHRATRKSDGLELVLKIQY
PGVANAIDSDMSLFKNMLKLTRMVPQTREFDQWFDEVREMMHREVNYHVEAATTRRFA
ERLKDDERYIVPTIVDEYCTDQVLCMTFERGVPINSPVMLSLPQERRNQLGEASLEIA
VREIFEWGEMQTDPNFGNYLVRLGNGQDIQDKIVLLDFGAIRQFDEHLLSVARNLIQA
GYQHDKHTMVQAMTGYEFFDAMPESIKPGMADVFLLATEAFSTPANNPDMPAGLMDEH
ERYDWKKSQLHSRVMQQASKSMASRYFSVPPKEFMFISRKFIGAYTFMTVIDAKTNVR RMIRNFA"
/product="Uncharacterized ABC1 family protein A1S_1644" /transl_table=11
CDS 168213..169505
/locus_tag="AUO97b_00307" /db_xref="SEED:fig|400667.82.peg.167"
/db_xref="GO:0004085"
/translation="MTNMINKAQGLGLSLITKLAGSDVLDQLKLRKFLERSLYQGSKA
GFRALSQTQKAFKPKQIPQQRLPQQKKNLFDLSLTEEQQMTSEAMSQFAQEVLLGLAH
DADQTAQFPESLWQYVEDLGLNYYALPEALGGVAAEQNIVSNLLIAEKLAEGDFSLTA
GLLSTFFSVINAITRWGSTEVQSMYLPCFAEDSDITATFAVQEATPAFNPYVLKTKASL
ENDQYFIEGEKTLVILGDLADVFLVSADFNGKPDVFVVQAGDTISIKSTPAMGLKAAE
TATLKFSHTPALRLGATDFDYTAFLDLGNLMWCAMAVGTCEAVKAYCIKYANERTAFG

EPISHRQSVAFMIADMAIEIDAMRMLILNAASLAEAGKPFHREAYLARLLCAEKSMKI
GTDGVQILGGHGFTKEHPVERWYRDLRATAILHSGLHA" /product="Acyl-CoA dehydrogenase (EC 1.3.8.1), Mycobacterial subgroup FadE24" /EC_number="1.3.99.2"
/transl_table=11
CDS 169527..170732
/locus_tag="AUO97b_00308" /db_xref="SEED:fig|400667.82.peg.168"
/db_xref="GO:0003995"
/translation="MNLQNPKKFKMMIDQAHDVAINVLRPISRKYDKAEHAYPKELDM
LASLIDGMNEGGEGINAGAAGAGKRGETDNEGVRNGTNMSTALGIIEMCYGDTGLLLS
MPRQGLGNSAIAAVANDEQLERFKGTWAAMAITEPGCGSDSAAIRTTATKDGNDYILN
GEKIFVTSGERADSVVVWATLDRKLGRAAIKSFVVPKGTPGMKVERLEHKLGIKASDT
AVISFTDCRVPAENLLGNPEIDVAKGFAGVMETFDNTRPLVAAMAIGCAKASLERIKE
IFKDQLDPNYSTPYLQTSNLAAQIYRMEAEWEAARLLMLKATWMADNKKPNSKEASIA
KAKAGRVGNEITLKCVELAASVGYNEDELLEKWARDSKILDIFEGTQQIQQLIIARRE LGKSSSELK"
/product="Acyl-CoA dehydrogenase (EC 1.3.8.1), Mycobacterial subgroup FadE23"
/EC_number="1.3.99.3"
/transl_table=11
CDS complement(170848..172071)
/locus_tag="AUO97b_00309" /db_xref="SEED:fig|400667.82.peg.169"
/db_xref="GO:0018685"
/translation="MNAPVNVQQELMPVPASMREIDRKRYLWMISPALPVIGLGILAG
YHFGPRPLKKVFALGGPLLLHVVIPAIDTIIGKDARNPTDEEIKLLEKDPYYSRLVKS
FIPLQYAANIYACYLTSRKDTSFIDKILLGISMGAINGIAINTAHELSHKHDRIDHIL
SHLALVPTGYNHFRIEHPYGHHKRAATPEDPASSRMGETFYEFWPRTVIGSFKSAIEI
EKNRLKRKGKEFWSLDNELLQGWGMSAAFHASMVGIFGKGTIPYLATQAFYGISLFEI
INYIEHYGLLRQKKENGQYERTMPEHSWNNNNVVTNLFLYQLQRHSDHHAYPTRPFQA
LRHFDEAPELPSGYASMLLPAMIPSWWFKMMDKRVFDHYKGDLNKANISPKRRAKIFK KFGVVDKSLEATQAE"
/product="Alkane-1 monooxygenase (EC 1.14.15.3)" /EC_number="1.14.15.3"
/transl_table=11
CDS 172261..173208
/locus_tag="AUO97b_00310" /db_xref="SEED:fig|400667.82.peg.170"
/translation="MDALSKFFDDIHLNKSEYIYIKAQGEWAFKTQDQSALLAYIVLT
GSVYIQLNVSEKITARAGDIILIPSGKAHHATDSKATASKLVDPVDITPLFNGHRHDP
IELGSSTSENTLLLCLRCQVDTYMAGPLINALPSIMHIQHENQTTPPEWLQIGLYFLA
VETQKIQPGHDKIIDHLVSILLIECIRDHIQQMSDQQNWLGALTHPELSNALSAIHSQ
PEISWTVESLAEQCCMSRSKFANLFNSIIGEPPLTYLQHYRFRLASQYLRTSNLSIQQ
ISNKVGYSSETAFSQAFKRVFDLSPKQYRQNYIGTNLDE" /product="Transcriptional regulator, AraC family" /transl_table=11
CDS complement(173247..175112)
/locus_tag="AUO97b_00311" /db_xref="SEED:fig|400667.82.peg.171"
/translation="MESFRTVIKGWLGKVLLVLFLTPLALVGIEGYFNRGNKADVAKT
VNGQDISKKDLETLTQRYKEQYLAAVKGDESLLNLPVIQAKALDILVSRNLLIQQAEK
LGISLSDAQIEQMLAQQPSLQENGQFSQKLYENYLRSIGMTSEGLIASLRQDHALKML
TSTFADYSLVSKVDLMQIANLQTEQRTLHLASIKLDPYKTGLTASNQEATDYYNKHQN
EFKQPASVDVDYVVLSPSLMAKPAPATDAELKQAYAKFVETQQKDAKRIVKHILITTD
ARDDAAAQKLAKDVYAKIQGGLSFAQAAAQFSEDPTSKTKGGLVEAYAPGVFSDAFDK
TVLSLKNGQISQPVKTQYGYHIIEAETQANQIPSFEAEKPRLIAEVEKNKVASVYSDT
VNSLNETIVGNDSLDAVVQQVKGTKIESLNGVTLATQNPYLSDPNVKIKLFNDDVKNG
DRNASSNIQLANGDTVWVKVRDYHAAGVKPLAQAMNEVKAKVIDEKARKAAQAKIATM
LTEFKTQPAEQVVAKNKVAFESAGVFTRSQGLKRAIERAAFSVPTPKPGMWSVTTANL
PNELVVVAVSNVNSAAVNAMPADQLQQLKQLYRQSRGQQILEDYSEYLKSHAKIK" /product="Peptidyl-prolyl cis-trans isomerase PpiD (EC 5.2.1.8)" /EC_number="5.2.1.8"
/transl_table=11
CDS complement(175244..175516)

/locus_tag="AUO97b_00312" /db_xref="SEED:fig|400667.82.peg.172"
/db_xref="GO:0003677"
/translation="MNKSELIDAIAEKGGVSKTDAGKALDATIASITEALKKGDTVTL
VGFGTFSVKERAARTGRNPKTGEELQIKATKVPSFKAGKGLKDSVA" /product="DNA-binding protein HU-beta"
/transl_table=11
CDS complement(175686..176141)
/locus_tag="AUO97b_00313" /db_xref="SEED:fig|400667.82.peg.173"
/translation="MDNANIETQAEEQGEKNKSKSKSSRKSALDFRKYTKQIWLAGLG
AFSRAEEEGNKLFDSLVKVGEELESKTGDLADTTVGKVTEKAKESVSDTKDKVEKILD
TSVNHSLNRIGLVTPKDLQHIEQLLVQLHIKVDALIEENQKLKAKLNEK"
/product="Polyhydroxyalkanoate synthesis protein PhaF" /transl_table=11
CDS 176352..176999 /locus_tag="AUO97b_00314" /db_xref="SEED:fig|
400667.82.peg.174" /translation="MSETQQRQKHPRLLLNIVLIILETFFSFILTHDGVLRLQAKKFI
ANKITIRINSYLPFFDFYVQFTEKGLLFDIHSYDRRIDLEINSTLVDLIKIFVFSNQR
SLNNMRIDGDAETKKELIDLISHLTLPKLLADWKQWFNAPDDEAEALASRKKIAPLLE
KIDQQRSEINSLHVEIKQYQNRIARLQQRQRNINLCFGIISLIFIVYFIYNLWVA" /product="putative membrane protein"
/transl_table=11
CDS 177110..177583
/locus_tag="AUO97b_00315" /db_xref="SEED:fig|400667.82.peg.175"
/db_xref="GO:0005554"
/translation="MRLTTRGRYAVTALLDLALQPTEQTITLAEIAARQSISVAYLEQ
LFAKLKRHGLVSSVRGANGGYHLARNADEITVLEIIEAVNETVDATRCDHKGNCQNGA
MCLTHDLWQELSHHIADYLAKITLANLISRDNVQTVALRQNVTGTDSALLSGTGI" /product="Iron-sulfur cluster regulator IscR" /transl_table=11
CDS 177585..178802
/locus_tag="AUO97b_00316" /db_xref="SEED:fig|400667.82.peg.176"
/translation="MKRPIYLDYAATTPVDPQVAERMMECLTFDGTFGNAASRSHAYG
WQAEEKVEYAREQVANLIKADPREIVWTSGATESDNLALKGVAQFYASKGKHIITSKI
EHKAVLDPCRELEEQGFEITYLEPEPQTGLITPEMVKAALRPDTILVSLMMVNNEIGT
VTDVAAIGELTRANKTFFHVDAAQAAGKVDIDLSTMKIDLMSFSAHKIYGPKGIGALY
VRRSPRVRLKAQIHGGGHERGMRSGTLATHQIVGMGEAFELAGKTMHAEQERIRKLRD
KLWNGLQDLEQVFLNGHPTQNVANYLNVSFNFVEGESLMMSLKDAAVSSGSACTSATL
EPSYVLRALGLSDELAHSSIRFSFGKYTTEEDIDHVLTITKAAVEKLRELSPLWDMYK EGIDLSTVEWAEH"
/product="Cysteine desulfurase (EC 2.8.1.7) => IscS" /EC_number="2.8.1.7"
/transl_table=11
CDS 178869..179255
/locus_tag="AUO97b_00317" /db_xref="SEED:fig|400667.82.peg.177"
/translation="MAYSEKVIDHYENPRNVGVLDKNSENVGTGMVGAPACGDVMRLQ
IQVNDNGVIEEARFKTYGCGSAIASSSLVTEWLKGKTLDEAQAIKNIDIATELALPPV
KVHCSVLAEDAIKAAIEDYRSKKSKA"
/product="Iron-sulfur cluster assembly scaffold protein IscU"
/transl_table=11
CDS 179286..179606
/locus_tag="AUO97b_00318" /db_xref="SEED:fig|400667.82.peg.178"
/db_xref="GO:0005554"
/translation="MIHLTENAATHISNYLKNRGKGEGIRVGVKTSGCSGLAYVLEFV
DDIDEHDQIFEQFGVKVFVDPKSLVYLDGLEMDYVKNGLNEGFEFNNPNKKGECGCGE SFTV"
/product="Iron-sulfur cluster assembly iron binding protein IscA"
/transl_table=11
CDS 179692..180210
/locus_tag="AUO97b_00319" /db_xref="SEED:fig|400667.82.peg.179"
/translation="MNHFELFNLPVALDIDLASLKSNFLSLQQQYHPDKAADKDQALI

KSSEINQAFKTLSQVDSRAAYLLALKKQDHHLDQSISDFEFLQSALELREQLDEATSS
EHLRTLRLEVQQWIDGLVREFKIDYSEEDWAEARDTVRKLRFFQRVLNDIDKAEDQLL DDEDSFDLDDDF"
/product="Chaperone protein HscB"
/transl_table=11
CDS 180249..182108
/locus_tag="AUO97b_00320" /db_xref="SEED:fig|400667.82.peg.180"
/translation="MALLQIAEPGQSSAPHQHRIAIGIDLGTTHSLVATVLSGKPKVL
NDVQNRRLLPSIVHYGDNTTHYGEEAKPFIIADPKNTIVSVKRFMGRSKADIKFQHPY
ELVGSENEMPAFETRAGRKTPVEISAEILKQLKARAEDSLQNPVNGAVITVPAYFDEA
QRQATRDAAQLAGLNVLRLLNEPTAAAVAYGLDQESNLATDRNYVIYDLGGGTFDVSI
LRFSQGVFEVLATGGHTALGGDDLDRLIVKWAKKQLNIDVLSDEDYAVFIVAARQAKE
QLSTQDSVELKLLEATLTLDRPTFESIIQVALDKTISVCKRVLRDAKLELTDIQNVVL
VGGSTRSYAVQKAVREVFAQEPLCTINPDEVVAIGASITANQLIGNSQDGSLLLDVTP
LSLGLETMGGLVERLISRNTAIPVARRQEFTTYQDGQTAMLIHVVQGERDLVEHCRSL
GRFVLHGIPPMTAGQARIEVTFQVDADGLLTVSAREATSGVQAHIDIKPSYGLSEADT
ERLLIEGFQHAEEDKNLRHLKETKVEAERELEALEQALKVDADLLDEKQLEALNSAKG
SLKAQLEGSDIQAIEQAVQQLKVHSDAFAALRMNRHIDHALKGTKLDDWSKSN" /product="Chaperone protein HscA"
/transl_table=11
CDS 182127..182465
/locus_tag="AUO97b_00321" /db_xref="SEED:fig|400667.82.peg.181"
/db_xref="GO:0005506"
/db_xref="GO:0006118"
/db_xref="GO:0009055"
/translation="MPRIKVLPHAQFCPEGAEFEVEQNANLCQSLLNKGIKIEHACDM
SCACTTCHVIVRKGFDSLEEMNDVEADLLDRAWGLEPDSRLSCQVKVVDEDLEIEIPK YTINHASENH"
/product="Ferredoxin, 2Fe-2S"
/transl_table=11
CDS complement(182512..183981)
/locus_tag="AUO97b_00322" /db_xref="SEED:fig|400667.82.peg.182"
/db_xref="GO:0004016"
/translation="MQLERFIDREPKQFAYFHRLMGYSILSLILVIYAFTSPNTNYQI
YIPPFFLFLLFVSSKLEHWLQYQFDKKTQKSVFFAIDAIVVAVTLAGVHLNLVPTFIA
LFALFYSAINSRISFAVICLTSLLGAIIFYLSTFFLFGFHPYFEATSQELTVITLLGL
VMFITIGNYYQHRWVKKISQQRQHYYDQMTRYIAFANQLSRYAPLQLWQSIMRGEAEA
KIEYKRKKMTVFFSDIQGFTELSETLIPDDLAFLLNDYLSHMTEIAKQYEATVDKFMG
DAILIFFGDPNSQGVEQDAKSCVEMAIAMRQQMKLLRERWKKMGYSALHIRMGISTGY
CHVGNYGATHRMAYTIVGRDVNLAARLQYAAEIDEILISDDTYQLIKSDFLCAPKTPI
YLKGIQGAVKTWQVMEKYTGNKSDHQQWFDYEYKGFHLVLNLEEVQNYEYPELVEVLE
KMIQRIKVQQTLVNTQGVAQLRLEDEVKPINIDEEQDQV" /product="Adenylate cyclase (EC 4.6.1.1)" /EC_number="4.6.1.1"
/transl_table=11
CDS complement(184034..184468)
/locus_tag="AUO97b_00323" /db_xref="SEED:fig|400667.82.peg.183"
/translation="MFSLHPQLAQDTFFVGDFPLSTCRLMNDMQFPWLILVPRVPGIT
ELYELSQADQEQFLRESSWLSSQLSRVFRADKMNVAALGNMVPQLHFHHVVRYQNDVA
WPKPVWGTPAVPYTSDVLAHMRQTLMLALRGQGDMPFDWRMD" /product="Diadenosine tetraphosphate (Ap4A) hydrolase and other HIT family hydrolases"
/transl_table=11
CDS complement(184669..188130)
/locus_tag="AUO97b_00324" /db_xref="SEED:fig|400667.82.peg.184"
/db_xref="GO:0003676"
/db_xref="GO:0003700"
/db_xref="GO:0004386"

/db_xref="GO:0005524" /db_xref="GO:0008026"
/translation="MFQQEISQLNLQQLKAGEKRWVGSLLGSSAALLLKEIATQHSSL
LVLVARNNQHVAQLESELEFYGVKPTIFPDWEILPYDRLSPHQDIVSERLAILSNMPQ
QGVLLISASTLAQRVAPYSWVLGEHFDIKVGQKFDLEQQKLRLVQAGYHLVDTVYDHG
EFAVRGSIMDIFASGQESPIRIDLFDDEIDSLKFFDPETQRTTTTLKSFTVLPAKEFP
LKEGRSVFRDRYSEFFPTANPKKNPIYQDVLDGIASPGIEFYLPLFFDKEQMQTQSMF
TTYLPKNCIVITTNDIDTDLTSFWKEVMRRYEDRRHNMDQPILAPEELFISPNHLLSA
LNQFPRMLVSAETIEEKAGALNLKTEQPPKLPVDPKKEKPFSAVKKYIDEANHPVLLV
AESAGRRESLKDALRSSLGEIPNVENFEQFQISQFAIAITNAPLDRGLLLTDQLTVIS
ENQLYEHRVVQRRRKRQQEVSEEFLIRSLTELSIGAPVVHIDHGVGRYAGLITLAIEG
QDYEFLQLDYAEDAKVYVPVTNLHLISRYSGGDPDLAPLHKIGTDAWSKAKRKALEQI
HDVAAELLHIQARRQSKPGFAFELDQSLYMQFASGFAYEETLDQANAIEATLYDMQQA
KPMDRLVCGDVGFGKTEVAMRAAFIAVQNGKQVAVLVPTTLLAQQHYESFKDRFADWP
VRIEVLSRFGSNKTHLKNIEDLANGKVDIVVGTHKLLQENVQFKDLGLMVVDEEHRFG
VRDKERIKALRADVDMLTLTATPIPRTLNMAFSGMRDLSIIATPPARRLAVKTFVQEH
TDASIKEAILRELLRGGQVYFLHNEVETIERAAENIRVLVPEARVAVAHGQMRERELE
QVMQQFYHKEYNVLVCSTIIETGIDVPNANTILIERADKLGLAQLHQLRGRVGRSHHQ
AYAYLLVPSIKHLKGDAEKRLDAIQRASTLGAGFMLATEDLEIRGAGELLGEQQSGSM
QAIGYGLYMEMLEKATKAIQQGKTPNFDAPLSLTAEINLHIPALIPDDYLGDVHQRLL
FYKRISNTDTQEKLDNIRMELIDRFGIPPQSVKHLFSVHQIRLKAEHLGITKIDINTQ
GGNIEFSPDTPVQAISIIQLMQKNPTYYRMEGGQRLKVMVQLEEYEKRIQFINDLLQK LMSELHA"
/product="Transcription-repair coupling factor" /transl_table=11
CDS complement(188265..188621)
/locus_tag="AUO97b_00325" /db_xref="SEED:fig|400667.82.peg.185"
/translation="MFKKLTQLFQGSKETPEQIYLQENQLSFDSERGPVIKDVVINEK
WSEHLEYFSNRKLQNFDNLPKLFQITPQINEKIDLEIATQRYVERLGNTQEKLLELKA IIQVLNQYYVMFLRGK"
/product="hypothetical protein"
/transl_table=11
CDS complement(188701..189633)
/locus_tag="AUO97b_00326" /db_xref="SEED:fig|400667.82.peg.186"
/translation="MRIQPVTSHPGENALFIVLGLAQGDSVVEKVKDFAGKFSALTRS
LSNRYPETKFNAILGFSSNAWDILFPQQAKPQELETFQEIKGPKYTAVSTPGDLFFHI
RADRQALCYELGSIINEQLGKVTYPIDEVHGFRYFDGRAIIGFVDGTENPEPVIAAQW
ALVGNEDPDFIGGSYAFIQKYTHDMEKWRALSDEEQERVIGRKKFNDIELDDDEKPLT
AHNVVSKAHDAEGNELKIMRANMPFSNPSKNEYGTFFIGYSRKFSTTRQMLENMFLGN
EQGNLDRLLDFSTAQTGTLFFVPTVDFLDSLGDE"
/product="Predicted dye-decolorizing peroxidase (DyP), encapsulated subgroup"
/transl_table=11
CDS complement(189779..190558) /locus_tag="AUO97b_00327" /db_xref="SEED:fig|
400667.82.peg.187" /translation="MVNHQIKVLQALASAIAEGGRGVTGTAFPKQPVRALKLYEFEGS
PFCRRVREVITLLNLDVEIYPCPKGGLKYRSIVKEIGGKLQFPFFIDENTGDKLYESQ
DIIHHLFKYYGKTDQTPDKFSNYPKMPYLAFAGTLLNGARGVWINKKIVNRAAPEHKL
ELWSFEASPYSRVVRSVLSELELPYILHSVAKERWQDMGPAILRLKPGKYEPLKEGKR
EKLLPIMQGKMQVPYLVDPNTGVKMFESAEIVKYLKKQYGS" /product="hypothetical protein"
/transl_table=11
CDS complement(190586..191092)
/locus_tag="AUO97b_00328" /db_xref="SEED:fig|400667.82.peg.188"
/translation="MSTVPFVTCDLLDDNPEKDLQVVTPSMDGKFFKSYGARQTFGGQ
VVTVKCFEDNSRVKELLATDGTGKVLVVDGGASMRCALMGDMIAESAVKNNWNGVVIY
GCVRDVDALATLDLGVHALAAIPQKSNRKGIGEVDIPLYFGGVTIQSGDFIYADNNGI VIAKEKLV"
/product="Ribonuclease E inhibitor RraA"
/transl_table=11
CDS complement(191105..191770)
/locus_tag="AUO97b_00329" /db_xref="SEED:fig|400667.82.peg.189"

/translation="MTISTKKAIVVGATGLVGLALVEQLEQTPEFEAITVVVRKESQL
LNTYKKVTQLVIDDFLLLNDEDVNGHTHAFSCLGTTLKIAGSKQGFYAVDYEINAHFA
DLIQDKHIHFLIVSALGANANSPIFYNKVKGELEDYIQSLGIEKLSIFRPSLLIGKRS
DVRFIEDLGQSLFKLIENKWTKPFKYKPVTAEQLAHTMVVAALTQIEAFKRYDNLSIQ QTR"
/product="Oxidoreductase"
/transl_table=11
CDS 191983..192249
/locus_tag="AUO97b_00330" /db_xref="SEED:fig|400667.82.peg.190"
/db_xref="GO:0003723"
/db_xref="GO:0003735"
/db_xref="GO:0005622"
/db_xref="GO:0005840" /db_xref="GO:0006412"
/translation="MANSAQAKKRARQNVKARKHNASLRSMVRTYIKRTLSAIAGGDY
AVATEAYKKAVPVIDRMADKGIIHKNKAARHKSRLNAQVKALAN" /product="SSU ribosomal protein S20p"
/transl_table=11
CDS complement(192306..193040)
/locus_tag="AUO97b_00331" /db_xref="SEED:fig|400667.82.peg.191"
/translation="MALDLLPKSMFEAIDLLPDASTPVTLFTRHSLRELVDGQGLAGY
DLQLTPQGRELAQEWGSYLIKNTDRVIQHCISSPIQRCVDTAALMIEGADGVSPEPNT
HTIEIVEQGLLVEPGSFVLDIQQAAPYFRKQGALGFINSFVNNALPGMKHPITGVLDV
LELIYQTHPKTKSGLSLAVSHDTIIAAIVAVISGNNQIQKADWPAMMEGLFVWFEGDE
FPNCKLKWIWRGKTYELDVSEFHKVQ"
/product="FIG00968626: hypothetical protein" /transl_table=11
CDS complement(193098..193955)
/locus_tag="AUO97b_00332" /db_xref="SEED:fig|400667.82.peg.192"
/translation="MFITTTRPNDALIEQLLPILEEASQILLQEYQNYSAGYEFTIEE
KHDDSPVTQADLKVNRFLLKHLAEITPELPVLSEESDYSARHAWSSCWMLDPLDGTKE
FIHERDEFTINLSLIEGRETIFSVIVVPCEQVVYLGYLKDLPFKYSFSQQQWYQYQKT
PYSTQAPVQIGLSHSSKNPKYQKFIEPIEKIRTVIRREAGSAYKFCMMLEGEIDIYPR
FHPTSEWDTSSGQGLLESIGGGLLTLTGKPFFEYNQRDTVLNEGFIAFRDQESKKIAFE ALAQSGVLD"
/product="3'(2'),5'-bisphosphate nucleotidase (EC 3.1.3.7)"
/EC_number="3.1.3.7"
/transl_table=11
CDS 194265..194633
/locus_tag="AUO97b_00333" /db_xref="SEED:fig|400667.82.peg.193"
/translation="MMLYGYHFSTIENNWEDLTPLNEFLQTFADDDGDVSQRDKESLK
EIIAKSDTALALAKEMGWDGSYTGCPYLFWLPSKNTQSFEYGFVFKQTSDNSTFVISP
IELAYLAQDEQVQTLSKNID" /product="hypothetical protein"
/transl_table=11
CDS complement(194687..195421)
/locus_tag="AUO97b_00334" /db_xref="SEED:fig|400667.82.peg.194"
/translation="MPFYLVYRINTYKIRDVKVQYFIPAMCANFKPLTLAQLQQLQLP
VVGFSYADEVYPGGTMPLLFKSPQGFEWREVMFGLVPKWAEDTNIAKHTYNARHETIF
QKPSFQEAALKCKFGVIPVTEFYESKYINDKPERWGVRRKDGQAFFIAAIYEICKMND
MIVRSASMLTMDAIDHPMMKDFHEPGDVKRSVIVIPHDRLDEWLSLETPDISSFIEGF
PVEEFECLHVPKEKIIKHTPQLSMFE"
/product="hypothetical protein"
/transl_table=11
CDS complement(195454..197184)
/locus_tag="AUO97b_00335" /db_xref="SEED:fig|400667.82.peg.195"
/translation="MTEQEKKTPLLHIENLRVSFKGEDKQYIETVKGISFDIPTNTTV
ALVGESGSGKSVTSLATMGLLPVGQSKIDEKSKIIFEGKDLLGLSRTEMRKICGKDIA
MIFQEPMSSLNPVFTVGNQIAEVLCLHMGMSRKQARQRVLELLKEVGIPSPETKIDAY

PNQLSGGQQQRVMIAMAIACEPKLLIADEPTTALDVTIQKQIIDLLESLRQRRQMSML
FITHDLALVGEIADQVIVMRHGEIREQGTAEQVLEQPKDVYTRALLYCRPQMSQRPYR
LPVTSDFMRQENNILVEQSFDVSEIPERKRGLNGDEHIILEVKDLKKSFYSRKGLFGK
EEFQAVKGVSFKLAKGKTLGLVGESGSGKTTVGLLLMRLHQASGGQALIEGKDILSLT
EKEFAKYQRKIQIIFQNPYASLNPRFTIGQILLEPMQIHGIGKDDAERKQIALGLLER
VNLPEQAYYRYPHEFSGGQRQRIAIARCLTLKPEILICDESVSALDVSVQAQVLNLLQ
DLQDEFGLSYIFISHDLSVVKYISDQVMVMNHGEVVEIANSDELYAHPQHDYTKRLLQ AIPQGIQHVS"
/product="Dipeptide ABC transporter, ATP-binding protein DppD (TC 3.A.1.5.2)"
/transl_table=11
CDS complement(197197..198339)
/locus_tag="AUO97b_00336" /db_xref="SEED:fig|400667.82.peg.196"
/translation="MLSVLSKRKLETQTAPASAGLWRLAMRRLRADKIAMASLIVVLF
YLIVLILSLTGVIASDWNKEVAVSYAPPTFIGADKTTEALKSTAAVDEELPENPVDPL
KDVIHQLKAEIKQAKSSGSAIDYYGVVDPLADDMKAIDQQLGGHLLDQQSELKSTLIF
GADKWGQDVLKKTIKGAETSIIVGLVSALLAVTLGTLLGAISGYFGGWVDDILNWFYN
IFTSIPYLLLVLAIAAVLQQKGILSIVLILGLTGWTGVYRLIRAEYMKHTAREYVLAA
KAIGVGHFRRMFIHIFPNVSHIALVQMSILVVSFIKSEVILSFLGFGVPVGVVSWGSM
LNEAQSELLLGKWWQLVAASVAMAVLVTAFSMFTDALRDALDPKLK" /product="Oligopeptide ABC transporter, permease protein OppC (TC 3.A.1.5.1)" /transl_table=11
CDS complement(198347..199285)
/locus_tag="AUO97b_00337" /db_xref="SEED:fig|400667.82.peg.197"
/translation="MLAYVIRRLWQMIPTMLGVVLLIFILFNWVGGDPAYILAGKMSN
PEQIENIRKQLGVDQPYYVQLWIFIKQILTFDYGASWSTGESVSQIILTRLGPSLTLL
IPLTILQTVISIILALAVAAVRGSLTDRMVMMLCTIGMSISILVYIIVFQYVLAYQLS
WFPVQGWSDSFTENLFKFSLLPILIMLVVSIAPTLRLYRSFVLDEVNQDYVRTARAKG
VGESRILSVHVLRNASIPIITDVMSNLPALLIGAFLIERFFGIPGIGREVIIAVERSD
FPVIKAITVYIAAATMIFNLIADLVYKVVDPRVQLK"
/product="Dipeptide ABC transporter, permease protein DppB (TC 3.A.1.5.2)"
/transl_table=11
CDS complement(199298..201310)
/locus_tag="AUO97b_00338" /db_xref="SEED:fig|400667.82.peg.198"
/translation="MKNNTLKMTTLCMLTIGISQFASAEATRATLPLLKAEQLPAWCD
SNLKKIQQEISSFEKTPVKNDAAAAPILAKWDKIFGHLEDFSGPIGLYSNVDPDAKLR
KAAEDCEIKINQFHTDIFQNSKLYNVIKNTKATDPIDQKYRQDILDQFEDTGVQLEPA
KRARMKAILDELTKLEQEYNRNIRDNPEKLEFTPEEMKGLPESYISGLKKNTKGNYLL
GFEYPEYRPFMELADNDDARKKYQIAFTRRGTEQNLKLLKQAIDLRYELAQLFGKASY
ADWALKDRMAKTPEAVNKFLAEVQKTVAPLERKEVEELRAFKAQTLKTPLDKTEITRW
SEAYWSEKLRKSKYQIDQEKLRDYFPTLAAQKWLFAISSDLYGIDFKPVKVKAWQDEV
EYYDVVDKKTGKLLGGLYMDKFPREGKYGHAAVWGVYGGSTLTNRLPISVLVTNFNRK
GLNSDELETFVHEFGHALHGILSNTRYASQSGTSVERDFVEAPSQMYEEWARRKETLS
KVADYCDPACPRVDDELIARLKAVHNYGRGLRYARQTLYAQYDMALHTADALKVKPLE
TWQKMEAATALGYVPTTEFPGQFGHLMGGYQAGYYGYMWSEVLALDMLSAYGDNLNNP
QVGQRYRQTILSQGSQKPAAELVKDFLGRDPDNKAFFNEITGQRVK" /product="Thimet oligopeptidase (EC 3.4.24.15)" /EC_number="3.4.24.15"
/transl_table=11
CDS complement(201332..203152)
/locus_tag="AUO97b_00339" /db_xref="SEED:fig|400667.82.peg.199"
/db_xref="GO:0005215"
/db_xref="GO:0006810"
/translation="MTENLKQKLKHCSVGLFFAGMALTGSISIFAESPADPNKVLRYV
FPTAETGFDPAYVHDLYSAHVLTSIFETLYTYDYLARPAKLIPHVATAMPEVSADGLT
YTIHIKKGIYFTADPAFKGKPRELTAYDYAYSFKRLLDPNLRSPNSWLLEDKIEGMNA
LVKAANKSGKFNYDQNVSGLQTPDKYTLVIRLVKPDYNFPLLLAHDPTGAVAREVIEK
YKDKAGFVMGHPVGTGPYMLSKWIPASRIVLKANPEYRGFTWNFNASSPWDEAIVKRL

KGKQMPQIGTIDIQVMEENQSRWLAFQRGEVDIIQLEGQLVSKAIKDGKLRPELAKEG
VQLSRIVDPEISYIYWNLKDPVVGGMSKEKIALRRAIAMSRSIDQEIKLVRNSDAERL
HFPVPPGVVGYDPQYRSSTPYSVKAANLLLDRYHYKKDASGWRTQPNGKPLVVEYKAR
NDSIGQQSAELWKKNFDSLHIRMVYKPMLFSDLLRSQKQCEGMFGSSAWIADYPDGDN
FMQNFYGPNTHMTNWSCGSIPEFDELYRQSQQVKPGPERDALYRKMTRLLEVYMPVQM
SYARYRNMLAQPRIIGYKKHPILHAEWMYFDIDTNMKSNH" /product="Dipeptide ABC
transporter, substrate-binding protein DppA (TC 3.A.1.5.2)" /transl_table=11
CDS complement(203171..204955)
/locus_tag="AUO97b_00340" /db_xref="SEED:fig|400667.82.peg.200"
/db_xref="GO:0005215"
/db_xref="GO:0006810"
/translation="MNLKLKFFKLAILSLAITSVPCAYAITPAQPNKILHVAYEAPDD
GFDMVKTTNFYSASIAEAIFEPLLKYDYLARPLQLVPNTVESLPKVEQDGKVYIFKIK
PGIYFTNDPAFKGKRRELVAEDYIYTIKRILDPKNRAPSVSFIDGKLQGADVVVAQAK
KTGKFDYAAPIAGLKAIDRYTLQFTLTRQDYNFPYILAYIAFGGVAKEVVDYYGDRIG
MHPVGTGPYMLSKYVPRSKVELVANPDYRGFVWNFKSTGTPWDNQLVKEMSGKKMPQI
GKVVVSIIEEQQSRWLAFQSGQLDFDKLTADAVPQALDGNQLKASFQKRGIKHFPYKE
PEMTYTMMNMRDPVIGGFSPEKIALRRAITLAYDQKESIKQAYKGQAVRAEMFIPEGV
NGYNPKYKSSVGYNPRLANKLLDYYGYKKAADGYRTLPNGKPLILKINTENSSASVIH
SELWKKNLDAIGIRADFKVSNFADNLKAATQCKYMIWSGAWIADYPEGDNFAQLLYGP
NAGQGNHACYQSKTYDALYTQAIHLPPQQRLPYYEKLNRQIEADNPWIIHVTRIRNWL
IRPQVQGFKPHPMMNTNWQYLDITPIKK" /product="Dipeptide ABC transporter, substrate-
binding protein DppA (TC 3.A.1.5.2)" /transl_table=11
CDS 205836..208436
/locus_tag="AUO97b_00341" /db_xref="SEED:fig|400667.82.peg.201"
/translation="MAQAAETEQSSTDKKPTKVVKVAVTGSSIKGVAAQSASPITIIK
GEDLANAGVTTVEEALTRVSSNQAGFSTDQNVGASNTDGSTANLRGLGSDKTLVLLNG
RRLAANPFGTSTVNLNIIPLAMIDRIEVLRDGASAVYGADAVAGVINFITKKTYQGVG
ISAGLLQPEHKGGDKQDISIFGGYGDLDEDGFNIFGVVDYRRTNGIMAKDRKISERGG
VLPELGLDGRSANTFTSNFYDPISGVSGNPYAQNGQCLGPAESAEKGLCYANTQALIG
IKPDVENVSVLGRGTYKVNDNLNAIGEYIYSRSEVITSVAPDPFSRGNLSTRVTLPSD
SPYYPGNGIVPSVKGLSGAPLELYLRSHAGNRIAKSINESHRLFAGLEGDVWGWDLNT
GVTYAKSEASDNFVTGQLNKTKLQDALNNGTLNPFGESSDPNIWNNLSVKGKYNEATL
ESTTADFSISRPIFTLPAGEIGFALGGSFTNQDWKQHINADLVRQAPSSGTDPSKPDN
SGDRDITSAFAELQIPITKTIEAQLAARYDDYSDFGDTFNPKFAIRWEPLKQLMFRTS
YSTGFRAPSLYEINAAQSKTYTGAKHNDPLYCPGGEVVEGKNKNDFCNTQFMKLQGGS
KDLQPEESKSFTAGFVFEPIKNLVLTVDYFNIKIDDLIAQVGEATIFGEPEKYSALFV
RNADGTIDYIKTNLFNSGGMKTEGFDISLNYLTPMTSTGRFGFGIDGTYVSKLDYRDS
KGEPWTGQVGLYEDPAVVRWKHTANINWAYENWKLVFEQQFVRGYDDQNQIGEEQYDH
HKVSDYTLYNISGTYKGFKNLELTAGIKNIFDEDPSASNVLDNFQYGYDPRYGDPTGR TYYLRGTYKF"
/product="TonB-dependent receptor"
/transl_table=11
CDS 208545..209261
/locus_tag="AUO97b_00342" /db_xref="SEED:fig|400667.82.peg.202"
/db_xref="GO:0005381"
/db_xref="GO:0006826"
/translation="MGMTFTDIENKSAKRLIGIAAVIFLHLLVAYILMSGLANNIQKP
AEKPVELQIIQDIKPPPPPKPEEPKPKEKPPEPPKMVEKVAKVPEPPKEVEKVATPVQ
KTTPVAQPTKVATPAPAAPSTPSPSPVAAPAPVAAAAPAPKPAGVTRGVSEGSAGCEK
PEYPREALMNEEQGTVRIRVLVDTSGKVIDAKVKKSSGSKILDKAATKAYSLCTFKPA
MKDGVPQQDWYEIEYPFVIE"
/product="putative TonB-dependent receptor" /transl_table=11
CDS 209302..210183
/locus_tag="AUO97b_00343" /db_xref="SEED:fig|400667.82.peg.203"

/db_xref="GO:0006810"
/db_xref="GO:0008565"
/db_xref="GO:0016020"
/translation="MMKKSTQPLVHFASASLIAACSLLPLSSVFAEETSNAPVATATT
EATSNTTTPTPPPKPATSETVKNPYGLEALWREGDLVAKSTLFILVLMSIGTWYIIIS
KLLQQGKVKRQGKEAEKHFWEATSLDNAAEGLEQSSAYRFIAEKGINSTKSHGGSLLE
RIDFNTWVSISIQRAIEKVQNHLGGGLAFLATVGSTAPFVGLFGTVWGIYHALTAIGI
SGQASIDKVAGPVGEALIMTAIGLAVAVPAVLGYNWLTRRNKAVMENVRSFGSDLHAV LLSGEINTNNSINRSIK"
/product="MotA/TolQ/ExbB proton channel family protein" /transl_table=11
CDS 210199..210621
/locus_tag="AUO97b_00344" /db_xref="SEED:fig|400667.82.peg.204"
/db_xref="GO:0005215"
/db_xref="GO:0006810"
/db_xref="GO:0016020"
/translation="MGMSVGSENDDEMIGTINTTPLVDVMLVLLIIFLITIPVVTHTV
PVKLPEEKNTPYATTPENIQLSVNKKGDIFWNESYVPNKEILLTKLQAVAQKRPQPEV
HIRGDQLTHFEAIDQVISTTKQAGIGKIAFVTTPPASP" /product="Biopolymer transport
protein ExbD/TolR" /transl_table=11
CDS 210641..211054
/locus_tag="AUO97b_00345" /db_xref="SEED:fig|400667.82.peg.205"
/db_xref="GO:0005215"
/db_xref="GO:0006810"
/db_xref="GO:0016020"
/translation="MGMNVGSNNDDDVMLEVNMTPLIDVMLVLIIMFIITIPAPNNAI
NINLPNGTPPPTNEKPPEVIDVRIDAAGKVFWNNQQVSDRKALETLFQGVVAKKDQDQ
IKLKPDQMAEYKNVAMVMATAQRLGVTKIGIVSSN"
/product="Biopolymer transport protein ExbD/TolR" /transl_table=11
CDS complement(211121..211336)
/locus_tag="AUO97b_00346" /db_xref="SEED:fig|400667.82.peg.206"
/translation="MIGKDHQAKGYGTLALQMAIDEMATKGAKRIRTMYKSSNNIAGK
LYKKMNFIETGEYDECGDIILELGISF"
/product="FIG00350853: hypothetical protein" /transl_table=11
CDS complement(211673..213838)
/locus_tag="AUO97b_00347" /db_xref="SEED:fig|400667.82.peg.207"
/db_xref="GO:0004474"
/translation="MTARIQKGKLAIAKELYDFIENEALPGSGLDSETYWKNFEQVVV
DLSPKNKALLAKRDELQAKIDEWHRNNKFELGAYKAFLTEIGYLLPEVEDFQITTENV
DEEIALLAGPQLVVPVRNARYCLNAANARWGSLYDALYGFDVISEEGGAEKGKGYNPV
RGAKVIEFAKNFLNEIFPLAQGSHADATKYAIEQNKLVVTLKDGTKTGLAHEAQFVGF
NGEEANPSEVVLLSNGLHVIIEIDANSPIGQTDLAGVKDLTLEAAVTTIQDLEDSVAA
VDAEEKVEGYRNWLGLMKGTLQESIEKNGKTIVRALNKDREIKNLIGGTTKLHGRSLM
LLRNVGHLMTNPAILVDGEEIFEGIMDALVTPLLSIADIRSENENKNSRKGSMYIVKP
KMHGPEEVAFAVELFERAEQALGLPAKSLKIGIMDEERRTSVNLKNCIAAAKDRTIFI
NTGFMDRTGDEIHTSMEAAPVVRKEAVKTQKWIAAYENRNVAIGLKCGLQGKAQIGKG
MWPKPDSMKDMLATKAAHPNAGASCAWVPSPTGAVLHAMHYHQVNVKARQDQLKAEEM
LSLDDLLTPPFATDTNWSAEEINNELENNCQGILGYVVRWVDLGVGCSKVPDINNVGL
MEDRATLRISSQHVANWLRHGIVTREQVEEVLKRMAKIVDEQNANDPLYKPMAANFET
NIAFQAASDLIFKGCEQPSGYTEPLLHAARLKLKGYTGD"
/product="Malate synthase G (EC 2.3.3.9)" /EC_number="2.3.3.9"
/transl_table=11
CDS complement(214300..214497)
/locus_tag="AUO97b_00348" /db_xref="SEED:fig|400667.82.peg.208"
/translation="MQQLMIMVTEVGKLEHTCNLLAEVNKGGKVLKVFDYNGNQLPIN
IDGTVTFNRRRWELPIKVDLK"

/product="hypothetical protein"
/transl_table=11
CDS complement(214594..214764)
/locus_tag="AUO97b_00349" /db_xref="SEED:fig|400667.82.peg.209"
/translation="MNSLKPVIPANLIQPCPNLNEIEGTTGKDLMIWSVDTVAKYNDC KARHGAIVKALE"
/product="hypothetical protein"
/transl_table=11
CDS complement(214733..215095)
/locus_tag="AUO97b_00350" /db_xref="SEED:fig|400667.82.peg.210"
/translation="MTWILNNKRWSLIIVLSVLYLIQIGYTNHLAGKINQADQKCLSQ
IQKIEKKNLEALAEKQNQINKVSADYEKVKAEQNTKVEYIEREVQKIVERPVYKSSCI
DDDGVQQLNELIKAGNTS"
/product="hypothetical protein"
/transl_table=11
CDS complement(215092..215640)
/locus_tag="AUO97b_00351" /db_xref="SEED:fig|400667.82.peg.211"
/translation="MTNKTKLFVIGSTIAASMGGFFIFGPSDQQVQATAAKEGYTAKP
TIPVKGDRPTIGNGTTFYPDGRAVTMNDPAITRKQAFEYLKFTMNKDARAFNKTLLNI
PISQAEYDLYLDFTYQYGIGAWSGSSMLKNLKVGKYKAACDSLLKYKFVAKRDCSIRK
NGCYGVWTRQVERHAKCIGVNS"
/product="Phage lysozyme R (EC 3.2.1.17)"
/transl_table=11
CDS complement(215624..215863)
/locus_tag="AUO97b_00352" /db_xref="SEED:fig|400667.82.peg.212"
/translation="MAEPATSTSTATYGLVTNLAGGGMVLYGGLSTTEWMAVVGGICA
FAGLIIQLWSTYRKDQRDEELHKKLMGEDSHDKQD" /product="hypothetical protein"
/transl_table=11
CDS complement(215916..216695)
/locus_tag="AUO97b_00353" /db_xref="SEED:fig|400667.82.peg.213"
/translation="MTQLFFQAKNFVSKRSLPKLSNVDDLLPNLEYEAYGHWVFENTS
ASLTDKVNNRLLALQSGATVQPTYTESGVTISTAVGNALVSDLSDTSAQSVTLTAVVK
CNNTQLAILLGNLVPSNSTSSSGLAGFASAGKGYLTVKPTAAAGSGGISSLTPASGYN
QTSNFFIAVSVDKATKKGIIYVQQNSAELTNEAVYTAPVYESALNKFAFGNNAYSISN
ATGNTATFAEGVIFDKALTLDEIKAVAARAKSRMKNRGIIF" /product="hypothetical protein"
/transl_table=11
CDS complement(216698..218554)
/locus_tag="AUO97b_00354" /db_xref="SEED:fig|400667.82.peg.214"
/db_xref="GO:0004308"
/translation="MADYLTPAKLENASLDCDSWDKFFHGTEFEDVVTRLGKVFPTLP
KVLKLFFDNGLFRPFQTEAQLLAYVPETNPSAAYAFDTKKLYSWDGSVWNDEGLSPLD
LSKSYTDEQFILAVQKSVFETLSQLFGLAKSDDEKIKSLFTDGAGKVVIGYDLEKDTG
IYGGMLEQVVEIVPGLKIYNDGRYLGLLGDSERGILIGYDMLNDLPVIAGLEELIAGL
VIPNQKPLVKAVNHMLWYGESFSVGQKGTPPISVQQPYSNLTFDSTVRLINGTATATK
PLVEDAGINNPPVSTSQGETCCSGAANYASRAMMLENGIDPNNHVIFASTAGHGSYRI
DQLEKGTAWYPTLINHVTQAMSIVGPDYKVQCINFIQGANDALSTVRTPKSVYKPKLK
QLRIDANADIKAVTQQSDDIKFIIAQMSYGTRAWSDQALSHLELVQEDESFCMATPMY
HFPYDADSVHLTNIGYKWISAYFGRAYKQLIVENRQPDFINPIVAQLIGDEIHVKFKV
PQLPLVIDTETLAVTTNHGFKVLVDGAATTISNITVQNDTVILKLSTEPTGTVKVRYA
LDYLGSGINLTEGASGNIRDSTDEVTIIDGEIKPLYHVCPHFELTAFTDKGI" /product="hypothetical
protein"
/EC_number="3.2.1.18" /transl_table=11
CDS complement(218615..221473)
/locus_tag="AUO97b_00355" /db_xref="SEED:fig|400667.82.peg.215"
/translation="MSLKRVVIVPDVYKRDTWAYADVEDVCAYLAQQFAPSLPSGTKI

YHGAIAESQNITPKNKRDIEKLQKLEGVFYVVVMPQEPISIAMWIYYAIVAITTAYTL
YTVLTMPKPPSPTAGSANNELAQRSNQPRINGRIPDIFGRLRSYPDLIAQTFTTYVDG
VEIEECLMCIGRGYYQILDMRDGDTDVANIAGTSVSVYDPFTSIVGIPIYRVGEEFNH
LPKYVVNSDSINGQTIEQPNTAEFETQNVWFQSPNLIRATGIDFTQYFAANDRILLSG
AVYGVQDVSLSGTIMVNENKMVIIESSTNIDNPNLFKGLLLTGALVDIETTTGTPPDQ
ITEIDTRDLSGQFDVSGVTKTVISGGFHYEITLSNPQRVNANWLHVNKSYTITAGASL
NHNANSITLDDIYTINSISSDTIALVNPSAINNEWDKLLTLPNQSTQGQDALVRFDSI
SSKYVGWFNLEMPEATQAVFNLFFPNGLFYQDSKGGVWEEGFTLVIELQSIDNSGNPV
GEVTTIQQNIRAKNKSQFGRTLYIDLPNAGSFRFRLSRTTATQAGKTQDTCKIKSVYG
MAESSIDHYGNVTVIRSKTVATDGALSLKERKLNCLVNRKLPINGTGPLQVTRSAGQA
LINLALDEYIGRRTIDEIDIDQINEEIEKVNAYFGSDRMSEFNYTIDDDNLSFEEIAG
MVASAAFCEVYRYGSVTRLKFEQPQENAVLLFNHRNKVPGTEKRTTRWDDKNYDGLEL
EYTSDKDDARIKYSLYWDNATKSTKEGPFAKNPLKITTTGIRNEEQAKTRAWREWMKV
LYKAFLCEFEALDESELLLRNDAILIADNRGDLTQDGEIRGVDGLFLTTSQPCFFENG
QDHYIYVQLPDKTVDMIPCVAGDDEYSIKLLRAPLIPIIINPSNYIGYTGYKLVKATE
AEAFMFMLDEITPKSPMTNNLIASNYDSRFYQHDHDFV" /product="Phage-related protein, tail component" /transl_table=11
CDS 221618..221749
/locus_tag="AUO97b_00356" /db_xref="SEED:fig|400667.82.peg.216"
/translation="MQGEIGSSKDLNSPSPFKNFFKREVKSSPVISSQASHANSLHV"
/product="hypothetical protein"
/transl_table=11
CDS complement(221787..222227) /locus_tag="AUO97b_00357" /db_xref="SEED:fig|400667.82.peg.217" /translation="MSHPDFESSFHYTSDDTYGVTVKHEQDGPDYFYQYQPMTIKRST
VSNNLDQKISLTIGDVDDKLILAADKSRLSSYWNVRPKVKWRLYRDDNLTSPLVTLQT
LEVASISKDDTGNCTFEAVAPELNKVKTGEIYDLERFPLLRGLI" /product="FIG00352949: hypothetical protein" /transl_table=11
CDS complement(222286..222774)
/locus_tag="AUO97b_00358" /db_xref="SEED:fig|400667.82.peg.218"
/translation="MTLNILNRCSTQSGYSVAYRSGVIAQELDGGAPRYKRRLKNAYH
TVNVRWVVLEAGFQYLDAFYNVWCDNPSQPFYANLKVNGPEYKPYECSFVPDSFQLNE
VEGKFFTLSAQLRVRPIVDSNLNKIIVETGNKGDLATLLNPLEKLVNKDAPKSMEGII ND"
/product="putative bacteriophage protein"
/transl_table=11
CDS complement(222776..226363) /locus_tag="AUO97b_00359" /db_xref="SEED:fig|400667.82.peg.219" /translation="MAANSLGRLTLDLLVQTAEFTGPLSQAERQARTASKNIASSFDI
ATFAAKTLGAVVAGVSVASLVEFANQTIETGSEIKKFAQLSNTSVREFQYYAKGAETA
GISMESFADKMKDMQDRIGDFQQTGGGPLADFFENIAPLVGVTIQQFQKLSGPQALQL
YYDSLQKVGASQNDMKFYMEAIISDSSLLIPLLQNGGEGFKKWGEAAEQANAILSEDM
IQNLALAKENLRLMDLQWQGLEARLINNVVPVVKAVANNMDDIKAITIALAAAIGSKL
VVQGAILAGTFTMAAIRGAAMELTLMGMQRQALGAATSMGVLRGVMGFLGGPAGLAML
AVQGVAAGAAFYYMKKSSDELDPSLTIQQKSVAELRAEYEKLDATQQRVLTRKATDTL
ETANQLYREQKNEMLGLIDAITRSSKVSENDKQIAEGLFERYRLGKIDANQLANAING
LATVSDSAKASIDNKAKAVMDEAGKVVKAQSILDVYTGKVQNNTKANNDNAKSIGEQE
KALLSLTQKQREALKDIQGQLDRESYIQASMRTGISREHAEYYADYKDNAGISYFKPL
SNLEVKLVEQGYKLKEQTKDREESEKKIEETKKRQLELTQKQALVVASGNETSRNMLK
VYQAFMNTGVFSDQQARYLTAEVGRENDFRSSKMFGSHKDKNNGFTNVGIMSWQKSRA
KDLMSMLFSKGLLDNKGAIKETQEALNAQALFLAKEVFTDPSYTKSKNALLSNAGYKE
LEKIFGKNYVRWDYEGRKIKAGPHHKKRDNYYNQLNTLLGDGTTTNISSAIQSFSKLD
DELANVAERLKNDKRTLVEYYDEWQKISNANDDKVNEIGRLYADDPVMRDRLLKLQAE
AYAKDVENYIKAQDEKVAAQRKVAWEILEAQYSVINASKAMDDQIRGLSSGADDIFAK
ATMSPNDYAMWSLANQKSNAQLELSNQRVAVEQGIMTSDSFSNDDERYQALLQAHEEY
LIQKNALDVQYDQQVKDLQKSQFETQLGLWSTLLGQAQNTWSQMTQSVKTAQGEQSSA
YKAMFLMQQSIAIASAIVSAHLAAVQTTADITLPFAGKVSAANAILAFGYANAAMIGA

QTIAGMAHDGIDNIPREGTWLLDGGERVLNPQQNKDLTNYLSNQKNSGPQVVINNMNG
SRVSQRQGADGKLYVDIDDVEGFMVGALSDPNSKVSKAMQHNYNAQRRR" /product="Phage tail, tail length tape-measure protein H" /transl_table=11
CDS complement(226423..226806)
/locus_tag="AUO97b_00360" /db_xref="SEED:fig|400667.82.peg.220"
/translation="MKKIILISALSSLAGCATTENFYSLQPSPLATYGQWSGAHKDIS
VATVQLNKDGTGVICQDFKGVAKLSAIKKIDDKIYTQDGSYWRIKEFNSEKLTLAYGA
GGSYFLTKDDSGNNISPECKIKLIK"
/product="hypothetical protein"
/transl_table=11
CDS complement(226845..226982)
/locus_tag="AUO97b_00361" /db_xref="SEED:fig|400667.82.peg.221"
/translation="MAHITRFKVKEGTDVDARDFMPHEERPENDDELSLEEYFEKYHS G"
/product="hypothetical protein"
/transl_table=11
CDS complement(227096..227512)
/locus_tag="AUO97b_00362" /db_xref="SEED:fig|400667.82.peg.222"
/translation="MARLTLEQTHQELGIGSYVEKPIRYRDKNGNEAGGEVLILIASH
DEIVKAPDVWKLKNKAELTIDQLKKALIFLTVYHEEGEKFFPTVEDTGRLSSEVIEAL
YKAADEVLDFSGKNSISNQTMSSGASSSSMELAEEQ" /product="hypothetical protein"
/transl_table=11
CDS complement(227512..227991)
/locus_tag="AUO97b_00363" /db_xref="SEED:fig|400667.82.peg.223"
/translation="MAKHVKSQKTQLFTVIAGTVVRFICPKRISFGQDSFSKIDVTCL
DADVKEYERGMRDPGEGAIGIDLDDENTSHDKLLEIAASGEKLQWYVGSSHSATPPTY
DATTGIDLPETRSWWSFEGYLNDAAPNDIEVDTVIGYEFTLVRTSGVKYTKRTMVTP" /product="Major tail subunit"
/transl_table=11
CDS complement(228061..228435)
/locus_tag="AUO97b_00364" /db_xref="SEED:fig|400667.82.peg.224"
/translation="MIKVPIFKLARADPAVRALLESNNILRVWRFGSAPEQPETPYVT
WQIIAGDPNSSLDSRPVSDSAIVQIDVYATDEDVVDQVAEAIRFAIELDCYVVRYGEA
DKDPVTGMPHYSFDVSWIVNRE" /product="hypothetical protein"
/transl_table=11
CDS complement(228435..228920)
/locus_tag="AUO97b_00365" /db_xref="SEED:fig|400667.82.peg.225"
/translation="MENEIQGLEPALRRMRAIGNDKTVKRIARKAMRQAMNIARDEAR
QKVKRLDDPTTPEKIWKEIVVQNGRSRNKNTLVMRVGVRGGARIPYTNNAQNRRSGRV
GKTYQTDGRVFYWRFLELGTSRQPATPFLRPALYENIEQITDKFVQVFNFELSVVLGA A"
/product="hypothetical protein"
/transl_table=11
CDS complement(228924..229280)
/locus_tag="AUO97b_00366" /db_xref="SEED:fig|400667.82.peg.226"
/translation="MPSITPKLKHRITIQKAIQTQDQNTGKLITSWSNFATIWAEVTD
LSTRDVVAAKAANSAIQARAKVRYSSTTKQIDSTMRVLFDGYFYKIDGNPMRDPDSRR EYLTINLSTGDKAWNG"
/product="phage head-tail adaptor, putative" /transl_table=11
CDS complement(229282..229569)
/locus_tag="AUO97b_00367" /db_xref="SEED:fig|400667.82.peg.227"
/translation="MITLERAKLQCRVDHDDEDVLFLEWIAQADEEIAIDIDRKIISN
ESERTSETDIVDCKKLDNARLIFIEYKYSRSLEGKPQAYWDILQPIREMGV" /product="hypothetical protein"
/transl_table=11
CDS complement(229566..229742)
/locus_tag="AUO97b_00368" /db_xref="SEED:fig|400667.82.peg.228"

/translation="MKEYEVLRPHFGDKDYKEGDIRTADPNVVRHLVENKVLREYQTK VDPPKPATRRNNSK"
/product="hypothetical protein"
/transl_table=11
CDS complement(229790..230962)
/locus_tag="AUO97b_00369" /db_xref="SEED:fig|400667.82.peg.229"
/translation="MTEKTLEQLAQEFQKHVDTVKGYAEEFKGKYEKNEQISQSAKDK
ADEALTAMNEMKNKVAEIEQKAARRGNGDVETKKQTMGGEFVETTEYKNAAEAQYRGI
QRVELKNTIGTTEVGKIIPATNLGLQLPNQMRLTIRDILAGGSMSGNVLEYVQMQDFT
NNAAVVAEGAPKPESAITFTDKDAKAVVIAHWLKVTTQMLSDAPALQSFIDNILRHGL
DIKLEKQILAGDGTNGNMLGLIPQATAYAPPAGAPATPNMFDVLRFAMLQVVLADDFA
NGHVLNPIDWALMETQKDANGNYIIGNPQSQAVPTLWGLPVVQTAAMDAGKFLTGAFN
TAAQYFERWGAAVQIGMQGDDFTSNKRTLLAETRGALAVYKPKSLVYGSYTPATGG" /product="Phage major capsid protein"
/transl_table=11
CDS complement(230955..231617)
/locus_tag="AUO97b_00370" /db_xref="SEED:fig|400667.82.peg.230"
/translation="MDRLKLTLEIKATQEGGFFSGYLAAFDNLDSHGDIIRKGAFAKT
LQEWKAKGKYPAIFWDHNPSEPIGIFTEMREDEKGLYVEGRLLIDDVPRAKATYALMK
VGAIDGMSIGYITKSYRRDPDSLIRELLELELVEGSIVAFPSNPETLISSVKSKLQDG
ELPSLPEFEKFLRESGFSKTQATVIASKGLRHLLSESEGENEKAKSISNALNILRGIS ND"
/product="Putative prohead protease"
/transl_table=11
CDS complement(231610..232836)
/locus_tag="AUO97b_00371" /db_xref="SEED:fig|400667.82.peg.231"
/translation="MSTTQEPGFWSRFWSRLTGNTQLKKGDSSYPFDSYLSPGGSVVT
PETALKLSAVWACVKLRAETISTLPLQLYDNNKRLATDHYLYRILHDSPNADMCASEF
WQVQVACVDLWGNAYNLITKDSSGKVIALEPLFPSGMVVKRNDLGAIDFHYTENGKTT
TYSEDQILHFKGFTLDGLVGLSAIQFFAQTIGMQFDANNQAQDWFKNGLKVGGFLETG
EQTLTKEQRERLRNHLSEFSKPENAGKYMVLEAGMKLSGSNSIRINPVDAQLLESRYF
GIEEICRAFGVPPQLIGHTNKASSWASSLEQTNRGFLTYSLNPQLVRYEQTITKRLFL
PSEKYKYRPKFAVEGLLRADSATRSGFYTNMIQNGVMTRNEVRDLEDLAPLPGGDELM VQMQMVGLKDQGKTSG"
/product="Phage portal protein"
/transl_table=11
CDS complement(232833..234533)
/locus_tag="AUO97b_00372" /db_xref="SEED:fig|400667.82.peg.232"
/translation="MTAMLPDWTTACPDWADRIVKGQSLMPCKPLFQDVADVALRTFN
SLKVVDVLDSPEMGEIVRKWVTEFVAAIFGAYDKKSKRRLINEFFLLIPKKNTKSTIA
AFIMLTAFILNSRLSAELIIILAPTKEVADNSFNPIRDAIKADPELDEMMTISEHTKTI
THQGTQATLKVVAADDKSTGGKKASWILVDELHLFQTMSNAGSMFREATGGLASRHEG
CLIWLSTQSKEPPCGVFKSKLDYARDVRDGKIINKKFLPLIYEFPDEMIESEAYKDPA
NFHIPNPNFGTSVDPEQLLDDFEKAKYAGEDDLKDFYAKRLNVQIGMNLRANRWAGAD
FWEKKEVVFDLDYLIEQSECITVGFDGGGLDDLFSMYAIGRDKKYHTLWRGWSKSWLH
PIALERRKENKQRMDDFIAAGELVIVENIGDDVAQAGVIAKRIFDTGKMPKQGFGLDR
LGMPSLVDGLLESGIPETALIAVKQGFELSGYGMTLERKLAAGTFIPAKQELVKWAVG
NAKGKISGNALMVTKQESGKGKIDPVIAMFNAAALMSSNPEPANRVDIDEYLEDVVIA "
/product="Phage head, terminase DNA packaging protein A" /transl_table=11
CDS complement(234656..234850)
/locus_tag="AUO97b_00373" /db_xref="SEED:fig|400667.82.peg.233"
/translation="MFGMLESLTKAAVSVAVAPVTAVVDAVMTPIDASEDGEVFQRTK
STLNNAAENFSDAVKPENKK"
/product="hypothetical protein"
/transl_table=11
CDS complement(234860..235342)
/locus_tag="AUO97b_00374" /db_xref="SEED:fig|400667.82.peg.234"

/translation="MALTEKMEKFALAIVDGKTNKEAAISAGYAEKTASAAGARLARD
PEIIVYIEMLKAQKEGRSLTSDSPKVKPKETLENSGEDEKPIEEFQFEGDDPLEFLTK
VMSFNGNKLNIRMQAAMALMPYKHGKVAEKGKKETKQDKAKEATKTGKYATLDNQLPS " /product="Phage terminase, small subunit"
/transl_table=11
CDS complement(235693..235851)
/locus_tag="AUO97b_00375" /db_xref="SEED:fig|400667.82.peg.235"
/translation="MVQEYQRKGLDIDWISIDLLNGVDARVNLNETPNIQEQVADATG TRTSSEES"
/product="hypothetical protein"
/transl_table=11
CDS complement(235961..236224)
/locus_tag="AUO97b_00376" /db_xref="SEED:fig|400667.82.peg.236"
/translation="MGDVMNFDLEQIKPETKYLIVKYDSRSFVSSHQQECFQKELKAI
AKQTGLSIAAIDKSVTLEQLSDEDLANLGLRRIGEQDDSEEHY" /product="hypothetical protein"
/transl_table=11
CDS complement(236217..236399)
/locus_tag="AUO97b_00377" /db_xref="SEED:fig|400667.82.peg.237"
/translation="MSSEPNLQDVVLKLIEQNNKLIEQNSLIVQINAEQSAQLSEVLL MLEDSEPAQRSGSLDG"
/product="hypothetical protein"
/transl_table=11
CDS complement(236389..236919)
/locus_tag="AUO97b_00378" /db_xref="SEED:fig|400667.82.peg.238"
/translation="MEPATFPINSYSGIVQVINYLNNNHSKAAAEGKPLVVRINQKED
DRSAAQNRLYWAWLEQIKQKTGNSKDDLHLLFKKKFLARIYVEGRQETAEKYMALQNF
KDVIQAFDGPKRRQLEKDYQVLVNTFIKDHLQSKKATIKEFTKYLDKINIYAHRDLGV MLIIPDDLKWCYQNEQ"
/product="hypothetical protein"
/transl_table=11
CDS complement(236935..237333)
/locus_tag="AUO97b_00379" /db_xref="SEED:fig|400667.82.peg.239"
/translation="MKTNQKGQADVVLVTLAFVAILIVIILLMFAWPHYKVWKQGMNG
QALLAEAEQSKMIQVQTARAELESAKLRAEAIKTIGQAAKDYPEYRKQEFIGAFGDAL
RDGKIQQIVYVPTEANIPVLEAGKRPVVDE"
/product="hypothetical protein"
/transl_table=11
CDS complement(237417..237776)
/locus_tag="AUO97b_00380" /db_xref="SEED:fig|400667.82.peg.240"
/translation="MLFVDLQVYEEILKSSHMVKLSQPTNENPLTFNGCELIPVERMQ
SEFKYLSHKDLASAIKQFNIMPTDLIVIRKAENTNRKEAANARRLGNETMYKHFYIDS SIVKAYQRHYENKNLEF"
/product="hypothetical protein"
/transl_table=11
CDS 238059..238181
/locus_tag="AUO97b_00381" /db_xref="SEED:fig|400667.82.peg.241"
/translation="MDLPLPSLLELECAAWLGAGAPHLMHGSLRTLANRLASRI"
/product="hypothetical protein"
/transl_table=11 CDS complement(238170..238340)
/locus_tag="AUO97b_00382" /db_xref="SEED:fig|400667.82.peg.242"
/translation="MDTNEAKKNLNKYSDELNRYQNLSRTGLSREEMLVIDRIIIRLK NKINNLRSMLNA"
/product="hypothetical protein"
/transl_table=11
CDS complement(238358..238570)
/locus_tag="AUO97b_00383" /db_xref="SEED:fig|400667.82.peg.243"
/translation="MYMPEMDLTKHIQLKEVLRQEALKAMDQMIEMQQLESAGNDFKP
FTQRNDIKPATTKTMKNRQKRLRKKK"
/product="hypothetical protein"

/transl_table=11
CDS complement(238746..239462)
/locus_tag="AUO97b_00384" /db_xref="SEED:fig|400667.82.peg.244"
/translation="MDELVTAAATIHAAQIQAYYALWAAVIGAIGIIFAAWYAWQSGI
KLQQHNNIIEAKREVYLDAITKYQQLANDLKLITYAPDKFHEVYLSNKKDFLISINKV
QLICDKANEYVVLDFKNNVDTKIIELYGFFEEFLESYYSLIGFKEKLELANKDLNNFL
TENPKLAQGRVLHTGIQLSATKIQNELAESRKEYEAAKIGYDFNLMQINEKIQNFEAD
LINKYNNFCNILRAELKIVN"
/product="hypothetical protein"
/transl_table=11
CDS complement(239553..240050)
/locus_tag="AUO97b_00385" /db_xref="SEED:fig|400667.82.peg.245"
/translation="MNNKPHVLQACNWKKYTIENWLEQFGAWINEDNAETYLGTRNTL
TYLIDSVEGVKRDARKRSLPQCKISTDEARAVSGLLRDLRMNPNPTLQEWLDFVVLYY
VHGLSEETIADISKCSRNAVRQDLKCGIAYIVGQRNTLRSKLTEKQAKVRKPKKTLDL APIVL"
/product="FIG00353189: hypothetical protein" /transl_table=11
CDS complement(240050..240220)
/locus_tag="AUO97b_00386" /db_xref="SEED:fig|400667.82.peg.246"
/translation="MNEKWTYKEMMALRCAYNHGVRTPETRAAACLYVKLGRNKLLDQ FKKESEAKGKVE"
/product="FIG00353438: hypothetical protein" /transl_table=11
CDS complement(240217..240819)
/locus_tag="AUO97b_00387" /db_xref="SEED:fig|400667.82.peg.247"
/translation="MKPEQFIREKGLDKCGDEFEQHFLSLPFSNSEAAQKCLDACDFD
VKQNAFIPNAKWFNNNDVDEGVIYCCMLNTAYMSFLKQQAKVEGLKATIKGNHGRIAE
LERLNRVKAQAILDLHQEIKELKASHHGEVIGHEVHLKKIKQERDELQKQVDACQKLL
NELKDAKAELVECDLYDKGVRCALYSVIQDLEQALKGGEA" /product="hypothetical protein"
/transl_table=11
CDS complement(241122..242423)
/locus_tag="AUO97b_00388" /db_xref="SEED:fig|400667.82.peg.248"
/db_xref="GO:0003678" /db_xref="GO:0005524"
/db_xref="GO:0006260"
/translation="MSFNSNIHDVNMEQCVLAALMTTSLSLESIGQELDAECFYSDRH
QQIYKAIVELSESNHPYDVVMVSNYLKGKNVLHLMGGEDYLIQLMQDAPSSFYNAESY
VTQLKKLKTHRKIEQIGFRIAAMAKDTTVPDAFIEAENLLSQIDKTEDGDMGASFGDA
LTSALAQMIEKSEKKTKNQLSGVRFNLVTLDKMLGTVQNGHFCVVGGRPGSGKSTLAQ
MMAIDTAMVKKEGVLFISAEMDKETLSNRMFSSLSSIPYDNLHNATLYDGLLKEYARY
REVYSGLPIWIEPKQKPSISEVRAYARRAKRRFAKAGVKLGCIIVDYLQLVRDPSKKD
RFQEVGSISRELKSMAKEFECPVVALVQLNRESEKGKKPKASDIKESGQIEQDADQII
LVNPLTDDKTLQPLGGYRINYCQKPTREKGGSPSSRAFRHV"
/product="DNA helicase (EC 3.6.4.12), phage-associated" /transl_table=11
CDS complement(242423..243307)
/locus_tag="AUO97b_00389" /db_xref="SEED:fig|400667.82.peg.249"
/translation="MHYYERNIGDYYRKAGRLNILQHGVYNLLMDACYDRESFPTLEE
AIEWVWAETEEEIDAVKFVLKKFFKLNEDGVYIQNHIKEELEKYRAFLAKQAENGKKG
GRPKKNPKNDSGNNGNDFDNSGFKNESQDNPNESELNPEKPKETQIKPKPSNHLTNEP
SNQENNICPPNGEPVPAEKPKENFKNEIQEVFEFWKVTFNKNNRTILDNQRKSKIQAR
LKEGYTVEDIKTAIVGCSKSQFHIEGNHTDLTLICRDATKLDHFLAMSNPAQVAIQPQ
IEDVQTIPAQYKVIEGDW"
/product="Primosomal protein I"
/transl_table=11
CDS complement(243307..243603)
/locus_tag="AUO97b_00390" /db_xref="SEED:fig|400667.82.peg.250"
/translation="MNEQILAQNSDCASPNDDEDQVLTQWQVDHDAYVETLDAYRKAH
TDLEKALGIEKDFDKTSHSAKEIIEDLRKNWHLYALINRFESAVINRLRAKDKL"

/product="hypothetical protein"
/transl_table=11
CDS 244041..244640
/locus_tag="AUO97b_00391" /db_xref="SEED:fig|400667.82.peg.251"
/translation="MRGTGAGKATVSSWIKGAVNPSATYIDSLAQVLKTTPTWLLTGQ
INDTASNMDNNVDINSMASVIYAPVLSWVQAGNWTDMDAVNIQECEKLPLVPGAGKKS
FYLEVKGLSNAPYFEEGEKICIDPDYVLEDIQTGEMVVVRCGNEATFKALVAEPNGYY
LKPLNPNWSEQIIPLKEDCILVGKYVGSFKPAKKFNFLT"
/product="Phage repressor protein cI"
/transl_table=11
CDS 244895..245332
/locus_tag="AUO97b_00392" /db_xref="SEED:fig|400667.82.peg.252"
/translation="MNQITNLSQQKPNLNDFRNLAFEVSCHLDQLAAFMLQASCLEEH
QDEIKASCMAKAVSKTSLIIFNKTLLIIDQMEELFKSQKLVEFKNSFVFVESAVFAIS
ETNLTLKHQANYFYGIFHVLKELEKDINDMDLNAEIEAEKAHG" /product="hypothetical protein"
/transl_table=11
CDS 245325..245597
/locus_tag="AUO97b_00393" /db_xref="SEED:fig|400667.82.peg.253"
/translation="MANLKVDLTDHFDAVCLAKNDLDWTLYALHEIKEKVKALKEEAA
KHGMHEAYFHSLEQFTGILEYTMTLRSDYWEDEEMRIQKQLDGNGE" /product="hypothetical
protein"
/transl_table=11
CDS 245600..246232
/locus_tag="AUO97b_00394" /db_xref="SEED:fig|400667.82.peg.254"
/translation="MRTSSQLFPENKIVTVDDLVTARSEAKNDMGDINALLSAIELSL
VEKLKDHNLSKFAFDKTFRLIDIAKTHVDLSQDYHNGELAQLTGGGKYQCTAVKASKS
LDTKPDGKPAKELKDRLKLSRKNAGITQEVLALSSGTNQSTISQLESGLMRSSTHIPA
IAKVLGVDAHWLQTGREDSASVKINQLCLAPANAAISKTLTEGFKNDGRR" /product="Transcriptional
regulator, Xre family" /transl_table=11
CDS 246327..246452
/locus_tag="AUO97b_00395" /db_xref="SEED:fig|400667.82.peg.255"
/translation="MEFYCATVSKLFPRNVHIMYDGKMNKAATQLYKEFENHLKA"
/product="hypothetical protein"
/transl_table=11
CDS 246455..246718
/locus_tag="AUO97b_00396" /db_xref="SEED:fig|400667.82.peg.256"
/translation="MKKIELNIISGTSDQIATEIFQKIIGPMVDEMNSQDKDSAKIFT
FSVMWLGMALYAAQFEPHNAKKTIQFSVDQFMATFDKFNKRPS" /product="hypothetical protein"
/transl_table=11
CDS 246730..247260
/locus_tag="AUO97b_00397" /db_xref="SEED:fig|400667.82.peg.257"
/translation="MTDLNKEGKVNLSFEQDNGAVWVFAGDSQFGTEISHLMMMHADE
YSEDELRVICHHAACEIDRLRAELEKAKAQAVPEWISVEDQMPESLRNVLVLLDANSA
KNQNQMVAHFIPKFTEEYHGDDDWYDYDEERACGYVKEGWYANTAYIGDEYSSYFIEE KVTHWKPLKEASESGS"
/product="hypothetical protein" /transl_table=11
CDS 247264..247746
/locus_tag="AUO97b_00398" /db_xref="SEED:fig|400667.82.peg.258"
/translation="MNAKILDPCCGSRMMWFDRQNPNVVYGDIRKEEHTLCDGRSLVI
EPDVMMDFRDMPFKDGQFTLVVFDPPHLVKAGKQSWLAAKYGKLSEDWREDIRKGFAE
CFRVLANGGVLIFKWNETQIKVSEILELTDQKPLFGHISGKRSNTHWITFMKIEVAEG "
/product="SAM-dependent methyltransferase"
/transl_table=11
CDS 247743..248012
/locus_tag="AUO97b_00399" /db_xref="SEED:fig|400667.82.peg.259"

/translation="MSEVKVKTCEFCDDGNGECIFPYYGLAPHIHTKPIGGTVFLDES
FPENFIPDGDGLGMYTHCLNCGGDGTFEGTQLEVKAESKEGLLRD" /product="hypothetical
protein"
/transl_table=11
CDS 248005..248256
/locus_tag="AUO97b_00400" /db_xref="SEED:fig|400667.82.peg.260"
/translation="MTEVKFVSMPASELAQVIEKACENAVTKVLAAQGDELLNITQLC
ERIPGLSYHSFKKLAKEHRLKDIKGRYSLTAVKAALQSH" /product="hypothetical protein"
/transl_table=11
CDS complement(248222..249181)
/locus_tag="AUO97b_00401" /db_xref="SEED:fig|400667.82.peg.261"
/translation="MATFQKRNGRVTATVRIKPHPAKSKTFDTLRDAKKWAQETEVRL
KNEKLEIFDHIIFKDALIEYRDTVSINKRGYEKERRKINFLLKAMYVDQPLIQVNKDF
LTEWREQRLLNVKGATIRREFILLSAFFTWCIEVKRWLSVNPLREIKFPSESPHRERV
ISDEEIEILLPFLSTEMRYIFLIALQTGMRLSEICNLKWEKIRLSKSYLILDLTKNGR
AREVPLSSQAVEIFKSIGPKKQGYVFSITSDDATDEFRDAKLEAGLEGFTFHDSRHTA
ATKIALKIPLLDLCKMFGWSNPRRAMIYYNPTSSEIAARLSQP" /product="Integrase"
/transl_table=11 CDS complement(249358..250500)
/locus_tag="AUO97b_00402" /db_xref="SEED:fig|400667.82.peg.262"
/translation="MLNLKSSHSTAFTPSSPAERYAEALASGQFMADEAQAQAVQELD
RVWKELLNRYKASKKAFRRFRRQTSPKGVYMWGGVGRGKTWLMDQFYESVPFRRKTRM
HFHHFMQHVHKELNKLSGQRNPLDIVADQIYKDAVVICFDEFFVSNVTDAMILSDLFQ
KLFVRGVTLIATSNIAPDGLYKNGIHRDRFIPTIEMVKKNCVVLNVDAGVDYRLRVLK
QAQLFKSPLGNEAQSWISERYTALTHTQTNSHEPIVINNRVVETLGHTEDVLWCEFSE
LCLKPRSPADFIEIANIYNTVLVSNVPHLTDFLSEGTRRFIYLVDEFYDRGVKLLLTS
QDSIIDIYQGEKLAFEIERTRSRLLEMQSDEYLHSEHRHIDATKTS" /product="Cell division
protein ZapE"
/transl_table=11
CDS complement(250584..251603)
/locus_tag="AUO97b_00403" /db_xref="SEED:fig|400667.82.peg.263"
/translation="MKRSALGLIYLIQGMRNAGIDVDSRLADIGIKVDSLDPSSTIHD
SLEWDIQQIISENVDPEKGLFIGQHYALAGYGPLLMLLVTSHDIQTALEKGIQYQKLT
HLFGTLSLQETENHIILNYLPVDLKTEIGLLRAQCEISGTYKFIQDIYTMMGLKMPDM
RIDLPFPKPSDPDTVAQYYAYYGDDLHFNSTHAAFSLSKNVLEIKIPSADIITHRIYE
AKCIKEIERLNEQDHQIPPIIQYVQDYLELQQGIIPSMAETAFALKMPERTLRHQLQQ
LNTSYKQIREQIIKNKALKLMEYKEYSIEVIAEALGYSEPAAFNHAFKRWFGQSPRQY GKEFY"
/product="Transcriptional regulator, AraC family" /transl_table=11
CDS 251764..253272
/locus_tag="AUO97b_00404" /db_xref="SEED:fig|400667.82.peg.264"
/translation="MLNAKQHAIQKTKIAIIGAGFGGLAMAIRLLQSQQSDFFILEKS
NDVGGTWRENRYPGAACDVQSHMYSLSFAPKTDWSKRYAEAPEIFDYIQDITNQYQIK
NYCKFNHEVTHVQYDEVRHIWTLDFANQPSLEAQFVVFASGPLHIPQIPHIQGIEKFK
GKVFHSSQWEHDYSLEGKSVASIGTGGSAIQYIPEIAKDVKQLYVFQRTAAWVIPRDE
RKYSNLSKALFKKSNLYRQIHRSRLYWSNESRVVPIVQPQIMKYGQKLAEAFIRFQVK
DKDLAKKLTPDFVMGCKRILISNKYFPTFNRKNVELVTEGIQEIKENSIITKDGKERP
IDCLIYGTGFVTDPRIYLKHFTCIGRNQIELKQAWKNGAESYYGIMTKNFPNLFQLVG
PNTVLGHNSIIFMIESQVNYILQLIDLVEKSGQHAIEIKPEVQDSFNERIQTQLQGTV
WQAGGCSSWYQAADGKNFSLWPTYTWKYWLDTRKVNPKDYLLLNKCSQSYAA" /product="Cyclohexanone
monooxygenase (EC 1.14.13.22)" /EC_number="1.14.13.22"
/transl_table=11
CDS 253322..254233
/locus_tag="AUO97b_00405" /db_xref="SEED:fig|400667.82.peg.265"
/translation="MQSISLIIQIFLVLAFGYFLGPKLSLNIRHFIFKILPYFSYILL
TSVALELTLALDQIDNPSTILPPALLIALTTSLGSFFTCLFAYTIFDKESVKGKISLQ

LFMNALKNIAKAFLALTIGVILGILLTQLHTHIPFNSWYLLLLFIFLIGIELAFTHFN
RTWLSWKILIVPLAAFIGSCLAGFLNYSLLGHEFTLNEILALGQGYGWYSMSGILFTQ
LHSAELGGIALLTDLFREIVAILLMYTMGWRFPRPAISSAGATSMDVTLAMVKQSCGT
HYVPHAMMSGLLLSLLAPLLITVFLNF"
/product="FIG00350684: hypothetical protein" /transl_table=11
CDS complement(254230..254913)
/locus_tag="AUO97b_00406" /db_xref="SEED:fig|400667.82.peg.266"
/db_xref="GO:0004590"
/translation="MSIIVALDAKSQYDALKIVEQLDPTLCRVKVGKELFTHEGPSVV
KKLQEENFEVFLDLKFHDIPNTTAQAVCAAADLGVWMVNVHASGGRKMMETCVERLKA
GNYQTQLIAVTVLTSMGREDLKDIGLDIEPVEQVKRLAKLTKESGLDGVVCSAQEAKI
LRELIGQDFSLVTPGIRPEGSNADDQKRIVTPKQAMLDGSTHLVIGRPITKAENPTEM LKSILASIA"
/product="Orotidine 5'-phosphate decarboxylase (EC 4.1.1.23)"
/EC_number="4.1.1.23" /transl_table=11
CDS complement(255094..255459)
/locus_tag="AUO97b_00407" /db_xref="SEED:fig|400667.82.peg.267"
/translation="MRYILIALLIIVFGYSLALVLQNPTELPVDLLFTQVPAMRLGLL
LLLTLALGIVVGLLLGVQVFRVFQKSWEIKRLRKDIDHLRKEQIQSAQLAAAEAAANV
RHEKTVVDIYPQDKNSTPL"
/product="hypothetical protein"
/transl_table=11
CDS complement(255484..255786)
/locus_tag="AUO97b_00408" /db_xref="SEED:fig|400667.82.peg.268"
/db_xref="GO:0003677"
/translation="MTTEALNKSDLIERIALKNPHLAEPLVEEAVKIMIDQMIEALSS
DNRIEIRGFGSFALHHREPRVGRNPKTGKSVDVAAKAVPHFKPGKALRDAVNESGK"
/product="Integration host factor beta subunit" /transl_table=11
CDS complement(255943..257616) /locus_tag="AUO97b_00409" /db_xref="SEED:fig|
400667.82.peg.269"
/db_xref="GO:0003723"
/translation="MTESFAALFEESELNLNVEKGAVIQGVVVNIDSDWVTVDTGLKS
EGIVDRAEFLNEQRELEVQVGDTVDVVVEALDNGMGQTVLSREKAKRAETWTKLEKIF
EDGEIVTGVISGKVKGGFTVDIGPVRAFLPGSLVDTRPIRDTTHLEGKELEFKVIKLD
AKRNNVVVSRRAVMEAESSADREALLAQLEEGQTVTGTIKNLTDYGAFVDLGGIDGLL
HITDMAWKRIKHPSEVVEVGQEVTVKVLKFDRERNRVSLGLKQLGEDPWLAIMSRYPK
GSIVKARVTNLTDYGCFAEIAEGVEGLVHVSEMDHTNKNIHPSKVVQIGDEVDVMVLE
VDEERRRISLGIKQTRANPWEEFAKSHEKGEKVSGTIKSITDFGIFIGLNGGIDGLVH
LSDISWNEQGEEAIRRYKKGDTVEAVILSVDAEGNRISLGIKQLNSDPFNDFLAANER
GALVKGTVTAVDARGATVKLADEVEATLKASEINRDRVEDATKFLEVGQEVEAKIINV
DRKSRSINLSIKAKDEAEEKEAVANLRTASASQDNGPKTIGDLIKAQMK" /product="SSU ribosomal
protein S1p" /transl_table=11
CDS complement(257721..258407)
/locus_tag="AUO97b_00410" /db_xref="SEED:fig|400667.82.peg.270"
/translation="MTVQIITIDGPSGSGKGTLAAKLAAYYQFHLLDSGALYRLLGLS
LHKHDLLEKLDSHLDECVNYARQLNIKFETSAEGTLVFLDGEDVTQTIRTERVGEYAS
KVAAIPELRQALFERQRAFAQTPGLVADGRDMATSIFPEANAKIYLTASAESRAERRV
KQLQGMGLDAKINDILANIQARDKRDMEREVAPLKPAADAYIIDSSELTIDQVFKLMV DYVNSRTVSN"
/product="Cytidylate kinase (EC 2.7.4.25)" /EC_number="2.7.4.25"
/transl_table=11
CDS complement(258415..258858)
/locus_tag="AUO97b_00411" /db_xref="SEED:fig|400667.82.peg.271"
/translation="MRNVITIQKEFNAPLSDVFNLLSKHAAYNTAFAPLQVVRVKDSA
DPERPDGVGSVRRMGFGVIKPLKEEITHLEENKRIEYKLIDNPLVKHHLGRIEFSEIT
PYITLVTYRIELTAKAPVVSKLILAQLKLAITLGFSRLAKAFASS" /product="hypothetical

protein"
/transl_table=11
CDS complement(258928..259404)
/locus_tag="AUO97b_00412" /db_xref="SEED:fig|400667.82.peg.272"
/db_xref="GO:0008270"
/db_xref="GO:0016787"
/translation="MQLAYEQAELAAEQGEIPVGAVIVSQNKLIGAGFNAPIGLSDPT
AHAEIQAIRAACESLKNYRLPEDATLYVTLEPCTMCVGALVHARIKHVVFGTTEPKAG
SLVSARQLLENGYYNHKFTFEHGCLHEKCAQQLSLFFKQRREQKKQEKQQKTSLND" /product="tRNA-specific adenosine-34 deaminase (EC 3.5.4.33)" /transl_table=11
CDS complement(259438..260559)
/locus_tag="AUO97b_00413" /db_xref="SEED:fig|400667.82.peg.273"
/db_xref="GO:0003860"
/translation="MNSPNVNSYHPDLVVEEAKNGWRIVRLNRPKSLHALDESIVTAL
LRVFEDFRDDEQVKAIWLDSTTPKAFCAGGDVRKLRQLVINQEVETANKFFQQEYALD
LLLHNYAKPVVVWGEGYVMGGGLGLFMAAPFRLVTPYSRLAMPEINIGLYPDVGASRF
LADRGQIGLFTGLTGSIMTAAGAYSIGWATHICEAQRDNVLQKVLNINWAHYPAGDFR
AIDDTLNSLHRPVAAGPLQNSLDVIHSVCRGSSFEQDYQAIVSLRDASSDWLRQASEN
LQKGSPSTAAITWLLWQWGKQVHSWDEVFDLEAQISEWKIRHPDFVEGVRARLVDKDL
SPEWKVCEDLTLRGILADDPPITTIDSWNALLRQYGVVS" /product="Enoyl-CoA hydratase/isomerase" /EC_number="3.1.2.4"
/transl_table=11
CDS complement(260559..261272)
/locus_tag="AUO97b_00414" /db_xref="SEED:fig|400667.82.peg.274"
/translation="MQLTEQQQDKLSKVQLEESWKRSLTPFLLSPYMDSLRDFLFQQK
QAQKTIYPPSKQIFNALNITPLDHVKVVILGQDPYHGPNQANGLSFSVQRGVALPPSL
RNIFHELHTDLGVPVSRHGDLTKWAEQGVLLLNSVLTVEAGQPTSHQKQGWEEFTDAV
IDVLNEQREHIVFILWGAYAQRKGQRINREKHLVLTAAHPSPLAANRGGFFGCKVFSK
TNQYLKQHGIEPIDWQLDA"
/product="Uracil-DNA glycosylase, family 1 (EC 3.2.2.27)" /transl_table=11
CDS complement(261323..261919)
/locus_tag="AUO97b_00415" /db_xref="SEED:fig|400667.82.peg.275"
/translation="MLIRKLFKFENAHVVRNCTSDRCKRSIHGHSYKVELLLKASKLD
HGQMVYDFGLLKGVIKDLFDSFDHAICFWEKDDPQYIDACKTFSARWISLPVSPSAEQ
FSRIFFYLAQQVLQSTVTQNGEGDVEVYSVIVHETDTGYAQSFLEDIQNEQMGLLNLE
GIIFSEQVQSEWADPNMYENLKQGIKFHNPHVNLQVEV" /product="6-carboxy-5,6,7,8-tetrahydropterin synthase (EC 4.1.2.50)" /transl_table=11
CDS complement(262149..263108)
/locus_tag="AUO97b_00416" /db_xref="SEED:fig|400667.82.peg.276"
/db_xref="GO:0009306"
/db_xref="GO:0016021"
/translation="MAVSYKHQQGVALLTILIMVALATILAASIAKHQNNTMENTAYL
MRQNQSLLYAKSAEAFFSELLIQDANNAGGVDHLKETWAQPMPPFSIEEGSVSGRLLD
ESGKFNLNNLTNGEGIVNEDAKSWFERLLVRVGLPAELSQAVIDWQDPNDEPTGPMGA
ESSYYEGLDPGYMAANTKFHRIEELKLVRGFDGKKFDLIAPYISALPENSKLNINTAS
PLVLASMDEKLDIGAIERELQTRQQNLKFFQNVDELWQLNAFSAVDSQKKNQVNSLID
VKSSFFQAQIEVVLNNRKRQFTSALMRKDKQVYVYSRSMAPFN" /product="General secretion pathway protein K" /transl_table=11
CDS complement(263108..263809)
/locus_tag="AUO97b_00417" /db_xref="SEED:fig|400667.82.peg.277"
/translation="MIKNKYIHIRSIASRLAARSSSARLTRASGFTLVELLVAIAIFA
VLSLLGWKIFDYLLKVRDRNAEHEVHLFELQDAYQQILRDTLQIIPLSANQGGQLHPA
LEIDNQILRFSKAGVTDPLKQGLSPFERIEYRYDADQKKLYRLKYTNLNTSNREQPLS
STLLNQVDQYQIMVLTPQEVTKWPEVNIDPTKPNELKKLPKGIKIQLTVAGVNYEWIY SLNQGDLSLSQEGNS"

/product="General secretion pathway protein J" /transl_table=11
CDS complement(263809..264189)
/locus_tag="AUO97b_00418" /db_xref="SEED:fig|400667.82.peg.278"
/db_xref="GO:0008565"
/db_xref="GO:0015627"
/db_xref="GO:0015628"
/translation="MKSKGFTLLEVMVALAIFAVAAVALTKVATQYTQSTSNAILRTK
AQFVAMNEVAMMEINQEWLQGTQSKQVTSQGEIWQIDKSAQSTISPNVQKIDLQVSLY
DPDKGKVQNGITHMVFFNYPVKAK"
/product="General secretion pathway protein I" /transl_table=11 CDS
complement(264179..264733)
/locus_tag="AUO97b_00419" /db_xref="SEED:fig|400667.82.peg.279"
/translation="MKSNLPFQSQKGFTLIEVMVVIVIMTIMTSLVVLNIGGVDQKKA
MQARELFLLDLQKINKESLDQSRVLALETHGETDVSPFSYELYEYHDQSTLQVQNIKN
RWQKYTEFKTRQLPAHVSFSVQPLDDQNYSKAKNTDLIGGQTPQLIWFGNGEAKTVKI
QFYLEQKPIGSEIQIDHLGKINEI"
/product="General secretion pathway protein G" /transl_table=11
CDS complement(264743..265351)
/locus_tag="AUO97b_00420" /db_xref="SEED:fig|400667.82.peg.280"
/translation="MDRQAQFRAREALIFQVAEQLLLENGEAGMTLDVLAAELDLAKG
TLYKHFQSKDELYMLLIIRNERMLLEMVQDTEKAFPEHLAFFMLHHLHHPERTVLFHQ
IEEKLSITAQGVHHLFNELYQVRKQRLRIIIRMTDHYLESIQSHMTTRDYLASIWSLT
HGAAAILNSSFYQRYLGSRDTLRVAYIDQALALPKQAVEQYA" /product="Transcriptional
regulator, AcrR family" /transl_table=11
CDS complement(265389..266162)
/locus_tag="AUO97b_00421" /db_xref="SEED:fig|400667.82.peg.281"
/translation="MFVDTHCHLTMLDLTPYNGDLDLALAAARAEGVSKFMAISVDLD
DHIALAEIAKRHEDVGYTVGVHPCEDVDTMARATTEYLTELAQSEKVWALGETGLDYY
HSTDFINEQKDCFARHIQASKNVKKPVVVHTRSAKHDTVDIIRAEQSTHGILHCFTED
WETAKAVLDCGYYISFSGIVSFKNAQDLRDVAKQVPLDRLLIETDSPYLAPVPYRGKT
NEPKYVPYVAKALSEVYDKPVEEIARITSQNFENLLKAI" /product="Uncharacterized metal-
dependent hydrolase YcfH" /transl_table=11
CDS complement(266180..266521)
/locus_tag="AUO97b_00422" /db_xref="SEED:fig|400667.82.peg.282"
/translation="MQPQMGGIIQVNIPDRATLQASYMGYVQGGGLFVPSKQKVKMGQ
EIFILATLPEQSQKIPLTGKVVWISHKQSGFKPQGFAIQLSGDKGVYYKAEAERILAG SMSLDRPSYTM"
/product="Type IV pilus biogenesis protein PilZ" /transl_table=11
CDS complement(266533..267468)
/locus_tag="AUO97b_00423" /db_xref="SEED:fig|400667.82.peg.283"
/db_xref="GO:0003887"
/translation="MWETLTTRFPNIGHGLLFYGKQGCGKHAFAKHFLAWVLCLNKQP
QGACGECSSCQWLKSDTHPNYVHITTDEENKKQNAKIKIEKIRDLLPFVQQTGEGWRV
VVIEPAEALNLASSNALLKTLEEPGERVVLILLADHYLKLPATIRSRLQHFALDRISY
TEATSYLNEHLAETASVPAELLLGLSNDMPLQAVEIATSDWFAKRQSFLEDWTKVVNQ
KNMPLFYSAKWQKELNFSDFMMLLEYLLGDLICVKLNQPQKNIDLQFDQLAQNYNLES
LFSLYEELQQSKRLVEQNVQTQLIIDQLFIKLMNV"
/product="DNA polymerase III delta prime subunit (EC 2.7.7.7)"
/EC_number="2.7.7.7" /transl_table=11
CDS complement(267525..268286)
/locus_tag="AUO97b_00424" /db_xref="SEED:fig|400667.82.peg.284"
/db_xref="GO:0008690"
/translation="MKHIVIPARFSSSRLPGKPLLLIHDRPMILRVVDQAKKVEGFDD
LCVATDDERIAEICRAEGVDVVLTSADHPSGTDRLSEVARIKGWDADDIIVNVQGDEP
LLPAQLVQQVAKLLVDKPNCSMSTLCEPIHALDEFQRDSIVKVVMSKQNEALYFSRAT

IPYDRDGAKRDEPTLHTQAFRHLGLYAYRVSLLQEYVTWEMGKLEKLESLEQLRVLEN
GHRIAIAVAEANLPPGVDTQADLDRLNNMPVESFE" /product="3-deoxy-manno-octulosonate cytidylyltransferase (EC 2.7.7.38)" /EC_number="2.7.7.38"
/transl_table=11
CDS complement(268296..269306)
/locus_tag="AUO97b_00425" /db_xref="SEED:fig|400667.82.peg.285"
/db_xref="GO:0009029"
/translation="MSLAQLIQNAWNKQSSWLIVLRPLSCLYRAGFLLNRYLYSSGFK
KVYTAPVPVIVIGNITVGGSGKTPLLIELVNYLKQHNVKVGVISRGYGGAGPFPMLVT
SASQAAEAGDEPALIVQSTGVPMAVGPNRQAAIELLLASTKLDLIISDDGLQHWALGR
QIEWIVLDQNRGLGNRKLLPEGYLREPVERLKTSTVIEHTFTPTTTLHMHLDAGQPYL
LNPSSATELSFNIQNNYHAVVGIGFPQRFYQTLKGLGVKQFQEHAFRDHHDYSIDDLL
FNDDQPIITTEKDAVKLLPLLEKHPEFKRPIWVVPVKAVLSTECYQVLNQQLQKLDIQ IS"
/product="Tetraacyldisaccharide 4'-kinase (EC 2.7.1.130)"
/EC_number="2.7.1.130"
/transl_table=11
CDS complement(269309..270946)
/locus_tag="AUO97b_00426" /db_xref="SEED:fig|400667.82.peg.286"
/translation="MNSATEVSVAKLIKFIIDAIQNASRADLDWFPLLIILLVFFRGL
GLFMGGYYTAVISRSLVFSIRQEVYAKLLRLPAQYYLDNSSGHITAKIMYNVEQLTAA
SSESLKTIVRDGMITLGLLGYLFYTNWRLTICIMVFLPVIGILVRKASKRMRKLSMQV
QDTMGDVNHVVQESINGNAVVKSFAGEESEQERFYKSSEENLKRGLKMVIVQNLNSPV
VQVVMACAMALIVWLALRPQILGNTTAGEFVAYITAAGLLSKPVKNLTDVNEKLQRGL
AAAHSVFELLDLTEEQNSGELKPQLQGAIRFDHVVLNYADGTQAIKDFSLDIRPGETV
ALVGRSGAGKTSLVNMLVRFQEVSSGQIYLDDLPIRDIELSSLRTQIAMVNQQVVLFN
RTVRENIAYGQLHNASDEDVIAAAKAAYAHDFIMNLPNGYDTVLGAQGLNLSGGQRQR
IAIARAILKNAPILILDEATSALDNESEHFIQQAFDEAMQDRTTIVIAHRLSTIENAD
RIVVMDRGQIVEQGTHQELLAKHGAYYQLHQRNFEDH"
/product="Lipid A export permease/ATP-binding protein MsbA"
/EC_number="3.6.3.25"
/transl_table=11
CDS complement(271033..271461)
/locus_tag="AUO97b_00427" /db_xref="SEED:fig|400667.82.peg.287"
/db_xref="GO:0005215"
/db_xref="GO:0006810" /db_xref="GO:0016020"
/translation="MKFKRSQVEDIHINLTPMIDCLLFILVFLLLSTTFNQPSRINLT
LPDAQGVPPKQYDHKIEVVVDSSGHYAVNGQALSTKDVADLSTAIKQIAQERRDFMFI
IAADAKASHQDVIRVMDVAGQLGFVNINISTKVPSRGFSE" /product="Biopolymer transport protein ExbD/TolR" /transl_table=11
CDS complement(271477..272112)
/locus_tag="AUO97b_00428" /db_xref="SEED:fig|400667.82.peg.288"
/db_xref="GO:0006810"
/db_xref="GO:0008565"
/db_xref="GO:0016020"
/translation="MWELVKAGGWLMLPLILCSILTVAISIERFVRLRRSQVLPKVLM
NDGSADISVVITNLEQDTNAQSTALGNILKAGFEHQEHGEQFARAQMEAMASQEISYL
EKNINFLGTLSAIAPLLGLLGTVVGIIESFLVIDFGSAGSPSLMIPGISKALITTAVG
MLIAIPALISYRYFQRVVQDYIAELEQQSTLFHASLFYKRSSVMQEHRRVG" /product="MotA/TolQ/ExbB proton channel family protein" /transl_table=11
CDS complement(272162..273049)
/locus_tag="AUO97b_00429" /db_xref="SEED:fig|400667.82.peg.289"
/translation="MSIKKRGLAKGRGLDALLGSIQKEKLQLEAQALDHGQLKQIDLK
LLKRGEYQPRRFIHEHDLQELASSIEKHGVMQPIVIRPVDDEAHPYEIIAGERRWRAA
QIAGLTEIPAIVRDLNDQVAIALALIENIQRQDLNPIDQALALQRFHDEFGLSHQEIA

DTVGKARTTVSNLLRLLSLADDIKEFMQQGQLDMGHARAILTLKGKEQLEVAKIVIEK
GLSVRQTEQLVRDWNEPKQEKEKAPVAPDIEQLTQKLSERFGANVKIDHNQKGKGKLV
IHYHSLDELDGILNICLPD"
/product="Chromosome (plasmid) partitioning protein ParB" /transl_table=11
CDS complement(273046..273828)
/locus_tag="AUO97b_00430" /db_xref="SEED:fig|400667.82.peg.290"
/translation="MAQIIAIANQKGGVGKTTTAVNLAASLAVLKKRVLLVDIDSQGN
ATMGSGIQKNDLLYSITDVLLGEVPIETAIQKAEVGYKVLGSNRELSGVELAIAEQEG
REFILKNALNEIRDSFDYIIVDCAPSLSLITVNALAAVDGVIIPMQCEYYALEGLADL
TQTIDRIQKALNPDLEIIGVLRTMYDARNALTRDVSAELEQYFGKKLYDTVIPRNVRL
AEAPAHGLPVIYFEKSSKGAVAYLNLAAEMLKKSKVKKGSKV" /product="Chromosome (plasmid)
partitioning protein ParA" /transl_table=11
CDS complement(273850..274482)
/locus_tag="AUO97b_00431" /db_xref="SEED:fig|400667.82.peg.291"
/translation="MHPFFQELQQGSQKLGLSLSDEALTLLLKYQDALVLWNKAYNLT
AIRDPKEMLVKHLLDSLSILKDLPAGRLLDVGTGGGMPGMIIALCQPERSCVLLDSNG
KKIRFLKQFIADLKLKNVIAVQTRVENQDTIDELGQFDVITSRAFASLTDFVEAARPY
LHEQSIIAAMKGLIPVEEMEELKQEFSCKVIELHVPRLDEQRHLLLLQRI" /product="16S rRNA
(guanine(527)-N(7))-methyltransferase (EC 2.1.1.170)" /transl_table=11
CDS complement(274570..275259)
/locus_tag="AUO97b_00432" /db_xref="SEED:fig|400667.82.peg.292"
/translation="MKAMILAAGLGNRMRPLTLYTPKPLLEVGGKPLIVWHIEKLKKI
GVTEIVINSAWLADKLISSLGDGSQFGVDIRWTREEEGLETAGGIINALPLLGTDPFI
LVNGDVWTTMDFEALRHIKLNDDLAHLVLVDNPKQHPDGDFTLLNGRAFTFDQDVKGE
NLTFSGVSVIHPKLFDGLEAGKRPLAPLLKQAMHNQKISGEKLKGAWVDVGTPERLME LDLQIREGFYD"
/product="Nucleotidyl transferase possibly involved in
threonylcarbamoyladenosine formation" /transl_table=11
CDS complement(275259..276272)
/locus_tag="AUO97b_00433" /db_xref="SEED:fig|400667.82.peg.293"
/translation="MNTQREQLIHTWLTSVLENDQFQIAYLAGDASFRRYARIQLQNK
TYMLMDAPPEKEDCVPFVTIDEFFAGHGVRVPQIVAKDLNQGFLLLEDFGDVLLSTLL
NDETVDQYYEQSFKQLIHLQSINGAEHFPAYSYEKLLSEMQLLTDWMLPSLDIHPTAE
QKKTIDDAFDFLAQAALAQPQVIVHRDFHSRNLMKIANEEELGVIDFQDAVIGADTYD
LISITRDAYVQWNAERVYQWFKVFYELLPASAKQNRSFDQFKRDADLMAIQRHIKILG
IFVRLFERDGKSGYLKDLPRVMWYLLEESRGYHELDDFMAFIHSSVMPKFIEKYGPYE VAA"
/product="Phosphotransferase involved in threonylcarbamoyladenosine t(6)A37
formation in tRNA" /transl_table=11
CDS 276393..278831
/locus_tag="AUO97b_00434" /db_xref="SEED:fig|400667.82.peg.294"
/db_xref="GO:0010033"
/db_xref="GO:0016044"
/db_xref="GO:0019867"
/translation="MKHQFKFNPLATAIFTLLCSGSIQSSYAESAGVVSNIDNNQLKA
SIKEAYPGQEFFQQYYVDKSAPEAQLRNNKYLSSAFCQGTWITPINPETKALDADKAT
SVVTADYGHYNPAGDSVLEGNVVIDQEGRTVRADKVTIDKTQTFAHAQGRVQLAQGGL
LSQSDEIDYNLKTQTGNLDNSFYISEQQHAHGHAGKIERTSPNVMVLNDATYTTCPPG
QKPGWKIQANKIELNQETGRGVTRGTKLYVKDVPVLAVPYFNFPIDDRRTTGILNPQF
GFSNDGGIELSVPVYLNLAPNYDATITPRYLADRGAMLQGEFRYLTDGFGAGQIWGGI
LPSDKEYDDKDRKDFHFLHNWDINDQWSTNLEYNYASDKDYFSDLDSSPISKTDLNLR
RAWELNYQHGIPGLKAQLKVEDFQTLDPQVKDADKPYARLPQFLLNYVTGNPLGLQYE
FNNDTAYFKKSINDNSAQESSGTRIYNQFATRYNYRTPAAFVIPEVSVRSIQTFYDKD
TQLNNPGGSENKSVVVPQFTLDTGLNFEREGKYLQTLTPRAFYAYAPYKNQDGYPNFD
STTASISYDQLFNPYRFYGHDRLEDNNFLSLGVSYSLFDTVGLERLRASVGQSYYFED
RRVTLKQGQDEFDTERKTGPVISLSSQLNQNFTIAANSAWMSNGDNAQRDFQVYYTGD

KGNLYNLGYFYRKDIPGRQDTYDQVVASFIQPIKDNWRIMGHVQYDMDNDVARELLLG
VNYESCCWGISVYGRSYYNDLDDPKTSDVSEKRAIMAEITLKGLGGLNNKLASLLENR VLGFNKINQSWTQR"
/product="LPS-assembly protein LptD @ Organic solvent tolerance protein precursor" /transl_table=11
CDS 278831..280141
/locus_tag="AUO97b_00435" /db_xref="SEED:fig|400667.82.peg.295"
/translation="MKTKHLKQFFKATTLAVLISSSMHSFAQPTDEVVAIVDNSVILK
SDLEQGMAEAAHELQAQKKEVPPQQYLQFQVLDQLILRQAQLEQVKKYGIKPDEKSLN
EAVLKVASQSGSKSLEAFQQKLDAIAPGTYENLRSRIAEDLAINRLRQQQVMSRIKIS
DQDVDNFLKSPQGQAALGNQAHVIHMRISGDNPQEVQNVAKEVRSQLAQSNDLNALKK
LSTATVKVEGADMGFRPLSDIPAELAARITPLQDGQTTDLISVRDGVHVLKLLERKQN
EQKALVPQYQTRHILIQPSEVVSPENAKQIIDSIYKRLKAGEDFATLAATYSNDTGSA
RDGGSLGWVTPGMMVPEFDKKMQEIPVGEISEPFQTQFGWHILQVTDKREKDMTHEYQ
ERMARQILGERQFNTEIDSWLREVRANAYVEIKDPSLDKKNLQK" /product="Periplasmic chaperone and peptidyl-prolyl cis-trans isomerase of outer membrane proteins SurA (EC 5.2.1.8)" /EC_number="5.2.1.8"
/transl_table=11
CDS complement(280260..281297)
/locus_tag="AUO97b_00436" /db_xref="SEED:fig|400667.82.peg.296"
/db_xref="GO:0004332"
/translation="MALISMRQLLDHAAEHNYGVPAFNVNNLEQMRAIMLAADATNSP
VIVQASAGARKYAGAPFLRHLILAAIEEWPHIPVVMHQDHGTSPDVCQRSIQLGFSSV
MMDGSLGADGKTPTTYDYNVDVTRQVVAMAHACGVSVEGEIGCLGSLETGMAGEEDGV
GAEGVLDHSQLLTSVEEAKQFVADTNVDALAIAVGTSHGAYKFTRPPTGDILAIDRIK
EIHAALPNTHLVMHGSSSVPQEWLKVINEFGGNIGETYGVPVEQLVEAIKHGVRKINI
DTDLRLASTGAIRRFMAENPAEFDPRKYFAKTVDSMKQICIDRYEAFGTAGNADKIRP ISLEKMVDRYK"
/product="Fructose-bisphosphate aldolase class II (EC 4.1.2.13)"
/EC_number="4.1.2.13"
/transl_table=11
CDS complement(281419..281661)
/locus_tag="AUO97b_00437" /db_xref="SEED:fig|400667.82.peg.297"
/translation="MNLKLTFAALLIVPMMLTACAKKEEAAQAGQDAASTAVADKVTP
EQQAAIDALDKPVLDEKNTDVVEGSASSETAASETH" /product="hypothetical protein"
/transl_table=11
CDS complement(281786..282973)
/locus_tag="AUO97b_00438" /db_xref="SEED:fig|400667.82.peg.298"
/db_xref="GO:0004618"
/translation="MNFQRMTDLNLAGKRVLIREDLNVPVKNGVITSDARLRAALPTI
KAALEKGAAVMVFSHLGRPVEGEPKPEQSLALVAAYLTEALGQEVKLFTDYLDGVEVE
AGQVVLLENVRFNPGEKKNNPELAQKYAALCDVFVMDAFGTAHRAEASTEGVARFAPV
AAAGPLLAAELDALGRAMQTPEKPMVAIVAGSKVSTKLDVLNSLSGICDQLIVGGGIA
NTFLAAAGYNVGKSLYEADLVETAKQIAAKVSVPLPTDVVVADASQINFEDFLGSLAA
AQAVIKKVEDVTANDMILDVGPETAKAFANILTTSKTILWNGPVGVFEVDQFGEGTKA
LSLAVAQSDAFSIAGGGDTLAAIDKYNVADQIGYISTGGGAFLEFVEGKTLPAVAVLL ERA"
/product="Phosphoglycerate kinase (EC 2.7.2.3)" /EC_number="2.7.2.3"
/transl_table=11
CDS 283112..283495
/locus_tag="AUO97b_00439" /db_xref="SEED:fig|400667.82.peg.299"
/translation="MSIHPDPQINRLNVLGEPLASCCFDPITGYFRNGFCHTAVTDLG
QHTVCAQMTSEFLNFSQKVGNDLITPLPEAGFPGLQPGDFWCICVTRWVEAYQAGQAP
PVKLHACHQAVLSYVPLDVLMEFAV"
/product="hypothetical protein"
/transl_table=11
CDS 283495..284088

/locus_tag="AUO97b_00440" /db_xref="SEED:fig|400667.82.peg.300"
/translation="MKQPKIILASSSQTRKDLMNRLGIEYICISPDIDESPCGEMHAD
DLAKRLAFTKAQVIAQKNRKAIVIGSDQVAWREHAPHDFIGKPLTIENAKAQLAANSG
RTVFFSTALSVQCLKKSFEHTLVEHYQVKFRNLSAPEIERYVDLDLPLQCAGSFKCES
LGISLFEKMIGQDQTTLMGLPMIQLCQILRQLNLQIP"
/product="Maf-like protein YceF"
/transl_table=11
CDS 284119..285282
/locus_tag="AUO97b_00441" /db_xref="SEED:fig|400667.82.peg.301"
/translation="MTEALTVLGGITAEQFLTEYWQKKPLLVRNAMPEIVGMLEPNDV
KELALEDHVTARLIRQKDKNPNEWHVKSSPLTKGDFQKLPKLWTLLVQAVDHYSFDIA
ELWKKFPFIPQWRRDDIMVSYAPKGGSVGKHFDFYDVFLVQGYGHRRWQLGQMCDAST
EFVPNQPLKLLPEIDIHFDEVLAPGDLLYVPPGLSHYGVAEDDCLTFSFGFRMPNISG
MIDRISDQFATDELLQNPVVDITRKNPPQIGEINTEELAYLRDLVLAQLKNSTVLDAA
LMSYMSEPKYPDNIPEPDEIEVEDLNAILSEGYELLLEPASRLLYTEQNGVLKFWGNG
EELPTVESFATQLKSIADGKSIPFNSELNNTDILENIVQLLNNSILMLLPPAE" /product="hypothetical
protein"
/transl_table=11
CDS 285620..286186
/locus_tag="AUO97b_00442" /db_xref="SEED:fig|400667.82.peg.302"
/translation="MSKKDDIITTALRLFNSYSYNSIGVDRIISESGVAKMTFYKYFP
SKEKLIEECLLLRNSLLQNSLTAAISKEDETNPLARIKAIFLWYSDWFNSEDFNGCMF
QKALEEVLKQYPSTHQPATLYKIWLTQLMQDLLIQYEIEESRPLALLLVNILEGMTIQ
AQVEHGSVKIDDYWKRVEKLIEFEKIAH"
/product="Transcriptional regulator, AcrR family" /transl_table=11
CDS 286661..286969
/locus_tag="AUO97b_00443" /db_xref="SEED:fig|400667.82.peg.303"
/db_xref="GO:0003700"
/db_xref="GO:0005622"
/translation="MKDGLQIEEMKNAADSVVGTLKSLANTDRLLILCHLAHEELNVS
QIEEKTQITQPTLSQQLMMLRKSDVVSTRRDGKQIFYSIKDDKMHSILNMLYQLYCIE "
/product="Transcriptional regulator, ArsR family" /transl_table=11
CDS 287392..288594
/locus_tag="AUO97b_00444" /db_xref="SEED:fig|400667.82.peg.304"
/translation="MLEFFSRLSLVTKIIIAIILGIGVALLFPTVTPYLSLFGELFIK
ALKSVAPILVFVLVLSSIANFQVGHSANLRPVLLLYVVGMLLAAFSAVIASLSFPSTL
YLNTVSHNNLQAPGSLADILKNLLLSFIANPVQAISEANFIGILAWAIGLGLAMRHSS
DTTKQVMQDVSHAVSAIIHKVIAFAPVGIFGLVAVTFADAGLATLESYAQLLAVLLGT
MLFVALVINPILVGLTIRGNPYPLVFKCLKESGITAFFTRSSAANIPVNLDLAERLGV
NPSTASVSIPLGATVNMAGAAVTITVLTLATVHTLGIHVDLATMIILSVVATISACGA
SGVAGGSLLLIPVACSLFGISSEIAMQVVAVGMIISVLQDSTETALNSSTDVLFTAAV DIRSRQNS"
/product="Serine/threonine:Na+ symporter SstT" /transl_table=11
CDS 288600..289280
/locus_tag="AUO97b_00445" /db_xref="SEED:fig|400667.82.peg.305"
/translation="MPFQSLPNWFQLGAFLLAINAGMINVLGLITLLHQSVSHMTGNV
SMLAMSLVNWQFEHIIYLVLVILCYVCGSFYSGFILGNNHFKLGRRYGLPLSLVSLFI
FLCWLLLPYFPKYGLLWACVAMGLQNAMVSHYKGTIIRTTHLSGVLTDLGLALGYIAR
GLKVENRRIILHLLIFIGFLSGGIIAALVHPYLKLQSFLLPAILSLTLSISYWAVYFH SSSSSNSD"
/product="Membrane protein"
/transl_table=11
CDS 289284..289820
/locus_tag="AUO97b_00446" /db_xref="SEED:fig|400667.82.peg.306"
/translation="MVKNALQAQLLKAGLVDNKKAKKLTKQAQHEQRTGQSNEAEIKA
NIEKAQKEKQAKDQALNLEKQQQLEEKALKASIIQMIKQHKIVDFAGDVAYQFIDENK

IKKVYLSQQVYNALVTGSLVIAKDQDQYAYLPKALAEKIDQKMKGFILINNSEKNEQT
TDEEDPYAAYVIPDDLMW" /product="Nucleoprotein/polynucleotide-associated enzyme"
/transl_table=11
CDS 290195..292042
/locus_tag="AUO97b_00447" /db_xref="SEED:fig|400667.82.peg.307"
/translation="MLKWFEKLVDPYPTKGLDEPLPTRFFPFVWQATEGVRPYLLLLI
ICTAGAASFEALLFSKIGQLVDWLSNSQPESFLSQHASNILILISVLFANILFVNIQS
IIKHQILYSTFPMRLRWRFHNLLLKQSLDFFHNDFAGRLSAKVMQTALAVREFWIILG
DMLAYVSIYFITVSIVLGAISPTLLIPLMVWLGLFLLSAWFFIPRLSKVSQQQADARA
VMTGRVTDAYTNIQTVKLFAHAGRESQYAKASMKEFMTTVYAQMRLGTLFEVSINLLS
AVLFVGVIGTAVWLWTQGLAALGVIAATTAMILKLNSMAEFMMWHMSALFENVGTIQD
GMQTLGKKINIQDKPDAKPLAVTQGEIVFKDVTFAYNNKNVIDHFNLHIKAGEKIGIV
GRSGAGKSTLIQLLLHFYHLKEGAILIDGQNIEDVTQDSLRANIALVTQDTSLLHRSV
AENIKYGRPDATDQDMQSAVHKAKAAEFIPQLVDLKGRSGYEAQVGERGVKLSGGQRQ
RIAIARVFLKDAPILILDEATSALDSEVEAAIQSSLNDLMVDKTVIAIAHRLSTIAQM
DRLIVLDEGKIAEQGTHEELIAKNGIYAQLWKRQTGGFLIEQKVVQGQD" /product="Efflux ABC
transporter, permease/ATP-binding protein Atu2242" /transl_table=11
CDS 292042..292482
/locus_tag="AUO97b_00448" /db_xref="SEED:fig|400667.82.peg.308"
/translation="MLYLEDLKIGDRFISREYEITLDEIKQFASHYDPQPFHTDEELA
KEDPIFKGIAASGWHTSAITMRLWTECMPIHGGLVGSESSLRWPRPTRPGDRIHVEAE
ISAITPSKTKLDRGIVSYVTQALNQHGDVLLISTTKIVVFKKNV" /product="Acyl dehydratase"
/transl_table=11
CDS 292591..293616
/locus_tag="AUO97b_00449" /db_xref="SEED:fig|400667.82.peg.309"
/translation="MKITSARQDQGIPGVYGLLLLDVVSRWGYNDETLFAPFHLTSEQ
LADPEYRISTPVANELVKHALNLTGESTLGYHIGTQMRISIHGFIGYAIMTAKDITEA
IALAARFIQLRLPFLQLYFSTFGPKATLQLQCDIELEPLRTEIVLGLTIGIMTMAKAM
TGIEDLAGDVDLDFPEPAGFDKYKSKLSSTIRFNQPHLISSFDKKYLGLKLINSDPIA
SQIAINQCEAELSALGERRRLAMRVRDILSNSEQHYLSIESVAEYLHMSDRTLKRQLA
AEGTSFSTLVDEVRYRHATSLLSRTDYSLEQIADELGYSDVANFSRAFKRWSGRSPSN WRKDPYL"
/product="Transcriptional regulator, AraC family" /transl_table=11
CDS 293655..294029
/locus_tag="AUO97b_00450" /db_xref="SEED:fig|400667.82.peg.310"
/db_xref="GO:0005960"
/db_xref="GO:0006546"
/translation="MNHPSELKYARTHEWVKIEGDLVITGITDHAQDELGDLVYVETP
EVGSQVTAGEQAGVVESVKTASDIHAPVSGTVVEVNTDLEDDPDFVNEDPYGKGWIYK
IKPDNIADVEKLLTNAEYEAGL"
/product="Glycine cleavage system H protein" /transl_table=11
CDS 294132..294830
/locus_tag="AUO97b_00451" /db_xref="SEED:fig|400667.82.peg.311"
/translation="MTKLVVFSGAGMSAESGISTFRDSNGLWENYDIQQVATPEAWER
NPALVQRFYNERRKNILEAQPNEAHQYIAKLQDYYDVQVITQNIDDLHERTGNQNVLH
LHGNIRLAKSSGPDAQYTTQFYEVNGWKLDLEQDFCPDGYPLRPHVVWFGEAVPAYEE
AIRLVQSSDIFIVIGSTLSVYPVAALVHEIPHYTKAYYIDPQADHSRVPPQYKLLNMT ATEGMHELFNQLTS"
/product="NAD-dependent protein deacetylase of SIR2 family" /transl_table=11
CDS 295237..296727
/locus_tag="AUO97b_00452" /db_xref="SEED:fig|400667.82.peg.312"
/translation="MSYFDPTLIMFMVYIVAMVLIGLFAYRATSDFSDYILGGRSLGS
FVTALSAGASDMSGWLLMGLPGAIYLSGLSEAWIAIGLIIGAWLNWLLVAGRLRVHTE
IQNNALTLPDYFTSRFDDQKKILRIFSAVIILVFFAIYCASGMVAGARLFENLFGMSY
TTAIWLSAIATISYVCIGGFLAISWTDTFQAGLMIFALLLTPIVTYLAIGDTTQFVTL
IETARPHAFNIISDLSVVAVLSSMAWGLGYFGQPHILVRFMAADSVKSIPAARRIGMT

WMILCLVGAVGAGFFGIAYFQQHPELAGVVSKNPETVFMELTKILFNPWLVGIILAAI
LAAVMSTLSCQLLVCSSALTEDLYKGFIRKNASQKELVWVGRIMVLAIAVLAIVLAGN
PDSKVLGLVAYAWAGFGAAFGPLIILSLFWKRMTLEGALAGMIVGAVVVIGWKNLFAS
TGVYEIIPGFICAFISIIVVSLISKAPNSDVTGRFEQADKLYKEST" /product="Proline/sodium symporter PutP (TC 2.A.21.2.1) @ Propionate/sodium symporter"
/transl_table=11
CDS complement(296761..297249)
/locus_tag="AUO97b_00453" /db_xref="SEED:fig|400667.82.peg.313"
/translation="MNGPLLSLDRTDLKILDILQTDGRISNSRLAELVNLSPTAVMAR
VQKLTKDEFILGYEAKLNPVKLNANFLVFVEVLLDKTTPNVLDEFIDAVVHYPEIVEC
HMVSGGFDFLIKLRSGSMEEFRRIAGQVLWQLPGVKETRSYPVMQVVKDSSKIKIKTK NK"
/product="Leucine-responsive regulatory protein, regulator for leucine (or lrp) regulon and high-affinity branched-chain amino acid transport system"
/transl_table=11
CDS 297237..297371
/locus_tag="AUO97b_00454" /db_xref="SEED:fig|400667.82.peg.314"
/translation="MGHSSEFSSKKWAKYSELFGVSSVMINYLNLSTQDEFTRAGWRK "
/product="hypothetical protein"
/transl_table=11
CDS 297374..301126
/locus_tag="AUO97b_00455" /db_xref="SEED:fig|400667.82.peg.315"
/db_xref="GO:0003842"
/db_xref="GO:0004657"
/translation="MNTMDTFHQMDKSHQGYITEFNDKSEFEQRINTAWRRAEPEAVE
ELLQAAAVSDDLDHKIYDLAFNLAHNLRERKTSSGKAGIVQGLLQEFSLSSQEGVALM
CLAEALLRIPDTATRDLLIRDKINQGNWKEHVGQSSLMFVNAAAWGLMLTGKLMETPK
QTSLSSVLTGLLARSGRGIIRKAVDVAMRMMGEQFVTGETIEEAVDHAKVLEDKGFRY
SYDMLGEAALTDHDAERYFNDYTQAIHAIGKASNGKGVYDGPGISIKLSALHPRYQRA
QIERVHKELYGKVFELARLAKQYNIGLNIDAEESERLEISLELLERLCFEPELANWKG
IGFVIQAYQKRCFFVVDYIIDLAKRSQKRLMIRLVKGAYWDSEIKKAQIEGMDDYPVF
TRKVHTDLSYIACAKKLLAAPEFIYPQFATHNAQTLATIYHLADPSKYYAGQYEFQCL
HGMGEPLYEQVVGPREQNKLGVPCRIYAPVGNHETLLAYLVRRLLENGANTSFVNRIA
DKTLKVEDLIQSPIYDIRNAAKLEGSVGLKHPSIPLPLDMYGTLRKNSKGYDLANDTP
LAALDSTAQELRNRIWQSHPLLANADSLEQGHSVAITNPAQNDEIVGYVQEADLKHVE
IALTAAEQTQSEWSNTPKDQRAQYLKRAADLMESRIQELMVLLCRESGKTYANAIAEV
REAVDFLRYYATQVENLPANTVIQPLGTVLCISPWNFPLAIFSGQIAAALVSGNCVIA
KPAEQTPLIAAQAVQILWEAGIPHGAVQLLPGRGETVGAQLSQDSRIDGIMFTGSTEV
AKILQKTVAKRLSENGQSIPLIAETGGQNAMIVDSSALTEQVVLDVVSSAFDSAGQRC
SALRILCVQEDSAATVIKMLKGAMQQLIVGNPAILKTDIGPVIDDEAKQTIDQHIQKM
KSKGYPVHQLMFGATSQTELDKGTFVVPTAIELPNLDDLQREVFGPVLHIITYKYGEL
EQLISRINAKGYGLTMGLHTRIDETIQTVIQHAEVGNLYINRNIVGAVVGVQPFGGEG
LSGTGPKAGGPLYMYRLMQHCSNKVLATPFAVKNEQTVFEGFTREVYQSLQNWAKQHL
PQANREIEPFGVGKFYELQGPTGESNQYIILPRHRVLSIADTEQDQLHQLLAIFAVGS
QAAVMPNSPLLAKHKQTLPKDVLDAITTIKNITTDDFDAVLHHGNREEIFSLQQEIAS
RSGAIVGITHVEPNESIPLERLVIERAISVNTAAAGGNASLMTMSE" /product="Transcriptional repressor of PutA and PutP / Proline dehydrogenase (EC 1.5.5.2) / Delta-1-pyrroline-5-carboxylate dehydrogenase (EC 1.2.1.88)" /EC_number="1.5.1.12"
/EC_number="1.5.99.8"
/transl_table=11
CDS 301232..301705
/locus_tag="AUO97b_00456" /db_xref="SEED:fig|400667.82.peg.316"
/translation="MIFFEPEQYQKRCTQLFNLYRKQISALLPSAQIEHIGSSAIPNA
FSKGDLDIYIEVLPDQFEFAIEQLKTLNFIEKQNTLRTNELCMLESLNNDDVAFQVVV
TGSIFTFFLTFRDKLIASPTLVNEYNQLKLQSTHLDHDQYRAVKSNFIERVLSHS"

/product="FIG00350668: hypothetical protein" /transl_table=11
CDS 301809..302894
/locus_tag="AUO97b_00457" /db_xref="SEED:fig|400667.82.peg.317"
/translation="MTSSYRQQLQAKIDRITTQFSEFTPPTLEVFESPEQHFRMRAEF
RIWHTENDMFYAMFERNDDGKQKTVVRIDEFPIADKSINDLMPLLLAELKANSLLSQR
LFEVDFLATLSGEMLVTLIYHRKLNQEWEQAAKALAEKLNIKIMGRSRGQKIVIGDDF
VVEEFELLNRSFKYKQIESSFTQPNAQVCKKMLQWACDAAEGSKKHLLELYCGNGNFT
LPLSLKFERVLATELAKSSVYAAQWNIEQNQIDNIQVARLSAEEFTQAYQGEREFRRL
QEAYIDIQSYDFGTVFVDPPRAGIDDETLKLLQGFERIIYISCNPDTLYENLKTLTQT
HRVTKFALFDQFPYTHHVESGVLLEKI"
/product="tRNA (uracil(54)-C5)-methyltransferase (EC 2.1.1.35) @ tmRNA
(uracil(341)-C5)-methyltransferase" /EC_number="2.1.1.35"
/transl_table=11
CDS 303143..303373
/locus_tag="AUO97b_00458" /db_xref="SEED:fig|400667.82.peg.318"
/translation="MSFWQKLFLADQQHNEHKNQTACVENDCSCKTNEQLLSALAQAT
DEDVIKGIKKVLISRGYSRKELNELTQKPSIH"
/product="hypothetical protein"
/transl_table=11
CDS complement(303409..304155)
/locus_tag="AUO97b_00459" /db_xref="SEED:fig|400667.82.peg.319"
/translation="MKILVLCIVNFIIFTQSALALEYRQIRNTTDDQFEVIEISNLEQ
LRLFLKNPQTDQYYKSFDNIQYQLKACEQLTFAMNGGMFHSGFSPVGLYIENGRENQP
LNEDKGWGNFFLQPNGVLAWNDKQAVILTTEQYKAKVFQPDYATQSGPMLVINGKINP
LFLANSDSKKIRNGVGIKNNKLYFVISKNRVNFYSFAQFFQKNLEVEQALYLDGSISS
LYLHKNNRNDKRFNMGPIIGWVDQADCRPK"
/product="Mlr0120 protein"
/transl_table=11
CDS 304342..304932
/locus_tag="AUO97b_00460" /db_xref="SEED:fig|400667.82.peg.320"
/translation="MQQTNKRYPLGAHLIVKHLGYSHHGIYAGRGRVIHYSGLAHFIK
KRPIEITSLDAFSHGKKIHVRSYDAPRYRGKVVVRRMRSRMHENHYHLIINNCEHLCS
WAITGIESSTQVERMMHRLTTIGYVSSIMSYMNGMMLTVATTCFALVLYIKKKLRDQA
KMNIPTYQFLREKAEKRNIDPTSPLVFIEPDKKETI"
/product="Cell wall-associated hydrolases (invasion-associated proteins)"
/transl_table=11
CDS complement(304978..305568)
/locus_tag="AUO97b_00461" /db_xref="SEED:fig|400667.82.peg.321"
/translation="MHLKTLSFGLAVALASTVTLAAPVDYKIDPTHTATVFSWNHFGF
STPSANFSDIQGVIKVDNAKPANSSVNVTIPLSSVNTNVPALDKEFQEEAWFNAAKYP
NITFKSTKVETKDKKHFKITGDLTVKGITKPVVLDAVLNKQGEHPMAKVPAIGFNATT
SFKRSDFGLGNYVPNVGDKITVNITTEATAASAAKK" /product="hypothetical protein"
/transl_table=11
CDS 305784..306191
/locus_tag="AUO97b_00462" /db_xref="SEED:fig|400667.82.peg.322"
/translation="MVNTAQAQSTSTPYRVTLTDPSGHQWFADEPTDKGGRDTAPNPV
QLLLSALGACTTITLEMYANHKGIKIEHVQVDLALNPNGDPEAGQNNIERKITLKGEF
TEDQHKRLLKVAENCPIHKLLTSNITIQTELNI" /product="Predicted redox protein,
regulator of disulfide bond formation" /transl_table=11
CDS complement(306246..308234)
/locus_tag="AUO97b_00463" /db_xref="SEED:fig|400667.82.peg.323"
/db_xref="GO:0004802"
/translation="MTTPLNERRIANAIRVLAMDAVQQANSGHPGAPMGMADIADVVW
REFLNHNPNNPQWANRDRFVLSNGHGSMLQYALLHLTGYDLSIEDLKQFRQLHSKTPG

HPEYGYAPGIETTTGPLGQGIANAVGFALAEKTLAAQFNKDDLKVVDHFTYCFLGDGC
LMEGISHEVCSLAGTLQLGKLIAYYDDNGISIDGEVEGWFSDDTEERFKSYGWQVLRV
DGHDADAIRQATVEAKAETQKPTIIICKTIIGLGSPNKQGKEDCHGAPLGKDEITLTR
EALGWTEEAFVIPADVYAAWDAKAKGNEAEAAWNEVFAQYQAKYPTEAAELLRRISGD
LPAEFSAQADAFIRETNAKAETVATRKASQNTLQAFGPLLPELLGGSADLAGSNLTLW
KGCQGVQENPAGNYVYYGVREFGMTAIANGVALHGGFIPYVATFLMFMEYARNAVRMS
ALMKQRVIHVYTHDSIGLGEDGPTHQPIEQIASLRGTPNLNTWRPADTVETAIAWKSA
LERKDGPTALIFSRQNLPFQTRTEEQIQNAAKGGYVLAQEKGELKAIIIATGSEVSLA
MEAYAQLEGVRVVSMPCAEEFMKQDAAYREAVLPAHIRARVAVEAAHVDYWWKFVGLD
GKVIGMTTYGESAPAKDLFQFFGITTEAVVAAVKELTA" /product="Transketolase (EC 2.2.1.1)"
/EC_number="2.2.1.1"
/transl_table=11
CDS complement(308492..308608)
/locus_tag="AUO97b_00464" /db_xref="SEED:fig|400667.82.peg.324"
/translation="MKINHFYIRVLIKCLNFKHLILLNINQEDLDYEVKSYK" /product="hypothetical protein"
/transl_table=11
CDS 308816..309925
/locus_tag="AUO97b_00465" /db_xref="SEED:fig|400667.82.peg.325"
/db_xref="GO:0004478"
/translation="MADQISDAILDAILKEDPYARVACETLVKTGAVVLAGEITTTAN
IDVEAVVRQTVNGIGYHHSDLGFDGSTCAVINMIGKQSPEIAQGVDRQKPEDQGAGDQ
GLMFGYASRETDVLMPAPISYAHRLMERQAELRRSGALPWLRPDAKSQVTFAYENGKP
VRLDAVVLSTQHDPEITQTQLKEAVIEEIIKPIIPAEMFHAATKFHINPTGMFVIGGP
VGDCGLTGRKIIVDTYGGMARHGGGAFSGKDPSKVDRSAAYAGRYVAKNIVAAGLADK
CEIQVSYAIGVAEPTSISINTFGTAKVSDELIIQLVREHFDLRPFGITRMLNLIQPMY
KQTAAYGHFGREGSDTAFTWEKTDKVEALKDAAGL" /product="S-adenosylmethionine synthetase (EC 2.5.1.6)" /EC_number="2.5.1.6"
/transl_table=11
CDS complement(310049..310297)
/locus_tag="AUO97b_00466" /db_xref="SEED:fig|400667.82.peg.326"
/translation="MLYQYHCACCDKVVASTDKSCPYCGSHHIRSPYGLWMFCVMACL
AVVVVFKVVHLYIQNHQDTPVQSTLLDVLNEGDKSTKQ" /product="FIG00351277: hypothetical protein" /transl_table=11
CDS 310556..310741
/locus_tag="AUO97b_00467" /db_xref="SEED:fig|400667.82.peg.327"
/translation="MTQLINKGGFRERANRSRKYQQSENKQVTLPSNKYQPQTKLQDN QNEMIQAKAGTSETSDD"
/product="hypothetical protein"
/transl_table=11
CDS complement(310804..311364)
/locus_tag="AUO97b_00468" /db_xref="SEED:fig|400667.82.peg.328"
/translation="MNLLLIVVLGAIVEIAVWIGVAQFISGWYVFFWFIIAFFIGLTL
IRNSTAGIMPQLQQMQMTGQLGNDPTVTKKLAYAFAGFLLMLPGLVSDVLALLILIPG
VQTAFRNIAMRTLAKRQQAMMNKMMGGMGGAGAGQNPFADLMRQMQDMQNQQGGGSQY
HDSSIIDGEAREVTPDHKKIEHKNKD"
/product="Protein affecting phage T7 exclusion by the F plasmid"
/transl_table=11
CDS 311763..312587
/locus_tag="AUO97b_00469" /db_xref="SEED:fig|400667.82.peg.329"
/db_xref="GO:0008800"
/translation="MNIKALLLITSAIFISACSPYIVTANPNHSASKSDEKAEKIKNL
FNEAHTTGVLVIQQGQTQQSYGNDLARASTEYVPASTFKMLNALIGLEHHKATTTEVF
KWDGQKRLFPEWEKDMTLGDAMKASAIPVYQDLARRIGLELMSKEVKRVGYGNADIGT
QVDNFWLVGPLKITPQQEAQFAYKLANKTLPFSQKVQDEVQSMLFIEEKNGNKIYAKS

GWGWDVDPQVGWLTGWVVQPQGNIVAFSLNLEMKKGIPSSVRKEITYKSLEQLGIL" /product="Class D beta-lactamase (EC 3.5.2.6) => OXA-51 family, carbapenem-hydrolyzing"
/EC_number="3.5.2.6"
/transl_table=11
CDS complement(312659..313204)
/locus_tag="AUO97b_00470" /db_xref="SEED:fig|400667.82.peg.330"
/translation="MNYSLDSNFRLIDCNEAEHAAAILEILNDAIVNSTALYDYVPRS
LDSMKTWFSVKRENGFPVIGVVDELGKLLGFASWGTFRAFPAYKYTVEHSIYIHHEHR
GCGLSKVLMQALIQRAQQAQLHVLIGCIDATNQASIGLHEKMGFTHAGTFKQVGFKFG
QWLDAAFYQLVLDTPFEPVDG"
/product="Acetyltransferase, GNAT family"
/transl_table=11
CDS complement(313201..313794)
/locus_tag="AUO97b_00471" /db_xref="SEED:fig|400667.82.peg.331"
/translation="MDDINIRIAHRIRELRLARGYTLDVLAARCQVSRSAISLIERGE
TSPTAVVLEKLANGLEVPLTQIFTNEAGNSSSQPLIRRNEQAEWRDPETGYIRRTVSP
PNLKLPFQIVEIEFPPHSRITYETNESSRVVQQQLWVVEGKIEIQLGETSYALGQGDC
LAMQLDQPVIYSNHSAQVARYILVVSNQLVPALKESL" /product="Transcriptional regulator, Xre-family with cupin domain" /transl_table=11
CDS complement(313850..313966)
/locus_tag="AUO97b_00472" /db_xref="SEED:fig|400667.82.peg.332"
/translation="MSNLKNAAMNGYFSVFKPKDLYCPPNFPYIHKEEFEII" /product="FIG00350203: hypothetical protein" /transl_table=11
CDS 314024..314569
/locus_tag="AUO97b_00473" /db_xref="SEED:fig|400667.82.peg.333"
/translation="MPLIIGIDPGSRLTGYGIIEKDGSKLRFVDAGTIRTETQEMPER
LKRIFAGVERIVKFHGPTEAAVEQVFMAQNPDSALKLGQARGAAIAALVNLDLQVAEY
TARQIKQSVVGYGAADKEQVQMMVMRLLNLTIKPQADAADALAAAICHAHASGSMSKL
TVLNALGGMARGRSRSSSRRR"
/product="Crossover junction endodeoxyribonuclease RuvC (EC 3.1.22.4)"
/EC_number="3.1.22.4"
/transl_table=11
CDS complement(314574..316034)
/locus_tag="AUO97b_00474" /db_xref="SEED:fig|400667.82.peg.334"
/translation="MQAVVYHSQDIQAWEQRWFAAQNSSLGLMQQAAWSITQQLIVLF
QKQNFKNIAVWCGQGNNAGDGYFIASYLKQAGFYVEIFAADLGESADLHHAVHFAKAN
HVQIHQGFEVNRTFDCHIDALFGIGLNRELNGYWQHAIQQFNGQAGLKISIDIPSGLH
ANTGSTLPCAIRADHTFTVLGLKAGLFTGQGKEYAGQIHLINLIPIDQELKPLAYFTP
THIKLPKRLAFGHKGSYGHVLVIGGHEQMGGAVIMAAEAAFHAGAGKVTVVCHRNHHQ
AILSRAPNIMLRDINDFDENGIKEILSQVDAVCFGMGLGRDDWAHQIYQQWFNYLNQT
SHLEVVLDADALWFLAKQPEKLSLHIYATPHPGEAATLLGCSTWQIENDRITAIYALQ
QKYAGQWVLKGAGSLILEDNLYICKQGNAGMGTGGMGDVLAGMIASLKAQFHKAVTLH
EIVTLHAQAGDQLAKQGMRGLQAYEMNQAITQVVNQ" /product="NAD(P)H-hydrate epimerase (EC 5.1.99.6) / ADP-dependent (S)-NAD(P)H-hydrate dehydratase (EC 4.2.1.136)"
/transl_table=11
CDS 316063..317151
/locus_tag="AUO97b_00475" /db_xref="SEED:fig|400667.82.peg.335"
/translation="MTSSNTSRIAVQQIDPHELKAWIKAQALDLGFADCVIAKPDAQE
QMPRFLEYLERGYHADMTYLEENLEKRADPTLLVPGTKSIICVRMNYLVESPKPRYVP
FEPNSAIIARYARGRDYHKVMRGRLKTLATRIREKVGDFESRPFADSAPIFEKSLAES
AGMGWTGKHTLLIHKKSGSFFVLGELFTSLDLPFDEPATSHCGSCSACIDICPTQAIV
EPYMLDVRRCIAYLTIEYKGIIAEELRAGIGNRIFGCDDCQLICPWNSFAKTASIPDF
NPRHGLDNISLLDIWQWDEATFLANTEGSPIRRTGYQSFKRNIAIGLGNAPYSKEIVD
QLNKGKSLHDEIVNVHIDWAIEQQLNQL"

/product="Epoxyqueuosine reductase (EC 1.17.99.6) QueG" /transl_table=11
CDS 317251..318240
/locus_tag="AUO97b_00476" /db_xref="SEED:fig|400667.82.peg.336"
/db_xref="GO:0004076"
/translation="MTLRNDWTREEIQALYEQPFLDLVFKAQQVHREHFTANTIQVST
LLSIKTGKCPEDCKYCSQSAHYDSKLEAEKRIAVEKVISEAKAAKDSGSSRFCMGAAW
RNPHERDMPYVLEMVREVKALGMETCMTLGMLNQSQAERLKDAGLDYYNHNLDTSREY
YSHIISTRTFDDRLNTLDYVRQAGMKVCSGGIVGLGESREDRIGLLHELATLPIHPES
VPINMLVPIEGTPLADVEKLDVIEWIRTIAVARIIMPHSYIRLSAGRESLSDSDQALA
FMAGANSLFSGDKLLTTPNAGEGKDQALFNKLGLTAEKPKPTVSDLSVDAMSA" /product="Biotin synthase (EC 2.8.1.6)" /EC_number="2.8.1.6"
/transl_table=11
CDS complement(318286..318498)
/locus_tag="AUO97b_00477" /db_xref="SEED:fig|400667.82.peg.337"
/translation="MSKDFKALTYIADDNIADTVTWLLHHQDVFESFHFDVLSQELSV
THAAGRDIIRVGTFLNATYGILVTSV"
/product="FIG00350416: hypothetical protein" /transl_table=11
CDS complement(318562..318912)
/locus_tag="AUO97b_00478" /db_xref="SEED:fig|400667.82.peg.338"
/translation="MNNMKYTYKPNYFFFAHKLVLFLKDYLLKHPTEQNTTFNLQTIY
DVFSHDLASSTTNLEGILNIADEYVLETEEGLLPLISSHSINLKNHVLSLEFSPKALT SLLSGRSLVNPKAA"
/product="FIG00351400: hypothetical protein" /transl_table=11
CDS 319358..319894
/locus_tag="AUO97b_00479" /db_xref="SEED:fig|400667.82.peg.339"
/translation="MKKFIFIAASSLFNIAAAQAADGTITINGLVTDNTCTIDTGDKN
LTVNLPTVSSQSLKNAGDVAGRTPFQINLTNCASVGKVATYFEPGATVDFNTGRLLNQ
ATSGAATNVNIQLLGSNNAVIPVIAKGASGAQDNSQWVNVSAGGNADLNYYAEYYATG
ASTAGTVTSQVKYTIIYQ"
/product="Fimbrial protein precursor"
/transl_table=11
CDS 319965..320705
/locus_tag="AUO97b_00480" /db_xref="SEED:fig|400667.82.peg.340"
/translation="MLFRGRKFFWGMACLQALIATTGHAEIVLHGTRVIYPSDAREVN
LQLSNNGTAPSLVQAWIDDGNAKSTPDEANVPFIITPPISRVEPSKGQTLRITALPNA
SQLNQNKESVFWLNVLDIPPRPEGKKQENNEVLTNNFLQLAIRSRIKFFYRPVSLKEN
INTLSEKIQWVKNGENLLIKNPTPFYITISSIFQEINHQKTDLLKQGLMLSPFSEDQI
KLKNSNITNMSFVYINDYGGRIEQVIRF"
/product="P pilus assembly protein, chaperone PapD" /transl_table=11
CDS 320865..323294
/locus_tag="AUO97b_00481" /db_xref="SEED:fig|400667.82.peg.341"
/translation="MNRENILKADFDTNFLVGNAQKIDIGRFKYGNPILPGEYSLDVY
INGQWLGKRKFVFKSTHSNENAKTCFTPDMLLEYGVKPEIFHHEVSSTYTCNDLDKWV
NDAFYQFDTSRLRLDISIPQVALQKNAQGYVDPRLWDRGINAAFFAYNASAYRIVNNN
HETNHAFMGTNVGLNLYDWQLRHTGQWKWQDHNEIQEKVSSYTSNNTYAQKAFPKLNS
VVTLGDYFTNNNFFDALPYRGINISSDDRMLPNSMLGYAPQIRGYAKTNAKVEVRQQG
NLIYQTTLTPGSFEINDLYPTGFGGELQVSIYETNGEIQKFSIPYASVIEMLRPKMSR
YSFTLGQFRDANINLKPWLVQGKYQRGINNYLTSYTGFQATENYQSFLIGSAFATPIG
AISFDATQSSAEFEQKPTLKGRSYRLSYNRLFTPTNTNLTLATYRYSTENYLKLRDSI
LIRDLQQQDIDSFSVGKQKSEFQITLNQGLPNQWGNFYLVGSWINYWNQPNRNQQFQF
GYSNQFKDLTYSISAITHEFDQENQNSGHETQYLLSLSFPLQFKKNTVNFNSSIAKDS
RTLGMSGYIGNRFDYGSSISYQDAGQTSLNINGTYRTNYTTIGASFGQSDTYQQEMIN
LNGSLVAHSQGILFGPDQVQTMVLVYAPQATGARVGNTPGLSINKQGYAVIPYVTPYR
LNDISLDPQGMPSTVELTETSHRIAPYAGSITKVNFSTKTGYAVFISTQTPNGGHLPF
AAQVFNQNNEIVGMVAQGSRIYLRTPLTQDHLYVKWGQSNSEECQLDYDIQSKILQEK QSIIMTEAVCK"

/product="type 1 fimbriae anchoring protein FimD" /transl_table=11
CDS 323291..324310
/locus_tag="AUO97b_00482" /db_xref="SEED:fig|400667.82.peg.342"
/translation="MKKMLIKSGLLITSFFIYNQTYANCTLSKGFTTVDIPMTIGTIV
VRPTDPIGTVLQKNTFTISPNNSTATCNRANDQITAALPLNYPVSSIGNNVYATNIPG
IGIRLYREAFDSTDFSGYYPYKRSLTPNTTYTLSPGYFVMEVIKTAATTGSGALVAGR
YSTYYVTGQQNRPFLTTTVLSSSPILIASSSCEIQNGIDTVVQLPTVMKSGFRAIGST
QGEKNFNLSILCNGGENNSGVPTSNTLSLSFDYISDTSNSQVINNSAAESTKANGVGV
ELLWNMNGANNPIRKGNKLDIGTVSSNQTVQYDIPLTARYYQTASNVTAGEVKANATV TIQYD"
/product="Fimbrial adhesin"
/transl_table=11
CDS 324887..325066
/locus_tag="AUO97b_00483" /db_xref="SEED:fig|400667.82.peg.343"
/translation="MLIIWKIFCILLLSLGAFGVGLGISYLDFFLFFIGLLFLLCFFL ALKQAREDAGSFPDE"
/product="hypothetical protein; putative exported protein" /transl_table=11
CDS complement(325106..325957) /locus_tag="AUO97b_00484" /db_xref="SEED:fig|
400667.82.peg.344" /translation="MIDWENLHYFSVFSEEKTLSAAARKLGVDHATVARRIAQLEDNL
KLKLVDRRPRTYILTSEGERLAKIVTRMMEETFSIERLAQAGQQEISGVVSVSLPPAT
AAHLVMPHLGKFYRQYPELQLRILGDVHYASLQHREADIAVRFGRPKDDYLIARKAGE
VPFGIYAAQEYLDAIQEENYEWISVEVGGVQGQKQQKLKQRLNYKEPVMVTNHPELQW
RATCGAVGLAVLPDFLGQQGHLVKLPDDDPMSAEIWLVIHEDLRSSPAVRVVMDFLLD CFESFRV"
/product="Transcriptional regulator, LysR family" /transl_table=11
CDS 326052..326981
/locus_tag="AUO97b_00485" /db_xref="SEED:fig|400667.82.peg.345"
/db_xref="GO:0016020"
/translation="MNKSMVYAYTGVVITMLFWGSAFNAMSYVIQYMPPLSAAAERFT
IASLGLFILFAAIGKLRWAALRQNLAIYLIIGIIGIAGFNLGCFYGLQTTSAVNGALI
MATTPLITLLLTILLDGEKLTLNKSIGVVFGLSGVLFVISHGHISTLLHLKIAIGDLF
ILLGGVSFCLANMLSRRYVKNATPLETTTFSMMSGALTLIILSVIFEHPSQAIAMAPI
TAHLAMGYVIICSTMIAYLFWFNGIQKLGAGRASIFFNFVPVFSMLVALLAGQSLNIW
QLIGTALVMLGVMSSGGFIQIKRPTLIAKPCTK"
/product="Permease of the drug/metabolite transporter (DMT) superfamily"
/transl_table=11
CDS 327094..327558
/locus_tag="AUO97b_00486" /db_xref="SEED:fig|400667.82.peg.346"
/translation="MNLDFTTIEKQAKLLKEEQEKLEQQDHDFQLALDKHRESLKNLF
KELFHDREIKTENGGQFCVVFGDFKISLLIETAKFENGVPVKLNSVNPIIVKFKKDKP
VAKAQFSDATQYLDSGFETSHYQYYYKHADKTQLVQFSELPVFFQAILDAEV" /product="hypothetical
protein"
/transl_table=11
CDS 327811..329226
/locus_tag="AUO97b_00487" /db_xref="SEED:fig|400667.82.peg.347"
/db_xref="GO:0015205"
/db_xref="GO:0016020"
/translation="MDSSAGKSKSGFVENRSIDFIPENERHGSIFAQFTLWFGANLQI
TAIVTGALAVVLGGDVFWSIIGLFIGQCFGAAVMALHAAQGPKLGLPQMISSRVQFGV
YGACIPIILVCLMYIGFTATGEVLAGKAIAHLAQVSNTTGILIYACFIIIFATIGYRL
IHWVGKVASVIGLIAFVIILTQILALSNISELLAVRHFSWSSFLLAVSLSASWQISFG
PYVADYSRYLPTKTSSTRVFWAVGLGSLIGSQISMMLGVFIAQVSNGGFVGNEVQYVV
GMNPIALVITFLYFSIAFGKVTLSTLNAYGSFMCIATIWNSFKPSAQISKLTRLTIVL
AMVIISTFIAVVGEHTFLSAFKSFILFLLTFFIPWSAINLVDYYFISKEECDVDALYD
PNGKYGRWNTIGIACYCIGILLQLPFIDSKFYKGQIAELLGGVDLSWLVGLLSTAVLY
YVLAKRAQIKISKALQLERIK" /product="Cytosine/purine/uracil/thiamine/allantoin
permease family protein" /transl_table=11

CDS complement(329284..329721) /locus_tag="AUO97b_00488" /db_xref="SEED:fig|400667.82.peg.348" /translation="MQGLKRRIVYVSSYEIIGMVISSVGLALLAGDSVEHTGPLSVMI
TTIAVTWNFIYNILYEKWEARQESKSRTVKRRIAHAIGFQITLVMFLIPLIAWWMNIS
LVAAFWLDVAFIIIIPIYTFIFNWTFDKLFGLPASAQPNTAQQ" /product="hypothetical protein"
/transl_table=11
CDS 329811..330710
/locus_tag="AUO97b_00489" /db_xref="SEED:fig|400667.82.peg.349"
/translation="MNFNSENIELFLTVLDTGSFSAAARKLNRVPSAVSMAIANIEAE
LGYHLFERTHRKVIPTAAALALEPQARIIAEQLRLLATHAYELSLGLESKLKIGVVSD
ANTELLFKAIKQLAEKFPLLNLEIITAPQDDILNLLYTENISLCLAGSNLNIKMRESL
QLVMTETVVATISAHHPFLKEQNKLNTIEELINIRQIVVASSELDMEDSRSIIGAMYW
KTNSLQTAINMVEAGLGWGNFPLSLVKEKIEQGHLVQLDFKNTKNHLPLSIYLIWLQD
KPLSKAARELVQIVQANLSQNCH" /product="LysR family transcriptional regulator
PA2758" /transl_table=11
CDS complement(330742..330930)
/locus_tag="AUO97b_00490" /db_xref="SEED:fig|400667.82.peg.350"
/translation="MNKTDLNSTQSETWQNAKFNFQSIRHRSVIMFKAMFFFSAGAMF
GYCYKHYDEKVIESNKSK"
/product="hypothetical protein"
/transl_table=11
CDS complement(331290..332522)
/locus_tag="AUO97b_00491" /db_xref="SEED:fig|400667.82.peg.351"
/translation="MSTSRVKLTKSFIDQLELTPAIYRDSEIIGFAIRVNNSYKTYIV
EKKVKGKSIRCKLGDYEKITLEDARILAQQKLKELTDSNSLSIKNNKILKNSLNEKID
QPYLKEAFQVYINHHELKERTLADYKEVIEKYLIDLSELKLIDITEQRIEEKYIQLSQ
YSYAKANLSMRVLRAVYHFSIKYYQNKNCEVIIPRINPVNLLKKKQLWEEIPPRRNYI
DVDNLTKWVQAIIEYKGRGQENETNKDFLLTLILTGLFRNECESLHWKNIDLEEGTLS
FINPYNNVYYKIYMGNFLWYLMKKRRIQNKGEWVFPSVKSESGHIINISKFRKKINEQ
CNLSFTFQDLRRTFYFVLNNLTNKPLASKRTDQYEEKLDVKNIAHAQDMRNRMNKFEQ
IILGPYRDELIKSININL"
/product="Phage integrase"
/transl_table=11
CDS complement(332954..333817)
/locus_tag="AUO97b_00492" /db_xref="SEED:fig|400667.82.peg.352"
/translation="MPKKFEDFNNPRKKALAGMKQSISAELWDKNLNFLNQLRAKIAK
HPVSHHPAIETLNNGEINKENLKRIHLEYRHAIVQTFTDALLMAQYQTKQLEPRLHAG
AKMFPRCLLSLNIFDEFGFRPGTDKDGYYQGNPEYAHYPLFEDVLDDFEVTEEERQSY
QPTEIAHQVREFLENSYKDYKAVSALLAVAEEEVILYSPPLRKATSAVGIAVEGGGYY
HVHGVSEDETAEAADDDHEEDLWFILMQACTEEDYTYLENLCLEYCDLWEKFWDVQME NSKSILKQNLA"
/product="hypothetical protein" /transl_table=11
CDS 334093..334653
/locus_tag="AUO97b_00493" /db_xref="SEED:fig|400667.82.peg.353"
/translation="MSKKEDIINTALELFNQIGYNATGVDKIIAESNVAKMTFYKYFP
SKESLIMECLNHRNINIQNSIYEKLSLHPAVSPIEKIHLIFNWYIDWVNSENFNGCLF
KKAFIEVSKQYTSIREPFQEYTNWLINLLNSLLVELDIKDPTPLTHIIISIIDGIIID
GTIDKDLIDPSKKWQYIEYLIKTENP"
/product="Transcriptional regulator, AcrR family" /transl_table=11
CDS 334839..335384
/locus_tag="AUO97b_00494" /db_xref="SEED:fig|400667.82.peg.354"
/translation="MKKFLGNSIFKAVGWTYDADPTILEKKQVIIGFEHTSNLDAILS
IALFQILDLKIHTLIKKELFKGPMKPILEKLGGIPVDRKASKDIVSQMVEKFQSSDTF
NLVIAPEATRAKDGSERKPIRTGFWHIAKAANVPIVLMYANARTKKGGILGKIYPTDL
QKDLETIKELYAQYDIDVKIN" /product="Acyltransferase family protein"
/transl_table=11

CDS 335547..336296
/locus_tag="AUO97b_00495" /db_xref="SEED:fig|400667.82.peg.355"
/translation="MAGHSKWANIKHRKAKQDASRGKVFTKYIREIVTAAKLGGADPA
SNPRLRAVVEKALSVNMTRDTINRAIQRGVGGEDNDDLKEVTYEGYGVGGVAVLVETM
TDNLNRTVPDVRHCFSKTNGNLGTAGSVAYLFTKRGEITFDDISLEDKIMDVALEAGA
EDIEVSEDEILVITSPETFGEVQDALAAAGLKSDNAEVVMSPSTKAEITDIDQAKQVM
KLIDMLEDLDDVQNVYTNVEFSDEVLAQLDA"
/product="Probable transcriptional regulatory protein YebC" /transl_table=11
CDS complement(336327..336797)
/locus_tag="AUO97b_00496" /db_xref="SEED:fig|400667.82.peg.356"
/translation="MELDRFDKQILEILSHEDLNLNELSERINLSVSSVHRRIKHLIE
ANIMGQLKREINFSKLGFTLHILLQVSLSKHDSETFDKFLSEIEAIPEVTNAFLVTGQ
SADFILELVARNMDDYSEILLRRIGKIDNVVALHSSFVIKEYNVFNCYNLLNKV"
/product="Transcriptional regulator, AsnC family" /transl_table=11
CDS 337128..338459
/locus_tag="AUO97b_00497" /db_xref="SEED:fig|400667.82.peg.357"
/translation="MWGMPVKNIKTLILAMSTISLTGVVMPAHAATDAEVDALRKEVS
ELKQLVQQLSNQQKVVTVVNTPTVPPTTITASSPVPQSKPGWMQMPDGQTQVKLYGNV
RVDATYDFKGSNGSISNGANYPLNKDDPRQDSLNVSAATTRIGLDLTRPTQYGDLTGK
IEADFMGGNGDNGSGTFRVRHAYMSLGKWLAGQTTSPFVNTDTSPETLDFTGAVGTGT
TRTVQVRYTQPINAQQKILVALEGGDVEKVSNAAGGSRFPALTTRYDFKTADNKGLLQ
LHGLAHENRVATKDSSSEEKFGWGVGIGGKYDITPKDSVVANYYHVKGDGRYLSYSNS
AYAYDTSTQDINLGEFDSAVIGYQRKWSPIFRSTFAVGGIQYKDDSVYANSNLTNTSY
NKEIYNALVNLIWNPVKNIDLGAEYTYGQRETFAGDKGDYSRINLLAKYNF" /product="DcaP"
/transl_table=11
CDS complement(338510..339253)
/locus_tag="AUO97b_00498" /db_xref="SEED:fig|400667.82.peg.358"
/translation="MPMIDIQHISKWYGDFQVLTDCTTSIEKGDVVVVCGPSGSGKST
LIKTVNALEPFQQGDIIVDGVSLKDPKTDLAKFRSRVGMVFQHFELFPHMTVLENLTI
AQVKVLKRSVAEAREKGLKYLERVGLLAHKDKFPGQLSGGQQQRVAIARGLSMDPICM
LFDEPTSALDPEMVGEVLEVMVQLAHEGMTMMCVTHEMGFAKRVSNRLIFMDEGKILE
DCPTHEFFDNLEARHERAKIFLEKIQAMH" /product="Glutamate/aspartate ABC transporter,
ATP-binding protein GltL (TC 3.A.1.3.4)" /transl_table=11
CDS complement(339267..339944)
/locus_tag="AUO97b_00499" /db_xref="SEED:fig|400667.82.peg.359"
/translation="MDFSLLHNPDVIAALKEGFIFTLTVTVLAMMGGILIGTPLAMMR
LSNNALASNFAKFYVDLFRGIPLIQVIFIFYFLLPKIFEFQSDTYWGPLFSSVVTFAI
FEAAFFSEIVRSGIQSISKGQVNAGYALGFTYGQSMRYVVLPQAFRNMLPVLLTQTII
LFQDVSLVYVISAPDFLGRADTLANTYGPETKATFYLIVAVVYFCSSFVLSQLVKRLQ KKIAIIR"
/product="Glutamate/aspartate ABC transporter, permease protein GltK (TC
3.A.1.3.4)" /transl_table=11
CDS complement(339947..340789)
/locus_tag="AUO97b_00500" /db_xref="SEED:fig|400667.82.peg.360"
/translation="MSVGSLNWTIFCAESNEYGVDKAAWAESVCKVIGSSSYNAYAEA
PTWLQMLGSGVFTMVWTAIVAFLIAFLLGSLLGVIRTLPNKPLAFIGNCYVEIFRNIP
LIVQLFFWAFVFPEFLPDSLSAGSGDVVAGGWWKNILNQQPAVIGVFALGLYTAARVS
EHVRAGINTVSRGQKFAASAMGFTTAQSYRYVILPVAYRTVWPTITSEAMNVFKNSAV
LYALSVLNFFAYTKTMREETSQDIIILILSTPVYLIITYTIKFIMAWIEKRMAVPGLG SGGK"
/product="Glutamate/aspartate ABC transporter, permease protein GltJ (TC
3.A.1.3.4)" /transl_table=11
CDS complement(340884..341777)
/locus_tag="AUO97b_00501" /db_xref="SEED:fig|400667.82.peg.361"
/translation="MKRSATSLIVSTVMLCTVGATSQIQAADTLAKIKSTGKIVIGHR
ESSDPISYVVAGKPVGYAVDICNQFANDIKKELKMPGLKVEYKAVTSSTRIPELLAGN

IDMECGTTTNSKQRQQQVGFSTNYYATEVRMAVKANSGIKSLADLNGKAVVTTQGTTS
DKYIKMNEKGQAINVQNIYGKDHADSFAMMASGRAAAFVMDDNILAGLIAKSSTPKAF
AIVGPVLSSEPYGIMIAKDDPKFKAIADRTVNNLWKTGQMDALYKKWFLSPIPPKNTN
LNMQQSSSYKKLKAHPTDAGI"
/product="Glutamate/aspartate ABC transporter, substrate-binding protein GltI (TC 3.A.1.3.4)" /transl_table=11 CDS complement(342314..343078)
/locus_tag="AUO97b_00502" /db_xref="SEED:fig|400667.82.peg.362"
/translation="MRVLYQFPLSHYCEKARWLLDHKELDYVAHNLIPGFHRAFAQLK
TGQNLLPILKDDHRWIAESTKIALYLDDTYPEHALLRRDEQLRQQTLKIDSLADELGV
HVRRWALAHTLAQGDHALEIMMGEQGYLRQFEKISKPFLKTLVKKNYKLEEELVSQSK
GRMDELINELNHYLIENQARYMVGDRLSLADISVCSMLAPLLEIKGTPWEREEDGEVS
PDWSNYQKYLLDLPLGQYVLRIYQTERNARVDWRGI" /product="Glutathione S-transferase"
/transl_table=11
CDS 343448..344659
/locus_tag="AUO97b_00503" /db_xref="SEED:fig|400667.82.peg.363"
/translation="MSNSLSKTTAYVQVIQNDQQAINAAYQVADFALEGRNTRDQQRL
LPHEQIESFSQKGLGGIRIAKKYGGAFVSNKTLAQVFRILSKGDANVGQIPQNQISLL
NLIEIMGTEQQKQFIFSEILAGKRLANGGPERNTHDSKTLKTTLTIENGKYFLNGEKF
YSTGTSFAHWLAIKAIHPEGHVVLVIVNRDAQGIEVINDWNGFGQRTTASGTVKLNQV
EVNPELIFDERLLTQVPTYRGAYSQLLQVAIDVGIAEAAFEDTLSTIHKARPIIDANV
EKASFEHYTLQEVGKLNILLDAAILLLDEAAEYLDELDQLQTVTDEQVAKASILVAEA
KVYANDAALQISEKLLELGGSRSSLNQHNLDQHWRNARVHTLHDPVRWKLHAIGNYYL NGHFPARHAWI"
/product="Acyl-CoA dehydrogenase; probable dibenzothiophene desulfurization enzyme" /transl_table=11
CDS 344672..345895
/locus_tag="AUO97b_00504" /db_xref="SEED:fig|400667.82.peg.364"
/translation="MTSYQSVQTKQTSASGQAHIIQNDAEALEIAKQFAEQFKKTAVE
RDAKRILPYTEIDALSQSGLWAITVPKQYGGAEVSSHTVAKVIALLSGADGSIGQIPQ
NHFYALEVLRNTGTETQKQRLYGEVLNGTRFGNALAEFKTKTSTHKQTNIRPHENGYL
IQGEKFYCTGSLFAHRIPTLVLDDAGREYLAFVKSGSQGLKLVDDWSGFGQKTTGSGT
VKFDQVFVDADDVIPFDTAFLQPTLVGPFAQIMHASIEVGIARAAFEESLQRVHQARP
WIDSNVETANQDPLTIYELGRIAVDVRASEVLLKQAAQSIDAAKIETTPESIAKASID
VAKVRAHSTDIALKASSKLIELAGSRGSQSQDGLDRFWRNARVHTLHDAARWKYYFIG NYVLNGVLPPRRGTL"
/product="Acyl-CoA dehydrogenase; probable dibenzothiophene desulfurization enzyme" /transl_table=11
CDS 345895..347307
/locus_tag="AUO97b_00505" /db_xref="SEED:fig|400667.82.peg.365"
/translation="MADFKAKEILLNAFDMNCVGHINHGLWTHPRDESHRFNELSYWT
EQAKTLENGLFDGLFIADITGVYDVYQNGIDLTLKESIQLPSHDPSTLISAMAAVTQN
LSFGVTVNLSYEHPYQFARRFASLDHLTQGRIGWNIVTGYLDSAERLIGQKGLKDHDA
RYEQAEEFLELCYKYWEGSWENDAVKKDKTRRVFTDPSKVHTIHHHGKYYQSEGVFQV
SPSVQRTPTLFQAGASPKGMQFATRHAECVFIGGDKPEKIREQVKKIRTLAEQQGRSA
NDIKVFVGITVVVAETHDLAVQKLNEYRQYASPEAGLAHYASSTGIDLSKFADDEAIP
YQKSNSIVSITEKFKEQQITKNDLKAQHVLGGRYPLIVGSGEEVAEYLIHLLDETDID
GFNLTRTVAPESHHDFIRLVIPELQQRGRYKTAYKTGSLRNKIFNRGDQLPEQHPVQA
FRCSPYNNSKNLTKIEEITA"
/product="Coenzyme F420-dependent N5,N10-methylene tetrahydromethanopterin reductase and related flavin-dependent oxidoreductases" /transl_table=11
CDS 347322..348152
/locus_tag="AUO97b_00506" /db_xref="SEED:fig|400667.82.peg.366"
/translation="MAQTASKKWLGIGLVVVVLFVALFLWNKHRQSGLNELVIGISPP
FAKPLQAAADEAKKQGLNVKLVEFSDWNTPNITLNHGDIDANFFQHQPFLDNAIKETG
FKLKAFAVGAASHVGLYSKKYKSLDELPQNARVVIPNDPVNQGRALLLLQQAKLITLK
DSNNHLSALKDVVSNPKNLQFIEVEGPQTARAIDDVDLAFGYPHYLRLAKTADPNSAL

LLDDNTNKRYAILFVVRDDYEDKGDKLKKFVEIYQNSPKVKAVLDAEIGPKLWFPGWK "
/product="Methionine ABC transporter substrate-binding protein"
/transl_table=11
CDS 348166..348999
/locus_tag="AUO97b_00507" /db_xref="SEED:fig|400667.82.peg.367"
/translation="MAIHFKQHKPWLLGITILIVVLGLVGYPYIKNKQADDVLTIGIS
PPYAELLQSVANEVEKQGVHVKLVEFSDWRAPNVAVQNGDIDANFFQQSVFLRNAVKE
TGYDLHAFGVGSGSHVGLYSKKYKSLEELPSKARVAIPNDPVNLARALILLHRAGLIQ
LKDINNELSTTQDIIANPKQLSFVEVEGPQTAHAYNDVDLIFGFPHYLKMAKVTDPHS
ALFLDPIDKKYAILFVTRRDYQDKNQKLATFVKAFQNSKQAQDILDKDFGKGMWFQGW K"
/product="Methionine ABC transporter substrate-binding protein"
/transl_table=11
CDS 349011..350081
/locus_tag="AUO97b_00508" /db_xref="SEED:fig|400667.82.peg.368"
/db_xref="GO:0005524" /db_xref="GO:0016887"
/translation="MVSFGSQVDFSLPHIKIRGLNKFYQSQGQKLHALKEINLDIPQG
KILGIIGKSGAGKSSLLRTLNGLEQVNTGSIHIHQQNIAELSHSELIQTRQRIGMIFQ
HFNLMSAKTVWENVALPLKVSNYNKADIDQRVNEVLALVGLADKSNYYPSQLSGGQKQ
RVGIARALVHHPEILLCDEATSALDPESTATILTLLKKINQELGLTIVLITHEMQVIR
EICDQVVVIDQGEIVEAGQVWSVFSRPEQQITQELLNLEQITLPFKISRLPDEDSTHI
IVKLKYEAETHQVPDIQELLARFKAPVNLYQSQVDIIQGHIIGSLLVGIPNVEIDLSS
IQQDALTAIAQFEVLGYARPAH"
/product="Methionine ABC transporter ATP-binding protein" /transl_table=11
CDS 350062..350721
/locus_tag="AUO97b_00509" /db_xref="SEED:fig|400667.82.peg.369"
/db_xref="GO:0005215"
/db_xref="GO:0006810"
/db_xref="GO:0016020"
/translation="MQDQLIDLLITGTVDTLLMVGASAFIAFLIGLPIAVILVSTSEH
GIHPSQKINQALGWVINITRSVPFLILMVALIPLTRWIVGTSYGVWAAVVPLTIAAIP
FFARIAEVSLREVDQGLIEAAQAMGCNRKQIIWHVLLPEALPGIVAGFTVTIVTMINS
SAIAGAIGAGGLGDIAYRYGYQRFDMQIMLAVILVLIVLVMLVQATGDALAQQLDKRK V"
/product="Methionine ABC transporter permease protein" /transl_table=11
CDS 350815..351906
/locus_tag="AUO97b_00510" /db_xref="SEED:fig|400667.82.peg.370"
/db_xref="GO:0005525"
/db_xref="GO:0005622"
/translation="MGFNCGIVGLPNVGKSTLFNALTKAAIAAENFPFCTIEPNTGIV
PVPDPRLDKLAAIVNPQRVLPTTMEFVDIAGLVAGASKGEGLGNQFLANIRETDAIAH
VVRCFEDENVIHVNGKIDPLDDIATINTELALADLETVAKAILRLTKVAKGGDKEAVA
TKAVLEKIQPLLDEGKPARAADLSDDERKLIRGFGLMTLKPTMYIANVAEDGFENNPH
LEAVKKLAAEENAIVVPLCNQIEAEISLLEDEDRAEFLEAMGMEEPGLNVVIRAGYKL
LGLQTYFTAGVQEVRAWTVKVGATAPQAAGVIHTDFEKGFIRAEVVAYDDFVQYNGEN
GAKEAGKWRLEGKTYVVQDGDVMHFRFNV"
/product="GTP-binding and nucleic acid-binding protein YchF" /transl_table=11
CDS complement(351952..352473)
/locus_tag="AUO97b_00511" /db_xref="SEED:fig|400667.82.peg.371"
/translation="MSNLEQKLSQYAAYHLNHQNILTHFIGIPLIVFSILCLTARAGI
DIGNFKVTLAMVLIAFSTIYYLFLDKVFGLIMLMILVAVYPLASQIAELSLGQWLAAS
IGFFVVGWAFQFVGHYFEKKKPAFVDDVIGLAIGPLFVLAEFIFMLGFRKPLHERILH EARSKREMMDLSH"
/product="FIG028593: membrane protein"
/transl_table=11
CDS 352581..353273
/locus_tag="AUO97b_00512" /db_xref="SEED:fig|400667.82.peg.372"

/db_xref="GO:0003700"
/db_xref="GO:0005622"
/translation="MDLIYINRLKENTWFSVLPEVFQKFILEYGKQITFEKNSYVFHA
QDEFDGIYTVLEGSISLGYVDVNGNEALSAIAEPIMWFGEISLIDHEPRSHDAIALKK
SIVLHIPAKPLNELLKEHPYYWYYFARLTSQKLRYVFLEQIAIQTRSISQRLAQRLLF
ILEGYGNHILIQDHHIQISQEQLANMLTTSRQTINHELSLLEKQNIIKIAFRKIEILD IEKLKVIAQFHS"
/product="Transcriptional regulator, Crp/Fnr family" /transl_table=11
CDS 353446..353781
/locus_tag="AUO97b_00513" /db_xref="SEED:fig|400667.82.peg.373"
/translation="MKKLSTILTAGVLAMLSVSAFACPKGTQLQGGTGPNHKGGKCVA
VHGKATAQKTKKEATKTKQEVKKDLTMQKHDAMTSATHAQHESHQMTHQMKQDAVKTA NSAKAATKP"
/product="hypothetical protein"
/transl_table=11 CDS complement(353854..354324)
/locus_tag="AUO97b_00514" /db_xref="SEED:fig|400667.82.peg.374"
/translation="MFKSKTRLVYIAILSASVFILQGCDNGNQSSDKKVEIKPAPKLT
NDATVYAKKAWTLINTVDHLVYEKQLSQINDNVRKPLRQLSTDWRINVKMTDSVTEGK
YALCRKALTSLDVWARETLENNSSVAQKQADYERDKQQCKEAIENPNLGNTSPK"
/product="hypothetical protein"
/transl_table=11
CDS 354426..355166
/locus_tag="AUO97b_00515" /db_xref="SEED:fig|400667.82.peg.375"
/translation="MSNQQQMAAQTATFWQGAKDSQAIVLTYLPVSFAFGVSASQFGF
SPWEAFFLSCSMYAGASQFLVVALLASGSSIWLTALTVIALDIRHVLYGPALYNLIPT
KLNLKKTAVWAWGLTDEVFASGMIQLSQRRQQWSESWMLGLSLFSWMSWASGSLLGGL
FADQVAHLPKFLQAALDFLLPALFLSFLLAAFERKHSLVVAVSLVVSALACYWINLSA
AIFIGILSGILAGLFKHYVLKQFDEVET" /product="Branched-chain amino acid transport, AzlC" /transl_table=11
CDS 355168..355509
/locus_tag="AUO97b_00516" /db_xref="SEED:fig|400667.82.peg.376"
/translation="MNLEIILVGIIVGIANFASRFGPFFVIQKLQGNQQRRGSIWLKI
ALGSIGISAISAMLVVATLPPLLETPNKSLAMLIGFMVLAGLYFKFKKIVPATLTAAI VYGLIYTYLPL"
/product="putative membrane protein"
/transl_table=11
CDS 355638..356027
/locus_tag="AUO97b_00517" /db_xref="SEED:fig|400667.82.peg.377"
/translation="MSQVKIYANERTIIQYRELLSHAIHQALIEELKYPVEKRFQRFI
SLKPENFIYPSDRSQHYIIIELSMFAGRSPATKKQLIQTLFRNIEEQCKISPQDIEIT
IFETPKENWGIRGKNADELLLNYQVNI"
/product="Tautomerase"
/transl_table=11
CDS 356139..357347 /locus_tag="AUO97b_00518" /db_xref="SEED:fig|400667.82.peg.378" /translation="MSVTLGTPLQSSAFKVLLLGSGELGKEVVISLQRLGVEVHAADR
YDHAPAMQVAHFSYVLNMADPAQLKQLIEKVKPNLIVPEIEAIATEVLLEIEANKTAT
VIPSAKAVNLTMNREGIRRLAAEELGLPTSAYRFADTLESFRAACDDIGYPNFVKPVM
SSSGKGQSRVKSFDEVDAAWEYAMQGGRVNQGTVIIESQIDFDFEITLLTVRAKNPET
GEIETHYCDPIGHRQDAGDYVESWQPQPMTPAALEEAKRIANKVTTALGGCGIFGVEL
FIKGDKVWFSEVSPRPHDTGLVTLASQFQSEFELHARAILGLPVNTARHSVAASAVIY
AGVDANNLSYSNLNVALAHPDTDLRLFGKPEGFKRRRMGVATARAENTDLARTLAKET ADQVSVQTNS"
/product="Formate-dependent phosphoribosylglycinamide formyltransferase"
/transl_table=11
CDS 357375..360038
/locus_tag="AUO97b_00519" /db_xref="SEED:fig|400667.82.peg.379"
/db_xref="GO:0008773"
/translation="MINTSPLLNYVSSHHDIKAINQWRTDVEKQLQDSYENGQSIREI

IKARSDLVDEALVFLWKHAELDQSKLGLFAVGGYGRREMLPYSDVDIMILSEDEISEE
NEKRISTFISSLWDVGNFKPGISVRTIQSCVEQAATDLTVATTLIEARLITGNTQLAK
WPRRIVSQTWTDKTFYDAKMAEQAKRYHQHNNTESNLEPDIKNAPGGIRDINQIGWIA
KRHFRVNRIYDLVHLGFISEFELAVLEEAESFLWEIRHHLHRLAKRDENRLLFDHQRE
IAAKFGYVRQEGQPVNYGVEQFMKRYYRTAQQVSTLNEMLLAYFSESVITPRLPNYER
KIEVVNDHFKIVDNKLAVQHHKIFAEHPSAILELFYILANRPDIEGIRARTLRLLILA
AKRINQSYRDNPEHQALFMSIIRSPYRLYDTLVAMKRYGVLGNYIPAFGQIMGLMQYD
LFHIYTVDAHTLLLLRNLNRFREPEFAKEFPVVSSVFQRLARQDIVFIAALFHDIAKG
RGGDHSELGAEDAIEFGRAHGFTERECKLIAWLIQNHLLMSLTAQKKDISDPDVVKDF
AEKLGDMEHLDYLYTLTVADINATNPKLWNTWRASLMRQLYTHARDVIRTGLGRPVDY
QMLIEDTKFAASELLVNNFALADVEKVWQELGDEYFIKESADEIAWHTQAILKHGDNP
EPLVLLRAHRKAAQDAVQIFIYTRDQPNLFATTVAVLDRMNLDVQDAKIITASTAFSL
DTYVVLDRFGTLLTDPEREETVKNALVKALSQPDQYPGLMQRRIPRQLRHFDIENTVD
VTLNEALQQNMVEISTLDHPGLLARVGGLFMMQGLDIHSARIATLGERAEDIFFVTKK
DGKPLNHEEVKLFSEKLKAALDEASNQICQH"
/product="[Protein-PII] uridylyltransferase (EC 2.7.7.59) / [Protein-PII]-UMP uridylyl-removing enzyme" /EC_number="2.7.7.59"
/transl_table=11
CDS 360064..361233
/locus_tag="AUO97b_00520" /db_xref="SEED:fig|400667.82.peg.380"
/db_xref="GO:0009016"
/translation="MNSSLSLLHPYPFEKLNQLFKDITPANLPLIPLSIGEPKHPAPE
FVKQAIIDNFNHLSTYPNSKGLPELRQSIADWLTKRFKLNSISAENHILPVSGTREGI
FSFVQALINREDAPYVVMPNPFYQIYEGAALLAGAKPYFINCTEENGYLGDFDAVPAE
VWEKTALLFVCTPGNPTGTVLSKEQFKKLIALSDQYNFVIASDECYSELWFDQAPTGL
LEVCAELGRDDYKNCIVFHSLSKRSNLPGMRSGFVAGDAALLKPYLQYRTYHGAAMPV
QHQLASIAAWNDESHVEENRKQYRAKFDLFQSELGHLLPLQKPDAGFYYWLKVDNDET
FAKMLMEKAHIKVLPGRYLSRDTEQGNPGANHVRMALVADLAQCEEVVKRLKAIL" /product="N-succinyl-L,L-diaminopimelate aminotransferase (EC 2.6.1.17), type 2"
/EC_number="2.6.1.17"
/transl_table=11
CDS complement(361294..361926)
/locus_tag="AUO97b_00521" /db_xref="SEED:fig|400667.82.peg.381"
/db_xref="GO:0005293"
/db_xref="GO:0006865"
/db_xref="GO:0016020"
/translation="MEVFLVIAFTHFLALLSPGPDFFLILTSLLQKGRRYTYGIVLGI
TLGNALILAGCLFSFMLLGNLSSILLLIFKWLGALYLAYLSFLCFNAARSTVLNFSTD
VNNLSDQAEDKHNKIKSLVLGLQSSLLNPKNIMFYSSLMLLIQHKFSLFQKLLMSVWM
VSVVLIWNILWVKLLANDRILIKIKRSATGLYYCSAVAFILFAVLLITYK" /product="Threonine efflux protein"
/transl_table=11
CDS complement(361991..362563)
/locus_tag="AUO97b_00522" /db_xref="SEED:fig|400667.82.peg.382"
/translation="MLHLDLAWLNQLYSEFQEQGLDLHIPQHKPLIIKDEFLYDAFTE
MNETLFDAQKLIFEKEQSLLHCLIHLLLPHFILEEIQKPQYLYKDFLNLIDVISSSEG
FISLEELAQRVGLSRYAIIRLFKANVGLTPHAFQINLKINQAREQLKKGVPLAELAVN
LGFSDQSHFHKAFKAHTGVTPRQFQLAAAQ"
/product="Transcriptional regulator, AraC family" /transl_table=11
CDS complement(362836..363321)
/locus_tag="AUO97b_00523" /db_xref="SEED:fig|400667.82.peg.383"
/translation="MSNIYQFEAELLEGDIKQFADYKGKVLLIVNTASKCGFTPQFAG
LEKLYEKYKDQGLEVLGFPCNQFGGQDPGSNKEIGTFCQRNYGVKFPMFAKVDVKGPE
AHVIFRYLTREAKGILGSSTIKWNFTKFLVGKDGSVLNRYAPTTKPEALEADIEKALA S"

/product="Glutathione peroxidase (EC 1.11.1.9) @ Thioredoxin peroxidase (EC 1.11.1.15)" /transl_table=11
CDS complement(363318..363737)
/locus_tag="AUO97b_00524" /db_xref="SEED:fig|400667.82.peg.384"
/translation="MGKVNKTDREWQRELSPEEYRITRQKGTEPAFTGQYWNTKQHGT
YVCRCCGAELFSSDAKYDSGCGWPSFFRPLNGSVIDEHEDLTHGMVRTEIVCHDCEAH
LGHVFEDGPQPTGLRYCVNSASLQLKTQEKNDEETYP" /product="Peptide-methionine (R)-S-oxide reductase MsrB (EC 1.8.4.12)" /EC_number="1.8.4.12"
/transl_table=11
CDS 363978..365408
/locus_tag="AUO97b_00525" /db_xref="SEED:fig|400667.82.peg.385"
/db_xref="GO:0004069"
/translation="MSQKKSVLFQQLLPVIKQYQQSGFTHEKIVELLKDQHDLNLVTV
KTFKSYLYRYAKVNPAMSKNTATLQSMPTSREIKKSSKLEHVCYDIRGPVLRAANEME
EQGHKIIKLNIGNPAPFGFEAPQEIINDVALNLPNAIGYVDSKGIFPARKAICQYYQQ
KGILNMHVNDVYIGNGVSELIVMAMQGLLDDGDEMLIPMPDYPLWTAAVNLSGGTAIH
YKCDEENSWYPDIADIESKITSNTRGIVIINPNNPTGSVYPRHVLEQIVALAKKHDLI
LFADEIYDKIVYDGIEHVAVASLAGDQLCISFNGLSKAYRIAGFRSGWMAITGDKSRA
ADYIEGLDMLASMRLCANVQAQYAIQTALGGYQSINDLIRPGGRLYEQRNIAWEMLNE
IPGVSCVKPEGAMYCFPRLDPNIYPIEDDEKLMLDLLRAEKVLLVQGTGFNWPTPDHF
RVVFLPAENELREAIGRLGRFLANRR"
/product="Alanine transaminase (EC 2.6.1.2)" /EC_number="2.6.1.1"
/transl_table=11
CDS 365874..367181
/locus_tag="AUO97b_00526" /db_xref="SEED:fig|400667.82.peg.386"
/translation="MKQKKLLKYIVVAILLGVLTGWICHHFFNEGEQLKQIASYFNIV
TDIFLRLIKMIIAPLVFATIVCGIVSMGKSTSIGSITLKSMTWFITASFVSLAIGMGL
ANFFQPGAALDLALPTAQQLSSSSLPTTTGFTLQSFLSHVFPRSIAEAMANNDILQVL
VFSIFFGSALAFVNQGKEKDSVIIRLTDELSKIMFRITDYVMMFAPFAVFAAIASAIT
VQGLGLIVDYGILIAEFYFGLLLLWIILFSVGAIVLKKDIFRLGKLIREPVTLAFATA
SSESAYPKVMDALNKFGVPKKVTSFVLPLGYSFNLDGSMMYTTFAVLFIAQAYNIDLS
FTQQILILLTLMVTSKGIAGVSRASIVVISATLTMFHLPEAGILLLLGIDQFLDMGRT
ATNVVGNSIATAVVAKLEGDKVADTEELPEPVLIKSTQEQTIA" /product="Proton/glutamate symporter @ Sodium/glutamate symporter" /transl_table=11
CDS 367866..368933
/locus_tag="AUO97b_00527" /db_xref="SEED:fig|400667.82.peg.387"
/db_xref="GO:0004067"
/translation="MLTKTVKSFGLAMGLLACSLPLYAKNNVVVVATGGTIAGAGASS
ANSATYTAAKVPVDALINAVPQIQDLANVSGIQALQVASESITDKELLQIARQVNELV
KKPTVNGVVITHGTDTLEETAFFLNLVVHTDKPIVLVGSMRPSTALSADGPLNLYSAV
ALAASDDAKNKGVMVLMNDSIFAARDVTKGINIHTNAFVSQWGALGTLVEGKPYWFRQ
SVKRHTNASEFNIENIKGDALPTVQIVYGSDSMLPDAYEAYAKAGAKAIIHAGTGNGS
VAKYIVPTLQNLHDKNGIQIIRSSRVPQGFVLRDAEQPDSKYGWVAAHDLNPQKARLL
AALALTKTNDAKEIQRMFWQY"
/product="L-asparaginase (EC 3.5.1.1)"
/EC_number="3.5.1.1"
/transl_table=11
CDS 369148..372711
/locus_tag="AUO97b_00528" /db_xref="SEED:fig|400667.82.peg.388"
/db_xref="GO:0003887"
/translation="MQFVHLGIYTEFSITESIVRIPDLVKTAVKDEMPALALTDLSNL
HAAVKFYNSCLKKGIKPILGSTIRLNDAQHRATLLAMSDIGWKGLTEIVSRGFIEGQQ
LSIPCVQKEWVLEQHQDIIVLLGQHSDVGQMLCSSNPQKAEPLLEEWIEKFGNRVYLA
LTRTDRPGEEDFIQEAAKLAAKYNIGVVAHNDVHFIEKEDFEAHEARVCIADGYVLAD

DRRPRLYSPEQYFKTSDEMIELFSDIPSAIENTYQIAKRCNVTLKLGTYFLPEYPIPD
GFTIDTYFEHLSKEGLEERLNYLYPVEKRGEDWPEIRKPYDERIDYEVGIILKMGFPG
YFLIVMDFIQWAKNNGVPVGPGRGSGAGSLVAYSLKITDLDPLRYDLLFERFLNPERV
SMPDFDVDFCIAGRDRVIDYVSRTYGREAVSQIATFGTMAAKGAIRDVARVLGKSYGL
ADRISKMVPTKPLGVDLATAIEMEPQLKDIVTNPSNPDNDDASEIWEMALKLEGITRN
TGKHAGGVVIAPGKITDFSAVLCDADGTNRVAQYDKDDVEAAGLVKFDFLGLRNLTVI
EDAIQNINKNRDSNDPLNISHVPLDDPKAYSVFADANTTAVFQFESVGMKRMLKEARP
SKFEEIIAFVSLYRPGPMDLIPDFIHRMHGGEFEYLHPLLEGVLEPTYGIMVYQEQVM
QTAQICAGYTLGGADLLRRAMGKKKPEEMVKQRQIFLEGAGQKGIDESTANHIFDYME
KFAGYGFNKSHAAAYALVAYHTAWLKAHYPAEFMAAVMSSEMQNTDSVVFLIDDCRNN
GLEVLPPSVNMSTYHFHASDDKTIVYGLGAIKGVGEQAMQSVIDSRRQQGPYTDLFDF
CHRIDLKKINKRTLEALIRAGALDCLGIERSSLMAQLPEAVQAAEQARSNRESGIMDL
FGEVEEVQRKPAKPVKPWSDEVRLKGEKDTLGLYLTGHPIDVYRQELKAFIPAKLNEI
TATRRGVTTVYAGLVLDVANFPNRVVIVLDDGTARIEVSCNHERFQRFKDIIQVERVV
VFEGEIYEREGFDRPMGRLNKAFSLNEIRQKRANSIQIKLTHDLLQPSLAKDLQNILL
PYCNVDMHQHIAIQLQIDQPYAQAELQLGPQWKVAPLDELLAKLRDYFGKDNIHIEYQ
VKSKAAKAAEPVRPQPVASPPVDMTIDDALDSYQSEVSQYS"
/product="DNA polymerase III alpha subunit (EC 2.7.7.7)" /EC_number="2.7.7.7"
/transl_table=11
CDS 372726..373565
/locus_tag="AUO97b_00529" /db_xref="SEED:fig|400667.82.peg.389"
/db_xref="GO:0003723"
/db_xref="GO:0006396"
/db_xref="GO:0008173"
/translation="MSLFDDTAVSKQLAQVRIVMVNTTLPANIGSALRAMKTMGLSKL
VLVSPKTYPHPDIQALAAGAQDLLEQVEIVETLEEAIKDCHLVFGTSARSRTIPWPLL
DVRPASKEAIKAAAQGQQIAIVFGREDRGLTNEELALANYHLTIPVNSDYGVLNVAQA
IQVVCYELRMSVLEQKEVASDAGQMPLAQGQSMQWDEPLVTQQQMEEFYPHLEKMLTE
IEFLDPENPRLLPLRLRRLFGRIQLDRMEYHLLRGIFSRVQALTSGKWKKASSDKEDQ SNA"
/product="tRNA (cytidine(32)/uridine(32)-2'-O)-methyltransferase (EC
2.1.1.200)" /transl_table=11
CDS 373558..374373
/locus_tag="AUO97b_00530" /db_xref="SEED:fig|400667.82.peg.390"
/db_xref="GO:0009001"
/translation="MLKQLKEDIQAVFARDPAARNTLEVLTTYPGIHALLLHRLAHEL
WKKDCKGTARFVSSFSRFATGIEIHPGAKIGKRFFIDHGMGVVIGETAEIGDDVTLYH
GVTLGGTTWNKGKRHPTLEDGVVVGAGAKILGPFTVGKGAKVGSNAVVTKAVPAGVTA
VGNPARYIYKDADKTKDKDEERRRDYAQSIGFAPYATTADQSDPILEGMRVLLDRIQH
NETRMNNLCQRLSELDPTFKKESQDEQPFSDEELKILEEVRRECGAQNKTSKT" /product="Serine
acetyltransferase (EC 2.3.1.30)" /EC_number="2.3.1.30"
/transl_table=11
CDS 374477..375418
/locus_tag="AUO97b_00531" /db_xref="SEED:fig|400667.82.peg.391"
/translation="MNFKPLAYILLATSSLTACTMAPVKEQKIEPFVFKEPELTPPFY
ALNPFNYDAPPAFEVALKEAAAQPVTKMVVTRQDDPTKTLTLDTNKLIVPTVNNSQRS
MKYAVLAGDNEIDVTEIDDFLQLVEGKARHYPPRFTDRQERKGFESKLKEVTQRLDTL
AANPDASFDILIRAFKASVLARNLDLGTAHTTKSLEYAQRLLKINPDDAEANFWFGFG
LSEGGGQREAIPYLDKAIKGNVQEAYLAAANNYIAMEQKKNAIQTLKNYKVKYPDEAE
VADRLIQEIEKQGRWNVWQVLTNPAMSSATPSAATKK" /product="FIG00350119: hypothetical
protein" /transl_table=11
CDS 375712..377541
/locus_tag="AUO97b_00532" /db_xref="SEED:fig|400667.82.peg.392"
/translation="MSGQEAKACTDSPDTCVNQLKNLPQILDEQLLSAASEMYLAKAM
ALSDSSECKISRFTKHKPTEEQKVIQNKYDECLDQQLSLLDKSIRYSYAYLFSTKRQP

TDRIFDNRQVQIRDFYNQAIAKMVSVYDLRYPQKNVVEPQIHIGKSVYSIDFEFHRQL
SGQKLEKLISSYNLNFSGLRTINRRDGFGSEFVAVFPSSGKEDINEYILDPLNYSYKN
GVNPNIHHARYLAATIVAEPKHAKTIQEIINDPEFVIRVYDPYRTDNINVAGKQYPLA
ANFSAPYGLWLAENNLGVAAYLSLIDRDQHLTMPHLYMLEPYNPNKKIIVLVHGLASS
PEAWIALTNDVMGDTVLRDNYQVWQVFYSTNMPILESRFQIYALLKQAFGSLNPSDPA
AHDAVLVGHSMGGIISRLLVSDADITKPALEMMTIRQQNRFKKHPMVTERLQMHSISN
FDRAIFLASPHRGTDYADRWFTLAARKIIRLPGAFLSAVATSLTTENLDIKDFLNNID
NGLIQNGPSDLSHQSKFMELTENINPHQGLIFHSIMGNITKSDDPNVITDGIVPYKSA
HLEGAKSEKVLPGGHSIQLTPQAVLELRRILREHLVEHGLYKP" /product="FIG00350720:
hypothetical protein" /transl_table=11
CDS complement(377595..379160)
/locus_tag="AUO97b_00533" /db_xref="SEED:fig|400667.82.peg.393"
/db_xref="GO:0006118"
/db_xref="GO:0009055"
/db_xref="GO:0015035"
/db_xref="GO:0015036"
/db_xref="GO:0045454"
/translation="MLDQNIKTQLKAYLERLESPIELVAALDESDKAAQIKELVTEIA
ELSDKVTARFDGNNTRRPSFGVAKAGEQPRVFFAGLPMGHEFTSLILALLQVSGYAPK
VSDEVLNQIKGLNLKANFDVFVSLSCHNCPDVVQALNLIAIYNPNTTATMIDGAFFQD
EVEQRKIMAVPMVFQDNEHIGQGRMTLEEIVAKLDTNSAEKDVAALNAKDAFDVLVIG
GGPAGATAAIYAARKGINTGIVAERFGGQVMDTMDIENFTSVQKTQGPKFAAEMEAHV
REYDVDIMNLQRVSKITGADQTANGLVEIELENGAKLESKTVILSTGARWREMNVPGE
AEYRTRGVAYCPHCDGPLFKGKRVAVIGGGNSGVEAAIDLAGIVEHVTLVEFDTKLRA
DQVLQNKLNSLPNTTVIMNALSTEVLGDGSQVTGLKYKDRSTDEEHVVELAGIFVQIG
LLPNTDFLKDSEVELTNRGEIIVNDRNETNVKGVFAAGDCTTVPYKQIIIATGEGAKA SLSAFDYIIRSGQ"
/product="Alkyl hydroperoxide reductase protein F" /transl_table=11
CDS complement(379338..379859)
/locus_tag="AUO97b_00534" /db_xref="SEED:fig|400667.82.peg.394"
/translation="MGAVLLTLAIIFTQLSLIAFGGGNTILPEMQRQVVDIHHWMTAQ
EFSALFAMAQAAPGPNMMIVPLVGWHVAGLSGLLVTSTAKFLPSSLITVFVMRGWSKF
KDKKWRRVLQLALQPVTVGTVLASAWIISEAAAINTLLIIMVVIATLLSLIKKVHPLH VLIAGAVFGVVLL"
/product="Chromate transport protein ChrA"
/transl_table=11
CDS complement(379859..380419)
/locus_tag="AUO97b_00535" /db_xref="SEED:fig|400667.82.peg.395"
/translation="MNHVEIAVQKDTVPTCTELFLGFLTLGLIGFGGVLPLARKVIVE
QRHWLSPEKFTELLGLCQFLPGGNIINLSVAIGMEFRGVRGAVSSLIGLIFAPTVIVV
LLHYVYEQFQDNLMVKHLFEGLGAAAAGLLVATGLKMLKPLLCNPLAICVVVAAIVSI
AFLKIPLLLTMLILLAFYSAIIWRRV"
/product="Chromate transport protein ChrA"
/transl_table=11
CDS 380521..381423
/locus_tag="AUO97b_00536" /db_xref="SEED:fig|400667.82.peg.396"
/translation="MLDLYKLHAFVVVVEERNITHAANRLFIQQPPLTRLLKKLEQEL
GTQLLIRQPRGIEPTEAGLALFKEAQLLLEHARHIPQLVQDVSQGKTGQLNIGFTSSA
GLHPLISLVLRSYREMYPAVQTKLEEAGSQKQLDWLISEKLDIAFLRAPISRDIGLKH
LHILNEPMVVALPIGHPLTQKKKISLSNLSEENFVLYRRSSGQGLYDNILYSCYQAGF
SPRIIQEAPRPTATLNLVAAGIGISIVPASMHNFWDHEIVYREFDDSIKLNAPIYLIT
RENENSAKVSNFIELMQKLLPTMT" /product="LysR family transcriptional regulator
STM3121" /transl_table=11
CDS complement(381515..382489)
/locus_tag="AUO97b_00537" /db_xref="SEED:fig|400667.82.peg.397"
/db_xref="GO:0006814"

/db_xref="GO:0008508"
/db_xref="GO:0016020"
/translation="MALGLAIGVFFPQASVSLNELSVDNVNIPIAIGLVLMMYPPLAK
VEYAALPEVFKDKKTLTLSLIQNWIIAPVLMFILAIVFLHNYPEYMTGLILIGLARCI
AMVLVWNGLACGDNQYVAALVAFNSIFQILFFSTYAWLFLTFLPPYFGVAGQVIDVNF
WTITHAVLVYLGIPFLMGFLTRLILVKSKGLEWYQTKFLPKISPISLIALLFTIVAMF
SLKGGDVVSLPLDVLRIAIPLTIYFVVMFFISFFMSKRMGNNYPRTTAISFTAAGNNF
ELALAVAIATFGLASPVAFTTVIGPLVEVPVLISLVTVSLWLKKKYYS" /product="Arsenical-resistance protein ACR3" /transl_table=11
CDS complement(382654..382983) /locus_tag="AUO97b_00538" /db_xref="SEED:fig|400667.82.peg.398"
/db_xref="GO:0003700"
/db_xref="GO:0005622"
/translation="MINQVDFFKCLSDQTRLNILKLVLNKQNICVCELTEQLELSQPK
ISRHLALLRTHGVLLDERKGQWVYYSLNPDLPVWALDILKVIENDESGTKVKQQDQSF ITTTCCD"
/product="Arsenical resistance operon repressor" /transl_table=11
CDS 383125..383496
/locus_tag="AUO97b_00539" /db_xref="SEED:fig|400667.82.peg.399"
/db_xref="GO:0008794"
/translation="MALIRHAGIEPIVIEYLQTPPSKDELIQLIKDSNLTVREAIRKN
VDPYKDLELEQADWTDEQLIEFMVQYPILINRPFVVTPKGTRLCRPSEMVLDILDSQN
LGYFAKEDGEVIIDEQGRRLK"
/product="Arsenate reductase (EC 1.20.4.1) glutaredoxin-coupled, glutaredoxin-like family" /EC_number="1.20.4.1"
/transl_table=11
CDS complement(383784..385658)
/locus_tag="AUO97b_00540" /db_xref="SEED:fig|400667.82.peg.400"
/translation="MTEINLILKNGKITTLDPQNPEVQAIAIADGKVVRTGTTDEVMK
LATPTSKVVDLNGRRVIPGLNDSHLHIIRGGLNYNMELRWEGVPSVADALRLLKEQAD
NTPAPQWVRVVGGWTEFQFAEKRLPTLEEINKAAPDTPVFVLHLYASALLNRAALDVL
GFNKDTPDPPGGKIVRNEKGEPTGLLLATPSAMILYSTLGKAPKLPVEDQVNSTRHFM
RELNRLGITSAIDAGGGGQNYPEDYDVIKQLHDQNQMTVRIAYNLFAQKAGQELDDYR
RWTEMTFPGEGDELFRMNGAGENLTWSAGDFEDFYEPRPDLPEKMEGELEAIVEHLAE
KKWPFRIHATYDESISRLLNVFERVNSKQPFATRFIIDHAETVSERNIERIGALGGGI
AIQHRMAYQGEIFVKRYGAEAAQATPPVKKMLELGVPVGAGTDATRVASYNPWVCLYW
LTTGKTVGGLPLYDEKNLLDRQTALKLWTKGSAWFSGEKDIKGSLTAGELADLVVLSD
DYFKVEAEDIQWIESVLTVLGGKVVYAGAEFKQDDPPLPPASPTWSPVKRFGGQWRLS
ENRNAPSNQSLQSQSALCACASSCGMHGHSHAWILDVPVNDKDKKSFWGALGCSCFAF "
/product="Exoenzymes regulatory protein AepA precursor" /transl_table=11
CDS complement(385989..386675)
/locus_tag="AUO97b_00541" /db_xref="SEED:fig|400667.82.peg.401"
/translation="MNKSLLEQLSPTNCQVIFIDHQPQMAFGVQSIDRQVLKNNTVGL
AKAAKTFNIPVTITTVETESFSGHTYPELLDIFPDAPLLERTSMNSWDDQKVRDSLAK
NNRKKVIVSGLWTEVCNNTFAFGAMLEGDYEIYMVADASGGTSKEAHDYAMQRMIQAG
VVPVTWQQVLLEWQRDWAHRDTYDAVMAIVREHSGAYGMGVDYAYTMVHKAPERTTSK HEVLAPVPAK"
/product="Amidases related to nicotinamidase" /transl_table=11
CDS 386871..387761
/locus_tag="AUO97b_00542" /db_xref="SEED:fig|400667.82.peg.402"
/translation="MDRLDCISTFVSVVEHGNFSSAARALDISRDLVAKRVCYLENDL
KTTLLNRTTRQMNLTGCGEKFYQHCKVILSEFEWATYEISYNQQYPEGELKLNTPMSF
SNIFLQHIISDFIEKYPSIKIDLFMTDQLLDVNQHKFDLTIRLDGSAPSLANSKILNT
YQRYLYATPEYFNQHGLPTSLEELKEHKFLIYYQDSRHKKLIFQKDQKEVSFNCFPVM
TCNNGELLLDMCLKDHGIVFLPEFIAEPYYKEGKLQKCLEDYTSQPLHLYVTWPNRKI
LSKKVKLFIDFLTHQIIPIK"

/product="Transcriptional regulator, LysR family" /transl_table=11
CDS complement(387800..388537)
/locus_tag="AUO97b_00543" /db_xref="SEED:fig|400667.82.peg.403"
/db_xref="GO:0003677"
/db_xref="GO:0003700"
/db_xref="GO:0005622"
/db_xref="GO:0006813"
/db_xref="GO:0008324"
/db_xref="GO:0009058" /db_xref="GO:0030528"
/db_xref="GO:0045449"
/translation="MPSNAPKKAVEMSNDEIDDHIYNAIVDAILNRQLAPGARLVEAP
LCEAFGVTRGVLRRVFVKLAHDKVIEIQPNRGALIAKHSTNETKEVFEARSMLEIATV
KKLAQKSHRLDLSELRSLVDQESHERLAGNWAEWIKLSGQFHLKLIEANQNTIMTSYL
QTLIARTSLLIGLYEIPKHNNCSADEHRAILDAIEQGDEKRATQLMEEHLEHYATTFI
EENSTVSAEQNLLNLFKNKRIETPQTN"
/product="Transcriptional regulator, GntR family" /transl_table=11
CDS 388945..390414
/locus_tag="AUO97b_00544" /db_xref="SEED:fig|400667.82.peg.404"
/translation="MNNTEAIIKSSYDAKLTNQDLAPLKKQTWGAYNIFAFWMSDVHS
VGGYVMAGSLFALGLNSWQVLLSLLIGIAIVQFFTNLIAKSSQQTGTPYPVICRATFG
VLGANIPAVIRGLIAVAWYGIQTYLASSAFLLVILKFFPEWSVYANVSTYGFLGLSYL
GWVGFMLLWLLQAIVFWSGMDSIRKFIDWAGPAVYVVMFAMAVWLIWKAGWQNIDLNL
SGVQYDGFAVVPVMIGAIALVVSYFSGPMLNFGDFSRYGKSFNAIKMGNFLGLPINFL
GFSLLTVVCIAATLPVYGKLITDPVEMVGKLDNTFVVILGSLTLMIATIGINIVANFV
SPAFDFSNVSPSKISWRMGGMIAAVGSIFITPWNLFNSPQVIHYTIDILGAFIGPLFG
VLLADFYLVKKQKIVVDDLYTLDSKGLYWYKNGYNHSAFYALIPASLIPILCVLLPQL
SGLANFTWFIGMGFGFVIYRFLNQPLAHAKSAMTKVKVQ"
/product="Cytosine/purine/uracil/thiamine/allantoin permease family protein"
/transl_table=11
CDS 390411..391145
/locus_tag="AUO97b_00545" /db_xref="SEED:fig|400667.82.peg.405"
/db_xref="GO:0047653"
/translation="MKIKIINPNTTQSMTDKIAASAKIVANSDTQIIAMSPQMGPATI
ESYYDEALSAVGVLEEIRQGELLDVDAYVIACFGDPALYAAREMTSAPVIGIAEAAMR
TASYVSTGFSVVTTLQRTVNMSWHLAHRYGATPFCKNVRACDIAVEELELPDSDAYRT
IRDECKKALAEDKSDAIVLGCAGMSDLCLKLQNELGVIVIDGVAAAVKQAEMLVQLNL
KTSKVGDWASPPVKNYTGILSGFGAH"
/product="Allantoin racemase (EC 5.1.99.3)" /EC_number="5.1.99.3"
/transl_table=11
CDS complement(391216..392097)
/locus_tag="AUO97b_00546" /db_xref="SEED:fig|400667.82.peg.406"
/db_xref="GO:0000908"
/translation="MNTVVANLNIEVIKPTIGAIIHDIDLNALNEQTTQQIQQALLDH
QVIFFRKQQLAPQAQADLARSFGTLHVHPIYPSIEDVPEVMVLDSWKQDLRDNELWHT
DVTFSKTPPLGCVLQAIKIPPVGGDTLWSSNTAAFKGLPLELQQKLRGLTATHDIRKS
FPLERFAHNEEEREKLLQTFKRNPPVVHPVVRTHPVTGEPLLFVSEGFTTHINELPEQ
ESEQLLNFLFEHATQEQFHLRWKWQDGDVAIWDNRCTQHKALFDYGDAHRIMHRATIN GDVPFYKEEQQPELAEA"
/product="Alpha-ketoglutarate-dependent taurine dioxygenase (EC 1.14.11.17)"
/EC_number="1.14.11.17"
/transl_table=11
CDS complement(392106..392975)
/locus_tag="AUO97b_00547" /db_xref="SEED:fig|400667.82.peg.407"
/translation="MNTKDNVYEYDKAELKPELNVQTENASFLSSFFEKHRTLAVSII
SVGSVVALWFLITALHVVPELFLPSPQAVWQKFISVSQEGFMKATLWQHLAASISRVF

LALIAAVVIGVPLGLWMGLNKWVRAVLDPLVELLRPIPPLAYLPLLVIWFGIGETTKV
LLIFFSILAPVIISSTHGVLSHQLNRERAALSLGASQSQVFWHVILPTALPHIITGIR
IGLGVGWSTLVASELVAADRGIGFMVQSAAQFLITDTVILGIIVIAIVAVSFELFLRW LQKQFSPWYGQQL"
/product="Taurine ABC transporter, permease protein TauC" /transl_table=11
CDS complement(392972..393766)
/locus_tag="AUO97b_00548" /db_xref="SEED:fig|400667.82.peg.408"
/translation="MSVLEAKHIHLTFPKQQKPVLQDINLTIEEGSLTVILGESGCGK
TTLLNILAGFQKPSSGDVLVNHEVVTGPDVTRAVVFQDHALLPWLNVADNVGFALQLK
GLKRAEIEKQVSAILKIVGLSHVEKANIWELSGGMKQRVGIARALISHAPFILLDEPF
AALDAFTRENMQQLVLDLWIQQNKSFFLITHDIEEALLLSNQLVLMTAHPGKIVETLH
LDFAQRYRQGEAIRSIKSDPQFIQLREQLFESLRAQKQSGKEALPT" /product="Taurine ABC
transporter, ATP-binding protein TauB" /transl_table=11
CDS complement(393776..394816)
/locus_tag="AUO97b_00549" /db_xref="SEED:fig|400667.82.peg.409"
/translation="MKDHEIMSTKTKPLIITAIVIVAIVAFIVWQNKKDNNTSASVRD
NKPVVIAYQTGVDPSKVAQANGDYEKHSQRTIQWKKFDAGSDVVNALASGDVVLGNIG
SSPLAAAASRNLPIEVFLITSKLGASEALVVSNKSGIKTPQDLIGKTIAVPFVSTTHY
SLLSALKHWNIPEDKVKIINLRPPEISAAWERGDIDAAYVWEPALSKAKASGTVLTDS
KQVGEWGAPTYDLWVVRKDFAEKNPDFLKAFVQTTLEQLEKYNQDPAAYVKDADNVQK
IAQLTGSDAKDIPLLLSGNIYLDHAQQKQTLDGEFAQNIFDTAKFLKGQGKVDQLKAD YKGNVNSSFLQP"
/product="Taurine ABC transporter, substrate-binding protein TauA"
/transl_table=11
CDS 395259..396443
/locus_tag="AUO97b_00550" /db_xref="SEED:fig|400667.82.peg.410"
/translation="MFKHSQILTKTLITTSLLTFSLSSHAALTFGNVEEGELKVSGTV
RAKYIYDFDSDPTTSKFSFNDAVLWLDYNSPKWIGRLDYRVYEYYGHLGDANWLTDAW
LGYKINDNSKIIAGLNPVPFGLGRFWGNTYYLGIANSAGWEDVHNLGVKYDFNDGINE
AQLAFYPTDGGTYRGKSKDSSRFSINPVNADDSVSQGTSTKEKNSIVVRGAHTFKNIL
GQENFSTQLGASAWYSTIENKRSGQDGDRQVYSVFSNTNYNQWNLQLLAGYQDIDNAD
TQYKDHLTLGGFDYSFNSATKGQIYSAELSYLFPQQFGPITAVRPYLNYSSYRKEQDG
FKDSTRFIPGIAFNYQKLTVQAELLMGKHDPYLGDSEGLAAGGSNDKWNKKAFVIFAY YF"
/product="hypothetical protein" /transl_table=11
CDS complement(396505..397971)
/locus_tag="AUO97b_00551" /db_xref="SEED:fig|400667.82.peg.411"
/translation="MSTQELDHNHRYEIKSPDDVSQLISKFAGKGKSNKVIWLALIGV
FIDAYDLTTLSFGIEQVISEFSLSPVMTGVVASAIVCGTIVGNLIGGWLTDKIGRYRV
FMADMVLFVIAAIVAGFAPNVWVLILARFIMGISVGIDLPVAMSYLSEFSKFNGSSNK
AARLAAWCPMWYAASSVCFGLVLALYFLLPPEHSNWLWRASLIFGAVPALLIIFIRGK
YLTESPIWLANQGDLKGAAQVLQESYAIHAVATVEKVKRTAEKKKREGFAVLFNKTYL
PRTIVAIVIHISVAFQYTTIAFFLPSILTRFFHTDVLTTITTTLGLNLLFAFTGGLLG
VVVASRFKSRHVLLTGFLLQFIALIALAFIGEPGNSFLLYTAIGMLGLWLFAEGFGPG
AQMMVYPTMAYPASIRGVGVGFNRAVTGVAQAIALFVLPIWMAGYKTDVYLIISIFAF
IPLIVIGLLIKFEPTAQDVDVIDEDKTELPLNNSPVID" /product="Uncharacterized MFS-type
transporter" /transl_table=11 CDS complement(398167..399558)
/locus_tag="AUO97b_00552" /db_xref="SEED:fig|400667.82.peg.412"
/translation="MTKRISFNAFEMNCIAHQSPGLWRHPQDRSVEYKDLEYWTDLAQ
ILEHGFFDGIFIADVLGIYDVYHQSAEHALTGAVQVPVNDPLQIVPAMAAVTKHLGFG
VTTSISFEHPYPFARRISTLDHLTKGRVGWNIVTSYLESGSKNLGLKTQVNHDNRYDI
ADEYLEVLYKLWEGSWEEGSVLRDRESGIFADYKKVHPIQHEGKYFTVPGIHICEPSP
QRTPVLYQAGASSRGQKFASQNAECVFIAAPSKIATKKVVQGIRQKLAQEGRDPYSVK
IYALLSIVTDETDAKAQAKFKEYQSYGSYDGALTLLSGWSGVDFSQYQPTDKVEYIQT
NAIQSLLDSYVNADPERVWTIEEIANWNSLGGNGPVLVGSAETVSDALQQWVEDTDID
GFNLAYILAHQTFADVVEFIVPELQKRGVYQTSYAQGTLREKLFGAGPYLPENHRGAK YRNLKELKLAEAS"
/product="Coenzyme F420-dependent N5,N10-methylene tetrahydromethanopterin

reductase and related flavin-dependent oxidoreductases" /transl_table=11 CDS complement(399735..401030)
/locus_tag="AUO97b_00553" /db_xref="SEED:fig|400667.82.peg.413"
/translation="MTNLNFEHHLGSLTHAALSQVSIISRVWGEGPSERYDELASNFR
PTFARIKEGALIREQQHILPYEQLQWLKDIGFTRLRLPKAHGGFDATIPELFALLIEL
AEADSNLPQALRVHFGFTEDVLVSKDKTFQQRWLSRIANGETVGSAWSEGGKESIDQF
ETHLYRDSDGKIRVKGKKYYTTGSLYADWVDVGVTDLKGESGSVVVRREDEGVEIVDD
WNGFGQQLTASGTAYFHDVLVDEAEILPDDDRFKYSAAYYQLVQLAIITGLGRAATYD
VSQAVAKRTRNYTHANAQFVKQDPQILQVVGQIRGAAYSAGAIVEKVAQSLQRAYLAA
FQNNEQLEEEQNALAELESAQSQTVITDLILNASTILFDALGASATDKDLGLDRYWRN
VRTLASHNPRVFKNRIVGDFSVNGTLPPYQWRIGEVAKQ" /product="Acyl-CoA dehydrogenase; probable dibenzothiophene desulfurization enzyme" /transl_table=11
CDS complement(401149..401967)
/locus_tag="AUO97b_00554" /db_xref="SEED:fig|400667.82.peg.414"
/translation="MFITSQLNIIHKIIKNQSISTLVIDQLEQTYVNLEATLLRAKVL
RDFSKTQTVYLIQSHIEPQQSSLAYLFSPFIFANLNKAAIYTTPATPPALNILNKYYQ
ADKKVVFKVDEVLESLKIYLDLELIEMGEAEFIYLNLIKALCRSDISTVFLITHLELD
LETLKQLEQFLKIKIHWVKVVKDEGLKDLDQLDMRKLLFKNKDETHVQLCVLFAQMNA
ALVGLCDTFTVSQMTHLIDDMFYSEHIFEKLSVYSEYMQTLLQSQHSLHKKQIA"
/product="FIG00350748: hypothetical protein" /transl_table=11
CDS 402621..404066
/locus_tag="AUO97b_00555" /db_xref="SEED:fig|400667.82.peg.415"
/db_xref="GO:0006118"
/db_xref="GO:0016020"
/db_xref="GO:0016491"
/translation="MSLGLTALELARIQFAFTVSFHIIFPATSIGLACFLAVLEWKWL
RTQNPIYKDLFKYWIKIFAVAFGMGVVSGVVMSYQFGTNWSEFSRVAGSITGPLLTYE
VLSAFFLEAGFLGIMLFGWGRVGPRAHFFATLMVAIGTCISMFWILSSNSWMQTPQGF
AIENGIIVPKDWLAIVFNPSFPYRFAHMGAAAFLVSSLLVVGTSAWHLVKGRRDELVK
KSFSMGLWMVLVTSCLQVVIGDNHGLNTREHQPAKLAAMEGHWETNHNEPMPLLLFAI
PDMKEERNHLEVGVPYLGSLILTHSLDGQVTGLKEFAPEDRPNSTIIFWSFRVMVGLG
VLMVTLSLIALWLRKRGKLYETSWFHKFAVVMGPAGYVAMLAGWITTEVGRQPWVVYG
IMRTKDGLSHTVSADQVGLSLFIFVVVYTIVFGSGIYYTLKLINKGPVFIDTPNMEAG
GVGHFKTPMRPLSAVDENIDSKQNSGENRHD"
/product="Cytochrome d ubiquinol oxidase subunit I (EC 1.10.3.-)"
/transl_table=11
CDS 404059..405063
/locus_tag="AUO97b_00556" /db_xref="SEED:fig|400667.82.peg.416"
/db_xref="GO:0006118"
/db_xref="GO:0016020"
/translation="MIDLSLIWVGIIGLGVLIYVVMDGFDLGIGIMFPFIKNSQERDV
MMNTVAPVWDGNETWMVLGGAGLYAAFPLVYSTVLSALYLPIIFMVIALIFRGVAFEF
RFKAHRTKHLWDLAFIWGSVLTSFLQGIILGAYIQGIKTENGIFAGGPFDWLTAFSIF
TGIGVVAMYATLGCGWLILKTEKGLQQRMYELMPKLIIALLIIFGAVSLYTPLTHPEI
ADRWFSLPNLFYFSPVPILVLLFVGLILSACKKQQDHKPFIYTLALVFLAFTGFVISL
WPNIIPPSVTIWQAAAPHSSQMFALVGALILIPIIITYTIVSYWVFRDKVRVGDEGYH "
/product="Cytochrome d ubiquinol oxidase subunit II (EC 1.10.3.-)"
/transl_table=11
CDS 405077..405211
/locus_tag="AUO97b_00557" /db_xref="SEED:fig|400667.82.peg.417"
/translation="MKKSSVKLSQTQWFILLWLGGFLALAVIAGLFKVILIYAAPYLK "
/product="FIG00350241: hypothetical protein" /transl_table=11
CDS 405310..406728
/locus_tag="AUO97b_00558" /db_xref="SEED:fig|400667.82.peg.418"

/db_xref="GO:0004069"
/translation="MYKSEQLAHSIRQLIESGTLNAHEKLPSLRDQVQRSGFSLMTVM
NAYQELESQGLIYSKEKSGYFVAEQIALKPLEQYSIVSLNSKIEINSLVFKYLKSIQH
ESVVPFGSAFPDSQLLAAPKLIQIMGQLARQRQSYDQTASLPPGNLALRKLIAQRYCM
QGIQTDPDDIVITSGGLDALNLSLQAVAKPGDYILLQQTVFYGAWQAAERLGLKVITI
PEHPQHGFDLEAFEQVIHTYPIKVCWLMLNSHNPIGFTVSDEIKYKIAKLLHEHQIYL
IEDDVYEELYYGGQKPLSMKYFDQQNLVLHCSSFSKTLGAGFRVGWVYAGKFSDHIQH
LQLMSTISVNALIQNALVEFLSHHHYEKHLRTLRLSLERYKKQFYHYLKQHLPAICEI
YYYPSGYFLWVKLPHKLDSMQIYEELIQQDIGVAPSPLFSVLPAQQHYLRINCSFEWN
EKIQAALDQVIKTIQQRVEASL"
/product="DNA-binding transcriptional regulator, MocR family /
aminotransferase domain" /EC_number="2.6.1.1"
/transl_table=11
CDS 406826..407743 /locus_tag="AUO97b_00559" /db_xref="SEED:fig|
400667.82.peg.419" /translation="MEIDEELTLKKIQIFLAFMRCGNLSKTAAEMQLSNVSVHKALHS
LESALRCPLFKNEGRNLIPLKSAYVFEERAQKIVQDIFITVNKTREAAGFAAKVLHLG
SLYSLTVNTIPNVISGLKLRRSELDIQLLLSSNQDLVKKLKATELDAIIVALNETTQD
DDFEILPMFSDDIFLAVNKDSKYAEFKDIDLSLLKEETFLTLTKGFATHNDSDIIFKK
AGFSPKVALQVNDIFTLISMVSSGVGFALLPGRISAVYESSVKLIPLKQQYHMQQEIG
LVFLKSKERDPNLLALIAECRMFASNFKR"
/product="Malonate utilization transcriptional regulator" /transl_table=11
CDS complement(407793..408557)
/locus_tag="AUO97b_00560" /db_xref="SEED:fig|400667.82.peg.420"
/translation="MVELLLKDLTHLGLITAFALIALIMWLSTKLSKYLTNGRVHASA
IAIVIGLILAWFGGKMTGGEKGLTDLALFTGVGLMGGAMLRDFTIVATAFEVQATEAK
KAGFIGAFSLFLGTILPFIVGCMFAWIFGYRDAVSITTIGAGAVTYIVGPVTGAAIGA
SSEVMALSIATGLVKAICVMVSTPLTAKFMGLDNPRSAMIFGGLAGTVSGVSAGLAAT
DRRLVPYGALTATFHTGLGCLMGPTILFFAVKALVG"
/product="Malonate transporter, MadM subunit" /transl_table=11
CDS complement(408550..408966)
/locus_tag="AUO97b_00561" /db_xref="SEED:fig|400667.82.peg.421"
/translation="MIIYGVGLLALCTLVGVIAGDLLGVLLGVKSNVGGVGIAMILLI
CIRLWMEKRGMMTVETEKGVSFWGTMYIPVVVAMAAQQNVVAALSSGHMALFAAVVSV
AVCTLTIAGLSRFNKASPLPKEEDTALNRIGGKVHG"
/product="Malonate transporter, MadL subunit" /transl_table=11
CDS complement(409015..409953)
/locus_tag="AUO97b_00562" /db_xref="SEED:fig|400667.82.peg.422"
/db_xref="GO:0004314"
/translation="MASIWVYPGQGVQRSNMLHDLPQTALVKEYLERASDALKEDVLM
LDRPAALKSTRAVQLCLLISGVVSSALLRAENLKPDYVAGLSIGAWSAAVVAEVLAYE
DAVRLVAYRGELMQNAYPTGYGMTALIGTDRAAVETWVKQIYEITPEVFVANINAHNQ
IVISGSFEAMTQVAVLAKQQGVVAKKLDVSVPSHCELLSQQAKQLAASMEGVTLKQPK
IRYLSGTTARTLSRPEQIGDDLAFNMSRTVDWESTIQAAWERGVRLQIEALPGTVLTT
LARRTFKEGTVLSFQGTRLDSIQMAMQKEQANSFSF"
/product="Malonyl CoA-acyl carrier protein transacylase (EC 2.3.1.39)"
/EC_number="2.3.1.39"
/transl_table=11
CDS complement(409966..410568)
/locus_tag="AUO97b_00563" /db_xref="SEED:fig|400667.82.peg.423"
/translation="MKLKLEAHDLLWGMTVDCLADDAPHWVREVLQRGDPVVVRRAIT
PVDQVAVGVRGQLRYQRYAAQMPRSLISRQLKPEALTRVDTQQFEHLAERLQSISSIM
KNFSECWGYTGSFGFELATGIETVTEQSDIDLLIRAEQPFAKKQAVELLENFQQAGLN
VDIQLQLPQGGLALKEWARNSRKVLLKRPDGAVLLENPWN" /product="Malonate decarboxylase
holo-[acyl-carrier-protein] synthase (EC 2.7.7.66)" /transl_table=11

CDS complement(410565..411392)
/locus_tag="AUO97b_00564" /db_xref="SEED:fig|400667.82.peg.424"
/translation="MNIAVDTLKASTRGAHWFKALTQAFKTVEGFPASIWVADGQLDQ
QAVRVIAVVQDSNNLFPRAKQGEVGLLEGWSLAKAVNEVIQADIHSETKRAILCLVDV
PSQAYGRREELLGIHQALAGAVDSYARARLAGHPIVSLLVGKSMSGAFLAHGYQANRI
IALKDSGVMVHAMGKESAARVTLRSVDELEQLASSIPPMAYDIESYATLGLLSELLSV
ENPEQPTNDDLLLAKQAIAQAFKEINAEQSRGLEQRLHGQNRQMSKKVRELLREQWL" /product="Malonate decarboxylase gamma subunit" /transl_table=11
CDS complement(411389..412240) /locus_tag="AUO97b_00565" /db_xref="SEED:fig|400667.82.peg.425" /translation="MTDIQSLLSKQDFIELSARERAKALLDEGTFRELLDPFARVMSP
WLVQQNIVPQADDGVVIAKGTIQEQPIVLISIEGLFQGGSLGEVGGAKIAGALELAVE
DNLKGIPTAAILLLETGGVRLQEANLGLAAIAEIQAAIVNLRQYQPVIAVVAGSVGCF
GGMSIAAGLCSYIVMTQEGRLGLNGPQVIEQEAGVQEYNAKDRPFIWSITGGNQRFAS
GLADAYVDDDRQKIRQRVIDYLTQGVPHQHRSSNYPFYLEKLQQVDTAEQITPAQVQA LYQGEQA"
/product="Malonate decarboxylase beta subunit" /transl_table=11
CDS complement(412233..412532)
/locus_tag="AUO97b_00566" /db_xref="SEED:fig|400667.82.peg.426"
/translation="METLHFEFVATEPPVKKALVGCVGSGDLEILMEPGEIGKASIQV
VTSVDGSAARWHNLFERIFTAQIPPAVNIDIHDFGATPGVIRLRLEQALEEIAHD" /product="Malonate decarboxylase delta subunit" /transl_table=11
CDS complement(412535..413419)
/locus_tag="AUO97b_00567" /db_xref="SEED:fig|400667.82.peg.427"
/db_xref="GO:0046917"
/translation="MMAQVQYQTLSDSQLLADMAVEALIDEVNLTPKPALVDRRGSGA
HDDLTLELMERSAKSLGPMFDAMAQAAKHHGKVCLALREDIGEIGRQGENTMMLATDG
VNTHRGAIWALGLMVTAATLARTNQQYLSAVELCQLAGQIAQLEDRFIPEQALSHGQQ
VQKKLGILGAKEQAQQGFPTIVNFGLKQLYQSRSKPMKEEFARLDALLAMMTDLTDTC
VLYRSGTNGLKRMQQGAQQILDLGGSSSLEGRRALHLLEIDLLRMKASAGGAADLLAA
TLFIDRVEQSSAKNKKEF" /product="Triphosphoribosyl-dephospho-CoA synthase (EC 2.4.2.52)" /EC_number="2.7.8.25"
/transl_table=11
CDS complement(413419..415098)
/locus_tag="AUO97b_00568" /db_xref="SEED:fig|400667.82.peg.428"
/translation="MEGSVQNKERLWTKRRHAKQLKLEMANQYTDGVVIPTQDIIKVL
ETLITPGDKVVLEGNNQKQADFLSRSLAQTNPDILHDLHMIMPSVGRSEHLDLFEKGI
ARKLDFSFAGPQSLRISQLIEDGLLEIGAIHTYIELYSRLVVDLIPNVVLSAGFMADR
QGNIYTGPSTEDSPALIEPAAFSDGIVIVQVNELVDDVSELPRVDIPASWVDYVVVAD
QPFYIEPLFTRDPKHIKPVHVLMAMMAIRGIYEKHNVQSLNHGIGFNTAAIELILPTY
GESLGLKGKICRNWTLNPHPTLIPAIETGWVESVHCFGTELGMEKYVAARPDVFFTGR
DGALRSNRMMCQLAGQYAVDLFIGATLQVDGMGHSSTVTKGRLAGFGGAPNMGHDPRG
RRHDTPAWLDMRLQGANETETYLARGKKLVVQMVETFQEGGKPTFVDRLDAIDVAKTA
GLPLAPIMIYGDDVTHLLTEEGIAYLYKASSQEERQAMIAAVAGVTSIGLTQDPKTTA
RLRREGLVVFPEDLGIRRTDATRELLAAKNIADLVTWSDGLYQPPAKFRSW" /product="Malonate decarboxylase alpha subunit" /transl_table=11
CDS complement(415208..416122)
/locus_tag="AUO97b_00569" /db_xref="SEED:fig|400667.82.peg.429"
/translation="MNSPVYLNALRAFEASARHKSFSAAAKELNVTPAAVGQLVRTLE
DWLGFPLFHRQTNGKNRLVATELAESVLPDIRAGFDKLTLALEKLKQDSLSGVLNIAV
SPAFAEKWLLPRIDHFQKQYPDIDIRLDINLKTVDFLEQKVDVGVRYGQGQWQGLEAV
KLMDEEVYPVCSPAFLNEHSFLLTPNDLKNITLIHDLSMEKHQQFPGWQSWLKSVGLN
DINISRGMQINSSSAVLQAAMEGHGVALARSVMVQKDIQTGRLIRLFPQINFSSPFSY
YLVYRPQYANLPKVKICLDWFFEEIHRI"
/product="Transcriptional regulator, LysR family" /transl_table=11
CDS 416308..416982

/locus_tag="AUO97b_00570" /db_xref="SEED:fig|400667.82.peg.430"
/translation="MLIHADFSQAVIIKPEDYHWVKSPRGEVDRVMLDRIGDEVARAT
SLVKYAPHTTFPEHRHHHGEEILVLSGVFTENQNQHYPAGWYLRSPHQSGHQPSSEEG
TLIFVKLMQIPMSETQALRINTNDPAQWQKIDSRLICTLIDASYEHTYLEKLKPQQLF
IEEAEGGIEILITQGQLVDKEMVFDAGTWIRLPPKTLAYFQAGLSGASLYVKTGHLAS AIAEYN"
/product="no significant database hits"
/transl_table=11
CDS 416992..418179
/locus_tag="AUO97b_00571" /db_xref="SEED:fig|400667.82.peg.431"
/translation="MLQNTMHRQVLILASSQSLFQTVSVMVMTIGGLAGANIANTPTL
ATLPIASMFLGTALMMFPASILMAHIGRRNGFLFGAFLGVLGGIIASIGIFYSSLMLL
ALGTLCVGAYQSFAQFYRFAASEVANDAFRSRAISWVMAGGIVAALIGPTLARFGGPL
FQHLEYIGSFLIISIISLVAMGILSSLHIADTVEQKSDFTAGRPWQQIVFQPTYLVAL
FGAITGYGIMILGMTATPIAMRHSHHELGSITTVIQLHVLGMFLPSFFTGNLITRFGV
LKIMFAGLLLFVCYIGFALSGIQFTSFAISLILLGVGWNFLFIGSTSLLTGTYSVAEK
AKAQAINDMTVFVVGLICSFSAGALLDLFGWKAMNMALIPWLVIAAASLFWLSQKREK QLA"
/product="Uncharacterized MFS-type transporter" /transl_table=11
CDS complement(418188..418781)
/locus_tag="AUO97b_00572" /db_xref="SEED:fig|400667.82.peg.432"
/translation="MSLTTPVELVGEHVILKPLDVTQAEALKEAVCDGELYNLWYTRV
PRPEQMTAEIERRLDLQKSGSMLPFYIEDRKTGRALGMTTLMHIEEAHRRVEIGSTWY
RKSAQRTPVNTECKKLLLAHAFETLNCIAVELRTSSLNVQSQAAIVRLGAKLDGVLRS
HQIVKDDILRDTHVYSILKHEWPAVRQNLEWLLNKPR" /product="N-acetylglutamate synthase
(EC 2.3.1.1)" /EC_number="2.3.1.1"
/transl_table=11
CDS complement(418824..419459)
/locus_tag="AUO97b_00573" /db_xref="SEED:fig|400667.82.peg.433"
/translation="MYIPDDFAENRLDVLHSLIAEHPFGVLFTHGSNGLDANHLPFEL
QANEGKFGMLHAHVSRNNSVWQDIKNGDEVLVVFQAGDAYISPNWYPSKHEQHRQVPT
WNYRVVHAYGKVTIRDDERYVRGVVARLTRTHEVSQPEPWKMSDAPKDYLEPMLKAIV
GIEIEITKLQGKSKLGQNKERRDILGAADGLLKSGHQTIANAMYSVAELNK" /product="Negative
transcriptional regulator" /transl_table=11
CDS 419584..421065
/locus_tag="AUO97b_00574" /db_xref="SEED:fig|400667.82.peg.434"
/db_xref="GO:0004069"
/translation="MAKVVELPSIAKIDKSQGKISAQLTQALREAVQSGDLQPGDPLP
SSRELALTLGVARGTIIEAYDQLLAEGVFDSQPRTGTYVSHALVKTSVIQHSSKENKP
NSISLTKSARSYAKVLDEFKPLPHVPFAVSVPVGRTQPSDIWRKFGNKYRSRGAGVPS
GYGEPQGVLSLRIAIADYIRRSRSVHCEPEQIVITPGIQQSLYICSQILFEAKDEVWV
EDPAYRGTTAIFEHAVQNIRMVRVPVDEEGIQVETGIKLSKHARAAFVTPSHQYPLGM
PMSLARRTALLAWAKQQKAWIIEDDYDSELRYNGQPFPSLQGMAPDQVIYLGTFSKVL
FPSLRLGYAVLPKELVAPFCGLRVLIDRHPPAADQHVLAAFIQEGYLERHIRRTRNVY
AEVREYIIGLIEKYIPQELAWLQPGDQGMHMVLWLAQHINDIEVASSAVDAGIAIKAI
SPTFSKERRRSGLIVGLGDFEPELMQQAIKKLSKIIQQHAKSM" /product="Transcriptional
regulator, GntR family domain / Aspartate aminotransferase (EC 2.6.1.1)"
/EC_number="2.6.1.1"
/transl_table=11
CDS complement(421073..421762)
/locus_tag="AUO97b_00575" /db_xref="SEED:fig|400667.82.peg.435"
/translation="MNIWSILCLIWTLAAYVVAKRIYQKHPKLWLSPAISVPVLTIIL
MTIFGISYQTYSEDTQWIVNLLGPATVAFAIPIYRYRETIRKNLGVLTIAIIVGMSVG
VMTAYKMAQWFHFSPEVTNSLMARSISTPFAMILAEDIHGSAALVSLFTVITGLVGMI
CGDAILAITKIRSNVANGAALGNAAHGFGTVRAQQRNSEEGVIASLTMVIAGILMVLI GPFAIHLWLML"
/product="CidA-associated membrane protein CidB" /transl_table=11

CDS complement(421776..422207) /locus_tag="AUO97b_00576" /db_xref="SEED:fig|400667.82.peg.436" /translation="MSTESASGTPSQPSLFVLVKQILILAGFWWVGYLLHQKLGVPVS
AGILGMFLLLLCLFFKIIKMDQVAMGATVVLGELLLFFVPVVVAVVQYKTLFMTEGWQ
IVLSIGVGTILVMLSTSLTIHYYNRLKAYLQARKRLQHKHI" /product="Holin-like protein CidA"
/transl_table=11
CDS 422318..423223
/locus_tag="AUO97b_00577" /db_xref="SEED:fig|400667.82.peg.437"
/db_xref="GO:0003700"
/translation="MLSLKSLQCFVTLVKTKSFTRTAEELYLTQPTISKILQQLEEQL
QVQLLVKPDHGRKRQIELTEIGERIYQHAVELLQAEQNIFLEIENYQQLKTGTLKLGV
PPLGSQLLTTALFDFHRQWPDIELAFMEVGSRGIEQALLNNELDVGVLLQPFDEQTFN
SIELCNYPLMVLLRRDATWATRKKINLEELQHQSFLMFPENFSLNSIILDACKQYGFY
PTIACRTSQWHLLADMVLQRMGIALLPQYYTDMLDPTLFAAVPLEKPNIQWHLVMAWK
KNLPASPAVQAWLSIVRQHFQHIKP"
/product="Transcriptional regulator CidR, LysR family" /transl_table=11
CDS 423343..423969
/locus_tag="AUO97b_00578" /db_xref="SEED:fig|400667.82.peg.438"
/translation="MNNEHFKLYTDSQFLSPYAFTVFVGLHEKQIPFEIATIDLGQQG
QFETSFVEKSLTAKVPVLEHNDFALSESSAILEYLEELYPDTAIYPKDIQARARARQI
QAWLRSDLVALRTERPTDVIFIQPTSTPLSEQGQKAAEKLFFVAEKLLASDAEFLFGS
WSIVDAELALMLQRLIQNGDAVPQRLKTYALQQWQRPTVQKWLALRHK" /product="Probable glutathione S-transferase (EC 2.5.1.18), YfcF homolog" /EC_number="2.5.1.18"
/transl_table=11
CDS 424003..424863
/locus_tag="AUO97b_00579" /db_xref="SEED:fig|400667.82.peg.439"
/db_xref="GO:0004364"
/translation="MSDSNYIPPQVWQWTGEKTDLNQPTSGARYEHTLPVGKHPLQLY
SLATPNGQKVTILLEELLTLGHKDAEYDAWLIKIGEGQQFGSGFVEINPNSKIPALVD
HSQNPPLRIFESGSILLYLAEKFKAFIPTDIAARTECLNWLFWQMGSAPYLGGGFGHF
YAYAPVKIEYAINRFAMETKRLLDVLDQHLAKNEYLAGSEYSIADIAAFPWYGGLVKN
WVYNGAEFLGVDQYKNVQRWADAILARPAVQRGRMVNRTFGDLATQLHERHDASDFET KTQDKLQNQS"
/product="Glutathione S-transferase (EC 2.5.1.18)" /EC_number="2.5.1.18"
/transl_table=11
CDS complement(424901..425821)
/locus_tag="AUO97b_00580" /db_xref="SEED:fig|400667.82.peg.440"
/translation="MDKFDTLQLFTRIVELGSFSQAADQLNIPRATASNAIKELENNL
QCRLLERTTRHVRPSLDGQAFYERCTQILSELDDAESSLRNVIAQPRGLLRVDLSGAH
ATKIVLPNIDQFHELYPDIELVISTGDRLVDLIREGIDCVVRAGRLDDSTLIARHLVD
MPQVICASPDYLNKHGTPEQPEDLLSHYCVNFFSTSGGRNYPFELMVNGKKQDYFLKS
WIAVNDAENYVLSALQGAGLIQVPHYHVANELKNGRLVEVLSEWESPKMSVSALYPQH
RQLSPRVRVFVAWLSELYTGYFANFEYPQK"
/product="Transcriptional regulator, LysR family" /transl_table=11
CDS 425947..426684
/locus_tag="AUO97b_00581" /db_xref="SEED:fig|400667.82.peg.441"
/translation="MNISRKTALVTGASRGIGRAIAERLAQDGFYVIVNYAGNKVHAQ
AIVEHIIEQGGQASAIQADVANEHEVSRLFQEAKAINGQLDVVVHSAGIMPMAKITPE
SLPDFDKVIHTNLRGAFLILAHAAETVPDGGRIITLSTSVIAKSFPAYGPYIASKAGV
EGLVHVLANELRGRNITVNAVAPGPTGTDLFYNGKTEEQVAAIAKLAPLERIGTPDEI
AGVVAMLAGPDGRWVNSQVIRVNGGFA"
/product="Oxidoreductase, short-chain dehydrogenase/reductase family"
/transl_table=11
CDS 426923..427432 /locus_tag="AUO97b_00582" /db_xref="SEED:fig|400667.82.peg.442" /translation="MNAKLNTETPQSFISNGSNTSFLAEDILAKAQQYAQEHELNFSG
SLSPVDAWQLVQQGEAVLVDVRTNEERKFVGYVPESIHVAWATGTSFNRNPRFLKELE

SKVGKDKTILLLCRSGNRSAQAAETAFNAGFEHIYNVLEGFEGDLNEQQQRNQKNGWR IHQLPWQQD"
/product="Rhodanese-like domain protein"
/transl_table=11
CDS 427469..428392
/locus_tag="AUO97b_00583" /db_xref="SEED:fig|400667.82.peg.443"
/db_xref="GO:0009001"
/translation="MTQWNINAVVQGLQQARHDWRQQQHRTKEFGGRELPSKEALKAI
LDDLCGILFPMRLGPADLRQETEDSYIAYTLNRVLTALHAQVQLALNYEAKRHLGHSS
PVQQPQELAQTIVQDFANTLPSIRRLLDGDVRAAYEGDPAAHSVDEVLLCYPGIFAII
YHRIAHQLYAQVPLLSRIISELAHSATGIDIHPGAQIGKGFFIDHGTGVVIGETCVIG
ERVRIYQAVTLGAKRFETNDDGGLKKDYIRHPIVEDDVVIYAGATILGRITIGRGSII
GGNVWLTHSVAAGSQILQSPNESYQKNHIAG"
/product="Serine acetyltransferase (EC 2.3.1.30)" /EC_number="2.3.1.30"
/transl_table=11
CDS 428633..429565
/locus_tag="AUO97b_00584" /db_xref="SEED:fig|400667.82.peg.444"
/translation="MAKNTDKAQLALGDHAARQLANATKTAPQLSTITPRWLTHLLQW
IPVEAGIYRLNRVNNTDDIQVACTQRDEATLPQTFVDYDPEPREYFLNGVSTVLDVHT
RVADLYSSPHDQIKEQLRLTIETIKERQESELINNPEYGLLASVTDDQRISTLNGPPT
PDDLDDLLRKVWKEPGFFLAHPDAIAAFGRECTRRGVPPPTVSLFGSQFITWRGIPLI
PSNKIPVEDGKTKILLLRVGEKRQGIVGLFQPGLAGEQSPGLSVRFMGINRNAIASYL
ISLYCSLAVLTDDALAVLDDVEVDKYHDYPVNYK"
/product="cAMP-binding protein"
/transl_table=11
CDS 429540..431501 /locus_tag="AUO97b_00585" /db_xref="SEED:fig|
400667.82.peg.445"
/db_xref="GO:0031071"
/translation="MTTQLTTSNLQGSSTGPEVTGVNPPANFPDESTIARLANEFFSA
VPSSSSSTSSSGLNLDLPVGAPPHPPQPGEPFSALSGRAPNIAFQKSLNPEYPEHIPN
LPDYPERRIVASAPVVGGANLPRPPFEPHLPQAAHSEPNVPRIEPSLPTGAGESPYYF
LNDFSAPNATEPSVKGVEDNYSVAQSFQLPHGDQLKSLLTEDRFATNTPPQSSASSAF
YFLDLPQSGQSYSHYQPSQPTFVASSAQPLDVHAIRRDFPILQERVNGRPLVWFDNAA
TTQKPQRVIDRIAYFYQHENSNIHRAAHELAARATDAYEHARNVVARFIGAPSSKEII
FVRGTTEGINLIAKTWGEQNIGEGDEIIVSHLEHHANIVPWYQLTKKTGAKLRVIPVD
DTGQIILEELPKLINERTKLLSITQVSNALGTVTPVAEVIKIAHAKGVRVLVDGAQSV
SHIPTNVQALDADFFVFSGHKVFGPTGIGAVYAKPELLESMPVWEGGGNMIQDVTFEQ
VVYQPAPNKFEAGTGNIADAVGLGAALEYVESLGIHNIARYEHDLLEYGQNALSSVSG
LRLIGTAHHKASVMSFTLQGYSTEQVGKALNQHGIAVRSGHHCAQPILRRFGVEQTVR
PSLAFYNTTEEIDLLVSVLHQLTRNRRSN"
/product="Cysteine desulfurase (EC 2.8.1.7)" /EC_number="2.8.1.7"
/transl_table=11
CDS complement(432107..432745)
/locus_tag="AUO97b_00586" /db_xref="SEED:fig|400667.82.peg.446"
/db_xref="GO:0005293"
/db_xref="GO:0006865"
/db_xref="GO:0016020"
/translation="MWQLYGHEFLMLALIHFMAVILPGPDFVITVRQSVRYGYLIGCL
TAIGIGVGISVHVFYTLVGIGFLIQQSEWLMSLIRTAGAAYLVYLGWQCLRSQPNTTI
DINGQTDSDAPSLFKAFTMGFLTNALNPKATIFFLAIFTTIVSTTTPMKVQVFYGVWM
CMVNAIWFMVVSVLFAQPIVRKRFLEFGVYFERVMGLLLIGIALRLIWGLFN" /product="Threonine
efflux protein"
/transl_table=11
CDS 432837..433772 /locus_tag="AUO97b_00587" /db_xref="SEED:fig|
400667.82.peg.447" /translation="MSTNQSPVLLNEMATFVKVVESESFSETARQLGTTPSAVSRAIA

RLERALGTRLLYRTTRKLRLSESGQQVYQRCLDMVNAAQAVMESSGQFHVEPEGVIRM
SVPKAVGHFMIHPHLPEFLERYPKIDIQMLLEDRYVDLIDDGVDLALRITEQPPGGLM
GRKLIDIDHLVCATPEYLSKHGTPNHPHDLKQHECIYLGEQASDSKWKFQQGNKSISV
AVRGRYAANHTGVRLSAALQHLGIASLPYFVARHALQQGTLVQVLPDWYFKTYYSGGL
WLLYPPTRHVQPKLSVLIQYLADKLAAEPTLFKLK" /product="Transcriptional regulator, LysR family" /transl_table=11
CDS 433849..433968
/locus_tag="AUO97b_00588" /db_xref="SEED:fig|400667.82.peg.448"
/translation="MEKVTKKLSHLQNLTTVYIYKKYCKNIIFISYGQVVDES" /product="hypothetical protein"
/transl_table=11 CDS complement(434019..434885)
/locus_tag="AUO97b_00589" /db_xref="SEED:fig|400667.82.peg.449"
/translation="MTTLTFQNVKHILAASLLSLGLVACDKGTQSTEKSADTTQTTSS
IEQIKKNGVVRIGVFSDKPPFGYLDAQGKNQGFDVEIAKHVAKDLLGDENKVEFVLTE
AANRVEYLKANKVDIIFANFTVTPERKEVVDFAKPYLKVALGVVSPKSHPITDVAQLK
DKTLLVNKGTTADSFFTKTHPEIKLQKYEQNTETFDALKDGRGAALAHDNLLVLAWAK
ENPNYTVGITNLGEQDLIAPAVKKGDKELLNWLNQDLEKLAKEGVIHQAYEKTLKPVY GDTINPKDLLVE"
/product="Lysine-arginine-ornithine-binding ABC transporter, substrate-binding protein ArgT (TC 3.A.1.3.1)" /transl_table=11
CDS complement(434968..435828)
/locus_tag="AUO97b_00590" /db_xref="SEED:fig|400667.82.peg.450"
/translation="MFKRNSIKILSSLAVAGVLLTACSKTPSTEQASNAQEDHSLEQV
KKNGVLRVAVFADDPPFGYVDSAGNNQGFDVALAKRVTKDLLGDEKKVEFIVTEASNR
VEFLKSNRADVVFATFTVTPERKEVVDFAEPYLKGAFGIVSPKAKPITDIAQLEGKTL
IVNKGTSSDTYFTKHYPKVNLLKFERNSDAFKALTDGRGDAISQDSTYALAWAAKNPS
YVAGIQSIGDQEFIAPAVKKGNTQLLNWLNTEIKQLRTSGEIKKIYDETLKPIYGDSV NPNVFLDVGQ"
/product="ABC transporter, substrate-binding protein (cluster 3, basic aa/glutamine/opines)" /transl_table=11
CDS complement(435859..436590)
/locus_tag="AUO97b_00591" /db_xref="SEED:fig|400667.82.peg.451"
/translation="MPLLSIQQLQKSFAQSHILQGIDLDVEQGEVVVILGPSGCGKST
LLRCINGLEPIQGGEIRLTGSGVLGKDVDWTNARQKIGMVFQNYELFGHMNVIDNILL
GPLKVQKRHRDEAEQVADELLKRVGLFERKLDYPRQLSGGQKQRIAIVRSLVMQPKVI
LLDEITAALDPEMVREVLDVVLKLANEGMTMLIVTHEMSFARKVADRIIFMDKGKIIE
QASPEEFFEQPKTERAKAFLNILSY"
/product="ABC transporter, ATP-binding protein (cluster 3, basic aa/glutamine/opines)" /transl_table=11
CDS complement(436593..437258)
/locus_tag="AUO97b_00592" /db_xref="SEED:fig|400667.82.peg.452"
/translation="MELDYLFQASHLERLMWGLWATAKIAFISVFFSAIFGTVFGVIM
TSKNIVVKALCRLYLEAIRIIPILVLLFVFYFGFATWFNWQFSAMWVCVVVFVLWGTA
EMGDLVRAAITSIDQHQRDSAYALGLTHVQALIYVIFPQSLKRVTPGAINLFTRMVKT
SSLAVLIGVVEVIKVGQQIIENSLLTVPSASLWIYGFIFILYFLICYPLSRLAAHLEK VWE"
/product="ABC transporter, permease protein (cluster 3, basic aa/glutamine/opines)" /transl_table=11
CDS complement(437248..437907)
/locus_tag="AUO97b_00593" /db_xref="SEED:fig|400667.82.peg.453"
/translation="MNWQYIHSVLPQFFSATLTTLKISIISIILAIIVGLICSILITY
RVRVLNRIAQFYIELSRNTPLLIQLFFLYYGLPKLGIKIDGFACGVIGLTFLGGSYMA
EAFRAGLQSVAKGQIDSAKSIGLKPTQIFRYVIFPQALAISIPAIGANCLFLIKESSV
VSAIAVVELLFVTKDLIGMDYKTTEALFLLIMAYLIILLPVSILTSYLEHRSRKVSHG T"
/product="ABC transporter, permease protein (cluster 3, basic aa/glutamine/opines)" /transl_table=11
CDS complement(438192..438476)

/locus_tag="AUO97b_00594" /db_xref="SEED:fig|400667.82.peg.454"
/translation="MNIQQKDDTKHGSFSLLDGETVAGEMTYTWAGESMLIIDATDVN
ENYRGQGVGRQLLDALVAFVREKDVKVIPLCPYAKSVFDKDPSIGDVLRK" /product="FIG00351059:
hypothetical protein" /transl_table=11
CDS complement(438620..439579)
/locus_tag="AUO97b_00595" /db_xref="SEED:fig|400667.82.peg.455"
/translation="MSTQHVSFQQASPNEQAVILIVDDVPENLGLLHESLDQAGYRVL
VTTDGLSAIEIAHRCLPDMILLDGNMPHMDGFESCIQLKASPITQFIPVIFMTGLSET
EHIVRGFQVGGVDYVTKPLNIEEVLARVKTHLAHAKLLQQQKQVIDATETAILALDVH
GKIVWKTQKASDLLNQYLPELEGFEAGLKAWFSDLEHEKDPKKLTSCSYSTPSQQLQL
LLLTPWETEPSVAKNYLIQIKTTTPALEMADILKYCPQLTQREAEVMHWLLLGKTNKD
IAEILELSPRTVNKHLEHVFEKLCVENRTAAVAHINSLVQEFH" /product="Two-component
transcriptional response regulator, LuxR family" /transl_table=11
CDS complement(439576..443031)
/locus_tag="AUO97b_00596" /db_xref="SEED:fig|400667.82.peg.456"
/translation="MPDSSRPQQAPQRVIKTRREYNIWVADESIEDYALRYAPTSVRK
WSPWTVTNTAISTVSFLAMEAIGATMLWQYGFSNAVWAAIVVCTIIFLTSWPISYYAA
KYNVDVDLLTRGAGFGYIGSTITSLIYASFTFILLAFEAAIMAMALELALGIPQVIGY
LISALVILPLVVKGIGFINKVQAITQPIWLLLLLLPWFFVLWKQPQILSSSLHFVGAV
SNSTDFNLYYFGAACTLIFSFVIQIGEQADYLRFLPQKEKNRTAWRFAVFLGGPSWII
FGFLKVLMGMLLMVLAFQLFTPVSELDNPTYLYWVAYQQFIPNPQLALILTLALVCLA
QIKINMTNAYAGSLAWSNFFARLTHSHPGRIVWLLFNVFIAIVLMEMGISHAVERILG
LYSNIALAWIGAVVADLIICKPLGLSPKGIEFRRAYLYDINPVGVGALLIASVLSMLS
YLGFFGLMAKGLASFIALGSAVLCVPIIAYLTKGKYYIARQPEKIQATSVANCVVCER
DYELADMAGCPAYNGTICSLCCSLEARCHDLCKPDARWSVQLKKAIWHYLPERWASRL
NSRVSLYLLLTLGLSIVLAVSLSLVYIQEKTYLETINAAAVPQLFTLFVKIYTILFLL
MSVAAWWLVLNDESRRNAEIETHQQTALLIDEIAAHEVTSKDLQDARIAAERANDAKS
RYVIGISHELRTPLNSILGYSQLLQKQEQLSEQGKMALGVISRSGQHLTSLIDGLLDL
ARIETGKISLNVVDVHFPNFIEQIIQMFQPQFEQKNLQFVYEIGENLPHYVRTDKKRL
EQVLINLLGNALKFTTSGTITLKVEYRFQTAYFEIRDTGCGIAEADLERIFNPFERGS
NVVQGGFTGTGLGLPIVKLLVDLLGGQLSVTSQLNQGSQFKIKFYLPSKEVVHPISNV
YSNQITGYQGERKRILVVDNEAVDRGLVANFLKPLGFMIEEAESGIDCLRRVPIFQPN
LILMDLNMPLMGGWETARLLRQNNITNVPILIISANAGEREVNPQDAVLSEDFMLKPI
DLNLLLSKIGDKLGLVWIDSKSETLLENNDVQALDVKVFKQEQVTVHTLIQQPENNEL
LDFPQSLQQLNDLIGQGYIRGIQQTLTQCRQNFPKHNELWEQLEQAIQKFDLKHAQRL IQDKQ"
/product="Two-component system sensor histidine kinase/response regulator
hybrid" /transl_table=11
CDS 443236..444852
/locus_tag="AUO97b_00597" /db_xref="SEED:fig|400667.82.peg.457"
/translation="MSVSEHPNLDTVQGTDSQKAVNETLEDYTLRYAPHSFRRWSPKV
VAITALGGIAYLADFSIGASIGMSYGTTNAVFSILFAAIIIFLTGIPLAYYAARYNID
LDLITRGAGFGYIGSVLTSIIFASFTFIFFALEGSIMAQGLLLGLGIPLWAGYLISTV
MVIPLVIYGMKALSKLQVWTTPLWLVLMIGPVAYLIYQEPTLVSQFATFTGHEGFAPV
DMAAIMLGAGICLSLIMQIGEQIDYLRFMPAKTKENSKAWWAAVISAGPGWVILGAIK
QIIGAFLGFYLLTKIPGVNSTEPVQQFNAAFHDMLPGWAALTLAVILVVISQIKINVT
NAYSGSLAWTSAYTRISKHYPGRIVFVIVNLAIALALMEGNMFAVLGKILGFYSNFAI
AWVVVVATDISINKYVLKLSPKEPEYRRDMLYNVNPVGMVAFLVSAGLSIAAFFGLLG
NFLAPYSPIIALVLAFVLTPIMGLLTKGKYYIKSHDDGVKEPRYDAEGTPVATVYHCR
VCEQGYERPDIMFSHKHNGTICSLCKTLDA"
/product="Putative transmembrane protein"
/transl_table=11
CDS complement(444910..445365)
/locus_tag="AUO97b_00598" /db_xref="SEED:fig|400667.82.peg.458"
/translation="MDRIDKKILAELQLNGRLSITELAEKVGLSISPCHRRVKALEES

GAIKGYRAELDPNLVGFEFSAIVFITLKEGDKQAVEKFENAVIEIPQIIQAQRLFGEP
DYLLHVVAKDLPAFQRLYDEKLSAIPSVQRLISTIVMKDVVPERLFPIG" /product="Transcriptional regulator, AsnC family" /transl_table=11
CDS 445522..446133
/locus_tag="AUO97b_00599" /db_xref="SEED:fig|400667.82.peg.459"
/translation="MAFNIFIAFWSVSILFIITPGADWAYAISAGIKGKVVVPAVAGM
LFGHFITILLVAAGVGLLVANNPTALMILTVAGSAYLLWMGINLLLTPPTPNKSGSEK
AQSWLKWATKGVYVSGLNPKVFLLFLALLPQFIDTTASWSVTTQIVAFGVVHIISCAI
IYLMVGYGSEAILKTRPQAAQLVGRFSGGLMVVIATCLLIGQI" /product="Transporter, LysE family"
/transl_table=11
CDS complement(446204..446815)
/locus_tag="AUO97b_00600" /db_xref="SEED:fig|400667.82.peg.460"
/translation="MPKKKEFEHGGARENAGRKAQFNEPTKVIRVPESQVNFIKNWLL
NNVKTNNQTDFTSKLKIQQVHPNNDKIYHIPLATERVAAGFPSPAQDDIEQALDLNEY
LIRNENATFIVKANSLSMLDAGIDIDDPLIVDRSIPAKSGDIVIALIDNDFTVKRLII
DTQFQPPKVWLKAENPDYQNIYIEEGQELVIWGVVTYNLKRMR" /product="Error-prone repair protein UmuD"
/transl_table=11
CDS 447098..447334
/locus_tag="AUO97b_00601" /db_xref="SEED:fig|400667.82.peg.461"
/translation="MKNQRDAVLGYADEQHAYYIRLIENDAVLGESYGIFCENPESEA
AESIMTTLFLKKSFWLNGSEFTDIVKGLHPLTHS"
/product="hypothetical protein"
/transl_table=11
CDS complement(447395..448498)
/locus_tag="AUO97b_00602" /db_xref="SEED:fig|400667.82.peg.462"
/translation="MDPERVREIASKGGRAAHASGNAHEFTSEEAREAGRAAHASGNA
HEFTSEEAREAGALSHKNDDRNGRGRSRYDDDEDDDRGRSSGRGRGRSRYDDDDEDDD
RGRSGGRGRGRSRDDDDEDDDRGRSGGRGRGRSRDDDDEDDDRGRSGGRGRGRSRRDD
DDEDDDRGRSGGRGRGRSRRDDDDEDDDRGRSGGRGRGRSRYDDDDEDDDRGRSGGRG
RGRSRRDDDDEDDERGRSGGRGRGRSRYDDDDEDDDRGRSGGRGRGRSRYDDDDEDDD
RGRSGGRGRGRSRRDDDDEDDDRGRSGGRGRGRSRYDDDDEDDDRGRSGGRGRGRSRR
DDDDDDDDRRGRSDGRGQNSRNQKRDAYGRFTS"
/product="General stress protein"
/transl_table=11
CDS 449049..449462
/locus_tag="AUO97b_00603" /db_xref="SEED:fig|400667.82.peg.463"
/translation="MMQPIHTHSTLQYIHISVPEILLSNIQIKNSWQDYNQEWSYRLD
PPHASHPFQRDLYIIKSKNIETEDIKKLLDNTVVKNSKNKDDLKNIVEAETVIKEILD
LSNYIPIENWLNDTGNRSIVESMIDKNKVKLLDII" /product="hypothetical protein"
/transl_table=11 CDS 449604..450482
/locus_tag="AUO97b_00604" /db_xref="SEED:fig|400667.82.peg.464"
/db_xref="GO:0004316"
/translation="MENTTNQYPSEVPAQVQPHQPGDQEKMHPEPEIIKASHKGSEKL
KGKVAVISGGDSGIGRSVAVLFAREGADIAVLYLEEDQDAEITKQLIEKEGQQCLLLK
GDISDPDLAKQNIDKVLQHFGKINILVNNAGVQYQQKEIESISNEQLEKTFKTNIFAM
FYLTKEAIPYMEEGDSIINTTSITSYQGHDELIDYASTKGAITSFTRSLSNNLMKQKK
GIRVNGVAPGPIWTPLIPSSFDAETVEKFGKDTPMGRMGQPSEVAPAYLFLASDDASY ITGQVIHVNGGQIVNG"
/product="Oxidoreductase, short-chain dehydrogenase/reductase family"
/EC_number="1.1.1.100"
/transl_table=11
CDS complement(450538..452676)
/locus_tag="AUO97b_00605" /db_xref="SEED:fig|400667.82.peg.465"

/db_xref="GO:0004096"
/translation="MNDNTKSTNKTSEMAGADAANKANTTQKTEQLDTVRDDATNEAL
TTNQGVKIADNQNSLRAGIRGSTLLEDFILREKITHFDHERIPERIVHARGVGAHGYF
QAYEGNERLTKAGFLTDPTIQTPIFVRFSTVQGPRGSADTVRDIRGFAIKFYTQEGNF
DLVGNNAPVFFVQDGIKFPDFVHAVKPEPDTEIPTGATAHDTFWDFVSLVPESAHAVI
WAMSDRAIPRNLRSIQGFGVHTFRLINAEGKSHFVKFHWTPKQGLSALVWDEAQKLAG
KDPDFHRRDLYEAIEQGFYPEWELGVQIVEEEDEMNFDFDLLDPTKIIPEELVPVTPI
GRFVLNRNVDNFFAETEQVAFCPGHVVPGIDFTDDPLLQARLFSYTDTQLSRLGGPNF
HQIPINKPVCPFHNNQRDGIHQHTIHKGQAAYQPNSIDGNWPAETPPAASNGGFESYP
EKISGHKLRQRSETFSDYFSQPRLYYKSLAPHEQKHVVDAYTFELSKVQRKHIRERQV
QQILANIDLDLARQVGANLGIEVPDLTLNYKKTAVEKSAKLSFLAFPPEDIQGRKVAV
LIHNMVKSDALEAIKNWAIKEGVILHLLAPSLAPVKDHKDNVIVADGMQMSEPSIAYD
AVIIPDGDNLNTVMQDGVARHYLLEAYKHLKPIVFLGNKSDLLEPLGLVADEGTLIED
EFQHVTEKFKNLIKAHRVWSREQIAAHVPA" /product="Catalase KatE-intracellular
protease (EC 1.11.1.6)" /EC_number="1.11.1.6"
/transl_table=11
CDS complement(452737..454146)
/locus_tag="AUO97b_00606" /db_xref="SEED:fig|400667.82.peg.466"
/translation="MPVVVKKRLLDLLQDNQQNYYELFVFFLDPDVSSFEKEKKARDF
LVKEIDKLEMKEIDFPSDINLIKAWCENDNKKNCQEFQAYLNRRQSGQDREYFKNVAQ
AFEFLIKVSPTKKVDGAWLYSSVHYWNDPIFHELIITYLEELGLGEPKANHVCIYDDL
LRSLGLDSFDLLLEDEYYHQAVVQLALGYAPPEFIPEIVGFNLGYEQLPLHLLISNYE
LAELGIDSKYFNLHITIDNIDNGHAYKAIKVIEDIYNKYRDKELFLTKLKRGFALNNH
GVSSSNIIKNLNTEDFVHRIFKRKALVGQLIHNETRQFGCKTINQWLSNPDDIAGLIT
HLTEHKWIKFNTDPEQSVFWRMINEENGKMFGVFNPVERQIIHDWIAGSGHSSSFLAY
SRELKNSQRIQDYLFSYISDGELDALQERVQQSSDLAIKICKLTPFLAPDSHHKSIGL
WSTRKYVELLFPYLGTFKN" /product="FIG00350446: hypothetical protein"
/transl_table=11
CDS 454462..454941
/locus_tag="AUO97b_00607" /db_xref="SEED:fig|400667.82.peg.467"
/translation="MFNKCCELLAKEKIKIAFFESASAGYLAYRFSLSPYSGDILIGS
LVSYDLRVKENTLHISEELIEKYTAESMQVTVEMINKGKELLHADLYVACTGLLKKGG
SETPEKPVGTFFYSIYYKNNFYNFRRVLRGPAFLKLRKLIYYVTQDIKLIILEDKRR" /product="CinA-
like protein"
/transl_table=11
CDS 455226..455447
/locus_tag="AUO97b_00608" /db_xref="SEED:fig|400667.82.peg.468"
/translation="MSTTNNQANQRNNQQQQQQNDNRNQQQHGNQQQNDQQQQNNQQ
QQQNDNRGQQQGSNQKDSGQQNSNNNQQR" /product="TPR-repeat-containing protein"
/transl_table=11
CDS 455553..455870 /locus_tag="AUO97b_00609" /db_xref="SEED:fig|
400667.82.peg.469" /translation="MTDQNTDHAQLVAGDHNYSDSRWRESYLTRPYYQEAQLTTPDLD
YDRDFSAAYELGHRARSESKEGTQFEDMEGSLQQKWEELKAESRLKWEHAKQAIKDAW DDM"
/product="FIG00350320: hypothetical protein" /transl_table=11
CDS complement(455915..456175)
/locus_tag="AUO97b_00610" /db_xref="SEED:fig|400667.82.peg.470"
/translation="MFVYFTDGTCINDSEIYGVKAKYEQNKYVVEVTIKPTHVLATTP
SVQFKKEVFDDPNLANQYLSHNFNMPPFFDAHNVMTIKKPIS" /product="FIG00351498:
hypothetical protein" /transl_table=11
CDS complement(456263..456691)
/locus_tag="AUO97b_00611" /db_xref="SEED:fig|400667.82.peg.471"
/translation="MPQYLKIAEKIYQNVKEKKGFTDDPMEDLNNLMIELRQEIKGTK
LKLLYNYIDFAEILIKPLQESKIKLDLSLIPKSKNTQEFVLWLAGFIERITTGGQEKL
PPIRAKITLPAYAYEETYPSQKKPVNNGEEIIEYFKKNQS" /product="hypothetical protein"

/transl_table=11
CDS complement(456961..457104)
/locus_tag="AUO97b_00612" /db_xref="SEED:fig|400667.82.peg.472"
/translation="MVRIFCAVSMVLAIAMGMSACQDRNSRNDGMHEASDPATSHDMN KNS"
/product="hypothetical protein"
/transl_table=11
CDS 458148..458363
/locus_tag="AUO97b_00613" /db_xref="SEED:fig|400667.82.peg.473"
/translation="MSKNEFNTRCVWAGHDLSACNCIYNSLEQHYSPQFVESLPKLSL
QNIDAKMNFVETMNQTMQQCQLSASKH"
/product="FIG00351861: hypothetical protein" /transl_table=11
CDS complement(458365..459270)
/locus_tag="AUO97b_00614" /db_xref="SEED:fig|400667.82.peg.474"
/translation="MSYFDINRSGELAIFIRVVELGSFSAVARACNMTPSAVSKLISR
LEKRLGVRLLNRSTRQFQLTSEGCQFYEQGIQILNGLDELEQSVTANHIPKGRIRIHT
SFSYWTHFLLPCISLFNQRYQEIELEAHLSDEVINLVEQRIDVAIRTGPLKSSNLVAR
SLGSTYKQYVCSPLYIEKYGCPEHPDELHEHQLLDVSYQRQNKTWLFKKATQEISLTP
SKVLKVNHGEAILQLALAGAGIAQLNEFQIRNALQQKQLVKILEDWNIHASEEFHAVW
IGHDKYVPNRVRTFLDFLVEHASIN"
/product="Transcriptional regulator, LysR family" /transl_table=11
CDS 459382..460620
/locus_tag="AUO97b_00615" /db_xref="SEED:fig|400667.82.peg.475"
/translation="MKLYQAPLFSLVLITSFAQADVENSQWITTWSASPQKVWNKDFV
FPTLIPEQVSNQTIRQMSKISLGGEAVRLVFTNQYGKQPLYIDQTTIGLANAATLKSK
STYPVYFSGQLKARILPGKQLISDPIQLPVPDHAQLVVNSFIQKPTMFKTFHWDAKQT
SWLISGNQTTNLTTPTDAKTTTARLLLSAIEVKPKQKAHVVAVVGDSITDGATATLDA
NTRWTDFLAKRLSAHQVAVINSGISGNRLLTDGMGDSVLKRLNSEVFQYAGVKTLIVL
VGINDISWPGTAFAPKQQIPSFEALTQGYRRVVSEAHKHNIQVIGTTLLPFSGALPNT
PLDNYYQPNKDQLRQRINHWIRTSHTFDGVLDLDEGLKDPKHPNRLNPIYDSGDHLHP
NDRGNQHMAELVDLDQIIKN"
/product="Lysophospholipase L1 and related esterases" /transl_table=11
CDS complement(460666..461952)
/locus_tag="AUO97b_00616" /db_xref="SEED:fig|400667.82.peg.476"
/translation="MMKILNTTVQKSLLAVSVTLLMSSAFAGQVNPEIEQLRQEIKEL
RAMLQINMEAQKSLAQKRVEQAAPVVTQAPPPIQQTLTKSGAEFNLYGYIRADASYQI
KGASSMYNNISGVPLENTPEESAQKDQLHSTVYVTRFGLNFKTPTAVGDVGGKLEMDF
FGAATRDQFRIRHAYLTFDKWLIGQTWSTFIAPEYYPETIDAGTFVGSALLRSPLVRY
SDNLTANTSFAVSVEDPKYTAATDPDNKMRLPALVGRLNHKFANGSMLSGRSFVAEKR
TNNDSEWAWGVGLGGKYQITPDTLLKADYYHVKGDGRFLLWSNSGYAIDDQNNMHSNE
FDTVSLGITHQFTPQIRSTLGYGYMKAKDDNRFAELQRTSTTQNKQLWQGWVNALYNP
YKPITLGIEYVYGERETFDNRNGIDNRFNMIASYDF"
/product="DcaP"
/transl_table=11
CDS complement(462193..462864)
/locus_tag="AUO97b_00617" /db_xref="SEED:fig|400667.82.peg.477"
/translation="MAKDFNSPKVVEGYDAHIRKLIPGYDVIHQQVDAILQSVLRPDP
HILIVGCGTGYELEYLLKRHPNWQFTAVDPSLTMLQKAQEQVEILGKSTQVTFVHGET
SALPLESCFDAALSILVAHFIADDIKPAFFKDIYDRLKEKAPLLTYDLMTCVNLQQLQ
ALRYLCLAQGLTEEQCQKMLERMAEDFFSLTTEGYIKLLEKTGFESVQPYTQILTYQG FMAKK"
/product="SAM-dependent methyltransferase"
/transl_table=11
CDS complement(462935..464629)
/locus_tag="AUO97b_00618" /db_xref="SEED:fig|400667.82.peg.478"
/db_xref="GO:0030729"

/db_xref="GO:0004467"
/translation="MQQERLSYASGPSTQPLLGMTIGEQFDQACQQYAEKEAIVSFHQ
NRRLTYKALQDEVNAFACSLLKLGLKKGDRLAIWSPNCVEWTITQFAAFKAGIILVNL
NTAYKSHELEYVLNKVSCKGLIIASQFKTTDYQELLTKIAPELTTCTDKVLSSERLPH
LKFVIKIDDQQHTGIYRFSDLLTTPTSEQLEQLKQLARQLQFDETINIQFTSGTTGNP
KGTMLTHHNILNNGYFVGEGIRLTPQDKVCISVPLFHCFGMVMGNLACVTHGATMVYP
SAVFNPLETLKAIHQERCTAAYGVPTMFIATLEHEQFNDFDLSSLRTGIMAGSPCPRE
IMQRVIDRMHMSEITICYGMTETSPVSVQSSVNDSIDKRVSTVGHVHPHVEIKIVDLE
GQIVPQGTLGELCVRGYSVMAGYWGEEEKTREVIDAAGWMHTGDIAEMDQDGFIKIKG
RIKDVVIRGGENLFPKEIEDFLYTHPDVSDVQVIGLPDAKYGEELCACIILHEHHQVD
EDSIRQFCKEHISHNKVPRYVRFFNEFPMTASGKAQKFKLQEIMRAELNLTGNVFD"
/product="Acetoacetyl-CoA synthetase [leucine] (EC 6.2.1.16)"
/EC_number="6.2.1.3"
/EC_number="6.2.1.16"
/transl_table=11
CDS complement(464740..465357)
/locus_tag="AUO97b_00619" /db_xref="SEED:fig|400667.82.peg.479"
/translation="MSYKRSSLMQERMEQNRKSILNSARKIISEGGFKDAQIQTIAEQ
AGVSSGLVYRYFDNKSQVLIEVLSDAINTELLVIDSITESDLSAKQKLHKAVATFVKR
ALNSPQLAYSLMFEPVDSTVEHERFRVKQLIKQSIKKILADGNASGEFVLDDLNTAAL
CVVGAMTYVVVEPLDPAQNTKFDHAHKDYFSKQIADFCVDAVQKK" /product="Predicted
transcriptional regulator LiuX of leucine degradation pathway, AcrR family"
/transl_table=11
CDS 465502..466674 /locus_tag="AUO97b_00620" /db_xref="SEED:fig|
400667.82.peg.480" /translation="MNLRSLNFGLDETLIALQDSVAAFCAKEIAPIAQQVDQDNKFPA
HLWKKFGDMGLLGMTVSEEYGGANMGYLAHIIAMQEISRASAAIGLSYGAHSNLCVNQ
INRNGNEQQKQKYLPKLISGEYVGALAMSEPNAGSDVVSMKLRAEQKGDHFVLNGSKM
WITNGGDADVLVVYAKTDPQAGPKGMTAFLIEKGMKGFSHGNHLDKLGMRGSNTYPLF
FDNVEVPAENVLGGVGNGVKVLMSGLDYERAVLSAGPLGIMDACLDVVIPYLHQREQF
GQALGEFQLMQGKLADMYSTWLACKALVYAVGAACDKADHDRSLRKDAASAILYAAEK
ATWMAGEAIQTLGGNGYINEFPAGRLWRDAKLYEIGAGTSEIRRMLIGRELFNETK"
/product="Isovaleryl-CoA dehydrogenase (EC 1.3.8.4)" /EC_number="1.3.8.4"
/transl_table=11
CDS 466699..468300
/locus_tag="AUO97b_00621" /db_xref="SEED:fig|400667.82.peg.481"
/db_xref="GO:0004485"
/translation="MNQLQSKINVRSEDFKTNQQAMQTLVTDLKQVAQRIALGGGENA
RQKHLARGKLLPRERIDQLIDVGTAFLEIGQLAAYNVYEDDVPAAGVIAGIGQVNGIT
CMIVANDATVKGGTYYPLTVKKHLRAQEIAEQNHLPCIYLVDSGGAYLPMQDEVFPDR
DHFGRIFYNQARMSSLGIAQIAVVMGSCTAGGAYVPAMSDETIIVRNQGTIFLGGPPL
VKAATGEVVSSEDLGGGDVHTRLSGVADHLAENDEHAIAIARNIVANLNKKPNELNKQ
IDEPLFDASELYGVVPSDARKPFDVREVIARIVDGSRFDEFKARFGSTLVTGFASLYG
MPVGIIANNGILFSESAQKGSHFIELCTQRNIPLLFIQNITGFMVGRQYENEGIAKHG
AKLVMAVATANVPKLTLIIGGSFGAGNYGMCGRAYSPRFLWTWPNSRISVMGGEQAAS
VLSTLKRDQIEQKGAEWSAQEEDEFKQPIREQYERQGHPYYASARLWDDGVIDPAQTR
QVLGLSLAAAMNAPIQPTKFGVFRM"
/product="Methylcrotonyl-CoA carboxylase carboxyl transferase subunit (EC
6.4.1.4)" /EC_number="6.4.1.4"
/transl_table=11
CDS 468310..469113 /locus_tag="AUO97b_00622" /db_xref="SEED:fig|
400667.82.peg.482"
/db_xref="GO:0004490"
/translation="MSYQFLQLEQQGQVAYVWLNRPELHNAFNTTVIEELHTCFKQIN
TRDDIRVVVLAGRGKSFSAGADLNWMKQAGQASSAENEADALKLAQMLDALATLKQPT

IARVHGIAFGGGMGLASACDICIASTDAKFATSEVRLGLAPSTISPYVIRAIGARQAS
RYFLTAERISAREAKQIGLAHEVADAEDLDKKVQEIIDALLLGGPHSQAASKQLIQMV
SNQTMSNDLLQQTAHHIAQVRQGSEAKEGLSAFLNKQQPAWVSNSNNNN"
/product="Methylglutaconyl-CoA hydratase (EC 4.2.1.18)" /EC_number="4.2.1.18"
/transl_table=11
CDS 469125..471116
/locus_tag="AUO97b_00623" /db_xref="SEED:fig|400667.82.peg.483"
/db_xref="GO:0004485"
/translation="MFEKILIANRGEIACRVIRTAKKLGIRTVAVYSDADANAQHVKL
ADEAVYIGHSPATQSYLQVDHIIQAAIDTGSQAVHPGYGFLSENDQFALACQQHNICF
IGPPVDAILAMGLKATSKALMEKAGVPLTPGYHGTNQDADFLKQQADRIGYPVLIKAS
AGGGGKGMSLVERSEDFLHALASCKREAKSSFGNDDVLIERYVIQPRHIEVQVFGDTH
GNYVHLFERDCSVQRRHQKVLEEAPAPQMPSEKLDAMRQAAIDAARAVNYVGAGTVEF
IVEQDGTAYFMEMNTRLQVEHPVTEMITGEDLVEWQLRVAYGEPLPKLQNELQIHGHA
LEARIYAEEPEKGFLPAIGKIDYLRYPTQNQYVRVDSGIVEGDEITTYYDPMIAKLIV
WGKNREAALIQMQNALSQFHVDGLGNNIAFLEKIVRSESFKQAKLDTNLIQREQNFLF
SPEEIKPELVVAAAFIEFLSQLNNNSSSQKQLWQAQPLWRLNIAYQHSIKLNYLNQNI
QIKFASNEDGFTAEYNGQSYPISGQLIDAHTASVQIGGTQQKLSFNQSQQGITLFQNG
QSYKFVYICQDFNQADSQADEGHLKAPMPGVVTQVLVSANHSVKKDDILMTLEAMKME
YTIRAPKDGVIVDSYFQVGDQVKAGDELVEFQPAQEEVA" /product="Methylcrotonyl-CoA
carboxylase biotin-containing subunit (EC 6.4.1.4)" /EC_number="6.4.1.4"
/transl_table=11
CDS 471113..472015
/locus_tag="AUO97b_00624" /db_xref="SEED:fig|400667.82.peg.484"
/db_xref="GO:0004419"
/translation="MSEFVKIVEVGPRDGLQNEKQALTVEQRLNFINDLISAGLKSIE
VGSCVSAKWVPQMAQSDELFKLLPQTTDVQFSLLTPNIKGFETAQAVGCKEVAVFTAA
SESFTRKNINCSIDESFEKFSDVMNAAKAHNIRVRGYVSCIVDCPYEGAIAPEQVVKV
VKRLYDMGCYEVSLGETIGTATPDRVQKVWQACLAELDSKVLAGHFHNTYGMAIANIY
QSLQQGIRVFDSSLAGLGGCPYAKGASGNVSTEDLFYLLSHMGFETGIDLEKLMQASQ
NISNVLNRKSLSNYANAYWQTKCA" /product="Hydroxymethylglutaryl-CoA lyase (EC
4.1.3.4)" /EC_number="4.1.3.4"
/transl_table=11
CDS complement(472265..473206)
/locus_tag="AUO97b_00625" /db_xref="SEED:fig|400667.82.peg.485"
/translation="MRKNLDGGLLHAMHAFLKVIDSGSFTAAAEQMDLTTAQVSRLIS
ELETRLGTKLIQRSTRQQALTDIGATYAERCRQVISMVDEAEAEVTGTASKPKGRLRV
LSMGSFGHHYVFPVMAEFCKHYPELTVEYTTSQYVPDLLAKGVDVSLYLTESLTDSRF
VARRIGTIFSVLCASPAYLEKHGVPKSPEDLKNHACLRLVNPSITPDWHLLNANGESY
QIDIGGQLIADNPELLLDVVQQDMGVALLPMFSALDAVQNGRLCHILPEWRSPDIGVY
TLLPSRHFIDAKTRAWLDWVEEYISPKIEMDASYFYK" /product="Transcriptional regulator,
LysR family" /transl_table=11
CDS 473411..476893
/locus_tag="AUO97b_00626" /db_xref="SEED:fig|400667.82.peg.486"
/translation="MNIATKVTPNIIATKDVSLEDKYVSDNGTAFMTGIQALVRLPLA
QTRRDAINGYHTAGFISGYRGSPIGNYDNFLWQVEGLLKSHNVVFQPGVNEDLAATAI
WGTQQANLSGQGKYDGVSSWWYGKGPGVDRSGDVLRHANLAGTSERGGVVALFGDDHS
CKSSTVPHQSEHVMIGCGIPIFYPTSVQQILDFGVHAIAVSRFSGLWTSMKLVSEIVE
TSASVHVDLDRVTPVIPEDVNFPEGGVNIRWPDHGIQQEERLYKYRLPAVLAYARANK
INTVTWPCENARVGIAASGKGYLDTIEALRILGIEDETAQQLGLRVYQVGLIWPLEPQ
GIREFAKGLEELIVIEEKRPILETQIKDELYSLPDDQRPRVIGKATDGKGEWSTSIEE
APLIGHYEYQPEPIAKMLAARFLKLDIPESLKVQIQSRIDLLLKAEQESKRVIDLAER
KPYFCSGCPHNSSTVVPEGSRALGGIGCHYIAVSLDRGTETFSQMGGEGVSWVGASPF
TNEKHIFANLGDGTYFHSGYLAIRQSIAAKTNITYKILYNDAVAMTGGQHVDGHLSVA

QLTRQLDAEGIKKQVIVTDEIKLLNEEDGIAPGVEIRHRNELDAVQRELREISGVTAL
IYVQTCASEKRRRRKRDAYPDPAERLFINTDICEGCGDCSKKSNCLSIEPVETALGTK
RQINQSSCNKDFTCAEGFCPSFVTVHTRDMKRPAKFEGISAGWPTSPIIPQLYDVPSR
IMVGGIGGTGVVTIGALLGMAAHLEGKATRVMDMAGLAQKGGTVYSYVQLAASDEQIS
STKIPAGQCELLIGADAVVAGSGAALSRLKEDALVIVNEDSTPTSEFIKSRDWYAPIT
DLIDRLRGRVRQGQLVSLPAARIATLLLGDSIYTNQLLLGMAWQSGRIPLLRESIEKA
IRLNGTAVEKNIEAFRVGCHLASDPTLAARLLAAMPKTKNPQTLAELVEDRSARLTDY
WNEAYAAKYRTLVELAAKKLPEELTGTIATQLYRVMAYKDEYEVARLLTGKSFKESIE
IQFGKGLRLTYHLAPPALLGGLKNVRKRAFGYWMRFPMMMLARLQWLRETFLDPFARQ
EERQHEQAWRDRYIAFVEALVEAPDNHNLAVAEQIAKLPAEVRGYGHIKMKAMDAAKQ RWDELTPTLLKVRN"
/product="Indolepyruvate ferredoxin oxidoreductase, alpha and beta subunits"
/transl_table=11
CDS complement(476955..477758)
/locus_tag="AUO97b_00627" /db_xref="SEED:fig|400667.82.peg.487"
/translation="MNAYYQLLNRTIGPQGEVIAHYCSTVHAQGAWNPHEQHMAPASG
VIAVELEQFSPRQDMRIGRISFDIFGLIAFGEFTIKTHVIRAGKTIELIEAELQAQGK
TCIVARAWCMCTQDSSEIAGLEDQPIHHPDTFSEWTGMKRWPGGFIQTLVTKTNDQHR
AGKGIVWLNNDKEMVEGQPTTDFAHLIGMVDAANGIVPRQEGEFTWGFPNLDLQIHMH
RYPQGKWLGLETVQQYGSDGIGLTSAVLHDVYGPFGRSEQILTLRKMIK" /product="TesB-like acyl-CoA thioesterase 5" /transl_table=11
CDS 478316..478942
/locus_tag="AUO97b_00628" /db_xref="SEED:fig|400667.82.peg.488"
/translation="MSYSFLSFVLTALVINLAPGPAMFYVMNQSLKYGIKVGLKAAAG
VEFGVFFYVLLSAFGLMFVFQQIPLLYKILQFTGALYLLYLAYVAWPKRKSIKSSNVP
SQNSTESTRYSFSKGMLINLSNPKIGLFFVSLLPQFISSDSHPAWQYFLLYGLLFNFG
GICVNMTIGVMARKLKSIIQKASWFDYVPSLLFLTIAILTIVRDALSS" /product="Transmembrane amino acid efflux protein" /transl_table=11
CDS complement(479171..479986)
/locus_tag="AUO97b_00629" /db_xref="SEED:fig|400667.82.peg.489"
/translation="MTTNNTQIQAVVFDWAGTTVDFGSRAPILAFMALFKDNKVEITV
EEARVPMGLEKRDHIAAVLKMPRVAAAWQEVYGQAATEADIDRLYKDFIPYQLNSIPK
CSALIPGALETFENIKKRGAKIGSNTGYASMMIGRLVKDAADQGYRPDCIVTASDVKY
GRPYPEMLWKNLIQLDVSDIKTVVKVDDTVVGIDEGLNAGCWTVGLAISGNEVGLSYE
EWSALSADEQIVLKDKAYQKMNASGAHYVIDSVADLPNVLDDIERRLGQGERP"
/product="Phosphonoacetaldehyde hydrolase (EC 3.11.1.1)"
/EC_number="3.11.1.1"
/transl_table=11
CDS complement(480023..481171)
/locus_tag="AUO97b_00630" /db_xref="SEED:fig|400667.82.peg.490"
/translation="MNMQPQLDMDLENKYLLLTPGPLSTSATVRCAMQKDWCTWDQEY
NSLVQDIRNRLVNLATSEPEKYTAVLMQGSGSFAVESVLGSAIPRDGKILIINNGAYG
ARMVQMARCLNIEVVELNYLETQTPDLLEIERVLQDKTISHVACVHCETTTGILNPIQ
DIGRLVKAAGKIWIVDAMSSFGGVPMDMAELEIDFLISSANKCIQGVPGFGFILAKRD
CLEACKGLARSVSLDLYDQWNTMEQHNGKWRFTSPTHVVRAFYQALLELETEGGVAAR
YQRYLRNQQTLSKGMQQLGFELVLGEESPQSPIITTFLYPTAETFSFQSFYETLKKSG
YVIYPGKVTDLNCFRIGNIGEVYVQDIEGLLAAIASYCENHLNQAVTV" /product="2-aminoethylphosphonate:pyruvate aminotransferase (EC 2.6.1.37)"
/EC_number="2.6.1.37"
/transl_table=11
CDS complement(481237..482391)
/locus_tag="AUO97b_00631" /db_xref="SEED:fig|400667.82.peg.491"
/translation="MNNAQFDLIVVGAGILGLSAAIQAQEQGLKVCIFEKNAKPVGAT
RRNFGMVGTSTLTHPEQQWRKYALETRSFYQRIQTETDISFEQRQGVYLANTALEWQV
LNEFAERANSYQIPVHLFSHDELVTQFSYLNPSQQFQGGLVFEEDYSVEPHVIGQRLL

AYAERNGVKIYTNACVVQTQYQKESCQVRLASGEAYRANKVLICHGEVIDVLYPDLLQ
SLNLKRCGLQMALTQPFHQNLNASLYSGLSISRYPAFEICPSHAELVKASQQGFIKEF
GIHILIKQNEFGELIVGDSHEYHSINEAPQFEQREEINEFIQTYCRQKLGLTLPPIQK
RWNGYYLTHEHELACVAEAEKNIFLVSAIAGKGMTTGAGFMKEILEQNIY" /product="putative
secreted oxidoreductase" /transl_table=11
CDS complement(482493..484202)
/locus_tag="AUO97b_00632" /db_xref="SEED:fig|400667.82.peg.492"
/db_xref="GO:0005215"
/db_xref="GO:0006810"
/db_xref="GO:0016020"
/translation="MLNEAASAVLKTQPRRKTEYSLRSNIALAMVAIALTLAIIAPLI
MLFETAFFDENQNFVGLENFYNYFASPALLSSVFNSVWVACAATVITVFLASIYAFAL
TNVNIKGKGFFKLVAFLPILAPSLLPSLALVYLFGKQGVFKPLLGDIQIYGPIGILIS
YCFWLFPAILMLMMVSFRSVDQRLIEASLSLGKNIWKTHYHVTLPAIRYGLISASLVA
FIYVLTDFGIPKVIGGSFNMMALDVYKQIIGQQNMSMGAVISILLLLPAVFVFIFDRI
QSKRHARFQAFQAKPYVSASNKKLEVVLGLFCGLVSGAILLIIFTAVLASFIQSWPYD
LSLTLAHYSFEYVDGGGWAAYFNSVRMALFSTVFGTALIFMVALLTERFKAHPLIKNY
VQALVLLPLAVPGLVLGIAYILFFNQQSNPLNVLYGTMTILVISTIVHYYTVPHLTLT
NAIKQIPLQLDQAAETLGTSKWKTFWKVYLPMCFPALCDVSVYVFVNAMTTVSAAIFL
YSPDTSLAAVAVLNMDDAGDTVAAVAMSILILTTSCVVKLIHWLFTRKIMARSQQWRE SSH"
/product="2-aminoethylphosphonate ABC transporter permease protein"
/transl_table=11
CDS complement(484196..485398)
/locus_tag="AUO97b_00633" /db_xref="SEED:fig|400667.82.peg.493"
/db_xref="GO:0005524"
/db_xref="GO:0016887"
/translation="MQIPSSLQSSLLGQNQKAELAGHLLSTLTTYKYPEHGPSILEVV
DIQKKFQHFQALTHINLDLKAGEFVSFLGPSGCGKTTLLRIIAGLEKPDYGKVIKKGA
DITLLSAEKRHCGIVFQNYALFPNLNVEENITFGLDKKKWDKAQRSQRVQQLLELIEL
PDIAKKYPNQLSGGQQQRVALARAIAPNPDILLLDEPLSALDAQVRLNLRQKIRSIQT
QLNLPTIMVTHDQEEALSISDRVVVMNHGVIEQIDTPHNIYYKPQTQFVAKFIGTMNF
LKATCAVPNQLEVLGFIPLNLEQQKLKAGQNYSIGFRPEAVELVDDFGSDKESLYLPV
KVLSTEFLGAKRRLFCAIHIDGIEQAKHLLQIEIENIKFKTLQELMFIKVPNQLIHVF DEHGYALC"
/product="2-aminoethylphosphonate ABC transporter ATP-binding protein (TC
3.A.1.9.1)" /transl_table=11
CDS complement(485421..486458)
/locus_tag="AUO97b_00634" /db_xref="SEED:fig|400667.82.peg.494"
/db_xref="GO:0005215"
/db_xref="GO:0006810"
/translation="MKQYVQQTIKLGTASVLGVVVAIQMGCSSATSKQSEEITVYTAV
EADQLKQYQEELHKAYPELKVKWVRDSTGVITAKLLAEQKNPQADVVFGVALTSLLVM
EKKDMLEPFKPEGVQNLKSEFVSQKSVPTWAGMDAWESAICVNKVELEKRKLPIPKTW
KDLTNPIYKNLIVMPNPASSGTGYLDVTAWMQIWGEKQAWDYMQALDKNISQYPHSGS
KPCKMAAQGEIPIGVSFGYPAFKLKAEDAPLEVVYPTEGLGWEMEASAIVKGTKKLSS
AQKFINWSVSKAANEAYAHNFSMVAYKGIENTTMDFPKNLPQMLVKNDFYWAAEQRER ILAEWSKRFEK"
/product="2-aminoethylphosphonate ABC transporter substrate-binding protein"
/transl_table=11
CDS complement(486549..487271)
/locus_tag="AUO97b_00635" /db_xref="SEED:fig|400667.82.peg.495"
/translation="MEQDQARIPKYIPIRDAIAHDIESGVLEAHAKLPSERVLSEQFQ
TTRVAAREALLALETDGLIYRLDRRGWYVRSPRIIYHPQSTKNFNQFVIEQGYEPSTE
VISSELTQATSWDAKHLKVEKGHPIYSVWRRRCINGRPVLVEYLRVNAELFPEFLTHD
LKQSITLLMANQYNCHITRADINLYPTALSEQQAKALHVNVGALGLYICRSNRNEEGV
ITDVDQEYWLHDVLDLHFEARA" /product="2-aminoethylphosphonate uptake and

metabolism regulator" /transl_table=11
CDS 488581..489810
/locus_tag="AUO97b_00636" /db_xref="SEED:fig|400667.82.peg.496"
/db_xref="GO:0008784"
/translation="MQFAHAAPLLTSHITNEVSPAPTANAWIEVDTQAFEDNIKRTNQ
LLNGKTQLCVVMKANAYGHSIDLLMPSVMKLNVSCIGITSNAEAAMVRKHGYKGKITR
LRTATDSEIINAHALNIEELLGNYDQATRISKWAEANNTTVHYSLALNSGGMDRNGLE
MSTAQGKEQAVAITKLPNLKIDGIMTHYAVEDEKYVRERLAQFNEQTAWLIKKAKLNR
KELTLHTANSFATVNVPESHLDMVRAGAIIYGDFPNHPEYKRMMAFKTQIAAVNNYLK
GSTVGYDQTYTLTRDSKLANLPVGYSDGYRRSFSNKAFVLVNGQKAPVVGRVSMNTVM
VDVTDIPEAKAGDEVVLFGRQGNAEVQSSDLTSLTGHILTEAYTPWSNSNPQVIKPIQ SPQQVILTNSKVAATAE"
/product="Arginine racemase (EC 5.1.1.9) @ Lysine racemase (EC 5.1.1.5) @
Ornithine racemase (EC 5.1.1.12)" /EC_number="5.1.1.1"
/transl_table=11
CDS complement(489959..491173)
/locus_tag="AUO97b_00637" /db_xref="SEED:fig|400667.82.peg.497"
/db_xref="GO:0018659"
/translation="MDILKTQVAIIGSGPAGLLLGQLLYKAGIDHIIVEQRSAEYVAS
RIRAGILEQVSVDLLKQAGVDQNLKEKGLPHSGIEILTNGELHRVDLAALTGGKQVTV
YGQTEVTKDLMAAREAAQLTSFYEAHHVQVKDFYTAPKVEFEYQGKTFQIQCDFIAGC
DGYHGVCRASVPEDKIKTFEKVYPFGWLGVLADVPPVADELIYVQSERGFALCSMRSE
TRSRYYLQVPLTDHVEDWSDEKFWDELKNRLDPESREKLVTGPSIEKSIAPLRSFVTE
PMRFGKLFLAGDAAHIVPPTGAKGLNLAASDIAYLSSALIEYYAEGSEHGINEYSEKC
LQRVWKAERFSWWMTHLLHRFETESEFDHKIKQAELSYVLGSIAGKTTLAENYVGLPY EIKQIDSFKHAS"
/product="P-hydroxybenzoate hydroxylase (EC 1.14.13.2)"
/EC_number="1.14.13.2"
/transl_table=11
CDS 491315..492130
/locus_tag="AUO97b_00638" /db_xref="SEED:fig|400667.82.peg.498"
/translation="MPSLDAFLEHPNSHEKIRQDDYIAGLAKGLALLEAFGTDRQRLN
VTQVAERTGISRTAARRYLKTLKYLGYLETDEHYFWLTHRVLRFSSSYLSSAHLPKVA
QSFLNLLCAQTSLTFSIVVLDDNEVVPIARSYLPQQDNLRVSPYGMHLGNRLPAHATS
TGKVLLAALPEEAQHTWVKNYGLKRLTPFTIVDESKFFEVLKGISLSDYCLSKEEHEL
GVIAIAVPVFNAQGQAVAALNCMSQTNRVQEDYLVQQILPLLRNTANELRNVI" /product="Pca regulon
regulatory protein PcaR" /transl_table=11
CDS complement(492186..492782)
/locus_tag="AUO97b_00639" /db_xref="SEED:fig|400667.82.peg.499"
/db_xref="GO:0006118"
/translation="MAKILVLKSSIMGEGSQTNRLIDVMLEHRKDQGLQDDITIRNLA
EMNLPVLDLEIFQALRGAENVNQDIQRIVALSDELIAELKSADLLVIGAPMYNLNVPT
QLKNWFDLVARARQTFRYTETYPQGLVEGVKAVVVSSRGGIHVGQETEAVTPYLKAVL
GLMGIHDVDFIYAEGLDMQAYRSNALDLASQQVKEFAI" /product="FMN-dependent NADH-
azoreductase (EC 1.7.1.6)" /transl_table=11
CDS 492881..493786
/locus_tag="AUO97b_00640" /db_xref="SEED:fig|400667.82.peg.500"
/translation="MQHELSAISIFVTVVEAGSFVKAAEQLHLTRSAISKNIARLEDQ
LGVALFKRTTRSLSMTDEGALFYEHSRRALSEIQNAAALLDQRKINATGRLRMSVPVL
FGQLFAAPLMVKFAQQHADLQLEISFNDRNVDLIEEGFDLCIRIGALPDTTQLVAKPI
GEHRMLLCASPDYLNKAGPVEHIEDLDQHATIADPHFGQMQKWRLQKENDEPLFIKPK
AKLLLNDLQAIKNAALAGAGIAWLPDWLIQNELQDGKLVQVLDAMSSTAFPIHLVWPT
LPFMPLKTRLAIDYLAQHLPPELNK" /product="Transcriptional regulator, LysR family"
/transl_table=11
CDS complement(493990..494562)
/locus_tag="AUO97b_00641" /db_xref="SEED:fig|400667.82.peg.501"

/translation="MQNKSNDNAPRHFNLTARILHWLMAAAILAMLFIGVGMTTSMTW
RPWLIDIHRPLGIAILLLVIIRLINRLCYPIPPLPPTVPRWQAFLAHASHWLLYILMF
SLPLLGWATLSAGNWPVTLFPGWDLPPIAPTNSMLYAWFRTLHGILAWLLFAVVIGHL
SAALLHAWIYRDGVFSSMALGSSSKKENID"
/product="Cytochrome b561"
/transl_table=11
CDS complement(494575..495639)
/locus_tag="AUO97b_00642" /db_xref="SEED:fig|400667.82.peg.502"
/db_xref="GO:0004096"
/translation="MNNLNFETLKKYWLRFVGIAVIAGALVLAFAWTAGLFGHRTTSK
TFLGDTLKNFDPGYRRAHGKGICFDGTYHSNGAAAALSKATVFAKSDIPAIGRFSIGS
GNPHAADNSTATVSMALLLSPADHSQWRMKLNNFPYFPTRNAEGFLAQQKAFKPVPST
GKPDPALVEAFLKEYPEARKSIEQKAKMPLTGSFSGAEFYVVNAFILRAANGQEQPVR
WSMRPHSKFISLTKEQRDQADHDFLFKDIKKRLAEGPLYWDLVLQLAEPGDPVNDPSQ
PWPKDRKEVIAGTLEVKNVTDQVNGACRDINFDPTLVPPGIELSDDPVLAARAAIYSQ
SYNARLREIGFGKATDAVGK"
/product="Catalase-like heme-binding protein" /EC_number="1.11.1.6"
/transl_table=11
CDS complement(495758..496384)
/locus_tag="AUO97b_00643" /db_xref="SEED:fig|400667.82.peg.503"
/translation="MKFLKFLTFSTILTLSAHSYATVGGGQKIEVLGYQQKEKKLYVL
RHYEDGRGRLPQLYYYLLNSKSPDKLIEVKSLYINPKTHKIDYDQDSTAFNKALNKIK
KNLTPLIVSNSKTVKIQTLKTHQNQVSSWFDPSGKITQYKTEYVVKSPSLQSKTHVAV
HYSKAIKISQNYSVPKHNKRLVVVKYLGVPEETGYDIEDPVLLLPIKK" /product="hypothetical
protein"
/transl_table=11
CDS complement(496586..497296)
/locus_tag="AUO97b_00644" /db_xref="SEED:fig|400667.82.peg.504"
/translation="MCQAQSLWLCCAFFSPKKGTGFFISTYNKNFYNGKTMPNLVLPT
RTLYVVNKAIDLFHHRGFHLIGVDRIVKESEITKATFYNYFHSKERLIEICLMVQKEK
LQEQVVAMVEYDLSTPAIDKLKKLYYLHTDLEGPYYLLFKAIFEIKNSYPKAYQTAMR
YRTWLKNEIYSQLRVLNADTSFNDAKLFVYMVEGTIIQLLSSDGAIEQEKMLDCFLNS
FVRNFSPCMFTHVDASKC"
/product="Transcriptional regulator, AcrR family" /transl_table=11
CDS 498270..499466
/locus_tag="AUO97b_00645" /db_xref="SEED:fig|400667.82.peg.505"
/translation="MLFYDFLKEGFDFYFQEFKKKNINNYDKKYHTNYISIKEFEKDI
KVNSENIFNSIKNNNYKFQSLYPIVIENKKELNKKPRLICIPTVRDRLIQMILIYYIS
IHLKNELAVLKSQDFSVSGVGILKARQKAKDLRNTKPYVLKTDISSFFDNIDRAKLLN
EIKDVMPPDILYLFQSIIYCDPSIPYEYNKDYKKLIYSKLRKGVRQGMPISPLLAYFY
LNDFDEWLIKKKYKHVRYADDLIFFLDSEKQCKEVYREVSQELLKLNLTLPTLEENTK
TQIISPKETVNFLGLDLRYENEKYNWYIPPHVIENVKYNLLHLTNIQNNLKMKLNFSK
TISRMEQIISGYQHCYSDADSLNLKDFNKILILEKQKAINLLFKNLGIDISTVHSQYL KYLLDN"
/product="hypothetical protein"
/transl_table=11
CDS complement(499519..500352)
/locus_tag="AUO97b_00646" /db_xref="SEED:fig|400667.82.peg.506"
/translation="MLNNIDTLLLSNSTNESHYKVALEEVSKLLENIQEQGDEDLSNF
LWKLKTILIIKDRYIKTFFLLKDKKHYEAWVLLERIEIDISFLEKNVDEDFIKKYKLD
FYKEIVESWQSLFPYKIFFSIGATIKQYTCSICGHVIRPRNKCIHKKGKLYNGKLCVH
VADGGCELKEISMVKNPVQKSCIPMLDYDYSAVDFISERLQSPFDYWKPFKTKKLIDR
SEFNTVDENDICPCKESKKIFKECCFNKEKIEFPHIHIHFSKSPPSNLATSLIKIGGK L"
/product="hypothetical protein"
/transl_table=11

CDS complement(500342..500839)
/locus_tag="AUO97b_00647" /db_xref="SEED:fig|400667.82.peg.507"
/translation="MISDQLLKVEETDLNFSTLKNEKPLILNRDVSVLILPSVRQNDA
FHTGTIDIFSYLQEELGENEVELFAFDEEYQELALHSKAHWLGVFLVAQVAVPIFTNV
MSDYISNELQAKSNDEIEVNIIIEKNNDKNIKVAYRGKAEHINSVLDKVNELSRDNEV NRHAK"
/product="hypothetical protein"
/transl_table=11
CDS complement(501018..501401)
/locus_tag="AUO97b_00648" /db_xref="SEED:fig|400667.82.peg.508"
/translation="MQKMENTAVDQFAELLGVQVLHRSFDEARCQLNVKDEHMNALGG
VHGGVIFSLADIAFAMACNAGDAPYTGLQADIRYMSGAKDKVLFATATKVGSSKKFAH
YEVVITDGLNNRVALFTSTCYRLAP"
/product="Acyl-coenzyme A thioesterase PaaD (Pse.pu.) (E. coli PaaI)"
/transl_table=11
CDS complement(501477..502082)
/locus_tag="AUO97b_00649" /db_xref="SEED:fig|400667.82.peg.509"
/translation="MPCYSIDGVIPVVSPEAFVHPTAVLIGDVIIEAGVYVGPFASLR
ADFGRIHIKQNANIQDSCTVHGFPQSVTLVEEMGHIGHGAILHGCRIGKNVLVGMNSV
ILDYAEIGENTIIGANSLVKTKDVIPANVLAMGSPAKVARDLSEQEKKWKTRGTQEYM
ELAQRCLNSMQEVQPLTSELDDRLTYKDFNSSNYQIKQDSV" /product="Phenylacetic acid
degradation protein PaaY" /transl_table=11
CDS complement(502069..503028)
/locus_tag="AUO97b_00650" /db_xref="SEED:fig|400667.82.peg.510"
/translation="MSAKIQQIIDSVVKNETLSGTSLISTIFGDSVLHRGGNISLASL
IQLLELFGFNDRAVRTSVFRLVKNDWLCSDKIGRTSFYRITDSSRSTYLQAEQRIYND
QMKEWDHYWDLILMSSLDTENKALLKKELEWLGFANISTNLMAYPGCNRIELQRLLVD
LNMSEQVVVFKAETLQLFNNSVDTIGRMLRTNWPIDELRQRYLQFLDIFREIGVLLMQ
ENEQLEPVQAFQIRTLLIHYYRRILLKDPALPLELLPTDWPAISARTLSMNIYKKVFE
PADEYFLSVAATAEGPMPNATAHYWRRFGGLVATNERLNHALL" /product="Phenylacetic acid
degradation operon negative regulatory protein PaaX" /transl_table=11
CDS complement(503088..504377)
/locus_tag="AUO97b_00651" /db_xref="SEED:fig|400667.82.peg.511"
/translation="MDSLTMDNVEQLTLAELRELQTKRLKQTLTFVYENSPVYKKKFD
DAGVHPDDFVTLDDLAKFPFTTKQDLRDNYPFGMFAVPQEQIVRLHASSGTTGKPTVV
GYTQKDINTWSDIVARCLRMAGLTAKDMVQVAYGYGLFTGGLGAHYGAERLGATVIPM
SGGQTEKQVQLIQDFKPTAIMVTPSYCVSIIEKLEQQFGTARNTSLKVGIFGAEPWTN
ELRREIEERLNIKALDIYGLSEVMGPGVAMQCLGEGEGLTIWEDHFYPEIINPETGEV
CKDGELGELVFTTITKEGLPIIRYRTRDLTRLLPGENLAMRKMDKIVGRSDDMLIIRG
VNVFPTQIEEQILQVPHLVPNYEILVTKKGHMDTLHIRTEMCHNCTIQANQLAQELMK
RIKTMIGISVTVEVVTEATLPRSEGKAKRVIDMRQIA" /product="Phenylacetate-coenzyme A
ligase (EC 6.2.1.30)" /EC_number="6.2.1.30"
/transl_table=11
CDS complement(504418..505626)
/locus_tag="AUO97b_00652" /db_xref="SEED:fig|400667.82.peg.512"
/translation="MEDVLICDFIRTPIGRYAGALSAVRADDLAALPIKYLKDKHPNL
PWNTVDEVFLGCANQAGEDNRNVARMATLLAGLPDTVPAMTVNRLCASGLDAVGLAAR
SIKAGEAQFVLAGGVESMSRAPFVQAKPTEAFSRTPEIYDTTIGWRFVNKQLKAQYGT
DSMPETAENVAEKYQISREDQDAFALRSQQKTAAAQQNGFFNDEILPVEITDRKKNVV
VVNRDEHPRETTLEALAKLKAPFKKEGGSVTAGNASGVNDGAACVLITNREFANTYGL
KPLARVIGIASVGVEPKYMGIGPVPAVQKILKQTGLTLDQMDVIELNEAFAAQSLACM
RELGLKDDDERVNPNGGAIALGHPLGMSGTRLVITATRELKKRGGRYALCTMCVGVGQ GVALILENLN"
/product="3-oxoadipyl-CoA thiolase (EC 2.3.1.174) @ 3-oxo-5,6-dehydrosuberyl-
CoA thiolase (EC 2.3.1.223)" /transl_table=11
CDS complement(505639..507177)

/locus_tag="AUO97b_00653" /db_xref="SEED:fig|400667.82.peg.513"
/translation="MTDLDLSRARIAVIGSGTMGIGIAQLAIVNGHTTVLYDLDAQKA
QQAVQGLGQTFKKLVEKSKLTQQQADEALARLTVVNQVEALRDADLVIEAVVEKKEVK
QSLFKQLAEICSAQTIFASNTSSISVTAISAGIAHPERVVGLHFFNPAPVMKLVEIVQ
GLKTPNSLCLALKNLMLDWKKIPVLTKSTPGFIVNRIARPFYAEGFRALQEQVTSYDQ
LDYALKQCGGFAMGPCELTDLIGQDVNFSVTQSVYQEFFYEPRYRPSLIQKELVDAGA
WGRKSKQGFYSYNEKNQYEAFQPQAVVAKPVDAHIQLKGTWSQLPAFLTRLGLKSGSA
SDDNILQIDDIDLRLSQGESANIHYLSRKVVLMDWHHDFEHAEALVLSHNELCETNDL
AKVEAYFAQFGMSVMWIKDHPALLTLRTIALLINEACEASLHGVASLEDIDNAMKYGV
NYPKGPYQWLTQMGGAYVLQTLNNLYALYGEEKYRASIYLKQYVAKTQNIATQYSRDM TEFA"
/product="3-hydroxyadipyl-CoA dehydrogenase" /transl_table=11
CDS complement(507180..507971)
/locus_tag="AUO97b_00654" /db_xref="SEED:fig|400667.82.peg.514"
/translation="MDYQNIIAEEKNGVGYLTFNRPKALNSFNVDMHREVAEVLNQWT
KNPEVRCVVISGEGRGFCAGQDLGDRVVDPNAEAPDLGYSIETYYNPLIKTIVNMPKP
VICAVNGVAAGAGANIALACDLVIAAKSANFVQAFCRLGLVPDSAGTWFLPRAVGHAR
AMGLALLGDKLPAETAKEWGMIWDVVEDAELKTKVTELAERLAKQPTFGLSLIKKAIH
QSSNNTFDEQVLLERDLQRIAGRSEDYREGVQAFMNKREPNFKGR" /product="1,2-
epoxyphenylacetyl-CoA isomerase (EC 5.3.3.18)" /EC_number="4.2.1.17"
/transl_table=11
CDS complement(507971..508744)
/locus_tag="AUO97b_00655" /db_xref="SEED:fig|400667.82.peg.515"
/translation="MQEFIKASTATDGVLLLTLNRPEKRNALNNATLQCLCELLEEAE
HNAQVKAVVLTGNAQCFAAGADLSELAAMDAVSLQLDIRPKLWQKIDAFSKPLISAVN
GYALGAGFELVLHSDMVVCGENARFALPEITLGMLPGAGGTQRLARLVGQQLTMRWAM
TGEMISAKQAEQHGICSQVVPTELTVEYAVQLAAKIAKQAPLAIRVIKQSIKSIHETT
LSQGLKFERQNFVWLAATKDRNEGINAFFEKRKPVFRGE" /product="2,3-dehydroadipyl-CoA
hydratase (EC 4.2.1.17)" /EC_number="4.2.1.17"
/transl_table=11
CDS complement(508753..509814)
/locus_tag="AUO97b_00656" /db_xref="SEED:fig|400667.82.peg.516"
/translation="MSQFVPLKVKSITPQTDQAICIAFDVVPEQQEQFQFQPGQHLTI
RHLTEAGEIRRCYSICSYAGKEDISIAVKKIDQGQFSNWANDHLKVGDVLEVMPPQGV
FFQKAAKMGGQNYLGVAAGSGITPILSIIKQVLFEQPEANFTLLYGNRSWKQTMFAEQ
IMDLKDQFKERFQLINIFSREFNDSELMNGRIDAEKLKQLFDYEVLETNFDHVFACGP
DEMMNAVENTLPNFGIAKERIHTERFHTGQARKRSVEADANRKEEKVNIILDGRELIV
SVAQDDESILDAALRAGADLPYACKGGVCATCRCKVLSGEVDMFLNYSLEEDEVEKGY
VLSCQTLPKGSNVRLSFDE"
/product="1,2-phenylacetyl-CoA epoxidase, subunit E (EC 1.14.13.149)"
/transl_table=11
CDS complement(509838..510338)
/locus_tag="AUO97b_00657" /db_xref="SEED:fig|400667.82.peg.517"
/translation="MQMIRHCIDQCWDVLQTVSDPEIPVLSVVDLGMIRGVEINDQQE
IIVRLTPTYSGCPATDMLKAQIVEAFTAEALTPVKVMVDLSEAWTTDWMSDAGKKKLQ
VYGIAPPEGLAHQCGTHVHLNDGVECPRCKSRHTRLLTEFSSTACKALYKCQDCLEPF DYFKCI"
/product="1,2-phenylacetyl-CoA epoxidase, subunit D (EC 1.14.13.149)"
/transl_table=11
CDS complement(510355..511110)
/locus_tag="AUO97b_00658" /db_xref="SEED:fig|400667.82.peg.518"
/translation="MNHSVLSKFLLHIGDSQLVLSQRLAEWCGHAPELEIDIALANIG
LDLLGQARNFLTLAGQYDEAKRDEDQLAYFRTEREFLNLLLCEQPNGDFAQTIVRQWL
MDHYHLHLFTALAQSSLPELSALAVKSLKEVKYHIRFSTSWMERLSLSTDEAHQRVQD
GLNNLWRFTAELFELNADEQALVAEGVIPDFSNEKEQWNQSIADELKRFELNVAVNGA
YRRGAKQGLHTEHLGYLLAEMQCIQRTYPGMTW" /product="1,2-phenylacetyl-CoA epoxidase,

subunit C (EC 1.14.13.149)" /transl_table=11
CDS complement(511123..511413)
/locus_tag="AUO97b_00659" /db_xref="SEED:fig|400667.82.peg.519"
/translation="MEDKNNWSLYEVFVRSKQGLSHRHVGSLRAPDDEIALQHARDVY
TRRNEGISIWVVRSELIKSSQPDEKAEFFDPSLDKVYRHPTFYHIPDGIEHM" /product="1,2-
phenylacetyl-CoA epoxidase, subunit B (EC 1.14.13.149)" /transl_table=11
CDS complement(511446..512384)
/locus_tag="AUO97b_00660" /db_xref="SEED:fig|400667.82.peg.520"
/translation="MENNYQKFEQNIANEITIEAKDQMPDAYRKTLIRQIGQHGHSEI
VGMLPEGNWITRAPTLKRKAVLLAKVQDEAGHGLYLYSAAETLGADRDDMMEKLINGK
MKYSSIFNYPTLTWADVAAIGWLVDGAAIVNQVALCRTSYGPYARAMVRICKEESFHQ
RQGFEAMMALANGTPEQKQMAQDAVNRFWWPALMMFGPSDKHSPNSAQSMAWKIKRFS
NDELRQKFVDNTVPQVLQLGLEVPDPDLKFNEETGHYEFGEIDWHEFNEVIAGRGPCN
HERIEARRKAWENGKWVRDAAVVYAKKQQLEANKVA" /product="1,2-phenylacetyl-CoA
epoxidase, subunit A (EC 1.14.13.149)" /transl_table=11
CDS 512650..514752 /locus_tag="AUO97b_00661" /db_xref="SEED:fig|
400667.82.peg.521" /translation="MLEQAHLEGTSTDTDFQQSGLQLPHLASYVYGTWHSSDEELRTV
YHAITGESIYAVSSHGIDMKRVVQYAKQIGSELANWTFHQRANALKQIAQHLLERKED
FYKLAYATGATRKDAWIDIEGGIQTLFAYSSLVRRELNDEKIITEDSWIQLSKNGTFG
AKHILSPKAGVAVHINAFNFPIWGMLEKIAPTLLAGVPCIVKPATDGAQLTQAVVKAI
EETHVLPKGSLQLICGQTYDLLEQLGPQDCVTFTGSAYTGQKLRNHPHLNKYSIPFSM
EADSVNSAILSPDANEETVDLFVREVFREMTTKAGQKCTAIRRAFVPENLLATVQEKL
TAKLAKVVVGDPQKEETTMGALASIKQKHDVAEKVAELSKDAKIVFGGNDSFTFNADH
PEKGAFFAPTLLVCEQPLQATNVHTTEAFGPVCTLMPYQNIEELADLVSRGEGSLVAS
VVKNNDENIEQIIQKIAPWHGRVHVLDAESAKESTGHGSPLPHLVHGGPGRAGGGEEL
GGIRAVKHYMQRTAIQGSPNSLTQITHSWTAGSKVNEDRVHPFKKSFDELVIGERLLT
ARRTVTEADIVNFACLSGDYFYAHTDKIAAAESFFGERVAHGYFIVSAAAGLFVDAAQ
GPVIANYGMDNLRFVEPVKIGDSIQVELTCKQKTPKPQKDPSQPAHGVVVWDIKVKNQ
RGELVATYDILTLVAREA"
/product="3-oxo-5,6-dehydrosuberyl-CoA semialdehyde dehydrogenase (EC
1.2.1.91) / 2-oxepin-2(3H)-ylideneacetyl-CoA hydrolase (EC 3.3.2.12)"
/EC_number="1.2.1.3"
/transl_table=11
CDS complement(514796..516175)
/locus_tag="AUO97b_00662" /db_xref="SEED:fig|400667.82.peg.522"
/db_xref="GO:0003941"
/db_xref="GO:0003941"
/translation="MFISVFDLFKIGIGPSSSHTVGPMRAAYSFVDDLLKQNDLQATA
RVQVKLYGSLSATGVGHATDKAVLLGLMGFDPEHIDTQVSTSLIEDVLENNAIQLNQQ
KTISFDYKHDVLFLDESLPYHPNAMELIAYNEAQEILYAETYYSVGGGFIVSQRQLAT
TQTETNDVKVPYPFHSAAELLSLCKTHHLSVSELMLENEKSWRSETEIRSKIMEIWKV
MQQCIHNGLINEGILPGGLNVKRRAKKIHDKLLNKKNSNLIVTTFHAMEWVNLFALAV
NEENAAGGRVVTAPTNGAAGIIPAVLYYYVTFSKDFSEDKVVRFFLSAAAVGILCKLN
ASISGAEVGCQGEVGSACAMASAGLAEILGASPEQVEHAAEIGLEHNLGLTCDPIGGL
VQVPCIERNAIAAVKAINAAQMALHDDDEHFVTLDKVIQTMKETGKDMSEKYKETSKG GLAVNAIEC"
/product="L-serine dehydratase, beta subunit (EC 4.3.1.17) / L-serine
dehydratase, alpha subunit (EC 4.3.1.17)" /EC_number="4.3.1.17"
/transl_table=11
CDS 516625..518196
/locus_tag="AUO97b_00663" /db_xref="SEED:fig|400667.82.peg.523"
/translation="MKSQFKKQLSLMDLTFIGLGAIFGSGWLFSASHVASQAGPAGIL
SWIIGGFAVLILGIIYCELGAALPRAGGIIRYPVFSHGPLQGYLLGSVTVIAFSSLIA
IEVVAAREYAAAWFPSLTAVHDGVRTPTTIGWLFQFALLCVFFALNYYSVKTFAIANN
LISALKFAVPVLVMVALLYHFKPANFSMTEFAPMGAHGVQGAVSAGGIIFAYLGLTPI

ISVASEVKRPQFTIPFALILSVVLATIIYVVLQVAFLGAVPTDMLANGWSGLSDKFPL
PYRDIAMLLGLGWLALLVVSDAIISPSGCGNIYMAATPRVIYAWSNSNTFFRIFTRVD
PKSGIPRYALWLTFALSVFWTMPFPSWDKLISVVSAALVLSYALAPVTVGALRRNAPE
LERPFYVKGFTVLGPLAFVIASFIVYWSGWNVISWLLGAQIVLFALYVIFKRYVPTQE
VSLAQQLKSSTWLLVYYILMILASYLGSFGDGASHLLAAPFDTLFVMVISLGCYYWGI RSGLPKALIKNDDEA"
/product="Amino acid permease in 4-hydroxyproline catabolic gene cluster"
/transl_table=11
CDS 518349..519929
/locus_tag="AUO97b_00664" /db_xref="SEED:fig|400667.82.peg.524"
/translation="MLNITGQNFIAGQRSSAGSKFVLSYDAATDEALPYQFAQATPEE
IDQAAQAAALAYPAFRQTTPEQRAVFLETIASEIDALDDQFIATVCQETALPEARIRG
ERGRTTGQLRLFAQVLRRGDYLGARIDLALPERQPLPRPDLRQYKIGVGPVAVFGASN
FPLAFSTAGGDTASALAAGCPVIVKAHSGHMATAESIANAICSAIEKCAMPKGIFSMI
YGQGVGEPLVKHPAIKAVGFTGSLKGGRALCDLAAARPEPIPVFAEMSSINPMILLPE
ALKVRGDKIATELSGSVVLGCGQFCTNPGLIIGIKSPEFSQFLDHFKAAMAQQPPQTM
LNKGTLRSYEHGLKELLAHDKIEHLAGQPQQGQQAYPQLFKADVSLLLEHDELLQEEV
FGPTTIVIEVESAEQLALALSGLRGQLTASLIAESQDFENFATLIPLLEEKAGRLLLN
GYPTGVEVCDAMVHGGPYPATSDARGTSVGTLAIERYLRPVCYQNYPDHLLPLALQNA
NPLGIARLVNGEMSKAAL"
/product="2,5-dioxovalerate dehydrogenase (EC 1.2.1.26)"
/EC_number="1.2.1.26"
/transl_table=11
CDS 520085..521374
/locus_tag="AUO97b_00665" /db_xref="SEED:fig|400667.82.peg.525"
/translation="MTTTLKKVVAASMVGSVAEWYEFFLYGTASALVFGELFFQQTGN
AIDGILAAFALYAVGFLARPLGGLVFGHYGDKIGRKKLLQISLIIVGITTFLMGCIPT
FHQIGYWAPTLLVILRLIQGFAFGGEWGGAVILVSEHSPDDRRGYWASWPQTGVPLGN
LVATLVLLLLSKNLSPEQFLDWGWRCAFWFSAVVVLIGLWIRKNVDDAEVFKEAQAKQ
QLLEKQQLGIIEVLKYHKKSVIAGIGARFAENILYYMVVTFSISYLKLVVHKDTSQIL
LLMFGAHLIHFFIIPFMGHLSDIFGRKPIYLIGAVLTAFWGFVGFPLMDTGNDWLIML
AIVLGLFIESMTYSPYSALMTELFPTHIRYTALSFCYQVAPIMAGSLAPLIALTLLKE
FNSSIPISLYLVAASLISIVSILLVKETKGRSLAFKD" /product="Uncharacterized MFS-type transporter" /transl_table=11
CDS complement(521441..522193)
/locus_tag="AUO97b_00666" /db_xref="SEED:fig|400667.82.peg.526"
/translation="MTLVQIPKLTTTLEDFLKNLESIEPLFDALSSVVFFIKNTEARY
VFVNQTLVNRCGLKDKTAILGKTSSEVFPHSLGKIYTSQDLQVIRRGKKLTEQLELHL
YAKNQSGWCLTYKEPLFDADGNLVGIAGISNDLNVPENTHPAFYKMVQVEEYIKKNYA
ETITLAHLTAIAGVSVAQLERYCKKIYHLTPRQMISKIRLQVATELLATDLPITQIGL
RCGYTDHSAFCRQFKLHTGMSPTLYRASIKNI" /product="Transcriptional regulator, AraC family" /transl_table=11
CDS complement(522238..523500)
/locus_tag="AUO97b_00667" /db_xref="SEED:fig|400667.82.peg.527"
/translation="MEKQYDVVVVGAGPAGLSAAIAAAESGGRILILDNNPQAGGQIW
RAGPIFPVPEMAQQKYQQIKAHSNIEFMFGAKIVAAPFAGQLLVERPHDSLNLSYRQL
ILCTGARELFLPFPGWTLPGVTGAGGLQALIKAGTPVKNERIVIAGSGPLLLASADTA
KKAEAQVLYVAEQAASSSVRKFALQLWRWPAKIIQALSLPYRLYQPDSYVVEAIGQER
LERVRLQTPKGIIEIECDRLACGFGLVPNTQLGQLMGCQIKDNAITVDDYQRTSQPQV
WAAGECTGFGGSELAMVEGSIAGYAAIGQNEKARQLFAQRQRYRLFAHLLEKSFNLRA
ELKTLARSETIFCRCEDVTFDKVAKRSSWIDAKLHTRCGMGACQGSTCATAAACLFDW
QLPNSRPPLLPTRVKSLMAMSTSPLNQK" /product="Putative oxidoreductase in 4-hydroxyproline catabolic gene cluster" /transl_table=11
CDS complement(523500..523730)
/locus_tag="AUO97b_00668" /db_xref="SEED:fig|400667.82.peg.528"

/translation="MIQLFINGQSVCVIEGTSVAAALAQAKSYSRLSVCGEKRTPFCG
MGVCQECRVNINGLRVLACQTLCQQDMKVETQ"
/product="Hypothetical, similar to sarcosine oxidase alpha subunit, 2Fe-2S domain" /transl_table=11
CDS complement(523731..524834)
/locus_tag="AUO97b_00669" /db_xref="SEED:fig|400667.82.peg.529"
/translation="MRTDAIVIGAGIVGAACAYELARQGLNVQVLDARVGGATAAGMG
HLVIMDDLEAELKLSHWSVQLWHELGHELSEDCAYRQTPTLWLASSPEEMQIAEEKYQ
RLTAQGVRCQLRNADEVHQLEPHLKQGLYGGLEVFDDGILYAPCAAEWLLKKFPHKVQ
VQQTKVIHIEENRVQLSDGTWLEAAHIVLANGIHATDFFPELPIEPKKGHLAITDRYP
ELNVKHTLVALAYAASTQATSGISVACNIQPRPTGQLFIGSSRQFNTVDPTVEPEVFT
RVLKEAVDYFPALADLNVIRAWTGFRAATPDGIPVIGQHPAFQSVYLAVGHEGLGVTT
ATGTAKLIASHICGLTFDIDPEPFLPHRFIGAA"
/product="D-amino-acid oxidase (EC 1.4.3.3)" /EC_number="1.4.3.3"
/transl_table=11
CDS complement(524831..525763)
/locus_tag="AUO97b_00670" /db_xref="SEED:fig|400667.82.peg.530"
/translation="MKTVKILDSHTGGEPTRLVLEGFPDLGTGDMESRRKILSEQYDH
FRRATMLEPRGNDVLVGALLCKPVNPKASAGVIFFNNTGYLGMCGHGTIGLVASLAHL
GKIQVGTHLIETPVGDVEATLHEDHSVSVRNVPAYRYKKAVEVDVEKYGKVTGDIAWG
GNWFFLINDHGQRVASDNLDQLTEYAWTVRQALTAQGITGKDGQEIDHIELFASDTEA
DSKNFVLCPGKAYDRSPCGTGTSAKIACLAADGKLEPGKLWKQASIIGSQFIASYEQA
GEYVIPTIRGEAYMSAEATLFMDENDPFAWGIQL" /product="4-hydroxyproline epimerase (EC 5.1.1.8)" /EC_number="5.1.1.8"
/transl_table=11
CDS complement(525932..526576)
/locus_tag="AUO97b_00671" /db_xref="SEED:fig|400667.82.peg.531"
/translation="MPSVSEVIVKYIREAIIAGHIAEGEPIRQDEIAKLFNVSKIPVR
EALKQLEAEGLVEFIKNRGAIVTRISEEELAQIFEVRVMLETKLIQLAVPNMTEETYK
KADEICNAFIDNMDIGSWTTLNWQLHACLYEPAQRPYMLGLIRSIYDKIERYLRLQMN
VSEGKSRANDEHKEIVKACKAKDVDLAVKLIEEHINGVCQSLYEHLPHHKNAKK" /product="Predicted regulator PutR for proline utilization, GntR family" /transl_table=11
CDS 527033..527941
/locus_tag="AUO97b_00672" /db_xref="SEED:fig|400667.82.peg.532"
/db_xref="GO:0016020"
/translation="MSTPTEAKFSLTYYTGFLSVLFAAFLWGTAGTAASFAKGISPLV
IATISVGFGGLIHTFLSLKAIKKDYRKLFDYKTKVLIAVIVSILCPFAFYSSVSLAGV
SIGTVISIGCAPLFSVMLEWLLDKRKLSLKWLISFILGFLGVVLLSYAGEHSHQHQGV
QSERLLGVFFGLLSSLSYATYSWIMKNLIHKGINSRAAMGVIFGISAVLLLPTLLITA
THLFNYPNNIWVALYIPIVPMFLGYLFFSFGLKRIPASQAMTLALVEIPVAALLAVVV
VGESLTMNSYLGLVLIFLCVIVLTKK"
/product="Permease of the drug/metabolite transporter (DMT) superfamily"
/transl_table=11
CDS complement(527999..528898)
/locus_tag="AUO97b_00673" /db_xref="SEED:fig|400667.82.peg.533"
/translation="MTPCTPNREPDFDALVKKGHELIEAGMSAVVYCGSMGDWPLLTE
AQRQEGVARLVAAGIPTIVGTGAVNSKEAVSHAAHAEKVGAQGLMVIPRVLSRGASPT
AQKAHFSAILKAAPSLPAVIYNSPYYGFATRADLFFELRREFPNLIGFKEFGGAADMR
YAAEFITSQDDSVTLMAGVDTQVFHGFVNCNATGAITGIGNALPKEVLQLVDLSKKAA
AGDAKARRLAQELSSALEVLSSFDEGTDLVLYYKYLMVLNGDKEYTLHFNETDALSES
QRKYVETQYELFRTWYRNWSAEI" /product="1-pyrroline-4-hydroxy-2-carboxylate deaminase (EC 3.5.4.22)" /EC_number="3.5.4.22"
/transl_table=11
CDS complement(529794..530228)

/locus_tag="AUO97b_00674" /db_xref="SEED:fig|400667.82.peg.534"
/translation="MKKIIFLGLALVSLTACSSVQHKDSTPPKIGSPNPASQYCVEQG
GKLEIRNEANGQVGYCHLPNGQVVEEWKLFRDNQANCVSEEAQKLVGLSGLTDDQIKQ
KTKSEIVRKVAPGQPMTMDYRSNRVTVTIDPTSKKITQATCG" /product="Putative hemolysin"
/transl_table=11
CDS complement(530390..530818)
/locus_tag="AUO97b_00675" /db_xref="SEED:fig|400667.82.peg.535"
/translation="MLTVGEVAKRSGVAVSTLHFYESKGLIKSIRTNGNQRRFTLVVL
RYIAIIKIAQKVGIPLDEIKQALSKYPMGSKLTAQQWKELSLDWRKNLDERIQKLTRL
RDEMDWCIGCGCLSLEQCPLRNPNDVLGQEGAGPRILERP" /product="Redox-sensitive
transcriptional activator SoxR" /transl_table=11
CDS 531835..532404
/locus_tag="AUO97b_00676" /db_xref="SEED:fig|400667.82.peg.536"
/translation="MESLIESNINFSIFFYNPNIHPVKEYLIRKEENIRFAEKHNIPF
IDADYDTDNWFARAKGMENEPERGIRCTMCFDMRFERTALYAYENGFSLISSSLGISR
WKNMAQINDCGIRAASHYPDIQYWDYNWRKNGGSSRMIEISKREEFYQQEYCGCVYSL
RDTNRWRMSNGRDRIKIGVKFYSNAMEND"
/product="Epoxyqueuosine reductase (EC 1.17.99.6) QueH" /transl_table=11
CDS 532718..533176
/locus_tag="AUO97b_00677" /db_xref="SEED:fig|400667.82.peg.537"
/db_xref="GO:0008080"
/translation="MIQNETIEVSPVQQQDYTQWLEYWVAYQNFYQVNLPLHITKMTW
DRFFDEKEPIYCAVAKNKERILGFVTYMFHRSTWAENNYCYLEDLYVSPEVRGRHIGK
QLIEYVQKQATEHSCERLYWHTQESNQTAQKLYDWIAQKPGVIQYRMSLN" /product="Histone
acetyltransferase HPA2 and related acetyltransferases" /transl_table=11
CDS complement(533256..534413)
/locus_tag="AUO97b_00678" /db_xref="SEED:fig|400667.82.peg.538"
/translation="MTSSLKKIALLKQGLEKNSPFAAGRQGTLEAIEHLGYVQIDTIS
VVERAHHHILWSRVPDYDLSHLNSLVRERQIFEYWYHAASYLPMKDYRYALPAMMSVR
KGESRYFNRGDQHLMNEILARVRAEGKIRLRDIDKKNKESLGNWWNTGPGRRAFEQLY
MQGDLMICERNGMEKVYDLTERCLPENIDLSLPTLYEYAQYLFNNTLRAHGAFTWKQL
VHLKKNDLKETMRVVLKEQIDAGVVSAIKLADGQNLYVDVTAIEQQLDTDFGLKILSP
FDNSLIHRDRLTSLFEFDYRIECYLPAAKRVFGYFCLPILYQNELVGRIDCKAHRTVK
ELEVISLHLEKTVKDKEHFFFELDQELKRFAAFNQCSTVNDKVVKLIRSKL" /product="hypothetical
protein"
/transl_table=11
CDS complement(534547..535725)
/locus_tag="AUO97b_00679" /db_xref="SEED:fig|400667.82.peg.539"
/translation="MKQSGAFLARPMLILTVALVGLNLRPFMTSIGPLVSKIRAGTGL
SLQGIALLTLLPMMLMGLIAFIGPIVQSMVGERRVVLGALLTICIGCTLRFLFPTAEG
VIASAIIIGLGVALIQAAFPGIIKREFSQHIGPMMGLYSAMLMGGGALGAWLAPVISN
ITGLWSLGLAFFTIPAVLALVSTFIFLPSIVSSTAHLSPVKTLLKKPRTWLLMLCFGL
VNGGYSSVVAWLAPSFQDHGWSISQSGKLLALLALSQAASALSLPILARQYKDRRPWL
WFTLFMQFAGFAGLAFIPEFASVLWAICLGIGLGGCFTLTLIVALDHFEEPNNAGALS
ALMQGGGFLLAAIPPWFVAVLHDFTGTYKAGWIFHLCSVILVIVLVVRLAPRSYNKIH "
/product="Uncharacterized MFS-type transporter" /transl_table=11
CDS complement(535725..536207)
/locus_tag="AUO97b_00680" /db_xref="SEED:fig|400667.82.peg.540"
/translation="MKANQEFLRQAIELAYNNIEKGGRPFGAVIVKDGKVIASGVNQI
LTTNDPTAHAELLAIRVASQVLGTANLEGCSVFASGHPCPMCMAAMRLAGIKTVNYAY
SNEDGAPFGLSTAEIYADLVKPFAEQSMKIEYVPVRFEDRTDLYVFWKNYQAKQSGLK "
/product="Guanine deaminase (EC 3.5.4.3)"
/transl_table=11
CDS complement(536223..536891)

/locus_tag="AUO97b_00681" /db_xref="SEED:fig|400667.82.peg.541"
/translation="MRKSLIWINLIIAMGALGFTNVVNAKDEYPLHMAAANDDIQLIK
HILSQKTLIDARDETGSTALMVATRANNIHAAHMLIEAGADVNAKDNIQDSPYLYAGA
QGYLKILRMTLMHGADLKSTNRYGGTALIPAAERGHVETVRTLIAAGVNINHVNNLGW
TALLEAIILGNGKSNYQQIVALLLKAGANPNLADKDGITPLQHARTRGYREIEKLLLV AGAK"
/product="Ankyrin repeat family protein"
/transl_table=11
CDS 537043..537894
/locus_tag="AUO97b_00682" /db_xref="SEED:fig|400667.82.peg.542"
/translation="MFDPRLLRAFVTIFESGSFTAAADKLHLTQSTISQQLARLEESV
GHSLIDRTARPLQLTASGEYLIGYAQRILTLQQEAEAVLKDPAGSIPIRIGLPEDMMS
TAMAEVFGLFAQEHRTIRLDVTSGLSRNLTERYRNGELDIVIVKESAASADCYASFPE
EMAWFESENAITPLSEPIRLVTFPQGGLYRDEMFETLERERRRWYIAFTGSSLESVLV
SVETGLGISLLPVSTTKGRRVRRYKSLGEASPMVVSIYTWEKAGLISQLVDHMTTVLH DRFQSGS"
/product="Transcriptional regulator, LysR family" /transl_table=11
CDS complement(537897..538850)
/locus_tag="AUO97b_00683" /db_xref="SEED:fig|400667.82.peg.543"
/translation="MMIFLIFFCFACLAIGLAFLMSYELRNKGRTFVLSLLPQGRRQL
NQVRQFAQTMNQAAAPEKLQSHWHLQQWWIVIAGFFLFASILIFAFTRPISSTRIEAE
YLKKTDPQIYALLNGEILSPPPEVDESLIEAAIVEATQLEQQYASQESGVINSSPIDN
TSFDGRAILDTSLVNRKWDKMNPRYKQRLLMVFKIMKEQYGYELVLLEGYRSPARQNM
LAGNPNTTRAKGYQSYHQFGLAADVAFKRNGKVVISERDPWAMQGYRLYGQVAESVGL
TWGGRWKSIQDYGHTEYRMPGLKKTQEMAEKLIAESLNDIS" /product="T6SS-associated
peptidoglycan hydrolase TagX" /transl_table=11
CDS complement(538858..539460)
/locus_tag="AUO97b_00684" /db_xref="SEED:fig|400667.82.peg.544"
/translation="MAQEKPTSIRVLQSNKKKSRSPVIYIIIGIIAGVFVSISLFLFL
SQPYSSDPTSVESKSNQDQELDQVLMTNSSAHDQAVSDQQEQTDPMSHDGFEQPKENE
LSNIFKPAPHKEAAPNPQRVSPFDAQLTGEQVVPKQQAVAKPVQPKLQVVQQKPATQT
KVVQAKPAEVNKAPVEQEADVEPPKASVDIKITRSPFAVN"
/product="T6SS component FIG01317386"
/transl_table=11
CDS complement(539473..540279)
/locus_tag="AUO97b_00685" /db_xref="SEED:fig|400667.82.peg.545"
/translation="MSQSTGAPSLFDDGQIGTGGNNNSQSQVKLQAINLIDLLHDGFY
LIFLIRNQYVPADPQRFREKILDLLNRFEQQAKKLQFSADDIHDAKYAFCALIDETIV
TQQDPSYFNLQNSWLISPLQLSLFGSQLAGYQFFEILEQLRSRGKERLAALEVFHYCL
LLGFQGKYRIESIESLNHLVARVGDEIDYLKGKKVAFSPFSAIPDQIRNIIHRELPFF
WILIFLLIFALLTFAGLRFMLNNQNDKALSNYQNVISAPQEQAHITIYLP" /product="T6SS outer
membrane component TssL (ImpK/VasF)" /transl_table=11
CDS complement(540297..541661)
/locus_tag="AUO97b_00686" /db_xref="SEED:fig|400667.82.peg.546"
/translation="MFKAEKVLWGEGLFLRPQHFQIQDTYHEQRLNHTVRAVVPFSYG
VQKLSFDEAQLSTHILALESVEMIWQDGEIYQAPAYDLLPEPILLDELNLRGEMVIYL
ALPVLQANKQNLIDQNQSQKGRYQSHLVETHDLFTNATEADISLLRRRAVFKLLDAHS
NPDHNLDGFLYLPIGKIKRQSSGAFEIDRKFIPPILHVDASETLVSNLKRTLNVIKAK
IKTIQTNNRENEQKLIEFRSGDIVSFWLVNALNTAHAGLSHFLQYPQIHPERLFFELL
RLAGSLLTFSTAYEVDQLPAYKHHNLQESFTQLDTILRDLLDTIISSRYISIALKEIR
PSYWVGSLETDKITRDTRLYIAVSSSAMQTHELIQIVPLRFKIGSTLDVEQRVVAALP
AIPLHHLIQVPTAIPVRSGVSYFEIEPHHELYQRMLDSETICIYVPAGFQDISIELIA VMNA"
/product="T6SS component TssK (ImpJ/VasE)"
/transl_table=11
CDS complement(541678..542775)
/locus_tag="AUO97b_00687" /db_xref="SEED:fig|400667.82.peg.547"

/translation="MSIDISELLKPINDSLLCGEDYSFSNEFHEIKKARTQDDLLLDQ
GDWVAERKQADWDFVAKSVSTLLIEKTKDIRLLTWVIEAWTHLNGFEGMVKGITLTHT
MLNQYWQDIHPIIEDDDLDQRIGLLQGLINQLPMLLKKVPLTNTAPYYNLLDYDNFLY
HENIRRKQTEEYESQSGPSELEQFDQAIFNTSKTFQYSNYQEFNSVLTEWNVLKQTLD
HLMGLDSPSFAAIDSAFETIHSTLRKIYKAEAFGTGLAPSQEEQAAVITTPSMENQVP
VQIVSDQPMFQPQAQTHLANREQAMKVLQEIADYFQANEPHSPVSYMLQKTIKWSQMP
LHEWLAQVIKDEHPLQMVQEMLGVQPKNEYE"
/product="T6SS component TssA (ImpA)"
/transl_table=11
CDS complement(542799..545480)
/locus_tag="AUO97b_00688" /db_xref="SEED:fig|400667.82.peg.548"
/db_xref="GO:0005515"
/db_xref="GO:0005524"
/db_xref="GO:0019538"
/translation="MMSSLKILITKLSTAARTALEKSANACVLQQNYEIEIEHLFLEL
LNQPLENDLKILLKKYKISADALADDLKETISQLPKGNTRTPIFAKSIVRLFEQAWLL
ASAEQNPVIRSGHLLVALLTAPDLYQIATRASSLFDLFPIDSMKHKFLEICEKSVEQQ
EESKKSSQADELEQAATPTAKTQKTPALDQYTINLTEKAKNGGIDPVIGREYEIRLML
DILMRRRQNNPILTGEPGVGKTAVVEGLALKIAQGLVPEALKNVHLHVLDMGLLQAGA
SVKGEFENRLKQVIQEVQSSAHPIILFIDEAHTLIGAGGQAGQNDAANLLKPALARGE
LRTIAATTWAEYKQYFEKDAALSRRFQVVKVEEPTEEVAVDMLRAMIPVMQKHFNLQI
DDEAIVTAVHASHRYISGRQLPDKAVSVLDTAAARVALTQNAQPVKLDQLKAQEHNLK
LEQQILENEHRQIPIHHERLENLKNQLESLQSEIQETQEQWQKELELVRKIQELEAQS
HSNESEAYDQINLIRKDLADIQGQQPLVFERVNSQIINEIISDWTGIPVGKMVNDEIK
QILTLEDKLGERVMGQDYALTQLVQGIKTSKAKLEDPNKPQGVFLLVGPSGVGKTETA
LALANELYGGEQHLITINMSEYQEAHTVSSLKGAPPGYVGYGQGGVLTEAVRRNPYSV
VLLDEIEKAHSDVQELFYQVFDKGTLEDGEGRVIDFKNTTILLTSNTGSSAIMQACLN
QPVEKWPTAEDLIEHLKPSLYKQFKPAFLGRMRVVPYFPLHDDLLVRIIKHKLGKITA
RIEKQYGTEVQYSDDLVELLLSRCTEVDSGARNVDNILNSTVLPALATEILVALADQK
LPKLIMIDAKDDEIQYLLDPVAKPAKKRTSKKLKSEV"
/product="T6SS AAA+ chaperone ClpV (TssH)"
/transl_table=11
CDS complement(545700..545963)
/locus_tag="AUO97b_00689" /db_xref="SEED:fig|400667.82.peg.549"
/translation="MASPYITIGCPTTGGGQVISGNSAFLIDGIAIACVGDKATCPAH
KTVSTIVSGDPNMQVMGKAAARVNDSLSCGCKLLPKQSLVNQG" /product="T6SS PAAR-repeat
protein"
/transl_table=11
CDS complement(545980..546747)
/locus_tag="AUO97b_00690" /db_xref="SEED:fig|400667.82.peg.550"
/db_xref="GO:0019867"
/translation="MNQKYQIKLSQFIKGCFIYAIANYALAQPIVVEGVVPNEASKQA
ILMKMQSVYGADQVVDKIQVRPVAAPNGWSDSVTRVITPDLKKVSQGKLSVNGTRFEL
TGKMLNPTEIQPTIQSFQSLVQPPYQLFSQLSVNQAEQKIIDDALKNRIIEFESGSAV
LTEAGQKILDEMAVALNKVGGKKVKIVGHTDSSGDANKNLKLSQDRALAVKNYLISKN
IPADHLSTEGLGSSKPVADNTSAEGRKKNRRIEFTVL"
/product="T6SS peptidoglycan-binding component TagN" /transl_table=11
CDS complement(546750..547709)
/locus_tag="AUO97b_00691" /db_xref="SEED:fig|400667.82.peg.551"
/translation="MHTLKTTPLYYGKSPARGDFLKTKGQYALIQVLDQWITEALEYA
MQSNMFKEAYSKLPSLDFFIANPQENMFLAANLLASEDSSGRAFPMVLSHLLEIDLPY
ENLLYAPYSYKHVLVDLFQNNRALRGIKDSDLLFGKLNHLPNQIQVLSERECVGFYEN
HTMHSFAQLMKLSIYELAQSMIGLGLLLQPILQNGTSRLNKVLILPINNPAYCYEIAA
FWVSIICRFLKQHNTEVLIGLLHREQPVLFFGFQGADILALSDIFTQNMDSKHWVSLI

QAQWIDTYLEQNAGLATLEQSLCQRQLSLNQGLKLFRQTFLGE" /product="T6SS associated component TagF (ImpM)" /transl_table=11
CDS complement(547747..551571)
/locus_tag="AUO97b_00692" /db_xref="SEED:fig|400667.82.peg.552"
/translation="MHTILGYLWQYITNPKAIIALSLFVALMSSYTSIPRHIFWALAS
AYALGLIAYGIYWLIQRKKHAVQGEELAQAIEKDTQAEYGKNKDKEELQLISQQMKES
IQLIRKSKLGDKKGNAALYELPWYMVIGNPAAGKSSAIYNSGLKFPFEETHQKMVSAG
LSGTRNCDWFFSTEGILLDTAGRYSVYSEDHSEWLGFLNILKKNRSKAPVNGLILIVS
IAELVSQSPEHSLKLAKNLRARIQDLTERLEVVVPVYLVFSKMDLIAGFTEFFEFYEA
QEYNQAWGATLPYEQNSSHNAVDLFEKHYNILYDGLKGVSSTHLSRRHSQNISPSVMT
FPLEFKTLKPALKSFIATLFEDNPYQFQPVFRGFYFTSALQEGVIESPMTEQIAQEFQ
LSQIAQSEVKDQLKTTPNHGYFLKGLFSDVILKDKDLVKQHVNTSKKRQRYIAFIGAL
AGVSIILGVWVWSYRNNQQLIAEVQADLNKVVQLEKSSGQQLSTQLEALLVLQRRLQQ
LDEFDENRPLKFSFGLYQGNELREKLKAEYLKGVKQIVLIPTQQNIAQYLQRVKNNEA
TLKANHTNVEIKQTAQTQQYLEPSDTNPQDAYNALKAYLMMSNPQYMDASHLSDQVTR
FWRSWLDANKGQMPRADMIQEAEQILSYAMTLANDKQFPILDADSQLVDQTRQVLLSV
IRGMPARDRVYNQIKMRAAVRFPALTVSQIVGDANKNIVLGSYALPGVFTQKAWNEYV
EKAMEEAADKPTDTKDWVLNSRQSDDLTFSGSPEQIRKQLTALYKQEYIAEWRKFLSG
IHYAKATQFAQQVKNIDVLGEPQNSPIRTLIERVAIETNWDNPVVQAELAAPQKGFIA
WFKRKVLNHDDKQLANQAVTNAQGPISQEYQMFYQLVRKRDDQQGKSLLDEYMTNLAL
VRSKFNELKNAGEIGPNAMTLVKQTLNEQTSVFNQTQKIVDEKMAVGFSEIDQQLLQK
LVVSPLTQAFESLITPTQDEINKLWVMQAYQPFTANLAKKYPFNSSASLQATSSEIGQ
ILGENGSISRFVKESLDPFVIRRGYTLTSKTWKDLGISLNPQFVMNFQRYVAPTNGMA
TGELNSQAPAAPATNQSNFQFYPIQNPQLLSYTVDIDGQRMTYENGVQQWVNFIWPNQ
GSIPGARITAVDLQGQTHTIFDEPGEYGINRLIDSAQRKEQNGGFEMLWRSKTDPSLF
VKMNFRLISSNSGSIGSSRGYSGMQLVDKVTADKAARVVSAQQAPAQAAAPAKTENPV SALAQPAAGVKP"
/product="T6SS component TssM (IcmF/VasK)"
/transl_table=11
CDS complement(551602..553014)
/locus_tag="AUO97b_00693" /db_xref="SEED:fig|400667.82.peg.553"
/translation="MIKKILLSFIAIFTVVSGIIIFYWRDVQYNPNKIDFFLYFLLLP
AIITLVILSPWLIYRVYKSYKEKKEQAANQSQDDDSQKPSAVIEQPLEQLDFNVYSAF
AIHALGENETIIEEIQNFKSPELDEQLLNSYGLPLLSYRIKDLAETNDVDIQYAASPR
QVRIMSLIRQQLEQNIENLYRLAEHLKRSILFYESHQIREYHMHPAWVDPNSEYEDVE
TPVVEVHRLNRLNLHIFLPEDLLHVWNDEQSNDLILEFFTEIGIISQKVHIEYHFLGE
RVAYQEFIHLLKRIQKKEHEVFLMLAVDSEIDQDLIDEKSWMVKDYIPAEFAASCLLA
DPSLKIEELEPDKNLKIVVGQEKAAKVLHTLNLNELPQYEGDEPYVLILSDQTDIKAV
KQLQQQFAQTSVEPHHYIYVKSSLGHTQHLVDIYGFMLSMHFPEHIVPFVFGENTVSA
HTFVQSVTENSEDDAMVLNS"
/product="T6SS FIG00350534: hypothetical protein" /transl_table=11
CDS complement(553011..554009)
/locus_tag="AUO97b_00694" /db_xref="SEED:fig|400667.82.peg.554"
/translation="MHSERWWQDSSVTAELFQRPKSFEFIQATRLLRHMPANDAALSW
SDHFKFETSFNLNFPATEIESLELVDERVHLTNLIVGLTGIQGALPYTYTNKIKQAPR
QQRAETKEFLSLFNHKLTSQYVESSITYHLPVRYEIENKNDYLDILHALNGYVRSQHQ
QQDLDEYFAEFSGLMQGQNNTVHALKTMLSCIFKHEITIKEFVQESFKLAGDQLTTLG
GSQPSLLGINTFCGETIQQIDGKIEIQIGPLKRQQYLKFLPHQELSLKLKKIVETWCS
PTLSIDLRLILDESEIQPVRLTQGQESGLGQGAFLMSRKPNTHNDETCYSLIGEQI" /product="T6SS component TssG (ImpH/VasB)"
/transl_table=11
CDS complement(553973..555784) /locus_tag="AUO97b_00695" /db_xref="SEED:fig|400667.82.peg.555" /translation="MIEELLPFYEKQLQEFGQQSREFAQKYPKIAQRLSLNQEQIDDP
HIERLIQAFSLIAARIDKKLTDSYDVFTRSLFEVMFPQYLRHFPACSVVSFEDINKIK
QLKEPHLVPHHTVLKSRSFKGVQCEFNTTQDVKLLPIHLSGLDFKTTPSAHMHLNQNA

TLSLKFEIFNNAHACLTDEKLPIYLDAISNFPLQVLDSIFKKGTSFAVKYGQTIVELS
KNPFELIGFAEQESLLPLDQHTHHAYRLLMEYFCFPEKFSYLKLDLDFLKRIPQHVSE
FELLIHFKLNLNDQAVVRNYSELNIANFKLFTTPVINLFEKYAEPQKIVHKQLEYPLV
TDAHHPEFYQVYSILEMNMVREKTNQEESYVPVLPFFAMSHYHNDTARFFYSVNYQKL
QSNFVEMGYSIISKNLNPFSTRSDFISTKLLCSNRDLPHEALGQSNNVLNLNDSSLAR
RAIVLKRPTKPYKFEQGQSEQWRVISHLSLNSLALMKGDAVSHVKELLALYNLPQSKE
NYLIIESIKKLEFSLTHKLVDGKPFPMFVRGVKAELQIDSSVFRGHSLYIFSQLLSRV
FNLKVQINSFVDLVVKDYSSQQELYQCTQNVGGKTLL" /product="T6SS component TssF
(ImpG/VasA)"
/transl_table=11
CDS complement(555801..556277)
/locus_tag="AUO97b_00696" /db_xref="SEED:fig|400667.82.peg.556"
/translation="MNLDRLYPYGFRSTLFDRLIPESEDYAKGLSIQQLRESVAKDLE
DLLNSRVAKLDHVIDDYPLVKKSILQFGIIDFVGLSTANPLDRDKICQSIEQSIAAHE
PRLRQIRVEMLLDGHNMGALCLSIQAYLNIHPLYEPVVFDALLKPTTQQYVISART" /product="T6SS
lysozyme-like component TssE" /transl_table=11
CDS complement(556357..556860)
/locus_tag="AUO97b_00697" /db_xref="SEED:fig|400667.82.peg.557"
/translation="MKDIYVEFRGKYKVDGESRDSEHKGWLEVNSWSHNIRQPKSATS
SSVGGHTAERVEHSDMVFVKDLDATSPKLWEACSAGYTFDEVQIDFYRANGDKRIKYL
QIKLKHVLVSSVTPTVNEEGVPTEAFGLKYAAVEWTYNQQDINGTAKGAVTKKWSLSN NTASYAA"
/product="T6SS component Hcp"
/transl_table=11
CDS complement(556910..558391)
/locus_tag="AUO97b_00698" /db_xref="SEED:fig|400667.82.peg.558"
/translation="MNNTQSAAMPLVENEEVNLLDSIVEQSRIARNEEEHSRAKSLIG
ELAKEVMAGTITVSENMTLSIDKRIAEIDALISKQLSQIMHNEQFQKIESTWRGLYYF
CQETPSNPLIKIRMLNTTKKELVKDFQGATDFDQSTLFKKIYEEEYGSFGGAPYSALI
GDFEFDRTPSDMYLLEQISHVSAAAHAPFISAASPSILGLESFTDIDRPRDVSKIFET
AEYVQWRSFRDSEDSRYVALTLPHVLGRLPYHPKEGTATEGFNFIEDVSGENHNEYLW
MNAAYAFGTRLTNAFDMHGWCAAIRGVEGGGLVEGLPVHTFKTQDGEVVFKCPTEIAI
TDRREKELSDLGFIPLVHCKNTDYAAFFGAQSTQKPKKYDNDTANANSALSSQIQYIM
AVSRIAHYLKAMMRDKVGSFASAGNVEAFLNEWLSQYVLLDDGASQEAKAQYPLREAS
VKVVEDPAQPGHYKSVVFLRPHFQLDELSVSLRLVTELPQSSN" /product="T6SS component TssC
(ImpC/VipB)" /transl_table=11
CDS complement(558384..558887)
/locus_tag="AUO97b_00699" /db_xref="SEED:fig|400667.82.peg.559"
/translation="MAKRESVQKKLQRIRPPRVQLTYDVEIGDAKEAKELPFVVGVMG
DFSAASELERTKLKDKKFINVDLDNIDEVMESLSPRAAFQVDNTLTEEGGKMAVDLTF
KSMEDFRPENVVQQVDPLKKLVEARERLTDLRNKISNSERLEDLLDEVLKNTDQIRKL SAEADHE"
/product="T6SS component TssB (ImpB/VipA)"
/transl_table=11
CDS complement(558904..559596)
/locus_tag="AUO97b_00700" /db_xref="SEED:fig|400667.82.peg.560"
/translation="MKVVLKRSILILFLTGNIIGLSYAQAAELEPVKLKKSCIKKNPL
AEGQNDPELLNIFKQVCDSDNTTQKNDLLAKAAMRFYQTNQPVKALNLASQLQSQNIR
GSLITDVLFLSGVSIANNSLQQMRNQEMRYLTNDITYPPAKQLVENIHTAMPAPDPSS
FKTNNSESSSTDNEKRNYKKQYATRNKVSASRTSNKRVVSPAKATAVKPKTAPAATVN KSKSSPFDPLIK"
/product="FIG00350028: hypothetical protein" /transl_table=11
CDS 560368..561336
/locus_tag="AUO97b_00701" /db_xref="SEED:fig|400667.82.peg.561"
/translation="MIRQNLAIILLSSITLTACAKSLNNTDEPQTQATKQNLSPEQQK
KLQQLLDKTKKNLVEIKGGTFMMGDFGEQKSPDKLPYDSDADSKPLHKVTLDNYALSA
TKATYADFDIYTEVTGQEKIGKFDELSIQDRLPNSAAGVNWQQARNYCQWLGKQLNLK

MDLPTEAQWEYAARNRGQYILYPTDNGKIDTGRNVWSFEQRQKQQNKLNAFRNIPELM
KFPATPLGLYDMITDNYEWMLDWYDPQYYSKSPEKNPQGPAKGSLKVVRSSIPSEGQS
LQMFGGFTFSRHAIHPIEDPKAAKDELTKKHHLDLNQNNSVRCAVN" /product="Serine/threonine kinase"
/transl_table=11
CDS complement(561366..564371)
/locus_tag="AUO97b_00702" /db_xref="SEED:fig|400667.82.peg.562"
/translation="MNPYTIFEYTIVFYNQRNKELPNVKYSIIFFCEDGTKKTYEGAT
NPKGQTKPIPLNQNGKLHIFVEGHETIFSPKKTIKPILAEGSNIVEVNDIRLKSNTSF
LTKAQYELMQKKTSKDLEELRKNAKAVKQKNFFNLSKIRPTFPLDLAEELNERINMSY
EEYRKKNTHIVKKTKYLKFKNYALYRFVDSAGNGIPIVDYQIFAQGQSRPLINARPGP
PDKKGYTQLVYTHLKSRVTYTLGSARKNSEWYEPITCVDKQTIYQIIFPTSIAVTQPD
INHKENMEARQKPPIVINPTTNEVLILPSAVYAEFDKKTKILSDAVREVHKSNANLIK
AIQSRDLDEIKELEKRLNINQEKAIEKINGEFKQTADLREVWVVETTGKTNEKISKYN
LKRRYLKVTEYEELKAKRRNPQTEVDVTANQYTVQQPAQIRSSFEKLSQQLLTVKGNA
GSDEKAVYNLIGGLGGEIAEEYKNSRDVTVTQEAQWMRMVAGASGEGSISAAPKGVSI
KTSGDMSAKWTLFEGVKEWRKFYPCETGWKLEYDNYDLGTIRFLIGAEISGFSGANLG
IAGNLSVDISHQGAAQVIKAVVRPPERSMSQMVDRNKKPMFQPAQGSLKIIGNNNQEN
AQNQGNISINAFAGVQIQGLLKGAVEWFKPKGDGSGEGEFVAIASAAAGGGVSAGVGA
QGQFQIGYDQTSGNFKILVAAHLCWGMGAKGVASFVVGTEHLLSYLGFIKSQVAHAGF
KTLLYINEAAFLLAAQVLAYCIGENHPVISDINRIAASYGDWIRRLDIDQGRYKTAQN
INSSSGKKELLYATPETKGILLYAVTHWTDRTAPIFDMNVKFSDMKIEFFPTRKTAVI
NIFKTCISTEEWENTIQHIHPRGNKLTQTQLGKVEGDIIRFLNYGNDEKYAEDIIRCL
NSGIEYKGTQINAWLQDYLKYRKGAKKIAGSLNYMLVKNQDDNRFKQLEIQQGVWGDS
EEITLIASNLNVLSPFDQDDTDKYETYNV"
/product="T6SS effector tse"
/transl_table=11
CDS complement(564379..567093)
/locus_tag="AUO97b_00703" /db_xref="SEED:fig|400667.82.peg.563"
/translation="MQFSNASLNNQVFLQRIEGEHTLNQGSVAELLCLSTNAHIALKQ
FIGCQVAVDQVTDTGQFFRTTGIITEASQGQSDGSLTIYNLTLKDPTALWHKRRNSRV
FMNKSVRDISEILFKEWQGKSPLFASSITLDTSGLTKDYDVRPFVMQSNESDYDFLTR
LWRSEGINWLVDESQLFVADPNASIQPQVLRLIDDNQNYQALERRSIRYQRSSATEQF
DTITQVKAERRLQPTSVHVQRWQADALQQEEGSGSVQGTQKHSEHYDNASLNLEDAWH
VSPAWMQDLNGKDQATASGNSQIEQLNQHINAYHHLSSKQFTVAGNVRDAQVGYWFEL
NDHPELDQHDSADKEFLILSKHYYNQNNLPKELQQQLERLLPQGKLKAAQLDSQNPEQ
RHFAELNVVRRNIKAVPEYNPLEHRPAAHPQRARVVGLEGESIHVDQWGRIKVRFLFT
RADDHSHDGGAGSNDNDTDSAWVDVLTPWAGAGYGARFLPRVGEIVVIDFFDGNVDRP
FVVGRIHEAERQPTQFDQKGQLPDTKKLSGIRSEEVDGKGFNQLRFDDTTGQISAQLQ
SSHAASQLNLGNLSHPKDKAESDGRGEGFELRTDQWGAVRAGSGLLISTHKQDQAQGV
HLDASEAKQQIEGGLNNAKALSEVAKNQQTDPLDMLENIQTFLEVLKQEDPKKAAEFQ
SAVMLLASPKSIAVSSNEDIHLSANGQLTQSAGDSINMSTQKNIVNHASQKISLFAAQ
EGARLFAGKGKVEIQAQGDGLDVIARKGIQITSTEDTVFITSPTEINLTANGSQVKLN
GSGIFPVTGGKLEVKAGQHLFMGGEQVTPPKKQENKPCYLTFEITDLDGKPAKNVEYI
AFRMDGSRQKGQTNAQGLTQRFETDGSEQISIHICDENASKYKLAAKG" /product="VgrG protein"
/transl_table=11
CDS 567308..567709
/locus_tag="AUO97b_00704" /db_xref="SEED:fig|400667.82.peg.564"
/translation="MSKQMDQVIQSVIDLHVLIENVFTGQNAEQSLTPLLNSFDQNFK
MVTVQGQSIGLAEVKNLFSQNIGKKPSLKITILKSTALYKFENYCWVQYQEHQQTNET
ETVRTSTACIKVEGEKCYWVYLHETLVPSSS"
/product="hypothetical protein"
/transl_table=11
CDS 567805..568749

/locus_tag="AUO97b_00705" /db_xref="SEED:fig|400667.82.peg.565"
/translation="MGYVAKEFTQKNKLILSICSFVIPICLWCAISYLPFVWHPQVQI
TSSGSVSYLQVGSRVDKNVFYLEAQNAVNRDSAPPQGILVNPIYLPAPHEVAKALVTA
FTTPPEQPDSPWFHQSLWHSIKLVFSAFFISSLIGIPLGILCGFSKRISLLIEPFVEF
FRYLPAPAFGALAVAILGINDAPKIAIIVIGTLFQQILIIANTTRMVDRSLIEAGYTL
GTNKLKSLFHVVIPAALPDIYRDLRVLLGWAWTYLIVSELIGTTSGITWFITQQARYQ
NFDNVYAAILIIGVIGLVCDWLLMKLGERVFTWKVGAK"
/product="Urea carboxylase-related ABC transporter, permease protein"
/transl_table=11
CDS 568749..569630
/locus_tag="AUO97b_00706" /db_xref="SEED:fig|400667.82.peg.566"
/translation="MNDSTFNAHEYFQKIYERPVILEAKQLSQVFKHGHSERTVLNKL
DLKIHKREFICVIGPSGCGKSTLSRVIAGLDPYHSGEVLVDGECITGPCPDRGMVFQG
YTLFPWKTVKENVMFGPQMRGASQMTAEAQAREWINIIGLEKYENQYPHQLSGGMKQR
VAIARALVNQPKILLMDEPFGALDPHTRQKMQKHLMDLWQNIDITIIFVTHDMDEAIL
LADRIVALKANPGEIKEIIEVNLPRPRSLDLMSTPEFKQLRARVDYLVHAEEDELDPA LQDLPKIPRMTQVSTEK"
/product="Urea carboxylase-related ABC transporter, ATP-binding protein"
/transl_table=11 CDS complement(569682..570704)
/locus_tag="AUO97b_00707" /db_xref="SEED:fig|400667.82.peg.567"
/translation="MIKKAFLSVSILSAVVLGGCDNTAKVPEAKQDAQASTNTKPITI
GYSDWPGWVAWQVAIEKGWLKEAGLNVEFKWFDYSASLSAFSANQLDAVLVTNGDNLV
TASGGTQGMMIMATDYSAGNDVIIAKEGINTIQDLKGKSIGVEKGLVDHLLLATALSD
HNIKANEVKLVNSATNQLPQVFNSPDISAIAVWQPVAGQALKAVAGSKIIYTSKDKPG
LIYDTLTVNMSHLTAHQEEWKKVIQVWDKTVKYINDPATHADAVKIMANRSGVDPKQY
ELMVSGTHLLDINANKKVFTKSQGFDSIYGSSYHVNKFNVENGIYKTEQNVDGLIYPT LIEQLK"
/product="Urea carboxylase-related ABC transporter, substrate-binding
protein" /transl_table=11
CDS complement(570701..572512)
/locus_tag="AUO97b_00708" /db_xref="SEED:fig|400667.82.peg.568"
/translation="MLKNLWTIQDWKTAYQNGQIHLKDLIGYVAEIQNDDHAWIEIAS
NEQMQAQIDILKDQNVADLPLYGVPFAVKDNIDVAGFHTTAACKEVQYLANQDAAVVA
KLKKAGAIVVGKTNLDQFATGLVGVRSPYGAVKNSFNPEYISGGSSSGSSVVVANGIV
PFSLGTDTAGSGRVPAGHNNIVGLKPTKGWFSTTGLIPACRTIDVISIFALHVDDAWT
VASLMQGYDETDEYSRVHPANVPNQFSKRKIAIPTQLEFYGDLESEKAFLVAIERVKK
LGYDVESIDFSAFNELAAALYNDAWVAERTVAVERMTTREKAHPVIAQIIAQADKFKA
TDALQAEYNRAVLARKINLALQPFDALMVPTAPTIYTIAEVEADPLTKNAHMGAYTNF
VNFADLSALALPNVLREDGLPSGVTFIAPAWHDQALANFAQLWQTETSLSLGKSTQHY
QKSINIQSNHSVQLAVVGAHLTGMPLNFQLTSRNATLLKKTQTAETYKLFALKNTTPP
KPGLQCDAAGTSIEVEVWDVPLANFGAIVAEVPAPLGIGNLKLKDGTWVKGFICEAYA
IQDATDISHFGGWRAYIQSLNQTAQSVVSKNVGEVSI" /product="Allophanate hydrolase (EC
3.5.1.54)" /EC_number="3.5.1.54"
/transl_table=11 CDS complement(572525..572959)
/locus_tag="AUO97b_00709" /db_xref="SEED:fig|400667.82.peg.569"
/translation="MAKQKLARISITVPESTLNALDQKIVEQNYESRSQAIVDMINKH
LIAEQAQANEVMVGTLTLLYDASQMTLRNQLVDLQQQFLAQVISSLHIQLDQQKILQV
MLMQGASSELRQISQQFIVLKGVIKGHLELMDAVMPPIQQND" /product="Nickel responsive
regulator NikR" /transl_table=11
CDS complement(572964..573089)
/locus_tag="AUO97b_00710" /db_xref="SEED:fig|400667.82.peg.570"
/translation="MNDVKPVVQPKQTLKAFLVELLSVRAGVYIKQNNQLPKTKG"
/product="hypothetical protein"
/transl_table=11
CDS complement(573252..575963)
/locus_tag="AUO97b_00711" /db_xref="SEED:fig|400667.82.peg.571"

/translation="MQNQKFKDLVKQELKNKTRSIKDLARFVQRNNGYPSYSVFLGAG
ASVTSGIPTGGSLVKQWREEIYKEVFDSNIESLKDNELEIINYLSKNEGIWYNPQNEY
SSLFERKFDLPSQRRRFVESIVDNKKPSIGYNYLINLVNEKAFNTIFTTNFDDLLNEA
FYHFSDVRPHVCAHDSSIGSLSVLSDRPKIIKLHGDYLFDDIKNTLKETESLENNTRE
KFIEFSKDYGLIIVGYSGQDRSIMDVLNYLVQQEEYLKNGVYWCFREDDEISHEVRKI
LWKDKVYCVKIDGFDQLFAELNNELGINLKQFSEKNSLKIDKCIENFIKDDFNLSSND
YIRKDLLKLKEKSNNQDISELIAKLNESNQNNNLSSIEFKQILEIQNLILTGSFNKTV
AKIDKLLQDSGCDLSSELTKFQENLFFMKIDCLEKEKKIEQANEAINSLIASDKFNLT
YHHKKILLINDSKKNIEYLLSILDLFPYSASIRNDICRSYLSYYLKNKVEPDLINVDK
CLNYIEESLNIDNSLDNLAYEIKVELLFIKKDLISKKTSSTLNRNKIIDETNDQIKEL
IVKVLTVSPKHQNSIDCLINYLIKSDSSVDFLKFYNQIFLNIDKSSKKQKRYIIKSFA
MLFNKRKMLERYQKDSDIKKEAVNFFSKPEFDSNIFYLLYKTMFMISLNIRDISFYNL
IEKCINHKNVYEIALELVSLLESLDLKDEVRSLDLIMKNHQDKLTPYEASLFSKNVSL
LLYGAKSDEFMDYLENTRKLCPSFSRYLIKYTYDLLLAEKFDQVLECLKNSSEIIQDL
DEDDRAVLKINEATAYKKINNGQLSNSIYEELKKIVSKSLSQEITISCKSLMDDQQTL
SNIKTLINIDHHFYYYFQQWPVISDNLKQQLRNNYEKHMIVTLDRAS" /product="hypothetical protein"
/transl_table=11
CDS complement(576091..576444)
/locus_tag="AUO97b_00712" /db_xref="SEED:fig|400667.82.peg.572"
/translation="MANTENKCEITMNGKTYPCHISMAMDLVGGKWKCVILYYLKDGP
KRFNEINQLMPTITEMTLSLQLKQLEQSGLISRTVYGEKAPIKVVYEMTELGQSFKPV LESLSVWAHTMINPK"
/product="Transcriptional regulator, HxlR family" /transl_table=11
CDS 576538..577074
/locus_tag="AUO97b_00713" /db_xref="SEED:fig|400667.82.peg.573"
/db_xref="GO:0010181"
/db_xref="GO:0016491"
/translation="MALLILAHPYYAQSIANKTIVNELIKTYPDLEVRDIFQLYPDYK
IDVSAEQEALLRHDTIILQYPMFWFNMPAILKLWFDEVFTYQFAYGSQGDKLKDKKVI
ISMTVGQKEANMVNDQENLIDSFLKSVQHSIQYTQMQLSGTFLLYDVSPLSGNPESKI
KLEAVEHGHKVLKHLTEA"
/product="Uncharacterized NAD(P)H oxidoreductase, YdeQ/YrkL/YwrO family"
/transl_table=11
CDS complement(577113..580718)
/locus_tag="AUO97b_00714" /db_xref="SEED:fig|400667.82.peg.574"
/translation="MFKKVLIANRGAIACRVIRTLKKLGIASVAVYSEADRDSLHVTL
ADEAFLIGPAPAHQSYLNIEKILEVAKQSGAEAIHPGYGFLSENAEFCDLCESQGIAF
IGPTSSQMLDFGLKHTARELAIKSNVPLLPGSSLLANEAEALTEAARIGYPVMLKSTA
GGGGIGMRLVWNETELKDAYQTVSYLAQVNFKDAGLYLEKFVQHARHIEVQIFGDGQG
KVLALGERDCSVQRRNQKVIEETPAPHLNEQQRDYLQKVAIQLMQSVNYRSAGTVEFV
MDTETQEFYFLEVNTRLQVEHGVTEQVFGVDLVEWMVTLASGDWSAPTEKLEPTGHSI
QVRLYAEDPIKQFQPSAGLLTHVKFDAQARVETWVETGSTVSSFYDPMIAKIIVTAAT
RDQAIQAMSASLADTQVAGIETNLEYLQNIIACDVFALGTQTTRFLNTFEWKTQKIEV
LQAGIQTAIQDVTGRLGYWDVGVPPSGAIDPLSLTVANQLLANEPNTAGLECTLQGPT
LKFHCDSQIVITGGDMPSKLDGELVPMWQTINVKKGQVLKCGIIRSGCRSYIGVKGGF
DVPEYLGSQATFTLGQFGGHAGRNLLIGDMLPITEYSAQSTTALAQAQIPSFTQNWEI
AVMYGPHGAPDFFTKQDIDTFFANEFEIHYNSSRTGIRLIGPKPEWAREDGGEAGLHP
SNIHDNAYAIGAIDFTGDMPIILGPDGPSLGGFVCPAVVIHSELWKIGQLKAGDKVKF
VPVSYAQAKALDQKYHQSLTATDTTQIDFNPAIQAEPDTLKDAILATLDGDAGLPSVT
YRPAGNSYLLVEYGELVLDLNLRFRIHALMQWVKDQNIQGIIDLTPGIRSLQIHFDSI
QLDQLELLQKLQQAEAELPDIQNMQVPSRTVYLPLAWEDSQTQLATERYTQIVRPDAP
WCPDNVEFIRRINGLASKQAVKDVVYSANYLVMGLGDVYLGAPVATPLDPRQRLVTTK
YNPARTWTPENAVGIGGAYMCVYGMEGPGGYQFVGRTTQMWSRYRKNANFEEGKPWLL
RFFDQVKFYEVSETELLQMREDFKAGRLQLRIEDGVLNLKEYNEFLKTHQDSIQAFKD

MQQANFEAERRRWHEAGLQEYISESLDAVDEGETVVIPEGGCAVESHMPGSIWKIECA
SGDIVEEGATLAVIEAMKIEIPIIAPERMKVETITIEKGQTVKTGQVLFTLAPVA" /product="Urea carboxylase (EC 6.3.4.6)" /EC_number="6.3.4.6"
/transl_table=11
CDS complement(580721..581374)
/locus_tag="AUO97b_00715" /db_xref="SEED:fig|400667.82.peg.575"
/db_xref="GO:0004047"
/translation="MTVLQSFEKAVLNEVCPAGEAWMCEVKKGQYFRIIDLEGNQAVD
TLFMSAENPTERYSAMDTLAINQQIYLEKGTKLYSNLGRPIAVIHDDNCGRHDTIGGA
CSCESNTVRYAHETYPMHSCRNNFMYALAKHPIAKKHGLNVRHIGPNINFFMNVPVTP
DGQLKFDDGISGPGKYVEVRAEMDLIVLISNCPQLNNPCNAYNPTKIQLVVREAEGV" /product="Urea carboxylase-related aminomethyltransferase" /EC_number="2.1.2.10"
/transl_table=11
CDS complement(581390..582130)
/locus_tag="AUO97b_00716" /db_xref="SEED:fig|400667.82.peg.576"
/db_xref="GO:0004047"
/translation="MNTASYHNNQAIWTELLPGGHHWSGRIQKGTILRFTSLGAQANV
SLFCVNAADVLERFNMPDSLKGQHTAYLKASNVLYSDLGRVMASIVRDDHGWNDALCG
PSRPEQIEKQFGTRTFQDARNDMYQNGLDSLLIEMCKYGLASQDLSATVNLFSKVAPD
ENGALSYASSDNTNQSIELRFEMDCLVFLSAAPHGLDTSPSYQPADIQLSLFKAHSLT
DSDICRDACSQNQRAFQNTARYYALSNI"
/product="Urea carboxylase-related aminomethyltransferase"
/EC_number="2.1.2.10"
/transl_table=11
CDS complement(582445..583608) /locus_tag="AUO97b_00717" /db_xref="SEED:fig|400667.82.peg.577" /translation="MRALTYHGAEDVRVETVADPIIQEPDDIILRVTATAICGSDLHL
YRGKIPKTEHGDIFGHEFMGIVEEIGPAVTAVKKGDRVIVPFVIACGECFFCQLDLTA
ACETTNTGRGAIINKKQIPPGAALFGYSHLYGGVPGGQAEYVRVPKANKGPFKVPGSL
SDEKVLFLTDILPTAWQAVTNAQVKRGSTVAIYGAGPVGLLSAACAKMLGAEKIFMVD
HNEYRLNYAAATYDAIPVNFDEVDAAEFIIQNTDNYRGVDAVIDAIGFEAKGSVIETV
LTNLKLEGSSGAALRQCIAAVRRGGVVSVPGVYAGPIHGFLFGDAFDKGLTFKMGQTH
VHQYLPQLLELIERGELTPETIITHRMKLEDAAEGYRIFNEREEDCRKVILLP"
/product="Uncharacterized zinc-type alcohol dehydrogenase-like protein YbdR"
/transl_table=11
CDS 583861..584292
/locus_tag="AUO97b_00718" /db_xref="SEED:fig|400667.82.peg.578"
/translation="MLKTQKINIVQEFDAPVDKVFAALSEHENLSKLFAPAKVTRISN
GKDARNGVGSARKMSIPFTPSFVETNLVYKENELIEYAITSGISPIKAHRGVMQFTDL
GNNRTRLNYTISFKGRVPFIGPIIKAALQNGVSRGLKKLKF" /product="hypothetical protein"
/transl_table=11
CDS complement(584353..584913)
/locus_tag="AUO97b_00719" /db_xref="SEED:fig|400667.82.peg.579"
/translation="MPNLEASFRALRVLHTAKDLFNQYGFHKVGIDRIIAESKVTKAT
FYNHFHSKERLIEMCLTFQKDGLKEEVFSIIHSYRELMVFDKLRKIFYLHANLEGLYR
LPFKAIFEIEKFYPTAYKVVVDYRNWLVTQIHGLLLTVKPTALMEDAHMFLFVIDGAM
VQLLSKEETDERDKLLDYFLRKLSEC"
/product="Transcriptional regulator, AcrR family" /transl_table=11
CDS complement(585917..587044)
/locus_tag="AUO97b_00720" /db_xref="SEED:fig|400667.82.peg.580"
/translation="MNTFINYYEILHVSQDAPVEIIRLAYKGLAQKYHPDRYQGNDAN
EKMQLINEALEVLTNENKRKDFDLKLNSFNQQKQKERDFQEYQKRKKYEDFQQQEKNS
RKFSKNDDKDQKEAKDFNININININISKGLYIFKPFLSLYNVVKRNSKKIIISLSILGV
AIILINTIISIYENTRYSENIVTEGPLPAEQADETVNTLYASTSESVSTPELVNTTNE
EPKVIPYRPDLMNKAVLSVINIQEESGIIGVIKEIHDCYVDKKEERLYCLFLDLTARM

LDLGASQTMGIIRDDYLMDERVLTRINNNYYIPNGIDSSTATEHVQRMNIELAEIFKE
KYQEKIKNMHSSDNLNKSVEEELSDINNDEVKTDQPSSNLI" /product="hypothetical protein"
/transl_table=11
CDS complement(587295..588515)
/locus_tag="AUO97b_00721" /db_xref="SEED:fig|400667.82.peg.581"
/db_xref="GO:0005247"
/db_xref="GO:0006821"
/db_xref="GO:0016020"
/translation="MKILPSSEYDQILKYSVYWLVISIVIGVVAGLASTLIFVAFDIS
NKVRSLHHWLIYFLPFVGFGIGYLIKKYGSPIERGTHLLIDEIHQPKSFIPKRMSPII
FITSILTQLFGGSAGREAPAVQLSGALIDHLSYILKISEDNRKICLIASIGAGFAGVF
GLPLAGAIYGLEITALGNLRYSAIFPCFVSALIASAIPELFEIVHPHVFYVISEFPAI
HFGTLMSLIAAGLIFGLVARFFIAAIHFASDVFYKYVRYLPLRTMVGGIVIMLLTLFT
AHQQYNGLGTDKIISSFYVPIEFYDFFNKTIFTAITLGSGFKGGEITPLFYVGATLGN
ALGAVLNLPISLLAGLGLVSLFSGASKAPLTSIILAVELFGMNVATYAIITCLLAALF SGNCGLYRRKKLMD"
/product="EriC-type chloride/proton exchange protein" /transl_table=11
CDS complement(588818..590539)
/locus_tag="AUO97b_00722" /db_xref="SEED:fig|400667.82.peg.582"
/translation="MMNTEKLLIANRGEIAVRIIHACRDMGIASVALYADDDIDSMHV
ELADEAWGLAGATASETYLNIPAIIEVAKKSKATMVHPGYGFLSERAEFAQAVIDAGL
KWVGPSPSAIEKLGDKIEARKIAASVGAPLVQGTQDPLNNADEVLEFAKQYGLPIAIK
AAFGGGGRGLKVAWKLEEVKELYESAVREAKAAFGRGECFVEQYLDKPRHVEAQVIAD
QHGNIVVLGTRDCSLQRRNQKLVEEAPAPFISDQIYQQILSSAKNICQAANYVGAGTV
EYLLSHDGKLSFLEVNTRLQVEHPVTEETSKVDLVVEQIRVAQGHELSIKETPKAQGH
AIEFRINAEDPARGFIPAFGVLSLFEAPFGNGVRVDTGVRTGSLVSSHFDSLMAKLIV
TGPTREVAIARAKRALKQFKIEGVASVLDFHRAVLNEPDFTDEFNVHTRWIENDFKQE
LKPTKRGIPNHQQPMLLSYIEIDGKLHRLGLPAGMFAQGPATAVQAQTAEPEVSVEHL
LAPINGVISAWKVENGEQVTEGQVVAIMEAMKMEVQVLAHRSGVIQIGAEKGATCHAE TAIASIH"
/product="Urea carboxylase (EC 6.3.4.6) without Allophanate hydrolase 2
domains" /EC_number="6.3.4.6"
/transl_table=11
CDS complement(590536..592119)
/locus_tag="AUO97b_00723" /db_xref="SEED:fig|400667.82.peg.583"
/translation="MRFLSVNADCFLIELASLEETLALYNKLQNTQLNGIKDLVPAAK
TILVFFNEIETNFKTLVASIQSLKIDSGFERSSQEVIVPICYNGEDLAQVAELQGLTV
ADMIRKHHQSVWNVAFIGFAPGFAYMSSPDKPFTDIPRLTVPRKKIPSGSLGLAGKYS
GIYPKDSPGGWQLIGTTTEKMWDLERKNPALLLPGMTVHFEDVTHRPTTVTVPQQITR
KAEPKQSVPLFKITAPSLQMLIQDEGRLNQTNIGVGVAGAMDLSAMHSANRIVGNPTD
TPVIEVLNGGLKAKMQHAGVIAVAGAISNIRVKFADGQTADFASYQPIDLDEGDEFQI
QPPTAGLRNYLAVRGGIDVEPVLNSASFDSLAVLGPEPLKLGDSIYQGQVKAANISVN
EVGKTDLPQAGEVVELDIVMGPRTDWFEQDSIELLCQQEWLVTNESNRVGLRLSGAQP
LTRKITHELESEGTCIGALQIPPSGQPVLFMNDHPLTGGYPVIASVAKHHWDLVAQIP
AGCCIKFKKIAEFTDFENE"
/product="Allophanate hydrolase 2 subunit 1 (EC 3.5.1.54) / Allophanate
hydrolase 2 subunit 2 (EC 3.5.1.54)" /EC_number="3.5.1.54"
/transl_table=11
CDS complement(592152..592958)
/locus_tag="AUO97b_00724" /db_xref="SEED:fig|400667.82.peg.584"
/translation="MYKDIKVDPAQLEAALDARLKIRAGFDKPTAGMAAGMTQVNMIS
VPRDWAYDFLLYAHRNPQSCPVLDVLEEGIYATKLAADSDIRTDFPRYRIWKDGEMVD
EVTDAREIYNAHPDLVTFLIGCSFSFETALQEAGIEVRHIHDDTNVPMYLSNIKCEPA
GRISGNMVVSMRPIPSHQISEAVKITARMPSVHGAPVHIGHPESLGIKDVNKPDFGDA
SRIEAGEIPVFWACGVTPQAAVMNSKIPFAISHAPGYMFITDIPDRAWIG" /product="UPF0317 protein
YcsI"

/transl_table=11
CDS complement(592970..593734)
/locus_tag="AUO97b_00725" /db_xref="SEED:fig|400667.82.peg.585"
/translation="MFVDLNSDLGESFGSWKMGNDDQILPVVTSANIACGFHAGDPLG
ILKTVRKAVELGVTIGAHVSYPDLVGFGRRNMDLSRDELIADVLYQISALDGLAKVAG
SKVQYVKPHGALYNTIAHDQAQAAAVIDAIKMYNPELVLVALAGSNLVEQARVAGLKV
VSEAFADRAYNSDGSLVSRRLEGAVLHDSAFVASRVVSMLKNGGVESIDGVFTPIQAD
TICLHGDTDGALEMSAAIKAELVKNNIEIRPFVNKA" /product="Lactam utilization protein LamB"
/transl_table=11
CDS complement(593769..595001)
/locus_tag="AUO97b_00726" /db_xref="SEED:fig|400667.82.peg.586"
/translation="MSLLKSSFLSDRQWAIVASIFMMATSAMGPGFLTQTAVFTVKLG
AAFGFAILISILIDYVVQQNIWRVVTLTQMRASDIANKALPGSGYLLAFLVILGGFFF
SVGNIAGAALGLNALFGLDTKWGGILSGALAILIFASKKATLAMDKSMIVLGLLKILL
IIIVAVIVMPPVGQAVQQTFAPDQIDFAIITTIVGGTVGGYICYAGAHRLLDKGAVGP
ENINEVAKAATKGIVVVGIMRYVLFLAFLGVVVSGAHIDLASHDANPAAQAFQYAAGT
FGFKLFGLIFWAAGISSTIGAAYTSVSFFTAFKKNLTPKQTNYTTITFIALALLLYVL
LGATPAGLLIFVGGFNGLVLPIGLTIFVYVAACRKDLMGNYNYPKWLLILGGLTCLLT
WWMGYMSFTTVFNYLTKL"
/product="Uncharacterized metal ion transporter YcsG, Mn(2+)/Fe(2+) NRAMP family" /transl_table=11 CDS complement(595369..596280)
/locus_tag="AUO97b_00727" /db_xref="SEED:fig|400667.82.peg.587"
/translation="MNLKNLEAFYWVVTLNSFNKAATKLQTTQPAVSQKITALENELG
FKVLDRSLRQFKLTHKGMTLFKYAEKFMRLETDLVAELTENQHLTGTIRLGVSETIVY
TWLVEYIEKVQQEFPKVSVEIVVDLTPNLQEGVRTGDLDMAFLLGPTLAFECIEQSLC
NFELSFLAAPSFKGGIGQMSFKALMSHTILTYPKITYPYKELKAKMKEKGIDEPLSIT
SYSLATLLRLAEQGLGIAVVPTLTALKEITDGRLEILNTPIRLKTFHFTSIYIHGNDV
ALKEKLTEVAVQVSESAMKRLNEQIKK"
/product="Transcriptional regulator, LysR family" /transl_table=11
CDS complement(596564..596881)
/locus_tag="AUO97b_00728" /db_xref="SEED:fig|400667.82.peg.588"
/translation="MNKMHVTLAVVVGLIIGGVVGAIGYSKTAARYDAMTTACVMVNQ
AVEHEILKPEQVKELGELTGQTLKKDYASVASKFKFSEKQLGNASEGSNCSQFIVGVN AGQ"
/product="hypothetical protein"
/transl_table=11
CDS 597236..598483
/locus_tag="AUO97b_00729" /db_xref="SEED:fig|400667.82.peg.589"
/db_xref="GO:0008800"
/translation="MKNFLTKKMYATRALFFAVGVMALNVSPIVISTTHAATEQKMDN
LSTKLSLIFADKPIEASLFSPQFLEQVPITQIQKIVDDLKVSLGALKNINVSNGSGTI
DFEKGELPVSISLNEQGQISTLWFSAPHFKTISLDEMVKGLHENAIGKTSLLVIVDNK
PVVVENDKTPMAVGSTFKLLVLKAYEDAIKKGELKRETIVSLKEKNRSFPTGVLQNLP
ANTPVNLELLAQLMIQISDNTATDSLIEVLKKPRIEALSPRNSPLLTTRELFQLIDPS
NEKFRNKFKTGTKSTRLEALAELDKLPLPSVSSIGKLATWQDAEWYMSANEICPLLES
VQDAPALNSSLNPLFKNLNWQKIGFKGGSEYGVINFSVIGKTQKGHKVCAVFTANGNE
PQPESKLAILFTGILQAVDSINH" /product="Beta-lactamase class A-like and penicillin binding proteins (PBPs) superfamily" /EC_number="3.5.2.6"
/transl_table=11
CDS 598642..599319
/locus_tag="AUO97b_00730" /db_xref="SEED:fig|400667.82.peg.590"
/translation="MKKVLLFDLDQTILNRNESLLKFLNWQVSYLNLVPHELKKSFIN
RFIELDNNGSVWKDIVYSQLIKDFNIKKYDTSELLQSYINDFNKFSTAFENAPKTIQN
LHAQGYTLGLVSNGKTPFQEKNFYALELTDYFSIIVISEAIGLRKPDPEIYLYTCNQL

DCKPSDCIFIGDNPKADIEGAKKIGMKTIYFHPTLTLHPSLSDASIHHYDELEETVRR LINPPHF"
/product="2-haloalkanoic acid dehalogenase (EC 3.8.1.2)" /transl_table=11
CDS 599451..599879
/locus_tag="AUO97b_00731" /db_xref="SEED:fig|400667.82.peg.591"
/db_xref="GO:0008080"
/translation="MEISLRFATPADLKAINERYAEIDFVPSHDDELIVLASISEKTV
GQGRVVGIDANSGELGGIYVFPGNEGLGIARKVVDFLIKNSDFSMLYCLPFAELEGFY
GSLGFAAVKDIAQVPEAILKKHEWCNSNYDKPVLLLERSK" /product="Acetyltransferase, GNAT family"
/transl_table=11
CDS complement(599974..600432)
/locus_tag="AUO97b_00732" /db_xref="SEED:fig|400667.82.peg.592"
/translation="MPKYLAVAEKVYKNIKKDDLFTNDLTQNLNCLISQIRKAIKGTE
FKLKFNYIDFDEQLTKPLSECKVKVDLSVMPKFENEGEYLLWLASFIERITDGGKPKF
PPISQLIPHGFKISKESFPVTVAPPKEEHADMIIKYFKSEDFMKTNKTPS" /product="hypothetical protein"
/transl_table=11
CDS complement(601037..601987)
/locus_tag="AUO97b_00733" /db_xref="SEED:fig|400667.82.peg.593"
/db_xref="GO:0003857"
/translation="MEGIKTIGVVGVGVIGASWTALFLYKGFKVKVYDPYPIDEELFK
KRIQANLSDLLALDQQTDSIHHLQDIFLNLELYNNLKDAVINVDFIQENAPERLDLKQ
NLYQEITSYCPEKTLIASSSSGLKVSDFQKDATHPERIFLGHPFNPPHLLPLVEIVGG
KLTDPQILKKASEFYQSLGKHPIVLNKEVKGHVANRLQAALWREAFSLVKEGVCSAED
VDIAITSGPGLRWALFGPYINMELANQKGFKEAIHHLGPPMTEWWNDMQNFQHSEETT
ELLEEQTKELLTHYKDIDLSQKRDKGLVDILKLRQQLELD" /product="3-hydroxyacyl-CoA dehydrogenase (EC 1.1.1.35)" /EC_number="1.1.1.35"
/transl_table=11
CDS 602199..602936
/locus_tag="AUO97b_00734" /db_xref="SEED:fig|400667.82.peg.594"
/translation="MYDWQDFEPFNFNMTNTFSDHKQVEVVSTFAELVNTHFQGNKNA
ICWYRNLVGDFKEIVNKLQLKENITEVSVEDLLALQLSEKGNLARTTILEDIELLSDF
GASPVLNLLKNYERDEELDFISTDVYSFHVDRSPIETDTFLCTYHGAASDIVPNDQVE
QKVLIPEIREKLKALHDGPEAEFESFLAEYFFDLHYQPKPDAQPINLGIGHIWRLAVD
HPTQKVLPCVHRAPVENDGEYRLLLIC"
/product="hypothetical protein"
/transl_table=11
CDS 603146..604174
/locus_tag="AUO97b_00735" /db_xref="SEED:fig|400667.82.peg.595"
/translation="MLLKVGELAKQTGLTVRALHHYDDIGLLQPSARSDAGYRLYTPK
DITRLHQIQALRGLGMSLAEIYTVLEDPNLALLPIIDRQIQAIDQRLAEQKKLRNQLG
QLKSQLINGEELDLEDWLNMLELIAMYEKYFTQEELEKLTFLQSGTKTHQEWQELTQA
VNTVFNAGESSSSETAQKLAHKWMKTLEQNTRTNPEWLVKLNNINSAEPEFQEKLGVL
PEVVDFLLRAFSESKLSIFARYLSDNEFAFLKENYGREMKKWPQLLVDIEKLIDAEVK
PNSEGAKRLAQQWLSMLQGYTGENPSTQEKIRTAMQNEPSLADGTWLKPVTLHFLEKA VAAFKHSA"
/product="Albicidin resistance protein"
/transl_table=11
CDS 604511..604948 /locus_tag="AUO97b_00736" /db_xref="SEED:fig|
400667.82.peg.596" /translation="MKKYLFLLLLSFYSIHSFASDKLHGSVWKTIDDATNQPRALVRF
SQDKNGVLSANIEKILVPSEANKCTMCEGTYKNKSLIGLTIVKNLKNVGQNKYDKGSI
LDPQSDKTYRFSVTVSPDGEKLTARGYIGISAIGRDQTWYRVK" /product="FIG00350439: hypothetical protein" /transl_table=11
CDS complement(605043..606347)
/locus_tag="AUO97b_00737" /db_xref="SEED:fig|400667.82.peg.597"

/translation="MKEIVLSDLINQQKVGPYQKFILATCLLLMIMDGYDIQSMAYAA
PLIIEEWGVQKSMLGVVFSASLFGLFVGSFLLSLLSDRFGRRPILLISTFIFSILMLL
TPHVGNIEQLTVIRFVTGIFLGGIMPNVMAYSSEIVPYQSRIFTMMVISCGYTVGAML
GGGISALLVPWGGWQAIFYFGGIIPLIIFFITFFKLPESLYFLSENSKNSSKILFWLK
KFYPALTFNSEIKIINNTEVQVKKSPLELFKNQRAFFTYSIWIISILNMISLYFLANW
LPTLAKESGLSLNQALLIGSTLQLGGTIGSVVMGLKIDKTGFYKVLIPVFLVAVISVA
LIGYSVSHIVLLFIIIFIAGFAIVGGQPAINALSASYYPVSLRTTGVGWSIGIARLGS
VIGPLFGGYLSQFLVITHLFVIAAIPSLFVIIMLMIQAKYRS" /product="4-hydroxybenzoate transporter"
/transl_table=11
CDS complement(606612..607268)
/locus_tag="AUO97b_00738" /db_xref="SEED:fig|400667.82.peg.598"
/translation="MCGFCMPVFSTHFKSDGISISLHTTRIYNGKTMPNLETSSKKLH
IIRTAIRLFTTHGFHTTGVDLIVKESEIPKATLYNYFHSKERLIEICIAFQKSLLKEE
VLAIIYSSRYCTPTDKLKEIVVLHVNSNSLYHLLLKAFFEIKVAYQQAYRMAIEYRKW
LTREILELIFSLETRALKPDANMVLNLIDGLMFGFLSTKSLDERDVVVEYFFKPTCLR "
/product="Transcriptional regulator, AcrR family" /transl_table=11
CDS complement(607812..608501) /locus_tag="AUO97b_00739" /db_xref="SEED:fig|400667.82.peg.599" /translation="MASKNFLSTLPRKIKENKGSIAICSILFIIPLFVFPTYPKNEWQ
YEIYQYITQIMHVTIGTKGPLPFFTILYSLYITIVMFIVGCVICYFFIRKYGINKAYQ
EEIYKFFFKAEFESSKKYRWLEKPLVKKTLVSSTFAGCFVMGIIHFIEDDITQQRPRR
RGGLIAFSYNYRVGVMFWEIVTTVFSIFPIFYFGLLFLYIINYFFRGLGTGKVVVLEK TSNPKFRTSKK"
/product="hypothetical protein"
/transl_table=11
CDS complement(609346..610182)
/locus_tag="AUO97b_00740" /db_xref="SEED:fig|400667.82.peg.600"
/db_xref="GO:0008415"
/db_xref="GO:0009244"
/db_xref="GO:0016021"
/translation="MFLAYLPWAIQWRLAKLFSKIAWTLLGSRRKTTLRNLGACFPEK
TSEQIEAKAKSVFTDTMIGIFEALNAWYCPAWFKKRVHIEGLEHLNDNRDRGILLLGT
HSTLLDAGGAACTLFFDMDVVYRPQNNPFMDFLIHRSRARVYKNQIAKDNMRSLIEKL
KNGHAIWYSPDQDFGLKQGVMASFFGIQAATVTAHRRLMDISNAVAIPLYFYRSGDIR
DPQYNVLVEPKLENFPSECEVNDAERVNKIIENQIRIAPTQYMWFHRRFKTRPAGEAK FY"
/product="Lipid A biosynthesis lauroyl acyltransferase (EC 2.3.1.241)"
/transl_table=11
CDS complement(610409..611596)
/locus_tag="AUO97b_00741" /db_xref="SEED:fig|400667.82.peg.601"
/db_xref="GO:0005554"
/translation="MDICFTLISIFSFFLIYINRISAGILLSQAVLLVFPVVFCLFFD
VATPDRPRVAHLFLPAGAILGYLNYRREPSFLQIVLILLSIGCFIFFSGSSFTLDSAI
PLSEDIRDHGGWIATCVATLMICISIYTMQLEIQIESSMEHNLRLALTKQQFELFYQP
QVNSSEKLIGAEALLRWHHPVLGYIPTKEFIPAAATFGLMPEIGEWVLAQACKVLQDW
SKKEETKDLTLSINISADHFMQSNFEHTVIGLVQRYDINPSRLILEITENIALNNCAS
MIEKMHFLSKHGIQLSLDDFGTGYSSLSYLQKMPIKQIKIDRSFVQAALDDKRSNKLV
TGIIKIGLDLNLRVLVEGIETAEQFSAFKSNGCTEFQGYLWGCPMSLNEFIKQLPIKI DKE"
/product="diguanylate cyclase/phosphodiesterase (GGDEF & EAL domains) with PAS/PAC sensor(s)" /transl_table=11
CDS complement(612266..613123)
/locus_tag="AUO97b_00742" /db_xref="SEED:fig|400667.82.peg.602"
/translation="MIETNSKQGMAFAAAFLSLVSVQIGAAIAKVLFPILTAEGVTLA
RLALSACFLWIVFKPWRQWNKQTNWKNLIIYGSILTLMNTLVYKAFSYMSVGIAISIE
VLGPLTVAILMSKNKLDYFWGGLSIIGLLLLPMGNANNNFSFIGMTYALLAAVGWGLY
VIYGSKVAQGGGSSVATGMFIAALFATPLGASHIIHIFDSYKILFTCLLMAMLASAIP

FILDMIAMKKLHPRVFGILLSASPAVSALAGWLILDERLNSFQVLGITIIMASCAGCS YFSYKRNKV"
/product="Putative DMT superfamily metabolite efflux protein precursor"
/transl_table=11
CDS 613095..613211
/locus_tag="AUO97b_00743" /db_xref="SEED:fig|400667.82.peg.603"
/translation="MPCLEFVSIIIFTALGMIEILILYNQYISSIFHGKTIK" /product="FIG00353610: hypothetical protein" /transl_table=11
CDS 613245..613712
/locus_tag="AUO97b_00744" /db_xref="SEED:fig|400667.82.peg.604"
/translation="MSEISLDNFDYAILRILQKDNKTSHREISEKIGLSAASVQRRIT
RMEEKEIILKNCAILNPLKFGEKITSIIEVRLSEDRSVVMDRAKLYFASVEEIQQCYF
VNGGVSFIIIMLSNNLSHFESLVRKHFADNEDVKTYRTLIVLDRVKVNFELNI" /product="FIG00351682: hypothetical protein" /transl_table=11
CDS complement(613875..614006)
/locus_tag="AUO97b_00745" /db_xref="SEED:fig|400667.82.peg.605"
/translation="MISALFMRNKPLKKRKIYFWFINQFQVFKNECNSRKVQQLDEL"
/product="hypothetical protein"
/transl_table=11
CDS 613987..616632
/locus_tag="AUO97b_00746" /db_xref="SEED:fig|400667.82.peg.606"
/db_xref="GO:0003677"
/db_xref="GO:0003684"
/db_xref="GO:0005524"
/db_xref="GO:0006259"
/db_xref="GO:0006281"
/db_xref="GO:0006298"
/translation="MNSAEIMADLSNHTPMMQQYLKVKKDYQHALLFYRMGDFYELFF
EDAHLAAKLLGITLTHRGKANGNPIPMAGVPYHSAEGYLARLVKAGRTVAICEQVGEV
TGKGPVERKVVRILTPGTLTDDALLTSYQSSNLVALCIHQNQIGFALLDLSAGIFKVQ
QQEYKPEQLPIELARLMPSEILIDEDLVDPNIIEQIKKHLDCPVTKRPNVDFNLNNAQ
KTLCDQFSVSTLSGFGLDPLPLAKAAAAALIHYAKETQKTALPHIRSILLEQSTDFIA
LDPITRRNLEIIEPLFEHGTSLFQLVNDCQTAMGGRLLSRTLMQPVRDTALLDARLDA
IEQLIQGYHESPVRMVLKEIGDIERVLSRVALGSARPRDLVQLRHACAQIPFLRNALA
PVVQAKKSKLLGQLDQELGDFKSLHQHLMAAIVENPPVLLRDGNVIAEGYDAELDELR
QIRDHAGQFLIDLEIKERERTGISTLKIGYNRVSGYYIELTRAQAEQAPADYIRRQTL
KNTERYITPELKSFEDKVLSSESRALAREKALFEALLENLRENIAHLQMMSSAIAQID
VIANFAHQARLNNWARPEFTPETGIKIQGGRHPVVEALSKAPFTPNDTFLDVQHRMAI
ITGPNMGGKSTFMRQTALISLLAYCGSYVPARAAKLGPIDRIFTRIGSADDLSTGKST
FMVEMTETSQILHHATNQSLVLMDEVGRGTSTYDGLSLAWACVVDLTKRVKCLCLFAT
HYFELTELGSEPGIDNYHVTAQELNGNLILLHKVQQGPASQSHGLQVAKLAGIPANVI
KEAQKRLRILEKQQQQHLQTSVQSDLFATLDSEVTPSTQVIEKVIEVEVSSPALDLLK
QIEVDNLTPRQALEQLYELKAALNS"
/product="DNA mismatch repair protein MutS" /transl_table=11
CDS 616652..617227 /locus_tag="AUO97b_00747" /db_xref="SEED:fig|400667.82.peg.607" /translation="MYRHVCSYFKENKISIFLRPLQYTEIDLIWQQINRRELITQLYL
QKQEHLELTDCFYDVKHWDAYHLENDPPKLKELYEQGALFIGAFDASEQLAGISVVSN
QTITDYPDAKLLQYFYVDADQQGKGIGAKLMQAAKESAKQLEAHQLYISATPSKRTVD
FYIKHGAVVLKHPDRQLWELEPEDIHLLCTL"
/product="hypothetical protein"
/transl_table=11
CDS 617671..617895
/locus_tag="AUO97b_00748" /db_xref="SEED:fig|400667.82.peg.608"
/translation="MINPDECIDCALCEPECPANAIFSEDELPEGQEVFIELNAELSQ
KWPNITQIGEQPADREEWNGKPDKLQYLEK"

/product="4Fe-4S dicluster fused with DUF3470" /transl_table=11
CDS 618076..618747
/locus_tag="AUO97b_00749" /db_xref="SEED:fig|400667.82.peg.609"
/translation="MVGCASSPQHTTTGKTSPSGKRIFIPQERVIIERPIPPKVEPAS
YRAWLNTGDHYERVREYEKFLARHDVAGIVPSFELLRSARDWQKCGRSEYAVPNRELW
NNSLSTLRVFKYLIAAKVLTDFEVTSVYRDLPLNECAGGASSSKHLFNSAIDFRIGPE
VPQPQDYAFIENTKFKLCQFWAQHGQSLNLGIGLYSSGQIHIDTQGYRTWGPDLTRNT SMCNF"
/product="FIG00351016: hypothetical protein" /transl_table=11
CDS 618887..619555
/locus_tag="AUO97b_00750" /db_xref="SEED:fig|400667.82.peg.610"
/translation="MQQMWTDIDEYIDSHLIPEDPRLDFALKNTDEHGFSNHLAVAPN
QGMFLQMLIQMNKCKRVLEIGTFAAYSTIWLGRALPEDGYLLTIEGRDTHAEMAQKNI
DHANLPVKVELKLGRAADVLSAIDPQSIEPFDFIFIDADKQGYPEYLELSLKFSKSGT
IIVLDNVIRAGDILNPENKKPSIEGIRETFKALENHPQILSCTALQTVGSKGHDGFAI AIVK"
/product="Uncharacterized SAM-dependent O-methyltransferase" /transl_table=11
CDS complement(619672..620514)
/locus_tag="AUO97b_00751" /db_xref="SEED:fig|400667.82.peg.611"
/translation="MNRVIACIDSSPCINAIADAAAWVAKATGRELVLLQILDYYPAS
YHLGEISGVIGFESNAMLLKELAELEQKQSELALDYSNNLLRHISDLIKEKHGITCSK
IQEKGDFLEQSFNLLHENDIVILGRVGERAAEKHKPIGSNVENFIRGANCTVLTIGEN
FKVPTRFIFAYEYSPTCQKMMRRIAQSDLLRTLQCHLVYVGDHPEILNEPEHYLKQAG
LDVVTVYRYGDVAQNILEYQQEHGIQIIVLGAFSHSKIHQFFLGSIATTIFRNATVPL LVAK"
/product="Universal stress protein UspA and related nucleotide-binding
proteins" /transl_table=11
CDS complement(620670..621281)
/locus_tag="AUO97b_00752" /db_xref="SEED:fig|400667.82.peg.612"
/db_xref="GO:0008925"
/translation="MTDSLLKYREQHKHRLNYMPWLYWTLKPKHRVWAEEWQKEYQAY
LMDMETVEIGENCFISPLAHIFAEPGRKIKIGDNCFIAADCSLHGPLEIGNEVAINHH
CILDGGRAGIKLHDQVRIAAYCHLYAFDHGMQLDRPLYQQPVRSQGIEIEKDVWLGAH
VGIKDGIKIGKHAVVGMNSMVTKDVEPYHIVGGNPAKFIRLRE" /product="Acetyltransferase,
CysE/LacA/LpxA/NodL family" /EC_number="2.3.1.79"
/transl_table=11
CDS complement(621274..621873)
/locus_tag="AUO97b_00753" /db_xref="SEED:fig|400667.82.peg.613"
/translation="MAFTIASPVTFEEEIKKSRFQAIAAPVENEQQVKEFLELNKDIS
TTHQCWAWKIGHNVRFNDDGEPSGTAGRPILATIEGNDLTNIIVMVNRWYGGIKLGTG
GLVRAYGGCAGQSLLLAERIELIAKKTIHFSCHFNEWAIFQYELTQQQIEYQESYTAI
GVDIEARLQVHQIEPLALKLRDVTRGREELKVEEELTDD" /product="FIG000605: protein co-
occurring with transport systems (COG1739)" /transl_table=11
CDS complement(621965..623155)
/locus_tag="AUO97b_00754" /db_xref="SEED:fig|400667.82.peg.614"
/translation="MSEQQQELTRSMNVSYSEPPLPRASLVIWIVGIGLVIFFIWAWF
FKLEEVSTGTGKVIPSSKEQVIQSLEGGILTKLNVQEGDIVQKGQILAQLDPTRFASN
VGESRSLLIAAQATAARLRAEVNGTPLVFPEIVAQDPKLVHEETALYESRRADLEQTL
SGLRQALQLVQQELAMTEPLVAKGAASEVEVLRLRREANDLQNKMNDARNQYYVKARE
ELSKANTDTQTQQQVVMGRNDSLQRAVFKAPVRGVVKEITVTTHGGVIPQNGKLMTIV
PIDEQLLIEARILPRDIAFIRPGQEALVKITAYDYSIYGGLKGKVTVISPDTIRDEVK
QDQFYYRVYIRTDSDKLYNKEGKAFGITPGMVATVDIRTGEKTVLDYLLKPFNKAKEA LRER"
/product="Type I secretion membrane fusion protein, HlyD family @ Type I
secretion system, membrane fusion protein LapC" /transl_table=11
CDS complement(623152..625293) /locus_tag="AUO97b_00755" /db_xref="SEED:fig|
400667.82.peg.615" /translation="MMTKINYQPWLQAVLTIAKHYRIEPSEERIRLQIDWNQNQNLDN
VLQLMTRQVGLNLRKAPFSLDLLNPWRLPVMVEFNDGQVGVIDKADTQGNVSIQFSGD

QGLSQSLSLDALKTTLKNVYILRPETSIPDARIDEYIKPYEANWFWSIVLRDWKRYVD
IMFASLIANVLALATIIFSMQVYDRVVPSQSIPTLWVLVGGVLIAALFEFTLRVSRVY
LSDIIGKRADLRVSDRVFGHALRIKNKDRSKSTGSFISQIRELEGVRELVTSTTISAI
ADFPFFFLFLVIFAIIGGKLFWVMLLVVPLMILPGILVQKKLAQLAQEGMRESSIRNA
ILVEAVQGIEDIKLLRAESRFQNQWNHMNEVSADIGMRQRKIVGTLMAWTQMVQGLTY
AIVVLVGCFAVMDGEMTTGALVACSILSSRMLGPIAQITGVLGRLQQAKVAKQSLDEL
MQRPIDQADRSHLVHKAVLNGDYELKNILFQYGEEDPRPSLAIRHLKIRAGEKIAILG
RNGAGKSTLLQLLSGMQVPTQGAVHLDGLDLSLIDPSDVRRDMGLLNQNAHLFYGTIR
ENLTLGAPLATDEDILRALVVTGALSFVQEKKEGLDYLVLEGGVGFSGGQKQALLLAR
LLIRQPNILLLDEPTAAIDDVAEKQLIDHLKGWLAHRTLVVATHRRAVLELVDRIIVV
NEGKIVMDGPRDQVLNQSTAQQKQVSQGGNS"
/product="Type I secretion system ATPase, LssB family LapB" /transl_table=11
CDS complement(625290..626756)
/locus_tag="AUO97b_00756" /db_xref="SEED:fig|400667.82.peg.616"
/translation="MLLSTLHVPVVIYAAKPSEQYHTLKENLSQLFVPKSSDDFKVAK
MHKLSNFKLDRSIVQEFPTVQLNDNRASSFSNLPSRTMTLQDAVKIAIQRSPDISQSI
ATLAGQNSGVDVAKAGYYPQLSGGITTGDLSSGERGRQLLTLSATQMLYDFGKVKSGV
DVEEAKVQVEQANVLVSVDTLALDVAQTIVNIQRYLQLNKIAEQQIAGIRRIQEIANL
RANAGVASQADPIQAQSYLQAAQSALIAQQSLLRQYQQHLRTLLGADVSHTGWIISDD
LVKQSDLYGEPEFNTIPKMIAAQAGVEVAKAQKQQTRLTRYPTLAVKGSVSQAINGKN
PNNDKDDGLYSSVMLEATSQFYQGGATASQVKMASYAEEAAKAKVNSVYMDVLDQIRT
SREQIENKQRQMQVLASRQATTVRTRELYQEQYKLGTRSLVDLLNAEQAIHSANSELE
SARYDIYSSIVQYIEATGRSRQAYGLNNISIQGFEVQP"
/product="Type I secretion outer membrane protein, TolC family"
/transl_table=11
CDS complement(627032..627778)
/locus_tag="AUO97b_00757" /db_xref="SEED:fig|400667.82.peg.617"
/translation="MQQALQQPQTLCIKHLAKNYSKRWVVKDVSFSMQSGQIVGLLGP
NGAGKTTSFYMVVGLVRMDKGEIHLDDLDMSDLAMHERARKGIGYLPQEASIFRKLTI
AENIMSILETRKDLNKQQRQQRLTELLNDFKISHIKDSLGMSVSGGERRRAEIARALA
ADPKFMLLDEPFAGVDPISVGDIKDIIQTLKDRGIGVLITDHNVRETLAICERAYIVS
EGSVIAEGTPQEILDNEQVRKVYLGDDFTV" /product="Lipopolysaccharide ABC transporter,
ATP-binding protein LptB" /transl_table=11
CDS complement(627778..628326)
/locus_tag="AUO97b_00758" /db_xref="SEED:fig|400667.82.peg.618"
/translation="MHQSLNLKRSSAFLKQAAVITFVALSSASTFALPSDRNQQISLV
ADRATYNEKTGLTTYTGNVVIEQGTMKLQADSIVATLNSKREIQTITAKGRPSKFQQQ
ISADKGIARGEGQTIVYNADTGIITLSGGAYLYQDGSSIRGNTLKYSMNKGDVEAQGS
SSNRVQIIIPPSSSKSFPGARD"
/product="Lipopolysaccharide export system protein LptA" /transl_table=11
CDS complement(628310..628858)
/locus_tag="AUO97b_00759" /db_xref="SEED:fig|400667.82.peg.619"
/translation="MDTRVLYIVAVVVAAVSGGYYYYSGKAKKLDIGSAKNMTYSAQG
VHLTQTDDKGNLYIRAEVRQLEQDMQQKTSKLDQLNATMYKLGKVDATFFAKQAYGYD
DNQKVVLSGDVVATKLAPQGTIEFQTDELTGYPKTRDIETNHQVTVQSPQGEFVSKGL
KANLNNGQYEFFNIRGKYAPKS"
/product="Lipopolysaccharide export system protein LptC" /transl_table=11 CDS
complement(628896..629435)
/locus_tag="AUO97b_00760" /db_xref="SEED:fig|400667.82.peg.620"
/db_xref="GO:0019143"
/translation="MASYALLEQARHLQALVLDVDGILSDGFVTLTNTGDEIKSFDIR
DGLGMKLAQQAGMKVIIITGRKSNIVEKRMSDLGVDLVFQGREDKGSALREACAQFNI
LPSDCLYMGDDWPDLSAFAIAGMSVTVPNGHEEVRRRADLVTQAMGGRGAVREVCDML
LIAKGIYQELLEKYLAVPH" /product="3-deoxy-D-manno-octulosonate 8-phosphate

phosphatase (EC 3.1.3.45)" /EC_number="3.1.3.45"
/transl_table=11
CDS complement(629439..630416)
/locus_tag="AUO97b_00761" /db_xref="SEED:fig|400667.82.peg.621"
/translation="MPNPTDFQSSALATLRIEQQAIDVLATQIDDRFNRACEILLQCK
GRVVITGMGKSGHIGRKMAATFASTGTPSFFMHPGEAGHGDLGMLVRGDVLIAISNSG
KSDEIMMLMPLIKHLGVPLITISRDDKGPMPQNADIALTLGESDEACPLGLAPTSSTT
ATLVLGDALAVALLEARGFTADDFARSHPAGALGKRLLLHVKHLMHTGDELPKVSPNT
PMNQVLYEISNKRLGLTTIVDEQDHLLGIFTDGDLRRLIDKQQGFDVNLPVSEVMTKK
PSTISQEARAVEALQQLNQKKISQFVVVDDQNKVIGVISMHDLIQAGVN" /product="D-arabinose-5-phosphate isomerase (EC 5.3.1.13)" /EC_number="5.3.1.13"
/transl_table=11
CDS 630630..632051
/locus_tag="AUO97b_00762" /db_xref="SEED:fig|400667.82.peg.622"
/db_xref="GO:0004817"
/translation="MQPFVLYNSEQRKKVEFVPRKEGHIDMYVCGMTVYDYCHIGHAR
VMVAFDYIIRFLRSQGWKVRYIRNITDIDDKIIKRANENGETIQQLTTRFIDAMNEDA
ANLGCLAPDEAPKATEYIDQMQNMIGNLVNKGAAYPASNGDVYFEVTKFEKYGRLSGR
KLEDMQAGASERVDVEVEKKHPFDFVLWKHAKENEPSWASPWGNGRPGWHIECSAMST
CCLGNHFDIHGGGSDLMFPHHENEIAQSEASTGEQYVNYWMHVGFINVDGEKMSKSLG
NFFTIRDVMEKFHPEVIRYFIVSSHYRSPVNFSDVALKEAKTSLTRFYHSFKAYQQVY
GQTTTEALDQSFIERFNNAMCDDFNTAEAMAVLFELNKELNRAVKEEQADQATVLYST
LRHLTNILGLVQHNVDDFLKSDIGQDALALSDAEIEDFIQQRVDAKKAKDFAKADSIR
QSLLEQGVVLEDTRQGTVWRRAD"
/product="Cysteinyl-tRNA synthetase (EC 6.1.1.16)" /EC_number="6.1.1.16"
/transl_table=11
CDS complement(632552..633181)
/locus_tag="AUO97b_00763" /db_xref="SEED:fig|400667.82.peg.623"
/translation="MVNTKQSNSVGNHTAQKEPSSNGNVSNSKPLQDISSTIDNARPD
LNSPPTNNNHVPDMNEGGENLVATGAGTLGGAAVGAAFGVVGGPPGAVVGGIIGGVVG
AIAGNDIAQTNNQKDDSNDWQEEDNYWRENYKKMPYYTENKNLEYDRDYRAAYRLGYE
NRVDNNAEINFSEVESKLKTKWEQVKGSSRLQWEDAKFAVEDGLKKIHP" /product="hypothetical protein"
/transl_table=11
CDS complement(633565..634158)
/locus_tag="AUO97b_00764" /db_xref="SEED:fig|400667.82.peg.624"
/translation="MKAILTKKIISCIAISGVLSFSAFEIMAANQQTINDGKNHSKIL
NENHENLTDSQIFKILSTANNGEIKQAKTALPKLKMDEAKKYAEMMIKEHSANEKNTQ
ALASRLQLISQTSNLSKSLQNDSDKIVSKLNQITSDTDKNYMMSQVKVHRKVLTIIDK
QLIPNTKSSELKNMLVQTRDAVAKHLKAAEDIFKNMK" /product="hypothetical protein"
/transl_table=11
CDS complement(634739..634885)
/locus_tag="AUO97b_00765" /db_xref="SEED:fig|400667.82.peg.625"
/translation="MASLGWKIELYFLLTSSLTLAKRGKEGEKVLVRVLNIMQGQRYI EICE"
/product="SECRETION ACTIVATOR PROTEIN"
/transl_table=11 CDS complement(634899..635165)
/locus_tag="AUO97b_00766" /db_xref="SEED:fig|400667.82.peg.626"
/translation="MELHYCETVKKAASGVMDARQHEVPAKELHDIANHLEEQQAKQL
YQELIKSAYSSKLFEDPLIKSKAVENFQTTWHEQCLAKELAKNM" /product="hypothetical protein"
/transl_table=11
CDS complement(635315..635761)
/locus_tag="AUO97b_00767" /db_xref="SEED:fig|400667.82.peg.627"
/translation="MANSTLMKEEPCPVARCVNLIGDRWSLLIVRDAFDGMRRFGDFQ
RSLGVARNILSDRLKKLVDVGVLEMQDASDGTAYQEYVLTPKGESLFPVIVALRQWGE

HNLFESGERHSLLIDKNTGQQIPFMVPVAADGKLLKASDTQVQKVK" /product="Transcriptional regulator, HxlR family" /transl_table=11
CDS 635873..637087
/locus_tag="AUO97b_00768" /db_xref="SEED:fig|400667.82.peg.628"
/translation="MKASLVSSTSSVDNNKTHSNLPRSVVLLFAIASGASVANVYYAQ
PLLDILASDFNVSHAVIGGVVTATQIGCALALVFLVPLGDLVNRRRLMTLQLLALISA
LLMVAFAHSTIVLLTGMLAVGLLGTAMTQGLIAYAASAAAPHEQGHVVGTAQSGVFIG
LLLARVFSGGINDIAGWRGVYFCAAIIMLMIALPLWRRLPHLNVQPVTMRYPQLLSSM
LKLLRQEKVLQVRGVLALLMFAAFNILWSALVLPLSAPPYNFSHTVIGSFGLFGVIGA
LAATRAGQWADRGYAQRTSLAALLILLLAWWPLSLMTYSLWALVIGIVLLDLGGQALH
VTNQSMIFRTRLEAHSRLVGLYMLFYAVGSGLGAISTTATYAYAGWLGVCSLGACVSL LALLFWWRTMKL"
/product="Uncharacterized MFS-type transporter" /transl_table=11
CDS complement(637082..638188)
/locus_tag="AUO97b_00769" /db_xref="SEED:fig|400667.82.peg.629"
/translation="MKNITEKSLFDVFTQHQAYLYRASSRSVNELYKFFNSETMVMLD
RLSNLMKELNETERFALAGGEYTTQHLKNIQLVISNWFNSISTVLPEIFTHSATALAI
YESNYMAKLFGKVIKDLEGEQLYQSIKRVPLAGGALVDESLAQISENALQRIEYAIRD
GMNTGMTPQQIIKRITGTKRLKYEDGLLNSTKIDIERIIRTLRSHISNQVYLYNFKNF
NFEYVRFVSVLDGRTSKICAAVDGSIWKLNDSAKRVPPLHPNCRSILVPVSKDGRLIG
NRSFVMDERKVRDIPKDERTQLMGQIDANISFKEFFNLTDNFFQKEWLGPKRYKLYKE
GQFDFDKFFDPKGRLYTLDELRELDERTFKILGL" /product="NAD+--asparagine ADP-ribosyltransferase" /transl_table=11
CDS complement(638286..638630)
/locus_tag="AUO97b_00770" /db_xref="SEED:fig|400667.82.peg.630"
/translation="MNENAELIKYLDIAENVYENMFEKYQITDNPITNLNRVMAEIRK
EAEQNDLKLKYTKIDFEECLTKPLSEREIKVDLSLLPRFEYRDEFILWLTNFIRNISV QKKFVKQKYYFN"
/product="FIG00350089: hypothetical protein" /transl_table=11 CDS complement (638924..639382)
/locus_tag="AUO97b_00771" /db_xref="SEED:fig|400667.82.peg.631"
/translation="MPKYLAIAEKVYKEIKKDNLFTEDTIQNLNCLISLIRKAIKGTE
FKLKYNFINFEECLNQFNKEKSVHIDLSLMPKFKNEDEFILWLAGFIERMTIGGKEKL
PPISNYIPKGFKIDQTEIPVAAEPSKEENAEMIINYFKSEDFIKNNKISS" /product="hypothetical protein"
/transl_table=11
CDS 639869..639988
/locus_tag="AUO97b_00772" /db_xref="SEED:fig|400667.82.peg.632"
/translation="MSFLRKKNKASFVLFIITLYSMLGFGLGFIIWEYFLQIP" /product="hypothetical protein"
/transl_table=11
CDS complement(640145..640960)
/locus_tag="AUO97b_00773" /db_xref="SEED:fig|400667.82.peg.633"
/db_xref="GO:0004735"
/translation="MLNIVQPNICFIGSSNLSLALIGGLVLKGFQREKINLIEEVKFE
NQIILKQKQHEVKKADIVVLLLDPKDLKAILAPLKKWLADKTIVSMMAGVNIQQLMSI
TGSKKIIRVISNPPVLTYTGTHVLIGSDYLEPLDKEVIETIYSATGRTYWANSESQSD
AIIALSGSGPAYFFYILDSMVKTGVSMGLDKQFALDLILQAASGAVEMVRKSNVQPSE
LCGKVTLANGITESALRMFELGNLSDDIRLALKAAYHRSKEISLEINAEITRH" /product="Pyrroline-5-carboxylate reductase (EC 1.5.1.2)" /EC_number="1.5.1.2"
/transl_table=11
CDS complement(641236..641463)
/locus_tag="AUO97b_00774" /db_xref="SEED:fig|400667.82.peg.634"
/translation="MDICIGGILNGKVRKNNENYFSIGNPHSDDVTEYHKQYFHLDGK
LLSFWVCNEINFQEASRIAESFFKKEQSFIK"
/product="FIG00352334: hypothetical protein" /transl_table=11

CDS complement(641667..641882) /locus_tag="AUO97b_00775" /db_xref="SEED:fig|400667.82.peg.635" /translation="MSNTTGTVKWFNETKGFGFIQTDEGKDVFAHFSEIQTQGFKVLL EGQRVQFTVTQGKKGPQASNITIVTNA"
/product="Cold shock protein of CSP family" /transl_table=11
CDS 642400..643017
/locus_tag="AUO97b_00776" /db_xref="SEED:fig|400667.82.peg.636"
/translation="MSGLLVFITIAFLTLSSPGPGVLFTVTNSINYGVRTALFGISGL
IIGMFIIAVISASGVGLIITSNPTIFTALKFIGAFYLMYLGYKNFIKKSPTQDLLETE
HNQKSIKKSKLFYQGLFASLLNPKTIVFFIALFPQFIDIKKEILNQFLVLSLTFCLIG
FLIHLVYANFSSIFKEKMLAGNNFSKLNKVSGCIFFLLAVLLITQ" /product="Threonine efflux protein"
/transl_table=11
CDS complement(643084..643629)
/locus_tag="AUO97b_00777" /db_xref="SEED:fig|400667.82.peg.637"
/translation="MNIEQYLDALIKREGGYVNDPLDHGHATKFGITQAVARSYGYQG
AMENLTLETAREIYRNQYWLEPRFDQVNLISALVAEELFDTGVNCGISFTKPLLQRAL
NLLNRQGKEGWVDLKVDGEYGPATLHALIKYIKNRGKEGEKVLVRLLNIMQGQRYIEI
TETNPKHEQFFYGWLANRISL"
/product="Lysozyme (N-acetylmuramidase) family, (EC 3.2.1.17)"
/transl_table=11
CDS complement(643667..644056)
/locus_tag="AUO97b_00778" /db_xref="SEED:fig|400667.82.peg.638"
/translation="MENFLTSNGLAAAFNLYGTVMMVVLAVSLVVAVVLIMRFPRSPQ
EWVVGLICTVVSSLTGGSFIIMKWNLHEWVTDIWGMITLGGFFFICGLPGWAIVRWIF
NFINKQEGKTIVEVMQDLKKAKDDIQS"
/product="hypothetical protein"
/transl_table=11
CDS 644620..645291
/locus_tag="AUO97b_00779" /db_xref="SEED:fig|400667.82.peg.639"
/translation="MALKDRLKDSRIKAKKTQAEVAEAVKMSQPAYQALESGKNLKSA
FLPLIAQFLGVDAYWLTTGNSEDSFRESDVFSPTVVNNDAADQYVWIEVVEASFSCGT
GESIEFHFDAINGKIPFPASFFKEKRVAQECMRIIKAKGDSMTDYIKDGDLVGIDISQ
TEVIDGEIYAVYFAGEGMIKQIFKEADGSLILHSLNEKFRDRRVTEENGKNFKVMGRQ FWRAG"
/product="Peptidase S24 S26A and S26B"
/transl_table=11
CDS complement(645443..645676)
/locus_tag="AUO97b_00780" /db_xref="SEED:fig|400667.82.peg.640"
/translation="MIVNIVNEWQIATAVNGNEINVRIVPHVRKQNSLDGYRWVEVGK
KIQLQTGEEIELNQDGKSFYAGFNQLYRLNTYC"
/product="fragment of transposase"
/transl_table=11
CDS 646183..647445
/locus_tag="AUO97b_00781" /db_xref="SEED:fig|400667.82.peg.641"
/db_xref="GO:0008855"
/translation="MSDPQLSLSEYLGTVQEVIRLTFDEPVWVRAEIRNLNVKGGHYY
LELAEKDADTDKVIASCKATIWKFSASKIVLKFERETGIELASDLNVLIKIRARFDPQ
YGFSVNIEEIDSSFTLGEIAKRYQQIVERLTQEGLIHKNKLLPTPFDIQNVLVIAPQN
AAGLGDFKKDADALERNGVCHFVYQSATFQGNTAAVSLMESLATGLKQWANNFNFPPD
LIVIIRGGGAVNDLAYLNDYELAALLCKRSVPIWVGIGHEKDRTILDEIAHRSFDTPS
KVIAGIRNHIVERVQEAVDNLQTIKLLSQHQITTYQSKSDQYLQHIKSSAQAQLNSAH
QFLEQIKERIQFVSQQQVKFSLSQTESLMREILLQNPKQIQAKGYAIVRSEGKAIRSI
HQITNSTITVDMQDGAINANITEVIPNE" /product="Exodeoxyribonuclease VII large subunit (EC 3.1.11.6)" /EC_number="3.1.11.6"
/transl_table=11

CDS 647438..647632
/locus_tag="AUO97b_00782" /db_xref="SEED:fig|400667.82.peg.642"
/db_xref="GO:0008855"
/translation="MNDKELTFKEGHDILKRNAELLESQESPDIDNLMKIVEESIGAY
KACKARIEAVQQALDETFKE"
/product="Exodeoxyribonuclease VII small subunit (EC 3.1.11.6)"
/EC_number="3.1.11.6"
/transl_table=11
CDS complement(647857..648081)
/locus_tag="AUO97b_00783" /db_xref="SEED:fig|400667.82.peg.643"
/translation="MKRLKVVIKDQFDQESIALAKVRPANDQKLELYKSVEHIEIDDD
IILPNMDLLYENPKDGKIYKLVGPFNEDEI"
/product="FIG00351239: hypothetical protein" /transl_table=11
CDS complement(648132..648725)
/locus_tag="AUO97b_00784" /db_xref="SEED:fig|400667.82.peg.644"
/translation="MVSFLFIGLVVTILLTPGPTNTLLASAGIQAGVKHSLKLIPAEV
IGYLIAITSWGVLLESVSHFIPWLPAILKLMSAGFILYLAFKLWMTSSEDIKLDRPLI
TPKALFLATLLNPKALLFASAIFPHTAWEIPHLYFIHIVVFLSLITPIAFLWIMFGTV
LISNKVTWLNQRNLQRIAAFILTFFAMPIGYSAISSF" /product="Transporter, LysE family"
/transl_table=11
CDS 649166..649495
/locus_tag="AUO97b_00785" /db_xref="SEED:fig|400667.82.peg.645"
/translation="MMNAYVRPHPDGFKSYLGKDQKTGLYTVRVGWTVYETNANGSVL
YIVKEESKIPLDVEKFKTDRPKIYDALLNEVQFQRRKQLAIDLQKSNIPSYDRKAYKK RRGFTGS"
/product="hypothetical protein"
/transl_table=11
CDS 649975..650331
/locus_tag="AUO97b_00786" /db_xref="SEED:fig|400667.82.peg.646"
/db_xref="GO:0005506"
/db_xref="GO:0006118"
/db_xref="GO:0009055"
/translation="MNSSQEPNQSLFNQASTSIQACIECMFACKHCASACLQEEHVHM
MRECIKHCLTCVETCQLCTSLELRHSELAEQAMQLCANACQLCAAECSKHEHEHCQVC AKACLACAQACQAYRA"
/product="Uncharacterized cysteine-rich DUF326 protein bsYhjQ/STM1261"
/transl_table=11
CDS complement(650924..651331)
/locus_tag="AUO97b_00787" /db_xref="SEED:fig|400667.82.peg.647"
/translation="MAYTASCLCNGIQLRINAELEPIMVCHCKQCQKAQGAAFAAITQ
VQKSDLEIVQGENLLQAYFASPNKKRVFCKICGSPVWSERLDKPDVVRLRVGLINEEI
STPVISHAFVNSQVKWYPICDTARQYPNGVENP"
/product="Gfa-like protein"
/transl_table=11
CDS complement(651360..651761)
/locus_tag="AUO97b_00788" /db_xref="SEED:fig|400667.82.peg.648"
/translation="MNIGQASKHSGISAKMIRYYEEIGLLEAAQRSASGYRIYSETDL
KTLNFLKHARELGFSSEQMKELISLWKNTDRHSAEVKALTVKHIAELKQKIAHLQEMV
NILQASADDCCGDQQASCAILDHIEKGVGVS" /product="Cu(I)-responsive transcriptional
regulator" /transl_table=11
CDS complement(651837..654308)
/locus_tag="AUO97b_00789" /db_xref="SEED:fig|400667.82.peg.649"
/db_xref="GO:0004008"
/translation="MDSKTTKLPAYSETLSIEGMTCASCVGRVEKALKKVENVEIANV
NLATEKAVVYSNQPIQREALVKAVERAGYDVPKAAPVELSIEGMTCASCVARVEKAFK
KVEGVQNATVNLATEQAWVQADNSVNVEDLIRAVKKAGYDAKASEKNQDEQLDKKASE

LDQLKKDLIISIVLALPVFILEMGSHLIPAFHMWVMHTIGQYNSWLLQFVLTTLVLVF
PGRRFYQKGIPALWRLAPDMNSLVAVGTLAAYSFSVVATFMPQVLPQGTVNVYFEAAA
VIVSLILLGRYFEAKAKGRTSQAIQHLVGMQPKTARIQRDGQIVEVAVAEVVSGTIVE
IRPGERVPVDGEVVEGHSYIDESMITGEPVPVEKREGQQVVGGTVNQNGTLNIRATAV
GSSSVLSQIIRMVEQAQGSKLPIQGLVDKVTMWFVPAVMLIAAITFFVWFIWGPEPAL
TFGLVNAVAVLIIACPCAMGLATPTSIMVGTGRGAELGVLFRKGEALQLLQEAQVVAV
DKTGTLTEGKPTLTDFNVQSGFERNQVLTLVASVEAKSEHPIALAIVQAAESEGLNLL
PVTAFNSITGSGIEAEVSGQKVQIGADRYMHQLGLDTNSFQAIAAQLGEEGKTPLYVA
IDQQLAAIIAVADPIKETTYAAIEALHKLGLKVAMITGDNRHTAQAIAKKLNIDEVVA
EVLPEGKVDTVRQLQKQYGRLAFVGDGINDAPALAQADVGLAIGTGTDVAIEAADVVL
MSGSLKGVPNAIALSKATMRNIRQNLFWAFVYNVALIPIAAGALYPAFGVLLSPMFAA
GAMALSSVFVLGNALRLKRFHAPVE"
/product="Lead, cadmium, zinc and mercury transporting ATPase (EC 3.6.3.3)
(EC 3.6.3.5); Copper-translocating P-type ATPase (EC 3.6.3.4)"
/EC_number="3.6.3.4"
/EC_number="3.6.3.3"
/transl_table=11
CDS 654481..654675
/locus_tag="AUO97b_00790" /db_xref="SEED:fig|400667.82.peg.650"
/db_xref="GO:0030001"
/db_xref="GO:0046872"
/translation="MKLLIENMTCGGCARGVTATIQDVDPNAKVDVDLATKIVTVESS
ESVDKITEALEEDGFPAKVQ"
/product="Copper chaperone"
/transl_table=11
CDS complement(654725..655684)
/locus_tag="AUO97b_00791" /db_xref="SEED:fig|400667.82.peg.651"
/translation="MELRHLRYFVAVVEEQSFTKAAEKLFIAQPPLSRQIQNLESELG
IQLFERGSRPLQTTPAGHFFYQHAVKLLSNAEEIKSMTKRIGLIERSMTIGFVGSLLY
GLLPRIIYLFRQQQPHLNIQLMELSTTEQLQALKEGRIDVGFGRLRISDPAVRRILLR
KERLVVAAHTSHPIAQRTEGVYLADLIDEKMFMYPTSPKPNFSTQLLNIFAEHSLVPK
NMHEIREIQLALGLVAAGEGICIIPASADTIRFPHLNYIPILDNGAVSPIFITARAMD
RSEDLQSLFDCIYQVYDLEGIPYQRTVFTLDQNPIDDSNGIDF" /product="Aromatic hydrocarbon
utilization transcriptional regulator CatR (LysR family)" /transl_table=11
CDS 655967..657349
/locus_tag="AUO97b_00792" /db_xref="SEED:fig|400667.82.peg.652"
/db_xref="GO:0018623"
/translation="MPRIPVINTSHLDRIDELLVDNIDTGEFKLHRSVFTDEALFDLE
MKYIFEGNWVYLAHESQIPNNNDYYTTYIGRQPIIIARNRNGELNAMINACSHRGAQL
CRYKRGNKATYTCPFHGWTFNNSGKLLKVKDPTDAGYSDCFNQDGSHDLKKVARFESY
KGFLFGSLNPDVPSLEEFLGETTKIIDMIVDQSEHGLEVLRGSSTYTYEGNWKLTAEN
GADGYHVSAVHWNYAATTQHRKETQAADNIRAMSAGSWGKQGGGSYGFENGHMLLWTQ
WANPEDRPNFPKADEYTEKYGEAMSKWMIERSRNLCLYPNVYLMDQFGSQIRVLRPLS
VNRTEVTIYCIAPKGEAPEARARRIRQYEDFFNASGMATPDDLEEFRACQAGYAGIAL
EWNDMCRGSKHWIYGPDDAANEIGLKPMLSGIKTEDEGLYLAQHQYWLKTMKHAIASE KEIADQGETA"
/product="Benzoate 1,2-dioxygenase alpha subunit (EC 1.14.12.10)"
/EC_number="1.14.12.10"
/transl_table=11
CDS 657346..657846
/locus_tag="AUO97b_00793" /db_xref="SEED:fig|400667.82.peg.653"
/db_xref="GO:0018623"
/translation="MSAQDKATLENIAQFLYREARFLDDEQWDDWLECYAPEASFWMP
AWDDDDRLTEDPQSEISLIYYPDRQGLEDRVFRIKTERSSATMPDTRTSHNIANVEVL
DRDGDKVTVRFNWNTLSFRYKTNYSYFGMSRYVIDFSGDQPKILSKYVVLKNDYINQV IDIYHL"

/product="Benzoate 1,2-dioxygenase beta subunit (EC 1.14.12.10)"
/EC_number="1.14.12.10"
/transl_table=11
CDS 657917..658933
/locus_tag="AUO97b_00794" /db_xref="SEED:fig|400667.82.peg.654"
/translation="MSNHNVALQFEDGVTRFINVAQGETLSDAAYRQKINIPLDCRDG
ACGTCRAFCESGSYDMPEETYIEDALTPEEAAQGYILACQCRPTSDAVFQIQASSDVC
KTEIHSFQGTLARVENLSDSTITFDIQLDEGQPDIHFLAGQYVNVAIPETGETRSYSF
SSKPGNRLTGFVVRNVPNGKMSEFLSKNAKTGDKMTFTGPFGSFYLRNVVRPVLMLAG
GTGIAPFMSMLQVLEEKGSEQPVRLVFGVTNDFDLVALEKLNELQAKFPWFEYRTVVA
SPESNHERKGYVTGHIESEWLNGGDVDVYLCGPVPMVEAVRGWLETENIKPANFLFEK FSAN"
/product="Benzoate 1,2-dioxygenase, ferredoxin reductase component"
/transl_table=11
CDS 658955..659740
/locus_tag="AUO97b_00795" /db_xref="SEED:fig|400667.82.peg.655"
/translation="MSNRQRFTDKVVIVTGSAQGIGRGVALQVAAEGGQVVMADRSEY
VEEVLTEIQRTGGDAVTVNADLETYAGAQAVVTKAIEHYGRVDILINNVGGAIWMKPF
EEFSEEEIIKEVNRSLFPTLWCCRAVLPAMIKQQAGVIVNVSSIATRGINRIPYSASK
GGVNALTASLAFEHAKDGIRVNAVATGGTEAPPRKVPRNANPLSQNEKDWMQQVVDQT
KDRTFMGRYGTIQEQVNAILFLASDEASYMTGSVIPVGGGDQG" /product="1,2-
dihydroxycyclohexa-3,5-diene-1-carboxylate dehydrogenase (EC 1.3.1.25)"
/EC_number="1.3.1.25"
/transl_table=11
CDS 659798..660991
/locus_tag="AUO97b_00796" /db_xref="SEED:fig|400667.82.peg.656"
/db_xref="GO:0016021"
/translation="MATLFKTLKNDWSISATVAGFLAVLISYSGPLIIFFQAAQRAHV
STDMMVSWIWGISIGAAISGIYLSIKYKTPVITAWSAPGTALLVTLFPNVSLNEAVAA
YITSAIVIFLIGVTGYFDKLLKWIPQDVAAGMMAGLLFQFGISLFTASDSMPLIVFSM
LIVFLVAKRLMPRYTMIWVLAAGVLLSLILGKMNPVDVSFNLAIPQWISPEWTWNSTL
NLAVPLILVSLTGQFLPGMAIMKLSGYDTPAKPIITATSIASLAVACIGGITIVLASI
TAALCMGKDAHELKEKRYIAGIANGIFYILGGLFAGSIVMLFSLLPKELVAALAGLAL
LGAIATNISVAIKNDGQRDAALITFLASASGMHFLGLSSVFWGICIGVIAHFILTPRS TPATN"
/product="Benzoate transport protein"
/transl_table=11
CDS 661460..662818
/locus_tag="AUO97b_00797" /db_xref="SEED:fig|400667.82.peg.657"
/translation="MDTSKVNINELIDKARFTSFHWKVLIWCLLIIIFDGYDLVIYGV
ALPLLMQQWSLTAVEAGLLASAALFGMMFGAMIFGTLSDKLGRKKTILICVTLFSGFT
FIGAFAKGPTEFAILRFIAGLGIGGVMPNVVALMTEYAPKKIRSTLVAIMFSGYAIGG
MTSALLGAWLVKDMGWQIMFLIAGIPLLLLPLIWKFLPESLAFLVKSNHSEQAKNIVS
KIAPQTQVNANTQLVLNESTTTDAPVRALFQQGRTFSTFMFWIAFFMCLLMVYALGSW
LPKLMLQAGYSLGASMLFLFALNIGGMVGAIGGGALADRFHLKPVITIMFIVGSAALI
LLGINSPQFILYSLIAIAGAATIGSQILLYTFVAQFYPTALRSTGMGWASGIGRIGAI
IGPVLTGALLSFELPHQMNFLAIAIPGVVAALAIFMVNLKASVAAQTPSTFNPQNTLT QQ"
/product="benzoate MFS transporter BenK" /transl_table=11
CDS 662863..664125
/locus_tag="AUO97b_00798" /db_xref="SEED:fig|400667.82.peg.658"
/translation="MELLHRFKPCNLAITTLFGLLTSSSLWAADTTQQSEDEWKFTLK
NAYINRDFDNDALKDTGSWSQAASLFYKSKMYDTPLIIADKPITIGADASVQYAVRLS
SDKHVADTVLPFNKETQSQASDFLKYGATLKLGYDKTLLSIGELWLDLPVTAVDASRQ
LLASYWGTNLKSQLSDQLYAEIGRVEKVSPRNEEDFKKFSFTANGMTKESDGLNYIDL
RYQFTPSLKGEYYFGNLEDLYNKHYVGLEHTWKQPNFALTSKFKYFNAKDDGNTFDID
AQNIGVLETLKVKNHTFGLGYQQIIGESAYPLPDGFLPETYFINWNATGFFKEDEKSY

HVMYGYDFKDYVPGLNAMVKYVYGHDFKAANGEKNHETESNVILNYAFQQPFLKGFAL
QYIRIDYNVKHGNDFGEDRLFVNYTKKF"
/product="Outer membrane low permeability porin, OprD family"
/transl_table=11
CDS complement(664226..665332)
/locus_tag="AUO97b_00799" /db_xref="SEED:fig|400667.82.peg.659"
/db_xref="GO:0004315"
/translation="MGIRITGTGLFHPTEIISNEELADSLNAYVEQYNQENAEKIAAG
ELEELRGSSAEFIEKASGIKRRYVIEKSGILDPTRLRPRLSERSNDELSIQAEWGVIA
AKQAMENAGVTAEDIDVVILACSNMQRAYPAVAIEIQSALGIQGYAYDMNVACSAATF
GLKQAADAIRSGARRVLLVNVEITSGHLDYRNRDCHFIFGDVATASIIEETTTKTGFE
ILDIHLFTQFSNNIRNNFGFLNRSEDAVVDDKLFRQDGRKVFKDVCPLVAKIINAQLE
KMQLTANDIKRFWLHQANANMNELILKYVAGKDADLSRAPIILDEFANTSSAGVIIAL
HRTGHEVDDGEYGVISSFGAGYSVGSIVVQKHVA" /product="3-oxoacyl-[acyl-carrier-protein] synthase, KASIII (EC 2.3.1.180)" /EC_number="2.3.1.41"
/transl_table=11
CDS complement(665427..669311)
/locus_tag="AUO97b_00800" /db_xref="SEED:fig|400667.82.peg.660"
/translation="MLNFQENLSVLNGLNIDQWVMARDRYRLNRLRKDKKANTAEIEK
LFEQSNAKVRQRFERLPKIKLNQDLPVTQYADRLIEAIQKHQVIIVAGETGSGKTTQL
PQIAMLAGRGLTGMIGHTQPRRLAARSVSQRIAEEVGEKLGESIGFKIRFNEQGSQDS
IVRLMTDGILLAELGNDRFLSKYDTIIIDEAHERSLNIDFIMGYLKQLLPKRPDLKVI
VTSATLDVNRFSNYFNNAPIFEVEGRSYPVEVRYRPISEMNIGGSDDDEFDDFEENLP
RAVVQAVEECFADAEEKGHPEHADILIFSSTEQEIRELQETLQKYGPRHTEILPLYAR
LAVSEQQKIFSPSGKGRRIIIATNVAETALTVPNIRYVIDSGFARISRYNYRSRVQRL
PIEAISQAAANQRKGRCGRVAPGVCIRLYSEEDFLSRPEFTEPEIKRTNLASVILQMQ
SLGLGELENFDFIEPPDFRLVNDGRKLLIELGALNEKKNELTKVGQMMARMPIDPRLA
RMIVGGAHFGVLKEILVVVSALAVQDARERPADKQMQADQKHALFKEADSDFLFYLKL
WDTLNPKGEAGMSENKRRQFAKQHFLSWLRLREWKQTHQQLVELAEGLKLSFNEKPAN
YENLHRALLTGLLSFIANKTDERNTFMAVRQQKAKVFPASTLHKTNTAWVMAFEMVET
SQVYLRTLAKIDPEWILLAARDLLKYHYFEPHWSKKAGIVNAYAQISLFGLIIEPKRL
VNFEKVDQPAAHEIFLRDALTTGNLGTVPPFLKHNLLKLEEVERVEDKLRRRDLVVDE
ETIYQFYASKIPEEIASRRSFEDWRATVEAKNPRYLYVEDDALWLNDRPTTQQFPDHL
HNGQLRLATTYRFDPSHDEDGATVQIPVQALPQVDENIWSWGIPGWRLDLIEALLKAL
PKDKRRNLVPIPDTARKLMQGIDAVHLRQHIFSYLAFALRGEQITEKDFSFERIDPYL
VPFIKVIDEKGKLIAKGRDLAELKARCRVETHRPVKQQKGEFKTFPENFTFEASQKVT
GVVVKQYQALVPTKNFAELEAKDESGVVIQTFNDQAEAIKQHREGIIRLVHMQLGDLV
RQLKKQISKPLALAYSPLGDKAKLEQMLVYATLQMAITTLPINAGEFNQLLEQVKKQF
LSYGQQALEQLSDIYIQWQQIRRQLLVLDPDVFGRSIDDIEDQLDLMSLGNFVYCQPV
DLWQEYPRYLKALLLRLDRLPNNLQRDHAAIDDVDPWMDKVFKFKNDPRIKELYLMLE
ELRISLFSQPMKTKMPISPTRLQKLWDRLGIS"
/product="ATP-dependent helicase HrpA" /EC_number="3.6.4.13"
/transl_table=11 CDS 669637..670200
/locus_tag="AUO97b_00801" /db_xref="SEED:fig|400667.82.peg.661"
/translation="MSLINTEVKPFQATAYHNGQFIEVNETNLKGKWSVVFFYPADFT
FVCPTELEDLADNYAEFQKLGVEIYGVSTDTHFTHKAWHDTSDAIKKIQYPLIGDPTW
TLSKNFDVLIESEGLADRGTFVIDPEGKIQIVEINAGGIGRDASELLRKVKAAQYVHS
HPGEVCPAKWKEGEATLAPSIDLVGKI"
/product="Alkyl hydroperoxide reductase protein C (EC 1.11.1.15)"
/transl_table=11
CDS 670393..670608
/locus_tag="AUO97b_00802" /db_xref="SEED:fig|400667.82.peg.662"
/translation="MANTAVVTSWKYNATSRHLKIFYSDGSGDLYHPVPEFVYDNLLR
STDKAAFVHKYLEFNLHFTRLSLVIAS"

/product="hypothetical protein"
/transl_table=11
CDS complement(670673..671260)
/locus_tag="AUO97b_00803" /db_xref="SEED:fig|400667.82.peg.663"
/translation="MPNTIFLQRVADLYDTFKQHDSQQSDRLQRYRNIEVESAQLISQ
LIRMQQAKSILEIGTSTGYSTLWLAEAAQATGGQVITVEIDAKRSAEAKRHVAELELS
EIVQFWVGDAADYLKAAQEKFDFILLDAERDAYENYWPDLKRLMKPKGGVLVVDNVIS
HAAEVNRFIALIKKDPDYMSSILPVGAGLCLVVTK" /product="O-methyltransferase"
/transl_table=11
CDS complement(671297..671515)
/locus_tag="AUO97b_00804" /db_xref="SEED:fig|400667.82.peg.664"
/translation="MSLQRGIYQHYKGNLYQVFSVAKHSETEEELVVYQCLYGDYSTW
VRPLDMFTETVQMSDGSVVPRFKLIQST"
/product="hypothetical protein"
/transl_table=11
CDS 671777..673342
/locus_tag="AUO97b_00805" /db_xref="SEED:fig|400667.82.peg.665"
/db_xref="GO:0006118" /db_xref="GO:0009055"
/db_xref="GO:0015035"
/db_xref="GO:0015036"
/db_xref="GO:0045454"
/translation="MLDQNIKTQLKAYLERLESPIELVAALDESDKAAQIKELVTEIA
ELSDKVTARFDGNNTRRPSFGVAKAGEQPRVFFAGLPMGHEFTSLILALLQVSGYAPK
VSDEVLNQIKGLNLKANFDVFVSLSCHNCPDVVQALNLIAIYNPNTTATMIDGAFFQD
EVEQRKIMAVPMVFQDNEHIGQGRMTLEEIVAKLDTNSAEKDAAALNAKDAFDVLVIG
GGPAGATAAIYAARKGINTGIVAERFGGQVMDTMDIENFTSVQKTQGPKFAAEMEAHV
REYDVDIMNLQRVSKITGADQTANGLVEVELENGAKLESKTVILSTGARWREMNVPGE
AEYRTRGVAYCPHCDGPLFKGKRVAVIGGGNSGVEAAIDLAGIVEHVTLVEFDTKLRA
DQVLQNKLNSLPNTTVIMNALSTEVLGDGSQVTGLKYKDRATDEEHVVELAGIFVQIG
LLPNTDFLKDSEVELTNRGEIIVNDRNETNVKGVFAAGDCTTVPYKQIIIATGEGAKA SLSAFDYIIRSGQ"
/product="Alkyl hydroperoxide reductase protein F" /transl_table=11
CDS 673516..673860
/locus_tag="AUO97b_00806" /db_xref="SEED:fig|400667.82.peg.666"
/translation="MNKLLVALGLAATVALVGCNKDKAPETGATTGEHLENAAQQATA
DIKSAGDQAASDIATATDNASAKIDAAADHAADATAKAAAETEATARKATADTAQAVE NAAADVKKDAQH"
/product="Outer membrane protein"
/transl_table=11
CDS complement(673924..674535)
/locus_tag="AUO97b_00807" /db_xref="SEED:fig|400667.82.peg.667"
/db_xref="GO:0004364"
/translation="MKLYYSPGACSLAAHIILNEINVDFDLERVNLKTHKTEKGADYY
EINPKGYVPALEINPGLILTENVAILPFLAQHDPKQDLIPPSGLGRAKVLEWLGYLNS
ELHDAYAVFFGAPLTNDEKTKAYAEIDRLLKYIDNYLAESDYDYLVNDNFGPADAYLF
VLTNWSNSIEHDLTPYKHIIALRNKVAERQSVQIAMRDEGLIS" /product="Glutathione S-
transferase (EC 2.5.1.18)" /EC_number="2.5.1.18"
/transl_table=11
CDS complement(674743..677181)
/locus_tag="AUO97b_00808" /db_xref="SEED:fig|400667.82.peg.668"
/translation="MKTFQLKTLSVFLSALGAFSYSSVAQAQLMFSQYVDGSSNKKGL
EIYNPDGTTVNLADYEIQQFNNGGTAKTATFRLQGTLASKQKFLVGRSELQTELGNKV
NQVAALSFNGDDAVVLVYKGTPVDRFGRIGERPEAGWGTAVSSLGNSFKRIETENPAL
SIDPTAVFDLDHSWTAWANRNDFTNLSGTTTQPPIETVSCSSSDTPIADLATSAQNQT
YTVRGVITADYRYANGFSGFYVQTPDTKARANVSNAIFVYIPNSSTVKGGQIGDEVIL
RGRLTSYQNQLQIDQLQQDIQTCNSNMANQVQPISLELPFSSLTGGSTHSPQRYQGML

VKLPQTLTVSENYNYGRYGELSLSLGRLYIPTNLYPALSPEAKALAQKNLLSKIIFDD
GYNNQNRTPWLPTNFSAANTLRSGYQLKNVEGILEYRFNGWRVQPVLGRTQPEVITQI
NPRQNVITKNANHIRVASFNVLNYDNGATGFPTERGANTQAEFDKQHRKIVSALKSID
ADVYGLMEIANNGYGPNSAIAHLTSALGPDWKYVIPENLDRLGSDVIAVAIIYNSKRV
KPLNKAVVLDLGDKNRTTLAQTFQAVRGNKTFTVIPNHLKSKGCSGVDANSSDADQND
GQGCWNPTRVKAVDQIVQWLAKNPTQVPKQNALLVGDMNSYAKEAPILAFEKANYKVL
LNDAKVGQGAQAYSYVFGVASDANGNGGAGNLDHAIADADLYPKVVRTFAWHINADEP
TVLDYNEEYKTDEQKALFYGEDAYRSSDHDPVIVDLDLNGKDSNQPNDNQKSPIFDFL SQLMEWISQLFKRS"
/product="Extracellular ribonuclease/nuclease fusion protein"
/transl_table=11
CDS 677327..678043
/locus_tag="AUO97b_00809" /db_xref="SEED:fig|400667.82.peg.669"
/db_xref="GO:0008176"
/translation="MSIDQLETQIAELDNLPEHREIVTFMRRSAPLNTSQRTALEQHR
DLILEYPVGDLRQHFEHPERPLTVEIGFGMGRSLVLMAKANPERNYVGIEVHVPGIAQ
CVYEAGMAELKNLRVLDADAIQVLREMPDNSINCVQLYFPDPWQKKRHFKRRFVIHER
MQLVEQKLELGGTFHAATDWEPYAEWMLDVLDNRPNLENLAGKGNSYPRPEWRPVTKF
ERRGIESGHKINDFIFKKIK"
/product="tRNA (guanine(46)-N(7))-methyltransferase (EC 2.1.1.33)"
/EC_number="2.1.1.33"
/transl_table=11
CDS 678215..678841
/locus_tag="AUO97b_00810" /db_xref="SEED:fig|400667.82.peg.670"
/translation="MLKILGRDNSINVRKVLWLCDELNLTYEREDWGIGFRSAQSPEF
LQLNPNGQIPVILDGDLVLWQSNSIIRYLANAYDKDHILYPTQAKQRAIIDQWIDWQA
IELNNSWTYAFMSLIRHSALHQDPNLLQQGIDQWNKQMQILDQQLAKTQAYVAGTEFT
LADIPIGLSVQRWKATPFDHPALKHVDQYFERLNQRKGFLKWGNNGQP" /product="Uncharacterized
glutathione S-transferase-like protein" /transl_table=11
CDS complement(678852..679631) /locus_tag="AUO97b_00811" /db_xref="SEED:fig|
400667.82.peg.671" /translation="MKISLLGSGRVAFHLAKALLAQGHHIAQVYARDFEKTKKFAEQI
QAKACQSLQEFQPSDLIILAVSDSAITELATQVHEIFPETLMVHTSGSTDIQVISNIH
EKAGVFYPLQTFSLERDVDWQATPLFVEATNKDDLVILSDLANSLSKRVYQYTSKQRL
TLHLAAVFACNFSNYCFDMAKQVVDAEQVDFGLLYPLILETAKKATENDPKQMQTGPA
MRGDQNILVMHQSLLAQANRDDLKQVYQLLSDGIVKRHHSS" /product="Ketopantoate reductase
PanG (EC 1.1.1.169)" /transl_table=11
CDS complement(679718..681133)
/locus_tag="AUO97b_00812" /db_xref="SEED:fig|400667.82.peg.672"
/translation="MSINPIELLKEKVSSTILNNQDGYLGEKTNALSKFYPILLSLLA
AKPDLIGQLKNSLAPSLSDLFSHNEQIKNTVLTHLSGTAPNNEIENTLNSALKPSLNA
ISDVAGNDQQSIVNYLRQHAETIRSYLPGWAVGLLAPLGIGAGLSSVTSSTAPPLAAA
TETTGKSRGFLPIIALIILGLLIAWLWRSCQHKEATPAPETKAASGVEAAAAPATLTL
STDDKGAVSQCQAGIGDQGFLATLQTQVKQVFSATKDCDVDTSQTYAAAFTDKDALAG
VLGALKGIPNASLEWVGDKITLKAGDAAALEALTAKVKALVPHTEVVAAAPETAEQSV
SNSLSASQTALTAIDPNNVDVNALVKALNLQIINFASGSSDIPADNKAILDQAATLLN
KVSGVKLDVGGHTDSTGNAAANKALSQRRAQAVVDYLVSKGVDASKLVAKGHGSEQPV
ADNTTEEGRFKNRRIEFSVAQ"
/product="Outer membrane lipoprotein omp16 precursor" /transl_table=11
CDS complement(681347..682582)
/locus_tag="AUO97b_00813" /db_xref="SEED:fig|400667.82.peg.673"
/db_xref="GO:0004151"
/translation="MSIVKIENVRVLDPIQKTDSVQTVYIQDGKLVAPIDQVEQTIDG
QGKWLMPTMVDLCARLREPGQQQHGTLKSEGKAARANGILHVITPPDSKPIVQDNGAL
IHGLIEKAWHDGGIHMHIIGAQTQGLNGKQPANMAGLKKGGCTAVSNANAAFENDDVV
VRTLEYAAGLGLTVVFYAEEPQLAKDGCAHEGFIASRQGLPMIPAIAETVAIAKYLLM

IEATGVKAHFGLLSCGASVELIRAAKAKGLPVTADVAMHQLHLTEQLIDGFNSLAHVR
PPLRSEQDKELLRQGLKQGVIDAICTHHEPLSSSAKMAPFAETQPGITAFDTYVALGI
QLINEGLFEPLEWVTKVTSAPAQVANMTARWQAEAGWVLVDPELSWTVSKDTILSQGK
NTPLLGQKLTGKVLQTFAV"
/product="Dihydroorotase (EC 3.5.2.3)"
/EC_number="3.5.2.3"
/transl_table=11
CDS complement(682595..683611)
/locus_tag="AUO97b_00814" /db_xref="SEED:fig|400667.82.peg.674"
/db_xref="GO:0004070"
/translation="MHIAALHQPSQVQLNQDGNLKHFLTIEGLSKENLTKILDTAQSF
LDDNNNLINRPLLEGRTVMNLFFENSTRTRTTFEAAAKRLSANVLNIDIARSSTSKGE
TLRDTLWNLEAMAADIFVVRHSSSGAAHFIAKDVCPKVAIINAGDGRHAHPTQAMLDM
LTIRRETKKPFEDLSVAIIGDIKHSRVARSDVAALQTLGCKDIRVIAPNTLLPVGFSE
YGDHVRLFNNMDEGITGCDVIIALRIQNERIDSPALSSQSEFYRMYGLNKERLSLAKP
DCIVMHPGPMNRGVEIDSSIADGEQSVILKQVTNGIAVRMAVLALSMQGQLQEQGLIE AIAL"
/product="Aspartate carbamoyltransferase (EC 2.1.3.2)" /EC_number="2.1.3.2"
/transl_table=11
CDS complement(683735..684079)
/locus_tag="AUO97b_00815" /db_xref="SEED:fig|400667.82.peg.675"
/translation="MKDMMGNGFIANAEAFTLATQIYVRMRRVTGRVIDAMYVVQNKD
YAKYVIALALEAEDDELKRCVERLMVLTDSIPEQSKKEMTVSIQTSEESEITAEDIYR AQVPHHYIGALR"
/product="FIG00350662: hypothetical protein" /transl_table=11
CDS 684318..686522
/locus_tag="AUO97b_00816" /db_xref="SEED:fig|400667.82.peg.676"
/translation="MNTSLRLNHYWITCADGLETLLQEEIEQLGTKVTERKAGRLIIE
GTLEHAYRICMWSRLASRVLLPIHTYELERTHDARDVAEELYEGAISFDWSLIFAPQS
TFAIRLHAEREIKVNTQFATLRVKDGVVDSFMEAVGRRPSIDTKQPEITLYVLAGKTE
HTYCLDLSGDSLHKRGYRRFMTDAPIKENLAAAILQKAKLQERNPEIVLDPMCGSGTF
IIEALMILTDRAPGLVRRFGFNGWHGHDRELWLSLKAEAAERHEKALEQPLPKFHAYD
ADWEAVKATRENIIAAGFEKLLGDIQIEERTLADWPDFGAENKTAFIVTNPPYGERLG
DKASNRSLYLGLSALLQKNFPNQYAAIIAAQIEQADVLAFEAPETLRLMNGKLPIYVR
FGTVKPEKVTQPFLANWQAQPVEMEEAQDFANRLQKNMSALKKWATKENIYCLRLYDA
DLPDFNLAVDLYGDRLHVQEYAPPKKIDPEKAKKRFNLALAAIRAVTGLNRDAIFIKT
RARQTGTNQYTKQSTANKRFIVQEGKAKILVNLTDYLDTGLFLDHRQMRLRIAQEARG
KHFLNLYSYTSTASLHAALGGAASTTSVDLSNTYLSWSKENFVLNGLTVDHADEQHMF
FASDCFEWLKEGHEQYDLIFIDPPTFSNSKKFHGTFDVQRDHVSLIKRAMNRLTSEGT
LYFSNNYRGFEMDEEIEALYDVEEITSETIGPDFKRNQKIHRAWKIQHPGLN" /product="23S rRNA
(guanine(2445)-N(2))-methyltransferase (EC 2.1.1.173) / 23S rRNA (guanine
(2069)-N(7))-methyltransferase (EC 2.1.1.264)" /transl_table=11
CDS complement(686549..686968)
/locus_tag="AUO97b_00817" /db_xref="SEED:fig|400667.82.peg.677"
/translation="MMNNKQGCVLGILSCIVVLPTNLWAAQPMNESALATQTGAVTAA
LPVIIAKAQDDAKKPEIEQRQVRQTLGDNYLQQVRWNSLRTSVLSPYQEQKQNQVIDG
KEKKKLEMVALPERADRFTRYDFESKNVHGEVIITVK" /product="hypothetical protein"
/transl_table=11
CDS 687234..687722
/locus_tag="AUO97b_00818" /db_xref="SEED:fig|400667.82.peg.678"
/translation="MLQQCCQLLEDRLLKVAFIESASSGYLTSQFSIHKNSGADILLG
GLVSYDPSIKISVLKVDPKLIETYTAESPQVTEEMCRLGQTLFQQADIVVSCTGLLKP
GGSETTEKPVGTFFICISYLGRIYHYHYFLSGHAEQKLKTLTQKVADSIIALISSNQH KS"
/product="CinA-like protein"
/transl_table=11
CDS complement(687779..690358)

/locus_tag="AUO97b_00819" /db_xref="SEED:fig|400667.82.peg.679"
/db_xref="GO:0005515"
/db_xref="GO:0005524"
/db_xref="GO:0019538"
/translation="MRFEKFTNRLQQALSDAQSLAMGKDHTAIAGIHILSTLLEEPSN
ISLLQQAGARLPELKQKLEQALKDAPTIANPTGDVNLNPEAVKALNLADRYAQKAGDE
FLSTDWVLLGLAETGETKNILSAVGVTPDSLRKVIENIRGSDKVMSNNHEDQRDSLNK
YTIDLTERALSGKLDPVIGRDDEIRRTIQVLSRRTKNNPVLIGEPGVGKTAIVEGLAQ
RIVNGEVPEGLKNKRVLSLDLGSLLAGAKYRGEFEERLKAVLKDLAKHEGEIILFIDE
LHTLVGAGKGDGAMDAGNMLKPALARGELRCVGATTLDEYRQYIEKDAALERRFQKVL
VDEPSVEDTIAILRGLKEKYATHHGVQILDSAIIAAAKMSHRYITDRQLPDKAIDLID
EAASRIKMEIDSKPEALDKLDRRLIQLKMQLEAVKKDEDAGSKAEVTHLEKQIAEVEK
EYNDLEEVWKAEKTLVEGTKQAQVELDKARIAFEKAQREGDLAEAARLQYGVIPELQK
QLEQDEVAEENEEPKLIRTKVTENEIAEVVSAATGIPVAKMMQGEREKLLHMEEFLHD
RVVGQDEAVVAVSNAVRRSRAGLSDPNRPSGSFLFLGPTGVGKTELTKALANFLFDSD
DAMIRIDMSEFMEKHSVSRLVGAPPGYVGYEEGGVLTEAVRRKPYSVVLFDEVEKAHP
DVFNILLQVLDDGRLTDSQGRVVDFKNTVIVMTSNLGSQDVRELGEGATDDEVRTIVM
NAVSQHFRPEFINRIDELVIFHSLKKAQIRGIADIQLDRLRSRLVDRDMSLTVDDSAF
DLLIDAGFDPVYGARPLKRAIQQQVENTLAQKILSGDFVAGDTILVKGENGHLVFDKL KLS"
/product="Chaperone protein ClpB (ATP-dependent unfoldase)" /transl_table=11
CDS 690713..691465
/locus_tag="AUO97b_00820" /db_xref="SEED:fig|400667.82.peg.680"
/translation="MAELPQDSRRPGGIAVIPLTSDITQVTFQHKPVLISQEGQQRYA
VFGIPLSTPLGSVQLDTNKAPIQIEVKSYPYAEQRLKVTNQDYVNPNQSQLDRYAQEA
KEQNDVYSSFTQSSWQALPKFIRPTSGKFTNSFGRKRFFNGEERAPHSGLDIPAPIGQ
KVVAPADGVVVQTGHYFFNGQTVLIDHGQGLISMFCHLSEIKVEKGQHIRQGETLGLV
GKTGRVTGPHLHWGMSLNNARVDPQLFLNTTH"
/product="Peptidase, M23/M37 family"
/transl_table=11
CDS complement(691462..691887)
/locus_tag="AUO97b_00821" /db_xref="SEED:fig|400667.82.peg.681"
/translation="MNIRLAQWEDTETICYLLKQMGYSQPLKVVQEQFQILSKDPNAI
TLVAADDLKVYGVLSLYFIPQIALQGDFAKICYLCVDEKMRSKGVGHLLVQEAERIAK
QRGCDRMELHSGIQRPLAHQFYLREGYVEAPKYFRKALN"
/product="Protein PhnO"
/transl_table=11
CDS complement(691981..692403) /locus_tag="AUO97b_00822" /db_xref="SEED:fig|
400667.82.peg.682"
/db_xref="GO:0006950"
/translation="MQTSVHWLENVAFEAKSESGHSVVMDGSPEYGGENRGPRPMELI
LMGLGGCASFDIVTILKKSRQDVTNVVCQLKAERADAIPAVFTKIHLHFVVTGKAVKE
KQVAKAVELSAEKYCSASKMLSDGGVEITHDFEIIEAA"
/product="Protein YhfA"
/transl_table=11
CDS 692842..693549
/locus_tag="AUO97b_00823" /db_xref="SEED:fig|400667.82.peg.683"
/db_xref="GO:0003700"
/db_xref="GO:0005622"
/translation="MTSNFSQLSTDALSPGQLPESVKALLKRAHINRYPKRTTIVDAG
TESKSLYLILKGSVSIILREDDEREIVVAYLNPGDFFGEMGLFEPNPQRTAEVRTRDV
CEIAEISYDNFHELSKQYPDLSYAVFAQLVRRLKNTTRKMTDLAFIDVSGRIARCLID
LSSQPEAMILPNGRQIRITRQEIGRIVGCSREMVGRVLKTLEDQGMIQTDGKAILIFD TSLEETPVTDEDYDDEE"
/product="Cyclic AMP receptor protein"
/transl_table=11

CDS 693710..694759
/locus_tag="AUO97b_00824" /db_xref="SEED:fig|400667.82.peg.684"
/translation="MQFKPLGHTGINLPEICLGTMTFGEQNTQQEAFEQLNYALEHGL
YFWDTAEMYSVPPKPETYGATETILGNWFAQHGQRDKVFLASKIAGPGFGGTHIREGH
TKFNSEHISQAIDGSLKRLQTDYIDLYQLHWPERHTNFFGTLAYGNQQAQNDYDTTPL
EETLLALQKEINRGRIRYIGLSNETPWGTMKFLQLAENLGVSKFVSVQNPYSLLNRTY
EIGMSEIAKYEGVGLLAYSPLAFGYLTGKFRNGARPANARVTLFPRFSRYSNPQSEWA
TEQYAQLAEKHGLSLTQLALAFIKQQFFVTSTIIGATNLDQLKENIQAFEIELSPEIL QGIETIHIQQPNPAP"
/product="Tas protein, an NADP(H)-dependent aldo-keto reductase"
/transl_table=11
CDS complement(694819..695604)
/locus_tag="AUO97b_00825" /db_xref="SEED:fig|400667.82.peg.685"
/translation="MLMNKKFVLSLCAAGAVTISGCATMADMAGADTATLNAQSAQSF
NKMVQEARGKGALDTSSSTYNRINAVFNRLKPYANQMNQTGQPFSWQLAVLKSDTINA
YVAPGGKVVFYTGIVNKLNLTDAEIAAVMGHEMTHALEEHAKSKIGAQALTDLALNIG
LSQINGGNVSQLGAAAAQLGTQVGVGLPYSRSLESRADQGGLMLMARAGYNPNAAITL
WEKMNKLEGSGSSFLSTHPSNAQRINDMRKNLPAALAIYNGRK" /product="Zn-dependent protease with chaperone function PA4632" /transl_table=11
CDS complement(695715..696032)
/locus_tag="AUO97b_00826" /db_xref="SEED:fig|400667.82.peg.686"
/translation="MKLQPTISKTIFYSFLFAIGGSPTLAMLIIYGQNYNPDFFMTAF
MSLSVLIACVLIPVVLIQNSYLLWKRKNPNLEEKVKTLSHVYLGLNIICLIFWIYLMT LSN"
/product="FIG00352600: hypothetical protein" /transl_table=11 CDS complement (696155..697474)
/locus_tag="AUO97b_00827" /db_xref="SEED:fig|400667.82.peg.687"
/db_xref="GO:0004019"
/translation="MGKNVVVLGTQWGDEGKGKIVDLLTDQAAAVVRYQGGHNAGHTL
VVGGKKTVLHLIPSGILRENVLCLIGNGVVLSPAALIEEMGILEAEGVPVKERLRISP
NCPLILPNHIALDQAREKKRGNAKIGTTGRGIGPAYEDKVARRAVRVADLVRGGAALE
EKLAEMLELHNFQLTQFYGVEAVKFEDVLALCNQWREVLAPLVIDVTKVLHDYRKEGK
AVMFEGAQGSLLDIDHGTYPYVTSSNTTAGGVSSGSGMGPLHLDYVLGITKAYTTRVG
AGPFPTELVYDAASDSGDPIGKHLGTVGHEFGASTGRQRRCGWFDAEILRRSVDVNSL
SGICLTKLDVLDGLEEVKICVGYENTDSGCVGSSDAVSFECLKPIYETMPGWSESTVG
LTSIDQLPANALAYVKRIEQLIECPIDIVSTGPDRAETIILRHPFSA" /product="Adenylosuccinate synthetase (EC 6.3.4.4)" /EC_number="6.3.4.4"
/transl_table=11 CDS complement(697539..698705)
/locus_tag="AUO97b_00828" /db_xref="SEED:fig|400667.82.peg.688"
/db_xref="GO:0003879"
/translation="MTISETWLLPDGVADVLPEQAQVIEKLRREAIDFLAVRGYQLVY
TPFIEYIESLSSLSESNQDLDLVTFKVIDQLSGRLLGIRADMTPQVARIDAHVRPVEG
VARYCYAGTVLHTKPQNFNATRAPLQLGAELYGHDSIEADVEMVDVMLGLIENAYTLQ
GAHLDLGHVGLFRSLVKYAGLSKNEEHELSDLYQRKALPELAEFTQNLNMGSDFYALG
RYASDLDALQAHLSADILKDTEFDAALNALKTTLEQIKNRWPALNVGIDVVELRSYHY
HTGLMYAVYAPNRAAPLAQGGRYDGIGEHFGRARPATGFSCDLYALGANQFAEIETVV
APKGTEADLLKAIANARSEGLRVVQLLGNDDLSSIPYATHQLVLQNGQWNIEKI" /product="ATP phosphoribosyltransferase regulatory subunit (EC 2.4.2.17)"
/EC_number="2.4.2.17"
/transl_table=11
CDS complement(698981..700006) /locus_tag="AUO97b_00829" /db_xref="SEED:fig|400667.82.peg.689"
/db_xref="GO:0004365"
/translation="MQRIAINGFGRIGRNVLRAWFESPKQFHFEIVAINDIADVHTLV
HLFKYDSTHGRFNGKVDITIENEKIYLNIQSNQRLLKVEVLQQKQPELLPWASLKIDV
VLECTGLFRSHADATRHLEAGAKRVIIGAAPFDHVDAAIVYGVNHADVKATDQIISSV

SCTTQALVPLVKIIDDAFGIETALMTEIHAVTADQSVLDHAHRDLRRARASGQNIIPT
TSSALGALKRVMPKMEDRIDGYSIRVPTINVAAIDLTFIAQSPIKVHHINELLIKASQ
TDYAEIMAVTDEPLVSSDFNHSPYSLIVDLTQTMVVGHQAKVFAWYDNEWGYANRLLD LCDSFKS"
/product="NAD-dependent glyceraldehyde-3-phosphate dehydrogenase (EC 1.2.1.12)" /EC_number="1.2.1.12"
/transl_table=11
CDS 700255..702912
/locus_tag="AUO97b_00830" /db_xref="SEED:fig|400667.82.peg.690"
/db_xref="GO:0004813"
/translation="MVVSTRLMTSAEIREAFLRYFETQGHTRVASSSLVPANDPTLLF
TNAGMNQFKDCFLGLEKRDYVRATTSQKCVRAGGKHNDLDNVGYTARHHTFFEMLGNF
SFGDYFKRDALKFAWEFLTSEQWLALPKDKLYVTVYHTDDEAYDIWNKEIGLAPERII
RIGDNKGEKYASDNFWAMGDTGPCGPCSEIFFDHGEHIWGGLPGSPEEDGDRFIEIWN
NVFMQFNRTADGVLHPLPAPSVDTGMGLERISAVLQHVNSNYDIDLFQHLLKAAANII
GIEDEGQPSLRVVADHARSCCFLIADGVNPSNEGRGYVLRRIIRRAVRHGNKLGATGT
FFYKMLQPLIEVMGQAYPELEARREVIEATLIREEEQFAKTLEQGLKLLEGELAQLKD
KTIPGATVFKLYDTYGFPTDLTADIARERGFIIDEAGFEVEMAAQRQRARDAGKFAVD
YNNIVKVEGETQFDGYTNTTGQGQIVAIYKDGVQVDEVSEGDEALIVLNQTPFYAESG
GQIGDTGIFKNETGIFEVQDTKKSGGAFVHQGIVTVGNLKTSQNVEAIVKADIREATA
RNHSATHLLHAALRQILGSHVQQKGSLVASDILRFDFANDQPVSFEQLQQVERLVNAE
IIANTAVTTELLDIETAKAKGAMMLFGEKYGDEVRVLSMGSVIDEKNFSIELCGGIHV
KRTGDIGLFKITSEGGVAAGVRRIEAVTGTKALEVVQKADHDIQHINSLLKAQKDQTV
ERVQANVELVSALQKQIEQLNQKLANFQAADLIDQVQTIAGRQTLITTVQGVDAKALR
NLHDSVKSKLENAVIVLAGVEGDKVSLLASVASQYTANLKAGDIIKHLATELGGKGGG
KPDLAQGGAPLNEKFGQVMAALPAWLEQK"
/product="Alanyl-tRNA synthetase (EC 6.1.1.7)" /EC_number="6.1.1.7"
/transl_table=11
CDS 703199..704404
/locus_tag="AUO97b_00831" /db_xref="SEED:fig|400667.82.peg.691"
/translation="MALTEVWLKANNGKARDKVEEIADRDSMSVRISPKGKIVFQLRY
RFAGKAERLDLGTYPHMSLKDARIKAGEMRSLLDKGMNPKVEVRVQQQKYIDASTFEE
VFNDWYESYCLKKKTSAQQIKNTFEQHVIPEVGDLPVDRITLQQWLALLEELADEVPS
IADRVLTNAKQVLKWAKKRQLLEVNVLSDIYAKEDLGIERNRGTRFLSDEEIKMVLMA
IEESNILPKNKIFLKLCLMFGCRNGELRKAKKTDFDLNRKVWIVPVVNNKTGKKTGRE
IIRPILPEMEALIVEAFEYSSCEYFLTNDSEETPMSHGSSNSLPAYLMERLRRHHDYY
MKHWSLHDLRRTARTNFSAFTSRDVAQLMIGHVMSGEQGTYDYYEYLPQQTEAYAKWI EKLKTITKT"
/product="Integrase"
/transl_table=11
CDS complement(704497..705480)
/locus_tag="AUO97b_00832" /db_xref="SEED:fig|400667.82.peg.692"
/translation="MNFEQAKTILIDLHLDSSSFNKSEIVDKAAYLFSLFEADFVQKT
NHETFYNLPYKEISVRIFKSNINEIQSNTLDSFANTVKEVLVDKYKKSSNDFEHLLKC
YEKFTHHIELAFIQKQFIQEASNDALIIAKRAEGIANEADAQAKSTIANYISILGIFA
SIIFTLFGGVNLIGSTVKLLETSSRLPYLVFIVSLLMICLLTLLNMMVKWVNSMSNLK
NALEKHSNAANQHLAHHCWKVWQWDFYTKSVSFFLVILLISLGGMYNVNKENLFSITT
ETTTKSVPNVEENTNKPATKLESEDKNKEITVVEKITLSNHPNEKSSDKEE" /product="hypothetical protein"
/transl_table=11 CDS complement(705485..705997)
/locus_tag="AUO97b_00833" /db_xref="SEED:fig|400667.82.peg.693"
/translation="MNNYKAMDIANYIIWYVNKDEDVPLGELTPLKLQKILYYVAANY
LKNNDELLFDEPFQKWQYGPVVKEVYREFKSANIYHISKPKSLIEESSDAPFGIHKRD
FKEELFLADKKFVETADPIIERLIRRKAFDLVEMTHREEAWSRYEPEINSGIQNLIYS LSELKKAKDI"
/product="Phage protein"
/transl_table=11

CDS 706409..706903
/locus_tag="AUO97b_00834" /db_xref="SEED:fig|400667.82.peg.694"
/translation="MSEIAPSIIQIKPYLTQGIVLSEALSIKQVVPSTHLLIPYALEK
INAGFPSPAQDYVDKALDMNEHLIKNETATFIVKVASLSMLNAGIDIDDELIVDRSLD
AKHGDIVIALIDNEFTVKRLMIDEKGQWLKAENPEYKNIYLQEGQELIIWGVVTHIIK MTRH"
/product="Error-prone repair protein UmuD"
/transl_table=11 CDS 706907..708205
/locus_tag="AUO97b_00835" /db_xref="SEED:fig|400667.82.peg.695"
/translation="MKHENKVFFLIDVNNMYVSCERVFDPSLNDRPVIVLSNNDGCAV
ARSNESKALGIKMGVPLFQIKDIVHQHNVIVLSSNYAMYAEMSRRFHTILASYVTAEE
VEPYSIDECFVDFTAYEKNFDLEKVGQQMRQQIWKWLGLPVCVGIGRSKTEAKIANHI
AKKNAGFNSVCDLVNMDPCNKEYYFSLIDVSEVWGVGRKHSKKLQSMGINTVLDLACA
EPREMQKRFSIVMARTIYELQGISCIEIEHTPPSKKQIIKSCSFGAKVTELIDLQEAI
AMHAQEACKRLRDDESLCGCLIVFVQSSPFDENVPFYNKSITGSFSQPTDSALDFVKA
ATKMVSHIYKEGIKYKKCGVILTGLEPKAGHTYDLLTDFEAIEKKEQLMKTLDNVHTK
FGKKKLGISTCYVPGRNWSMSRDKLSKNPFKWDELLTILN" /product="Error-prone, lesion
bypass DNA polymerase V (UmuC)" /transl_table=11
CDS 708243..708755
/locus_tag="AUO97b_00836" /db_xref="SEED:fig|400667.82.peg.696"
/translation="MNSFLDALEMAIETENWYSALFLTLTLPDICGKIDEPNANSSKR
YINWYNNYLLEEYTIRIEVGHIKREAPLITGKDFYALRCAVLHAGSDTLTEQFIQETL
EKVNFTIPHTNGTRIHKTRVQNVLYIQVDQLAKEMINAVRLWINHIASDSIKIQKLEN MVTIKMYSLF"
/product="hypothetical protein"
/transl_table=11
CDS 708897..709610
/locus_tag="AUO97b_00837" /db_xref="SEED:fig|400667.82.peg.697"
/translation="MNKPTPKIYRTTNWPTYNRALINRGNIAIWFDPKTQWYAQPKSQ
HGRNQTYSDTAIQCCLMIKSIFRLSLRMVTGFVQSLIKLCGLDWTAPDYTTLCRRQKH
IDIAISYQKSSNGLHLLVDSTGLKFLGEGEWKRKKHQPEYRRQWRKLHIGIDAETLQI
RAVQLTTNNISDSQVLGDLLGQIPLDERVDSVYTDGAYDTKCCRRVISDRQAYAVIPP
RKNAKPWKDTKVHSQERNE"
/product="Mobile element protein"
/transl_table=11 CDS 709692..709829
/locus_tag="AUO97b_00838" /db_xref="SEED:fig|400667.82.peg.698"
/translation="MHCIKLLGDKLYSRNFDSQVNEIHARVAVLNKFTELGRPHTQVV T"
/product="Mobile element protein"
/transl_table=11
CDS complement(709935..710477)
/locus_tag="AUO97b_00839" /db_xref="SEED:fig|400667.82.peg.699"
/translation="MNIEQYLDELIKREGGYVNNPADRGGATKYGITQAVARTNGFKG
NMKDLPLEVAKAIYRKNYWTAPCFDQVNIISSAVAEELLDTGVNCGTGFAKPLLQRAL
NLLNNQGKAGYADLEVDGVYGSETLRALKIYLAKRGKEGEKVLVRVLNIMQGQRYIEI
CERNPSQEQFFYGWIANRIS"
/product="Lysozyme (N-acetylmuramidase) family, (EC 3.2.1.17)"
/transl_table=11
CDS complement(710520..710909)
/locus_tag="AUO97b_00840" /db_xref="SEED:fig|400667.82.peg.700"
/translation="MEPVSTSGFTALLKFYGVAIVVALAVGLVAAVVLMTRMPRSPQE
WAVGLICTVVSSLAGGSFIIVKWGLHEWVTDIWGMIALGGFFFVCGLPGWALVRWIFN
FINKQEGKTIIEVLKEVKKAKRDIENS"
/product="hypothetical protein"
/transl_table=11
CDS complement(710973..713462)
/locus_tag="AUO97b_00841" /db_xref="SEED:fig|400667.82.peg.701"

/translation="MGGLFGSTTISTTDTRINSMRIQQSAYGLCQPLVYGKTRVAANM
FWYGDFTATPHTTVQKSGGKGGGTKTSNTTFSYSASLMLGLCENQIKKIGLIWVDKEQ
YVPKQEGSIILDPIDQLKFELFDGNNNPPWGWLVSKHPEQAINYPYLGYVAVANYEMG
NSASLSNHNFEVISTITLSDTIDDANPADVIEDFITHPRHGAAPNLNIADLEEFRTYC
RAANLLISPAFTEQRPAYETINEIVEAVNCAVVPSPDGLKIRSFGDSAITGNGVTFTP
DLTPVYHLTDDDFIGDDEPVRVRRSRDTDAYNHVQIEYINRYNQYNTETTEAKDQANI
EMFGLRTEDPVECHYFCEPKIARHAAQLRLQRLLYVRNEYEFDLGWKYCRLEPMDILT
LTESGLGLDKFPVRITRIEEDESGMLTVTAEELSIGSRSAIEYDSQVSNGYQGGNEEP
GNVNAPSIFEPPLDLTDGKNQVWVAVSGGANWGGCNVWVSLDNTTYEMIGTIYGSARY
GQLVTTIDADDTTLQVELNTASQIFSGTLEDAQADQTLCKVGDEYFNYQVATLNGSGL
YTLSDVLRGRFDDAQSHNAGEPFVRLDKAIFKYPYNEGLVEKQIFLKFTSFNGLERKE
QTLDEVTAYSYTLSGGRPAGVKGLSLQSPFVGTTFKVQWQSSTGADGYRVQVWSNGTM
IRQVDTTNTDYSYSIEEAKQDGLGRAYTIRVASKNGDQVSTFAELSISNPVPPVLLNV
YTAATVDSITVNWVPSEVPDLKDYAVWLSPTPNFDPTQMPPTWTGTDLTTTFGGLQPT
TPYYIRVAARDVWENTVWNYTNQITQSTSEA"
/product="hypothetical protein"
/transl_table=11
CDS complement(713463..713885)
/locus_tag="AUO97b_00842" /db_xref="SEED:fig|400667.82.peg.702"
/translation="MKNLEAVKEALTWLGTPYHHQGRVKGVGVDCGTLICEVYEKVGL
MDHLDPRPYPPDWHLHQMGQRYLELILGVCDPVEGPPQPGDIVLYHFGKCISHGAIVI
EWPQVIHSYLHQGVIIQDGTKGSLARRIAGFFRMKRLK" /product="hypothetical protein;
putative cell wall-associated hydrolase (invasion-associated proteins)"
/transl_table=11
CDS complement(713886..714656)
/locus_tag="AUO97b_00843" /db_xref="SEED:fig|400667.82.peg.703"
/translation="MSFLFFKTKDFSSAHSLPQLKDVSDLIPQYENNAYGHWLFGGGP
SSLVDVVNGRILTLQNGATVQPVYGDKTITLSTAIGNALLTDLVDSSAQSMTLCSVVK
CSTTSLAILLGNLVPSSSTLSSGLAAFASAGKGYLTVKPATAGGPGGISSLTPPSSIV
QTSNFFIATSVDKNTKKGIIYLQQLGVESSNESSYTAVSYESSANKIGIGNVAYTGSS
NTATYSEAIIFDNALTLAEIQNVALRSKDRMANRNISF" /product="hypothetical protein"
/transl_table=11
CDS complement(714660..716462) /locus_tag="AUO97b_00844" /db_xref="SEED:fig|
400667.82.peg.704"
/db_xref="GO:0004308"
/translation="MPLPNADQFVGQNVTESGFKQAQTQLIQFLGDEVPTNEKLVNTF
ATKAIADSKTTLMPIDYKITVTSDPDETKNGDYTWNGTELVKSPYDPVDKSRQFTSEE
VGKVSADVKEKFEKIKLSTSSESTSVIPVLVDKDNKTLIGYDTEKDQIAAGRLQEQVL
ENLPNLKKSEDIGKIAVLTDSTHKILIGYDTEKDQAIIAGLELPNQRPLVKAVNHILF
YGQSLSVGATATTILSTSQPYFNVTFDTGPRKDSAANSVIPLIEQFNNPSSDGYDNRG
ETCCSGAANYASRAMMLENGIDPKDHVIFASTAGHGGYRIDQLEKGTDWYNFFIEHVS
EAKRLNGEDYKVQVVCWVQGENDAVSSVQTSYEVYRQKLLKLQSDASADIKAITGQTD
EVKFITYQMSYAARTWEKQALVQLHLCQQSDKFLMATPMYHMPYAIDNIHLTNVGYKW
LSAYFGRAYKQLVVDNRKPDFINPKVAQLIGDEIHINFDVPKVPLVLDTTTLALTTDH
GFKVLVDGDATTIVSVTVQDDKVVLKLSEPPTGVVKVRYALDHLGAGISLTGGASGNL
RDSTADEILIDGVLKPLYHVCPHFELTAFIDKGI" /product="hypothetical protein"
/EC_number="3.2.1.18"
/transl_table=11
CDS complement(716519..717376)
/locus_tag="AUO97b_00845" /db_xref="SEED:fig|400667.82.peg.705"
/translation="MRQASPKLIALLDADQFIMADLYTITTIQGIEYRYTSYDVNLTV
QGKEFRADGPIISREGTSLSLGIEVDNLSITIEATENTKFGDVPIAQAFHNGILDGAR
FKLERIFMDMNTPTDTSAGTLVLFEGRIVEPELNRYEINASVVSDVDNLKLQMPRNLY
TPGCLNTLFDSACGLLSADFAVNTTIGTNSTPNRILCDLSQPQGWFTQGVVEFLEGAN

IGIKRTVRLHEAGSLILTLPLLKMPEIGEAIRVYPGCDKRLDTCTNRFNNRARFRGAP FVPVPETSI"
/product="FAD/FMN-containing dehydrogenases" /transl_table=11
CDS complement(717373..718026)
/locus_tag="AUO97b_00846" /db_xref="SEED:fig|400667.82.peg.706"
/translation="MSNVLFPELPGLEWDTSITPMFNTKIMTSINGRELRASFQASPK
YEISLSYAFLRENKGRKELQQLQGFYLERRGAFDSFLYKMPDDNEFSCTFIGDGTTTT
FQLYKDMYTSQLPLGNTEEQIVGEVDPNMWNQTPAKTMWNTDQEKLMWNNATAQITSD
GKYVLSQPIEEGVEVTVTGTFYYRCRFKDDTQQYVNFMHKLWKAGKVELIGSLGNKI"
/product="hypothetical protein"
/transl_table=11
CDS complement(718037..721075)
/locus_tag="AUO97b_00847" /db_xref="SEED:fig|400667.82.peg.707"
/translation="MANNNRVEVHVGAKTSELKEGMQDAEKIVSDSAKKIESTGHNID
FKLDLSNLRSELNGFASNLSDKFKTVGNDIKSSLTNGLSLVRGGFFLGIGQEIARSAA
EAVAAIPDLVSAVGKASKELEIQARLANSNTLEFQEWAFAAKKVNVEQDKLSDIMKDV
NDKFGDFMQTGGGEMADFFEKIAPKVGVTAQQFKGLSGPQILEKYYQTLQKANVSQAE
MTFYMEAIANDATLLAPLLDNNSQKLKEYAKQAHDLGVIMSEDAIAATKEFNTSLETV
QTTLQGVLTRIAAQAAPSLTELANQFLTFAVDSKDAIDDSIKSIIGIFESLFSILSEQ
FTTIGAIWSDLTGSIGDDANKQIGFMDAISVVLRALGVVVTGFQVGVQSAFAIIRAVV
VTVCQALIIAFNGLMAGFDMVRSTIQYGLDVLQVKFQTFGSVVNNILHFNFSGAKAAW
EGGLSQLGSITDRYTNQMKGRMAELKNSWNAGATTAANSLVTAGKRILEVTTAGNQKI
TNYVFKDPTKPIEPPKPPKLGLGTAPPNPKLGIGTGEKDDKGGSKSSAKSKAEQEAKE
RQRQAEQAAKALADIRYKYASEEKKVALDLQKALDEIEKSKMTADEKAAAKVKAEKDA
SDKIIAIRLKEFEEYKKAREEQIDNYQQQAQRLYEIEAARIQAEFDAKKISNVRKVQL
EKQLEDQLREIKRQGLLERLALENEQTNITGKQGNQNQITNNISDLETDQKVADTKSM
GLISDAEMKDFEAKFGGFTSRLSNLWDQGIQSLMNGTLTWSNATKAVLADMGAFALQT
ATKELQGWLRIQAIKLARKLGFVGAETAAEASGQAAQTGATIAGEATRTSVTAAGGLA
RLGLKAAEAIKGIMMSAWEAMAGAFKAMVAIPYVGPILAVGAGAAAFGLVAGLAGKIK
SARGGYDIPSGVNPVTQLHEDEMVLPSQHANTIREMGKALRNGASFGAAAVAEGGGAG
ATINISAIDAKSIQRLLKSNGRAVASGLQSYARGFGKNGK" /product="hypothetical protein"
/transl_table=11
CDS complement(721170..721670)
/locus_tag="AUO97b_00848" /db_xref="SEED:fig|400667.82.peg.708"
/translation="MALTNCKECGAQVSTQAKNCPSCGAKVKKRSLLKWIFLGFVILF
IIGIIAGGGEGSSSSSSTRELSPKEDALKNTVLDYDWSKGGFDSVMLVDFKIKNNSKY
DIKDITVECEHYSNSKTKIDSNSRVIYEIVKAGETKTVKQFNMGFIHSQAASSGCGIT DLVVIQ"
/product="hypothetical protein"
/transl_table=11
CDS complement(721741..721968)
/locus_tag="AUO97b_00849" /db_xref="SEED:fig|400667.82.peg.709"
/translation="MGKDYDYVRNEMDLPRLRALSAYQQSNPPAHIGIQRLCRILEAF
MGIDETPQAITVSDDDEDDMLEVLSNFPQGG"
/product="hypothetical protein"
/transl_table=11 CDS complement(722004..722318)
/locus_tag="AUO97b_00850" /db_xref="SEED:fig|400667.82.peg.710"
/translation="MQFKQVDNPRGNSKEIAGQTWIFAPAPLGTIERFQEQLSSNNVP
ASVIVDMAHVCLKRNYPDITREYVSDELLDMGNMEEVLALVTKTSGLEYTGKPAGESS GE"
/product="hypothetical protein"
/transl_table=11
CDS complement(722325..723092)
/locus_tag="AUO97b_00851" /db_xref="SEED:fig|400667.82.peg.711"
/translation="MAQYLFGAGKIFATPIQDVYGQPISNPTPVEVGVMQSVGVDISY
DLKELFGRGQFAVDAARGKGTIKCKASFGRINGTLLNSIFFGGVVAEGGIETVSQTIN
GEVIPAGGTVTPVVPNSGTYVKDLGVTDAKAIPLKRVASAPATGQYSVDAATGAYTFA

AADAGKTVFINFRYSAMVAGAKSITVSNLDMGYTPEFAVDLQRDYKGKFMHMNFFRCT
SNKLGFSSKQDDYDIPEFEFQPMADDLNRVFKIDLSE" /product="hypothetical protein"
/transl_table=11
CDS complement(723155..723538)
/locus_tag="AUO97b_00852" /db_xref="SEED:fig|400667.82.peg.712"
/translation="MITVSRRLRHFNHVTPEQRPAMFITQGNQQEVPVHGLDSKVELA
AEVYLYIHESDTTKPPSSQMNIFIDRVREAIQPDHPDFSEYQTLGGLVEHCWIEGTIE
VYEAVENMLDDQAIAIIPIRILTTN"
/product="hypothetical protein"
/transl_table=11
CDS complement(723591..723854)
/locus_tag="AUO97b_00853" /db_xref="SEED:fig|400667.82.peg.713"
/translation="MGAGGALVPYAPAHEFGLNGALGVKAHLRTIKQAFGRPISPVQV
NIKAHSRNVRFRELRFMRDSLDIVAKIVPKNIDAAIERGIAGG" /product="hypothetical protein"
/transl_table=11
CDS complement(724081..724272)
/locus_tag="AUO97b_00854" /db_xref="SEED:fig|400667.82.peg.714"
/translation="MPFKYQAPEGYKPTKLVIAGQNLDIKNGVLESDSDIIHILKPLG
FERYVEVVEPKKSTASAKE"
/product="hypothetical protein"
/transl_table=11
CDS complement(724282..724665)
/locus_tag="AUO97b_00855" /db_xref="SEED:fig|400667.82.peg.715"
/translation="MDVQTFREKFSTDSSLMSLPDAKIQDALEEADLIVSQIEFGALK
ERAVGLYAAHILKVGISSGNGAAFGTASSMTIAGQSVSYSRSSKEAFYDLSMYGQRYL
ALKNSIPIDDEGTNPNRLGVGAFVV"
/product="hypothetical protein"
/transl_table=11
CDS complement(724675..725058)
/locus_tag="AUO97b_00856" /db_xref="SEED:fig|400667.82.peg.716"
/translation="MKYSYIYSGLQAAFVFSGIAVLPTGSPTLVDEEAHKKLSKNKFA
KHLIDIGELEVQEIPDDEPKTTGKTGGRGGKGGKQNDSAGDAAKTAEEAALAAVKAEL
TALEVTFSDDETLEQLQAKLAQAKE"
/product="hypothetical protein"
/transl_table=11
CDS complement(725073..726047)
/locus_tag="AUO97b_00857" /db_xref="SEED:fig|400667.82.peg.717"
/translation="MSKLAAMKLRLTPVAQMVQANIGDAFNIDALAQLFVKLEEFNEM
GPQLQQVMDYAKYIPVKPVNAVYGGGEILSRKKGVGMGKDHSGTGNDIPVAEVEYDTV
QLPVKVGTISYMYSVFELQAAQKLNLALEADKVEAARLAAEKHLSNIAWYGNALTGVK
GFLNQTGVTIVTAQHNWATATIEEVLSDFNASLADAEDLVDGDVSVQPDTYLMASNQY
LHLSTRVVADSGGKTFLKFIEENNIFASQGKPLTIRGLGRSNGKGTAGADRSIIYRRD
PSCIQMKCDDVTFLAAQPVGVDIKVPGHYKYQGVWLKRVDSLRYLDHV" /product="Phage protein"
/transl_table=11
CDS complement(726053..726523)
/locus_tag="AUO97b_00858" /db_xref="SEED:fig|400667.82.peg.718"
/translation="MVKQYDAVPGMKFHLIGPEDILSLPMAGTGLVNDGDVVVRSTDG
KTFSAVTGATNTKFGIIVRHGVGKTGKTADGKEAYKATDVAPVMTIGSIYVKVTAPVT
DINAKVVYVKTANGTTAAPLGSLSPTATDGTELPNASWETISNEQGLAAVRLRGA"
/product="hypothetical protein"
/transl_table=11
CDS complement(726537..727739)
/locus_tag="AUO97b_00859" /db_xref="SEED:fig|400667.82.peg.719"
/translation="MTKHIYQLKIGDFAPSESTRSFTKEGYLKCVNVRLAKAPQVRQY

YAYEFPSLEGYTADQVINVYTPAEELFKPEAIQSFNGVDATDYHPPKNEINASNWKDY
HIGYCENVRQEGDYLVGDLLIKDKISIDLIQSNERLEMSLGYGALLIVEQGTAPDGTP
YQARFINFIGNHVALVKYGRCGGDCRIGDKQQTPPKGNKSMEVIVNGIRFNIGDNTPL
ADALKQQQEQLENMKAAKLKVGDKQFSIGDELGAIQAVVDQLHAEKTALEQKVGDLEK
NQMTPEKLEQAAAERAAVIADAKALVPTVKTEGCTCEQIKRDVIAAKAGDALVTALMG
NVSVGDAKPEQIDTTFRALCAVKGTQPSNPVGDALHQQQSVKAGDGDPAGGGDEKTYS KENAYKTI"
/product="Mlr8006 protein"
/transl_table=11
CDS complement(727982..728281)
/locus_tag="AUO97b_00860" /db_xref="SEED:fig|400667.82.peg.720"
/translation="MKRKKFSKKRFYRRLQAQKFAKGGFVTGGDFTPLWLMSCCDGFP
NLAKAAGKAAEKFQEVVESLKGLQPPNIKPIKTNIFIDGVDFGSAKDFSVTYSRT"
/product="hypothetical protein"
/transl_table=11
CDS 728370..728921
/locus_tag="AUO97b_00861" /db_xref="SEED:fig|400667.82.peg.721"
/translation="MNHLNYLWALIGANSGQLQTLLAVIGLIFAVIAALYAKKQIKLS
QDQRLFELKLSILSAAYECKDLIYEIKHKNNALKSEFSKMLQAQNLTLEDKLDGFDYN
YHEYFKKQLDLLTTPEQVINELITGLSDEKQNPSLEELERYLKHLTTSKGRIYYAHNG
YLRRIEELKQKNDIFSQLKYPHS"
/product="hypothetical protein"
/transl_table=11
CDS complement(728893..729426)
/locus_tag="AUO97b_00862" /db_xref="SEED:fig|400667.82.peg.722"
/translation="MATKVVTDTQKEVDKQIAEHTKTIIGVDLTPFYRAADIQDEVDL
NITANVSLIKSIPQQYADKLEVLITNALQNGQTNEELAKAIKQLGLSTDYRARLIASD
QMGKINGQINQARQLSMGVETYTWQTAKDERVRPDHQHKQGKTFRWDSPPEGGHPGQP IRCRCTALPNYEDILID"
/product="Plasmid related protein"
/transl_table=11
CDS complement(729650..730984)
/locus_tag="AUO97b_00863" /db_xref="SEED:fig|400667.82.peg.723"
/translation="MAETKKPDAIGDAGAYTNFVSNIGTERDKASHGSFVKKVIPDEQ
LEAVYQHWLAKRIVNRPASDMLRAGWFFEGIQDNDLLKLKEACKAFNLDGVLLSSLVL
SRLYGVCYVLLGTVDGGDLDQPFDLNKLGIGRLEFFTVLKKKYIEADTSKYLSPKEAG
GLLKQPEFYKLKLDGKSTQRIHHTRLYKFGHADVVNEEPVSVLQEVYEDLLDHAAVKK
ASASLVHESKIDVIRTPNLVDKIKEDMKSVAERFLSVGLLKGLNGMIVLDKEEEYDSK
SYSFGGLPDLMREFSIQAAGAADMPYTILFGQSPAGMNATGEHDTRNYYDSIATKQTW
SLKPFMMKLLRVIVQTTFGRQIPSLDVVFNPLWQLDAKVRSEVEKANAERDSKYLEMG
IITEPQIAKQLVIDGVYSVIDEAHIKELETMVKLNDNDNSDPETTPPAGEET" /product="probable
bacteriophage protein STY1048" /transl_table=11
CDS complement(730993..732519)
/locus_tag="AUO97b_00864" /db_xref="SEED:fig|400667.82.peg.724"
/translation="MNSRTNIDPVKTKAKRIKCEKEHLFFTRAFFLPRMGFKFSVNWH
HEYIADKIDEVIAGKVKNLVINVPPGSGKTELLTNLIARGIARNARSRFLYLSFSQSL
VEDVSATARNIVKSEDFQNLWPVKISTSTDAKSSWKTTVDGYDAGHVYSASMGGQVTG
RRAGTLADEGFTGAIILDDPLKPEDAFSQTARRKANRKILNTVNSRKAKSDTPIILIM
QRLHVEDPTNFVLTGNVPGEWEQISIPALIDDEYISKLPEKIQRKIPRDVERDAKGRQ
SYWPLKESLQSLLQLEQGGQDKDGATVSRYTFASQYQQAPKKLGGDLVKAEWFPRYLE
LPVLKWRAVWADTAQKVKKHNDFSVFLCAGLGYDNNLYIIDVKRGKWEAPELLKEAKA
FINKHKDSNTKIGKLRYMAVEDKASGTGLIQSISRETTLPIRAIQRDEDKLSRTMDVI
LYVEDQRVWLPANAPWLLNYIEEIEGLTADWSHDHDDQWDPTIDAINDSLAKKPTVFD "
/product="Phage protein"
/transl_table=11
CDS complement(732497..732973)

/locus_tag="AUO97b_00865" /db_xref="SEED:fig|400667.82.peg.725"
/translation="MAALKKEVKLFIVRSLAVFNTPTETAELVNQEYGIKVTKQQCEK
YDPTKRAGENLSEELRKDFEKTREMFLGKPEAIPIANLAVRLQRYESQYQKHSRNRVA
ALSILKQAAEDIGGKYTNKTEITGAGGGPLQSENITYVTATDEQVRQAIDELENEY"
/product="hypothetical protein"
/transl_table=11
CDS complement(733032..733637)
/locus_tag="AUO97b_00866" /db_xref="SEED:fig|400667.82.peg.726"
/translation="MDQAEEEEALRLIPAPDLKNWVVANFLTLGGPLHNPDHDHIAEM
LHDSEGFLAFAWASSAYTRAKRMVLGQCEKVMFQQGGWKKARQEQQMRDWFGFVPVYL
ITIDASFCEKANDSEFCALLEHELYHIGVERDSDGEIIYSDHTGLPKHYLAGHDVEEF
IGVVKRWGANENVKRLIEVAKNPPFVSDLDISKCCGNCVIT" /product="hypothetical protein"
/transl_table=11
CDS complement(733642..734070)
/locus_tag="AUO97b_00867" /db_xref="SEED:fig|400667.82.peg.727"
/translation="MDGKDYFWLTRKKEPKTKPKSRPLPKAKEKYLEAEETLFQELEE
HRIGYRRKFQFESTKNWRFDFYIVKLNLLIEIAGSPWAVGRGGSKIANALCKYDLALD
RGYVFERLEPHQIESGYAINWIKSELARIEDGTNKTISSN" /product="hypothetical protein"
/transl_table=11
CDS complement(734083..734274)
/locus_tag="AUO97b_00868" /db_xref="SEED:fig|400667.82.peg.728"
/translation="MTEFQKITHEIRQLQIELNHTGSCTTKGLTEEEIAHLDERFFLA
IAKQNKLIARLNNKPEGFL"
/product="hypothetical protein"
/transl_table=11
CDS complement(734736..735470)
/locus_tag="AUO97b_00869" /db_xref="SEED:fig|400667.82.peg.729"
/translation="MLVEKFDFIELLRLAIAQSEGKGKITKHVVLGEIALLPAGAKKW
AELLLERVDFERIAEITETKKIYETRIINGKESKKRIGEIPGKVEIKKGEINSADFFR
IRNVLAGKIHREMIKKNFKPNNCQGDLSNVAKGIAEVVLRGRLFTKAMCGHCQGLGKL
ELFNEKGYPSGSKFCDKCSGTGKRPYTLHEKITIAKLKVSKSGYSERYEPYELIAEAC
IENWENTIRTSLARSFHFEPEEISLA" /product="hypothetical protein"
/transl_table=11
CDS complement(735481..735882)
/locus_tag="AUO97b_00870" /db_xref="SEED:fig|400667.82.peg.730"
/translation="MSSVSIAEYRKLFPIKKNKKRRSAKQVARQPSVGEMVLATHLRA
CKIGFEQEYKFHPKRKWRADFLITGTKILIEVEGGIWSGGRHTRGKGYIGDMEKYNSA
AMMGFTVLRFSTEQVKSGMAIQQIEILMKGK"
/product="hypothetical protein"
/transl_table=11
CDS complement(735882..736097)
/locus_tag="AUO97b_00871" /db_xref="SEED:fig|400667.82.peg.731"
/translation="MSEFEGKSGKWAWEIQKEQQANLVELRSSIENLVQKYKHDAHAS
SLFGDQDKARVYNCFANQLKNLLKGGA"
/product="hypothetical protein"
/transl_table=11
CDS complement(736090..736491)
/locus_tag="AUO97b_00872" /db_xref="SEED:fig|400667.82.peg.732"
/translation="MKLTKQQRAELKQKFGGHCAYCGELLGDKWHADHIEAVKRDLIH
VGGGKLITGEMTRPQNDTLENMNPACVPCNTNKSSMPLEGWRKMLTHYRDVQLLRDST
HARHLLRFGLIEIKTKPVTFFFENYKGASHE"
/product="hypothetical protein"
/transl_table=11
CDS complement(736488..736757)

/locus_tag="AUO97b_00873" /db_xref="SEED:fig|400667.82.peg.733"
/translation="MDCKTKLNPVLWIAKYLDQLNQVTQRNNRMLAEVREIQAKLEKK
NKFMCKHCHEVNTIDFKKLPSQAAVVRGMAVIDQEFDGMKVEHSR" /product="hypothetical
protein"
/transl_table=11
CDS complement(736892..737641)
/locus_tag="AUO97b_00874" /db_xref="SEED:fig|400667.82.peg.734"
/translation="MHEITLNEVRQLIASLRTVYAAQFNKQFPTSGESAIPLSVVEQI
ALKTLVGVQQNQFNNALARLLTAGGRFMPSFAEFRTWCIGESWMSPEEAWSRACKFTT
DRTVVITQITKYALDEVMYLIEAGQMRAAQDNFFGTYNVMVAKAQLKGRQQEFYTPPL
QLEHKEPEHTPVSNDEAQKHLKSLMERLKINGRKPAPVQKLKAKEKEPELAKELGPDP
FDNPHEYAEMCRREGMPIPRNILQLIEGANV"
/product="hypothetical protein"
/transl_table=11
CDS complement(737634..738584)
/locus_tag="AUO97b_00875" /db_xref="SEED:fig|400667.82.peg.735"
/translation="MNYYQHHIGDFNNATRHLSLIERAIYRDLLDMYYDTEKAIDASS
IDRLARRLQCTTEEQKEALKYVLDEFFILEEGVYRNNRCEREIAEYHGKKKQASEAGK
ASAAKRAAKKKGSSNSDSSKDDQASNENSTVVENPLNEEQTDVQPTNNHKPLTINQEP
IIDSSSNAREENSQFTPIQFAQYQIDDHKRYSMREFISEYSEFQYDFISLAQQRFVSV
PEIDLRTMIQNFGDWYFANESSSLNTPSIWLVKWFSWVQNNEKQVAANRKKQEQITST
GQKPQESGYFANLFEEQSESQIVDVTPAKKFPMIEEVGHA" /product="Primosomal protein I"
/transl_table=11
CDS complement(738581..739141)
/locus_tag="AUO97b_00876" /db_xref="SEED:fig|400667.82.peg.736"
/translation="MLVSRGLTRVISDLAQMGYDAQWARFSASNFGAPHIRDRIWIVG
HATSKGFSKWSTPTMGQSGSIEKPKRSSCEIPNSQSIGWEQAWEGEQSSKERLSRCCG
ESSNSDCFNEQRLITSDSDQERWQEQDKRSARSSCNELTWWEVEPELGRVADGVANRV
DRLKAIGNGQVSIVAKCAFEYLGGAK"
/product="DNA-cytosine methyltransferase (EC 2.1.1.37)" /EC_number="2.1.1.37"
/transl_table=11
CDS 739110..739400
/locus_tag="AUO97b_00877" /db_xref="SEED:fig|400667.82.peg.737"
/translation="MTRVSPLETSIGEFSTNTYLGRTSPIIRAISDQSPERSPSIPAP
FPADEMSWHGKPPDITSTIPCHGFPSKVVMSDQIGKASRIPSFCRCAKTCAA" /product="hypothetical
protein"
/transl_table=11
CDS complement(739492..739779)
/locus_tag="AUO97b_00878" /db_xref="SEED:fig|400667.82.peg.738"
/translation="MQTNLSNQEQIIQSWFEPALHTLKALIKKCEENLEQVKADTKNA
AVKRDDFKDVLVRQHRITYNHAEEIIKSLGRAGRIRFLGSTYIQLNVQETK" /product="hypothetical
protein"
/transl_table=11
CDS complement(739830..740330)
/locus_tag="AUO97b_00879" /db_xref="SEED:fig|400667.82.peg.739"
/translation="MSEINLSPEAKTAIYKMIHQSQGVTPQEIANVLGDSYKSVLNYA
NPNMESHLPSIKKLEAMIQFTRNPALVKAWAHMLGYVLVPANQVDEKGHEVSIVETLL
HININNGQTNQQVHKVLEDGVVTPAELADTEEILEEMENHIRQLREALKSEAATYISK VKKEKA"
/product="hypothetical protein"
/transl_table=11 CDS complement(740385..740633)
/locus_tag="AUO97b_00880" /db_xref="SEED:fig|400667.82.peg.740"
/translation="MTTPHEAFNNAVTFAGSISALARKIGVTPWAASKWNPEKIPEDR
CLKIEEITQGQVKAEQLRPDINWEYVRKNLKKQNQSVS"
/product="Phage repressor protein cI"

/transl_table=11
CDS 740636..741427
/locus_tag="AUO97b_00881" /db_xref="SEED:fig|400667.82.peg.741"
/translation="MTPKNLLKVERNILPKIELVQLKIDSKILPTVEKKPILMEDAKY
KDFADRLNALMKAKDSPIKTINELKNAIGVSYEMARRYTLGSAKPRIEKLQTLADIFG
VEISYLDHGTKLDNNIDLSDKVGFEGRRVPVISWVAAGSFTPIETVLKDTEIEEYLPP
NKRCGKNGYALKVVGYSMAPTFLPGDRIYVNPDIQTFDLKTDDLVIVACAGDSEATFK
RLIIEGEGTSKFLEPLNPDWPDKIIKLSEDCRLVGKVVGLYRDIY" /product="Phage repressor
protein cI"
/transl_table=11 CDS 741655..742095
/locus_tag="AUO97b_00882" /db_xref="SEED:fig|400667.82.peg.742"
/translation="MNVKVNSFNSFAFVSMAALAISGGSLVACQLQPAFQAKEAPSLF
TPKTQPSTYSVLTAKITGKHSGVAVIKLDSFRLNVSFDFEAHPDSYGVPGSEFTAVEI
TQLTVNEITDVNGKSYNDFTEFEDIRNINDLLKGFIERNKLVEA" /product="hypothetical protein"
/transl_table=11
CDS 742098..742421
/locus_tag="AUO97b_00883" /db_xref="SEED:fig|400667.82.peg.743"
/translation="MTNFKKHPDGYISFLGRDDKGLYSVRIGWQVYASNANGSVLYKV
KDGVKTPLNVAKFQTDYPKVWNELTQEIDFQRRKQLAIKLRETNIPTYDRKAYKQKRG FTGSR"
/product="hypothetical protein"
/transl_table=11
CDS 742433..743200
/locus_tag="AUO97b_00884" /db_xref="SEED:fig|400667.82.peg.744"
/translation="MTTENSKDNLHIWNAVKQTPTNFLKKIEFGYLKGKSDINPQWRL
MAMTQAFGPVGHGWTYRHVRLWSETAPDGTIMAFAEVAVKTKIDGVWGEEFFGNGGSA
IVEVQKGKLVAIDEGYKKAVTDALGVAFKAIGVAADVYLGNFDGSKYLYNYDYAYLEQ
NASTPAGQNTNQNNQSTAQGGNQKPPRTQDQLYQDALKAIKDAPDTNILNAAIKKFKG
TTYEAGINRACQARADQMGWAPKNNPQQVQQQQSLHH" /product="hypothetical protein"
/transl_table=11
CDS 743215..744174
/locus_tag="AUO97b_00885" /db_xref="SEED:fig|400667.82.peg.745"
/translation="MSNLLTAAEAFAALQKGKTVLCRPIGDMLDFSDLDQFPASVFGK
PGFEFCIKIETIELAGITFTKPLTIDEYEEGQEVYVISTYSPTVYVLDFKTNALIDSI
NSGFVQRDAENAKLQLKAFSKALGIEINNDLSVIRLGEEPKKQRGKKSKAEKPIEVIS
AEIQPTIVITEQTNVTTSEDLLVPETNEPKVDPEYQQTLDTLLQRVKESKTPAEVNAV
YRYTRTWDDEQMKPILLATHKRLEELEKDKASANEPPSLMVQIQTAPDLTTLDALEID
VAARDPQIQPKLMGYIRKRRYELENPTPTQPEADPDYLLVDGF" /product="hypothetical protein"
/transl_table=11
CDS 744176..744427
/locus_tag="AUO97b_00886" /db_xref="SEED:fig|400667.82.peg.746"
/translation="MKDQYKKVSQKHMLGFMYYLQLLGYVIVRQGMDQAMFLTKHYAV
PVAWRRITIDYHNRLNKPAQQLYREFVEWTKEEYAVMVA" /product="hypothetical protein"
/transl_table=11
CDS 744428..744835
/locus_tag="AUO97b_00887" /db_xref="SEED:fig|400667.82.peg.747"
/translation="MTGNERIPFESQFKTTEIFKREIAIRKNDILAFSETMNGYFNIV
TNDAWQLWNKAKAETVPDTPTPSVTLTCAELKEAFDFGAPDGEKDQFQMETEMTIKWL
QDGYDGEGYYCWYADLPEEGCIKLGVSESGAEG"
/product="hypothetical protein"
/transl_table=11
CDS 744832..745116 /locus_tag="AUO97b_00888" /db_xref="SEED:fig|
400667.82.peg.748" /translation="MSKVIGEVNLNPSRIEGTPDQVAVHFFEKIICPSTDELLKNNPE
AAKVFAYHIFGLALSQLAEFHSTKSLDKAVTVTLHNLLRQLKKERNELRN" /product="hypothetical
protein"

/transl_table=11
CDS 745120..745377
/locus_tag="AUO97b_00889" /db_xref="SEED:fig|400667.82.peg.749"
/translation="MSGLKVKTCNFCDDGNGECIFPYYGLAPHIHTKPIGGTEFIDVS
LPENFSPDGDGLGIYTHCLNCGGDGTYEGIQLEVKAESKEG" /product="hypothetical protein"
/transl_table=11
CDS 745415..745594
/locus_tag="AUO97b_00890" /db_xref="SEED:fig|400667.82.peg.750"
/translation="MFHITKRTLNRWEINTPWGIPFPAPALSSEGGTMKRYLATDVMK WEEECQQKKQLKKAI"
/product="hypothetical protein"
/transl_table=11
CDS 745804..747084
/locus_tag="AUO97b_00891" /db_xref="SEED:fig|400667.82.peg.751"
/db_xref="GO:0004072"
/translation="MALIVQKYGGTSMGTPERILNVARRVKRWHDHGHKVVVVVSAMS
GETNRLLALAKAITETPDPRELDQMVSTGEQVTISMLAMALNSIGVEAKSYTGRQVGI
KTDSAFTKARIESIDTDVMTNDLDAGRVIVVAGFQGFDANGNTTTLGRGGSDTSGVAI
AAALKADECQIYTDVDGVYTTDPRVAPKAKKIDRISFEEMLEMASLGSKVLQIRSVEF
AGKYQVPLRVLSSFDNDDDGAFDDEFKENVGTLITTEAEDTMEQPIIAGIAFNRDEAK
LTILGVPDEPGIASKILSPVSDANIEVDMIVQNVEEDGTTDFTFTVNRVDLAKAEKIL
NETAKNIGAREVSTRDDIVKVSIVGVGMRSHAGVASKMFTALADEGINILMISTSEIK
VSVIIDEKYLELAVRCLHTAFGLDREHGESSARA" /product="Aspartokinase (EC 2.7.2.4)"
/EC_number="2.7.2.4" /transl_table=11
CDS 747331..747585
/locus_tag="AUO97b_00892" /db_xref="SEED:fig|400667.82.peg.752"
/translation="MLILTRRVGETLMIGDQVSVTVLGVKGNQVRIGVNAPKEVSVHR
EEIYQRIQHERAMHEHLQHLDQDYQVSYEDDNYAQKNFNR"
/product="Carbon storage regulator"
/transl_table=11
CDS complement(747655..748221)
/locus_tag="AUO97b_00893" /db_xref="SEED:fig|400667.82.peg.753"
/db_xref="GO:0004523"
/translation="MFVAGVDEAGRGPLVGSVVAAAVILDPNNPILGLNDSKKLTEKK
REKLFIEIQEKALAWSIAEATHAEIDELNILQATFLAMQRAVEGLKTQPQKVIVDGNQ
IPKGIIIPCEAIVGGDASHAEISAASILAKVTRDRQMAELDQQYPLFGFAKHKGYPTK
AHFEAIAAHGVIDQHRRSYAPVKRALGL"
/product="Ribonuclease HII (EC 3.1.26.4)" /EC_number="3.1.26.4"
/transl_table=11
CDS complement(748287..748676)
/locus_tag="AUO97b_00894" /db_xref="SEED:fig|400667.82.peg.754"
/translation="MKKLMLAVAPVVLALTACATTTGTGTGGASAQTTTQQLGVAALK
VAVNAKCITEINNVAAWKTATKYMTAEQRDSIQTNVCGCVSEKAPNSVTAVELAAAAL
DVNARATIVNQVVSKTVNACVAEALQK"
/product="putative lipoprotein"
/transl_table=11
CDS 748916..749473
/locus_tag="AUO97b_00895" /db_xref="SEED:fig|400667.82.peg.755"
/translation="MPASTSQYYTRTAQILHWVMAIIFITAWVIGFYSGNFLSYDVDG
SFKGDVITLHKNIATTIIFLVVIRLLWRYTHPAPQLPDTMSPTMKTLAHIGHLLLYVI
LVALPVTGCLFSWSAGHPAPVLYLFEIPRLVQDNPELLAIVKPLHIYISWFAGLLIAG
HVLAALKHHFVDKDNVLNSMTKQPK"
/product="Cytochrome b561"
/transl_table=11 CDS complement(749517..750515)
/locus_tag="AUO97b_00896" /db_xref="SEED:fig|400667.82.peg.756"

/db_xref="GO:0004000"
/translation="MNQVELIQALPKAELHVHIEGTFEPELMFAIAQRNQIQIPYKSV
EEVKQAYNFHNLQSFLDIYYAGANVLVHEQDFYDLAWAYFEKCAEDRVVHTEMFFDPQ
THTDRGIAFATVINGLKRACADAKDKFGISSQLIMCFLRHLSEEAAFETLEQALPFKE
DIIAVGLDSSEVGHPPAKFERVFAKAREEGFLIVAHAGEEGPPEYVWEALDLLKVNRI
DHGVRSEEDEQLMARLINEKMPLTVCPLSNLKLCVVKDMKDHNIRRLLQKGVHVTVNS
DDPSYFGGYMNDNFFAIQQALDLTNDELKQLAINSFEASFISDEEKQKWISEIQKI" /product="Adenosine deaminase (EC 3.5.4.4)" /EC_number="3.5.4.4"
/transl_table=11
CDS complement(750627..751946)
/locus_tag="AUO97b_00897" /db_xref="SEED:fig|400667.82.peg.757"
/translation="MAAPNSSAGWLERLFKLSDNKTTFRTEVLAGVTTFLTMCYIIIV
NPLILSETGMDHGAVFVATCLAAAIGCLVMGIIANYPIALAPGMGLNAYFTYSVCMGM
GVPWQTALAAVFVSGVVFLAISFFKIREAIVNAIPMSLKFAIGGGIGLFLALIALKNA
GIIVANQATLVGLGDIKQPTVLLALFGFLLIVILHQLKVRGAIIISILVVTAIATALG
LNEFKGVVGEIPSIAPTFMQMDFEGLFTASMVGVIFVFFIVDLFDSTGTLVGVSHRAG
LLQDGKLPRLKKALFADSTAIVAGAALGTSSTTPYIESASGVAAGGRTGLTAVVVAFL
FICCLFLAPLAQSVPGFATAPALLFIGVLMIQGITHIDWDDITEAVPAFLTIVFMPFA
YSIADGIAMGFISYALVKLFTGKAKTVPYMVWIIAALWVFKFIAFGG"
/product="Xanthine/uracil/thiamine/ascorbate permease family protein"
/transl_table=11
CDS complement(752269..752397)
/locus_tag="AUO97b_00898" /db_xref="SEED:fig|400667.82.peg.758"
/translation="MRIIQEAEKNLQTIERNPNRVSHSNLASYNLQKYISDKKVFF"
/product="FIG00352497: hypothetical protein" /transl_table=11
CDS complement(752865..754415)
/locus_tag="AUO97b_00899" /db_xref="SEED:fig|400667.82.peg.759"
/translation="MVTKPRTNTRTRKTQEVAHIYDTFIVGAGISGLAAAIKLNEAGL
TNFKIIEKASRVGGTWRENTYPGCGCDVPSALYSYSFAPSAKWSHLFARQPEILSYLE
DVSREFDIESLIEFNTELLKAEWDNVKNVWKLETSSGLYIAKTVLFATGPITEAQIPR
LEGLDTFTGEMFHSAKWNHDYDLTGKRIAVIGTGASAIQFVPQIQPKAKELFVFQRTA
PWVLPKPDTDLGELSKSIIAKYPKIQATWRQTVAGTLNVINFGLRNPKALKPVNFAAK
QLLKLQIKDPVLRKNVTPNFDIGCKRILFANNYYPALQAPNTTLIPHGLVKVEGNTVV
AANGERHEVDVIIWGTGFEVSHPPIGKRVHNEKGECLAELWKDSSPEAYLGTNIENVP
NAFLMLGPNVLVYDSFIGLAEAQLQYIVDGLLQMKAKGIVKLSVKSEIIKKHNELVQK
HLQTTVFNSGGCKSYYLDANGRNFAAWPWSLKKLKQRLHHLNLEEYEVIYEADLVKVK PKVEEVAG"
/product="Cyclohexanone monooxygenase (EC 1.14.13.22)"
/EC_number="1.14.13.22"
/transl_table=11
CDS complement(754439..755269)
/locus_tag="AUO97b_00900" /db_xref="SEED:fig|400667.82.peg.760"
/translation="MILFGKKDQPSRNAYAVVTGAGSGIGRSFAVELAKRGGSVVCAD
INLEAAEETVKLIEQLGQKAFAVQCDVGQAEQVQTLADQAEQLLDHPVTLIINNAGVG
LGGKFDEMSLEDWKWCMHVNLWGVIHGCHAFVPKFKKLGYGAIINVASAASYTAAPEM
SVYNVTKAGVLALSETLSAELRKFNIKVNVLCPTLVPTNIIKNGRIPGRYSKLADHAL
MNYAMTTSDAVAALTLNRLDRGQLYTTPQIDAKLFWLMKRTSPTLYAKFLGLSYQLFK "
/product="Oxidoreductase, short-chain dehydrogenase/reductase family"
/transl_table=11
CDS complement(755335..756297)
/locus_tag="AUO97b_00901" /db_xref="SEED:fig|400667.82.peg.761"
/db_xref="GO:0004029"
/translation="MVAQAIRNIISPKSVIAQYQKIDFSNKGTIPIRHLNIGFDGKEI
DIEFYVNNQFATLFFATLSVFLTYGEDLVIETARHHREFIQDPILKQRVTSLIGQEAI
HSKLHNEFNDAIKELEYPVDFYRFLGENFFKYVFLKFPQPLKLSLMAGIEHFTAVLAE

YMMKHEKNFYYTDDAKSRALWMWHMLEESEHKDIAYDVYQVLNGNYPLRATGFLMAFI
TILGLIPTATLFVPLLRKPKELLTRKFWKDARRGVNLIFSPKDGVFGSTIGQIFDYLR
PDFHPNDHDTTEYLEYYKKKLLNEGGAIAPYFVKEFTPPVRAVS" /product="Predicted metal-dependent hydrolase" /EC_number="1.2.1.3"
/transl_table=11
CDS 756808..757530
/locus_tag="AUO97b_00902" /db_xref="SEED:fig|400667.82.peg.762"
/translation="MFDMRLSHERGAAHHGWLQSKHTFSFANYWDPKQVGFSDLLVIN
DDTVAPSKGFGTHPHRNMEIISYVLEGALEHKDSMGTGSVIVPGDIQLMSAGRGVAHS
EFNHSAQENVHFLQIWVVPNEVNTTPGYQQLHISEEDKRGKLHLTISPKGGEKTLSIK
QDINIYSGLFDGEETAEFAIPEGRYVYLHVAKGRIDVNGKTFNTGDAARIRDGGKLSF
TNGDNAEILIFDMRPEEIPTMP"
/product="Pirin"
/transl_table=11
CDS complement(757595..758218)
/locus_tag="AUO97b_00903" /db_xref="SEED:fig|400667.82.peg.763"
/translation="MSTRKPRQARAKVTVDTIIEAGFIAVALHGPSGTTTRHIAEIAG
VSVGSLYEYFKNKEEIYDAMNHYFVSEILDMIKELTPTILELELEPVIEMIFYTFSDL
LKKNNDRYLTVLRYAGELQYDKYIPKIEQALMEVIMKYMMHNPKYLKINNLPVITYIC
INSGIFNVARHLILPNPFISFDEMVQGLTTMIMSYINTEMARSEDQS" /product="Transcriptional regulator, AcrR family" /transl_table=11
CDS 758744..759703
/locus_tag="AUO97b_00904" /db_xref="SEED:fig|400667.82.peg.764"
/db_xref="GO:0004029"
/translation="MISTTVKKSLNKIKFYREIELPDFASKKSIPIRHLNIGFEGKEI
DVEFYMNNQFATMFFATLSVFLTYGEDLVIETARHHREFIQDPVLKQRVTSLIGQEAI
HSKLHNEYNDALKDVEYPVDLYRFLGENFFKYVFLKFPQPLKLSMMAGIEHFTAVLAE
YMMKHEKNFYYSDDAKSRALWMWHMLEESEHKDIAYDVYQLLNGSYPLRASGFFLAFF
TILGLIPATTLFIPVLRKPQEMLTRKFWKDARRGVGLIFGPKDGVFGSTIGQIFDYLR
PDFHPNDHDTTEYLEYYKDKLLKEGGAIAPYFVKEFTPPVRLS"
/product="FF domain protein"
/EC_number="1.2.1.3"
/transl_table=11
CDS 759766..760599
/locus_tag="AUO97b_00905" /db_xref="SEED:fig|400667.82.peg.765"
/translation="MILFGKKKVKPSQKAYAVVTGAGSGIGRSFAIELAKRGGSVVCA
DINLEAAEETVMLLEQQGAKAFAMHCDVGNPEHVTNLAETAEILLSHPVTLVINNAGV
GLGGKFDELSLEDWNWVMNINLWGVIHGCHAFVPKFKKLGYGAIINVASAASYTAAPE
MTAYNVTKAGVRALSETLSAELRKFNIKVNVLCPTLVPTNIIKNGRIPGRYSKLADHA
LMNYALTTSDDVAKLTLNRLDQDELYTIPQIDAKLFWLMKCASPSLYTKFLGTSYKLF K"
/product="Oxidoreductase, short-chain dehydrogenase/reductase family"
/transl_table=11
CDS 760623..762146
/locus_tag="AUO97b_00906" /db_xref="SEED:fig|400667.82.peg.766"
/translation="MTAQMKTNASAKKAVNSPSHIYDTFIVGAGISGIAAAIRLDQVG
YTNYKIIEKAGRVGGTWRENTYPGCGCDVPSALYSYSFAPSAKWSHLFARQPEILSYL
EDVSNEFNITSKIEFNNELLNAAWDESRHLWVLDTTTGQYLSRTVIFATGPITEAQIP
RLEGLDTFTGEMFHSAKWNHDYDLTGKRIAVIGTGASAIQFVPQIQPKAKELFVFQRT
APWVLPKPDTDLGEFSKSIIAKYPAIQASWRKSVALTLNAINFGLRNPLALKPVNVLG
KQLLKLQIADPELRKNVTPNFDIGCKRILFANNYYPALQAPNTTLIPHGLVKVEGNTV
IAANGERHEVDVIIWGTGFEVSHPPIGKRVFNEKGQRLNDLWKNSSPEAYLGTNIENV
PNAFLVLGPNVLVYDSFIGLAEAQLDYIVDGLLKIKNKGISKLNVKADVIKKHNDLVQ
KHLQTTVFNSGGCKSYYLDANGRNFAAWPWSLKKLKQRLKKLDLKDYEVTYQMSKTH"
/product="Cyclohexanone monooxygenase (EC 1.14.13.22)"

/EC_number="1.14.13.22"
/transl_table=11
CDS 762258..763133
/locus_tag="AUO97b_00907" /db_xref="SEED:fig|400667.82.peg.767"
/translation="MKEGQSSRTAEAAAALRANHFKNTTNPVFSDPYAFEMTSKGWKK
LLSTPLIVKLMNSPILNRTLGLLSGQVVGRSRYAEDLLNQAVQHNIEQYVLVGAGLDS
FVLRKSQHYPTLKIFEVDHPDTQAAKQSKLKKLGELPSTVEFVSINFEKESISEALAR
SRYVRKTPAFFSWLGTTHYLKPDTTLQTLKSIAEFAANGSEVVLDYSTDFQELKGIER
LGSMGVAQFTHLLKEPLLGQFKPSDLHQAVEKMGFEVVEDLSGEAITERYFYNRADNI CHTSATRLLHLRLKK"
/product="O-Methyltransferase involved in polyketide biosynthesis"
/transl_table=11
CDS complement(763145..764095)
/locus_tag="AUO97b_00908" /db_xref="SEED:fig|400667.82.peg.768"
/translation="MESGISYQIILQGTGLSQQQLLDPNMVVTGHQELQLIHNLVEQL
GDRPTLGLEVGTRYHFTTFGPLGMALMSSASIREALDLALTYFTLTFAFTRFDVSDTP
QGIRIEIDEQDIPSSVRQFIIERDAAALITVQRDLVQDNFCQYLAFSFQPKGDAEPYR
NLFGVMPEFGAAKSFVILDPQKVDQKLLMANELVLHTAEEQCRQILEKRQSQNKFTTL
VQQQLMSMRGEMPSMDIVADRLHLNIRTLRRHLASEGMTFIQIREEVRQVLAEQYLAL
PKASVEQIAEWLGYAEPASFIHAYKRWHGKTPHAMRLNSQ" /product="Transcriptional
regulator, AraC family" /transl_table=11
CDS 764295..765782
/locus_tag="AUO97b_00909" /db_xref="SEED:fig|400667.82.peg.769"
/translation="MFNSSSNSNKNVRIAIIGTGFGGLGLAIRLKQAGFEQFTLFEKA
ADVGGVWRDNTYPGAACDVPSHLYSFSFEQELGWKNRYGTSQEIYQYLRHCADKYDLR
PHIKFNTEIASAEFDALQGIWHIQSTTGEQFEAEFLVGAVGQLNRPAYPKLKGIENFK
GKAFHSARWDHDYDLTGKRVAVIGTGASAIQFVPEIAKQVAHLDVYQRSAPYVIPKPD
RVYQPLEKKAFRKLPILQSLDRALQYGHHEIRLLAFTTSLNEMPLVEYIFQRHIRKVV
KDGKLRHRLMPDYPIGCKRILISNHWYETLTQPHVDVIDTGIQEITEHGILDTEGKLR
EVDAIIYGTGFTATEFMAPMQITGLDGRTLKDTWKNGAEAYLGLTVNGFPNFFMLYGP
NTNLGHNSIVYMIESQVNYILDALRTTLKNNLLFTNIRAEIQHEFNQNIQEKMKKTVW
AQGCTSWYQTADGKNTNNWPGFTLEYRQRTRRFDIENYTQVSAIS" /product="Cyclohexanone
monooxygenase (EC 1.14.13.22)" /EC_number="1.14.13.22"
/transl_table=11
CDS 765827..766681
/locus_tag="AUO97b_00910" /db_xref="SEED:fig|400667.82.peg.770"
/translation="MKSFKNKVAAVTGAGSGIGQAIAIALAKQGCHLALSDISEAGLA
KTVELLAPYSVKVTTQKVDVAKRDEVATWAKAVVDEHGQVNLIFNNAGVAIGSTAEGV
SYEDLEWLIGINFWGVVYGTKEFLPYLKQSGDGHIINISSMFGLTAQPTQSAYNASKF
AVRGFTESLRQELDMQNAGVSATCVHPGGIRTNIAKAARMNNSVQSLGMDPLKSQDAF
DKLLRTPADDAAQQILEAIRKDRRRLLIGADAKAVDVIQRILPQGYQKIFAAATAWQT RLLNKSAK"
/product="Short-chain dehydrogenase/reductase SDR precursor" /transl_table=11
CDS 766754..767647
/locus_tag="AUO97b_00911" /db_xref="SEED:fig|400667.82.peg.771"
/db_xref="GO:0008800"
/db_xref="GO:0030655"
/db_xref="GO:0046677"
/translation="MKQLLAWTIRTTLRPALSPKTPLKLQRFCSDAASAIVLGPRGYQ
TKKQIIAQVPTLHIRPKTIKSGRGILYLHGGGYVVGSSKSHAKLAAQVGHIAQAQVWL
PEYRLAPEYPSPAAIEDIVAVYKALLAYGQDPKKLVIAGDSAGGGLSLSTVIALRDAG
LPLPAALVLLSPWVDLSLSGNTIKTHAAQDAMLSEGWLAWCAKNYCGQKSATDPTCSP
LYADLTGLPPILIHVGTEEVLLDDAKRLAEQTEKYGIPTNLRVYDQVGHVFQFHAGIL
KESNDSIECIGQFIDKHTESI"
/product="Lipase/esterase"
/transl_table=11

CDS 767676..769346
/locus_tag="AUO97b_00912" /db_xref="SEED:fig|400667.82.peg.772"
/translation="MTINNYDYDYLIIGSGFGGSVSACRLTEKGYSVAVMEMGRRWKA
EDFAKNNWNTRRWIWRPGMKLFGYFNMRFFRHVTIICGNAVGGGSITYANTLLVPPEH
IWDEGTWADAADWKNEMPQHYAEAERMLGVTDNKIFGPADHMLKKMGEAVGVGHTFKP
TRVATFFPPEGEEGGKTYPDPYFNGEGPDRGTCTACGGCMTGCKHNAKNTLDKNYLYF
AEKNGAKVYEETKVVDVKPLNGKADGSDGYEVTTECSSSWFNKQRRTWRVRNVIFSAS
SLGTQEMLFRLKQSGSLPNISDDLGNRVRTNAESILGVRFFGKDVDMSKGVAIGSSIY
IDHDTHIEATRYQSGSDAMGLMCTYMAKGKPGWTRIFFWLWALICHPFIFLRMSNPVS
FARQTLIFLVMQTADASINMRLKRNWFWPFGKVLSSEGKKLPVYIPQANAFTEKVAKI
FNGHPMTTITEILFNVPFTAHCMGGCAIASSPERGVVDGQNRVFNYKNLYVVDGSMLG
ANLGVNPSLTITALAERAMSYIPAKHTLEEQAYTQTHNEVLEAAKVSA" /product="Cholesterol
oxidase (EC 1.1.3.6) @ Steroid Delta(5)->Delta(4)-isomerase (EC 5.3.3.1)"
/transl_table=11
CDS complement(769406..770686)
/locus_tag="AUO97b_00913" /db_xref="SEED:fig|400667.82.peg.773"
/translation="MKKTPLWVGVCLCPLFSQVVHAEFIADSKAELTLRNFYFDRDYK
KDPYPYTAARDWAQGLIFKGQSGYTEGTVGFGVDVLAMAGFNLMGSRADDYARSGLLP
VNTDNSRDDYYGKIGITGKAKFRKNELFVGDLVPQLPTIFSSPARLFPQTYRGIRFVS
NEIPNLQLEGFYVDEVRQRDSIRYTDVGTDNINHRFNKAATTDSFYTLGGSYQLKDYR
LRAYHAELKDIYQQQFLGFNGKQPLNDQLNFLSDVRFFNSEETGSKKIGEVDNRHISG
LFGLNYQNHTVSLGYMQSFGSTGLPFLSGTESPVVLDFMSSDYSNKDEKVYSIRYEYD
FKNARIGDVSLNGLRFMTRYAKGEDIDLLQYGDQRFKEDSLEFDLGYKIPEGKLKGLG
MRARFSHYRNDMPTNMTFHSANETRLNVDYTFKF"
/product="Outer membrane low permeability porin, OprD family => putative
vanillate porin VanP" /transl_table=11
CDS complement(770718..772064)
/locus_tag="AUO97b_00914" /db_xref="SEED:fig|400667.82.peg.774"
/translation="MERDVLSEINHNNMSRYQWFIILICICLNIIDGFDVMVMAFTAP
SVSAEWSLSGAQIGLLLSSGLFGMAAGSIFLAPLADKIGRRLLILVCLVIAGLSMLGC
ALVQSHGMLAVLRFITGIGVGGILASSNVLASEYANARWRSLAVSLMSTGYGIGATLG
GVLSLALIEHLGWRSIFLAGGITTIVMLLISAWLLPESLDYLLAKRPKQALERINATV
NRMGLTALKALPYYGQNNQKNTGDIGKLFSGQQGFQTLCLWFAFFLVMFGFYFVMSWT
PKILISMGMSPEQGVSTGILISIGGIFGAAIIGLLASRMKIFYALSLFLGLTAVCVFL
FVAVSSQVSIALIVGLLLGTLINGCVAGLYSISPTIYDAEIRSRGVGYAIGFGRIGAI
LSPTVAGIFLDKGIAPATLYAYYGVVFILAIFLILSLGSAFYRSKKEQNFSLKTAP" /product="Putative
hydroxycinnamate transporter" /transl_table=11
CDS 772556..773632
/locus_tag="AUO97b_00915" /db_xref="SEED:fig|400667.82.peg.775"
/db_xref="GO:0018489"
/translation="MFIKNAWYVACRPEEIQDKPLGRTICGEKIVFYRGKENQVAAVE
DFCPHRGAPLSLGYVEDGNLVCGYHGLVMGCDGKTVSMPAQRVNGFPCNKAYAVVEKF
GFIWVWPGEKALADEAKLPTLEWADHPEWSYGGGLFHIQCDYRLMIDNLMDLTHETYV
HSSSIGQKEIDEALPVTKVEGDHVITERYMENVIAPPFWQMALRGNHLADDVPVDRWQ
RCHFFAPSNVHIEVGVAHAGHGGYHAPKDKKVSSIVVDFITPETETSHWYFWGMARNF
QPDNAELTDQIREGQGKIFTEDLEMLEQQQQNILNNPHRKLLMLNIDAGGVQSRKVID
RLLAEENKTPPETSTQKFPNIRII"
/product="Vanillate O-demethylase oxygenase subunit (EC 1.14.13.82)"
/EC_number="1.14.13.82"
/transl_table=11
CDS 773645..774589
/locus_tag="AUO97b_00916" /db_xref="SEED:fig|400667.82.peg.776"
/db_xref="GO:0005506"
/db_xref="GO:0006118"

/db_xref="GO:0009055"
/db_xref="GO:0016491"
/translation="MDVIIQKIHQLTPTIRAFELVTANGTELPSFEAGAHIDVHLKNG
LTRQYSLSNCCSEKHRYVIGVLHDANSRGGSRCIHTEYREGDHLKIGEPRNLFEIHPQ
TKQAVLFAGGIGITPILSMAYRLKSANIPFELHYFVRSHEMIAFYGNLTEHFGDQVHF
HIQDQPDTECNMAEVLGQASPDKHLYVCGPTGFMQFVMSSAEQAGWHSEQLHQEHFVA
QKVDTSNDEAFTIEVKGTGRRIEVLPEQTATEALIANGFNIPVSCEQGICGTCITRVV
EGIPDHRDLFMTDEEHALNDQFTPCCSRAKTKHLVIEL" /product="Flavodoxin reductases (ferredoxin-NADPH reductases) family 1; Vanillate O-demethylase oxidoreductase (EC 1.14.13.-)" /transl_table=11
CDS complement(774633..775313)
/locus_tag="AUO97b_00917" /db_xref="SEED:fig|400667.82.peg.777"
/translation="MSSGQQVLLKLRKMIISGELEGGARIAEIPTAELLGVSRQPVRI
AFRLLEQEGLLIKNPTRGYTVREISGKLVHDALEVRGVLEGLAAKTLAEQGLSEDQKK
TLQHCIQETEKLFNEKDEFGDAELERYHHFNVIFHDTIIEGAQNVALIQALAKNNQLP
MASAQAITYDQNQALSEYRRLHYAHLQHCSIFDALVNRQAGRAETLMREHSSVVILGE TLRNVLSA"
/product="Transcriptional regulator for ferulate or vanillate catabolism"
/transl_table=11
CDS complement(775432..775890)
/locus_tag="AUO97b_00918" /db_xref="SEED:fig|400667.82.peg.778"
/translation="MVRSNLVQKKVEDEPKLSYMIARVDRIISKHLTEHLKELEITLP
QFTALSVLASKSNLSNAKLAERSFIKPQSANKILQDLLVNGWIEKKPDPTHGRRILIT
LTESGIEKLNKCNEVVLKVEEKMLEGIDINLAYLIRNNLDIMVNNLKNFK" /product="Transcriptional regulator, MarR family" /transl_table=11
CDS complement(775961..777193)
/locus_tag="AUO97b_00919" /db_xref="SEED:fig|400667.82.peg.779"
/translation="MEKISGQPSRAKITLLLCFAIAIFEGFDLQSMGVAAPRMRAEFM
LDNAQMAWAFSAAILGTLPGAILGGRFADIVGRKKILIFSILLFGIMSLLTAYAANFS
LLLLIRFCTGLGMGGALPMMITLASEAVPDQHKGTAVSVMYSGIPFGGLLTSVVAMSL
AGDAEWRHIFYIGGIAPILLIPLIMRFLPESNDYLQRKVQAQQTTPFFEVLFAKERRM
STIQLWISFFCTLVVLYFLLNWLPLLMGAQGLSKLQANYVQMGYNVGGILGSVLMGIL
LDKLRMSFVIKLIYLGILFSLCCLAISPTVALLALSAVGCGLFIVGGQSALYGLAAMY
YPTEMRGTGVGAAVAIGRIGSFAGPLMAGFLLSLGQSSTIVIGSSIPVILIAAISALL
LVKKPKQPVHLVQSSGAR"
/product="Putative hydroxycinnamate transporter" /transl_table=11
CDS 777447..778280
/locus_tag="AUO97b_00920" /db_xref="SEED:fig|400667.82.peg.780"
/db_xref="GO:0004300"
/translation="MSYENRWETVDVKVEDGIAWVTLNRPEKKNAMSPTLNREMIDVL
ETLELDQNAKVLVLTGAGDSWTAGMDLKEYFREVDTQPEIFQERIRRDSCRWQWQLLR
MYSKPTIAMVNGWCFGGGFSPLVACDLAIAADEATFGLSEINWGIPPGNLVSKAMADT
VGHRASLYYIMTGKTFSGKEAETMGLVNKSVPLAQLKAEVTELANCLLEKNPVVLRTA
KNGFKRCRELTWDQNEDYLYAKLDQCIHRDTENGRQEGLKQFLDEKSIKPGLQSYKRT G"
/product="Trans-feruloyl-CoA hydratase (EC 4.2.1.101)" /EC_number="4.2.1.17"
/transl_table=11
CDS 778318..779769
/locus_tag="AUO97b_00921" /db_xref="SEED:fig|400667.82.peg.781"
/translation="MQNVQLLIHGQSVDASNQATFERISPIDGTVATQAAAATLEDVD
RAIDSAQQAFKVWSKLSPTERRLRLLKAADLMDQKTEQFIETGMQETGSTATWYGFNV
HLAANMLREAAAMTTQIDGSLIPSDVPGNLAMGVRVPCGVIVGIAPWNAPVILATRAL
AMPLACGNTVVLKASEACPATHRLIGEVLHEAGLGEGVVNVITHAAEDAPQIVERLVS
HPAVKRINFTGSTKVGKIIAETAAKYLKPVLLELGGKAPVVVLNEADINEAVNAVAFG
AFFNQGQICMSTERVLVQDKIADQFIEKLIEKTRSIQAGNPTSKDNVLGVLESRRAAN
RIQHLLEDAQNKGADLPLGIHIEDTTMQPTLVLNIKPDMLLYREESFGPVCTVQRFSS

IEEGIALANDSEFGLSSAVFSQNISQALEVAKQIDSGICHINGATVHDEAQMPFGGTK
SSGYGRFGSKVSVAEFTELRWITIQTQSRHYPI"
/product="Vanillin dehydrogenase (Hydroxybenzaldehyde dehydrogenase) (EC 1.2.1.28)" /transl_table=11
CDS 779860..781737
/locus_tag="AUO97b_00922" /db_xref="SEED:fig|400667.82.peg.782"
/translation="MQMNATQSQDRERFVKLGQHDIHYHYKDQTLYISPKEQLKPYPQ
KLTDRLIHFAQTKPDHIFAAKRNAQGEWVKLSYAEVLQRAWHIAQALHDRNLSQERPL
VILSGNDLEHLTLSMGAMLAGVPFSAISPAYSLISQDFGKLKHVFEVLTPGMVYASDG
QAFAKAIQACITSDIEVVTNKGIVGDQICTSFQSLLDTSVTNVQEFYQTLDENQIAKF
LFTSGSTKLPKAVPTTHLMLCVNQQMLLQTFPEFEETPPVLLDWLSWHHTFGGSHNVG
IALYNGGTIYIDDGKPVAGKFDETIRNLKEISPTVYLNVPKGWEELTEALEKDEELRD
RFFAKVKILFFAGAALSEAGWNRLDKIAQQHCGEKIRIMSGLGMTETAPSCAFTTGPR
VMAGFIGYPAPGCEIKLVPCGDKLEFCVRGKHVMKGYWRLKADQQSTIFDDEGFFHTG
DAVRLVDINDPTKGLMYDGRIAEDFKLNTGTFVNVGTLRNKVLIQGNLLIQDVCITGS
NLNAVGFLIFPKLEACAQYAGLKLGDHSATEILQHPKVQQWFRQFLTTFNKDATGSSN
TVSMLYLMTEPPQLDAGEVTDKGNLNQSSITKRRAALIDELYQKQTDNPLIIRVPTLK Q"
/product="Trans-feruloyl-CoA synthase (EC 6.2.1.34)" /transl_table=11 CDS 781812..782951
/locus_tag="AUO97b_00923" /db_xref="SEED:fig|400667.82.peg.783"
/db_xref="GO:0004085"
/translation="MPHDTEFELFRDSYRRFLKEQVAPYYEQWEHDGLIPRDLWLRLG
ENGFLCVDVPEEYGGYGAPVHYSLMLVQETAQAGFASLAVAIGGQNELVSPYLQNIGT
EEQKRYWLPKMVTGEVVTAIAMTEANAGSDLQAIRTQAILENDHYRINGSKTFISNGL
HADLIVLVAKTDSQAKAKGISILLVDAALDGVKKGPSLQKIGLHAQDTAELFFDDVCV
LATQRLGEEGQGFAYLMQELPRERLSISMMALGAILGAIELSKDYVLERKAFGQPLSQ
MQNTRFVLANAQIKAKAAQAFVDQCAALYQQHQLSVTQVAALKCFITDVQCEVIDQLL
QLFGGYGYMQEYPISRFFVDARVQKIYGGTNEIMKEIVARELLGK" /product="Acyl-CoA dehydrogenase"
/EC_number="1.3.99.2"
/transl_table=11
CDS 783033..784283
/locus_tag="AUO97b_00924" /db_xref="SEED:fig|400667.82.peg.784"
/translation="MSKLWMYSTLMLSGSVWAGSFIDNSSVELTTRNFYFDRDYQEQS
AYPAAKDWTQGFILKANSGYTEGTVGFGLDVLATAGFKLDADAEHGGTGNLPRDTRTN
EPADSYGEIGVTAKAKMSQTELRIGTLMPMNPVLVASPARLLPQTYRGISLTSKDIKD
FDLQAAYLDKVNHRDSTNYEKIKISGVNGRFKSAETDGLYYLGGNYQFNPALKLTAFY
MDVDDLYNQTMVGALHQHKINDTTNFSSQLRYYRSRDDGQAKAGLVDNDLYHAHFELK
HQNHKFIFGTFQHHGDTAFPYLTGGETGLLIDTWPGEFLNPKEKAYSFRYEYDFKDYV
PGLRFMTRYTTGHNIYAPNLGGTNLKERETDFDLGYTIQSGWLKNLGLRARYAIYDNN
MLSTANIKPVNETRLNIDYTWKFK"
/product="Outer membrane low permeability porin, OprD family => putative vanillate porin VanP" /transl_table=11
CDS 784340..784876
/locus_tag="AUO97b_00925" /db_xref="SEED:fig|400667.82.peg.785"
/translation="MKKVVTLLCGISLIALTHAATQYNPPPIQLEPLAKFSVDLNAPV
WELGTTSDAGKRRIIPITGGTFEGKSLKGRILNNGADWQIVDSKGLAIIDTRYLLETD
DGALIYLQTKGYRHGSAETLKQLAQGKDVDPKNYYFKITMQFETSSPKYSWLNQTVAV
GSAMRLGKAVIYDAYTVK"
/product="hypothetical protein"
/transl_table=11
CDS 784917..785306
/locus_tag="AUO97b_00926" /db_xref="SEED:fig|400667.82.peg.786"
/translation="MDQKTLEHLPPIHQHLGGHNIHWNDQNTELTIHYTALESFTNPR

GTVEGGMICAMLDDVMGLFAYLANDRKPATTINLTMDFLRPCAVGEVITKCRFIKQGK
TVLNLESEAWQNNKMIARSTANFLVLD"
/product="FIG00351372: hypothetical protein" /transl_table=11
CDS 785340..787091
/locus_tag="AUO97b_00927" /db_xref="SEED:fig|400667.82.peg.787"
/translation="MNYTNNKRILTHSALVLAMLGALAGCGSDNDDTTAPSTTKPPQL
SPAVGVKMTGSCSDLLGFQYNNTVISSATLQETGTLTVANKPIGAHCLVKGYMDQHVS
PVDGQTYQIGFEMRLPIDWNGRFLYQGNGGTDGNLVPATGQVGSGGPLTNALHDGFAI
ISSDAGHNASQNPMFGLDPQARINYGYGAITKLTPMAKNLIKAAYGKLPDRSYAGGTS
NGGRHAMIAATRLGDQYDGILASTPGFHLPRAAAAQLYTAQQLRRVATDENDLSTALT
LPERKVLAKAILDRCDALDGVTDGLVQDVEACRTAFDIHKHVTVCSSTRDGTCLSTEQ
IDVLANIYRGPVNSAGQALYATQPFDPGLVGSNWASWKFESSVGTARDPVAVGILFQV
PPDPTVTQNSRQFALNFNFDTDYPKLSATNDVYTESSMSFMMPPDELNLDKLRNHGGK
MIVVQGTADGVFSVDDTQNWYDQLLQHYKNDAKGTAPEFARFFRVPGMNHTRDGIATD
QFDALTALVNWVEYGQAPDKIIATARGAGNPSGQVNTEIPQDWAPDRTRPLCPYPLIA RYNGQGDSEKAENFSCK"
/product="Chlorogenate esterase"
/transl_table=11
CDS complement(787156..787869)
/locus_tag="AUO97b_00928" /db_xref="SEED:fig|400667.82.peg.788"
/translation="MSSAIKPRSTKKLSQIIVEQLIEKIQSGEFKVGEKIPTELELIE
TFEVSRSVIREAITELRSLGFVETRHGIGTFVKEQTVEQNFLLSNASLETINDIISLL
ELRISLESEAVFLASERRKQIHIDKMKAALEDFERHISSDANDGTVKADYDFHIAIAE
ASENQYFVDFLKYLGEKIIPRARVKSIEQSPENREEYLKAVHHDHVNIYNAIVDQDGL
LARQMMRAHLSKSIKKFKQ"
/product="Predicted D-glucarate or D-galactorate regulator, GntR family"
/transl_table=11
CDS complement(788113..789699)
/locus_tag="AUO97b_00929" /db_xref="SEED:fig|400667.82.peg.789"
/translation="MSKNNGRQFINGQRVAVNAPALESIGAKDYQPTGYVFSQASLEE
VDQAAQAAHNAFLVYSQTTQEQRASFLEEIARQIEAMGANLQEVASLETGLPLARLQG
ETGRVTGQLRLFAELLRRGDFYGARIDVALPERKPLPRVDLRQYKVGVGPVAVFGASN
FPLAFSTAGGDTVAALAAGCPVVFKAHSGHMATAELVAEAIEKAIEVCGMPKGTFNMI
FGGRIGANLVEHPLIQAAGFTGSLEGGMALYNLAQSRPQPIPFFAEMSSVNPVVVLPE
ALHSRGEQIAQDTVASFNMGCGQFCTKPGLILGIKGPEFDRFITALAENTQKAVPQVM
LNEGTLKSYRTGIDALINEAGFEVIASGQEPELVSQAKAHLFKADQSVLLSGNPKLQH
EVFGPMSIVIAVEDEATLLKGIEALGGQLTATIIGDENELSKAGELLDLLTRKAGRVL
FNGFPTGVEVSDAMVHGGPFPATSDARGTSVGTGAIERFLRPVCFQNTPQLLLPDALK
DSNPLQITRLVNGQLSQAQI"
/product="2,5-dioxovalerate dehydrogenase (EC 1.2.1.26)"
/EC_number="1.2.1.26"
/transl_table=11
CDS complement(789730..790641)
/locus_tag="AUO97b_00930" /db_xref="SEED:fig|400667.82.peg.790"
/db_xref="GO:0047448"
/translation="MDALELKNIISDGLLSFPVTDFDQNGDFNKSSYAKRLEWLAPYG
ASALFAAGGTGEFFSLTGNEYSEVIKTAVDTCKGSVPIIAGAGGPTRQAIEQAKEAER
LGAHGILLMPHYLTEASQEGLIEHVKQVCNSVDFGVIFYNRSVSRLNLDSIQKLTEMC
PNLIGFKDSSGQIDMMTAVTQTIGDRLSYLGGLPTAEVFAAPYKALGCPVYSSAVFNF
IPKTAMEFYNALRSDDFETTNRLIKDFFLPLIKIRDRKSGYAVSMIKAGAKIVGHDAG
PVRPPLSDLTQADYEDLAALIATLGPQ" /product="5-dehydro-4-deoxyglucarate dehydratase
(EC 4.2.1.41)" /EC_number="4.2.1.41"
/transl_table=11
CDS complement(790804..792135)
/locus_tag="AUO97b_00931" /db_xref="SEED:fig|400667.82.peg.791"

/db_xref="GO:0008872"
/translation="MATSTPIVKSIRAIPVAGHDSMLLNLSGAHGPYFTRNILIIEDN
SGNIGVGEIPGGEKILATLNDAKALVEGQPIGEYKNLLKKIQQTFADRDSSGRGNQTF
DLRTTIHVVTAYESALLDLLGKYLNVNVASLLGEGQQRSEVEVLGYLFFIGDRKQTSL
DYATSTQQHEWYKVRHEKALTPEAVQRLAEASYDRYGFKDFKLKGGVLQGEQEAEAVT
AIARRFPDARVTLDPNGAWFLDEAIALGKHLKGVLAYAEDPCGAEQGYSSREIMAEFK
RATGLPTATNMVATDWREMSHSIQLQAVDIPLADPHFWTLEGSVRVSQLCKMYNLTWG
SHSNNHFDISLAMFTHVAAAAVGNVTAIDTHWIWQEGTDQLTKAPLEIKDGKIQVPTA
PGLGIELDWDRINRAHELYKLKGLGARNDADAMQFLIPNWTFNNKKPCLVR" /product="Glucarate
dehydratase (EC 4.2.1.40)" /EC_number="4.2.1.40"
/transl_table=11
CDS 792421..793779
/locus_tag="AUO97b_00932" /db_xref="SEED:fig|400667.82.peg.792"
/translation="MDNLQTNIAVVTQQRAKHTKTRYYILAMIFLVTAFNYGDRATLS
MAATPMSHELGIDSVTMGYIFSAFAWAYVIGQIPGGWLLDKFGARRVYFWSLFLWSLF
TVLIGFTDILGDTATIITSLFILRFLVGLSESPAFPGNSKIAAAWFPTKERGTAAAIF
NSSSYFSTVLFAPLMGWLVATVHWQSIFWVMGGLGIILSFIWLKVIYSPTNHPTVNPE
EVKYIASEGALLDMGENSQNAKKEKITWSKVKQLLASRMMLGIFIGQYCVNTLTYFFL
TWFPVYLVKERHLSILEAGFAAVAPALCGFVGGILGGLISDKLIRMNYSLSFSRKLPI
VVGFLVSTSIILCNYVDSQAAIVFFMSLSFFGKGIGSLGWAVMSDVAPKEMVGLSGGM
MNAFGNTAGIVTPIVIGYILASTGSFNLALTYVGIHAIAAVICYTVIVGKIQRFELKP TS"
/product="D-glucarate transporter"
/transl_table=11
CDS 793850..795397
/locus_tag="AUO97b_00933" /db_xref="SEED:fig|400667.82.peg.793"
/db_xref="GO:0008867"
/translation="MLKPLLIRISPLDNVAIVVNDGGLPSSTYIEEYQLTLVDEVPQG
HKVLLEPLKQGEAIVRYGEIIGYVNKDLPAGSWVNEAVTQMPEAPELDALELATRPDP
KLPPLTGYTFKGYKNKDGSVGTKNILGITTSVNCVEGIVDYVVKIIERDLLPKYPNVD
GVVGLNHLYGCGVAIDAPAAIVPIRTIHNLALNPNFGGEIMVVSLGCEKMQPERLLNI
PKENKYIPLANEDIIQLQDERHNGFESMVNHILTVAEQHLEKLNQRTREEVPASELVV
GMQCGGSDAFSGVTSNPAVGFAADLIVQCGGTVMFSEVTEVRDGIHLLTPRAANEQVA
KDLIREMKWYDDYLAAGQVDRSANTTPGNKKGGLNNIVEKAMGSIAKSGRSPIVEVLA
PGQRPTKKGLIFAATPSSDFICGTQQMASGITVQVFTTGRGTPYGLAAVPVIKMASRN
NIANRWYDLIDISAGDIAIGKKTIEEVGWELFEMILRVASGEQQTWSDRWGIHNSLAV FNPAPVT"
/product="D-galactarate dehydratase (EC 4.2.1.42)" /EC_number="4.2.1.42"
/transl_table=11
CDS 795523..796419
/locus_tag="AUO97b_00934" /db_xref="SEED:fig|400667.82.peg.794"
/translation="MQNQTASNLPSTQLGKALLCLMTSALLFSIMGVCIRFASQTVDN
ATVVFFRNAVGLFIFIPMLFKQGLDFIKTDKLWMHTWRSIVGLAAMYGFFYAIANLKL
SNAMVFSYSSPIFIPLIAWLFLKEKITKSMIFAAVIGLIGVLFVAKPDQGLFNALSFI
GLGACFLSAMAFVTVRALTSTEPPERIVFYFCVFGSLISSIPMFWHWRIFTWHELALL
IAAGLLANISQLFMSYAYSLAPAGQIGPMNYIAIIFAGIWGFVFWHELPDLFSIIGIF
IILFAILLCNPFLQKKLLSRLK" /product="Permease of the drug/metabolite transporter
(DMT) superfamily" /transl_table=11
CDS complement(796435..797505)
/locus_tag="AUO97b_00935" /db_xref="SEED:fig|400667.82.peg.795"
/translation="MPKNLYKNANNVSEGNYDNTPLGANTYLLGLTSLFDEVGSQGHS
ISDVLSGTNISPDAMNQSSLYISQVQKIQLFQNIQKLSHDPLIGLRAGQKQRLSDFGV
YGYALYSSRNFRQAVELGIRHIKLAGPILKKSFYIRDDVAVFEGQDIIALGKLLPLVC
EFWFSSMQTLIERVLEGPFQAKKLLLPYPAPDYAEEYEKVFHCPVQFNAGVMQWHFDL
KWLDVPCPNANPITADMCKSFCERMLGAAELEEPELVKMIRLILLDGTGLFPSAQEMA
ERLHISKRTLHRRLAGFGLTYQELLDETRRRLADEYLRETTLTIDEIAERIGFSDTSN

FRKAYRHWTGISPNEYRIQYLN"
/product="Transcriptional regulator, AraC family" /transl_table=11
CDS 797635..799095
/locus_tag="AUO97b_00936" /db_xref="SEED:fig|400667.82.peg.796"
/db_xref="GO:0004029"
/translation="MESSTLKEYSGSATEHMELIFSKQRANFEADPYPSLEARRTKLH
QLKKQIIRYQDALAAAINADFSSRSLDESKLLDLLGSVLEADHAIHHLRRWMRPSKRR
TELLFLSNRLSVQYQPKGVVGVIVPWNFPVYLALGPLIAALAAGNRVMIKLPEITPNT
NAMLRRMLAEVFNEDEVAVFGEEITDPARFTTLPFNHIVFTGSPAIGKVVMRAAAENL
TPVTLELGGKSPAIVSRNYPLADAAKRITHGKATNSGQICVAPDYALVPKESIDEFVD
AAKSSFIKMFGQSIENNENYTSIVNDRHLKRIQDILTDAQEKGARIIPCDTYSFDQQG
RRMPVQIVLNCTPDMRIMKEELFGPILPVVAYDSLDDAITYVKSDERPLALYCFTHSS
VERDRILRETHSGGVTINDWGWHVVNHDAPFGGIGNSGMGSYHGEEGFRELSHAKTVF
IRHRFFPTQLFHPPYGTFLQKLAIRFFLKKGDPNIK"
/product="Aldehyde dehydrogenase (EC 1.2.1.3); Probable coniferyl aldehyde dehydrogenase (EC 1.2.1.68)" /EC_number="1.2.1.3"
/EC_number="1.2.1.68" /transl_table=11
CDS complement(799269..799607)
/locus_tag="AUO97b_00937" /db_xref="SEED:fig|400667.82.peg.797"
/translation="MKLNMKKILIIIALLAPLMLITNYIANRLSKKNEIYIDQVLKQD
VELHNKYGDITEYNLRKAGKSFSGGGDEKSYYYYTYSVKGNITSGLIKLKLFENDQKK IDGYTIEFIK"
/product="hypothetical protein"
/transl_table=11
CDS complement(799810..800256)
/locus_tag="AUO97b_00938" /db_xref="SEED:fig|400667.82.peg.798"
/translation="MNNQKIVIDYLDKNNELSKELIWGEMYKTDQYFVRSIPLFAPNL
AFGDLIQVEIDDETLYFDDLIKPSNNSTLRIVFFNNDIKCIEKILTTLESYLCGWEGF
ESRRYYAINIPKKVDYALIKKYLDDRKDFFDYEESCLSDKHLGDSF" /product="hypothetical protein"
/transl_table=11
CDS complement(800328..800738) /locus_tag="AUO97b_00939" /db_xref="SEED:fig|400667.82.peg.799" /translation="MFEITPYVGASLIKFGMKREEVRKVFSEYEPRVFGENSSTDGYD
ELGISLDYDNKDGVVALVFCEPAQVVFKGIDLFKLSASEAYKLMASLDKDIAIDRDGL
TSFKFGIDFYEPNYEEEPFLPVEAIIIFIEGYYD"
/product="FIG00353111: hypothetical protein" /transl_table=11
CDS complement(800964..802427)
/locus_tag="AUO97b_00940" /db_xref="SEED:fig|400667.82.peg.800"
/db_xref="GO:0006810"
/db_xref="GO:0016020"
/translation="MSKNKGKQFLEGDLLGSQHMTEQEEEKLQRSLTNRHIQMIAIGG
AIGTGLFMGSGKTLSVSGTSIVLTYLIIGFFFFFVMRAMGELLLANTNFKTFADFATA
YLGPWAGFFLGWSYWCNWIITAIADVIVIGGYMQFWYPDLPVWIPAFTSLAILTILNF
VAVRLFGELEFWFSLIKITAIILFILAGIYLIGTGFTSPNGVKASMSHLFETESMFPY
GVTGFLAGFQIAIFAFVGIELVGTTAAETKDPHKSLPKAINSIPLRILLFYVGALVCI
IAVTSWAKISPEKSPFVEMFTLVGLPIAAGLINFVVATSALSSANSGIFATSRMLYGL
ALDNDAPKSFSKLSKRKVPIRGLVFSMACVTVGTSILFIVPNVMTAFTIISALTSILC
IFTFMLIVLSYIYYRKKSPELHIQSKYKMPGGLFMAWMTMLFLVFTIVILALDHDTLI
ALECSPIWFIGLFIAYKYKKKKMLQLNILKAQKVDYI" /product="D-serine/D-alanine/glycine transporter" /transl_table=11
CDS complement(802827..803921)
/locus_tag="AUO97b_00941" /db_xref="SEED:fig|400667.82.peg.801"
/db_xref="GO:0008791"
/translation="MMIIRYIEPKDVNDLYLLAQKAGFGLTSLQPNMEKLAARIERAC
KTVAGELPKADQVYLFVLEDTKLNKVVGVCGIEVALGLKEPWYNFHVGTQVHASEPLS

VYNALPTLYLSNDHTNCSELCTLFLDPDYRLNKNGKFLSKVRFLFLSAFRQYFEETIV
AEMRGYSDANGQSPFWNAVGHKFFNIEFTKADYLSGVGQKAFIAELMPRHPLYVDMLP
DDAKAAIGIVHPNTRPAYNLLLEEGLRYKGYVDIFDGGATLQADIENLRAIKESQSVA
VQIEQPENIAVGDEAYIVANDDYENYRAILVYSQPHQNILQLTKEQAKQLNVENGSLV
RVLSVQVKHVSNPEVVNKLKETEHFRMAVN"
/product="Arginine N-succinyltransferase (EC 2.3.1.109)"
/EC_number="2.3.1.109"
/transl_table=11
CDS complement(803918..805153)
/locus_tag="AUO97b_00942" /db_xref="SEED:fig|400667.82.peg.802"
/translation="MNNFAVSRGDFNEWMVPVFAPANFIPVRGEGSRIWDQENKEYID
FAGGIAVNALGHAHPVAVNALTEQAKKLWHVGNGYTNEPVLRLAKQLTENTFADKVFF
CNSGAEANEAALKLARKVGLDSGVAGKSGIVAFNNAFHGRTLFTVSAGGQPKYSQDFA
PLPNGISHVAYNDLEAAKAVINEQTCAVIVEPVQGEGGVIPADIEFLKGLRELCDQFG
ALLIFDEVQTGVGRTGALYAYMNYGVIPDVLTTAKALGGGFPVGAMLTTDKFAPHFSV
GTHGTTYGGNPLASAVAGAVFEFINTLEVLEGVKERHDYYKNALNQLNEKYEVFKAIR
GSGLLLGCVLKDEFAGKAKDINNLAGEEGLLSLIAGPNVVRFTPSLIVPFADIDEGIK
RFERALARFVEMQKDEQAA"
/product="Succinylornithine transaminase (EC 2.6.1.81)" /EC_number="2.6.1.81"
/transl_table=11
CDS 805289..805711
/locus_tag="AUO97b_00943" /db_xref="SEED:fig|400667.82.peg.803"
/translation="MDNIDQIILGLLKDNARISVTELAEKVHVSRATIKKRMEYLESS
GIITGYTVRFKPNAERNVIRAWMSIMVEGTKAQSVIKELRLESAVECLHKTNGKWDIL
VELRSDTLENFDKVLERIRNISGIYNSETSILLASHKT" /product="Transcriptional regulator,
AsnC family" /transl_table=11
CDS 805743..805907
/locus_tag="AUO97b_00944" /db_xref="SEED:fig|400667.82.peg.804"
/translation="MSQLTAELIHFPFIKKTLYGRSLNLKGHPHDERILTILNKFLIF LNNQNQKLKI"
/product="hypothetical protein"
/transl_table=11
CDS 806029..807459
/locus_tag="AUO97b_00945" /db_xref="SEED:fig|400667.82.peg.805"
/translation="MAKTQLQHFINGEYVASKGNDFFDLVSPVTGEVYAQSPNATEAE
VDAAYAAAKEAFKIWGRSTPSTRQKALLNLADAIEANADRLIEAQSRNTGQLKHLIAS
EEVGASADQVRFFAGAARLLDGTATGEYLEGLTSSIRREPVGVVGQVTPWNYPLMMAV
WKIAPALAAGNTVVLKPSDTTPESTLLLAEIAAPFFPKGAFNVVLGQAQVGSKVVSHK
TPALVSITGSVRAGLQVAASAAANLAKAHLELGGKAPVLVFEDADLDKAVEMIALTGY
FNAGQDCTAATRVIVAESVHDEFLAKLVEAAKNTRFGDPDDQDALYGPLNNANQLKNV
KAFIDNLPAHAKVETGGKQADRPGFYFEPTVISGLKQHDEAIQNEIFGPVITVQKFTD
ENDAIEKANDVEYGLASSVWTKDHARATRLSRELDFGTVWINTHIPLTAEMPHGGFKK
SGYGKDLSGYGFEEYTRVKHVMSSNE"
/product="4-aminobutyraldehyde dehydrogenase (EC 1.2.1.19)"
/EC_number="1.2.1.19"
/transl_table=11
CDS 807488..808258
/locus_tag="AUO97b_00946" /db_xref="SEED:fig|400667.82.peg.806"
/translation="MNKQEMKDLVTKAHHELFNLHDTTALDRYFSEDFIEHSPLVANG
ISGLRQLVEDCPDMQHEAVRVLADGNLVAIHGRFQGLDENPLVGFDIYRVKDGKIVEH
WDGLVAEAAPNVSGRTQLDGPTEIVTHHDPEKNREIVTSFFKKSLIDGNYEAFKEYTD
GDQFIQHSPDIGDGVKAVIDFLNNIRNEGQGLVYSKTHRSIADGQFVLTHSEGSIAGN
RHAYFELWRVDNGKIVELWDAIPAVPEDEQAVHGYGVF" /product="FIG00923150: hypothetical
protein" /transl_table=11
CDS 808318..809037

/locus_tag="AUO97b_00947" /db_xref="SEED:fig|400667.82.peg.807"
/translation="MSAYTIIFAPIAQATRSEQVVERLENAIISGLLKSNEQLPNEAD
LARLMGVSPITVREALNTLRVKGLIDTRRGRNGGSFVCELPSDLLLNQHPLRQASNEY
LADLGEFHSAILSHSAYLAAQRTTEYELNKIKELIDQFEQAVEADTRAQLDLRCLLTL
TSFAQSSRLANQELTIQAEWAPLIAVLYQDDDFHREVVKQYHHLLSSFIDNNENEAVI
QARKIVSMLTDQMLRYKLSSE"
/product="Lactate-responsive regulator LldR in Enterobacteria, GntR family"
/transl_table=11
CDS 809103..809810
/locus_tag="AUO97b_00948" /db_xref="SEED:fig|400667.82.peg.808"
/translation="MTQHLDVEELQNLLKTVVDETTSITETLASKASKILSKHASEKN
ADIKLSTSERAALQKEIKKALSQSHYSQGTGFASYSPATQDEQDYWTLEWWYKKEDQL
QQAKLENYQNAQRFLDFRSFEWFHKPAQNKSPCIHGPYVDYICNGAYTITIAHPVMIH
DHFIGVIATDILVSALEKLLMPKLKNIKQKAVIMNDSSRVITSNDISIRTGTLFKDKT DQQFFSQPCQSFQLVVI"
/product="hypothetical protein"
/transl_table=11
CDS 810005..811336
/locus_tag="AUO97b_00949" /db_xref="SEED:fig|400667.82.peg.809"
/translation="MENKASSLKKLSLWQVTIIGIAYMTPMTVFDTFGIVSGITNGHV
PLAYLLALGAMLLTAWSYARFSQNSEKSGSAYSYTAESLGPKSGFFVGWCSLLDYLLL
PLINVLLAAIYLTALIPSLPYWFWVLVSAGLVTLVNCFRIRLLANLSLVFVFAPIILM
VIFVYLVVRGIGSTQGYEHVLTLAPLWHGQQSLLPLVAGASVLCFSFLGFDAVTTLSN
ETKQPTKNIPRAVMLTTLAGGAIFFTAAWFIQLYFPNNVRFHHPSEALPEIVLYVGDA
LFQSVFLCGQIMNTVASGLASHASASRLLYIMGTDNIFPKKYFGTIHSSLGTPFFSVL
FVGLISMSAIFLDLAQVVSLISFGALVAFTAVNFSVFMKFYIKDKQRSGFKNKFLNLF
LPLLSVISIICLWLNLDSSSLTFGCFWLALGILLFIYKTIKKQSIAISNAY" /product="Putrescine importer"
/transl_table=11
CDS 811356..812801
/locus_tag="AUO97b_00950" /db_xref="SEED:fig|400667.82.peg.810"
/translation="MKIISKPQLLKDVEYKPSQSLQVGKEWSWLNPKHAQFDTSTING
TLVDNPANYYESSLSNWHTFDSLNADIECDVVVIGGGLLGSSTALHLAEQDVDTVLLE
KNRIGSAASGRNGGQLTPGLARWEAQEMADRLSYEDAKKLWHFTSTEAMQLIDDISEK
YQLNFDRKYGHITAAVHEGHLVGLTQGADARKYLGEYHTRIVGKHELMDYIKSDYYTG
GLIDELGGQIHPLALNRGLIYGFCQNGGSVYEQTEVISIEEKADGIYVHTANAVVKAK
KSVVLAVHHASFKLLSEQNNTTIPFYTYVATTAPLELDTKELLPFGHPVYDTQFQIDY
YRPVFNNRLLFGGQGTGTCWGPEKTLNYLEHRIHTVFPQIKNLEMDFVWSGTTDLTVN
GAVDSRKFGNKFPIYAVHGWSGHGVAQTVRIGKAIANDFVGQSNDFEMLSKIDHQNII
FGRTLAPVVIPLAKSMYGLGAMINPGKMVSF"
/product="Oxidoreductase"
/transl_table=11
CDS 813009..814304
/locus_tag="AUO97b_00951" /db_xref="SEED:fig|400667.82.peg.811"
/translation="MGGAVGTGLFLGSAQVIQSAGPSIILGYAIVGLVAFLIMRQMGE
MIVEEPVVGSFSYFAQKYWGRFPGFLSGWNYWVVYILVAMTELTAVAKYVHYWWPHIP
AWVSVLFFFVLVTCLNLGNVKFYGESEFWLAIIKVAAVISMIVFGLYLLLTAGSDSIA
SFSNLWQHGGFFPHGFSGLFYMLAFLMFAFGGIELIGMTAAEAENPEKSIPQAINQVI
FRILVFYVASLAIIMSLIPWNQLDLGGLDKSPFVMIFSQLGIGWAAHLLNFIILTAAL
SVYNSGMYANSRMLYGLAVQGNAPKVFAKVSKQGVPTSAVIFSSILIFGCVLLNYFIP
EEALSYLMYMAVAALVLNWAIISFTHLKFKRSMKLENKVAKFPALFSPLSNYIVLTFI
GMILYIMWTQGFEKSVILIPIWIAFMFGLYTILSWKKSL" /product="Aromatic amino acid transport protein AroP" /transl_table=11
CDS 814688..815047
/locus_tag="AUO97b_00952" /db_xref="SEED:fig|400667.82.peg.812"

/translation="MEKTLQCPKCGSTEIETRDHDKLLKTTGGVLLTAAGTTAGTVGG
AATGATVGAAIGTVAGPLGVIVGGTVGTFVGAISAGITGGVVGNIFGKKAGVMIDKNI FQDYRCLKCKYRFKQKK"
/product="hypothetical protein"
/transl_table=11 CDS 815281..815751
/locus_tag="AUO97b_00953" /db_xref="SEED:fig|400667.82.peg.813"
/translation="MKKLLLLSLVSIFSSYSFAEDNSSIYRGLGTGPEDFLKFQTLKQ
KGFLAKKPNVSRADYNDIYKLKKPYEFMGQNVVLISDEYMSEYVGCCVSEGWGAVFKQ
TTNLKLIEKFAKKNQCNIAPIETDSTYYGFKISSLPKGNYYELSYRERDLDESQ"
/product="FIG00352142: hypothetical protein" /transl_table=11
CDS complement(815883..816434)
/locus_tag="AUO97b_00954" /db_xref="SEED:fig|400667.82.peg.814"
/translation="MPNLETSSKKLHVIRTAINLFTTYGFHTTGVDLIVKKSGIPKAT
LYNYFHSKEGLIEMCIAFQKSLLKEEVLAIIYSNRYCTPTDKLKEIVVLHVNLNSLYH
LLLKAFFEIKGTYPQAYRMAIEYRKWLIRELFDLIFSLETRALKPDADMVLNLIDGLI
FGFISTNSLSERDEVLERFFKIT"
/product="Transcriptional regulator, AcrR family" /transl_table=11
CDS 816481..816621
/locus_tag="AUO97b_00955" /db_xref="SEED:fig|400667.82.peg.815"
/translation="MGEKKAQQSHKDCAWHISSFIVNASFLSRNDLKHENPTGYQAAL NF"
/product="hypothetical protein"
/transl_table=11
CDS complement(817760..818638)
/locus_tag="AUO97b_00956" /db_xref="SEED:fig|400667.82.peg.816"
/translation="MKKLFSVLFSASVLTLTACNKQPAQTENTAAAKDKTESVRTIKL
VSTGSDTDVWKYVATLPETAQAGIKLEVTNLTDYVVLNTSVASGEQDVNAFQSFNYLA
AYNASNKAKVAAVATTYLEPMGIYANKVKTVDEFPQGATIAIPNDTANEARALTLLQS
AKLIKLKPDFDPVKGTTNDVVENPKNLQLKPIQMTTAVRVKNDVDAIVLGNTLALEGG
LNVMKDAIFREPIDQSTKLYVNLLGVAEANKNDPIYTKLGELYHLPKVQKFVNEKFGG TKVEVNKPVSEFADIK"
/product="Methionine ABC transporter substrate-binding protein"
/transl_table=11
CDS complement(819100..820848)
/locus_tag="AUO97b_00957" /db_xref="SEED:fig|400667.82.peg.817"
/translation="MLETDVLIVGSGPAGSSAALMLSTYGIRNIIITKYRWLANSPRA
HYISQRTMEIVRDLGISDEVIAKAAPKEVMGDVVYCTSLTGDEIGRFPYGMNHPERMS
EYAKASPCEQCDLPQHLFEPILLSNAASRGSHPRFDTEYLSHVQDEDGVTVTVLDRLT
KQTYLIRCKYLIGADGASSQVVKDLDLPMAGKMGKSGSISIIFKADLTKYVSHRPGYL
WWIIQPGANIGGIGLGLLRMVRPWNEWQIVWGYDIEKEEPSLTNEEAIAICKQLIGDN
DIEISISSKSLWTVNEMYAEQYSKGRVFCMGDAVHRHPPSNGLGSNSSIQDAYNLCWK
LAFVLNGKAGRGLLNSYNDERAPVGERIVKRANQSVREFSTIFSALDLDGKYSTETMQ
KSINALSESNEKGAVKRRDLRSAIKYKAYEFAALGVETNVRYNSSAIIGAEHNIENEL
DSCRDEDLYYYPSFTPGHKLPHIWLVKNHRKISTLDLIGNGKFTLFCWLQGDKWKLAV
DNLNRQLNLDIKVIQVGPKRDVEDSYGELSRSWTNDENDCILIRPDGYIAWQSNVIES ADNELYSAISSILSWN"
/product="FAD-binding monooxygenase, PheA/TfdB family, similarity to 2,4-dichlorophenol 6-monooxygenase" /transl_table=11
CDS 821304..822122
/locus_tag="AUO97b_00958" /db_xref="SEED:fig|400667.82.peg.818"
/translation="MEKQEIISIVDRYFSNPISKVTNSPTHKLKNNTPTENENSFFFL
HDFGTYELEAKFNYCDGFPQKIALLIHGGNSDLSSLSPLENLLRSKNIGTLSFNLSGL
NNTSETKSSLSQNINESMVFYRLLKKVDIVIGYSLGGYIAINLAKKFQIDRLILLCPA
IYSDQAVKKMYGTEFKDEISKPYSFYSSSIWLFLMEYKREITIVYGELDGIKTDNGKS
KGFYEINDITNYSPIPSEIVQTLKKISMRKANFNFIIEKDKDHYLLKNIRNYNI"
/product="hypothetical protein"
/transl_table=11
CDS complement(822304..823731)

/locus_tag="AUO97b_00959" /db_xref="SEED:fig|400667.82.peg.819"
/translation="MSVEQSSITIPNIRTPKNIALITIIILSFALAFYAKGLPIEEVG
LIGLLPIFALALLTVIGLDIVLAVIIAIIISVIMTSTSVFELGGILAKSTGSFIATVG
LIIMLGAGVGEVANRTGAAKELVKFIVNKVGLSSIPRVKLGIMISSVVICGSLGTMAG
GNAIIVAVIIPIAAAVGLTPPTVALLLMTAGSVGLFIGPFTPSTVTILELGGLTYPQY
LSTCAIPMSVVTLLTGWFMSGYIQKWTFGKYTYNENFLEPSEKIESASELKRSKRAAI
VFIGTIIVMAILGIIFKAGFSFAIIVMLTVATLTGLVAGFSPKQILEALYEGCGKLVW
MFILYWLYNPILVLVEKLNAYQTLLDNATPYLVDLSPAMLCFGIFLFNIVGHIPGAAV
AQMTFTNKIFGPILLANGVPPVAVTAVLLSSSQVDWFGPFPSSDMFGQMGLARSSQLK
YMLYGGWAIVIANILLFAVLFQILN"
/product="Putative permease"
/transl_table=11
CDS 823883..824725
/locus_tag="AUO97b_00960" /db_xref="SEED:fig|400667.82.peg.820"
/translation="MLFGSLTEAASSLCISQPAASKLLKHAEQRLGFALFKRVKGKLQ
PTKEAYTLYDEIKPIYEKINELNKLTDNLARNTQGQITLGCLPSLGLSLIPKLTAKFL
KKYPDVFVNIGTDHTQALEQKLLRREIDIAVTFQPHTQHGIIATPVLEIPLVYIDSQP
ISSNQDIHSIDENRWIQPYADSLMQVVKKYRTFNKSIVNVQTYYMASELVREGLGCSI
TDIYTAAHSLPNHMIHETKPSLSTTIYILKNANVSTNKATEDFIELIKREIASMTPSI NQKL"
/product="Transcriptional regulator, LysR family" /transl_table=11
CDS 824806..826047
/locus_tag="AUO97b_00961" /db_xref="SEED:fig|400667.82.peg.821"
/db_xref="GO:0008718"
/translation="MKKHVVVIGGGVIGLSTAYALIQAGHKVTIVEANDKIGCQTSFA
NGGQLSYRYVAPLADAGVPFQALQWLGKAHSPLNFKLNFSPLQWSWLFQFLMACNRKD
NIVNGNHILRLSLYSQSIMSKWRDEIDDFDWVKSGKMIIHRNEKAFKKAATGIDPTVQ
KVLSSEETVQLEPALKGIESTLVGSIYTPDDETADCYKFCLALYKSLEKSSNFTCLYA
HEAKVFFVENNKITAIETNQGRLEADEFVVAAGNGSTAIFNMVGISVPVYPLKGYSLT
LPFPEKANVVPNMSVTDYANKIVYAKLGNQLRIAAMVDIGYQDIYIRKERINALKDTV
RTTFPDLLGVNTAQEWSGLRPSTPTGLPILGRTKYLNLWANIGQGSLGFTLAAGCAMV
ISQLISQATSPIILDGLSLEK"
/product="D-amino acid dehydrogenase (EC 1.4.99.6)" /EC_number="1.4.99.1"
/transl_table=11
CDS 826049..826408
/locus_tag="AUO97b_00962" /db_xref="SEED:fig|400667.82.peg.822"
/translation="MSHDIQRIDTNPRLSGCVLHNGLVYVSGQVPTNLDADAKQQTLE
VLAKIDALLEQSGSDKSRILTAQIWIKNIAEDFAVFNECWEAWLPDGAAPARAAIQAD LARPQVLVEVMVTASIK"
/product="RidA/YER057c/UK114 superfamily, group 2, YoaB-like protein"
/transl_table=11 CDS complement(827264..827458)
/locus_tag="AUO97b_00963" /db_xref="SEED:fig|400667.82.peg.823"
/translation="MSVSQMFQDFLSNIKINNEEQITIRYQEITKALNQNFRNKEFQT
DNCLQVGSYGRWTTLKVFLT"
/product="hypothetical protein APECO1_2271" /transl_table=11
CDS complement(827823..838016)
/locus_tag="AUO97b_00964" /db_xref="SEED:fig|400667.82.peg.824"
/db_xref="GO:0004035"
/translation="MLTKLSQNHMNQISALRIQAENGQIGYHIIYSTLASLLQSDYSY
TSSDPTVLWLRGATEANAGRGSLSELIRVYSNTQAQLRYGENISNSLMQQASNEVAKN
LLKNLFGEGDNGVADYAIIPDINKIAADDATAVGKIIFNRDLGDTSAEKQANSAWSGT
LLFTQLTSDQSFRLMSKGEDGLKVDSLNDWRDVLYAYVSYEAGLKAARSEFISEDLKQ
EKTDIDILGNTFYGYVTGEKTFIDYLSTLATGSDSQGLLSAFNLINSVGVNKFLDMLM
GVVLGDSIIGTTNDDNFFANAKEFFKSSVLSLETQTLNQLTLDNLIDLAENDANIRAA
LNALSMVSIETSPAVKEKFDLYNPIIGYGDITPEWIEDRAKALQYFYEYNKNAGLYDH
PEFNFIDIATGIELKNPSILGVSKPSKHDYIFGSEDNNIDIQGGDGNDHIYGGGGDDE

IYGGEGDDYLEGGHGSDELHGGNDNDKLVGMDGDNYLYGDDGNDLLLGGADKDELYGG
SGNDYLNGGSGADLLDGGTGNDILVGGPNWLIAEGGEGNDIIFADPNKVSNLERVDEL
SGGAGNDIFYGSIGPSNIKGAEGNDIIYSANGQDIITGGDGNDIIYGYSGEDTTESTI
RNNLSGGTGNDLIYGSVNSDFIEGGVGTNILEGKESVDNYIITENEGLGLGTQYIIDQ
DGRIQINLATLAVGTYDENIRAWYSSNNLYIIRKLGEGTDKEIISIHKIGDEKNTIYI
EGWGKNGDFGLVFSEITPTNLPSFDNLSTLNNGNNIAYNQTSVNGASGNDYLSGTTGN
DIIDGANGNNFINGLDGDDTLKGGDNQDIILGGVGKDKIYGGDGEDLLISSGKDTIVA
SSFATYLLEKYNNQVNPNATLADLTNLITNDASSHWDINFNFTKQWSEETQSYTYFFT
ENGVITPFLLPGINIFSDIASNTLNLTSIYSLLETDDGGALGADTVYGGLGDDFILGS
GSVDNLYGDENSDHLYGRGGDDYLYGGTGKDVVYGGAGRDYISGGDDDDKLIGGYEAD
VIYGGEGNDNITGDLLNLFGTTAPPTSADSTRYGNDLIFGGKGEYQIWSNGGDDIIFG
GTDKDYIDGGEGNDYLYGDNEDQLIDIDGKEKDDVFGGKGDDFLFGGAGNDQLYGGKD
NDILYGGANDDLIKGEDGNDIIYGGDGNDIFLFGGEGNDILYGEDANDKIHGDSGDDI
IFGGTGNDNLQGGQGSDLYVFSIGDGTDTIVEEVSDIASLNYQNFIYFTFNSDQIRGV
IRDGYDLIIKYDQYNQDDQVIVKDYYKVRNTNNHSYLDSKGIFDQIEISEVRFEDGVV
WDTAKIMEMAPPPRENELPPDPLDGVAYFIDALVTRGDITLQGKTIITYAFPANDLSN
HQYYTPEQVIAVGKALNKFAEILNIQFVQSEMGSGDLKFYLDDLSSADASAAAGYASA
QTGEIHLNSLIFEVSSDLGEGSNGFEVLLHEISHALGLKHPFEAPVLPELENNQNNTL
MSYTSNGVNDTNLKLYDLASLQYLHGVNQNVHVGNDTYTFADKYIWDGNGIDTFNAFT
LTQNVYIDLNAGGWSYIGQKSQSILDEGQTFIGYGTVIENAIGGIGDDTLISNAANNL
LQGGAGKDTYIFNGNFGHDQIIDVDHNNKIIILNNGFDNLNLVLAEDTILLLNSNSSI
QLDINQFESIIINGITYLKNDFIANFGGYISVNHDYFLESNQNNVVILGTGDFHIVGN
DLDNKIIGNSGNNTISAGDGNDTLQGGFGNDEINGGTGDDTLIGGLGNDVLYGGLGND
QYIFKDNDGDDVIVDSDGLNKIYLNHSLSNQISLHVISDGDLTITYGENSNILIKDYF
NYSEQFELVFSNNEVWSKDQIGEKLKSEIFGTSGNDVLAGEVYLKNKFIALQGDDKII
GGNKDDLIYADIVDNSDSDLKGNYTIINEDGIISLMYYEKGNDIIIGGKGNDSLFGGR
GNDTYIFNRGDGVDKIYDFYTYLDNSDEMNAKVTELNWGIKLSDGVFEDFARQDGKYN
RIKFQDVLKEEVLLILVGNDLEITYGMNDKIIITNFKDTMSTRYGFSESYSIAELEFS
DGTIWNYKKIIDNISKVEIFGTENNDEIHSEDILNTIYAKGGNDNIITGNKGSVTYGG
DGDDNISGGTGKNIIFGDSGNDNIYNYGEGSLFGGDGDDRLSGGDRSYLDGGNGYDIY
NISEDSNVHGNYKITDADGLGLISLNSSVLWAYLSNNSGSMSRTLLQANYDINSGILN
FTKDGQNYFSIIGLYNKSDFEKIGNLDIYIPGVRYYNMITGEFSFETSATNTKLKDLI
NGTYTQYNYTDSNDSISDLIHSSNISGYYNNHYIYAKDGNDTIEIMEVTSTVYGGNGN
DSITLKSGDAYGGEGNDAIAINNGIAYGENGDDLIIIQAGDAYGGDGNDSITILTDAS
YGGDGNDVLMGSGQFYGGKGDDTLIANNDSAYMNGGEGADILQGGSGNDTYIVDELDI
LQLENETGGYDTIVYQYDVGNIDLANSQFEAIELFGFSDSYLLGNTANNSLIGNSGNN
YLDGRTGSDYMAGGAGDDYYVVDVTDTIITDEDGNTRIIDGDQVAEDFDAGTDTIELW
QDARFIGQDENGNPVLTNNYRLLEDNIENLVLKGSAKTAFGNGLDNIITSNAQDNYID
GLGGDDTYIYTKGGGTDTYSFYDDLSATNTLQIQGYNSSDVFAQKYGNSVYLSFKGTS
DHIWLSNYYVADTESNTYKMDQIEFDSGTVWSSSDIDTLVNRAANNHAPTVNAAIPII
TSNQGAVFSYKFASNVIIDQDAWDYLSYKITLTTKDSNGQYEPIPSWLSFDVTTQTLS
GTPPSSVVGNLSFFYWGTDMYGYGTGTSFTLKVNPPNQAPTVLNPIADQSVTDAKAFN
YTVPSTTFKDLDGDTLTYTATLEDGSPLPPWLTFNASTNVLSGTSPDYAGALNIKITA
KDTANQTVSDIFKLTFVVQNLTVNGTSSIDTLYGASGNDTITGLAGNDVLYGQSGNDT
LNGGTGNDTMYGGKGDDIFVIDSSTDIVNENVNEGIDTVQSSITYSLGNNVENLTLTG
TTAINGTGNALNNTLLGNSAINTLTDGAGDDYLDGGAGNDKLLGGLGNDVYVVDSTTD
TITENTNEGIDTVRSSVTYTLGNNLENLTLTGTTAINATGNALNNTLIGNSAVNTLTG
GAGDDYLDGGAGNDKLLGGLGNDVYVVDSTTDTITENTNEGIDTVRSSVTYTLGNNLE
NLTLTGTTAINATGNALNNTLIGNNEVNILNGGAGNDILDGQGGNDQFTGGTGSDTLI
YQLLIASEATGGNGTDSWSDFTVGNVSTNMNADKIDFGDLLINFTGNYNTSSLDPYIK
TLLSGSNTQIYIDRDGSGASYNSTLLLTLNNVNTNLNDLLNNQQVIV" /product="Hemolysin-type calcium-binding region" /EC_number="3.1.3.1"
/transl_table=11
CDS complement(838006..838596)

/locus_tag="AUO97b_00965" /db_xref="SEED:fig|400667.82.peg.825"
/translation="MKIKNYTPTKGFIWILLLVVFIAWVVYKCVPLTNEEREGELRRL
MEAKNRRLAQEFDAITDTDRARLPKYDSRKFILIKRNKRFWLIPKEYYGVDGLNVIWP
DTVNDLLNKKWKNEFGYGTFFRISMYSKQYYDGDLNTFNYVLCTSKINRFKWNGILIR
IYNAHFINITDEQYLDVCLTTLKILNVKIKELHFVN" /product="hypothetical protein"
/transl_table=11
CDS complement(838593..839357)
/locus_tag="AUO97b_00966" /db_xref="SEED:fig|400667.82.peg.826"
/translation="MHIQFTVEDRDPSTATGPIQVGWFLTEEKASILYEPPYRMRSQS
QTKHAKSASRCPAVLQLESRYFVVNCPFDFHLRFSRDAQGKPSIVNVLGEQSPVRTNK
LRELITLVNENEWRTPQVPILQLKLPYCFIADEVVYITQLEAFGYYRKHPLPGTIFGG
RFPIHIWPRPLMWAFEWYDTSKDLILKRGEPLFYCQFDGYDPSRTMKLIQAEKTPELM
HYMDQISGVVNYVNQTFSLFAEVEKVRPKSLLKVEK" /product="hypothetical protein"
/transl_table=11
CDS complement(840053..840169)
/locus_tag="AUO97b_00967" /db_xref="SEED:fig|400667.82.peg.827"
/translation="MRLQRQAADLNNQIQDIRHQYEVNAREELAKINTEIES" /product="hypothetical protein"
/transl_table=11
CDS complement(840939..841550)
/locus_tag="AUO97b_00968" /db_xref="SEED:fig|400667.82.peg.828"
/translation="MKIKNYTPTKGFIWILLLIIFIAWLVYKCVPLTEKDQDALIHSN
MERERIRLAEEFDSYTQEDFARLPKFDSRKYFLIKRSGRFWLIPREYQGDSGFKIRWP
TDVNKLLAKKWKNDFDRDYAFNVFMYSPQYYNRTTDYWGRKIYNNASCQPKPYVGKFK
WNGVLVRIYDSYHRNIKDEQYLDVCLTALKILNEEVKEIYFVN" /product="hypothetical protein"
/transl_table=11
CDS complement(841840..843870)
/locus_tag="AUO97b_00969" /db_xref="SEED:fig|400667.82.peg.829"
/translation="MTSYANLLKPLHLGFTTIKNRVVMGSMHTGLEDRFFNYPKLAAY
FEERAKGGVGLIVTGGISPNRQGWLAPAGGTMNSLFDIPQHRLVTHAVHKHGSKILMQ
ILHAGRYGYQPFVVSSSAIKSPISPFKPRQLSEKNILSTIEDYAQCADIAKKAGYDGV
EIMGSEGYLINQFLSRHVNQRTDCWGGEIENRMRFPVEIVKAIRAKVGEKFIICFRLS
LLDLVHDGNTMQEVVTVAKALEKAGVTLLNTGIGWHEARVPTIVTSVPRAAFVDYTAH
VKQHISIPVIASNRINMPETAEEILATGKADMIQMARPLLADAFWVNKTATNRVDEIN
TCIACNQACLDHAFKNKRVSCLVNPRAGHETELVYLKTKQPKRIAVVGGGVAGMSAAT
VAASRGHAVTLFEANADVGGQFNFAKVVPGKEEFHETIRYFKVQLQKTGVDVRLNTRV
NREQLEREGFDEVIVATGVVPRALKIEGSNSPQVLSYAQVLQGAEVGRKVAVIGAGGI
GFDVSEFLLKPPHQPQPQPLAEWQCEWGVDPDPNYVSEGGMQPPVVEPAIREIYLLQR
KTTPLGIGLGKTSGWVHRAQLKKHGVRMLRGVQYKAVTDEGLWIEHNGQDQLLRVDTV
VVCAGQESVKDLMPKEGESTIANYHIIGGAKLAAELDAKRAIKEGAELAARL" /product="2,4-dienoyl-CoA reductase [NADPH] (EC 1.3.1.34)" /EC_number="1.3.1.34"
/transl_table=11
CDS 844122..844430 /locus_tag="AUO97b_00970" /db_xref="SEED:fig|
400667.82.peg.830" /translation="MDNAIGIYAVEDKRIAQLFAIEYLGLSNDARFSIKFKDDFVYVE
LYQCSVNWDRIGYLYTLPSENFIKIDHMQWLSSESIIPTKVEPVNPHDFKTFIQQRSK "
/product="hypothetical protein"
/transl_table=11
CDS 844652..845995
/locus_tag="AUO97b_00971" /db_xref="SEED:fig|400667.82.peg.831"
/db_xref="GO:0004055"
/translation="MTDNATILQHVPVGKKVGIAFSGGLDTSAALLWMKQKGAEPYAY
TANLGQPDEDDYDAIPKKAEQYGAVKARLIDCRLQLALEGIAAIQCGAFHISTGGVPY
FNTTPLGRAVTGTMLVTAMKEDDVNIWGDGSTYKGNDIERFYRYGLLTNPNLKIYKPW
LDQNFIDELGGRAEMSQFLIDNGFDYKMSKEKAYSTDSNMLGATHEAKDLEYLNAGIK

IVDPIMGVAFWKEEVEIKPEEVTVRFEEGVPVALNGQRFDNPVELILEANRIGGRHGL
GMSDQIENRIIEAKSRGIYEAPGMALLHIAYERLVTGIHNEDTIEQYRINGLRLGRLL
YQGRWFDSQALMLRETAQRWVAKAITGEVTLELRRGNDYTIMNTESPNLTYEAERLTM
EKGDSMFTPMDRIGQLTMRNLDITDTRAKLGIYTDAGLLSIGQGSAVPQLDNKKK"
/product="Argininosuccinate synthase (EC 6.3.4.5)" /EC_number="6.3.4.5"
/transl_table=11
CDS complement(846054..847316)
/locus_tag="AUO97b_00972" /db_xref="SEED:fig|400667.82.peg.832"
/translation="MANRGNVHQLLKSKEEIEYLVTLHTHRYANMPLPKQLEKKFWCL
NLDRTRQNISKFLASGVLTYLIFILLALPIDYLVIGVPYITLDFIHCLLSAMNIALAL
LLFWVFAKFTKLSEHFYLAACGIVFLTIIINAMLLLSVGNMALKNQSMLLLSFLYMLG
FILSGIKPLHMLCVGLLAAFLVFAFLILLDVNCDYIALGRALFGSCILGFSISSMLIS
RERSLFLNNQLAEINEQILRIEASELLHLSQQDALTQISNRRTFDEMFDFFYYRANQE
KRPLAVLFIDIDFFKNYNDFYGHQMGDKVISSIAAAIKNSIRHVDFVARYGGEEFVVL
LPETPAQGAYSVAANIYKAIERQAIPHAASLVSKYVTISLGFTVYTGGLKMGQDRLIH
AADQALYRAKQLGRNQIYYQPLQVAEIA"
/product="Phytochrome-like protein; Cph2"
/transl_table=11
CDS 847466..848500
/locus_tag="AUO97b_00973" /db_xref="SEED:fig|400667.82.peg.833"
/db_xref="GO:0004151"
/translation="MNSITLLQPDDWHAHLRDGLALKRTVPDLAKQFARAICMPNLVP
PVKTVEEALAYRERILAHVPEGNNFDPRMVLYFTDHTSPDEVRKIKESEHVNAIKLYP
AGATTNSDNGVSDIRKVYAVIEQLEEHQVPLLLHGEVTHNHVDIFDREKRFLDEVLSP
LLKQFPKLKVVLEHITTSDAAHFVLEQDRNVAATITPQHLLFNRNDMLVGGIKPHFYC
LPILKRQTHQTTLLEVATSGNPKFFLGTDSAPHAQNAKENACGCAGCYSAPNAIELYA
QAFDQVGKLERLEGFASHFGADFYGLPRNTSTITLVKEDNLVPESFDYLDNQKIIPLH AGKTLQWRKV"
/product="Dihydroorotase (EC 3.5.2.3)" /EC_number="3.5.2.3"
/transl_table=11
CDS 848485..849147
/locus_tag="AUO97b_00974" /db_xref="SEED:fig|400667.82.peg.834"
/translation="MEKSVTQENSVPVIGQRFRGFLPVVVDVETAGFNAQTDALLEIA
CIPIVYNAQGQFVPGPAFHAHINPFEGANLDRRSLDFIGIDPFNPMRIAMAEDERTAL
RRIFKSVNEVRKQQHCTHAVLVGHNAHFDLGFLQAAIARSGTKNQNPFHSFSVMDTVT
LSAVMFGQTVLAKACIQAGIEFDGKEAHSALYDTQKTAELFCYILNKLSPYLLDSLVA AS"
/product="Ribonuclease T"
/transl_table=11
CDS 849425..851539
/locus_tag="AUO97b_00975" /db_xref="SEED:fig|400667.82.peg.835"
/db_xref="GO:0004872"
/db_xref="GO:0005215"
/db_xref="GO:0006810"
/db_xref="GO:0016020"
/translation="MDLKLRLLSLSIAQLCCIPATFADTNNSSTTLATIKIKAQQASD
QTYKVDSSSSATRSEIALQDTPQSVSVVTQKVIEDIGATRLVEALDLAGGVTRANNFG
GQGLTGFNVRGFTSGEFYRNGFPINRGYPNAPDSNTIERVDVLRGPSSSLYGRGDPGG
TFNLISKTPNSEQQTTLGAQLNSEGLYRTTVDTTGTIPNAENIGYRLNVIAEGGDSYR
DHVESKRYGIAPVIQWQATDATKVTFEADILRNQHPLDRGHTRYPTQKSFNSSPETYL
WETGKYYNRLYNDNNMTQLRVEHDLGNDWKLNAGVQYLNGKLHGYAVEANGIQNDGET
LGRNYNYRELKWQDTDAQINLTGNFQLLGLAHTLVTGLEYENYDYKSYIIRSSEDIGS
YSINIYNPVLGQPLPELNTVTTHDRENLKTTALFVQDQLELNERLSALLGLRFEHYEH
DYQDLRPGKPNWNTSHDAFIPRLGLVYKASDDLSLYGNAAKSFKPNTGASHSGEGFDP
EEGMAYELGFKWLALNNMLSVDSAIFYANKENVLTNDPLFPNYKVAAGEVRSRGIELN
IAGQITPAWKIIGGYAYTDAEVTKDNTLQKGTALANIPKNSFNLLNIYEFQDGPLQGL

GLGINQKYIDKRAGQTANSTYIMKGYAVTDLVSYYQATPKLRLNLDVKNIFDKVYDES
AFNLYAYPGESRTVQLGMSYTF" /product="Ferrichrome-iron receptor"
/transl_table=11
CDS complement(851598..852842)
/locus_tag="AUO97b_00976" /db_xref="SEED:fig|400667.82.peg.836"
/translation="MSQIAQPITIRNTTFKNRIIKGAMSEALANYEGQPNDLHLGLYS
AWAKGGLGCAITGNVMVNVEAKNEPGVVVIETERDLERLKEWAAVGKQYGMVQLIQLS
HPGRQCPKGLNKETVAPSAVPFSPALATMFGTPRALKHEEILDIIKRFATAARICEKA
GFEGVQLHGAHGYLISQFLSPLTNKRTDQWGGSIENRTRFLLEVYKAVREATSDNFII
SVKLNSADFQRGGISEEDVISVFKAVDEAGIDLIEISGGTYEAPAMAGAKVDKRKAST
IAREAYFLDFAEKIRQHVKCKLMVTGGFRTVEGMNAALASGACDFIGIARPLAVETDL
TDRLIAGQDVRYAVKPIKTGLPFVDKMAIMEIIWYAAQFKAIGQGKKPNPKLSPLIVF
LNYAKGNIKAVVQGRVNSRKSA"
/product="2,4-dienoyl-CoA reductase [NADPH] (EC 1.3.1.34)"
/EC_number="1.3.1.34"
/transl_table=11 CDS complement(853028..854617)
/locus_tag="AUO97b_00977" /db_xref="SEED:fig|400667.82.peg.837"
/translation="MHLTQENNLNVDARLLNIENLSISTQDQILLEGLNLHLQARDTL
AIVGESGSGKTISCLAMMGLLPEKLKTSGRIIIDSKNILEIDEKTQTHIRGRQIAMIF
QEPSTALNPLHRVEKIVGENFILQGGSKAKAQQQVIQLLKDVGIANPEYILKRYPHEL
SGGQKQRVMIAAALALQPKILIADEPTTALDVILQTQILELLKSLQSKYGMALILISH
DLNVVRRYADELIVLHKGQVVEQGQLQQIFKNPASIYTQQLLDHDLGFAERYSTSHKI
LSVQQLNVKYPIRRGFFNRIQGYTNAVENVNFELAQGEALGIVGESGSGKSSLALALV
RLIKSEGQINFLGQDINRLEQKALRKIRSDLQIVFQDPFGSLNPRMTVGQIIAEGLKV
KAIADKEIKQQVESALIKVELAEGFQHRYPHELSGGQRQRVALARALVVQPKLLILDE
PTSALDRTTQKAMIQLLRRLQQTEYLSYIFISHDLNVVKALCHKVMVLRHAKVMEYQP
TELLFNAPKTEYTKQLIAASC"
/product="Oligopeptide ABC transporter, permease protein OppB (TC 3.A.1.5.1)"
/transl_table=11
CDS complement(854595..855605)
/locus_tag="AUO97b_00978" /db_xref="SEED:fig|400667.82.peg.838"
/translation="MSFIVRSRLQKFRQHKLGWASFVLFSLILMMSLAAELIANDKPL
LVKYEDAYYLPVFKTYPETTFGGVFETEADYKDPAVQELINHKGWAIWPLIRFSYQTP
NLDLAVPVPSPPSKQNWLGTDDQGRDVLARILYGLRVSLLFGLALTGCAALLGIFVGA
IQGYYGGWVDLLGQRLVEVWNGLPMLFMVMILVSMFTPNVYWLFFIMLFFSWTVLVGI
VRAEFLKARQLDYVRAAQALGVTDRAIIFKHILPNAISSSLSQLPFILTTNIIALTAL
DFLGYGLPPDAASLGELLLQGKNNLDAPWLALSGFFSLAFVLSLLIYIGEAARDAFDP RK"
/product="Oligopeptide ABC transporter, permease protein OppC (TC 3.A.1.5.1)"
/transl_table=11
CDS complement(855605..856657)
/locus_tag="AUO97b_00979" /db_xref="SEED:fig|400667.82.peg.839"
/translation="MGSYILKRLLLMIPTLFIILLINFVVVQIAPGGPVEQAIHEVES
GLGAGRILGTEMYYQGAKGLSPEMVEQIKAQYGFDHSPVERFLLMLKGYLTLDFGQSF
FKDKSVVELLWEKMPVSISLGLWSTLLIYLISIPLGIKKAKQQGTWFDRSTSLLLVVG
YAVPSFVFGILLIVFFAGGSYFQWFPLQNLVSDNFYQLSWFGKITDYLWHMTLPLITM
VLGGFASLTYLTKYSFLEELNKPYVLAAYAKGLNSQQVLYKHVFRNAILVVLAGLPEA
LAGIFFVGNLFIEIIFHLDGVGLLGFEAIVQRDYPVIFGVLFFFTLFSLLLRLICDVL YQWIDPRIHFDAQGGK"
/product="Oligopeptide ABC transporter, permease protein OppB (TC 3.A.1.5.1)"
/transl_table=11
CDS complement(856687..858522)
/locus_tag="AUO97b_00980" /db_xref="SEED:fig|400667.82.peg.840"
/translation="MQKLYSQLSLLVGLSVFSQVIFSAQQTTPYIAIHSKPKYLAMAA
MPYANPNAPKGGVLSQAAQGTFDNLNSMNGKGNATEGVNYLFDTLMTQSLDEVGVLYP
LLAEKVSYDPIKTQSVTFYLNPKARFSNGLPVTAEDVKFSFDTYQTKSNFGLQMYLAD

LAKTEVINPHQVKFTFKSSHNPKMPFVVASLPIYSKADWQKRDFTRISLQPIIGSGPY
VVEKIDAGRSISYKRNPNYWAKDLPINKGRYNFDHLKYVYYRNWDIAFEGFKSGQYTL
HEETNPKKWVTDYHFPAVKAGLVTQYKFRHHNPIATESYVFNTRRKPFNDIRFRQALT
YAYDFEWQNKALFYGQYQRLQSYFENSDLAATGRPSNNELAILKPLLPKLSPVMQKAV
LADWKYPASDASGFNRQNLLLARQLLIEAGYKIKEGQLYTPEGKPVQIEFLIQQDGKQ
RTLMPFVRNLKKLGININLRQVDAPQYLERTRRYDFDMTTMNLPQSLNPGNEQAQFWG
SAAAVQDGNYNYAGIRNPVIDEVISKLVTARDREQQITYTHVLDRLLRAGYYQIPTYG
KGDYWYAYWNMYQQPKVKPVLSAGIEYWWSNANQAKKVAQYLHQQ" /product="ABC transporter, periplasmic substrate-binding protein yejA" /transl_table=11
CDS complement(858538..861753)
/locus_tag="AUO97b_00981" /db_xref="SEED:fig|400667.82.peg.841"
/translation="MYKPTTFVWQPSAASLFKITVLSSALAALGITTGCSSTPQSAKT
SKTKQVSGAGYLDASSLDSLEDLLSATDMRAVEGDRLLILKHGDVWKRMAVGFKMDLN
HWDPRIEAQRSWFISRQPYLDRLSARASRYLYHTVKEAERRGLPTELALLPVIESSYD
PAATSSAAAAGLWQFIPSTGRIYGLQQTGMYDGRRDVVESTRAAYEFLGSLYNQFGSW
ELALAAYNAGPGRIQQAINRNQAAGLPTDYWSLKLPQETMNYVPRFLAVAQIIKNPRA
YGVSLPPIANRPHFREVTLSAPLSLNEIASVTGLSRAELYALNPGYRGETVDPASPMR
ILIPADISPSVDNKLKGMKAGGSSGWWASVTSPSKPTTTTSTSVTVRTTPSTPAQPVR
PSTPAKTSSSSVTVKTATPRGSDALAAFAASADVPSAPRIPVAVTPAANIKPVRTEPP
ISATEREKILAAVRAEGEKETVDQALEPQATQAEKDQVVAELKALAPQGTEIVDPYDG
KIKLTAIQTSQSVAEQQGKEVSKGFAYPKTLAEDATLANSEDAQRNKDKPYIKTDTDV
VVVQPKGKRSTYTVQPGDTLAVIAMKNGVNWRDVAKWNQIDPEKTLFVGTSLYLYDAK
PQEAETTAKSAAKPDVYVVQANDSLTGVANQFNLSVKQLAEYNDLSVTDGLFVGQKLQ
LKEPKGNRAAKVEPKAIQASTRRIATKSYTVKRGEYLKLIADRYALSNQELADLTPGL
TAGSNLIVGQKINVPAKEITVDEVDDSKASGKYEKLAAGPSYKTESYKVQRGDTLSSI
ATKSKISLAELAELNNLKANSHVQLGQTLKVPAGASVPDQYVVQSGDSLNAIAAKYNL
QTSYLADLNGLSRTAGLRAGQRLKLTGEVETTSKVSAKNTKEETPETYTVKSGDSLGN
IANRYHLQLDYLAALNGLSRNSNVRVGQRLKLTGDLPTVETAKTDTAKSSPKAVVAGK
NTEKYTVKAGESLNAIASRAGISVRELAEMNALKANANLQRGQNIVIPKTVVEYKVKR
GDTLIGLASKYGLETTLLAELNNLTPSTQLRIGDIIKVPNL" /product="Membrane-bound lytic murein transglycosylase D @ repetitive LysM domain" /transl_table=11
CDS 861986..863365
/locus_tag="AUO97b_00982" /db_xref="SEED:fig|400667.82.peg.842"
/db_xref="GO:0003887"
/db_xref="GO:0003676"
/db_xref="GO:0004523"
/translation="MSQTITLYVDGACRGNPGLGGWGAYVITEQDEHKLFGGEPDTTN
NRMELTAAIEGISFCPPDAQLIVWTDSNYVKQGITEWIHGWKKKNWKDVKNPDLWQKL
DAVCAGRNIEWNWIKGHAGHAGNEMADQLANLGADKTAQELKQPQSATVSQDIKKPEQ
DWLLDDPFGFDLVETTEDETVTLEQTQEVEMIVVDEEMNVVEAVEQEEQQQNTNIHPQ
IVMTEAKLKLQGPRQLILDTETTGFYFQDGDRIIEVGAIEMINRKLTGSSIHIYINPE
KPVGDSVQVHGITDEFLVDKPKYAEIADTLFEYLKGAEIIAHNATFDMNFLDMEFKRV
GLPALSEVCEVTDTLALAKNKHPGQKNSLDALVRRYEIPARDRTFHGALLDAEILSDV
YLAMTGGQVSFDMDALSQSESDQQKTTKARVQIELPVIYPSDEELNIHETWVKEFEKK HGEPCLFAK"
/product="Ribonuclease HI (EC 3.1.26.4) / DNA polymerase III epsilon subunit (EC 2.7.7.7)" /EC_number="2.7.7.7"
/EC_number="3.1.26.4"
/transl_table=11
CDS 863499..865142
/locus_tag="AUO97b_00983" /db_xref="SEED:fig|400667.82.peg.843"
/translation="MSEHNSIQFDPTALLIIKNEIDNSIKLVEGAVSTLIEEQALPFG
IDDALEQFKQCTQVLRLIDIPYLAKITQYSTELMQKIMANPERINTDDVVALSEGTTM
VKRYIEFICLREVEVPQFLLDTLNNLEKALNKPLTSSGGQQIASKLSTVSLELPLPEVL
INERTQFIHQLYKLSLHQFLNKTESARDFQAFKLIGSYLVSMAQGQPSQQYWQLVNSA

FSHIDELVLNDARLRVFINLENAISLFLASPEGFEANLTALADILSIVIGQEDQLAQQ
IRSQLNIGHEFLTDTQLKALSQHLYGPDFDTMQTVSQLILSEMNKVRNDIEYNYQNMS
PEKAQQLQSNLMQLAHTFKLLNLNEAASELSQQASSLSQINILSNENYAQQLMKSILS
AMNAIGILVRHYSSNRLQIRVNNTNISLDRLDEAHQTLLNETKNLTDFVCQSLTLYAN
DQTQNIEAIAGSLKELAGAAEFLGSTVQQNALLETAKFVQKQIDQNQPFNHDQIHCIF
NVLAGLDMLVDNLKNKQPVLQSMFDVALLSSQQLQKKAA" /product="FIG00350110: hypothetical protein" /transl_table=11
CDS 865142..865885 /locus_tag="AUO97b_00984" /db_xref="SEED:fig|400667.82.peg.844"
/db_xref="GO:0000210"
/translation="MSELSLAYIFHHQQLLVDQNLQLPKVEKLASDLLFTHDEQVIAR
DLLSEEAIPEGLQLVPIRQLISNWSKEQFLQASRAVQLLEWRRNHKFCSHCGHATEVH
PTEYAMVCPSCRYHQYPRVNPCIITIITKGDDEILLAKSIHNKTNMYGLIAGFVEVGE
TLEEAVQREALEEVGLKLKNIQYMSSQPWPFPSNLMIAFRAEYESGEISLQEDEIADA
QFFKFDQLPEIPFKGSIAHAMITQITQAK"
/product="NADH pyrophosphatase (EC 3.6.1.22), decaps 5'-NAD modified RNA"
/EC_number="3.6.1.22"
/transl_table=11
CDS complement(865908..866771)
/locus_tag="AUO97b_00985" /db_xref="SEED:fig|400667.82.peg.845"
/translation="MNLIEQLQSKIEQARTLYKEFRLYDLGSFALNRFTPKDGFEQVL
NVRYGLKPRHRLDIYRSTKRLAHRPLIVFVHGGAWQHGNKRDYLFIGEAFTKEGYDVA
VINYQLAPKNIFPSYVDDLTQALNYLHQNQEKLEISTENIVLMGHSAGAFNVMSAVYH
PKPNTIQCLGNIKAIFGLAGPYHFDYKGDPLAEDAFDQSISYKEVMPYYFVNQNHIKH
YLLMAENDQLVKKENTFDLDQALRQSGNHSHIAVIPKTGHITILASLSSFVSHYFRTK RTILHFLDEVF"
/product="Esterase/lipase/thioesterase family protein" /transl_table=11
CDS complement(866775..867482)
/locus_tag="AUO97b_00986" /db_xref="SEED:fig|400667.82.peg.846"
/translation="MLKRIAVTALCVASLSGCASFISGGTGTAPVGTDSGVRSLGQVF
IDSSIKRTANINLYKLDQRFKQSRINIESFHSTVLLTGQVPDPYLKQLAEDNVKAMSD
VKAVHNYITVGNKVSYNTIMQDAGVTANTRALLMKAPVVSDSKVLVHTEDGVLYVMGR
LNTAEINDLNNVLQNVGNVTKIVTLIDNIDLAPAPAASTASATTTPVINNVLAQPTVQ TPVAIDPDQTDPASSAQ"
/product="Uncharacterized protein YraP"
/transl_table=11 CDS complement(867563..867964)
/locus_tag="AUO97b_00987" /db_xref="SEED:fig|400667.82.peg.847"
/translation="MLVAQQLGQWAEQTALKLLKEQNYEWVASNYHSRRGEVDLIVKR
GNELIFVEVKARGQGNYGQACEMVTLSKQKKIIKTAMRFLQRYPSYQDFYCRFDVICF
DFPQKIAKTVQQDFSKFHYDLQWIENAFTLD"
/product="UPF0102 protein YraN"
/transl_table=11
CDS 868158..868994
/locus_tag="AUO97b_00988" /db_xref="SEED:fig|400667.82.peg.848"
/translation="MSAQLFVVATPIGHLDDMTFRAIDILKSVSVVAAEDTRQSAQLF
KHYNISTPLTACHDHNESNKIEQLVQKLLAGENIALISDAGTPLISDPGFKLVRAAQE
NGIRVVPVPGACAAIAALSAVGLPSDRFSFEGFLPSKASQRISQLEKLKNETQTLIFY
EAPHRILECVKNMAEVFGENRPVGFAREITKTFETIKKMTLKDLVSFIENDHNQEKGE
IVLVVGGAPEKTDLEQEKLDELLKRLLQDLSVKAASQLAADLTGIKKKVAYQRALELT QS"
/product="16S rRNA (cytidine(1402)-2'-O)-methyltransferase (EC 2.1.1.198)"
/transl_table=11
CDS 869089..869475
/locus_tag="AUO97b_00989" /db_xref="SEED:fig|400667.82.peg.849"
/translation="MTESLGKLGPHEGQELELLLSGKKPIAYFYELLPIEFIKHLEQG
SLSMISKDIETSLSLPFSIMLIYKDASLADLNELMLCIEKSLKETQLEDRLELDRRIG
QLLGYSTQDIEFYIQHISNRHLKTKI"

/product="FIG00351276: hypothetical protein" /transl_table=11
CDS complement(869478..870368)
/locus_tag="AUO97b_00990" /db_xref="SEED:fig|400667.82.peg.850"
/translation="MLDSKQCEAFLAVAEVGSFDAAGEHLCITPSAVSLRVQALEKYL
GQILIIRGRPCVLTQAGQTLLQHLRHTRLMEQNLLQGLMGKSSESEFYKIALASNADS
LATWLLPSIQQTLFKEKIVLELKIDDQSHTHTLLETGQVNACITAEEQVMAGCLAQPL
GKMRYKMLASAEFVNRWFSGGINREVLRKTPAVIFNHKDLMHSEVLLKGYGLPMQSYP
YSFIPATDAFVKAIQLGLGYGMVPELQVQPLLENGTLIDIMPEAQLDVPLYWHHWKRQ
SKQLDVLTETIVESAKQILR"
/product="Transcriptional regulator ArgP, LysR family" /transl_table=11
CDS 870470..871078
/locus_tag="AUO97b_00991" /db_xref="SEED:fig|400667.82.peg.851"
/translation="MVMLSLSVFFKGLGIGSGLIVAIGAQNAFVLKQGLKQQYVFWLC
LICALSDSILIACGVLGFAEIMTASPILITVAKYLGATFLFVYGAKAFYAAFKTTQGM
ELDSSQKQTLTQALITCLAFTWLNPHVYLDTIVLIGSVATQLEDKISFALGSILASWI
FFFSLGYGARFLKPLFINPKAWKILDFMIGCVMWSIAISLLI" /product="Arginine exporter
protein ArgO"
/transl_table=11
CDS complement(871136..871777)
/locus_tag="AUO97b_00992" /db_xref="SEED:fig|400667.82.peg.852"
/translation="MAGCGCASTCSPTKKASPRFRKALWIALVINALMFVVEIVGGYK
AQSVSLWADALDFAGDAANYALSLVVLSMSLYWRATAALVKGITMAAFGFFVIAKVVW
SFFHGVSPEPMVMGAIGVIALIANVSVALMLYAFRDGDANMRSVWLCSRNDSIANIAV
VLAAVGVFGTGTIFPDLFVAFVIAYLGVSSGYAVIKQSLQERKQSKLMLGSEA"
/product="Cobalt/zinc/cadmium resistance protein CzcD" /transl_table=11
CDS 871843..872244
/locus_tag="AUO97b_00993" /db_xref="SEED:fig|400667.82.peg.853"
/translation="MSLSIGQLSKKADVPIDTIRYYEKVGVLDRIQRSENNYRVYTDQ
TLADLLFIKHCRELDISLSDIKTLKEMKAQPKRACTEVDNLVNKYLIEVSEKIERLLL
LKETLIDLKQHCSTDRTVDECGILKELQNHP"
/product="Transcriptional regulator, MerR family" /transl_table=11
CDS complement(872299..872550)
/locus_tag="AUO97b_00994" /db_xref="SEED:fig|400667.82.peg.854"
/translation="MTDINDYEDAEDSAVDEDEAKAAESGAADEEAGNLTDADLAEAE
TLTVTAKQKQRQALEDEVAAFLARGGRITEVPPDEHSDR" /product="FIG00350187: hypothetical
protein" /transl_table=11
CDS 872577..872693
/locus_tag="AUO97b_00995" /db_xref="SEED:fig|400667.82.peg.855"
/translation="MHHRYGLRDLRNLAISLHLAKSQIGQIRIFSDRIGNDI" /product="FIG00350608:
hypothetical protein" /transl_table=11
CDS complement(872760..873992)
/locus_tag="AUO97b_00996" /db_xref="SEED:fig|400667.82.peg.856"
/translation="MSEILDVVIVGGGLVGGLTALLLAQGGVQPTVLDAVPILDVGKT
LSVMNPRVLALSQATIHLLKTVNVWDDLARQMPYTGMQVWNLNGYGEINFGHESVQRP
IPEQSLGSMVEPSVLNVAIQQKMLEQLTDYRTQVRVTRIEQGVGCWHIQLADGTTLKT
KLLIGADGANSFVREQAFIDLDVLDYKQAAISCAIKTSKPHHYVARQIFLPTGPLAYL
PMASLEESENGYWQSIVWTLPDDYADEYSALSDAEFMQLLTRESQHMLGEVLDVRSRA
QFPLKARAAKQYVKDGLALIGDAAHVIHPLAGQGVNIGCLDAAVLCDALLHDLGRGVW
AHEQTLMRYEHRRKGQNDAMMHSMSAIGWLESSELFPLIWARNVGLKQVEQISFLKER
FMQQANGLGALQNTQYSR"
/product="2-polyprenylphenol hydroxylase"
/transl_table=11
CDS complement(873989..875197)
/locus_tag="AUO97b_00997" /db_xref="SEED:fig|400667.82.peg.857"

/translation="MQQQVIIVGGGMVGLSLSLMLAKANIAVKLLEAVKYPNYDDQNV
APYHSSFDARNTALSRRSVQIYQKLGLWDALQQHATPILQVHITEQGSFGKARLVAEQ
EKVESFGQVIENAWLGRVLLTQVRQQPLIELIDGVQVTALTQDAEQVHIEAQRGDELL
KLESKLLIAADGRDSFCRQAIGVGVDVHDYDQVAIVTTVQTSKPHENVGFERFSALGP
LALLPLPGEYRRSVVWPVKKGTESEWLGDENDQHFLSALQQTYGDRAGKFEKTGKRFS
YPLSQVLAHKQAVGRVILMGNAAHTIHPVAGQGFNLCLRDADVLLRYLVNQLSASDDI
GNPDNLLAYEQARLSDQQRVIKFCDTVVRGFSNQNPLLKLIRNTGLIAFDVIPGVKPL VANYAMGLKA"
/product="2-polyprenyl-6-methoxyphenol hydroxylase" /transl_table=11
CDS complement(875229..876551)
/locus_tag="AUO97b_00998" /db_xref="SEED:fig|400667.82.peg.858"
/db_xref="GO:0008451"
/translation="MKLPQADFQHRRNRLAEKMGPNSIAIIATREEMYRNRDADYKFR
ADSSFFYLTGFAEPEAVAVIETFDDVTDYSYSLFCRERNREMEIWNGYRAGIDGAIEE
YEADEAYAIDLLDEEIIEKLLNKERLYYRIGHNAAFDARVSQWIKKANAEHRHEAAPA
QLVQLDRIVDEMRLIKSPQEIELMQIASTISAKAHTRAMQTVRPGMMEYALEAELNYI
FGQNGCVPSYNSIVGGGANACILHYVENNQALKDGDLVLIDAACEYEFYASDITRTFP
VNGKFSPEQKALYEVVLASQYAAIDAVRIGNSYREPHEVAVKILTEGLVDLGFLKGDV
SELIETEAYRQFYMHGTGHWLGMDVHDVGSYKKDDDWRQYEEGMVVTVEPGLYIAPDD
ETVDKKWRGIGIRIEDDVVATSKGPRVLTADVVKDIADIEHLMAQAKA" /product="Xaa-Pro
aminopeptidase (EC 3.4.11.9)" /EC_number="3.4.11.9"
/transl_table=11
CDS complement(876619..877251)
/locus_tag="AUO97b_00999" /db_xref="SEED:fig|400667.82.peg.859"
/translation="MQDDISGWTEWNAHFEGIEEISSPSELHGLLTGIVCVTEAPTRE
EWTQILTTLNVPELNEEALALLTDEAEDVAHALSEDELDYLPMLPDDEHLLQDRVQAL
SDWCAGVVLGFGLASGHVRTDERELIEHLQDVAAVEFEDSDNDEEGESSYEELYEFVR
LIPVSLSIGRKKVTVAESSLLKNFYAKSKTSTVGTADQNIVEMFTPHRPS" /product="FIG001590:
Putative conserved exported protein precursor" /transl_table=11
CDS 877316..877876
/locus_tag="AUO97b_01000" /db_xref="SEED:fig|400667.82.peg.860"
/translation="MLEQLQRLQAHIGVLKTRLHHLESENSALSEAKELAETEHHAQV
VQKNSIITKKQEEIETLTEQLTQLQGQFQQLNQDANTLAERYSRLEKSTTDLKNRFQE
ILAERNELRVTKEKLQSQQRQTQQELHDLQQDRDRLLQKNELAKAKVEAIIQRLAILG
TAQDQHAQEIQQLAHPNAEAGEETQS"
/product="hypothetical protein"
/transl_table=11
CDS 877873..878157
/locus_tag="AUO97b_01001" /db_xref="SEED:fig|400667.82.peg.861"
/translation="MSEQVMVELRLIEQTFRLATTSDKREELERAAELLNEKFNDMRR
SAPRVEHNKLVIMVALQLTQEVLSLNKSLQEYAHCERLLQTILEDVEQIV" /product="Z-ring-
associated protein ZapA"
/transl_table=11
CDS complement(878570..878776)
/locus_tag="AUO97b_01002" /db_xref="SEED:fig|400667.82.peg.862"
/translation="MEARQQKVPAQDLQQIADGLADEKAKQLYQDLISSAYAAKVFKT
SFFKRQAIEDFQAGWYEECLRRNE"
/product="FIG00350276: hypothetical protein" /transl_table=11
CDS complement(878963..879076)
/locus_tag="AUO97b_01003" /db_xref="SEED:fig|400667.82.peg.863"
/translation="MQILKFLQSFNTVGTYLTLASILLVVMIIYFYVINPA" /product="FIG00351046:
hypothetical protein" /transl_table=11
CDS complement(879184..879525)
/locus_tag="AUO97b_01004" /db_xref="SEED:fig|400667.82.peg.864"
/translation="MKLKILTMMLCVALLSGCTKQAESEAPQIDYKAQFEESDRKIGE

FLDQLDNPNTPQEVKVKILCHDYPDVYKKQYMPALIKVSPKPYTEEKLLSDLKSATDY YKGTLGIKCNE"
/product="FIG00350136: hypothetical protein" /transl_table=11 CDS
879767..880351
/locus_tag="AUO97b_01005" /db_xref="SEED:fig|400667.82.peg.865"
/db_xref="GO:0030272"
/translation="MNELSFIRKNLRSRRRALTQFEQKQAQLNVLHCLNHLPIFHSSK
KIGLYLHAFGEIHTDLLIKLCFKKNKQVYLPMICSMNQRLVWVKINKNQYLSRRFSHH
PLGMKEPMATRGKHVSQLDLLLMPLLACDHYGTRIGMGGGYYDRTLASAKHKPYRLGL
AHQFQFIEHTLERQSWDQPLDGLLTPQHFYYFKR" /product="5-formyltetrahydrofolate cyclo-
ligase (EC 6.3.3.2)" /EC_number="6.3.3.2"
/transl_table=11
CDS complement(880462..881241)
/locus_tag="AUO97b_01006" /db_xref="SEED:fig|400667.82.peg.866"
/db_xref="GO:0016021"
/translation="MNKTIQSLVLAAFAGFAVTTYANEQAQQQEIHFPAIEKSYLKQV
KRYEYPDVARLDTGLNKDQIRALLGNPQFSEGLFAVKVWNYVLDIRVPNTHQYQRCQL
RIDFDKHYLAERLSWKGEACEGLVVLGENNQAPAHSTVIADRIASVLFAFDRFDASAI
EEGTNSVVKIAEQIKKSPTITPIIVSGFTDPLGKFSYNQELSSKRANTVAKLLVQQGV
DASRIQIQANSQTDIYKQCSSNNSSQLIQCLAPNRRVNISW"
/product="Outer membrane protein A precursor" /transl_table=11
CDS complement(881299..886920)
/locus_tag="AUO97b_01007" /db_xref="SEED:fig|400667.82.peg.867"
/translation="MNKVYKVIWNASIGAWVATSEIAKSKTKTKSKTLNLSAAVLSGV
ICFAPNAFAGTNTEGGIGQGTSISGTTSCREGSANTANQKDIAIGCGAQTQDRTGSNI
ANRNNPYNNSTGAYAGAMKQGGAISVGTGAVVEKGLGTAIGSYATTQGISGVAIGTGA
LSSGNTALAVGRQSAATADFSQAIGNVAAATGKGSLAIGHSATAEGYRSIAIGSPDIE
NADPVAGQAGAAYQPKMATKATGKDSIAFGGGAVATEENALAIGAFSESKGKKSVAIG
TGAKAQKDNAVVIGDQAEASFEGGVAIGKGARSEAENSIALGKDSKASQATGESFLTK
QSAPTGVLSIGDIGTERRIQNVADGAADSDAATVRQLKAARTHYVSINDNGQPGGNFE
NDGATGRNAIAVGVNASAAGREAMAIGGNAQAIGSGAIAMGSSSQTVGRGDVAIGRNA
STQGAEGVNSNQSVAIGDQTKAIGDQSVAIGADVIAKGNSSVAIGGDDVDKIARDTEL
SNTYTEITGGTLQAGKYPTTEANHGSTAVGVQAVGTGAFSSAFGMTSKATGDASSAFG
VMSNASGKGAAAFGAVAQATGDGASAMGINSLASGTNSTAIGSGNKPGEGANATGNSS
AAIGSGAQATGDNSAAIGKGAEATNENAAAVGGGAKATGKNAAAIGGGAIADQENAVA
VGQGAQSLVEGGVALGARSKVEAKNSVALGQDAVATEATGTSFLTNRDASQSNGVISV
GSAGKERRITNVEDGSADSDAVTVRQLKNVDSRVNQNTSNIGKNTQNITNLNQKLDDT
KTNLGNQIADTNKNLNDAKKDLGNQITDTNTKLNTTKDQLTTQINDTNTELNNTIGNT
KTELNTKIDNTKTELENKGLNFAGNSGADVHRKLGDKLNIVGGAAASTPAAKTSGENV
ITRTTQDGIQIELLKDSKFDSVTTGNTTLNTNGLTIKEGPSITKQGINAGSKQISNVA
DGINAKDAVNVDQLTKVKDNLNGRITDTNNQLNDAKKDLGNQIADTNKNLNDAKKDLG
NQITDTNTKLNNTKDQLTTQINDTKTELNNTIGNTKTELNSKIDSTKTELENKGLNFA
GNSGADVHRKLGEKLNIIGGAAASTPAAKTSGENVITRTTKDGIQIELLKDSKFDSVT
TGNTTLNTNGLTIKEGPSITKDGINASGKQITNVADGVNAKDAVNKGQLDNLAAKQNA
TDDAAVKYDDAKTKDKVTLKGKDGTVLDNVKAGHISSTSKEAVNGSQIHNISNSIKNS
IGGNTVVNPDGSLTTNNIGGTGKNNINDAISEVKNTATKAKTTVTEGDNIVVKETVNK
DGSTNYEVSTKKDLTLNSVTTGDTVLNNNGLTIKDGPSITKDGVNAGGKKITDVANGV
IAQNSKDAVNGAQVHHISNSIKNSIGGNTVVNPDGSLTTNNIGGTGKNNINDAIKSVD
EKVTNGVNDLTQKGLNFGANDQKTTQGKAVHRKLGDTINIVGGADAKTAEDKTSGENI
ITRTTEDGVKIEMLKDVKFDSVNVGGHVLNQQGLIIKGGPSITVNGINAGGKQITNVA
DGINAKDAVNKGQLDKQINEVKDQIGKDIGKLSDHAVQYDKDKNGNVDKSSVTLGGGE
KGTNLKNVADGKVAEGSKDAVNGGQLWNVQNQVDKNSNDIKNIQNNIDNISNGKAGLV
QQQKPNGEITVGRDTGGTSINMAGKEGDRVVQGVKDGEIKAGSNQAVNGGQIHKISES
IKNSIGGNTTIDPKDGSITTNNIGGTGKNNINDAIGTLNQSNQELGNKITNLGDQLQQ
VFYDTNKRIDDVEKKANAGIAAAMALENAPFVAGKYTYAVGAAYHGGENAVGVTLRKT

SDNGRWSITGGVAAASQGEPSVRVGISGVIN"
/product="Cell surface protein"
/transl_table=11
CDS 887698..890127
/locus_tag="AUO97b_01008" /db_xref="SEED:fig|400667.82.peg.868"
/translation="MSELIMNEKTDLEPQVPSVLPLLALRDVVVYPHMQIALFVGREK
SINAVDVARNSDNLVFVVAQKDSLTEEIDHDNLYQYGTVAKIVQVVNHENDENCIKVL
IEGLHRSKLKKIIDEDSYLTAEHELSPMTINVDKATQETRLQELRNLFAQYAEAKLRN
ARELVAAANKIEDLLQLMFFVATRVPLNIEIKQKFLEYDEFEAHLQELMNYLMNQSAE
QQIEQTLHDSVKRQMEKNQREYFLNEKMKVIQRELSDMNGGAEDDVAEIEKRLAEADL
PEHVRKKAEAEFRKLKAMQPASSEAAVVRNYLEVILDTPWNKASKVSINLNKAQEILD
ADHYGLDDVKDRIVEYLAVQSRVKKLKGPILCLVGPPGVGKTSLGESVAKATGREFVR
MALGGVRDEAEIRGHRRTYIGAMPGKIVQSLTKVGVKNPLFLLDEIDKMAQDYRGDPA
SALLEVLDPSQNSKFNDHYLDLDLDLSEVMFICTANSMNIPEALLDRMEVIRLPGYTE
DEKVNIAERYLVPKAIKNNGLRPKELTIHEEAIRDIVQRYTREAGVRNLEREVSKIAR
KVVKEAVSKKSKNLQLDVTSANLPEYLGPHKFDFGMAEDEAQVGRVNGLAWTSVGGEL
LTIEVAAVKGKGKFITTGSLGDVMKESITTAMTVVRTRADELGIEASRFEETDVHVHL
PEGATPKDGPSAGLALTTALVSAFTGIAIRPDIAMTGETSLGGRAMRIGGLKEKLLAA
HRGGIKLVFIPQDNVRDLAEIPDNVKEGLEIKAVKSIDEILPLALTSMPKPLPKTPIV KPVEGSKAARH"
/product="ATP-dependent protease La (EC 3.4.21.53) Type I"
/EC_number="3.4.21.53"
/transl_table=11
CDS 890429..890908
/locus_tag="AUO97b_01009" /db_xref="SEED:fig|400667.82.peg.869"
/translation="MKIRILTIGQKMPAWVLTGFEDYFKRIQPFVQTQVIELPMAKRG
KNDSEADILKYCQIEGESILNALKPNETLIALEVGGRELSTEKLADTMKQWMLEGNDV
ALAIGGPDGLSDQVRKAAAWHWSLSKLTMPHPLVRILLIEQLYRAMSINHNHPYHRA" /product="23S rRNA (pseudouridine(1915)-N(3))-methyltransferase (EC 2.1.1.177)" /transl_table=11
CDS 891021..891797
/locus_tag="AUO97b_01010" /db_xref="SEED:fig|400667.82.peg.870"
/translation="MELQTLPGLFISHGSPMLALDPEQVGPALHRLSVNLPTPKAIIV
MSAHWESKALEVSSSTRPETWHDFRGFPPELYEIRYSAPGQPELAEEILKLLADAHFV
AHANSTRPRDHGVWMPLLHMYPEADIPVVEISLPMGLTAQEIYRIGQTLAPLREKQIL
LIGSGSITHNLAELSWQGDNSKVPEWASTFRNTVVSKLSHQDYDGVLEWQTLPFVKRN
HPSLEHFAPLFFAMGTGHRFTIVHSSFTMGSLGMDIYRFD" /product="hypothetical protein"
/transl_table=11
CDS 891967..893076
/locus_tag="AUO97b_01011" /db_xref="SEED:fig|400667.82.peg.871"
/translation="MKIKYLILALLPFSLMACQTVSNTQAPIVSEQQQNLATTLSEYA
WTYQNVKASKPLILNFNADGKLAINTGCNGQGGTWKVEGNQLVTSPLASTMMACQDDL
MKQEQLSNSIFSEAKLPIEISNNNGQVILSVTDKAGQKHIFQGEKATNTQALTDYSWS
YQPENTKKPIVLNFTNDRLSIDTGCNRQGTTWKVENNTIVTTDVMSTMMACEPALMKQ
EQFSSSLFQKRAIPFELNTTNVDQPTLTVTDAQGQKYTFTGKMTPEAKYQSEGKTVFL
EVAPETKSCTGVAPQTCLQVREVKYDDKGVKTYADKNWSLYYGQIEGFEHNPNQRVIL
RVKRFEVKNPAADQSSQADVLDMVVEQELVKKPKK"
/product="putative lipoprotein"
/transl_table=11
CDS complement(893132..894520)
/locus_tag="AUO97b_01012" /db_xref="SEED:fig|400667.82.peg.872"
/db_xref="GO:0004354"
/translation="MYCTNLIHYYAEDRALKYNNLNEFLNYVQARDPHQPEFLQAVEE
VMTSLWPFIEKNPEYAEQGLLERLVEPERVIQFRVSWMDDQGQTQVNRAFRVQYNSAI
GPFKGGMRFHPSVNLSILKFLGFEQTFKNSLTTLPMGGGKGGSDFNPKGKSDAEIMRF
CQALMIELYRHLGPNTDIPAGDIGVGAREVGYMAGMMKKLSNDTACVFTGKGISFGGS

LMRPEATGYGTVYFAEEMLKTRGQSFAGKTVSISGSGNVAQYAAEKAMFLGAKVVTLS
DSNGTVYLKNGFTDELLAEVMELKNIQRGRISEFASKHGFEYFEGKTPWHIPVDIALP
CATQNELTGEDAKTLIANGVICVAEGANMPSTLEAVEHFIEAKILYAPGKASNAGGVA
TSGLEMSQNAIRLGWTHAEVDERLHAIMKDIHANCVRYGTKEDGTVNYVDGANIAGFV KVADAMLAQGIY"
/product="NADP-specific glutamate dehydrogenase (EC 1.4.1.4)"
/EC_number="1.4.1.4"
/transl_table=11
CDS 894807..895598
/locus_tag="AUO97b_01013" /db_xref="SEED:fig|400667.82.peg.873"
/translation="MNPSISLIREIDALLPQTQCGLCGHRDGCLPYAKSIAEGEEANK
CVPGGQPVADALAQLLNRPLLPAETSVWPVQADGRPQRMKAIIREDECIGCTKCINAC
PVDAIIGSGKLMHTILTDLCTGCELCIPPCPVDCIDLVEDTQALPSEQQRIAEQNDLR
QRYYAHIQREEKRRIHRKGPVVRAEIDTQLFAQFSQALDDLPSIQLIEKPKEAIVSDA
KTTIELAKIRTQIKKLEKQLSVREDAKKRLQLDELQQQLIQLQEV" /product="Iron-sulfur cluster-binding protein" /transl_table=11
CDS 895599..896291
/locus_tag="AUO97b_01014" /db_xref="SEED:fig|400667.82.peg.874"
/db_xref="GO:0003906"
/translation="MAVKNMTKKQIQIFFERLREQRPSPQTELKYSSPFELLIAVMLS
AQATDVSVNKATDKLYPVANTAEKIYNLGVDGLKEYIKTIGLYNAKAENVIKTCKILM
EQFNGEVPSNRKDLEALPGVGRKTANVVLNTAFGQPTMAVDTHIFRVGNRTGLAIGKN
VLEVEHRLVKVIPKEFILDAHHWLILHGRYCCIARKPKCSECVVADVCNWPDRFEFGA QKQIAVKNIEAE"
/product="Endonuclease III (EC 4.2.99.18)" /EC_number="4.2.99.18"
/transl_table=11
CDS complement(896321..896458)
/locus_tag="AUO97b_01015" /db_xref="SEED:fig|400667.82.peg.875"
/translation="MKIILILVVLLSLALLLFLLYQSYKMLRKVQKQAKEERKQQKRL K"
/product="hypothetical protein"
/transl_table=11
CDS complement(896530..897183)
/locus_tag="AUO97b_01016" /db_xref="SEED:fig|400667.82.peg.876"
/db_xref="GO:0004017"
/translation="MRIILLGPPGAGKGTQAQLICKRYNIPQISTGDMLRAAIREGTE
LGLKAKSVMESGGLVSDELIIGLVKERIAQPDCVNGCIFDGFPRTIPQAEALEKEGIS
IDHVIEIDVPDEEIVKRLSGRRQHPASGRVYHVVYNPPKVEGKDDETGEDLVQRPDDQ
EETIRKRLASYHTETEQLVGFYQGRAASGENAPTYDKLDGLRTIEDVQKDLFNILDK"
/product="Adenylate kinase (EC 2.7.4.3)" /EC_number="2.7.4.3"
/transl_table=11
CDS complement(897270..898472)
/locus_tag="AUO97b_01017" /db_xref="SEED:fig|400667.82.peg.877"
/translation="MALKMVFYPIKFWGFHLGPLPVGWQGIVPRKAGRISGIITDNTL
SKLGSLREFLEAMDPEDMARIIGEQVGFELEHLIDEVMIDRNAVLWENLPYSIKRRIY
AQAHKQLPGVLRELVTELTMNVESLVDMREMVVSQMEGDRRLMVRMFLKVGQKEINFI
WHISALIGMFFGVFQMVVWFVVPWHWTVPFWASIWGFLTNWIAIWMVFNPIEPHYIRY
PQIFRLTKDRKFPWIVPVLRIGTYNVQGAFMKRQEEVSEVFSSVVTEDLITLKSIMTE
MMYGSRKDKTRRIVKRHINEIMETPLVRTSLQLSLGPREYARLKTDLIDRSIEITMGP
VCDPALNASRAQKIFQMFKERIRELTPKEFQNLLRPAFQEDEWILIVLGGVTGFIAGT IHLFVAFL"
/product="FIG00969471: hypothetical protein" /transl_table=11
CDS complement(898644..899162)
/locus_tag="AUO97b_01018" /db_xref="SEED:fig|400667.82.peg.878"
/translation="MRKIYPLLAVLTVSSWAHAQDYSFLPEAKLADHKASTWNVGAGF
TQKLLHLNGEWVNPYGIAYAKVGAFLNGDKTAGGQVGFRYPYYLTGTDKNGYYIGFYA
GHLDSKSVDGDYKSRLGAGVDLAYVLLNSERISTFSVGIGAAEKLTDQNGDVAADTKP QIQFSYSLSFGL"
/product="hypothetical protein"

/transl_table=11
CDS 899399..901417
/locus_tag="AUO97b_01019" /db_xref="SEED:fig|400667.82.peg.879"
/translation="MKQHFPLKDIQQEKRIYRGRIFFAVGLVIICLLVLASRYAYLQI
FHYDEFSTASDKNRIRLQPLPPARGYIYDRNGVLLADNYPVFTATLSKADVENVDTVI
EQLQPILELTQEDVDRFKSRIKTARKTERVAIKLNLTETNIAKFSEVKYKFPGVRIET
QMTRYYPHGDLFAHVIGYVGRINDKELKSIDKDLYAGTNLIGKIGVEKSYEDLLHGTP
GYESVEADAHSNILRHLGRKDPTRGNDLYLSLDYGLQVVASQQLAGRRGAIVAIDPRT
GEILALVSSPSFNPNLFVTGINHKDYSSLRDNIDQPLYNRAVQGVYPPGSTIKPMEAM
GGLHYGIVDWATAISDPGYFHLPGDSHKFRDWKKTGHGIVNMHKAIIMSCDTYFYILA
NQMGIDQMNQWMRQFGFGQKTGVDLPSESEGLYPNPEWKMRTRKSKWMKGETISVSIG
QGAFTATPLQLAMATAITANHGSHVVPHVLRATHGAKPFTVRNAPDGKINFNGTDEDW
VKMREAMIDVIQSGTGRGIRTPLYQIAGKTGTAQVKSIAQGKRYNEAALSERQLDHGL
FVGFAPADKPEIAIAVIWENGRHGGSAAQLAKPVFDYWLLTRKKNPIRPANHQVNGGL
MTAGIKPGELPSGNESASSTPATSAPTSAAASTPQATPTRPATNEVDE" /product="Peptidoglycan
D,D-transpeptidase MrdA (EC 3.4.16.4)" /EC_number="2.4.1.129"
/transl_table=11
CDS 901417..901968
/locus_tag="AUO97b_01020" /db_xref="SEED:fig|400667.82.peg.880"
/translation="MKNQLRIIGGEWKRRVLPFASIEGLRPTPDRVRETLFNWLMWDI
QNAHVLDICTGSGALGFEALSRGAASVYMIEPDKMQARFLKDNIQLLKAQNCHLINAT
AQQALPRLKEQFDVVFLDPPYSLNLWQELAHLADTHIKNNGYIYVEADRDLSQLHLPA
TWQQVKTTKAGTVHAGLYQKVSE"
/product="16S rRNA (guanine(966)-N(2))-methyltransferase (EC 2.1.1.171)"
/EC_number="2.1.1.171"
/transl_table=11
CDS complement(902010..902579)
/locus_tag="AUO97b_01021" /db_xref="SEED:fig|400667.82.peg.881"
/translation="MNFIKKWSVGLLSMLPALAMAHPGHDHVHSGFMAGFIHPFTGLD
HLVMALGFGVLLWSAAKQWKIAGVITLSITLVIGFLVGAQGLVPANVAEYGIVASLII
TAIALWTKSNRILPIAAALFASFHGMAHGVELAHAGHIVALVTGMVAGMALIYCGGLA
LGAVFTRYVPYGKKIVGACAALVAVIGLS"
/product="Nickel-binding accessory protein UreJ-HupE" /transl_table=11
CDS complement(902589..903203)
/locus_tag="AUO97b_01022" /db_xref="SEED:fig|400667.82.peg.882"
/translation="MTERSPLRVGIGGPVGSGKTALTLNLCRALRDKYNMAVVTNDIY
TKEDSNFLTRNEAMSPDRIVGVETGGCPHTAIREDASINLAAIDDLCEKFDGLELIII
ESGGDNLAATFSPELSDLTLYVIDVAGGEKIPRKGGPGITKSDLLIINKTDLAPMVGA
NLDVMDQDAKRMRGEKPFLFSNMKTQDGLEEIIQFIEKQGLFKA" /product="Urease accessory
protein UreG"
/transl_table=11
CDS complement(903227..903892)
/locus_tag="AUO97b_01023" /db_xref="SEED:fig|400667.82.peg.883"
/db_xref="GO:0006807"
/db_xref="GO:0016151"
/translation="MSTHNAAQLLQLLTLSSTALPVGAYCYSQGVETAIDIGLIHDES
SAIAYFEEVLEMLLVRFELPVLKRLMQHYDDPDQFLIWAHLYKASRESRELLAESQQL
AFSLNAWIKDVLKKTVEVKKQFGFVPVYAQLCGRLELKDVDVLTAYTFTVLENQVLAA
VKTVPLGQMAGQRILWHLHGRVPQAVEKALKLKDEQLSSALPRYAMLSMKHETQYSRL FRS"
/product="Urease accessory protein UreF"
/transl_table=11
CDS complement(903879..904361)
/locus_tag="AUO97b_01024" /db_xref="SEED:fig|400667.82.peg.884"
/db_xref="GO:0006461"

/db_xref="GO:0016151"
/db_xref="GO:0019627"
/translation="MKIYTQRLEDISPDQAFETVELTFDTRQKSRFRAALASGVDIGA
DLPRTGILRSGSYIATQEGDVLRVDAKPERLMKVTAQTEFDLLKAAYHLGNRHVPLML
TPTALYFEPDHVLAEMVEGLGLAVTETDHPFEPESGAYAQHSHDHRLSPIKALHHVHS "
/product="Urease accessory protein UreE"
/transl_table=11
CDS complement(904431..906131)
/locus_tag="AUO97b_01025" /db_xref="SEED:fig|400667.82.peg.885"
/db_xref="GO:0009039"
/translation="MKMSRRAYAEMFGPTVGDRIRLADTALFIEVEQDLTTYGEEVKF
GGGKVIRDGMGQSQLLADEVADTVITNALIVDWWGIVKADVGLKNGRIWKIGKAGNPD
IQPDITIPLGAATEVIAGEGQILTAGGIDTHIHWICPQQVETALMSGTTTMVGGGTGP
AAGTSATTVTPGPWHIATMLQAIDDLPMNIGLLGKGNLSLPDPIREQIKAGVIGLKLH
EDWGSTPAAIDNCLSVADEYDVQVAIHTDTLNEGGFLEETLAAFKDRTIHTYHTEGAG
GGHAPDILKAIGQSNVLPSSTNPTRPYTINTIDEHLDMLMVCHHLDPAIAEDIAFAES
RIRRETIAAEDILQDLGAIAMMSSDSQAMGRVGEVILRTWQTAHKMKVQRGPLEGDNE
FHDNNRVKRYIAKYTINPAITHGLSHEIGSVEVGKLADLVLWKPAFFGVKPSMIIKGG
MIAAAPMGDINASIPTPQPVHYRPMFGAYPRGVHNTCITFLSQVAIDEKVAEKLNLKK
LISPCKNTRSITKADMKHNTYCPVMQVHPETYEVRADGELLTCEPADVLPMAQRYFLF "
/product="Urease alpha subunit (EC 3.5.1.5)" /EC_number="3.5.1.5"
/transl_table=11
CDS complement(906144..906602)
/locus_tag="AUO97b_01026" /db_xref="SEED:fig|400667.82.peg.886"
/db_xref="GO:0008080"
/translation="MLIRKATKDDTDTIVAIIAPYVDQVIANDEGRSRFKPEIIQTIF
GREDIHYFVGEIDQQIVGIVAYMEPSHLMHFFIKKTHLKLGLGRQLWDFIEEKIKNEN
IDIEKITVNSSFYAQDIYEKFGFIVSGDAAEKWGIRFIPMTKYYTLPSEK" /product="Histone
acetyltransferase HPA2 and related acetyltransferases" /transl_table=11
CDS complement(906592..906912)
/locus_tag="AUO97b_01027" /db_xref="SEED:fig|400667.82.peg.887"
/db_xref="GO:0009039"
/translation="MIPGEVITPETDIELNVGRETLKVVVANLGDRPIQVGSHFHFYE
ANDALQFDREAVKGFRLNIAAGTAIRFEPGQSREVELVALAGKREVYGFAGRVMGRLD ENVD"
/product="Urease beta subunit (EC 3.5.1.5)" /EC_number="3.5.1.5"
/transl_table=11 CDS complement(906925..907227)
/locus_tag="AUO97b_01028" /db_xref="SEED:fig|400667.82.peg.888"
/db_xref="GO:0009039"
/translation="MELNPTEKDKLLIFTAGLVAERRKARGLKLNYPEAVAFISAALL
EGARDGMTVSELMHFGTTLLKREDVMDGVPEMIAEVQVEATFPDGSKLVTVHQPIV" /product="Urease
gamma subunit (EC 3.5.1.5)" /EC_number="3.5.1.5"
/transl_table=11
CDS complement(907329..908204)
/locus_tag="AUO97b_01029" /db_xref="SEED:fig|400667.82.peg.889"
/db_xref="GO:0006807"
/db_xref="GO:0016151"
/translation="MNRIQQTFQTSSAPFWYAQLELGFCYENSRTIMSHRKHYGPVRV
QKMLWPEKTGVCHAIIVHPPAGIAGGDHLTFQIETEGQAHAVITTPGAGKWYRTNGKQ
AFQHIYLNVKDDSILEWMPQETMLFDGALAHSETDIHLEQTASFIGWDMLVLGRQARA
ENFVQGSYHNQFKLWRKNKLLVADTLYFEGGDRWLSSCLGMNNQAVMASFWAVPPEKF
RSSFYLEQHIELIRELIMRMDVPVTLTLLEDVLCARFLGNDVRRCHDAFAAIRAKLRR YWFDLDEEFPRIWKT"
/product="Urease accessory protein UreD"
/transl_table=11
CDS 908162..908302

/locus_tag="AUO97b_01030" /db_xref="SEED:fig|400667.82.peg.890"
/translation="MVLKKSEKFVEFDSFWLIFDNGFFDKQNMHQKQKVVITNLETIF LF"
/product="hypothetical protein"
/transl_table=11
CDS 908414..908722
/locus_tag="AUO97b_01031" /db_xref="SEED:fig|400667.82.peg.891"
/translation="MLVPFVFLTGCIFGQSSEVKRAEKLLNNFQCKNIETSEISTSSI
NSYYQQSLAVSKEKATSYVESYKNGEELFAMPLDEVVEQQYQLYKAACDSLGGVSAQP "
/product="FIG00350481: hypothetical protein" /transl_table=11
CDS 908926..909405
/locus_tag="AUO97b_01032" /db_xref="SEED:fig|400667.82.peg.892"
/translation="MKKSLLAIALMSTLLVACNKHENKTETTSDASTPVQTAQSNNNE
AVDTAHTAENSLDWDGKYKGTLPCADCEGIKTELELKDDKTYELTETYLGKGDANPFE
THGKFTFDKDNTSVITLDDKAQNRKFFIGENTATALDMEGKKVEGSLAEHYVLKKED"
/product="Lipoprotein"
/transl_table=11
CDS complement(909468..911072)
/locus_tag="AUO97b_01033" /db_xref="SEED:fig|400667.82.peg.893"
/db_xref="GO:0004451"
/translation="MTTYQTAIDAVRELKAKFGNTWADISPEDAARMQLQNRFKTGLD
IAKYTAAIMRRDMAEYDADSSKYTQSLGCWHGFIAQQKMIANKKYFGTTSKRYIYLSG
WMVAALRSEFGPLPDQSMHEKTSVPALIEEIYTFLRQADAKELNDLFRALNKAKEAGD
SAKVAEITAQIDNFETHVVPIIADIDAGFGNEEATYLLAKKMIEAGACALQIENQVSD
AKQCGHQAGKVTVPHEDFIAKIHALRYAFLEMGVEDGVIVARTDSEGADLTQKIPVVK
EPGDIASKYISYLDTQEIDISEAQEDEILIKRDGKLHRPTRLASGLYQFREGTQIDRV
VLDCITSLQNGADLLWIETPTPNVQEIAHMVNRVKEVVPNAKLVYNNSPSFNWTLNFR
QQAYDRWVAEGKDVSAYDRAKLMSAEYDNTELAAEADEKIRTFQADAAREAGVFHHLI
TLPTYHTAALSTHELAQGYFGDQGMLAYVAGVQRKEIRGGIACVKHQAMAGSDIGDDH
KEIFAGENALKAGDDAKNTMNQFSAH"
/product="Isocitrate lyase (EC 4.1.3.1)" /EC_number="4.1.3.1"
/transl_table=11
CDS 911661..912650
/locus_tag="AUO97b_01034" /db_xref="SEED:fig|400667.82.peg.894"
/translation="MNLERVDLNLLIYLDVLLREKNVTRAAEQLGVTQPAMSNILRRL
RNLFNDPLLIRSSEGMTPTERALELQPRIRDALSDLSMILEPRTEFRPYTSNRVFRIM
TSDYAEATLVPRLVKALRSEAPNVVLDFLTPSDVSYRDMEQGKVDLAINRFNEIPQSF
HQVLVWRDSFSCILNDKHPAVTHLNLKSYLDAQHIWVSKTGMGVGFGVNPDKQGGLGW
IDQALERIGQRRKISVFTRHYQMPALLAQNVDLIATLPTRMARLQAQNPKLVIKDPPF
YIPEFELKMAWCPLLHHHPAHRWLRQLILFVARQMIEEENREFLTNNSQFSHY"
/product="Transcriptional regulator, LysR family" /transl_table=11
CDS 912774..914099
/locus_tag="AUO97b_01035" /db_xref="SEED:fig|400667.82.peg.895"
/translation="MSLFQNIIIIVLLIIAAGFLSLTEIALAGARKVKLKILAEAGEE
RAQKVLDLQEQSADFFAASQIGLNAVAILGGILGEAAFRPFFVNLVDRFYEGPWTQTI
GFALSFTLVTSLFILFADLMPKRLAMIAPEKIAISVINPIQIFIKICKPLAWGINAIA
NLLFRLFKVNTTREDNITFDDISAVMDAGAQAGVLQKQEHHFIENVFELEERTVPSSM
TTRENVVYFTLNEHEDSIRQKLAEYPYSKFLVCNENIDQVIGYVDAKDILVRILNNQS
LTQLNESTIRTVLMIPDTLTLSELLDRFRSTKEKFAVVINEYALVVGVITLSDIMITV
MGDWVTPMEEEQQIIKRDNNSWLIDGSTPIEDLKHALEMDELPDEENYETLAGFMMYR
LRKIPRPADFVEFGGYKFEVVDVDHFKIDQLLVTRVLEKNDLPSPPDEN" /product="UPF0053 inner membrane protein YtfL" /transl_table=11
CDS 914653..915990
/locus_tag="AUO97b_01036" /db_xref="SEED:fig|400667.82.peg.896"
/translation="MLTLLGVGMILCFMYLIMSKRLSPIIALILIPFIFSLIAWGLGH

YFESLSHIELKGLSEMMLDGIKKLAPTGVMLLFAILYFALMIDAGLFDPPVKWILKKV
KGDPLKITLGTVFLTTLVSLDGDGSTTYMICVAAMLPLYQRLGMNTLIMTALMLLCSG
VMNLTPWGGPTARAASALQVDPSHIFVPMILPMVVSIGWLFFLAYLYGRYEQKRLGVI
ELESHHGDNITVSKDPEATRPHLRKVNMVLTILLMIALIKGILPMSVLFMLAFCIAML
INYPDVDMQKKRIAMHADSILAVVGLIFAAGIFTGILSGTGMVEAMSKEFVAMIPTSM
GPYLAPITALVSMPLTFFMSNDAFFYGVLPILAEAASHYGISPEAIARASIVGQPVHL
LSPLVPSTYLLCGLAKVDFADHQKFTFKWAFITCMVLMGTALVIGVFPLFSTM" /product="Citrate/H+
symporter of CitMHS family" /transl_table=11
CDS complement(916037..916549)
/locus_tag="AUO97b_01037" /db_xref="SEED:fig|400667.82.peg.897"
/translation="MFEQSPESLSDIEILDILQSMKKDKLDTEANEIIRNGGKAGRQE
AHKLALVALNTNFEEKFVEAVTLALGLNTAQAKKIRYKKDRIRILKARGIDYLAIDGA
ETAQVLAQISQAIVREDAIVTHDLHNIFPFWKEGFPMVQFDNAYKILQEDISLHFHAF LDAMIEYVNK"
/product="FIG00350904: hypothetical protein" /transl_table=11
CDS 916772..917320
/locus_tag="AUO97b_01038" /db_xref="SEED:fig|400667.82.peg.898"
/translation="MNTKITYTAFTGSTLIASDSLVELAKKLKALPKTTENILIFNDQ
TGQQIDLDLSGSEQELQQRYAEPEEIKKVGRPKLGVISREITLQKKHWDWLDQQSASA
SAVIRKLIDKELNNPNSEGNIMLAKQAIDRFMSAMLGNMPNYEEATRALYQGDRDIFL
KMIQSYPKDVREYLTLKTQNIF"
/product="hypothetical protein"
/transl_table=11
CDS complement(917373..918986)
/locus_tag="AUO97b_01039" /db_xref="SEED:fig|400667.82.peg.899"
/db_xref="GO:0004020"
/db_xref="GO:0004781"
/translation="MSHQSDLISQDILAYLKQHEQKDLLRFLTCGNVDDGKSTLIGRL
LYDSKLIYEDQLQAVTRDSKKVGTTGDAPDLALLVDGLQAEREQGITIDVAYRYFSTE
KRKFIIADTPGHEQYTRNMATGASTADLAIILIDARYGVQTQTRRHTFIASLLGIKNI
VVAINKMDLVEYSSERFNEIQVEYDAFVSQLGDRRPANILFVPISALNGDNVVNPSAH
TPWYKGQTLMSILESVEINRESSKHEFRFPVQYVNRPNLDFRGFAGTVALGEIKVGDE
IVALPSGKRSTVKEIVTFDGNLEQAVAGQAVTLTLNDEIDISRGNVLVRAGEQPLISR
SVRASVVWMNEHPLVKGKLYNIKIGTQTVPAKVTNINYRVNVNTLEHTQVEELELNAI
ADVVVEFDAPVVFDRYQDSRYTGSFIFIDRLSNVTVGAGMVEAAVEWTAHSNPVTAED
RAARLGQKPAVIGVSAQLIEKAQALESLLIQQGVVAIAKTSLTAEQLVLLRETGVVII
TTSVDGIDTEVTAETIEEAVEKIVELVRL"
/product="Sulfate adenylyltransferase subunit 1 (EC 2.7.7.4) /
Adenylylsulfate kinase (EC 2.7.1.25)" /EC_number="2.7.7.4"
/EC_number="2.7.1.25"
/transl_table=11
CDS complement(919017..919925)
/locus_tag="AUO97b_01040" /db_xref="SEED:fig|400667.82.peg.900"
/db_xref="GO:0004781"
/translation="MTESRLTHLKQLEAESIHIIREVAAEFENPVMLYSIGKDSAVML
HLALKAFYPAKLPFPLLHVDTGWKFKDMIAFRDNMAKTHGFDLIVHQNKEGREAGINP
FDHGSSKYTDIMKTQALKQALDKYQFDAAFGGARRDEEKSRAKERVYSFRDSKHRWDP
KNQRPELWNLYNGKVNKGESIRVFPLSNWTELDIWQYIYLENIQIVPLYFSAVRPVVE
RSGTLIMVDDERMRLKEGEVPQMKSVRFRTLGCYPLTGAVESEADTLPEIIQEMLLAT
SSERQGRMIDHDEAGSMEKKKQEGYF"
/product="Sulfate adenylyltransferase subunit 2 (EC 2.7.7.4)"
/EC_number="2.7.7.4"
/transl_table=11
CDS complement(919918..920049)
/locus_tag="AUO97b_01041" /db_xref="SEED:fig|400667.82.peg.901"

/translation="MDYLSRKIGVVMALVPPHGLLSGGVIPLLDFMGKMDREGRVHD"
/product="hypothetical protein"
/transl_table=11
CDS complement(920308..921750)
/locus_tag="AUO97b_01042" /db_xref="SEED:fig|400667.82.peg.902"
/translation="MFLRKTLSIALLATASSAVFAQGLVLNNDDLRTDLNWLNQQGVI
NISTSTWPLSGDEIQRALSQAKVTHPAQQKVINSVLNALKADNNTVKVGAFAESDIKN
IPQAFGDNQKSQYQGSLEFNAGGENWDAKIRVNAEKDPQIDSGHDVNVEGSYVAGKLW
NQWIVAGQIPTWWGPGHDGSLIRGDASRPVYGVTAQRAVQNAFETKWLSWIGPWQYQA
FAGQLDDYKAVPDAKLIGLRLTAQPLPYLELGASRTIQWGGDGRSESFSSLWDAIKGN
DNVYGDTENPSNQLAGFDGRLLLQPLLNIPVSLYGQYVGEDEAGYLPSKKMYLAGVDY
SSSYNDMPYQLYAEWADTRTNGDVKSISYTHSVYKDGYYQHGFPLGHAMGGDGQMYSV
GGDIRFDVMNRLSGRAMVVKVNQSNLAINKAFPKDDEIKALDLTWTHYIKPDLPLKIN
GWVSDSDLEGNDAGASIGVEIPLERKMFGF"
/product="K30 capsule biosynthesis cluster, partial sequence"
/transl_table=11
CDS complement(921940..923469)
/locus_tag="AUO97b_01043" /db_xref="SEED:fig|400667.82.peg.903"
/db_xref="GO:0004824"
/translation="MTQQNAQSTSEPTISENDLIAQRHAKLKQIQDVAKETGKSPWPN
TFKREHYAADLQEQFKDQSKEQIESAEHVYVKVAGRVMLNRGSFMVIQDMTGRIQLYV
DRKGLPKDTLETIKGLDLGDIIAAEGYIGRSGKGDLYVHLEGFELLTKSLRPLPDKFH
GLNDTEVKYRKRYLDLIVNEETRKTFEIRAKVVAGIRAFLTNERFMEVETPMMHVIPG
GASARPFETHHNALDMPLFLRIAPELYLKRLVVGGFERVFEINRNFRNEGVSTRHNPE
FTMIEFYQAYADYKDLMALTENMLEKLAIDILGTTDVPYQGEVFSFKGPFKKISMFDA
ILENNPQFTPENVGDREFLAKFVREELKEEVKPGFGLGKLQTIVFEETVETKLRQPTF
ITEYPAETSPLARRNDDNPHITDRFEFFIGGRELANGFSELNDPIDQAERFQAQVAEK
DAGDDEAMHYDAEFVEALEYGLPPTAGEGIGIDRLVMLFADAPSIRDVILFPHMRRKE G"
/product="Lysyl-tRNA synthetase (class II) (EC 6.1.1.6)" /EC_number="6.1.1.6"
/transl_table=11
CDS 923727..924215
/locus_tag="AUO97b_01044" /db_xref="SEED:fig|400667.82.peg.904"
/translation="MKILFLHGLDSSRESTKFHAILHPEKFCIDVDYRNLSYASVKDF
YHQAIKTIKPDLLVGHSLGGYWALKTAAQHKLAVIVANPSLTPNFRNDYPHLCDEDLD
HSNPKMAYLELGDEQLDMYQVQEKLSPYMTIETYDGGHHRLAYPSRLNDLITKIHKKY FA"
/product="FIG00350948: hypothetical protein" /transl_table=11
CDS complement(924285..924722)
/locus_tag="AUO97b_01045" /db_xref="SEED:fig|400667.82.peg.905"
/translation="METLIYEVEINAPIEKVWDVLWNSETYNAWTKFFAPDSSMRTDW
KVGGKTYFLDGEGNGMVSTIEEMKKPEVLIFKHLGMIKDGKEDLESEEVKGWSGSLEK
YFLSQNGDTTTVKVELATLPEYIGMLKSGFVQGFEAVKQMAEK" /product="2-oxoglutarate
dehydrogenase complex, dehydrogenase component" /transl_table=11
CDS 925166..925330
/locus_tag="AUO97b_01046" /db_xref="SEED:fig|400667.82.peg.906"
/translation="MKKYQCIVCGWIYDEAEGWPQDGIAAGTKWEDIPDDWTCPDCGV SKADFEMIEI"
/product="Rubredoxin"
/transl_table=11
CDS 925423..926604
/locus_tag="AUO97b_01047" /db_xref="SEED:fig|400667.82.peg.907"
/db_xref="GO:0015044"
/translation="MHPIVIIGSGMAGYTLAREFRKLNPEQELVMICADDAVNYAKPT
LSNAFAGKKAPEQIPLGDAAKMSAQLNMRIEPFTWVKEILAERHELVLEKDGVISQQP
YSKLILAVGANPIRLAIAGDGSDDIHVVNSLIDYRSFRENLAQRKDKRVVILGAGLIG
CEFANDLLHSEYDVTVIDLAPQPLGRLLPSHIASAFQQNLEEAGVKFALGTTVEKVSK

INNGEDYAVTLANGQTLVADIVLSAIGLQPNISLAQSANIQTSRGVITNSLLETNQAD
IYAIGDCAEVNGTLLPYVMPIMQQARALAKTLSGQQTNVHYPAMPVAVKTPAAPLTVL
PAPVDVDVNWETEEFDDGMLAKAIDNEGTLRGFVLLGATAGKQRLTLTKLVPDLIPAQ A"
/product="Rubredoxin-NAD(+) reductase (EC 1.18.1.1)" /EC_number="1.18.1.1"
/transl_table=11
CDS 926659..927603
/locus_tag="AUO97b_01048" /db_xref="SEED:fig|400667.82.peg.908"
/translation="MNSALQFSISPYTPFMQETKVNLGNGIELHVEVGGKPEHPTILL
IMGLGAQMLFWPDFFCKSLIDQGFRVIRFDNRDIGLSSKVRHQGKRLNTMKLMGRFAL
GLRNQGAPYTLYDMADDVSMLLDRLGVSRAHVIGASMGGMIAQILAAKYPEKVEKLGL
MFTSNNQPFLPPPFPKQLLSLIGKPESRDEEGIVNHSLKLFQLIGSPGYINHIEAVQT
ARKLYQRSYYPAGVLQQFLAILCTGSLLQLDREIKQPTLVLHGSRDRLLPPSHGKAVA
KAISGAKFELIDGMGHDIPAHFIPQLSGLFAHHFKSSY" /product="Hydrolase, alpha/beta fold family" /transl_table=11
CDS 927610..928518
/locus_tag="AUO97b_01049" /db_xref="SEED:fig|400667.82.peg.909"
/db_xref="GO:0003700"
/translation="MAALPSLRQLSYLVTLSETLHFTEAARRSFVTQSTLSGGIMELE
RLLGGVLVERDRQNVRLTPLGEQVVARARVLLADAQDLMRLSREMSEPLTGDLHLGVI
PTIAPFILTRLLDEVHQQLPKIQLHLHEAQSEKIVERLEHGNLDMIVLALPFDTRSLK
VAEISKENLYLVCSKQDTNALQANSLDDLDLSRLILLEEGHCLRDHVLSACPIGERKN
DNRLKASSLPTLVEMVSSDLGFTLLPEIAIENSMIKFNDQITAKPIEDAPSRTLALVT
RKSTPLQSEFDVLLQIMQKITTNLHE"
/product="Hydrogen peroxide-inducible genes activator => OxyR"
/transl_table=11
CDS complement(928548..929138)
/locus_tag="AUO97b_01050" /db_xref="SEED:fig|400667.82.peg.910"
/translation="MQGFIKSLFIRIFLGLMPVTAYADQKCYGDIIVFQKFDDQMIYP
YFIHPSREKNKKNETQNKTIVIQKRKLTQEELLAQKKIDDNQLKKIEEKKNIEKKKQL
DAAVAKEILENGEKKWMVQVALAPNQNKANMIQSQLQAKGYKVVTSPTTKGIRVMVDP
ANDYAIAQIIREKIIADDSLDLRSAWVFKWASLTAQ" /product="hypothetical protein"
/transl_table=11
CDS complement(929378..931456)
/locus_tag="AUO97b_01051" /db_xref="SEED:fig|400667.82.peg.911"
/db_xref="GO:0008976"
/translation="MNTAITATTPTEYSYNDRYINRELSILDFHLRVLEQAVDPLHPL
LERMNFLLIFSRNLDEFFEIRVAGVMEQFALGNESRSPDGLTPRQVLQKISETAHAAI
ERQYRILNEEILPKLREEDICFLRRGELTPAQSAWVKKYFQEQVAPVLTPISLDPAHP
FPRLVNKSLNFIVTLEGKDAFGRQIDLAVVPAPRSLPRVVRLPDELTGGKEHHVMLSA
IIHEHVSDLFPGMTATGCYQFRVTRNADLALNEDVEDLAKALKGELSSRRFGRAVRLE
VTQNCPQHIYEYLLEEFDLNEEQLYKVDGPVNLARLVSNFKRPHLRYDSHTPVVPKVF
KKTESIFSAMQKQDILLHHPFESFAPVIQLLREAARDPQVLAIKQTLYRSGADSEIVQ
VLAEAARNGKEVTAVIELRARFDEESNIEVANVLQEAGAVVVYGIVGYKTHAKMIMVV
RRENNKLVRYVHLGTGNYHAMNARIYTDYGLMTTDKDLCEDVHRIFQELTGMGKMAKL
KKLLHAPFTLHAQLINFIDEEIANAKAGRKAQIIVKVNALTEVQLINKLYEASQAGVQ
VDLIIRSICCLRPGLPNLSENIRVRSIVGRFLEHTRVYYFSNNGDARIYCSSADWMDR
NLFNRVEACFPIEDPALKKRIYQQGLLNYLQDNQQAWLLQGDGTWVRAQPAAGEKLHN AQRELLETFK"
/product="Polyphosphate kinase (EC 2.7.4.1)" /EC_number="2.7.4.1"
/transl_table=11
CDS complement(931561..932238)
/locus_tag="AUO97b_01052" /db_xref="SEED:fig|400667.82.peg.912"
/db_xref="GO:0003824"
/db_xref="GO:0008658"
/db_xref="GO:0009252"

/translation="MKAFIVRVLLIFIGAILFIQLWIFSSLVWWRTHEVDTTMFMRID
YWSDTSEPIIHEWLDYDDISDNFKHAILAGEDAKFIHHHGFDWDGIRFALERNNEQGE
VVAGGSTVSQQLAKNLFLYNKRSFIRKGQETVATWMMERMWSKRRILEVYMNSVEFGK
NLYGVEAAAQYYYGKSAKNLTREQAAFLAALLPDPKYYQDHRNDRKLQYRKRVILRYM NSTQIPE"
/product="Monofunctional biosynthetic peptidoglycan transglycosylase"
/transl_table=11
CDS 932315..933124
/locus_tag="AUO97b_01053" /db_xref="SEED:fig|400667.82.peg.913"
/translation="MTQSQPPHINRKLNVQAWWLTALLISINVGLFIWQIISGVDISD
PAIKDALHWGADFTPLTFSGQPERLFTSMFFHFGIVHLMLNMGALYIFGNVAEVLLSR
SYFIILYLLAGVFGSLLSSYINIQNGYELLQHFDQSLLPHVSAGASGAIMGLGTALTV
LSLFPPLPHQVRILNKKVYLVTMAINLIFGFVATGINNAAHVGGMIMGALLALIWYLS
YRTQFKSLLKFLGLLGGLLITVGFYVYCNQLNSPLLPLWHEVLIQNPEILP" /product="FIG056164:
rhomboid family serine protease" /transl_table=11
CDS complement(933126..935585)
/locus_tag="AUO97b_01054" /db_xref="SEED:fig|400667.82.peg.914"
/translation="MRNLLKPLLLQSLRTGGLYLLIIALSLAISATTALKFSNDQVKN
AVSLQAAQMLAADLVLSNNEPIDAKWQKRADQLGLKQTNVTMFSSMAHTQDQFVMVNV
KAIEPAFPLRGKLEIEPIARGIQPGEVWLSQRAADLLKVKLGDMVSIADGAFRFSGVI
VRDSNQELGFSGFSPTVIIHQADIAKTHAIQTGSRIDYRLLMSGQPEQVQSFSRQFKQ
QHQPANEQEETIGLRLRDASESNSRLLRPLENLDTFLQLANILTILLCGIAIALTSQR
YVQQNQDHIALIRCLGASKFQILWAYIGLLCVVSAISIVIGSLLGIMMGYGLLELMLQ
LIPQLELSFSVVGFLLGPLPIAIFTSVIVLLGFILPSIWELLNTPPIRVIREQAKSRK
SLFFMFFAGITSLVIFSLVLSENLMLSIWVLTAIILLCILLYTVVWLLLRALKKMKTR
LSFYIRSASQSALQITALALGLSLITVLSVLRTDLLERWQQQLPEGTPNQFVYGLPPF
DLKAFEQQLKQNGWQSTPLYPNIRGRLVAKNDVPFSEEAIKQNNALRRELNLTQSSAL
PNDNVITKGQAEFNAVGQVSVESKTAESLGIQIGDRLTFSLPEGTLQAKVINFRSVEW
ESFSPNFFFIFSPKTLDENAGSYLGSFYVPKQDQPKMIHTIQQFSNTVFIDVDRILDE
VKRLMNVLVKIVTVLAALVGFSGILVLIACLNLLMDERRREVALLRSFGLSKNKMKQM
LSLDIGFLGLLAGIVACCFAEVISAIASYKMNMALQWHIEIWLILPFAMMLLCALIGR
YRLGYLCNLPPLQSLRELNQI"
/product="ABC transporter, fused permease protein" /transl_table=11
CDS complement(935589..936281)
/locus_tag="AUO97b_01055" /db_xref="SEED:fig|400667.82.peg.915"
/translation="MPQPIIAAHQVTQTIQINQNQFTILKNIDLDIYEGEQVAITGRS
GSGKSTLLGILATLDQPSEGEVWLCGQAVHKLTEEQRAQIRMQHIGFVFQSFQLLPQL
SALENVMLPLRLRKDFDYKIAEKKALDWLHRVGLSRQAEQTPKVLSGGEQQRVAIARA
LIAEPQIIFADEPTGNLDGQTAQEIETLLFDLNRELGTTLILVTHDRNLAALCQRHVE LDDGRLKTVQGG"
/product="ABC-type antimicrobial peptide transport system, ATPase component"
/transl_table=11
CDS 936389..936949 /locus_tag="AUO97b_01056" /db_xref="SEED:fig|
400667.82.peg.916" /translation="MFVSAKTILILGDSISAGYGIDPEQGWVQLLQKRLDQQYPKQHK
VVNASVSGETTSGALARLPKLLQTYKPEVVVIELGGNDGLRGQPPQMIQKNLSQLIQL
SQKQKAKVILFGMKIPPNYGTAYSKAFENNYKTVSQKYQVKLLPFFLDGVAGQKQLMQ
NDLIHPNAQAQSKLLNLAYPYIKGAL"
/product="Arylesterase precursor (EC 3.1.1.2)" /EC_number="3.1.1.2"
/transl_table=11
CDS complement(936982..937596)
/locus_tag="AUO97b_01057" /db_xref="SEED:fig|400667.82.peg.917"
/db_xref="GO:0004089"
/translation="MPTAQEVLDRLKAGNQRFVNGNTSLAKTLSHHERAEMAKDQNPF
AIVLGCSDSRVPAEMVFDQGLGDLFVIRVAGNVVAPSQVGSVEFAAERYDCPVVVVLG
HSHCGAIQATIDTLMNPEHPPSSNLMSIVNRVRPSVEILMQTDLKNDLKKLSCHAVRS
NVFASVNQLRHGSAVLENLIAKGELIVVGAEYSLETGEVTFFDF" /product="Carbonic anhydrase,

beta class (EC 4.2.1.1)" /EC_number="4.2.1.1"
/transl_table=11
CDS 937881..938906
/locus_tag="AUO97b_01058" /db_xref="SEED:fig|400667.82.peg.918"
/translation="MTRLSASIKASMKKTATALTLACSLLSVMSISQAQAADNIDVSF
QTILQQERNWAGLQSKSLKVGDITWSYSEGGSSTKPTLLLIHGLAGSRDNWNRVAHYL
TTNYHVIIPDLPGSGETIVSQDFDYSVPNLAEKLRRFVEAANLKGPIHIAGHSLGGSI
ALLYAGQYPFETKSLFLVDSGGIFRSANTIYLKDPTYLKQLLVSKKGDFNYLLKQTMF
NPPFIPKEFLQAQEKLMINQAPQTQKLVDQLIALNKVYTPDSFAVLTKTIDAPTLILW
GKQDKIINVEVANELKRLLKNAQPPVILENVGHMPILEAEQLVIQQYVPFLLKVETNQ SSKTTTP"
/product="Lipase"
/transl_table=11
CDS 938933..939940 /locus_tag="AUO97b_01059" /db_xref="SEED:fig|
400667.82.peg.919" /translation="MSTQPIIEKLIEAHLDFLDEQFAQAQVIQQEFEQFYHWLGSRQL
QHLWTFEQVQQLIQKQILDTPASDFLIEQIAEHIRFALIHPANDTATVEDVIPVLTID
RIAQYVASKEEHRKKLIKTIVNNPAFSALLTQLIQQTMHDYLDESMSKRVPGVGRFMK
MGKSVLETVTDSNLDNTINHYLQKNILKLSQMSERVLNQHFDNDKLYHFQANVWHKVK
TSPLSVLKNYIEVQDLTKTVGLGHEIWDHIRQTDYLKQQVHDGIYTWYVRNQERNFDL
LLRDLNIDENLVKHELTELLVPVLQQLVTTGHLRRRARVYLEKFYYSEKALEILNNKG A"
/product="FIG00350364: hypothetical protein" /transl_table=11
CDS complement(939963..942227)
/locus_tag="AUO97b_01060" /db_xref="SEED:fig|400667.82.peg.920"
/translation="MSKRIIQSVLSVSVLASMMSMAFAAQNEQEQAEQTLEKPAEPVK
LETIFVTAEEQVKQSLGVSVITKEDLEKLPVRNDISDYVRRMPGVNLTGNSATGQRGN
NRQIDIRGMGPENTLILVDGKPINSRNSVRYGWKGERDTRGDSNWVPAEAIESIEVLR
GPAAARYGSGAAGGVVNIITKKVTNETHGSVEFYTSQPEDSKEGSSNRVGFNVSGPLI
KDVLSYRLYGNYNKTEADDVDINKSIGSTAAGREGVKNKDISGRLAWQATDQQTVLLD
ISSSKQGNIYSGDSQLNANAEADAILSQLIGKETNTMYRDSYALTHEGDWSWGKSKLV
AQYDKTHNKRLPEGLAGSVEGKINNLDDKATSRLETLRFNGEANIPFEYYLPQVLTVG
TEWVEDRFKDNVSTTQGKDSSGSGYGDQLAKGDRSKMESRIASAYIEDNLKVTDSTDV
VLGLRFDDHSKSGSNWSPSLNITQRLNDYFTLKGGVAKAYKAPNMYQNAEGYLLSTNG
NGCPANIESRCLLQGNGDLKPETSVNKELGIQFQKDIVNASLTWFRNDYKDKIVAGTH
VVGTVDGSSTNANTGAVTNTKWNILRWENTPKALIQGFEGSLGLDFGDIRWTNNFTYM
MDSKDKQTGNPLSLVPNYTINSIFDYDITDQLDVNFVFTQYGRQKSRQFAENRLESGI
GSGGANSALKPSTVKSYSTAGINVGYKFSDQISTRVGVSNLFDKQILRDSNSISQTYN EPGRAYYASLKYSF"
/product="TonB-dependent receptor; Outer membrane receptor for
ferrienterochelin and colicins" /transl_table=11
CDS complement(942397..943035)
/locus_tag="AUO97b_01061" /db_xref="SEED:fig|400667.82.peg.921"
/translation="MSTENTNTAVAEEIPNLLITPSAQEYLHELLAKQNTPGIGVRIF
VEHPGTPRAECCMAYSAPEEVVPTDYKQDYPDFPAYIDAPSIPYLLDAVIDYNKDRFG
GQLTFRAPNSKVPRVGPDASIEERITYVLQAEINPGLAGHGGNCSLVEVQDDPEHGLT
AVLKFGGGCQGCSAIDVTLKQGVETTLKEHIPELQRVVDQTDHTQAEGAYFK" /product="[4Fe-4S]
cluster carrier protein NfuA" /transl_table=11
CDS 943193..943810
/locus_tag="AUO97b_01062" /db_xref="SEED:fig|400667.82.peg.922"
/translation="MLHLPFNHTITLIIITVIVSLLAFSNQNVMNRLIFWPPAMQRGQ
FDRFITHGFIHADGTHLLFNMITLFFFGSIIESFYRQYFYDMGFVLFYLGGLIFAILP
SYLQHKNDANWASLGASGAVSAVLFAYILFQPWKLIFVFFIPVPAIIFAVLYVAYSIW
AGKRGNTHINHSAHLWGAAYGVIVTILIEPRVIPHFLDQLTRLPF" /product="Rhomboid family
protein"
/transl_table=11
CDS 943811..943939
/locus_tag="AUO97b_01063" /db_xref="SEED:fig|400667.82.peg.923"

/translation="MHIFEKSPIWAFLFFSFIYQDKFSDIFLIFFDQFLVLIAKTI"
/product="hypothetical protein"
/transl_table=11
CDS 943981..945657
/locus_tag="AUO97b_01064" /db_xref="SEED:fig|400667.82.peg.924"
/db_xref="GO:0004065"
/translation="MKYKALLSSLSIALFSLSLAGCNDNDSQETAVSTPKQPNILFIM
ADDLGYSDLGAFGGEIHTPNIDSLAQEGRILTDYHTAPTCSPTRSQLISGTDHHLAGI
GAMAELTPDHLKGQPGYEGYLNERSLSIAQVLKDNGYRTYISGKWHLGLTPETNAHAK
GFDHSFTLLQGLDHHFKQAPSAFKRNSTYTEDGQIIPVSALPDDFFSTNYFTDKLLSY
LESGKNSGKPFFAYAAYTAPHWPIQAPAEYREKYRGVYDVGYDAIRNARIARQKQLGI
IPVNFEAAEPIATQNAPQKYGKWDELTTEQKALEARKMEIYAGMVENLDANVGRIIQY
LKQNNLYDNTLIFFVSDNGAEGFVRGSYGSESGFDNSVANVGTPTSYHYIGPRWAEVS
AAPFHLWKDTAGEGATTAPAIVKLPNQKKAEETNHSFASVLDVFPTLLDYAHIPVPQG
QYKGRTINTPSGYSWKSVLENKSQTIRPANFSFADELHGNKYAKQGEWKIALQGRPEL
GTGSWELYNIATDRGENHNVAQLYPAKVQELLAVYQKYTEQNGVQEYNAK" /product="Arylsulfatase
(EC 3.1.6.1)"
/EC_number="3.1.6.1"
/transl_table=11
CDS 945654..946661
/locus_tag="AUO97b_01065" /db_xref="SEED:fig|400667.82.peg.925"
/translation="MKLKLVLSTTIGLLFITGCSKSEHNADTKTVLPKHAIQSAALGS
IEQCKSYTGLPTDWLKNTTAGMVFIPDGHFNFGSEKAYPDELNFGKKQREVKGFWIDQ
TEVTVAQFASFVKATGYITDAEKQKQAAVFSPDPHHPQQWWQLKTGYTWKTPNGKNGT
LPNPHEPVRYVTKNDAEHYAVWLGRDLPTELEWEYAAKANSPTDTPLYQAPTDEHQHP
QANYWQGAFPFENLNQDHFTGIAPVGCFKPNGFKLFDMIGNVWEWTSSPYQGAHDQHM
GNYSALRQQEMSSTQYVIKGGSFLCAENYCSRYRNSSRYPQDFDLAATHVGFRTILRS P"
/product="Sulfatase modifying factor 1 precursor (C-alpha-formyglycine-
generating enzyme 1)" /transl_table=11
CDS 946771..946935
/locus_tag="AUO97b_01066" /db_xref="SEED:fig|400667.82.peg.926"
/translation="MKTLLLCAAAIFTMSLSACAVHTPEGSIVIDPDSPYYGHPGGFC PPGQAKKGRC"
/product="FIG00351968: hypothetical protein" /transl_table=11
CDS 947472..949019
/locus_tag="AUO97b_01067" /db_xref="SEED:fig|400667.82.peg.927"
/translation="MSSQSPAEGLQFPIHASIKKQSTSLTGQEILSEALSIVDNKTAQ
QILAEKNWRKNYPIYFKALVKHGITNSNNPITIAKQGLHKAHHLFDYYRDGKHYLLKD
ALHIPTSTPLNTVKFKGESEAAPEWYVPYKGQKLSGQSLLDQIQKWENAGIIEPSHAK
ALREAAAHPEWFDLSDRTMVLFGAASEAGPLPWLARWKANIVAIDLPNPRVWGKILNT
IQQGNATLIAPSIEKIDSSAKASALRDKLGANLLTQIPEIAQWLVQFPQKLDLAAIAY
LDGEKHVRVSMAMDSIMQYVSEHKPDTSLMFMCTPTDVYAVPKEVAEAAQEKFKSRSQ
LQKMAVKGVSTLSLKRFFQAPYQDLITSENGKTYGIADCLVVEQGPNYALAKRIQQWR
ATLARHQGQRVSINIAPSTTTHSVTKNPLLKAAFNGAELFDVEAFSPETTNAIMAALW
IHDLRNDSSVANPETVLDHPLELMMEGANHGGLWRVAYLARTALPFAAIYGFAAEKLP FRKFSKK"
/product="hypothetical protein"
/transl_table=11
CDS 948970..949086
/locus_tag="AUO97b_01068" /db_xref="SEED:fig|400667.82.peg.928"
/translation="MALQPRNFHLGNFLKNNEVESPILELGFFVQLIALAMR" /product="FIG00351134:
hypothetical protein" /transl_table=11
CDS complement(949063..950397)
/locus_tag="AUO97b_01069" /db_xref="SEED:fig|400667.82.peg.929"
/db_xref="GO:0008721"
/translation="MKTVQLDQLKQQFPLIQTLQDYQETFWFNPHRYPLNEALAKVGL

TEQDVKEAEARLARFAPYLAKVFPETQAQHGKIESALVKIADMQQALSLQKHKTLIGK
LWLKKDSHLPISGSIKARGGIYEVLAHAEKLAIEAGLLKLEDDYSKLDQDSFRTFFSK
YQIAVGSTGNLGLSIGIMSAKLGFRVSVHMSADARQWKKDKLRSLGVNVVEYASDYGV
AVEEGRKAAEQDPFCFFIDDENSTTLFLGYAVAGLRLKQQFEQKQIKVDADHPLFVYL
PCGVGGGPGGVSFGLKLAFGEHVHCIFAEPTHSPCMLLGVYTGLHDQISVNDIGLDNI
TAADGLAVGRASGFVGRAMQQLIDGYYTIHDECLYELIALLNQTENIQVEPSAAAGMM
GPYYVQTTPDYLALHQLSAEKLQHATHVVWATGGGMVPPDEMQKYLTHSQSN" /product="D-serine ammonia-lyase (EC 4.3.1.18)" /EC_number="4.3.1.18"
/transl_table=11
CDS complement(950560..952095)
/locus_tag="AUO97b_01070" /db_xref="SEED:fig|400667.82.peg.930"
/translation="MPVFLTIIIATLIYIGFQVSADLAHVPPLSLYSVILLSTALLIA
LGFEFVNGFHDTANAVATVIYTNALPAPVAVMWAGFCNFLGVMVASGAVAYGIIALLP
VELIMNMGSGAGFAMVFALLIAAILWNLGTWFLGIPASSSHTLIGSILGVGIMNHLLS
ASTGVTTSGVDMDQVIKVGKALLFSPLIGFAFAAIVFLLVKTIFKRQLELFQPPEGNK
PPPAIIRAILIFTCTGVSFAHGSNDGQKGMGLIMLILVGLVPLAYSLNKNLDTQQVQS
FHQLSSQTAVLLNQNQPELTDEKARAVLTKYIQTKQQTPEVVPALASMTDHLGERVSS
YSNLKDIPEAAISEIRNDMYLSTTTFKRLDKADALPKMDDSQKKLVKDYRSSLDSFLQ
YIPNWVKVAVALALGLGTMVGWKRIVVTVGERIGKHHMTYGQGMSAELVAMSTIAAAD
GLGMPVSTTHVLNSAVAGTMVANKSGLNFNTVKTILSAWVFTLPATICLSGGLYWLFL QFV"
/product="Low-affinity inorganic phosphate transporter" /transl_table=11
CDS complement(952650..953612)
/locus_tag="AUO97b_01071" /db_xref="SEED:fig|400667.82.peg.931"
/translation="MTEPNSISCTQLAETYNISHDSVNRFLEREDYTPHDLYQEAIQH
IDNNKLIVSIDDTVLDKPYSQHMDLVSYFWSGKHHRSVKGINLITLYATDQNGQNIPI
NFRIYDKSESKTKNDYFMDMLSEVLSWGAKIQFITGDSWYSSTGNLKTIRKYGIRFMF
GIDCNRKVSPEKGQLFQLRLLPDFHQGQVVWLKDFGFVQLFKTQLKEQQRFYIVHQDE
DDLLSFEGFHELHSSHWKIEQYHRVIKQVCHIEKFQVRRSKLILNHIFSALMAYVEIQ
KNQFEGIFENVYRWQKKLFRPIIKDFIDDFILDKNHLLPQRIYK" /product="hypothetical protein"
/transl_table=11
CDS 953760..957446
/locus_tag="AUO97b_01072" /db_xref="SEED:fig|400667.82.peg.932"
/db_xref="GO:0008705"
/translation="MSTLATLKALLAKRILIIDGAMGTMIQRHKLEEADYRGERFADW
AHDLKGNNDLLVLTQPQIIQGIHEAYLDAGADIIETNSFNGTRVSMSDYHMEDLVPEI
NREAARLAKAACEKYSTPDKPRFVAGVLGPTSRTCSISPDVNNPAFRNISFDELKENY
IEATHALIEGGADIILIETVFDTLNCKAAIFAVKEVFKQIGRELPIMISGTITDASGR
TLTGQTAEAFWNSVRHGDLLSIGFNCALGADAMRPHVKTISDVADTFVSAHPNAGLPN
AFGEYDETPEQTAAFLKEFAESGLINITGGCCGTTPDHIRAIANAVKDIAPRQVPETV
PACRLSGLEPFNIYDDSLFVNVGERTNVTGSKKFLRLIREENFAEALEVAQQQVEAGA
QIIDINMDEGMLDSQNAMVHFLNLVASEPDISRVPIMIDSSKWEIIEAGLKCVQGKPV
VNSISLKEGYDEFVEKARLCRQYGAAIIVMAFDEVGQADTAERKREICKRSYDILVNE
VGFPAEDIIFDPNVFAVATGIEEHNNYAVDFIEATGWIKQNLPHAMISGGVSNVSFSF
RGNEPVREAIHSVFLYHAIKQGMTMGIVNAGQMAIYDDIPTELKEAVEDVILNQNQGE
SGQAATEKLLEVAEKYRGQGGATKEAENLEWRNESVEKRLEYALVKGITTYIDQDTEE
ARLKSKRPLDVIEGPLMDGMNVVGDLFGSGKMFLPQVVKSARVMKQAVAWLNPYIEAE
KTEGQSKGKVLMATVKGDVHDIGKNIVGVVLGCNGYDIVDLGVMVPCEKILQTAIDEK
CDIIGLSGLITPSLDEMVFVAKEMQRKGFNIPLLIGGATTSKAHTAVKIDPQYQNDAV
IYVADASRAVGVATTLLSKEMRGAFIEEHRAEYAKIRERLANKQPKAAKLTYKESVEN
GFKIDESYVPPKPNLLGTQVLKNYPLATLVDYFDWTPFFISWSLTGKFPKILEDEVVG
EAATDLYNQAQAMLKDIIDNNRFDARAVFGMFPAQRTDADTVSVFDEAGQNVTHTFEH
LRQQSDKVTGKPNLSLADYIRADREQQDYLGGFTVSIFGAEELANEYKAKGDDYSAIL
VQSLADRFAEAFAEHLHERIRKEFWGYKADEQLSNEELIKEKYVGIRPAPGYPACPEH
SEKAVLFDWLGSTDKIGTKLTEHFAMMPPSSVSGFYYSHPQSEYFNVGKISQDQLEDY

AKRKGWTLDEAKRWLAPNLDDSIA" /product="5-methyltetrahydrofolate--homocysteine methyltransferase (EC 2.1.1.13)" /EC_number="2.1.1.13" /transl_table=11
CDS complement(957527..958531)
/locus_tag="AUO97b_01073" /db_xref="SEED:fig|400667.82.peg.933"
/db_xref="GO:0004802"
/translation="MSQLANPPKKKLTTSAMIASIAAEGQPTKPAPFGHALNALAKER
DDIVGLSADLSKYTDLHIFAKENPDKFFQMGMAEQLLMSAAAGFAREGFVPFATTYAV
FASRRAYDFICMAIAEDNLNVKIVAALPGLTTGYGPSHQATDDIAIFRAMPNLMVIDP
CDALEIEQAIPQIAAHNGPVYMRLLRGQVPLVLDKYNYQFKLGKAQVIKPGKDVLIIS
TGLLTMRALEAAEELEKEGLEVSVLHVPTIKPLDEQTILDEVAKGGRPVLTAENHSVI
GGLGEAVGSLLLRNSQHPRFDMIGLPDAFLDAGALPTLHDRYGISTEAVKEKIKSHLK "
/product="Transketolase, C-terminal section (EC 2.2.1.1)"
/EC_number="2.2.1.1"
/transl_table=11
CDS complement(958542..959387)
/locus_tag="AUO97b_01074" /db_xref="SEED:fig|400667.82.peg.934"
/db_xref="GO:0004802"
/translation="MLNVDKAGQIAELQQRAYHIRQHALRMGQVQGQGYVGQALGAAD
LLAVSYFHAMKYQPENPEWEGRDRFYLSIGHYAIALYAALIEAKTIPLEELETYGADD
SRLPMSGMASYTPGMEITGGSLGHGLGIAVGACLGLKQKKSDAFVYNLLSDGELNEGS
TWEAVMSASHWKLDNLIAIIDVNNQQADGHSSEILAFEPIVDRWQSFGWYTQRVDGNN
MEALLEAFDQARNHEGACPRVIICDTKMGKGVSFLESREKTHFIRVDEHEWDDALNIL THYGQ"
/product="Transketolase, N-terminal section (EC 2.2.1.1)"
/EC_number="2.2.1.1"
/transl_table=11
CDS complement(959407..960708)
/locus_tag="AUO97b_01075" /db_xref="SEED:fig|400667.82.peg.935"
/translation="MDTVFSSEVDTTIRKSAYRKIAFRLMPFLMLCYFCAYLDRVNVG
FAKLQMMSDLQFSEAVYGLGAGIFFIGYFLCEVPSNIVLHKVGARRWIARIMITWGIL
SGCFAFVQTEWQFYTLRFLLGVAEAGLAPGLLLYLTYWFPSYRRARMTVLWFIAIPIS
GMIGGPLSGLIMDRMSGVHGWFGWQWMFVIEAIPTVLVGLLVLAVLKDSVQEANWLTQ
DEKNLVKQELAQDNQHKEGHASVKEFIADKRLWLLAGIYFCVVMGQYAITFWLPTLIR
NSGISDNWHIGLLTSLPYMCAIVVMILAGRSGDHFQERRWHLIIPMCAGAIALTFATL
FASNLTLSLICLCIAASGVLTASSLFWMLPTNFLGGVSAAAGIAAVNSFANLAGFCSP
YLIGWVTTNTGSNAIGMFLITAVLIFGASLVLRVPAKLVNR" /product="Uncharacterized MFS-type transporter" /transl_table=11
CDS complement(960784..961533)
/locus_tag="AUO97b_01076" /db_xref="SEED:fig|400667.82.peg.936"
/db_xref="GO:0004316"
/translation="MLLQGKVALITGAASERGIGRATAEIFAQQGAKVIIVDLDLAQS
QNAAKALGEGHIGLAANVANEEQVKAAVEQALQHYGKIDILINNAGITQPIKTLDIQR
SDYDRVLDVSLRGTLIMSQAVIPSMKANGGGSIVCLSSVSAQRGGGIFGGPHYSAAKA
GVLGLAKAMAREFGGDQIRVNSLTPGLIQTDITGGLMNDDRRHDILAGIPLGRLGKAQ
DVANAALFLASDLSAYLTGVTLDVNGGMLIH"
/product="Oxidoreductase, short-chain dehydrogenase/reductase family"
/EC_number="1.1.1.100"
/transl_table=11
CDS 961688..962593
/locus_tag="AUO97b_01077" /db_xref="SEED:fig|400667.82.peg.937"
/translation="MDSSENNFNQMPSIKTLQAFEQTARFGNVAKAAENLNLTPSAVS
HQIAKLEEMIGQSLFIRGARGVTLTPSGERYFQDVTTILHNLMLATEKAADKSPKDSL
YIHSSPSFGLLWLLPRLESFKEKFPMIQVNLSCSYENLHFTRDKIDIDIRHGLPNWTG
VEIRTIRNEKLEVLASPKLLERSPVHKPQDLLKKELILSRSTLVTWPQWFAHQGLSIP
EFPYTLSFDRSYMSLEAATHGLGFVLESNLLTQNYLESGKLVRVFADELSIPISAHHL

VYPRTHQFIPKIELFLNWIHDELAG"
/product="Transcriptional regulator, LysR family" /transl_table=11 CDS
complement(962605..963051)
/locus_tag="AUO97b_01078" /db_xref="SEED:fig|400667.82.peg.938"
/translation="MNHPNSILKFSLGLNSLWFDVDSQHIHLEQGLGFINSKSISKGV
IDELAIEKMIYFIEEIIESQPLRNLNIGGVAVTADTAMAQLSELFFSAKKQISRIELE
HGFNDFIERLSYYTNQMAPNQLYVFTYFVFVREMMHHLRVEEIQII" /product="hypothetical
protein"
/transl_table=11
CDS 963243..964388
/locus_tag="AUO97b_01079" /db_xref="SEED:fig|400667.82.peg.939"
/db_xref="GO:0004034"
/translation="MKKLVILGVTVYSFAQLANAATLNVKPYGTTQDGQKVDLYTMSN
NNGVSVSFISFGGVITQILTPDAQGKQNNIVLGFDDLKGYEVTDTKEGIHFGGLIGRY
ANRIGNAKFSLDGKTYNLEKNNGPNSLHSGNPGFDKRVWQVKPLVSQGETVKASLKLT
SPNGDQGFPGKLDVEVIYSLSDQNEFKIEYKAKTDQPTVVNLTNHSYFNLSGAGNNPY
GVLDHVVQLNADRILVTDQNSLPTGEIASVAGTPFDFRTPKAIVKDIRANNQQLAYGY
GYDQTWVINQKSQGKLNLAAIVVDPKSKRTMQVLTTEPSVQMYTANHLLGNIVGANGV
LYRQADALALETQHFPDSPNQPAFPSTRLNPNQTYNSITVFKFGVQK" /product="L-arabinose-
specific 1-epimerase (mutarotase)" /EC_number="5.1.3.3"
/transl_table=11
CDS complement(964540..965775)
/locus_tag="AUO97b_01080" /db_xref="SEED:fig|400667.82.peg.940"
/translation="MFAIKQLSLSVFLVSTLTLVACGGGGGGSSTDSSSTSSKSNPNE
IPTLSSNPLINYTTTFEQNLTIINEKNNGISTQLVPSNGSSKSVIEPSYSVSTYGNSG
LSFYYKLDKNQFVRVEYDKLSGKVINARIDTEKSSLTCRNDKWTPEGCEGISMIYDSR
TGDSTLLFNNKKFEDSYYTDSSSNNKKVTYLVTLNGTVKGSISSKPLEFTDFEKTHNG
EITVNGKKMDILRAYMRDTQLVVQFTNGESLYLLTGKSSGLVNTYYTGEEILSSAAAM
GTLKKLDNDRVNVEFNNVYFNSKTGSNSKIASGNITLINSYAKIDISNVGKIDTNEVY
AYELSNKEITYFITSTTGHGYITVQGQNPVNIQINGYSCTLGDCKNVLISKDGSYIQL
NGTKLGDVSVTGSIRTNIH"
/product="hypothetical protein"
/transl_table=11
CDS complement(966047..967267)
/locus_tag="AUO97b_01081" /db_xref="SEED:fig|400667.82.peg.941"
/translation="MTSSIQLKDGIANTVANDQSQGMIIKIVGAVAVAHLLNDLIQAV
LPAIYPMLKANFSLSFAEVGLISFVYQITGSLLQPWIGLYTDKHPKPYLLPLGMVVTF
CGIILLAFSPSFAVLLCASALIGVGSATFHPEASRVARMASGGRFGTAQSTFQVGGNT
GTAIGPLLAALLIVPFGQHAVAGLVIFALLAIWVLFGVSRWTVNHAKNQVAARATQAH
TKLHGRKLIIALGTISVLMFAKFTYIASISNYFTFYLIQKFHISIQTAQLHLFAFLAA
VAVGTFAGGPIGDKIGRKAVIWVSFVGMAPFALMMPYANLFWTTVFSIIAGLVLSSAF
AAMVVYAQEAVPGRVGMIAGLMFGLMFGVSGIAAAGLGHLADINGIEWVFGLCSLLPL LGFATALLPNTKVK"
/product="Fosmidomycin resistance protein"
/transl_table=11
CDS 967548..968162
/locus_tag="AUO97b_01082" /db_xref="SEED:fig|400667.82.peg.942"
/translation="MCALWIPANVEHSVISLSHVKLNTALVEVNAAALMGQHCFIIRV
SNLLHELLIRLNEIEREETPNTATSQELSRSLQILIFEEIHRANLLPIQIPWPKDKRL
LKICQELLHTPDSSKDLTDWADDIGASSRTLMRMFQKETGLSYRAWVQQMHIALALSK
IANGESIAQISESLGYTNPSAFSAMFKRHLGKTPQQFRSAAMSL" /product="Transcriptional
regulator, AraC family" /transl_table=11
CDS complement(968129..969451)
/locus_tag="AUO97b_01083" /db_xref="SEED:fig|400667.82.peg.943"
/translation="MKTPLPDYLANVIEACDIDNSGHLADYIPELANANPNRLALAMS

TVDGEIYSVGDDDVEFTIQSISKPFVYAYVLQQLGIDAVLAKVGVEPSGEAFNEISLG
KDGRPKNPMINSGAITVHSLIQVKHGLHSAEILRRFMSELAGRELSFDESVYDSEVKT
AYRNLSIGYMLRTVGILETDPVDIVNGYIRQCAIMVTVKDLVRMGSVLANGGVDPKTG
KRLLNRSVVRQVLSVMMSCGMYDAAGDWLSTVGIPAKSGVAGGILGVLPGQVSIAAFS
PRLDEHGHSVRGIDILERLSRDMGLHLMEGTPSAQTIVQSHYRTGKDASLSVYVLRGV
LKFTEAEMLLRILQDETTDQSTIVIDLTQISLIHDVGKRMFLEGVDRLIDDGHTLVLV
DPEQRLDHARTAKDRELHVYHDFDDLLEKHEIRSKKKTYSDIAALLNC" /product="Glutaminase (EC 3.5.1.2)"
/EC_number="3.5.1.2"
/transl_table=11
CDS complement(969630..970112)
/locus_tag="AUO97b_01084" /db_xref="SEED:fig|400667.82.peg.944"
/db_xref="GO:0042586"
/translation="MSVVLPVAKRGEDILKLIAAPVSANELNSNWLYQLADAMHATML
ERNGVGIAAPQVYISKRIIIVASRPNPRYPDAPEMNAVVMVNPEILEFSSETCLGEEG
CLSVPDERGQVERAEMVKVKYLTLQGEAVETIFHGFPARIVQHEVDHLNGILFVERIS "
/product="Peptide deformylase (EC 3.5.1.88)" /EC_number="3.5.1.88"
/transl_table=11
CDS 970347..971645
/locus_tag="AUO97b_01085" /db_xref="SEED:fig|400667.82.peg.945"
/translation="MDQELTLQDANYLNKEDKRTLALSSLGGALEFYDFVIYVFYAKI
ISDLFFPSTLSPFWAMLNTYGIFAAGYFFRPLGGVVMAHFGDLVGRKKLFSLSILLMA
LPTLFIGILPTFENIGYLAPLLLLLMRVVQGIAIGGEIPAAWTFVSEHVPERKIGLAN
GLLTAGLSLGILLGALMSLWISLNFSEGQIHDWAWRIPFIVGGIFGLVALYLRTYLKE
TPVFKAMQARKEISKEMPVKQVLKTHKTAVAIGMLFTWFLTGCVVVVILAMPNLLIGS
FGFERAQTFEMQSAAIVMQMVGCILAGYFADRFGCGKVMMVGALAVALTAAVFYNSLG
HAAHSTIFGLYMLLGLCSGTVGMVSSSMVKMFPAPVRFSGISFSYNLSYAIVGGMTLP
LVHWLSQYSDIGAMYYIFALTILAFVTAFVFWNKFEKNRY" /product="L-Proline/Glycine betaine transporter ProP" /transl_table=11
CDS complement(971708..972904)
/locus_tag="AUO97b_01086" /db_xref="SEED:fig|400667.82.peg.946"
/translation="MKKTSLIMAFIAPLTLGVSVNAFAKDSLLEWKSDDGTDSLKLGG
VVRLNQRYEDWEGPNRGFGKLHFDIFRVDLKGKFDDAYINASYMVQDQQKTSIEKAYV
GYNLDANNSIEAGLVYKPFTIYPYPQNGWTYHIPFFLGYGNNIAPGINWNYHNKDWDV
KLGYYPQMLETNLRYSPESATYDDLAGNAFPSQKAYQNEKRHQFNTRIVRKLETDFGK
QEFGVSGAIAQLHNDITDKDGKYYAVGLHTENNYKRFNLQSSVIHYQYDAKNPDGVNS
DMTLMGANGLTPAYFIASEGTIASLNLAYTLPVTNMGKLKAIKFYNDYSYLDKKRDDW
SDSQMNTTGMMFIASPFMVWADYTWGKNSNIIGGSTNSTGYTSATSPNSDKWLYRINF NIGFTF"
/product="FIG00350354: hypothetical protein" /transl_table=11
CDS complement(973060..974700) /locus_tag="AUO97b_01087" /db_xref="SEED:fig|400667.82.peg.947"
/db_xref="GO:0003984"
/translation="MTERVNVGEAIARVLEAHQVDSIYGVISIHNLPIADAVGRREKM
RFVAARGEAGAVTMADAHSRFKGLGVALTSTGAGAGNAVGSLIEAMNAGSPVLHLTGQ
VEREYLDRDASFIHETKDQLTFLRASSKAAFRITSPDNAVGVIREAIRVATTVPMGPV
SVELPIDVQAAEIDLPLNLGPVKALELPQAEQAEIDLLVSEIKKAKRPIFWIGGGTLN
SVAEVKAIADLGIPVVSSTHARGVLPDDHPRSLGAFHNSAGVEQLLKDADLMVVVGSR
LRSNETKTYSVEFPENIIQVDANPVAQQRNYKIRNFICGDAKDVLTRVLEQLQETSKI
DADYDAAVVAAKQAAIDALRKQIDQYALICDHLRAALPQDGIFVRDITMSGSTWGSRL
FPVQAPNQNIHSLAGAIGLGLATAIGASIANPDKKVIGLVGDGGLMLGIGEIATMVQE
NTNMVLMIMNDGGYGVMRGIQNNYFGGRQYFNELHTPDYKLLGESMGVKSWKVGSADE
FKTVIQEAVAFEGPTVIELDMNSIGPLNFAGPPQKKLY"
/product="Thiamine pyrophosphate-requiring enzymes" /EC_number="2.2.1.6"
/transl_table=11

CDS complement(974739..976205)
/locus_tag="AUO97b_01088" /db_xref="SEED:fig|400667.82.peg.948"
/db_xref="GO:0004029"
/translation="MAKEIYIAGEWRLGRGAVIQSLFPADQSVNAELSTATLEDVNEA
IEKADQAWRQPSWRNSLPHERARILYKVADIIEARVDELAKLQTRDNGKPLTETRGLV
MSAAATARYVAAACETLNDELTTQRAPDFMTMSVHEPVGVVAAITPWNSPIASEVQKL
APALAAGNAVVLKPAEATSLIALELAKIFEEAGLPKGLLSVLVGRGSIIGDAIAQHPL
VRKISFTGGTTTGRHLAHIAADKLITTSLELGGKSPTIVLPDADVELAAKGVAYGIFS
SAGQACIAGSRLFIHSSLYDQFLTRLVEITKGLRVGHPEQAGVHLGPLVNDKHLQSVD
RYVQLAKSEGGQVLIGGEALTTGDYAKGSYYLPTIITGLNNSAQTCQEEIFGPVLVVM
KYDNEQDLIAQANDSCFGLAAGIWTESYRKAWRIARALEVGTVWINTYKKFSISAPFG
GFKDSGIGREKGRLGILSYMQQKSIYMGLNEQPNPWCD" /product="Aldehyde dehydrogenase (EC 1.2.1.3)" /EC_number="1.2.1.3"
/transl_table=11
CDS complement(976219..977010)
/locus_tag="AUO97b_01089" /db_xref="SEED:fig|400667.82.peg.949"
/translation="MKKLMMIGFGAMAAEVYAHLPQDLQLKWIVVPSRSIEKVQSQVS
SDIQVISDIEQCDGTPDYVIEVAGQAAVKEHAQKVLAKGWTIGLISVGTLADSEFLVQ
LKQTAEKNDAHLHLLAGAIAGIDGISAAKEGGLQKVTYKGCKSPKSWKGSYAEQLVDL
DHVSEPTVFFTGTAREAAMKFPANANVAATIALAGLGMDETMVELTVDPTINKNKHTI
VAEGGFGQMTIELVGVPLPSNPKTSTLAALSVIRACRNSVEAIQI" /product="Aspartate dehydrogenase homolog"
/transl_table=11
CDS complement(977026..977820)
/locus_tag="AUO97b_01090" /db_xref="SEED:fig|400667.82.peg.950"
/db_xref="GO:0004316"
/translation="MSFQVEGKVAVVTGGSSGIGLAAVEILVAEGAKVAWCGRDEERL
NASKHYILEKFPHANIFTKACNVLQKEEVQQFAKEVKLNLGNVDMLINNAGQGRVSNF
ENTQDEDWMKEIELKYFSVLHPVRAFLDDLKQSANASITNVNSLLALQPEPHMIATSS
ARAALLNLTHSLAHEFTQYGVRVNSILLGMVESAQWKRRYETRSDLNLSWEEWTGNIA
KNRGIPMQRLGRPEEPARALVFLASPLASYTTGSAIDVSGGFNKHL" /product="Oxidoreductase, short-chain dehydrogenase/reductase family" /EC_number="1.1.1.100"
/transl_table=11
CDS complement(977836..978660)
/locus_tag="AUO97b_01091" /db_xref="SEED:fig|400667.82.peg.951"
/db_xref="GO:0047570"
/translation="MMTLIEKLAEFPVKTVNVQGQQQAYREAGQGEYLVLLHGISSGS
ASWVNQLEVLSHHFHVIAWDAPGYGKSDELLTDQPNATDYAKRLAGLFDALKIEKAIV
VGHSLGALQGSAFAALYPERVKHLVVANLAQGYQRHDEQTQIQVFEKRPKMLKELGAK
GMAQSRGPHLIYKQEPQALALVSEVMQQLTLQGFTHASYLLAYDEIRNYLTDLKVPCT
VIAGQQDQITPALGIQELAQELQLEQRFVIEDAGHLSYVDQPQAFNQIMLTIQEQS"
/product="Hydrolase, alpha/beta fold family" /EC_number="3.1.1.24"
/transl_table=11
CDS complement(978697..979218)
/locus_tag="AUO97b_01092" /db_xref="SEED:fig|400667.82.peg.952"
/db_xref="GO:0004476"
/translation="MTTQQFESWKQPEGASLEDWLNTRIARFETRKYDFEALKFQADY
DPKYRRAQMRYMGTGATGVANDSNTVPAENFTFSTMVLPPQCEGPLHIHHDVEEVFFM
LRGEIDLFIEHNGEKFQTRLKERDLISIPPGIYRGLFNPGQEDALMCVMLGTGKPTIP NYPPEHPLSQIKR"
/product="Mannose-6-phosphate isomerase" /EC_number="5.3.1.8"
/transl_table=11
CDS complement(979242..980465)
/locus_tag="AUO97b_01093" /db_xref="SEED:fig|400667.82.peg.953"
/translation="MSNEEKSGLKHWVPAFVLMVCVVLAFFDKISIAVLFSDPHFQSS

MGIAEDKAKLGWLMTSFLLAYGFSSVFLSFLGDIFNPKKMLFWSVTSWGLLMLCMGFT
TSYSGMLILRVLLGLAEGPLFALAYTIVKQTYTDRQQARASTMFLLGTPIGAFLGFPI
TAAVLAHHDWHTTFFVMAALTLIAILSIVFGLRNLQLKKTVELEGESKRTNFKGHIAN
TKVLVSNSAFWLVCLFNIALMTYLWGLNSWVPSYLMQDKGFNLKEFGVYSSFPFIAML
IGEVVGAFLSDKLGRRAIQVFSGLLLAGIFMYVMVIMTEPLLIIAAMSLSAMAWGFGV
AAVFALLARVTTSNVGATAGGIFNGLGNFASAIAPVLIGYIVMQTHSFNLGITFLAAV AVIGSLFLVPLLKRY"
/product="D-galactonate transporter"
/transl_table=11
CDS complement(980508..981719)
/locus_tag="AUO97b_01094" /db_xref="SEED:fig|400667.82.peg.954"
/translation="MEKIVIVGAGQAAGWAVSTLRQNGYVGEIHVVSNEDHVFYERPP
LSKQVLSKEASYESLNLFSPEQVQEFNIQWHKPEIATKVDREQKQVHLESGQILPYDK
LLIATGSRARVPVNTWQFIPNVVTLRNVRDCERLAEILKNAKNVAVIGGGWIGLEIAA
TARKQGKDVHIFEYGDRLCARSVSPEVSAFLKNMHEAQGTKIHLDSKSLHLVEAPDQK
VEVVNHPQHSQLFDCVVVGAGAEIAKELGVHAGLDVKDGIVVNCFGQTSDQDIYAAGD
VAIHPGLGYCIQSWANAQNQAIAAAKSMLGIETEYTDIPWLWSDQYHFNIQILGTYQP
EKTKEVVIRQGGEDQVSYLYLDHENRLLNMIAINDSKLIKLAKRWMQANTVLDPKLLA DAEFNVMKLKP"
/product="Ferredoxin reductase"
/transl_table=11
CDS complement(981749..982678)
/locus_tag="AUO97b_01095" /db_xref="SEED:fig|400667.82.peg.955"
/translation="MITGIEILKFGVEDRAASNKFLADFGLSQSTSDIEGTDLYETQN
GSKIYLFNCDDERLPTAIEQGSTLREVTWGVESAQDLEDLAARLADVDGFKRTENTVQ
CLDPNGMTIRFEQSFVKEVPELKTEGINQYGNIQRVNAASPVYEKGQPVAIGHVVFFT
PDLATTENFYIEKVGFHLSDAYKNRGAFLRCRGEGYHHDLFLLSVPNKPAGLNHVAFV
VRDIHEVIGGGLHMNRSEWSTFIGPGRHPISSAYFWYVNSPLGGAFEYYTNDDYLTEE
WQPRVEEHRLELFTEWAIEGGLDDKTRRQVKPV"
/product="Glyoxalase family protein"
/transl_table=11
CDS complement(982690..983454)
/locus_tag="AUO97b_01096" /db_xref="SEED:fig|400667.82.peg.956"
/translation="MANVALLQGKKVLVTGAARGLGRDFAQAIAEAGAEVVMADILSD
LVQQEAQALQQQGLKAHAVTVDLANADSIENTVAKSVEVLKGLDGLVNCAALATNVGG
KNMIDYDPELWDRVMNINVKGTWLISKACVPHLKQSAAGKIINVASDTALWGAPNLMA
YVASKGAIVAMTRSMARELGQFNICVNTLSPGLTLVEATEYVPQERHDLYVNGRAIQR
QQLPQDLNGTALYLLSDLSSFVTGQNIPVNGGFVFN" /product="Oxidoreductase, short-chain
dehydrogenase/reductase family" /transl_table=11
CDS complement(983447..984514) /locus_tag="AUO97b_01097" /db_xref="SEED:fig|
400667.82.peg.957" /translation="MNAIVSTSQSAEEYLELGLRDQWHPVLASWEVAANPVGITRLGE
NIVVWRDAEGQVHALEDRCPHRGARLSLGWNLGDRVACWYHGVEVRADGVVADVPAVH
ECPMTGTKCLKSYPVIEQHGAIFIWFGIDANEQPAPLEFPEQLASEEWSSFLCQADWK
VNHQYAIDNVMDPMHGTYLHCTSHSMAEGERTAEMKHRTTENGFVFEKEGQTGVNFDW
VEYGSTGASWLRLSIPYRKQFGPGGEFWIVGYATPIDANNTRVFFWRCRKVSGWQRNV
WRFLYRNHLEQLHWDVLEQDRIILENLAPNARAREFLYQHDVGLARLRRLMKKEAEKQ
LKKIAALNASNRIEMQEEAHG"
/product="GbcA Glycine betaine demethylase subunit A" /transl_table=11
CDS complement(984531..984830)
/locus_tag="AUO97b_01098" /db_xref="SEED:fig|400667.82.peg.958"
/translation="MKTFNEKLASWIHATPATEAGINNIQVPGQSELLVWQTRYKEPT
LYEQDFVKNLIQAFSAGATELDEVVSALNQQGFRCESGDEWTSESFTEEMQRLGY"
/product="FIG00351215: hypothetical protein" /transl_table=11
CDS complement(984858..985163)
/locus_tag="AUO97b_01099" /db_xref="SEED:fig|400667.82.peg.959"
/db_xref="GO:0005506"

/db_xref="GO:0006118"
/db_xref="GO:0009055"
/translation="MSWISVCQQGDVSEDEPKAVEVEGKKIGVFFVDENYFAIENVCP
HAYALLTEGFIEDQTVECPLHEAIFDIQTGELKSGPGCRNLCTYPVRVEGQDIQIQL"
/product="Ferredoxin"
/transl_table=11
CDS 985437..986249
/locus_tag="AUO97b_01100" /db_xref="SEED:fig|400667.82.peg.960"
/db_xref="GO:0003677"
/translation="MSLEIEEDSQESETTKNDDRYLVPGLVRGLAILQAFNQQAQEMT
ITEIAEILEVNRSSAFRLIYTLEACGYLRKASQKTYALDSKVMELGFNSLSKLSLLDL
STPLMKELRDQTKLAVHLTVLEGTHIVFVNNVQSMGTFTSNISLGTRWPAHATVIGQM
LLSDLPEAEVRQRYRNFNDWDSYSELTPTSLKALLQKLNYVKTQKSMVSWGHYNHDMA
ACAAPIFKQSNGKMVAVLSVSCPITTYDEKTFKEDIAALVIATADKISKFIY"
/product="Transcriptional regulator, IclR family" /transl_table=11
CDS complement(986296..986595)
/locus_tag="AUO97b_01101" /db_xref="SEED:fig|400667.82.peg.961"
/translation="MLLSLDAYKQQQFDQIAAKIIAEPAKYIDFNSVSDFYNAAWLKD
FPQGTQVSATGLDDGAEEFYAVVQFKQQYLKFDIKENHSTLSFQNMNGETFKCNF"
/product="hypothetical protein"
/transl_table=11
CDS complement(986610..987191)
/locus_tag="AUO97b_01102" /db_xref="SEED:fig|400667.82.peg.962"
/translation="MSPLEIFAVLISLIGVGLTVIRNMWCWLFNFFAFVLYAYLFYEF
KLYGETILQFFFMVVNFYGFYYWLKGKQQDHDIRIEPIAVQTAIIQMIIAAVGGLVFG
LVLKNFTDAAVPMLDSQLAAFSLLATYWTSRKHIATWILWVFVDIVYVGMFIYKDLFL
TSALYAAFVLMAAYGWYQWEKVKRKQNLGAGQL"
/product="PnuC-like transporter linked to homoserine kinase and OMR"
/transl_table=11
CDS 987438..988226
/locus_tag="AUO97b_01103" /db_xref="SEED:fig|400667.82.peg.963"
/db_xref="GO:0004300"
/translation="MSNQEGKVSRETRGHIFLIGLDRAAKRNAFDSHMIKDLSLALTE
YENNDALRCAIVFAHGDHFTAGLDLVELQPKLVTGVFDFSENEINPWGTVGRKLSKPL
IVAVQGYCYTAGIELFLNADIVIASENTQFAQMEVQRGILPFGGATARFTQAAGWAKA
MRYLLTGDSFDAKAAFDMNLITEISPEGTELKRAIELAEHIAQAAPLAVKATLASARE
AVNEGYEVAFNQLQKHLQPLLTTEDVQEGVFAMLQKRPPVFKGK" /product="Enoyl-CoA hydratase
(EC 4.2.1.17)" /EC_number="4.2.1.17"
/transl_table=11
CDS complement(988329..988748)
/locus_tag="AUO97b_01104" /db_xref="SEED:fig|400667.82.peg.964"
/translation="MKTNSLIIDLRRQLPWHKRYASTTSTAMMWAAWLLLWRPFVFIW
VLAELQKTHLVHRLFSAFGVGIEHGITALIACAVGLLLWSHVLPERRVEGSELNMKQT
DDYARYFDLPVQEIEQGRSQKITVVHHNEFGKIIRVE"
/product="Biofilm PGA synthesis auxiliary protein PgaD" /transl_table=11
CDS complement(988831..990090)
/locus_tag="AUO97b_01105" /db_xref="SEED:fig|400667.82.peg.965"
/translation="MTIFAAIWSHALKFVFYYPLFMSYLWMIGAIIFYWKERKAPPYD
QPAPLESYPKVAVLIPCFNEGDNAEETITHALKLDYPHFEVIAINDGSSDNTGEVLDR
LAEQHEKLRVVHLAQNQGKAMGLQAGSLMTDAEFLIGIDGDALLDPHAAKWMVRHFLQ
NPTVAAVTGNPRIRTRSTLLGRIQVGEFSSIVGMIKRAQRTFGRLFTVSGVITAFRKS
AVHQVDYWSPNMLTEDIDITWKLQRAGWDIRFEPNALVWILMPETLNGLWKQRLRWAM
GGAQVLIKNIDVFTKPKLNFLWPLMFELCLTLVWSYLMLAMALLWLVHFILPVPALAV
VSSPFLPYGSGILLGATCLIQFALSKWMDSRYEPHLGKNYFWMIWYPLVFWLITISAT

IVAFPKVLRRGDEKRARWVSPDRGIRG"
/product="Biofilm PGA synthesis N-glycosyltransferase PgaC (EC 2.4.-.-)"
/transl_table=11
CDS complement(990104..992098)
/locus_tag="AUO97b_01106" /db_xref="SEED:fig|400667.82.peg.966"
/translation="MITRFASPLLNLTLGLALAGLSGMALANPPKIDASTLTVIGYHE
ITDTKNALIPQYAVTTQQFTEHVDWLQKNGFHFITVDQLIRAHQGKAALPTKPVLLTV
DDGYQSFYQNAYPVIKAKKIPVVLAVVGSWLEPKAGQKVDFSGEEIPRDKILSWGELK
EMQDSGFVEIASHSYHLHRGITGNPQGNSEPAATTRFYDVKTKTYENDSQYQARIYND
LKKNNQLLKEHGIRSPRIMVWPYGRYNMQTVQIAKKLGMPITITLDDGADHAKQSLQN
MSRILVEGGMSTNDLAQEIKNRELNLTDNNRPQKIMHIDLDYIYDPDPQQQERNLGHL
LDRINAIGVNTVYLQAFSDPDANGSADMVYFPNRHIPMRADLFNRVAWQIQTRTPVSR
IYAWMPLLAWELPKTDPVSKDLVVTEQAKAGEHLNMGYIRLSPFSPQARQTIREIYQD
LAKSASFNGILFHDDVTLSDYEDASPDALKAYAKQGLSTNLAKIRENDQDLQKWTAYK
TKYLDDFAMQLVEDVRQYEPFLLTARNLYAQVALKPYAENWYSQSLEESLRRYDFTAI
MAMPYMEQVDNADQFYKDMIDRVKKYPNGIKKTVFELQATNWRNNEKVPSTEMAATIH
SLYQQGAMHVAYYPDDPIKDHPDVNVMHKAFAEKSSRLVP" /product="Biofilm PGA synthesis deacetylase PgaB (EC 3.-)" /transl_table=11
CDS complement(992335..992676)
/locus_tag="AUO97b_01107" /db_xref="SEED:fig|400667.82.peg.967"
/translation="MITELEIIDLKVGEGKEAVKGALITTHYTGWLEDGTKFDSSLDR
GNYFECVIGTGRVIKGWDQGIMGMKVGGKRKLMVPAHLAYGERKMGKIIPANSNLIFE IELFDVKTRDD"
/product="Peptidylprolyl isomerase, FKBP-type (EC 5.2.1.8)" /transl_table=11
CDS complement(992763..993293)
/locus_tag="AUO97b_01108" /db_xref="SEED:fig|400667.82.peg.968"
/translation="MALKATIYKADLNIANMDDHIYGDYQLTLALHPSETLERLMVRV
MAFARFASDSLEFTKDLFETDEPALWQKDLTGQLETWIEVGLPSEDKVKKASARCKTV
AIVAYGSQVEDWWKRNSKIKTLSNVQVWQLAPESTAELEQLCERTMQLQLNVMDNEWT LLSDKGQANVVWTQLQ"
/product="YaeQ protein"
/transl_table=11
CDS 993482..994744
/locus_tag="AUO97b_01109" /db_xref="SEED:fig|400667.82.peg.969"
/translation="MWQKLKRSLFLQVLCALFLGIAVGLAFHDFSLALKPLGDGFISL
IKMLIAPIVFCVVVLGIYGANDIKKMGKVGAKTILYFEVVTSIALILGIIVAYVFKPG
VGMNIDITHLDAKDLSTYTERAHDMHTGSEFLLNIIPKTFTAAFANGDILQVLLIAIL
FGISLLLIPKHLAELVRQALEAFSEVFFKIMGLIIRLAPIGVFGSVAYTTAKFGLDSL
LQLGYLVLLFYVTCILFVVIILGSILKLTGLNIFKFVGYFKDELAIVLGTASSDSVLP
QIMKKLKNLGIKDSTVGLVIPTGYSFNLDGFSIYLTLGVIFIAQACNINLSMHDLLAI
LLVSLITSKGAHGIPGSALVILAATLSVIPSLPAIGLVLLLSVDWFIGIIRACTNLIG
NCVATVVIAHWEKDIDHAQAKQVLNIKN"
/product="C4-dicarboxylate transport protein" /transl_table=11
CDS complement(994782..995780)
/locus_tag="AUO97b_01110" /db_xref="SEED:fig|400667.82.peg.970"
/translation="MKLIAQKTVPTHIISGFLGAGKTTLLQHLLSQKPKDEVWAVLMN
EFGQIGVDQQLLPQDQGYAVKELLGGCLCCSSQLPMHIALARLLSEQKPDRLFIEPTG
LGHPSQLFDQLTEPHWQNSLAMRALVTVVDGSRLHDTNWVKQNLYEDQLKAAQIVVVS
HADTMTFEDEQALVELKEEYQPYQQQWLKTEHGQLELAQIDVPARLTERSIQPLLKLQ
KQMTEAEVVKEIHQLPYHYVETAQGYMVAGWKFPKRWKFEFYALLDVLCAQQDWLRIK
GIFNTDQGWKTFNFNPEQFNYKSGEEGIDNRIEMISQHEHDWLAFETELLACRIEQ" /product="Metal chaperone, involved in Zn homeostasis" /transl_table=11
CDS 996279..996479
/locus_tag="AUO97b_01111" /db_xref="SEED:fig|400667.82.peg.971"
/translation="MKLAKTLLATTLALTAASTFAASKHDQAHNTAGEEKVVVSTQEQ
ANTANAASDAVGSASEAAPATR"

/product="hypothetical protein"
/transl_table=11
CDS complement(996590..997105)
/locus_tag="AUO97b_01112" /db_xref="SEED:fig|400667.82.peg.972"
/translation="MPTLDEMTTVTDDQSELTMSVLMTPDMANFSGNVHGGTILKLLD
QVAYACASRYSGSYVVTLSVDKVNFKEPIYVGELVTFLASVNHVGRTSMEVGIRVEAQ
NIQKRTVRHTNSCYFTMVAVDEHGKPREVPKLNLDTEWKRCRFEAAEHRKVLRLQENH NPSCSMYKKTS"
/product="cytosolic long-chain acyl-CoA thioester hydrolase family protein"
/transl_table=11
CDS complement(997367..997981)
/locus_tag="AUO97b_01113" /db_xref="SEED:fig|400667.82.peg.973"
/translation="MVSPYLHSFALFFSLLNPFLMSIYMIGLIRHSETKVFNKALIQG
SLIAYIVFMLFAWGGEAIFSKYLNVRFEAFQIFGGLIFLVIGYRYVFQGADTIGEMRG
APEHLAGTIAMPFMIGPGTISAAVVTGIEMSIGAAALVIGFTMFLTCSILILMKFSHD
HLRYKHAKYIDRYFDIVGRLSALLIGTIAVDMIINGVTGLIHKV" /product="MarC family integral
membrane protein" /transl_table=11
CDS complement(997993..1000059)
/locus_tag="AUO97b_01114" /db_xref="SEED:fig|400667.82.peg.974"
/translation="MATDNPRAVDDLTLSKSKKDPLNRVVFYISALIILIFSLVTFLF
NDFANRTINTVLAWVTSTFGWYYLLAATLYMVFVIFIACSRYGEIKLGPKHSKPEFSL
LSWSAMLFSAGIGIDLMFFSVAEPLSHYMQPPVGVGQTYEAARQSMVWTLFHYGLTGW
CMYALIGMALGYFSYRYNLPLTIRSALYPIFGNRINGAIGHSVDTAAVIGTIFGIATT
CGIGVVQLNYGLHVLLGLPENIWMQFALIAIAVGISIISVTSGVNKGLRILSEINIYV
SIGLLLFILFLGNTEFLLNALVQNIGDYLTRFPTLALQSFAFEQPKDWMSSWTLFFWA
WWIAWSPFVGLFLARISRGRTIREFVCGTLIIPLLFTLTWLSIFGNSALHSVIFEGNQ
TLAATVLSNPAHGFYDLLAQYPGFTFIASVATVTGLLFYVTSADSGALVLGNFTTKLT
HIDNDAPRWLSVFWAIAIGLLTLAMLMTNGITALQNATIIMGLPFSFVMFLVMAGLYK
SLRLEDYRHASRSMNAAPVVGNVDILNWKQRLTRVMHHPGTTETRRMLDTVCKPAVQA
VADELIKQGMNVDVQQTPLEDEEALYHLDITIHLEEEQNFVYQIWPVRYSAPNFSQRV
KRGKQFYYRLETYLYEGSQDNDLVGYSKEQVINDILDKYERHMTFLHINRISPGNRPL FPDPQA"
/product="High-affinity choline uptake protein BetT" /transl_table=11
CDS complement(1000177..1001799)
/locus_tag="AUO97b_01115" /db_xref="SEED:fig|400667.82.peg.975"
/translation="MVLKSDGYSSDHIRLNRFVFWSSAVSIGIFGLLFVLFPEKSQFW
LTYVQEQVNHFFGWYYMLVIVLCLGFVAWLAFSKVGQIPLGKDHDKPEFGYLAWTSML
FSAGIGIALLYYGVAEPVDHFLRPPEGEAGTIQAARNAMTYSFLHWGIHGWVLYALLG
VTLGYFAFRQDLPLALRSALYPIFGERVHGLVGDFVDGFGILATVISLVTNLGIGALV
LVSGICYLIPEIPDNSATLITAVLIMMFVATVTTVIGIEKGLAWLSRINLRLLYALLL
FVFLTGPTNHLLNGLVQNTGDYLNNFMGKSFDMYLYNQKASGWLGSWTVFYWAWWIAW
APFVGMFIARISKGRTIREVVLGVCLIPLGFTLAWISIFGNTAIHLILNQKQKVLGDM
VLSDPALSLFKLLEYLPFNPYIAGIVVVICFVLFLTPVGSGTLMIANLSSKGGTSDSD
SPIWLRIFWSVVITIVSIGLLLAGSFNSMQSAVVLCGLPFSVIILLYMFGLAKALKQD
DFDPNVAKKQLAISKDISIPSNCDQSKVTEVL"
/product="High-affinity choline uptake protein BetT" /transl_table=11
CDS 1002065..1002688
/locus_tag="AUO97b_01116" /db_xref="SEED:fig|400667.82.peg.976"
/translation="MTKRRVKPEHVRREEIMNAALDVIYEVGLSNTTIAQIAKKAELS
TGIVSHYFGDKQGLINTCMQEMLNVLRRKTEQYRAEADSHPESQIKAIIDSNFDISQV
NEKAMRVWLDFWSASMHVPDLSRLQKINDQRLYSNLKFYFLKLMDEQQASVAARGLAA
LIDGLWLRGSLSRHNEFDSELARSIAYDYVKTQLQFANKPLQERQNE" /product="Transcriptional
regulator BetI, TetR family" /transl_table=11
CDS 1002681..1004153
/locus_tag="AUO97b_01117" /db_xref="SEED:fig|400667.82.peg.977"
/db_xref="GO:0008802"

/translation="MSDVQVHQLYIHGRYVEATSGKTFNSINPANGEIIATLQQASEQ
DIEAAVKSAQQGQKIWAAMTAMERSRILRRAVDILRERNDELARLETLDTGKAYSETS
TVDIVTGADVLEYYAGLATAIQGEQVPLRESSFFYTRREPLGVVAGIGAWNYPIQIAL
WKSAPALAAGNAMIFKPSETTPLTALKLAEIYTEAGLPDGVFNVVQGAGREIGQWLTE
HPVIEKISFTGGVETGKKVMASAAGSTLKEVTMELGGKSPLIICEDADLNRAADIAVM
ANFFSSGQVCTNGTRVFVPKSLLVDFEKAVVERVKRIRIGDPMAEDTNFGPLTSFPHM
EKVLSFIESGKQQGAKVLIGGGRATEGELAKGAYVLPTVFSDCTDQMAIVQEEIFGPV
MSILSYETEEEVIQRANDTTFGLAAGVVTQDISRAHRIIHQIEAGICWINTWGESPAE
MPVGGYKQSGVGRENGLTTLGHYTRIKSIQVELGDYQSIF" /product="Betaine aldehyde
dehydrogenase (EC 1.2.1.8)" /EC_number="1.2.1.8"
/transl_table=11
CDS 1004249..1005907 /locus_tag="AUO97b_01118" /db_xref="SEED:fig|
400667.82.peg.978"
/db_xref="GO:0008812"
/translation="MNIKEYDYIIIGAGSAGNVLAARLTEDKDTTVLLLEAGGPDYRL
DFRTQMPAALAYPLQGRRYNWAYLTDPEPHMNNRRMECGRGKGLGGSSLINGMCYIRG
NAMDLEQWATHKGLENWTYADCLPYYKKAETRDIGGNDYHGDSGPVSVATPKNGNNVL
FHAMVEAGVQAGYPRTDDLNGYQQEGFGPMDRTVTPKGRRSSTARGYLDMAKGRPNLT
ILTHATTNKILFNQKQAIGVEYIIGADQNNLQRALVKREVLLCAGAIASPQILQRSGV
GQSTFLKSMDIDVVHDLPGVGENLQDHLEMYLQYKCKQPVSLYPALKWYNQPAIGAEW
LFNGTGIGASNQFEAGGFIRSSDEFKWPNIQYHFLPVAINYNGSNAVKEHGFQAHVGS
MRSPSRGRIKLKSKDPFAHPSILFNYMSTEQDWREFRDAIRITREIMHQPALDPYRGD
EISPGKHLQTDAELDDFVRNHAETAYHPSCSCKMGEDEMAVVDGQGRVHGMNGLRVVD
ASIMPLIITGNLNATTIMIAEKIADQIRGREALPRSTAPFYVAS" /product="Choline dehydrogenase
(EC 1.1.99.1)" /EC_number="1.1.99.1"
/transl_table=11
CDS 1006419..1008059
/locus_tag="AUO97b_01119" /db_xref="SEED:fig|400667.82.peg.979"
/translation="MKKFLKYLLVLIILILIAGIVFLFRPIASKQVQTAKDEPVVDAV
LVGGGIMSATLGTYFTELEPNWQIRMFERLDQVAQESSNGFNNAGTGHSGFMEMNYTE
EKNGKMEIAKAEKVASQFEVAKQFWSYQVKQGVLAEPKTFINPVPHIAFVWGDNVKFL
EKRYAAMIQSPLFKGMKFTEDPAVIKQWAPLVMTDRDPTQKVAATRMEVGSDVNYGSI
TKQLVNHLNQNPNFKLQTSTEVTGISQNDDKTWTVSFKNLKTGKTDHVKTRFVFIGAG
GAAVKLLQLTGLPEAKQYAGFPVGGEFLITDNPAITAQHTAKVYGRAELGAPPMSVPH
IDTRYIDGKKYVLFGPFATYSNKFLKNGSQLDLLASTNKSNVLPMTTVGLENLDLVKY
LVSQVMMSDEDRLNELRKYYPDAKAEDWRLSQGGQRVQIIKKEPGKPATLQFGTEIFA
SKDGAVTALLGASPGASTSPYIMLNLLEKAFPQQTEGKWNQKLHEIVVSYKQDLSKDP
VLLDKVRQYTSSTLGLNYTSPFKAANDETAAAPVAKAN" /product="Malate:quinone
oxidoreductase (EC 1.1.5.4)" /EC_number="1.1.5.4"
/transl_table=11
CDS 1008247..1008621
/locus_tag="AUO97b_01120" /db_xref="SEED:fig|400667.82.peg.980"
/translation="MWMIGGTAIYVSSAVLFVITAYSFKLGKYQKLCSVLNVLMSALA
LYFSTAHLFFSPIQFFIFLPALFFVVLAFTQVSKVRSLSKVLILISLLIWSGVHFTQL
EQLKAYYQTQHTGESWQQYGAL"
/product="hypothetical protein"
/transl_table=11
CDS 1008838..1009716
/locus_tag="AUO97b_01121" /db_xref="SEED:fig|400667.82.peg.981"
/db_xref="GO:0008898"
/translation="MKILDGGLGRELARRGAPFRQPEWSALALIEAPETVKEVHLDFI
NAGAEVITTNNYAVVPFHIGQERFETDGVRLIKVAIEQAKNAVKESGKNVKIAGCLPP
LFGSYRADLFQPEQAKNLAEPIINTLAPEVDFWLAETQSCLKEVETVHALLPQDGKDY
WVSFTLQDEIKQEQALLRSGENMQQVADFIKQSNAKAVLFNCCQPEVILQAINEIKGL

IPESVQIGAYANAFPPQDESATANDGLDEIRKDLDAPAYLAFAKQWQQAGASLVGGCC GIGPEHIAELSQFFKE"
/product="Homocysteine S-methyltransferase-like protein"
/EC_number="2.1.1.10"
/transl_table=11
CDS 1009722..1011056
/locus_tag="AUO97b_01122" /db_xref="SEED:fig|400667.82.peg.982"
/translation="MSAAKIGLLSLTALVFSSMVGSGVFSLPQNMAQVANGSALLVAW
LITGVGIIFLALSLLHLTRQRPDLDGGIYNYAREGFGDLIGFCSAWGYWLCTTIGIVG
YVVIAFSGVGMFTDSKDHVIFGEGNTLYSLIGSSIFVWLVHWLVSRGIKEAAIVNLLA
TIAKIIPMVVFIFFTFIAFKFDLFKLNLNDFSLKVPLWQQVKDLMLITLWVFTGIEGA
VVLSSRAKNRHDIGKATILGVLLALSFYVMVTVLAYGVVSREALAGMHNPSMATILQQ
LIGLPGTIIITIGLIISVSSSYLSWTLFATEIPYLAAKNGAFPKLFLKNNQNNVPISS
LWLTTIVVQGSLIAVYFFNKNYTQLLLISSVMILLPYFLVSAFYLKESIRHKRTPHIA
IASVASIYALWLLYAAGLDLLLLSGVLYLVGLVIFSISYFEHKKSLKQNQLN"
/product="Arginine/ornithine antiporter ArcD" /transl_table=11
CDS complement(1011112..1013025)
/locus_tag="AUO97b_01123" /db_xref="SEED:fig|400667.82.peg.983"
/translation="MTKSGHYLVLAIMGLLSGCQVIHLKESNLSSALKSKNESILTDD
TLSHQTQNLLYLVKESETSCLQNFNVCLNKIQGLSENSSREERYAALSEIYLAKALDV
GRSSQCNATLKSNSCVEQELALFDKSLRYSYVYLFDSEESPFDRVFDHRQNQVRIFYN
VALSKLMTTYFNYLNTLHFPPLLKADGHEYHVNFDHAVDVQHIEVDTFRSSYNMNFSG
FNTVNRKDGLGAEFIVGRKEHDVNHGFILDPDNFYAHQSNPNIHLPRFFPVTAIAYPK
QKATADQVIDGAELEIAMFDPYRQDRVKVEGVDYPLTANYSAPYGLWLSKYNLGAAGY
WSLINKEANLIMPHLYMLEPFNPNKKIIVFIHGLASSPEAWVSLTNDIMGDAELRQNY
QVWQVFYSTNMPIFESRFQIYSLLNQAFQNVAKDSYAAHDAVLVGHSMGGVISRLLVS
DADVSDLAMQKMNEAQLKRLKENPVIRERFQFKDLPYFKRVVFVSAPHHGTDYADRWF
TQIARRIIRLPADFFIAVEMRDEKNTKLRKGLIENGASNLSRSSNFMQLTQAIQPSSN
VVYHSIMGNINGTTDKSKMSDGIVPYQSSHLGGEQSELIIKGGHSIQTSPEAILELRR ILRLHLKQSQPSK"
/product="hypothetical protein"
/transl_table=11
CDS complement(1013174..1013710)
/locus_tag="AUO97b_01124" /db_xref="SEED:fig|400667.82.peg.984"
/translation="MKGGAGLWIKLAAIPLFIVTVVITKLLIDRSQTKHKTLSYLFLF
EAIFLFAFMAAGLYFEPFKNADSITVAITGGLGLIALAIRNTSSKTLIKNISPSTMMT
GNTTQLGIDIANLLKNNNAANRASLLKSASIVIGFVIGALMGAVLYVYFDFWSVAPFI
LPILYFSYLASQQKFKQA"
/product="DUF1275 domain-containing protein" /transl_table=11 CDS complement
(1013707..1013880)
/locus_tag="AUO97b_01125" /db_xref="SEED:fig|400667.82.peg.985"
/translation="MPETSIQQETQSTWAICRDPVLLTMVGGAIDTIGFIALFGFFTA HVTGNLVLAGLRW"
/product="DUF1275 domain-containing protein" /transl_table=11
CDS 1014201..1014476
/locus_tag="AUO97b_01126" /db_xref="SEED:fig|400667.82.peg.986"
/translation="MSLYNFSVFLGGLLFVSIASAGYKSHAHRDFYSKCVAAGHSRAT
CLCIYHRLERQYSPKLMHKLGKLSLQSPEVPRDFVKTMLRTMQQCQS" /product="FIG00350011:
hypothetical protein" /transl_table=11
CDS complement(1014490..1015380)
/locus_tag="AUO97b_01127" /db_xref="SEED:fig|400667.82.peg.987"
/translation="MNYELWKIFLDAVELGSLSKVAMLHNTNQPQISRQMNELEAQCG
GRLFNRTGRGVELTDLGQHLLPKIRSWLNVTDQLNNEILHSTDTPIGTVRIASLPSTA
HPLLSTLYKVLQEKYPLIKLSVREGQGVHLEKWLEDGSVDLAILYRFNPTPKQGDIYL
TQADTYLVGCEGDALTQASEVDFKAINQLPLVTFCRPSNWRNFLDHMAYENGVELNIV
FEADSISLQTHLVSESRMYTMLGPQALKKASQYTSIQAAKIINPALKRYISLSISKHG
YLTPACKAVMEEIRNLADLL"

/product="Transcriptional regulator, LysR family" /transl_table=11
CDS 1015568..1016512
/locus_tag="AUO97b_01128" /db_xref="SEED:fig|400667.82.peg.988"
/translation="MSLDLRGLTPAPVTPFTRDGQVDHDAIQRLGSWLGSIDGVKGLV
VLGHAGEGTFLSQKEQMQVIESFVKSVDDKIPVIAGITLEGTSVAAEEAKRAVSAGAS
AGLIYPSHGWLRFGYQKGAPQDRYKAIYEESGLPSILFQYPDATKATYDLDTLLDISA
QPGVFAMKNGVRNMRRWDVEIPIIRRERPDLQVLTCHDEYLLHTMFDVDGALVGYGNI
APELLIEMIKAGKAKDYAKARAIHDQLLPVTRNVYHRGSHMEGTVALKHALVARGILD
HATVRSPLLPLADGAEQEIHDAMRQAGIGKVDLTQAVA" /product="Dihydrodipicolinate
synthase family" /EC_number="4.3.3.7"
/transl_table=11
CDS 1016685..1018070
/locus_tag="AUO97b_01129" /db_xref="SEED:fig|400667.82.peg.989"
/translation="MTNLADVEQISHHQKNAEIIARLERLPVTGRLQFMRITIGIATF
FDAYTVLAIAFALPQLITEWHLTPAYVGAIIAAGYVGQLVGAIFFGSLAEKVGRLKVL
SFTILLFVAMDISCLFAWSGMSLLIFRFLQGVGTGGEVPVASAYINEFIGAEKRGKFF
LLYEVLFPLGLMFAGMAAFFLMPIYGWKVMFIVGLIPSLLVIPLRFFLPESPRWLASK
GRFKEADQVVKSFEDGAIKSGKTLAEPVVKEINPKGMAKTDWRELFRGIYRKRTFTLW
GMWFCVYMVNNAMVTWLPSLYKQHFGLSLQTSLGYGWITSGVGVIASIICALMIDKVG
RRPWYSAAFFLAIIPLMSLSVLGAKSAMQVVIFATISYAILQTISFSLYLYAAELYPT
RLRAMGIGFSTAWLRAGSAIGPVMVGLVVGGYGIQYVFSVLAVVALIGGLVTFLFAIE TKGQVLEKLSP"
/product="Uncharacterized MFS-type transporter" /transl_table=11
CDS 1018149..1019495
/locus_tag="AUO97b_01130" /db_xref="SEED:fig|400667.82.peg.990"
/translation="MNKYLLWGCIGLGSVSNAYAELPQEFGKLELKLNTRYWNDEGTA
HPTLANPSPSSSEYEQSALGLELNYQSPYIWDWLGVDGSLYAVTKLFDSGKPSAQLLE
VENNGKLDQNFATLGQLYIKAKLGDKGEIKLGRQLHDSLLLKSTNNRAVPDTFSGASG
QFQLNDQLQTYMAYYDRWKPRSMDSFEKLVTENDERIDYVGLLGAKYQYKNWSLNAEY
LNSKDYLKKYGAIAQYKLPLHGLVWTFNTGAFFSRDDGKLFKCGAETELDCVKGQDIN
NRGNGYFIDVNLAKNNLEGGIAVSKFDGFWIEDNFAVHSARNSVLTQDHGTNPFPTSA
TIGPDFTNNDETAVALRLKYNWKDYVKGLKTEAKYIYGFGAHQSNISSDIEGKERYFD
FTVSYALPWVKNLDVRYSFLHYESKFENANLGEKINGMSRKGWDQHRVFINYRYTF" /product="PhaK-like
protein"
/transl_table=11 CDS 1020619..1021347
/locus_tag="AUO97b_01131" /db_xref="SEED:fig|400667.82.peg.991"
/translation="MAKERYLEGDIKSLIFNHLINNNLLGKNDTIINEFIIGNYTRRV
DLALIKSQKLHAFEIKSEADNLSRLEGQIIKYLEYFDKVTVVAASKHISKILSLAPPN
VAIWEIRDKKIIILRRGKAKNITDKLRFLEYLRVSELLQIARQEKLVFQSNRRKTLEK
GLIYLPTHKLREYSIKYLKKRYQPTTDLFFQEINKANYASLENLSLLRINRNNKVPNN
STKNINDLLNVLEELEHERELSLL"
/product="Protein cII"
/transl_table=11
CDS complement(1021356..1022462)
/locus_tag="AUO97b_01132" /db_xref="SEED:fig|400667.82.peg.992"
/translation="MFFDTFNYIPILGLKPADMAALEELPEKDKDLLLPLLPLKRWTT
STSLDKACNRIEKAFGIRKILIDIDKDFLVECRSKDTPIAKEFCSLGDSTNGYKNWID
LFKRKTNYLPVLQLEDLAEFTSQLNGFIELNLPIALRIEMKGKDKVETENFKFIIRNI
LPLANKINSLLIILDYTDYDRTDLLNYSESASLIIGLNKYFPNAKFSISGTSFPYSFA
GSYKGEIPIYERLIYNKVQNYLEDMKIIYSDRASTRAYSIDGGGGTPLPRIDYPLKND
WRFVRKEFITDDENEKDYLYQEAAIEVINSDYWDENLHLWGTQMIERTALRDPYGINS
PQKATSARINIHLYNQLHYMDNLSDLDTDEDWVD"
/product="hypothetical protein"
/transl_table=11
CDS complement(1022471..1022971)

/locus_tag="AUO97b_01133" /db_xref="SEED:fig|400667.82.peg.993"
/translation="MNISPEWTRIGTQNQNIIQKYQNELPVKLGALASELGLIVKKAT
LPANISGEIKEENGQVIIRINRHDANSRQRYTLAHEIAHFLLHKHLLKDGIADDVLYR
SALSNEIEAEANRLAADIVMPLAAVRDLWKKHNPDAPNKEITYERIADELGVSVIALK IRLEKI"
/product="hypothetical protein"
/transl_table=11
CDS complement(1022984..1023586)
/locus_tag="AUO97b_01134" /db_xref="SEED:fig|400667.82.peg.994"
/translation="MKSKIYLVFILFFSSLTNSYADEAKLALSNISGSNQALAPFEKN
KLENLNKEELLSIILKQDKSIDDINKKYESLKLELASQKEANTNLEFPVWISIVLGCV
AILVTVLGVIVAILSIFGYQKIKDSATKIAEEKTLETATTVAKDVVNDQLQGIASTEV
ARLIAEGELREELERAVDMILRRNRDTQNATGFNKYPELD" /product="hypothetical protein"
/transl_table=11
CDS complement(1024357..1026342)
/locus_tag="AUO97b_01135" /db_xref="SEED:fig|400667.82.peg.995"
/db_xref="GO:0003840"
/translation="MNKRYSFLFSSLLVVGLLQGCGDDSSSDQPKDDSNTTPPNLIVD
NDPTSCSKLAQDGSSVVVGSNQNGDPAAPEGASGYRLGNTVKYADKYMVVANTPLAVK
AGCDVLKAGGSAVDAAVAVQAVLGLVEPQSSTIAGSGFMMYYDAKTKQVTAYDGRETA
PAAANEYYLIRQNIYDPNSPPPVPSARRSGRSIGVPGVMRLLEQAQKEHGKLEWKQLF
GEAIGLADNGFRIPGRLADAIASNASNLALDADAMRTYFHSDGSPRKVGEIMTNEAYA
RTLEALASKGANALHTGPIAQAIITEAGRPEGDDPAKTPITPSLMTLQDLSNYQVIKR
DPICTTYRDRYYVCTMPPPSSGGIAVAQTLGILENFDMSLYPPKNPENEGGVPDVMGV
HLVSEAERLAYADRDKYVADTDFVELPAQGIPSFIDKNYLKQRAALINPNQSMGVAPA
GNFNTAAGVDTTVEHGTTQFTIVDAYGNVVSMTSTVESSMGSFHMVDGFLLSNQLTDF
SANPYDSTGALVANRVEGGKRPRSTMAPTLVFKGTTPDEFYMATGSPGGGTIIQYVVK
TLVGALDWNLNAQQATSLVNFGATNSKNTNIDSSNVQLSLVDLIEGLKAKGHGISNTA
QTSGISTIMKVNINNQSKYAGGVDPRREGIVLGNGAL" /product="Gamma-glutamyltranspeptidase
(EC 2.3.2.2) @ Glutathione hydrolase (EC 3.4.19.13)" /EC_number="2.3.2.2"
/transl_table=11
CDS complement(1026646..1028169)
/locus_tag="AUO97b_01136" /db_xref="SEED:fig|400667.82.peg.996"
/translation="MKTQTPFAELSGGRLLLAAFVIALSNFMVVLDTTIANVSVPHIT
GNLAVSSTQGTWVVTSYAVAEAICVPLTGWLAGRFGTVRVFIFGLIGFTVFSFLCGLA
TSLEMLVFFRIGQGLCGGPLMPLSQTLLMRIFPQEKHAQAMGLWAMTTVVGPILGPIL
GGLISDNLSWHWIFFINLPVGIVCVLAAMRLLRVAETETISLRIDTVGLGLLILWIGA
LQLMLDLGHERDWFNSTSIVILALTAAIGFVVFLIWELTDKHPVVDVKVFRHRGFAIS
VLALSLGFGAFFGSIVLIPQWLQMNLSYTATWAGYLTATMGFGSLTMSPIVAKLSTKH
DPRALASFGLILLGIVTLMRAFWTTDADFMALAWPQILQGFAVPFFFIPLSNIALGSV
LQQEIASAAGLMNFLRTMAGAIGASIAVTVWDDHAKVARSEMVSNLHTTEVQNTLLQN
GFTADSTLGIISNLVDKEAITMSANHVFLLFAIVFVFAGLVIWLCPKPKQVSGMPSH"
/product="Multidrug efflux system EmrAB-OMF, inner-membrane proton/drug
antiporter EmrB (MFS type)" /transl_table=11
CDS complement(1028176..1029327)
/locus_tag="AUO97b_01137" /db_xref="SEED:fig|400667.82.peg.997"
/translation="MTDAQSNVQETVPTTSASDDNMQNKRKKFLGFFALILLIAAILY
AIWALFLNHSVSTDNAYVGAETAQITSMVSGQVAQVLVKDTQTVHRGDVLVRIDDRDA
KIALAQAEAELAKAKRQYKQTAANSSSLNSQVVVRADEINSAKAQVAQAQADYDKAAL
ELNRRAQLAASGAVSKEELTKAQSAVETAKAGLELAKAGLAQATSSRKAAESTLAANE
ALIQGVSETSTPDVQVAQAHVEQAQLDLERTVIRAPVDGVITRRNIQVGQRVAPGTSM
MMIVPLNDLYVDANFKESQLKKVRPGQPVTLTSDLYGDDVEYHGKVVGFSGGTGSAFA
LIPAQNATGNWIKVVQRLPVRIALDPKELAEHPLRVGLSMEAKVDLSAK" /product="Multidrug efflux
system EmrAB-OMF, membrane fusion component EmrA" /transl_table=11
CDS complement(1029597..1030130)

/locus_tag="AUO97b_01138" /db_xref="SEED:fig|400667.82.peg.998"
/translation="MGVYHAFKFRQRMSDEKKNWTGNIQLGAKTDLNVVLNQLCKAFN
SCPFFKHSGMIMRVVDGHIEGYVEMQPNLIGNLAFQVLHGGVAATLLDSIGGIVAMEH
LYRRSTPETLPETIKQVTRLATVDMRVDYLAPGRGKYFIARAEVLRLGRKGCTMRMTM VNDEDKQIAAGIASYAY"
/product="putative protein PaaI, possibly involved in aromatic compounds catabolism" /transl_table=11
CDS 1030237..1031250
/locus_tag="AUO97b_01139" /db_xref="SEED:fig|400667.82.peg.999"
/db_xref="GO:0004655"
/translation="MTYTFNRPAFPATRMRRIRKNDQLRAMVSETQLTTNHLIYPVFV
LPGQNQTQDIPSMPNIQRLSADLLLKKAERLLELGVSKLALFPVTPQEDKSLTAEAAW
REDGLVQTTCRLLKKELPEMVLITDGALDPYTTHGQDGIIDETGYVLNDETVECLIKQ
ALSHAEAGAEVIAPSDMMDGRIGAIRQALEANGHIYTNIMAYSAKYASSFYGPFRDAV
GSASNLKGGNKYNYQMDFANRAEALHEIALDIQEGADMVIVKPGMPYLDVVREVKDTF
GIPTFIYQVSGEYAMLAGAIQNGWLSDSVILESLMCCRRAGADGIWTYFAETAAEKLK EMN"
/product="Porphobilinogen synthase (EC 4.2.1.24)" /EC_number="4.2.1.24"
/transl_table=11
CDS 1031276..1032391
/locus_tag="AUO97b_01140" /db_xref="SEED:fig|400667.82.peg.1000"
/translation="MHIAIIGAGISGLMSALELVEQGCTISIFDQQQAGQAASWAGGG
ILSPMYPWRYVHAVNQLAQFGKASYQAWNQKLYPVTGIDFEIHDTGMLIFDEEDFDIG
LSYAEQHQEPMQRCEYLQRDALEQVNPHISEQFQEAIYFPELSNIRNPRVLQSLISYL
KQHPNVEFFEHTPVKKLIQQGDVIQALQTEDGRKHTADHFVITSGAWSHYWNSQLQLD
IPVEPVQGQMLLFKTPAHWLPTMCMNRVMYLIPRMDGHIVCGSSMAHRGFDTSTDETT
QHNILEACLEMVPELADFPIVHRWAGLRPSSPNGVPYIGKMPEMDNLWANFGHFRNGL
CMGAGSAQLLRQLMLGQPTLVDAKAYSPERLQNKILA"
/product="Glycine oxidase ThiO (EC 1.4.3.19)" /EC_number="1.4.3.19"
/transl_table=11 CDS complement(1032383..1033288)
/locus_tag="AUO97b_01141" /db_xref="SEED:fig|400667.82.peg.1001"
/translation="MNKNVLITGASGFIGTHLIRFLLQKNYNVIAVTRQAGRESDHPA
LQWVQKFEDISTRQIDYVVNLAGANIGEKRWTESRKKHLIESRVNTTQKLYAWLKQSQ
IFPEVIVSGSAIGYYGIDAQEKWTEVCTEQSSPQPIFMSQLCQEWERAALADAQQNTK
IIRLGVVFGQGGGILPKMLLPIRLNLVGQIGHGRQPVVWVHIEDVLNAIEFIFKHPQS
AQIHNVVAPENVTQKVFVEQAAKVLNKKPMLSAPSTVFRCLLGEQSQLILNGQYVKPE
ALQAEGFEFAYPQLKIALENILASG"
/product="Cell division inhibitor SulA"
/transl_table=11
CDS complement(1033377..1033829)
/locus_tag="AUO97b_01142" /db_xref="SEED:fig|400667.82.peg.1002"
/translation="MTKLRSYFLWFFALCIVLTLIVGVIAALLPASVGGILTAVPYLG
AMIFVLFRFLKKERRAPTVPEKKKFTLGFTLIFWGYNLCGVLFGLFLFSRKDPEILQN
FMLYLKQPQFLSIMVIMLLMLAIPLYLITYWFYGKQAQRMANKMFNVS" /product="FIG00350345: hypothetical protein" /transl_table=11
CDS 1034209..1034586
/locus_tag="AUO97b_01143" /db_xref="SEED:fig|400667.82.peg.1003"
/translation="MNNIAYFEIQSSNPARDAQFYQAVFGWQFKLDESLPIEYYRIET
PSIMGGLLKRPAQTPPMEYGTNAFTCSVQVENFDEVAAKILAQGGIVAMDKFAIPGRA
WHGYFVDLDHNVFGIFQVDENAA"
/product="Glyoxalase family protein"
/transl_table=11
CDS complement(1034654..1035145)
/locus_tag="AUO97b_01144" /db_xref="SEED:fig|400667.82.peg.1004"
/db_xref="GO:0006950"
/translation="MNFENKMTIHKYEVKIQWEGNTGTGTSSYRSYKRDFSIQHSQKT

TIQGSSDPAYLGDVTRWNPEDLLVASASACHKLWYLHLCAVNHIHVVSYVDHALGFME
DTDPVKRGHFTQIILRPEVVLEKGADQELAAKLHEEAHHECMIANSVNFPITCEASFS FAE"
/product="OsmC/Ohr family protein"
/transl_table=11
CDS 1035292..1036560
/locus_tag="AUO97b_01145" /db_xref="SEED:fig|400667.82.peg.1005"
/db_xref="GO:0016787"
/translation="MSVSMSVHFLHTSDWHLGQFFYNHSRHYEHQQFLSWLLTQIQEK
QPHALLIAGDIFDVINPGSQAQKQLYQFLADAHRIAPHMQTLMIAGNHDSGYRIEQVE
PLLEKYNAKTVGVVRWNEDKTLDLDRLLLPIYNQNQDIVAWCLALPFLRSAEITGFNE
HTTNSKNAIAYLHQQLIAEAKRRKTPDQALILMSHAHMQGGETSDSERPIIIGNEEAL
STTLFEDAVDYVALGHLHKPQKVGQPHIRYSGSPIPLSFSEINYKHQVVEVKIDPSQD
TDSRLQFEAVEIPRCIQLHRIRGELNEVLQQLKALPHGVIENIDHREYVDIEYYSLTP
PQPNLRQQFEAALPPDRYRLVRISRQYVNKDATNSNTTQHIALEPPTPEKLFQNIWEK
QGYSADDAVLKDFLSLVQEAQKHLENDASH" /product="Exonuclease SbcD"
/transl_table=11
CDS 1036570..1040166
/locus_tag="AUO97b_01146" /db_xref="SEED:fig|400667.82.peg.1006"
/db_xref="GO:0005524"
/db_xref="GO:0005694"
/db_xref="GO:0051276"
/translation="MKILSIRIKNLASLADEHFIDFESAPLAHAGLIAIVGKTGAGKS
TILDAMCLALFNRVPRLKDSDGKLKDVDGSELLTNSPLTVLRRGTGHGFAELCFIAQD
QKRYLARWEIKRARENPNGKLQSVQRHLKCLTDGVVLADKAKAVDEKVKQITQLSFEQ
FTRAVLLAQSEVTAFLKARDSERGELLEYLTNSSIFAKIGELAFRKTADIAKQRKQLE
EFLGHIEILSDEEIAAFTEQYQQAEQNYQQLEQQKHVLDKQQQWFERKAKLEQEVQAK
QQQFQTQQNHHQQLASEREQLKRLEVFSEIRPQVFQQAQNLQTLQQLEPQIQQAQTKF
NELVQIFETGQKQYQLAEQQLKQTLDFEQQHQHALNQVRQSIQERAFIADEYKKCKEK
RQVIEQNLSPLQQQQNAVQQQVAQLEQNKIHLQQQLLQTQQYAVLDKGLSAHLHQLGQ
FIQNYQAIEEQLGNPTLAHQKLSEAKSELEQLTASLGTVEQIELKLEQQRKDKEQKLA
QVTQLDLIQQKIKIYHELYAELQHFSEKYTQASTQEEQLKTVCQLAEQDYQTAKNERE
KLQHILQQQRLLHTENIEQLRANLKEGEACLVCGSIHHPYRVDNSAVSKALFDLQQQQ
EQQAIALEQTKFNAWQTQQHALTQCHAELEQVQKYLAQLQTKQSSLQQELKQAFNLNQ
LHIELNQAPEQILQTLNELRQATQTAISLFDSENARLTQAIKQHNQLIQTIQRNESLL
NTAQQWQQQVQHIVECLSETEQHAWQQASSQTAKQTWAILDARAKQLEQQEQLSQRFE
QQQQELKMLTASLEQMTKQIDEIDQNLQEITLKGQQNNEKAVSLIQQMTGRSDIKPHE
WLIEHDAKRQHQQTAYHEAKQRFEQTRQHFEQQKQALDQLKHQYQHTEQHQQQIDGQI
QNWLKAHTDFQASDLTALIQINSAQEQDIRNRLNHAERLLSEASSALKTMQEQLSEHL
QTQPDIEYEKLVTLIQDNIAKLKAQLEVRDGLKLKLELHQQNLAKQQKYAEQIQNIQQ
EEHRWSKISGLIGDAKGKEFRDYAQQYHLDILVEHANQQLAMLSQRYTLKRLDQSLSL
AIIDHDMDGETRSVASLSGGESFLTALALSLAIANMASGSMKIESLFIDEGFGTLDAS
SLHMVMNALDQLQNQGRQVILISHIQEMHERIPVQIQVKPLGAGASTIEIVG" /product="Exonuclease
SbcC"
/transl_table=11
CDS complement(1040368..1041060)
/locus_tag="AUO97b_01147" /db_xref="SEED:fig|400667.82.peg.1007"
/translation="MNYLQDARQHVLQQIRTACELAQRAPETVQLLAVSKTHPSERLR
EMYAAGQRAFGENYLQEALDKIDALQDLDIEWHFIGHVQRNKTKHLAEKFDWVHGVDR
LIIAERLSNQCGDDQAALNICLQVNIDGQESKDGCAPEDVAELVAQMSQLPKIKLRGL
MVIPAPDNTAAFADAKKLFDAVKVQHAHPEDWDTLSMGMSSDLEAAIAAGSTMVRVGT ALFGARDYSQKG"
/product="Pyridoxal phosphate-containing protein YggS" /transl_table=11
CDS 1041187..1042224
/locus_tag="AUO97b_01148" /db_xref="SEED:fig|400667.82.peg.1008"
/db_xref="GO:0005524"

/db_xref="GO:0005622" /db_xref="GO:0006810"
/translation="MDITELLAFSVKNGASDLHLSAGMPPMIRVDGEVRRINLPALEH
KDVHRLVYDIMNDKQRRDYEEKLETDFSFEVPNVARFRVNAFNQNRGAGAVFRTIPSK
VLTMEDLGLGQIFKDICDYPRGIVLVTGPTGSGKSTTLAAMLDYINENRYDHILTVED
PIEFVHQSKKCLINQREVHRDTHGFNEALRSALREDPDIILVGEMRDLETIRLALTAA
ETGHLVFGTLHTTSAAKTIDRVIDVFPAEEKDMVRAMLSESLQAVISQTLLKKNGGGR
VAAHEIMIGIPAIRNLIRENKVAQMYSAIQTGANHGMTTLDQSLKGLVARGVISPQTA RTAAKQPESFL"
/product="Twitching motility protein PilT"
/transl_table=11
CDS 1042252..1043370
/locus_tag="AUO97b_01149" /db_xref="SEED:fig|400667.82.peg.1009"
/db_xref="GO:0005524"
/db_xref="GO:0005622"
/db_xref="GO:0006810"
/translation="MDFNDLLNLMVEKKSSDLFITDGVAPSMKINGQIVPISKNSLSG
EVIGQLLHSIMSEKQRKEFADTRECNFAIMNREKTARFRVSAFQQRDMPGMVLRRIET
KIPSIDDLQLPPVLKDLSMTKRGIIIFVGATGTGKSTSLASMISHRNHNSKGHIITIE
DPIEFIHEHAGCIITQREVGIDTDSFEIALKNTLRQAPDVILIGEIRSREVMDYAIGF
AETGHLVLATMHANNANQALDRIIHFFESDRHSQLYMDLSLNLKAMIAQQLIPTPDGN
SRRAAIEILINSPLISDYIRKGEIHEIKDLMKRSRELGMQTFDQALFDLYKAGQITYK
DALKHADSPNDLRLTIKLADEGPDQLSDGKQHLTFDRQ"
/product="Type IV pilus assembly ATPase component PilU" /transl_table=11
CDS complement(1043440..1043877)
/locus_tag="AUO97b_01150" /db_xref="SEED:fig|400667.82.peg.1010"
/db_xref="GO:0003700"
/translation="MPISNQDLRKAGLKVTLPRIKILELLENSKQHHLSAEDIYKTLL
EQGEDVGLATVYRVLTQFEAAGIIQRHHFENNHSVFEIMQEDHHDHLVCQNCNKVIEF
TNDVIEKEQHSVAEQHGFTLTGHSLNLYGYCNEPECQEALRKK" /product="Ferric uptake
regulation protein FUR" /transl_table=11
CDS 1043989..1044387
/locus_tag="AUO97b_01151" /db_xref="SEED:fig|400667.82.peg.1011"
/translation="MQKLVLTLFVTSLLAGCSIFGVYKVDIPQGTPLTKAQASQVQVG
MNFQQVRFLLGSPTVTDPLNPQRWDYIYNYIPGTYAKKAKIPAAHGQHLKIYFDGTGT
VTKIEGLETIPESQPGLPASKEAILTAPPL"
/product="Outer membrane beta-barrel assembly protein BamE" /transl_table=11
CDS complement(1044442..1044765)
/locus_tag="AUO97b_01152" /db_xref="SEED:fig|400667.82.peg.1012"
/translation="MERTQQVWVAFAVPEQQFLISVPFEEGMTAAQAVQASGLSNQVD
LPEPLQLGIFGVKVETVAVLHAGDRVEVYRPLTINPKDIRRKRAAKNPVGRYIKGNRF KQSAQ"
/product="UPF0125 protein RatB"
/transl_table=11 CDS complement(1044765..1045808)
/locus_tag="AUO97b_01153" /db_xref="SEED:fig|400667.82.peg.1013"
/translation="MAFCLSFVIMATLAPTMGIQIDRQIDFWLLWFGTMLLLALPVCY
LEIALAKRSKTTALNALSSLTREADSSPKWRVVGWLAVVFIPFLAGNVLSTASNILVA
QFAPSISGQIIFAGLAVAALVLSFIPRQILILLMTLGVIASIVLANTMGSTLQPWHWT
SVEFKEWGNATVLALVASGLGLGLYWQNSVGAVQAQEGATKTVLPIWLAQLIAVVAFG
FFSLQAQLPVLTWIFTGVMTSALFVQLAREQLAQRQLMPVLQWVIIVVAIAVWAVPEV
HNLFTLILMLWGLLICLIYAVFAGWIMKISHLRKSMNFSNELFYNLWRIAVRIVLPLS IIVAMIAVIGQSI"
/product="FIG00351543: hypothetical protein" /transl_table=11
CDS complement(1045985..1046440)
/locus_tag="AUO97b_01154" /db_xref="SEED:fig|400667.82.peg.1014"
/translation="MKMKWASEYNTGIDVIDEQHKRILDYINEIDDVKDDEDRRRIKD
VLDNIIDYTQSHFTFEESLQEEADYKYRVPHKRVHDLFIKKIELYRERFEMGHTIEAE
LQEILAKWLINHIQHDDADYVGAVKENMMGIIREKEKKKGKNWFARFFS" /product="Hemerythrin

domain protein"
/transl_table=11
CDS complement(1046693..1047523)
/locus_tag="AUO97b_01155" /db_xref="SEED:fig|400667.82.peg.1015"
/translation="MNCATPTDITFSFRGFSQRAGITSDHSDGFGIAFFEDKACRLFV
DNQSAVESPIADLIRNYPIKSRNVIAHIRKATQGKITLENSHPFIRELWGRHWIFAHN
GDLHDFNPPLSGRFTPVGNTDSERAFCYLLDQLVEVFGYEEPTLEQIFEVLEKISPQI
AEYGTFNFCLSNGQALFSYAITKLHWLVREYPFNNAHLIDLDVEVDFSQVTTPEDRVA
VITTEPLTHNETWIAYQPGEMILFQHGKPIKKAITQVERLIREEKNPELKRITRADQY "
/product="Putative glutamine amidotransferase YafJ" /transl_table=11
CDS complement(1047650..1048432) /locus_tag="AUO97b_01156"
/db_xref="SEED:fig|400667.82.peg.1016"
/translation="MLEKFNQYRQTWTLPLNRHKANNQTQFRFEWVDNLKELQDVQRF
RAQQFSKQFGIQFEDNLDQDIYDFGCEHAVLREKWTGEIVAYTRLKLFQGHEIGQSYS
ANEFDIVPHLSHLPNVLEIGRTCVHPRFRNGKALSTLWLNLAPKVLWSMRAKYLMGCV
SIHLQDNLARAYYTHRQIQQLPDTKTIDIRSKKIYEPEYPEFSFPQDERMPKLFQMYL
SMQSKLSKDAFFDAEFNCLDYFVFLEVNKIATSFVMNKMAQR" /product="FIG00350997:
hypothetical protein" /transl_table=11
CDS complement(1048553..1049461)
/locus_tag="AUO97b_01157" /db_xref="SEED:fig|400667.82.peg.1017"
/db_xref="GO:0003991"
/translation="MPQDQHLGVDKAKILIEALPYIQRFSGKTLVVKYGGNAMTDPEL
ESSFARDIVLLKTVGLNPIVVHGGGPQVDSFLKQLGRESDRIDGMRVTDEATMEVVEM
VLGGSVNKSIVNLINKHGGRAIGLTGQDGNLLRARKLLMEKQEEDGSIKHIDLGMVGE
VTGVKTDVLEMFTQSDFIPVIAPLGVDEKGNTYNINADLVAGKVAEALGAEKLILLTN
ISGVLDENKNLLTGLTTQEVDRLIETGVIYGGMIPKVGCALDAVKGGVVSAHIVDGRV
PHATLLEIFTDHGVGTLISNRTQTTH"
/product="N-acetylglutamate kinase (EC 2.7.2.8)" /EC_number="2.7.2.8"
/transl_table=11
CDS complement(1049476..1050894)
/locus_tag="AUO97b_01158" /db_xref="SEED:fig|400667.82.peg.1018"
/db_xref="GO:0004615"
/translation="MNVRHSFPKNIFRAYDIRGKLSYLTTDVVRSIAYGLAQQYKQAE
QTQLIIGYDARLTSPAYAHLIEEILVEQGLNVTNIGCCSTPMMYYIAREFGGNGIMVT
ASHNPKSDNGIKWILRGEPPSPEMIQQVGEFAQTYVPTHTISLLELSTPQFNSEFCKK
YQQAIFNDIQLKRPLKVVLDGLHGSAGHCSKLVLEKMGCEVIALRTTPNGEFPDHAPD
PSHAAHLKELRKTIIEQGADIGIALDGDGDRVVLLDEKANILTADRLLSLFAQMCLEQ
QPDKEIVFDVKCSLMVQRTVERLGGKPKMIRTGSSFLRAYLSQSNGNAIFGGEYAGHY
VFNDGRGFGYDDGLYAALRVMEYFTESSAATISDLFSNYPERCCTEDTYIGTHHSDPK
HVLQDIEILSHRLGARISKIDGVRLDFDDGFGIIRASNTGEYFTVRFDADNPLRLKEI
QQKFIDMLQERYPQIAQELSEA"
/product="Phosphoglucomutase (EC 5.4.2.2) @ Phosphomannomutase (EC 5.4.2.8)"
/EC_number="5.4.2.8"
/transl_table=11
CDS complement(1051094..1051546)
/locus_tag="AUO97b_01159" /db_xref="SEED:fig|400667.82.peg.1019"
/db_xref="GO:0004170"
/translation="MKVQVKLLDPRLGKEWPLPSYATAGSAGLDLRACLDEAIEIEPG
QTVLVKTGMAIYIHDVNFAGLILPRSGLGHKHGIVLGNLVGLIDSDYQGELMVSVWNR
GQTTFRLEPGERLAQYVLVPVVQAEFEQVEEFEETLRGAGGFGHTGKQ" /product="Deoxyuridine 5'-
triphosphate nucleotidohydrolase (EC 3.6.1.23)" /EC_number="3.6.1.23"
/transl_table=11
CDS complement(1051571..1053610)
/locus_tag="AUO97b_01160" /db_xref="SEED:fig|400667.82.peg.1020"

/translation="MSSASQLNNKQEEAMKYTQGPLLVLAGAGSGKTSVITRKIAYLV
QHCRIPAHRITAMTFTNKAAREMKERVTKLLSREEAKGLSVSTFHTFGLNLLRLELKN
LPLKANFSILDADDCKRILMDLMHRDNLSGAESKELIAKAMKKISDWKNDLILPEQAH
STCETPEDVQFAHLYQLYERNLRAYNAVDFDDLIVMPTRLLQENAEVRDKWQNRVRYL
LVDEYQDTNTAQYILVKLLVGVMGQFTAVGDDDQSIYAWRGAKPENMALLKQDFPNLH
IIKLEQNYRSTSRILKAANCVIQNNPHIFDKKLWSDKGHGEVIRIITCRNDDDEAERV
VKDLLTHKLMNGKNWKDYAVLYRGNFQARVLETQLRQMQIPYKLSGGTSFFARAEIKD
VMSYLRLIINPEDDSAFLRIINTPKRAIGPVTLEKLGLFAQENNLSLLGASSDQRLSM
VLPKKAETQLHEFADFISTFTRELLEDDEPVPKVRQMMNEAGYIDYIREQSATPAQEK
SKLDNIENLFSSIQNLINRAEDVDEKNIESVIRKLVLLDMLEQQQEEEDTDKVNLLTL
HAAKGLEFPYVYIMGLEEELLPHKNSIAAETIEEERRLMYVGITRARQGLTLTLAEQR
KNGGQMKQMTPSRFLDELPQDELEWLGRKKKIAANVDPKEQAQQYLANLKALLKR" /product="ATP-dependent DNA helicase Rep"
/transl_table=11
CDS complement(1053804..1054457)
/locus_tag="AUO97b_01161" /db_xref="SEED:fig|400667.82.peg.1021"
/translation="MRALVISTVVGAAVVLSGCQTTGNNLGGVEYDKAALGTLIGAAA
GYGISKSNANSSRQNNRAAAIGAVLGAAGGLYLDQKEKKLREQMAGTGVEVGRNPDGS
VQLIMPGSITFDTNKSNIKPNFYATLDKVAQTLAEDNKSAILVTGYTDNTGNDSINIP
LSQARAQSVKNYLAGKGVPSSRIDAQGYGSSNPIADNSTASGREQNRRVEISIYAKQ" /product="Outer membrane porin, OmpA family" /transl_table=11 CDS 1054702..1055628
/locus_tag="AUO97b_01162" /db_xref="SEED:fig|400667.82.peg.1022"
/translation="MSTAPNKQSALKKITRGLTVGTVITGSTFFHGPPVLALGLTKLF
KKSSKIDETNIQITNSWLGVNNWLIDHVLPNLKWDISIDEDLDLNMQGRYLMTCNHQS
WVDTTVNQYFGLTRMPLTRFFTKWELIFIPFVGQAFKILGFPMMKRHTKEQIAKNPEL
KDRDMMEARKACEQLLSQPFTLLNYLEGTRFTEEKHDQQKSPYKHLLKPKAGGLALAL
NILGDKIDALVDMTIVYPDGVPGYGDFWLGDVSKIAVNLRKIEIPDWVLGGNYEDDPV
YRERFQQWVHELWTEKDQLIEKMKAQYATTNL"
/product="Probable acyltransferase PA0834" /EC_number="2.7.7.19"
/transl_table=11
CDS 1055645..1056691
/locus_tag="AUO97b_01163" /db_xref="SEED:fig|400667.82.peg.1023"
/translation="MQNSSGKQTKYHRVDSSSLSFKLFLVYDIFMVFIIIFNLFCLAA
NFFLMSSIGAWFFEHIHLPQVLSFYRTHLHPWVITSEAWFIGFLIIELLVRWGIAIVY
KHHKRWFFFPFIHWYEILAIIPQLRFLRLFRAGIIAYRLHELGYPVIPESWRKTGLFY
YRVVMEELSDRVVITVIDGIRYELETSSSHKQIIHDLVNHHREQFTVTLTALLQESLA
TALKEQKPAITRAVGQIVDQAIEDTPELTQLLRLIPLVGGRIEQQIQSIGQRLGENIS
AGLIEPLIEGSAAHPNSTYQLISQKISHINIDNHELEQLVESVVFETLEAVRKQVKVK QWQQTLAEYDRIKE"
/product="putative membrane protein"
/transl_table=11
CDS 1056813..1057535
/locus_tag="AUO97b_01164" /db_xref="SEED:fig|400667.82.peg.1024"
/translation="MADIRITGRMVNFSRITFDTNDHDVIRQQLSNILNEGSYQGTVV
IIDSTVEQELIALIQLLVSMGLQPMAVIDGILGDEARAIQFPVLPADQPLQRIKPTAE
QVAIVEKPTSAQASVETKKPLNNNAVAHITSYHDEILRTGQSLVQDQGDIILKAGMNS
GSEVIASGNIHIYGTVRGRVIAGAGGHAAARIFCQSLEAELVSIAGTYCVADDIPKHV
VKKPVHIYLNEKQELEFEALEL"
/product="Septum site-determining protein MinC" /transl_table=11
CDS 1057606..1058418
/locus_tag="AUO97b_01165" /db_xref="SEED:fig|400667.82.peg.1025"
/translation="MAKIVVVTSGKGGVGKTTTSASFATGLALRGHKTVVIDFDVGLR
NLDLIMGCERRVVYDFVNVINNEARLQQALIRDKDIENLYILPASQTRDKDALSDEGV
ARVIDELSQEFDYIICDSPAGIERGAILAMYHADEAIIVTNPEISSVRDSDRIIGMLD
SKTKKVEHNEGRIRKHLCITRFNPERADRQEMLTIDDISKDILRVPTLGVIPECPSVL

QASNEGKPVILYSETKAGQAYDDLVARFLGEDRPYRHITAQPKGWLARLFGA" /product="Septum site-determining protein MinD" /transl_table=11
CDS 1058421..1058693
/locus_tag="AUO97b_01166" /db_xref="SEED:fig|400667.82.peg.1026"
/translation="MAGFWSKLFSSEEKPSSAQTAKDRLKVIVASEQGLGRRLSQDKI
DQMKKEIMQVVSRYVSGVGEQHIQMQVRSEANIEMLEMNINLPEER" /product="Cell division topological specificity factor MinE" /transl_table=11
CDS 1058777..1059064
/locus_tag="AUO97b_01167" /db_xref="SEED:fig|400667.82.peg.1027"
/translation="MALSQPATFNEEWSDERVFAYLNQLPPNGVNADFHVLYHAFKHM
RPNDYERLLTKFVADGRDINATNPEGERIHDVIAKFPRQSAPFLEVLAKFA" /product="FIG00350596: hypothetical protein" /transl_table=11
CDS complement(1059107..1059727)
/locus_tag="AUO97b_01168" /db_xref="SEED:fig|400667.82.peg.1028"
/db_xref="GO:0005293"
/db_xref="GO:0006865"
/db_xref="GO:0016020"
/translation="MLVALITLAFIHFCALITPGPDFFLVSQTAVSRSRKEAMLVVAG
ITAGVMFWASLALMGLNIIFEKMAWLKQGLLIAGGLYLCWLGYQMLRSAFSKNQQEEV
TTVELPKAPYLFFMKGLLTNLSNPKAVIYFGSVFSLFLANPQLDQVHWLLFIIVSVET
ILWFCFVTFIFSLPSFRAAYRNFSKWIDGISGGIFTVFGIFLIGNR" /product="Threonine efflux protein RhtC"
/transl_table=11
CDS complement(1059877..1062837)
/locus_tag="AUO97b_01169" /db_xref="SEED:fig|400667.82.peg.1029"
/translation="MFLATVTYTPFDPGWMHISSDTQQVSNASGIAGAWIADLLFGFL
GWASLLIPIFLFVEAIQVWWPHSFLNRPFRYAAQFFLILVVSSLLYLHWNVPADTLDN
AAGGIIGYELGQSLSQLLTIYGATLFLLVFGVVLFTLAFGVQWSKTWVTLKAMPGYLQ
DLFYKNVSPNESAYDLTTQPANKAATVKVAEMPKAEEVTDEVELNAQPEKAQSKASST
RHDEIAERLFADVLAKEQSQPETIEEKTTPQPENFERTLEQAQQLKKDSERLVATGEV
WRALQRDDVSHKQEIDALLRAADDSSEQPSTHEQFQQTVYQAKQNQPSNTHQGLDWND
DEIFDELLAAVPNSKTATDVHTPFVQDHHVEPEVTPQAPQTLVESSVPVSNLNQSPKN
LSNEEVFDDFDDLLIDEDIPPATQPIKASSYAQSSAFVKAPIQTTIGAEKLSKEEFIE
AWQETAGKPQDDLEDEFDFDAPLTDASGRPMSRAMQVAKKRLDLPTLPGFELLDKVDP
NKKVNFTEEQLSRLSELLEIKLQEFNVKAQVVEAQPGPVVTRFELDLAPGVKASKVTN
ISRDLARSMSMASVRVVEVIPGKPYIGIEVPNSAREMVRLIELLETPAYRDPSALISM
AMGKDISGNPVLTDLAKAPHMLVAGTTGSGKSVAVNSMILSMLLKYTPDQLRLILIDP
KQLELANYNDIPHLLTPVVTDMKDAVSALNWCVNEMERRYKLMSFLKIRKLSDYNRKV
EEALANGEDLIDPTWKPSDSATQERAPRLTPLPSIVIVADEFADMIMQVGKKAEEMIT
RLAQKSRAAGIHLLLATQRPSVDVITGLIKANIPTRVALRVNSKIDSRTILDAGGAED
LLGHGDMLFLGPGKIEPERVHGAFISDDEVNRICDAWRERGEPDYVDEILTPFDEEPA
SRGFEEGDGGSDRDALYDQCVSFVLETRKASTSSLQRKFSLGYNRAARIIDQMEENGI VSAMGANGKRDILV"
/product="DNA translocase FtsK"
/transl_table=11 CDS 1063179..1064126
/locus_tag="AUO97b_01170" /db_xref="SEED:fig|400667.82.peg.1030"
/db_xref="GO:0004791"
/translation="MSARHSRLIILGSGPAGYSAAVYAARANLKPTLIAGLQLGGQLT
TTTEVDNWPGDPEGLTGPVLMDRMQAHAERFGTELVYDHINEVDLNVRPFVLKGDMDE
YTCDALIIATGATAQYLGLDSEQKFMGQGVSACATCDGFFYKNQNVMVVGGGNTAVEE
ALYLSNIAEHVTLVHRRDSLRSEKILQDHLFAKEKEGKISIVWNHEVEEVLGDNTGVT
GVRLKSTKDDSKQEVQVQGLFIAIGHKPNTSMFEGQLNLRDGYIQVQSGTSGNATATS
VAGVFAAGDVADSIYRQAITSAGSGCMAALDAEKYLDNL" /product="Thioredoxin reductase (EC 1.8.1.9)" /EC_number="1.8.1.9"
/transl_table=11

CDS 1064247..1064978
/locus_tag="AUO97b_01171" /db_xref="SEED:fig|400667.82.peg.1031"
/db_xref="GO:0008914"
/translation="MKTLPLSQYIFPDPEESDPDGHGLICIGADLSPSTLYEAYTHGL
FPWFNEDEPICWWSPEPRCIIYPQNYKPSKSLIRNMKKYDYTITVNRAFEQVIRSCSL
PRSYANETWISEDIIEGYCGMFDAGYGYSVEVWQEEQLVGGLYGVTIGKGCFGESMFS
TQTDVSKMAFYTLMLIGQENQLPWVDCQLVNSHLISLGACTLSRQEYLKSLQDVIIHP
SINWKKYQERVFSSKTIALNAKLME" /product="Leucyl/phenylalanyl-tRNA--protein
transferase (EC 2.3.2.6)" /EC_number="2.3.2.6"
/transl_table=11
CDS 1064997..1065812
/locus_tag="AUO97b_01172" /db_xref="SEED:fig|400667.82.peg.1032"
/translation="MKSYHPKSLLNDLQYYITPPHDCSYLENKSARMVFLDPIHRIDV
VTLSELSRLGFRRSGDFVYRPECHLCRQCLSCRVPVADFQMNSMQKKAWKRNQDLTMT
VLPTRQASQIHYDLYERYINERHADGDMFPPSLDQFEKFLVHSCTDSFFLELWKDNRL
ISVSTCDLMDDGLSAVYTFFDPDEHRRSLGVYSILNQIEYVKTLGLEYVYLGYWVPHS
AKMNYKSQYTPLELLLDGQWRRLNRSLSPEEINQLGNSLMTTLPSEWNNLIIK" /product="Arginyl-
tRNA--protein transferase (EC 2.3.2.8)" /EC_number="2.3.2.8"
/transl_table=11
CDS complement(1065857..1066312)
/locus_tag="AUO97b_01173" /db_xref="SEED:fig|400667.82.peg.1033"
/db_xref="GO:0008080"
/translation="MIRTMQASDIDSVAQIEKLVQTHPWSRQQFVESLNSYQCTVIEL
NNKVVGFCILQPVLDEANLLLMAIDPQMQGKGLGYQLLDASIERLENHPVQIFLEVRE
SNKAAIGLYEKAGFHQIDVRRNYYPTQEGGRENAIIMVKSCTDDFASLF" /product="Ribosomal-
protein-S18p-alanine acetyltransferase (EC 2.3.1.128)" /transl_table=11
CDS complement(1066421..1067296)
/locus_tag="AUO97b_01174" /db_xref="SEED:fig|400667.82.peg.1034"
/translation="MNAKVNISNRAGASFPVRRMDFEFAQVPRYWANYDAGLTHFMTA
LSALFPEGEQFFVNSTRAVRNDPKLADPALQKEISAFIGQEAMHSKEHLAFNASAQAY
GYDVRKMEKQTGKVIKVGTAVVTKLMKPFGYTKEMIDLTGTCALEHFTSTIAAELLQN
PEIQAMFQDDTMYHLWMWHAVEENEHKAVAFDVYTNMYGQGPKAYFMRSTALIIAMAL
IFATQSYFTAKLLKTDDKLTWKDTKYMLKFMYGSKGFMTRQIPELLDFLRPKFHPNDS DTTALLATWREKLGL"
/product="FIG00350000: hypothetical protein" /transl_table=11
CDS complement(1067533..1068414)
/locus_tag="AUO97b_01175" /db_xref="SEED:fig|400667.82.peg.1035"
/translation="MNAKVNITNRAGASFPVRRMNFDFNDVPEYWMNGSAGLTHFMTA
LSALFPAGEKFFIDSVRAVRYHPAIKDNEELQKEISAFIGQEAMHTQEHVNFNASAQK
FGHDVETLEKFTDTAIQTARKTFAKLVKPFGMTQEMVDLTATTALEHFTATIASQLLV
NTHIQELMTDKTMSTMWYWHAIEENEHKAVAFDVYEGVFGKGVKAYALRTSSLVFAMA
LIFAIQSSFVVRLLKQDHKLNLDELFVIYKYGYSPSKGIITGMAKEMLAYFKPGFHPN DLDTVSLLKTWKSKLGL"
/product="FIG00350000: hypothetical protein" /transl_table=11
CDS complement(1068618..1069808)
/locus_tag="AUO97b_01176" /db_xref="SEED:fig|400667.82.peg.1036"
/db_xref="GO:0005525"
/db_xref="GO:0006412"
/translation="MAKAKFERNKPHVNVGTIGHVDHGKTTLTAAIATICAKTYGGEA
KDYSQIDSAPEEKARGITINTSHVEYDSPTRHYAHVDCPGHADYVKNMITGAAQMDGA
ILVCAATDGPMPQTREHILLSRQVGVPYIIVFLNKCDLVDDEELLELVEMEVRELLST
YDFPGDDTPVIRGSALAALNGEAGPYGEESVLALVAALDSYIPEPERAIDKAFLMPIE
DVFSISGRGTVVTGRVEAGIIKVGEEVEIVGIKDTVKTTVTGVEMFRKLLDEGRAGEN
CGILLRGTKREEVQRGQVLAKPGTIKPHTKFDAEVYVLSKEEGGRHTPFLNGYRPQFY
FRTTDVTGAIQLKEGVEMVMPGDNVEMSVELIHPIAMDPGLRFAIREGGRTVGAGVVA KVTA"
/product="Translation elongation factor Tu" /transl_table=11

CDS complement(1069903..1072041)
/locus_tag="AUO97b_01177" /db_xref="SEED:fig|400667.82.peg.1037"
/db_xref="GO:0005525"
/db_xref="GO:0006412"
/translation="MARQTPITRYRNIGISAHIDAGKTTTTERILFYTGVSHKIGEVH
DGAATMDWMEQEQERGITITSAATTCFWSGMGNQFPQHRINVIDTPGHVDFTIEVERS
MRVLDGACMVYCAVGGVQPQSETVWRQANKYKVPRLAFVNKMDRTGANFFRVVEQMKT
RLGANPVPIVVPIGAEDTFTGVVDLIEMKAIIWDEASQGMKFEYGEIPADLVDTAQEW
RTNMVEAAAEASEELMDKYLEEGDLSKEDIIAGLRARTLASEIQVMLCGSAFKNKGVQ
RMLDAVIEFLPSPTEVKAIEGILDDKDETKASREASDEAPFSALAFKIMNDKFVGNLT
FVRVYSGVLKQGDAVYNPVKSKRERIGRIVQMHANERQDIDEIRAGDIAACVGLKDVT
TGDTLCDEKNIITLERMEFPDPVIQLAVEPKTKADQEKMSIALGRLAKEDPSFRVHTD
EESGQTIIAGMGELHLDIIVDRMKREFGVEANIGKPMVAYRETIKKTVEQEGKFVRQT
GGKGKFGHVYVRLEPLDVEAAGKEYEFAEEVVGGVVPKEFFGAVDKGIQERMKNGVLA
GYPVVGVKAVLFDGSYHDVDSDELSFKMAGSYAFRDGFMKADPVLLEPIMKVEVETPE
DYMGDIMGDLNRRRGMVQGMDDLPGGTKAIKAEVPLAEMFGYATQMRSMSQGRATYSM
EFAKYAETPRNVAEGIIAKFQAGGKKGDDE"
/product="Translation elongation factor G"
/transl_table=11
CDS complement(1072220..1072690)
/locus_tag="AUO97b_01178" /db_xref="SEED:fig|400667.82.peg.1038"
/db_xref="GO:0003735"
/db_xref="GO:0005622"
/db_xref="GO:0005840"
/db_xref="GO:0006412"
/translation="MPRRRVVAAREILPDPKFSSQTIAKFMNHVMQDGKKSIAESIVY
GALERVQEKNKVDPVEFFETTLEKVRPMVEVKARRVGGATYQVPMEVRPSRRTALAMR
WLVDAAAKRSEKTMALRLAGELLDAAEGKGAAIKKREDVHRMAEANKAFSHYRF" /product="SSU
ribosomal protein S7p (S5e)"
/transl_table=11
CDS complement(1072850..1073224)
/locus_tag="AUO97b_01179" /db_xref="SEED:fig|400667.82.peg.1039"
/db_xref="GO:0003735"
/db_xref="GO:0005622"
/db_xref="GO:0005840"
/db_xref="GO:0006412"
/translation="MATTNQLIRKGRTTLVEKSKVPALKACPQRRGVCTRVYTTTPKK
PNSAMRKVCRVRLTSGFEVSSYIGGEGHNLQEHSVVLIRGGRVKDLPGVRYHTVRGSL
DCAGVKDRNQSRSKYGAKRPKK"
/product="SSU ribosomal protein S12p (S23e)" /transl_table=11
CDS complement(1073425..1074750)
/locus_tag="AUO97b_01180" /db_xref="SEED:fig|400667.82.peg.1040"
/translation="MTKFNNNQSDNLFSQVFGVAKKLSSTGLNILQQSQIGEVSKLVE
PLSNGKTVEGSARNKSPFEVEQYESPQQMLREHLPKVTRQVFGRHFRKVNGIATFISP
DWNEKISSYLFDWLNDFSSKSTLTEKILEEAGAKDLFELTKDTSRSQRLSQALIEQNK
LIASIQGAITGVSGMVGAAVDIPVSLVLVLRTIYQTGRSHGFDLTEATDQDVVEFIFK
EVDISLIAEKQTLLLALKALRNMLETQDIQQFQQVLGSSNDIETLKSWLVDENGEFKW
NWLNKVPQLAVVGKFTPVAGAVLSAVYSWKLQEDVGHKAQAIFGAARHYLNEHPNEHL
SPLQAYYAAVTLIQKASPRLLNVGENGSVHATQHHKIENHDVISKVSVVVKSNTSEKS
EEKVQENVHQGIEHLAEKHVVEHEHSEQKPALEPESEENDDVIEGQKYS" /product="FIG00351038:
hypothetical protein" /transl_table=11
CDS 1075047..1076276
/locus_tag="AUO97b_01181" /db_xref="SEED:fig|400667.82.peg.1041"
/db_xref="GO:0004315"

/translation="MKRVVITGMGINSCIGNSLEEVTHSLKNGISGTRFNPTYAELNF
KSHVSAAAEQDFDNIDRKLKRFMGVCAMYAYNSAVAAVEHAGLKAEDLADNPRYGIAG
GSGGGSTASVVEMTELLETKGARKVGPFFVPRNMTNTITANVGVAFKLQGIAHSIASA
CATSADAIGYAYNLIALGKQDLMLAGGGEEDHWSQSLLFDAMGALCSKYNDTPETASR
PYSKDRDGFVIAGGGGFVVLESLEHAQARGANILAEVVAYAANSDGADMVAPSGEGAT
RCILMALEEAKQHGVDKIDYVNTHGTSTPAGDVTELKAMERAFGEGKVPPLSSTKSMT
GHSLGAAGVHEAIYSVLMLQNDFIAPNINVTELDEGTEGFDIVLEKRDTKLNTVMSNS FGFGGVNACLIFKKWDA"
/product="3-oxoacyl-[acyl-carrier-protein] synthase, KASI (EC 2.3.1.41)"
/EC_number="2.3.1.41"
/transl_table=11
CDS 1076295..1076747
/locus_tag="AUO97b_01182" /db_xref="SEED:fig|400667.82.peg.1042"
/translation="MDAPSDAFLWVKALHIIAVVCWFAALFYLPRLYVYHAMSDDATS
HQRFEVMERKLYRGIMWPSMIATLITAHFLVDWGDATRHYHEATWFYLKVGLVALLVI
YHLVCGYYRKKLIGNAHYKSHKFWRFFNEMPTLILFAVVILVVVKPQF"
/product="Protoporphyrinogen IX oxidase, novel form, HemJ (EC 1.3.-.-)"
/transl_table=11
CDS 1076918..1077778
/locus_tag="AUO97b_01183" /db_xref="SEED:fig|400667.82.peg.1043"
/translation="MKSLAFIHRNDTRFAVGDFYPVLSVFSYHELGNTLSPFLLLDHS
GPGKIAPSMKRRGVNDHPHRGFETVTLVYAGELEHKDSSGGGGFISAGDVQWMTAASG
VIHRELFSEEYSRSGGPFEMIQLWVNLPAANKMNSPRYQSLKKAEIPMVKLENEAGFV
RIIAGQFNHVIGPALTHTPITVLDVELHAGQQAAFPAQSGETALVYLRSGRAQFQKDE
DVLEEQGLAVMSNQGEHFSITALQSCKLLILTGQPITEPINGHGPFVMNTYDEILQAY DDIKNGRFAK"
/product="Pirin"
/transl_table=11
CDS 1077893..1078429
/locus_tag="AUO97b_01184" /db_xref="SEED:fig|400667.82.peg.1044"
/translation="MFTLNAFKLVIYRQFIIFTLFIFRHLKSYPIRFLTPEEKALAQR
VFGSLLDCERPKIIATRYLPWQAHGILMAPNGNIYVNLSDYSSNYALESKFIQGIFIH
ELAHVMQYQRGIHVLLKGALLQSAYYLSFKYYNPYKYTYHPQKAFSSYNIEQQGEIAR
DICSGKLPNIICLPQLDL"
/product="FIG00351361: hypothetical protein" /transl_table=11
CDS 1078814..1080439
/locus_tag="AUO97b_01185" /db_xref="SEED:fig|400667.82.peg.1045"
/db_xref="GO:0008795"
/translation="MKSFKVALAQFSPHIGNIDSNTQKMIEQANQAKKQDADLIIFPE
LSVIGYPAEDLLLRPNLNKRMQKAFAQLSEVKDIVMVFGFVNQTEDGQRYNSAAVMKD
GQVLGVFNKHNLPNYGVFDEKRYFQKGHQHLVFEYLGHKFGVLICEDIWSINTVKQLS
QLNVDTVLVLNSSPYEVGKPQHRKQTLSELAKQLHLNIVYVNQVGGQDDLIFDGTSFV
SNQNGEIALQAPSFKEDLYIAEFDRDTKLYKVVESAPALETFAEIYQGLVMATRDYVE
RSGFPGVILGLSGGIDSALTLAIAVDAIGAERVQAVMMPYTYTSQISVEDAAEQARRM
GVTFGIAEIHSIVNSFMQTLYPFFGNSPADATEENLQARARGTLLMGLSNKFGNLVLS
TGNKSELSVGYCTLYGDMVGGFAVLKDVYKTIVFELAKYRNSLSETPVIPERVITRPP
SAELRPDQKDQDSLPAYDVLDAILYAYIEEDLGQADIIAKGFDKEVVEKVIRLVDRNE
YKRRQGAIGPRITSRAFSRERRYPIVNGWTAND"
/product="NAD synthetase (EC 6.3.1.5) / Glutamine amidotransferase chain of
NAD synthetase" /EC_number="6.3.1.5"
/transl_table=11
CDS 1080432..1081460
/locus_tag="AUO97b_01186" /db_xref="SEED:fig|400667.82.peg.1046"
/translation="MTEQKNSSKTTALAQALLLEQVEFFKKQLSIENSPIYFRQFIQL
FMQHADEIKLYEVVDLEQLQAVVKRYAFEMQLGAGLLEFIGEIAQRIYLSAMKSPVQL
QDLVSDHQFEMWLSKFLEMEHIPHYLNQFLRTSPSVQQLCQYIATSTLEQKLPKFLTA

SRVDDYHFEWQHKLKKFSFLQQQRLEHKLETWIASFIHEQLTELSLLSAEDLESLVRH
IWEDIRHKKIYEFMKQLTPLDVEEFFVLIYEYWKELRQSQFMQGLILYGVEVFYDFYK
DQSLFEVLSAIGLSETDLQTEALRFYPKVMDAFNEHGILEPLLQALLAPFYQSSKTLD IIEKHFSE"
/product="FIG00350262: hypothetical protein" /transl_table=11
CDS complement(1081738..1082694)
/locus_tag="AUO97b_01187" /db_xref="SEED:fig|400667.82.peg.1047"
/db_xref="GO:0005506"
/db_xref="GO:0006118"
/db_xref="GO:0009055"
/db_xref="GO:0016491"
/translation="MASHYEMFPAVVTRVEQLTPLIKRFTFKRQDGQNFPRFSGGSHI
IVKMNEQLSNAYSLMSCTQDLSTYQVCVRKDVEGKGGSVFMHDQCNEGCEIQISEPKN
LFPLAETGNKHILIAGGIGITPFLPQMDELAARGAEYELHYAYRSPEHAALLDELKQK
HAEHVFSYVDSEGSMLNLDELISSQPKGTHVYVCGPKPMIDAVIDCCNKHRYRDEYIH
WEQFASTVPEDGEAFTVVLAKSNQEIEVQSNQTILQAIETLNIDVECLCREGVCGTCE
TAILEGEAEHFDQYLSDAEKASQKSMMICVSRAKGKKLVLDL" /product="Carnitine
monooxygenase, reductase component CntB" /transl_table=11
CDS complement(1082742..1084193)
/locus_tag="AUO97b_01188" /db_xref="SEED:fig|400667.82.peg.1048"
/db_xref="GO:0008911"
/translation="MSSLQSTELFQQQAYINGQWLAAQSNATVPVSNPATGEEIGTIP
NMGAAEATQAVEAAYTALQSWKALTAQNRADILLAWHKLVLDHTDELALIMTIEQGKP
LAEAKGEVRYAASFIQWFAEEGKRVYGDVIPTVNNQQRFIISKEPVGVVAAITPWNFP
IAMITRKAAPALAAGCTVVIKPANETPYCALAIAKLAEKAGIPAGVINVVTGKSQEIG
SVFTSHEKVKKLTFTGSTPVGRLLMQQCSSTIKKLALELGGNAPLIVFDDADLDKAVQ
GAIFAKFRNAGQTCVCANRIYVHDNIYQAFAEKFVQEVQKFKVGNGLEDGVHIGPLIN
EKAVLKAQQLIDDAVSKGSKIACGGKQHALGQTFYEPSVLTNVDRTMEIVQEEIFGPV
APLIRFTDETDVVAQANDTIFGLAAYVYSENISRLWRVSEQLEYGMVGMNATAISNEV
VPFGGVKQSGVGREGSKYGLEEFMTIKYMCLGL" /product="Succinate-semialdehyde
dehydrogenase [NAD(P)+] (EC 1.2.1.16)" /EC_number="1.2.1.22"
/transl_table=11
CDS complement(1084258..1085373)
/locus_tag="AUO97b_01189" /db_xref="SEED:fig|400667.82.peg.1049"
/db_xref="GO:0018623"
/translation="MSAVEKLPEDFCANPDVAWTFPKVFYTSSQVFEHEKEAIFAKSW
ICVAHGSELAQPNDYITRKVIGENIVIIRGKDSVLRAFYNVCPHRGHELLSGSGKAKN
VITCPYHAWTFKLDGSLALARNCDHVESFDKENSSMVPLKVEEYAGFVFINMDENATC
VEDQLPGFAERLNQACGVIKDLKLAARFVTETPANWKVIVDNYMECYHCGPAHPGFAD
SVQVDKYWHTTHQNWTLQYGFARSSEKSFKLDPSVTDPEFHGFWTWPCTMFNVPPGSN
FMTVIYEFPVDAETTLQHYDIYFTNEELTQDQKDLIEWYRNVFRPEDLNLVESVQRGL
KSRGYRGQGRIMTDKQRSGISEHGIAYFQHLVAQYHQ"
/product="Carnitine monooxygenase, oxygenase component CntA"
/EC_number="1.14.12.10"
/transl_table=11
CDS complement(1085416..1086390)
/locus_tag="AUO97b_01190" /db_xref="SEED:fig|400667.82.peg.1050"
/translation="MTTYEQATYALSEEMQSLVYWSSIYSPADADIDSVRAAYDAMCR
HYTLPRDDKLDVEDQVIANEEHPVPVRVYLPKTNRPESGWPCVLYLHGGGWMVGGLDS
HEFITSYLCKDLNAVVISVDYRLAPEHRFPAAFEDCLAVYHWLKQHGSAWQIDSENIV
LAGDSAGGNLAAALAVELQHSGLQAQGLVLVYPCLTTAFDTPSAQKHAHAPLLTTEDM
HFYLKEYAPDSQDWQDLRLAPLLATDFSDMPTSFVAVAEYDPLSDDGYFLTQKLEQAG
IPNEFHLGKGLLHGSLRLMRDCPEVQQLYQNMLSAIRRMFGSAEKTSI" /product="Lipase"
/transl_table=11
CDS complement(1086402..1088054)

/locus_tag="AUO97b_01191" /db_xref="SEED:fig|400667.82.peg.1051"
/translation="MDMDNQNTKIYTDKFLAVTSLLFVFISVAGLAIYSQESIKIAAT
WMQWTTSVFTTPVLLFAFLAIIFTFGLAFSKYGKIKLGEGKPQYSTMSWIFMFILSGI
GSSTLYWGFLDWAYYYQTPGLSLPPESAEALKYSVAYSFFHSGLSAWAIYALASISLC
YSYHVRKNKGLSLASVIEAVTGFKSTGVVGRLVDLMFLLCMFGALTISLVLTAVTFTN
ILSQLTGIPNTFMTKVIIILAVSVLFALSSYVGMDKGMQRLSHMVCLGVVLFAIYVLC
FGPTQFILNNSLMSFGLMATNFVDMSLFTDPMGDGKFTREWTVFYWLWWISYAPGVAL
FVTRVSKGRTIKEVIFAMVIGGSVGLWFIFGVFENYSVYSFIHGAVNVPQILSQQGGE
VAIGQLLSLLPAGKLMMWIFLGIMVVFLAAHMDAVGYAVSATCTRGLSEGQDPSPNAR
LFWCVMLTLVPIAMIFSKAPLDTMKTATIVTALPFIVIILIQTYGLVKWLIQDYAKVP
SHLIEQQGYDEQEIGLNQTQDEHAKRMQLELASSIKLDRKTS"
/product="Betaine/carnitine/choline transporter (BCCT) family"
/transl_table=11
CDS complement(1088075..1089187)
/locus_tag="AUO97b_01192" /db_xref="SEED:fig|400667.82.peg.1052"
/translation="MAKRYKVATIAGDGIGLEVLPEGIKVVKAAAEKHGIELQLDAFD
WASCDYYLEHGKMMPDDWFETLQGYDAIYFGAVGWPDKVPDHISLWGSLLQFRRGFDQ
YVNLRPVRLMPGVKCPLVGKKPGDIDFYVVRENSEGEYSAIGGKAFEGTDREFVLQEA
VFTRHGVDRILRYAFEFANQRDAKKITAATKSNGIAVSMPYWDERVDAMAKQYPQIQA
DKQHVDILAARFVLQPERFDVVVASNLFGDILSDLGPACTGTIGLAASANLNPERKFP
SLFEPVHGSAPDIYGKQIANPIAAIWSGAMMFEFFGEEDERCIQAGQDIMQAIENVLI
NGPKTADIGGQAKTYEVGDAIASCVAQTKMDVFAME"
/product="D-malate dehydrogenase [decarboxylating] (EC 1.1.1.83)"
/EC_number="1.1.1.83"
/EC_number="1.1.1.93" /EC_number="4.1.1.73"
/transl_table=11
CDS 1089291..1090271
/locus_tag="AUO97b_01193" /db_xref="SEED:fig|400667.82.peg.1053"
/translation="MNNLPNLSDLKVFCTVAKLKSFVESAEELGTSPAFISKRINILE
NTLSCKLFHRSTRHVSLTEDGKLILERVSNILEEFDEVCELVNNPLSTPTGRMDIISS
FGFGRKHIAPILSKLMMLYPKLQIHFDTIDNTKDLIQHSIDLDIRIGNEIAPNMIAKK
LASNFRILCASPSYLLKHGVPDSIEDLQTHDCLTISERDQSSVLWKFRHASEDVSVHI
KPRFVSNNGEIIHQLAIEGHGIIFRSIWDVADELITGQLQHILPEYWQDADVWAVYPS
RLKSSSKLQTCILFIQNELLNRLQHVQNLSRGQLISPAVKREPPPEKVFL" /product="Transcriptional regulator DmlR, LysR family" /transl_table=11
CDS complement(1090321..1091505)
/locus_tag="AUO97b_01194" /db_xref="SEED:fig|400667.82.peg.1054"
/translation="MKRSLLFLGILSLTAGAQVSAADLSWGDPTLDSGQFKVSGAVRS
RYLHKDYVVGANEGSQNDDWRLTDIKLVLGYENPNWIAGADARCYQYDRLCDAIFLKK
AWVGYKLSDQQRVTAGLQPVDFGFGEFWGSSYYETLLNTVGLEDIDNLGIKYKFADDK
YNLTLGFYPTDGGNYKGTSKDSSRYSGNFVEADDLTIGTNIKEKNMWIARASRKFELD
KAQKFSTELGGSVWYSDLENKRTDEDGHRKSWNVFAQTQYQAWQWMFLAGKQDVTNGD
NLLPNSSTIGAFDYPYQVANKGKYLVNEINYTFAQPFHKLENIKPYISHSRFFKDEDG
YKDSERLIAGVYFNYKAIGIQGEYIMSKNDPMVGGGANGLAQGSSNDWDKLFYLSIGY YF"
/product="FIG00350547: hypothetical protein" /transl_table=11
CDS complement(1092178..1092366)
/locus_tag="AUO97b_01195" /db_xref="SEED:fig|400667.82.peg.1055"
/translation="MGEVIYEINPDLCTECVGHHDQPQCQLFCPVDCIPKDPQHEETE
EQLLDKYKRLIAQKSTSN" /product="Uncharacterized ferredoxin-like protein YfhL"
/transl_table=11
CDS complement(1092443..1092850)
/locus_tag="AUO97b_01196" /db_xref="SEED:fig|400667.82.peg.1056"
/db_xref="GO:0004595"
/translation="MFDEVVVAIAIGHHKNPLFSLEERVALAQSSLGHLSNVEFVGFD

GLLVNFFKEQKATAVLRGLRAVSDFEYEFQLANMNRQLDPHFEAVFLTPSEQYSFISS
TLIREIARLKGDVTKFVPQAVVEAFERKHQQGW" /product="Phosphopantetheine adenylyltransferase (EC 2.7.7.3)" /EC_number="2.7.7.3"
/transl_table=11
CDS 1093070..1093546
/locus_tag="AUO97b_01197" /db_xref="SEED:fig|400667.82.peg.1057"
/translation="MAKATVVKKHNGGTIAQNKRARHDYFIEEKFEAGMSLLGWEVKS
LRAGRMSLTESYVIFKNGEAFLFGAQIQPLLSASTHIVPEATRTRKLLLSRRELEKLM
GAVNQKGYSCVPLACYWKGHLVKLEIALVKGKQLHDKRATEKERDWQRDKARIFHK" /product="tmRNA-binding protein SmpB"
/transl_table=11
CDS complement(1093588..1094139)
/locus_tag="AUO97b_01198" /db_xref="SEED:fig|400667.82.peg.1058"
/translation="MSLPEKHLSIVYHSPYGHTAKVASAIASGAEVMGVKVHVMNIEH
IDWDVLDASQGIIFGSPTYMGSVTADFKKFMDSTSKRWKNRLWQGKLAAGFANSGGLS
GDKLAVLQQLNIFAMQHGMLWSGLPLMTTGHTETDLNRLSSCLGLMTQSDNAPVEITP
PQGDLDTAKWFGEYIAGLLYRLN"
/product="Multimeric flavodoxin WrbA"
/transl_table=11
CDS complement(1094360..1095097)
/locus_tag="AUO97b_01199" /db_xref="SEED:fig|400667.82.peg.1059"
/translation="MEFVQGLPQGVFVGQTRVHHPLALPAEQTELTGFNLALHVKDEA
QRVQQHRMMLLDEFAQFGVKKMTWMTQTHSTICHTVNEQIPFTALIGDGLITQTKGHA
LMMMTADCLPVVLGNAEGTEIANLHAGWRGLAGGIIENTVAAMQSPPTWAWLGAAISQ
PCFEIGAEVKTAFCSKYPELETAFIDGAAPNKFHADLYAIARFILQSLGVEKVLGGDQ
CSYQQQDEYFSYRRDAKTGRMATFVFM"
/product="FIG00003370: Multicopper polyphenol oxidase" /transl_table=11
CDS complement(1095108..1096160)
/locus_tag="AUO97b_01200" /db_xref="SEED:fig|400667.82.peg.1060"
/translation="MTSAQSSNTNFSETDFNLLEDSEDADNHTSDTTATRLSLQVQLD
ESYLGQRIDQVAAMVWSEFSREKLKQWMKEGHLLVNGNIVKPKYRCEGNETLTLNVEL
EAQTNSQPENIPLDIVYEDEDILVINKPVGMVVHPGAGNTSSTLVNALLYHSPKSAEL
SRAGLVHRIDKDTSGLLVVAKNLEAQFSLSKQLGDKSVYRIYDLVTYGNIIAGGTIDE
PIKRHPVDRVKMAILPGGRDAVTHYNVKERFRDFTRVQAQLETGRTHQIRVHFSYIGH
GLVGDQVYMNRVRIPAGASELLIETLRGFKRQALHAAKLGLKHPRTGEEMLFEAPWPE DFTQLVNVLRTENAAY"
/product="LSU rRNA pseudouridine(1911/1915/1917) synthase (EC 5.4.99.23)"
/EC_number="4.2.1.70"
/transl_table=11
CDS 1096232..1097389
/locus_tag="AUO97b_01201" /db_xref="SEED:fig|400667.82.peg.1061"
/translation="MSLPRYKITMLALSLGVASAFVGCSSNPSKKEVVDTGPQSSEQA
YFDKAQKALDRGQYLDATKSLEAIDTYYPTGQYAQQAQLELLYSKFKQKDYEGAIALA
ERFIRLNPQHPNVDYAYYVRGVSNMEMNYDSLLRYTSLQQSHRDVSYLKVAYQNFVDL
IRRFPSSQYSVDAAQRMKFIGQELAESEMNAARFNVKRKAWIAAAERSQWVIEHYPQT
PQVPEALATLAYSYDQLGDKATSQQYIEVLKLNYPSLVNKNGTVNMRAARKEGNWINR
ATLGILGRESKSVTPDTTTSSEAEPKRSLLNRVSFGLIGNSGKEETEETTNTPVEAPK
SERSWTNRLSFGLLDKPKPKAAEGATIAPATSSSEAPSASPADNEADDAAQ" /product="Outer membrane beta-barrel assembly protein BamD" /transl_table=11
CDS 1097548..1099413
/locus_tag="AUO97b_01202" /db_xref="SEED:fig|400667.82.peg.1062"
/db_xref="GO:0003676"
/db_xref="GO:0003677"
/db_xref="GO:0003896"
/db_xref="GO:0006260"

/db_xref="GO:0006304"
/db_xref="GO:0008270"
/translation="MAIPQHTIDQILDRTDIVDLIGQRVKLKKTGRTYSGCCPFHQEK
TPSFHVYRDKQYYHCFGCQANGNAIRFLMDIDNRNFIDVMKELSSNTGVELPKDNTDN
KKLSYTRQVTKPSTPPKTVAEPTTQAPIEDQYNTFEPVYFDDPFAQFEQPFSFDEPVQ
EGNLYDLLENVAQFYEHQLPTSQKAKNYFKQRGLSDQTIQFWRLGYAPEDWQHLEKAF
PYDIEGLKQLGLIRSSDSGRDFDLLRDRVIFPIRDPKGRVVGFGGRALNDEIKPKYIN
SPDSEVFHKNQLLYGLYEGRKLKANDWLMVEGYMDVIALQQYGITGAVATLGTASNTE
HLNILFKQNNRITIAFDGDVAGQKAARRTLELALPLLNDGRELKFFVLPNDHDPDSLI
RREGVENFQKLLQQAPLLSDFVFAHLTGQHDISTPEGKSLVMGELRELTELLPKQGSF
RYLLTQSFREKLGLGKRFTPQISHDASLSFNIQTKDEDFAIAILMHHPFLYIHFEGLR
AYIPQDELLAKILAILNRIFDELPDDQELATYYVLGACSSYCHEIADIMQRTNIQALT
QAPEVADKLAKEYALGLQERYLRQKLKSKISLVESRNLRQQLNELTKQISLRLLS" /product="DNA primase DnaG"
/transl_table=11
CDS complement(1099410..1100693)
/locus_tag="AUO97b_01203" /db_xref="SEED:fig|400667.82.peg.1063"
/db_xref="GO:0008883"
/translation="MSFFALGVNHQTASVELREQIAFNAERLSNLLAEQRHHESLKDL
VVVSTCNRTEVYAMAEDAESLLKWLADANNIDVKQLIHHVYRYENAQAITHLMRVASG
LDSLMLGEPQILGQVKSALALSKEAQTVSPELNSVFEYAFYAAKRVRSETAVGSHAVS
MGYAVAQLALQVFSKPEKLTVMVVAAGEMNSLVAKHLAEMGVAKMIICNRSRERADQL
AQEIAHQVEVEIIDFSDLAENLYRADVVSSCTGSLHQVIAYADVKTALKKRRYQQMLM
VDLAVPRDIDPKVESLDGVYLYGVDDLQSVIDENLAQRRQAAVEAEVMVNQLATQLIT
HQKVKEAGSTIHAYRQHSEEISQRELTHALEALHHGGNPEQVLQQFAHRLTQKLIHPT
SMLLREAAKAESPDYFEWLQQHLQDVFDHERKPKR" /product="Glutamyl-tRNA reductase (EC 1.2.1.70)" /EC_number="1.2.1.70"
/transl_table=11
CDS 1100854..1102491
/locus_tag="AUO97b_01204" /db_xref="SEED:fig|400667.82.peg.1064"
/translation="MSSYVYARPVQETYAVHSFDQALQQSMVAEFALAYDDIPTALHN
YTVLAIKSNSTTIKQRALDVALEYNDLQAALNIATHWVVQEPKDVPALFYLSHIALKT
HEYQLAAETLDKILKIDPTADLEQILASIAPENAQDREVLLTALRSSAEKDNPSILAL
IANLEAQNGQLEPALSTINRALRRRPKVTSFILMKANLLIALSDQEGALKWYAKSSRK
YKKNLDIRLAEVRYLIQINQPQLALEKLEKIIESNPHAEEALFIAGLTSIDLKQYDKA
EQYLVDLRNSAKYQNEAYYYLAINAERKQHYETAKAYYRLVDGSLYIVSRRNLIAIYD
KQENLHDALRFLTQERVNYPQHASFLYQAQAEILKKMGNKKAALNLLDEAIKNLPDDP
ELIYAEVLLLDPYTDRDKLDKTLKQLLQLEPNSPTYLNAYAYTLALQNRRLKEARQYA
EQALEYAPEQASILDTLGYIAFLQNDYEAAAEALGKAYELSHNINIGIRYAKALYMQG
SLTQFSAVLQQLKQKHANDPQLQQLDALILPTSAKKS" /product="FIG140336: TPR domain protein"
/transl_table=11
CDS 1102496..1103074
/locus_tag="AUO97b_01205" /db_xref="SEED:fig|400667.82.peg.1065"
/translation="MSKLAQLCTAICGSSVLFLTGCQHFTQPKAAVTHQVQDEKHFNL
QGKIGVRTPQQTGSAFFTWIQQQDNFDIELSGILGVGKTQIQGKPGEVTLNSAKTGLI
TAASPEELLERATGWQAPITHLTSWILAKPATLSAQISKDNANRVSQLIEDGWTVNFS
YDGEQTLPNKLVLKQALAEDKENRITMVIQNR" /product="Outer membrane lipoprotein component of lipoprotein transport system LolB" /transl_table=11
CDS 1103093..1103926
/locus_tag="AUO97b_01206" /db_xref="SEED:fig|400667.82.peg.1066"
/db_xref="GO:0050515"
/translation="MIRVPSPAKLNLFLHITGRRENGYHELQTIFQLIDLYDWMTFTP
ISEDEIQIEGLGEVQLEQNLIYRAAQILRPHAQNPCGLHIKIEKNIPMGAGLGGGSSN

AATTLIVLNQLWQCGLTEEQLAQFGVKLGADVPIFIYGLNAWAEGIGEHLSFIDLDQK
QFIVLKPDCFISTQLLFSQKTLTRDSKPTTFCAYQLEPSNFGNNFEPLARELYPEVEE
AMQYLDQFGHAKLTGTGACVFAEVTDEMNVDDILKHAPCKAYLVHSLKESPLRHFKVA S"
/product="4-diphosphocytidyl-2-C-methyl-D-erythritol kinase (EC 2.7.1.148)"
/EC_number="2.7.1.148"
/transl_table=11
CDS 1104400..1105350
/locus_tag="AUO97b_01207" /db_xref="SEED:fig|400667.82.peg.1067"
/db_xref="GO:0004749"
/translation="MPNLVVFSGNAHPQFAQKVVSHLHIPLGAASVGQFSDGEISVEI
TENVRGKDVFIVQPTCAPTNDNLMEILVMADALRRASAGRITAVIPYFGYARQDRRPR
SARVPITAKVVADMLTTVGIDRVVMIDLHADQIQGFFDIPVDNIYGTPALLADLRQQQ
HDNLMVVSPDVGGVVRARAVAKQMGDIDLAIIDKRRQKANESQVMHLIGDVKDRDCVI
VDDMVDTAGTLCKAADALKQFGARRVVAYATHPVLSGKAIENLRNSVIDELVVTDTIP
LSEEALNLGKIRQVSVASMVAETIRRINNEESISAMFDSL" /product="Ribose-phosphate
pyrophosphokinase (EC 2.7.6.1)" /EC_number="2.7.6.1"
/transl_table=11
CDS 1105450..1105746
/locus_tag="AUO97b_01208" /db_xref="SEED:fig|400667.82.peg.1068"
/db_xref="GO:0003735"
/db_xref="GO:0005622"
/db_xref="GO:0005840" /db_xref="GO:0006412"
/db_xref="GO:0008097"
/translation="MANFVLNAQARAEDKQGKGASRRLRRESLVPAIIYGGNAEPVAV
TLELRELVKALESNAFFEEVVEIKVGDKVENVKIQALQRHPAKNTPMHADFKRA" /product="LSU
ribosomal protein L25p"
/transl_table=11
CDS 1105767..1106348
/locus_tag="AUO97b_01209" /db_xref="SEED:fig|400667.82.peg.1069"
/db_xref="GO:0004045"
/translation="MSNISLIVGLGNPGSEYAQTRHNAGFWFVEQLADKYGITLKNDP
KFHGISGRGNIEGHDVRLLLPMTYMNRSGQSVVPFSKFYQIAPEAILIAHDELDMNPG
VIRLKTGGGHGGHNGLRDIVPHIGPNFHRLRIGIGHPGSKERVSGHVLGKAPSSEQSL
MDGAIDHALSKVKLLVQGQVPQAMNQINAYKPA"
/product="Peptidyl-tRNA hydrolase (EC 3.1.1.29)" /EC_number="3.1.1.29"
/transl_table=11
CDS 1106480..1106860 /locus_tag="AUO97b_01210" /db_xref="SEED:fig|
400667.82.peg.1070" /db_xref="GO:0004068"
/translation="MLSRLLKCKIHRAVVTHAELHYEGSCAIDGVLMDLAGIREYEEI
HVWNVTNGKRFTTYAIRGEDNSGIISVNGGAAHQADVGDLVIIATFGDFTEAEANAHK
PRLVYANPDNTVNHTANCIPVQVA"
/product="Aspartate 1-decarboxylase (EC 4.1.1.11)" /EC_number="4.1.1.11"
/transl_table=11
CDS complement(1106916..1107476)
/locus_tag="AUO97b_01211" /db_xref="SEED:fig|400667.82.peg.1071"
/translation="MKKYFSLMLAIVTVLFFAGLVMKNEWHLHQSKSIFIKLKPVDPR
SILQGDYMALAYELNLQSLKALTGSESEALDQVIFNHASVPAKVILDSRNRVIRTILD
PNSLSNEQNLILKNPDNHYQSLYPASRSFLFAEGLAHCYEKAKYAEFKVNAKGEAILF
DLRGEELQPLNCKQQQSWWKGTARSL"
/product="putative membrane protein" /transl_table=11
CDS complement(1107476..1109257)
/locus_tag="AUO97b_01212" /db_xref="SEED:fig|400667.82.peg.1072"
/translation="MHIRDYYPFRNTLFIQHLHIFSYVFMALSILYLIAANWLMLPDS
IQLIIPPVILLITAWVSVKKTLSEGVRQTLHGICGLMVGLSLAVIGQVYQTGADSYLL

FLIWTLLLLPWLYRPNIGIFALICITSQLTLFLFFKQTFWAEKFPYLYLFALNLLSLV
QFWICQKKYTALRFIFIAWFAVISITGMIQFLSSENLPYLISAFFLGIIAFYYFFNKD
DQLCASLMAAVLGVTATIWLVDGINQLFKDSNEFIFLLIAGIIFTWFALISYFLIKIF
RQSRFYIIPLAIGAWLAGLALAAFTLVFWDTISLIIGIIFVAVAITLLTKSQSYFIRQ
FAYCLFVSGQTAFLFHLGSETDQILWVLIAQIFILCISYFLKPHWFFILIQMLATYGI
AVIYLLQMDHSLWSLNSTQTYLNLVLLNYLVFSSVLLIGSKAVVSYKRSIFLCTLVVI
WVSSFFDTFIGLALVDSADQSLWFLYALPCVWLLCFSFFFLYRQLHGITFFAFLVFGI
LLIALGYFEVFILFVILTWALKNKDRIVYGVTLVVFAFVLWQLYYSLQLSFLAKSASI
LVSGIILLALYGLLMKEAKINCIEGEK"
/product="hypothetical protein"
/transl_table=11
CDS 1109675..1110334
/locus_tag="AUO97b_01213" /db_xref="SEED:fig|400667.82.peg.1073"
/translation="MSSLIQPELFFSALSPAEQRIQQALEDIRQGKPVLVMDDFDREN
EADLIVAAETLTVETMARMIRDGSGIVCLCLTEELADHLELPPMVSQNSSQFHTAFTV
TIEAAQGVTTGVSAKDRVTTIKTAIKDGAVASDLNRPGHVFPLRARNGGVLTRRGHTE
GTIDLARLAGLKPAGVLCELTNPDGTMASGIQVLAYAQTHHLTVITIEELVQYRQQHG I"
/product="3,4-dihydroxy-2-butanone 4-phosphate synthase (EC 4.1.99.12)"
/EC_number="4.1.99.12"
/transl_table=11
CDS complement(1110400..1111074)
/locus_tag="AUO97b_01214" /db_xref="SEED:fig|400667.82.peg.1074"
/translation="MAIKHILIDLDGTLTDPKVGIHTSIRYAMDKLGYPLAADLNIDW
TIGPPLKASLAKLLATQDDALAEQALLAYRERFSVIGLFENEVYPSVAETLKALKAEG
YHLFVATAKPTIYAKRILDHFDLSQYFVQIYGSELTGERTNKAELIHYILEREQLNPE
ECLMVGDRQYDVLGARHNGIEAVAVTYGYGTPEELTQAQPKAKITKFSELLDYVEEQA AQKKVS"
/product="Phosphoglycolate phosphatase (EC 3.1.3.18)" /EC_number="3.1.3.18"
/transl_table=11
CDS 1111172..1111939
/locus_tag="AUO97b_01215" /db_xref="SEED:fig|400667.82.peg.1075"
/db_xref="GO:0008972"
/translation="MRPTVLCFSGLDPSGGAGLQADIEAIGQSGAHAAIACTALTIQN
SQQVFGFEATSKELLLAQANAVVGDLPIKCVKSGMLGTTDNIAALAEFLRAHPDYQYV
LDPVLVANSGGSLGDQTTLVKAFVELIPLATLITPNTVELRALTGVTDLDQATQKLFE
MGAKAVLVKGGHEDTPNFIKNSLYIDGELATSSTCPRLEGEYHGSGCSLASFIAGRLA
LGDSLKIAVQHAETWLFGVLKNAETPVPNGQKIPKRF" /product="Hydroxymethylpyrimidine
phosphate kinase ThiD (EC 2.7.4.7)" /EC_number="2.7.4.7"
/transl_table=11
CDS complement(1111985..1112458)
/locus_tag="AUO97b_01216" /db_xref="SEED:fig|400667.82.peg.1076"
/translation="MGLFDFVKGIGKKNTAPAEPQAAPATPAEPSAQEIANKLLGLIK
SLGLGVEGLSVTYNGSTDTAIIKGQVQSQADKEKIILIVGNVDHVAQVDDQMTVATPE
PESKFYTVKSGDNLSKIAKEFYGDANQYQKIFEANKPMLKDPDEIFPGQVLRIPQ"
/product="Uncharacterized LysM domain protein YgaU" /transl_table=11
CDS complement(1112649..1112885)
/locus_tag="AUO97b_01217" /db_xref="SEED:fig|400667.82.peg.1077"
/db_xref="GO:0048037"
/translation="MSDIEQRVKQAVAEQLGLKAEEIKNEASFMDDLGADSLDLVELV
MSFENDFDITIPDEDSNEITTVQSAIDYVTKKLG"
/product="Acyl carrier protein"
/transl_table=11
CDS complement(1112970..1113704)
/locus_tag="AUO97b_01218" /db_xref="SEED:fig|400667.82.peg.1078"
/db_xref="GO:0004316"

/translation="MTQERKVALVTGASRGIGAAIAQQLIQDGYFVVGTATSESGAQK
LTDSFGEQGAGLALDVRNLDEIEAVVSHIEQNYGPVLVLVNNAGITKDNLLLRMSEDD
WDDILNIHLKAVYRLSKRVLKGMTKARFGRIINISSVVAHFANPGQANYSAAKAGIEA
FSRSLAKEMGSRQITVNSVAPGFIATEMTDALSEDIRKKMSDQVALNRLGEPQDIANA
VSFLASDKAGYITGTVLHVNGGLYMA" /product="3-oxoacyl-[acyl-carrier protein] reductase (EC 1.1.1.100), FadG" /EC_number="1.1.1.100"
/transl_table=11
CDS complement(1113701..1114687)
/locus_tag="AUO97b_01219" /db_xref="SEED:fig|400667.82.peg.1079"
/db_xref="GO:0004314"
/translation="MSAKRLEQVALATKTAFVFPGQGSQKVGMLAELAEQFSGVGQTF
AEASDALGFDLWHIAQSGEGLDQTENTQPVLLTASIALWRLWLDLGGLAPKYLAGHSL
GEYSALVAAGSMSLADAVKLVNLRGKLMQNAVPQGEGAMAAILGLADDKVVELCQSVS
ASGQGSVEAANYNSQGQVVIAGSTASVQQVMALAKEQSAKAIALPVSVPSHCSLMKPA
AEKFAEALEQTAIELPTIPVIQNVNAGIATDVAQLRQALTAQLYQSVQWTRTMQSLQD
QGIQYIVECGPGTVLSNLAKRLPNIEKAFSIDSKSKMEDALNAVLVAEGKIA" /product="Malonyl CoA-acyl carrier protein transacylase (EC 2.3.1.39)" /EC_number="2.3.1.39"
/transl_table=11
CDS complement(1114840..1115025)
/locus_tag="AUO97b_01220" /db_xref="SEED:fig|400667.82.peg.1080"
/db_xref="GO:0003735"
/db_xref="GO:0006412" /db_xref="GO:0015934"
/translation="MAVQQNRKSRSRRDMRRSHDALTENALTVDQATGETHRRHHVTK DGFYRGRQLFAKAADAE"
/product="LSU ribosomal protein L32p @ LSU ribosomal protein L32p, zinc-independent" /transl_table=11
CDS complement(1115100..1115660)
/locus_tag="AUO97b_01221" /db_xref="SEED:fig|400667.82.peg.1081"
/translation="MSANTFPAQIEPFKWAEQGFTWSGQLPLSRFARIAREAVGSIDN
QLINIDCKLSMDAYHRIVWLDGHMETKVPMECQRCLEPVEMPIVSDFHIALVDDESLI
ERLDEDADFIVLGESESSTKGDFDTPASINLLALLEDELLLLMPLSPKHDACEHKHQP
TIQNVVEEKRDNPFDVLAGLKGKLNS"
/product="FIG01269488: protein, clustered with ribosomal protein L32p"
/transl_table=11
CDS 1115757..1116419
/locus_tag="AUO97b_01222" /db_xref="SEED:fig|400667.82.peg.1082"
/translation="MHLLQPQPEVNTSLLVCTHSTHQNSLLPDMVQLSPWPSSKLESE
QVDWLILHFNHWFSHHNVTLVRGEFEPEYFPANEHEPAKIQFAHGFFNSALHEISHWT
IAGAKRRLLPDLGYWYAPDGRTKEQQDLFEQVEIKPQAIEWLFAQSFGRKFRVSLDNL
TGDGGDGRKFKDNVYAQVQRYFSGEAKLPADAARFIECICQCTRAGAALQLNEFKREL LD"
/product="Translation elongation factor P Lys34 hydroxylase" /transl_table=11
CDS 1116509..1117126
/locus_tag="AUO97b_01223" /db_xref="SEED:fig|400667.82.peg.1083"
/translation="MLHLHVHPENPQQRLIEQAVERIRAGDVVVYPTDAAYAIGCQIG
NKNAMERIAQIRGLGPKHQYAIMCCDLSDIATYAKVDNATYRLLKANTPAITTFILPA
TSEVPRRLMHPKKKTIGLRIPSNPICQALLKELGEPLLTSTLILPGQEDPLDDPYDIE
NQLAKRIDVFIDGGFGTLTTTSIVDLSGEHPEIIRRGVGDVSAFE" /product="YciO protein, TsaC/YrdC paralog"
/transl_table=11
CDS 1117355..1118146
/locus_tag="AUO97b_01224" /db_xref="SEED:fig|400667.82.peg.1084"
/translation="MNQSLHQSMEDVPHIRVLDEWHDTIPEDLYIPPAAFEILLEQFE
GPLDFLIYLIQKNGFDLIQLDIAPIATQYLTYMDSMKSLNIELTADYMVMAALLADLK
SRLLLPKPTVVSIEKDPKQDLIDRLETYLRIKQAAERLGQMPVLDRDTFSANVSLGHI
APVYEGYDVSALHDALFCVFNRPEPVTHTIAQEPVMLEERIAYIEEKLESGDVLSFKE

LLNPQQGRMGMVVTFMAVLELTRQQKIKIIATGVEAPLAIQGSSI" /product="Segregation and condensation protein A" /transl_table=11
CDS 1118143..1118739
/locus_tag="AUO97b_01225" /db_xref="SEED:fig|400667.82.peg.1085"
/translation="MSSFEQNNNDLSLADIHHEVLLQLEAIIFASEAPVSIARLKEAF
NNQYNKQQLRQLLQQLALLQHGRSIELIETAQGFRFQVRSKYRNIIAQIWPERPTKLS
PSLLETLAVIAYHQPVTRADIEQIRGVSNNSQILRTLFDWNWIKEAGFRDLPGRPALL
ITTPQFLNAFGLASLGQLPPLQNAKEAFMALDANAPKS" /product="Segregation and condensation protein B" /transl_table=11
CDS 1118781..1119704
/locus_tag="AUO97b_01226" /db_xref="SEED:fig|400667.82.peg.1086"
/db_xref="GO:0003723"
/translation="MSEKLQKVLARIGLGSRRYMEEVIAAGRVSVNGRVAQVGERIEP
GDELRIDGRKVQFQIEDEIRRRVLIYYKPEGEICSRNDPEKRPTVFDHLPQIANDRWV
MVGRLDINSTGLLLFTNDGELANRLMHPSNEIEREYAVRVMGEVTPQLRQNMVNGVEL
EDGPAKFESFSEIGGEGINRWYQVVVKEGRNREVRRIFESQGLKVSRLLRTRYGTVIL
PRELRTGRWMELDKTDIDNLAKSVELKPRQGTGLFGMAKRRTERMTEKPMAARRGGYL
RQQRRDDEKEAPANTGNQRKSTGFNRGFKKF" /product="LSU rRNA pseudouridine(2605) synthase (EC 5.4.99.22)" /EC_number="4.2.1.70"
/transl_table=11
CDS complement(1119757..1120413)
/locus_tag="AUO97b_01227" /db_xref="SEED:fig|400667.82.peg.1087"
/db_xref="GO:0004141"
/translation="MSGQIYFVSGIDTEIGKTYATGFLAKLWTEQGKKVITQKLIQTG
NADISEDIQKHREIMGQGWFQEDHDKLTMPEIFSYPASPHLATRLDNREIDFQKIENA
TQTLAERFEIVLLEGAGGLMVPLTTSLLTIDYVAQHQFPVILVTSGRLGSINHTLLSL
EALKSRGLKLKALVYNLKDESKDPLISQDTSNYLKNHLAIHFPEAEWIELAKMNELKN "
/product="Dethiobiotin synthase BioD (EC 6.3.3.3)" /EC_number="6.3.3.3"
/transl_table=11
CDS complement(1120414..1121163)
/locus_tag="AUO97b_01228" /db_xref="SEED:fig|400667.82.peg.1088"
/translation="MSLNKNLVAQRFAKAGQSYSKHAIVQKQICQNLTNLLKQFCPSA
MSRVFEIGCGSGNLTRLLVESFQIENLVLNDLYAEVQQHFNHEEHVKWLIGDVETLEF
PQQLDMIVSGSALQWMQDLPRLLQHCYAALNEQGWLCFSTFGPKNLIEIKELTGQGLN
YWNLENWNSALTQAGFEILHLAQSETQLYFDSPKAVLQHLKATGVTATAQHRWTKQSL
QQFYQDYDRFKHTEGYSLTYHPIYCIARRMK" /product="Malonyl-[acyl-carrier protein] O-methyltransferase (EC 2.1.1.197)" /transl_table=11
CDS complement(1121160..1122317)
/locus_tag="AUO97b_01229" /db_xref="SEED:fig|400667.82.peg.1089"
/db_xref="GO:0008710"
/translation="MSLLDHFATELDELKRQGNFRQFTQNVQHGRFITIQNKTMLNLA
SNDYLGLAADIDLRQEFLDCYPLERSYFSSSSSRLLTGNFDEYEQLENSLSQAFGGRV
ALLFNSGYHMNIGILPAVADSKTLILADKLVHASMIDGIRLSGAQYVRYRHNDLQHLE
QLLQKYHDDDQIERIIVVTESIFSMDGDETDLTALVQLKKRFAKTMLYVDEAHSIGVR
GQQGLGCAEQYNVIQEIDFLVGTLGKALAAVGGYIICHPIIKDYLINKMRPLIFSTAQ
PPIVMAWANFIFKKVLMAQSQREHLKNISQYLQQAVVQKGYVSPSTSHIIPVIVGESQ
AAIDKAKQVQESGFYAMPVRPPTVPKNSSRLRISLTSMVEQHELEALVSFL" /product="8-amino-7-oxononanoate synthase (EC 2.3.1.47)" /EC_number="2.3.1.47"
/transl_table=11
CDS complement(1122319..1123599)
/locus_tag="AUO97b_01230" /db_xref="SEED:fig|400667.82.peg.1090"
/db_xref="GO:0004015"
/translation="MTDNFDLEHIWHPYTSMTQPLPTFKVKRAYGATIELDDGRTLID
GMSSWWCAIHGYNHPELNQAVTDQLQNMSHIMFGGLTHDPAIELGKILLKITPPSLDK

IFYADSGSVAVEVALKMAVQFWTAQGQPQKTNFITTRSGYHGDTWNAMSVCDPVTGMH
QIFGTSLPNRLFVAAPQTKFHEEWNQEDIAELEQAIQQNHENLAALIIEPIVQGAGGM
RFYHPEYLRQAKALCEKYHLLLIFDEIATGFGRTGKLFAWEHAQVEPDIMCLGKGLTG
GYMTLSATLTTKHVAETISRGEAGVFMHGPTFMANPLACAVALKSTQLLIEQDWQANI
KRIEQQLSQYLMPLNQLDYVADVRVLGAIGVVELTFNVDMKTLQQQFVERGIWIRPFG
KLVYVMPPYVITQQELSDLLEHLVEVVKTMQGAH" /product="Adenosylmethionine-8-amino-7-oxononanoate aminotransferase (EC 2.6.1.62)" /EC_number="2.6.1.62"
/transl_table=11
CDS complement(1123690..1124424)
/locus_tag="AUO97b_01231" /db_xref="SEED:fig|400667.82.peg.1091"
/translation="MKLQNKILLITGWGVGIEPLQSLKLGLTKAGCDTQLINIFNVLD
GGFHDQLDFLTDIDVVVGWSLGGQLATYLIDFFYKQTGQTKTLITLASNPCFVANTSW
HTAMPADVFSQFKASFLQNPQATLKRFYYLITLGSSQAKQDWLSVQNIANPPSNKLLL
EGLQMLEQLNLVDNLKTYPGRQLHLFAEQDNVIPCKIVENFKDIATENMSYKLIKDAT
HAFPYLQVERTIEEICQFLTIHQQSS" /product="Pimeloyl-[acyl-carrier protein] methyl ester esterase BioH (EC 3.1.1.85)" /transl_table=11
CDS 1124781..1125632
/locus_tag="AUO97b_01232" /db_xref="SEED:fig|400667.82.peg.1092"
/translation="MNSQSIISDGREKDTYFVTPNDIINTLPTDKNYCLFLDIDGTLA
PFQIHPEHSFIPNTTLEVIKKIIELNIPVIAVTGRDVETASKLLQSIELPIAGLHGLD
IYFDSDTYIRPDLSDINFQKLKEDIINSCEKYPDLLIEDKGHSIALHYRKNPELENNA
IYIMQQIKYFYPQLKLNRGKFVVELLPKQADKGKAIQTILNHLNLPLIHPIFIGDDLT
DESGFIFINQQFGTSIKVGSGETEAQYRLKDINSVSNFLFFFLEKIKKLYVKNSQDQN GEQICLN"
/product="Trehalose-6-phosphate phosphatase (EC 3.1.3.12)"
/EC_number="3.1.3.12"
/transl_table=11
CDS 1125620..1127050
/locus_tag="AUO97b_01233" /db_xref="SEED:fig|400667.82.peg.1093"
/db_xref="GO:0003825"
/translation="MSKLIVLSNRISMPSGKASAGGLAVAVQDALNDSNGIWLGWNGQ
QITDTEAPEFDQAYSHGIDYITCPLTHQQYAQYYCGFANKVLWPAMHDRDDLIEYNAE
EYNTYQKVNRLFAEKLQQIAQPDDLIWVHDYHFFSVARHCRELGMQNKIGFFLHIPFA
SLNIWRKIPVALQLVQDLCQYDVIGLQTQTDQNTCMQTCMGLLEAQKILSDRISYKKR
QILIKSYPIGVQPELIQRQAQQAFHTPYVFNFEDIPRQKTIIGVDRIDYSKGLLERFN
AFATFLETNPEYHGLVRHLQVATPSRTDIPAYQRLYQRFKAKLELINEEFAHEDWRPI
DCCFDTVQHDSLMHIYRRSDICWISSLRDGMNLVAKEYIAAQDPENPGVLILSKYAGA
AEQMTQALIVDPLDRAAMMDSLKTALEMSKAERINRYEQLIEGLAATDLSDWRNNFLK
DLENSPTFTKSKLSLQEKYQPTYHVL" /product="Alpha,alpha-trehalose-phosphate synthase [UDP-forming] (EC 2.4.1.15)" /EC_number="2.4.1.15"
/transl_table=11
CDS complement(1127118..1128311)
/locus_tag="AUO97b_01234" /db_xref="SEED:fig|400667.82.peg.1094"
/translation="MLFSKPAVLDTATLLSTQRLATRLSFFSLGFAISAWAPLIPYAQ
QRLNLNHANFGLLLLCMGIGSMIAMPATGALVKRWGCRPLIAVATILLMVLLPSLTIW
HSLVSMAVALFIFGTAAGSLGVAINLQAVVVEKHSLRALMSSFHGMCSLGGLIGAMLV
TALLAIGLSPLMSTLSVVMVLLVVSFVAIPSALTTFEQDEQGAAEITDAPKKSSRPNG
TILLIGVMCFIAFLSEGAAMDWGGIYLTSKYQLNPAFAGLAYTFFALSMTSGRFAGHI
LLKQWGEKTIVTYSAIVAALAMVTIVMAPVWQVVVLGYALLGLGCSNIVPVMFSRVGR
QNDMPKAAALSLVSTIAYTGSLSGPALIGLIGQWTSLTTVLSGVAVLLTMIAILNRFT LVKAK"
/product="Membrane protein mosC"
/transl_table=11
CDS complement(1128607..1129071)
/locus_tag="AUO97b_01235" /db_xref="SEED:fig|400667.82.peg.1095"
/translation="MRGNPEVIDYLNMLIGGELAARDQYLIHSRMYEDWGLNKIYERI

DHEMQEEASHADAIIRRVLFLGAKPNMHREDINVGTDVVSCLKADLALEYHVREKLAT
GIKLCEEKGDYISRDMLRQQLSDTEEDHTYWLEKQLRLIELIGLQNYIQSQI"
/product="Bacterioferritin (EC 1.16.3.1)"
/transl_table=11
CDS complement(1129325..1131361)
/locus_tag="AUO97b_01236" /db_xref="SEED:fig|400667.82.peg.1096"
/db_xref="GO:0003911"
/translation="MAITSVIEQMRQLIQLIAKHNHAYYVMDQPTISDSEYDHLFHQL
KALEEQYPELVQADSPTTKVGGQALSKFESVTHVVPMLSLGNVFNQEDLFAFARRVEE
RLPNQKVQYEVELKLDGLAISLWYENGVLVRGVTRGDGETGEDITQNVKTIRNLPKVL
HSEKYEIPRLLEVRGEVLMPKSGFEKLNADQEAKGEKTFANPRNAAAGSLRQLDPNIA
AARPLAFYAYGIAQCEPNHGLTTMHDSLQWLTELGFQIAERQYLCNSIQEVQQRYEQI
QQERPNLQVEIDGMVVKVDDLKQQQQLGFLSREPRWATAYKFPAQAALTTVEQIDWQV
GRTGTLTPVARLNPVFVGGVTVSNVTLHNIGEIHRLDVRIGDTVSVYRTGDVIPKVEK
VWPEFRPAEAEVVHLPESCPVCASPVVMPEGEALARCSGGLYCAAQRIEAIRHFVSRK
AMDIEGLGDRWVESLLRLDLLKDVADIYHLHEHRETLLGIEKMGEKSVQNLIDAIEAS
KKTTLARFIYALGIRGVGETTARMLANTFQTLEALKAANVEALKKTPDVGDITAEWIA
DFFLAPHNIEVLDRLIAAGIHWDAPTAPTRQPLNGESWVLTGTLEQMTRDQATQMLQA
LGARVSGSVSSKTKCVVAGEKAGSKLEKAAKLGIPVMNETDFLSLMAGYGQTLS" /product="DNA ligase
(NAD(+)) (EC 6.5.1.2)" /EC_number="6.5.1.2"
/transl_table=11
CDS complement(1131456..1132493)
/locus_tag="AUO97b_01237" /db_xref="SEED:fig|400667.82.peg.1097"
/translation="MEINTIIGIVVAIIIMLIGLRMILKKPNHAEPSLDSDLHINPES
NQPVIPRHVRDQLEQPEVTVASAAVAARVEPTLSEPAQSEEKGTKELEQASQAQTVQT
QVPVEKTPVEVEEVKAEENTVSPTVSENSSVELVDTVSAEPEVVSSSEPKVAEGQPKT
EPELSLNPNIETAEIAEFEGESNILDVHLHEQQRFDDESALAMAEQIIALNVYPNPRR
ALSGDKALKVLLKYGLRYGEMSCFHRYENTDEPSALMFSVLRMTDNGPAGFDLETLST
EQVQGLAFFLALPNSKAVTGFDMMTSIAGLIAREIDGKVYDENNLEFTPQLKEHWRHH VIDYRPAQATA"
/product="Cell division protein ZipA"
/transl_table=11
CDS complement(1132495..1135944)
/locus_tag="AUO97b_01238" /db_xref="SEED:fig|400667.82.peg.1098"
/db_xref="GO:0005515"
/db_xref="GO:0005524"
/db_xref="GO:0005694"
/db_xref="GO:0051276"
/translation="MRLSSLKLSGFKSFADSTTLNFKANRTAVVGPNGCGKSNVIDAI
RWVMGESNARQLRGGSMQDVIFTGTSKRKPVGVASVELRFDNTYGKLGGAYNAYNELA
VRRQVTREGKSEYFLNGTRCRRRDITDIFLGTGLGPRSYAVIEQGMINRLVDAKPEEM
RIFIEEAAGVSRYQARRRETLQHLEHTEQNLSRLEDIALELKSQLKTLKRQSEAAVQY
KTLESQIRTLKIEILSFQAEKSVRLQEEYTVQMNELGETFKLVRSELSTIEHDLESTS
ALFQRLIQQSSPLQQEWQQAEKKLSELKMTLEQKQSLFQQNSTTLVQLEQQKAQTKER
LQLSELQLETLNSQLEEQTEALTAIEHTAAEAEQSFAGLQSQQRQAQQQFEQVKAQVE
KQQQQKMQMSAQIEQLGKNVQRIEQQKETLQHQANQIQSQVHEDEQGELEQLQQQLCR
EISTLEAEIEQYVQRIEQAQQAHQVNKNQQQTLKTEIQVLLSEQKNLSQLVAKQSPKQ
NQDTLRLMQALELTEQGKPHAQIIEKFLAKWLQAHIVETEQPFQEGIARQLKPSEQSS
KFKGNLTCLNDWIASPQLSIFSNVAIAQDLSHALNEQKQLQYGQSILTLDGYHVGQDW
VIALMYDDESQSAQGMLSHRIRLDEIEQVLQKQQPELQALDQIIVQQKDELGQLQVDL
QQKQQVIKQKQKDLQQLDVQIAKQQTAAQAFLLQKQQLKDQLAQLDTQLEEDAMQKDD
LEIDLHALAMKLETILPDYKTLQFQVEELTEQLEEQQQVLQQQQQEREILRRNSTQTT
QQIELLEKDISFLQSQYQQITAQMEQAKKFVDPIQLELPNLESEFQQQFAQTEKLQKT
WNEWQIELNSVQEKQQTLTDQRHQYQQQDEKLREQLEAKRLAWQAAKSDREHYQEQLK
ELNAELQTGLKIDLTEHQQKLEKVQKQFEKIGAVNLAASQEFEEVSQRFDELSHQIQD

LENTVTQLKDAMKSIDQETRKLFMSTFDQINQELQNLFPKVFNGGEASLSLEDDWQSG
VKLMARPPGKKNSSLALLSGGEKALTALALVFAIFRLNPAPFCVLDEVDAPLDDANVQ
RYCNLVKELSEHVQFIYITHNKLAMTMATDLLGVTMPEPGTSKLVTVNLEQAKEYGLV SES"
/product="Chromosome partition protein smc" /transl_table=11
CDS complement(1135955..1136650)
/locus_tag="AUO97b_01239" /db_xref="SEED:fig|400667.82.peg.1099"
/db_xref="GO:0003677"
/db_xref="GO:0003700"
/db_xref="GO:0005622"
/db_xref="GO:0006813"
/db_xref="GO:0008324"
/db_xref="GO:0009058"
/db_xref="GO:0030528"
/db_xref="GO:0045449"
/translation="MNLEETESLSEQIVKYISEQIISGELVEGERIQELRIAKELDVS
RGSVREALLLLERTHLIEIFPRRGAIVSEMSAQQVKALFDTNMMLLGHIVQRISETWR
AHEADQLQLLLEQLLEHVKAGDIEKFYDAIFQYLAEQQDMVGNPYLMKFYKELLPSLR
RSYFLTLNTSKRELQEAFALFKLVTDAILIRKSQQAALFMEDFCRHLRNLVLESLTRM KQIELAWARRSRR"
/product="Transcriptional regulator, GntR family" /transl_table=11
CDS complement(1136900..1137703)
/locus_tag="AUO97b_01240" /db_xref="SEED:fig|400667.82.peg.1100"
/translation="MELIIYLLIGAIAGFTAGLFGVGGGLIIVPILYVVFTQLHYDPA
VIMHIAVGTSLATIIVTSFSSVSAHHKKGAVLWPVFRNLAPGLVIGSFLGAGIADLLS
GQHLQLLIGVFAVVMAYRMFKGAHVVVDPTRQLPSTPMQFMAGGGIGIASAIFGIGGG
SLTVPYLNRHGVVMQKAVATSAACGLPIAIAGAIGFMWFGAKEHISVPNTIGYIHIYA
FIGISVMSFVTAKLGAKVAHALSPQMLKKCFACLLVVVGSYFIYKGFMN" /product="Uncharacterized
UPF0721 integral membrane protein" /transl_table=11
CDS 1137734..1138486
/locus_tag="AUO97b_01241" /db_xref="SEED:fig|400667.82.peg.1101"
/db_xref="GO:0004077"
/translation="MDLETRQLQQLLSAKNQLPEVVLLKATTTSTNDDIREIAQKGIT
TGLVCSAQQTQGRGQHQRQWISPEGNIYLSTLVQTRTPLDGRLALEVALNILQIPQLQ
SLSLQVKWPNDLYSTQGKWGGILVEPLSQHQAIVGVGINLKTPPVTDSDQPITSLEDL
GLEQMSRLELISELYVAIQNAARWFDHGCYNLAGRFNHHAAWLNQLVQFEHSQGLIYG
RFVGISNEGAVILETPEPQQFYQGRLRLQTTQ" /product="Biotin--protein ligase (EC
6.3.4.9)(EC 6.3.4.10)(EC 6.3.4.11)(EC 6.3.4.15)" /EC_number="6.3.4.15"
/transl_table=11
CDS 1138502..1139236
/locus_tag="AUO97b_01242" /db_xref="SEED:fig|400667.82.peg.1102"
/db_xref="GO:0004594"
/translation="MKSLWLDIGNTRLKYWITENQQIIEHAAELHLQSPADLLLGLIQ
HFKHQGLHRIGISSVLDTENNQRIQQILKWLEIPVVFAKVHAEYAGLQCGYEVPSQLG
IDRWLQVLAVAEEKENYCIIGCGTALTIDLTKGKQHLGGYILPNLYLQRDALIQNTKG
IKIPDSAFDNLNPGNNTVDAVHHGILLGLISTIESIMQQSPKKLLLTGGDAPLFAKFL
QKYQPTVETDLLLKGLQQYIAHYPKD"
/product="Pantothenate kinase type III, CoaX-like (EC 2.7.1.33)"
/EC_number="2.7.1.33"
/transl_table=11
CDS complement(1139286..1140695)
/locus_tag="AUO97b_01243" /db_xref="SEED:fig|400667.82.peg.1103"
/db_xref="GO:0003723"
/db_xref="GO:0005525"
/db_xref="GO:0006605"
/db_xref="GO:0006614"

/db_xref="GO:0016020"
/translation="MFDTLTERLTQSLRNVTGSGQLTEDNIKDTLREVRMALLEADVA
LPVTREFIAKVKEEALGQEVMTQLSPGQAFVKIVYDELTKMMGEANETLDLSAKPPVV
VLLAGLQGAGKTTTAAKLARFLKERQKKKVMTVSADVYRPAAIKQLETVSAEVGAGFI
PSDPSEKPIDIVNRAIEQAKIQFADVLIVDTAGRLHVDEDMMDEIKELHAAVKPTETL
FVVDAMTGQDAANTAKAFNDALALTGVILTKTDGDARGGAALSVRAITGKPIKFLGMG
EKLDALEPFHPDRVAQRILGMGDVLSLVEEVERKIDKEKAEKMAKKLQKGGSFNFEDM
LMQFEQMKKMGGMMGFLDKLPGMSSAGIQQAIEQANPEKQVKKMEAIIQSMTIKERRN
PDLMNPSRKKRIAAGCGMDVAEVNKLIKQQAQMAKMMKKFANPSGMSKMMRSLGNMQK
QFGGGGGMGPLFGNNDQKK"
/product="Signal recognition particle protein Ffh" /transl_table=11
CDS 1140836..1141645
/locus_tag="AUO97b_01244" /db_xref="SEED:fig|400667.82.peg.1104"
/translation="MISLPLVYTILALIAYTTSFWYLFIRLMSKREPNPWLFAFVTTL
ALLLHGTVLCHAMLTPSGINYDVFNLVSFTSGLMLLLSLVFSTFRPIVPLNLLGIPVA
AIGLILGFAFSRPDQFIEQHSLGLDTHIILSLSAYAVLLMATIHAILIWFQNRELKKK
QKKRIWVNLLPSIQAMESLLFDLIITGFILLTIALAFGFLTVDSFFAQHLAHKTVFSI
ISWFIYGSLLIGHYKLGWRGQKAIRFTLIGFALLAIGFIGSKFVLEMILGR" /product="Inner membrane protein YpjD"
/transl_table=11
CDS 1141730..1142674
/locus_tag="AUO97b_01245" /db_xref="SEED:fig|400667.82.peg.1105"
/db_xref="GO:0008033"
/db_xref="GO:0016491"
/db_xref="GO:0050660"
/translation="MKLILAPMEGLTDPIMRDTLTSVGHFDWCVTEFIRVTDSILPDH
IYYSFCPELKNDGKTAAGTPVHVQFLGNNPEMLAANAAKVVELGAPAIDLNFGCPAKT
VNRHRGGSVLLDEPDVVHMLIKAVRDAVPAHIPVSAKMRLGYLDENHTMENAYAVEDA
GASWLTVHARTKADGYTPPAYWEKILPIKEALKINVIANGEIWTNADARACQQQSGCE
DLMIGRGAVTTPDLTQCIRQNMDEALFTWEQLVDLQIRFLNGQAKTEIGMVGRYKQWL
GMMTKAYPQAKELWDQVKRIKALDEIVQQLEQTSSSRI" /product="tRNA-dihydrouridine(16) synthase" /transl_table=11
CDS complement(1142754..1143707)
/locus_tag="AUO97b_01246" /db_xref="SEED:fig|400667.82.peg.1106"
/translation="MKNKNIGCWLLLAGSSSVCAMQPLDDQSLAAATGQNGLTLGIQA
DQVKFNQVALIDTNGIASTSYNSKAGLIIAGNSTNPVPGIEFIKAAVSANPSFNIAID
TDAGGGNPFLNLAVTMGSDVNGIRLLPFSVYLAPSTSLSSPSDYALTSYAPKSIFSSG
TTVNTGVKELIRSTGNLDINFVQTNKPRLNIQLGHAAQSVMVKFGGAIQSICSTASGC
PITLVSDNTGATFGFKFAGTNASTGFVLDGFYAGVDPTGLTIGNIGVSSKFDASLNNV
TLGNLGTQSTTTFNNLPNGSMGSFGVTGASVTDFKMKVSGF" /product="hypothetical protein"
/transl_table=11
CDS complement(1143869..1146280)
/locus_tag="AUO97b_01247" /db_xref="SEED:fig|400667.82.peg.1107"
/translation="MNKNKESHGSKFILNTLTASMLLVSGQVFALEALTDADLSAVNG
QDGISIQTTYNEINIDNAYWDDHAGTSNSADQVLRAQAGGIKVQKSNASTQTLGTNYR
LDVGSNPTTGKTGLDFSLQSNPSLITVNSFKVCNSSSICSPTMGQLAIQTSSPLSLAL
TTQDGLFNANSQSSMRLGLNNANIYLGQVDARTQLNQLILKNFNFNFVGKGAMLIDPN
RGLVLQTNTGSNTAAVGQTPNSTYGYVDFNRVADSASGLTAGTYVDSSGKVTNSGLNI
EVMLSSNVDKSNPYALDTTNSPQNSKGLIRLGASGRMVNSYLQVRGMDGSSDKTTLGT
ANTASGTGSSNSILGNSGIAFRMKGEFTKDNDSMLGSDGKATTLEIGGAGLNAYGFEF
GNLTGLNSATRGYFDSGNIYLNLADTKTLLMPNNATLNAIRLGSGTLTTAADYQHNIH
RDTVTNPFSLILAMRGAEFQAFSRRGRFTTSANVAAANQFADNGLNNQWGLALPFYNL
NANAAVYGVDAPANSAYYYTKDANGKPIQNLVATSGTTSRLGFGIAVGTTGRDAGGTK
TTSILLIDGSPNANNAGNPTDYYMGLRNIDMFLKGNGTIGLENGSLNIGLKDMLLALS

TEIAAGYLPGAKYKTCPATGSCTSPIDNFAKNNDVLFGLKLRLGGDLNLSIVPNSSIA
DGSALTVLGDFTMPATATGNTVQISDPIDGSAIGFDNITGKLAFNTALVVGKDTASGL
GKVGVNTAVYFNPDKSIDGALRVKDINFYPPSTGAGARLGELAITGGRLNSSFSIVPR NGAFN"
/product="outer membrane autotransporter barrel domain" /transl_table=11
CDS complement(1146313..1147038)
/locus_tag="AUO97b_01248" /db_xref="SEED:fig|400667.82.peg.1108"
/translation="MKKITALLFALCATVTHAELQTLDNDALQAINGQAGADLSLMLN
LNQTSNGIFDNGSGGVCENVEFCHIGLAINKRFVQADTTKPSGWAENSDSGNKLWLVF
KGVQGTLNIQKMGLDGADLKYLDKSNTEIIKPAIQLSFSAAQPILIRNFGFNALSIEQ
DNFTSSTTTQGSSANMYDYAYFKKPTYAATANSANQQVATATPSAYDYGRETGFMGLM
MSGNMAIQGKVMVFSCDGSHPRC"
/product="putative signal peptide"
/transl_table=11
CDS complement(1147054..1149087)
/locus_tag="AUO97b_01249" /db_xref="SEED:fig|400667.82.peg.1109"
/translation="MTTLNYTVRFQKTVLASLIGLFISQSSFALEELSDAGLSETTGE
GIAILPQNTYMVFRGAGANETTNQILTDRTKDTGYINYVPVGPLSMTAADTNKNGTID
SGDRAVGKADIFLYGLALSKSDNDTNTRLASTEAAAAISSWGTAVNPWIFKVATENSV
PNFSATNCSGAADPTCQVTYLALEAPLYEVGTRDTAGLDAYKLKLGLWSDIFVRNPNK
INGATDQFNYGDSNGLIGTSTDASRANRLRLQAIWNNFSLNGSRLQLFQTLGGATSAG
GMSPFYNDTLGFAGVIRLNSGDASNLRATITANTPTSTVGPWVNRYSTQYTGAPSNNS
PSSDWLYRIRSQTTTITSTGSWTAPTDSTMNNVLRLSTRESGTGQGNLITPAINGGLA
PTFDANEGLYLYNPNINLVLGSLYQPLVLSSDGKNFSLELARIPNKPEIYKKIYTDYT
GNDSSYLGSTCNVYQCGKNVTLGGRTYQGSSATHSSISIGSTVYNATTNTLEAFKGNN
TQDAVGISFGKLPTGTVAATTQTRNFYQLQNQERRVNSYTCSLIFTCYDWQYRTATGW
TGNAGSGLRFDSQGANWANIDSTAYYNPTTNTTGYTTTDAGNGAQFVVPNGTPLPDAL
YNNARWYTTTPNADINTYKLSGAQISSSVSNNMGSAVIDGVLIQHLKLTTKGL" /product="FIG00350393: hypothetical protein" /transl_table=11
CDS complement(1149306..1150646)
/locus_tag="AUO97b_01250" /db_xref="SEED:fig|400667.82.peg.1110"
/translation="MRQFTSLQVAILALGSLCFSSAYAGSTLVPMTDAELSATRGQAL
MSMSYIAPNDSANLEKLRDSSSNVGFFKLSLDADIELNTNIRKLQLGCGGANGAGACD
IDIDNLSLSGLSNTNDGRASSSAKITNPFIEFAIKNPNSTALREVTGLRVSAEAIEGL
LTFGTENSQTKNGINSLSGYMEVAQAGGTVKVNPVNGLRPADVNNTQISGEACGYLAC
VPFQTYDYTLNLTTDAAGTSTDKLTGTLFLPQQTITGKRISVAPLKATATVSGINVSG
NIKAIAAGLLNLDKSVTGSINNLAVNVDISEDLGFFHKANLNGSPVSLSLQGNDIRWP
GTKSVAERGWWLELSNPIDIGDITPAESVSIANKTIEETLALVSQKLSTGTRGNTKVD
CGDLGLLSCIGGSTINIGNLNLAGATPVQMSLSNLVLKNQTFATNCHGADVKFC"
/product="hypothetical protein"
/transl_table=11 CDS complement(1150943..1151512)
/locus_tag="AUO97b_01251" /db_xref="SEED:fig|400667.82.peg.1111"
/db_xref="GO:0008829"
/translation="MAIKSDRWIREMSEKHGMIEPYAENQVRFDKNGEKLISYGVSSY
GYDVRCAREFKVFTNVHSAIVDPKNFDEKSFIDIESDVCIIPPNSFALARTIEYFRIP
RNVLTVCLGKSTYARCGIIVNVTPLEPEWEGHVTLEFSNTTNLPARIYAGEGVAQMLF
FESDEICETSYKDRGGKYQGQTGVTLPKT"
/product="Deoxycytidine triphosphate deaminase (EC 3.5.4.13)"
/EC_number="3.5.4.13"
/transl_table=11
CDS complement(1151627..1152220)
/locus_tag="AUO97b_01252" /db_xref="SEED:fig|400667.82.peg.1112"
/translation="MISWLFIGLVATILLTPGPTNTLLASSGVQVGLKKSAKLIPAEV
LGYVISISAWGMLIGKVSATLPLLPPILKLLSACYIIFLAIKLWHTANQQLELDQPTI
RPRELFCATLLNPKALLFASAIFPVAAWTNFHIYLIHMLAYLALITPIALFWIFLGSV

LAANKVRWLNQSNLQRTASLVLVSFSIPISYSALLNL" /product="Transporter, LysE family"
/transl_table=11
CDS 1152577..1153143
/locus_tag="AUO97b_01253" /db_xref="SEED:fig|400667.82.peg.1113"
/translation="MIMAKQFFQSWLPSPEKVSNMKFLRIFGQKTLNPVLWYVNRKSI
TRAIFVGTFFGLLPIPFHSVFIVMAVLLFEVNLPISLMLAWLSNPLTLAPILYIGFWF
GAHIYHVHMINKKMLLGVLHQISRWITHFGHGHIDLSLAKILISGLIVEAFVFATILY
IVTEVFWRWMVIRNWKNRTGHKKQEDAL"
/product="COGs COG3216"
/transl_table=11
CDS complement(1153174..1154046)
/locus_tag="AUO97b_01254" /db_xref="SEED:fig|400667.82.peg.1114"
/translation="MQFKYSLGVISLCSALFLVGCNDDNSSVSQTPTVQEQKQKLQPI
IIQGALPVESERMASKLENKTVEKIGGWTFWKGTYNGYPMIISKTRMGMSNSAAATAL
AIERYKPIAIINQGTAGGHDPDLHVYDIVLGKYATNIGAFRTPKQPLGGGSNSLTWVE
AFDVLPTEESEPEPIAIRKFEGDQELLMAAHKVRYDKGEVVEGTIGSADVWNNELDRI
QFFHERYGTSVEEMEAASVAQIASQLNVPFLGIRILSNNITNNGAYDPGTGEACQDYV LNVAEEYMKSKLPK"
/product="5'-methylthioadenosine/S-adenosylhomocysteine nucleosidase related protein VF1653" /transl_table=11
CDS complement(1154192..1155421)
/locus_tag="AUO97b_01255" /db_xref="SEED:fig|400667.82.peg.1115"
/translation="MSWLSSLKSVFSPAQEVKEEEIQTVLQNYLLPDSKDALKERISQ
LQVQGRVLQLTINTFPDEKEYLQQIHDDLAGALQKCGIEELNLHVVQQKRPTQESSGQ
GCSSKAPKENSNLPPVLDASPKSEPDPNNPPIQKAAPQQRDVPLHPRIKNVILVSSGK
GGVGKSTTTVNLALALQKMGLKVGVLDADIYGPSIPTMLGNAGKTPLIESENFVPLDA
YGMAVLSIGHLTGDNNTPVAWRGPKATGALMQLFNQTLWPDLDVLMIDMPPGTGDIQL
TLAQRIPVTGSIIVTTPQNVALLDATKGIELFNKVGIPVLGVVENMSTHICSNCGHEE
QIFGIGGGDKLSEQYHIPLLGRLPLNAQIREHADQGKPSVIAMDDAADSYIDIAKAVW QQIEKIPQRARDDKRIF"
/product="[4Fe-4S] cluster assembly scaffold protein Mrp (=ApbC)"
/transl_table=11
CDS complement(1155567..1156067)
/locus_tag="AUO97b_01256" /db_xref="SEED:fig|400667.82.peg.1116"
/translation="MTTENKLDELKAKAADAKVQGEKALDDLKENVKEKQTAGKEAVA
DKVDELKTKAADAKVQGEKALEDLKENVKEKQAAAKEAVEDKASDLKGKLDDAQHSLQ
DKFDHLRTEAAHKLDDAKAKAAELKEEAATKFDELKTQATAKFDELKKTATEKLNKLK NHDSAE"
/product="hypothetical protein"
/transl_table=11
CDS 1156353..1158416
/locus_tag="AUO97b_01257" /db_xref="SEED:fig|400667.82.peg.1117"
/db_xref="GO:0004825"
/translation="MRKILVTNALPYANGPIHMGHLLGYIQADIWVRAMRAMGHDVTY
VCADDAHGTAIMLRAEANGISPEEQIANVQKEHIRDFDGFGVHFDHYDSTHSDANKAR
STDIYIKNREAGNIAVRPVTQLFDPEKGMFLSDRFIKGTCPKCKSEDQYGDSCEVCGT
TYNATELLNPRSTLSGATPVEKSSDHYFFKLPNFAEYLQKWTRDEGRLPLSIANKLDE
WFEAGLADWDISRDAPYFGFEIPDAPNKYFYVWVDAPIGYMSSFENYIKTKRPDLNFD
DFWKKDSQNEVYHFIGKDIVYFHALFWPAMLEGANYRTPTGLFVNGFLTVNGQKMSKS
RGTFIKAETYLQHLNPEYLRYYFASKLSDKVEDSDLNLDDFVQKVNSDLVGKVVNIAS
RCAKFINSSFNNTLSSTCAESDLVQSFIDAGDSIAAAYEAREFSTAIREIMALADRAN
QYIDEKKPWALAKQEGQEQQVLDVCSVGINLFRQLAVYLAPVLPTLAQQVQDFLKLES
FDFESRKQILVSHEIAQFQPLMQRVDPKAVAAMVDASKESLGTPAPQATKAAKKEKSA
EKKAAPTPAVGEAEIIGIEDFLKVDLRVAQVVEAGTVEGSDKLLQLTLDVGEAEPRNV
FSGIRSQYAPEDLKGKLVVMVANLAPRKMRFGISNGMVLAAGNGEGIFIISPDSGAKP GDKVS"
/product="Methionyl-tRNA synthetase (EC 6.1.1.10)" /EC_number="6.1.1.10"
/transl_table=11

CDS complement(1158545..1158673)
/locus_tag="AUO97b_01258" /db_xref="SEED:fig|400667.82.peg.1118"
/translation="MRTTSKQQPSNERAMSIAGSPVYEKDYKIPDLQANNHEQSNY"
/product="FIG00350235: hypothetical protein" /transl_table=11
CDS complement(1158896..1159519)
/locus_tag="AUO97b_01259" /db_xref="SEED:fig|400667.82.peg.1119"
/translation="MFESLIPLFSIAMALMLGAISPGPSFIYVAQNSISKSRKHGLFT
ALGTGTGAALFGFLAVMGLQAVLLAVPSAYLILKIGGGLYLLWLAFKIIKHAKEPIAM
ENDAKSKMTYKQAYRYGLITQLSNPKIAVVLASVFTALLPKEIPNYYYVALPLICFMI
DAGWYSCVAMLLSSEKPRKMYLKAKTGVDRVAGSIVSVLGLKLIFFK" /product="Threonine efflux protein"
/transl_table=11
CDS 1159698..1160267
/locus_tag="AUO97b_01260" /db_xref="SEED:fig|400667.82.peg.1120"
/translation="MSKRQKIAAHNRDELLNAAEECFRIHGINVPLQVVIDHAGVGRA
TFYRNFCDRKALISALLERAITQLEQKAAHFQQFEDGLFRLIEGHIAQLPKLAILQDF
WRVIDRQDPIMLKIYERRNNALKPLIENAIEQKLCRADLTADDYAMVTAMLGSSFQGH
EPEEQTRLAKRAIELLFHGIQVQKIRGIE"
/product="Transcriptional regulator, AcrR family" /transl_table=11
CDS 1160264..1161907
/locus_tag="AUO97b_01261" /db_xref="SEED:fig|400667.82.peg.1121"
/translation="MSKTPRYLETPPDWTPEEQPTLPGSPAAIAHSVPKRFLYFFIGL
FVAISASLSNGFITANLPLIQGEYGLTPSEAAWLPAAYVMANVSSNLILFKARQQYGL
RVFSEIGLVIFIAVLVLHIFVHTYEMALFARVVAGLAGAPLSSLGMYYTMQAFKKADM
AKGIYIAFGFQQLGVPLAWIISPFLVSTDSWSVLYTFELGLALCCLAMVVSLKLPRSL
RIYVFEKQDFFTFALLAPGFACLCIVLTQGPILWWFNSEWLAYVLIAGFSLVVLGLLY
EHYRANPLIMTRWLGASSTLRFIFGAFAIRLLMSEQSYAAVNFLKTMGMGPDQFVPLY
VVILVGILSGTLFSALTFSRERIIFHLLFAEFLILIACGLDYHLTSDVRPSNFFASQF
LVGFAGGLFIGPLLLIGFAQTLAKGPSYVVTFIVLFSATQTFGGLVGSSFFSTYQQHR
TQNYQVEIIKDLEQADPLVAQRLSTYQRSAAITTNDPALQTAQSMRSLNQVVTREAQV
RAYNDVIALNGIFAVALLLWGGFNIARSKYLQRRGISRP" /product="Inner-membrane proton/drug antiporter (MSF type) of tripartite multidrug efflux system" /transl_table=11
CDS 1161948..1163075
/locus_tag="AUO97b_01262" /db_xref="SEED:fig|400667.82.peg.1122"
/translation="MSDDQNKPEQTPAQTSNNEPAPAPAPAPASKLIPTKRSTLLWML
GVLIIGILVILWAWRIGPFATSVQQTDNSYVKGKTTILSSQINGYVKDVLVKDFDHVK
KGQVLMHIDATTYDQKVAQAASGVEQAKNTLANQTQSIAQKQADIVAAQAKVEQVRAQ
YELSLAQLRRYQQLGNSGAASKSEQDKAAADAENNLAALKQAEANVLVAKEALKTAQV
AEAGLEAQVSSAKAQLDQAQTTKDYSVIVAPMDGQLGEVNPRVGQYVAAGSQLLYLIP
QQTWVIANFKETQIANMRIGQKAWFTVDAMKHKKFTGHVEQISPAAGSEFSVLKPDNA
TGNFTKVVQRIAVRITIDPNQEGMEHLRPGMSVITSVDTSS" /product="Membrane fusion component of MSF-type tripartite multidrug efflux system" /transl_table=11
CDS 1163140..1164021
/locus_tag="AUO97b_01263" /db_xref="SEED:fig|400667.82.peg.1123"
/translation="MFDHIAQPISYQHIELSFPVHLDIKRLDLVHPQISGNKFFKLKY
NFLAAKEQGLSSILTFGGAYSNHIAATAYAAHLFGLKSIGIIRGEELAGKPLNPTLAK
AQSLGMQLHFVSRHEYRLRDDANYLQQLQQQFPQTYIIPEGGTNELAVQGCQEILSQY
DLDQYDVICCAVGTGGTISGLIERSAAHQKVLGFSALKGDFLQQAIRQWTKKQNWSLT
DVYCWGGYAKTSPELFAFIENFEEQYTVPLEPIYTGKMMFGLFDLIKNNYFPANTRIL AIHSGGLQADIRNKPHT"
/product="pyridoxal phosphate-dependent deaminase, putative"
/EC_number="3.5.99.7"
/transl_table=11
CDS 1164139..1165338
/locus_tag="AUO97b_01264" /db_xref="SEED:fig|400667.82.peg.1124"

/translation="MSNSHVDVIVLGAGASGLMLAAHAAKRGRSVLILEKANKIGKKI
LMSGGGKCNFTNLYVEPENYISHNPHFVISALSRYSNWDFIGLVCDYGIAYEERKHGQ
LFTLNGAKEILEMLVSECNKTNKVEIQTHCEVGQVDPLEQGGFSVHTNQGRFSCESLV
VATGGLSIPTLGGSGFGYELAQKFGHTVFPTRAGLVPFTFSDHIKEVTTRLSGNALDV
TLTNSKNSFTEALLFTHRGLSGPSSLQLSNYWNTGESFKIDFFPSQDLATYLKDKKKA
QPKVLLRTLLNEFTAKSIVQELQQLIWPEQSETPIGNISDAQLDHIAEQLHGFTVKPS
GTEGYRTAEVTLGGIDTTEVSSKTLESKKQKGLFFIGEVLDVTGHLGGYNFQWAWSSA YAAAQYV"
/product="Uncharacterized protein YhiN"
/transl_table=11
CDS complement(1165351..1165521)
/locus_tag="AUO97b_01265" /db_xref="SEED:fig|400667.82.peg.1125"
/translation="MKKPGRFLAPVVIAGITVGFLASAYKFVFSKSESSKQTTSHKHD SEKSSNDSKASN"
/product="hypothetical protein"
/transl_table=11
CDS complement(1165650..1167326)
/locus_tag="AUO97b_01266" /db_xref="SEED:fig|400667.82.peg.1126"
/translation="MNEFFNGPRGAQFDAMYYWDQFHPILAAIVILLVGWIIALLVAA
GVKKLLQKVNTNAKLSSATGRTPNIEGLISKVVFWFILILAVVAALNVLNISGVSGPF
SNMVNQVLVYIPNIIAAVVVGFIGWIVARLVRAGVTNVLSRTQLDDKLSSEVGVGGIS
QNIGEILYWLVLLLFLPIVLSILGLTGLLIPVQNMVNDAVAFLPNIFIAGVIVFVGYI
LAKIVRGIVEGLINSLGVQSQAEKIGLFKNSNVGKFLGSFVFAIIIITTLIVAFEALG
IETISQPATAMLNEILQAIPNIIAAGLILLVAYIVSRFVGRLVAELIAGTGVDEIPAK
LDVQRFLGQTKVSNAVGWLIVFFTMLFAVSEAADRLGFDQISGLIAMFIHFGANILLG
AVILVIGFWLANVVANVVQRGEYNSSRWLGSLVRVLIIGLVLALGLRAMGIADSIVNL
AFGLTLGAVAVAFALAFGLGGRQPAERLLSDLLDKAKKEANQPNPLYQPPTNNKTTGS
SSTVSTPSTTTTAPSTAVSSDAKPADSAQVNPSQPPVNKPFGSTGENDEI" /product="Small-conductance mechanosensitive channel" /transl_table=11
CDS complement(1167554..1167781)
/locus_tag="AUO97b_01267" /db_xref="SEED:fig|400667.82.peg.1127"
/translation="MHDFSKPPHVTSPEETAKKRRLPVYILIIVGLFLLALVVYMVAD
HSKTPKEEAAFQHETIPQVISGTSDLKTYEG"
/product="FIG00350985: hypothetical protein" /transl_table=11
CDS 1168074..1168493
/locus_tag="AUO97b_01268" /db_xref="SEED:fig|400667.82.peg.1128"
/translation="MYRQQSVTLDLDTDVTHQCYLHQTRDDHLIGIIEFNKPSYELKW
GDLEYFRRRTEEFSVMPFPECINAMIVDIRNINVFLDNEVPILPWRLLEEDCPVRLVV
PQDRLEHYAGLFEPTWLSTDVDSAIAEIREFMDMFVH" /product="FIG00351014: hypothetical protein" /transl_table=11
CDS complement(1168544..1169305)
/locus_tag="AUO97b_01269" /db_xref="SEED:fig|400667.82.peg.1129"
/db_xref="GO:0004324"
/translation="MSIEKFSVEKVLSVHRWTPTLFSFTMTRPSHFKFTAGQFARIGL
KVGDELVVRAYSVVSSPFDETLEFFSIVVPDGAFTSNLQHLKVGDELYLEKIPYGYLT
LARYQQPLPHDLWLLATGTGLAPFLSMLQDFETWSNYQKINLVYSVRTAAELAYVDRI
QEIAETFGEGHSGFKFIPIITRDPSAPLHERLPVLIENGELEKVAGLELSPASSHVML
CGNPQMVDDTKEALKCRGLTMNRRGEGNIAVENYW" /product="Ferredoxin--NADP(+) reductase (EC 1.18.1.2) @ Flavodoxin--NADP(+) reductase (EC 1.19.1.1)"
/EC_number="1.18.1.2"
/transl_table=11
CDS 1169392..1170279
/locus_tag="AUO97b_01270" /db_xref="SEED:fig|400667.82.peg.1130"
/translation="MRMTLRQLAVFVAVAQEGTVTKASDAVRLTQSAASMALADLEDG
LGAPLFDRLGKRLQLNDLGRFLLPQALEILGRCEAFEQAAKGELQSIDLRIGATLTIS
DYLMPDLMANFLQRHPQAHLQLQVGNTRQMIEAVNQFQLDLALIEGSCHLPQLQCIHW

RDDELAVCCSPDHPLTRLNRPLTPQDFHDVEWILREEGSGTREVFDNAILQDLPDANI
RLTLGHNEAILKIVAGGLGMSCISKLAIEPLSEKGQLVILDTPFWQLTRPLYMLVHRQ
KYQGPGLKAFLKFCDEDQA"
/product="Transcriptional regulator, LysR family" /transl_table=11
CDS complement(1170291..1170638)
/locus_tag="AUO97b_01271" /db_xref="SEED:fig|400667.82.peg.1131"
/translation="MVLHYRQQAQQRASHEKVQLLIQQQKTIIEAQRAALGKLPDVQL
SEKTKKALALTSEKVPERVNDETSAFQCDGREYCTQMHSLEEARWFVRNCPNTKMDGD RDGEPCENDSRWH"
/product="Cold shock domain family protein" /transl_table=11
CDS complement(1170904..1171197)
/locus_tag="AUO97b_01272" /db_xref="SEED:fig|400667.82.peg.1132"
/translation="MKQEFYTGRVKQYNADKGFGFISTTEGDIFFHISDFPASEGEPK
RNEKVRFLAADNRGKFKAVKIERIDPNPAKTKKTKIADHNKAITSELLSNFRR" /product="putative
cold shock protein"
/transl_table=11
CDS complement(1171314..1171949)
/locus_tag="AUO97b_01273" /db_xref="SEED:fig|400667.82.peg.1133"
/db_xref="GO:0004845"
/translation="MAIQEIRHPLIRHKLGLLRRADISTKNFRELAQEVTMLLTYEAT
KDLPVVDCEIEGWAGNVTTQRIAGKKITIVPILRAGIGMLDGVLNLIPSAKVSVLGLE
RDEATLEVRTYYKKLVPDVANRIAMIIDPMLATGNSLVAAIDVLKASGCKDIRVMVLV
AAPEGIAKVETAHPDIQLYTASIDNGLNEHGYIVPGLGDAGDKIFGSVQKD" /product="Uracil
phosphoribosyltransferase (EC 2.4.2.9)" /EC_number="2.4.2.9"
/transl_table=11
CDS complement(1172050..1173546)
/locus_tag="AUO97b_01274" /db_xref="SEED:fig|400667.82.peg.1134"
/db_xref="GO:0008137"
/translation="MNFTVSFSTLMPLAPVMIVALTAVVVMLLISIKRNHNLIATTSV
VGLNLAALYILLELFGGKFVPANVMGMFMVDPFTMFYQFMILVASLACCTLSHAYIET
YKDNREELYLLLLASVAGAMLMVASSHYASFFISLELMSIPVYGLLAYTYQRSQSLEA
GIKYLVLSATASAMLLMGMAYIYAYTGSLSFYDSVQALFGAIKQPMVLLGLALIIFAV
AFKLSLAPFHKWTPDVYAGAPAPMATFLATAAKVATIGLFVRYLLASGAIMVNSLVTV
LTIIAVLSILVGNLLAVRQVNLKRILGYSSIAHFGYLLIALISMTYASLGSVTVYVVT
YVLTTIGAFGAVALMSSPYNNVDEAQSLADYRGLFWRRPVLTATLTVMMLSLAGIPLT
AGFIGKFLVVMAAVTTQHWFLAAMIIVGSGIGLYYYLRVMVVMYMTPPETPRIDADAH
WGQKVGGLMVLAAAALVIILGVYPDPMINLALKAEILSPLHFMLSQQQ" /product="NADH-ubiquinone
oxidoreductase chain N (EC 1.6.5.3)" /EC_number="1.6.5.3"
/transl_table=11
CDS complement(1173549..1175147)
/locus_tag="AUO97b_01275" /db_xref="SEED:fig|400667.82.peg.1135"
/db_xref="GO:0008137"
/translation="MENNLILPALILVPFIAGFTCWLVDKLDKTLPRWIALIGMLITL
GLGLALWQSGTYSYELGSKVPTWTAEFYLPWIQSLGIGIHLAVDGLSLLMVLLTALLG
VLAVGCSWGEIQKNVGFFHLNLLWSLGGVIGVFLAIDLFLFFFFWEMMLVPIYFLIAL
WGHKGSNGRSRVYAATKFFLYTQIAGLVMLIGILGLVVYGYMMTGMIGFDYNYLLAVA
NRLPAGFAYGFMICFFIGFAVKLPVFPLHGWLPDAHAQAPTAGSVDLAGILIKTAAYG
LLRFVIPFFPAASAQFADIAIIFGLIGIFYGAWCAYQQTDMKRLLAYTSISHMGFVLL
AIYAGNILTFQGLMIMMLAHGLSSAALFIMSGQIYERLHTRDLRLMGGLRGQLQYLPF
FLMFFVAALVGVPGLGNFIGEFLILMGSFNKYPVFTIIASISLVFAGLYGLILIHRAL
FGEPNSEQKQHYTSPLKDLSAREVSILMICAIGLVWLGIYPQTFLDVSHSSMQWLVNS
YVPVQEVVETVQQTATQLDHVEIQ"
/product="NADH-ubiquinone oxidoreductase chain M (EC 1.6.5.3)"
/EC_number="1.6.5.3"
/transl_table=11

CDS complement(1175149..1177038)
/locus_tag="AUO97b_01276" /db_xref="SEED:fig|400667.82.peg.1136"
/db_xref="GO:0008137"
/translation="MSYLYLTVLFPLIGFILLAAGRDKLSENVAAIIGVGSVGLSALF
ALIAGMAFTSSGQQVFVQNLWTWFNVGGFAPGISLHLDGLSLLMMGMITGVGFLIHIF
ASWYMRGEEGYARFFSYFNLFVASMLLLVLGDNLALLFLGWEGVGLCSYLLIGFYYAN
PANGWAAIKAFTVTRVGDVFLLIALFLLYQQFGTLNTQYIVEHAAEVMTKSSSLTIWT
ALMLFLGAAGKSAQIPLQTWLADAMAGPTPVSALIHAATMVTAGVYLCCRLFSVFELA
PEVMQFISVTGAVTLLVAGFAALVQTDIKRILAYSTMSQLGYMFMAVGAEAYQAGLFH
MLAHAFFKALLFLSSGAVILAYHHEQNIFKMGGLFYKNKFLFACFAIGGGALAAIPFL
TIGFFSKDAILGEVWVQGQSIALYNSLYWAGVAGAFLTSIYTFRLIWVVFFGKENTHY
HEIKGATYWAPLGILAVLSTFVGAALKAPVANILNTAKIPEFHVAEQFVEGMHGAEHL
AVGIALVGLAVGIGLFAFAYGAVKSFAKTSLGAGLAHICRTAFGFDALYDIVFVKPYL
FFAKLFGRDPIDSLWLVLPALVKGGNSFTSSRQTGSLREYASSMSLGVVVLLMILIVI QVVGK"
/product="NADH-ubiquinone oxidoreductase chain L (EC 1.6.5.3)"
/EC_number="1.6.5.3"
/transl_table=11
CDS complement(1177035..1177343)
/locus_tag="AUO97b_01277" /db_xref="SEED:fig|400667.82.peg.1137"
/db_xref="GO:0008137"
/translation="MGQIPLEHGLIVATILFALGFYGVMVRRNLLFMLMSLEIMMNAA
ALAFVLAGSVWAQPDGQVMFILILTLAAAEACIGLAIVLQFYHRFHHLDVDAASEMRG "
/product="NADH-ubiquinone oxidoreductase chain K (EC 1.6.5.3)"
/EC_number="1.6.5.3"
/transl_table=11
CDS complement(1177343..1177864)
/locus_tag="AUO97b_01278" /db_xref="SEED:fig|400667.82.peg.1138"
/db_xref="GO:0008137"
/translation="MMWPFYLMALVAIVSTVRVVTNTNPVHALLSLIVSLLAVAGIFM
IVGAPFAGALEIIVYAGAIMVLFVFVVMMLNLGQHTVEQERKWLSSDAWAYPALMSFL
LGLLLVWMLGGEYTTISAPLGAQVIGPKVVGQALFTHYLLLVEVAAMLLLAALVAAYH LGKREPGAEEEKE"
/product="NADH-ubiquinone oxidoreductase chain J (EC 1.6.5.3)"
/EC_number="1.6.5.3"
/transl_table=11
CDS complement(1177861..1178355)
/locus_tag="AUO97b_01279" /db_xref="SEED:fig|400667.82.peg.1139"
/db_xref="GO:0008137"
/translation="MVFTHGFRKRDTILYPEVPAEEIVPPRYRGRIILTRDPDGEERC
VACNLCAVACPVGCISLQKAEKEDGRWYPEFFRINFSRCIFCGMCEEACPTTAIQLTP
DFELGEYVRQDLVYEKENLLISGPGKYPDYNFYRVTGMAINGKEKGQAQKESAPIDVR SLLP"
/product="NADH-ubiquinone oxidoreductase chain I (EC 1.6.5.3)"
/EC_number="1.6.5.3"
/transl_table=11
CDS complement(1178422..1179438)
/locus_tag="AUO97b_01280" /db_xref="SEED:fig|400667.82.peg.1140"
/db_xref="GO:0008137"
/translation="MNQEIIRQTPLWAENWPIAYAVVQAVVILLVVVLVAALMSFIER
RLLAWWQDRYGPNRVGPGGMFQIVADMLKIMFKEDWTPKFADKLTFRLAPAVAMATAV
LSFMVIPVSPALGVADMSIGLLFFMAMAGIAVYAVLFGGWSSNNKYSLLGGLRSAAQT
ISYEVFLGISLMGVVAIAGSFNLREIVEAQRDVWFIIPQFLGFLIFVVAGVAVTHRHP
FDQPEAEQELAEGYHVEYGGMKWGMFFVAEYVNVVLISALIVTLFFGGWLAPFNLEIP
FVPPVFWFVIKTAFFVMMFVLARGSLMRPRYDQVMNFGWKICLPLALVNLLVTGAVIL MNQA"
/product="NADH-ubiquinone oxidoreductase chain H (EC 1.6.5.3)"
/EC_number="1.6.5.3"

/transl_table=11
CDS complement(1179442..1182126)
/locus_tag="AUO97b_01281" /db_xref="SEED:fig|400667.82.peg.1141"
/db_xref="GO:0008137"
/translation="MATIHVDGKSYEVNGSENLLQACLSLGIDIPYFCWHPSLGSVGS
CRQCAVTQYANPEDTRGRLVMSCMTPASDNTYISIEDKEAVDFRESIVEFLMTNHPHD
CPVCEEGGHCHLQDMTVMTGHDRRRYRFTKRTHYNQELGSFIAHEMNRCIACYRCVRY
YKDYAGGTDFGVYANASRVYFGRPESGTLESEFSGNLTEVCPTGVFTDKTHSERYNRK
WDMQYAPSVCHGCSSGCNISPGERYGEIRRVENRFNGSVNQYFLCDKGRFGTGYVNRA
DRPRQPQFRNGETVATVSVDQALDQTIAKLEGKKVLGIGSPRASLESNYALRELVGQA
NYSTGVAQKEQSLVELAASIMQTEGIYNPNMREIESYDAVLILGEDLTQTAPRMALSV
RQAAKNKAREMAAKTRTPDWLAEPVQRIGQEAKSPIYILAATQTRLADVATGEVVASP
NDIARLGFAVAAAVKGETVNGLDEDAKAFAQTIADTLKAANKPLIIAGTSLQDASIME
AAAELTQNLGSKAGLSLVVPEVNSMGLALFGGLSLEQAFAQDYDTLVIVENDLFRRLP
AAQVKAALDKAETVIVLDHSETETVKQADIVLSAASFAEGDGTVVSQEGRAQRFYQVY
DPSYYKPEYAIKESWRWLHAIETGLKGKPIDWTVLDDVIETIVKNVPVLEAIQDVAPD
AGYRVHGLKIAREPRRYSGRTAMRAPLSVHEPKQPTDPDSALTFSMEGYVGPQKASSL
VPFAWAPGWNSPQSWNKYQDEVGGHLKGGDSGVRLFDRLAKRPARSYVAPTAVVANPE
SFRLVPMHHIFGSGEFTIKTPAMETRIPEAVFAVGEQDATRLNLQEGQRITVKAGETT
VSLPVQVIEYLPTGYIGYPVGLAPTVSLAEPVSVALGV" /product="NADH-ubiquinone
oxidoreductase chain G (EC 1.6.5.3)" /EC_number="1.6.5.3"
/transl_table=11
CDS complement(1182138..1183469)
/locus_tag="AUO97b_01282" /db_xref="SEED:fig|400667.82.peg.1142"
/db_xref="GO:0008137"
/translation="MNTEPKPIYGDGNPETHPLTWRLSKQESVRSADDYEALEGYAGF
KKALGMKPAEVLEVIKAATVKGRGGAGFPAGIKWSLMAPNDGGPRYLICNADEMEPGT
FKDRLLMEKLPHQLIEGMLIAGYTLEATQGYIFIRGEYIEAAQYLNEALEQIRAKGYL
GDNILGSGWNFELHVHTGAGRYICGEETALINSLEGRRANPRTKPPFPQVAGAWGRPT
IVNNVETYNNLPAIMLRGPEWYIGLSAGKSKDPGTKIYGASGKVKFPGLWELPFGTTA
REVIEEHAGGMRDGLKLKAWLPGGASTDFLPADTIDLPMDAETIMKAGSRLGTCLLMV
VDETQCMVSLTRNLEEFFARESCGWCTPCRDGLPWAVKALKALETGEGKKEDIDHLQE
LTRKLWIGKTFCAHAPGAMEPLMGALKHFRSEFEAKVTDRPVVQTSNVEQA" /product="NADH-
ubiquinone oxidoreductase chain F (EC 1.6.5.3)" /EC_number="1.6.5.3"
/transl_table=11
CDS complement(1183466..1183975)
/locus_tag="AUO97b_01283" /db_xref="SEED:fig|400667.82.peg.1143"
/db_xref="GO:0008137"
/translation="MMILTDKKPRVNVEGILTAEEIHEIEHHIGHYPYPRAASLDALK
CVQRRNGWVDDAQMNAIAQLLTISVADLEGVATFYNRIYRQPVGRHVILLCDSIACFL
MGAETLAEAFQRELGIQFGQTTQDGRFTLLPICCLGNCDKGPTLMIDEDTHGLVEVTS IKQLLEKYV"
/product="NADH-ubiquinone oxidoreductase chain E (EC 1.6.5.3)"
/EC_number="1.6.5.3"
/transl_table=11
CDS complement(1183989..1185776)
/locus_tag="AUO97b_01284" /db_xref="SEED:fig|400667.82.peg.1144"
/db_xref="GO:0008137"
/db_xref="GO:0008137"
/translation="MAETDIAMPESTPVDSRPAFAIVEELKTKFGENFYVQATFEEFP
TVWVERARVQEVLMFLRKVERPYVMLFDLSAMDERLRQHRDGLPASDFTVFYHLLSLE
RNSDIRIKVALNENDLNLPTATNIWPNANWYEREAYDMFGINFEGHPMLRRILLPTYW
EGHPLRKEYSARATEYTPYMQDKAKQDFEQEHLRFVPEDWGLKRGNADEDFMFLNLGP
NHPSAHGAFRIVLQLDGEEVKDCVPDIGYHHRGVEKMAERQTWHSFIPYTDRVDYLGG
CAQNMPYVMAVEQLAGIKVPERAQVIRVMLNELFRINNHLLYCGTAIQDAGGMTPVFY

MFADRQKVYDIVEAITGYRMHPAWFRIGGTAHDLPNNWQKLVKELLDWMPKRLNEYYT
AAFKNSVFIGRTRNVAQYDAKSALAWGVTGTGLRATGIDFDVRKYRPYSGYENFDFEV
PVEYEGDAYARVLVHFREIEQSLKIIKQCLDNMPSGPYKADHPLAVPPPKDKTLQDIE
TLITHFLSVSWGPVMPAGEASFMTEVVKGASTYYLTSDKATMSYRTRIRTPTFTHLQQ
IPSVINGSLVSDLIIYLATIDVVMADVDR"
/product="NADH-ubiquinone oxidoreductase chain C (EC 1.6.5.3) / NADH-ubiquinone oxidoreductase chain D (EC 1.6.5.3)" /EC_number="1.6.5.3"
/transl_table=11
CDS complement(1185860..1186537)
/locus_tag="AUO97b_01285" /db_xref="SEED:fig|400667.82.peg.1145"
/db_xref="GO:0008137"
/translation="MKYTLTRANPDADQYPLQDRQIVTDPLEEEVNKNVFMTRLEDVL
HTAVNWGRKNSLWPFNFGTSCCYVEYATTLTGVHDLSRFGAEVIRASPRQADLMIVAG
TCFVKMAPVIQRLYEQMLEPKWVISMGACANSGGMYDIYSVVQGVDKIIPVDVYVPGC
PPRPEALIQALMLLQDQIQLERRPLSAVIGDDLQPVYKPKMMPERDRKNAQRIAVKNL RSMDEIK"
/product="NADH-ubiquinone oxidoreductase chain B (EC 1.6.5.3)"
/EC_number="1.6.5.3"
/transl_table=11
CDS complement(1186544..1187095)
/locus_tag="AUO97b_01286" /db_xref="SEED:fig|400667.82.peg.1146"
/db_xref="GO:0008137"
/translation="MSAITPYDWAIIAFVIGVTFLCVFMLTVPLLLGGKSWGRAKQEQ
FESGVVSAGGARIRLSAKFYLVAIFFVVFDLEALYLYAWSTSVREVGWLGYTTVVIFV
VDLLIALVYAFSVGALSWAPADRRKLAGEKIKVGSPTMNIAEITRFNSIEELVTDPTG
QIPAQSSGRVKSKTTPALSSEKE"
/product="NADH ubiquinone oxidoreductase chain A (EC 1.6.5.3)"
/EC_number="1.6.5.3"
/transl_table=11
CDS 1187523..1188944
/locus_tag="AUO97b_01287" /db_xref="SEED:fig|400667.82.peg.1147"
/translation="MPYSLREFSFSTSLKFFLLFTIIITLCCFIGIASRPLSFLAFFW
PANSVFLGLLLRFPQTRQIGSFAGAFTGYMIADLVNGTPLFLTLILTISNYLYVVTTL
ALAILFNRHIKAAYQGYSYLLLFALCGIGSITGAFFAATFVPMFNTKFMIGSFWAEFG
YWVTSELQNALLLLPIILNIPPYLKIKNHLKGNRIYIKAIDFLPLFAVLISIGVSYYD
SGPGSLLYPIAALIWCAIRYKPIIVALITSFTSAYIIYHVSGHYLLLYPQDYLGNTIS
IRIGLIMMTVAPLTVSSISALHSELIQKLQHAIAHDELTSSLTRRQFLQSVRLLQEKV
QKENKTSAFFMLDVDHFKKVNDSYGHLIGDRALQTCVTTIQNILHPHDLLGRFGGEEF
AIFIPDTTREAAFELAERIRIQISQQPIYVYGKLPIFVQVSIGISLYSPSYHRSIESL
FKEADDALYQAKRQGRNRVFISL"
/product="Putative Heme-regulated two-component response regulator"
/transl_table=11
CDS 1189037..1189390
/locus_tag="AUO97b_01288" /db_xref="SEED:fig|400667.82.peg.1148"
/translation="MNLEYTHKPDYYLFAQLLVRHIESYIHKHPDADNAIFDLRDVYE
IFRQDFASTTTNLEGILHIADSYRVETLNGDQPLIQKYQIDAKNNSLLIDFNTDALNS LRSGKPILEPDATQL"
/product="FIG00349934: hypothetical protein" /transl_table=11
CDS complement(1189413..1191005)
/locus_tag="AUO97b_01289" /db_xref="SEED:fig|400667.82.peg.1149"
/translation="MVAVFGYLLVQIINYQRAQEYRESLTDGISYVISEGVARQPGKQ
QKIDWISDASDLLELPIYYTDASKVELSRTEKKRIEAQKSVVRYDASNSIAYVIIGLR
DDPQHYLSIKVDKITERQMKALPIFVLDYLMFYPGQEQEYLAKIQKHFSYPINIQNIQ
DVNLDSEQIGRLRQDQSVMLYKDSATVRGTTISIVSPIPNHPAQVLVLGPVPMFNWMP
LQLSAGITLFSLFLLSLGVYGLILPLERKIRQVRYALNRMKSGDLSLRVPIEGSDEMA
NLASSYNNMSDHIQRLIEAQRELMRAVSHELRTPVARIRFGTEMLAEEDDYNHRMHQV

DMIDKDIEALNTLIDEIMTYAKLEQGTPSLDFAEIVLFEVLDQVAVETEALKTQKEIE
LIPPPLYVKVDAERRYLHRVVQNLVGNAVRYCDNKVRITGGIHNDGMAFVCVEDDGPG
IPEQDRKRVFEAFARLDDSRTRASGGYGLGLSIVSRIAYWFGGEIKVDESPSLGGARF
IMTWPAHRFKQPPLKTNKKAPA"
/product="Two-component system sensor histidine kinase" /transl_table=11
CDS complement(1191095..1191811)
/locus_tag="AUO97b_01290" /db_xref="SEED:fig|400667.82.peg.1150"
/translation="MSQEEKLPKILIVEDDERLARLTQEYLIRNGLEVGVETDGNRAI
RRIISEQPDLVVLDVMLPGADGLTVCREVRPHYHQPILMLTARTEDMDQVLGLEMGAD
DYVAKPVQPRVLLARIRALLRRTDKTVEDEVAQRIEFDDLVIDNGGRSVTLNGELVDF
TSAEYDLLWLLASNAGRILSREDIFERLRGIEYDGQDRSIDVRISRIRPKIGDDPENP
KRIKTVRSKGYLFVKETNGL"
/product="Two-component transcriptional response regulator, OmpR family"
/transl_table=11
CDS 1192344..1195178 /locus_tag="AUO97b_01291" /db_xref="SEED:fig|
400667.82.peg.1151" /db_xref="GO:0004748"
/translation="MSVITSTPGQIQVIKRTGDVAAFDAEKISVAIGKAFLAVEGQQS
ADSSRIHDRITQLTEMVLNTFTRRLPSGGTIHIEEIQDQVELALMRTGEHKVARAYVI
YRDQRASARKDTNSNHHPTLQVTDANGQLQPLDLSALQATVNRAAEGLEGIDVQAIID
ETVKNLYNGVKESDIATTMMMATRTRIEQEPNYTYVTARLLRDELVSTGLAFLGLPAD
TAENNALEAFLKKGVELDLLSPDLLKFDLEKLAAAIQPERSNQFTYLGLQTLFDRYFI
HSNGVRFELPQLFFMRVSMGLALNEQDKEERAIEFYNLLSSFDYMASTPTLFNSGTLR
PQLSSCYLTTIGDDLYDIYGAMRDNAMLSKWAGGLGNDWTPVRALNSYIKGTNGKSQG
VVPFLKVANDTAVAVNQGGKRKGAVCAYLETWHLDIEEFLELRKNTGDDRRRTHDMNT
ANWVPDLFMQRVFEDGEWTLFTPSETPDLHDLTGAEFAERYAYYESVAKEQNMLHKKV
RAKDLWRKMLSMLFETGHPWITFKDVCNLRSPQQHVGVVHSSNLCTEITLNTNQDEIA
VCNLGSINLVQHVKGGVLDREKLARTVKTAVRMLDNVIDINYYAVPQAKNSNLKHRPV
GMGIMGFQDALYEMGMAYGSDEAVNFADESMEVISYYAIQTSSDLAVERGAYSTFKGS
LWDQGILPIDSLEIVAKSRPERMFEVDRTQRLDWDSLRAKVQKDGMRNSNVMAIAPTA
TISNICGVSQSIEPTFQNLYVKSNLSGEFTVINPYLVRALKERGLWDTVMVNDLKHFE
GSVQKIARIPEELKAIFATAFEVEPRWIVDAASRRQKWIDQAQSLNLYISGANGKKLD
ITYKMAWLRGLKTTYYLRALGATSAEKSTINTGALNAVKPATVEAAAPAAAPVVEAKK
PEAVEEDGFTQAAPVPMACSIDNPDCEACQ"
/product="Ribonucleotide reductase of class Ia (aerobic), alpha subunit (EC
1.17.4.1)" /EC_number="1.17.4.1"
/transl_table=11
CDS 1195234..1195368
/locus_tag="AUO97b_01292" /db_xref="SEED:fig|400667.82.peg.1152"
/translation="MFFHLSHNYMLGVGILIFAFIFFYTPMVYAFFKIRKQQRKQNKL "
/product="FIG00351687: hypothetical protein" /transl_table=11
CDS 1195506..1196789
/locus_tag="AUO97b_01293" /db_xref="SEED:fig|400667.82.peg.1153"
/db_xref="GO:0004748"
/translation="MSILSWDDFEDDSQKPAAPEHKSAPVNPEKTPDSQNVSAAQPSS
QSVAAPQSAGTHSVRSTNPTDSMARASEALEHLDVAPGLEELEMGAQRVQVDDKAMIN
CRADLNQLVPFKYEWAWQKYLDGCANHWMPQEVNMNHDIALWKSENGLTEDERTIVMR
SLGFFSTADSLVANNLVLAIYRHITNPECRQYILRQAFEEAIHTHAYQYCIESLGMDE
GEVFNMYREIPSVARKAAWGLKYTQSLSDPTFHTGTPENDQRLLRNLIAFYCVLEGIF
FYCGFTQILSMGRRNKMNGVAEQFQYILRDESMHLNFGIDMINQIKIENPHLWTAEFQ
QEVIQMILEGTMLEIEYARDTMPRGVLGMNASMMEEYLKFICNRRLSQLGLPEQFAGV
TNPFAWMSEMMDLRKEKNFFETRVTDYQTGGALSW" /product="Ribonucleotide reductase of
class Ia (aerobic), beta subunit (EC 1.17.4.1)" /EC_number="1.17.4.1"
/transl_table=11
CDS 1197624..1200497

/locus_tag="AUO97b_01294" /db_xref="SEED:fig|400667.82.peg.1154"
/db_xref="GO:0004035"
/translation="MFIKSILSSITSIIPLPENSNTSSNLGNGSGDGLLNGISSGNGE
HNYGIGNGIADDASITAPITIPLNLSGNSITLIGNSSSSVNSSPTTTSNNVNDNDVT
NNGNGSTSGSGTGNGSGDGLLNGAASGNGEHNYGIGNGIADDASITAPLSIPINLAGN
SITLIGDSSSSSVNNSATNTSNTVNDNDTTYNGNGSGTGNGSGDGLLNGIGSGNGEQN
YGIGNGIADDASITAPITLPINLSGNSITLIGNSSASSVNSSPTTTSNTVNDNDTTYN
GNGTGDSGVSALGGSGNGSGDGAGNGIASGNGEHNYGIGNGNGDDVDITAPITGVLNI
SGNSFTLIGNSSSSSVNTAPTTTSNTVNDNDTIDNGNSGGTGSGSGNGSGDGLLNGAA
SGNGEHNYGIGNGNGDDVDITAPITGVFNFSGNSFSLIGNSSSSSINTAPTTTTNTVN
DNDVTDNGNDGGGLVGGSSGNGSGDGLLNGAASGNGEHNYGIGNGNGDDADFTFPLTG
VLNFSGNSLSGFGSSSSDSVNVAPTTATNTVNDNDTIDNANTGGLGGGSGNGSGDGLL
NGAASGNGEHNYGIGNGNGDDADFTLPFTGGLNILGNALSGIGGSSTDSINISPTTTS
NTVNDNDTTNNGNTSGGVIGSSDSGNGSGDGLLNGISSGNGEHNYGIGNGNGDDVDVV
APITTPLNVLGNSFSFIGGEGTGDILGPITGVIGGIGGDGDILSPITGIIGGIGGIGG
DLGDNPLTGIIQSGIDVLQNLESLKTGLINTGIDTIAGTIIGVFPDAEHPVGDFADLG
KLLFETSRDSVNGTLEAISDLAGADLEGASGSITGVIDTLITNGSTASTIIQHIVGDD
LVTENGGLLGSITTIIGGVDSGDGGLLGGLDGLININYGDSDNSNSVDVEDILGNILG
SVGSNQGIAVGEPDPTGGSLIHTISLNTVNQLTDQLLHALPTV" /product="hypothetical protein"
/EC_number="3.1.3.1"
/transl_table=11
CDS complement(1200607..1201206)
/locus_tag="AUO97b_01295" /db_xref="SEED:fig|400667.82.peg.1155"
/translation="MSETLPDPRKRPRQARSVATFEAILEAAARILESLGFAGFNTNA
VAELAGVSIGSLYQYFPSKDALIVELIRRERAKLSNHIVEVIQQSDAADLKDKLKLII
QAAVQHQLSRPQLARTLEFASELIGKDIEESEHQHELETIISDLFKRSGVSHAQTAAQ
DVIALSKGMINAAGIVGESDLNHLQQRVEKAVFGYLDLD" /product="Transcriptional regulator,
AcrR family" /transl_table=11
CDS 1201328..1201657
/locus_tag="AUO97b_01296" /db_xref="SEED:fig|400667.82.peg.1156"
/translation="MSRTYTFFIHLRALPAWLALARQKRAEFSAQKLEPIFEKYPTVK
VRWYDVEAFSTKASDIAVFETTSLQDYYFVIDAIRDSEFCTVPYFEFVEIIPAIEDGY VEYESSL"
/product="Darcynin homolog"
/transl_table=11
CDS complement(1201739..1208116)
/locus_tag="AUO97b_01297" /db_xref="SEED:fig|400667.82.peg.1157"
/db_xref="GO:0004035"
/translation="MGSSLIIQANELYSTVNDLNSAISQGDGKGIDQQIAQLSLISTG
LGFTIEFAKIAKSPEAVAGLTGLKYQVDATLTGAALTQAMINIDQGNYSGAIANTLAA
MSTATGLVALAVDKPGLSTALNGLSGFLGASSNVAEKGPQIADALANALSEIQKMQHD
GTFPELDPGLMAGFAGEVLEGWSKEFYSWFPDDWNPWKNIDRNSDGVKKYHIYDPVVL
DLDGDGIETIAANKWAGVLFDHDNDGIRTSTGWVGSDDGILVLDRNGDGVINDGSELF
GDSVTLKDGSQASNGYAALAEFDSNGDGKVNAEDTNFEQLKVWRDLNQDGVSQTNELF
GLTELGIQSLNLNHTNTDTALGNDNILAQTGSFETLDGQSHIMGDVNFSFNSIYSSFN
NAIELTAEQQQAANLQGAGRVRDLREAAALSPELAETLKNYSNAETKAEQYDLLDQLI
LNWAKTDPDFKEANEVRMVSWVKTANEGIALTPGQAAIVTLAAPLSKESQAMLDKIHH
ILPILDAFSGSKPSQIYYENEADIKNVFNIINNAYVSLKEGIYTGLLYQTRLDDYLAE
IELNLQATDPTELFNYSGIQEKLEQIFIINPEKAFTDLSDLVNYHPDILTNWLPEGKT
LLIQFLNEVIEQGLWDDLASKVDLKILEQSGFIIGTKLDDTLNGTKNNDTLNGGWGND
KLNGGAGQDTLIGGSGNDVMTGGDYEKDIYIFQSGHGQDIINDKSYDWTNLQYFNDVK
FEGANFTDAQFLRVGNDLVIKAFGTDDSVTFKNYLDQNDSYSRDFNFIFADKTVSASE
ITGLTMQLNGTDGNDVLNGWKGKDVLIGGLGDDTLTGYTGDDLLDGGEGKNVLNGGDG
NDTLITGSGNDVLNGDNGNDTLNGGAGNDTLNGGAGQDTLIGGSGNDVMTGGDYEKDI
YIFQSGHGQDTINDKSYDWTNLQYFNDVKFEGANFTDAQFLRVGNDLVIKAFGTEDSV

TFKNYLDQNDSYSRDFNFIFADKTVSTSEITGLTMQLNGTDGNDVLNGWKGKDVLIGG
LGDDTLTGYTGDDLLDGGEGKNVLNGGDGNDTLITGSGNDVLNGDNGNDTLNGGAGND
TLNGGAGQDTLIGGSGNDVMTGGDYEKDIYIFQSGHGQDIINDKSYDWTNLQYFNDVK
FEGANFTDAQFLRVGNDLVIKAFGTEDSVTFKNYLSDSDSYSRDFNFIFADKTASTTE
LTSLTIQLNGTDGNDVQNGWKGKDNLLGGLGDDTLNGLAGDDWLEGGEGNDTLNGGEG
NDLLEGGAGNDKLTGGAGQDTLIGGSGNDVMTGGDYEKDIYIFQSGHGQDTINDKSYD
WTNLQYFNDVKFEGANFTDAQFLRVGNDLVIKAFGTEDSVTFKNYLSDSDSYSRDFNF
IFADKTASTTELTSLTIQLNGTDGNDVQNGWKGKDNLLGGLGDDTLNGLAGDDWLEGG
AGNDILNGGEGNDLLEGGAGNDTLNGGAGQDTLIGGSGNDVMTGGDYEKDIYIFQSGH
GQDTINDKSYDWTNLQYFNDVKFEGANFTDAQFLRVGNDLVIKAFGTEDSVTFKNYLS
DSDSYSRDFNFIFADKTASTTELTSLTIQLNGTDGNDVQNGWKGKDNLLGGLGDDTLN
GLAGDDWLEGGAGNDILNGGEGNDLLEGGAGNDTLNGGAGQDTLIGGSGNDVMTGGDY
EKDIYIFQSGHGQDTINDKSYDWTNLQYFNDVKFEGANFTDAQFLRVGNDLVIKAFGT
DDSVSFKNYLNDNDSYSRDFNFIFADKTLTTADVMNIVIPLNGTDGNDVQNGWKGKDN
LLGSLGDDTLNGLAGDDWLEGGAGNDILNGGEGNDLLEGGAGNDTLNGGAGQDTLIGG
SGNDVMTGGDYEKDIYIFQSGHGQDTINDKSYDWTNLQYFNDVKFEGANFTDAQFLRV
GNDLVIKAFGTDDSVSFKNYLNDNDSYSRDFNFIFADKTLTTADVMNIVIPLNGTDGN
DVQNGWKGKDNLLGSLGDDTLNGLAGDDWLEGGAGNDILNGGEGNDLLEGGAGNDTLT
GGIGADTAIFKILAGFGNDATGGNGVDTWSDFNISQGDKINISELIIGPASKENLNQF
VSFEKSGSTVTLSLDRDGNDINYSATKLLILNNQTNVNSLNDLIKADVFIV" /product="Iron-regulated protein frpA"
/EC_number="3.1.3.1"
/transl_table=11
CDS complement(1208205..1208516)
/locus_tag="AUO97b_01298" /db_xref="SEED:fig|400667.82.peg.1158"
/translation="MKKEIMSKSDVRGFVGLFLGLTSYSIFMFYLLAKRSKGINYFDD
LYSVNKLVVYFLVFLQFILLRQAKKYVKQNKTSFVNFLWGIGAFIGGTLLASFFFTIT L"
/product="hypothetical protein"
/transl_table=11
CDS 1208751..1209029
/locus_tag="AUO97b_01299" /db_xref="SEED:fig|400667.82.peg.1159"
/translation="MALAREKRAEFSAQKLEPIFEKYPSVKVRWYDVEAFSTKASDIA
VFETTSLQDYYFVIDAIRDSEFCTVPYFEFVEIIPAIEDGYVEYESSL" /product="Darcynin homolog"
/transl_table=11
CDS 1209131..1210426
/locus_tag="AUO97b_01300" /db_xref="SEED:fig|400667.82.peg.1160"
/translation="MDTKKKGDIFENFVFDYFSALIQKGYMGLNPSHTKIYKQKKYYS
KDREDYIIFDVAIEVFLFDQKEPYLVILIECKNYEKNVPVDDIEEFSTKVSQVGKHYT
KAICITKNGFQKGALNVAKNRKIGLWRITANDNHEVIFNRTINRIKNNDEIISNALTS
ENLQDISATNIFIQTPLRFTSIPKDLIYDFLSLQKDVSPKEFFTQHRVNSYSKTIPYK
SKKELSILAENLFDKFYKNDEIDLNEIINTLGYKLIEIDTPEHPNIIGKLETKLKTIT
IFGVGFYSQHQINFAKAHEIAHIFLNHSKYIISENFSPAMESHSIDIEITQNIDRLEF
QANYFAGCLLLPENRLKATLNYYLRLYGIRNRGFSPLYIDSQPCNFENYSRIILPMTG
IFNVSQETMKIRLTELGLAQFAFQPTSTHKVTRSYIDRF" /product="hypothetical protein"
/transl_table=11
CDS complement(1210423..1210974)
/locus_tag="AUO97b_01301" /db_xref="SEED:fig|400667.82.peg.1161"
/translation="MPTLEISSKKLQVVQTAIQLFTTHGFHNAGVDLIIKEAKIPKAT
FYNYFHSKERLIEMCIAFQKSLLKEEVLSIIYSSRYYTQKDKLKEIIVLHTNLNSLYH
LLLKAVFEIKRLYPQAYRMAVEYRKWLHRELFDLIFSCETRAFKFDADMVLNLIDGLL
LQVLSSNSVDERDVVLERFWGRA"
/product="Transcriptional regulator, AcrR family" /transl_table=11
CDS complement(1212069..1212560)
/locus_tag="AUO97b_01302" /db_xref="SEED:fig|400667.82.peg.1162"

/translation="MVQATDFRNAMSLLTSAVSVVTTAGMSGRFGFTASAVCSVTDTP
PTLLVCMNQAASSHAHFLDNKILTVNVLGAHHEHISKAFSSKLTPEERFKHGEWIELE
TGAPVLEDALVSFDCEIEQIQQVGTHTIFICPIVAIKQSQHDQSLVYFNRAYHQVGQT ETV"
/product="NADH-FMN oxidoreductase"
/transl_table=11
CDS complement(1212746..1213789) /locus_tag="AUO97b_01303"
/db_xref="SEED:fig|400667.82.peg.1163" /db_xref="GO:0003871"
/translation="MKKLLPTSTAGSLPKPSWLAEPEKLWSPWKLENEGLVEGKKDAL
RLALHEQKLAGIDIVSDGEQTRQHFVTTFIEHLNGVDFEKRETVRIRDRYDASVPSVV
GTVSRQKPVFVEDAKFLRQQTDQPIKWALPGPMTMIDTLYDGHYKSREKLAWEFAKIL
NQEAKELEAAGVDIIQFDEPAFNVFFDEVNDWGIATLERAIEGLKCETAVHICYGYGI
KANTDWKKTLGSEWRQYEEAFPKLQTSNIDIISLECHNSRVPIDLLELIRGKKVMVGA
IDVATNQIETPEEVANTLRKALQFVDSDKLYPCTNCGMAPLSREVARGKLNALSAGAE IVRRELTGFDLTA"
/product="5-methyltetrahydropteroyltriglutamate--homocysteine
methyltransferase (EC 2.1.1.14)" /EC_number="2.1.1.14"
/transl_table=11
CDS complement(1213811..1214797)
/locus_tag="AUO97b_01304" /db_xref="SEED:fig|400667.82.peg.1164"
/translation="MSKEFTFTIKSICFDENYRPADNTRITTNFANLARGMSREENLR
NTLRMIDNRFNALAHWDNPKGDRYGVELEIISVEMNIDAENRTSGLPLIEILKTNIVD
KKTNERIEGIVGNNFSSYVRDYDFSVLLLDHNKNKSTFSTPEKFGELHGNLFKSFINS
ETYKANFKKAPVICLSVSTSKVYHRTENEHPVLGVEYKQEDYSLTDEYFQKMGLKVRY
FMPPHSVAPLAFYFTGDLLSDYTNLELISTISTMETFQKIYRPEIYNANSVAGKSYQP
SLKHQDYSLTRIVYDREERSRLAIEQGKFVEEQFIKPYQAILEQWSANYAPN" /product="hypothetical
protein"
/transl_table=11
CDS complement(1215164..1215946)
/locus_tag="AUO97b_01305" /db_xref="SEED:fig|400667.82.peg.1165"
/translation="MTQSAVSKQIYNLEEALNTTLFDRKNKTLVMTKQGEILLSCCHQ
VFGQLDECLIQLSQPKTENKQLVLSCEPTLSMKWLIPRLSKFKKLGHDFEVVLLTAGG
VVDFEKDNIDIAIRRNDFDWGEHIYSEKIADEYIALVQATNPSETNDVLISTSRPNFF
KNMSRIAELKQSLKGYTKVELEHFYLCLEGCLAGLGATVISIYMIEKELEWNLLKKNS
PVVRDNSAYFLLSHSAFEEDPRKTLFLEWLKAEMNESQHKLL" /product="Transcriptional
regulator"
/transl_table=11
CDS complement(1216066..1216185)
/locus_tag="AUO97b_01306" /db_xref="SEED:fig|400667.82.peg.1166"
/translation="MGLYIYEDRYRYLKYFSSSKIGFNKDDFYFEQKLKNKNR" /product="hypothetical
protein"
/transl_table=11
CDS 1216292..1216900
/locus_tag="AUO97b_01307" /db_xref="SEED:fig|400667.82.peg.1167"
/db_xref="GO:0005293"
/db_xref="GO:0006865"
/db_xref="GO:0016020"
/translation="MAEFIAVILITILAVISPGADFAIVTKNSYLYGRKIGVFTSLGI
SLGVLVHVTYTLVAVAFVMTYTPQILNIVKYIGALYLIYIGYKTFTQKPVLGSAALTA
IGTFQAIKYGFFTNALNPKTTLFVISIYTQIVSLTTPKTILLAYGFFMSFAHFVWFSL
VAILFSSMLLRQKMLAKQVQINRVIGSILCVLGVILLFTKFQ" /product="Threonine efflux
protein"
/transl_table=11
CDS complement(1217002..1218057)
/locus_tag="AUO97b_01308" /db_xref="SEED:fig|400667.82.peg.1168"
/translation="MTYIYKNPAGMTDSEKTEHIKKIVTAEGVALRKRYPILNHQNAI

GAVILFFSLAGMITTAVLYINHQLSAWFAIPIIAFFASLTHELEHDLIHWMYFRKKPW
AHHLMMGLVWLARPSTINPWKRRELHFNHHKNSGTEVDLEERALTNGEQWSIRRLIAI
GDNGLAVLFRIISASNWTVRKVIFKRAFMAYFPLGIIHWSLWYIFLGFHAVDAVLSWA
NAPIAWSATTLNIMHVVNILTVVWVAPNVLRTFCLHFVTSNMHYYGDVELGNVIQQTQ
VLKPWWMMPFQLFCFNFGSTHAIHHFVVKEPFYIRQMTAPVAHKVMRDMGVRFNDVGT FKRANRWNINDLSESKS"
/product="Fatty acid desaturase"
/transl_table=11 CDS 1218243..1219250
/locus_tag="AUO97b_01309" /db_xref="SEED:fig|400667.82.peg.1169"
/translation="MPQTATALASWVKAIQKALEKAGVDSQPLLEQAGLDIQALSNPD
ARYSLKQTANLWKLAVQKTQDSCFGLKVASQVNQNTFHVLGHSLITSTTLKEMFLRII
RYFRVVTDIPELEFYGQGNNHYFVIHVPDEVQPESIDAFISVFIRSSRALQGSVFSPL
RIELRRSEPEDLETFQSILKAPIVFNAPKDMIVFDTATIEQPLEGANPTLTQEYDEII
NRYLARFDKENILARVKVKLIEKLSTGDFQQQDVAKSLGLSIRSLQRKLSAENTSYSE
LLDQTRHELALSYIKNSSHNITEIAYILGFTDVSSFTRAFRRWTDLSPIHYREKYLAC S"
/product="Transcriptional regulator, AraC family" /transl_table=11
CDS complement(1219309..1220676)
/locus_tag="AUO97b_01310" /db_xref="SEED:fig|400667.82.peg.1170"
/db_xref="GO:0050118"
/translation="MNKIIKNGMFLGLSTLISGYGTLLHAEASPTNLPAVSQKALTII
DGQTEWLNKVYKDIHEHPELGFMEKRTSAIVAKELKSLGFDVKTGIAKTGVVGILKNG
EGLTVMYRADMDANTVEEATGLPYASKVRVKLDDGTETPVAHMCGHDAHVTWMLSMAK
TLVALKKEWSGTIILVAQPAEEPITGAKAMVTDGLWTKYNLPKPDYFFAVHTTPAPVG
VVINAPGPRMAGTDQLDVLFKGVGGHGSLPMLTKNPISMAANALNQYQTIMGNAVDPQ
QAAVLSIGSIQAGNSNNVIPSTALVKMNLRWFDPKVRETMLNNIKSMSEGAAQMQGMG
KDQMPTFTFKGSSTPVINNKEFSARLNGPLKALLGDKHVLTEYPAVMGSEDVHHLLGE
QKDIPFNFMFIGVADPVVFANAVKQGKPVPYIPHSPNYIVDLKALPVGAKVTTVSMLE LLTKK"
/product="N-acetyl-L,L-diaminopimelate deacetylase (EC 3.5.1.47)"
/EC_number="3.5.1.47"
/transl_table=11
CDS complement(1220710..1222095)
/locus_tag="AUO97b_01311" /db_xref="SEED:fig|400667.82.peg.1171"
/translation="MNFVVNKKIFILGIASICTSTLHAAAFDKTGQSVAAFLQPGNYF
EASLGITDADLSGVESGTNPSHHSILDIANTDYRPTAALKLQLAPQYSFGLLYDQPFG
ADTEYSGVNGFVATPKDTVMLPGITTTGLASATLGHAPTGNAKSEFDIQNLSFILGYQ
PTKNWNLYAGPVYQTFKSQVELRGNVFSLYNGYDFHTKTVSDLGWLAGLAYQIPEKAV
KVSLTYRSAITHKVNATENAPLIDMLSTQQGRDLVNQHLDFMISIGQMTPQKKAYLNQ
IIANLPEQEDTGTTKFKSPDSVNLEFQTGLRQGTLLFGSLRWVNWSDLALQPYRFGEI
SKVVGVLSTPSRPEGFNLVRYYDDQWSLNLGLGQRFSPKWLGSVSVGWDSGAGDKVST
GGPTKGYYNLGVGAQYSPTSRYFISGGVKYYWLGDAKAQLGAQAGSDYYVGEFKDNHS VSYGLKIGYKF"
/product="Outer membrane protein E"
/transl_table=11
CDS 1222394..1223323
/locus_tag="AUO97b_01312" /db_xref="SEED:fig|400667.82.peg.1172"
/translation="MEIKQIKYFLTVVESGSIGKAAQKLDVGASAISQQISKLEQELS
IRLLQRNAFGVTPTPAGLAFLKHSQLILRQVNFAIDAAHSARLSGHVSLGFPPTITAL
IGIPLMKLMSERYPDIRLEIVETLSGNLIQSINSRQLDIAIIFTNEIDKQWSIQPLVR
EQMYLMARKEVLEKYGLSECIKSGIIQSQQIGDIPLVLPRQRHSLRKLLEHKIGQLNV
VYEIDGLHLLMDSLIHLDLASVRPGSACLQEYKHKLQLLKVVDPEVERINYLVSLAEE
ELSPAALAAKSAIKACVKELIQNQIWPCSEIIL" /product="Transcriptional regulator, LysR family => TcuR" /transl_table=11
CDS 1223602..1224912
/locus_tag="AUO97b_01313" /db_xref="SEED:fig|400667.82.peg.1173"
/translation="MDVVNNRRATFKKILNVTSGNFLEQYDFFLFGFYATYIAAKFFH
SDNEYVSLMMTFTVFAAGFLMRPLGAIFLGAYVDKVGRRKGLIVTLSLMAIGTILITF

VPGYETIGIIAPILVVIGRLLQGFSAGVESGGVSIYLAEIATDKNRGFITSWQSGSQQ
IAVVFAALLGYWLNTILTHAQVGEWGWRIPFLIGCLIIPLIFLFRRTLEETEDFKAQK
THPSSKEIFSTLVSNWRIVLAGMMMSAMTTTTFYFITVYTTVYAKRTLEMSVTDSLLA
TVFVGLSNFFWLPMGGLLSDKIGRRPVLVGITTLAIFTSYPVLSWLVSDISFSNLLIT
LAYFSFFFGMYNGTMVATLAEVMPKRVRTVGFSLAFSLAAAIFGGMTPMACTFLVENT
GNASTPAFWLMLAAVCSLISSLYLCRGSKQKDPDAIAEPAKVSA" /product="MFS-type transporter => TcuC"
/transl_table=11
CDS 1225006..1225800
/locus_tag="AUO97b_01314" /db_xref="SEED:fig|400667.82.peg.1174"
/translation="MKKSPIFITICLAGAGLGSTFANADTLEVISSGGFYSSMEKLIP
IFEKQTGHTVHLSSGSSMGESPTAIPNRLNRGERFDIVVLAGPELSKLAEKGYVDPNS
QSALVNSSIGMAVPKGAPKPDISSAAKFEKVLLNAKHIGYSASASGTHLEKDVFPSFP
PVEYKVISSKAEKVVGDRVAKRIAEGQFDIGFQQISEIKPFTGKYGQVELVGPIPAPY
QKVTVFAAGIGKNSEHAKVAKELIKFVKSPQATQIIEEQGLEQVKQ" /product="Hypothetical ABC transport system, periplasmic component" /transl_table=11
CDS 1225932..1226861
/locus_tag="AUO97b_01315" /db_xref="SEED:fig|400667.82.peg.1175"
/translation="MELKQLKYFIAVIESGSLGKAAKNLDIGTSALSQQISKLESELS
IRLLQRTALGTVPTPAGLAFFQQVQLALRHLDHAVDSAHSSRLSGHVTVGFSPSVAAV
IGTHFLKLMRNRYPDIKVRLIEGLSGDLKALVNARQLDVAIIFSDDVDPQWAIQPLVK
EQMFLISPKEVLNQYHLESCIDNQTITHSQLKELPLILPSQRHGLRLLLEQKIHQLKV
AYEIDGLHLLMESISQLEMATIQPAGASLNSRQDLCLLRVVEPAIERINYLISLSEEE
LSPATLATKVVIRACVANLINEKRWPGAELINT" /product="Transcriptional regulator, LysR family => TcuR" /transl_table=11
CDS 1226965..1228371
/locus_tag="AUO97b_01316" /db_xref="SEED:fig|400667.82.peg.1176"
/translation="MHDVIVIGGGNAALCAALTARESGASVLVIEAAPKEWRGGNSQH
TRNLRCMHDAPQDVLVDAYPEEEYWQDLLKVTAGKTNEHLARLVIRSTATCRDWMRKH
GVNFQPPLSGALHVARTNAFFMGGGKALINAYYRSAEKLGIEILYNTKIKDLKLNQGH
FEAAIAEDGRVFKAKSCVLAAGGFESNLEWLREAWGQNENGEWPADNFIIRGTRFNQG
NLLKFMIDQGADIIGDPSQSHCVAVDARAPLYDGGICTRIDCVSLGIVVNNKAERFYD
EGEDFWPKRYAIWGRLVAKQPQQIAYSIIDSKAIGRFMPPVFAGVKANSIQELAEKIG
LDAKTLCHTVEEFNQACVQGTFNHTVLDDCTTENIIPNKTHWAVPLDQPPFYAYALKP
GITFTYLGLKVEDDAAVRFNNKASRNLFVAGEMMAGNVLGQGYTAGVGMSIGTTFGRI
AGKNAAHYALSQGVEHEC"
/product="Tricarballylate dehydrogenase, TcuA" /transl_table=11
CDS 1228361..1229494
/locus_tag="AUO97b_01317" /db_xref="SEED:fig|400667.82.peg.1177"
/translation="MNASTKALIPVVQLSDHEQEVQRALQICNACRYCESFCAVFAAM
TKRLEFNQADIHYMANLCHNCGACLHACQYAPPHEFGVNIPKAMAQVRLETYQEFATP
QPLGRLYKSVGIPFVSTLTLIFFFCMLAVVWWKGTNLFAGYQGNFYAIFPHNFLALLF
GATFTVAIVLLGIGVSKFWRQTSKVIHGKVEKPDLVQATQNVLTLKYLDGGHGKGCNE
QDDRYTQIRRVFHHLTFYGFMLCFAATGVATLYHYFLDLHAPYPIFSLPVILGTLGGI
GLVIGPSGLLYLNLKRHPLHGDREQRPVDRGFIFLLLIVSASGLVLMAFRNTPYMALL
LIFHLATVMTFFITMPFGKFAHGFYRSASLLKFAIEERISKKS" /product="Tricarballylate utilization protein, TcuB" /transl_table=11
CDS complement(1229599..1230828)
/locus_tag="AUO97b_01318" /db_xref="SEED:fig|400667.82.peg.1178"
/translation="MGALQGIRVLDLSRVLAGPWCGQILADLGAEVIKIERPRVGDDT
RMWGPPWMPDQDGNMTRESGYFQCTNRNKYSVAVDITTPEGQALIYEIAKTADVVIEN
YKTGSLAKYGLDYASLSELNPNIVYCSITGFGQDGPRAEEPGYDFIIQGMSGLMSITG
EPDDVAGGGAQKVGVAVVDIQTGLYSTIAIQAALIARSHTGRGQYIDMSLLDVQVAAL
ANQGMNYLASGKAPQRMGNQHPSIVPYQTFKAKDKEFIIACGNDKQFKDLCIAIGYPE

LLENEKFVRNQDRVKYRNELIPLLSEHFLRKTAKEWVDMIHALKVPVGVINSIEDALA
EPQIVYRNLVVNIPHRLNENFQTIGSPIKLSDTPVEYHHAPPELGEHTAQILSRFKTA EELAALKEKGIIDGKIA"
/product="CaiB/BaiF family protein"
/EC_number="2.8.3.16"
/transl_table=11
CDS complement(1230881..1232101)
/locus_tag="AUO97b_01319" /db_xref="SEED:fig|400667.82.peg.1179"
/translation="MLFAYFAMVVDGIDIMLLSYSLTSLKAEFGLTTFQAGALGSASL
AGMGIGGILGGWACDKFGRVRTIANSVTFFSVATCLLGFTQSFEQFMALRFIGALGIG
ALYMACNTLMAEYVPTTYRTTVLGTLQTGQTVGYIAATLLAGAIIPDHGWRVLFFLTV
VPAFVNIFLQRFVPEPKSWQLTKIESLQGNRQPKERVVAEKPKSSSIYKQIFNNFKHR
KMFLLWMTTAFFLQFGYYGINNWMPSYLETEVHMNFKNLTSYMVGSYTAMILGKILAG
YLADKFNRRAVFVFGTIASAVFLPIIIFFNTPDNILYLLITFGFLYGIPYGVNATYMA
ESFSTDVRGTAIGGAYNIGRVGAAIAPATIGFLASGGTFTMAFIVMGAAYFVAGVLPG LFIKDRQYDPQRQS"
/product="Uncharacterized MFS-type transporter" /transl_table=11
CDS complement(1232425..1233645)
/locus_tag="AUO97b_01320" /db_xref="SEED:fig|400667.82.peg.1180"
/db_xref="GO:0004361"
/translation="MNHMVTTKKAKVQFNWQDPFLIEQQLSPDERMIRDAAHAYCQER
LQPRVLEQFRHETTDPSIFREMGELGLLGPTIPEQYGGAGLNYVSYGLVAREIEYVDS
GYRSMASVQSSLVMVPIHEFGTEEQKQKYLPKLATGEYIGCFGLTEPDHGSDPGSMIT
RAKKVEGGYRLTGAKMWITNSPIADVFVVWAKEVSAEGNVGDIRGFILEKGWEGLSAP
AIHGKVGLRASITGEIVMDNVFVPEENAFPEIRGLKGPFTCLNSARYGIAWGAMGAAE
FCWHTAHQYTMDRKQFGRPLAANQLIQKKLADMQTEIALGLQAALRFGRMKDEGIASV
EGTSLIKRNNCGKALDIARMARDMMGGNGISDEFGVARHLVNLEVVNTYEGTHDVHAL ILGRAQTGIAAFSN"
/product="Glutaryl-CoA dehydrogenase (EC 1.3.8.6)" /EC_number="1.3.99.7"
/transl_table=11
CDS 1233891..1234838
/locus_tag="AUO97b_01321" /db_xref="SEED:fig|400667.82.peg.1181"
/translation="MRRSIPSIQGLICFESAAKHLSYTYAAQELCITQSAVSRQIQQL
EDFLGVELFIRTRHGVELTIAGQQYFKSIKPYLLGLEKCTADVISHKGLGGTLKLGVV
PTFATRWLLPKLHLLNQKHPEITVHLETSTKPFLFNDNIFDAAIFAGTQQQIDHWPGI
QAHYLMDEEIVPVCSPRLIEKYFPDAVMINENTYDLSPEDLLKIPLLQQTTRPSIWQE
WFLTHHMQHPKPFDGQRHELFSMLAVAASHDMGMALIPQMLIESELQKKELVIVSNKK
LKGSRKYYLIHSSQDISPTIQKFVEWIYQELEQLKTNTN" /product="Transcriptional regulator, LysR family" /transl_table=11
CDS complement(1234932..1236107)
/locus_tag="AUO97b_01322" /db_xref="SEED:fig|400667.82.peg.1182"
/db_xref="GO:0003939"
/translation="MKSVLCHQATLQVVDQPNLTPAKGQVLLEVVRCGICGSDLHMQH
HCDHMHDLAARVGFNGLAKSTDPFVLGHEFCGKVLDYGPKTRHKFKADTLVCAMPLLN
VDQHGYLSTGLSPNASGGYAEQVLAQSSLMFEVPNGLDADSAAMTEPMAVALHAVRRS
RVKTSEPAIVIGCGPVGLGVILMLKAAGVKTVVASDFSPNRRKLAEQCGADIVVDPKE
TSPFANWKEFGLLGNVSDAINMGMGLFDKLQATRLPWWHGWRMIDKLGALPKRPVIFE
CVGVPGVLQQIIEGAPLFSRIVGVGVCMQSDKIEPALAINKELEIQFVLGYTPLEFRD
ALHMIAEGKVNCSPLITGVVGLEGVTNAFEALRDPEQHAKILIDPKRSGSDIQLMSH" /product="zinc-binding dehydrogenase"
/EC_number="1.1.1.14"
/transl_table=11 CDS 1236485..1237504
/locus_tag="AUO97b_01323" /db_xref="SEED:fig|400667.82.peg.1183"
/db_xref="GO:0004000"
/translation="MQTDKKKSMITKTLPLTDIHRHLDGNIRIQTILELGQQYHLDLP
AYDIESLRPHVQVMDNQPDLLSFLSKLDWGVKVLASLDACKRIAFENMQDAAQQGLDY
VELRFSPGYMGMTHQLPLEGVVEAVIAGVKEGSQAYGVKANLIGIMSRTFGQEACEKE

LNALLAHKNDIKALDLAGDELGFPGNLFIDHFKKARDAGWHITVHAGEAAGPESIWQA
IEELGAERIGHGVKAVQDLKLLDYLAKHQIGIESCLTSNIQTNTVPSLAEHPLKTFLE
HGVLATINTDDPAVEGIEIQHEYLTAAPLAGLSPEQIYTAQENGLKIAFLSEQEKDEL RQKYQ"
/product="Adenosine deaminase (EC 3.5.4.4)" /EC_number="3.5.4.4"
/transl_table=11
CDS complement(1238202..1239266)
/locus_tag="AUO97b_01324" /db_xref="SEED:fig|400667.82.peg.1184"
/translation="MSDATLIANDAKNIVQAHLDRLGEPKDNRLSEEAKQQKFAQIEA
ELKKRDAVLVAHYYCDPDVQELAEKTGGCVSDSLEMARFGRDHAASTLVVAGVKFMGE
TAKILSPEKTILMPTLEATCSLDLGCPVDEFTAFCDQHPDHTVVVYANTSAAVKARAD
WVVTSSCAVEIVEHLDSLGEKIIWAPDQHLGRYIQKKTGADMLLWDGACIVHEEFRAR
GIANMKALYPDAAVLVHPESPESVVEIADAVGSTSQLIKAAQTLPNERLIVATDRGIF
YKMQQAVPDKILVEAPTAGEGATCRSCAHCPWMAMNELDGILEVLQKGDQEIHVDLAL
AERAKMPLDRMLAFSASLKR"
/product="Quinolinate synthetase (EC 2.5.1.72)" /EC_number="2.5.1.72"
/transl_table=11
CDS complement(1239413..1240633)
/locus_tag="AUO97b_01325" /db_xref="SEED:fig|400667.82.peg.1185"
/db_xref="GO:0004358"
/translation="MAVGDVTMPQMHVVKGVKIGSTEAYVRYPNRRDLVIFEFAEGSN
VAGVFTQNAFCAAPVHVSKAHLAEGNPRYLVINTGNANAGTGPTGLKNAQDTCAKLAE
LAGVNSFEVLPFSTGVIGEQLPMERLLAGLQPALNSVQDSAWSDAASGIMTTDTVPKG
ASEQFELDGITYTMTGISKGAGMIRPNMATMLSFVATDAPIRRDLVQKLLKTTVEHSF
NRITIDGDTSTNDSCIFVATGQAGGAEITSESDVRYAKVLEVLARVMNRLAQLIVRDG
EGATKFITVAVEGGANTQECCDIAYSIAHSPLVKTALFASDPNWGRILAAIGYAGVKD
LDVSKIQVWLDDVQICKDGGAAEDYTEEAGARVMAQTEITIRVDLGRGQAKDTVYTCD LSYDYVKINADYRS"
/product="Glutamate N-acetyltransferase (EC 2.3.1.35) @ N-acetylglutamate synthase (EC 2.3.1.1)" /EC_number="2.3.1.35"
/EC_number="2.3.1.1"
/transl_table=11
CDS complement(1240770..1241249)
/locus_tag="AUO97b_01326" /db_xref="SEED:fig|400667.82.peg.1186"
/db_xref="GO:0003908"
/translation="MLLSYKYINSPVGQLKLVANENALVAVLWDNENPKRVRLAELVE
QLEHPVLLKTEQQLQEYFEGKRREFTLPLDFEGTAFQKQVWAALLTIPYGETRSYKDI
AVQIGNEKAVRAVGAANGKNPISIIAPCHRVIGAGGALVGFAGGLDKKDILLRLEQK"
/product="Methylated-DNA--protein-cysteine methyltransferase (EC 2.1.1.63)"
/EC_number="2.1.1.63"
/transl_table=11
CDS complement(1241261..1241581)
/locus_tag="AUO97b_01327" /db_xref="SEED:fig|400667.82.peg.1187"
/translation="MAWAILILAGVFEVIWAYSMKMSEGFTRLTPSIITVVFMILSVV
LLSISMKTLPLGTAYTVWTGIGAIGSFLVGIFFLHEPIGAMRMIAAVLIVSGLVLMKI SSPS"
/product="small multidrug resistance family (SMR) protein" /transl_table=11
CDS complement(1241731..1242672)
/locus_tag="AUO97b_01328" /db_xref="SEED:fig|400667.82.peg.1188"
/translation="MALEKQENQINVNKSCSGCGCSNVAAQDFSVASEPRVNKTQWVS
YYHIPRMDCAAEEQMVRMALASYAEHIQELYFDLPARHLAVYHSTQDEKITQCLQRLG
LGAELQQTQPHYEPVEKPKKVRPSSSTNAAQKQVLQWLLLINGVMFVIELMAGIIASS
TGLIADSLDMFADAAIYGIALFVVGKNLDAQLKAAHLSGWFQFGLAIIVLIDVLRRFI
YGSEPVSILMILIGGLALAANISCLYLMSDHKESGAHMKASYIFSANDVIVNLGVIVA
GILVAMTGSRYPDLIIGLIIVLFVLNGARKILQLKNE"
/product="Co/Zn/Cd efflux system component" /transl_table=11
CDS complement(1242755..1243510)

/locus_tag="AUO97b_01329" /db_xref="SEED:fig|400667.82.peg.1189"
/translation="MRITKLFSQMICCGAFSGISVFAFAQEPLTHDHLREHGGQIYQA
TKIDTGWTQNEEGEGSFSSELETWVGTDENKLFIKAHVEKAESEDANYGGSVLYSRNV
ADYWDVQAGVNYERLQREDEKQDRWAGVVGLHGMAPYFFETDAYLYVGEDQLWKLSLE
TERDLLFTQKLIGKPYLKADVVLQDESRYASKTGLSSLQAGFQTRYEINKKVMPFVDV
GYGYEKGLKQTAWQTGTDSEHGWYYGAGLTLKF"
/product="Copper resistance protein B"
/transl_table=11
CDS complement(1243497..1245431)
/locus_tag="AUO97b_01330" /db_xref="SEED:fig|400667.82.peg.1190"
/db_xref="GO:0005507"
/db_xref="GO:0016491"
/translation="MSHKTISSLVAVFGLLVSPWTLAAIKEYHLNINEQQVNVTGKPL
KRITVNGKFTAPLLEFEEGDEAVIHVHNQLKNQDTSLHWHGLLLPGLMDGVPAFNNFK
GIAPNGDFVYRFKVKQNGTYWYHAHSKGQEQDGLYGPLVIYPKGKIPVVAHEKTDRDY
VVMLSDFHDSSSDSIMKNLKKSAEYYQNRRETVSDILKQVKTQGLKATWQDRSMWNQM
RMLKTDMSDVTGYTFLVNGKTPQQNWTGNFKAGDKVRLRFINASAMSFFDVRIPNLKM
TVVSADGQPVKPVAIDEFRIGTAETYDVIVEPKQPNYQIEAESIDRSGFAIGTLHNEN
TQAVGPVQMPTPRPRALLTMDDMGMSHGGGSNEHAGHQMNMQHDMSAMPEMKKETTQA
NHDHTMMNMDHDMKNMSSDGHDHSMMHMTHDMSAMSEMNHDMQAMSASEHDHAMMNMN
HEMPAQSENKPKKDEPVYGWANASTPAGMKALQYSDLQSLTPQPDTRAPERELVIRLG
GNMERYIWTINGKKFSDTTPLQVKYGERIRLKFVNDSMMAHPMHLHGMFMQLENGQQA
ENLPNKHTIIVPPGKTVTALLTADALGEWAIHCHLLYHMSAGMMNKLIVAQVNDGQQP
AQSQPTVQSENEKGASHAHH"
/product="Multicopper oxidase"
/transl_table=11
CDS complement(1245472..1245870)
/locus_tag="AUO97b_01331" /db_xref="SEED:fig|400667.82.peg.1191"
/translation="MMTLQHLRKYAWFVFLMTLVIGWSGVSIAQTPMQHDLHTMPCHS
EQMKHHSMHIQADEQTTCHQSMAQVSHQHGEHCQDCNPVHCQMVNLALQQAVPDIESS
FIPIPSQFSSIYQAQHLAGYWQEILRPPKA"
/product="hypothetical protein"
/transl_table=11 CDS complement(1245968..1246789)
/locus_tag="AUO97b_01332" /db_xref="SEED:fig|400667.82.peg.1192"
/db_xref="GO:0050129"
/translation="MTVKILAVDMDGTFLNSKKQYNKARFLKQYEQLKQNNIRFVVAS
GNQLAKLVTYFPEINHEIAFIAENGAHVVDAGQELAFAHLSKEQFIEILNAIDPVYTS
KMVICGKQSAYVHSSMNAEDYAKVARYFEKLTVIDDFYALDDLVCKITFTAQENESFT
IFEHFQKQSFVKDKVLVPVSSGFNFIDLILPDQHKAHGLKLLLQKWQVQPDQVVAIGD
NNNDIQMIKAVGYGFAVENAVEALKAVAPYTTANNEQEGALQVIDLVLQHQPPFA" /product="Hydrolase, HAD superfamily"
/EC_number="3.5.1.68"
/transl_table=11
CDS complement(1247146..1247832)
/locus_tag="AUO97b_01333" /db_xref="SEED:fig|400667.82.peg.1193"
/db_xref="GO:0004750"
/translation="MSKPYLIAPSILSADFARLGEDVDKVLAAGADVVHFDVMDNHYV
PNLTFGAGVCKALKNYGITAPVDVHLMVSPVDRIIGDFLEAGADIITFHPEASHHIDR
SLQLIKSGGAKAGLVFNPATPLHYLDYVLDKVDQILLMSVNPGFGGQKFIPMTLEKLR
EARKIIDASGRDIRLEVDGGVGPANIREIAEAGADMFVAGSAIFGQPDYKAVIDQMRA ELAKVGSVDA"
/product="Ribulose-phosphate 3-epimerase (EC 5.1.3.1)" /EC_number="5.1.3.1"
/transl_table=11
CDS 1248017..1248289
/locus_tag="AUO97b_01334" /db_xref="SEED:fig|400667.82.peg.1194"

/translation="MKSTTHLLTNDGKRIAKRLVNHWKHKFEVQETEQDFKILMPTAT
ITLAPETEQLNVAIDSQLDDHNHLEKVVLDHLNRMAQQEFQVEWQH" /product="hypothetical
protein"
/transl_table=11
CDS complement(1248293..1249138)
/locus_tag="AUO97b_01335" /db_xref="SEED:fig|400667.82.peg.1195"
/translation="MQLLQKNRCFEGEQRIYQFDSQLLKGSTKFGVYLPPAALEGKPC
STLFYLAGLTCTEETFAIKAHAQHMAAQLSLILITPDTSPRGEQVAVGDHWDLGQGAG
FYINATQAPWSEHYQMESYLIDELYPLILKSFPVNAQQVGIFGHSMGGHGALTLAFKY
PEKFKSVSAFAPICAPTECAWGEKAFSHYLGTDRDSWLAHDATALVSKNGAVFNEILV
DQGLDDQFYEQLHPEKFKQACLKAGQPLTLRQHAGYDHGYYFIQTFMDEHLQFHAIQL EKVAG"
/product="S-formylglutathione hydrolase (EC 3.1.2.12)" /EC_number="3.1.2.12"
/transl_table=11
CDS complement(1249107..1250558)
/locus_tag="AUO97b_01336" /db_xref="SEED:fig|400667.82.peg.1196"
/translation="MIDLKPSINFWHDFKSNQLAGMWLFLGSRRSLQIVHPSILQLIV
WGVLGGCTNSLYSWLVAGQIGDFNSQGLIGYALWPFIALIVGIFLSQRINQPRLMLVP
ALLWLVLDTNILLIQCLIQYLGSNGYLNFIPDAIYNGILPGLFVALFVWQSLAVIWVF
SRELKWPWWERALVMVATIATMVVWQLSVKDQPIWKVEDSPPTFAEDAFYAQSKLLHD
ALEQVQYGELAKSHWYFLGVAGDSYQDVFKSEVVRIKEQFDTRFGTVGRSMMLVNNPD
TRTEIPIASKTSIELALRRMGQQMNRDSDVLFLYMTSHGEQNHFEIENAPLDLGQVDP
KWLRETLDKAGIRWRVIVISACYSGSFIPALQSPDTLIITASAADKTSFGCNNEADYT
YFGRAFFDLAMREQSSMKDSFNAAKQTVTKWEVAQGIEPSEPQWMIGKNMELMLPQLE
KYLFPQPNTGSVNIAQTQTEAKNDATPAKKPLL"
/product="MORN repeat family protein"
/transl_table=11
CDS complement(1250695..1251486)
/locus_tag="AUO97b_01337" /db_xref="SEED:fig|400667.82.peg.1197"
/translation="MHIYFEVDFQEQAQHYQAVLYSRGVTVDLQPIEKLNARFLRLNP
DLALCVDENGLWLSANGMKMQPDWKAEIPRLKRATLKSEMIARACQLGEKPVLVDATA
GLGHDSLLMAYLGAQIQLVERHPILFTLLEDSKAQAQHDPFLSQFMDRIQLIFADSAS
YLQQLDQEEKTVDVVYLDPMFPQRDQNQQAIKKQAQVKKQMQLLHLLLPEDGEMDLGD
HLLELAKKVAKRVIVKRPRHAIFLANQEPAHQWQGDACRFDAYFQ" /product="16S rRNA (guanine
(1516)-N(2))-methyltransferase (EC 2.1.1.242)" /transl_table=11
CDS complement(1251498..1252163)
/locus_tag="AUO97b_01338" /db_xref="SEED:fig|400667.82.peg.1198"
/translation="MKLLALETANEQCSISLIDETQELFFQLDTRAKAQTQTILPMIE
QGLQQTGLDVAGLDAIAFSRGPGSFSGVRINAAVAQALAWSQDLPVIPVSTLQALAQA
AYRLKGLEQVTAVLDARMNEVYIASFVLDEQGIMQCIDEEKLMNYEQAAAYAKHCLIG
SGAKLLQTDAEYQTITATAQDIASIARVYAAQKQWVDAEHALPVYLRDDAWKKIADQG KAN"
/product="tRNA threonylcarbamoyladenosine biosynthesis protein TsaB"
/transl_table=11
CDS complement(1252296..1252826)
/locus_tag="AUO97b_01339" /db_xref="SEED:fig|400667.82.peg.1199"
/db_xref="GO:0008415"
/translation="MMYSYQGHHPKALHEPWDGWIAPTATLIGQVELGRQVSIWFGAV
VRADNCVVRIGNFSNIQENSVLHTDAGLELNIGEYVTVGHKVMLHGCTIGDNSLIGMN
AVILNRAVIGKNCIIGANALIPEGKVIPDNSVVMGSPGKIVKTLDEEGAAKIRLSALH YAEHFKSYADLKEFHF"
/product="Carbonic anhydrase, gamma class (EC 4.2.1.1)" /transl_table=11
CDS complement(1252846..1253463)
/locus_tag="AUO97b_01340" /db_xref="SEED:fig|400667.82.peg.1200"
/translation="MEEHLLTQKLQQRLRFSTRIQEAQAAVLIAITNENNPKVLLTRR
SIHMNNHAGEVSFPGGKRDPSDTSNIVVALREAQEETALNPFDVQLLGDLPMQRARSG
LSVKPIVGLIPPEVTLIPQPTEIDRIFFVPLQQLIETRPTPYEVRYAHQSLYFPSLQI

DNEIIWGLTARMLIALFKYGLGYQKEWPFLLNAPTFGMPKFSHKK" /product="Uncharacterized Nudix hydrolase NudL" /transl_table=11
CDS 1253587..1254135
/locus_tag="AUO97b_01341" /db_xref="SEED:fig|400667.82.peg.1201"
/translation="MSFCVACGHKTEQKIPLGDHKVRRVCTHCGNIHYENPKVICGAL
ALWEDKVLLCRRAIEPRYGLWTLPAGYMELFETMEQGAARETREEAEAEIEIEQLYCM
YNIPRIGQIYVLFKAQLKDGLFGAGEESIECRLFEEHEIPWGELAFPSVEHTLRHYFE
DRKQQVFPTHLETLGTRLDHTG"
/product="MutT/Nudix family protein"
/transl_table=11
CDS complement(1254191..1256122)
/locus_tag="AUO97b_01342" /db_xref="SEED:fig|400667.82.peg.1202"
/translation="MNKRILWPFALTAIALMLNGCGGGSSNINEDPNSGGDGNLTSGS
CKPSVSGNSSRDSNCLEFALDYPVEGINFDCSTALGQHFVTTLSQGTVTGTCKVGDTA
RFYLQGKGDKQIFLGSVKLDTIGKYTSFVNNGDQKTSLPIYLRLLDLASGITGKAPIT
LDKNDETIKVAIALVKIFQSIGLENDDNLIGDIQPTEITDEKKELLSKLSQSITAADF
QSGAYASLLKPWLDVSQISDEEAYKLVVQTTNLSNAGTYQALDVRNIFLFRGCNLNSY
EECEKNTSTALRDNVNSMYLLSDRQGYSFGYGLQWSGIRKDLTDPLDLRRKVKPVKMN
INAQNAWLHPISREIQSNTPLRLSTLDNPNEDMQIYQGKLFNDYAIAGSEVAYKSLTN
LSTGNPQHYGRWRQNLGGESYNGGVDIYKGNKISFLESSVFKTSGNVKTGESYIFPLY
ATLTFNFEQTGAQPVNLGIVIDEHGDIRTDIKPNATITDMSGQCATVADSNLIDSLGV
QQYRIGSTAATINNPINSDRSVYIRMILANPKFANIDGAIVGLSFIGVSAGTAKLNLY
NLLANKIDNNSINLNNGAKGLASWYNPHAATQASYNALENVTPTDEEKALAQRIAGTV
TIKLADQSIPACKAIKIKS"
/product="FilF"
/transl_table=11
CDS complement(1256142..1257404)
/locus_tag="AUO97b_01343" /db_xref="SEED:fig|400667.82.peg.1203"
/translation="MVKQYKRMFRPKFAIAALCIAIANISLSYADGFYTIIGPDGRPM
IVPSKKVETRNSNSKLEITKPVIESRSVNTPIQEIPQGNIQNSKGNKKVEQEATKILN
TAPTAPTAPTAPTAPTAPTAPTAPTAPQKNTKTVSVVNDDLKTSTEKNKIIEGGNVPS
VSNNEGQKKLVLVPPPIKSTSQAAPSSGSSVDKSKFTIIDGVEYVNNEYLEGQEFNIE
GKKRFYLMPDGLGRTESVERKKGVSRSTLEQLFKRHTQDQDSTVVLAPTYMRLSSQEL
EEAFEQDKCFIKGYKKSIKTLSTNKEVNLWPRKPLKEKFEYELVQLNPSVQHLKVMSF
ASSNEKPLYYWPLVVFLDDKGCILEGASGFKSKSYPSTMLQHASIQGVLKVPPSAHYI
MMTPLSSAVDVTEKELTNQGQIQISVLR"
/product="FilE"
/transl_table=11
CDS complement(1257398..1259068)
/locus_tag="AUO97b_01344" /db_xref="SEED:fig|400667.82.peg.1204"
/translation="MQVKYPKLCPLTAAIVISGFSSFANAQLGQNLAVDIRSLSMGNA
VTADPPGISAIHFNPAALAKIDGLQTDVQGILANFDIQREFSAPPGYNVFGYSDDPIV
CNDGPEVSVGICTDFKGPVRGDVEYASLYVPILKKMVDLGAGYPLVAPTAGIAYKPPG
SKVTYATAMYAPLVAGFGAENGNPGNYMGQQVAVERITYLSPSFGYQVNDHLSIGASF
GMSYNAIALNTDLRFPNEMVGVLRMIDDVVCRPFKNNKEFFTDLILLGICNTQEGMNP
FNKMGALQVSLEQSLSPSYNLGLLWEPTDNFSFGMVYQSSAKMRLKGKYLINNAKAPQ
QLIAGLNSSTTGSVASGVLQLPGYIPNVESGLVAMDLEYPAHFQAGIKYKLLPDLQVN
FDVGWTDYSAWNKFKFEFDRQISLLQVARLLSNDVTPASLALPLKFTSPWSWGIGLEY
SATDRLKLRAGYEPRASSIPDDKRNTMVPINNAQLFGLGVGYRFDTDTDLDLSVGFLR
SRDNIPACTSTLSNKCGVDNILLNPYAGLDVKTNTKVTVLGLSYRTKW" /product="Long-chain fatty acid transport protein" /transl_table=11
CDS complement(1259075..1259842)
/locus_tag="AUO97b_01345" /db_xref="SEED:fig|400667.82.peg.1205"
/translation="MALGDDNSRISRLRIREDAQHTLTNSFTAQYGVLDNLTFSATLP

LVAKSDIASDSTTAGLGDISFGARWEPFPLKQGRLPLVLFGNVSTKTGDSPYEIGLDE
LSTGKGYYSVGVGASTRKYIDPVVLFASVSANYGFKESGLNQARGSRILESFDPGISG
GFSFGFAYSFNYDVSLTMSYQQSFNFNSEFQFNNGESYKSPDQSSAMMAFALGVRVSP
DTIVNGTVGFGLTEDAPDVSLGLSFPLDILGFNKKQK" /product="FIG00973752: TolA-like membrane protein" /transl_table=11
CDS complement(1260311..1261144)
/locus_tag="AUO97b_01346" /db_xref="SEED:fig|400667.82.peg.1206"
/translation="MLEIALGSALIYYFATEAFEIEKKPPGTVYYTETADSRNLSFHR
NHIEPVTIKPAVEDQFRGIVRQAYDYSCGSAALTTLLNGYVGTSLTEQQTMSGLLQYG
EYQRIIERRSFSLLDMKRFVTAIGLESGGYRGEFSDLVKLGQPAIVPISYAGFKHFVV
YKAYKDGRVYVADPALGNISFDEGRFKEIWDNNTLFVISVPESQRKDLLALKEADMRH
VEDATVNKYAFVDIQYPTFNYERLANKASTMRRVLDDDNKSPTYGKPIETYMRLYYKR K"
/product="Bacteriocin resistance protein; peptidase C39" /transl_table=11
CDS complement(1261441..1262280)
/locus_tag="AUO97b_01347" /db_xref="SEED:fig|400667.82.peg.1207"
/translation="MKMFTKLALVSSLAISANAMAMQSMDDAALSAATGQDGINIGIA
LGADGITIDKLYLHDNDGLATATGITGASGTAGAISIGGVTLKQTGTDPLLNLAIDTN
GASGTNGAFLNVAATVGAVDVHVGSIGVGTSGTVNETTATRGITETSPTEIITGLDLS
LGKITANVQLGAAPQGAMIKVNSSLQGGLNISNLGIKDAAGGGTIKLDKIMVRGANAT
GDLDVNAGISVKTTGLEVKNNSTQAMNVYLQGVHLGTAASIGDVEVQGLNIANSTITI SGH"
/product="Type IV fimbrial biogenesis protein PilY1" /transl_table=11
CDS 1262599..1263987
/locus_tag="AUO97b_01348" /db_xref="SEED:fig|400667.82.peg.1208"
/translation="MPPLPNSHMFKMSHLKKKISLESYYSAADILKKLEELTGLVYLQ
DDNNPVIGFLPKNYLIVRNNQNFLFTKTDLNKYKFSSYIENLLDFTQFQSQELKNSNR
EKISFKGGYIGFISYDFSAHQFINNQSHKQPSLFIGSYYSFIKKINNEWYFFSDENNA
FEIFNYLSALLNKPSSKLQKNNFKLEQNIHPRWSKNTYLTAFHKIQEYIKAGDCYQIN
LTQEFKANFTGSLLNKADELWNLTNAPYSGYLKLTEFELLSCSPELFIEFNVDKQIKT
RPIKGTMPRYENKKQDLLSKQTLKNSKKDQAENVMIVDLLRNDLSIYANTGSVKTTKL
FEIESFNQVHHMVSEITATLKNDINPMQMLLTALPGGSITGAPKIRAMQIIEELEGAP
RGAYCGTLGYFNFDGTGRWNILIRSFQQYQNQLSIWAGGGITIASEAEAEYQESLDKI SAMLNLMNSTAE"
/product="Para-aminobenzoate synthase, aminase component (EC 2.6.1.85)"
/EC_number="2.6.1.85"
/transl_table=11
CDS complement(1263991..1265076)
/locus_tag="AUO97b_01349" /db_xref="SEED:fig|400667.82.peg.1209"
/db_xref="GO:0004400"
/translation="MTVSTAQMRFWSPEVRELEPYVPGEQPKIQNLLKLNTNENPYPP
SPKVVEAVQAVLHEQADVLRLYPDPDATALKQAIAKQQNIDVSQVFVGNGSDEVLAHI
FKAFFLQDEPILYPDITYSFYPVYSQFFGTKTKEIPLNENFEIDVRDYTQPNGGVIIT
NPNAPTSIALSLAEIEQVLQANPDRVVVIDEAYVDFGAESAVSLINRYENLVVCQTTS
KSRSLAGLRVGFAIAQSHLIAALETVKNSFNSYPIDRFAIAAAVASFEDQVYFEEQCQ
KVITSREKLVRDLTVLGFNVLPSKANFIFATHSQHDAGQLAQKLREQGIIVRYFNKPR
INQFLRITVGTDEQNARLVQTLKQDIL" /product="Histidinol-phosphate aminotransferase (EC 2.6.1.9)" /EC_number="2.6.1.9"
/transl_table=11
CDS complement(1265146..1266435)
/locus_tag="AUO97b_01350" /db_xref="SEED:fig|400667.82.peg.1210"
/db_xref="GO:0004399"
/translation="MRRLSTQDQNFKQVFAELLAFETVNDPNLVQTVDQIIADVRQHG
DDHVLKLTQQFDRHPAHQFSDLELSQQQLKAAFDGLNQEIREALQLAADRIRTFHEAQ
KQDSWTYVDDLGNTLGQKVTPLDRVGIYVPGGLASYPSSVLMNAVPAHVAGVPEIIMV
VPAPNGELNPLVLAAAYLAGVSRVFTIGGAQAVAALAYGTQTVPRVDKITGPGNRFVA
AAKRAVFGQVGIDMIAGPSEILVYAEGVNNAEWLAMDVLSQAEHDTVAQAIFITPDEA

LLEAVESAIEKHLNELPKAEIARTSIENRGALVLVKDRAEAVDLINQVAPEHLELCLD
DAEAMSQDIRHAGAIFMGRYTPEAIGDYCAGPNHVLPTSGTARFSSPLGVYDFQKRSS
LIMCSKDGVKTLAKTADVLAVQENLDAHARSARYRYQ" /product="Histidinol dehydrogenase (EC 1.1.1.23)" /EC_number="1.1.1.23"
/transl_table=11
CDS complement(1266541..1267224)
/locus_tag="AUO97b_01351" /db_xref="SEED:fig|400667.82.peg.1211"
/db_xref="GO:0003879"
/translation="MNDVRNDDPNFNVMGNFDHGLTLALSKGRILKETLPLLATAGIN
LLEDPEKSRKLIFPTTHKQVRILILRASDVPTYVENGAADLGVAGKDVLMEHGAQHVY
ELLDLQIAKCKLMTAGKVGMERPKGRLKIATKYVNLTRQYYASLGEQVDVIKLYGSME
LAPLVGLGDYIVDVVDTGNTLRANGLEPLEEICKVSSRLIVNKASFKRKQVLLNPIIS QLEQAVQSR"
/product="ATP phosphoribosyltransferase (EC 2.4.2.17) => HisGs"
/EC_number="2.4.2.17"
/transl_table=11
CDS complement(1267224..1268480)
/locus_tag="AUO97b_01352" /db_xref="SEED:fig|400667.82.peg.1212"
/db_xref="GO:0008760"
/translation="MDKFLITGGVKLEGEVRISGAKNAALPLLAAMILADSPITLTNV
PNLKDVNTLVKLIGGLGVTISYENDTVKADTSTLDNQFAPYELVKTMRASILVLGPLL
ARYGNAKVSLPGGCAIGSRPVDQHLKALEALGAHIEVENGYVHATVDGRLKGGEVVFD
MVTVGGTENILMAAALADGVTTIRNAAREPEITDLAQMLIKMGAKIEGLDTDTLVVTG
VESLHGCEYAVVADRIETGSYLAAAAITGGRVKTTHTDPSLLEAVLDKFEEMGAEVTR
GDDWIELDMLGKRPKAVSFRTLPHPEFPTDMQAQIMAVNAIGRGFATISETIFENRFM
HVPELSRMGANIQVEGHDAVVTGVEKLQAAPVMATDLRASFSLVLAALVAEGDTLIDR
IYHIDRGYEHVEEKLQGLGAKIKRVS" /product="UDP-N-acetylglucosamine 1-carboxyvinyltransferase (EC 2.5.1.7)" /EC_number="2.5.1.7"
/transl_table=11
CDS complement(1268487..1268738)
/locus_tag="AUO97b_01353" /db_xref="SEED:fig|400667.82.peg.1213"
/translation="MNSEQLTQILKEAFPEAEVVVSGQAGKFDLRIVDDQFEGKRTVA
RQQAVYAPLNTYIASGEVHAVTIRAMTKDEWRKASLFGA"
/product="YrbA protein"
/transl_table=11
CDS complement(1268877..1269215)
/locus_tag="AUO97b_01354" /db_xref="SEED:fig|400667.82.peg.1214"
/translation="MQITIRGHHLAITPAIEENIKAKFNQLTKHLDQVNSMQIKLTKD
HQIDKRSHKGSSNHVAEAIVRLPGIELFAQATADDMYTSIKKLTEKLKKQLLKYRKMQ CTYSQVAVSI"
/product="Ribosome hibernation promoting factor Hpf" /transl_table=11
CDS complement(1269384..1270832)
/locus_tag="AUO97b_01355" /db_xref="SEED:fig|400667.82.peg.1215"
/translation="MKLSVGLKVANSLSLTPQLQQAIRLLQLSSLELEQEIQIQLDSN
PLLEKVEDESLAESLSTLEHKETDDLTTELNADHLPDDLPVDTEWDDIYTHQSTALGT
PEFEEREDNRQVHLTLKEHILEQVNLLHFSKIDQLIAYCIVDALDDKGFLDAELEEII
LAVQHLLSEMDIDEEVEEDEVLVVLKHIQRLDPIGIGARNLAECLKVQLEFLPRETEY
LKEARSLLQYYELLIANDLNKLLKQTGLSKEQLKFAVDLLKTLKPYPGMDFEKQESEY
QIPDVVVAKKDLHWQVQLNPDVMPKLRINSFYSSMIRRADQSDDNLYLRNQMLEAKNF
IKSIDERHKTLLKVATCIVEHQKAFLEIGPEAMKPLVLRDVAEEVELHESTVSRVTTN
KYMLTPRGLFELKYFFSSHVGTTTGGEASSTAIRAMIKKLVSNENPRKPLSDNAIAAL
LKEEGIEVARRTVAKYRESLHIPSSSERKVLI"
/product="RNA polymerase sigma-54 factor RpoN" /transl_table=11
CDS 1276980..1277879
/locus_tag="AUO97b_01356" /db_xref="SEED:fig|400667.82.peg.1217"
/translation="MNYSNLLLSSLLTVGLYTSAHAQENLTEESAASSEDVTSTQTQH

SRPHLQIIKDLKGIKVKDLKINANAAQPDTVKDPLQSLNRPIYSFNDMLDRHVLRPVA
VEYREKTPEDVRGSYRQFRKNLGEPWNAVNQLIQGRPGRAAKTLGRFTINTLTTLGLA
DPASRLGLPPEEESFGVTLGYYGVPSGPFLMLPFFGPSTLRDGVGLAVDAQARPQKYI
MDDQDGLYWSTNLLQAVDTRAQYLDLDQTIQGDQYAMIRDLYLQRKAFQIAEKKGDSA
DVSFIDDDESEDVPEDNTDKTEK" /product="Outer-membrane-phospholipid-binding lipoprotein MlaA" /transl_table=11
CDS 1277995..1278963 /locus_tag="AUO97b_01357" /db_xref="SEED:fig|400667.82.peg.1218" /db_xref="GO:0004721"
/translation="MYFIKPTRSIPFVEQALEALPDLQVIHIDDLDLYDPTIIAIADV
QDFLTYKWRLPTIVIAFEHEGSALAQAWEAGALAGWVWNQLPKDLNKALTRIDAQYKR
NQDSRDLPSAAELQKRLLPNPIDLLNYEVETFFQPSAYLSGDWYDYWKLNDKEVLFYL
ADVSGHGVTSSLLTSWMAAFHGRSKTPRQLIKKLNGMLVQENIEKHITIVVGILNLET
HSLRWSSAGHYPPPIIFEPNQPPKILTTSSFPLGLTDELEVEEHVCTLNRHARFIICS
DGALEPFDGGLNDQFQQLVQHLQNQSFQAPDHVADDIAIFSLCRMN" /product="Serine phosphatase RsbU, regulator of sigma subunit" /transl_table=11
CDS 1279066..1279587
/locus_tag="AUO97b_01358" /db_xref="SEED:fig|400667.82.peg.1219"
/translation="MSTGHVEYASLNGTHIFKLIGEVRAHSCISLDKLLNRIEQQENV
VGAIVDLTQTTFIDSTVLGILAKLGLKLKQTHHIQAVMLSTNPDITTLANSMGLGQVF
VILNYCGDPNVCTLTLTDEHITHNAMLRTVLDAHKTLMKLNANNQNMFEPLVKQLQKE QDTLEQVSDKQNA"
/product="Serine-protein kinase RsbW (EC 2.7.11.1)" /EC_number="2.7.11.1"
/transl_table=11
CDS 1279614..1280438
/locus_tag="AUO97b_01359" /db_xref="SEED:fig|400667.82.peg.1220"
/translation="MTLVSVVQMNSQDDIESNFQVIESLIQQSKVQNASLIVFPENFV
CFAAGKQRETAEQFESIQQRLEKLAHQYQIWIVAGTLPCPFRPDGSIIQDGRVRTVSL
CISPERTEARYDKIHLFDVQVGDAVGGYQESRFFEPGTDVVVTSTPFGNIGLMVCYDL
RFPELALTLRQQGAHLLTAPAAFTYTTGQMHWQLLLQARAMDSQCYVLGAAQQGWHGE
KRQTWGHAGATDSRGQVLSMIGYEGNGLITVPFDLAAQELVRTSMPLMMHRKLIHY"
/product="FIG003879: Uncharacterized subgroup of the nitrilase superfamily"
/EC_number="3.5.1.4"
/transl_table=11
CDS 1280453..1280830
/locus_tag="AUO97b_01360" /db_xref="SEED:fig|400667.82.peg.1221"
/translation="MWKYLFSIFCLGANIHCYATDFGTTNNFVSPNLQLKQNVLPPTP
KNIPLPAFGQRIIGWGTGAEGARQRLENIQPADVSMIKKQGTTLEMITAWQDFYEQEQ
QRNENNPTAKYRARLMKKIADLW"
/product="hypothetical protein"
/transl_table=11
CDS 1280990..1281427
/locus_tag="AUO97b_01361" /db_xref="SEED:fig|400667.82.peg.1222"
/translation="MDQDCQNLKLENQLCFLIYSTNLALNQLYRKLLTPLGITYPQYL
VMLVLWEKDEITVSEIGSKLFLESSTLTPILKKLEALQLLNRTRSKEDERQVIITLSE
KGKKLKEQAVNIPTHILEASSCDTATLLGLKDQLTQLRTNIAK" /product="Organic hydroperoxide resistance transcriptional regulator" /transl_table=11 CDS 1281515..1281946
/locus_tag="AUO97b_01362" /db_xref="SEED:fig|400667.82.peg.1223"
/translation="MSLEKVVYRAKAKATGGRDGRATSSDGVLDVQLGVPKEMGGAGG
AVTNPEQLFAAGYSACFLGALKFVANRDKFNISKDAYVEGEVGIGPIPTGFSIEVTLN
VYLIGMDREEAEKLVAAAHIVCPYSNATRNNIDVTFNIVTE" /product="Organic hydroperoxide resistance protein" /transl_table=11
CDS complement(1282001..1283491)
/locus_tag="AUO97b_01363" /db_xref="SEED:fig|400667.82.peg.1224"
/db_xref="GO:0005524"
/db_xref="GO:0005622"

/db_xref="GO:0006810"
/translation="MQILKQIQVPYSFAKRHGVLFRYDGDQVFIVRRQNTEKIALQEA
RRILGKPAHYQLCTEQEFNSLLSTSYAGDTGESQQVAAGLEDHPDLLSLADQVPETED
LMDQEDDAPIVRLINALLSEAIRVGASDIHIEAFEKKLSVRLRVDGQLREIVQPRREL
APLLVSRIKVMAKLDIAEKRVPQDGRISLRLAGREVDVRVSTLPSSHGERVVMRLLDK
QAGRLNMTHLGLMANDYERLTQLVHRPHGIILVTGPTGSGKTTTLYAALSDLNDNTRN
ILTAEDPIEYQLEGIGQTQVNTKVDMTFARALKAMLRQDPDVVMVGEIRDLETAEIAV
QASLTGHLVLSTLHTNTAIGAVTRLKDMGIEPFLLSSSLIGVVAQRLVRTLCPHCMTW
READTFEKQVFQHISHEPSLKLPEAQGCDQCSHLGFNGRTAIYEIVPIDEPMRRLIHG
NAAEFELENHARQYSGSIRDDGLRKVLSGKTTLEEVLRVTNEASEA" /product="General secretion
pathway protein E" /transl_table=11
CDS 1283588..1284409
/locus_tag="AUO97b_01364" /db_xref="SEED:fig|400667.82.peg.1225"
/translation="MFENNEYQPHPQNSLKWWGEQHFEINQSKAWQFGSMLFRLTRGI
KEWRIEYYRPTLQDDNEQDWHIITDANFGFPHSVQVERYMFRKTNPKFLLMPRLADRS
VVIKPVDPIYIPAGQRGTLYISTPLWIAGLVESQHEPLFEIPVIQPKDTWFGPNAQQG
EICYATSVDGRTDLNLLTPRAFRAVTPIDFHNTSNHQLRFDRMNVPVTALPLFYSEST
GRLWTSQIKVYYEGSDRPARIRIENRTPPLAGEVTYVHPPRSPGGALFNMFDSFF"
/product="hypothetical protein"
/transl_table=11
CDS 1284419..1285336
/locus_tag="AUO97b_01365" /db_xref="SEED:fig|400667.82.peg.1226"
/translation="MADSSIPKDIADSLKSIFTNINTERISEILVGVVLCFIGFVLAR
IISNTFIRTVGSRFNAHQRLVWRRGIFYFIFLLFIMTSLKEAGFKLSVFLGAAGILTV
ALGFASQTSASNLISGLFLIGEGSFEVGDTIQITLIRGNTIEGEVISIDLLSVKLLTL
DNVYIRLPNEQLIRAPVHNLSKYPIRRIPITLAINFHEDIIKVREVLLNVAANYPLVL
DDPKPAVTVTAFRESSIELLFAMWCRQENSLKVRDEMQERIRNGFLDNQIEIPVPKMG
LIDRPSTAFAEDDIDQYSNQKELKREPKA"
/product="Potassium efflux system KefA protein / Small-conductance
mechanosensitive channel" /transl_table=11 CDS complement(1285326..1288109)
/locus_tag="AUO97b_01366" /db_xref="SEED:fig|400667.82.peg.1227"
/translation="MIIVLIALTLFLAISIGFKFQSAVVALSIALTVILVQSLSSLHQ
RLTHLEQQFSHQTTPLYSIHMQRTVIYTAALVALIAYINHWMWIAGGALLVLLLCLIQ
TISAFHTRLLFLETAKQTVNINESIHTEVITPVLEQVDPNNITEPALPETIALNQKEQ
TVRTQTIAWWQPAVDWMIHGNPILRVAVAVLMVGVVLLLRFASEHWQLSLGVKLGFIA
IAGGVLTAFGYALQKKNQLFAVALQGVGLAVVFLTLVFSHHFGVITSLTTASILFTIL
LAITVFLSLKQQAIYLAILALGMAYAAPLVIPQYRPDVTFLFSYYLVINLAVAAVNFI
QPWKILNQIAFFATMFIGGSAIAFYAEPAKFDTLDWILWLHIALFIWLSVRYSQNISR
VSEHEKQEGIRLPPLLDVGLIFSVPVLGFTLHAYLVHESTQALTIGAAVLAGTYAVLT
FWIKKTHPQLSVLAKSFFILAVAFFALIFPLAKGAHWTAIGWVAQGTALIVWGVTERY
RLSRYIGVILVLLSSLALFYQVWANEEFPTLSTSIYAIAQFISAFYLLQYNSKEQRYF
SASMFSGIFLCLGMYAGAVAGVEIMAWHHHALSPYLIFAVALIAIFSAIVHYKLRVQW
QSLQLILISLLLLLVLGEAFMSQVFTLFKWVDSLQQTTFLVATIILGSLFIMAQPQSD
ESGYLKVWAGLSWLALAIVGVTIFPQMPVVALAFVPVIYSLWAYKIHKTTLLHQIPVW
CLSLIWLLVVSVDVHSAEYLYFVPLINLTDFFSILVFAGLLFIIYQHAFDQDKSLEWT
FKITTILVGLLVFSSVVVRGLHHYWATPLWSASIWTNGVVQLSLTLLWVILAFILTTY
SSRKMIRQLWFVGAALLGIVVLKLILLDLSQSATLTRVISFIGAGGVMLIIAYLAPLP PSSSVQKNQEPKL"
/product="hypothetical protein"
/transl_table=11
CDS 1288288..1291059
/locus_tag="AUO97b_01367" /db_xref="SEED:fig|400667.82.peg.1228"
/db_xref="GO:0003887"
/translation="MPPFVLVDGSYFLFRAYHALPPLTTSTGLHTNAIRGAISAIQKL
MRRMQPTHMAVIFDTPEPTFRHKLSPIYKGDRPTMPEELSQQIPYLHALIKAQGIPLY

SLPGAEADDIIGTLAKRAVKEGHHVLISTGDKDMAQLVNDYVKLEDSFKDRVLDTDGV
FEKFGVWPNQIIDYLTLMGDASDGIMGVPGVGAKTAAKLLTEYGTLDNIIANVDQLKG
KLSQNIKDNLDNIKLDHQLASIVCDLPLELDWHELKLTDPNVDALRNLYTELEFRNQL
QSLDHPNNPNSSSYKQQASQTIVQNEVKPAQTIETEDQASLTSQDDQLGTANYHTVLT
QEAWDQLWQRMQNADHFAIDTETTSLDYRIAEMVGFSIAFDAKDAYYIPLAHNYENAP
QQLNREQVLAQIKPVLENEAVKKIGHHLKYDAHIFANHGIELKGWYFDSMLASYVLNA
AATRHGMDDVARVYLSHLTTTFEQIAGKGAKQKTFNQIEIEVAAHYAAEDAHVTYRLY
EVLSNKLKAHPELENILYNIEMPVARILSGMEEDGIRLDHAFLDKLSVEFAKTMEDLE
NQAMEIAGETFNIASPKQVGEILFDKLGIKGGKKTATGQYSTSESVLEKIEHPLTEII
LEHRGLAKLKSTYTDRLIEQSHNDTHRVHTSYHQALTATGRLSSSDPNLQNIPIRRQI
GRQIRKAFIAPEGRVLLAADYSQIELRLMAHFSQDDALVHAFQHGQDVHRRTAAEVLG
IAIEDVTNDQRRQAKAVNFGLLYGMSEFGLIRQLGFTREESQNYIKQYFQRYPGIYEY
MQRTRQVASEQGFVETILGRRLYTPDIDARNVMVRKAAERAAINAPLQGSAADIIKLA
MIEVDKILPKDQAKLLLQVHDELVFEVDSNIADELSKQIADVMQSVLEISVPLVVEVG QGKNWDDAH"
/product="DNA polymerase I (EC 2.7.7.7)" /EC_number="2.7.7.7"
/transl_table=11
CDS complement(1291119..1291688)
/locus_tag="AUO97b_01368" /db_xref="SEED:fig|400667.82.peg.1229"
/translation="MGANSALIFGILINVAILLVFFRFLMQLAAVSPYNPVVLSTVKA
TKIVDIFGRIFPTVAKGRFNLAALVLLIILYLLKIFGVMYLSGSMPNSPVHLVILTFV
TMIQDLIRFCRYLIFATIILSWVVMFTQSRSPYIEVIQDLAEPLLAPFRRLLPNMGMI
DLSPILAFLALYIAEILMNEVAKVLLTGL"
/product="Cell division integral membrane protein, YggT and half-length relatives" /transl_table=11
CDS complement(1291710..1292540)
/locus_tag="AUO97b_01369" /db_xref="SEED:fig|400667.82.peg.1230"
/db_xref="GO:0004735"
/translation="MATAVNCNICFIGGGNMAQALIGGLISRGLPPTRITVSDPVEQI
RQLLQEKEVHVTQDNTAAIQNADVVVLAVKPQVLANVLRPLKGLLSDKLVISIIAGAE
IKTISNLIDSERIVRVMPNTPALVQTGAHGIYATDVVGASDRELTSQILAATGLTIWV
NSEAQIDAVTAVSGSGPAYFFYLMESMIRAGKNLGLDEKVATALTLQTALGAAQMAIT
SSNTPSELRKNVTSPNGTTQAALEVFDRAQISQNIQSALAAAQKRSQELAQELSDSAK "
/product="Pyrroline-5-carboxylate reductase (EC 1.5.1.2)"
/EC_number="1.5.1.2"
/transl_table=11
CDS complement(1292518..1293792)
/locus_tag="AUO97b_01370" /db_xref="SEED:fig|400667.82.peg.1231"
/translation="MLLLHLMAQIFPQKIRAIYIDHQLQDQSAEWAEVVATQATILNI
PYIIQKVQIANGNLEAQARQARYQAYQQHLQENEILLLAHHQQDQAETVILRLLSGAG
VDGLAAMQAIDYRKDMTIWRPFLDLTREQIALWTAQLEVKYIDDPMNYDTHYDRVWCR
EALWPFLTSRFPKMQQALSRTSYLMQDASEILEEVLKDDWQYSGSADYLDLTKLYELS
FARQRQLLSAWMKGQGQYRPAFEMVERLRTEVIESKSDAQAALHWNQFYYVRYQNILY
RLSREVYLAETLNPVVAELEHSFKLEERWQGAAGLFHVECKKIGLSHSLLNKKLKIIR
RQGGEKIHLYGRVGQWPLKKAIQEAHILPWLRHTIQILVLDNVMLGVFTPKGFWLAQS
PYCEEGGWQPDLISHSCNLVNGEYSYGNSSEL" /product="tRNA(Ile)-lysidine synthetase (EC 6.3.4.19)" /EC_number="6.3.4.19"
/transl_table=11
CDS complement(1293858..1294679)
/locus_tag="AUO97b_01371" /db_xref="SEED:fig|400667.82.peg.1232"
/db_xref="GO:0003989"
/translation="MKKATQSKAWTTVQIARHPERPQFLDYVGEIFTEFDALHGDRLF
GDDGAMVGGLARFDGQPVMVIGQHRGRSTREKLKHNFGMCNPEGYRKSQRLLDMAERF
NLPVFTFIDTMGAYPGVGAEERGQAEAIATSLAQLSSLKVPVIATVLGEGGSGGALGI
GVADRVIMLSHSIYSVISPEGCASILWKTAEKAAQASEALGLTADKLQSLGIVEYVVD

EGEGAHLDPERVMQNLKVVLKQALDELLPMDANERCEARYQRLMKFGSENLGMAS" /product="Acetyl-coenzyme A carboxyl transferase alpha chain (EC 6.4.1.2)"
/EC_number="6.4.1.2"
/transl_table=11
CDS complement(1294698..1296218)
/locus_tag="AUO97b_01372" /db_xref="SEED:fig|400667.82.peg.1233"
/db_xref="GO:0004309"
/translation="MSDFLIDEELLAAIDMGSNSFHLAIARVDHGEVKKVASMSEKVQ
LAAGLDENKNLTEAAQQRGLACLARFVGRLGSVQPNRLRIVATNALRQAKNGHEFIQK
AAEILPKPIEIIAGREEARLIYLGVSHTMANGGRRLVVDIGGGSTEFIIGEEFEPIYT
ESLQMGCVAYTKAYFADGEITQKAFDKAVVAARKELSAIATTYKMEGWDTVVGSSGTI
KACRQIMVNMGLSDEQENITREGLHKLKDKLLKFKNISEIDFEGLREDRRAVLPAGLA
ILYAVFEVLEIERLAYSDGALREGVMYDLLGRFKHEDIRDRSVQALMGRYNADPKQAE
RVVNTAQYLFDSVAKSLNLTSEDSDLLRRAAYLHEIGLAISHGGYHRHGAYLLQHSDI
PGFSQIDQNHLSHLVAHHRRKLRNDVKNEVLKAGGHKLVYLSLLLRLAVLLNHSRSDQ
MLPAIELTIINDQQWQLSVSGDAKQWPLLVADLHDEQEQFKHWDIELNIQSEKFID"
/product="Exopolyphosphatase (EC 3.6.1.11)" /EC_number="3.6.1.11"
/transl_table=11
CDS 1296498..1296824
/locus_tag="AUO97b_01373" /db_xref="SEED:fig|400667.82.peg.1234"
/translation="MSATIVNTTDDNFQADVLDAETPVLVDFWAGWCAPCKAIAPVLE
DLSSEYAGKVKIVKVDVTSCEETAVKYNIRNIPALLLFKNGEVVAQQIGAVPRSKLVS FIDENV"
/product="Thioredoxin"
/transl_table=11
CDS 1297146..1298414
/locus_tag="AUO97b_01374" /db_xref="SEED:fig|400667.82.peg.1235"
/db_xref="GO:0003715"
/db_xref="GO:0003723"
/db_xref="GO:0005524"
/db_xref="GO:0006353"
/db_xref="GO:0015986"
/db_xref="GO:0016469"
/translation="MNLTELKKKPIGELIKIAEFMGLEGMARNRKQDIIFAILKRHAM
NGEEIFGDGVLEILSDGFGFLRSAAGSYLAGPDDIYVSPSQIRRFNLRTGDTITGTIR
PPKEGERYFALLKVNQINYDTPENSRNKILFENLTPLFPTEQLVMELGNGTTEDLTAR
VVDLVAPIGKGQRSIIVAPPKAGKTMLLQNIAQSIVRNNPEVFLIVLLIDERPEEVTE
MERTVRGEVVASTFDEAPARHVQVAEMVIEKAKRLVEHKKDVVILLDSITRLARAYNT
VIPSSGKVLTGGVDAHALERPKRFFGAARNIEEGGSLTIISTALIETGSKMDDVIYEE
FKGTGNQEITLDRRIAEKRVFPAMNIKKSGTRREERLMDEDKLRKVWILRKLLHPMDE
LAAMEFLLDRMKETKTNDDFFDQMKRKAST"
/product="Transcription termination factor Rho" /transl_table=11
CDS 1298829..1299080
/locus_tag="AUO97b_01375" /db_xref="SEED:fig|400667.82.peg.1236"
/translation="MLFKKFAVAAAVAATLAFVGCSKKEEAPAAAAASEAVAAASEAT
AAASEAAVAVDAAASETAAAASEATTAASEATAAASAAH" /product="conserved hypothetical protein; putative exported protein"
/transl_table=11
CDS complement(1299251..1299547)
/locus_tag="AUO97b_01376" /db_xref="SEED:fig|400667.82.peg.1237"
/db_xref="GO:0003677"
/translation="MTALTKADMADHLSELTSLNRREAKQMVELFFDEISQALIAGEQ
VKLSGFGNFELRDKRERPGRNPKTGEEIPISARRVVTFRAGQKFRQRVGNEQID" /product="Integration host factor alpha subunit" /transl_table=11
CDS complement(1299544..1301925) /locus_tag="AUO97b_01377"

/db_xref="SEED:fig|400667.82.peg.1238" /db_xref="GO:0004826"
/translation="MKISENWLRTWVNPAIDSDTLSDQLTMLGLEVDELAPVAKPFTG
VVVGEVLTVEQHPDADRLRVTTVNIGSGEPLQIVCGAPNVRAGMKAPVATIGAILPGD
FKIKKGKLRGVESQGMLCGASEIDLEDKIDGLLELPADAPVGVNIREYLKLDDNVIDI
SITPNRGDCFSIRGIAREVAVINQLQMNEPEIKSVDATITDEKKVVINTDGAPRYLGR
VIKNVNVKAATPEWMEQALARSGIRTHSILVDITNYVLMELGQPMHAFDLAKIEGTVH
VRQAKPKEKLQLLNDQEVELQDDVMVIADDQKALAIAGIMGGLASSVTDDTTDIFLES
AFFAPLAIAGRARRFGLHTDSSQRYERGVDFELPLIAMNRASQLIQELAGGEFGPITV
AEKSDLLPKREAIELKQAQVDQLLGYKVAAEFITDALTRLGCEVTVQADGEWSVVPPS
HRYDMAIYQDLIEEVARIDGYDNIQISLPSMDVQLAKYQDRFEIAQLRQTVVTLGYQE
AISFSFADAKLEKQLNPQVSPLMLANPISSDLAAMRSTLLSSLIPCVQYNLNRQQSRV
RFFELGLRFDYQNANSIQDLKQIPTLALVAVGSREPESWHAKPQPMDFFDFKGEVEEI
LAAGRVKVEYVRSERPWLHPGQSAEILVDGQSIGYLGRLHPSLENELDLSTTWVAELD
QAAVLQSYVSNFTELSRFPSVRRDIALLISDNINVRDIQQLIEKTGGELLDSTWLFDV
YTGQGVEEGKRSLAFALLWQHPSRTLEDAEIKSGMDNIIQVLENTYQATLRAS"
/product="Phenylalanyl-tRNA synthetase beta chain (EC 6.1.1.20)"
/EC_number="6.1.1.20"
/transl_table=11
CDS complement(1301959..1302939)
/locus_tag="AUO97b_01378" /db_xref="SEED:fig|400667.82.peg.1239"
/db_xref="GO:0004826"
/translation="MSLEALTTEALAAIAAAQDLVALDQVRVQFTGKKSQLAEQSKAL
GKMDPEERKVQGAAIHAVRETINNALTERQTALQQAALAQKLASETIDITLPGRGQRV
GTVHPVTQVQERICQFFTKAGFTVATGPEVEDDYHNFEALNIPGHHPARAMHDTFYFD
ANHLLRTHTSGVQIRTMETSQPPIRIVCPGRVYRCDSDQTHSPMFHQIEGLYVAENTS
FAELKGLLINLLNEFFEKDLKVRFRPSYFPFTEPSAEVDIMDERGRWLEVLGCGMVHP
NVLRAAGIDPDKYKGFAFGLGVERFAMLRYGINDLRMFYQNDVRFLRQFA" /product="Phenylalanyl-
tRNA synthetase alpha chain (EC 6.1.1.20)" /EC_number="6.1.1.20"
/transl_table=11
CDS 1303097..1304098
/locus_tag="AUO97b_01379" /db_xref="SEED:fig|400667.82.peg.1240"
/translation="MALNQIWKQQLTLVTYGNEYLRQNLSFSQWRQHHIFDQHSFQFR
DLISQHLLAQHFQVWLEALKKQGTTQISLHLSTILNEEQNPNSNVELLPYTHFIVSHQ
NNKKIAWIFGHELAEWYSSDNEFEPPLTQRSDLRLETFWRFELNEKLSKKVEADLKAP
QWDEIANFMETELFRSKYAAGFEEPEFQKKYFDGSTHQISVEHQPTLALIPESYPADC
AHQLLYRTQALSDYLEQKKRTANDPLGEDLDQEQIINLRNFTHKTDDLFAKLIVKAAN
HYQTAQLKPVEKPSPFDATNESAAHLKVPHHKAGGTGVIKLIVLTIIICALAYYFGL"
/product="hypothetical protein" /transl_table=11
CDS complement(1304105..1304608)
/locus_tag="AUO97b_01380" /db_xref="SEED:fig|400667.82.peg.1241"
/translation="MKPLNDTDIRIASFKDALSIAEVHVQSWKETYTGMIKQEILNKL
NVLDKQQLWKEISRSPDHRLFIYTENGVVKGFLDGYLNPENNIAEIRAFYLLGEIQRK
GVGRALFQKFYQCALNQGYAFIRLEVFNKNPSRFFYEKMGAKLTGEAELPEFGLGIIE LFYEWQL"
/product="Acetyltransferase, GNAT family"
/transl_table=11
CDS complement(1304667..1305035)
/locus_tag="AUO97b_01381" /db_xref="SEED:fig|400667.82.peg.1242"
/db_xref="GO:0003935"
/translation="MMAQKAKLFNDKEIFAQLQVKHPNEAKQLGRKVRNYDEQIWREK
RFDIVVQANFAKFSQHPELKKFLLATKDRILVEASPVDKIWGVGMAQDHPHIQDPSLW
QGLNLLGFALIHVRELLLTE" /product="Uncharacterized protein COG3236"
/EC_number="3.5.4.25"
/transl_table=11
CDS complement(1305032..1305220)

/locus_tag="AUO97b_01382" /db_xref="SEED:fig|400667.82.peg.1243"
/db_xref="GO:0003935"
/translation="MIDEKFLNDLIQKIQQGHQFKYLYFWGHTPKKANLIDKSCFSQW
FPAQFNVEDIEYFTLSTI"
/product="Uncharacterized protein COG3236" /EC_number="3.5.4.25"
/transl_table=11
CDS complement(1305311..1305670)
/locus_tag="AUO97b_01383" /db_xref="SEED:fig|400667.82.peg.1244"
/db_xref="GO:0003723"
/db_xref="GO:0003735"
/db_xref="GO:0005622"
/db_xref="GO:0005840"
/db_xref="GO:0006412"
/translation="MARVKRGVVAHRRHKKILARAKGYYGARSRVYRVAFQAVIKAGQ
YAYRDRRQKKRQFRALWIARINAGARQNGLSYSRMIDGLKKAQVIIDRRVLADIAMHD AVAFAALAEKAKGALAA"
/product="LSU ribosomal protein L20p"
/transl_table=11
CDS complement(1305682..1305876)
/locus_tag="AUO97b_01384" /db_xref="SEED:fig|400667.82.peg.1245"
/db_xref="GO:0003735"
/db_xref="GO:0005622"
/db_xref="GO:0005840"
/db_xref="GO:0006412"
/translation="MAKLKTRRGAAKRFKATANGFKRKQAFKRHILTKKSAKRIRQLR
GCVMVHVSDVASVRRMCPYI"
/product="LSU ribosomal protein L35p"
/transl_table=11
CDS 1306125..1307375
/locus_tag="AUO97b_01385" /db_xref="SEED:fig|400667.82.peg.1246"
/translation="MQKHDAFAALRYRDFSIVTINQFCLTLAILIQEIIVAYSLYQIT
KDPLTLGLIGLAEAIPFIALSLWGGYFADKFNKQVIMKICLFFSVPLPLVLWLLFHLH
GLGHISVNFLSWGIYAVIFGLGTIRGFYNPSATSLKPFLIPRELYANGATWTTIGWQS
GVIIGPMLGGFMLAYLGRETSLFSVAGLLSICFILINLLHKRSFPKIETANVIESLGD
GFRFIWKTKIVLWAISLDLASVLFGGVIALLPIFAEDILKVGPEGLGYLRAAPSIGAL
ITMIALTRFPPTQHAWRNMLLAVAGFGVFTILFAFSNNMWLSLFALAMTGACDSISVV
VRQTILQIFPPENMRGRVAAVNGMFVSSSNELGAFESGLAAKYLGTIAATIFGGCMTL
AVVTFCWLKTNDLFKVDITKSSED"
/product="Uncharacterized MFS-type transporter" /transl_table=11
CDS complement(1307415..1307843)
/locus_tag="AUO97b_01386" /db_xref="SEED:fig|400667.82.peg.1247"
/translation="MLSYFLLFLSAFGAATLLPLQSEAVLAALLLKNQHSAFLLILIA
TFGNVLGSCINWWLGLKIEQFKHKKWFPVSEKRLEQAQRFYHKYGFYSLLLSWTPVIG
DPITLVAGLFKENFWRFLFMVLIAKAGRYIFIYWVITGFI" /product="probable membrane protein
YPO3302" /transl_table=11
CDS complement(1307896..1308516)
/locus_tag="AUO97b_01387" /db_xref="SEED:fig|400667.82.peg.1248"
/db_xref="GO:0004364"
/translation="MIDLYYWGTPNGHKITIALEEMGLDYTLYPINILENDQFQPDFL
KISPNNKIPAIVDQDGPRGEPISVFESGAILQYLGRKTGLFYPIDEQERVEVEQWLMW
QMGGLGPMLGQNHHFNRFAPEKIPYAIDRYVNETKRLYGVLNKQLIGQKFVAGEYSIA
DMAILPWILRYEWQGIQLEDYPYVQEYIVRLTARPAVQKALSIKVI" /product="Glutathione S-
transferase (EC 2.5.1.18)" /EC_number="2.5.1.18"
/transl_table=11
CDS complement(1308722..1309060)

/locus_tag="AUO97b_01388" /db_xref="SEED:fig|400667.82.peg.1249"
/db_xref="GO:0003743"
/db_xref="GO:0006413"
/translation="MDYNKHLFDLKQKQKDAKKKQHQVQVKEIKLRPATDVGDYQVKL
RAILKFLEEGNKVKITLRFRGREMAHQQLGLAQLQKIEADVAELGVVEQAPKMEGRQM GMLLGPKKKK"
/product="Translation initiation factor 3"
/transl_table=11
CDS complement(1309279..1311201)
/locus_tag="AUO97b_01389" /db_xref="SEED:fig|400667.82.peg.1250"
/db_xref="GO:0004829"
/translation="MPIITLPNGDQKSFDHPVSVMEVAQSIGPGLAKNTVAGRVNDRL
VDACDLITEDSTLQIITPKDEEGLEIIRHSCAHLVGHAVKQLFPEAKMVIGPVIEEGF
YYDIWMPRPFTLDDMAAIEERMKKLIDQDYDVIKKMTPRDEVIKVFTDRGEEYKLRLV
EDMPEEKAMGLYYHQEYVDMCRGPHVPNTKFLKSFKLTKISGAYWRGDAKNEQLQRIY
GTAWADKKQLAAYIKRIEEAEKRDHRKIGKALDLFHMQEEAPGMVFWHANGWTIYQVL
EQYMRKVQQDNGYQEIKTPQIVDFTLWEKSGHAANYAENMFTTHSESRNYAVKPMNCP
CHVQVFNQGLKSYRDLPIRLAEFGSCHRNEPSGSLHGIMRVRGFTQDDAHIFCTKEQI
GKEVADFIKLTLDVYKDFGFEEVQMKLSTRPEKRVGDDALWDLAEKSLADALDAAGLE
WELQPGEGAFYGPKIEFSLKDCLGRVWQCGTIQCDFNLPVRLDASYVTEENERDQPVM
LHRAILGSFERFIGILIEHYAGFMPPWLSPVQACVMNITDSQAEASEQVVAKLKENGL
RAISDLRNEKIGFKIRERTLERIPYLLVLGDREVEEGTVNVRTRSGKNLGTMSVDAFI DLVKSAVAERGRYIVE"
/product="Threonyl-tRNA synthetase (EC 6.1.1.3)" /EC_number="6.1.1.3"
/transl_table=11
CDS 1311613..1313241
/locus_tag="AUO97b_01390" /db_xref="SEED:fig|400667.82.peg.1251"
/translation="MVSAYNELPRTPANFVALSPLRYLERAAYIYPDQASIIHGNRQI
SWKQTYQRCRQFASQLQQLGITKNDTVSVLLPNVPAMIEAHFAVPMAGAVLNTLNTRL
DAKTIAFMLEHAETKVLLVDPEFVNLAREALSLIPNQHIIVIDVADEEYEGENQFLGS
FEYEEWIAQGDANFEWHLPQDEWDAISLNYTSGTTGNPKGVVYHHRGAYLNAASNILA
CGMKPRAVYLWTLPLFHCNGWCFAWSIAASGGTNICLRKVDPELVMQLIAKHKVDYFC
GAPIVLSMIINLPKEKQPIIEHHVEVMVAGAAPPVAVIEGMRNIGINVNHVYGLTETY
GPSALCASQAGWSDLSITEQAQLHSRQGVPYPLQDSMRVLDPETMQPVPNDGETMGEI
MFRGNIVMKGYLKNPKATEEAFAGGWFHTGDLAVCHPDGYAKITDRSKDIIISGGENI
SSLEVEDVLYKHPAVLTAAVVAKPDERWQEVPCAFIELKTGASVTPEEIIEHCQKELA
RFKVPKDVVITEIPKTSTGKLQKFILREWAKERA" /product="3-methylmercaptopropionyl-CoA
ligase (EC 6.2.1.44) of DmdB1 type" /transl_table=11
CDS complement(1313301..1313837)
/locus_tag="AUO97b_01391" /db_xref="SEED:fig|400667.82.peg.1252"
/translation="MARRPKRYTEEDFDSLEGRASKTEQKKAVQRMAALGEQLAQLSI
KQIQKLPVDERLIDALLEVQNISSFEARRRQFQRVGKLLRNEDETVILSYLTPQQGAK
KQAQLMRWVDRMIEQGDPAINEFSKIYNASERHTLRQHVLRINRDKTQQVAEADLEAT
KMKFINYVQQVALLSDQG"
/product="Sodium-dependent phosphate transporter" /transl_table=11
CDS complement(1313840..1314109)
/locus_tag="AUO97b_01392" /db_xref="SEED:fig|400667.82.peg.1253"
/translation="MIDTTVDVINKLGLHARASGKLIEVTTKFRSSIQIGKGDHLVDA
KNIMSLLMLGAGKGTTLRLVIDGTDEEQALNEVQALFAAKFYEAD" /product="Phosphocarrier
protein, nitrogen regulation associated" /transl_table=11
CDS complement(1314106..1314957)
/locus_tag="AUO97b_01393" /db_xref="SEED:fig|400667.82.peg.1254"
/translation="MKRILIVTGQSGSGKSSALQVLEDLGYYCIDNLPLALLPEIVAK
LDHENNLEQLALGVDVRSTRADMQEFDHVFEQLQKHGTVDVIYLTTQDQDLIARFSAS
RRPHPLANRFKSLLQCIHEEKQLLIPIQFRATVHIDTTDKSVHDLKHILLSKLGQSDN
LIVILQSFGYKHGIPLDADYVFDVRHLPNPHWDLELRRFSGLDEPVKQFLEASPQANE

MFEDILHFLKKWLPAFAEGHRHYMTISIGCTGGQHRSVYMVDRLKQALEAEWSVQVLH REMKHWS"
/product="RNase adapter protein RapZ"
/transl_table=11
CDS complement(1314980..1315828)
/locus_tag="AUO97b_01394" /db_xref="SEED:fig|400667.82.peg.1255"
/db_xref="GO:0004592"
/translation="MKTETTIQGLAASLNPARAARKIIGFVPTMGNLHEGHLTLVREA
KKLCDVVVVSIFVNPTQFGPGEDFDNYPRTLEQDSRLLADVGCDIIFAPSVEQMYGTQ
PRLTNISVSQITDDLCGSSRPGHFDGVALVVTKLFNIVQPNYAFFGQKDYQQLAVIRQ
FVQDLNIPLEVIGVPIVRAEDGLALSSRNGYLTPEQRQVAPVIYQGLKQAEEQLHQGK
DLQQVLADLKTLLTDNGFVVDYVEARQPNLLAASQFDRDIVLFVAAKLGGTRLIDNLQ VAFTPQ"
/product="Pantoate--beta-alanine ligase (EC 6.3.2.1)" /EC_number="6.3.2.1"
/transl_table=11
CDS complement(1315832..1316641)
/locus_tag="AUO97b_01395" /db_xref="SEED:fig|400667.82.peg.1256"
/db_xref="GO:0003864"
/translation="MISLSDLRKFKAEGRKFSCLTCYDASMAKAMELAEIDTILIGDS
LGMAIQGRDSTLPVTVEDMAYHTAAVRRGNQHALIMTDLPFMSYATLNDALQNAKTVM
QAGAQMIKIEGGAWLSETVQVLTRNGVPVCVHLGLTPQSVHVFGGYKLQARTREAADQ
LIADCTAVVEAGAAVLLLECVPAQLGQEIAELFPNTPVIGIGAGNATDGQVLVVQDML
GLTFGRVARFVRNFMKEQSGETAILDAFKAFHAAVQDQSFPAKEHTFQVEL" /product="3-methyl-2-oxobutanoate hydroxymethyltransferase (EC 2.1.2.11)" /EC_number="2.1.2.11"
/transl_table=11
CDS complement(1316687..1317169)
/locus_tag="AUO97b_01396" /db_xref="SEED:fig|400667.82.peg.1257"
/db_xref="GO:0003848"
/translation="MTVTTYIGLGSNLGDSRQILSEAITKLKSLGEVKVSKLYQSPPM
GPQDQPNYLNAVAQLKTELLPLDLLDQLQRFEQEAGRVRLRHWGERTLDLDLLIYGNE
KIQNERLTVPHVGILQRDFVVIPLLDLDAGLHINDQPLKNLELIQQPTLTVLADESWA " /product="2-amino-4-hydroxy-6-hydroxymethyldihydropteridine pyrophosphokinase (EC 2.7.6.3)" /EC_number="2.7.6.3"
/transl_table=11
CDS complement(1317166..1318629)
/locus_tag="AUO97b_01397" /db_xref="SEED:fig|400667.82.peg.1258"
/db_xref="GO:0004652"
/translation="MQTLRASKCGLSTAQLPSSILDVIDSLTKAGYEAYIVGGGVRDL
MLGLNPKDFDAVTNATPSQIKEVFGRRCRIIGRRFELAHVYSGRELIEVATFRAPPKK
AVTSASGMILRDNNWGTIEQDFARRDFSINTLYYQPRKGIVLDFCKAIDDVKSKTLRL
LGDPVQRFEEDPVRMLRTLRFAAKLNFKIDPAILDIFDVEMTQLLRDVSPHRLYDESQ
KLFTMGHLARVLPMLIDFGVWKQLFADIRPDLTPFIQRAAKNTDQRIQVGKTINPAFF
YAVLLWQPFLERCDFYLSKGVVPAEARAQAGLDVLKRQATRTVIPRFAETFIREVWEM
QTRLLNPKPQQIEALASHARFRAGFDFLLLREKSGDDTTQGMGSWWEAYQEMSNDEKE
AAISQYNRQKAKSRRKVAAEPVESNKVDTEIEPLVDVPEPRSRRGKKERARQEQSIDR
FIEKSSAAQTNVMSDHPILKRKRVQRDLSQVIFGPTQ"
/product="Poly(A) polymerase (EC 2.7.7.19)" /EC_number="2.7.7.19"
/transl_table=11
CDS complement(1318754..1319116)
/locus_tag="AUO97b_01398" /db_xref="SEED:fig|400667.82.peg.1259"
/translation="MLCLGGISPSVYAQSFDKNFQEWKAKQQMYDQKLNISKFSHSNG
SKISQTKNFNDSTGQIHLNQANVNEFQQLKGIGEKKAQAIVEYRQKNGSFKNIDEIKN
VKGIGPAIFEKNKSRLAL"
/product="Late competence protein ComEA, DNA receptor" /transl_table=11
CDS complement(1319133..1319939)
/locus_tag="AUO97b_01399" /db_xref="SEED:fig|400667.82.peg.1260"

/translation="MDKLLKIMQELREKCPWDQEQTPMSLTKYAIEEAYEVEAAIRQG
DINEIRNELGDLLLQVVFQSQMFSEQGAFNFQDVVEAISEKLVRRHPHVFQADQFNNL
TPEQVSELWKQIKQQEKQGKPQSRLDEIKHGPALSQAQEIQKNVAKVGFDFETVEDAY
TKLEEELDEFKQALKNQNSDEIQDEFGDCLFSLVNVGRKLGISSESSLLSTIHKFRSR
FAFIEEQAIKQQRTLEDMTLSEMDELWNQAKRQLKSGEKPHAIQHEILEK" /product="Nucleoside triphosphate pyrophosphohydrolase MazG (EC 3.6.1.8)" /EC_number="3.6.1.8"
/transl_table=11
CDS 1320051..1320824
/locus_tag="AUO97b_01400" /db_xref="SEED:fig|400667.82.peg.1261"
/db_xref="GO:0004316"
/translation="MKHSILISGAAQGIGAEIARVFYQRGYKVGIYDINESLAQNLAQ
ELGPNACAGYLDVSDYSQWQHILKEFTDWAGELNILVNNAGILYSGAFENIDIKSHQR
TININVNGVINGCHAAFPYLKQASFARVINLSSASAIYGQADLISYSASKFAVRGITE
GLDVEWKKYGIRVLDVMPLFVQTAMVNNMDAGSIQNMGIDLTPNDIAQQVLSLVEKKA
HFWTPTHTPVGIKTKLLYQLSTISPQFLNRLTNIYLSRK" /product="Oxidoreductase, short-chain dehydrogenase/reductase family" /EC_number="1.1.1.100"
/transl_table=11
CDS complement(1320869..1323175)
/locus_tag="AUO97b_01401" /db_xref="SEED:fig|400667.82.peg.1262"
/db_xref="GO:0008893"
/translation="MVTVREQLPEQLTELSEETTVEHAAETQVGLASWLDRVREILDG
AELKQLEEVAHLTLQKELDSTVNHRSNTFATGVGMADILAHLHVDEDTLSAAMLYRSV
REDITDIEEVKRKFGEQVYNLVKGTLAMGKLSELIEKNKRLEDHFNNNQREHLSGIYK
MLISVTEDVRVVLIKLAERTYSLRELANSSRERQERVAREILTIYSPLAHRLGIAQLK
WELEDLAFRYLAPDRYKEIASLLNEKRLEREHYIQFVIDRLKSELAAHGIEAEITGRA
KHIYSIYRKMKSKNLSFDQLYDIRALRVLVNSVPECYHSLGIVHQIWRHIPHQFDDYI
TNPKANGYRSLHTAVIAENKSLEVQIRTHEMHDEAELGVCSHFNYKEGSKNTDHSFNH
RLHSLRAVLEHYQERNETTVHQNEDETEGFDQLQDFEGFEKIYVFSRDGDIKELPRGS
TVLDFAYHVHTEVGNKCYAARVNQRYVPLTYTLKTGEQVEILTKKDREPNRDWLVNSL
GYIKTARARDKLRHWFRQQDRSKNLEVGRELLNKELSRLAIHPKSIDLNDYSSHFNVK
TGDDILVSLVSGDISLHALINQINRQMHLDQDEPELVLKPTLNPRASHTLSAHGILID
GLDNVELHIAQCCQPVHGESIAGYITLNRGVSIHKVLCSDYQRMIKQEPERAVEADWE
MQPTRGQSVQIVVEAYDRRGLLKDLTQVIFSDQINIRQVNTISEADGIANMKLLIEVK
GLAQLSRLLARLEQQPGIISARRMIQGV"
/product="Inactive (p)ppGpp 3'-pyrophosphohydrolase domain / GTP pyrophosphokinase (EC 2.7.6.5), (p)ppGpp synthetase I" /EC_number="2.7.6.5"
/EC_number="3.1.7.2"
/transl_table=11
CDS complement(1323206..1324597)
/locus_tag="AUO97b_01402" /db_xref="SEED:fig|400667.82.peg.1263"
/translation="MKQQAKSRKPQQPEYIFQVETLSHEGRGIAHYGSHPDHPADKHG
KKVFIRYALPGETVKAQITHEAKRLEEAEMVALLAEPSANRVEAVCPHYGICGGCSMQ
HIHPDEQIRLKQNVLQSHLQHFAGIQPEQWLEPIRSLQSDYRRRARIGVRYLPKQDRL
ILGFREHHSNRLTSIHTCSVLDKKLSDSLPELRNLLQSLKGKVHIGHVELAKGDHETS
LLVRHIEKLNNADVNQLRQFALHKGWQLYLQPKGPESLRRIDEEQGAMRLHYALNAFD
VNFAFSPLDFTQVNATVNEQMVQLACELLQLQQGERVLDLFCGLGNFSLPLARCVGAK
GVVVGVEASEEMVQRATDNAKRNNLVQASFFSQDLTKDFSHHSWANQGFDALLIDPPR
AGAYEIMQYVPNFGAKRIVYVSCNPATLARDAGVLVQHGYQLKKAAVMDMFTHTEHVE SIALFEKIQEIND"
/product="23S rRNA (uracil(1939)-C(5))-methyltransferase (EC 2.1.1.190)"
/transl_table=11
CDS complement(1324607..1325425)
/locus_tag="AUO97b_01403" /db_xref="SEED:fig|400667.82.peg.1264"
/translation="MRFPTLVFDIETLTDLKAGAHLYHLDLPETDVEQALTKIRRQES
GMDFQRLPLHEIVCISGLWIDESGFRLFSFSREHYSEAEILQKFLSIFDKRHPTLVSW

NGSQFDLPVILFRAMYHGLSAPGLFDQGELDSQKRFNNYQNRYHHRHIDLMDVMAMFN
GRNFQKLDDVACILGLPGKRGESGYHVPEYVRTEQWLKLTSYCEGDVLNTWFIYLRWL
LLKGQMNVDEHNHWISSSIEYLQTMPQQADFLEVWQRTSKHTEFTSHYFNPLNF"
/product="Polysaccharide biosynthesis protein WlaX" /transl_table=11
CDS complement(1325448..1326362)
/locus_tag="AUO97b_01404" /db_xref="SEED:fig|400667.82.peg.1265"
/db_xref="GO:0004124"
/translation="MSNTQPDFLADEFLLDHYVGKTPLVRLQRLACHTQATVLAKLEG
NNPAGSVKDRPAYNMIMQAEKRGQIKPGDTLVEATSGNTGIALAMVAAMRGYKMKLIM
PGNSSQERKDAMRAYGAELIEAPNMEAARDMALQMQAEGLGLVLNQFGNPDNVEAHYL
TTGPEIWKQTGGKITHFVSSMGTTGTIMGVSKYLKEQNPDIQIIGLQPSEGSNIAGIR
RWPQEYLPTIFEPKRVDQIMDIPQIEAEKTARRLAREEGISAGTSSGGAVWASVKIAE
ENPDAVIVCIICDRGDRYLSTGLFSVQD"
/product="Cysteine synthase B (EC 2.5.1.47)" /EC_number="2.5.1.47"
/transl_table=11
CDS 1326535..1329342
/locus_tag="AUO97b_01405" /db_xref="SEED:fig|400667.82.peg.1266"
/translation="MSNFNKTLSKRLRLNHAYGQLIALIFVPIMILTCVGAFLVLTET
SRSAKQQQLHHASAILARYNQIAKDLYTLVELQPDEYDHAQHIMQSMFSEKNLKRAAL
IDSNGQTYLSIGYRDNRYWPNFTQNNNFFGPISYNHNNIYGVRIIDTAGKPPVWLLIE
MDNQPLELARYRILIALVITGLMTLLLLLLCLNFYSRRWIAPMYEIRMQLQRLNADTL
DQHIVINSSGELRLLQRDIANVVKRLHFSFLELKEHTEQTEEDLRRTLDTLEVQNITY
RQARDQAISSNQAKSVFLANISHELRTPLNSIDGFIHLLLRQQNLSNEQNLYLQTIRK
SSAHLLALINDVLDFSKIDAGKLELETAPFDLEEAVFDVMDMLSPLAAQKHIAMAFYY
ADNIPQQVIGDALRFKQILTNLISNAIKFTPDGEIIVRVRMEHDDIGQCLLHFSVQDS
GIGLSGTDRKKLFESFSQGDASVTRQFGGTGLGLAISKQLVHLMHGQIGFEDNQERAP
TEKGSTFWFTAQFAVDEEHEIEHPHFEHLQVVSYLAHPATASVLRYYLENYQVPHIET
QSILDLFSRLKHLDQKDNTWLIVDHSGDTEALLKEIRSRYQGNLAVYGYQMTLEPNML
TEYRARPLYQPLSRSGLIQLLSDQPIFEEEQQDFNGQGLHILAVDDHLPNLIVLEALL
GELNVKTTKALSGQEALNIIQERIDQKLKPFDLVFMDIQMPVMSGIDTTRAIRSLEST
LDGEMQLPIIALTAHALADEKQKLLKVGMNDYVTKPIQMEQIIQILTQWTKNNFTAQN
LAKDPHVVAEALDPEILNWQQSLQLAANKEDLAQDLLKMLVDSFPTELEEMQQLIELE
DFPQLEHVLHRLYGATRYVGTPKLQQVTGDFEQFISTLRKERRRADDGFIEEVMRRFD
ELGLVIKEVESAAHQILVIPD"
/product="Signal transduction histidine-protein kinase BarA (EC 2.7.13.3)"
/transl_table=11
CDS 1329439..1330227
/locus_tag="AUO97b_01406" /db_xref="SEED:fig|400667.82.peg.1267"
/db_xref="GO:0004300"
/translation="MALLRIEKNNGIATVYLNRPDKRNAMSFALLKELVTTAKAIQKD
RDIRCVILTGEAQVFSAGIDLSDLNNPKNSAFAIWELVKPGQSLFQKAFLIWQNLPVP
VIAALEGYCFGAGMQLALAADIRIAHPDTKMSIMESRWGLVPDMGLTRSLKGLIGVDL
AKELTLTARVFDGNYAKDIGLVTHLSENPLEKANAIASEMLQRSPDALTAAKRVLDAM
EHQPEKSLRLEKIWQLKLLLGKNSKLARKKDKHPEVKFLPRQYR" /product="Enoyl-CoA hydratase
(EC 4.2.1.17)" /EC_number="4.2.1.17"
/transl_table=11
CDS 1330238..1331104
/locus_tag="AUO97b_01407" /db_xref="SEED:fig|400667.82.peg.1268"
/db_xref="GO:0008679"
/translation="MNFDRNTPIAFLGIGLMGSRMASRLIQAGFQVAVWNRTASACEE
LIDIGAHALDLSNIGHYPLILTCLADDKAVQAVFDQIQTNLKAGQVIVDFSSLSVAAT
KALAQAAASQDVIWIDSPVSGGTAGAEQGTLVIFAGGDAQTIEALSPVYNVLSQRVTR
MGDTGTGQATKICNQLIVAANSALIAEAVALADRAGVDTTLLAPALAGGFADSKPFQI
LAPRMATHTFEPVQWKVQTLSKDLNNAVTLANNVNLDIPVAQKALLQLQTHQKNGFAE KDLATMIQVVEQ"

/product="3-hydroxyisobutyrate dehydrogenase family protein"
/EC_number="1.1.1.60"
/transl_table=11 CDS 1331121..1331915
/locus_tag="AUO97b_01408" /db_xref="SEED:fig|400667.82.peg.1269"
/translation="MGRLAVNLSMIFTEVPLIERFALAHAEGFEHVEIQFPYELAISD
IQDQLERYNLNLCLINVPAGDLMQGGNGLAGIPGQEAAFREALQLAIRYATALKVPRV
NILAGKQPQDSDLLPCLKTLASNLKLACDLLTEHDIEPVFEMINGTDMPRFLVQNIAQ
AQEMLEAVNHPALKMQYDCYHMAMMGEDVLEGLQENIHQIGHIQFADCPGRHEPDTAQ
IPYEQIFSWIKQSAYEGYIAAEYKPKNGSNQSFTWKKKYFSDDVNI" /product="Hydroxypyruvate isomerase (EC 5.3.1.22)" /EC_number="5.3.1.22"
/transl_table=11
CDS 1332045..1332398
/locus_tag="AUO97b_01409" /db_xref="SEED:fig|400667.82.peg.1270"
/translation="MNTSNKDIQACLKVVHDALVDVKAKDILQLDVSSISNVADAIVI
ASGTSTRHVKALADNVAEEARKAGFRPIGVEGERDAEWILIDLGFVVVHVMLPTARKF YDLESLWRTAPESVA"
/product="Ribosomal silencing factor RsfA"
/transl_table=11
CDS 1332649..1333470
/locus_tag="AUO97b_01410" /db_xref="SEED:fig|400667.82.peg.1271"
/translation="MKILITGANTGIGFATAEQLVKQGQHVILACRNPQKAQEAQNKL
RSLDQGQVDVVSLDLNSLELTQKAAEEITDKYGSLDVLINNAGLFSKTKQLTVDGFEQ
QFGVNYLGHFLLTQKLLPVLKQSPQARIIHLASIAHWVGSIKPNKFRAEGFYNPLFYY
GQSKLANLLFSNALAEQLTDSSITSNALHPGGVASDIYRDLPKPVYAAMKVGLVPTSV
PAKLITEMATGDTWQNRNGEYVSAHMPDWKSSHAKNQQLARDLYQQSMTLVEKFL" /product="Retinol dehydrogenase 12 (EC 1.1.1.-)" /transl_table=11
CDS complement(1333524..1334978)
/locus_tag="AUO97b_01411" /db_xref="SEED:fig|400667.82.peg.1272"
/db_xref="GO:0008750"
/translation="MEFIQANANWLYLVGAILFILTLRGLSGPKTAIQGNRYGMIAMA
IAVVTTFFVANNPVIWMIGGAMVLGAIVGIARARTVPMTQMPETVALMHSLVGLAAVL
IAVAAILHNNQLTALFAQNEAALTAAGVQHAHMSKVHLFELFVGCFVGAITFTASVFA
YGKLAAKKWAKTIFGGWVKPVQALIFVAMLACGFYFFTTGNMTAFWAMTALALAFGWV
WIAPVGGGDMPVVVSLLNSFSGWAAAGIGFTLENNMLIVAGSLVGSSGAILSYIMCKA
MNRSIINVLFGGAMGGAAVSTAAKGEQVQRNYRSGSADDAGFLMSNADSVVIVPGYGM
AQGRAQNAVKELCEILKEQGVRVRFAIHPVAGRMPGHMNVLLAEADVAYEDILEMDEI
NSDFPATDVVLVIGANDVVNPAAKDDPGSPIYGMPILEAHKARTIMVIKRSMATGYAG
LDNDLFYNEKTMMIFGDAKKVVEDMTKAINGTGH"
/product="NAD(P) transhydrogenase subunit beta (EC 1.6.1.2)"
/EC_number="1.6.1.2"
/transl_table=11
CDS complement(1334991..1335305)
/locus_tag="AUO97b_01412" /db_xref="SEED:fig|400667.82.peg.1273"
/db_xref="GO:0008750"
/translation="MVETITIFVLAIFVGYYVVWGVTPALHTPLMAVTNALSSIIVVG
AMLQTVGLPVLGVDANVAFQSVNVVSVLGAIAVFLASINIFGGFAVTARMLEMFKPKQ KK"
/product="NAD(P) transhydrogenase C-domain of subunit alpha (EC 1.6.1.2)"
/EC_number="1.6.1.2"
/transl_table=11
CDS complement(1335317..1336444)
/locus_tag="AUO97b_01413" /db_xref="SEED:fig|400667.82.peg.1274"
/db_xref="GO:0008750"
/translation="MQIGIPTETVVGENRVAATPETVKKLISAGHSVVIERGAGVKAA
YIDSAYEQVGATITDDAYTGSQIILKVRAPQGGEIQKLAANTTVVAMFDPYRNTELDQ
FANQQVSAFALELLPRTLSRAQNMDVLSSQANLAGYKSVLLAAAEYQRMFPMLMTAAG

TVKPARVVIMGVGVAGLQAIATAKRLGAIVEATDLRPTAKDQVESLGGKWLDVPMSEE
EQQRAADAAKNGYGWMPGEQYIKDQAAIVDKAVSNADIVITTALLPGRDAPRLIKAET
VAKMKPGSVILDMAVETGGNVEGSKVGETVVTENGVKILGIPNIPATVATEASALYAR
NVFNFVETLFDKDKNFAINQEDEIQKALLVTHGGQVLLKRG" /product="NAD(P) transhydrogenase N-domain of subunit alpha (EC 1.6.1.2)" /EC_number="1.6.1.2"
/transl_table=11
CDS 1336682..1337608
/locus_tag="AUO97b_01414" /db_xref="SEED:fig|400667.82.peg.1275"
/db_xref="GO:0016020"
/translation="MKISLSSFQIGSFFALCSAFLFSTKAIFIKQTYALSPLVDGTVL
MALRMLSALPFFLLICWFNRHHNQGIKKKDWLILIFAGLLGYYFASWLDFMGLMFISA
SLERIILFLYPTLTVIASSLLYKQKLDVKSLFAIFLSYGGTVLVMLQEHNNAPIQGNF
WLGTSFVFASAVAFAGYLLLTPPLIKKFGSWNFTGLALTVACIGTLTHYTLSTSHPFQ
LLLQLPSSVIWYGIGLGFLVTVLPTVMLMQSIERLGASQSAMIASIGPVLTILLAVAF
LNEHLNIWQWVGCFLNIVGVMMITLRKKRLKH" /product="Drug/metabolite exporter"
/transl_table=11
CDS 1337676..1338083
/locus_tag="AUO97b_01415" /db_xref="SEED:fig|400667.82.peg.1276"
/translation="MNKEIFKIINPSTLYDPIPNAYSHVAVVSNFNNIIHIAGQGGED
QQGQLSSHFSEQVLQAFENINYALVAANAELTDIAVLRILVVDHSVEKHEILIKIMKD
LWKNHPFPACTLIPVPRLALEHMLIEVEATAYT" /product="RidA/YER057c/UK114 superfamily protein" /transl_table=11
CDS 1338102..1339286
/locus_tag="AUO97b_01416" /db_xref="SEED:fig|400667.82.peg.1277"
/translation="MSTPEDISQNKPLLWLMAAACGLCAGVNYYCQPLIHSIQQDFAV
TQAQAALTVTFAQVSYALGLLFIVPMGDIVNKAKAIPFLMMLCAVGLFTSAFAVNLPM
LWIGTILAGLFSVAAQVLIPLATMTVKPEKTGEIIGFLMSGLLVGILLSTSLAGLFSN
LFHWKVVYAISGILMLLLAYTLKSRLPYVMRMKLNYGQIFVSMAQLLKEEKRLLLRAL
TGAFAFASVSILYSTIALLLTSAHHLPDLFIGLVPLVGIFGALSTRYIGKYADQGYTR
LLTWMGCGLFLVSWICFYFGQILLSAYVIGFALIQLALALVHTSNQSIIFRLRPDAKS
RINAIYMTTYFIGGAAGSALGIFAWNRGGWTMTCLAGVGLVVFCILFSMIDAFLQKKQ MA"
/product="Uncharacterized MFS-type transporter" /transl_table=11
CDS 1339435..1340439
/locus_tag="AUO97b_01417" /db_xref="SEED:fig|400667.82.peg.1278"
/db_xref="GO:0008033"
/db_xref="GO:0016491"
/db_xref="GO:0050660"
/translation="MQSLQSLQPRISVAPMMDWTTKDYRFFARLFNPNVVLYTEMVTT
GAILFGDAKRHLDYNAQEHPIVLQLGGSTPQELATCTKMAEDWGYDEVNLNVGCPSDR
VQNNKIGACLMAEPDLVAECIHSMQKAVNIPVTVKHRIGIDDMQSYEEMLHFVDTVAA
TGCTHFVVHARIAILKGLSPKENREVPPLRYEDVYRLKQERPHLTIEINGGIKTFAET
QAHLQHVDGVMIGREAYHNPYLLAELGQLWNLEAPDRFDIMEQMLPYIEQRLAEGAPL
SIITRHILGLFQNLPGARKWRQALSGGNAKTLTDVENAIHNMQAAIIRTEEYLKEHQF "
/product="tRNA-dihydrouridine(20/20a) synthase (EC 1.3.1.91)"
/transl_table=11
CDS 1340774..1341283
/locus_tag="AUO97b_01418" /db_xref="SEED:fig|400667.82.peg.1279"
/translation="MIIYDGQVADNYMYQDSNQAAIVVSHSTPSLPYPFTMKPNNHST
ETNTPPAIDVEKFVAKLESITSRRSKARCARSIRIALESAGADVENHPIAASDWGDTL
KKIGYKEINPAFDEPQEGDIYIIHRTRNHIYGHIAGYTGSEWVSDFKQSSYDVYKDDN VTYTYYRLG"
/product="hypothetical protein"
/transl_table=11
CDS complement(1341291..1342190)
/locus_tag="AUO97b_01419" /db_xref="SEED:fig|400667.82.peg.1280"

/translation="MVMLTLKQFQYFIKIVEEGSFIAASEKLFIAQSALSRQMKLLEE
EIDFLLFDRTDKRVKLTVAGEVFYKKIKDNLLYLNEIIDLSKSVSEGKNRQIKIAHSS
SIVVDNTKVQILKEVSLTQQLSFEINTLSSEQQVLALLNGEIDIGLIRPPVRHSLDGI
NTLKLYEEPLMVAVHIDHAKFSKSEKVYLKDLKDECFVSTPHAERGGLSYLVANLCLA
AGFTPQKASIQSRKISQLQLVAANLGVGIVPAEFQQILPANVKLIPLEDSLSLSEVLL
VYRKDHDEIIQHCAELIHQMFLS"
/product="Transcriptional regulator, LysR family" /transl_table=11
CDS complement(1342473..1343234)
/locus_tag="AUO97b_01420" /db_xref="SEED:fig|400667.82.peg.1281"
/translation="MTDLQQDEYDRRFAGVAKIYGDSSFQHYEQSHVMVIGIGGVGSW
AVEALARTGIGEITLVDMDVVAASNVNRQLPAMTSTLGCEKVEIMAERCRQINPRIKV
NIIDDFLTPENVAELLNPVPDIVLDCIDDVKAKLALMLHCRFNKIPLIVSGGAGGKLD
PLKIRVADLSKTEQDPMLAKLRSQLRARGICKKPKEKFGITCIYSIDNPFSSADVCPS
AGLRCGGYGSAVVVTSSFAMIAVAEVLKKLDLKKA" /product="Dinucleotide-utilizing enzymes involved in molybdopterin and thiamine biosynthesis family 1"
/transl_table=11
CDS complement(1343569..1346325)
/locus_tag="AUO97b_01421" /db_xref="SEED:fig|400667.82.peg.1282"
/db_xref="GO:0003994"
/translation="MAKYSNINSFNALQTLTVGSSNYQIFSLTQAEKKLGDIAKLPKS
LKVLLENLLRFEDQHSVKTEHIYALAEWLKTRTSDQEIQYRPARVLMQDFTGVPAVVD
LAAMRAAMAEAGGDPEKINPLSPVDLVIDHSVMVDHFADDQAFEENVQIEMQRNGERY
QFLRWGQSAFNNFSVVPPGTGICHQVNLEYLAQAVWLGEDNGQTFAFPDTLVGTDSHT
TMINGLGVLGWGVGGIEAEAAMLGQPISMLIPEVIGFKLTGKLQEGITATDLVLTITQ
MLRQKGVVGKFVEFYGDGLADLPLADRATIANMAPEYGATCGFFPIDEVTLGYLKLTG
RQSDRIALVEAYSKAQGLWRNPGDEPVFTDTLSLDMSTVQASLAGPKRPQDRVLLSEV
PKTFNALMELTLKPAKEAKERLENEGGGGTAVEATKANIQHESPSCVIEGQEYPLNHG
DVVISAITSCTNTSNPSVMLAAGLLAKKAIEKGLQRKPWVKSSLAPGSKVVTDYLLAA
GLTPYLDELGYNLVGYGCTTCIGNSGPLPEPIEDAIQCHDLNVASVLSGNRNFEGRVH
PLVKTNWLASPPLVVAYGLVGNIRTDLTTQPIGQGKDGQPVYLKDIWPSQAEIDAVLQ
KVNTDMFHKEYAAVFDGDESWQAIQIPISKTYEWADDSTYIRHPPFFEGIGEPPKPIK
NIEQARILAVLGDSVTTDHISPAGNIKKDSPAGRYLQEQGVEPKDFNSYGSRRGNHEV
MMRGTFANIRIKNEMLGGEEGGNTIHVPSGEKLAIYDAAMRYQQEHTPLVIIAGKEYG
TGSSRDWAAKGTNLLGVKAVIAESFERIHRSNLVGMGVLPLQFVDGQTRQSLNLTGHE
VISIRGLSDGIQPHEILEVDVKGPNGVASHFNVLCRIDTLNEVEYFKAGGILHYVLRN LIAS"
/product="Aconitate hydratase (EC 4.2.1.3)" /EC_number="4.2.1.3"
/transl_table=11
CDS complement(1346450..1347262) /locus_tag="AUO97b_01422"
/db_xref="SEED:fig|400667.82.peg.1283"
/translation="MHLFDTHTHFDVADFDEDRHQLALEAKKVGVDALVLIGFLQSRF
DELVQTHHQLKQWENVPASYLAPGLHPFYIEQHKPEHLSHLEQILQQEDCVAVGEIGL
DTFLKEHKQPDIYAKQKQYFADQLDLATQYQKPVLLHIRKAHGDVLALLKAHKFKLGG
IAHAFSGGVEEAKGLIKLGFKIGVTGQITNPNAKKLHTVVQTIGAEHLVIETDCPDMT
PLCCQTSTEQRTRNTPVNLPYVLKSLAVNLNMAESELADLLWKNSLSALKLS" /product="Putative deoxyribonuclease YjjV"
/transl_table=11
CDS complement(1347262..1348203)
/locus_tag="AUO97b_01423" /db_xref="SEED:fig|400667.82.peg.1284"
/translation="MLTSKASLHLTLLASAIFLVACQPKSDPKESEDQQKPAVVEQKP
VELTLKGETVPSKVTLPDCDGKTCPEFTVERLQSNFPFIDKIIDQQVLKALGQILEIA
EPDAKAAQADKKTEASAAATTEQQDSFDAQVQRYANSFIDLDNELKALSSNHQINLLV
KPKIIQSQGKVVTVVVNSSSYLGGAHGSAAQQYYNFDLKKEKQVKLEDLLRPEKKAAL
EKLAHEAFKAWVTDSKLANSVSEYEQAWPFKLTENFLLGDQGLILQYGEYEIGPYVVG
LPRLVIPYDQLQEVLKEEYLPQPKAKPASTPAVKSAS"

/product="putative lipoprotein"
/transl_table=11
CDS complement(1348291..1349133)
/locus_tag="AUO97b_01424" /db_xref="SEED:fig|400667.82.peg.1285"
/translation="MNLSKKTIIALVVAAIALILAICVFVWKKPQSSSSSQENSASTQ
DHPVEKAEVLPYLNLTETKANYAVPFCEKKNCIDVDIQTIKTQDAWLNSWIEKNQAKV
IQAQINLKKDLSLQQAINAYVKKSDEWQDKYSKNRAYELHIRTRIASQRNQYVLLQLA
LDSKQEEITIKDRYYFFVADRKLHKNLTLLDVLKKDQQPTVHQIVQTAYQDWLKKQTA
DVKKQAPKTLYWGQADWFFDGEGVGLHYQANQITKDAPQLDIYLTTEQTKKILQPQVY EQMF"
/product="hypothetical protein"
/transl_table=11 CDS complement(1349203..1350795)
/locus_tag="AUO97b_01425" /db_xref="SEED:fig|400667.82.peg.1286"
/db_xref="GO:0005525"
/db_xref="GO:0006412"
/translation="MSFKDELAAQVAQRRTFAIISHPDAGKTTMTEKLLLWGKAIQVA
GMVKSRKSDRAATSDWMEMEKERGISITTSVMQFPYKGHTINLLDTPGHEDFSEDTYR
TLTAVDSALMVIDGAKGVEERTIKLMEVCRMRDTPIISFVNKMDREIREPLELLDEIE
NVLNIRCVPITWPLGMGRDFAGVYNILEDKLYVYKAGFGSTITDIEVRDGYDHADIRE
KVGELAWASFEESLELVQMANEPLDRELFLQGKQTPVLFGTALGNFGVDHVLDAFMNW
APEPKAHPTQERMVEAKEEGFSGFVFKIQANMDPKHRDRIAFMRICSGKYEKGLKMNH
VRIGKEVRISDALTFLAGEREHLEEAWPGDIIGLHNHGTIQIGDTFTSGENLHFTGIP
HFAPEMFRRVRLKDPLKSKQLQKGLKELSEEGATQVFMPQISNDLIVGAVGVLQFDVV
AYRLKEEYKVDCVYEPVSVNTVRWIHCDDEKILNEFKKKAHDQLSIDGGGHLTYLAPS
RVNLQIMQERWPDIEFRSTREH" /product="Peptide chain release factor 3"
/transl_table=11
CDS complement(1350965..1352032)
/locus_tag="AUO97b_01426" /db_xref="SEED:fig|400667.82.peg.1287"
/translation="MSITVNDQEIDWQKWLDLEITDENEFRDHHLLKQDYHENFDELV
NGLYSVTDGFSNYKNEKKFGGYIAAGHQRLRDTLDLMSLQYSAGGDIGFIKELYPYLL
HWTEEYAETSHLYNLSPDAGGRYVWHISLGTEDYWYIALRLICFGLLTGYADQMYQIM
PIIDYVEATPEGQEKDGLVERLVAPFVTDRGTPPDEARRHLPYRKLIKVFNAAPEQRP
ALMLQYLESWYEASRREPYHDQHPQTDLRSGVSYYGYWSWEAAAVTWLLEIDDTLYRD
HEFYPKDLVDFARSQSNMVSDEEQSEHIKVKGGEVCIKTGYWITPARPDTRLYFTQGT
TLPILSETDWGEVYWYWDGEN"
/product="hypothetical protein"
/transl_table=11
CDS complement(1352159..1353019)
/locus_tag="AUO97b_01427" /db_xref="SEED:fig|400667.82.peg.1288"
/translation="MNQEDIDYFYEKYGQPIDKVEVTEDIIKKYRGKLPESILEQWHL
FGFAGYLNGLYWITNPDDYAEVIYDWLEETPLPDDDVYHVLARSAFGELLIWGERNYG
RYYIKTMEGILHDNGEQLESAEFYGSDFFFLPKKNYLDYTDKNGNKLFDRAVKKLGVL
KADEMYAFEPALALGGEESLQHLTKVNLPVHMKLLKQVTPLRLRTFEDLTAALYGTSY
SVDDLTSGQDAESQYQESVQAGEVCPRTGYWTTPAQPNTRHYCKKGEVLPEIKEQDWG EVYWYWDGEN"
/product="hypothetical protein"
/transl_table=11
CDS complement(1353034..1354623)
/locus_tag="AUO97b_01428" /db_xref="SEED:fig|400667.82.peg.1289"
/db_xref="GO:0008887"
/translation="MPDPQNQQFWSKIAERPTEIQQQFWYGAEKLAKYLNDNPAMYKG
YMTYDTSKPDPNLSWWEKILFYSGQAHYNEAVTHYNTSKVIADQGSWLWGMLKGDFNK
DPSMSQIIVGGLISLIPVADQVCDIRDLIANFITLSDEKARTKDNYMALALTSVGIIP
EVGSAIKTIVKSSRAKDVSKVKLFKFMEYFEEALTKLKIKCPWGKAPEAWLRSRPWKG
IATQAFNTLKNNIDRILSVINKCLIRFNGALKVALAKFQATLNHVLNTIKKYIDQLCD
EVDKAIGRLLPQQPLAMAHAGGGGKNAPTGRYEANVSSGKKTEASHRQKKETPPPPRK

MKPKHVVEPCLEPKKGLRNSWGKKTGKSQAELDAEFDRQLKRQEEGLNRLTVEEYQQN
RQLYEKYKRAGTGTQQQRIREDMQRQLEESEYKKLKTEQPQLSKGQIERMAENNAKKT
LEGLDVLHNPDMQLGGFDVKYDPKKPPTLDDFGHSGVNRSIGSQMAAKKRLANMDAAA
AQAKAKGMGKDAMDVELKRCK"
/product="hypothetical protein"
/EC_number="2.7.1.31"
/transl_table=11
CDS complement(1354630..1357395)
/locus_tag="AUO97b_01429" /db_xref="SEED:fig|400667.82.peg.1290"
/translation="MYLQFSDASLNSQVFLQRIDGQHYLNQGMTAELICLSTNAHIPL
KTFIGLQVAVDQVTDRGSFFRTTGIITGASQGQSDGALTLYKLAISDPTYLWHKRRNS
RVFMNKSVKEISEILFQEWQGKSPLFASSLTLDLSGLKQTYDVRPFVMQLNESDYDFL
TRLWRSEGISWLIDEAELTVASNTDNIQPQKLRLIDDNSQYQTLTRRTIRYHRSSATE
QFDSMTSLIAGRHLQPSSVFVQRWQSDVLQQTDGAGSVQSKHEHSSNYDNQSLNLEDA
WHFSPAWMQDLNGEDGATSASNQQLEKFNQNLSAYHDAQSKQFVAQTTVRDTQVGYWF
ELNEHPEIDLHSGADKEFLIIGKHYYNQNNLPKDLDQQIQTLTTQSHWQSSHTKERQG
NQLILQRRHIKTVPEYQPLQDRPQASVQRARVVGPEGESIYVDQWGRIKVRFLFTRAN
DHSHDGGAGSNDNDTDSAWVDVLTPWAGAGYGARFLPRVGEIVVIDFFDGNIDRPFVV
GRIHEAERHPAQFDQKGQLPDTKKLSGIRSEEVDGKGFNQLRFDDTTGQISAQLQSSH
AVSQLNLGNLSHPKDKAESDGRGEGFELRTDQWGAVRAGKGLLVSTHKQDQAQGTHLD
AADAKQQIEGGLNNAKALSEVAKNQQTDPLEMLENLKTFIEQIEEKDQDKAATFKQAL
MVLTAPNSIAVASNEDIHLSADGQLSQNAGDSISLSTQKNLIAHAQNKISLFAAQQGA
RLYAGKGKVEIQAQGDGADLIARKGIQIISTEDKIEINASKEIVITSGGSQIKINSSG
IFPVTGGKFEVKAGQHVFMGGAKADYVLPSLPTQEIKTDDLLLEYLHSDGTPVKGADY
EVLLSDGSIRKGKLDASGKAIVSGVPAGRAKIQYGEDQSKDEFPALEVDDWFTQLGSS TKTGKEE"
/product="VgrG protein"
/transl_table=11
CDS complement(1357834..1358703)
/locus_tag="AUO97b_01430" /db_xref="SEED:fig|400667.82.peg.1291"
/translation="MIMSKKFEDFNNPREKALQGMKDSIPASQWEENLQFLKQLRNKI
AKLPVSKHPAIEILNNGYLDKQTLTRIHLEYRHAIVQIFTDALLKAQFLTKQLEPKLH
SGAKMFPRVLLSLNILDEFGFRPGLDKDHYYLGNPEYAHYPLYEDLLNDYGLTEADRR
NYKPSKIADQVRTFLEASYDRYINVVALLAVAEEEVILFSPPLRQATKFVNIDVEGGG
YYHVHGVSTDETAEAADDDHQDDLWFALAQAITKDDYESLTQLCLDYCALWTEFWDAQ IADVQFIEEKKLA"
/product="hypothetical protein"
/transl_table=11
CDS 1359028..1359600 /locus_tag="AUO97b_01431" /db_xref="SEED:fig|
400667.82.peg.1292"
/translation="MSKRETIIITTAMTLFNQKSYTSIGVDKIIAESKVAKMTFYKYFS
SKEVLIEECLRRRILEVQTSLLDKVNSADDPLNKLKSIFNWYIDWINTEDFSGCLFKK
ATIEVLQLYPSIKKQVNKYREWIYSLVLSIFLELEIEDPKVLSSLFLNIIDGLIIDGT
INKPEINSEETWSYINKLIELETKQKYEAA"
/product="Transcriptional regulator, AcrR family" /transl_table=11
CDS complement(1359817..1360380)
/locus_tag="AUO97b_01432" /db_xref="SEED:fig|400667.82.peg.1293"
/translation="MPYSDLSFRALSVLHKSRYLFNKHGFHNVGVDRIVREAEVPKAS
FYNYFHSKERLIEMCLHFQKDILKEQVRSIIYIQKDLILREKLKEIFFLHTNLDGYYH
LLFRAIFEIERLYPAAYQVVVQYRHWLTNEVYKLLLTVKKDATKSDSDMFLFTLEGAI
IQLLDETRGDTRELLFTYILKGIFLKD" /product="Transcriptional regulator, AcrR
family" /transl_table=11
CDS complement(1360987..1363041)
/locus_tag="AUO97b_01433" /db_xref="SEED:fig|400667.82.peg.1294"
/db_xref="GO:0005554"
/translation="MGHVDYDSTLIVGSFIAAGAICYIVISMEQLIFKQTYKKIEPFI

LLLNGLLLAAAISIVHIVGMHAYHLFEAASSNVPLITLAFGISAVLSSVAIWLTSRFT
LPIFRLILSSVIMGIGISASYYVSMLGWNIDIYKKDYTSFLILFSVLIAMSGSGLAFL
LAYKLKESERHRISLKLAFAVMMTLSIMGMHYTALISTNITDKFVSQYQAEHDLLLFT
IILVTCLVLVASFIVAVLEQRLNQRNLELRKANKELANLSIQDNLTKLPNRLYLVDYA
EVLLSDHRYKDQKIAFLYIDLDRFKSVNDAFGHHIGDQLLIQMANRLHWQLNEKCKLL
RIGGDEFLLIAENTDTEEAMQLAEKVLHLIQDSYQISGKEINISASLGIALFPEHGQN
VQDLLMNADAAMLMSKEQGRNTYTVFSYSTHQQETRSQSKLINDLYKAVEEKQFVLYY
QPKFNTNLEICGVEALIRWNHPSLGMLTPHMFIGGAEKTGLIIPMGYWALEQACQQIQ
EWERSNTQFYPIAVNLSALQFENKKLFSTLEGLLKKYQIQPHHLIIEITESTAMRHID
LSIRACERLRDMGIRVAIDDFGTGHSSFLYLKDLPVDELKIDRGFIVDLKPGSKEEVI
LESIIHLAKKLGLTVTAEGVETQQQVEILTRLGCQQFQGYLLGMPVNVGQLIQSQGAH FV"
/product="GGDEF domain protein"
/transl_table=11
CDS complement(1363372..1364388)
/locus_tag="AUO97b_01434" /db_xref="SEED:fig|400667.82.peg.1295"
/db_xref="GO:0004455"
/translation="MQIFYDKDCDLSIIQSKKVAIIGYGSQGHAHALNLKDSGVDVTV
GLRAGSASWKKAENAGLKVAEVPAAVKQADLVMILTPDEFQSQLYRDVIEPNIKEGAT
LAFAHGFSVLYNQVVPRKDLDVIMVAPKAPGHTVRSEFQRGSGVPDLIAIHQDASGNA
RNVALSYASGVGGGRTGIIETSFREETETDLFGEQAVLCGGAVELVKMGFETLVEAGY
APEMAYFECLHELKLIVDLMFEGGIADMNYSVSNNAEYGEYVTGPEVINEQSREAMRN
ALKRIQSGEYAKMFIQEGALNYPSMTARRRQNAAHGIEQTGAKLRAMMPWIQANKIVD KEKN"
/product="Ketol-acid reductoisomerase (NADP(+)) (EC 1.1.1.86)"
/EC_number="1.1.1.86"
/transl_table=11
CDS complement(1364421..1364912)
/locus_tag="AUO97b_01435" /db_xref="SEED:fig|400667.82.peg.1296"
/db_xref="GO:0003984"
/translation="MRHIISVLVENEAGALSRLVGLFSQRNYNIETLNVAPTEDPTLS
RLTLTTYGDDHKIEQITKQLNKLVEVVKVVDLSEGSHIERELMLIKVKALGASRAEIK
RTADIFRAQIVDVTPTTYTIQIAGTTEKLDGFIDALAENTILEVVRSGVSGIARGEKV LAI"
/product="Acetolactate synthase small subunit (EC 2.2.1.6)"
/EC_number="2.2.1.6"
/transl_table=11 CDS complement(1364912..1366636)
/locus_tag="AUO97b_01436" /db_xref="SEED:fig|400667.82.peg.1297"
/db_xref="GO:0003984"
/translation="MELLSGGEMLVRALADEGVEHVFGYPGGAVLHIYDALFQQDKIN
HYLVRHEQAAGHMADAYSRATGKTGVVLVTSGPGATNTVTPIATAYMDSIPMVVLSGQ
VASHLIGEDAFQETDMVGISRPIVKHSFQVRHASEIPAIIKKAFYIASSGRPGPVVVD
IPKDATNPAEKFAYEYPEKVKMRSYQPPSRGHSGQIRKAIDELLTAKRPVIYTGGGVV
QGNASALLTELAHLLGYPVTNTLMGLGGFPGDDPQFIGMLGMHGTYEANMAMHHADVI
LAIGARFDDRVTNNPAKFCPNSKVIHVDIDPATISKTIMAHIPIVGAVEPVLQEMLAQ
LKQLNVSKPNPEAIAAWWSQINEWRKVHGLKYENPSDGTMKPQQVVETLYKVTNGDAI
ITSDVGQHQMFGALYYKYKRPRQWINSGGLGTMGVGLPYAMAAKLAFPDQQVVCITGE
ASIQMCIQELSTCKQYGLNVKILCLNNRALGMVKQWQDMNYEGRHSSSYVESLPDFAK
LMEAYGHVGIQINHADELESKLAEAMAINDKCVFINVMVDRSEHVYPMQVAGQSMRDM WISKGERI"
/product="Acetolactate synthase large subunit (EC 2.2.1.6)"
/EC_number="2.2.1.6"
/transl_table=11
CDS 1367141..1367476
/locus_tag="AUO97b_01437" /db_xref="SEED:fig|400667.82.peg.1298"
/translation="MNLSYLKTSLYTAVTTAILLCSSQSQAQQYYKWMDQSGSTHYTT
TPPPKGAKHLNKVSTYGSQPLLKNPTSNSEQPSQDKDKVVQEVTNVAVEKGAPAVPVP PAPSVSAPR"
/product="FIG00349950: hypothetical protein" /transl_table=11

CDS 1367663..1370287
/locus_tag="AUO97b_01438" /db_xref="SEED:fig|400667.82.peg.1299"
/db_xref="GO:0004823"
/translation="MTISHIDPEYQANTIEPSVQQDWENRKVFKVADTVEGKHRYILS
MFPYPSGKLHMGHVRNYTIGDVISRFYRLKGETVLQPMGWDAFGLPAENAAIAHKVAP
AKWTFENIAYMRDQLKKLGLSVDWDREFATCTPEYYHWEQWLFVQLYKKGLIYRKLST
VNWDPVDQTVLANEQVENGRGWRSGALVEKRDIPMYYFRITDYAQELLDDLDTLQDGW
PQQVLTMQRNWIGRSTGMEITFPSANTEIYADGLTVYTTRADTLMGVTYVAVAAEHPL
ALKAAENNPELAAFIEECRMGSVAEADLATAEKKGMATGLFVKHPVTGEELPVWIANY
VLMSYGSGAVMAVPAHDERDFEFANKFNLPIKQVIDAKGADDADYSATEWQEWYGSKE
GKLVNSGEFDGLEFQAAFDAFLAKLEPQGLANSKVQFRLRDWGVSRQRYWGCPIPMIN
CDTCGQVTVPEDQLPVVLPTDVVPDGSGNPLNKMPEFYETKCPCCGGDARRETDTLDT
FVESSWYYARYASPDFTGGMVKPEAAKNWLPVNQYIGGVEHAILHLLYARFFHKLMRD
EGVVQGNEPFTNLLTQGMVLADTFYREAENGKKTWFNPADIELERDEKGRIISAKYSG
DGQEVIIGGQEKMSKSKNNGIDPQAIIDQYGADTARVFMMFAAPPDQSLEWSDAGVEG
ANRFLKRVWRLVASFLEKGNSATAIDKANLSKDAQDLRRKTHETIQKVSDDIERRHAF
NTAIAALMELLNASNKFEAKDDNDVAVEREAITTLLTLLAPFAPHLSQTLLAQFGTDL
TEATFPEVDASALTRNTQTIVVQVNGKLRGKLEVSVDISKDELLAQAKALPEVQQFLT
GPTKKEIVVPNKLVNLVV"
/product="Leucyl-tRNA synthetase (EC 6.1.1.4)" /EC_number="6.1.1.4"
/transl_table=11
CDS 1370315..1370824
/locus_tag="AUO97b_01439" /db_xref="SEED:fig|400667.82.peg.1300"
/translation="MHLAQRLAAVVLTLGLSAGLVGCGFHLKGTNPTATPLVYKKLSL
ELPAKTDDLETQLKVYLTANGVQLSNDNDAYVLRVLEYTPRRQLLNGKLTEVLLRLTV
TFQIEDRQGNKITEPRTLTAARSYQYDLATVNTENQQESYLQRIVIDDLAQQITRQIS ANRLPKAQP"
/product="LPS-assembly lipoprotein LptE"
/transl_table=11
CDS 1370844..1371833
/locus_tag="AUO97b_01440" /db_xref="SEED:fig|400667.82.peg.1301"
/db_xref="GO:0003887"
/translation="MKLDYLQALKRIPEARGAWILHGQEPLLEQNLLDTFRKSWQQQD
IERQRYDISSVSDWKNVFNALNSLSLFSQQLAIEVHGNIKPDANGLKQLKSYIQHNET
NLLLIVLPKQDSSSLKSAFFQVVEANGVVVALTANYPQDRQRILAAEAEKLEIQLDND
AWQWLMQHHEHNLLAAKNSLMRVRDTFPDQKLIQIEQLYACLQDQSRYTTYDLSDALL
EGNLAQSIKIFQYLIGSGEPESLILWTLSKEMRLLMQLFEQPHNALQLGIWKTKVSLY
QQALRRLNPQQFLGWSALLLQIDAAIKGMSNENAQHLMQQAIAELCGKTLFIH" /product="DNA
polymerase III delta subunit (EC 2.7.7.7)" /EC_number="2.7.7.7"
/transl_table=11
CDS 1371941..1373281
/locus_tag="AUO97b_01441" /db_xref="SEED:fig|400667.82.peg.1302"
/translation="MPKIKPIKLVIIVVCIAIIAVLAWKFLKPKQQQPQYITAEVTRG
DIENNVLATGTLDATKLISVGAQVSGQVKKMYVQLGDQVKQGQLIAQIDSTTQENSLK
TSDANIKNLEAQRLQQIASLNEKQLEYRRQQQMYAQDATPRADLESAEAAYKTAQAQV
KALDAQIESAKITRSTAQTNIGYTRIVAPTDGTVVAIVTEEGQTVNANQSAPTIVKIA
KLQNMTIKAQVSEADIMKVEKGQQVYFTTLGDETKRYATLRQIEPAPDSISSESNSTT
SSTTSSAVYYNALFDVPNTDGKLRIDMTAQVYIVLNSAKNALLVPSSALSSKQFSGQR
KQGQSADKASSTPSAERKHQGNGVRLERLNLTPEQKQLIEQGKATLSVVRVLQADGTT
KPTQILVGINNRVNAQVLAGLKQGDQVVIADSSENSAASANSGNNRRRSGPMGM" /product="Macrolide-
specific efflux protein MacA" /transl_table=11
CDS 1373284..1375278
/locus_tag="AUO97b_01442" /db_xref="SEED:fig|400667.82.peg.1303"
/translation="MTKQALLEVSNLVREFPAGESTIQILKGIDLTIYEGELVAIVGQ
SGSGKSTLMNILGCLDRPTSGSYKVNGQETGKLEPDQLAQLRREYFGFIFQRYHLLGD

LSAEGNVEVPAVYAGVTPADRKQRATALLTELGLGTKTQNRPSQLSGGQQQRVSIARA
LMNGGDVILADEPTGALDSHSGVEVMRILRELNAAGHTIILVTHDMQVAKNATRIIEI
SDGEIISDRPNVPDQSLEEVKSDPDAAPALQNKQKKGKSISAWRSTLDRLSEAFQMAL
LSMNAHRMRTFLTMLGIIIGIASVVTVVALGNGSQQQILSNISSLGTNTITVFQGRGF
GDNSKTANFKTLVPADADALMTQPYVSAVSPMVSTSKTMRYQQNEANATINGVSNDYF
DVKGLVFKDGQTFDQRSVRDRSQDVVIDTNTQKQFFSDGTNPIGQVVLLGSVPARIIG
IVEPQTSGMGSDDTLNVYMPYTTVMSRMLGQAHVRNIVVRINDKYSTSAAENAIVNLL
TQRHGAQDIFTMNSDSIRQTIEKTTSTMTLLVSAIAVISLVVGGIGVMNIMLVSVTER
TQEIGVRMAVGARQSDILQQFLIEAILVCLIGGVLGVLLSLGLGQLINKFAGGNFAVA
YSTTSIVAAFVCSTLIGVVFGFLPAKNAAKLDPVAALSRE" /product="Macrolide export ATP-binding/permease protein MacB" /transl_table=11
CDS 1375289..1376695
/locus_tag="AUO97b_01443" /db_xref="SEED:fig|400667.82.peg.1304"
/translation="MSFSMTKLSAALLLTSSLVGCAAVVKTPYQAPAVQVPGSFQYDK
AKAKTASVEQYSDRWWTLFGDTQLNQLVTNVLERNSDLAVAGITLQQARLQADLTANK
QGLRTSSSVSTGHSFDLNSGDDSAKGLSMSAGVSYELDLFGKLARQTEASKWEALATE
QDLQATGQSLIATTAKLYWQLGYLNERYATAQQSLATSQKLYQLVQTQYKAGAVSGLD
LTQAEQSVQSQKASLSQIEQQLVETRTAIAVLLHEPLQQLNIQEPQRLPRTALPAIGA
GLPADILSRRPDLQAAELRLRKALATKDATKASYYPSISLTSSLGSSSTSLTELLRNP
ALTLGASLSLPFLQYNDMKKDIAISNLDYEKAIIQYRQTLYQAFADVENALSSRTELD
KQVALQERNVELAEKTERLTEVRYRYGAVALKTLLDAQQTTRTARLSLVETKQSQYNA
YVTLMQALGGSPVKELPQ"
/product="Efflux transport system, outer membrane factor (OMF) lipoprotein"
/transl_table=11
CDS complement(1376765..1377631)
/locus_tag="AUO97b_01444" /db_xref="SEED:fig|400667.82.peg.1305"
/db_xref="GO:0004318"
/translation="MQFRKTLNIGIMLKIVLSEIGMTQGLLAGKRFLIAGVASKLSIA
YGIAQALHREGAELAFTYPNEKLKKRVDEFAEQFGSKLVFPCDVAVDAEIDNAFAELA
KHWDGVDGVVHSIGFAPAHTLDGDFTEVTDRDGFKIAHDISAYSFVAMARAAKPLLQA
RQGCLLTLTYQGSERVMPNYNVMGMAKASLEAGVRYLASSLGVDGIRVNAISAGPIRT
LAASGIKSFRKMLDANEKVAPLKRNVTIEEVGNAALFLCSPWASGITGEILYVDAGFN TVGMSQSMMDDE"
/product="Enoyl-[acyl-carrier-protein] reductase [NADH] (EC 1.3.1.9)"
/EC_number="1.3.1.9"
/transl_table=11
CDS complement(1377705..1378436)
/locus_tag="AUO97b_01445" /db_xref="SEED:fig|400667.82.peg.1306"
/translation="MQSNNPILTRVETVSDYSQPMTVQGAIQKSVMLTIIAAAVGVAL
FFYAAFTANVGIAYAASIVGAIGGLVLALITTFKPTTAPTLAIPYALFEGAFLGGISF
TFQLKYPGVPLQALLATFVTTLVMFGLYKFQIIRATEKFKSVVISASLAIFIVFIVQM
VMRLAFGSSIPYIFESNWLGIGFAAFVAVIASLNLILDFDLIETNAAYRAPKFMEWLC
GIALLATLVWMYISFLRLLGLLSDD"
/product="hypothetical protein"
/transl_table=11
CDS complement(1378592..1379158)
/locus_tag="AUO97b_01446" /db_xref="SEED:fig|400667.82.peg.1307"
/translation="MSSTLNTRLIWIDLEMTGLDTDNDQIIEIATIITDDHLNVLAEG
PVLAIHQPDRILNAMDEWNTRQHGQSGLIERVRRSKLTARDAELQTLEFLKKWVNPKV
SPMCGNSICQDRRFLHRLMPELEQYFHYRNLDVSTVKELSKRWRPEIMSGLKKNASHL
AMDDIRDSISELKYYREYFFIMNTDGKD"
/product="3'-to-5' oligoribonuclease (orn)" /transl_table=11
CDS 1379284..1380342
/locus_tag="AUO97b_01447" /db_xref="SEED:fig|400667.82.peg.1308"
/translation="MALIRKRRLTEQQQRRIEKQHKTRQEEVDTSQDLDGLVVQHYGR

QLEVQALSVPDHHPEKPQVAEGEPEPFWKPIELNSVWRCHTRTNLELLVTGDRVKWQA
DPNTGLGIITAIHPRTSLLTRPDRYHKVKPVAANISLIVIVFAPLPEPAPTLIDRYLV
ACADANIPALLVLNKSDLLTENDPILDMLKEYENLGYEVMICHSKGDISALSQRLDGE
TVAFVGQSGVGKSSLINVLIPDAEQKTNIISENSALGQHTTTSTRLINFGKNGALIDS
PGIREFGLWHLDLDKIRMGFPEIEAHLGSCQFRNCTHTHEKNCGLKQAVEAGEILPRR
LDSFLRLIDEIQEAQQKN"
/product="Ribosome small subunit biogenesis RbfA-release protein RsgA"
/transl_table=11
CDS 1380440..1380856
/locus_tag="AUO97b_01448" /db_xref="SEED:fig|400667.82.peg.1309"
/translation="MERWLEFMGNHPILFGTLGVLIVLFFIFEGQRNGRKISPQSLGI
LVKAKNALLIDLRDSKDFREGHISGSRNIPYSQIASHADELKASDRPLVFICNLGQVA
GSALQKVAHHDSYRLDGGISNWKAQGLPLVKSKPKA" /product="Rhodanese-related
sulfurtransferase YibN" /transl_table=11
CDS 1380868..1381125 /locus_tag="AUO97b_01449" /db_xref="SEED:fig|
400667.82.peg.1310"
/translation="MAANVIVYSTSVCPYCVRAKQLLERKGVAYKEVNLSVEAPEVRA
ELMQRTNHRTVPQIFINDQFIGGFDQLYALEREGKLDELLA" /product="Glutaredoxin 3 (Grx3)"
/transl_table=11
CDS 1381153..1381611
/locus_tag="AUO97b_01450" /db_xref="SEED:fig|400667.82.peg.1311"
/db_xref="GO:0015031"
/db_xref="GO:0051082"
/db_xref="GO:0051262"
/translation="MSEEQQVQPQLALERIYTKDISFEVPGAQVFTKQWQPELNINLS
SAAEKIDPTHFEVSLKVVVQANNDNETAFIVDVTQSGIFLIDNIEEDRLPYILGAYCP
NILFPFLREAVNDLVTKGSFPQLLLTPINFDAEFEANMQRAQAAAVEGQA" /product="Protein-export
protein SecB (maintains pre-export unfolded state)" /transl_table=11 CDS
complement(1381679..1382335)
/locus_tag="AUO97b_01451" /db_xref="SEED:fig|400667.82.peg.1312"
/db_xref="GO:0000287"
/db_xref="GO:0006633"
/db_xref="GO:0008897"
/translation="MSKRKIEVYTQALSQLLLKSKQDFPDFRSFNHYKKQQIKSVRNR
LIAEKLRLTSMELEFAKHEHGKPYLLNHTLHFNHSHSQQYYALALSERIKDIGIDVEE
LDRKVRLDSLAQHAFHPDEYATWQSLEQDREYWFKVWTTKEAVLKASGLGIRLDLNTL
NTQAHPTNHGGLCSHELIGTFAYQNFVLGNMILTVAWRSEQSCRGFQFPSIQIHSLDR "
/product="4'-phosphopantetheinyl transferase (EC 2.7.8.-)" /transl_table=11
CDS complement(1382359..1382793)
/locus_tag="AUO97b_01452" /db_xref="SEED:fig|400667.82.peg.1313"
/translation="MLNKKLLFVAMLSAGFTATVQAADEVHHLNFKEAVDRAVADGTL
DGSVKFYLKGTKSGGKVIQKDAVTNKKTNGFGKSAATSCDWVLRSALIQMQNNAKAQG
ANAVTNIVSYFKANETTSTTTYDCYKGTMMAGVALKGDVVKF" /product="Excinuclease ATPase
subunit"
/transl_table=11
CDS complement(1382805..1384031)
/locus_tag="AUO97b_01453" /db_xref="SEED:fig|400667.82.peg.1314"
/translation="MKRVVVTGMAGITSLGETADDIFARFEAAKSGIRYMPEWEQYVD
LRTKLAGPVETFHIPKHFNRKVTRGMGRVALMSVVCAETALQNAGLLGHEILSSGEAG
VAFGSSAGSVDAVGEFASMLLHQSMSKINATTYIRMMAHTSAVNMTVYFGLKGLTLPT
SSACTSGSMAIGQAYEAIKYGKQQVMIAGGAEELSAAGAAVFDVLFATSGMNDQPEKT
PRPFDAKRDGLVIGEGAGCLILEEYEHAKARGAHIYAEVIGYGSNTDGQHVTRPESEM
MGRCMELALKDASVEAKDIAYVNAHGTSTDQGDVAESQATAKVLGYKPISSLKSYFGH
TLGACGAIEAWLSIEMMNRGRFIPTLNLDEIDPLCGELDYIVQQPRNLDADIIMTNNF AFGGINTSLIFKRVKQ"

/product="FIG138576: 3-oxoacyl-[ACP] synthase (EC 2.3.1.41)"
/EC_number="2.3.1.41"
/transl_table=11
CDS complement(1384031..1384756)
/locus_tag="AUO97b_01454" /db_xref="SEED:fig|400667.82.peg.1315"
/translation="MTRRILVTGSSRGIGKAIALQLAKAGFDVTVHARSRQAEAEQVV
QEIQALGQNSHYLMFNVNERQTVQQILEQDVEQHGAFYGVVLNAGLTHDGAFPALTDQ
DWDEVISTSLDGFYNVLKPLIMPMIHLRKGGRIVTLSSVSGIMGNRGQVNYSAAKAGL
IGATKALALELAKRKITVNCVAPGLIETEMVTDEVKEHALKMIPLQRMGQVDEVASVV
KFLCSDEASYVTRQVISVNGGLI"
/product="3-oxoacyl-[ACP] reductase (EC 1.1.1.100) FadG2, NOT involved in fatty acid synthesis" /EC_number="1.1.1.100"
/transl_table=11
CDS complement(1384780..1385205)
/locus_tag="AUO97b_01455" /db_xref="SEED:fig|400667.82.peg.1316"
/translation="MKLDAIQYIPHEQPMVFIDHLTEVAEGRAQAELTITPELMFCEA
EGLPTWASIEIMAQTISAYSGWQGQQSKQAPKVGFLLGTRKLNLPIPYFALGSRLIIQ
IEQQYFHEGLGQFSCEIQYAEHCIQATLSVFEPTESPFE" /product="3-hydroxydecanoyl-[ACP] dehydratase (EC 4.2.1.59)" /EC_number="4.2.1.60"
/transl_table=11
CDS complement(1385205..1386407)
/locus_tag="AUO97b_01456" /db_xref="SEED:fig|400667.82.peg.1317"
/translation="MKHLPSANAAPMAVGIQFSVGLSALGSELNQIKQALQEPQQILT
LRNDLIADHDVWVGQYTHPLCSSVPDALQSVDSRNLRFALTALSKIETELKAYTASFE
NKRLAIVVGTSTSGIADNELLLKQYFQGQTDLSISHYPQEMSCLAKALQLYLGWEGPA
YTISTACSSSAKALAAGQRLLHADLADVVLVGGVDTLCKLTLNGFNSLESLSEHICQP
CGLNRDGINIGEAAAFFVLSKEQAPVMLMGAGESMDAWHISAPHPEGKGAALAMQCAL
DMAHISAQEVGYINLHGTATPQNDAMEIKAVRQVFGDYPVALSSTKHKTGHCLGAAGA
IEAFICEQVLKDQNWLPLHQNVEIDPDLADQNYVQEAELKQPIRYVMSNSFAFGGSNI SLVFGVKP"
/product="3-oxoacyl-[ACP] synthase (EC 2.3.1.41) FabV like"
/EC_number="2.3.1.41"
/transl_table=11
CDS complement(1386404..1386949)
/locus_tag="AUO97b_01457" /db_xref="SEED:fig|400667.82.peg.1318"
/translation="MQRKWATILLSSFIFLNGCQVMPHAKGLQSPLWQAQTYQRQDQV
EVQWKQQSFSFLLYQQQKGAVLDMVALSLTGQQLFKLQFDGHRVHVEQRIDQMRLLPF
EFVVRDLLFATYPQFSHLGQQAVEMKQQADKQVVYIEKQPVLQVKQGQGSIELINEQV
PYTMTLSSIENTLQTEESSTP"
/product="hypothetical protein"
/transl_table=11
CDS complement(1386952..1388247)
/locus_tag="AUO97b_01458" /db_xref="SEED:fig|400667.82.peg.1319"
/translation="MSTIEKIDVVIIGAGPSGSSAAALLRKKGYTVTVIEKQHFPRFS
IGESLLPQSMAFLEEAGLLETVREHVEQYAFQFKNGAAFLRGKQRSFYDFTEKFTDGP
GTTWQVRRADFDNLLAKQAQAYGADIRFGHEVLDVDVASEQPVITVLDEEQQRYQIQT
KFLLDASGFGRILPKLLDLESPSNFPVRRALFTHIEDGIADDPEFDRNKILITVHEKD
HRAWYWLIPFADGRASFGVVAEQDFFEKYTVEDAEDNELDAMFKRILADEPSLSYVLR
RANFDTPVRTLVGYSANVKHLADRNYALLGNAGEFLDPVFSSGVTIALKSSSLAVPLV
ERVLQGQQVDWLEEYEKPLRRGIQVFRAYVESWYEGEFQDVVFSQNQNEGIRRMISSL
LAGYAWDEKNPIHKNAKRRLSTLAEYCREGLVQEQVFEG" /product="FIG022199: FAD-binding protein"
/transl_table=11
CDS complement(1388277..1390592)
/locus_tag="AUO97b_01459" /db_xref="SEED:fig|400667.82.peg.1320"

/translation="MRFSNWQNKFTALWLIALLLVAVGLGVTWLNKDIHIQTNIFALL
PKVQQDPELARTQQYMNEQLNNKVFVVVDAKDEAAIQQATHFLTVQAKQSQLWQPLKP
QLDFDQFAKQLYQHRAGLLSAQDQALLQKKDYAALTEQSLMQMMSPGMPVTAESLKQD
PLLLFPRYAMQLATPSQQDIEMEQGFATIHHEQGISRLFVLQLTQSPYNIDYQEQTST
WIEQTKQKLAAMGLKSHWTGTILFSNFGTQSAKQEISTIGVGSTLGLIFLVWFGFRSL
RPLATEFIAVSTGSFVAFAVTHWMFGEIHLMTLVFGASLIGVCVDFSFYFMAMQSQHR
TLGGFQILKPLLPSLFMGLMTTLVAYIFLSFTPFPGFKQIAVFSIVGLTAAWISSVLL
LPRLPALNAQPAIKALSWIGQARLWFQQRTKIRYGLIAVILAVGTASLFYLKSNDDIR
NLQGMDASLKQQDQVVREQFGQQQSSDYFVVRAASANEMQQQEQQLIGHLQQLQTNGK
ISAFQALGQWIPPLAEQQNNLQLLRNIPKATLQQYAQSMSLNVTDVLQWQQQLATQPL
LDFSVFKDHPLAFLQPQANERLVMVTGVKQPDALKQLQNEHIHFQQPITAMSQMFAEH
RVQAQHLLIYALIGLAVGLALIYGLSSIVPLVLPVSLALLSTFAVQAWLGVEINLFSI
MATFLIIGIGVDYAIFYRHGHDHPQVVGMALFLCMMSTLLGFGLLSFSHTYAIHCFGL
TVLFGVIFSFIYATLLTPADEKHIVNLQDHS" /product="FIG021862: membrane protein, exporter" /transl_table=11
CDS complement(1390567..1391196)
/locus_tag="AUO97b_01460" /db_xref="SEED:fig|400667.82.peg.1321"
/translation="MSKFIKTLGLGAAVWMSSLSAVYAAPTQITQIFQQLSQSPTVRA
DFEQQKKLPSLNKTFVSNGSLLFSKSYGVAWQIKRPVQAVLIVTPTKLVQKTQRTFSQ
IQVDQSPYSSVATLFLQLMSGNEQALAKNFNVVSANYSPAGWSMSLTPKSSLFKKLFV
RVDAQGQQYVNQIVITEKANNLTTIRFSHQTSQPTTLTAAEHAIFQLAK" /product="FIG027190: Putative transmembrane protein" /transl_table=11
CDS complement(1391196..1391624)
/locus_tag="AUO97b_01461" /db_xref="SEED:fig|400667.82.peg.1322"
/translation="MHADVIIEIPFHDVDTMNVVWHGHYLKYFEIARCKLLDQFHYNY
MQMKESGYAWPVIESHVRYAYGIEFEQKIRVRAILKEWENRLKIDYLIFDAESGKRLT
KGYTTQVAVNIEAREMCFQSPQVLFDRLNAWSEFQPKRNA" /product="FIG002571: 4-hydroxybenzoyl-CoA thioesterase domain protein" /transl_table=11
CDS complement(1391634..1393169)
/locus_tag="AUO97b_01462" /db_xref="SEED:fig|400667.82.peg.1323"
/translation="MRVLTVGEQPLSIEDVVSVARGECQVALPETSEWRELIQKGADF
LDQLLEEEGVIYGVTTGYGDSCLVEVPLHQVNELPLHLSRFHGCGLGENLDVITARAV
VVTRLCSLARGYSGVSIALLERLVWLLNENVIPVIPSEGSVGASGDLTPLSYIAGTLV
GERDVYIDGQIVPVAQVYAEKNMLPLTLRPKEGLALMNGTAVMTAIACLNYKKAEQIA
LASTLITALNVLALEGNPSHFDEVLFAQKPHPGQQHIAKQLRDWLNSEVQTAHQSPRL
QDRYSLRCAPHIIGVFEDSKVWLRQFIENELNSSNDNPLIDPVGMRVLHGGHFYGGHI
AQAMDSLKIMIANIADLMDRQIAQLVDHKMNHGLPRNLTGATAERLPLNHGFKAVQIG
VSAWTAEALKNTLAASIFSRSTECHNQDKVSMGTIAARDASRVITLTQQVIAALCCAS
VQAIHLKGLTTQLSPTLQAFMQWTLQSFAFVEEDRPLQTELQQIVDRLDQLELFNQPE FMG"
/product="Putative histidine ammonia-lyase protein" /transl_table=11
CDS complement(1393171..1394097)
/locus_tag="AUO97b_01463" /db_xref="SEED:fig|400667.82.peg.1324"
/translation="MTDSASPKWNDIKERGGMLPLMLMLWFYRFGGRWLCKFVMYFVI
MWYWLFAVTARQASLLYLQKLHHFAGSQSPFNHQPNWTNSYAHFMQFGECILDKIEGW
LGNIPEQELQVFGHENFSQHYQKGALIIVSHFGNIELLRAIKSEHPQKINVLVYQKHA
TKFNEFLKKINDKADVCLLSVDELGIETAMLLQDKMQQGEWVIVAADRVPVQSDRVQH
VDFLGESAAFPQGAWILANLLKVPVIAVFCYRVQQQFQVHIHSIAEQINLPRANRIES
MQTITKTYVAVLEQHCLRAPYQWFNFYNFWTK" /product="Lysophospholipid acyltransferase" /transl_table=11
CDS complement(1394100..1394840) /locus_tag="AUO97b_01464"
/db_xref="SEED:fig|400667.82.peg.1325"
/translation="MQSNYGFVVPVYNHPHYLADLISYLDSFHLPIVLVNDGSDDACS
QILKALAEQYSQIHLIEHDVNKGKGQAVMTGLRAAYELGLSHVLQLDSDGQHCWDDIP
KFIEQSRQHPDAMVIGQPYFDASIPKKRLYGRYITHFWVWLNSLSFEIKDSMCGFRVY

PLESTIKVLNSAKFQPRMGFDSEILVRLKWENTPFINVPTPVVYPEQGISHFHAWRDN
LGLSKAHATLFMGMLIRLPKLLKQKLQG"
/product="FIG143263: Glycosyl transferase"
/transl_table=11
CDS complement(1394843..1396501)
/locus_tag="AUO97b_01465" /db_xref="SEED:fig|400667.82.peg.1326"
/translation="MYCHFQKFIDSAQPLCIDGTLNSTSFNQFWQDVALQAAAIAQME
NLTWALWEADSYEFLVLFFAALLAKKQILLPPNRVRELEQDFSAQQIYFLKRQEIGEV
VPLSLTLDDTFLEQAQLYFYTSGSTGEPKKIPRTLRQLLNEVQGLSQSFSFDEHATAI
ATVSHQHIYGLLFKLLLPLARGRSFYNPQMAFPEDVVQAQKQLETMGLSNYLISSPAL
LKRWTTDVVLQDCQMVFSSGGKLESGVRPLLNRPIIEVLGSSETGGIAHRAKDEDAWT
AFSNVAIRIEDQQLMVKSNHAYENDWITTGDGAAWTDATCQIFKLLGRTDRIVKLEEK
RLSLDAIEQSIQALDVVKQCHVLVFEHEQRQMLGCIVVLTEQAREQLQQQGKLAFVSH
LKQQLKDGLETIAIPRQWRFLSQLPQNTQSKLNKNYLKTLFKPMLQPVVLSQSQNSDD
HIWELEFPPELACFKGHFPTQPIYPGVGQIGFLQHFAKSIWSDLSWCQGYEQLKFQNL
IRPYAVVQLKLARKEHKVSFELRDSEHILASGRLLFALQTEVED" /product="FIGfam138462: Acyl-CoA synthetase, AMP-(fatty) acid ligase / (3R)-hydroxymyristoyl-[ACP] dehydratase (EC 4.2.1.-)" /transl_table=11
CDS complement(1396511..1397041)
/locus_tag="AUO97b_01466" /db_xref="SEED:fig|400667.82.peg.1327"
/translation="MTALFVLYPFIVGWSLSHGQFVWVSALLIGLGVIRLFSKGNSLL
WPLTGFAIVCGSLSLLSHDHAWLKLYPVGMSLGALIIFALTLIKPPSMIERFARLVEP
DLPASGVQWTRQVTKIWCVFFFCNALIALSTVLFTSTQVWVIYNGFISYVLMGILFLG EFILRKRHQRLHSSNH"
/product="FIG017861: hypothetical protein"
/transl_table=11
CDS complement(1397062..1397310)
/locus_tag="AUO97b_01467" /db_xref="SEED:fig|400667.82.peg.1328"
/translation="MLSQEQVLTKLREWMEDLFEIEPETVQLDSNLYSDLDVDSIDAV
DLVVKIKELTGKQVKPEDFKNVRTVLDVVTVIQNMTAE"
/product="Acyl carrier protein"
/transl_table=11
CDS complement(1397322..1397582)
/locus_tag="AUO97b_01468" /db_xref="SEED:fig|400667.82.peg.1329"
/translation="MSNLADELKQMIIDVLALEDITIADIDTEAPLFGEGLGLDSIDA
LELGLALKKRYNIHLNAESDETKQHFKSIQSLVALVEAQQKS" /product="Acyl carrier protein"
/transl_table=11
CDS complement(1397584..1398378)
/locus_tag="AUO97b_01469" /db_xref="SEED:fig|400667.82.peg.1330"
/translation="MLNLKNLKQKSNYCWRVAATGFSFASFGLGGIAIASVIAPVLNA
TTSDPQKRQQRAQNVIKHSFKGFTDMMVKLGIMTYSVEGLEKLQNSRQELVIANHPTL
IDVVVLIGMMQQANCVVKQSLWSNPFTKGPVQSAGYILNAGSQQFVEDCVKRLKENNA
ASLLIFPEGTRTEKGMTLNEFQRGAANIAIRANVPIRPVIITCIPSTLTKNEKWYHVP
SQPFHIEVKVLDAVQVSDLLDDLTVGPKQVRQLSRSFYKIFDEELS" /product="FIG018329: 1-acyl-sn-glycerol-3-phosphate acyltransferase" /transl_table=11
CDS complement(1398372..1398986)
/locus_tag="AUO97b_01470" /db_xref="SEED:fig|400667.82.peg.1331"
/translation="MVTLHLAHLTLTHAQPSYAALECIPAMQRRRLSPLAKLALNTAI
SSLDGRSADYIVWVSKYGDEAKTLNILQDVLNDQTPSPTQFSTSVHNAISGLYSILCQ
DDTPSTSLSCSWTEGLIEAYALLKSMPEIKRVLVVAYDEPLPNIYAEAINFPAYAMAA
VVTLEKPNLQITAWTHIDEADAPAFAHFWQDADQLTSAFGWNKC" /product="3-oxoacyl-[ACP] synthase"
/transl_table=11
CDS complement(1399183..1400592)
/locus_tag="AUO97b_01471" /db_xref="SEED:fig|400667.82.peg.1332"

/db_xref="GO:0005525"
/db_xref="GO:0005622"
/translation="MKPVIALIGRPNVGKSTLFNQITKSRDALVADFAGLTRDRKYGD
ATYQNKSFIVVDTGGIGESEGGIDNYMAEQSKTAINEADIIIFVVDARAGLLASDEQI
ARELRTLGKKIYLVANKVDGVHAEAALVEFYKLGMGEPLQVAASHGRGVQQMLEDVLQ
DIPEDENPEEHDKDTGLRLAIIGRPNVGKSTLVNRLLGEDRVVAFDQPGTTRDSIYIP
FEREGRKYTLIDTAGVRRKGKVDEMIEKFSIVKTLQAMKDAHVVVVVVDAREGIVEQD
LHLIGYALEAGRAMVIAINKWDNMSEYDRKQCKLDVERRFDFIPWARIHLISALHGTG
VGELYPSIHRAYESANLKVSPAKLTQILNDATDQHQPPTVQGRRIKMRYAHMGGQNPP
TIVIHGNKVDKTPADYRRYLENVFRKVYKLEGTPVKIEFKTSENPFEGRKSQVDERTA
ARRRRYIQKFKKAEKKFKR"
/product="GTP-binding protein EngA"
/transl_table=11
CDS complement(1400780..1401925)
/locus_tag="AUO97b_01472" /db_xref="SEED:fig|400667.82.peg.1333"
/translation="MNQKFKLPLAIAIASAVLVGCSSNKVKEAKPNPLPKLTESNKSL
VPVFSRSVSSTNKADPLRLQLDASEGVVFTLDPKGEVAAYRGKQRLWEKKVSKLGLSS
GVEAAEGIVVVGNSKGQLFALDQATGEQKWTAQLSGALLSPSLVQSGRVITIANDGTV
FAHDVSSGQQVWAYKLPNVQFSLRGQPSPVSLDPRTVLIASANAYVYAIDTISGIPRF
QRRVAVSEGRSDIQRLIDIDGDPVVAGQLMVTTSFQGQVTVTDLASQRVVWSEDSSST
KRPEVYDNKVFVSSTDGKLTAYDLTTGEQLWQNDSLLNRHLSNPVVLGSDLIVGDLDG
VLHLIDPTTGKLIGRSKTSGEVNTLRVIENQLYVSTRKGDLSIWQNR" /product="Outer membrane
beta-barrel assembly protein BamB" /transl_table=11
CDS complement(1401925..1402623)
/locus_tag="AUO97b_01473" /db_xref="SEED:fig|400667.82.peg.1334"
/translation="MSLSDEEQFDSLKSFAKKYGSAMISGILIALIAFFGWEYWQKRN
LATSQTETAKVQQLMDEANATADNPNALASITASADKIVKDDIDSVQAIQTQFVLAKL
AYEKQDYAAAEKALKKVENSKVKDEGLIQVVKLRLADAQLAQNKYDEALKTLSGDVDP
AFKATVEELRGDIFVAKKDIDSAKKAYQAAWDSLLERKQERQILQIKLESVGVLVEDP QIERPILETQVEES"
/product="UPF0070 protein YfgM"
/transl_table=11
CDS complement(1402652..1403944)
/locus_tag="AUO97b_01474" /db_xref="SEED:fig|400667.82.peg.1335"
/db_xref="GO:0004821"
/translation="MSSIVAIKGFNDVLPTQTAAWRRLEQHLASLMDAYGYQQIRLPI
VEQTGLFKRAIGDATDIVEKEMYTFFDKGNPPESLTLRPEGTAGCVRALVEHNLLRGA
TPRVWYMGPMFRYEKPQKGRYRQFHQFGVETFGVATPDIEAEVILMTARLWKRMGVAH
MVQLELNTLGEKEERTEYRNALVAFLNEHKDALDEDSQRRLTTNPLRILDSKIESTQK
ILENAPKLYDFLKEDSLSHFQQLQDYLTAAGIKFVINQKLVRGLDYYNKTVFEWTTTA
LGSQGTVCGGGRYDGLVGQLKGKADQSVPAVGFGMGMERLLLLIEQVEQAEIVRDCEA
FLVAEPAYQSKALVLAEQLRDQLEAANSNIRIKTGSQGSMKSQMKKADQAGAVYAIIL
GEREWEAQQLAIKELATAEQSQVALAELVPFLIEKFTK" /product="Histidyl-tRNA synthetase
(EC 6.1.1.21)" /EC_number="6.1.1.21"
/transl_table=11
CDS complement(1403941..1405056)
/locus_tag="AUO97b_01475" /db_xref="SEED:fig|400667.82.peg.1336"
/translation="MIENPIKRRPTRKIRVGSVYVGGDAPISVQSMTNTETCDVDATV
AQIERCVDAGADIMRVSVPSMEAAEAFGAIRKRVSVPLVADIHFDHRIALAVADYGAD
CLRINPGNIGSDQKVREVVAAARHHGISMRIGVNAGSLEKDLQKKYGEPTGQALLESA
LRHIDILDRLDFHEFKVSVKASNVFLTMDAYRLLSQQIDNPLHLGVTEAGIYRTGTVK
SAIALGGLLMEGIGDTMRISLAAEPEDEIKIGFDILKSLGLRSNGINFIACPSCSRQE
FNVIQVMQALEERLEDIRTPMDVSVIGCKVNGPGEAKEADIGVVGAAPRSLVYRNGEK
SHLIDTNQLVDEIETMVRQRVQELEEAKSKEIIRSSS" /product="(E)-4-hydroxy-3-methylbut-2-
enyl-diphosphate synthase (flavodoxin) (EC 1.17.7.3)" /EC_number="1.17.7.1"

/transl_table=11
CDS complement(1405078..1405869)
/locus_tag="AUO97b_01476" /db_xref="SEED:fig|400667.82.peg.1337"
/translation="MEINPNSQQPTGSSLPTSALGNIQRPGEYLRQIRVSQKKELEQV
SSDLNMPLKTLNALEQDDYKSLPEATFIKGYYRSYAKYLNTDATAIIQRFDEIYANDT
GLLPNHALNNSPIKIMGKLPGSNSDRNKKWLKRALLAILIIAVVSLIVMGVQKWTSNK
EDAELPKANQSNVEVLPMKGNTSATVGDQLVLNFNRPTSVHIVDATGKVLATGRQAST
LTLNGESPFQIRLDDATAVSLSLNQEQISLSPYTVNGKAEFRLSR" /product="Cytoskeleton protein RodZ"
/transl_table=11
CDS complement(1405860..1406627)
/locus_tag="AUO97b_01477" /db_xref="SEED:fig|400667.82.peg.1338"
/translation="MGVAVALLASGCQTSQTVKKDPEKAVKVRTQLAAEYIRSGDLDS
AKRSLDQALSVDSRDATANMMMGILLQQEGSKPNLEKAEHYFKRAISSEPDNAQAHNN
YGTYLYQMERYNDAIEQFRIAGATLGYDQRYQALENLGRIYLKLGDIASAEKTFKQAL
LANRDSYISMLELAEIFYLQQQIPAATQMYEQYVRTVGQKNQGARALWIGLRVARANA
DKMGMQVLVNQLRALFPESPEYQRYLQLQYSTEAVWK"
/product="Type IV pilus biogenesis protein PilF" /transl_table=11
CDS complement(1406676..1407908) /locus_tag="AUO97b_01478"
/db_xref="SEED:fig|400667.82.peg.1339"
/translation="MSSAVVVSSENLDGQQQSSSTPASPAAEKVNLLGMSRAELEKFF
EDIGEKKFRAGQVMKWIHQYFVTDFAEMTNISGKLRAKLEQICEIKAPEVVHRHYSKD
GTRKWVFRVGEGSGSLVETVLIPAEDKTGSRKTLCISSQVGCALDCSFCSTGKQGFQR
DLTPDEIIGQLWMANYSYMEEVPVAERERSVTNVVMMGMGEPLLNYDAVLSSMHIMLD
DFAYGMSKRRVTLSTSGVVPKIDQLAKDIDVALAISLHAPNDELRNELVPINKKYPLA
QLIAACQRYIAKDGNESARKHVTIEYVMLEGVNDQPEHAQQLLKLLKNLPSKINLIPF
NPFPHAPYGRSSRNRIISFQKTLSDAGFVCTIRQTRGDDIDAACGQLVGQVADRTRRA
EQWQKKVAQRQEILRTQG"
/product="23S rRNA (adenine(2503)-C(2))-methyltransferase @ tRNA (adenine (37)-C(2))-methyltransferase (EC 2.1.1.192)" /transl_table=11
CDS complement(1408031..1408462)
/locus_tag="AUO97b_01479" /db_xref="SEED:fig|400667.82.peg.1340"
/db_xref="GO:0004550"
/translation="MAIERTLSIVKPDAVSKNHIGEIFARFEKAGLKIVATKMKHLSQ
ADAEGFYAEHKERGFFGDLVAFMTSGPVVVSVLEGENAVLAHREILGATNPKEAAPGT
IRADFAVSIDENAAHGSDSVASAEREIAYFFADNEICPRTR" /product="Nucleoside diphosphate kinase (EC 2.7.4.6)" /EC_number="2.7.4.6"
/transl_table=11
CDS complement(1408576..1408776)
/locus_tag="AUO97b_01480" /db_xref="SEED:fig|400667.82.peg.1341"
/translation="MGLRWTDTIDIAIELSEAHPEVDPQWIRFTDLHAWVCALPDFSD
DPNKSTEGLLEAIQMAWLDEVR"
/product="Protein IscX, believed to be involved in assembly of Fe-S clusters"
/transl_table=11
CDS complement(1408876..1409427)
/locus_tag="AUO97b_01481" /db_xref="SEED:fig|400667.82.peg.1342"
/translation="MKFKNAKIKILDLDLRGCLYLNNFSHSQRVALFFKIISRAGDGP
FWYLMLAIVWGMQGITYSLQIIYLLLGGSVGTAIYKFLKHKTTRPRPYQVHQVIVLGE
RPLDHFSFPSGHTLHAVMVTIVLGYIQPVLLAAMLPFMVLVALSRMVLGLHYPSDVIV
GALIGAAVASLIIFVAPLLGIAL"
/product="putative membrane-associated phospholipid phosphatase, PAP2 superfamily" /transl_table=11
CDS complement(1409452..1410738)
/locus_tag="AUO97b_01482" /db_xref="SEED:fig|400667.82.peg.1343"

/translation="MQGSYATALLKQQQLPESFQFYFKQKQHLSNTQELHQLHELVRP
RLKIAIVTETWPPEINGVALSLLQLCQGLQKQGHKILLVRPEQKAKCHDFLPEQECLV
MSQAIPKYPTLQFGWPQYLKVSKAFEKFVPDVVHIVTEGPLGLTAMQAAKAKDIPVSS
GFHSPFQDFSRFFDLAFLVKPIQKYLCWFHNNTQVTCVPSRDTEEALRGFGITCPLVV
VGRGVDTTRFSPKHRSESLRQQWGVDSDTRVMLYVGRLSPEKEVQLIVESYAAMQNIQ
QQKTKLVVVGDGPDLARLKALPEAKNVIFTGSLRGHDLAVAYASADVFVFASQVETFG
NVVLEAMASGLPVIAYDYACAHQYLIHGVNGWLSPLGHKNHFIQQIYQLPSIQQLREM
GIQACHKVQQSGWQLPVQQLEQAFYQVVKEPSADFT"
/product="Glycosyl transferase, group 1 family protein" /transl_table=11
CDS complement(1410889..1413072)
/locus_tag="AUO97b_01483" /db_xref="SEED:fig|400667.82.peg.1344"
/translation="MKLQTIACAVAIATGGLFFSHTMNEARAATNTAAVSQSIQPTQE
QALVARQLATLVDRQHYLNMRLDANTSNRILDMYLDSLDPDHSLFLDAEVQNYKKLYG
SNFGASLKAGNLTGPFAIHQQYRERLKQFYEFMLAELKKPQNLKQPNTFIEVDREKAP
YFKTSAEQQNHWRKMLVSQLINLTISREEEQAKQKALKENPSLADGQDLTGPEDLTPA
QTLTKRYTRQLERISRVKSDDVLDKTLNAMLATYDPHSNYYPPIDAIELNRQTTLQLE
GIGVSIRPERGNEDYTKIETIVEGGPASKSGQVKSGDRIVGVAQEGGKMIDVVGWSSS
EIVGLIRGKRGTKVTLKLLGAGASMSQARNVTLVRDVIQEEDAGVRSRTVEVTRDGKK
HLLGVIEIPSFYFDYRSRRAGQQYRSVSEDTANAFEALKAKKVEGIIIDLRNDPGGSL
EEVARMLGQVIKSGPVVQIRDGNGNVSVFEDNDGGQQIYTGPLAVLVNLASASASEIY
SAAIQDYERGIIIGSTTTGKGTAQVQLDTLAYGQATLTQRKFYRVTGGSTQNKGVVPD
IKLVDIYNEEFGERKSKNALKWDTIPTAPFKREGSVQPYVAKLSQLSEQRVAVDPQFK
YLNKRTAIAKVTSDQKQVVLDIDKRRAELLSLEKQTLDAENERRIATGQKPFPNWESY
QASLDALAESRAKMKANQRPALPEEETFVNEAANVLMDYAKLQNR" /product="Tail-specific
protease prc" /EC_number="3.4.21.102"
/transl_table=11
CDS 1413265..1414284
/locus_tag="AUO97b_01484" /db_xref="SEED:fig|400667.82.peg.1345"
/translation="MIGALMLDIAGTELTQEDIELLQAPQVGGMILFARNIESPQQVR
ALTDHMRQVRPDILIAVDQEGGRVQRLKSGFTLLPAMGRFGELYITQPQKALELAEQC
GWLMATEVLAVGIDFSFAPVLDLNAISDVIGDRGFSKNIEDIAPLAGAFMRGMKKAGM
ANTGKHFPGHGSVKADSHVAAAIDSRSYDEIYNHDMQSFIKLMPELDALMPAHVIYDQ
VDPNPAGFSSFWIQEVLRNRLKFNGVLFSDDLSMQAACVAGGADARIQAALAAGCDMG
LVCNDRSAACTALEGIANLELPNQERLERMRGRIPQIQVGETLSLGNDWQAVKTAIEE FKNSI"
/product="beta-N-acetylglucosaminidase (EC 3.2.1.52)" /EC_number="3.2.1.52"
/transl_table=11
CDS 1414610..1415452
/locus_tag="AUO97b_01485" /db_xref="SEED:fig|400667.82.peg.1346"
/translation="MTQHLSKHRHISFTAHYTGYIWYQMGISHEALATSKGKSLAYLV
HPLESWAEKYVGGSMRTTLKQRHTMLDHDLEKLIQENPDLQVLEIACGLSPRGWWFRQ
HYPSISYRELDLPDMAQTKQNALQQIEKNAPEVLSVDLFTEAFAQAFEVFDSNRPLVV
ISEGLINYFDKDLLKQLIQSIAHYGASFKKLHYLTDLYPEPVKNKLANIIWNSSKLLK
LMSRSSFSFHFKTPLEVKNFFKDAGFSQVNVEQPQIFFEQVSKDSDEEHLGDLVWTIH AQLK"
/product="Tetracenomycin polyketide synthesis O-methyltransferase TcmP"
/transl_table=11
CDS complement(1415506..1416819)
/locus_tag="AUO97b_01486" /db_xref="SEED:fig|400667.82.peg.1347"
/db_xref="GO:0003979"
/translation="MNSIIQMDSEINQAYAGFGFFDIYHRDSFKQPARTTWIDGWKIE
YMAIADPQTIHKTPIVIVGGAFQNFNSYKYCVEQLFESGPVILIDLPSMGANQQITNR
DTGISAGTLELPDLSEMLGRWLDIIGIQKVSVMGMSLGSVVASCFAYHRPDLMDRMIL
MGVMQKTRKSWRMILEESLKLMQENRMEEFGQAVILYLVNHAKLDKTRMSPTAKKLFF
RQMAEFTGTERERYEINCNRLLRLTDVPIPECKTLVAAGQYDSFTLPHENANFALQCP
DMEFALIANADHVPQLQRRKETMSLFTSFLKGESIQNLDGIIPMTREQMQNMERRGEE

RIPVLQPKTKLSHRECETEVPVTIVDVTFFGMYLKLDDVAQLEFVNEHPRDLALHLED
EEGAFSIECLIFEATEQGVRALFKHGSFELADRLSRFIARQKQAA" /product="Putative hydrolase"
/EC_number="1.1.1.22"
/transl_table=11
CDS 1417119..1418384
/locus_tag="AUO97b_01487" /db_xref="SEED:fig|400667.82.peg.1348"
/db_xref="GO:0004350"
/translation="MQDSIEQYMQKVGQQARDASRVLTSASTSLKNHALSAIYTALEN
NQAAILAANQIDMEKGRSNQLDSALLDRLELTPARFKGMLQGLKDVIVLVDPIGEITD
LAYRPTGIQIGKMRVPLGVVGMIYESRPNVTLEAASLAIKSGNAIILRGGSEALESNK
AIAEAVKHGLKVAGLPEHSVQVIETSDRGAVGHLITMAEYVDVIVPRGGKSLIERVTN
EARIPVIKHLDGNCHVFVEAQADLQKALPITLNAKTHRYGVCNAMETLLVDEKIAEVF
LPHIAELYAEKQVELRGCPETRRILGSSVKPATEEDWYTEYLGPILAVKVVSGIDEAI
DHINKYGSHHTDAIVTENYTLARQFLARVDSSSVVVNASTRFADGFEYGLGAEIGIST
DKIHARGPVGLEGLTSQKWIVLGDGQIRQ"
/product="Gamma-glutamyl phosphate reductase (EC 1.2.1.41)"
/EC_number="1.2.1.41"
/transl_table=11
CDS complement(1418491..1420119)
/locus_tag="AUO97b_01488" /db_xref="SEED:fig|400667.82.peg.1349"
/translation="MNQLNNLQHKFPVVDGIPENVRLPSQIHQRVSLVDGELKLWAGA
TKKTLSPIWIQQPDGNLEQVELGSYPVMGEKESDEALEAAVRAYNNGRGEWPMMKVSE
RIACMQNFIQRMVEQRDLIIKLIMWEIGKSLADSEKEFDRTITYMRQTIDALKDLDNA
NSRFVIAEGTIGQIRRTPLGVVLCMGPYNYPLNETFATLIPAMLMGNTIIFKPPQFGT
LLFEPLLEAFRDSFPKGVINTIYAPGSLVVPHLLASGQINVLALIGSSKVADHLKKQH
PKSHRLRAILGLDAKNAAIILPDADLDLTVKECLLGALSFNGQRCTALKMLMVHRSIA
DEFVNRLTTELAKLKVGMPWEKGVSITPLPGMHRTAYMTEVIEDAVAKGAKVVNPEGG
EFCKTMFYPAVVYPVTEGMRLYREEQFGPVVPVAVYDDIETVLDYVTTSDHGQQVSIF
GSDPAQIGHLVDTLVHQVCRVNINCQCQRGPDVFPFGGRKDSAEGTLSVHDALRAFSI
RSMIAAKQTDDSKGMLASMVSEHYSKFVNTDFIF" /product="Glyceraldehyde-3-phosphate
dehydrogenase, putative" /transl_table=11
CDS complement(1420292..1420804)
/locus_tag="AUO97b_01489" /db_xref="SEED:fig|400667.82.peg.1350"
/db_xref="GO:0046316"
/translation="MIVIAMGVCGTGKTLIGELLSERLACEFLDGDTLHSAANKSKMS
QGIPLTDEDRLPWLQAIRQAIEAKQRDGETAVFTCSSLKRMYRDILRGQDQNVKFVYL
KGSYELLQQRLAERSGHFFDPALLQTQLDTLEEPDVNEAIAIDIALTPEQIIEQVIQK LGVTDSVCRG"
/product="Gluconokinase (EC 2.7.1.12)" /EC_number="2.7.1.12"
/transl_table=11
CDS complement(1420822..1422180) /locus_tag="AUO97b_01490"
/db_xref="SEED:fig|400667.82.peg.1351"
/translation="METVQGGMLLVYTLIAIVALIVMIAKFRIYPFLVLIIVSLGLAL
AVGMPMDGIVKSYEAGTGKTLGHLAIVIALGTMLGKMMAESGGAERIAITLIKWFGEK
HIHWAMMFIALIVGLPVFFEVGFVLLIPIAFNIAKRTGKSLLIVGLPMVAGLSVVHGL
IPPHPAALLAVQAYHADIGKTIMYSLLVGVPTAVVAGPLYALWINKYVKLPENNPLAK
QFVEADANNTRELPSFGITLFTIMLPVALMLVGSWADVFFAPKTFPNELLRFIGTSDI
ALLIAVLVSFITFGTMQGFNREQIEKFCGGCLASIAGILLIVGAGGGFGGILRDSGIS
NEIVSTALKANLSPLLLGWLVAALIRLATGSATVAMATACSIVAPIAAAAGVVVRPEL
LVLATGSGSLIFSHVNDAGFWLIKEYFGMTVGQTLKTWSVLETIISVLGLSFTLLLSA VL"
/product="Gluconate transporter family protein" /transl_table=11
CDS complement(1422376..1422996)
/locus_tag="AUO97b_01491" /db_xref="SEED:fig|400667.82.peg.1352"
/db_xref="GO:0008675"
/translation="MSKIEDVVKLGPVIPVLAFDSAEQGEHVSRALHAGGVKVLEITL

RTAAGLAAIERASQLADDIVVGVGTITKPEHCAQAKKAGAKFGVSPGLTKDLHLAAQD
AGLPLLPGVMTPSDLIQAIELGYDIVKFFPAQQAGGVEMLKAFYGPFPNLRFCPTGGI
TAETAPDFLKQPNVVCVGGSWLTPKPVVAAQDWAEITRLAQIASQL" /product="4-hydroxy-2-oxoglutarate aldolase (EC 4.1.3.16) @ 2-dehydro-3-deoxyphosphogluconate aldolase (EC 4.1.2.14)" /EC_number="4.1.2.14"
/EC_number="4.1.3.16"
/transl_table=11
CDS complement(1423017..1424870)
/locus_tag="AUO97b_01492" /db_xref="SEED:fig|400667.82.peg.1353"
/db_xref="GO:0004456"
/translation="MDLPNPVLAKVTERVIARSQKTRSAYLQRIEHAQGKFPARGALS
CANLAHGFAGMEDNEKLIIKVGREPNIGIVSSYNEMLSAHAPYKTFPDIIKTAARENG
GVAQFAGGVPAMCDGITQGNAGMELSLFSRETIAMGTAIALSHNMFDAALCLGVCDKI
VPGLLIGALQFGYLPTIFVPAGPMSSGLSNDDKAKIRQQFATGQVGRDALLEAESAAY
HGQGTCTFYGTANSNQMLMEVMGLHLPSAAFVHPHTPLRDALTAEAAKRVLDLTAERG
NYTPIGHVIDEKAIINGIVALLATGGSTNHTLHLIAIARAAGILIDWDDFDELSAVVP
LLAKIYPNGKADVNHFQAAGGVAFLIRNLLEAGLLHNDVTTVAGKGLQHYTKEPKLID
GKLTWVDGVVQSLDDKVLRSIDAPFQPDGGLRLMQGRLGRGVIKISAVAPEHRKVKAP
AIVFDSQEAVQAAFDRGELHRDFIAVVRFQGARANGMPELHRLTPVLGVLQDQGFHVA
LVTDGRMSGASGKVPAVIHLSPEALLNGPIGKVQTGDMLIIDAEAGVLDIELDEQTWQ
SRPVAQPEHQAENEVGFGRELFGVFRAAAAPAEHGASVFGALVGEEPQGQI"
/product="Phosphogluconate dehydratase (EC 4.2.1.12)" /EC_number="4.2.1.12"
/transl_table=11
CDS 1425633..1426835 /locus_tag="AUO97b_01493" /db_xref="SEED:fig|400667.82.peg.1354" /db_xref="GO:0008776"
/translation="MATSVLVINCGSSSIKYALVSERREDRIYGLAENLGAADARIKG
ITVGGEPLELSIPYADHAKALETLLARLANYKPQAIGHRVVHGGSLTKAELLTPEIVE
RIRAATPLAPLHNPAHLIGIEATMRLFPELPQVAVFDTAFHQTMPAHAYRYAIPKFLY
TEHNVRRYGFHGTSHAYVSDRGSELAGKFQKGGWLTAHLGNGSSTCAVWNGQSVDTSM
GLTPLEGVVMGTRSGDVDPSLHSFLAKNLGWDLAKIDKVLNNESGLLGLSQLSNDMRT
VIEAAENGNEDACLAIEVFSYRLAKSLAALSCGLPSIDGLIFTGGIGENSAYIREKTL
AYLPHFGLQLDKDQNNNLKRGTEGRIDSGTGPQIWVIPTDEEGRIAKETRQVVEVSQN TSPEVSLA"
/product="Acetate kinase (EC 2.7.2.1)"
/EC_number="2.7.2.1"
/transl_table=11
CDS 1426879..1429023
/locus_tag="AUO97b_01494" /db_xref="SEED:fig|400667.82.peg.1355"
/db_xref="GO:0008959"
/translation="MNTILLIPTGEGVGLTSACLGMIYALDCNGIKAGFLKPFSQVDQ
ENLDRTTSLFGHLFQSKTVQSISHEKLTQRIAAGEVDELLEEAVSLHRSIAADNNVII
VEGLLPNGQDHFASELNANLAQALDAEVVLVSTADIQNPRKTAEKVEAHLRQFGGATS
NRTAGVLFMRTRGLSEETAQIPVAFDPSLRPTEDIAKFTAELQKYNRYFGSSDLPIIG
LVPFSNTLSVPRTLDIASVIDGQWVNQGEAKTRRILHSSLIASSIEYELNKFIAGELI
ISASERTDVLLASSLATSNGIPLAGLVLTEREAPAPKLLEFCQSAIKQGLPILHTHLN
TLETAQRLSDFGNEIPTDDTERAEQVTRFVSSHIDVEWLKQHSNNGATPRLSPSAFRH
ELVQKSIAAKKRIVLPEGDEPRTIEAAAICQARGIAHCILLAKPEAVHDVAKARGIEL
PAGLEIIDPDQIRDQYIAPMVELRKGKLNDLQAKEQLQDTVVLGTMMLALNHVDGLVS
GAVHTTANTVRPAFQLIKTAPAYSLVSSIFFMLLPDEVYVYGDCAINPDPTAEQLAEI
AIQSADSAKAFGIDPRIAMISYSTGTSGTGADVEKVQQATQIAQQRRPDLLIDGPLQY
DAASVESVGRQKAPDSRVAGRANVFIFPDLNTGNTTYKAVQRAANVVSVGPMLQGLNK
PVNDLSRGALVDDIVFTIALTAIQAEQQAAAK"
/product="BioD-like N-terminal domain / Phosphate acetyltransferase (EC 2.3.1.8)" /EC_number="2.3.1.8"
/transl_table=11

CDS 1429252..1430778
/locus_tag="AUO97b_01495" /db_xref="SEED:fig|400667.82.peg.1356"
/db_xref="GO:0004333"
/translation="MTTIIKQDDLITSIKDALQFISYYHPQDFIQAMSRAYDREENKA
AKDAIAQILINSRMCAEGHRPICQDTGIVNVFLEVGLDVKFDLTMSLDDAVNEGVRQG
YLENSNVLRASVLADPAFGRKNTKDNTPAVIHYKLVPGNKVDITVAAKGGGSENKSKL
AMLNPSDSIVDWVLKTVPTMGAGWCPPGMLGIGIGGTAEKAMMLAKEALMEEINMDEL
LRRGPENKIEELRIEIFEKVNALGIGAQGLGGLTTVLDIKIKDYPCHAAGKPVGMIPN
CAATRHAHFQLDGSGVAHIQAPKLEDYPSVTWDASQSKRVNLDTITQEEMDSWKPGDT
LLLSGTMYTGRDAAHKRMVEMIDNGEELPIDLKGKFIYYVGPVDPVRDEVVGPAGPTT
ATRMDKFTRKVLEHTGLFGMIGKADRGPTAIEAIKDNKATYLMAVGGAAYLVSKAVRE
AEVVAFADLGMEAIYKFVVEDMPVSVAVDVNGTSIHAVAPKIWQAKIGKIPVIDAAAG "
/product="Fumarate hydratase class I, aerobic (EC 4.2.1.2)"
/EC_number="4.2.1.2"
/transl_table=11
CDS 1430920..1431474
/locus_tag="AUO97b_01496" /db_xref="SEED:fig|400667.82.peg.1357"
/translation="MTIRPILKNLSISLCLSCITTSLFASPAKLSSVKELMQMSQIDY
LLKESINELTPYYDQQAEQIISNITGIKTLGPKEKEAAKKLGLLLKDSSNQLISSPKT
TQALQDIYLKTYTEEEIQANLKFLKTPEGQSITRKNVQIMGQISEYMMELGQQTFNDP
KARDHMQEEMLKIIAPLMKDKEKS"
/product="hypothetical protein; putative signal peptide" /transl_table=11
CDS complement(1431499..1432215) /locus_tag="AUO97b_01497"
/db_xref="SEED:fig|400667.82.peg.1358"
/translation="MRYLIIALSCAALLAGCQSSSHLEKNSGELTATLPKVDVDKEPG
LLDQYRLGGFTVGGWVNQKLGQTFAPVKPQNEQAAVVYLYRPDTKWNRQEIAAINLFV
NGKRIPSLLHNHYYWIELPAGTYRISASRPLLGIHFQEPKYIDITVDAGASYYLKYDE
ENKMDRSEHTGPFMLMQDNIGRREIAFTELKSSSYNFVAEDASGKIRHKPQELKPAKY
NEKSDVHLIKPFKLWNPLTW"
/product="hypothetical protein"
/transl_table=11
CDS complement(1432363..1433676)
/locus_tag="AUO97b_01498" /db_xref="SEED:fig|400667.82.peg.1359"
/translation="MSEHPQGQKHCSFCGKTQSEVGKLIAGEDAYICNECVDVCLDLV
QTSQQVEAGDWASKALPKPHEIRAALDQYVIGQDLAKKTLSVAVYNHYKRLKVGQSGH
VSKDVEIAKSNILLIGPTGSGKTLLAQTLARLLDVPFAMADATTLTEAGYVGEDVENI
VQKLLQKADYDVEKAQKGIIYIDEIDKITRKSENPSITRDVSGEGVQQALLKMIEGTV
ASIPPQGGRKHPQQEFIQIDTSNILFICGGAFAGLEKIVQQRQEKGGIGFTADVKNKD
ETKKLAELFRQVEPTDLVKFGLIPEFIGRLPVIATLEELDEEALMQILTEPKNALTRQ
YQYLFNMENVDLVFEDSALRAVAKRALERNTGARGLRSILENVLLETMYDLPSRTDVG
TVFINEAVINGEAEPVYKSERQPKEAVTHESVAKADLKVIDSKSA" /product="ATP-dependent Clp
protease ATP-binding subunit ClpX" /transl_table=11
CDS complement(1433778..1434383)
/locus_tag="AUO97b_01499" /db_xref="SEED:fig|400667.82.peg.1360"
/db_xref="GO:0008462"
/translation="MYVPTIENALVPVVVEQSSRGERSFDIYSRLLRERVIFLTGEVE
DNMANLIVAQMLFLEAENPDKDIHLYINSPGGSVTAGMAIYDTMQFIKPDVVTYCMGQ
AASMGAFLLNAGAKGKRYCLENARVMIHQPLGGFRGQASDIEIHAREILFIKERLNRL
MAEHSGQDYDTIARDTDRDNFMTAQAAKEYGLVDQVLSKRP" /product="ATP-dependent Clp
protease proteolytic subunit ClpP (EC 3.4.21.92)" /EC_number="3.4.21.92"
/transl_table=11
CDS complement(1434576..1435910)
/locus_tag="AUO97b_01500" /db_xref="SEED:fig|400667.82.peg.1361"
/translation="MQVTTEAVSGVARRLNVSVPTSRINEQFEARLKRTAKTVKINGF

RPGKVPANVVRREYGASIYQEVVNDIIRDSVFEAIQQEKINAVGMPNIEKVEHKEDAL
VFEATVEVYPEVEVKAFDGLEVERKTAEIKDADVDTMIENLQKQRQTWAVTKGMAKKD
MQVTFDFEGSIDGEKFEGGSAEDFKLVLGSGRMIPGFEDGIIGMKAGEEKVIDVTFPE
DYQAENLAGKAAQFKITVKQVEKPKLPEIDAEFLKIFGVSEEEGIEKLKADVRKNMER
EVRNGLRNQVKQAAFDALVAANEIEVPAAMVAQEIDRQRQQMVQQFTQQFGGAGAQSF
DKSMLPDELFKEQAERSVKLGVLVSKVLADAKLEVDQARVDAYIDDMASSYEDPTEVI
EYFKNDAQQRAQIEAVVLEDQVVDHILASAKVTDKAVSYEDLLKEQQARRMG" /product="Cell division trigger factor (EC 5.2.1.8)" /EC_number="5.2.1.8"
/transl_table=11
CDS 1436403..1438613
/locus_tag="AUO97b_01501" /db_xref="SEED:fig|400667.82.peg.1362"
/db_xref="GO:0004872"
/db_xref="GO:0005215"
/db_xref="GO:0006810"
/db_xref="GO:0016020"
/translation="MAQEAVSQLPTIHTKATQEESLKVDQSANSKFVAPLKDTPKSVS
ILSQKLIKDTNSNTLLEALRYEPGITLGAGEGGTPFTDMPYIRGYSGQSSIYVDGVRN
TTSQNRDMFAIEQVEVIKGSSSALGGGGSVGGSINLIPKVAHEGDVYQGSVQSGTDNY
RHIQLDANKDFGNGIAGRVVIMGHENEKAGQSNGAEYKRVGIAPSITFGLDTATRGTL
SYYYLRSNDEPDAGIPFNNANPAKPPVGVTVTPGDGKPVDVKAGTYYGWKARDFDKRE
NHIGTFKLEHDFNENLTLSNIATYNKSKSDYVYTNADDSKGNIYRGTVARRALSRILD
TDAYSDQLSLRGKFNTGSLKHSFNVGTEWSFQETDQGVHTFTNAAGETTSTILDSNIQ
NCTSAAAVANGWCTSLNNPGNGAFTDKRGSITAQSTTRSHNVGIYALDSIEFNPQWLL
NLGVRWDKFETEKKYNKDVDGRTPHKAGDKLESDTDYFSYQAGLVFKPTEDGSIYLSY
ATSANPVGVLAEGDTGSDSISDSGTASASANALKPEEARTFELGTKWDLFNNRANLTA
AVFRTEKQNTRIQIDPTTTANAGKSKVDGFEISLNGKITDKWDVSTGYSYLDSEITEA
AYNAVAQEGKPLPFVAKNSATLWSTYRVMPQLTLGAGVEYRDQVFVNTTAPKYLPTYT
IYNAMAKYDVNKNVNLQLNINNISDKRYFTSAHAAHYAFEGNGRNAVLAINFKY"
/product="Ferrichrome-iron receptor"
/transl_table=11
CDS 1438719..1439402
/locus_tag="AUO97b_01502" /db_xref="SEED:fig|400667.82.peg.1363"
/translation="MIHHIPNVLNKEQVAEFRKLMEEANWVGGKVTAGTLSASVKRNQ
QLSEQDPLTHHLSDIVIKAIWQNPLFQAAALPHKIIPPLFNRYDEHESFGFHVDNSIR
LIRGTAEQIRTDLSCTLFLSEPDEYEGGDLVIEDTYGYHEVKLPAGDVVLYPSTSLHE
VSSITAGTRFASFFWVQSLVRDDSKRHLLFSLDESIRELRKSHGDSYSEVMKLTNIYH NLIRMWSEL"
/product="PKHD-type hydroxylase YbiX"
/transl_table=11
CDS 1439861..1441558
/locus_tag="AUO97b_01503" /db_xref="SEED:fig|400667.82.peg.1364"
/db_xref="GO:0003852"
/translation="MMLADPSKKYRRMYQRVDLPDRQWPNNEITKAPIWMSTDLRDGN
QAIFEPMNMEQKFKMFKMLVKIGFKHIEIGFPSASQIDFDFTRMLIEENHIPDDVYIE
VLVQARDHLIERTFEALAGAKRAIVHIYNSNSPTFRQKVLNVDVNGAKQLAVNAAQKV
KEYAAQYPETDWIFQYSPECFSATELEVAKEVCDAVTEIWDARPDHKVILNLPATVEV
STPNVYADQIEWMHRNLARRDGVIISVHCHNDRGCGIAASELAIMAGADRVEGCVFGN
GERTGNVDVAAIALNMYTQGVAPELDFSNINEVIATVEECTGLPVHPRHPYAGDLVFT
AFSGSHQDAIKKGFEYQKNEEIWDMPYLPIDPKDLGRDYDAVIRVNSQSGKGGIAYLL
ESNYNVVLPRRLQIEFSQVVQQYTDENGTEISAKQIWTLFKDTYVEVKNHHYTVKNYK
LSDINGTQIIELEIDVEGETQQLRGEGNGPISAFLNALQLPIDVLNYEERSISSGANA
KALTLIELQVKGTGRGSFGAGVHDNTVTSSIEAIIACTNRLIDQGVLSTDQVVAAAV" /product="2-isopropylmalate synthase (EC 2.3.3.13)" /EC_number="2.3.3.13"
/transl_table=11
CDS 1441641..1442801

/locus_tag="AUO97b_01504" /db_xref="SEED:fig|400667.82.peg.1365"
/db_xref="GO:0004414"
/translation="MSFPADSVGLVTPQKFQFEEPLHLECGRVLPRFELMVETYGTLN
ADKSNAILICHALSGHHHAAGYHHEDDKKAGWWDSCIGPGKAIDTNKFFVVALNNIGG
CSGSTGPTSPNPENDNRPYGPDFPLVTVRDWVKTQAMLSDRLGISVWYAVVGGSLGGM
QALQWSVDYPDRLQKCVVIASAPKLSAQNIAFNEVARQSILSDPDFHHGRYLENDSYP
KRGLILARMVGHITYLSEEAMKQKFGRDLKSGKFMYGFDVEFQVESYLRYQGEQFSRN
FDANTYLIMTKALDYFDPSREYGHSLTEAMSKTKCQFLIVSFTTDWRFAPSRSQEIVD
ALITNHKPVSYLDIDAEQGHDSFLFPIPLYVKTLRAFLGGEEHLKSTSLEAS" /product="Homoserine O-acetyltransferase (EC 2.3.1.31)" /EC_number="2.3.1.31"
/transl_table=11
CDS 1442801..1443394
/locus_tag="AUO97b_01505" /db_xref="SEED:fig|400667.82.peg.1366"
/translation="MRIDQQLAEKWIKPGSSVLDLGCGDGELLAHMSQKHQIRAYGLE
IDQEKIAIAVSRGLNIIQQDLNLGLSRFADQSFDYVVMAQALQAVDAPDVLLRDMVRV
GKQAIITFPNFAHWKTRSFLALKGMMPVSDALPYMWYNTPNIHLCTFKDFEALCAENQ
IQIINRLAVNGNQQGSLLSKHVPNLFGEVAIYRVSAL" /product="Methionine biosynthesis protein MetW" /transl_table=11
CDS 1443436..1443978 /locus_tag="AUO97b_01506" /db_xref="SEED:fig|400667.82.peg.1367"
/translation="MQAHADYIAQPQSVASQAARFSTMGIHDLQKAAQAGQAGAQFYL
GTRYQYGKDVAKDDKQAFAWFKAAADQGLSPAQLNVGRMYADGIGVKKDEAMARKYFE
KAASNGDNRASYNLAMMEEQKKNYVGAYQWYELSTRDGMLDNKVISLSEGKKTALAAN
LTQEQIRMARDRADKWIQAQ"
/product="hypothetical protein"
/transl_table=11
CDS 1444147..1444770
/locus_tag="AUO97b_01507" /db_xref="SEED:fig|400667.82.peg.1368"
/translation="MSTPHWFDQGSLVLASNNKGKVAEFEKLFEQLKLPVEIIPQGRL
NIPDAIEDGLSFIENAIIKARHASKISGKPAMADDSGICVPVLGGAPGIYSARYAGEH
GDDAANNAKLLNDLLPFRKNGEVIEGMFVCVLALVTHAEDPLPQIFQGIWHGEILEAP
RGENGFGYDPLFWLPELQVSSAELSKEEKNKISHRGQAMQLFRESLV" /product="Nucleoside 5-triphosphatase RdgB (dHAPTP, dITP, XTP-specific) (EC 3.6.1.66)"
/EC_number="3.6.1.15"
/transl_table=11
CDS complement(1444963..1445838)
/locus_tag="AUO97b_01508" /db_xref="SEED:fig|400667.82.peg.1369"
/translation="MALLLPVLAFITGLLLAQTSISETVKPILSAWLARLLIPIVIIY
NMVFYQSGSLSLMLFSFGSAFIIFFAYLALFKDRLLALCVSYTNMGWLGFPFAMALFG
PEATAPIVALYIGMSLFGNAWAVTAVTTAPQSKLNILKKVLTSPPCISIFCAVVLRLL
GAQHIESPAIHWVYEVAKFAMTFAGMCVLGMWLRRTRVYTKDLIYSTKAQAFKVACGF
VLCGLSYRFLPIPNINQHIGVMFLMFCLPPAANIVALETHYQGTGRSAAYIASGTIVS CVCIAIYAVFLHVAL"
/product="putative membrane protein"
/transl_table=11
CDS 1446091..1446309
/locus_tag="AUO97b_01509" /db_xref="SEED:fig|400667.82.peg.1370"
/db_xref="GO:0008565"
/db_xref="GO:0015031"
/translation="MAGLSIWHVVIFAIVVILLFGTSKLKNIGKDVGGAVRDFKKSVR
EEDEAASLNSPRTIDAQVKTSESTSVKS"
/product="Twin-arginine translocation protein TatA" /transl_table=11
CDS 1446321..1446758
/locus_tag="AUO97b_01510" /db_xref="SEED:fig|400667.82.peg.1371"
/db_xref="GO:0008565"

/db_xref="GO:0015031"
/translation="MLDVGMTELLCFAIIAILVLGPEKLPEAARFAGRWYVRLKRYIT
NLQNEIDQELRLSEFRKEMQEELNRIEALERKVQQQLDEIQKQQVSESLEVTETAKTT
QKPIWKCTPISGHYKVPYLTKVTSLAAQTDISETSPVELKIAV" /product="Twin-arginine
translocation protein TatB" /transl_table=11
CDS 1446755..1447525 /locus_tag="AUO97b_01511" /db_xref="SEED:fig|
400667.82.peg.1372"
/translation="MNQLPSTAVNSQENLDQMPIMNHLVVLRRHLFRIVGVTLFLFFC
LLPFRNHTYQLLSEPLRLQLPASSSMIATDVTATFMAPFKLNLFVALMLAMPFILYEI
WSFVRPALYQKERHLALPLLIGSIILFYAGVAFAYYITLPAILHFFISVSPETVAPMT
DINSYLSFCLKLFLVFGVTFEIPIATLLLILIGVVNTQSLAEKRRFIIVGCFFVAMFI
TPPDAISMVMLAIPMWLLFEIGLLFGKILEKRKTHSAE" /product="Twin-arginine translocation
protein TatC" /transl_table=11
CDS 1447699..1449879
/locus_tag="AUO97b_01512" /db_xref="SEED:fig|400667.82.peg.1373"
/db_xref="GO:0004035"
/translation="MTTASNHPTRRDILKWFTGIPFLPLGAMATAATLAGCNDSDNDS
SIVTPSKPINFKNATFTSMPAPSTVADIATTACASKLAISWEDGSKTEYQLAYKPFFL
TGTEVPDGKGGKVIAGGYYDINNKPIIDKSVAGKERQFFSDCPDGSSLISFKQATGKD
FTDADKKALGVTGNPVFHVVQFEYLSKDQNGGDTYGKLSSPIAVLTLDQDPKTGHLTL
IKYHNVDTSSAHGLWITCGASLSPWGTHLSSEEYEPDAFDQKLGQSLSTLKAFSKNIY
GDENIANPYNYGHLPEITVNADGTGRVTKHYCLGRISHELVQVFPDNRTVLMGDDYTN
GGLFMFVADKEKDLSAGTLYVAKYTTVLSDTTTGQISWIRLGHATSAEIENLIKSGIK
GTDIFESVLQIAKYPSDATIAEKEAIDAGDKGTPEQQALAKAAKERLKLQQAAQKAEL
EAQGFKFTYLSKTGVYLKLKDNSDRTKLAAAFLETHRYAAYMGASMALTKNEGTTVNI
ADKKAYSALANIVDSMVEGGSGYLAEHNVKFPKITAGGILEHTLTGSQKDSSNVAINS
EWVPSQSNLLIKGKDISFDSLGNTADPEQIASPDNLKFSEKLRTLFIGEDSGNHLNNF
LWAYNVDTKQLIRILSTPAGAESTGLHAVDEVNGWTYIMSNFQHPGDEWNRFYKEKDG
VRTGISADLLAQIDTAINTNYSNKFAAAVGYITADPIAPSVVKK" /product="Alkaline phosphatase
(EC 3.1.3.1)" /EC_number="3.1.3.1"
/transl_table=11 CDS 1450087..1452420
/locus_tag="AUO97b_01513" /db_xref="SEED:fig|400667.82.peg.1374"
/translation="MTELTPYHEDQELDNNTSDNIHFRDILEQHISRRSLITKAASGA
VALTLASTLTGCNDNDDDSGSNNGGTKPVDPNKKPEKLTFTPVAKNLNDIVTVPEGYE
ANVIYALGDSINPRVGDWDDNNIPSGPSFQFRSGDCHDGMHYFGLNTSTNRFDETVSA
QGLLVMNHEYINQTFLHPKGPTKVDGRRPEDEVIRETNAHGVSVVHIKKDPTTQKVEI
VKNSIFNRRITASTVMEFAGAAAGSSLLATRFSPAGRQTRGTHNNCGNGYTPWGTYLT
TEENFIGYFQRSGSDEYARTDAEKIALKRYGLGVKKDEPYLYEKDEKGAPKKDKDGKI
IYLKDKNGELIPNVDEQGRQIYLGTSSRYGWETAIGQVESQDLYDRWNADVKAAQATQ
DYRNGPNTFGWMVEIDPFDGRQNPVKRTSLGRFAHEDSACRAVVGQPLAFYMGDDSRG
EYIYKFVSTAVWDTKDINGGYTAGDKYMNAGKLYVAKFNNDGSGQWIELAYGKNGLNE
SNTTYPFKSQADVVTFARLAADSVGATKMDRPEWCTVNPVNGEIYVTLTNNSNRGKDY
ATDAANPRNYTDLYAGTKEQKGNVNGHIIRFKETDDKTTAETFKWDIYLFGAEASMAS
NINLSGLTDNNDFSSPDGMWFDPRGVLWIETDDGAYTDVTNCMMLAALPGQIGDGGTA
TTSNGQQTITGAKVTDATLRRFLVGPKQCEITGIAMTPDYKAIFINVQHPGEDSPSYA
KPESNWPATQKDPSNKTARPRSATVVITRKDGGVIAG"
/product="Predicted phosphatase"
/transl_table=11
CDS complement(1452464..1453243)
/locus_tag="AUO97b_01514" /db_xref="SEED:fig|400667.82.peg.1375"
/translation="MCIVALAWHVLDDMPLCLISNRDEFYHRPTALLHQWEHTPIIAG
QDLQSGGTWMGVTPQGRWAVLTNFRDGRDKKQYETSRGHLVQAFLESDLLPIRFAQQL
EQQQQNFAGFNLFMGTADQAVYMSNRGEAPQVLANGVYVVSNGLMSDHWEKTRHLRTR
FTQEFLPMHQHKTEELSLQHAAWDILEDERKVTTALLPNTGIQPEMEELLSSTFIQSP

VYGTRCSNFLRMTNNEWIWLEKTQQGEHSGQIVQQQVLLNR" /product="Uncharacterized NRDE family protein" /transl_table=11
CDS 1453337..1454155
/locus_tag="AUO97b_01515" /db_xref="SEED:fig|400667.82.peg.1376"
/translation="MLTYPNIDPVAIHLGPLQVHWYGLMYLLAFLCAWGLASYRAKQR
DGWTSDMVSDLVFYGALGVVLGGRIGYVLFYEFDKFLENPIWLFQVWTGGMSFHGGFL
GVMIAMLFWCKKYQKTWFQTLDFVAPCVPTGLMFGRIGNFIGGELYGRAVTDPNYPFG
MIFPTDPLHLVRHPSQIYQALCEGLLLFIILWWFSSKPRPRMAVSALFLMGYGVARFV
MEFFRQPDADQGFILFGWMTKGQILTVPMLLIGLWMMWYAYQKKIYDWGPQKNS"
/product="Prolipoprotein diacylglyceryl transferase" /transl_table=11
CDS 1454229..1454588
/locus_tag="AUO97b_01516" /db_xref="SEED:fig|400667.82.peg.1377"
/translation="MLEFWFNTQVPTFKKLIILIITLLICIGLYQYQPLPLDTILMFT
GTGVIFLICRYFKLHFAQSNPTGLLYRLFTWVPIALLFALIFVKTMPNLLIWGVQGIA FMALAAFIFSPQSLFNK"
/product="hypothetical protein"
/transl_table=11
CDS 1454599..1454961
/locus_tag="AUO97b_01517" /db_xref="SEED:fig|400667.82.peg.1378"
/translation="MLAYWYNAPRHIKLIFIIVVCAAIYAANQVQPLSPTYTVLSLVL
GFGLHFGQYLQTKIPEHSSYKLKLQFLLRIYPLIIVAIIMWLLPPQHNWIATLQALGF
VLVGFFLVSIYQNRAKRF"
/product="hypothetical protein"
/transl_table=11
CDS 1455084..1455926
/locus_tag="AUO97b_01518" /db_xref="SEED:fig|400667.82.peg.1379"
/db_xref="GO:0004799"
/translation="MRAYLDLLQHILDNGGDKGDRTGTGTRSVFGHQMRFDLSKGFPL
LTTKKVHFRSIVIELLWFLKGDTNVKYLQDNKVTIWDEWATAEQTARFGRPEHELGPV
YGHQWRNFGATKNADGTYNQDGFDQIKWLINEIKTNPNSRRLIVSGWNPNEAGQVALP
PCHTLFQFFVQDNKLSCQLYQRSADVFLGVPFNIASYALLTHMIAQVCGLDVGDFVWT
GGDTHLYANHFEQAKLQLTREPLPLCQLKLNPDVKDIFDFKFEDIEIVGYESHPAIKA PVAV"
/product="Thymidylate synthase (EC 2.1.1.45)" /EC_number="2.1.1.45"
/transl_table=11
CDS 1455971..1456480
/locus_tag="AUO97b_01519" /db_xref="SEED:fig|400667.82.peg.1380"
/db_xref="GO:0004146"
/translation="MAWQNVEVVHVVAMDKNHCIGKGNALPWHISADLKHFKEITQGG
VVIMGRKTLESMGRALPNRVNWVITRDINWHFDGVKIAYSIEDALNAALEDAKNTEKQ
ALFIIGGGEIFKQTLSIADRLELTHVDLDVQGDAHYPTIPSEFHKTASEQQVDEKSGT SFEFATYKK"
/product="Dihydrofolate reductase (EC 1.5.1.3)" /EC_number="1.5.1.3"
/transl_table=11
CDS 1456495..1457550 /locus_tag="AUO97b_01520" /db_xref="SEED:fig|
400667.82.peg.1381"
/translation="MHNIGTREFIIALLFGALHSLFTLFIVLSSIWVCLALWVQQPFG
WLGSRIIIGIWIAFALSMAGLYFNGHIISRRTDVLIYLLAFACSLVWYFSITARQDRD
WNPEVANMLSYEKHGDVITLHNVRNFNWHPDGTYDVRWETRTFDLNQLNGINIITSYW
MGPQIAHTLVSFEFKNQQPLVFSIEIRKEKTEEFSAIGGFFRKYELSLIASDEKDIVY
TRSNIRKEQVYNFPVNMPRSEQKALFLEYLKKADELRAEPKWYNTLTSNCTTLIFDMV
QAINPYQLPKDYRLIASGYLPNYLYDLKALDQNISLKQWYQIAHINPRTEHFEQFADQ SSEHFSQIVRQGLPKVK"
/product="putative membrane protein"
/transl_table=11
CDS 1457651..1458172
/locus_tag="AUO97b_01521" /db_xref="SEED:fig|400667.82.peg.1382"
/translation="MQQALFGGGCFWCVEAVFLQIRGVEKVTSGYAGGHTTHPTYEQV

CQGDTQHAEVVLIDFDEQQVTYSQLLDVFFATHDPTTLNRQGNDIGTQYRSVIYYFNE
EQKQAAEHTIQTLKDDDLDIVTELSPTPTFYPAEDYHQNYYEKNPSQGYCNFAIPPKL LKLHSKFQHLMKN"
/product="Peptide-methionine (S)-S-oxide reductase MsrA (EC 1.8.4.11)"
/EC_number="1.8.4.11"
/transl_table=11
CDS complement(1458233..1458637)
/locus_tag="AUO97b_01522" /db_xref="SEED:fig|400667.82.peg.1383"
/db_xref="GO:0005215"
/db_xref="GO:0006810"
/db_xref="GO:0016020"
/translation="MAFQLGEDHDSGMNEMNLIPLIDIMLVLMIIFLVTATVANPSIP
LTLPKTTAEIIDPPPKAITISINANGEVAWDTQVISLDELQKRFQEAGQGAVKPTVQL
RADKESKYDTVAQVMSRASEAGLSDIAFVSEN"
/product="Biopolymer transport protein ExbD/TolR" /transl_table=11 CDS
complement(1458637..1459257)
/locus_tag="AUO97b_01523" /db_xref="SEED:fig|400667.82.peg.1384"
/db_xref="GO:0006810"
/db_xref="GO:0008565"
/db_xref="GO:0016020"
/translation="MNFSIYWQHADAVSKTLYFILLAMSIATWTVFILRLMGTRQLKQ
QAYAQLSQAITSLKAKLQPLSFEQRKAVAEQALLRQISAEKANADKGISVLGTIASIA
PFVGLFGTVWGIFHALVAVGKSGQAGLAQVATPVGEALIMTGLGLAVAIPAVLAYNIC
VRANRGLAHDLQDQAHSLLIDTMLQQDSTVKSDVKTAQQSYVGGQA" /product="MotA/TolQ/ExbB
proton channel family protein" /transl_table=11
CDS complement(1459302..1460057)
/locus_tag="AUO97b_01524" /db_xref="SEED:fig|400667.82.peg.1385"
/db_xref="GO:0005381"
/db_xref="GO:0006826"
/translation="MSQSSAALNSPNPMKKKVITALIAVAIGHIGVLWAVSHIKPVEL
KPIEKKPLQVRFVKIQEQPKPLPPKPKEPPKKPEPKKEVKEVKVVEKPVTPPKKVEKI
QQVKKAETPKETVKTEPKVEPKIVTPTTTVTEKVVEKPKPVVQEAPKAQPAPDPSPKR
VSIGGSGVQWSRSPRLTVSPKDLQGEARSVVVLIEADEKGKITNVRVTRSSGIPSLDD
KVVRAVRSAKFKPYMENGVAYPIKAEQPFDLTP"
/product="putative TonB-dependent receptor" /transl_table=11
CDS complement(1460449..1461312)
/locus_tag="AUO97b_01525" /db_xref="SEED:fig|400667.82.peg.1386"
/db_xref="GO:0008864"
/translation="MNMTTANTARLLITCEDKPGIVQAVSSFLYHQGANITALDQYAT
EAQGGRYFMRVEFELDHLQSRKDALIQTFAANVAERYGMQWRLAFVNDIKKVGILVSK
VDHALLELLWRHARGSLPCEITHVISNHEDLREAVENFGIPFTVIKVTKDNKAEAYAQ
IHEMMQGNDLLVLARYMQILSEDFVSKWEMKIINIHHSFLPAFVGANPYKQAYEKGVK
LIGATAHYVTADLDQGPIIEQDVERVSHDYNVEQLRELGEDVERNVLARAVKWHLEDR IIVDGNKTVVF"
/product="Formyltetrahydrofolate deformylase (EC 3.5.1.10)"
/EC_number="3.5.1.10"
/transl_table=11
CDS complement(1461387..1461902)
/locus_tag="AUO97b_01526" /db_xref="SEED:fig|400667.82.peg.1387"
/translation="MHQYQVETLEMLSLPEDRPLTTNDKINYFQVLSGQLWNYRFIHR
DVYHLVESNEDFKKIYPRFAGQVMQQGQRIYQAFVDAGLMKMTPSEIEALIINLWIVL
TNWTNFLYMSGHISDNNHLEEKWVWQALRQMVFLEGPYLMGESRATYEQLLESLGPSD LFASLSSLKDE"
/product="Transcriptional regulator, AcrR family" /transl_table=11
CDS 1462192..1463640
/locus_tag="AUO97b_01527" /db_xref="SEED:fig|400667.82.peg.1388"
/db_xref="GO:0004029"

/translation="MNSQTKTNPETQVPYDVKHLHDLLEQQKIAYLRHPVPTAKERID
RLARLKRVLVKYQDQIAEAINLDYGNRAIMETKIGELLTCLEQIKYYSKNLTGWMKPS
KRHISVLHQPAKGWVQYQPMGVIGIITPWNYPLLLSVGPLICALAAGNHAMIKISSAS
PNFGQVLENALSEAFPQELVAVVNGGGVISDAFSHLPFDKMIFTGSTSVGKTVMAAAA
QNLVPVILELGGKSPALVHASVDMKDVAQRIAVGKLWNAGQTCVAPDHIFLPRGKTAE
FIENFKLIVAGMYPHFRNNQDYTSIINDKQYNRIQGYLENARDQGARIIEINPQNEIL
DDVRKIAPTLVTGVTTAMDIMQNEIFGPVLPILEYDQIEEVIEFINSRPRPLAMYYFD
YDQARADYISQHTHSGHFGINMVITHVAQDDLPFGGIGASGMGKYHGPEGFFGLSHER
SVMSNPKLYSLKYILPPFNKPIHRFISKTLLR"
/product="Aldehyde dehydrogenase (EC 1.2.1.3); Probable coniferyl aldehyde dehydrogenase (EC 1.2.1.68)" /EC_number="1.2.1.3"
/EC_number="1.2.1.68"
/transl_table=11
CDS 1463808..1464044 /locus_tag="AUO97b_01528" /db_xref="SEED:fig|400667.82.peg.1389" /db_xref="GO:0003735"
/db_xref="GO:0005622"
/db_xref="GO:0005840"
/db_xref="GO:0006412"
/translation="MSKVCQVTGKRPVVGNNVSHANNKTKRRFEPNLHHHRFWLESEK
RFVRLRLTTKGMRIIDKLGIEKVVADLRAQGQKI"
/product="LSU ribosomal protein L28p @ LSU ribosomal protein L28p, zinc-independent" /transl_table=11
CDS 1464057..1464212
/locus_tag="AUO97b_01529" /db_xref="SEED:fig|400667.82.peg.1390"
/db_xref="GO:0003735"
/db_xref="GO:0005622"
/db_xref="GO:0005840"
/db_xref="GO:0006412"
/translation="MRDKIRLVSSAGTGYFYTTTKNKRTMPEKMEIKKFDPKIRQHVI FKEAKIK"
/product="LSU ribosomal protein L33p @ LSU ribosomal protein L33p, zinc-independent" /transl_table=11
CDS 1464360..1466069
/locus_tag="AUO97b_01530" /db_xref="SEED:fig|400667.82.peg.1391"
/translation="MPHDVDLIILLAVGFGVALFFGYIAARLRLPPLIGYLIAGIIIS
PNTPGVEADIHLANQLAELGVMFLMFGVGMHFSLNDLLLVRRIALPGAILQIAVATLL
GIGVSMLWGWSFGSALVFGLSLSCASTVVLLKALGDRGLLDSVNGKIAVGWLLVEDLV
MVLVLVLLPATAVLLGGKAPAGAEGNIWLTLGITLLKVIGFIAFMLIVGKRVVPIIMQ
FVARLGSRELFTLTVVAAAVSIAYGSYAIFGVSMALGAFFAGMVVKESDFSHRAEEET
LPLREIFSILFFVSVGMLFDPHILIERPLHILAVIAIIMVGKTLAAMALVLFFRYPIN
TALTVGASLAQIGEFSFILATLGVSLGLLTLEAQNLILAGALFSITLNSFIFSAIEPV
QRWIRERSHLARLLERSGDPLAMLPDEVDQSYLRDQVVIVGYGGVGRRITENLINQNI
KVVIAEENREIVEKLRQANIAAVSGVATEPGVLIQAHIMHARLLVISPMDILDIHRIV
DIAKQLNPQIQVLICAESKEEAAVIRDEHIGEVFYAKEEMAKNMSHHILNQIELAHQS THH"
/product="Inner membrane protein YbaL, KefB/KefC family" /transl_table=11
CDS complement(1466106..1466384)
/locus_tag="AUO97b_01531" /db_xref="SEED:fig|400667.82.peg.1392"
/translation="MSIQQLKELFGNQEAVLELVQLEGGELALRNAGSEKEPLVKIQF
SDELKAILGEQTPIVAQHMIQAALFGLLEKQVNEWQAEVVDEQPKFLS" /product="hypothetical protein"
/transl_table=11
CDS complement(1466497..1467030)
/locus_tag="AUO97b_01532" /db_xref="SEED:fig|400667.82.peg.1393"
/translation="MAILEQESLVMSRVARYHADRTNQKLYFARLACQQAEQTDHVQQ
VQAYREAAVFHLHGAMLAFLQELVRYYRLNDLQPTLKSIEEHMAAKGQVSPEVSVLQQ

LAKDGFVAELKRAYRMCQYAPEPTEPTPEEETSSNLIIKVTQGPQSWLPDVKILREWH RELSHLIDGFRNEMVEF"
/product="hypothetical protein"
/transl_table=11
CDS complement(1467117..1467284)
/locus_tag="AUO97b_01533" /db_xref="SEED:fig|400667.82.peg.1394"
/translation="MQQFLALSVVAPNGTRIAQRIKTLEVRSWVPAQLPLKDLFIVEN QNFLINDGDEG"
/product="FIG00352841: hypothetical protein" /transl_table=11
CDS complement(1467292..1467531)
/locus_tag="AUO97b_01534" /db_xref="SEED:fig|400667.82.peg.1395"
/translation="MKILVRNLDRSVTEAEVLELFKAYGKVESCVVVTDKDTGKSKGF
GFVEMPNPREAIKAIKGLNTLKVKGYGIRVKAAEE"
/product="RNA-binding protein"
/transl_table=11
CDS complement(1467605..1469584)
/locus_tag="AUO97b_01535" /db_xref="SEED:fig|400667.82.peg.1396"
/translation="MSLAKLINELNAQQKKAATTKAQNCLVLAGAGCGKTKTIVARAA
YLIDQGLPANQIQILTFTRRAASEIVARVEQHVGAQAKGLRASTFHTFCMYLLRRNPQ
AFGLTQFSIIDRDDQLLMFRLLRGKDKDNVLPKAAELCDLYSYARNTQSKLSEALLKQ
LPEAYEYKSQIAELMKTYEQRKRERNFLDYDDILSIVAVHLQNSEALTNWVAGFCQAL
LVDEMQDTNPLQWALLQPLIGKVKLFCVGDDAQSIYGFRGADFENIHSFKERIPDAVI
HTLDLNYRSTQEILDLSNWLLAHSPIDYQKQLQAHRGKGQKPQLHLFGNEFEEANWIA
QDLIERHQQGANWYDHMVLVRSGYSARYLEGAFIAAEIPYRFIGGVKLLESAHVKDVL
SLLRIVSNPQDDLAWMRFLTLWDGIGDVGASKLAQELMSISEVEERCQRLERHGKVPL
QAILILKQLDVLQQHVEASIGLALDALSEQLEQNYKTKDWSRRARDFDLVKQLARKHA
SLGEFLEEYVLEPISISEIEKAGDDDLVTLITIHSAKGAEQKVCYVPHVSPNQYPYAR
AQGDFDEVEEERRVLYVALTRAENELILTKQNLNTWSQDTYDEQGRKTESYFLNDLPA
HLVQAKIHRQVPQPFAKKQWNTSRTVNLGFGIDLD" /product="ATP-dependent DNA helicase
UvrD/PcrA, proteobacterial paralog" /transl_table=11
CDS complement(1469701..1470273)
/locus_tag="AUO97b_01536" /db_xref="SEED:fig|400667.82.peg.1397"
/translation="MKTVGNDLVRNQLHADRKWYLILGFVLIIFGLVLFSSLPFATFS
VVFLFGILMMVGGVLHLIAALSVFKSGSRWLWALFGVLYLMAGYYAFSTPVTTAVVLT
SLLSIALIIAGVIRTINAFLLRPIAGWGWTLFSGLLTLATGILILVSKNSPFWVLGMF
LAVDILFQGINFLGLASAIKHLPSSSKTVS"
/product="HDED PROTEIN"
/transl_table=11
CDS complement(1470411..1471169)
/locus_tag="AUO97b_01537" /db_xref="SEED:fig|400667.82.peg.1398"
/translation="MWKNIRILCLLIVLLIVAVQAWRDQNQDWNQPIVVVLHPINADG
LQTTQTYIHQLQNTDFQALKSYLSEWSQHYRGQSANFKIRLGQQLQQRPPEVPQNAGI
FHVVWWSLKFRFYAWRQQQPEDNGASLKLYLNYYDPNYQKVLVHSTALEKGRIGSVNL
FATRGQALQNQVVIVHELLHGFGAKDKYDLKTGQPIYPLGYAQPEKVPLYPQKRAEIM
GGRTPLSEQTSKMPSDLQETVIGLPTAQEVGWLK"
/product="hypothetical protein"
/transl_table=11
CDS complement(1471257..1473893)
/locus_tag="AUO97b_01538" /db_xref="SEED:fig|400667.82.peg.1399"
/db_xref="GO:0003917"
/translation="MANTSRSASQSTASSASAAHKRALVIVESPAKAKTINKYLGSQY
IVKSSVGHVRDLPTGGSKATEKKPAARTKLTEAEKEQKANQALINRMGVDPEHGWQAH
YEVLPGKENVVAELKKLAKDADAIYLATDLDREGEAIAWHLREVIGGDDSRYHRVVFN
EITKNAIQEAFKQPTRLDLNRVNAQQARRFLDRVVGFMVSPLLWEKIARGLSAGRVQS
VAVKLVVEREREIRAFIPEEYWQVFADTKAKKDDIRLEAVKQAGKTLKLKNKAETDAL
LDVLKGAEYKVAQREDKPTKVNPSAPYITSTLQQAASTRLGFSVKKTMMLAQRLYEAG

FITYMRTDSTFLSDDAVSMVRAHIESQYGEKYLPAKPNRYGNKAGAQEAHEAIRPSNV
ALTGDQLAGVERDAQRLYDLIWRQFVACQMTPAEYLSSTLTVEAGNVELKAKGRTLVF
DGFTKVRGANKSDDDIILPAIKVGEILKLEKLDPSQHFTKPPARFTEASLVKELEKRG
IGRPSTYAAIISTIQERGYVKLENRRLFAEKMGEIVTDRLDESFNNLMNYAFTADLEG
QLDRVATGERNWKELLDTFYGDFKKRLTNAQGEQGMRRNQPVEVPAVHCPECSRPMQI
RTGTTGVFLGCSGYNLPPKERCKGTLNLTPVESLAALSDDDSAETADLMSKHRCPKCG
TAMDSYVIDGGRKLHVCGNNPDCDGYELEEGEFKIKGYDGPTIPCDKCDGEMQLKTGR
FGPYFACTSCDNTRKVLKNGQPAPPRVEPIKMEHLRSTKHDDYFVLRDGAAGLFLAAS
KFPKIRETRAPKVAELRSVADQLDPKYQFILQAPDVDPEGNPTIVKFSRKNQSQYVGS
ETPEGKQTKWSLIYQDGKWIEG"
/product="DNA topoisomerase I (EC 5.99.1.2)" /EC_number="5.99.1.2"
/transl_table=11
CDS complement(1473929..1474378)
/locus_tag="AUO97b_01539" /db_xref="SEED:fig|400667.82.peg.1400"
/translation="MDNIGIWVTVIIVLFVLGSIFGLRVSPREKALGIMRDKARKMGL
HPRVIAAPEWTHVPMATEKRASMVAYYSVLIPDARLPLMRARVIDGKLKVVQGDEKFN
DSPIALKGIYAIDMQANCVGLYWDEESDLKATQLDEMKAYLLALAEA" /product="hypothetical
protein"
/transl_table=11
CDS complement(1474526..1474903)
/locus_tag="AUO97b_01540" /db_xref="SEED:fig|400667.82.peg.1401"
/translation="MAKARHSSFDCSPGCSVEAAISLIDGKWKCVILWHLLNEGTLRF
NEIRKRVPSITQRMLTNQLRELEQDGIIHREVYPQVPPKVEYCLTDLGRGLEQILLAL
KTWGDAHLDQFGKLTTLETEKSA"
/product="Transcriptional regulator, HxlR family" /transl_table=11
CDS 1475003..1476013
/locus_tag="AUO97b_01541" /db_xref="SEED:fig|400667.82.peg.1402"
/translation="MKAVAYQKAGPITLPEALVDIELDTPVAKGHDLLIRVQAVSVNP
VDTKIRKNVSAEQSGWKVLGWDAVGTVEAIGDKVTQFKIGDVVWYAGALNRQGSNSEL
QLVDERIVGHKPKTLEVTEAAALPLTAITAWEMLFDRLQIPKTAPANTTILVIGGAGG
VGSITIQLLKQLTNLTIIATASRPETKEWVEQLGADYVLNHRQPLAAQIKQLGLSAPL
YVFSTTETDQHLSDIVELIAPQGHFGLIDDPDQLDIKPFKSKSVSVHWEFMFTRSMFQ
TEDMEKQSELLNEVSKLVDEGKIKTTVTQVLSPINAENLTRVHEQIESGSTKGKIVLH GF"
/product="Zinc-type alcohol dehydrogenase-like protein" /transl_table=11
CDS complement(1476061..1476306)
/locus_tag="AUO97b_01542" /db_xref="SEED:fig|400667.82.peg.1403"
/translation="MTKPPYHDDQASFSAPIEDLQVRIAFLDDLVEELNQQLAIQTLE
IADLKKQMQLLYQRVESADLSEGVAPFDPLTNKPPHY"
/product="Protein SlyX"
/transl_table=11 CDS 1476339..1478249
/locus_tag="AUO97b_01543" /db_xref="SEED:fig|400667.82.peg.1404"
/db_xref="GO:0005524"
/db_xref="GO:0016887"
/translation="MAYITLRDVQLAFGGPALLDGANFNLERGERVCLIGRNGEGKST
LLKLIEGSLLPDSGEVSVQNGLTVSMLAQDVPMSSGKVADIVAEGAGEAAAVLKAYHE
ASDACVLGDMEACDRMGSLQHKLDQLDGWALENKVNTILGKMELDPNADLADLSGGRK
RRVLLARALLTQPDVLLLDEPTNHLDVESIEWLEKFLLDQNNLTLLFISHDRSFVDRL
ATRIVELDRGILRSYEGNYSRYLDLKAQQLEAEEKQNALFEKKLAEEEAWIRQGIKAR
RTRNEGRVRALKALREESKARRFQQGKVNMGVQEAQRSGKLVFDIEHLSVSYDRQTLI
RDFSAIVMRGDRIGLVGDNGVGKTTLIKAILGEVQHEGVVKTGTQLEIAYFDQLRNQL
DLEKSVSENVSEGSDFVDVNGQRRHILSYLQDFLFSPERARTPVKALSGGERNRVLLA
KLLLKPSNLIVMDEPTNDLDMVTLELLEEMLSEYKGTLLLISHDRAFMDNVVTSTWVF
DGKGGIAEYIGGYQDYLIQRPNERVVDQKGDVKKAQAKAEAEKNAAQTNVKKVKLSYK
DQRELEQLPAEIEKLENEQAELSDKLADGSWFVTDADAATKASQRLAEIEELLLEKLE RWDELEQMSKGN"

/product="Bis-ABC ATPase Uup"
/transl_table=11
CDS 1478395..1479027
/locus_tag="AUO97b_01544" /db_xref="SEED:fig|400667.82.peg.1405"
/db_xref="GO:0005293"
/db_xref="GO:0006865"
/db_xref="GO:0016020"
/translation="MLDLSQILAFGLICLAMVLTPGPNMIYLISRSISQGKIAGFISL
GGVAVGFVFYMLCASFGITALVVAVPYAYDTIRIAGAMYLLWLAWKALRPNAAPIFNI
KDLAVDSPLKLFLMGFLTNLLNPKIAIMYLSLLPQFIHPQQGSILAQSIQLGTIQIFV
SVSVNALIVFSAGSIALFLQKKPLWASIQRWVMGTVLAGLAVRILLEHRR" /product="Threonine efflux protein"
/transl_table=11
CDS 1479147..1480082 /locus_tag="AUO97b_01545" /db_xref="SEED:fig|400667.82.peg.1406" /db_xref="GO:0008415"
/db_xref="GO:0009244"
/db_xref="GO:0016021"
/translation="MSQKQPYTPGEFQWSFLLPKYWGVWIAITFLMLLAILPWAIQWR
LAHGLANLAWKYLKSRRKTTIRNLEVCFPEWSPEKVQQQAKQVFVDMMLGIFETLNAW
YKPYWFKNRVTIEGLEHITNAQAQGKGVLLLGTHSTLLDAGGYVCAQYFEPDVVYRPQ
NNPLLDMLIYRCRGTIYKAQIDHDDMRGLIRHLKEGDAIWYSPDQDFGLKQGVMAPFF
GVPAATVTAHRRLLKISKAVAVPLYFYRHGDVQNPKYHILIEPAVDNMPSEDEVDDAT
RVNKIIENQLRIAPTQYMWFHRRFKTRPEGYEEIY"
/product="Lipid A biosynthesis lauroyl acyltransferase (EC 2.3.1.241)"
/transl_table=11
CDS 1480348..1481448
/locus_tag="AUO97b_01546" /db_xref="SEED:fig|400667.82.peg.1407"
/translation="MKVMQLLPELNSGGVERGTLEIARALVAQGHQSLVVSNGGRLVS
QLEAEGSTHLTLPIHKKSLSSLWQIRPLRQLIEEHQPDIVHVRSRVPAWLTHFALRKI
PANKRPHLISTVHGFYSVNRYSAIMTQAEKVIAVSDSVVKYITDHYKNCPPQDIVRIY
RGIDPAAFPHNYQPSAQWFNQVFNDFPELENKFLLCLPGRITRLKGHESLIELMQQLH
SQYPQLHAVIVGGADVKKQAYLSELQNTIQSKGLADKITFVGHRSDIREWLAFSDIVL
SLSNQAETFGRTALEALSVGTPVIGWNRGGVAEILSHVYPQGLVEAENEKALLETVKH
HIEQPQTVAPVTMFSLKDMCDQTLELYQSVLN" /product="Glycosyltransferase"
/transl_table=11
CDS complement(1481511..1482797)
/locus_tag="AUO97b_01547" /db_xref="SEED:fig|400667.82.peg.1408"
/translation="MKNFKFSLAWQILIALILGIVVGAVLHNQPEIKDSIVNNVLTPL
GKIFISLIKMIVIPIVFSTLILGIAGVGSTKSLGRLGFKTILYFEIITTIAILVGLFA
ANIFHPGSGIDMSQLVQTDISQYKHTTEEVQSQSHGIIQTILSLIPTNIISSMAHGEM
LPVIFFAVLFGIGLSSLPATTKDPLLNVFHAVSETMFRVTHIIMKYAPVGVFGLIAVT
VANFGFASLIPLGKLVVLVYGAILFFALVVLGITAKMFSINIFTLFKILKDELILAYS
TASSETVLPRIMQKMEAYGAPKAISSFVIPTGYSFNLDGSTLYQSIAAIFIAQLYGIE
MSISQQVILVVTLMITSKGIAGVPGVSFVVLLATLGSVGIPVEGLAFIAGVDRIMDMA
RTALNVVGNALAVLVISKWEKQYDYEKAAAYEASLK" /product="Proton/glutamate symporter @ Proton/aspartate symporter" /transl_table=11
CDS complement(1483487..1484608)
/locus_tag="AUO97b_01548" /db_xref="SEED:fig|400667.82.peg.1409"
/translation="MIYIEVLAVVVIWLSFWSLIPRDEWWFRGADFPRLQILFIGLIA
LIGMLFWPTEWDVWRELLLAVLIAAIAYQLKMVLPYTLFWKKQVKKVKQHQLDPTKQI
SLIVSNVLTPNDKYHLLLEHVRTLKPDLLLTLESDTTWENALKEIEKDYPYRVPVPLD
NLYGMHLYSRLPLKNTEVKFILSNEIPSIHTTVILRSGMPVQLYCLHPKPPSPTEAKD
STLRDAELLIVGDQIKDLDESCIVMGDLNDVAWSRTTRLFQRISGLLDPRVGRHFINT
FHADYPLLRWSLDHIFHSTDFGLVKMQRLSHIGSDHFPVYVVLQTGRIFEEIHEELEQ

TQADEEEAQAAIQEGIAKAEKEEKIVTDEIAQPYKEKNI" /product="hypothetical protein"
/transl_table=11
CDS 1484798..1485916
/locus_tag="AUO97b_01549" /db_xref="SEED:fig|400667.82.peg.1410"
/db_xref="GO:0004073"
/translation="MKVGLVGWRGMVGSVLMQRMVEENDFAHIEPFYFSTSNAGGEAP
SFGGKTAPALMEATDITSLKQMDVIITCQGGDYTSEVFPKLKATGWDGYWIDAASTLR
MTDDAIIVLDPVNLNVIKDGLAKGTKTFVGGNCTVSLMLMGVGALFQNNLVEWMTAMT
YQAASGAGAQNMRELLTGMGYLYNNTKTLLDDPKSAILDIDRQVAELQRGEGFPSANF
GVPLAGSLIPYIDKQLESGQSKEEWKGQVETNKILGNSQIVPIDGHCVRIGAMRCHSQ
ALTIKLKKDVPLDEIEDMIRNSNQWAKVVPNTREASMTDLTPVAVTGTLTVPVGRLRK
LNMGKEYLGAFTVGDQLLWGAAEPLRRMLRILVEYKSS" /product="Aspartate-semialdehyde
dehydrogenase (EC 1.2.1.11)" /EC_number="1.2.1.11"
/transl_table=11
CDS 1486065..1487429
/locus_tag="AUO97b_01550" /db_xref="SEED:fig|400667.82.peg.1411"
/translation="MTAYNKLKIAIFTIMSSPSLYAITLDPIQIQSAPGELLYAEMNF
QQADPNTPLQVSLATQDDLNGLGVTHQPPGNLNFYIRQNGQGSGVIVITSSRPIIDPE
LNIVVKVSEGNATRLQHIKTVLKPSPIKKAAINESPLSPQFIVNEKDIALNLPESTQY
TPTENTSTVAETTSNEHNLNISTGTAPALNSSSPAPVAPFTEKPSTQTLQPTIATMNT
ESSAVEKQPSTSLKSSDGNQTTTVKKAAQSKTAQQKNVLSKNTPKKQKISAYKGPAPT
GKYVVQRNESLWSIANRIAAQTKQPVAKVMRDIQAQNRHAFIQGDVNRLRQGSSLNLT
TNTTKASHLKPKSSEAHLAKPSSGRAKYRLQQAEMSIVAENSPNSAHGSAKKSTQQSQ
NNTELAVKVMTTRKKTVTLQRNVTKLNQTLSLKDQRIQLLNARLAELQQQLQAQQQTH KQKH"
/product="Tfp pilus assembly protein FimV"
/transl_table=11
CDS 1487489..1488748
/locus_tag="AUO97b_01551" /db_xref="SEED:fig|400667.82.peg.1412"
/translation="MLYVIPFIILLVVAVILKKRENSQKQEATSPKNINRKSGKKASA
KSSKSSREKTKAKVIEENTPAIPQSNPVPEALRHNIQQLIQEKQFSAAEAQVNQALKK
DNTQHELYLLLLEIHIAQKDEFAIQQLISHIRSLGLNEIAAQAETRQKEYESSRQPDA
IDFPQAQTYEEPKNTDTTAQFDELTTSSSEASFDDLQKDYTPVKQEPAVEIEPLEFNF
SFEQTSATENTNQPAQQPELSSTQETNELADLEFSFDLAPLHETEEKSQAVEVKADQE
NSINALDFHLDLNPSSSETKSVEQAPSLDEIKLIEQTPLEATSITPLEFSLDEPALVT
APEIEIQNNEVVNERVTQTQIEDPLLEAFPKLKQINENELDLKLAEQYIKFGANQAAR
NLLQGDEQKFNTEQQQHAKNLLNRIAS"
/product="hypothetical protein"
/transl_table=11
CDS 1488807..1489778 /locus_tag="AUO97b_01552" /db_xref="SEED:fig|
400667.82.peg.1413"
/translation="MKKIALFYMGGTFGCIGEPLAPMPYDQFLPQLEKVIPPHLTVDC
FAAPNIVDSSACTAPDWLRLIQRIQQLQLEGYQHFVVIHGTDTLSYAAATLARFLGQS
CHIVITGSQYPLLNIQGDNTREFTDAIENLYLALEQVIALPVGAYLAFHHQVFHAQTA
LKTHTTELDAFSGLSSNVEFTPQQNELIVQDTQIEKAASFQILNWMMQPIATQYLVQQ
LRHLLPAPPHFLVLQGFGTGNIAVNDEFLATLDELYARGCVPILATQVTFGGIDQRYA
ISAWAKTAKIVINDTHSHADLYAKALQIYLKYPTPEQWLNHWNENLH" /product="L-asparaginase I,
cytoplasmic (EC 3.5.1.1)" /EC_number="3.5.1.1"
/transl_table=11
CDS 1489793..1490590
/locus_tag="AUO97b_01553" /db_xref="SEED:fig|400667.82.peg.1414"
/db_xref="GO:0004730"
/translation="MQRYAVGIEFSGIQYRGWQTQQPGVASVQETIERVLSKIADEPI
TLHGAGRTDAGVHATNMVAHFDTNAIRPERGWIMGANSQLPKDISIQWIKQMDEEFHA
RFKATARRYRYVVYNAPHRPALLHKQVTHIYQKLDVQKMIKAASKFEGTHNFETFRAA

ACQSNQPVRHVKHCRLFEHGRYLVLDIQADGFLHHMVRNIMGCLLEIGQGMYEIDHID
AMFAAEDRKAAGITAPPDGLYFIQCYYPEQFDLPHPPLGPHWLNLPE" /product="tRNA pseudouridine (38-40) synthase (EC 5.4.99.12)" /EC_number="4.2.1.70"
/transl_table=11
CDS complement(1490610..1491635)
/locus_tag="AUO97b_01554" /db_xref="SEED:fig|400667.82.peg.1415"
/translation="MEGHCVGQLTDASVVLRFGYQAIRRAGLPTEEILTKAGVALNQV
DTNARTPLSAQYAFWTAAQEVSKDPDIGLHLGEHLPLYRGQVIEHLFISSETFGEGLK
RALAYQRLISDAFDAKLVVEDGRCYLTNGEQVGADNLVNRHFSECAISGVLRFFKFIT
EGQFHPIFIDFNFSEGASEDEYFRVYGCPVSLGQKETRLYFDPAILDFQLWQAEPELL
QLHEQLAIEKLQELARYDLVGEVRRAIGSTLESGETTLETVAAQLNITPRRLRTQLSE
ANTSFQQILSDYRCRLAKKLLANTNESVERIVYLTGFSEPSTFYRAFKRWTNETPVEY RKRKQHR"
/product="Transcriptional regulator, AraC family" /transl_table=11
CDS 1491884..1492105
/locus_tag="AUO97b_01555" /db_xref="SEED:fig|400667.82.peg.1416"
/db_xref="GO:0003723"
/db_xref="GO:0003743"
/db_xref="GO:0006413"
/translation="MANKEELIEFEGVVTETLPNTMFRVRLENGHEVIAHISGKMRKH
YIRILTGDSVKVEMTPYDLTKGRITYRAR"
/product="Translation initiation factor 1"
/transl_table=11
CDS complement(1492157..1492525)
/locus_tag="AUO97b_01556" /db_xref="SEED:fig|400667.82.peg.1417"
/translation="MKKIVFAFLCFGITTAYADNCDSARNTYDDIYCTNKIYASADAD
LNKNYQALRAKLNTVQRNTLKRSQLAWIRQRDAECTDSNRNSVDVQCRLQTTQERNHW
LQERLRECQTVGCKTSRLSE"
/product="FIG00351133: hypothetical protein" /transl_table=11
CDS 1492736..1493101
/locus_tag="AUO97b_01557" /db_xref="SEED:fig|400667.82.peg.1418"
/translation="MLLNINGCASHPRQSVDLQEYLKNFIGKSSTTIQQDLNLRGLGF
QVARTPQKTSDQLIYTILRPLSIPIPMVSNVDMRGGSVPIQSGNLSGNSYDINFNCKV
IFKLKNDIAESIQYEGKAC"
/product="FIG00350926: hypothetical protein" /transl_table=11
CDS complement(1493155..1494234)
/locus_tag="AUO97b_01558" /db_xref="SEED:fig|400667.82.peg.1419"
/db_xref="GO:0003862"
/translation="MSKQILILAGDGIGPEIVGAAEKVLNKVNEKFNLSLTWEHGLLG
GAAIDAHGEPYPAVTSEQAKKADAILLGAVGGPKWDTIERSIRPERGLLKIRSELNLF
ANLRPAILYPQLADASSLKPEIVAGLDILIVRELTGGIYFGQPRGIRELENGEKQGYN
TDVYSESEIKRIAKVAFELAGLRGGKVCSVDKANVLEVTELWKQTVTDLQQANYPNIQ
LSHMYVDNAAMQLVRAPKQFDVIVTGNLFGDILSDEAAMLTGSIGMLPSASLDENGKG
MYEPCHGSAPDIAGQNVANPLATILSVAMMLRYTFREEAAAKAIEDAVGQVLDQGLRT
ADIMSEGMTKVGTAEMGEAVVAALA"
/product="3-isopropylmalate dehydrogenase (EC 1.1.1.85)"
/EC_number="1.1.1.85"
/transl_table=11
CDS complement(1494266..1494775)
/locus_tag="AUO97b_01559" /db_xref="SEED:fig|400667.82.peg.1420"
/translation="MDTVRIKQAEQENLGQNAAIYCSGAKSCEFERLNDITVVDAQTR
RISNQAIHQGIVRLNGSVLSQSNSLYLSVPAKQYEVVIRYYPISPDRAETIHVIHQFK
ANHRYMFKMYRDKSNRSGSLLNVSVPDPLCVDLEQDGRVIRRFCRPFDVTTGLGEFLE QKKLTPRQS"
/product="hypothetical protein"
/transl_table=11

CDS complement(1494969..1495616)
/locus_tag="AUO97b_01560" /db_xref="SEED:fig|400667.82.peg.1421"
/db_xref="GO:0003861"
/translation="MKAYTVEQGIVAPLDRANVDTDLIIPKQFLKSIKRTGFGDNLFD
ELRYLDEGYPGQDNSVRPKNPDFVLNQPRYQGATVLIARTNFGCGSSREHAPWALNEY
GFRTVIAPSFADIFFNNCFKNGMLPVILPEDIVDQLFKECAAQEGYQLTIDLAAQEVR
TPTGEAFKFEVDPFRKHCLLNGLDDIGLTLQNADAIRAYEEKTKQVRPWVFQEIN" /product="3-isopropylmalate dehydratase small subunit (EC 4.2.1.33)"
/EC_number="4.2.1.33"
/transl_table=11
CDS complement(1495635..1497053)
/locus_tag="AUO97b_01561" /db_xref="SEED:fig|400667.82.peg.1422"
/db_xref="GO:0003861"
/translation="MAGKTLYDKLWDDHVVTQRDDGSCLLYIDRHLLHEVTSPQAFEG
LQLAGRQPWRLSANVATPDHNVPTSKKERDQGIAGIEDDTSRIQVQTLDDNCKAFNIV
EFGINDIRQGIVHVVGPEQGLTLPGMTVVCGDSHTATHGAFGCLAHGIGTSEVEHVLA
TQCLVQKKSKNMLVRVDGVLGKGVTPKDVVLAIIGKIGTAGGTGYAIEFGGQVFRDMS
IEGRMTVCNMAIEAGARVGMVAVDEKTIEYVKGRSYAPKGEQWDQAVAYWNTLHSDDD
AVFDAVVELNGTEIEPQVSWGTSPEMVIPVSKAVPTLEQAKDDVQRNDWTRAYQYMGL
NAGQALADIQLDRVFIGSCTNSRIEDIRAAAEVVKGRKVAPSIKQAMIVPGSGLVKQQ
AEKEGLDKIFLEAGFEWREPGCSMCLAMNADKLQPGEHCASTSNRNFEGRQGNGGRTH
LVSPAMAAAAAIAGHFVDVRSF"
/product="3-isopropylmalate dehydratase large subunit (EC 4.2.1.33)"
/EC_number="4.2.1.33"
/transl_table=11
CDS 1497291..1497545 /locus_tag="AUO97b_01562" /db_xref="SEED:fig|400667.82.peg.1423"
/translation="MVMANIQKFVVNNQRTLKKTLSYYIMHITVAMLVAYFVTGNIWM
ALTLSMLEPTVQAFAFFFHEKVWAAKDRRISALSENETTA" /product="Uncharacterized membrane protein SO_4740" /transl_table=11
CDS 1497663..1498523
/locus_tag="AUO97b_01563" /db_xref="SEED:fig|400667.82.peg.1424"
/translation="MNLAAFEAFVKVMETGSISVAADQLFITQPAVTKRIHSLEEYFG
VKLFESAGRGVQATHAAHSLLPKVKSWLNELRDIHHTLSHEQTQIQGRLKIGTSHHIG
LHHLPAPLKHFVQQFPQVTLDVHFVDSEQAHEQVLAGDLELAFLTLPPSGDERLNYIT
IWNDPLVFVAAPFHPLAQKKNLTLQDLIEYPSLLPAAQTYTAQITLAEFEKQGLKPKI
TMSNNPLESIRMLVSIGLGWSVLPKTLLNQDMQQLDLNVEMNRQLGMVWHPARTQSRA MQELIKMMQE"
/product="Transcriptional regulator, LysR family" /transl_table=11
CDS complement(1498528..1499178)
/locus_tag="AUO97b_01564" /db_xref="SEED:fig|400667.82.peg.1425"
/db_xref="GO:0004647"
/translation="MKLALFDLDHTLLNTDSDHSWGEFLVNEGLVDPVHHRQMNDKFY
EDYKAGQLDPYAYNEFVFGFLTKHDNNYLTELHQLFMEKVIRPQMRPKGFDAIKKHQD
LGHTIVGITATSDFITAPIFREFGITEILATNAEVADGKYTGKVAGLACYQKGKLARL
EAWLDGRSVSESWAYSDSINDRFLLEYATHAIAVNPDDRLEKLAQEHNWEIQDWSI"
/product="Phosphoserine phosphatase (EC 3.1.3.3)" /EC_number="3.1.3.3"
/transl_table=11
CDS 1499361..1499846
/locus_tag="AUO97b_01565" /db_xref="SEED:fig|400667.82.peg.1426"
/translation="MIDSEGFRPNVGIILANDDGQVLWAKRIGHNAWQFPQGGIQFGE
TPEQALFRELREEIGLLPEHVQIIAQTKGWLRYRLPHRYIRSDSDPVCIGQKQKWFLL
KLTAPAKNIQLNLADPPEFDEWQWVSYWYPLGQVVNFKRDVYRKAMVELCTQLPVQQL P"
/product="Adenosine (5')-pentaphospho-(5'')-adenosine pyrophosphohydrolase"
/transl_table=11

CDS 1499932..1502226
/locus_tag="AUO97b_01566" /db_xref="SEED:fig|400667.82.peg.1427"
/translation="MSNMQLDTLRRIVQEINASVSLHESLDIMVNQVAEAMKVDVCSI
YLLDERNQRYVLMASKGLNPESVGHVSLQLGEGLVGLVGQREEIVNLDNAPKHERFLY
LPETGEEIYNSFLGVPVMYRRKVMGVLVVQNRLPQDFSEAAESFLVTLCAQLSGVIAH
AHAVGNIDVFRKPSNGPAYKTFQGVSGAGGVALGRAIILYPPADLGSVPDREAEDISD
ELRILDQAISSVRSEIRSLDEKMHDSLMAEERALFSVFLRMLDENALPAEIKELIRDG
HWAQGAVRRVIEKHTALFAQMEDDYLRERVSDLKDLGRRILAYLQEEDSSHRELSPDS
ILIGEEISTAALVELPVDNIAAIVTSEGAANSHMVIVARALGIPTVVGVTELPVNTLD
DAEMIVDAYQGRVFVNPPRRLRQRYKEIQKEDEQIAKDLKQYETKEAITPDGVSVQLF
VNTGLMIDVVRGVQRGAQGVGLYRSEIPFMLRERFPGEEEQRAIYRQQLSHFANKPVV
MRTLDIGADKDLPYFSIEEENSALGWRGIRFTLDHPEIFSAQIRAMLKASIGLNNLHI
LLPMVTTVSEVEEVLYLLERDWIAVQEEEQVKITKPKIGIMVEVPSVLLQIDEFAELV
DFFSVGSNDLTQYLLAVDRNNPHVANVYSHFHPSILRALTRLVKECHKYQKPVSICGE
MAGDPLSAILLMAMGFNTLSMSSSNILRVRKAICHVPMSDAQKLLDDVMKMNNPLIVK
SWLEYYFKTHGLADMVKSNRLVSV"
/product="FIG001592: Phosphocarrier protein kinase/phosphorylase, nitrogen regulation associated" /transl_table=11
CDS 1502742..1504898
/locus_tag="AUO97b_01567" /db_xref="SEED:fig|400667.82.peg.1428"
/db_xref="GO:0004096"
/db_xref="GO:0004601"
/translation="MSNESKCPFSGHNSKPQVTVGGGTANLHWWPNQLRVDLLNQHSE
RSNPLGKDFNYRQEFKKLDYYALKADIKNVLTDSQDWWPADWGNYTGLFIRLAWHAAG
TYRMGDGRGGAGRGQQRFAPLNSWPDNASLDKARRLLWPVKQKYGQKISWADLFILAG
NIALESSGFRTFGFGAGREDVWEPDNDVNWGDEKEWLAHRNSEALAGSNLAATEMGLI
YVNPEGPQASGDPRSAAPFIRATFGNMAMDDEEIVALIAGGHTLGKTHGAAPADHVQA
DPEGAPIEQMGFGWANSYGTGVGKDAITSGLEVIWSQTPTQWSNYFFENLFKYEWVQE
RSPAGAIQWVAADAEAIIPDPFDPSIKRKPTMLTTDLTLRFDPEFEKISRRFLNDPQA
FANAFARAWFKLTHRDMGPKARYLGPEVPAEDLIWQDPLPAASATPSSASIADAKAKI
VALGLSAGELVSLAWASASTFRGGDKRGGANGAHIALSPQREWEVNKKAVETLTQIEE
LKASTQLSLADLIVLAGNVGVEQAAQAAGFNITVPFAPGRVDALQSQTDVESFQLLLG
LADGFRNWKKQGVNTPAEVLLIDKAQQLTLTAPELTALIGGLRVLGTNWDGSQHGVFT
QQVGVLSTDFFTNLLDMSNVWAPVDSTSEVFEGKDRKSGTVKFTATRNDLVFGSNSIL
RALAEVYAQADGKEKFVQDFVAAWTKVMNLDRFDLA" /product="Catalase-peroxidase KatG (EC 1.11.1.21)" /EC_number="1.11.1.6"
/EC_number="1.11.1.7" /transl_table=11
CDS complement(1504965..1506017)
/locus_tag="AUO97b_01568" /db_xref="SEED:fig|400667.82.peg.1429"
/translation="MKDKPTLTYQLPTQSCWTHTWIKPPFCHPELGHENDTRFYNQQP
RSPARGSKELLRWLVTRESYTWNVDIQNEYDVRVNTPIALPQNRPHADLDDWQVWFVG
HATVLIQIGPYNFITDPVWCDYASPLHGRGPRRVCPAGFALEQLPTIHGVLLSHNHYD
HMDLATLEWLHKKFAMPIYTGLGNGFYLPKEFHVIEMDWWQEIPYHELRIVYTPAQHG
SGRGLRDQNKALWGGFSVLAKTGHCFFAGDTGYAGHFKQIHTRYGAPRVALLPIGAYE
PRKLMSYVHMNPQDAVHAHLDLQAHRSVAIHYRTFQLTDEGREDPEHALAQAIKASSK LANPFYCIREGHKLTV"
/product="Outer membrane protein romA"
/transl_table=11
CDS 1506754..1507653
/locus_tag="AUO97b_01569" /db_xref="SEED:fig|400667.82.peg.1430"
/translation="MELRHLRYFITVAEELNFSKAALKLYTAQPSLSQQIKDLEEDVG
VRLLNRTKRKVELTEEGEVFLEHARLTLAQADKAVAMARQVSKAKQQLLRIGFVPIAE
MKIFPYVLPNLRLQHPDLTIELSSLNNAEQLKALKRGELDITFTRDNTENDEIQSQFV
LTEPLIFLLPRNHPLAKYDRIPVKALHGIDFIIPAAEQSNTLHNTILEFTKTHGIDLN
IVQKADSILLNINSIGSGLGCTILPAYVAPLGVSNTVVRPLDVELPSLDLFVSYRKNT

ESVGVKRFIDQLNKVFHLDKNLD"
/product="LysR family transcriptional regulator STM3121" /transl_table=11
CDS 1507917..1508594
/locus_tag="AUO97b_01570" /db_xref="SEED:fig|400667.82.peg.1431"
/translation="MVGLFQHLGRSVQNRPHFFIALIFGVVVASLLAWLTSWKWSTIL
LASWNGSISLYLLHVWKLMRSADHSQMQQQAKKQDESKWVIMLIVLLAITMSLVAILV
QLSQLPSGHYEKLGHVALALLTIISAWLFMHTVFALHYAHDFYMALSRNEENGGLDFP
GTEHPTYPDFLYFSYIIGTSAQTADVSITNKHMRLLNLFHAVLSFGFNTTILAICINV AAGFLTN"
/product="Putative transmembrane protein"
/transl_table=11
CDS complement(1508654..1509259)
/locus_tag="AUO97b_01571" /db_xref="SEED:fig|400667.82.peg.1432"
/db_xref="GO:0004364"
/translation="MSLKLYTNNESRGVVIDWLLVELGVECERIEVAYRTEMKSPEYL
KLNPFGKVPVLVDGDVVIYELGAICAYLADKFSDKGLAPALDDPKRGLYYRWLFMMAG
PWEAAGVDKALGIEIAPEQKMFVGYGDYNDAYQALVQGLSEANPYVCGEQFTAADVSV
GAMLLWQLKIKTIESHPAITRYVETIKQREGLKQSSMGQLL" /product="Glutathione S-
transferase (EC 2.5.1.18)" /EC_number="2.5.1.18"
/transl_table=11
CDS complement(1509309..1510004)
/locus_tag="AUO97b_01572" /db_xref="SEED:fig|400667.82.peg.1433"
/translation="MSRSIRLLNLLQQLRESRYPVTAQVLAERLGISIRSIYRDIDSL
RAQGVRIDASAGLGFQLKEDILLPPVTFNENEAEALFLALEWLKDVPDQAMKSAAVSI
YSKLYAVLPEHRKYLIDDMTLGVTHYWHPVDEALVEQVRLAIRKQVKIKVDYCDEHKR
ISQRSLWPFALGYLHDKVVLAAWCELRQAFRHFRIDRIHDLQMSEEIYPEFKKHLFQR WWKQEMQRTTDKN"
/product="Transcriptional regulator, DeoR family" /transl_table=11
CDS complement(1510078..1510752)
/locus_tag="AUO97b_01573" /db_xref="SEED:fig|400667.82.peg.1434"
/translation="MSLKTELVIFDWDGTLYNSVGQIVASLQHAAEEHKLTLTDEAAK
SIIGLGLPEVMQTLFPEVPDLHDSILKAYGDHYIANSTNDAWFEGISELLHDLKAQGL
KLAVATGKNRRGLDRVIAKTQSTHLFDVTRAANETRSKPDPLMLQEILTVTGVSVEQA
VMIGDSSYDLEMAQRLGMPRIGVGYGVHSVEVLQQFQPLTIAKDVPELHNFLREYAKL STVDVA"
/product="Similar to phosphoglycolate phosphatase, clustered with ribosomal large subunit pseudouridine synthase C" /transl_table=11
CDS complement(1510749..1511681)
/locus_tag="AUO97b_01574" /db_xref="SEED:fig|400667.82.peg.1435"
/translation="MNSTQQWQSVTWFEVDEHQAGQRIDNFLFSRLKGVPKSRIYRLI
REGQVRVNKKRIKAETKLAIGDQIRVAPIRYEQKDETAVPVSDSVAQGLLSRVVYEDE
GLLVVNKPSGIAVHGGSGVAYGLIEALRAATGKKYLELIHRIDRDTSGLVMISKKRST
LKLLQDMLREHKIRKTYAAIVKGQVSLDKQLIDAPLFRYELANGERRVRVSKEGKPSK
TEWVVAERFKNATLVHASPLSGRTHQIRVHGLSIGHPLVGDDKYGHNTAYTGPEARRL
CLHAMRLDIPGYPTIEAPLPEDMTQLLEALRVAK"
/product="LSU rRNA pseudouridine(955/2504/2580) synthase (EC 5.4.99.24)"
/EC_number="4.2.1.70"
/transl_table=11 CDS 1512101..1512217
/locus_tag="AUO97b_01575" /db_xref="SEED:fig|400667.82.peg.1436"
/translation="MSIPYQTSNKEVTSRPMTVKVINQCTAHPPVKQKGIPI" /product="FIG00352464: hypothetical protein" /transl_table=11
CDS 1512404..1515736
/locus_tag="AUO97b_01576" /db_xref="SEED:fig|400667.82.peg.1437"
/translation="MKRMLINATHAEEVRVALITGNRLYDFDLENRTREQKKSNIYKG
HVTRVEPSLEAVFVEYGAGRQGFLSMREIANSYFQADPRQTSNIRELITEGTELLVQV
EKEERGNKGAALSTFISLAGRYLVLMPNNPKGGGISRQISGSVREELKEILASLNVPR
GMSVIVRTAGIGRTQEELQLDLQHLLDLWAQIQGTASSGPSPMLVHQEAGVVTRAIRD

YLRDDVAEILIDSEQAYNEAYNFVKAVMPRQLDKLKTYTLNEPLFAHFGIESQIQTAY
EREVKLPSGGSIVIDQTEALVSIDINSAKSTRGHDVEETALNTNLEAAEEIARQLRLR
DIGGLVVIDFIDMTKERNQRMVEAKLREATQSDRARIQFGQLSRFGLMEMSRQRLRPS
LEEATGYVCPRCHGTGMVRDLRSLSLSIMRKVEEIALRERHGEVQVEVPVEIAAFLLN
EKRHSLVYLEQTSGVRVTVLPHPHLETPHYEIAYNPDGFAPSSYERTEATRSSEKELG
YESSEWHLEEADHGHAHVTATASTHAAAQKKANHATQPVAQPSAQKAASPCAWLENLF
VQKQAQTVDQSRSAQNAAAAIEQMVNTGAVSRGQFGQVAVPAVAEVAPVQSNNAYISQ
SPVKQDVREHVEKDDKSQQQRQNNKKRKHKEQREQHHQSHEQQHQVHEEVVQLSRQEQ
RELKRQQKRQQQQDQQHQNNDVQHTENAVPRRDRNNQQRPNRPNRHRDPSVLNENQNT
PVVVDEKQIKVDVIDAPKHDVMNTALIINVDQGQSEIVALTPERRHVERVETTSTEVA
QEPTPAPVVAEKAAVVETKEEAQPSQEAAQPQIKRASNDPRMRRRQQREAKHAKAATP
SIAPSQIPTLAQHTIGSLIRHVYGEDCTVLIEQFGLVPTFNRALQKFAEQYASTLVVE
VTAETEEKKPVTRDAELPSHKPAEEAEPAPVLPLTPPQAPAPRVANDPRERRRLAKLA
AEQAFEQVKQQHSAQEEVATPAPVAEETVAAPTAETQATVEPAQQPLELNQSTEVVQP
EAAPAEEKATEETVAEAPAAKEPAPSKAASKAKAAAEETVAPAEATTDTESEDVKADK
DKPSRPRRPRGRPPKKANPVAE"
/product="Ribonuclease E (EC 3.1.26.12)" /EC_number="3.1.26.12"
/transl_table=11
CDS 1515929..1517770
/locus_tag="AUO97b_01577" /db_xref="SEED:fig|400667.82.peg.1438"
/db_xref="GO:0030729"
/db_xref="GO:0004467"
/translation="MSQTTQTDIITLGDVATRLPSFIPKVPHILNGLKQAYLRTSNTP
TGLGIAFEKAVKRNPKGTALLFEDQSYSYEALNEWANQISHYYLSLGARKGDVIAVMI
ENRSELIATIVGLAKIGVTIALVNTSQVGKVLAHSINLVKPIAVIAGEEVRAAIDEIR
QDLNVPKDRFHWFADQATRQNSGTAPEGYVNLADQIDQFPKFNPSTTRSVTGNDGLFY
IYTSGTTGLPKAVIFKHSRWTLAYGTYGHILNLGPDDVMYVTLPLYHATGVVVCWCGV
IAGSATLAIRRKYSTSAFWKDVQKFNASAIGYVGELCRYLMDAPVTELDRNHRVTKMI
GNGMRPNIWDKFKQRFGVKEVLELYASSEGNVGFSNIFNFDNTVGFSPTPYAIIQFDK
EKNEPIRDKNGWCQKVKAGEVGLLIGKITSRSPFDGYTDPEKNKSVIWKDVFKKGDSY
FNTGDLVRDIGFRHAQFVDRLGDTFRWKGENVSTTEVENMVCEYDKIAEAVVYGVEIP
NTNGRAGMAAITLADGEELNDADLTEMVTVFKKCLPTYAVPVFLRVQKKVETTGTFKY
QKNKLKEEAFNPSKTSERLLALLPGASCYCDITTEIFDNIQAYKYRF" /product="Citronellyl-CoA synthetase"
/EC_number="6.2.1.3"
/EC_number="6.2.1.16"
/transl_table=11
CDS complement(1518239..1519135)
/locus_tag="AUO97b_01578" /db_xref="SEED:fig|400667.82.peg.1439"
/translation="MDRLQQFYIFTEVAKRQSFSEVANQLDLPRSTVTSAIQQLETHY
GVRLFHRTTRKVSLTQDGQRILPECQNLLFDYEQLEQLIQTQKQHYRGTLKISMPSRI
VHQVIIPELPDFYHRYPDIHLQLNSSDDLTDLIEKGIDCVVRVGELDNSSLIARFIGH
LVMVNCASTQYLQDYGIPEQLNDLEQHKLINYAGAVGEKQGEFIYQGGTVMMDSALSV
NNTEAYIAAACAGLGIIQLPYYDVQDKIEQGILVEVLSAYSAPSLPLNILYPNRSYIP
KRLEVFMNWVGEVLNRKCIVIF"
/product="Transcriptional regulator, LysR family" /transl_table=11
CDS 1519248..1520003
/locus_tag="AUO97b_01579" /db_xref="SEED:fig|400667.82.peg.1440"
/translation="MTTHSLKDKVVLITGGAKNLGGLISRKFAEQGAKLAIHYNSSET
QADAEQTLADVKALGADAILIQADLTDIDNIEKLFLDTKQHFGGIDIAINTVGKVLKK
TFLETTEQEFDSMSDINSKIAYFFIQSAGRHLKNGGKICSIVTSLLAAYTGFYSTYEG
LKAPVEHYTRAASKEFGDRQISVTAVAPGPMDTPFFYGQESAEAVAYHKSASALGGLT
KIEDIEPLVRFLVTDGWWITGQTIFANGGYTTR" /product="Oxidoreductase, short-chain dehydrogenase/reductase family" /transl_table=11

CDS complement(1520055..1521209) /locus_tag="AUO97b_01580"
/db_xref="SEED:fig|400667.82.peg.1441"
/translation="MAVLNPASVPLSLYIHMPWCVRKCPYCDFNSHAVPDGALSAELE
QTYLKALVADFETQVEMAQGRSIHSVFIGGGTPSLISAKGYVWLFEQLKSLLNFEDDC
EITLEANPGTLEHDPFAGYLEAGINRLSIGVQTFNTDHLQKLGRIHSANNAMDAIEQA
RRAGFKRVNVDLMHGLPEQTLEQALYDLKTAVEHGATHISWYQLTIEPNTVFFRTQPV
LPQDEVLEDIQEQGEAYLKANGFINYEVSAWRKEQPSVHNLNYWQFGDYLAIGAGAHG
KVTRPDGVYRFQKTRLPKDYLAKVPAEHLQMKKIEADELPFEFMMNALRLNDGVKAEL
YEQRTGLSLDDLNDLLTSLRSRELLVEDSGRLACTEQGHIFLNSVLEEFL" /product="Oxygen-
independent coproporphyrinogen-III oxidase-like protein YggW"
/transl_table=11
CDS 1521330..1521788
/locus_tag="AUO97b_01581" /db_xref="SEED:fig|400667.82.peg.1442"
/translation="MKFSSQLFLILPLAMGIGVAMAFQTAINAQLREQLHSPLQAALL
SFFIGTIVLAIMVFFQSAEKPSLSEISNIPWLLWTGGFLGVYAISMSIYTAPKLGFLT
FTGLVVFGQLVISMLLDHFGWLGTEKTPVTWQRFLGGVVIFVGVLLTLQR" /product="Integral
membrane protein"
/transl_table=11
CDS 1521873..1523168
/locus_tag="AUO97b_01582" /db_xref="SEED:fig|400667.82.peg.1443"
/translation="MLPILITQFAQAGFGLIDTIMAGHLSAADLAAIAVGVGLWIPVM
LLFSGIMIATTPLVAEAKGARNTEQIPVIVRQSLWVAVILGVLAMLILQLMPFFLHVF
GVPESLQPKASLFLHAIGLGMPAVTMYAALRGYSEALGHPRPVTVISLLALVVLIPLN
MIFMYGLGPIPALGSAGCGFATSILQWLMLITLAGYIYKASAYRNTSIFSRFDKINLT
WVKRILQLGLPIGLAVFFEVSIFSTGALVLSPLGEVFIAAHQVAISVTSVLFMIPLSL
AIALTIRVGTYYGEKNWASMYQVQKIGLSTAVFFALLTMSFIALGREQIVSVYTQDIN
VVPVAMYLLWFAMAYQLMDALQVSAAGCLRGMQDTQAPMWITLMAYWVIAFPIGLYLA
RYTDWGVAGVWLGLIIGLSIACVLLLSRLYLNTKRLSQT" /product="Multidrug efflux
transporter MdtK/NorM (MATE family)" /transl_table=11
CDS complement(1523203..1524453)
/locus_tag="AUO97b_01583" /db_xref="SEED:fig|400667.82.peg.1444"
/translation="MTTAQIELEELSVGADYENVANRFRPIFEKIAQGAIQREKERIL
PFEPIQWLKEAKLGAVRIPRKYGGDGVSLPQLFQLLAELAEADSNIVQALRGHFAFVE
DRLVAHKEHSQEVWFQRFVQGDLVGNAWTEIGNVQIGDVVTRVTKDASGNLVVNGEKY
YSTGSIFADWIDLFAYDEVNDRHVIAAIYRHETGVSVIDDWDGFGQKTTGSGTLKVHQ
VHLPASHLIPFDQRFKYQTAFYQVVHLATLTGIARAAVETFSQEIRERKRIFSHGNGD
LVRHDPQVLQVVGKASAQAYASEAITLKTAEALQKAYESHFAESEVKEHQFNVDAELE
SAQGQVVISNLVLDLTSQLFNALGASASSQVKQLDRFWRNARTVSSHNPLIYKEKVIG
DWEVNRTDLPFVWQIGASPRAKTA"
/product="Acyl-CoA dehydrogenase; probable dibenzothiophene desulfurization
enzyme" /transl_table=11
CDS complement(1524486..1525733)
/locus_tag="AUO97b_01584" /db_xref="SEED:fig|400667.82.peg.1445"
/translation="MTSLNSSYGRSQAVPLSVFEQSKDAHIIRSDEEAIEIAKQLAQE
FAQEASDRDRERRLPLNEIQQFSQSGLWAITVPKQFGGAQVKYRTLAEVIKTIASVDP
SLAQLPQNHLAFIEHLRLDAKPEQQQFFFNLVLRGIRFGNAFSEKHSKTVADLTTTLR
KTEHGYEINGKKFFATGALLAHWIPIVAVNELGQPYAALVPRETSGVNIINDWSGFGQ
RTTASGTVELNQVPVREEYLVPIYQAFERPTPAGAISQFIQAAVDAGIARGAIEETIQ
YVKNHSRAWIDSGLEKASDDPYTIANIGDLKIKLLAAEAVLDLAGDAIDQAIANTTEE
HVNQATLLVAEAKVLTTEIAILAANKLFELSGTRSTLSELNLDRHWRNARTHTLHDPV
RWKYHIVGNYYLNGVQPPRHAWS"
/product="Acyl-CoA dehydrogenase; probable dibenzothiophene desulfurization
enzyme" /transl_table=11
CDS 1525991..1527289 /locus_tag="AUO97b_01585" /db_xref="SEED:fig|

400667.82.peg.1446"
/translation="MAKNASSPGKRFMKLAGMTASIATKTVSNSIRNLTADEEQKLAA
KTKLFQDIGLQIADTLGEMKGAVMKVGQIASQYKDIFPPEVAKAISKLQRQAPAMPFA
AIQQQVERELGKPLNVAFKSFEQEPFAAASIGQVHKAVLPSGEQVVVKVQYPGVDEAC
ESDLKQVRLALRLMGVLKIDKKLQDQLFAEIQDSLSAELNYEIEAQNLEVFKTFHSKL
DDKIIIPTVYKDYSSRRILTLSLEQGDSIETASSWPIEIRNTIGRRLIRALGQEMFFL
KRFHCDPHPGNFAFRQDGSVIIYDYGSVKTLSNEIVYSFKRLVNAARHEDIDLIETEL
LELHSLAEKGKFPSDLYKLWIEVLLRPLTTTYDFAENSSHHDGMLLVKKSLKYWDVFK
PSPDTLMVNRTISGHYWNLIHLKVHDNLNDLFEELVPLSN" /product="Uncharacterized ABC1 family protein A1S_0392" /transl_table=11
CDS 1527391..1527645
/locus_tag="AUO97b_01586" /db_xref="SEED:fig|400667.82.peg.1447"
/db_xref="GO:0003735"
/db_xref="GO:0005622"
/db_xref="GO:0005840"
/db_xref="GO:0006412"
/translation="MRKDIHPAYQQVLFHDTNADVYFLIGSTIQTKQTKEYQGQVYPY
VTLDISSASHPFYTGEVRQASNEGRVASFNKRFARFNRKS"
/product="LSU ribosomal protein L31p @ LSU ribosomal protein L31p, zinc-independent" /transl_table=11
CDS 1527898..1528230
/locus_tag="AUO97b_01587" /db_xref="SEED:fig|400667.82.peg.1448"
/translation="MAQELLAQLQAGTAKFSDVLAYIEARYQHTPTAFQNGAQHNAAT
ENQGSAKVFSFAKLQGLDQVQTLSLFAEHYASVLATPEGTDHQNIRQFMQNGWDGVKF EGQALTEK"
/product="Type III effector HopPmaJ"
/transl_table=11
CDS complement(1528733..1529113)
/locus_tag="AUO97b_01588" /db_xref="SEED:fig|400667.82.peg.1449"
/translation="MYYPLLSIALGSVLGAWLRWFLGLKLNPIYPQIPLGTVTVNLVG
GFIIGFAMAYFAHSDLSPNYKLFVITGFCGALTTFSTFSIEIVTLLQSGKWGMAMLAI
SIHLIGSLIFTCLGLAAYYWVAGH"
/product="Fluoride ion transporter CrcB"
/transl_table=11
CDS complement(1529349..1529759)
/locus_tag="AUO97b_01589" /db_xref="SEED:fig|400667.82.peg.1450"
/translation="MSQASPVYQGSCLCQGIRYEIKGDIGDIIQCHCQRCRKSNGTAF
ATNAPINSADFKITQGEDLIKKFAVNGVYRWFCSECGSPLISSRDAQPELYRLRIGTL
DTPLDQKPTMHIFAASKAEWECIHDDLPQYDERP"
/product="Gfa-like protein"
/transl_table=11
CDS complement(1529837..1531042)
/locus_tag="AUO97b_01590" /db_xref="SEED:fig|400667.82.peg.1451"
/translation="MSNKRIAIIGAGPAGLMAAEVLSQYAYEIDVFEQKPSAARKFLM
AGKTGLNISHAEPLPQFIERYDHTEWLKPWVEKWDAVWIQKWMKSLGIESYVGTSGRI
FPVEMKAAPLLRAWLKRLAEQQVKFFYRHRCINLQGTTLTLENQNKQNTETFTQQYDA
VVLACGAVSWSQLGSDGAWQQWMDQSSIEPFQASNAGVLHTWSAFMESCFGEPLKRIN
AWVEPSQVTHGDIVITHYGLESGVIYKLGRALREQQKQGKSLNLFLDLLPDVELTQLV
KKLQASKKQSLTNIWRKAGLDTAKINLIREVVDKALWSQPETMAQKMKALQIKLDGFR
PIEEAISCAGGVKQDALTESLELKQTSGVFCCGEMLDWDAPTGGYLLTACFATGRAAG EGVHSFLEK"
/product="NAD(FAD)-utilizing dehydrogenases" /transl_table=11
CDS complement(1531032..1531664)
/locus_tag="AUO97b_01591" /db_xref="SEED:fig|400667.82.peg.1452"
/translation="MQIRQFQAQRMQAPRDFTPIENTPICVEIGAGKGKHALLFSGQN
PQHTLYAIERTREKFLAMQKQHGLEPRDNLIPVHADALPWVVHALYPAQVEHFFILYP

NPEPHNPAQRWLNMPFFEFLLSRLKTGGTITLASNIPEYIEEAEQQLLNVWKLPYQKE
KIAQTSARTHFEIKYLERGELCQQLIMTKPEGYNTRFDEFAPLQGQIHVE" /product="Uncharacterized methyltransferase Pcryo_1403" /transl_table=11
CDS 1531758..1531952
/locus_tag="AUO97b_01592" /db_xref="SEED:fig|400667.82.peg.1453"
/translation="MPRTFPCPRCGEPSVWEGNEFRPFCSERCKLIDLGAWANDEYRL
PTQDAPQQDKGSQHEDDYED"
/product="DNA gyrase inhibitor YacG"
/transl_table=11
CDS complement(1531949..1532593)
/locus_tag="AUO97b_01593" /db_xref="SEED:fig|400667.82.peg.1454"
/translation="MLQVKIVPVTAFAQNCSLVWDSETKEAVLIDAGGDAAVLKKEVE
ALGLKVKALWLTHGHLDHAGAVGELAKEWSVPVVGPHKEDQFWLDMIQEVSARYGFPI
PQPVKVDQWLEGGEVLKLGEDEFEVRFAPGHTPGHVMFYNKNHGLLWTGDVLFKGSIG
RTDFPRGNHEQLIESIQRECFSLPDETQFISGHGPMSTIGYEKQFNPFVAGKAG"
/product="Hypothetical metal-binding enzyme, YcbL homolog" /transl_table=11
CDS complement(1532617..1533633)
/locus_tag="AUO97b_01594" /db_xref="SEED:fig|400667.82.peg.1455"
/db_xref="GO:0004325"
/translation="MSFEQKPKVTVILANLGTPDEATVPAVRRFLKQFLSDPRVIEIP
KFIWWIILNLFVLPFRPKRVAHAYASVWSTDSPMREIVFEQTQRVQAYLERENKQFDL
TVLPAMTYGNPGIDAVLEKLSAHPQEHVILLPLFPQYSATSTAPLYDAFAKWIPTQRN
LPGLTIIKDYYQHPMFIQALAESVLAYQEQHGKPEKLLMSFHGIPQPYADKGDPYADR
CRITAKLVAEALHLKDVEWAISFQSRFGKQEWVKPYTDQLLQDWAKQGVKSVQVLSPA
FSADCLETLEELAIQNAELFQQAGGGSYAYIPALNSDQAHIDLLAGLVQANLDALTHT LAHR"
/product="Ferrochelatase, protoheme ferro-lyase (EC 4.99.1.1)"
/EC_number="4.99.1.1"
/transl_table=11
CDS complement(1533707..1534711)
/locus_tag="AUO97b_01595" /db_xref="SEED:fig|400667.82.peg.1456"
/translation="MLDTRYQIFISTSGREMQPERMVLSQTLVNMGFFAWGLEYRNPL
TTTLARRQIDESDYVVLLLGSQYGEQSISGASYFSLEYEYALSRAKPIVVFMHEQPES
RDMHLQETHPQLKEKFLAFRKKLLHEANHIFYFKSPRDLELAVRLNMPLMVEQYMGQG
WVPARQAHHLEDEINRLKSKVLQLEQQLSESSIQANEVAPQDVFAFEYQIQAFQDGNF
KELKRQRQMTWSQLLSILAKHFETAMPEENFGTCLNEYLNQDGLEDARQELPRAHAVA
GAQINRKALFRIKKQMQSQGWIVPTKIDQQHSLWKVTTKAQKLLLSYKWNHRSTRLKA "
/product="hypothetical protein"
/transl_table=11
CDS complement(1534832..1535698)
/locus_tag="AUO97b_01596" /db_xref="SEED:fig|400667.82.peg.1457"
/db_xref="GO:0008881"
/translation="MTAIQPLFTELEPMPKALADAPIGIFDSGIGGMSVAAEIAKYLP
NERIVYYADTAYVPYGPRSDEEIRELTARAVDWLYRQGCKIAVVACNTASAFSLDHLR
EHYGEHFPIVGLVPALKPAVLQTRSKVVAVLATPATFRGQLIKDVVEKFAVPAGVKVM
TLTSLELVPCVEAGQQMSPVCLNALREVLQPAVEQGADYLVLGCTHYPFLNEAIHHLF
DNQFTLVDSGLAVARQTARILIKNELLCDQIRQNVARIECYVSGNNADALQPVLQNMI PQELTWTLHNLS"
/product="Glutamate racemase (EC 5.1.1.3)" /EC_number="5.1.1.3"
/transl_table=11
CDS complement(1535747..1536358)
/locus_tag="AUO97b_01597" /db_xref="SEED:fig|400667.82.peg.1458"
/translation="MVNQNNSPSDMGKIPLSDDKNLMNIAQYLKGVQQSHKRSILPLE
QWHPKHCGKMDLTVKANGEWWHEGQLIKRQALLDLFTKVLWKEEGKFYLKTPVEQIEI
EVEDEPLLVNQVDQIEISGDEFLQLTTTNQDVILVDAEHPIFMREYAGELRPYVHVRF
GINALIQRQAFYHLVNYGTLVENDKGETILQLKSGNLHLQLGT" /product="hypothetical protein"

/transl_table=11
CDS 1536545..1538257
/locus_tag="AUO97b_01598" /db_xref="SEED:fig|400667.82.peg.1459"
/translation="MEHIERESMEFDVVIVGAGPAGLSAAIKIRQLAIENNLPDLSVC
VVEKGSEVGAHILSGAVLEPRAINELFPNWKEEGAPLNVPVTEDKTFFLLSETTSKEA
PHWMVPKTMHNDGNYVISLGNVVRWLGQKAEELEVSIFPGFAASEVLYHEDGTVKGIQ
TGDMGIGKDGEPTHNFTPGYELHAKYTLFAEGCRGHLGKRLIAKYNLDKDADPQHYGI
GIKELWEIDPAKHKPGLVMHGAGWPLSETGSSGGWWLYHAENNQVTLGMIVDLSYENP
HMYPFMEMQRWKTHPLIKQYLEGGKRISYGARAVVKGGFNSLPKLTFPGGCLIGDDAG
FLNFAKIKGSHTAMKSGMLCGEAVFEAIAAGVEKGGDLAVARVTEGEDLFVKELTSYT
DKFNNSWLKEELYNARNFGPAMHKFGQWIGGAFNFIDQNVFKVPFTLHDLKQDFAVLK
TVDATSFKPTYPKPDGKLTFDRLSSVFISNTVHEENQPAHLKLTDPSIPVNVNLPKWD
EPAQRYCPAGVYEIMENDDGSKRFQINAANCVHCKTCDIKDPSQNITWVTPEGGGGPN YPNM"
/product="Electron transfer flavoprotein-ubiquinone oxidoreductase (EC 1.5.5.1)" /EC_number="1.5.5.1"
/transl_table=11
CDS complement(1538344..1538505)
/locus_tag="AUO97b_01599" /db_xref="SEED:fig|400667.82.peg.1460"
/translation="MGFLLILDLLFGKSVWYFDLARNFGFLGLFLLGTFIFYKRNLNL NVAKIPPFE"
/product="hypothetical protein"
/transl_table=11
CDS complement(1538868..1539125)
/locus_tag="AUO97b_01600" /db_xref="SEED:fig|400667.82.peg.1461"
/translation="MSEQPISPTVQLNTRGLRCPEPVMMLHQAIRKAKSGDVVEVLAT
DPSTSWDIPKFCMHLGHELLLKEEVLDEQNHKEYRYLVQKG"
/product="tRNA 5-methylaminomethyl-2-thiouridine synthesis sulfur carrier protein TusA" /transl_table=11
CDS 1539310..1540149
/locus_tag="AUO97b_01601" /db_xref="SEED:fig|400667.82.peg.1462"
/translation="MQEEPSPSWGMRGLRKWLGTAPETRDELLKLVQNSRRFLEPDTV
AMLEGVLDLPATKIREVMTPRTAMISMQEDDQLLDILHVLVESAHSRFPVFSADQPDN
VVGILLAKDLLPFLTEPNTKLDIGSLMRQPLFVPESARSDQVLRMLKHTQTHIAIVID
EYGSTAGLVTLEDILEEIVGEIEDEHDTADEDAQYIVPDNDHTIANAWMVQALTPIEH
FNTVLDADFSDDEVETVGGLLLQEIGLVSDLQGQTVELGNWLFTIVEADARTIHLIRA VRQ"
/product="Magnesium and cobalt efflux protein CorC" /transl_table=11
CDS 1540146..1541705
/locus_tag="AUO97b_01602" /db_xref="SEED:fig|400667.82.peg.1463"
/translation="MRAYFEKLLDSSQQQKQLPLIFPLLIALFSGAVFSFALAPYYWW
WLAILSPALLYATLHNRSAKQAFAIGWSYGFGLWFVGAFWLYTSIHIYGDTNAFLSVC
MIAVMALVMGLFTAFQTWIYRRFFPETPLTFAPLWIIFEWAKTWVFTGFPWLFVGYAF
TERLLDGYAPLFGIYAISFVVIVLACALVEVLRKRIFWVIPAALLVLGAWGASYIQFV
QPKAAKPLSVSLIQGNIPQDLKWLTEYQVRTLEIYAGLTQSEWGRDLIVWPESSIPLF
QTDIEPFLDAMDAQAKKNHTAWVTGIPYWDVAKSHQVGSPLYYNSIMASGSDSSGLYK
KQRLVPFGEYIPLSGLLSWVLPAMQNDISMSGFTRGESDQKPLLIKGHALAAAICYEV
AYPNLTRRNAEDSDFLVTVSNDAWFTGTAGPWQHLQMVQMRAKENGRWFIRATNTGIT
AFIDQNGHITEQAPIDKEFVLRGDLPAMQGQTLYNRLGDYPILGFAILLLVLGWIYRP RKVDVSYKSRR"
/product="Apolipoprotein N-acyltransferase / Copper homeostasis protein CutE"
/transl_table=11
CDS complement(1541707..1542045) /locus_tag="AUO97b_01603"
/db_xref="SEED:fig|400667.82.peg.1464"
/translation="MEQIEWNDFLKVELRVGKIIQAEVFEKARKPAYILHVDFGEELG
VRKSSAQITKFYQPQDLIGKLVVAVVNFPKKQIGPIQSECLVTGFHNADGDVVLCIPE FEVPLGTKLL"
/product="Protein secretion chaperonin CsaA" /transl_table=11
CDS complement(1542190..1542534)

/locus_tag="AUO97b_01604" /db_xref="SEED:fig|400667.82.peg.1465"
/translation="MKAFLVLDELNQFHWAMLKSVLLILALLPIAEVSLKLWLSTEGS
SQIMIGFFALSIVSAWLMVSFFTALKTSVWQTKQMASKYEQLLFKAYRYVPMVFLSSL VAYLSLQLSIAF"
/product="FIG00350389: hypothetical protein" /transl_table=11
CDS complement(1542801..1543382)
/locus_tag="AUO97b_01605" /db_xref="SEED:fig|400667.82.peg.1466"
/translation="MKVKNIQSEIAMKQHSESVVSNQFEPTCHSEHSSVSDVCSSSAF
GLATRSSRARMKRVSGFTLIEVMVVIVILGVLAALIVPNVMGRSEKAKIDTTQITLKG
VAGALDQYKLDNGHFPTMQEGGLDALINQPATAKNWMPGGYVKGGYPKDSWKNDIQYV
VPGKDGHPFDLYSFGADGKEGGEGNDADIYYQP"
/product="General secretion pathway protein G" /transl_table=11
CDS complement(1543406..1544611)
/locus_tag="AUO97b_01606" /db_xref="SEED:fig|400667.82.peg.1467"
/db_xref="GO:0009306"
/db_xref="GO:0016020"
/translation="MPAYQFTAIDASGKQQKGVLEGDSARQIRQQLRDKAWTPISVDP
VEQKDKHQSHGLFQKKFSAYDLALMTRQLSVLVAAAIPLEEALRAVSRQSEKAHVQNL
LLSVRSKVLEGHSLAQGMQQSGRFPDLYIATVAAGERSGHLDLILDQLSDYTENRFAM
QKKVQGAMIYPIILMLMSFGIVMGLMTYVVPEIVKTFDQSKDALPWITVALMKASDFI
RHAWVFIILFAIVGVVAFVRFLKTTAGHYAFDRLTLKLPLFGKLSRGINAARFASTLS
ILTRSGVPLVDALKIGAAVTNNWVIRDSISQAAERVTEGGNLGTQLERSGYFPPMMVQ
MIRSGEASGELDRMLERASTMQDREVTTFISTLLALLEPLMLVLMASIVLVIVIAVML PIVNMNNMI"
/product="General secretion pathway protein F" /transl_table=11
CDS 1544760..1546988
/locus_tag="AUO97b_01607" /db_xref="SEED:fig|400667.82.peg.1468"
/translation="MTITPKPDSVVYRVRVAVPVHLYDTFDYTLTKAQYERAQVGSRV
AISFGRQNLIGIITEKVDPQETFTGHFQLKAISELLDDEPILDEQVLNLLTWSAQYYQ
FPIGEVMQTALPALLRQGKPMDVLFHLWKIIPCDNAEALLKRSIKQQDAYQILKLHPA
GTTENILNLSGVETATLKALQKKGLVDCTLEPHDFSPSSVQLAQMPLTLNEDQKKATQ
HVLNAQHQYQAFLLDGLTGSGKTEVYLHIMHEVLKQGKQVLVLVPEIGLTPQTISRFK
SRFNCDIALLHSGLNDSKRLQAWQQAQTGKASIILGTRSAIYTPLPRLGLIILDEEHD
LSYKQQEGFRYHARDVALYRGHLQGCPVLLGSATPSIDSYHLVETGKLTALQLNQRAG
HALLPKMHLIDLKIVKKQHGISQPLIEQIKNTLARKEQVLIFLNRRGYAPVLVCESCG
WQANCPHCDAHFTLHTQPYSYLHCHHCGTVHRLPDHCPECQQKSLKTLGAGTAKVEEH
LQELFPDHDVIRVDRDSTSRVGSWQKIYDRIQQNKPSILLGTQMLAKGHHFPHVTLVA
ILDIDAGLLSVDIRAPERTAQLIVQVAGRAGRGEHKGHVYLQTLRPDHPLLTTLIEKD
YRAVAKQTLAERKVALLPPYRYTVLIRAESKDRDYTLHFLNEAAEQLRQIAGDIVDIW
GPIPAPMERKAGRYRAHMVILSADRARLHFYLRQWWAQLVHAPRQHQLRLSIDVDPQE FS"
/product="Helicase PriA essential for oriC/DnaA-independent DNA replication"
/transl_table=11
CDS 1547065..1548906
/locus_tag="AUO97b_01608" /db_xref="SEED:fig|400667.82.peg.1469"
/translation="MSFLLQATIFLGASLILVPLGKRLGIATVLGYLFTGILLGPSVL
NIAHDPEAIMELAEFGVILLMFLIGLELRPQRLWEMRDSIFVMGSLQVVITGIILMLT
VLLLFQQHLAASFVIGFALALSSTAFVLQLLTEKQQLNTTYGQQSFSILLFQDIAAIP
LLAVIPLLAGTESTHHGIAYFAAIIATFTGLFLFSRYVMRPFFRFVAKSGATELITAV
GLFIILAVVLLMDTLGISTTLGAFLTGVLLADSEFRHELEASIAPFKGLLLGLFFMTV
GMTTQLSLLIEMPWLIIGGAIGLLVIKTLVLTAIARYKKYSWNNSLLLGTCLAQGGEF
AFVILSLAKSEKILTDALLEPVTLIVTLSMVLTPVIYWIMACKIIPLFHKELPPEYDE
IPQQDNPIIIAGFGRFGQIIARIARLQHLGFTAIDNNLHQVDFVRRYGGKLYYGDVTQ
PDLLRSAGIEKAKVFILAIDDVEDSMNVARHLRLNYPNLKLLARARDRHHVHLLRDLG
VEHIWRETYLSSLGMAYRALRELGISDEQAYNNIEIFRDYDEKLLIQQQSIYTDEQKV
FETHRNALAELEHLFESDAQQATSKHDVNLKRHLQPNRIDITRDHEE" /product="Inner membrane protein, KefB/KefC family" /transl_table=11

CDS 1549072..1549947
/locus_tag="AUO97b_01609" /db_xref="SEED:fig|400667.82.peg.1470"
/translation="MSDLRQRFYIEDFPVRGEVVHLEEALKTILAQRDYMPAVQILLG
EMLSATALLASTLKIKGRISLQIQANGSFKWAMAECNHLGEVRALADYETDPRFGEAT
DSSTVLAALVNPVLFINIEPEHGERYQGIVPLDQANLAGCLMQYYDLSAQIPTRIVLA
STAQRSGGLLIQLLPRHDEEEQKLVDEDLWPRLTMLTETLKSEELTELSANEILYRLY
NEEEVRLPEVEHLKFGCTCSKERCAKALIQIGVDAVHETLEAQNPIQMDCQFCNTRYE FTAEEALGLFGEHLS"
/product="33 kDa chaperonin HslO"
/transl_table=11
CDS 1550085..1550696
/locus_tag="AUO97b_01610" /db_xref="SEED:fig|400667.82.peg.1471"
/translation="MNYFDYFLFIFSVIIMIATPGPVMILVASVGLKGGYKKALETIF
GTNLASLVLIFISVLVLKGVLGINENYLNIIRVLGCLYIGYLGFSILKEVIQAPHPEA
IQTVSAQNGGFKKGFLVGISNPKDIIFFSAFFPQFVSISPHLDLSLTILTLTWIVLDF
LTLSLVYIFFRRLSNSHLYPKILGLCGLLLLVIAVYGLYQTFT" /product="Efflux protein, LysE family"
/transl_table=11
CDS 1550847..1551803
/locus_tag="AUO97b_01611" /db_xref="SEED:fig|400667.82.peg.1472"
/db_xref="GO:0006457"
/db_xref="GO:0031072"
/db_xref="GO:0051082"
/translation="MAKNYYEELGVKREASADEIKKAYRKLARKYHPDISKEKDAEEK
MQAINVAYDTLSNPEKKAEYDQMLDHPQGFNGFGQGAAQGGFDGARFYRQGFTGGEQA
DFSGFEDLFGRFGTGFGGGQQQYQRQQRSYRGEDQHASIEVDLDIAYHGSTQHITLQI
PTINAYGEPEVQRKTLQVKIPKGMKEGQQIRLSGQGQSGINGGANGDLYIEIQYKDTD
RVHVEGSDVYLTVDVAPWEAALGQGIEVKTPAGPLHVNLPKNAKQGQQLRLKDKGIPN
KTPGHLYLILNIVFPPAHSEKEKEAYQQLAEAFASFEPRSSH" /product="DnaJ-class molecular chaperone CbpA" /transl_table=11 CDS 1551815..1552144
/locus_tag="AUO97b_01612" /db_xref="SEED:fig|400667.82.peg.1473"
/translation="MTTIHYREIVCDGHTFSAEVVDEQCTFDLQQFAQACGQSPDWIL
QLIEYDILAIDTPPEKHQFMGEDVIRARKAYRLQRDFDASLAAVAVMLDLIDEVQQLR KQLKHFH"
/product="hypothetical protein"
/transl_table=11
CDS complement(1552391..1553602)
/locus_tag="AUO97b_01613" /db_xref="SEED:fig|400667.82.peg.1474"
/translation="MQVYYFYRHQSDGYVFLSREKHTEHILEFFWIDSVRSDVVSQNE
EWQQKIQQLADVSINEFHMRDIANIEHPCAFDTLEEYDLLIFRKLVTPDDEIKNGESH
ESVFGLATTPISFIFTPKVLISVREQGNKSIENYIQRLENILCKTLEEQNKTRKLPNS
PVDLCLRLLNSMVDGYLDIRSPLTRRVEHWQQQLLQGNRRFKQWHQLFHENMAFQQVE
NLCEEQIETLQEFRDEIVENYHHVIGEKTHSSQDLLLVRLNDLMSHVERIQKHTLRLR
SAIQSAIDLHFSAIANQTNENMRILAIITAVFAPLTLLTGIYGMNFEFIPGLKSPVGF
WIMLGVMLLSTILLLYYFYRQHLVGRGEKSVIDLLAQQHRQDRMNLFWFLEYEPIKQT LKEVEKITRLK"
/product="Magnesium and cobalt transport protein CorA" /transl_table=11
CDS complement(1553687..1555780)
/locus_tag="AUO97b_01614" /db_xref="SEED:fig|400667.82.peg.1475"
/db_xref="GO:0004654"
/translation="MSMFNIVRKEFQFGQHQVVLETGRVARQANTVLITMGGVTVLVA
VVAAPTAKAGQDFFPLTVNYQEKQYAAGRIPGGYGKREGRASEAETLISRLIDRPIRP
LFPEGYYNEIQVTATVVSSDKTMEADIAAMLGTSAALAIAGTPFRGPIGAARVGLING
EYVLNPNFEQMAQSDLDLVVAGTESAVLMVESEAKELSEDQMLGAVLFGHDEMQIAIQ
AINEFAAAAGAKPSDWVAPAHNEELRAKLKEAFEAKISEAYTIAVKQDRYAALDALHA
EAVAQFVPEEDVDGIADEVDYLFEDLKYRTVRDNILSGKPRIDGRDTKTVRALDVQVG
VLERAHGSALFTRGETQALVTTTLGNTRDALMVDTLAGTKTDNFMLHYNFPAYSVGET

GRESGPKRREIGHGRLARRGVQAVLPAADRFPYVIRIVSDITESNGSSSMASVCGASL
SLMDAGVPLKAPVAGIAMGLVKEGERFAVLSDILGDEDHLGDMDFKVAGSANGITALQ
MDIKIEGITEEIMEVALNQAFAGRMHILNEMNKVISRARPEISMHAPTFEVITINPDK
IRDVIGKGGATIRQITEETKAAIDIEDNGTVRVFGETKAAAKAAIAKIQAITAEVEPG
KIYDGKVIRIVEFGAFVNIMPGTDGLLHISQISNERIANVTDVLKEGQEVKVQVQDVD NRGRIKLTMKDIEQA"
/product="Polyribonucleotide nucleotidyltransferase (EC 2.7.7.8)"
/EC_number="2.7.7.8"
/transl_table=11
CDS complement(1556032..1556301)
/locus_tag="AUO97b_01615" /db_xref="SEED:fig|400667.82.peg.1476"
/db_xref="GO:0003735"
/db_xref="GO:0005622"
/db_xref="GO:0005840"
/db_xref="GO:0006412"
/translation="MALTNADRAEIIAKFARAENDTGSPEVQVALLTAQINDLQGHFK
AHKHDHHSRRGLIRMVNQRRKLLDYLNGKDHERYTALIGALGLRR" /product="SSU ribosomal
protein S15p (S13e)" /transl_table=11
CDS 1556337..1556486
/locus_tag="AUO97b_01616" /db_xref="SEED:fig|400667.82.peg.1477"
/translation="MNQLSATDCRASLQQVAYFKVSIYGLQNSFLLPVILDTDISPLT LHTWP"
/product="hypothetical protein"
/transl_table=11
CDS 1556514..1557812
/locus_tag="AUO97b_01617" /db_xref="SEED:fig|400667.82.peg.1478"
/translation="MKIFSTNTCPVPNDLESVIHHKDEVPAELGGMSEQQVQKIWKSI
ESLYKTGNYPLITFCLRRQGKILLNRSIGYAQGNSPAGLQKDALIATPDTPVCLFSAS
KMITAMLIHLLDEKGEINLLDPVSYYIPEYGVNGKRRATIFHLLSHRGGIPYVDGDVT
PELLFDKDEILRRLYAARPVSPAGNHLAYHAVTAGYILGEIIERVTGQDLRQFVHETI
EKPMDMPYFNYGLKPEYRAEVALNCATGLHPRLGTDHYLNHVLGGGLQLAVDVTNDSR
FMDTICPAGNIYTSAEQAGRFFEMLLSGGSYQGKQILSEKTIFRATLPTTGVNIDRTL
LIPMRYALGPMLGSNPVGLFGPMTGQAFGHLGFSNILCWADPERDISVSLLTTGKSVV
GTHLPALANLLYQISTQCPRIPRDQRRSLFGSDSHETDLV" /product="hypothetical protein"
/transl_table=11
CDS complement(1558529..1560280)
/locus_tag="AUO97b_01618" /db_xref="SEED:fig|400667.82.peg.1479"
/db_xref="GO:0008854"
/translation="MDNLNSPEQSDLSWLMTWSTFLNQAPFTAQTQAPEAAYFLQQLI
EASLQGDSCIEISPEQIETLGQLVISAEQAKSQVAPCVHDGQGLALYRYWNLEQRLAE
QIRRLKQQPIQPVSCEEHLDLLTDPHQRAALQMVTRQSLSIITGGPGTGKTYTLARII
AVLSQAIPHIRVAMAAPTGKAAQRMQEALQNSFNDPKLLESGLMSDELRNQSTQTIHR
LLGMGHSQTPRFNQKQPLPYDVIVVDEASMLDLNLATLLFEAVPESCRIILLGDANQL
ASVDVGAVLADLQQIEALSENRVHLQTSRRFAEGALIGQVARFIQMQTGQIDHEQVLQ
QFEIDIVQVSELRSIPLEADMPDVVQLEYLPEESPIALETYYQKLMVGFQGYVQSLKA
YLQDDRSTEHIENIVKSFDDYRILTAVRHGPFGLQQLNQYAERWLQQQLGIVTLGGWY
VGRPVMMTYNDYQLGISNGDIGLCFEHRTQPQQFEVYFPSLNKWVAANRLPKSIQSAF
ALTIHKSQGSEFTHTAVVLDQAAKNLLSQELIYTAITRAKKVVSLLVDPEALLQSFTV RTTRKSGLVEKVNRLVR"
/product="Exodeoxyribonuclease V alpha chain (EC 3.1.11.5)"
/EC_number="3.1.11.5"
/transl_table=11
CDS complement(1560389..1564087)
/locus_tag="AUO97b_01619" /db_xref="SEED:fig|400667.82.peg.1480"
/db_xref="GO:0008854"
/translation="MNSRVQVSYQPIIDIEFSGLHLIEASAGTGKTYTLSSLMVRIFL
EKYLPGQVIATTFTRAAAAELKSRIRARLVETHRYLDAKRSLTEKEILLQAEQESDLL

LQHILKHFATRIAYACERLKLVIDQLDELFVGTLDSFSQKLLREFAFESGKIERAQIT
DDAKTYSRQLIHDVLREWIQSQPQTVIDALYLAGELKSVDSFVKLVEDSLNFSSAHFK
LPEKPTIQFEQLAQLKQLAAEIDISLLEPYYSLDGEHYKHVSGTIFRNGAFNKLFSEC
LPQLLQVLKQSDSILVFDGSLAIQRELVFKFLGQLADQKVFKKCPAEISDGFYQHPCI
QQIQQLFGVLKKYAEQFDQLHIYLKAYLCVEVKKRLPQVLQNKGETTFSQQIRTLSEA
LKGEQGQRFAVFVQARYPLILVDEFQDTNQDQDDMLASIWRHPERYQKGCMIMVGDRK
QAIYGFRGGDMLTFLNAYKDIQAKHGREYKLIHNHRSVADLVEVVDALFQRQIDFGEQ
VQYDPIRAGTRPHPVLIDQNQPNPHPLRWLMLKDKETEAQQVAWKIRDLLNQSHAGQL
YFQKDAQTQTLNEDDIAVLSRNHDGLDKVQFELERLGIRVNRPSKRSVFDCTIAQDVG
ALLTAILHPYDEAKVKRALISRLFAMDLKQLLQLEQTAEGLSQFMTGFDTIRELWSAQ
GFLVAWQQCLNQFGIWKNLVAVQSKDNERVVVNLRHLTEILSQHSEKYQGAQNLYHWY
LKQLQSPLDREWELERRLSSEAGVQLMTIHQSKGLEFKIVFLLGADKPFRENNKTLNF
STQDITVPESAQTLTQRVVAIADKTYLNEIELKQHEERALAEQNRLWYVALTRASHRV
YALLQDTDGKSVSGLAFWKNRAEPFQHRCCTDEIILEQPPAARHLNQHINIIEIQAQH
FPDQRFYSRGKTSFSYLAQHLRHKVGTDLLASQSHEAVLAEDELDQVISVEAPAAQPI
NWIKSNFPRGTLAGNFLHEIFEHIDFQCSDEWVSEIRRRFKNDYSSLWQDLLIKYQES
FPEEQEAEYSLYHAVAEWLQEILSTPLYQGFRLNRLQPEHYLSECPFYLALSDRVLAM
KRIQQLFAEYGMEMPELLEARSARYLNGSIDLVYFDGQRYHIADYKSNYLGENLADYS
VESIAQSMSLASYWLQAGLYLVALHRYLKVKMQNYDIEQHLGGATYLYLRGMNGEAEQ
GYYYWQPSTEFVLRLDAILGYFAEDKIA" /product="Exodeoxyribonuclease V beta chain (EC 3.1.11.5)" /EC_number="3.1.11.5"
/transl_table=11
CDS complement(1564091..1567726)
/locus_tag="AUO97b_01620" /db_xref="SEED:fig|400667.82.peg.1481"
/db_xref="GO:0008854"
/translation="MGIHVIQSQRIDVLLQGVLASTSQPSTHPLQVLKAQHFIVPSPA
IEQWLIQKLAEQQGMSANYQFHQRVRGFQWYAYQQVLTAHKEQVRKANIPRLIFKWRV
HQALQEFIQPDVMSIDSSHPLHSIVQRIYDSADRLEQGVEKQLKKQKMLYWVAEQVAD
LFSNYMVYRGQCQRGCENMCTCPNNWLAAWGQGRSLDIEKYIAHKDKEVSAFTLQQTQ
ELERWQRWLWQQHFHDDFIQMQQIDELFWQELEHPERQKKALSRLPQQIVIFTLLDLP
PSQLQFLRRLGQYIDVLILHYNPSQEYWADSVDPLWKQRYDLGVKERFIAKNPQATDA
EIADFFNKFTLNFNAEARESRHPLLTRLGKQARDHFSLLSNLSTGEEGKWVDAFVDDF
PESLLGKVQSDILHLVEPQAKQYELAPNDDSIQIHICHSTLRQLEVLKEQLIGWLAKP
HEQPRRPNDILVLVPNLAEVEPLIRSVFPATATEQGVHLPVKIAGIASLDALNAWRAV
IGRIHLMQGRFSFDEFADWLGLHATQQRYALEYAQVERILNLLADAGFKRGLDAEHLK
RSLCDGDDDYRYSFKFALERLALGIAIPEHATFNQVLSYAKVQPGDFELIGTLIQIYQ
DLNERRDWLIMHEQKKSHTVEYWLQILSQDIIEFEQAGVAALKTAREIVKKQERMLTL
ASYYAETETGTLRKITLPLPYILDEIQRTLENQTAQAEPTGQITFAQIGQIRPLPYRL
IVMLNLDAGQFPNRDTHVPFDLMDALRQQLGDRSRLEDDQGAFLDALLLAQENLWLFY
NGFDVNDGEVRDPSSILQEFREHLALIVKPEPNAPDREVIEGIEIPTQLKQLYHLHYL
QPFDPKGFMAENRYIRYQDHWFNVAMQIQQASGVLKPWANISYPLEIPDMLVLDSHQW
IQDVTFPARLYLKTLGVENLGNVSVLDQNEPLLLDGLGRYTIRHFLQQNEQQAQPEVL
LDQLPVGKVQYSAWQQGIFEQECLLERLHHYAPAVTQTTQRVWRIAKQLHMNITVPKS
ETQDWVSMEASSARAKRRAKVWLEYLLWLAYLNEGSAGTERRRIVVFSDQTVICKGIS
SEQARQYLQPWFKIWRYAQQQPLVLPAALLLKPLEKAKAYQWETTNPTEKIKLDEKSY
AELLKYWNETGTFTSVDMTQNEACKLHQDWRFILQEQDAQALLQHACDEFAYDLYHPV FQFQHSE"
/product="Exodeoxyribonuclease V gamma chain (EC 3.1.11.5)"
/EC_number="3.1.11.5"
/transl_table=11
CDS 1567898..1568404
/locus_tag="AUO97b_01621" /db_xref="SEED:fig|400667.82.peg.1482"
/translation="MKIDNIPDYIDPSLNLEQIRDECQELIQKRAYVSAGAAIVPIPF
FDVVVDLGILSQLIPDINSRFGLAPEHVSVYDPKTKTIHWDELRKRGFQFSGFVVART
TVKKTFNGFFGKIVTKQVTKFVPLGGQLVAASLGYFMMKKIAQTHLNDSYNLAKRIQQ KSRGTVVN"

/product="hypothetical protein"
/transl_table=11
CDS complement(1568427..1569845)
/locus_tag="AUO97b_01622" /db_xref="SEED:fig|400667.82.peg.1483"
/translation="MSEQQPKFEIRDEEQLSVDLIEAQYALKESRGKPNAKSTLILMS
GIELAGKGEAVKQLREWLDPRYLRVKADAPRVLTDSEAFWQSYSAFIPTEGQIVVMFG
NWYSDLLVTATHVSEPLDDARFDAYVENMRAFEQDLKNNYVDVIKVWFDLSWKSLQKR
LDKIDPSEQHWHKLHGLDWRNKKQYDTLQKLRRRFTDDWYIIDGEDEKQRDQFFAQYL
LQHMRQLPEHETEVKGKWQQAKIPESLLKPAQEKMDKAEYKKELDKLSKKIADTMRFD
KRNVVIAFEGMDAAGKGGAIKRIVKNLDPREYDIHCIGAPERFEARHPYLWRFWNRIN
EAEKITIFDRTWYGRVLVERVEGFASPIEWQRAYDEINRFEKDLFDSQTIVVKIWLAI
SKDEQEQRFKAREETPHKRFKITAEDWRNRDKWDDYLKAAADMFERTSTEYAPWYIVA
TDDKYTARLEVLRAILKQLRAD"
/product="UDP-galactose-lipid carrier transferase (EC 2.-.-.-)"
/transl_table=11
CDS 1570053..1570760
/locus_tag="AUO97b_01623" /db_xref="SEED:fig|400667.82.peg.1484"
/translation="MSKLAAFDELLQQYKTFNYHDNPVLAQRLQDVQTWLKERMKETH
HDFFNLPEHKLMAQYFLNRLYGGPEFDALAAQIERLLKYAHKAEKVLPENAIKTGTKS
VSLAVLATQLDEQVAMQLLEDYPADTVLTDEIMRLTLIKLDQAEARYQQLALLDDLGA
ALDKYMRSFMMFTAFKMCKGIAQKYHFELMYDFIQDGFSAMKPLKSAEAFIKTFTEKE RQIVEKVHSGHPNPFRV"
/product="hypothetical protein"
/transl_table=11 CDS 1570909..1571856
/locus_tag="AUO97b_01624" /db_xref="SEED:fig|400667.82.peg.1485"
/translation="MSNENQKPTSPTEQAQSSDKVSPFLSSPLPQGTPQGQQQSLQQT
LTDTPVSGSVPKYNLPRGAGNTGNVGETTHFGYSTVRTEDKAQKVAEVFHSVASKYDL
MNDLMSFGIHRLWKRFAINMSGVRRGQHVLDIAGGTGDLAKVFSREVGPQGHVVLSDI
NESMLNVGRDRLIDAGCTNVDFVLANAETLEPFADNSFDLVTISFGLRNVTDKDAALA
SMFRVLKPGGRLLVLEFSKPVFEPFSKLYDLYSFTALPIMGKLVANDSESYKYLAESI
RMHPDQRTLKGMMENAGFQNCDYHNLTGGIVAVHRGFKL" /product="2-methoxy-6-polyprenyl-1,4
-benzoquinol methylase (EC 2.1.1.201)" /EC_number="2.1.1.163"
/transl_table=11
CDS 1571868..1572536
/locus_tag="AUO97b_01625" /db_xref="SEED:fig|400667.82.peg.1486"
/translation="MWSILALGAVERIIHHLIDLDALTRIQLNQLQGKMLRVIIDSPQ
LSVDVFFDQEKVRLEPTVTGHSEKPSIFEQRPFDPQFKISEATATLHVKDVVELIKLL
LSDPDQIGNIPLQGDYHLLQDIQKIMQQAEPDLAAHLSKWVGPQLAHEIGKIQLAPKH
LKRSLQSHLFFAEDALKEDSGLFAPRWEMDDLNQATRKLNQDIDRLEAKLQQLNAQLQ PTQD"
/product="Ubiquinone biosynthesis protein UbiJ" /transl_table=11
CDS 1572549..1574168
/locus_tag="AUO97b_01626" /db_xref="SEED:fig|400667.82.peg.1487"
/translation="MIPHVSRLLELWRIAAHYRLDTLFPADELPVKAKHALNIIKMHP
AAWSSRERKNPLKLKEALEDMGPLAIKLGQLLSTRRDLIPPEILAQLVLLQDQVKPFD
GEVAKQRIQDSLKADVNTLFARFDDQPLAAASIAQVHTAALHDGREVVVKVTRPDIRS
QILQDFEILAWLGNTLESRLEAARALHLSEIIQDYRQIILNELDLSIEADNTRRMRHY
FTGSTMMYVPEVYMDTKDVMVAERITGVPISDTATFDRLGMDRALLAEKGLTIFFTQV
FRDNFFHADMHPGNVFVETINPSNPRFIALDCAIMGELSKHDQMTVARMLLAVMNSNF
MQLIQIVHQAGWIPPGTDQDALAREMRRTVGPMVSKPMDQLDFAGILIQVMDIARRFH
LEIPPQLMLLLKTLVHVEGLGTDLYPQLDIWKLAKPILTEWVKSNMNPVKNVKELGQQ
LPDLLLGAQDFPSLIIDSLNGLKNQSAWQDRQLREIQQLRLQMEHQQRRSWMFGSTML
IFLTIAIISPWFVSIILIVLSSLLALWRVLK"
/product="Ubiquinone biosynthesis regulatory protein kinase UbiB"
/transl_table=11
CDS 1574293..1575606

/locus_tag="AUO97b_01627" /db_xref="SEED:fig|400667.82.peg.1488"
/translation="MKTPHFAIIGAGTAGLATAILLAREGNHVTIFEQVDELSPVGAG
LLLQPAGLAVFEHLGVLDKALTLGAKVTGLEGQLPDKRLLVNSHYREASTNLYGLGIH
RATLCHVLTQKLSEYSSQITWCMNHSVESFEEHNDEVRLFGSHQNQKFDACFDGLLIA
NGARSQLRPKAWVKVDKAYPWGAAWSIVPECQVLDSEILHQFYDRSKIMMGILPTGAI
PTEPQQRLSSVFWSLPPPQLQSFLQDEQAKQAWLKQVSERWPKVAEWLKEILYNSQTQ
PKWLSANYRDVVMTQFGQGRIGVIGDAAHAMSPQLGQGANMALLDAWAFSQSLRHAQK
NQNIDWSLLWQHYHQLRGSSTQFYQFLSRLLTPLYQSDHWWAGGLRDLVFPWMYQIPY
FQKEMAITISGLKTGLFRHLEYDQVAQLPKESSAFHLKSESLTDF" /product="Oxidoreductase"
/transl_table=11
CDS 1575760..1576533
/locus_tag="AUO97b_01628" /db_xref="SEED:fig|400667.82.peg.1489"
/db_xref="GO:0004635"
/db_xref="GO:0004636"
/translation="MNNTQWLDEVKFNEQGLIPAIAQHHQTGRILMVAWMNRESLALT
AEKNQAVYFSRSRQKLWHKGEESGHFQTVYEIRLDCDGDVIVLQVEQHGGIACHTGRE
SCFYRKLTPQGWEIVDAQLKDPTAIYGDNAKTESHDHAHTTEQVDVLAHLGQLMQERK
QAEADTSYVASLYKKGINKILEKVGEEGVETIIAAKDYATQNTESNLNDLIYETADLW
FHSIVMLGYFDLNPQLIIDELGRRQGLSGLVEKANRNKV" /product="Phosphoribosyl-AMP
cyclohydrolase (EC 3.5.4.19) / Phosphoribosyl-ATP pyrophosphatase (EC
3.6.1.31)" /EC_number="3.6.1.31"
/EC_number="3.5.4.19"
/transl_table=11
CDS 1576543..1578045
/locus_tag="AUO97b_01629" /db_xref="SEED:fig|400667.82.peg.1490"
/translation="MSNIEKPKATYEQAIAIDNARLGQSFKVIAYAGTGKTTTLQMIS
DAMPERRGMYLAFNKAIAGEAQNKFHRNVDCRTFHSLAFRSVPRGVTDKLRLPRLSPS
FIAKEYRLEPITLRRMMGGRYEKYVLMPSRLASLVANAVSYFCSTSSQYPAPRHIQAP
NWLHPDDITALQTHLYPAVERRWLESIDPNHQAGIGHDIYLKLWALSEPNIPADYVLF
DEAQDADPLMLGILLRQKSTQVIYVGDAHQQIYAWRGAVNAMQQLPLPESRLTTSFRF
GEAIADVANALLGGLNETVPLLGNPNQKSSVVNKPHTKMRDAILCRTNARAMELLLAG
LVHGDKVSLQADHQKLNRFVDAASLLKQGKRVTDVPELAWFNSWHDVHEYCETNEGSD
IKPLVKLVDDHGTDPLKKALAKITPLEQADYVISTAHKAKGLEWNRVHIEDDYQFKIN
GLEHKITDEELRLLYVACTRAKVSLNIHHLYDLIQQLKLRAPLSLRQSVG" /product="Putative DNA
helicase"
/transl_table=11
CDS 1578069..1579451
/locus_tag="AUO97b_01630" /db_xref="SEED:fig|400667.82.peg.1491"
/translation="MKLESIQFKHIALFHDLKIQFQYDKQPITLILGEQATGKSMLLK
HIYHALTWFPARLKDARTPGIVMPDQDISHSRLQSKIEVSIQIPPEFGQLPETTSTQQ
TETSLCSWKLFKTLNSSGVGISQVETQQLDQTITLYHQVTRKDPLQGLPLLAYYPSER
FVNDINLLNKNLPGITQSISAYDISFIPFTTFARFFEWLREISDIENAQAAHVVQRII
SKQSNSQDQEEILRQLQLELAGHPKQLPSPNLYALKSALKIIFPELEDFYLQYHPKLQ
LMAHYKGEVLAFQQLSNTTKTWIALVGDVVRRLCLLNPLSLDPCLEGEGVLLIDQIDS
QLDSAHCSEILNRLHRAFPRLQIIVTGSREELLEHAAAYQCFKLEHGKISPLDLNTTQ
QQLEHLYTLLQRDEALTPHIDPLPLDTEPATAQIDSLYQQIQNLNEQQKNELLRMIHA GDTSEETSSL"
/product="ATP-binding protein"
/transl_table=11
CDS 1579778..1582615
/locus_tag="AUO97b_01631" /db_xref="SEED:fig|400667.82.peg.1492"
/db_xref="GO:0042773"
/translation="MLPIIILLPLILGTTLVSLLQRFSRGVTALGAIGVSLTSFGLLL
TQAKTVLGGASIQQSWDWLPQLGINLSFRLDALGLLFSLLITGIGTLIYIYAYYYLGP
KNSLSKLYLLLMLFMAAMLGISLSNNLIIILLVFWELTSISSFLLVGYWSHYEAAQRGS

RMALTITGMGGLAMLGGFVLLGQITGTYEINDIVGMKDLIQSHYLFVPTLLLILLGTF
TKSAQFPFHFWLPNAMAAPTPVSAYLHSATMVKAGIFLLARLAPIFIGAALYHNIVTF
VGLFTLCMAACFAIFKEDLKGLLAYSTISHLGLIVCLLGIGSPLAVAAAIFHIINHAT
FKAALFMIAGIIDHETGTRDLRKLSGIWQLLPFTATLTMITAASMAGVPLTNGFISKE
MFFTELLASLSGSTVIFASIIATLAGIFAVSYSIRLVHGVFFDGPVGKQVPNKNAHEP
PIGMRAPAILLATLCILVGIFPALLVEPLVNSVTRASLMQPDFAGTHLAIWHGFNAPL
IMSIIALVGGTLFYFALAKDGMIRKIDLDPRLGRLQGRILFDLFLKHLLLTSRKIKQK
TENGSLQSYLFLIIAFSIVMVTLPLFNQGLTTGTRELTHAPWIAVVLWLTLFSGCWMM
LWFHHERIKAVLISGAIGLVVTMVFVTMSAPDLALTQITVDVVTTVLLLMSLSLLPQL
TPYESRRSRRWRDAALAIIGGLGIGWITWLILTRDHNSISWFFAQQSLPLGGGSNIVN
VILVDFRGFDTFGEITVLGIAAIGALCLMDGMRVHGTTMTQGLTYRFNPSPLMFRITA
SWVLPLALVVSVYIFMRGHNYPGGGFIAGLITSMALIIQYIALGQDQAEQMLKAQSGR
LYEIWIGSGLTIAGLTGIAAWFWARPFLTSAHVYVELPVLGKLHLASAASFDLGVYIT
VVGATMLLISVLGDSRHSSMSGPVPHGEKSS"
/product="Na(+) H(+) antiporter subunit A / Na(+) H(+) antiporter subunit B"
/transl_table=11
CDS 1582612..1582980
/locus_tag="AUO97b_01632" /db_xref="SEED:fig|400667.82.peg.1493"
/translation="MISLEFLLASAIGLLVATGIYLILRARTFPVVLGLAVLGYAVNL
FLFAMGRLQVSSPAILTETTNITDPLPQALVLTAIVIGFATTAFIVQLALRSRYESGS
DHVDAKEDISPTYDPREDEP"
/product="Na(+) H(+) antiporter subunit C"
/transl_table=11
CDS 1582980..1584791
/locus_tag="AUO97b_01633" /db_xref="SEED:fig|400667.82.peg.1494"
/db_xref="GO:0042773"
/translation="MFDFLSFWQANTPIFSILIPAFTGFILLLLGNPGAGALKEDWRQ
PWRRGISLISAIAGLITAVSYLLVANTGQITVYQLSEWSAPFGIVLILDRLSAFMLVL
TYALAVPVLWYASENWDTRGRYFHAIFHFLLMGLCGAFLTGDLFNLFVFFEILLMASY
VLLLHGQGKPRFQLGVHYVIINLLASALFLIGLGMIYGSVGSLNMADVSRLLPTLDAD
QHKLAVAGALLLFVVFGIKAAMLPVGFWLPKTYAVASTPVAAIFTIMTKVGIYAILRV
NGTVFNDVLSQEIFKSWLLPIGLVTSLYGVIGAIGADRLRRFVGFMVLSSIGTLLIAI
AMSNTQAWSGALFYLVHSTLIGAAFYLFCGWMTSQRGDFKDHLKIAPRIKQEKAAMLT
YFLIAMMLAGLPPFSGFFGKVFILQATVETAYQGWVIGIVLLVSLLSIIALTRVGFIL
FWRASPPEEDPINPAYIIYRALPEKAPPRNDTVIYILLAGLVAYVAFASPVLHYTTNT
AQQIQDHALYQQSILKTDQNGEVISVQPYDSTYLPETKYGGEVEDHNAYLIPDIISKD
TFNGEHISEYKHRQIQQQEKLQTPTIADESQLKPMEP"
/product="Na(+) H(+) antiporter subunit D"
/transl_table=11
CDS 1584794..1585321
/locus_tag="AUO97b_01634" /db_xref="SEED:fig|400667.82.peg.1495"
/db_xref="GO:0006812"
/db_xref="GO:0008324"
/db_xref="GO:0016021"
/translation="MQLSKLLDRWFPHPFVSFLIIIFWLMLAHSLDASDLLMAVVLGL
VIPRLVRPFITRTPHIHWKPAIKLCFVVLWDIIVSNFRVAKLVLGSPKKLHPKWYRVP
LETEHEQVNSLLAMIITTTPGTVSAGIDQERGDILVHSLSTENTESDVQDIKQRYEAP LMEIFDVKSKSEDNA"
/product="Na(+) H(+) antiporter subunit E"
/transl_table=11
CDS 1585393..1585593
/locus_tag="AUO97b_01635" /db_xref="SEED:fig|400667.82.peg.1496"
/db_xref="GO:0006811"
/db_xref="GO:0015075"
/db_xref="GO:0016021"

/translation="MVMGPSIVDRLLALDTLFLNATCLIIVLGIYWTTTSLFEGALLV
AMLGFVSTAALARYFTTGHVID"
/product="Na(+) H(+) antiporter subunit F"
/transl_table=11
CDS 1585606..1586010
/locus_tag="AUO97b_01636" /db_xref="SEED:fig|400667.82.peg.1497"
/translation="MQITMEIIVSIFLVFGAFFMLVGSIGMVRLPDLFMRLHAPTKSS
TLGLGSFLIASMIFFAFQGRFGFAELLITLLAFITAPVSANLIAQAALHLRLRSLSGE
VPEAIERPLPWDRYKIGQRFSQNKPPMEPPQE"
/product="Na(+) H(+) antiporter subunit G"
/transl_table=11
CDS complement(1586067..1586468)
/locus_tag="AUO97b_01637" /db_xref="SEED:fig|400667.82.peg.1498"
/db_xref="GO:0006364"
/translation="MAGGQRLKRMADSVQRELSELIRQELKDPRLGGLVTISGVKVSP
DLGYADVYVTVMGRELSDDQNEVAHRETLDILNKASGFLRQELSRRIKTRITPRLRFH
YDKTNAYGNYMFGLIEKAVQDLPKRESDDEE" /product="Ribosome-binding factor A"
/transl_table=11
CDS complement(1586468..1589167)
/locus_tag="AUO97b_01638" /db_xref="SEED:fig|400667.82.peg.1499"
/db_xref="GO:0003743"
/db_xref="GO:0005525" /db_xref="GO:0006412"
/db_xref="GO:0006413"
/translation="MTDKSIKELALSVGRPVEKLLEQAREAGLPQRTADDIITTEQQD
TLVNYLKKVHGQESGNTGKIALKRKTTSTAKVASTSGKAKTINVEVRKKQVFAKPNPE
QIAAEAKARAEAEAKARAEQQAREAAEQKARLQTEQKAKATLDAMRAAHQQDSAAQSA
PKAAVVVKKRGGGTVKPAPKPAETLEQKKAREAQTAQLKATEEAARRKAAEEAQQRTL
EQMRKMASKYSNDDATATIRVIDDSPLASGLVGQAYEDSFNQEDREIKRGGATTNPRA
GKKGGRRGQEEQSFVNHNKRGLKSSQANKHGFEKPVKKQVYDVEIGSSIVVADLAQKM
AIKVREVIKTLMKMGELVNQNQTIDQDTAALVVEEMGHNPVLVSDTQAEDNLLEAAEE
ARGEQTTRPPVVTIMGHVDHGKTSLLDRIRRSKVAAGEAGGITQHIGAYHVETDKGII
TFLDTPGHAAFTSMRARGAKATDIVVLVVAADDGVMPQTAEAIDHARAAGTPIIVAIN
KMDKESADPDRVLNELTTKEIVPEEWGGDVPVAKVSAHTGQGIDELLDLILIQSELME
LKASAEGAAQGVVIEARVDKGRGAVTSILVQNGTLNIGDLVLAGSSYGRVRAMSDENG
KPIKSAGPSIPVEILGLPEAPMAGDEVLVVNDEKKAREVADARADREREKRIERQSAM
RLENIMASMGKKDVPTVNVVLRTDVRGTLEALNAALHELSTDEVKVRVISSGVGAITE
SDVILAESSEAVLLGFNVRADTAARQKSDQDGIDIRYYSIIYELIDDVKDAMSGKLAP
EHRETILGVAQVREVFRSSKFGAAAGCMVMEGVIHRNKPIRVLRDDVVIFQGELESLR
RYKDVVDEVRAGMECGLAVKGYNDIKPLDKIEVYDVQMVKRSL" /product="Translation initiation
factor 2"
/transl_table=11
CDS complement(1589178..1590662)
/locus_tag="AUO97b_01639" /db_xref="SEED:fig|400667.82.peg.1500"
/db_xref="GO:0003676"
/db_xref="GO:0003723"
/translation="MGREILTVVETVSNEKGVSREAIFEALEQALVAATKKKFYEGTN
SEEAQLRVEIDRRTGDYRTFRQWTVVADEDHEMPACQDAISDVDPAKWSIGDVRELEV
ESIEFGRIAAQIAKQVIVQKIREAERALVADAYESKVGELIYGEVKKQTKDGFIIDLG
DNAEAYLAREEMIPKEILRPKQRVNAILYSVNREGRGAQLLLSRAKPEMLIALMKKEI
PEISEEIIEIKAAARQPGVRAKIAVKTNDHRIDPVGACIGMRGTRIQAVQQELNGERI
DVVVWSDDPAQYIASALEPADVSGIVLDEDAKSADIIFATNDQLARAIGSQGQNVRLA
SDLTGYKLDMMLEEEYRARQQNEAQQYLDMFVSRLDIEEDLAMALVEMGFTSLEEIAY
VPAETFDEIELDAELVELLQSRAKEAALTDALKQQENIQEPSADLLNMEGMTSEIAYS
LAARGIITVDDLADQATDDISDIEGLGHEKAGQLIMKARESWFN" /product="Transcription

termination protein NusA" /transl_table=11
CDS complement(1590700..1591224)
/locus_tag="AUO97b_01640" /db_xref="SEED:fig|400667.82.peg.1501"
/translation="MKLSNKSQALYDMIAPAVEACGVDLWGIEFLPQGKRSLLRIYID
RPVDENAEPVINEDGEVEQGRGIGVEDCVRVTQQVGAMLDVHDPISGEYALEVSSPGW
DRPFFQLEQLQGYIGQQVALRLIAAVENRRKFQAKLLAVDLENEEIQVEVEGKHVLDI DSNNIDKANLIYQD"
/product="Bacterial ribosome SSU maturation protein RimP" /transl_table=11
CDS complement(1591866..1592195)
/locus_tag="AUO97b_01641" /db_xref="SEED:fig|400667.82.peg.1502"
/db_xref="GO:0009306"
/db_xref="GO:0015450"
/db_xref="GO:0016021"
/translation="MHSFVLVVHIILAVLMIALILVQHGKGADAGASFGGGGAATVFG
ASGSGNFLTRVTAILTALFFVTSLTLAVFAKKQTTEAYSLKTVQTTAPAQTTSPETSP NAPKTGQ"
/product="Protein translocase membrane subunit SecG" /transl_table=11
CDS complement(1592208..1593002)
/locus_tag="AUO97b_01642" /db_xref="SEED:fig|400667.82.peg.1503"
/db_xref="GO:0004807"
/translation="MSGSTITPWVVGNWKMNPMRANANQLIEEFKQLLQQNQIADENC
HVGVAPVSIALTTVQAQLQDAARTVYTVAQDVSRVAGTGAYTGEVSAELLKDSQINFV
LVGHSERRDIFGDNVEILKAKLQNALNAGMTVIYCVGESLEQREQGQAEQVVLQQICD
IAPVVTAEQWQNQVVIAYEPIWAIGTGKTASPQDAQAMHAKIREGLCQLTPAGSNIAI
LYGGSVKAENAVELAACPDINGALVGGASLNAASFYQIVQAFAQSK" /product="Triosephosphate
isomerase (EC 5.3.1.1)" /EC_number="5.3.1.1"
/transl_table=11
CDS 1593293..1595005
/locus_tag="AUO97b_01643" /db_xref="SEED:fig|400667.82.peg.1504"
/translation="MSALHTSPKFTGFFRRLVEEKHVSAATMQTALDAAKRAKQDTVA
YLIEEVHLSPSLLAETISAEFAEPYFDLDVYDTSQIPKDLVDQKLILKHRILPLIQRG
QILYVATSNPSNIEAIDAIRFNSKLLVEPVIVEHHKLEKVLGQHFAEESSFDFNDEEF
DLDVNLDGPTAQDDEEEAPQGDEAPIVKYINKLLIDAIRMGASDLHFEPYEKSYRVRY
RVDGVLRQIANPPLQLANRLASRLKVMSQMDISEKRVPQDGRIKLKLSKSKAIDFRVN
SLPTLFGEKLVLRILDPSSAMLGIDALGYEEDQKALFMEALDKPQGMLLITGPTGSGK
TVSLYTGLNILNTESSNISTAEDPVEINLEGINQVNVNPKVGLTFAAALKSFLRQDPD
IIMVGEIRDLETAEIAIKAAQTGHMVMSTLHTNSAPETLTRLRNMGVPSFNIATSVNL
VIAQRLARRLCSQCKIPADIPKQSLLEMGFTEQDLAHPDFRVFQPVGCPECREGYKGR
VGIYEVMKVTPEISKIIMEDGNALEIAAASEKLGFNNLRRSGLKKVMQGVTSLQEVNR VTSE"
/product="Type IV fimbrial assembly, ATPase PilB" /transl_table=11
CDS 1595035..1596261
/locus_tag="AUO97b_01644" /db_xref="SEED:fig|400667.82.peg.1505"
/translation="MAVKKAQMMPTFAYEGVDRKGVKIKGELPAKNMALAKVTLRKQG
VTVRNIREKRKNILEGLFKKKVTTLDITIFTRQLATMMKAGVPLVQGFEIVAEGLENP
AMREVVLGIKGEVEGGSTFASALRKYPQHFDNLFCSLVESGEQSGALETMLDRVAIYK
EKSELLKQKIKKAMKYPATVIVVAIVVTIILMVKVVPVFQDLFASFGADLPAFTQMVV
NMSKWMQEYWFIMIIAIGAVIAAFLEAKKRSKKFRDGLDKLALKLPIFGDLVYKAIIA
RYSRTLATTFAAGVPLIDALESTAGATNNVIYEKAVMKIREDVATGQQLQFAMRVSNR
FPSMAIQMVAIGEESGALDSMLDKVATYYENEVDNAVDGLTSMMEPLIMAILGVLVGG LVIAMYLPIFQMGSVV"
/product="Type IV fimbrial assembly protein PilC" /transl_table=11
CDS 1596261..1597121
/locus_tag="AUO97b_01645" /db_xref="SEED:fig|400667.82.peg.1506"
/translation="MQDIIAYFIQNFTALYIAVALVSLCIGSFLNVVIYRTPKMMEQD
WQQECQMLLNPEQPIIDHEKLTLSKPASSCPACQQPIRWYQNIPVISWLVLRGKCGHC
QHPISIRYPAIELLTMLCSLVVVMVFGPTIQMLFGLVLTWVLIALTFIDFDTQLLPDR
FTLTLAALGLGINTFNIYTSPNSAIWGYLIGFLCLWIVYYLFKVITGKEGMGYGDFKL

LAALGAWMGPLMLPLIVLLSSLLGAIIGIILLKLRNDNQPFAFGPYIAIAGWVAFLWG DQIMKIYLGG"
/product="Leader peptidase (Prepilin peptidase) (EC 3.4.23.43) / N-methyltransferase (EC 2.1.1.-)" /EC_number="3.4.23.43"
/transl_table=11
CDS 1597123..1597719
/locus_tag="AUO97b_01646" /db_xref="SEED:fig|400667.82.peg.1507"
/db_xref="GO:0004140"
/translation="MAFILGITGGIGSGKSAATQWFESQGIQVVDADIVAREVVEKGQ
PALQKIQQTFGDWVLQPDGSLDRRALREYIFQNPQARHTLEQITHPAIRQSIIQQLQN
RKSPYVILVSPLLFETNQHELVNHTLLIDASEQTQIQRASQRDGQNQEQIQKIIAAQM
PRERKRELANDIVFNDGLLEHLYQQLEPLHQSYLKRAN" /product="Dephospho-CoA kinase (EC 2.7.1.24)" /EC_number="2.7.1.24"
/transl_table=11
CDS complement(1597716..1598633)
/locus_tag="AUO97b_01647" /db_xref="SEED:fig|400667.82.peg.1508"
/db_xref="GO:0016020"
/translation="MSPRTQGYFFVLVTMCIWGGFTLFARLNAQWHISAWDIAALRFA
IAFLILMPILIYKKDLAFLWSKHAVILALIGGVIYCLTVYTAFLYAPAAHAAIFLNGC
IPICTAIAAYILFRQPFDKHTWVSLAIMITALALMSALMLQSSTSAFGIGDLLLFISA
IWWGIFTVLLKQWKLSAWHSMASVAIWSAIIYLPIYLLFLPKHFMDVDPLHLAVQSIF
HGVFVVIIATLTYVAAIQRLGAFKTGSIVTLAPFIAAILAVPLLGESLSPSIVVGLVG
MGIGALQPWRWWFRQDSLTQKIKQQNTQN"
/product="Permease of the drug/metabolite transporter (DMT) superfamily"
/transl_table=11
CDS complement(1598669..1599418)
/locus_tag="AUO97b_01648" /db_xref="SEED:fig|400667.82.peg.1509"
/translation="MAKPEYYYGVHSVESLLELEPERVLTLFTLKGRDDQRLQKILQL
AEPFGISVQKASRDSLEKLAGLPFHQGVVAAVRPHPTLNEKDLDQLLAETPDALLLAL
DQVTDPHNLGACIRTAAAMGVQAVIVPRDRSASLTPTARKVAAGGAEKVKFIQVTNLA
RTLAHLKETTHVRVIGTMLDENALPIQKCDFSGAVVIVMGAEDTGLRPITQAQCDQKA
YIPMSGNLQSLNVSVATGMALYEACRQRSEV"
/product="23S rRNA (guanosine(2251)-2'-O)-methyltransferase (EC 2.1.1.185)"
/transl_table=11
CDS complement(1599520..1599846)
/locus_tag="AUO97b_01649" /db_xref="SEED:fig|400667.82.peg.1510"
/translation="MSSTQFDHVTVIKKSNVYFGGACISHTVQFEDGTKKTLGVILPT
EQPLTFETHVPERMEIISGECRVKIADSNESELFRAGQSFYVPGNSVFKIETDEVLDY VCHLEG"
/product="hypothetical protein"
/transl_table=11
CDS complement(1599923..1601233)
/locus_tag="AUO97b_01650" /db_xref="SEED:fig|400667.82.peg.1511"
/translation="MKKTLSLPKAPIGMINRHKKTNPAEMNKILNQHFNAFKQAAAQG
DYVKAYQHVKKAVSLVPSHPGALSDLAYTELRLRRYDDAYQHYMQAIKASGSNVNTNL
YDGLTEVCHHLNKKEEKIKFGRLAISTKKELTKNEPTLNIPTHKPVPFSPNPQENIIV
FSLFGANPRYCETSILNTKLAQEIYPEWTCRFYVDESVPELVQQRLQANGAQVVHVSP
TQKQLSGLFWRFLVMDDPVVKRFLVRDADSIVSYREKAAVDAWLKSDQWFHLMRDSYS
HTELILAGMWGGCTGIFHNIEAHIRDYVATGRYPDNRVIDQHYLRYCIWPTLKQSVLV
HDSQQFDENSADFPVYDLTSMQNDQDNFHVGMDNGSPVVTTAVNHPTAEKVYWILFDE
NHDEVCRYDAIVSKSRNIEVNLPHAFAKKIQLQQWKLQVYPYEN" /product="TPR repeat"
/transl_table=11
CDS 1601348..1603015
/locus_tag="AUO97b_01651" /db_xref="SEED:fig|400667.82.peg.1512"
/db_xref="GO:0005524"
/db_xref="GO:0005694"

/db_xref="GO:0051276"
/translation="MLTHLTLINFALADHLAIDIEQGFNVLTGETGAGKSLLLDALSA
CLGERTDTNYVRYGSDKADVTAVFTYQDNSPEAKWLKEHELDDDSGEIHLRRVIFATG
RSKAWVNGRPSSLSELKELGRLLVQLYSQHSQQQLLEPPYPKHWLDRYSNFYAEANDV
REAYSTWQRNIRQHQAALDAQATRLQRIATLELQIEELEEIIQTDYKEIEQEFDRLSH
HEHIMQDCSYSLNALDEAEQNITQEMSSIIRRLESHAGRSEQLSEIYNSLLNAQSEID
DATSNLRQFIDRQSFDPERMEELNSKLEVFHRLARKYRTQPETLKEEYETWQSELEQL
HQLEDPETLAEQVEKSHQEFLEKAQHLDNIRREAAAPLAKQLTEQVKPLALPEAHFEF
KFEPLEQPNAEGLSFIQLLFTANKGIPPQPLARVASGGELSRIALVMQVMNAEKTEAE
VLVFDEIDVGISGGTAEVVGRLLADLAQHVQLLCITHQAQVAAQSDQHLLVKKQQTDP
ASSTIVQLDENQIISELARMSGGVEINETTLQHAKQLRQLKFQASST" /product="DNA repair protein RecN"
/transl_table=11
CDS 1603142..1603696
/locus_tag="AUO97b_01652" /db_xref="SEED:fig|400667.82.peg.1513"
/translation="MTKQYMTHRCLIAPPEMADDFFANTVIYLARHDEEGAQGIIINR
PAGIQIKELLNDLDIDADNVNPHEVLQGGPLRPEAGFVLHTGQPTWHSSIAVGENVCI
TTSKDILDAIAHNEGVGRYQIALGYASWGKNQLEDEIARGDWLICDADMDLIFNLPYD
DRWDAAYKKIGVDRTWLASEIGHA"
/product="UPF0301 protein YqgE"
/transl_table=11
CDS 1603696..1604136
/locus_tag="AUO97b_01653" /db_xref="SEED:fig|400667.82.peg.1514"
/translation="MTATQSKSIMAFDFGTQKMGMAIGQSAIESANPLPLFVMKDGIP
NWDQLLKIVKEWQPDLFIVGLPLNMDDSESELSTRARKFARRLRHQTNIETWMVDERL
TTREAREELGFYQEQGRAKKLSADSFAAALLIQSWYRNPVGLTP" /product="Putative pre-16S rRNA nuclease YqgF" /transl_table=11
CDS complement(1604193..1605194)
/locus_tag="AUO97b_01654" /db_xref="SEED:fig|400667.82.peg.1515"
/translation="MNQLELKKVIRPVILIVALMIAVYTIVHLWNYYNAAPWTRDGRV
RGDVIQVSSDVAGLVTEVLVQDNQTVKKGQVLFKIDVSRRALDVEQAKSDLAKAKAAY
AQAQAGLAQAKANLIKSSTNIKLAEKNANRYSNLMDGAISKQEQDQVFATRDQSHAEH
EQLQAAIQQAEATIKQQQALVEAATSNLHLAELNMHRAAVVAPADGTLSNFDLRPGNY
VQVGQAVAALLDRKQLYVVGYFEETKLDRIHIGDEATVQLMGDRQKIKGHVQGIASGI
EDRERSSSSKLLANVNPTFSWVRLAQRVPVKIVLDEAPKNELAFVSGRTATVHIIEK"
/product="Tetrapartite efflux system, membrane fusion component FusE-like"
/transl_table=11
CDS complement(1605234..1605440)
/locus_tag="AUO97b_01655" /db_xref="SEED:fig|400667.82.peg.1516"
/translation="MGEFNVYGIYVPALLVQAIFAYICFRWLSPLTNKWIAQGWIALP
SIFNLCFYLLLLLVIHQIFVGLGA"
/product="Tetrapartite efflux system component, FusD-like" /transl_table=11
CDS complement(1605433..1607532) /locus_tag="AUO97b_01656"
/db_xref="SEED:fig|400667.82.peg.1517"
/translation="MLLKQILAFRPSKLDLIFAIKTFIAGMLALFVSFELDLINPMWS
IGTVLIIANPYSGMVSSKCVYRLLGTVGGAIIALTITPHLINTPWIFTVVLSLWVGFA
LYVSLLDRTPRSYAFMLAGYSTAMIVYNAITYIDQYNIFDIALARVLEISIGVISSAV
VSATILPMHIGSAIKQRVTKTLKDTENLFANLLNADQQQNNTQLLAAITRDTTDIHAL
AVHLSYEKGELHGMTKPLQEMLHQMSMVVANLVALSERIKQLQELNFIESHSDKLKQL
SGHVIHFLEQKDQMTDENILQLPDEFENDFLNLNDSASEHQQVLLAAMKMDVRHFISN
VLAVKVLWQRIKQGNKEIPDNITPMTTTYPSLHRDHGVAVRGGISAVLITFIVTGVWI
LSGWKAGFMMAQIGAVTACILTALDNPVPVLRIFIWGSVASAVLVFIYAFGIFPHVTT
FWELGLVLLPMFLFAVSMMANQALMPVGMVLGINTMMGLNLHNAYSMDAVSYLDSSFG
MVMGVLVSLVVIDVVRAMSPDTSANRILALHYRAMRQAIYIPYGIEFKVHLRGMLDRV

GVLNTKMVQSEEIKKSIQQALVESSAIIDLSRLQELANQVPTSSELAHHIGRLQQDLD
ELFRAKENEKGESDVLVQHIHQTLFELKQLASNIEDMTLRQRLFISLNNIAYSMCHVS SNQMNADRTLTGATAHG"
/product="Tetrapartite efflux system, inner membrane component FusBC-like"
/transl_table=11
CDS 1608028..1608801
/locus_tag="AUO97b_01657" /db_xref="SEED:fig|400667.82.peg.1518"
/db_xref="GO:0003677"
/translation="MAISSFGKILTVLDLFSVSRPVINVDIICEELGLSKPTSYRYLK
ELVSADLLKRINGTSGDYTLGSKIAVLDYVSRTTDPLVQISTPFMRNIVERTELCCLL
TYLNDDYCIDIHHEIFKDTELLSYGRGCPRPIYVGASPKTMVSHLSKQRMHDYYERFQ
HELSESGFAVDEPSFIQRMRKIKKQGFYFSQGEIDPNVSGLAVPVRFSSKEVPLALTL
VASKNRFDFLNIEKLIEILQENAALIEQRFMELSEKGEI" /product="Transcriptional regulator, IclR family" /transl_table=11
CDS complement(1608996..1609607) /locus_tag="AUO97b_01658"
/db_xref="SEED:fig|400667.82.peg.1519"
/translation="MTLDLFSPEPCSNLLPYDGEVQDYGCILTAEEAEQYFHYLYHHL
AWRHDEAKLYGKHFITPRKVAWYGDEHYRYKYSGVFRDSLPWDKALAQLKQQVEQILS
EKFNSCLANLYEDGTQGMAWHSDSDVSLARTTTIASLSFGATRKFSFRHIQTKEKVEM
WLQPGQLIVMRGETQQYWQHRLNRSTKILQPRINLTFRQFKFS" /product="Alkylated DNA repair protein"
/transl_table=11
CDS complement(1609604..1610458)
/locus_tag="AUO97b_01659" /db_xref="SEED:fig|400667.82.peg.1520"
/db_xref="GO:0003908"
/translation="MQMLSSRQFAAIATIIEYLYEHLDQQPSLEDVATYMNLSPSYIQ
RQFQEWVGISPKKFVQYMSLQQAKYYLMQQRSLLDTALNTGLSGTGRLHDLFIQLEGM
TPGEYKQQGDGVELNYSVETSPFGDLLVVSSDKGICSVRFVDHSKNIQEIVKQYFPKA
QLKNHSPIWHQQITQWFRQDFSEHLQQKLPLNLAGTPFQLQVWEALLTIPEGQLRTYQ
DIAEQIGKPKAVRAVATAIGQNPIAYLIPCHRVIRATGMVGEYHWQKGRKLALLAWEM AKQQGEIA"
/product="ADA regulatory protein / Methylated-DNA--protein-cysteine methyltransferase (EC 2.1.1.63)" /EC_number="2.1.1.63"
/transl_table=11
CDS complement(1610582..1611169)
/locus_tag="AUO97b_01660" /db_xref="SEED:fig|400667.82.peg.1521"
/db_xref="GO:0008444"
/translation="MTTGRILNIPNILTLARIALIPVFLVIAYWPPAIGIGEHVGSMT
RHIILTAIFVLAAVTDWFDGYLARTLNQTSAFGRFLDPVADKLMVAAALIVLVQWKPT
IAMAFAAIVIISREITVSALREWMAELGARTSVAVSTVGKYKTAFQMIAISVFLLNWQ
PLEVLAYALLYTAVILTLWSMFIYLKAAWPYLKQP" /product="CDP-diacylglycerol--glycerol-3-phosphate 3-phosphatidyltransferase (EC 2.7.8.5)" /EC_number="2.7.8.5"
/transl_table=11
CDS complement(1611288..1612082)
/locus_tag="AUO97b_01661" /db_xref="SEED:fig|400667.82.peg.1522"
/translation="MSLLHLWEDVKQNEWIDIPAGWLQGRTVFGGLVAGLLMQKACSY
IQDSNKRLLSCSVTFVGPVQQGAARLTIEVLREGKSVTTLEARLWQDDAVQTILVASF
GASRASSIEVKQEPVVPAYVSPENLSVIPFAKHMPECFQHFEVCWAEGNYPVTGSKHP
DFGGWSRFSPEQHANRQMTLSDLIVLMDIWPPGVLPMFKQVAPASSLSWHITYVHPVR
HQLCDWFKYKVTTEYAAEGYSTEYAYLWDQNDHLIAILRQTVTVFT" /product="TesB-like acyl-CoA thioesterase 1" /transl_table=11
CDS complement(1612203..1613930)
/locus_tag="AUO97b_01662" /db_xref="SEED:fig|400667.82.peg.1523"
/db_xref="GO:0003677"
/db_xref="GO:0004518"
/db_xref="GO:0005622" /db_xref="GO:0006281"

/db_xref="GO:0006289"
/translation="MLGKEGELLYVGKAKNLKNRVSSYFVKTIEHPKTQALVARIYDI
ETLVTRSETEALLLEQNLIKQHRPPYNIMLRDDKSYVYIFVSADKPYPRIASGRGKGK
HQIGKFFGPYPSAYSARDTLLVLQKLFNVRQCENSYFSQRKRPCLQYQIKRCSAPCVG
LVSPEDYKEDVNNSIRFLQGDTKELNQELIAKMEQAAADLEFEKAVFYRDRLSLLREV
QAQQAVFKVKGEADILAIAYQAGVTCVQIMHVRNGRMLGGKSYFPDMLGDDLGQMLSD
FMANFYFQVADEVPSELIVNTALPDRKELEEALAQQFGKKVQIKSSVRETRAEWLELA
EMNVQHAIKGQLSNHLELNERFHQLEQVVGRPIDRIECFDISHTMGEAPIASCVVFDQ
GGARKRDYRQFAIQDITAGDDYAAMRQALTRRYKKAMLPDLLLIDGGKGQLHMAMEVM
QELGLEAFMVGVSKGEGRKPGLETLHFTDGTKIQLPEDSKALHLIQQVRDEAHRFAIT
KHRAKRDKRRSTSVLEAIPGLGPKRRRDLLTHFGGIQGVLKASEKELTVVPGLGEVMA RTIYKILHE"
/product="Excinuclease ABC subunit C"
/transl_table=11 CDS complement(1614136..1614666)
/locus_tag="AUO97b_01663" /db_xref="SEED:fig|400667.82.peg.1524"
/translation="MAHAHHAAHAYVHHIKVRKEESSQGDLKTKAMLSKASSGNKKLH
RPASKRHKSEDQNIDLHKFSSKLESMSAKHSKEKCAKNIRIALQAAGADVSKHPVAAS
DWGQTLEKNGYKKIKPAFNRPQEGDIYIIERTSGHTYGHIAGYTGNGWFSDFRQKTYA VYKEKDVKYSYYRLDS"
/product="hypothetical protein"
/transl_table=11
CDS complement(1614839..1615753)
/locus_tag="AUO97b_01664" /db_xref="SEED:fig|400667.82.peg.1525"
/translation="MIKWIILAIFVISALYIQHRGKVRHSFYRQFFDHSTLLAPINFL
MYIFSKVPNQPYIDTQHFKDLKVLDENWEMIRDEAKALYDQGGIKASSTYNDLGFNSF
FKTGWKRFYLKWYESSHPSAAELCPKTTALLKTLPTIKAAMFTELAPDSRLVRHRDPY
AGSLRYHLGLITPNDDRCFIDVDGERYSWRDGQSVVFDETYIHYAENKTDQNRIIFFA
DVERPMKVKWMERFNHWFGRNVMTAASSPNETGDQTGGLNKAFGYVYQIRIKAKALKA
KNRKLYYFLKWFLMLGIFFLIFVRPYVF" /product="Fe(2+)/alpha-ketoglutarate-dependent dioxygenase LpxO" /transl_table=11
CDS 1616325..1618478
/locus_tag="AUO97b_01665" /db_xref="SEED:fig|400667.82.peg.1526"
/db_xref="GO:0003857"
/db_xref="GO:0008692"
/db_xref="GO:0004165"
/db_xref="GO:0004300"
/translation="MIHAGNAITVQMLADGIAEFRFDLQGESVNKFNRATIEDFKAAI
AAVKANNDIKGLVVTSGKSTFIVGADITEFGQNFAQGEKAIVDWLMPVHEIFNSFEDL
DLPKVAAINGMALGGGFEMCLVCDYRVMSEAAQVGLPEIKLGIYPGFGGSVRLSRLIG
IDNAVEWMAMATPKKPAAALKDGAVDAVVAADKLLDAATDLVKQAISGRLNWKAKRQE
KLEAVKLNPLEQMMAFNTAKGAVLAKANPAQYPAPKLLLDSLQAGASLARDEALKAEA
EGFAKAAVTPQAEALIGLFINDQVVKKASKQHEKGAHPVNQAAVLGAGIMGGGIAYQA
ASKGTPIIMKDIGNPQLALGMKEANNLLTKQVERKKMKPAQMGETLARIRPTLSYEEF
KEVDIVIEAVTENPKVKEIVLAETEKNVRENTIIASNTSTISITRLAKALQRPENFVG
MHFFNPVHMMPLVEVIRGEKTSEEAIATTVVLAQKMGKTPIVVNDCPGFLVNRVLFPY
FGAFDLLVKDGADFQQIDNVMSKFGWPMGPAYLIDVVGIDTGVHGAEVMAEGFPDRMK
PDYKGAIEAMYEAKRLGQKNDVGFYKYELDKKGKKAKTVDPTAYEVIAPFVTGEKREF
DNQEIIDRMMLALCNETVRCLEDNIVATASEADMAMIMGIGFPPFRGGPCRYIDQTGV
AEYVALCDKYAHLGKAYEAPQMLRDMAANNKKFYG"
/product="Enoyl-CoA hydratase (EC 4.2.1.17) / Delta(3)-cis-delta(2)-trans-enoyl-CoA isomerase (EC 5.3.3.8) / 3-hydroxyacyl-CoA dehydrogenase (EC 1.1.1.35) / 3-hydroxybutyryl-CoA epimerase (EC 5.1.2.3)" /EC_number="5.3.3.8"
/EC_number="5.1.2.3"
/EC_number="1.1.1.35"
/EC_number="4.2.1.17" /transl_table=11
CDS 1618491..1619663

/locus_tag="AUO97b_01666" /db_xref="SEED:fig|400667.82.peg.1527"
/db_xref="GO:0003988"
/db_xref="GO:0003985"
/translation="MATLNPRDVVIVDGVRSAMGKSKNGMFRNVRADSLSAELVRALI
ARNQFDVNEVEDLIWGCVNQTLEQGMNIGRNIGLLAGLPKTVAGQTVNRLCGSSMQAI
HTAAAQIATNQGDIFIIGGVEHMGHVGMMHGIDLNPEASKHYAKASNMMGLTAEMLGR
MNGITREEQDTFGVESHRRAWAATQEGRFKNEIIGVEGHDANGFKILCDIDEVIRPDA
NLEAFKALKPVFDPKGGSVTAATSSALSDGASAMLLMSAERAQALGLKPRAVIRSMAV
AGCDAAIMGYGPVPATQKALKRAGLSIADIQTVELNEAFAAQGLSVLKGLGLYDKQDI
VNLNGGAIALGHPLGCSGARITTTLLNVMEQQDTQIGLATMCIGLGQGIATVIERV" /product="3-
ketoacyl-CoA thiolase (EC 2.3.1.16)" /EC_number="2.3.1.9"
/EC_number="2.3.1.16"
/transl_table=11 CDS complement(1619714..1622635)
/locus_tag="AUO97b_01667" /db_xref="SEED:fig|400667.82.peg.1528"
/translation="MSTQLIAHLKKKCEADDNVKILLSQWEFDQKLVGKALENIGGFY
PHFSNHNASHSQQILVNIERILGNDIDLLTATDTWLILEAAYWHDVGMLVDAKSAKDV
HGNEDFQFMVKTIAHDKGHDLHTFCLAYEQSDWVGAIGSEDHPFDGVEKYRQLIAEWF
RRNHDKRIGGLVEDPFEFLNITSPRTELLPKRIYRYLGKICMSHGMNFTDLMEKLPFK
QTGLGTEDCHPRFIGCLLRLGDLFDLDDNRFCPVMAKHVSHQPRLSKAHEDKHLSLRE
FQLDERTVKLVAECPNEMSYVETQNWFGWIREEFQNQMSQWNLIVPNFKFGSLPTIEQ
LDVKMEVKYENGIRKEKILLHNKPMKFTIDEKNGIELLQGANLYKNKLSIYRELIQNS
IDSTMLRVWLENKDELIDLNPYDQKYLNIIKNYQLIIDLEKVKDIESSLDSVWELSIK
DKGIGISQNDLFYMQKMAGSDKNLEKKRIINEMPLWMRPSGKFGIGLHSAFLLLENTL
EDTDKKIKFKTCSYFNNENLEVEMFSPLGGKQGFCFINKIQDNLKHEYGAEFLIKIKR
KFILSYRGLYRKKSPVRWSREFIDDNWKKNLIDNIYRNIVLNSPINPKKDIEDILWDA
DRCVAFKFDILSSNDFFGFRGQNTSFRSKSYHKFLCGIYIDYYNLDPSKALNISRDTW
NLKFVNKIEEDIFYYMGEYVRKIKNENTSLLRKLYYNKLSIDCVDDGQWKDFVFKFSG
ENKSINDLNKIEEFYIISNSDYPDNGYNSEFISSINKVKFFVVEERVALQIKLLVEEF
SNEVFPEHRALQMVDNQFLHYRCFYFSKRKSKPLSKVLDKKLYWGEIIKKSIEENINY
SIEMCFKAMQVFTKLQLKNDYYYGTKTWYSYLMGVSVIRDVEIISDYKILCIKEGEEE
VVINPYQVVDNRIELTNLEELSALLVSYLNIDMSEYQKILHDYKRLQLKIEKDLGIYI Q"
/product="Chaperone protein"
/transl_table=11
CDS complement(1622746..1623237)
/locus_tag="AUO97b_01668" /db_xref="SEED:fig|400667.82.peg.1529"
/translation="MNFLKQSAFIAVFAFMGLPTVQAEEVATLPTIRVMAESELREEV
GFVPFQEDKKVRQALQHREYKTHNDIQNALVPEGMTTVDYQPVMAQPDLSQLSPFLRE
YILAVAGGIQSSDPTNGLFEMLKPLQINRSNVDAYREGTMKVNLDDILKLQQQIQDGL KGR"
/product="hypothetical protein"
/transl_table=11
CDS 1623513..1624229
/locus_tag="AUO97b_01669" /db_xref="SEED:fig|400667.82.peg.1530"
/translation="MNFKILNQNILMSCLLASFFLTGCVSNRIIGENTRELFTKYDSG
YEWITLTEDDVIAFGKPSVSLPNEPEDSIVIAGKKYSYVISKGGNDFIQLISQLNPQY
IQISNELDFNAPASNSNHFYGHIRFSYTPPNGKLNEKESALFKQYGVIPCDYEKTLEL
HKFELNLRGTVYPPVNNLKKITPLSKPYHVRIQYLHDKSGKVPLSTKEKLASLPLLPF
TLTFDIIALPAKVMGIIYQP"
/product="FIG00351808: hypothetical protein" /transl_table=11
CDS complement(1624298..1624855)
/locus_tag="AUO97b_01670" /db_xref="SEED:fig|400667.82.peg.1531"
/translation="MRQIFVLHGYSASINDHWFLDLKHQIEDENTTVTLIPFPDSEHP
DVDAWQKVLDEQIPAVNENTYFVAHSLGVITLLHFLQRHDYQNIGGMILVSGFSGPIS
DSSPLSPYITKSKVDTNYFRDIKKKLVYLSDNDDLVPPQLTIQLAKDIDAPYITVPNG
GHFLGREGYTEFPQLVDSLKEMLEN"

/product="hypothetical protein"
/transl_table=11
CDS 1625015..1625380
/locus_tag="AUO97b_01671" /db_xref="SEED:fig|400667.82.peg.1532"
/translation="MNLAKVSELTKVSPRMLRYYENLGLIQPHRASNNYRSYTQKDIE
NIKKIKILNDAGMNLKDIHVLLPCFDLDERVFTLCPVVQEKLQTELKHVSDQLNKLQT
SQSLLQSFLAKGKAESTNI"
/product="Transcriptional regulator, MerR family" /transl_table=11
CDS complement(1625402..1626160)
/locus_tag="AUO97b_01672" /db_xref="SEED:fig|400667.82.peg.1533"
/translation="MRRVLIAIASLVVITSIVFFTYQHIKQQQAKDSSQMFDVVMTEK
MNQLYDQAQDWTKPVQLDIHDKRLAGHYKQVSEFLLSYWVQNVNARNEYLRELKAAKL
DTFLNVDRLDHDKKQKYAETEKMLADVRRASDKYQSEYEKIHKTFLAKIQELSVDKEM
RQILEIKLGAQQKADQDHAIFMIELQILDKAEEMFKLLKTYPWQKKDQMILFHENAQV
KKFNALYQDVLKLNAKIEKIKKKNVAALEGELKE"
/product="hypothetical protein"
/transl_table=11
CDS 1626338..1627042
/locus_tag="AUO97b_01673" /db_xref="SEED:fig|400667.82.peg.1534"
/translation="MRVDVWSDVVCPFCYIGKKRLEHVAAEAGIELDIHWHSFELDPD
APAKHDTSNTERLAKKYGRTYEEMEEMERNIAAMAASEGIDFQWQKANSGNSFNAHRI
IHLAQSKGLGNEAKEAFFHAYMTEGLAIGEREVVEEIASRIGLDNAEVEYVLDTNELA
DFVRHDEKIAHEQLNVTGVPFFVFDQRIALAGAQPREVFLQVLEQAQLKANAENIEQD DAAVCNDETCDIPQNK"
/product="2-hydroxychromene-2-carboxylate isomerase/DsbA-like thioredoxin domain" /transl_table=11
CDS 1627401..1628540
/locus_tag="AUO97b_01674" /db_xref="SEED:fig|400667.82.peg.1535"
/translation="MKTFHIITYRQLFALLTLGFGSVCMAHAEVNSSSTQQVNGLSSG
AQAEENKDENLLDQIPRWIDATPTIFPEQSNEPIVPPTEQTEDQTWFDRKQKKIRNWA
DRTSGKIDNWFGEVDPQKPASATIRVMIDNYWNEYDNYEIKPRIRGKIKLPTLEKRLS
VVFGDDSLDDEFNNSPANINQNPNQDPNKKLDGKRTRDDNSSIALRWSNFSKKLPFET
DADLGIRSGDDIYVRLKASRDWQLRNDFKFYAEQIYRYGIDSENYLRTNLELTHARPN
QPILSNQFSLTYADDQDDDLTWENRLFREHSFFANNRFNYGIYTGGYYNDNDLRLNSW
GPFVSWRQPVLREWFFVQGDLNYFNDHREDRNHYVSTFLRLEALF" /product="hypothetical protein"
/transl_table=11
CDS 1628662..1629564
/locus_tag="AUO97b_01675" /db_xref="SEED:fig|400667.82.peg.1536"
/translation="MNQFSSFSFEYRSTALLCIYSLGMLVLIPHIQLFPISLKHSRST
RRPLYSSVVTLKTKSQSVSFLRSPCEVETDHSSDTALIQDLMAVFGNDTLWSRAVQHK
HPYKTFGKIKKKASTPHVSENNPLTASWNTISTWMPFENDLDLGSNSESDVYVQFLAK
KKWNLRHNISLDTEQIFRYGSTSRNYTETTLNLTQKMQDNTLFSNTFNVNKSGKEDYN
WGNWTFQQFEFVKDNRLTYGIYSGGVYANNDIRLNSWGPYISWRQPIWRDWIFMQNDL
NFFNYLDNDPKHQLSVQMNLEANF"
/product="hypothetical protein"
/transl_table=11
CDS complement(1629648..1631567)
/locus_tag="AUO97b_01676" /db_xref="SEED:fig|400667.82.peg.1537"
/db_xref="GO:0005524"
/db_xref="GO:0006457"
/db_xref="GO:0051082"
/translation="MSEQASQNYSFQAEVAQLLHLVTHSLYSNPEIFLRELISNASDA
CDKLRFEGINHPEYYENDPNLHVRISLNKEDKTLTISDNGIGLSQQEAIDNLGTIAKS
GTKDFMSKLTGDQKADAQLIGQFGVGFYSGFIVADKITVESRRAGLDASEGVRWISGG

TGEFEVQQIDKASRGTDIILHLRDDALDYLESYKVKQIVNKYSDHISLPIEMQKEVWQ
EEEVAEGEEPKGGQMVKTDEWEAINSASALWTRNKSEVTEEQYVEFYKNLTHDFEAPL
AWAHNRVEGSTEYTQLLYIPSKAPHDIFTREAKAGIKLYVKRVFIMDDADNLIPNYLR
FVQGVVDSADLPLNVSRELLQESRDVKTIREGNARRVLTLLDGLAKSEDEKDQEKFKT
FYTEFGSVLKEGLGEDFGNRERILKLLRYATSTNDEVTTSFADYKARMKEGQKAIYYV
TAESLAAAKNSPQLELFKKKGIEVLLMAERVDEWAMNFVHEFDGTPLKNVSKGAVDLG
DLQDAEEKKALEQAAEQFKPVVEKLSDSLKAKTKEVRVTTRLVDSPACLVTSEGELSP
QLIRMLKQAGQAVPEIKPILEINPEHPLVKKLEGSEQFDDLANVIFDQAVIAEGGLPE DPAAYVKRINSLLLK"
/product="Chaperone protein HtpG"
/transl_table=11
CDS complement(1631670..1632026) /locus_tag="AUO97b_01677"
/db_xref="SEED:fig|400667.82.peg.1538"
/translation="MMNRKFAMGLALFSGLAFTHSLQAASFSCNAAKTKTELSICKNR
SLNDADVKMATTYQIVLHALPMGGRDNQKDTQQQWLKKRNACAANVSCISKAYQQRQK QLDTILQDRVLSHGPF"
/product="hypothetical protein"
/transl_table=11
CDS complement(1632207..1632788)
/locus_tag="AUO97b_01678" /db_xref="SEED:fig|400667.82.peg.1539"
/translation="MFKKALVIALMGVSSFTFAGNWQVKFGGSVIAPSEDTTTALGVV
KADHEYAFTPSVEYFFGQSPFSAELLLATPVNHDVLLDGQKVARIKQLPPTITAKYHF
KNSTRFTPYIGIGATAFIPWDEQGVADKVKEDFGVAGQIGFNFQPADAKNWGVFVDVR
YADISPEVTLTNGAKFDLDINPFVYTLGYSYKF"
/product="Outer membrane protein W precursor" /transl_table=11
CDS complement(1633113..1634537) /locus_tag="AUO97b_01679"
/db_xref="SEED:fig|400667.82.peg.1540"
/translation="MNAVVIAIAVMFLLSLARVSVVLTLVVSAIVGGLVAGLSLSQTV
EAFNAGLGDGAEVALAYAVLGAFALALSKSGLPDLLAHKLIGLLGMEAGKSRETKVKY
LLLGILLIAAIFSQNLIPVHIAFIPVLVPPLLRVMNHLKLDRRAAACVLTLGLVGTYI
FLPVGFGAIFLEQILMGNINKIGAAYGLHVERGMMPIGMAIPVLGMVLGTLVAVFISY
RKPRIYADRSISPVTTIDLDKPLRATAQDETADLKADAEELKHEAEQGPVIAKKTIFM
ALLAIGLTLVAQLYSGSMILGGLVGFAVLSTAGIFKWQDADDVFVQGMRMMALVGFIM
ISAAGFASVMTHTGDINQLVKGVVEIIGDNRALAAFLMLCIGLFVTIGIGSSFSSVPV
LAVIYVPLCVQFGFSPLATIALIGTAAALGDAGSPASDSTLGPTAGLGVDGQHDHIWD
TVIPTFIHFNIPLLIFGWIAAMVL"
/product="Histidine permease YuiF"
/transl_table=11
CDS complement(1634642..1635514)
/locus_tag="AUO97b_01680" /db_xref="SEED:fig|400667.82.peg.1541"
/translation="MQPKIYAVTTSDQQTLCAKAWGDEKNTPLVLVHGYPDNQEVWES
VIEHLINDFYIVTYDVRGAGLSSIPKRIRDYRLERLSLDLQEVVDEVIPNRQFHMAAH
DWGSIQSWESATEPKFKGRLLSYTTISGPCLDHAALFLREKFKTSPAQILKILTKSWY
IGAFHLPLIAPTVWSLFSPEKWGKILKDLERKDNLPLNSNISSDGKYGINLYRANFIP
RLTSPRQRYAQCPVKAIILKYDTFVSPEYITEAMPKWVENFEYVELEANHWAILSQAE KVAAHIRQFIDSQS"
/product="Putative oxidoreductase"
/EC_number="1.1.1.122"
/transl_table=11
CDS 1635638..1636135
/locus_tag="AUO97b_01681" /db_xref="SEED:fig|400667.82.peg.1542"
/translation="MAILKKLQNIPSISKFLFNHYPPYRGAGIYIEVMNLELCHVRVK
MPLTWKNQNLVGTHFGGSLYSMVDPFYMLILMHHLGSKYIVWDKAAEINFLSPGRSTV
YADIRVDLAEIEQIRELAENYAPVLRTYHLNIFDESGVRIAEVQKTLYIRRKKAKPST NKISA"
/product="hypothetical protein"
/transl_table=11
CDS complement(1636164..1636520)

/locus_tag="AUO97b_01682" /db_xref="SEED:fig|400667.82.peg.1543"
/translation="MNKKIGLISTVILSTVMFTGCQNMSPSDQRIGAAAAGGALGGGL
GNHVGGGIGAGLGAAVGAGVGSNTQGGSKQTTTKSAIGAGIGSVVGKAIIGGDTGAAI GGAIGGGAGAAIEEKK"
/product="hypothetical protein"
/transl_table=11
CDS complement(1636734..1637099)
/locus_tag="AUO97b_01683" /db_xref="SEED:fig|400667.82.peg.1544"
/translation="MKKMMMIAGVGLMSTTLFVGCQNASPESKRIGSAAIGGGAGGAV
GKHVGGNIGAGLGAALGAGVAANAKGSSSKTVRNSAIGAGVGAILGGAVIGGDAGAAA
GGALGGSAGAAYEEKKGKY"
/product="Glycine-rich cell wall structural protein precursor"
/transl_table=11
CDS complement(1637266..1641459)
/locus_tag="AUO97b_01684" /db_xref="SEED:fig|400667.82.peg.1545"
/db_xref="GO:0003899"
/translation="MKDLLDIMRKKTDSDGHAPVEFDRIRIGLASPEMIKSWSHGEVK
KPETINYRTFKPERDGLFCAKIFGPVKDYECLCGKYKRMKYKGVICEKCGVEVTTAKV
RRERMGHIELASPVAHIWFLKSLPSRIGLLLDMTLRDIERVLYFESYVVTDPGMTPFE
KYQLLNDEEYFTALEEHGDEFVAKMGAEAVQDLLKDIDLEAEISRLREEIPQTTSETK
LKKASKRLKLMEAFKDSNNKPEWMVMNVLPVLPPDLRPLVPLEGGRFATSDLNDLYRR
VINRNNRLKRLLDLAAPDIIVRNEKRMLQESVDALLDNGRRGRAITGSNKRPLKSLAD
MIKGKQGRFRQNLLGKRVDYSGRSVITVGPTLRLHQCGLPKKMALELFKPFIFAKLQA
SGQATTIKAAKKMVERETPEVWDVLASVIRQHPVMLNRAPTLHRLGLQAFEPILIEGK
AIRLHPLVCAAFNADFDGDQMAVHVPLTLEAQLEARALMMSTNNILSPANGEPIIVPS
QDVVLGLYYITRDAVNAKGEGMVFADTHEVNRALATGQVAIHARVKVRVHQTVINENG
EREQQTIIVDTTPGRCLLWEVVPEGLSFDMINLEMTKKNISKLINSCYRKLGLKDTVI
FADQLMYLGFRQATRSGVSVGMEDMLIPPTKHTIIDKAETEVREIEQQFEQGFVTAGE
RYNKVVDIWARTNDQVAKAMMDNLSYTLVKNKQGEDEKQKSFNSIYMMSDSGARGSAA
QIRQLAGMRGLMAKPDGSIIETPIKANFREGLTVLQYFISTHGARKGLADTALKTANS
GYLTRRLVDVAQDLVITEPDCGTSGGLVMTPFIQGGDVIEPLRDRVLGRVTAEDVRRA
SDDEVVLPRGTLIDEKIAAQLEEAGVDEVKVRSVIACESTFGVCAKCYGRDLARGHLV
NPGESVGVMAAQSIGEPGTQLTMRTFHVGGAASRTSAANSVQVRNKGTVRFHNVKTVQ
HAKGHLVSVSRSGEIGIADELGRERERYKLPYGASILLKDGELVEAGGIVATWDPHTH
PLVTEVAGKARFSQIADGVTATSKTDDATGMTTVEILPVTARPASGKDLRPAIVLDTT
DGGEQFYFLPQNTIVTVRDGETIGVGDVIGRVPQESSRTRDITGGLPRVADLFEARKP
KEHAILAEVSGIVSFGKETKGKNRLVITPDDGSEIYEELIPKWRQINVFEGEHVNRGE
TISDGPQNPHDILRLKGEVALTNYIVNEVQDVYRLQGVKINDKHIEVIVRQMLRKVDI
IDGGDTSFIKGEQVDYIRVVQENQAVLAQNKFPAKFERQLMGITKASLSTDSFISAAS
FQETTRVLTEAAVTGKEDDLRGLKENVVVGRLIPAGTGLAYHLERRRQEAEAAEHALH
NDFSEVDQAFSQALNSEQF"
/product="DNA-directed RNA polymerase beta' subunit (EC 2.7.7.6)"
/EC_number="2.7.7.6"
/transl_table=11
CDS complement(1641546..1645634)
/locus_tag="AUO97b_01685" /db_xref="SEED:fig|400667.82.peg.1546"
/db_xref="GO:0003899"
/translation="MAYSYTEKKRIRKNFGKLPQVMDAPYLLSIQVDSYRTFLQDGKS
PKNREDIGLQAAFRSVFPIESYSGNAALEFVEYSLGKPEFDVRECILRGSTYAAPMRV
KIRLIIKDRETKSIKDVREQEVYMGEIPLMTENGTFVINGTERVIVSQLHRSPGVFFD
HDKGKTHSSGKVLYSARIIPYRGSWLDFEFDAKDLVYVRIDRRRKLLATVVLRALGYN
NEQILNLFYEKVPVYLDMGSYQIDLVPERLRGEMAQFDITDNEGKVIVEQGKRINARH
VRQMEAAGLTKLSVPDEYLYERITAEDITLRDGEVIAANTLLSHEVMVKLAEGGVKQF
NILFTNDIDRGSFVADTLRADLTRDREEALVEIYKVMRPGEPPTKEAAENLFNNLFFS
SERYDLSPVGRMKFNRRLGRPYEVGTDQKSREVEGILSHEDIIDVLRTLVEIRNGKGE

VDDIDHLGNRRVRSVGEMTENQFRVGLVRVERAVKERLSQAETDNLSPQDLINAKPVA
AAIKEFFGSSQLSQFMDQNNPLSEITHKRRVSALGPGGLTRERAGFEVRDVHQTHYGR
VCPIETPEGPNIGLINSLSVYAKANDFGFLETPYRKVIDGRVTDDVEYLSAIEEVGTV
IAQADSAVDKDGNLTEEFVSVRHQGEFVRMPPEKVTHMDVSAQQVVSVAASLIPFLEH
DDANRALMGSNMQRQAVPTLRADKPLVGTGMEANVARDSGVCVIANRGGVIEYVDASR
IVIRVNEDEMVAGEAGVDIYNLIKYTRSNQNTCINQNVIVNLGDKVARGDILADGPST
DMGELALGQNMRVAFMTWNGYNYEDSILLSERVLQEDRLTSIHIQELSCVARDTKLGA
EEITADIPNVGEAALSKLDESGIVYIGAEVTAGDILVGKVTPKGETQLTPEEKLLRAI
FGEKAADVKDSSLRVPSGTKGTVIDVQVFTRDGLEKDDRALAIEKAQLDSYRKDLKEE
YKIFEEAARERVIRLLKGQESNGGGSTKRGDKLSEDLLSGLELVDLLEIQPADEAIAE
RLTQIQVFLKEKSAEIDEKFAEKKRKLATGDELTTGVLKVVKVYLAVKRRIQPGDKMA
GRHGNKGVVSNILPVEDMPHDANGVPVDIVLNPLGVPSRMNVGQILETHLGMAAKGLG
DKIEKMLKEQRTVLELREFLDKIYNKVGGEQEDLDSLTDEEILALAGNLRAGVPLATP
VFDGAEESQIKDLLELADISRTGQTVLFDGRTGEQFDRPVTVGYMYMLKLNHLVDDKM
HARSTGSYSLVTQQPLGGKAQFGGQRFGEMEVWALEAYGAAYTLQEMLTVKSDDVEGR
TRIYKNIVDGNHYMDPGMPESFNVLTKEIRSLGINIELKNGD" /product="DNA-directed RNA
polymerase beta subunit (EC 2.7.7.6)" /EC_number="2.7.7.6"
/transl_table=11
CDS complement(1645940..1646311)
/locus_tag="AUO97b_01686" /db_xref="SEED:fig|400667.82.peg.1547"
/translation="MALTNEEILNAVAEKTVLELVELISAFEEKFNVSAAAVAVAAPA
GGAAAAAEEQSEFNVELTSFGANKVAVIKAVREATGLGLKEAKDLVEGAPQVLKEGVS
KEEGEELKKKLEEAGATVTLK"
/product="LSU ribosomal protein L7p/L12p (P1/P2)" /transl_table=11
CDS complement(1646354..1646860)
/locus_tag="AUO97b_01687" /db_xref="SEED:fig|400667.82.peg.1548"
/db_xref="GO:0005622"
/db_xref="GO:0042254"
/translation="MALLIEDKKQIVAEVSEVASKAFAAVVADYQGLSVEQLTTLRVE
ARKLGVTTRIVRNTLAKRAFQGTQFDILNDNLVGPTILGFSTSEDDMGAAARLFEEFA
KTNKAFELKAAAFDGKVYQGADVSVIANLPNQEKALTMLASVLQAPISKLGRLITALK EKNESEAA"
/product="LSU ribosomal protein L10p (P0)"
/transl_table=11
CDS complement(1647180..1647875) /locus_tag="AUO97b_01688"
/db_xref="SEED:fig|400667.82.peg.1549" /db_xref="GO:0003735"
/db_xref="GO:0005622"
/db_xref="GO:0005840"
/db_xref="GO:0006412"
/translation="MAKLTKRQKAIAAAVEANKVYTLEEAVQVLNSLPAAKFKESLDI
SVNLGVDPRKSDQVVRGATTLPAGTGKTVRVAVFAQGAQAEAAKEAGADVVGFDDLAE
SIQGGNLDFDVVIAAPDAMRVVGKLGTILGPRGLMPNPKVGTVTPDVAGAVKNAKSGQ
ARYRVDKAGIIHAAIGQVGFDAAAIRQNVETLVADLKKLKPATSKGVYIKKITLSSTM GPGLTVDVNNVSN"
/product="LSU ribosomal protein L1p (L10Ae)" /transl_table=11
CDS complement(1647879..1648307)
/locus_tag="AUO97b_01689" /db_xref="SEED:fig|400667.82.peg.1550"
/db_xref="GO:0003735"
/db_xref="GO:0005622" /db_xref="GO:0005840"
/db_xref="GO:0006412"
/translation="MAKKIDGYIKLQVPAGKANPSPPIGPALGQRGVNIMAFCKEFNA
ATQKVEPGLPIPVVITVYNDKSFTFIMKTPPASILLKKAAGIQKGSSVPNKTKVGKLT
RAQLEEIATTKEPDLTGADLDARVRTIAGSARSMGLEVEL"
/product="LSU ribosomal protein L11p (L12e)" /transl_table=11
CDS complement(1648416..1648949)
/locus_tag="AUO97b_01690" /db_xref="SEED:fig|400667.82.peg.1551"

/db_xref="GO:0003711"
/translation="MKRWYIIHAYSGYEKQVMRSLKERIQRSAVADSFGDVLVPTEEV
VEMKDGKKRKSERKFFPGYVLVEMEMNDDTWHIVKECPKVLGFIGGTPEKPAPITQRE
ADAILARVRNTGEAPRPKTMFEPGEELLVIDGPFTDFKGVVEEVQYDKSRLTLTINVF NRPTQVELEFRQVEKTI"
/product="Transcription antitermination protein NusG" /transl_table=11 CDS
complement(1648957..1649397)
/locus_tag="AUO97b_01691" /db_xref="SEED:fig|400667.82.peg.1552"
/db_xref="GO:0006605"
/db_xref="GO:0006886"
/db_xref="GO:0016020"
/translation="MSNDKSRDALSDAPIPQRNNSAEVVRSGSPLDIVLWVIAIALLL
SATMVNQHLPAYWAPANDVWVRVGVIFACIVVALGLLYATHQGKGFVRLLKDARVELR
RVTWPTKQETVTTSWQVLLVVVVASLVLWCFDYGLGWLIKLIIG" /product="Protein translocase
subunit SecE" /transl_table=11
CDS complement(1649609..1650799)
/locus_tag="AUO97b_01692" /db_xref="SEED:fig|400667.82.peg.1553"
/db_xref="GO:0005525"
/db_xref="GO:0006412"
/translation="MAKAKFERNKPHVNVGTIGHVDHGKTTLTAAIATICAKTYGGEA
KDYSQIDSAPEEKARGITINTSHVEYDSPTRHYAHVDCPGHADYVKNMITGAAQMDGA
ILVCAATDGPMPQTREHILLSRQVGVPYIIVFLNKCDLVDDEELLELVEMEVRELLST
YDFPGDDTPVIRGSALAALNGEAGPYGEESVLALVAALDSYIPEPERAIDKAFLMPIE
DVFSISGRGTVVTGRVEAGIIKVGEEVEIVGIKDTVKTTVTGVEMFRKLLDEGRAGEN
CGILLRGTKREEVQRGQVLAKPGTIKPHTKFDAEVYVLSKEEGGRHTPFLNGYRPQFY
FRTTDVTGAIQLKEGVEMVMPGDNVEMSVELIHPIAMDPGLRFAIREGGRTVGAGVVA KVTA"
/product="Translation elongation factor Tu" /transl_table=11
CDS complement(1651477..1652970)
/locus_tag="AUO97b_01693" /db_xref="SEED:fig|400667.82.peg.1554"
/db_xref="GO:0004049"
/translation="MTTLAQFEQLKAAGYNTIPVYRQRLADTETPLSVFARFKDQTQA
YLFESVEGGENWARYSMIGLGESTVFSCNAGVLSIQHADGSVTQQNCLDPFQYIREFQ
KQFKVPTAKLLPDLPSFTGGLVGYLGYDAVRYIEPRLKNVPAADPITLPDLWLMLSKT
VIVFDNLKDTLFLIVHADTEQSNAYEDAQKKLDQLEQLLATPVSLQARPHTPPHFESI
TGKAKFLETVEKVKEYIRAGDVMQVVPGQRMVSDFDGEALQVYRALRHLNPSPYLFLV
QGQTITDKKPFHIVGSSPEILSRLENGIATVRPLAGTRPRGKTKEEDIALEKDLLSDE
KEIAEHLMLIDLGRNDVGRVSKIGKVQVTDQMVIERYSHVMHIVSNVQGEVRDDIDAL
DVFKATFPAGTLSGAPKIRAMEIIDEVEPVKRGVFGGAVGYLGWHGEMDMSIAIRTCV
IRDKKVYVQAGAGLVADSNPESEWNETQIKARAVIKAVELSSNGLIL" /product="Anthranilate
synthase, aminase component (EC 4.1.3.27)" /EC_number="4.1.3.27"
/transl_table=11
CDS complement(1653079..1653747)
/locus_tag="AUO97b_01694" /db_xref="SEED:fig|400667.82.peg.1555"
/translation="MSVAQLSKRDLILFDLDGTLVDSAADLYRSMNLSLQSLSWPLVT
EAQIREWVGKGASKLCESVLLHIFGKLDVEQHKVLLQKFVEVYGAELCVNTQIYPGVP
EFLKHCQTLNIKMACVTNKPVKLAQGLLDALELSSYFQVVLGGDSLPERKPHPLPLLH
CMESLKISASQSLMIGDSSNDIEAARRAGIDCIVVSYGYNHGENIYDCQPQQVVDSLA ELIV"
/product="Phosphoglycolate phosphatase (EC 3.1.3.18)" /EC_number="3.1.3.18"
/transl_table=11
CDS complement(1653813..1654442)
/locus_tag="AUO97b_01695" /db_xref="SEED:fig|400667.82.peg.1556"
/db_xref="GO:0004721"
/translation="MTWKLQAITGEFTGQEINVDRDMLVGRHQDADLLLQAAEISRRH
AALLLKDQALWVQDLNSSNGTFVNDIRIEQEKQLHDGDIVQFASLKFSVFAPAQENTD
LPEIEVEPVQTAPIQDLSDQGMPSIAERAAETEVSRDGMPQRVSVPKPAPIPEGVDIH

AQPEQTPVAIEEPISRVTEEKEQQKNASIGLVTIIILVILAVLAWLFFK" /product="Serine phosphatase RsbU, regulator of sigma subunit" /transl_table=11 CDS complement (1654483..1656759)
/locus_tag="AUO97b_01696" /db_xref="SEED:fig|400667.82.peg.1557"
/db_xref="GO:0009306"
/translation="MALLNHQRPLWALLAAAPLIATVTSSAYAQTWKINLRDADLTAF
INEVADITGKNFAVDPRVRGNVTVISNKPLNKDEVYDLFLGVLNVNGVVAIPSGNTIK
LVPDSNVKNSGIPYDSRNRVRGDQIVTRVIWLENTNPNDLIPALRPLMPQFAHMAAIA
GTNALIISDRAANIYQLENIIRNLDGTGQNDIEAITLQSSQAEEIITQLEAMSATGAS
KDFSGARIRIIADNRTNRILIKGDPQTRKRIRHMIEMLDVPSADRLGGLKVFRLKYAS
AKNLSEILQGLVTGQAVSSSNNSNNSSNSSNPINSLIGNNQNSGSNTSGSNGASISTP
AINLNGNSNSSNQNNITSFNQNGVSIIADNAQNSLVVKADPQLMREIESAIQQLDVRR
QQVLIEAAIIEVSGDDADQLGIQWALGDLSSGIGLLSFSNVGASLSSIAAGYLSGGSA
GAASAIANGANKGNGATLGLGNFDNSRKAYGALIQALKTNTKSNLLSTPSIVTMDNEE
AYIVVGQNVPFVTGSVTTNSTGINPYTTVERKDVGVTLKVVPHIGEGGTVRLEVEQEV
SAVQDSRGQAADLVTSKRAIKTAVLAEHGQTVVLGGLVSDDTSLSRQGIPGLSSIPYV
GRLFRSDNRSNVKRNLLVFIHPTIVGDANDVRRLSQQRYNQLYSLQLAMDKNGNFAKL
PEQVDDIYNQKMTPPSIASKPKNYQQVPSGGKSSTITTPVAVEPTVQKQTLQLPDPEI
NRTKNTVTTTTLRPSTAP"
/product="General secretion pathway protein D / Type II secretion outermembrane pore forming protein (PulD)" /transl_table=11
CDS complement(1656801..1657637)
/locus_tag="AUO97b_01697" /db_xref="SEED:fig|400667.82.peg.1558"
/translation="MKVWIDKLQQLQWQKLDRLSVVVLAILILWLCWKLASFFWLVIA
PPQLMQFDRVELGSQQPQIPNISTFSLFNEPSANAAQESVNLELQGVMVGYPNRFSSA
VIKIDNTAERYRVGETIGSTSYQLAEVYWDHVVLSQGNGSTRELQFKGLPNGLYQPMT
PDASQQSATPSQPTEPMNTAQQALGQAIQQMQGNREQYLRDMGVSGNSGEGYEVTERT
PTALRNKLGLRPGDRIVSLNGQTIGQGQTDVQLLEQARRAGQVKIEIKRGDQVMTIQQ NF"
/product="General secretion pathway protein C" /transl_table=11
CDS complement(1657637..1658380)
/locus_tag="AUO97b_01698" /db_xref="SEED:fig|400667.82.peg.1559"
/translation="MKKKSKQWKWWFFALIAFLIFIILQIPATWLISKFSKNNQTVHN
VSGNIWQGQADWHRGALRGTIHWKTRPLDLLLLRFAADVDIHSGNTQLTGIMAYGFGK
KVIVRDMNGQIAPETLKQVVNWQWPVNSIQLKDIQFNYKKEQGFAAVDGQLHWGGGAL
IYNIGDRQDRMNMPSLSGQLTDQNGQLQVDIRDQRNQKMANLLLDANMMLDVQLTQRL
LLNVPSYDGKAGLDTFVISSRQPLLQGGN"
/product="putative general secretion pathway protein [KO:K02463]"
/transl_table=11
CDS complement(1658711..1659037)
/locus_tag="AUO97b_01699" /db_xref="SEED:fig|400667.82.peg.1560"
/translation="MKPDISELSVEELKRLQEEAEALIASKKDQAIEDAYNQIIEIAE
NVGFSVEQLLEFGAQKRKKTTRKSVEPRYRNKNNAEETWTGRGKQPRWLVAEIEKGAK LEDFLI"
/product="Putative DNA-binding protein"
/transl_table=11
CDS complement(1659232..1659672)
/locus_tag="AUO97b_01700" /db_xref="SEED:fig|400667.82.peg.1561"
/translation="MKELSVYPVIYDQKVAWGDMDAFGHVNNVQYYRYIESSRILYME
NVNILTPNIYTVVASSQCKYLKPVFYPDVLKVGVRVEELRKSAFRMAYILWSESQQQI
VATGEAVMVCVNKMTGLKCEIPSEVKSKIIELEKSVGHDLIHLA" /product="putative 4-hydroxybenzoyl-CoA thioesterase" /transl_table=11
CDS complement(1659680..1660873)
/locus_tag="AUO97b_01701" /db_xref="SEED:fig|400667.82.peg.1562"
/translation="MKHLLWVYFLISLVLFAALALFSYGYGMGYVYIYWRQLQLQTNV
WGLVLTFVVMSFIAQVIWLWIKRYSSREQRKRENIFQFKNLHPYEQLGIVWLLEAAED

QRVFIERVFTQSGLLKNIIDAKFLVLSGDYQKALEALDQSPPMAFELAELQRIEIFLA
QNEAERALTHLEFLYQHQLSPWLEEIETAYQQRLTALWGQLALQQPWVYLRSMKYGLL
DAEHRDLWLQQLLQQFDQASIDDLQALQQRYLDLESEIQTRPYSSKLLWLKLLARMPE
MSIQHEALTLHLLKEQFDPDVFYLWFQQQLLKQVPDYADVEEKINQFESQYMNLPVLT
FAKWHVYMATGRQAEAEILLSLYPDNILMSYLRIKSTLKEDDELIKQLNLIFENDANF LKFKI"
/product="hypothetical protein"
/transl_table=11
CDS complement(1660883..1661722)
/locus_tag="AUO97b_01702" /db_xref="SEED:fig|400667.82.peg.1563"
/translation="MRKLVSIIILLGIAWVAKLSYDMWQISRTVPELQQSLLQSEQQY
ANLNDQLVALQRQIQNQPSHNSKTTPLATTEVVQTGIAPTVLIKQKLELIQFAIDQQQ
FIYAVDHLTQLQQTLPQYGIAPALQHSLNQALEQDKQAIQQFVLAQNQRHQLIDDLLQ
NIDKNIQQALKQPKFEMDQSEAVSWWKKWFRIEKVETPSINLMNRSVVLKEVQLRLLI
AEQALNQGKMAEYQNELQTVMQKLNELPDATSQQLKNRIAKVAHLSVVPVPKLSTLGL IGS"
/product="hypothetical protein"
/transl_table=11
CDS complement(1661731..1662498)
/locus_tag="AUO97b_01703" /db_xref="SEED:fig|400667.82.peg.1564"
/db_xref="GO:0004852"
/translation="MLFINTRPDSRAAELTHALQKKGFQVESLPLLELVAQPFSESLS
QLYQQLTGAQAIVVVSPTAVDVGMQYLTQSGLKIDELKYIQWIAVGQATANRLHDYGI
EAHVPEVETSEGMLNLPLLHRMQKSGSVAFWRGEGGRQFMMETLQQQGIQILNFVLYR
RQCPQASYSTFKHLTQNSNYNLDKWLVLITSEASWKYWLELCLKNKAQPDCVYLVLGP
RLFQLVKDYRDQQQAHFHIIQLENLSPSGMIHHIQAL" /product="Uroporphyrinogen-III
synthase (EC 4.2.1.75)" /EC_number="4.2.1.75"
/transl_table=11
CDS complement(1662503..1663447) /locus_tag="AUO97b_01704"
/db_xref="SEED:fig|400667.82.peg.1565" /db_xref="GO:0004418"
/translation="MFDQPMLEFMKTLKIATRQSPLALWQAEHIRARLQELHPDLTVE
LVKFVTQGDKILDTPLAKIGGKGLFVKELEAALLDGRADLAVHSMKDVPMALPEGLTL
AVICEREDPLDAFVSNQFEKFADLPQGAKVGTSSLRRKSQILKQRPDLQIIDLRGNVG
TRLAKLDDGQYDAIILASAGLKRLGLAERIRHCIEPSVSLPAVGQGALGLECRADDQE
VLALIQPLLHPETDVCVRAERAFNAYLEGGCQVPIAGYATLQDGKIHIEGRVGSVDGQ
TLLRAELTDEANNAQQLGENLARNLLDQGAGDLLKALY" /product="Porphobilinogen deaminase
(EC 2.5.1.61)" /EC_number="2.5.1.61"
/transl_table=11
CDS complement(1663521..1664261)
/locus_tag="AUO97b_01705" /db_xref="SEED:fig|400667.82.peg.1566"
/db_xref="GO:0000156"
/translation="MKVLICDDEPLAVERLSRLVSKMGHEVVATAHHGQDALEQARLH
HPDVILLDIQMPGMNGLVCAEQLSQLNPRPAIVFCTAYDQHALEAFKSQAQAYLLKPI
DPQELEQTFKQLTQLTQAQLSALPKPELLQDLKTQRHQIAAKTYRGVELIPVENIYYF
LADQKYVTVRHKNGSVLIDETLKELETEFADQFIRIHRNALVSIAYLDGLELVSSGQY
QVRLRGLDERLSVSRRHLSTLRERIHQL"
/product="Positive alginate biosynthesis regulatory protein" /transl_table=11
CDS complement(1664322..1665461)
/locus_tag="AUO97b_01706" /db_xref="SEED:fig|400667.82.peg.1567"
/db_xref="GO:0000155"
/db_xref="GO:0005524"
/db_xref="GO:0007047"
/db_xref="GO:0016021"
/translation="MWSWWLSKIKKSISLAEAQQTKLFSTAYRNQNSSYFFTKIDRWQ
HLLELIIASNVLALVLALAEAQSWHALNGSRLLQYIVFINWVILSFFALVEHFQDFFS
QLQQKYALIIGFVMLQAIVVVTTIISNFFQFGVLKKTSVIAQDWSIYFTNVPLYLSYG

ILLGAFCLRYLYVREQWLHQQYAELNARIQAMQARIHPHFLFNSLNNVVSLITIDPDK
AESMLISLSRLFRASFQELKLVSLHEEIELSKQYLMIEQIRLGERLKIEWKVEIEPLQ
LKQVTIPLLTLQPLLENSIFHGVEPMMGKATIGVLVEILQNQVSIVITNPYTHDTINS
RKGHGIALENVKQRLKAYYGNSVRFQVYKGEALYTTIMSYQYQSK" /product="Alginate
biosynthesis protein AlgZ/FimS" /transl_table=11
CDS 1665452..1666885
/locus_tag="AUO97b_01707" /db_xref="SEED:fig|400667.82.peg.1568"
/db_xref="GO:0004056"
/translation="MTTSSNPPNSAAPNQTSGMWGGRFSEATDAFVAEFTASVQFDQR
FYKQDIAGSIAHATMLAKVGVLTEAERDDIIEGLSTIRAEIEAGTFEWRIDLEDVHMN
IESRLTQRIGITGKKLHTGRSRNDQVATDIRLYLRDEIDDILGLLERLQKGLLGLAAK
NVNTIMPGFTHLQTAQPVTFGHHLLAWFEMLVRDTERLQDCRKRVNRMPLGSAALAGT
TYPIDRAYTAELLGFEAVSENSLDAVSDRDFAIEFNAAASLIMMHLSRMSEELILWTS
AQFKFVNIPDRFCTGSSIMPQKKNPDVPELIRGKSGRVFGDLISLLTLMKGQPLAYNK
DNQEDKEPLFDAIDTVRGSLMAFADMIPALVPNVEIMREAALRGFSTATDLADYLVKK
GVAFRDAHEIVGKAVALGVAEEKDLSELTLEQLQQFSDLITADVFDKALTLEASVNAR
DHIGGTSPKQVEAAIARAHKRLEQLYA"
/product="Argininosuccinate lyase (EC 4.3.2.1)" /EC_number="4.3.2.1"
/transl_table=11
CDS 1666895..1667164
/locus_tag="AUO97b_01708" /db_xref="SEED:fig|400667.82.peg.1569"
/translation="MSRQVFCRKYQKEMEGLDFAPFPGAKGQEFFENVSKQAWQEWLQ
HQTTLINEKRLNVFEPEAKKFLEEQREKFFNNDESVEKAEGWKPE" /product="FIG001341: Probable
Fe(2+)-trafficking protein YggX" /transl_table=11
CDS complement(1667480..1668202) /locus_tag="AUO97b_01709"
/db_xref="SEED:fig|400667.82.peg.1570"
/translation="MSPSNPVLSHHISSQFNEDLQDVNTKFMTMGGLVEQQVANAIHS
LLDTDANLAIDVQFKDNAVNQYERDIDEGLTLILARRHPAAIDLRMVIAMSKANTDLE
RIGDEAAKIARIAQNLCEEGGSPRGYMETRHIGNQVRVMIHDALDAFARLDADQALRV
LLADADIDREYQSATRTLMTYMIEDPRHIARVINVMWVLRSLERIGDHARNIAEQVIY
MAKGFDARHTKIEEIEAKVHEK"
/product="Phosphate transport system regulatory protein PhoU"
/transl_table=11
CDS complement(1668311..1669642)
/locus_tag="AUO97b_01710" /db_xref="SEED:fig|400667.82.peg.1571"
/translation="MLVAGLWSFTSSSFALDLVETYERAKLNDPTWQANQQQFEADQL
NLGLATGALLPTVTLSGNITRNRQTVKRSNFPGVDQEGLSDALVSNTSTTKQATLSAR
QPLFRMDAWEGYKQVKTSVALSEITLKLQKQDHVLNVAEAYFNVLRQQALTAAYLQEE
KALLEQLNMMNAKLKEGLVARSDVSEANAQYQNARANRIATNVQLLLAQEQLSEYIGP
YQDKLAVLRSDFIFQKPYPAQLDEWLGLAQQQNLKIQQARLQKRYAEDQRRVEKAALY
PQIDAVASYGYTKQTPETLISTDGKFDQVGVEMNWNLFNGGRTRTSIKKASVELNKAQ
AQLDAAIRRANVDVKSAFMQVDTDRAKLEARKAAMDSSALVSQASKASYNEGLKSMVD
VLLAQRNAFSAKQDYLNAQYDYLLNVLRLKAAVGQLGEKDLVELNSWLTYQ" /product="Outer membrane
channel TolC (OpmH)" /transl_table=11
CDS complement(1669738..1670643)
/locus_tag="AUO97b_01711" /db_xref="SEED:fig|400667.82.peg.1572"
/db_xref="GO:0016020"
/translation="MISLIKNSSYGGTIAILVASFLWGTTGTAAAFAPTLGPLAIGAV
AMGGGGLLQALIASQAIREHRQFIGRNISIILLGVVAVGIYPLAFYSSMHYAGITIGT
VVSIGSAPLIAAVLERFFDRKVLSKTWFLSFVLGVVGVSLLSMGESHTAQESTNEWNK
IFGIFLGLVAGATYSLYSWAAKRLIDRGIAAKAAMGMIMGGGAVILLPTLLITGGGLL
QSSTNLLVAGYMMLIPMFLGYLLFGFGLKTVNASKATTLTLFEPLVAAIFAIVLVGES
VSWIGWVGMFLIFICILMLSKPENT"
/product="Permease of the drug/metabolite transporter (DMT) superfamily"

/transl_table=11
CDS complement(1670636..1671217)
/locus_tag="AUO97b_01712" /db_xref="SEED:fig|400667.82.peg.1573"
/translation="MNDKTARQKILDAAATLFYNDGITATGINTVTAKADVAKMSLYN
NFSSKGELVDAYIAARHQEWLDLYQKRLEKTKTAKEAILAVFDAYQDHAEFAYEKGFR
GCGLLNAAAEFPANSSGRNAVRQHKEQVEAIVAEHLNHLLKDSQRVSYIASQLSFLLE
GSMARAGLEGSSRQLQLARQMAEHILDRECQHD" /product="Transcriptional regulator, AcrR
family" /transl_table=11
CDS 1671343..1672407
/locus_tag="AUO97b_01713" /db_xref="SEED:fig|400667.82.peg.1574"
/translation="MMSMCSALLLAGCSKTSEQDQTVSKEQKSTAQKATEQRDSSCLQ
IMVAMHTINQNSKIEDLNQINEKLKTCVPSLKNEEQLKLIEASTAMYQRFLKQDYTDK
TARAFEAFGYAVLEQKQDLKKVIQSQKKLFAQLSPRDQYLLQHEGQAYIDLLYQGEGM
FTYRRQPNYLVDVFSKALPADQKEFLSRMAKDNQNIFYNDGALAVSWKELTERALFWE
KFIQKYPKSYFINDAKLLFNEYRYFIFFGLDNTPVSNEYAPNTWFDPDALQQIRFLST
QSQSSLAKPAQQFLKFIATPVDERNKQFKIDLTDENGQKKSTYQIVHEQLEQLLKFDS
PWNTEVYRDCHIDAVCIDTN"
/product="hypothetical protein"
/transl_table=11
CDS 1672666..1674543
/locus_tag="AUO97b_01714" /db_xref="SEED:fig|400667.82.peg.1575"
/db_xref="GO:0009228"
/translation="MNQLTNLSSAEISAQHEQDAKDLTRILPASKKVYIEGSRPDIQV
PMREISLTDTPTGLGGEHNPPIMVYDTSGVYTDPNVQIDLNKGLPSVRQKWIEERNDT
DVLSGLTSKFGQERLKDIRTADIRFAHIQNPRRAKAGKNVTQMHYAKQGIITPEMEYI
AIRENQRQREAVDMRQHPGQNFGAKNLKEITPEFVRQEVAEGRAIIPANINHPELEPM
IIGRNFLVKINANIGNSALGSSIDEEVAKMTWATRWGADTIMDLSTGKNIHETREWII
RNSPVPIGTVPIYQALEKVDGVAENLTWEIFKDTLIEQAEQGVDYFTIHAGVLLRYVP
LTANRLTGIVSRGGSIMAQWCLAHHEENFLYTHFDEICEIMKAYDVSFSLGDGLRPGC
IQDANDEAQFSELKTLGELTHRAWEHDVQVMIEGPGHVPMHMIKENMDLQLEVCKEAP
FYTLGPLTTDIAPGYDHITSAIGAAMIGWYGTAMLCYVTPKEHLGLPNKKDVKDGIIT
YKIAAHAADLAKGHPGAQVRDNALSKARFEFRWDDQFNLSLDPDTARSMHDETLPKEA
HKSAHFCSMCGPKFCSMKITQNVRDYANNLTNSDSEVEEGLKAMKEVYQEQGQKLYHK V"
/product="Phosphomethylpyrimidine synthase ThiC (EC 4.1.99.17)"
/transl_table=11
CDS 1674619..1675245
/locus_tag="AUO97b_01715" /db_xref="SEED:fig|400667.82.peg.1576"
/db_xref="GO:0047631"
/translation="MSIVERPSYTSKDVEVTSREPLFSGFIQVEKVSLRHRLFNQSEY
TPVLQRELVHRPEAAGVLLYNDQKQQFALIEQFRVGALDDSHSPWQLEIIAGVLDGNE
SPESCIRRESLEESGCEVQDLEHLFSFYPSAGACSELFHLYVAETNLPAVGGVFGVDN
EGENIQLHLFSYSEIQTLLNSGRLRNAPVIMALQWLAQHSKTIINPKR" /product="ADP-ribose
pyrophosphatase (EC 3.6.1.13)" /EC_number="3.6.1.13"
/transl_table=11
CDS 1675250..1676062
/locus_tag="AUO97b_01716" /db_xref="SEED:fig|400667.82.peg.1577"
/translation="MTFQVSTLSQKNHVIIQITDTHLLEYPQLEFVGMNPEESFHAII
QQILKQHPEADAIIHTGDLAQAPTPITYKRYINFMQTLGLPFFQTLGNHDNVDHFPLH
NENHQQPVVVGLGNWRVIMLNSAVKGKVDGHLSSEQLENLANLLEQFADHPVLLACHH
HPFAMKSKWIDHHKLQNSNALLDTLSPFQNVKALVCGHVHQDSLNIWQGIEFFSTPST
SVQFKPFSNDFALDQNAPGYRYIRLNNDGSFETKVFRLENFKTRINTDISGY" /product="3',5'-cyclic-
nucleotide phosphodiesterase (EC 3.1.4.17)" /EC_number="3.1.4.17"
/transl_table=11
CDS 1676273..1676809

/locus_tag="AUO97b_01717" /db_xref="SEED:fig|400667.82.peg.1578"
/db_xref="GO:0008270"
/translation="MANDNHNQVLDEHTEVVVEGDKASAKRARKVKPKTSDVGSTASL
FGIAPYQPKKNEEYMSEGQLEHFRQILQAWKAELMSEVDRTLNTMQDESTALPDVNDR
ATQEEEFAIELRTRDRERKLIRKIEQSLEAIKNEDYGFCETCGIEIGLRRLEARPTAT
LCIDCKTLAEIKEKQNNG"
/product="RNA polymerase-binding transcription factor DksA" /transl_table=11
CDS 1676812..1677795
/locus_tag="AUO97b_01718" /db_xref="SEED:fig|400667.82.peg.1579"
/db_xref="GO:0004818" /db_xref="GO:0005524"
/db_xref="GO:0006424"
/translation="MTVKNSPAEFASAFAQQLNRLPEKYSFSLAQRRNNVPLNRCSYS
GRFAPSPTGPLHFGSLITAVASYCDARAHQGRWLVRVEDTDIPRIYPGSEDHILASIE
AFQFDPDAEIIFQKDRLDIYESVLDQLKKEGLVYACQCTRKMLGSNAIYAGTCRDLQL
DFQHQAIRVKVQDQPICFDDRLQGLHCSNLEHDLGDFVLKRRDGIINYQLAVVVDDYL
QGITHVVRGADLLDNTERQIWLGQLLGYPKLSYMHLPLAMNDQGQKLSKQNLAHALDL
TKAPELLQQAIQALGQPQVDLDRPEVMLKQAVTQWNVDLIPHGQQLCGTYL" /product="Glutamyl-Q
tRNA(Asp) synthetase"
/transl_table=11
CDS complement(1677845..1679041)
/locus_tag="AUO97b_01719" /db_xref="SEED:fig|400667.82.peg.1580"
/translation="MAGLAQTTIQKINHWYERILPKWPAEVTPRNVLIFCVVALLCIG
SVMVASASMPYAEYMHENPFHYVIRHGISIVAAGVVAYLTYRISLNTWFKNTFPLWLL
TMVLLLAALAVGSEVNGSTRWIKIGGFTLQPTEVAKVMMAIFTADYVVRRAKEVRTHW
KGLLRLSGVMAITVGLIIAEPDLGATVVIVMMMVGVFFLAGAPPTQFLIMLGAIVTGI
VFLILFEPYRFQRLISFTDPWADPLGVGYQLSNALMAFGRGEWFGTGLGHSVQKLSYL
PEAHTDFMLAVLGEEFGFFGISIVIGLSFLMLACCIKIGHRALKHHYLRAGYLAYGIS
IIFLLQILVNAGMNMGLMPTKGLTLPFISYGGTSLMMCAAMISLILKIDASTQEVNPE REESNF"
/product="Peptidoglycan glycosyltransferase FtsW (EC 2.4.1.129)"
/transl_table=11
CDS complement(1679066..1680412)
/locus_tag="AUO97b_01720" /db_xref="SEED:fig|400667.82.peg.1581"
/db_xref="GO:0008764"
/translation="MLIQRGGLKVVAGLGISGVSAVNFLHEQGYQVAVTDSRPTPPGH
DQIPAGVKTSFGQLDQELLLQAEEIILSPGLAPQLPEIQAAIAKGISVVGDIQLLRRA
TDVPIVAITGSNAKSTVTTLIGLMAKDAGKKVAVGGNLGRPALDLLKDQPELLVLELS
SFQLETTSHLNAEVAVVLNMSEDHLDRHGNMLGYHQAKHRIFQGAKKVVFNRDDALSR
PLVPDTTPMQSFGLNAPDLNQYGVLRDADGTLWLACGLQRLIKSSDLYIQGMHNVANA
LACLALGEAIGLPMESMLETLKQFKGLEHRCEYVKTVHDVRYYNDSKGTNVGATLAAI
DGLGAAIEVKKGKVALILGGQGKGQDFSPLRSSIEKYAKVVVLIGEDAPVIEQAIQGA
TKILHAATLKEAVELCQRETQAEDVVLLSPACASFDMFKSYNDRGQQFVACVNSLV" /product="UDP-N-
acetylmuramoyl-L-alanine--D-glutamate ligase (EC 6.3.2.9)"
/EC_number="6.3.2.9"
/transl_table=11
CDS complement(1680573..1680824)
/locus_tag="AUO97b_01721" /db_xref="SEED:fig|400667.82.peg.1582"
/translation="MLQYLIIAVLVIWSAVVVFKKVFPKTANSAFSALSVFCEKQGWA
TLAKWLKPKMTFGCAGGCGCSSEDKPTNTVQEIKPVKWQ" /product="hypothetical protein"
/transl_table=11
CDS complement(1680845..1682698) /locus_tag="AUO97b_01722"
/db_xref="SEED:fig|400667.82.peg.1583" /db_xref="GO:0001882"
/db_xref="GO:0005525"
/db_xref="GO:0005622"
/db_xref="GO:0015093"

/db_xref="GO:0015684"
/db_xref="GO:0016021"
/translation="MSDALRIALVGNPNCGKTSLFNHLTGTRQKVANYAGVTVERKVG
HFQLPSGRSVRVLDLPGTYSLNATSPDEAITRDVCLGKIAEEGQQDAFLCVVDATNLK
LHLGLVLEMIELGRPILLVLNMMDEARRRGIQINTQKLSQRLGVPVVETVAVRNAGIE
NLLHALDQEKYSVPQTELSGLTGTHHQKIDMIFKDVVHYVDQEDKRTDFLDRIFLHPV
LGLLSLAIMMFIVFQAVFAWAAPFMDGIEGFFGWLGEVVGSVITQPLLHSLVVDGIIA
GAGGVVVFLPQILILFFFILVLEESGYLPRAAFLLDKLMFQAGLSGRAFIPLLSSFAC
AVPGIMATRSISDPRDRFTTIMVAPLMTCSARLPVYALLIAAFIPEKMVWGIFNLQGL
VLFGLYMAGIVSALCVSFLMKFFQKDKSQHALLLELPSYRFPDLKSVGIGLLDRAKIF
LKRVGGIIFALSILLWFLCTFPQPPEGATLPAIDYSFAGMLGHLIQPLFAPVGFNWQI
CIALIPAMAAREVVVAALGTVYALSATDEDAVAQGLSHLISADGTGWSLATGLSLLVW
FIYAPHCLATLATVRRETGSWKHVAAMTTYLFGLAYFASFVTYQIASHYWG" /product="Ferrous iron transporter FeoB"
/transl_table=11
CDS complement(1682695..1682958)
/locus_tag="AUO97b_01723" /db_xref="SEED:fig|400667.82.peg.1584"
/translation="MRLSALKVKQAATITKVNRIEVESEQQDLVAIRLESLGFVPGTR
VEVITKGVFGGDPILIQIGFTRFALRKAEAEKIEIQVEEGVPA" /product="Ferrous iron transporter-associated protein FeoA" /transl_table=11
CDS 1683102..1684022
/locus_tag="AUO97b_01724" /db_xref="SEED:fig|400667.82.peg.1585"
/translation="MINKKPRIPSPVQIPEHLSFLQGFRDYLVAQTVSPHTRNAYLSD
LIQCSELHKKNRLPDWTSDDISDVLIELTKVGKSPRSIARCLSALRQFYKFLREQKLR
SDNPVATHHSPKIGRALPKDLSEEDVEALIQAPDITTALGLRDRAMFEVLYACGLRVS
ELLNLRLELINLKQGYLRITGKGNKERLVPLGQYACDWVERYLNEARPQLYKSSTDYL
FLTQHGGIMSRQNFWYAIKHYALQANIQAELSPHTLRHAFATHLLNHGADLRVVQMLL
GHSDLSTTQIYTHVAQVRMQQLHEKHHPRG"
/product="Site-specific tyrosine recombinase XerD" /transl_table=11
CDS 1684169..1684984
/locus_tag="AUO97b_01725" /db_xref="SEED:fig|400667.82.peg.1586"
/translation="MSFTRSQIFLACALASSLLFSACSKENKPQKEDALTATAPATGE
ASTIIERNAKQRLIETLQKQFKNANINVKILDIKPTEVPNLYWVNLEGMTSVYTTSDG
KYLIQGDVIRLGGKELHNIGDSLQASENKKHLAALKNEDLIVYPAKGGKAKHVIYVFT
DASCPYCHKLHEHLAEINEKSIEVRYIAWPRGEQFMPTMEAVWCSADRKAAFDQAVQG
ISIPPAQCKNPVREQYQLGLNMGVNGTPAIYNVDGEYLGGYLTPDELIKRLDK"
/product="Thiol:disulfide interchange protein DsbC" /transl_table=11
CDS 1685229..1686530
/locus_tag="AUO97b_01726" /db_xref="SEED:fig|400667.82.peg.1587"
/db_xref="GO:0004412"
/translation="MKPVRLAILGLGTVGGGALKLLQENAAEIKRRTGREIQITHVGT
RRPRPDLELEGIKQSADLLDIVRQPDVDVVVEVMGGIHPAYEIIMEAIKHGKQVVTAN
KALLAEHGNELFKAAEDNAVQIAYEAAVAGGIPIIKVIREGLAANHIEWLAGIINGTG
NFILTEMREKGRAFDDVLKEAQELGYAEADPTFDVEGIDAAHKLTILASCAFGIPLQF
DKVYTEGISKITAQDVKYAEELGFRIKHLGIARRAEKGIELRVHPTLIPDEQLIANVN
GVKNAVLVQANAVGPTLYYGAGAGAGPTASAVVADVIDIVRDISYTEDGAGTIPQLAF
EALTNMPILSREEMTTGYYIRLNAEDQTGVLADVTTILSRAGISIDAIMQQSRLKDLI
PIVILTDPIVESKMDDALAQIQALPAIRGEIVRIRLESLDS" /product="Homoserine dehydrogenase (EC 1.1.1.3)" /EC_number="1.1.1.3"
/transl_table=11
CDS 1686586..1687725
/locus_tag="AUO97b_01727" /db_xref="SEED:fig|400667.82.peg.1588"
/db_xref="GO:0004795"
/translation="MSNANRYTGLVDRYRDRLPVSATTRAISLGEGNTPLIKLENIPR

IIGKDVEIYVKYEGLNPTGSFKDRGMTMAVTKAVEEGSKAIICASTGNTSAAAAAYAA
RAGIKAFVLIPEGKIAMGKMAQAMMYGAITMQIRGNFDDGMRLVKEVADQAPVTIVNS
INPYRLQGQKTIAYEIVEALGRAPDYHCLPVGNAGNITAHWMGYTEAVANQPADQFEQ
VVYDAATDQFTGPKPEGLPTMVGYQASGAAPFLRGAPVEKPETVATAIRIGNPQSWNH
AKAVVRDSKGWFDELQDSEILEAQRLLSMYEGVFVEPASAASIGGAIRDIKAGKIAEG
SVIVCTVTGNGLKDPDTAIKQCADAVMLSIDATMDQVKDSILSNM" /product="Threonine synthase (EC 4.2.3.1)" /EC_number="4.2.3.1"
/transl_table=11
CDS complement(1687834..1688841) /locus_tag="AUO97b_01728"
/db_xref="SEED:fig|400667.82.peg.1589"
/translation="MSILLSLSSTSFAELVNNPSSGSTGTASLNWTAADASQLLNDED
DEPTPQGSTSVTTTLRGSNAPRVITSAPKVAPIRDTVGYNAQPSVSARAALVMDAQTG
EVLYSKNTNASVPIASITKLMTAVVTADARLNMSEEITLEQIDFAGAGGKNSSSTLRV
GDKMNRAEVLLFALMKSENPAAAALARTYPGGRAAFVAAMNAKARALGMNATHYYEST
GLDPRNVSSARDLGILASTASQYGLIRQFSTTPTYDFNLGYRVLKSNNTNALVRNGGW
NINLSKTGYINEAGRCVVMHTTVNSRPAVIVLLGEPSTQARNNDATNLLGWLSNLPKR I"
/product="D-alanyl-D-alanine carboxypeptidase (EC 3.4.16.4)" /transl_table=11
CDS 1689093..1689728
/locus_tag="AUO97b_01729" /db_xref="SEED:fig|400667.82.peg.1590"
/translation="MITVLVVDDHELVRTGICRMLEDHADVEVIGQAESGEEAIAIVR
QQHPQVVLLDVNMPGIGGVETTRRLLQTAPETKVIAVSGLAEEPYPSLLLKAGAKGYI
TKGAPIAEMVRAINKVMQGGKYFSADIAEQLASSYLSDTQQSPFDSLSEREMQVAMMV
VNCISAQEIADKLFVSVKTVNTYRYRIFEKLGIDSDVKLTHLAIRYGLIKP" /product="BarA-associated response regulator UvrY (= GacA = SirA)" /transl_table=11
CDS 1689784..1691307
/locus_tag="AUO97b_01730" /db_xref="SEED:fig|400667.82.peg.1591"
/translation="MGTWYGLYRLIIAVSLNIILVLTDAQTDNSLQQPALYSYTLLGY
SLLSLIQLLCFKFIATQATRQLILFFIVDIICLSLLTFSVGEPNLQLSLLYVIAIFTS
AILLSARMSLLITLLAVIAVIYQRFVGSLFDYNNLNTIGNSALLAFLFFVVHRIGQIA
VQRFKLLEALTFHQSIELYQLQNINRYILEQIEEGYLVLDENYDIVVSNPAACSLLGI
PPQFANEKYPLAKWHADLFEILKFGDLKEGDRFIFESRLSAYSINIKVQHLLVPQQTL
TLLILQDAQQINQQAQQLKLAALGQLSASIAHEIRNPLAAIVQANELLKDSDPEQQNT
LRHMIGKQTKRIDSIVQDTLGLARSERTHPIQIDVKHFIDTLLEEDLFDVKHSIQLKI
SDSSLKFLFDEKQLRQVMINLVRNALRHNATDSPYITINIHSQTNKIYIDVIDYGEGV
SKRDISQLFKPFFSTEINGTGLGLYLSHSFCEANHAKLTYVEQKQGACFRIECPIIY" /product="Sensor protein"
/transl_table=11
CDS 1691332..1692753
/locus_tag="AUO97b_01731" /db_xref="SEED:fig|400667.82.peg.1592"
/translation="MAEQQPLVLLVDDEEDLCLLMQMTLARMGIKTHLAYRVEQAKQL
FTQFHYDACLTDLNLPDGSGLDLVKHVSQNYPNTPIAVLTAYGNMDIAIAALKAGAFD
FVSKPVNQVHLDQLLQKALNRQKVEHDAAENALENKLLIGRSLPIQQLRIAIKKIARS
QAPVFVTGESGTGKEVVANLVHRLSNRSEGPFIAINCGAIPTELMESELFGHKKGSFT
GATQDKQGLILSAHGGSLFLDEIAELPLSMQVKLLRAVQEKKIRPVGSDQEIDVDFRV
ISASHQDLDLLVRQGKFRQDLFFRIHVMDLILPPLRERGEDVLLLANHFIQKICMEWE
TPSKQLTEAAKTYLLQQHFPGNVRELRNMIERAITLSEDATIDISHLHPAPLRANISN
PFVSATQNIQTTVAAPQVVKKLPSEGLERYLENIEKDILINALNMTHWNRTLAAKKLG
MTFRSLRYRLKKFGLDTETEQEV"
/product="Helix-turn-helix, Fis-type"
/transl_table=11
CDS complement(1692757..1693941)
/locus_tag="AUO97b_01732" /db_xref="SEED:fig|400667.82.peg.1593"
/translation="MLQHNIYKAFFVSVLMCCSQLVFSAPVVKEKLSPLHPDSPESEE
SYAEVPIESIQQFVQIYGIVRDNYVDEKSDDALFLQAIKGLVSGLDRYSRYLSAEEYR

QLIQYTEGDLASVDFVLSPESHVHKWMIRDLKTGSDSYKLGLRNGQTILKIDNQELKN
LTHDQVLGLLYGSIGSTLQVQTEESNSPISLVRNKKIETDIEPVMLHNQVLVLKIRVF
QQDTANEIKRLIEENSSSRLKAVLIDLRNNPGGLLSAAVESADLFLNHGIIVSTKSRS
EGNQQFQALPGNDFQNIKVGILINHRSASAAEVFTAAMKEHQRAWVMGEKSYGKGVVQ
KLFPLPSGAALQMTVSHYYTPNGNMIEGQGIQPNQTYPLPPEMKEEVYLDRVADLLLK RK"
/product="Carboxyl-terminal protease (EC 3.4.21.102)" /EC_number="3.4.21.102"
/transl_table=11
CDS complement(1693957..1695504)
/locus_tag="AUO97b_01733" /db_xref="SEED:fig|400667.82.peg.1594"
/db_xref="GO:0004619"
/translation="MTDATAGKIPHVLVIMDGVGHREAIEDNAFLAAKTPNLTAMKAK
HPNSLISGSGEDVGLPDGQMGNSEVGHMNLGAGRVLYQDFTRITKDIRTGAFFEHEVL
VDAVEKAKAAGGAVHIMGLLSEGGVHSHEDHIVAMCELALKRGAKVYLHAFLDGRDTP
PRSAQPSLEKLDALFAQYEGKGRIATMIGRYFAMDRDNRWDRVEQAYRLLTEGEAVRT
ANTAVEGLELAYAANENDEFVKATRIGESAKVQDGDSVVFMNFRADRAREITRAFVEK
DFAGFERKVVPNLSKFVMLTRYQASIDAPVAYMPEELKNSLGEYLSSLGKTQLRIAET
EKYAHVTFFFSGGREDEYPGEKRILIPSPNVATYDLKPEMSAYEVTDELVKAINSGEY
DLLVVNYANGDMVGHTGVFDAAVKAVEAVDTCLGRVYEAVMAKKGHMLITADHGNVEQ
MQDYESGQVHTQHTTELVPFIYVGPTQATIAEGGVLADVAPTILNLMQIPVPAEMQGR NLITLSA"
/product="2,3-bisphosphoglycerate-independent phosphoglycerate mutase (EC
5.4.2.12)" /EC_number="5.4.2.1"
/transl_table=11
CDS complement(1695630..1696700)
/locus_tag="AUO97b_01734" /db_xref="SEED:fig|400667.82.peg.1595"
/translation="MLARRIVAKYVTKTTALAMFGTTIVLSVLQILFTYLGELGELKP
DYNAWQAFIYVLWGAPRYLYEILPISALIGAVIGLGALASNSELIVMRSVGISLWRIV
GWVMRSALLLIVLSFALSEWVVPYTNEKAQSVKSHRSVAALGEVKGYWSREGQRFIYI
DYANSQGNLRDIQVVDFNKDYHLQSLINAEQGKFIQDGQWTLQKASQMDILTDGNSIL
NNHDQQSLGLALQPKYVHMVTLDPEDLSPSQLISFMRYMDEYSQVPKTYQLAFWQKVA
SPFSLITLVLVACSFIFGPLRQQSMGFRLVIALFIGLGFYYLQDFLGYASLVYAPSPA
WFVLMPIILMFGAGSYLLYRAR" /product="Lipopolysaccharide export system permease
protein LptG" /transl_table=11
CDS complement(1696700..1697800)
/locus_tag="AUO97b_01735" /db_xref="SEED:fig|400667.82.peg.1596"
/translation="MIIRRYLVKQVVSTSLVVIALLTLIMMGGRLIKYFGVAAQGRLD
ASILFSIIGYRLPEFLTLILPLGFFIGLMLVFGRLYVDHEMAVLNGSGVSRHQLARLL
IPMTLVYMVCQSVLMLWMTPWGLREFEKLTTTQAVRTGFDLVRPREFISSGPYTIYAG
SLSEDRKNLKDIFFYQRAAKEGKPDVMILAKEATRVEVANDTANVVDLVQGRRYEIFP
GQPKYTQAEFQSYRLRLENDKDVKFESGDVEALSTSKLFSKANDPVVRSELGWRLFGP
FTIIIALMLSVALSEVSPRQGRYYRLIPSIFIFASLIVLMIAIKTRISKGELDIWAYP
VALLVYAIAAALFSRKQKLGPKIKKQIKRVKL" /product="Lipopolysaccharide export system
permease protein LptF" /transl_table=11
CDS 1697944..1699392 /locus_tag="AUO97b_01736" /db_xref="SEED:fig|
400667.82.peg.1597" /db_xref="GO:0004178"
/translation="MKFTTYTTFPEQTSNESLWILVDSEQLQSNLNTYQINNLESILT
ATQFKANFNETLPLFGQLSTQPHSQLLGLGKAAELQAAKLAKLAQTIIKSAQNKFKHI
AIDIAALPVEYHYLFALSLTQAAYGYDEFKSKKNEFVLQQVDLISSQTSLDENQLALV
HAVQSGQSYARDLGNRPGNICFPEYLAEQALALAAEFPDLLKVTVLNEQQMADLGMYA
FLAVSKGSERPGRIVTLEYQAQLEQAPVVLVGKGVTFDTGGISLKPGLGMDEMKFDMC
GAASVLGTIRALCEARLPIHVVGAIAAAENMPSGKATRPGDIVTTMSGQTVEILNTDA
EGRLVLCDTLTYIKRFNPAVVIDIATLTGACVVALGKVLSGLFSPDDTLAAELQQAGE
QSFDRVWRMPVIDDYQELLDSPFADIANIGGPHGGAITAACFLERFTRDYRWAHLDVA
GTAWLSGSAKGATGRPVPLLMQFLANRVSTNG"
/product="Cytosol aminopeptidase PepA (EC 3.4.11.1)" /EC_number="3.4.11.1"

/transl_table=11 CDS 1699385..1699792
/locus_tag="AUO97b_01737" /db_xref="SEED:fig|400667.82.peg.1598"
/db_xref="GO:0003887"
/translation="MAKVSFYLFETSEERQVDSACRLCRKILLKHSQIWWYCPDVQLQ
QTLDEKLWSFDPTSFIAHGVDQVDAAVCISAKQPSEQGWLVFNFNNHALEQVNHFSHI
IEIVENNEAAKQIGREKFKMYRRLGIEPRTFKL"
/product="DNA polymerase III chi subunit (EC 2.7.7.7)" /EC_number="2.7.7.7"
/transl_table=11
CDS 1699834..1700469
/locus_tag="AUO97b_01738" /db_xref="SEED:fig|400667.82.peg.1599"
/translation="MALNVGQDFKKRWLNAPEAVRHAYQQDLARICDLLEPQTPIQLW
VLNDEKAQLESQQNIEKAYADLKAELIEQARIRRQLALEKALADKRAKEAAYAAELQA
DEVRKFSEQTEALQALRSHLEQEVAEHTARYQKNPETPAIDYSSGTKLSITDDQILSE
LESVRVRLELEAESLIEQAVTVFRAKLHAAAQEEIEYILKNSNFSDEKIEK" /product="hypothetical
protein"
/transl_table=11
CDS 1700600..1700818
/locus_tag="AUO97b_01739" /db_xref="SEED:fig|400667.82.peg.1600"
/translation="MDIPHQISTQIEQLNQGEQWTFSAQELYMSHNDFNSLSILLTRA
SEKGEFSITRTQHNKPWVGTHSVTLTKH"
/product="FIG00349975: hypothetical protein" /transl_table=11
CDS complement(1700877..1701536)
/locus_tag="AUO97b_01740" /db_xref="SEED:fig|400667.82.peg.1601"
/translation="MFTGIIESLGKVESLQSVGGDVRLRIQTDLDMSDVHLGDSIATN
GICLTVIEWGDNWYAADVSRESLNRTTLGNWKVGQRVNVEKAMLPTTRFGGHIVSGHV
DGVGEITLVREDARSIYFEVTAPVELAKYLAEKGSVTVDGISLTINHLRGNILSLNLI
PHTAERTNIGTWQVGSKVNLEVDVLARYIERLLLGDKAAEQKTESNISMAFLAENGFL K"
/product="Riboflavin synthase eubacterial/eukaryotic (EC 2.5.1.9)"
/EC_number="2.5.1.9"
/transl_table=11
CDS complement(1701579..1702871)
/locus_tag="AUO97b_01741" /db_xref="SEED:fig|400667.82.peg.1602"
/translation="MNSEPSVYHKRRHAARTTDEYLFHQLVPYLGNKRRLLHLILEAL
EITGTLNSKKKNPPIFADFFAGSGVVSRLARQNGYRVIANDWEPYSHALNHAILACVD
APAFKELGGYQKAIDYLNRLPEVKGWVTHNLCPRNDDVYDPSRDRLFFKRRNGMRIDA
IRQQIATWQAQGAINDVEMSALLAPLLYSASFVSNTSGVFKSFHQGWGGRTQTALERI
ESLLWLTPSRFCEIGDRKRPAAEMWCVDAQHLANQMSGFEVDVAYLDPPYNQHAYSSN
YHVLNALTLWDQVDLPPPDTKGYKSGIDRAWRKERPSPYNSSKHAKDAYEKLLSTINA
RYILTSYSTDGNIEPKDLLMANLERGKVTLLTQDVPRYRVSKQRQSERARVLEFIVIT
DTHAKPGPPLRQLLGQLYHFAELGGVDTSGNSTQLALW" /product="DNA modification methylase
(Adenine-specific methyltransferase) (EC 2.1.1.72)" /EC_number="2.1.1.72"
/transl_table=11
CDS complement(1702868..1703953)
/locus_tag="AUO97b_01742" /db_xref="SEED:fig|400667.82.peg.1603"
/db_xref="GO:0008703"
/db_xref="GO:0008835"
/translation="MSELKQDQYWMQQAIELAKRGLYSTKPNPNVGCVIVKDNQLIGE
GFHPKAGQPHAEVFALREAGEHAQGATAYVTLEPCAHYGRTPPCAEALVKAQVKKVVV
ACPDPNPLVAGKGVQILKNAGIEVEIGICEDLAAQLNQGFLKAMSTGMPYVRLKVASS
LDGRTAMASGESKWITGSAARQDVQHWRAISGAVITGIDTVIVDDCQLNVRSLHNIDI
ETVAQPKRVILDRRGRLPLTAKILENPETVMVMGPYRQELADLGVIQLEIQPLKTLLQ
TLSKQYQIYDVLVEAGATLSTAFLQEGLVDEMISYVAPTLLGQSARAMFNADFEYMAQ
QLRFKLLDVTQLDQDIRLRLIPTQEKV"
/product="Diaminohydroxyphosphoribosylaminopyrimidine deaminase (EC 3.5.4.26)

/ 5-amino-6-(5-phosphoribosylamino)uracil reductase (EC 1.1.1.193)"
/EC_number="3.5.4.26"
/EC_number="1.1.1.193"
/transl_table=11
CDS complement(1703969..1704427)
/locus_tag="AUO97b_01743" /db_xref="SEED:fig|400667.82.peg.1604"
/translation="MHCPFCNAADSKVIDSRLAAEGCQIRRRRECVSCGERFTTFESY
EVVMPRVIKSNGKNEPFDEAKLRRSLMHALQKRPVTQEQIETVLSDIQLQIRRLGERD
VKSRTIGEIVMQSLFALDHVAYVRFASVYQDFQDVEAFRRQIEQMQQREH" /product="Ribonucleotide reductase transcriptional regulator NrdR" /transl_table=11
CDS complement(1704586..1705983)
/locus_tag="AUO97b_01744" /db_xref="SEED:fig|400667.82.peg.1605"
/translation="MKKMLMALSLTGALLGGTAAWAEETVTTPTSEVTATSTSEAPAT
IAAAPAAEETPAAPTPTAKLDTGDTSWILISTALVLLMTIPGLALFYGGMVRKKNVLS
TMMFSLSAAILVSLLWVIAGYSIAFSGTGAYFGDLSKAMLNGVAFDALSGTIPESLFV
IFQMTFAIITVAILSGSIADRMKYSAFMAFIAIWVLVVYAPITHWVWAADGWLFKAGA
LDFAGGTVVHINSGVAGLVAAYMLGKRIGLGRESMAPHNLTLTVIGASLLWVGWFGFN
GGSALGAGARASMAILVTQVAAAAAAFSWLVVERMIRGKASVLGGASGAVAGLVVITP
AAGFVGVGGALVMGLIGGVVCFWGITALKRLLKADDALDAFGLHAVGGIVGAILTGVF
YSDEIIKATNVALAPTFAGQLWVQVEGVLATMVYSGIATFIILKVIDLIIGLRVNSDD ERMGLDLSQHGERIE"
/product="Ammonium transporter"
/transl_table=11
CDS complement(1706041..1706379)
/locus_tag="AUO97b_01745" /db_xref="SEED:fig|400667.82.peg.1606"
/db_xref="GO:0006808"
/db_xref="GO:0030234"
/translation="MKLVTAIVKPFKLDDVREALSDIGVQGITVTEVKGFGRQKGHTE
LYRGAEYVVDFLPKVKIEIAISDEMVDAVIESITRVASTGKIGDGKIFVTNLEQVIRI RTGETGPDAV"
/product="Nitrogen regulatory protein P-II, GlnK" /transl_table=11
CDS 1706659..1706886
/locus_tag="AUO97b_01746" /db_xref="SEED:fig|400667.82.peg.1607"
/translation="MLETLLQAILEQIDEPKKDLEKNLRALLNETVEKLDLVSKQEIE
RQKTALHLANQRLEALQKQVSLLEEALKNKK"
/product="hypothetical protein"
/transl_table=11
CDS 1706952..1707809
/locus_tag="AUO97b_01747" /db_xref="SEED:fig|400667.82.peg.1608"
/translation="MSFSKIYTRGLLGLHAPLIEVEVHVSQGLPSLTIVGLAEAAVRE
SKDRVRSAIINSGFQFPTKRLTINLAPADLPKDGSRLDLPIALGILIASGQLPENCTE
DFEFIGELALDGHVRPVSGALTLAMACQQAQHKIVLPVDNSQDISHLPNLECYPVNHL
KEVCEHFLGTQKLAHAQPSAHSNEMPYKFDLADVKGQLRPRRALEIAAAGGHSLLFKG
PPGTGKTLLASRLPSILPPLSTQETLEVASIYSISNTPHTFGQRPFRAPHHTASAIAL VGGGCQFHK"
/product="AAA+ ATPase superfamily protein YifB/ComM, associated with DNA recombination" /transl_table=11
CDS 1707892..1709688
/locus_tag="AUO97b_01748" /db_xref="SEED:fig|400667.82.peg.1609"
/translation="MPVNNDKITTIDCFPNDNISRVVYRYGGLVRNEDNDSTNPFVEV
LLIEIRKSDQWLFLDKCSTFLVPVLDLDAVQHGSIWDGNVLTDQSYRFSGKLITKRFS
FDFTNNKPKNIKLTDRIPNTSDYYIPLKNYFLPNKVDYVLQGYPFTKYTNDSYRKVNH
CLMHSNDGTQVITSSIHVLHSLFVNRKDIRGLLLGTSSQSIINRFLESYTTEIVDDNV
EYKIKIRKPYEDIGETAIIFLANLALNPYVQTIVDKIQRSMEITEFNHLQQGTGKRYP
IVFPPHPTKLFLEAEGIWLDDNKTRFFITRVKKFDPINDHMIDVNKDLTNTISKPDEK
NPRPREKSQNKNEHINTQKPPSRTSGEYRKRSEVETGNTQGILKYSFNEPTDDPIEVG
ASNQPYSDKSEEVETSSDEPYGNQNTKIKKSETTDTPPSRDERFDIQYIIQSLQELAS

EMGSPLEHLSAINEHGEMIASINLLQIKKLVPEPKHPSWIDYDKGRKLLFLKLDLKDQ
NGFSYLIDIHKNKNHEAFCAFLIFTQNKLTSEQIKKICIELENAKGIKKWALYCDSFI
HKMIAIKHLYATPDEWKNRFKNLFISLQKDKS"
/product="hypothetical protein"
/transl_table=11
CDS 1709983..1711470
/locus_tag="AUO97b_01749" /db_xref="SEED:fig|400667.82.peg.1610"
/db_xref="GO:0005215"
/db_xref="GO:0006810"
/db_xref="GO:0006813"
/db_xref="GO:0008324"
/db_xref="GO:0015137"
/db_xref="GO:0015746"
/db_xref="GO:0016021"
/translation="MLSTIREQWFSNIRGDVLAGIVVALALIPEAIAFSIIAGVDPKV
GLYASFCIAVVIAFVGGRPGMISAATGAMALLMVTLVKEHGLQYLLAATILTGFIQIL
AGYLKLGALMRFVSKSVVIGFVNALAILIFMAQLPELTNVTWHVYAMTIGGLAVIYLF
PYIPVIGKLLPSPLICIVLLTLFALFIGLDVRTVGDMGQLPDTLPIFLLPDIPLNLET
LAIILPYSLGLAAVGLLESMMTATIVDDLTDTTSDKNRECKGQGVANVASGFLGGMAG
CAMIGQSIINVKSGGRTRLSSFSAGVFLLIMVVFISDWLKAIPMAALVAVMIMVSIGT
FNWDSLRNIRQYPLSSNIVMIVTVVVVVATHNLAYGVLVGVLLSALFFANKIERYMAI
QSVFDESESTRTYTVTGQVFFSSADKFTSAFDFKEALSKVVIDVSQAHFWDVSAVAAV
DKVVIKFRREGTEVELIGMNEASKTLVDKFGIHDKPDALDRLNSH" /product="Sulfate permease"
/transl_table=11
CDS 1711483..1712334
/locus_tag="AUO97b_01750" /db_xref="SEED:fig|400667.82.peg.1611"
/translation="MSKIIACIDGSLVTNTVCDYAAWFSDKLNSPIKLLHVIDKPKAK
APQDLSGAIGLGSRETLLKELVELEERKGKIELEHGQILLREAKNYLLEKFSIDAQSF
QRHGSVLETIMGMEDDIRVLVMGKHGNETEHDSSKIGTHIENVVRALHKPVLITSAPF
SPPKSFLIAFDGSQTARKCVERIASSPLLKGLAVHLVYVGNPNSEMQNQLSWAKEQLE
SQGFNITSNTLDGEVDKAIINYAEQHQIDLIVVGAYGHSKIRQFFIGSTTTKVITSAN KPVLLLR"
/product="UspA-related nucleotide-binding protein" /transl_table=11
CDS 1712774..1713145
/locus_tag="AUO97b_01751" /db_xref="SEED:fig|400667.82.peg.1612"
/translation="MGQAKQRGTLEQRVKQAQEQKDDFYGTERSVAEVLAELGLPKES
TVKGYVIHLPEKDEFVAAINDNNISFSVAYARTPELAMIYDEPQKAISDAKKISKHKL
LVCVLFETSNQYMINDIWANY"
/product="hypothetical protein"
/transl_table=11
CDS 1713254..1713373
/locus_tag="AUO97b_01752" /db_xref="SEED:fig|400667.82.peg.1613"
/translation="MIFIFMSLKLYCVNTAFKASLGDFLGGGDNLINTWKSTL" /product="hypothetical protein"
/transl_table=11
CDS complement(1713520..1714950)
/locus_tag="AUO97b_01753" /db_xref="SEED:fig|400667.82.peg.1614"
/translation="MTENVIEHDCHGCNQSVSFIKKRYKGKKYCSTCYARIFKKRLCP
SCGDFARLPRDDEQAICNECIKKQPCIRCNQTNKPIGKLTEYGVVCNSCSVYFRPIEP
CERCGTPSQKLTRISPFNDDLRVCPKCATRDYETCPSCQKHRLLESDVSGQRTCKKCR
DKPQKSCKACHCMIAAGCADLCDDCYWHQNLWNKFDQNQKVFESSDLKQQYENYIGWL
EKKVGSHKAALYINKHTHFFIKTEIDWNQSVPTPKQLLVRLRSSGLRKFELVMQWLEE
VHDIRIDMDNKKSCSERDQMEKLVQRILQPSLAYDVVLEYKNKLEEKIKRGETSIRSA
RLAVKPAVALMLSMEGESAQLPNLEHVKAYLAEYSGQAAALTGFINFLNENYGASIDY
LKLKKSDFLKTKQKKKLEMELIALTQTDLNDSELILSWVRNGLRYFHQLPYIDALKIK

TEMITEIEDGFTVVLNGQYYWLPKTQ"
/product="FIGfam110555"
/transl_table=11
CDS complement(1714943..1716058)
/locus_tag="AUO97b_01754" /db_xref="SEED:fig|400667.82.peg.1615"
/translation="MSQPEQCWYIRTPIQQGEIFSSWLIRSALDVGCSPMVLIEALWG
KWRALTIDLDKGVDAERFDALLSHSMESKQKIQQSMLSSVVSQIQPNYDSNQNIPWVL
SLGTRNRSNTSGRQVCVECLKSHENPPYLRLMWRIGWHCSCVEHQLSLIDHCPECGVT
IQPFKADMEHGCLAICTTCGFDLRRCEESKNINLNALNFQNKAEQVLKQKIGFYNQSP
VTTQVWFEIARSWLSEIRFLVNTPNKNVIQLFESFDVNLHLSHPVTPLAFEYLSTQER
IVLLSMLDQIMDIPCDLLVQRSKEYGVSRANFWDKRKKLPVQLQQMKDLMIKPTRRYP
VSRAAITVTKPKSKATVQRQWLNLLRRSNNSGAMHID"
/product="Mll9366 protein"
/transl_table=11
CDS complement(1716088..1717008)
/locus_tag="AUO97b_01755" /db_xref="SEED:fig|400667.82.peg.1616"
/translation="MEKYEHLAEHVLEIMQLSDSERIETLFTDRWIGYKKAITIMNTL
TDILNRPRKLRPECLLIVGDSNMGKTTIIHEFARQYYTKTVSDADMDLLSVTKPVLPI
LAPAKANVKELYINILNHFFVPFRASDPEAKLRNQAVHLMRKYETKMLIIDEIHNCLT
GSAKLLPEVMNTLKNLSNELSLNIVGVGTREAITILHTDPQYASRFDVVNLPKWELNQ
DFLRLLVSYVRLLPLKKQSNLASKEIATLIFEVSGGNLGDLNRLLVECAKEAILQGDE
EITYDIVHKFKWLKPTEGLRNIRNINLSLS"
/product="TniB NTP-binding protein"
/transl_table=11
CDS complement(1717013..1718923)
/locus_tag="AUO97b_01756" /db_xref="SEED:fig|400667.82.peg.1617"
/translation="MIESNAVSIDRSRITLKPNVVVGYEGNPYKIVNVLNANDIVISS
LDSVRSLQVNSKSLTVFEGENTLTENLNKGDKDISNEAWQIALQRYEIIKPLIEYSTT
ELVENRANEYDVNRSTLWKWLRDYRENNSLMTLIPKKRGWTTEKSRLSPQVSNIIKQA
INDEYLNAKKPSISKTIEIVKAECSRLQLEAPHENSIRRRIEALNDYQVTKARLGSKA
AIDKFKAAAGSFPNADYPLAYVQIDHTPLDIEIVDDEFREAIGKPHLTLAIDVFSRMI
VGYYLSLEAPSTTSVAMCIASCILSKKRKLIELDIDAEWQVEGIMDSVHTDNGPDFRT
NHISKACLKYGIHWEYRPIGGARFGGHIERMLGIVNLEMHVLDGTTFSNVQQRGTYDS
AKQACMTLKELEYYIVYWITKVYHQKKHSALGTSPIVKWEEGVWGTKTTAGTGLKERV
SDEDTLFIDFLPEFEATIQRTGVQKDNLFYFADCLRQWVNSIDPEDNNRKRKRKFLFK
RDPRDISQIWFYEPYSNTYFKVPTAKREIPPISLFEYKQVQNYLKSERQDVQNQDEIY
KAILHLRDQLNQARSLTRKQRRSNQRKKENAKAITQLSEQNQSKKAVVSESLQTSDDL WSTPLTAFDDLR"
/product="TniA putative transposase"
/transl_table=11
CDS 1719335..1720297
/locus_tag="AUO97b_01757" /db_xref="SEED:fig|400667.82.peg.1618"
/translation="MTEPNSISCTQLAETYNISHDSVNRFLEREDYTPHDLYQEAIQH
IDNNKLIVSIDDTVLDKPYSQHMDLVSYFWSGKHHRSVKGINLITLYATDQNGQNIPI
NFRIYDKSESKTKNDYFMDMLSEVLSWGAKIQFITGDSWYSSTGNLKTIRKYGIRFMF
GIDCNRKVSPEKGQLFQLRLLPDFHQGQVVWLKDFGFVQLFKTQLKEQQRFYIVHQDE
DDLLSFEGFHELHSSHWKIEQYHRVIKQVCHIEKFQVRRSKLILNHIFSALMAYVEIQ
KNQFEGIFENVYRWQKKLFRPIIKDFIDDFILDKNHLLPQRIYK" /product="hypothetical protein"
/transl_table=11
CDS complement(1720288..1720740)
/locus_tag="AUO97b_01758" /db_xref="SEED:fig|400667.82.peg.1619"
/translation="MKKLSDEYLPVRKAQTVYGSISGNYAFRGEKTIWFESTLERDFI
LKQEFNNNVIDVIGQPVVIPYITELGNQSTYTPDFLVQFSSSNCDDFEDFPVPMLIEI
KPKKKLVEDWDKLKPKFRAAHRFAADKGWKFKIISETRLYVGLTHYHL" /product="FIGfam050825"
/transl_table=11

CDS 1720865..1721548 /locus_tag="AUO97b_01759" /db_xref="SEED:fig|
400667.82.peg.1620"
/translation="MPLYSIAVNDSGSHPKPGEITLSHLGVLFLDELPEFDRKVLEVL
RQPLESKEIIISRAARQITYPANFQLIAAMNPCPCGYAFNQDSRCQCSPESIKRYQNR
ISGPLLDRIDLHIDVPPLKAQELQDPTPAEDSTTVRQRVIYAYEQQMQRQGCLNQALS
PKQLEQHAVLDSTSQRMIEMAQQRLNLSARAYHHVLRVARTIADLAQSEIIQSPHLTE ALSYRGNHS"
/product="AAA+ ATPase superfamily protein YifB/ComM, associated with DNA
recombination" /transl_table=11
CDS complement(1721585..1722997)
/locus_tag="AUO97b_01760" /db_xref="SEED:fig|400667.82.peg.1621"
/db_xref="GO:0004069"
/translation="MLRPWQIHFRIDEREEKTVFLKLTNLISQEILSGRLVQGTMLPG
SRSLSKELGINRKTVQAVYEELEAQGWLVTRPRKGTFVADVLPEKKPHIEPRADRKVS
NKPIIQQVAQRPHNDGVPDPRLIPYELFSRAYRHALIKITQNQYMGYGDPRGMAELRQ
ALKQMLSMERFMNVAEDEICVVRGSQMGIFLASRALPNRQGVIVVEELYYPSAVKAFQ
SNGFQVVSVKLDDQGIVIEDLERILKEHSVAAIYTTPHHQYPTTVTMTMSRRLQLLEL
SKKWGFYIIEDDYDHEFHYDSRPMPPLASLPHSELVIHVGSLSKVFAPGIRLGYIVAA
PTIIQTITEDILLIDRQGNNITELALADLMQRGEIKRHIRKMKKIYQLRRDHALAEFQ
RVFAESIEIQPPAGGMALWVKFQKSFTQDQLIKLKELNIDTEQKFKQKECANHCIRFG
FAALSEKEITSLIETLNEAL"
/product="Transcriptional regulator, GntR family domain / Aspartate
aminotransferase (EC 2.6.1.1)" /EC_number="2.6.1.1"
/transl_table=11
CDS 1723109..1723885
/locus_tag="AUO97b_01761" /db_xref="SEED:fig|400667.82.peg.1622"
/translation="MKTASVNKHDLMQHAQQDMQKWISDLDLTDRQKLALTCRILFDH
GHDAGLAGQITCRSENKETFITQRFGLGFDEITASNLLEVNQDLEPINQEGMANPANR
FHTWIYKEHPEVNCIIHTHPTYVAALSMLQIPLMISQMDVCALYEDCSFIGHWPGVPV
GNEEGILISSALDKNRAVLLSHHGQLVTGKTIEEACNLALLIERAAQLQLLAMSAGQI
QPIPHALAAEAHDWVSTDKRNKVNFAYYARKALKKHSDCI" /product="Ribulose-5-phosphate 4-
epimerase and related epimerases and aldolases" /transl_table=11
CDS 1723901..1724779
/locus_tag="AUO97b_01762" /db_xref="SEED:fig|400667.82.peg.1623"
/translation="MKLNGIIGYPVTPFSDDNTINLPVLKQMLDLLIENGCDAIAPLG
STGESAYLEWDEWCLVAKTSIESINKRVPVIIGISELTTNQAIKKAQIAQSYGADALM
VIPVSYWKLTDQEIFEYYQAIAQATTLPIMIYNNPATSGVDMSPELMVRMFNNIANIM
MVKESSGDIQRMHKIYELSKGELPFYNGCNPLALEALCAGASGWCTAAPNLLGQRPQQ
LIEYVKNGELEQAQKSFYQQLPLLRFIVSGGLPKTIKAGLQLKGVAAGAPRKPLTKAT DIELNQLKQLLSEIEN"
/product="Dihydrodipicolinate synthase family PA0223" /EC_number="4.3.3.7"
/transl_table=11
CDS complement(1724822..1725643)
/locus_tag="AUO97b_01763" /db_xref="SEED:fig|400667.82.peg.1624"
/translation="MMKFALKAVTFGVLTAGSTMVMAEDAPSFYGITATGSVAATTDY
RFRGVTQSSNNPAIQGGFTFSHKSGAYLSLWGSSVDFGVTGNSTETDVALGYTNTLKL
SDTLAPTYDVGIIHYGYIGAGHSFDRNGIGYDGKNGLDFNEVYGKLTFANSLLKGDAI
SVGVNYSDDYWANSKQFWYFNIGYSAPIADTGFTGLASVGYNKFKNKDALAVVAGGPG
KDDSYIDYKVGVNYNILGIVAELDVVGTDISTSGMTDAQKKPYDTGLVFSLTKTF"
/product="hypothetical protein"
/transl_table=11
CDS complement(1725700..1725870)
/locus_tag="AUO97b_01764" /db_xref="SEED:fig|400667.82.peg.1625"
/translation="MFSLYFTQTNYSYFIVIYKNNYLEDFYFSRCISFLVSKLMKCSD TKYELGTAIAKA"
/product="hypothetical protein"
/transl_table=11

CDS 1726568..1727842
/locus_tag="AUO97b_01765" /db_xref="SEED:fig|400667.82.peg.1626"
/translation="MLKAQKLTLAVLISAAIISSAQASEQSEAKGFVEDANGSILFRT
GYISRDKKDGRADNSSFAQTAIVNIDSGFTPGIVGFGVGVVGDGSFKIGENKNAGNNM
IPQHNDGSAYDHWARGGANVKARFSNTTVRYGTQVLDLPVLASNTARLVPEYFTGTLL
TSHEIKDLEVVAGKFTKNQYSDQIATDQNGLDRAVVWGAKYKFDDQISGSYYGVDVKD
KLDRHYVNVNYKQPLANDSSLTYDFSGYHTKFDKGANLSYATGPADEDKTNNIWAISG
TYATGPHSVMLAYQQNSGNIGYNYGVNQDGGQSVYLPNSYLSDFIGNDEKSAQIQYSL
DFGKLGVLPGLNWTTAYVYGWDIKTSNGADDSNESEFFNQVKYTVQSGFAKGSSLRLR
NSIYRADNAYTTDYMPDTNEWRIFLDIPVTLF" /product="Outer membrane low permeability porin, OprD family" /transl_table=11
CDS complement(1727949..1728476)
/locus_tag="AUO97b_01766" /db_xref="SEED:fig|400667.82.peg.1627"
/db_xref="GO:0004427"
/translation="MSYSNIPAGKDAPNDIYVIIEIPANAAPIKYEIDKDSDALFVDR
FMGTAMFYPANYGYVPNTLSEDGDPLDVLVVTPHPVAAGSVIRCRPVGKLNMEDDGGI
DAKLIAVPHEKLSPLYKDVQEYTDLPPLLISQVEHFFSHYKDLEPGKWVKISGWEGAD VAKAEVLKAIEAAKK"
/product="Inorganic pyrophosphatase (EC 3.6.1.1)" /EC_number="3.6.1.1"
/transl_table=11
CDS complement(1728604..1729002)
/locus_tag="AUO97b_01767" /db_xref="SEED:fig|400667.82.peg.1628"
/translation="MHGISSIIYIILVACLLPYAFTVIAKFTGGFQPVDNQNPRDFLA
NTKGIAARANAVQQNSFESLPLFLTAILMAEYMVVPEYFILRLGWAYIILRVIYGICY
LSNWATLRSIVWFLSLLCPIFLLIVTIKLT"
/product="hypothetical protein"
/transl_table=11
CDS 1729109..1729492
/locus_tag="AUO97b_01768" /db_xref="SEED:fig|400667.82.peg.1629"
/translation="MQQPDQKELFEVLEKQKNNQLQVDRIFKIVLPIVAFLLSVICAN
FNWQSTLGTFVILTVTFMAVGVKRLPLWHWLIVITIYCLVDNYLSYGQLDVTHLRLQL
GTMLVFVGMVGIGRPYVDRWFMKNH"
/product="hypothetical protein"
/transl_table=11
CDS complement(1729531..1731210)
/locus_tag="AUO97b_01769" /db_xref="SEED:fig|400667.82.peg.1630"
/db_xref="GO:0004467"
/translation="MEKIWFAEYQKTGIPETVALPAENTSLVDIFERNFQKFGSRDAF
IFMDKAMSFNELELASRKFATYLQNLGLAKGTRVAVMMPNVLQYPVVALAVLRAGLVL
VNVNPLYTARELEHQLNDSGAEVLVIIENFASVYQSILGKTPVKHVVVASVGDMLGTL
KGTLVNFVLRKVRKQIPAWNIPGHVKFNSALNKENPSNYKRPTLTLSDTAVLQYTGGT
TGVSKGAELTHRNLVANLLQCDGIFQSKFGANDGAKGDRIVCALPLYHIFAFMVCAMY
GMYKGQANILIPNPRDLPAVIKELRKYQPSFFPAVNTLFNALVNNEEFKQLDHSNLKM
AMGGGMAVLPSTAEAWKKITGTTIIEGYGLSETSPVATANPPASTEFSGTIGIPLPLT
EVAILDDDGKEVPLGEQGEISIRGPQVMKGYWNRPDETAKVMTADGFFRTGDIGVMDS
RGYVKIVDRKKDMILVSGFNVYPSEIEEVIAKHPKVLEVAAIGVPDEKSGEVPKLFIV
KKDPSLTTEEVLNFAKENLTGYKRPRYVEFMDELPKSNVGKILRKDLRKPA" /product="Long-chain-fatty-acid--CoA ligase (EC 6.2.1.3)" /EC_number="6.2.1.3"
/transl_table=11
CDS complement(1731508..1733727)
/locus_tag="AUO97b_01770" /db_xref="SEED:fig|400667.82.peg.1631"
/db_xref="GO:0003677"
/db_xref="GO:0003916"
/db_xref="GO:0005524"
/db_xref="GO:0005694"

/db_xref="GO:0006259"
/db_xref="GO:0006265"
/db_xref="GO:0006268"
/translation="MTSLAHHATENRSVAEFTEQAYLNYAMYVIMDRALPHISDGLKP
VQRRIVYAMSELGLKSSGKPKKSARTVGDVLGKYHPHGDSACYEAMVLMAQPFSYRYP
LIEGQGNWGSPDDPKSFAAMRYTEAKLSAYSELLLSELGQGTSEWQDNFDGSLKEPIT
LPARVPNILLNGTTGIAVGMATDIPPHNLREVVKGTIALIRNPQTSDEKLAEYIPAPD
LPTKAEIITPPEELLKIQTTGRGSYRMRAVYTIEKNEIVITELPYQVSGSKVITQIAD
QMQAKKLPLVVDVRDESDHENPTRLVIVLRSNRIDAEAVMSHLFATTDLESSYRVNLN
MIGEDGRPQVKSIRRILLEWIEIRKKTVTRRLQYHLNRIEKRLHILAGLLIAYLDIDT
VIRIIREEDQPKPVLMEHFNIDEIQAEAILELKLRHLAKLEEMEIRHEQDELSAKAAI
IREQLENPESLKNLIIGELKEDAKKFGDERRSPIVARAEAVQIKEQDLMPAETVTVVL
SEAGWVRAAKGADVDAENLNYRAGDQYLSHAVGKTNQRVYFLDETGRSYALPISNLPS
ARGLGDPLSSKLSPASGVSFIQVYLDDDESELIAASSAGYGFKTQTKQLDTNAKAGKT
FLTVPDKAKALPLISAQNMTHLAVLSSAGRLLILDLAELPNLNKGKGNKLIQLEGKEQ
ILSMTTLNLDEIIQVVAGQQHLKLKGDDLQKYMGKRASKGQLLPRGYQKANKLLIQR" /product="DNA
topoisomerase IV subunit A (EC 5.99.1.3)" /transl_table=11
CDS complement(1733881..1735200)
/locus_tag="AUO97b_01771" /db_xref="SEED:fig|400667.82.peg.1632"
/translation="MDLVSRIQNLPIGKFHYTLLWVVGLGWMFDAMDTGIIAFIMTTL
VKDWSLTPAESGWIVSIGFVGMAIGAVCSGALADRFGRKTVFAMTMAIYSIATALCAF
APDLKWLLIFRFIVGLGLGGQLPVAVTLVSEYVPAHVRGRFIVLLESFWGLGWLVAAL
ISYFIIPKFGWHIAFLIGGLPLIYILVIWKKVPESIPYLINRGRIEEAHALVQKLEAE
ACVQIVHHIEVVPVAMRQKVSFKQLWSGQFARRSLMLWLIWFGIVYSYYGIFTWLPSL
LVKQGYDIVKSFEYVLLMILAQLPGYLAAAWFVERLGRKITLAAFIGFCALSAYFFGQ
ADSVNSIMFWGCLMSFFNLGAWGVLYTYTPEQYPANIRAFGSGWASAMGRIGGIVAPM
VVTHMMVAKNGFNHVFMMFTVVLLAVALVILILGEETQGKRLESIGL" /product="Niacin transporter
NiaP"
/transl_table=11
CDS complement(1735283..1735927)
/locus_tag="AUO97b_01772" /db_xref="SEED:fig|400667.82.peg.1633"
/translation="MLLTVIYIIAITAEAMTGALSAGRRSMDWFGVVLIACVTALGGG
SVRDVLLGHYPLTWVKHPEYLVLTCCAAFVTIVIAKWMRHLHNIFLVLDALGLIGFTI
IGCQIALQMGHGFVVSAVAGVLTGVSGGILRDILCNDVPFVFRRELYASISFVAVICY
WVCLDLGLSLELTVISTLVFGFTLRLIAIYFGLEMPKFIYKDDDAESASSTDEH" /product="UPF0126
inner membrane protein YicG" /transl_table=11 CDS complement
(1735973..1736440)
/locus_tag="AUO97b_01773" /db_xref="SEED:fig|400667.82.peg.1634"
/translation="MTQQTCPCGKGSYAECCEPLHLGTTKALTAEQLMRSRYSAFALQ
QIDYIVQTTALGQQTALDKEAIAEWSKQNQWLGLEVVNANKKLDKTHAQVEFKAHYHD
GKSAQIHHEVSHFVFHQQQWFFLDPTVDMQVTMKQPCICGSGKKIKQCCAQFL" /product="UPF0225
protein YchJ"
/transl_table=11
CDS complement(1736512..1738104)
/locus_tag="AUO97b_01774" /db_xref="SEED:fig|400667.82.peg.1635"
/translation="MIFEWMSDPSAWVGLATLIVLEIVLGIDNLVFIAILAEKLPPEQ
RTKARIVGLILALGMRLILLASIAWVVTLTQPLFHIFSHPFSGRDLILLFGGVFLLFK
GTMELHERMEGKQLHKEDNPVHAAFWMVIVQIVVLDAVFSLDSVITAVGMVKELSVMM
VAVVIAVGIMLWASRPLMDFVNKHPTVVILCLGFLMMIGFSLVVEGFGFHIPKGYLYA
AIGFSILVEMINQTMRRNQEKLVTTTDLRYRTASAVLRMLGSKNSGSTSDAQTADKED
VLATQAFADEVFDEENGAYHSVLVQGVLGLSERPVKSVMTPRPELEWIDLDEEPEVLK
ERLMSMTHSRLIVARGELDNIEGIALTHKVLNGFIETGVIDFQKHLREPVIVHENAQV
LMVMEQLRQAPLQMAIVLNEYGSIEGIATPIDILEAIAGEFPDEDELDAAAESLEDGS
LILEGSTDIRHVSLLLGRDLVDESEQYSTLSGYLLFHLGRLPENGETVEADGYRFEVV

TMEGHKIEKVHIVSIEQDESKD"
/product="UPF0053 inner membrane protein YoaE" /transl_table=11
CDS 1738431..1739054
/locus_tag="AUO97b_01775" /db_xref="SEED:fig|400667.82.peg.1636"
/translation="MNSVLTMAVNDSPITSVKTTQPLIALYCGSRAGNNPIYREKAIE
LASGIAEQGFGLVYGGASIGLMGQVAESVLAHGGEAVGVIPEFMLDYEVAHAGLTELH
VVETMHERKAMMAERASAFIALPGGLGTFEEILEIATWGQLNQHQKPMILYNVNGFYN
ALIAQLDHAVQEGFLPLQHRAKLIVCEEIDQIYNVIKNLKMPKRFVV" /product="Lysine
decarboxylase family"
/transl_table=11
CDS 1739252..1740643
/locus_tag="AUO97b_01776" /db_xref="SEED:fig|400667.82.peg.1637"
/translation="MNATPTHQALQPEYRLLVLLVSIGFFMQALDTTIVNTAIPAMAH
HLNEDPLRMHSVVVAYVLSVAACIPLSGWLADRFGVRNTFLSAIIIFTLASLGCGLSQ
SLNELLFFRVIQGVGGALLLPVGRLSLLKIIPRTQFLAAMSLMSLAGLLGPLAGPTLG
GWLVEVATWHWIFLINIPMGVLGILMTLKVMPNVKEPTVKTFDLIGFVLLVVAMIGWS
LGIEHLASPEYSKWFSISLLVVGVIATLWYAYHSHTHQNALFRSRLFRNKIYAIGILG
NFFARFGGNALPFVLPLMLQVAFGFEPFIAGLMMIPLVLGSLFSKPIVRPIIQKMGYR
RFLLINTTLVGLCIASFAIMTVDTPTWFRALHFFLFGTLNSLQFVGMNTLTLKDLPQQ
DASSGNSFLSMIMMLSMSIGVALAGTLINIFTDYYGTAHVTTAFHVTLICLGCINIIT ALIFWQIPKNTPV"
/product="Uncharacterized MFS-type transporter" /transl_table=11
CDS 1740721..1741479
/locus_tag="AUO97b_01777" /db_xref="SEED:fig|400667.82.peg.1638"
/translation="MTKLAKTFSAPRYNTLIGIVLACVMGVLTYFGLFYQQKAIAQDY
PVQGFDVSHHQENINWKKISPQKFQFVYLKATEGGDYKDPKFQENWLKAREHGFHVGA
YHFYRLCRDGKTQAENFIATVPNKVDALPPVIDLEYDSNCINSHTKEQLLKEIGIMHD
SLKHHYGKQPIFYISKTFYHIVLIGSFPNTPLWVRDYEGKPELKDKRKWLFWQHSNQG
KIEGMTKPVDLNVYEGSVKEWHQFLQQQGIVKAQ"
/product="Lyzozyme M1 (1,4-beta-N-acetylmuramidase) (EC 3.2.1.17)"
/transl_table=11
CDS 1741481..1742536
/locus_tag="AUO97b_01778" /db_xref="SEED:fig|400667.82.peg.1639"
/db_xref="GO:0003887"
/translation="MRKIIHIDMDAFYASVELRERPDLRHLPVVISSHHPRAVIAAAS
YPAREFGLRSAMSMSQARKLCPQVVIIEPNFEKYRAISAQIHSIFQQYTTLIEPLSLD
EAYLDVTENLKQIASATEVAMHIREDIFRQTGLTASAGVAPNKFLAKVASDWNKPNGL
FVIKPSQVASFIQDLPLKKIPGVGKVTQEKLQQLELHTLGDLQKIEEAVLVHHFGKYG
QQLYLYAQGIDNRPVQAERARQQISKETTFDSDFTLAQCQPYWHGLAEKVWQSLEKKQ
LNARGVNIKLKLKNFQTLQHSKSFKNPIHSQQDLIQVLFLLLNEMHIPENFQFRLIGI GVYQLQTKADDFQLSLW"
/product="DNA polymerase IV (EC 2.7.7.7)" /EC_number="2.7.7.7"
/transl_table=11
CDS 1742857..1743210
/locus_tag="AUO97b_01779" /db_xref="SEED:fig|400667.82.peg.1640"
/translation="MTVKRLHVTSRYCEVAISGNLVHLAGQLADDTSVDITAQTQQTL
DNIDRLLAEAGTDKSHILSVMIFLKDIDKDYAAMNTVWDAWISKEHPPARTCVESKLY APDVLVEMTVVAVLP"
/product="RidA/YER057c/UK114 superfamily, group 2, YoaB-like protein"
/transl_table=11
CDS complement(1743356..1743856)
/locus_tag="AUO97b_01780" /db_xref="SEED:fig|400667.82.peg.1641"
/translation="MYYLITILLLFLTVVMAAKSLSRKHQQDSALKRRAVLSSHEQMT
LTRLQTVLPKFTVLAHVSFDALLTTKYAHTRLKYQNMTADFVVLDQNYQVVVVVMLEE
SGLRRAHQQYERRLLQLAGYRVLSYSTVPDYHELRRDLLTPESEMATLLATPQLERIS NAFVKS"
/product="hypothetical protein"
/transl_table=11

CDS complement(1744170..1746548)
/locus_tag="AUO97b_01781" /db_xref="SEED:fig|400667.82.peg.1642"
/translation="MSTAYTMQTAPKALFDYDKYWASCFEPAPFLPMSREEMDQLGWD
ACDFILVCGDAYIDHPSFVSGVIGRVLEAQGFRVGIIAQPDWTNVESFRVLGKPTIAW
GVTAGNMDSMINRYTADRKIRSDDAYSPNNEPNKRPDRAATVYCQRCREAFPDVPVLL
GGIEGSLRRIAHYDYWSDKVRRSILMDSKADMLMYGNGERAIIDVMHRLAKGEKIHEI
TDVRGTAFIINKHNKAAKAKFVEIASNDVDTIGRVDPIINPYVMTEDIDGCEIEKDKG
NTLAQYQGFQKEIVANPIVREGDQLDENTQIVQLKPAQSKAIKHKLPPRELAVIRLPS
FEEVANDQVLYAHANRILHLETNPGNARALVQRHGERDVWINPPPIPLTTEEMDYVFD
LPYARLPHPAYGDARFPAFDMIKFSVNIMRGCFGGCTFCSITEHEGRIIQNRSEDSIL
REIEKIRDTAPNFTGIISDLGGPTANMYRLHCKDPEIEKNCRKPSCVYPGVCQNLHTD
HAPLVQLYRKARAIKGVKKILIGSGLRYDLAVLNPEYVKELVQHHVGGYLKIAPEHTE
QGPLSKMMKPGIGTYDRFKQMFDRFSKEAGKEQYLIPYFIAAHPGTSDYDMMHLAIWL
KKNGFRADQVQTFYPSPMATATTMYYSGKNPLAKVARYTENVDIVKGEKRRRLHKAFL
RYHDPNNWPLLREALKEMGRADLIGNSKQHLIPTYQPKGAEGEYKSARKKNSTVAGDS
VKRSGQTVQNKNQHKRPQKGQVLTQHTGLPPRETGEKRPFGGKSKPKSKARG" /product="UPF0313 [4Fe-4S] protein YgiQ"
/transl_table=11 CDS complement(1746763..1748022)
/locus_tag="AUO97b_01782" /db_xref="SEED:fig|400667.82.peg.1643"
/db_xref="GO:0008889"
/translation="MSKKTLIALLCCASLGLTACNDDNDQDQTSPTENVTEPTLISFA
KLPVETYAAGPDSGAYVKGANGIYPPFKGQPVQGFSAALKNEDGSYMAMADNGFGTQD
NSSDFLLRIYKIKPDFKTKTKGTGQVTVQSFIQLRDPNKLIPFNIINGNTPERLLTGA
DFDPESMQRTSDGTYWIGDEFGPYLLHFSADGVLLDAPIPLPNPLNPTQELRSPQNQF
NKAQINFVEPLVQQSGGFEGMAISPDGQYLYPLIEKPIKSIRETERQLLISQFDIKKK
AYTGKYYWFQLDSKATNIGDFQLFNDKEGIIIERDATQNNLGGYKKLIRIKLNESGQL
VSREDLVDLIKIANPNMLYGTARAGDIGMGQVFAFPFETIEDVIIENGTTLTVFNDNN
FPGSTGRNAKLADDNEIIQIRLPKALF"
/product="Glycerophosphoryl diester phosphodiesterase (EC 3.1.4.46)"
/EC_number="3.1.4.46"
/transl_table=11 CDS complement(1748170..1748574)
/locus_tag="AUO97b_01783" /db_xref="SEED:fig|400667.82.peg.1644"
/translation="MDIGEVAKKAKVTTATLRFYEEKGLIKSIGRQGLRRQYGKQVID
QLALIALGRAAGFSLNEIATMFGQDGKPDLDRQKLKDKAEQLEQMAKRLHFISQGLEH
AAVCPAENHMECPTFQKFLKAAIAGEYQPTKL" /product="Transcriptional regulator, MerR family" /transl_table=11
CDS 1748656..1749141
/locus_tag="AUO97b_01784" /db_xref="SEED:fig|400667.82.peg.1645"
/translation="MNTSTLFFQALCLGIGATIVMDIWLLILKIFKVPTLNFAFLGRW
VGWIFQGKLVHQSIAKSTPIKGEYLLGWIAHYSVGIIFALSFLLIVGSTWLDHPQFYS
ALLFGLVTVLIPFFIMQPAMGSGFASSKTPHPFLNCLKSLLNHSVFGCGLYLTAKLFQ I"
/product="FIG00350655: hypothetical protein" /transl_table=11 CDS 1749158..1749724
/locus_tag="AUO97b_01785" /db_xref="SEED:fig|400667.82.peg.1646"
/translation="MKTVAIIYHSRQGHTQFIAKQIQIGVLSHHNIKADLLNAEDLIE
RPGILIQYDGLIWGSPTYLGGVSSKLKQLMDATGPLWKKQSFKGKLAAGFTVSSLPAG
DKQSTLISIFTFCMQHGMLWVGNPILPEQHQGVAYTQAANRLGSWSGLMAQAEQASNA
DGFDEGDIKTAQQFGENFALTLNAYQGT"
/product="Tryptophan repressor binding protein" /transl_table=11
CDS 1750030..1750638
/locus_tag="AUO97b_01786" /db_xref="SEED:fig|400667.82.peg.1647"
/translation="MKFDYWFCILLTSSLAQDHFAPSQLQRLSLKFFIIALMTIMTGC
TSLGPNSGSFSWRSSKLSSGLQKAYSVPPSTANRLSPMIIQSADRYNVPPLLLAAVIR
QESSYNSNARSPTGAIGLTQIIPSYWQQTCAGNLYDESTNIQCGAYILNHYYQTADSW

FKATAYYNVGPTGYERSFWTRHKAKKYARSVKRHQKTLKSAL" /product="Lytic transglycosylase"
/transl_table=11
CDS complement(1750694..1751692)
/locus_tag="AUO97b_01787" /db_xref="SEED:fig|400667.82.peg.1648"
/db_xref="GO:0004124"
/translation="MSTDPQFPTPNNLGIAVYSNNAEAIGNTPLVRINRLIKTGATVL
AKVESRNPAFSVKCRIGAALIADAEKRGVLKEGMHIVEPTSGNTGIALAFVAAAKGYS
ITLTMPASMSLERRKVLKALGANLVLTEPAKGMKGAVDEAVRLATEQPEIYFLPQQFE
NPANPQIHVDTTGPEIWQATGGQVDILIAGVGTGGTITGISRYFEQVQNKPLYSVAVE
PAESPIITQTKKGENITPAPHKIQGIGANFIPKNLDLDLVDEVIPVSSEEAIQWARNC
ATQEGILVGISSGAALAAAAKIAERPENAGKTIVVILPDSGERYLSSVLFEGLFDE" /product="Cysteine synthase (EC 2.5.1.47)" /EC_number="2.5.1.47"
/transl_table=11
CDS complement(1751899..1752441)
/locus_tag="AUO97b_01788" /db_xref="SEED:fig|400667.82.peg.1649"
/translation="MQFKIIAAQDLDRNQRHQIAELCFSAFDEDPWTQYAFMQKAIHV
VGILNNQIVSHALWTDRVFTINGSSDVKTAYVEYVTTDYTMRGKGLATQLLKYLVEIL
THLEYELAALQPEDEAFYEKLGWTVWKGNLSIKQDTCSYLTDEYEIMLYPLNIQMKDQ
LSNSSEEDTICADWREGELW"
/product="Acetyltransferase, GNAT family"
/transl_table=11
CDS complement(1758421..1759017)
/locus_tag="AUO97b_01789" /db_xref="SEED:fig|400667.82.peg.1651"
/translation="MRKAPQQQRSKIIVDHILEATQQCIAQYGLLHTTTPKIAEKSGV
SVGSIYQYFENKNQIIEELLRRKSELLGQQLKELVIQQGNIPLELLIPLAIELGFNAL
KADHGFFIEVLKHWHDYSHSQAAQILEKHFFEVGLYTFSRNPHQWDFEQVKHKSFVII
NSTLFTMMRYVSQNNFLISEQQLKRELSSMILAYLSQK" /product="Transcriptional regulator, AcrR family" /transl_table=11 CDS 1759105..1760319
/locus_tag="AUO97b_01790" /db_xref="SEED:fig|400667.82.peg.1652"
/translation="MQMISPTRLASFNDMQDSNFFTQFLNICCEKPVQPGYTEYVSLQ
HALYEGDIEMDKVIDWVMQNPKDHRIIFEKILFQGRDELSELIPTELENFFNYIEQKP
EWLDQRQIDEAVKFTHRLGINNGFILRDLSLMAGYLYPGFNQPLILTGALKKEAGTRL
AETTKWWVDITEPQGLSRLSAGFTSTIYVRFIHALVRRQLKKSDRWDSEVWGVPLNQF
DLAMTNLAFSSVVLLGIRALGIWPTKQEAKSFLHFWRYVGWLMGIDEKWLIQSEPEGW
RLLYWMQFAHPRSDHSSVELGSSLSKEPFERKYLHLRSLQQKLAYRQHLELTQFFIGK
KRMKLLGLPQQSASWFAYYLIVRNLLLYNGAKLSPKVEKFLSKSGRNIQKLGLTLYQN QGKAKTLASMHQ"
/product="hypothetical protein"
/transl_table=11
CDS complement(1760389..1760595)
/locus_tag="AUO97b_01791" /db_xref="SEED:fig|400667.82.peg.1653"
/translation="MTNKLSPEQSWWGWKLLIVASILSVLFMIIFYLAINNEPDYMPS
QKKMQMMESHEQMNHAEMHSSSAQ"
/product="hypothetical protein"
/transl_table=11
CDS complement(1760649..1762724)
/locus_tag="AUO97b_01792" /db_xref="SEED:fig|400667.82.peg.1654"
/translation="MPHSKFLLQPLWVAMLAVSHSGLVFAESEKNDADTNTLHSLAPI
VVTAQQGNDANGLIVHADPKQPIQPVPATDGADYLQSIMGFNSIQSGGTNGDVTFRGM
FGSRIKILTDGTENLGACPNRMDAPTSYISPESYDRISVIKGPQTVQYANTGSAATVL
FERQPEKLTSEKPYRGQASVLLGSYGRIDHNIEAAVGDEKKYIRLNANRSESNSYQDG
DGNTVPSAWKKWNADVALGFTPDENTWVEITGGKSDGESLYAGRSMDGSQFARESLGL
RFEKKNITDVIKKIEGQVNYSYNDHVMDNFRLRIPPMTHDMMTHQMVVNPSEMQVTRR
TLNTRFAMTSEWGKLNVITGIDSQQNHHAESMKSLMMDMPLTTNMKFQSYGAFGELGY
QLSENSKLVTGARLDQVKIDALKLNDDRSETLPSGFIRLETQLPEHNAKSYIGLGYVE

RVPDYWELFSTAHGNSGMPKPTFNDLDTEKTLQLDMGYQHQHGAFNSWASAYVGLIND
YILMSYHNHPTSGGHGHGSSFSAGAKNVDATIAGAEAGIGYQFTDRIQADLSAMYAWG
KNTTDDKPLPQISPLEGRLNIRYVADKYNLGLLWRAVAEQNRVSLHQGNIVGYDLKPS
KGFSTLSLNGSYNLRKDIDVSVGIDNVLDKTYTEHLNKAGSAGFGFASEEQFNNIGRN YWVRMSMKF"
/product="TonB-dependent receptor"
/transl_table=11
CDS complement(1762797..1763225)
/locus_tag="AUO97b_01793" /db_xref="SEED:fig|400667.82.peg.1655"
/translation="MVFRSGLLLACAAVFLQIAVFLQPLLPKQYQIAPVCETITRALL
KPKAQTEAMSHAMHHSHHQHHIEQAQVPDHHDHHDANHQCQYCTVYANLVLPPEFGVK
EVLVRIQVRLVAYQQAFRHVYFALQRLFLLPQGRAPPLFA" /product="hypothetical protein"
/transl_table=11
CDS complement(1763262..1763792)
/locus_tag="AUO97b_01794" /db_xref="SEED:fig|400667.82.peg.1656"
/db_xref="GO:0042586"
/translation="MALLPILSFPDPRLRTIAKPVEEVTDEIRQLAADMFETMYAAPG
IGLAASQVDRHIQLIVMDLSESKDEPMVFINPKVTPLTEETQPYEEGCLSVPQIYDKV
DRPSRVKIEAINLEGQAFEIEADGLLAVCIQHEMDHLNGKLFVDYLSPLKRQRAREKV EKIVRQREREKVAVKR"
/product="Peptide deformylase (EC 3.5.1.88)" /EC_number="3.5.1.88"
/transl_table=11
CDS 1763915..1765069
/locus_tag="AUO97b_01795" /db_xref="SEED:fig|400667.82.peg.1657"
/translation="MKKVFNGMVNFHALGIKKHLLALALCTGVAIGTIAEVHATSPNH
NPPSLKSNAPNVYVVKRGDTLWDISGHFLNKPWRWPEIWASNQHVKNPHWIYPGDRLL
LCSLDGRPLVGKDEGDGCVGIIRRYTGQTTNLQPQVRVEALNNSVPVIPLEHIKQWLE
NSTILPADSITNTPYIVGTADQRVLAGKGQTIYARGQGLINGQRYAVYREGEPYYFTD
NKGKKHSLGIELLQVASGVAVSSEKDITTLELTDSYNAEVRRGDRVMPEEQATLPTLF
YPVDAKQVTDGGKIIRVMGSIGRAAKNSVVTLDRGTTQGVQVGQVFDITQQGESIRDP
KTKEVIQLPGQQIGSLMVFRTFDQLSYAYVLESDLPIKVGSSIQPPQFND" /product="Uncharacterized
protein with LysM domain, COG1652" /transl_table=11
CDS 1765090..1766220
/locus_tag="AUO97b_01796" /db_xref="SEED:fig|400667.82.peg.1658"
/translation="MLTQLSSHHYHTLKVWYLVQHSLVSFKKIIDYFGNCEKATQPDG
LAKWSSLGLHANHLKRVSEFQTPQGQTQFEQLVQQVVQHTDFILTPDDSGYPTQLLPY
TDHPPIIFGKGQAQALLQPQIAIVGSRKPSPHGRQVAYDFAYYLSEKGFFISSGLAYG
IDEAAHQGASAHQRTIAVTGTGLDTTYPAQNKKLAEHILAQNGAIITEFLPGTPPLQQ
HFPRRNRIVSGLSLGVLVVEATLKSGSLITANKAAEQGKTVFAIPGHIYSEFHQGCHQ
LIREGAILIDHPEQIIEDLALPTQWQSQQQNQTEEANTNTPEIPEHLIDLYQSLDWVG
QNIDQLVIHHNIPVSELTSSLMELELLGLCMQQSGLYLRCRS" /product="Rossmann fold
nucleotide-binding protein Smf possibly involved in DNA uptake"
/transl_table=11
CDS 1766271..1766840
/locus_tag="AUO97b_01797" /db_xref="SEED:fig|400667.82.peg.1659"
/translation="MITTSVTEAAECLQQGQVLAYPTEAVWGLGCDPFNEQAFQKILE
LKQRPIEKGVILLAGHISQVEHLLTSLPQTTQQEIIDCWTNHQPSERATTWLLPADQH
IPSWIKGEHPLVAVRVTTHPLCVALCNAFHGFIVSTSANPSGQEPAHSLQDACQYFGS
QLNYLNGDLGQSQQPSRIINALTGEVIRP" /product="Threonylcarbamoyl-AMP synthase (EC
2.7.7.87)" /transl_table=11
CDS complement(1767232..1768152)
/locus_tag="AUO97b_01798" /db_xref="SEED:fig|400667.82.peg.1660"
/db_xref="GO:0016020"
/translation="MSQVMKYHKWAFIFPILAVLIWSLNIVVTRYVSDYISPVSISFY
RWLIAFIILTPFMFLSVWRQRRLVIPYLPKLAVLSAFGMVLYQGLAYTAAHYTSATNM
GIVNAFIPIFTIFVSLAILKDVPNRFAVFGSILSFAGLLYVMSQGNINKLLSGGGHLG

DALMIIAVFFYAFYGVFLKKWQLPLPIMTSLYVQIGFSVLFHLPLIFWFGLDALNAQN
APSAIYAGVFASLIAPLVWMLAVQQLGPNRTSIFMNLVPVFTAIIASIWLSEQWTIYH
TFGGIMILVGIIMAQKKVNSKKSDLLPEQN"
/product="Permease of the drug/metabolite transporter (DMT) superfamily"
/transl_table=11
CDS complement(1768258..1768797)
/locus_tag="AUO97b_01799" /db_xref="SEED:fig|400667.82.peg.1661"
/translation="MLLFWEHGYEATSISDLTHALEITAPSLYSAFGDKAGLFYKSID
YYLAHEACPIETIFLEAKTAKIAFELYLYDNVKRLVQPNKPAGCMLVVAAMNCSDATQ
EVQQNLLDKRIKTKEKLLKRLEQGVEHGDLPINAPLQEMTDFYATVIQGLTIQARDGA
STEQLHKVVEHAMKTWTLF"
/product="Transcriptional regulator, AcrR family" /transl_table=11
CDS 1769045..1770259
/locus_tag="AUO97b_01800" /db_xref="SEED:fig|400667.82.peg.1662"
/translation="MDQSTTSKARPMTSTQGSWFAILAVAIAAFALVTSEFLPVGVLN
SVAADLHISVGTAGLIITVPGIMAAIAAPLLPVSVKQLDRRYVLILLTAIMVIANTIT
AFAENFHVLLLSRLILGISIGGFWATAIALSGKLAPANLPIAKATAVVMAGVTFATVL
GVPIGTWLSEFYGWRSAFGITAAIGLVVLVLQLIFLPKLLPESAIHIRDLPALLRTPK
ARSGMLIVLLIGLAHFCAYSYLAPFFKNVAGFNGTTISSLLLLYGIAGIFGNAFAGYS
GNLNVRYTLAFVGTCFAIVFFGFPIFAIHEFGAIVLTALWGFAFGAFPTSANIWMFVH
APHAVEKGMPLFVGMFQVMIATGSLLGGYVVDHFNENTLIYGVLSFVALALISTFTLA KGLNNPKATCEN"
/product="Transcription regulatory protein opdE" /transl_table=11
CDS 1770281..1771045 /locus_tag="AUO97b_01801" /db_xref="SEED:fig|
400667.82.peg.1663"
/translation="MKNEDQQFSLLESIEVHEFLYEKEDIVRPHASLWGDFNFSLNGI
LEIQVEEQIYLSPPSYGLWIPPQTVHQSTHIDHEIHYICIRLHPRLCSILGDICRCFS
IQPFFRTLVLQILEQQKQTEKPEYLEHLLQVLFDQLQQASAYSHYLPQTRHPVLLPIL
EKLSDPLLFNLSLQQLLQNFSVSDRHLLRLSQQELQLSLSEWRNRAKIVYAIHQIRQG
TPIKRLAYDLGYQHSSSFIEFFKRYTGQTPVQIRNN" /product="Transcriptional regulator,
AraC family" /transl_table=11
CDS 1771168..1771713
/locus_tag="AUO97b_01802" /db_xref="SEED:fig|400667.82.peg.1664"
/translation="MTQSVYHIPVKAISGETVDLDQYKGKVLLIVNTASKCGLTPQYE
GLEKLYQAKKDQGLEILGFPANNFKEQEPGSDEEIQQFCSLNYDVHFPLFSKISVAGE
DKHPLYQALTTAQPERIGEGPFRERLEGLGIPTNPAPEVLWNFEKFLVNKNGEVVARF
APNLTADDEQIVKAVEAELAK"
/product="Glutathione peroxidase (EC 1.11.1.9) @ Thioredoxin peroxidase (EC
1.11.1.15)" /transl_table=11
CDS 1771859..1772458
/locus_tag="AUO97b_01803" /db_xref="SEED:fig|400667.82.peg.1665"
/translation="MKKVLQGAFFPFVIFASGMLLLGCDQAQDHSKEQESVQTNDQIS
EQADDSQQEITATQNKTELKNGNVFYIVRDAANLQLKAGDYIEKLKDTQLDVEQAIQD
KDQHELKTTVTTLKAQLEGLNQALLGLDIRSQEVENIRQSLLQANQQALSMPLLNGKL
EQINFDQIEKQLNTIQMDMVKLAAMIMAGDEKSDSKTDS" /product="hypothetical protein"
/transl_table=11
CDS complement(1772507..1772989)
/locus_tag="AUO97b_01804" /db_xref="SEED:fig|400667.82.peg.1666"
/translation="MKFKYWLPILVSTAFVGCASVPQANPQLAQQAKQLTAPTNGNAV
IYVYRSNNIVGSALKKDVWVDGECLGETARGIFFYKEVAGNQEHTVSTESEFSPNHLK
FKTEAGKKYFVQQYIKPGVFVGGANLKLVDDAQGQKAINEYRLAEAGKCSKATIALVN "
/product="hypothetical protein"
/transl_table=11
CDS complement(1773138..1773557)
/locus_tag="AUO97b_01805" /db_xref="SEED:fig|400667.82.peg.1667"

/db_xref="GO:0046933"
/db_xref="GO:0046961"
/translation="MATMQCDVVSVKESIYSGAVTMLIAKGAGGELGILPGHAPLVTL
LQPGPIRVLLENGTEEIVYVSGGVLEVQPHVVTVLADTAIRADNLDEAAILEARKNAE
QLLANQKSDLDSAAALAALAETAAQLETIRKIKNRAQ"
/product="ATP synthase epsilon chain (EC 3.6.3.14)" /EC_number="3.6.3.14"
/transl_table=11
CDS complement(1773575..1774969)
/locus_tag="AUO97b_01806" /db_xref="SEED:fig|400667.82.peg.1668"
/db_xref="GO:0046933" /db_xref="GO:0046961"
/translation="MSSGRIIQIIGAVIDVEFERTSVPKIYDALQVDGTETTLEVQQQ
LGDGVVRTIAMGSTEGLKRGLTVTSTNAPISVPVGTATLGRIMDVLGRPIDEAGPVAT
EERLPIHRQAPSYAEQAASTDLLETGIKVIDLLCPFAKGGKVGLFGGAGVGKTVNMME
LINNIAKAHSGLSVFAGVGERTREGNDFYHEMKDSNVLDKVAMVYGQMNEPPGNRLRV
ALTGLTMAEYFRDEKDENGKGRDVLLFVDNIYRYTLAGTEVSALLGRMPSAVGYQPTL
AEEMGVLQERITSTKSGSITSIQAVYVPADDLTDPSPATTFAHLDATVVLSRDIASSG
IYPAIDPLDSTSRQLDPLVVGQEHYEIARAVQNVLQRYKELKDIIAILGMDELAEEDK
LVVYRARKIQRFFSQPFHVAEVFTGAPGKLVPLKETIRGFKGLLAGEYDHIPEQAFYM VGGIDEVIAKAEKL"
/product="ATP synthase beta chain (EC 3.6.3.14)" /EC_number="3.6.3.14"
/transl_table=11
CDS complement(1775000..1775869)
/locus_tag="AUO97b_01807" /db_xref="SEED:fig|400667.82.peg.1669"
/db_xref="GO:0046933"
/db_xref="GO:0046961"
/translation="MANLKEIRAKVASIKSTQKITRAMQMVAASKMRRAQERMAQGRP
YADNMRRVIAHLVQANPEYKHRYMVDRPVKRVGYIIVSSDRGLAGGLNINLFKKVVQH
VKAQQEQSIEVQFALIGQKAVSFFKNYGGKVLGATTQIGDAPSLEQLTGSVQVMLDAF
DKGELDRIYLVSNGFVNAMTQKPKVEQLVPLAPAEEGDDLNRTYGWDYIYEPEAEELL
NGLLVRYIESMVYQGVIENVACEQSARMVAMKAATDNAGQLIKDLQLIYNKLRQAAIT QEISEIVGGAAAV"
/product="ATP synthase gamma chain (EC 3.6.3.14)" /EC_number="3.6.3.14"
/transl_table=11
CDS complement(1775947..1777491)
/locus_tag="AUO97b_01808" /db_xref="SEED:fig|400667.82.peg.1670"
/db_xref="GO:0046933"
/db_xref="GO:0046961"
/translation="MQQLNPSEISALIKQRIGDLDTSATAKNEGTIVMVSDGIVRIHG
LADAMYGEMIEFDGGLFGMALNLEQDSVGAVVLGNYLSLQEGQKARCTGRVLEVPVGP
ELLGRVVDALGNPIDGKGPIDAKLTDAVEKVAPGVIWRQSVDQPVQTGYKSVDTMIPV
GRGQRELIIGDRQTGKTAMAIDAIIAQKNSGIKCVYVAIGQKQSTIANVVRKLEETGA
MAYTTVVAAAAADPAAMQYLAPYSGCTMGEYFRDRGEDALIIYDDLSKQAVAYRQISL
LLRRPPGREAYPGDVFYLHSRLLERASRVSAEYVEKFTNGAVTGKTGSLTALPIIETQ
AGDVSAFVPTNVISITDGQIFLETSLFNAGIRPAVNAGISVSRVGGSAQTKIIKKLSG
GIRTALAQYRELAAFAQFASDLDEATRKQLEHGQRVTELMKQKQYAPYSIADQAVSVY
ASNEGYMADVEVKKIVDFDAALIAYFRSEYAPLMKQIDETGDYNKDIEAAIKAGIESF KATQTY"
/product="ATP synthase alpha chain (EC 3.6.3.14)" /EC_number="3.6.3.14"
/transl_table=11
CDS complement(1777538..1778074)
/locus_tag="AUO97b_01809" /db_xref="SEED:fig|400667.82.peg.1671"
/db_xref="GO:0046933"
/db_xref="GO:0046961"
/translation="MAELLTLARPYAKAAFAYASEQGATDNWSNALQVLSAAVQDEAF
SAYLNRPELTPAEQVKLFAKVLGEDQSQAVSNFLTLLADNDRLVLLPEIAAEYEQLKS
QNNNNVDVVIESAFPLTAEQEQLLKSALEKRFNSTVTVSVEVKPELIAGVVIRAGDQV
IDDSALNKLEKMRTRLLA"

/product="ATP synthase delta chain (EC 3.6.3.14)" /EC_number="3.6.3.14"
/transl_table=11
CDS complement(1778087..1778557)
/locus_tag="AUO97b_01810" /db_xref="SEED:fig|400667.82.peg.1672"
/translation="MNINLTLIGQAIAFAFFVAFCMKFVWPPLINAISERQRKIADGL
NAAEKAKADLADAQAQVKQELDAAKAQAAQLIEQANRRAAQLIEEARTQAAAEGERIR
QQAKEAVDQEINSAREELRQQVAALAVTGAEKILNQQVDAEAHNAMLSQLAAKL" /product="ATP
synthase F0 sector subunit b (EC 3.6.3.14)" /transl_table=11
CDS complement(1778596..1778841)
/locus_tag="AUO97b_01811" /db_xref="SEED:fig|400667.82.peg.1673"
/translation="MELTLGLVAIASAILIAFGALGTAIGFGLLGGRFLEAVARQPEL
APQLQTRMFLIAGLLDAVPMIGVGIGLFFIFANPFVG"
/product="ATP synthase F0 sector subunit c (EC 3.6.3.14)" /transl_table=11
CDS complement(1778926..1779801)
/locus_tag="AUO97b_01812" /db_xref="SEED:fig|400667.82.peg.1674"
/translation="MAAEEHALTSTEYIKHHLTNMTYGKMPDGTWKLAETAEEAHSMG
FTAIHLDSMGWSIGLGVIFCLLFWIVARAANAGVPTKFQSAIEMIIEFVDSSVRDTFH
GKSRLIAPLALTIFVWIFLMNLMDLIPVDWIPQVAAFVGANVFGMDPHHVYFKIVPST
DPNITLGMSLSVFVLILFYSIREKGVGGFVGELALNPFNPSNPVAKALLIPVNLILEL
VTFLARPISLALRLFGNMYAGELIFILIALLPFWIQWALSVPWAIFHILVITLQAFIF MMLTIVYLSMASEKH"
/product="ATP synthase F0 sector subunit a (EC 3.6.3.14)" /transl_table=11
CDS complement(1779906..1780304)
/locus_tag="AUO97b_01813" /db_xref="SEED:fig|400667.82.peg.1675"
/db_xref="GO:0015986"
/db_xref="GO:0016020"
/db_xref="GO:0016469"
/translation="MSQTSRLIDRRLAKALVFLQACMIPVSALVAWSIKDMTAALSAA
LGALVCWLATSYFAWQSFRTAGARASKQVLSNMYRGMLGKFAIMIVGFILILSNVKPL
SPLALLFGFILVQAMSWVAPFWVSRLQKRV"
/product="ATP synthase protein I"
/transl_table=11
CDS complement(1780474..1781250)
/locus_tag="AUO97b_01814" /db_xref="SEED:fig|400667.82.peg.1676"
/db_xref="GO:0005488"
/translation="MVVSTHPIYLIAKEITKGVEEPQLLLQGQSGHDVQLTPAHRKAI
NDATLVIWLGKAHEAPLNKLLSNNKKAIALLDSGILSILPQRNTRGAALPNTVDTHVW
LEPNNAVRIGFFIAALRSQQHPENKAKYWNNANTFARNMLQAAQAYDSSSNGKPYWSY
HDAYQYLERSLNLKFAGALTDDPHVAPTAAQIKYLNDSRPKAQMCLLAESFTTKGQYQ
KLGSITFQPVDESMNNEDNFVTAWKKLAIKTDKCVLNTQK"
/product="Zinc ABC transporter, substrate-binding protein ZnuA"
/transl_table=11
CDS 1781453..1781938
/locus_tag="AUO97b_01815" /db_xref="SEED:fig|400667.82.peg.1677"
/db_xref="GO:0003700"
/translation="MHEHHDSLHGVHDHHNVASRLAEAETLCTAVGARLTPLRKEVLE
LILNASGPMGAYDLLARIKSQTDRPAAPPTVYRTLDFLLEKGLIHRLTSINAYIPCCH
PREGHQAAFLICTECHTVKEASSQGLTQQLDQLAALDDFAAQHSIIEISGKCQQCRTA R" /product="Zinc
uptake regulation protein Zur" /transl_table=11
CDS 1781920..1782699
/locus_tag="AUO97b_01816" /db_xref="SEED:fig|400667.82.peg.1678"
/db_xref="GO:0005524"
/db_xref="GO:0016887"
/translation="MPHSSLNPVLIQLEQVSVHRDQRDILKKVDFALHENEIVTLIGP
NGAGKSTLIKVLLGILKPSSGRVISNKKLKMAYVPQKFNPSHSLPLRVQDLLDLEKCS

PALRQEIIQDTGIAKLQQSKVQQLSGGERQRVLLARALLRKPDILVLDEPMQGLDIQS
EAELYEYVRSLPERYGCAILIVSHDLQWVMQGTQRVVCLNKHICCSGLPESIQQHPEY
QAIFGTQRVFYQHHHDHCAHGDAAHPCPHNSRPHIHPEPEA"
/product="Zinc ABC transporter, ATP-binding protein ZnuC" /transl_table=11
CDS 1782703..1783512
/locus_tag="AUO97b_01817" /db_xref="SEED:fig|400667.82.peg.1679"
/db_xref="GO:0005524"
/db_xref="GO:0006810"
/db_xref="GO:0016020"
/translation="MMEWLQLLLPAWIMGTLLVFLTAPLGCLMLWRRMSFFADTMAHG
TLLGVAIAGALSLPLWLGVAGTAILLVAILWALHDNRLPNDALLALCSATLLCSGLLF
IQHVPSLRPELLSYLFGDLLSIDWPDLPIFTLIIAVSLAILYRYWQPQIQIAIDPDIA
ISEGVNAKWQRLIFMLLLALFTVLALRAVGSLLMGALLVIPALSARLLAHSPKQMVIW
AFVLAQLGVSVGLWSSAALNISTGLSIVLCMALSFALIFMVQKIKNQTQAA" /product="Zinc ABC
transporter, permease protein ZnuB" /transl_table=11
CDS complement(1783514..1784140)
/locus_tag="AUO97b_01818" /db_xref="SEED:fig|400667.82.peg.1680"
/translation="MSLQVWFAYMLACWVISISPGAGAIASMSSGLNYGFRHGYWNAI
GLQIALLIQIMIVAAGVGVLFATTPLAFQAVKWFGVAYLLYLAYLQWRAPVKDIEIQH
EKKDKSVSALLFNGFVVNISNPKAIVFLLAVLPQFLDLSKPQWIQYLIMAATMVTIDL
IVMAGYTGLASKVLRLLRSPKQQKYLNRGFAVMFSCAALLLSTVHQAA"
/product="Homoserine/homoserine lactone efflux protein" /transl_table=11
CDS 1784299..1785219
/locus_tag="AUO97b_01819" /db_xref="SEED:fig|400667.82.peg.1681"
/translation="MTAQSVILPLPSDHARFIVLRLKDLTIAELKEQLEALFETRDRL
ITQHSDAQIKTAVAFGPELWKQLYSQSPAGFKQLEPENGAFNMPVVPADVLIHIASMR
SDICFALSQAFFEGIKDKVEVLDERVCFRYFDGRDITGFIDGTENPQFPDDRAETALL
PESAGVFADGSFIFAQRYAHDLEKWKKLKVDAQENVMGRTKLESIELDDDVKPENAHV
ARTVVEDEEGEEMEILRHSLPYGDGRGDQGLFFIAYTKDLSIIDAMLKRMFGTSGDGI
HDRLLHFVTPLDGAYYFAPSEELLEEILEA"
/product="Predicted dye-decolorizing peroxidase (DyP), YfeX-like subgroup"
/transl_table=11 CDS complement(1785265..1786962)
/locus_tag="AUO97b_01820" /db_xref="SEED:fig|400667.82.peg.1682"
/db_xref="GO:0016619"
/translation="MISGHPSKHPLYIPYAGYTLLELPLLNKGSAFTQEERSNFNLHG
LLPHIIETIEEQSQRSYQQYCAFNDDINKHIYLRNIQDTNETLFYHLIENHLEEMMPI
IYTPTVGEACQRFSDIYRRHRGVFISYPDRDVIDDILQNVNKNNVKVIVITDGERILG
LGDQGIGGMGIPIGKLSLYTACGGISPAYTLPITIDVGTNNPQLLNDPIYMGWRQPRI
SGDEYYDFVDMVIEAVKRRWPKALIQFEDFAQKNAMPLLEKYRDKICCFNDDIQGTAA
VSVGSLIAASRAAGKQLKDQTIAFLGAGSAGCGIAEQIVAQMVSEGLTDAEARARVYM
VDRFGLITENQPNLLDFQRKLAQKAEVVDQWGNIEEVISLLDVVKNAKPTVLIGVSGQ
PGLFTEEIIKTMAANCERPIVMPLSNPTSRVEAVPADIVEWTEGKALIATGSPFAPVN
HHGKLYNISQCNNSYIFPGIGLGVVASGAKRVTENMLMASSSALADCSPLLKDPQADL
LPPLGEIQQVSKVIAFEVAKAAMADGVAVTISDDLLNQKIDQSFWKPEYRKYKRIPF" /product="NAD-
dependent malic enzyme (EC 1.1.1.38)" /EC_number="1.1.1.38"
/transl_table=11
CDS 1787458..1789248
/locus_tag="AUO97b_01821" /db_xref="SEED:fig|400667.82.peg.1683"
/db_xref="GO:0004814"
/translation="MNTAIQAALDHAVQTLQQEGVLPSDWNNSSNLTRTKDRSHGDFA
SNIAMIGSKAAGMKPRDLAEKILAVLPEVADINKAEIAGPGFINFFLNADQRFAILDQ
IQAQKESFGRSQSNAAKKIQVEFVSANPTSSLHVGHGRGAAYGMTVANLLEATGAKVD
REYYVNDAGRQMDILATSTYLRYLELLGQNLVFPKNAYQGDYVKEIAQGIIDKDGDAY
VREVANVYKDVPEDVQYAEELDSEGNKVVLSGDKEKHIDGLIANSQQLLGEGYRVFHQ

AALHAILDDIKDDLADFGVTFNQWFSEASLSAKIDEALETLDQRGFLYEKDGNIWFKS
TEFGDEKDRVVKRRNGQTTYFASDIAYHLNKLQRGYTDLVDIWGSDHHGYISRVKAAI
DAMGYDSKKLTVLLVQFVSLWRGGEMVQMSSRSGQFVTLRDLRKEVGNDAARFYYVMR
KSEQHIDFDLDLAVSQSKDNAVYYIQYAHARICRMLEKAASTGLQFEVSAARSHAARL
SLDAETEILAKLAAYPDVVLRAANAYEPHQVGNYLKELAALFHGWYNEHKVLSDDAEL
TQARLLLSINVQQVLRNGLELLGVSAPEAM"
/product="Arginyl-tRNA synthetase (EC 6.1.1.19)" /EC_number="6.1.1.19"
/transl_table=11
CDS 1789402..1790028
/locus_tag="AUO97b_01822" /db_xref="SEED:fig|400667.82.peg.1684"
/translation="MFGKTQRGVSERPNKPKKPLIPKWLGTLVAILAVLCIAVMLMLW
KPWQPVSSKNQITSEHYKEEETNKDYRFYDLLPQQQVTPIPEQAIPESKNQGTAMIVE
APSTAQPAASEPASVSTDDTTDSTAAIPQQPNYILQVRSYTDPDQADARRAEIILNGL
SADVIKSSENGKTWYRVISGPYDTQEAALAAQQTLQHSGIDSIVIKRK" /product="Cell division protein"
/transl_table=11
CDS complement(1790072..1790671)
/locus_tag="AUO97b_01823" /db_xref="SEED:fig|400667.82.peg.1685"
/db_xref="GO:0004364"
/translation="MDYDIHFYQRLPNYSAPETLKCIHPLGKAPILTDDEQVIAESAV
ILEYLQQRYDQKQQFKPKQAQDLQQYIYWMHYAEGSLMPLLVMTLVMNNVNKHVPWLI
QPVAKKITEGVKANFVRPRMKDHISFLENYLAEHEYFAGDFSFADIQMSFPLEALQSR
LQGKYPHIQAFLHRIQQRPAFQKARQKGLGSNERNCADI" /product="Glutathione S-transferase (EC 2.5.1.18)" /EC_number="2.5.1.18"
/transl_table=11
CDS complement(1790853..1791260)
/locus_tag="AUO97b_01824" /db_xref="SEED:fig|400667.82.peg.1686"
/translation="MTAVIASSKAKPLAEQWKGEITSGRHQYFCDEPEKLEGHDQGPA
PYDLLTGSLAACTLITLRMYAKHKGYDFGEYSVEIDFHTNREHEEHIERRIVFKELPN
EELQQKILTICSKTPVTKTLLRSLDIHTVLVTA"
/product="hypothetical protein"
/transl_table=11 CDS complement(1791257..1792204)
/locus_tag="AUO97b_01825" /db_xref="SEED:fig|400667.82.peg.1687"
/translation="MSNNNDYDISDSKDCEKFANQFIQEFPIRSAEIGQGTVIKRALP
SRQKRMIGAWCFLDHAGPVTFPAGNGLDVGPHPHIGLQTFTWMIEGTMMHTDSLGSKQ
LIRPKQVNLMTAGHGISHTEVAPDTETQMHAAQLWIALPDHKRNMDPKFEHYPDLPVV
EKDGLEFTVLVGEYLETTSPVVVHTPLVGVDLIATQDTKTRIPLNPEFEYGFMALDGV
AHVNGHELTADNMVVLDTGLNEIEIEVKKGNRVLLIGGEPFETPILLWWNFVARTMDD
LKEAREQWVNHDVRFGEIPDYVGARLEAPVLPDQMRASK"
/product="Pirin"
/transl_table=11
CDS complement(1792361..1792666)
/locus_tag="AUO97b_01826" /db_xref="SEED:fig|400667.82.peg.1688"
/translation="MKQHQYHVTVQHLKDAKGEVSTYTERLEFYTGNHDDIFEIVEKL
KNAEFFDDQTTKSFAVGLKLFSEVMLENRDHPLFQEFLPQFGQFMKNLKQQVKTQSP"
/product="hypothetical protein"
/transl_table=11
CDS complement(1792727..1793254)
/locus_tag="AUO97b_01827" /db_xref="SEED:fig|400667.82.peg.1689"
/translation="MRQQSEHHHAEHNDHHHGHHRSGRRGRLFEAGRMKLLVLHLIQQ
SPKHGYEIIKEISDLVGDGYTPSAGTIYPTLTSLEEMNLINLLDVERKQYQITEFGEA
YLNEHQDKLSGLLEKLRLRREIHSNDQLIEIHRAMENLKTALRLKLNTTELKQEQIYQ IAEKIDQAAVAIGRL"
/product="Transcriptional regulator, PadR family" /transl_table=11
CDS complement(1793473..1794261)

/locus_tag="AUO97b_01828" /db_xref="SEED:fig|400667.82.peg.1690"
/translation="MSDFIQLEYLQEKLQQLLAKSLFAIYLYGSAVDGGLGPESDLDV
MVVVTQPLTSALREQLAQELLKISQPVGELQRPLEVTILLKDEIQSGNYPLSYEMQFG
EWLREELKEGGTLNSQKDPDISILLRKARFHHTVLFGPALDQWAPEISDQELWQAMSD
TYPEIVAHWDEDADERNQILALCRIYFSLVMKDIASKDNAARWVMSQLPPEQKFVLQR
LIQEYRGEISKQNWQEEHYALQPIVNFLSSKIEEQFEQKRNLIT" /product="Aminoglycoside 3''-nucleotidyltransferase, putative" /EC_number="2.7.7.47"
/transl_table=11
CDS complement(1794327..1795895)
/locus_tag="AUO97b_01829" /db_xref="SEED:fig|400667.82.peg.1691"
/translation="MTTNTQITEDRILILDFGSQYSQLIARRVREAGVYSEMYAFDMS
EEDIRAFKPNGIILSGGPESVHEEGSPRAPQVVFELGVPVLGICYGLQTMSEQLGGKV
EPGTVHEFGYAEVDIVKRDQLIGNLQDRENQLHVWMSHGDKVSQIPEGFTITASTPSC
PVAAVSDETRRFYGVQFHPEVTHTAKGEELLSNFVHKICGCGGLWTPEHIIDLRVEQL
REQIGNEKVLLGLSGGVDSSVVAALLHKAIGDQLTCVFVDNGLLRLNEGDQVMQMFAE
NMGIRVIRADAEARFLNALAGVTDPEAKRKIIGREFIEVFAEEARKLDGVKFLAQGTI
YPDVIESAASKQGKAHVIKSHHNVGGLPDDLAFELVEPLRDLFKDEVRKLGTTLGLPH
SMIYRHPFPGPGLGVRILGEVKKEYADILRLADDIFMQELRDSGWYDKTAQAFAVFQP
VKSVGVVGDGRRYAWVIALRAVETVDFMTARFAHLPYELVDKISTRIMNEIKDVSRVV YDVSSKPPATIEWE"
/product="GMP synthase [glutamine-hydrolyzing], amidotransferase subunit (EC 6.3.5.2) / GMP synthase [glutamine-hydrolyzing], ATP pyrophosphatase subunit (EC 6.3.5.2)" /EC_number="6.3.5.2"
/transl_table=11
CDS 1796271..1797500
/locus_tag="AUO97b_01830" /db_xref="SEED:fig|400667.82.peg.1692"
/translation="MLQTMIADFQQHFWLYVSIPFMSGLIGYVTKVIAIQMMFSPLEF
KGIKPFFGWQGIVPRKAEKMATTAVELMTAKLIKPEEIFARLDPKRIAKEIEKPLMAA
AEDITREVAQEYQPGLWEGMPEFARQRLIRRVQSKAPEIVEHIMSEVQRDVNRYFDIK
HLVISNLLKDKRLLNNIFKRVGKQEFKFFSNVGFVFGFGIGVIQMLCWIATQGKYPWM
LPLFGGFVGFFSDWIALQMMFRPLYPKKILGYTWQGLFIKRQNEVAADYAALISKQLL
TSRHMMEELFSGTHSARVIELVNRHVKQEIDMQAGVIRPLVVYAIGGEKYQNMKTQVA
ERIMQQLPETMKYVESYAEDAMNIRNTLIERMQKLTPSEFEGMLRPAFKEDEWALIAV GAVLGFVVGELQIQFML"
/product="hypothetical protein"
/transl_table=11
CDS complement(1797559..1797996)
/locus_tag="AUO97b_01831" /db_xref="SEED:fig|400667.82.peg.1693"
/translation="MFNPNVVVKNIVTQPGADSFISLVARLLIAYIFLAAGWGKITGY
AATAGYMEAMGVPGGLLPLVILVEFGGGLALLFGFQARFAAFGLGIFSILTAFLFHQG
GADAAAQYNNGIHFMKNLAMAGGLFFLMLHGAGRISLDHAIEK" /product="Uncharacterized membrane protein, DoxX/SURF4 family" /transl_table=11
CDS 1798190..1798831 /locus_tag="AUO97b_01832" /db_xref="SEED:fig|400667.82.peg.1694"
/translation="MELLDFILHVDQHLAEFITNYGTWIYGILFLIIFVETGLVVMPF
LPGDSLLFAAGALAASTGTMDPWLLGILLFIAAVLGDTVNYHIGRFIGPRVFEMNSRF
INKQHLIKTHEFFARHGGKTIIFARFIPFARTFAPFVAGAGSMNYKFFLTYNIIGAFC
WVASFVTLGYLFGGHPLVKDNFTHLIFGIIIISILPGVIGFIRQKLKNRKANV" /product="DedA protein"
/transl_table=11
CDS complement(1798839..1799429)
/locus_tag="AUO97b_01833" /db_xref="SEED:fig|400667.82.peg.1695"
/translation="MIESWFFVLAMLAVLLIPGPTNALLATAAHSQGLSKTFWLIPME
WLGYAYGISFWAVFIHLADPVWPALLLILHITSVLYVFWMAFRLWKTTHLQQFSQNHR
NIRPRQLFFSTLKNPKSLLFAAGIFPAETWNSPENFLMVLAAFTLILIPAASFWMFFG
RALLTGSMKKIKADHLYKGSAMLLILCMLPVVFRFF" /product="Transporter, LysE family"

/transl_table=11
CDS complement(1799645..1800031)
/locus_tag="AUO97b_01834" /db_xref="SEED:fig|400667.82.peg.1696"
/translation="MKGLYIVPLAAATLLLGACATTVKPTYVSPTQYQSLSCQQLQSE
YNRIQQYIDNGVQTPKSTGMGVGLGLGGGWGRGGWGFGPSISVNMGQSSSTKNTELSR
VLGQQEAIVQAAQFKNCPIIVRKKTN"
/product="hypothetical protein"
/transl_table=11
CDS complement(1800042..1800884)
/locus_tag="AUO97b_01835" /db_xref="SEED:fig|400667.82.peg.1697"
/translation="MLTRVVFNQKGGVGKSSITVNLAAISAKHGLRTLVIDLDPQANS
SQYLLGEEATYAADKAVLEPNIENFFEDVLGNNQQKGLIGNALGSILKAPRNKDLDSF
VHSTSFAKLDVLPASPTLGALEHALESKHKIYKLRDSIQSLIGRYDRIYIDTPPAFNF
FTLSALIAADKVLIPFDCDVFSKRALQTLIENVLETQDDHNDRLEIEGIVVNQFQSQA
KLPREVVQQLKDEGLPVLNSMLPPSILMKESHQKNLPLAHLAPEHKLTQSYEALFSEI EPKR"
/product="ParA-like protein"
/transl_table=11
CDS complement(1800961..1801353)
/locus_tag="AUO97b_01836" /db_xref="SEED:fig|400667.82.peg.1698"
/translation="MDAHLLTKLIHMTAVAAALMVFVLRASTLFIGVQGEQPNPAGRK
VLVALQHLSFTVVFITGAILLVMKNFQVQPWFYAKIILFLVLLSSLMKAFKKDDTILL
AQRRAGLVISAIAFVAIIILVIVKPVFA"
/product="hypothetical protein"
/transl_table=11
CDS complement(1801365..1801673)
/locus_tag="AUO97b_01837" /db_xref="SEED:fig|400667.82.peg.1699"
/db_xref="GO:0030528"
/translation="MSLEQQLIERLQTLAPSHLEVINESAGHGGYFPGKESHFKVIVV
SEEFNGLRLVQRHQKVYALASDLMNPGQIHALAIHAYLPSEWQGQAPASPECAHAPKS "
/product="Cell division protein BolA"
/transl_table=11
CDS complement(1802196..1802960)
/locus_tag="AUO97b_01838" /db_xref="SEED:fig|400667.82.peg.1700"
/db_xref="GO:0000287"
/db_xref="GO:0006633"
/db_xref="GO:0008897"
/translation="MSDIAQVQVYVCPVSRVPDNHLILSHYLSFLNAAEKLRYDQYHS
HAARLFLISRVLMKSVLSDKLGILPHEVIIQLQPNGKPFVRGNKAIYFNLSHSADLIV
FAVTEKREIGVDVERMNHEFEWRRVDSVLAPSEIEWIQQNEWTNPTSVYQRFFQIWTL
KESYIKCTGEGMSRHLKKLNFHVSPEHIQFCDLTNNDVQRTEQYHFKSYIYNYSFIFS
LCLQQSHCLESFNIDCFQLLPYISTHRITLEPCEHN" /product="4'-phosphopantetheinyl
transferase (EC 2.7.8.-)" /transl_table=11
CDS complement(1802953..1804818)
/locus_tag="AUO97b_01839" /db_xref="SEED:fig|400667.82.peg.1701"
/translation="MDNLNNAKKDNFSRKTILVTGAAGFIGSRLIVELLREGHQVIAA
LRNAATKKDKLLGFIATQGLADPSISFVEYDLSRDFKLDSLLSDAQTKIHVIYHLAAS
FNWGISKAEAERTNIKSGLALIEWAATLKQLERFIWIGGYRVAAPPQESADELYCKHG
GYEASKILGHQAFIEACKRLNVPWTAINPATVIDGFYNYGDMQCIGIADMIDKLYQGR
LLALPGGRDTFLPLCNMQYIVSFLIRTMSYPETIAQEYMLLDPATPKFHRLVYLAAEH
LGVSSPHLQIPKVLLEKLPEVLLDGSKEQLSFISTEHYHVAGAEAMAAKMGITDLISI
MPYFSRWIDRLVFTRFGRMQVPTPNSFSYQNRYWTEIYTKQPVGSEKASALFLHGIPF
DSACWSPIINKITYDQVAMMDLPGLGRSGSYEMMEDNNHQFIDTAAELLAPNSVVIAH
SLGCLFALNLAKKYPDKVARLILISPYFVQAQAAKMFQIQAISKLIFRFVPKDVIAKD
LHPEGQQNLSVRYAMDSLRRMSVSKHISEYMHHINLPQQRAAQTALLSELRSKIEIIV

GEKDPIVTPISPEIPVHIIAGAGHNPHVTHVEAVYDYLTPVLTKYISTTAQLSDV"
/product="hypothetical protein" /transl_table=11
CDS complement(1804872..1806101)
/locus_tag="AUO97b_01840" /db_xref="SEED:fig|400667.82.peg.1702"
/translation="MLLQHTYADDSEMSFVSQNSARLDVWSSDRDLSSLKNIGQASFW
SNGTLKFNPAFKLHYDIVVSDETDHTVNPDQVSKVNRNLRELYASYNWDQTFYLDLGR
QIVVWGRADGINPTDNLSPRDYTRLVPDETDQRLGNDALKLTYIPESGTNKWTALWYP
RSRSDVIPLRQIAGVQYDIDRKSRPAFAMRWDYSVDGLDVGASYFSGLDHMPDLSVLS
ASPTGEATLQLKNNPMSVYGFDFSYNHNDLVWRGEAAYTKTDSNGSEDLIHKKNNLTV
VMGPEWSLKNSTLSLVGVYKHTEDFKSADNLTNLLVREVYRYQQTISDQYEQDQYGAM
LRYALNLLNDNLKLEFTGFYFAPEPNELLRVRINYNLNDHWQLNAGGDRFWGRNDTVL GQFRDNSLVYAQVRYNF"
/product="hypothetical protein"
/transl_table=11
CDS complement(1806143..1809793)
/locus_tag="AUO97b_01841" /db_xref="SEED:fig|400667.82.peg.1703"
/translation="MVTIEHAFLIPAKIDKVFNYLANPANDAGWQLSCKHSELLDSTP
RVGSKYEIGFSFIGREMSFKGEITHLVPNELYAFKVVEGPFHYTGTYRFKPHPEGTWI
EWVFEAEPGSFFGVLPPALLKKMVLAQFKKDVDNLQALAQKGEAYESVGNENKPTLET
NKPPHKTQQMMERYARWILSHRRIVLTVVMLLTLALAYLASGVKIIIDPDALAPKGHP
YITSTKLIEKKFGSKYMVVIGITPKKGDIYQPQVLEKVKRITEEVDNAPGVVRSTMMS
LAARQAKGIEANAEGFDAKKLLPSSSVTQEDIDHLKKLLALNPTYMNSVVSKDQRTAA
ILLELEESPEGFQKMMGPINKIVESEQSKDMTISVGGNPVYLDKAEDYSKRINILFPI
AVLVIGLLHFEAFRSKQGLILPLVTALLAVAWGMGMMGLFKQPMDIFNSPTPILILAI
AAGHAVQLLKRYYEDFDRLIAQGMEPKAANSEAVVQSLVRVGPVMVLAGGIAAAGFFS
LLTFNIPTIRSFGIFTGIGIISTLVIEMTFIPALRSMLPPPSVVKVKRKGLPIWDWIP
NRIGDVILSVRPRMMLMTAIAAMGIFLAIGTSRIVVDNDSRNFFSRDLPMQQDDRFLN
QSLGGTNSLYIMVDTKVRDGIENPEILKAIDNTEKFANSIPEVGKTISIVDYIKRMNQ
AMNADQPQAFQVPATKDIVAQYLLLYSMSGEPTDFESYIDTTQRYAKITILLKTGSNH
RIKEILESLKTYMAGQLGDKAVVSFGGDVTQTIALTETMVHGKLMNILQISFAVFFIS
ALVFRSISAGLIVLTPLLFSILAIFGVMGWLDIPLNIPNSLISAMAVGIGADYAIYFL
YRLREILREEGGDIKDAIRKTLSTAGKASLFVATAVAGGYGVLSLSQGFHVHQWLAMF
IVIAMLFSVFATLIMVPTMILILKPRFIFSSKKKSIPVAQTVVTSLLLGTALTMSMPK
TSHADEVQDIVNRSDDASKFLSSTASAKFILTSKNGEQRVRLTKNMTKLAGNTQNNMR
LTEFISPADVQGTTTLLIENAKGSDSMFVYLPALKKVRRLASANKGDAFIGTDFSYGD
VLGYKLSDWKYTKLADGKFNGKDCYMIEATPINNTVKSDFGYSKRRMCILKDNFVTAT
IDIWDTAGKPLKHIEFTDIRPYGKVKPRWQAMKSMAKNLQTQHMTQVIVNDFAAEKTL SDKLFSPQSLEK"
/product="Exporter protein, RND family"
/transl_table=11
CDS complement(1809818..1813777)
/locus_tag="AUO97b_01842" /db_xref="SEED:fig|400667.82.peg.1704"
/translation="MNNLARLEPEVLSRHAISAEQLGIWYIQRFEPTCSAYNMVVAFD
VKVNQSLGNKPIEILEAVMHDYPLLRVSMPANDQGIEQLIWDRVYPNIIFSDARHIEA
SDLTQLVEQDTKQPFDLTQPPLWRIHCYERGQNHYVIAFVIHHALMDFWSIGLLLRDV
SKRFGLVAESDAVNGIEFAQYADKQQSNVIDDTDESLLFWKNALKHAPHVHSIPLDYP
RPAVQQHKGSSLAFRVPESVSSGLINLAKDYEITLFGLVLSGFYILLHKLSNENNLVI
ATPVAGRLERSLRNALGQFVNTIAIHIDIDADQTLRQFTQQVQEQLRQSLKHQKVAFS
RVVEAVAPKRDGSINPLAQIGMFWERLGGMDEFKELLLPIQTPATLVGQDLTLGSFPV
RQQEGQLDITLEMGGEYQGELVGVLKYNTDLFSAQSAENMVQLLQAVLAEMVAHPERK
IVELDIAPDYKDGIQFEALRGKVTDYAQHDLLGMILKQIVERGENHALTSNDHTVSYR
ELGQHIAGIAEYLRAHGITQGDRVGLMLDRTALLPAAILGIWAAGAAYVPLDPNFPTE
RLQNIIEDAEPKVILTQTELMDGLNVSVPRLDINQAGVVALEQVRETLAFGDIAYVMY
TSGSTGKPKGVRIGHPSIINFLLSMNDRLQVTTETQLLAITTYAFDISILELLIPLMY
GGVVHVCPREVSQDGNQLVDYLNAKSININILQATPASWKMLLDSEWSGNAGLTALCGGE
ALDTILAEKLLGKVGCLWNVYGPTETTVWSSAARITDAKCIDLGEPLANTQLYVLDEQ

QRLVPPGVMGELWIGGDGLAVDYWHRPELTDAQFRTLPSLPNAGRLYRTGDKVCLRTD
GRLTHHGRLDFQVKIRGFRIELGEIENVLKQIDGITDAVVLVKTTAGNDQKLVAYVTG
QELDIAGLKKTMQIHLPAYMVPSAFIRLDEFPMTANNKLDRKAFPEPIFEQSNDYVAP
RDPIEIELCTTFEQILSVKRVGIHDDFFELGGHSLLAVKLVNHLKKAFGTELSVALLA
QYSTVERLGEIIRENKEIKPSIVIELRRGTSEQPLWLFHPIGGSTFCYMELSRHLNPN
RTLRAIQSPGLIEADAAEVAIEEMATLYIAEMRKMQPQGPYFLGGWCFGGAIAYEISR
QLRQMGQQVTGIVMIDTRAPIPENVPEDADDAMLLSWFARDLAAPYGKKLTIPAQYLR
ELSPDQMFDHVLKEAKAINVLPLDADPSDFRLYFDTYLANGIALQTYFPEPEDFPILL
VKAKDEQEDFGESLGWDQLVKDTLTQVDLPGDHSSIMYAENVVAVAQTIDQMYPIPA"
/product="Polyketide synthase modules and related proteins" /transl_table=11
CDS complement(1813774..1814034)
/locus_tag="AUO97b_01843" /db_xref="SEED:fig|400667.82.peg.1705"
/db_xref="GO:0048037"
/translation="MNKDKAYWSAIIRTLVAKEMRVEPETIDPDQKFTSYGLDSIVAL
SVSGDLEDLTKLELEPTLLWDYPTINALAEYLVSELQQGVAS"
/product="Acyl carrier protein"
/transl_table=11
CDS complement(1814031..1815824)
/locus_tag="AUO97b_01844" /db_xref="SEED:fig|400667.82.peg.1706"
/db_xref="GO:0004085"
/translation="MSLTSNLRQFQSAARLEQILGSVHDTNNDFSWSAAIERDEKEQY
PQRAVDFLNAVGMNRFYVPSELGGRMEIGEELLYLHRVLARRDLTINSTYSTNAWSVI
VWIGGNANQCQNIANRILSGAAPALAYSEKAHGADLLSSEVTAQPSDSKYVLNGEKWS
INRATLGDSLSLIAKTSNDRGPRSLSAFWLDKRHMASKGTYSLNRLPTVGMRGLDISG
IGFNNAEIHKDALIGEEGQGLELGLKTLQVTRAYCSSFSLGAGDTMLRIATEFAIERE
LYNKKVISIPLVREQLATAYAYLLAAEVLSLVGARGLHVCINQFSTWSPIVKVLVPEY
VESLAKITSSVLGSRFFLRNAYADGMFQKAFRDHLIVSVFDGSSTVCLDSLSFQLKSA
NKGRSKKADHLNQAEAKARYRQLYDLQVETGAIDFRELEIFNRDGDLVMESLETIIEM
LNDSNVTVGLSAETLATLHERANQLLVEQRSLDQKIQDYFSNSEQSKEFETMRFSLAR
DYAELFARIAVLGFWVFNRHGLRPALQNGAWLIIFLNAAEGQTAPPMTSLRESTLADL
LDRISTNHMLSVIDFALAPRDAKPVKKEITP"
/product="Acyl-CoA dehydrogenase"
/EC_number="1.3.99.2"
/transl_table=11
CDS complement(1815857..1817746)
/locus_tag="AUO97b_01845" /db_xref="SEED:fig|400667.82.peg.1707"
/translation="MAVMCTDNIIATLESRAKLNGNKIAFEYLGRQSGGIQSLTYADL
NERVQHQAQILMSIVDSGDRAVLLFEPGLDFIVSFFACLKAKIIAVPVSLPFNRNGFS
NILNIMNDCEPKIVLTTKKILELAGLITLKAQNENLILHVVDAELQRDLVEKDFPLIT
EKDICFLQYTSGSTGWPKGVIVTHKNIMANEVMIAEAFGTQPEDVGLTWLPVYHDMGL
IGSVLQTVYVGLTCYVMSPLDFIRKPLKWLQFISEKGVTISGGPNFAYELCLHRITDE
QAAALDLSRVRVLFNGAEPIKAHVMQRFMEKFNTQSRLRLDTFLPCYGLAEVTLLVSG
VTGPLNAISLDRDKLNAHLVQTSQDQTAVQVVSCGKISKHIDCRIVNPHTRKEVAAHE
VGEIWLAGESVTAGYWQKPEVNQETFEATILNEAGQPSQVHYLRTGDMGFIKNGDLYI
TGRLKDVIIIRGKNYYPQDIECSVEMAHPAVRKGCVAAVNFADSEGVTVVLEIKKKSL
DKTLNFETIRTQVKEQVAADIGLPVDGVYLLHQGRINKTTSGKIRRRKIKEQIERGQL
VCLTHPNRRTVIARNYVKNGLDVIRDREQRGQLIRYSSNLLNSMIKEREVRTLIFNRL KSALF"
/product="Polyketide synthase modules and related proteins" /transl_table=11
CDS complement(1818363..1819079)
/locus_tag="AUO97b_01846" /db_xref="SEED:fig|400667.82.peg.1708"
/translation="MESWQEDLLSAFLVVKNEYQLFEIVKSTASRLGFDYCAYGMQSP
LSIAEPKTIMLNNYPEAWQKRYVERQYVKIDPTVQHCMVSLQPLVWSSQSAKTQAEKD
FWEEARSYGLNVGWAQSSRDFIGTRGMLTLARSNDQLSEKEQKAQYTNMYWLTQTVHS
SIAKIVNDVEFAKFNLYLTNREKEALRWTAEGKTSAEIAQILGVTERTVNFHLSNSMQ

KLNVNNKISAAIRAVMLGLL"
/product="Acyl-homoserine lactone-binding transcriptional activator, LuxR family" /transl_table=11
CDS 1819890..1820255
/locus_tag="AUO97b_01847" /db_xref="SEED:fig|400667.82.peg.1709"
/translation="MNENQLMSYPLQHIISIVESRHTEASQIFYYGFTEWATSQTPAL
STGWDWELIENNGITTVKRVGLPRSNIMIVDVSGMDIGFDINETLLEKKIDTLFWEPF
IYAQINTSLTKSSLSQTFS"
/product="FIG00351083: hypothetical protein" /transl_table=11
CDS 1820334..1820885 /locus_tag="AUO97b_01848" /db_xref="SEED:fig|
400667.82.peg.1710"
/translation="MNIIAGFQNNFSEGLYTKFKSYRYRVFVEYLGWELNCPNNEELD
QFDKVDTAYVVAQDRESNIIGCARLLPTTQPYLLGEIFPQLLNGMPIPCSPEIWELSR
FSAVDFSNPPSSSSQAVSSPVSIAILQEAINFAREQGAKQLITTSPLGVERLLRAAGF
RAHRAGPPMTIDGYSMFACLIDI"
/product="N-acyl-L-homoserine lactone synthase, LuxI family (EC 2.3.1.184)"
/transl_table=11
CDS complement(1820978..1822261)
/locus_tag="AUO97b_01849" /db_xref="SEED:fig|400667.82.peg.1711"
/translation="MVGTTIEWYDFFVYGAAAALIFNKLFFPNLDPLTGVLAAFATYA
VGFIGRPLGGLVFGHFGDKIGRKSMLLLTLMLMGIPTVLIGLLPTYESIGYWAAIGLV
ILRFIQGMAMGGEWGGAVLMAVEHAPEGGKGFWGSLPQASTGGGLMLASIALGLVSLL
PEQALFSWGWRLPFLASIILLAVGWYIRVKVPESPDFEKVKQQSEEVKVPAFQVFKNH
PKQLITIILARAAENAWFYIASTFTLAYTTTQLGIPRQDILFATICGAAVILFMTPLC
GHLSDKVGQRNMFMFGLCVLALYSYPFFSMLNTKDPVLVWTAIVLAIGVVFPIMYAPQ
AQLFARQFPAEIRYSGISISVQLAGVLGGGLAPMIATKLLSIGQGNPYLIMFYIGSMA
VVAIISTSFMPRDWSYKKDETYYKKANLNLKTKID" /product="Uncharacterized MFS-type transporter" /transl_table=11
CDS complement(1822450..1823475)
/locus_tag="AUO97b_01850" /db_xref="SEED:fig|400667.82.peg.1712"
/db_xref="GO:0003860"
/translation="MNTENHLMIEQQGKLGIITLDRVTHLNALSLDMIKSIGAQLELW
RNDAAVQAVLIKSNSPKAFCAGGDIRYLYDSYKNGTADYKGYFSAEYKMLDILREYAK
PVIVMLDGYVLGGGFGLAQACHIVVSSEKSRFAMPETAIGFFPDVGATHFLSRLDDIG
VYMAITGEQISSSDALYLDLIDYHVPSEQLQALQDALVEAPSLTKEGIEHIITRFITH
PAESELKQLAEGIRKHFGYQHLDEIEQSLENEQDESLKTWASKMLSILQQRSFIAKQT
SLKLQHLGRGLSLQQCMQLERDLQDIWFEHGDFIEGVRALIVDKDKQPRWQERNPELE QILEKLS"
/product="3-hydroxyisobutyryl-CoA hydrolase (EC 3.1.2.4)"
/EC_number="3.1.2.4"
/transl_table=11
CDS complement(1823488..1824261)
/locus_tag="AUO97b_01851" /db_xref="SEED:fig|400667.82.peg.1713"
/db_xref="GO:0004300"
/translation="MQWQSILLEKRNGVGLITLNRPQALNALNSELISEINQALDQLE
KDRGIGCIVLAGSEKAFAAGADIKEMADLAFPDIYLDDFFHLADRIAQRRKPLIAAVS
GYALGGGCELALMCDFIYCADNAKFGLPEVTLGVIPGIGGTQRLTHAIGKAKAMEMCL
TARQMGAVEAEQSGLVARVFAKEELLEQTLQAAEKIAARSLTANIMLKETINRAFEVN
LTEGLRFERRMFHSIFATFDQKEGMQAFVEKRKANFKNQ" /product="Enoyl-CoA hydratase (EC 4.2.1.17) => degradation of branched-chain amino acids and alpha-keto acids"
/EC_number="4.2.1.17"
/transl_table=11
CDS complement(1824283..1825410)
/locus_tag="AUO97b_01852" /db_xref="SEED:fig|400667.82.peg.1714"
/db_xref="GO:0004085"

/translation="MQFTEEQLLIRDMAKSFAQEQIKPNASDWDRDGTFPKDTLAQMG
QLGFMGMLVSEQWGGSDTGNLAYVLALEEVAAADGATSTIMSVHNSVGCVPILKFGTD
EQKERFLKPLAQGEMIGAFALTEPHTGSDAAAIKTRAVKDGDYYILNGAKQFITSGNN
AGVIIVFAVTDPSAGKKGISAFLVPRETPGYEVIRVEEKLGLHASDTCQIALTDVRIH
KSLMLGKEGEGLKIALANLEGGRIGIAAQAVGLARAALEEATRYAKERITFGKPIFEH
QTIAFRLASMATEIEAARQLVHYAARLKEAGQPCLNEASMAKLFASEMTERVCSSALQ
VFGGYGYLRDFPIERIYRDARICQIYEGTSDIQRLVIARSL" /product="Acyl-CoA dehydrogenase,
short-chain specific (EC 1.3.8.1)" /EC_number="1.3.99.2"
/transl_table=11 CDS complement(1825422..1827068)
/locus_tag="AUO97b_01853" /db_xref="SEED:fig|400667.82.peg.1715"
/db_xref="GO:0003987"
/translation="MDYQNAVQAFDLESTAKQMLSGSLDALNACYECCDRHADGDKIA
LYWQGKDGRKEQYTFRELKEWSSQFANFLKAQGVKTGDRISGLLPRTPELIVTILAAW
RIGAVYQPLFTAFGPKAIEHRIQLAQSKLVVTDMGNRSKLEEIEKCPAIVTVADAQGT
PLKAGDFNFWNEVKQQSDQCDLVMRSIQDPFLLMFTSGTTGPAKPLEVPLKALIAFGR
YMQDAIGLTEEDSFWNIADPGWAYGLYYAITGPLFLGHATLFYEGGFSTDSLCQIVKD
YKVNNLAGAPTAYRMMMAADPAQMAPLKGQFRVVSSAGEPLNPEVIRWFKQVLDAPIY
DHYGQTEVGMVVCNHHGLKHEIHAGSAGFPSPGYRVAIVNEQGEELPPDTPGILAVDI
SQSPMMWFGGYKESRKSPFVGHYYLTGDTAELHADGSMSFVGRSDDVITTSGYRIGPF
DVESALLEHDAVIEAAVIGVPDPDRTEVVKAFVILATGVQPTEALAEELSQFVKRRLS
AHAYPRLVEFVSELPKTPSGKIQRFLLRNQEIAKQQAKAG" /product="Acyl-CoA synthetases
(AMP-forming)/AMP-acid ligases" /EC_number="6.2.1.1"
/transl_table=11
CDS complement(1827153..1828043)
/locus_tag="AUO97b_01854" /db_xref="SEED:fig|400667.82.peg.1716"
/db_xref="GO:0008442"
/translation="MNIAFIGLGNMGGRMAQNLLKAGLKVYGYDLSEIAIQHFAEAGG
VVCDSPQDAAKQADVVITMLPAAKHVKEVYLGENGVLEVLKAGSLCIDSSTIDPQTIK
DIAAVAHSKNIKICDAPVSGGTIGAQAGTLTFMVGADEQTFNEVKPVLSHMGKNIVHC
GDVGAGQIAKICNNLILGISMAAVAEGMALGVKLGIDPQALAGVINTSSGRCWSSDVC
NPWPHINENAPASRGYQDGFATQLMLKDLGLAVEAAGQVKQPVLLGGMVQQLYQQMCM
RGNAHLDFSSIIQQYLPQEA"
/product="3-hydroxyisobutyrate dehydrogenase (EC 1.1.1.31)"
/EC_number="1.1.1.31"
/transl_table=11
CDS complement(1828055..1829572) /locus_tag="AUO97b_01855"
/db_xref="SEED:fig|400667.82.peg.1717" /db_xref="GO:0004491"
/translation="MNTIQKIELDTAKLLINGQFIESKTQEWQDIVNPATQEVIGRVP
FATVEEVDAAIQAAQDAFASWRQTPIQARMRIMLKLQDLIRANMKEIAQVLTAEQGKT
LADAEGDIQRGLEVVEHACSVGTLQMGEYVEGVARGVDTYTLQQPLGVCAGITPFNFP
AMIPLWMFPMAIVCGNTFVLKPSEQDPLSTMMLVELAIQAGIPAGVLNVVHGGKEVVD
RLCTHKDIKAISFVGSTAVGTHVYNLAGQHGKRVQAMMGAKNHVVVMPDANKEQTLNA
LVGAAFGAAGQRCMALSVAVMVGDSKQWIQELVEKAKTLKVNAGHEPNTDIGPVISKR
AKARVIDLINSGVEQGAELLLDGRNVQVQGYESGNFVGATIFSGVNTDMRIYKEEIFG
PVLSIICVDTLDEAIALINANPFGNGVGLFTQSGAIARTFQNLIDIGQVGINIPIPVP
VPFFSFTGSRGSKLGDLGPYGKQAVQFYTQTKTITSRWFEDSHEVGGVNTTISLR"
/product="Methylmalonate-semialdehyde dehydrogenase (EC 1.2.1.27)"
/EC_number="1.2.1.27" /transl_table=11
CDS 1829738..1830619
/locus_tag="AUO97b_01856" /db_xref="SEED:fig|400667.82.peg.1718"
/translation="MKVDWDHLQFFLVLARTKTLTNAARIIGVEHSTVARRIQALELA
LGTTLFKREATGYELTLEGMALVPRVEQMEQAFLQIEKPHQPLQGRVRIGTPEGFGTA
FLARLLAEFSIQYPLLTIDLIPVPKMIKLSHREADIVVSIERPTSGPYIITRLSDYCL
KIYGSQNYLAQNPPIRRLEDLTQHRFVNYIDDLVYSPELYCLERLPLKLNANFRSSSI

LAQQIAVSAGAGLAILPKFLADDKPELEEVLEQQVRFTHTFWMLTFVDLQHEPRIKLV WDYLRKQADKYQHLLVD"
/product="Transcriptional regulator, LysR family" /transl_table=11
CDS complement(1830677..1832107)
/locus_tag="AUO97b_01857" /db_xref="SEED:fig|400667.82.peg.1719"
/db_xref="GO:0006810"
/db_xref="GO:0016020"
/translation="MTMVHHSDEASSPDHLQRKLSNRHLQLIAIGGAIGTGLFMGSGK
TISLAGPSILVIYMLIGGMFFFLMRALGELLLANLHYKSFVDMAYDLIGPWAGYYIGW
TYWLGWVLVGIADLSAVINYLSFWLPEGASFSPMQQAMISAGCVLFVLGLNLLTVKLF
GEVEFWFALIKILAIIGLIGVGGYMILTHFQAPHGDVVSISNVWSHGGLFPKGVSGFL
AGFQIAVFAFIGVELIGTTAAETKDPQTNLPKAINAIPVRIILFYVLALFVVMSVTPW
NHIRADKSPFVELFLNAGIPISAIIMNLVVLSSVMSSMNSGVFSTSRMLFGLSKDGQA
PSVLGRLSKRAVPSNGLIFSCIFIMGGAVLQYFVPNTMEAFTLASSLCVILFISVWIL
IMACYLRYRKIRPELHAASTFKMPGGVLMAYAVIAFFLFTLVILALEPDTLKALYVSP
VWLVVLSVTYYVFYKPRMRKLGQEIF" /product="D-serine/D-alanine/glycine transporter"
/transl_table=11
CDS complement(1832478..1833917)
/locus_tag="AUO97b_01858" /db_xref="SEED:fig|400667.82.peg.1720"
/db_xref="GO:0006810" /db_xref="GO:0016020"
/translation="MTTARHSDMENSPDHLQRKLSNRHLQLIAIGGAIGTGLFMGSGK
TISLAGPSILFIYMIIGGMFFFLMRAMGELLLANLHYKSFVDMAHDLIGPWAGYYLGW
TYWLGWVLVGIADLSAVINYLSFWLPDGASFSPIQQAMISAGCVLFVMGLNLLTVRLF
GEIEFWFALIKILAIIGLIGVGGYMIFSHFQAPQGAVASISNVWSHGGLFPKGTEGFL
AGFQIAVFAFVGVELVGTTAAETKDPQKNLPKAINAIPVRIILFYVLALFIVMSVTPW
DHIRADKSPFVELFLNAGIPVSAIIMNLVVLSSVMSSMNSGVFSTSRMLFGLSKDGQA
PGAFGRLSKRAVPSNGLIFSCTFIMGGAVLQYFVPNTMEAFTLASSLCVILFISVWSL
IMVCYLRYRKLRPELHEKSTFKMPGGIWMSYVVLAFMLFTLVILALEPDTLKALYVSP
VWLIIILGVTYHVLYKPRMKRLGRELVNDH" /product="D-serine/D-alanine/glycine
transporter" /transl_table=11
CDS complement(1834079..1834438)
/locus_tag="AUO97b_01859" /db_xref="SEED:fig|400667.82.peg.1721"
/translation="MSNSDIQKINTNEVMSAVTVFNKVVYLSGQVPKNTEQDVAGQTR
EILATIDELLALANTDKSRLLSAQLYLKNLSDFSTVNAIWVDWLKGCVAPSRATIQAD LVNPNWLIEIAVTAAQK"
/product="RidA/YER057c/UK114 superfamily, group 2, YoaB-like protein"
/transl_table=11
CDS complement(1834449..1835552)
/locus_tag="AUO97b_01860" /db_xref="SEED:fig|400667.82.peg.1722"
/db_xref="GO:0008784"
/translation="MPRPITAVIHRQALQNNLAVVRKAMPNSKVFAVVKANAYGHGIE
RVYEAFKAADGFALLDLDEAKRIRALGWTGPILLLEGIFSPQDLFDCVQYQLSFTIHS
EAQIEWVEQHPYPAQFDVFLKMNSGMNRLGFKPQHYVQAWERLNNLANVAKITHMMHF
SDADGDRFGQQGIDYQITAFEEIVKDLPGERSVSNSAAILRYQDQLKSDYVRSGIMLY
GSSPDYPTHSIADWGLQPTMSLRSEIISVQHLEPNESVGYGSNFVAEQPMTIGIVACG
YADGYQRISPTGTPVLVDSVRTRTVGRVSMDMLAVDLTGIESAKVGSEVVLWGQSSTG
VVLPIDDVAVSSGTVGYELMCAVTARVQFINQV"
/product="Alanine racemase (EC 5.1.1.1)" /EC_number="5.1.1.1"
/transl_table=11
CDS complement(1835573..1836838)
/locus_tag="AUO97b_01861" /db_xref="SEED:fig|400667.82.peg.1723"
/db_xref="GO:0008718"
/translation="MRVIVLGSGVIGVASAYYLARQGAEVTVLDRQSGPAEETSFGNA
GQISPGYSTPWAAPGIPFKAVKWMFQHHAPLAINLDGSMWQLQWMAQMLKNCNPQSYA
VNKERMMRVAEYSRDCLRELRKDTGIHYENRAKGTLQLFRKEAQMEAVQRDISVLEEC
GVSYELLNANELGRVEPALANAQDKLVGGLHLPNDETGDCYLFTNALAQIAKELGVNF

QFNQNVEKLIVEGDQIKGVQVNGKILTADRYVLAFGSYSRDFLKPLDLQLPVYPVKGY
SLTIPIVDPAFAPQSTVLDETYKIAITRFDQRIRVGGMAELSGFNLGLNEDRRATLQM
VTQDLFPGGDMEQASFWTGLRPMTPDSTPIIGATRFKNLFLNTGHGTLGWTMACGSGK
LISDIVLNHKTDISTDGLSIQRYSHAHAA" /product="D-amino acid dehydrogenase (EC 1.4.99.6)" /EC_number="1.4.99.1"
/transl_table=11
CDS 1836975..1837442
/locus_tag="AUO97b_01862" /db_xref="SEED:fig|400667.82.peg.1724"
/translation="MRPLDRIDRMILDILQREGRIAISELASRVNLSTTPCSERVKRL
ERDGIIMGYYARLNPAYVDRNLLVFLEIKLSAKSGDVFDQVARDLVEIPEVLECHLIS
GEFDYLVKARLKEMSAYRRLLGDLLKKLPASASSHSYVVMEEVKETLYLDVSK" /product="Leucine-responsive regulatory protein, regulator for leucine (or lrp) regulon and high-affinity branched-chain amino acid transport system" /transl_table=11
CDS complement(1837483..1838628)
/locus_tag="AUO97b_01863" /db_xref="SEED:fig|400667.82.peg.1725"
/translation="MSLEKNIERLQSIDALRGLVIIIMLLDHVRETFYLHKQVTDPMD
VTVTEPALFGSRLLAHICAPVFVLLTGISAFLFQSKKQDLQQTRAFLLKRGLFLIVLE
LTLVNFAWTATFPPEVIYLQVIWAIGISMVVLACCVSLPLPVLAGVALVIIFGHNLLD
SVHFSQGILQNIWFVLHERGWLEFAGIKLRTSYPVLPWIGVILLGYVLGQFFNSKYTA
KQRSRTLLSLGLASIGLFVLLRFINVYGDQPWQHFESLQLSLMSFFNLTKYPPSLLFI
LLNVGIGLLVLVAFERMQQYSFLKPLVVFGSVPMFFYLLHLYVLKLMYVFALSVWGAN
YGNYLSVNHVWMLWLITIVLSFALYPAVKWFSKFKHQNKHISILKYF" /product="putative membrane protein"
/transl_table=11
CDS 1838704..1840803
/locus_tag="AUO97b_01864" /db_xref="SEED:fig|400667.82.peg.1726"
/db_xref="GO:0004872"
/db_xref="GO:0005215"
/db_xref="GO:0006810"
/db_xref="GO:0016020"
/translation="MRHKLGLLSSLGLSFISTLTWGEETSTVLETIRVQAESTREDVS
QNSSATKFTHDVLDVPFNRAYLSKQIMEQQDVQRIDDALTLVSGVFHQNSFGGGFWDN
YSFRGFSTDPNLGAAMIRNGLSVNRGINAPKDVVNIESLEFLKGPMAALYGRGETGGL
LNLNSKKPQWESESELNLRANTQEQYRISLEHTAPINDELAYRLAVAHEDNQSFRDHV
SSERWFFSPQLTWKISDQTQLDFDSEFTEHKGTFDRGVSTVNHQFVMDPKTFTGEPDD
GDLKIKDYFYQLRLSHEFNPDWKLNSAVSYKDAKMVGFATEPRRMQADGRTLERQRRY
RDYTSEDVLAQTELLGKIDTSWARHEILLSTELGQLDYKQNQLRRNHDSNSPTTVNTI
DIYQPEYGKYLPNLTPFTDTKERQRYFAFNVQDQIFFNDQWSVLFGNRFDQVEQDFKN
HIKQTEDNQTLHQNSPRFGVNFKASEQWAFYTNYGRSFAMNSGMNRNGQTFAPEKGKS
YEVGTKYKINDQSVLSLALFKMKKQNVLTTDPIDKDFQTAAGELSSKGVEFDLNSQIN
DRWFVNANYSYTDAQIEKDQDLAKGARLSNVPKHQGSVSTNYEFLQDGARKAGVGANL
TYVGERSGHNLDNGFNLPSYTLVNLNGYYAPSDRLRYQLNINNLFDKTYYVSSYSDLW VQPGEPLNASISAQWKF"
/product="Ferrichrome-iron receptor"
/transl_table=11 CDS complement(1840848..1841228)
/locus_tag="AUO97b_01865" /db_xref="SEED:fig|400667.82.peg.1727"
/translation="MSLSQKEIAVRFLELAAAGEVDEAYGNYTAPNFKHHNPYYAGDK
TSLKEGMRESAVETPNKVLEVQHVIEDGALVAVHSKLEMQMNNKLTILAVVHICRFEN
GKIAEFWDIGQIQPDPLVNENGMF"
/product="putative membrane protein"
/transl_table=11
CDS complement(1841257..1841631)
/locus_tag="AUO97b_01866" /db_xref="SEED:fig|400667.82.peg.1728"
/translation="MNRDYEQFPDDDNGNVLWQMVEDGDDLTELHEIEFSIAFQDQQN
AEQCAMHLLYQEQKISLFQDDSVEPNEWIITIFVTMEPEYSDIVDLEQWFSTIAEQFN

GEYDGWGCMAYVFDEEEDDILQ"
/product="hypothetical protein"
/transl_table=11
CDS complement(1841742..1842407)
/locus_tag="AUO97b_01867" /db_xref="SEED:fig|400667.82.peg.1729"
/db_xref="GO:0004730"
/translation="MPLNDNFVYAPPQDPLSILFEDDDLIVVDKPAGLLSVMGRLPEH
HDSAYLRVLEKFPLAKVTHRLDMATSGLLMFAKHRDAEVAVSKMFQARTVKKHYIALV
QGQVKQEGSVEVPLITDWENRPRQIVHFELGKHAKTLFQPLVYDATTDQSRVLLEPVT
GRSHQLRVHMMHIGHPIMGDKLYHPEPKRFHLNRMALHAAYLAFQHPLKGTDVVIESQ VPF"
/product="LSU rRNA pseudouridine(746) synthase (EC 5.4.99.29) @ tRNA
pseudouridine(32) synthase (EC 5.4.99.28)" /EC_number="4.2.1.70"
/transl_table=11
CDS complement(1842518..1843219)
/locus_tag="AUO97b_01868" /db_xref="SEED:fig|400667.82.peg.1730"
/translation="MENKNQCRQCAVCGKSFPLKDLVSGEVIRNVISDEILKDHPDWS
YSSFICRADLADYRIKYVQSLLRSEKGELSNLENEVIGSMQRHELISRNTESDFDQNW
TFGEKLADKIASFGGSWAFLICFALFLTGWIVLNTVVIVVRPVDPYPFILLNLVLSCL
AAIQAPIIMMSQNRQEAKDRLRSQHDYQINLKAELEIRHLHEKMDHLLSHQWERLAHI QEIQLDLLAEMNKKR"
/product="Acid-resistant locus arl7 (Fragment)" /transl_table=11
CDS 1844020..1844721
/locus_tag="AUO97b_01869" /db_xref="SEED:fig|400667.82.peg.1731"
/translation="MNNNIIIFGYGTGISKAVAHKFGKEGYKIGLVARNAQKLEKAIL
ELKAQGIEAYAFACDLAVLEDIPNLIKRIKDQLGEIKNIHWNAFHDIEGNILETPPLE
LTKSFHIRVSSYIATVQACLGDLEKNHGSILSTNGIFAFDAVGIDLVAKEYSSLATTA
AAQYKTTNLLAHSLADSNVYVSQVIVNGFVDGTSGAQDKTYTVHPETIAGQFWYLHQH KQETVSFCGEAIQAA"
/product="Short-chain dehydrogenase/reductase SDR" /transl_table=11
CDS complement(1844810..1845052)
/locus_tag="AUO97b_01870" /db_xref="SEED:fig|400667.82.peg.1732"
/translation="MVYFNLQGVDFKFEEQDPDILKQPSEVSGIRSKDLPDLLKEIPL
IKKDIYLIIKHDERIKDSVRSILFKEYKIDTSEYEK" /product="hypothetical protein"
/transl_table=11
CDS complement(1845332..1845652)
/locus_tag="AUO97b_01871" /db_xref="SEED:fig|400667.82.peg.1733"
/translation="MAGCGHEYTIEPERLPVAYVGKAYNQQLNISGGRVIPYSFEVET
NFPSDMNISISPIDEHEADAYNNLKISGVPKYKGTFTIHIYAGFYATGDGKLDKTYEF IVNE"
/product="hypothetical protein"
/transl_table=11
CDS complement(1845708..1845914)
/locus_tag="AUO97b_01872" /db_xref="SEED:fig|400667.82.peg.1734"
/translation="MLDAYIDKIENNKIDVSVEFCSNYDNRSCEPINISSLKKINLEC
QAIFSAMIGNVSKSEKFPIVWEEK" /product="FIG00352080: hypothetical protein"
/transl_table=11
CDS complement(1846612..1847442)
/locus_tag="AUO97b_01873" /db_xref="SEED:fig|400667.82.peg.1735"
/translation="MDNVIDANKDIKKQATELLKKAHGKVSNVPPVIPVKLTTYTHSL
TIFIGGAADKYVFTPGDVPVGWGDFRPIGPTYFVGNYVNNYFATIKGGSKNAFVKYYG
YEEAYVSDTRELTVNTNKDKKLNAFNDVKNFLKTYPQTQVNIVGHSLGGWNAAGLAEI
LHKNNICKVNVLITIDPVGEILSKIGLGSRTSIYYSRPKPVFNLWISISCDPKSYEWN
NDLIADLGGQWSSYPSSNSSYYYVTRYSHADFRLMMQEKIVNNLSVQDILSKELSRIK "
/product="Hydrolase, alpha/beta fold family" /transl_table=11
CDS complement(1847444..1850182)
/locus_tag="AUO97b_01874" /db_xref="SEED:fig|400667.82.peg.1736"
/translation="MERLGLVSQNRAVHIQFSNQSLNQQVFLQRIEGEHTLNQGSVAE

LLCLSTNAHIALKQFIGCQVAVDQVTDTGQFFRTTGIITEASQGQSDGSLTIYNLTLK
DPTALWHKRRNSRVFMNKSVRDISEILFKEWQGKSPLFASSITLDTSGLTKDYDVRPF
VMQSNESDYDFLTRLWRSEGINWLVDESQLFIADPNASIQPQVLRLIDDNQNYQALER
RSIRYQRSSATEQFDTITQVKAERRLQPTSVHVQRWQADALQQEEGSGSVQGTQKHSE
HYDNASLNLEDAWHVSPAWMQDLNGEDQATASGNSQIEQLNQHINAYHHLSSKQFTVA
GNVRDAQVGYWFELNDHPELDQHDSADKEFLILSKHYYNQNNLPKELQQQLERLLPQG
KLKAAQLDSQNPEQRHFAELNVVRRNIKAVPEYNPLEHRPAAHPQRARVVGLEGESIH
VDQWGRIKVRFLFTRADDHSHDGGAGSNDNDTDSAWVDVLTPWAGAGYGARFLPRVGE
IVVIDFFDGNIDRPFVVGRIHEAERHPTQFDQKGQLPDTKKLSGIRSEEVDGKGFNQL
RFDDTTGQISAQLQSSHAASQLNLGNLSHPKDKAESDGRGEGFELRTDQWGAVRAGSG
LLVSTHKQDQAQGVHLDASEAKQQIEGGLNNAKALSEVAKNQQTDPLEMLENLKTFIE
QIEEKDQDKAAAFKQALMILTAPNSIALASNEDIHLSADGQLNQTAGDSINLSTQKNL
IAHAQNKISLFAAQQGARLYAGKGKVEIQAQGDGADLIARKAVQVISTEDKIEATAAK
EIVLTAGGSQVKITGSGIFMTTSGKFEVKAGQHLFMGGGSVNSSLPYLPEQGKQKYGV
WFDVMDKQGNKLKPGTEYIIFDEHDKEIERGKLDRTGLVKLETEEPNKQYKIHVVN" /product="VgrG
protein"
/transl_table=11
CDS 1850467..1851159
/locus_tag="AUO97b_01875" /db_xref="SEED:fig|400667.82.peg.1737"
/translation="MKIKKSAFYIFTMIFSLHSSAFAEPSVADVQYRAQQGQPIAQYH
LGIMLLTGEQGVVKNYEQAFKWLTAADQNGSVGAKYSLGMMYFTGTGVEKDMKRAFEY
FAKAADKGHAKAQYNLGVLYDRGEGTAQNYEQAFEWYSRAAEQGYPPAEYNLAHLYKK
GHGVAQSDEQALKWYTKAAEHNESDAQYNLAQMYLNGEGTPKNLQLAKKWFQQAADAG DSDAKEMLKSLD"
/product="hypothetical protein"
/transl_table=11 CDS complement(1851169..1852395)
/locus_tag="AUO97b_01876" /db_xref="SEED:fig|400667.82.peg.1738"
/db_xref="GO:0004315"
/translation="MERVVITGMGINSCIGNTLEKVTESLKAGRSGIRYNEVYAKLNF
RSHVSATAEMNFENIDPELKKHMGVCAMYAYNSALDALQHAKLDVTDIANNSKYGVIG
GTGGTSTASIIDMLHTLKEKGTSEVSPDLAPRYLTSTISSNLSRAFNLKGVSQSIASA
CATSADAIGYAYHLIAAGKQDLMLAGGGEEDHWSQTAIFDAMGALCSKYNDAPETASR
PYSVDNDGFVIAGGGGMVVLESLSHAQKRGAKILAEVVGYAANSDGVDMVAPSGEGAT
RCIKLALAEAKQNGSDTIDYINTHGTSTPAGDIPELLAIERAFGGEKVPPLSSTKSMT
GHCLGAAGVHEAIYSILMLQNNFIAPSINITELREEAKKFDIVQTSRETELKTVMSNS FGFGGVNTCLVFRKWE"
/product="3-oxoacyl-[acyl-carrier-protein] synthase, KASI (EC 2.3.1.41)"
/EC_number="2.3.1.41"
/transl_table=11
CDS complement(1853715..1854224)
/locus_tag="AUO97b_01877" /db_xref="SEED:fig|400667.82.peg.1739"
/translation="MTVILVKPHLRFKDSYNDYINELADEDRHPLTTEFDHTDFESFL
NKLRDYEKGESLEEGHVPNITYWLVEDNEVIGASNLRLKLNEQIEYCGGHIGLGIRPS
KRGQNFGSKLLELTIQEAWKLGLSELHIHCYKYNLASAKTIQANNGRLHSEITLNQVV QRYVVYKTL"
/product="hypothetical protein"
/transl_table=11
CDS complement(1854630..1855205)
/locus_tag="AUO97b_01878" /db_xref="SEED:fig|400667.82.peg.1740"
/translation="METILGLCIGVGLSAACGFRVFVPLLVMSIATMMGWFEPSKGFE
WLALPSVCLALGVATVCEVAAYYIPWVDNALDTVATPAAMIAGTLATMAVSSGEMSQF
ASWAAAIIVGGGTAGVVQMSTVAARGVSTATTGGLGNFIVATGEWIGAILLSVSAMLV
PALVAIVVLIAVIWVVRWIRHKKQEQAHTPL"
/product="hypothetical protein"
/transl_table=11 CDS complement(1855791..1858259)
/locus_tag="AUO97b_01879" /db_xref="SEED:fig|400667.82.peg.1741"
/translation="MEKNDTFLSDLLTTYKKLKLSDANEAETRKKLIDVVIENVLGWT

TDDISYEERVSEDGETTYADYIIRTADVSILIEAKRLGSTFYTVPEKKKVKLSTFSED
DLTGKAIKQAREYCRKKSIPFAAVTNGSQWIIFPAVRTDSVSFSDSTALVFDSIERIL
GEESSHFRGLLSRDGVIEGNLALELIGRKTDQFDERRLNKFFKGISIKKENPIYPLIE
GAVVSAFSDSIVDNDNSILEKCYVKNADRKKFDNRIEMHLQKREPLFNSQPKRPMRKR
ESSSLVDSINKASHNKKPLAILILGTVGTGKTTFLQYTRRISSASFFEQKEKQPYPHW
IEIDFRTFSKNENPLDFIYDKLFDYIKTDKFFCDYDRSIKFAYEKEINSLKSGPLFLL
ANSDEELKKRITDLLIADYQAIKPYVDKLIGYASKLSPIFLIIDNVDQFEDDFIQSEI
FSDSIAIASRLGINLIIAMRESTFINHRSSPTFDAFDFDPLHIEPPEIPAVLSRRFFL
TGKLLEGKSGEFIALNGANFKVEDLSVFIDIVKGSVLGTEVGEKIDVLSNHDVRLALR
MTREFLARGYTDPAKALITHKPEKRYTLPKQEAFRSILLGNQSVYSEEYSVIGNPFDS
RLNRSNTELLRIFVLSALVRKGSSNGQGYIEGQIIRDSLKTIGFSETDTLAILKDLCT
LRFAHTKSHGKADINSSYFASRLGGHIIKSLIGDFTFIENILMDTFISEKDQWEQLKS
ISSSIQEERDIVRKINQRIDRIKIFYELMISQYSILLSEATKRGLDPIWLSNPLIEMK
DTLWSNCDKAYASANRIYGENAQK"
/product="hypothetical protein"
/transl_table=11
CDS complement(1858369..1860975)
/locus_tag="AUO97b_01880" /db_xref="SEED:fig|400667.82.peg.1742"
/db_xref="GO:0047547"
/translation="MNTKYRKPLAGTQLEYYDVRQAVEDIQPGAYEKLPYTSKVLAEQ
LVRRADAENLTAYLTQLIERRQDLDFPWYPARVVCHDILGQTALVDLAGLRDAIAEKG
GDPSKVNPVVPTQLIVDHSLAVEYGGADPDAFAKNRAVEDRRNEDRFHFIEWTKTAFK
NVDVIPAGNGIMHQINLEKMSPVIQARDGVAFPDTCVGTDSHTPHTDALGVISVGVGG
LEAENVMLGRASWMRLPDIIGVELVGQRQAGITATDIVLALTEFLRKERVVGAYLEFF
GEGADSMSVGDRATISNMTPEYGATAAMFYIDQNTIDYLRLTGREDAQVALVEQYAKE
IGLWASEMTKAEYPRVLRFDLSTVTRNIAGPSNPHARVSTSDLKEKGIAGVVENRSDG
LMPDGAIIIAAITSCTNTSNPRNTVAAGLLARKANELGLVRKPWVKSSFAPGSKAAAL
YLEEAGVLKDLEKLGFGIVAYACTTCNGMSGALDPAIQQEIIDRDLYATAVLSGNRNF
DGRIHPYAKQAFLASPPLVVAYAIAGTIRFDIEKDALGHDKEGNPIYLKDIWPSDAEI
DALVKQAVKPEQFRKVYIPMFDLGEVEQAKSPLYDWRPQSTYIRRPPYWEGALAAPRT
LANMRPLAILGDNITTDHLSPSNAILMDSAAGEYLHKMGVPEEDFNSYATHRGDHLTA
QRATFANPKLYNEMVRRSDGTIKQGSKARVEPEGEVMRMWEAIETYMNRKQPLIIIAG
ADYGQGSSRDWAAKGVRLAGVEAIVAEGFERIHRTNLVGMGVLPLEFKPGVNRKTLKL
DGTELYSVIGNIAPRSTLTLVIERATADGKEEILEVPVTCRLDTEEEVSVYEAGGVLQ RFAQDFLEGQIA"
/product="2-methylcitrate dehydratase (2-methyl-trans-aconitate forming) (EC 4.2.1.117)" /EC_number="4.2.1.79"
/transl_table=11
CDS complement(1860975..1862132)
/locus_tag="AUO97b_01881" /db_xref="SEED:fig|400667.82.peg.1743"
/db_xref="GO:0050440"
/translation="MSSNETTTGFKPKKSVALSGQVAGNTALCTVGRSGNDLHYRGYD
ILDLAAGSEFEEVAHLLVHGKLPNKAELKAYKAKLKALRGLPAALKTALEQLPPSAHP
MDVMRTGVSVLGCLTPEHEDHNEAGAKDIADKLMASLGSMLLYWYHFSNNGRRIEVET
DDDSIAAHFLHLLHGEKPCEEWIQAMHTSLILYAEHEFNASTFTSRVVAGTGSDMYSA
ITGGIGALRGPKHGGANEVAFVIQQRYDNPDEAEADIRKRVENKEVVIGFGHPVYTVS
DPRNEVIKKVAHDLAQAQENTKMYLIAERLEAVMKEVKNMFPNLDWFSAVSYHLMGVP
TAMFTPLFVIARTAGWSAHVIEQRQDGKIIRPSANYTGPENLEFKPLAERG" /product="2-methylcitrate synthase (EC 2.3.3.5)" /EC_number="2.3.3.5"
/transl_table=11
CDS complement(1862402..1863286)
/locus_tag="AUO97b_01882" /db_xref="SEED:fig|400667.82.peg.1744"
/db_xref="GO:0046421"
/translation="MAKQSAGQLFRDAVAQEKPLQVVGTINANHALLAKRAGYKAIYL
SGGGVAAGSLGLPDLGISNLDDVLTDVRRITDVCDLPLLVDADTGFGASAFNIARTTK

SLIKFGAAAMHIEDQVGAKRCGHRPNKAIVTQEEMVDRIKAAVDARGDDSFVIMARTD
ALAVEGLQAAIDRAGAYIEAGADMLFPEAITELDMYKQFAQKTGVPILANITEFGSTP
LFTTEELASADVSLALYPLSAFRAMNKAAETVYETLRKEGTQKNVVDIMQTRKELYER
INYYAFEDYLDNAFAKKK"
/product="Methylisocitrate lyase (EC 4.1.3.30)" /EC_number="4.1.3.30"
/transl_table=11
CDS complement(1863279..1863989)
/locus_tag="AUO97b_01883" /db_xref="SEED:fig|400667.82.peg.1745"
/db_xref="GO:0003700"
/db_xref="GO:0005622"
/translation="MQDLTFAPEPLQSQGRTLTEHVFKQIQTAIVLGQIPAGSKISEP
ELARTYGISRGPLREAIHRLEGQRLVERTAHVGARVVSLSLQQFKELYQIRASLEGLA
CKLAAQHIDKKQILALRDVLRMHAEDENFKAGKGYYLQEGQDDFHYCIIKSSGNKTLE
KMLCDELYHLIRMYRIQFSNTPDRPSKAWDEHIRILDAIAEGDGELAELLMHRHINAS
YKIIEQTLLQAQGEHNNG"
/product="Propionate catabolism operon transcriptional regulator of GntR family" /transl_table=11
CDS 1864505..1865719
/locus_tag="AUO97b_01884" /db_xref="SEED:fig|400667.82.peg.1746"
/translation="MFQHIPPYAGDPILSLMEQFNADTRSEKVNLSIGLYYNEDSIVP
QLETIIEAQKRIEPKNGKTKLYLPMEGFKPYREAIQALLFGANSPAVKAGRAVTIQTL
GGSGALKVGADFLKTYFPNSDVWVSQPTWDNHVAIFNGAGIKTHFYPYFDAETRGVDF
DGMLSTLKTLPEQSIVLLHPCCHNPTGADLNPAQWDQVIAVLKDRNLIPFLDIAYQGF
GDGMEEDAYAIRALDQAGLNFIVSNSFSKIFSLYGERVGGLTFVCDDAEAAQCTFGQL
KATVRRIYSSPPTTGAWLVDEVLNDAELNQQWQGEVKEMRERIIKMRSILKDELTKAL
PDRDFSYLVNQKGMFSYTGLTAEQVDILREEYAIYLVRSGRICVAGLNMNNVYTVAKA MAEVLAKSVEAA"
/product="Biosynthetic Aromatic amino acid aminotransferase alpha (EC 2.6.1.57)" /EC_number="2.6.1.57"
/transl_table=11
CDS complement(1865768..1867474)
/locus_tag="AUO97b_01885" /db_xref="SEED:fig|400667.82.peg.1747"
/db_xref="GO:0047051"
/translation="MQTFSPQAVIEHLQNIVGRSYVLTDDQSTRQYRQGRRFGEGKVL
AVVVPGTLLEQWQVLQAAIEADCIVIMQAANTGLTGGSTPYGDDYDRPVLVMSTRRLK
GIQVIHDGKQVICLPGATLDNLEQILKGYNREPHSVIGSSCIGASVLGGVCNNSGGAL
VRRGPAYTELALYAQVNAAGQLELVNHLGVNLGSTPEEILTRLEKQQYQAVDILDDNE
KQASDHRYAHDVTQVDEDTPARFNADPSRLFEASGSAGKVCVFAVRLDTYEKVESSVF
YIGSNDADDLTAIRRYLLTSLPSLPIAGEYIHRDAYLIGEKYGKDTFLFIEKFGTANV
PKAFAMKDKVDGFLEKFKIKGLTDQILQAITFFLPSHLPKRMTEYRDRYEHHLVLRVE
NNSKAQTEQFLKEYFAVHQSGNYFVCSEEEGRKAFLHRFAIAGAAIRYRDTHRSEVED
IVALDIALRRNDREWVEQLPAEMEKKIIHKLYYGHFFCHVFHQDYILKKGNDPLEMEH
QMWKLLDARRAEYPAEHNVGHLYIAKPALANFYQKLDPTNSFNVGIGHTSKLKYWGKA KS"
/product="D-lactate dehydrogenase (EC 1.1.1.28)" /EC_number="1.1.2.5"
/transl_table=11
CDS complement(1867766..1868917)
/locus_tag="AUO97b_01886" /db_xref="SEED:fig|400667.82.peg.1748"
/db_xref="GO:0004460"
/translation="MIISSGNDYRAAAQRRLPPFLFHYIDGGAYAEYTLKRNVQDLSE
IALRQRVLNDMSALSLETKLFNETLSMPVALAPVGLTGMYARRGEVQAAMAADKKGIP
FTLSTVSVCPIEEVAPAINRPMWFQLYVLRDRGFMRNALERAKAAGCSTLVFTVDMPV
PGARYRDAHSGMSGPNAAMRRYMQSVFHPHWSWNVGLMGRPHDLGNISKYLGKPTGLE
DYIGWLGSNFDPSISWKDLEWIREFWDGPMVIKGILDPEDAKDAVRFGADGIVVSNHG
GRQLDGVMSSARALPAIADAVKGDLAILADSGIRNGLDVVRMLALGADTVLLGRAFVY
ALAAAGGQGVSNLLDLIDKEMRVAMTLTGAKSISDINADCLVQAIKQGL" /product="L-lactate

dehydrogenase"
/EC_number="1.1.2.3"
/transl_table=11
CDS complement(1868914..1869666)
/locus_tag="AUO97b_01887" /db_xref="SEED:fig|400667.82.peg.1749"
/translation="MRISDQVVMKLQALIEERHMKKGDRLPAERQLATSLGVSRPSLR
EAIQQLNSQGVLISRRGDGTYIQQLPEQWPQQLIVNPISNLIEEDPLYRFDVQEARLV
LEGGTAWYAALRSTPEDRAKIHHYFNEISRHQNAGDSAQAAVADAEFHLAIAEASHNV
VLIQMMRSLFDLLQYNVLLGRKKVYNDPVNGDLLSEQHFQVMDAIDRKDPEAARQAVC
GHIEFVINHVRSLDEDEARQKRANRLNRVDSK" /product="Lactate-responsive regulator LldR in Enterobacteria, GntR family" /transl_table=11
CDS complement(1869686..1871347)
/locus_tag="AUO97b_01888" /db_xref="SEED:fig|400667.82.peg.1750"
/db_xref="GO:0015129"
/db_xref="GO:0015727"
/translation="MLNMWQQLYDPLNNIWLSSAVALIPIIFFFLALAVFRLKGSIAG
TGTVIIALLIALFFYQMPGQMAFASIIYGFFYGLWPISWIIIGAVFLYKISVKTGQFE
VIRSSILSITEDQRLQMLLVGFAFGTFLEGAAGFGAPVAITAALLVGLGFKPLYAAGL
CLIVNTAPVAFGAMGIPIIVAGQVSGVDTMEISQMVGRQLPFLTIIVLFWIMAIMDGW
RGVKETWPAVLVGGGAFAIAQYLTSNFIGPELPDITAAIASLVSLTLLFRVWKPKHIF
RFEPEAGQTLTQQPTTVQRYSIGKIAKAWSPFAILTVMVTIWSVKPFKALFAKDGALE
HWIFKLEVPYLHKLVEKMPPIVSEMKPYEAIYKFDWFSATGTAIFIAAIITVIFLKMK
ASEAVTTFGETLNELKTPIYSIGMVLAFAFIANYSGLSATLALALSHTGHAFTFFSPF
LGWLGVFLTGSDTSSNALFSALQATTAQQIGIPEVLLVAANTSGGVTGKMISPQSIAI
ACAAVGLVGKESDLFRFTVKHSIIFTVFVGIIITVQAYLVPWMIP" /product="L-lactate permease"
/transl_table=11
CDS 1871728..1873098
/locus_tag="AUO97b_01889" /db_xref="SEED:fig|400667.82.peg.1751"
/db_xref="GO:0004615"
/translation="MTTLTCFKAYDIRGKLGTELNEEIAYKIGRAYGQIYKPKTVVVG
CDIRLSSEALKQATIRGLNDAGVNVLDLGMTGTEEVYFAAFHLDVQGGIEVTASHNPM
DYNGMKLVRENARPISADTGLKEIQALAETNNFEEVSQKGTTQSYNILPEFVDHLLTY
IEPAKIRPLKLVVNAGNGAAGHVIDAIEEKFKALNVPVEFIKIHHEADGTFPNGIPNP
ILIENRDSTRNAVLEHKADMGIAWDGDFDRCFLFDEKGQFIEGYYIVGLLAQAFLIKQ
SGEKIVHDPRLVWNTFDIVDEYKGVAVQSKSGHAFIKDVMREHNAVYGGEMSAHHYFR
DFAYCDSGMIPWLLTIALLSETGQSLSTLVENMIAKFPCSGEINFKVADTQTTIQKIF
DFYADQNPQIDRTDGVSLDFGAWRFNVRASNTEPLLRLNIESRADRQAQPMQYYVDEL TGLIQN"
/product="Phosphomannomutase (EC 5.4.2.8)" /EC_number="5.4.2.8"
/transl_table=11
CDS complement(1873142..1874158)
/locus_tag="AUO97b_01890" /db_xref="SEED:fig|400667.82.peg.1752"
/db_xref="GO:0003978"
/translation="MAKILVTGGAGYIGSHTCVELLEAGHEVIVFDNLSNSSKESLNR
VQEITQKSLTFVEGDIRNSGELDQVFQEHAIDAVIHFAGLKAVGESQEKPLIYFDNNI
AGSIQLVKSMEKAGVYTLVFSSSATVYDEANTSPLNEEMPTGMPSNNYGYTKLIVEQL
LQKLSVADSKWSIALLRYFNPVGAHKSGRIGEDPQGIPNNLMPYVTQVAVGRREKLSI
YGNDYDTIDGTGVRDYIHVVDLANAHLCALNNRLQAQGCRAWNIGTGNGSSVLQVKNT
FEQVNGVPVAFEFAPRRAGDVATSFADNARAVAELGWKPQYGLEDMLKDSWNWQKQNP NGYN"
/product="UDP-glucose 4-epimerase (EC 5.1.3.2)" /EC_number="5.1.3.2"
/transl_table=11
CDS complement(1874151..1875821)
/locus_tag="AUO97b_01891" /db_xref="SEED:fig|400667.82.peg.1753"
/db_xref="GO:0004347"
/translation="MSKSIEKFPKELVSPIAQLHSLVEKNSKLHIKELFAAEQDRFQN

YSVKFDQLVFDYSKHRITKSVLEQLFALAKTKQLTHWIERLFSQDKVNCTEQRAAMHW
ALRLPSEYSKFPELTKQVHTQLQRMYALVEKIHAGQYRGATGEVIQDVVNIGVGGSDL
GPQMVTHALCDFKVKTAKPLNVHFVSTMDGSQLSDLLHQLRPETTLFIISSKSFGTID
TLSNAQTVRQWLEKALGKHDRVVKSHFIGVSTKAEKMTEWGIAPENQLLLWDWVGGRY
SLWSCIGFPIALTIGIDGFQQLLAGAHAVDEHFQNTSFEQNIPVLMALLGIWNNNFLN
IQTHAVLPYDGRLKYFAAYLQQLEMESNGKSVQRDGQKVELDTCPIVWGEVGPNAQHA
FYQLLHQGTQAVSCDFIAPIQRYNADHFTYVENAEALIEQHHLALSNCLAQSRLLAFG
NEALDSAELKNLPIYKQYEGNQPSSTLLLKELNPYSLGMLIALYEHKVFVQSVIWNIN
PFDQWGVEKGKQIADQLLPILNGAQNDLSALDASTRGLIKILLGKVDG" /product="Glucose-6-phosphate isomerase (EC 5.3.1.9)" /EC_number="5.3.1.9"
/transl_table=11
CDS complement(1875818..1877080)
/locus_tag="AUO97b_01892" /db_xref="SEED:fig|400667.82.peg.1754"
/db_xref="GO:0003979"
/translation="MKIAVFGTTLHAGVMAALLAEYGNQIYWCTSVTCEENISILSYQ
DQEVNHYLNKQRKAGFLKESPFSEIPLDIEVYLFCFSPTQIELALKTVEKLSERPIVH
PRLMINGSTFGLHGTNQLKQHLPKDEWVYFPDVIQEGNAINSVLNVKHVIVGVESSYA
QDTMQELLRPFFRFSYQYLFMPILDAEFTKLSISGMLATRISYMNDLAMVAEKLGIDI
ANVKHGIAADTRIGAAYLSAGVGFGGENFSHDILTLSSTVSGTGAKSRLLEQVWAINE
QQKEILFRKLWNYYHCDLSGKTVAIWGASFKENTSSTHNSPIHILLAALWAQGVKVRL
HDPQALDEIATTYGDREDLVLCADQYEAAQGAHALCLVTAWKQYWSPDFKQLQQVMQH
PLILDGRNIYDPAYVKAKGFAYEGVGRL"
/product="UDP-glucose 6-dehydrogenase (EC 1.1.1.22)" /EC_number="1.1.1.22"
/transl_table=11
CDS complement(1877196..1878071)
/locus_tag="AUO97b_01893" /db_xref="SEED:fig|400667.82.peg.1755"
/db_xref="GO:0003983"
/translation="MIKKAVLPVAGLGTRFLPASKSIPKEMVTVVDRPAIEYVVREAV
EAGIEQIILVTHSSKASIENYFDRNFELETTLEQKKKFDLLAEITQIVPEHVSVVSVR
QPQPLGLGHAVLCAKSVVGQDDFAVLLPDVLVKDSSGKNDLSRMIARYNSSQAAQIMV
EAVPDHLVDQYGIVDVKHSPNEGESIAMQGIVEKPAVGSAPSNLSVVGRYVLPAKIMQ
LLENTPKGAGNEIQLTDAIAMLQETDTVEAYRMQGQTFDCGSKIGYLKAVLHYGVEHP KLGNDFKQLIQELKL"
/product="UTP--glucose-1-phosphate uridylyltransferase (EC 2.7.7.9)"
/EC_number="2.7.7.9"
/transl_table=11
CDS complement(1878096..1878716)
/locus_tag="AUO97b_01894" /db_xref="SEED:fig|400667.82.peg.1756"
/translation="MKRLVDIVISLIALTVLSPIFLIVAYKVRKNLGSPIFFYQERPG
KDGKLFKMIKFRSMKDAFDAQGNPLPDEARITPFGQKLRSTSLDEMPQLINVLKGDMS
VVGPRPMLKDFVALYSPEQARRLEVRPGMTGLAQVSGRNELDYEERFKCDVWYVDNHN
IWVDFKIMFKTVKVMLKREGINAPGHVGPSLFKGNDTQENIDSSVK" /product="Lipid carrier : UDP-N-acetylgalactosaminyltransferase (EC 2.4.1.-)" /transl_table=11
CDS complement(1878729..1879556)
/locus_tag="AUO97b_01895" /db_xref="SEED:fig|400667.82.peg.1757"
/translation="MFSVLSSIYHKEQPEHFNTCMESIWDNQTLKPTEIVLIEDGPLT
PELDQVIAHWQKKLGNVLRVTKLEKNVGTGKAKNIGLQECNYDIVSIVDTDDIYVPER
FEKQIKFLQQNPEISIVGGQIFEFIEDIGNPVGMRKVPLSNEELRNYAKKQSPFNNMT
ITYRKSHILEVGGYQHHLWMEDYNLFLRVIAKGYKIENLPDVLVYARIDNGMHGRRKG
LQYIKSEKQLLDLKKQLKLQNPLYANMLFLVRSAFRLLPANLLGTIYNTFLRKDIKK"
/product="hypothetical protein"
/EC_number="2.4.1.266"
/transl_table=11
CDS complement(1879563..1880597)
/locus_tag="AUO97b_01896" /db_xref="SEED:fig|400667.82.peg.1758"

/db_xref="GO:0009058"
/translation="MKILFLINSLKSKSGSERVAVELANKMAAIGSYNITLLNRESIK
ENAAYPIADNVEVIALSGSLFEFYKKLKKQISSNNYDVVVVHNMGKLSLLCAFVPNIK
KLVVLEHVSFISRPKKIQILSKFLYRNIDQVVTLTQNDKEQFDKFHSNVIVIPNFSPF
SIASQPQNNGKQIVTIGRLTDQKNYIHLLQAWKKIYQSIPDWQLNIYGEGEQEEMLQD
YIKQQSLKNVSLKGSTSNVKEVYEQSSFFVMSSKYEGLPMVLIEAQSFGLPIVSYNCP
YGPSDVIRDSKNGFLVEDQNVDELAAAVLKVALSPQLLEQFSQSSLLNAKKYQPEQIL KIWIEKVLEG"
/product="Alpha-1,4-N-acetylgalactosamine transferase PglH (EC 2.4.1.-)"
/transl_table=11
CDS complement(1880601..1881641)
/locus_tag="AUO97b_01897" /db_xref="SEED:fig|400667.82.peg.1759"
/translation="MSLKIRKELLIFLILSSFLSLLVGFRGNTNDTITYYMIFKNIGS
YDLTNFSLFYNETGVEIGWGLYSKIISLFSDSPVVLFTIFSFFTFFTFYRISRLVEIK
FLYVMLYYLPTGFFMMQQFMQIRQGFAIPLVIYGSVLYLSGKKYISLVFFILAILFHQ
SSLAFILIFISYLFFNNFLKINTSVFKFFIINILILVFGFIVARFILLDAAMDYFQRL
EAYSTTDYAESVGFFSLSNIKFYIEFFLIFLLLNNRLLLNKFIVFFVFIFTVGLSLRV
AFYDFGILSGRLSNTFLLIEVFLMPMLLSSRLNKIYLYIYFLLYFLVIFYVTWTFQAS EFIKNNYFLPLS"
/product="Capsular polysaccharide biosynthesis protein" /transl_table=11
CDS complement(1881724..1882815)
/locus_tag="AUO97b_01898" /db_xref="SEED:fig|400667.82.peg.1760"
/translation="MKILHAAYMKDASSGIVNQMYWEQEAAKELGISWKSILFVQNPN
GFQNNEIVTVCNDIMGGELKKWFKAKKSYYSWLEGIMQEYDVLIIRHSLCDPFEPFFI
RKMIKPVYIMHHTFEIDELYSYNYGVKTKIKSLMEIYFGKKSISYSKGIVAVTKEIFL
YENSRVNGKFKKEIVYPNGIISQHDIILDDRNDDIPEIVFIASYFNGWHGLDLLLHNL
SSINDDFILHIVGDVSVEDELLAKKDSRVILHGHLNNIEISQLMSKMWIGLASFGLFR
KGMKEACTLKVREYLNYGLPIYSGHKDIFPEEFEYYKFGEPKFVDILSFAHLMRKVSK
SDVKQAAIKFIDKKILLKELYEQLLEEKS" /product="hypothetical protein"
/transl_table=11
CDS complement(1882907..1884070)
/locus_tag="AUO97b_01899" /db_xref="SEED:fig|400667.82.peg.1761"
/translation="MKIGLTKFNYPEIRCVTTSQENDYINLKKYNIYYYFNNIPVIKN
YFHRFLFKPISVKNVDVIHSFNDICLTNNKWVVTFETMLPRFLDILSNHKNLNPEYIY
NDEINKYLEVVARDNCLGVIALSKSAKKIQSDILKAYPKVRDKIEKKMFVLYPPQKIY
TTQHEIEEKVLKPLKLIFVGNDFYLKGGAECILAINELLEEGIISENEIMLTVVGILN
RTHNYSFGIYQDDSDFSKNINTIIMNRKNIKIYSNVDNNKVIEMIREHHIGLLPTWAD
TFGYSVLEFQACGCPVISTDVRALSEINNNDIGWLINVDKNKYGEIVVDSYSKKDLCR
RTIIDQLKKHILSAYENPNVVINKGVESLNRIKKEHSIDYYNDKIKSVYTIGI"
/product="Glycosyltransferase"
/transl_table=11
CDS complement(1884247..1884813)
/locus_tag="AUO97b_01900" /db_xref="SEED:fig|400667.82.peg.1762"
/db_xref="GO:0008925"
/translation="MKKKLRKLIYFILPCIRLFLNLFFKRKYLEGRFFSKSLSGYVWA
VRSIWVKNILRLSKPTPFPTALTCSISNPYNIHFHPDDLNNFQSPGTYFQNFSAHIYI
GKGSYIAPNVGIITSNHDLADLESHTEGKDVVIGDKCWIGMNSVILPGVVLGNGTIVA
AGAVVTKSFKQGNIVIAGVPAKIIKEIK"
/product="Maltose O-acetyltransferase (EC 2.3.1.79)" /EC_number="2.3.1.79"
/transl_table=11
CDS complement(1884810..1886162)
/locus_tag="AUO97b_01901" /db_xref="SEED:fig|400667.82.peg.1763"
/translation="MRLNAQGGFLKAVSVLVGGTAFAQGLAIIALPFITRLYSPSDFS
IFAVYASILGILTVASCLRFEIAIPIPSEDEEAAILVILALISNLAISILTGLLIWVF
HVEIISLLKKPNFNHIIWLIPLGVFFSGIYTALQYWATRKKNFTVIARTRVVQSISGV
ATQIILGFWGTAALGLALGQIIKVSFGIGKLAKNFWKESEVFIRNITIIKLYNVFKKN

DQFPKYSTLDSLANSAGAQLPIIIIATLALGSEAGYLMIAMQVLAVPIQLIGGAVSQV
YLAHAPAAVQEKTIAHYTTNILENLFKYGVSSLIFIGLVSPILAKYIFGYEWEKVGLI
ISWMIPWFAFQLIASPISMIMHIVGRQKQMLLLTLTGFILRIGILYGQFYLNPTYLLE
AYAISGGIFYGICYIVFSNSAGLEVKDHILLVKKVGFLLIFITSLGVMVNITLRVLGL "
/product="O-antigen flippase Wzx"
/transl_table=11
CDS complement(1886197..1887276)
/locus_tag="AUO97b_01902" /db_xref="SEED:fig|400667.82.peg.1764"
/translation="MIDFIDLKAQQNRIKDKIDAGIQNVLTHGQYILGPEVIELEEKL
ASYVGAKHCITCANGTDALQIAQMAFGIGPDDEVITPGFTYIATAETVALLGAKPVYV
DVNPKTYNLDSEKLEAAITPRTKAIIPVSLYGQCADFDVINAIAKKYSIPVIEDAAQS
FGATYKSRKSCNLSTVACTSFFPSKPLGCYGDGGAIFTNDDELAKVIRQIARHGQDKR
YHHIRVGVNSRLDTLQAAILLPKLEILDDEMQARQRVAEVYNRLFNEVGIHTTPYIEA
HNTSAWAQYTIQVDNRAEVQEKLKAQGIPTAVHYPIPLNKQPAVADSDIHLPIGDAIA
EKVMSLPMHPYLAIDDQLKIVKAFG" /product="Glutamate--UDP-2-acetamido-2-deoxy-D-ribohex-3-uluronic acid aminotransferase (PLP cofactor) (EC 2.6.1.98)"
/transl_table=11
CDS complement(1887278..1887856)
/locus_tag="AUO97b_01903" /db_xref="SEED:fig|400667.82.peg.1765"
/translation="MSFYQHETAIVDNGAQIGEGSRVWHFVHVCGGAKIGKGVSLGQN
VFVGNRVVIGDHCKVQNNVSVYDNVTLEEGVFCGPSMVFTNVYNPRSLIERKDQYRDT
LVKKGATLGANCTIVCGVTIGAYAFIGAGAVVNKDVPAYALMVGVPAKQIGWMSEFGE
QLDLPLQGQAQAICSHTGAVYQLDGTTLTKQG" /product="UDP-2-acetamido-3-amino-2,3-dideoxy-D-glucuronic acid acetyltransferase (EC 2.3.1.201)" /transl_table=11
CDS complement(1887853..1888803)
/locus_tag="AUO97b_01904" /db_xref="SEED:fig|400667.82.peg.1766"
/translation="MKKFALIGAAGYIAPRHLKAIKETGNTLAVAMDVNDSVGIMDSH
FPEAEFFTEFEEFEAYVEDQKLKGEKLDYVAICSPNYLHAPHMKYALKNGIDVICEKP
LVLNSEDLNMLAEYEKQYGAKVNSILQLRLHPSIIALRDKVQAAPADKVFDVDLTYLT
SRGKWYLKSWKGVDNKSGGVATNIGVHFYDMLHFIFGKIVKNEVHYRDEKTASGYLEY
ERARVRWFLSIDANNLPENAVQGEKLTYRSITIENEELEFSGGFTDLHTQSYQRVLEG
KGYGVEENRAAIETVEVIRVSPIIENPENPHPLLAKVKKA" /product="UDP-2-acetamido-2-deoxy-D-glucuronic acid dehydrogenase (NAD+) (EC 1.1.1.335)" /transl_table=11
CDS complement(1888834..1890129)
/locus_tag="AUO97b_01905" /db_xref="SEED:fig|400667.82.peg.1767"
/translation="MQLADLRIAIIGLGYVGLPLAVEFGKKGPVVGFDINQNRIDELK
SGKDHTLEVSPEELQKAEQLSFSANLDDLKTSNFFIVTVPTPVDQVNRPDLTPLKKAS
ETVGQALKKGDIVVYESTVYPGATEEVCIPILEKVSGLKFNQDFFAGYSPERINPGDK
VNTLTKIKKITSGSTPEVANTVDAVYASIITAGTHKASSIKVAEAAKVIENTQRDLNI
ALVNELSVIFDRIGIDTLDVLEAAGSKWNFLPFRPGLVGGHCIGVDPYYLTHKAEEVG
YHPQVILAGRRINDNMARYVARNTIKLMLQNGIDVPRSKVGVLGVTFKENCPDIRNSK
VADLIKELEFWGAQVVVADPWADAEEVKHEYGVELGTVNAQNPVDSLIVAVGHSEFRS
LSASELRSYVKAEKPVLADVKSLFDRTQMSDVGFTVFRL" /product="UDP-N-acetyl-D-glucosamine 6-dehydrogenase (EC 1.1.1.136)" /transl_table=11
CDS 1890490..1891590
/locus_tag="AUO97b_01906" /db_xref="SEED:fig|400667.82.peg.1768"
/translation="MKYCQFFSVLALSLSAASCAVTSGLQTYDIPSEGVYKTDLGTTV
NVVKISQETLPAIQPAQIDYQRDYASLFKNQQSIYRLSPGDVLSIQLWAYPEITPPVN
NISNEQSIQANGYPIDQSGYIQFPLVGRYKAAGKTLAQVNRELHSQLARFLKNPDVVV
RVVSYEGQRFSVQGSVTKGGQFYLSDQPVSIYTALGMAGGVTTTGDNTYIQLIRNGRT
YNLNTIDLEKAGYSLHKLLVQPNDTIYVSTRENQKIYVMGESGKNQALPMRDQGMTLS
DALGESLGINPNSASASRIYVVRTNPNDRTTEIYHLNLMSLGDFGLANQFRLRSNDIV
YIDATGLTRWQRVVNQIIPFSNALYNIDRLGQ"
/product="Putative polysaccharide export protein YccZ precursor"

/transl_table=11
CDS 1891595..1892023
/locus_tag="AUO97b_01907" /db_xref="SEED:fig|400667.82.peg.1769"
/translation="MQIKNILVVCIGNICRSPMAEYFLKQQYPQLNIESAGISGLIGH
QADEKAQLCMQRLGIDMQPHIARKLNAELIKKADLILVMSSNQQKHIEQTWPFAKGKT
FRLGHWQNKNVPDPYQHEQAVFDETCQLIQQCVADWKPYI"
/product="Low molecular weight protein-tyrosine-phosphatase (EC 3.1.3.48) => Etp" /EC_number="3.1.3.48" /transl_table=11
CDS 1892043..1894229
/locus_tag="AUO97b_01908" /db_xref="SEED:fig|400667.82.peg.1770"
/translation="MNQQNTNTEDTIDLKELFFSLIAQWKLIALCVILSVVCALLYLR
VTPDTYSVDALVQVEDSKGASAALLGDLSQMIEQKSPAQAEIEILKSRLVLGTVIKDL
HLNIQVSSTENTLTHRLLSDADYKTEYTTKSVIFKDGLKSFDIRQFEIPAYYLDKNLL
LDFDKQSLRLTDPVTEEVILTVPLNQVNHVAGPHGLWKVAIFTKDQFDATYNIKSLSL
PIAVNAISANYAVAERGKLTGVLGLNYQGQDKEHITKVLNAILATYSAQNIERRSAES
AQTLKFLDEQLPDLKKQLDDAERQFNKFRQQYNTVDVTKESELYLTQSITLETKKAEL
EQKQAEMAAKYTAEHPAMREINGQLAAINKQIGELNSTLKQLPDVQRQYLQLYREVEV
KTQLYTALLNSYQQLRIAKAGEIGNVRIVDTAVEPVEPIKPKKLLVLILSVFVGGFIG
ALIALLRNMLRTGIKDSGQIENEMDLPVYATVPRSPIQESRIKILKKKKSIPILAVKN
SDDIAIESLRSIRTAIHFALANAKNNIIMIAGPSPEVGKSFISTNLATIFAQGNKRVL
LIDADMRRGYMHKYFDVDVKPGLSELLSGQADLQKVLHKTQVANLDVITRGKSPTNPS
EILSSNQFKELLEQLQSQYDHIIIDTPPVLAVTDGIIISQYTGVNLIVARYAKSQMKE
LELTVNRFEQAGVKVNGFILNDIQRASAGYGYGYNYAYAYKAQKED" /product="Tyrosine-protein kinase (EC 2.7.10.2)" /EC_number="2.7.10.2"
/transl_table=11
CDS 1894422..1895144
/locus_tag="AUO97b_01909" /db_xref="SEED:fig|400667.82.peg.1771"
/translation="MSKALPIAVAVVLGGAALVPVYYATQHPTTEVGRKADKNASPIQ
KISYVLGYEVAQQTPPELDTKAFVQGIHDARNKQPSAYTQEDLKAAVAAYEKELQQKM
QHQDKPEQAGTAADSADAQFLAENKTKAGVKTTASGLQYIITKEGTGKQPTAQSIVKV
HYEGRLINGQVFDSSYKRGQPVEFPLNQVIPGWTEGLQLMKEGGKATFFIPSNLAYGP
QELPGIPANSTLIFDVELISVK"
/product="FKBP-type peptidyl-prolyl cis-trans isomerase FklB (EC 5.2.1.8)"
/EC_number="5.2.1.8"
/transl_table=11
CDS 1895195..1895890
/locus_tag="AUO97b_01910" /db_xref="SEED:fig|400667.82.peg.1772"
/translation="MKKISLVIAASTMSLSVFAAAPITNKSPAKDQFSYSYGYLMGRN
NTDALTDLNLDIFYQGLQEGAQNKTARLTDEEMAKAINDYKKTLEAKQLVEFQKQGQQ
NAQAGAAFLAENAKKSGVITTKSGLQYQVLKEGTGKTPKATSRVKVNYEGRLLDGTVF
DSSIARNHPVDFQLNQVIAGWTEGLQTMKEGGKTRFFIPAKLAYGEVGAGDSIGPNST LIFDIELLQVLPK"
/product="Peptidylprolyl isomerase, FKBP-type (EC 5.2.1.8) / Macrophage infectivity potentiator" /transl_table=11
CDS complement(1895936..1897477)
/locus_tag="AUO97b_01911" /db_xref="SEED:fig|400667.82.peg.1773"
/translation="MALWRSTFIVSAMTMLSRVLGLVRDVVLLNVFGAGKDFDTFVVA
FRIPNFFRRLFAEGAFSQAFIPVLTEYKTGRAHAEVQILISRVFGCLLTVMTLLTFVA
MVLAPAIIYMYAPGFHNDPEKFDLAVSMFRLTIPYLMFMSLTAFASSILNSYGSFASP
AFSPVLLNVAMIAGAWWLTPYMAEPIKALGWSVVAAGILQLAIQIPELWRKNLLIPPK
VDFKHEGVERILKLMLPALFGVSVTQINLLLNTIWASFMQDGSVSWLYSAERMTELPL
GLIGVAIGTVILPSLSARHAEQDQAKFRSMIDWAAKVIVLVGLPASIALFMLSTPIIQ
ALFQRGEFDLRDTQMTALALQCMSAGVISFMLIKVFAPGFYAQQDTKTPVRVGLMSVA
ANAILNVVFIGFFKLINWHAEHMALALASSGSALVNAGLLYFYLHKRNIFRFGAHWKK
LALQYGLANLAMIAALWFGLNWYNGELSQWIRVAEVVGLCVVGVIAYLIGLLLTGFRP RDLKH"

/product="Peptidoglycan lipid II flippase MurJ" /transl_table=11
CDS complement(1897559..1898128)
/locus_tag="AUO97b_01912" /db_xref="SEED:fig|400667.82.peg.1774"
/translation="MKQITPYEVIDGQLKGARQVPSPNFNQRPAGTEIQMIVVHNISL
PPSQFGGGYIEQFFQNKLDWSVHPYFQTIEGMQVSTHLLILRTGEVLQFVNFNDRAWH
AGRSSYLAKVECNDYSIGIELEGSDDLPFEDVQYEVLTDVVTAIRQAYPEIKNHIAGH
SDIAPGRKTDPGPYFKWQHFRQLLAQKKT" /product="1,6-anhydro-N-acetylmuramyl-L-alanine amidase" /EC_number="3.5.1.28"
/transl_table=11
CDS 1898300..1899145
/locus_tag="AUO97b_01913" /db_xref="SEED:fig|400667.82.peg.1775"
/db_xref="GO:0004514"
/translation="MSIPQSLLEQSIQINIQQALQEDIGDGDITAMLTPEDEQATATI
ISREDMVLAGQPWVNALISAYDNTVQVTWLKQEGDRVAANEAFLKLAGSARSLLTVER
PALNFIQTLSAVATKTAEYVQHLEGLNTKLLDTRKTLPGLRIAQKYAVTVGGGQNHRL
GLFDAFLIKENHIMAAGGIAQAIAKAHQIAPGKPVEVEVETWDELNQALEAGADIVML
DNFSQQQMIDAVKHVAGRCKLEASGNITIENLREVATTGVDYISMGVLTKDVKAVDLS MRFNA"
/product="Quinolinate phosphoribosyltransferase [decarboxylating] (EC 2.4.2.19)" /EC_number="2.4.2.19"
/transl_table=11
CDS complement(1899142..1899309)
/locus_tag="AUO97b_01914" /db_xref="SEED:fig|400667.82.peg.1776"
/translation="MLQGFIVLLAVVTFVLMAADPRDSGAQATQSAWQKTQAADMTKS QKNTNNDADFI"
/product="hypothetical protein"
/transl_table=11
CDS complement(1899714..1901882)
/locus_tag="AUO97b_01915" /db_xref="SEED:fig|400667.82.peg.1777"
/translation="MNRREFLLNSTKTMFGTAAIASFPLSIQKALAIDAKVESGTIQD
VKHIVILTQENRSFDNYFGTLKGVRGFGDRFTIPMTEGRKVWEQYDANKKKVLPYHLD
SRLGNAQRVSGTPHSWSDGQAAWDNGRMSDWVAYKKPQSMGYYKKQEVEYQFALANAF
TICDAYHCAMHAGTNPNRKFIWTGTNGPTGAGVASVVNEFDGIGPSTEGYEWTTYPER
LLQAGVKWKVYQNMPDNFTDNPLAGFKQYRRANEQSGQPVSNDTLTCPAYDEKIDATQ
PLYKGIANTMPDGGFLGTFKADIAQGKLPQVSWLVAPATYSEHPGPSSPVQGAWYIQE
VLNALTENPQVWSQTVLLVNFDENDGFFDHVPSPSAPSKDINGVVYGKTTLTDQQVSY
EYFNHPAVATSKSQPKPDGRVYGPGVRVPMYVISPWSRGGWVNSQVFDHTSILQFLEK
RFGVQEPNISPYRRAVCGDLTTAFNFKTPNLLPVAELDGKKTKAEADAIRVAQALLPQ
VPVPSQQQFPQQEMGIRPSRALPYILHTSAKVDAAQKTVKLMFSNTGKQAAVFHVYNR
LDLTVIPRRYMVEAGKQLDDVWNTTNGQYDLWVLGPNGFHRAFKGNLSQANQTQALPE
IRVCVEECDANLYLKVRHDGNKSVKLNVKANAYLPNKTWMIETNSSEKELVWDMSEFG
GWYDFTVTLADDATFSRRFAGRIETQEDSISDPYMGYLES" /product="Phospholipase C (EC 3.1.4.3)" /EC_number="3.1.4.3"
/transl_table=11
CDS 1901923..1902060
/locus_tag="AUO97b_01916" /db_xref="SEED:fig|400667.82.peg.1778"
/translation="MQLLVGGVVGDELSELLSSLQALSMSAKDVINKIESFRIGFFQT V"
/product="hypothetical protein"
/transl_table=11
CDS complement(1902172..1902888)
/locus_tag="AUO97b_01917" /db_xref="SEED:fig|400667.82.peg.1779"
/db_xref="GO:0009022"
/translation="MRIDQRALDQLREVKITRNYTRYAEGSVLVEFGHTKVLCTASID
NSVPRFLKGQGQGWVTAEYGMLPRSTHSRCDREAARGKQTGRTQEIQRLIGRSLRAMV
DLKKLGENTITIDCDVIQADGGTRTASITGAAVALVDAMNVLLAQKKIKQDPLKGLVA
AISVGMYQDEVLLDLCYEEDSNCQTDLNVVMTQAGEFIEIQGTAEDKPFTRAQSNAML

EMAEKGIAELIKKQQEALGW"
/product="Ribonuclease PH (EC 2.7.7.56)" /EC_number="2.7.7.56"
/transl_table=11
CDS complement(1903047..1904195)
/locus_tag="AUO97b_01918" /db_xref="SEED:fig|400667.82.peg.1780"
/translation="MNMALKVSMASKSAHLTPEQIEEFGRRVEQIRQDVMQSLGEQDA
KYIYKVRNFVRYTEIASRGMLMFGGWIPPVWLLGTGLLGISKIVENMELGHNVMHGQF
DWLNEPSLNGNTYDWDTIASGDDWRETHNYVHHTYTNIVGKDHDVGYGILRVSDQQKW
EPRHLFNIPLALQLMFFFEWYVGVQNLHLEDALVYKTKSWKQVWKDAAKVRKKATRQI
LKDYVFFPVISGPMFLPVFAGNVVANIIRNLWSSAVIFNGHFTEDAETFEPDNTDTET
KAEWYLRQIRGSSNFSGTEWLHFMSGNLSHQIEHHLFPDMPANRYKEVAPKIKALCAE
YGINYNEANFMRQFWSVWVRLAKCSLPNHTTAKVMQTLEKLKAKFKFA" /product="Linoleoyl-CoA desaturase (EC 1.14.19.3)" /transl_table=11
CDS complement(1904220..1905245)
/locus_tag="AUO97b_01919" /db_xref="SEED:fig|400667.82.peg.1781"
/db_xref="GO:0005506"
/db_xref="GO:0006118"
/db_xref="GO:0009055" /db_xref="GO:0016491"
/translation="MNATLNYQPHWVREDFVDFVLQKINATWAWKRVLAEVTAVQPLH
TDMVLIKLKPNRNFNFEQVCAGQSILLTLLIDGVYQQRSYSIIEVTTQGEIALGIKVQ
GLVSRAAQLLHVGECVEISQPQGDFTLHQGQQPAILIASGSGITAIYSLLQQALKQQL
EQIHVIYFNRAEIFHAELKALAEQYPQLQYHFFNTTEQKQHLTESLLQKLVPDFAQTA
TYICGHHGMMQQANEIYAQKGAQSRLHQEYFQPLQVTGTHAAQPVIFRRAQHEFLAET
NLLSSAEQAGLRPQHGCRMGVCNKCSCTKVSGVTQNLLTGEIEDQPNRPIKLCVSQAL SPVTIDL"
/product="Flavodoxin reductases (ferredoxin-NADPH reductases) family 1"
/transl_table=11
CDS 1905419..1906057
/locus_tag="AUO97b_01920" /db_xref="SEED:fig|400667.82.peg.1782"
/translation="MSIRDERKQQSRQALLDAALHLSTSGRSFSSISLREVAREVGLV
PTAFYRHFQDMDELGKELVDQVALHLKSVLHQLGQSYLQHKSAKTQTSIELFVQAVNH
SPKQWQFMIAERWGGSETVRAAIAREIEFLIEDLTTDLTKLENFKHIQNPQDLNVLST
ILTNMSFTWAMTWLNLAKQYQGEQLKQQQTAFIENASTQVRLLFRGIANWER" /product="Unsaturated fatty acid biosythesis repressor FabR, TetR family" /transl_table=11
CDS 1906194..1906841
/locus_tag="AUO97b_01921" /db_xref="SEED:fig|400667.82.peg.1783"
/translation="MNLDRHTQFLMRKEKILSVAEKLLLENNQEITLDELVAELDIAK
GTLYKHFRSKNELLLELIIQNEKQILEISQKYNTDFKEYAPRYMLHHLLTPGKTILLH
QLEENLTMTESKLKHLFEELYEVRQQRILAIKDITESYLKSLNYDMSIRDYLSYIWSL
TYGASLLLNSTYYQRSIGSRQKLINLYINQALSLPTQTTNFDALNFQIEDENSQN"
/product="Transcriptional regulator, AcrR family" /transl_table=11
CDS complement(1906919..1907536)
/locus_tag="AUO97b_01922" /db_xref="SEED:fig|400667.82.peg.1784"
/db_xref="GO:0015035"
/translation="MKKLVLGGLSAAIMAVSGSAMAADFVAGKDYTVIANPGKVEVPG
KIEVREFFWYGCPHCFKLEPHMQTWLKQIPSDVRFVRTPAAMNKVWEQGARTYYTSEA
LGVRKRTHLPLFHAIQVNGQQIFDQASAAKFFTRYGVPEQKFNSTYNSFAVTAKVAES
NKLAQQYQLTGVPAVVVNGKYVVQGEDGKVTQVLNYLIEKERKAK" /product="Periplasmic thiol:disulfide interchange protein DsbA" /transl_table=11
CDS 1907716..1908429
/locus_tag="AUO97b_01923" /db_xref="SEED:fig|400667.82.peg.1785"
/translation="MSQLNVDLQEIAKFEALAAKWWDQHSEFRPLHQINPLRLNWIDE
RAGGLAGKKVLDVGCGGGILAESMARRGADVLGIDMGEAPLAVGRLHAQQENVQNIEY
RQIPVEELAQEQAGQYDVVTCMEMMEHVPDPASIVKACQTLVKPGGHVFFSTINRNPK
SYLFAIIGAEYVLRMLPKGTHDYHKFIRPSEMAHDIRNAGLTLKEMTGLHYNPITKRY

WLAPNVDVNYMVHTIKTGA" /product="3-demethylubiquinol 3-O-methyltransferase (EC 2.1.1.64) @ 2-polyprenyl-6-hydroxyphenyl methylase (EC 2.1.1.222)"
/EC_number="2.1.1.64"
/transl_table=11
CDS 1908429..1909127
/locus_tag="AUO97b_01924" /db_xref="SEED:fig|400667.82.peg.1786"
/translation="MKAVLFDLDGTLIDTAADFIRIIQQMCRDARRPVVEADLIRTQV
SEGARAMVKLVYPELDVTNPIFLAHRQNFLDLYGNNIVVDTDLFEGMYPLLEELEAQR
IPWGIVTNKPRGLSESLLEKLNLTERCAVLVCPEDVSKTKPDPEPMYLAAKQLNIPAN
EIIYVGDHPRDIDAGRNADMYTILAAYGYLPIEHRDDLNAWQADSIVNTVTELHELLR QKLPALQENQRMIS"
/product="Similar to phosphoglycolate phosphatase, clustered with ubiquinone biosynthesis SAM-dependent O-methyltransferase" /transl_table=11
CDS 1909193..1909939
/locus_tag="AUO97b_01925" /db_xref="SEED:fig|400667.82.peg.1787"
/translation="MKYSEYQPRPDLLKDRIILITGAGDGIGRAAALSYALHGATVVL
HGRTLNKLEVIYDEIEGLGAPQPAILPLQLSSASDRDYDFLVSTLEKQFGRLDGILHN
AGILGERVELAHYPAEVWDDVMAVNLRAPFALTQALLPLLQKSENASVVFTSSGVGRE
ARALWGAYSVSKVAIEAVSKIFAAEHTYPNIRFNCINPGATRTAMRAKAYPEEDPKTL
PTPESIMPAYLYLMGEDSLTLNGQSIDAQD"
/product="Short chain dehydrogenase family protein" /transl_table=11
CDS 1910157..1910567
/locus_tag="AUO97b_01926" /db_xref="SEED:fig|400667.82.peg.1788"
/translation="MKKLFTILALCITVLTTSMASFADPPFDRGHGPKGPKGGPRGEW
NDRGHKFDRDDNGDRVRDERRMREERGFERLKQHRWQPGYVMPQHYRGNGYKVDYKDN
NLPKPDRNQQWYKINNDYILVDTDSNSIVSIRGF"
/product="Putative transmembrane protein"
/transl_table=11 CDS 1910790..1911110
/locus_tag="AUO97b_01927" /db_xref="SEED:fig|400667.82.peg.1789"
/translation="MKKITTAIILSMSGLIATTAMAAPHDHYHHQGYETKTVKVIHKD
DHANIWRAGQVVPHQYQHPRYVIDYRSHQKLTKPGRYQKWYKVNGNYVLVNEQSHKII KVIR"
/product="hypothetical protein"
/transl_table=11
CDS 1911231..1912586
/locus_tag="AUO97b_01928" /db_xref="SEED:fig|400667.82.peg.1790"
/translation="MPTKSLEKSHNNTQHYVHWFRHSAPYINAHRDKTFVLMFGGEAV
RDPNFEHIIHDIALLHSLGIRLILVHGARPQINHNLKEKGIETPFYDNCRITTRESLS
CVMSAVGSIRLEIEALLSMGLANSPMYGARIDVVSGNFVTAKPYGIREGMDFQLTGEV
RSIDTDAIERHLDNNNIVLLGPTGYSTTGEVFNLRAEEVATKTATYLKADKLIFLGEQ
QGLPNEDGQLLRELSPHQLDYYIQQYQTTHPTLTLHLQEAKKASLSGVHRVHLISYSY
DGALIEELFTRDGVGTMITDAHYEEVRMAHIQDVGGLMNLLRPLEEEGILVYRSRERL
EQEINQFAVIERDGMILACAALYPIPTEFNETRSAEIACVAVHPSYRKSNRGSQILQF
LEEKAKQQGIRQLFVLTTRTAHWFLEHGFHQVSVDDLPNARQALYNYQRNSLVFKKLL P"
/product="N-acetylglutamate synthase (EC 2.3.1.1)" /EC_number="2.3.1.1"
/transl_table=11
CDS 1912920..1913888
/locus_tag="AUO97b_01929" /db_xref="SEED:fig|400667.82.peg.1791"
/translation="MALFNSTSWAKYSVIAASLAGVMMLGGCAKKEEHTTTTLNIGYQ
KYGILPILKARGDLEKALKDQGVQVKWVEFPAGPQLLEGLNVGSVSLGEAGEAPPIFA
QAANPNLVYVANQPAAPKAEALLVQKDSPIQSIKDLKGKRVALNKGSNVHYLLLKVLE
ANHLSLSDIQPVYLPPSDARAAFEKGAVDAWVIWDPFFAAAEHQIQARVLATGENLVS
NHQFYLADRKFAENNPKVLNTVVQTLNQTTEWVSTHQDEAAKLLEKPTALEFDVLKTS
ISRMGFGVQPISDKVAQEQQYVADAFYAQKLIPNKLTIQEAILKLK" /product="Alkanesulfonate ABC transporter substrate-binding protein SsuA" /transl_table=11
CDS 1913960..1914943

/locus_tag="AUO97b_01930" /db_xref="SEED:fig|400667.82.peg.1792"
/translation="MQHQPLFNKSQWTQLVKRFSAVAVLGLTIAAPAWAIDPTVKELR
IGFQKSSINFAIAKQQKLFEQEFPNAKITWNEFPAGPQILEALAVGSLDVGVTGDTPP
VYAQAAGKPLYYIAYEAAKPLASAILVPKNSQLKQLKDLKGKRIALQKGSSSHYLLVQ
AVRKAGLKWSDITPIWLTPADARAAFQKGAVDAWAIWDPYYASAQLEDQARVLASGKG
LSPNYTFYLAAPNFVKQYPKAVPGLIKQINQADKWVQSHQAETASAIGQSTGLKPATS
DLFIKRRPRPSSAAPLNSKVIAEQQQLADIFTQQGIIPKPISIKQAVWGAK"
/product="Alkanesulfonate ABC transporter substrate-binding protein SsuA"
/transl_table=11
CDS 1914970..1916145 /locus_tag="AUO97b_01931" /db_xref="SEED:fig|
400667.82.peg.1793" /db_xref="GO:0008726"
/translation="MKIFWFIPTHGDSRYLGTSKGARQVDHAYMKQIAVAVDNLGYEG
VLIPTGRSCEDPWITAASLIDATQHLKFLVALRPGVTTPALAARMAATFDRLSNGRVL
LNLVTGGDEAELRGDGLYEDHSTRYQTANEYVKIWREILTRSHTGEAFTFHGERLQVD
DAKLLYPPVQKPYPPLWFGGSSDVAVELAAEQVDTYLTWGEPPAAVKEKIEHVRAKAE
AKGRKLTYGIRLHVIVRETNEQAWAAANELIQYLDDATIAAAQKKFAQMDSVGQQRMA
ALHGGNRDKLEVSPNLWAGIGLVRGGAGTALVGDPETVAARIQEYADLGIDTFIFSGY
PHLEESIRFAELVFPLLPIETRAKLAQPNLTGPFGEIIANNYVPDEKKQNSSVKEPA" /product="FMNH2-dependent alkanesulfonate monooxygenase (EC 1.14.14.5)"
/EC_number="1.14.14.5"
/transl_table=11
CDS 1916142..1916939
/locus_tag="AUO97b_01932" /db_xref="SEED:fig|400667.82.peg.1794"
/translation="MSKDINIVSRGTKQVGQRLLPWIFPIVLLVVWQIASSSGLLENR
ILPAPTAVVSAFWHLLISGELWQHVKVSAGRALLGLFIGGGLGLLLGLLNGSSRFAST
LLDTTLQMIRNIPALALIPLVILWFGIDETAKLFLVAVGVFFPIYINTYHGIRSVDPQ
LIEMGKSYGLSRWQLYKEIILPGAMPSILVGLRFALGLVWVLLIVAETISAQAGIGYM
TMNAREFLQTDVVLVGILLYALLGKLADVLAQLLERYLLRWHAGYQK" /product="Alkanesulfonate ABC transporter permease protein SsuC" /transl_table=11
CDS 1916953..1917753
/locus_tag="AUO97b_01933" /db_xref="SEED:fig|400667.82.peg.1795"
/translation="MTNLNLNINQNDVELIPENHPESQEPVVGAEILIEQLYKFYGEV
KVLEDLNLHIQPGEFLAIVGRSGCGKSTLLRLIAQLEKASFGEIKFKSARHLREGITN
DDIRVMFQDPRLLPWRNILQNVQLGLPKSQHALAEELLEKVGLKEKAGQWPSQLSGGQ
RQRTALARALSHTPRILLLDEPLGALDALTRLEMQSLIERLWKEQGFTAILVTHDVSE
AVQLADRIILLDKGQIAQSFQVNLPRPRKKSITFAQLEQQVLDAVLAT" /product="Alkanesulfonate ABC transporter ATP-binding protein SsuB" /transl_table=11
CDS 1918014..1918634
/locus_tag="AUO97b_01934" /db_xref="SEED:fig|400667.82.peg.1796"
/translation="MQQMEQKKSTQKRNQLLAAALDVFSLYGFSGASLDEIAQLADMH
KSNIFYYYENKESLYVEVLTTVLQKWLAPLQTLEAELEPAEALSHYLIQKIELSRSQP
KASRLFALEIIQGAPHILPILKGPLKKLFKRKAKVIQTWQEQGKISPDIDPELLILNI
WGLTQNYADFATQMEMVTGKTLRNRSMFQRSIEHTVHMMLYGVLPR" /product="Transcriptional regulator of pyrimidine catabolism (TetR family)" /transl_table=11
CDS complement(1918669..1919235)
/locus_tag="AUO97b_01935" /db_xref="SEED:fig|400667.82.peg.1797"
/db_xref="GO:0008930"
/db_xref="GO:0008782"
/translation="MNTDTALIMALPNESKGLFEQAGIEVHYSGIGKINAAFKAFEVI
QKTGCKTLINLGTAGSSSFNRHDLVEIKTFVQRDMDVSPLGFEVGVTPLDDHLAAEIH
LQTHFADLPKGICGTGDSFETGQPKVACDVVDMEGYALAKVCHKLGVRLISVKYITDG
ADDTAHLDWEENLLLGAQKLLALYQNHF" /product="5'-methylthioadenosine nucleosidase (EC 3.2.2.16) @ S-adenosylhomocysteine nucleosidase (EC 3.2.2.9)"
/EC_number="3.2.2.16"

/EC_number="3.2.2.9"
/transl_table=11
CDS complement(1919316..1920371)
/locus_tag="AUO97b_01936" /db_xref="SEED:fig|400667.82.peg.1798"
/translation="MGTGVVSMILIQLPFAKSFLFMLATLLWQFNIVLFSVFSVLYLL
RWLLFPHEAKQIFNHPNMSLFLGAIPMGLATILNGFLSFGIGLYGEVAVHIAQALWYI
DVFLALAIAWIVPFCMFSRQNHQLHTMTAIWLLPVVACEVAASSAGMLVAHLPTDVHS
FHLLLAGYVLWGISVLPAFAILTILMLRMALHQLPEKEVAISSWLSLGPIGTGALGLL
LLGEQAQRVFVNVGFPELAHVFQALGVVGSLVLLGFGLWWLGLAILTTLRHAKTGIPF
NLGWWGLTFPFGVFILALFNLGHQIEVVFLQYVAVAFSILLLVMWSGVMKKTLAGAYS GKLFFSPCLVALQQRMQ"
/product="C4-dicarboxylate transporter/malic acid transport protein"
/transl_table=11
CDS 1920658..1921659
/locus_tag="AUO97b_01937" /db_xref="SEED:fig|400667.82.peg.1799"
/db_xref="GO:0003919"
/db_xref="GO:0008531"
/translation="MKLLRLNALSPNFQLPQTAVTIGNFDGVHLGHQAMIAQLKKIAA
AQGLKTLVMIFEPQPLEFFKGYDAPPRINSLREKVEYLAELGVDYIAVAKFDQHFRSM
SASEFADLLKDKLNAQALVLGDDFHFGKDRQGNSEFLKNYGFQVTNLHTIELEGERVS
STRIRQVLQVGNLALAAKLLGRPYSITGRVQYGDQIGRTLDFPTINVRLNRHKPCLNG
IYGVEVICETTSLTQKVLQDNPEKPGIAGYHENSLYGAGHVGTRPAIQQEHPEWRLEV
HFPDVSANLYGLFMRVTFLHYLHGELNYPSLEALKAGIDNDVQQLRQYRNDTTQFPF" /product="FMN
adenylyltransferase (EC 2.7.7.2) / Riboflavin kinase (EC 2.7.1.26)"
/EC_number="2.7.1.26"
/EC_number="2.7.7.2"
/transl_table=11
CDS 1921721..1924558
/locus_tag="AUO97b_01938" /db_xref="SEED:fig|400667.82.peg.1800"
/db_xref="GO:0004822"
/translation="MSDKQTTENAVDYKATLNLPDTQFAMKANLAVREVKWLEEWYTD
NIYQQIRASRIGKKKYILHDGPPYANGTIHLGHAVNKVLKDIIVKSRTLAGFDAPYVP
GWDCHGLPIELKVEEKVGKVGEKVDAATFRKHCREYALKQIDLQRTDFKRLGILGDWD
NPYLTMNYKQEADIVRALGLIQKNGHIQPGLKPVNWCMDCGSALAEAEVEYEDKKSDA
IDVGFGVVDLKDLSARVNVDVQDPTDIVIWTTTPWTLPANQAVALHAEIEYQLVQVTT
ERGKQNFILAKDLVASAIERYKLENPVVLADFTGSALENLTLQHPLLTDRQVPVILGE
HVIATSGTGAVHTAPGHGADDYKVGLQYNLKVENPVGGNGVYLSTAPIFAGEHIYKAN
PKIIEALGAVGRLWAHQPIKHSYPHCWRHKTPIIFRATPQWFISMDQKGLRDGALNAI
ENDIEFVPNWGKNRIESMIEGRPDWCISRQRTWGVPIPFFVHKDTNELHPRTPELIEE
VAKLIEKEGIDAWFNRDAKDFIGEDAEQYNAVRDTLDVWFDSGTTHYAVLEQRDELES
PADLYLEGSDQHRGWFQSSLLTSMAIHNRAPYKALLTHGFTVDENGRKLSKSLGNYIP
LEEIIKQLGADGLRLYVASSDYRYEIAASKEIFSRVSDSYRRIRNTLRFLLANLNGFK
PSTDALAVDNLIALDQYILQRANEVQQTIIAAYEEMNFHVVCSALTSFCINDLGGFYL
DIIKDRQYTTKADSQARRSAQTALYHIVQAFVRWMSPILSFTAQEAWRLIPEQTDKYV
FTAEWYDLPVSSKANLLSEADWQTLISVKSAVNKQIESARNAKLVGSNLSAKVELWAN
ESLQTVLNQLADELRFVLITSQVVVHPFAEQGEATEMEGLRVQVSAAEGEKCARCWHV
LPDVNTHADHPGLCGRCIVNVTGRGEVRKYA" /product="Isoleucyl-tRNA synthetase (EC
6.1.1.5)" /EC_number="6.1.1.5"
/transl_table=11
CDS 1924551..1925081
/locus_tag="AUO97b_01939" /db_xref="SEED:fig|400667.82.peg.1801"
/translation="MPNSQAKKGLFQFYPHNLIWLGLSVLAIVLDQWTKWIASTHLNY
ADPVPVLPFLNWTLLHNYGAAFSFLSDAGGWQRYFFTSLAGLVSILFVFWLLRMPKKM
VVLPVAIALILGGALGNLIDRITLGYVVDFIHVYYQNHHFPAFNIADSAITLGTILLL IDTFFLEKQRPKNSDA"
/product="Lipoprotein signal peptidase (EC 3.4.23.36)" /EC_number="3.4.23.36"

/transl_table=11
CDS 1925083..1925565
/locus_tag="AUO97b_01940" /db_xref="SEED:fig|400667.82.peg.1802"
/translation="MTEIIQPNEEIRITDGSKVDLHFSVAIENGVEIDNTRSREEPVS
LTIGDGNLLPGFEKALLGLRAGDRRTVHLPPEDAFGPWNPENIQTFDTVKFEQRPIPG
HMIEFEDKAKATLFGVVKSVNDDITEIDFNHPLAGKNITFEVEIFKVTPAGQQGIKIM "
/product="FKBP-type peptidyl-prolyl cis-trans isomerase SlpA (EC 5.2.1.8)"
/EC_number="5.2.1.8"
/transl_table=11
CDS 1925700..1926242
/locus_tag="AUO97b_01941" /db_xref="SEED:fig|400667.82.peg.1803"
/translation="MLVYIIVGSVREGRTAIKIANWLETTIPSFNLKDFQTEVIDLKE
WNLPLFSGSHPPATGIYDQPKQQEWADKIAQADAFIFISPEYNHGYSPALKNALDYVG
KEWSGKPAAFIGYGSTNGSRSISQIRQVTSSLSIVDPNAVIEIRDIFKRNKDEKFEAN
EFEVKGLQSIVEKLQKYHLS"
/product="Putative reductase"
/transl_table=11
CDS complement(1926288..1926599)
/locus_tag="AUO97b_01942" /db_xref="SEED:fig|400667.82.peg.1804"
/translation="MKINSFSPIPEYDEELSLPELIYWACLGDLEQVKQLLIDGEDPN
QTDDEGYSALQAAAENDHLEIVKLLVEKGAHVTYKSEYTALQLAEMAGNTDIVNYLKS L"
/product="FIG00350053: hypothetical protein" /transl_table=11
CDS complement(1932487..1933044)
/locus_tag="AUO97b_01943" /db_xref="SEED:fig|400667.82.peg.1806"
/translation="MKKLLMLLCLGVSSHSVWANTLVEMQTNLGNIEIELYDDKAPMS
VNNFKGYIKSGFYKETIFHRVIPGFMAQGGGMTANMQEKTTRAPIKNEAGNGIANTRG
TLAMARTSNPDSATSQFFINVADNNFLNRSPGNPGYAVFGKVVKGMDVVDRIVQAPTS
NYGMHQNVPKQPIKIVDIKIKNFQK"
/product="Peptidyl-prolyl cis-trans isomerase PpiA precursor (EC 5.2.1.8)"
/EC_number="5.2.1.8"
/transl_table=11
CDS complement(1933188..1934258) /locus_tag="AUO97b_01944"
/db_xref="SEED:fig|400667.82.peg.1807"
/translation="MTAFNQKIQTLLDKGQGPAARALDRLPRLAQESLAKILGYSYQY
PDLDSFTKCMMAVQIKQGRVGFIGSDPIESRRAFDAQMQAIRQKPTDIELVEDIRLPL
QSGTIFARHYHPAPNKKLPLIVFYHGGGFVVGGLDTHDEVCRLLAKYAKVQVLSIDYP
LAPEVSPQHLIQSCEDALAWVYQNRRQLKILKSRIAVAGDSAGGNISTVVAQRSVNKA
YAPQAQLLIYPTVDFKSRHPSFYAYNEGLVLTNTDIDYVTQYYATHHNVELDDPIISP
TYGNLKKNPPAFVITAGHDVLHDEAKIYSHKLRQNGVKMHYEEFPDQTHGFINLTPIS
RKAKRYTIEISKNFRKFWDKNS"
/product="Esterase"
/transl_table=11
CDS 1934469..1934795
/locus_tag="AUO97b_01945" /db_xref="SEED:fig|400667.82.peg.1808"
/translation="MSAKLVVTLLATSLLTVGCVAYTDDPYYRGGYGYHDHDDDRYDR
NDGRRYNEWERKRWEERKRLYEQQRKDIREQQKDRREWEKRHREWEKKRLEDRDHDHR DYRHDD"
/product="hypothetical protein"
/transl_table=11
CDS complement(1934969..1935199)
/locus_tag="AUO97b_01946" /db_xref="SEED:fig|400667.82.peg.1809"
/translation="MKKLGLISVVLAGVAFTAVGCASEAGLNASGSAQTSASPSGVQA
GVGVDAGVSGQAQPGQVDAGASGSVSGSATAQ"
/product="hypothetical protein"
/transl_table=11

CDS complement(1935591..1936457)
/locus_tag="AUO97b_01947" /db_xref="SEED:fig|400667.82.peg.1810"
/translation="MRLFKLSASALATATALTMAQSAFAQDLKIQSFLAKPEHFGVTS
TLIEGDKEVLLVNAQFSKSEALRIAANILDSGKTLKTIFVSYGDPDYYFGLDVFKQYF
PNVQIIATPETVKHIQDTQALKVKYWGPQMGANAPSQIIVPQAYTAKTLKLENESIEI
KGKKELTYLWVPSAKAVVGGIPVSSGIHLWMADTPKTKDRVEVIQSLESIKALQPKIV
VPAHMVEGAPQGLDAVNFSINYLNSYEKAAKATKNATELSKLMQKQYPTLQSVDSLEL GAKVVKGEMQWP"
/product="Metallo-beta-lactamase superfamily protein PA0057" /transl_table=11
CDS 1936565..1937458
/locus_tag="AUO97b_01948" /db_xref="SEED:fig|400667.82.peg.1811"
/db_xref="GO:0003700"
/translation="MDTLKAIQVFVSIAQHGNLTKAAEHLNYSRAMVSRYLEHLEHTF
STRLFQRNTRKISLTPAGEKALLYCENILQQQQLLQELAAPEQHNGTIRFTCGLFLFE
LGVAECIRQFKRQHPHIQFDVYLTESTMDLIDAQVDLALRITQKVADGLIARPVCQIE
SVFCAHPDYLKQHSPLLHPRQLIQHECIAHHSQHQFWTLFDNEQQPQNYPLNVTFKSN
DVNALYDMCLHAQGVAMLPTLLVEKDLKEKRLKALFKNFTAPELSLSVVYASRQHLPR
ITQEFIAFMIENLDFYLKPQN"
/product="Transcriptional regulator clustered with PA0057, LysR family"
/transl_table=11 CDS 1937589..1938176
/locus_tag="AUO97b_01949" /db_xref="SEED:fig|400667.82.peg.1812"
/db_xref="GO:0000774"
/db_xref="GO:0006457"
/db_xref="GO:0042803"
/db_xref="GO:0051087"
/translation="MFKNPFKRRSSMANEQNEQAQDIQNEQVEQSNEQTQAEGVEQAN
DVTVESLQAQITKLEENLKLEKARTANAVYEAQKSVERIQRESEKHKETVLEKFAKEL
LDSVDNLERAIQAAGDEETPVLEGVKLTLKSLLTTLEKFGVVEADTQNGFNADLHQAV
GIDPNAKANEIGTVLQKGYTLNGRLLRPAMVMVGQ"
/product="Heat shock protein GrpE"
/transl_table=11
CDS 1938330..1940270
/locus_tag="AUO97b_01950" /db_xref="SEED:fig|400667.82.peg.1813"
/translation="MAKIIGIDLGTTNSCVAVLEGDKVKVIENAEGARTTPSIIAYKD
GEILVGQSAKRQAVTNPKNTLFAIKRLIGRRYEDQAVQKDIGLVPYKIIKADNGDAWV
EVNDKKLAPQQISAEILKKMKKTAEDYLGETVTEAVITVPAYFNDAQRQATKDAGKIA
GLDVKRIINEPTAAALAFGMDKKEGDRKVAVYDLGGGTFDVSIIEIADLDGDQQIEVL
STNGDTFLGGEDFDNALIEYLVEEFKKEQNVNLKNDPLALQRLKEAAEKAKIELSSSN
ATEINLPYITADATGPKHLVINVTRAKLEGLVADLVARTIEPCKIALKDAGLSTSDIS
DVILVGGQSRMPLVQQKVQEFFGREPRKDVNPDEAVAIGAAIQGAVLSGDKNDVLLLD
VTPLTLGIETMGGVLTPIIEKNTTIPAKKSQVFSTAADNQPAVDISVYQGERKMAQQN
KLLGNFQLGDIPPAPRGVPQIEVSFDINADGILKVSAKDKSTGKEQSIQIKANSGLSD
AEIEAMIKDAEANAEEDRKFEELAKARNEADALISSSNKAVKDLGDKVTEDEKTAVNT
AVSELEAATKENDVEAIKAKTEALQNILMPITQRAYEQAQQAGGAEGFDPNAFQGGDA
GQQKADDGVVDAEFTEVKDDKK"
/product="Chaperone protein DnaK"
/transl_table=11
CDS 1940601..1941518
/locus_tag="AUO97b_01951" /db_xref="SEED:fig|400667.82.peg.1814"
/translation="MKKFLWWGISLYFSISLQVAAFAQSNLSNISFNQPLKYRIDYID
PRFELTKEQFIQIGKEAAEIWQKETGKTYFIYDSQAELTINLVLDNQQATKNERKNSI
NELLKQQEEWREKNKAILLFKQQIDQETSLLNKEKENLNYKFEQYQKDVSLFNQGNYG
QFTQSSLNKRQAELSQLSLELQTKFSQHSNKIEMLNRQIKQINQQQNLLNQSIEQFNL
STTSGSKTFHKGLFSQNQIQIYGFTSFDDLRLTLAHEFGHALGLKHTDDPKSLMYPLL
REQDIHNFKLTNSDLDLLATLYGSNDENH"

/product="Membrane-type matrix metallopeptidase-1" /transl_table=11
CDS 1941705..1942610
/locus_tag="AUO97b_01952" /db_xref="SEED:fig|400667.82.peg.1815"
/translation="MRLTSLLLGMAFAFSNFQANANLATPLHTQVQQKNVQHLTYKIA
EVDPRFGLSQEQLIQITQQAADIWKEGTGKNYFTYDPNAKLEIRLVYDDSQNRSAERQ
KIASQFKQDQQRVIDEQQQIKQLKQNLSQTQSDLENKKQILNEKLKNFDQQMMQFKEG
KLAPEYTAKSLSKTQKDLQKQTVALKKDIAAYNQQAADLNKKVTHFNQINDEFNQSLN
QFKQNAQADVFKKGIYNGKQIMIYEYSSIDDLRLTIAHELGHALGLKHSDQPGALMYS
VRKDSDKKSNILTDADRDLLSALPQ"
/product="Membrane-type matrix metallopeptidase-1" /transl_table=11
CDS 1942724..1943098
/locus_tag="AUO97b_01953" /db_xref="SEED:fig|400667.82.peg.1816"
/translation="MSYYHILIEVNDHISTIEQTRDIELFDIIELKPYLHSILLPYFN
EQEIELEDENIEYKDILHLEVKQTLLPIEHLIEEEQKQLPSDTDVTITAYEIFNDRDL
SQDVTSVIFDILEAVKLDQSIV"
/product="hypothetical protein"
/transl_table=11
CDS complement(1943148..1944440)
/locus_tag="AUO97b_01954" /db_xref="SEED:fig|400667.82.peg.1817"
/translation="MRRFSCIVGSVFLAMTQAQAELVINGSTISSNATVPVTNSDTYT
PNNNFQSCLANLRSQAIAAGVSGSTYDRYTQNLTPDYSVIDKLNYQPEFSTPIWDYLS
GLVDEERVALGKQKLVQHRDVLNRASQVYGVPAETIVAVWGVESNFGDISGKYPLLQA
LGTLSCEGRRQSYFRTEFFATMRILQRGDLTEDQLKGSWAGAFGHTQFMPSTYERLAV
DFDGDGRRDLVSSTADALASTANFLNKAGWQTGMPWGFEVQIPAGMSIDGEGRRSKKP
LSSWSARGVTRIDGSPLIQGPLSGSTPAGLMAPAGPSGPVFLVFKNFDAIYSYNAAES
YGLAIAHLSDRLRGAGPFVSSWPTDDPGTSRAERREIQQFLINRGYDIGAVDGLIGDK
TRVAIRQEQTRLGLNPTGRAGQQILRAFRQEQARKMMQ" /product="Membrane-bound lytic murein transglycosylase B" /transl_table=11
CDS complement(1945129..1946250)
/locus_tag="AUO97b_01955" /db_xref="SEED:fig|400667.82.peg.1818"
/db_xref="GO:0004638"
/translation="MDKTIGIFGGGQLGRMMAQAALPLNIQCTFFEANTDCPAGVLGQ
VFSSQDEQGLKQFIESADVFSLEFENTPVADVDVLTQTKTLHPPRIALATAQNRLSEK
ALFDELEIPVAPYRAVDSLESLKKAVAELGLPIVLKTATGGYDGKGQFVLRSEDQIDT
AWAELGPAKSLVAESFVKFSREVSIIAVRGQNGEVKTWPLAENHHHNGILSHSIVPAP
NSEALQPVAQDYITRLLNHLNYVGVLTLELFVTEQGLCANEMAPRVHNSGHWSIEGAV
CSQFENHIRAVAGLPLGSTDVVRPTVMINIIGQHPKTKDVLALNGAHLHLYNKSERAG
RKLGHITLMPVDSNELTNLCRQLAKILPEPLALTDDMTI" /product="N5-carboxyaminoimidazole ribonucleotide synthase (EC 6.3.4.18)" /EC_number="4.1.1.21"
/transl_table=11
CDS complement(1946260..1946772)
/locus_tag="AUO97b_01956" /db_xref="SEED:fig|400667.82.peg.1819"
/db_xref="GO:0004638"
/translation="MNAVATDTQPLVGIIMGSQSDWATLEHTANMLKQLGVPFEAEVV
SAHRTPDRLFEYAETARDRGIQVIIAGAGGAAHLPGMCAAKTDLPVLGVPVKSSILNG
VDSLLSIVQMPAGIAVGTLAIGPAGATNAAIMAAQILGLTRPEIAKNVADFRAAQTDK VASNNIPGQV"
/product="N5-carboxyaminoimidazole ribonucleotide mutase (EC 5.4.99.18)"
/EC_number="4.1.1.21"
/transl_table=11
CDS 1946757..1946969
/locus_tag="AUO97b_01957" /db_xref="SEED:fig|400667.82.peg.1820"
/translation="MRPRSLAFPKRKNGKNSTCTKPSMREKDEANIIAVFSLTPKPKF
MWKKHDSDGFNNDLSENPISDNFTGF"
/product="FIG00353701: hypothetical protein" /transl_table=11

CDS complement(1946956..1947303)
/locus_tag="AUO97b_01958" /db_xref="SEED:fig|400667.82.peg.1821"
/translation="MSPTVLAFVLLIISNCFMTLAWYGHLKFLHHAPFWQAILFGWTI
ALLEYSFMIPATRLLSDQGWTLGEMKITQEVVTLIVFVPFMVLLFKQPFKLDYVWAML CLFGCVYFVFRSQ"
/product="Uncharacterized membrane protein PA1577" /transl_table=11
CDS 1947560..1948933
/locus_tag="AUO97b_01959" /db_xref="SEED:fig|400667.82.peg.1822"
/translation="MHLHILGICGTFMGSLALLARDLGHKVTGSDSNVYPPMSTQLEN
AGIELMQGYDRSHLQPHPDLVIVGNAMKRGIDAVEYMLNEGLPYISGPQFLADHVLQG
KHVLGVAGTHGKTTTTTMLAWVLDQAGLNPGFLIGGVPLGFSESARLGGGKYFVVEAD
EYDSAFFDKRSKFVHYHPKTAILNNLEFDHADIFDDLAAIQKQFHHLVRTIPSEGRII
APITETHIDEVLEMGCWTPVIRTSLEANEKAALSAELISIDGSHFKVLENGNVVGEVK
WSMTGQHSVANALATIAAAQHVGVSLEKACEALSNFGGVKRRMELLGTINGIEVYDDF
AHHPTAIDTTLDGARKRLGERRLWAIIEPRSNTMRMGSHKDGLAHSARLADEVIWYQP
EGLDWDLQPVIEAATNHAQVSRSLDEIIERIVNEAGEGDAVVIMSNGGFGGLHQKLMS ALKAKAA"
/product="UDP-N-acetylmuramate:L-alanyl-gamma-D-glutamyl-meso-diaminopimelate
ligase (EC 6.3.2.-)" /transl_table=11 CDS complement(1949004..1950254)
/locus_tag="AUO97b_01960" /db_xref="SEED:fig|400667.82.peg.1823"
/translation="MRVRGVNIKVLTCWHFIRERYFMTTQEKQKKLSLRPLSPRDPEQ
PHRAATPLELLFDLIFVVAIATAGQQLHHAIIENHLWHALPSYLMVFFALWWAWMNFS
WFASAYDNDDALYRCLTFVQIVGSLVMAAGIPDVFHSQDFDIIIVGYVIMRLALVTQW
LRAAKHDPERRITAYRYAIGIVLVQIGWLVANFAHALSIPLFLLLVVVELFVPIYAEK
YSPTPWHPHHIVERYALLTIIVLGESIVGSFNAIRNALAAQSINIPAVFLMIGGLMLM
FAMWWAYFDRSEQHHHVKGVRPFVWGYGHYFVFVSVAAVGAALAAAVDVTTHHAHISD
LYMGVIVAVSVVLYTSCIWILYEFQCLSGITKWFYPITALIILCIPFICNNVGYSVFA
MGIVYTLRLFISNKFMENIEPKQV"
/product="Integral membrane protein"
/transl_table=11
CDS 1950390..1950986
/locus_tag="AUO97b_01961" /db_xref="SEED:fig|400667.82.peg.1824"
/translation="MSLETLNLSLFHVINGAQDASQFIIRLAIFFANDLLYILLFILT
FLWFYGDQDLKNRVIKSVFLTCVSLLVGYVISLFYHHARPFVMGVGTTFIEHAPTASF
PSNHMLIFSTIALSYLFAQRKIIGVILLFLSFVVAWSRIYLGVHFPLDMLGAFIVALL
VNTAGYYFWNAYGTSLTAFCIHLYQIICKPLLDRGLIK" /product="Undecaprenyl-diphosphatase
BcrC (EC 3.6.1.27), conveys bacitracin resistance" /transl_table=11
CDS 1951304..1952260
/locus_tag="AUO97b_01962" /db_xref="SEED:fig|400667.82.peg.1825"
/translation="METIGNLWLYIAFFGIVIVMLLIDFLGFKQKQGQDVSIKQAAYW
SVAWVSVAALFGGGLWLYLQQTVGVTLANQKTMEYFAGYLLEKSLAIDNVFVWLMIFA
AFAIPAALQRKILLYGVLGAIVLRTLFIFIGAWFVQEFSWVLYIFGAFLVYTGFKFLK
GQDEETNIEDIKLLKWLRNHMRITPQLEGDKFFVRQNGLLWATPLFLVLILVEASDVI
FAVDSIPAIFAVTSDPFIVLTANLMAILGLRAMFFLLAGAATKLHYLPYGLGIILLFI
GAKMLLLDVFHMPIWISLSFIVIVLAITAYLSLRHNKKQLQS" /product="Alkaline induced inner
membrane protein Alx" /transl_table=11
CDS 1952449..1952814
/locus_tag="AUO97b_01963" /db_xref="SEED:fig|400667.82.peg.1826"
/translation="MRSKKINLVVVPLLLTACSHNDGPLVQDVYNNQYDCARDWNNET
CNQENSSSGHGSSYVGSRYLGPAYYQNNREVSYHGNTLHPYSNLSIGRPIISSTAKSA
SSSSPIRGGFGRSGFSFGG"
/product="hypothetical protein"
/transl_table=11
CDS 1952836..1953996
/locus_tag="AUO97b_01964" /db_xref="SEED:fig|400667.82.peg.1827"
/translation="MKRKIFTPRPDWQVEHQNIGFDYFNLPSLDGSIYWSEGVAYEFT

LKQIEQLEDAANELHQMCLSVVGDLIQRGDYPAYFQIPEVAINHIEHSWKQNAPMLYG
RFDFAYDGRNIKMLEYNADTPTGLLEASVAQWLWIEQVSGIVNRDQFNSIHEDLIKRW
KTILPRGSHVHFAACQEAGREDWGNLEYLMDTAYQAHLQVSELSMENIGWNGQDFVDL
HNQPIQNIFKLYPWEWIWEEAFSQYIHPQTQWIEPCWKMLLSNKAILVELWKCYPNHP
LLVETHTYDPQQSLSGKWVKKPILAREGANIRVLQDGHDQGAASGSFYFDDYDKYGYV
VQKWVDTPLFSGQLPTLGLWMVGHQCAGMSIREDCYDIIGNDAHFAAHYFVE" /product="Putative
glutathionylspermidine synthase" /EC_number="6.3.1.8"
/transl_table=11
CDS 1954286..1955341
/locus_tag="AUO97b_01965" /db_xref="SEED:fig|400667.82.peg.1828"
/translation="MNAIPTLTPLAMPCNSSFEAKDWEILSTHWYPVARIQDVSNAPQ
RVTLLDINMALYKTESGEIHLVRDICPHRGVPLTKGWVDGEEIVCPYHGLRYNTEGKC
TQIPAQPELTKISDRFSLTKFPVVQRYGLIWTSIHGRDVTQANIPVLDTWDDAEHQAI
LPPFVDIGGSSGRQLEGFIDVAHFAWVHHNAFANRDNPIVPKYHTERTNYGLKTVYIS
NVSNYPHELKHLEPEGFLWKRTFEVYPPFSAVLTVDFPEGGILKILNACCPISSNKTR
LFVPLTRNFDQTGDLEKVYAFNAQIFAEDQDMVESQKPEELPLDLMMEAHFEADRSST TYRRILAEWGLSKRYTV"
/product="Hypothetical protein with Rieske (2Fe-2S) region" /transl_table=11
CDS 1955398..1956723
/locus_tag="AUO97b_01966" /db_xref="SEED:fig|400667.82.peg.1829"
/db_xref="GO:0008892"
/translation="MSLIANTTVVRGRFLDIQQTVSEPTDIPNQVRYLEDGVLISEHG
KIKWFGAWADAQQHLPAGVEVQHYPEQLIVPGFIDTHIHFPQTEMVGAYGEQLLSWLN
TYTFPTEIQFQDKTYASEIAQFFVQELLKHGTTTALVFCTVHPESVDALFEAAERVQM
RLIAGKVLMDRNAPEALCDTPETAYSDTKALIEKWHGKGRALYAITPRFAPTSTPEQL
ERAGQLKQEFPDVYVHTHLSENKDEIAWVKSLFPEQAGYLDVYQHYGLTGKRSVFAHC
VHLEDEEWQCMHDTQSAIAFCPTSNLFLGSGLFPLKKTWEKQVKVGLGTDIGAGTSFS
LLQTVNEAYKVQQLQGDKLSAYEALYHATLGGAKALDLQDQLGNFNVGKEADFVVLNL
KPTALQELRQSKSKSVEDSLFALFTLGDDRNIEATYIYGNRAYQKETAK" /product="Guanine
deaminase (EC 3.5.4.3)" /EC_number="3.5.4.3"
/transl_table=11
CDS 1956937..1957464
/locus_tag="AUO97b_01967" /db_xref="SEED:fig|400667.82.peg.1830"
/db_xref="GO:0004422"
/translation="MTVAMSIMISTEEIQAKVKELGEQINSHYANSDKELVLIGLLRG
SVIFMADLCRTITKPHELDFMTVSSYGGGTTSSRDVKILKDLDGEIRGKDVLVVEDII
DSGNTLSKVVEMLQTREPNSIQLCTLVSKPSRREIDLEVKFLGFEVEDKFIVGYGLDY DQKYRHLPFIGEIGL"
/product="Hypoxanthine-guanine phosphoribosyltransferase (EC 2.4.2.8)"
/EC_number="2.4.2.8"
/transl_table=11
CDS 1957637..1957888
/locus_tag="AUO97b_01968" /db_xref="SEED:fig|400667.82.peg.1831"
/translation="MWSLIVAIVVGFFAGLIARALHPGDDKAGFIVTTLIGIAGSLLA
TYGGRLLGLYGENSAAGFIASVIGAVIILFIYNLIARKS" /product="hypothetical protein"
/transl_table=11
CDS complement(1957941..1958639)
/locus_tag="AUO97b_01969" /db_xref="SEED:fig|400667.82.peg.1832"
/translation="MIKKLGILALFSLTTSLSFANVDTVRENIKKQYPNLKISNIQKT
EMPGLYSANLDQQIIYLGEDGQHMFVGSMIRLKDQKNLTKDLVLGQNSIDWKQLPLKD
AIKTVKGNGQHVLAVFSDPNCPYCKQLEPELDKLKDVTIYTFIYPLKPQSIVVSRQVW
CAPNQSYSWKKLIEQGVKPMVASCTNPIDRNLELGKKLGFNGTPTLIFANGFKLVGAR SAEEIQAVWKELGL"
/product="Thiol:disulfide interchange protein DsbC" /transl_table=11
CDS complement(1958735..1959730)
/locus_tag="AUO97b_01970" /db_xref="SEED:fig|400667.82.peg.1833"
/translation="MGLNFMQDYSVSRHHQHQFDEGNPLAQKRILIATILTASMMVLE

VFGGWFFNSMALLADGWHMSSHMLALGLAYFAYRAARHYSNDHRFSFGTWKIEILAGY
SSAILLMVVAIFMAFQSVQRLFNPVEIFYNEAIPIAILGLVINLICAWLLHDDGHHHH
HHHHHHHHHHHHHEHGHHHHDLNQKAAFLHVVADAVTSVFAIVALFAGKYFGWDFLDA
LLGILGAILVAKWSFGLMKETGKTLLDAEMDHPVVDEIREVIAEFPKHVEITDIHVWK
VAKGKFSCILALETDDISLNADQIRDALSIHDEIVHISVEINTLKPVYVPRETLA"
/product="Cobalt/zinc/cadmium resistance protein CzcD" /transl_table=11
CDS 1959773..1960036
/locus_tag="AUO97b_01971" /db_xref="SEED:fig|400667.82.peg.1834"
/translation="MSHIGQDKKIVNRVKRLKGQINSIENAIEQPESSCIDILQQVAA
IKGAINGLMSELMEQHLHHHVLKGAEVDQNELDEFLKVLKRYG" /product="FrmR: Negative
transcriptional regulator of formaldehyde detoxification operon"
/transl_table=11
CDS complement(1960083..1961438)
/locus_tag="AUO97b_01972" /db_xref="SEED:fig|400667.82.peg.1835"
/db_xref="GO:0005525"
/db_xref="GO:0005622"
/translation="MHSQTTIAAIATPPGRGGVGVIRLSGPKAYDIAQKLTQKNLPEA
RMAGFRKFYDADGSIMDEGIVLCFPNPHSFTGEDVVELQGHGGPVIQNALLGRLFELG
AIAAKAGEFSMRAFENGKMDLVQAEAIADLIDATSQAAARSAVRSLQGAFSTKINTVL
EKLIYLRLHVEAAIDFPEEEIDFLADGKILALLEDVQQSVHAVQTSARQGQLLREGLQ
VVIAGKPNAGKSSLLNALAGVERAIVTDIAGTTRDVLHEKISLNGLPITLTDTAGLRE
TGDIVEKEGIRRAIKEIEQADLLLLVYDLNQGDDPLKLAQEYFSEHIEPRRLMLIGNK
CDLTGQSAEISDYQGFRHITVSAKQEMGVQGLVDAITAHAGFHPEEDTFIARTRHLDA
MKRTQLYLAEAREQLVVFNAGELVAESLRLAQNALGEITGDFSADDLLGKIFGSFCIG K" /product="tRNA-5-carboxymethylaminomethyl-2-thiouridine(34) synthesis protein MnmE"
/transl_table=11
CDS complement(1961529..1963289)
/locus_tag="AUO97b_01973" /db_xref="SEED:fig|400667.82.peg.1836"
/translation="MQQWARFAILGAMFVTAYLLILAWQKDYGNAATAPQQQAAAVSS
HEVSADLPNAKTATVASDVPQANIAQSQTTDATAPVNQQLISVQTDLYHLWINPKGGD
IVRIELLSHDKSKDSNQPFVMLENDAKRTYVAQSGLIGLNGPDSSRGGRPLYAVEKTS
YTLADAKGVQDQSGHSEKVLTVPMVFKTPEGVEIIKTFTFTQGQYPIVVKHQVVNRSQ
QNWQGQMFGQLKRDNSEDPGKSSQGIFTLGTFLGGAWGTPDAHYNKLKFANFSEEKLN
TEAKGGWVAMVQHYFVSAWIPGNLKLTQANGQPYVAKLESRQSADHMNIIGFTSPVIN
VPAGTSMEVDAKLYSGPKVQSELKDLAVGLNQTVDYGWLWPIAKLLFLGLQFFHSIVG
NWGWSIILLTIMVKMILWPLSSKSYRSMAKMRVIAPEMQRMKEEFGEDRMRFSQEMMA
LYKREQVNPLSGCLPLLLQMPIFLALYWVLMESVELRHAPWLGWIQDLSAMDPWFILP
LIMGATMFAQQMLNPQPADPMQAKVFRIMPIMFTVFMLFFPAGLVLYWIVNNSITILQ
QWFINKSVEKSRISKAESAS"
/product="Inner membrane protein translocase and chaperone YidC, long form"
/transl_table=11
CDS complement(1963295..1963615)
/locus_tag="AUO97b_01974" /db_xref="SEED:fig|400667.82.peg.1837"
/translation="MVRILRWFIRLYQIAISPLLGPRCRYIPTCSQYALEALQTHGAI
KGVWLSSKRICRCHPWGGSGYDPVPPKAIRFISFHQIDSQTHHVAVPFRDRLMKQNLS NHLG"
/product="Membrane protein insertion efficiency factor YidD" /transl_table=11
CDS complement(1963626..1964018)
/locus_tag="AUO97b_01975" /db_xref="SEED:fig|400667.82.peg.1838"
/db_xref="GO:0004526"
/translation="MALYSFSTDLRICCAADYKSVFDGALFKVHQPHFLFLAKLTEQP
NSRLGIVVAKKKVRRAHERNRIKRLARESFRLHQPKFGSDIDVVVMPKVGIETITNAE
LYQQLDFAWQKLQRLAKKHSKVVPTSQN"
/product="Ribonuclease P protein component (EC 3.1.26.5)"
/EC_number="3.1.26.5"

/transl_table=11
CDS complement(1964048..1964182)
/locus_tag="AUO97b_01976" /db_xref="SEED:fig|400667.82.peg.1839"
/db_xref="GO:0003735"
/db_xref="GO:0005622"
/db_xref="GO:0005840"
/db_xref="GO:0006412"
/translation="MKRTFQPSELKRKRVHGFRARMATKAGRQVLARRRAKGRHSLTV "
/product="LSU ribosomal protein L34p"
/transl_table=11
CDS 1964853..1966250
/locus_tag="AUO97b_01977" /db_xref="SEED:fig|400667.82.peg.1840"
/translation="MLWTDCLTRLRQELSDNVFAMWIRPLVAEEVEGILRLYAPNPYW
TRYIQENHLELISILAEQLSEGRVRQVEILVDSRPGSILSSSEQPATTTAALQTAPIP
QPAKVKREPEPVANTAVSSKSSKKKLLNPQFTFSLFVEGRSNQMAAETCRKVLTQLGA
SQHNPLFLYGPTGLGKTHLMQAVGNALLQAKPNARVMYMTSESFVQDFVSSLQKGKVE
EFKKNCRSLDLLLVDDIHLLAGKEASLVEFFYTFNALLDESKQIILTSDRYPKELTEL
DPRLVSRFSWGLSVGVEPPDIETRIEILLKKAENSGVDLPRNCALFIAQQVVANVREL
EGALNKVVAISRFKGAPIDLDVVRESLKDVLAIRARTISVENIQRVVSEYFRIPLKEL
IGPKRTRIYARPRQLAMGLARELTGDSFPEIGMAFGGRDHSTVMHACEKVVSLREEDP IFDEDYKNLLRLLQS"
/product="Chromosomal replication initiator protein DnaA" /transl_table=11
CDS 1966348..1967496
/locus_tag="AUO97b_01978" /db_xref="SEED:fig|400667.82.peg.1841"
/db_xref="GO:0003887"
/translation="MRLKIAKESLLNVLSHVVGAVERRHTLNILSNVKIQTNAQALTI
TGSDLEVELVASTALSEGACLEAGETTVPARKLMEICKSLPTAALIDLQITEDQRCIL
KSGNSRFVLGTLPAEDYPLLTTENSQGTQVQVTQRELKRLFEKTAFAMAVQDVRFYLT
GTLLEIDENQLRAVTTDGHRLALCEILASSTSSQLVQAIVPRKAVGELQRLLSIEDEQ
LTLLIGRELLNVTINTPSRDKEQGDITVRFTTKLIDGKFPDYRRVIPRGGDKHVLIGH
DVFKQSLQRVAILSNEKLRGVFLNFNQDSLQLRANNPEQDEAIEDLAIQYQSAPLEMS
FNAQYLLDVLGVLDGDDVNMSMTEANQSVLVQDPAHPDQTYVVMPMRV" /product="DNA polymerase
III beta subunit (EC 2.7.7.7)" /EC_number="2.7.7.7"
/transl_table=11
CDS 1967511..1968593
/locus_tag="AUO97b_01979" /db_xref="SEED:fig|400667.82.peg.1842"
/translation="MHLTRLNIERVRNLKTVALHGLQPFNVFYGANGSGKTSILEAIH
LLATGRSFRTHIPKNYIQYEADDAIVFAQSATEKIGMQKLASGEQLMKVNGDTVATQG
QLAKLLPLQHIDPQSTDIIDHGAKPRRQLLDWLMFHVEPEFYFAWQYYSRALKQRNTL
LKTRRNLSLADLEPWNKMLSDYGEILHSQRLSIVEQWNVYFQNDLSQLLPDLEIELEY
SPGFHTEQGLMQDLLNQHQKDIERRYTEYGPHRADLRLKTPFGHADDVLSRGQKKLLI
IALKLSQIAMLHASNKETVVLLDDLTAELDLTAQQRLIERLSQLGSQVFMTTLDHASV
KKHLHDLSISYQLFSVESGQVSLAAS"
/product="DNA recombination and repair protein RecF" /transl_table=11
CDS 1968646..1971114
/locus_tag="AUO97b_01980" /db_xref="SEED:fig|400667.82.peg.1843"
/db_xref="GO:0003918"
/translation="MSSESQSASQTEQTNEKAYDSSSIKVLRGLDAVRKRPGMYIGDT
DDGTGLHHMVFEVVDNAIDEALAGHCDEIIVTIHEDESVSVSDNGRGIPTDIHPEEGV
SAAEVILTILHAGGKFDDNSYKVSGGLHGVGVSVVNALSSKLHLTIYRAGQIHEQEYH
HGDPQYPLRVIGETDNTGTTVRFWPSAETFSQTIFNVEILARRLRELSFLNAGVRIVL
RDERINLEHVYDYEGGLSEFVKYINEGKNHLNEIFHFTADADNGIAVEVALQWNDSYQ
ENVRCFTNNIPQKDGGTHLAGFRAALTRGLNQYLENENILKKEKVNVTGDDAREGLTA
IISVKVPDPKFSSQTKEKLVSSEVKPAVEQAMNKEFSAYLLENPQAAKSIAGKIIDAA
RARDAARKAREMTRRKSALDIAGLPGKLADCQEKDPALSELYLVEGDSAGGSAKQGRN

RKMQAILPLKGKILNVERARFDKMISSQEVGTLITALGCGIGREEYNPDKLRYHKIII
MTDADVDGSHIRTLLLTFFFRQMPELVERGHIYIAQPPLYKLKKGKQEQYIKDNDALE
TYLISNAIDELALHISADAPAITGEALAKVIQDYQVSQKSLQRLTLRYPASLLDALLE
VDAFKADQNHDQAYVQQWADQVREAVQRLQPSLRPEITLETFERENAQGEKSAHYWPR
VTVYIHNLPHAYLLDAGLLNSAEYARLLKNSKSWFKLIEDGAYLQKGDRRIQVANFHQ
VWQHILQDSRRGMMIQRYKGLGEMNAEQLWETTMDPENRNMLQVTIDDAIEADRMFSC
LMGDDVEPRRAFIEENALNADIDA"
/product="DNA gyrase subunit B (EC 5.99.1.3)" /EC_number="5.99.1.3"
/transl_table=11 CDS 1971152..1971544
/locus_tag="AUO97b_01981" /db_xref="SEED:fig|400667.82.peg.1844"
/db_xref="GO:0005506"
/db_xref="GO:0006118"
/db_xref="GO:0009055"
/db_xref="GO:0020037"
/db_xref="GO:0042597"
/translation="MMSQKILASVLISCAALVSHSAFAADLEADMKTLAKSTKAFAEA
KDIDNAKQQLVIMRQAALSSKLSLPHKLEGLPPENVQVKEYQAGLDQLVAEIDRVTVF
VDKGQLDQAKTEAINLVNIRNENHKKFR"
/product="Soluble cytochrome b562"
/transl_table=11
CDS complement(1971630..1972187)
/locus_tag="AUO97b_01982" /db_xref="SEED:fig|400667.82.peg.1845"
/translation="MNFIDFITNFEQFLPILIQEYGAWVYAILFLIIFSETAFVFMFF
LPGDSLLLTVGALCSVVELMHLGYMITLLTVAATLGYIVNYSIGRHFGNRIFEAKSRF
IKKEYLNKTNRYFLQHGGKTILLARFIPFARSFAPLAAGSSNMSYGKFLIYNVAGAIL
WICILLTAGYLFGHALIQVTDFVEN"
/product="DedA protein"
/transl_table=11
CDS complement(1972438..1974369)
/locus_tag="AUO97b_01983" /db_xref="SEED:fig|400667.82.peg.1846"
/db_xref="GO:0005524"
/db_xref="GO:0016887"
/translation="MIQFDQVSLRRGGRVLFQKASMQLHPGWKIGLTGVNGAGKSTLF
AALLGSLGADEGSLTRPSVWTVAHMAQEVKALDMPAIDFVLSGDEEYWDIQNKLAQPE
QLTDSDLAKLHGRFDEIHGYSAPSKAAQLMAGLGFLEHQLRLNVASFSGGWRMRLNLA
RTLMSRSDLLLLDEPTNHLDLDAILWLEDWLKAYEGTLILISHDRDFLDAITDHILHI
ENQELTLYTGNYSTFETTRSERLAQQQQAFEKQQEARAHLQKFIDRFKAKATKARQAQ
SRIKQLERMQQLAPAHVDTPFTFSFREPTKMSSPLLTLENASIGYGDKKIAEKIKLQI
TPNSRIGLLGMNGAGKSTLIKSLVGDLPLLAGERKASELLNIGYFAQHQMDALDGHAS
PMLQLARIADKQISEATLRSFLGSFGFSGERMDTPCESFSGGERARLALALIVWQRPN
VLILDEPTNHLDLDMRHALSMALQDFEGAVVLVSHERQLIASVCDELLLVHGGKCTEF
EGDLQDYAKWLREARQQQINAQTAVAQNNSSSAAPAPAKVDKEAQRKEAARRREQTRP
IRKNIEKVESQIEKLQPRLAQIEEALADPSLYEANRKEDLLKLMNEQTELKAKLEQYE
EQLLELMMELEEMEASFEN"
/product="Bis-ABC ATPase YheS"
/transl_table=11
CDS complement(1974449..1974625)
/locus_tag="AUO97b_01984" /db_xref="SEED:fig|400667.82.peg.1847"
/translation="MTFLSLVLLVSSSFINQVLNRFYFSHIFLNVAYLAQNLDIFRRI ALIGISETMQSQYL"
/product="hypothetical protein"
/transl_table=11
CDS 1974626..1975630
/locus_tag="AUO97b_01985" /db_xref="SEED:fig|400667.82.peg.1848"
/translation="MKKRLGIVSAVICYAMGTTHAGQNVCVFDLLGKSGESYKMMEEW

ALAAKSWQADITLIPFQDEALVDRRLREGKCDAAYMTSMRARNYNKFAGSIDAIGGVT
SNAIAQKAISYVLDQRNKHRMLTTQNGTNYEVVGIVQIGLAYLFVRDRNLNSIEKVRG
TKFAILQYDAAQKIMVESVGAQPIPSEITDFVKKFNSGQVEAIAAPAYAYKPLEIGKG
IGANGAMFTFPVVNVTGDLIARSDKFPANFGMKSREWFIKQLPQNFAMVKRLETGIPP
KIKLNFSAEDKLKYQKILREGRLGLTKQGVYDATMMSVLKRARCTIERTNFECTLTGE "
/product="AdeT"
/transl_table=11
CDS 1975886..1976893
/locus_tag="AUO97b_01986" /db_xref="SEED:fig|400667.82.peg.1849"
/translation="MKKIVLAMAAASVVGFSASAQAASTVCVFDLLGKAGDSYKMMEE
WALAAKGWGTEINLVPRQDEAVADNDFKAGKCDAVAMTAMRARQYNKFAGSIDSLGGV
PNNQIAQRAITYVLDARNAAKMTTNLGGKKYEVAGISPLGSAFIFVRDKNINSVEKAA
GKKFAVLGYDDAQKIMVQRVGAQAVLSDISNFAAKFNNGQVDMVGAPAYAYKPLELNK
GLGANGVMWNFPVLHVTADLVLRADKFPAGFGQKSRDWFVKQLPKSFAMINRLEAQIP
GKYKMNLSAEDKLKYQKMLRDGRMDLTKRGIYDAGMMSVLKKARCSVDKANFECSMPG E"
/product="AdeT"
/transl_table=11
CDS 1977236..1978240
/locus_tag="AUO97b_01987" /db_xref="SEED:fig|400667.82.peg.1850"
/translation="MRFSKTLLLAMGLTTISFAAEAKQTICVFDFVGKNGDVYALMKD
YQLAAKNWGADIELKVNQNEAVIAEDFKAGKCDGISVSGMRGRQFNSFTGSLDAIGAI
PDLKLAVKVMQGLASQTFSKYMVNGRYEVVGVIPVGDAYLLVNDRSINTVAKAAGKKI
AVLDYDEAQKIMVQQVGAQAVSADVTNFGAKFNNHQVDIIGAPAAAFKPLELQKGLGT
KGAIVNYPILQVTGNIIIRPDKFPAGFGQKSREWVAGQLPRAFTILGKMKADIPQKYW
MEVPAADKPGYQKLMRESRINLTQRGVYDKRMMKLLWQFRCKQDAKNFECGLQDENYK "
/product="AdeT"
/transl_table=11
CDS 1978365..1978700
/locus_tag="AUO97b_01988" /db_xref="SEED:fig|400667.82.peg.1851"
/db_xref="GO:0005554"
/translation="MNAQALVLTDNAANKVRQLRDSEGNDDLMLRVYVTGGGCSGFSY
GFNFAESVNEDDAEFVNGDVKMLVDSLSYQYLVGSVVDYVEGLEGSRFIVQNPNATTT CGCGSSFSI"
/product="Iron-sulfur cluster insertion protein ErpA" /transl_table=11
CDS complement(1978756..1979601)
/locus_tag="AUO97b_01989" /db_xref="SEED:fig|400667.82.peg.1852"
/translation="MDLTKRIHQYTRGAWAFNVIDSGPIDGPLVVLLHGFPETAHSWE
QTSELLHQHQFRTFAIHQRGYSLNARPQSRFHYALSELTEDVAVFIKGLGQPVYLIGH
DWGSVVASSVAMKYPQYIQHLTLVSVPHQAAFLKACLSSNQLLKSYYFAVFQLPILPE
LLFKKMPKVARKFLKSSGMTEQQIKIFETEFIKENRLATALNWYRGFFWEKPENPFKA
IDVPTLFIWGKHDIAVTEKSAKLNSGYFKNNYKAIFMDASHWIPYQNAPELVQCFLES IHDQA"
/product="Epoxide hydrolase-related protein" /EC_number="3.3.2.9"
/transl_table=11
CDS complement(1979729..1980856)
/locus_tag="AUO97b_01990" /db_xref="SEED:fig|400667.82.peg.1853"
/translation="MSAIYIGVMTGTSMDGVDIVAASFEPLQLHATLTVPFEPELRDE
LMALTLPDDNEIDRMGKADVALAKMIGHGINSLIEKHQLDRNQIKAIGSHGQTIRHRP
EHGFTLQIGDPNIITEITQIPVVSDFRRRDMAAGGQGAPLVPAFHQALFQHPSIHRVI
LNLGGIANVSMLPANNPDGVFGFDTGPANILMDAWCHRHTGHPYDENGDWAAYGHPIR
SLLDRLYAHEYFSKEPPKSTGREDFNIDWLDDQLIDWRNDLTYDELEDTPENIQATLL
KLTVRAIQKAIYRSNMETGEVYVCGGGAYNSYLLEQLRWRLRKHNWSVQTTDVLGLSP
TWVEATAFAWLAMRFVDGLSGNLPAVTGASDFRILGTITAV" /product="Anhydro-N-acetylmuramic
acid kinase (EC 2.7.1.170)" /transl_table=11
CDS 1980918..1982150
/locus_tag="AUO97b_01991" /db_xref="SEED:fig|400667.82.peg.1854"

/db_xref="GO:0004831"
/translation="MCLGFVMSNFLPAEEQLALIQRGTHEIISEEDLLKKLKENRPLK
IKAGFDPTAPDLHLGHTVLINKLKTFQDLGHEVTFLIGDYTAMIGDPTGKSATRPPLS
REQVEANAKTYQEQVFKILDPNKTKVRFNSEWFNQKSAADLIQLASQQTVSRMLERDD
FTKRYNNHQPIAIHEFLYPLVQGYDSIALEADVELGGTDQTFNLLMGRTLQSRYGQES
QVCITVPILEGLDGVNKMSKSLGNYIGVFDAPGAMYQKVLSMPDSLIERYFDLLSFKS
LDEIKALLDEIAAGRNPQEVKRILALELVERFHDAEAAANAHKSAGNRITEGEVPADT
PEVTISRGEFGGEIFIATILRVAGLNPNAAAAKDAVARGAVKVDWNVVDASFSVKENG
TFIIQSGKKAIARVTFTD"
/product="Tyrosyl-tRNA synthetase (EC 6.1.1.1)" /EC_number="6.1.1.1"
/transl_table=11
CDS 1988457..1989056
/locus_tag="AUO97b_01992" /db_xref="SEED:fig|400667.82.peg.1856"
/translation="MIIYGDVDSGNCYKIKLLLSLLNIHHRWIHVDILNKDTQTAEFL
SLNPNGKIPVLVLDDGRVLSESNAILGYLAEGTELIPADPYMKAKMYQWMFFEQYSHE
PFIAVARFINKYLGLPPERIEEYHKLQPKGHKALSIMNKALVEHDYLVGNKFTIADIA
LYAYTHVAEEGGFDLKLYPNIQEWCQRIQKCPKYVSMVE" /product="Glutathione S-transferase,
unnamed subgroup (EC 2.5.1.18)" /EC_number="2.5.1.18"
/transl_table=11
CDS complement(1989114..1989839)
/locus_tag="AUO97b_01993" /db_xref="SEED:fig|400667.82.peg.1857"
/translation="MRSKIFFLGLMMKILKLSFLALGMGLSGFAQADFIGVKGDVGYW
FYDGKANMSSQSPEDQDLDRKGSAQLSLAFEHPIPFIPNAKIRYVNLDTQTKSETLGQ
ANYNVDLDHSDFILYYELLDNIVSVDAGLGATVLNGDITAYTGKRVDIDKTYPIAYLS
GEVKLPFTGLSAKGEATYTNFDDAKITDALVEAKYKFADNLLIDLGLTAGYRILNIDL
DDYDNNDLKFEFKGPYVGLEAHF"
/product="hypothetical protein"
/transl_table=11
CDS complement(1990281..1990424)
/locus_tag="AUO97b_01994" /db_xref="SEED:fig|400667.82.peg.1858"
/translation="MLKDYLFLLLLLIVIPHAKKEPKNTFVFVENKMHLLSIFIPLTN KQK"
/product="hypothetical protein"
/transl_table=11
CDS 1990435..1991913
/locus_tag="AUO97b_01995" /db_xref="SEED:fig|400667.82.peg.1859"
/translation="MTDTRENWTSRSGFIIAAVGSAVGLGNIWRFPYVAYENGGGAFL
IPYLLALITAGLPLLFLDYAVGHRSTGSPPKAYRALFKGGETLGWWQVCVCIIIGLYY
ASVLTWAGSYVYFSIGQMWGSDPEGFFFNTYLQTTKATGFDLQFVGHLFWPIVGIWAL
TLIILYGGVKKGVELSNKIFMPLLFILFTILVIQSLRLPGAVQGLNAFFTPNWSAMMD
YKVWLAAYGHTFFSLSVGFGIMVTYASYLKPKTNLTGSGLIVGFANASTEILAGIGIF
AALGFMAHAAGTEVKDVVSGGIGLAFIAFPKIISSLGSGADLFGLLFFSSLFVAGISS
MVSILEVPIAAMQDKLKWGRKKAVTIIGGGSALVSIILFSSVNAIKLVDIVDHFINNI
GIIGGALLSIISVAWFKRSALKELRDHVNRISTIQLGKGWDFTLTVITSLILLTTLCM
TVFNLIKNGYDTYSMSLQGVFGWGSVIFCAVVAIVLSKMKDR" /product="Sodium-dependent
transporter, SNF family" /transl_table=11
CDS 1991926..1992069
/locus_tag="AUO97b_01996" /db_xref="SEED:fig|400667.82.peg.1860"
/translation="MNTSAIVMLIISMVFVWGGLALSILHLTKHPEELDEVLEEVKDQ HTL"
/product="hypothetical protein"
/transl_table=11 CDS complement(1992113..1993417)
/locus_tag="AUO97b_01997" /db_xref="SEED:fig|400667.82.peg.1861"
/translation="MNQVTSNSSRFNLRAQVVQTLLKVQQGQSLASILNTQLNQVAER
DRALFHELVLGTLRQWFALKSISLPLLSKPLNNETVETCLYVGLYQVLCTRIAAHAAI
SETVDATKQLGFPALSGIVNAILRRATRETEDFEQGLQQAHGLPSWLFKRLKKDWGEQ

TEALCQSLKQVAPLTLRVNQRHIGRDAYLAKLQSLDIQARACLFSEAGIVLEQSVQIT
QLPGFEQGWFSVQDEHAQLCATLLPDLNNKTVIDACAAPGGKTAHLLEKFKPAQLIAI
DQDPSRLVRVTENLNRLALDQSHTEILAADATKWNPVQPVDCIVLDAPCSATGVIRRH
PDIRLLRQSSDIAQTIELQKQILEHMWQQLKVGGTLLYITCSILKSENEQQMINFFTE
HPDAKEVKIEADWGIEQVHGRQLLPEAQSGDGFYYCKIQKIA"
/product="16S rRNA (cytosine(967)-C(5))-methyltransferase (EC 2.1.1.176)"
/EC_number="2.1.1.176"
/transl_table=11
CDS complement(1993414..1994376) /locus_tag="AUO97b_01998"
/db_xref="SEED:fig|400667.82.peg.1862" /db_xref="GO:0004479"
/translation="MKIIFAGTPEFAATALAALLKTSHEIIAVYTQPDRKAGRGQKLT
PSPVKQLALEHNIPVYQPLHFKASTEEGLAAQQELAALGADVMVVAAYGLILPQAVLD
TPKYGCLNIHGSLLPRWRGAAPIQRAIATGDDETGITIMQMAAGLDTGDMMYKTYCPI
TSEDTSATLHDKLAAQGATAICAVLESEETLQKYLAEREVQDESLTVYAHKLVKSEAR
IDWSMNAVQVDRNIRAFNPWPVAFIQLDENNALRVWNSTISSQSKVNAQAGEIIAIDK
QGVHVACGENTFICLTSVQWPGGKALNAQQIAQTQKLHVGQILP" /product="Methionyl-tRNA
formyltransferase (EC 2.1.2.9)" /EC_number="2.1.2.9"
/transl_table=11
CDS 1994725..1996554
/locus_tag="AUO97b_01999" /db_xref="SEED:fig|400667.82.peg.1863"
/db_xref="GO:0004160"
/translation="MPDYRSKTSTHGRNMAGARGLWRATGMKDEDFGKPIIAVVNSFT
QFVPGHVHLKDLGQLVAAEIQAAGGVAKEFNTIAVDDGIAMGHDGMLYSLPSRDLIAD
SVEYMVNAHCADAMVCISNCDKITPGMLMAAMRLNIPVVFVSGGPMEAGKVKFRGDEK
AIDLVDAMVVAADDSYTDEEVAEFERSACPTCGSCSGMFTANSMNCLTEALGLSLPGN
GSIVATHANRKKLFLKAGQLIVELAKRYYEQNDASILPRSIATKAAFKNAMTLDIAMG
GSTNTVLHLLAAANEAEVDFTMDDIDELSRRVPVLSKVAPAKQDVHMEDVHRAGGIMA
ILGELDRANLLDVSVPTVHEKTLKDALDKWDIIRTEDPDVYEFYRSSPGGVPTQVAFS
QNRYYSTLDGDREKGVIRNAEHAFSKDGGLAVLYGNIALDGCIVKTAGVDESILKFTG
SARVFESQDAAVEAILGNEIKAGDVVVIRYEGPRGGPGMQEMLYPTSYLKSKGLGKDC
ALVTDGRFSGGSSGLSIGHVSPEAAEGGAIGLVEDGDTIEIDIPNRTIHLNIDDATLA
HRRTVQEAKGWHPKEERKRKVSKALKVYAMHTTSAAKGAVRIL" /product="Dihydroxy-acid
dehydratase (EC 4.2.1.9)" /EC_number="4.2.1.9"
/transl_table=11 CDS 1996868..1998157
/locus_tag="AUO97b_02000" /db_xref="SEED:fig|400667.82.peg.1864"
/translation="MSLLRRWFDPIRSSWFYQKPSRQAVLPTENGLSIYLRLDDVYSY
LAVQQLSQLDEILSDELKPLKIIISHTASEPPNSMTHEEWQHYCLNDAKILANQHRFA
FDEFPEIPSPESLKQAAVILKRTPLQGQNFLHLLEDIFHMLWQQQYGKLRTLHAMAVK
HQVPQHFPERIFTDEPVRAAYFEFGGRKYHAVDDLLRLTRRLKQQKLLTGNPIFLINH
IEWREHLINDAEALNEIQALHPELDVFIALEDPMSWLLLAYIKEELADYYDIQLKVYP
LSYRGRDWFDWSLATRVSKRTEVAFTPFCRPTEESTLGMAKLFYSVPENQQLDTIFTI
LQAVWTKGKDLSFQKHFQQLQQQLGIEQLTEQDIQSVLAQNDALCQDKHQPDLPVLEL
RIDGQSYVFNSLYRVWMIESIFSNVLEEKYKTASTSN"
/product="DSBA-like thioredoxin domain protein" /transl_table=11
CDS 1998257..1998967
/locus_tag="AUO97b_02001" /db_xref="SEED:fig|400667.82.peg.1865"
/translation="MMVFAFAGMIKGMIGLGLPAVSMGLLTIAMSPFQAASLLIVPSM
VTNVWQLFAEGHVWAFIRRFWTLLAGIVVGSIWSFLPTLSQSHGQSSEILLGCMLALY
GLYGLCVKKLPHLGKHERWLSPIIGYIGGAVTVATGVIIIPIVPYLQSLHLKRDELVQ
ALGLTFTVSTICLAVFLHHNPMSGISLDYRLSFAALLAALIGMWIGKKIRYRLNEQIF
RRIFFIGLMSLGLYMILH"
/product="putative membrane protein"
/transl_table=11
CDS complement(1998976..1999866)

/locus_tag="AUO97b_02002" /db_xref="SEED:fig|400667.82.peg.1866"
/db_xref="GO:0003700"
/translation="MRFDFFDLQLVLHIVSTGSLTKGADRSAISLQAASERIKKLEQY
FETPLFIRQTTGVELTTAGHAFVEHARQLLAQKDQLEHEMQRFRQPTTEALTLWCNSS
AQSEYLPTLLPQYLVLHPEMNIDLHEAESSEIVDALTQGIASLGLVSSFFNTRHLQTK
EFASDPLVLICPVTHELAPYKELNLVDALSYGFIGLKSHHSLQQSIETQAKLLGFNIQ
YRLRLPNFGAIAEVVAKGVGIAIMPARAAQRLQSDYDFHSIQLLGAWANRKLLLATQS
FNQLPSSYQQFADFLLQHRP"
/product="Transcriptional regulator BDGL_002924, LysR family"
/transl_table=11
CDS complement(1999946..2001241)
/locus_tag="AUO97b_02003" /db_xref="SEED:fig|400667.82.peg.1867"
/translation="MSNWFPKWQPYQGDVDHRPVSTNEYLPPVQSAILGIQHAFAMFG
ATVLAPLLMGFNPNLAILMSGICTILFFLITGGRVPSYLGSSFAFIGVVAAATGHITG
SGANPNLSIALGGIVACGIFYALIGFIVMLTGTRWIEKLMPPVVTGAIVMIIGLNLAP
VTIKGVAGQPFEMWMALITVLCMGSIAVFTRGLLQRLLLLVGLILAYVIYAIATNGLG
LGKPIDFSQISQASWFGIPNFSHPTFDTKAILIIAPVALILVAENLGHIKAVGAMTGE
NLTPQLGKAFVADGLATTLSGSVGAPGMTTYGENIGVMAVTRVYSTIVFVIAGIFAIF
LGLSPKFGAVISTIPSAVLTGASIVVFGLITIAGAKIWIENKVDFSNNKNLIVASVTI
ILGAGNFELLFGNFNLGGIGTATFAAIILNWLFSLKDKT" /product="Pyrimidine permease"
/transl_table=11
CDS complement(2001542..2004226)
/locus_tag="AUO97b_02004" /db_xref="SEED:fig|400667.82.peg.1868"
/db_xref="GO:0008964"
/translation="MVQQIDAPLREDVRLLGNLLGETLKQHAGQELFNQIEQIRALAK
GARDGQAEAEKQLEQLFLELPDEELLPLTRAFSHFLNFANIAEQYHVVRSRRQAEFDP
DANSPNPLVHLFKKFKDKNISTEKLFQQICDLKIELVLTAHPTEVSRRTLIQKYDDIN
ACLSQLDQQKLTPRERQNALANLKQQISSAWQTDEIRQHRPTPVDEAKWGFATIEQTL
WNAVPKFIRELNELVQDNCQQNLPLHIAPVRFASWMGGDRDGNPNVTHQITQEVLWLS
RWQAADLYLRDIENLRWELSIQSCSEEMVQAIGSPHPEPYREYLRATRERLKATRHWL
AQRLQGLEADDSNVIKSKDELLQPLLLCYRSLIDSNLPEIANGQLLDFIYRVNCFGIE
LLKLDIRQESGRHRQAISAITEYLGLGNFESWTEQARQNFLIQELQSKRPLLPKYINE
PEGSLIGHPDVQEVFATMRTLADQPPESLGAYIISMAEYPSDVLAVLLLQKEAGIQHP
LRVVPLFETLKDLDGAATTMNTLFNMHWYKQHIQGKHEVMIGYSDSAKDAGFMSANWA
QYRAQEELTAIARKHGVQLTLFHGRGGSISRGGAPTQQALFSQPPGSISGAIRVTEQG
EMIRFKFGLEGIAMQNLEIYTAATLEATLLPPPEPKAEWRELMNRMTDHSVKVYRQTV
RENPHFVKYLRTVTPELELQMLPLGSRPAKRKVSGGIESLRAIPWVFAWTQIRLMLPA
WLGTGAAINEVIADQQKATLDEMLQQWPYFQTLIDMLEMVLSKADANIALYYESHLTE
DEDLKVLGNQLRQRLKDAVETLLTLKDESKLLSDNEVLDQSMQVRKPYLLPLHLLQAE
LMKRRRDYLAERQAEHTPVDHALMVSIAGIAAGLRNTG" /product="Phosphoenolpyruvate
carboxylase (EC 4.1.1.31)" /EC_number="4.1.1.31"
/transl_table=11
CDS complement(2004397..2005020)
/locus_tag="AUO97b_02005" /db_xref="SEED:fig|400667.82.peg.1869"
/translation="MQTVNQSGRPKDLEKRARILQAAKAIFLKSGYHGTSMNLIAQEA
GVTKLTVVYNHFQDKANLFICAITETCEETLGTKPFELDASADFYQTLYIVCSRALQII
YSPEALKLEHVLFELAAEQSPLALQFFNASHTRLQNQLVEFFQKAAQLGFIQADDPLY
QTELLLTLLLGVRHHKVLLRIIPVPNAQEIDRFIRHAIDLFLLRYRH" /product="Transcriptional
regulator, AcrR family" /transl_table=11
CDS 2005275..2006270
/locus_tag="AUO97b_02006" /db_xref="SEED:fig|400667.82.peg.1870"
/translation="MVTQPSTTLHEQKSYAGDVQARQQTALAFRVGGQVTARYVDVGD
RVRVGQVLAKLDVADAQLQLNAAKAQLENAQAAAKTASDELKRFQQLLPINAVSRSQY
DTVKNQYDAAQAALQQARSNYEVSANQTGYNQLVSNKNGVITARNIEIGQVVAAGQAA

YQLAIDGEREVVIGVPEQAVSEIKVGQAAWITLWSKPNERFAGYVREVSPAADQSRTF
TVKVALKEGQSAIQLGQSARVFFSSTQTNVMSVPLSSVSATDNQPYVWVVNANQTLRK
VPVTIGAYGRDSVPVLSGLTPNDWVVIGGVHLLRDKQKIHPIDRENRAVKIQGAK" /product="RND efflux system, membrane fusion protein" /transl_table=11 CDS 2006273..2009398
/locus_tag="AUO97b_02007" /db_xref="SEED:fig|400667.82.peg.1871"
/translation="MKFNLSEWALNNKGIVLYFMLLLGIIGAISYSKLSQSEDPPFTF
KVMVVQTYWPGATAKEVSTLVTDRIEKELMTTGQYDKIMAYSRPGESMVTFVAKDSLT
SAQIPDVWYNVRKKVNDIRHELPSGVQGPFFNDEFGDTFGNIYVLTGKDFDYALLKEY
ADRLQLQLQRVKDVGKVELIGLQDQKIWIEISNTKAVQLGIPVSAIQEALQKQNSMAS
AGFFETGTDRIQIRVSGQLQSVDDIKKMPLLVGDKTIQLGDVADVYRGFSQPAQPRMR
FMGDNGIGIAVSMRKGGDIIALGKNLETEFAQLQKTLPLGMKLQKVSDQPVAVQRSIH
EFVKVLAEAVIIVLLVSFFSLGFRTGLVVAFSIPLVLAMTFAGMNLFDVGLHKISLGA
LILALGLLVDDAIIAVEMMAIKMEQGYSRIKAAGFAWKTTAFPMLTGTLITAAGFLPI
ATAQSSTGEYTRSIFQVVTIALLVSWVAAVLFVPYLGEKLLPDFTKTGHQAPWYVRLW
ARITKKPQPQTVAISQDHHYDPYQSSFYLRFRKMVEFCVTYRKTVIATTVGIFVLSVL
MFKMVPQQFFPPSNRAEILVDLKLEEGASLTATEQAVKKVEQFLSKQKGIDNYVAYVG
TGSPRFYLPLDQQLPQASFAQFVVLASSLDDRDEIRRSLETQIKQLLPQVRTRVSLLE
NGPPVGYPLQYRVSGEDLNLVRKEAQQVARVISENPNTTNVHLDWGEPSKIISIQIDQ
DRARQMGVSSLDLANFLNASITGSAIEQYREKRELIEIRLRGDKAERVEVASLASLAV
PTANGTTVPLAQIAKIEYKFEDGLIWHRNRLPTITVRADIRTNLQPATVVGELAESMD
KLRAELPSGYLIEVGGTVEESARGQSSVNAGMPLFLAVVMTLLMIQLKSLSRATIVFL
TAPLGLIGVVLFLLLFNKPFGFVAMLGTIALSGMIMRNSLILIDQIEQDRQAGHPTWE
AIIDATVRRFRPIILTALAAVLAMIPLSRSIFFGPMAVAIMGGLIVATLLTLFFLPAL YAAWFKVKKTA"
/product="RND efflux system, inner membrane transporter" /transl_table=11
CDS 2009538..2009915
/locus_tag="AUO97b_02008" /db_xref="SEED:fig|400667.82.peg.1872"
/translation="MGQDHIEQHRRYIVISYAFMFLALFTVIFAAFAYLVARKVAVVD
DAEVWIHAHALWIMRNGILFLLMSVFAVVWFIPLFFFAWDSNLWVTASTVAGVVFSAI
AWLFLLNAWLKGLSKYLKNKAVF"
/product="hypothetical protein"
/transl_table=11
CDS 2010020..2011132
/locus_tag="AUO97b_02009" /db_xref="SEED:fig|400667.82.peg.1873"
/db_xref="GO:0006457"
/db_xref="GO:0031072"
/db_xref="GO:0051082"
/translation="MAKRDYYEVLGVSKTASDDEIKKAYRKLAMKYHPDRNPDNAEAE
EKFKEASEAYEILSDSEKRSMYDRMGHNAFEGGFGGAGGGFGGFSAEDIFSQFGDIFG
GAFGGGGRQQRQRRGSDLRYVMELTLEEAVKGVKKTITFTAPAPCDVCDGKGSKNPKD
VETCKTCHGSGQVRMQQGFFSVQQTCGTCRGQGKIIKNPCHACHGSGVADRQQTLEVT
IPAGVDNGDRVRLSGKGEAIRDGQAGDLYVEVVVREHEIFQRDGADLYMDVPVSIADA
ALGKEIEIPTLEGRVSLKIPEGTQTGKLFRLRGKGVRPVRSSMVGDLLCRIVVETPVN
LTSRQRELLKELQASFDGEDSASSPKKKSFFDRLFD"
/product="Chaperone protein DnaJ"
/transl_table=11
CDS complement(2011247..2011594)
/locus_tag="AUO97b_02010" /db_xref="SEED:fig|400667.82.peg.1874"
/translation="MHSKIIGLIAIMGIISSVAFAEPAIQPGETLESLSKARITTNVN
TAATPTAQTSDANAEVKVEDIDPIIEEKTEEALKAAVQPAPQATVEAIVAPVASSELN VSVDDIDVAHPTE"
/product="hypothetical protein"
/transl_table=11
CDS 2011779..2012600
/locus_tag="AUO97b_02011" /db_xref="SEED:fig|400667.82.peg.1875"
/translation="MSAAPRIGILGAGGRMGRTLIQAVQQAGYQLAAAVERPESSLVG

TDAGELAGIGSVGVKVSGSLADVLKDCDVIIDFTAPAATAQHLKLCREAGVAMVIGTT
GMSDEQKAELDEAATHTPVVYAANYSVGVNVSIKLLELAAKVFGDTVDIEVIEAHHRH
KVDAPSGTALMMGEAIADTLGRNLKEVAVYGREGHTGPRDRQTIGFETIRGGDIVGEH
TVMFIGEGERVEVTHKATNRMNFAAGAVRAAAWVVGREARKYDMKDVLGLNDVQV" /product="4-hydroxy-tetrahydrodipicolinate reductase (EC 1.17.1.8)" /EC_number="1.17.1.8"
/transl_table=11
CDS 2012652..2013305
/locus_tag="AUO97b_02012" /db_xref="SEED:fig|400667.82.peg.1876"
/translation="MNKKQIALTTLLCLSSLYTGAASTDKAKLSLDRNHIKVWTYQKA
DNPVFQYKAETTFDVPLERAVAVILDVDRAAQWVPYMGKVQMLSQDEKKGEFTLYMVL
DFPFPLKDRDVVVKGKMSKAANGLITIKNTAVNSNYPIQPDVVRLTRYEGDWTFQKLA
NNKVKVTTSGYADPAGAIPLSFVNMFVQQQPYQMLLKMKREVMNPIYEQPKLPEILK"
/product="Collagenase and related proteases" /transl_table=11
CDS complement(2013346..2014536)
/locus_tag="AUO97b_02013" /db_xref="SEED:fig|400667.82.peg.1877"
/translation="MNTQSNTAFSLLALAIGAFAIGTTEFSPMGLLPNIANDLGISIP
TAGMLITGYALGVMLGAPFMTLRFGGFARRNALIFLMAIFTVGNLIAAFSPNYMSLLG
ARLITSLNHGAFFGIGSVVAASIVPAHKQASAVATMFMGLTIANIGGVPLATWVGQNI
GWRMSFLAISVLGIITMLALWKALPQGMVAQKPNVKAELKVLTRTPVVLALLTTVLGA
GAMFTLYTYIAPSLTEFTHASPTFITFMLVLIGVGFSIGNHLGGRFADLSINKTLIGF
LVLLIVMMVTFPILAQSQIGAAIALVIWGAATFALVPPLQMRVMSVAHEAPGLASSVN
IGAFNLGNAVGAAAGALVLDLGWGYSAVSFAGALLAGLGLLLVLFQIKRESSPAQTLQ QCSD"
/product="Inner membrane transport protein YdhP" /transl_table=11
CDS complement(2014541..2015344)
/locus_tag="AUO97b_02014" /db_xref="SEED:fig|400667.82.peg.1878"
/translation="MNIPRFGLGTFRLKEQAVIDSVKNALDVGYRAVDTAQIYENEAA
IGQAIAESGVSRQDLFLTTKIWVDNFAQEKFIPSLKESLQKLRTDHVDLTLIHWPAPD
LGVSIPEIMQLLLEAKQQGLTKQIGISNFNIALTQQAIDTIGVEHIATNQIELSPYLQ
NHKLVNFLQEKNIDVTSYMTLAYGKVLQDPVLAEIAAAHKATTAQIALAWALQRGFAV
IPSSTKRENLISNLKAQDIELTAAEMQMIAELDRNSREVSPEPWAPVWD" /product="Methylglyoxal reductase, dihydroxyacetone producing @ 2,5-didehydrogluconate reductase (2-dehydro-L-gulonate-forming) (EC 1.1.1.346)" /EC_number="1.1.1.274"
/transl_table=11
CDS 2015465..2016367
/locus_tag="AUO97b_02015" /db_xref="SEED:fig|400667.82.peg.1879"
/translation="MKSTIEELVAFITIVDTGSFVAAAEHLKQTPSGVSRSLTRLEAK
LDVTLLERTTRKLKLTQEGQQFLIKARKILNELNAAEEELQKSDQGTAGLIRVDSATP
FVLHVIAPLMHKFRECYPDIEIELNSNDQVIDLLQHKTDVAFRFGELNDSSLHAKLVC
KSRLYIVASPDYLALKGTPTQPEELEQHDLIGFTKPTYINSWPIKVGDEYFFAQSKIK
ASSGETVRQLTIRGHGIARLSEFEIWKDMEEGRLIALFEDKIEHQYQSIHAVYYQQEH
LPKRIRLFIEFLAEQLKDGFKNAL"
/product="Transcriptional regulator, LysR family" /transl_table=11
CDS complement(2016351..2016725)
/locus_tag="AUO97b_02016" /db_xref="SEED:fig|400667.82.peg.1880"
/translation="MKLKVILLFTIVLAGCQPQPKNEQHRHTVCQSLIEGYLKMTNQQ
DYKMEQRTDDETSAISHYQYKRNSSNEVVMVNSVYSKLYFSCQEQQKSYFLSQHSAQG
QTTPILEVHIPTDSYTTFRERF"
/product="FIG00349978: hypothetical protein" /transl_table=11
CDS 2016816..2018240
/locus_tag="AUO97b_02017" /db_xref="SEED:fig|400667.82.peg.1881"
/db_xref="GO:0004069"
/translation="MTFQYQVLANQLAHRIYQDELKPHQKLISLRDFARQQGISLSTA
KSCYELLEARGLIYVKPKSGYFVVARTPSSPIPDSPDFLSLPRHVSNLELHNQIQEAA
LQSHLVPLGSIQLTPHFIPVEGLRRSIQRALKNCQPQDFLYCNKQGHEQLRKALSDHW

REDGIYIAPEDIFITNGCMPALSLVIQKLTEVGDSILIPTPTYNGHLQLLASLKRQIV
EIPADHRGIDLERLESLMQQGLAKVCLMTANYQNPLGYCFSNAEKQKIAELAAEYQCF
IIEDDIFGECGYSSERPLPIRYWDREGYVVWCGSVSKSLSSAYRVGWFCLTTKLEHLK
LELLVSNIGVNTPLQLGLADFIYSRGYREHLEQLRPNLMRQVEEYRSCILRAFEGIPI
ALSQPEGGYALWIQLPKSVDSLALYYTAQAQGITVVPGYVFGEDERYRHFIRLNAGHE
LTADVRQAIMSLADWSRQQMQTVS"
/product="DNA-binding transcriptional regulator, MocR family /
aminotransferase domain" /EC_number="2.6.1.1"
/transl_table=11
CDS complement(2018243..2019271)
/locus_tag="AUO97b_02018" /db_xref="SEED:fig|400667.82.peg.1882"
/db_xref="GO:0004022"
/translation="MSNNTIQAYAAMQAGEKLVPYKFDAGELQPHQVEVKVEYCGLCH
SDISVLNNEWHSTVYPVVAGHEIIGRIVALGSEAKGLQIGQRVGIGWTAESCQACDEC
IGGQQVLCTGENIATIVGHAGGFADKVRAGWQWAIPLPEDLDPESAGPLLCGGITVFD
PLLKHKIQATHHVGVIGIGGLGHIAIKLLKAWGCEITAFSSNPDKTEELKAMGADHVV
NSRDTEAVKAQKGKFDLLLSTVNVTLNWRAFISTLAPNGSLHFLGLTLEPVPVSVGSL
IDGAKSVTGSPTGSPAALRQLLKFAARKKIAPQIELFPMSQLNEAIERLHSGQARYRI VLKADFAE"
/product="Cinnamyl alcohol dehydrogenase/reductase (EC 1.1.1.195) @ Alcohol
dehydrogenase (EC 1.1.1.1)" /EC_number="1.1.1.1"
/transl_table=11
CDS complement(2019310..2019870)
/locus_tag="AUO97b_02019" /db_xref="SEED:fig|400667.82.peg.1883"
/db_xref="GO:0008725"
/translation="MKTQRCGWCSDDPLYIAYHDEEWGKPEHDEARLFEMLCLEGQQA
GLSWITVLKKRESYRQHFFNHPIADIAVFTDDFLETKLSDAGLIRHLGKLKAIRDNAI
AWQALKAQVGDVSKWLWQFVDATPQHNDVADYRKAPAQTELSLKLSKTLKKNGFKFVG
PTTCYAFMQAVGMVNDHENDCQFKAS"
/product="DNA-3-methyladenine glycosylase (EC 3.2.2.20)"
/EC_number="3.2.2.20"
/transl_table=11
CDS complement(2019901..2020146)
/locus_tag="AUO97b_02020" /db_xref="SEED:fig|400667.82.peg.1884"
/translation="MSEALINRLVEFAESGNQQKIILNGNSYQGWIMEISDDALLIST
GFSDKVGKDFWLKFEDLTQAELYYWDTRPNEWVPFKL" /product="hypothetical protein"
/transl_table=11
CDS complement(2020156..2020692)
/locus_tag="AUO97b_02021" /db_xref="SEED:fig|400667.82.peg.1885"
/translation="MRRLVSHLSLGFLIILLAACASAPKKPAPLSPAQVSKLKSMQLP
SRLPIPVKGVDRDELKDTWGAARSQGRSHEGIDILAPRGTKVYSVTEGLIADLRNNNL
GGKVIWILGPAGTWHYYAHLDGHKRGLNVGDYVKKGDLIGYVGNTGNARYTSPHLHYG
LYLDGKGRGAVNPYSYLR"
/product="Peptidase, M23/M37 family"
/transl_table=11
CDS complement(2020740..2021774)
/locus_tag="AUO97b_02022" /db_xref="SEED:fig|400667.82.peg.1886"
/db_xref="GO:0003677"
/db_xref="GO:0005622"
/db_xref="GO:0006284"
/translation="MPEFSFSDALLNWFDQHGRHDLPWQVADDPYKVWVSEIMLQQTQ
VKTVLQYFDRFMERFPTVEALGYATWDEVAPYWAGLGYYARARNLHKAAGLVAQQGKF
PETLEEWIALPGIGRSTAGALMSLGLRQYGVIMDGNVKRVLARFFAIEDDLSKPQHER
EMWKLAEELCPTQRNHDYTQAIMDLGATICTPKKPLCLYCPMQAHCQAYQQGLEQELP
FKKPKKTPPVKTADVLIIQCEDEWFWQQRQAHGLWGGLFCLPILENEHERLKLSQQFK

LQPQPQTFQISHSFTHFTWLLNAHVFHVEPDQKEHLAIELEGQWLSPEQAIAKGVPTA MKKLISTSRS"
/product="A/G-specific adenine glycosylase (EC 3.2.2.-)" /transl_table=11
CDS complement(2021915..2022277)
/locus_tag="AUO97b_02023" /db_xref="SEED:fig|400667.82.peg.1887"
/translation="MTDKKCPYCDFDEYDIIDKNEFAVILPDPNPLSKGHCVVTPLRH
VSSFFDITSKEHQGLLTLLEIARHETQLRHQPDGFHIGFNDGEVFGQTSDHFHIHVIP
YYKNQPLKLDQRWGVQKD"
/product="HIT family hydrolase"
/transl_table=11
CDS complement(2022463..2023200)
/locus_tag="AUO97b_02024" /db_xref="SEED:fig|400667.82.peg.1888"
/translation="MSTAIKTREIQYTAPDGSHLIGYFAAPESETPVPGVIIGPEWWG
RNEYTEQRARELAEHGYAALAIDMYGDKKVTTTAAQAYEWMMQTFEDLDTVTDRANAG
LQTLAAQPEVNSEKLAAVGFCYGGKVVLDLARSGAPLKATATFHGTLAPKAPAQKGNI
QGEVLVLHGELDSMVTLEDVANFEKEMQAAEVKHEVVVLKDAKHGFSNPLADERAKAN
GVDLGYNSEAEKQGLAKMYALLERTLK"
/product="Dienelactone hydrolase family protein" /transl_table=11
CDS complement(2023372..2023530)
/locus_tag="AUO97b_02025" /db_xref="SEED:fig|400667.82.peg.1889"
/translation="MFRWAIIFAVIALIASLLGFGGVAGLSKDFAVILLVIAVILAVI GFISRGRT"
/product="UPF0391 membrane protein YtjA"
/transl_table=11
CDS 2023725..2024183
/locus_tag="AUO97b_02026" /db_xref="SEED:fig|400667.82.peg.1890"
/translation="MSTETSLRPLFWKNYTLEQLTQAEWEALCDGCGLCCLVKLEDED
THEVAYTKVACKLLDCNTGRCSDYPNRQQQVPDCLQLTPETLKTIHWLPSSCAYKRLN
EGKNLPSWHYLNTGSRQSVVKAKKSVAGRCIPETDVHEDDIEDYVVRWVR" /product="UPF0260 protein
YcgN"
/transl_table=11
CDS 2024296..2025465
/locus_tag="AUO97b_02027" /db_xref="SEED:fig|400667.82.peg.1891"
/translation="MRRLAAPLLCSSFFLLMACGSNNTNSKNPEQNTTAKTEQTSAAK
QTYQVEAVAHFNEPWAITSLPDQRLLVTERQGKLKIFNPQNKQVLDVQGVPAVNYGGQ
GGLGDVALHPDFAKNHWIYLSYAAKGQGGSGAVISRAKLDLSNPNQPKITDIKQIWQQ
VPKVSGQGHYGHRMLFGADGKLWVSSGERQKFDPAQNMKSNLGKILRLNDDGSAALGN
PFYEQGGVTAEIWSLGHRNPLGMAFDRKGQLWVVEMGPKGGDELNIITKGENYGYPIV
SNGDHYSGQPIPDHHTRPEFKAPEIDWTPVISPSSLIIYRGQQFPAWQNKALIGGLSS
EAIVVVDLEHKPVKEVQRLEMKQRIRGLHEAQDGSIWVIEDGPNARLLKLSKKPS" /product="PQQ-
dependent oxidoreductase, gdhB family" /transl_table=11
CDS 2025571..2026836 /locus_tag="AUO97b_02028" /db_xref="SEED:fig|
400667.82.peg.1892" /db_xref="GO:0003887"
/translation="MSDTHIPLPERLRPRDLSEIIGQDHLLGEHAPLRQMIDQGHLPS
IIFWGPPGVGKTTIALLLAQAIDRPFVSLSALNTGVKELREVIAESGDLLTPVVFIDE
IHRFNKSQQDALLGAVEKGKITLIGATTENPSFEVNSALLSRCQVYTLNSLDSEAIQT
LLNNALQNDKFLKERYIHIEEYDALLQFAAGDARKALNLLDLIASTFEPEVENTINNA
VVVKVAQQNIARYDKSGEQHYDLVSAFIKSIRGSDPDAALYWMARMLKGGEDPVFIAR
RMLIAASEDIGNSNPNALLLAGECFRSVQAVGMPEARIILGQTAVYLATSAKSNSTYL
AINKALELAEKTANLPVPLHLRNAPTKLMKQQGYGINYLYPHDYPEHFVLQDYLPPEL
KGTKLYESARNKREVEGERLQQRRWQQEQ" /product="Replication-associated recombination
protein RarA" /EC_number="2.7.7.7"
/transl_table=11
CDS complement(2026852..2027691)
/locus_tag="AUO97b_02029" /db_xref="SEED:fig|400667.82.peg.1893"
/translation="MDHELEEEKVDQTLSWREKMAVEEHEKLSPRLVYEIIRRDGAEE

LDRPTAALIFSGIAAGLVISFSFVFKAIIASYIPTDAIWTDLITNFGYTIGFLIAILG
HMQLFTENTITTVVPLFKPFTLDKLRAVGRLWGIVFLCNIIGTALASLFFLTTDLFTP
NIDKALDELAHHVGSFSAIQNLLKGVMSGLLIAALVWMLPSVSNKFLVIFFMTYLIGL
GDFTHVVVGSTEMSYLVWQGEASLGEYLFNFLIPTTIGNIIGGTGVFTLLIYGQVTEE LEQ"
/product="Putative transport"
/transl_table=11
CDS complement(2027977..2028180)
/locus_tag="AUO97b_02030" /db_xref="SEED:fig|400667.82.peg.1894"
/translation="MELFKPEKRLMNHPIHFGENPLVILSNFSHSALKQGWSQAEVET
VISEASQGDYMKLIRTLRAYTLF"
/product="FIG00350259: hypothetical protein" /transl_table=11
CDS complement(2028373..2029092) /locus_tag="AUO97b_02031"
/db_xref="SEED:fig|400667.82.peg.1895" /db_xref="GO:0004639"
/translation="MLKQTLLYTGKAKSVYETDNADHLILVFRDDASAFNGEKIEQLD
RKGKVNNRFNAFIMEKLAEAGIETHFEKLLSPTEVLVKKLQMIPVECVIRNYAAGSLC
RRLGVEEGKELTPPTFELFYKDDGLGDPMVNESQAIALGWATAEQLEQMKVLTYKVND
VLKALFAEGNMILVDFKLEFGVFHDRIVLGDEFSPDGCRLWDKDTKKKLDKDRFRQGL
GGVVEAYEEVAARLGVDLSDI" /product="Phosphoribosylaminoimidazole-
succinocarboxamide synthase (EC 6.3.2.6)" /EC_number="6.3.2.6"
/transl_table=11
CDS complement(2029129..2029734)
/locus_tag="AUO97b_02032" /db_xref="SEED:fig|400667.82.peg.1896"
/translation="MMQLRLGLVLAVSALSLAGCGRFALNNHSLDYKNAKQLAPLEYP
ADATVRPATPLYPAPTVDQLAIDHAPKFENKRGNRFALPRPEPLQTDTTADASAQTGS
ALGRPQLVTDGNKNPLLKIDGSTAEIWQYTKATLSTLNYNVIAQGNNQATIKVNDNTY
VLKLTGVGSSHSLALFNPDNTFASPDVAAEVLNQIYQNWPA" /product="hypothetical protein"
/transl_table=11
CDS complement(2029747..2030640)
/locus_tag="AUO97b_02033" /db_xref="SEED:fig|400667.82.peg.1897"
/translation="MTQQAQTIQGSIVAIVTPMLKDGGVDWKSLEKLVEWHIEQGTNS
IVAVGTTGEASTLSMEEHTQVIKEIIRVANKRIPIIAGTGANSTREAIELTKAAKDLG
ADAALLVTPYYNKPTQEGLYQHYKAIAEAVELPLILYNVPGRTGVDLSNDTAVRLAEI
PNIVGIKDATGDVPRGKALIDALNGKMAVYSGDDETAWELMLLGADGNISVTANIAPK
AMSEVCAVAIAKDEQQAKTLNNKIANLHNILFCESNPIPVKWALHEMGLIDTGIRLPL
TPLAEQYREPLRNALKDAGII" /product="4-hydroxy-tetrahydrodipicolinate synthase
(EC 4.3.3.7)" /EC_number="4.3.3.7"
/transl_table=11 CDS 2030970..2031614
/locus_tag="AUO97b_02034" /db_xref="SEED:fig|400667.82.peg.1898"
/db_xref="GO:0008936"
/translation="MKMNKQPQNSALVVVDVQNGFTPGGNLAVADADTIIPTINQLAG
CFENVVLTQDWHPDNHISFAANHPGKQPFETIELDYGSQVLWPKHCIQGTHDAEFHPD
LNIPTAQLIIRKGFHAHIDSYSAFMEADHTTMTGLTGYLKERGIDTVYVVGIATDFCV
AWTALDAVKQGFKTLVIEDACKGIDLNGSLEQAWQTMQQQGVVRIQSTDLLNEC"
/product="Nicotinamidase (EC 3.5.1.19)" /EC_number="3.5.1.19"
/transl_table=11
CDS 2031669..2032607
/locus_tag="AUO97b_02035" /db_xref="SEED:fig|400667.82.peg.1899"
/translation="MSALLRFTQFVQKTFALWVIIFAALALWQPEFFVWLKAYIPWIL
GIIMLGMGMTMTVDDFKGVLQSPKAVLIGVVAQFVVMPGLAFILCKLFNLPPEIAVGV
ILVGCCPGGTASNVITYMAKGNVALSVACTSVSTLLAPVLTPAIFYLLASQWLKIDAA
SMFISILQVVLLPIVIGLILRTWLKRQVESYIQVMPLVSVIAIVAIVAAIIGGSKAAI
LQSGLLILAVVILHNGLGYLLGFAAARFFKLPYADSKAIAVEVGMQNSGLGVALAAVH
FAASPITAVPSAIFSLWHNISGPALATYWASKHKQE" /product="Uncharacterized sodium-
dependent transporter YocS" /transl_table=11

CDS 2032786..2034129
/locus_tag="AUO97b_02036" /db_xref="SEED:fig|400667.82.peg.1900"
/translation="MNQIFKLFIPMLSSNLIVVLSGLIDIAYVGHFSNDHVAALSVTL
SLYTVVYVIGMGILQGVMLKLSEAYGCHDIPRIRQIFIQGVWLMLGSALVSIALLYLF
RDLPKLFGASEHVVLITKNCIWALVFILPAHLLMRLFTALSQVTENAYKVLLSDSLFF
ILKIILSYLLIYGVALFSIPPLGAIGALLATIIVRWFMLTVYYLFFLEKKYIFIEHPE
RSVEDSWQSLRMILKIGIPTAIMALMDVIAFCSVAILILPFGSVVSAAHQIVASTGGF
MFIFPSSIASAYSILVSRTMGSGNMKEADKIARQSIWVIMAVSGSLSLCLWVERGLIV
TFYSDDLAVQILAASLLLIVCMSHFLDGLMTLLTSLLRCWDVSILPMLIFLVVVLCFG
LGGGWWMAYKGLHWGSHSISAMGIYGFWSMAAISYLVACVLSAFCFRFRKQLRGF" /product="Multidrug resistance protein"
/transl_table=11
CDS complement(2034150..2034620)
/locus_tag="AUO97b_02037" /db_xref="SEED:fig|400667.82.peg.1901"
/db_xref="GO:0004731"
/translation="MLLISACLIGEPVRYDGRSCSHTTLKQLFLNKKAHALCPELLGG
FTTPRLPAEIVGGTGQDVLDGKAKILDSSGLDVTELYLKGAYRTLDIARQIQATCVVL
KENSPSCGSQKIYNGTFQGEKITGVGITTALLQRHGFEVISENEIEEWLTAHPL"
/product="Uncharacterized protein YbbK" /EC_number="2.4.2.1"
/transl_table=11
CDS 2035142..2036197
/locus_tag="AUO97b_02038" /db_xref="SEED:fig|400667.82.peg.1902"
/db_xref="GO:0003868"
/translation="MDILENPLELCGFAFIEFVSKENELDPIFETIGFSKVAKHKSKK
AYLWRQGNINIIILNYQPESYASFFFNEHGPSACAMGFKTRDAAKAFKKAVELGAEPMY
SKVGPMELNIPAIKGIGGMPIFLVDRDIYENDFVFFDDAQRNPEGAGLKEIDHLTHNV
YKGRMEYWANFYEKIFNFQEIRYFDIKGEYTGLTSKALTAPDGMIRIPLNEDSDKGNG
QIAEFLADFNGEGIQHIAFICDDLISTWDKLKGLGLKFMTPPPNTYYEMLEERLPEHG
EPTEELKQRGILLDGSTKDGQKKLLLQIFSENMIGPVFFEFIQRKDDDGFGEGNFKAL FESIERDQIRRGVLEAK"
/product="4-hydroxyphenylpyruvate dioxygenase (EC 1.13.11.27)"
/EC_number="1.13.11.27"
/transl_table=11
CDS complement(2036264..2037043)
/locus_tag="AUO97b_02039" /db_xref="SEED:fig|400667.82.peg.1903"
/db_xref="GO:0003677"
/translation="MIEEKISGGVQSLEVGLSVLNALLEHNQPIILKDLSSKLEMHPA
KVHRYLVSLIRMDYAKQLEDGQYALGDQAWRLGLNCIQHSDTLQLVQHLIYDLQSKIG
CGIQISKWSPKGPVVVQSIESNHPISIVTKVGSIMPLVNSAAGRLFASYLPEHFVRPL
LETEWQKHQELGLNIAPHNWEEFLELKETIRQQQMSAAKGDLLTGINAVGLPVFNSNQ
GIEFCIVALDSEMFLPVDPQSKIIETLKNEINSINQYIKSR" /product="Transcriptional regulator, IclR family" /transl_table=11
CDS complement(2037342..2037890)
/locus_tag="AUO97b_02040" /db_xref="SEED:fig|400667.82.peg.1904"
/translation="MTFAIKKIHHVAYRCKDAKETVEWYKKMLNMDFILAFAEDHVPS
TKAFDPYMHLFLDAGQGNVLAFFELPTQPEMGRDENTPQWVQHIAFEVEDLNALLAAK
KHLEDNGVKVLGITNHGIFHSIYFFDPNGHRLELTYNDAHAEEKIAKITEEMKTEMLE
EWSKTKRAPHHTHFLHEEELGA"
/product="Glyoxalase family protein" /transl_table=11
CDS complement(2037908..2038444)
/locus_tag="AUO97b_02041" /db_xref="SEED:fig|400667.82.peg.1905"
/db_xref="GO:0016034"
/translation="MVKNEQQSEDYLKLNRSALVPTLVDGDLTLSQSLSILEYLDEQY
PETKLLPNDVKERAKIRAFAQAIACDIHPLNNLRVLKYLKNDLNVSDEQKNYWYQHWI
LEGFQTLEQQLQDSNGQFCFGQQATIADCCLIPQVYNAKRFKIDLSAFPKIESIYQHC

LSIPAFYNAAPEQQPDWE"
/product="Maleylacetoacetate isomerase (EC 5.2.1.2) @ Glutathione S-transferase, zeta (EC 2.5.1.18)" /EC_number="5.2.1.2"
/EC_number="2.5.1.18"
/transl_table=11
CDS complement(2038527..2039834)
/locus_tag="AUO97b_02042" /db_xref="SEED:fig|400667.82.peg.1906"
/db_xref="GO:0004334"
/translation="MEQKLNSFIEVSPQSDFTIHNLPYGIFSRTAEGERQVGVAIGDW
VIDLAALEKHGLLKLSDQDTYFNQPTLNKFIESGKANWSKVRKTLQSLLSVDNLTLQE
NEALRQEVLVKQDSVTLHLPIQVPGYTDFYSSKEHATNVGCMFRDPKNALLPNWSELP
VGYNGRASSVVVSGTHVVRPSGQIKLPNEERPVFSATRKLDFELETAFIVGKPTQLGQ
PIAIEDAWDHIFGMVLLNDWSARDIQQWEYVPLGPFNAKTFASSISPWVVTLEALEPF
KVEGPKQEPKPLAYLQENIANTYDINLSVEIQSPKSTQPDVICRTNFKYMYWSMAQQL
THHTIGGCNVQVGDLMGSGTISGSTPDSYGSLLELTWNTTKPLTLANGETRGFLQDGD
TLIMKGHCEKNGIRIGFGEVRNTVLPALTFDFAETSEPNYEAV" /product="Fumarylacetoacetase (EC 3.7.1.2)" /EC_number="3.7.1.2"
/transl_table=11
CDS 2040193..2041581
/locus_tag="AUO97b_02043" /db_xref="SEED:fig|400667.82.peg.1907"
/translation="MSTQEQGNLKHGLSNRHIQLIALGGAIGTGLFLGISQSIKLAGP
SVILGYAIAGFIAFLMMRQLGEMVVEEPVSGSFSHFAYKYWGPFAGFMSGWNYWVLNV
LVCMAELSAIGLYIQYWWPQIPTWASALTFFILINGINLLHVKFFGEMEFWFSIVKIL
AILAMIGFGSYLLATGTAGPQASISNLWALGGFFPFGIEGLVMAMAVIIFAFGGIELF
GITAAEARDPDKTLPKAVNQIIYRILIFYIATLFVLFSLFPWNQMAEGGSPFVMVFAS
LDSQGVATLLNFVILTAAVSVYNGTSYCSSRMLLGLAQQGNAPKALARINKRGIPTNA
VLVSAFVTVLCVLLNYIFPEKAFGLLMMLVVAAIVINWVVISWTHLKFRKFMQRQGQT
TKFPSFMYPFSNYLCMAFMLGILVVMSLTPDMRVAVMMIPGWILCLMVAYQFKRRKIK AEQPVHALEADM"
/product="Aromatic amino acid transport protein AroP" /transl_table=11
CDS 2041745..2042428
/locus_tag="AUO97b_02044" /db_xref="SEED:fig|400667.82.peg.1908"
/translation="MKRYLGLLVLSLAACSTQTPQPTITHRSPSLPQPSKTVVKIKQQ
PEDYVVWIATADHRQSVQAYKQFLKQKGLADLVPDHELLRSARDWQKCGVEPYAVPPR
EIWSNIVPTLNILKALVDEGVINDFEVTSVYRALALNRCAGGADASRHVFNAALDFRI
GPEQPSDLDQFNIQQTKTKLCQFWATKGQAFNMGLGVYASGQIHIDSQGFRAWGPDHH YRTSICQGL"
/product="hypothetical protein; putative signal peptide" /transl_table=11
CDS 2042571..2043836
/locus_tag="AUO97b_02045" /db_xref="SEED:fig|400667.82.peg.1909"
/translation="MISLNIIQRDQAMHTAIANSFAKLPVTVLSGFLGAGKTTLLNHI
LNNREGRRIAVIVNDMSEVNIDAALVREGGAELSRTDEKLVEMSNGCICCTLREDLLV
EVRRLADEGRFDQLVIESTGISEPLPVAETFTFEDEDGNSLNEFARLDTMVTVVDAFN
FLKDYSSVDSLQQRGESLGEQDERTVVDLLIDQIEFCDVIVLNKIDLIDQAQQDQLIA
ILRTLNPRARIEIAQFGKVALDKILNTQLFDFDEAVQAPGWLKELRGEHTPETEEYGI
SSFVYRARRPFHPERFYDLVQAEWPGVVRSKGFFWLAAEPTLAYSWSQAGAMARHGLA
GYWWAAVAEEDWPSDLASIEQIKKNWDARTGDARQELVLIGMQMDEQALIQRLDSCLL
SDEEMALGPQVWQSWSNPFKQDDETMLIA"
/product="Zinc-binding GTPase YciC"
/transl_table=11
CDS complement(2043870..2044982)
/locus_tag="AUO97b_02046" /db_xref="SEED:fig|400667.82.peg.1910"
/translation="MRNYKIDILRGISILLVLLHHFNIPYKLKDTCLGFDFLGEAFST
VIARNGNYGVTMFFVISGFLITHHTLKRDKQFSAINLKHFYIRRAARILPCLVLLILG
VSILGSFDLKPFMNQAPNGIEVSYPLTIFAALTFWMNILIIKFGWVNYALGVLWSLSV
EEVFYFVFPLLCLLTRSNKVFITVLVGVILYGPYFRSLHFGEESGAYLYHYFSSFDGI

AVGCLTAIFSHKYQPNWTYKKPLSWLIILSMTALYLYAPIKEVSTWGISLFALATGLL
ILCFQHPSETQAHSLFTKVMVWLGQRSYETYLFHLVVLGLMKVAFIPAQTDSSIKIML
LIGYLILTFIISAAIEKYYSTPLNSYIRKVFIKDKS" /product="Acyltransferase 3"
/transl_table=11
CDS 2045335..2046087
/locus_tag="AUO97b_02047" /db_xref="SEED:fig|400667.82.peg.1911"
/translation="MPRTSKQEHTELSIEADSPVPLYQRVKQLISQKIYEGSWAVNKK
IPSESELVNQLGCSRMTVNRALRELTTEGLLVRIQGVGSFVAEGQGRTALFQINNIAD
EIIARNHKHHAEVLVLEQVYANAEQSVLMQTREGQRLFHSIIVHYENDVPVQVEDRLV
DAALIPDYLEQDFKTITPNAYLMAKAPVTEGEHIVEAVLASPQECKWLKITKAEPCLL
IRRRTWSNKQLISSARLIYPGSRYYLEGKFNP"
/product="Histidine utilization repressor"
/transl_table=11
CDS 2046084..2046659
/locus_tag="AUO97b_02048" /db_xref="SEED:fig|400667.82.peg.1912"
/translation="MIELIRADQYTKMLWKNGAGFTLEIARSQGEADFEWRISMADVT
TSGPFSLFPNKQRIISVLDGQGTVLHVDDLPAKKLKQGDIFAFYGESQVQSELVDGAI
RDLNLIYDPAKFHARFQWLNEAAEQAFISSADLIFIFNQGGETEVNVDDDFVQLAAHE
TLKIEKNASVTSINFPKKQLKSCYIIELIQR" /product="Stresses-induced protein Ves
(HutD)" /transl_table=11
CDS 2046911..2048587
/locus_tag="AUO97b_02049" /db_xref="SEED:fig|400667.82.peg.1913"
/db_xref="GO:0016153"
/translation="MTTTTTKFRDVEIRAPRGTELTAKSWLTEAPLRMLMNNLDPDVA
ENPKELVVYGGIGRAARNWECFDKIVDTLKNLETDETLLVQSGKPVGVFKTHKDAPRV
LIANSNLVPHWANWEHFNELDAKALAMYGQMTAGSWIYIGSQGIVQGTYETFVEAGRQ
HYNGDLKGRWVLTAGLGGMGGAQPLAATLAGACSLNIECQQASIDFRLRTRYVDEQAT
DLDDALARIDRYTKEGKAISIALHGNAAEILPELVRRGVRPDMVTDQTSAHDPLNGYL
PVGWTWDEYRERAKKEPEAVVKAAKQSMAKHVQAMLDFQKMGVPTFDYGNNIRQMAKE
EGVANAFDFPGFVPAYIRPLFCRGIGPFRWAALSGDPEDIYKTDAKVKELIPDDEHLH
HWLDMARERISFQGLPARICWVGLGLRAKLGLAFNEMVRSGELSAPIVIGRDHLDSGS
VASPNRETEAMQDGSDAVSDWPLLNALLNTAGGATWVSLHHGGGVGMGFSQHSGVVIV
CDGTDEAAARIARVLTNDPATGVMRHADAGYEIAINCAKEQGLHLPMITQ" /product="Urocanate
hydratase (EC 4.2.1.49)" /EC_number="4.2.1.49"
/transl_table=11
CDS 2048675..2050213
/locus_tag="AUO97b_02050" /db_xref="SEED:fig|400667.82.peg.1914"
/db_xref="GO:0004397"
/translation="MELLIQPGKLTLADLRQAYLNPIKVKLDESASSAINASVACVEQ
IVNEGRTAYGINTGFGLLASTKIAPEDLEKLQRSLVLSHAAGVGEALDDAMVRLIILL
KANSLARGFSGIRRKVIDALLALINAEVYPHIPLKGSVGASGDLAPLAHMSLVLLGES
KARYKGEWLPAVEALKIAGLEPISLAAKEGLALLNGTQVSTAYALRGLFEAEDLFAAA
TVCGGLSVEAMLGSRAPFDARIHEVRGQRGQIDVAAAYRDLLTDSSEISRSHEECGKV
QDPYSLRCQPQVMGACLTQIRQAAEVLEIEANAVSDNPLVFAEQGDVISGGNFHAEPV
AMAADNLALAIAEIGSLSERRISMMMDRHMSQLPPFLVANGGVNSGFMIAQVTAAALA
SDNKALAHPASVDSLPTSANQEDHVSMAPNAGKRLWYMADNVRGILAVEWLGACQGLD
FREGLKSSPKLEQARKILRDQVPYYSEDRFFAPDIEQASELLASGCLNELLIPKLLPS LSEV"
/product="Histidine ammonia-lyase (EC 4.3.1.3)" /EC_number="4.3.1.3"
/transl_table=11
CDS 2050278..2051690
/locus_tag="AUO97b_02051" /db_xref="SEED:fig|400667.82.peg.1915"
/translation="MSQNSTLQRGLNTRHIRFLALGSAIGTGLFYGSATAIKMAGPSV
LLAYIVAGIAIYIVMRALGEMAVHNPVSGSFSHYASQYIGPLAGFTTGWTYVFEMVIV
AIADVTAFGIYMGFWYPDVPRWIWILSLIMFLGAINLIHVKVFGELEFWLSIVKVSAI

VAMILGGLGLMFYGFHADHSSVVPGIQNLWIYEGFMPHGIAGLVACLSVVVFAFGGIE
IIGITAGESQDPKTTLPKAINAVPVRILLFYVLTIFVLMSIFPWNQIGSQGSPFVQIF
ENLGIKSAANILNIVVVTAAISAINSDVFGAGRMLYGMANRGQAPRIFQKLSRNGVPW
MTVVVMAGVLLIGVVLNYLIPENVFVIIASIATFATVWVWLMILLSQVAMRRKLSTAE
IKALDFPVFGWPYAPAFAIGFMVFILVMMGYFPDSRPAIYVGITWLALLTIAYRIWVK
PEQSLGKESEPVQQNIEMES"
/product="Histidine transport protein (permease)" /transl_table=11
CDS 2051742..2052947
/locus_tag="AUO97b_02052" /db_xref="SEED:fig|400667.82.peg.1916"
/db_xref="GO:0050480"
/translation="MKKLWQNCHIATMQNGQYSYIEDAAIVTEGHLIHWIGKQQQLPA
DTYSETVDLNGAWVTPGFIDCHTHSVFGGNRSVEFEKRLQGVSYAEIAASGGGIASTV
RATREASEEQLLNSALKRIRCMQQDGVTTIEIKSGYGLNYENERKMLRVIRQIGEKLP
MTVKSTCLAAHALPPEYKDQSDAYIEHICTEMLPKLHAEGLVDAVDAFCEHLAFSPAQ
VERVFKTAQSLNLPVKLHAEQLSSLGGSSLAARYHALSADHLEYMTEDDVKAMAASGT
VAVLLPGAFYLLRETQYPPIESLIKHGVRIALSSDLNPGTSPALSLRLMLNMGSTLFR
LTPEQALAGVTIHAAQALGLEQTHGSLEQGKVADFVAWDIEHPSEIVYWLGGDLPKRV VQHGQEVIF"
/product="Imidazolonepropionase (EC 3.5.2.7)" /EC_number="3.5.2.7"
/transl_table=11
CDS 2052958..2053878
/locus_tag="AUO97b_02053" /db_xref="SEED:fig|400667.82.peg.1917"
/db_xref="GO:0050415"
/translation="MNHTFKWQGRHDGEGEAHQRIHHIVNTTQHAEFALIGFSSDEGV
KRNKGRVGAADAPDAIRTQLANLPIHRPVSIVDLGTVTCEYGNLEQAQSELAEQVANS
LEHGLKPVVLGGGHEVAFGSFSGLFQYVQAHASDKKIGIINFDAHFDLREAEHATSGT
PFLQAARLSEQHQKQFNYLCIGVANHANTKILFDTADALNCSYLRDHEVNIFNLNNVL
AVVDAFIEKVDCLYVTIDLDVFAAAVAPGVSAPAVKGIDLATFEAIFKHIQETGKINL
LDIAECNPKFDLDNRTAKLAAYIVYQYLFS" /product="Formiminoglutamase (EC 3.5.3.8)"
/EC_number="3.5.3.8" /transl_table=11
CDS 2054132..2055958
/locus_tag="AUO97b_02054" /db_xref="SEED:fig|400667.82.peg.1918"
/translation="MKNRTLHFACISVLFWMSHSSDAATPEINTNTIEKSQSKQKSDV
SVRVKVLNQLLQEQWEYTLKNSPETATVLGDLRYNDRWTEFSKNQIEKDKKTTQNFLK
RFEAIDSTGFSATDQLNKDLMIYQLKETLKNYDLKLYEMPFNQMWGLHLQFPGFISSI
PFDNAKQYQDYIARLKQIPLILDQGIQLAKQGQKDGLIPPKYLLEKVAKQIDSIATPA
GKDSVFASPLKQFPKNISKAEQECLSREILQAIDQNVRPAYQKLGTFIQKDYLPYGRQ
HEGIWSLPNGDELYRFYVENNTTTLESPETIHQLGLKEVARIEAEMLKIAKAQGFNDL
KSFQQSLKTNPAVFPKSREEILEIYRGYIAQMQPELPKLFGLLPKNKVEVLPVEQYRE
KEAAGAEYHQGTPDGSRPGQVYVNTGDFSERSKISMEATAYHEAIPGHHMQIDIAQNL
PNLPMFRKQPNHTAYIEGWALYAEQLGKDVGFYKDPLSDYGRLSSELFRACRLVVDTG
VHYKKWTRQQMIDFMREHSALDEPDIQAETDRYIAIPAQALAYKMGQLKILELRELAK
QELGDRFDIKAFHDMVLNAGTLPLNILDARIKNWIKEQKAAA" /product="hypothetical protein"
/transl_table=11
CDS 2056057..2056695
/locus_tag="AUO97b_02055" /db_xref="SEED:fig|400667.82.peg.1919"
/translation="MEKNLKEYQAIIFDLDGTLADSFHFFLSVLNQLSAKYKFKSVSL
HEVDYYKQLTPREIMKEMNVSRWKLPWIAKDFIRLMKERDEEIQLFEGMRDHLIELHK
RGYTLAIITSNSKENCQNVLGKELCELFSHIDGGSSIFGKAKRIKRVLNILDVSKNQA
IYVGDQTTDGEAAHKAGIDFAAVGWGYTSAEKLKTIQPKVVLNDLATMKEFF" /product="Hydrolase,
haloacid dehalogenase-like family" /EC_number="3.1.3.18"
/transl_table=11
CDS 2056719..2057519
/locus_tag="AUO97b_02056" /db_xref="SEED:fig|400667.82.peg.1920"
/db_xref="GO:0008881"

/translation="MNNNNNPIGMIDSGLGGLSLFKYIRQALPNEDIIYFADSKYVPY
GDRESDWIVSRTTHLISNLVTHGKCKAIVIACNTMTAVAVETIRAQINVPLIAIEPAV
KPAVAMTLSKHIAVLATATTVKGKNLKSLIETYAQDIKVSLVPCIGLAEKIETGKAHT
AEVKDYLKNILAPLVEQKVDTIILGCTHYPFVSDTIQEIVGRDIQIIEPSEAVTAQLI
RQLNQYHLSSESPNEGNHIIWTSSDPLEVADVTFSLLGTRLPVETTDF" /product="Glutamate
racemase (EC 5.1.1.3)" /EC_number="5.1.1.3"
/transl_table=11
CDS complement(2057506..2058129)
/locus_tag="AUO97b_02057" /db_xref="SEED:fig|400667.82.peg.1921"
/translation="MISLELFITFLITTIIFAYIPGPAMLYTAAQTLSRGRKSGLMAA
FGIFLGGCFHIIAASLGLTTIFQIIPKLYDIIKILGALYLVWLGIKLIRSTSSPTVQQ
NVENGQLSLKQSILVEVLNPKTAIFYIAFLPQFINTSLDFPVWSQFIILGLIVNLIFV
SADVVCVFLANYISQKLTKSEKPQKSMSVMGGLIFMGLGVFLGFKNQ" /product="Threonine efflux
protein"
/transl_table=11
CDS complement(2058361..2059575)
/locus_tag="AUO97b_02058" /db_xref="SEED:fig|400667.82.peg.1922"
/translation="MANGFPGGNFRIINKETGACIASAYGGQTHGLQDAVTARGETGV
LPYSHTNDRLFTVHDKVKNIDEELWYFDSRVYNGGHRENKLYNKVKDIRSNWVLGARV
KTNAIAYKLLDKDLEHFLSLLPKQDEETLAHLKTSDCLDIVGLLETFILSNGEETIKS
ELVGKLDETKLNEYLNAVLAHESIHRIEQKFYKYAESPLLGLNIPGLSDLPNNPFAQQ
NEQLYKIIFASVQLDVLDHKDWTSDSEFRLYLRHDNIDALLKNMKKESIAELREKAYI
EYANTQDLKKEAIKVLLELRKTLDLHTNEKNMETVKKILKVSLKSNESFEQLQTALNS
TNGKEIKPVNWHSGDIFLQGAGCSGTTNWAFKDGYIFVEGQPDAVLTHLWGSSVGISQ RSNDPNQIWELK"
/product="hypothetical protein"
/transl_table=11
CDS 2060577..2060699
/locus_tag="AUO97b_02059" /db_xref="SEED:fig|400667.82.peg.1923"
/translation="MKLHKFILILNLVVGAYSITNDDANAQQEFKRICLSGEHM"
/product="FIG00350582: hypothetical protein" /transl_table=11
CDS complement(2061100..2062938)
/locus_tag="AUO97b_02060" /db_xref="SEED:fig|400667.82.peg.1924"
/db_xref="GO:0004360"
/translation="MCGIVGGVAERCVTEILIEGLKRLEYRGYDSAGVALLNKQQILR
ERRVGKVINLEDAVADQQLTGTIGIAHTRWATHGKPTENNAHPHMSGKVAVVHNGIIE
NYQELKDDLQALGYVFTSQTDTEVVAHLVAEALKNTDSLLEAVESVVPQLKGAYALGI
IHSDYPDELITVREGSPLVIGVGIGENFISSDQLALLPVTNRFIYLEEGDIARLTRTS
IEVFVKGERVERPVKELDATVSSASKGEYKHYMLKEIYEQPEAIKQTISQALDGNNLR
DDFLKDAEADFSKLQSVQIIACGTSYHSGMIAKYWFEQLIGVPCQVEIASEFRYRSPV
IVENTLYICISQSGETADTLAALRETQKRAKANNIDIQTLTICNVATSSMVRETDHHL
LTLAGPEIGVASTKAFTTQLAALMLLILKIGQVKQRISNVMIEELARELWHSPKVILD
TLKNDPEILRLSELFVEKQHCLFLGRGTHYPIALEGALKLKEISYIHAEGYAAGELKH
GPLALVDTEMPIVILAPNDEMLDKLKSNMEEVQARGGELFVFADENSGVVEKDRQHVV
HIPAVNEWLAPIIYSVPVQLLSYHVAVLRGTDVDQPRNLAKSVTVE" /product="Glutamine--
fructose-6-phosphate aminotransferase [isomerizing] (EC 2.6.1.16)"
/EC_number="2.6.1.16"
/transl_table=11
CDS complement(2062951..2064270)
/locus_tag="AUO97b_02061" /db_xref="SEED:fig|400667.82.peg.1925"
/db_xref="GO:0019134"
/db_xref="GO:0003977"
/translation="MRSQLPKVLQPLAGRPLLGHVIKTAKQLLAENIITIYGHGGDHV
KKTFAQENIQWVEQAEQLGTGHAVQMTLPVLPKDGISLILYGDVPLVRQTTLEQLIEA
SNKTGIGMITLHVDNPTGYGRIVRQDGKIQAIVEHKDATEAQRQIQEINTGIYCVSNA

KLHEWLPKLSNENAQGEYYLTDIVAMAVADGLEIASIQPELAFEVEGVNDRLQLAALE
REFQKQQAKELMQQGVTFADPARFDLRGTVKVGHDVRIDVNVIIEGDCELGDFVEIGA
GCILKNTTIAAGTKVQAYSVFDGAVVGENTQIGPFARLRPGAKLANEVHIGNFVEVKN
TTIGLGSKANHFTYLGDAEIGAESNIGAGTITCNYDGANKHKTTIGDAVFIGSNSSLV
APVTIGNGATVGAGSVITKDVAEQSLSFERAQQISKANYQRPQKLKK" /product="N-
acetylglucosamine-1-phosphate uridyltransferase (EC 2.7.7.23) / Glucosamine-1
-phosphate N-acetyltransferase (EC 2.3.1.157)" /EC_number="2.7.7.23"
/EC_number="2.3.1.157"
/transl_table=11
CDS complement(2064332..2064643)
/locus_tag="AUO97b_02062" /db_xref="SEED:fig|400667.82.peg.1926"
/db_xref="GO:0008962"
/translation="MSVVGIYICGQTAKVINVHDDGRIVWDEFAGQSITFLPLLYLQQ
INWIWVITGFILFRIFDVWKPWPIRVIDRQVHGGFGIMLDDIIAGVWAALCTLIIIHL S"
/product="Phosphatidylglycerophosphatase A (EC 3.1.3.27)"
/EC_number="3.1.3.27"
/transl_table=11
CDS 2064690..2064815
/locus_tag="AUO97b_02063" /db_xref="SEED:fig|400667.82.peg.1927"
/translation="MGTKSKAKAEPKVPGALGARPDPKPTPQKTMQRLNQDMSLK"
/product="hypothetical protein"
/transl_table=11
CDS complement(2064825..2065742)
/locus_tag="AUO97b_02064" /db_xref="SEED:fig|400667.82.peg.1928"
/db_xref="GO:0009030"
/translation="MAEFSIIDQYFNRQSHPDVALGIGDDSALITPPPNQQLVICADT
LVAGRHFPLETSPHAIGWKSVAVNLSDIAAMGAKPHSILLAISLPQVDHEWLEGFSQG
IYDCCNQFGVALIGGDTTQGPHLTITVTAMGWIETGKAVLRSGAKVGDYVCVSGQIGD
AAYGLQHLGHPLQQRLDYPTPRCKLGEELKGLASSMIDVSDGLAQDLGHILKASKVGA
RLILEKLPVDPVLQQLEEQQRWQYALAGGDDYELCFTITPQNYEKLLQKQLDIKITMI
GQIVEQTKLTFEHLGSDYPLQIHGYQHFA" /product="Thiamine-monophosphate kinase (EC
2.7.4.16)" /EC_number="2.7.4.16"
/transl_table=11
CDS complement(2065758..2066207)
/locus_tag="AUO97b_02065" /db_xref="SEED:fig|400667.82.peg.1929"
/db_xref="GO:0003723"
/translation="MSQTLQAAYAAKRKARRFAVQGIYEWQMSHNPVHEIEARTRAEN
AMHKVDLNYYHELLTQVIAQHEDLDALLIPVLDREIDALDGVELATLRLGAYELRDHL
EIPYRVVLDEAIELAKHFGGADSHKYINGVLDRLSSTLRSAEKQQAK" /product="Transcription
termination protein NusB" /transl_table=11
CDS complement(2066211..2066681)
/locus_tag="AUO97b_02066" /db_xref="SEED:fig|400667.82.peg.1930"
/translation="MAIRRIEGLLHLASEGRYAILVGRFNSFVVEHLLEGAIDTLKRH
GVNEDNITVIHAPGAWELPIVAKKLATSNQFDAIIALGAVIRGSTPHFDFVAGECAKG
LGVVALESSLPVINGVLTTDSIEQAIERSGTKAGNKGSEAALTAIEMVNLLKAI" /product="6,7-
dimethyl-8-ribityllumazine synthase (EC 2.5.1.78)" /EC_number="2.5.1.78"
/transl_table=11
CDS complement(2066693..2067814)
/locus_tag="AUO97b_02067" /db_xref="SEED:fig|400667.82.peg.1931"
/db_xref="GO:0003935"
/translation="MPLSRVEELVADIRAGKMVILMDDEDRENEGDLVIAATHVRPED
INFMITHARGLVCLTLSRERCKQLNLPLMVDQNGAQHGTNFTLSIEAAEGITTGISAA
ERAHTIQAAVAAHAKPTDIVQPGHIFPLMAQPGGVLHRAGHTEAGCDLARLAGLEPAS
VICEIIKEDGTMARRADLEIFAEKHGLKIGTIADLIHYRMTNEQTVERLDQRTIQTEY

GSFELYRYREIGNPDIHLALVKGEPKEGVTTVRVHGFSPVRDLLKLNKADGEPAWNLD
RALQTIAASDRGVLVWIGQDHLQDLGPALDDLTKPKPVKSNAALSHQYQTIGVGAQIL
RDLGVEKMKLLSSPLRFNALSGFNLEVVEYVTADQITTK" /product="3,4-dihydroxy-2-butanone 4
-phosphate synthase (EC 4.1.99.12) / GTP cyclohydrolase II (EC 3.5.4.25)"
/EC_number="4.1.99.12"
/EC_number="3.5.4.25"
/transl_table=11
CDS complement(2068054..2069343)
/locus_tag="AUO97b_02068" /db_xref="SEED:fig|400667.82.peg.1932"
/translation="MIAPRQGLFASLLIISFALHTFLLVIATTHQLNENRASQGQLMT
SQLVADSLSELEPANTVSLALIANRYATNPSVASIRILDANKQVLATSGMSKTRQGEI
FVRDALQNEKKVGSIEITLIQPSIGEILRTQWLAILASLFLHVLLWLAYRAIARPTRS
EYLARINEENRLKHEIQELTQALALEKQNTVTLVAQAQQQAKAKPIVRSQPEKSLEST
DQNTLALNIQFYDPKQLLSSVNQSVSVPYFKLCQLFLNKSIELCTKHYHLKATDIDVV
DEFHAEGATLAISTSHPHAVECLLMVGTVFQLLSDVLYKRYREDKRFALQTRSAVCNA
VEAMQIDAKEAAQRLAQHLHAKESALYLDNEQLKAIQDSYQLVAMPNPSNVMTRHAFM
INGMNAECAELAQNIRTEILMGKKSIPQNDSPSSAAS" /product="hypothetical protein"
/transl_table=11
CDS 2069421..2070644
/locus_tag="AUO97b_02069" /db_xref="SEED:fig|400667.82.peg.1933"
/db_xref="GO:0004647"
/translation="MREIIILISFLGPDQPNQFTRLMQVLSVHSLQILDVGQAVIHNQL
TLGIVVASENETATALAMKEILILAHDIGLTVRFKPISGAEYDQWVSEGGRTRYIVTA
LAPELTAAHLQAVTQIVSSQGFNIETVTRLSGRVDLEKDSTLPRRACVQFGLSSGPTL
DAQAMRAACLLLSSELNIDVAVQEDNAYRRNRRLVCFDMDSTLIEQEVIDELALEAGV
GEQVAEITERAMQGELDFQQSFRARVALLKGLDASVLPKIAERLTITEGAERLISTLK
ALGYKTAILSGGFQYFAEYLQAKLGIDEVHANVLDVQDGVVTGEVKGVIVDGARKAEL
LRELANKLGISLEQAMAVGDGANDLPMLAIAGLGVAYRAKPLVRQNANQAISSVGLDG VLYLLGMHDKDLSRA"
/product="Phosphoserine phosphatase (EC 3.1.3.3)" /EC_number="3.1.3.3"
/transl_table=11
CDS 2071054..2071389
/locus_tag="AUO97b_02070" /db_xref="SEED:fig|400667.82.peg.1934"
/translation="MVTANFAAIAGLSLIAVALVAVFFSPYRRWLGFMLAGMFFWGLL
EVVRFGVQVTFEMPVTYSYLTALSLAMVMVTFVLLREDKQAQKALANRQYIEHTPVYE DDQQQCSSR"
/product="hypothetical protein"
/transl_table=11
CDS 2071644..2072123
/locus_tag="AUO97b_02071" /db_xref="SEED:fig|400667.82.peg.1935"
/translation="MKLSSIPVVKLPLVDVSTDPLDLLVAGLALRMKQLARTSPKFIE
LVHDRAFRIQIGTDLGVARQIIINHGHIDTVAGSPEKADFILQFADSEQGVKTLLKGD
PTAFMTGMQDGSIKMEGDFSLLVWFNQAAKLIPPKLPKPVKDKVRQARAFIKEKTGR"
/product="Superfamily II DNA and RNA helicases" /transl_table=11
CDS complement(2072163..2072606)
/locus_tag="AUO97b_02072" /db_xref="SEED:fig|400667.82.peg.1936"
/translation="MRALIQRVLEAKVEVDGQTTGEIKKGLLVFLGIGRDDTLATGQK
LIDKILKYRIFDDEQGKMGWNVSQANGGILLVSQFTLMAQTQKGLRPDFGPAMPPSDA
KALYEQLVEYTRSQFENVQTGIFAADMKVHLINDGPVTFNLEVEA" /product="D-aminoacyl-tRNA
deacylase (EC 3.1.1.96)" /transl_table=11
CDS 2072807..2074330
/locus_tag="AUO97b_02073" /db_xref="SEED:fig|400667.82.peg.1937"
/db_xref="GO:0004096"
/translation="MFKRTFLFTIIAATLSTVYAAPLTKDNGAPVGDNQNSTTAGANG
ATLLQDVQLIQKLQRFGRERIPERVVHARGTGVYGEFVSTKDLSDLTLASLFKAGTKT
PVFVRFSTVIHPKGSPETARDPHGFAVKFYTQQGNWDLVGNNLPVFFIRDAIKFPDFV

HAMKPDPVTNVQDPNRIFDFLQSQPWSINMLTYVYSNLGTPESYRTLDGFGVHAFKLY
NDKGEYKYVKFNWRSQQGVKGLNLEQLREVQGRDWSHLTNDMYKNIYAGNYPKWDLYI
QVLDPKDLDKFDFNPLDATKIWPNELVPEFKVGTLTLNRMPKNFFQETEQSAFAPGNL
IPGIEPSEDRLLQGRVFSYSDTQLYRLGANYQQIPVNRPRVAVNNNNQEGFMNMGQTE
SHVNYEPSQIEPKPATEKARAVQTPLEGTVMQHAIQKQQPYKQAGDLYRSYSALEKKD
LIRNLAADLGQVKNVETKTVMLSYFYKADADYGTRLAKAVGVDLKMVQAKAAQLKDE" /product="Catalase KatE (EC 1.11.1.6)" /EC_number="1.11.1.6"
/transl_table=11
CDS 2074460..2074945
/locus_tag="AUO97b_02074" /db_xref="SEED:fig|400667.82.peg.1938"
/translation="MIYFRSVFLATCLLCSSIGASVYAAMPSSQDNGSAEVIELFFSA
AKVGNVEVLQEFLSHGFPIDVQDHSGYTALMMASYYGQKDAVKVLLEQGANRCLRDKR
GHTALMGAIVKAEWGIAKQLRQVDCDANAAKTGLLTAEQFAIQFGQQQRLKDIQPSTE K"
/product="AnkB protein"
/transl_table=11
CDS 2075137..2076300
/locus_tag="AUO97b_02075" /db_xref="SEED:fig|400667.82.peg.1939"
/db_xref="GO:0004516"
/translation="MLQVVLHKFPQTHSVYHFRCRNLEDTVYPLVDILDDLNEQLDHL
CNLKYKEDELQYLRKLRFIKSDFVDYLELFQLKRRFIHASIDEEGRLDIRIEGPMVQA
MMFEIFVLAIVNELYFSRIKTDEVWAEGERRLQAKLELIQQYEKAQQPNDPPFLVSDF
GTRRRYSFEWQKHVVAAFHNTVPNVFRGTSNVLLAKELNITPIGTMAHEFLQAFQALD
VRLRDFQKAALETWVQEYRGDLGIALTDVVGMDAFLRDFDLYFAKLFDGLRHDSGDPY
EWGDKAYAHYRKLKIDTKTKMLTFSDGLNLPKAWELHQYFKDRFQVSFGIGTNLTNDM
GQTPLNIVLKLVECNGQSVAKISDSPGKTMTDNDTFLAYLRQVFQIEELDEAI" /product="Nicotinate phosphoribosyltransferase (EC 6.3.4.21)" /EC_number="2.4.2.11"
/transl_table=11
CDS 2076451..2077299
/locus_tag="AUO97b_02076" /db_xref="SEED:fig|400667.82.peg.1940"
/translation="MTLPDFKLDLLIEAEELVPYLGNEYIRIVDLSRASVYEQLHVPH
AIHLQPKWLVAQHEEATGVLPDEAGLQALIEKLNISPNHHVVVYDDEGGAWAGRLIWN
LHCLGFTRTSLINGGIHAWLGAGLPTTAEVEELPRVDNLIQIDLSHAAQFRVNYEELR
KQVEQESVQLWDCRTSDEYSGVRLAARRGGHIPNALHFEWSTALNRQNHLKLHPLERT
RQRLEQLGFDLKQPVVVYCQSHHRSGLAYILARLLGWEAKAYDGAWSEWGNRLDSPII TGESPS"
/product="Thiosulfate sulfurtransferase, rhodanese (EC 2.8.1.1)"
/EC_number="2.8.1.1"
/transl_table=11
CDS 2077296..2078147 /locus_tag="AUO97b_02077" /db_xref="SEED:fig|400667.82.peg.1941" /db_xref="GO:0004609"
/translation="MSFTSRLKKELFIKAQNLVPQHQLSRVVGKVAASENPILKAAVI
HAFKTKYGIDLSIAEQGNALKYKSFNDFFTRALKDGVRLVDENPDSIVSPADGAISQI
GKITAGEVFQAKGQSFSVEKLIGDPQLAQPFQEGEFATVYLSPRDYHRVHMPFSGTLT
ETLYVPGELFSVNQVTAENVPGLFARNERMVCLFDTELGRMAVVLVGAMIVAGIETVA
TGKVKPSGRIELQHHELKLEKGAELGRFYLGSTAIILFEKDKIEWEKRYKAESVVVMG ERMGHTL"
/product="Phosphatidylserine decarboxylase (EC 4.1.1.65)"
/EC_number="4.1.1.65"
/transl_table=11
CDS 2078368..2079564
/locus_tag="AUO97b_02078" /db_xref="SEED:fig|400667.82.peg.1942"
/translation="MIRKSNSLLKILFSSICLISGSAMAAPITDLSKQIVSEKPGLIL
SIAQQDVTDLFPHADQRLLVNYRSRGVNDEPIVASAFILLPKGTPPKNGWPILAWAHG
TTGVADTCAPSGDYAGGPVHSYQEVASKALDGWLARGYAVVAPDYQGLGTLGGHPYMN
AKSQLHTVVDAVRALHMLKPKDFNQQWLVMGHSQGGAAAITVAAYGQKDAPELDLKGA
IALAPGGYQYEGIAEYVLSHPNPEPSVAAFFPIVLLGAQAAEPSIIPENLVSPEMGNV

LTQARSRCLSELQSDLKKSPSSIFRPNADLRPLLSYLKQQSIENMVPTVPLMIVQGSK
DHLVDPRGTYAYYQQLCKLKKPTIYQTIDGGDHRDALRQSNTFATDFLNAIEKKQAKS YCSNFK"
/product="Secretory lipase precursor"
/transl_table=11
CDS complement(2079634..2080992)
/locus_tag="AUO97b_02079" /db_xref="SEED:fig|400667.82.peg.1943"
/translation="MYEPYPVPEQVREQKLSRYSSLWTFAKQDLRLLLFFLIIAGLVG
LGIGYFWSCIFIAFVVFFTLQLRSLYLVNDWIANNPYDVPPNLNGIWGALLFNVYRAQ
RQERIVQAEMVGLIDRAQSSLVALAEAVVLIDDQHQIEWWNPAAERLLGISPLDRGRN
LLTILRQPTFIEYFNNIDQAPDGIKLHSNLDDDRYVQVKLTRFGGESRLLVAYDVTRM
HNLEQMRKDFVDNISHELRTPLTVLSGYIETFTDQEDINPRWKRAFDQMQSQTKRMNA
LVNDLLLLSNLENNKKIAKNQIIEMPSLMNQLFDDAQAYNADYGHTLNLHIDSHCDLI
GSDMEIASAFSNLITNAIKYTPKGGTITIGWHDDGEHAYFSVQDTGIGINPKHLPRLT
ERFYRVDSDRSRQTGGTGLGLAIVKHVLMQHGAYLDVQSKENEGSTFTAVFPKERLYN MT"
/product="Phosphate regulon sensor protein PhoR (SphS) (EC 2.7.13.3)"
/EC_number="2.7.13.3"
/transl_table=11
CDS complement(2081002..2081712)
/locus_tag="AUO97b_02080" /db_xref="SEED:fig|400667.82.peg.1944"
/translation="MKDDYILIVDDELPIREMIHTSLDMAGFQCLQAEDAKQAHQIIV
DQRPALILLDWMLPGGVSGVDLCRRLKRDENLAEIPVIMLTARGEEDHKVQGLDAGAD
DYMTKPFSTRELVSRIKAVLRRANALSGEKTIDANGLILDPVSQRVSFGNSILDMGPT
EYRLLAFFMTHPERAYTRAQLLDQVWGGNVYIEDRTIDVHIRRLRKVLEPFGVDRFVQ
TVRGTGYRFSTRADLAAG"
/product="Phosphate regulon transcriptional regulatory protein PhoB (SphR)"
/transl_table=11
CDS complement(2081968..2082639)
/locus_tag="AUO97b_02081" /db_xref="SEED:fig|400667.82.peg.1945"
/translation="MATNDSTSKKKTKTVSKERQFKGLSLSERKEARREKLIEAGIAT
YGTLGFFSVTVKDVCQEAKLTERYFYESFKKSEDLFQTIFLKMIEELQQNLMQAVIKA
APDPEQMVDAGLRALLSTLKDDPRLARIVYVDAILVQELHNQATIQETLAQFDRMIQA
FVMLTMPQIQHNENELSLIATGLNGYVTQIAVRWVMGGFKQSLEQVLAACRIVFLALL AKVSK"
/product="Transcriptional regulator, AcrR family" /transl_table=11
CDS 2082836..2083669
/locus_tag="AUO97b_02082" /db_xref="SEED:fig|400667.82.peg.1946"
/translation="MKNFKNKVAAITGAGSGIGQQLAILLAKQGCHLSLSDINEKGLE
KTVELLKPYSNITVTTKKLDVSDREAVKQWAQETVQDHGCVNLIFNNAGVALGSTVEG
ATYEDLEWIVGINFWGVVYGTKEFLPFIKQTQDGHIINISSLFGLTAQPTQSGYNATK
FAVRGFTESLRQELDIEKCGVSSLCVHPGGIRTNIAKAAKMSDSLSSLGMDPTKSIQN
FDKLLRTPPEEAARQILDAVLKNKRRLLIGSDAKILDAFQRLFPTGYQRASTIVTKWM K"
/product="Putative short-chain dehydrogenase" /transl_table=11
CDS complement(2083726..2085093)
/locus_tag="AUO97b_02083" /db_xref="SEED:fig|400667.82.peg.1947"
/translation="MPPRQSLIHQQNLWKTFFVFLLPLIATNILQNLSGTINTIFVGQ
MMGVDAIAAVSVYFPILFLLLAFIIGISTGTTVLVGQAWGAQDIEKVQSVVGSTLFMT
IIGGIITAIIGVFFAHDILELLGTDPKVMHLSLPYVQWMLAGSPLLFVYIIYTSILRG
VGDSTTPLLALALTSVIGVIITPILLKGYFGFPALGIIAPAIASIISYIAILIFLAIY
LNKKKHPLRPNRQLLQHIRHNSALSKIILRLGVPTGIQMITTSMAGLVIVGLVNRFGA
HATAAYGAVNQVLNYIQFPAISISIAASIFAAQAIGAGKSDLLARVTRTALGMNFLFT
GALIALGYLFSKYLMALFITDPTVVVLGQQLLFIVLWAILFFGAGAIFASIMRASGTV
TVPMLINISAILFIEMPCAYWFSSMWGLKGIWVAYALAFVSLCIIQGLYYQFIWKKKK IKVLI"
/product="Na+-driven multidrug efflux pump" /transl_table=11
CDS complement(2085200..2086354)
/locus_tag="AUO97b_02084" /db_xref="SEED:fig|400667.82.peg.1948"

/db_xref="GO:0004069"
/translation="MLQLQSKLPAQGVTIFSTMTAMAQRLGALNLSQGFPDFPAPPAL
LEALSQATLAGHNQYPPGDGILALREQLALQFQQRDQLQLDPVTQITITPGATIAIFC
AIQACINTGDEVIIFDPSYDSYGPSVELAGGKAVHIALQAPDFKVNWQQVKDRINDKT
RMIVVNTPHNPTGTIWSEQDWLELIALIQDKNIVVLSDEVYEHLVFDGQQHFSALHFS
ELRERSFVIGSFGKTFHVTGWKTGYCVASPDLMRLFRQIYQYVNFCGTTPCQIALAQY
MQQHPEHIQELSQFYQTKRDRFNQAIHNSRFLLKPSQGTYFQNLDYSQIRPDLNDVEM
CQFLAEQHKIVAIPVSVFYQQAPESLRLIRFCFAKNDETLQKAGEILNQC" /product="Methionine aminotransferase, PLP-dependent @ Glutamine-dependent 2-keto-4-methylthiobutyrate transaminase" /EC_number="2.6.1.1"
/transl_table=11
CDS complement(2086382..2087692)
/locus_tag="AUO97b_02085" /db_xref="SEED:fig|400667.82.peg.1949"
/translation="MNMAEEVHHAPNLKRSKYFATMALIIVVVLWIGLLIANRFLPEY
TDLIHILMLGAEAGVVGGLADWYAITVLFRNPFGKLPIPKFLRDHTEIIPRNKARIAE
SMGRFVQENFLSPQVVERSLEKTDLSLAIGQWLASPQNNTQVVQLIQQTVPKVFEFVS
QEQIANFVQNNSVQWVRNTQVNKLASEMLRAVLENDFHQDVLQRGLDIAHEWVVQNPD
KTRELTRKMFKALGVWSLAKGASWIGIDVQQRTIDSLVEKVESMLADHEHPWRQEIET
IAHSLMLELANPESIASQRLNSGKDALLDSPQVLNFISGAVTILCDAIKEDLMKEDSG
IAMNLRAAIQQLGENLVQNVKVREVLNKEMTGLAMNFTDQYSHKIIRYVSERIHEWDS
REMIGKIENEVGGDLHMIRVNGVVVGAFIGLTLGVIRAAIESIL" /product="Putative inner membrane protein"
/transl_table=11
CDS complement(2087706..2088269)
/locus_tag="AUO97b_02086" /db_xref="SEED:fig|400667.82.peg.1950"
/translation="MKYLSLCLALGTAITLSACSTVPSKPKTFDQLGQFSAYPLNAQT
FRISFQADPNMSYGAAEEITLVKSAQTTVQKGFRFFKVLNDPSNQSQKPPRQAVVYPS
APSFYPYGYYRRYPGFWHDPFYDFPQVVNIDPVQVSYTIECYRDQKQAPQDAFDATLI
LKSIGQKYGLSPTGELLPPPEPPVKQK"
/product="hypothetical protein"
/transl_table=11
CDS complement(2088329..2088868)
/locus_tag="AUO97b_02087" /db_xref="SEED:fig|400667.82.peg.1951"
/translation="MNDQDILNFWFSPEHRSLWFAKSEDFDAKIRDQFTDVHRQATRA
ELWSWRKTPEGRLAEIIVLDQFSRNIYRDQPESFAYDGLALALSQEAISLQLDAQLNP
EQRSFLYMPFMHSESKLIHEFALKLFQRLGNEINLSFEKKHKVIIDRFGRYPHRNAIL
GRVSTPEETEFLLEPNSSF"
/product="hypothetical protein"
/transl_table=11
CDS 2089037..2090614
/locus_tag="AUO97b_02088" /db_xref="SEED:fig|400667.82.peg.1952"
/db_xref="GO:0004357"
/translation="MSQPTTPSQSVIPAWVDSSLLQGMLRGIERESLRMQSNGFLSQE
LHPKALGSALTHPKITTDYSEALMEFITSPQPTIGDALHELTDIHAVVHRHLENGEKL
WPLSMPCMLDDNEERIRLAQYGTSNIGRFKTLYRRGLGIRYGRRMQTISGVHYNLSFP
DSLFSALQAHETDEKLKALSLQDYRSHRYFGLIRNFIRLTPLVMFLVGASPSVCRCFL
TGHEHHLLPLIKGSYYLPYATALRMGRLGYQNSAQKSLGIHYNNLTGYLDGLQKAVHS
PYPPFSRLGLNDASGEPLQINDHVLQIENEYYSLVRPKQVPQAGETPSQALKNRGVGY
VELRAVDVNPYSAIGVNETTAGFLEALALYCLLSDSPELLCPEQDLIEKNQTEVVNRG
RAPNATITDLNGSYHIEDWARQHISKMQDCARLLDQTYATDLYSSALAVMQERIDEVD
ETLSAHVIDDTLKHGGTWSFGSHMAQLHAETYEKHEISAETLQYFEQLAVQSLQQQEQ LEQDSQISFDQYLEQYR"
/product="Glutamate--cysteine ligase (EC 6.3.2.2)" /EC_number="6.3.2.2"
/transl_table=11
CDS 2090629..2091144

/locus_tag="AUO97b_02089" /db_xref="SEED:fig|400667.82.peg.1953"
/db_xref="GO:0006118"
/db_xref="GO:0015035"
/db_xref="GO:0016020"
/translation="MRLSYRLVSGLLVLASIVGMSFALYLEHVKGLEPCPLCIFQRVG
LMAMGFVALIAFLHNPVSNAIKRFYAFLAGVAILWSVGVAGRHVWLQHLPPDQVPSCG
PGLNYLIDALPMKTVLQEVLSGSGECAAIDWTFLGQSLPVWSLAYFLLLLLVCLWQLF RFYPVFKTAKK"
/product="Periplasmic thiol:disulfide oxidoreductase DsbB, required for DsbA reoxidation" /transl_table=11
CDS 2091309..2094626
/locus_tag="AUO97b_02090" /db_xref="SEED:fig|400667.82.peg.1954"
/translation="MLFNIFSVLEKIGLNAQKRAIHVQFSNELLNHQVFLQRIEGQHQ
LNGGLMAELICLSTNAQIALKQFIGVQVAVDQVTDSGQLFRTTGIVTEASYGQSDGAL
TLYKLTIEDATNLWHKRRNSRVFMNKSIVEITEVLFKEWQEKSPLFAASLSLDLGGLS
QNYDIRPFTMQHNESDYDFLTRLWRSEGVSWLIDESELFVPHFTAPIQGQKLRLIDDN
NQYQALARRSIRYHRSSATEYQDSITGFVAVRSLQPTAVHVQRWQPDALAQEEGNGSV
VTTHTHSDNFDSATLSLEQAWHVSPAWMQDLKGEDQATASSSSQLEKLNQHFTDMHAS
RAKYFKAYSSVRDTQVGYWFNLREHPEIDQHEGADQEFLIIAKNFYNQNNLPKDLHQQ
VSQLLTQSRWDQHGYDDIERQGNELTLIRRQIKTAPEYNPEQHRPIAYPQRAKVVGPE
GETIHVDEWGRIKVRFLFTRSDDHGHDGGAGSNDNDTDSAWVDVLTPWAGEGYGARFL
PRIGEVVVIDFFDGNIDRPFVTGRIHEAQRSPTKFDVKGQLPDTKKLSGIRSQEINGS
GFNQLRFDDTTGQISTQLQSSHAATQLNLGNLSHPKEQETSQGRGEGFELRTDAWGAV
RAGKGMLISTYAQEQAIADHLEAAQAQSLLSQGYESMKMLSEVAAKQQTDALNVINRL
PKFIQSLELKTTGQALNSTVNLFKEGINNDPIHALKDCGGFIQDIGALGGNAKGVVDE
FNSFFSDAKDAVENLKAFIENVEEHGPDIVKGKLASIKDRIHKNPFESIQEVGKVLAN
VETKDFDLMSTCGTFSKGSKLEVSPSKALSSLQGFMEGYTQGLESSSDTKQQEQGKIF
RQALMLLASPNGIALTTPENIILQASQDIAESASGSINLSAQKNIIGHAQDKISLFAA
QKGLRAYAAKGKLELQAQDDAIEAIAKKVIKLISTEDKIELTSPKEIVLTAGGSQLKI
NANGVFSTTGGKFESKAGQHLFTGGAKVSYEVPELPSSGPFTKAFHFVSLEGEKIENA
IIQVYEKINDSYKFKIGSKIDKSIFKLEESEENIEYKALVGFDDWVSWFEEEEEEEEG EEVEEIDLGEHYNPFDK"
/product="VgrG protein"
/transl_table=11
CDS 2094640..2097069
/locus_tag="AUO97b_02091" /db_xref="SEED:fig|400667.82.peg.1955"
/translation="MSNIHIVKKGDTLWGISNKYHIKLNELIEINGLYGRKKNLLKIG
QQIYLKKDNVKKYDTTLVIKIYDLKWEPITHGKLLLEYDDKCHLATSNDKGVIEDIQI
EDALKGIKISFYTLKNKFELIAHHKTLPLGKKILKLSSRAMMIKGNTYKVEGIPRTNT
KTIEKELKVNSKKVQHSSGASGASGASGASGASGASGASGASGASGASGASGASETRI
EGGLPAVTVAPVYSEENLYLHPDNEKYRKIIIDAAKKYNLAPQALAAKINAEAGTKKG
SQEWNPQAQASTSSAVGLTQFLSGTWYEICTVSTYKDTLLQQYVIKKKLISNFKENMA
LKLISNTDKAKLKALGTDPTFSVDSAAAYARANINIVKKIYSKIDSLEPGDLAKVGYV
AHHDGPTGFGKIVEATNDPSWSKLQGQVCPKNNPNCDTYLNYKERFSNGRDAYRWWLC
TEYTDSKINVNRFTLSPKDGKKFKNARTMEEIIVFLGGKPLVRPSRDFEKKGTTDNSK
LNDNINIVITSLVNNKVQANISYFVKSKYGLKPHSTNVNGHEIIHAKKGDKIEILFND
QVIASINAINDKEEFKINFPSKNITNASTEKQSSNSEQKLNFNRWRNPLNGICQIRRF
GYNSLPLKANASSYNEKNLEQATKIASRFNRTSYRASGIHQGVDLEADNGVNIYPVCV
GTIAKVIPAYPGYGKTIILECDVNDLPSSKKVLAKNLDTIYFIYAHLNSINVSEGDSI
TTLDTVLGKTGNSGNAGSMTRIEDGSHLHFEVRSEIMKSMGKSGMSYRLDPFPWLDNC MTTENGVKVNR"
/product="Cell wall hydrolase"
/transl_table=11
CDS 2097074..2097880
/locus_tag="AUO97b_02092" /db_xref="SEED:fig|400667.82.peg.1956"
/translation="MIKYKLCLALLLGASTVANAEFNIENNPNQPVYIKLGYQNVLNS
SFIYTDVSKNVDLKKNIVTVPYGEVTVLLNPVLNNNNEIKGKDYYFINYQPKILLGKI

QYKLPFVISDVGAIGTSIIYNDNNISIFSIESYANPVSNNNSKKFKSDIYIFNKKNLG
IYNPSFLSINVKLKSLNDASMKFTKLSSIKYNKNDKSYQVVYDFQKVTEDFTSTGKVN
YEKFPIQYSFSLIPDKNKPFLIKDIIEKRKGDKNIISKIENNDMYSYSLK" /product="hypothetical protein"
/transl_table=11
CDS 2097996..2098721
/locus_tag="AUO97b_02093" /db_xref="SEED:fig|400667.82.peg.1957"
/translation="MNKYFKSTVFTILMIGSIEGSYADFGFIQDKDGYVNVRENSSLS
SKVISKLNNNEIVSCAMDEGTNNFCLVNASNGVTGFIYKNRINNFSGYTSIKLSQYSR
EKAVYNDKNIIVEVYAKKAILDPKLYKNFKGEYKYFNNKQFFGTDGNLPNNDFLQLDK
IIVKEKDNRIEISRTELEQYFFPKDGIDEGKNELADFKIYYLNNNIFILNTFNDGGVA
AYNIVLNLKNGKIVTNKAWKVEI"
/product="hypothetical protein"
/transl_table=11
CDS 2098791..2099462
/locus_tag="AUO97b_02094" /db_xref="SEED:fig|400667.82.peg.1958"
/translation="MIYKKLVFVCLLGLSVCTIQYPIEGAYANSFEIQKVNNNQSIES
TEKIRKIKKSLDNKIYLLLDSDEGCIVSEKPLNKDIVIIYKEDEKFKYDIVNPILKKD
SSDCVSELAFVTDEPNYFYNINKRSKELVRGYGFDLNKDQVLYKNNKIRGVDLIGNGI
PNIITECFSNEGAHFNILGGYNNEEKLLHRYTYLDMNLQASCTENEEIYSKGLLDLRN KYREN"
/product="hypothetical protein"
/transl_table=11
CDS complement(2099597..2100253)
/locus_tag="AUO97b_02095" /db_xref="SEED:fig|400667.82.peg.1959"
/translation="MKEIVVFDLDGTLLSGDSTRTWLTDKLKSNIFRFIGALIVTPIA
LPLMKFKKYKSKGASLYLWIATYSLNEQELEYSFKNFSKNINETTFSSLYWFEQGIAE
VKDHLANGRIVFIATAAPEKLAKVLLDSINLNVQIIGTPLQNKLGGWVSGIHCRAEEK
VKRLNKLDIKQPWFATYSDDIEDDLPILIGAKLPYLINGDNNKIKNIKIKNLKMLNWF "
/product="hypothetical protein"
/transl_table=11
CDS 2100606..2100752 /locus_tag="AUO97b_02096" /db_xref="SEED:fig|
400667.82.peg.1960"
/translation="MKEKNNELYFFKVQSGEKLGKVVFKEGKYYLYSDDVLDIKDNLF YKDK"
/product="hypothetical protein"
/transl_table=11
CDS complement(2100817..2102160)
/locus_tag="AUO97b_02097" /db_xref="SEED:fig|400667.82.peg.1961"
/translation="MPSLTPLHETYCKWDRTPPSQADVLEGLAQLFSTSLNGVHELIQ
VINIEVLKNAFGISAQNAKNFQKRPAVQKAYQFSYGALQKYGSCFLAPGLRRIIEKFP
TLHEKPLTPTLHFLVGVLNGIFGDYLLKQHSPLALPMVLYDHYGAIQQGELAGRIVIL
VHGLCMNHLTWSNAHYGGIGERLLAQRDHNTMLYLNYNTGRRISANGRSFSNLLEDLV
QRNPRITSIDLIGHSMGGLVSRSALFYGKQNMYQWIHMVENLVCIGSPHHGAVLERFG
FALQDRLGAFPFVGLIGQLVNIRSNGILDLRHGSVRDDDWEYMDARIGMMDDNRAPAP
LPSHINTFLVAGTLEFEKVRNKALTVIGDYLVSVKSALGEHPNPRFQLKVPDSHKAVF
YGLNHFEIQYHSSVAEQITRWLYPHVNDYVQDGIQTHIIDMPNYTLEDLEGIVET"
/product="hypothetical protein"
/transl_table=11
CDS 2102465..2102953
/locus_tag="AUO97b_02098" /db_xref="SEED:fig|400667.82.peg.1962"
/translation="MHLPDLNRSPEQVVAQVSELIESLQEVALVGSSLGGFFATYFVA
KYNIPAVLINPAMQPWKLFDELFQVESMPYKVNDQWSIDHVQLRQLEQLELKQPENAD
KILVLLQQGDEILDYRQAQRYYSAATPSSLILTDAHGNHAMEDFEEKLPLVIEFFAHT IK"
/product="Esterase YqiA"
/transl_table=11

CDS 2102968..2104851
/locus_tag="AUO97b_02099" /db_xref="SEED:fig|400667.82.peg.1963"
/db_xref="GO:0003676"
/db_xref="GO:0003677"
/db_xref="GO:0005524"
/db_xref="GO:0005694"
/db_xref="GO:0006259" /db_xref="GO:0006265"
/db_xref="GO:0006268"
/db_xref="GO:0006304"
/translation="MTQYTAQSLEVLSGLDPVRRRPGMYTDTSRPNHLAQEVIDNAVD
EALAGHADKICVTVYKDGSLSVEDNGRGMPVDIHPEYGQSGIEIILTKLHAGGKFSTD
NYQFSGGLHGVGISVVNALSTRVEVEVQRQGNLYQMAFEQGEPVAPLAVLEGKAPKRA
TGTTVRFWPEAKYFDSPKFALKALKHNLKAKAVLAAGLKITYIDQINDEKIEWQFENG
LVDYLMDELQDREILPNPAFVSSGQADRAACEFAICWNVEGGEQVQESYVNLIPTAQG
GTHVNGLRSGVTEALREFCELRNLLPRNLKLSAEDVWDGVNYILSLKFQEPQFSGQTK
ERLSSREAANIVLNIAKDAFALWLNQHAEIAMQLAEMAISKAGRRLKAAKKVERKKIV
SGPALPGKLADCVGQTREESELFIVEGDSAGGSAKQARDKNFQAIMPIRGKILNTWEV
SSDEVLASQEVHDIAIAIGVDPGSDDLSELRYGKICILADADSDGLHIATLLCALFVK
HFPALVEEGHLYVAMPPLFRIDIGKDVHYALDDEELETILKNVKGNKNPQITRFKGLG
EMNAIQLRETTMDPNTRRLVQLDLDDAHLTAGLLDKLLAKKRAADRKQWLEQKGNLAD ITV"
/product="DNA topoisomerase IV subunit B (EC 5.99.1.3)" /transl_table=11
CDS complement(2104973..2105476)
/locus_tag="AUO97b_02100" /db_xref="SEED:fig|400667.82.peg.1964"
/db_xref="GO:0004848"
/translation="MIMKNIQIQPLTIENFQPFGEVICCDGHDFFHINDAHTERYHAL
VETEFEGEAKAGISIFRNIKASVLPMEISMLERHPKGSQAFIPLQEQKFLIIVAPALD
ENTPDISKLCAFVSDGKQSINYRAGTWHHPLLTFEAPSDFAVVDRIGGGTNCDIFQFP HPIKITV"
/product="Ureidoglycolate lyase (EC 4.3.2.3)" /EC_number="3.5.3.19"
/transl_table=11
CDS complement(2105473..2106483)
/locus_tag="AUO97b_02101" /db_xref="SEED:fig|400667.82.peg.1965"
/db_xref="GO:0004037"
/translation="MATLHAPAFELPEILNTKTNLADARIGAQVIECSDDFFAEAKRM
LQFEAPIFVEDKFDDHGKWMDGWETRRKRHAGYDWCIVKLGVSGKISALDIDTTFFTG
NYPASASLEACYAPNGDLTGAKWQSILENTELGPSQHHIFMVNNDAIFTHIRLNIFPD
GGVARLRVYGDVHIQVTDHEQTLDLLALENGGRVIAYSDAHFGHPRNLINPGRGVNMG
DGWETKRRRAPGYDWCILALGKSGKIEKIEIDTAHFKGNFPAEVSIQAVYLENATDAQ
LIPQSMFWSYLLEAQPMQMDHIHEYVNEILQYEKVSHIRINMIPDGGISRVRLWGKIA KS"
/product="Allantoicase (EC 3.5.3.4)"
/EC_number="3.5.3.4"
/transl_table=11
CDS 2106683..2107840
/locus_tag="AUO97b_02102" /db_xref="SEED:fig|400667.82.peg.1966"
/db_xref="GO:0018658"
/translation="MNVVIIGAGMGGLTTGIALKKFGHQVRIFEQTEKILPVGAAISL
WSNGVKCLNYLGLTEKIAKLGGQMDDLAYVDGLTGDVMTQFSLRPLIEEVGQRPYPVA
RADLQNMLMDEFGRDQIYLGKKMVGLEDKADFVEVHFADGSSTQADLLIGADGTHSMT
RAYVLGQQVQRRYAGYVNWNGLVEISEDLAPAQQWTTYVGEGKRASLMPVADGRFYFF
LDVPLPAGLENNRDEYKKLLKQYFADWCQPVQQLIERLDPQKTNRVEIHDIEPFTQFY
KGRVVILGDAAHSTTPDIGQGGCQAMEDAIYLARSLQINTLGLEDALRRYQNKRNERA
NELVLRARKRCDVTHMKDETVTQAWYAELRREQGGHIMQGIISNIVGNPLD"
/product="Uncharacterized oxidoreductase" /EC_number="1.14.13.1"
/transl_table=11
CDS complement(2107893..2108618)

/locus_tag="AUO97b_02103" /db_xref="SEED:fig|400667.82.peg.1967"
/translation="MQLKQLAATCALLSATAMVQAKPIWQDFSVTGLYGENYEVVDDK
ETTITLEYAAKVKYADVFFFMDRMRGEDDYKSTYFELSPRLSLGEVSGKKLTYGPIKD
VLISTTWESNTQNGNNFDNFLYGFAVDLDIPYFQYANLNFYRANNEKTDDDYQMTFVY
GIPFKIASEDFLVDGFLDWSTAEDDHASELNWTTQWKWNVGKHISPDTRLYLGIEHSV
WNNKFGIKGADENNVSALVKYHF"
/product="Nucleoside-binding outer membrane protein" /transl_table=11
CDS complement(2108740..2110107)
/locus_tag="AUO97b_02104" /db_xref="SEED:fig|400667.82.peg.1968"
/translation="MNHQQKNAISPEHEYLGIAKSFAYGLQHVLTMYGGIVAPPLIIG
TAASLSAAQVGMLIAAALFVGGLATLIQTIGTKYLGAKLPLVQGVSFAGVATMVAIIT
TGGGLPAVYGAVIAASLIGLCLAPYFSKIIRFFPPVVTGCVITIIGLSLLPVAIRWMM
GGNNKAPDWGSVENISLALLTLGIVIILNMLPQASIRRLSILLAIVAGTTLAYFMGFG
DFSQVSSGAWLQFPHLFAFGLPTFELSAILSMLIVTLVIMTETTADIIAVGDIVGTEV
DSKRIANGVRADMFSSAIAPIFGSFMQSAFAQNVGLVAITGIKSRFVVAAGGVILIIL
GLLPVMGRLIAAIPMPVLGGAGLVLFGSVAASGIRTLAKIDYNDQKNLIIVATALSAG
MIPIINHEFYAHFPVWVQTLFHSGISSTCIFAILLNLLFNHLPSFRSSRTPHLSQTIN TRNTH"
/product="Unknown substrate:proton symporter, NAT/NCS2 family"
/transl_table=11
CDS complement(2110132..2110455)
/locus_tag="AUO97b_02105" /db_xref="SEED:fig|400667.82.peg.1969"
/translation="MISTHILDTNLGKPAAQVEVKLFYAATHELIATAITNQDGRVSD
FGIKNLGKGAYQLEFEVAPYFSSQQIKTFFPRVCIQFFIEDINQHYHIPLLISPFAYS TYRGS"
/product="5-hydroxyisourate hydrolase (EC 3.5.2.17)" /EC_number="3.5.2.17"
/transl_table=11
CDS complement(2110452..2110955)
/locus_tag="AUO97b_02106" /db_xref="SEED:fig|400667.82.peg.1970"
/translation="MFLAEFNQAPPDQLKTLLNNCVHIPVWAEKIISERPYSSTSVLL
GYAEQLSHTWSWQEIQAALATHPKIGERQAKKQLNAKEQNFSAQEQAGISLDEQTQQA
LLQGNIEYEQKFGFIFLIKAAGLSSDEILKKLHQRLQNNLAIEKTIVHEQLAAIALLR LSQEVQA"
/product="2-oxo-4-hydroxy-4-carboxy-5-ureidoimidazoline (OHCU) decarboxylase
(EC 4.1.1.97)" /transl_table=11
CDS complement(2111055..2112020)
/locus_tag="AUO97b_02107" /db_xref="SEED:fig|400667.82.peg.1971"
/translation="MSESYLSGAELIEQIQKSPYSRDLIGYHGQPPEVKWPNNAKVAV
QFVLNYEEGGEKHIEHGDEGSEQFLSEIIGAASYPAKHMSMDSMYEYGSRAGFWRIHS
EFQRRKLPMTIFGVAMALVRNPYIVEAIKQADYDVVSHGYRWLHYQETDLEVEKQHME
QALSVLENLFGNKTIGWYTGRDSPNTRQLLAEFPQIQYDSDYYGDDLPFWTTLTDTAG
ASRPHLIIPYTLECNDMKFCSPGGFNSSEQFFQYLKDSFDVLYAEGETAPKMMSIGMH
CRILGRPGRFKALQRFLDYIEMHDRVWVCRRQDIAEYWYKNHINQ" /product="Uricase (urate
oxidase) (EC 1.7.3.3)" /EC_number="1.7.3.3"
/transl_table=11
CDS 2112273..2112623 /locus_tag="AUO97b_02108" /db_xref="SEED:fig|
400667.82.peg.1972"
/translation="MNIEIRTDKNIHNSERLITYVRAELTQEFQRHSERITHFSVHFS
DENGDKGGDKDIHCMIEARPSGLKPVAVHHKAGNIDASIHGAIEKLKRSLEHTFEKKE HPRGGQPEFIDDEV"
/product="Ribosome-associated inhibitor A"
/transl_table=11
CDS 2112776..2113327
/locus_tag="AUO97b_02109" /db_xref="SEED:fig|400667.82.peg.1973"
/translation="MVMLTTQQQALIKAIEELELAQVQKLLAEGLDPNFIDPEQGPPV
SIICDGIFKWWEDVSEAYEAGTPLSQEEKQQALQVYLDILEALIQAKANVHLWDAEEF
YGPLWDAASSACAPAVQRLLDEKVDPNTRDEEGLTILSSISQLFFDCDFDEIDWSEAL
QEERETLELLRRHGAKMSKELTT"

/product="FIG00350877: hypothetical protein" /transl_table=11
CDS 2113340..2114107 /locus_tag="AUO97b_02110" /db_xref="SEED:fig|400667.82.peg.1974"
/translation="MRTFQILTFALTAVAAGSTFAAEFSFDRPGSGFGTGITPVGKLA
WEQGLPSATYTETTVDGVKEKTVTVNEDMLFRTGLADGLELQLGWQGPAWTQTKRAGK
KTDNSGFGDVSIGLKKAIDLNDENLTMAVLAEAVIATGNDEFTAHDDIYSLSSAVAYK
YNDLLDTSITMRYEVQNSNWAVTAIPTINYKIAGKLSGYSEFVYRKAESQDYQYGLGT
GLVYAVNNRTQLDANIGVDLEGQDKSYKGGLGMAFLF" /product="FIG00350252: hypothetical protein" /transl_table=11
CDS 2114136..2115950
/locus_tag="AUO97b_02111" /db_xref="SEED:fig|400667.82.peg.1975"
/db_xref="GO:0047134"
/translation="MKKLISQGIIYFLVAATFVLSQFVFAEDKLLSPEQAFSFSVESP
QSHTAKLSWQIQPNYYLYQHKFTVQQGNQPLTLNLPKAVSQYDENYGQSQVYYHQVNF
QIKTKPSEHYTVTWQGCAKDRICYPPQTIEFQTDADGLVGLQNQAGAAPKRLLDLANT
QASSSTESLENQSSTTQAERAAPEIAQDQVWSAKLIEHSLWYGVLLFLGLGMLLAFTP
CSLPMLPILTSLIIRDHKGLKAWTIALTFVCSMALVYAGLGLIASSAGLNFQRWLQQP
ATLIAFSLLFVVFALNLFGLFELRLPQTWLNRLDRIQSMQQGGTLVGAGVMGVVSALL
VGPCMTAPLAGTLLFISQTQNQWQGALLLFCLGFGMGIPLLIASVLGAKFLPKAGDWM
HQIKVIFAFLMLGLSLYFIRPLLPDWGMQVLSLLLGLAFIIYALYQFFWKKGQLQWLY
VLLLLLVTPFVVYNQYQHIQNRSLQNSEHMAEWHVATTAADFQKILAAAPADRAIVVD
VYADWCVACQPIEHRILKAPEVQMALAPYYLIKLDLSHYDQSHEALLKQWNILGPPTY
LFLNKQQQEIRSLRLTGAFSQQELLQQLASLSKAKDSN" /product="Cytochrome c-type biogenesis protein DsbD, protein-disulfide reductase (EC 1.8.1.8)"
/EC_number="1.8.1.8"
/transl_table=11
CDS complement(2115942..2117135)
/locus_tag="AUO97b_02112" /db_xref="SEED:fig|400667.82.peg.1976"
/translation="MKIRLITRPINKIKQVILDFIHHNHIINWTPLQKSALMLTLACA
MNFSWILWKGYILITPSIWQWANLSLVESQIWLNLLTLFLLAVLIIPCYRYKDQAWAQ
KIVPLISVQAFMLMLCHDGYLIGSISPATMVGYVGTIGVGLVLFERKIVYSAFIPATI
ILAICTYLSMTGVISYAPLFNFTAMRHAQTNPFWLGSMLFFIMPILVACFILFEILLT
QWRQRETYIQRLSQIDPLTNVLNRRSLNTHLEALHEQQFDYAVILLDIDHFKKINDIY
GHHQGDQVLIEIARCLSENLRNEDIIGRFGGEEFILLLPHTDIIQAEKIAERCRQALQ
ELTIFNNQNIQIHVSASFGISSSAFANDPYLVIRQADQALYAVKASGRNQVRTFHQLP QIKSI"
/product="GGDEF domain protein"
/transl_table=11
CDS complement(2117311..2118222)
/locus_tag="AUO97b_02113" /db_xref="SEED:fig|400667.82.peg.1977"
/translation="MNIPNVLKYYFTETFLKTAIRKPSQLNLPPTALRPMLEQLCRAF
PKQKNVTVRPIRLAGIKGEEIKAQDSATQLIFHIHGGAFFLGSLKTHHAFMTDLAART
QMQIIHVDYPLAPEHPYPEATEALYDVYQSLLVQGIQPKDIILSGDSCGANLALALCL
RLKEQPELMPSGLILLSPFLDLTLTSESLRFNQKHDALLSLEALQIGIQHYIGNHIAA
DDPRVSPIFDNLEGLPPTLVQVGSKEILLDDAKRFREKAEEAGVKVHFKLYTGMWHNF
QMFNAWFEEAKQAMADIAEFAHELDKT"
/product="Esterase (EC 3.1.1.-)"
/transl_table=11
CDS 2118372..2118809
/locus_tag="AUO97b_02114" /db_xref="SEED:fig|400667.82.peg.1978"
/translation="MKKIALALGLVGMAAFANAADPLNGTVWKTIDDQTNKPKAVVKF
TEQKDGTLTATIQNILTPGEENACTKCEGPYKNKPLKGVTIVHNLKNVGGNSYENGTI
LDPKSGKTYKLKGELAEGGKKLKLRGYIGVSALGRNQTWIRAN" /product="FIG00350325: hypothetical protein" /transl_table=11
CDS complement(2118847..2119197)

/locus_tag="AUO97b_02115" /db_xref="SEED:fig|400667.82.peg.1979"
/db_xref="GO:0008794"
/translation="MLKIYGIKNCNSMKKAFDALQAKGLSYEFHDYKKQGIDADTLKI
WLKEIGQDTVLNKKGTTWKKLSEEEQTRALSSEDHLIEALIAQPSLIKRPVLQTSKGY LVGFDETAYQNLSA"
/product="FIG138056: a glutathione-dependent thiol reductase"
/EC_number="1.20.4.1"
/transl_table=11
CDS complement(2119653..2120477)
/locus_tag="AUO97b_02116" /db_xref="SEED:fig|400667.82.peg.1980"
/db_xref="GO:0008853"
/translation="MIPKDQYPSDVKILRVVSINVNGLRSSVTKGLLEWLEQSDADVV
CMQESRITHEQWTEKFRPEGWHTHLFPAERAGYAGTAIYSRLPFVSIKDGLGFELADS
QGRFISAEFDLGLSHPVHIASLYLPSGSSGEEAQARKDLFLGEYAKILKQWRDENKSI
IICGDYNIVHKRIDIKNWSGNQKSSGCLPHERAWLDHIYDELGYVDTFRVVRTEAELY
SWWSNRGQARAKNVGWRIDYHACSPDWKARTVNAWVYKDQWFSDHAPVIIDYKIQE"
/product="Exodeoxyribonuclease III (EC 3.1.11.2)" /EC_number="3.1.11.2"
/transl_table=11
CDS 2120516..2121166
/locus_tag="AUO97b_02117" /db_xref="SEED:fig|400667.82.peg.1981"
/db_xref="GO:0004588"
/translation="MTTPVSFHPQAFIELALSRGVLKFGEFTLKSGRVSPYFFNAGLL
NDGEALSLLAQGYADKLTQCENVDVIFGPAYKGIPFVAATAVALSQVHNKSVPWGFNR
KEAKDHGEGGVLVGAAVEGKKVWIIDDVITAGTAIREVVTILKNAGATIAGVLVALDR
QERGQGELSAIQEVQKELEIPVHALITMKDLMDYLEAKGEKEALANMQAYREKYGI" /product="Orotate
phosphoribosyltransferase (EC 2.4.2.10)" /EC_number="2.4.2.10"
/transl_table=11
CDS 2121407..2121601
/locus_tag="AUO97b_02118" /db_xref="SEED:fig|400667.82.peg.1982"
/translation="MTKQPEWIIRGKSIKQLIKELESFEDQNLLVELSFDDGETSKPI
SLVSKIDGKCMLLNCENSTE"
/product="hypothetical protein"
/transl_table=11
CDS complement(2121845..2124034)
/locus_tag="AUO97b_02119" /db_xref="SEED:fig|400667.82.peg.1983"
/db_xref="GO:0004872"
/db_xref="GO:0005215"
/db_xref="GO:0006810"
/db_xref="GO:0016020"
/translation="MLRISIPSPQMKRLFPLSLLSLMILNIQGANAEELIDSSSKAAA
TMPTIKIEAMSELDPIKSYIDYDKANVTRNGLDKKDIPQTVDTIDVQKYKIYGSNDLS
VMLQGTPGVSTSYDMRGDGITIRGFGADTGDIYRDGIRESGQVRRSTANIERIEILKG
PASVLYGRSAGGGVVNMVSKFANFDSKSSVGAYAGSYDNYGTTADINQVLNDNLAVRL
TGEYSEAGSFRSGIENKIEMFSPSFTYKNDDGKLTWTTQYTYDKLGRVPDRGPTRNNL
PAGTSIKMGFAQDGDYVDDILQVVRTDVNYEYAPDWNFHWAASYRQAEQNFDHFYFGN
YCGLDGKNSKNEACTKKGYIDQIYYWQQTSNKTTTNTFDIKGKFKTGQLEHQIMVGTD
WTYEQREPRLANKTQNGSAIYGYVNPITGEREYSRGNGPLKISQHNYNEGTTYGVFMQ
DLIGLNDQLKLMMGLRYDYFDFSTTNKIKNEHRNVKDSTFSPNVGLVWQPVPEHSFYT
SYSKSFAPFGGQMGVNQVTGSTDVAKMDKEPQYNEQYEVGVKSEWFDNRLNTQFSVFD
IRKNNIRYKPNPDSEPEVWATAGQHQSRGLEFSFIGRVLDNVFVRGGYGYTDAKVKED
KQNPEREGNYLANTSKNTGNLFVRYLPTEQWYTEVGVTYVGSYYPNINNQVKMEGFNR
VDAAIGYSADPWNVTLAVNNLTNKEYWRSDSMPGTPRNVLLRLNYQF" /product="Ferrichrome-iron
receptor"
/transl_table=11
CDS complement(2124264..2124476)

/locus_tag="AUO97b_02120" /db_xref="SEED:fig|400667.82.peg.1984"
/translation="MKLYRYLTGPDDSAFCARVTKALNHGWELYEAPTMTFNGSQVIV
GQAICKTIDENYDPEMDILDVLKNNA"
/product="Hypothetical protein in cluster with mesaconyl-CoA hydratase"
/transl_table=11
CDS 2124637..2125581
/locus_tag="AUO97b_02121" /db_xref="SEED:fig|400667.82.peg.1985"
/db_xref="GO:0004363"
/translation="MRVLVVMDPIETVNLKKDSTMAMLWAASRRGHELGYALQQDLYI
DQGKAYGLISPLKVFEDYNHYYELGEKKKESIAAYDVVLMRKDPPFDMNFVYTTYVLE
QAEREGSWIINKPQSLRDCNEKLFATQFPELQVPTLVTSQQSLIREFITEYGDVIVKP
LDGMGGMGIFRLYQDGVNIGSTLEMLTELGTRPIMAQRYIPEIVEGDKRILMVNGEPI
PYCLARIPQNGEVRGNLAAGGLGQARPLTENDKLIAAKVGPFLREKGLVFVGLDVIGN
YVTEINVTSPTCIREIDAQFGTSIADDLFDVLEAGRPA" /product="Glutathione synthetase (EC
6.3.2.3)" /EC_number="6.3.2.3"
/transl_table=11
CDS 2125819..2126916
/locus_tag="AUO97b_02122" /db_xref="SEED:fig|400667.82.peg.1986"
/db_xref="GO:0050511"
/translation="MTDSQQSKPKHVMMMAAGTGGHVFPALAVAKQLQQQGCQVSWLA
TPTGMENRLLKDQNIPIYQIDIQGVRGNGVIRKLAAPFKILKATFSAMRYMKQLKVDA
VAGFGGYVAGPGGLAARLLGIPVLIHEQNAVAGFTNAQLSRVAKVVCEAFPNTFPASE
KVVTTGNPVRREITDILSPKWRYDEREQADKPLNILIVGGSLGAKALNERLPPALKQL
EVPLNIFHQCGQQQVEATQALYADAPANLTIQVLPFIEDMAKAYSEADLIICRAGALT
VTEVATAGVAAVFVPLPIAVDDHQTANAKFLADIGAAKICQQSTMTPEVLNQLFTTLM
NRQLLTEMAVKARQHAQPNATQHVVDLIQKM" /product="UDP-N-acetylglucosamine--N-
acetylmuramyl-(pentapeptide) pyrophosphoryl-undecaprenol N-acetylglucosamine
transferase (EC 2.4.1.227)" /EC_number="2.4.1.227"
/transl_table=11
CDS 2126928..2128376
/locus_tag="AUO97b_02123" /db_xref="SEED:fig|400667.82.peg.1987"
/db_xref="GO:0008763"
/translation="MSPTTAANQAKKLIKVPEMRRIKHIHFVGIGGAGMCGIAEVLAN
QGYKISGSDIKASKTTQQLEENGIKVYIGHEAENIKNANVLVVSTAIDPENPEVKAAI
EQRIPIVRRAEMLGELMRYRHGIAVAGTHGKTTTTSLLTTMLAEENLDPTYVIGGLLN
STGVNAALGESRFIVAEADESDASFLYLQPMAAIVTNIDADHMDTYEGSFDKLKDTFV
QFLHNLPFYGLAVVCGDDANIREILPRVGRPVITYGFNEDNDIRAIDVEQDGMRSHFT
VLRKGREPLRLTINQPGLHNVLNALAAIGVATDEGVSDEAISRALKGFSGVGRRFQVQ
GEFELGEGNVKLVDDYGHHPKEVEATIKAARQSHPDRRLVMLFQPHRYSRTRDCFDDF
IEVLSQVDQLLLLEVYPAGEKPIVGADSRTLARSIRLRGQVEPILIDPVEGNLQNIMQ
NVLQPNDLLLTQGAGNVGAISVELAQHHLYVK" /product="UDP-N-acetylmuramate--L-alanine
ligase (EC 6.3.2.8)" /EC_number="6.3.2.8"
/transl_table=11
CDS 2128414..2129340
/locus_tag="AUO97b_02124" /db_xref="SEED:fig|400667.82.peg.1988"
/db_xref="GO:0008716"
/translation="MSNATKFGKVAVLLGGKSAERAVSLDSGQAVLDALLRSGVQAEA
FDPQDRSVTELVNYDRAFIVLHGRGGEDGQIQGVLEWLNIPYTGTGVQGSAIGMDKVK
TKQIWQGSDLPTAPYRIITKETDLDSVIAELGLPVIIKPVHEGSSVGMSKVEKAEDFA
AAIEKATQHDAVVMAEKWITGREFTISFLNGQPLPVIRLQPPADVAFYDYEAKYQRND
VEYGIPCGLSETEEKKLQALCLRAFQAVGAEGWGRIDAMQDEQGNFWLLEVNTVPGMT
SHSLVPKAAKAVGYSFDELCVAILEQTLEGTA" /product="D-alanine--D-alanine ligase (EC
6.3.2.4)" /EC_number="6.3.2.4"
/transl_table=11

CDS 2129343..2130197
/locus_tag="AUO97b_02125" /db_xref="SEED:fig|400667.82.peg.1989"
/translation="MAQLPASMRRKRAAITSIHEKPPTRKQKLANAGGWVLLVIAFLV
LAVGIYGLYKVITDATVAKLEVVGSTTPVETQQVMRYVQPIVKDNYFTSDLEQIRDKA
LEISWVDRVVVSRAWPNGIRVRVMPRHAIARWGTGRLLSDGGDVFSEAEPRLHPELPL
LHGPVSQSKMMMRRYNEINQLFHPVNLRLKELYLTERMTWFMQFDSGLRIIVDQDQTM
NKLQRLSHLAQSDLKPVWPKISAIDLRYRNGLSIQWKNATPPKIVNGQFVVTSDDTSI AGGTKAKP"
/product="Cell division protein FtsQ"
/transl_table=11
CDS 2130258..2131520
/locus_tag="AUO97b_02126" /db_xref="SEED:fig|400667.82.peg.1990"
/translation="MSEAVPSVVAIDIGTHKVSVLIGKIHAPDNIQVIGMATARNRGM
NKGKIVSLDKVIAAIKNAVAEAENMAECRIHSAWVSIPSTELQSFYASGRTPVANPAH
VITTNEVVRALELAKASHVTSDYYLASAVPLGFELGDSSEWVQNPINMTAHSMTGHYQ
LMMMPIATMQNLDRAMKGANIGVEKMVVSCLATAEASLLKDEKEYGVCLVDIGAGITN
LAVYLDGRLALARTLQRGGEHVTRDIAAVLQTTTEEAERIKILHGCVDLSAVKPDHMI
QVQGIDGPQTISRIELAEIIIARYEEIFSQIREELEQSGAIHGLYHGVVLTGDACQIE
GMVSLARRMLGVSAHLGNPPLQVYADDQHQAALRRSMYATAAGLLMFSQSELQEAVEE
PEEGNDRSVWERMVNGWNAFNSKLKAIF"
/product="Cell division protein FtsA"
/transl_table=11
CDS 2131685..2132860
/locus_tag="AUO97b_02127" /db_xref="SEED:fig|400667.82.peg.1991"
/translation="MASFEFIEDELNDGNGQARFTVFGVGGGGGNAVQHMVQSDIQGV
KFVCANTDKQALDCMNAPFKIQLGEQSTRGLGAGANPEVGQVAAEESREIIRQHLEGT
DMVFVTAGMGGGTGTGAAPVVAEVAKEMGILTVGVVTTPFNFEGRRRQKSAERGIEAL
EAHVDSLIIIPNQRLLSVYGDISMKDAYKKADDVLLNAVRSIFDLVVNRGHINLDFAD
LKTAMSTRGYAMMGAGLGRGEDRARQAAEQAIRSPLLDNVNIINAKGVLINITGGDDI
TLRETEIITDVVNQIVDLDEGEIFYGTVFDPDARDELRVTVIATGLTRNAADAEPRKR
NTVSHTSTQSAQSVDEDDVPAINKRQNAENDVNNAPSSTPRSSPMSIQDYLKNQQRK" /product="Cell
division protein FtsZ" /transl_table=11
CDS 2132971..2133873
/locus_tag="AUO97b_02128" /db_xref="SEED:fig|400667.82.peg.1992"
/translation="MVKQRTLNRVVKASGIGLHSGQKVMINFIPHTVDGGIVFRRIDL
DPPVDIPANALLIQEAFMCSNLVTGDIKVGTIEHVMSAIAGLGIDNLIVEVSASEVPI
MDGSAGPFIYLLMQGGLCEQDAPKKFIKILKPVEALIDDKKAIFSPHNGFQLNFTIDF
DHPAFAKEYQSATIDFSTETFVYEVSEARTFGFMKDLDYLKANNLALGASLDNAIGVD
DTGVVNEEGLRFADEFVRHKILDAVGDLYLLGHQIIAKFDGYKSGHALNNQLLRNVQS
DPSNYEIVTFNDEKDCPIPYVSVT" /product="UDP-3-O-[3-hydroxymyristoyl] N-
acetylglucosamine deacetylase (EC 3.5.1.108)" /EC_number="3.5.1.108"
/transl_table=11
CDS complement(2133971..2134411)
/locus_tag="AUO97b_02129" /db_xref="SEED:fig|400667.82.peg.1993"
/translation="MSKPDNVFQQTGKHIKTGNLTFLTTQVAQWQRLTKIIQPLLPQP
EQWQVVCYQNGSLIITGENQAMISQLSYLQSQYVSKLSQLEGLKDLQRIQVRLRNKTI
PVTTSSEPSKSIPPETQEMLRSAADFVSDPKLSQALLRLASNKK" /product="Zn-ribbon-containing,
possibly RNA-binding protein and truncated derivatives" /transl_table=11
CDS 2134519..2135211
/locus_tag="AUO97b_02130" /db_xref="SEED:fig|400667.82.peg.1994"
/translation="MHTRRILLAFSLAASAASVAFADYQNINQSTDSDRLEQLSKTLS
QGSYTHPDDLDLPASAKVSVTLREKTVELNNDSLAKKYGTTTAKNSFKTSSSNPYSWL
VSHPLPDTVRVSSNFGGRTMGGRAEHHGGLDMAAPSGTPIYATGPGIVTKSGWGTGYG
QYVEINHGNGYLTRYAHASRLMVRVGDQVSAGDHIANVGCTGRCTGPHLHYEVVKDGQ RKNPSTYLAMLP"
/product="Peptidase, M23/M37 family"

/transl_table=11
CDS 2135462..2138179 /locus_tag="AUO97b_02131" /db_xref="SEED:fig|
400667.82.peg.1995" /db_xref="GO:0004739"
/translation="MAFYGDSDAQETQEWQDAFDSVLQHMGTERAAFLLEKLYQQAIA
KHVPIQRLNTPYLNTISVEEQPAMPGDQDMERRIRALIRWNALAMVLRANKTGDDLGG
HLASFASSATLYDVGFNHFFRANSDNFGGDMIYYQGHCAPGIYARSFLEGRLTEEQLS
NFRREVGGNGLPSYPHPYLMPDYWQFPTVSMGLGPIMSIYQAHIQKYLMNRGLIKEED
RKVWAYLGDGEMDEPESLGAISLAGREKLDNLIWVVNCNLQRLDGPVRGNGKIIQELE
SIFRGAGWRVIKVVWGRHWDPLLAKDTSGALKARMEEAVDGDYQRYQVKGGAYTREKF
FGKYPEAAELVKDLSDEDIDNLNRGGHDPYKVFAAYAEAMKAKGQPTVILAKTIKGYG
LSEEIEAVNKTHQIKKMQIDSLRYVRDRFNLPFTDEQLEELPFYRPSENSPEMKYMKA
RREALGGYLPARRRESESLAIPDLSVFDAVLKGSGGKEQSTTMVMVRLIAALLKEKAI
KDRVVPIVPDEARTFGLEGMFRQLGIYAAHGQKYTPEDQEQLMHYREAKDGHMLQEGI
NEAGAMSAWAALATSYSTNNLPMIPMYMYYSMFGFQRIGDIAWAAGDAQAQGFLLGAT
AGRTTLNGEGLQHQDGHSHILANTIPNCVSYDPCFGYELAVIVHDGLQRMYVNQERVF
YYLTVMNENYEHPAMPEGVEEGIKRGMYLFEKDEKATVQLLGSGVILREVIKAAKILR
DEYQIHSNVWSVTSFNELARDGMACEEYNRLHPLAEEVKESWVSKQLRGTEGIVVSAT
DHMRAYSEQIRAYLPDGRPFVALGTDGYGRSDTRANLRSFFGVDAAHIVVATLKKLAD
EGEVDARLVKDAISNFELDTDRPVAWAPQAHPEVQPVAEYNETQTGEGN" /product="Pyruvate
dehydrogenase E1 component (EC 1.2.4.1)" /EC_number="1.2.4.1"
/transl_table=11
CDS 2138182..2140170
/locus_tag="AUO97b_02132" /db_xref="SEED:fig|400667.82.peg.1996"
/db_xref="GO:0004742"
/translation="MQIKTPDIGVDKANVAEILVKVGDRVEVDDSIVVLESDKATVEV
PSTSAGVVKSILINQGDDVTEGVALIEIEAEGAAQPAPEPTPAPAAEKPAAPAPAQQT
QASAQPAAATSSATVEVTVPDIGVEKALVGEILVKVGDQIDVEQSIVVVESDKATVEV
PSSVAGTVESIQVKEGDTVKEGVVLIQVKTAAASNAQAEAPATTPAPAPAAAAEPVAA
KQKTVAAAPAQSGSVDINVPDLGVDKAVVAEILVQVGDKVDVDQSLVVVESDKATVEV
PSTVAGIVKAIHLQAGQQVSQGVLLATIEAEGQAPAAAPAAKAEAAPAPQAAAPKAAA
PVATQSAPAASTSGTDKLTKEQEAENAKVYAGPAVRKLARELGVILSQVKTSGEHGRV
VKEDIFAYVKSRLTAPQAAPVAQATAAPAGLPSLPDFTAFGGGEVKPMTRLQQVSVPQ
LSLNNYIPQVTQFDLADITELEAWRGELKDGFKKQGVSLTILAFIAKAVAHLLKEEPY
FAGHLADDQKSVLLRNEIHMGIAVATPDGLTVPVLRNPDQKSIKQIAVELGELSKKAR
DKKLTPKDLQGANFTITSLGSIGGTAFTPLVNWPQVAILGISPATMQPVWNGKDFDPR
LMLPLSLSYDHRVINGADAARFTNKLTKLLKDIRTLLI" /product="Dihydrolipoamide
acetyltransferase component of pyruvate dehydrogenase complex (EC 2.3.1.12)"
/EC_number="2.3.1.12"
/transl_table=11 CDS 2140319..2140591
/locus_tag="AUO97b_02133" /db_xref="SEED:fig|400667.82.peg.1997"
/translation="MLGFLKIMLPLLLVVFLLMGIWVSILHFPWEWMTYVVMGIEVFV
ATLILPLEYQTGTSSSHIGFISISACFAVLLGIARLFVWTWVKFFA" /product="FIG00351517:
hypothetical protein" /transl_table=11
CDS 2140840..2141415
/locus_tag="AUO97b_02134" /db_xref="SEED:fig|400667.82.peg.1998"
/translation="MQEFLISTSIVALAEMGDKTQLLALLLSARFRKPIPILIAILLA
TLINHGISAVLGQWITTVLSPEILVWVLAVGFIGMAFWMLIPDELDDETASINKWQKF
GVFGATFILFFLAEIGDKTQIATVALAARYDSIFWVMLGTTLGMMIANAPAVFIGNKL
AERLSIALIHKIGAAIFFIVGVSTLVQHYFF"
/product="Putative transmembrane protein"
/transl_table=11
CDS complement(2141474..2143630) /locus_tag="AUO97b_02135"
/db_xref="SEED:fig|400667.82.peg.1999" /db_xref="GO:0004872"
/db_xref="GO:0005215"

/db_xref="GO:0006810"
/db_xref="GO:0016020"
/translation="MTQRFAFSTSKTLLAVAIFTSMTTSHAEETAEQQNSTNVLPTIS
IQAQKENPTSYVATKANSALKSDAPLFKTAQSVSVVTREQLDQKQARTLTDALEGVAG
VEAGKLGRRGWDDFIIRGQTSSDSVYVDGLRVGQNTYVATELSGMDQVQILKGPASIN
FGLVAPGGMVNLVTKRPEAESFARASMTYGSYSLKEGTFDLNYSPNNSEKGAFRLNGR
ISDQDDPTDYVYFKNFYISPSYNFDLGDNTDLSVSASYQHREYIRQQGLPVIGTLKNN
PNGPIDRSLYIGDPNFGKYEADVYRTGYTFKHTFDNGWNFNQNFAVQKTEMDGKAVFA
RTGSNFWAKDKQGEIDYTTISRRNNSRHQVIDNLSFAIDNRLNKQFDLYGMQHDINIG
VDAFQEKSDYTNDKYDIGDLNIYNPVYGQNVTLKQNVRDINRLKYLGLYLRDRIQLND
QLLLSLSGRQDWAQTQTTSLVTGNASKQSDNAFTGSASVMYTLNDIVAPYVSYATSFT
PNSGTDVNSNPFKPEKGKQVEVGMKLQSPGQRIQGAIAWYDLKRQNVLVTDTANSGYK
VQRGEQLTRGIETELSAEILEGLKLTAAYTYTIDAEISKDANASNVGKALDNIPEHAY
SLSARYKFDPSSKLGWYVGGGFRGETYKTVDGLDVHVPGYTVFDTEAGYDAERWGAQL
AIRNLFDKDYYAGALNENLVTLGNPRQINFTVKFNY" /product="Ferrichrome-iron receptor"
/transl_table=11
CDS 2143859..2144656
/locus_tag="AUO97b_02136" /db_xref="SEED:fig|400667.82.peg.2000"
/translation="MGFEKTTSQILFDNGVHKCISFTSLVKGEGIQANQFLIIDHERA
AVIDPGGDLTYVPLTMELNKYTRLKNLDYVMASHQDPDIITSMPRWLVYTDAKVVASK
LWARFLPHLNSSFMSERMKGNWEDRLIELPDRGQVIQLGESKLVIVPAHFLHSVGNFQ
FYDPVAKILFSGDMGASIVDDASQPLKDFDSHVMKMKAFHQRYMCSNKVIRLWVNMVR
QMDIEMIVPQHGSPFIGKEMINQFLDWIETLPCGVDLMTEQIFSCPT" /product="MBL-fold metallo-hydrolase superfamily" /transl_table=11
CDS 2144748..2145113
/locus_tag="AUO97b_02137" /db_xref="SEED:fig|400667.82.peg.2001"
/db_xref="GO:0005506"
/db_xref="GO:0006118"
/db_xref="GO:0009055"
/translation="MLSQVATPDECLRCGACCAHFRVSFYWAEAELMEEHLVEPLTPV
YSCMRGTNQPEPRCQALTGEIGKEVGCSIYAVRSSTCREVQIADEQCNKARLAHQLIP
LIQVSPADSENDHDYDQVS"
/product="Ferredoxin"
/transl_table=11
CDS 2145249..2146715
/locus_tag="AUO97b_02138" /db_xref="SEED:fig|400667.82.peg.2002"
/db_xref="GO:0003938"
/translation="MLTIVQEALTFDDVLLLPAYSTVLPKDVSLKTRLTRGIYLNIPL
VSAAMDTVTESRMAIAMAQNGGIGILHKNMDIAAQAAEVRRVKKFEAGMVKDPITVSP
ETTVRELIAITSANNISGVPVVKDGKVVGIVTGRDTRFETNLEQPVSNIMTGQDRLVT
VREGESKENIQALLQKHRIEKVLVVGESNELKGLITVTDFRKAESYPNSCKDDLGRLR
VGAAVGTGADTPSRVEALVEAGVDVIVVDTAHGHSAGVIERVRWVKQNFPQVQVIGGN
IATGDAALALLDAGADAVKVGIGPGSICTTRIVAGIGMPQISAIDSVASALKDQIPLI
ADGGIRFSGDMAKAIGAGASTIMVGSLLAGTEEAPGEVEFFQGRYYKAYRGMGSLGAM
AGATGSADRYFQDSKAGAEKLVPEGIEGRVPYKGPMGNIVHQMMGGLRSSMGYTGSAV
IEDLRQNAKFVKITSAGMSESHVHDVTITKEAPNYRVG" /product="Inosine-5'-monophosphate dehydrogenase (EC 1.1.1.205) / CBS domain" /EC_number="1.1.1.205"
/transl_table=11
CDS 2146865..2148202
/locus_tag="AUO97b_02139" /db_xref="SEED:fig|400667.82.peg.2003"
/db_xref="GO:0008966"
/translation="MSYFGTDGIRGKFGQMPITPEFALKLGFAAGKVLKRTSPKNKPL
VVLGKDTRLSGYILESALQAGLNAAGVYVHLLGPLPTPAIAHLTRALHAHAGIVISAS
HNPYFDNGIKFFSSEGKKLPDSLQEEINKELEKDLFIEDTANLGKSVRVTDANGRYIE

FCKSTFPYHFDLNNLKIVVDCAHGAAYSVGPSVFRELGAKVVALYNEPDGLNINENCG
STHPESLQKAVVEHGADLGIAFDGDADRVVMVDKFGNLIDGDHILYILATQAKNKPAG
VVGTVMSNMALEVALEKANVGFVRAKVGDRYVLQALEENGWVTGGEPSGHILTLDKST
TGDAIIAALQVLTVMVEQNKALHELVHDFKLYPQVLVNVRLEQMLDPYSIPALVAEFN
KAEEQLKGRGRILIRKSGTEPVIRVMVEGDNEQEVKTLAEHLANAVRSQAQVA"
/product="Phosphoglucosamine mutase (EC 5.4.2.10)" /EC_number="5.4.2.10"
/transl_table=11
CDS 2148213..2148869
/locus_tag="AUO97b_02140" /db_xref="SEED:fig|400667.82.peg.2004"
/db_xref="GO:0004733"
/translation="MSDVIKDLSELRLSYEQGELYETQVASNPHEQFLGWFNHALAAN
LHEPYAMSLATASASGRPHVRTVLLRGATEAGYDFYTNYDSQKGIDLAENPYAELLFY
WPSLERQVRVGGHVVKIPEQESTDYYHKRPRDSQIAAHISTPQSGKIESRELLQQRFQ
DLQQQVQSREVLDKPEFWGGYRLQPDYYEFWQGRPNRLHDRLSYEKIDGQWTLHRLMP "
/product="Pyridoxamine 5'-phosphate oxidase (EC 1.4.3.5)"
/EC_number="1.4.3.5"
/transl_table=11
CDS 2148870..2150567
/locus_tag="AUO97b_02141" /db_xref="SEED:fig|400667.82.peg.2005"
/translation="MTKIQIKQRPLLTRPEQFQGVPPFIAEILARRGVQSEQELELKL
KNLLAPELKGLDAAVALMDQAIDEQKKIVIVGDYDADGATSTALMMLVLRDMGAQVDY
LVPDRFKYGYGLTPAIAELAHHTYQPDLLITVDNGISSHAGVDAAHALGMQVIITDHH
LTTKETPLAEAVVNPNQLGCEFPSKALAGVGVAFYVLANLASLRNRQGKSTSKVTQYL
DLVALGTYADVAVLDYNNRILVDAGVKRIQQHQCRVGILALLDIAGREATSIRAQDLG
FVLGPRINAAGRMESMRIGIECLLADTMETAYPIAQQLNQLNIDRRQIEGEMKQQALS
ALDSLQLSQQDIPAALVLFEENWHQGVIGIVAGRLKEQFHRPTIVFAPDEDGIHIKGS
ARSIDGVHIRDTIEQVAEQHPELVSHFGGHAAAAGLTIRKENFEAFKTVFNDCVAAMD
ESIFQATLWTDGELPVSALQLDTLNWIEQLGPWGQKFPLPQFEGHFKVIDFRWLKETH
LKLRLAIDQYSFDAIAFNTAGRFEFDPMRDHVHLVYEIDRNVFNGNVSLQLRIVHLN" /product="Single-stranded-DNA-specific exonuclease RecJ" /transl_table=11
CDS complement(2150615..2151382)
/locus_tag="AUO97b_02142" /db_xref="SEED:fig|400667.82.peg.2006"
/translation="MKKLAIASALLSALAVSGTANAYQAEVGGSYNYLDPDNGSSVSK
FGVDGTYYFNPVQTRNAPLAEAAFLNRASNVNAHVNYGDNSGTKDTQYGVGVEYFVPN
SDFYLSGDVGRNEREIDNTNIDSKVTTYAAEVGYLPAPGLLLALGVKGYDEKDGKDGA
DPTVRAKYVTQVGQHDVNLEAYGAFGDLDEYKVRGDYYIDKTLSLGVDYYNNDLTDKD
EFGINAKKFLNQQVSVEGRVGFGDNDNTYGVRAAYRF" /product="hypothetical protein"
/transl_table=11
CDS 2151938..2152870
/locus_tag="AUO97b_02143" /db_xref="SEED:fig|400667.82.peg.2007"
/db_xref="GO:0003747"
/db_xref="GO:0006415"
/translation="MAKEKGELENVINVLDGLSTQLEDAKAMLDLAVEADDESLLEDV
QSELSTAEEELAKLEFRRMFSNPMDPNPCYVEIQAGSGGTEAQDWASMLLRMYMRWIE
RHGFKAELMEVSDGDVAGIKSATIRVEGEYAYGWLRTESGVHRLVRKSPFDSGNRRHT
SFSAVFISPEVDDNIEIDINPSDVRTDTYRASGAGGQHINKTDSAVRLTHIPTGIVVA
CQNQRSQHANRDHAWKQLRAKLYELEMQKRNEAAQALEDSKSDIGWGSQIRSYVLDDS
RIKDLRTGVENSNTGAVLDGDLDRFIEASLKQGL"
/product="Peptide chain release factor 2 @ programmed frameshift-containing"
/transl_table=11
CDS 2152969..2153100
/locus_tag="AUO97b_02144" /db_xref="SEED:fig|400667.82.peg.2008"
/translation="MNIEKNRYEYRQNTILCFRNAEQSFVRLQDVLSLWILMQLAKP"
/product="hypothetical protein"

/transl_table=11
CDS 2153070..2153372
/locus_tag="AUO97b_02145" /db_xref="SEED:fig|400667.82.peg.2009"
/db_xref="GO:0003700"
/db_xref="GO:0005622"
/translation="MDIDAISKALANPLRRQILQWLKEPEHYLPVEECGGSFEKGVCA
GHIERLGKVAQSTMSNHLSVLQQAGLIQVQKYGQWSYFSRNEALIQQYIEHLKQTL"
/product="Transcriptional regulator, ArsR family" /transl_table=11
CDS 2153401..2154459
/locus_tag="AUO97b_02146" /db_xref="SEED:fig|400667.82.peg.2010"
/translation="MAELNTPLTVGDFEIKNRLVMAPLTRARSGESRVPNDLMVEYYQ
QRANAGLILTEATVIGSKTVGYADTPGLWSQEQAQAWNKIIEAVHAQGSKIVVQLWHV
GRISHPELLDGDIPVAPSAIQPAGEVSLLRPKRPYVTPRALSIEEIQEIVAQYKHSAE
LAKAAGFDGVELHAANGYLIDQFLQSNTNQRDDEYGGPVENRARLLLEVVDAFIEVWG
AGRVGVHIAPRGDSHDMGDENPLATFGYVVEQLSQRNVAFIFSREYEAADSISPQLRK
KFNGVWIANENLTPESAKRILREGQADAVSFGKAYIANPDLLQRLEQGLPLNELQPTT
LYAKGAEGYTDYPALEAS"
/product="Xenobiotic reductase B"
/transl_table=11
CDS 2154628..2155920
/locus_tag="AUO97b_02147" /db_xref="SEED:fig|400667.82.peg.2011"
/db_xref="GO:0005529"
/db_xref="GO:0005975"
/db_xref="GO:0009058"
/db_xref="GO:0016740"
/translation="MATPFWYNTALHLLKPFYRWRIKRRAESLELYQQECLERFGPFE
APKNVKAIWFHAVSVGETNAAQPLIEYYLKLGQPVLVTNTTKTGQARAKSLFLKEPYL
DLFQAVYLPVDQKPLLKKFFELYQPKLLALVETELWPNLIDQAKLQHVPCLLLNARLS
EKSAKGYGKVSGLTAGMLKQLDWVLAQDSATRQRYVELGLDEHKSQVVGNIKFDIHAP
EAFIKQAAQLRQQWYLENRQVVTIASTHAPEEQQILEALAPYLNSDRELVCIVVPRHP
ERFDEVFEICQNLNLITHRRSMGQSIHASTQVYLADSMGELWLWYALSQVCFVGGSLN
EPGGGHNILEPMVLNVPTVVGPRYFNFQTIVDEFIDENAVLIAQDAQQVVDIWLACLA
EPEATEQLVAQAHKVLQRNQGSLQKHIGVINRYLAEKS" /product="3-deoxy-D-manno-octulosonic
acid transferase (EC 2.4.99.12)(EC 2.4.99.13)" /transl_table=11
CDS 2155917..2156624
/locus_tag="AUO97b_02148" /db_xref="SEED:fig|400667.82.peg.2012"
/translation="MNIVLLEPEDVQSDTWSIHSKRQLQHLREHLDITVGQNLKVGIR
NGARYITEIVSMNEHEVRIRPICEELLPAKLPVHLIVALPRPKVLRRLIMDSVTLGVE
KISLIHSYRVDKSYWQTPFLQQIDNYVTLGLEQAGDTIVPEIQLYKRFKPFVEDVLPS
LITQETPAYVAHPYAEQRMPINISHACSVVVGPEGGFIPYEVDLLIKNGCQAMNLGNR ILRTETSISYILGRLFS"
/product="Uncharacterized methyltransferase PA5071" /transl_table=11
CDS complement(2156604..2157584)
/locus_tag="AUO97b_02149" /db_xref="SEED:fig|400667.82.peg.2013"
/translation="MADEQKSLTDVAKNTGELLQEAGTKTTQTVLAHTEKYHDAYTTI
DKIVDSFWERVPYICIAIVVFLIFWLLTKLFKFFVRKTLENRSYTRQNLVLVLNRVGS
TAILFFGFLIALVIMIPGFTPGQLMSALGIGSVAIGFAFKDIFQNLLSGILILLSEPF
RIGDSIVVNSLEGTVEDIQIRATFLRSPDGRRIVIPNATVYTSALTVNTAYQQRRCEF
VVGIGYDDDEQKAKQIILDILNNDRNVLSQPAFSVNVTALADFSVNLTVRWWVDTTET
DISSSISNIQAQVKQAFNENDIDIPYPIQELKMVSNPPALNPETSAKQAT" /product="hypothetical
protein"
/transl_table=11
CDS complement(2157697..2158272) /locus_tag="AUO97b_02150"
/db_xref="SEED:fig|400667.82.peg.2014"
/translation="MFAETPSSRPPQLPFKDSPFSIHGRFNRMSYLGGYGLIYLVTIV

GYFILASFLGSFQLSSDLFNFEFYSSLSGISALMVWAFWLFLIYLNIILVVRRLHDLN
KSGWMGLLLFIPVVQFFFMLYLLLASGTAGTNQYGPVRPSTFIEKLMAWLILIAILIS
LISTVGFFYYFSGTDTIQTPTQILQKGTEYF"
/product="hypothetical protein"
/transl_table=11
CDS 2158567..2160516
/locus_tag="AUO97b_02151" /db_xref="SEED:fig|400667.82.peg.2015"
/db_xref="GO:0003987"
/translation="MNEIYPVPEEFKKTARTVEADYFKRYQHSIENPDEFWAEQAKIV
DWIKPFTQVKNTSFDKDNFKIEWFADGELNVSANCLDRHLKEHPHKPAIIWEGDHPSR
HKIVSYKELHDEVCRFANVLKKYGIGKGDRVVLYMPMVTEAAIAMLACARIGAVHCVV
FGGFSPDSLASRIEDSQAKLVITADSSLRAGKLLPLKENVDLALALPGTECVENVIVV
YRNANPIEMKPGRDLWYHLIIMEVDANCPPEPMKAEDPLFILYTSGSTGKPKGVLHTT
GGYLVYVASTFKEVFDLKQDDVYWCTADVGWITGHSYLIYGPLANGTTTLMFEGVPQY
PTWARLGHVVDKHKVSILYTAPTAIRAMMREGDSYVRESNRSSLRLLGSVGEPINPEA
WNWYYNVVGEGRCPIVDTWWQTETGGILIAPLPGATALKPGSATRPLFGIQPAIVDGE
GNELEGAAEGNLVIKDSWPGQMRTIWGDPDRFIEAYFSTFKNTYFTGDGARRDEDGYY
WITGRVDDVLNVSGHRLGTAEIESALVSHEAVAEAAVVGMPHDIKGQGICTFVTLQAG
VPESEELRKELISWVRKVLGPVASPDALHWAPALPKTRSGKIMRRILRKIAANELDSL
GDTSTLAEPAVVDQLIATVYPDRQK"
/product="Acetyl-CoA synthetase (EC 6.2.1.1)" /EC_number="6.2.1.1"
/transl_table=11
CDS complement(2160563..2161105)
/locus_tag="AUO97b_02152" /db_xref="SEED:fig|400667.82.peg.2016"
/db_xref="GO:0008908"
/translation="MKQALLVIDVQNDYFKNGKMELVGPDQALDKIKQLEQYFNEKDL
PIIYVQHINPPQASFFQENTEGVLLHPELSAGSDSLIVTKHYPNSFLETNLKELLKAH
QIEQLVITGMMTHICIDSGTRTAKELGYQPILIGDATATRDLSHNGKTVKAEDVQTAF
LAALGMFASVQNTADFLAKS"
/product="Isochorismatase (EC 3.3.2.1)" /EC_number="3.3.2.1"
/transl_table=11
CDS 2161211..2161624
/locus_tag="AUO97b_02153" /db_xref="SEED:fig|400667.82.peg.2017"
/translation="MNIELDPIDLKIIALLKQDSRLTNKEIGQRVHRTGQAVGARIAQ
LMDAGVIKNYTIAVHYRHKQFIHLHLNEQRSFEEIENLVKQYEQIDECFKVMGNACYM
IVSHFEPAELNAFIEVLSKWCRYSVETVIREVEKA" /product="Transcriptional regulator,
AsnC family" /transl_table=11
CDS complement(2161716..2162828)
/locus_tag="AUO97b_02154" /db_xref="SEED:fig|400667.82.peg.2018"
/translation="MTQLQQPIKFAYWVPNVSGGLVVSNIEQRTDWSYDYNVRLAQAA
ENNGFEYALTQIRFTAGYGAENQHESVSFSHALLAKTEKLKVIAAILPGPWKPALAAK
QLATIDYLTQGRIAINVVSGWFRGEFDAIGEEWPEHDERYVRSEEFIRTLKGIWTTDN
YSFDGKYYQFQNYTLKPKPLQKPHIEVFQGGSSRAARDMASRVSDWYFTNGNTVEEIQ
KQVEDIRNKAKANNHQVKIGVNAFIIARDTEEEAKAVLQEIIDKANTEAVNAFGDATR
EAGAASPEQEGNWAKSTFEDLVQYNDGFKTNLIGTPQQIAERIVALKNVGVDLILSGF
LHFIEEVEYFGEKVLPLVRQLELQQEKEVEIQAKSA"
/product="Coenzyme F420-dependent N5,N10-methylene tetrahydromethanopterin
reductase and related flavin-dependent oxidoreductases; sulfonate
monooxygenase" /transl_table=11
CDS complement(2162873..2163610)
/locus_tag="AUO97b_02155" /db_xref="SEED:fig|400667.82.peg.2019"
/db_xref="GO:0008752"
/translation="MTGIASSKPLNIVAVSGGLNTPSKTESLVQAILDELSEAIDIKV
HFIKLSEIGPLLGGAFYRNQLPQRVQDDLAAVEAADALIVGTPVYRASFTGLFKHFFD

FVEQTALVDVPVLLAASGGSDRHALVLEHQLRPLFSFFQAQTLPIGVYATDRDFTPEY
TVKSEQLSDRITLAVARALPILEWAPAKGKRAEVVKPKSQQANQELGINKQIEQEEVL
PSAAVPNLDAAESRLHHKSGKSLTHVA"
/product="FMN reductase (NADH) (EC 1.5.1.42)" /EC_number="1.5.1.29"
/transl_table=11
CDS 2163987..2164637
/locus_tag="AUO97b_02156" /db_xref="SEED:fig|400667.82.peg.2020"
/translation="MNILIVDDHPLFRHALIQAVRYSLPQAQIHETASVDEFYERLEN
GAEPDLVLLDLNLPGASGFSALVYVRAQYPSIPIIVVSAHEEASIIQRAIAHGAMGYI
PKSAHPSHIGEAIRQVLDGEIWLPPNLPSNINLDPRAADETALAERIQSLTPQQFRVL
MMVAEGLLNKQIAYELDVSEATIKAHVTAIFRKLGVQNRTQAVLAISALNIEDKKI" /product="Two-
component transcriptional response regulator, LuxR family" /transl_table=11
CDS complement(2164680..2165447)
/locus_tag="AUO97b_02157" /db_xref="SEED:fig|400667.82.peg.2021"
/translation="MNISDNTPQGQLSSQQFYRQYSANALLPELDWQSIFPHSKLSEA
HIKALNTIYQCAVPLALNVFHDLNFDVFAPAAYHPQGLGLFDKLAQQEENFLKVLTAE
SENLDHETRHQIWSMLLRGGAVLVFKAWLGHVKLGENQLDMTQFDELSDLLFIKTPPH
QLAERLHVDEKAKLDHIFLMYGNDIFLDRFNSLETAALFVDLGVYDAAFLSLRDDRVA
EYLKSKGYVTQEQIDDLQCALNPLYCDSLTPKQDCLA" /product="hypothetical protein"
/transl_table=11
CDS complement(2165841..2169338)
/locus_tag="AUO97b_02158" /db_xref="SEED:fig|400667.82.peg.2022"
/translation="MNSWLIIGVLTLYIALLFVCAFFGEKHASRLSTRGRMFLFSLTL
GVYCSSWTFYGATGAAVREGVIFLPIYLGPLLFVWFGYDIWRRLGRVRQHHAISSIAD
FVAARYGKSGVLASLVTILAVIAIIPYLALQLRAIALSTSVIMEQSSHIHTTTNSVLL
LTSVLAILAMIFGTRQIANTEQHGGLMLAVAFESFVKLFALLCVALFFIFESPANLSQ
MTSDVNQTFQEVQLFGVPETFWIQTLLAGLAIICLPRQFHVAVVELRDEKHIRGARKW
FAVYLILTILAIIPIASWALHAAPKFLTVPDVAVLSLPLSYNQEWLALLAFLGGFSAS
TGMLLVSSVALSIMLSNDLIMPALWRFKLLSRHDKHLPKVLKFSRRISIVAVMLLGFL
FFHFFNDINQLSVFGLLAFSAVAQFAPALIGGLYWRGGSRQGVYAGLIIGFLMWSYTL
LFPTILRSLPEAYQETAQSILLFGPFGINALRPESLFGFESFAPLTHGVIWSLGLNIV
LYIWISKIYRPSVAEQIQAESFFYYETKPLPSPHTSTDLTYLPHDAARLKVGDLFALA
KRITGEQPTTQAFENFCEQNHLTLNPNSIANGMWWRFTEQYLAGIIGAASARTLLTTA
MINNGLALGQVANILDQASQWQRFNQNLLMTMIDHMTQGVSVVDKNMCLVAWNNQYLK
LFGYPKDLVYVGCPIADLIRYNAERGECGPGSVEEHVRKRLHWMKVGSAHEFERIRKD
GRVIQMRGNPIAGGGFVTTFADITAFRANEAVLEARVKDRTQQLADALTEQQLAREQA
DMANMSKSRFIAAASHDLLQPMHAARLFSTVLEQSISTEEDRQTLQQLDRALYGAESM
LSALLDIARLEGGTIQPKRQPYPLHDLLSDLELQFKSIAAQRNISLKVHDVSFWIDTD
PQWIRRIIQNFVSNALRYTAKGRVVVGVLRSAQQSGHIRIGVWDTGPGIAEEQRIKLF
QEFERCGHLSPWGEQGLGLGLAIVQRMTGLLNYPVHVYSELGKGSCFMIEVPIVEPPK
VATPAVQAVPLKTKAYRILCLDNDETILEGMSSLLGRWGYQVFKATEPEQALEIIQKE
NIQVWLIDQHLNHNQLGVDFITQNRQEDVPVALITADSHPELPQQLKELNIVLLKKPL
KPASLRAWLSGLKIASTTD"
/product="Two-component system sensor histidine kinase" /transl_table=11
CDS 2169522..2169854
/locus_tag="AUO97b_02159" /db_xref="SEED:fig|400667.82.peg.2023"
/translation="MDQSQVEQILRNPKFQKMVKKKSALSWTLTIIMLVLYVGFMLLV
GYNKEFLMSSFSGGVTTWGIPLGLGIIVLSFLLCGVYSYIANNTLDQLSEEALKEVEA ITHEKGLH"
/product="Inner membrane protein YjcH, clustering with ActP" /transl_table=11
CDS 2169856..2171571
/locus_tag="AUO97b_02160" /db_xref="SEED:fig|400667.82.peg.2024"
/translation="MKWNSLKAKALLAAASLYSTVAMASPDLGEAEQQATNWHAIIMF
IIFVGFTLFITKWAAKQTQSAQDFYTAGGGISGFQNGLAIAGDYMSAASFLGISAMVF
SSGFDGLLYSLGFMVGWPIVLFLVAERLRNLGKYNLSDVVSFRLEEKPVRTLAAFSSL

VVVAFYLIAQMVGAGQLIKLLFGLNYNIAVVIVGLLMMAYVIFGGMLATTWVQIIKAV
MLLSGATFMAFMVMKAVGFSFSNMFTQSIEVFSKVHDVSFEDAAKIMGPGKLAANPID
ALSLGLALMFGTAGLPHILMRFFTVKDAKEARKSVVVATGFIGYFYLLTFIIGFGAIL
FVSNNPQFLDVAKMAMTGKLELVGGNNMAAVHLSDALGGDLFMGFISTVAFATILAVV
AGLTLLGASAISHDLYANVFKKGQTTPASELRMSKIATLGLAIFAMILGILFEKQNVA
FMVGLAFSVACCANFPILVLSMFWKGLTTRGAVIGGVVGLVTAVVLIILSKAVWVDTL
KISDTPINPFNGPALFAMPLSFLCCWFFSVTDSSARAKKERASFDAQYVRSMTGIGAS GASDH"
/product="Acetate permease ActP (cation/acetate symporter)" /transl_table=11
CDS 2171741..2172433
/locus_tag="AUO97b_02161" /db_xref="SEED:fig|400667.82.peg.2025"
/translation="MRSIIEFWSSFRLLPSAWLLLVQFILLILAMLTFDNEAYRTLTW
ALGVLALLIIAKVIRQTPMFTFLGLAFVSGAIIASVFILLGYSNLTIQIFAHSCEALA
YFSAAYGLFRYMFADRYLTRDELFAAAAVFTLLAWGFAFLYNICQLLIPNSFQNPNHI
HHLQTWLDLLFLSFSLQSATGLSDLMPLSPPARVLAMLQMFCGVMYLALIVTRLIALQ YIKHTPKKNSDK"
/product="Potassium channel protein"
/transl_table=11
CDS 2172599..2174566
/locus_tag="AUO97b_02162" /db_xref="SEED:fig|400667.82.peg.2026"
/translation="MPSKQASWFANVNPNVFLGTVIIIALFLAVVVIAPSSFELLTQQ
LKQWITDSFSWFYVLSVAFFLILLIYIACSAIGRIKLGPDHSQPEYSNGSWFAMLFTA
GMGIGLMFFGVAEPVMHYVNPPSGEPQTIEAAQQALRVTFFHWGLHAWAIYAVVGLAL
AYFAYRHNLPLKTRSALYPLIGKKIYGPWGDSIDIFATIGTVFGVATSLGFGVTQINS
GLHYLFGIEQSTTTQVLLIIFVSILASLSVFLGLDKGVKRLSELNLVLALILLVFVFI
AGPSIYLLQTTIQNTGQYISNLFTMTFNLYAYQPNGWIGGWTILYWAWWISWSPFVGM
FIARVSRGRTIREFIVGVLLIPTGFTIIWMGFLGNAALFSIIHEHQTTLIQAVQQDSS
VALFEFLGHLPWSGVMNILATILVVLFFVTSADSGALVTDYLTAKTENSPTWQRLFWT
VLMAVLAIILLLVGGLAALQSSIIMSALPFTVVMLLMSWGLIKALHLDVTKMIAIQEA
RITPRAIHNPRSWQQRLGLIMHYPHSEEEVKQYIEKQVSRAFENIQHEFRRRHLEVSI
SEVEDGMQLRVDHHHEINFIYKVVSRETVPPSFLIGRTEAEDGQYFQAEVFLREGGQN
YDVMDWTQEDLIQDIIDQYERHLHFLNIVRS"
/product="High-affinity choline uptake protein BetT" /transl_table=11
CDS complement(2174633..2175532)
/locus_tag="AUO97b_02163" /db_xref="SEED:fig|400667.82.peg.2027"
/translation="MKKLGLATAVLLAMTGAHAYQFEVQGQSEYVDTTANDKNFTGDV
AGTFYLKNVDTAKGPLAEAAFLNQASSVSLGYSYQQYDQNNVNYHIGTYGVKGEAYVP
TPYLPVYASATYNHTDVDGKNNNSKDDNGDRYALEVGAMLLPNFLMTVGYTSVANQFA
LDNFGIIGNGIYSAVNQTAAIQNDQDAVTARAKYVGPIDGTNMAIGFEAAGAFGQENQ
YGLKTDLYLTPKLSVGATFVGNDGEADIKDNNLGEFRQAWGGNVNYFITPALAVGASY
MKADVKKSSYDTQTIGLNAKFRF"
/product="33-36 kDa outer membrane protein" /transl_table=11
CDS 2176525..2178231
/locus_tag="AUO97b_02164" /db_xref="SEED:fig|400667.82.peg.2028"
/translation="MSGLRSEHISNRLFFFMLVIILSLLFMAVPLIVSSYQEYLKTKQ
ALVEIKSLRSIAEVANKVSKERAPANKLMSSNAADFLKNQKNLKEYRLSVDRQLNETI
HILKEEGYTDLANTLDTKFRDDLKQARAVVDYYVATPEQARSSIQFDNAILAMFGAWD
SCREVLQNLMLQLKSKDSQINNYFTLIVVLTDMRDQAGRTASNVIAPVTFKETIPNDN
RARSLQTQQQARYLWALVDTLQSEQAKTPEYHRLYSRVKTEFLDKGIPIVGQLLDESQ
KHQAYHLNGTELTDQIVDKFTTVIDLQKFLLNKSVLIASQENYLAKKKFFLTLLISSI
SLAAALLTLIYAKRRVFEPLIQARTLILDLSHSTDYPIDELKPKKGEFISLFEAIDRL
QRMLKQRDAFEFQLKNIANTDPLTGVSNRLALSEYLKIVESYPQKFSQTCLMIIDIDR
FKHVNDQYGHIVGDQVIRRIADQLKANVRASDLIVRYGGDEFLILLEQVQFLDARLLA
EKIRIAISLEEIDLSGTQEKLHVSVSIGFAIGATSWMELLENADRSLLRAKARGRNVV EG"
/product="GGDEF domain protein"
/transl_table=11

CDS complement(2178265..2181099)
/locus_tag="AUO97b_02165" /db_xref="SEED:fig|400667.82.peg.2029"
/db_xref="GO:0005524"
/db_xref="GO:0016887"
/translation="MSQSHIRIRGARTHNLKNVSLDIPRDKFVVITGLSGSGKSSLAF
DTLYAEGQRRYVESLSAYARQFLSQMEKPEVDSIEGLSPAIAIEQKSTSHNPRSTVGT
ITEIYDYLRLLYARVGTPYCPEHDLPMVAQTVSEMVDAVKNLEEGTALMLLAPVVRER
KGEYSNLFEQLQGQGFVRARVDGEIIDIDTPPELDKKKKHTIEVVVDRFKVRDDLGNR
IAESFETALRLGGDIAVLSWMNGEHPDRVFSAKHSCPECDRAVAELEPRLFSFNNPFG
ACPVCDGLGTRSHFSPEKLIPNPELAISEGAIRGWDRQRPYYYSMLQKVADHFGFQLD
TPWNQLDKDTQKKFLQGTGKEKIDLSYIDERGRKHTRVQPFEGILPHLERRYRETESN
YVRDDLAQYLSNAACDACGGSRLNEISRHVRVKDKTIADITRMSIGDAESYYQGINLE
GAKGEIADKIFKEIRERLHFLVSVGLNYLSLARSAETLSGGEAQRIRLASQIGAGLMG
VMYVLDEPSIGLHQRDNDRLLQTLIRLRDLGNTVLVVEHDEDAIRAADHIIDIGPGAG
VHGGVVIAEGTYDELAKHADSLTGQYLSGKLKIEVPKQRTQPPKPEEKIKLSGAAGHN
LKNVDLTIPLGIMTCVTGVSGSGKSTLINRTLLPLAATQLNGATTLTAEKFDSIDGLQ
HLDKVVDIDQSPIGRTPRSNPATYTGLFTPIRELFAQTPEAKARGYSAGRFSFNVKGG
RCEACEGDGMIKVAMHFLPDMYVPCDACHGKRYNRETLEVGYKGKNISDVLEMTVEDA
AEFFSAIPVIHRRLETLTQVGLGYIRLGQAATTLSGGEAQRVKLARELAKRDTGKTLY
ILDEPTTGLHFHDIAKLLDILHELRNKGNTIVVIEHNLDVIKTADWVVDLGPEGGSGG
GMIIAEGTPEQVAEVEISHTGRFLKPMLKQ"
/product="Excinuclease ABC subunit A"
/transl_table=11
CDS 2181396..2181998
/locus_tag="AUO97b_02166" /db_xref="SEED:fig|400667.82.peg.2030"
/translation="MKRLNKSETTRQHILDTSFELVLHKGFVGVGLQEILKACDVPKG
SFYHYFASKEAFGCALLEQYMANYKVRMEQLWQHSEQSAHALLMALWQAWIDDPVHGS
WAENCLIVKLAAEVSDLSEDMRQILNDGVHKLTQRLALLLKEGQQEGSIPKHIEPLKT
AQVMYQLWLGAALLTKLSQDKAHLHLALETTQQLLKPIEE" /product="Transcriptional
regulator, AcrR family" /transl_table=11
CDS 2182002..2182979
/locus_tag="AUO97b_02167" /db_xref="SEED:fig|400667.82.peg.2031"
/translation="MRSIIHRNFGEPVDVLEQADMPKPEPKAGEVRIKTIMSPIHNHD
VWTVRGSYGYKPTLPAIGGSEAVGIVDALGEGVEHVQVGQRIAVAAVHGSWAEYFIAP
AQGIIPLNNEIDDETAAQLIGMPISALMLLDFVNVQPGQWLIQNTANGAVGKTVAMIA
QARGFPVINLVRRSDAIAEMQALGIQHVVATDQPNWKEQVKQIHGDQPLIAGVDSIGG
TASGEMLNLLSENSLLVSFGSMTGETMQISSGDLIFKQATVKGFWASVVNKEMPAARK
KELIVELLTLATQKKLILPVEGVFSFDEIKTAAQRATQGARQGKVLLKP" /product="Putative
oxidoreductase SMc00968" /transl_table=11
CDS 2183044..2183397
/locus_tag="AUO97b_02168" /db_xref="SEED:fig|400667.82.peg.2032"
/translation="MIGQILIALIAILHVYILVLEMFLWDKPYGMKAFGNSTEKAKLT
KVMAQNQGLYNGFLAAGLFWSLLADAPFATSIANFFLGCVLIAGIYGGLTASKKIIYI QAVPALIALVAVNFL"
/product="Protein of unknown function DUF1304" /transl_table=11
CDS 2183450..2183791
/locus_tag="AUO97b_02169" /db_xref="SEED:fig|400667.82.peg.2033"
/translation="MWALFLKCMLGAGVVLIISILSKSKAFYIAGLVPLFPTFALIAH
VIVFQQKGAEALQKTALFGLWSLIPYAIYLVAVYVLATRMSMWSCLGVATVCWVVAAA GLIYGWQLFQS"
/product="GlpM protein"
/transl_table=11
CDS complement(2183841..2184515)
/locus_tag="AUO97b_02170" /db_xref="SEED:fig|400667.82.peg.2034"
/db_xref="GO:0050334"
/translation="MLFSQELWQRNLNLYQKILDLPFNQELANGTLDKEAFCHYVIQD

AQYLVAYGRVLAVAAAKAFDADDIMQFSDAAKIAIVVERSLHDDFMKNFGVTKEEFKN
TPLTLAGHHYTSFLTATAWSESYPVVLAALLPCFWIYAEVGKDIVSKSVPNNPYQAWI
DTYAGEEFHTAVRNVIATVDKVAARCDADTLEKMHAAYTMGAKLEWLFWDSAYHQRQW LGLDQI"
/product="Thiaminase II (EC 3.5.99.2) involved in salvage of thiamin
pyrimidine moiety, TenA subgroup with Cys in active site"
/EC_number="3.5.99.2"
/transl_table=11
CDS 2184778..2185860
/locus_tag="AUO97b_02171" /db_xref="SEED:fig|400667.82.peg.2035"
/translation="MKHFRFSIFFTVVCLALSAYWGFTHGPEAGVSTMLKALTITAIL
AVMEVSLSFDNAVVNASVLRNWDPFWKMIFLTVGILIAVFGMRLIFPVVIVAVTADMG
VIEVAKMALNDPKTYSERLMAHHAEISAFGGTFLLLVFLNFFFDDEKETHWFRWLESK
LASLASVPAMSVFLALIALIVMAGYVDDHQRLAVTMAGIWGIVVYIGVEVLSHLLGGE
PEIDENGNAVLQDGTGAASGVVKAGLGGFLYLEVLDASFSFDGVIGAFAITSDVVVIM
LGLAIGAIFVRSMTVYLVEKGTLDAYVFLEHGAHYAIGALAFIMIASGTGVHVPEVVT
GLIGVAFIVWAVIASIQYSKREQQSP"
/product="Integral membrane protein"
/transl_table=11
CDS 2186010..2187371
/locus_tag="AUO97b_02172" /db_xref="SEED:fig|400667.82.peg.2036"
/translation="MNALERRSTFALSSIFALRMLGLFMIIPVFSVVGQSYQYATPAL
IGLAVGVYGLSQAILQIPFSLLADRFSRKPLVVFGLLLFAIGGAIAGLSDTIYGVIIG
RAIAGAGAVSAVVMALLADVTREEQRTKAMAAMGMSIGLSFVVAFSLGPWLTSLVGIS
GLFFVTTIMGLIAIAMLLLVPKVTRHHRNYQQGYMAQLKQVIQMGDLNRLHVSVFALH
LLLTAMFIYVPSQLIEFAHIPLASHGLVYLPLLVISLFFAFPSIIIAEKYRKMRGIFL
TAITGIIAGLLLLIFGYQSKYVLLAGLGIFFIAFNVMEALLPSWLSKSAPIQSKATAM
GVNASSQFLGAFFGGTLGGQLLMLHNTAIGWSVLAGIAIIWLLISFGLAQPRYLSSIV
LPLPQVQQVNEWTTQLLAIRGIEEVVVMPDQQVAYIKVDKQSLDDASRRDLTQLFGKE VAI"
/product="Inner membrane transport protein YajR" /transl_table=11
CDS 2187423..2188004
/locus_tag="AUO97b_02173" /db_xref="SEED:fig|400667.82.peg.2037"
/db_xref="GO:0003697"
/translation="MRGVNKVILVGTLGRDPETKTFPNGGSLTQFSIATSEVWTDKNT
GERKEQTEWHRIVLHNRLGEIAQQFLRKGSKVYIEGSLRTRQWTDQNGQERYTTEIRG
DQMQMLDARQQGEQGFAGGNDFNQPRFNAPQQGGGYQNNNQGGGYGQNNGGYGGQGGF
GNGGNSPQGGGFAPKAPQQPASAPADLDDDLPF" /product="Single-stranded DNA-binding
protein" /transl_table=11
CDS complement(2188045..2188368)
/locus_tag="AUO97b_02174" /db_xref="SEED:fig|400667.82.peg.2038"
/translation="MSQQLLIIVGIALLALGTYSIRFAGFHLGAKFSFSEKYQVLLSN
GATVLLCAIAVTTTFFEGQHFAGFARIFGVGLALFLVWKKAPLLLVICLAAAGTALVR LLGIE"
/product="Putative inner membrane protein"
/transl_table=11
CDS complement(2188365..2188946)
/locus_tag="AUO97b_02175" /db_xref="SEED:fig|400667.82.peg.2039"
/translation="MSLGSLAASYGLALWIPLCLSIFVLAGTAEFIFIGFLAVGSSPI
AAAIAGLLVNLRHLPFGIAVNELIRGKFSQYFGSHIMNDESVLFGMAQPDFDTKKAAY
WLCGIGIMLSWPLGVTAGYFIGSAIPDPKTFGLDAIFPAILIALTFSALKNKVTRKAA
FAGSTLALITTPFLASGLPILISLFGLIWGRKK" /product="Branched-chain amino acid
transport protein azlC" /transl_table=11
CDS 2189129..2189677
/locus_tag="AUO97b_02176" /db_xref="SEED:fig|400667.82.peg.2040"
/translation="MSQPIEIIAKGLTRERQRAGLSLAEVARRAGVAKSTLSQLEAGQ
GNPSIETLWALCVALNIPFARLMEEPSNQVQVIRCGDGPTVSSEIANYKAILLATCPP

HARRDVYLLIVEPGEDRLSEPHPVGSVEHIIVVEGRALVGLIDEAVELGVGDYICYPA
DQKHIFRALETGTKALLISEQN"
/product="Transcriptional regulator"
/transl_table=11
CDS 2189983..2191425
/locus_tag="AUO97b_02177" /db_xref="SEED:fig|400667.82.peg.2041"
/translation="MAAHQNGSEANHSQSLKHGLKSRHLTMISIAGVIGGSLFVGSGS
IIYNTGPVVFLTYALGGLLVWFIMRMLGEMAVLNPDSGSFSTYADRAIGRWAGFSIGW
LYWCTLAMLMGWEAYVAGKILNNWFPFIPIWVYMTVVIVALVVVNLQNVKNYGEFEFW
FALIKVIAIVVFLVIGSLAIMHLWPWGNPAASGISNLTSQGFMPNGGSSVITALLGVM
FAYIGAEIVTVAAAESANPSKEIRKASNSVVWRIILFYVGSMFVAVCLIPHNNELLKD
STWGTYSVTLSALGIPGARHIVNFVVLTSVCSCFNSALYTCSRMLFSLSKRGDAPKSF
GSVNSKSSPWVGVIVSCFFSVIAVILTATESMNVYDFFMLTTGAATLYVYLTIAYSQL
RMRKKLEAEGVKIDFKMWMFPYLTYVVIFAIIGAILTMLIEGTYFKEVIYTTALFGII
VFFGLLSEKLGWGKERRATHLTHSHEEKLV" /product="gamma-aminobutyrate (GABA)
permease" /transl_table=11
CDS complement(2191490..2192989)
/locus_tag="AUO97b_02178" /db_xref="SEED:fig|400667.82.peg.2042"
/translation="MRSLLGDHLLQRLQQDTEGKLHQRLFRCLRGAIIDGVIQPKTRL
PASRDLASEIHVSRNTVLSAYEQLQAEGYLEARTGHGTWVAEKLPESFLNTQNKKKVV
SSPNIQSSYALSQRGLNLLGYAAASPHQWGAFVPGAPDVTEFPHHIFSRIQARLSREP
DINRLIYSNAGGCIELRSALADYLRVARSVQCDADQIIITEGIHQAIDLVSRALSDMG
DKVWIEDPAYWGMRNTLRINGLDIQPMPVDAEGIIPEENPAVPPKLIFVTPSHQYPLG
SHLSLDRRRQLIQIARQHNSWIVEDDYDSEFRFSGQPYPSLQGLENNAPVLYMGTFSK
TIYPSLRIGYLVVPKPLFSPLRIVAAELYRGGHLLEQKALAEFIREGHYEAHIRRMRL
LYGKRRDYLVSLIQRYLGPEFIHEYDEAAGLHLVLKLPNSCDDVAIAATALERGVKVR
PLSQYYMQSHAHAERGLLMGFACVNEKDMVMAFGVLLQCLREAGVPTLN" /product="Transcriptional
regulator GabR of GABA utilization (GntR family with aminotransferase-like
domain)" /transl_table=11
CDS 2193146..2194438
/locus_tag="AUO97b_02179" /db_xref="SEED:fig|400667.82.peg.2043"
/translation="MDTQHSALNARKQQATPRGVGVMCQWYAEKAENATIWDKEGNQF
IDFAGGIAVLNTGHRHPKVIAAVTEQLTKFTHTAYQVTPYESYVALAERINERAPIAG
PAKAAFFTTGAEAVENAVKIARCYTGRHGIITFGNGFHGRSFMTMAMTGKTAPYKRDF
GVMPAGVFHARYPVPAKGISVDAAIESVEDIFSEDIAPHDVAAIVLEPVQGEGGFNVV
PAEFLKRLRAICDKHGILLVADEVQSGFARTGKLFAMNHYETKADLITMAKSLGGGFP
ISGVVGRAEVMDAPNPGGLGGTYAGSPIAVAAAHAVIDAIEEENLCDRANELGAELVT
TLKDIQQATVDVVTDIRALGSMVAVELETAEQAKVVQNYAMENGLLLLTCGKYGNVIR
FLYPLTIPAEQFRQGLDVLKEGFATLKAGSAKAMEQSA" /product="Gamma-aminobutyrate:alpha-
ketoglutarate aminotransferase (EC 2.6.1.19)" /EC_number="2.6.1.19"
/transl_table=11
CDS 2194435..2195883
/locus_tag="AUO97b_02180" /db_xref="SEED:fig|400667.82.peg.2044"
/db_xref="GO:0008911"
/translation="MIQNNLQYLLQHPDISLEAPASNDYIEVNDAATGETLAWVKTYD
RAGVEAAINRSAKAQAAWKKQTALARADVLLAWYNLMLEHKENLAQILTAEQGKPLAE
ARGEIGYAASFIRWFAEQARRVDGEVLTPTLPNQRLLVIKQAIGVTAAITPWNFPAAM
ITRKAGPAIAAGCSMLVKPAEQTPLTAYALEVLALQAGLPADVLINISGDAVEVGKTL
CESDIVRKLSFTGSTQVGRILMQQCAPTIKKLSLELGGNAPVLVFDDANLEQAVQGIM
ASKYRNSGQTCVCANRIYVQDGIYDALAERLVEAVSKLKVGDGRQEGSTQGPLIDEDA
VAKVQSHIADATEKGATVRIGGQRSALGGTFFEPTVLTGVTQDMKVSKEETFGPLAPL
FRFKTEDEAVAMANDTEFGLAAYLFTQSTARQWRVGEALEYGMVGINTGAISNEVAPF
GGVKQSGLGREGSKFGIEEYVEMKYLCVDLSE" /product="Succinate-semialdehyde
dehydrogenase [NAD(P)+] (EC 1.2.1.16)" /EC_number="1.2.1.22"

/transl_table=11
CDS complement(2195988..2196962)
/locus_tag="AUO97b_02181" /db_xref="SEED:fig|400667.82.peg.2045"
/translation="MLKHSNLEFKWVERTLISSFDDFYYFYLVVKHGGFSAASEAENI
SKSKLSRRIIDLENKFNVSLIQRTTRHFKVTDLGQEFYEECCKVIAQVECAENVLLKQ
KSEPQGLVKVSCPPLMMHFQIRKILNQFLKTYPKVEISLELTSRRVDILHDDIDLAIR
TNFEPNEDSNLIVRDVIKTDHCLVASPELLQGRTIKHYSELQDFPSISLGTQKQNYIW
HLCNIRNGESVDVPYQPRIKSNDLAGVYYAVMDGLGIADLPFLTVESEIRKGTLVHIL
PDWKSNIGMLQLVYVSRKGQRLVVEKLIETLIDELRRLPESHEGYLPT" /product="Transcriptional regulator, LysR family" /transl_table=11
CDS complement(2196983..2197609)
/locus_tag="AUO97b_02182" /db_xref="SEED:fig|400667.82.peg.2046"
/translation="MAQDYVRLDKDNAAVLLVDHQAGLLSLVRDIDPDKFKNNVLAVA
NAAKYFNLPTILTTSFETGPNGPLVPELKEIHPDAPFIPRPGQINAWDNEDFVKAVKA
TGKKQLIIAGVVTEVCVAFPALSALAEGFEVFVITDASGTFNELTRDAAWDRMSKAGA
QLMTWFGMACELHRDWRNDIEGLGTLFSNHIPDYRNLISSYNHNTSQK" /product="Nicotinamidase family protein YcaC" /transl_table=11
CDS 2197887..2198759
/locus_tag="AUO97b_02183" /db_xref="SEED:fig|400667.82.peg.2047"
/translation="MKKFLGAYQNNHMHWVGDGFPVYNLFSYDRLGQTLSPFLLLDYA
APYNFSPTTEQQGVGSHPHRGFETVTIAYQGEVTHKDSSGGGGTIKTGDVQWMTAGAG
VLHEEFHSPEFAEHGGLFEMVQLWVNLPSHSKMTPGKYQAIEAKDIPDIALDEHGSHL
RVIAGEYADAKGAATTFSPLNVWDGKLVKGQKHTLYVPEGHTTLVVVLEGAVVVNDTN
RLEGKTVAILSREGVEFSLNAEEDTKFLVLTGQPLNEPIEGYGPFVMNTKAEIMEAIN DFNRGKFGSIMQEG"
/product="Pirin"
/transl_table=11
CDS 2199199..2200497
/locus_tag="AUO97b_02184" /db_xref="SEED:fig|400667.82.peg.2048"
/translation="MDIAIIGSGMAGLAAARILKDAGHTITIFEALPGRGMDSHSIEF
DGGIIDAPLRVMNPHLWKNTLSLAAHLGIKTFPVRTYMSCSWLFEDRTETWLTTSRSR
IGNFPIINNRKGIQQYGWRLVKGMLQLKTAIHQFFKSKNQDITLAEFINQNDIEEVFW
HGVVMPVLYTICTCNPKTIGDWPAKNLLEFLRHLTDGDMLLRMKGGTPAFVDSLIKGI
DIHSGSAIKKVELQGEKVLVENEKGDQGTFDRVIVATPTSKLEEFLNPEQFAEDMNLL
KQFRFEQGDLVIHTDATVMPPRRKDWSVLSYMMDRKFTRQQFTVWMNAVEPSLVGKNP
VFQTWRPVVDIDPKKVISKVTLTRAVVDSQTIALNKELQQRHLEPNRKVFYCGSWSCD
GLPILESAVTSAMHIAEILGAPLPFVGLKPKVEVAPELGY"
/product="Amine oxidase, flavin-containing" /transl_table=11
CDS 2200502..2201329
/locus_tag="AUO97b_02185" /db_xref="SEED:fig|400667.82.peg.2049"
/translation="MKAKVRAALSRFIPHKYAIDASSLGDSEELAWTNLGFWKNTQTY
REACRQLADHLAQAVNLNSKDHLLDLGCGQGASLLHWLQHYHPKSLNAVELQASCVNK
IQKLIPEISQIFCGSFLNLKQFEFKQRFDVVLCIDAAYHSHLNSFLDSVTPILNSKGR
LGFHYLMRADSCQNMTAFQEQKYRYLLKAADVTWNTLLTEEILRTTLEQQGFTDIKIE
DLSEQVLLGFSQYIQNQQAQSHNRGLANFKIQMTAKLCQQLYQNGYVRYIQITAIKK" /product="SAM-dependent methyltransferase" /EC_number="2.1.1.95"
/transl_table=11
CDS 2201338..2201763
/locus_tag="AUO97b_02186" /db_xref="SEED:fig|400667.82.peg.2050"
/translation="MSVKYQGSCLCGAVSYQSDAEPRYSFNCHCRDCQKATGSAYAPI
AFFHQSELKISGELKYFETLGSSGRPIKRAFCPTCGSQLFGLPELVPEMISIRAGTLD
DPSLYHPKAEVFVSQTYAWDMLNPNLKHFEKMIEKKKEG" /product="Gfa-like protein"
/transl_table=11
CDS complement(2201992..2202375)
/locus_tag="AUO97b_02187" /db_xref="SEED:fig|400667.82.peg.2051"

/translation="MKLSFILPALFLVFPLTTYAQETTSQPQIQTTANPPICLIEGTP
SAEHYKSIKRIKAAKGTYGGFEEVKPKLANVARKYGADVVINYHQSQRFGFWPWRIVR
PVLTGTAVKWNTPFNCAALGGKEIR"
/product="hypothetical protein"
/transl_table=11 CDS complement(2202886..2204130)
/locus_tag="AUO97b_02188" /db_xref="SEED:fig|400667.82.peg.2052"
/translation="MNSSSTPSTLNTEATSVLSREWIHKGTKAYKHAGFALFLVGFAS
FSLIYCVQPLLPAFSQSFQISPASSSLALSLTTAFLAISIVLSSAFSQAIGRRGVIFT
SMLCAAILNIVSMFTPNWHSLLIARALEGLLLGGVPAVTMAWIAEEIAPEHLGKTMGL
YIAGTAFGGMMGRVGMGILVEYFSWRTALGLLGAICFICSIAFLKLLPVSRNFVQKKG
LNLGFHMQMWRAHLSNTKLLRLFAIGFLLTSVFVTLFNYATFRLSGAPYSLSQTQISL
IFLSYSFGMVSSSLAGSLADRFGKKTMMMSGFALMIVGSLMTLLSSLFGIIIGIAFIT
TGFFITHSLTSSSVGAESKQAKAHASSLYLLFYYMGSSIVGSAGGWFWLHGGWSAIVG
LTVVLSLIGIFLAVYTSHAKAH"
/product="Uncharacterized MFS-type transporter YnfM" /transl_table=11
CDS 2204296..2205189
/locus_tag="AUO97b_02189" /db_xref="SEED:fig|400667.82.peg.2053"
/translation="MAVAEEKNFTKAAQRLNMSQPPLSMQIRDLEEELGADLFIRSPH
GVELTEAGLAFLNAIQPVQQRVEDAANLVKQVANGEVGQLRLGFTGTSMLNPLIPKCV
RYFQQQYPKVNLKLEEANTLLLIDLLLEDRLDVAIIRSPRNMPDSLILQELLAEPLIA
ALPSESFNLDDVSQEIDLTALRELDFIVSPPSVSAGLFDAIKQACHERGFQPKIGQNA
PQIVSILSLVSANLGVSLVPESTKQLQIQGVAYRSLKAPVPTVGLGLAYKKHAPSQMA
INFASVVQVACHHANEIDEIG"
/product="LysR family transcriptional regulator STM3121" /transl_table=11
CDS complement(2205186..2206073)
/locus_tag="AUO97b_02190" /db_xref="SEED:fig|400667.82.peg.2054"
/db_xref="GO:0016020"
/translation="MNNLIIGTLFALCAAALNASIGVISKLLMHSGLNPQDIAFLKTI
IAFFFLSVFLFKVPVSQKIAYISSTPSKLSVFTQIAICAFLGIFSLFFFETIAYNHGA
AANVVVVLMASAAISALFFGRLILKESIYINSLIGTLLAVAGISIISWKGENSLLMLI
NASIAGTGYGLFSVLVKKFKLNGGLFLTKYLLLFGSVYLAVPFLVNLHAIHFNIDIVL
GLIGLAVLPTILGFFCTTKALSYMSAAKVQVTELSEPIFAALMAWVFIHEQPTLSFLY
GAIFIISGIFVMNRAPKSN"
/product="Permease of the drug/metabolite transporter (DMT) superfamily"
/transl_table=11
CDS 2206302..2207045
/locus_tag="AUO97b_02191" /db_xref="SEED:fig|400667.82.peg.2055"
/translation="MIKSIITGCAVLTLSALAVTHAEFSYEQELQQSCNKVKQYASLG
KKFYDQKQYKKALENFQNQASWTAFCKANADETTVKFTDRDIEVANNNVGLAYAKLGQ
PLWARAWFMLDEKSKSSQFNLKQLPQPKASNDLSGEYVRYSGFGEWDHITVSRKQGQY
AISYSGVYMGLRSLIYGPNMGEFDTKMPTNAKQTIYKDQDCRIQLDFKSNAKLGNYIQ
VKQSEGESGCGFGHNVYADGTYLKVESGK"
/product="hypothetical protein"
/transl_table=11
CDS 2207070..2207462 /locus_tag="AUO97b_02192" /db_xref="SEED:fig|
400667.82.peg.2056"
/translation="MKNIFAPLLLVSTAIFSAHIYAEPTSTCKEVKFQKADQAWSGHY
YLHGVMEVGSELLLQPDGKFQWMLAVGALDQYAEGTWWKNGDCIGLKPEAKFKKDLSI
FPESLTISDKSLDAIWEGGRQQGTYSKD"
/product="hypothetical protein"
/transl_table=11
CDS complement(2207508..2208143)
/locus_tag="AUO97b_02193" /db_xref="SEED:fig|400667.82.peg.2057"
/translation="MIDLYYAHRIDPNIPVEDTIGAMADLVKQGKVRYLGLSEASAET

IRKAHAIHPIAAVQHEYSLLTREFEQTHLQTIRELGISLVPYSPLSRGLITNTLDVNN
LDENDFRRQLPRYQGDNWKNNQSLAQAFSEFAQSKNATAAQLALAWILAQGDDIIPIP
GTRKIERLVENAGAVDLHLTAADLAEIEAIIARYPNMGARYNAQQLAAVNH"
/product="Oxidoreductase, aldo/keto reductase family" /transl_table=11 CDS
complement(2208162..2208509)
/locus_tag="AUO97b_02194" /db_xref="SEED:fig|400667.82.peg.2058"
/translation="MKYRTLGQTGEKVSALGLGCMGMSFAYGASDDTQSIATLEKALD
LGINFWDTADMYGNGANEVLLSKVLEKHRDKVFLATKFGFRYKEDNLNPKNSLESYID GSPEWIKVAVENS"
/product="Oxidoreductase, aldo/keto reductase family" /transl_table=11
CDS complement(2208621..2209523)
/locus_tag="AUO97b_02195" /db_xref="SEED:fig|400667.82.peg.2059"
/translation="METIHDFYRRFSLTRDSDYSVPSTSFGHFNVFQRDACSFLTPYS
RRDYYKISLVLGTGELHYANRWIRVDRPALLFSNPMVPYAWEINSPEQAGWFCLFTEE
FVNQESRQSFLKDSPLFKVDGDPLYFLNDQQVEEISFIFKKMMNEINSDYIHKYNVLR
NYLHLIVHEAMKMQPTQQFEKHSNASVRICHLFFELLERQFPIDSPHFQLKLKTANDF
AYGLSVHVNHLNRAVKEATGKTTSEHISSRITQEAKALLQHTSWNISEIAYSLGFEYS
AYFTNFFKKNTGFSPKKFREDCVA"
/product="Transcriptional regulator, AraC family" /transl_table=11
CDS complement(2209646..2210356)
/locus_tag="AUO97b_02196" /db_xref="SEED:fig|400667.82.peg.2060"
/translation="MAFLAQTQATNAEWQLLRLYDSQNAKLLLGVAVIVAGGTCFWVP
SEASSGSLLCTSILQLQPRRIVTTAIGRDLLHAEIKQKGRILRKYDQWIMICSRQFSE
AQGRLAELSDIARLNEYQHQYNKERSVNETPDWASLIQQQKIIVLEADQQIVSIVRLG
IETARLVSIGGTYTFPAYRRKGFAERVLQFAVNQIVATGRIAHLIVDVDNTPAVTLYR
QMGFECLESSYIAYPEYL"
/product="hypothetical protein"
/transl_table=11
CDS 2211136..2211687
/locus_tag="AUO97b_02197" /db_xref="SEED:fig|400667.82.peg.2061"
/translation="MKNLEASFRALRVLHTAKNLFNQYGFHKVGVDRIIADSQIPKAT
FYNYFHSKERLIQMSLTFQTDALKEEVFSTIYSYRELMVFDKLKRIFFLHADSDGLYR
LPFKAIFEIEKHYPSAYKVVIDYRNWLIQEIHKLLLTVKPAATVEDAHMFLFVVDGAM
VQLLSSNQTDEREYLIDYFLSRV"
/product="Transcriptional regulator, AcrR family" /transl_table=11
CDS complement(2211760..2212176)
/locus_tag="AUO97b_02198" /db_xref="SEED:fig|400667.82.peg.2062"
/translation="MEYKEISKKTIGHLYSAHSSIRSSDLDPKLIALAELYVSQINGC
AYCCAFHSKELRDFGIAQEVIDQIPGYKHSKAFTEKQKVVLEFASAITYLNDPKQIEA
SRESLAQHFSEKEIVDLTASISLMGALNRLRITLGD" /product="FIG00350685: hypothetical
protein" /transl_table=11
CDS 2212288..2212602
/locus_tag="AUO97b_02199" /db_xref="SEED:fig|400667.82.peg.2063"
/translation="MKDNISGCTVEEAMTVLGGRWRMLIISFLLERTMRFNELRRAIP
NISQRMLTLELRFLEEEGFILREVYAEVPVKVEYSLTEEGRKLEKLVDVLKEIGGWLK SR"
/product="Transcriptional regulator, HxlR family" /transl_table=11
CDS complement(2212682..2213344)
/locus_tag="AUO97b_02200" /db_xref="SEED:fig|400667.82.peg.2064"
/translation="MTFILAIQLEDSIVIAADTRSTISYQDNGSYLYQDGVQKFFPWE
NGIITGTGELNLIQNVMLSFNNFTKQNIHELPNLLKKSKLIREQEISHSQLEITKLLY
STYSKNGAQIYSIQPNKLEEYEVIRCEKNEILLWTFNPDIRPIINALTELYSSLKPLK
VFNNMADCIYYYLNQISPIFRKQAQHDSMMSKEFNFFFQTKDEYFFGEFLNNSSNTLN RK"
/product="hypothetical protein"
/transl_table=11
CDS 2213424..2213786

/locus_tag="AUO97b_02201" /db_xref="SEED:fig|400667.82.peg.2065"
/translation="MAGGSQIIINKNGITLITPAKFEVKAGQHLFKGGAEVGVNIQGL
PAYEAYNEKFQMLLPSGEPMKKVDYKISTDGNEYISQADDKGRSKRIHTSKEENLKLD
LNWISFEADSADNNGDAK"
/product="PUTATIVE VGR-RELATED PROTEIN"
/transl_table=11
CDS 2213783..2214934
/locus_tag="AUO97b_02202" /db_xref="SEED:fig|400667.82.peg.2066"
/translation="MSGSLDWDSNTNTTENSCKTVQCDCLTFWEIYQQEAIKRVSNLK
SANHYKLEPGYEARARRIASVYARIYLEQEISGTKLFKGRYYWMGLGAFASKTVAAIF
KHGLTAWGYKWAPVISGALGGVLTAPVINSMIRDPVHSFAKGNLWLFMDIAPWHYAWS
ASSTSFKTCMKARKVSGFKHIKKQVENLPWSNSLSVIKQLGWTDQIEEGFGYLPDIEN
TFKRVGKTRNDNFKTASKNLFDHLMAIAVQEQYNILQELVWKEWKVQKQASLQRFTGL
PDSTLVLTSDYGVDAVKPNKQGQYKGRHAGVLSQLPETVYSEPLEGTQVEVYDSRMKW
IKVAAAKYHRLMQSDKGRDFLEKELETIAGWGNSKAEFKVGKDSNDGKI" /product="hypothetical
protein"
/transl_table=11
CDS 2215014..2215799
/locus_tag="AUO97b_02203" /db_xref="SEED:fig|400667.82.peg.2067"
/translation="MNQQAYTLHMGEQGIQDFSKYSNGNVDNHPAGVSFRELQFSPPN
LGKITIENGSNTLSIEHVFSVLGTQYDKNEGIQVLDIDAGLTKEEFATPEQVYQSYVA
LMKRINQAGWKNYFFTDAPRIAKEDNIKHLSKSRDVIDPSYIFSFEEWKNIINNSPTK
SLGYRLYANGIILDISLKRTAKNERNEEQYMLRFSFQSVRYNERDSIPDSDKMTPSEF
EQAFKKRLIENKKYREKEERKAKAEGYHIDEEYTDPDVWSYVK" /product="hypothetical protein"
/transl_table=11
CDS complement(2215876..2216082)
/locus_tag="AUO97b_02204" /db_xref="SEED:fig|400667.82.peg.2068"
/translation="MRHELREMFIKGCDDKIEKKGDNVGLSFYAFFKNKNDNPELLME
AAHWWIMEHKLDHFEKAIKIKQLV" /product="Msl2237 protein"
/transl_table=11
CDS complement(2216110..2216325)
/locus_tag="AUO97b_02205" /db_xref="SEED:fig|400667.82.peg.2069"
/translation="MRFNELQRCIGNISFRTLSNTLKELERDELIIRKEYPQIPPKVE
YRLSERGQSLIPLLDMMCQWGERNMNS"
/product="Transcriptional regulator, HxlR family" /transl_table=11
CDS 2216563..2217120
/locus_tag="AUO97b_02206" /db_xref="SEED:fig|400667.82.peg.2070"
/db_xref="GO:0010181"
/db_xref="GO:0016491"
/translation="MKKTLVIVAHPHIDQSVINKRWVEELEKYPDQFTVHQIYQAYPD
GVIDVAKEQALVEEHENLVFQFPVYWFNCPPLLKQWLDEVLAYGWAYGSTGDKLKNKK
FMLAVSAGAQEKVYSEQGEYKLTLEQIFSSFELTALYVGAVYQPLYAFYGLDTNPSPD
DAIPTHQEIDNSAVQYVQKLLALSE" /product="Uncharacterized NAD(P)H oxidoreductase,
YdeQ/YrkL/YwrO family" /transl_table=11
CDS 2217293..2218249
/locus_tag="AUO97b_02207" /db_xref="SEED:fig|400667.82.peg.2071"
/translation="MFSSDLTDYVIRQLGRTKNKRYEAYVVSRIIHLLNDFTLKFVTQ
QFVRLSNKKIALTDLYFPQLGIHIEVDEGHHFLRNSKMEYSLNQIDEPLYSISQTESD
AMREEDIISITGHKIFRVNVYKNQQGKPQNLESIHQQIDKIIEEIKTAKNKLIKASTF
KEWNIETEYNPQTYIDLGRISLADNVVLKTTKDVCNCFGYNYKNYQRGGALHPYEKDT
LIWFPRLYENKDWINTISPDGLTITEKSTDETITLKKLEEWKNGPQKRIVFARVKDNL
SSRAMYRFMGLYEFQKADLKDGAVWKRVKCEVQTYSPKETKC" /product="hypothetical protein"
/transl_table=11
CDS 2218243..2218701

/locus_tag="AUO97b_02208" /db_xref="SEED:fig|400667.82.peg.2072"
/translation="MLKPRIFITIGLLLAGLSLFAVLEAYEKKTNVEKEQASRVAISF
FNSLSNGDSATAYKYVWSGENLNIRNAEIPQIYKDSKVLEVLKVRYDSAKNRPDYYQQ
FYKMISLTIKIKTVHADIAGNPAGTYIVFVTIVKKDPKSNWLVTELGSGG" /product="FIG00353375:
hypothetical protein" /transl_table=11
CDS 2218783..2219124
/locus_tag="AUO97b_02209" /db_xref="SEED:fig|400667.82.peg.2073"
/translation="MNIAPFPFQITNWEEIEVTEYSGETGKAYWKTQFFGEEGNKIRV
RLVEYSANYLADHWCEKGHILFCLEGELESRLKDGRTFTLTSGMSYQVGDNSEPHQSF SLNGAKLIIVD"
/product="hypothetical protein"
/transl_table=11
CDS complement(2219183..2219845)
/locus_tag="AUO97b_02210" /db_xref="SEED:fig|400667.82.peg.2074"
/translation="MKKVAVILSGCGYLDGSEIRESVLTLLALDTVNIEYQIFAPDEP
LFHVIDHVSGEINMTERRNILQEAGRIARGKISSLDQLNENEFDGLILPGGFGVAKNL
STFAFKGAEARVHGTVASILKAFHQSKKPIGAICISPALLALTFGELHPTITLGSDLN
IAKEIEKTGSIHHVCQTSDCVVDKQNLFVTTPAYMDDQANLKDIYTGITSLVNTMTAL AN"
/product="Enhancing lycopene biosynthesis protein 2" /transl_table=11
CDS complement(2219935..2220747)
/locus_tag="AUO97b_02211" /db_xref="SEED:fig|400667.82.peg.2075"
/translation="MKNLISLAKKSLVVHSNLNLPFAVYSAYQEQKITNVPISKPLLI
IVLSGFKELGKTENIKCFAGSFIFLANSSSIDMRNIPDQEEYFALLIEFDFEDFNDLP
QNAGTSQNYVQGQLNPLLESALHQFIDFSLVAPPSAFKFRKRELLELIYYSGYTAIAK
LNMNKSLSERVQTLISYHLSTELTAEFIAAQLFMSSSTLRRKLKSEGNSIQDIRNRVR
LGHGLHLIQTTNNSIGNIAFECGYQSQSRFTEQFKNLFGITPRELRKTKMLT"
/product="Transcriptional regulator, AraC family" /transl_table=11
CDS 2221092..2222969
/locus_tag="AUO97b_02212" /db_xref="SEED:fig|400667.82.peg.2076"
/db_xref="GO:0006813"
/db_xref="GO:0015079"
/db_xref="GO:0016020"
/translation="MQNTAKKATLPATALAALGVVFGDIGTSPLYALKESFHAAHGLG
IQPENVLGILSIIFWCLMLIISIKYVAIVMRADNNGEGGIMALLALNLRKAKIADNKK
IYMIAIGFIGASLFFGDGIITPAISVLSAVEGLSIATDVFDPFIMPIAIAIIVTLFLV
QKHGTAFVGKFFGPITLVWFLSLGILGIHSVIQTPVVLGMFSPHWAIQFIYHHPIMTF
FVMGAVVLTVTGGEALYADMGHFGPVPIRLAWFFVVLPCLVLNYAGQGALLLRDPAAI
ENPFYLLVPQWALYPMIIMATMATVIASQAVISGVFSLARQAIQLGYLPRLSIKHTSE
SEEGQIYVPFLNWLLLIAIIILILIFKTSSNLASAYGLAVTLTMLCDTILVAVFIYSA
WKWSLPKVLLLIIPFFILESVLVGATSLKILSGGWVPLLIGAIAVTILMTWKRGRELT
FAKLEHDTLSLDLFVKSIGNSVHWVPGDAVFMTGTPNVVPHAMLHNIKHNKVLHQRNI
LVTVVIEDVPFVAPEERITTETLAEHFFRIKIFYGFKDEMNVPKALMQAYEQLGLEYD
LMHISFFISRDRIVHSVGDGMSPWREKLFISMQRNTSPVSDFYQIPTNRVVELGSQIE I"
/product="Kup system potassium uptake protein" /transl_table=11
CDS complement(2223025..2223468)
/locus_tag="AUO97b_02213" /db_xref="SEED:fig|400667.82.peg.2077"
/translation="MIHSKIIGIGVITGLCSMAALAEPAVQPGDTLESLSKVKVTTTV
NGQPGSISDLVNSGQVQQVSAAPTSAAPTGAPIPADVPPAPPAGGEMAPPAAPTDAVP
PAPNQAAPAPQDPSTPPPAANPNQSADPMAKDGALPADAPMPQAQ" /product="Chromosome
undetermined SCAF14611, whole genome shotgun sequence" /transl_table=11
CDS 2223724..2224803
/locus_tag="AUO97b_02214" /db_xref="SEED:fig|400667.82.peg.2078"
/translation="MASKKRSKGTNSFSLSEHFGKIILACIAAGSFAIAFGSEKISQW
FSPLSTNSTCLNQFYREVPPALNKESLKKDSYPLCFNGFNVLYSGISKTPLWSAEHLD
AERLSVKIKREDNFHEETRVPQKHRALLSDYRGSGYDRGHMAPNGDMPNKESQSDSFS

LSNMVPQAPKNNQEVWRKLEEATRAIVTKQKQDVYVVTGPVFEGKRLKTIGQGVIVPT
AVYKAVYMPKTGAIGAYYAPNNNSQQVKVVSVCYIEEKLGINLFPQLTEQQKRNVYRL
PLTASQVKPNQKLDYLHWDGESQCEQDLSAEQIQALQDQFKKQKTGSSEPMEAKVPSI
DEETRNAIVKQLVEALVNYFLQIMK"
/product="DNA/RNA non-specific endonuclease" /transl_table=11
CDS 2224900..2225499
/locus_tag="AUO97b_02215" /db_xref="SEED:fig|400667.82.peg.2079"
/translation="MFASMFLFSFAMSITPGPVNTVILSTSLNHGLKRSLPYISGATI
GFTLLLIFMAFGLQSVLTQFPVVLKILAVCGTLFVCYIGIKIILSAANISISSAPVGH
MLIPNFKDGFLLQWLNPKAWLACVSGITMFTSIENPQSLPIFIIIYFFTCYACLFFWG
LCGDKFSVVLNQGNRLKYFNILMGAFLILSALLILIDFF" /product="Transporter, LysE family"
/transl_table=11
CDS 2225545..2225787
/locus_tag="AUO97b_02216" /db_xref="SEED:fig|400667.82.peg.2080"
/translation="MFKPFEKGTESSSIEDLTLENDVDCVSIYGNLQITKDKVGLEQA
KALQSFLNDVVAALEKEELPEKIERPAEQEINNPFL" /product="hypothetical protein"
/transl_table=11
CDS 2225878..2226000
/locus_tag="AUO97b_02217" /db_xref="SEED:fig|400667.82.peg.2081"
/translation="MSFVTFAHSKVRHYLYNYNLSDRGICGCAVNPYPQQLPYK"
/product="hypothetical protein"
/transl_table=11
CDS complement(2225966..2226655)
/locus_tag="AUO97b_02218" /db_xref="SEED:fig|400667.82.peg.2082"
/db_xref="GO:0005215"
/db_xref="GO:0006810"
/db_xref="GO:0016020"
/translation="MNKYFAEFLGTFWLVFGGCGSAVLAAAFPELGIGFAGVALAFGL
TVLTGAYALGHISGGHFNPAVSVGLWVGGRFDVKDLIPYIVAQVVGATAAAFVLYIIA
QGQAGFSGVGGFATNGFGDLSPNKFGLGSAFIIEVVLTAFFLIIILGATDRRAPAGFA
PIAIGLGLTLIHLISIPVTNTSVNPARSTGVAFFAETAALSQLWLFWVAPILGAVIGA IIYKVVAGDKD"
/product="Aquaporin Z"
/transl_table=11
CDS complement(2226788..2227792)
/locus_tag="AUO97b_02219" /db_xref="SEED:fig|400667.82.peg.2083"
/translation="MPQLKNYTGSVYGGLGHLLKAYCEANGLAIPEQLEQIQNLERFD
YVVWRDLLEVIYKLDPKPGLGLEIAKHVQPKHLGIIAYLALSCDNLGEALIRYHDFHR
LIYDGSPLVVELQGRYGSIRWEATELHPTQLTDEIAIALVIQFLRLFMSHEDVQLHEV
RFLNAAPKNIRVYEQYFQCPVRFSQPRTELLIPITEIHKPLSSADHTLQQLLVQQAQA
LLEQLPNSTQLDQRLQHSILTGLQKNQYQIEYIAAQLGLSVRQLQRHLQQQSTTFQER
VQQVRFMLATEYLKDPHLSLQEIALLLCYSEQSAFQRAFKHWTKQTPQQWRDQFLDKE "
/product="Transcriptional regulator, AraC family" /transl_table=11
CDS 2227902..2228540
/locus_tag="AUO97b_02220" /db_xref="SEED:fig|400667.82.peg.2084"
/translation="MLKGFIAGIAVANAFEWVAHKYILHGVHRAGQPRYSPVPKSMES
HWAHHREVRKQQFHDDCYVEGVGNWRTKNELISLAVVATVSSAIFYPFSKGMALAAWY
SAGNYYYIHRRAHLEPDWAKRKIPWHYDHHMNSNQDANWCVTKPWFDYVMGTRVVSSA
DLKEQNPLGIRLPTALARPLSQAVEKIFPAKWVEKKEQPKLVSDVNAIEGAA" /product="Fatty acid
hydroxylase family like" /transl_table=11
CDS complement(2228582..2229340) /locus_tag="AUO97b_02221"
/db_xref="SEED:fig|400667.82.peg.2085" /db_xref="GO:0000105"
/translation="MLAKRIIPCLDVDNGRVVKGVQFLDIRDAGDPVEVARRYNEQGA
DEITFLDITATHHGRDTTYRTVERMAETVFVPLTVGGGVRKVEDIRALLNAGADKVSI
NSAAVFNPEFVQEASQHFGAQCIVVAIDAKKTGDNKWEIFTHGGRKPTGIDAIEWAVK

MADYGAGELLITSMDADGTKAGYDIALMRAINDRVTIPTIASGGVGNLQHLADGILQG
GADAVLAASIFHFGQYTIPEAKQYLAEQGIEMRL"
/product="Imidazole glycerol phosphate synthase cyclase subunit"
/transl_table=11
CDS 2229453..2230403
/locus_tag="AUO97b_02222" /db_xref="SEED:fig|400667.82.peg.2086"
/db_xref="GO:0004413"
/translation="MSVYTPLSLDEVRTFAAPYGLEVLELNPIQGGIQNTNYFLVDVN
RKQYVLTVFEELDAQGAGELIPVLEQLGTHDVPVAVPLKHSGQAVHFIAGKPAQIAPR
LMGHHPMQTTVAQVAAIADAQAKLHVALQDFPLEREYRRDHQYWTGVAEQLKPNMTQD
DQTLLEQVYQAFNAKTAQYSNRPTGFIHSDLFRDNTLFEGEQLQGILDFYELNQDELL
FDIAITINDFCTEYPAAHLNSDKVDAYLEAYQKVRALTSDELECLDVFLAMAACRFWS
MRLQVAQKNKEEGRTGDDILQKDPQEMRAMMQDRLSHVKV" /product="Homoserine kinase (EC
2.7.1.39)" /EC_number="2.7.1.39"
/transl_table=11
CDS 2230416..2231045
/locus_tag="AUO97b_02223" /db_xref="SEED:fig|400667.82.peg.2087"
/translation="MRDQGRLVEWFDEKGYGFIQPDDAEKERVFLHIKDFARPGPRPI
IGCALEYLVILDERGRFRAQQVTYLKASQTRKALKPAKTQSSFQASPWSAMQMGIVFY
FVLMGIMSFIHILPAYTLLFVLMMNVLSYWLYSQDKEAAQLGNRRVPEQTLHIVSFLG
GWPAAWWAQQKLRHKTQKQPFRKIYFCTIFFHLLLILWLISPLNVLRGS" /product="Cold shock
protein of CSP family / DUF1294 domain" /transl_table=11
CDS 2231082..2231474
/locus_tag="AUO97b_02224" /db_xref="SEED:fig|400667.82.peg.2088"
/translation="MNSSIDQDPNRTLTLILYVLYIVAIFTGGLLAIIALIINYVKRS
DVQGSIFASHFTWQIRTFWWYLAWNIIAFLPFIFLFFTGENTDLFAGVAISSTVFCVG
VIAAAWIWIVYRAIRGLIALNDNRPMYQ"
/product="Putative transmembrane protein"
/transl_table=11
CDS 2231478..2231789
/locus_tag="AUO97b_02225" /db_xref="SEED:fig|400667.82.peg.2089"
/translation="MSIEHVHLSIKSGQSEAFLKAFEQAKHIVYPMEGFNSVQLIKHA
TDPHHYILMIFWDEIENHTEGFRKSEAYQEWKKLLHPFYDPMPTVEYYEPSILLKKND I"
/product="Antibiotic biosynthesis monooxygenase" /transl_table=11
CDS complement(2231815..2232717) /locus_tag="AUO97b_02226"
/db_xref="SEED:fig|400667.82.peg.2090" /db_xref="GO:0016020"
/translation="MLPTYLSSSKVPQLALILITVIWGGTFLTVQYALNFSSPMFFVG
CRFAVAALTLLLISLKSMKGVTLKDLGAGSVIGLVIAAGYGTQTIGLQTIPSSESAFL
TALYVPLVPILMWLIFRKTPHIMTWVGAALAFTGLVLLTGNGFKQISLSFGQLLTVLG
AFAIALEIIFISYFAGKVNLRRVTIIQLGVASLLSFTIMPIVGEHTIPAFSWPLVLIA
VALGLASALIQFVMNWAQRVVDPSRAAIIYAGEPVWAGIIGRIAGERLPVIALFGGAL
VVLGVLVSELKIKLPIKKNKRVRE"
/product="Permease of the drug/metabolite transporter (DMT) superfamily"
/transl_table=11
CDS 2232816..2233367
/locus_tag="AUO97b_02227" /db_xref="SEED:fig|400667.82.peg.2091"
/translation="MSQSSSVLQHVGTNIRSLRDERSLSQQDLADRAGVSRRTIAALE
TGQVNISLAKLDAIAAVLGVDFRTIVSAPELKEHALVNALAWQGEKEESKATFLASVP
SRSQVELWTWSLAVGESYVAEADAEGWQELIYVLEGELTIQFTDSSKTIAAGSSFIFA
SSVTYTYINSGSQVLKFIRNVVY"
/product="Transcriptional regulator, Xre family" /transl_table=11
CDS complement(2233435..2234166)
/locus_tag="AUO97b_02228" /db_xref="SEED:fig|400667.82.peg.2092"
/db_xref="GO:0003949"

/translation="MLIIPAIDLKDGKCVRLKQGRMEDDTVFSDDPVATAQHWVNEGA
RRLHLVDLNGAFAGTPIHKPVVEAIAKAQPELPIQIGGGIRSLETIEHYLEAGVTFVI
IGTKAVQEPEFVEEACKRFAGHIIVGIDAMNGMVATDGWANVTDVKATDLAKRFADAG
VSSIVYTDIARDGMMQGVNVEQTVNLAQYSGLPVIASGGVTNLDDVRNLKGQPGILGA
ITGRAIYEGTLNLREAQLLLDENRL" /product="Phosphoribosylformimino-5-aminoimidazole
carboxamide ribotide isomerase (EC 5.3.1.16)" /EC_number="5.3.1.16"
/transl_table=11
CDS complement(2234261..2234902)
/locus_tag="AUO97b_02229" /db_xref="SEED:fig|400667.82.peg.2093"
/translation="MSEASALLDKTSIQQLPPFENLNYQNIVVIENSEQCKTIEEELK
AATFLGFDSESKPTFQVGEVSTGPHLIQLATEHKAYLFHVNSSTLKFLQPILSNPKQI
KVGFGLKNDKHIFHKKGIKLESCIDLAKGFSHFGFKQQMGVQKAVALLLGQYLAKSKK
IGTSNWARKPLTSQQISYAAADAYAALLVFLELKKRKVLPIHISEKIQTALQG" /product="3'-5'
exonuclease domain protein" /transl_table=11
CDS complement(2234989..2235537)
/locus_tag="AUO97b_02230" /db_xref="SEED:fig|400667.82.peg.2094"
/translation="MNSPFENQPLQQDSPFKANGRFGRLSYAAWTFLFTLVLVTAILL
IMFTFGFTNPEGTPDFSTPGLICIAILYIVGIYINFVFTIRRLHDRNNTGWLSLLMLV
PVVNIGLAIYLFCAKGTEGPNDYGPKRPTPSWERVLGWIYIALIPLALIFGVIAVIMA
PTYEGYVEKSESIVIGSPSQSE"
/product="hypothetical protein"
/transl_table=11
CDS complement(2235691..2236308)
/locus_tag="AUO97b_02231" /db_xref="SEED:fig|400667.82.peg.2095"
/db_xref="GO:0003824"
/translation="MTRIALLDYGMGNLHSAAKALEYVGATVDVTNDPKLIAQADKIV
FPGVGAMRDCMQGMREAGIDEVVRKAAFNKPVLAICVGMQALLQSSEENGGVDALGIF
DGVVKHFPQMEGLKVPHMGWNQVHQMDPSHPMWNNIEQDARFYFVHSYYVEPKDENLV
AATCEYGVNFCTAIHKDNLFATQFHPEKSHTAGLQLLKNFVEWNI" /product="Imidazole glycerol
phosphate synthase amidotransferase subunit HisH" /transl_table=11
CDS complement(2236308..2236901)
/locus_tag="AUO97b_02232" /db_xref="SEED:fig|400667.82.peg.2096"
/db_xref="GO:0004424"
/translation="MTQRISEVVRNTNETKIRVRLNLDGTGQGTLNTGVPFLDHMIDQ
IKRHGLFDIDIHCDGDLEIDDHHTVEDCGITLGQAFAQALGDKKGLRRYGHFYAPLDE
ALSRVVVDLSGRPGLFMDIPFTRARIGTFDVDLFSEFFQGFVNHALMTLHIDNLKGKN
SHHQIESVFKALARALRMACEIDPRAENTIASTKGSL" /product="Imidazoleglycerol-phosphate
dehydratase (EC 4.2.1.19)" /EC_number="4.2.1.19"
/transl_table=11
CDS 2237004..2237387
/locus_tag="AUO97b_02233" /db_xref="SEED:fig|400667.82.peg.2097"
/translation="MPITVHAYSSLDNSEIRDQLERLYDTSPEFGDGHDAIEQLEQDL
QQYTTLYTAEFNTKIIGAIWCSGQGESKVLEYIVVHPANRGRGVAERLVEETCRIEEA
KGVKIFEPGCGAIHRCLAHIGKLHS"
/product="hypothetical protein"
/transl_table=11
CDS complement(2237942..2238373) /locus_tag="AUO97b_02234"
/db_xref="SEED:fig|400667.82.peg.2098"
/translation="MFIIRQFTNADLDDVVNLWESCGLTRPWNNPETDIFRKVSQQDD
LFLVAIKDEQLIGTLMGGYDGHRGWINYLAVHPHQQRLGIATALVQQLEKRLMARGCP
KLQLLVRKDNLNVLNFYEQLGYDEVEAVCLGKRLISDNPHD" /product="Acetyltransferase"
/transl_table=11
CDS complement(2238504..2240018)
/locus_tag="AUO97b_02235" /db_xref="SEED:fig|400667.82.peg.2099"

/translation="MSLSRIRLASLHDKVISAEQAAQFIENGMTVGMSGFTRAGEAKA
VPQALVELAKKNPLKITLITGASLGNDLDKQLTEAGVLSRRMPFQVDNTLRRAINNGE
VMFIDQHLSETVEQMRNQQLKRPDVAVIEAIAITEDGGIIPTTSVGNSASFAIFAEKV
IVEINTSLSESFEGLHDIYIPTYRPTRTPIPLTKVDERIGTTAIQIDPAKIVGIVFND
THDSPSTVTPPDDETQGIANHLIAFFEKEVAEGRLPKNLGPLQAGIGSIANAVLTGLK
DSNFEDLIMYSEVLQDCTFELIDAGKMKFASGSSITLSAKCGEKVFGNIEAYKDKLVL
RPQEISNHPELVRRLGIIGINTALEFDIYGNVNSTHVCGTKMMNGIGGSGDFARNAHL
AIFVTKSIAKGGDISSIVPMVSHVDHSEHDVDILVTEQGLADLRGLAPRERARAIIDN
CVHPLYRDALNDYFDRACAKGGHTPHLLREALSWHANFEETGQMLQPTPVAKSA" /product="Acetyl-CoA hydrolase/transferase family protein" /transl_table=11
CDS complement(2240210..2241667)
/locus_tag="AUO97b_02236" /db_xref="SEED:fig|400667.82.peg.2100"
/translation="MSLEPIPPQTFTDYVAYSERKRTRWERFLDKIKPRSAAMRTTIL
VLLVVLFSLFMSLWFFWRTLYLPEIQQHARYLAVELELVNNPNIRIFHRDSEVDVDTW
LRNRVGIEYVTNPKEYPSVRDKIIAEFFTNQVEQKLANEIGAKDVTVYFQFKPSPRIW
IQTPEMHGNWVREPLKTYANYSPELLVGWVVGVPLVSAIIILILVRQMNRPLRRLQNA
ANEYSKSGTAPYLDTNHGPLEIRQVNQAFNRMVYTLEQTERERRIMLAGISHDLRTPL
TRIRLTAEMLPDEFLREGLVYDVDDMDAILNQFISYMRDGSDEELKDTNINILLQELV
VQFKPLDIRFNMQDVPIIPARSLSLKRLIANLINNAKRYGAEPIELSAKVENEHILIT
VADHGEGIPEDQIEELMQPFVRGNSARTIQGSGLGLAIVKRIVDIHQGEIQIHNREQG
GLEVIISLPIPKPAPEENTTHNPLEKIKQTLSERF"
/product="Osmolarity sensory histidine kinase EnvZ" /transl_table=11
CDS complement(2241685..2242449)
/locus_tag="AUO97b_02237" /db_xref="SEED:fig|400667.82.peg.2101"
/translation="MSLVVPAEHPETVHNETDRVERILVVDDDVRLRTLLQRFLEDKG
FVVKTAHDASQMDRLLQRELFSLIVLDFMLPVEDGLSICRRLRQSNIDTPIIMLTARG
SDSDRIAGLEAGADDYLPKPFNPNELLARIRAVLRRQVREVPGAPSQQVEVVSFGPWS
LDLSTRTLTREGQIVTLTTGEFAVLKALVQHPREPLTRDKLMNLARGREWGAMERSID
VQVSRLRRLIEDNPARARYIQTVWGVGYVFVPDGAE" /product="Two-component system response regulator OmpR" /transl_table=11
CDS 2243105..2245453
/locus_tag="AUO97b_02238" /db_xref="SEED:fig|400667.82.peg.2102"
/translation="MTDLVQQLASELAVRPNQVEAAIRLIDEGASVPFIARYRKEVTQ
GLDDTQLRQLDTRLAYLRDLYERREKVIQSLQEQNKLNDDLLARVNAAETKNALEEIY
APYRPKRTSKSFKAKEAGLGPIAEKIISEQIDPTEALAGFSHEDYPDVESQLDAIQHI
LIDDWAQNIPLTTELKATFAKTAVLKSTVASEEKKEVGKKFRDYFEFSEGLNKLPSHR
LLAMLRGRQENVLGLKVDGEDDAPLARIETEYNLDQVQPQTRQDFLKQTAKLFWLGKV
RPQIEHSLLTEKRLAAEAEAMQVFAENLRHLLLSAPAGSRTTLGVDPGIRTGVKLAVV
SEAGDVLAHSTIYPFAPKEDKEGSIAELERLCREYNVELIAIGNGTASRETEAVVAEM
MAANTDLKLTRVTVSEAGASVYSASELAAAELPELDVSIRGAVSIARRLQDPLAELVK
IDPKSIGVGQYQHDVNQTGLAKTLDAVVEDCVNAVGVDVNTASPAILAYIAGLNKAIA
QQIVEYRKEHGRFDTRQALKNVPRLGERTFEQAAGFLRIQNGSEPLDASAVHPESYGL
VEKIVAAKATTVKDIIGNTEIIRQVKAEEFVDDKFGLPTIQDVLAELEKPGRDPRPEF
RTAKFREDITEVAQLTEGMQLEGVVTNVTNFGAFVDIGVHQDGLVHISELANEFVSDP
HKVVKPGQIVQVRVMQVDVERNRVNLSMRAEGAAPVKAQRPPRREQNNEQRSERKPQG
KRPQPRKDQVERPQRSKQEKPQEQKIGGFGALLLQAGIKGSK" /product="Transcription accessory protein (S1 RNA-binding domain)" /transl_table=11
CDS 2245825..2247945
/locus_tag="AUO97b_02239" /db_xref="SEED:fig|400667.82.peg.2103"
/db_xref="GO:0004089"
/translation="MALPLCLGIALASGAPIISGIIAGIVGGIIVGLLSGSHISVSGP
AAGLTAVILVQLDQLGGNYAAFLLCIVFAGILQILFGVFKLGFFANFIPNNVILGLLA
AIGAILIVTQLPYLFGLTDFSWKEVWTATPDTFIQKFDAGAALIGLLSLFLILAWDSS
PLKKLALPSALIAVVLAAVFNFVLISIGSSWAVQSNNLIQLPNILQAPEEVLVFPDFS

YLAEPLIYTGAITLAIVASLETLLNLEAADKLDPQKRSSPPNRELWAQGAGNIVSGLI
GGMPVTSVIVRSSVNANTGARSKCSTIIHGVLLLLTILFFVPLMNMIPLSALAAILIV
TGFKLTHPKLFKQLYQKGWRQFLPFIITLVAILLTDLLTGILVGLFTSSAFILYGNFN
KGVRVYKEKHLHGIVTRIELPSQVTFLNRSALISALEHVHKDQQLIIDATQCDSIDPD
IYQVIQDYQNETAVKRQVDLKLIGFKQHYEEVDDAVLDIYISTRDLQRKLSPQQVITL
LKEGNERFVKNERLQRDIYRQIRVTADEGQHPIAAVLGCMDSRAPTEMIFDVGIGDLF
SLRIAGNIAGQKVLGSLEFACQAKGSKVILVLGHTDCGAVTSACQLRLQQKQISDVKE
MPHIQYVLGPLMHSVESVYDIMQPRELNKTFVNQVTAMNVHYNIQYIINNSTVLKDLL
DRGEIAIVGAIYDVKTGHVEFLDA"
/product="Na(+)-dependent bicarbonate transporter BicA / Carbonic anhydrase, beta class (EC 4.2.1.1)" /EC_number="4.2.1.1"
/transl_table=11
CDS complement(2248013..2248903)
/locus_tag="AUO97b_02240" /db_xref="SEED:fig|400667.82.peg.2104"
/db_xref="GO:0004316"
/translation="MNKKLETLFQEKVQGKVILVTGASSGIGLTISNKLADAGAHVLL
VARTQETLEEVKADIESRGGQASIFPCDLNDMEMIDQVSKEILASVDHIDILINNAGR
SIRRAVHESYDRFHDFERTMQLNYFGAVRLVLNILPHMIQRKDGQIINISSIGVLANA
TRFSAYVASKAALDAFSRCLSAEVHAHKIAITSIYMPLVRTPMIAPTKIYKYVPTLSP
EQAADLIAYAIVKRPKRIATNLGRLASMTYSVAPGINNMFMSIGFRLFPSSDAAKGKK
AEKLNWVQKAYARIFPGEHW"
/product="Oxidoreductase, short-chain dehydrogenase/reductase family"
/EC_number="1.1.1.100"
/transl_table=11
CDS complement(2249177..2249545)
/locus_tag="AUO97b_02241" /db_xref="SEED:fig|400667.82.peg.2105"
/translation="MTIFISGVLAASVSFKIDLDITENIHIPVWAQREYRWDQIMTTP
ALFITFASGLIIAIHGQWFSSKWLPAKIVFVLILAGIHGFQAKLLRQIANGINVRKKQ
ATLIILICTISIIVLAIIKP" /product="hypothetical protein"
/transl_table=11
CDS 2250235..2251182
/locus_tag="AUO97b_02242" /db_xref="SEED:fig|400667.82.peg.2106"
/translation="MHTVAILALHNFIPFDLGIPCEVFGFVRLLNGETGYCVKVCGEA
EYVNTGAFTLQAHFGLDHLIDADTIIIPGIKDPKEPLNPKVKTAILKAWQKGARIASI
CSGAFVLAETGLLDGYRAATHWMLAEELAIRYPKVKVEPKVLFVDEERLITSAGASSG
LDMCLHLVRRDYGQSVAAQVAKLAVAPLNRDGGQAQFIQYEIPETRDSLATLLEWMNT
HLDDKIDIKLLACKANMSPRTFARRFREQIGTTPLQWLLTMRIQRAQELLETSSQSIE
EIALCCGFNSPVTFRTRFKNSVGVSPSTYRARFISYLSS" /product="Transcriptional regulator, AraC family" /transl_table=11
CDS complement(2251304..2252581)
/locus_tag="AUO97b_02243" /db_xref="SEED:fig|400667.82.peg.2107"
/db_xref="GO:0003961"
/translation="MTYKDETLAIHAGYTPEATTKAVAVPIYQTTSYAFDNTQHGADL
FDLKVQGNIYTRIMNPTTAVLEQRLAALEGGIGALALASGMAAITYTIQTITEAGDNI
ASVSTLYGGTYNLFAHTLPKQGIEVRFFDYQKPESLRNLIDDKTKLVFVESIGNPLGN
IIDLEEISKIAHEYGVPVVVDNTVATPALLKPFQYGADIVIHSLTKYIGGHGNSIGGA
IIDSGKFPWGKYPERFKVLNTPDPSYHGVNYVEALGEAAYIARARVVPLRNTGAAISP
LSVFLILQGLETLNLRMERHTENAQRIAEYLQNHPKVKWVNYAGLTDHPQHQLAQKYV
KGKPSAILSFGVQDGREGGTRFIDALQLFTRLVNIGDAKSLACHPATTTHRQLNEQEL
KAAGVSEDMVRLSIGIEHVDDLIADLEQALSSV" /product="O-acetylhomoserine sulfhydrylase (EC 2.5.1.49) @ O-succinylhomoserine sulfhydrylase (EC 2.5.1.48)"
/EC_number="2.5.1.48"
/EC_number="2.5.1.49"
/transl_table=11

CDS 2252918..2254540
/locus_tag="AUO97b_02244" /db_xref="SEED:fig|400667.82.peg.2108"
/translation="MVPPKREILKDISLSFFPGAKIGVLGLNGAGKSTLLRIMAGVDK
DFSGEARAQPGIKIGYLEQEPPLDPTKDVRGNVEDGVREALDALERLDQVFAEYADPD
ADFDALAKEQEKLESIIHAWDAHNLNNQLEIAADALNLPAWDADVTKLSGGERRRVAL
CRLLLSKPDMLLLDEPTNHLDAESVSWLERFLKDFPGTIVAITHDRYFLDNVAEWILE
LDRGHGIPYQGNYTSWLEQKNARLEQEQKQEESFAKALKKELEWVRQNAKGQQKKNKA
RMERFEELNSREFQQRNETSEIYIPPGPRLGNKVVEVENISKSFGDRLLYENLSFTVP
PAAIVGIVGPNGAGKTTLFRMMTGEQKPDTGTVTLGDSVKVAYVGQIRDTLDDNKTVW
EEVSGGLDILKVGDYEIASRAYIGRFNFKGQDQQKRVGELSGGERNRLQLAKILQQGA
NVILLDEPSNDLDVETLRALEDAILVFPGTVMVVSHDRWFLDRIATHILSFEDEQPEF
YTGNYTEFEAYRRAKLGDDAQPHRKKYKKISG" /product="Energy-dependent translational throttle protein EttA" /transl_table=11
CDS complement(2254583..2254963)
/locus_tag="AUO97b_02245" /db_xref="SEED:fig|400667.82.peg.2109"
/translation="MNMKTIKHGLNQLFAFENLPADMHSQAEDEIEQGLRFEELAIQD
HERMQNWLDITPNDRNAMWCYLRAALRGNADASFKLGIGYLNGQFGLDKNYTEAEKWL
NKAASQGHPDAERRLQEAFSKLAF"
/product="hypothetical protein"
/transl_table=11
CDS 2255194..2255313
/locus_tag="AUO97b_02246" /db_xref="SEED:fig|400667.82.peg.2110"
/translation="MIIQNKKIEKIYKYQTKEIFLNKTSLRAGFVFRLVRVLI" /product="hypothetical protein"
/transl_table=11
CDS complement(2255306..2255998)
/locus_tag="AUO97b_02247" /db_xref="SEED:fig|400667.82.peg.2111"
/translation="MYKTLTTCLLVTCLGLSGCATSILSSALPNETTEVQTTILKTDQ
IIALGQAVKNQQEQGVVFIGQDFNYLMTEGSSEFLNIIKNIPVNQRTLIIPSPLLLEM
DDPTHFHGELKFQYNIPASKLSDQQKENLKNLGFKNHFMVMENQTQLLYPYAIIRFKG
QIYQTSPTLKVQQTIPTPYPIALQQKDEITKKHPFKRVTRMALYPLAMAFDIVTVAPS LILSDLRGDFTK"
/product="putative lipoprotein"
/transl_table=11
CDS 2256147..2256545
/locus_tag="AUO97b_02248" /db_xref="SEED:fig|400667.82.peg.2112"
/translation="MPRSAIFMSVLFCLLIFQSLWNIAAAFCAHENQEKALHHFGHHA
ALNVYQTSSHTHVEHTETLDAAHKAPLNLQDHHDHLPTCFHVVMTEVAKQAQTPVLHV
HELSQIYHWSNSYQSPHLSALKPPPVLTPL"
/product="Cation efflux system protein"
/transl_table=11
CDS 2256723..2258015
/locus_tag="AUO97b_02249" /db_xref="SEED:fig|400667.82.peg.2113"
/translation="MANAASDIQQGSYTQKAAFSFEQALARAQSYQTQQGVWQAQQQM
AEAQLKQSRLWANPSLSIEQTGLQSDQEKELAIGISQPLDIFGQRKAAQHLAKVEMSK
VDLAEQRYKAELELIVKYFWSQVALLELEKSLIGEQLAVSQENLSASEKRYQAGSIAQ
VDVDRVRMSHLENQRLYQQVDLKLQVAKQQLANLWDGDSNQFQLSQSSNQLWVLAADV
DTGQDRQNNLLERSFQLDALVQQATIQQLKAKARPQPTVTLGVNNTRSPEQRTENQIR
LGVEIPLNLFNRQQYGIKIAQAKQELSQRQQSFYRQQNQVDIETLMSELKGLHIQFKQ
LNDQQVPLAVQVQQKTLQGFRLGKFAVTDVQQATMQLQDVRLRKVELLKQAWQNSIEI
QSLRLGLEPEQIMAKDALMQLNQRAWQQAQTFPTQAGE" /product="Cobalt/zinc/cadmium efflux RND transporter, outer membrane protein CzcC" /transl_table=11
CDS 2258018..2259235
/locus_tag="AUO97b_02250" /db_xref="SEED:fig|400667.82.peg.2114"
/translation="MSANLKKNSQWLWVGVIAAITAILIGLLVLNSKNKSNSSEPSEG

HGHAEEEGEEHHDEGEKPLLLTAQQMQEQNLKIEQAELGEVPQLQTYPAKLVVNTDRQ
AHVSPSFSGRVEAVYVELGQQVKKGQALASLLVPDLVDQQANLQIAQSNLELARQDYE
RERSLWSQGISAKQDYQRAYNAYQQAQIQVKAARSRLSAFGAGSGSAGRYTLTAPIAG
MVSNKDIVVGENVQLADQLFIINQLDQLWLEFILPSNANINVQPNQQIEFKSLQTGNT
FSAQVQSLTTEADAQTGRLQVRAKVLANSSELRPNLMVNVELNAGSTQKVTRVKAQAV
QQVEGKDVIFTPKMVKTGFEFEPVTVQLGQRSKDGQWVEVVKGINPSQRYVAEGSFLL KSELEKGEAEHGH"
/product="Cobalt/zinc/cadmium efflux RND transporter, membrane fusion protein CzcB" /transl_table=11
CDS 2259225..2262383
/locus_tag="AUO97b_02251" /db_xref="SEED:fig|400667.82.peg.2115"
/translation="MDINKPELPKPEGLFDRVIQFAIQNAIWVMLFVLAWIGVGIWSY
QKLPIDAVPDITNTQVQINTQANGYTALEVEQRITYPIENAMAGIPNLEQTRSISRYG
LSQVTIIFKDGTDIYWARQLINQRLQEADGQLPESVDPIMSPVSTGLGEIYQWVVKAK
SGAKKADGTAYTAMDLREIQDWIVRPQLQRVKGVAEINSIGGYNKTYIVSPDLKRLQQ
LQISINEFQTALQENNENRGAGFIEENGEQLTVRVPGMLSSVEDIQNITVSTKNGLPI
RVVDVANVSIGHDLRTGAATYNGEETVLGIAMMMMGENSRTVAQAVDAKIKEIQSTLP
KGVEIETVYDRTSLVNKAIATVQKNLVEGAILVIVILFIFLGNFRAALITACVIPLSM
LFTLTGMAEQNISANLMSLGALDFGIIVDGAVVIVENCIRRLAEAQHALHRPLTRSER
FKEVFLAAKQARRPLIFGQMIILVVYLPIFALSGVEAKMFHPMAMTVVMALLGAMILS
VTFVPAAVALFVTGEVKEKETRWMQLLKQKYRDVLDQAYQLKIVVVSFALSILVLTGV
LATQMGSEFAPQLSEGDFALQQMRSPSTGLEQSLRMQENTEKLILKNFPEVKAVFART
GTAEVATDVMPPNISDAVILLKPHDQWPNPKETLAELRSRMEAFLATLPGNNSEFSQP
IELRFNELISGIRSDIGVKIFGDDMQILNEQAQALAQKVQKISGATAVKVEQTSGLPV
LSVEINRPLAAQYGLSAKAIQDIVAASVGGQNVGQILQGDRRFDFEIRLEDQQRTIQN
LAQLPIQLPNGGLIQLQDVAKVERTSGLNQVGRENGKRRVIITANVEGRDLGSFVQEL
RTTLAKEQLPAGYWLEYGGQFENLASAAARMKIVVPLALAMIFILLMAVFHNVKESLL
VFSGVPFALSGGLIALWLRDIPLSMSAGVGFIALSGVAVLNGLVMLSFIKELREKFDI
QTATWNGAILRLRPVLMTACVASLGFIPMALATGTGAEVQRPLATVVIGGIISSTILT
LVLLPVIYRWMNENKTKIVEHS"
/product="Cobalt/zinc/cadmium efflux RND transporter, transmembrane protein CzcA" /transl_table=11
CDS 2262516..2263472
/locus_tag="AUO97b_02252" /db_xref="SEED:fig|400667.82.peg.2116"
/translation="MGGHHGHDHSHAVVTEGNAKKLTIALALTTTFLIVEVIAGLITQ
SLALLSDAAHMFTDAAALAIALVAIQISKRPADNKRTFGYQRFEILAALFNALMLFVV
AIYILYEAYIRFSQPPEIQSVGMLIVATIGLVINLISMKILMSSANNSLNVKGAYLEV
LSDALGSVGVIIGAIIIYFTNWYWIDTLIAVLIGFWVLPRTWVLLKQSINILLEGVPE
EVDIEKLRADLLSLNGVESIHQLKVWAITSKNIHLTVHLFAPEADRNKLYQDAVEMLS
HEHGIGEVTLQIEDDAEINCQHIAQHASHEHNDNDKTHSHQH" /product="Cobalt/zinc/cadmium resistance protein CzcD" /transl_table=11
CDS complement(2263491..2263829)
/locus_tag="AUO97b_02253" /db_xref="SEED:fig|400667.82.peg.2117"
/translation="MQYRCPQCQSVKIMPVSQGVNSAKPVVPKSLVILVPAIFVLLLL
VVISIFMWIFGNGAGSSLQIATVVAFVVTVGAGFMFWRDLPDFKLSMQAFMQSQKHWK CRDCNHEWEI"
/product="hypothetical protein"
/transl_table=11
CDS 2264103..2265050
/locus_tag="AUO97b_02254" /db_xref="SEED:fig|400667.82.peg.2118"
/translation="MGQEDDNKSKPTTTSTVSGNPDPIKLSFSRRLLHKTLQHAQDAV
GFASDTTRNLAEVADAALDALDQVTSHTKRGVVGMTNLAAGTVRDMVSETSETARQVA
LIMARTFLGKSTLNLYLPEMLLNSQIRKRIDRSEIDDIKVQCGNDRFQVEIDGHYHRF
LYRLSLDFRVLECRIGQEKFLRLRQMDESLDVQIRHGGTLSNWAVRRIGRVGFEVINM
LPIPSLINHLIRDIPGIQRDGHRMWFIDLEQAGFIDFINNRSWMVEKLLNLTDFSILP
GLNILRESRELVQQLVDQFEIKGLRVQSGRLEIQVGIAN" /product="hypothetical protein"

/transl_table=11
CDS complement(2265458..2266204)
/locus_tag="AUO97b_02255" /db_xref="SEED:fig|400667.82.peg.2119"
/translation="MPFYGDLMTKYQLSNQLDLNTLLPKTFNFHLPIHLHHQPSTPKE
HTPFIPHLPLSPNQPAQSLSERLYLTSQLVKDRVLKEMVVLLNNFPKLHESLIQQFLI
ETYMYLSNPDFMYEVHQRILKQMHDDEDCIVVAHSLGSVIAYHLLSDPSYQFSVQRFI
TLASPLSFRVIQSKLPTPIERPKCLKGDWYNFYSKDDFLTAFPLSEAPFNFTPPIINQ
EIFTFANQPHEIVGYLQHHAVVKTIIEPFQ"
/product="hypothetical protein"
/transl_table=11
CDS complement(2266462..2267880) /locus_tag="AUO97b_02256"
/db_xref="SEED:fig|400667.82.peg.2120"
/translation="MQPSDKQLDAQHQNSTAPLKRAMSTRHLVMLSLGGAIGTGLFLG
SGEVISQTGPIGAIIAYILGGAIAYMVMLCLGELAVHMPVSGSFGAYARKYISPSTGY
MISWMYWLTWTATLGTEFTAAALLMQEWFPQISMWIWTLIFAVTIFGLNISSTRVFAE
SEFWLALVKVITVIAFIVLGLLAIFGLIPFHGAQTAPLFSNLTAQGWFPHGFLPIFTT
MLIVNFAFSGTELIGVAAGETKDPAQNVPKAINAAIWRLLIFFVGTIIVISALLPFQL
AGLGGDNVSNSPFVTVFNYIGIPYADDIIRFVIITALLSAANSGLYAASRMMWSLSEQ
KLLPGVFATLSKSGTPIVALVVTMFGAIPGLLSEQFAPETIFKNLLGVAAFTMVIVWM
SICLSQFNFRRQWYRSGHTVSDLKYAAPLFPIVPILGFLFCFITCLSMAADPEMRAGF
IGCLIFTALCYLSYFIFYRNKA"
/product="Uncharacterized amino acid permease, GabP family" /transl_table=11
CDS complement(2268246..2269754)
/locus_tag="AUO97b_02257" /db_xref="SEED:fig|400667.82.peg.2121"
/db_xref="GO:0004818"
/translation="MKVRTRIAPSPTGFPHVGTAYIALFNMCFAKQHGGEFILRIEDT
DQLRSTPESEKMILDSLRWLGLNWSEGPDVGGPHAPYRQSERMGIYKQYALELVEKGH
AFYCFATAEELDQMRAEQQARGETPKYDGRGLKLSQEEVARRLEAGEPHVIRMKVPEE
GVCKFNDLLRGEVEIPWAQVDMQVLLKTDGLPTYHLANVVDDHLMEITHVLRGEEWLP
SAPKHQLLYQYFGWEMPTLCHMPLLRNPDKSKLSKRKNPTSINYYRDIGVLPEALLNY
LGRMGWSMPDEREVFTLQDMMDNFDIQRVSLGGPIFDVEKLNWLNGQWIKGLTPGQLL
DRLLTWKSDRSTLEDIAAAIQPRINLLSEAVNWAGFYFNHMPQITAEMFESKKLTQEQ
VRQSLQFAIWRLESQFTWNNDTVSQTLMDLANQMGIKLRDFMPTFFIAIAGSTSSTPV
MQSMVTLGPDLTFARLRHALEIVGAPSKKEVKNWEKLNESLKLPKNEATSEA" /product="Glutamyl-tRNA
synthetase (EC 6.1.1.17)" /EC_number="6.1.1.17"
/transl_table=11
CDS complement(2269871..2270170) /locus_tag="AUO97b_02258"
/db_xref="SEED:fig|400667.82.peg.2122"
/translation="MKIHYGKALSLGLVLSFASQLSTAAVLKDTPSKSLSDKQSSLIE
KALDQQKRNTIVLPSTDDELKVLNTIRVTPTQNFLAAQHERFSRFVQAIFQPHTS"
/product="hypothetical protein"
/transl_table=11
CDS 2270603..2271568
/locus_tag="AUO97b_02259" /db_xref="SEED:fig|400667.82.peg.2123"
/translation="MGLAVSAVQAATTFLNVSYDPTREFYQEYNQAFGKFWKQRTGQD
VDFKQSHGGSGKQARAVATGLEADVVTLALANDIDEIVKAGFIQPNWQKEFPNNSAPY
TSTVVFLVRKGNPKNIRDWNDLTKPGVEIITPNPKTGGAPRWIYLSAWGYALKQPGGN
DAKAKELVKKLYHNVKVLDSGARGSLTTFAERGIGDVLLSWENEALLATKGLDKDKYE
IVYPSISILAEPSVAIVDKTVDKDGNRTLAKGYLNFLYSPLGQELAAKHYFRPRNPQV
AAKYAAQFPKIKLFTINDVFGGWAKAQKTHFANGAIFDQIYDGKQ" /product="Sulfate-binding
protein Sbp"
/transl_table=11
CDS 2271791..2272714
/locus_tag="AUO97b_02260" /db_xref="SEED:fig|400667.82.peg.2124"

/translation="MSHISVLLFETVESLLADRTTGVYIDATFGRGGHTRLLLSKLDE
NARVYAFDKDPQALEVAAALAQEDPRFTIIHASFADIKEKMQEIGVQSVDGIMADLGV
SSPQLDQAERGFSFMQDGPLDMRMDNSKGLTAAEWLLEVEEEDLANIIYQYGEERYSR
RIARAIKQAGKLDTTAQLAEIVKTAHPKWEKHKHPATRTFQAIRIAINKELDDIEVFL
PQAVDLLKPKGRLSVISFHSLEDRLIKQFIQKESTLAEDSGWGMPQQQVDTRRLKKIS
RVRASEEEVKANPRSRSAWLRVAERLEQKGA"
/product="16S rRNA (cytosine(1402)-N(4))-methyltransferase (EC 2.1.1.199)"
/transl_table=11
CDS 2272714..2273046
/locus_tag="AUO97b_02261" /db_xref="SEED:fig|400667.82.peg.2125"
/translation="MKSSDEIETTENKVVKKVVVYAVMVALVFISAMMVVFQVFEYRH
DYRELSSYMRERDDLNAEWGRLLIEQQTFGATAQIGTRAVTQLRMFSPPAAETVVISL PMTSEQNK"
/product="Cell division protein FtsL"
/transl_table=11
CDS 2273058..2274890
/locus_tag="AUO97b_02262" /db_xref="SEED:fig|400667.82.peg.2126"
/translation="MVDKRTKQTRKKQQSISEKPSLAFDMWRFYLLWAVVLLCFVVLI
ARAFYVQVINKDFLQNKANANILRTERIEAMRGVISDRHGVPLAISSPIMKIVIDPRD
YFETKHLYDQITAELKQDPNNRKLKRQLPDKNLNLDELADVVGVDRADLKKQMNARPR
SRYLVLKKEVPPQQADLIMKGNFQGVYAEKTYKRYYPQPQPNAQIIGLTNSEGQGIEG
LEMQLNKQLSGVDGEQKIIRDKRGNRLKVSEVIREGEPGENITLSIDSRLQYIMYREL
TAAGVANNARSATAIAVDVKTGEILAMTSWPSYNPNDKNGLSNKDAMRNRGAIDMFEP
GSTMKPFTISAALETGQYTPNTIVNTSPGSMRLGWHTIRDTHNYGALTVSGVIIKSSN
VGSAKIALSLPKETLPSFFNRVGFGKRSAVRFPGESSGLVLPVNKLNSSQIGTMAYGY
GLNATILQLAQGYAMLANHGVKMPLSLHKLDQPPKGEQVLNPKIADQVLLMLEQVTMP
GGTAKQANIPGYRVGGKTGTAHKLRADGKGYSNNEYRALFAGVAPISDPRLAVIVVVE
NPQGRYYGGLVAAPVFARIMQESLRLMNVPLDKPLNTPENPIRR" /product="Cell division protein
FtsI [Peptidoglycan synthetase] (EC 2.4.1.129)" /EC_number="2.4.1.129"
/transl_table=11
CDS 2274894..2276393
/locus_tag="AUO97b_02263" /db_xref="SEED:fig|400667.82.peg.2127"
/db_xref="GO:0008765"
/translation="MTVSFQEIHPIEIDAQWPQQPFHGFSLDSRKVETGQIFIALTSY
SQPEKTRTFAEAALANGALAVISETELGVANEWVCPDVRQRMGEWQKRYLQQADVVKP
LRIIAVTGTNGKTTISRLIAELISSQQQRCAVMGTTGNGILPNLTPSTHTTLDALQLQ
NALHDYAKQGATFASLEASSHGLEQGRLNGCDIEIAVYSNLSRDHLDYHGTLEAYAEA
KARLFQFNSLKVAVINLDDAHADLMIKSAQNNPAQPKILTYSLTQNTADYYITDLSYS
LAGATFNLVSQQGSFAVESPLLGHFNVENLIAALIAAEQAGFDLQALVNFVPKLIGAP
GRMQVIRDDERLFVVDYAHTPDALIQVLKTLKRHVSNQLWAVFGCGGDRDRGKRPLMT
QAALDGANPVILTSDNPRTEDPEQIFADMKQGIDFSGHRMHEIHDRREAIKFVAEQAQ
AGDIVVIAGKGHENYQEINGVRHWFDDVVEVRSAIDAQHHTVDAAYPAQ" /product="UDP-N-
acetylmuramoyl-dipeptide--2,6-diaminopimelate ligase (EC 6.3.2.13)"
/EC_number="6.3.2.13"
/transl_table=11
CDS 2276402..2277802
/locus_tag="AUO97b_02264" /db_xref="SEED:fig|400667.82.peg.2128"
/translation="MHTSTTSTVPLEPWTAQQLQQATQGYWHKDQIPQTEIKRILTDS
RHAESGDAFLALKGERFDAHNFVAQVAANGCQVAIVERPIDAEIAQLVVADTRLALGQ
LGAYRREQNAQLKVIALTGSSGKTTTKEMLGSILSRLAPTLITRGNLNNDLGVPMMLL
ELRKEHQYAVMELGANHQGEIDYTSKIVQPHVAGILNIGTAHLGEFGGRDGICRAKSE
IYRHILPQGVAIIPQQDDFTAEIREAAKSHQIMSFGAGGDVFATEIELLPQSANFQLH
TPQGSSFVRLPFAGEHNVQNATAAVAFALALGVSLEDIVKGLAQAQGAKGRLNFIQKA
PHLFIDDTYNANPTSMRAAAQVLLQQNGIKVMVMGDIGELGDSSWQEHHDLGRDLAEL
PLDHIVAVGQFASAALEGAGLHSTKLKAFQTQAEALPFLINLIQTHQPQSMSFLFKGS RFTHMETLMADLMEKL"

/product="UDP-N-acetylmuramoyl-tripeptide--D-alanyl-D-alanine ligase (EC 6.3.2.10)" /EC_number="6.3.2.10"
/transl_table=11
CDS 2277803..2278921
/locus_tag="AUO97b_02265" /db_xref="SEED:fig|400667.82.peg.2129"
/db_xref="GO:0008963"
/translation="MLLWLFEQLAGYHSSFQVVRYLTLRSLLSVLTSLTIGLVLGPIM
IRKLQALKYGQAVSSFAPENHAKKMGTPTMGGILILLSIGISTLLWADLSNPYVWIVL
GVMVVFGAVGWADDWIKIRYKDNAGLPARKKFFWTSVASLGAGIALYLIATQQSNAEY
TANMLDLLIPFFKNLSIPLSIVPLGLAFIVFTYLVINGASNAVNLTDGLDGLAIMPVV
MVATGLGVFAYLSGDIRFANYLHIPYVKYTSELVVICSAMIGAGLAFLWYNAHPAQVF
MGDVGALALGAMLGTIAVMVRQEIVFAIMGGVFVMEAVSVFLQIGSLRMRNKRVFLMA
PLHHHYEKQGWKETQVVIRFWIITIMLVVLGLMTLKLR" /product="Phospho-N-acetylmuramoyl-pentapeptide-transferase (EC 2.7.8.13)" /EC_number="2.7.8.13"
/transl_table=11
CDS 2278982..2279314
/locus_tag="AUO97b_02266" /db_xref="SEED:fig|400667.82.peg.2130"
/translation="MTKVAGLDQLSVINQKVVGEGEVLPQVVLKDGSQVQTGTVATML
HNIELYNAGQRGQIEEELKIAIPTLVKVGLFDLFEVDEWIKGTNAGRTFVGMHAKAYL QQKEQENN"
/product="hypothetical protein"
/transl_table=11
CDS 2279335..2280141 /locus_tag="AUO97b_02267" /db_xref="SEED:fig|400667.82.peg.2131"
/translation="MCPVCRQRLELVSKTWRCEQGHSYDIAKQGYVNLHVVQHKHSKN
PGDTPESVDARRAFLQGGYYQPLQQAVVHLLKDLKAKMVLDIGCGEGYYTSAMQQVVE
QCIGVDIAKNAVQRAAKLNDKVTWVVGTGATLPVIDQSMDVCTSLFSPIPQTEILRVL
KDDGYLIVVTPATDHLYAMREALFEQVNPHTPQKFVEQLQDLFELKEQQVIDAPLVLD
QQALKNLIAMTPYAYKASPERRMQLEQKAHLQVTASFQIYLFQKRNKKAI" /product="23S rRNA (guanine(745)-N(1))-methyltransferase (EC 2.1.1.187)" /EC_number="2.1.1.51"
/transl_table=11
CDS complement(2280159..2282624)
/locus_tag="AUO97b_02268" /db_xref="SEED:fig|400667.82.peg.2132"
/db_xref="GO:0008955"
/translation="MYLYIAPTLPEMSSLKKAPLLKPLQVYTADNQLIAEYGGKLSIP
VEYKQIPPNFIHAFLAAEDSSFFEHSGISFKGLGRALSESVTGSDVQTGGSTITMQVA
KNYYLSPERTLKRKLTEIFLARKIEQNLSKEDILSLYVNKIFLGKNAYGIAAAAKIYY
NKSINELSIAQMAMIAGLPKAPSKYNPVVNPERALERRNWILGRMLQLGYISQAEYQK
AVAEPINLNMPNRDLNNIHPYAGEMVRSELVKHFGEQAIDSGYKVYTTINAKRQAIAE
KAVQDGLEAYDRRHGWRGAEAHDKPLSEFRAYANTYPAQVTKVNSSSFEALMQDGSTV
TVQWSGMSWARPYRNANSVGAAPSRASQIVKVKDIVRLRPNEAKTAWSLVQVPKVQGQ
LIAINPNDGSIEAIVGGYNFYQSKFNRALQGWRQPGSTIKPFLYALALERGMTPYSMV
NDSPITIGKWTPKNSDGRYLGMIPLRRALYLSRNTVSVRLLQTVGIERTRQLFMDFGL
QEDQIPRNYTIALGTPQVLPIQMATGYATFANGGYRVQPHFIQRIEDAYGKVIYEAKP
EYACIPCINAPETTDDAQVTTPDDQVVEVTNKELEQKEKTTKQLNLKQTDKNNSQYRQ
AQRILKSSSAYDMANILRDVIEHGTGRAALKIGRSDLGGKTGTTNDAKDAWFAGFNGK
LVTVTWVGFDQPTTLGRREYGGIAALPIWINFMGQALQGTPAAWVRLEKDAQAPISRD
KQEVTTEVGDKKTYRAAPPLARPLYRPAPPQPKTTNNDFDDLPGEEIVIPSKTTPPAM
KPSQGAAPKREKDELENLINQIE"
/product="Multimodular transpeptidase-transglycosylase (EC 2.4.1.129) (EC 3.4.-.-)" /EC_number="2.4.1.129"
/transl_table=11
CDS 2282913..2283935
/locus_tag="AUO97b_02269" /db_xref="SEED:fig|400667.82.peg.2133"
/translation="MGVDISSTSVKLLELSVKNGKYWVESYALMPLPENSVVEKNILN

PEAVAEALERAMNLANPQTTHAAIAVPTSTVIHKTIEMDADMSDDEREVQIRVDAEQY
IPFPLDEVSLDFEVLPDRLANPNRVNVLLVATRTENVETRVEVLELADLNPKLADVES
YAVERAFSVFADSLPMGANTIGILDIGHTMTTLSVMQNGKIIYTREQVFGGKQLTLEI
QSRYGLSLEEASRAKKDRSLPDDYEIEVLDPFLDAVVQQAARSLQFFFSSSQFNEIDH
ILLAGGNANIPGLAKLLQQKLGYRVTIANPFLQMGFSPQVDVQKIENDASSLMVACGL ALRSFD"
/product="Type IV pilus biogenesis protein PilM" /transl_table=11
CDS 2283935..2284576
/locus_tag="AUO97b_02270" /db_xref="SEED:fig|400667.82.peg.2134"
/translation="MATINLLPWREELREQRKKQFITLCFGVVVLGITTVFGGWFYFD
HKLDDQEQANQLIMSTNQNLDQQLKTLNGLQEQRDAIIERMKLIQGLQSQRPVVVRLV
DELVRVTPPAMYLTKFSRTGDKFTIEGKAESPNTVAELLRNLEASPWYRNAFMNSFLA
NEEKKDKAASSLLPRVEDHYGSFVVTVDLGEMGVATTDDSAKPSTGESVGAAK" /product="Type IV
pilus biogenesis protein PilN" /transl_table=11
CDS 2284573..2285313
/locus_tag="AUO97b_02271" /db_xref="SEED:fig|400667.82.peg.2135"
/translation="MSQDELQELSLEQLSAKKKKFNLDKFLQQFNTLDMNNYGSWPIS
VKITCWIFIFFAVLALGYFIVIQPKLQAIDNAQAQESNLLNEFREKDSKLRNLQQYQV
QLQEMQANFNQQLEQLPKETEIPSLVEDINLTGVNSGLKFKNIRLEDEVKQEIFIEQP
IAIEATGDYHAFGAFVSSIAALPRIVTMHDFTVDVSPAKDNKSDIPVLNYSIKAKTYR
YVGTTDTSEQTGKAPAASASTNTTVQPK"
/product="Type IV pilus biogenesis protein PilO" /transl_table=11
CDS 2285321..2285851
/locus_tag="AUO97b_02272" /db_xref="SEED:fig|400667.82.peg.2136"
/translation="MMKIKQITLYVLACAVLVGCDSRIDAVNQEMANIRNQPPLPIEP
APIFTPVPQFNYAAHQLKSPFMPSSLAAELKIMAGKRVYPNFSRAPQPLESYALEALN
MKGSMRNNRGQILALIQTPDQQIERVQVGNYMGMNQGRITHISPTQIDLVEIVPDGRE GYVERPRTLVLIGPAP"
/product="Type IV pilus biogenesis protein PilP" /transl_table=11
CDS 2285919..2288084
/locus_tag="AUO97b_02273" /db_xref="SEED:fig|400667.82.peg.2137"
/translation="MNHVFRQFSMGAVAIAIMQAASAQVSMTNIVPMQIAGQGTEIRV
MFNGLPPQPQAYQLENPSRLILDFDKAQQGLKQSKISVATNEASSVDVTSDDQRSRLT
VNLKDAGAFTTRVEGNTFILKINSAQTSNKPLPVVSAQPQGVSNIGFQRGSQGEGLVV
VDLLGSNTPVDVQQQGSKVVIRTIGTKIPTHLARRLNVNDFATPVSSIDAYNDKGAGV
ITIQSSGSYEYMAYQAENKLTISLKRPQDKNVTSLYKTPNYSGNKLSLDFQDIEVRRV
LQLLADFTGINMVAADSVQGNITLRLKDVPWDQALDIILKTKNLDKRRNGNVIWIAPV
AELIKAEEEEAKAVAQSVKLAPLQTEYIQLKYAKAGDIMGLITQGSNNSNGLQHTSGG
GTSTSTNLNTGVDSLGNNVGSLLSPRGTITQDDRTNTLIINDTAQSIDQIRKMIDLLD
VQVKQVMVEARIVRASTSFTKELGVKWGILSQGITNNNNLLVGGSETTLWNLREPKKD
ETTGGYKYTIERPDNLNVDLGVSNPAGSIAFGLISMSDFMLDLELSALQADGYGEVIS
TPKVMTADKQTAKVATGQQVPYQSTTNSAAGSTATTSFKDALLSLNVTPSITPDGKIQ
MKLDISKDSVAGAAPNGELILNKNNINTNVLVDNGETVILGGVFEQTTMNSQTKVPFF
GDIPVVGRLFRKDVKSDDKQELLIFVTPRIVNDTLARNH" /product="Type IV pilus biogenesis
protein PilQ" /transl_table=11
CDS 2288096..2288665
/locus_tag="AUO97b_02274" /db_xref="SEED:fig|400667.82.peg.2138"
/translation="MKAIEIGGALPSKAFETLPNIYLVGPMGAGKTTVGRHLAELLGR
EFLDSDHEIERKTGATIPWIFEKEGEVGFRTRETVVLNELTSRKALVLATGGGAITQA
PNREFLKQRGIVVYLYTPVELQLQRTYRDKNRPLLQVENPEQKLRDLLKIRDPLYREV
AHYTIETNQGAARDLAQKILQLILSNKLK"
/product="Shikimate kinase I (EC 2.7.1.71)" /EC_number="2.7.1.71"
/transl_table=11
CDS 2288694..2289776
/locus_tag="AUO97b_02275" /db_xref="SEED:fig|400667.82.peg.2139"
/db_xref="GO:0003856"

/translation="MQTLHVELGERRYPIFIGSQLDPKQLLEPYIHGQQVMIVSNVTV
APLYLSHYQEALESLGKTVATCILPDGEKYKDIQHLNLIFDALLEAGFNRDCTVLALG
GGVIGDMAGFASACFQRGVYFVQVPTTLLSQVDSSVGGKTGINHPLGKNMLGAFQQPQ
VVLADMAQLNTLPERELSAGLAEVIKYALLGDEDFLVWLEENMDGLVARDADLLAEAV
YRSCAHKARIVANDEKEQGERALLNLGHTFGHAIESYLGYGTWLHGEAVATGMVMAAD
LSQRLGWISNEDVARTKKIIQRANLPISCPQIPLDDFLGYMAHDKKVLNGQLRLVLLK
QLGQAVITKNFDVELMKQAILANQHG"
/product="3-dehydroquinate synthase (EC 4.2.3.4)" /EC_number="4.2.3.4"
/transl_table=11
CDS 2289792..2290667
/locus_tag="AUO97b_02276" /db_xref="SEED:fig|400667.82.peg.2140"
/translation="MTVSRTLWQNVQQYIWLVGGIVCLFLAFIFWVITDNKKLVEVEK
SADSDAPVQIQPEKVATTPNLGALADEVRPLDLTTRTVASGEHEPEFRGTKFINENKK
QWTLELFRASDEDIIKNFLKSRTDRNKFIYFRLSGEQQAEQYVLAYGTFKRSEDAVQQ
LTQINLQLPDSVKPQPQQFSSYAPLVNDLGADEMKGGNNQLYEVRLRPAALPRIDESL
LMSAPASSTLNTQPKPASTNSATKTTIVRRDAAGNVVDVQQSNSNIDQPNKPVQTRVN ENQPKNATNEGTRGN"
/product="hypothetical protein; putative signal peptide" /transl_table=11
CDS 2291128..2295603
/locus_tag="AUO97b_02277" /db_xref="SEED:fig|400667.82.peg.2141"
/db_xref="GO:0004355"
/translation="MPSPNTVAPAQGLYQPDEFKDNCGFGLIAHMKGESSHHLVETAI
HSLSCMTHRGGIAADGKTGDGCGLLLAMPKQFFRDEAKKLSDITLSEIFAVGTVFLNI
DPALAQHSKNILTKEIESEGLRVLAWRIVPTNNDALGEIALQSLPAFEQVIVNCPMGV
TEVEFNRKLFLARRRAEQQLQNDPLFYVTTLCSTVISYKGLMMPAAIAEFYTDLADER
LKSHIVVFHQRFSTNTLPRWPLAQPFRYLAHNGEINTITANRNWALARTPKFENPLLP
GLTELNPIVNRTGSDSSSLDNMLEILVGGGMDLFRALRMLVPPAWQNVETLDADLRAF
YEFNSKHMEAWDGPAGLVIQDGRHAICMLDRNGLRPARWVITKNDYITLASEIGVWGY
EPEDVVSKGRVGPGQILVVDTLTGKVLDTKDVSNHLKNMRPYREWLRDHAIRLNANPE
LEEQLVDKGLTGDALKAAQKMFMVTFEERDQLLRPIAESGQEAVGSMGDDTPMAVLSR
QVRHVTDYFRQQFAQVTNPPIDPLRESIVMSLETCLGREQNVFEQGPEHADRIIISSP
VLSNSKMQQIRSIERAGYEAVDIDLNYAETEGLQAAITRICEESAQAVRDGKTLIVLT
DKNIRQGYLPANAALATGAVHHHLIKTGLRTDANIMVETGFARDPHQFAVLLGFGATA
IYPYLAYDVINDLIAKGELLGDPIHAQANFRKGIEKGLLKVLSKMGISTVASYRGGQL
FEAVGLSSEVVDQCFLGVPSRIQGATFVDLENDQKKLAATAWSNRKPIDQGGLLKFVF
GKEYHAFNPDVINSLHKAVRSGKYEDFKEYAELVNNRPIATIRDLFKLKTTNPIPVEQ
VESVEAILPRFDSAGMSLGALSPEAHEAIAIAMNTIGGRSNSGEGGEDPARYGTIRNS
KIKQIASGRFGVTPAYLTSAEVLQIKVAQGAKPGEGGQLPGGKVNGLIARLRYSVPGV
TLISPPPHHDIYSIEDLSQLIFDLKQVNPKAMVSVKLVSEPGVGTIAAGVAKAYADFI
TISGYDGGTAASPLSSIHHAGSPWELGLSEAHQALRVNDLRGKVRVQTDGGLKTGLDV
IKAAILGAESFGFGSTPMIALGCKYLRICHLNNCATGVATQQDHLRQEHYIGEPQMLI
NFFHFIAEETREWLAALGVASLKDLIGRVDLLEVLPGETEKHAHLDLSALLTSHPAAE
GKAQYCEVEGNAPFDKGVLAERMVEEMLPAIESSAGGQFNFNVVNCDRSIGARVSGEI
ARRYGNLGMEAHPVVMNLTGTAGQSLGVWNAGGLHIRLEGDANDYVGKGMAGGRISIF
PPKGSPFQTQNTAIIGNTCLYGATGGKLFAAGTAGERFAVRNSGAFAVIEGAGDHCCE
YMTGGVVTVLGKVGHNFGAGMTGGFAYVLDLDNDFVDYYNHELIDLTRISTESMEDHK
EFLLRIIDEHIKETGSAWAYKIRNEFDFYSRKFWLVKPKAANLQSLLKTTQADPQ" /product="Glutamate
synthase [NADPH] large chain (EC 1.4.1.13)" /EC_number="1.4.1.13"
/transl_table=11
CDS 2295674..2297095
/locus_tag="AUO97b_02278" /db_xref="SEED:fig|400667.82.peg.2142"
/db_xref="GO:0004355"
/translation="MAERLNNDFQFLDVPRQDPEKKDITVRKAEFVEIYKPFTSETVT
NQTHRCLGCGNPYCEWKCPVHNYIPNWLKLIAEGQIFQAAELCHQTNTLPEVCGRVCP
QDRLCEGACTLNDGFGAVTIGNAEKYINDTAFALGWRPDMSHVKWTGKKVAIIGAGPA

GLGCADILARGGVKPVVFDKRPEIGGLLTFGIPEFKMEKDVMKRRREIFTGMGIEFRL
NTEIGKDVTIDELLAEYDAVFMGMGTYTYMKGGFPGEDLDGVYDALDFLISNVNRCQG
WEKDPSEYISMDGKKVIVLGGGDTAMDCNRTSLRQGAHDVTCAYRRDESNMPGSAREV
KNAYEEGVKFLFNRQPIEIVGENGKVTGVKVVTTQMGEPDSRGRRSPEPIPGSEEVLP
ADAVLLAFGFRPSPADWFGSVNINLDGSGRVVAPEQQEFKFQTSNPKIFAGGDMVRGS
DLVVTAIWEGRQAAEGILDYLSV"
/product="Glutamate synthase [NADPH] small chain (EC 1.4.1.13)"
/EC_number="1.4.1.13"
/transl_table=11
CDS 2297269..2298156
/locus_tag="AUO97b_02279" /db_xref="SEED:fig|400667.82.peg.2143"
/translation="MNIAGMTAEKSYLNRRKALGITVRRLEFNPQAIKRYYFANSPLM
SHFLTALSSTFPVGEQFFVNSVRNVRDKVSDPQLQAQIAAFIGQEAMHSKAHGEFNEA
WRRDDYNLDRFQNWLNERDKYLRTISPKLQLALTCAFEHFTALLGGYILQHPELLKTL
DQDALKLWVWHAIEEIEHRSVAFDVYQQVYGDDRIRRLLMRSVTTGFASLAFYGTTRL
FWQDKWKSLSKIGGNLFGLYLLAKMLIQLTPEYFAYYKKDFHPSQKDYSHMVDYWKSY
LADEYQMTSFQEEKNPRPS"
/product="hypothetical protein"
/transl_table=11
CDS 2298287..2298877
/locus_tag="AUO97b_02280" /db_xref="SEED:fig|400667.82.peg.2144"
/translation="MKTFKQLAIATTLASTLLFSGCGYNTLQVKDEAVTAAWSEVQNQ
YQRRADLVPNLVNVVKGYAKHEEQVLTEVTQARANVAGLKVDKEVLEDPALLEKYQQA
QSQLTGALSRLIAVSENYPDLKANTQFQELQVQLEGTENRIAVARNRYITTVQDYNAY
VRQFPQAVTAKVIGMHPKANFSAEASAQQAPKVSFD" /product="LemA family protein"
/transl_table=11
CDS 2298899..2299981
/locus_tag="AUO97b_02281" /db_xref="SEED:fig|400667.82.peg.2145"
/translation="MRNTILLQPKQIRVFVITLIVLCCGVLFQNAAWSENNIATATDE
TDTVVAGKIIQQQQAQASKPQINSQAASSVTPQTQVANDMADGESIRGLPTLNQPVID
QANVLSETEKQQLSQKILNLYQQGKAQIGIVIVPTTGQEDIFDYALRVGEKWQLGSSK
RDNGLLIAVAINDHRIQILTGYGLEGVLPDIVASRIIRNQITPYFKQAQYAQGLNAGL
DEIGRILNLDPEVAQQAAQDLKERQHQAAEEQQAKQTTLTMALFILVAGVVGSFIVGR
PLSASTAGVAAAVAGFVNGAGVVMSLLLGFGIFFLLITSIAQLILQAILSGAGGGGRG
GGFGGGGGGYGGGGGRFGGGGASGSW"
/product="Beta-propeller domains of methanol dehydrogenase type"
/transl_table=11
CDS 2299975..2300535
/locus_tag="AUO97b_02282" /db_xref="SEED:fig|400667.82.peg.2146"
/translation="MVTTSETTQIITQPKIEEYVEPSLKRWFKHFCYLPASSKYFSKQ
DKQAIAEAVQQAEHGHVGEIQVVIEGHIPCSQAYRQNTRLRAQQLFAELGVWDTELNS
GVLLYLNLCERTVEIIFDRGIRSATTEQIWQQICKSIVEQLQQKQYQKAVVTGVQQIG
EVMSRFYDENMPDQLNELGNAPIIIN"
/product="FIG004694: Hypothetical protein"
/transl_table=11
CDS 2300877..2301341
/locus_tag="AUO97b_02283" /db_xref="SEED:fig|400667.82.peg.2147"
/db_xref="GO:0007155"
/db_xref="GO:0009289"
/translation="MNAQKGFTLIELMIVVAIIGILAAIAIPQYQKYTARSQITAALA
EISPGKTQFELALSEGTANSVNNNPAAIGLKSTTNNCSAVNVTANGTTGTIACTLQGS
ATITGGVLTLTRSADVAAASGVNANVGGWTCSITKGTTDISSVIAPKGCTII" /product="Type IV pilin
PilA"
/transl_table=11 CDS 2301587..2303119

/locus_tag="AUO97b_02284" /db_xref="SEED:fig|400667.82.peg.2148"
/translation="MGAFAAGLSILAVLLNQNIKTPRAQLLILPFILIPLVQWSFGLI
LDFSTALLSTLYLLGFWFMILIGYNLSLDEKKREQLFSGFCVLVLTVAVGTSFIAIYQ
WLNLESNFIYMLNLIGNRPYGNFGQPNNMATFLIMGLLGCLFLYEKNKATVWLLFPSA
LIILFTIALSQSRTSWVVFPFLLIYWIVKLFGKQKRFGFIQGFLWCAGFFVIAEVILP
FATSLIEAWSSTDVTQASSLVERASSGYLRFNIWTQMLLAVQQHPWLGYGWNQTSVAQ
MSAYALFPTTEWTTSAHNILLDLIIWNGIPIGIVIIAYFACWFLWLNQQAKETISIIA
IMMVCTVLIHAMLEFPQRYAYFLLTCGFLLGIIQAQTPVLKGIVLNKQVLRLIWGISV
ILLVAIWRDYNVYVTNSNLLFKNKQPNAEILGSNQIFILTQFEQRLKWIEMKPETTLS
DADLAVWGNFVKNKATPYNLRKYAQLLAYNGKVEQAEQQIFILQHLYRQQITLAELLK NK"
/product="Lipid A core - O-antigen ligase and related enzymes"
/transl_table=11 CDS complement(2303163..2303627)
/locus_tag="AUO97b_02285" /db_xref="SEED:fig|400667.82.peg.2149"
/translation="MKGNRDVINQLNQVLYHHLTAINQYFLHSRMFNDWGIEQLGSAE
YKESIRQMKHADKIIERILFLEGLPNLQHLGKLYIGQHTEEVLQCDIRKVKENIEAIQ
KAVALAETEQDYVTRDLVQEILEKEEEYWDWLDTQIDLIGSVGIENYIQSRM"
/product="Bacterioferritin (EC 1.16.3.1)"
/transl_table=11
CDS complement(2303872..2304066)
/locus_tag="AUO97b_02286" /db_xref="SEED:fig|400667.82.peg.2150"
/translation="MYVCLCRGITDQDIKDAIENGAESYREIRDLLDLGTCCGRCAPE
ARAIISEELAEIAARISVAA" /product="Bacterioferritin-associated ferredoxin"
/transl_table=11
CDS complement(2304276..2304659)
/locus_tag="AUO97b_02287" /db_xref="SEED:fig|400667.82.peg.2151"
/translation="MSRQVIHTENAPAAIGTYSQAILVGNTLYLSGQIGLDPYSMELV
DGIEAQIRRVFDNLKAVCEAAGGTLADIAKLNIFLTDLSNFQLVNQIMGEYFAQPYPA
RAALGVASLPKGALVEMDGIVIINQ"
/product="RidA/YER057c/UK114 superfamily protein" /transl_table=11
CDS complement(2304780..2306885)
/locus_tag="AUO97b_02288" /db_xref="SEED:fig|400667.82.peg.2152"
/db_xref="GO:0008893"
/translation="MPGEEVSQAKQQLKLIIDPYLSVSEVEKVLAACDFGDLAHTGIT
RKSGEPYILHPIAVSCILANMRLDPETLMAALLHDVIEDTQYTKDDIIERFGQTVAEL
VDGVTKLSQSSDKEYNKAASFRKILQATLQDPRVIIIKLADRYHNMTTLGALRPDKRA
RIAQETFDIFVPMARLVGMNEMADNLENLCYQNLDLDMFDNVQNALLQTKPERCKYQS
IWEQNLAELLHNYHIQGRIKKKNNNIELLRHFVKNEMDLQELTHSHAFEIVLQSIADC
DRLVAALKENFQVIQYQDHIRRPLPGGNQSLMIKLKGEKTTLSLTIQTELMRKAARFG
VVLGENAPQTCRSAIQASMQNLNTLIDGECAKTTFNDLLDYLHQEKIWVYTPHGQLHE
LPQGATVVDFAYSASLFLGNHAVGAKVDGEIKPLSTPLVSGQVIEIITDVLATPNPDW
LSFINTQKARRALQHVLKDQDIEEQRLVGAQALSRALKLFNRSINDLSDADWLDLLQW
RHIDNKDALFEQIAVGDLLPQLVANHLFANDKHPRAENSDRLIQGTEGIDVKYAHCCN
PILGDPIQGHLTRRGLIVHRIRCHNLLHEQHLHPENIMPLQWKADDVDDVRFTAYLAI
YMAMNDEQVSDLIYQCRKNNAGVEMVHSNEQRTFVNIVVNNRKHIAKVIRDLRMHYGF
PRIERLDAPAPQMEISKVS" /product="Guanosine-3',5'-bis(diphosphate) 3'-
pyrophosphohydrolase (EC 3.1.7.2) / GTP pyrophosphokinase (EC 2.7.6.5),
(p)ppGpp synthetase II" /EC_number="2.7.6.5"
/EC_number="3.1.7.2"
/transl_table=11
CDS complement(2307094..2307372)
/locus_tag="AUO97b_02289" /db_xref="SEED:fig|400667.82.peg.2153"
/db_xref="GO:0003899"
/translation="MARVTVEDCLDHVDNRFELVLVASKRARQLARQGMEPTVEWDND
KPTVVALREIAVGHVTKEILKQREQDYQTSSLDLALSTNSLNLEGFSF" /product="DNA-directed RNA

polymerase omega subunit (EC 2.7.7.6)" /EC_number="2.7.7.6"
/transl_table=11
CDS complement(2307445..2308074)
/locus_tag="AUO97b_02290" /db_xref="SEED:fig|400667.82.peg.2154"
/db_xref="GO:0004385"
/translation="MSGLLFVVSAASGTGKTSLVKALLDRVSNLHVSVSHTTRGQRPG
ELDGVHYHFTTKDEFLDQVNQGGFIEYAEVFGNYYGTSQATVKQQLAQGHDVLLEIDW
QGAEQVRKLFPESKQIFILPPTQFDLRQRLSNRGTDSVEVIEHRLSCAVEDMQHYVNF
DYIIINDDFNKALHELEAVITANRLVLSQQAKRHQNLIQDLITPQPKQE" /product="Guanylate kinase
(EC 2.7.4.8)" /EC_number="2.7.4.8"
/transl_table=11
CDS 2308197..2309147
/locus_tag="AUO97b_02291" /db_xref="SEED:fig|400667.82.peg.2155"
/db_xref="GO:0051745"
/translation="MEIVLANPRGFCAGVDRAIAIVNRALECFNPPIYVRHEVVHNKF
VVDDLRQRGAVFVDELDQVPDDSIVIFSAHGVSKAVQQEAERRGLKVFDATCPLVTKV
HIEVTKYAREGTEAILIGHEGHPEVEGTMGQYDKLKGGDIYLVEDEADVAALEVRHPE
KLAFVTQTTLSIDDTAKVIDALRAKFPNIQGPRKDDICYATQNRQDAVRDLAEKCDVV
LVVGSPNSSNSNRLRELAERMGKAAYLVDNADQLEQSWFNDTCKIGVTAGASAPEILI
KQVIQRLQDWGAQAPKELEGREENITFSLPKELRIHVTQA" /product="4-hydroxy-3-methylbut-2-
enyl diphosphate reductase (EC 1.17.7.4)" /EC_number="1.17.1.2"
/transl_table=11
CDS 2309317..2309790
/locus_tag="AUO97b_02292" /db_xref="SEED:fig|400667.82.peg.2156"
/translation="MRGIIPQEGFTLVELMVTIAVMAIIALMAAPSMSNLLESKRLDA
NQRDLINTLSEAKSQAILGRQNVSVNLNSTASNTPSSLNWKTASNNTLELKNIAADGT
QSSLTTTTLAFNANGVVANITQDTLLSICNSRINKKKVIILTKLGTLVFKAEETC" /product="Type IV
fimbrial biogenesis protein FimT" /transl_table=11
CDS 2309790..2310344
/locus_tag="AUO97b_02293" /db_xref="SEED:fig|400667.82.peg.2157"
/translation="MHHYKQKAQAGVGLLEVLVALILLAIGVLGYVALQLRAMDASSE
ALSKSQAILVMRGLAENIRTNSTQASQYPTFVRSYSNYTSDTPAPTSCFNSLCTASQL
AQFDAYQAARNANQLGMRITMSNCPGVTNTMVQQRQCLFVFWGKTAPVITTNGTNTSV
DVSSCMSNNGVYVNNSTCLMMEAY"
/product="Type IV fimbrial biogenesis protein PilV" /transl_table=11
CDS 2310345..2311346 /locus_tag="AUO97b_02294" /db_xref="SEED:fig|
400667.82.peg.2158"
/translation="MAQSFISNKEQGFTLIELIVALALGLILVAAATQLFIGGLLSSR
LQKANAEIQDSGIFGLEYMARDIRLLNYGNVVNPQLTDTTPWGGIVLTGSTATNANNI
NFIPKVDTNTYIPEALLSRGAGDTVSTVNNHWKGLSNIQNSSNAEVQSDQLTIQFIAP
TNMTNCEGVNVLAGDLIVQRYFLRVDNNGSSQQDYALACDANTPAVSATAQPDIVNGL
GDAGQIILPRIDHFHVLLGAKNAAGNFAYYTIPQYRVAAQAARDASPAVAAPRILSIQ
ISVLARSTNNAQNKAIDPNQSFLMIDQNVHAADNRTRFLRRVYSVTIALRNAMGETI" /product="Type IV
fimbrial biogenesis protein PilW" /transl_table=11
CDS 2311343..2312161
/locus_tag="AUO97b_02295" /db_xref="SEED:fig|400667.82.peg.2159"
/translation="MTHLNYKNIKGRSKETGATLIVVLIILLIVISVGVLAIRVAIVS
LKVATNSQVGQLNFQSSDTPLELIVQMNPTTLTNITNVIGAALKAHESNPGAEYNFCY
KPTSTSVSFAQTRGASLLRAGSANNAVVEDGGVAGFCDLTSDYGSNRQAVVTQVAVSI
PTDAMTDIPGSNLPRDINLSEGTQLPKSMLSTQRIRVITTAFLPAYASTSLETLQSDC
LSTSSAKISDNFDSALTDKQTLADCLANHNVPFSTQVQEFNYTNKLTEITAPGS" /product="Type IV
fimbrial biogenesis protein PilX" /transl_table=11
CDS 2312173..2316027
/locus_tag="AUO97b_02296" /db_xref="SEED:fig|400667.82.peg.2160"

/translation="MKLKLKNFKPNNLWYAVCSSSMIFTWLMTSSVVQASDLQIYASP
TAGKKTIVMMLDTSGSMTNNSYGENRLAMLKNGMNAFLASNNPVLNDTRVGLGNFSAN
GDSRSGQILVAAAPLGDASTLNTVGSQRYKLKQAVANLTAGGSTPSAHAYAEAAAYLM
GTTTYSETNYAIRKDSYIKRVRRSDNRTEYSYCTNYRDSQIDTANLWQPCRSNSYWSS
WSTNNPGVDNATAYDTSSDWTYYYTYYYTTFNYAVANADSGIPKSKSNDTASNPNIVV
DRNATNSNVVYQSPLPAVANRQSCDGQGIYFLSDGEPNNTTNTRSASVMSTALGSTFG
ADFNCSGGLSNTTADSGWACMGEFAKRLFDKTKNPAGVSIQTAFVGFGSDFSSLSSSD
VKNACRLSSRTQSDRKGDDACSPNQSTNAVAAPGYGNGGFFPTQSSQGVTDSVIAFIN
NLDKVPLEPLTTGAISVPYDALNPKNLQEYGYLRAFEPNPANTYLTWRGNLKKYHVVL
SGANAGAFEANSGGLVYNASGAFRTGTKDYWNSSTYTDGGKVFLGGSYANVPLPIAGQ
PETRDAEGNITKYYYAVQSKIRNLFTDVSAVAADGSLTKISTSGTNLLKIPAAPPEGT
NPFDTVANTASYVLGKFDPSTGQNILKAFPISLKLKILNYLGYSTDINATTLPPSLVT
SNEPYLSMGGSIHSLPVQLTYNGTLDDNGNLTSAREQSILYGTMEGGLHIVDASSGIE
QMVFVPADILNDSVASKALVVGQSDASAPAHGMDGAWVSDPAYNITTVGSGSSAVSKV
TAKQMNIYGGMRMGGSSYYGLDVLSPTSPKLLFRIGADQTDYSRMGQSWSKPVLANIR
YNGSIRRVLIVGGGYDQCYEKPNITLTDACFTNGKAKGNAVYIIDAKTGQRLWWTSDT
GSNTDNANMKHSIVSRISTLDRDADGLVDHLYFGDLGGQIFRVDLNNNQTKTNSTYSS
FGVRVVRLANLATNDSTYDGTNDYTGGNAPRFYEPPTVTIHDYGIHTFITVGIASGDR
STPLDVYPLTGREGMTPASALSGRPVNNVYGIIDRDFVKKNLMSLTDNQLETKDITRT
GLRKNPQILRTGETRVAQIFFPTTGVGKGGWYRSLSSTSDGTEKANNSFRIKGGLKAF
EEPMAITGNLIIPVYDPQGTGIVAADPCLPRVVGETDRQTYCLPFGACLNPDGTINSN
KENPSGFQTKTGSNCPAGVSECNTNVIGAGIRNITFVPKRDEPPVTNSCGKLQLSGNE
NGTGEWQCTSHLLPVRWYERYR"
/product="Type IV fimbrial biogenesis protein PilY1" /transl_table=11
CDS 2316040..2316522
/locus_tag="AUO97b_02297" /db_xref="SEED:fig|400667.82.peg.2161"
/db_xref="GO:0007155"
/db_xref="GO:0009289"
/translation="MNKYSILRFNQGVTLIELMVVIVIVAIFASIAIPSYQSYSRRAT
ASAAKSEILKLAEQLEQHKSRNFTYRGFTTTSITLPRGGYTIQITDDTTTGNLLTNAA
ANGQTWVIKATTTDSRNFNFIAKNSGLRCQSLTATAVDNDCGGTVTCNVCETNSENWQ "
/product="Type IV pilin PilA" /transl_table=11
CDS 2316519..2316944
/locus_tag="AUO97b_02298" /db_xref="SEED:fig|400667.82.peg.2162"
/db_xref="GO:0007155"
/db_xref="GO:0009289"
/translation="MKNGFTLIEIMIVVAIIAILAAIATPSYLQYLRKGHRTAVQSEM
MNIAQTLESQKMVNNRYPSNTTIQSIYGSNVSPLQGKALYTLAFTTLNDSTWVLTATP
IANTSQAGDGIICLNDQGQKFWAKGATDCALSASSSWTE"
/product="Type IV pilin PilA"
/transl_table=11
CDS 2317091..2317342
/locus_tag="AUO97b_02299" /db_xref="SEED:fig|400667.82.peg.2163"
/db_xref="GO:0003735"
/db_xref="GO:0005622"
/db_xref="GO:0005840"
/db_xref="GO:0006412"
/translation="MVVIRLARGGAKKRPFYQIVVTDSRNARDGRFIERIGFFNPTAQ
GQAEKLRLDADRFAHWVSQGAQPSERVASLAAQAKKATA"
/product="SSU ribosomal protein S16p"
/transl_table=11
CDS 2317362..2317910
/locus_tag="AUO97b_02300" /db_xref="SEED:fig|400667.82.peg.2164"
/db_xref="GO:0006364"

/db_xref="GO:0007046"
/translation="MTPTQNVPEDRIQIGQLRSAYGLNGWLWVYSNTEPMSNMFDYLP
WYIETKAGWQIVDVKRWKPHGKGLVVALKGVSDRTGAESLVGANIWIAKSQLPKADVD
EYYWSDLKGLTVLGLDDDEQEVNLGQIHELFETGANDVMVVRATPDSIDSEERMIPWH
KDVVQRVDLEAGRIYVNWGVDY"
/product="16S rRNA processing protein RimM" /transl_table=11
CDS 2317956..2318696
/locus_tag="AUO97b_02301" /db_xref="SEED:fig|400667.82.peg.2165"
/db_xref="GO:0009019"
/translation="MFFAVITLFPEMFEAITAYGISGRAAKRDIVQVTCINPRDFAEG
NYRRVDERPFGGGPGMVMMAEPLAKAINHAKQLASQAGCVHVPVVYMSPQGKTLNEQA
VQQFVDYDGLIVLCGRYEGVDERLIQHYVDQEWSIGDYVLSGGELPAMVLLDSIIRRL
PNVMSDEQSAIQDSFVDGLLDCPQYTKPDQFEGLDVPEILKSGHHANIEKWRFLQRYQ
RTLERRPELIEKVALTKQQKKWLSDEQG"
/product="tRNA (guanine(37)-N(1))-methyltransferase (EC 2.1.1.228)"
/EC_number="2.1.1.31"
/transl_table=11
CDS 2318904..2319272
/locus_tag="AUO97b_02302" /db_xref="SEED:fig|400667.82.peg.2166"
/db_xref="GO:0003735"
/db_xref="GO:0005622"
/db_xref="GO:0005840"
/db_xref="GO:0006412"
/translation="MSGKHPLVQAIENSQLKTDLPEFAPGDTVVVQVKVKEGDRERLQ
AFEGVVIAKKNRGLNSAFTVRKISSGVGVERVFQTHSPVVAKIEVKRRGDVRRAKLYY
LRDLSGKAARIREKLPARKA"
/product="LSU ribosomal protein L19p"
/transl_table=11
CDS complement(2319324..2320298)
/locus_tag="AUO97b_02303" /db_xref="SEED:fig|400667.82.peg.2167"
/db_xref="GO:0004806"
/translation="MKRNLIFFCAAILSGLSVSATHATNAEQVKSSFVYSTYAQTKYP
LVFNHGMAGFNRVGTDTLGLDYWYQILPDLARNGGNVWATRVSPFNSTEVRGEQLAQQ
VEEIIAITGKPKVNLIGHSHGGPTIRYVAGIMPEKVASLTTIGAPHKGSPMADVILNV
EGTPLSGLATLVNWFSAAITWAGGLDPNSYPHDSLAGAHSLSTQGSAQFNAQFPMGVP
TTSCGEGAYQEKGIYMYSFSGNKALTNPLDPFDMALTGSSLVVDPFGDNDGLVSRCSA
KFGKTIRDDYNWNHLDEVNQVMGIRSIFAADPVSVYRQHANRLKLQGL" /product="Lipase precursor
(EC 3.1.1.3)" /EC_number="3.1.1.3"
/transl_table=11
CDS complement(2320380..2321414) /locus_tag="AUO97b_02304"
/db_xref="SEED:fig|400667.82.peg.2168"
/translation="MQRMQKKIWWCVLGFFILSLIACVYWLSPDSENTSPQPNQDEAK
SLVATSQSTLNSPLNENTYLSKSQQDTQVNCQLKIDSSQHLVVNSQTRDCFEYFITQY
GESNLQQVKTHFEKFIQDQYLEPARSQIIDLWTRYLKYREQLAQIQPPQSKQQDQNYF
QKIFSSIQDIRKRFFSASEIEGLFSTEDIYQNYTLDRMKILEDSSLSEIEKAKKLKER
FEQLPEDWQENLQELSKLDDLHTLTKQIKARNGSAEELRQMRTALVGAEATQRLETLD
IQRNAWQQRVTGYLNQRDEVLHSNMSDSAKKQAIQQLRQQQFSSSQEQLRLRTFETVH DQGGELPFNY"
/product="Lipase chaperone"
/transl_table=11
CDS 2321568..2322473
/locus_tag="AUO97b_02305" /db_xref="SEED:fig|400667.82.peg.2169"
/translation="MTTKNQKISRRHISGVFLLNKPLGLSSNAALQKVRWLYRAQKAG
HTGALDPLATGLLPICLGEATKFSHYLLDSTKRYQTTVRLGQTTTTGDVEGDILQERP
IPVLSKELIEQTLEKFRGDIQQVPPMYSALKKEGKPLYELARKGIEIEREARPVTIYA

LELIEFTENSITLDVTCSKGTYIRVLGEDIGEALGCGGHLTMLHRIQTGHFELIPSYT
IEYLESLTEEQREALLLPVYAPIDHFLKIQVPEGREKYFCNGLESNIDHSAEAEVLVF
SGERCLGLAEITDKKRLVPKRILNL"
/product="tRNA pseudouridine(55) synthase (EC 5.4.99.25)"
/EC_number="4.2.1.70"
/transl_table=11
CDS 2322493..2323269
/locus_tag="AUO97b_02306" /db_xref="SEED:fig|400667.82.peg.2170"
/translation="MGMEWDVILSLIFFAFTAGAIDAAVGGGGLIQIPGIMSTFPNMS
TATVIGTNKVSSIFGTASAAYTFAKKVKLQWKLLAVIAICALISSFAGAACLSLIPQS
VLRPFVFVMLIVIAIYTLVKKNFGQVHTEQKITTKMLVLAGIGSLAIGFYDGIFGPGT
GSFFIFFFIRFLQVDFLHASALSKIGNFMTNLAALSFLIPTGHAILHIGLMMAAANVL
GSIVGVRTALKYGSGFVRIIFLILVSILICRLGYQMFVTG" /product="putative membrane
protein"
/transl_table=11
CDS 2323479..2323922
/locus_tag="AUO97b_02307" /db_xref="SEED:fig|400667.82.peg.2171"
/translation="MNIFEALRESHENQRNLSEQLIQTHGLTEERKELFDALKNELYA
HSVAEDRYLYIPLMFDDVGLDITRHALSEHHEMDELVEQLEKTDMSSPSWLATAKQLS
EKVHHHLKEEEHKFFQQAGKILKDSEKETLGNKYLKEYKKYKKQQ" /product="Hemerythrin domain
protein"
/transl_table=11
CDS complement(2323930..2324790)
/locus_tag="AUO97b_02308" /db_xref="SEED:fig|400667.82.peg.2172"
/translation="MQNLPIIAVLYMILSMVSYQISASFAKQLIAALDPLTVTILRLC
FAAVIVAVMFRSWKIWSRLPYLKWRDLLKYCASLGLMNILFYTSLGKLPQGIAVGLEF
IGPLGLALVSIKQKSEYIWVAFAMLGIALMVPWQDPTQHHFSYVGAACALGAGLCWAF
YIYFGQKVVTQNIGMHALTIGITVSALALLPIGIWYNGPALLDTQYWGKALLIAVLAT
AIPYALDLMALKQLNKLTYGTLTSLAPALAALTGFLLLHEEISMLQWVALACVMLASI GVTLRSKTAS"
/product="Putative DMT superfamily metabolite efflux protein precursor"
/transl_table=11
CDS complement(2324881..2325765)
/locus_tag="AUO97b_02309" /db_xref="SEED:fig|400667.82.peg.2173"
/translation="MSSPSQKSQLHALGFLILAMLSVQSSASLAKVLFQNFPVLTVSA
MRLCIGAFILAFIFQIWKINFSQVRWKAILSYGFALAGMNALFYLSLERLPIGIAVAF
EFIGPLSVALYHAKQKYDFVWVTFAIIGLILLFPFNQAHSLDWIGVLFAMSAGACWAL
YIIAGQKPSGISGNHTVCLGMTVGACILLPITFFSGSIGQVIELPYFFYFIGLAVLAS
ALPFSLEMLALRNLTPLSFGTLMSLEPAVAALSGFIFLGEYLLWNQWLALATIITASV
GCTITTQRARQQREKTVG"
/product="Threonine/homoserine exporter RhtA" /transl_table=11
CDS complement(2325864..2327096)
/locus_tag="AUO97b_02310" /db_xref="SEED:fig|400667.82.peg.2174"
/db_xref="GO:0004617"
/translation="MSQHLSLPKDKIRFLLLEGVHQNAVDTLNAAGYTNIDYRKTALE
GEALKEAIKDAHFIGIRSRTQLTEEVFEAANKLIAVGCFCIGTNQVDLKAAMARGIPV
FNAPYSNTRSVAELVLAETILLLRRVPEKSAACHRGGWDKSAVGSFETRGKTLGIVGY
GSIGSQLSVLAESMGMHVIYYDAVTKLPMGNARQVGSLDELLATADVVTLHVPDVPST
RNFFTKEQFAKMKEGAIFLNAARGTCVVIEDLADAIKSGHLAGAAVDVFPKEPKANGE
EFQSPLRGLDNVILTPHVGGSTMEAQANIGLEVAEKFVAYSDKGMTLSAVNFPEIALP
LTAGQHRLLHIHKNVPGVLSKINNLFAEQGINISGQSLMTKGDIGYLVMDVDASASQE
ALDMLHEVEGTIRVRVLF"
/product="D-3-phosphoglycerate dehydrogenase (EC 1.1.1.95)"
/EC_number="1.1.1.95"
/transl_table=11 CDS 2327476..2328885

/locus_tag="AUO97b_02311" /db_xref="SEED:fig|400667.82.peg.2175"
/translation="MNAPVALTPELLTQLTAIVGENRIKTDADSLENWGKDHTKHFNP
NPSVIVFPSTTEQVQEVVKLANQFNIAITPSGGRTGLSAGAVATNGEIVISMDKMNQI
LEFFPADRMVRVQAGVVTEQLQNYAEEQGMYYPVDFASAGSSQIGGNIGTNAGGIKVI
KYGMTRNRVLGLTVVTGKGDILRLNKGMIKNATGYALQHLFIGGEGTLGLVTEAEIKL
ERQPQNLQVLVLGVPDFDAVMPVLHAFQKDIDLTAFEFFGELAMQKVLDRGHVQRPFE
TQCPFYVLLEFEAPYEPIIDKAMEIFEHCMEQGWVLDGVMSQSLDQVESLWRLREDIS
ESIAPFIPYKNDISVLITHVPAFIREIDAIVQENYPDFEICWFGHIGDGNLHLNILKP
ENLTKDEFFAKCQVVNKYVFDTVKKYDGSISAEHGVGMTKKPYLEYSRSAEEIEYMKA
LKKVFDPKGIMNPGKLFDL"
/product="D-2-hydroxyglutarate dehydrogenase (EC 1.1.99.2)" /transl_table=11
CDS 2328981..2329349 /locus_tag="AUO97b_02312" /db_xref="SEED:fig|
400667.82.peg.2176"
/translation="MQRLFLISALCAGLMTGCATTSKLIPFVGSETPMQQVLKTQPEI
VKEHNNTSVQQVFNRVESPTVSRITVIQTGLMDDSVSAIRTEYLFKLVDEKWQLQNKQ
KSYQCARGKGSKGFQMQLCS"
/product="hypothetical protein"
/transl_table=11
CDS complement(2329394..2329708)
/locus_tag="AUO97b_02313" /db_xref="SEED:fig|400667.82.peg.2177"
/translation="MLTIIAEIRTQSGGQHRQNVLNAFQKIIPTVLAEDGCHGYEPLV
DHISNASFQNKDPDIIVMLEKWESVAHLEAHLATPHMQAHHEAVKDDVVDVKIKILES GV"
/product="Uncharacterized protein YgiN"
/transl_table=11
CDS complement(2329722..2330309)
/locus_tag="AUO97b_02314" /db_xref="SEED:fig|400667.82.peg.2178"
/translation="MSNILVINGAKQFAHSNGELNDTLTTLATNHLTELGHEVKVTRA
DSDYNAEAEVEKYLWADVVIYQMPGWWMGAPWTVKKYIDDVFTTGHGSLYANDGRSRS
DTSKKYGSGGLIHDKKYMLSLTWNAPMEAFDDADQFFHGVGVDGVYLPFHKANQFLGM
STLPTFIVNDVIKMPDVDSYVEAYKAHLNTLFAAA"
/product="Modulator of drug activity B"
/transl_table=11
CDS 2330534..2330899
/locus_tag="AUO97b_02315" /db_xref="SEED:fig|400667.82.peg.2179"
/translation="MKKIGLLMMYNLLILSACTPNTNPPTPQANSPIQAVLLDQPNLL
NNASNLSISQQMNALEDPSNAQVTILQTEPSEDAVSKTRTEYLLKKDQTVWKIVNKKQ
SYQCTKGEETADFQVNPCP"
/product="hypothetical protein"
/transl_table=11
CDS 2331269..2332498
/locus_tag="AUO97b_02316" /db_xref="SEED:fig|400667.82.peg.2180"
/translation="MKNIQTTALNRTTLMFPLALVLFEFAVYIGNDLIQPAMLAITED
FGVSATWAPSSMSFYLLGGASVAWLLGPLSDRLGRKKVLLSGVLFFALCCFLILLTRQ
IEHFLTLRFLQGIGLSVISAVGYAAIQENFAERDAIKVMALMANISLLAPLLGPVLGA
FLIDYVSWHWGFVAIALLALLSWVGLKKQMPSHKVSVTKQPFSYLFDDFKKVFSNRQF
LGLTLALPLVGMPLMLWIALSPIILVDELKLTSVQYGLAQFPVFLGLIVGNIVLIKII
DRLALGKTVLIGLPIMLTGTLILILGVVWQAYLIPCLLIGMTLICFGEGISFSVLYRF
ALMSSEVSKGTVAAAVSMLLMTSFFAMIELVRYLYTQFHLWAFVLSAFAFIALWFTQP RLALKREMQERVAQDLH"
/product="Multidrug efflux pump MdfA/Cmr (of MFS type), broad spectrum"
/transl_table=11
CDS complement(2332535..2334685)
/locus_tag="AUO97b_02317" /db_xref="SEED:fig|400667.82.peg.2181"
/translation="MNNLTQLFNRFKANSILIYCLQILIVLTGTTLGLLWLGHNELIV
PVTLGAIAAALTDFDDRLSLRLRNLLYVCLLFFTVSTILGFLAPYKFLFILYLSISSA

CFILLGALGQRYATISFGTILLSIYSMFGLGEYAHWYQQPTYFVYGALWYGLTSILFF
ILKPTLPLQDKLSQIFEEISALLQAKARLFDPDNKENVEQLLFELSLQNTQVAQSLNQ
IRSSLLTRLKASRVSNKSIYWLNLYFFARDIHEQATSNYLHYEHIQQNFSRSDLIFRF
QKNLRLQAQACHDLAQCILHNQQYQSSQESQIGLNHLESSLKDWIQQHPQNFEVKNLK
LIFNNLKGMHEQFEQLQYAQPFNETLSQNKQEHLNLLDDDIQGFSDLVLKIRQQLTPQ
SALFRHAVRIAVVFAAGYAISLLPFAQHGYWILLTSLFVCQITYFATKSRLKLRTIGT
LLGVLLGIPILYFVPSIEGQLILTIICGVSFFYLRQKKYALATLMATLMVLLIFNLKG
AGYSIILPRLIDTLLGCFIAWLAVNFIWPDWNFRNIPNNIKKSSQATFDYFNVIVEQY
QHGKNQDIEYRRIRRAAHNAQIELSNMISSLSAEPNPNPELIHYAFRYLVYSHSQLSY
VAALGSQRQKIDDQQVLQLLLDCQQILKQSLFEQALVNFNFLEQTLKQIQSLITHEHF
SENYTLVLKQMSLLLETLPELLSLKGKLLEHEIK"
/product="hypothetical protein" /transl_table=11
CDS complement(2334878..2335453)
/locus_tag="AUO97b_02318" /db_xref="SEED:fig|400667.82.peg.2182"
/db_xref="GO:0004784"
/translation="MPVFNKIGAVCALTALLTVGCSTVSKTVSDTAHALTTMHTKKVV
DVNEVTANGIGKKIGTISFQDSDKGLIITPALADLPSGTRGFHIHENPSCAPAVKDGK
PGAALAAGSHYNPNQAPHHGTPTTGHLGDLPALVVDNTGVATTAVIAPRLKLADIQGR
AIMIHAGGDNYSDSPLPLGGGGARIACGVIK"
/product="Superoxide dismutase [Cu-Zn] precursor (EC 1.15.1.1)"
/EC_number="1.15.1.1"
/transl_table=11
CDS complement(2335569..2336687)
/locus_tag="AUO97b_02319" /db_xref="SEED:fig|400667.82.peg.2183"
/translation="MHPYAPTLQRVLLFGLFFILIFLGFNILKYFIVPVLWAAIIAYM
TWPIYLRIQHFFGENRNNLNATVMISLVILIVGVPFTCAIFILQHEGRNLYFDLQRQV
FSGHLSVPDFIRNLPFIGKEISRTLNEINTDPNSTIQNIAAWLQSHLSYGRVLLNEIS
KNIVKLCFAILSLFFFYRDGHTILTQVSKALEMVIGPRIHHYLDTISETTRAVVYGVG
LTAIAQAVLAGLSYFVAGVPNPMVLTIATFLLALIPFGTPVSYLGVGLWLFSQGQTME
AIGVILWGVLIVSSSDNVIRPLVISGATQIPFLLIMFGVLGGIASFGLVGLFIGPVIL
AVLLAIWREWLHETIDPEPMPKTTMIYDSDDDLPPSKN" /product="Uncharacterized UPF0118
membrane protein" /transl_table=11
CDS complement(2336810..2337454)
/locus_tag="AUO97b_02320" /db_xref="SEED:fig|400667.82.peg.2184"
/translation="MKTILIANQKGGCGKTMTAITLATALAQKGYKVALADSDNQKSS
LQWLKQRPDNVAVIQSLDWRQEKSIGDAPKNLDYLIIDAPGALSGDHAEQLVSEAHAI
ITPLQPSFFDIDSTRRFLKHLQDIKRIRKGKVQILLLANRVKPNSASSKDIQQFFEKI
EHEPIAWIAERSAYGSLAMQGLSVFDKPQKNFIAIQSQWQPLLNKLIEDKSEWF" /product="ParA-like
protein" /transl_table=11
CDS 2337613..2338080
/locus_tag="AUO97b_02321" /db_xref="SEED:fig|400667.82.peg.2185"
/translation="MSVWLAMLIGAGIGIIITTLILSNTRNGRIKAEYEQYIAELELE
HQQALAQAQKRSVNTSRAVLKGKMAEQFAPVLPEFQYLPSDAKFMGDPVDYIIFDGYT
DFRDGDGTADDIQIILLDVKSGGARLTKGQQAIAQAIKEGKVRFETLRIDFED" /product="hypothetical
protein"
/transl_table=11
CDS 2338220..2338783
/locus_tag="AUO97b_02322" /db_xref="SEED:fig|400667.82.peg.2186"
/translation="MKKQRLMIALLATLGMGSAFASTSSEYNIANGLLPVEKDQSISV
LQPFQGNFRILGSKIYHNDEQAKFSPIDYAVSWGLFAQPEIARHISVKQYDRYLNWKI
AKLPVPAEQAMQMVSNMHIIPANPEIAQQIKQVKRGDLVYLKGDLVEIKDKDLVWKSS
LTPTDTGDGACELFRVQSIHWVEKQNI"
/product="hypothetical protein; putative signal peptide" /transl_table=11
CDS complement(2338833..2339366)

/locus_tag="AUO97b_02323" /db_xref="SEED:fig|400667.82.peg.2187"
/translation="MLKKTPHIILMSLLSSSLLLTACKKADEPAKTEQPHTSSSTDQV
LEKLNERPVKKFPATVDDAHDIALLEDYDRRFTEMSDEMENELAKMHEAGTLTAEFEQ
KRTLDNVRSALTMLKDLDLKTEQGRYIQGLLYQYWENQEKNFSDKQASKDEQVKQLAD YLQAQNQLKYWKASQQH"
/product="hypothetical protein"
/transl_table=11
CDS complement(2339382..2340176)
/locus_tag="AUO97b_02324" /db_xref="SEED:fig|400667.82.peg.2188"
/translation="MTQSLHPAAQKGFSSAAELYQQVRPNYPVEIVSWLQDRLQIHEN
STVIDLGSGTGKFLPYLKQTHANIIAVEPIGEMLQQLQQAYPDIKTIQAFSHNIPVND
QQIDAVICAQSFHWFDNIETLTEIHRVLKPQGHLGLVWNQRDEGTDWVKALADFLLPL
EGDTPRYHSERWKKVFEEQDLFTFDNLNTFSQSQHGTVEQVVSKRLLSTSFIAAMTEK
DQLQLKAQFEKIVYDFTGLGPQDQIDFPYVTYAYHFQKSTSSNHRE" /product="SAM-dependent methyltransferase"
/transl_table=11
CDS 2340304..2341581
/locus_tag="AUO97b_02325" /db_xref="SEED:fig|400667.82.peg.2189"
/translation="MDKQIIFEDEHIRAIFLQGDSNTLVLSFGDLITRASGLSINAEK
SLIKYQYNVIGVMPKQKSWFPKASMVELAKVILPIIQRFKNIVGYGGSMGGYAAIKYS
NLLNMNRIIAFVPQYSIDPEQVEDRRYAEFFDAVANKDMQIQPQDVDASREYIIVYDP
YFSIDREHYLKIKEILPSLHTIHLPFTGHEALSVLASSSLLHDFIEHEFDETYFYQQV
RKIKKQSKFYYRNVLANVLTYHDSMLLKILRQNDFQLDERYLDNPLKQAVTRSLIKTK
QATEQDFQKLGIKIQYRQQAENHKKGLKNQFEAILVFNLINLKLETYAVDVVLANPSY
LVPIVAEQTGVAHIELNNEIYLLGMNDRKIIKLFKQGDALTSDMSPFVIKQYSDFFAL
SYKQLNLNCDEQGVCDYVEGSVQPSQQFVLTSY" /product="hypothetical protein"
/transl_table=11
CDS 2341995..2343428
/locus_tag="AUO97b_02326" /db_xref="SEED:fig|400667.82.peg.2190"
/translation="MQSLNYRNNDASQHGSSAPLKRAMSTRHLVMISLGGAIGTGLFL
GSGDVISQAGPIGAIISYFLGGLIAYTVMLCLGELAVHVPESGSFGEYARRYIGPGTG
YMITWLYWLTWTATLGTEFTAAALLVQEWFPSTSVWAWTLFFGALVFFLNVSSTRLFA
ESEFWLALVKVITIICFIGLGLLAIFGFIPYQGHETAPLFSNLTAHGWFPNGLFPIFA
TMLIVNFAFSGTELIGVAAGEAENPAKTVPKAINAAIWRLLIFFVGTIVVISSLLPYH
LAGLSANDVSSSPFVTVFNYIGIPYAEDIIRFVIITALLSAANSGLFAASRMMWSLSA
KNQLPKIFAKLTPSGTPIIAIIVTMFGAIPGLLSEQFAPETIFKNLLGVAAFTMVVVW
IAICISQFNFRRQWYKSGKTVKDLRFAAPLFPLTPIVGGLFCIVTCISMVFDPSMQVG
FICCIIFILLCYVSYFIFYRKKDIAQN"
/product="Arginine permease RocE"
/transl_table=11 CDS complement(2343676..2343831)
/locus_tag="AUO97b_02327" /db_xref="SEED:fig|400667.82.peg.2191"
/translation="MEIKKILKLKMIKKLPFNNKDLINKKFQKILKISINFHNIYSVS LIQNKDI"
/product="hypothetical protein"
/transl_table=11
CDS 2344464..2345735
/locus_tag="AUO97b_02328" /db_xref="SEED:fig|400667.82.peg.2192"
/db_xref="GO:0004354"
/translation="MSDLSYVNEHSDAWQTYLAQIDRVAPYLEHLADYIDTLKRPKRA
LIVDVPIVMDDGSIQHFEGYRVQHNLSRGPGKGGVRYHPNVDLNEVMALSAWMTIKTA
VLNLPFGGAKGGIRVDPRKLSNRELERLTRRYTTEIGHIIGPQKDIPAPDVGTNANIM
GWMMDTYSTSQGHTVTGVVTGKPVHLGGSLGRVKATGRGVFVTGREVAAKINLPIEGA
KVAVQGFGNVGSEAAFLFVESKAKITHVQDHTGTIFNADGIDLVALRDHVNANQGVGG
FAGAQAIADEDFWTAEVDIIIPAALEGQITVERAEKLKAKLILEGANGPTYPKAEDVL
VERGIVVVPDVVCNAGGVTVSYFEWVQDMASYFWSEEEINERLDKLMVQAMDDVWNTA
NKNACTLRTAAYILACERILKARKERGIFPG"

/product="NAD-specific glutamate dehydrogenase (EC 1.4.1.2); NADP-specific glutamate dehydrogenase (EC 1.4.1.4)" /EC_number="1.4.1.2"
/EC_number="1.4.1.4"
/transl_table=11
CDS 2345880..2347094
/locus_tag="AUO97b_02329" /db_xref="SEED:fig|400667.82.peg.2193"
/translation="MEQPISVTRSNFNDWMVPVFAPANFIPVRGEGSRIWDQENKEYI
DFAGGIAVNALGHAHPVAVNALTEQAKKLWHVGNGYTNEPVLRLAKQLTENTFADKVF
FCNSGAEANEAALKLARKVGLDSGVAGKSGIVAFNNAFHGRTLFTVSAGGQPKYSQDF
APLPNGISHVAYNDLEAAKAVINEQTCAVIVEPVQGEGGVIPADIEFLKGLRELCDQF
GALLIFDEVQTGVGRTGALYAYMNYGVIPDVLTTAKALGGGFPVGAMLTTDKFAPHFS
VGTHGTTYGGNPLASAVAGAVFEFINTPEVLEGVKHRHQYFLDALNTLNAEYQVFDEI
RGKGLLIGCVLKAEYAGKAKDITTWAGEEGLLALIAGPNVVRFTPSLIIPEQDIDEGI ARLKRALAKLAK"
/product="Succinylornithine transaminase (EC 2.6.1.81)" /EC_number="2.6.1.81"
/transl_table=11
CDS 2347113..2348144
/locus_tag="AUO97b_02330" /db_xref="SEED:fig|400667.82.peg.2194"
/db_xref="GO:0008791"
/translation="MMIVRHAEFRDLEDIYKLAGKSGVGLTSLPQNMDTLSARISRTR
NTLNGNVHKSEQGYLFVLEDTEAQRVIGVSAIEVAVGLIEPWYNFHVGTQVHASKALN
VYKSLPTLFLSNDRTGSSELCTLFLDPERRENQNGKFLSKIRFMFIAAFKQYFEKKLI
AEMRGYSDENGCSPFWDAIGHHFFNMDFSTADYLSGIGQKVFIAELMPRFPVYVDLLP
KDAQEVIGKMHPHTLPAYHVLESEGLRYQGYVDIFDAGPTIEANIDELRAVKESQLLN
VKITNEATVGKTQYLVANDNYHDYRAMLLKLDLVDNTLNLTHEQAEKLGVQEGHAVRV LSLNPMEVS"
/product="Arginine N-succinyltransferase (EC 2.3.1.109)"
/EC_number="2.3.1.109"
/transl_table=11
CDS 2348144..2349613
/locus_tag="AUO97b_02331" /db_xref="SEED:fig|400667.82.peg.2195"
/translation="MSNSLFINGVWLQGSGTTFVKTNPVDNQTVWSGAEATASDVEKA
CQAARTAFPAWARLPFAERVAIVEKFAALLEENKKHLAEVISQETSKPFWETLTEVQS
MIGKVAISIRAYHQRTGESSTEMADGVASLRHRPHGVLAVFGPYNFPGHLPNGHIVPA
LLAGNTVVFKPSELTPWTAEETVKLWQQAGLPNGVMNLVQGGRTTGEALAASHEIDGL
LFTGSANTGYHLHKQMAGAPEKILALEMGGNNALIIDEIENMDAVVNLTIQSAFVSAG
QRCTCARRILVKNGAQGDAFIQRFVEVAKNLKVGRWNDEQQPFMGGVISSAAATNMMN
AQQKLLELGAKSLLPMTRPQEDSSLLTPGIIDVTGVAGIPDEEYFGPLTTIQRYDSFD
EALKIANATRFGLSVGLVSPKRELFDDLLIEARAGIVNWNKPLTGASSAAPFGGVGAS
GNHRASAFYAADYCAWPMASLESESLTLPEKLSPGIVLP" /product="Succinylglutamic semialdehyde dehydrogenase (EC 1.2.1.71)" /EC_number="1.2.1.71"
/transl_table=11
CDS 2349631..2350974
/locus_tag="AUO97b_02332" /db_xref="SEED:fig|400667.82.peg.2196"
/translation="MKGYEVNFDGLVGPTHHYAGLSFGNEASTKNRNNLSNPKLAAKQ
GLLKMKALADMGMKQGVLAPHERPHVPMLRRLGFTGDDISVVAQAMRYSPELLSSLSS
ASPMWTANAATVSPSADSQDERVHFTAANLNNKFHRSIEAETTSQVLQAIFKNERHFV
HHEALPQVALFGDEGAANHNRLGGDYAKRGVQVFVYGQQHLNNGLPGPKRYPARQTRE
ASEAIARLHRLDEAHTVFVQQNPDVIDQGVFHNDVIAVSNQQVLFHHQHAFLNQDQAF
AEIRQKMASIGEDFISIEVPENRVTVDDAVATYLFNSQILTRPDGGMTIVVPEESRQN
AAVWSYLNDMIQMGTPIDAIQVYDLRESMRNGGGPACLRLRVALNETELNAVNPKVLM
NDQLFMTLNQWVDKHYRDRLAQEDLADPHLLMESRMALDELTKILGLGSVYPFQK"
/product="Succinylarginine dihydrolase (EC 3.5.3.23)" /EC_number="3.5.3.23"
/transl_table=11
CDS 2350987..2351961
/locus_tag="AUO97b_02333" /db_xref="SEED:fig|400667.82.peg.2197"

/translation="MQDFLALTLQGEQPATREGKQANFSWRWLGEGLLECTPHAQYDK
AVVLSAGVHGNETAPIELLSHLCTDLFAGRLKLAVRLLLVLGNPYAMRQGKRYVHDDV
NRMFCGGYKNLPVTEESKRAEVLEQTVATFFQESSSQAKRYHYDLHTAIRASLLPTFA
LFPYQTHSYDADLTASLEAADLDALVYHNAVGKTFTHFTSENFKAASATLELGKALPF
GQNDLSQFASIDEVIRNVVSEQALPVRNKPKIRVFQVSDSLIKKDEEFHMNLSAEAPN
FSTFTKGEIIATQPSGNYVVEQDQVWILFPNPNVKIGLRAGLVLTETI" /product="Succinylglutamate desuccinylase (EC 3.5.1.96)" /EC_number="3.5.1.96"
/transl_table=11
CDS complement(2352024..2352785) /locus_tag="AUO97b_02334"
/db_xref="SEED:fig|400667.82.peg.2198"
/translation="MKMMKTAIVTASVLASASIFAQSAGVNAGASAQVNVQPGGLVSG
VANTVKNTAHTVGNTAKHAGHVAADTTVKATKKTTGKVTELSSKAATGTKHVASEAVT
GTKHFATEAATGTKNLATKAATGTKNLAVEAKADTKAHLDAVKTKVAEKQADQKEFTA
EKQADAQARVDAVKARVAQNQAEQKEFVADTKADAQAKLNAAQPAHGVNAQTGVNVGV
KVAGINANANVNAGAQATTQKGEKKSFIKGLFGTN" /product="hypothetical protein"
/transl_table=11
CDS 2353463..2353687
/locus_tag="AUO97b_02335" /db_xref="SEED:fig|400667.82.peg.2199"
/translation="MFNSALHETNSPQPDTSSSLSKLKEPTQPKNVSLNSNHSQSVSA
DTVPFYDTKSDPSIQPIDPEKDPANEIKAK"
/product="hypothetical protein; putative signal peptide" /transl_table=11
CDS 2353723..2355078 /locus_tag="AUO97b_02336" /db_xref="SEED:fig|
400667.82.peg.2200"
/translation="MQSKTLFLLGLCTIFSTTVSALPLDSKGAKQRLSQAAKQKVFQG
KIDLDALVSNDSNDLIVEYNIPSSSVPSGLERRSYIATNKKNLQTRFNRAGGVQVLRD
YNNLPLAFYRISNREALVSLLNDPNVKAVYPNRINRTTTTESLPLINQPQANTNGFTG
EGSSVAVLDTGVNYLHSDFGCTAVNTPSSTCRVVYSFDSAPDDGTLDDDGHGSNVSAI
VSKVATKTKIIGIDVFRKVRSQGKWVSTAYDSDILAGINWAVNNAQTYNIKAVNLSLG
VPGVKYTSECSDSSYGTAFANARAAGVVPVVASGNDAFPDGISSPACVAGAVRVGAVY
DSNIGGVSWGNPVKCSDPTTAADKVACFSNGGSLVTLLAPGAMITAGGYTMGGTSQAT
PHVAGAIALLRANSVSPTESIDQTISRLKATGKPITDSRTGLVFPRIDLLAATNGLTV N"
/product="Peptidase S8 and S53, subtilisin, kexin, sedolisin"
/transl_table=11
CDS complement(2355177..2355608)
/locus_tag="AUO97b_02337" /db_xref="SEED:fig|400667.82.peg.2201"
/translation="MNSQKLLFSTLCCLALTACATTAPLSSYPQAQVLEQDTNIEAYP
TTTGNVAKLIKHDKQACMIEFTGYLGGGQVTEHWIFNNKGLISAQSFTEQYPDSSGIL
SQATNSTTKSSTTFNIQDPEIQNNFKKLQSNFSKDNLAKCD" /product="hypothetical protein"
/transl_table=11
CDS complement(2355703..2356122)
/locus_tag="AUO97b_02338" /db_xref="SEED:fig|400667.82.peg.2202"
/translation="MKAIKILCITSSILVSSSLFAETPQPQQVNEATSKTMPYGDNPS
LGRVLLYKTGKGIQNLGDSIQGASEKTSNKISEKWKDTKEFTAEKAEVVQQKADTAKV
FTEQKIEQAKQNITSSRNGENIPIEQGELSKSSTTAN" /product="hypothetical protein"
/transl_table=11
CDS complement(2356271..2357137)
/locus_tag="AUO97b_02339" /db_xref="SEED:fig|400667.82.peg.2203"
/translation="MDITQLKMFQTVVECGTMAKASLQLHCVPSNITTRIKQLEEELG
APLFYREGKSLKLTPSGEIFLDYCHKILALCDEAKRSIHPDAPPSGPLKIGAIESSAT
TRLPNLLAKYHQRYPEVSIQIITGTWKQLLVDISQHKLDGAIVAGNIEFPMLNKLSIY
QEDMVLIASHSLGEIRCQEDLIGKEIFMWTEGCPYRAALEEWLKLKNISLPITSITSY
ATIIGCVSAGSGVALVPKSIYEQYKNLAHIQSYKFEYLAFMQNHFFWHKNVSNHKARD MFIELIKSEFCL"
/product="Transcriptional regulator, LysR family" /transl_table=11
CDS 2357246..2358139

/locus_tag="AUO97b_02340" /db_xref="SEED:fig|400667.82.peg.2204"
/db_xref="GO:0016020"
/translation="MAISLAAETQPPAFKLYLCIAVTVFCWGYSPIGVHSALHSYTPQ
HIALLRFLIASVFLLLLVMKKGIQPLKWKDVPALAVLGFFSVTLHHLLINTGQQYVTA
VASSILSQSIPLFTFIISALLFKEQISFKQWLCILLGLCGAVLVVSGDHGFGMPSPYS
LLIVLAAIAWGLYFNLYKKFALNYDALSMMCYIIWFGTLPLLFYSAHLPSEILHATWQ
ANMSVVLLGIFPSAMAYVLWGYVLKNMPLTIASNFLYCSPIVAMSLAVLFLNEKPSII
VLLGGVIIVGSLVWMNWGRKK"
/product="Permease of the drug/metabolite transporter (DMT) superfamily"
/transl_table=11
CDS complement(2358142..2359257)
/locus_tag="AUO97b_02341" /db_xref="SEED:fig|400667.82.peg.2205"
/translation="MPTLFFYTILPLFIFLSVLGKYTYAQQPSTTLMTREECKQDLDF
IAYFLLHNDAGINALAWSNYPSFIQKVLDIQTKKSVQAQTVQECLNAIKPFLTSIRKG
HLWVSGKPMIEDVFQNHENIQPPLKVTTKQLSNLTTYIHIPSFDEDIHTQLENIIQEN
QDDILQKPYLIIDVRNNNGGFDSAFKPLLMLMGDTRYWLQSPQIYTTSANIQSWKEIE
RLISTSSDKKFLRDIIEKMELKKAGWVNMFDQLEVSEFIQGYKNMAYPNKVIVLVGEN
CGSSCEQFVLYAQQNPNVTTMGQRTYGALDASNVREKQTPSGKILLSYATTFVFRHRQ
LNIDTVGIPPDILLPPPQNHQTSEAEIDLAKRFLEQQ"
/product="TSPc, tail specific protease"
/transl_table=11
CDS 2359473..2359652
/locus_tag="AUO97b_02342" /db_xref="SEED:fig|400667.82.peg.2206"
/translation="MASGRRENRVGGVILVLSSSIQEAEEIMKNDPFYIHDVADYDFM WFEPSKSLEEIKEFV"
/product="hypothetical protein"
/transl_table=11
CDS complement(2359724..2360554)
/locus_tag="AUO97b_02343" /db_xref="SEED:fig|400667.82.peg.2207"
/translation="MKNLNLLLISTLLFTGCATTKSIQYPAAGKQTSLSVSSEQLSSM
TESGAGDYFIKDSQILVGDSSNASASPVSGMFGLIGVGVAMAIDKNANSSAIEKSSLN
QPIKFDQLIHQKITDSLTQNKTDPTLKLLSLNQASEIKIVPYSRLSFREKPIVKVNFG
LKTQFKNAADNNADTKRLYHYVSKDRISLSEWEANNNLLFMKKADQAFSALSQVFTLD
LQHQLNLKAFDENKQKSCKTNADTSLVSFIQTPDNLCVGVVKTSKGNVFQNNIYIVEQ "
/product="hypothetical protein"
/transl_table=11
CDS complement(2360778..2362847)
/locus_tag="AUO97b_02344" /db_xref="SEED:fig|400667.82.peg.2208"
/db_xref="GO:0004820"
/translation="MSKHTVLFELGCEELPPKSLKTLRDALQAETVKGLNEAGLDFAS
VEAYAAPRRLALKIVDVDAAQADTQKRFDGPAVQAAYDAEGKPTKALEGFMRGQGITV
DQLSTFQAGKVEKVCYLKDVKGQSLDALLPQILQTALDNLPIAKRMRSAASRTEFVRP
VKWVVLLKDDQVIEATIQDHKAGNVTYGHRFHAPEAVTLAHANDYLAALEKAYVVANF
EKRQATIQEQVKKLADEVNATAIVPADLLDEVTSLVEWPVALRATFEERYLAVPQEAL
ITTMQDNQKYFCLINAEGKLQPYFITVSNIESKDSTQIIEGNEKVVRPRLSDAEFFFL
QDQKQPLASRKEKLANMVFQAQLGTLWDKSTRIAKLAVALSSITGANPADAEKAALLA
KCDLTSELVGEFPELQGIAGTYYARIEGENTEVSEALGEQYLPKFAGDVLPKTKTGTT
IALADRLDTLVGIFGIGQAPTGSKDPFALRRSAIGILRLIIENELDVTIEELVNLALQ
GYGDIVKDHDKTRADAVAFLEGRYRAKYEDQGVAVDVLQAVQALAPKSPLDFDKRVNA
VNHFRTLPEAAALAAANKRVANILAKEAAPEGSVIEANLVEDAEKALFAELQAVTPVV
EPLLAAKDYTAALSKLAALRAPIDAFFDGVMVMADDADLKANRLRLLAQLRNLFTAVA DVSVLQG"
/product="Glycyl-tRNA synthetase beta chain (EC 6.1.1.14)"
/EC_number="6.1.1.14"
/transl_table=11
CDS complement(2362847..2363824)

/locus_tag="AUO97b_02345" /db_xref="SEED:fig|400667.82.peg.2209"
/db_xref="GO:0004820"
/translation="MSRAISHIDTFQGLILALQNYWAEQGCVVLQPYDMEMGAGTFHT
ATFLRALGPETWNAAYVQPSRRPKDGRYGENPNRLQHYYQFQVVLKPNPDNIQQLYLD
SLKAIGIDTLTHDIRFVEDNWESPTLGAWGLGWEVWLNGMEVTQFTYFQQVGGVECYP
VTGEITYGLERLAMYLQGVDSVYDLVWTKGQFGTVTYGDVFHQNEVEQSTYNFEYAPV
DKLFELFDFYESEANRLMEAKLPLPAYEQVVKASHTFNLLDARGAISVTERQRYILRV
RTLARAIAQSYVQARAELGFPMAEPHLRDEVLAQLKAQAESEAAKAEKN" /product="Glycyl-tRNA
synthetase alpha chain (EC 6.1.1.14)" /EC_number="6.1.1.14"
/transl_table=11
CDS 2364194..2365378
/locus_tag="AUO97b_02346" /db_xref="SEED:fig|400667.82.peg.2210"
/translation="MNRSLFIIFATIALDAIGIGLIFPILPLLLQDMTHSTHISIYMG
ILASLYAAMQFIFSPLLGALSDRWGRRPVLLISLAGSAVNYLFLTFSHSLILLLVGRI
IAGITSANMAVASAYIVDVLHENNRAKYFGLINAMFGAGFIIGPVLGGFLSEYGLRLP
FFAAAILTGLNLLSAYFVLPETRRVTSEGKQLSTLNPFKIFAGISSIRGVLPFVMTFF
IFSAIGEVYGVCWALWGHDTFQWSGFWVGLSLGAFGLCQMLVQIFIPSHASRLLGNRN
AVLIGIACSCLALAVMAFAQSGWMIFAIMPIFALGSMGTPSLQALASQKVSADQQGQF
QGVITSTVSLASMIAPLFFSTLYFQFQKKWPGAIWLSVILIYLITLPIILYSTQPVEQ QR"
/product="Tetracycline resistance, MFS efflux pump => unclassified"
/transl_table=11
CDS 2365552..2366814
/locus_tag="AUO97b_02347" /db_xref="SEED:fig|400667.82.peg.2211"
/translation="MSDRIREKLQILADAAKYDVSCSSSGSDRKNKDKGLGDASHSGI
CHSYTEDGRCVSLLKILFSNVCIFDCAYCVSRRSNDVQRAAFTVQEVVDLTINFYRRN
YIEGLFLSSGIFKSADHTMERMLQVVKKLRLEENFNGYIHLKTIPGASPELIHEAGLY
ADRMSINLEMPTEIGLKTFAPEKSHQEVQKDLGLVRDRLIQLKDERQIIKHVPKYVPA
GQTTQMVVGAHQESDQDVLFMADKHYKEFKLKRVYFSGYIPINTENNYLPAVGSAPPL
LRENRLYQSDWLMRFYGFEVNEIVNEKHPNLDLDVDPKLSWALRHPEQYPVDLNRADY
QMILRVPGIGVKSAKKIVQARRFGKIHIDLLKKLGVAYQRAKFFIRCEDSPKFQKELS
SSFIRQQILTQGSSKYVQQLSPQLSLGF"
/product="Biotin synthase related domain containing protein" /transl_table=11
CDS 2366817..2367614
/locus_tag="AUO97b_02348" /db_xref="SEED:fig|400667.82.peg.2212"
/translation="MFNEEVAYYFDGSMVGLLSCVFRAFQFKELQVRLCLNDAAQHGL
FADKIEVSNNEQHAERVWAALQKKLSSSSLKQFYFAYLSESLDAYQHLFNYCIYVFSS
HSSVEKDYSHPSVLAIAQWTKKVGREKHRMEAFIRFKKTKDELFLSLVRPDFNVLPLI
QPHFKRRYQDQRWLIYDEQRKFGLYYDLREIHEVSLEASDVDRNLKNGMSQSFQLELD
EQEVLYDQLWKDYFKSVNITERQNIKLHVQYLPKRYWRYLNEKLIEY" /product="Domain often
clustered or fused with uracil-DNA glycosylase" /transl_table=11
CDS 2367747..2368847
/locus_tag="AUO97b_02349" /db_xref="SEED:fig|400667.82.peg.2213"
/translation="MSVIQPEWLQILKPNFKVLPLKERLLCGVGALCGLAISSLISWY
VLGGINAWYIAPMGASSVLLFAVPTSPLAQPWNVIVGNTLAGIIGVACAQWIPDLTTA
FSVAVGFAIFLMMTTDSLHPPSGAVAITAVLGGAAIHKLGFYFVFYPVLLNSLLLLGF
AVFFNRLIGRHYPVTAHLNERSKDPTPTQKVSIQPKDIEYALEHHTELLDISQYDLEK
IILEAQEHANERMVNQYTCQDIMSRDVIKLHENDDIHQALDKFKQENLMSLPVVNADN
HLVGTLALYEVVEWFKGATDPRNSWQHYVRQIMSRRVVTVLPTQPIQDLVPYFVEKSF
NYIPVVEERKLIGIISRADMIAALQQQLNRKL"
/product="Putative transmembrane protein"
/transl_table=11
CDS complement(2368917..2369171)
/locus_tag="AUO97b_02350" /db_xref="SEED:fig|400667.82.peg.2214"
/translation="MANSGLYRSNRQSMIAGVMGGIAERFGWNANLLRIIFVLVSIMS

AAFPGILVYLVLWLVIPKKQPEQVQGYQQPVRTVREEQIH" /product="Uncharacterized membrane protein YvlC" /transl_table=11
CDS 2369425..2370072
/locus_tag="AUO97b_02351" /db_xref="SEED:fig|400667.82.peg.2215"
/translation="MSLKWSGLLIKHLGVAAILCMGLSACDVNHADSKDHSAVATTSR
HEDSLIKSTTKVKTLDDAAGRTFLPEGVPDQTSKLSEADQQYAGRYHTRISCEDNFAD
CETGEAEYILNLLPDGTAYWNVVHFGRVGSKDGSKSAVINQLCPSLKWKVEPAAHELT
IQCPLSDVNFYFDIDAKQRLVVNLEKLFYSDFGKNREFLEQNYFVPTKAYVLDKE"
/product="hypothetical protein"
/transl_table=11
CDS complement(2370144..2371967)
/locus_tag="AUO97b_02352" /db_xref="SEED:fig|400667.82.peg.2216"
/translation="MPAYKAPLHDIRFLMNEVLDYPAHYKTLSNGESADPDTVDMILE
GAADYCENVLSPLNQSGDEEGCHFDNGEVKTPKGFKEAYDQFVMGGWQGLSYPEEFGG
QGLPMSLNLIKSEMMGTANWSFTMYPGLSSGCMNTILQFGTDEQKQTYMPKLVEGTWS
GTMCLTEPQCGTDLGQVKTKAEPQADGTYKISGTKIFISAGEHDLTENIIHIVLARLP
DAPAGTRGISLFIVPKFIPTADGGVGERNTVSCGSIEHKMGIRASATAVLNFDNATGY
LIGEVNKGLHAMFTFMNTARIGTAVQGIAHAELSFQGALPYAKERMSMRALSGKKDPD
KVADAIIHHADVRRMLLTQKAIAEGGRSMIYYAAQLADKMTDALTRGDQAAFEDYDDK
LGFYTPILKGFLTELGLEAANHGMQVYGGHGYIKEWGMEQIVRDARISTLYEGTTGVQ
ALDLIGRKVLLSSKGKVVREYTAEILKFCAAHARNKYLRRFAWDLTKLCAQWNTLTVR
IMLAARKDRDIVSSASVDFLMFSGYVMMAYFWAQQAVVASEKLEAGNGQETPEFYKAK
IKVADFYFDRLLPRAQGHAESMVTTSRTLTSLPAEHFSFDY" /product="Acyl-CoA dehydrogenase"
/EC_number="1.3.8.7"
/transl_table=11
CDS 2372225..2374528
/locus_tag="AUO97b_02353" /db_xref="SEED:fig|400667.82.peg.2217"
/translation="MHLKYLSLCLFSLGLTACAQHGVRPQVASASLEQKAIQSVNAMY
EYPSYDYRGNFKITVDPSQIKQNVKAENTAKLDAELQKKVDQYLREQKVALSKAQKQT
LYAAIANEQGDWGLTSSARSEKINNILINLLNDLQFSYDGSIHYRQKMGSFNLTARYE
KPTLLVQAKLPMVLDLENYKFYINYFGLMPYLVNKDNQNNLAYVDFSKYKAFFKNVDK
KKFIEYLKASSAVSYRLAEPQNLQRVSLTEADRKAGAVERIRLKTTVEQLLLEVDLFG
QVNEKYLQKSVLGLDEEKLAETLAAEIAASDAKKGTAGKEEQKVSSDDAAAVSQQLYS
LVNAHLGNTSTSEDEEVESASSEEASDVAVAEAEQTSENEEVVVLTEDQCIELKSLKN
PVALGDINYCQIYGIDVLDQSDTSIQKAQIKSRQDALKQTFEVYNQNQFINDEAFKVL
WLKHKDEIEQALPKQRNPITIDVALDDKGRAVNMDYDVDYTPAEFKHRFNIKADMQIL
NYGKATSIDQQQLKQAKSVAEASKGSMLENIIKGFSEKLGQSDVSEHPVGTHSDVQDL
DANLAILADKTYDATHAYDKTYKAVFIAKLTAEKPSYIKYYSVQQLQEIAEVYAYWFS
DEDTYNPQGKALERITALQKKHHLEQDDQFDHELGRAVDHIVLTTIQGKTGREAWQRL
QKQYKQPAQLFSKQYQLEFEKQNGVSAEEKHLLSETADILGNVYVAAHKKQLSEKTIQ
NLKPEHNEFIDYEIFREVYKQMVAARK" /product="hypothetical protein"
/transl_table=11
CDS complement(2374576..2375364)
/locus_tag="AUO97b_02354" /db_xref="SEED:fig|400667.82.peg.2218"
/translation="MTKQFAVIGNPIEQSRSPELHHAFAEKTGVDLNYQKRLAPLDGF
ESSMRSFFAEGGSGMNVTVPFKEQAFALCDVLTERAQIAKAVNTLWMENGKLHGDNTD
GQGLVAAIQALEWNLENTTILILGAGGATRGVIYPLVQAGTQKIVIANRTLARAEQLV
DDLKTAVPQAQLQAISLNDLEGDFDIVINATSASLSGDALQLPEKLQFKYAYEMAYGK
PSSFLDQAKQRNVPYSEGFGMLVGQAIEAFYIWNGVRPQLKDFL" /product="Shikimate 5-dehydrogenase I alpha (EC 1.1.1.25)" /EC_number="1.1.1.25"
/transl_table=11
CDS complement(2375538..2376488)
/locus_tag="AUO97b_02355" /db_xref="SEED:fig|400667.82.peg.2219"
/db_xref="GO:0004109"

/translation="MQHPTSTDIQRVREFLLDLQARICAGLEQQEKAGGGTAEFIIDD
WERPEGGGGRSRVLQNGTVIEKGGVMFSHINISKLPASATERHPQIAGAKAQALGVSL
VIHPKNPNIPTSHANVRLFVAEREGQDPIWWFGGGFDLTPFYPDDQDVLNWHQAAYDL
CKPFGDNVYAEHKKWCDDYFYLKHRDEQRGVGGLFFDDLNCWDFETCFKYIQAVGNGY
LNAILPIFEKHREQPYTEAQREFQLYRRGRYVEYNLVYDRGTLFGLQTGGRIESILVS
LPNLAAWSYRPEWDEDSPEKRLTDYYLKPRDWLGLEEKVA" /product="Coproporphyrinogen III oxidase, aerobic (EC 1.3.3.3)" /EC_number="1.3.3.3"
/transl_table=11
CDS complement(2376616..2377218)
/locus_tag="AUO97b_02356" /db_xref="SEED:fig|400667.82.peg.2220"
/db_xref="GO:0003935"
/translation="MPIEFIATSKLPTAFGEFNISVFQDPVTGEEHVALSKGLENPPT
GPVLVRVHSECLTGDAFASLKCDCGPQLQATQKLINEAGQGVILYLRQEGRGIGLTNK
IRAYALQDQGHDTVDANLLLNLPADARRYDMCSIMLDHLKVKEVKLITNNPLKIQALK
DQGINVVDRVPLTVGRNPFNEQYLKTKRERMDHLYQKDDF"
/product="GTP cyclohydrolase II (EC 3.5.4.25)" /EC_number="3.5.4.25"
/transl_table=11
CDS 2377522..2379426
/locus_tag="AUO97b_02357" /db_xref="SEED:fig|400667.82.peg.2221"
/db_xref="GO:0008661"
/translation="MLYTEIPTQRPVTPLLDAIDHPQQLRQLEHSQLLQVADELRQYI
LYAAGQSGGHFGANLGVVELTVALHYCFNTPNDRLVWDVGHQAYPHKILTGRREQITT
IRAKNGLAAFPAREESVFDTFGVGHSSTAISAGLGMSLARRYQKDPCEVVCIVGDGAM
TAGMAFEAMNDAVAHDADLIVVLNDNDMSISCSTGGFAKHLAAIWEKGHLVNVNEHGE
AYIQPHPKWTYNSRLHQSATDAADNLFKAIGFDYFGPFDGHDVTQLVQVFNALKKRKG
PRLVHVVYTKKGKGFAPAEADPITYHAIGKINAASGGKTPPKYSDVFGEWLCDEAAQDE
RLLAITPAMCEGSGMVKFAKQFPQRFFDVAIAEQHAVTLAAGMACEGLKPVVAIYSTF
LQRGYDQLIHDVALQNLDVTFGIDRAGLVGEDGPTHAGAYDYAYMRTVPNMVIMAPKD
ENECRQMLHTAYAYNGPAAVRYPRGAGVGVEIQKEMTVLELGKAEIVAEIKANSDEQI
TVLAFGSRVMVALEAAEQFAQKHDVSVCVVNMRFVKPLDEQMIRDLAEHTHLFVTVEE
HAIMGGAGSAVNEFMAQEQIVKPIINLGLPDSFLHQATHNQMLQDCGLDAKGILNSIE RAWLKVNQVV"
/product="1-deoxy-D-xylulose 5-phosphate synthase (EC 2.2.1.7)"
/EC_number="2.2.1.7"
/transl_table=11
CDS 2379549..2380379
/locus_tag="AUO97b_02358" /db_xref="SEED:fig|400667.82.peg.2222"
/translation="MEPMVVMAARAAQTVGQELLKAHQNRHKLDLQVEEKGIDGPVTR
VDRYLEQLTIDTLRKSYKNHSFLGEEFGLQEGKGHDADWCWVIDPLDGTQNFINGFPH
FCISIAVQHKGVTQHGVIYDPVRDELFSASRGRGAVMNQRRIRVNVKDSLENTFLAVG
HPYRAKRAGEIVSYAEQHFASLLAVTQAGAQIRRGGSAALDLAYVAAGRFDGFFELGL
KPWDIAAGELIIKEAGGVVVDARGGNDSLENGQVIACSLKMLKPLMQTVVPAWDKAAK "
/product="Inositol-1-monophosphatase (EC 3.1.3.25)" /EC_number="3.1.3.25"
/transl_table=11
CDS 2380789..2382636
/locus_tag="AUO97b_02359" /db_xref="SEED:fig|400667.82.peg.2223"
/translation="MSKTFAEFSLHETLQQALEGLGFTNPTPVQEQSIPAALEGKDLL
VSSQTGSGKTAAFLLPTLHNLAGQDTFVPFKERMKAVTQPSILVLCPTRELAQQVSQD
AIAFVRHMKGVRIAAIMGGMPFGKQIQQLKGAQVVVATPGRLLDLVNRRQLKLDKVDA
LIVDEADRMLDLGFSEDLEAISDLAANRGQTLMFSATFADRIIRLAERMMNEPERIAI
ETGHSTNTDITQTLHWTDGFEHKKKLLTHWLADETLDQAVVFASTQEDTDMLAEELAE
AGHSVVALHGAMPQTVRNRRLRSIREGRAKILVATDVAARGLDVPTISHVINFGLPMK
HEDYVHRIGRTGRAGRTGQAITLATYRERGKIRALEDYLEARLNVSEIEGLEPSPPPA
RPRREGGRGGNGGRDGRRGGGFGGGRRFEGEGNFKRREGGRDDRPRRSFDDKPRGERP
AFGGEDRPRREFNSDRPRREGGFEDRPKRSFGGEDRPRREFNSDRPRREGGFEDRPKR

SFGGEDRPRREFNSDRPRREGGFNDKPRFDSNDDNRGNRVDYKPRRENGFGDRPQRSF
GGEDRPRREGGFGDRPKRSFGGEDRPRRAVREEHFNQESRGERRRKFDR" /product="ATP-dependent RNA helicase NGO0650" /transl_table=11
CDS 2382743..2382913
/locus_tag="AUO97b_02360" /db_xref="SEED:fig|400667.82.peg.2224"
/translation="MRKSKRKESPLAWLSKFLFQHGLPYLFIALALCNGIYFAYALLT GHFVDQFQVLDQ"
/product="FIG00349961: hypothetical protein" /transl_table=11
CDS 2383028..2383858
/locus_tag="AUO97b_02361" /db_xref="SEED:fig|400667.82.peg.2225"
/translation="MIAIMNNKTPLSTQSLIEVKNLSFNRGERVIYDNISLNIRRGQI
TAIMGPSGTGKTTLLRLIGGQLVPDQGEVLLDGKDIAQMSRQELFAARARMGMLFQSG
ALFTDMSVYENVAFPIRAHTKLSENLIAELVALKLESVGLRGTEQLMPTELSGGMNRR
VALARAIALDPDLIMYDEPFAGQDPIVKGVLTRLIRSLREALDLTTIIVSHDVPETLS
IADYIYVVAEGKIQGEGTPEELQAYASPFVKQFLTGSAEGPVEYQFSHQAYLDNEVRP "
/product="Phospholipid ABC transporter ATP-binding protein MlaF"
/transl_table=11
CDS 2383855..2384631
/locus_tag="AUO97b_02362" /db_xref="SEED:fig|400667.82.peg.2226"
/translation="MNTIAWLGRLVIERIRGIGVAALMLLQIIFSLPSAGGFGRFVYQ
MHRVGVMSLLIITVSGLFIGLVLGLQGYSILVNVGSESMLGTMVSLTLLRELAPVVAA
LLFAGRAGSALTAEIGSMKQSEQLASMEMIGVDPLKQIVSPRLWAGIVSLPMLTVIFA
AIGIVGGKLVGVDFLGVDEGSFWSGMQNNVQFGHDVVNGIIKSIVFALLCTWIAVFQG
YACDPTPEGIATAMTRTVVYSSLCVLGFDFVLTAVMFGGI" /product="Phospholipid ABC transporter permease protein MlaE" /transl_table=11
CDS 2384631..2385311
/locus_tag="AUO97b_02363" /db_xref="SEED:fig|400667.82.peg.2227"
/translation="MKSRTSELAVGIFVIIFGIALFFLAMKVSGLVGTNLSDGYTMKA
QFDNVNGLKPRAKVTMSGVTIGRVDSITLDPVTRLATVTFDLDGKLTSFNAEQLKEVQ
KNALDELRYSSDYTQATPAQQKTMEQQLISNMNSITSIDEDAYIMVATNGLLGEKYLK
IVPGGGLNYLKRGDTISNTQGTMDLEDLISKFITGGGAGKVAAGSSSAEEKAPASTDS SAQPSFVE"
/product="Phospholipid ABC transporter substrate-binding protein MlaD"
/transl_table=11
CDS 2385338..2385979
/locus_tag="AUO97b_02364" /db_xref="SEED:fig|400667.82.peg.2228"
/translation="MNTLFKQTLTASILSTMIAGTAFAAPSEAPPDFIKRVADGLISR
LKADHAKLQNNPALVKTIVRQNLDPYVDSQAFTRIVMGTYATNQYSTAAQRAQFETNF
RNTLIENYGSAFAKYTNQTYTMRPYKVTAGKNPVVTLDFNHNGEKIPVSFQLADKGSQ
WKIRNINVSGIDLGLQFRNQFAATVKRNGGDLNKAIATFQPDADAAVNQNKQK" /product="Phospholipid ABC transporter shuttle protein MlaC" /transl_table=11
CDS 2385991..2386278
/locus_tag="AUO97b_02365" /db_xref="SEED:fig|400667.82.peg.2229"
/translation="MVQYLNQELVVSGKIDFENAEQQYQAGLAIIKKQTSFPLIVDLK
QLEHGNTLALAVLVQWLRQTPQKSGLHFKNVPEKMLKIIQACHLQEDLHLV" /product="Phospholipid ABC transporter-binding protein MlaB" /transl_table=11
CDS complement(2386235..2386357)
/locus_tag="AUO97b_02366" /db_xref="SEED:fig|400667.82.peg.2230"
/translation="MDLKRLLPQILYIKKHLNLGAFLSLLIKPNVGLLANGRLE"
/product="hypothetical protein"
/transl_table=11
CDS complement(2386348..2387361)
/locus_tag="AUO97b_02367" /db_xref="SEED:fig|400667.82.peg.2231"
/translation="MLEAFYATERGSLEDATINGGFDLHPELVWLDLIAPSQEEQQWV
LDAYNQNLPTLKSLEDISSSARFYRDDDGILHISTYFLTKNKNYQVDGDAEDSSHMLA
MVQTVAFILHKDRLFTLRGEKLVAFRAFRARARRNDYEMDYKDPTWILLGLLEAKLDE

LADILEDIHKDLEKYSTEVLNNHQREQILDLDDMITRLAQQEDMLGKAQLCLIDLRRV
LTFLSRPRALGSHIYDADIRELSEDVRSLVEHDAFLFQKVRFLLDTTSGFINTEQNDT
IRRFSILPSMLAPPMLIASIYGMNTDVLPFAHGTTSFIIVLLIIIGFFIGPIIYFRWK KWI"
/product="Magnesium and cobalt transport protein CorA" /transl_table=11
CDS 2387519..2388100 /locus_tag="AUO97b_02368" /db_xref="SEED:fig|
400667.82.peg.2232"
/translation="MNSICIFCGSSLGSNPIFQQIAQLTGEAIAKQGKTLVYGGGRSG
LMGVVADSALQAGGQVIGVIPRALVDRELAHPGLTKLYVVENMHERKTKMADLSDGFI
ALPGGAGTLEEIFEQWTWAQLGIHQKPCAFLNVAGFYEDLLKMIQGTVDNGFSQARFV
DKLIASDKIEDILQQFEQYQAPAPKWTNADVQP"
/product="Lysine decarboxylase family"
/transl_table=11
CDS 2388115..2388516
/locus_tag="AUO97b_02369" /db_xref="SEED:fig|400667.82.peg.2233"
/translation="MKTITVAAAVILNEQNELLLVRKRNTQAFMQVGGKLEPNEAPES
AIQREILEEIGSPCVIEQFIGRFETAAANEPDHKLISHLYLVRLKQSPQIAAEIAEMK
WVKFNDSETKLAPLTKEIVIPWCEQNLSITL"
/product="Nudix hydrolase family protein PA3470" /transl_table=11
CDS complement(2388519..2389154) /locus_tag="AUO97b_02370"
/db_xref="SEED:fig|400667.82.peg.2234"
/translation="MFKFLNPQYLFQLLSPCLLCEIGTREKYSLCKECWEQLPWLKQT
IQRNDQSVLVACHYAYPINRIIQQFKYEQKLHYQTLLAEVLQQLKFPKVQAIVPMPIS
KQRLTERGFNQSLLLANLLSKQLKIPVWQPVQRLNEHSQKGLSRLERFENIEQQFVAL
TQEKRRYRRVLIIDDVITTGSSIHALSQALKQLGCTSIHASCLAAASSTSY" /product="Competence protein F homolog, phosphoribosyltransferase domain; protein YhgH required for utilization of DNA as sole source of carbon and energy" /transl_table=11
CDS complement(2389147..2391192)
/locus_tag="AUO97b_02371" /db_xref="SEED:fig|400667.82.peg.2235"
/db_xref="GO:0003676"
/db_xref="GO:0004386"
/db_xref="GO:0005524"
/db_xref="GO:0008026"
/translation="MTSVHQLQGVGSASAALLEKLNIFTTDDLLFHLPRDYEDRSTII
PMNQLVVGRSYLLEGEVKSVDFPPGKRKSMAALIQDEFGKVTLRFYHIYKNLTDKIKP
GNRLRIFGEVRVGARGLELYHPEIQLINEHTPLPKTQLTAIYPSTDGLTQAKLREYVK
QALKHHSDALPELLPKQYTNGYALKEALHYIHEPPVDANMIQLAQGSHPAQQRLIFEE
LVAHQISLLTRRAYIRQIASPAFPSSKVLAKKLLEALPFQMTNAQKRVSKEILNDLKQ
HQPMLRLVQGDVGAGKTLVAAVAACHALEADWQVALMAPTEILAEQHYLNFKRWFEPL
GITVAWLSGKQKGKARAHAEQQIKEGHAELIVGTHALFQDNVEFAKLGLVIIDEQHRF
GVDQRLALRNKGAEQLTPHQLVMTATPIPRTLAMSAYGDLDTSIIDELPPGRTPIQTV
TIPLDRREEVLHRIASNCREGKQAYWVCTLVEQSETLDAQAAEATYQEMKERFPELNI
GLVHGKMKADEKQAVMQAFKNNELQLLIATTVIEVGVDVPNASIMVIENAERLGLSQL
HQLRGRVGRGAKASFCVLLYKPPLSQNGQERLSILRESNDGFVIAEKDLELRGPGELL
GTKQTGDMGFRVARLERDDHLLSQAHYVAQQVLKDYPEQADALLKRWLPEAPRYAYV" /product="ATP-dependent DNA helicase RecG (EC 3.6.4.12)" /transl_table=11
CDS 2391213..2392028
/locus_tag="AUO97b_02372" /db_xref="SEED:fig|400667.82.peg.2236"
/translation="MHILFIGYGKTSQRVAKQLFEKGNQITTISRSEKTDPYGTHLVQ
DIFTLDLSEIAPVDVVYILLSPDDSTVEGYQHTYVDSIEPIRQALKSHPVKRLIVVSS
TRVYGENSGETIDDHSEIHPNDAQGHILHNMELLWQKYFPSQCVIVRPTGIYGASIDR
LKRMAEHTQTYPNIHYSNRIHIDDLARFLAFLADFEKPHKSYLVANNAPVPLHEVLLW
FQSQLDLPLLTLDSAHVSGKKIYAKHLFETGFQLEHPICFNDYLLCLNAHGTN" /product="Nucleoside-diphosphate-sugar epimerases" /transl_table=11
CDS 2392174..2393094

/locus_tag="AUO97b_02373" /db_xref="SEED:fig|400667.82.peg.2237"
/translation="MWCAYDPAGTQGDITRRLNDIRLYAQQYQVKFKVVSYQKEQQAI
QAFDEGKCSGLAASNFNTYQYNHFMGSTSGIGLIPNNRTARNLLQLLNHPTIEKRLIN
KDYEAVGMIPVGTANMVMKTKKISKVAQLRGQRIGVLANNPPQQALVRSVGAQPVYVD
LSNAIAQFQQNKIDIMPAPVYGLLPYNLQKDFGPDTQVINFPLAYFGVNIIIKPQAYP
ANFGRKIRAWFVQNSQLLTNRAIQWENHLPAYYWVDVSFYEKQSYDIMVAKIRNQYVR
SGYYDAYFVELMKRLRCIDDPRYFECPMSR"
/product="AdeT"
/transl_table=11
CDS complement(2393138..2395711)
/locus_tag="AUO97b_02374" /db_xref="SEED:fig|400667.82.peg.2238"
/db_xref="GO:0004366"
/translation="MSKSGFGQMYRRLSSKLLDLVVTPHVLGEVPTEPVSATTETEAK
TERQKVVCYVLQNHSRSNALVVDGETRRLNLKPALDPLVIGTHQEKASVLFLQHNDEN
NLLNPPPHAFPPRLIKLIEVLQANPELDIELVPVTVLWGRSPDKEDSWFKLLFSDTWA
TPSTVKQLVNIGLHGRQSYLEFHEPQSLRDLVEYAQKHYPNLSPATYIVSTLNDYLDR
QREVVLGPDLSDRRNVMQSVLKSRDVQEAIRRESIRGKISMLEAERRAIGYVNEIVSD
YSHSAVRFADLALTRLWTQLYDGVEVHNFSTVRELAKDYEIVYTPCHRSHIDYLLLSY
VIYKRGLMVPYIAAGDNLNLPFVGQLLRGGGAFFIRRSFRGNGLYTSVFKEYLYSILS
RNTPLEYFIEGGRSRTGRLLPPKTGMLAMTVHSHLRGRAKPIVFVPTYIGYERLMEGS
TYVGEMQGKPKEAESIFGILKTLRKIERIFGKVHVNFGEPVFLDDLLKQHNAENVYIE
KNDDPVPPAVSEAVNSSANAILENINRAVVINPVSLLSIILLATPKHTLDEEICIKQL
EAYRNLASNFPYDQRTEVTPLSGKEIIAYGLKLKLIKRVQHALGDIIAIEDNQAVLLT
YFRNNILHAFVLPSLIASLVEHNGKISRADLSNVIYTLYPFLKAELFLKWKSSELKEQ
IEQYADALVQSNLIQQDDEGNLISSAPNTEEHNQLVVLATPVKQSLERYYMTLALITQ
RGSGNISAKQVEELSHLLGQRLSVLYEFNSPEFFDKALFQSFIKVLTQQNYIRTNDQG
QIEFDDNFRQMAAGAQLVLDEVTLQMLQHITTFTDEELKEALEAMASQQAKRRLKRKK A"
/product="Glycerol-3-phosphate acyltransferase (EC 2.3.1.15)"
/EC_number="2.3.1.15"
/transl_table=11
CDS 2395987..2396859
/locus_tag="AUO97b_02375" /db_xref="SEED:fig|400667.82.peg.2239"
/translation="MNALTQELVELLTLEKLEENIYRGISRNLVGKRVFGGQVLGQAL
RAASYTTDRPAHSLHAYFLYGGDINAPIIYEVDRLRDGKSFVSRQVRAIQHGRVIFSA
MVSFANPEEGLNYQHPEPDYPAPEALKSESELKEGILNFVPENVRASFMRERHVEIRP
IDPVNPFQPQPEAPFNAHYIRTHDRIPKQLDDISLHQAIVAFYSDFTLMTTALRPHGL
SYISPSLQCASIDHAIYFHRPLRADEWMLYDMEATVSAASRGLNFGRMWQNGQLVCST VQEGLMRLREIETQ"
/product="Acyl-CoA thioesterase II (EC 3.1.2.-)" /transl_table=11
CDS 2396993..2398231
/locus_tag="AUO97b_02376" /db_xref="SEED:fig|400667.82.peg.2240"
/translation="MNLNTQILIAAILGLAFGFLLTMYPDTAFVKHSLYGLGILSSVF
IGLLKMLLVPLIFSSIVVGVSNLQAGGQLGPVWKITFLCCITTTTLALILGISCAHLF
EVGRGVDISIFQASMQSYQTPDSLNPATFFTNFIQNTLINPFKAFSDGNVLAVVVFAL
FMGVALVHGGERFKSIRVLSHQFFEMMMMLVGWVMKLAPLGIFALLAKLMATEDISVL
SRLAEFAAVVTGTTIFHGAVVLPLLLWIFGRMSPWTFFKGTRTALITAFATSSSSATM
PLSMKCAQENLGVRPQTAGFVIPLGTQLNMDGTALYEAAAALFVANLVGLDLNLTQQI
VVCLTAMIASLGAPGIPSAGMVTMIMVLQSVGLPAEAIAILLPIDRLLDTVRTVVNVQ
GDMMISVVVDRYTKDKDISI"
/product="Sodium:dicarboxylate symporter precursor" /transl_table=11
CDS complement(2398274..2399221)
/locus_tag="AUO97b_02377" /db_xref="SEED:fig|400667.82.peg.2241"
/translation="MKLLSFLKNKALGSSIDYKILPRKVKFDWKDTPVDWIPNQPFAS
YFINEINNILPAGEFWFCRLYNKVLPRITDEKLKQDVQAFIRQEAMHANAHTSANKEY
LSARNIDIQRNLDIMNYLFTTALADKPFDKEVPQFLQKQWDLFRLGVIATVEHMTCVL

GKYALYNKRWEELGADPEMVDLVKWHGSEEIEHRTVAFDLYRHLGGGYIPRYYLSLAV
IVLVLGLWVDGAAHIMKQDPRFADAAKSRFFPAWVALEWYKISRKDNQVLPNPIWLIA
QQIDYLMPWYDPVKEGSTEDAVSYLSQSPAAKRAELQAA" /product="hypothetical protein"
/transl_table=11
CDS complement(2399348..2400124)
/locus_tag="AUO97b_02378" /db_xref="SEED:fig|400667.82.peg.2242"
/db_xref="GO:0005525"
/db_xref="GO:0005622"
/translation="MRRSEKSKDTKARLAPKQKISYEKKPDPAITAYAVQSLNWLRQA
EFLMSAPKLALCVEDSGYEIAFAGRSNAGKSSAINALTNQKQLARASKKPGRTQMINF
FSLGNPDQRLVDLPGYGYAAVPEDMKRVWQKELENYLIHRKSLQGLVLLMDIRHPLQH
FDMMMLEWAYSRHLFVHILLTKADKLNRGPANKVLLEVKQQLKKMKLDFSIQLFSSLN
KLGLEELASVMAGRLHFTLEQQPEFDVDAIPEASDEDAEE" /product="GTP-binding protein EngB"
/transl_table=11
CDS complement(2400233..2400670)
/locus_tag="AUO97b_02379" /db_xref="SEED:fig|400667.82.peg.2243"
/db_xref="GO:0005554"
/translation="MQLNKFTDYALRILMYVARPSDAPYTIAEIAQDLYVSQNHLVKV
VHFMGKQEWIITIRGKGGGIRLNPKALDLKLGTIVRILQGDHQIVECNTPPCVLRSHC
GLKGILDQALESFYQSLDQYTLGEVLQQPKSASDHSPIAFLQL" /product="Nitrite-sensitive
transcriptional repressor NsrR" /transl_table=11
CDS complement(2400756..2401520)
/locus_tag="AUO97b_02380" /db_xref="SEED:fig|400667.82.peg.2244"
/db_xref="GO:0008941"
/translation="MTPQQIELVKSTVPVLREHGVTLTTYFYKRMLNNNPELKNVFNL
DDQTSLRQPRALAAAVLAYAENIENPTVLAKAVERITTKHVSLDIQPDQYAIVGDNLL
HSISEVLNVPFESELIEAWKQAYLQLADILIGVEKQKYEQLESLKGGWAGWRSFEITQ
IDPLESGKRFTLKATDHEDVLTSPANAFISVKVQVPNQQLEQPKAFKFTEAQEDNTYH
FDVQPEEDHTEFSVSNILLEHYRVGDQVQVSAPLTL" /product="Flavohemoglobin / Nitric
oxide dioxygenase (EC 1.14.12.17)" /EC_number="1.14.12.17"
/transl_table=11
CDS complement(2401666..2403213)
/locus_tag="AUO97b_02381" /db_xref="SEED:fig|400667.82.peg.2245"
/translation="MGTPIVIAKNINDTSKEIVLLSQFANRHGLIAGATGTGKTVTLK
VLAESFSRIGVPVFLADAKGDVSSLAKAGEASDKFNERLKLLQIDSVPFAANPVVFWD
LFGQQGHPIRTTISEIGPILLARILNLNDTQEGVLSAVFRIADDQGLLLVDFKDLKAM
ITFVSEHAAEFKAEYGNLSPASLGAIQRNLLALADQGGEQFFGEPALNILDFIQTDAN
GHGYINILAADKLMNTPKLYATFLLWMLSELFEQLPEVGDMDKPKLVFFFDEAHLLFD
NASQALQEKIEQVVRLIRSKGVGIYFITQNPLDLPESVLGQLGNRVQHALRAFTPKDQ
KAVKTAADTFRSNPDFKVDEAITELGVGEALISCLDEQGTPQIVERGWVMPPYSSFTP
ITPEERQTLISQSIIAGVYEQAVDRDSAYEMLQNKVAEREQQKQDAELAKQQAKEQET
LAKQQAKEQERLAREQQKEEERAARERAKLTQDIVGTFAKSAARSLGGSTGQKLVRGL LGSLFGK"
/product="Uncharacterized protein YjgR"
/transl_table=11
CDS complement(2403257..2404447)
/locus_tag="AUO97b_02382" /db_xref="SEED:fig|400667.82.peg.2246"
/db_xref="GO:0004121"
/translation="MKKHNNFQTQLIHAPRKAPQFIETVQPPLFRASTIIFKSTSHLF
DRHWTDPYDYSYGTHGTPTTFTLGDNIAQIEGGLYCLLAPSGLSAINLVNSAVLATGD
EVWVPDNIYGPNLEHLNYLKDQYGINVQVYNPIDVSSFQPSDKTKLLWLEAAGSVTLE
FPDLKALVKKAKAHGVLTALDNTWGAGLAFNAFDFSDEHLSVDLTIHALTKYPSGGGD
ILMGSVVTRDEKLHHRLFRMHAILGISVSGDDTAQIQRSLAHMSLRYEQQSNNAKTLL
TWLKEQSQFAQVLHPSDKASPGHQYWQEICSTGRSAGLVSVVFKSDYDISAVRRFCDA
LKLFKLGFSWGGPVSLVMLYDLKMMRKLENTHLQQGLLVRFCIGLEDPQDLIQDIENA LKQM"

/product="Cystathionine beta-lyase (EC 4.4.1.8)" /EC_number="4.4.1.8"
/transl_table=11
CDS 2404615..2405343
/locus_tag="AUO97b_02383" /db_xref="SEED:fig|400667.82.peg.2247"
/translation="MKSHLRVHYFQHIAGEGFGSCYSFLKAHHATITATEFFALPVDL
PLEIEALPHIEDVDLLLIMGGTMSVNDEANYPWLKIEKRWIRRYLAAGKPAIGLCLGG
QLIANALGAAVSRNRYQELGWSTVQRVPNLPKESFLLPEKINVMQWHSETFEIPKGAI
HLAENSVCRNQMYQIGSNVLGFQFHPEMTPKVLNLLLENEQELSIFKGEYVQSLDELH
HCDIQKFEQGNQLLNRAIEFVVNQ"
/product="Glutamine amidotransferase, class I" /EC_number="6.3.5.2"
/transl_table=11
CDS 2405595..2405906
/locus_tag="AUO97b_02384" /db_xref="SEED:fig|400667.82.peg.2248"
/db_xref="GO:0003735"
/db_xref="GO:0005622"
/db_xref="GO:0005840"
/db_xref="GO:0006412"
/translation="MSNQRIRIRLKSFDHRLIDQSAQEIVETAKRTGAQVCGPIPMPT
RIERFNVLTSPHVNKDARDQYEIRTYKRLIDIVQPTDKTVDALMKLDLAAGVDVQIAL G"
/product="SSU ribosomal protein S10p (S20e)" /transl_table=11
CDS 2405964..2406602
/locus_tag="AUO97b_02385" /db_xref="SEED:fig|400667.82.peg.2249"
/db_xref="GO:0003735"
/db_xref="GO:0005622"
/db_xref="GO:0005840"
/db_xref="GO:0006412"
/translation="MAIGLVGRKCGMTRIFTDAGVSVPVTVIEVDPNRITQIKTLETD
GYQAVQVTTGERRESRVTNAQKGHFAKAGVAAGRLVKEFRVTEAELEGREVGGTIGVD
LFTVGQIVDVTGQSKGKGFQGGVKRWNFRTQDATHGNSVSHRVLGSTGQNQTPGRVFK
GKKMAGHLGDERVTVQGLEIVSVDTERSVLVVKGAIPGATGGDVIVRPTIKA" /product="LSU ribosomal protein L3p (L3e)"
/transl_table=11
CDS 2406614..2407216
/locus_tag="AUO97b_02386" /db_xref="SEED:fig|400667.82.peg.2250"
/db_xref="GO:0003735"
/db_xref="GO:0005622"
/db_xref="GO:0005840"
/db_xref="GO:0006412"
/translation="MNLKTVSGSAVELSEVAFGREFNEALVHQVVTAYLAGGRQGTRA
HKSRADVSGGGKKPFRQKGTGRARAGSIRSPIWVGGGKTFAARPQDWSQKVNRKMYRG
AMQCILAELVRQDRLVLVEEFAVAAPKTKELLAKLNDLNAARALIVTDAVDENLYLAA
RNLPHVDVVDATAIDPVSLIAFDKVVMSVAAAKKIEVELG"
/product="LSU ribosomal protein L4p (L1e)"
/transl_table=11
CDS 2407213..2407533
/locus_tag="AUO97b_02387" /db_xref="SEED:fig|400667.82.peg.2251"
/translation="MNNERIYQVLKGPVFSEKAQVLGDTAGVQVFKVDINATKLEIKK
AVEKLFGVEVVKVNTTITKGKTKRFGRTLGRRSDVKKAYVTLKAGQDVEMADLGDTAE SAAE"
/product="LSU ribosomal protein L23p (L23Ae)" /transl_table=11
CDS 2407545..2408369
/locus_tag="AUO97b_02388" /db_xref="SEED:fig|400667.82.peg.2252"
/db_xref="GO:0003735"
/db_xref="GO:0005622"
/db_xref="GO:0005840"

/db_xref="GO:0006412"
/translation="MPIQKCKPTSPGRRFVEKVVHDHLHKGAPYAPLVEAKKRTGGRN
NNGHITTRHVGGGHKQHYRIVDFKRNKDGVPAVVERIEYDPNRTAHIALLKYADGERR
YIIAPKGLRAGDKVQSGNDAPIRPGNCLPLRNMPIGSTLHNVELKIGKGAQLARSAGA
SVQLLGRDGSYAIIRLRSGEMRKVHVECRAVIGEVSNQENNLRSLGKAGAARWRGVRP
TVRGMAMNPIDHPHGGGEGRNKGIQPVSPWGQKAKGYKTRTNKRTTKMIIRDRRVK" /product="LSU
ribosomal protein L2p (L8e)"
/transl_table=11
CDS 2408383..2408658
/locus_tag="AUO97b_02389" /db_xref="SEED:fig|400667.82.peg.2253"
/db_xref="GO:0003735"
/db_xref="GO:0005622"
/db_xref="GO:0005840"
/db_xref="GO:0006412"
/translation="MPRSLKKGPFVDAHLFAKVEAAVASNSRKPIKTWSRRSMILPDF
VGLTISVHNGRNHVPVIVTEHMVGHKLGEFAPTRTYRGHGVDKKSKR" /product="SSU ribosomal
protein S19p (S15e)" /transl_table=11 CDS 2408670..2408999
/locus_tag="AUO97b_02390" /db_xref="SEED:fig|400667.82.peg.2254"
/db_xref="GO:0003735"
/db_xref="GO:0005622"
/db_xref="GO:0005840"
/db_xref="GO:0006412"
/translation="MEVTAKLRGAAISAQKARLVADLIRGKSVAHALNILNFSNKKAA
VLVKKALESAIANAEHNNSLDVDDLKVSTIYVDEGMSLKRIMPRAKGRADRITKRTCH ITVKVGV"
/product="LSU ribosomal protein L22p (L17e)" /transl_table=11
CDS 2409001..2409753
/locus_tag="AUO97b_02391" /db_xref="SEED:fig|400667.82.peg.2255"
/db_xref="GO:0003676"
/db_xref="GO:0003735"
/db_xref="GO:0005622"
/db_xref="GO:0005840"
/db_xref="GO:0006412"
/translation="MGQKVHPIGIRLGVVKRHNANWYANPKQYAEYLLKDLQVREFLT
KKLKNAMVSNILIERPSGAAKVTISTARPGIVIGKKGEDIEKLQRELTNIMGVPAQVS
INEIDRPDLDARLVAEAIASQLEKRVMFRRAMKRAVQNTMRAGAKGIKVEVSGRLGGA
EIARTEWYREGRVPLHTLRADIDYATMRAETTYGTIGVKVWIFRGEILGGMKQVMNPA
PAEERPAKRGRGRGEGQERRGRRGDRAADKGE"
/product="SSU ribosomal protein S3p (S3e)"
/transl_table=11
CDS 2409757..2410170
/locus_tag="AUO97b_02392" /db_xref="SEED:fig|400667.82.peg.2256"
/db_xref="GO:0003735"
/db_xref="GO:0005622"
/db_xref="GO:0005840"
/db_xref="GO:0006412"
/translation="MLQPKRTKFRKVHKGRNTGLAHRGSTVSFGSIAIKATERGRMTA
RQIEAARRTISRRIKRGGKIFIRVFPDKPITEKPLEVRMGNGKGNVEYWVCEIKPGKI
LYEIEGVNEDLAREAFALAAAKLPFKTTIVTRTVM" /product="LSU ribosomal protein L16p
(L10e)" /transl_table=11
CDS 2410170..2410367
/locus_tag="AUO97b_02393" /db_xref="SEED:fig|400667.82.peg.2257"
/db_xref="GO:0003735"
/db_xref="GO:0005622"
/db_xref="GO:0005840"

/db_xref="GO:0006412"
/translation="MKTKDLREKSVEELKALLDEQQLNQFRLRMAKATGQLGKSHEVQ
VARKTIARIKTLLTEKQGNGQ"
/product="LSU ribosomal protein L29p (L35e)" /transl_table=11
CDS 2410364..2410621
/locus_tag="AUO97b_02394" /db_xref="SEED:fig|400667.82.peg.2258"
/db_xref="GO:0003735"
/db_xref="GO:0005622"
/db_xref="GO:0005840"
/db_xref="GO:0006412"
/translation="MSEKTVRTLTGKVVSDKMDKSIVVLIERRVQHPLYGKSIRRSTK
LHAHDENNVAKIGDVVTIKESRPISKTKAWTLVEVVEAAAE"
/product="SSU ribosomal protein S17p (S11e)" /transl_table=11
CDS 2410714..2411082
/locus_tag="AUO97b_02395" /db_xref="SEED:fig|400667.82.peg.2259"
/db_xref="GO:0003735"
/db_xref="GO:0005622"
/db_xref="GO:0005840"
/db_xref="GO:0006412"
/translation="MIQTETMLDVADNSGARRVQCIKVLGGSHRRYASVGDIIKVTVK
EAIPRARVKKGDVMNAVVVRTKFGIRRPDGSVIRFDDNAAVILNNNKAPIATRIFGPV
TRELRTEQFMKIISLAPEVL"
/product="LSU ribosomal protein L14p (L23e)" /transl_table=11
CDS 2411092..2411409
/locus_tag="AUO97b_02396" /db_xref="SEED:fig|400667.82.peg.2260"
/translation="MAKIKKGDQVIVIAGKEKGKQGTVLSVSEDRVKVEGLNLVKKHQ
KPNRVTGAEGGIVTQEASLHISNVAILNATTQKADRVGYQVIDGVKTRVYKSTGESVA VAK"
/product="LSU ribosomal protein L24p (L26e)" /transl_table=11
CDS 2411425..2411961
/locus_tag="AUO97b_02397" /db_xref="SEED:fig|400667.82.peg.2261"
/db_xref="GO:0003735"
/db_xref="GO:0005622"
/db_xref="GO:0005840"
/db_xref="GO:0006412"
/translation="MARLKARYNDELKAKLQEELSIKNVMEIPRITKITLNMGVGAAA
TDKKLLDGAVADMQLIAGQKPVVTLARKSIAGFKIRDGWPIGCKVTLRGDQMYEFLDR
LISIAIPRIRDFRGFSAKSFDGRGNYSMGLKEQIVFPEIDFDKIDRIRGMDITITTTA
RTDDEGRALMRAFGFPFK"
/product="LSU ribosomal protein L5p (L11e)" /transl_table=11 CDS
2411971..2412276
/locus_tag="AUO97b_02398" /db_xref="SEED:fig|400667.82.peg.2262"
/db_xref="GO:0003735"
/db_xref="GO:0005622"
/db_xref="GO:0005840"
/db_xref="GO:0006412"
/translation="MAKKGMINRELKREKTVAKYAAKRAELKATIANVNASDEERFEA
MLKLQALPRNASPVRLRNRCGLTGRPHGYFRKFGLSRNKLRDTVMQGDVPGVVKASW" /product="SSU
ribosomal protein S14p (S29e) @ SSU ribosomal protein S14p (S29e), zinc-
independent" /transl_table=11
CDS 2412288..2412683
/locus_tag="AUO97b_02399" /db_xref="SEED:fig|400667.82.peg.2263"
/db_xref="GO:0003735"
/db_xref="GO:0005622"
/db_xref="GO:0005840"

/db_xref="GO:0006412"
/translation="MSMQDTVADMLTRVRNAQMAKKQTVSMPSSKLKVAIANVLQQEG
YISNVEVAQEETKSTLTITLKYFEGKPVIEMVKRVSRPGLRQYRGKDKLPSVKQGLGI
AIVSTSKGIMTDRAARAAGIGGEVIAFVS"
/product="SSU ribosomal protein S8p (S15Ae)" /transl_table=11
CDS 2412697..2413230
/locus_tag="AUO97b_02400" /db_xref="SEED:fig|400667.82.peg.2264"
/db_xref="GO:0003735"
/db_xref="GO:0005622"
/db_xref="GO:0005840"
/db_xref="GO:0006412"
/translation="MSRVAKAPVTVPNGVTVTQNGRQVEVKGSKGTLSFNLHALVELK
QEEGKLQLAPAKESKDAWMQAGTARAVLNNLVKGVSEGFERKLQLVGVGYKAAVKGTV
VNLNLGYSHPIDYALPEGVTAETPTATEIILKSANKQLLGQVAAEIRAYRSPEPYKGK GVRYSDEVILRKEAKKK"
/product="LSU ribosomal protein L6p (L9e)"
/transl_table=11 CDS 2413245..2413595
/locus_tag="AUO97b_02401" /db_xref="SEED:fig|400667.82.peg.2265"
/db_xref="GO:0003735"
/db_xref="GO:0005622"
/db_xref="GO:0005840"
/db_xref="GO:0006412"
/translation="MNEKKQSRLRRAKSTRLHIRALGATRLCVNRTPRHIYAQVISAD
GGKVLAQASTLDASLRSGTTGNIEAATKVGALIAERAKAAGVTKVAFDRSGFKYHGRI KALADAAREGGLEF"
/product="LSU ribosomal protein L18p (L5e)" /transl_table=11
CDS 2413598..2414095
/locus_tag="AUO97b_02402" /db_xref="SEED:fig|400667.82.peg.2266"
/db_xref="GO:0003735"
/db_xref="GO:0005622"
/db_xref="GO:0005840"
/db_xref="GO:0006412"
/translation="MAKVEQNEGLVEKLVAVDRVAKVVKGGRIFSFTALTVVGDGNGR
VGFGRGKAREVPAAISKALEAARRNMITVDLAGTTLQHPVNARHGASRVYMQPASEGT
GVIAGGAMRAVLEAAGVHNVLAKCYGSTNAANVVNATFKGLRDMTSPEKVAAKRGKSV EEIQG"
/product="SSU ribosomal protein S5p (S2e)"
/transl_table=11
CDS 2414102..2414278
/locus_tag="AUO97b_02403" /db_xref="SEED:fig|400667.82.peg.2267"
/db_xref="GO:0003735"
/db_xref="GO:0005622"
/db_xref="GO:0005840"
/db_xref="GO:0006412"
/translation="MKTIKVTQTKSSSHRLKNHKLCLQGLGLRRIGHTVEVQDTPSNR GMINKVYYMVSVEE"
/product="LSU ribosomal protein L30p (L7e)" /transl_table=11
CDS 2414282..2414722
/locus_tag="AUO97b_02404" /db_xref="SEED:fig|400667.82.peg.2268"
/db_xref="GO:0003735" /db_xref="GO:0005622"
/db_xref="GO:0005840"
/db_xref="GO:0006412"
/translation="MTLRLNELAPAEGAKREHRRLGRGIGSGVGKTGGRGIKGQKSRK
SGGVRPGFEGGQTAIYRRLPKFGFTSQIALKTAEVRLSELSKVEGDIVSLETLKAANV
VRRDQIRARIVLSGEITRAFTVQGVALTKGAKAAIEAAGGKVEE" /product="LSU ribosomal protein
L15p (L27Ae)" /transl_table=11
CDS 2414729..2416087
/locus_tag="AUO97b_02405" /db_xref="SEED:fig|400667.82.peg.2269"

/db_xref="GO:0009306"
/db_xref="GO:0015450"
/db_xref="GO:0016020"
/translation="MSMSPSSSGHVNMMKGQPFHVKYREIIRRMSFLIGALLVFRLGA
HIPVPGINNAALENLFHANQGTILGLFNMFSGGALERMSILALGIMPYISASIIVQLM
STVIPSLEALKKEGEQGKRKINQYTRQGTLLLAVVQAVGMCAGLIGQGITLSSGLAFY
IPAVTSLVAGTMFLMWLGEQITERGIGNGISMIIFAGIVAGLPKLIMQSVSSVDNGQT
SLIGLVIFGLLSLGVLAAIVFIEKAQRRIPVNYAQKQQGRRIFTAQQTHLPLKINMAG
VIPAIFASSLLLFPASLGQWVGSADPNAGLIKRSLQDLALVLSPGQPLYLVLFGALII
FFCYFYTALVFSPKEVSENLKRSGAYVPGIRPGEQTARYLDHILNRLTFIGAIYITVI
CLMPMILQSSFGIPFYLGGTSLLIVVVVVMDFMAQLQAHLTSHQYDNQTLMRKTTAHP KG"
/product="Protein translocase subunit SecY" /transl_table=11
CDS 2416108..2416224
/locus_tag="AUO97b_02406" /db_xref="SEED:fig|400667.82.peg.2270"
/db_xref="GO:0003735"
/db_xref="GO:0005622"
/db_xref="GO:0005840"
/db_xref="GO:0006412" /translation="MKVQASVKKICGSCKVIRRNGVIRVICSAEPRHKQRQG"
/product="LSU ribosomal protein L36p @ LSU ribosomal protein L36p, zinc-dependent" /transl_table=11
CDS 2416332..2416688
/locus_tag="AUO97b_02407" /db_xref="SEED:fig|400667.82.peg.2271"
/db_xref="GO:0003723"
/db_xref="GO:0003735"
/db_xref="GO:0005622"
/db_xref="GO:0005840"
/db_xref="GO:0006412"
/translation="MARIAGVNIPDNKHAVISLTYIFGIGRHTAKNILAAVGITETTK
IRELDDAQLDAIRAEVAKVPTEGDLRREISMNIKRLMDLGCYRGLRHRRSLPVRGQRT KTNARTRKGPRKPIKK"
/product="SSU ribosomal protein S13p (S18e)" /transl_table=11
CDS 2416708..2417094
/locus_tag="AUO97b_02408" /db_xref="SEED:fig|400667.82.peg.2272"
/db_xref="GO:0003735"
/db_xref="GO:0005622"
/db_xref="GO:0005840"
/db_xref="GO:0006412"
/translation="MAKDTRTRKKVTRTVSEGVAHIHASFNNTIVTITDRQGNALAWA
TSGGQGFRGSRKSTPFAAQVAAEVAGKAALDYGLKNLDVLVKGPGPGRESAVRALGAV
GYKINSITDVTPIPHNGCRPPKKRRV"
/product="SSU ribosomal protein S11p (S14e)" /transl_table=11
CDS 2417107..2417733
/locus_tag="AUO97b_02409" /db_xref="SEED:fig|400667.82.peg.2273"
/db_xref="GO:0003723"
/db_xref="GO:0003735"
/db_xref="GO:0005622"
/db_xref="GO:0005840"
/db_xref="GO:0006412"
/translation="MARYIGPKCKLSRREGTDLQLKSGVKPFDVKTKKANKAPGQHGQ
ARGGKQSEYSLQLREKQKVRRIYGVLERQFSNYYKEAARVKGATGENLLKLLESRLDN
VVYRMGFGSTRAEARQLVSHRSITLNGRRVNIASIQVKAGDVIAVHEGAKQQLRIKNA
IELAAQRGIPAWIEVDHSKLEGTFKAAPDRSDLPAEINESLIVELYSK" /product="SSU ribosomal protein S4p (S9e) @ SSU ribosomal protein S4p (S9e), zinc-independent"
/transl_table=11
CDS 2417751..2418758

/locus_tag="AUO97b_02410" /db_xref="SEED:fig|400667.82.peg.2274"
/db_xref="GO:0003899"
/translation="MTRTANEFLTPQAIKVEAVSGTSAKVILEPLERGFGHTLGNALR
RILLSSLPGAAVVEVEIEGVEHEYSTLEGLQQDIVELLLNLKGLSIKLFDQNEAYLTL
EKQGPGDITAADLRLPHNVEVVNPEHLIGTLSATGSLKMRLKVSQGRGYETSDSRFPE
GETRPVGRLQLDASYSPIKRVSYTVENARVEQRTDLDKLVIDLETNGTVDPEEAIRKA
ATILQQQIAIFVDLQKDQTPVAQEPREEVDPILLRPVDDLELTVRSANCLKAENIYYI
GDLVQRTEVELLKTPNLGKKSLTEIKDVLASKGLQLGMRLENWPPASLRMDDRFAYRS R"
/product="DNA-directed RNA polymerase alpha subunit (EC 2.7.7.6)"
/EC_number="2.7.7.6"
/transl_table=11 CDS 2418777..2419154
/locus_tag="AUO97b_02411" /db_xref="SEED:fig|400667.82.peg.2275"
/db_xref="GO:0003735"
/db_xref="GO:0005622"
/db_xref="GO:0005840"
/db_xref="GO:0006412"
/translation="MRHRNSGVKLGRTSSHRKAMFQNLANSLFEHELIKTTLPKAKEL
RRVAEPLITLAKNDTVANRRLAFARTRNAATVGKLFTVLGPRYKERNGGYLRVLKAGF
RAGDAAPMAYVELVDREVNTSAE"
/product="LSU ribosomal protein L17p"
/transl_table=11
CDS 2419336..2420826
/locus_tag="AUO97b_02412" /db_xref="SEED:fig|400667.82.peg.2276"
/translation="MEKQVDVLIIGAGISGIGVAAHLSKNSPQRQFEILERRESFGGT
WDLFRYPGIRSDSDMSTFGFNFKPWRKANVLADGASIKGYLGEVIDEFKLKEKIHFGH
RVLSANYDSASKKWHVIVEDNKKKQQTWVANFVVGCTGYYNYDQGYAPTFPNQEAFKG
QLIHPQHWPENLDYKGKKVVIIGSGATAITLVPAMSKGGAGHVTMLQRSPTYIASVPS
IDFIYEKTRRFMSEEAAYKFTRARNIGMQRAIYALSQKHPKTVRRLLLKAIEMQLKGK
VDMKHFTPSYNPWDQRLCVVPDGDLFKILREGKASVETDQIEKFTETGIQLKSGKHLD
ADIIVSATGLQVQIMGGVQATIDGEPVKSSEHMLYNGVMLSDVPNMAMIIGYVNASWT
LKVDIAADYICRLLNYMDKNNYDEVIPSGDKSVMEEDTVMGSLSSGYISRAADVIPKQ
GKQAPWQVTNNYLEDRKSLKNAKFDDGVLRFHKHSEVTTRKPKLVS" /product="Monooxygenase,
flavin-binding family" /transl_table=11
CDS complement(2420876..2422048)
/locus_tag="AUO97b_02413" /db_xref="SEED:fig|400667.82.peg.2277"
/translation="MQSGAIRPIPNMLPRHLFNEEHEAFRETVRKFYEKEVVPNIEKY
EKQQHVDRDLWNKAGALGLLCTTMPEQYGGSGVDRLYSMILIEEQAYAMDSSTGFSLH
SDIVANYINNFGNEEQKQKWLPKMATGETVTAIAMTEPGTGSDLQAVRTTAVLDGDEY
VINGSKIFITNGYLCDMAIVVCKTGNSDKGSANLSLIIVEADRTGFTKGKPLNKIGMK
GQDTCELFFDNVRVPKENLLGMEGMGFIMLMKELAWERMLVAIICQAGAEAAFAHTVQ
YTKDRKAFGKPIGAFQNTRFKLAELRTEIDFCRTYLDRCMELQLDEKLSVEAAAAAKY
KISDMFSKVVDECLQLHGGYGYMMEYPIARAYIDHRANRIYAGTNEIMKELISRTL" /product="Acyl-CoA
dehydrogenase"
/EC_number="1.3.8.1"
/transl_table=11
CDS 2422236..2422493
/locus_tag="AUO97b_02414" /db_xref="SEED:fig|400667.82.peg.2278"
/translation="MSEEMTLEERKVIYRARRGLKEIDVYFDPYVKNYYLKADPAEKA
LFAELVEQEDPDLLDWFMEVSEPPRTELRDFIYKLKHYVHG" /product="Succinate dehydrogenase
flavin-adding protein, antitoxin of CptAB toxin-antitoxin" /transl_table=11
CDS 2422500..2422889
/locus_tag="AUO97b_02415" /db_xref="SEED:fig|400667.82.peg.2279"
/translation="MINHLNFKLKYSRFSIIFQFFIGLSLAVLFYQLMPLLWWLVVIS
LLFINFIFFLKRPQLAQIAYLDKKLWSLAYHSQNTISRVKILKIIDYQIFIVIYFEGH

KKLTSIIWFDQMSLTEWKKLKTLEKLY"
/product="FIG00350107: hypothetical protein" /transl_table=11
CDS 2423659..2424744
/locus_tag="AUO97b_02416" /db_xref="SEED:fig|400667.82.peg.2280"
/translation="MSKVQKICQGLAVSFLAASVLSGCSYVVKSGANVALGFTEKHVV
PPILAMQDAEMVCNTGSALTPAIMSTKGMGADPTRVAVLMYAASGVCAENQALEQELR
YLRASKAGQVTEAQDARIAQKRWAAIAAERQYAGYKLFADRWESKYKYHLGDSCPTMR
SDIDQTVYLLGMISGLQAVTNDINSGGSVNVPKDIAAIVERGMVCLDNQKFWGAPNAT
RAVIWTLLPGANEGKPDPYETLKQSVQIGEQKGVRLSHALYAVAAQASGDDAKIRDAL
KTYAASQADDKPVNPNFKLIDSMAGLIVRGISDRYWTEHTGVRTGDDGMSHFWDEKNE
GSAELDELFDGGAASEAPADTKTAPAQ"
/product="FIG00350353: hypothetical protein" /transl_table=11
CDS 2424823..2425389
/locus_tag="AUO97b_02417" /db_xref="SEED:fig|400667.82.peg.2281"
/translation="MKDHVLNKKVLLVAGCISVSACLQVFPDYFIYWRESLLGEFWRL
WTAHWVHVGWIHFFLNMMAFACLPFIFPHTKVWHILSLLFLLPPFISLVFYFYLPNID
AYAGLSGVLHGLYTAVALVYLQYRKERNFAFLVLGLIVAKLIWENTFGQTGTAQLIGS
PVLTEAHLYGAIGGAIFGGCYWLIQRFK"
/product="Membrane protein, Rhomboid family" /transl_table=11
CDS 2425577..2427772
/locus_tag="AUO97b_02418" /db_xref="SEED:fig|400667.82.peg.2282"
/translation="MQEYQEKRSGLGLLGYIWNEWISTFIILILLLMTLIIGTGEMIH
GQLLRIGERLYGDPATGMQYSFLRAEPEKPTCDRHPNIDAQVKEQMKANASDDFASFF
GAASESDVRASLLAAQQQCEEKYQAYDKTIKYIEANPGVRTYRAIETGFFGIFKFGTE
NRAILLVFMVLISAITASLKYHHIGLRNPATKIDYKVYSLFMLIGNALLSFSVISQYR
SVINSGVTPTYETVTIYWIWMILFVVLTLISLYQLFVSPPPKREGGNIGLALLSVPLY
AYMALITGIVFTFFMDYPMGQGIYLGLLVEFSGIFLNLALFIWAGMLLTQTRVMDLFL
NVLRPWNLAPETLTWLILIAAAVPTAYTGASGIFVIAAGAIIYKEVWNAGARRQYALA
VSAMSGSLGVVVRPCLLVVLIAMLDSRHVTSDELFGHGIYVFWLTAFIFLGVSLLLAE
EKFRVNSPKVALPGMARAFVPVIPYILITVVVLAFYKYALDTGMNEFTAPVILPLVLI
AMILYDKMVMPKDAPALNIHEAIVREHEKKSPFLQTHDPHSSQFRLGFGGALRFATSE
TVGHIGALIILMALSASVGGLIERSEVVELLPTHLGSIYISLACIALLLAIIGMCTDP
FGAVILVAATIAPVAYENGIHPIHFWMIVLVAFEFGYVTPPVALNHLLTRLSVGDDEV
NAADAEAKEKYTSFYYRYERWLLPIIVLFSSLVLVTYAPLILKLFGWYK" /product="C4-dicarboxylate transport system permease large protein" /transl_table=11 CDS complement (2427921..2428352)
/locus_tag="AUO97b_02419" /db_xref="SEED:fig|400667.82.peg.2283"
/translation="MPLQRKQNSKLLWILIGGLSILVVLFFAAQWFFKKTEISAPMVD
PQPASVPAPEKTTNPSESSEPVVQTQTANSQQLVNEDVLKQPIPAQESLAKEEVSKLN
DIHRQLQDQEATLKAQHADADQLIKLKEEQVKLLEAQVKQQ" /product="hypothetical protein" /transl_table=11
CDS complement(2428364..2428573)
/locus_tag="AUO97b_02420" /db_xref="SEED:fig|400667.82.peg.2284"
/translation="MKKRFIAGAKCPKCEAIDRIVMLTTAEDEWIECIECGYSENRPT
HIDEPETPAIPDEIGVIQFKPRRSD"
/product="Cytoplasmic protein, probably associated with glutathione-regulated potassium-efflux" /transl_table=11
CDS complement(2428614..2430653)
/locus_tag="AUO97b_02421" /db_xref="SEED:fig|400667.82.peg.2285"
/translation="MTLQQATLPTPRFDQITLDNLKQDIQQAIQAGQEFLNTLTAAPS
ETDQQLAVLEQIDALENNLSESWGVLSHLNAVMNNAETREVYQSLLPALSEYYTQLGQ
HTALYQTYQLIHDGQSYSTLSPAQQSAIRLALRDFKLSGVALEGEEKKRYAEISARLS
QLSSDFSNHVLDATQAYFKPLTEDQLKGLPESNVELLKQYGKQRELDQAVATLDFPAY
FAIMTYADDRALREELYKAYVTRASDQSEQTEFDNSKIMEEILSLRQEMAKLLGFNNY

AEYSLASKMAPDVKTVHDFLVDLAEHARTPALQEVAELQAIAKQDGIDELKPWDTTYY
SEKLKQQQFNLSQEALKPYFPAPKVIQGLFQIVQRLYGINIVEREAPVWHPDARYFEL
EDQGEVIGGFFFDLYARTGKRGGAWMSGFRSRMQTVHGLQKPICYMVCNFTPPVGNQP
ALLTHDEDITLFHEFGHGLHHMLTEVDNISVAGTHGVAWDAVELPSQFMEFWSWDKES
LDVLSEHIESKQTLPQELLDALLNARFFQSGMQTLRQLEFALFDLTIHTKTPALNSEQ
IQQTLNDIRKQYAVVPTVDYNRFQHSFSHIFAGGYAAGYYSYKWAEVLASDAFDRFEN
EGIFNTQTGKEFRQAILAVGGKDTALEAFVNFRGREPKIDALLRHQGWTNDNKTA"
/product="Oligopeptidase A (EC 3.4.24.70)" /EC_number="3.4.24.70"
/transl_table=11
CDS complement(2430802..2433219)
/locus_tag="AUO97b_02422" /db_xref="SEED:fig|400667.82.peg.2286"
/translation="MTKNWVDPEAKAEAERYDNPIPSRTLILDTLEQLQTPQSHAELV
DHFHIQDQKSIEALSHRLSAMVRDGQLMKDGFKFQLVADQPTYEATVYINSKGLGTAH
IDGQDDLLLPERELRLVFNGDRVRVRQTSVDRKGKPWGFITEVIQHRVKQLIGKLSVH
DGEYFIQPNNPNQHQPIPLEKELVEHAKANVGDMLRVAIDTYPTREEFATAHIIQSMA
DKADTEIIIPQTILEFGLPYEFPDEVIKEAESFKEPAEKDREGRVDLRDLALVTIDGE
DARDFDDAVYAEKRPGGGYRVVVAIADVSHYVRLDSALNEEAEERGTSVYFPHFVLPM
LPEALSNGLCSLNPHVDRLCMVCDLKLSRTGRVTGYEFYPAVMHSKARLTYTQVGQYF
EGATDAIPKDRDIHKSLNTLFQLYQILKNLRVDRHAMEFETIETYMTFDELGGIKEIL
PRTRNDAHKLIEECMLLANVAAAEYALEHDIPMLYRVHEAPEFSRIQKVKDFVKLLGL
PFPDQPTQADYQKVIEATKDRIDAPSIHAVLLRSMMQAYYGAKNAGHYGLAYEAYTHF
TSPIRRYPDLLLHRAIKAHLKQKPYPLSGAALDDAGEHFSQTERRADEASRSVTTWLK
CHYMQQHLGDEFIGIVSAVTEFGLFVTLKDLYVDGMIHISQLGDDFFIYDQASQNLIG
QNRGQVFGLGDEVKIQVAGVNLEERKIDFQLIKQVTHAGRVVRSRAPRTTKTSTQATE
EVFGKPSVSTDGEKPVRKKKEKGKPSSYSKKSGKKTSTKAEAKPEKVKKKAKPKKKKS NAKSKAD"
/product="3'-to-5' exoribonuclease RNase R" /transl_table=11
CDS 2433882..2434475
/locus_tag="AUO97b_02423" /db_xref="SEED:fig|400667.82.peg.2287"
/db_xref="GO:0004647"
/translation="MNLALFDFDGTIIYNDTFSLFLKFSLSKKTQILGGIRLAPYIAG
YKLGWVTDKTIRTKLCQVGYVGYDAASLIYKGQEFAKKVIPSCIRENALERILWHKQQ
GDQVVVVSASLSVYLESWCKSLDLDVICNQLEIDNGILTGCFIDGDCGYLEKVNRIKN
KYDLTQYSIIYAYGDTPNDYAMLELAHRKYYKWQEIN" /product="Phosphoserine phosphatase
(EC 3.1.3.3)" /EC_number="3.1.3.3"
/transl_table=11
CDS complement(2434535..2436901)
/locus_tag="AUO97b_02424" /db_xref="SEED:fig|400667.82.peg.2288"
/db_xref="GO:0047632"
/translation="MFLLKNLVSSISKVTQDLGNIVSITPVVNTGSSVNVNVSDINIA
NVSTTGLLSNVISTVTDTVSHTTTDLVSNVVGTVTGTVGSTNPIDTVTNIIGGVTGGV
VGGTSPISPVIDVVQGGIDILQGVESLKTEIINTGIETVADAVIGVLPQAEHPVSEIA
DLGTLTFETSRDTVNGALEVVSDLVGGDIQGATESATGILDTLITNGTTATGIVTGVV
GSVTDIIGGVTGGVDGNPLEVITDIIGGVTGGVDGNPLEVITDIIGGVAGGVGGDNPL
GVVTDIIGGVTGGIIGGGTSPISPVIDVVQGGIDILQGVESLKTEIINTGIDTVADTI
IGVLPQAEHPVSEIADLGTLTFETSRDTVNGALEVVSDLVGGDIQGATENATGILGTL
IQNGSTATGIVTDILGGVTGTIGGVTGGIGGDNPLGVVTDIIGGITGGIGGDNPLGVV
TDIIGGVTGGIGGDNPLGVVTDIIGGVTGGIGGDNPLGVVTDIIGGVTGGIGGDNPLG
VVTDIIGGVTGGIIGGGTSPISPVIDVVQGGIDILQGVESLKTEIINTGIDTVADTII
GVLPQAEHPVSEIADLGTLTFETSRDTVNGALEVVSDLVGGDIQGATESATGILDTLI
TNGTTATGLVTEIIGGVTGTIGGVTGGDSPLGLVTDLLGGLTGSVGGDSPLGVVTDLL
GGLTGSVGGDSPLGVVTDLLGGLTGGVTGGTDNPIGIVTDIVGSLTGGVTGEGSLDVI
SNLLGGLTGGSLLGGVTSTVSSVTNTTHTIVPTSLLTDNFLENSFNTV" /product="hypothetical
protein"
/EC_number="3.5.3.12"

/transl_table=11
CDS complement(2437690..2438382)
/locus_tag="AUO97b_02425" /db_xref="SEED:fig|400667.82.peg.2289"
/translation="MSFFAKLFGFKQKTTAQEDVALKAAQELEPSNETSFIELIKNRR
SIYAIDNNLSQSNDEIEKLIQEAIRHSPSAFNSQSSRAVILFGQSHHKFWNTVLEVLK
TIIPAEAVSGTEQKIQSFAAGAGTVLFYEDQSVIKSLQEQFELYADNFPIWSEHSTAI
AQFAVWNVLAEQNIGASLQHYNPIIDEKINTSFNIPAEWKLRAQLVFGSIEGAAGEKA FIDDESRFKTFG"
/product="Nitroreductase family protein"
/transl_table=11
CDS 2438607..2439104
/locus_tag="AUO97b_02426" /db_xref="SEED:fig|400667.82.peg.2290"
/translation="MTEQKSALSLRYYLNLEESQDGFGLVTFGKKQFTRFLTPVISIA
IIIWGLYLGFSGVGRYYVALGVFFLVMQGLMRYWLLPMMFKRQFVRYQFGKSEQGIDL
YQDYFELYAAGRKQAVQYSEVQTFAKGKLTYMLELKNKTVIIVPKRAFAQASDQTIFE NTFKK"
/product="hypothetical protein"
/transl_table=11
CDS 2439144..2439785
/locus_tag="AUO97b_02427" /db_xref="SEED:fig|400667.82.peg.2291"
/db_xref="GO:0003824"
/db_xref="GO:0008152"
/translation="MKTRPSKIVCVGRSYADHVKELNNAMPGRPVLFIKPPSSLIGLD
EGISWNPAWGSCHFECEFVLRIDQPLKGETDPQKVLQAIGAVTLGLDLTLRDLQNELK
DKGQPWERAKAFDGSCVLADWVDVSEITDWEETRFTFHINDELRQDGNTALLMFNIAY
LLVDINQVFSLEPGDVIMTGTPAGVGPLNAGDQLKMTLKGATQDFVWETFVKE"
/product="Fumarylacetoacetate hydrolase family protein" /transl_table=11
CDS complement(2439862..2440656)
/locus_tag="AUO97b_02428" /db_xref="SEED:fig|400667.82.peg.2292"
/translation="MKKSAQRVQDFQEIPAHNTIPDLLWFRVRNAPAETVYPQHAHAW
GEFIYAFSGVLEVNIDQINYLTPPPYGIWLPPYTKHSGINRNEVTHATLYVHESLCQK
LPQKAGILLTSPLVPALLDHLRQNPQDETQVEYIRLLQVLLDQLMQAELVGSYLPTTN
HPALAKILNDLHQCPADNSTLQELAERINMTERTLARYSQKELGMSLHEWRQRLKVMK
AMTMLNQGKTIESIAFDLGYASASAFIYMFKRWMGFTPDQFRKLYQ" /product="Transcriptional
regulator, AraC family" /transl_table=11
CDS 2440762..2441676
/locus_tag="AUO97b_02429" /db_xref="SEED:fig|400667.82.peg.2293"
/db_xref="GO:0016020"
/translation="MEERKNLDTRASALMVLLCMVLGLQQVVLKIAAPDISPIMQIAL
RSGLSAVLVLPLLWRDHSIHLFSYRQCKAGALVALFFSLEFYFVTQALKFTSTSHTVV
LLYTAPIFVALGLHWKFPTERLNRAQWGGITLAFMGIILTFYPTTTATNAPALAQVLL
GDLYALMAGLAWALSTIIVRLSPLAQAPATQTLFYQLFGCFVLLLLIAFLTNQTTIHF
TTLAILSLSFQTLIVSFASLLLWFWLLRNYLASRLGIFSFLTPLFGVLFSILLLEEKL
EIKFIFGSILVLSGIVIMSLKQGKANSS"
/product="Permease of the drug/metabolite transporter (DMT) superfamily"
/transl_table=11
CDS complement(2441697..2442944)
/locus_tag="AUO97b_02430" /db_xref="SEED:fig|400667.82.peg.2294"
/translation="MLERFLKKLPFYNKTWFQFILFVIKRFEADRCREQAGSLTYTTL
FAVVPMLTVFLVIISSIKALEPARQQLQHLIYSNFLPKSTIAFDKALNAFTEKSSNLT
VIGVLFLFVTTVLMLTSIETVFNRIWRVKETRSGIVGFMRYWTIISLGPIILGSAFVI
SSTVASMNLLSNNFTGYQLDGAFLLWLISFVLTILGFFILYWTIPNRTVPLYAAAIAA
CLSAALFETLKNLFSFVMSNFTSYEIIYGAFAAVPIFLLWVFLSWNIVLLGVEVSFAL
TAFHSGKEQKRHPVLMLLDILELFYKKQKLGESVSDKEALEILGRGEVGRWPAYVLLL
EEQNLVKRTDKDEYVLVRNLSQVDFWSFFTALPYPLPLRQDVSNVHDDDEWMEKIGPA
LVESNDYLAAKLSIPLSTIFEEK"

/product="Inner membrane protein YihY, formerly thought to be RNase BN"
/transl_table=11
CDS 2443232..2443825
/locus_tag="AUO97b_02431" /db_xref="SEED:fig|400667.82.peg.2295"
/translation="MQPYILVLYYSKYGSTKEMAHLIANGVESAGMNVKIRTVPNIAT
VVTEAEPSIPEEGDIYCTLEELANCAGLALGSPTRFGNMASEMKYFWDQTTSLWLNGA
LHNKPACVFTSSGSMHGGQESTLLTMLPPLFHHGMMILGLPNSIPALSNTKTGGTPYG
ASHVSGPRHDQSLSQDEKILCEAQGKRLGEIVKKLHS" /product="Flavoprotein WrbA"
/transl_table=11
CDS complement(2443878..2444453)
/locus_tag="AUO97b_02432" /db_xref="SEED:fig|400667.82.peg.2296"
/translation="MHALEQKILTEGIVLSDQVLKVDAFLNHQIDPVLMQQIGKEFAA
RFKDAGITKIITIEASGIAPAIMAGLELGVPVIFARKYQSLTLKDDLYRAKVFSFTKQ
TESTIAISNKHINSSDKALVIDDFLANGQAALGLIDLIHQANAEVVGVGIVIEKSFQP
GRDLLLEKGYRVESLARVQSLADGTVTFVKE"
/product="Xanthine phosphoribosyltransferase (EC 2.4.2.22)"
/EC_number="2.4.2.22"
/transl_table=11
CDS complement(2444526..2445239)
/locus_tag="AUO97b_02433" /db_xref="SEED:fig|400667.82.peg.2297"
/translation="MATKFLKSFSIATGISTTLLIAGFSSHALAMSPFQANYQFTYNN
KGMGSATRALSQQGNNWTYQFTAKAGGIASASETSKFSFANGKIGSQSFSRTSKILIH
NNTMSINFNPGNKTISTKKDDKARSFAWQAGVLDELNAELQIREDLKNGSLKSSYPLA
DAKEVENRRFVKQGNEKIKTSYGTFDTVKVVLQHKKPERSTIFWLAPKLDYLPVKVSH
IDGKTSYGLLLTSYTGKTN"
/product="Enzyme involved in the deoxyxylulose pathway of isoprenoid biosynthesis" /transl_table=11
CDS complement(2445397..2446068)
/locus_tag="AUO97b_02434" /db_xref="SEED:fig|400667.82.peg.2298"
/translation="MQTLFSSLVKRLHKVYRKAEAFIEEERDFTLSQTFLNATLERYV
TNNVEFLDDLHADLYHDWLRLYATMHVKGLETTLSVDLKLIQMEFNKEQQLIVFEQIS
NTQIVEAKYKNFWQKIAVKAAIFYYHKILKKDPLGMILERFDVAQEKDDLIFLDLNRW
FAKKASIIETLGKVHINYARVREAELVVFGNVNLAALLFRDQDDDFEDELGDTEITPI QQKDV"
/product="hypothetical protein"
/transl_table=11
CDS 2446299..2447183
/locus_tag="AUO97b_02435" /db_xref="SEED:fig|400667.82.peg.2299"
/translation="MLKKRSLSIEPFYYWWQDRVFIAAIILAILLHIFVLLIHFAMPA
PSEQSTKEIAISIHPTEDVIKHADFLAQADQQGSGAFRAAHRMSSNSPAPMPSDASTG
EAQLETLEKVQQQRELKFEEKVLMTVLSWQKQAEESQRKKALEQLQSQFQAKAAMVAS
LEAQYLQRQQDFSRQQKIKTVDGIQAKKDASAAYLDKFREKVELYGNRYYPEEAKQQQ
LKGEVRLMVILNAQGGIRAIRLLESSGHPVLDEAAKASVRRGAPFGRFDVNMKDISEL
RIIRTWRFDPAEAEFEVH"
/product="TonB, C-terminal"
/transl_table=11
CDS complement(2447273..2447749)
/locus_tag="AUO97b_02436" /db_xref="SEED:fig|400667.82.peg.2300"
/translation="MKISLSLQQDFQSPELELKRAQLKKIIETTLRHVGYKEDCEIGI
ACVDLEESHQLNLQYREKDKPTNVLSFPSDIPEEVLPMLDALPLGDLVICIPVVLQEA
LEQKKTAQNHFAHLLVHGVLHLLGYDHETSDEDAEEMEGLEIEILAKLNIANPYQE" /product="Metal-dependent hydrolase YbeY, involved in rRNA and/or ribosome maturation and assembly" /transl_table=11
CDS complement(2447759..2448847)
/locus_tag="AUO97b_02437" /db_xref="SEED:fig|400667.82.peg.2301"

/db_xref="GO:0003676"
/db_xref="GO:0005524"
/db_xref="GO:0016036"
/translation="MTAAIRRTVTFPEISMERLKSILGAYNGHLKQIEQRLDVKITHR
GDVFYIDGEIDAVGRAEALLQRLYEETEASQQISADLLHLLIQSSQTERNFELVGEEM
DEHDAPMDVYFQTRKGRINPRGANQKRYVQRILQSDISFGVGPAGTGKTYLAVAAAVD
MLERNEIQRILLVRPAVEAGEKLGFLPGDLTQKIDPYLRPLYDALYEMLGFEKVAKLI
ERQVIEVAPLAYMRGRTLNHSFVILDEAQNTTPEQMKMFLTRLGFGSRAVITGDITQV
DLPRGQQSGLAHALRVLENIKEIHITRFHSRDVVRHQLVQKIVEAYEGWDGEQQRLNA
EARAERKARQEALIAENDTAADLQHQDA"
/product="Phosphate starvation-inducible protein PhoH, predicted ATPase"
/transl_table=11
CDS complement(2448885..2450336)
/locus_tag="AUO97b_02438" /db_xref="SEED:fig|400667.82.peg.2302"
/db_xref="GO:0003824"
/db_xref="GO:0005506"
/translation="MTVQTFIPNGAKAASENTVTQPTHTTDVSIKKLYIETQGCQMNE
YDSHRMADLLGDSHGYVLTNNPNEADILLMNTCSIREKAQEKVFSELGRWRKLKEQNP
DLVIGVGGCVASQEGDNIQKRAPYVDMIFGPQTLHRLPQMLDQHHAQVEKPKKEKIKL
VDISFPDIEKFDFLPEPRVEGFKAFVSIMEGCSKYCSFCVVPYTRGEEVSRPLDDVLA
EIAGLAEKGVREISLLGQNVNGYRGETFEGGICTFPELLRLVAEIPGIGRLRYTTSHP
LEFSDELIQCYEDLPQMVSHLHLPVQSGSNDVLKAMKRNHTIDVYIDKIAKLRKIRPD
MHLSSDFIIGFPGETDENFAKTLQFIKDLDFDHSYSFVYSKRPGTPASDLPDTTPEHV
KKERLAQVQQVIKQSSIEKTDAMLGKIERVLIEKVSDQDPNILVGTADNTRLVTFVGD
ASWVGRFAEIEITEIKTLNLVYGELLNLEPDVA"
/product="tRNA-i(6)A37 methylthiotransferase (EC 2.8.4.3)" /transl_table=11
CDS 2450625..2452568
/locus_tag="AUO97b_02439" /db_xref="SEED:fig|400667.82.peg.2303"
/translation="MKNKYAHRSWLGAVCLLGCSLSYAAEEQFNDALNAANSGNTALL
DQYQLAMQNDVLGYYPEYWKLNTNLGFQSPTSIVSFAQRYPQSAMAEKLAADYVEEKV
KQADFASAQPILPYVSNPDQAENCALAQVRAKSGDALVFAEYKDVWLATESQPESCIG
LGRMMLSSPLMSTQDKQQRLWVQLRAGLSGQALATAQTLGLNLSLAQLNQIQANPLNY
LWSAPKTNDVDYAYLIFALGRLANNDLGNAFANVQRVAQGTPESVQKYLYRTVAYIGG
TTVMKNNFNREVLQYFDASYGYPLSPEEAEIYARQAIRFSAWESLIRAIDSMSVSQKQ
EDRWQYWLARATEQRGDSNSKNTAQRIYKKLAESGDDYHNLLAKDRLGVRYNHQPYND
EPTASDLRRLDQNIHFNRAFTLRRINANPTYTNREWNWAVRQAYLQHDDGLLLAAAKR
AHDMGWYDRAIYAADRTTNKHNDTYRYVTPHKTNVVSHSYNAGIDPAWAYGLMRQESR
FVTSARSHVGAGGLMQIMPDTAKLIARQMGETYNPAALSEMNTNIRYGTFYLSMIQGQ
LSNNPVLATAGYNAGPNRARRWQPDYQPIEADQYTETIPLLETRDYVKHVMTNATHYG
VILGQGAQSLIQRMKVIPTRSSP"
/product="Soluble lytic murein transglycosylase (EC 4.2.2.n1)"
/transl_table=11
CDS 2452749..2453288
/locus_tag="AUO97b_02440" /db_xref="SEED:fig|400667.82.peg.2304"
/translation="MHVQMTPAVCALDPSKQKQRKCLEGYSLTITGLMPETTRTDCST
QSSATLSPLQAKVVARVMPDNNARVQLWRSVGGCVPMNASQYFRTIINFAERLKIPAN
LTSSTNVEMQQSTLRQQFTKLNPSLPQNGIRFTCQLSRSDVVLTEVKVCYTVNGQYKQ
CSNHVVSNCPSEITIKGSY"
/product="Ribonuclease T2 family protein"
/transl_table=11
CDS 2453500..2454486
/locus_tag="AUO97b_02441" /db_xref="SEED:fig|400667.82.peg.2305"
/db_xref="GO:0030060"
/translation="MKQPVRVAVTGAAGQIGYSLLFRIASGEMLGKDQPVILQLLEVP

VEKAQQALKGVMMELDDCAFPLLAGMIGTDDPKVAFKDADYALLVGSRPRGPGMERAD
LLKVNGEIFIGQGQALNEVASRDVKVLVVGNPANTNAYIAMKSAPDLPAKNFTAMLRL
DHNRALTQVAQKAGVAVADIEKLTVWGNHSPTMYADYRFATANGESLKDKINDPAWNK
DVFLPTVGKRGAAIIEARGLSSAASAANAAIDHMRDWALGTNGKWVTMGVPSDGSYGI
PEGVMFGFPVTTENGEYKIVQGLEIDEFSRERINFTLNELEEERAAIADMVK" /product="Malate dehydrogenase (EC 1.1.1.37)" /EC_number="1.1.1.37"
/transl_table=11
CDS 2454649..2454894
/locus_tag="AUO97b_02442" /db_xref="SEED:fig|400667.82.peg.2306"
/translation="MFEQQPTLQLLFEQLGLDSDDASIENFIRTHQLPAEQKLHEASF
WSQGQSDFLKSHWEKDDEWIVVIDELNEQLHEDSVKK" /product="FIG00351533: hypothetical protein" /transl_table=11
CDS complement(2454943..2455470)
/locus_tag="AUO97b_02443" /db_xref="SEED:fig|400667.82.peg.2307"
/translation="MCIETKAIVSRIGETDQLYLTENTPELALERAELRMQLVTLSRV
RQEQIHFLQEAIVLLEQARMEYEEMPMSLYLNLSMHLAKAYMLYFELNKEKRFALIAQ
QILKPLAHHQHGDIYFFLAYASAAQQESALTRHWLTKYLSTAQCDLELLHEHPIFNPV RHEPWYKNLIKLRTH"
/product="hypothetical protein"
/transl_table=11
CDS complement(2455538..2456143)
/locus_tag="AUO97b_02444" /db_xref="SEED:fig|400667.82.peg.2308"
/translation="MHSQPLPDCWNLDQILDDLNAHGFAIVNQAYSAEYHTQVAKECS
HHFDEFREAGIQNGVVSTIRSDHILWINESLPVAEQHVETLSSFCQHLNQAFFLGIKE
VEAHFACYNPGEFYALHRDNPQQKNDRIMSTVYYLHPEWQDDWGGQLRLQDKNNIWHI
ITPEPNRLVIFQSNLLHEVLVSKQQRLSITAWLRSGNSIWV" /product="SM-20-related protein"
/transl_table=11
CDS 2456261..2457118
/locus_tag="AUO97b_02445" /db_xref="SEED:fig|400667.82.peg.2309"
/translation="MNYRHHFHAGNFADVMKHVLLLQLLNRLNAKDKPYRYIDTHGGA
GKYDLSQAPAQKSGEFLTGIHRLVQLSDMEKRQAPEAIQQYLKLVEELRAQEGKGSYP
GSPWFALQGMREIDKATIFEMQRDVFQQLRHNIHDKRAGLHERDAYEGLLAVIPPKEK
RGLVMIDPPYELERKDFPQLVELLQSAYKKWPTGVFAVWYPIKDRAMIERFEKKMFKT
GIRRQLICEICVWPDDTPVGLNGCGLLVINPPWQFSEQADQALQWLFPHLRMQETGGH AAVRWLVGE"
/product="23S rRNA (adenine(2030)-N(6))-methyltransferase (EC 2.1.1.266)"
/transl_table=11
CDS 2457160..2457993
/locus_tag="AUO97b_02446" /db_xref="SEED:fig|400667.82.peg.2310"
/db_xref="GO:0003882"
/translation="MTNAPKPEQDLTHNEHSFDGITFEVVEEEDNPEGQKVKRRGIYL
WPNLITTAALLSGFYSIIASMNGEFAQAIYAIFLAALFDGLDGRVARAIGAQSAFGEQ
YDSLSDLLAFGVAPAMLMYSWSLHDLGRIGLACCFVYTACAAFRLARFNVQIGVVDKR
YFIGIASPLAAIIIISLVWVARDYPFIFDLRDIAIQTINAVIMVVVGLLMISNIKYYS
FKQMDRKRVPFVVMLPVVLIFAAITYNIPMGILIVSIIYALSGFVTTLLAKKNNETIK T" /product="CDP-diacylglycerol--serine O-phosphatidyltransferase (EC 2.7.8.8) @
Archaetidylserine synthase (EC 2.7.8.38)" /EC_number="2.7.8.8"
/transl_table=11
CDS 2458012..2459079
/locus_tag="AUO97b_02447" /db_xref="SEED:fig|400667.82.peg.2311"
/translation="MPKLISEKNQLQQLSLNSNGGVVYQPARGFFKVVYQGLILPNLP
APLRYFNYISLIGQPRIPLCYNANGIVTSAVDTATVLVSNSLHSVGHLKTYSIRRQCQ
LNPSRYQFDKTDLIEWQIPRVQLRRIDSEMSCDLIVQTPSDISNSSALQWGISDYWSI
LCHCEGEILYKGQKYEVNGLGRFKHARALHLPFLSLCFYTCQIINLNETTQVTLSQIR
NQWNIILFSRLDIQELGKPPVTFTEDVNFHIHRVYPKVETPHGREMYLPREFSWQCKK
NGKVIFELYGESRGDYKFGLAAGYIGSFRYQLSWNDQCLQGEGGYCEYIDCRPLRWQE

KNQNEKMLDKLLLLQPCLYKK"
/product="hypothetical protein"
/transl_table=11
CDS 2465011..2465361 /locus_tag="AUO97b_02448" /db_xref="SEED:fig|400667.82.peg.2313"
/translation="MEYKGSCHCGQVKFAAEGELTEALSCNCSICQKKGSLLWFLPTN
QVTVTLDSPDILANYTFNKHVINHHFCKNCGIHPYAQGIDAQGNSIFAINVRCIDDID LEKIKINYFDGRSV"
/product="glutathione-dependent formaldehyde-activating, GFA"
/transl_table=11
CDS 2465381..2465944
/locus_tag="AUO97b_02449" /db_xref="SEED:fig|400667.82.peg.2314"
/translation="MMTKKQLTSKQIDMLLELLKNRFEKNVHRHKEIDWMNVRAKLIE
SPDKLWALQEMENTGGEPDVIAYDQQKDEYLFIDCSVESPKGRRSLCYDREALESRKD
HPPKNSAIDLVQEIGAELLTEEQYHQLQQLGEFDLKTSSWLATPEEIRKLGGALFADR
RYGRVFIYHNGAQSYYAARGFRCCLRV"
/product="hypothetical protein"
/transl_table=11
CDS 2466081..2467244 /locus_tag="AUO97b_02450" /db_xref="SEED:fig|400667.82.peg.2315" /db_xref="GO:0004085"
/translation="MQLNPIYYNEQHIAFADNVRRFVQKEMTPFVNEWDEAETFPREL
YKKAAEIGLLGLGFSEEYGGIPDADPFYSLLAGIEMAKAGSGGVHISLMVHTIGAPPI
QHFGSEELKARVLSGIISGEKISALAITEPGGGSDVAALQTKAIRDGDNYIVSGEKTF
ITSGIRADYYTVAVRTDPTKTGAEGISMLLIDAHSEGITKTPLKKMGWWASDTAHLHF
DQVRVPASNLLGQENAGFKVIMNNFNMERFFLGVVAYGYALICYEEALEWAQQRKTFG
KRLIDHQVVRHKLVDMATQLTSTRALLEETAWKMTQPKLQGPELVAQISMLKNVATRT
MQFCADAAVQTLGGMGFMRGTKSERIYREVKVNMIGGGAEEIMKDLISKQLGY" /product="Acyl-CoA dehydrogenase"
/EC_number="1.3.99.2"
/transl_table=11
CDS complement(2467276..2467875)
/locus_tag="AUO97b_02451" /db_xref="SEED:fig|400667.82.peg.2316"
/translation="MIATSIEQIPYIPCFDDSPRGRLLHGAAYLFHKQGYDKTTVREL
AQFIGIQSGSLFHHFKSKDDILAHVMEQTIIYNLARLNDAATQSTDPEQQLRALIKAE
LISITGDTGAAMAVLVYEWFALSKEKQDYLLKMRNEYEQIWLDVIEKLRTQGKVKHDA
FIWRRLIGGAISWTVTWYKSEGKVKIDELTEMVWEMALK"
/product="Acyclic terpenes utilization regulator AtuR, TetR family"
/transl_table=11
CDS 2467910..2468041
/locus_tag="AUO97b_02452" /db_xref="SEED:fig|400667.82.peg.2317"
/translation="MEIFTKFHRISDYLPNLEDLVNITITFCALQTKRLLGNVEADF"
/product="hypothetical protein"
/transl_table=11
CDS 2468062..2469867
/locus_tag="AUO97b_02453" /db_xref="SEED:fig|400667.82.peg.2318"
/translation="MANNQQDDHRVVKIGCASGFWGDTNTAAFQLVHLTDINYLVFDY
LSEITMSIMAKAKMVEPKHGYALDFVSRVMAPLLKKIAEKKIKVISNAGGVNPLACRD
ALQKIIKEYGLDLKVAVVLGDDLLPKHEQLKSQNIQEMFSGEALPEQVASSNAYLGAV
AIRDALDLGADIVITGRVVDSAVVLAPLLHEYQWPLDDYDKLAQGSLAGHVIECGAQC
TGGNFTDWQLVQGFDNMGFPVVEVSEDGSFVVTKPQGTGGLVSTATVAEQIVYEIGNP
QAYLLPDVIADFSHVHLEQVGEHRVRVTGAKGQAPTTQYKVSATYPDGYRVLVSFLIA
GREAPQKAQVIADAILTKCERVLAMRSVPPFSEKSVEILGIESTYGDHAQTLNSREVV
VKIAVKHMFKEACMFFASEIAQASTGMAPALAGIVGGRPKASPVIKLFSFLIDKNQLN
IEIDFDGQRHAVEIPQGVSTEQLLTLTAGENAVYQGDEIEVPLIEIAHARSGDKGNHS
NIGVIARKADYLPWIRAALTEQSVASYMQHVLDAEKGRVIRYELPGLNALNFMLENAL

GGGGVASLRIDPQGKAFAQQLLDMPVKVPAHLLEK"
/product="Terpene utilization protein AtuA" /transl_table=11
CDS 2469888..2470799
/locus_tag="AUO97b_02454" /db_xref="SEED:fig|400667.82.peg.2319"
/translation="MSYQSIFRPDAFANKVIIVTGGGSGIGRCTAHELAALGAQVVIT
GRKIEKLEKVSQEIIEDGGLVHFIVCDNREEEQVKNMIAEVIEKFGKLDGLVNNAGGQ
FPSALENISANGFDAVVRNNLHATFYLMREAYNQWMAKHGGSIVNMTADMWGGMPGMG
HSGAARSGVDNLTKTASVEWGKSGVRVNAVAPGWIVSSGMDNYSGDFAKVIIPSLAGN
VPLKRMGTESEVSSAICYLLSDAAAFVSGVTLRIDGAASQGTRMYPLAEATNSQSYNG
FHRAFIPEIFKKDKTEKVEQSNNSGSE"
/product="Citronellol dehydrogenase; Citronellal dehydrogenase"
/transl_table=11
CDS 2470810..2472423
/locus_tag="AUO97b_02455" /db_xref="SEED:fig|400667.82.peg.2320"
/translation="MTILQSEITVGGEQYQKNKEALLAQLSEVRAIQQKSIDKSYAAK
PKFDKKGKILPHERVRLLLDADSPFVELCGLVGYNMHDDKDGSEAGGGVIAGIGFVSG
VRCLVSASNSAIKGGTMSPMGVQKTLRLQKIALEQKLPLITLTESGGANLNYAAEVFT
YGGMTFANQARLSAAGIPQLAVVHGNATAGGAYQPGLSDYVIVVRKQTEMLLAGPPLL
LAATGEVATAEELGGAEMHAQVAGTAEYLAENDADGIRLAREIFEHLNWKEQIKPVEV
TYKEPRYDTEELLGVIPSDPKQPFDMKEVVARIIDDSDFLEFKQEYDDLTVCGWAKIG
GLHIGIITNNGPITPQGAAKAAQFIHLCEQTNRPLLFLHNTTGFMVGTDAEQNGIIKH
GSKLIQAVANCTVPKISIVVAGSYGAGNYAMCGRSLSPDFIFAWPNSHVAVMGSAQAG
KVLRIVAEGKQKASGQEPNPQMLDFLEQSTAMKLEQQSTALFNTAMLHDDGIIDPRDT
RKLLIFLLQTIYEAQQRELNPTRFGVSRF"
/product="Geranyl-CoA carboxylase carboxyl transferase subunit (EC 6.4.1.5)"
/transl_table=11
CDS 2472466..2473623
/locus_tag="AUO97b_02456" /db_xref="SEED:fig|400667.82.peg.2321"
/translation="MKFTAEHEALRRTARQFVENELNPNIPEWEEAGCFPIHDVFKKM
GDLGLLGICKPEENGGLALDYSYNLVVAEEIGRAACGGVPLAIGVQTDMATPALARFG
SKELRDEFLTPAIAGEYVASIAVSEVHAGSDVAAIKTTAKKDGDDYVINGSKMWITNS
LQADFFCLLANTSDDKPHVNKSMIIVPAKTKGISFSEPLNKLGMRSTTTAQVYLDNVR
VPQRNLVGVEGMGFMMQMMQFQEERLWACANAIGGLENLIQKTIAYTKERTTFGQPLI
NNQYIHFRFAELMTEVEALKALTYQACEQHIAGEDVTKLASMAKLKAGRLSREVADSC
LQYWGGNGFMWDNPASQLYRDGRLGSIGGGADEIMLGIICKLMDILPKKQK" /product="Citronellyl-
CoA dehydrogenase"
/transl_table=11
CDS 2473634..2474470
/locus_tag="AUO97b_02457" /db_xref="SEED:fig|400667.82.peg.2322"
/translation="MTLSASLQAIDIDDSIQLEQDGSILYLWLNRPESRNAMNLNMVN
AIQQVFTAIRDDLSIRAVIIRGEGGTFCAGGDIKDMAALRVEATNVGSLQPYTNFNRR
FGAMLEQVEAAPQTVVVILEGAVLGGGFGLACVSDVAISRDNAQFGLPETGLGVIPAQ
IAPFVVKRIGLTQARRLALLGMRFEGHTALSVGVVHQIAHNEIELEQALQETIQHIKR
AAPQASRVTKALLHRTLNEPLNELLDDAAQQFAQAVGGAEGQEGTMAFIQKRLPNWAD GS"
/product="Isohexenylglutaconyl-CoA hydratase (EC 4.2.1.57)" /transl_table=11
CDS 2474489..2476429
/locus_tag="AUO97b_02458" /db_xref="SEED:fig|400667.82.peg.2323"
/db_xref="GO:0004485"
/translation="MKFSKILVANRGEIVVRVMQTAKAMGYQTVAVYSDADRNARHVQ
EADEAVYIGASKVSESYLSIAKIIEACKKTGADAVHPGYGFLSENTDFAQACIDNQIT
FIGPTASAIELMGSKRLSKIAMIEAGVPCVPGYEGDRQDLEYLATQAEQIGFPIMVKA
SAGGGGRGMRLVQQASELFEALQTARSEAENAFGSGELILEKAVIAPRHVEIQVFGDT
HGNYVYLFERDCSIQRRHQKVVEEAPCPVMTPELRQKMGEAAVAAAKACAYVGAGTVE
FLLDASGAFYFLEMNTRLQVEHPVTELITGLDLVEWQLRVANDEQLPLKQQELTLNGH

AIEVRLYAEDPRQDFLPQTGKVLCWKPAALPNVRIDHGMLATDEVSPFYDPMVAKVIA
YGKTREDAIRLLARAVDDSVLLGVNSNKQFLVNLLRHPVVVAGDTNTAFIQQHFQNDP
SLHYQVLSLETLAIAAALFSQSKGTAVWQTGLGVPLPLKLQTADQQIQLQLSSVNNTF
TVQFCDQVVCIDVLEKTPEQLVYLIDGVRRRVQYVLDDDQLYLDRDNGNVLIRNMTYA
APEATDVAGDGKIRAPMDGAVVNILVNEGDQVVKGQTLLILEAMKIQQQIKSDVDGVV
DEILGQQGQQVKKRQMLFSIQI"
/product="Geranyl-CoA carboxylase biotin-containing subunit (EC 6.4.1.5)"
/EC_number="6.4.1.4"
/transl_table=11
CDS complement(2476534..2477190)
/locus_tag="AUO97b_02459" /db_xref="SEED:fig|400667.82.peg.2324"
/translation="MAFDLVQYFAEQIKIQKPQLLNQYPANEKNKLIDEVNILTLGKL
ISLWRQDDNKIYHEIKTADPLYIQEVARHLTTSKHNKSVLKNSELEQSISEILALQLT
ELNQLDETGGFGQSGLKELILGQVEHLSGQAEDWVWSTNHLTELIGSKPVEQEELSLD
ATMKEFNQMVHQAQPHHEDLHVEEQPIETFIPGWSKVIAPLVALAILGYLYCMYTQLV "
/product="hypothetical protein"
/transl_table=11
CDS complement(2477208..2477636)
/locus_tag="AUO97b_02460" /db_xref="SEED:fig|400667.82.peg.2325"
/translation="MSEQTIELTPTRPYLARAIYEWICDNQLTPYLLVDATQPHTDVP
QQFVKDGQIVLNIVPHAVHQLLISNDAITFSARFGGVSKDIYVPIQAVLGIYARENGQ
GLFFDPEEYANVAPLEDKLDSETQETSEPTKKKPTLRILD" /product="Stringent starvation
protein B"
/transl_table=11
CDS complement(2477643..2478296)
/locus_tag="AUO97b_02461" /db_xref="SEED:fig|400667.82.peg.2326"
/translation="MSVENTSIQGITLYSHADDFRSHWIRFLLAEKQIKYQLIVTDHE
DEDLASLNPYNQLPMLVEQNLKLFSAPIIAEYLDDRYRQNKLYADAPMARAEQRQYIW
RLENDWFKLADHMLRHADTLNIEQKQKAQKELRDTLISLTPLFQHFPYFMSENFSILD
CMLAPIFVRLNSMGIDLPKQQCRPIFLYCKRIFSRPSFVKSMTPQEKTRYNELLNME"
/product="Stringent starvation protein A"
/transl_table=11
CDS complement(2478467..2478853)
/locus_tag="AUO97b_02462" /db_xref="SEED:fig|400667.82.peg.2327"
/db_xref="GO:0003735"
/db_xref="GO:0005622"
/db_xref="GO:0005840"
/db_xref="GO:0006412"
/translation="MATNYGTGRRKTATARVFLSAGTGKLVINNRTLEQYFGRETARM
VVRQPLELLEATEKYDLYITVKGGGIGGQAGAIRHGITRALIAADETLKPVLRQAGFV
TRDAREVERKKLGLRKARKRPQFSKR"
/product="SSU ribosomal protein S9p (S16e)" /transl_table=11
CDS complement(2478866..2479294)
/locus_tag="AUO97b_02463" /db_xref="SEED:fig|400667.82.peg.2328"
/db_xref="GO:0003735" /db_xref="GO:0005622"
/db_xref="GO:0005840"
/db_xref="GO:0006412"
/translation="MKTLSAKPAEVQHDWFVVDATGKTLGRLATEIARRLRGKHKTSY
TPHVDTGDYIIVINAEQVQVTGNKALDKKYYRHTEFPGGLKETNFEKLVAHKPEEIFE
RAVKGMLPKGPLGYAMIKKMKVYAGSEHPHAAQQPQVLDI"
/product="LSU ribosomal protein L13p (L13Ae)" /transl_table=11
CDS 2479476..2480450
/locus_tag="AUO97b_02464" /db_xref="SEED:fig|400667.82.peg.2329"
/db_xref="GO:0050570"

/translation="MIVLPLYVTSGEPAGIGPDICLSLAKRVDERPVVVLADRNLVQQ
RAQKLGLDVKFLEYSGQAESSLQGELYIEHVPLETIVVDGQLNAANAAYVLEQLRRSA
DYAMSGKSVGVTTAPVQKSVINDAGILFSGHTEYYQEFAGVERVVMMLATKTLRVALV
TTHLPLRDVADAITKERLHQVIDILLHDLKTKFKINDPRVLVCGLNPHAGEDGYLGRE
EIDTINPVLESYRSQGVKLSLSLPADTLFTPEHLKNADAVLAMYHDQGLPVLKSQGFG
EAINITLGLPFIRTSVDHGTALSLAGTGLAKSSSLNVAVDLALSLAAS" /product="4-
hydroxythreonine-4-phosphate dehydrogenase (EC 1.1.1.262)"
/EC_number="1.1.1.262"
/transl_table=11
CDS 2480474..2481286
/locus_tag="AUO97b_02465" /db_xref="SEED:fig|400667.82.peg.2330"
/translation="MYQINALNPKDEGHKARKRFGQNFLHDQRVIAKIVRSVNPRTGD
NIVEIGPGLAALTSPLIGECDALTVVELDRDLAAGLPERVPHPERLTIVEADALKYDF
SQLVKDGRPLRVVGNLPYNISTPLLFHLLEFGSQVKDMHFMLQKEVVERITAEPNTKE
YGRLSVMIQYYCQPTFLFEVPAGAFNPPPKVTSAVFRLVPYEQKPITAKDEKALARLV
AHVFTQRRKTLRNSLKGMLAEDGFEKAGVDPMARPETLTLAEFVALADQMVA" /product="SSU rRNA
(adenine(1518)-N(6)/adenine(1519)-N(6))-dimethyltransferase (EC 2.1.1.182)"
/EC_number="2.1.1.182"
/transl_table=11
CDS 2481287..2482126
/locus_tag="AUO97b_02466" /db_xref="SEED:fig|400667.82.peg.2331"
/db_xref="GO:0016787"
/translation="MTKRYNYVIGDVQGCFEALKALLKEIRFDPDQDFIWFAGDLVAR
GENSVGALRFIKKLADRGAAATVLGNHDLTLIAGARGLKEIKEKDRTRDVIDAVDGDD
LIDWLRKQPLCLFPNEQTILTHAGVPCIWDAQKTAALAKEVEAVLAHDDLSVLDAFLA
EMYGSKPDLWTDDLTGNERLRCITNYLTRMRLTNAEGALEFSFKDTLDAPMPEGYLPW
FEFPSKAAQTHQIFFGHWAALEGRTISEHIQNVDGGCVWGKNLIAYRLEDQQAFSVSN PVM"
/product="Bis(5'-nucleosyl)-tetraphosphatase, symmetrical (EC 3.6.1.41)"
/EC_number="3.6.1.41"
/transl_table=11
CDS complement(2482169..2482612)
/locus_tag="AUO97b_02467" /db_xref="SEED:fig|400667.82.peg.2332"
/translation="MGKFFVGALLLVGINTATFAADITGLWQTIDDKTGTPKAHVEIR
KEANGTYAGKIVKITPRPGYTPKEICDNCPAPYTNKPILGLDIATGLKQTDNSTYTGG
KILDPNTGKVYGLKAKLSANGKRLHMRGYLGVSALGRNQIWIRLE" /product="hypothetical
protein"
/transl_table=11
CDS complement(2482697..2483371)
/locus_tag="AUO97b_02468" /db_xref="SEED:fig|400667.82.peg.2333"
/translation="MFMILYGFLITLVGYLCAKPLNRRFPQVPLLVFGMFIVIGLLSI
LKVPYEEYRLYVNDLFSHLLGYVTVALAIPLAAMRYDDLPLKSVIGILCFASISAVAL
PMGLAYLLHMSEPTILAFATRAVTTPIALNIATLLHAPETLVTLIVILSGVIGAAFSP
FLLKHIHDERASGLALGLAAHAIGTAQAWQRGPVAGRYAAFGMALNGVFTAIWLPTFM LSFGIH"
/product="LrgA-associated membrane protein LrgB" /transl_table=11 CDS
complement(2483382..2483753)
/locus_tag="AUO97b_02469" /db_xref="SEED:fig|400667.82.peg.2334"
/db_xref="GO:0016021"
/translation="MDKIKSIDWAAWVGTLLLIIGFREGSVVVMRAFGHPELGNLVGL
ISLLIVLLIWRKFKKIPLRLVDTNNKVMKESGFAFLPICAGSLIMLVHMGKEIPLFLF
VLVVSTLIPLWVYAKMAKHWL"
/product="Antiholin-like protein LrgA"
/transl_table=11
CDS complement(2483796..2485130)
/locus_tag="AUO97b_02470" /db_xref="SEED:fig|400667.82.peg.2335"

/db_xref="GO:0005525"
/db_xref="GO:0005622"
/translation="MEYVDRHQGGERTILVSVSVQLLDDLDAEEFALLAQSAGADILE
HIKVQRNKPDPKFFIGSGKVDEIAEQVQDLEASLVIFDHALTPAQERNLEKILKCRVI
DRTGLILDIFAQRARTHEGKLQVELAQLKHLSTRLIRGWSADFEQQKGGIGLRGPGES
QLETDRRLIRVRITQLKDKLEKVHQTRMQGRAARQKASIPTVSLVGYTNAGKSTLFNI
LAKSDVYAADQLFATLDPTLRRLEWDGIGTVVLADTVGFVRNLQHDLVESFKATLEET
LEATLLLHVIDSSSHDMLDQIEAVEGVLKEIGADAPVLRVYNKIDLSGEEAKIIYSEP
HVPDRVYVSAHSGLGLDLLRQAVQECLMGQLQHFSLVLKPAYGKLRTQLYALNVIQSE
HYDDEGLLHIDIRIAPHKLEQLIRQAKLPIDEILGERASQFKRPLEEFEIKH" /product="Ribosome LSU-associated GTP-binding protein HflX" /transl_table=11
CDS 2485334..2486143
/locus_tag="AUO97b_02471" /db_xref="SEED:fig|400667.82.peg.2336"
/db_xref="GO:0003841"
/translation="MTQTQSIVNSTLKKFSKIGLYGKKVTSATAAISEGFYLVYRHGL
YKDPNNPVNTRYVQYFCRRLCQVFNLEVQVHGTIPREPALWVSNHISWLDIAVLGSGA
RVFFLAKAEIEKWPILGNLAKGGGTLFIKRGSGDSIKIREQITEFLKQNIPVLFFPEA
TTTDGHSVKRVHGRLLGAAIEAQRPVQVCLICYVNRHGELDTVAPFIGEMSFVEHIQR
VLEMPKVTAHLLTLPPISVEGHDVKSLTQEVQEKMVEGLAQLHKKVLKPKS" /product="Acyl-CoA:1-acyl-sn-glycerol-3-phosphate acyltransferase (EC 2.3.1.51)"
/EC_number="2.3.1.51"
/transl_table=11
CDS complement(2486144..2487769)
/locus_tag="AUO97b_02472" /db_xref="SEED:fig|400667.82.peg.2337"
/db_xref="GO:0003824"
/db_xref="GO:0008152"
/translation="MAQSFHSKQLQTHQLAKGFLIKASIVVCSSFAVALTGCSTLPKH
SPEPIQYARDIDTSQTSLSKIITPLREKNPNLTGYHLLNDPLEALAARLRLIDKAEKT
LDLQYYIWDNDKVGALALHAIIRAADRGVKVRLLIDDNNAKKMEGVLLALSQHKNIEV
KLFNPYRFRKYRAMDMILDLKRINRRMHNKSFIADNQVALIGGRNMTNQYYNVSDSYQ
FSDVDVMLVGAAVDDIVNSFDDYWNHEYAYSVQSIVSAQQHRLRYESLKQQLDAHYQQ
ATVQNFLDLTSRSHAFDKWLDRNIKFDWVKAEVVKDSPDKIRSKAKKEEHLNFQLINH
LEKPESNVDLISAYFIPEKQGAKILSTLAKEGVEVRVLTNSFKANDVAVVHAFYGKYR
KELLKNGVQLYEFLPTPDKRDLNKNTDELATKAKVNMKGLSRSSLHTKLMALDEQVFI
GSFNFDPRSAYLNTEIGVILDSPSLAKTIHHTMDENLNKYAYKLKLDPNNHIYWQQET
PKGPVIYKKEPEMKWWQKAGMKLLSWLPLEGFM"
/product="Cardiolipin synthase (EC 2.7.8.-) phosphatidylethanolamine-utilizing, bacterial type ClsC" /transl_table=11
CDS 2487946..2488887
/locus_tag="AUO97b_02473" /db_xref="SEED:fig|400667.82.peg.2338"
/translation="MKPGFINHVLKTIHPFKAFVHLLAFYLSYLLIPLWFFNLYAGWI
GYYSIHETIFIFLLSGILIYARFIEPHMVRVDVHQYRLNPDRRFAKPVKVALIADLHI
GLFSGHERQLKIIVKKLNEQQPDLVVVAGDWTYEPEDKLVQELSVLKDIKAPVYSVPG
NHDEQYPGPPIQQLLKDALHYNDVMDIEGKIVEFDEFRLIGIGDLWAGKTDMRSMPDL
PQDKPWLILSHNPDTVDMVPKLPNRPLMLSGHTHGGQVELPWVTNYIMKKVSILGHKR
GFYSHEHADVFVTVGTGMVGIPLRFRVPPTIDIIELV" /product="Phosphoesterase"
/transl_table=11
CDS complement(2488935..2489414)
/locus_tag="AUO97b_02474" /db_xref="SEED:fig|400667.82.peg.2339"
/translation="MNTRSLKLSFIALLCIALAGCLSFGPLKYRQVKMLKKEGFVLTN
EGWTLGLPERLLFDFNDATLKQSHEAELTRLANQLNKYDLNKLKIVGHTDDVGNPEYN
QKLSEERAQSVANLFLTHGFKKENIYVIGRGSTQPYVPNTTNENRAINRRVAIVIIP" /product="Outer membrane protein"
/transl_table=11

CDS complement(2489429..2490529)
/locus_tag="AUO97b_02475" /db_xref="SEED:fig|400667.82.peg.2340"
/db_xref="GO:0005554"
/translation="METYAKQNLQLLSHTLLERIQPAVVFNDKITIEQILNEYTNDHS
IRAIHIYDSEHHLIAQSFKLSSQTSVLEKWFDHWFLNEPVHLTIYHHQQNVGELTLFG
SSEKILQFLKMIMVGLAIAMLFIVCALWWSVNLTYRQIMQAIYPLTNIAQIVSEQKAY
NLRFPYNRIKEFQDLNTVFNELLEEIQIWDNQLQKENRKLSFQAHHDQLTLLPNRHYF
YQILLDMFEDKDFDNKSALLFIDNNNFKSINDQFGHLAGDEVLKEMAKRLQTRLRHQD
FIARLGGDEFAVILHSITHADHLISIAENLLESCKEPLHLNDQTIYFSFSVGIALSQF
ASSPEDFIMQADQAMYKAKTSEQHWFIYKPEN"
/product="Diguanylate cyclase/phosphodiesterase domain 1 (GGDEF)"
/transl_table=11
CDS complement(2490658..2491119)
/locus_tag="AUO97b_02476" /db_xref="SEED:fig|400667.82.peg.2341"
/translation="MFATSGPAYANSKHNFYVLTLSILSYSKWENVSTPTLCVIDNAS
ITSTFQSYIQQLSYNYRVQTVNAKDFSKSHCQAVYFSTTPPQQQQNLIQNYPYRSLLS
LSINNPECEVGSIFCLYNQNNYTTFKVNLDALSHSKVHIDPRVLLLAKNAE"
/product="Uncharacterized protein YfiR precursor" /transl_table=11 CDS
complement(2497242..2498615)
/locus_tag="AUO97b_02477" /db_xref="SEED:fig|400667.82.peg.2343"
/db_xref="GO:0004148"
/translation="MFDLIIIGAGTAGISAYKEAVKYTNNLLIINDGPWDTTCARVGC
MPSKVLISTANRMHDIQNAQEVGLSASADINTDQVMEHVRTLRDRFTKATVKDVEQWP
TEHKISGKAHFIDAKTIEVNGKRYQSKSFILAVGSTPNYDQSWKQELGDRLITTDQIF
ELNTLPKSIAIIGSGVIALEIAQAIHRLGVETTIFARSKRIGIFTSPKLQQLAQEELS
KELNFLFETLPHEVKSTSDGVILNYKIDEKEESIQTEYVLSATGRSSLLDTLKLENID
KSFKDIKLLPVNAKTKQLDDYPIFIAGDAYTSTPLQHEAAHEGKKVVHNCLNYPQLNS
VKTLPPLGIVFSHPEMAIVGQSYKQLKDGGVDFVTGEASYERQGRAIVLGKNKGAIEI
YIERENQKLLGAELFTEASKHMAHLLSWIIGEELTLNDILEKPFYHPTLEEGLRTALK HARRQLK"
/product="Dihydrolipoamide dehydrogenase (EC 1.8.1.4)" /EC_number="1.8.1.4"
/transl_table=11
CDS complement(2498701..2499894)
/locus_tag="AUO97b_02478" /db_xref="SEED:fig|400667.82.peg.2344"
/translation="MKLTQGVCGLTLAVFLSACGGGGDGYYNQNSDSGSTTTPTTPST
DQELANSVLNSVKQEGKYLFGAYDLNNESLSKGYIDHALDTFAQGPLKLTLDIRKVDL
TKYNDHYREKCFINSTTDYRACYVFKGEEIKSLLTGYNDWDFDITADDLKNIKLASDN
ISPNLQEYQSNTRIYIFENENKDKNFQDVTITGAFAYPFQQSWGLQQTKQKRFVLINS
TTSDYKITTTTMVQDPQTGQPTEKEVTTGAMSVYKDNTSTDGDLYVIQGGSGFNVLIN
DNPNVPFAEPISFVVNSTPGNTNNATYRVRLGKNQVLSLPNITGIEGSRVENETPTTN
IQKFNGSIYLEGKNIFTFKQDLNGSTLDFKHVINGVSIEGQSTNQNGVVSTTLKSPFA QKFTF"
/product="hypothetical protein"
/transl_table=11
CDS complement(2500252..2500587)
/locus_tag="AUO97b_02479" /db_xref="SEED:fig|400667.82.peg.2345"
/translation="MSENVGFAGQIGPEHVGQVVEKGFKSIINNRPDMEGGPEQPTSA
QIEEAARQAGLDYVYQPVVAGQITELDVRTFANHYNELPKPVLMFCRTGNRSNNLYQL AKQMDLLDD"
/product="Protein tyrosine phosphatase"
/transl_table=11
CDS 2500780..2501676
/locus_tag="AUO97b_02480" /db_xref="SEED:fig|400667.82.peg.2346"
/translation="MHAYTVEPLYVPCDQEMIAADFYIPKTNNKSAVIIMAHGFAGLR
QFKLIQYAQRFAQAGYAVILFDYRYWGGSTGKPREMISINSQLEDWKTMIQYASTCKF
IDNRRIVLWGTSLSGGYALSLASELKNIQAIMVQIPYVDGAETAKLYPLQRYPQALKL
SSQDYMGSKMGLNPRRLPVVDQYKLCFMPTADSYYGYLSIVNPDYYWSGEVPARVFFN

LMRYRPIQLVRQINIPVLFIAAQHDSLIPIESSREAATNIAPFVSYHEWDMKHFDIYH
GSWFEKAVTTQLEFLHQHIGVM"
/product="Hydrolase, alpha/beta fold family" /transl_table=11
CDS 2501677..2502114
/locus_tag="AUO97b_02481" /db_xref="SEED:fig|400667.82.peg.2347"
/translation="MIIVCASCGAKNRVPEEKLTAQPSCGQCHQPLLPLAPIELNEQN
FSNYITNSDLPILIDLWAEWCGPCKMMAPHFAQVAKQNPRVIFAKINTEESPRLSQAF
NVRSIPTLVLMNKTTEVARMSGALRAPELQQWLDQQLQTNFGS" /product="Thioredoxin 2"
/transl_table=11
CDS 2502116..2502538
/locus_tag="AUO97b_02482" /db_xref="SEED:fig|400667.82.peg.2348"
/translation="MSEAYTKPEGILSLQTIAMPADTNWSGDVFGGWIVSQMDLAGAI
HAERFSRGRCATISINQMTFLVPVKVGFVISCYTKILKVGNTSIQMQIEVWDSHDDSR
EPIRITEGVFTFVAVDVKGNKRQIPEEAKQKFLASQES"
/product="Acyl-CoA thioesterase YciA, involved in membrane biogenesis"
/transl_table=11 CDS complement(2502578..2503072)
/locus_tag="AUO97b_02483" /db_xref="SEED:fig|400667.82.peg.2349"
/translation="MSQSNRLLKLVKATSKFPKGIRSTLWSKAFGRIVPMVGTANIRY
QEVDVNHVTVRLENQKNMQNHIKGVHAAAMALLAETATGFLTGLHIPDNRILLIKSLH
VDYLKVVEGGLTATATLSTEQQKYIADHEKGELVIPVTVIDDAGNEPIQCQMLWAWLP KRKK"
/product="hypothetical protein"
/transl_table=11
CDS complement(2503507..2504493)
/locus_tag="AUO97b_02484" /db_xref="SEED:fig|400667.82.peg.2350"
/translation="MLTQNQLIESQANAAIAQLPYGPTTCINSLQQTFQKILKIKAIY
SLQTDEDGQKRMGLNQAGQEFPSNISIIKDSPFTNGRMGHNKMSPFLEASSCFFNQSG
LLFIPPNQELNQDFILNSIQYFNNSLLTREYDQQLNHWNEQFNEGEQRYDQFMKDLKK
PLKGCDMNEVICVYPLNQHLFSNVAFSSQSTFSESIDGEITRLICQSKENAVCGVLVK
REITVNYQLICRYLVFTECQFINDPCSFTNLRFMEEVKRLVEPNSQNQVVLASTPLNG
FLLSNRFIKKSSDYKEQMKRLKTYVVGTDKFIRVGGTHPTFKVLFSKFEVKQ" /product="hypothetical
protein"
/transl_table=11
CDS complement(2504495..2505634)
/locus_tag="AUO97b_02485" /db_xref="SEED:fig|400667.82.peg.2351"
/translation="MTEFNAKKLRTLICENQVTLAPSDVCQSLFLYSDFRLDRMLDIP
HFVLECNKTQLPAATVKQTDQGREYFEYEDWFRPFIKILDELINTNPYFFKHFDLCEE
TKCFFKTLNAMDSPPNLYGLFERNLRGRNPSNLKQQIELLNEFIELLREELNSPTVKE
VIQNREKQHKENFTSCKLLISKLFQKYGQLWVLPVDFAYTPDQQDLQHNHPTDLKQRF
HGPYQFERLKQQIDQLLENRRRNKTLKQIKGYLFYFGHSLKKGVFVKVIFFLDGNTCQ
TEDYVKYLSKYWSRLTNQQGCTYFCDSDSSQSPTGISLIKEMDKEQRENLMLSIQYYC
QLEQFFRLKYLGENSYKRLQMSQPPVISKGKASGLNQNLNVKKGR" /product="hypothetical
protein" /transl_table=11
CDS complement(2506201..2507256)
/locus_tag="AUO97b_02486" /db_xref="SEED:fig|400667.82.peg.2352"
/translation="MGFPSDHQEDKTNNEKIPYIIEQLGIYERYILDRYSHQKRDYID
PISIDPNHIVSNVNNEAFKYEYLYDKGEHTRVITPYDDYLYLRDELIKNPFEIGKMPE
ELLLSYLFCLCKDTREILWNGDIQACLSQIEKIEKVNKLLQGLLKNASYLLFSPEDCG
ITNREMMYFYPLGQQARYVADPDFGEDMIELFNKNKSSQLNEWHRTLFIKVIKEISCE
KNKKWNAVNAIVKDPIVKTQFREIMKDQPKQNLDYALAGRRDYKQLYSQAKDRLEKEL
KKNAWLNSYASNTERRSHAQERLKHLDMLIAEQETLEKNFKLGKYTFIKRNSYSDDVI REWIENDEDFIKEFIQA"
/product="hypothetical protein"
/transl_table=11
CDS 2507670..2508389
/locus_tag="AUO97b_02487" /db_xref="SEED:fig|400667.82.peg.2353"

/translation="MSDLNVLKILATNIGISGVDTLAMGLGSAIKNSVTEIQNYTKQT
SDAIYYLQVKTFLETAELDKDDVNTFFQNNPDNQRLGAEIFKILEQTYIENQSQMMAR
AFSLYVQKKIEKPILDQLIYIITNLNQYLINKLEEYLPEDAITIKEFDLLHESIGLDS
IFNPDGNYTRDEIAYDRKISSFFSRKYKNVPQALINFEFYKPIDMAITMDLETLPQQE
YEPTRFFLWFVTHILKDSISK"
/product="hypothetical protein"
/transl_table=11
CDS complement(2508745..2509236)
/locus_tag="AUO97b_02488" /db_xref="SEED:fig|400667.82.peg.2354"
/translation="MKLDLTDKERLFLSNQYEILGTLKKDDHYLLLAEQLREGHKWLY
QHNFDALYENLPDEDAELVLNILQVYEALKDSYDALTDKSGISIQEVSFAGFDGNREP
ELMGFVDALNKAHRFSSTIDAGVLNSHSPKADRYQNMIQKWKELDKSNTLSKEQILYI LSR"
/product="UPF0304 protein yfbU"
/transl_table=11
CDS 2509506..2510603 /locus_tag="AUO97b_02489" /db_xref="SEED:fig|
400667.82.peg.2355"
/translation="MQLSIFEHYKQLLKNAKKYKIPPREKTFFDTAIRNHYENPTTEL
LEFFLNPTESHDLGDLFWKGFCDVLQQEASLSKLDLGNIVKLEREYATHQGNRIDLWI
ETDTCFILLEAKIYHHQNNPFQDYIQFAQSKNQSKNKQIVGVILSIAGKSEKKGWLGL
SYQQIVNSIRPYLAEQMLANPMNKWNLFAREFLLHLDSYYRIKNLDMNRVQFILDHYK
EIEELQRLRTSTISEVVDSLSQQLNEMIDGYESENKYESWGGIRFYNKAWGNKSNTTL
LIKQEDGQTVIKVITYILNLSLELEEEAFSILGTQTDSRYLDQKVENYRRGTERWLCI
YWRSPENNLTAITDLLFEKVKLLDTIERTLK"
/product="hypothetical protein"
/transl_table=11
CDS complement(2511225..2512430)
/locus_tag="AUO97b_02490" /db_xref="SEED:fig|400667.82.peg.2356"
/translation="MNHYITKPNLMSTAGQWSLDDLEALQLLDQIQQDYACYEVNNDE
GDLVEFRLPSHPETPCFIDRLQLGIHCHQQRPLASLRTTKDIQGNKKLSVQHAKDLPI
QLVSALPSIFSMPLSDTYTVSVELETIHSVIQDLSDAERLVLGTFLSNSAYTNEQAQT
YVDALNQFQNLLNERMSSAKVRKRLHNRKASRLKTKEKMIELVHALLHVHSKILVVRV
DLGIMRSPESLINHHISSNQWKSEQDSQFILKALERLNENARMNRMKHALGYIHRIEY
GPQKGYHVHAYYFFEGQDHQADITWGQYIAKQWKHVTNNQGSVFICNMKKEDYRYCAL
GMLDYSDVEMLKNLEQSFDYLCKNDQYFQFTTQKRIRGFRISQTPVIPDVKMGCPRKH DQSHLRMCG"
/product="hypothetical protein"
/transl_table=11
CDS complement(2513219..2513386)
/locus_tag="AUO97b_02491" /db_xref="SEED:fig|400667.82.peg.2357"
/translation="MPTPMNPHEQAYFDQFKEDREEQIEQIMIEKGYSRVEAEATWEK DYEEAISTDDD"
/product="hypothetical protein"
/transl_table=11
CDS complement(2513491..2513709) /locus_tag="AUO97b_02492"
/db_xref="SEED:fig|400667.82.peg.2358"
/translation="MFRLKCLGGPLYGQEYFHAHDEFIFKDKQTGKQTLYRKQTLDFT
PPQEFFVAESISKTVAYNLALQLIKRPY"
/product="hypothetical protein"
/transl_table=11
CDS 2514611..2514757
/locus_tag="AUO97b_02493" /db_xref="SEED:fig|400667.82.peg.2359"
/translation="MKHPLIGLYFTIVIGYWLYLIWQYPIYSGSYSLGRALVWPADFL RWLF"
/product="FIG00353281: hypothetical protein" /transl_table=11
CDS 2514828..2515853
/locus_tag="AUO97b_02494" /db_xref="SEED:fig|400667.82.peg.2360"
/translation="MLRASDNIYFAPAIPYKKLQGAMSYLPQGIHPDEILMLIDDTVF

GSAKAGLCVTATGLFYKESFGDEAVYLFKSIHHVEADIGVINHGIVLNRIETLTFTQL
DKGTVRTLASFLNEVCQGETETDRAPPQIDAELKVIIDLFAYFITFNMGKWNPESSHA
ISKHFVKLNDEASQHYIKRLLTEHPNFEYEELLHRFAELKDVLAYKLRTEMIEQLVYA
MALGQVEQNQADLFMTHLCRVSNVSKAVFPDLVKIIYQCLADEMNQSTTSTFNGGQLQ
ACKLLDIQPNSMTEQNLQSAYRKKMAEFHPDKYQNLPESVRQLIESQAQQLNEARALL KSYLDNN"
/product="hypothetical protein"
/transl_table=11
CDS 2515941..2516633
/locus_tag="AUO97b_02495" /db_xref="SEED:fig|400667.82.peg.2361"
/translation="MIKNSTNSTKKIPPLYWLLTVVMMAVLIAGGILFNQHQKRQTEL
QLAQLALEQSKVQNQSATTQAASEPVESGFTGIESMVENPESESAHSQAEDEVVAGAI
AAVDQAKAQDTSLGNLSSEVDGEYHEGKTLSSNASRYGLKPIKSAPADLKSTIPNIDQ
MLASHLMNVWGRSSPDQVSKRVEGWSQQGNTMTYRTAWDSDRYQCTWYVVSWDKNSNQ VLHEGYQPCFGH"
/product="hypothetical protein" /transl_table=11
CDS 2516692..2517021
/locus_tag="AUO97b_02496" /db_xref="SEED:fig|400667.82.peg.2362"
/translation="MNPFKLIACGILSLSLSSIAFAKTEQITLKANVYYGEESVVFPT
TKGEVILNSYAIPAKVVPQVKPFKKGQCLEIKSKHGFFKDTGDGQYIESIQPCSKKGL ATPKVTR"
/product="hypothetical protein"
/transl_table=11
CDS 2517236..2517409
/locus_tag="AUO97b_02497" /db_xref="SEED:fig|400667.82.peg.2363"
/translation="MQLSKRSPIHPLVGGFVGLVVGGMFLLYVQHQDNTVSLHYQCEQ CLQHFEIQQSSFS"
/product="FIG00350529: hypothetical protein" /transl_table=11
CDS 2517464..2518513
/locus_tag="AUO97b_02498" /db_xref="SEED:fig|400667.82.peg.2364"
/translation="MAHQVETMAFVGQTPWHGLGNQLTRNQPLEVWAQQAGMDWRIES
SNVSYMTKNERGQNIIMPYEDQRVLYRSDTHAPLSVVSQRYQEVQPMEILNFYKDLTE
QSGFELETAGVLKGGRKFWALATTGQSTALKGKDVSNGYILLATACDGTLATTAQFTS
VRVVCNNTLAIALRGQNSSAGVVKVPHSTKFDAEKIKQQLGISVHAWDEHMYEMKQLS
QRKVTQQEAAAYFDAVFNNTNLNIAEQDESIIQFYRNVATPKQINSSKTDNKTEPNGR
AMSKVMTMFNGHGRGAELASAKDTAYGLLCSITEFVDHERRAISKDHRLDSAWFGVGA NLKQRGLEQALRMVV"
/product="Mycobacteriophage Barnyard protein gp56" /transl_table=11
CDS complement(2518779..2519660)
/locus_tag="AUO97b_02499" /db_xref="SEED:fig|400667.82.peg.2365"
/db_xref="GO:0005507"
/db_xref="GO:0006825"
/db_xref="GO:0006878"
/db_xref="GO:0016021"
/db_xref="GO:0042597" /db_xref="GO:0046688"
/translation="MLPETWIYATWLAKVVLYLGIAFVVGGAFCYFLLGRYVEIKQTI
LKYITIGAGLGLISSILGFFLLIGSFANTGLSGMWDSNYINILINTPTGHVHIIRSIS
FALLLLLMIIKLSKGTIQVSKIEGVIFLILLTPIVFSFSQLGHVTNLPLFAQFLLSIH
VLVMSLWMGSLYPLWRTSKKITGLPLKERMHLFGRIAAFVVGILLACGASIALLLIKD
FNTLLNTPYGHGFIIKIAFVLSILLLAAFNKWYFTPRLQDPKFARQLGYAILFEMSLG LMILLMTGYITTVVGID"
/product="Copper resistance protein CopD"
/transl_table=11
CDS complement(2519728..2520108)
/locus_tag="AUO97b_02500" /db_xref="SEED:fig|400667.82.peg.2366"
/translation="MKIFQNKVSVLRNVLVGATMVVAASSAFAHVKLESATPAINASV
ASQPQNITLNFGEEVMLMNVKLLDAQRKDIPLNYKITHDMKKSFEVAVPKLKKGKYTV
VWTTMGADGHNMNGEYNFTLKAAK"
/product="Copper resistance protein CopC" /transl_table=11
CDS 2520387..2522744

/locus_tag="AUO97b_02501" /db_xref="SEED:fig|400667.82.peg.2367"
/db_xref="GO:0004008"
/translation="MDHHHEDKNTGKRLIDPVCGMAVTQASKFYEKIDSKTFYFCSEK
CKLKFENDPLFYINEEGKSSPTKKSMNRAEVDSNEITNSAHVNTSTIYTCPMHPEIRQ
NQPGNCPICGMTLEPLLPNLDEADENPELKDFKRRFWYTLPLTVIVITLAMFGHKLNW
FEIKVQTWIELLLTLPIVFWAGWPFFVRCWQSILNRSPNMWTLIGIGTGAAFIYSVVG
TLAPQVFPASFISMGRVAVYFEATAAIISLTLLGQVLELKARSQTSSAIKSLLGLAPK
TARKINEDGTEEDIPLSHVHVGDLLRVRPGEKVPVDGIITEGSSSIDESMLTGEPVPV
TKRIGDQVIGATMNMNGSLVIRSEKVGSSTVLSQIVQMVAQAQRSKAPMQRMADQVAG
WFVLVVIGISILTFFGWGIFGPEPSWVYGLINAVAVLIIACPCALGLATPMSIMVATG
RGASNGVLFRDAAAIENLRKIDTLIIDKTGTLTEGRPSFDKVIALSGYEENEVLRLAA
SLDQGSEHPLADAIVQAARERNLVLTTPTSFESGSGIGVKGELDGQQLSLGNTALMEQ
LGISVDTYIPQAEQLRAEGASVMHLAVDGTLVGLLAVSDPIKVSTPEAVHALKNSGLR
IIMATGDGLTTAKSVGAKLGIDEVYGEVKPADKLELVNKLQKEGRMVAMAGDGINDAP
ALAQADIGIAMGTGTDVAMNSAQVTLVKGDLRGIEIARSLSEATIKNMKQNLMFAFLY
NALGIPLAAGILYPFTGWLLSPMIAALAMSLSSASVIGNALRLRS" /product="Lead, cadmium, zinc and mercury transporting ATPase (EC 3.6.3.3) (EC 3.6.3.5); Copper-translocating P-type ATPase (EC 3.6.3.4)" /EC_number="3.6.3.4"
/EC_number="3.6.3.3"
/transl_table=11
CDS complement(2522822..2524195)
/locus_tag="AUO97b_02502" /db_xref="SEED:fig|400667.82.peg.2368"
/translation="MTHKLFNAISFRIAIIFSLSTVIILLIMGLVIHQLVMHHFETQD
RTQLEGKIQLLENLLEQNPTNTHELNTYLKDALVGHHDLIVQIERPTGQIIFSSAPAV
IKTQTLVKSKQAPWIEWEIQKKIYRGLVYKKSFNNISIPSAQIIVGIDTSEHQHFLND
FRRQLLYIGVVGTICLMFLGWFAAWRGLRPVQKMAKVAEGISAQHLSERLAVDNTRTE
LKSLAIAFNDMLDRLETAVEKLSDFSSDLAHEIRTPINNLMTQTQVCLSRSRDINTYQ
EVLFSNLEEFERLARMVSDMLFLAKAEHGLHLPNLQQVSLVKEISALFDFYDAIAAEK
GMSLEQSGQGYVKGDPSMLRRALSNLLSNAIKYGKPDSIIKINCRQNIDTTIFTIENE
SSPITSEQLNRLFDRFYRTDASRQRVEEGTGLGLAITKSILDVHGATIQADYNNGRIV FKITFKN"
/product="Copper sensory histidine kinase CusS" /transl_table=11
CDS complement(2524185..2524868)
/locus_tag="AUO97b_02503" /db_xref="SEED:fig|400667.82.peg.2369"
/translation="MRILLVEDEQKTGDYLKQGLSEAGYITDWVTDGLTGKHQALSEE
YDLIILDVMLPKLDGWNIINDIRKSGKSMPILFLSARDQIDDRVKGLELGADDYLVKP
FAFAELLARIKSLLRRGQQKEDSNLITIADLELDLRKRRVTRAGQRIDLTAKEFALME
LFMRRRGEILPRALIASQIWDMNFDSDTNVVEVAIKRLRNKIDSNFTPKLIQNIRGMG YVLEVEDDS"
/product="Copper-sensing two-component system response regulator CusR"
/transl_table=11
CDS 2525017..2525367
/locus_tag="AUO97b_02504" /db_xref="SEED:fig|400667.82.peg.2370"
/translation="MKTLFRRSLTTFLCIGSLFAGTQSWAAEAKQTSTVSTAVQADIK
EQGKCQKPCDMKNDKTTQQDHSQHDHSKMSDMSQMDHSKMMNMDPSKMGNMDHSNMMN MDHSKMNMSNTSNK"
/product="Copper resistance protein B"
/transl_table=11
CDS 2525449..2527419
/locus_tag="AUO97b_02505" /db_xref="SEED:fig|400667.82.peg.2371"
/db_xref="GO:0005507"
/db_xref="GO:0016491"
/translation="MSKKLSHVFLIGIGLFSSTWVMAAVKEYDLTIAEQTVNITGKPL
KRITVNGKFVAPLLEFEEGDDAVIRVHNKLKNQDSSIHWHGLILPGIMDGVPGFNQFD
GIAPNKTYEYKFKVRQNGTYWYHSHSKGQEQDGLYGPLVIYPKNKVPLSAGEKADRDY
VVLLSDFHNSTSGQIMSNLKKEADYYQNRRETVFDVFKQIKKDGLKATWQDRSMWNQM
RMLKTDMSDVTKYTFLMNGKTPEQNWTGNFKEGERVRLRFINASAMSFFDVRIPNLKM

TVVSADGQPVKPVPVDEFRIGTAETYDVIVEPKQANYQIEAESIDRSGFSIGTLHNEN
TLPVKNILMPKPRPRSLLTMEDMGMGHDMSSMEGMNHDMSSMGSNSKDQSMSGMNHDM
SSMEGMNHDMPSKGSNSKDQSMSGMNHDMSSMEGMNHDMSSMGGNSKDQSMSGMNHDM
SSMQGMTHNMEMNTAMTSPTQVKNGENVYGWANASTPAGLKALQYSDLQSLTPQKDTR
APEREIEIRLGGNMERYIWTINGKKFNETEPFKVKYGERIRLKFVNDSMMAHPMHLHG
MFMQLENGQDASNMPNKHTVIVPPGKTVTTLLTADELGEWAIHCHLLYHMSAGMMNKL
IVANVDSNSSDSKDVVAQPINNDKGVNQHAHH"
/product="Multicopper oxidase"
/transl_table=11
CDS 2527406..2528326 /locus_tag="AUO97b_02506" /db_xref="SEED:fig|
400667.82.peg.2372"
/translation="MHTTKKLYSKTLVSLALLGLTSVAFANDPSTGNQVNSSQSESMR
VLLKEPGGLGYAERNTYQKTTSGNDGDNSTGFETVISNGQNHDKHLKEHGGQIFQTTK
FENEWTVDEDGKGALGSKIETLIGTDENRLFIEANLDKSESHDPKYDVSALYSRNVAP
FWDLQAGVRYSEDKNNSNSDRVDGVIGVLGLAPYFFETQAYLYGGENNFWGASFEVER
DFLLTQKLITQPYLEVDAVFSDDSNYAAKSGLSELKTGIKTRYEITKRIRPFIDVAYQ
YEKGQKATTMQEATESEKGWKYGAGIELVF"
/product="Copper resistance protein B"
/transl_table=11
CDS 2528914..2530218
/locus_tag="AUO97b_02507" /db_xref="SEED:fig|400667.82.peg.2373"
/translation="MKFYKEKLSRRVRWPIGVICFSLVYGQVYAQPLTLDTALKLAEQ
YAPTLRVNSAQIEGAENVVLASGILPNPKLFVGLENYPISGDASWSVTQDGMTMQKIG
VMQDFPNRAKRQAEVELAKAELGSVTAQTEILRIELRQKVASAWFKRFYLERKLALYD
ELFSENRLLSQMTQTQVTSGRTMVADALDPKQEATVLLDQKDDLLRDLNKAKLELHRL
ISAEPTENLTQIETLPDQAPSIHLDSSKLYHHLHQHPELKAFQAEKKTAEAKLKQAQA
LQKPDWGIELAYQHRAREFGDMIGVQLTTELPVFSKRRSGPLVQAALAEQNRIIADQE
VQYREHLTMLDDGLSDLDALDQQIRRAMQISIPLAKQKVGLQLASYQAGKSNLSDVIT
ARQSLLEQRLRLIDLQQQQAVTKAQLYFAFEEPTLNYVEEQP" /product="Copper/silver efflux
RND transporter, outer membrane protein CusC" /transl_table=11
CDS 2530218..2531717
/locus_tag="AUO97b_02508" /db_xref="SEED:fig|400667.82.peg.2374"
/translation="MLRNKLAVTVIGVATISLVAGGGAGYWIAHQKDNPQSTLAGQQQ
DAKVLYWYDPMKPEQHFDKPGKSPFMDMQLVPKYADENTAMTDESRPTVKIDPSLQQN
LAIRYGTVEQAVIGNAMFTNGILQANERQTAILQTRASGFVQRVYGHAVGDMVTEGSP
IADISIPEWTGEQTEFLAVLRTGDRSLIQASRQRLQLLGIPQNVIHQVERTRRVQSNI
TLQAPISGFIDSLEVRNGMALAMGQTLATIKGINPIWLEAAVPEKQIAGIKRGMSVEA
NFAAFSQKVTGKVIDILPTLDTTSRTIKVRIELPNPSGQLKPGMFASVNIINNPQSSL
VVPEQAVIRTGTRNVVIVGREQGRFKPVVVQLGQSDGNKIAVLQGLKAGQRVVISGQF
LIDSEANLQGILDKLNTGQPITSSQTIKRSTYQGLGKVEKVTNQDITISHQAIPELGW
GAMTMIFKQPVQPFTRIQQGDQVNFSFTKVGNSYVISDISKMSMASMKN" /product="Copper/silver
efflux RND transporter, membrane fusion protein CusB" /transl_table=11
CDS 2531731..2534877
/locus_tag="AUO97b_02509" /db_xref="SEED:fig|400667.82.peg.2375"
/translation="MIARIIRFSILNRVFVLLAALILAAWGVWAVKNTPVDALPDLSD
VQVIVRTNYPGQAPQIVENQVTYPLTTTMLSVPGVKTVRGYSFFGDSFVYVIFDEHTD
LYWARSRVLEYLNQIQGKMPANAKSSLGPDATGVGWVYEYALVDPTGQHDLSQLRSIQ
DWFLKYELKTLPNVAEVATIGGMVKQYQVVLDPSKMAALGITQNNVIEAIQKANQETG
GSVLEMAETEYMVRASGYLKTLEDFRQIPIKTNSFGIPVTLGDVATIQLGPEMRRGIT
ELNGQGETVGGVVILRAGKNARETITAVKAKLAELQQSLPKGVQVVPVYDRSKLIDRA
VENLSHKLIEEFIVVALVCGLFLWHLRSAMVAIVSLPLGILSAFLVMYYQGLNANIMS
LGGIAIAIGAMVDAAVVMVENAHKHIETWQHEHPNQVLETQARWNIIAHSASEVGPAL
FFCLLIITLSFIPIFTLQAQEGRLFSPLAFTKTYAMAAAAGLSITLIPVLMGYWIRGK
LPSEQRNPLNRFLIKIYRPMLDQVLTHPKTTLFGALLIFLISLFPLTRLGGEFLPNMD

EGDLLYMPSALPGLSAAKASELLQQTDRMIKTVPEVATVFGKAGRAESATDPAPLEMF
ETTIQFKPRSEWRSGMTPDKLIKELDKAVQVPGLTNIWVPPIRNRIDMLATGVKSPIG
IKISANDLQDIDRVAQQIEQVAKQVPGVSSALAERLTGGRYVDVDINRMQAARYGLNI
NDVQQIVSSAIGGENIGETVEALARFPINVRYPREIRNSLEALRNLPIVTESGQQIVL
SSVANIQITDGPPMLKSENARPSGWVYVDVQGRDLASVVQDLKQSIDQKVKRSSAMSI
SYSGQFEFMERANARLKVVIPITLMIIFLLLYLIFRQVQDAVLIMATLPFALIGGIWA
MYLSGFHFSVAIAVGFIALAGVAAEFGVVMLFYLKQAVEHAQQNLSSSTAPLTEQQLN
EAIRTGAVLRVRPKAMTVAVILAGLIPILLGTGTGSELMSRIALPMVGGMISAPLLSM
FVIPAAYQLLIKRKLSTH"
/product="Copper/silver efflux RND transporter, transmembrane protein CusA"
/transl_table=11
CDS 2534911..2535273
/locus_tag="AUO97b_02510" /db_xref="SEED:fig|400667.82.peg.2376"
/db_xref="GO:0008565"
/db_xref="GO:0009306"
/db_xref="GO:0016020"
/translation="MLKFMKIIALVTTIAALSACSKNEASEQKKADAPMEQMNKESST
ATTAQAVGVITAIDTKENILTLDHEAIPAINWPAMKMGFKVADPSLLNGLTVGDKVDF
ELKTESENQIIVAVKKSS"
/product="CzcABC family efflux RND transporter, membrane fusion protein"
/transl_table=11 CDS complement(2535345..2537174)
/locus_tag="AUO97b_02511" /db_xref="SEED:fig|400667.82.peg.2377"
/db_xref="GO:0001882"
/db_xref="GO:0005525"
/db_xref="GO:0005622"
/db_xref="GO:0015093"
/db_xref="GO:0015684"
/db_xref="GO:0016021"
/translation="MNSKNIALIGNPNCGKTSLFNTLTGTRQKVANYAGVTVERKEGF
FKLPSGESVRVLDLPGTYSLKPTSLDEEVTRAVCLGELEGEVLPDIYVCVVDATNLHL
HLSLVLEIRALNRPMLLVLNMMDEVKKRGISIDINKLSELLGIPVIEAVAVKTKGVQD
LLNQLDKKNLFVTPYHSDLNHFDQIKNITKQVILKNDNGDARTAFLDKIFLHPVLGLV
ILTFTMFVMFQAVFIWAQPFISLIENSITWLGDTIGPLITHPLLRSLIVDGVIAGSGS
VLAYMPQILILFFFILILEESGYLPRAAFLLDKLMSKAGLSGRSFIPLLSSFACAIPG
VMATRSISSERDRLATIMIAPLMTCSARLPVYALLIAAFIPNKLVYGWLSLQGLVLFG
LYMAGIVSALCVSLFLKLVRKDKTESIFIFELPTYRLPDIRNVALGLYDRATIFLKRV
GGIIVALSILLWVLVTFPQPPEHATMPAINYSLAGQLGHFIHPIFAPIGFTWEICIAL
IPAMAAREVVIAALGVIYAMSGDEDTVTQTLLSQISGPDGWGLATGMSLLVWYIFAPH
CLATLATIRRETGSWKQPVIMATYLFALAYFFSFITYQLISRL" /product="Ferrous iron
transporter FeoB"
/transl_table=11
CDS complement(2537164..2537424)
/locus_tag="AUO97b_02512" /db_xref="SEED:fig|400667.82.peg.2378"
/translation="MNLFQVKPNDFVKIVDLKKGNQYLDKNDPVLERLELLGFRIGET
LQVLAKGLFGGDPVLVQIETSRFALRKNEAERIFVEIVSNEQ" /product="Ferrous iron
transporter-associated protein FeoA" /transl_table=11
CDS complement(2537436..2538743)
/locus_tag="AUO97b_02513" /db_xref="SEED:fig|400667.82.peg.2379"
/translation="MGTSMSGCGCNKENSCEDTKQNQNESETNSGNSKDLTAEESKSS
CCGNELPQQSKPDEKDPCASTQPCSTQSNSCCTPDSISSPETVNDNEDVATFISDYKI
PKMDCSAEEQMVRMTLSSLVNVKRLVFDLPNRNLKVMHNGKSNEITAKLEALGFGAKL
NKTEAYVNKEHAKEISTFLIPKMDCSAEEQMVRMALSPVNEIKGLTFDLPNRKLKVFH
NDGLNKITSKLEALGFGAKLESTATSVGEMPEKADTSEQAKVLKILLAINAALFVIEF
ISGLIAASTGLIADSLDMFADAAVYGIALYAVGKAAKYQVKAAHFAGWIQLFLAITVI

VDVIRRFVLGSEPESLLMIVIGFFALIANVACLYLISGHKDGGAHMKASWIFSANDVI
VNLGVIIAGFLVALTGSAYPDLVIGIIVSLFVLNGARKILALK" /product="Co/Zn/Cd efflux system component" /transl_table=11
CDS 2538996..2539211
/locus_tag="AUO97b_02514" /db_xref="SEED:fig|400667.82.peg.2380"
/translation="MNLIEIKKLLNYKDLPNLNCSDVNELIDSHINDVEENIRNQQKL
IQQLLEIRKTCDGLCTVEKCGVLKKLA" /product="Transcriptional regulator, MerR family" /transl_table=11
CDS 2539356..2539481
/locus_tag="AUO97b_02515" /db_xref="SEED:fig|400667.82.peg.2381"
/translation="MQTIWLVGYGEWTGMTSATLIGVSRTARAVVDEIVVYLAID"
/product="monooxygenase, putative"
/transl_table=11
CDS complement(2539626..2542313)
/locus_tag="AUO97b_02516" /db_xref="SEED:fig|400667.82.peg.2382"
/translation="MNIEICTPNSATTKDKGDLLEKLCKKMLEAQNYLVTEEVRKTGS
ELDLLCEHKVSGKKIYVECKAYRDKKIDAPIIRQLFGTVVFENYSEGWLIGTSEFSKD
AKGFCEELPSRPLGDRIVVYSSTDIVESLQASKIISSIPREHLEQHLDINSIGEWFLL
ITTFGNFWVSTILSAGIPTNAVCYYAKTGVLVEDQELLDNIASTDSSLSKLDFSKIIN
TKKEMKPLFDSQIEVVEVQRGEDWNDYRPARPQDFVGRTKDIQEIFDFFKKIRTNEIN
TRIFAITGNSGLGKSSLIITLSEKAKNRHQKQKLFLYAIDVRAAKSPEYIYSALLKTL
QEAQIKGFGDPTIKLQITNVNHPLESESIAKYLNSLERSKQLVVLVFDQFEELFSKPE
LFELFNNATNILLDAASLKTNLCIGFAWKTDSTTHSEHPAYFFWHRLSDYRIVRKLNP
FSVRESNAVINKFEEAIGEKVHSDLRHNLIVSSQGYPWLLKKLCIHLHEKILSGQKQE
ELLENKLDISSLFASDLEELNPNEVRALKFIAQRAPVDLVDTIDTCGEDVVTNLLHKR
LIIKSGIRLNIYWDIFREYILTESIPIISLRYLPSNDFLAIWNVAQHLTGTPISIPEL
EIKTNLSDGTIQNIGTDLLTFGIAVRENSQYSLSEEILATGNSMESILSLMREKFKKH
IITLSLKDLPNGTNITLAFFIDLMKSIYSNNDYADKTWRYYAIRMCKWLEITGFLSRS
IETYTWFYKDIGSVKTSVSNVRRQQSSFFIPRNSPQLTINIYNELKGKKISEYKNGSS
NAKAVEIFKRYNLVDDDNKIRDVEDIESYLYDAISNELSIQETILIKKLYSGTKKLTA
SFVGQLLRDKHDIKWAEGTVEYAGKKLNSWASWYENFDL" /product="hypothetical protein"
/transl_table=11
CDS 2542862..2543191 /locus_tag="AUO97b_02517" /db_xref="SEED:fig|
400667.82.peg.2383"
/translation="MKTYNFIFTYAMSQSGYPSDKKCESIKKLENKIGNRQIEKWTKL
DKVENTFIGELVLHSCSISEKSEEAKRIVRTVFEEMMFEIEVYSDVTFTIAMLVDGLG EYLEFNA"
/product="hypothetical protein"
/transl_table=11
CDS complement(2543419..2543799)
/locus_tag="AUO97b_02518" /db_xref="SEED:fig|400667.82.peg.2384"
/translation="MQLRQKKPQTNVTIALDKSLIDLYLRFGEYAFAEHKDKKYIVVA
RIGFKKQKNGYGTALIKELCNFGEKFGYEYLQIECPNQDCQAFMKKLGFKDEFYLPID
RLKKSIQEYELLKQTRNQSLMSSS"
/product="hypothetical protein"
/transl_table=11
CDS complement(2544019..2545296)
/locus_tag="AUO97b_02519" /db_xref="SEED:fig|400667.82.peg.2385"
/translation="MLKDTQVKNLKPQDKPYRKLDADRLYIEVRPSGKKVWIHKFSLN
KKEGSITYGEYPSISLAEARYYHARDRALLAKGINPVAHRNETARLALETEQETFKKY
GDEWKEKHKIDKSEDYKKLIEYGLKTDIYPVIGHLHPKDVTSKHVLLIINNTMERIRT
STRSQKLKPSTGETTAIVNKTFIGMIMRSCIMKGLAEVDPTYAVKGVIKRQKINHARP
LSMKEIRELKERIPLYNGAQTTRNALNFLFYTMLRTIEVRRLEWAWVDWENKTITFPE
RTFQEIIEGKRAMKNNKIHLLPLSDQAFSILKEQYTITAKCQFVFSSPMQLTLGKPEK
MLGRSTLNFALDALKMNDVSPHDARATASTYLNELGSDDRWIEKQLSHTDNDKTRATY

NHAKWLRNRRSMLQWYADFLDGKAEMPVHEEVT"
/product="Integrase"
/transl_table=11
CDS complement(2545456..2545944)
/locus_tag="AUO97b_02520" /db_xref="SEED:fig|400667.82.peg.2386"
/translation="MPSFDIVSELELFEVNHAVQNTQKEIATRFDFRGHDVSIELNEK
NKEIKISTESDFQCEQVYNMLENHFYKRKIDVQALDPQKATASGKNFVQVIKLKDGLD
SDTAKKINKAIKESGIKVQSSIQGDKIRVTDKKRDTLQQVMAFLREQQFGLPLQFNNF KD"
/product="UPF0234 protein Yitk"
/transl_table=11
CDS 2546036..2546350
/locus_tag="AUO97b_02521" /db_xref="SEED:fig|400667.82.peg.2387"
/translation="MIRKQEITTFEFPENAVIWDVRDSSAYAEGHVKGAVSRPINDLN
ADILNQVAADQPIYILCGGGSKAPRAAEKLEGLDASREYVVLMGGTRAARDAGLPLEQ GA"
/product="Rhodanese-like domain protein"
/transl_table=11
CDS complement(2546402..2548273)
/locus_tag="AUO97b_02522" /db_xref="SEED:fig|400667.82.peg.2388"
/db_xref="GO:0004808"
/translation="MNKSNRIQTAELDWEFIDGIEVPISKQFGDVYFSKDNGLLETRH
VFLNGNDLTERLANLQDFEYFSVGETGFGTGLNILALWQLWQQVRPNNHSHLHAISVE
KFPLSKADLIRALNVWDELKPLSKQLIEQYPLPLAGCHRLSFPEERFSIDLWLGDAQD
IFPSMVKTKAVNAWFLDGFAPSCNPDMWEQNVLNNIVRLSDYGTTFASFSVAGVLKRG
LKAHGIDISRPRGFGHKREMLKAVWKAPVSEEILLEPVSDTLHFTQQHIAVIGAGIAG
LSTAWAFAQRGHQVTLFERTAPLSGASGNPLALLNPKLCPIEQSHEHLMTLSWQHALN
FYKNFQAFHPIQIQQMALKNAQDLLDLADQYPADIVTQQTSSLESDYPHILLTEAGAV
SPHQLRAEILQHPSIELKIANITTFVSFENKVQLKSGNETTLEADHVVVCCARESAAL
FENYPLLKPIRGQVSWVDNSFATLPLNEAYSYGGYCMQLNTSELILGASFYPNRDDQE
VLLDDHVHNFELIHSVFPHYAKQLPPVEQWQGRASVRAQSPDYFPVVGKMQKESRIST
FAGLGSKGFLFAPLCSEVLVAQILGEACPVPKSLLQKLDAQRFQKKVKPKKPYFKSQ" /product="tRNA (5-methylaminomethyl-2-thiouridylate)-methyltransferase (EC 2.1.1.61) / FAD-dependent cmnm(5)s(2)U34 oxidoreductase" /EC_number="2.1.1.61"
/transl_table=11
CDS complement(2548297..2548776) /locus_tag="AUO97b_02523"
/db_xref="SEED:fig|400667.82.peg.2389"
/translation="MQLATKSFLGLCLICSTAGAVEPAATPATPPTAASTQNAKPAPT
SATVAKPATTTAPTSTQPLTAEQIAKNKQQQDAKIKALVQDQASRLKQLEHANLEALA
QNQELQLKNDNLSVQVQVLQSERSAQMFLYGAVTLAGGVLLGFFIASYVYTKRRRQW"
/product="hypothetical protein; putative signal peptide" /transl_table=11
CDS complement(2548781..2549083)
/locus_tag="AUO97b_02524" /db_xref="SEED:fig|400667.82.peg.2390"
/translation="MPFFVLSCTDNEGTLEKRLAVRPQHIERLQKLDDEGRLVAAGAM
PKDPNDPQAGFYGSTMIVEFDTREALDEWLKEEPFLKEGIYSHVDVKPFNKAFPKG" /product="YciL protein"
/transl_table=11
CDS complement(2549118..2549732)
/locus_tag="AUO97b_02525" /db_xref="SEED:fig|400667.82.peg.2391"
/translation="MKALLDFVPLIIFFYLYKTVDPADTQHPLLKLIGSAGGVDNNNI
LVATTGLIISMLVVYGALFIMQKFRLDKQQWIVLFMTVIFGGITLMLSDDFYIRLKAA
ILNLVFAGAFLVSPWFGKDRKPLIQRLFDPILALSEKGWKNLNFAWAAMFVVMSGLHV
FFAFLFHGGKYWGEFTAFGDMIVMFSFIIIQFIILRKHFKSPDA" /product="Intracellular septation protein IspA" /transl_table=11
CDS 2549777..2550628
/locus_tag="AUO97b_02526" /db_xref="SEED:fig|400667.82.peg.2392"

/translation="MQGIDLHTHSNISDGTLSPELLVQAAVDVKLHTLALTDHDTMDG
LQRARNAAQPHDLQIITGVEISSQWSRPSTKKSYGVHIVALNMQAEAPILEALEQQKR
IRAQRAEVICELLKKCIGFDIYQDVLDKVENQPDRITRTHIAKTLVEKNVVSRPQQAF
DRFLKEGKRAFVKFEGLGLKETIEVIHNSKGFAVLAHPTRYDLSATNTRYLIELFASC
GGDAVELPPPVDPLSTRQMIDRMIDQHHLAISIGSDFHGENMPWIKLGNTPKPASNQQ GIWERFI"
/product="FIG00031715: Predicted metal-dependent phosphoesterases (PHP family)" /transl_table=11
CDS complement(2550636..2551547)
/locus_tag="AUO97b_02527" /db_xref="SEED:fig|400667.82.peg.2393"
/translation="MIVREQPSIFKVLFSWRGTILPKILPSLGVVMLISAIIGGVEYI
NLYRFPEIPLVGFTLIGVVLSIFLGFKNTACYDRWWEARKLWGVLIATSRHFDRDCRI
LTQARRERVIQHVIVFANVLRDRLRRQTANPTELIQTSGMSQQALTQLYQQNNAPQYT
LSLIQWELLQALKEGEISDIIYAQMNKNVADLSVVQTGCDRIANTPIPFAYSVLLNRT
VYFFCFMLPFSLGSLLGLATPLLVGILAYTFLGLDALSTEIEEPFGTQSNDLPLDAMV
RSIEIELLGTLGKPTPPPIQAQDNNLL"
/product="probable membrane protein STY1534" /transl_table=11
CDS complement(2551609..2552316)
/locus_tag="AUO97b_02528" /db_xref="SEED:fig|400667.82.peg.2394"
/db_xref="GO:0006281"
/translation="MNTSIKNWPEQERPRERLLQQGPQSLSDSELLAIFLRSGSRQHS
AVELARLLIQQFGSLNAVFDASYNELAQFNGIGATKYSQLLAVKELGRRYLDYHFSQT
ELSLHSSHLVLDYLRYELKGEKQEVFAVLCLDAELRKLHFKKLFFGSVQHCAVSVNQT
LRYALQQHACQLVIAHNHPFGSPQPSPEDIKLTQQLEQACQLVEIRLLDHFIISPEGS FSFAEQQLLNPTSIAVQ"
/product="UPF0758 family protein"
/transl_table=11
CDS 2552429..2553682
/locus_tag="AUO97b_02529" /db_xref="SEED:fig|400667.82.peg.2395"
/db_xref="GO:0004633"
/db_xref="GO:0004632"
/translation="MSFDLSVIPHKNIVLAVTGGIAAYKSAILVRRLKDYGFDVRVVM
THGAQAFITPLTFQALSGNPVHTELLDPGAEAGMGHIELARWADLLLVAPASCDTLAK
FANGLADDLLSTLFLATKAPVWVAPAMNQQMWAAKATQRNLQTLVEDGVHVIMPDAGE
QACGDVGLGRMPEPEEIARQVAGYFHQAQRAIAEKFGLLAGKRVVITAGPTREAIDPV
RYISNHSTGKMGFALAAACYAAGAKVTLIAGPVSLDTPNGVQRVNIASARQMLDVSME
SLETGCDIFIATAAVADYRVAEVAEHKIKKAGDELNVSLVKNPDIVATIAAQEKRPFM
VGFAAETQNVEEYAAGKLVAKKLDMIACNDVSRADIGFASDENAMTVFFAESYHMKKR
ELEKASKQEISQQLVEAISDALRRK" /product="Phosphopantothenoylcysteine decarboxylase (EC 4.1.1.36) / Phosphopantothenoylcysteine synthetase (EC 6.3.2.5)" /EC_number="4.1.1.36"
/EC_number="6.3.2.5"
/transl_table=11
CDS complement(2553746..2554711)
/locus_tag="AUO97b_02530" /db_xref="SEED:fig|400667.82.peg.2396"
/translation="MMTKVTQQPSKQYGRPDDAKIIPFMKIAKPAAIFSILITLASIF
FIATKGLNLGLDFTGGVSAELNYAQPANQAEVIQALDKAGFKDAVVQTLGSNKDLLVR
MPVQDIKVEDLNNAITKAVQLPNNAAEVHKVDSVGGQVGNELYVRSAGAVALALILML
IYVTIRFEFKLAMGAVMSLFHDIIIIIGAFALFQWPFDLTVLAAVLAVIGFSLNDNIV
VSDRIRENFRKIRGATPREIVDIALTETLRRTIHTSMTLLLVVLAMMFLGGDGLHWFS
VAMFVGVFVGTYSSIYIGTAFALWRGLNRQDFIVQVKPEFEDEIP" /product="Protein translocase subunit SecF" /transl_table=11
CDS complement(2554720..2556621)
/locus_tag="AUO97b_02531" /db_xref="SEED:fig|400667.82.peg.2397"
/translation="MRYPAWKYLLILVVLVVSTLYALPSLYPDEPAVQISGAKAGTQI
DQSVVQKAEQILKTANIASHDNTFTNNAALLRVSSSEAQLKAKEVLRRDLGDQYVVAL

NLAPTTPEWLQKIGAKPMKLGLDLRGGVHFLLEVDMDKAISQRMETSATDLRRQFREN
KIKFNNLALNNNVITVQFADNADRDAAMDFLRRNGNEYTQQALASTTGSILKLTYTDV
RRQEIQAYAVNQNLTTLRNRINELGVAEALVQSQGSNRIVVELPGVQDTAEAKRVLGR
TANLEFRLVSDLNDQYIDPYTGKYNGQPLPPGTEVFAYQSLDSGRQLLLQRNRILTGE
RVQNASSGFSQDSGGAEVNITLDSAGGKLMSDATRNAVGKRMAVLFIENKQKISYVTD
PKTGAQTEVRTPYTESVVINAATVQAVLGSTFRITGLSSPQEASELALMLRAGALAAP
MYFVEERVVGPSLGQENIDKGVLSTQIGFLLVAIWMVVFFRLFGLIANFALVFNLAMI
LTIMSWIGASLTLPGIAGIVITIGMAVDANVLICERIREEMLWGASPKQAIVAGYDRA
YNTIFDSNLTTFLVAFILFAIGTGPIKGFAVTLMIGIICSMFTAITVTRAIVQIIYGK RRNLKKLSI"
/product="Protein translocase subunit SecD" /transl_table=11
CDS complement(2556673..2557002)
/locus_tag="AUO97b_02532" /db_xref="SEED:fig|400667.82.peg.2398"
/translation="MSLFISTAHAAPAAAQSPGLLPNILMIVVFVAIFYFLIWRPQAK
RAKEHRSLIESLGVGSEVVFAGGLMGKVTKLEGDYAVVELNRGVEVKIQRASVISVLP EGTLNNL"
/product="Protein translocase subunit YajC" /transl_table=11
CDS complement(2557101..2558234)
/locus_tag="AUO97b_02533" /db_xref="SEED:fig|400667.82.peg.2399"
/db_xref="GO:0008479"
/translation="MKFEKLGQSGRARRGRLTLEHGVVETPVFMPVGTYGTVKGMLPR
DIEDIQAQIILGNTFHLYLRPGLEVIKQHGGLHDFIKWNKPILTDSGGFQVFSLGAMR
KIKEEGVTFRSPIDGSKVFLSPEISMEIQHVLNSDIVMIFDECTPYPATHEEAQKSLQ
LSLRWAKRCKAHHHDELKNKNALFGIIQGGMYEDLRDESLNGLLEIGFDGYAIGGLSV
GEPKEEMIKVLDYLPNKMPHDKPRYLMGVGKPEDIVEAVRRGVDMFDCVMPTRNARNG
HYFVTDGLVRIRNSKYRHDQGPLDPHCDCYTCKNFTRAYLFHLEKCGEMLASMLGTIH
NLRYYQRLTEGMRDALDNGTFDEFVQDFYARRGLEVPPCPVDE" /product="Queuine tRNA-
ribosyltransferase (EC 2.4.2.29)" /EC_number="2.4.2.29"
/transl_table=11 CDS complement(2558410..2558979)
/locus_tag="AUO97b_02534" /db_xref="SEED:fig|400667.82.peg.2400"
/translation="MTGLFIFLGIFVVLIAWGIHIRNNIVRYFNATKRAWAEVANFER
QKVKTLEALEITLNQYTQFEKSTLEQVTALRQQILSLNVDHTDIAQLQQIEKMSKELI
RSLNVVVENYPELKADTLYSKMMDDIQEQNENVGAAISIFNRNVEVFNNQIEVFPNNI
INTMTLSKKTIRPFRDNLVTDNFDYKPNF"
/product="hypothetical protein"
/transl_table=11
CDS complement(2559065..2560093)
/locus_tag="AUO97b_02535" /db_xref="SEED:fig|400667.82.peg.2401"
/translation="MPIDTAQQALHIRRKKNTQVFRNIARLWDAGQKSQTDQELLNAL
HPWREDHNLRFVNTLSYLLSICSITTLLFGYFFHPHIQYIWSLLWVFLTGFLAYLLYE
PQKPLTEVIHYLEQRMTALRYGLKFQQLPAYLPIQAQPILVLSKLKQHFPLFNRGNEA
NEITEFASVTWNDGITDHQVLLFKYHYVNNLPILQDQNSDKKIVKEIHKDLWGAFIFQ
IPALGIAVSNQRSRFFEPYLSEWQSSDILINQELSIFGTDQHQLAKEMSPSLTLKLHD
FFQHFSGDLIYHHEEQILCYLGEQDLFQTTSKRSEIHDIPALRGHLRTMTMPQYEKFQ QFMLNLIK"
/product="hypothetical protein"
/transl_table=11
CDS complement(2560247..2561284)
/locus_tag="AUO97b_02536" /db_xref="SEED:fig|400667.82.peg.2402"
/db_xref="GO:0003824"
/db_xref="GO:0008616"
/translation="MQLSDFSFELPDELIARYPLETRSASRLLHLDAKGQYHDHMFTD
IIDLFEEGDLLVLNDTKVMKARLKGKRATGGAIEILVERMLNHTTAYCHIKASNSPKA
GAELFVGADNIPVIVRGRHENLFVVEFSQPILPVLEQYGQLPIPPYFNREAEEIDTER
YQTVFHNPEKIASVAAPTASLHFDEELLAKLDQKGVKKTFVTLHVGAGTFMPVRTDDI
TNHVMHSEWCDVPQETIDLILATKARGNKVIAVGTTATRALESAAQAHGGKIAAWTGD
TQIFIYPGYEFCIVDRLITNFHLPESTLLMLVSALSNRENILAAYEHAVKDRYRFFSY GDAMLIDKLEV"

/product="S-adenosylmethionine:tRNA ribosyltransferase-isomerase (EC 2.4.99.17)" /transl_table=11
CDS 2561707..2563008
/locus_tag="AUO97b_02537" /db_xref="SEED:fig|400667.82.peg.2403"
/translation="MAKQVRPLTDTKCASLKPKDKEYVEADGGGLYLRVRPTGAKSWI
FRYTNRGNDKREKITLGPYPALSLAKAREKKHEFLRMIAEGVDPKEQLSILLAKKNNV
NTLENVVRVWLDAYAVRKPLSEDTKNKQLRKFENHLFPKFKDKAIEQITLRDLKDALN
VIYDHSPDNAQRIRASLIQVYSYAVQHSYIQTNIARDLEDMDLSARKNHRATFRSLDL
IPQLIRRIKADSGNPLTKLCLLLGLHTFLRSSEIRFARWNEIDFEAEIWRIPPRRRLI
EGVKHSDRGAKMKEEHLVPLSKQSIEILEKVYQYSGDCDFVFPSLNNKRIFISENTPN
DALRRMGYTKEEISFHGFRALARSALGEMSIFSRDALEKQMSHQERNETVGAYTHIAE
YLEEREKIMQIWSDWLSAIENGDYISPHEYGRHLRLGARVG" /product="Integrase"
/transl_table=11 CDS 2563290..2563832
/locus_tag="AUO97b_02538" /db_xref="SEED:fig|400667.82.peg.2404"
/translation="MSETSEEKKKRKLIAELRDHQWLYQYSLFPRLEGQNRDEDKSNA
ETTILRGLDDFRDRLRRKTPNIGILFELRKLNVARLRNFVTRYRNKNFIQIYITFRTT
KEIDEQMLKEIAEDTYSDRVNILERSINEEDIIKSISTIRNENLYDFTPYYQIIGKKF
KRYSCIHRSSFIRKEEILQC"
/product="hypothetical protein"
/transl_table=11
CDS complement(2564784..2565017)
/locus_tag="AUO97b_02539" /db_xref="SEED:fig|400667.82.peg.2405"
/translation="MEESPLILFGLRLRELRKIKGISQERLALESGIGRSYLGEVERG
KRNIALLNIYKLARALNIEPSQLLEKPELDQNQ" /product="Transcriptional regulator, Xre family" /transl_table=11
CDS 2565174..2565950
/locus_tag="AUO97b_02540" /db_xref="SEED:fig|400667.82.peg.2406"
/translation="MGNNFHSRKKSFSTSSKQQGKAGLILLIIVVLIVFLASLAISSS
WNTDEYSPIVTQTESENNNITTTKSKEMEVPIPISDSSDKGKNFLVSKESKHNGFIVI
YKRIGVEQTLFNKYEINCKTEKMRVLGEGDSIDNIKNYSEKGSWFTPVSEASTGDTFN
FICGRDKKINERREDSIKQSFGNLQYKIEPEAVINSKIDKDILSQLILAVLVNGYKCD
TVSSAIPYIRGGGYTMQCNNYQYEYEIEDKGGDIVVTVLN" /product="hypothetical protein"
/transl_table=11
CDS complement(2566005..2569757)
/locus_tag="AUO97b_02541" /db_xref="SEED:fig|400667.82.peg.2407"
/translation="MNHTLNIPLPSTEQDFESMCSHIFGKLYSCTLPAMYGRRGQKQY
GLDILIHENDHFDQNSRIGIQCKHVNKLTYDGLSGDSILKEVAKADSGIQKIKLLVIA
TTKESDVSLQNSVSNLSDERVKKGLFPIIIMSWNDISNYINKDQDLLKYYLTDNKIIN
AFYKNIENNIKYEKFQEALEQLKNNTFTDSFNISEIYKQLLLKAFCYYNLEDKSQFNN
VLNKLEEYEWLDEKYIYLKILQLKEKNHQLALDKLKNELELRPESLYLLTLDAYFKIF
DKETNIEIDQINPSIQNTVKVKKFFMMKYSQSDKNIEKVNDIYNTLDASDKKIPSIYL
TYITSKLNNYYFSKTELSKEELIQAIKILEPYQQKFWEIEIPAFKKLAITILAATYFH
LEYFDKIIILYQFIIHTQILIDNQAINIFLETSYKKNRDDLFIELCNKYFHLLENEKK
IEILEKLIQLKKFDFVERELSTLEIDIEPKQYLKSLLWVNKLDEIDFLTKISEENALS
FSSPISLIVIAQKLKNDIKNIELFESFNQKIKDLVDKNQDDEYYSLYFFQTNQYSDAI
KYLEKLYQKHQDSKFSFYLFESYIKVKNFKKAKYLFEKHKNSFLSFDNFIDLCHEMAK
ETLDWSLCEDLISEYENEYSTQGWLWALKIIITKNLSSSLQFQRLIRDIPENLEGPPN
TICLIAVQEVNANFYAKAKNRIKHLWRKNLNDVSVEAEIFKVLNSFINLKVSEKHPFF
EEELTNAQIGTAITYKLKNMTETKIIDFDESLPRDNEFISYESELAKNLIGKKVGDKF
TIQGTFGFENEYEITEITPILLYLWRKFLNKSGQADNPFNFATSFNVGSKDEGITDLL
SKISTITKKRTESIEYNIKIYQEHPLTIGGLAKQLNIDLVQLVYEWHYGNYTLLRTID
ERYISEEIERENFFKNRSSVKKVVLDAFTLIELFYFDSFFIVEEFEEVYLSTNTYQLL
IYLQEKYKDESQNLNIQGRMGFNNNSPIFINHDQNYINNIYSDISSIIHHIEKNTNFL
IEHAYGDGNINELNLIIDNFITTEENSCLRLSKELNIPLISFDARLRALANQLKIQTI

NMDDLVMLSEYKNNFFAENRFIKSRTISNSYLNSRNLQNFFLSDFKNFYSFLHVFFKE
ISSFSNEEAIHYYINMLKKISGDKISIVLEAVNHINMIFFSFMFHQKKENFDLIILRE
LLSPYMQPTIINMSSFTNYLDEILIVDINKITLNLALEKPTILLKS" /product="hypothetical protein"
/transl_table=11
CDS 2570204..2571094
/locus_tag="AUO97b_02542" /db_xref="SEED:fig|400667.82.peg.2408"
/translation="MENKNYHWLILFVILIAIITAWLSFPIFFEWLITKHFHINPEDY
GKKFGAVGDTYGSLNTLISSIALCAVAYSTWLQVTSLKETREVNAKQLTLAKQAHDEQ
MIESQNAIFATKFYSLLNFKKDKLNALTIQKRVKNDENEWRLAQEPGMQAIEIIANEF
IKLNRKNNKLYIGVKGDDLFDAYRAVCSELNYGSVSSLVSYFYIYEDLCQLIRKSKIS
DEDRKFYKSVLSSSMTQAEQILLLWICPMFKIDIEDSEIFTLIACTEVFKEFAFEFHK
SSHFKSVKWKDVFSKIQTPA"
/product="hypothetical protein"
/transl_table=11
CDS complement(2571112..2571327)
/locus_tag="AUO97b_02543" /db_xref="SEED:fig|400667.82.peg.2409"
/translation="MHSTLDVNNYKKMMAEKILEEIEYPPENLQNLFFAFSKMKVDDG
LKEQEFSTIINTQHYQVVNIKCAVHAK"
/product="hypothetical protein"
/transl_table=11
CDS 2571600..2572868
/locus_tag="AUO97b_02544" /db_xref="SEED:fig|400667.82.peg.2410"
/translation="MTQRLELHQVEQLTACKISLLLGLNAEQNYIEQFFRFSLRLLKC
QKALLTFNQEPYFWHHCPDGMTAISFKPSRHLKQCFAKQQVIHHNHPSYQNLINYLKE
LNIECGRALAVHLVQPDQTSMGFAVFFDDDENCFEDDQIQLLLDYCSSFMQQVELKFN
YEELNELYEQQVALNSSKTKFFSIISHDLRAPFHGLLGFSEVLAKERETLDESSIQNI
ADYLYDTSQSTYNLLESLLTWAMAEGGRFVYHPINFKLRQVSNIVCDVLHTLALKKNI
ELVNAVSEDLKIYADINMMTSVIQNLVSNALKFTDVDGSGKVFIEAKQVGTNVEITVR
DTGLGMTEQQMANLFHPRITASFKGTAGEKGAGLGLSLCKRFVEINQGKISVTSQKGV
GTTFKVLLPSAQEVPEHHIEQQNTSEAKLV"
/product="Two-component system sensor histidine kinase" /transl_table=11
CDS 2572937..2575687
/locus_tag="AUO97b_02545" /db_xref="SEED:fig|400667.82.peg.2411"
/db_xref="GO:0008882"
/translation="MNAEQLQKTLRASQYAEQVLGLHQAVLEQDYQIDQFAAPLSTEQ
IYQLVDITLDGIGDEITWMRALRILRSRLMFRWIWQDANQLTDVVTLTRELSDFADAS
ICAAKAFARVALVAKHGEPLSYSGKVQDLIVVAMGKLGAQELNLSSDIDLIFAFDEQG
ETNGRKCIDVQQFCILWGQKLIYLLEHITADGFVFRVDMRLRPWGDGSALAISHAALE
KYLSQHGREWERYAWIKARVVTGGKEGQDLLEMTRPFVFRRYVDYTAFAAMRDMKAMI
EREVLRRHIEDDIKLGAGGIREIEFIVQVFQLIYGGSKRELQDRQCLVSLEHIGEAGL
LEKQAVLELEDAYLFLRRVEHAIQALNDQQTQLLPEETDLRQRIIDTLGFTSWNEFID
VLNEKRQKVKVQFKQLIEDTSSNAATENFGQLEQQLDEVLDDNAKNLIHEFWHGHALK
KLPSNAVQRLKTFWPHLIEAILQSEQPQTALLRLMPLIESVMRRTVYLVMLIESKGAL
QRLVKMATVSPWICEELTQYPVLLDEFLSMDFELPKRKDLEDSLRQQLLRIEIDQVED
QMRVLRLFKKSNVLTVAASDVLAESPLMKVSDALTDIAEVSVNATLNLAYQTVAKRHG
YPKDVEGKRCSLDYKAFAVIGYGKVGGIELGYGSDLDLVFIHYLDEQADTDGTKSITG
FEFAMRVAQKFMSLMTTQTLDGRVYEIDTRLRPSGEAGLLVTSLKAYEHYQLKSAWLW
EHQALVRARSIAGDEALCQKFEIFRREILTQSRDEAYVREEVLKMRQKMKDHLGSSSE
QKKHGIFHLKQDAGGIVDIEFMAQYAVLAWSGTKPDLAHYSDNVRILEDAAKADCLSS
EDATALIRAYLSERAESHRLALANQSMQVSAADWRSTRAVVCNLWQRLIDPTASVELD SE"
/product="Glutamine synthetase adenylyl-L-tyrosine phosphorylase (EC 2.7.7.89) / Glutamate-ammonia-ligase adenylyltransferase (EC 2.7.7.42)"
/EC_number="2.7.7.42"

/transl_table=11
CDS 2575712..2576638
/locus_tag="AUO97b_02546" /db_xref="SEED:fig|400667.82.peg.2412"
/db_xref="GO:0004084"
/translation="MNLADRDGFIWQDGQLIDWRDAKIHVLTHTLHYSMGVFEGVRAY
ETPKGTAIFRLQDHTKRLLNSAKIYQMKVPYDQAALEQAQIDVVRENKLASCYLRPLI
WIGSEKLGIAATNNTIHAAVAAWAWGAYLGDEAMAKGIRVKTSSFTHHHPNVTMCKAK
ASGNYTLSILAHQEVAHSGYDEAMLMDPQGYVCQGSGENVFLIKDGVLHTPDIAGGAL
DGITRQTVMTIAKDLGYEVVERRITRDEFYIADEAFFTGTAAEVTPIREYDDRQIGAG
CRGPITTEIQKTFFDAVQGKNPKYEHWLTYVK" /product="Branched-chain amino acid
aminotransferase (EC 2.6.1.42)" /EC_number="2.6.1.42"
/transl_table=11
CDS 2576706..2577596
/locus_tag="AUO97b_02547" /db_xref="SEED:fig|400667.82.peg.2413"
/translation="MHIVYVSDGKAGHRSQALGLFQAMQRQQANATFEEVSINDLPIF
SLIKALFSSKKSLFQQAPDFIFGVGSHTHFRVWLLGKIFKKAKTIILMKPNLPTVWFN
YAVIPEHDGIPANNRVIVTRGALNPIRNEKRHQASRILIALGGSSKRHQWNQEKVLLS
VQKIVENNPDAEIILTTSRRTPAEFIDILRQQSFAQHLQIFPVEQTPQGWIFEEMQKA
EAVWVTEDSVSMVFEALTAGCRVGVIAIDRLKDDRITHSIDQMIESQLISQNTCVVQL
PQPYAFKEADRVATYLLAKN"
/product="DUF1022 domain-containing protein" /transl_table=11
CDS 2577619..2578647
/locus_tag="AUO97b_02548" /db_xref="SEED:fig|400667.82.peg.2414"
/translation="MKILHIANFGFNKQGAHFYCTDRKISAGLIENGHFVYDFSFRDM
ARMGTIFKTKKLGAKWANKEVLKVVNNIEPDLVLIGHSDLMSPDVLRQIKESYPKTKI
AFWYVDPLYLENKLDFVKAFSPYLDAIFCTTGGEYLQKLKQPHLSVAYMPNVGHRNVE
TLQQFSNSKTDKTFIFCGVVYKEPEREKFLKDLAEALQQGHVNYQYYGCFEQPGVYGK
AYYKVLSETKMGLNYSRRNDVTLYSSDRIVQLTGNGLLTFSPRIPGFEKLYTEQEVVY
FDDQFDLAKKIQFFDQNPEQAVKVAKEGWEKTRKSFNAKRITQFMVEVTFKQPLSEDY EWSHEVYA"
/product="probable hexosyltransferase"
/transl_table=11
CDS 2578647..2579399
/locus_tag="AUO97b_02549" /db_xref="SEED:fig|400667.82.peg.2415"
/translation="MWFLYVAVGLLALAALLYVLGNYTFWLPFRSSKLPRIVMLHQVT
PKDAASGMNMPPEKFEQLLTLLTRKGAIFCFVSELEQYRNQKNVVALSFDDGFMDNYL
YAYPLLKKYNAKATIYLATQIEGIEKLTHEQIHEMANSGLIEFGAHTQHHVNLLKLDD
QTAYDEMLQSKRDVEALVGKCPSFAYPFGRFNEKHQKMAQEIGFKNAVSTRKKIEAYE
ADNQFNIPRVSTHGAMNALQMRIALAKGRYKL"
/product="Carbohydrate Esterase Family 4"
/transl_table=11
CDS 2579396..2580163
/locus_tag="AUO97b_02550" /db_xref="SEED:fig|400667.82.peg.2416"
/translation="MNKIPCSVYIVTLNCGAWLERTLQSVSEFDEVIILDSGSTDNTY
EIAKRFENTQISHQDWQGYAGQKSLALAKCRNDWVLNLDGDEVLSEELKQEIVETIQQ
NKLDALITPINDVFLGVPNSKHTKKHAKVRFFRRSKGHYDLANKVHENVIVDGETQRA
QHDIYHYGESSIFVKVEKNNQYSNLKALEKYQKGKSPSIAKLVLVMPVTFMKSYFIRR
SFLNGWRGFVNSMINAFYAFLKEAKLFEQTMNKDKNK"
/product="Putative two-domain glycosyltransferase" /transl_table=11
CDS 2580171..2581205
/locus_tag="AUO97b_02551" /db_xref="SEED:fig|400667.82.peg.2417"
/db_xref="GO:0047265"
/translation="MKIVQVLATSGGVGGLEQHTFNLVNELALNHEVHVIAHPCYVDK
FSQQVHFHAIDFARSRWNIFLLWQLKNLIQQIQPAIVHAQAGKAAELIARIKPFLSGP
KFITTVHGTKKNKSAYLAGDAVIAVSQALTQGIPESKAHVVYNGVYPQPVLTTENKEK

LLQSIQKDFTELDTSKRVVMCIGRLEPVKNISLLIESMQQIDANLWIVGDGSLRASLE
KQVSELNMQNRVAFLGFRTDARDLVQLADIVVLSSDREGFPLVMVEALQADKAMASTK
VNGVIEWLPEQYLAEIGDTQGLAKAIEYALQPEAQNDFGPLYAKAKAELTVPAMAEQT LNIYKGLLKT"
/product="Poly(glycerol-phosphate) alpha-glucosyltransferase (EC 2.4.1.52)"
/EC_number="2.4.1.52"
/transl_table=11
CDS complement(2581202..2582095)
/locus_tag="AUO97b_02552" /db_xref="SEED:fig|400667.82.peg.2418"
/translation="MLITRGLQKKWYKYQQAKKAKTFDSHWYMRFGWLEQPFTTLEQL
DSLFEIHSPRKFTFADSFYAHENGRHFIFFEEVDDQHPVGFLSVLEVFKDGTYTPPET
ILKLDYHLSYPCVFKIDSTWYMIPESSANKTIELWKCTDFPMKWEKHSNLMENIEAVD
STPFYHEGLWYLFTSTRRDCKKFGDRLDLFFTEDILNPNWQEHPMNPACRGSQQFRMA
GKPFIYKGQLVRPSQDSLKRYGGNIELKAITQLSPAAYEEKLLEVVLPDWNQADDGCH
TINVEDNFVVLDAIRLTPKNN"
/product="Bll3360 protein"
/transl_table=11
CDS complement(2582114..2582899)
/locus_tag="AUO97b_02553" /db_xref="SEED:fig|400667.82.peg.2419"
/db_xref="GO:0009103"
/translation="MKGSNERQEKIKQQHQSLQSVIHDIQIDFEFFEAIYGKKLPNEY
LTFLNLSRQFAGLCDHELGPSELGCWLSHMIIWQRLAQGDYAAYDRIIIIEDDVIFQT
EHIQQKLHSLLETNPAFAFLGGHSEPSRRRIRGYVSNDELYFNMTGPKDLYTATYAYS
LTRETAQDFVNKQIKKLTYIDDWKYLLEGDISTPFYYCFEHDDEMQSHIASDRKTYMK
KPNRFKKNFNKMKNDLISRVISLFIFKKIIRLAYFLQSAQTQK" /product="Beta-1,4-
galactosyltransferase"
/transl_table=11
CDS 2583109..2584236
/locus_tag="AUO97b_02554" /db_xref="SEED:fig|400667.82.peg.2420"
/db_xref="GO:0008761"
/translation="MIKVLISFGTRPEAIKMAPVIQCLKKDNRFDVKVLVTAQHREML
DQVLEVFKITPDFDLNLMKANQSLNQLSANILQNMDSIYQNFRPHILLVHGDTLSAFV
VAQSAFYNQIDIGHVEAGLRTYNLKSPWPEEGNRQLISKISNLHFAPTELAAECLKKE
NIQLDNIYITGNTVVDALLSVSDQILPLPIKYNGSKWNLDEKIKKILITGHRRENFGQ
GFINICQAIKDLALKYPAIEFIYPVHLNPNVKDIVFSYLNDIKNIILLPPQSYLEFIN
LMKHSYLILTDSGGIQEEAPSLGVPILVMRDTTERQEAIDAGTVKLVGTETASITFWV
ETLLENSELYHQMARANNPYGDGTASIKIKQILLDKYSHTI" /product="UDP-N-acetylglucosamine
2-epimerase (EC 5.1.3.14)" /EC_number="5.1.3.14"
/transl_table=11
CDS complement(2584238..2585011)
/locus_tag="AUO97b_02555" /db_xref="SEED:fig|400667.82.peg.2421"
/translation="MIPRIIHYCWFGPNKIPEQLVKYMESWRLFCPDYEIKLWNEKSF
DINSHPFTLSAYNQKKYAYVSDYVRAYALHNFGGIYLDTDVELKENLDIFLQHEAFTG
FEGKGSPFTAVWGSIPNHSLTKRILEYYHERIYTAEESTNTFSVSEILREKFFIDPLN
NNLQVGKDSINTIHVYPSTHFCLDLPKNFATHHFYGSWLPDRTKSYKENAVEIYFNEQ
LESLENITHSKNFLKTLATRIKIKDLFKLIRYFIQYKLK" /product="Putative
mannosyltransferase involved in polysaccharide biosynthesis" /transl_table=11
CDS complement(2585072..2585287)
/locus_tag="AUO97b_02556" /db_xref="SEED:fig|400667.82.peg.2422"
/translation="MAMRPDVRRHALVVIVFSLAQWFFMRYILANELFNLTTNQRILY
FCVSSLVGALLIFVALIYMVLKGNADK" /product="hypothetical protein"
/transl_table=11
CDS complement(2585427..2587205)
/locus_tag="AUO97b_02557" /db_xref="SEED:fig|400667.82.peg.2423"
/db_xref="GO:0004815"

/translation="MMRTHYCGSLTEAQIDQTVTLCGWVHRRRDHGGVIFLDMRDRDG
LVQVVIDPDTPEAFATADKARSEYVLKITGRVRRRYEGTENPNMVSGQIEVLGKEIEV
LAASETPPFPLNDDTINVSEEHRLKYRFLDIRRPEMLERLRFRSKVTNLIRNYLDDHG
FLDVETPILTRATPEGARDYLVPSRVQNGSFYALPQSPQLFKQLLMVGGIDRYYQIAK
CFRDEDLRADRQPEFTQIDIETSFLNDDDIMDLMEGMTIKLFNDLLGVKFEKFRRMPY
SEAMRDYASDKPDLRIPLKLVDVADLMQEVEFKVFAGPAKDPKGRIAALRVPGAGSLT
RSQIDEYTKFVGIYGAKGLAYIKVNEIEKGIEGLQSPIVKFIEPIVMQLLERVGAENG
DIVFFGADKAKIVNDAMGALRVKIGHDLNLATCEWAPLWVVDFPMFEETDDGKWTSVH
HPFTLPKSSVEDVKSNPGEALSVAYDMVLNGTEVGGGSLRIYTLEMQKAIFEALGISD
EEAEEKFSFLLNALRYGAPPHGGLAFGLDRLVMLMTGATSIRDVIAFPKTKTAECPLT
QAPAPVEANQLRDLGIRLREQQKKEA"
/product="Aspartyl-tRNA synthetase (EC 6.1.1.12) @ Aspartyl-tRNA(Asn) synthetase (EC 6.1.1.23)" /EC_number="6.1.1.23"
/EC_number="6.1.1.12"
/transl_table=11
CDS complement(2587376..2588140)
/locus_tag="AUO97b_02558" /db_xref="SEED:fig|400667.82.peg.2424"
/translation="MPFTISHALIAPPLAKLSRNTLPIAALAIGSMTPDLYRLFTMQS
GMLTHQWKGLFYPNLALGLLFCAIWYLLYRPVVYRFFGIQHDLKLDSFRHFFSFFIGI
IIALLIGIATHLIWDGLTHSDFRTFIFQDFLSQTVHLFGRPYPLHRLLQLGSSALALP
FLGWMSIHYYQRHKQHWKVNYKIKIFAWCLFALSTLLGLFSFWDYARYIPHQVWHSDT
YYFTGRSINEFMQGWLSTFSLGCLLFLFLDRQQRMG"
/product="Putative membrane-spanning protein" /transl_table=11 CDS 2588070..2588198
/locus_tag="AUO97b_02559" /db_xref="SEED:fig|400667.82.peg.2425"
/translation="MGNVFRLNLASGGAISAWEIVKGIRKESFSHHQKVESIETCH"
/product="hypothetical protein"
/transl_table=11
CDS 2588276..2590351
/locus_tag="AUO97b_02560" /db_xref="SEED:fig|400667.82.peg.2426"
/translation="MLFYKNILTLSILAAISIPVFAAENENVEKLETIRIKAHPLEQT
SKDFAVADTVVDQKHLTEGAATIGDALNSEVGIYANQFGAGSSRPVIRGQDGPRVKVL
QNSSENVDVSTLSPDHAVTVDPVLAKQVEVIRGPSTLLFGAGTVGGLVNVIDNKIPTQ
MPENGYEGQVGLRYNTGSDEKLASAGVTVGLGSQVALRVEGLTRDANNYIAPNYIHEG
EKERRVDNTFAQGDSVNVGLSWIYDRGYTGISYSNRRDQYGLPGHSHEYETCSAHLTG
RPHLHCDAHEHDHEEGEEAHAHEEHEHEHGGPWIDLKSERYDFKTELNDPFAGFQKLR
AQASYTDYQHDEIEEGAIATRFQNKGYDGRIELVHNPIADWEGVIGTQLGQQKLNLTG
EEAFMAPTTTKKWSVFALEHKQWKAVHFELSARADQQEIDVDDNSKQDFDGSAFSYAG
AANWEFAPNYKLSFVASHQERLPLAQELYANGAHFATNTYELGNDQLSKEKSNNVELG
LHFDNDKLDYHLHVYHNWFDDYIYAQTLDRYKDFRLVQYTQDKARFYGAEGEIGYQIT
PMYKISAFGDYVRGKIDAEGNAPRIPAGRLGTKVDADFGDGFSGSAEYYHIFNQDKIA
AYETETEGYNMLNLGVAYSGQYGAKTDYRVYLKANNLLDDTVYQHASFLSNIPQVGRN FTVGVDFSF"
/product="Zinc-regulated outer membrane receptor" /transl_table=11
CDS 2590427..2591890
/locus_tag="AUO97b_02561" /db_xref="SEED:fig|400667.82.peg.2427"
/translation="MRIFQRIHQKLNWSGRRYMAVILCVVAIAYLASAIYHTVKPLPQ
GINFSGKLRHADVKFLADKTYIDANGQQQVDQHIFDEILKMIDEAKTTIVVDMFLFNS
EVGDSKLKQRPLMQELTDALISKKRQNRQVQVVMITDPINSVYGGLSPEHYRQLRQAG
VDVIETNLAPLRASNPFWSGFWYICCQNIGNNPEKGWLPNPFGDEKITLRSYLNLFNF
KANHRKTIVVDTDAGWKSLVTSANPHDGSSRHSNVALMVNGATATDVLQTEAAVAQMS
GSSSPSLILGDFEKDVSKPRVQVLTEGAIYEAVLKLINTAKPKEHLDLSMFYLSERKI
IKALKSAQERGVIVRVLLDPNKDAFGRQKNGIPNRQVASELHAAGIPIRWCDTHGEQN
HSKMILKYNAQQAELIVGSANFTARNLKNYNLETDMLVVGKVQDQVFKDAQNYFNTSW
SNLQGRQMSVDYAKYADESKVKYWIYRFMEWSGLSTF"

/product="Phosphatidylserine/phosphatidylglycerophosphate/cardiolipi n synthases and related enzymes" /transl_table=11
CDS complement(2592085..2592531)
/locus_tag="AUO97b_02562" /db_xref="SEED:fig|400667.82.peg.2428"
/translation="MSEISFERLHQFFCKVPSVQEARIMAHGADGEHAWWFKFSIDVE
HPLAWQTVQELGHVLNYLSTNERLPTLFFPVSPPPYMNGEAKDFLSWVIQCNHPEFSP
DVVCDWLEARLPNPVDDESQWKIKTDLSEIEKLDDKALDQLIPPAP" /product="hypothetical protein"
/transl_table=11 CDS 2592696..2593079
/locus_tag="AUO97b_02563" /db_xref="SEED:fig|400667.82.peg.2429"
/translation="MKKPIVLVLSTLMLGMSATAMADSNHRWDNYHGKDHNRYEQRHD
YRYDHRNDHRAPVWSHSERGNHYGRYVAFKRGDRIPVEYRNSRYYVSDWRAHRLYAPP
RGYHWVKTPNQYLLVDSKHHIYRVR"
/product="Putative transmembrane protein"
/transl_table=11
CDS complement(2593118..2593600)
/locus_tag="AUO97b_02564" /db_xref="SEED:fig|400667.82.peg.2430"
/translation="MPDPKKFERTLDRFGHYAVEAFHYLALFIIGCMIAWSAAHTVFE
ILTVKKYASIDDILLLFIYLELGAMVGIYFKTNHMPVRFLIYVAITALTRLLISDIQH
DHKADLDQVIITGSILILALSILVVRFASWHYPSVMKEKHSKTPTSLKTPQPQDDELA "
/product="hypothetical protein"
/transl_table=11
CDS complement(2593719..2595500) /locus_tag="AUO97b_02565"
/db_xref="SEED:fig|400667.82.peg.2431"
/translation="MPIYNAPLADMKFILNDVFKAEQFWQANEKLAHVDAATAEAILE
EMAKFAQNVTHPLNRTGDEEGARYENGEVFTPAGFKDAFRQYAEGGWIGLGADEEWGG
QGMPKMLTVLADEMLFATNPSFMLYPLLSVGAGMALSSYASQEQKETYLPKIYSGEWS
GTMCLTEPHAGTDLGIIKTKAERNEDGTYNITGTKIFITGGDNDLAENIIHLVLAKTP
DAPAGSRGISLFIVPKYLVNEDGSLGDRNHVGPGSIEHKMGIKASATCVMNFDGAKGY
LVGKENEGLAAMFVMMNYERLSMGIQGLGASEFAYQNAAQYATDRLQGRSAAGVQSPS
KPADSILVHGDVRRMLLNVRANNEASRAFAVYVGQQLDITKFSTDAEAVKKANDRVAL
LTPIAKAYLTDTAFQATLDAQMVFGGHGYIREWGMEQCIRDLRIAQIYEGTNGVQSQD
LIGRKTIKCGGAFIAEYITEIRDFANDLDTDLNFIKDATLDAATEIEAITQFIIEQAA
ENVEFPNSAAVDYLHAVGLLSFSYMFAKIAAAAKDKSGDFYQNKLALAQYFVERILPD
IDARIAKIKAGSDLIMGFSEDYFTNQA" /product="Acyl-CoA dehydrogenase 2 [fadN-fadA-fadE operon] (EC 1.3.8.7)" /EC_number="1.3.8.7"
/transl_table=11
CDS complement(2595668..2597470)
/locus_tag="AUO97b_02566" /db_xref="SEED:fig|400667.82.peg.2432"
/translation="MPQYKAPLRDMQFVLHELLNAEEHYAKLPDFQGNVSRELVDQYL
EAAADFCENELSPLNQVGDREGCTWNDGVVTTPTGFKEAYQKYIELGFPSLSAEEQYG
GQGLPNSLGIAISEMVGSSNWAWGMYPGLSHGAVRTLEHHGSEEQKNTYLPNLVSGVW
TGTMCLTESHAGSDLGIIRTKAEPNADGSYAISGEKIFISAGEHDMAENIIHIVLARL
PGAPKGTKGISLFIVPKFNVNADGTLGERNAVRCGSIEHKMGIHGNATCVINFDQAKG
YLIGPENRGLNCMFTFMNTARIGTAVQGLSASESSFQGALTYAKERLAMRSLSGPKAP
EKEADPIIVHPAVRNMLLTQKAFAEGGRALVYLLAQYADIVEKGETEEERKFADNILS
LLTPIAKAFLTETGSESAKHGVQVFGGHGFISEHGMEQIVRDTRIACLYEGTTEIQAL
DLLGRKVLQTQGAMLRDFTKIVHKFVEANKDNAAMKEFVEPLAALNKEWGDLTMQIGM
RAMQNPDEVGAAAVDYLYFSGYVTLAYLWARMALVAQEKLAAGTTDVDFYNAKVTTAR
FYFKKILPRVRSHVDVIAGGLDPLMALDAEHFAF"
/product="Long chain acyl-CoA dehydrogenase [fadN-fadA-fadE operon] (EC 1.3.8.8)" /EC_number="1.3.8.7"
/transl_table=11
CDS complement(2597760..2598140)

/locus_tag="AUO97b_02567" /db_xref="SEED:fig|400667.82.peg.2433"
/translation="MKMTAKIALFSAAIVTMGSLAACQSTTQPPKPEHGMMQDGPRDG
HHHRMKHREFTPEQKAAWEQHRAERKARFEQIQKACEGKAVGQTVNVQVGDKTLEGTC
NLRFEPKRPQPPVNAPAPVATQAK"
/product="hypothetical protein"
/transl_table=11
CDS 2598374..2599930
/locus_tag="AUO97b_02568" /db_xref="SEED:fig|400667.82.peg.2434"
/translation="MQKNFAKYVADVEMQKLDRLITNLGNVYTVYHDWGNAIQAQILQ
IEGTAAPDDYDRLSQWWLRRQYDIAIQQHSFDEHTLINISPDAGTVNKNSIFSDEELR
TLEQNLPSEYQPFEGLRFPLSANQFRPSDDKNKSKKGSLKDIETQNGKKRFIALPDRL
GLSSRLSLYDAKHQFVVGEPPSSDQMSYRPIMVNSQVVGYLGLKPVLDKEDALSINFF
SNQKRYLLLIYALTLLSSLVAALLMATYFKKPIQRLLNATNELTKGNYQHQVVIKRND
ELGDLSTQLNHLAEILHQHEESRRQWVADTSHELKTPLAVLQAQIEAMQDGIRKATPE
HLDAMMRQVSSLKKLTQDLADLAQADAQQLKCYLSSVNPWEVILQEVENFRPKLEQAG
LEIEVEGEGASLLLDRDRFKQIIVNLIGNSIRYTETGGKIHIHTSQTPQEWTLYVDDS
PFGLSDEQLSRLGERFYRVDDSRTRSTGGTGLGLALSCKIAQALGGTLSFEHSPLGGL RCVLTFKKQS"
/product="Sensory histidine kinase BaeS"
/transl_table=11
CDS 2599950..2600636
/locus_tag="AUO97b_02569" /db_xref="SEED:fig|400667.82.peg.2435"
/translation="MKHIMLVEDEVELAHLVRDYLEAAGFEVSMFHDGQDAYTSFQQR
KPNLMILDLMVPRMDGLTICRKVREQSDLPIIMVTARTEEIDRVLGLNMGADDYVCKP
FSPKELVARVQAVLRRLERKAEPEQNDSFRIDKAQQRIWYQQKSLSLTPTEFRLLELF
LEHVGQVYSRAQLLDHINPDSFDVADRVIDSHIKNLRRKITEVAETGNRHEWIQAVYG VGYRFEYPEE"
/product="Response regulator BaeR"
/transl_table=11
CDS complement(2600700..2602142)
/locus_tag="AUO97b_02570" /db_xref="SEED:fig|400667.82.peg.2436"
/translation="MQFNPLYSSLNPKLFHIQQPSPLRGAKAGHFNLALAEELQWTDE
EKQAWVEICSGQRTFSEFPPLAMVYAGHQFGQWAGQLGDGRGLLIAQILNKKGQTIDL
HLKGAGPTPYSRMGDGRAVLRSVIREYLAGHALNALGVASSHAVGFTSSTQGVQREKL
ELGAMLLRTSECHIRLGHFEWINQYAPELLAEFTQKCIEWHYPECQQEEQPILAFAKA
VIRNTAIMIAKWQLVGFAHGVMNTDNLNITGSTLDFGPYGFMERFRPNWINNHSDYQG
RYTYQNQPSIAHWNLWTWLNNLIPLAEPEHKEQFKEALAACLEEFEPTFIEHYTTGLC
QKMGLPHFHKDSTECGLSFLRILQAEQLDYTQSFIRLQNKEYKALRDDCLDIRQFDAF
LTQYQNIREYQDIDELDANMQQVNPIYILRNHMAQRAIEAAERDDFSEVDRLFKLLNH
PYTKQSDLEKPEDLGPLPSDVPDVAVSCSS"
/product="UPF0061 protein YdiU"
/transl_table=11
CDS complement(2602256..2603428)
/locus_tag="AUO97b_02571" /db_xref="SEED:fig|400667.82.peg.2437"
/translation="MTSAPLKAPINWTASITLIGLPILAAIIIPIYAYYYDFSVSAWV
SLFFLLALSSMGITAGYHRLWAHRAYEASLPLKILLMIGGTFAVQNSILFWASGHRTH
HRHVDDVDEDPYSIERGFWYAHMGWMIRDHSPSEPDFKNAPDLLNDKLVMFQHKYYAL
MVVAVHVGILGLVGWATGDLWGVVLLGGLLRLIISHQVTFFINSLCHMFGKRPYTDEN
SARDNFWLAIATWGEGYHNYHHIFQYDYRNGVKWWQYDPTKWLIWTCSKIGLAKNLRR
IPSFNIKKAELAMKFKYAEQDLAIYGHDVNADISQMKQRIAQEYEAFTHTLNDWAKLK
EQELQAKKAAMAEKIHKMDHKLKVDFQLLEHRLSHHRECLETLMRNIKKNTNVVPD" /product="Fatty
acid desaturase (EC 1.14.19.1); Delta-9 fatty acid desaturase (EC 1.14.19.1)"
/EC_number="1.14.19.1"
/transl_table=11
CDS 2603637..2604167
/locus_tag="AUO97b_02572" /db_xref="SEED:fig|400667.82.peg.2438"

/translation="MKIQRLRKKNSIDIARKLPLKCLFIAISLVLAACQSTPQPNNHK
GQVKPTHRVQTPVVKKKVSPDGVQDIDWQITQINGHKAKFFNQWPTLSLNSTVKTVTG
HTGCNRVFGRYTFDFSQKKLDMEVNAGHSSCDGALAQEAELVDSLQRIKRFQLVGNTL YLLDQSGQRLIQAEKK"
/product="hypothetical protein"
/transl_table=11
CDS complement(2604195..2604908)
/locus_tag="AUO97b_02573" /db_xref="SEED:fig|400667.82.peg.2439"
/db_xref="GO:0008889"
/translation="MRIIGHRGARREAPENTLGGFQHIKNLGIRGVEFDIRQLQDQEL
VVIHDDNFLRTAGVDQIVEQSTLAQALSFDHRQNWPNWPTSEPTPTLTDVLNLLDNFD
HIEVEVKAVRDMALAEKLVQKLETELQGFEKVVTITSFDLQILTALRDINSQFKRGLL
VELPVGATAIELAHQYGCCHIGWHDQLATDEMIQLSHQAGLNISVWTVNDVERAKRLQ
DLDIQGLITDIPTTMLEAL"
/product="Glycerophosphoryl diester phosphodiesterase (EC 3.1.4.46)"
/EC_number="3.1.4.46"
/transl_table=11
CDS complement(2604981..2605559)
/locus_tag="AUO97b_02574" /db_xref="SEED:fig|400667.82.peg.2440"
/db_xref="GO:0008817"
/translation="MGHRLSKIYTRTGDSGTTGLGDGSRVAKDDLRIAALGDVDELNA
IIGVLRAQITDSQVANKADWDKSLSLIQHWLFDLGGEVCIPNYNLLQPVCIEFLEKEI
DRMNEDLPMLKEFILPSGSLSCSYAHQARAVCRRAERSLMSVQTRDQNIQATALQLLN
RLSDWLFVASRALQRAEGGQEVLWQKNINEMI" /product="ATP:Cob(I)alamin
adenosyltransferase (EC 2.5.1.17)" /EC_number="2.5.1.17"
/transl_table=11
CDS complement(2605656..2607542)
/locus_tag="AUO97b_02575" /db_xref="SEED:fig|400667.82.peg.2441"
/db_xref="GO:0004872"
/db_xref="GO:0005215"
/db_xref="GO:0006810"
/db_xref="GO:0016020"
/translation="MEHVMSKSFQPTRLVGAIAIAMGCSPVIFAEDATDATQLDPIVI
TASKSAEKASEVPARISVISKEEIEKNPALNLSDILQKDASIYVKQNGGIGQLADLSL
RGGQTGQTLLLKDGARLNSQNGFGPVNPEFINLTDVDQIEILKGPASVQYGSDAISGV
INLISKKPTKSGINLTGIYGENNTYKSIINADYVDASGFYAQVGGQRLESDGTRILSI
QDKNDKAGFDQKGYHAKIGYQQNQIDTSISISENQGTNVYYDSTLNSNTGIRDFKNQL
INWLGSYQVNPDIKLNARYSNIKNTRVSTDTDWQTYKLTSSHYNTETNESDLNAVWKF
MEHQNILAGVTYSKSEYDASSTPEAIRSTNSTGYYLQHQYQKNGLNTQTGIRLEDNDW
FGTHTVGQIAVRYQLLPLTSIYTNVGTAFKAPTLEHLYGSYGSSFYSNPNLKPEKSVS
YEIGLDQKLNYGLSAYISGYSTDVENLISSGKIDGKTTYINLNKAKIQGGEIGLKWVL
DNLFANAEYVYSKAQNKETKLDTPYRPRQTYTLTVGYDDGLYGVNAAVVARSKANTSS
ANIKVPGYATVDLNAFWNISSNVKLFTNIQNIGDVENIVVNDFGSEYINGGRQASVGV TFRY"
/product="Outer membrane vitamin B12 receptor BtuB" /transl_table=11
CDS 2607899..2608558
/locus_tag="AUO97b_02576" /db_xref="SEED:fig|400667.82.peg.2442"
/db_xref="GO:0004640"
/translation="MSQSNQKRTRAKICGITRKQDVQAVVAAGADAIGFVFFPPSPRN
VTVEQAEELAKLVPPYVQMVGLFVNASVTEIQDVLDCVPLDVLQLHGDETPEQCQEIA
RHCKRRWYKAIQVKPDLDVVAEIQKYQAAGASAVLLDAWHPELKGGTGHQFDWSKFPK
LDIPLILAGGLTPDNVIDAIHTTGAFAVDVSGGVESAKGIKDQQLIERFMQGVQRGSA K"
/product="Phosphoribosylanthranilate isomerase (EC 5.3.1.24)"
/EC_number="5.3.1.24"
/transl_table=11
CDS 2608542..2609771

/locus_tag="AUO97b_02577" /db_xref="SEED:fig|400667.82.peg.2443"
/db_xref="GO:0004834"
/translation="MDQQNNVIDYTQFPDANGHFGIHGGRFVSETLMAALEDLENLYS
RMKNDEQFLAEFDRDLAYYVGRPSPLYYAERWSKELGGAQIYLKREDLNHTGSHKVNN
TIGQALLAKLSGKKRIIAETGAGQHGVATATIAARLGLECVVYMGAEDVKRQAMNVYR
MRLLGATVVPVQSGSKTLKDAMNEAMRDWVTNVDTTYYVIGTVAGPHPYPQLVRDFQS
IIGREARKQILEQAGRLPDALVACVGGGSNAMGLFYPFLNDASVKMYGVEAAGFGIET
GKHSAPLNAGHVGVLHGNRTYLMSDEQGQIIETHSISAGLDYPGVGPEHSFLKDMKRV
EYVPINDTEALQGFRDLTKIEGIIPALESSHAMAYVSKLAPTMSKDQIIIATVSGRGD KDLMTVARIDGVEMVEM"
/product="Tryptophan synthase beta chain (EC 4.2.1.20)" /EC_number="4.2.1.20"
/transl_table=11
CDS 2609781..2610293
/locus_tag="AUO97b_02578" /db_xref="SEED:fig|400667.82.peg.2444"
/translation="MPSLFMVMLGGRHARANTEVHDVVMAVGDTLEEVYPQLKQAWFG
EAQGLHIDAWAKLSGVSSQGQNYQIHFTDVAPQPDDLKLYLINLGGYNAREFGELHRY
EFVVAPNTVVAKQLGKQFIDRQWQKAHTDRVIDIDDCLAIDCVAGRYIHLIKGDFAAT IWENTYLTVG"
/product="hypothetical protein"
/transl_table=11
CDS 2610331..2611326
/locus_tag="AUO97b_02579" /db_xref="SEED:fig|400667.82.peg.2445"
/translation="MDLIQENGQPRYGRFESVPSTIHVQRYIYKTPYGKVLKGWRKQL
KYKKFKFCGIQHKHYSIGLAIADIGWVGHGFFYIYDHETGQVIEWNAIQPLGHKTYLD
EQPLFNQSYFSKSPYQFDIQHANGVRYIRVTKYGEICLSARIFCAGTDILSLCTPTGI
NGWTYTQKQTTLAVEGMFINKQNQVVQFDEKTFASLDDTCGFLRPETAWFWLSCNFWD
EQGRRIGLNLASGVNESFGNENCLWINGVLFPLTDVLFEKVNDELWQISSLDQKLNLQ
VHIGWCRYENINLRMIGSQFNQWQAKISGTIEHETTDIINCKEQYGLLEQHYAKW"
/product="hypothetical protein"
/transl_table=11
CDS complement(2611335..2612210)
/locus_tag="AUO97b_02580" /db_xref="SEED:fig|400667.82.peg.2446"
/translation="MNAHVSYQVEPVVRKDLDFHLNEAPRFWFNNDPFLTRMFDALSL
TFPDGERYFIECVRMFRDKIDDPELQKRVADFIKQEAQHGIAHDKMNQLMKEQGMPVD
QFTTTLKKIFRFELTKRSPQYNIAMTAAAEHLTALMAETFYSHKKTLENAHPYVRALF
AWHAIEEMEHRDVAFDVMRQVGEVPESTRRFVLVLTTVLMFGFTLYRTNIMLKCDGFS
PRERLLMNLKGLPWFFGKNGTLTAMKKQYMDWFKKDFHPSQHPVIRQYPVWIETLEKT NDPIAAGEAFWQAGL"
/product="Predicted metal-dependent hydrolase" /transl_table=11
CDS 2612331..2613380
/locus_tag="AUO97b_02581" /db_xref="SEED:fig|400667.82.peg.2447"
/translation="MQEFTISNGYFYLWNTYLKEKGIDWQTLNLSEIQTRQVQNILSA
SIDAQSSLDLFIELLEITETRLHVTNLALEMAACITPANFGVLGYMASRSDTLGQVVE
YIAKFHRLVIDGAQVVPIQIEQDASKIRLYWPLVDDSNILLSELTLAAMVQLAKQIAP
VHEFLLQNVEFVHRARGAVVHYQRFFQCKLSFERPYYALTFASESLNVRPQHADPTLV
QLLVKQAEEALVQRKIHADLVQQIHWIVQEYFKHKQQAPKIEDIAIELNVSVRSLQRQ
LQALNTSFKLILDAERMKRCEILLSQNESLTAIADQLGYSDQSALARAYKNLTGQTLL TRKKQLQLIQKNATK"
/product="Transcriptional regulator, AraC family" /transl_table=11 CDS
2613625..2614428
/locus_tag="AUO97b_02582" /db_xref="SEED:fig|400667.82.peg.2448"
/db_xref="GO:0004834"
/translation="MSRLATRFEKLQSQQRKALVSYVMAGDPQPQVTVPLLHKMVAAG
VDVIELGLPFSDPMADGPVIALAAERALAAGTNTLDALNMVKEFREQDQETPVVLMGY
LNPVEVIGYEKFVSYAKQCGVDGLLLVDLPPEESKEFGAILKQHDMDQIFLLAPTSTD
QRIQHVANQASGFIYYVSLKGVTGAATLDTSEAAARIEKIKGMTNVPVGVGFGISDAA
SAKAMGSVADAVIVGSAFVKSFATLAADEAVEQTVNKVKELRAALDELV" /product="Tryptophan
synthase alpha chain (EC 4.2.1.20)" /EC_number="4.2.1.20"

/transl_table=11
CDS 2614425..2615321
/locus_tag="AUO97b_02583" /db_xref="SEED:fig|400667.82.peg.2449"
/db_xref="GO:0003989"
/translation="MNQEVKSGKVLSPSTPWTQRPVPGIEVADEQQTLKATFTEPTIE
CPECHALVTRTAISFNAYVCPQCDEHLRMKARDRLNWFFDNVVAELGQEFSAKDPLKF
VDSKPYPDRMREAQTKTGETEALIAMQGNLNGVDMIACAFEFDFMGGSMGTVVGDRFV
KAAELAIEKRQPLICFAASGGARMQEGMLSLMQMARTSAAIQKLKDAGLPYIVVLTHP
VYGGVTASLAMLGDIHIAEPKAMIGFAGKRVIEQTVRETLEEPFQRAEYLLDHGVVDQ
IVHRHALRDTVSRLVSKLMNLP"
/product="Acetyl-coenzyme A carboxyl transferase beta chain (EC 6.4.1.2)"
/EC_number="6.4.1.2"
/transl_table=11
CDS 2615318..2616604
/locus_tag="AUO97b_02584" /db_xref="SEED:fig|400667.82.peg.2450"
/db_xref="GO:0008841"
/db_xref="GO:0004326"
/translation="MKQQAPLSTDSLDTWLNYWSHVHVTGIDLGLERVIPVAEKLGVT
KPNAKVFTVAGTNGKGSTTTTLASILKAQGYKVGLYQSPHIYRFNERVKLGGVEVDDQ
SLIDAFVQVDQARRECDLSLSFFEATTLAAFVIFKQQQCDVWVLEVGLGGRLDVVNVI
DPNIAVITNIGLDHTDWLGDTIEKIAFEKAGIIRPHIPVIFAGEQSLPQAIQDKANSS
QATLYAINRDYFYKLADDGKHWYFASEGTTLRLPLGTLAIENIAGAVAAVLNSGLEIS
QLALEEGITNARLAGRFEVREVNGKTIIFDAGHNPHGVEFLLKQLRNFLEYNKQYTEV
VAVFSMLADKDIKSVVDLLKPTVLHWKIAELNVPRAAPIQQLNDALQGQTIQQFGNIK
EAFKSALEQTNNNQLILACGSFHTLEAIWEYLEECQ" /product="Dihydrofolate synthase (EC
6.3.2.12) @ Folylpolyglutamate synthase (EC 6.3.2.17)" /EC_number="6.3.2.12"
/EC_number="6.3.2.17"
/transl_table=11
CDS 2616595..2617665
/locus_tag="AUO97b_02585" /db_xref="SEED:fig|400667.82.peg.2451"
/translation="MSMNNKQRWMGGVVLLGGGVLLAALLLKGNEEIKQVDVQPQTST
SPKLQAKPKQSAQEGQMVQLQPLAVDVETEKRLLEEQRRSREKAVAEQEARAAEFLAM
QQQAEADAARKAAAEYAAINARRAAAQESSDNIPPEVAGSENKAKGQQTDTKKSVDPA
KADADKKAAEAKRLAEADKKAAEAKRQAEADKKAAEAKRQAEADKKAAEAKRQAEADK
KAAEVKRQAEADKKAAEAKRQAEADKKAAEAKRKAEAEKKAEAEKARELLENGDKKWM
VQVALAANQANADAVVSKLRAKGYKVTTSPTSKGIRIMVGPAKDRDTADTTRKKITSD
ASLNMKSAWVIDWVPLDQRKSD"
/product="hypothetical protein"
/transl_table=11
CDS complement(2617685..2618263)
/locus_tag="AUO97b_02586" /db_xref="SEED:fig|400667.82.peg.2452"
/translation="MKIFSLIAFAVCLSLQGCMQHESIAHEHRPHDWGALVSQTHNFY
QISNDVFRSEQPDAAMIPELKHHQIGTVINLRAKASDDLVFKNENFNLVHIPINTWAI
DRQDLLEVMQQIQLAKQNNQRVLLHCYHGSDRTGASVAMYRIIFENWAIDDAVKEMKQ
GGYGYHIIWKNIDRLFTPKNVKWIQQQLSNPS"
/product="Protein tyrosine/serine phosphatase" /transl_table=11
CDS complement(2618260..2619090) /locus_tag="AUO97b_02587"
/db_xref="SEED:fig|400667.82.peg.2453"
/translation="MQKTKIMIHKIHHLKCGSMCSVCAPLFGQRGWKAEIVCHCLLVE
TDRGLVLIDTGFGLQDYLHMQQRLGSLVKRLGKIEPNLEFSAIQQIQKLGFHPKDVQH
IFVSHLDFDHAGGISDFPHATVHVLATEYNAAQLPNFKGKLRYRTNQYKQHRYWNFVE
YQQGEKWFNLEKVKGLPLFQDEILMVPLLGHSAGHCGIAIKQQNQWLLFCGDAYYSHL
ELNPVNKLRSLTLLEKTFAEDNEKRLINLKKLQHLAQHEPTIEIICAHDPHELRRYQT "
/product="MBL-fold metallo-hydrolase superfamily" /transl_table=11

CDS complement(2619377..2620018)
/locus_tag="AUO97b_02588" /db_xref="SEED:fig|400667.82.peg.2454"
/translation="MTLRLGDTAPDFQQESSEGTINFYDFLGDSWGILFSHPADYTPV
CTTELGYTAKLKDEFEKRNVKAIALSVDDVESHKGWINDINETQNTTVNFPIIADKDR
KVSELYGFIHPNASETLTVRSLVIIDPNKKVRLIITYPASTGRNFNEVLRVVDSLQLT
DKHKVATPANWQQGEDVVIVPSLKDEEEIKQRFPKGYTAVKPYLRLTPQPEQD" /product="Alkyl
hydroperoxide reductase subunit C-like protein" /transl_table=11
CDS 2620426..2623149
/locus_tag="AUO97b_02589" /db_xref="SEED:fig|400667.82.peg.2455"
/db_xref="GO:0003676"
/db_xref="GO:0004386"
/db_xref="GO:0005515"
/db_xref="GO:0005524"
/db_xref="GO:0016020"
/db_xref="GO:0017038"
/translation="MLASLIGGIFGTKNERELKRMRKIVEQINALEPTISALSDADLS
AKTPEFKQRYNNGESLDKLLPEAFAVCREAAKRVMGMRHYDVQLIGGITLHEGKIAEM
RTGEGKTLMGTLACYLNALSGEGVHVITVNDYLAQRDAELNRPLFEFLGLSIGTIYSM
QEPAEKAAAYLADITYGTNNEFGFDYLRDNMVFSLAEKKQRGLHYAIIDEVDSILIDE
ARTPLIISGQSEDSSHLYTAINTIPPKLRPQKEEKVADGGHFWIDEKQRSVEMTEIGY
ETVEQELIQMGLLAEGESLYSATNLNLVHHVSAAIRAHFLFQRDVHYIIHDGEVIIVD
EHTGRTMPGRRWSEGLHQAVEAKEGLTIQPENQTLATTTFQNYFRLYKKLSGMTGTAD
TEAAEMKEIYGLDVVIIPTHRPMIRNDQNDLIYLNRNGKYNAIIQEIMNIRQQGVAPI
LIGTATIEASEILSSKLKQAGIHHEVLNAKQHEREADIIAQAGSPNAVTIATNMAGRG
TDIILGGNWKAKLAKLENPTPEDEARLKAQWEQDHEDVLQAGGLHIIGSERHESRRID
NQLRGRAGRQGDPGVSRFYLSLEDDLMRIFAGDRVVAMMRAMGLKEDEAIEHKMVSRS
IENAQRKVEARNFDIRKNLLKYDDVNNEQRKIIYSQRDEILAENTLQEYVEEMHREVM
QAMIANFIPPESIHDQWDVEGLENALRIDLGIELPVQEWLEQDRRLDEEGLVERISDE
VIARYRQRRAQMGDESAAMLERHFVLNSLDRHWKDHLAAMDYLRQGIHLRGYAQKNPE
QEYKKEAFNLFVNMLGVIKTDVVTDLSRVHIPTPEELAEMEAQQQQQAEAMKLSFEHD
DVDGLTGEVTASQEALNESATEQQTFPVPESRNAPCPCGSGLKYKQCHGKI" /product="Protein
translocase subunit SecA" /locus_tag="AUO97b_02590"
/transl_table=11
CDS 2623280..2623723
/locus_tag="AUO97b_02591" /db_xref="SEED:fig|400667.82.peg.2456"
/translation="MKKVLQPLFLTVLAVPSFVFAAETASAPQTTTVDKVLAAKGPTS
AQAVVKQENTTVISPRTGIRYTLGNTGNRPIILQTAAIAPANSANINRIVASNPALSP
KSQEKAKVALIGEAAVAGAAAVAANNVTAPAVPNSVTPANPASLN" /product="putative signal
peptide"
/transl_table=11
CDS complement(2623790..2625100)
/locus_tag="AUO97b_02592" /db_xref="SEED:fig|400667.82.peg.2457"
/translation="MESTSATAAPVVATNSKTRVLFASLVGTTIEFFDFYIYATAAVI
IFPHLFFPASSGSAAVLQSLATFAIAFIARPIGAALFGHLGDRIGRKATLVAALLTMG
ISTVCIGLLPTYAQIGIVAPLLLALCRLGQGLGLGGEWSGAVLLATENAPEGKRAWYG
MFPQLGAPIGFILATGSFLLLSAAIPEQAFMQWGWRIPFIASAVLVIVGLYIRLKLHE
TPAFQKVLDKQKEVNIPFKEVVTKHTGKLILGTIAAICTFVVFYLTTVFALNWGTTKL
GYARGEFLELQLFATLCFAAFIPLSAIFAEKFGRKATSIGVCIAAAIFGLFFSSMLES
GNTLIVFLFLCTGLAIMGLTYGPIGTVLSEIFPTSVRYTGSALTFNLAGIFGASFAPL
IATKLAETYGLYAVGYYLTAASLLSLIAFLLIRETKNVDVNNQI" /product="Uncharacterized MFS-
type transporter YhjE" /transl_table=11
CDS complement(2625235..2625864)
/locus_tag="AUO97b_02593" /db_xref="SEED:fig|400667.82.peg.2458"
/translation="MLETYDPKEELFNAYSHGAGVVMAVIASVFLIIKGSYLSTAQWI

GLWVYAFSLILVFTSSTLYHFAQTQNLRYWYKKLDHTAIYYLIAGTYTPFLSIAIPTQ
KAHILLIALWSIAAIGTLFKLVFIHRFQKISLLAYVLMGWLAVFVMDDMRTYLSKDTL
MLLITGGLAYTVGALFYALKKVRYTHAIWHIFVLLGAGAHFLAIYWYVV" /product="FIG01964566:
Predicted membrane protein, hemolysin III homolog" /transl_table=11
CDS 2626003..2627214
/locus_tag="AUO97b_02594" /db_xref="SEED:fig|400667.82.peg.2459"
/translation="MSVFSFEQEQQFFHEIKQMLNQQTFERLILSQYKGELTQLEKIT
FRVVELHGKKQLSALYHHTTQDVTKNYSFEDGLEQIAVLITQCKQANLFSTHQEIQLK
KNKKKAMLNMGKKQSMTTAPTVQAHDREKQRYVQQGSAFLKELGITDEKAQIIPSMAR
KWKQINKFIEIFASAYEQIDAEQKELNIVDFGSGKGYLTFALYDYLQEQQKVPLITGV
ELRRNLVEFCQNVADKVHFNHLDFFEGDVRSYQPEKLDVMIALHACDIATDFAIHTGI
RLNASMIMCAPCCHKELRPQLHSPEVLQPMLQFGIHAGQQAEMLTDTLRALLLKAYGY
ETKVFEFVSLEHTSKNKMILATKRKNVTQPDAKIMAQIQALKEMYGIKKQTLELLLQD QLPIENIGCKC"
/product="SAM-dependent methyltransferase"
/transl_table=11
CDS 2627227..2627646 /locus_tag="AUO97b_02595" /db_xref="SEED:fig|
400667.82.peg.2460"
/translation="MYKVIAGSWTQFEEAAKYIREQVFIQEQGIEPEDEWDDFDATAV
HFMVYNKEQPIATARLLPQHSVGRVAVLMPYRKQGIGKILMQHIIEYARQHKLPYLKL
SAQTYVTAFYEALGFKVQGEVYQDCGIPHIDMTLALS"
/product="GNAT family acetyltransferase YjcF" /transl_table=11
CDS complement(2627676..2628491)
/locus_tag="AUO97b_02596" /db_xref="SEED:fig|400667.82.peg.2461"
/translation="MRGAFTSGVLDAFLQQRFNPFDLYVGVSSGSTNVANYLAGQQGR
TLTFYIDHSLRPEFIDYKRFFKGGDLLDLKWMWEIGEQEHPLDQQSLFAKNPDFYMVL
THAKTGHAEYLRAGKDNLLNALRASSSIPVLTRHPVDIMGEPYFDGGVADALPVRWAA
QQSGVKKLLVLRTRPKNYFKASSRGDQFLAKYAFKHHYGFANSLRNRCARYNASVEFV
RSSNPEQQILEVCPPPLKNMAGRLTTNPKKLRYSYEVGLETGLQAIENWNAMK" /product="Serine
protease"
/transl_table=11
CDS complement(2628552..2628806)
/locus_tag="AUO97b_02597" /db_xref="SEED:fig|400667.82.peg.2462"
/translation="MSDKNSSESAILGWKFVLIVGVLSAIFLGFFYLAMSNEPDYMPG
AQRKAQQHEMQQKAEKSTDQQTQHDNMPEMDMQEHQHSHQ" /product="hypothetical protein"
/transl_table=11
CDS complement(2628971..2631106)
/locus_tag="AUO97b_02598" /db_xref="SEED:fig|400667.82.peg.2463"
/db_xref="GO:0003857"
/db_xref="GO:0008692"
/db_xref="GO:0004300"
/translation="MSAIQYEKNADNIVILTLDSTGQSANTMNAEFRDSLDEVTQKLK
AETELSGVIFRSAKKTFFAGGDLDELIQVQPEHATDFFNMVEKLKGHLRTIETLGVPV
VAALNGTALGGGWEIALSCHHRIAINDPKSKFGLPEVTLGLLPGGGGIVRMVRLLGLQ
NAFPFLMEGKQFGVDKAKSLGLIHDTAENEQELLDKAIAWIKANPKSQQPFDVKGYKI
PGGDPKTPAVAQVLAIAPAMLKDKTKGCYPAPEAIMAAAVEGAQVDVDTALRIESRYF
TQLATGQISKNMIGTFWHGLNAIKSGASRPADIAKWQATKVGVLGAGMMGAGIAYATA
IKGIPVILKDVSVENAEKGKAYSQKLLDKRVSQGRMTAEKRDQILSLITATASAEDLQ
GCDLIIEAVFENQELKAKVTQEAEQYLAPNGVMASNTSTLPITGLAQASKDDKAFIGL
HFFSPVDKMQLVEIIKGKNTSAETLAKTYDFVQQIGKTPIVVNDSRGFFTSRVFGTFI
QEGMRLLAEGVHPARIEMAALKAGMPVGPLAIQDEVSLTLTEHVASETRKALQAEGKE
LPKTLVDEVIHTMIHELNRKGKAAGAGFYDYPENGKKHLWEGLNRWQKDHDISEQDMI
DRILFVQALDTLRCYEEGVLESVIDANVGSIFGIGYAPWTGGAIQFLNQYGIDKAKKR
AEELAAKYGERFTPPTLLKTKAEQKQNIQ"
/product="Enoyl-CoA hydratase [isoleucine degradation] (EC 4.2.1.17) / 3-

hydroxyacyl-CoA dehydrogenase (EC 1.1.1.35) / 3-hydroxybutyryl-CoA epimerase (EC 5.1.2.3)" /EC_number="5.1.2.3" /EC_number="1.1.1.35"
/EC_number="4.2.1.17"
/transl_table=11
CDS complement(2631127..2632332)
/locus_tag="AUO97b_02599" /db_xref="SEED:fig|400667.82.peg.2464"
/db_xref="GO:0003988"
/db_xref="GO:0003985"
/translation="MSEAYIIDAIRTPRGKGKKDGSLYEVKPITLLTTLLNELQQRHQ
LDTSKVDDIVLGCVTPIGDQGGDIAKTAAIAAGWNDDVAGVQINRFCASGLEAVNMAA
MKVRSGWEDLVVAGGVESMSRIPMGSDGGPWALDPETNLKSSFVPQGVGADLIATLDG
YSREDVDNFAVKSQQKAAAAQANGYFDQSVVPVKDHSGVVILEKDEFIKGNTTLEGLA
KLNPSFEMMGQMGFDAVALQKYPEAQKVNHVHHAGNSSGIVDGAALVLLASEKAVKEQ
NLKPRAKVLATALVGTDPTIMLTGPAPAARKALEKAGLTIDDIDLFEVNEAFAAVVMR
FINELNVPAEKVNVNGGAIALGHPLGATGAMILGTLLDELERQNKKRGLATLCVGGGM GIATIIERV"
/product="3-ketoacyl-CoA thiolase (EC 2.3.1.16) @ Acetyl-CoA acetyltransferase (EC 2.3.1.9)" /EC_number="2.3.1.9"
/EC_number="2.3.1.16"
/transl_table=11
CDS complement(2632504..2633514)
/locus_tag="AUO97b_02600" /db_xref="SEED:fig|400667.82.peg.2465"
/translation="MSRDTISIHFVNAALTGVKRLGMDVETLLSHVGIEAELLRQPKA
RISPEQYTRFIKMLWMVTQDEHVGFDVQPRRLGTFAIMCQLIIHAKTLGQALELSSQF
YKLFGDEWSVTLERDKHEARLVPLIPKTLDPDHFITESMLMIWHGLASWLIERRLPLE
RVHFSYPRPAHADEYDALFFAPVMQFDAPRTEITFAADYLDLPIRQDEKTLEEFLKAA
PAQLLVKFKNTNSLTSRIREVLKSQIGEEMPTLNDVASMLYLSPQTLRRRLAAEGKSY
QGVKDALRRDAAIHLLLNPELTLEDVAQQVGFSETSTFHRAFKKWTGVTPGLYRQLHG YH"
/product="Transcriptional regulator, AraC family" /transl_table=11 CDS 2633725..2634843
/locus_tag="AUO97b_02601" /db_xref="SEED:fig|400667.82.peg.2466"
/translation="MTTALNGLKVLDFSTLLPGPFATMYLADMGAEVIHIESPSRPDL
IRIMPPYANGQATAHSYLNRNKQSIVLDLKDAASIDLIKAKISEFDIVIEQFRPDVMR
RLGLDYATLAEINPRLIYCSITGYGQTGSYKDRAGHDINYLALAGIAGYSGRQDSGPP
PLGIQVADIAGGSLHAVIAILAAVVERSRSGIGQYIDISMTDCVASLNSMAASATLAA
QVEQAPEQGMLNGGIFYDYYMTQDGRYLSIGSLEPQFMAGLSAALDLPVLLQKGASLD
EQDRQEVKQAIQEKIKAKTFKEWHEIFANLDVCVEPVLSLSEALNSPLAQQRGWIVNV
PLQNNTPDTEPQIACPIKFSRSQMRYSFVGQKLGEGQW" /product="Alpha-methylacyl-CoA racemase (EC 5.1.99.4)" /EC_number="5.1.99.4"
/transl_table=11
CDS 2634978..2636231
/locus_tag="AUO97b_02602" /db_xref="SEED:fig|400667.82.peg.2467"
/translation="MKTHKSLMVSCLSVLSITLFVQHAQAAAAFDPNGSWMLGDWNGQ
RTALQAQGYDFSFGYTGEYAGILDSKQTSTHGSAYTGQLALGSHLDLGKILGWQDTEA
QITLTYRDGQSLSEHSPALAGHQSSVQEVWGREQTWRLTDLWIKKKFLDQKLDVKVGR
FGEGEDFNSFDCDFQNLALCGSQVGNWVGDQWYNWPVSQWAMRVKYNLQPDLYTQVGV
YEYNPENLERGKGFNLSTDGSHGAIIPAEVVWSPKLGVQSMPGEYRLGYYYSTADAKE
IADSTKTSHKQGVWVTAKQKLFQPADQTDRGLTGFVNLTFHDSDTNKVDNMQNIGLVY
KGLLNQRPQDELALGVARIHINDDWSDVQAKEYDTEYNTELYYGIHATNWLTIRPNVQ
YVRHVGALKNGDNTWVGGIKFSTAF" /product="Carbohydrate-selective porin OprB"
/transl_table=11
CDS complement(2636236..2636367)
/locus_tag="AUO97b_02603" /db_xref="SEED:fig|400667.82.peg.2468"
/translation="MCQKTYRKMTSPIRLKQNSGIKKKTEGQDTSINDLTLDNPFSE"
/product="FIG00351548: hypothetical protein" /transl_table=11

CDS 2636405..2638810
/locus_tag="AUO97b_02604" /db_xref="SEED:fig|400667.82.peg.2469"
/db_xref="GO:0008876"
/translation="MNQPSSRSGLTTFTVIIIGLLALFLLIGGIWLATLGGSIYYIVA
GVLLLIVAWQLYKRASAALWVYAALMLGTIIWSVWEVGTDFWALAPRLDILGILGLWL
LVPAVTRGINNLGSSKVALSSTLAIAIVLMVYSIFNDPQEINGEIKTPQPETAQAVPG
VAESDWPAYGRTQAGERYSPLKQINDQNVKDLKVAWTFRTGDFKTDNDSGETTNQVTP
IKIGNNMFICTAHQQLIAIDPATGKEKWRFDPKLKTDKSFQHLTCRGVMYYDANNTTE
FATSLQTKKSTSTQCPRKVFVPVNDGRLVAVNADTGKACTDFGQNGEVNLQEFMPYAY
PGGYNPTSPGVVTGSTVVIAGSVTDNYSNKEPSGVIRGYDVNTGKLLWVFDTGAADPN
AMPGEGTTFVHNSPNAWAPLAYDAKLDIVYVPTGVGTPDIWGGDRTELKERYANSMLA
INASTGKLIWNFQTTHHDLWDMDVPSQPSLADIKDKSGKTVPAIYVLTKTGNAFVLDR
RNGQPIVPVTEKPVPQTVKRGPQTKGEHYSKTQPFSDLNLAPQDKLTDKDMWGATMLD
QLMCRVSFKRLNYDGIYTPPSENGTLVFPGNLGVFEWGGMSVNPDRQVAVMNPIGLPF
VSRLIPADPNRAQTAKGAGTEQGVQPMYGVPYGVEISAFLSPLGLPCKQPAWGYVAGV
DLKTHEVVWKKRIGTIRDSLPNLFQLPAVKIGVPGLGGSISTAGNVMFVGATQDNYIR
AFNVTNGEKLWEARLPAGGQATPMTYEINGKQYVVIMAGGHGSFGTKMGDYLVAYALP DNK"
/product="Glucose dehydrogenase, PQQ-dependent (EC 1.1.5.2)"
/EC_number="1.1.5.2"
/transl_table=11
CDS 2638977..2640620
/locus_tag="AUO97b_02605" /db_xref="SEED:fig|400667.82.peg.2470"
/db_xref="GO:0004783"
/translation="MYLYTDFDQQLINQRVAQFRDQTERYLAGKLSEDEYRPLRLQNG
LYVQRYAPMLRIAVPYGLMNSKQLRKIAEVSTQYDRGYAHVSTRQNIQLNWPALEDVP
EILAELATVQMHAIQTSGNCIRNTTTDQYAGVVAGEIADPRPTCELIRQWSTFHPEFA
FLPRKFKIAVSALEEKDRAATAFHDIGVYIVRNEAGEMGYKIMVGGGLGRTPIIGSVI
REFLPREDLIAYLEAVLRVYNLHGRRDNKYKARIKILVKALTPEVFAQKVEAEFEHTR
ETLKIQPEILKKLDEEFTPFDYQDLEDEDFTALFAEHPKFKQWFNINTHAHKVKGYRI
VTISLKRAGIAPGDMTTEEMNFIADLADKYTFGELRTTHEQNIALADVPQKDLFDVWQ
ALEQHNMARAHIGFITDIISCPGGDFCSLANAKSIPIAEAITRRFEDLDKVYDLGHLD
LNISGCMNACGHHHVGNIGILGVDKKGAEFYQITLGGNSDHDASIGDILGPSFAAEAV
PDVIEEVLNTYLDLRTEGERFVDTYRRVGIQPFKERAYA"
/product="Sulfite reductase [NADPH] hemoprotein beta-component (EC 1.8.1.2)"
/EC_number="1.8.1.2"
/transl_table=11
CDS 2640613..2641098
/locus_tag="AUO97b_02606" /db_xref="SEED:fig|400667.82.peg.2471"
/translation="MLNTALPVLYKDGTIADNTYQIIAEDGVIPQGDVVLTTAQLDQL
ANIQGKKALYYVTVNDSPEDHTFPLSELDAIFIEFAGFGDGRGYSFAALLRRQGFQGEL
RATGDVFKDVLNYLKRSGFDSFVIKEGKDVQEAAAGLQDFTHPYQASTAVPKASYQTG A"
/product="Oxidoreductase probably involved in sulfite reduction"
/transl_table=11
CDS 2641181..2641534
/locus_tag="AUO97b_02607" /db_xref="SEED:fig|400667.82.peg.2472"
/translation="MFALISKINPGAWWLIFAIATDVLSTFYSAKGNGLVNKTAQGIA
LVLYIISFACAAIALKYMQAGILYVLWSGVGVLATAFLAKIFLGQNIDVAGWIGIGFI TVGLMIIAQYSNIDV"
/product="quaternary ammonium compound-resistance protein" /transl_table=11
CDS complement(2641577..2642020)
/locus_tag="AUO97b_02608" /db_xref="SEED:fig|400667.82.peg.2473"
/translation="MQLNHYLNFQGQAEQAFNFYKSVFGGEFAMLSRYSDIPPNDGVT
LSEAQKNLVLHVSLPINEYTVLMASDVIDQFCTPNSVFTQGNNHYIAINLEKDEQEKA
KQLFDALSVNGKIEMPLEKTFWGALYGAFTDQFGVKWMVNCQLDT" /product="PhnB protein;
putative DNA binding 3-demethylubiquinone-9 3-methyltransferase domain

protein" /transl_table=11
CDS complement(2642147..2643322)
/locus_tag="AUO97b_02609" /db_xref="SEED:fig|400667.82.peg.2474"
/db_xref="GO:0003988"
/translation="MTDIVIVNGARTAMGGFQGSLSGLTAPELGAVTIKEAIARAGLQ
PTDVEEVIMGCVLPAGLKQGPARQAMRKAGLPDSTGAVTINKLCGSGMKAVMQAADMI
KAGSAEIVVAGGMESMTNAPYVLPKARAGYRMGHGEIKDHMFFDGLEDAETGRLMGSF
AQDMANTRGYTREQMDDFAIRSLKRAQTAITEGYFKDEIVPVTVSTRKGDVIVDQDEQ
PLNAKIDKIPSLKPAFAKDGTITAANASSISDGASALVLTSSEVAAQRGLQSLAKIIA
TASNSQHPSEFTIAPVGAIEKVLKKAGWNAQDVDLWEINEAFAMVTMCPIDDFKLDPE
KVNIHGGACALGHPVGSTGSRIILTLIHALKRTGGKKGVAALCIGGGEATAVAIEIL" /product="3-ketoacyl-CoA thiolase (EC 2.3.1.16) @ Acetyl-CoA acetyltransferase (EC 2.3.1.9)" /EC_number="2.3.1.16"
/transl_table=11
CDS complement(2643431..2643946)
/locus_tag="AUO97b_02610" /db_xref="SEED:fig|400667.82.peg.2475"
/translation="MFKSPGFTTVELTITLVILVIMSVIAIPLYHQFMASVELKNTPR
ILTIHIQKAKYDAIIRHKNVVLCPSSDQLVCNTNWDNRLISFVDNNRNLQHDLNEELL
TSIDLNHHYGSMKLQRFGKKQNSIIFQGSSGLPIESNGSFIYCSYDQLKNFKLILSKM GHVRLEELKNC"
/product="Type IV fimbrial biogenesis protein FimT" /transl_table=11
CDS 2644328..2645398
/locus_tag="AUO97b_02611" /db_xref="SEED:fig|400667.82.peg.2476"
/db_xref="GO:0016021"
/translation="MKLSRIALATMLVAAPLAAANAGVTVTPLLLGYTFQDSQHNNGG
KDGNLTNGPELQDDLFVGAALGIELTPWLGFEAEYNQVKGDVDGASAGAEYKQKQING
NFYVTSDLITKNYDSKIKPYVLLGAGHYKYDFDGVNRGTRGTSEEGTLGNAGVGAFWR
LNDALSLRTEARATYNADEEFWNYTALAGLNVVLGGHLKPAAPVVEVAPVEPTPVTPQ
PQELTEDLNMELRVFFDTNKSNIKDQYKPEIAKVAEKLSEYPNATARIEGHTDNTGPR
KLNERLSLARANSVKSALVNEYNVDASRLSTQGFAWDQPIADNKTKEGRAMNRRVFAT
ITGSRTVVVQPGQEAAAPAAAQ"
/product="Outer membrane protein A precursor" /transl_table=11
CDS complement(2645523..2645681)
/locus_tag="AUO97b_02612" /db_xref="SEED:fig|400667.82.peg.2477"
/translation="MKIKEMPKIKVRNQVALSPLLHKGGMHETEKPRAQHRRNRQDTK QQLKKGVW"
/product="hypothetical protein"
/transl_table=11 CDS complement(2645685..2646890)
/locus_tag="AUO97b_02613" /db_xref="SEED:fig|400667.82.peg.2478"
/translation="MGIQKILNEEAQYGVPVKIFTQDIDSESIEQLKKMAQLQFIYPH
IAVMPDVHVGKGATVGSVIPTKHAIIPAAVGVDIGCGMNAIRLSLKASQLPDNLSRLR
DAIERKVPVGFALHKQVKAKASSIIPLEKRLEPIIKKHPGLVRMLRQFEATWQKQLGT
LGGGNHFIELCIDENQDVWVMLHSGSRGLGNVIGTYFIELAKKEAQHRFGHVPDKDLS
YFAEGSQSFNDYVEAVEWAQEYAFENRKEMMRLILEAIRPLLPSFQMTKEAINCHHNY
VSQETHFGENLFVTRKGAIRAGLDELGIIPGSMGARSYIVKGKANPESFCSCSHGAGR
KMSRSKAKVLFNQQDLIEQTQGIECRKDSGVVDEIPSAYKDIDEVIANQADLIEVIHT LKQVLCIKG"
/product="RNA-2',3'-PO4:RNA-5'-OH ligase"
/transl_table=11
CDS complement(2647166..2648626)
/locus_tag="AUO97b_02614" /db_xref="SEED:fig|400667.82.peg.2479"
/translation="MSETHAPEKLQRKLGARHLNMIAIGGSIGTGLFLASGSTIANAG
PGGALLAYSLIGIMIYFLMTSLGELATHTPTSGAFFTYGSRYVEEGFGFALGWNYWYN
WAITVAFELVAVQFIMKFWFPDVPGFYWSALFLVIIFMINAMTVKGFGESEFWFSMVK
VIAIVAFIIIGIAMIIKIMLTPGVATFGNWTYKEAPFVGGLQAMIGVAMIAGFSFQGT
EMVGVAAGESKDPKKTIPLAIKQIFWRILLFYILCIFIIGTLIAYDDPNLLQAAATEN
IALSPFTLLYEKVGFAFAASLMNAVILTAILSAGNSGMYSSTRMLFDMAQQGRAPKWF

AKLDNRGVPMNALYATTFIAAFCFLTTFIGEKQVFNWLLNMSGMCGFIVWLGIAISHY
RFRKGYIAQGYKLEDLAYRAKFFPFAPWFAFILCSVIILGQNYQAVLGGKIDWIGLLS
TYISLPLFLVIWLGYKWKNKTKLIPYDQMDVKPEQD" /product="Lysine-specific permease"
/transl_table=11
CDS complement(2648648..2649661)
/locus_tag="AUO97b_02615" /db_xref="SEED:fig|400667.82.peg.2480"
/translation="MDPRSEVILRQQDYLKGRVLLINAPNDALVSQLPTEIDASVWTW
NYADYQGFLNAGATAHFSVEFPLQEFDQAIIFVPKSKELLNYILHVVMSHLKIDQSVF
LVGEKKGGVERAAKQLQSFGKILKLDSARHCQLWHLKIEKTEKIKPLESWLKTYTVQV
NEQELTICALPGVFSQTHLDVGTAVLLPYLNQVKSGRIADFGCGAGIISCYLAKANSS
NIIHALDIDAFALQSTEMTFSRNGIGSDQLRLQPVTGIADAPTELDAIVSNPPFHQGI
HTNYDASEGLCQNAKKHLKASGELWIVANRFLNYPILIEKHFGQCEIKTDLQGFKVLY ACA"
/product="16S rRNA (guanine(1207)-N(2))-methyltransferase (EC 2.1.1.172)"
/EC_number="2.1.1.52"
/transl_table=11
CDS complement(2649724..2651082)
/locus_tag="AUO97b_02616" /db_xref="SEED:fig|400667.82.peg.2481"
/db_xref="GO:0005215"
/db_xref="GO:0006810"
/db_xref="GO:0016020"
/translation="MSNQPSQDRLDLVYGLDDRPKPFVAFLAAFQHLLAIIVPIVTPG
LLICLALGVPRNETNMILSMSLVISGIATFLQCKKVGPFGAGLLIVQGTSFNFIGPII
GIGSAMVAAGTPVNQVMAAIFGVVIAGSFIEMGVSQILPWVKKLITPLVTGIVVLLIG
LTLIKEGLISMGGGYQAMQDHTFASADNLIMSCTVLAIIIVLNRIRIVWIKSSAILIA
LVIGYILAGFMGYLDFSGLKDAPVIQIPTPMHFGLSFSWGLFIPMAFIYLVTSLEAIG
DVTATSKLSNQPVDGPQWMQRIKGGVLVNGANSLLAGLFNTFPSSVFAQNNGVIQLTG
VASRYVGIWIAALLILLGLFPAVAGVIQAVPQAVLGGAVMVMFGAVAASGINILSSIH
LDRRALLIIAISLALGLGVAQVPQILEHLPELFKNIFSSGVATGGIAALILNVVLPET HK"
/product="Xanthine permease"
/transl_table=11
CDS 2651261..2653096
/locus_tag="AUO97b_02617" /db_xref="SEED:fig|400667.82.peg.2482"
/db_xref="GO:0005525"
/db_xref="GO:0006412"
/translation="MSDIKNLRNIAIIAHVDHGKTTLVDKLLQQSGALGDRAGEIERV
MDSNALESERGITILAKNTAITWLDKRTDTTYRINIVDTPGHADFGGEVERVMSMVDC
VLLLVDSQEGPMPQTRFVTQKAFARGLKPIVIINKVDKPSARPDWVIDQVFDLFDNLG
ATDEQLDFPIVYASGLRGVAGPAPEELAEDMTPLFETIVDIVEPPAVDVDGPFQMQIS
SLDYNSFVGVIGVGRIQRGSVKLNTPVTVIDKEGNTRNGRILKIMGYHGLERIDVDSA
SAGDIVCITGIDALNISDTICDPKNVEALPPLSVDEPTVSMTFQVNNSPFAGKEGKFV
TSRNIRERLDRELIHNVALRVEDTDSPDRFKVSGRGELHLSVLIENMRREGFELGVSR
PQVIIKEIDGEKQEPYENVTFDVEEQHQGAVMEQMGHRKGEMTNMEVDGKGRIRIEAT
VPSRGLIGFRSEFLTMTSGTGIMTSSFSHYGPLKQGTVAKRQNGVLISMVQGTCLGYA
LFSLQDRGRLFAKPQLEVYEGMIVGINSRSDDMVVNPTKAKQLTNVRASGTDDALTLT
PAIEFTLEQALEFIEDDELVEVTPKSIRIRKRYLTENERKRNRDK" /product="GTP-binding protein
TypA/BipA"
/transl_table=11
CDS complement(2653054..2653185) /locus_tag="AUO97b_02618"
/db_xref="SEED:fig|400667.82.peg.2483"
/translation="MTLSLLSALPVSNKKPLNEFSGFSFLFKKNYLSRLRLRSFSVK"
/product="hypothetical protein"
/transl_table=11
CDS 2653352..2653783
/locus_tag="AUO97b_02619" /db_xref="SEED:fig|400667.82.peg.2484"

/db_xref="GO:0005216"
/db_xref="GO:0006810"
/db_xref="GO:0016020"
/translation="MSIIQEFKEFAIKGNMMDLAIGVIIGGAFGKIVDSLVKDIIMPL
ITVITGGGVDFSQKFIVLGANPNNLQSLDALQKAGINVLTYGNFLTILINFLILAWVV
FLMVKLLNKLRRDKNEPEAPAATPEDIQLLREIRDELKKQA" /product="Large-conductance
mechanosensitive channel" /transl_table=11
CDS 2653926..2654279
/locus_tag="AUO97b_02620" /db_xref="SEED:fig|400667.82.peg.2485"
/translation="MNQLFVYGTLCPNKANAHILEQIGGNWTKASVRGVIHILDWGPD
KGLKALELDPEADWVEGYLFSTEKLAENWQMLDDFEGFQYQRVTADIKLESGEYVKAW TYQVNMQAKASHNYK"
/product="hypothetical protein"
/transl_table=11
CDS complement(2654290..2654580)
/locus_tag="AUO97b_02621" /db_xref="SEED:fig|400667.82.peg.2486"
/translation="MQTAIVRHILVKDKDLAEQLKKKLQSGADFAKLAKQYSTCNSAK
RGGELGEVKKGQLVPVIDKVVFTAAERVLQGPIKSQFGYHLLEVKFRMTGLR" /product="Peptidyl-
prolyl cis-trans isomerase PpiC (EC 5.2.1.8)" /EC_number="5.2.1.8"
/transl_table=11
CDS complement(2654600..2655424)
/locus_tag="AUO97b_02622" /db_xref="SEED:fig|400667.82.peg.2487"
/db_xref="GO:0008534"
/translation="MPELPEVETTKTSLFPLLNQKVLSVEVRNPSLRWPIPDNVQKLV
GQRLIGLNRRSKYILAEFEQDQMLWHLGMSGSFRLCQPNDELRKHDHLIIQFEDQQLR
YHDPRRFGCILWLNPETQGKLIDTLGPEPLSTDFHAEYLASKLKNKSVGIKIALMDNH
VVVGVGNIYATESLFNVGIHPAQPAGDLTMQQIEKLVVEIKRILKSAIDLGGSTLRDY
SNAMGENGYFQQTLLAYGRAGEMCVNCETTLENLKLGQRASVFCPQCQPLKKLRKP"
/product="Formamidopyrimidine-DNA glycosylase (EC 3.2.2.23)"
/EC_number="3.2.2.23"
/transl_table=11
CDS 2655500..2656066
/locus_tag="AUO97b_02623" /db_xref="SEED:fig|400667.82.peg.2488"
/db_xref="GO:0003905"
/translation="MSDILPLSWFQRETSEVAYDLIGCVLCKRQPDGQVIRCTISETE
AYLGVRDKACHSYNGKRTARTEVMYRHGGTIYVYLIYGMYEMLNIITQTEGVPEGVMI
RSAFLNSASTKKEYKLLAGPGKLTRYLGIDRTLKGQTLGEESGLWIEAATTQPEVVLT
PRIGIDYAEEAKDWPERYCWKDHPSLSR" /product="DNA-3-methyladenine glycosylase II
(EC 3.2.2.21)" /EC_number="3.2.2.21"
/transl_table=11
CDS 2656199..2658403
/locus_tag="AUO97b_02624" /db_xref="SEED:fig|400667.82.peg.2489"
/translation="MSLLRLDRLHYCILMSMGCISSPLVWAEDLNSDVAKLPTLHVEA
TRTDTGYLQTPASVFRIEAPQVDSSSQVNLTEVVKGIPSLQIRNRENYAQDLQLSMRG
FGARSTFGVRGIRLYVDGIPATMPDGQGQTSNIDLSSLDHVEVLTGPFSSLYGNSSGG
TILTSTKEGQGKDSIELSYSGGSHDKSRAGLVLQGGAKGANEPSYIISSSYFDTDGYR
EHSGAEKVLNNAKLSWNLDDGSKINWVTNYVKIHADDPMGLERKDWQANPKQIAPYVK
KWGFNARKDIEQTQTGITWFKPINDQHELYAMAYLGNRQVTQYQSIPQGQVVVENGKP
VYTGQKSPKHAGGVIDFERNYYGADFRWTGKELLPNTTVSIGVAFDAMDEERKGFENF
NADGIYGVKGNLRRDEDNTLWNIDPYLQASWQFLPTWRLDTGVRYSNVHYKSKDHFTS
GPDEYGTVNGDDSGKTDYEKVLPSAALSWQILPELMAYVSYAKGFETPTFTEMAYHTD
ISKSGFNFGLKPSTSDTYETGLKSQNLLGDFTLAVFQTKTKNDIVSAGNLGGRSTFRN
ADKTLREGVEFAWNKKLWRDLTATASYTYLDATFDANVPEKLDQDNKVLASAIPTGNA
IPGIAKNQAYASLAWQPSHGLYGGVDVQYMDKVYVNDTNSDAAPSYSVTSANVGYAWV
MGDWKVNSFARVDNLFDKKYAGSVIVNDGNSRYFEPADGRNWSAGLRVIKQF" /product="TonB-

dependent receptor"
/transl_table=11
CDS 2658413..2659522
/locus_tag="AUO97b_02625" /db_xref="SEED:fig|400667.82.peg.2490"
/translation="MAKLFETVNFDSLQLVNRIVIAPMCQYSATDEGEITYWHEQQWA
NYALSGAGLCIVEATAVQAEGRISYADLGLWNDQQRDQIKTLLGKVKTLSPMPFGIQL
AHAGRKASTEKPWLGKGQIAKDQPHGWQTVAPSTSTFSVHDAAPHALTIAEIKQIQQD
FAAAAKRAVEAGFELIEVHAAHGYLLHQFLSPIANQRTDEYGGSLENRMRMTLEVLQA
IKLAVPEGYPVGVRLSATDWMDGNEQWDIESTVGLSKALEQLGAAYIHVSSGGLHEHQ
NITIGAGYQVPFAEQVKKHVAIPVIAVGLITDPQHAEQILENQQADAIGLARAMLYDP
RWPWHAAATLGAKVKIASQYLRCQPHGLKQLFDSF"
/product="FMN oxidoreductase"
/transl_table=11
CDS 2659588..2660511
/locus_tag="AUO97b_02626" /db_xref="SEED:fig|400667.82.peg.2491"
/translation="MVFQVILPILILLLMGYCSVLIKLVTPEQIKALSAFVIKISLPA
FLIQSLANKNLQDIWHPAYFIAYGGGSLILFFLAFILYRKYFKNSLTHSAVISMGASM
SNTGFMGTAILTMLMGSHAAIYISLTLIIENLLIVALMLILAEAGLHQQQRLLPLLKK
TFINLLKNPVIIAILVGMSCILLDIHLPSKLGQALELLGRTASPLALFAIGGSLVGIG
ITALNFQSILLAGFNVILMPVVILGLFLLLPNVSQEMLYAGTLLAALPMPIAFGIFGQ
AYGLNERALTPLMISTIIGFMGVSGLIALWW" /product="Auxin efflux carrier family
protein" /transl_table=11
CDS complement(2660545..2661369)
/locus_tag="AUO97b_02627" /db_xref="SEED:fig|400667.82.peg.2492"
/translation="MKKLLIASLIAISSSSIFAADTTTSAAKNDVFKQAEQLYAAKNY
SAAFQEVQRLAQTGNAQAIYNLGYMTQMGQGTAKDNAKALKYYEDASNKGYAQASYTL
AQIYETGELGVAKDSNKFSQYIQKAAAQGSDDATVKIATILFAQKKPQSHQIALQKLA
PLIRKGNYPAIQVKALYDISQGVENKNPLMKRQGIEALQTIAQKGYAPASMALATMMA
NGNIIPQNLPQAKQIFTELAKQNVPNARESLASVEKIIAEKNKQAAAAPTQPAPKK"
/product="hypothetical protein"
/transl_table=11
CDS complement(2661552..2662262)
/locus_tag="AUO97b_02628" /db_xref="SEED:fig|400667.82.peg.2493"
/translation="MKTKLATLLLSLAFFGSAHADVKTLEQNLKTNYPDLPVKGVYQS
PVAGIYEVYTSGRIVYTNQDAKYFFVGNLVDIKQQKNMTEERIAELGKIDVKSLPLNQ
AIKYVKGKGERTLYIFSDPDCPYCQRLEQNMVGVDNVTVYVFLYPLTSLHPNAEKVSN
QIWCSKNPSEAWTNYMLNRKLPAAGKSCTSPIQKNIALGQKLNIDGTPTLFLQDGQRI
SGVPSDAKQIEALLQSVK"
/product="Thiol:disulfide interchange protein DsbC" /transl_table=11
CDS complement(2662408..2663130)
/locus_tag="AUO97b_02629" /db_xref="SEED:fig|400667.82.peg.2494"
/translation="MSLLAFSSYALNGVDAQKFLQGQVTVDTERLAENETRYTAICDL
KGRIHFGLWLKKNNAESFDIIVTQDQAEEFAKHIKKYGAFSKMTLSEQGAVFPKVVNG
HTEFSSTETDISEWQKQAIVTGQAWIAQATEHEFQPQELRLHQREGVNYDKGCYLGQE
IVARLWFKAKPKHWLHLVQGTGDAPAPATQLHNDVEVVNSTQTTDGYIALVVAKPAAL
QELGLQVLDLPEALSGDVARPQ"
/product="tRNA-modifying protein YgfZ"
/transl_table=11 CDS 2663347..2663796
/locus_tag="AUO97b_02630" /db_xref="SEED:fig|400667.82.peg.2495"
/translation="MATGTVKLHRVFKAPAERVYRAFLDPDALVKWMAPHGFTAKVHS
FDAKVGGSYKMSFTNFSTGSTHSFGGTYVELVPNELIRYNDQFDDANLPGTMQVTITL
KTVLVLVGTEIHITQEGIPEVIPVEACYLGWQESLSLLKLLVEADIPDQ" /product="Ligand-binding
SRPBCC domain protein family" /transl_table=11
CDS 2663938..2664294

/locus_tag="AUO97b_02631" /db_xref="SEED:fig|400667.82.peg.2496"
/translation="MNIQIKRIYDDVSGDDGKRILVDRLWSRGISKENAHLDVWLKEI
APSTELRKWFHAESAEHWDEFKQRYLKELETNPAVKELLEIVSHHKVTLLYSAKDVEH NHAIILKEYLLEKSKS"
/product="DUF488 family protein SAV0238" /EC_number="2.1.1.107"
/transl_table=11
CDS 2664385..2664729 /locus_tag="AUO97b_02632" /db_xref="SEED:fig|
400667.82.peg.2497"
/translation="MSLPNCPKCQSEYTYQDGDLLICPECSHEWKEGEALVTEEQEII
KDANGNVLADGDSVTVIKDLKIKGSSSVVKVGTKVKSIRLVPDASDGHNIDCKIDGIG QMKLKSEFVKKA"
/product="Protein PhnA"
/transl_table=11
CDS 2664941..2665282
/locus_tag="AUO97b_02633" /db_xref="SEED:fig|400667.82.peg.2498"
/translation="MAGWYEITQANDGQYRFVLKAGNGEIILNSELYKAKASAVNGIE
SVQKNSSDDARYDRLTAKNGKPYFNLKAANHQIIGSSQFYASEASRDKGIESVKNNGS STTIKDLTVQA"
/product="UPF0339 protein YegP.N / UPF0339 protein YegP.C" /transl_table=11
CDS complement(2665336..2667051)
/locus_tag="AUO97b_02634" /db_xref="SEED:fig|400667.82.peg.2499"
/translation="MRASRFLFATLRETPNDAEVISHQLMLRAGMIRKLASGLYTWLP
MGTRVLKKVDAIVREEMNRSGAMEVFMPVTQPASLWEESGRYEQYGPELLRFKDRHDN
PFVLGPTHEEVITDLARNELKSYKQLPVNFYQIQTKFRDEIRPRFGVMRSREFIMKDA
YSFHATQESLQETYDVMYDTYSRIFTRLGLDFRPVQADTGSIGGSASHEFHVLAASGE
DDIAFSTESDYAANVEMAEAVLVGERAAPTQEFKLVETPNQKTIADVCQFLNADPKQS
VKALLVQGVADEKGNVPVVALFLRGDHELNEIKAEKHPLVAAPLAFATEEQLQAFGLT
AGFTGPQGLVEKGITVIVDRAASVLSDFVAGANEADKHAVGVNWERDAQITEVFDLRN
VVEGDPSPDGKGTLQIKRGIEVGHIFQLGTKYSEALGCKVLGEDGKPFTVTMGCYGIG
VTRVVAAAIEQNYDDKGIIWPQAIAPFEIAIVPMNAHKSPRTLEAAEALYAELQAQGF
DVLLDDRNERPGVKFSDLELMGIPHRIVIGEKGLDAGTFEYKGRRDAEASNLTKEELL AKLAR"
/product="Prolyl-tRNA synthetase (EC 6.1.1.15), bacterial type"
/EC_number="6.1.1.15"
/transl_table=11 CDS complement(2667110..2670208)
/locus_tag="AUO97b_02635" /db_xref="SEED:fig|400667.82.peg.2500"
/translation="MWFTRISVKYPVFTIMMMLSLMVLGLASWKRMTVEEFPNIDFPF
VVVTTQYAGASPEAVESDITKKLEDQINTISGIKQITSRSSEGLSMVIAEFNLDTSSA
IAAQDVRDKIAPVIAQFRDEIDTPIVQRYDPSSSPIMSVVFESNSMSLAQLSSYVDKK
IVPQLKTVSGVGNVNLLGDAKRQIRIKVHPEQLQSYGIGIDQVINTLKNENIEVPAGT
LQQKNSELVVQIQSKVIHPLGFGDLVIANKNGSPIFLKQVATVEDTQAELQSSAFYNG
RTAVSVDILKSSDANVIQVVDKTYQTLEKLKAQMPAGLNYKVVADSSKGIRASIKDVV
RTIIEGAVLAVLIVLLFLGSFRSTVITGLTLPITLLGTLTFIWAFGFSINMMTLLALS
LSIGLLIDDAIVVRENIVRHTELGKDHVTAALDGTKEIGLAVLATTLTIVAVFLPVAF
MGGLIGRFFYQFGVTVSTAVLISMFISFTLDPMLSAHWKDPVKKKESRLQRFFNYISN
LLDGLTHIYEKLLKLALRFRFITVIIAIVSLVVALGLSKMIGTEFVPTPDKGEIRIQF
ETPVDSSLEYTQAKLHQVDQIIRQFPDVVSTYGVVNSEVDSGKNHAGLGVTLKPKQER
SADLTTLNNEFRDRLQSVAGIRVTSVAAAQDSVSGGQKPIMISIKGSDLNELQKISDR
FMAEMEKIDGVVDLESSFKEPKPTLGVHINRVLASDLGLSVSQIANAIRPLIAGDNVT
TWEDRDGETYDVNIRLNENKRVLPQDVQNLYLNSNKTNANGQNILVPLSAVATTQEKL
GASQINRRDLEREVLIEANTSGRPSGDIGQDIDKMQKAFKLPAGYTFDTQGANADMAE
SAGYALTAITLSIVFIYIVLGSQFNSFIHPAAIMASLPLSLIGVFLALFLFRSTLNLF
SIIGIIMLMGLVTKNAILLIDFIKKAMEDGISRYDAILQAGKTRLRPILMTTSAMVMG
MVPLALGLGEGGEQSAPMAHAVIGGVITSTLLTLVVVPVIFTYLDDLKNFMLRQTRRF MS"
/product="RND efflux system, inner membrane transporter" /transl_table=11
CDS complement(2670224..2671342)
/locus_tag="AUO97b_02636" /db_xref="SEED:fig|400667.82.peg.2501"
/db_xref="GO:0008565"

/db_xref="GO:0009306"
/db_xref="GO:0016020"
/translation="MRTTHLTPSTFFKLSLISLSLILAACNQQEVPKTTASEAKKIEL
IPQDLIPVKEGSLAAQTAFTGTIRAVQQSSIQAQVSATATAVTANVGQKVQKGQVLVR
LNNQDNAARLAQAQANLASAQAQAELARNLMNRKQRLLNQGFIARVEFEQSQVDYKGQ
LESVRAQQANVDIAKKADRDGIITSPISGVITKRQVEPGQTVSVGQTLFEIVNPDQLE
IQAKLPIEQQSALKVGSSIQYQIQGNSKQLHAILTRISPVADQDSRQIEFFASPKEAI
DSLSIGAFINGIILHNDSNQGQTIPLDSIQNLQHDPFVWVIRNQTIQKVKIRVVEQRY
NENIALVQGLQSTDQVSRIQFEDSDINKKVTLTTEKNK"
/product="CzcABC family efflux RND transporter, membrane fusion protein"
/transl_table=11
CDS complement(2671402..2672001)
/locus_tag="AUO97b_02637" /db_xref="SEED:fig|400667.82.peg.2502"
/translation="MSVSIGIASCGALFFSSPTLAANAAPKAVWYRYYDQHGIANIST
SVTPAHIRYGYEALDQNMQVIKRNHPYNSEADLNQSSQRALSARYREEDNKLKRAYSN
SKTATIKRDQALLAIKKQINYQQDQLSQLQQDKILFKRQEMEYFRKGEQVPERHKTLI
EKNLMNINKVKQTLEILSKTYNRTNKDYENIIERLKKLE" /product="hypothetical protein"
/transl_table=11
CDS 2672323..2672706
/locus_tag="AUO97b_02638" /db_xref="SEED:fig|400667.82.peg.2503"
/translation="MEDAFQNLKVMVIDDSKTIRRTAETLLQREGCEVITAVDGFEAL
SKIAEANPDIVFVDIMMPRLDGYQTCALIKNSQNYQNIPVIMLSSKDGLFDQAKGRVV
GSDEYLTKPFSKDELLNAIRNHVSS"
/product="twitching motility protein PilG"
/transl_table=11
CDS 2672730..2673092
/locus_tag="AUO97b_02639" /db_xref="SEED:fig|400667.82.peg.2504"
/translation="MARILIVDDSPTETFRFKEILTKHGYDVLEASNGADGVTLAKAE
QPDLVLMDVVMPGVNGFQATRQITRDEDTKHIPVVIVSTKDQATDRVWGKRQGAIDYL
IKPIEEKQLIDVIKQFLN"
/product="twitching motility protein PilH"
/transl_table=11
CDS 2673153..2673689
/locus_tag="AUO97b_02640" /db_xref="SEED:fig|400667.82.peg.2505"
/translation="MAANGFIELLRLSKRGNKNYASVQNEAQRWSGIAFEMRGQYFVA
PLGEVSEVIYPPKYTPVPNTQSWVKGLANIRGRLLSVSDLAHFISGQRSSFSSTQKVL
CISHHDQYVGLVVDQVLGIQHFNKKSFFSESLDLEENLKEYCQGYFHQHNQHWHVFLF
SRLLQNPHYMNASTKFIN"
/product="type IV pili signal transduction protein PilI" /transl_table=11
CDS 2673736..2675814
/locus_tag="AUO97b_02641" /db_xref="SEED:fig|400667.82.peg.2506"
/translation="MGFKLKKKSGNRGATGKSSVNFIAKIQSKIEQFASLFGESEKSK
PILYAALLCLVCALISLAYLFYNVPRHNQLIRSLGELRLLSQTISKQATEATESGSQE
AITKLQQSKKDFDENLLEIENIHGKSTDEYQKVEKIWGEVSKNIDLISSHQRVLNQLY
DTNISISETVPEIQAEYNLMVDQMARQGLPSSQVIIAKNQVFIAERILRSINSVLSGT
DGNVSTSDFSVDIETFGTYLNAELNGNAELGVDRIADPALRESLESIKSEYDKVLKSA
AATVLKNGSQIVNVRQASSQIFSKSDVMLESLGHLSDVARKNWLTLFLGAVLILSLGG
LIFSVFKLITLRSVMDKQRVARLQEEYDRNQNAILRLLDEIADLADGDLRSYATVSED
FTGAIADSINFAIDQLRDLVSRIHETSQEVARYTQDTQSITNQLAEASEHQAQEIAGA
STAMNEMAQSIDQVSANASESAEVAQRSVQIASNGAQVVNRSIEGMDTIREQIQETSK
RIKRLGESSQEIGNIVSLINDIADQTNILALNAAIQASMAGEAGRGFAVVADEVQRLA
ERSASATKQIETLVKTIQTDTNEAVISMEQTTTEVVRGANLAKDAGIALDEIQKVSGD
LAKLIASISDAAKLQSASASHIATTMTVVQEITSQTTTATFDTARSVSELANMAESLR ESVTDFKLPD"
/product="type IV pilus biogenesis protein PilJ" /transl_table=11 CDS

2675961..2680481
/locus_tag="AUO97b_02642" /db_xref="SEED:fig|400667.82.peg.2507"
/translation="MKEILKTLTEAMTLPEDSYSEQDAEFLEIFIEEIEEIFVDLQPL
INKWMQSENIATLTEIRRHFHTLKGSGRMIGAKSSAELAWTVEDTLNRVINQSLQLTP
TIQSYVQLVFKFYFLKLVDNFKQKRAHTLDFRPLILLGQQLQQQQSLEPALEELLQLS
NTLMAETVTGLELDYIEQDSLAEPIIQATHTEIEINLDETLTLFMEEAEEHLATIHQF
LDQELHQYDSYNALIRALHTLRGSSAMAQVETIFEASTKVEHLFKILLQEELSSHSEE
ILLLQEYREFVRDSLELLSRYSSSEQLEARLQQFNQFWDSYVEQHGDRTDPLMPSHGL
VSQLLQLDVSELLDAELDFEKGIRNEFPDYLERLSEQADLLLQHTHSQAMLGLHEYTS
QLKESYEVLLDKPALLQSDYIFEIYQKAHQQLIQLFDALAAGQRVGVVKQHQSILEEL
KLYTQYTPDISNDLPQQDSTSFEPISNIEQEPEVVATVENFSDSVDWAVLGQSVQQDR
QYISSTQVNRNFDADLLDIFLEEAEELLEGIDTDLNIWVGEQENFAALNNLMRYLHTL
KGGANMVQATYLGLIAHELESIYERLIQKQLVATSDLIDFIRLVQDDLADRLQVMREQ
QLDYAAPYTINALKRAGQNSNFQPLPVVEAFDTESEIFSEQEVISEIIIDEIPVELEP
ALAELETHHDQVFDTAVTELATPVEMITTVTSQESEIAADEQDIEAVVEQTFLEEATE
LLEMAESLLKQWFEQRTNRSILLQLQRAVHSLKGGARMVGLEAVQAIAYQLENAFEQF
ALHHFNSNIYDHLLESAIAWLKDAIFNHNYQHFDGLQQSLENIQFFETTIQIPTKLTR
ADLFSSEPVMTFIQGDGTEPPPMMGAWEQTERLDQNNEMIRVSADLIEKMIDLSGENS
INRSRIEMDLSQFSHTLVEMELAIQRLADQLRRMEGELETQIIAKHGIEHSRYTDFDP
LEMDQYSSLNQLSKSLAESASDLVDFKNTLSDKIRDTESLLLQQSRIQAEIQEGLMRT
RLVPFSRLLPRLQRIVRQTSTALNRPAELFVNNTEGELDRNILERLVTPLEHMLRNAI
DHGLEDRMQRQQANKPETGRIELNIQRQGTDVVVVFSDDGQGIDVEKVRQKALLAGLI
KPEQDLEQQDILQLIFHPGLSTAEQVTQISGRGVGLDVVQSDIKSLGGHVSVESVYGQ
GTTFTIRVPTTVAVSDALMVKVADQQFAIPLAQIDRIIRVSPASLEQYFESPQELFEY
ENKRYPLRYLSEFVGNQPIPRLNGVMYSLPVLMIKANNGQTVALLVDQLIGSRAQIVV
KPIGQQFSSIGAIAGATILGDGQVCLILDGQNIARQIQSTRRHKQLSEAVYRQRESDE
RRLIMIVDDSVTVRKVTSRLLERQGYDVVTAKDGVDAIEQLENIKPDLMLLDIEMPRM
DGFEVLNLVRHHDMHQYMPIIMITSRTGEKHRERAFSLGVSQYMGKPFQEEELLENID ALLVASESEVKS"
/product="twitching motility protein PilG"
/transl_table=11
CDS 2680478..2680942
/locus_tag="AUO97b_02643" /db_xref="SEED:fig|400667.82.peg.2508"
/translation="MKGSVNTSLIANMDQQELQHLITVSTGFIDAYIIECNDQVPMLL
PQNIVLSAMDVKNGIKHIEWHDVKLPVYSVHNTIDQHAVALVIEGEDISQRFALICKT
MPVSTRLRISEIVDEMDEHEAEQHPAVFKYVRMQDQRYYIPNLEYIEKLLGL" /product="hypothetical
protein"
/transl_table=11
CDS complement(2680978..2682114) /locus_tag="AUO97b_02644"
/db_xref="SEED:fig|400667.82.peg.2509" /db_xref="GO:0009014"
/translation="MNHSDTLSLSLELLQQPSVTPIDHTCQTIMADRLAKVGFHIEPM
RFGDVDNLWARRGTEGPVFCFAGHTDVVPTGRLDAWNSDPFAPEIRDGKLYGRGSADM
KTALAAMVVASERFVAKHPNHKGSIAFLITSDEEGPAVNGTVKVIETLEKRNEKITWC
LVGEPSSTHKLGDIVKNGRRGSLNAVLKVQGKQGHVAYPHLARNPIHEASPALAELCQ
TVWDNGNEYFPATSFQISNIHAGTGATNVIPGALEVTFNFRYSTEVTAEQLKQRVHEI
LDKHGLQYEIVWNLSGLPFLTPVGELVNAAQTAILNVTGTETELSTSGGTSDGRFIAP
TGAQVLELGVLNATIHQINEHVDVHDLDPLTDIYEQILENLLAQ" /product="N-succinyl-L,L-
diaminopimelate desuccinylase (EC 3.5.1.18)" /EC_number="3.5.1.18"
/transl_table=11
CDS complement(2682240..2682383)
/locus_tag="AUO97b_02645" /db_xref="SEED:fig|400667.82.peg.2510"
/translation="MMKKVLVASVMVAFVLTGCNTFKGFGQDVSKAGDAVTNTAQKTE NKM"
/product="Entericidin A/B family protein"
/transl_table=11
CDS complement(2682482..2683390)

/locus_tag="AUO97b_02646" /db_xref="SEED:fig|400667.82.peg.2511"
/translation="MNIASIKTSYFEPWLQFSHPMVRQLAFTIASPNLLSQLPCRLTI
RHNFQLHTDQNWEQHFQNYLPRLKQLDAAPEPLLQFMSRLKSTRLGLRFENLLWFWLQ
EDQYHPYQLLGHSIQKIDGAKTLGELDFLVLNQNTQEIEHWEVALKYYLGEGQLNLEQ
WIGLNRQDTLSKKLYHFTDKQFQFSEALGFKVQQRFAVLKGQLYLPLNLPQEQPIPEW
VNLKRRLGTWGTEIPKSDFYRLERHEWLCPHQKASSNTAYWWTDGLYCQNINETRFYM
FRHPALLSLKPSLIKNNGVLQETTSL"
/product="hypothetical protein"
/transl_table=11
CDS 2683495..2683863 /locus_tag="AUO97b_02647" /db_xref="SEED:fig|
400667.82.peg.2512"
/translation="MRTLFWRTLVVIFITLGIIGAILPGMPTTVFLILAAWAASKGWP
QMDAWLLNHPKYGPTLRDWRANGTVPRKAKWIATIMMTASGILMLFTSAPFWVKVFTD
TTMLVVAIWLWLRPEPKLKD"
/product="Inner membrane protein YbaN"
/transl_table=11
CDS complement(2683852..2683971)
/locus_tag="AUO97b_02648" /db_xref="SEED:fig|400667.82.peg.2513"
/translation="MKNYGKACLAPKLKDYQSSLLYAQFHCLSCAKIKRLSIF" /product="hypothetical
protein"
/transl_table=11
CDS 2684014..2684517
/locus_tag="AUO97b_02649" /db_xref="SEED:fig|400667.82.peg.2514"
/db_xref="GO:0004355"
/translation="MSQFKLEDADILIDLANQTLSLPKHNKFYVVSTGKNGIGEQENT
GKTPRGWHRVAKKFGMQSPKNAVFIARQPTGEVYSSELAAQFPERDWILSRILWLDGL
EEGFNKGEGHDTMQRYIYIHGTPDKEPMGVPMSHGCIRMRNEEIIELFDLVSEDALVY LSEQSLT"
/product="L,D-transpeptidase"
/EC_number="1.4.1.13"
/transl_table=11
CDS complement(2685572..2686357)
/locus_tag="AUO97b_02650" /db_xref="SEED:fig|400667.82.peg.2515"
/translation="MQVSKWIRIFLAVIGSILFLDGLFLAFLNKIHVGTLVPLVLGAF
FCLYALFYYHLERFFFYHYRLHTLWRFGWLCFWIWLIGLGYFFNFIKENKDASQNLPA
VKAIIVLGSGVENGEPSAILAKRLDTAAPVALSQPQAKVILTGGLDFSEKEPEALVMS
RYLKQHFHITKDRMILEDKSTSTELNLKNSKPLLEQAHISVNQPIAIVTSDFHTPRAA
AIAKKQGYTQVYMVAAETPLITRYNAWLREYFAYASGWLLNEY" /product="Integral membrane
protein"
/transl_table=11
CDS complement(2686484..2689030) /locus_tag="AUO97b_02651"
/db_xref="SEED:fig|400667.82.peg.2516"
/translation="MFKKFLFQIHWFLGISAGLILSIMGVTGAIYSYDQQILKWVNTD
SYVVQAQSSPKLTPAQLYQHFTTIQPEIKINSITIAKDPTASSVVNIEKEGERRGYNM
MVNPYTAQVLPEVQGRKLLLLIQQIHRNLTAGEFGKQITGACALMLIYFVLSGLYLRW
PKKHSARQWLAVKPKLKGRNFIWDLHAVVGTWVIVFYLLFACTGLYWSYDWWRSGMFK
VLGVEQPKMQGHSGSGRNKDQLPKIQLDNAQLITALNQTWSGFNNQIGRDYSTLTVNL
PKKDDGKIELSFVDATPQHERARNQAVYNYKTANIEKMELYEDKKLNQKIMSSMLPVH
RGSFFGPVYQFVAMLASLAMPLFFVTGWMLYLKRRKQKKLTQAARQSLAGHYIDQNAK
PWLITYATQTGVAEQLAWSTATSLQEAHQPVQVKSVQQLTEADLQQHEQILFVISTYG
TGEAPDLASNFAKKLLKTNLELQHVKYAVLALGSKEYPDTYCSFGHTVDEWLKNNGAK
AFFDIIEVDNANPADIQNWNQALVKATKLDLHAVNIEKVFDNWTLQQRDLLNPNSLGQ
PAYNIELTASHEAIWQAGDIAEIQPGNSPERINKFLQHHHILKNAVVDSLQVSIEKAL
WNKDLTGEIEPFANLDHLLEQLPTLPTREYSIASIPSQQVLRLVVRQQYDESGNLGLG
SGWLTQHTEINQNVALRIRTNESFHLIDDNRPIICIGNGTGIAGLMSLLHTRTRHNYT

ENWLIFGERQRAHDFFYASTIEAWQTMGMLKRLDLAFSRDQEQRVYVQDIIRQNAAEL
INWIERGAVLYVCGSIDGMASGVDQALIHILGEEQVDELRQQGRYRRDVY" /product="Uncharacterized protein PA4513"
/transl_table=11
CDS complement(2689188..2689763)
/locus_tag="AUO97b_02652" /db_xref="SEED:fig|400667.82.peg.2517"
/translation="MTAKTLYAYTLQPVPYEVSEAEQRNAQLLIWRSTNKISTKIWAI
MGVILALAILGLIFIKNYSTVICWVAIVCVVLYYLGRKYGLEWYVKRKMNEFPVQEIK
GIRLGVQPHGIVMRQQMGLQEGVGTIGWKNIYEWYNTPDFILVNFKVKDPKGQEQQGA
YILPKRMDSKNFSFNTIRKHLKESVGEPKTL"
/product="putative membrane protein"
/transl_table=11
CDS complement(2689864..2692038) /locus_tag="AUO97b_02653"
/db_xref="SEED:fig|400667.82.peg.2518"
/translation="MKSWFKRLKRATYNTSFMYNVRMLIAFSGTAFVPYFLGHQLATI
PLTLGVVAAGLSDIDDRFSVRIMNLIYTYIGFFITAASVQLLFPYPMLFALGLIASCI
GWILLGSLGRRYATISYGCLVVSVYTMLGVHLFDQWYIQPALLVAGAAWYGLIATISF
LLFPVRQLQDKLAASYASLGDFLFAKSNLFDVDMTPASYQQSMIDLSLENGKLITIFN
DLKTALLTRLKGDRGQKGTRRSLHYYFVAQDIHERADSAHIDYQKLAKIFQHSDILFR
FQRILAIQGKACQELNECILQRKPYIHNKRFKQSFENLRLSLVKLRDDQQYDLLWVNA
LFALYRNLKSIDSQLLNLETEQHIQSDKVKQAENQLKDDDLKGWNDIVVRIKQNLTPE
SVLFRHAIRVSIVLFIGYVFIQMTHIEYGYWIMLTALFVSQPNFNATKRRLRLRIVGT
LVGIIVGLSIVFLVPSVEGQLVMLILSGVLFFELRSKQYAQATAFITILALINFNLDG
SAVAAAIPRFVDTLIGCALAWFGVTFIWPDWKFRRLPRSIRRSLQAQCNYLAEVVKQY
HEGHNHALNYRIVRRAAHNTDAEVASLISTLATEPDFDPTRKSDIFEFLCLNHTFLSY
IAALGAHREKIQDQAVLNVLDQALDDIQGALLRDEMPDLTAQNMLQTIRQRLSQNDED
DQKSLIILQQLSLMLSVLNRFSMLKQRLSHDLDNDASELASL" /product="Putative efflux (PET) family inner membrane protein YccS" /transl_table=11
CDS 2692166..2693380
/locus_tag="AUO97b_02654" /db_xref="SEED:fig|400667.82.peg.2519"
/translation="MSEQTAQRQYSIGWIMLLALLTALGPLSIDMYLPALPQMAHDFG
VSTQMVANTLPAYFFGLAIGQLVYGPLSDRIGRKKPLYFGLALYAVASLFCVLATNEW
SLIAARILQALGGCVGVVMARAAIRDRLDVQGSAQAFSSMMIVMGLAPILAPMIGAWI
LIWFPWQAIFIALLIVGAICWLCVHFFFKETLAVDKRLKLSLYQVATLYGAIFKDASF
RLPMFAGCLTGAALFCYISSAPAVFMDQYGLNQQEFAYVFGLNAFGIMLMSSLNKHLT
TRVEITKRLKAGSMVQVTGASIVLIAGLISAAPLWLVMLGLFLAISGIGLTGPNAMAL
AMSKQGARAGTASAIMGSMQFACGLLGGVLLNFLIWSASLNMGVMMVLFTLSGFIVVL KAIRQAKSQSLS"
/product="Multidrug resistance transporter, Bcr/CflA family" /transl_table=11
CDS complement(2693408..2694112)
/locus_tag="AUO97b_02655" /db_xref="SEED:fig|400667.82.peg.2520"
/translation="MNLEQVRLVDEAITSRHSVRAFLSTPIEPEVIKDILRVASRAPS
GTNTQPWKVYVVTGHKRDEMVERVCAAQIEVSKNPELAEQYKETFAYYPEKWVSPFID
RRRENGWGLYGLLDIKKGEKEKMAAQQLRNFKLFDAPVGIFFTVNKAMGIGSKMDIAM
MIQNVMVAAKARGLDTCPQAAWNHFHPLVLDILGAADDEELVCAIALGHADPEHIVNT FITPREPVENFAVFLD"
/product="Nitroreductase"
/transl_table=11
CDS 2694623..2694826
/locus_tag="AUO97b_02656" /db_xref="SEED:fig|400667.82.peg.2521"
/translation="MSLPLINKQFMKQGLAITIATMKMNVTHATTEPQEIQQLGTDLN
ALFFNEKEKGTDSRVMTMACYSL" /product="FIG00351427: hypothetical protein"
/transl_table=11
CDS 2695009..2695149
/locus_tag="AUO97b_02657" /db_xref="SEED:fig|400667.82.peg.2522"
/translation="MFSGAMNLFYLKGAFSHVTFKGLVLGMMLLKLVTVIAIQVTLKV FP"

/product="hypothetical protein"
/transl_table=11
CDS 2695249..2696724
/locus_tag="AUO97b_02658" /db_xref="SEED:fig|400667.82.peg.2523"
/translation="MNEQLNATLMSWVQFFNEPLWDFLVIFLLAVGIFYTVLTGAVQI
RMFLQSIRVMKSSRTEGEDEHGLTPFQAFVTGLASRVGVGNIAGVAIAIAIGGPGAVF
WMWVTAVLGMSSAFIESTLAQLFKVRDSNSKQFRGGPAYYITQGLRSKTFGVIFALAL
IFTYGFVFNSVQINAIANASSHAWGWDKANLIAHLGGVDLEISWVGLALVVMVALAIF
GGIKRIAKFAEMFVPLKAGLYLSVALYIALSNYAILPDVLKLIVTEAFHFNAAAGGFF
GAAVSMAMMQGIKRGLFSNEAGMGSAPNAAAASDVKHPVNQGLVQMLGVFVDTFIVCT
STAIIILVSGVYQDAGFVGVELTQRALETQVGHWGADFLAVLLFLFCYSAVLGNYAYA
ESNVQFINNNPKVMFIFRIFVLVMVYFGAIGSVPLVWSMADLFMGIMATINLVAILLL
TPMARTLLKDYRAQLKQGIKEPVFKINKYPELKKKVDSDIW" /product="Sodium:alanine symporter family protein" /transl_table=11
CDS 2696893..2697864
/locus_tag="AUO97b_02659" /db_xref="SEED:fig|400667.82.peg.2524"
/translation="MKQLKLWVLGLSMVLAGCQTTSHLSALKPQTAEAYSRFVDQPFA
QLKKQHKKPVVALVLGSGGARGYAHIGVIEVLEQHGIRPDFIVGTSAGSIVGAIYASG
KTPDELRDTALKMKAADVRDISIGLKGFFDGKKVEDYVNQQVNNLPLEKMKIPMYVVA
TELKHGTKTVFNYGNTGQAVRASASIPSMFVPTKIGKSEYVDGGLVSPVPVEVARDLG
ADVIIAVDILAQPIYTETSNVWGLFNQNINIMQGRLAAEELQYADVVIQPDLREKAHI
FDVKGREATMKAGIDAANAKLSDIQFAIDEKIAEQNLSTDGLSAQTQ" /product="FIG00613342: Bacterial patatin-like phospholipase domain containing protein"
/transl_table=11
CDS 2698062..2699297
/locus_tag="AUO97b_02660" /db_xref="SEED:fig|400667.82.peg.2525"
/db_xref="GO:0004794"
/translation="MIQQQVKNEFPTLEDIHAAAERLDGLVVKTPFVFSETISKTLGA
EMWLKFENLQFTASFKERGALNKLLSLSEQEKQHGVIAASAGNHAQGVAYHAQRTGVT
ATIVMPKSTPNVKVQRVREYGARVILHGQDFSEAAAEMHRVAQEESLTIIHPFDDAEI
IAGQGTIALEMLEAVPDLDILVVPIGGGGLISGIAIAAKSINPKIKIIGVQSVVYPSM
AKLLCNYQLAVSMGSTVAEGIAVKTPGELTTQIAKHFVDDIVVVTEDMIEEAIALLLN
IEKTVCEGAGATGIAAIMSRPDLFLGHKVGVVLSGGNIDTRVMVSVLQRHLTRTGRMV
RIRVELPDNPGALARLTAIIAEQGGNIYELRHERFAATSRAKESAVSVDIELKSAPDL
EPLIQAMQLEGYIVRKEEI" /product="Threonine dehydratase, catabolic (EC 4.3.1.19) @ L-serine dehydratase, (PLP)-dependent (EC 4.3.1.17)" /EC_number="4.3.1.19"
/transl_table=11
CDS 2699534..2700727
/locus_tag="AUO97b_02661" /db_xref="SEED:fig|400667.82.peg.2526"
/translation="MFKSFFPSPRYFFISAVIWIALNMVLWYSGGSTWGSFIGFHQGY
HSEQLPIGVSRFWSPTFIWFYIWFLVSTAIFAGFWRIISNHPWQRWSVWGSAFILFNI
WFGVQVSVAVNAWYAPFYNLIQAMLDHGGGDINKLYSGTVTFLLIAMVGVTLAVINAF
FASHYVFRWRTAMNEYYTEHWAKLRHIEGASQRVQEDTMRFASVLEDLGVNFIKAIMV
LIAFLPILFELSKQVKVLPIVGEIDHSLVWAAIVWAIFGTVLLMVVGIKLPGLQFNNQ
KVEAAYRKELVYGEDYEERAQPKTLKELFSKVRFNYFRLYLHYSYFNLVSIWYLQLDI
LYSLVVLFPSIAAGKMTLGLITQISNVFDKVRESFQYLITSWKTIIELLSIYKRLRTF EDTLN"
/product="SbmA protein" /transl_table=11
CDS 2700996..2703044
/locus_tag="AUO97b_02662" /db_xref="SEED:fig|400667.82.peg.2527"
/translation="MKSILLRSALTIALMSSANLVHAELVTTSQIKKISGIDQQYIDS
SISEKNDFYAHVNGIWLKNTEIPADKANWGAFNQLRELSISQVHGIVEELSRKKWADG
SLEQKIATLYASFMDEKGIEKQGIIPLQAELNQIDSLKNKKQIAVLMAHFARINVNSP
IGLGIEQDMKKSTEMVAGLEQSGLGLPDRDYYLKDDKKFIETRAQYLKYIEKTLSLAG
DSQAAQHAQEILKLETQLARIQWSNVQNRDLAKRYNKYKLADLYKLTPAFDWQGYLTA

TELKGKIDTIQVNQPDYFQGLNTIIQNTPLDIWKAYFKLHLINNFSPLLNSAFVDNKF
DFYNKTLRDITEQEARWKRGVQLVDKVLGDGIGKLYVEKYFPAEKKQQMELLVQNLIR
AYDQSITELDWMSPATKIQARKKLSHMSIKIGYPKKWRDYSDLHIAKNDLVGNIIRAS
EFRYQYELNKLGKPVDRDEWYMKPQTVNAYYNPSLNEIVFPAAILQPPFFNINADDAV
NYGGIGAVIGHEISHGFDDQGSQFDELGNMRNWWNAEDHQHFKAKTQALIEQYNRYEP
IAGYHVNGELTLGENIADNSGLAIAYKAYQIALGGKPAPVIDGQTGEQRFYMGWAQVW
RAKTREAQAIIYLKTDPHSPDKVRGNGALMNQKPFYEAFKINEDDQMYLPPEKRVTIW "
/product="Peptidase, M13 family"
/transl_table=11
CDS complement(2703086..2703835)
/locus_tag="AUO97b_02663" /db_xref="SEED:fig|400667.82.peg.2528"
/translation="MKKLNFLLWATLVSLNSTAYAEVKSFTPHFPKFYSSATTRKADN
QFYALGEAKFLNGVAVPFYGVTTQNPIEDGLLKSFEKCTPKSCSFNFKLDAQHAKQLK
LLALPEVGVVLVPRNWQDIQANAGANGTGFALIMSPDQKQAIKLYDSSFCVGCGLPNA
TLYFPELLKESLENEYGGLKDPKNLINIVHPSKKVAFFSYQIPQVNNKTHGIAKYDDE
DTFNYKEIQVTLDKSQQFLVGPILNFYNATH"
/product="hypothetical protein"
/transl_table=11
CDS 2703960..2705027
/locus_tag="AUO97b_02664" /db_xref="SEED:fig|400667.82.peg.2529"
/translation="MKNILSICCLAVISSYSLAQDIKGISFSHQEWEISCSNTGTCKA
AGYQNEENGDNPASILLTRKAGPKQPVQIEFALSDYEQSIPANQLKNIHFYINGKDLG
MVGVDGTELPIMGKLNSSQVNALLQQSKQKTEIVFKNAQHKWKISDAGMTAVLLKMDD
FQKRIGTIGALVKKGSANENQVLMPEPKLVVKRIKTSTKPYLTLQPKNKQYQAIHRSL
MAAKPNPKEDGFCEGIYGGNSDGVEPQKIELYKLTNKKVLATTLCWRGAYNEGYGAWV
LDGSLNGKAAFVTESASDFDSGIMSSAQKGRGIGDCWASEEWVWDGKSFVHTKDMWTG
MCKGLAAGGVWELDRIESVIK"
/product="DUF1176 domain-containing protein" /transl_table=11
CDS 2705111..2705629
/locus_tag="AUO97b_02665" /db_xref="SEED:fig|400667.82.peg.2530"
/translation="MSRHLYAIARRKFSHLSRSICVAATVLGATQIAMAGPTVDQLSD
CLVKATTASDKTTVLQWTFTALAAHPDLKAFSNVTPEQKDQLDQKLAQVLQRIIVEQC
SAQTKAVIQAEGVKAVGEAFQQLGQSAGEDIVKDPAVKQQLQGTLRYIDLNKLVTTFL TPEIWNKLGITR"
/product="hypothetical protein; putative signal peptide" /transl_table=11
CDS complement(2705708..2706286)
/locus_tag="AUO97b_02666" /db_xref="SEED:fig|400667.82.peg.2531"
/translation="MSKKILMLVGDYAEDYETMVPFQFLTGLGYTVHAVCPNKKNGEH
IATAIHDFEGEQTYSEKRGHNFAINYDFDAINTEDYVGLVIPGGRAPEYLRMNERVVE
IVREFDRVKKPIAAVCHGAQLLAAADVLKDRLCSAYPACAAEVKLAGGQYADIAVTEA
VTDGHLVTAPAWPAHPAWLAQFVKVLGAKISI"
/product="Intracellular protease"
/transl_table=11
CDS complement(2706458..2707927)
/locus_tag="AUO97b_02667" /db_xref="SEED:fig|400667.82.peg.2532"
/translation="MAEAQKLKLIDGWEVVIGIEIHTQLATNSKIFSGSSTEFGQDPN
TQASLVDLAMPGVLPVLNEKVVELAIRFGLGIDAYIDQASVFARKNYFYPDSPKGYQI
SQMDNPIVGLGHIDIQLEDGTTKRIGVTRAHLEEDAGKSVHDQFEGMSGIDLNRAGTP
LLEIVSEPDMRSVEEAVAYIKAIHTLVRWLGISDGNMAEGSFRADCNVSLRRPGQPFG
TRCELKNLNSFRFIEQAINVEIERQMEILEWGGEIDQETRLFDPNKMETRSMRSKEEA
NDYRYFPDPDLLPVIIPDEQIEAIKATMPELPAARRARFIADFGVTEYDAHVLTLSRE
MADFYEAVVAAAGGAKQGKIAANWVMGEFSGALNKAGLELTDSPVSAEQLGGMIARIV
DNTISGKIAKQVFGFMWESEGKSADDIIAEKGLKQETDTGAIEAIIKDVLAANEKMVE
EYKSGKEKAFNGLVGQVMKASKGKANPAQVNELMKKLIG" /product="Aspartyl-tRNA(Asn)
amidotransferase subunit B (EC 6.3.5.6) @ Glutamyl-tRNA(Gln) amidotransferase

subunit B (EC 6.3.5.7)" /EC_number="6.3.5.6"
/EC_number="6.3.5.7"
/transl_table=11
CDS complement(2707927..2709405)
/locus_tag="AUO97b_02668" /db_xref="SEED:fig|400667.82.peg.2533"
/translation="MTDLHRLSIRELAEGLSQAKFSSRELTEHYLKRIAKIDPQVKSY
VTVTSEQALREADAADAALKAGNATALTGIPLAHKDIFCTKGIKTTAGSKMLDNFISP
YDATVVEKTKAAGLVTLGKVNMDEFAMGSTSESSYVGATSNPWALDHVPGGSSGGSAA
AVAADLAPFATGTDTGGSIRQPASFCGLTGLKPTYGRVSRFGIIAYASSLDQAGPMAR
SAEDCAYLMNVIAGHDAKDSTSVKKEVDDYVANLNGTSVKGLRIGIPKQYFNVAGLDA
DVKARVEESLKKLEEMGATLVEIDLNMTEAYVPTYYLIAPAEASSNLSRYDGVRYGYR
CENPADLMDLYKRSRSEGFGPEIQRRILIGTYALSAGYYDAYYVKAQKVRRLIQQDFL
KAFENVDVIAAPAAPTTAYKIGASLDPVEMYLGDIYTIAVNLAGLPAINAPVGFDKDN
LPVGLQLIGNYWSESQLLSIVHQYQQNTDWHTKRAAIAEENA" /product="Aspartyl-tRNA(Asn) amidotransferase subunit A (EC 6.3.5.6) @ Glutamyl-tRNA(Gln) amidotransferase subunit A (EC 6.3.5.7)" /EC_number="6.3.5.6"
/EC_number="6.3.5.7" /transl_table=11
CDS complement(2709459..2709773)
/locus_tag="AUO97b_02669" /db_xref="SEED:fig|400667.82.peg.2534"
/translation="MSTSDAQHSADLNAQTVSAIANLARLSLNDTQSAEYAQSLNKIL
GMMETLKGIDTEGVEPLKSPFDNPQPLRADVVTESNHRDEYQAVAPATQDGLYLVPRV IE"
/product="Aspartyl-tRNA(Asn) amidotransferase subunit C (EC 6.3.5.6) @ Glutamyl-tRNA(Gln) amidotransferase subunit C (EC 6.3.5.7)"
/EC_number="6.3.5.6"
/EC_number="6.3.5.7"
/transl_table=11
CDS 2709931..2710947
/locus_tag="AUO97b_02670" /db_xref="SEED:fig|400667.82.peg.2535"
/translation="MFSPDLAIDLGTANTLIYAPGRGIILNEPTVVAIRHSGSQKIVA
AVGLDAKQMLGRTPANISAIRPMKDGVIADFEVTETMLNQFIGKVHEKRLFPPAPRVV
VCVPCKSTLVERRAIREAVFNAGARDVRLIEEPMAAAIGAGMPVEQACGSMVVDVGGG
TTEIAIISLQGCVYADSLRIGGDVFDEQIINYVRKAHGCVIGETTAEIIKKEVGMAVS
DGTTLEIEVRGRNLAEGVPRAITVTSDEITQAISDPLQSIVSAVKSALEQTPPELSSD
IAERGIVLTGGGALLRNLDKLLAQETGLPVVVAEDPLTCVTRGGGKVLEFFDNPNHDM LFVG"
/product="Rod shape-determining protein MreB" /transl_table=11
CDS 2710964..2711821
/locus_tag="AUO97b_02671" /db_xref="SEED:fig|400667.82.peg.2536"
/translation="MQPNIFSRQPPSFRSFIIAVITCLVVLFFDWRMPYVIQPARDVL
YAAYNPIYALASYPVLSREWLNQQTKSETQLRRENTAMQAELLQAQVRLQKLSELSAE
NTRLRGLLDTPLIIDGRMEIAEVIGTDADPLRHIIIINRGAMDHLKVGQTVLDDKGIM
GQIINVYPHSSRVMLLSDKEHSLSVRLERTGMRAIVSGTGDLGRLKMEYVPTSANIQV
GDKVLSSGLGEHFPAGYAVGTVAKIRRHNSGEFAEIDVTPAAQLATGHHVVVLFSDSL AKEQPYADR"
/product="Rod shape-determining protein MreC" /transl_table=11
CDS 2712009..2712299
/locus_tag="AUO97b_02672" /db_xref="SEED:fig|400667.82.peg.2537"
/translation="MGIFTDLLLDAPLGLNALSFVIVTFITRFLIRERRILTFGNLWT
IATLVIIAHLAFMWVTQTMGGIHFSIARHWQPLMTSILTWPVVYYCLAKWRN" /product="Rod shape-determining protein MreD" /transl_table=11
CDS 2712335..2712847
/locus_tag="AUO97b_02673" /db_xref="SEED:fig|400667.82.peg.2538"
/translation="MQQLGLNFEIYSPDIDESVHEGELVHQYVERLAREKAQAVLNIF
PDSVIIAADTSLGLDSQIIGKPDSKQHAFDIWKQLSGRWHDVFSGICIATQQQILSQV
VQTQVEFASLTTQDMEDYWATGEPVGKAGAYAIQGIASQYIPKIQGSYSNVVGLPLYE FSQLFKRVKT"
/product="Septum formation protein Maf" /transl_table=11

CDS 2713006..2714415
/locus_tag="AUO97b_02674" /db_xref="SEED:fig|400667.82.peg.2539"
/db_xref="GO:0003723"
/translation="MALIENGTVNELYVERTVKRGLVGNIYKGKVVRVLPGMQAAFVD
IGLSRTAFLHINDMVWPRSQPTPNVFELLHPGQILTVQVMKDMLGTKGARLSTDLSIP
SRYLVLMPYGNHIGVSQRIESEEERDRLRNIIESIQAEHNLPGSVIVRTAAEGVDEAE
IAQDMCYLSKLWEYIQRKQVDVAVPSLIFEELPLPQRIIRDLASEETAKIYVDSREIH
GKLREFVDEFVPNMQSRLIHYPGERPLFDLYNVEEDIQKALQTRVALKSGGYLMIDQT
EAMTTIDVNTGSYVGGRSLEDTVFKTNMEATQVIARQLRLRNLGGIIIIDFIDMQEAI
HREEVMRQFEKMLERDHAKTKITQVSELGLVEMTRKRTRESLEHLLCESCPTCQGRGF
VKTAETVCYEIFREILRYTRAFESTSGFTVVAHPSVIDRLLTAEAPAVADLEHFINRV
IKFQVENLYTQEQYDIILS"
/product="Ribonuclease G"
/transl_table=11 CDS 2714555..2714728
/locus_tag="AUO97b_02675" /db_xref="SEED:fig|400667.82.peg.2540"
/translation="MNVSENLISNGLKHVLESTPVNSCYMLNDQGKEVPITTAMIRSV CHQLLNQCRAIKK"
/product="hypothetical protein"
/transl_table=11
CDS complement(2714758..2716143)
/locus_tag="AUO97b_02676" /db_xref="SEED:fig|400667.82.peg.2541"
/translation="MRPLHPIDFIFLSLEKRQQPMHVGGLFLFEIPENAPETFVHDLV
EEIRQSKSIPVPPFNNRLNGLFWDEDEEFDIDHHFRHIALPHPGRIRELLVYISQQHS
SLIDRAKPLWTCDIIEGIEGNRFAMYFKIHHAMVDGVAGMRLIEKSLSKTPQEKHVVP
LWCVESKRTKRLKVPKPSTSKIKSILGGIKSQLEVTPKVMQELSQTIFKEMGKNPDYV
STFQAPVSILNQRVSASRRFAAQSFELSRLRKISKALGVTINDVVLAVCSGALREYLI
SHNSLPKKPLIAMVPASLRTDDSDVSNRITMILANLGTHKDQPLERLEIIRRSMQNSK
QRFSRMTANEILNYSAVVYGPAGLNIMSGMLPKRQAFNLVISNVPGPREPLYWNGAKL
DALYPASIVMDGQALNITMTSYLDKLEVGLTACRNALPKMQNLLTHLEDEIQRFEEII GEKQLKHHSAS"
/product="Wax ester synthase/acyl-CoA:diacylglycerol acyltransferase"
/transl_table=11
CDS complement(2716315..2717049)
/locus_tag="AUO97b_02677" /db_xref="SEED:fig|400667.82.peg.2542"
/db_xref="GO:0004604"
/translation="MTVIPTIDIVDALAAEYAAKSPREILELALSQQGEIAISFSGAE
DVVLIDMASRLGKPFRVFSLDTGRLHPETYQFIETVRKHYNINIEICFPDAEAVQSMV
NEKGLFSFFKDGHQECCGIRKVQPLRKKLATLDGWITGQRKDQSPGTRTEIPVVQADA
GFSGPGKQLIKYNPLANWSSADVWSYIRMMEIPYNPLHERGFVSIGCEPCTRPVLPNQ
HEREGRWWWEEATQKECGLHAGNLKK"
/product="Adenylyl-sulfate reductase [thioredoxin] (EC 1.8.4.10)"
/EC_number="1.8.4.10" /EC_number="1.8.4.8"
/transl_table=11
CDS 2717234..2717851
/locus_tag="AUO97b_02678" /db_xref="SEED:fig|400667.82.peg.2543"
/db_xref="GO:0004413"
/translation="MEIVCLDLEGVLVPEIWINFAKKTGIKELEATTRDIPDYDVLMT
QRLNILKQHGLGLKDIQEVIAEMGPLPGAKEFVEWVSNHFQLVILSDTFYEFAHPLMK
QLGWPTIFCHKLETDENGMITAYKLRQPDQKRESVKALHGLNFRVIAAGDSYNDTTML
GEADHGFLFDAPANVIAEFPQFPAINGYDALKEAIRSVSQRNIPA" /product="Homoserine kinase
(EC 2.7.1.39) @ Phosphoserine phosphatase (EC 3.1.3.3)" /EC_number="2.7.1.39"
/transl_table=11
CDS complement(2717919..2719430)
/locus_tag="AUO97b_02679" /db_xref="SEED:fig|400667.82.peg.2544"
/translation="MKLKHLSTAMILATLPATGVFAAALDRSGQSMSAFFQPGNYFEA
GISVLDPDVAGKEAGSSATRRDIGDMANDYYFPSAALKLQINDQFSFGLLYDQPFGAD

AEYSGNNVFVSNPGSDTILSQKALGDLATSSIQKLVQASGSAFTPALIEVTKVTGGDP
TKPTQTEILGALQQVAAGGNTTVGAGLTALQKTQAAINAANNYLGTGGTKVKVDTQNL
SFVFGYQPTKNFNFYAGPVLQTVKGNVSLRGQAYSLYNGYDANIKETTGAGWLAGAAY
QIPEIALRASVTYRSEIDHKVNIDENLSILNFPGLTSVLAGLDVPASKLQAINSSGKT
TITTPQSVNLDFQTGIMADTVAFANVRWVNWKDFSIQPYKFGKVSEAVGGLIGRPNGF
NLVEYSDDQWSVNAGVGRKLNDKWAGNVSVGWDSGAGNPVTTLGPTEGYWNVGLGVQY
SPTPQTFIAGGVKYFWLGDAKAQTGAQAGSDEYVADFSDNNAIAYGLKLGYKF" /product="Outer membrane protein E"
/transl_table=11
CDS complement(2720028..2720726)
/locus_tag="AUO97b_02680" /db_xref="SEED:fig|400667.82.peg.2545"
/db_xref="GO:0010181"
/db_xref="GO:0016491"
/translation="MNILIVHAHPEPLSFTTSLKTTAQQTFKKLGHQVEISDLYAMQF
NPVASKEDFLELNQPDYFNYALEQRNATKQQLLATDIQAEIDKVKRADLIILNFPLYW
TSVPAILKGWIDRVFVSGLFYGGKRFYNHGGMAGKKAMLCLTLGGRTHMFGENSIHGP
IEHYLSPIQRGTLAYTGFEVLPPFIAYHVPYISQEARETILEDYVTYLTHLDQLEPLK FPKLEEFDEKLYPL"
/product="NAD(P)H oxidoreductase YrkL @ Putative NADPH-quinone reductase (modulator of drug activity B) @ Flavodoxin 2" /transl_table=11
CDS 2720890..2721924
/locus_tag="AUO97b_02681" /db_xref="SEED:fig|400667.82.peg.2546"
/translation="MTPELPGTYINLLAEIVKRRGVSCEQFLEGSGVTPEQLKKPYWY
VEFNTLNNLLEHAIQLTHEPALAGYLALEMKASCYGSVGMAAMVAANLGEALKIFEQF
IGSRCDAFKPELKQEQNDVYWSIHQPLKSFQLSSDATIFLLLGFIHIAKHLTGLSALG
TVQLNMPEPIGFSKIKDKLGVSFLFDQKHNIWIFPKEYLMQPVLTADPMLAPLLKAQC
KKDIDKLKLRSGWKAEISQVIKKLLINSQGEILKVKQVAQMMNMSERSLQRYLAIENT
SFSALVDLTRKQYAQELLQNSLMSIESVALSLGYADTSHFTRAFKRWMGVTPKYYQTQ LKLKNLEIRE"
/product="Transcriptional regulator, AraC family" /transl_table=11
CDS complement(2721967..2722959)
/locus_tag="AUO97b_02682" /db_xref="SEED:fig|400667.82.peg.2547"
/translation="MNAQVSVTDLFSREEIQELTEPSDAYGAWAVASTWAVIGGTFAS
LIMMWDYLPSWGKLLACMLALAVLAGRQLCLAILMHDASHKSLFKNKKINDFVGDWLC
ARPIWNDLQKYRVHHVRHHAKTSTPDDPDLSLVAGFPVSKKSLTRKFLRDLTGITGLK
FSLGRVLMDLDVMKWTVANDQIWLDRSDKNFVDYAKSIAKNSTGAIATNLLLYGVLKA
CGQQRFYWLWPLAYLTPFPLFLRIRSMAEHAGMQTSNTALTNTRTTRASWIARSFVAP
IHVNYHMEHHLMASVPYFKLPRMHKILRERGHVPTPPSYFEVIHTLSSKQELTN" /product="Fatty acid desaturase" /transl_table=11
CDS complement(2723066..2723866)
/locus_tag="AUO97b_02683" /db_xref="SEED:fig|400667.82.peg.2548"
/translation="MKNTKINARHLIIDLFLSSAYPQLSIKQILVAAKLFNLSENGIR
VATTRLLNEGMIQSVERGIYQLSPSAKDWAKVILNRKNGIKQTKEWQQHYLAVFTGTL
GRIDRTALKKRERALRQFGFKELETGIYIRPDNLAYSFEKTCDELVLAGLEKEAKICI
IQHFDSTTLKQIFSLWDTKQLEKNYEMYSRDITHWLENYAHSPLNDAAVKALLLGRET
IALLMNDPLLPAPFVNESARNQFAQDVQQLDAIGQKLWKKIYEQELNL" /product="Phenylacetic acid degradation operon negative regulatory protein paaX" /transl_table=11
CDS 2724222..2725040
/locus_tag="AUO97b_02684" /db_xref="SEED:fig|400667.82.peg.2549"
/db_xref="GO:0050380"
/translation="MENFEVIKALFLGFVEGLTEFLPISSTGHLILFGHIIDFHSDGG
RVFEVVIQLGAILAVCWLYRQKIINLIKGFFSGDVESRHFAISVLIAFFPAVIIGVLA
VDFIKSVLFSPIVVAIALIVGALIIFWVESKQFEHKTDDATKITFKQALLVGLAQCVA
MIPGTSRSGATIVGGMFAGLSRKAATEFSFFLAMPTMLGAATFDLIKNADVLTSDNMV
NIGVGFVAAFIAALLVVKALVLFVERHTLRVFAWYRIVLGVIILIAAMFFNLSA"
/product="Undecaprenyl-diphosphatase (EC 3.6.1.27)" /EC_number="3.6.1.27"

/transl_table=11
CDS 2725336..2726094
/locus_tag="AUO97b_02685" /db_xref="SEED:fig|400667.82.peg.2550"
/translation="MQEPSIVLVIQGVKRAYIGKDIFKFQQGQCLFISIAIPFDCDTL
VEREPMLAIAIKFEPQMMAELATKMDKEHRPLIEDFDNKEIKLSCGLNIIEMNSVISD
VALRLLNLLRSKQDTAILGEQVKRELIYRVLQAGGSDFIQNLSAIMSRSGVIYTICEI
IQRDYYRNLTVQELAKQAGMSVSLFHQAFKKVTNYSPLQYIKITRLHKARDLILNNQM
GVAEAAYEVGYVSASQFSREFKRLFGVPPKLSTN" /product="Transcriptional regulator, AraC family" /transl_table=11
CDS 2726141..2727139
/locus_tag="AUO97b_02686" /db_xref="SEED:fig|400667.82.peg.2551"
/translation="MNLPTTMKIVEITVPGGPEVLKLQDSNVPVPQADEVLIEVKAAG
INRPDVLQRMGLYPMPKGVTQIPGLEVAGVVVAVGEQVQQFKVGDKVCALTNGGGYAE
YCAVTATQVLPIPENLSFTQAAAIPETFFTVWANLFDIGRLKKDETALIHGGASGIGT
TALAICHALGIKTFATVGSEDKVEALSDLTTAINYKTQDFEQEILNHTQEQGVDVILD
IVGGSYFQKNLNLLKRDGRLVIIGFMGGRIAKEFDLQKLILKRATITGSTMRARNSQE
KAQIAQSLHEHVWPLLEQGKCLPQIYKTYAFSDVQSAHACMEQGDHIGKIVLEMNA" /product="Quinone oxidoreductase (EC 1.6.5.5)" /EC_number="1.6.5.5"
/transl_table=11
CDS 2727342..2728217
/locus_tag="AUO97b_02687" /db_xref="SEED:fig|400667.82.peg.2552"
/translation="MNTPATNDKNPTPDLAEDNAFFPSPYSLSQYTSPKTDYDGTTYP
TPYAGNKKVLMIATDERYIQMQNGKFFSTGNHPVEMLLPMFHLDNAGFEIDVATLSGN
PAKLEMWAMPKQEQVVLDTFQKYADKLKNPLKLADILENVVGENSPYAAVFIPGGHGV
LAKIPHSLEVKKVLKWAVEQDKFIITLCHGPASLLAAAVDEQPENYIFKDYQICVFPD
SLDKGANIDIGYMPGALPWLVGENLEKLGVKILNTGITGQCHRDRKLLTGDSPLASNN LGKLAAETLLAEVKD"
/product="D-lactate dehydratase (EC 4.2.1.130)" /transl_table=11
CDS complement(2728380..2729672)
/locus_tag="AUO97b_02688" /db_xref="SEED:fig|400667.82.peg.2553"
/translation="MGGAIGTGLFLGSAQVIQSAGPSIILGYAIGGLIAFLIMRHLGE
MIVEEPVAGSFSHFAYKYWGKFPGFLAGWNYWMLYVLVAMSELTAVAKYINYWWPHIP
AWTSVLFFFVVITAINLTNVKFYGESEFWLAIIKVAAVISMIVFGLYLLFTADVGSSI
SFSNLWSHGGFFPNGFSGLFYMLAFLMFAFGGIELIGMAAAEAKDPKKTIPKAINQVV
FRILIFYIGSLAILLSLVPWNQLDLGGLDKSPFVMIFSQMGIGWAAHLLNFIILTAAL
SVYNSGMFANSRMLYGLAQQGNAPKIFKKVNKQGVPVPAVLLSALLIFGCVLLNYFVP
EDALSHLMYIVVGALVLNWAMISMTHLKFKSAMKKLGQKTHFPALWAPFSNYLVLGFI
AVVLYIMWSQGFKESVMMIPIWIVLMFILFKVLNAKEQ"
/product="Aromatic amino acid transport protein AroP" /transl_table=11
CDS complement(2729848..2729973)
/locus_tag="AUO97b_02689" /db_xref="SEED:fig|400667.82.peg.2554"
/translation="MEISIHACNDRIKQNHKKKSLSKNIFLEDFSTNLTRLKASL"
/product="hypothetical protein"
/transl_table=11
CDS 2730115..2731302
/locus_tag="AUO97b_02690" /db_xref="SEED:fig|400667.82.peg.2555"
/translation="MSFVPQIKIPATYMRGGTSKGVFFKLDDLPEKAQVAGQARDQLL
LRVIGSPDPYGKQIDGMGGATSSTSKTVILAKSTRPDHDVDYLFGQVSIDQAFVDWSG
NCGNLTAAVGSFAISNGLVDADRIPENGLCTVRIWQKNIQKTIIAHVPITNGQVQETG
DFELDGVTFPAAEVQIEFLDPADDGEEGGDMFPTGNVVDQLDVPEIGSFQATFINAGI
PTIFLNAEDLGYEGTELQDHINGDAAALARFEKIRAYGAVQMGLIKDISEAAARQHTP
KIAFVSKPKNYTASSGKNVSENDVDLLVRALSMGKLHHAMMGTAAVAIGTAAAIPGTL
VNLAAGGGEREAVRFGHPSGTLRVGAQAELTNGQWVVKKAIMSRSARVLMEGWVRIPG DSF"
/product="2-methylaconitate isomerase"
/transl_table=11

CDS 2731462..2732373
/locus_tag="AUO97b_02691" /db_xref="SEED:fig|400667.82.peg.2556"
/translation="MSSLSQVNADVLKQAQQRIQQDLLTTLAAFSIPEPLKSAVHHAV
MLGGKRVRPALCYATASLQDNPNFAAARRAAVAVELIHCYSLAHDDLPCMDNDLLRRG
QPTCHVAFGEDTALLAGDILQSMAFEVLGSRLFDEQGQGTDAAIVLKQIQILATASSK
MVCGQVLDLQAEAKQISQDELENIHRNKTGALIQAAIMMGAVTIFSGTDQAIPKLRQY
GQAIGLAFQVQDDILDITSSTETLGKTAGKDEQVQKSTYPALMGLEQAQIYAKELHDQ
AFEALAHFGENAKELVEISQFLLARTN"
/product="(2E,6E)-farnesyl diphosphate synthase (EC 2.5.1.10)"
/EC_number="2.5.1.90"
/EC_number="2.5.1.29"
/EC_number="2.5.1.1"
/EC_number="2.5.1.10"
/transl_table=11
CDS 2732433..2732801
/locus_tag="AUO97b_02692" /db_xref="SEED:fig|400667.82.peg.2557"
/translation="MNGEQLHQLAIEAARNLPFSEQTHPFGPEYEVFKILEKMFMLTV
EVSGVKMINVKCDPYKSQEYQELYPFIIPGYHMNKKHWISIKPHKNLTSDFLRDLIRD
SYDLVVKKLPLKDQKRLNNQ"
/product="hypothetical protein"
/transl_table=11 CDS complement(2732870..2733724)
/locus_tag="AUO97b_02693" /db_xref="SEED:fig|400667.82.peg.2558"
/translation="MPVGTIIVLAFLAFVAVTIFKGVRIVPQGYKWIVQRLGKYHSTL
NPGLNFVIPYIDDVAYKVTTKDIVLDIPSQEVITRDNAVLLMNAVAYINLTTPEKAVY
GIENYTWAIQNLVQTSLRSIVGEMDLDDALSSRDHIKAKLKAAISDDISDWGITLKTV
EIQDIQPSSTMQAAMEAQAAAERQRRATVTKADGEKQAAILEADGRLEASRRDAEAQV
VLAEASQKAIEMVTSAVGDKEIPVAYLLGEQYVKAMQDMAKSSNAKTVVLPADVLNTI RGIMGKHN"
/product="Protein QmcA (possibly involved in integral membrane quality control)" /transl_table=11
CDS complement(2733753..2734214)
/locus_tag="AUO97b_02694" /db_xref="SEED:fig|400667.82.peg.2559"
/translation="MEFVVEPWHWFVLGILLILSELILPAFAALWFGIAAIMVCFLYW
LFPDISLTTQIVLWIVLSVLCTLLWFKFIKPLSIDKTKAGLPREATIGQIGMVIQTGL
THDQIIVRFPMPILGADEWNCRSTTPVKVGDRVRVIDISGNDLIVQPHSTS" /product="Putative activity regulator of membrane protease YbbK" /transl_table=11
CDS complement(2734320..2735237)
/locus_tag="AUO97b_02695" /db_xref="SEED:fig|400667.82.peg.2560"
/translation="MKIAQFASIGFISLALFGCDKFSKTPTPTTSVKAREPVIALALG
GGGAKGFSHIGVIKVLESHGIKPKIVTGTSAGSFVGSIYASGQTPYQLQNLALKLQES
DIRDLTLNRQGIILGQKLQDYVNRNVGNKPIEKFPIRFAAVATRLDNGQKADFIKGNA
GQAVRASCSIPNVFVPATIGKQKYVDGGLVSPIPVKTARDMGADIVIAVDISARPAGN
RPANMWGLLDQTINIMGQQSINEELKQANVVIQPKVGHLGTLDLKSSNEAILEGEKAA
QGQIRQIETAIANFKKSPAAFKPAPRPKF"
/product="FIG00613342: Bacterial patatin-like phospholipase domain containing protein" /transl_table=11
CDS complement(2735305..2735931)
/locus_tag="AUO97b_02696" /db_xref="SEED:fig|400667.82.peg.2561"
/translation="MEQHFPNLPSQVPQRGNALSRALFKKLFLAQGWTIEGEVPNFPK
AVAIISPHTSNIDGWYGFLAIFGLGIQITVLGKDSLFKPPFKRVLDWAGVIPVKRDSA
NGLTEQVVATIQQYDKIWIGMAPEGTRKKAKKMKSGFYHIAAKANIPIVMFSFDYDRK
TIHCLDYLTPSGNYDEDLAKIFERYEGKISPKNRNWLAEPLQNLVKKT" /product="1-acyl-sn-glycerol-3-phosphate acyltransferase" /transl_table=11
CDS 2736059..2737306
/locus_tag="AUO97b_02697" /db_xref="SEED:fig|400667.82.peg.2562"

/translation="MFRQQNLYLLLFSLYWAQGLPVGFMTHALPVILRAQGVSLAHIG
GFGLLMLPWSIKIFWAPWVDRHAISHLGHYRSWILPAQFLTVGVLCILSFFPIQVLDQ
PLYLFIFFITLLFMNLTGATQDIATDALAVNLLQHDQQHWGNTFQVVGSRLGFIVGGG
AVLWCLDWLSWQPTFLLLAALVFINTLPILLFKEPSHTSHSPHQYSQPSLVTKIKSYL
GYFSQNKELRSWLIVLITFKVADGLAGPLLKPLMVDMGLSFTQIGIYITMLGAIAALL
GALIAGWMLKYISRSTALMAFSILKIMSLGGYAYLAYAYEQKVKINVWLIYAVNALED
AFAAMLLVVMLTLVMHYSRKNYAGTDFTFQVSVMATVSGGLYSFSGVIADVLGYFHYL
IAIVSIAVLFLIPIYLWKAAKTV"
/product="AmpG permease"
/transl_table=11
CDS 2737852..2739156
/locus_tag="AUO97b_02698" /db_xref="SEED:fig|400667.82.peg.2563"
/translation="MDLFLNRKSFVVKSLAITVTALMMSGANAATSDKEEIRKLRQEV
EALKALVQEQRQVQQQQQQVQQQQQVQLAEVKAQPQPVAAPVSPLAGFKSKAGADVNL
YGFVRGDANYIIEGADNDFGDVSKSDGKTHDKLRATAKTTRLGLDFNTPVGDDKVGGK
IEVDFAGSTTDSNGSLRIRHAYLTYNNWLFGQTTSNFLSNHAPEMIDFSTNIGGGTKR
VPQVRYNYKLGPTTQLFVSAEKGDSTTSVTGDSIKYSLPALTAKITQGYAEGRGSASA
RVLVENYKSQLADDDKTGWGVAVGTDFKVSDPLKLFADASYVVGDNSYLYGSNSPYAV
DGNSIEQNEFVAVQVGGTYKILPNLRSTLAYGAQFSDDGTDYARLNASANEKVQQAWI
NFIYTPVKPIDLGVEYVNGKRDTFDGKSYKDNRVGLMAKYSF"
/product="DcaP"
/transl_table=11
CDS 2739388..2740989
/locus_tag="AUO97b_02699" /db_xref="SEED:fig|400667.82.peg.2564"
/translation="MFNLIIAIWLGAILNIGFYHQVHTLTPYFGVKAILFLAATLIIL
VATYYAVLQILNWKWTAKIFAILLIFIGGFSSYFVNTLGVIISPDQIQNMVQTDVSEV
TDLISLRFVLWTIFFVILPIFLITQVKFKQEKVSRLLLKKVFSLVASFAVVGVLLFTY
YVDFAAIFREHRDLKGMISPQNSISSLMSYYHKKAPKKNLPLVIYGQDAHQIQRVQKN
LPKLMILVVGETARAESFSLNGYAKNTNPELSKQDIFNFSQVSSCGTATAVSVPCMFS
GMPRVDYDEQLASHREGLLDIAKRAGYQVTWIDNNSGCKGACDRVEQYQIPENLKRKW
CKDGECYDDILIDSLKQYLATIAKDDDRPRLIVLHQVGSHGPAYYKRAPEAYQPFKPT
CDTNAIQGCSQTELLNSYDNTIVYTDHVLSQMINTLKEISKYQTGLWYLSDHGESTGE
HGLYLHGSPYAIAPSQQTHVPMIMWFSESWKQHNLAQVNCLSQQTKQKLSQDNLFPSL
LSLLDVKTQVINPQLDMLHSCAHVN"
/product="Lipid A phosphoethanolamine transferase, putative" /transl_table=11
CDS 2741004..2741678
/locus_tag="AUO97b_02700" /db_xref="SEED:fig|400667.82.peg.2565"
/translation="MTKILMIEDDFMIAESTITLLQYHQFEVEWVNNGLDGLAQLAKT
KFDLILLDLGLPMMDGMQVLKQIRQRAATPVLIISARDQLQNRVDGLNLGADDYLIKP
YEFDELLARIHALLRRSGVEAQLASQDQLLESGDLVLNVEQHIATFKGQRIDLSNREW
AILIPLMTHPNKIFSKANLEDKLYDFDSDVTSNTIEVYVHHLRAKLGKDFIRTIRGLG YRLGQS"
/product="Two-component transcriptional response regulator, LuxR family"
/transl_table=11
CDS 2741704..2743038 /locus_tag="AUO97b_02701" /db_xref="SEED:fig|
400667.82.peg.2566"
/translation="MHYSLKKRLIWGTSIFSVILGCILIFSAYKVALQEVDEILDTQM
KYLAERTAEHPLKTVSSKFDFHKTYHEEDLFIDIWAYKDQAHLSHHLHLLVPPVEQAG
FYSHKTAQGIVRTYVLPLKDYQIQVSQQERVREAFAWELAGSMFIPYLIILPFAIFAL
AAIIRRGLKPIDDFKNELKERDSEELTPIEVHDYPQELLPTIDEMNRLFERISKAQNE
QKQFIADAAHELRTPVTALNLQTKILLSQFPEHESLQNLSKGLARIQHLVTQLLALAK
QDVTLSMVEPTGYFQLNDVALNCVEQLVNLAMQKEIDLGFVRNEPIEMHSIEPTVHSI
IFNLIDNAIKYTPHQGVINISVYTDPDHYACIQIEDSGAGIDPENYDKVLKRFYRVHH
HLEVGSGLGLSIVDRATQRLGGTLTLDKSLELGGLSVLVKLPKVLHLHETRA" /product="Two-component
system sensor histidine kinase" /transl_table=11

CDS complement(2743039..2743638)
/locus_tag="AUO97b_02702" /db_xref="SEED:fig|400667.82.peg.2567"
/translation="MYLIQPWIDSTGHFFNRDNWYLERLNHEIVKQIITAVYVIFFGL
WLASFKLKKLKPFRWQYGYMFFVSMLGSSIVGLIKSQSAHACPWNMVHPNTLGSYIWD
FSAKHGHCFPGGHASAGFILMTGYFVYRLEQPKRAYFYLFSGILLGFMMGWAQMMRGA
HFLSHNLWTGWVVWAINILFYAICIHRFEFEKQVKQELT"
/product="Lipid A 4'-phosphatase LpxF-like, putative" /transl_table=11
CDS complement(2743890..2745077)
/locus_tag="AUO97b_02703" /db_xref="SEED:fig|400667.82.peg.2568"
/translation="MQNVDLFFLLLGAVLVLAMHAGFAFLELGTVRHKNQVNALSKIL
TDFALSAIAYFFVGYYISYGQHFFHEGTVLSNDHGYNLMRCFFLLTFAAAIPAIISGG
IAERAKMRSQAIATLALVALVYPFFEGMVWNGNYGLQKWLETTFGAAFHDFAGSVVVH
AMGGWIALAAVILLGARSGRYKKDGRVSAHPPSSIPFLALGSWILIVGWFGFNVMSAQ
RVDAISGLVAINSLMAMVGGTITANAIGKNDPGFLHNGPLAGLVAICAGSDIVHPVSA
LVIGGAAGAMFVYLFTYTQNKLKVDDVLGVWPLHGVCGAFGGIAVGIFGQKWLGGLGG
VSFLSQLIGTALAIAIALAGGFIVYGILKATIGIRLSQEDEFRGADLSIHKISANSEE GMF"
/product="Ammonium transporter"
/transl_table=11
CDS complement(2745380..2746264)
/locus_tag="AUO97b_02704" /db_xref="SEED:fig|400667.82.peg.2569"
/translation="MTFTQLEIFALIAELKSFTATAEKLGISQSAVSHALKSLEQQWG
INLISRSQSDIELTTTGQQLLTHVKELLSISDTLEKEVAAIHGLNEGTLRIGSFGASS
SIYLLPEILEAFRQRYPKIEIYIDEGEDKEVAQWLLERRIEVGFLIMPDERFDTFPLI
EDRFVALIPSGYPLAQQLYIDPAQLENCSFIMTMAGSRHQVELILKNFNVKPDIKFYV
SQILSIVSMVHNQLGISIVSDMVITKELLSLYPNVVKRPFKPNLKRSIGLAVKNKKHI
SPAVKAFIEVAQEVWSDH"
/product="Transcriptional regulator STM2281, LysR family" /transl_table=11
CDS 2746381..2747286
/locus_tag="AUO97b_02705" /db_xref="SEED:fig|400667.82.peg.2570"
/translation="MNSIVLSLFPLVALIASGYLFKKYRFFSDEFWAGAEKLNYYILF
PALLFSTLSTAKIDLQSLSTAIIAMLIVVLAVTAFLYILRMFWHIPPARFGVHVQSMV
RFNTYIGLALVTSLFKSEGMAILAILLALCIPLVNVISVLALTSKEHMAIKPVLIALL
KNPLIASCVVGAAVNALHIPIWEGFTQFIKLFSVSSLPLGLLCVGAALQFMQIKKDIV
VLAADTFARLLAVPALAYAVCTWFGLPSLQTQILVIFFALPTASASYILTKVLGGDSQ
LMAAVISFQTLCAAVTLPLVIWWIS"
/product="Auxin efflux carrier family protein" /transl_table=11
CDS 2747415..2748431
/locus_tag="AUO97b_02706" /db_xref="SEED:fig|400667.82.peg.2571"
/translation="MSFDLKVSLQQRSEPSAQQKKLNRLIDKIEQQKVSLSTWQNAQA
EIQQYIRQKLMPVYSDLHEVLFQQLEQLWNMLHSHEFSKADMQQLDEKIAQLAQMLKR
SKMLSAEQLELVKQIDTFYQQHAGESVKKNKKAQPIEFEYEESADQGSEFEEDFEKYT
AEQQQLREQAKRQRQQQKREQAEQMAAQSLKTVYLKIAAMIHPDREQDETKKEEKTEM
FQQASQAYEKQDLFYLLKLQLQLEQNKGLGAKDLSAEQLRFYKLALDAQSQQLESQIA
EILDSFQLAKNVKAEHVHISDVYKAIDADCAELKQQLKWEKERLKHMKKVSGVEMLLG HGVL"
/product="DnaJ domain protein"
/transl_table=11
CDS 2748569..2749153
/locus_tag="AUO97b_02707" /db_xref="SEED:fig|400667.82.peg.2572"
/translation="MNEKFIQLSKELKHRPILEKALGYFPNLPLVAIDCGCGAGNESA
YLLSKGFNVHAFDISIDAQKVCTERFKDDSKFIFYHESFEDFNFPSASLIIASSSLFF
CNPSYFYSVIKRMINALSEGGILVVDLLGIYDEWVIREPTKFIGFTYQDIADLFLCDF
NLLFHKEINENLPLLEGYIKTWHLHMLILQKSDF" /product="SAM-dependent
methyltransferase"
/transl_table=11 CDS complement(2749210..2752089)

/locus_tag="AUO97b_02708" /db_xref="SEED:fig|400667.82.peg.2573"
/db_xref="GO:0004832"
/translation="MTDAQTAQNIATTYDPTEIEKKWYKTWEEQGYFKPSGHGESFCI
MIPPPNVTGSLHMGHGFNNAIMDALTRYNRMMGKNTLWQPGTDHAGIATQMVVERQLG
LQGVTRHDLGREKFIEKVWEWKEQSGGTITKQIRRLGSSVDWSRERFTMDEGLSNAVK
EVFVRLHEDGLIYRGKRLVNWDPKLQTALSDLEVESKEEKGSLWHFKYFFEDKSVKTQ
DGKDYLVVATTRPETLLGDTAVAVHPEDERYAHLVGKNIVLPITGRLIPIVADDYVEK
DFGTGCVKITPAHDFNDYELGKRNSLPIINIFNKNAEVLGEFEYIAKAGEQISKTITA
PADYAGLERFEARKKLVAQAEAEGWLDQIQPYDLKAPRGDRSGVIIEPLLTDQWYVKI
APLAEPAIEAVQDGRIKFVPEQYSNMYMAWMRDIQDWCISRQLWWGHRIPAWYDADGN
IYVGRSEEEVRAKNNITADVELKQDEDVLDTWFSSALWTFSTLGWPEQTPELKTFHPT
DVLVTGFDIIFFWVARMIMMTMHFMKNEDGSSQIPFKTVYVHGLVRDGEGQKMSKSKG
NVLDPLDLIDGIDLESLVAKRTTGLMNPKDAAKIEKSTRKEFPEGINAYGTDAVRFTF
CALANTGRDIKFDLKRVEGYRNFCNKIWNATRFVLMNVEGQTVGTDARPDLWELPEQW
IISRLQKAEAAVHQAFATYRLDLAAQAIYDFIWNEYCDWYVELTKPVLNDAEVSEERK
AEVRRVLLAVMEASLRLAHPLMPYLTEEIWQTLAPMLGQGGPTIMTAQYPIPEQAKIN
EQAEADMQWLQGLIGAVRNIRGEMGLGNARLLPVLLQNISNAEREQITRIEALFKALA
KVESIEFLGKDQEPPLSSSSVVGHASVFVPMKGLIDPKAELARLQKDLDKIQKQHDQI
ANKLANEGFVSKAPAAVVEGEKAKLAEFAAQLDKVKANMEQIAAL" /product="Valyl-tRNA
synthetase (EC 6.1.1.9)" /EC_number="6.1.1.9"
/transl_table=11
CDS 2752286..2752963
/locus_tag="AUO97b_02709" /db_xref="SEED:fig|400667.82.peg.2574"
/translation="MSYKVFLKISDSTYTQFASIREKLHAGVRESQSKVLGDVLSDLS
CEIIEQVFSVLLKDEQDNSTMTQKQRYESEKVLQQILDTFRKYMPWSVSFFGNERLLP
LVDYMTSLMKEREQEVYITYPITPQLVQQAQTLTEQIHEGNMQSVEKAFQTLIQIVDL
GVTSLVREPKKRLKFNLVVDKTLNGVINMTTHLGYKRLEKLGTQVDQTTATHYINHFL AFMHQAA"
/product="hypothetical protein"
/transl_table=11
CDS 2752989..2753795
/locus_tag="AUO97b_02710" /db_xref="SEED:fig|400667.82.peg.2575"
/db_xref="GO:0004316"
/translation="MAKLDTLQNKVVWITGASSGLGKALAGEFALQGAQVILTSRRFE
ELEEVRVGLLHPDQHLSVVADITNQEQVQEAYEQILKAKGRIDWLINNAGLSQRALIQ
DTTMATERAIMEVDYFSQVALTKTVLPTMLKQKSGRVVFVSSVAGLLGTQYRASYSAA
KAAIHMWANSLRAEVADQGVEVSVIFPGFVKTNVSFNALNGAGQPQGHQDEAIENGLE
ADVFAEQSVKALMQGQEYIVVGGTKEKLGVMVSRMSPKLLYKMIRKTKVK" /product="Oxidoreductase,
short-chain dehydrogenase/reductase family" /EC_number="1.1.1.100"
/transl_table=11
CDS 2753850..2754284
/locus_tag="AUO97b_02711" /db_xref="SEED:fig|400667.82.peg.2576"
/translation="MDQDYHRILEVITRFQLVFDQKNWDAFDELLADQLEVDYLQFRG
EPLCVVSCHEYKGSRQQALSHLRLQHNLSNPLIRIEQDQAWLECNYQIYRFSENDYFH
SFGRYYFTLAKQQGIWKITGICQHLTKNIGNPRIHFSAMGQC" /product="hypothetical protein"
/transl_table=11
CDS complement(2754281..2754715)
/locus_tag="AUO97b_02712" /db_xref="SEED:fig|400667.82.peg.2577"
/translation="MDNLIFQLVIFLILFSIGWAFGRHIERKHLNELLEKEQQFAHIR
IDTNRFATSDQLGHFISSNVVISHDYFKYVLASIKNILGGRLTSYESIVERARREAIV
RLKQQAQSVGANHIMGVRLSTTELGMQGGMVEVFAYGTAVKD" /product="hypothetical protein"
/transl_table=11
CDS complement(2754723..2755073) /locus_tag="AUO97b_02713"
/db_xref="SEED:fig|400667.82.peg.2578"
/translation="MLLSNLESVPGHQILKQLDVVYGSTVRSKHVGRDLMASLKNIVG

GELTGYTELLEESRQEAMQRMIAKAQQLGANAIVGIRFSTSNIAQGASELFVYGTAVV VQPQTPHLPDPFSA"
/product="UPF0145 protein TM_0763"
/transl_table=11
CDS complement(2755183..2756637)
/locus_tag="AUO97b_02714" /db_xref="SEED:fig|400667.82.peg.2579"
/translation="MQKVWSISGRSIAVSALALALAACQSMRGPEPVVKTDIPQSYAY
NSASGTSIAEQGYKQFFADPRLLEVIDLALANNRDLRTATLNIERAQQQYQITQNNQL
PTIGASGSAIRQVSQSRDPNNPYSTYQVGLGVTAYELDFWGRVRSLKDAALDSYLATQ
SARDSTQISLISQVAQAWLNYSFATANLRLAEQTLKAQLDSYNLNKKRFDVGIDSEVP
LRQAQISVETARNDVANYKTQIAQAQNLLNLLVGQPVPQNLLPTQPVKRIAQQNVFTA
GLPSDLLNNRPDVKAAEYNLSAAGANIGAAKARLFPTISLTGSAGYASTDLSDLFKSG
GFVWSVGPSLDLPIFDWGTRRANVKISETDQKIALSDYEKSVQSAFREVNDALATRAN
IGERLTAQQRLVEATNRNYTLSNARFRAGIDSYLTVLDAQRSSYAAEQGLLLLQQANL
NNQIELYKTLGGGLKANTSDTVVHQPSSAELKKQ"
/product="Efflux transport system, outer membrane factor (OMF) lipoprotein"
/transl_table=11
CDS complement(2756637..2759813)
/locus_tag="AUO97b_02715" /db_xref="SEED:fig|400667.82.peg.2580"
/translation="MAQFFIHRPIFAWVIALVIMLAGILTLTKMPIAQYPTIAPPTVT
IAATYPGASAETVENTVTQIIEQQMNGLDGLRYISSNSAGNGQASIQLNFEQGVDPDI
AQVQVQNKLQSATALLPEDVQRQGVTVTKSGASFLQVIAFYSPDNNLSDSDIKDYVNS
SIKEPLSRVAGVGEVQVFGGSYAMRIWLDPAKLTSYQLTPSDIATALQAQNSQVAVGQ
LGGAPAVQGQVLNATVNAQSLLQTPEQFKNIFLKNTASGAEVRLKDVARVELGSDNYQ
FDSKFNGKPAAGLAIKIATGANALDTAEAVEQRLSELRKNYPTGLADKLAYDTTPFIR
LSIESVVHTLIEAVILVFIVMFLFLQNWRATIIPTLAVPVVVLGTFAVINIFGFSINT
LTMFAMVLAIGLLVDDAIVVVENVERVMSEDHTDPVTATSRSMQQISGALVGITSVLT
AVFVPMAFFGGTTGVIYRQFSITLVTAMVLSLIVALTFTPALCATILKQHDPNKEPSN
NIFARFFRSFNNGFDRMSHSYQNGVSRMLKGKIFSGVLYAVVVALLVFLFQKLPSSFL
PEEDQGVVMTLVQLPPNATLDRTGKVIDTMTNFFMNEKDTVESIFTVSGFSFTGVGQN
AGIGFVKLKDWSKRTTPETQIGSLIQRGMALNMIIKDASYVMPLQLPAMPELGVTAGF
NLQLKDSSGQGHEKLIAARNTILGLASQDKRLVGVRPNGQEDTPQYQINVDQAQAGAM
GVSIAEINNTMRIAWGGSYINDFVDRGRVKKVYVQGDAGSRMMPEDLNKWYVRNNKGE
MVPFSAFATGEWTYGSPRLERYNGVSSVNIQGTPAPGVSSGDAMKAMEKIIGKLPSMG
LQGFDYEWTGLSLEERESGAQAPFLYALSLLIVFLCLAALYESWSIPFSVLLVVPLGV
IGAIVLTYLGMIIKGDPNLSNNIYFQVAIIAVIGLSAKNAILIVEFAKELQEKGEDLL
DATLHAAKMRLRPIIMTTLAFGFGVLPLALSTGAGAGSQHSVGFGVLGGVLSATFLGI
FFIPVFYVWIRSIFKYKPKTINTQEHKS"
/product="RND efflux system, inner membrane transporter" /transl_table=11
CDS complement(2759826..2761076)
/locus_tag="AUO97b_02716" /db_xref="SEED:fig|400667.82.peg.2581"
/translation="MMSAKLWAPALTACALATSIALVGCSKGSDEKQQAAAAQKMPPA
EVGVIVAQPQSVEQSVELSGRTSAYQISEVRPQTSGVILKRLFAEGSYVREGQALYEL
DSRTNRATLENAKASLLQQQANLASLRTKLNRYKQLVSSNAVSKQEYDDLLGQVNVAE
AQVAAAKAQVTNANVDLGYSTIRSPISGQSGRSSVTAGALVTANQTDPLVTIQQLDPI
YVDINQSSAELLRLRQQLSKGSLNNSNNTKVKLKLEDGSTYPIEGQLAFSDASVNQDT
GTITLRAVFSNPNHLLLPGMYTTAQIVQGVVPNAYLIPQAAITRLPTGQAVAMLVNAK
GVVESRPVETSGVQGQNWIVTNGLKAGDKVIVDGVAKVKEGQEVSAKPYQAQPANSQG
AAPNAAKPAQSGKPQAEQKAASNA" /product="RND efflux system, membrane fusion
protein" /transl_table=11
CDS complement(2761101..2761727)
/locus_tag="AUO97b_02717" /db_xref="SEED:fig|400667.82.peg.2582"
/translation="MPYLLLCIGCVFLGLGVLGLFVPSLQSLDLLTVQTLSHHRLDYL
NTITTFLARVGGMPFVCFLSFLVCIYLAWYKKYITVIFISLGVIGSITMGWLLKWCVN
RPRPPEAYHIVESYGASFPSAHSVYASTLACLAMIMLCHKHNINSPYIVLISCLWFVC

MGLSRIYAGVHFPTDVLAGWGIGFIWIALLWLWLLQTQSRLSRKQIYF" /product="putative membrane-associated phospholipid phosphatase, PAP2 superfamily"
/transl_table=11
CDS complement(2761912..2763972)
/locus_tag="AUO97b_02718" /db_xref="SEED:fig|400667.82.peg.2583"
/translation="MHINFSEHFFKIQKQLENPQAVIDENILIDLVNALRPSDPQDTD
EIEQKIQAFIDSLLLTPTAPALLQTFLLRLINQYKQVSLYADSGILSLDGFWNQLGQR
LGGHFLPLIEDASQLKILIGKIFYLESDSIWLNNVDDKDWATLFGLIGQSNSNVDEKH
AIQREMIKAITVLSYRISGIGLYPEFINAQPELTEYESPFLVQNREIIEFIEKYKKQD
ISSNDIAVLPPPDASQAFVMLEQCRDVVLKIRRATKRIGVSLSLTYLLSLLEQCLDRI
ELLLYLVVDDSEGRYVSLGNLISDLTKAHYSEKSVRSLLSTTSELIAFQVTENASRTG
EHYVSTDTKGFWGMYKAAAGAGVIIACMASLKILAARITMAPLMQAFTFSMNYSLGFI
LIHVLHFTVATKQPAMTAAALAATVQQRKGSKTAQIAELAALIINIIRTQFIAILGNI
SIAIPTAALITFAWQFYLDEPLLTHTKATYLLHSLNPFTSLAVPHAAIAGVCLFLSGL
IAGYFDNMAVYRKVGPRLKAHRRLRNLFGQERLNRFAEYIERNLGALAGNFLFGVMLG
SMGTIGFILGLPLDIRHIAFASANFIQGLMTINGSPDIGLIIVSFLGVLCIGLTNLFV
SFTLTIIVALRARRVRFEQWKPLAKLVMTHFLTRPSDFFWPPKQPLELEENAQANSGK KAEH"
/product="Probable site-specific recombinase" /transl_table=11 CDS complement (2763987..2764964)
/locus_tag="AUO97b_02719" /db_xref="SEED:fig|400667.82.peg.2584"
/translation="MVIDFKQDILAPVATDFAAMDHLINEGISSKVGLVMSVSKHVVE
AGGKRMRPIMCLLAARACDLDNMQNAQRLAAIIEMLHTATLVHDDVVDESGLRRGRPT
ANATWNNQTAVLVGDFLIARAFDLLVDLNNMTLLKDFSTGTCEIAEGEVLQLQSQHQP
DTTEETYLKIIHGKTSRLFELATEGAAILAGQEAYREPLRLFAGHFGNAFQIIDDILD
YTSDAETLGKNIGDDLMEGKPTLPLISALAHSTGEEHAIIRRSIATGGVDQLPKVIEI
VQKSGALDYCQRRAQEETEAALQALSILPDTPYRQALINLTRLALHRIQ" /product="Nonaprenyl diphosphate synthase (geranyl-diphosphate specific) (EC 2.5.1.84)"
/EC_number="2.5.1.90"
/transl_table=11
CDS 2765408..2765719
/locus_tag="AUO97b_02720" /db_xref="SEED:fig|400667.82.peg.2585"
/db_xref="GO:0003723" /db_xref="GO:0003735"
/db_xref="GO:0005622"
/db_xref="GO:0005840"
/db_xref="GO:0006412"
/translation="MYAVIQSGGKQHRVVEGETLKVELLKAESGATITFDDVLMVVNG
DNIQIGAPVVAGAKVTAEVIGHGRHDKIRIIKMRRRKHYRKQQGHRQWFTELKITGIS G"
/product="LSU ribosomal protein L21p"
/transl_table=11
CDS 2765738..2765995
/locus_tag="AUO97b_02721" /db_xref="SEED:fig|400667.82.peg.2586"
/db_xref="GO:0003735"
/db_xref="GO:0005622"
/db_xref="GO:0005840"
/db_xref="GO:0006412"
/translation="MATKKAGGSTKNGRDSNPKMLGVKVYGGQTVTAGNIIVRQRGTE
FHAGANVGMGRDHTLFATADGVVKFEVKGQFGRRYVKVETV"
/product="LSU ribosomal protein L27p"
/transl_table=11 CDS 2766236..2766931
/locus_tag="AUO97b_02722" /db_xref="SEED:fig|400667.82.peg.2587"
/translation="MNMLRKTMCAITLSASVLAPVMSTTAFAAPVAAPEQQAIGNLVN
QLSGLQRLTADFEQTTKANTKTVAQKKGLSAQHMNQTFKGSMKVERPGKFYWETVSPS
KQTIVTSGKTVWIYDPDLQQAVRQSLDDQVADTPALLLSGNTNQIMKSYRVTQPDKSK
LYFTLFPKKEDGAFQSLTISFGANKAPTLMVLQDSLGQTTYVRFKNVKVNASIPASTF NFTPPKGTDIIDQ"

/product="Outer membrane lipoprotein carrier protein LolA" /transl_table=11
CDS 2767059..2767847
/locus_tag="AUO97b_02723" /db_xref="SEED:fig|400667.82.peg.2588"
/translation="MQLYDKKIIFILPMLAAMVAVSGCQPKSTAKDEQLASSASVKEQ
KQAEVPVIQAKVVPVKLPKPKVCLEDGCTEYDFQTVQTNQKWINDYFTNRIKKADPNA
FANLADKPVEVPEGEPSSSQSAIYVRYLGQNYNLATFAFQTYSYSAGAAHGMSHQEFV
NFDLLNKKHITVEELIKPDVEKQLVDALFDANTNWLQEHNISREKLQLSDNFYYGANG
IVFVYPIYELASYAEGMSELTLPYFEAAKYIKPEYLPSVPNYNL" /product="Chromosome
segregation ATPases"
/transl_table=11
CDS 2767966..2769237
/locus_tag="AUO97b_02724" /db_xref="SEED:fig|400667.82.peg.2589"
/db_xref="GO:0004828"
/translation="MIDPKLLRNNIEVVNAALAKRGVQLDVQEWASLETRRKDLQSKT
EKLQAERNAGAKQVGQIKKSGGDASEIMARMQAIGDEIKAAEVALSELQNEIEQKALS
IPNLPDESVPAGKDENDNVEISKWGTPRQFDFEIKDHTDLGEWMGGLEFETATKLTGS
RFSVLKGPLARLQRALTQFMLDTHTLKNGYTEAYVPYLVNADSLRGTGQLPKFEEDLF
KLQGEKEYYLIPTAEVPVTNFVRDEIIDADRLPLKYAAHTPCFRSEAGSYGRDTRGLI
RQHQFDKVEMVQIVKPETSMQALEELTAHAEGILQALGLPYRKILLCGGDMGFGATKT
YDLEVWVPSQNTYREISSCSNMGDFQARRMKARYRMDQKKTELVHTLNGSGLAVGRTL
LAVMENYQREDGSIEIPEVLRPYMGGATFID"
/product="Seryl-tRNA synthetase (EC 6.1.1.11)" /EC_number="6.1.1.11"
/transl_table=11
CDS 2769305..2770678
/locus_tag="AUO97b_02725" /db_xref="SEED:fig|400667.82.peg.2590"
/db_xref="GO:0043115"
/db_xref="GO:0051266"
/db_xref="GO:0004851"
/translation="MDIFPISLKLQQQRCLIVGGGHIALRKATLLAKAGAIIDVVAPA
IEDQLLQLITTTGGVSFIEAFAEKFLSTPYRLVIAATNDAEVNKTVFEQCEARNLLVN
SVDDIPHCRFMVPAIIDRSPLIVSVASNGTSPVLSRQIRTQLETSIPHGMGKLAEFSG
KWRNQVKEKISNPDERRIFWENLYASPLKEQVFNDNLDVADSMLEQALQEWKAPKGEV
YLVGAGPGDPELITLKALRLMQQADVVIYDRLVSAPILELCRRDATKIYVGKARSNHS
VPQEGINALLVDYAKKGKRVCRLKGGDPFIFGRGGEEIQELFQAGVPFQVVPGITAAS
GCSAYAGIPLTHRDYAQSVRFLTGHLKEGSPELPWNELVYENQTLVLYMGLVGLERIC
EQLIAHGQRPDMPVALISKGTTPEQKVVVGSLADIASKVTEHQIHAPTLTIIGEVVRL REQLQWN"
/product="Precorrin-2 oxidase (EC 1.3.1.76) @ Sirohydrochlorin ferrochelatase activity of CysG (EC 4.99.1.4) / Uroporphyrinogen-III methyltransferase (EC 2.1.1.107)" /EC_number="1.3.1.76"
/EC_number="2.1.1.107"
/EC_number="4.99.1.4"
/transl_table=11
CDS 2770755..2771228
/locus_tag="AUO97b_02726" /db_xref="SEED:fig|400667.82.peg.2591"
/translation="MIHVVLYEPEIPANTGNIIRLCANTGAQLHLVKPLGFELDDKKL
KRAGLDYHEWARMQIWDNIELCLADLKAKGVEHVFPLTTKGSATPHTVDLNRPVALLM
GPETRGLPEHVRLMFPQEQWIRLPMAENSRSLNLSNATAVIVYEAWRQQGFKKLG" /product="tRNA (cytidine(34)-2'-O)-methyltransferase (EC 2.1.1.207)" /EC_number="2.1.1.207"
/transl_table=11
CDS complement(2771278..2771658)
/locus_tag="AUO97b_02727" /db_xref="SEED:fig|400667.82.peg.2592"
/translation="MNECLKYNIFQQHCPARLFFEKIADKWVLLILNILEHETQHFNL
LKKNIEGISPKVLSQKLKMLERDGFIERKIQDTSPIRVDYSLTPLGQNVAAMAYQLKE
WAETNIEQVLAAQIIYDEKILEQA"

/product="Transcriptional regulator, HxlR family" /transl_table=11
CDS 2771775..2772335
/locus_tag="AUO97b_02728" /db_xref="SEED:fig|400667.82.peg.2593"
/translation="MSNSNIAVVYFSGYGHTKVVAETFANEINAQLIQINQEGNITDQ
DWETLNNAKGIVFGAPTYMGTAPWQFKKFADATSKVWFTRGWQDKVFAGFTNSASLNG
DKQVTLIQLQTLASQHGGIWVSLGLLPANTKDATREDVNNLGGSVGLLVQSPSDASVD
EVPTGDLETAKVYAKRVQAIVNKIYG" /product="Multimeric flavodoxin WrbA"
/transl_table=11
CDS complement(2772375..2773760)
/locus_tag="AUO97b_02729" /db_xref="SEED:fig|400667.82.peg.2594"
/translation="MTNLSGTKPTAKLQKNLVLWHIIIIGLAYIQPLTLFDTFGLVSE
RSGGHVPTSYIFALVAILLTSISYGHMIKRYPSSGSAYTYAQKSIHPNVGFMVGWSSW
LDYLLSPLVNIILADIYLRALFPEVNNWLWVIGLTALMTVINLFGARFVARFNSTIVV
IQLGVIFYFVYQVYILLTQGVYADGTLNPDKAELWSLTPFWNDMTSISALIGGATVLC
FSFTGFDALSSLAEETKDAEKTLPKAIFSTALLAGIIFIVSTYFIQLYFPSNPNIYFK
PLDESQPVMVAAIGSIAFKTLVLYFAVVAVMASGISAHAGVSRLMYVMGRDGVINKKL
FGHISPRLHTPTYNILIVGVVALSAGFLDLEHIVNLISFGALTAFSFVNFSVISRYAL
RDGKNKSIKDIINYIIVPTLGFASVFLMWLEIDKLALQIGLAWAVFGVGYLAYKTKGF KYNAPQHNEHE"
/product="Uncharacterized amino acid permease, GabP family" /transl_table=11
CDS complement(2774026..2775210)
/locus_tag="AUO97b_02730" /db_xref="SEED:fig|400667.82.peg.2595"
/db_xref="GO:0004022"
/translation="MAKPYYEFFCPVKVIAGNAALEHIPFELATLGAKRPLIITDKGV
RANGLLNPIEAAFSTTDAVIGHVFDDVPPDSSLEVVRLAAEAYRTNKCDAIIAVGGGS
VIDTSKATNILVSEGGDDLLQYSGAHNLPKPLKPFFVIPTTSGTGSEVTMVAVVSDTQ
KNVKLAFASYYLMPHAAILDPRMTLTLPPHLTAMTGMDALTHCVEAYTCMAANPLSDA
YASAGIKKISENLFNVLENPNDSQGRLELAQASTMAGIAFSNSMVGLVHSLGHALGAV
AHLPHGLCMNLFLPYVLEYNKQVNGQKIGELLLPLAGADIYAQTPATQRADRAIATIL
AMRDRLFSLTKLPRTLRETGKVTEAQLDEVAEKALNDGSIIYNPKEASLKDLREILQK AW"
/product="Alcohol dehydrogenase (EC 1.1.1.1)" /EC_number="1.1.1.1"
/transl_table=11
CDS complement(2775212..2775745) /locus_tag="AUO97b_02731"
/db_xref="SEED:fig|400667.82.peg.2596"
/translation="MISTKAVKPTLQLAYVKLMMDVVGRGLVMASQVDSEIQEEVSKF
PVHFVLSMNVFPNGPAFFARVTEDKQLELLKRAEKKPDLTITFKHVTHAFLVFSFQES
TAQAFANDRMIADGDVSAAIRLVRCLNKMESLILPKLVASLAVKEYPTELTLKEKFNQ AANIYLKVAKSYFKRSA"
/product="hypothetical protein"
/transl_table=11
CDS complement(2775826..2776479)
/locus_tag="AUO97b_02732" /db_xref="SEED:fig|400667.82.peg.2597"
/db_xref="GO:0003824"
/db_xref="GO:0006464"
/translation="MSLDKPTLIIRQWNDLQDYQSKFESMKNLTNQRDENTADELWLL
QHHEVLTQGQAGKPEHILIPSNIPVVQTDRGGQVTWHGPGQLVAYFMFDLNRLKWNVR
TLVSFAEQFMIDVLKKYNIEAYAKPDAPGVYVDGRKIGSLGFKIRRGRSYHGLALNLD
CALTGFQTINPCGYAGLEMVRIQDLVTPYPSFEQLCQDFIEYIKATGYFNDPEVKIE"
/product="Octanoate-[acyl-carrier-protein]-protein-N-octanoyltransferase (EC 2.3.1.181)" /transl_table=11
CDS complement(2776663..2776950)
/locus_tag="AUO97b_02733" /db_xref="SEED:fig|400667.82.peg.2598"
/translation="MLDRTPSRELREDLWVFPMDYPIKLIGDAGEELRIAVVDILVKH
FPDFDQTTLKVQESRTGKYHSLTAQLRFDELEQVHALYADLAACPQIRTAL" /product="Proposed lipoate regulatory protein YbeD" /transl_table=11
CDS complement(2777048..2778934)

/locus_tag="AUO97b_02734" /db_xref="SEED:fig|400667.82.peg.2599"
/db_xref="GO:0003677"
/db_xref="GO:0003700"
/db_xref="GO:0006352"
/db_xref="GO:0016987"
/db_xref="GO:0030528" /db_xref="GO:0045449"
/translation="MSDMHSPTSQVAALISRGKEQGYLTYAEVNDHLPDSITESEQIE
DIIQMLQDVGIPVHERAPESDDTMFGESADATDEVAEEEAAAVLASVENEPGRTTDPV
RMYMREMGTVELLTREGEISIAKRIEEGIRDVLNSIAYWPNAVEVVLKEYNDFLTGER
RLADILSGYLDPETDEDIPEVLEDVEELEEEDESSTKSTKEVKLDDDDEEEESEGDDD
SEGDSGPDPEVAKVRFAELEAAWAQTKAVIEKHGRNSPEANEALESLATVFMMFKFTP
RLFDIISEMIRGTHEQIRANEREIMRYAVRRGRMDRNQFRTSFPKQESNPAWLDEQIA
KAPAEIKAHLEKVRPDVLAFQQKIADIEKELGLNVSEIKDISKRMAIGEAKARRAKKE
MVEANLRLVISIAKKYTNRGLQFLDLIQEGNIGLMKAVDKFEYRRGYKFSTYATWWIR
QAITRSIADQARTIRIPVHMIETINKINRVSRQLLQEMGREPTPEELGERLEMDEVKV
RKVLKIAKEPISMETPIGDDEDSHLGDFIEDQNITSPIDAATSEGLKEATREVLENLT
EREAKVLKMRFGIDMPTDHTLEEVGKQFDVTRERIRQIEAKALRKLRHPSRSEHLRSF LEND"
/product="RNA polymerase sigma factor RpoD" /transl_table=11
CDS complement(2778931..2779083)
/locus_tag="AUO97b_02735" /db_xref="SEED:fig|400667.82.peg.2600"
/translation="MILAQYSKNSREFKDIFYLTVTDQSPNISSKLCYHARFFTATDS TKSKQR"
/product="hypothetical protein"
/transl_table=11
CDS 2779293..2779499
/locus_tag="AUO97b_02736" /db_xref="SEED:fig|400667.82.peg.2601"
/translation="MSSTDFELDDNYGDDDDVGFDESSGKISAKESLEKRRLIDDLLA
QRRLERELKDFDYDLDDDDLDDED"
/product="FIG00350425: hypothetical protein" /transl_table=11
CDS 2779575..2780099
/locus_tag="AUO97b_02737" /db_xref="SEED:fig|400667.82.peg.2602"
/translation="MSALDLDQLSEYLDGDHNEYGLDFAATHGFLCAIAVGPQFDRWL
DELFEGNQKKVPAEIIQQIKVWLESIRQDLANENGIEFPFEVEEADVESSLGDWSVGF
VDAMFLNEEAWFAPEYEEQLVDLTLPIMVFSGIDEEDPQMESFRRNGQLMDELAEEIP DNLNELYLMYHTPN"
/product="hypothetical protein"
/transl_table=11
CDS 2780325..2781446
/locus_tag="AUO97b_02738" /db_xref="SEED:fig|400667.82.peg.2603"
/translation="MNIIFKITSVLSVILLVACGDENSNSVSEEKIAQSTQNQIVYKL
ISGTAFSTSALNGANVTAICKDGLGFKNKVIVNAQGQWQGEIDSSKFPCRLEAKAEGQ
TYHSYIDHEGRVNINPLTDMVVAYASNQVPVTWYQSGSMTTEKLNSANSALVAELIKK
GYGINNDMDLFTAEMKANNPIHQAIQELLEAIQNNRNIKDYDSLLMLIKDGNLSQVPK
KIEFLNNSVAKVFGFNKDACQVLPKTENTEQYNKCSDKVIDDFSESNLVATDSDEKCI
LVKQGNKVSFTKGNQTVSALLDKEQEDGMDFTFDEGELEIIDLIINTGPYTDINTYSQ
IGLSFSSDGKLRAVVGKSPIVSSMNCASPEFKKLMELYK" /product="hypothetical protein"
/transl_table=11
CDS complement(2781487..2782419)
/locus_tag="AUO97b_02739" /db_xref="SEED:fig|400667.82.peg.2604"
/translation="MNATVEQLAPVEQQATAGWVVAALYQFKEVQDPADLQQRLLDLV
KTINLCGTLIVAGEGINGTVAGDREAIDTIHQFLLNEGFNAMEYKESHSSDKPFRKMK
IKLKKEIVTLGVEVKPRDLVGHYLDPKEWNELIARDDVILIDTRNDYEYKAGTFKGAI
DPKTETFREFPEYVKKELEQHKDKKIAMFCTGGIRCEKSTSLLLQEGFKEVYHLKGGI
LKYLEETPPDESLWEGECFVFDGRTAVTHGVEEGANIKCHACGWPLTPEESALPSYEH
GVSCLYCIDKTTEKQKAGFRMRQSQIAAAKRKRL"
/product="Rhodanese domain protein UPF0176,

cyanobacterial/alphaproteobacterial subgroup" /transl_table=11
CDS 2782531..2783016
/locus_tag="AUO97b_02740" /db_xref="SEED:fig|400667.82.peg.2605"
/translation="MSSDHRMLSLTPCAGRCSTVFGDQVCRGCRRFNHEVIQWNTYTA
EQRLAVWKRLDDQLDQILVPMLPQANLQHVEGFILGKRVRLMEGASKGRKLYHALKIC
EKNKHLAHESGLGISEAQVKEVWTEFERRILALAKASYELAWLRADGISHHLLNMFEE E"
/product="hypothetical protein"
/transl_table=11
CDS complement(2783087..2784361)
/locus_tag="AUO97b_02741" /db_xref="SEED:fig|400667.82.peg.2606"
/db_xref="GO:0004108"
/translation="MSEATGKKAVLHLDGKEIELPIYSGTLGPDVIDVKDVLASGHFT
FDPGFMATASCESKITFIDGDKGILLHRGYPIDQLATQADYLETCYLLLNGELPTAEQ
KVEFDAKVRAHTMVHDQVSRFFNGFRRDAHPMAIMVGVVGALSAFYHNNLDIEDINHR
EITAIRLIAKIPTLAAWSYKYTVGQPFIYPRNDLNYAENFLHMMFATPADRDYKVNPV
LARAMDRIFTLHADHEQNASTSTVRLAGSTGANPYACISAGISALWGPAHGGANEAVL
KMLDEIGSVENVAEFMEKVKRKEVKLMGFGHRVYKNFDPRAKVMKQTCDEVLEALGIN
DPQLALAMELERIALNDPYFVERKLYPNVDFYSGIILKAIGIPTEMFTVIFALARTVG
WISHWLEMHSGPYKIGRPRQLYTGEVQRDIKR"
/product="Citrate synthase (si) (EC 2.3.3.1)" /EC_number="2.3.3.1"
/transl_table=11
CDS 2785356..2785718
/locus_tag="AUO97b_02742" /db_xref="SEED:fig|400667.82.peg.2607"
/db_xref="GO:0000104"
/db_xref="GO:0006099"
/db_xref="GO:0006118"
/db_xref="GO:0016020"
/translation="MGQVLAVNLRSPVAIASILHRLSGVIVFLLVPVLLWILDKSLSS
PEGFDYVKNVVFGNILVRFVVWVFVAGLIFHFIAGVKHLLADLGFAEELQSGRIAATI
SLILSVVAIIAAFVWIMF"
/product="Succinate dehydrogenase cytochrome b-556 subunit" /transl_table=11
CDS 2785718..2786083
/locus_tag="AUO97b_02743" /db_xref="SEED:fig|400667.82.peg.2608"
/translation="MKSATGLTGSGSRDWFIQRVSAVVLAAYTVVLFGWILCKGGFDY
QQWAGFMMTLPMKIFSLLAILSLIAHAWIGMWQVFTDYVTTRQMGPSAKGLRLVLTTA
VIIAVFVYAIWGIQIFWAN"
/product="Succinate dehydrogenase hydrophobic membrane anchor protein"
/transl_table=11
CDS 2786096..2787931
/locus_tag="AUO97b_02744" /db_xref="SEED:fig|400667.82.peg.2609"
/db_xref="GO:0000104"
/translation="MGAINPKEDYSNIQNLTFDAVIVGGGGSGMRASYQLAQAGLKVA
VLTKVFPTRSHTVAAQGGIGASLGNMQEDNWHFHFYDTVKGSDWLGDQDAIEFMCREA
PKVVYELEHLGMPFDRNADGTIYQRPFGGHSANYGDKPVPRACAAADRTGHALLHTLY
QSNVKMGTQFFVEWIALDLIRNEAGDVLGVTAIDQETGNIAVFQAKATLFATGGAGRV
YRASTNAYINTGDGLGMAARAGIPLQDMEFWQFHPTGVAGAGVLLTEGCRGEGAILRN
KDGEPFMERYAPTLKDLAPRDFVSRSMDQEIKEGRGCGPKGDYILLDMTHLGADTIMK
RLPSVFEIGKKFANVDITKEPIPVVPTIHYQMGGIPTNMHGQVCLPEPGTDNYTKPVK
GFYAIGECSCVSVHGANRLGTNSLLDLVVFGKAAGEHIIDYVTKHHGDEYAPLPTNVL
EQTLARVRKLDESTSGENAQEVADAIRDIVQDHAGVFRTQALLDKGVKEILALEPRVR
NIHLKDKSKVFNTARVEALEVENLYEVAKATLISAAARKECRGAHTVVDYELPADHPT
YSYGRRDDEWMKHTLWYSSDNRLEYKPVRFKPLTVDPIPPAPRTF" /product="Succinate
dehydrogenase flavoprotein subunit (EC 1.3.5.1)" /EC_number="1.3.99.1"
/transl_table=11

CDS 2788012..2788656
/locus_tag="AUO97b_02745" /db_xref="SEED:fig|400667.82.peg.2610"
/db_xref="GO:0000104"
/translation="MQTFKLELTDKHRMLLDALLALKVQDETLTFRRSCREGICGSDG
VNINGKNGLACLQNLNDLPEKIVIRPLPGLPVVKDLVVDMNQFYDQYDKIQPFLINNQ
PAPPKERLQSPEEREHLNGLYECILCACCSTSCPSFWWNPDKFLGPSALLNAYRFIID
SRDTATAERLARLDDPFSLFRCKGIMNCVSVCPKGLNPTKAIGHIRNMLLDQAG" /product="Succinate dehydrogenase iron-sulfur protein (EC 1.3.5.1)" /EC_number="1.3.99.1"
/transl_table=11
CDS 2789227..2792067
/locus_tag="AUO97b_02746" /db_xref="SEED:fig|400667.82.peg.2611"
/db_xref="GO:0004591"
/translation="MQEVADALRLDTELSADSAAYIEELYEQYLTSPTSVAEDWRQYF
DKYPKGDQPHSAVREQFLLLGRNANRVQPVVQSTVSTEHERRQIGVLQLIAAYRNRGH
QKAKLDPLGLAKREDIPDLDLSAHGLTKSDLDTVFNTGNLEIGKSEATLAEMIEAMEA
IYCGSIGVEYMHIVDTKEKRWIQQRLESARGKFNFSNEQKKGFLERLTAAEGLEKYLG
NKYVGAKRFGVEGGESFIPMVNEIIQRAGAVGCKEVVIGMPHRGRLNLLVNIMGKNPA
DLFGEFEGKALHKKGSGDVKYHQGFSSNVMTPGGEVHLALAFNPSHLEIVGPVVEGSV
RARQVRRRDIGGDDVLPVIVHGDAAFAGQGVNMETFQMSQTRGYTVGGTVHIIVNNQV
GFTTSDPRDARSTEYCTDVAKMIQAPIFHVNGDDPEAVIFATQLAHDFRHEFRKDVVI
DLFCYRRRGHNEADEPSGTQPLMYQVIAKKATTRTLYADQLVQEKVLDRAEADQMVED
YRADLEAGNHVANALILEPNTKMFVDWTPYLGHEYTDDWDTSFDINRLKELGEGMSKL
PEGFVMQRQVQKVIEDRVKMQTGETPLNWGAAETLAYATLLDEDYLVRITGEDVGRGT
FSHRHAKLHNQVDGSTYIPLCHVKENQPRFAIYDSLLSEEAVLAFEYGYATTIPKSLI
IWEAQFGDFVNCAQVVIDQFIASGETKWERVCGLTMLLPHGFEGQGPEHSSARLERFL
QLCAEDNMQVITPTTPAQIFHALRRQAVRPIRKPLIVMSPKSLLRHKLATSTLEELAH
GSFQTVIDEIDQINKSDVTRLVLCGGKVYYDLLEKRREQNLTNVAIVRIEQLYPYPEQ
RLAEILAAYPNVKELVWAQEEPKNQGAWLFIAPRLYDDILKSGKQIRISFAGREASAA
PACGSPYLHAKQQAQLINDALAIEAEQSGDSQ"
/product="2-oxoglutarate dehydrogenase E1 component (EC 1.2.4.2)"
/EC_number="1.2.4.2"
/transl_table=11
CDS 2792067..2793263
/locus_tag="AUO97b_02747" /db_xref="SEED:fig|400667.82.peg.2612"
/db_xref="GO:0004149"
/translation="MATEIKAPVFPESVADGTIATWHKKVGEPVSRDEVICDIETDKV
VLEVVAPADGSLVAIIKGEGDTVLSDEVIAQFEAGAGAAAAAPAAVEQAVAQTQAGAA
PVVERNETVSDQAPAVRKALTESGIAASDVQGTGRGGRITKEDVANHQAKPAANVTPL
SVAVGERIEKRVPMTRLRKRVAERLLAATQETAMLTTFNEVNMKPIMELRKQYKDAFE
KRHGARLGFMSFFVKAATEALKRYPAVNASIDGDDIVYHGYYDIGVAVSSDRGLVVPV
LRDTDRMSYAEVEAGIAAYAAKARDGKLSIEEMTGGTFTITNGGTFGSLLSTPILNQP
QTGILGMHKIQERPMAVNGQVEILPMMYLALSYDHRMIDGKEAVGFLVAIKELLEEPA KLILDL"
/product="Dihydrolipoamide succinyltransferase component (E2) of 2-oxoglutarate dehydrogenase complex (EC 2.3.1.61)" /EC_number="2.3.1.61"
/transl_table=11
CDS 2793326..2794759
/locus_tag="AUO97b_02748" /db_xref="SEED:fig|400667.82.peg.2613"
/db_xref="GO:0004148"
/translation="MSQQFDLVVIGGGPGGYEAAIRAAQLGFKVACIEKRIHNGKPSL
GGTCLNVGCIPSKALLDSSHRYEDTVHHLADHGITTGEVNFDLAKLLARKDKIVDQLT
GGIDQLLKGNGIEWLKGTGKLLAGKKVEFVPHEGETQILEPKYVILASGSVPVNIPVA
PVDQDIIVDSTGALNFPEVPKRLGVIGAGVIGLELGSVWRRLGAEVVVFEAMDAFLPM
ADKALSKEYQKILTKQGLDIRIGAKVSGTEVNGREVTVKYTQAGEDKEQTFDKLIVCV
GRKAYAEGLLAEDSGIKLTERGLVEVNDHCATSVEGVYAIGDLVRGPMLAHKAMEEGV

MAVERIHGHAAQVNYDTIISVIYTHPEAAWVGLTEEQAKEKGHEVKTGQFGFAVNGRA
LAAGEGAGFVKFVADAKTDRLLGMHVIGPAASDIVHQGMIALEFVSSVEDLQLMTFGH
PTFSEVVHEAALAVDGRAIHAIQRKRK" /product="Dihydrolipoamide dehydrogenase of 2-oxoglutarate dehydrogenase (EC 1.8.1.4)" /EC_number="1.8.1.4"
/transl_table=11
CDS 2794907..2796073
/locus_tag="AUO97b_02749" /db_xref="SEED:fig|400667.82.peg.2614"
/db_xref="GO:0004775"
/translation="MNLHEYQAKALLKEYGMPVQEGILATNADEAVAAFEQLGGKFAV
MKAQVHAGGRGKAGGVKVAKSKEDVIEFANNIIGTRLVTYQTDANGQPVNSIIVAEDV
YPVERELYLGAVVDRSSRRITFMASTEGGVEIEKVAEETPEKIIKVEVDPLVGLQPFQ
AREVAFALGLKDKQIGQFVKIMTAAYQAFVENDFALFEINPLSVRENGEILCVDAKVG
IDSNALYRLPKVAALRDKSQENERELKASEFDLNYVALEGNIGCMVNGAGLAMATMDI
IKLYGGQPANFLDVGGGATKERVIEAFKIILADTSVQGVLINIFGGIVRCDMIAEAII
AAVQEVNVTVPVVVRLEGNNAELGAKLLDESGLKLISANGLSDAAEKVVAAVKA" /product="Succinyl-CoA ligase [ADP-forming] beta chain (EC 6.2.1.5)" /EC_number="6.2.1.5"
/transl_table=11
CDS 2796088..2796978
/locus_tag="AUO97b_02750" /db_xref="SEED:fig|400667.82.peg.2615"
/db_xref="GO:0004775"
/translation="MSVLINKDTKVLVQGFTGKNGTFHSAQALDYGTKVVGGVTPGKG
GTTHLDLPVFNTMKEAVRETNADASVIYVPAPFVLDSIVEAVDSGVGLIVVITEGVPT
IDMLKAKRYLETNGNGTRLVGPNCPGVITPGECKIGIMPGHIHQPGRIGIISRSGTLT
YEAVAQTTKLGLGQSTCIGIGGDPIPGMNQIEALQLFQDDPDTDAIIMIGEIGGTAEE
EAAEFIKSNVTKPVVGYIAGVTAPKGKRMGHAGAIISGGQGTAEEKFAAFEKAGMAYT
RSPAELGSTMLQVLKEKGLA"
/product="Succinyl-CoA ligase [ADP-forming] alpha chain (EC 6.2.1.5)"
/EC_number="6.2.1.5"
/transl_table=11
CDS complement(2797394..2798407)
/locus_tag="AUO97b_02751" /db_xref="SEED:fig|400667.82.peg.2616"
/db_xref="GO:0004830"
/translation="MSNVDQRPIILTGDRPTGQLHLGHFVGSLRSRVGLQDSHHQHLL
LADAQALTDNADNPDKVRRNILEVALDYLAVGIDPTKTTICVQSCLPALNELTMLYLN
FVTVARLERNPTIKSEIQMRGFERDIPAGFLCYPVAQAADITAFKATVVPVGEDQIPM
IEQTNEIVRRVNRQIGQDLLPECKALLSNMARLPGFDGKAKMSKSLGNTIVLNASDKD
IKKAVNAMYTDPNHLRIEDPGQVEGNIVFTYLDAFDPNKEEVEELKAHYRRGGLGDGT
VKKRLEGVLKELITPIRERREELAKDPDYIMDVLRQGTDKCRIITQQTLDEVKDGLGL FKF"
/product="Tryptophanyl-tRNA synthetase (EC 6.1.1.2)" /EC_number="6.1.1.2"
/transl_table=11
CDS 2798615..2799538
/locus_tag="AUO97b_02752" /db_xref="SEED:fig|400667.82.peg.2617"
/translation="MASGLLLLLDDIATILDDVALMSKMAAKKTAGVLGDDLALNAQQ
VTGVRADRELPIVWKVAKGSFVNKLILVPLALLISVIAPWLINPLLMLGGLFLCYEGV
EKVLHTITHKKASTSEEAQKEFDSEELDLATFEKDKVKGAIRTDFILSAEIVVISLGT
VATATLMTKVSVLSIIAVVMTIGVYGLVGMIVKIDDLGLYLTKQAASFKQTIGRGLLA
FAPLLMKLLSIVGTIAMFLVGGGIINHTIPLLHHVTENTVDHVETIPAVGNIIGALTP
TLINFAIGIVAGAIVLLIVSLIQKMWPKASS"
/product="Inner membrane protein YedI"
/transl_table=11
CDS 2799568..2800467
/locus_tag="AUO97b_02753" /db_xref="SEED:fig|400667.82.peg.2618"
/translation="MRWKGRRVSTNVEDRRGGGGVRAGGISIIGLVVAFVAWKFFGVD
PQQAYQATQQVTASQQSNATAPESLTAEQKEASDFIRTILADTEDTWTPIFKQLGKTY

TPPKLVLFSGMIQSGCGTAQSAMGPFYCPADQKVYIDTEFFKDMREQMGISGEQNQTE
LSRQDQAGDFAQAYVVAHEVGHHVQTLLGISSQVQQARAQVSQREGNQLSVRQELQAD
CLAGIWANHNQQRTQFLEQGDVEEAMDAAQKIGDDYLQKRATGQVVPDSFTHGSSEQR
MHWFQVGLKTGDISQCDTFNNSI"
/product="YpfJ protein, zinc metalloprotease superfamily" /transl_table=11
CDS 2800705..2802207
/locus_tag="AUO97b_02754" /db_xref="SEED:fig|400667.82.peg.2619"
/translation="MGNFFLLQAFTVVFALSIATTIFNKRILGFPQAIGVPLVSAIFV
FILQWGASLLNGNQFITINIHNIEEAVRHIDFYDFLINGVICFILTSSALKFKISDLR
SYWKQISILATIALVICAVLFGSLLYGFQLLVGHHVPILVLLLLGAALGATDPIGIKG
VLSSIRAPHHLIVKLEGESLFNDAMCIALFMTLLNVLQGEHFSLVATLEALLYEIVVA
VGIGWAFGIGTLRLLRGKHEMESLILTTALLASGAYLVALFAHASAPIACVIGGLIVG
NKWKEIREEREIREVNHFWHTVEGIINSFLFTLIGLELFILDLNFSLILGGIVAFFIL
HISRFAANFMSFAFFPTMRKTKSYNGSLTILSWGGVRGGISLALILAVANIPALSPYS
SILIGYTFISVLLSGVVCGLGLPAVMNAFYYNPNEETEGLKGFYQKLCHKMNRRGFQY
IVGEDALGRETITIFNPDVIVDQKTEDGIATMHDPNKLQEVKRFESPDNF" /product="Na+/H+
antiporter, NhaP family"
/transl_table=11
CDS 2802667..2812017
/locus_tag="AUO97b_02755" /db_xref="SEED:fig|400667.82.peg.2620"
/db_xref="GO:0004375"
/translation="MPEIQIIAKDNHKTLVTTEGTSAKLSEASVVLVKVAASDVLVVN
REGTNAVIRLKNGETIVIEGFFSGTAEPKDNSLVFQDENGQLIWAKFKDAENDADADS
DADADADSDVEPQALLGEDLPAALPAEAPQELVSDVIYQPISSIEPLLYHDAGVNPWL
WAAIPLVAGGIIAAASNHDSNDDSSAPADTTPPSTDGVTFSVDPVTSDNVINASEASG
NVTITGVLKNIPADAANTAVTVVINGVTYNATVDKAAGTWTVSVPGSGLVADADKTID
ATVTFKDAAGNSSSVNDTQTYTIDTIAPNTDEVDFSVDSVTADNVVNASEAAGEVTIT
GVLKNIPADAANTVVTVVVNGVSYTATVDKAAGTWTVNVPGSGLVADTDKTIDATVTF
TDAAGNSSTVSDTQLYTLDTTAPDVDGVNFTVDSVTADNVINASEAAGNVTITGVLKN
VPSDAAATAVTIVINGVTYTATVDSAAGTWTVNVPGSGLVADTDKTIDATVTFTDAAG
NSSTVSDTQTYTLDTTAPTVVLEDVSTNDNTPALTGTVSDPSATVVVTIDGVDYPAVN
NGDGTWTLADNTLPVLTDGSHTVTVTATDAAGNKGTDTGVVTVDTAAPNTGTVNFSID
SVTSDNVINASEASGNVTVTGVLKNVPADAANTVVTVVINGQTYTATVDSTAGTWTVS
VPGSELTADADKTIDAKVTFTDAAGNSSSVNDTQTYTLDTTAPDAPVINPVNGTDPIT
GTAEPGSTVTVTYPNGDTATVVAGPDGSWSVPNPGLNDGDEVEAIATDPAGNPSLPGT
AIVDAVGPNTDGVNFTVDSVTADNVINASEASGNVTVTGVLKNVPADAANTVVTVVIN
GQTYTATVDSTAGTWTVSVPGSGLTADADKTIDAKVTFTDAAGNSSSVNDTQTYTIDT
TAPNAPVIDPVNGIDPITGTAEPGSTVTVTYPDGSTTTVVAGPGGTWTVPNPGNLVDG
DEVTAIATDPAGNPSLPGTAIVDAVGPNTDGVNFTVDSVTSDNVINASEASGNVTVTG
VLKNVPADAANTVVTVVINGQTYTATVDSTAGTWTVSVPGSDLTADADKTIDAKVTFT
DAAGNSSSVNDTHTYTVDTVAPNAPVLDPINATDPVSGQAEPGSTVTVTYPDGTTATV
VAGTDGSWSVPNPGNLVDGDTVTATATDPAGNTSLPGTGTVSADITAPVVALDDVLTN
DSTPALTGTVNDPTATVVVNVDGVDYPAVNNGDGTWTLADNTLPTLADGPHTITVTAT
DAAGNVGNDTAVVTVDTTAPGVSFTDASTNDTTPALTGTIDDPTATVVVTVDGVDYPA
VNNGDGTWTLADNTLPSLIDGPHTVSVTATDPAGNVSLPATGTVTISSSSILAFDNTD
HAVLSPQPSLVGDDVSLGSTSYLVLTSVAGLDLQLGGNSLGFTVAAGHEGDVTFQYSG
LIDAAVLSDYKLVVQKFNTTTNQWESIHGDANSSLISLHLLGIGTGNVPGAVLDGLDA
GQYRAFLAYDGLLGLGVLGTLSATMDDYDLSVAGGYEIGNAEGNVITDPDPTTGQVDQ
VTANTYVSSVNGHPIDADGETFAGTYGTITFYQDGSYVYVPNADGSGVGQTDVFTYTL
TDSVTGATGQANLNIVFDSIRAADNLVEVELNPQYQLVGTETDSAFYGVLLNVGNIVD
LQLLTVDTVDFTIGAGQEGVATFNFNSLIGASALGDYNVVLQKYNDVTGQWEAVNGTG
DRSLLNLTLLGNTPTAQIGGLTEGEYRAFLSFNGLVGGAVAVTLNGSVDVYNPAVITG
YDVVAAHGNLISDPNTNGDVDIATPNSIISEVNGVAVSASPTEIIGTHGTLLIYANGD
YTYTPNADSAGLGQVDQFTYTLLDPASNTTSQATFYVHLDSKVVDMNWDAADPSQPAT

VTITAVDDAVNAAIAAEPHLIEDDRALGSATYLALLSLAGINLQAPLPFVNSTVEFNV
GAGETGTATFKYSSLINEGALGDYQLVVQKFNTATNRWESITGSSEASLLNLSVLGIG
VNATPGVVVEGLDEGQYRAFMTYNGLYGKSILGTLSGTMDVYDPNQIDFTGLASEGNV
ITGLGVGTNADAVTGYTIVDSVTVNGVTTNVDPNTGTIIQGQYGTLQIFANGDYIYTP
NNTNANLGLVDHFTYTLADPLGGNISASLDFTIGNSTPVIAVDDLAVAVVNPEYLQIG
NDVAVGNTTYLALLTLTDNFDFQAGGQSVNFTLTDATLNDVTFNYSALISASLLADYV
LVVQKFDTATNQWVAVNGTGDADLLSLAAFGGNSVTLEGLAAGQYRAYMTYAGSGVGV
SLLGTLSVQKDVFDATNITGYSTQVAEGNVIHDVGLNGHADTASGFSTVTSVEFNGTA
FAVNATGVTTIVGDHGTLSIYANGNYSYQPNGEAASLGQVDQFTYTLSDGLNTSQATL
YLHIDSDAVDMTWNTSDPSQPAVITVTPVDAIDNVVSAGVDIVPQGELGVAVGSATYL
ALVGITENLNVSLLGTPSVAFTIDAGHEADVKFAYAPVLSLSLFNDYKVVLQQKGADG
AWHNIDGGSSTGLLNIGLLGNGGIGVTVPDLGQGEYRAFMVYTGLGVGILGTMSVVKD
DFDYTVAPTNTAVVADGNVLTDDVTTLTTQVTTVTSEVVGALPQTVGTDTVINGAYGT
LVISTNGHYTYTPNTTDLSAIGKVDSFTYTIRDVLTGATDTATLHVQVGSPDVTIAWD
AANPANDGVVQLTANPDHVVTTTDFSNAADLPVDVASPVVSVNLIGSNSVVSDQFVVG
AGTVANIDLSAVYTAQPLASLLPTVSYVIQSWNGTAWVNTIYSGSQTALGSVATIQAG
SVAFQDTVEHLAAGTYRVQYTLTGVSLGATSLDTNVSTTTVHLDTYRSDWISGNVLAG
DNIGGVADTGILSHEGAKLQVWDNAQNAYVDAVGQTINTGNGVLIMQSNGEYEYRPND
VTTTTQLASTDSINYKIVSVTGNVESQSTLTIDLTHTDYNLLYTSTSANDTFTTGTGS
DTVIYQVLNNVAIGNGGNTGGNGVDTWTDFHVGNVATDKQADLIDIRGLLDGAQTDAN
IGEYLNVTVSGGNTTIQIDRDGLTGLFPANNFTTLLVLQGVTTTETELLNNGQILY" /product="Glycine dehydrogenase [decarboxylating] (glycine cleavage system P protein) (EC 1.4.4.2)" /EC_number="1.4.4.2"
/transl_table=11
CDS 2812166..2813821
/locus_tag="AUO97b_02756" /db_xref="SEED:fig|400667.82.peg.2621"
/translation="MAVFLDFKRQLKLWLEHIVHHVSDLQEETILFISFGPKDHRCSV
WHSEKTVLTQATLQLFNFIDDQFSPDQLPDYIKIDVAYNLEKQSWNQIEQQVHHQFHN
NHYRRGIGFDDSCSVAFLEQEIYGKAIIRGLSYDKPNFFDETNLNYAIKQKYRATKPE
IKLKSLQEVWTFDTYATFYENGQFINLASRYDANGIRAIASNKKQHFRDLIEKNAAFL
HSQIQENGKFIYGYFPAYDRDIRNYNTVRHCTSLYALLETFEVQDKPEYWPKIVAAIQ
YALTTFYKEKDPITAFMIDGKEGELEIKLGANAAAILMLTKYQEITGKDDYLKYAEKL
AHAILELVDPDGLTTHVLNYPNYDLKEKFRIIYYDGEAALALLRLYQINQDKRLLDTV
KLMFENFIHKRYEKYHDHWLSYCTNELTKICPEEKYFIFGLNNYLKHFIFIRNRKTTY
ATLLEMLMAAYKMVNRLKEQGHNALFEQAYMSELKKLIIFRAEFQTTGFFYPEIAMYM
ARPDKILHAFYIRHDRFRVRIDDQEHNLSGYIAYVKDFEGGEI" /product="poly (glycerol-phosphate) alpha-glucosyltransferase" /transl_table=11
CDS 2813818..2815542 /locus_tag="AUO97b_02757" /db_xref="SEED:fig|400667.82.peg.2622"
/translation="MTYPLFEIKLLTALDHAQFEEAIWAFEIDGDISTLLLIDYALEQ
FHQKKVQADEMYRVPEQKIKKIGEQNLGLEKNESYTFAELLQFLIFTQANDVKDALSN
MLLGSVEQTQLILSKRAEYYQLALRAPNQLKNLFLLVKHIYSYPAELKKLFFIRTVSF
KNKVYQPITPLLAHPVLTSVLYISHDFRQIYITYSEYNRSIGFFSFLDGIHRLEHLVP
YYHYFQEGHVEAKKYSSQTGIINILGDTYFGEMYTEKRKSRGQTDALQQYGYHYSFEK
IQPFLGKNDINIANFEAVFSLENQSPLKDKKPFVLKADAKKTLEEFKSIHLNYLVLAN
NHLKDYGEQGLAYTLQQLDQASISYIGAGLNQKDAHNYFEITFETKHYAIFNGYWHRD
MAYLDYDFYALGSRSGVACLNGVLLEQIMRYKQAHPERKIIVICHWGVDFKPITKDQT
KLATILTQAGADLIVGHGAHTIQPIQIINQKPVVFNIGNAVFNSDGEYEQQNALPFGC
IARLDLAKDLLRLYPIYTNNLQTFWQPYPVDAEDFSKASTYMTSLLTPENYMASQDNL GRYLEVKF"
/product="CapA protein"
/transl_table=11 CDS complement(2815539..2816255)
/locus_tag="AUO97b_02758" /db_xref="SEED:fig|400667.82.peg.2623"
/translation="MKKILLLISGTLLCVSCTTRPPLLSERGPSISADGRPIVPVTFV
FTTNTPQATKFDNYQQMRKEIKILNKYYVDDKNNKIFKFKLHRYIPYEEFSKLHCDLK

QQINQPYPISTETIPASVNTCFPKRTASKEVIVFIYDAYSTKWKFEDVTSRAFRNNGK
PFILLDWNRLNYNIQAGSVHEMGHVFGLKHVCAPKATKRTPTNIMTSAECKLGSGGLR
NLGFTPVQLQTILSTYNQYP"
/product="hypothetical protein"
/transl_table=11
CDS 2816349..2816666
/locus_tag="AUO97b_02759" /db_xref="SEED:fig|400667.82.peg.2624"
/translation="MTTQYELHRQILEVIALIPYGKVATYGQVARMAGLPKHARLVGY
VLKHLDADHQVPWHRVINSQGKISLSKFNEKGKNIQQLKLETEGIYLLNGKVNLKQFA WQP"
/product="DNA base-flipping protein" /transl_table=11
CDS complement(2816722..2817159)
/locus_tag="AUO97b_02760" /db_xref="SEED:fig|400667.82.peg.2625"
/translation="MLYQHILVPIDGSETSMVAMKEAIKIGKALNSKITVVQVMALDP
IIADLYVKTGQTNELIERTRTYLLDILEQAKQQFLQEGLSVETKLLEGFVVHEEIIQA
AQELNADLIVMGSHGRTGVRKLVLGSVAQKVLGESHIPVLIVR" /product="Universal stress
protein"
/transl_table=11
CDS complement(2817281..2817913)
/locus_tag="AUO97b_02761" /db_xref="SEED:fig|400667.82.peg.2626"
/db_xref="GO:0008811"
/translation="MAEKLIGSPVRHWCEFEFISKTVKNPNIHIKGNYSYYSAYWDQG
FERCVVRYLHDKPSTPDKPIDQLYIGNFVCFGAECVIMMGGNQLHRPDWISTFPFDTR
SFLAAGDTIIADGCWIGSRAMIMQGVKIGEGAVVATGAVVTKDVPPYAIVGGVPAKII
KYRFPQEQIEKLLALKIYDLDEKKVLKMREYLQTDDIDALTAYLKKLQSE" /product="Xenobiotic
acyltransferase XAT family" /EC_number="2.3.1.28"
/transl_table=11
CDS complement(2818026..2818418)
/locus_tag="AUO97b_02762" /db_xref="SEED:fig|400667.82.peg.2627"
/translation="MRTAIFPQDRHALYEQHGYSAAIQSKDLLFVSGQVGSLEDGSPV
PDFEKQVIQAFKNLAETLKAAGCTFDDIIDVTTFHTDPERQFESIMKVKKQIFTQKPY
PNWTAVGVTWLAGFDFEIKVIARIPHNN"
/product="RidA/YER057c/UK114 superfamily, group 7, YjgH-like protein"
/transl_table=11
CDS 2818558..2819145
/locus_tag="AUO97b_02763" /db_xref="SEED:fig|400667.82.peg.2628"
/translation="MSVREQKMAETRKKLIEVARRAFAEYGYADTSMDKLTAEAGLTR
GALYHHFGDKRGLFAAVVDQIDSQMAEYAQQHREQPDDLWEGLLLEGQTYIQNALNPE
FQRIVLRDGPAVLGDPAHWPSQNRCLQSTRECVEQLLAAERIKIVDPEAAAVLLNGAA
MNAALWVASSEYPEQVLPEALNAFQIFASGFLKDA" /product="Transcriptional regulator,
AcrR family" /transl_table=11
CDS complement(2819226..2819702)
/locus_tag="AUO97b_02764" /db_xref="SEED:fig|400667.82.peg.2629"
/db_xref="GO:0003677"
/db_xref="GO:0003711"
/translation="MQRYPMTPEGKIALEKELQHLKTVERPRITQAIAEAREHGDLKE
NAEYHAAREQQGFCEGRIQDIEGKLGAAQVIDVKDLEQNGRVVFGVTVTIENLDTEER
KTYKIVGDDEADFKINKISVNSPIARGLLGKNEGDEVKITTPQGEVEYEVVSVEYI"
/product="Transcription elongation factor GreA" /transl_table=11
CDS complement(2819790..2823020)
/locus_tag="AUO97b_02765" /db_xref="SEED:fig|400667.82.peg.2630"
/db_xref="GO:0004088"
/translation="MPKRTDIKSILIIGAGPIVIGQACEFDYSGAQACKALREEGYRV
ILVNSNPATIMTDPSMADATYIEPITWQTVAQIIEKERPDAVLPTMGGQTALNCALAL
DENGVLEKYGVELIGATKEAIEKAEDRKLFDEAMRKIGLECPKAAIAETMEEALEIQS

RFGFPVIIRPSFTMGGSGGGIAYNREEFLEICERGFDLSPTHQLLIDESLIGWKEYEM
EVVRDKNDNCIIVCAIENFDPMGVHTGDSITVAPAQTLTDKEYQLMRNASVAVLREIG
VETGGSNVQFGINPKDGRMVVIEMNPRVSRSSALASKATGFPIAKIAAKLAVGYTLDE
LKNDITGGVTPASFEPSIDYVVTKIPRFNFEKFPQAEPVLTTQMKSVGEVMAIGRNFQ
ESVQKALRGLEVGVSGFDEKIEAGATNVKETILKELKVPGPERIWYVADAFRHGFSLD
DVFEATKIDRWFLIQIQDIVNTEAQVKTLGFGDLTAENIRSFKRKGLSDLRIANLMGI
SQKQFRKQRWNLGVYPVYKRVDTCAAEFESGTAYMYSTYDEECEANPSNRDKIMVIGG
GPNRIGQGIEFDYCCVHAALAMREDGYETIMVNCNPETVSTDYDTSDRLYFEPVTLED
VLEIVRTEKPKGVIVQYGGQTPLKLARALEEAGTPIIGTSPDAIDRAEDRERFQQMIQ
RLQLRQPNNSIVKSAEEGISEAAKVGYPLVVRPSYVLGGRAMEIVYNEDELKRYLREA
VQASNEAPVLLDHFLDDATEVDVDCVSDGKDVVIGGIMQHIEQAGIHSGDSACSIPPY
SLSKEIQDEMRRQTIAMAKELGVVGLMNVQFAVKGNDIYVLEVNPRASRTVPFVSKCI
GESLAKVAARCMAGQSLESQGFTKEIIPNRFAVKEAVFPFAKFPGVDPMLGPEMKSTG
EVMGVGQTFGEAFYKAVLGSNDRLPGLPTEGEVKHAFLSVRESDKKYIADIAKKLVEY
GFKLVATGGTYQVLKEAGLECESVNKVTEGRPHIVDRLKNGEIHLIVNTTEGKQAQYD
SAMIRRAALQGKVYYTTTINGAEAVCQAFAVKLPMDVYRLQDLTVG" /product="Carbamoyl-phosphate synthase large chain (EC 6.3.5.5)" /EC_number="6.3.5.5"
/transl_table=11
CDS complement(2823035..2824174)
/locus_tag="AUO97b_02766" /db_xref="SEED:fig|400667.82.peg.2631"
/db_xref="GO:0004088"
/translation="MSTPAILALADGTIFKGTSIGATGSTTGEVVFNTAMTGYQEILT
DPSYAQQIVTLTYPHIGNTGCNEEDVESGRIHKVWANGLIIRDLPLLHSNFRSEQSLS
EYLQAHNVVAIADIDTRKLTRILRDKGAQNGCILAGENITEEEAIAKARAFGGLNGLD
LAKECCDPTGFEWSEGSWVLGKGFTQPELKYHVVAYDYGVKTNILRMLADRGCKLTVV
PAETPAEKVLALNPDGVFLSNGPGDPAACDYAIEAVKTIVETTTLPVFGICLGHQILA
LASGAKTVKMNHGHHGANHPVQNLEDGTVMITSQNHGFAVDAETLPANLKATHKSLFD
GTLQGIHRTDKPAFSFQGHPEASPGPHDCAPLFDHFIELIEASKQ" /product="Carbamoyl-phosphate synthase small chain (EC 6.3.5.5)" /EC_number="6.3.5.5"
/transl_table=11
CDS 2824487..2825074
/locus_tag="AUO97b_02767" /db_xref="SEED:fig|400667.82.peg.2632"
/translation="MSDNQVSKDIAENTSSVAETVKTEVAEAKEAVAKTTTRAKKAVS
TKATQVKEVVAEKTEQVKEVAHEATSQVKEAIAETQEQVKAVINETEEKIEAETQELN
QNIHNQFAQIKQDILQRLDVIKAQFNTSQEGLNELKNTLKAEINALIEDLTKVTKELK
GDISQISLKHKDSLTESLKRTKNNAVEAWNKARDH" /product="hypothetical protein"
/transl_table=11
CDS complement(2825133..2825669)
/locus_tag="AUO97b_02768" /db_xref="SEED:fig|400667.82.peg.2633"
/translation="MASYELSAFDRFHAVSVVGAIEQQAPYTLNVGFWIRDPNQLIIW
PQEVAAHPRQDFLWEETCFEIFIGVHDEDFYREINLSPSQAWQTYQFEEYRYPESMPP
LVAYDIELNHLKRTHYGLNVSLDLSQFMQQHRLKWANLYLGLTAVLKTKDGMQHYAMQ
HSGKHADFHNKRDWLHEF"
/product="hypothetical protein"
/transl_table=11
CDS complement(2825684..2826007)
/locus_tag="AUO97b_02769" /db_xref="SEED:fig|400667.82.peg.2634"
/translation="MAALSIHERKRLRQIGHVLNPVVMIGGQGLTDAVIEETLRALND
HELIKVKIAGEDREARAAVIDAIVEATGAEAVQKIGKIVLLYKKAAKQNQHLSNLVRH AHLAN"
/product="RNA-binding protein YhbY"
/transl_table=11
CDS 2826207..2826857
/locus_tag="AUO97b_02770" /db_xref="SEED:fig|400667.82.peg.2635"
/translation="MATRITNQKLSKSSRAWMREHLDDPFVKKAQKEGYRARAAYKLL

EIQEKYKLIKPGMTVVDLGAAPGSWSQIAGKLVGSKGLVIASDILPMDALPDVTFLQG
DFREEAVFEKLLNILNGRQVDIVISDMAPNTSGNRAVDQPRQIYLCELALDFAQKVLG
PNGQFVVKVFQGAGFDEFRKQVVDSFDVLKTAKPAASRARSKEVFLVGQGRKKALQ" /product="23S rRNA (uridine(2552)-2'-O)-methyltransferase (EC 2.1.1.166)" /transl_table=11
CDS 2827091..2828887
/locus_tag="AUO97b_02771" /db_xref="SEED:fig|400667.82.peg.2636"
/translation="MKYSDFVAAVNAGQIKQVTIDGLNISGEKTNGSQFETVRPQVED
TELMPSLNKQNVVVEGTAPQRQGILMQLLIASFPVLLIILLFMFFMRNMGGGAGGKNG
PMSFGKSKAKMLSEDQIKVTFADVAGCDEAKQEVVEIVDFLKDPAKFKRLGATIPRGV
LMVGPPGTGKTLLAKAIAGEAKVPFFSISGSDFVEMFVGVGASRVRDMFEQAKRHAPC
IIFIDEIDAVGRHRGSGTGGGHDEREQTLNQMLVEMDGFEGNEGVIVIAATNRVDVLD
KALLRPGRFDRQVMVGLPDIRGREQILNVHLKKLPSVTGVDVKVLSRGTPGFSGAQLA
NLVNEAALFAARRNKNTVDMHDFEDAKDKIYMGPERKSMVLREEERRATAYHEAGHAI
VAEILPGTDPVHKVTIMPRGWALGVTWQLPEQDQISHYKDKMLNEIAILFGGRIAEEV
FIQQQSTGASNDFERATKMARAMVTKYGMSDKMGVMVYEDENQNGFFGNVGSRTISEA
TQQQVDQEVRRILDEQYKVARDILENNKDIAHAMVKALMEWETIDRDQIRDIMEGREP
QPPKVYIAENPVSAFEPPKDGPSTPPPLPAMN"
/product="Cell division-associated, ATP-dependent zinc metalloprotease FtsH"
/transl_table=11 CDS 2829019..2829870
/locus_tag="AUO97b_02772" /db_xref="SEED:fig|400667.82.peg.2637"
/db_xref="GO:0004156"
/translation="MQLISLPKQILQCRQLQLDLSQPHVMGILNVTPDSFSDGGKHNQ
LDQAVDHALSMIEQGATIIDIGGESTRPGASEVAVEEEVRRVVPVVEALSHHNVILSI
DTSQPEVIRAAKAAGAHIWNDVRALTRPNALKTAVELDIPVVIMHMRGEPTTMNQLDQ
YTDVTLDVMQELQQRIDEALAAGVKKHNIIVDPGFGFAKNAQQNLKLLKEFWKLNELG
YPILSGLSRKRFIGEALQGAPADQRAVGSVTGHLLSIQQGASIVRAHDVKEMHEAILV WKAMQQA"
/product="Dihydropteroate synthase (EC 2.5.1.15)" /EC_number="2.5.1.15"
/transl_table=11
CDS 2830005..2830319
/locus_tag="AUO97b_02773" /db_xref="SEED:fig|400667.82.peg.2638"
/translation="MFEDLLLPMFDDEYYPDILVAELKQLIEQFAKKVQKPALAEQDI
YRYAHQTVNEINEMKPQFEDLDSSLDDSAADYIAEAMMMVVQDAGYLDLEMEELVMNR EW"
/product="hypothetical protein"
/transl_table=11
CDS complement(2830320..2830685)
/locus_tag="AUO97b_02774" /db_xref="SEED:fig|400667.82.peg.2639"
/translation="MPDENNAMIRILIFISALLFFIRFANAEDIFNGTILFKNNDWHF
VRCSITQDDYLIEAPQEIFTQFKELQQQQKNYWVSVLAEVNEQQNGNLILKIEKIDEV
HLDETCHLLEALKNFENRE"
/product="FIG00350883: hypothetical protein" /transl_table=11
CDS 2831022..2832785
/locus_tag="AUO97b_02775" /db_xref="SEED:fig|400667.82.peg.2640"
/translation="MSEKISRRELIQKSLLGFGALSIPVAFTGCNDGSDDESPEIQAD
FLHGVASGDPLQDKVILWTRVTPRVSGVRLKVVWEISTDNQFKQNLKTGMVETSKTDD
FTVKVDAAGLQADTVYYYRFKFGNKVSPVGQTKTLPTSTNKVSFAVCSCSNYPAGYFY
VYREMAKQNVDVIIHLGDYIYEYGADGYATEDAIKLGRNLPADNNKEIIKLDDYRKRY
ALYRQDKDLKAAHQRHPFIVIWDDHELANDAWREGAENHQSNEGAFSDRKLAALQAYF
EWMPIRPVSSTDHLNIYRQFNFGSLVQLTMLDTRIIARDKQLAYADYMTTTGLDIAKF
QADLTNPVRTLMGYMQRDWLVDKLKQSTATWNVVGQQVLMSKMWIPAELLASLGQITS
GGTSPDALAKMNAQITELVALKLRLQQNDPTLTAQEKARIMTVAPYNLDAWDGYYAER
EFVYDKLAEFNKKIIVLAGDTHNAWASYLYSQKGKYVGVELATSSVSSPGLEKYLSIP
LAQLQQFEFAFTTLIDELVYCNLNQRGYLLVTLDQAQVHSEWRFVDSIKNAEYQIDSS
RQKDIVLDLNLMPLKQGQKTA" /product="Phosphodiesterase/alkaline phosphatase D"
/transl_table=11

CDS 2833209..2834057
/locus_tag="AUO97b_02776" /db_xref="SEED:fig|400667.82.peg.2641"
/db_xref="GO:0004477" /db_xref="GO:0004488"
/translation="MALVLDGRALAKQIEENLLVRVEALKAKTGRTPILATILVGDDG
ASATYVRMKGNACRRVGMDSLKIELPQETTTEQLLAEIEKLNANPDVHGILLQHPVPA
QIDERACFDAISLAKDVDGVTCLGFGRMAMGEAAYGSATPAGIMTILKENNIEIAGKH
AVVVGRSAILGKPMAMMLLQANATVTICHSRTQNLPELVKQADIIVGAVGKAELIQKD
WIKQGAVVVDAGFHPRDGGGVGDIQLQGIEEIASAYTPVPGGVGPMTITTLIRQTVEA AEKALG"
/product="Methenyltetrahydrofolate cyclohydrolase (EC 3.5.4.9) /
Methylenetetrahydrofolate dehydrogenase (NADP+) (EC 1.5.1.5)"
/EC_number="1.5.1.5"
/EC_number="3.5.4.9"
/transl_table=11
CDS 2834298..2834966
/locus_tag="AUO97b_02777" /db_xref="SEED:fig|400667.82.peg.2642"
/translation="MSCTFQLTKAPEHLLQALHEVIPHCELMAQQLPETPISLWLIPP
VFPTDRLDDEVIRRIWNDTPYWIFCWASGLAMAQWILAEPHHVKDKVVLDFGAGSGVV
AIAAKMAGAKRVICCDIDQVSLNACRENALLNDVELEYLDDLYKAEQVDVLLAADVLY
DQYNRFFLDEFLKFAPEVWVADSRVKNFSHPKYEKIEERSATTWPDLDEAKEFRNVSF YKTL"
/product="hypothetical protein"
/transl_table=11
CDS complement(2834989..2835411)
/locus_tag="AUO97b_02778" /db_xref="SEED:fig|400667.82.peg.2643"
/translation="MNKSILCLALSSMFILTACQTTPRQYNGSTGYQIENQTKTSATL
AYTLAGRSNQQLDERKLQRACQNVLGAQKIYKLSILSINEIPNPAKDEHYGIQLGETR
ASFGLSNTPSLNNGEDYATRQALEARPSTLKVVRYTCS" /product="hypothetical protein"
/transl_table=11
CDS complement(2835528..2836847)
/locus_tag="AUO97b_02779" /db_xref="SEED:fig|400667.82.peg.2644"
/translation="MVDQPSTATTPHSNLDTKTRLKSILGGSAGNLVEWYDWYVYAAF
TLYFAHAFFPKGSQTAQLLQAAAIFAVGFLMRPIGAWIMGIYSDRKGRKAGLTLSVTL
MCIGSLLIAVTPSYESIGVFAPLLLVIARLIQGLSVGGEYGASATYLSEMAEKDRRGF
FSSFQYVTLIAGQLTALCVLLILQMILTEEQLHDWGWRVPFFIGALLAIVVFRIRRGL
LETQSFKNAQAETDQPKSGMFALFKHYPKEAFTVLFLTAGGTLAFYTYTTYLQKYLVN
TSGFTKPEATQITTLALFIFMCLQPLAGALSDRIGRKPLMIAFGVTGVLFTYILFDTL
ANTHNYWTAFWLCLGGLVMVTGYTSINAVVKAELFPAHIRALGVALPYAIANTLFGGT
AEFFALSFKEAGHESWFFIYVSIMIFISLLIYIFMKDTKHHSKIKEH" /product="Dicarboxylate MFS
transporter"
/transl_table=11
CDS 2837234..2838742
/locus_tag="AUO97b_02780" /db_xref="SEED:fig|400667.82.peg.2645"
/db_xref="GO:0004370"
/translation="MSNCPKKYIVAFDQGTTSSRAIVLDHDANVVSIAQREFTQIYPQ
PGWVEHDPMEIWATQSAVWVEALAQAGIKSEQVAAIGITNQRETTIVWDKKTGRPIYN
AIVWQSRQTTEICNQLYKAGWQEYIRKTTGLVIDPYFSATKIKWILDHVEGSRERAER
GELLFGTVDTWLIWKLTNGAVHVTDFTNASRTMLFDIEKLEWDEKLLQALDIPRAMLP
EVRSSSEVYGYTHTISGQEVGIPIAGIAGDQQAALFGQMCVESGQAKNTYGTGCFLLM
NTGKKIVRSEHGLLTTIACGASGEVNYALEGAVFNGGSCVQWLRDELKVIKNAKDSEL
YATRVKDNNGVYVVPAFTGLGAPYWDPTARGAIFGLTRGASIEHIIRATLESIAFQTR
DVLDAMQQDAEEELRTLRVDGGVTENNFLMQFQADILTTPVERPIMKETTALGAAFLA
GLATGFWQDLHELRNKSAIEKVFEPKMPYEQAELIYKGWLKAVKRSQNWAED" /product="Glycerol
kinase (EC 2.7.1.30)" /EC_number="2.7.1.30"
/transl_table=11
CDS 2838897..2840414

/locus_tag="AUO97b_02781" /db_xref="SEED:fig|400667.82.peg.2646"
/translation="MKVQPNDYSKIYDLAVIGGGINGVGIANDAVGRGLSVFLCEKDD
LASHTSSASSKLIHGGLRYLEYKEFRLVREALAEREVLLAKAPHIIKPMRFIMPHRPH
LRPAWLIRAGLFFYDHLGKREKLLGSNLIYFKEDSPLKPAITRGFEYSDCTVDDARLV
VLNALQAKEKGAKVVTRTRCVKAYRQQELWHLELQSGAEFYQIRARALVNAAGPWVEE
IISKNISLSSPYQIRLIQGSHIVVPKLYDCHKAFIMQNEDRRIVFAIPYLEKYTLIGT
TDQEYTGDPQKVEITDVEIDYLLTVTNSHFKKQLKRADIVSQYSGVRALCDDESDNPS
AITRDYTLALQAEDKTTPLLSVFGGKITTYRKLAEAALEHLAPFFNDMAEEWTADEPL
PGAENWTTLEGLINQIKTRISGISDSLANRWAHAYGTRVWNMLKERNAIEQLGQHFGH
DLFECEVRYLCEYEWAHTAEDILWRRSKLGLAFDEKQVKVLEAYLSERRLKDDAA" /product="Aerobic glycerol-3-phosphate dehydrogenase (EC 1.1.5.3)" /EC_number="1.1.5.3"
/transl_table=11
CDS complement(2840456..2842288)
/locus_tag="AUO97b_02782" /db_xref="SEED:fig|400667.82.peg.2647"
/db_xref="GO:0004613"
/translation="MTTVNAPEFVRHPKLIAWVEEIANLTKPAKIEWCDGSEEEYQRL
IDLMIANGTMQKLNQEKHPGSYLANSDPSDVARVEDRTYICSQNKEDAGATNNWEDPA
VMREKLNGLFEGSMKGRTMYVVPFSMGPLGSHIAHIGIELTDSPYVAVSMRKMARMGK
AVYDVLGTDGEFVPCVHTVGTPLAEGQKDVAWPCNPEKYIVHYPETREIWSFGSGYGG
NALLGKKCLALRIASVMGREQGWLAEHMLILGVTNPQGEKHYIAAAFPSACGKTNFAM
LIPPAGYEGWKIETVGDDIAWIKPGEDGRLYAINPEAGFFGVAPGTNTKTNPNCMATL
HKDVIYTNVAVTDDGQVWWEGLSKEVPANLTNWKGQPHVNGEKAAHPNARFTVAAGQC
PSIDADWENPAGVPISAFIFGGRRADTVPLVSEAFDWVDGVYKAATMGSETTAAAVGQ
QGIVRRDPFAMLPFAGYNMADYFDHWLNLGAKVSEKAEASGNKLPKIFNVNWFRRDAE
GNFVWPGFGQNMRVLEWIIDRCEGRANAVETPIGFVPTYEDLNWEGTEFTKEQFDLIT
NQDKDQWVTEIESHTELFNKLGERLPKALKERQAALLEAVKTGF" /product="Phosphoenolpyruvate carboxykinase [GTP] (EC 4.1.1.32)" /EC_number="4.1.1.32" /transl_table=11
CDS 2842689..2843618
/locus_tag="AUO97b_02783" /db_xref="SEED:fig|400667.82.peg.2648"
/db_xref="GO:0004143"
/db_xref="GO:0007205"
/translation="MKPLSIIYNEKSGFHASKHEDVYEQLMTVFTEYGFEIQVFELNE
NTLFDDLINNVIHRHSQNENTGVVVAAGGDGTLNAVATKLKNTSIPMGILPLGTFNYV
AKVLEIPLDLLEAAEVIATGKPRSVHVAAINDHIYLNNASLGLYPLFIKKRELYNKYL
GRLPLHAYTSALDVLLRENKSMKLSITVDGKKYPVKAPLIFFGNNQLQLCDMKLRIAE
CAAQGRVAGVVITKSDKLSLLNMLWQWVQGKVEDAQDVYSFCADHVIVECAKKTKLTV
ALDGEIIEMKPPLNFTVEKNALNIMVPNVTTSV"
/product="Transcription regulator [contains diacylglycerol kinase catalytic domain]" /transl_table=11
CDS 2843599..2844357
/locus_tag="AUO97b_02784" /db_xref="SEED:fig|400667.82.peg.2649"
/translation="MLLHLSDLHFGAEKKECLEAIRDFCIQQQPEVIVVSGDITQRAK
YEQFFKCRQYLDSLNIPYLVVPGNHDIPLYHVWNRFFSPFTRYRYFFGELEPTLETEN
FYIVGVNSIRRRYHTRGHISIEQIQATYERLDRGPKNKIKLVVFHQPFYTPPDNKHGI
KDCPVLGKIALEKWSTTGLFGMLHGHLHKTAIYDLTQIYSLKIDHPIFDIHAGTATST
RLYHHNPNSFNTISNEGKIQHYWFNEQLAEFTSY"
/product="Probable DNA repair exonuclease"
/transl_table=11
CDS 2844543..2844833
/locus_tag="AUO97b_02785" /db_xref="SEED:fig|400667.82.peg.2650"
/db_xref="GO:0005524"
/db_xref="GO:0006457"
/translation="MSNIRPLHDRVVIRRVEEETKTAGGILLPGSAAEKPSQGEVIAV
GNGQITDNGVRALDVKVGDKVLFGTYAGTTVKVNGEELLIMKESDILAVLEG" /product="Heat shock

protein 10 kDa family chaperone GroES" /transl_table=11
CDS 2844892..2846526 /locus_tag="AUO97b_02786" /db_xref="SEED:fig|400667.82.peg.2651" /db_xref="GO:0005515"
/db_xref="GO:0005524"
/db_xref="GO:0044267"
/translation="MSAKDVKFGDSARSKMIAGVNVLADAVKVTLGPKGRNVVIDRSF
GAPHITKDGVTVAKEISLKDKFENMGAQLVREVSSKTNDIAGDGTTTATVLAQAILNE
GIKSVTAGMNPMDLKRGIDIAVKTVVENIRSIAKPADDFKAIEQVGSISANSDTTVGK
LIAQAMEKVGKEGVITVEEGSGFEDALDVVEGMQFDRGYISPYFANKQDTLTAELENP
FILLVDKKISNIRELISVLEAVAKTGKPLLIIAEDVEGEALATLVVNNMRGIIKVCAV
KAPGFGDRRKAMLQDIAILTGATVISEEVGMSLEQATLQDLGTAHKITVSKENTVIVD
GAGDAAAIAERVQQIRAQIEESTSEYDREKLQERVAKLAGGVAVIKIGAATEVEMKEK
KDRVDDALHATRAAVEEGVVAGGGVALVRAVNALEGLKGANEDQTAGINILRRAIEAP
LRQIVANAGDEPSVVINAVKNGEGNFGYNAATGEYGDMLEMGILDPAKVTRSALEHAA
SVAGLMLTTECMITDIPEDKPAAPDMGGMGGMGGMM" /product="Heat shock protein 60 kDa family chaperone GroEL" /transl_table=11
CDS complement(2846701..2847075)
/locus_tag="AUO97b_02787" /db_xref="SEED:fig|400667.82.peg.2652"
/db_xref="GO:0004143"
/translation="MDSYSPYKGKSGLKRILNATSYSISGFKAAYQNEAAFRQIVLIN
VVLIPVSFFLDVTRGEHALMIIVCLFAIIVELFNSAIEAVVDRVSLEKHQLSKNAKDM
GSAAQFVALSIIVATWLIILFG"
/product="Diacylglycerol kinase (EC 2.7.1.107)" /EC_number="2.7.1.107"
/transl_table=11
CDS 2847215..2848144
/locus_tag="AUO97b_02788" /db_xref="SEED:fig|400667.82.peg.2653"
/db_xref="GO:0050129"
/translation="MGSLHISYAEPASAQQVPGYYHHQFGNYRITSLLDGTIYLDPKL
FKNLSQAEKTKILTKYAAVNEKGIQTSVNAFLVDDGKSLTLVDSGAASCFGPQLGSIA
KNLELAGYQLANVKTVLLTHLHPDHVCGIAQNGKAVFPNATIYAHEREADYWLNPANE
KTVPADKKENYLGTVKNVKAALAPYQAKKAFKTFKDGDVIQGFEVINTQGHTPGHHSF
RLKSKGQQIVFVGDIVHSHSLQFDAPKTGVDFDVNSEQAINTRLKMFAEISNKQQWVA
APHLPFPGIGHVYKVNAEQYQWIPLYFNNSLDK"
/product="MBL-fold metallo-hydrolase superfamily" /EC_number="3.5.1.68"
/transl_table=11
CDS complement(2848188..2849330)
/locus_tag="AUO97b_02789" /db_xref="SEED:fig|400667.82.peg.2654"
/db_xref="GO:0008889"
/translation="MFRKALLCLSLISLVGCNDDDKTETTPTTPEYQLPKILVVGHRG
ASALRPEHTLASYQKAIDDGADFIEPDLVSTKDGVLVARHENEIGGTTNVSTLSQFAD
RKKTKNIDGVDLTGWFTEDFTLSELQQLKARERIPEFRPANTAYNDLYPVPTLEQIVE
LAEANYKKTGKIIGLYIETKHPTYFKNQNLAMEDTLLKTLAKYKYTRDIAPVYLQSFE
VSNLKYLKNELDLHKSLKHAQIIQLYDSKTSRPADFVESGDTKTYADLATAQGLKDVA
KYANGVGPSKGYILTFNNDGSYKTSTFIADAHTAGLKVHPYTFRPENNFLPAPLKCSP
DKPAERCPSGALKEFEAYFKAGVDGVFTDDPALGREAVTNFEKAAK" /product="Glycerophosphoryl diester phosphodiesterase (EC 3.1.4.46) periplasmic (secreted in GramPositives)" /EC_number="3.1.4.46"
/transl_table=11
CDS 2849594..2852689
/locus_tag="AUO97b_02790" /db_xref="SEED:fig|400667.82.peg.2655"
/translation="MLSKFFIQRPIFANVLAIIVMAFGIFSVMNLPVERYPDIAPPKI
TVSANYSGADAQTVEQSVTQILEQQIQGIDHLLYFSSSSDSSGRSRITISFDNGTNPD
TAQVQVQNSISGVIRRLPDEVQRQGVTVSKSLGDTFMVIGLYDSTGKTGNIELSDYLT
THVVDNLNRIEGVGETDVFGSQYAMRIWLNPDKLKQYNLMPSDVANAITAQNTQVAAG

AIGDLPVIDGQYLNTKVTAGSRLKTVEDFKNIVVKSNKTASYVYLKDIARVELGAENY
QSFNTINGYPAAGLGISLSSGANAIQTSKLIHQTLDQLTTKLPAGYKIVYPRDNTPFV
QESIKEVVKTLVEAIILVILVMFLFLQSWRATLIPSITVPVVILGTFAVLYVLGFSIN
TLTLFALVLAIGLLVDDAIVVVENVERLMHEQHLSPKEAAIESMGEISGALVGITLVL
TAVFIPMSFLGGSIGVIYRQFSITLVAAMALSLIVALILTPALCALILKPNPQPQRWA
VWFNQKIEQLKNQYIKLVQTSIHYSKSVIVIFVALIAVFTLFYNGLKSGFIPKEDQGI
LSVQIKLVDSAPIYQSQKIGEQVRQYFLTQEDKNVDLVLIRYGRNYSGTGQNLAQGFI
ALKPWDVRTGKENSAEAIQKRAMKYFSHFNNAQINVTLPASVNGLGQTDGLDLWIQDL
NGQGQDFLDSAFRQLQAQSKNYSSFENFDKQSTNSKANLNIKIDQKQALANGLQLSAI
NNTLSSAWGGTYVNDFIDRGRIKRVMIQGDAEFRSKPEDLYNWSVRNDQNEMVPFSSF
ANFSWGGAPEIVKRYMGYSALQLQADVASDSSSGQAMKDVEQLVNQQKDIGLAWTGLS
FEEQKSTNQAVWLYLISAGFIFLCLAALYESLSIPAAVMTSIPLGVGGSVIFSYIFGL
PNDVYFQIALLTTIGLSCKNAILIVEFAALAQEKGKNAIQAALEGASLRLRPILMTSL
AFGAGVIPLVFAQGAGAVSRQEIGISILGGVMFGTVLVLFFIPVMYVLLRSLFKSKAS T"
/product="RND efflux system, inner membrane transporter" /transl_table=11
CDS 2852762..2853337
/locus_tag="AUO97b_02791" /db_xref="SEED:fig|400667.82.peg.2656"
/translation="MPQEKKLTQQQVIALERDLAKFATMMDSAVRIPFTKQGIGADAA
LSTIPIAGDVAGFALTCYAIYKAKQIGVPQHKLTPVIKLAVVDAVVGFVPVAGTIFDI
FIRPSRKALDVVHTHIREEYAIQSDIHVVHPYLHEKLEKKQQQSAFWRNPVVSWLWLH
IPDLLGAFVLLLIGLAVWWGASYLWGLYQTM"
/product="hypothetical protein"
/transl_table=11
CDS complement(2853388..2854293)
/locus_tag="AUO97b_02792" /db_xref="SEED:fig|400667.82.peg.2657"
/translation="MMRIGLFLLTNLAVLVVAGIILSLFGVGSYHGAGGLNLGNLLVI
CFVFGMVGSLVSLFMSKWMAKKTTGTELIDPNAPRNQAESWLLQTVAELSQRAGINMP
EVGIFPSYQSNAFATGWNKNDALVAVSSGLLERMNKDELRAVLAHEIGHVANGDMVTL
ALIQGVVNAFVMFFARVVGDFIDRNVFGRQDNEAPGMGYFIITMVLDIVFGILASAIV
MWFSRYREYRADEAGARLAGKQAMISALLRLQAETELPDQMPKEMKAFAIAEGKEQGF
SLAALFQTHPTIEQRVAALHQLDCP"
/product="Protease HtpX"
/transl_table=11
CDS complement(2854551..2855327)
/locus_tag="AUO97b_02793" /db_xref="SEED:fig|400667.82.peg.2658"
/translation="MYIYQDKNGSTLLTNRKSYDHSLKKVKVTYYPDSNIHSYSNWGT
SEASVLPSYSKNKNAFDHIIKQAAQQHGVSEGLIKAVMHTESGFNVNARSPVGAQGLM
QLMPATARRFNVSNAYDPQQNIFAGAKYLSWLLKRFNGNTQMALAAYNAGEGNVDKYG
GIPPFRETQDYVRRVTSRYQNLYSSGVGLSSFSNSSISAQAINQPAIPHSTSTQVSAQ
PIKYSSSRQIVTLPDGTYTDAPTGTYVTNNATAIAHIRIE" /product="Soluble lytic murein
transglycosylase and related regulatory proteins (some contain LysM/invasin
domains)" /transl_table=11
CDS 2855918..2856823
/locus_tag="AUO97b_02794" /db_xref="SEED:fig|400667.82.peg.2659"
/translation="MYAPVESNEGFNFKPELPTSSAYYRLLKKLRRQVGHAIRDFNMI
EDGDKVMVCVSGGKDSYTLLDILLQFKRIAPINFDIVAVNLDQKQPGFPEDVLPRYME
ENNIPYYILEKDTYSITKRLTPEGKTYCAVCSRLRRGSLYGFAQEIGATKVALGHHRD
DIIATFFLNLFHGGSLKAMPPKLLSSDKKNILIRPLAYVEEKDIIKYAELRKFPIIPC
NLCGSQENLQRAMINEMLREWDRQYPKRLHSIFGALQNVSPSQLADRDLFDFEVLDSQ
RELDFKDPEELKKRLDVVNLSFAAE" /product="tRNA-(cytosine32)-2-thiocytidine
synthetase TtcA" /transl_table=11
CDS 2857030..2857413
/locus_tag="AUO97b_02795" /db_xref="SEED:fig|400667.82.peg.2660"
/translation="MPAFLTDDWFATVEKLTAEAGDLNLPPALANLAINLVVTDASGN

TELALDGGKIQKGLSSNAKTTLNMDAETLRKVFLEFDMAAAMQAFMTGKIKVQGDMSQ
LMALQTAKPSQEQKDLFKKVLEQTA"
/product="hypothetical protein"
/transl_table=11
CDS complement(2857505..2858452)
/locus_tag="AUO97b_02796" /db_xref="SEED:fig|400667.82.peg.2661"
/translation="MVAVESPTTVFKEDQFLHGFGYDLARNYAQSLNVKLDFKIVTDN
ATALKWVQQGKANLAMTTASLSSIENKGLMSFSASCGDIVNLQKNGLNPNLSWVFKQA
DDPLTQTASGFVCQSKQNGLTQQLASFYNRNVVKPEAWSTIQRDLSARIPIYKASFKQ
SAAQYDLDWHLLAAIGYQESYLKPESVSPTGVRGLMMLTNSTARAMGVSNRNDPAQSI
QGGAKYYDLMLSEYDDIPFPDRNWYALVAYNMGPGAVNQIQKRLQAQGKDPNQWVNLY
NYLQSNKTRNGRYKQAVQYVTRIRAYLEHIKTAQTRINI" /product="Transglycosylase, Slt family"
/transl_table=11
CDS 2858990..2859484
/locus_tag="AUO97b_02797" /db_xref="SEED:fig|400667.82.peg.2662"
/db_xref="GO:0003677"
/db_xref="GO:0003711"
/translation="MKSNLITRSGHDKLVAELKQLWHEERPEITKKVNWAASLGDRSE
NADYQYNKQLLRKIDRRVRYLGKRLEELKIVDYSPEQEGKVYFGAWVDIENEEGEQKT
LRIVGVDEIYDHHPQHISIESPMARALLSKEVDDEVEVHTPLGKKLWYINAIRYEKPK NDEI"
/product="Transcription elongation factor GreB" /transl_table=11
CDS complement(2859743..2860171)
/locus_tag="AUO97b_02798" /db_xref="SEED:fig|400667.82.peg.2663"
/translation="MSQHIITPKEIPLAFQTAWNKHDMQAFAALFDKDATFVNRFGHY
VKGVDEIIAMHQPIHETIYRDSTLENELIDLIPMSEDICISHFWSRLTAGVAHPQGPH
QIDTLILTVLIKKNHSWYIQALENVTLTNPRTGETILRNI" /product="hypothetical protein"
/transl_table=11
CDS 2860880..2864878
/locus_tag="AUO97b_02799" /db_xref="SEED:fig|400667.82.peg.2664"
/translation="MNQFVTNTKNVIRGKYHPEFLQNEVLSDIFAHTAQTLPDKTALI
EADKTLSYGELYQQALIMAQHLALKGVKPGHIVGLWLPRGIELLKAQLAICLSGAAWL
PFDMDTPADRIAVCLEDAEAVGMITTDEWYEHLAEVPQTKWTNTELQKPLSESVSLAK
TTPDQPAYIIYTSGSTGKPKGIVITQKNICHFLRSENSILGIQEQDKVYQGFSVAFDM
SFEEIWLSYLVGATLWIAPKSLVSDPERLCQTLKQEQITVLHAVPTLLALFPEDVPNL
RIINLGGEMCPDSLVDRWALPHHQMFNTYGPTETTVSASLELLERGKPVTIGKPLPNY
GMLVINSERELLEQGETGELCIFGPSVAQGYLGRPDLTADKFIENPWAMSIEEELLYR
TGDLAKIDEFGQVHCLGRADDQVKIRGFRVELGEIEAALCDIDGIGTAAVILRPEDGI
DQLIAFIAPEIDAKQAIEIKELRHNLSQRLPPYMVPNRFEIIEEVPRLLSGKIDRKAL
KARPLTSVVDRSESDQPQNPAEEILFEILNRLFPNMPIKLDSDFFDDLGGHSLLAAVL
ISNLREHAEYSHLTIQNLYQARRVGAIAALMLEQPEPTLFDSQIGQDNPRNQTYKWLC
GIAQLVTIPVLISINILQWLAPFFTYHYFTGGTRDSIPYAIALSLLVYVSVIMSSFVL
SITVKRLLMLGIGAGRYPLWGLTYFRWWLADRISNISPVYLLSGSTLLNLYLKALGAK
IGHDVTISSVHIRMPSLLTIEDGVSIGSQVNLENAKVEHGHLVLGSIHLKQDSYVGSY
AVLEENTVLEKQAHVNALTSIEYDTVVPEGEIWDGTPAQKIGHIDEQAKLPERPKLSF
IRKIAEYGYYGVSALVIACLFFIPIFPSFLLVDWLDVNVFNINPNNHLQIALYYFILA
IPASAMMMMITAVISSGLRKIALPRLETGTYAVHGSTYYRKWFAAQILETSLQTLHGL
FATIYAPTWFRMLGAKVGKNTEISTATGVIPEMLTLGEESFIADAVMLGDEEIKGGWM
SLKATKIGNRSFVGNSAYIADGTVLPDNVLIGVQSKTPDNREMYDGQTWFGSPALLLP
AREAAEKYPDHLTFKPSIKRRLMRGFIEGLRIVLPAALAIGVGYMIVLDVIDVINNYN
IETGLVALTLAGLLYGVGCFLIVALLKWILIGRYQPRSAPMWTMFVWLSEGITSLYES
VAIPNFLNYLRGTPMLPFFLRILGVRIGKDVYMDTADITEFDCVSIGDRAEFNSFSGP
QTHLFEDRIMKIGQVNVGNDVVVNARSIILYNANVSNHAVLGPLTLVMKGENIPAKSA WIGSPAVPWVHK"
/product="Polyketide synthase modules and related proteins" /transl_table=11

CDS complement(2864948..2865454)
/locus_tag="AUO97b_02800" /db_xref="SEED:fig|400667.82.peg.2665"
/db_xref="GO:0008080"
/translation="MNITIRDEQPEDIKNIEALTKAAFLNEEHSSHTEQFIVNSLRKH
KQLTISLVAIEDDLIVGHVAISPVTITSGDTGWYGLGPISVCPKKQGIGIGSLLMNAA
LQKLKELGGKGCVLLGDPNYYSRFGFKSYPDLNLPDVPNEYFQALSFINHIPSGEVTY HDAFTATE"
/product="Acetyltransferase"
/transl_table=11
CDS complement(2865488..2865622)
/locus_tag="AUO97b_02801" /db_xref="SEED:fig|400667.82.peg.2666"
/translation="MLSDCSLDVFKKLKAKRLIKSKNSQPYQITLLGLQTVRPQLDNR "
/product="UPF0386 protein YjhX"
/transl_table=11
CDS 2866024..2866443
/locus_tag="AUO97b_02802" /db_xref="SEED:fig|400667.82.peg.2667"
/translation="MKKTIFSLIFATCAAFSNSIQAEEVYYQQEDIAYAFELDSIEFY
LPSKSQFKITATRKLVGVPETKFYTGDFDKDNIAQFHMAFDCSHKTIQILDFMIGDKN
NGELKYKSDKKSEVMKATGDNDLAMLNLVCENAKTIK" /product="hypothetical protein"
/transl_table=11
CDS 2866539..2867321
/locus_tag="AUO97b_02803" /db_xref="SEED:fig|400667.82.peg.2668"
/translation="MIPVQTFSVFAIFLLFIAPLIGVVLTLGLLVKALFFWRNKAQKP
VRFFSQKTLFITLVALICDLWAGYLFYMTESVKYEIALKQHYKERRSHFVLSQDAQYG
ELLIPKGSLISRYDAFDNGEPQVPFNLRGLQAVRFPHPIRVAGMWVTAMEPPRMELAW
DQHVGPVMRFDPKEENGYGKWVYDTKRPTIACSRGDIVLLEIPLIDYDIQKEFGKPEP
DGANARFRPSEWGVQECEKGQEPIKVSPAYTGTAPKKPWFYK" /product="hypothetical protein"
/transl_table=11
CDS complement(2867499..2867759)
/locus_tag="AUO97b_02804" /db_xref="SEED:fig|400667.82.peg.2669"
/translation="MEITDQEATNLIIGVKNAAIAIEKEYKPLGISIWQNNGVPASQS
IGHVHFHVAGTLEKGGTEWEDVPELSLEETEKIAVRLRSYFK" /product="putative histidine
triad protein (HIT domain)" /transl_table=11
CDS complement(2867939..2868580)
/locus_tag="AUO97b_02805" /db_xref="SEED:fig|400667.82.peg.2670"
/translation="MEYSAPKTKIKIIKGDLLDQDEHLVIGICNTFDTETPHIVSKKS
LLGQSIDRLFGGDIKKLDELLNQALVGKSFENREPVKDGKNKQYPLGTVAIVEHSPRL
IFFLAYCQMSDKNEAFGTVDSVWKSLLSLWTAQSERGNQHPISIPVIGGGQARMSSVL
PAQDSIKLIVLSFMFASRKHKITDELRIIIQPNDYDRLDRMELQSFLASLRPS" /product="hypothetical
protein"
/transl_table=11
CDS complement(2869510..2869818)
/locus_tag="AUO97b_02806" /db_xref="SEED:fig|400667.82.peg.2671"
/translation="MKKLKYQYQQSVKTPHSHEFFLNYFPNMKSVYYKKFSTKQEKSF
YSIHCTEYKDYLLKLRVARGSGLTDAWDDLPTYVTKVAKSWKHNSKRAHQYYKDHEQK "
/product="hypothetical protein"
/transl_table=11
CDS complement(2869994..2870599)
/locus_tag="AUO97b_02807" /db_xref="SEED:fig|400667.82.peg.2672"
/translation="MNNFWTIFFTITGWIIGLVSIFYAYFSGKKSDEKFSNVLNKLAA
QPIELLSILSSPNENKVVELYDFQNRVNEPYVLSKFDIDNDGEDEIFIQSGAGPYSCK
LEIYNYIQDPDEPTLQLMDTIVVSTTAGYIFNDIDNDGILEIITNDNSIKANKPYVQG
FRDRVIFKFIDKKIQEISRLELYTEEEMESMLESFEKENFS" /product="hypothetical protein"
/transl_table=11
CDS 2871328..2871906

/locus_tag="AUO97b_02808" /db_xref="SEED:fig|400667.82.peg.2673"
/translation="MPNLVLPTRALYVVNKAIDLFHHRGFHLIGVDRIVKESEITKAT
FYNYFHSKERLIEICLMVQKEKLQEQVVAMVEYDLSTPAIDKLKKLYFLHTDIEGPYY
LLFKAVFEIKNSYPNAYQTAVRYRTWLKNEIYSQLRTLKPDASFTDAKLFLYMVEGTI
IQLLSSGGVDERERLLDYFLGLSDLSRFKIES" /product="Transcriptional regulator, AcrR family" /transl_table=11
CDS complement(2871986..2872849)
/locus_tag="AUO97b_02809" /db_xref="SEED:fig|400667.82.peg.2674"
/translation="MNTALKSDQKIFRRNVELTYDHHKSYSFYYEENPVVTSLFVVLS
AMFPAGEMFFIESIRNVRNQIKDEKLLEDIKAFIAQEAFHSREHKMLNNHLIHSNYPE
VVEIEAQTKARLDKLRQLSAVEQVAATVVMEHYTATLARLLLTDSLIKAKTTQESRNL
WEWHALEELEHKSVAFDVLNAIGGNSSKNRKVALARVAKLITPIIFKYWIKILKRKDI
NFTLKQLKDGIYLGFGGINRVGILSKAFVDMLDVRAENFDPLNMQTEQLEAEFREKLF GQAGFLRDYLS"
/product="FF domain protein"
/transl_table=11
CDS 2872958..2873575
/locus_tag="AUO97b_02810" /db_xref="SEED:fig|400667.82.peg.2675"
/translation="MVTIRKPKQNRAINTFDSIVGAGFISVMKNGLDHTTVLKVCEIA
GVGSGSFYEYFKNKEALFVEMQQHFIAEISKVIYGVIPEILDLEIPEAIRTIFLKIGE
FLAKDNNKYFYWLSVSSKYSTEASHVKAVKAINVLAIEYAMKHPEVLKIKNLKMMVYI
LIHSSIALTMNFFTSENMGFTFEELVDCLINITMSYIEDDRNKVA" /product="Transcriptional regulator, AcrR family" /transl_table=11
CDS complement(2873690..2874295)
/locus_tag="AUO97b_02811" /db_xref="SEED:fig|400667.82.peg.2676"
/translation="MNVIDALRKRRAVKRFDPAFQLSEDDKKQLLQEVLANAPSAFNL
QHWRPVIVEDAELRQKIRAIAWDQPQVTESSLLIVLCAKVNTWEVDAKRVWDGASPEV
QDIMVGAIDQYYRDRPQTQRDEVMRSAGIFAQTLMLLAQEHGLDSCPMDGFDFDAMAK
LINLPEDHVVCLMIAVGKSASEPYPRVGKLPYDDVIIKNTF" /product="Oxygen-insensitive NAD (P)H nitroreductase (EC 1.-.-.-) / Dihydropteridine reductase (EC 1.5.1.34)"
/EC_number="1.5.1.34"
/transl_table=11
CDS complement(2874333..2875115)
/locus_tag="AUO97b_02812" /db_xref="SEED:fig|400667.82.peg.2677"
/translation="MSVQSKVLITGASSGIGSVYADRFAQRGYHLILVARDTNRLDKI
SKDLQEKYGVQVEFIQADLSNDQDICKIEDVLKNDADIEILVNNAGIALNGNFLTQDR
NEIEKLLTLNMTAVVRLSHAMSQSLIRKGKGAIINLGSVLGLAPEFGSTIYGASKSFI
QFFSQGLHLELKDHGVYVQAVLPSATKTEIWERSGIDLSQVPPLMDVNDLVDAALIGF
DRKETITIPVLKDENQWNNFEKSRMTLLPNFSSAEVAQRYKN" /product="Oxidoreductase, short-chain dehydrogenase/reductase family" /transl_table=11
CDS 2875238..2875804
/locus_tag="AUO97b_02813" /db_xref="SEED:fig|400667.82.peg.2678"
/translation="MKVSKTQVKENRDKIVEKATQLFRSKGYDGVGIAELMSSAGFTH
GGFYKHFSSKTDLVTITAKYGLEQVLKRIEGLNLTQFIQMYVSRTHRDNRDQGCTLTA
LSCDAARQPDEVKVEFEEGIEQLIEFIMNERAKQVSLETEELRKQAINILAQSVGAIA
LSRACPDQSNLSDEILESCRESLLKLAD"
/product="Transcriptional regulator, AcrR family" /transl_table=11 CDS complement(2875801..2876958)
/locus_tag="AUO97b_02814" /db_xref="SEED:fig|400667.82.peg.2679"
/db_xref="GO:0008887"
/translation="MKVVIAPDSFKDSLSALKVAQAIAQGWQAVFPHAETILCPMADG
GEGTIEAVLEVCDGQWSEKTVVGPLGQPVQAKWGWLEKQKIAMIEMAQASGIQLVPPS
ERDACHSTTFGTGQLILDALEAGAKDIILTVGGSATNDGGTGLLSALGAVLLDANQKV
LPAGGLALSHLSKIDLTHFDSRIQHTRFLLAADVTNPLCGPNGASHIFGPQKGASPAQ
VQLLDAALAHFADVTAQLLGFDKRDEAGSGAAGGLGFAAKSYLNADFKAGVKVVAELN

QLEHKISNADWVITGEGKFDQQTLSGKTVFGVSQIAKAHNVPVIVIAGTLGEDYQALY
EHGVSAAFSLANGPITLEHACEHAAELIYERTVDIARLIQFSQTNLKDKKT" /product="Glycerate kinase (EC 2.7.1.31)" /EC_number="2.7.1.31"
/transl_table=11
CDS 2877353..2879761
/locus_tag="AUO97b_02815" /db_xref="SEED:fig|400667.82.peg.2680"
/db_xref="GO:0016491"
/db_xref="GO:0030151"
/translation="MDNSEEQKHIQENNGFARIEPYTHPAGGWGALLSVARNLKRQDI
LGKGSITLLNINQPTGFDCPGCAWPEKKDAHAFNFCENGAKAVAFEATSKTVTPEYFA
GHTVSWLSEQSDFFLEDLGRLTDPVRYDAATDKYVPISWDDAFKLIAQHLHALDHPDQ
AAFYTSGRASNEAAFLYQLFVRSFGTNNFPDCSNMCHETTSVGLLDSIGLGKGTVTLE
DFDLADAIFSFGHNPGTNHPRMLGTLREVSKRGGNIIAINPIKERGLERFQDPQAPLE
MMTNGSTPISRYYFQPKIGGDYALMLGILKHLHEWDKKALASGKPSVFDRNFIAVNTV
GFDEMIAEVERTEWADLYKHSGLSPEHLEKLAKLFLESERSIFCWGMGITQHRHGTAN
VHMLANLLLARGQIGRPGAGLCPVRGHSNVQGDRTMGINELPSPKLLDNIDRVFGIKS
PRKNGYGVVETIKAMAEGDIKVFIGLGGNFAVATPDTAYTQEALRKCNLTVHVATKLN
RSHLVCGKDALILPCLGRTEIDEQLHGPQAITVEDSMSNVHLSAGRNTPISKNILSEP
DIVARMAEAVLPESQIKWKWYIESYDRIRDSIADVFDEFHDFNLRVYKPGGFHLEHPA
NQHIWNTKSGKAQFLITPLEEVYADKENQYAAAYTESKVYTLMTTRSHDQYNTTLYGL
DDRYRGVFGQRRVLFMNQADIDEAGFEANQWVDIESVFSDGVKRIVHSFRIVPYNIPR
GSLAAYYPETNPLVALSSHDKYAKIPASKSVPVILHPGNAPEHFNLATAVDPEDANKK VSSF"
/product="Putative formate dehydrogenase oxidoreductase protein"
/transl_table=11
CDS 2879840..2880631
/locus_tag="AUO97b_02816" /db_xref="SEED:fig|400667.82.peg.2681"
/db_xref="GO:0008863"
/translation="MTPHAYGVETLPVQRFNKTESENVLDHIVQEYPIALVYNGISHA
VMMATPTNVDLFAKGFSLSEGILTSLKELYTLDVHQNELGITVEMTISSRAFAALKEH
RRMMVGRTGCGLCGIESLEQFQLDVPKITTHASKIWLEQIPSAISHLKTQQEITALTG
GAHAAAWVVDGQIVALFEDVGRHNALDKLLGYLAQENYDTSLGFVVMTSRASYELIRK
CAQLNIGLLASISAPTSMAIQLAKQSGLTLASFCRGDGFVLYTEI" /product="Sulfur carrier protein FdhD"
/EC_number="1.2.1.2"
/transl_table=11
CDS 2880751..2881143
/locus_tag="AUO97b_02817" /db_xref="SEED:fig|400667.82.peg.2682"
/translation="MSWVQSIKEWAKRLKKQIIMLWFASRHPQMPWLPKIIAVVAVAY
AFSPIDLIPDFIPILGFIDDAVILPILIWLAVRFTPQQVIFDAEQQAKEWLDEHEKRP
KNYLVAVLIILIWLTLAVMAYFYFGGGL"
/product="putative membrane protein"
/transl_table=11
CDS 2881248..2882315
/locus_tag="AUO97b_02818" /db_xref="SEED:fig|400667.82.peg.2683"
/translation="MKIVADENLAFTDYFFSEFGDIQHKAGRTLTHTDVQDAEALLVR
SVTAVNESLIQNTALKYVGSATIGTDHLDIQALEKQGITWANAAGCNAQAVAEYVITA
LLHLDASLLEQQEKFTLGIVGLGNVGKRLAYMAQLLGWKVIGFDPFVQLDSIENVSFQ
TLLQQANAVSIHVPLTKKGEHATYHLFDEKAFAALQPNTILINSARGPVVKEAALIED
IQRTQRKVVLDVFEHEPVISEELLNMLALATPHIAGYSLEGKARGTQMIYEAFCQKFG
YDINKRFETQLPACEDYFSRHDLKAVLKQKISQIYDIAQDDANIRACVKEGKVEQKAF
DLLRKNYPLRREWAAHGGPQA"
/product="Erythronate-4-phosphate dehydrogenase (EC 1.1.1.290)"
/EC_number="1.1.1.290"
/transl_table=11

CDS 2882312..2883286
/locus_tag="AUO97b_02819" /db_xref="SEED:fig|400667.82.peg.2684"
/translation="MSLSYQPTCSIDALKARAKLYTQIRQFFAERGVMEVETPVVSQA
GVTDVHLASVQALRHINGKLQTQYLQTSPEFAMKRLLASGSGPIYQICKVFRDDEHGR
KHNSEFTMLEWYRPGLDLKALMHETADLLQTCLQHRFGEVRPFILSYKHAFQDRLDIN
PLQATLKQLKDTANRVGLNLDLGDDRLAYMDLLFSHFVEPSLGFDAPVFLTDFPPEMA
SLAKVKLDEDGEEVAARFEVYIEGLELANAYDELLDADVLRSRFEADNAERAKQGLHV
MPLDEHLLAALPHMPECSGIALGVDRLLMVATDQLKIEKVIAFPAEIA" /product="Translation
elongation factor P Lys34--(R)-beta-lysine ligase" /transl_table=11
CDS complement(2883345..2884325)
/locus_tag="AUO97b_02820" /db_xref="SEED:fig|400667.82.peg.2685"
/translation="MEVHQRGLITGILMAALAISPLAEAKRAGGGKSHGMARSTTSSQ
SYQQPRQVTPVQQPATTAPQRSGPGVGSMVAAGVAGAAVGAVAANALADDKSTSAEHQ
VTQPGNEQAVQAQEEKKGGIPGWLWVLLAAAVAFFVFRKLGSKKKLAANNPYAPNNGG
QNPFGRSAAPTNTGDNTNIFGQSVGGGAATQAPFGSATTNSGNQLPDGSEPASFLRVA
RQRFNHIQSINSASNIEEIRRYLTPELYNSMYNDIMENQDQDVAEFSNLNAMVVDSAT
ENGQYIVSVRFTGTVSEDLNSLPQPFTEIWHFVKPVGSQQDWVVAGIQQA" /product="hypothetical
protein"
/transl_table=11 CDS complement(2884408..2885796)
/locus_tag="AUO97b_02821" /db_xref="SEED:fig|400667.82.peg.2686"
/db_xref="GO:0003678"
/db_xref="GO:0005524"
/db_xref="GO:0006260"
/translation="MSKVKTVYRCEQCGADHLKWAGQCSECGEWNCLNEVKLEPTTAH
RARPKVGGYAGQVANITTLNKVSVSHETRLPTGISEFDRVLGGGLVTGSVVLIGGDPG
IGKSTILLQTATHMASAKSSALYITGEESLSQVALRAQRLDLPTDQLKVMAETCVERI
CEVLEQERPVVAILDSIQTLYTETLQSAPGGVSQIRESAALLTRYAKNSGTALFIVGH
VTKEGALAGPRVLEHMVDCVLYFEGQSDSRYRMIRAVKNRFGAVNELGVFGMTDKGLR
EVANPSAIFLSRYDEAIPGSIVMISREGTRPLLVEVQALVDDAQGQPRRVALGLEQNR
LNMLLAVMHRHGGVQTTGQDVYVNIVGGLKITETGSDLAVLLACASSLRTKALPQQLA
VFGEVGLSGEIRPVPNGQERLKEAAKHGFKYVILPRGNAPQKAIPGVQVIAVARLHEA LTEAMQLSDELT"
/product="DNA repair protein RadA" /transl_table=11
CDS 2886128..2886241
/locus_tag="AUO97b_02822" /db_xref="SEED:fig|400667.82.peg.2687"
/db_xref="GO:0006810"
/db_xref="GO:0016020" /translation="MYLEIISNTDKNWLAEKLSKNIHLKDTCKWCDIVFLI"
/product="D-serine/D-alanine/glycine transporter" /transl_table=11
CDS 2886286..2887692
/locus_tag="AUO97b_02823" /db_xref="SEED:fig|400667.82.peg.2688"
/db_xref="GO:0006810"
/db_xref="GO:0016020"
/translation="MSNQNLSDPHEQGELHRSLSNRHLQLIAIGGAIGTGLFMGSGKT
ISLAGPSILFIYMIIGLMVFFVMRALGELLLSNLTYKSFIDFSTDLIGPWAGYFVGWT
YWLCWITIGIADLSAIIYYLQFFNNGLPFSPVEGAMISVAAIVFIMGLNLLTVRLFGE
LEFWFALIKILAIIILIAVGLWMIFTGFTSTTGEVASFTHLWANGGFFPTGVHGFLAG
FQIAIFAFVGVELVGTTAAETKDPERNLPKAINSIPIRIIIFYVLALLIVMSVTPWNR
IDPAISPFVNLFSQAGVAAAAILMNLVVLSSVMSSMNSGVFSTSRMLFGLSREDQGPK
AFGKLNRRAVPANALYFSAACLLLGAALQYFVPNTVEAFTLATTLSTILFICVWLLII
WSYIRYYTTRPELHAKSTFKLPGGLFTCWITIIFFIGMIYVLSLEPDTFKALKVSPIW
FVILIIGYFFFYKPRHKK"
/product="D-serine/D-alanine/glycine transporter" /transl_table=11
CDS 2887833..2888057
/locus_tag="AUO97b_02824" /db_xref="SEED:fig|400667.82.peg.2689"
/translation="MRRVICLISLLSSSILLSACGQSGALQLPSDPNYDKRAKYLLYR

NKEQKQVVQQDEQTEQSVESSAAQPQTTSP"
/product="Lipoprotein"
/transl_table=11
CDS 2888080..2889330
/locus_tag="AUO97b_02825" /db_xref="SEED:fig|400667.82.peg.2690"
/db_xref="GO:0008836"
/translation="MSFTRINGVLHAEQCSLEQLAQQFGTPLYVYSKAAFEKHYLDMD
RAFDFIDHQICFAVKSNSNIAVLNVLAKLGAGFDIVSGGELARVLKAGGDASKIVFSG
LGKTEADIETSLNVGIACFNVESYAELDRIQKVAARLGKKAPISLRVNPDVDAKTHPY
ISTGLKENKFGIPSDAVYETYQYAASLPNLEIVGIDCHIGSQLTETKPFVDALDRVIV
MIDELKKLGINLKHIDIGGGLGVCYKDETPPSVEEYANSMKPALEKLGLKVYMEPGRS
ISANAGVLLTKVDLLKPTTHRNFAIIDAAMNDLIRPALYEAWMDIQPVVPRTDTEEKT
WDLVGAICETGDFIGKDRSLALQENDLLTVLGAGAYGFVMSSNYNTRGRAAEVMVSGE
KSYLIRERETVESLWEKERLLPEE"
/product="Diaminopimelate decarboxylase (EC 4.1.1.20)" /EC_number="4.1.1.20"
/transl_table=11
CDS 2889336..2890181
/locus_tag="AUO97b_02826" /db_xref="SEED:fig|400667.82.peg.2691"
/db_xref="GO:0008837"
/translation="MLLEFTKMHGLGNDFMVVDLISQRAYLDTATIQRLADRHFGVGF
DQLLIVEPPDVPEADFKYRIFNADGSEVEQCGNGVRCFARFVHERHLTNKTNITVQTK
AGIVKPELGQNGWVRVNMGYPKFLPNEIPFVADEPEALYTLELANDQNISIDVVNMGN
PHAVTIVPDVLTADVAGIGPQVELHKRFPERVNAGFMQVIDDKHVRLRVFERGVGETL
ACGTGACAAAVSGMRRGLLANSVEVELAGGKLQIEWQEGDVVWMTGPTTHVYDGRLDL RYFQG"
/product="Diaminopimelate epimerase (EC 5.1.1.7)" /EC_number="5.1.1.7"
/transl_table=11
CDS complement(2890268..2890891)
/locus_tag="AUO97b_02827" /db_xref="SEED:fig|400667.82.peg.2692"
/translation="MTTILIPYLIAITLLTLTPGLDTTLIIRTATIEGKSKAFQAALG
ISLGCIAWGIVVACGLGALLMASDLAFNILKWMGAAYLAWLGLNMILKPRSQLADIQD
NHSNRSTSENWFIKGFFGNLLNPKVGIFYISFLPQFIPAQASAVTWVMGLVMIHVVIG
VLWSSLLILAMQPLSRYLKQPKFVKYMDRITGSIFVLFALKLALSKR" /product="Transporter, LysE family"
/transl_table=11
CDS 2891027..2891941
/locus_tag="AUO97b_02828" /db_xref="SEED:fig|400667.82.peg.2693"
/translation="MDFALQLLSMWLKERKIQNQSEHTITAYKRDVKSFLEFCELKKV
DLRNVEASDLREYLAQRVEQDQLSSSSMQRHLTSIRQFMKWAEQGKYLEINPTDDFKL
KRQPRPLPGMIDIEMVNQILDQPMPEKPVDQQLWLRDKAMLELLYSSGLRLAELQGLT
IKDIDFNRQLVRITGKGNKTRIVPFGKKAKESLLNWLKIYNIWKGHFDQNASVFISQR
GGALTPRQIEKRVKLQAQRAGVNVDLHPHLLRHCFASHMLSSSGDLRSVQEMLGHSNL
STTQIYTHIDFDHLAQVYDQAHPRATKH"
/product="Site-specific tyrosine recombinase XerC" /transl_table=11
CDS 2892378..2893127
/locus_tag="AUO97b_02829" /db_xref="SEED:fig|400667.82.peg.2694"
/db_xref="GO:0006118"
/db_xref="GO:0009055"
/translation="MKALVAVKRVVDANVKVRVKPDNSGVDLTNVKMSINPFCEIAVE
EAVRLKEKGTVSEIVVVSIGPKEAQEQIRSAMALGADRGILVETTDEIGALEVAKILK
GVVDAEKPELILLGKQAIDDDSNQVGQMLGALLGAGQGTFASEVKVDGGKVQVTREID
GGLQTVELALPAIITTDLRLNEPRYAALPNIMKARKKPLDTKSPADYGVTAGTKLKTI
KVEAPAERKAGVQVKSVDELVEKLKNEAKVI"
/product="Electron transfer flavoprotein, beta subunit" /transl_table=11
CDS 2893145..2894077

/locus_tag="AUO97b_02830" /db_xref="SEED:fig|400667.82.peg.2695"
/db_xref="GO:0006118"
/db_xref="GO:0009055"
/db_xref="GO:0050660"
/translation="MSILVIADHNNQTLNGATLNVVAAAQKIGGDITVLVAGSGAQAV
ADAAAKVAGVSKVLLADNAAYANQLAENVAGLVADLAKGYKYVLAASTTTGKNILPRV
AALLDVSMITDIISVESANTFKRPIYAGNAIATVQSDEAIIVGTVRGTAFDPVAAEGG
SAAVETVGEVKDAGVSKFVSEEIVKLDRPELTAARIVVSGGRGVGSGENYHKVLDPLA
DKLGAAQGASRAAVDAGFVPNDFQVGQTGKIVAPELYIAVGISGAIQHLAGMKDSKVI
VAINKDEEAPINSVADYWLVGDLNAVVPELVSKL"
/product="Electron transfer flavoprotein, alpha subunit" /transl_table=11
CDS 2894443..2897157
/locus_tag="AUO97b_02831" /db_xref="SEED:fig|400667.82.peg.2696"
/db_xref="GO:0003918"
/translation="MSVSEIRPIAIEDELKHSYLDYAMSVIVSRALPDVRDGLKPVHR
RVLYAMHELGNDYNKAYKKSARVVGDVIGKYHPHGDSAVYETIVRMAQDFSLRYLLVD
GQGNFGSIDGDSAAAMRYTEVRMTKLAHELLADLEKDTVDWEDNYDGSERIPEVLPTR
VPNLLINGAAGIAVGMATNMAPHNMTEVVNACLAYADNPNISIEGLMEYITGPDFPTG
GIIYGKSGIVDAYRTGKGRLHIRGKYHFEEDEKTGRTTIVFTEIPYQVNKARVIERIA
ELVKEKKLEGISELRDESDKEGMRIAIDLKRGENAEVVVNNLFLNTQLENSFSINMVC
LDNGQPKLMNLKDIIAAFIRHRQEVVTRRTMFELRKARERGHILEGLTVALANIDEII
ETIKTSANPAEARERLLAGEWAGGGVVALLEKAGAISVRPDEIEGEDPNRPFGLSDSI
YRLSPTQVGAILELRLHRLTGLEQDKLHAEYTEILGQIAELTAILNDFNLLMGVIREE
LAQVLQQYGDARRTEIVESRVDFCREDLIPEEQVVLTVSQTGYAKTQPLSDYQAQRRG
GRGKSATSMKDDDFIQHLIVASNHATVLCFTNVGKVYRLKVFEVPQASRGAKGRPIVN
LLPLDATETVTAILPLTEFPENHYVFMATASGTVKRVELEQFANIRSNGLRAIELNEE
DTLIGVAITDGNQQIMLFSNEGKAIRFAETDVRAMGRTAKGVRGMRVSFASSTLSEED
ADVENDDSDDNDDSADSSLVSRIVSLVVVPETGEVLCASANGYGKRTPVNDFPTKKRG
GKGVIAIKTSERNGELVGAVSIDETKELLLISDGGTLVRTRAAEVAMTGRNAQGVRLI
RLSEEETLVGVVSIEAVEDEEELLEGEVDTTETDSEEAVSNNEDTSEE" /product="DNA gyrase
subunit A (EC 5.99.1.3)" /EC_number="5.99.1.3"
/transl_table=11 CDS complement(2897208..2898854)
/locus_tag="AUO97b_02832" /db_xref="SEED:fig|400667.82.peg.2697"
/translation="MQQWLRSSKFLLIFLPIWLFVLSWIRPLSVPDEGRYGDISRTMF
ESGDWLTPRIDGLPFMHKPPLLHWLSSMFMELFGVHVWVLRLVPVLAGTLMLVGLFLF
VKKHISENVAQLTVIILATNLLFFGSSQYINHDLLLASWITISVLCFVDFTISARKSI
LFLGYISGAAAFLSKGLIGVLIPGMILLPWLIYTKQWRKIPSLLNPLAILLFLLIVSP
WLYLVQSKYPQFLHYFFIDQQFNRFSSKEFNNKQPWCFYLMILFVSFLPWLFASRFTS
IKTIFKDYKSCSLLALLVWWFVSVTVFFSIPPSKLAGYILPAVPPLAIFFALVMNKVL
ESSNKTRLQTWGIPVFTVLVGIGVNATPHFIRAHQLFIQNQAIFIYLIGALLIVLPLV
LVGLYKKQKLNYLTYIFISLIVLCSAVPFAVRILDTKNNVGQTDFAEYIAPSTKIVFY
NYYFYDVPFLLQLKQPVYIVNQWDTVHSDSASLEIKDGLLFEPQLKKYLWSEQQLQDA
LMQKQDLIVVSQPHNFATKDPSVKTLHYRNYDVFIFHPSK" /product="dolichyl-phosphate-
mannose-protein mannosyltransferase family protein" /transl_table=11
CDS complement(2898864..2899244)
/locus_tag="AUO97b_02833" /db_xref="SEED:fig|400667.82.peg.2698"
/translation="MFAHILQLARFTTTGTAAAFTHYSVAMLLFKKHVALQYANLCAF
LLAYWVSYFGHRVFTFKAQHLTHRQTLPKFTLVAGLGFLFNESFLLLSNLYFNVPVST
LVCTAIVLTSMVTFLLNRFFAFQH"
/product="FIG00351671: hypothetical protein" /transl_table=11
CDS complement(2899244..2900224)
/locus_tag="AUO97b_02834" /db_xref="SEED:fig|400667.82.peg.2699"
/translation="MNIKPFLSCVVPAYNEAENLKTFIPALANSLKQQNLSYEIIVVD
DGSKDDTIPILQTMIEDYPLVVLELSRNFGKEAALSAGLDHVTGEVALLIDADFQHPF

EAIPTMINLWKNGYDMVYGIRNRTTESWLKRVLTQAYYRILNLSSPIDIPESAGDFRL
LDAKVVEAIKQLPEKNRYMKGLYAWVGFKSIGINFSEQERQHGHSSFNLKSLFNLAMS
GLTGFSDLPLRVCIYLGAILALGAMSYGVWIIIKTLIEGISIPGWATLAAGMTLLGGI
QLLFIGILGEYIGRIYAEVKNRPKYIISTEHSRTKQEHSIAHQENTFSSF" /product="Glycosyl transferase, family 2"
/transl_table=11
CDS complement(2900221..2901015)
/locus_tag="AUO97b_02835" /db_xref="SEED:fig|400667.82.peg.2700"
/translation="MAKVCYCADDFAMNAEISDAIIQLIEQGALQATSCMTQSDLWET
AAAKLKPFSDRVDIGLHLNLTHAFASGNLVLPLPMLIVRAWSASLNRELITQCIEEQW
NLFVSVLGKQPDFIDGHQHIHQFPFIRDILLQLLKEKKFTGWIRNLQQPINTPHYRFK
TRMLSALGSNSLAKACQTYHFNQNGQFAGIYDFKLTNYGELNQYWLANAKDHLLIMCH
PALAQSNDQDPIQHARIQEYQYFSSAQFQLDCQQYGIQLTRLGAMQ" /product="hypothetical protein"
/transl_table=11
CDS complement(2901280..2902371) /locus_tag="AUO97b_02836"
/db_xref="SEED:fig|400667.82.peg.2701"
/translation="MKEFLKAYVQTFKDIVSNSSVFTTLILSVILYSFFYPTAYKAER
AESIPIVIVDEEQSLLTSQVIGQTANSPHVKIVDVTANFLEAEQMVREQKADGILLLP
SNLTQSLRRGETGGIGLYLSTTNFLKTKEIGLGLATSIEATLKEYIERFGQRTHFQPA
LSIHQMPLFNTLSGYGSYIFPAVASLIIHQTIVLGLAMLVASYREQHEKITPIRFAGI
FASIFTIGCLGSFYLFGFTLWFNDYPHGGNFVGLLVAVPIFISCVIGLGMLIGSLLDM
PERAGHIIVFSSVPLFLLTGAAWPHQAMPEWLQWFAWCLPSTHGVQMFVQLNQMGVPL
NVAVPKLIFLATIGAIFLITAYSRLKVSK"
/product="Efflux ABC transporter, permease protein" /transl_table=11
CDS complement(2902368..2903498)
/locus_tag="AUO97b_02837" /db_xref="SEED:fig|400667.82.peg.2702"
/translation="MFASILRELNYLKSNKWDLCMVTLIPAFIIFLFSSMFYAGKAEH
LPIAIIDQDQSELSRNIGKYLSLNHTVDVKTITTSSSEAERLLNETKIWGYVFIPDGA
EKRFVKAQDAQISIAFNQSFFSVGNTISSAMLVSTLNALADYAGQNYLANNIPYLDVP
TAHVKISTLYNPSMSYEFYLEPFMVPAVLHLLLCCCVAFAIGQEIKRGTLTQWVNRES
FIQGLLAKNLLYSFIFCFWTWLWMFWLVEIRGWFVAGSLSFLLAAQFLLYFSYALISS
TVILATKNLSKTFGFIAVYGGSSLSFAGVTLPLNNAPIFTKFWSLIIPYTPYAKLQTE
QWVIGSPLFISMSPFLILIGYCLIYFFLSCLLLKKLVQGATS" /product="Efflux ABC transporter, permease protein" /transl_table=11
CDS complement(2903502..2904623)
/locus_tag="AUO97b_02838" /db_xref="SEED:fig|400667.82.peg.2703"
/translation="MNQDSAHSEQNSPVADTAENHQNQQNTQTDNTQEVKTSKSDSQH
PKNSKLKLIIAVVVIALLGLILFGLWKSYQSKPIELQGRVEAETVHVSTKVPSRIEEI
YVQEGQKISKGQPLVRLVSPEIEAKKQQALATLQSALAFQSTVDRGSQQENIDTLYAN
WQSMKAQANLAKTTYQRGENLYRQGVISRQRRDEMLAAQTSAQELSEAAYQQYARAKR
GSTSQQKSTADAQVEIAKAAVSEAQALEAETRLLAPISGTVSKTYGKPSELVAMGVPV
VSILEDDDLWVSLNVGENQYASVYKNKTLEGFIPALNQSVMFKVKNIDAEGEFATIKT
TRQTGGYDIRSFKLHLVPERPVKDLKVGMSVLFKIKEEK" /product="Membrane-fusion protein"
/transl_table=11
CDS complement(2904636..2906135)
/locus_tag="AUO97b_02839" /db_xref="SEED:fig|400667.82.peg.2704"
/db_xref="GO:0019867"
/translation="MLYKMPTHQYRQSKRPFLFKPNYLALVLGVLIATPIYAQSLSYA
EAEQQALKSSYSTQANQALQQASQLEAEAVKGLGLPRVDLNVRAYAFHSETDVPLGSF
KQKLENDLSQGLNDKLSQWNNVIPSDVLGQVQEGSNQIIHDGINRFPDYANLTVEDQV
VRPSISVVMPLYTGGLTTSAKKIANIQAQRSELSSQQQQDIQRFEVVQSYFNVQLQQQ
LVASSLFNFNAMQKHYSNALKLEQQGFISKGQRMQFEVARNNAERTLQNAQANLNASQ
FNLNNLLHQQNNADLSTPLFVNTVRSQSLESLLSSYSQKSSLVQKMQLDTQLANANIQ

AQQAAKKPSLFAFGEYSLDENENWIVGVMAKYNLFSGVDKNKNIHAAELKRYASELMT
ERTKQEIEALLNKSYNELNSAQQSHTLLQRNISAAQENLRIQELSFREGMGTATQVID
AQNALSALKTEMALNAYKYVMSLATLLQSHGSMDQFKAYVTQPHTDYIR" /product="Efflux transport system, outer membrane factor (OMF) lipoprotein" /transl_table=11
CDS 2906503..2907582
/locus_tag="AUO97b_02840" /db_xref="SEED:fig|400667.82.peg.2705"
/db_xref="GO:0004648"
/translation="MRAYNFCAGPAALPTAVLEKAQQELLDWQGKGLSIMEMSHRSAD
YVAVAEKAEADLRKLMNIPENYKVLFLQGGASLQFSAIPLNLLGKNNKADYIHTGIWS
EKALKEAKRYGDINVVEAGIKVDGKFAISEQSEWNLSDDAAYVHYADNETIGGLQFAG
VPDVKAPLVCDFSSSILSAPLDVSKFGLIYAGAQKNIGPAGLTIVIIRDDLLDQAKAE
IPSILKYADQAKNGSMVNTPSTYAWYLSGLVFEWLLEQGGVDAIHKVNLEKAQLLYGY
IDSSDFYNNPIAIPNRSIMNVPFTLADEALEKQFLKEAEANHLLNLAGHRSVGGMRAS
IYNAVPLEGVQALIRFMDDFAKRNG"
/product="Phosphoserine aminotransferase (EC 2.6.1.52)" /EC_number="2.6.1.52"
/transl_table=11
CDS complement(2907820..2908761)
/locus_tag="AUO97b_02841" /db_xref="SEED:fig|400667.82.peg.2706"
/translation="MKNQNSPEVITVNDQNFGSHGEHWNLLTSRPETDVPKWLGLALD
APVMPMGLCQNEDEMDQSFWLIQGPQGQKVTINQIIAVENQKPRALKTAFPSFDSPYQ
YNAQIERIITCDSATQAVLSLKLNKSTTIYAFDNLFSVNRCQYDKTQTYQVQFNAWAY
ELESVPAGETIVVDDPASIKHHRALNAILTEHNGIAPENLQELINEWQPKTKEDQEPV
TVDFSKMVAYLYGENLGQEDEAWFQGNIVGKTSMSFMGAEYTLYDVTLVLEDNLPAIL
VRIATKNDLYKNFNIGEYIRGNIWIQANIYAKNSETN" /product="hypothetical protein"
/transl_table=11
CDS 2909036..2909503
/locus_tag="AUO97b_02842" /db_xref="SEED:fig|400667.82.peg.2707"
/translation="MLPFKLWVDADALPKILREVILRASDRYQLEVIFVANQNVGITP
SVRIKSLQVLSGADQADQEIVNRMSENDIVITQDIPLAAQVIEKGGIAIHPRGEVYTT
ANVKARLHLRDFMDTLRGAGVQTGGPPPISERDKREFSSALDQTILKQKRKTA" /product="Protein YaiI"
/transl_table=11
CDS 2909514..2910422
/locus_tag="AUO97b_02843" /db_xref="SEED:fig|400667.82.peg.2708"
/db_xref="GO:0016020"
/translation="MPSQTNLASTYVLLVFIWATTPLAIVWSVSDLHVMWALLLRFYI
ALPLSVIVLLVLKTSFPTHKQALHSYFAGSFSLIGSQIFTYAATQYLSSGMIALMFGL
APIMAGLIGRFAFGQHLFKLQWLGMAVAVCGLAIICLNGSNQHVHPFGIVLMLVSVFV
YSFSIFWVKKVNAKIQPMAQATGSILVSSILASCLIPFIWQYAPTHLPQAKSLFALIY
TVLMASLVAMFCYFKLVQNIKPTTLSLTTVITPMLAMIIGAVLNHEQLSIMVFVGAFI
ILFGLFLYFYKDIQANRNFAQHIKSK"
/product="Permease of the drug/metabolite transporter (DMT) superfamily"
/transl_table=11
CDS 2910760..2911905
/locus_tag="AUO97b_02844" /db_xref="SEED:fig|400667.82.peg.2709"
/translation="MVGLTLGVAVLITVLSVMNGFDRELKNRVLGMVPQATVSSTQIL
TDWPELVKRVENHPHVTGVAPFTQLQGMLTAQGQVAGIMVTGIDPKYEKNVSIIQNHI
VAGSLDSLKKGEFGIVLGKDMADSLGLRLNDSVTLVLPEATPSPAGVVPRFKRFKVVG
IFSVGAEVDSMVGYIALYDASTLLRLPDGAQGVRLKLDDIFAAPQVADDIVKNLPSNF
YATNWTYTHGNLFNAIQMEKTLVGLLLVLIIVVAAFNIVSSLVMVVTDKKSDIAILRT
LGASPSMITKIFMVQGTVIGVIGTVAGTVLGVILALTISDIISWFNNVLGLNLFDAYF
VHYLPSYLRWQDVTIIVIVSLLLSFLATIYPALRAAKVQPAEALRYE" /product="Lipoprotein releasing system transmembrane protein LolC/LolE" /transl_table=11
CDS 2911898..2912584

/locus_tag="AUO97b_02845" /db_xref="SEED:fig|400667.82.peg.2710"
/translation="MSKVVLEAKDIYKHFDDGKSKVEVIKGLSLQVEAGQFVSIVGAS
GSGKSTLLHVLGGLDQPTKGQVFLNGQRFDNLGEAERGFQRNQYLGFVYQFHHLLPEF
TALENVAMPLMLRADSQYKSVKAQAEYLLDRVGLSHRMDHKPGELSGGERQRVALARA
LVTKPAVVLADEPTGNLDRKTAVGIFELLTDLKKELNMAMLIVTHDEQLAQAADSILH MEDGLWVNGS"
/product="Lipoprotein-releasing system ATP-binding protein LolD"
/transl_table=11
CDS 2912670..2915102
/locus_tag="AUO97b_02846" /db_xref="SEED:fig|400667.82.peg.2711"
/translation="MFKIILLGWIGGIALMGIDFPLIMQYEKVGEALLLLAFIFYLYK
RPMFVDRPFLKAVFCLLCTTSLFMVGYHYAEKALIERLEQRETDTRNLDIIVYINHLS
EEKDNKVQQTAQVLNLSKEPVNWLLYLKSNNQNLLKNNQNLELGHYYRISGKTRPAHS
YATPGAFDQEKWFIQRNIVSSFNVRYIEPLSLDEIYRLGYQQHLKEQQSFSNSFRLNI
EKLRLTFRQILNSSSLQQKGLILALLTGDESLLSDETQLQFKQLGISHLLAISGPHVL
IFAIMLSWACHQFISRYYPQIYLWKPKQILMAVPCCLGVLIYTAFVGFEIPALRTLLS
VFIFIGFLLLKQPIKPFTLLVYSASLLLLIDPFSVFSAGFWLSYGACFILLRIYQTIA
QLPEQHFLSLSSKMIFMSKVLIESQGKIFIALSPLTLLFFQQISWVAPLTNIIAVPIV
GGVIVPLNIMAACAWFVIKPFGNMLFHFNDMLLSILLSCLGLLEKLSLPLQGISLTPL
SLLAISFAIIILFLPKGILPKTWGILCCLPLVIMNKTSQQIQLNILDVGQGQAIFLQH
SEQNWLIDTGGSYDEKIFSIGQNVVVPFLRQQGVRQLDHVVLSHLDQDHSGAFPLIQQ
EIPVKQLISNEQLPNDLKQPFQYCHQGQQWHYPELDIQILWPKEKDLAFVASNQNQYS
CVVYLQFKKVGGYQNFLIMGDAGWEAEYELLKDYPNLKIDVLVLGHHGSKHSSAYDFL
ATLKPKLAIASAGFDNRYGHPSQQVIARLKALHIPLKSTVEQGTLSFVLENHKIVLHD RRLDRLWLSRGF"
/product="DNA internalization-related competence protein ComEC/Rec2"
/transl_table=11
CDS complement(2915055..2916014)
/locus_tag="AUO97b_02847" /db_xref="SEED:fig|400667.82.peg.2712"
/db_xref="GO:0008415"
/db_xref="GO:0009244"
/db_xref="GO:0016021"
/translation="MNYQLLKTFSRQPIQFGRFLARLLAGLVNTLKITRTSKSIELNL
RIALPYLTPQQRIAITEKAVRNELTSYFEFLSIWGSSNSKNISRIHRIEGEHFFHEAL
AAKKGVVLIVPHFGTWEVMNAWCAQFTSMTILYKPVKNADADRFVREARSREQANLVP
TDESGVRQIFKALKQGETTVILPDHTPNVGGDMVNYFGVPLASSNLSAKLIQKTKAKA
LFLYAIRNENDGFTMHIEPMDEKIYEGTADDGTYVIHQAIEQLIYQYPEHYHWSYKRF
KANPALDNIYNIDPTEALKIVDRLKAEALKTSTQPEPIQTSVM" /product="Lipid A biosynthesis lauroyl acyltransferase (EC 2.3.1.241)" /transl_table=11
CDS 2916171..2917187
/locus_tag="AUO97b_02848" /db_xref="SEED:fig|400667.82.peg.2713"
/translation="MSDWPPKPQNEPITPKQNGPEWQILEKAVLASVEEQRRSRRWGI
FFKTLTFIYLLFIIVLMGKGCSTTKDGSTASSSSAHLAVVDIIGTIDASSNQAVNSED
TNKALKRAFEASNSKAIALNINSPGGSPVQSDEIWQEIRYLKKEHPDKKVYAVIGDMG
ASGAYYIASAADEIIVNPSSLVGSIGVIMPNYGITGLAQKLGIEDRTLTAGTNKDILS
MTKPINPVQRQHIQSVLDNVHTHFINAVKEGRGKRLKSNDPAIFSGLFWTGEQAIQLG
VADRSGNITSLMRELKLDNKVDYTIERNPLQSILGRMGSEMGKGLSESVAERLQTSQD AKLQ"
/product="signal peptide peptidase SppA, 36K type" /transl_table=11
CDS 2917206..2918015 /locus_tag="AUO97b_02849" /db_xref="SEED:fig|400667.82.peg.2714"
/translation="MSVKPVIHFAHANGVPSMVYQKLFDQLKDEYDVIYVPLIGPDKR
YPITNHWPFLVDQVIDSIVRQAKGRKVIGLGHSLGSVLTLMASFRRPELFSQVIMLDP
PLILGRYSFAFHMAKLFKPKIVDDMTPAGLSARRREHWESREQAAALLRPKGFYKDFD
EDCFQAYIDYALKEDSVRGGVTLTISREDEVAIFRKNPSWWWLPMPKPKMPVHLVVGK
ESAFLKRGFPQMAKRKMGIPFSVVEGGHMFPLEHPIDTVNYIKELIHRNSR" /product="Predicted hydrolase or acyltransferase PA1621" /transl_table=11

CDS complement(2918079..2918708)
/locus_tag="AUO97b_02850" /db_xref="SEED:fig|400667.82.peg.2715"
/db_xref="GO:0004644"
/translation="MMKIAVLVSGNGSNLQALIDANLSGQIVGVLSNKADAYALERAQ
NANIATAVISHKDFPSREDFDEAMHQQLIAWQADVVILAGFMRILTADFVDKWQGKML
NIHPSLLPAYKGINTHQRVLNTGDRLHGCTVHFVTSELDAGQAIAQSAIEVKEHDNVA
SLAERVHKLEHFIYPQVAEWLCNGQLAWKNGQAYFRNQILEHPIRFASW"
/product="Phosphoribosylglycinamide formyltransferase (EC 2.1.2.2)"
/EC_number="2.1.2.2"
/transl_table=11
CDS complement(2918705..2919775)
/locus_tag="AUO97b_02851" /db_xref="SEED:fig|400667.82.peg.2716"
/db_xref="GO:0004641"
/translation="MSNSTSTPNTGLSYKDAGVDIEAGDALVDRIKSVAKRTTRPEVM
GGLGGFGALCKIPKGYEEPVLVSGTDGVGTKLRLALNLNRHDTIGQDLVAMCVNDLLV
CGAEPLFFLDYYATGHLNVDVAANVVTGIGKGCELAGCALVGGETAEMPGMYEGEDYD
LAGFAVGVVEQSKIIDGSKVKSGDVLIGVASSGAHSNGYSLLRKILDVKNVDLTQVID
GRPLADVAMEPTRIYVKPVLELCKQVDVHAMAHITGGGLPGNLPRVLPNGAQAVINEA
SWEWPELFKLLQREGNVERFEMYRTFNCGVGMVIAVDANDAEKAIEVLNAQGEKAWKI
GHIQENAESVEGADEKIRVIFE" /product="Phosphoribosylformylglycinamidine cyclo-
ligase (EC 6.3.3.1)" /EC_number="6.3.3.1"
/transl_table=11
CDS 2919911..2921104
/locus_tag="AUO97b_02852" /db_xref="SEED:fig|400667.82.peg.2717"
/translation="MQDRILRRIFLLAGIVLLVWVLYLLKPVVIPFIGAFFLAYLFSP
LVEVLVKIKIPRWLAISIVFIGIGVTLTVALWYLVPLIWKQLVYARDSIPAGIHWINA
ELLPWISSTFHVQQMEIDTDQMSKAVMDYVQTNYSADSIQAVLLKLAQSGLNFIQIGG
TVVLIPIIAFYFLLDWDRMLQNLRRLIPRPYEATTLQIVRECHSVLGAFVKGQFLVML
LLGVVYAVGLQLIGLEVGLIIGMVAGLASIIPYLGFAVGIIAAVIASLFQFGMDWTHL
LLVGVVFMIGQAVEGYILQPFLLGDKIGLSPVAVVFAVLAGAQLAGFLGMLIALPVAA
VIVVLLKHLRENYERSSFYAPSSTLLIQEGNIEQLHVETENTKLDLEVKKQQDTDTPN LSDQK"
/product="Putative permease often clustered with de novo purine synthesis"
/transl_table=11
CDS 2921125..2921823
/locus_tag="AUO97b_02853" /db_xref="SEED:fig|400667.82.peg.2718"
/translation="MRQLQLDIEPQLDARISDFSGPGWGHVVDAVRQLHAGLVNRFYI
YGGAGTGKSHLLSAICDSYLEVGKSAIKVSLLELLDAPIEAITCLEHYDLVALDDIES
ISGVPHWQKAVFHLMNNHEGQLVFSSRVAPIELKLELPDLQSRLTQAVSVKVPSGSLY
ADRYALVTSVMARRGIHFDQQIVDYLLLHGPHQASILLQTVAQLEKLLKGEKTKLSNA TLRQIYALIDEYQQ"
/product="DnaA inactivator Hda (shorter homolog of DnaA)" /transl_table=11
CDS 2922141..2923994
/locus_tag="AUO97b_02854" /db_xref="SEED:fig|400667.82.peg.2719"
/translation="MLKRSIAFALLAAAGHAYSADIEVTTTIDEDVDNTVCSLREAVE
LINKRNSSDSTVVASVKDGYHGCGNKDSSSNIILQRDKEYTLNSRITITAPLTISTAK
NDSTLVDTDQPGSHNATIKMAGTDQLFKIDDESVEKLSFAVTLSDLNLQGARSSNTIL
EGGLIYNHEQLTIRNSRLINGYATRGGAIYNAGNLSNTLNTAGTVTLVNNLIQNNKAA
EGGILYSEMPLYFITQSVLRDNEVTTADNALFYTQTKFSDESTGGYLTSRAIGISNST
IFHNKGGFIANIRDGMFVNNITMIKNDKGLFLEAPQGNASVSNSILVGNTINCQASST
DKAIIQSNLVISDCNRNATSKLPNILFPANEKLIAGNNDEGICDVTEKDGLLCPFNTP
KDSFLGFFKPRLLESYNTLADSLIINKGHLYSDASSVGLAGCEKFDQRGKRRSGYDEL
CDLGAIEYILNSEISPVGQDIRYGQVAKFSILGSLSESDLVTPATCKRLFGERFDGKD
WQPGCLKIIQNKDTPISKGTLILDQSGNVTYTPNGNWHGADIFSIQVVTSVTRFNEGV
DFQYISIPTTISQAPLSGIEDKSVSTGGGGSIGGGFLLSLFSLIALRRLKS" /product="Rhombosortase"
/transl_table=11

CDS 2924009..2926456
/locus_tag="AUO97b_02855" /db_xref="SEED:fig|400667.82.peg.2720"
/translation="MKKYKKVLGIITVVAAIPLMAAENSTIIKVNTYADEDLDNDLCS
LREAIITAEKSKSYHGCNLPAINTQYTIQLESGTYNLEKGELVPKNSLIIQGTNLIDW
TKTGILSGEYPAPKAIETRINAGGKFRIFNTAVGKQPLVLNNIILENGKASDVGGAIY
AGSNVNLQNTQILNSQAPRGGAIYLGAANSELIIFHSLLQGNGLNTGVGSVIAMSCMF
NNLYTTHKITFDSNSIIKNGTSSSLSTFEFCGAPTITFENNTIAQNTANLTNGTILKF
TGDADPQQNQKTNLSSTSSLKLKNNTIVDNTANTVFLYDKLGEKDLIFNVLAFNSGSY
ACRYLLGPATDEKDARILLKFNALKLSGSNEKCDMPSETLPSGHTNIDVSSKVLTQLL
ERQESTESTSFLPLYYPKNNQQSDDLVDVDLKNEGGCSSFDQRRLIRTTSSSTDQTID
NSNNKCDIGSVEQNKLAIITTEGDATSSIVNSSLTELLNKYQLGIDQTKADLANPSFT
LQEAALKINLSTFENLFKQTKENLKYRGIYVDLKNYYSDFNNGNINKVNIIPYEDINH
VIIPFKADNYDIDVKVIGKGQTLETATEDEAKSLKCEWNPSLKQIILYRTDDTPSGDD
FEFCKYTVILKSNRNITATGIIKASLVNIPPVSENTTITFKYLANEIIPLNLLKYAND
EGDGPTNTLITKPNKPQFADLPIYLPSKVSEDGIFKVVKADREGPCPGKDSKNTCYGG
NIYIQAKNVFNTFNDTLTYYVYDADGTISAKAGTINLVNTATTTDDSRGGGGGGSIGI LSIASLLSLIAYRRYRK"
/product="RTX toxins and related Ca2+-binding proteins" /transl_table=11
CDS complement(2926546..2926935)
/locus_tag="AUO97b_02856" /db_xref="SEED:fig|400667.82.peg.2721"
/translation="MVAKKLALILCSLSFLQTSFAGFERFWIFSKDANTQVDDTWNAL
SEEEQRAVIKRYQTLKELPSNQSANLQQRMDWFNQLPDAEKQKMREAWQKMSTQERND
LRQRMLKASADERTAIREEYINKYLEH"
/product="hypothetical protein"
/transl_table=11
CDS complement(2926925..2927245)
/locus_tag="AUO97b_02857" /db_xref="SEED:fig|400667.82.peg.2722"
/translation="MNHDDFTKKVTSKLDSLAHHHKNKTVVMNHVLDHIQQKPASYYS
VWKMTGFALAAAITGFVILPNSVDMLDKPQNQVVVNPKLSPQMVEDLEMLMVLGEDKV QHGS"
/product="hypothetical protein"
/transl_table=11
CDS complement(2927235..2927849)
/locus_tag="AUO97b_02858" /db_xref="SEED:fig|400667.82.peg.2723"
/translation="MDLAPKKSRSEASSAHASTAEQRLKVFMQDVTGRALVMMESATQ
GQQGIAMDLVQEAFIALHKSYADRSTEEWYPLFYTILNNKLQDWRRKEARRANPFSFF
KKVSLDNDDEEIIDIVDEKAINPADFLDQAVTIEEIQEAIAKLPVRQQQAFMLRAWEG
FDTHTTAQIMNCTEGSVKTHYHRAIQGLREYLAHLNPMGGSNES" /product="RNA polymerase ECF-
type sigma factor" /transl_table=11
CDS complement(2928009..2928785)
/locus_tag="AUO97b_02859" /db_xref="SEED:fig|400667.82.peg.2724"
/translation="MAVIFLESKDNAKIKHLRGLIELNSARKKHQQTVLEGTHLCLAW
LQQQKKLFSLFTTEQALEHPDLKKIIELHQGHVFIISEVLYKDLSTLGNTLPCLGIID
LPKTASTIDYSVDTLILENVQDPGNVGTLLRSAAAANIKQIICTQGSASLWSPRVLRA
GMGAHFSLSCFENFQLTDILPKFDIPVFVTSSHRSTSLYSKDLSKPCVWILGNEGQGA
SDYALEHAQSVTIPQPGGQESLNVAIAGSICFFEMVRQRQ"
/product="RNA (nucleoside 2'-O)-methyltransferase" /transl_table=11
CDS complement(2928842..2929813)
/locus_tag="AUO97b_02860" /db_xref="SEED:fig|400667.82.peg.2725"
/db_xref="GO:0042132"
/translation="MSNLTLSQFLQQEKGNLTPELAQVIDTIAATCKTIDQALQKGAL
AGILGSAGNENVQGETQKKLDVISNDYLIDALKVHPHVGGLASEELDDFTLAQENGEY
LVLFDPLDGSSNIDINMCVGTIFSILPAKNAVTQAQDFMQAGTQQVAAGYVLYGPSTM
MALTVGNGVAFFTLDPETQTFLLTTENVQVSADTQEFAINASNQRHWEQPVKQYIEEL
LAGKTSVREKDFNMRWVACMVGDVHRILCRGGIFLYPYDLKDPKKAGRLRLMYEANPM
SMLIEQAGGASTTGRVRILEIEPTELHQRVPVIIGSKNEVERVTSYH" /product="Fructose-1,6-

bisphosphatase, type I (EC 3.1.3.11)" /EC_number="3.1.3.11"
/transl_table=11
CDS 2929996..2930184
/locus_tag="AUO97b_02861" /db_xref="SEED:fig|400667.82.peg.2726"
/translation="MTTKKFDATPTAGEPINLEEISKESVQEAWKDYEAKPEYKAFNK
HDLIESMQHSEEEQNKSS"
/product="hypothetical protein"
/transl_table=11
CDS complement(2930239..2930802)
/locus_tag="AUO97b_02862" /db_xref="SEED:fig|400667.82.peg.2727"
/translation="MKSIKYLAFPLLSAALVMTGCASRKPATTATTGTTNPSTVNTTG
LSEDAALNAQNLAGASSKGVTEANKAALAKRVVHFDYDSSDLSTEDYQTLQAHAQFLM
ANANSKVALTGHTDERGTREYNMALGERRAKAVQNYLITSGVNPQQLEAVSYGKEAPV
NPGHDESAWKENRRVEINYEAVPPLLK"
/product="Tol-Pal system peptidoglycan-associated lipoprotein PAL"
/transl_table=11
CDS complement(2930830..2932110)
/locus_tag="AUO97b_02863" /db_xref="SEED:fig|400667.82.peg.2728"
/db_xref="GO:0015031"
/db_xref="GO:0042597"
/translation="MKTSRKHLLALTLLTALSPLAPTAAFAQLHLEIAKAPDQAPKIA
IVPFNNDNGLYPIVETDLNRSGRFTSSSKNLPANAAINQIQASDWQAAGIPYVVTGQI
KQTADGFEVHYQLYDVQKQQYLLNELLNVPASRIRQAGHIVSDAIYQALTGIPGDFSG
RIAYVLRNPATPAERYTLQIADTDGEQPKTVLSSRDPILSPAWTPDAKKIAYVSFETK
RPAIYLQDLSTGTREVLTSFKGLNGAPSFSPDGKSMLFTASMNGNPEIYQMDLSTRQV
KRMTNDSGIDTEARYTPDGKAFIFTSDRGGSPQIYRYDFGNGSVKRLTFKGSFNARGT
LSADGKKIALVHRPSGSNYKVAIQDINTGIVNILTPTSLDESPSFSPNGQMVVYATRE
GNRGLLSIMSTDGRFRMNLPSEQGEVREPAWAPK"
/product="Tol-Pal system beta propeller repeat protein TolB" /transl_table=11
CDS complement(2932204..2933607)
/locus_tag="AUO97b_02864" /db_xref="SEED:fig|400667.82.peg.2729"
/translation="MNKNQKPPFKQNAIALSFTLGVHAIAIVGLLFLGLSKPPEQPKK
LTTVLVKPEDLPPPLAKEVEQPTVAENQAEEVLSPIVDETLSQNLPAAPPPPTAQQLA
AQKQKAEQAQQAKLAEEKRKAEEAAKAKQAAEQQRLEEAQKQQAEAKRQAEAKARAEA
EQKRKAEQNAKAEADAKARQKATEEAKRKAETEARLKREAQKAENAKLQAQQEAKRKA
EADAKAKQQKAAEDAKRKAEADAKAKQQAADNAKRKAEADAKAKQQKAAEDAKRKAEA
DAKAKQQKAAEDAKRKAEADAKAKQQKAAEDAKRKAEADAKAKQQKAAEDAKRKAEAD
AKAKQQKAAEDARRKAEIEAEEKAASAKKAQEEAAQKKGEAKKIASSAKRDFEQKIRR
AWDVPTGSSGKTVSVRFTLSDSGSVSSIVITRSSGDDALDASIKAAIQASAPYPMPSD PDARREARSVTSTFRAQ"
/product="TolA protein"
/transl_table=11
CDS complement(2933611..2934063)
/locus_tag="AUO97b_02865" /db_xref="SEED:fig|400667.82.peg.2730"
/db_xref="GO:0005215"
/db_xref="GO:0006810"
/db_xref="GO:0016020"
/translation="MAIQRSGRFERIKKPLKSDMNVVPYIDVMLVLLVIFMVTAPMIT
SGIKVDLPQANNHPIESKDMPAMVTIGKDGNIYLQYEDYKDNEPLSLEKLESVLSEAQ
KQAEQENKQLTVLVNGDKTRPYGEVVSLMASLQEAGLTQVGLLTEPLK" /product="Tol biopolymer transport system, TolR protein" /transl_table=11
CDS complement(2934063..2934761)
/locus_tag="AUO97b_02866" /db_xref="SEED:fig|400667.82.peg.2731"
/db_xref="GO:0006810"
/db_xref="GO:0008565"

/db_xref="GO:0016020"
/translation="MATNIESTLHISDLILQASPVVQLVMLILLLASIFSWYLIAKLH
MSYKKARQDDEHFQKMFWSGAELNTLYNNAQLNSKRSGLEDIFYQGLSEFFKLKKRQA
PTSQMIEGTERILRVGLSRDQGSLEYGLGTLASIGSVAPYIGLFGTVWGIMNAFIGLA
AVDQVTLATVAPGIAEALIATAIGLFAAIPAVLAFNHFTAKSESVYSDRALFAEEMIA LLQRQSVGSSQEDA"
/product="Tol-Pal system protein TolQ"
/transl_table=11
CDS complement(2934785..2935192)
/locus_tag="AUO97b_02867" /db_xref="SEED:fig|400667.82.peg.2732"
/db_xref="GO:0003824"
/translation="MANHFEFNIRVYIEDTDAGGIVYHANHIRYMERTRTEWLRAAGV
DHYWHQKDYNFVVYKIDVKYMRPILMDDLITVTARVVSCKAASFVLQQNIYRGEILLA
SGEVELACLSKDLMPRRLPEEIRDLIRKELEQD"
/product="Tol-Pal system-associated acyl-CoA thioesterase" /transl_table=11
CDS complement(2935403..2936737)
/locus_tag="AUO97b_02868" /db_xref="SEED:fig|400667.82.peg.2733"
/db_xref="GO:0050118"
/translation="MYKKTALIFPLALISQLSLADWVKDAIQQNENQVIQLRQHIHEY
PELGNMEFKTSALVQKELKSYGIQVRTGYAKTGVIGILKGSNPGPIIALRADMDALPM
EEKSGVPFASKQKAIYQGKETYVMHACGHDAHTAMLLGAAKILAANKDKILGTVIFVF
QPAEEGGADIDNFTHGDQIGSRKMIADGAFKDYKPEAIFGMHVMSGMKSGHLYYKDGA
ILNSADHLRIQVNGKQVHGSTPWLGRDPIYASAQMINNLQSLISRRIDLTQGMGVISI
GNIQGGTAGNVIPEQVNMIGTIRSNNEQIRENILKSLPTLVENNAQANDVTAKVEIAP
YAPVTMNNKALTQLIQPTLAKTVGDSNLHVLNHNASASEDFAYYGKLMPSFFVFLGAT
PENQDLSQAAPNHNPSFIVDDKALKTGTELHIRFVLDYPNIAKQVQAAWKQS"
/product="Amidohydrolase family protein" /EC_number="3.5.1.47"
/transl_table=11 CDS complement(2936942..2937946)
/locus_tag="AUO97b_02869" /db_xref="SEED:fig|400667.82.peg.2734"
/db_xref="GO:0003677"
/db_xref="GO:0005524"
/db_xref="GO:0006281"
/db_xref="GO:0006310"
/db_xref="GO:0009378"
/translation="MQDRLISGTEKPEDHFDRAIRPTSLADYIGQPVVREQMEIFIGA
ARGRGEALDHTLIFGPPGLGKTTLANIIAREMGGNLKSTSGPVLERAGDLAAMLTNLE
EGDVLFIDEIHRLSPVIEEILYPAMEDYQLDIMIGEGPAARSIKLDLPPFTLVAATTR
AGLLTSPLRDRFGIVQRLEFYSVEDLTHIVSRSANLMDVPITVEGAEEVARRSRGTPR
IANRLLRRVRDYAQVKGTGEVNHEMAQRALDMLNVDKAGLDTLDRRYLSMLLERFDGG
PAGVEALAAAMAEDSGTLEDVIEPYLIQQGYVMRTARGRIATNQSYLQFGMTPPEPKN "
/product="Holliday junction ATP-dependent DNA helicase RuvB (EC 3.6.4.12)"
/transl_table=11 CDS complement(2937955..2938554)
/locus_tag="AUO97b_02870" /db_xref="SEED:fig|400667.82.peg.2735"
/db_xref="GO:0003677"
/db_xref="GO:0005524"
/db_xref="GO:0005622"
/db_xref="GO:0006281"
/db_xref="GO:0006310"
/db_xref="GO:0009378"
/db_xref="GO:0009379"
/translation="MIGCLIGEVFALEAPTVLLNVNGVGYEIDTPLSTFCQLQKGQKV
TLWTHLVVREDAQQLYGFSDAQEKTIFRTLLKVNGVGPKMALGILSTLSVELLVHTIE
HDDVNTLVKVPGVGKKTAERLMIELRDRFKTLAQGTSSAAALPQIQFVSNSPVAEAEA
ALQSLGYKPLEAQKAVAAVKADYTESADIIRAALKSMMK" /product="Holliday junction ATP-
dependent DNA helicase RuvA (EC 3.6.4.12)" /transl_table=11

CDS complement(2938646..2939986)
/locus_tag="AUO97b_02871" /db_xref="SEED:fig|400667.82.peg.2736"
/translation="MEQMRWLELLSAVRLGSKKSSTELARSPFHKDYDRIIFSQSFRQ
LNRKTQVHPLAQHDGIHTRLTHSLEVSCIGRSMGMLAAEKIKDELPVWISPADVGAII
QAACLAHDIGNPPFGHAGEYAIREWFDDASHNDFLKKLTPEEEADVRQFEGNAQGLRL
LTRIDYHPDDGGMRLTYATLGAYLKYPWLSKTIASQGDRPSHQRAKFGCYQSEKEILK
QIAEQLGLIQLGEYHYCRHPLTYLLEAADDICYALIDLEDGIILNMLNYQEVEPIFLN
LIADYGQPEELSLPSSTWQQKISALRGRVMKRLVDEVTTAFAKHHYEIITGQLKGSLL
QYCSPDIAAGIETAKNLAREKIFEHPQKSGLEIIAHQSLQTILDAFVPLTMPHKSLSF
KEQRLMSILNLYGARFSNNHYSNIMQVLDIISKFSDHQAYSLAQELQGNKIGLF" /product="dNTP triphosphohydrolase, broad substrate specificity, subgroup 3"
/transl_table=11
CDS 2940296..2944129
/locus_tag="AUO97b_02872" /db_xref="SEED:fig|400667.82.peg.2737"
/db_xref="GO:0004642" /db_xref="GO:0004642"
/translation="MFIVAGAAAHSSFKKAQLLTRLSSISSVQSFDSQWVYLFDQALN
EQQHQSALQLLNDGESFELRQPASDEIQILVTPRVGTISPWSSKATDIFKNCNTPVHR
LERGLLFTLKGVSEISNEVKQVLHDRMTETVFAQIEDAKALFSETAPKPLNSIDILGQ
GKEALVKANNEFGFALSEQEIDYLTEAFTRLGRNPNDIELMMFAQANSEHCRHKIFGS
EWTIDGEKQPLSLFQMIKNTYKESPTDVLSAYKDNASVIVGYDTMRFYPKADENGHFV
YKYKSQAAHILMKVETHNHPTAISPFAGAATGSGGEIRDEGATGRGGKPKAGLTGFTV
SNLNIPGFEQPWEENYGKPSRMASPLQIMIEGPLGGAAFNNEFGRPALNGYFRTFEQN
VNGEVKGFHKPIMIAGGYGNIRPDHVEKDAIQPGDLLIVLGGPAMLIGLGGGAASSVD
SGTMGESLDFASVQRENPEMERRCQEVIDTCWRLEDFNPIVSIHDVGAGGLSNAMPEL
VNDHELGAVLNLRKIPSLEPGMSPMEIWSNEAQERYVLAIRPESLEQFESICARERCP
FAVLGEATEARHLTVEDPLFQNNAVDIPMQVMLGGTPRMQRSYETIERKGNEFDASKV
DLKDAIFRVLKNPTVASKSFLITIGDRSITGMVARDQMVGRWQIPVADAAVTTTSLQG
FTGEAMAMGERPPVALLNPAASARLAVAEAITNIACANIEQISDIKLSANWMAAAGQK
GEDQALFEGVKAIGMEMCPALGIAIPVGKDSLSMRTTWNDNGEDKAVTSPMTGVITAF
APVADVRKTLTPELKNLEDSVLVRIDLSKGQFRLGGSILAQVYKAIGSVTPDVDSFDD
FKAFFALIQDWNNRGLIQAYHDIGDGGLLATVAEMMFASRLGVALEDQTTAGLFAEEI
GAVVQIKAADWEALQAEVAASSLKDAIAVVGRVNNTDQLSVNGLTLERAELQQAWSEV
SHQIQRLRDNVETADQEFALITDKSHQGLIAKPTFDLNEPIEAPFINSRRPNMAILRE
QGVNGHIEMAAAFDKVGFNTIDVHMSDLLAGRVSLSDFEGLVTCGGFSYGDVMGAGGG
WAKSVLFNAKLRDQFEQFFHREGTFSLGICNGCQMLSQLAPLIPGAEHWPRFHRNMSE
VFEARSVNVRVEKSVSVLLDGMEGSILPIAVAHGEGRVVASEADIASLNAANQVALRY
VDSHGNPTQHYPLNPNGSPEAITGVTSKDGRATIMMPHPERTFRAIQHSWSPEEWTED GAWLRMFRNARKFIG"
/product="Phosphoribosylformylglycinamidine synthase, synthetase subunit (EC 6.3.5.3) / Phosphoribosylformylglycinamidine synthase, glutamine amidotransferase subunit (EC 6.3.5.3)" /EC_number="6.3.5.3"
/transl_table=11
CDS 2944212..2945315
/locus_tag="AUO97b_02873" /db_xref="SEED:fig|400667.82.peg.2738"
/translation="MKSKIHFREFALLMALLMSIVSFSIDAVLPALGEVGRVFELKNN
NQTQWVIIGIFSGMTIGQLIAGPLSDAIGRKRILFTGIIIYFLGSLLCFTTQSFEWFL
VGRFIQGIGVSGPYVASISIVRDKYSGAQMARIMSLIMMVFMVAPAIAPSLGQLIIHF
FGWRDIFVLYMIYATVVGAWVALRLEETLLPENRLPMRLQAFQDGFKEVVSNKTTMSY
LLCAGFCFGGFIGYLGTSQQIFMQQFGKTGQEFSAYFAVLAGVMGIASFTNSKIVMKF
GMRPICIYGFLGLCLISLIFLGIQLIGVTVAFWMFMLYACILFLLFGTLFGNLNAIAM
EPMGHVAGMASAIIGAASVFEQYSSKLSFIHNQ" /product="MFS family multidrug efflux protein, similarity to bicyclomycin resistance protein Bcr" /transl_table=11
CDS complement(2945405..2945980)
/locus_tag="AUO97b_02874" /db_xref="SEED:fig|400667.82.peg.2739"
/translation="MAQLKQNSDSSNYLITRIDIARVLEQEPLLTANGFGHADTYHES

FYKRHTFHDSKAEYIQHFHASQEILRNSIDECQRCCMYLQHLKKLKSVRYNLGSYGLK
HSVERYHRKLNQFNDAYVSNGALICAAIHMGFSIMRKDHLSPNVWIFASVQSDIIVWE
RLLEEQKSFLSFTQQRLFEKVSKNTDQISIL"
/product="hypothetical protein"
/transl_table=11
CDS complement(2946010..2946126)
/locus_tag="AUO97b_02875" /db_xref="SEED:fig|400667.82.peg.2740"
/translation="MQNLVFPSGLSVQRAKKRAKELVKSKQKTKMMHLMQFL" /product="hypothetical protein"
/transl_table=11 CDS complement(2946677..2947315)
/locus_tag="AUO97b_02876" /db_xref="SEED:fig|400667.82.peg.2741"
/translation="MWSDIESNIDFLNYSEASEIIINVLQNPQMLPISIGVFGSWGTG
KSTILNLIKNNLEHIENHDDYVIIRFDAWLYQGFDDARASLLEVINTEISKLVKQDEK
LYEKALSIGKRINKLRALGWAAEGTALAFGVPTFGLLSKAIDSIGNGINGSAGDKDAQ
HVTEAINKGKESLKDILNEEKSSAPKEITALKNEFEDLLESLKALLHKYVSI" /product="Phage T7 exclusion protein"
/transl_table=11
CDS complement(2947700..2948260)
/locus_tag="AUO97b_02877" /db_xref="SEED:fig|400667.82.peg.2742"
/translation="MALITLDFNETFLEIMQTSFQHHDLAKFFLADPNPALKTINIPN
KNVLHQAHYLLNLPPAQSSIDLLHLEGAALELMSLLLDQHSKSNQIPAPIHKSIMILE
SEFEQKITIRTLAKRVGINECGLKRLFKEYTGKTIGHYLLETRMKHAQILLKDGVSVE
NVANQVGYSSVQYFKQIFEQHFGYSC" /product="Transcriptional regulator, AraC family"
/transl_table=11
CDS 2949003..2950178
/locus_tag="AUO97b_02878" /db_xref="SEED:fig|400667.82.peg.2743"
/translation="MNYMSLGFDGTSHRAQEDIEASCFLTQNYHLYTSERVETIRLAA
ENQQQLLDTIGAAFKHVTQDQDSNPVVIGAIPFDTTQPSMLHFYRQHQKQNTASFTQF
EHQQQFGSFKLNSQKRLVSKQQFANAIDQALIQFDHNNLEKIVLSQAVDFELSHQQSP
YDLVHTLAAQNQHAFSFVIPVEDNAHLLGASPELLISKQGHIVKSNPLAGSRPLSEDD
RINQKNIEDLYASTKDLHEHQIVVDSVFKNLKPFCQKLSVSEQPEILKTTTMLHLSSV
FEGQLKNEQTNALALALSLHPTPAICGSPTQLSKHFILEHEGYDRNYFAGLVGWMDAD
GNGEWAVTIRCGLLSERNLRLYAGAGIVKGSEAELEWNETEAKMQTLLKTLNATCET"
/product="Isochorismate synthase (EC 5.4.4.2) @ Isochorismate synthase (EC 5.4.4.2) of siderophore biosynthesis" /EC_number="5.4.4.2" /transl_table=11
CDS 2950270..2950899
/locus_tag="AUO97b_02879" /db_xref="SEED:fig|400667.82.peg.2744"
/translation="MAIPKIQSYPLPFKQDYPAAKVKWKIEKNRAALLIHDMQDYFIN
FYGEDSPLIQEVINNIAQMKAWCVENHIPVFYTAQPVKQNDEDRALLNDMWGPGLTAY
PEQAEIVQKLAPTQHDVVLTKWRYSAFYHSNLQNQLKEMGKDQLIICGVYAHIGVLQT
AAEAFMNGIQPFMVGDSVADFSLQDHLFALQYVHRNLGVVEDAQSVIAS" /product="Isochorismatase (EC 3.3.2.1) of siderophore biosynthesis" /EC_number="3.3.2.1"
/transl_table=11
CDS 2950936..2951727
/locus_tag="AUO97b_02880" /db_xref="SEED:fig|400667.82.peg.2745"
/db_xref="GO:0008667"
/translation="MKEAETQEKSPTLTHSADTKIAWVTGVNQGMGAEIMQRLIAQHI
YVVGFDLSTNNIVESDRYEVHQCDVRDAVQISSLCQKLLKTSPPDYFINTAGVLHLDE
HDTLPEDAWLQTFEVNTFAPFYFLKHLSPYFREKRNGNIVMVSSNSAHVPRMKMAAYG
ASKAALTSFSKTVGLELAEYGIRVNIVSPGSTATPMLRQLWKDASGEKQTIHGNLAQY
KVGIPLQKIALTEDIAHAVMFLISDQASHITMHDLVIDGGATLGQ" /product="2,3-dihydro-2,3-dihydroxybenzoate dehydrogenase (EC 1.3.1.28) @ 2,3-dihydro-2,3-dihydroxybenzoate dehydrogenase (EC 1.3.1.28) of siderophore biosynthesis"
/EC_number="1.3.1.28"

/transl_table=11
CDS 2951741..2954245
/locus_tag="AUO97b_02881" /db_xref="SEED:fig|400667.82.peg.2746"
/translation="MQLQQEITADIHHVLQIDSIQIQPEDNLIEHGLHSLAIMQLVDH
FEKKYNTALSYADFAMSPTVHDWQSLIQENTKSVDAVQSEQPKQNSILPAWLDTLPTQ
TVALSDMQYAYWAGRETEGVSAHLYMEFEGTDLNLEQLQCAFEQLIQRHPMLSASIRD
GQQIIQSVDHQEIQIEDLTQLDSTAIEEHLKAKRKRLSHQQLDIAKGQVLDVQLTLLP
EHRHILHIDSDMSAIDPQSFLVVVEDLSALYQGEERADLNLDGIGYFNYLAQRRHDVN
LQEKIQQDQQWWQSQLDAIAPAPLLPVIAEGLRQDPHQFERLIHIFNADERARLQSLA
QTQGVSVGSLCLSLFAQTIANWAAQPEFRLNLPAFIREPYGQNIEHVVGDFTQISLLS
LKLNPAETLLETTKRIEHDVNQIHEHYRYGGIHVLRDLSKHRQQLEISPIVFTSALDE
GEIFSEQLQRTLGQPVWCISQGPKVDLDVQIAYFNQGLAVNWDIRSHAFKPNVIQGMF
DHYIQSIQHVLTVGSSVLTQSLNALSQQLAPHNQQPKQVKVQLPAPLFAKFPQTNANT
PYRVVNGLDTDCPAWVLGRLVLSFDHSNAHTTDPSNQSEWIETGIQAYSDDQGRLHLF
EQSHQLILKNGQCIEVKNIEDQIAQLSFIKQVKVYTLQDQSKTTIVAVLNLHQKQSSA
DLLKAYAEVLPAYLIPDHNYVVDEIQSFTATLSSEQLNGLLTKYEPLEQSDASQLEET
TVLEKVVTLMMAKIIGIPQSQANSHLDFFDEGGDSLLATHLVAALNQYFQNADISVVD
IFTQRNAKNIAALLEQKLPDTAQRIAEVFMQVIEGK" /product="Non-ribosomal peptide
synthetase modules, siderophore biosynthesis" /transl_table=11 CDS
2954249..2957485
/locus_tag="AUO97b_02882" /db_xref="SEED:fig|400667.82.peg.2747"
/translation="MNSTPLTAMQRAYLLGKSKMFPLSQSSMHDFQEFHGRMDVETLT
QRLTQLIALYPALRTTIDEMQLMQVIEPEINLNQQLKVYDLSHLNSMEATIQLEILRD
EYRHFSHDLSTPPWQIAWIQLPETFEEQTVVLTSFDGLIIDGYSISVILNSLFDVNQD
LNLLDKDQNLDAQQNHSSFFDATEKDRDFWHNALSQVEELHPLPWANALESIDTPVYA
RQTVVIEKAKFDAVSKLAGQYKVFPNALLTTLVLDVISHWTEEQKLLVSMPVSHSALH
KRVGNSSSFIVVDYAYCPDLGFVDQVKDTQKQILQAMAHSSYSGIELSKYLIKHLQES
IVLPVAVTNGLSWVKLSESSAMRYVSGQTQTPQLALDIRLSFSAGSDLVIDLDYIEQA
LSPAMVQDMAEALSKRLHDLNQLETELKQPKRFSHVATLDDDAVIADDYAHVEVGDYL
ANIQRNLFEQIPDKTALIYADQTISYAELGANVAKVRRALDLAQIRQHQVVAICLRKS
PEHIYAVLACALSGIIWLPVDMDSPSLRQQYILSNSHADLAISTAPVEGLLTLNIDEI
LKDSAHGQLHSGSAESAFESEIIWQHRHDASPAYYLYTSGSTGTPKCVVLNNRATAHV
LQETIDFWCINQNDIHLAATPFHHDMSIFDLMAPLSVGGSLVVPTLEEAKSAVAWADL
IERYQVSIWCTVPAMADMLLTSADPKQLQSIRLINQGGDYVKPSVVQKLREILPNTRL
ISIGGPTETTIWSIWHEITPEDVDVIPYGKEMVHNRYYILNPWGEFCPPGVVGQLYMS
GINLANGYLLDGKITQKDFVLLTLPNGEVHRVFRMSDKGYLREDGNIIFAGRDEGYLK
VRGVRIAASEVENALLKHPQITDCVVTTCTNPVYEGNELVAIYTTQEAALNSIRPSEL
RQFLQDYVPNSHIPSRWVSLAQFPITRNGKIDRKALKQTAQDFLYQTYAQTASSKNTQ
VFDSKMNLQKDSPKYHRHSKQSDLARPSIAQEVIEHLQRSLPSQQESSDVLLDTEILA
LGMTSGHLSRLAKHLSQQLNTAIQLSDLAKCKTVGEIVHNIESQLDQK" /product="Siderophore
biosynthesis non-ribosomal peptide synthetase modules" /transl_table=11
CDS 2957498..2960713
/locus_tag="AUO97b_02883" /db_xref="SEED:fig|400667.82.peg.2748"
/translation="MSHQQSIMSDELFNLFASKLQDIGLSDQKAASTPTTSNLQDSLQ
SAIVETQAQPQVSFAEERAWMMHQQDPLASSGPFILALRLTGKIDIARLSRAIEKLYV
GQSNLNLRYDLDDEGVLTKFHAEEASTALQIHVVQSETDAIQYLLSWLQQPIDLAKQP
AIQFSLLPQSNDEVILGILGHHILLDDAAWQPIFTQLSQYYEQIDSTGVVVNQSHQMP
LQPKMKDSTSALQYWKNTFPTGLTTMTWPKFCLATQVQAPVTEYGKHRDFDYVQKAVR
CYSHVHVGKIQQLSQQAQASIFHTVVALFGSYLNQLLGQNSLDLFVPIVEQYDASSLD
KIQSSSNILPIRIARADQSKHPQTAQDKINNVIANVRNQILQGMSNNVSIEQILSATK
TKRNTIPNVLITQFVEPSQYLQLEGVTVETIAIPPIHSDYDLTLAFQVKGDEIHFELT
TGEKLSKSISPLLLEQWLDFLDADIATWTEADLIFAVTTQQELQPQTNSQAQSSSQHI
ENSVSSKKIDISAIQLILQEFQAVLMNSNLQVDDNFFDFGGHSILATRVIGKLQSQHG
LVIKIADFFNAPTARELAQYVQQSNALSEDNQVVADDRDIVAPLTLLQKAFMGFSDQG

RDAMYNIPFAFRFSEDVDEQAFYAAFLDILKRHHALRSIFVRADQGDIFQTVIPMQQL
EQHPWFWGSEHQIAQSAQQILRAEANHSFDLMHEFPIRVRFMKDEQGQHILSMLLYHI
AVDEWSSGILMRELIQAYTSRVAGQAPVWEQTPRQFHEYAMAQDDSTLQAHIQYWVDT
IGKIKKQPPLLPAYATAHDEQDQTLDTDVAGAAVEFGFTAFQVEQLYALAREYRSSLF
YVVYAAMVLASHYLGAGRKILTGTSVACRQDPLYQDTMGYFTNVVMHNTHLDENLTIK
DLVQQVQNNIFKAQEYADIPFAIVEENIRAEGEEWTESPYEVYVQLHAQNALTGSFQL
NSGHEIGFELIEPERDTAKFGLHYEIYEEPSSEHERLRVVINYRVNRYNDTQIQLIRQ
VAHQVLSRFIDIDTQSDESMNIDVRDIRRQLSSIQLPEMAV" /product="Siderophore biosynthesis non-ribosomal peptide synthetase modules" /transl_table=11
CDS 2960725..2962344
/locus_tag="AUO97b_02884" /db_xref="SEED:fig|400667.82.peg.2749"
/db_xref="GO:0008668"
/translation="MFIEQDIQLGYVPFPKSRAQQYRDEGCWKSQTHFQLLASLKDRF
AQRVAVIQDDKQLTYQQLYDYAIHYGTYLKQQGIRETDFVLLQSPNVIEVFIVIFGLY
AIGARPVFCLHGHGSYEIENIARQSRAVGFLKLCGSANESTATDVCEEFSKPNFKLWF
RESIVSRSSIEASLPQLQGVAPAFNLRAQSESEDIAFLQLSGGTTGLPKLIPRTHADY
IYSIEKSVDVAGLTQDTKQLVVLPVMHNFCMSSPGFLGVFYVGGTVVLSQLTHPRVCF
ELIEKYQIQQVSLVPAIATLWLNAESLKDYDLSSLQVVQVGGAKLLPSLAEQIIDTLQ
VKLQQVYGMAEGLVNFTHLDDSDQITIQTQGKKLSHLDEIRIADQDGNALPINAIGHI
QTRGPYTINGYYNLPEINQRAFTQDGFYKTGDIGYLDENLNIVVTGREKEQINRSGEK
ITPSEIEEFILQYPSVKDVCVIGVSDDYLGERIKAIIIPKLDDSEINLKNIRKFLISK
NIAHFKIPDEIEVVADFKYTHVGKVNRQKLG" /product="2,3-dihydroxybenzoate-AMP ligase (EC 2.7.7.58) of siderophore biosynthesis" /EC_number="2.7.7.58"
/transl_table=11
CDS 2962358..2963698
/locus_tag="AUO97b_02885" /db_xref="SEED:fig|400667.82.peg.2750"
/translation="MNSQHIYDIVGIGVGPFNLGLACLTQPLNELSTIFFDSKDEFDW
HSGIMPEGSTLQIPFIADLVSFADPKNNYSFLNYLKLHNRLYQFFIRESFFILRAEYN
LYCKWAAEQLENVHFKSFVERIDYDESRQLYTVRVKQPQGEMKVVTKNLVLGTGTTPI
TPKFCQGYPEQIQSSADYLRHKKDYLTKKSITIVGGGQSGAEIYYDLLSEIDQHGYQL
NWLTKAPHFFSMDLGKLTLEYTSPDYTSHFYSLDEDKRDQVIGSQNALYKGIELSFVN
RIYDLLYQKSLHQPIPTRMMPNCALDAVEQQSNHLNLTFKNSDINKRFKLESEVLILA
LGYEYKIPECLTPIRTLINWDSKGRIALNWNYSINDDNTIFAQNIGIYSHGFTVPDLG
MGCYRNAIIINTILGREVYPVEKRIAYQEFAPTTEEIVTPVKTTAKSHSTELSF" /product="Siderophore biosynthesis protein, monooxygenase" /transl_table=11
CDS 2963708..2964868
/locus_tag="AUO97b_02886" /db_xref="SEED:fig|400667.82.peg.2751"
/db_xref="GO:0008792" /db_xref="GO:0004586"
/translation="MNFPHYFQSNIQEYLDDLLKTKATPFLVLDLSVIEKHYLELKQG
FPNADIYYALKANPANEVIERLCRIGSSFDLASVNELKKVQAFGVDASRLAFGNTIKK
RADIQYFYEQGIRLYVTDSDADLNNIAQYAPGSKVFFRLSIDGSETADWPLSGKFGCN
AECVIRLAVKAKELGLIPYGISFHVGSQQRKIDLWDSALAETKAIFDVLVNDHQIHLK
MLNLGGGLPAQYVQQTEAFASYSKQINAALEMFKPYQLEQIILEPGRSLVGNAGILVS
EIVLISQRPQSGTKRWVYTDVGVFNGLIETLGEAIQYPIVCKKTGQSDDIILAGPTCD
SMDIMYQNKKYELPLSLEIGDHLYWLSTGAYTTSYSSIEFNGFPPLQYYALN" /product="Pyridoxal 5-phosphate (PLP)-dependent ornithine decarboxylase (EC 4.1.1.17)"
/EC_number="4.1.1.19"
/EC_number="4.1.1.17"
/transl_table=11
CDS 2964950..2965576
/locus_tag="AUO97b_02887" /db_xref="SEED:fig|400667.82.peg.2752"
/translation="MMTISKALPNFFEYAEHQTSYALRDVILPKDLSLLVKWMHQPHI
IPQWQLNKPELELAVYFERMLCDDHQHLYIVQIDGKDVGYLEIYEAKRDRLALYYDAK
KDDLGWHVLLSEDAVGQGHFRAVMRMLSFLIFEHSNAEKVVGEPDQTMSVYEKIRADI

ALEPQGTIQLQEKTAMLYHCYREKFYQQCGHYYKTFKRKKADKCLVEN" /product="Siderophore synthetase small component, acetyltransferase" /transl_table=11
CDS 2965942..2966121
/locus_tag="AUO97b_02888" /db_xref="SEED:fig|400667.82.peg.2753"
/translation="MDQEWVFIDGSYIRVHQYASGARHGFERAIGQSRGGRITKIHLA TDANGLPIDFKITGG"
/product="transposase, IS4 family protein"
/transl_table=11
CDS 2966096..2966449
/locus_tag="AUO97b_02889" /db_xref="SEED:fig|400667.82.peg.2754"
/translation="MILKSLGGDVHDSQVAKQLIDIVGEATYLIADKGYDAEHIRICA
KNKNMISIIPLRSNSKRSNKEFDKYLYKLRHLVENAFARLKHFRAIATRFDKLARNYK SMIRIACIFIWCKAK"
/product="Mobile element protein"
/transl_table=11
CDS complement(2966610..2967479)
/locus_tag="AUO97b_02890" /db_xref="SEED:fig|400667.82.peg.2755"
/db_xref="GO:0000287"
/db_xref="GO:0006633"
/db_xref="GO:0008897"
/translation="MCNTNTLRHADVNIAKQYAQHKKLMFKDECLEYSILDIEPQSIH
QIRILNMNEQRLHCLYSQYKIFMPFHVTRASLKRKSEFFIGRLAAQFALATLGDYTNF
IIKKGERGEPLWPRYIRGSISHSMSNCTHGIAIATAAKHPTQFTDGITQSNSQYETQI
LGVDVEIKQNIEIFKENPNILNSLMSAQEHKQLIPFLPQLNCLHLIVFSAKESLIKAV
YNKYQTLIFFEAIHCIGIQPSTNTLSFYIPQISSELSTGQIIEVNYLESEHEIITYCC LPISSNQRVHNDA"
/product="4'-phosphopantetheinyl transferase entD (EC 2.7.8.-)"
/transl_table=11
CDS complement(2967494..2968144)
/locus_tag="AUO97b_02891" /db_xref="SEED:fig|400667.82.peg.2756"
/translation="MIALEYPGHGRKIQQPLSNNPDELAKQFTQEILSYGDVPFVLFG
HSVGASLIWKIEEKLQQANADQNLILKIVSARPSPRFQSIIHRYSEISDEQIVQELKL
YNNFPDEILNHPSALEFFLKIIKNDFILSDNLLKKSIQKSIIPLMAIFGEEDPYINNH
DIMQSWQDLTEEWFGAYSVRGDHFYFLNPEILQEVAELIQNKIEYIAKKSLAKPVA" /product="Iron aquisition yersiniabactin synthesis enzyme YbtT @ Thioesterase in siderophore biosynthesis gene cluster" /transl_table=11
CDS 2968478..2970685
/locus_tag="AUO97b_02892" /db_xref="SEED:fig|400667.82.peg.2757"
/db_xref="GO:0004872"
/db_xref="GO:0005215" /db_xref="GO:0006810"
/db_xref="GO:0016020"
/translation="MSSTYQLKRSALFLSLVLLSSQYVLAGDNLTDLNIQTLDATENS
KQNHSVEQSPSEQQLPTIVVSADQDQTLSAGQISKTSQVGMLGDKQDLDIPFSITSYT
SKYIADQQATSVADALRSEPSVRSVFSKGGIGEYFNIRGLYTQSHELAWNGLFGLLPH
NRVPTEFLERVDVFKGTSALLNGMSIGGAVGGVINVVPKRATSEDITRLTTTYKSDST
FGAHIDVGRRFGQEDQFGVRVNALKSLGDKALDGQEENRYLGSAAFDYKGEKFRASLD
LYDINEKLDGGQPLMVSFATSVVPKAPDSKLNHQPGSYSHTKTRGAIAHVEYDFAPEW
TAFASAGTKRQNGVGVIANNALGNAAQPNGDYMAVSRMNANNTNVDAGELGVRGKFNT
GAIKHNVVISANSLDQDTHMGLVMGTPWASNIYRPTRPALIASPPTYTPKTLDTTLTS
FAFADTLSILDDKYQLTLGVRHQNVKSQSYSILTGLPSSPYYDESAVTPMVGFVVKPW
DNTLSLYANYIEGLSQGGTIIDPSNPLVAEKTFKPYKSKQYEIGTKWDMGKFKNTLSI
YQITKNDLAQDANRNYSESEQRNRGIEWTTAGEIVKDIRLLGGVSYLEAEWKKTAGGL
MDGNDVKGIPHWQSNLGLEWDVQPVEGLTLTANTTYTGSLYANDTNTQKIPDWFVLDL
GGRYATNIAKHNVVFRGGVDNVFDKKHWAGGWNGYVGVGGDPRSYKLSMQIDF" /product="Ferrichrome-iron receptor"
/transl_table=11
CDS 2970805..2971953

/locus_tag="AUO97b_02893" /db_xref="SEED:fig|400667.82.peg.2758"
/translation="MSAAFAILRQSGIALDQLAMLNLIAIPMLGKILYAPFVDSFRLF
FQGQYRSWLIFAQFCMMILLVICGWLDIQRQFSIVLILFIVYTLFMSIQDVAIDGLAS
KIFQASERQFANSIQYASNLLGNIIGGGLLLILYPWLQWKGALTVLAVLTAITCIQLL
FYREPEDIYANPHRTITAQFKSLGQQLKVFIYKYRCWFLLLFIYPIGFTAVFGILNPM
LVDAKWSLVDIGFAIKIFGSIVGVFSAFLATLLINRLQRNKALITLTIIQGLTLLLFI
PLTLGYTSKPLVYLAIFGYFIANPGVMATLSTMIMDRASSMPAKATFFSLQLSLVVFM
GFAYSALGLALAQRIGYTAVVMLTFFCALAVAILAWKILPADAEHLAH" /product="AmpG protein"
/transl_table=11
CDS 2972005..2973129
/locus_tag="AUO97b_02894" /db_xref="SEED:fig|400667.82.peg.2759"
/translation="MAHFTTIKDQDRKLDIMDHVNQDHRKELLIIAQTYGKNNDIHDA
VIADIYEEGILLNTQVPSSSIKQDLFIHFELKGDLEEQILYLAYNSFAKQKIEFSNNA
KQFFEVVAKSQVTPNITRLTIKSQIPLPNYYAGYAYGFILKVVEKVPVVQQSTSNKSD
TLKNLFDRGFLWLMKHLSAPKRQKLMESMNKDVRLYTLRKAFAHGSNGEDLHFGYVDV
FTHGASPGSQWVQQLNTGSLISSRTETDDKHEHLHEGQAVLIADETAFPALAGILDFW
KNPIPPIVILLSADAVDFAYFDDFSFPENTNLQKITGAVHEQGSQTVELLKTFDQLDQ
VWGALENNAAKQIRHYFRNERKLAGKNNHIKGYWRLSKAH" /product="iron-chelator utilization
protein, putative" /transl_table=11
CDS 2973148..2974503
/locus_tag="AUO97b_02895" /db_xref="SEED:fig|400667.82.peg.2760"
/translation="MNMLKDISLPILKLALPLILIQICQASLGVINTMVAGRYHYIDL
AGIGLGSNMWTPVFILFTGILYVLVPKISAMQQANKAEIATLIQQGKKVAFLLSIFGF
ILIQLLALLCPWLIENPQVASIAKQYLHFVAFGVPGLVYMTLFRFISEGHSLLKPIIL
TYLILLFCDALLCVVLVKGIGFISEIGGAGTGLATAISAYIACFCMYQLVAKAVPELK
TNKAKVNIQQSLQLLKQGLPIGITLVLQILALATLAFFASQLGAKAIAAHQIVINIAM
LIIMIPIAISSATTIRVAYFSATHDHQSKLATSAAVAVITLIYGVVIAGLLVAFAEPL
TGLFSNDAEVLNIAAGLMSFIAVFLIFDAIQTVASGVLRGLQQFMQPLFVILFCYWLV
IIPLSYLMGVRGWLQERANVDVIWLVLTCGISLAAILLVIQSYRYLKKGELPVGETIS T"
/product="Multidrug resistance protein norM" /transl_table=11
CDS complement(2974707..2976824)
/locus_tag="AUO97b_02896" /db_xref="SEED:fig|400667.82.peg.2761"
/translation="MRYLDRLSLGLGRKVPVILQTESSECGLACLAMIAGYYGYESDL
LTLRQKYPISQKGATLTTLVKIANKLHLTTRPLKLELDELNQLRLPCILHWDLNHFVV
LKSVSATKITIVDPAFGEKVFTYDQASSHFTGIALELWPDSNFEEKKDKTSIRIFKLF
GEIRGLWKSLGQILILALVLEVFSLVSPFFMQWVIDHAIVSADLNLLTTLAIGFGLLM
ILSSLISLLQAWVVMHMATTLNVQWKANIFHHLVNLPTNFFQKRHLGDIISRFGSIDA
IQRTLTTSFITAILDGLMTIFTLILMFLYSTKLAWIAVITMLLYAVIRWIWYYPLRRA
TEDQIVHAAKQNTHFMETMRGIKTIKQFEKQDYRQATWLSLFVDQINAGLTTQKLNLM
FGFVNTLLFGLQNIIIVWLGATLVIEGEFTVGILMAFLAYKNQFGGRVSSLIDKYVEV
KMLSLHGERLADIVLTDQEKTDSYSFVPELSEQQKTGIDVKSLRFRYSEDESWIIHGI
NFNIPEGQSVAIVGPTGCGKTTLMNLLLGNLTPEHGEIKIGGQSIKNIGSNKLRNFIA
YVAQDDVLFAGSILENISFFDDGAQQDWVEQCAKMASVHDEIMAMPMGYQTLVGDMGN
ILSGGQKQRILLARALYRKPKILFLDEATSHLDIIKEKEINNMIKSLNITRIIIAHRP
ETISSVDRIIALNNGQIVSDQML" /product="Bacteriocin/lantibiotic efflux ABC
transporter, permease/ATP-binding protein" /transl_table=11
CDS complement(2976828..2978093)
/locus_tag="AUO97b_02897" /db_xref="SEED:fig|400667.82.peg.2762"
/translation="MSSNLFRKEALDAKQAKWTGTIILSRPFSFTYLTICALCIALVI
IAFAIWGSYTKRSTVQGQLIPQSGLIQVYTTQSGTILKKNVHEGQTVKKGDILFTIST
ASYGEQGSIADALAKQTQLKEQSIRNEITRMRFIHQDEKRTINNQIGLLNGTLVKVEN
LIANQRERVNLAKKNQQRYGNILHENAISHEEFEARKIDYLDQLAQYESLQREKVSLE
KQLTEQQISLSGLENRQNNQIEQLERLLSSNTQELIEMQSRQHIAIQANSSGVIGTIN
AEVGQFVDLSKPLLTVLPENTALIAQLYVPSRAIGFVKEGDQVLLRYQAYPYQKFGHA

KAQVLSVAKTALASQDLQTIGIISPQEQLNNEPVYLVRAKLDKQMVKAYGNDMPLQVG
MTLEGDIMHEQRKLYEWVLEPLFSITGKL"
/product="HlyD family secretion protein"
/transl_table=11 CDS complement(2978102..2979250)
/locus_tag="AUO97b_02898" /db_xref="SEED:fig|400667.82.peg.2763"
/translation="MLLNFSLDKDIFKNDFLYKKPYLFKSAIDSSGISWNDVNELYSR
GDISHRDFKLMNGYEVPKKEYIESYECLGVIEYRCITSVLYKYLRNGATLVRNRISNE
PFVDQISKQIATFAEARTLVGGYAAFSSKSSYKSHWDTRDVYAVQLLGRKRWILRKPN
FEFPLYMQQTKNFPDIKEPEEIYMDVILEAGDILYIPRGWWHDPLPLDEETFHLAVAT
FAPTGFEYMRWLQNIMPGILDCRKNFTNFENDVEMIDSFSHQVAEIIKDKNYYQSFMV
HHLAEQSVPSMLSLDILGNGKINHLNHNQKLYLNASILYYFDEGFVIINGNKINIDGI
SLSLIQYLFENPYSTVKDVLDQFNEYSLEKINKLLFQLSIENVIELVM" /product="hypothetical
protein"
/transl_table=11
CDS complement(2979524..2979862)
/locus_tag="AUO97b_02899" /db_xref="SEED:fig|400667.82.peg.2764"
/translation="MRELTLKELDLISGAAAQVPVMVGGAVIGAVGYAGAVIGGQEFS
WAELGLAVTGGAIAPMAGGAKVATMVTKELLHSMNSAFAAGVLSGALGNQPDKEKEDG NGYCEDGGNY"
/product="hypothetical protein"
/transl_table=11
CDS complement(2980034..2980387)
/locus_tag="AUO97b_02900" /db_xref="SEED:fig|400667.82.peg.2765"
/translation="MSLCTSRGAVHDFELFKRNLNQIHVGVFILADKGYQRIYTVYPN
SLLPLKAKKHCKLYPELKIYNQEINKRRIGIEHVFGSLKTFKILAERYRNRGKRLSLR FNLIAGIYNLELTKK"
/product="Mobile element protein"
/transl_table=11
CDS 2980736..2980900
/locus_tag="AUO97b_02901" /db_xref="SEED:fig|400667.82.peg.2766"
/translation="MGYSTLDKWIRETNPAGSSKRQLSPEQQRILELEKEVKQLREAN DILKKRMCTF"
/product="Mobile element protein"
/transl_table=11
CDS 2981112..2981339
/locus_tag="AUO97b_02902" /db_xref="SEED:fig|400667.82.peg.2767"
/translation="MHDLGYSMSERTVGRMLKKLGLRSRIARKYKHTTDSNHRLPTAP
NLLERQFTVIQPNTVSATLVQAQATITLIYN"
/product="Transposase"
/transl_table=11
CDS complement(2981576..2983162)
/locus_tag="AUO97b_02903" /db_xref="SEED:fig|400667.82.peg.2768"
/translation="MHIKSLPLDTLITGIGRLFKYENKPWFINLWGESEETKKGYYTS
FSHMHLLTKRRIINSTKNEHRKSGFHLKFTCPPPAEWISFAQSKSQFHFFGFDALATF
SNEAQTVKNIHIELPQLELARAFFFQNAYLTRSALELNVLAEDFDIQNRADHYLINVL
PSCEGSLALTHFNKPGFRRFLAYLLLNENIRASYESIAQQCQAFERINNTQRTWDFSF
IPPNLTDVNIEAYGYYDKLTNTFKINEIIGFSGLYAHIDKPVYFHHDKFSGASKKSGN
TSTIPPKPNHAEPKLNDEEEATPSNKPTIIDSPTTLLDFDDPFETKKVADKTGSKNDV
IVDDAREYIEELIGDVNTDEPGIGGTVKAGDFEGAKDQTNDAHLYLDRFSAFIQMLYK
LEENYGIQYSLTLKTLPEVEGFTKHLKTDNNPRCIAEVHFLYQGQHFILLEVDTSDNA
TRLSTQLLTIKNMNCWEEDYEKLRKFVVQKTLNWPNSFIKKIAIQHIRFNHPRIDDSQ
KVSIEDLEGWATRIYIGLNK"
/product="Transposon Tn7 transposition protein tnsE" /transl_table=11
CDS complement(2983152..2983796)
/locus_tag="AUO97b_02904" /db_xref="SEED:fig|400667.82.peg.2769"
/translation="MTPKDILQYVSSISANQYFVKRPTSTDNIPPVKIEEMRQKWQDI
VLLHGVTSGRKIKSGQATYIWLYRNDQNWLLTFNSKHLSKPQVRKNKVNWPIRDFSIT

KELFKILYRSNDDLACPRMSKSWFLNQLSKGNSISKNLHQLPLSSKFLSTYSEDTIAY
QIRRITHAMIRLGYTEYSTKDRWRILRLAGLSKERITQEAQIFLNMICEKIYAY" /product="Transposon Tn7 transposition protein tnsD" /transl_table=11
CDS complement(2983893..2984762)
/locus_tag="AUO97b_02905" /db_xref="SEED:fig|400667.82.peg.2770"
/translation="MSRGNIAIWFDPATQWYAPSKGKQGRNQTYSDAAIQCCLMIKSL
FRLSLRMVTGFVQSLIKLCGLNWIAPDYTTLCRRQKHIDIVINYQKSSDGLHLLMDST
GMKFLGEGEWKRKKHGPEYRRQWRKLHIGIDAKTLQIRAVQLTTNNVSDSQVLGDLLD
QIPQDERIDSVYTDGAYDTKQCRQVIADRQAHAVIPPRKNAKPWKDTKSSSLERNELL
RTVKRLGRTLWKKWSGYHRRSLVETKMHCIKLLGDKLSARSFDSQVNEIHARVAVLNR FTELGRPLTQVTP"
/product="Mobile element protein"
/transl_table=11
CDS complement(2984833..2985729)
/locus_tag="AUO97b_02906" /db_xref="SEED:fig|400667.82.peg.2771"
/translation="MWNFPTPLPDELIYSTVARAGLHHGIQSPKQLLDEVFQDRKVVA
TVDLPSHLNAIVHLLQRTDRFVLIDLIYKHTMFGLYAPFVQESHRLKAITLMTEQASG
SVHLMFGLNASRVPNKNKFHYCQICIQQQRETYGEYFWNRAWFVPNLPICLKHNCSLL
SQDYIQQQHRHLFLPLLPNQTQDLPEPYFKEDFILGQMSVELLQLDPQPSPSHEQWTK
FYLEIADAKNCRRGSQVRHEQTYEQVINKFKYVYLNSKNLGINPDQDTSWLKTIFRKH
RKAFSFLGLCCTNLSVKGFSAI"
/product="Transposon Tn7 transposition protein tnsD" /transl_table=11
CDS complement(2985745..2987394)
/locus_tag="AUO97b_02907" /db_xref="SEED:fig|400667.82.peg.2772"
/translation="MNNLITDAEYHDTGVHAYKGNPFIEALPPIQEPFEDTKILISKV
TPTLDDLKQKRVIRAHNIHQITGSFFQPLAMHMLLSERISLMIRGGYVGRNPNTGKLQ
QHLQNGYERLQKGDLDAKRFDGAESTAHSMAIIGCSGSGKTTSLRRILNTYPQVIFHP
TLNRHQIVYLKIDCSHDGSLKELCLNFFRAIDKVIGSNYEKKYGMKRHGVETMLALMA
QTANMFALGLLVIDEIQHLSRAKSGGSEKMLNFFVTMSNTIGIPVLFVGTPKARDIFD
LDLRSSRRAAGLGSIFWNPISQYLPDQKSQNPEWIAFTNTLWKLQYLQKRDPILSDEI
RDTWFDLSQGILDIVVKLFILAQLRALATGAEKITVELLKRVYEDELKLVHPMLSALR
SGIPERIAQFSDLAVPDMDKKIIHLIQKISASTVESEEDRALKQLSNDDQRRIYLMLK
DEFPSDSIIFAIQKIVELKPQISRQQLLASIIQAMETDNTTSQVTVTKKKNQRIGLKQ
WNELEDTDLRHIFSMNSTSPESIYTQLNHHGLILQMSDLLS" /product="Transposon Tn7 transposition protein tnsC" /transl_table=11
CDS complement(2987391..2989481)
/locus_tag="AUO97b_02908" /db_xref="SEED:fig|400667.82.peg.2773"
/translation="MWRTNEVVKFDESHYRILFFQSGEIIWINMEETKGVPSLVLEEQ
LSQWIDEKRLFRSSDPFEYLKSIEPPINSVAFEKRAQAYQIIEPIIYDKGCFNEEIRA
KRVRLVEQKNIATKATIYKYLRRYWQRGQIPNALLPDYRNAGAPGKPRTLAGTKKSGA
KRKFGDGTGIKVTPEIERLFRLIAESELLNDKKINITSAHRKFEELFVQHYPHIKQAD
IPTRRQFDYFYKREYQLPQRIEARTPVLSFQKDIRPLSGTATANTLGPGSRYEIDATI
ADIYLVADDDRSKILGRPTLYVVVDVFSRMVVGFYIGFHNPSYVVAMQAIVNACSDKV
DLCKMFGIDIQPEQWPTIGLPDAILADRGEMMSHQVERLIYGYNVRIENAPAYRGDAK
GIVERYFGTLQAEFKPYAPGVVKGNRIKKHGESDYRLEAALPITAFTKMIIKTILYRN
QHHVLDTYDREADFPPDLPSIPLHIWNWGIQNRTGHLRIVEPTPFRIMILPRKKVSIS
TSGVNLWGLYYIGSEILREGWLHRSKEVSRPQDLQAAYDPSNVDKIYLFPKHDSTIYW
ECSLSTKSRQFSGKSFWQVWEIQDQEKNLHAIGKDNSKAARRELDQFIRDTIAEAKKM
ATNSGVSNRQRLSEIKSNKKSARENEKLSDNYSKTDKHPKDKGTIISFKPKQDESSYP QFVPGFFDDEDDQS"
/product="Transposon Tn7 transposition protein tnsB" /transl_table=11
CDS complement(2989465..2990274)
/locus_tag="AUO97b_02909" /db_xref="SEED:fig|400667.82.peg.2774"
/translation="MSRAPRFFTQEQISKRITSGHGQGMGKDYQPWLTIQDVPSHGVS
HRIYSHKTQRVHHLLSNLELYVFLILDWSSSVQDIREQFPLNLDDTKEICLEHGLRHP
SIQGSDQVMSSDFLIDTNNKNKPQFVIQVKPLKELLKERTIEKLEIERRYWALKEIPW

SIVTEQNIPTTARKNLEWLSPFVTEHIDPDIWDKLHLISYYFSKKPSVSIIKLCKEVD
VQEGTTAGTTLQYLRILIANRLIKFDIQQPFQKLKGSDLEFDLEVSYVANQ" /product="Transposon Tn7 transposition protein tnsA" /transl_table=11
CDS 2990419..2990562
/locus_tag="AUO97b_02910" /db_xref="SEED:fig|400667.82.peg.2775"
/translation="MSLILASIIGQLYNGTLIPMTCGFVILCGLAFMMTVYENRYLKK SNV"
/product="MFS family multidrug efflux protein, similarity to bicyclomycin resistance protein Bcr" /transl_table=11
CDS 2990681..2990809 /locus_tag="AUO97b_02911" /db_xref="SEED:fig|400667.82.peg.2776" /translation="MYLTPYAWIAVGSIFAFSVTTIKIGDQNDEKQKSHKNDVHKR"
/product="hypothetical protein"
/transl_table=11
CDS 2990766..2990888
/locus_tag="AUO97b_02912" /db_xref="SEED:fig|400667.82.peg.2777"
/translation="MKNKNHIRTMYTRDKGWRLFGLALAIQILFIGANYLAMMS"
/product="FIG00352577: hypothetical protein" /transl_table=11
CDS 2990957..2991274
/locus_tag="AUO97b_02913" /db_xref="SEED:fig|400667.82.peg.2778"
/translation="MLKLIYYVPESHLESTKQSIFAAGAGGIGNYEQCAWQVKGIGQF
KPVKGADPYIGELGELEQVDEWRVETIVLEEKASAVAKALKASHPYEEPAFEFIQIIE VDF"
/product="Bsu YqfO NIF3/CutA domain" /transl_table=11
CDS complement(2991271..2992071)
/locus_tag="AUO97b_02914" /db_xref="SEED:fig|400667.82.peg.2779"
/db_xref="GO:0004300"
/translation="MTLSCIQQPHQHLQANLEGGVLTLAINRPEAKNALYGELYLWIA
KALDEADQNKDVRVIVLRGAEHDFTAGNDMKDFMGFVQNPNAGPAGQVPPFVLLKSAA
RLSKPLIIAVKGVAIGIGVTILLQADLVFADNTALFQIPFVSLGLSPEGGASQLLVKQ
AGYHKAAELLFTAKKFNAETALQAGLVNEIVEDAYATAQATAQHLTALPLASLKQTKA
LMKHDLDQIIECIDHEAEIFMQRVQSPEMLEAVQAFMQKRKPDFSQFN" /product="Enoyl-CoA hydratase (EC 4.2.1.17)" /EC_number="4.2.1.17"
/transl_table=11
CDS 2992181..2992771
/locus_tag="AUO97b_02915" /db_xref="SEED:fig|400667.82.peg.2780"
/db_xref="GO:0047631"
/db_xref="GO:0000309"
/translation="MYTFDYLVFIGRFQPFHLAHMQTIEIALQQSRYVILALGSAQME
RNIKNPFLAIEREQMILSNFSLDEQKRIRFVHVVDVYNDEKWVKQVKSLVNGVIEPNS
KVGLIGHFKDESSYYLRLFPEWVMVELDSLKDSISATPMREAYYQGKIKTDAFPKGTI
QFLEEFQNTPLYLQLQQKFLQADHSNIEETVSSHKE" /product="Nicotinamide-nucleotide adenylyltransferase, NadM family (EC 2.7.7.1) @ Nicotinate-nucleotide adenylyltransferase, NadM family (EC 2.7.7.18)" /EC_number="2.7.7.1"
/EC_number="3.6.1.13"
/transl_table=11
CDS complement(2992842..2993411)
/locus_tag="AUO97b_02916" /db_xref="SEED:fig|400667.82.peg.2781"
/translation="MNLLKVCTLLPCSFVIAACSVNSSSTPPTLSSSTPQVQKSSIEI
SCQDLQNPAYRQAVLNAINQIRQDSRQCGQQYFSAANPLSWNNNLYQGANAHSKDMAN
NNFLGHVGSTGLDLRARLKKYHMLSKANGENVASGQKTLNEVMAKWIASPLHCSNIMN
PRYTEYAIACASDQSAKQRSYWTQQFAGF"
/product="Uncharacterized protein, YkwD family" /transl_table=11
CDS complement(2993537..2993998)
/locus_tag="AUO97b_02917" /db_xref="SEED:fig|400667.82.peg.2782"
/translation="MSENIQLPNQVFPTTHGEVNLAEVDNDWLIIYFYPKDSTPGCTT
QAVGFSCLKDQFDALGARIFGVSRDSVKAHQNFTEKQALTIDLISDKEEVLCKHFDVI

KEKNMYGKKVMGIERSTFIFHNKTLVKSYRKVKAAGHAEQVLEDLKALQTA" /product="Thiol peroxidase, Bcp-type (EC 1.11.1.15)" /EC_number="1.11.1.15"
/transl_table=11
CDS complement(2994016..2994609)
/locus_tag="AUO97b_02918" /db_xref="SEED:fig|400667.82.peg.2783"
/translation="MQTGNVIGLIVMLEEQGQRSRSHAMPLDIIRAQAQAIGLPVFMA
SSSWNDYENKFINLLNEAKQKGAEVLVTGDLDMPEHGCWHDRVTKTVGLKLGMPLWLR
PHREVVEEFIQLGFQSVVVTVNLKLGMKVEDLGQVLSLEYIQELENRGIDPCGEGGEF
HTTVIDGPIFNKAIPVRKLDIVYHEEYAFLPLELDQI"
/product="COG2102: Predicted ATPases of PP-loop superfamily" /transl_table=11
CDS complement(2994793..2995209)
/locus_tag="AUO97b_02919" /db_xref="SEED:fig|400667.82.peg.2784"
/translation="MKLLKSNLILSSLFSAAALMAGSAQAAENPVQAAYKSTNVKGAL
INVCKDQTAKGGKLSSAEVSKFCSCQVEAQGKVTEAQKWEIQSAINAKKSPSSLAFVQ
QQNRDLKACFGPQLTTKLEKLTEEAMKAAQQQAPKK" /product="hypothetical protein"
/transl_table=11
CDS complement(2995223..2995894)
/locus_tag="AUO97b_02920" /db_xref="SEED:fig|400667.82.peg.2785"
/db_xref="GO:0005554"
/translation="MRPRAIVLLSGGLDSTTCLAWAQARYECIAISFMYGQRSTTELD
AAKALAQRAGVEHRVINIDLANLGGSALTDHNIAVPEQLQEGIPVTYVPARNTIFLSY
ALAAAEVFNAEAIVIGINAVDYSGYPDCRPEFIDAFANMARLATKAGVEGKPLKFETP
LLHLSKANIIRLGIEHGVDYSQTVSCYQADAQGRACGKCDSCRLRKEGFIDAGVTDPT RYIPQ"
/product="7-cyano-7-deazaguanine synthase (EC 6.3.4.20)" /transl_table=11
CDS complement(2995935..2996645)
/locus_tag="AUO97b_02921" /db_xref="SEED:fig|400667.82.peg.2786"
/db_xref="GO:0003824"
/db_xref="GO:0005506"
/translation="MASLRSSAIPVSDPASGLRITEIFYSLQGEANASGLPTVFIRLT
GCPLRCSYCDTTYSFEGGERLSLEHIIETAEKYQTPYICVTGGEPLAQPNCLILLQRL
CDAGFDVSLETSGALDVSRVDPRVSKVLDLKTPTSGEEHRNLISNLDHLTPRDQIKFV
ICNREDYEWSKQQVEQYQLQTKVSTVWFSPAFAVEKGAVGLPRLARDLAQWILDDKLP
VRFQLQLHKLLWNDESGR" /product="7-carboxy-7-deazaguanine synthase (EC 4.3.99.3)" /transl_table=11
CDS complement(2996724..2997545)
/locus_tag="AUO97b_02922" /db_xref="SEED:fig|400667.82.peg.2787"
/db_xref="GO:0008666"
/translation="MSQLSTIIEQAFEDRANFTAADCPSEIRQAVEEAIAGLDNGTLR
VAEKINGEWVVHQWLKKAVLLSFKLNDNKPIESCDLRFYDKVETKFSGWTEEQFKAAG
VRVVPPAVARRGSFQAKNVVLMPSYVNIGAYVDEGTMVDTWATVGSCAQIGKNVHLSG
GVGIGGVLEPLQANPTIIEDNCFIGARSEIVEGVIVEEGSVISMGVYIGQSTRIYDRE
TGEIHYGRVPAGSVVVPGNLPSADGKYSLYAAIIVKKVDAQTRAKTSLNDLLRAD" /product="2,3,4,5-tetrahydropyridine-2,6-dicarboxylate N-succinyltransferase (EC 2.3.1.117)"
/EC_number="2.3.1.117"
/transl_table=11
CDS 2997840..2998568
/locus_tag="AUO97b_02923" /db_xref="SEED:fig|400667.82.peg.2788"
/translation="MKVLRVLVTTTALLAAGAAMADEAVVHDSYAFDKNQLIPVGARA
EVGTTGYGGALLWQANPYVGLALGYNGGDISWRDDLSINGTKYDVDMDNNNVYLNAEI
RPWGASTNRWAQGLYVAAGAAYLDNDYDLTKRSSDGTIKINGTNYSYNGSVNGQLSYK
NDIAPYLGFGFAPKINKNWGVFGEVGAYYTGNPTVELDKQGTFVNAAGGNADADLRAE
ENKIRNDDKYKWFPVGKVGVNFYW"
/product="Outer membrane protein G1b"
/transl_table=11

CDS complement(2998660..2999583)
/locus_tag="AUO97b_02924" /db_xref="SEED:fig|400667.82.peg.2789"
/translation="MNFQQLRIIRETVRQNFNLTEASAALYTSQSGVSKHIKDLEDEL
GVQLFVRKGKRLLGLTEPGQSLLSIVERMLVDAENIKRLADDFNKVDEGTLTIATTHT
QARYVLPPIVNQFKKLFPKVHLILQQASPVEIAEMLLQGEADIGIATESLTTEENLAS
IPYYQWQHSIITPQDHPLTQLDKIDLDALSEYPLITYHGGFTGRSKIDKAFEDAQIDA
DIVMSALDADVIKTYVELGMGVGIVNDVAYDAERDYRLKQIKTDIFGVNTTWIAVRKG
HLLRGYGYEFISLCSPDADIKALKKIAYPDE"
/product="Alkanesulfonate utilization operon LysR-family regulator Cbl"
/transl_table=11
CDS complement(2999655..3000716)
/locus_tag="AUO97b_02925" /db_xref="SEED:fig|400667.82.peg.2790"
/translation="MSIQVKNIEKHFGAFHALKNISLDFPEGELVALLGPSGCGKTTL
LRIIAGLESADGGQVLLEGEDATNVHVRERQVGFVFQHYALFRHMTVFDNIAFGLRVR
PRATRPSEAEIKKRVTRLLDLVQLGFLADRYPAQLSGGQRQRIALARALAVEPRVLLL
DEPFGALDAKVRKELRRWLRNLHDELHITSIFVTHDQEEALEVADQIIVMNKGNVEQI
GSPREVYEKPATPFVFDFLGQANRFEGEHASGIIRIGNDRIELPTTVQAPQGKVIAFA
RPDELHIHAQPQANTIEATFVREVWIAGKVVAELQDRNGRLIEIALSSEAAKQHAFKP
NQTVWVSASQLHLFADQVA" /product="Sulfate and thiosulfate import ATP-binding
protein CysA (EC 3.6.3.25)" /EC_number="3.6.3.25"
/transl_table=11
CDS complement(3000728..3001612)
/locus_tag="AUO97b_02926" /db_xref="SEED:fig|400667.82.peg.2791"
/translation="MNLHTDSNALALKLQSRDATREPTWVRYTLITIALIFFLSCLML
PLILVFVEAFKQGLEVYVQALIDPDTLSAVKLTLLTAAIAVPINVIFGVAAAWAVSKF
QFKGKAILTTIIDMPFSVSPVIAGLMIVLIFGAQGWLGGWLMEHDIKILYAVPAIVLA
TIFITVPFVARELIPLMEAQGTEEEEAAIVLGASGWQTFWKVTLPNIKWGLIYGVILC
NARAMGEFGAVSVVSGHIRGETNTLPLHVEILYNEYTFSAAFAVSSLLAFLALLTLIL
KTWLELHQDKPEPHSTDS"
/product="Sulfate transport system permease protein CysW" /transl_table=11
CDS complement(3001609..3002400)
/locus_tag="AUO97b_02927" /db_xref="SEED:fig|400667.82.peg.2792"
/translation="MGFTLAYVSFIVLIPLAAVFIKSFGIGWDGLWEILTSERILKSL
QLSFSSALIAAFINVVFGLLLAWCLVRYNFPGKRLVDALVDLPFALPTAVAGIALTSL
YAPTGWIGQYLEPLGIQVAYTPIGITLALVFIGIPFIVRTVQPVLSDIETELEEAASA
LGANRWQTITKIILPILLPALFTGFALAFARGVGEYGSVIFIAGNQPFKTEIAPLMII
SRLEEYDYAGATTIAAVMLVLSFIILFVINLLQAWANRRTGRNVT" /product="Sulfate transport
system permease protein CysT" /transl_table=11
CDS 3002441..3002566
/locus_tag="AUO97b_02928" /db_xref="SEED:fig|400667.82.peg.2793"
/translation="MIFPQREKPAETSKRINKDKRRGIKLGIKQISKRNVSLPDQ"
/product="hypothetical protein"
/transl_table=11
CDS complement(3002523..3003131)
/locus_tag="AUO97b_02929" /db_xref="SEED:fig|400667.82.peg.2794"
/translation="MTHTVIVPGVGGSEHQHWQSWLQRQLVSSSRVEQKNWDRPVLSE
WIEQFVNTVQAVQAPVQIVAHSFGCLTSVAALAEHPELQLQVKKLILVAPANPARFGE
AGFARHSLTDYKDYFHQLKINVPATLLISENDPWLDFFDALQLAKSWQLTPINLGQVG
HINVASGFGPFPDLLNHLLPEDSMCSHSSIDPVKTHFSLKFA" /product="Hydrolase, alpha/beta
fold family" /transl_table=11
CDS complement(3003162..3004169)
/locus_tag="AUO97b_02930" /db_xref="SEED:fig|400667.82.peg.2795"
/translation="MRFSQLKVGVIAALLSVSSFAAQEFLNVSYDPTRELYTDFNKQF
GTYWKQRTGQDIEFKQSHGGSGKQARAVIDGLNADVVTLALAADIDEIAEKAKLLPTD

WQKKLPQNSTPYTSTIVFLVRKGNPKQIKDWGDLIKPGVEIITPNPKTSGGARWNYLA
AWAWAKHQAGGNDAKAQEFVRQIYKHTKVLDSGARGATTTFAERGIGDVLLAWENEAH
LAIREQPGKFEIVTPSLSILAEPPVAIVEKNAAKKGNLTIAKGYLNYLYSPAGQEIAA
RNFYRPRNAAVLKKYSNVFKPLKLVTIDKEFGGWTKVQKQHFDNGGVFDQIVKINSAE K"
/product="Sulfate and thiosulfate binding protein CysP" /transl_table=11
CDS 3004433..3005245
/locus_tag="AUO97b_02931" /db_xref="SEED:fig|400667.82.peg.2796"
/db_xref="GO:0008696"
/translation="MWCFKNGQPVETIPLLDRAFHYGDGCFTTIRVFQNKIELKARHW
ERLKLACQKLSLVADFELIEQSLQHLQNQNLVLNGTLKIVISRGEGDRGYSLPKHAAD
IYIWFYPKALEQFQPDFIQCGVLNQALGLTMPSLVGLKSLNRLEQVLLKKEADQQGWV
EALVTDVQGYIVEGVSSNCFIRLNDRWITPELRYNGVHGVMRAEILARMQHYGIACEV
RIIELDEVPQIQSLFFCNALHPMRVVTQISEQILESQACLNLFHTLNLNQIH"
/product="Aminodeoxychorismate lyase (EC 4.1.3.38)" /EC_number="4.1.3.38"
/transl_table=11
CDS 3005247..3006317
/locus_tag="AUO97b_02932" /db_xref="SEED:fig|400667.82.peg.2797"
/translation="MPKPPVNAKAKNAKKKGKKTASNFPKKLVLIGCFIVLISIFAIL
WSSLFKAYPIEGKKQMLSITSGETYSGFIDRLAKENKIHFPIVLKLYQKFMIHDSMKA
GVYEIEQGMSVRQVLEMLSDADNAQMNRVLVIEGTTFKQLITALKNDKNVKNTILDLP
DDQLMKALGIPYHHPEGLFAPNTYFFAKGETDKKILTDLYHRQMKALDAAWAKRAPNL
PYKDKYEALIMASIVEKETSLDSELTQVSGVFVRRLKLGMRLQTDPTVIYGMGANYKG
NITREDLRTPTPYNTYTINGLPPTPIALPSQKAIEAALHPDDSNNIYFVATGNGGHKF
TADLQAHNQAVQEYLSVLRSKK"
/product="Murein endolytic transglycosylase MltG" /transl_table=11
CDS 3006327..3006926
/locus_tag="AUO97b_02933" /db_xref="SEED:fig|400667.82.peg.2798"
/db_xref="GO:0004798"
/translation="MFISFEGTEGVGKTTLIRKIHQHFEEQGKQVVLTREPGGTPLAE
QIRSMLLAVNHDENMSHDTELLLIYAARAQHLQQVILPALESNKIVLSDRFTDASFAY
QCSGRGLSQDKLQLLNQNFVSRMPEVTFWLDAPIELGMNRARERGALDRFEQEKLSFF
TKVREGYETLWKVEPERIKRLDATQSPDQVFEQALQYLA" /product="Thymidylate kinase (EC
2.7.4.9)" /EC_number="2.7.4.9"
/transl_table=11
CDS 3007065..3007481
/locus_tag="AUO97b_02934" /db_xref="SEED:fig|400667.82.peg.2799"
/translation="MKSSKEEIVAFLEKEFPQSLEHCTIESVTSKAAVVYYHVDQRHQ
RPGGTISGPTMMTLADFALYIAILGEIGIIGLAVTTNFNINFLRKPAGDQDLRSECKL
MKVGKSLVVGEVWIYSVGQEEPVAHVTATYSIPPRP" /product="Phenylacetic acid
degradation-related protein" /transl_table=11
CDS complement(3007535..3009184)
/locus_tag="AUO97b_02935" /db_xref="SEED:fig|400667.82.peg.2800"
/db_xref="GO:0008734"
/translation="MRMDMPNQQTIHHFDVIIVGSGGAGLSLALSLPSHFNIAVLAKA
ALTEASTFYAQGGVAAVLDETDSIQQHINDTMIAGAHLCEMDAVQHTVEGGRPSVDFL
LKQGVQFTLDEDEQLHLTREGGHSQRRIIHSADATGKAISTTLVERAKERKNITIFEN
YIAIDLITSHKLGHTEQANRAIGLYALDESTEKVHTFLAPFTALACGGAMKAYLYTSN
PDIATGDGIAMAYRAGCRVANMEFNQFHPTCLYHPQARSFLITEAMRGEGAYLRLPDG
ERFMLRFDDRAELAPRDIVARTIDHEIKRLGIRHVWLDITHKSPEFIKEHFPTLYARL
LELGIDITKDMIPVVPAAHYTCGGVVVDSNSQTDIEGLYAIGETSYTGLHGANRMASN
SLLECFVYGMSAAKDIESKFDKNFKLPEVPTWDDSQVTNPDEDVVILQNWDELRSTMW
NYVGIVRTTKRLERALHRIEMLKREITEYYQDYRVSKNLIELRNLVLVSEMIVRCAMH
RKESRGLHYTLDYPETSSEIRKTVLTPPNFAVEQPLVNTSV" /product="L-aspartate oxidase (EC
1.4.3.16)" /EC_number="1.4.3.16" /transl_table=11

CDS 3009409..3010785
/locus_tag="AUO97b_02936" /db_xref="SEED:fig|400667.82.peg.2801"
/translation="MKSRYLQQGMYAAVFTVAAVQANAAVDFSNLVEQVSPAVVSVNV
VKKMTQDELLQQQVPEILKRFFGNQVIIPQQQGPQEKTAYGSAFFISKDGYLLTNHHV
IENASRISITLNDRREIDATVVGSDERTDVALLKVNGTNYPALRVGNVDRLRVGEPVL
AIGSPFGFDYSASAGIVSAKSRNMSGETSVPFIQTDVALNPGNSGGPLFNQNGEVVGV
NSRIFSGTGGYMGLSFSIPIDVAMDVAEQLKTKGKVTRSYLGVMMQDIDRNLADAYKL
PKPEGALITQVSPNSPAQKAGLRAGDVILKLNGASVLRTSDLLYALNKVQPNQTVQFE
VLRDDKTRNISATLATAPDETPATGNQASASKGPVLGMSIRDLAVPEKNALGIKGGIY
VQDVRRGGLASLSNIIPGDVIIQVNNTQILNSQDFAKVVSNLPKNTVARVGIIRQGQR AMLGLRIQ"
/product="Serine protease precursor MucD/AlgY associated with sigma factor RpoE" /transl_table=11 CDS 3010906..3011361
/locus_tag="AUO97b_02937" /db_xref="SEED:fig|400667.82.peg.2802"
/translation="MSSIFDLHIQVQPQHIDALGHVNNVMYVQWMQDVAAAHVETLGV
GVTKYLELKHAMVAVEHHVQYRKAAFEGEEIVLRTWLDDINALYSFRQYAFFRPSDQA
ILFVGNTKWACIEIASGRPKRMSPTFTQAYKPLDSSINPLDFTVSYSSN" /product="FIG002571: 4-hydroxybenzoyl-CoA thioesterase domain protein" /transl_table=11
CDS 3011375..3011923
/locus_tag="AUO97b_02938" /db_xref="SEED:fig|400667.82.peg.2803"
/translation="MERLFSIETDRLILNELKEDDLKFIFELFSNSIVLEFYDLKAFT
SLEEAEALIEKMHKKWDQQLGFRHAIRHKHDGIIFGTCGVNSIKQINDDFGVVIGYEL
HPDAWGVGYMQEALIGLIQYLKQEQLFRKKIKYIWAEIFEKNVRSQQVVNRLGFELIG
TTVNHTVNTLGGDLLKFELKLY"
/product="Acetyltransferase, GNAT family" /EC_number="2.3.1.128"
/transl_table=11
CDS 3012107..3013924
/locus_tag="AUO97b_02939" /db_xref="SEED:fig|400667.82.peg.2804"
/translation="MAQAKKSVDIKNIRNFSIIAHIDHGKSTLADRFIQMCGGLQDRE
MQAQVLDSMELERERGITIKAASVTLYYTHPNGQEYQLNFIDTPGHVDFSYEVSRSLA
ACEGALLVVDAAQGVEAQSVANCYTAIEQGLEVLPILNKIDLPQAEPERVIHEIEEII
GIEATNAPTCSAKTGLGVEGVLETLVDVIPAPTGDREAPLQALIIDSWFDNYLGVVSL
VRIKDGRIRKGDKMLVKSTGQTHIVTSVGVFNPKHTETGVLEAGEVGFVIAGIKDIFG
APVGDTITLSTTPEVASLPGFKKVKPQVYAGLFPIDASDFEPFREALQKLQINDSALF
FEPESSDALGFGFRCGFLGMLHMEIVQERLEREYDLDLISSAPTVVYEAVTKKGDTIY
IDSPSKMPDGSVVEDLREPIAECHILVPQEYLGNVMTLCIERRGVQKDMKFLGNQVSI
TFEIPMAEVVMDFFDKLKSCSRGFASLDYNFIRFESSSLVKVDVLINGEKVDALAMIC
HRNDARHRGIALVEKMKDLIPRQMFDVAIQAAIGAQIIARSTVKAMRKNVLAKCYGGD
VSRKKKLLAKQKEGKKRMKQVGSVEIPQEAFLAVLKVER" /product="Translation elongation factor LepA" /transl_table=11
CDS 3013995..3014822
/locus_tag="AUO97b_02940" /db_xref="SEED:fig|400667.82.peg.2805"
/db_xref="GO:0006508"
/db_xref="GO:0008236"
/translation="MDFDFNLILVPVTLILFAVWLLDKLVFKQRANKGRENENFVITW
AYDFWPVLAVVLVLRSFLYEPFNIPSDSMVPTLETGDFILVNKFDYGVRLPIVNKKVI
DIGEPKRGDVIVFRYPPQPTISYIKRVIGLPGDHIVYDHGQLIINGQKIPKVPTQFSR
EKDALDTPTSIYHKETIGDHTFTMRELEGVNVARQAPFINYVDNGKYANQDGLYWEVT
VPKGHYFAMGDNRDQSADSRFWGFVPEENLTGRAFYVWMHKEPGFHLPSFNRNGKID" /product="Signal peptidase I (EC 3.4.21.89)" /EC_number="3.4.21.89" /transl_table=11
CDS 3014846..3015220
/locus_tag="AUO97b_02941" /db_xref="SEED:fig|400667.82.peg.2806"
/translation="MRKAQQGTSYLAILFGVVIFAIAVKAVLAVWPAYWDDRVINNQI
TELMQQSSADITPQKFMTQMDQRLEMNNIRDLHFKEIAEVFNQPSLTVKKKYEVRKPF
LLNIDLVLTFEKSFDKTSVQAK"

/product="hypothetical protein"
/transl_table=11
CDS 3015417..3015884
/locus_tag="AUO97b_02942" /db_xref="SEED:fig|400667.82.peg.2807"
/db_xref="GO:0004525"
/translation="MRATLVRQEALGKIATDLQLSRCLILSTGELKSGGHHRESILAD
TVEAIIGAIYLDSSDLNLLKDIVLKWYTPYLDHIEPTDQLKDPKSRLQEYLQARKKPL
PVYEVVDIQGDAPHQHFKVECLVDGLSKIHGEGSSRRFAEQAAAAEILKLLEQ" /product="Ribonuclease III (EC 3.1.26.3)" /EC_number="3.1.26.3" /transl_table=11
CDS 3015890..3016918
/locus_tag="AUO97b_02943" /db_xref="SEED:fig|400667.82.peg.2808"
/db_xref="GO:0003676"
/db_xref="GO:0005525"
/db_xref="GO:0005622"
/translation="MSMHSDQINPDSNENQDPNNLIDQFFSSKGVTIPSDFKSGFVAI
VGRPNVGKSTLMNHLLGQKLSITSRKPQTTRHKIIGIDSREKMQAVYVDTPGMHKKEV
RAINKMMNRAAHSALRDVNLVLFVIDAHKWTQNDDLVLEKLKNADMPVILVINKADTF
GDKREILPLIQERAKLMNFAEIVPVSALRGANLEHLSETIEKYLPYQPPLYSFDQITD
RSERFLASEIIREKIMRQLGEELPYDLTVQIESFKTEEATVNEKTGRLKPACTYIDAT
IFVDRPGQKAIVIGEKGAKLKTIGMDARKDMEKMFEQKIMLTLWVKVKGGWSDDERAL KSLGYSDI"
/product="GTP-binding protein Era"
/transl_table=11
CDS 3016982..3017137
/locus_tag="AUO97b_02944" /db_xref="SEED:fig|400667.82.peg.2809"
/translation="MLQGCVYKLVTVPVGIAYKTTKGVVKGTAAVVGAVIPDGDDDDK KEKESEE"
/product="hypothetical protein"
/transl_table=11
CDS 3017147..3017860
/locus_tag="AUO97b_02945" /db_xref="SEED:fig|400667.82.peg.2810"
/translation="MMRNEVLHGYMIHHRKYREKSHIVHLFTQEYGRVDGILRQTPPP
QYQPIRLQATGKSELKNFTKLEILNQPVFFFGDAFFAGFYLNEIVLRLCPLEEAMPQT
FEQYQLTLLQLQQLSSHENPDLFLRQILRQFEHILLAELGYAIDFSIDSSQKEILPQQ
FYQFQVTEGFAPVVQASRSSLSGKAILSMLNYEQGQDFSHEQLQLLGKLYRQMITSLL
GDRPLKSRQLWIQNTQTQS"
/product="DNA recombination and repair protein RecO" /transl_table=11
CDS 3017874..3018599
/locus_tag="AUO97b_02946" /db_xref="SEED:fig|400667.82.peg.2811"
/translation="MAALLGVNIDHVATLRQARGTAYPDPVEAALICEQAGAEGITLH
LREDRRHIQDDDVRRMRPLLKTRMNLEMAVTDEMVEFAKEIQPQHVCFVPERRQEVTT
EGGLDVVGNFEKVKAATQALAAIGCDVSLFIDADFAQIDAAIACGAPTIELHTGAYAD
ATTEQEQQEELARIVKGVEYAASKGLVVNAGHGLNLKNIAPIAAIPQIHELNIGHSII
AESVFVGLVQAVKDMKTAIQAAG"
/product="Pyridoxine 5'-phosphate synthase (EC 2.6.99.2)"
/EC_number="2.6.99.2"
/transl_table=11
CDS 3018606..3019250
/locus_tag="AUO97b_02947" /db_xref="SEED:fig|400667.82.peg.2812"
/translation="MSSINYDELMQPVLAFLGCQTPKEWLDEAVNNLDILMQDHANCE
KKAAGTAMNLMFRYSFFTDLQVKLAQLVREEMLHYEQVLEFMTKRGQEWKGLSAGRYA
GGLRKEIRTYEPEALIDVLVIGAFVEARSCERFYALAPRVDDELGRYYRYLLKSESRH
FEDYLALALDVAKTAKMKDPEEDIQQRIEHIREVEKNLILTPDDTFRFHSGIPA" /product="tRNA-(ms[2]io[6]A)-hydroxylase"
/transl_table=11
CDS 3019544..3020302

/locus_tag="AUO97b_02948" /db_xref="SEED:fig|400667.82.peg.2813"
/translation="MLALVTGASAGFGYSISKKLIESGYKVIGCGRRAEKLEELQKQL
GENFYPLVFDMTDTAENINKLFKELPNEFQIDQIDLLVNNAGLALGLEPADKADLDDW
YTMIDTNVKGLVTVTRLILPSMVKKKSGLIINMGSIAGTYPYPGGNVYGATKAFVEQF
SLNLRADLAGTGVRVTNIEPGLCGGTEFSLVRFKGDQEKANSLYDKKNPILPEDIANT
VAWIASQPPHININRIEMMPTTQTFNPLKVVEVE" /product="NADP-dependent 3-hydroxy acid dehydrogenase YdfG (EC 1.1.1.381) @ 3-hydroxypropionate dehydrogenase (EC 1.1.1.298)" /transl_table=11
CDS 3020796..3021224
/locus_tag="AUO97b_02949" /db_xref="SEED:fig|400667.82.peg.2814"
/translation="MKTLTVASIFSNFDFYQRNYLNILNQPESYYTPVEDAWIDAFPF
KKQDLYLGDLLQLWFSSKWDVHTSLKVLKCSKILDQSKPLYIFQLEGELFLGKNKVLA
WSTQHHEVIELQLKNIWAPYVVAKTCDRPDIPYLIKKAAV" /product="FIG00350937: hypothetical protein" /transl_table=11
CDS complement(3021239..3021676)
/locus_tag="AUO97b_02950" /db_xref="SEED:fig|400667.82.peg.2815"
/translation="MKNPMEMTGLEFLQAMIEGHIPPASISKTIPMQPTEISEGSVTF
KAQADHNHLNPLGGVHGGFAATVLDSVTGCAVHTMLPAGVGYGTIDLNVKMCRPLPQN
QVLIATGKVINLSKNLGISEGKITDEEGKLYAYATATCMIIRP" /product="hypothetical protein"
/transl_table=11
CDS 3021862..3022272
/locus_tag="AUO97b_02951" /db_xref="SEED:fig|400667.82.peg.2816"
/translation="MNGQCLCGETAFEVELKNHDVHVCHCSMCRRQTSGVIMTVDVVK
GSLKFIQQKHLSVFNSSEWGERGFCNACGTTIFWRTKDQSYCNINVFSLNEPVKDLKL
DMEIFIDSKPDFYNFQNDTKKLTEAEVAALFNLE"
/product="Gfa-like protein"
/transl_table=11
CDS 3022637..3024337
/locus_tag="AUO97b_02952" /db_xref="SEED:fig|400667.82.peg.2817"
/translation="MIDTKPLIAIDLGTSNSLVGIFENGQSKLINNPYGMKTTPSAIA
LDEQGQILIGQAALELRSRGKEVLTSFKRLMGTSKRLKLGQQSFSAVELSSLILKSLK
QDVEQALQCEVEEAVITVPAYFNDIQRQATISAAELAGLKVSRLINEPTAAALAYGLG
QTQDSCFLIFDLGGGTFDVSIVELFDGITEVRASAGDNYLGGDDFVQLLMKQYWKQHA
TIFGYTENAIPFDIETALRARAQHSLHVLSKEAKTDLIFKWEDKEATLEITQEEFAVW
AEPLLLRLRRPLERALRDARVLPQQVDQIIMVGGATRIPVVRKLVTKLFGRFPSTSVQ
PDEAIVRGACVQAGLKAKDVSLKEIVLTDVCPFSLGIAVENDEQFSPILERNIVIPAS
KVNTYTAMNKGQREIIVKIYQGEHRLCKENIFLGELNVPLPPNNDYLSIEVRFSYNPN
GILEVDIEVPSTGEKLQKVIVNHQNVMSTEQIEQARQQLQELKIHPRDTLMNKSLLLR
AERLFSEYTGDLRLQIGERTQQFNYILNLQDPRQIREAQQHFEAFLNEIEDLSLFEEY "
/product="Chaperone protein HscC"
/transl_table=11
CDS complement(3024389..3026350)
/locus_tag="AUO97b_02953" /db_xref="SEED:fig|400667.82.peg.2818"
/translation="MTAWQRLGIEPTVNQREIKKAYAKQLKLIDQDTQPEEFILLREA
LETAQFEAEHLIYENEDLDNSSSIFEENNSFNDQNLNSDDLNDQTLENKLKRLKHHIA
IQDINFNIRDELIHFNNEIEAYKDKSISSHFRDQILDLLRKNDLDDFCYIFTEKDAEN
IVTNHQKIIEKEKTEYSVSSNECIQSDALSIPVILEEVSNALWNKDISDITFDKFNYL
LSQQYDIPLSQQIEIKDQLQAPLAEIEIDDLQPEYFRFLELWHETYPDDIHQYNPSYY
SSLLQEKLNSFLSRYKLLKKLPNEHFELLNELSGEKKFHPLKMLKLRKSLNKISKNYS
TVEVIDKLQLSITQTNPNYLFLKSLSNFWKFTIINILFITITFLFFNSLIAPDNQIIV
NLLITACISLLYIFILQPILNTRILGHPGYERTLGYYLKYWCLSGFILCSLTPWLPDL
PHQLLSYGWFISTTILLSTLLLDATPYMNRLEQSAFINIDKWIINIGLISLSIFLISV
FLIIGEPDHIWFIIYSLIPICFLFYPESFRPLFYIFGYNKEDTNLTDKQIMYRSIGII
FFRLFWVFGFSYLFIKSSSQPFMYAAGLILGSILITGFSAKNLSSTLKYLTYIALCIA

TLYTILFPLILIYYLYQSYQAKKIILSSC"
/product="hypothetical protein"
/transl_table=11
CDS complement(3026573..3027802)
/locus_tag="AUO97b_02954" /db_xref="SEED:fig|400667.82.peg.2819"
/db_xref="GO:0004069"
/translation="MDVRLSDRVNAIKPSPTLAVTNKAAELKAAGKNVIGLGAGEPDF
DTPQHIKDAAIEAINNGFTKYTAVDGTPGLKKVIIAKLKRDNNLDYQPNQILVSCGGK
QSFFNLALALLNKGDEVIIPAPFWVSYPDMVIIAEGTPVIVKCGEEQRFKITPEQLEA
AITPNTRLVVLNSPSNPTGMIYSKAELEALAEVLRRHPQVFVASDDMYEPIRWEDEFY
NIATVAPDLYDRTIVLNGVSKAYAMTGWRIGYAAGPAKIIGAMKKIQSQSTSNPTSIS
QVAAEAALNGPQDVLKPMIEAFKRRHDLVVNGLNDIKGISCLPADGAFYAYANIRPLI
RAKGLKSCTEFSEWLLEETGVAVVPGDAFGLGGFMRISYATADEVLVDALARIKKAAE SIEGVDAAIASIEAEKA"
/product="Aspartate aminotransferase (EC 2.6.1.1)" /EC_number="2.6.1.1"
/transl_table=11
CDS 3028010..3028189
/locus_tag="AUO97b_02955" /db_xref="SEED:fig|400667.82.peg.2820"
/translation="MTEQQPKQKKALVKLPFPMPNESNQAGDAVHNQVRPKPEQYADR TWMPPRGTRRSMGKR"
/product="hypothetical protein"
/transl_table=11
CDS complement(3028224..3029660)
/locus_tag="AUO97b_02956" /db_xref="SEED:fig|400667.82.peg.2821"
/translation="METLDSSIFDLTPIPMWIEDFSEVKQLFDLWRNQGVENLYEFLS
QNENLVVECAHKIKIIKVNQKVLDLFEAKNQEELCANLNLIFKKEMFEAHIHELEALW
NGKTHFSSTTINYTLSGQRIDVQLRGAILPGSETTFDRILITTEDITPYQNALRQEEK
NRRLAESMFIYSPTSLWVEDFSRIKNRIDQLRLLGIEDFRTFLDVHPEFVRQCIEDIL
ILDVNQSTLDLFKAPDKTTLLKNTHKIFAEEMVETFREQLIELWQGNIHHKREAVNYA
LDGQIRNVLLQFTVFPGYEETWGLVQVALTDITARKKAENYLEYLGQHDVLTKLYNRA
FFTEELNRLNRSIIRPVSSIFLDMNGLKETNDQLGHDIGDGLLRRVGNILNQAIMNTA
YTASRIGGDEFVILMPGADEANVLMMLQTIQELFNIDNQYYSSHPISIAIGHATTKEN
EQVEDMLKRADHHMYQKKKSYYQEILLP"
/product="Sensory box/GGDEF family protein" /transl_table=11
CDS complement(3029829..3030620)
/locus_tag="AUO97b_02957" /db_xref="SEED:fig|400667.82.peg.2822"
/translation="MTYQYHDESIVTELPEDTVFVFGSNMAGQHGSGAARVASQHFGA
VEGVGRGWAGQSFAIPTLNEHIQQMPLSQIEHYVEDFKVYAKNHPKMKYFVTALGCGI
AGYKVSEIAPLFKGIHHNVIFPESFKPYVEEDAVSQFPTLTQKMVQSFINDEVIFYFN
HGSESFEDALDKTDLSRAEKAIALIVLNEELYPRDRYGRGRDHELRDILGKLNGKIFN
IHGNSEGAMIFVSVIVALMELYDFDEQDFIKLWRGEKNIDHPINR" /product="hypothetical
protein"
/transl_table=11
CDS 3030800..3032821
/locus_tag="AUO97b_02958" /db_xref="SEED:fig|400667.82.peg.2823"
/db_xref="GO:0003676"
/db_xref="GO:0003677"
/db_xref="GO:0004386"
/db_xref="GO:0004518"
/db_xref="GO:0005524"
/db_xref="GO:0006289"
/translation="MNENQPFDLVTHYQPAGDQPQAIEKLVNGIEKGFRNQLLLGVTG
SGKTYTMANVIAQTQRPTIVMAHNKTLAAQLYGEFKAFFPNNAVEYFVSYYDYYQPEA
YVPSSDTFIEKDAAINDHIDQMRLSATRALLERRDAIIVASVSAIYGLGDPNAYMQML
LHVVQGDRVSRDEIIRRLVEMQYTRNELEFLRGTYRIRGEIIDIFPAESDQHAIRIEL
FDDEVDSIRWFDPLTGKMVRKVPRVTIYPKSHYVTPKDNLTRAIDTIKDELQDQLKFF

REHDKLLEAQRIEQRTRYDLEMMQQLGYTNGIENYSRHLSGRPAGEAPPTLFDYVPED
ALLIIDESHVTVPQIGAMYKGDRSRKENLVNYGFRLPSALDNRPMKFEEWERIVPTTI
FVSATPARYELEKSEQVVEQVVRPTGLIDPEIEVRPVLTQVDDVLSEINIRKNLNERV
LVTTLTKRMAEDLTSYLKEYGVKVAYLHSDIDTVERVKIIHELRTGVFDVLVGINLLR
EGLDMPEVSLVAILDADKEGFLRSERSLIQTIGRAARNVKGKAILYADTITDSMRKAI
DETERRRAKQIEFNEQHGITPRSAVRQAVKEIDTGEVLSDDQIDEKVLEQAQALSADE
RHILSDPKLFSKHITKLEKEMLKASKDLQFEQAARIRDEIVRLKAQMLQ" /product="Excinuclease ABC subunit B"
/transl_table=11
CDS 3032915..3033502 /locus_tag="AUO97b_02959" /db_xref="SEED:fig|400667.82.peg.2824"
/translation="MAFRNNVPQASWRLAKIALGGIVLTGLAVGTYAYAQQKPLPTVD
KVELDRYLGVWYEVARKPAFFQKKCAYNVSATYTLNENGNIVVDNRCYDNQKQLQQSI
GEAFVVNPPYNTKLKVSFLPEAVRWIPIIRGDYWILKLDEDYQTVLVGEPSRKYLWVL
SRTPHPHKEVVDEYLNYAKTLGFDIRDIIHTEHKE"
/product="Outer membrane lipoprotein Blc"
/transl_table=11
CDS 3033614..3034558
/locus_tag="AUO97b_02960" /db_xref="SEED:fig|400667.82.peg.2825"
/translation="MLKGVILSVMASLTFGVLYFYTQLLGQLDSEQTFGWRIIATLPF
LTLFMWWSGDLSHIKNIYQRILAKPSLLLLLITTSVLTSVQLWLFLWGPMHGRGLQVS
LGYFLLPLVLVLAGSVLYGEKISKFQWIAIVLAIIGVGHEIFRLGSIAWETALVAIGY
SAYFLLRKKIKTDNLGGFWWDLLIIMPVAIYLTHTGLLPYSKFLDQPTLGLVIAGLGV
LSAIGLGCYILASRYLPLVVFGLLGYLEPVLLALASLVLGESIGKEEWFTYLPIWCAV
FVLVLEGAFHLYQQQQKAKNLQLNIEKYQKRLKNDAHD" /product="Uncharacterized inner membrane protein RarD" /transl_table=11
CDS 3034775..3036232
/locus_tag="AUO97b_02961" /db_xref="SEED:fig|400667.82.peg.2826"
/db_xref="GO:0047100"
/translation="MSKDTIIALHAEHQGRWKNREEIAERMIALIGQLYREKNIVVSV
YGRSLINRSVIQILKTHRRTRVVDVELSVVNTFPILEALAKVENIGSAEVDIGKLAVE
YKEKGGDVDAFVAQAVESIKGSATSEQPKDVVLYGFGRIGRILARLIISQSGLGRGLS
LKAIVVRKSSDGDLAKRASLLRRDSIHGTFAGTISVDEENEAIIANGNFIKVIYATSP
SEVDYTQYGIENALLIDNTGKWRDAEGLSQHLKCPGVARVVLTAPSKGEMKNVVFGVN
NSDILDEDKIISAASCTTNAITPILKVLDDKYKVLNGHVETVHSFTNDQNLIDNYHKA
DRRGRAATLNMVITETGAAKAVAKALPALKGKLTGNSVRVPTPNVSLAILNLTLDKEV
DREEVNEYIRQISINSNLQGQIGYTNSTEVVSSDFIGSRTAGVYDAQATITSGNRLTA
YVWYDNEVGYSCQVLRIAEQMCGVSYKKIPAETNA" /product="NADP-dependent glyceraldehyde-3-phosphate dehydrogenase (EC 1.2.1.13)" /EC_number="1.2.1.13"
/transl_table=11
CDS 3036382..3037116
/locus_tag="AUO97b_02962" /db_xref="SEED:fig|400667.82.peg.2827"
/translation="MQQFKRLLIVFFFIIIANICGVLIAIWVFKHFNIYIPLKNQAVA
IDLQEPLQVQVKVKDALNVDVKGRVNANIPINEKLNIPLTQTLTPRVYFDNMVPISTV
IPVKETLKVEQSLPIDTKVQVRVLGKDITLPLKGNIPLQLDVPIDIQVPLDQKVHLKF
DAPVKTVLKENLHIPLNAQLKTNIPIQGSLNVPVKTALNASVDVKNTLPVKIEHGELK
IPLSSMSLNRVKSGEDHSSPVQNQTK"
/product="FIG00351097: hypothetical protein" /transl_table=11
CDS 3037128..3037802
/locus_tag="AUO97b_02963" /db_xref="SEED:fig|400667.82.peg.2828"
/translation="MFGSLKSILISFFLIMILTAILFWFYLNIQASVVVSAHESDIRL
PESLETKIHVGNNLQVQSIGKLDTSIDIDRQISVPLKGRYLADLEFEVETPITVSVDY
ATTLKVDQVMPLETTTDLIYKNKLLPRFPLKLNIPIKLDIPFQLKRSYTIPVKIAFNG
PVYFEFDESINLPIKHQFNPSLNVNDAMEMGNISSFNAIMYNSVQHTKANLDMKMDLP LRNVHP"

/product="hypothetical protein"
/transl_table=11
CDS 3038019..3039239
/locus_tag="AUO97b_02964" /db_xref="SEED:fig|400667.82.peg.2829"
/translation="MRFVDEAVITVEAGDGGNGVASFRREKFVPFGGPDGGDGGRGGS
IYIQADDDTSTLVDYRYTRKFRAERGKNGAGANCTGRGGEDVVLKVPVGTTIVDTDSG
DIIGDLVEDGQRVMVASGGEGGLGNTHFKSSTNRAPRKCTTGTKGEFREIRLELKVLA
DVGLLGMPNAGKSTFIRAVSAAKPKVADYPFTTMVPNLGVVDADRHRSFVMADIPGLI
EGAAEGAGLGIRFLKHLARTRILLHIIDVQPIDGSDPAHIAKAIMNELAKFSPTLAKL
PIVLVLNKLDQIAEESREEWCQHILDELQWTGPVFKTSGLLEEGTKEVVYYLMDQIEQ
QREREVEDPEYAAEVRAFREQLEAETREQTIAAKEAYRAMRKAQRLESMMDDDDDFDD DEDDGDVESIYVRD"
/product="GTP-binding protein Obg"
/transl_table=11
CDS 3039255..3040388
/locus_tag="AUO97b_02965" /db_xref="SEED:fig|400667.82.peg.2830"
/db_xref="GO:0004349"
/translation="MIEVVDGQRKLSECKRIVVKIGSSLLTANGQGLDLDAISHWAKQ
IADLHNAGHEIILVSSGAVAEGMVRMKLASRPTDLPSLQACAAIGQMGLIHTWSSVLE
NHSIRTAQVLLTHDDLADRRRYLNSCDALQNLIDWRVIPVINENDTVSTDEIRFGDND
TLAAMVAGQVHADLLIILTDQQGMFDSDPRHNPDAKLLSTVRAMDDVLFEMAGGGGVL
GRGGMVTKVRAARLAAKSGCPTLIASGESDNVLSRVMAGEMLGTLFTTDKDRMTAHQQ
WLAAHLQTAGRLVIDDGAVEAIKLKHRSLLPVGVKTVEGHFDRGDVVECVDKQGKRVA
VGRVNFSSRSAEIIKGLSSDKVYQVLGEARSLEMIHRDHMAIY" /product="Glutamate 5-kinase (EC 2.7.2.11) / RNA-binding C-terminal domain PUA" /EC_number="2.7.2.11"
/transl_table=11
CDS complement(3040415..3041071)
/locus_tag="AUO97b_02966" /db_xref="SEED:fig|400667.82.peg.2831"
/db_xref="GO:0004647"
/translation="MYATSETNKTIKNLALFDFDGTLCSKDSFTGFIFYALSKRHIVK
QGLKILPWIQAYYLNFYPAHAMRAKLFRSMFRDTPAIELQRLGEEYAQELVSALSPEI
FTQLQQHQLLGDQVVLISASIDIYLAPLCKLLDIELICTEAQIKNGMMTGYYSTPDCS
SEQKKLRIREQYSLKHYQRIYAYGNSSEDLDMLSLATHPFMVGEDRILPSLTPQKKLA "
/product="Phosphoserine phosphatase (EC 3.1.3.3)" /EC_number="3.1.3.3"
/transl_table=11 CDS complement(3041038..3042186)
/locus_tag="AUO97b_02967" /db_xref="SEED:fig|400667.82.peg.2832"
/translation="MSLAACGDKAWWSNNDEPELESQQIKRLIPSRVHDRESWAKDID
DIMKDLDIPKTKQNVCSIVAVVDQESNFVANPQVPGLGQKAVEEVSTRLNEKFEDKLG
KTIGGTIAGYFEEVLRTQPSPDNNYMSQMRKVKTEKDLDLLYREIFDFMAKHYHVSAL
TGAAKLVGQDIGEKMNPITTLGSMQVHINYAKANKRSSMNTAALRDDLYTEYGGLYYG
IHRLMVYPADYDKAIYRFADYNSGMYSSRNAAFQKMLKELTDKDISLDGDLLLYTKDG
DPRATQSESEKELITVFASNNVLVTPRQIRDDLKLEKEKKFESTQTYIALTKLYKSKT
GKEPLYAIMPQVVISGPKLSRDYNTNWYATRVNGRYETCMQRAKRIRL" /product="putative lipoprotein"
/transl_table=11
CDS 3042330..3043355
/locus_tag="AUO97b_02968" /db_xref="SEED:fig|400667.82.peg.2833"
/translation="MYIGPYQLSNNLIVAPMAGVTDRPFRTLCKYFGAGHAVSEMMTA
DKTLRMTKKSLYRANFDGELAPISAQIAGSDPEQLAEAARYQVANGAQIVDINMGCPA
KKVCNKLAGSALLQDEDLVARILDAVVAAVDVPVTLKTRLGFLNGQENILRVAKRAEE
AGIAALALHGRTREDMYLNTARYELIKHVKELIHIPVIANGDIDSPEKAKYVLDYTGA
DAIMIGRAAQGRPWIFREIAHYLKTGEHLAAPNIEEVKEVLLGHLSELYQFYGEYSGC
RIARKHIAWYTKGLRSSNEFRQNMYKVETTAEQALVVESYFNQLLAEGNLMSDVQVEQ VNLLDTH"
/product="tRNA-dihydrouridine synthase DusB" /transl_table=11
CDS complement(3043399..3044445)

/locus_tag="AUO97b_02969" /db_xref="SEED:fig|400667.82.peg.2834"
/db_xref="GO:0004318"
/translation="MSLLQQLAIKHPIFLAPMAGVSTPELAAEVSNQGGLGSLGLGAN
TPQSAREQILKTQALTENPFQVNFFCHQSTELNVEKAKLWLDYLRPHFEKFGAQPPQE
LHCIYPSFLDNDDFLNVVLETKPKAVSFHFGIPHPHQIKALKEAGILTMVSATNLIEA
QAIEAAGIDIIIAQGIEAGGHRGIFNQTFDGAIKTSDLVQLIVQHCTLPVVAAGGIMT
GLQAKHMLGLGAAAVQLGTAFVQCQTSNASAEYRKALFSKPVTQISASLSGRPARGIL
NHWHIKIDSPTRPVQPEYPYTYDLAKQLNALASKHQDYGFGAFWAGSNVAQIRELEAP DLVNQLVVEMLDSE"
/product="Nitronate monooxygenase (EC 1.13.12.16)" /EC_number="1.3.1.9"
/transl_table=11
CDS complement(3044557..3045258)
/locus_tag="AUO97b_02970" /db_xref="SEED:fig|400667.82.peg.2835"
/translation="MAFPHHTEHHAIQRSGWLRASVLGANDGIISVTSLIMGMAASGA
SSHTLFITCIAGLISGATSMAAGEYISVKSQEDIEKSDLAIEAKELKKYPQKELDELT
QIYISRGLSKELAKEVAIQLTTHDALGAHARDEIGIHENTAANPIQAALSSAASFSFG
AFFPMLAILFSPEHLIMPSVLITGIAALTILGALSSYFAGTSKIKGSLRITLWGILAM AFSSWIGSLFNVTPL"
/product="Nodulin-related protein CC_0717" /transl_table=11
CDS 3045511..3046731
/locus_tag="AUO97b_02971" /db_xref="SEED:fig|400667.82.peg.2836"
/translation="MKNIKTINSITHSILLMGAVISSLGMTGISSVYAQEQVDPAYKP
GNPIYDKWDRFYKIEQSQPQEAEKILVELSKLTPTDIKVWKSLTYLQIRLEKREEALQ
SLRQARNLAPQDDTLKLQEAYLLNQQKKDREALVLFKELSSSSDPEIAAKATQAVKNL
SGGEVKPYFKDIYFAPSYESRYDDVIFPLKMRYGKNIDNGRAQVYAFLNLNRDTQSQG
GVRPEIIDENAATLGLGANYQPWTSIPVRAYVEVGGSYDLIDRNRKRFRESVVGGVTG
YQEWYSQSNCDHSLCLDNYFTDLYGNVATYSREDYNVIGDLRLRTGLNLYKGESGTVQ
AYVKLHGLADSEDEYYNNLFEYGPGISWQPFNYQPIKLRVERLYGNYFKDVPVNTKDH YNNTRVELVFYKDF"
/product="hypothetical protein"
/transl_table=11
CDS 3046788..3046928
/locus_tag="AUO97b_02972" /db_xref="SEED:fig|400667.82.peg.2837"
/db_xref="GO:0008761"
/translation="MAPVILLAKQDSRFQVEVIFTGQHDELVRDAIDFFGVEIDHRLK KL"
/product="UDP-N-acetylglucosamine 2-epimerase (EC 5.1.3.14)"
/EC_number="5.1.3.14"
/transl_table=11
CDS 3046925..3047872
/locus_tag="AUO97b_02973" /db_xref="SEED:fig|400667.82.peg.2838"
/db_xref="GO:0008761"
/translation="MNAGQSLNQLLIHGLTQLENIYTDGQKRDAIVIQGDTTTVLAAG
LVAFSMKIPVAHVEAGLRSYDLDHPFPEEGNRQLVSRITKWHFAPTEQSKRNLLNEQI
PPSLITVTGNTVVDAVYLGRKLIAEKTGLKNQLEPYGIELKQNDKVVLITAHRRENFG
EGIQNICNAVEYLAKQHLDLHFIWPVHLNPAVHNVVHDKFKSHAQIHLVKPLDYPSLL
AVIDRSTFILTDSGGLQEESPSFNKPVLILRDTTERPEVVEVGGGVLVGTNQQKIIEE
AEKLLTDSQHYQKMAHVENPFGDGRAAQRILDEIARTYN" /product="UDP-N-acetylglucosamine 2-epimerase (EC 5.1.3.14)" /EC_number="5.1.3.14"
/transl_table=11
CDS complement(3047880..3048785)
/locus_tag="AUO97b_02974" /db_xref="SEED:fig|400667.82.peg.2839"
/translation="MKKQIPIKITAFIILIICSVVGYAGLFKNHGQPPIIEGIFWQPD
NDTTPPKGNWHYLGINTFVPQWSVVESKSWWKNSNLPQWEKAIDLQKIKQQPWAKNLI
LGLAGEYNEHEARANVVALGEKSAQIIQEQNDASLKGYYFPVEADPTWLRVSTLGHVL
EKLPSPIWVSVYSGESEPENYDLWVKSWLPQQAGVFFQDGVGVGVRTPEQARRILDQL
EQTLGKDKTVIVLEAFRTKKNGQFRAAYPWEIISQIKAYEGKKIYIFDGPHYMGRWSV
YIVGLWYRLVYGSTPVTINESENSK"

/product="hypothetical protein"
/transl_table=11
CDS complement(3048763..3050607)
/locus_tag="AUO97b_02975" /db_xref="SEED:fig|400667.82.peg.2840"
/translation="MKKTFDLYQTAQVLLASTSFLLAMSTHAYTWKLDDISTSTKYTV
EPHFFYIGKGAEWQNIQFSSDYSTENDTTVLVKYDDKLIYNTTLNGDGQFKFNIPASQ
SGFHRLDFIIQQYAKPTTLNQTKETFCTEDIDHLTYLSNSQLDFTPVRNEYRLKDLPD
ALFNPQARRPTPFVGILKYNRKEITEASMLGRLVSSWSSVTPIEWVDSGQTNLNPETN
FIIEVIRSASPLKGGALVQIAKPAEGVPTLSITYHTPQELTAAINGLINPTYVQQLDT
GSAIFPTTISAPAWAQFKKIDTLADLGIQDFRLNHAEKNLFLDFPAVWQPTDILQGQI
ALRIQSGLLQGSNITAWIDGGLAGSMKTADLASDPVNRQFNIFAKEISNTTNFSLKLE
NSVIANSQCLPNAHGSLWVDTAKSTVKLPHKLKNGVAALSMTLATKPTIAIDDQSGAL
NIAIILGQVAKNMLLTQAPMPVNLVRFSPTSPQAVNVRVNKEIYQQQVLMHQNIIYAP
AAANGFIVSYNNNRFDVITDSENGAQTFMHLWGKIQHKIPNNVTKMLVSENGNIYVLQ
KLIVGNQKAPLVQQSSFFLLVVIISAIMIIVIFLWYWLRRNNEKTDTN" /product="hypothetical
protein"
/transl_table=11
CDS complement(3050608..3051858) /locus_tag="AUO97b_02976"
/db_xref="SEED:fig|400667.82.peg.2841"
/translation="MAPIDILFLFGFLGIWIPQAFWAWLSYQAWKYSKTAEKELQNLP
IPERWPVLSVLIPAYNEGVVIEDTLHAIAQQDYPAESYEVLLINDGSKDNTLEIAENL
AKIYPCIKIVNVPKGMGGKGKSRTLNNGLPHAKGELIVVYDADSTPEPDCVRLLAQTL
LADKKLVAVNGKVRTRNWQDSILTRFIAIEFIFFQWIFQGGRWQRFELSTLMGTNYVI
WRDALETLGGFDEKSLVDDTEMSFRIFIGQKRIKWVPYAIGWQQDPPSLSVFVKQRSR
WTQGNFYVTRKYLPVALRTPFPIGIEILNNIMCYILFVPALFWSHITLTLGLLDIAGI
SVPGPFTLLWGLSFCLYVAQMWFTLSLEKVKPELYFYSVLSYVSYSQIFLFIVFKAAF
DMLKNKIQGNSLQWYKTERSKEKK"
/product="Glycosyl transferase, group 2 family" /EC_number="2.4.1.83"
/transl_table=11
CDS complement(3051845..3052741)
/locus_tag="AUO97b_02977" /db_xref="SEED:fig|400667.82.peg.2842"
/translation="MSNLLNICGIVIASSQYPDATLQQFYRQYYHCEIKAEQIKAEVQ
SPSDLSMFFPYQDTWWPVFTIDQISSESFQKFIHNGIRPGIILPDEVFGFPHYFLLKE
AVSQGAIPIALFKTEQPQYFAAKATFSTAIGLRPMAAFVSTGWDENLISQPAGSYIIQ
LNSANLPLPSREVRQGQHFFYSAKGFNGHVSGYEIIINPPADLPLSNIRYPQLGISWN
FNNIDYESTPEHVSTNLIGYIFIILSIVVVPLDLILTTTYPDLLGTFGSYISWISLVV
GAILLLLLISSIIRRVRKNGSN"
/product="hypothetical protein"
/transl_table=11
CDS complement(3052741..3053481)
/locus_tag="AUO97b_02978" /db_xref="SEED:fig|400667.82.peg.2843"
/translation="MQQKHPGRTLVLWIIAAHLFMGMVAYEWLPVSFRGSWIFILLLL
ISGAFLEVGLSLIVGLVAFIGVAVYFLLDLGAQTHIERQLLLLFIIPLIPLFLSAVRH
NIEINVQKYRDIQSYDRNYRRDIFPLSALKYFQRSFFKLLDHHNVQDYEVFEIRILNR
SLIKEMLGDDVWKTTQNEMIKILSQHEPNEIVFQFADDDLENIYSIMIRQQKTEEDPV
FLKKLKEITTLRLEVEYKIVPIPPEEMS"
/product="hypothetical protein"
/transl_table=11
CDS complement(3053675..3055117)
/locus_tag="AUO97b_02979" /db_xref="SEED:fig|400667.82.peg.2844"
/db_xref="GO:0003913"
/db_xref="GO:0006281"
/translation="MSNAYQLIWFRQDLRIHDHAALWHASQQGPSLGLVILSPEQWKQ
HADAPIKIGFYLRQLRTLQKELAALHIPLVIQVIPYWKEIANFISDFSKKYNIENVYA

NIEIGVNELKRDKTVQDYLNRHGKELFLFHDRTIFPLRSIRNQSQQPYQVFSAFKKAC
YTKLDTSGLPQCYPVPNKQNEVPLDFLQGKNIDLDEIEKLFCSSLSKEQQDLWPVGEQ
YALEQLDQFIEESVCHYKVERDFPNIQATSKLSPYLNIGILSIRQCLQALFRNQHGNF
HLVNEGQQTWLDELLWREFYQHILFDFPHVSKHIPFKKDTQKIKWNHNPEHLTAWQMG
QTGIPIVDAGMRQLLQTGWMHNRVRMITAMFLCKNLLIDWRIGEQWFMQHLIDGDLAA
NNGGWQWCASTGTDAVPYFRIFNPITQSKKFDPEGEYIRQWVKELAHLDNKTIHEPYS
SKTDLGLNYPKPIVDLKETRLKAIETFKNI" /product="Deoxyribodipyrimidine photolyase
(EC 4.1.99.3)" /EC_number="4.1.99.3"
/transl_table=11
CDS complement(3055221..3055703)
/locus_tag="AUO97b_02980" /db_xref="SEED:fig|400667.82.peg.2845"
/translation="MTAIANNHVVSFHYKLTNAEGETLDQSQGEPLAYLHGAGNIIPG
LENALTGKTVGDKFTVNVPAAEGYGEYNPDLVQEVPAQMFQGVDNIQPGMQFQAQTDD
GVQIVTVKAVEGDNVIVDANFPLAGQDLTFEVEIVEIREASQEELDHGHVHGAGGHHH "
/product="FKBP-type peptidyl-prolyl cis-trans isomerase SlyD (EC 5.2.1.8)"
/EC_number="5.2.1.8"
/transl_table=11
CDS complement(3055839..3057125)
/locus_tag="AUO97b_02981" /db_xref="SEED:fig|400667.82.peg.2846"
/db_xref="GO:0003954"
/translation="MTQNLHHIVIVGGGAGGLELATQLGETFGKNGKAKITLVDQNLT
HIWKPLLHEIAAGSLNPHEEQTNYFAHAEKHHYEFVLGTLTEVNKDKKIIDLIPPQVS
KEQNTHIQQLSYDTLVLALGSVSNDFNTPGVRENCHFLDSRKQADIFQQDLLHLYIEA
QNQPDQRTLNIGIIGAGATGVELAAELIETTKNFYRYGLKKIHPNQVKITLIEASERI
LPALSEKTAEHSAKQLQRMGVEILTKHRVEKIDEHCIYFSNGNQLNSDITVWAAGVKA
PKVLESLSDFERDNINRLMVYATLQTYSDPNVFAFGDCAHCQLDARHPPLGPRAQVAS
QQASFLVDAMAARLNGRSQPMFTFNDKGSLVSLSRNKAVGELLGDVSVQGYIAKTMYV
SLYRLHQATIHGYTQAGLLTMKDLLTRRVRPKIKLY"
/product="NADH dehydrogenase (EC 1.6.99.3)" /EC_number="1.6.99.3"
/transl_table=11
CDS complement(3057225..3058544)
/locus_tag="AUO97b_02982" /db_xref="SEED:fig|400667.82.peg.2847"
/db_xref="GO:0009002"
/translation="MKFFLSLFTLFSIFCTTLTNAALLNIAPESVEAAAWTIVDTQSG
QIIAEHNSHVQRAPASLTKMMVAYIALKEIKAGKLKLNEVITATPVVSVVQWDESQMY
LKAGEQISVDQLLAGLIVMSANDAAVTLAEKISGDVPHFVQRMNQEAQALGMKDTHFS
NPAGITMPDHYTTAHDLSLLSQAVIHQTPEYLHYSKMPSFSYNQRFHHATNLALKYDP
SVDGLKTGYTKAAGYNLALTASRPSFSPNLPQRRLLVIVLGTPSAVKRAEIADKLMNL
AYAYTRDEVVIPEQKLIAELPVIKSTLKMFKVETKQPTIVTTSLYAEPTPIDLNTFDY
ATQRIQVLDSNQQPKVIAPLETTQTRVNIQLNEQKLTAPLMKAMNLATVSIYQNNQLI
RSLQIENDVHIEEANIFQRIMMWFSSLFSIFSSSEHSAAKLYPIDSH" /product="D-alanyl-D-alanine
carboxypeptidase (EC 3.4.16.4)" /EC_number="3.4.16.4"
/transl_table=11
CDS 3058717..3060420
/locus_tag="AUO97b_02983" /db_xref="SEED:fig|400667.82.peg.2848"
/translation="MLHYQIEFDDYKQHLVHVTIRFLANPNQELWLPTWIPGSYLIRE
FSKHIEAVKAYDEAGRMLNIKKTEKNRWRLFNTDHELITVEYDVYAYDLSVRGAYVDQ
TRLYINPACVCLGLEGQEQSACELEIFLPDELKHFQLATGLASKSLVKGRFTLKADHY
DQLIDSPFELADQTRFSFETHGIEHEFVISGTHATNVDRLKTDIEKICAAEIDLFGSA
PFKNYTFMTMATGNSYGGLEHCNSTSLITPRDDLPKSNEPTEPSKDYQRFLGLCSHEY
FHSWLVKFIRPENFANYNLHQEGYTSLLWIFEGFTSYYDDLILLRSGVISQKSYLDLL
KSQIDRYLQNPGRFVQTVAESSFDAWIKFYRQDENSNNAGTSYYNKGALVALCLDLGL
RLRGSSLDALMRRLYENTQNGVQVNERTIFDLCEELTGDSWLEQINHLINTTEELPLD
QLFPEFGLSYTLKNDKTLAYGLKLADKPEGVLVQNAHREGAGAKAGISANDVIIAIDG

LKATTKLIEKYAKQKGNYSVLAFRRDELLSFEVTSDSINLTEVELIIEDQSKADKWLK A"
/product="Aminopeptidase N family protein, contains PDZ domain"
/transl_table=11 CDS 3060821..3063058
/locus_tag="AUO97b_02984" /db_xref="SEED:fig|400667.82.peg.2849"
/db_xref="GO:0004450"
/db_xref="GO:0004450"
/translation="MAGEKSTIIYTLTDEAPLLATYSLLPIIETFTKPAGIEIVKSDI
SVAARVLAEFADYLSEEQKVSDNLAELGRLTQDPDTNIIKLPNISASVAQLTACIKEL
QSKGYAIPDYPENPATEEEKTIKARYGKCLGSAVNPVLREGNSDRRAPAAVKNYAKKH
PHSMSEWKQWSQTHVSHMEEGDFYHGEKSMTLDRPRNVKMELITNSGKSIVLKPKVAL
QEGEIIDSMFMSKKALCDFYEKQLDDCREAGILFSLHVKATMMKVSHPIVFGHCVKIY
YKEAFEKHGKLFDELGINVNNGMAGLYEKIETLPTSLREEIIEDLHACQEHRPALAMV
DSAKGITNFHSPNDIIVDASMPAMIRAGGKMWGADGKQYDAKAVMPESTFARIYQEMI
NFCKWHGNFDPKTMGTVPNVGLMAQKAEEYGSHDKTFEIPEAGIANITDLDTGEVLLT
QNVEEGDIWRMCQVKDAPIRDWVKLAVTRARNSGMPAIFWLDPYRPHENELIKKVQKY
LKDHDTTGLDIQIMSQVRAMRYTLERVVRGLDTISVTGNILRDYLTDLFPIMELGTSA
KMLSIVPLMAGGGMYETGAGGSAPKHVQQLVEENHLRWDSLGEFLALAVSLEEMGIKE
NNARAKLLAKTLDEATGKLLDNDKSPSRRTGELDNRGSHFYLSLYWAEALAAQNEDAE
LKAKFAPLAKALAENEQKIVAELAQVQGKPADIGGYYAVDPAKVSAVMRPSATFNAAL STVQA"
/product="Isocitrate dehydrogenase [NADP] (EC 1.1.1.42); Monomeric isocitrate dehydrogenase [NADP] (EC 1.1.1.42)" /EC_number="1.1.1.42"
/transl_table=11
CDS complement(3063176..3063964)
/locus_tag="AUO97b_02985" /db_xref="SEED:fig|400667.82.peg.2850"
/db_xref="GO:0004730"
/translation="MKIVILNKPYDVLSQFRKDEAHMTVSDFVDDPTLRIAGRLDMDS
EGLVFLTDHGGLNQFITNPANKKYKTYLVQVDGDVTEEALEQLRKGVELNDGMTLPAK
AIKVSEPEWLWDRDPPVRYRASIPTSWIEISICEGRNRQVRRMTSAVGFPTLRLIRTK
IGTIDLVQLGLQPGETKEIEPLLYPDFKDVPAEQPYRSRSYVKKPGGTGGKPMVRKNK
DGSAKKSGTKRIWQMDESEKPRRKTNGTTRPNTKAPRGRSRNSR" /product="LSU rRNA pseudouridine(2457) synthase (EC 5.4.99.20)" /EC_number="4.2.1.70"
/transl_table=11
CDS 3064165..3065421
/locus_tag="AUO97b_02986" /db_xref="SEED:fig|400667.82.peg.2851"
/db_xref="GO:0004450"
/translation="MGYQKIVVPADGDKITVKADLSLNVPNHPIIPFIEGDGIGVDIT
PTMKKVVDAAILKAYGGKRSIEWMEVYCGEKANKIYGTYMPEETFEALREFVVSIKGP
LTTPVGGGIRSLNVALRQELDLYVCVRPVRWFQGVPSPVQHPELTDMVIFRENSEDIY
AGIEWKADSEEAKKVIKFLQEEMGVTKIRFPEGCGIGIKPVSKEGTQRLVRKAIQFAI
DNDKPSVTLVHKGNIMKYTEGAFKEWGYELALDRFGGELIDGGPWVKIKNPKNGKDII
IKDVIADAFLQQILMRPADYSVIATLNLNGDYISDALAAEVGGIGIAPGANIGGAIAV
YEATHGTAPKYAGQDKVNPGSIILSAEMMLRDMGWIEAADLIIKGISGAIAAKTVTYD
FERLMPGATLLRCSEFGDAIIQHMED"
/product="Isocitrate dehydrogenase [NADP] (EC 1.1.1.42)"
/EC_number="1.1.1.42"
/transl_table=11
CDS complement(3066014..3066424)
/locus_tag="AUO97b_02987" /db_xref="SEED:fig|400667.82.peg.2852"
/translation="MSNNNYIDELLKLLAMFAFIGVLLLAFKCQTIFALDMTTSYEVS
VRIVIYIVTAAILGFLTRNHIEFTTQFLIAVPFAYFWLEPILDYKAIQTIPDVPFYLS
GHGQSLGLLIVIIFCFALWVFKETSSNSLESQNV"
/product="FIG00350424: hypothetical protein" /transl_table=11
CDS complement(3066651..3066776)
/locus_tag="AUO97b_02988" /db_xref="SEED:fig|400667.82.peg.2853"

/translation="MKTLLFPLFLVAFVLIIQALDQSYQKKIGIPKTAKHPEFMQ"
/product="FIG00351435: hypothetical protein" /transl_table=11
CDS complement(3066773..3067951)
/locus_tag="AUO97b_02989" /db_xref="SEED:fig|400667.82.peg.2854"
/translation="MSTFYRMSLLVFLATCLGIGIFRFSYTALMPIMIVQYGWTHDFA
SYLGSANLLGYLVGALLALFSIKEKSIPALIITSAVLGSLSLLCCSFIHMSLAWFVLW
RTISGIAGGLLMILAPSFAMKNIAISQRLKVNFLGFSGIGAGVLSATLIIPALKNMPV
QYVWEIFAAISIIGSLVLFQLLRHPPTESVQTANQAPQNFKHSRLGLVIIIAYMCSAF
AYIPHSLFWMDYLSGTLHFPLQQRNLFWVLYGVGSCIGAFVAYCLSRLIGYLQAIKCL
YGVYCLAVALPFFSHATALLTLSSFLTGLLTPATVFISSYSLLQIYEQRYQKIWSLAT
IGFASTQLLGGVVISTLHHLHWSYAHLFLMGGIVLLFALILLVRTRSSIPTTLGETKS "
/product="Uncharacterized MFS-type transporter" /transl_table=11
CDS 3068048..3068929 /locus_tag="AUO97b_02990" /db_xref="SEED:fig|
400667.82.peg.2855"
/translation="MLLKSLEFFLAVAESGSFSLAAQKMCTVQSNITSHVKKLEEELG
VILLNRHNPVTLTNAGHQLHSYAVQMIALHNKAKTIFREGDIDPNETIRLGSMETTAA
IRLPQLLNECMQQHPNLPLELQTHPTGYLLNAVQQGEVDCAFVASKYVIPELYSFPVW
QEQLVLVCPKQQIDFPDIATLAKTRFIAFRQGCSYRHAIELFLNDHSVPPSQIMEMGS
LDGIVSCVELGVGFALLPKSYLNKVADKVSLSCFEINTESAHFTTYLVAQPPETWGSN LKQFICFIEQQFIQKCA"
/product="Transcriptional regulator, LysR family" /transl_table=11
CDS 3069127..3070521
/locus_tag="AUO97b_02991" /db_xref="SEED:fig|400667.82.peg.2856"
/translation="MNIPLIINIVVFVGLIFLLAQTRKTDWSLSKKVLAGLILGVVFG
LGLHLIYGGGHPILAESISWFNIVGNGYVKLLQMVVMPLVFVSILGAVAKLHQASSLG
KISFYTIGTLLFTTLIAALVGVFVTNLFGLTAKGLVQGAAETARLTAIQTDYVGKVTD
LSVPQFILSFIPSNPFAELTGANPTSIISVVIFAVFLGIAALNLMKDDAEKGQRILTA
IQTLQSWVMKLVRLVMTLTPYGVFALMTKVVASSNVADILNLGGFLVASYLGLAIMFV
VHAIILTVTGVSPLKFFKKVAPVLTFAFTSRSSAASIPLSIEAQTRRLGVPESIASFS
ASFGATIGQNGCAGLYPAMLAVMVAPTMGINTLDPMWIASLVGIVTLSSIGVAGVGGG
ATFAALIVLPAMGLPVTLVALLISIEPLIDMGRTALNVNGSMTAGVVTSQFMGQTDKA IMNSEDEIELTQHH"
/product="L-cystine uptake protein TcyP, sodium:anion symporter family"
/transl_table=11
CDS 3070670..3071485
/locus_tag="AUO97b_02992" /db_xref="SEED:fig|400667.82.peg.2857"
/translation="MKMYQVDAFTKELFRGNPAAVIVGKEWPDADLMQKIALENNLSE
TAFVKIIDSENYEIRWFTPTVEVDFCGHATLASAFVLFKDFTEKKTLNFHVRNLGLFV
VHQDEDGKIRMDFPIRKAQQVEDYPAILRQALTKPFKAVYQNVQAYIVEYESVQDVLD
EQPDMNLLKALGKRTAITTTAMDVAITSKADHQYDCVSRYFAPVAGIDEDPVTGSIHT
AIAPLWAEKLAKNNIVAYQASSRGGVLYCNVLNQERIEISGYGKLYMQAEIFP" /product="Phenazine
biosynthesis protein PhzF like" /transl_table=11
CDS 3071608..3074370
/locus_tag="AUO97b_02993" /db_xref="SEED:fig|400667.82.peg.2858"
/translation="MFMQFKQLTLSLCIFGLSAASFAQTTLVKSKQNVEEYKLDNGFR
VVLAPNDKENKIFINTIYLTGSLNDPQGKSGLAHLLEHLAFKGTQNVKGEEFQRRLDQ
YTLMTNASTDYYSTKYTNIVRPEKTALDQVLYLESERMDKLVLQEKFVPSEIEIVKRE
REVRMDQPFAVLMDQMWKSAYGNQYLGRLPIGDLPELKSIKMPELNQFYRSWYAPNNA
VMVISGKFDKTDVLKTIDQYFSPIPARAVPKPVQVPVLDSTKLKNREFVVKKGSDLAK
FHIYMDGKNTKIQPELAFAPMLYTMQPSGHLYQNMVETGVSTDVQASTWLDQDFNVVF
LGAIYSPKNDPKKVESALLSGIEKGKPFTETELNRVKSLMKTQGELVNKDAVALGSRL
SDYTVADGQWDQYFKDLDSIDKVKLNEVNQTLKQFLVAGHRIDGDILPTPEDQKKALQ
LDAKDKPKTLDQTDVKAEPLKDPKVYQQEVAEFLKTSKQYAESNEKKIIRGKLKNGMK
YALFPVETRDDRTYATITMDFGTEKSLFDKGTVVDLTSYLLLRGSDKYSLQDIADKSI
DAGGAAYASADGNGMTINIQSKTEKFDEFFKFVLEVMKNPKFEQSQFDLIKSQSLSSL
DRPYTEPDVVAGLTLSRLVEEYQPGDLRYHFEPELAKKQLKNVTQEQVKELYQHFFAM

NHAQIAITGAFDAQKMKKLLNQEFGSWNSKQPYEKILIQHVDFPAQQVHVLSEQREFG
SYQSVLALPVGKNHPDASALILMNYILGESQISSRLAQELREKNALVYGFGTGLQLDR
DTNVGALSISANYTAGRSAQVSASVHKVLNDLITHGITEQELAAAKADIMKKRVTALE
DERNIHGMLNLELESGKTLLDRVKHDQELTKLTVADVNAVIKKYIKPSNLVEVMADQY GQAQKQ"
/product="Zinc protease"
/transl_table=11
CDS complement(3074428..3074811)
/locus_tag="AUO97b_02994" /db_xref="SEED:fig|400667.82.peg.2859"
/translation="MSQKINATDITEEEALNAVFFERADEFIKQANDFCRPPKGQKPD
PAELRGQVSAAMLFATARFNTWVAANNFKDGNEMRDAKDQVMSYLLQQFQMMLEDNFD
EYCEQFDNYLRFRKNEDFHTHKHDH"
/product="hypothetical protein"
/transl_table=11
CDS 3074915..3075547
/locus_tag="AUO97b_02995" /db_xref="SEED:fig|400667.82.peg.2860"
/translation="MQDAIAVQSLKTDIALLRQHIFPPQYLEHVEGLPIYYGLQEEVV
AYYQQWKDLIERAQELFQPFMEDELPDAIHLPSHLNLPLFFFHVDRIRINKTRAKESK
TFRGVASLIEKCGQFDTDQIFTMQEWLQSDDTAALVAHREFIDLRTYVFQHGQSEYTR
SRFYTNGIVLGVEPHFKLVDARDKPRKQRSDSYSDPLADNGVWKVFGKYR" /product="hypothetical
protein"
/transl_table=11
CDS complement(3075583..3077046)
/locus_tag="AUO97b_02996" /db_xref="SEED:fig|400667.82.peg.2861"
/db_xref="GO:0004016"
/translation="MVEVELKFQLPESKKKTVQQYLKKHKAQHIHLQAKYYDTPDRLL
AKNGMALRLRKENDLWVQTFKAAGQSHLHRVEEEVFLGKCDQEPDLNLELYKDNKAVT
DLITQALGSEVEKLSLQFETDVQRTYHVFEADDTAIEVCLDDGAVKTATAQSKICEVE
FELKQGAVKTLIQFAQQWINRYELWLDVRSKAERGNLLALGQAASPAVHAKNLNLDKN
ISAEQALKKIVENCLGQFLPNMAAIADGVAEAEHIHQARVSLRRLRSALKHFSAWSDE
LNPVWEEQIAELFRQLGDTRDEDAIRTEILPMIVQHGSPELLLPISEQPLKDLATLFK
SADTIKLILALLAFAYSEEDNQNGKGKLKKQIGKSLDKLHHQVVSNADHFTELEVTEQ
HRTRKKAKQLRYCIEFISSLYPRKNIQQFLKQLQPVQNTLGLYNDLFIAEDLFNKAIE
HDPHFWFALGWVKAKQPYLQNQSAEALQKFKQAKTFW"
/product="Adenylate cyclase (EC 4.6.1.1)" /EC_number="4.6.1.1"
/transl_table=11
CDS 3077218..3077595 /locus_tag="AUO97b_02997" /db_xref="SEED:fig|
400667.82.peg.2862"
/translation="MNAPVNFNHETQPEFELPIKNYQEFYRFYLTEHRNIMSRRLHAV
GSSIGLYFFSKAIRKKQAKYALYGLVSGYACAWVGHFMFEKNKPASFKQPLYSFISDW
RMLSDVATGRLSLIDRKHDKIPS"
/product="FIG027190: Putative transmembrane protein" /transl_table=11
CDS 3077711..3078322
/locus_tag="AUO97b_02998" /db_xref="SEED:fig|400667.82.peg.2863"
/db_xref="GO:0004789"
/translation="MRGLYLITNDDPIQLLLEKLDAALATRQIAILQYRRKKVDKADQ
PAEVEQIKQLCEKYQVPFVINDDLKLAAQFGLGVHLGQSDGEITDAKSQLPEGVIIGR
TCLNSLELAQKAIADGATYVAFGAVYATATKPEAGNVGIEVIKQAAAQYDLPICAIGG
LTVENSKPVIEAGADLCAVISDILGRSTAEIPARVQAWAQLFS" /product="Thiamin-phosphate
pyrophosphorylase (EC 2.5.1.3)" /EC_number="2.5.1.3"
/transl_table=11
CDS 3078365..3079663
/locus_tag="AUO97b_02999" /db_xref="SEED:fig|400667.82.peg.2864"
/db_xref="GO:0042286"
/translation="MSLSPKQEQLFKQASKHIPGGVNSPVRAFNGVGGTPVFIEKAKG

AYLWDVDGKRYVDYVGSWGPMILGHAHPDIIKAVQTAAEDGLSFGAPTVHETTLADII
CEIMPSIELVRMTNSGTEATMTAIRLARGYTGRDKIVKFEGCYHGHSDSLLVKAGSGL
LTKGEGEPTSKGVPADFAKHTLTLPYNDIAALKECFAKFGHEIAGVIIEPVAGNMNMV
KPIDGFLQAIRDVCDEYKSVFIIDEVMTGFRVALGGAQSVYNVKPDLTTLGKIIGAGL
PVGAFGGKREIMECIAPLGGVYQAGTLSGNPLAMRAGIEMFKHLRQPDFYSKLSAQLE
KLLAGLQAAADEAGIPFKTQQAGAMFGLYFTDQEDITSFDSMLACDIEAFKKFFHGML
KRGVNLAPSAFEAGFISSAHSDEDIEFTIQAAKETFAEMK" /product="Glutamate-1-semialdehyde 2,1-aminomutase (EC 5.4.3.8)" /EC_number="5.4.3.8" /transl_table=11
CDS 3079725..3080429
/locus_tag="AUO97b_03000" /db_xref="SEED:fig|400667.82.peg.2865"
/translation="MFYSLPRKIQLAASTSNWPIESTQSILLLVGLDDLEKISDWAHQ
PLADHLEMLSKRAQALEIPVMMIQSSQLQQAMLQLGQHLSSNTQAQVIMAGNLSPLFK
QVMQLVLSITDYVAVVNDAILASSLEQHIQWIEKISFDHIQHINTQTLMRLWSLSAPS
SQVLSDKGILLAVAEQVGRHPMEIHPEIDLRNYGLDASGVNYLVELWRANGASLTVDE LMQTPTLQHIMQLLKR"
/product="Isochorismatase DhbB"
/transl_table=11
CDS 3080562..3081200
/locus_tag="AUO97b_03001" /db_xref="SEED:fig|400667.82.peg.2866"
/db_xref="GO:0004730"
/translation="MSDFLSEHLIYRDDDFMVIHKPSDILSVPGKGDLHDSVLTRLVA
IEPRTLLIHRLDRDTSGILVFGLSKRGQSAIARQFQDRQTSKIYEALVAGHLEGEGTV
DIPVVYDPSRPPLHIVDASHNKPALTHWQAIEHFQIQGQPVTRVKLTPITGRSHQLRV
HMQYLGHPIVGDTLYATPEQLLVPRLCLHAKQLSFNHPVTGESLTFNCPVPF" /product="LSU rRNA pseudouridine(746) synthase (EC 5.4.99.29) @ tRNA pseudouridine(32) synthase (EC 5.4.99.28)" /EC_number="4.2.1.70"
/transl_table=11
CDS 3081354..3084182
/locus_tag="AUO97b_03002" /db_xref="SEED:fig|400667.82.peg.2867"
/translation="MQQYAIGQRWLSDTETELGLGVLIDVDERSVSILFPKSDETRVY
ARNNAPLSRIIFNTNDEVQDQEGQKWIVESVEDRHGVLRYNVVRTLENGEQERKALNE
TRIGAQIQLSKPLDRLLASQIDYKEWYDLRIEAMLMQAQMQTSPLRGLVGARVGLIPH
QLYIAHEVGKRFAPRVLLADEVGLGKTIEAGLIIHQQLKTGRSERILILVPDSLQYQW
MIEMRRRFNLQFSLFDLTRTASIKEHDPELNPFLTEQCIIASVDLMVDHDDLREQALE
AGFDLLVVDEAHHLMWSEEEGGNDRYDLVEELAENTAGVLLLTATPEQLGVESHFARL
RLLDPQRFSSLERFLDEEAQYQQTAQVAEVLMSDEPLTQEHLTALEGLLGHSIEDQPE
QRFRAIHELLDRHGTGRILFRNTREAIQGFPGRDCQPAPLPAPADWSKDGKLREQMWP
EEAQIDGSWMQNDPRVPWLMEILRKELKHKKVLLIARSGPVVESLESALRLHAGIRTA
MFHEGMSLLERDQAAAYFAEESYGAQILLCSEIGSEGRNFQFASDLILFDLPANPDVL
EQRIGRLDRIGQENRIQIHVPYLMGTAQERMFRWYNEALNIFSNISPTAQTLQENFIV
ELKDCLLADQGQTFEDLLEEVNVQRQALEAELQAGRDRLLEYNSCRPIVAQEIVQALE
DYDDNTTLPMFVKRFMSSTNIDFDEQSNGTVIIRPTDQMQVQGLALDEEGMTATFYRD
QAQMREDAQYLTLEHPFIESVMEMIRTQSFGSTNVAVLKSNALKQGSVLLEVWFKVDV
VAPKSLNLPSSLPKQLIRVLLSENGQDLSEKIDPTILKPYLHHLDGNSCRQVVKARRE
VIEERYKQALDIAKEGLPQLQQQAKEHYGNKWQYEIDRLTYLKQFNPSIRQDEIERLQ
KLQKEGLSLLDGLTVTPEAIQVLVVVKP"
/product="RNA polymerase associated protein RapA" /transl_table=11
CDS complement(3084238..3085254) /locus_tag="AUO97b_03003"
/db_xref="SEED:fig|400667.82.peg.2868"
/translation="MQSLSNRQSLIASYILMGIFLLSVIPLHLLASFFAGFLIYEIII
SLSSIVERYIDGQRARIFISILLSIITISLIAFFITSLISFVVYDLKGVGLHSINGKV
DQTLLHLQAEIGKYLPGYIPDSVTELKDEIFAFMKDNVTTLKNASSDILHNLVTMVMG
LIIGILVAIHGFHRRTPQPVFKSLLIQRIQKLSISFRNVVFAQIKISAINTLLFILFA
FVLLPIWGVHLPFAKTLTILTFMFGLIPILGNLISNTLTFIAALTISLGLAGVALLYL
VLVHKLEYFINAKIIGHKINANAWEILLAMLIFESIFGLSGLIAAPIFYAYLKLELKD ARLI"

/product="putative membrane protein"
/transl_table=11
CDS complement(3085421..3086116)
/locus_tag="AUO97b_03004" /db_xref="SEED:fig|400667.82.peg.2869"
/translation="MTQTSLSQSPNDTTSSQDVIEIKTVTRSVGRKATITKEELFQAA
LNLIGPQKSIASLSLREVAREAGIAPNSFYRHFKDIDELAISLIDRAGIVLRKIIRQA
RLRASLQDSIIRSSVEIFLQQLDADEGNLSLLLREGFTGSASYKAAVDRQLNFFQQEL
QEDLIRLERLNNNHLYHPELVAKAITQLVFHMGAKVIDLPIDQRAEVAEQTMIMIRMI LEGARHLDEAKIR"
/product="Unsaturated fatty acid biosythesis repressor FabR, TetR family"
/transl_table=11
CDS 3086257..3087327
/locus_tag="AUO97b_03005" /db_xref="SEED:fig|400667.82.peg.2870"
/db_xref="GO:0005506"
/db_xref="GO:0006118"
/db_xref="GO:0009055"
/db_xref="GO:0016491"
/translation="MQAIEKRNSLFNSLTQAVFDKNMANFWLQKINPLWSVQHGLVQI
VKKEFVAHDTVSLTLKCNRLVKMGAAGQHHPVIVEIAGRRYERTYSLTQIDAEHLRLT
VKKVADGIVSNWFIAESKIGDVFELGQPYGDMQQNIQTPKLIMLAAGSGITPMLSLIT
AIKQSQQLEKTQVQLLYWVKQRSDAAFTEYFEQVAEQNPNFSYQVFYTQETPNDERLN
AEHLALVDGIENSTVYACGPSGFVSTVEQLFEKAPTVLTEAFSLTNESSADDIGYVNV
TLTQSNKVIAIPKGQSILVSLEHEGLKPTHGCRMGICNKCVCSKTQGSTRNLLNGSQN
TEPSQLLKICVNSAQSDLVIDL"
/product="Flavodoxin reductases (ferredoxin-NADPH reductases) family 1"
/transl_table=11
CDS 3087340..3088434
/locus_tag="AUO97b_03006" /db_xref="SEED:fig|400667.82.peg.2871"
/translation="MNMPVKVEYFKNPKNRDLTPAELEAFAKELDQIKQEVLDDLGEK
DAKYIRRVYAAIRYSSFLGRACLFAGWFPPAWILGTGLLGFSKIMENMELGHNVMHGQ
YDWMNDPKFNGQTYEWDTVGTSDNWRQTHNYKHHTYTNIKGIDDDIGYGLLRLFPEQR
WKPGFLLQPIYSIPFCLLFQWGVAIQNLEIGRVLYKRKTKAQFLEELKPVNKKIGKQL
FKDYVFFPLIAGPAALPVFTGNLVANGLRNIWTFSIIFCGHFTKDAEVFPKSVLQEES
RGHWYMRQIRGSSNLTGSEAFHILSGHLSHQIEHHLFPDIPARRYRQMAPKVEAVCKK
YGLNYNNASFVKQFGQVVGRIVKYAFPFKK"
/product="Linoleoyl-CoA desaturase (EC 1.14.19.3)" /transl_table=11
CDS complement(3088501..3089076)
/locus_tag="AUO97b_03007" /db_xref="SEED:fig|400667.82.peg.2872"
/translation="MNKPLLLVFHPDLSQSKVNRALVATLQDQDKIDIVDIQKLYNGS
LDKMNDGAIEAQRLISASHIILQFPIYWYSVPPIFKAWMDAVLTRMFYLFYEQEGQHI
EGKPLYLHITVGNLSSAYQKLGQNGFTLDELMAPLKATARRCGLIWHPPHVIFNANQL
NENDITEQTSLSIKQFEQWGIFNNNSIIPSY"
/product="Uncharacterized NAD(P)H oxidoreductase, YdeQ/YrkL/YwrO family"
/transl_table=11
CDS 3089196..3090092
/locus_tag="AUO97b_03008" /db_xref="SEED:fig|400667.82.peg.2873"
/translation="MNKQISWDDQKFFLVVLEEGSFSAAARKLGVTQPTVRTRIEHLE
HQLGTVLFSRSVHGLKPTEHALALEVPARAMQRASDYFMRTASAPLNLIAGSVRVSVS
DFLGTMVLPQLLSKFRLLYPEVNIELSLNNSSANLLEQEADLAIRMYPPVQEALVAKK
IGEVPLGLYAHRDYIQRKGNPTCLEDLRQHDLIGPDRNLLEREMIRMLLPELKNEQFK
IRTDSHPAQFSAVKAALGIGFIQCPIANQCQELIQVIPDLEVHRYDLWLVIHEDFRQI
PKIRTLFDYLAQALGNYLIGKI"
/product="Transcriptional regulator, LysR family" /transl_table=11
CDS complement(3090211..3090912)
/locus_tag="AUO97b_03009" /db_xref="SEED:fig|400667.82.peg.2874"

/translation="MRTLAISTLLLGTVLSPMVFAQDDNALHYNIVNVQAEASRQVSN
DEMHATLYIEKSNKQPAELSNQINQLMNQALATSRKYPQVKVETGAQSTYPIYDNDSN
KLKEWRGRAEIRLESKDFKAASQLINELQQSFQTQSINFSVSDEQRKKVENELMVEAS
KNFQQRAQMLTQAWNKSQYSLVTLNLNTNNYFPQPVMRASLAKFAAAEAAPAQDMAAG ESKITVNANGSIQFK"
/product="probable periplasmic protein NMA1059" /transl_table=11
CDS 3091380..3092753
/locus_tag="AUO97b_03010" /db_xref="SEED:fig|400667.82.peg.2875"
/translation="MSVTSVNPATNATNEYYLTRQSQMESNVRSYPRKLPLAIAKAQG
CWVTDVEGTQYLDCLAGAGTLALGHNHPAVIQSIQDTLASGLPLHTLDLTTPLKDAFT
EALLAYLPGGKEEYCLQFCGPSGADATEAAIKLAKTYTGRSSVISFSGGYHGMTHGSL
AMTGNLSAKNAVNGLMPGVQFMPYPHEYRCPLGLGGEAGVDALTYYFENFIEDVESGV
TKPAAVILEAIQGEGGVVTAPVKWLQKIREVTEKHNIVLILDEVQAGFARSGKMFAFE
HAGIEPDVVVMSKAVGGGLPLAVLGIKRKFDAWQPAGHTGTFRGNQLAMGTGLVVLET
IKEQNLAQNAQERGEFLQAELKKLATEFPCIGNVRGRGLMIGVEIVDERKPADRIGSH
PADSQLAAAIQTACFNNKLLLEKGGRNGTVIRLLCPLIITQEECVEVIARFKKAVAEA LVAVRGA"
/product="Diaminobutyrate--2-oxoglutarate aminotransferase (EC 2.6.1.76)"
/EC_number="2.6.1.76"
/transl_table=11
CDS 3092756..3094288
/locus_tag="AUO97b_03011" /db_xref="SEED:fig|400667.82.peg.2876"
/translation="MVDFAEHRKALLCNDAQSIADYESAMGEAVKAVSAWLQNEKMYT
GGSIKELRSAISFQPSKEGMGVQQSLQRMIELFLNKSLKVHHPHSLAHLHCPTMVMSQ
IAEVLINATNQSMDSWDQSPAGSLMEVQLIDWLRQKVGYGSGQAGVFTSGGTQSNLMG
VLLARDWCIAKNWKDENGNPWSVQRDGIPAEAMKNVKVICSENAHFSVQKNMAMMGMG
FQSVVTVPVNENAQMDVDALEKTMAHLQAEGKVVACVVATAGTTDAGAIDPLKKIREI
TNKYGSWMHIDAAWGGALILSNDYRAMLDGIELSDSITLDFHKHYFQSISCGAFLLKD
EANYRFMHYEAEYLNSAYDEEHGVPNLVSKSLQTTRRFDALKLWMTIESLGEELYGSM
IDHGVKLTREVADYIKATDGLELLVEPQFASVLFRVVPEGYPVEFIDSLNQNVADELF
ARGEANIGVTKVGNVQSLKMTTLSPVVTVDNVKNLLAQVLAEAERIKDAIASGNYVPP ID"
/product="L-2,4-diaminobutyrate decarboxylase (EC 4.1.1.86)"
/EC_number="4.1.1.86"
/transl_table=11
CDS 3094565..3095995
/locus_tag="AUO97b_03012" /db_xref="SEED:fig|400667.82.peg.2877"
/db_xref="GO:0004029"
/translation="MQQANMLNEFKLIIGGQLCSGEQGELEIINPATGLTAAHCAKAS
VAQVNQAVSAAKQASKAWQLVSHDERKSILNKIADGIEKHAEMLAELVVLEQGKPLAL
AQMEVQGAIGWTRYAASMDLPVEVIEDSETKRIERHRQPLGVVASITPWNWPLMIAVW
HIMPALRAGNVVISKPSEYTPLSTLRLCEIIQQEVPAGVISIVVGAGEIGEALSSHPD
VQKVVFTGSTRTGQHIMAGAAQQLKHLTLELGGNDAGIVLPDANIDEIAAKIFNMAFL
NAGQTCAALKRLYVHESQYEALSQKLADIANAQVVGDGMASSTTFGPVQNQMQYNKVK
ALIAEAIAQGAKALSGQQQLPEQGYFIAPTILTEVSDSCRVVQEEQFGPVLPVLKYTD
INDAIARANDSEFGLGGSIWSSDLKAAQTYATQLQCGTVWINTHAEVLPHAPFGGWKM
SGLGAEFGLEGLLENTIGQTVHISKI"
/product="Aldehyde dehydrogenase (EC 1.2.1.3)" /EC_number="1.2.1.3"
/transl_table=11
CDS complement(3096067..3096552)
/locus_tag="AUO97b_03013" /db_xref="SEED:fig|400667.82.peg.2878"
/translation="MDKFDWQIIQALQKNGRLTNQEIGDLIGLSASQCSRRRQLLEQK
GVILGYTARIQQQALGLEVTAMIHINLRTHEAKSQQAFQELIEAEDNIQDAFSISGDA
DFILKVVAENLEQLSQFVTERLLSYNFIGHIKSYIVLKKIKEQNHFLIKPKSMSYHHS Q"
/product="Transcriptional regulator, AsnC family" /transl_table=11
CDS 3096687..3098408
/locus_tag="AUO97b_03014" /db_xref="SEED:fig|400667.82.peg.2879"

/db_xref="GO:0004737"
/translation="MFIEIGEFLNLRLKQMGIQHLFGVPGDFNLSYLEQVEADPQLEF
IGNCNELNAAYAADGYARINGFSALATTYGVGDLSAINGIAGAYAENVPLIHISGIPP
LHAVQKGTLIHHTLVDGNYDNIMNCMKEFTVAQTRLTPANAASEIDRVLRQCFLERRP
VHIQLPGDITHVKIEVSERPLDLSYPAVEPELLQSVVSKLCDIIENAQSPALLIDNEA
SVFGVTSLLNDLSQKCSIPFAGMNTAKNIMDEGSPRYIGTYVGGASQPHVKNIIEQSD
CLIGVGARFTDVGSAVFTHQIETKNYIEIKSYGLNIFGQDFPGIEIGQLLVELNKKVA
PRKSSIPLLEKQPQKVIEAPAQQKLSQDVLWNYISGFLKEDDVIIGEVGTSNSALSGI
KLPATAKYIAQPLWGSIGYTLPALLGSLLAAPERRQILFIGDGSFQLTVQELSTIIRH
GLKPIIFLLNNGGYTIERLIMGENAAYNDVQNWKYTEIPAVFNGKKGHTTYVVETAGQ
LKSVLDNVQQNDQLTFIELKLPAMDAPVSLKKFASVIARFDYGDRGYEILKERSQPVK CKDAISF"
/product="Pyruvate decarboxylase (EC 4.1.1.1); Indole-3-pyruvate decarboxylase (EC 4.1.1.74)" /EC_number="4.1.1.1" /transl_table=11
CDS 3098549..3099928
/locus_tag="AUO97b_03015" /db_xref="SEED:fig|400667.82.peg.2880"
/translation="MDANNKHELKQGLSNRHIQLIALGGAIGTGLFLGLSQTIKLAGP
SILLGYAIAGIIAFLIMRHLGEMVVEEPVSGSFSYFANKYWGKMAGFMSGWNYWVLYV
LVSMAELSAIGTFIQFWWPEIPTWLTALFFFILINGINLVNVRFFGESEFLFSCIKIV
AILSMIGFGAYLLLSGSAGPQAGVANLWQHGGFFPHGIHGFIMALAVIMFAFGGLELI
GIAAAETKKPETTIPKAVNQIVYRILIFYIGAIGILLCLYPWNMVAEGGSPFVLIFQS
LNSNGVANVLNFVVLIAAISVYNSCIYCNSRMLHGLAEQGNAPAILKKVNSRGIPVPA
AIVSASITAVCVVVNYLIPGQAFQLFMMLVVAALVINWLMISVTHLKFTKAMKLQKRQ
TKFQSIMSPWSNYLTISFVCFILIIMAMTPDMRLAVILGPIWLAVLAIMYFFKYRKKM VAMPEVQSN"
/product="Aromatic amino acid transport protein AroP" /transl_table=11
CDS 3100384..3101415 /locus_tag="AUO97b_03016" /db_xref="SEED:fig|400667.82.peg.2881"
/translation="MRLNPRQIAIALAVTGAAVTTTANAARDTIQIAGSSTVLPFASV
VAEEFGNTFPQFKTPVVGSGGSSAGLKQFCQGVGDNTIDVANASRKIKSTEIEACNKA
GVKQIQEIKIGYDGIVFASSSSKAAYKLKPYHVFAALAAQLPSKGKLVPNPYTNWNQI
DKSLPNEPITLVIPASNHGTREVFQEKMVEAGCEAYEYFKSLDKDAQKKACSTFRKDG
RVIEIAGDYTETLARLKTSPSAVGVFGLSFYDMNRDKLRVATVNNVVPSEKTVLNGTY
PVSRPLYFYVKGEHLKSVKGLPQYVEYFLSKKATGKGSKAERDGLIAMSDAERAKVLA DFKAGKTVK"
/product="Phosphate ABC transporter, substrate-binding protein PstS (TC 3.A.1.7.1)" /transl_table=11
CDS 3101516..3102895
/locus_tag="AUO97b_03017" /db_xref="SEED:fig|400667.82.peg.2882"
/translation="MNLLLIGVLLALVAIAYQLGLRKSRHLAGKGSNSATLHSRPGYY
GALVGLWCGIPAFLILIIWNLVEPSILNHIIFNNVPASVTASLDEAARSVLVDRVQAI
ASGFGVSDHPAAYELAAAQQYAKFQTIGSFAKFAVVVCAALLGLVWAKKKISQEYRAR
NQVERAINVGLILCSGVAILTTIGIVMSMFGEAMHFFHFVSPIDFFFGTEWNPGFSTS
GNAEGSYGLLPLLWGTLMVSGIALLVAVPVGLMIAIYLAEYASPWLRSWAKPTIEVLA
GIPTIVYGVFAMMIIGPFFKAAGAYVGIEINATSALTAGFVMGIMIIPFVSSLSDDII
TQVPRALRDGSLGLGATKSETIRQVVLPAALPGITGAFLLAVSRAVGETMIVVLAAGN
SPLLHANPFEAVSTVTVTIVKQLTGDTDFASPQALVAFALGLTLFVITLGLNIVALYI VRKYREQYE"
/product="Phosphate ABC transporter, permease protein PstC (TC 3.A.1.7.1)" /transl_table=11
CDS 3102892..3104289
/locus_tag="AUO97b_03018" /db_xref="SEED:fig|400667.82.peg.2883"
/translation="MSTSNTSSPMDQNVFDPQAAAQARDRRKKVIEKSLAKRHRKEKA
FRFAGLSAVVIGLAFVALLFGSILAKGLPAFWQTSMNVPVYFDPKVIDAGPVPVRTQG
ETPAHYQERYVDWQTKMGMVDWDSLIVNGMIAKDPSLASQRDYLSSLYASSEAYRLRD
MVFADPSLIGKKENLTFLGDANVDVWLKGNIDRSLPDDQQQLDPEIRKLADDLKAKGV
LENTFNTTLFKNPDSRSSPAISGLAGAFMGSLFMMLIVIIISIPIGVASAIYLEEFAP
KNVITDIIEVNINNLAAVPSIVFGLLGAAIFIGWMHLPLSAPFVGGLVLSLMTLPTVI

ITTRASLKAVPPSIRQAALGLGASKVQTVFHHVLPLALPGIMTGAIIGVAHALGETAP
LLLIGMSAFVASVPTTPFDQATALPVQVYLWQGNELRNFFEGRTAAAIIVLLALMIGL NSLAIWLRKKFEVRW"
/product="Phosphate ABC transporter, permease protein PstA (TC 3.A.1.7.1)"
/transl_table=11
CDS 3104303..3105205
/locus_tag="AUO97b_03019" /db_xref="SEED:fig|400667.82.peg.2884"
/translation="MNTIDITNSLEKDKLVNTEQSQLTDTQLHHGTSYVSHFEPHASK
KPNSTEIKISAQDAHVYYGDFEAIKGIDLDIYQNEVIAFIGPSGCGKSTFLRTLNRMN
DTIDGCRVTGKITLDNKNIYDPNLDVVLLRAQVGMVFQKPNPFPKSIFDNVAYGPKLH
GLARDKYDLEEIVENSLRKAGLWEEVKDRLNQPGTGLSGGQQQRLCIARTIAVSPEVI
LMDEPCSALDPIATAKVEELISELSDQYTIVIVTHSMQQAARVSDRTAYFHLGDLIEV
NSTEKVFTQPDHQLTEAYITGRFG"
/product="Phosphate ABC transporter, ATP-binding protein PstB (TC 3.A.1.7.1)"
/transl_table=11
CDS 3105352..3106314
/locus_tag="AUO97b_03020" /db_xref="SEED:fig|400667.82.peg.2885"
/translation="MLFHLPKLPAEIRVSHLNARVNEQRKKIAQTTASRLELLQLAQQ
LAKEAKIRRKNNQKIFVLDFKGDIQASAVENLREEITLILATAKAGRDRVVVRLESPG
GMVHGYGLAAAQLVRLRDAGFHLTICVDKVAASGGYMMACIANEIISAPFAVVGSIGV
VAQVPNFNRLLKEHNVDFELYTAGQYKRTVTMFGENTPEGKAKFEEELQQTHVLFKHF
VEKYRPQLNVDKVATGEHWYGQDALDLNLVDKLQTSDEYLLALLPQHDVYVINTRKKA
TLGEKLGLQAAQMADSLIPAVMNKVADTLAKANSTLVQMRDTKF" /product="SohB protein,
peptidase U7 family" /transl_table=11
CDS 3106623..3108779
/locus_tag="AUO97b_03021" /db_xref="SEED:fig|400667.82.peg.2886"
/translation="MDVLQDGQLNKVVLNQPTIIQIGVSQKDIASMEKQGGSLVIHLK
NGETIILENFFNEATNTTEHSLVFPTEQGKFVEAQFDAQGKVIDYRGLNHVTDLAYTS
TSPSTATMAVDNDPSFSMGNVLKAGLAVLAAEGLYLWAFDNDDKDDSPVNVPTTAKPT
EVKLADDAVTVTGKAEANAKIYIKDLSGNVIASGNADASGNFTIKLDKPLTDGNKLNV
FAQNGGGKESIGAAITGTKDTIAPDAPQAQLSEDGSILDGKTEPDAMVFIYDASGKFI
DTTKANSDGKFSYKFTPPLTSEAGGKVVAVDKAGNESEPTKIIAGKDTFAPDIPLVEV
NKEGTMVEGKTEANAKVQIKDADGKVIGTGTANAEGEFQITLSSALKDSQKATIIVED
AAGNTSKPLEIKSGYDTIAPDKPTAQINADGTTVTGTAEANAKIQITIGTKVIGTGTA
DANGKFTIAISPALTDNNKAKVTAIDGAGNSSVAFEVTGTKDTTPPDKPTFNSVYNDN
GTTLKQIIPNSETDDTTPKFTVKVESHASLTIYDNGVAISMIEIGDLGKNTTWSFTLN
KELSLGKHTITLIQKDAAGLPSEESASFTFYVVAPKVASTSEVVSSDVNEVVPSVTDS
VGLNTLKLAQTTTNEMTSQQQKSVSLHDLLKSSSTDASDPIAKILSVTDSKPVVNEQS
EVNNIASDVTTNLEQLLPNTTSSPLQNILEQTYPVV"
/product="Large repetitive protein"
/transl_table=11
CDS complement(3108827..3109468)
/locus_tag="AUO97b_03022" /db_xref="SEED:fig|400667.82.peg.2887"
/translation="MIEKEFKVKHYIALGLLFIAIIISGIQPLEFEAYLLHQAGTVFM
VMLLFIIFKKIGLDFLSFTFYLLFLLIHIIGAHYLYSYVPYNDWIQQVFHFNLDQYMG
WSRNMYDRLVHLAYGVLLYPLIYRVFQVWLPTARPFSLFLLVVQFVMASSVFYELIEW
AIAVGLSPEQAENYNGQQGDMWDAHKDMLLATIGAILYGLFALITSPKLNNKP" /product="Integral
membrane protein"
/transl_table=11
CDS complement(3109551..3110939)
/locus_tag="AUO97b_03023" /db_xref="SEED:fig|400667.82.peg.2888"
/db_xref="GO:0004018"
/translation="MNALTALSPLDGRYASKCDALRPFLSEFGLIHARVTVEVRWLQA
LSNRPEIVEVAPFSAETNAALDAIVSNFSEEDANRIKEIERTTNHDVKAVEYFLKEKI
AGIAELQNAGEFIHFACTSEDINNLSHALMLKNGREVLVSSMKQILNAISALATTHAE

QPMLSRTHGQTASPTTLGKEMANVAYRLARQIKQFENVELLGKINGAVGNYNAHLSAY
PNVDWPAHSQAFVESLGLTFNPYTTQIEPHDYMAELFDALRRFNTILIDFNRDVWGYI
SLGYFKQKLKEGEVGSSTMPHKVNPIDFENSEGNLGIANAVLAHLGEKLPISRWQRDL
TDSTVLRNMGVGFAQSLIAFDACLKGIGKLELNANRLNEDLDQAQEVLAEPIQTVMRR
YNVEKPYEKLKALTRGQAMTRDMMVDFVNGNELAQVPSEERARLAELTPATYTGNAAE QAKQINELISKI"
/product="Adenylosuccinate lyase (EC 4.3.2.2) @ SAICAR lyase (EC 4.3.2.2)"
/EC_number="4.3.2.2"
/transl_table=11
CDS complement(3110994..3111728)
/locus_tag="AUO97b_03024" /db_xref="SEED:fig|400667.82.peg.2889"
/translation="MVELPFQQSQALNVRQNRALALAGVFQATQLTHMTAMTGQQSIG
ESGNFYFELLIKASLNIRPTTNNNTVQTLDFFNQLADISLGLKTLENCITQPFSNAPK
SRLPKMRSAKLPMSYAMSLLQLEKKVYSNPEYVAIIEKAQQKILKQLSFFDNNYLHPS
ILANLAQTYVDTAGQINPRILVRGNAEAFKDTNHTNRIRACLFTGLQMAHLWRQLGGS
SWNMIFSKRKLLQDIQALARLQYQVI"
/product="High frequency lysogenization protein HflD" /transl_table=11
CDS complement(3111742..3112875)
/locus_tag="AUO97b_03025" /db_xref="SEED:fig|400667.82.peg.2890"
/translation="MQQRVIVGMSGGVDSSVSAALLLQQGYQVEGLFMKNWEEDDGTE
YCTAMEDLADAQAVADKIGIKLHTANFAMEYWDRVFEHFLAEYAAGRTPNPDILCNKE
IKFRAFLDHAMTLGADFIATGHYARRAETAYNSKGEAYAPLLRGLDNNKDQTYFLHAV
HGREINKTLFPVGEIEKPEVRRIAEELDLATAKKKDSTGICFIGERRFNDFLKQYLPA
QPGKIVLDNGKEVGEHHGLMYYTLGQRGGIGLGGMKGASEGAWFVLHKDVANNRLVVG
QGHDHPLMQSTQLWSEAIDWVAGEQNIPAEGLRCTAKTRYRQPDQACTVFIDENSEHG
VRVEFDEPQRAVTPGQSVVFYSDEVCLGGGVIHHTNAPTPNFI" /product="tRNA-specific 2-thiouridylase MnmA (EC 2.8.1.13)" /transl_table=11
CDS complement(3112880..3113368)
/locus_tag="AUO97b_03026" /db_xref="SEED:fig|400667.82.peg.2891"
/translation="MAAWTPHVTVATVVEKDGRYLFVEEHSEGFVHTVFNQPAGHVEC
SETLTEAAIRETLEETGHHIDIDALLGIYTYTPPMFPDRTYYRFCFLAHVTHVESDPK
LDTGIVSAVWMTLDELKESARARSPLVIKAIEDAMKGQHYPLALIYEHPFSPSLTSHL DA"
/product="Nudix-like NDP and NTP phosphohydrolase NudJ" /transl_table=11
CDS complement(3113488..3114033)
/locus_tag="AUO97b_03027" /db_xref="SEED:fig|400667.82.peg.2892"
/db_xref="GO:0008415"
/translation="MAKNIRPYLDHHPQIDPSCYIDEMSVVVGDVKLAENVSVWPFAV
IRGDVNSIQIGKNSNVQDHCMLHVSHKNDAKPNGSPLIIGEDVTVGHHVTLHGCTIGN
RVLVGINTVILDDVVIEDDVMIGAGSLVPPRKVLKSGYLYVGSPVQQVRPLTEKELAF
LPYSARHYVKVQNNHKDNQIN"
/product="Protein YrdA"
/transl_table=11
CDS complement(3114133..3115281)
/locus_tag="AUO97b_03028" /db_xref="SEED:fig|400667.82.peg.2893"
/db_xref="GO:0009002"
/translation="MTRKSAIAALLLLPSFSYAATVLSAPPELNNKSYVLMDYETGQI
LASKNENEKLAPASMTKMMTSYIIEQKLLKGELTENEQVRMNESAWCKGSSSESCMYV
PLNGTATVLEMLRGIIIQSGNDASKAMAEHIAGNEGTFAHMMNQEAKRIGMTNTQFIN
STGMPAEGHYSTAKDMAVLAQHIIKDSSKYYPIYSEKEFTFNGIKQGNRNALLYTDPS
VDGLKTGHTDEAGYCLTTSSKRGPMRLISVIFGTPSMSERADQTRALLAWGFANFETA
NVQPANQVLAKAKVWFGKENEVQVGLAENFNVTMPKGKADGIKTQLVVQPNLNAPLQK
GQVVGKLVASLDGKVIAEKPLVALKPVEEAGFFARLIDHIKQFFSNLF" /product="D-alanyl-D-alanine carboxypeptidase (EC 3.4.16.4)" /EC_number="3.4.16.4"
/transl_table=11
CDS complement(3115408..3115830)

/locus_tag="AUO97b_03029" /db_xref="SEED:fig|400667.82.peg.2894"
/translation="MFMKKSLVQSLSVVLLMTMATVGYAADKKKTAEKKTENENVVEV
TPSKGTTPEELAAIQVLSEICPSLIGKKDAEFAQGYERLVKDYLPNEADPVAALEKRS
KDKSFKKVLKEARNDAKAAGNEQNTLVCQDVKAYQSQN" /product="hypothetical protein"
/transl_table=11
CDS complement(3115978..3116301)
/locus_tag="AUO97b_03030" /db_xref="SEED:fig|400667.82.peg.2895"
/translation="MCTQAVFADEGISTQEADSLIKDDIASTQVLQEICPTFVGANRK
LETNTQKIIAMYLSGYSNKSITFSALQNDSEYKTLLSEARQAAKEMDHHEQHELCEEV VNYKD"
/product="hypothetical protein"
/transl_table=11
CDS 3116452..3117219
/locus_tag="AUO97b_03031" /db_xref="SEED:fig|400667.82.peg.2896"
/translation="MNILIANDDGVFAPGIQALADALKPLGRVVVVAPESERSGFSSA
LTLDRPLRPIQIAEDVWAVNGTPADCVYLSMNGLFDFEFDLVVSGINSGANLGDDVLY
SGTVGAAFEGRLMKQPAIAVSLAGPDVRSYDHKDDYAQAAKWVHDFIAKGLPALPPRH
IFNINIPDVPQLKGTQITYQGRRAQSKPITSHVDPRGRQVYWIGLAGEAVTDPQRIAS
QIQSDFFAVANSFVSVTPIQMDATNYAVLEDLQASLG" /product="5'-nucleotidase SurE (EC
3.1.3.5)" /EC_number="3.1.3.5"
/transl_table=11
CDS 3117385..3118164
/locus_tag="AUO97b_03032" /db_xref="SEED:fig|400667.82.peg.2897"
/translation="MKIMMLSVAVGFTVAMAGCASKPQINNSSRYAMAPNYYTVRSGD
TLSGIAMRYGLDYLSIAEMNGIAPPYRIYVNQSLRLKKSSSPRTVSTQVMAQPEPIKR
QTIALPTTTAPTTTTPPPATVAPSTNTTVGASIPSSSLRWVKPNNGPVIQGFNLANNV
KGIRYGGNQGDPIYAAADGQVVYAADGLKEYGNLVLIKHIDGYISAYAHNSKMMVKSG
DNVTAGQKIAEMGSSGASRVMLEFQIRLDGKPINPANLLPN" /product="Murein hydrolase
activator NlpD"
/transl_table=11 CDS 3118266..3119195
/locus_tag="AUO97b_03033" /db_xref="SEED:fig|400667.82.peg.2898"
/translation="MLDQLRAMGVFACVVEKSSFSGAARELGITTSAVSQQIRSLENE
MDVILLHRSTRKLSLTEAGQAFFSSCQEMLAAAERGKIRINELRDDLIGDLRIATTPD
LAVQHIIPALSHWMSAHRGLSVHFEVGHRYIDLIEERIDIAVRMSSTAVEESDSVIPM
AFVDQILVASPSYLNQTSPILHPNDLNNHELLSINAMNDSRSFNFKHAKTGEKLNIEM
ASRLQSNNLQVAKALCQQGHGIARILYLDAQKELKNGSLIEILPDWKLPAFTLYAEIA
KHDQQPMKIQRCVEALKQYFSQLAGGRAMQIVR" /product="Transcriptional regulator, LysR
family" /transl_table=11
CDS complement(3119253..3121871)
/locus_tag="AUO97b_03034" /db_xref="SEED:fig|400667.82.peg.2899"
/translation="MTQKLDQVTSSTLSLAKTNTTNVTINAFPSAFEQPDIKMTLDKT
ALKPEQLTHIPNFENIPASTKRIKPLNNFLGQDRAKASVEAGISLPYSGYNIFAVGTA
GLGKRTMIKRLLQQHAKTMPTPDDWVYVYNFKQARQPIALRFPAGQGTKFQQTLHQTW
QIILKQLERRFSAETYHNRIERIRQETGDEQQQALVELTKEGEELDLKLISRNEKHGF
VPVQLKDNQVQELTQTEIDALNSKQRAEIAANIRYMDKKLERLGLHLGDLEDDARDKV
SVLNRDIATQVVMPRMDLILNKYGQVKGLEDYLKQYAQDIIDNVELILEQEEDDFAPA
MFNRVPARYQANVIVSNKPNSGAPVIFEDFPTHYNLLGHVEQLTHNGTITTDFTLIRP
GALHQANGGFLMLEAEQLLEQPYAWQGLKRALKSGQLKLSSLEHMLTLTGSISIEPAA
IPLDIKVVLMAEPEIYYEILEVEPELGSVFKIRADFTDTLQRNDENEQAYMQLIADYV
QADKLLPFDRSALAALLTDSSRQAEDQSSLSLHASTLGDLIREAHHHAFKANDKMVTD
QHINLALQHRQYRLGYLRELYWQDLSRGTQLIETSGHRLGQINALSVIHYADVEFGLP
SRLTASVYQGGGDILDIERSVELGGSLHAKGVLLMSSFLKAHFGREQILHFSAALAFE
QSYGQVDGDSATVAELSALISAISQLPIDQSWAITGSMNQLGQVQPIGGVNAKIEGFY
DACKLQGLTGKQGVIIPRQNMQHLMLRQDVIDAVRMGQFHIHAIDTIDQALEILMARP
VGTLDKKGKYSKHSIYAAVMEQLEYWQAIEDGAEVEEEPKKKKKNKKKIKKHEELTAD DLPQETQEEVATLKQK"

/product="ATP-dependent protease La (EC 3.4.21.53) Type II"
/EC_number="3.4.21.53"
/transl_table=11
CDS 3122038..3124227
/locus_tag="AUO97b_03035" /db_xref="SEED:fig|400667.82.peg.2900"
/translation="MENKKRMTTQTSGWVSAFKAFLDRRALIMLFLGFSAGIPILLIF
SSLSLWLGEAGIDKSAVTFFSWAALGYSFKFVWAPLIDELPVPVLTKALGRRRAWLLI
SQCLIVLAICTMAFADPALGHSYLVQMAAGAVLLGFSAATQDIVIDAYRIELAETEMQ
TVLASTYNAGYRIGMIVAGAGALFLAAHLGTAKGNYIYEAWKTTYLTMAAVMLVGIIT
TLLVREPKVDRIYKNYKSSDYYRLVLVFFIAVISFVLTYIYSGQVTEAISKAGNIQDS
AALFGLEALRFLTATGVAILAGYLLVKIGVVNQQMAKETWIAPILDFFKRYGVKLALV
LLFLIGFFRISDIIAGVISNVFYQDLNFSKEQIAEAVKVYGVIFSLVGGFLGGLLAQR
MNIMKLMFVGAVLASSTNLIFIGLVKSGKPLDMVDVQIGNNHYKVKPDEVGYWKLKVP
SSSFNGTQQIDVKAGYALQDQQAETIVRAQPLVSTHSESVPLQILPLMGSDQISANSS
EGSVVIRGQYFGKALSPTQKIVVNLDGQNFDAKMTDQKGVFNAAIDMQKLLSSPSKQL
TASIVDGNQKILSIAHHYEVASNSVPKPELDVNIDPLPYIDTQSGQEVEISGKVIKPY
SSLWLYFAIVVDNMASGLAGAAFIAFLSSLTSVSFTAVQYAIFSSLMTLTPKLLGGYS
GTIVSNIGYPNFFLMTTMIGIPILILVVWVGKLLKDHQTHDIEKAGE" /product="AmpG permease"
/transl_table=11
CDS 3124230..3124631
/locus_tag="AUO97b_03036" /db_xref="SEED:fig|400667.82.peg.2901"
/db_xref="GO:0004462"
/translation="MRMLHTMLRVGNLEQSLKFYTEVLGMKLLRKRDYEEGRFTLAFV
GYGDEENNTVLELTHNWDTSSYDLGNGYGHIAIGVEDAYKACEEIKARGGKVVREAGP
MKGGVTVIAFVEDPDGYKVELIQQDANARNN" /product="Lactoylglutathione lyase (EC
4.4.1.5)" /EC_number="4.4.1.5"
/transl_table=11
CDS 3124771..3125736
/locus_tag="AUO97b_03037" /db_xref="SEED:fig|400667.82.peg.2902"
/translation="MLKFLALDRFTILLVGMVLLATFFPVSGQAAQYFNILTTVAIAI
LFFLHGAKLSREAVIEGILHWKMHLLVFAITFFIFPAIGLLAKPILLPLLGQQLYWGF
LFMCFLPSTVQSSIAFTSVAKGNVAGAVCSASFSNLVGMFITPVLVSFFILGQSQHGF
DPTSSIIQITLLLLVPFVLGQLLRPYVFPYMAKVPSIVKAFDQGSILMVVYGAFSGAV
VAGLWHQVSWKTLLLLTIACSVLLTIIMLLALYLPRVFGFNRADQITVFFCGSKKTLA
SGVPMAQILFIGQPLGMIVLPIMIFHQIQLMVCGVIANYLAKSQD" /product="Sodium/bile acid
symporter family" /transl_table=11
CDS 3125930..3126154
/locus_tag="AUO97b_03038" /db_xref="SEED:fig|400667.82.peg.2903"
/db_xref="GO:0003735"
/db_xref="GO:0005622"
/db_xref="GO:0005840"
/db_xref="GO:0006412"
/translation="MRADIHPKYEKLVATCSCGNVIETRSALGKETIYLDVCSACHPF
YTGKQKNVDTGGRIDKFKQRFAGMSRSIKR"
/product="LSU ribosomal protein L31p @ LSU ribosomal protein L31p, zinc-
dependent" /transl_table=11
CDS complement(3126217..3128325)
/locus_tag="AUO97b_03039" /db_xref="SEED:fig|400667.82.peg.2904"
/translation="MRNSLLSKRLKRTEIRLLIIDDNQLRYNQILNLLSGNDYQVNAL
LLDDLKSFEKQLNTSWDVIIFGRAYDLKIEQTLSLVQASEQPQLPILLLQPDDYQAQQ
YQSYIQKGIYDVVPLEPLDYFYITLVRALSYSRLLQTEQRLLAELDTIHTHTQTLVDN
SHKAVAIFQEGIHIKANTEYLNLFGFQQEEDIIGLPILDVLQPNDLNIFKQRFKKISL
GQFDQARFEIQSQHQAISGNNALKLEFIPSQEEDAVQLTIDYQVDLTAPANSAPSEKS
VGLSAAWPQINRHLSTHPAQANAVVLFRLAQCPERIFQQGWQTLGQYFNQLHGFLKDH

AQMPIFQVDLGSYVGILQAENSSVLNSQLTSLQALQKEHLLKVNEQNFSVQLKLGYAF
LNQPLTENSFSSLLNGAEKQDLPRVTPQLEVMPTVEKTVSPSIEAVPLDLTIEHFDHT
PQSSLLQQLRQKLAQGEIHLKYQQIYDKHDQETHNYEVTSGFIAGEQWYDLNDLSDLK
EDAELSIQLDRWILVEACKQLHHFIAQYPKAKLIINLNIDILLKDKTFPELISKLLTI
IGSKIESPLILQFSEQALQPYLPIAQQHIARLRQHGAEISIRDFASSMYSDSILQQLD
IQLVKLQSSKTDLLSSDQGLNNLQEKILNYLTVKPIGILLAHLNDMNLFANAWNVEAR
FLQGDYFQKKLDRLTDVQDQ"
/product="FOG: PAS/PAC domain"
/transl_table=11
CDS complement(3128454..3130064)
/locus_tag="AUO97b_03040" /db_xref="SEED:fig|400667.82.peg.2905"
/translation="MNNALSDIFHNQTALTRERLSFCEANIKDLNEWVTTLSIMQLGD
TSKALFTAILELNELNCSETLRFDLIQTLHPTINNVLASLEKNFFNQGVISTDRNEHI
IELAMLLRCYFASIYLNIAQTSHEQLQKQKFSLFSFKQKKNLQTARILATFHALQQLT
NLLYQQHMLYSEPVKGQWLIAHQLYETAVQYKYHLTNINHIQGVQHPLANITQAYAQL
ILLDIFNTNQIRQSEIQALFQCSFDWARMIQILPKDTASTKYVVDPTKDHPPVYNKKQ
SSNFNPSIFISTQALLEHVTATMHKNAQYMSKNEKIYLSPALKFHVQTILGTTAQRRH
ERYEYNAALQICFGLSTAHFYLSKAKNFEETLQLSNNYNLQSESKVLSNLEKKEQQTS
SYQRLSRESKTIYQTTVLDISVNGYRIKWSGEAPKNLRTGEYILVKETLHGQWRGGVI
RWIKQSTEKSLELGLEILSQAMFPCAVHIQAERHTRNYHPALLLQTQNLEEIQNTLIL
PGSQIFREQQTIHLRLGTEEIKVYLLKA"
/product="Molecular chaperone"
/transl_table=11
CDS complement(3130083..3131099)
/locus_tag="AUO97b_03041" /db_xref="SEED:fig|400667.82.peg.2906"
/translation="MINYLHQEQNWQSQLSDLITDPLELLNLLELSTDQLLSGAILAS
EKFKLRVPRAFVGKMNAKDPLDPLLLQVLPHHLELEEHPEFVTDPLGEEAANQLPGVL
HKYKSRFLLTLTGACAVHCRYCFRRHFPYQENLPKNEDWLNIKNYIESNPDINEVILS
GGDPLTLSNRKLALWLERLSSLKQVKILRIHSRVPIVIPNRIDEELISLLKNSRLRII
LVVHSNHASELDDFTCSKLLQLSEHHITVLNQAVLLKGVNDSAQTLTDLSYRLFEARV
MPYYLHVLDKVKGAQHFDLIPSEIDAIYQDVLASLPGYLVPKLVREIAGEKNKTPLFG ATTF"
/product="Lysine 2,3-aminomutase (EC 5.4.3.2)" /transl_table=11
CDS 3131173..3131742
/locus_tag="AUO97b_03042" /db_xref="SEED:fig|400667.82.peg.2907"
/translation="MANYSTNDFKPGLKVMLDSNPCSIMENEYVKPGKGQAFNRVKLR
NLKTGKVLEKTFKSGDTLEAADIVEVEMNYLYNDGEMWHFMDPESFEQIAADKTAMGD
AAKWLKDDSNETCTIMLFNGVPLNVNAPNFVVLKVVETDPGVRGDTSGGGGKPAKLET
GAVVRVPLFVQQEESVRVDTRTGEYLERA" /product="Translation elongation factor P"
/transl_table=11
CDS 3131909..3134005
/locus_tag="AUO97b_03043" /db_xref="SEED:fig|400667.82.peg.2908"
/db_xref="GO:0009267"
/db_xref="GO:0016020"
/translation="MDTMQAKSTLPSKLVWSLVAIIGAISFGMLALSRGEHVNAVWLV
LAAACVYSIAYRFYSLFIATKVFELNPRRLTPAHRLADGLDYVPTNKYVLFGHHFAAI
AGAGPLVGPILAAQMGFLPGTIWLLVGVVLAGAVQDFLVLFISTRRDGRSLGEMAKQE
LGPFAGIVVMLGALGVMIIILAVLALVVVKALAHSPWGVFSIAATIPIALFMGVYMRF
IRPGRIAEVSIIGFVLMMLAIVYGGHVAADPYWGEFFTLTGTQLTWCLIIYGFIASVL
PVWLLLAPRDYLSTFLKIGVILGLAVGIVIALPDLKMPAVTHFIDGTGPVFSGSLFPF
LFITIACGAISGFHALVSSGTTPKLVDNEVNIRMIGYGGMLMESFVGIMAMICATVLD
PGVYFAINAPAAVLGTTVESAAEAVRNLGFVVTPEMLTVLAQEVGESSILSRTGGAPT
FAIGMAHIISEIFNSRAMMAFWYHFAILFEALFILTAVDAGTRACRFMVQDTVGIVIP
AVKSSGSFFGNLLGTAVAVGGWGFFVYQGVIDPLGGVNSLWPLFGVGNQMLASMALIL
GTVILFKMKKEKYVWVTIIPTIFLFITCMTAGWQKIFHENPKIGFLAQAHKFSDAIAR

GEILKPAKTIAEMQTIVMSNQINAALCGFFMIVSIVMIIASIGIVRRALASPTPTVNE APAVYTDPEVVTTRGE"
/product="Carbon starvation protein A"
/transl_table=11
CDS 3134008..3134310
/locus_tag="AUO97b_03044" /db_xref="SEED:fig|400667.82.peg.2909"
/translation="MNFKFAKNGTVIIAKIIKMTVLSQKEILLSPKNWSRIATLWQRL
QQSFRLMVGVPDYQTYLEHMKAHHPDLTPMDAQTFYRHCVDARYPSAGGTLKKCPC"
/product="Uncharacterized proteins YbdD and YjiX" /transl_table=11
CDS 3134820..3136766
/locus_tag="AUO97b_03045" /db_xref="SEED:fig|400667.82.peg.2910"
/db_xref="GO:0004035"
/translation="MVTYGNDQNGLWLKFDNFTSNDIRQIEYINNVLYVTNYSGTYYS
LNFNSLAKARMIFSDGVEKQVLFTSGGNGLNISTANSEWIVGTNSVDQISSGAGNDRL
FGGVGDDIYQFSKGDGIDKVTDYSGNNTIQFMASNLADLSIQGNGSNLEVKYSDTDAV
LIEGNVSNFKFLDGTTATLDQLLTNKTITLTGTKGNETINGFASNDIINGLEGNDTLN
GNAGNDTLNGGAGDDRLYGGTGNDLTVGGTGNDWLASGGGDDTYQFSKGDGNDTINDY
SGKNTIQITDVSAKDITFTTNANNELVINYSATDSITLKAYINNYILSDGVVQTYQQF
LTGKTATWNGTDNADNFSGTLPNDVITGGKGNDTLNGREGDDTYRFSLGDGIDTINGD
YSGNNTIVFTDVKSTDVQYSFDKTTLNIQYSDSDIVKLTGYLASNNKNFSIQFADGVT
IDRAQLDTTVGVNYWVRALVATTNSNPDFKYIFPSTAPDYLLDANGQAPAWWSPFSQA
QQDFMTQVYQKASEFSALTFTLTDNASQLNTVAAYRNSTAATAYAQYPSGAFVGSDVA
FGPDFWFDPAGTWVTASYPHELGHALGLRHTFEGSTISSFSDEEDTRKWSVMSYDRTG
MSYDGSFAPFDVAALQAIYGVNSS" /product="Hemolysin-type calcium-binding region:Haemolysin-type calcium binding related" /EC_number="3.1.3.1"
/transl_table=11
CDS 3136777..3138105
/locus_tag="AUO97b_03046" /db_xref="SEED:fig|400667.82.peg.2911"
/translation="MFDSTKGVLIWDGAGVDTINASNATQAATIDLNQGGWSYIGEKS
PHISSANQLSINLGTQIENAIGGAYDDILVGNNLSNRLEGGAGNDTLKGNWGDDVLLG
GAGNDTLDGGKGADSLIGGTGDDTYYVDNVGDSIVENTSEGNDKVISDINYTLGDNLE
NLTLNGADNLNGNGNALNNVIIGNAGNNNLNGYAGNDTLNGGAGNDTLDGGAGNDTLD
GGIGIDTLIGGLGDDIYYVDNVNDVIIEKINEGFETVNSTVSYALDSGNALNNLNLLG
TDNIDGTGNGNGNTIVGNSGNNILSGGTNNDVLNGGAGNDILNGGKDNDTLIGGLGSD
TALYKLLNTNDVTGGNGADVWKDFKVGNTLTDSEADKIDISELLINYANGSTVNSIFS
YLSLDLNGNNSILSIDRDGAGTVYNSTSFLTLNNVNTTLNTLWDNYQILV" /product="Hemolysin-type calcium-binding region" /transl_table=11
CDS 3138339..3139205
/locus_tag="AUO97b_03047" /db_xref="SEED:fig|400667.82.peg.2912"
/translation="MTTQKINARTLITDLLLASEGKIISIKEMILAAKLFGISENGIR
VAVTRLASEGVIVSAERGFYQLSEQSLEWADVMLNRRAGLKETKAWNQKYLCVLTGTL
GRVDRTALKRRERALKKYGFRELEPSVYIRPDNLVISLEDMLEHMKSIGLESGAKMCH
INSFDQITTGMIKDLWQTENLNKNYEKYSHIIQHWLSRVPALGLEDAARESLLLGRQT
ISLLMNDPLLPEPFVNVEARDQFTADVKKLDEVGQRLWKQLHELTFGTAATSLLNLEL PAVADVTPKILN"
/product="Phenylacetic acid degradation operon negative regulatory protein paaX" /transl_table=11
CDS 3139235..3140119
/locus_tag="AUO97b_03048" /db_xref="SEED:fig|400667.82.peg.2913"
/translation="MPLYQSALAKDIFKDQIIIVTGGGSGIGRCTAHELASLGAQVIL
TGRTEEKLQQVKQEIIEDGGKASYRVCDNRDEEKVKQTVSSIVEEFGQINGLVNNAGG
QFPAFLETMSANAFDSVIRNNLLGTFLMMREVFNQSMVKTGGSIVNMTIDMWSGLPSM
GHSGAARAGIDNLTKTSALEWARAGVRVNAVAPGWVMASGLDSYKDGMKAMIPTVSSK
IPLKRMATESEVSALMCFLLSPAASFITGMNVRIDGGASLVSPFFPLIDHEKSKPFEA
FHRDGIPNVIKEMNQSKL"
/product="Citronellol dehydrogenase; Citronellal dehydrogenase"

/transl_table=11
CDS 3140358..3141377
/locus_tag="AUO97b_03049" /db_xref="SEED:fig|400667.82.peg.2914"
/translation="MNTRVSVTELFSREEIKELTEPSDIHGLWAVGSTWAVIGATFAG
VGLTWSYLPWWGKLLICLAALAVLAGRQLALAILMHDASHNSLFKTKWMNDYLVDWLC
ARPIWNDVHKYRTHHTRHHAKTSTPEDPDLTLVAGFPTTKTSLTRKFLRDLVGITGFK
FTVGRILMDAEVLKWTVANDLTWLPRENRQWYDYPKVFLKNSSKAIIANASIFGILWA
FGRPELYLLWPLAYLTPFPLFIRIRSMAEHAGTQISNNALTNTRTTKAGWLARAFVAP
IRVNYHMEHHLMAAVPFFKLPKMHQMLRERGHVPEPPTYVQVIHMLSSHQTNQHPLNS VEQGE"
/product="Fatty acid desaturase"
/transl_table=11
CDS 3141377..3142369
/locus_tag="AUO97b_03050" /db_xref="SEED:fig|400667.82.peg.2915"
/translation="MITDKQTRISDFLSREEISQFSQRSNLWGLWCIFSVWLIIGLAF
TVMALAIDYLPWYFAVPVICIGTVVLGGRHLALAIVMHEAAHYTLFKSRWMNDVFANW
ASAKFIWNDVHKFRAHHMVHHANTTASFDPDRPIYEALPVTKKSLIRKFGRDLVGLTG
IKFFIGRILMSAGVLKWSDATGERADTRGWPWHRYITNFIKDFWPTFVTNLGLYLILA
VTGHAWLFLIWIIAYAIPFTVFVRIRAMAEHACLELVPDTLRNTRTTSAGWIARMTVA
PIRVNFHMEHHLMASAPYYRLPKLHKLLYSRGKVPKPPSYLDVVHLCSSKPIEK" /product="Fatty acid desaturase"
/transl_table=11
CDS 3142412..3143242
/locus_tag="AUO97b_03051" /db_xref="SEED:fig|400667.82.peg.2916"
/translation="MGILAKVFDRGAHFPVRHMSFNFNQSQATLFKDNPWGEYWLATL
SAMFPAGERFFVHSVRNLQNQAKTERLQKDIAAFIGQEAMHAKEHEHLNQLIQSSGLD
TEPVSANVEWLMARLKKYFTPTQQLAITSAAEHFTAIIARQIMRRSDLQERLSSNETL
KNIWLWHAVEESEHKSVAFDLYQDVSGGNFIRLLVMPFVTLYMAGIMAAGTVYLGITH
FSAWKLLHLKEFNALILGKNGFLSTLWKDYLDYYKLDFHPEKHDSKALEERTKAQLGF "
/product="hypothetical protein"
/transl_table=11
CDS complement(3143304..3143984)
/locus_tag="AUO97b_03052" /db_xref="SEED:fig|400667.82.peg.2917"
/translation="MSTPANFNGQAPVIDPNNVAMLLIDHQSGLFQTVKDLTVPELRA
NVVTLAKVATLCNVPVITTASVPQGPNGPLIPEIHQFAPHAQYVARKGQINAWDNPDF
VEAVKATGKKQLIIAGTITSVCMAFPSISAVAEGFQVFAVIDASGTYSKMAQEITLAR
VVQAGVVPIDTAGVCSELQKTWHRSDAADFAAAYSAIFPPYQLLIESYQKAQDVVKNN EMLDSERT"
/product="Nicotinamidase family protein YcaC" /transl_table=11
CDS 3144167..3145087
/locus_tag="AUO97b_03053" /db_xref="SEED:fig|400667.82.peg.2918"
/translation="MQDLNDLYYFAKIVEYGGYSAAARALDIPKSKLSRRVALLEERL
GVRLLHRSTRQFSVTELGQQYYQYCHAILIDAENAQEFIDLNQSEPRGTIRVSCPPAL
LHHHMGEFLTKFMLQFPKIKIYIDVTNRNVDVLNEGIDIAFRVRYPPLKDSGLVMKKF
VTSDQCLVAHPRVLSKQPIPQTLDELHQFRTIGLGNAEQQSVWVLHHQEHGTTHFQHD
PDLIVNDMTGVLQAVIHGLGIAPLPIMMAHEYILRKELIVVLPEWKPTSGIVHAAYAS
RKGLLPAIKRLLDFLEEQFNQLDKDLLGYR" /product="Transcriptional regulator, LysR family" /transl_table=11
CDS 3145336..3146259
/locus_tag="AUO97b_03054" /db_xref="SEED:fig|400667.82.peg.2919"
/translation="MYKKNIGGLINALALVIYSSSVMAADNSSSFVKLEFTVTKPFLF
FSQTSGNTCGGVLIRNDVVLTAAHCTAAVWGDSANQGYTLGGMVKYGASFLSQVQAVS
FKKDEGWVEIPDVDIALIRLSHPIDNNSGATVAKIPAACSQTEARQNIGRGDVTAYLG
RSSTGAALPENLYGTSYVNIRDVGPSPLARGIYYKATASMNAGDSGGPWLSDDNTLIG
INNGIAGTDKYGAVICQYTHQINDVLVKWSAAGNQTGAVTPPTTPPSVEPTTQPPVTN
PPTTSQPTTEPQTTDNVVFSFFNWLASLFGF"

/product="Trypsin beta precursor (EC 3.4.21.4)" /EC_number="3.4.21.4"
/transl_table=11
CDS complement(3146329..3147834)
/locus_tag="AUO97b_03055" /db_xref="SEED:fig|400667.82.peg.2920"
/translation="MNKLVKPSTPTQLVDVLIIGAGPSGLSTCIKLKEQGIKNVVILD
MASRIGGTWALNDYPGLFCDVPSEMYSLGFAPNPNWSRTYAPQAEIRQYLEDVAYKFE
IVNQIKLNTEVTHAEWNNGLNRWLISTRDGKKYSAKIFVPATGFIGEAKMPSFPGQEK
FKGTMFHSGKWNHEHDLTGEKVAVIGSGASAIQFLPAIQPKVGHLYSFQRTPSWVLPK
PDFAVPNIVKVTFKKAPILEKIIRESALWSLEPSLPIFMNENIMRKLHALGKLNIFLG
VKDPEMRKALTPTHTLGCKRPMFSNNWYTALAKPNVTVLFQGLSHITENGVVGEDGKE
IKVDTIIFGTGYAVAEPAIYKIIKGAAGRSLSEVWQRQPRAYMGMSIHGFPNMFMMLG
PNSQNTVGSVMWTAEQQSVYIAKMVKLIQQQRLDRIEVKADVQKRFNDRIDKRLAKMP
VRPDVCKSYYLDDAGRNHIIWPEFGFVIKHKLHHVNLKDYDATTKVIKVVA" /product="Cyclohexanone
monooxygenase (EC 1.14.13.22)" /EC_number="1.14.13.22"
/transl_table=11
CDS complement(3147890..3148810)
/locus_tag="AUO97b_03056" /db_xref="SEED:fig|400667.82.peg.2921"
/translation="MIQVEQLTLNRDGLTLHGTLYGEADASLVVLIHGFPDTPHSWDH
VYPLLVKAGYQVFVPWLRGYTQDSVNRQAHYGLASALADLAAWLELLKAKERLKNKAH
LVGHDWGAAIAMSFASGQSEALHSMSLLAVPPIPNLKDIVKAIPNFPRQLYMSSYMLL
MQSRFAEQVLSRNNAGFVEKIWRKWSPTWNFTESDFSPTRAAFQNPNIAWAATRYYHN
LFAVLNSDNLRANRHLLSPVTTPTLALAGLDDGCMNIRLHQALAQTSYFPQGIESVYV
PGCGHFLQAEQPNIIAQYLIQHFQKTDRST"
/product="PROBABLE EPOXIDE HYDROLASE EPHC (EPOXIDE HYDRATASE) (EC 3.3.2.9)"
/EC_number="3.3.2.9"
/transl_table=11
CDS complement(3148819..3149766)
/locus_tag="AUO97b_03057" /db_xref="SEED:fig|400667.82.peg.2922"
/translation="MSTFIDHHIVARKVQFDFSQTPRHWVPNDVLTTHVLNSMHVLLP
AVEHWFCRLANKTLPYVEDKNLKADIRGFIAQEAAHANAHKGAEIYFQTHGIDPTPFK
DFLNWFFKDGFMGDTPFGIYGPFKRYPKQWLAFRMGIIAGLEHYFCFFGTWALDAEGL
EGADPAMLDIVRWHGAEEVEHRTVGYDAYRALAGDGVKGYLGRQLSMGFAFAAMVGFW
LGSTVYLCHLDGTKEAQKIAKKNPLALVWLFQKTAKKKKSLPDLGMILTALKGWSKLS
YHPEHDGDVEKALAYLAQSPAAQLAAEAYAKALSSKMKS" /product="hypothetical protein"
/transl_table=11
CDS complement(3149789..3150829)
/locus_tag="AUO97b_03058" /db_xref="SEED:fig|400667.82.peg.2923"
/db_xref="GO:0045552"
/translation="MKALVTGSAGFIGSHIVRTLLNENYEVRVLHLPQEKLTNLEGLD
VELIAGDITDPAKMDEAVSGCDLVFHTAAIYALWLPKPELMRKVNVEGTRTVLNAAKK
AGVKRVVYTSTGACFAGQPKGIQATETSPFALGATGDAYVLTKFEAHQVALQFAAGGL
DIVIVCPTGPIGPQDIAPTPTGKLLLTIATMPALAVPGAINNMVDVRDVAKGHVLAAQ
KGKTGETYILGNRDLDGVAMAKTVHQLLGIWRPVMTIPSVVEGVSSQLAGHAALWVTE
HITHKAPLVTPSAAKIGQLGTSFNCTKAVQELGLPQTPIEIAVRDALQWFAANGYVKS RSLIKKIESISI"
/product="Hopanoid-associated sugar epimerase HpnA" /EC_number="1.1.1.219"
/transl_table=11
CDS 3150994..3152013
/locus_tag="AUO97b_03059" /db_xref="SEED:fig|400667.82.peg.2924"
/translation="MNPSLPGTYVSLMADVVQKLNVSPEELLEGSGVSVEQLLEPFWY
LDFNIFNNLLNRAVELTNEPALGIYLGLQMTVSCHGSVGLAAMVSQNLGEALNVMEQF
IGLRCPALKPRLEIENDLAFLYLDQPMTNFKMGIVGMTFLMVGFAQMSNAITGQKLEI
KAELNYPEPAFLEQVQELLPCEIQFNQPFNRLVFSENFLALPLIMADPLAARLAREQC
KHDLNKLAAKRGEKHPLTALVRELLFDEVEGFFKMKDVAEKLHLTERTLQRQLAKEGV
TFQILLDQVRERHAKKLLNHHEYSISYISEKLGYSEVTHFTRAFKRWTNQTPKQYRNL REEQE"

/product="Ornithine utilization regulator" /transl_table=11
CDS 3152452..3153855
/locus_tag="AUO97b_03060" /db_xref="SEED:fig|400667.82.peg.2925"
/translation="MQKKLPIPDNLPNHLTDIFYHINISLPKGDLKETVKLDVAINQL
NARDVYALQDYPLHNLSEYAGWAINSEITQTASIESPLFIANWYYWGQQLYRSINPYK
LDLKTPSLLEIPAYTKLPEPLDSVIPEQDERLDLSDTDAKKITGSIPAMYGVIPKGSI
FKQGDVILKKDERLTPEKLIILRRAGIKELTIYKKPKILIVSMHSFDKEEMSEESLYV
KDVLKTWGYEQVEIKSLKPERYDAAFNSLKKDNNSALDESLTTSRDDYIKFFEEQTMN
FDVILCCTYQNSRSALFQLEKLPIFDQSSMSAALNTRCMPGNEIKILKGKDRTPPSRE
TVQIYDENGNHKGMRVVVTEDKATIINLPGDIQDIAILMHAFVKYILNKHISDFFDHA
YFKGKTDIEIVPDVTNRKLLWGHYKAHESGEYVLEIIEQQPYQIEPSLSANCIVMVPF QEKVIAKSNDLYFMKID"
/product="MoeA"
/transl_table=11
CDS 3154550..3154672
/locus_tag="AUO97b_03061" /db_xref="SEED:fig|400667.82.peg.2926"
/translation="MIIENIISLIDEQLYTLDSQKEIKKSELKIFYSDECTQYL"
/product="hypothetical protein"
/transl_table=11
CDS complement(3154697..3155725)
/locus_tag="AUO97b_03062" /db_xref="SEED:fig|400667.82.peg.2927"
/translation="MASTPRITNYDEVLGLPGFLEQNLITPENFDSVFSDYQIRDRDM
HCCLVSDRGLCNHKHNFGYVIKLKDGSLSIMGNDCAERKFGANGGIIQGISLYENTKE
AEKKLGKVFDFAIRIDEYIERLRKIEVNLKIIEDYYFNLKLNIGSRNYLKLNERYKAA
NTKVEVKTYKLGKPDPDNPNEETLIYMATHTIGHISSLNIFDDRNFKEFEKKVFKLRE
ALRDALKSHNQIIEGEKIQPAALRKRVNTILSQLNSFDEVETTINTRVSDIEKFRRTD
PEILCYLTDDYEESKIFASLSMAFHGIENMKEETYLRSLEIKYSTAFRCHRIKAADHS VMRRSIFY"
/product="hypothetical protein"
/transl_table=11
CDS 3156547..3157110
/locus_tag="AUO97b_03063" /db_xref="SEED:fig|400667.82.peg.2928"
/translation="MPNLVLPTRALYVVNKAIDLFHHRGFHLIGVDRIVKESEITKAT
FYNYFHSKEQLIEICLMVQKEKLQEQVVAMVEYDLSTPAIDKLKNLYFLHTDLEGPYY
LLFKAVFEIKNSYPNAYQTAVRYRTWLKNEIYSQLRLLKPDVSFTDAKLFLYMVEGTI
IQLLSSGGVSERESVFGCFLRGLTSCK"
/product="Transcriptional regulator, AcrR family" /transl_table=11
CDS complement(3157342..3157464)
/locus_tag="AUO97b_03064" /db_xref="SEED:fig|400667.82.peg.2929"
/translation="MSNHLMNENYGDCYREYGGVVDVLKLNNHAHPMVNNFLNF"
/product="FIG00350175: hypothetical protein" /transl_table=11
CDS complement(3157546..3158232)
/locus_tag="AUO97b_03065" /db_xref="SEED:fig|400667.82.peg.2930"
/translation="MNAYLHRSEDRGHVKAGWLESYHSFSFGSWYNPKYMGVSALRVI
NDDRIDAHNGFGTHSHDNMEILTCVLDGAISHRDTMGNEGQIKAGEWQLMSAGTGVAH
SEINNTDTSVHLLQIWIIPDVKEAEPNYQQISLDPRNQPNEWHLIAGPDANAPMHIRQ
DAEVKSAVLEKDRELPVETSKRVNYVHVISGEVRIGEYEVKAGDALVFEDNAVIHANE DSQFIWFDLP"
/product="Pirin"
/transl_table=11
CDS complement(3158477..3159445)
/locus_tag="AUO97b_03066" /db_xref="SEED:fig|400667.82.peg.2931"
/db_xref="GO:0004794"
/translation="MNNLTLPNYEDVAAAAERIKDFINKTPVLTSRTVNNEFEAEVFF
KCENFQRVGAFKFRGAMNALLQFNETQKKAGVVAFSSGNHAQAIALSSKILGIPATII
MPKDAPAAKMAATREYGGNIVEFDRYTEDREKIGKEIAEKNGLTLIPSYDHPHVIAGQ
GTAAKELFEEVGDLDYLFVCLGGGGLLAGSALSARQLSSNCKIYGVEPALGNDGQMSF

RKGEIVHIDTPPTIADGAQTQYLGKLTFPIIQQKVDDILTVTDEELINAMKFFAERMK
MVVEPTGCLGFAAARNLKDELKGKRIGIIISGGNVDISKYAEFLSA" /product="L-threo-3-hydroxyaspartate ammonia-lyase (EC 4.3.1.16)" /EC_number="4.3.1.19"
/transl_table=11
CDS complement(3159561..3159719)
/locus_tag="AUO97b_03067" /db_xref="SEED:fig|400667.82.peg.2932"
/translation="MFITIAQCISPNDVITVSNPRVKYANINLDFLSNFKLNVNQNLI SLCYQIKK"
/product="hypothetical protein"
/transl_table=11
CDS 3159699..3160322
/locus_tag="AUO97b_03068" /db_xref="SEED:fig|400667.82.peg.2933"
/translation="MGNRDKHLLTQLDTIAKGLSETLAPFCEVVVHDLTNPEHAILSI
HNNLSGRQVGDPATELGLARIMSPEFPNLITNYANQFADGRPAKSTSIGIKDEEGQYV
AALCMNIDLTLFRGMQSALEHFTKIETDSIIENLEPNGTEAIRRYIDQFASKHATTPR
MLKLNERKQLIQELKNNGLLEIKKAVETVAQHLGISRASAYLYVKET" /product="YheO-like PAS domain"
/transl_table=11
CDS complement(3160319..3161179)
/locus_tag="AUO97b_03069" /db_xref="SEED:fig|400667.82.peg.2934"
/translation="MTKIAEKSKQEYGDLLKEKDHLQDMEQLEMTIVSIQTPYPSIVR
IQGKINTLQPELWQAPNLAIRLIVSNPPEGQPISRVYTVRSFNPINAQIEIDFVKHED
LSPAMEWLNSAQVGTKIGLIGPRPHFIPNFTAKKHVVMFADDTAVPALYSILKQWELG
ISADIFIESFEKDIASQLPELEHVKIHSFHKEHHTSQKGLLLKAAFALEHYENITIWA
ACERNEARALRQFFLEDQQLNKNDVRIAGYWRDGVSSSELDKLRAQHYQEHIQQGKTL NEYDDLDLAN"
/product="Iron-chelator utilization protein" /transl_table=11
CDS 3161572..3163419
/locus_tag="AUO97b_03070" /db_xref="SEED:fig|400667.82.peg.2935"
/translation="MLKDLSQNLGQMNDKNNNVENFLKQIYENIFDKNSNRTALIFED
RSISYAEVGTQVAKVMYALRKQDLATGSVVAICLRKSPEHIYTALACALTGIIWLPVD
MDSPPSRLNYLLTNSRADVVVSDSSIAGVRNLNINEILSATTEYEPSFNAEINRRPAY
YLYTSGSTGTPKCVVLNNQATENTLQQTISEWKITTDDVIMAVTPFHHDMSVFDVFAS
MAVGATLVVPSFEQSKNAVVWANLVDRCKVTIWSSVPAIVDMLFSVAQKVQLQSLRLI
AQGGDYIKPSLIAKLRQQLPNARLFSLGGPTETTIWSIWHEINEQDQEIIPYGKALEN
NRYFILDENLKPCQMGEVGTMYMAGLNLSNGYLLDGEINYKDFVDIQVSENETQTAFR
MSDQGYFREDGNIIFAGREEGYLKIKGVRISAAEVENTLTKNPYIHNCVVISCVHPTT
ETQELVAVYTLENKYKTTRLNSPELKNFLKIHLPSSHIPSKYLAVETIPLTRNGKIDR
KAVQEIAQGKIYVTSTLSANSSKTQSSIAEVGDSVLSVFRECIADNQAEKMDIFYRSE
ILDIGIRPKQLMSIAQKLSQKLNTQIDFYTLVSCKTIQDVVNKVEQQIK" /product="Siderophore biosynthesis non-ribosomal peptide synthetase modules" /transl_table=11
CDS complement(3163490..3165517)
/locus_tag="AUO97b_03071" /db_xref="SEED:fig|400667.82.peg.2936"
/translation="MLQLQQPEQVNRRFNLWKLNQGLDIQLLIKAIQDIIKTTPDLNV
RYTFSDEGDLYKYPFDDHSACLEVKKSNAEQVFKQVAALKAQAWNAEFHPPFFTSLIE
TEQDYFLILALHPILDESYQKSDFIQAIQNRYQQYSPNNVPLVLTEIDISNHLASSTK
APEQPNQTYVSEIILEEFRNTLAEPEMSQHDDFFDFGGHSLLATRIIGNLLNKHGIEI
QFNDFFKSPSAADLAQYAFVKSAKTEKTTLQSVDKAPLTLAQDFLWQAYSAFDFSPIY
NLPFAVEFLEEINEDVFFQAFTDIVERHAGLRTIFNSANGQTYQQVIPTSELKKFKWF
WNSAESKDATLASEASYKFDLTRELPLRIRLIQNAKGRQTLSFLVHHMVIDEWSLNTI
MADLAHAYLARSNAQAPNWKAPAQSILDFSLLQQKQGINQDHLNYWTNLLRGATKGLN
LPVSEHELNAEKGKPPVQWLELKFAPEMYEKLLAFSRQHSSSIFAVLYTAIANALQQQ
GNLKDIVIGTSASGRTDPEFFDTVGYFTTMVAHRTQFSPSDSFQSLLHNISTMINTSM
AYADIPINHIQNALGMSADEGLLFDVFIHIHSNNALNGALKTPQGQDLPYRQILPERD
ESMFGLHFEIMENVIDGQHQLSMIITYQAHRFPTATVQSICEKIKATLAQI" /product="Non-ribosomal peptide synthetase" /transl_table=11

CDS 3166018..3166149
/locus_tag="AUO97b_03072" /db_xref="SEED:fig|400667.82.peg.2937"
/translation="MRIFNHERLISPSNVEQIFPRFGLMGLNLQFASALLRSSGARH"
/product="hypothetical protein"
/transl_table=11
CDS 3166149..3167090
/locus_tag="AUO97b_03073" /db_xref="SEED:fig|400667.82.peg.2938"
/translation="MRFGLFCIFIILCICSLLLGAEQIQWSTLFSFSHESWLTLTASR
IPRLITIVLTGIGLAVCGVILQHIVRNKFVEPETSGGLDAAKLGILVSLTLVPVTSTL
SKMIFAIIFCFIASLIYIAIIRRIRFRNTVLVPVIGLMYGSVLSALAEFYAYRFNILQ
SMQGWLLGDFSKIVQGHYEVIYIIFPIVALTYLFAHRFTVIGMGEEMASSLGLSYAAI
AAIGLILVAITVSTTVITVGAIPFVGLVVPNLVALKYGENLAKTLPIVALGGASLLLI
CDILGRSIIYPFEVPIGLTAGGVGGIIFLILILREFR"
/product="Iron transport protein"
/transl_table=11
CDS 3167090..3168037
/locus_tag="AUO97b_03074" /db_xref="SEED:fig|400667.82.peg.2939"
/translation="MRITKLWMIYLIVICLAVCFLFLNSGLDFDYVIPNRLLRLATIV
IAGICVAFSSIVFQTLVGNRILTPSIMGYEAIYLLWQVLLLFFWGTHGLSQLGVNGNF
FISIVLMLLYSWVIHKWLLPYGRNDVFLLLLLGLVLTMVVGTITQFIQLKINPGEFSV
FQGLSYASFNRSQPETLLYASLAVVIVLFLGRKALPVLDVLILGREQAISLGIHHHRY
VSFYLALIAILVAVSTSLIGPTVFMGVFIANITYALTRSYKHKLTLPMGCAITIAIFI
AAQILVEHVFNYKTTVSILVNLVCGIYFLALTVRTRGVA" /product="Iron compound ABC uptake transporter permease protein PiuC" /transl_table=11
CDS 3168034..3168804
/locus_tag="AUO97b_03075" /db_xref="SEED:fig|400667.82.peg.2940"
/translation="MIHVKNINKSYGNKPILTDVNVEFPTGQVTSLIGPNGAGKSTLL
MMMARLIEPDKGEIFLNHQNIADIKTAVYAKHVATLRQAPGFNLRLTVEELVSFGRFP
YSRGVLTEQDHQVVDEAIEFLSLEPLRKSYIDEISGGQRQMAFLAMTIAQQTDVLLLD
EPLNNLDMKHAVQIMRALRRLCDEQGRTVILVIHDINFATNYSDHIVALKNGKLHFSG
LTEQVITEAQLNDLYELDFEITYSERGCMCNYFNSSGA" /product="Uncharacterized iron compound ABC uptake transporter, ATP-binding protein" /transl_table=11
CDS 3168811..3169779
/locus_tag="AUO97b_03076" /db_xref="SEED:fig|400667.82.peg.2941"
/translation="MNWKKKYGGVALIIAAAVTLQACDQKVADTTQASQKLAEPITVK
HALGTTVIDHLPQRVAVLDMNEADFLDQLNVPIMGMPKDYIPHFLEKYKKDAQIQDLG
AIVQPNMERIYALKPDLILMTPLHVNQYQELSKIAPTIHYDINFNNSESNHIGLVKDH
MMTLGKIFNKEDLARQKVSELDEQVKQVQAVTANRPERALVVLHNNGAFSNFGIQSRY
GFIFNAFGVKPASGVVDTSLHGQPISSEFIKKADPDILYIVDRTAVMEHRSNINAASV
ENPLLRQTKAWKNGRVIFVDADAWYTTAASPTSLKIVMEDVKKGYQ" /product="Iron compound ABC uptake transporter substrate-binding protein" /transl_table=11
CDS 3169892..3172192
/locus_tag="AUO97b_03077" /db_xref="SEED:fig|400667.82.peg.2942"
/db_xref="GO:0004872"
/db_xref="GO:0005215"
/db_xref="GO:0006810"
/db_xref="GO:0016020"
/translation="MNNVSYNRNFKTGNDQRINHRSFVLNGVASAQMALRLGYALGTV
FVLCASNTYATVIDNSTKTLEQQQAAQTNVAALPAITVKAEQDDTYAGGQVSSKSSVG
FLGNKTAMETPFNTIAYTEKYIADQQAKDITDVIAKTDPSIFSTGAYGVIAESYAIRG
FRSSSVAAGRDTSMGGLYGIAPLYRASPEMFERIDVLKGPSAMLNGMPPGSTLGGTVN
LVPKRAGDDPLTRFTTTYMSDSQFGGHLDIGRRFGENKEFGVRFNSVYRDGEGPIKEQ
EKTTHLFSLGLDWRGDNARLSADLYTSRDHADGINRGLTILPGVNLPKAPNPDTLLNP
TWTVYNTRDTGGMIRGEYDLNDKWMAYATAGMSKTEYNTLGAAKTEIQNEAGDIKFNI

AHLGFKYERKSAEVGLRGKFDTGAVKHALALNATHYRETDDEAGIRQGFPEGDRVTNI
YNPNWGSKPNRVPIAPIFSNKENLTSVGLADTMSVLQDQVQLTLGLRYQNIVTESLNG
QGVRNINGRYDKNAITPAAAILVKANDHVSLYANYIEGLSSGGTVPTGQNYENEGQIF
SPFKTKQMETGIKIDSGNFTNTFSVFQIKSRNSYVIKSTDEKIKSIYGADGEQRNRGV
EWGFYGSPVDSIRVMGGLTYIDPKITKTRIASELGNVVAGVPKVQAKLGIEGDVSYIP
GLTLTANATAASKQYIYNDNSTSVPGREVYGIGARYNTKIAQFPVTIRGNIDNLTNKS
YWSMPQFTSLMLGAPRTYLLSATIDF"
/product="Ferrichrome-iron receptor" /transl_table=11
CDS complement(3172262..3173572)
/locus_tag="AUO97b_03078" /db_xref="SEED:fig|400667.82.peg.2943"
/translation="MKKIDITGIGIGPFNLGLAALLSHHPEIKSVFLERKPEFRWHEG
LLLQGTTLQVPFFADLVTMANPCHPLGYINYLHQHDRLHQFYYYDSFLIPRREYDHYC
RWASKQLPDCRFGENVSHVEYDAKLDKFIIESESASGEKQKYISQNLAIGIGTKPRIP
KWLETCKHPLVKHSAEFAKIQEQLKQCKQVTVIGSGQSAAECVLALFNSLTPEQVKAG
ASIRWITRSAGFHPMEYSKLGQECFTPAYMQYFQSLPRDKRRDIAASQGLIYKGISFS
TIGDIYDVLYERSVAGEKSGLSLYTSCEVESVKELESGSLRLTCLHTQLDQRCELETD
ALVAATGYVHDWPEWFQNMKGSILAVDDKNDCIVQEDFTALRCDQGQGRIFIQNAEIF
QQGVGSPDLGIGATRNSVIINQLLGRNAYRIPKQSSFQHYGLPK" /product="Siderophore
biosynthesis protein, monooxygenase" /transl_table=11
CDS complement(3173620..3176562) /locus_tag="AUO97b_03079"
/db_xref="SEED:fig|400667.82.peg.2944"
/translation="MQLVIIFICLYDAETRLICQPSYRSMCELTSMQAACWFGRSGNA
TLGGVAAHLYAEFDGQFIDLQKLHLALQRLYKEHPILRLSLSADGIANIMAEKAQQIL
EVDDFSKLSDHEIEQMLMQKREQWTHQKLDLSQGQTARFSVSVLKNNIFRFHVDTDMI
AIDPSSFLNLMEDLSLFYEDPEISFSRPPNFFDWYQKVRTDPDLKKLSQRDRLWWKER
LPQISPAPSLPFIHQEFKTAKSDRLSTWLSPKERTALQQLAREQRITVTNLILGLFAY
TLGHTTKDHSFRLNIPTFWREPVLKNVEGTIGDFANLVILDVDMKGITTLAAFCKQIA
NQMLELLEHSHYSGVNVLRDLSRYHGSAQIAPVVFTAALDLENDNLLSERVRRVFGSM
NWVISQGPQVALDAQVAHVDDGILVNWDIRLDALPKEWITNLFESFIHLLKNLAAHPE
QLNTQIISPAQNTSSDRTSQKPLNALQQAYLLGRTQALPLGGVAMQEFRQYHGKMDIV
LLRQRLAEMVRRHDSLRTYIDKNRLIQYVSDQVSVNLKEIDLTTWEPERASHHIQSYK
NSYTHELFDLNQSPWNITVFLLKNNLLTIFVRFDALILDGRSIASLMLELFDGQQHDI
QTQIEENEVENSLSVHHADMAYWERKFSKLSAIPALPWKTPLQHLPTSRYQRNSLVIE
KDQFKQLSKIGAKHSLFKNSVIMAIILEVLSHWNTDKSLCVAVPTLPLYAGPFSNSST
FIAVEWKASDQFAEQANRLQTEVLEGLQHLSFSGVDLARLLFEKVGTAPVLPIVITNG
LSWPVLSESHPIQQLDGLTQTPQVAIDIRFSTRNDGALIFDIDYAQEAFPPNMIDDFL
DALQLAIKQIIGSEIFSFDLSNFFSELQNKRLYFKNNESDHSSIPIENNAKQQNQLLD
IYLEVIGHPPNMEVDNSTHFTHLGLRPHHLKVVSKRINETYAIQLSPVQLIQCRNIAD VEKLLTPH"
/product="Non-ribosomal peptide synthetase modules, siderophore biosynthesis"
/transl_table=11
CDS 3176724..3178352
/locus_tag="AUO97b_03080" /db_xref="SEED:fig|400667.82.peg.2945"
/db_xref="GO:0008668"
/translation="MKKQLIEFVRWSPERAQHYRNKGYWIDQPLTRILTVGVQSHPHS
PAIICGERQLSYIELDQLSTNLASRLAEKGLGKGDTALVQLPNVAEFYIVFFALLKAG
VVVLNALYSHRQYELNAFIKQIQPKLLIGSRQHEVFSNNQFIDSLHEVNLSPEIILML
NHQATDFGLLDWIETPAETFVDFSSTPADEVAFFQLSGGSTGTPKLIPRTHNDYDYSV
RASAEICGLNSNTRLLCALPAPHNFMLSSPGALGVLHAGGCVVMAPNPEPLNCFSIIQ
RHQVNMASLVPSAVIMWLEKAAQYKDQIQSLKLLQVGGASFPESLARQVPEVLNCKLQ
QVFGMAEGLVNYTRLDDSDEQIFTTQGCPISSDDEIKIVDEQYKEVPEGEIGMLATRG
PYTFCGYYQSPEHNSQVFDEDNYYYSGDLVQRTPDGNLRVVGRIKDQINRGGEKIASE
EIEKLILLHPEVIHAALVAIVDEQFGEKSCAFIVSRNPELKAVVLRRHLMELGIAQYK
LPDQIKLIESLPLTAVGKVDKKQLRSILNTFTTS" /product="2,3-dihydroxybenzoate-AMP
ligase (EC 2.7.7.58) of siderophore biosynthesis" /EC_number="2.7.7.58"

/transl_table=11
CDS 3178370..3179239
/locus_tag="AUO97b_03081" /db_xref="SEED:fig|400667.82.peg.2946"
/translation="MAISKISTYLMPERESYPNNKTDWQLDPSRAVLLIHDMQRYFLN
FYDAESELIKTVVNHLVQLRSWAHQNNVPVVYTAQPYEQPAADRALLNAMWGPGLPAS
TIDQQKIIDQLSPAEHDIILTKWRYSAFKRSDLLERMQNWNRDQLIIGGVYAHIGCMV
TAIEAFMSDIQPFLVGDAVADFSEEEHRLALKYVSSRCGQVVDTESVVGQVATGITRP
WLEQKVQQLIEEDELDPEENLILYGLDSLRIMQFSSELKAQGINISFEELGRTPTLSN WWSLVDARQRIAG"
/product="Isochorismatase (EC 3.3.2.1) of siderophore biosynthesis"
/EC_number="3.3.2.1"
/transl_table=11
CDS 3179357..3180508
/locus_tag="AUO97b_03082" /db_xref="SEED:fig|400667.82.peg.2947"
/translation="MILSPADQERIETFWNYCLKHQYFNIGYPESADFDYSALFRFFK
FSINNCGDWKDYSNYALNSFDFEKDVMAYFAEIFQIPFEESWGYVTNGGTEGNMFGCY
LARELFPDSTLYYSKDTHYSVGKIAKLLQMKSCVIESLDNGEIDYDDLIHKIKTNKES
HPIIFANIGTTMTGAIDDIEMIQERLAQIGIMRRDYYIHADAALSGMILPFVDHPQAF
SFAHGIDSICVSGHKMIGSPIPCGIVVAKRQNVERISVDVDYISTRDQTISGSRNGHT
VLLMWAAIRSQTNLQRRHRIQHCLKMAQYAVDRFQAVGIPAWRNPNSITVVFPCPSEH
IWKKHYLATSGNMAHLITTAHHRDTRQIDSLIDDVIFDLQGASKRTVGF" /product="Histidine
decarboxylase (EC 4.1.1.22)" /EC_number="4.1.1.22"
/transl_table=11
CDS 3180606..3180761
/locus_tag="AUO97b_03083" /db_xref="SEED:fig|400667.82.peg.2948"
/translation="MQLKFLKKYFLYDFLIHAFLKPKMTILAGQLKRDFILSTAFSMN IKRDKHV"
/product="FIG00352701: hypothetical protein" /transl_table=11
CDS 3180754..3182364
/locus_tag="AUO97b_03084" /db_xref="SEED:fig|400667.82.peg.2949"
/translation="MSDLKLSHLMTDSSLAPALKPAFPKLFAGTLAAVFSGLAILTAL
WGMIQLIGYLSSYWVIFSAVFWIVGAALAALASWLVHDAEAGFSARLRRQIAHHLIRL
PNTTLSKQGDQNLKRLVSDDIATLHHMVAHLPSEIAIFAVIPLVSIVLLIALVGPMAL
WVLLPGAIASLYYLIIVPHVSKRDGAARMQVMLDIIGSADDYARGIRDNRIYGQQSGA
LTAYTQSAQNFTQNMVSWVSKVATLAAVATALLQAVATFAIAYLVAYQYDSMTLAATL
FFSLAIVTPSLRLGHGLDYVAAGRAAATRLSAFLKEPVLSSGNLQQIEEDVTLEIVNA
SFAIENQKVFDRLSYSFPKNSMTAITGPSGVGKTTLLRALAGLAVLQEGVVQLAKTDI
LQLHEKLRHEKVLFIPQGGDVLPTTVRENLSLSAINVSDEQLEQALLKAQLKVDLNAD
ATLLSGGEKQRIGIARAFLSSAPIILLDEPTSALDQTNATRIILELSHLAKNQNKTIV
MVTHDLALAAHADNRLVLKGPTDSGKSL"
/product="Transport ATP-binding protein CydCD" /transl_table=11
CDS 3182361..3183956
/locus_tag="AUO97b_03085" /db_xref="SEED:fig|400667.82.peg.2950"
/db_xref="GO:0005524"
/db_xref="GO:0006810"
/db_xref="GO:0016021"
/db_xref="GO:0016887"
/translation="MNTAAKLNKDQIFSMLPRAAKQKLIIVGIGWIIVAAIEAIAYTV
LAFAIVNQWSPSWVLISAIAAILVTVIVTRAGFLTGVRLAGDLFAELGKSLARAKLSW
FTNEQRAQIKTIAGQGIPGFMSIPAHQLQTFLHAPFMPLFLTIGIGLLAGAGVAFVAL
GLLLLSLFAQFLAQRALMHIDKKRNTADLAASQATLELVDHVELLRTAAGPEHAVQRL
EHRWEEQEETLVRINRAAAFASFIATLASVLPIAGMATYLVLAGVNHAALVLALLILI
GRAAAPLGELATAGLHINDLFAALRNFNQITHPPELPEPLHPQIPTGHHISVQGISYA
PALENISLDIALGSRVWVTGPSGSGKSTLLELLTRFDDPQRGKITLGGIALDQMKYED
LVSNIAYVTQEPILFTGTLAENIRLGKPDATDDEIEKAARDAALGAMIDRSSEGINQS
VGKQGTALSGGERQRVAIARALLKNAPILILDEATSALDEETEQEIVTTIRALSSTVI

FVTHRDSAIWQPTQTINLASISV"
/product="Efflux ABC transporter, permease/ATP-binding protein"
/transl_table=11
CDS 3184029..3184763
/locus_tag="AUO97b_03086" /db_xref="SEED:fig|400667.82.peg.2951"
/translation="MNKHQYNMFCLPPAGSSASIYHPWKKRISDNIRIIPIEYSGHGI
KINEPLIDDPDLLAMQIANEIQAYSDTPFILFGHSVGGGLIWKVLNYLNEKPIIDQLR
LIVISSRPEHHYIQHMRYKHELTDEKIIDELKRYNNFPDEILNNQDALIFFLKIIRND
FYLSDQLLSEKIHKTEVPIVAFYGKQDPDIPNKRMMDAWQQHTENWLGSIEFEGDHFY
FLNPETRVKMLENIAAIVETLTVNIE"
/product="Putative thioesterase"
/transl_table=11
CDS 3184774..3185529
/locus_tag="AUO97b_03087" /db_xref="SEED:fig|400667.82.peg.2952"
/db_xref="GO:0000287"
/db_xref="GO:0006633"
/db_xref="GO:0008897"
/translation="MNTKKQLSRGANSCLDEINIQYEKLTAFGLDIHKVQLSQIKEIP
QLDHIYQELNIFLPPNIKSSRFIRQLEFLTGRLAAKYALQRFNLQDSIVHQGKQGEPL
WPEGVMGGISHVGSKKSCHAIAYVRNNTLKEKIFGIDIESQKHHIFFQKKDEFYDVFL
HKNEQEEIEKLLKDQAYLYLIIFSAKESIIKAFYLKYKQIIDFKNIKFKALDGTFLYF
YLRQESLIEITLEVKVYFFHTNNEIITISCIEN" /product="4'-phosphopantetheinyl
transferase entD (EC 2.7.8.-)" /transl_table=11
CDS 3185654..3186823
/locus_tag="AUO97b_03088" /db_xref="SEED:fig|400667.82.peg.2953"
/translation="MMQTQISVEQNLDHIGQKKFSCFSTKNYYLEVNEFVKTLNTPAA
NTALLNEEIAKCFEEIKKQGHQNPVLIGAIPFDITKKSSLNFYADHKKITQASSTINS
LKNRNNIEIINKSLLIQRQKFEGTVQLALDAFEKKKVEKVVLSQAVEYEFNRHQEPEN
LLAALLKQNPNAYNFVIPVEHNNYILGASPELLLSKQDRIVTSNPLAGSRPKSQISEK
NFVSQQELNHSEKDQHEHRIVVENIIKNLATHCVELKVSEFPDILETSTMLHLSSEFQ
GVLKQQAPDALNLALELHPTPAVCGSPTHVAKQFILDHEGYDRHYYTGLVGWMDAEGN
GEWVVTIRCGLLSENKMRLYAGAGIVEGSDAEAEWLETEAKMQTMLNILNTKIAV"
/product="Isochorismate synthase (EC 5.4.4.2) @ Isochorismate synthase (EC
5.4.4.2) of siderophore biosynthesis" /EC_number="5.4.4.2"
/transl_table=11
CDS complement(3186944..3188191)
/locus_tag="AUO97b_03089" /db_xref="SEED:fig|400667.82.peg.2954"
/translation="MFVRSLLAMSLSCIIANVALAASTTEQPLNPNKVSAPVEDPIDP
LAVDAASTVKAQAQTQITAQEQNDLNRASTTLQNLQKAEDQNADSGIAASTPVATAKT
TTATASTATSAPAVTWTLDSLNNAEWYENIGKGQLPVYARAHVMLNNAHASPGAIDGM
SGKNTLKAIASFQQMNGLSPTGELTKETWDALVAKQNKPAFIEYTITDADLKGPYAQS
IPSDYALQAKMKGLYYTRVSEMLGEKFHIDEAFLKKINPTATFKKVGEKIIVPNVRND
LPEDIHLIIAHKGAKQLYLFNSRNQMIASFPATIGSTDTPSPTGTYKVVGVARNPWYS
YSPSNFVQGNNLKPLSLPPGPNAPVGNIWIGLSKKSFGIHGTPNPSLISKTASHGCIR
LTNWDANDLGNKVRSGVTVKFLE"
/product="L,D-transpeptidase"
/transl_table=11
CDS 3188491..3189564
/locus_tag="AUO97b_03090" /db_xref="SEED:fig|400667.82.peg.2955"
/db_xref="GO:0004853"
/translation="MTTLKNDRFLRALLREPVDTTPIWMMRQAGRYLPEYRETRSKAG
DFLSLCKNTEFACEVTLQPLRRYDLDAAILFSDILTIPDALGLGLYFETGEGPKFHKT
VRTEQDVANLPKLNAKADLDYVMNAVSTIRSALGGQVPLIGFSGSPWTLATYMVEGGS
SKEFRFTKQMMYAQPEVLHALLDHLADSVIDYLNAQIDAGAQAIQIFDSWGGALAHRE

YVEFSLNYMKKIIAGLQREKDGRRIPVIVFTKGGGQWLEPMITTGADALGLDWTTPLN
TARNVVSGRVALQGNLDPAVLYGSAASIEKAVKAMLDDAYANGEKTGYVANLGHGITQ
WVDPAQPKIFVDTVHEYSAKYLG"
/product="Uroporphyrinogen III decarboxylase (EC 4.1.1.37)"
/EC_number="4.1.1.37"
/transl_table=11
CDS 3189571..3190365
/locus_tag="AUO97b_03091" /db_xref="SEED:fig|400667.82.peg.2956"
/translation="MQYLFLPLQFIDKAVSAALFGIFLLIAFALTASMGSHEYMGFYR
YDYLLIYALIIQICLLYLKLESWAEAKVIALFHVMAMVMEIFLTHPAIASWQYPQPAV
FKILTVPLFAGFMYSAVGSFFARSIRLLQVSFEKLPSFGSMLLLAFFSYINFMSKFFV
PDIRYILFAISVFIFWKTKLYFQLNEHKFKIPMLPVLFTLAFLIWIAENISTFYKIWL
YPSQVEAWHMVGWGKLGSWYLLLLLSLVLVLKILGHRDNQGNWNLR" /product="hypothetical protein"
/transl_table=11
CDS 3190785..3190985
/locus_tag="AUO97b_03092" /db_xref="SEED:fig|400667.82.peg.2957"
/translation="MAVGQGESLNVLAELMQIKPQDKDRFFKVSKANFGEIYSTNNQN
TLQVLASLQNVMAKDEVLKAYV" /product="hypothetical protein"
/transl_table=11
CDS 3191056..3192939
/locus_tag="AUO97b_03093" /db_xref="SEED:fig|400667.82.peg.2958"
/translation="MKSVVLIFASLAVNIAYSAETNPSIQNYWSIAEQKKLDQDITWQ
RLMYANKNQKSEVTYAGYFLSENGNNNLKEELKADISALFIPTQDNQSIRCKFPARSQ
WLIQQLGIQENELPQVKCSEFENWIGQIKPYKATLIYATDFMGNPSSMFGHTLLRLDP
KDQQQLNLVSYAVNYAATVAGNDNWSYAWKGLTGQYPGEYSLMPYYRKVKEYGDFESR
DLWEYELNLSPEETRFLVSHIWEMQHVSFPYYFVSDNCAYRLLGLVDLVKPESHLQEK
FNYASIPMETIKAMQQQGLTKAPVYRPALETQLLAQAHQHGASLAKVAHQLAMKPIKE
SSETLKSFSPSDQAKILEMAYDDLYLQFIGRKVEESFAQPQLRQLLALRSQIDLDKQR
QEPKRPSTEPTQGHNARNVSLKLGEVQGDKFIEIGHRQAYHDLIDPQGGYRAGTQLLF
LNGNAQWRDDHLKLERLDLLEVNSYNPIQPFKTPLTWGFNLGWRQEAVHDGVYSDEKQ
HGVASFNAQVGYSLADYERKHICYGQVQTYVQAGSNLDKGWRVGVGPTLGCMNQWLEK
FNTVVQVELPYWEDQNQWNLRLNTQWQYAINSNNAIRFNWDYEKQNHLDWMKSSLGYV WFF"
/product="putative outermembrane protein"
/transl_table=11
CDS complement(3193007..3194158)
/locus_tag="AUO97b_03094" /db_xref="SEED:fig|400667.82.peg.2959"
/db_xref="GO:0008800"
/translation="MRFKKISCLLLSPLFIFSTSIYAGNTPKDQEIKKLVDQNFKPLL
EKYDVPGMAVGVIQNNKKYEMYYGLQSVQDKKAVNSSTIFELGSVSKLFTATAGGYAK
NKGKISFDDTPGKYWKVLKNTPIDQVNLLQLATYTSGNLALQFPDEVQTDQQVLTFFK
DWKPKNPIGEYRQYSNPSIGLFGKVVALSMNKPFDQVLEKTIFPALGLKHSYVNVPKT
QMQNYAFGYNQENQPIRVNPGPLDAPAYGVKSTLPDMLSFIHANLNPQKYPADIQRAI
NETHQGFYQVNTMYQALGWEEFSYPATLQTLLDSNSEQIVMKPNKVTAISKEPSVKMY
HKTGSTNGFGTYVVFIPKENIGLVMLTNKRIPNEERIKAAYAVLNAIKK" /product="Class C beta-lactamase (EC 3.5.2.6) => ADC family" /EC_number="3.5.2.6"
/transl_table=11
CDS complement(3194320..3194874)
/locus_tag="AUO97b_03095" /db_xref="SEED:fig|400667.82.peg.2960"
/db_xref="GO:0003934"
/translation="MSMQQSYANILTAVGEDLNRPGLKDTPVRAAKAFSYLTSGYSKT
LEEVTNNAVFPSDNHEMVLVKNIEFYSLCEHHLLPFYGRVHVAYLPEGKVLGLSKFAR
ITEMFARRLQIQENLTQQIAEAVAEVTGARGVAVVIDSAHMCMMMRGVGKQESTTRTV
SFVGDFKTDKEARREFLSAVPESY"

/product="GTP cyclohydrolase I (EC 3.5.4.16) type 1" /EC_number="3.5.4.16"
/transl_table=11
CDS 3195131..3196630
/locus_tag="AUO97b_03096" /db_xref="SEED:fig|400667.82.peg.2961"
/translation="MLERPITFFFRGGQQQVENIVPTMTVLQFLREYTQTGKTRQTGT
KEGCAEGDCGACTVVIGELVNDNLQLRSVNACIQFLPTLDGKALFTVEDLHSLLPVPD
GTLHPVQQAMVDMHGSQCGFCTPGFIMSLWSMYENEQQSPSKDKISDYLSGNLCRCTG
YRPILDAAQKAYDYPRVVLQRQKVIDVLKEIKALPALHLNNHSQQFFAPKTLQDFAVL
RLQLPQARIVAGSTDVGLWVTKQGRDLGDMLYIGQVEELKKIVVTDHALTIGANVSLS
DALIKISDFYPDFQELQRRFASMPIKNAGTLGGNIANGSPIGDSMPALITLGTRLILR
VGEQTREIALEDFYLDYQKTALQLGEFVEAIVIPLREGQTRFKFASYKIAKRFEQDIS
AVCAAISCELDNDDVTHNVRIAFGGMAAIPKRTKYAEAILEGQQITAELIVQAQEALS
QDYQPLDDGRASSAYRLHVAKNCLQRFYVEKILSQTLTRVNDLIAMVEI" /product="Xanthine
dehydrogenase iron-sulfur subunit (EC 1.17.1.4) / Xanthine dehydrogenase, FAD
binding subunit (EC 1.17.1.4)" /EC_number="1.17.1.4"
/transl_table=11
CDS 3196632..3199007 /locus_tag="AUO97b_03097" /db_xref="SEED:fig|
400667.82.peg.2962"
/translation="MNQTIDLNISKKSAKAGDSIPHESAHLHVTGQATYIDDLPELEN
TMHLAVGFSNCAKGKISKFDLDAVRQAEGVYAVFSAKDIEVENNWGPIVKDDPIFAEE
QVEFYGQALFVVVAESYQQARQAVRLAKIEYVPETPILTIQDAIEKESWVLPPVEFSH
GEVEQAFQNAAHQLSGAIELGGQEHFYLEGQISYAIPQENQSLKVYCSTQHPTEMQLL
ICHALGMNMHQVSVESRRMGGGFGGKESQSAQWACIASLAAKKTGRPCKLRLDRDDDM
SATGKRHGFAYEWSVAFDDTGILQGLKVQLASNCGFSADLSGPVNERAICHIGNAYYL
NAVELRNLRCKTNTVSNTAYRGFGGPQGMFVIENIIDDIARYLGCDPVEIRQRNFFAE
QPGGGRDRMHYGAEVRDNVAPKLVAELLQSSDYAKRRQTIHAFNQNNDIIKRGIALTP
LMFGISFNAVHYNQAGALVYVYMDGTVAITHGGTEMGQGLYTKVRQVAAHELGLPIDS
VRLIATDTSRVPNTSATAASSGADLNGKAVQNACIKIRERLAKLAAEISQSEADQVQF
EDSMVSTANGHSWTFPDLVQRAYMARVQLWDSGFYKTPEIHYDQVNHLGRPFFYYAYG
AAVSEVAIDTLTGEMKVLRADILHDVGQSINPAIDIGQIEGGFVQGMGWLTTEELYWQ
PQGPHAGRLFTHAPSTYKIPTSVDIPHIFNVKLFNNQNQADTIYRSKAVGEPPFMLAL
SVFSAIRQAVQAAIPENAPLVLNAPATPEEILRAIAIGRGQALAARPQAKM" /product="Xanthine
dehydrogenase, molybdenum binding subunit (EC 1.17.1.4)"
/EC_number="1.17.1.4"
/transl_table=11
CDS 3199014..3199997
/locus_tag="AUO97b_03098" /db_xref="SEED:fig|400667.82.peg.2963"
/translation="MQHLQWWTDFVAWQADALPMVLVTVVRADGSTPREVGATMLLRF
NLQQQWQQSDTIGGGHLEFQAIDIAKAMLKESETCVRRIERFNLSARLGQCCGGVMWL
LFEKILPAEIASDLSGSLQAWRAGKHIWRNASYQYASTWQTMDMEQQPSFEFHSGSSE
EWQFSQQIQPNTMQVLICGAGHVAEAIVRCLLPIGVRVKWIDPRDDIFPSDLLEQAYC
ITTDTLDAEIEHFDRSGAVLILTHDHQLDLQLCFTALKPTAKPFAYVGMIGSKSKRAI
FEKRMQVRGYTSETLQRLVCPIGIDGISSKRPATIAVAVVAQLLQVFDVKK" /product="Xanthine and
CO dehydrogenases maturation factor, XdhC/CoxF family" /transl_table=11
CDS complement(3200008..3200703)
/locus_tag="AUO97b_03099" /db_xref="SEED:fig|400667.82.peg.2964"
/translation="MSNKDSSLSKEQQKLLKQFKKQISNPHSSKIANQPESKKSQAES
EELEDTHLFKQAVAGVKPINNSNIADVKIPRAKKVDAQTLAKRAAAEGGRDTEHAELS
DTQAALNPVASQATLSYRIATLQHKVFEDLKAGKMRWFEAVDLHGCTIEQARDAVLQI
IQMAKDENQNVIKIVHGKGPEAVLKTYVNGWLRQHRDVLAFVSAPENQGGTGAVLVLL KRAEKNPKFEQKK"
/product="ortholog of Bordetella pertussis (BX470248) BP2475"
/transl_table=11
CDS complement(3200713..3201519)
/locus_tag="AUO97b_03100" /db_xref="SEED:fig|400667.82.peg.2965"

/db_xref="GO:0004425"
/translation="MINIQNTILGKIVDRKHEELAARLKQRNLQDVEELAKAATPVRG
FANALQHKRPGVIAEIKKASPSKGIIRADFNPAEIAQQYEQAGAACLSVLTDVDFFQG
ADENIAIARNHCALPALRKDFLVDPYNVVEARALHADCILLIVACLSDQQLEEMSKTA
FEHQLDVLVEVHDEEELERALKLSEQCLLGVNNRNLKTFDVDLNTTIRLKKLLPASRL
LITESGIATPDDVRMMQEHDIHSFLVGESFMKQPRPDQAFTALFGQPQTV" /product="Indole-3-glycerol phosphate synthase (EC 4.1.1.48)" /EC_number="4.1.1.48"
/transl_table=11
CDS complement(3201529..3202578)
/locus_tag="AUO97b_03101" /db_xref="SEED:fig|400667.82.peg.2966"
/db_xref="GO:0004048"
/translation="MNIQQALNHITKNIHLTQPQMEEIMRSIMQGEATEAQIGALMMG
LRMKGESIDEMTAAARVMREFAIKIDVSDIKHLVDIVGTGGDGQNLFNVSTASSFVIA
AAGATIAKHGNRGVSSKSGSSDLLEQAGIHLDLDMQQTERCIREMGVGFLFAPNHHKA
MKYAAGPRRELGIRSIFNLLGPLTNPAGVKRFVIGVFSDELCRPIAEVMKQLGAEHVM
VVHSKDGLDEISLAAPTTIAELKDGEITEWTLNPEDVGIESQTLNGLVVADATASLKL
IKDALSKNKSDIGEKAANMIALNAGAGIYVAGITKTYAQAVAFAQDIIYGGQALEKMS VLAEFTKTLKQSQAD"
/product="Anthranilate phosphoribosyltransferase (EC 2.4.2.18)"
/EC_number="2.4.2.18"
/transl_table=11
CDS 3202934..3205666
/locus_tag="AUO97b_03102" /db_xref="SEED:fig|400667.82.peg.2967"
/translation="MYLNNLKYVGALKNKRSFIFVFVALFHPLESYAQEALEQPQNQA
KPDKVVRVAVTGSRISKAQKDGPTSVTVITAADIEKQGFSNAFDALNNLTQNTGFVQG
ADYGNTFTPAANAISLRGLGPNHTLTLINGHRVADYPVPYDGSVNFVNLANIPTAIID
RIEILNGGASAIYGSDAIAGVVNIILKKKTDGTQFNIKAGGTKEGGENLRLQLSGSKT
LDKLSLVYGIELSGREPIWAADRDFMKSRTRLGEKPDTIVGRKNADTGKYLSIGGCQA
FNGLFNGSVNNIGQAGADNCASGLARPTYWTVQTQNRSQNGYLGLDYELNDKTQLFAD
FLIGANQIENNTRSPIWTSLAATSGYFLNQDSGNYEIWNRRFAPEELGGVERINKKWK
ELSSNLNFGIRGDIGETSWSYEAAYNGSIYTSQLNRQGLLRSNVDEYFLGQQLGTDAD
GIPIYSPHLDRLSRPLTASEFESISGTTKEKDKSWAQSLTLSANGDVFKLPAGTAKLA
AVAEIGRQGFSIKPDQAVENGEFYNTSSSGEYSGTRTRQALGTELFLPLAKPLNLTLS
GRYDRYALSDNSIDKLTFGSGLEFRPHSTLLVRGNYATSFRAPDMNYLFLNKQKGYFE
KTTDYLRCSQTGQSLDKCTFKDYAPGANYTLTGNKELKPEEGKSYGAGFVWSPTNKFD
ISVDYWDIKIDNLVRNLSDDKILRLEADCRLGNQDINSAACIDALARVERNPANAVVD
PNVIKNINIVPINAASDHTRGIDFTSRWRWKTDSFGNFLWTINYSRVLEHEYQQSKDH
EKDDYLKDLSSLDWRDRFNTSLSWSFGDWASTVLVNRYGKIPNGDQTAYLSPTYLVNW
SGTYQISPKASASIIINNLFDKVKRDDTGGWPYYPVGSYSPFGRQGWLEFNYKF" /product="TonB-dependent receptor" /transl_table=11
CDS 3205746..3208445
/locus_tag="AUO97b_03103" /db_xref="SEED:fig|400667.82.peg.2968"
/db_xref="GO:0004179"
/translation="MLVKPSFKKRLLLTTLLSLSATQIFASNNSEQIPIGKLPEWVVP
ESYDLDFKIDPAQKGYTGKTTIHLKLAQATDHIWIHGKSLTVKDVNITSAQGTKTKAK
YEQASEIDGVSKIKFAKTLPAGQYQLVLDFNAAYDQQLDGIYKIEFEGKPYVMTQMEA
ISARQSFPSFDEPRFKTPFNIRLTIPSKYSGFANTQQTSEQIEKSGWKTLSFAQTKPL
PTYLLALAVGPWQLQKGPDIGATSWRKQPIQLRGIAPDAKAEKMQQALSETPAILKTL
EDYFAFGYPFDKLDLLAAPDFAAGAMENPGLITFRDYLMLLDKDSPVSFVQNSFNVNA
HELAHQWFGDVVTMPWWDDLWLNESFATWMQSKITQKLHPEFNADLERIADTADAMKS
DSLVSVRRIRQPILSNADIQTAFDGITYQKGAAVLNMFESYLGEEKFKQGVRNYINKH
QYGNATANDLISALAEQSGQGERFTRAMKSFLDQPGIPLLNTSLQQEGNKVFLNVKQS
RYLPVGSKGDARSLWGVPLCVRYEVPNAGSKVQCELVDQAEAKIELKGASLGSWYIPN
ADAAGYYQFSLPQKEFTRLTAATEKLSNTEQLAYAYAISAAFNHGDINLLAVVDAAKK
FANSNSRQISTALFSQLSTIHRHVLKTEAEREHFRKVLANLYLPKLNQLGYVSKTGES

AEDSLWRSELVRFLALDIQVPEVRTQLLKQSDALFAQKQLNFAQVTPELLPTILAVRV
QEKGQPAFDRLSGELQRVTQPTQRLAILTALGSANQEATRQQARQLILNPRVKVGEVR
TVVNSINNYGDEQGGLWSWFKVNHDAVFDRLGKSSAGRFPAMFSGAACTQQQAAQLND
FFAPRTKELVGVERGLKQTKERIQLCESLVAKQDGSIVQQLKL" /product="Membrane alanine aminopeptidase N (EC 3.4.11.2)" /EC_number="3.4.11.2"
/transl_table=11
CDS complement(3208542..3209126)
/locus_tag="AUO97b_03104" /db_xref="SEED:fig|400667.82.peg.2969"
/db_xref="GO:0004049"
/translation="MLLMIDNYDSFTYNIVQYFGELNQEVKVVRNDQVTLEDIERWQP
KYLVIGPGPCSPSEAGISIPAINHFAGKIPLLGVCLGHQSIGQAFGGKIVRAKTVMHG
RLSDMYHSNKGIFSNLPSPFSATRYHSLVIDQETLPDCLEVTCWTNEADGSMEEIMGV
KHKTLPVEGVQFHPESILSQHGHQIFKNFLDIYA"
/product="Anthranilate synthase, amidotransferase component (EC 4.1.3.27) @ Para-aminobenzoate synthase, amidotransferase component (EC 2.6.1.85)"
/EC_number="2.6.1.85"
/EC_number="4.1.3.27"
/transl_table=11
CDS complement(3209422..3211224)
/locus_tag="AUO97b_03105" /db_xref="SEED:fig|400667.82.peg.2970"
/db_xref="GO:0008451"
/translation="MNDLTVPEKLAKLRELMTNQSIDTLVVMSADPHMSEYLPDYWKA
RQWLSGFSGSVGTLVVTQNFAGLWADGRYWVQAEQQLAGTGFQLQKLTSDESSTHLAW
IEKNLPAGSVISVNGQTLSIQQFKALENTAKQRGFKLETQQDLIGSIWSNRPELPLEQ
IHLMPEGLNALSRKEKIQAIRETLKTKAIEGHFISSLDDIAWVLNARGQDVEYNPVFL
SHLYISAQQAVLFIDSNKVDLTTQQAFKADGIEIRDYEDTAKFLSNISDASVLLDPAK
VSIFHEQAIAKDIQVVYDINPSTLFKSRKHESEIAHIRHAMVKDGVALCHFFNWLEKA
LHQGQRISELTIDEKITAFRAQQEGFIGPSFSTIAGFNANGALPHYRATEEHYSFIEG
DGLLLIDSGGQYVDGTTDITRVVPVGTPTEQQKRDYTLVLKCHIALAKTIYPEGLAAP
LLDSICRHTLWQHGLDYRHGTGHGVGFALNVHEGPQVLSYYAPIHAYSKLREGMILSN
EPGLYHEGQYGIRIENLVANKLHSGFEKTYGDFLEFETLTLCPIHLDCIVVDMLTDEE
KDWLNGYHQTVQERLAEHLSGDVLDWLIYNTRKI"
/product="Xaa-Pro aminopeptidase (EC 3.4.11.9)" /EC_number="3.4.11.9"
/transl_table=11
CDS complement(3211221..3212762)
/locus_tag="AUO97b_03106" /db_xref="SEED:fig|400667.82.peg.2971"
/db_xref="GO:0005215"
/db_xref="GO:0006857" /db_xref="GO:0016020"
/translation="MKYDFEQSDKAFFGHPKPLQTLFFTELWERFSYYGIRPLLVLYM
IAMVNDGGLALDRPTAAAIVGLFAGSMYLMTVFGGWVADNWLGQARAIWYGSIIIALG
HLSIALTSVFDQSFFYLGLVLIVLGTGLFKTCISVIVGTLYKANDARRDAGFSIFYMG
INMGSFIAPLITGLLAKDHGWHLGFGIGGIGMLIALLIFRFMAMPQLQTFNELRQEAN
SCSKPVVENKNAPKIVFSFLFAVAVVIALTFLGIIHINPVAVATYLTVGISIGIIAYF
AYLLLFLNLEQHEKFKIIICFVLLAASALFWSAFEQKPTTFTLFAQDYTDRIVFGFEI
PTVWFESINALFIIIFAPVAAWLWAKLGKANKDPSYISKFIIALLFAAGGFLLMSLAS
HFAIHGGVVSPFWLVGTLFLLTIGELCLSPIGLSTMTKLAPDVIRSQIMGLWFTGTAL
GNLMAGLIGGQVSADGINHLPSLFMRCVLALVIGAIVLFLLKKPMNKLMDKSTNKVQP DLETI"
/product="Di-tripeptide/cation symporter"
/transl_table=11
CDS 3213497..3214912
/locus_tag="AUO97b_03107" /db_xref="SEED:fig|400667.82.peg.2972"
/db_xref="GO:0004356"
/translation="MSMANKVLQLIQESGAKWVDFRFTDTKGKEQHVTYPADSIDEDT
FEDGKMFDGSSIAGWKGIEASDMILRPDAETGFIDPFFAEPTVVVTCDVIEPSTGQGY

ERDPRSIARRAEEYLKSTGIGDTAFFGPEPEFFVFDEVKWDIDMSGARHTLIAEEAAW
STGKDYESGNSGHRPRVKGGYFPVPPVDSAQDMRAEMCAKIEDIMGPGRVEVHHHEVA
SCQLEIGVSFNTLVRKADEVQQFKYAVWNVAHQYAKTATFMPKPMVGDNGSGMHVHMS
ISKDGKNLFAGDEYAGLSEMALYFIGGIIKHARALNAITNPSTNSYKRLVPHFEAPIM
LAYSARNRSASIRIPYVSNPKGKRIEARFPDPMMNPYLGFAALLMAGIDGIQNKIHPG
EAADKNLYDLPPEEEAKIPTVAHSLDMALEALQADHEFLLKGGVFTKEMLDAYIELKT
EDVRRLNTTTHPVEFDMYYSL"
/product="Glutamine synthetase type I (EC 6.3.1.2)" /EC_number="6.3.1.2"
/transl_table=11
CDS 3215038..3215547 /locus_tag="AUO97b_03108" /db_xref="SEED:fig|
400667.82.peg.2973"
/translation="MRKRQPVLKQEKTLNPELKTWLYASGSLTQQLTELGGGKFSVKP
FKEHFQRLTFADSQWMNMPHTHTSWVRETYLYGSDVEPWVKAKSIFPIQSLQKKARIF
QHIGSKPIGLFLFQRTTPLCDRRVIRLPEGWTRQSCYTWHGCKFIVQETFLPAFEAFL YQQHDKELL"
/product="Chorismate--pyruvate lyase (EC 4.1.3.40)" /EC_number="4.1.3.40"
/transl_table=11
CDS 3215544..3216422
/locus_tag="AUO97b_03109" /db_xref="SEED:fig|400667.82.peg.2974"
/translation="MTTATGTTWRQRLEAYYYLCRFDKPIGTELVFWPTMWALWVASK
GIPNLGILFVMILGVIFMRAAGCAINDFADRKVDGHVERTKTRPLATGVIRAREAVYV
FLGLVLASACLLFFLPIETFYWSFGALFLAFIYPFMKRYTNLPQVFLGAAFSWSIPMA
FTAVGKTPDLTCWLLYFGNLAWTVAYDTQYAITDREYDLKIGVKSTAILFGRYDIQII
ALLQAISLVLIGTALYLENILFPWAIIALVVVAFDFVYQTIKTKDRNPQVCFWAFRHN RWVGLIIFLGIFLNFI"
/product="4-hydroxybenzoate polyprenyltransferase (EC 2.5.1.39)"
/EC_number="2.5.1.39"
/transl_table=11
CDS 3216533..3217990
/locus_tag="AUO97b_03110" /db_xref="SEED:fig|400667.82.peg.2975"
/db_xref="GO:0004622"
/translation="MKRSKIALAITLSISALFLTACNDDDDNYTGIDTNKTYVSESNY
AIDKVDNASSIKVMTYNMTNVQGKTATATAMVLFPKAIQPKDGYRVVVWEHGTVGVGD
GCAPSKNAINPRFKILAETLLAAGYVIIAPDYEGLGTPGVHPYLNLSSEAKSALAAVK
AAKEHYGTQLKGDWMSIGQSQGGHASLGTAEFANTDISYKGAVAGAPASSLGTIIQIY
IDPEMNQDSNFAVSKLDQAIRARRQIDAAIAAGQMSPNDPLALAVPTIDKTAEGYAEL
LAYAAFASAGIKAQQPDYDLKAIFTSGAADIAELAYGRTGDDGACLSYPAPDTANGLQ
EKFKAGILAFLADPTHQITQYGIDLNKFKTDQVVQNFLKATQPATNATAEKVIKTPVF
IIQGEKDQAVLPVVTQGLFANMKANALKFFPQAGYDKGYQLTIVPNATHTQAIVCQNA
NAVDFIQAKMSADTGIVLTDAQKDASQSPHCTGKF"
/product="SpnM"
/EC_number="3.1.1.5"
/transl_table=11
CDS 3218102..3218302
/locus_tag="AUO97b_03111" /db_xref="SEED:fig|400667.82.peg.2976"
/translation="MSQQSVEKKSVPWLLMGLGLLIPFLIIGILFLAAKSDAQNKKQY
EQQRLEMEKRIAEKEAANSDKK"
/product="hypothetical protein"
/transl_table=11
CDS complement(3218312..3218755)
/locus_tag="AUO97b_03112" /db_xref="SEED:fig|400667.82.peg.2977"
/translation="MFESSHLFFIILGCLSTCIFLLVCLRPYLFPKQKFFARPVITNF
ETQMFIRLKQSFPSYHVLAQVAFSALITSNDYKIRSQFNRKVTDFVLLDENMEVIAII
ELDDPTHLEKVEEDRLRDQMLIEAGYIVRRYTSIPSVRQLQKDIY" /product="hypothetical
protein"
/transl_table=11

CDS complement(3219372..3219998)
/locus_tag="AUO97b_03113" /db_xref="SEED:fig|400667.82.peg.2978"
/db_xref="GO:0004784"
/translation="MTTITLPALPYGYDDLAPHISKETLEYHHDKHHNTYVVNLNNLI
KGTDLEGKTLEEIIKATAGDASKAGIFNNAAQVWNHTFYWNSMKPNGGGKPTGAIAAK
IDEAFGSYEKFAEEFTAAATTQFGSGWAWLVADEVNGKLSITKTSNADTPLAHGQIAV
LTIDVWEHAYYIDFRNLRPKYIATFLENLVNWDYANAKLAGQPAGVEK" /product="Superoxide
dismutase [Fe] (EC 1.15.1.1)" /EC_number="1.15.1.1"
/transl_table=11
CDS complement(3220144..3220902)
/locus_tag="AUO97b_03114" /db_xref="SEED:fig|400667.82.peg.2979"
/translation="MANLHEIIQWCDQTLKAAEFKDYAPNGLQIEGSTEVKRILCAVT
ASEDAIDAAIAQNADLLLVHHGYFWKGEPYPITGMRGNRIKKLIQNNISLVAYHLPLD
AHPSLGNNIAIAEKLNLKNLEPLDLTEKHPIGNIGYLEQALSVEEFKAKLQNSFDFKV
IHLPAEKQSIQKVGFCTGGAQDFIAKAALQNCDAYISGEVSERTFYEAKELGVHYFAC
GHHATERYGVQRLAQAISKQFAIESEYFELNNPI"
/product="GTP cyclohydrolase 1 type 2 homolog YbgI" /transl_table=11
CDS 3221096..3222271
/locus_tag="AUO97b_03115" /db_xref="SEED:fig|400667.82.peg.2980"
/translation="MRRTFTWLPWVLLIIVIASFIAWQKYHQPKPTVAVDGVQMPAEK
VEPLVDTSRTGGVVSYSAAVKVAAPAVVNIFTTQKVKQNHPLLSDPVFREFFGNQVPE
QQQQNENSLGSGVIVRADGYILTNNHVIAQADQIVVALHDGRRAEATIVGTDPDTDLA
VIKIDLDKLPVLPFKLSGNEVGDVVLAIGNPFGVGQTVTQGIISATGRSDLGINTYED
FIQTDAAINPGNSGGALIDVAGNLIGVNTAIFSQSGGSLGIGFAIPAKVCQQVLNSIL
KDGRVVRGWLGISLLPPTQDDVLAPKQQVGVVVADVLKNGPADAAGIKVGDKIVQVNN
EPITSASHLINYVALQAPNSEINVLVEREGKQQNYVVTVGERKAQNTQSQYIPLPRR" /product="Outer
membrane stress sensor protease DegS" /transl_table=11
CDS complement(3222321..3223559)
/locus_tag="AUO97b_03116" /db_xref="SEED:fig|400667.82.peg.2981"
/translation="MQVYLVGGAVRDYLLGHPYQEKDYVVVGATPEHMLAQGFQPVGK
DFPVFLHPETKEEYALARTERKSGQGYHGFQFFTDTTVSLEDDLIRRDLTINAIAMDQ
DGKIYDPYGGQNDLENKILRHVSEAFAEDPLRVLRVARFAARYFPYGFQIAPETLQLM
QTMADSGELDALTPERVWKETSRALMENHADIYFQTLRNCGALKHLFPEIDALFGVPQ
RPEYHPEVDCGIHTLMSLQQACKSNYSLDVRFAVLVHDLGKALTPAEELPRHIMHEER
GIKPVTQLCERLRVPTQTKQLALSVCKEHLKCHQIMSLKPGTLWRLLQRLDVLRRPER
VEAFVQACECDAKGRLGLEDRPYPQAQYMREAMQIVRSIKVQDLPENIKGAEIGEMLI
QYRIEALAEFKNQHQSLSHS"
/product="CCA tRNA nucleotidyltransferase (EC 2.7.7.72)"
/EC_number="2.7.7.21"
/transl_table=11
CDS complement(3223651..3225921)
/locus_tag="AUO97b_03117" /db_xref="SEED:fig|400667.82.peg.2982"
/db_xref="GO:0004473"
/translation="MDDQSLKQAALNYHEYPNPGKISVTPSKQLVNQRDLALAYSPGV
AAPCLEIERDPSAAAKYTARGNLVAVVSNGTAVLGLGNIGPLASKPVMEGKGVLFKKF
AGVDVFDIEIAENDPDKIVDIVASLEPTFGGINLEDIKAPECFYIEKKLRERMKIPVF
HDDQHGTSIIVGSALLNALQIVNKKIEEIKIVASGAGAAALSCLDLLCALGVNKENII
VADSRGLLTTSREGLDESKKRYVQDIKATQLHEVMAGADMFLGLSAAGILTKDMVKQM
AENPIIFALANPDPEILPEHAHEVRPDVIMATGRSDYPNQVNNALCFPYIFRGALDVG
ATTINEDMKIACVHAIARMAHVEADAATYGEKSASFGRDYLIPRPLDQRLILEIAPAV
AKAAMDSGVATRPIEDFSAYRQKLSEFVYNSAFLMKPIFAQAKTDPKRIAYAEGEDER
VLRAVQIAVDEDLAKPILVGRTAVIEANIKKLGLRLQHGVNIEIVDQEQNPLYEEFWK
DYYQTMQRKGITVEYAQREARRRSTLIAALLVKFGKADGMLCGTYSSYNIHLDFVRNV
IGLKEGMNNFFTLNALMLEDRNLFIADTYVNTNPTAEQLAEMTILAAEEVRRFGITPR

VALLSHSSFGSDQTDSSAQKMREVYRILSEQAPELEVEGEMHGDAALDENIRHFAFPG
SRFKGSANLLIMPNLDAANISFNLLKATSGNNVTIGPILLGAAKPVHILTPTATTRRL INMTALTVAEIQQQEK"
/product="NADP-dependent malic enzyme (EC 1.1.1.40)" /EC_number="1.1.1.40"
/transl_table=11
CDS complement(3226158..3229007)
/locus_tag="AUO97b_03118" /db_xref="SEED:fig|400667.82.peg.2983"
/db_xref="GO:0005554"
/translation="MSGLQEEYLQHQTKIEQLRLSIQYIRWYLVSLILLCLISAIIFF
CYFTSYITNFIWSIGFTILTLTCLLFVPKLLTHYRDIHFIKKTDLVLQGICLASGILM
GINIIVLHFSLPDTLISVSDVHILIALFITSAHIIGLTFLTQQPRYFYLLFIPSTVPL
IIGQFTHSQITQLPFYFAYNLAFVATLLCAYATQRNYQKISQLLFKNKQLVQVAEQHT
QWAEELCVQLQQEVNKSKDIEAQLQFNNHLLEQKVRERTYDLTKMNERLESHHHNLAF
AHETAGIRPWDWDIEKQKLEITFFDQQKQTQNTPLHLKSILERIHPNDRKLFDERLKE
HLEGKSDHFNITFRILRRNGSWRWIHDVGRVISRDPKTNKALRMVGMTRDIHQEKKDQ
EHLRLSAIVLEQAAEGIFILDEKLNYIEVNPYYEKLTGFSKSELLSQQLFNITINQKL
QQQQFHDSITQQLLKTGEYMGQFDEKFTSGKSVYLWLHINAVKDEYNQIINYIGFARD
LTDQKRQEQHLSYLKNYDSLTHLPNRFYYYNQLHQYLVNPSYSFKNLALIRINIDRFR
AFNEFLNNDSGDELLKQFAQRLRLTNINAILVAYLNGDDFAIIYEISPIHPNIEQYCQ
NILQALNAPFYIDEQEYFITASIGVACFPEHGRQIDHLNNHAEQALSEAKRLGGNTIS
YYCNKTTNPYKTADLEQELRKAIQNDEFVVYYQPKINLNDKSIKGFEALIRWQHPEKG
LVMPNMFIPFAERSSLISDIGKVVLDKVGKQLQEWKKAGYADVRVSVNIVAQQIHRGL
LLSDLDEVLDTYHLDGSNLELEITESALLDNTDNVKKLLHSIKERNISIALDDFGTGY
SSLAYLTEYPIDVLKIDRAFVSKIGDQKQEAIVNAMIAMGKSMGLKLVAEGVETEEQV
IYLQKQQCDFLQGFFFSRPLHPNQILQYLQTNTHI"
/product="diguanylate cyclase/phosphodiesterase (GGDEF & EAL domains) with
PAS/PAC sensor(s)" /transl_table=11
CDS complement(3229142..3229864)
/locus_tag="AUO97b_03119" /db_xref="SEED:fig|400667.82.peg.2984"
/translation="MNRFFIETELTVGSTIQLTESVFHHWVRVLRAQLQEQATLFNGQ
GGEYLATLTEINKKNAFVTIENFNPVNRNAPFKALLGQVMSKGDRMDYAIQKATELGV
SKIQLLTSERCEMRLKYDRDQKKLDHWQAVAIAACEQCGLNLVPEVLAPISLHEWLNS
SDLPVSKFVLAPEKEQKDVLAGIQPELALLIGPEGGLSDNEITQANQAGFMNWCIGDR
VLRTETAPVVALSILNYRFLSI"
/product="16S rRNA (uracil(1498)-N(3))-methyltransferase (EC 2.1.1.193)"
/transl_table=11
CDS complement(3229883..3230719)
/locus_tag="AUO97b_03120" /db_xref="SEED:fig|400667.82.peg.2985"
/translation="MTKRTPISFEFFPPKTDAGAEKLRIVHQELQQLNPEFFSITYGA
GGSTRERTLAAIEDFNGKGTPVAPHLSCIGDDKTRIAELLDLYKAQGIDRIVALRGDL
PSGQVGLGELPYAQDLVRFIREHSGDHFHIEVAAYPEMHPQAQSLDSDIQRFIEKVQA
GANAGITQFFFNPDSYFYFIERLEKAGINIPVAPGIMPITNASNLIRFADGTGAEIPR
WIRKQLQAYGDDSESIKAFGHEVVVKLCERLIAGGAPSLHFYSMNQVEPTRQLVVDLG LN"
/product="5,10-methylenetetrahydrofolate reductase (EC 1.5.1.20)"
/EC_number="1.5.1.20"
/transl_table=11
CDS complement(3230791..3232173)
/locus_tag="AUO97b_03121" /db_xref="SEED:fig|400667.82.peg.2986"
/db_xref="GO:0004013"
/translation="MNAVNASFTDYKVADISLADYGRKEIKLAEAEMPALIGLRKKRYA
ASKPLAGAKILGCIHMTIQTAVLIETLVELGAEVRWTSCNIFSTQDHAAAAIAARGIP
VFAWKGETEEEYVWCLEQQINVNGQPWDANMILDDGGDLTALVHEKYPALLERIHGIT
EETTTGVQRLIEMWKDGTLKVPAINVNDSVTKSKNDNKYGCRHSLNDAIKRATDMLLS
GRRALVIGYGDVGKGSAQSLRQEGMIVRVTEVDPICAMQACMDGYEVVSPYKNGVQTG
KKEDINHDLLGNTDLVVTTTGNYHVCDAAMLDSLKAGAVVCNIGHFDTEIDTAYLRGY

KWVEVKPQVHQVYRSEDENNYLILLSEGRLVNLGNATGHPSRVMDGSFANQVLGQIHL
FQEKFADLPASEKAAKIRVEVLPKKLDEEVAAAMVAGFGGVLTQLTQEQADYLGVAVE GPFKSDAYKY"
/product="Adenosylhomocysteinase (EC 3.3.1.1)" /EC_number="3.3.1.1"
/transl_table=11
CDS 3232395..3232931
/locus_tag="AUO97b_03122" /db_xref="SEED:fig|400667.82.peg.2987"
/translation="MALNIPESLANLEANCGLFAVWMVLQHHGVQIDVAELIKVCRYD
DEGTYTIALAVALKKLGFEVSFHSDHDPNISPSERMSYKEAKVLRIPTGPALSYQDIQ
TETQNGKMVIVYYDTLEGVGNQSLIYSIDQNEICFFDSFEPMSAAVFENQRKAEGICR
QTVVIGDRNLNIHSTKLN"
/product="hypothetical protein"
/transl_table=11
CDS complement(3232970..3233353)
/locus_tag="AUO97b_03123" /db_xref="SEED:fig|400667.82.peg.2988"
/db_xref="GO:0047575"
/translation="MSKQDYENGLKVRTEVMGEAFVKRAQENTVPFTQPLQDWINEHA
WGSTWQREGVLPRKYRSLITLAMLTALKSPTELKGHVRGALNNGCTVEEIQETLLHSL
PYCGAPATQEAFRAALEVIQEYQQQ" /product="4-carboxymuconolactone decarboxylase (EC
4.1.1.44)" /EC_number="4.1.1.44"
/transl_table=11 CDS complement(3233457..3235922)
/locus_tag="AUO97b_03124" /db_xref="SEED:fig|400667.82.peg.2989"
/db_xref="GO:0004085"
/translation="MIFLLFVLSIVIQLVAIWAIFFFNLNRVAGSVITIAVAILTAFI
LTPWALLLGIPLIALSIIILFKPLRFNLITQPAYKTLANSMPSISTTEQEALEAGTSW
WEKELFMGAPDWSQFEKYPYPSLSPEEQSFIDNEVEVLCSMLDEWEIHHQLKDLPPEV
WQFIKDKGFLGLIIPKSFGGKEFSSFAQSRIMSKIASRSLTAAVSCMVPNSLGPGELL
MHYGTDAQKQQYLPGLAKGEEIPCFGLTSPEAGSDAGAIPDSGVVCYGEYEGAQVLGL
RMNFSKRWITLAPIATVVGLAFKLYDPEGLLGDKTKNEYGITCALIPANHAGVKIGAR
HYPGSPFMNGTVEGEDVFIPIDWIIGGQENAGKGWRMLMECLGVGRGISLPALATAAG
EMSYLTVGAFARIRQQFNISVGKFEGVQEASSEIASDAYMLEAFRYLVTCGLNQGGTP
AVMTAMAKYYATETMRKVVNHGMDIAGGRAIQLGPRNFLALTYQAIPIAITVEGANIL
TRSLMIFGQGSMRCHPYLFEELQLLQSDDKANAVQKFDDLLFKHLAYTFNRGARSFAY
GWTGGSSDAPQSADQFTASYYKTINRFSANFSLVSDMALGLLAGDLKRKEMLSGRLAD
IHAHLFIATAILKYYEAGQKTEAEQLHAKLALQKAFLNIQEAFWGLFDNFPAKLPAAF
VKWICFPLGRVISKPDDELKQQVAELMMEEHTFREQLKHHVYYSTEPNDVTGRLEHTF
QMLRTIEPLWDKFKKAESKGKFTGLTFEENIAQAIKEGFISESEAQQLLQYNAIRFDS
MLTDVFDEKLNKDLPLSNPHQIV"
/product="Acyl-CoA dehydrogenase, short-chain specific (EC 1.3.8.1)"
/EC_number="1.3.99.2"
/transl_table=11
CDS complement(3236206..3237219)
/locus_tag="AUO97b_03125" /db_xref="SEED:fig|400667.82.peg.2990"
/db_xref="GO:0003824"
/db_xref="GO:0005506"
/translation="MSEHRKPEQGVKLRGAEKVARIPVKVVPTVEVPRKPDWIRVKMT
APEEVQRIKTTLRAQKLHTVCEEAACPNLPECFGGGTATFMIMGDICTRRCPFCDVAH
GRPNALDPDEPRHMAETIANLKLKYAVITSVDRDDLLDGGAQHFVDCIKEARALSPNT
LLEILVPDFRGRMDIALRIMTECPPDVFNHNIETVPRLYKAMRPGSDYQHSLNLLKMF
KEYCPDIPTKCGLMVGIGETEEEVIALLDDLRAHDVDYVTIGQYLQPSKQHAPIDRFV
TPEEFERYAEHGRKLGFRNIWSAPMVRSSYFADRQYYGEPVPEVRRKVDPAKKIAVQA IEA"
/product="Lipoyl synthase (EC 2.8.1.8)"
/transl_table=11
CDS 3237416..3238828
/locus_tag="AUO97b_03126" /db_xref="SEED:fig|400667.82.peg.2991"

/db_xref="GO:0003957"
/translation="MPRKKEVVSGNFVKYDAVVLGSGPAGEGAAMKLAKAGKRVAIVD
VRDQLGGNCAHVGTIPSKALRQTVSSIIRYQRDPMFQKVGEWKQFTMKQVLRNAHKVI
QQQVDTHTRFYDRNKIGVFHGRAYIQDKNTVLVFSHEGIKETIICKQIVIATGSRPYH
PQGLDFDHPRVFDSDKILDLDYSIQKIIIYGAGVIGCEYASIFIGLDHKVDLINTQQK
LLSYLDDEIADALSYHLREQGVLIRHNEQMDHLETFDDHVVLHLQSGKKIKADAILWC
NGRSGNTEGLGLENVGLVPNSRGQLSVNDQYQTEVENIYAAGDVIGWPSLASAAYDQG
RCAGANMSGEDAKPVRDIPTGIYTIPEISSIGKNEQELTEEKIPYEVGQASFRHLARA
QITGDTVGELKILFHRDTLEILGIHCFGNNAAEIIHIGQAVMHSPNNTLKYFVETTFN
YPTMAEAYRVAALNGMNRLF"
/product="Soluble pyridine nucleotide transhydrogenase (EC 1.6.1.1)"
/EC_number="1.6.1.1"
/transl_table=11
CDS complement(3238948..3239082)
/locus_tag="AUO97b_03127" /db_xref="SEED:fig|400667.82.peg.2992"
/translation="MTHNAVLLKTIMTFLILALLVFSIVLWKQTPDLFEYFNQAFCAH "
/product="FIG00352575: hypothetical protein" /transl_table=11
CDS complement(3239072..3240286)
/locus_tag="AUO97b_03128" /db_xref="SEED:fig|400667.82.peg.2993"
/translation="MTKTSHQFSQSLIYMLIGSAIILALSLGVRHGFGLYLVPMSHEF
GWGQHVFSLAIAMQNLIWGAVQPFTGAIADKYGSKVVVTVGGLLYTLGLLLMAFSSSV
LILNLSLGLIIGLALSATSFTVLLSAVGRAAPPEKRSMAMGIASAAGSFGQFIMLPST
LLLLKTVGWSSALMVSALLIALIIPLAWMLKGPSNQTPKAIAQPQLTFKQVLHIARKH
KPFWWLALGFLVCGFQVVFLGVHLPGYLIDHGFDAATGTVFLALVGLFNIVGTYGAGW
LGDRFSKPKLLMALYGSRGIAIIAFLLLPLSTYTVYAFGIIMGLLWLSTVPLTNGIVA
NMFGVKYLSMLSGIVFFTHQVGSFFGGWLGGVNHDLTGNYNAIWLCSIALSILGVIVH FFVNEEAVIHDA"
/product="Uncharacterized MFS-type transporter" /transl_table=11
CDS complement(3240461..3241807)
/locus_tag="AUO97b_03129" /db_xref="SEED:fig|400667.82.peg.2994"
/translation="MSSNINLDAKKATEIKLFSFSTPAMRAFHMTWLAFFVCFFAWFA
CAPLMPVIKGEFGLTKDQIANINIAAVAITILVRLAVGPLCDKYGPRITYTTLLLLGS
IPVFGVAAANSYESFLFFRLLIGAIGASFVITQYHTSIMFAPNVVGTANAAAAGWGNA
GGGATQALMPLLLGAIVMFGVEQTMGWRVALIVPGVMMVIVGVLYWKLTQDCPQGNFK
EVRAQGIEVGSGKKGGMAILMQAARNYRVWILFLAYGACFGIEIFIHNVAAMYYVDQF
KMDLKTAGLTAGIFGLLALFARALGGIISDKVALKKGLDGRTKVLVLMILGEGLFLIL
FSQMNVVALAIISMTVFALFTHMACGATYALVPFIDRNALGGVAGIIGAGGNVGAVAA
GFLLKGVLDIQTCLFILGGLVMVAGCSVILIRFTTEHKEKEKILFEQALLERNSAA"
/product="Nitrate/nitrite transporter CHU_1319, NarK/U family"
/transl_table=11
CDS complement(3242220..3243338)
/locus_tag="AUO97b_03130" /db_xref="SEED:fig|400667.82.peg.2995"
/translation="MMKKSLFCMTAALFALPTVTSAASPYFSLKDGDGFKRFSVSAGW
LHAMPQGSGNPVNINTSVAEGTKSKVGDVSTKAVLDAIDQSKPSGQFWHSTISLLDKF
TDTLPSSLAGTAEINGLSQWEQQGSGLEAADVDTVGLMFNYNFTDNLSLEIKGGIPPK
VDINGKGNIYAPLSGKATPLKDGSVIGGIIGDGIAKAGGDIPLKQDIHITDLSQGGKA
ATARAWLPAVELHYQFGKTGVNKFRPYIGAGVMYAYFNDLKMNSGIEADLIQAGHMIQ
NIHDNKAGAALDRKTSSANPIVKLEATDTFAPIVTVGATYDFNPNLFAVGSVSYSKMN
SEAKIYVTDQNTGKELIQANSKIDIDPLITYLGVGYRF"
/product="Outer membrane protein W"
/transl_table=11
CDS complement(3243543..3244370)
/locus_tag="AUO97b_03131" /db_xref="SEED:fig|400667.82.peg.2996"
/db_xref="GO:0004239"
/translation="MNSTYTAPRRLIKTPDEIEKMRIAGRLAAEVLDMIKPHIKAGVS

TLELDTICRNHIENVQHAIPACVGYGGAPGRPAFQHSICTSVNHVVCHGIPSENKILK
NGDILNIDVTVIKDGYHGDTNMMYIVGGETSILANRLCKVAQEAMYRGMATVRDGSYL
GDIGHAIQKYVESERFSVVREYCGHGIGTVFHDEPQVLHYGQAGTGMRLEAGMTFTIE
PMVNAGVWQTKLLGDKWTVVTKDHKLSAQYEHTILVTKTGIEVLTARPEEDLSRFNQ"
/product="Methionine aminopeptidase (EC 3.4.11.18)" /EC_number="3.4.11.18"
/transl_table=11
CDS 3244580..3245332
/locus_tag="AUO97b_03132" /db_xref="SEED:fig|400667.82.peg.2997"
/db_xref="GO:0003735"
/db_xref="GO:0005622"
/db_xref="GO:0005840"
/db_xref="GO:0006412"
/translation="MADYNVSMRDLLQAGAHFGHQTRFWNPKMRQYIFGARNKIHIIN
LEHTVPALNDALNFANQLASKKNKVLFVGTKRAASNIIREQAQRAGQPYVDHRWLGGM
LTNWKTLRQSINRLKDLQTQSQDGTFAKLTKREALERTREMEKLERSLGGVKNMGGLP
DALFVIDVDHEAIAIKEAKNLGIPVIGIVDTNSNPDNVDYVIPGNDDAIRAVTLYASA
MADAILAGKEYAQSQANAQAKGDDAAKDASEA"
/product="SSU ribosomal protein S2p (SAe)"
/transl_table=11
CDS 3245470..3246345 /locus_tag="AUO97b_03133" /db_xref="SEED:fig|
400667.82.peg.2998" /db_xref="GO:0003746"
/db_xref="GO:0006414"
/translation="MTAITASMVKELRDRTGLAMMECKKALTEANGDIELAIDNLRKS
GQAKAAKKAGNIAADGAITIVQDGNKAILVEVNCQTDFVAKDENFSNFAHTVAAAALA
AGETDAAKIAELKLADGQSVEEARIALVQKIGENIQVRRAKIVEGEQLAIYKHGLKIG
VVVSYTGDADTGKGIAMHVAAFNPVAVNAEAVPADLIAKEKEIAEAKALESGKPANIV
EKMVTGSVEKYLNEVALDRQMYVIDNEKKVADVLKATGTNVANFVRFEVGEGIEKKAE LSFAEEVAAAQAAAK"
/product="Translation elongation factor Ts" /transl_table=11
CDS 3246411..3246896
/locus_tag="AUO97b_03134" /db_xref="SEED:fig|400667.82.peg.2999"
/translation="MTNKKNLGIGGIIVLLIAAYLGLDLSGHKNNSTPSSSIPANQRT
ETTFSNDGVDTIKAAYERRQSNIQVQGSGRVKAILREDNDGSRHQKFILILKNGLSIL
VAHNIDLAPKIEDLRKGDIVEFNGEYEYNAKGGVLHWTHHDPQNRHENGWLKHNGRIY Q"
/product="hypothetical protein"
/transl_table=11
CDS 3246994..3247818
/locus_tag="AUO97b_03135" /db_xref="SEED:fig|400667.82.peg.3000"
/translation="MHREQADYLHLRELGGLELLKAHYHQTQFSKHTHEGYCIGVIEE
GAQSFFRTGQLHVAPKGDIILVNADEIHTGSSAVESGWRYRAIYPTPEMLAEVSQDFF
EQGHGAPWFPQAVIQDLGLSQQLCLLFDLLEQKDNFLLKETMYLSTLACLMVRHGKSR
HTFCELPEAYSKVLKVKDLLADMPEKNFSLQELADMVGLSAWHFLRQFKKHVGLPPHA
WLVQARLQKARQLLKQGVQIVMVAQQCGFSDQSHFNRHFKKAMGVTPTQYVASLNL"
/product="FIG229276: Transcriptional regulator, AraC family" /transl_table=11
CDS 3248042..3248629
/locus_tag="AUO97b_03136" /db_xref="SEED:fig|400667.82.peg.3001"
/translation="MAIHAGLSFYKALAMSGIVFAGAAQLVSLSMVMEGASLLTIYVT
IFFLTAQHFIYALTLRNDVSILPLSKRLTLGFLLTDELFAVSVPNEKKHPQYLFGAGL
CFYLFWVVFSLVGILLATALPNLLNYHLDFSIIAIFVAMIVPMCKGKPVMAGILMTCV
SGFVLKYFHIEGAILISGLLGMFIAVIVENKMGEK"
/product="FIG006238: AzlC family protein"
/transl_table=11
CDS complement(3248989..3249441)
/locus_tag="AUO97b_03137" /db_xref="SEED:fig|400667.82.peg.3002"
/translation="MKSISEWFDEYSESHQNPINKKIHWVCVPAILFSIIGIIAHFSA

LLTALLVVLTLIFYARLDLVLAVAMAALLVVMAWLIYVLPVGVGFYIALFVFAWIGQF
YGHKVEGKKPSFFKDLQFLLIGPVWCMDAYLAKILPHWKSRQKKLIAQ" /product="Putative PRS2 protein"
/transl_table=11
CDS 3249623..3249772
/locus_tag="AUO97b_03138" /db_xref="SEED:fig|400667.82.peg.3003"
/translation="MSSKILGYLFVILRETNPQKNLIFMKNGKIIEKYSTHLVLKRVK QLFLD"
/product="hypothetical protein"
/transl_table=11
CDS complement(3249998..3250615)
/locus_tag="AUO97b_03139" /db_xref="SEED:fig|400667.82.peg.3004"
/translation="MQLSLKYILALTASLSMAPLHAEMMQSSSLNSDVDGPNANLAAR
VLSKDTQNFNSRFSNINSLSITERSGDQIRRQAIAAKIEIPEDEPSVIEKLNTVASNT
VRKFSQTGMASWYGRQFHGRKTASGETFDMNALTAAHRSLPLNCYIRVTNKDNGKSVV
VKVNDRGPFHGNRVLDLSYGAAKRLGITNAGTAKVSIERVDGPTS" /product="Septum-associated rare lipoprotein A" /transl_table=11
CDS complement(3250906..3251907)
/locus_tag="AUO97b_03140" /db_xref="SEED:fig|400667.82.peg.3005"
/translation="MLSQFVNKTLKVLALCTGLISTSHFAQANDFVTHPNYLNFKQKA
MSTYGLSGEQIDAAMNGAKNLPNIINIMTRPGESKPWYDYRSMFLVEGTIQRGVRFKN
QYADALNRAEQQFGVSQAVILGILGVETGYGANKGSFITRDALATLAFGYPRRAEYFG
DELAALIAWTYKEGYPTNSIVGSYAGAIGFPQFMPSNISKYGVDFDGNGHIDLRNSAE
DAIGSIANYLAKQGWQRDQPIGFMARYTGSNPESIIAKDLTQPTPYGALKNQGISPLN
PLVKIDDLDMVNVIQLQDYNGPIYYLTYPNFQVITTYNKSRMYATAVWLLGTEVASR"
/product="Membrane-bound lytic murein transglycosylase B" /transl_table=11
CDS complement(3251927..3253006)
/locus_tag="AUO97b_03141" /db_xref="SEED:fig|400667.82.peg.3006"
/translation="MQPSRWQQLHIDPWLCLFLFLNALLGLTVLYSASAQDVGLVSKQ
AMSFGIGFLVMISLAQIPPKVYQAFSPYFYLFGLFSLIGVMVFGEVRMGAQRWIDIPG
FGSVQPSEFMKIGMPMMVAWFLARKPLPPSFSQVILSLMLIGVPFLLIAEQPDLGTSL
LVLASGIFVLFLSGLSWRMIGAAAACAAIVIPIAWEFLLHDYQRQRVLTLLDPEADAL
GTGWNIIQSKTAIGSGGFSGKGFLEGTQSHLHFLPEGHTDFIIAAYSEEFGLIGVLIL
VILYSAIIFRTFQIGLQSFHNYGRLVAGAFGLSFFVYVFVNAGMVSGILPVVGVPLPF
MSYGGTAIITLMATFGLVMSIHTHR"
/product="Rod shape-determining protein RodA" /transl_table=11
CDS complement(3253198..3253848)
/locus_tag="AUO97b_03142" /db_xref="SEED:fig|400667.82.peg.3007"
/translation="MPFFGFTPSEGLLNDIQTGIANKNSSEPLYPLRDKIALQLNEEI
IENVLTQLVQHFPASEKRDTAEKLAGYVKSTVAVLLKQLLSKAPNDVVKQSVEFSEKS
LFKDPQGQYKVGVALDAGLVTNLKHNFAELQAGNDINKAALAELYKQFGDAMVRHFMS
DFNKTLDLGMIKRKAADIGAAAVTKAVHIAIDKLIPSLNRTELKAMAEYHDGLFFN"
/product="hypothetical protein"
/transl_table=11
CDS complement(3253872..3254684) /locus_tag="AUO97b_03143"
/db_xref="SEED:fig|400667.82.peg.3008"
/translation="MSVEQSLLGKETQYPTSYQPDVLFPIARAQSREKYAHIEGITQG
KDWWHVFEISWLNAHGIPQVAIGRITLPASSPNLIESKSLKLYFNSLNFTQFDSTQSF
IETVEKDLSAAAGAKVELTLFQVDDLEISKPQGICIDDLMPERLEQHPDATLLKLDES
DEEIEVELYSHLLRSNCPVTGQPDWGTVFIRFKGKKPCYRSILAYIISYRQHNGFHEQ
CVEQIFADIWQNLQPEKLMVYATYTRRGGLDINPCRVSDLTWMPKPIRLARQ" /product="NADPH-dependent 7-cyano-7-deazaguanine reductase (EC 1.7.1.13)"
/EC_number="1.7.1.13"
/transl_table=11
CDS complement(3254681..3255454)

/locus_tag="AUO97b_03144" /db_xref="SEED:fig|400667.82.peg.3009"
/translation="MNFNQLQIALWTLVRKEVRRFLRIWPQTLLPPAITMSLYFVIFG
NLVGSRIGEMGGVSYMQFIVPGLIMMAVITNSYANVSSSFFSVKFQKSIEELIMSPVP
LHIILWGYVIGGICRGVLVGAIVTAMSMFFTDLFIHNWFVTIYTVLITSVLFSLGGFI
NAVYAKSFDDISIIPTFVLTPLTYLGGVFYAISALGPFWQKLSLINPIVYMVNAFRFG
ILGHSDVNVSFSLIIVTLCCAVLYGIAYYLLARGSGMRE" /product="Uncharacterized efflux ABC transporter, permease protein YadH" /transl_table=11
CDS complement(3255451..3256386)
/locus_tag="AUO97b_03145" /db_xref="SEED:fig|400667.82.peg.3010"
/db_xref="GO:0005524"
/db_xref="GO:0016887"
/translation="MTDALVLRDLSKTYRNGFQALKGINLTVPEGEFYALLGPNGAGK
STTIGIISSLTKKTSGTVEIFGHNLDTHPSLAKQQLGVVPQEFNFGQFEKAFDILVTQ
AGYYGIHRKIAEKRAEHYLEKLGLWEKRNIQARMLSGGMKRRLMIARAMMHEPKLLIL
DEPTAGVDIELRRSMWDFLTEMNENGTSIILTTHYLEEAEMLCRQIAIIDRGVIKEDT
SMKSFLNQLSEESFIFDLAEPIAPLQLNIIGVKFNLIDSSTLEVTMDKAHTLNDLFQL
LESQGIRVRSMRNKSNRLEELFVKMVEKNLEGAAK" /product="Uncharacterized efflux ABC transporter, ATP-binding protein YadG" /transl_table=11
CDS complement(3256680..3257363)
/locus_tag="AUO97b_03146" /db_xref="SEED:fig|400667.82.peg.3011"
/translation="MIEKIKQAASFLYWGKMMAGFYRTNLGRVALQQRNIALNAKQRR
LLLLIDHEDFQTLNTEFKKRIAPPELIQQLIDLKLIAPISENDSEFTEQIPLSESPTT
SLEVKAQQKSTIDESESADLTGEIKVSLEPSCHSSNIENTQPPIPVQQLTFEEIQLLM
KQSLSQYCGLMAKPLIQKIEQIKNLQELKMCQMQWITSLQESRIPPHELAHTLHSINY SIQLIQQKN"
/product="hypothetical protein"
/transl_table=11
CDS complement(3257460..3258164)
/locus_tag="AUO97b_03147" /db_xref="SEED:fig|400667.82.peg.3012"
/translation="MFKGPDMQAIILDTETHTLNGLPIEIAYAPIEINAGKLTLDKSK
LFDQLYQVGTPISYAAMAVHHILESDLENQPHYKTFKLPDTTTYIIGHNIDYDIAAIA
RCGVDVSHIKPICTLALARKTWPDAEAHNISALIYMISQGSSKARELLKGAHRADADI
ILTANILMHIVYHLNIQDIEELYRVSEEARIPTTINFGKHKGTAIAELPKDYIQWLLR QDELDVYLRKALESAF"
/product="Exodeoxyribonuclease X"
/transl_table=11
CDS 3258396..3259625
/locus_tag="AUO97b_03148" /db_xref="SEED:fig|400667.82.peg.3013"
/db_xref="GO:0004622"
/translation="MKMKLLMTSVLSTSLFLVACGGGSSDDGPATTNPSGTPTNNIQN
PVVKVEAYTSTNLGSVAAESSILTYKMLGQSGQEVQATSLVFTPNTPQPVGGWPIVVW
AHGTTGVADACAPSKAALADSTKDLISKLLAAGYVVVAPDYEGLGTPGIHPFLNVKSE
AFSITDAVVAARNYLSQRNLLTSKKWVTVGHSQGGHAALGAAQYASRAQLDYKGTVAV
APASNLGSILVDGEAQVANAPIDIKIGTYAQLDTYTALVTAGIRNTQPSFDYGQVFTS
QISSIAAQAENLCSGPLYGAFYEGMSNYAKDHNGTLDGFTRTQPNFMAVPLVKTFLDK
DSQPLQVKVTTPIIIYQGIADPTVPKLATDLLISNATAVGTKINNYVTGNWDHGTAMS SNVDNIVGNVQTLLSAQ"
/product="Lysophospholipase (EC 3.1.1.5)" /EC_number="3.1.1.5"
/transl_table=11
CDS complement(3259649..3259810)
/locus_tag="AUO97b_03149" /db_xref="SEED:fig|400667.82.peg.3014"
/translation="MNFIIKQNEVSSVLNKNGSSFIKNEPLFINQLKSSFKAYILNLS IVNFSLQQY"
/product="hypothetical protein"
/transl_table=11
CDS 3259871..3261124
/locus_tag="AUO97b_03150" /db_xref="SEED:fig|400667.82.peg.3015"
/db_xref="GO:0004372"

/translation="MFANISISEFDPELAQAIASEDERQEAHIELIASENYCSPAVME
AQGSKLTNKYAEGYPGKRYYGGCEFVDVIEQMAIDRAKELFGADYANVQPHAGSQANS
AVYLALLNPGDTVLGMSLAHGGHLTHGAKVSFSGKTYNAVQYGLNAETGEIDYEEVER
LALEHKPRMIVAGFSAYSRVVDWQRFRDIADKVGAYLFVDMAHVAGLVAAGVYPNPVQ
IADVTTTTTHKTLRGPRSGLILAKANEEIEKKLQSAVFPGNQGGPLMHAIAAKAICFK
EAMSDDFKAYQQQVVKNAQAMAEVFIARGYDVVSGGTDNHLFLLSLIKQDVTGKDADA
WLGAAHITVNKNSVPNDPRSPFVTSGIRIGTPAVTTRGFGEAEVRELAGWIADVIDSK
GDEKVIADVKAKVEAVCAKFPVYAK"
/product="Serine hydroxymethyltransferase (EC 2.1.2.1)" /EC_number="2.1.2.1"
/transl_table=11
CDS complement(3261165..3262616)
/locus_tag="AUO97b_03151" /db_xref="SEED:fig|400667.82.peg.3016"
/translation="MVITSKQNWLLSSLMGSLLLAGCSLAPEYQPAKVIVPVKFKESD
PKLEDNNWKIAQPADQQTRGEWWRIYNDTQLNELEQQAIAGNQNLKAVAANIQASRAL
RSAAQAERLPSIDAGFGPTRQKPSPASLGLDDNAHTSAQTLWRAQANVSYELDLFGRV
ASSVNAATADLQQQEALYQSALLALQADVAQGYFLIRQLDTEQAIYNRTIKLLGETRD
LMQLRFKNGLVSELDVSRAQTELATAQTTALNIARNRASAEHALAVLLGKPPADFNLA
VQPLTANSIRLPAGLPSTLLERRPDIAAAERAMAADNARIGIARAAFFPKLSLTGALG
YESSSLSELGKWSSRTFLLGPVAGTILSLPLFDGGQRKAGVAQARAAYEESVANYRQT
VLNAFREVENGLSDQRILDQQIQAQNQALSSSRHANQLSHLRYREGAISYLDVIDSDR
TILQQEQLAAQLKGSQIIASINLIRALGGGWSS"
/product="Efflux transport system, outer membrane factor (OMF) lipoprotein"
/transl_table=11
CDS complement(3262626..3265805)
/locus_tag="AUO97b_03152" /db_xref="SEED:fig|400667.82.peg.3017"
/translation="MNISKFFIDRPIFAGVLSVLILLAGLLSVFQLPISEYPEVVPPS
VVVRAQYPGANPKVIAETVASPLEESINGVEDMLYMQSQANSDGNLTITVNFKLGIDP
DKAQQLVQNRVSQAMPRLPEDVQRLGVTTLKSSPTLTMVVHLTSPDNRYDMTYLRNYA
VLNVKDRLARLQGVGEVGLFGSGDYAMRVWLDPQKVAQRNLTATEIVNAIREQNIQVA
AGTIGASPSNSPLQLSVNAQGRLTTEQEFADIILKTAPDGAVTRLGDVARVELAASQY
GLRSLLDNKQAVAIPIFQAPGANALQVSDQVRSTMKELSKDFPSSIKYDIVYDPTQFV
RASIKAVVHTLLEAIALVVVVVILFLQTWRASIIPLLAVPVSIIGTFALMLAFGYSIN
ALSLFGMVLAIGIVVDDAIVVVENVERNIEAGLNPREATYRAMREVSGPIIAIALTLV
AVFVPLAFMTGLTGQFYKQFAMTIAISTVISAFNSLTLSPALAALLLKGHDAKPDALT
RIMNRVFGRFFALFNRVFSRASDRYSQGVSRVISHKASAMGVYAALLGLTVGISYIVP
GGFVPAQDKQYLISFAQLPNGASLDRTEAVIRKMSDTALKQPGVESAVAFPGLSINGF
TNSSSAGIVFVTLKPFDERKAKDLSANAIAGALNQKYSAIQDAYIAVFPPPPVMGLGT
MGGFKLQLEDRGALGYSALNDAAQNFMKAAQSAPELGPMFSSYQINVPQLNVDLDRVK
AKQQGVAVTDVFNTMQIYLGSQYVNDFNRFGRVYQVRAQADAPFRANPEDILQLKTRN
SAGQMVPLSSLVNVTQTYGPEMVVRYNGYTSADINGGPAPGYSSSQAEAAVERIAAQT
LPRGIKFEWTDLTYQKILAGNAGLWVFPISVLLVFLVLAAQYESLTLPLAVILIVPMG
ILAALTGVWLTAGDNNIFTQIGLMVLVGLACKNAILIVEFARELEMQGATAFKAAVEA
SRLRLRPILMTSIAFIMGVVPLVTSTGAGSEMRHAMGVAVFFGMIGVTFFGLFLTPAF
YVLIRTLNSKHKLHSAAVHEAPLASPHDH"
/product="RND efflux system, inner membrane transporter" /transl_table=11
CDS complement(3265812..3267029)
/locus_tag="AUO97b_03153" /db_xref="SEED:fig|400667.82.peg.3018"
/translation="MSFSRKQFALSAIFVAILATGGSFMLLHENADAKAAPTAAQQAA
TVDVAPVVSKTITDWQEYSGRLEAIDQVDIRPQVSGKLIAVHFKDGSLVKKGDLLFTI
DPRPFEAELNRAKAQLASAEAQVTYTASNLSRIQRLIQSNAVSRQELDLAENDARSAN
ANLQAARAAVQSARLNLEYTRITAPVSGRISRAEVTVGNVVSAGNGAQVLTSLVSVSR
LYASFDVDEQTYLKYISNQRNSAQVPVYMGLANETGFTREGTINSIDNNLNTTSGTIR
VRATFDNPNGVLLPGLYARIRLGGGQPRPAILISPTAVGVDQDKRFVVVVDAKNQTAY
REVKLGAQQDGLQIVNSGLQAGDRIVVNGLQRIRPGDPVTPHLVPMPNSQITANATPQ PQPTDKTSTPAKG"

/product="RND efflux system, membrane fusion protein" /transl_table=11
CDS 3267227..3268258
/locus_tag="AUO97b_03154" /db_xref="SEED:fig|400667.82.peg.3019"
/translation="MDLFHAMRVFNKVVETNSFSLAADSLGLPRASVTTTIQALEKHL
QVRLLNRTTRKISLTPDGAVYYDRTARILADVADIESSFHDAERGPRGQLRIDVPVSI
GRLILIPRLRDFHARYPDIDLVIGLNDRPVDLVGEAVDCAIRVGELKDSSLIARRIGT
FQCATAASPIYLEKYGEPTSIEDLQKNHKAIHFFSSRTGRNFDWDFVVDDLIKSVSVR
GRVSVNDGDAYIDLALQGFGIIQGPRYMLTNHLESGLLKEVLPQWTPAPMPISAVYLQ
NRHLSLKVKVFVDWVAELFAGCPLLGGTALPFDQKCEFACDKETGHEYTIRTLVEQHN IAEAYTLKT"
/product="Probable transcription regulator protein of MDR efflux pump cluster" /transl_table=11
CDS 3268433..3269218
/locus_tag="AUO97b_03155" /db_xref="SEED:fig|400667.82.peg.3020"
/translation="MASFMKKIFVSTTLVLAAVATNVQAKDWKVIRFGTESSYAPFEY
KTPDGKLTGFDVDLGNAICAKLKAKCVWVENSFDGMIPALKAKKFDGILSSMTVTDER
AKQILFSSKIYNTPTRMVAKKGSPLLPTPTSLKGKRVGVQQGTIQETYAKTYWAPKGV
SVVPYPTQDLIYQDMMSGRLDATLQDAIMVDGAFLKQPKGKNFSFAGGNVVDVKTLGV
GAAIGLRKEDADLKANIDKALAAIIADGTYKKLEKKYFSFSIY" /product="Histidine ABC transporter, substrate-binding protein HisJ (TC 3.A.1.3.1)" /transl_table=11
CDS 3269310..3269999
/locus_tag="AUO97b_03156" /db_xref="SEED:fig|400667.82.peg.3021"
/translation="MSFSGYGPLLLSGTWMTIQLALLSLLLSVIIGLIGASSKLSNIK
ALRYIATAYTTLIRSVPDLVIMLLLFYSLQLGLNQITEALQMDQIDINPFVAGVITLA
FIYGAYFTETFRGAFQSVPRGQIEAAMAYGMTPWQVFHRVLFPQMMRFALPGIGNNWQ
VLIKATALVSIIGLTDIVKITQDAGRSTMQLFFFSIVAAAIYLAITTVSNLILMWLER HYSAGVRKGQL"
/product="Histidine ABC transporter, permease protein HisQ (TC 3.A.1.3.1)" /transl_table=11
CDS 3269996..3270709
/locus_tag="AUO97b_03157" /db_xref="SEED:fig|400667.82.peg.3022"
/translation="MIEILQQYWQSYLWTDGLQMTGVAMTAWLLVLSIGIGFLLAVPL
SLARVSENIWLRGPVWLFTYVFRGTPLYIQLLIIYSGIYSFDYIHEHQTLDAFFREGI
NCTILAFALNTGAYTTEIFAGAIRSIPHGEIEAAQAFGMSKWKLYTRIIIPSMLRRAL
PFYGNEVILMLHATTIAFTATVPDILKIARDVNAATYDTFSAFGIAAVLYAVLAFILI
WIFRKLEKRWLAFLKPSNH"
/product="Histidine ABC transporter, permease protein HisM (TC 3.A.1.3.1)" /transl_table=11
CDS 3270719..3271495
/locus_tag="AUO97b_03158" /db_xref="SEED:fig|400667.82.peg.3023"
/translation="MEQVAKLVIRDLHKTFGDHEVLKGISLDANAGDVISIIGSSGSG
KSTLLRCINFLEQPSNGTIKLNGEKLRTVFDKKGNLKVVEPKQLQKMRTQLMMVFQHF
NLWSHMTVLENVIEGPIHVLGVKRSEAEERARKYLRKVGLPESVESKYPAFLSGGQQQ
RVAIARALAMEPEVMLFDEPTSALDPELVGEVLKVMQGLAEEGRTMIVVTHEMGFARH
VSNQVIFLHQGKIEEQGHPDEVLNNPKSERLKQFLTGSLK" /product="Histidine ABC transporter, ATP-binding protein HisP (TC 3.A.1.3.1)" /transl_table=11
CDS complement(3271568..3271897)
/locus_tag="AUO97b_03159" /db_xref="SEED:fig|400667.82.peg.3024"
/translation="MSYLYLAIAIACEVIATSALKASQGFTVPIPSIITVVGYAIAFY
LLSLTLKTIPMGIAYAIWSGAGIILISAIGWIFYKQHLDVAACIGLTLMIAGIIIINV FSKNTHL"
/product="small multidrug resistance family (SMR) protein" /transl_table=11
CDS complement(3272133..3272396)
/locus_tag="AUO97b_03160" /db_xref="SEED:fig|400667.82.peg.3025"
/translation="MALLITSDCINCDMCLPECPNTAIFEGSKVYQIDPLRCTECVGF
YDAPTCKAVCPIDCIKPDPAHIENKVQLLEKFKGLNLLDESIS" /product="Uncharacterized ferredoxin-like protein YfhL" /transl_table=11

CDS complement(3272399..3273769)
/locus_tag="AUO97b_03161" /db_xref="SEED:fig|400667.82.peg.3026"
/translation="MKTELLSPAGSLKNMRYAFAYGADAVYAGQPRYSLRVRNNAFDH
ENLKIGIDEAHALGKKFYVVVNIQPHNSKLKNFIRDLEPIVAMQPDALIMSDPGLIMM
VREHFPEMQIHLSVQANAINWATVKFWKDYGLSRVILSRELSLEEIEEIQQHVPDIEL
EVFVHGALCMAYSGRCLLSGYMNKRDANQGACTNACRWDYKLHEAQEDLNGDVIPVTQ
LNTPEKSCCSSGQSETTSVQTLLVQRNDEEMFAAEEDEHGTYFMNSKDLRAVQHVDRL
TKMGIASLKIEGRTKSYFYCARTAQIYRKAIDDALAGKPFDPSLMTQLEGLANRGYTE
GFLRRHVHSEYQNYADGSSHFDYQQFCGEVVERHGDYIRIEVKNRFVVGDSLELMTPQ
GNIPFNLTEMRDLKGNTITDAKGSGHFVEIPLSQDVDISYALLIRNLPHAKENITAST LAYSAS"
/product="Uncharacterized protease YegQ"
/transl_table=11
CDS complement(3273826..3275223)
/locus_tag="AUO97b_03162" /db_xref="SEED:fig|400667.82.peg.3027"
/db_xref="GO:0004069"
/translation="MGSRLPSIRDCAQHYQVSINTVKEAYRKLEDQGLISVRPQSGYY
VCHTPSNLPDLEERYISEEKVPLSGISCFLAQIVKKQMDPDYVNLALACPSGENFYPI
ERLKRLTAQVLRTQPDIFKSYTLPPGSIRLRTQIARRSLQLGMFLSADDIVLTHGTME
ALSLAIMATTQAGDSIALEIPTFHNLYPLLQNLGRKIVEVPTSPHTGMCLDALEELLK
SQSAQAILTIPTGHNPLGFSMPESNRRRLAELANQFQIPVIEDAMYAELQLTEPLLPN
IKAFDQNGWVLVCGSYTKTVAPDYRIGWLEAGRFRNIVQQLKFTTTVAEPALLTETLG
LFLENGGYDLHLRQLKKRYQQQIDTIKSYICRYFPVGTRVSRPQAGFILWLELPESID
TLELFHQAMDEKILCMPGILCSADKRFSHCLRIAACFELNPKMLNALMRLGELAKNML PVQKAIDQIPTNSVG"
/product="DNA-binding transcriptional regulator, MocR family /
aminotransferase domain" /EC_number="2.6.1.1"
/transl_table=11
CDS 3275522..3276391
/locus_tag="AUO97b_03163" /db_xref="SEED:fig|400667.82.peg.3028"
/translation="MSVVAFKQVDVFTSQAFKGNPVAVIMDASTLTSEQMQAIANWTN
LSETTFVLPAIDSQADYQVRIFTPQSELPFAGHPTIGTAYALLEAGLIKAKEGRLVQQ
CAAGLVTLTVNDSNHISFELPKPKITPLDALQTEKLAEILKCKINTQWNAALVDVGAR
WVVLQAVNAEAVLASQPDLNALKQFSLDMKVTGSTIYGFYEESNEHKRIEVRSFAPAY
GVNEDPVCGSGNGSVASFIRYHGILPAQNDVVLSSQGQALGREGQLQLELHQDKILVS GAAVTCIDGTIKL"
/product="Phenazine biosynthesis protein PhzF like" /transl_table=11
CDS complement(3276446..3277225)
/locus_tag="AUO97b_03164" /db_xref="SEED:fig|400667.82.peg.3029"
/db_xref="GO:0004324"
/translation="MAAFNVERITHVHHWNDTLFSFKTTRDASLRFKNGQFVMIGLEV
NGKPLMRAYSIASANYEEELEFFSIKVQDGPLTSILQKVQVGDEILVSKKPTGTLVHD
DLLPGKNLYLLSSGTGLAPFLSIIRDPETYERFEKVIVVHGTRYISELAYQDLILNEL
PNNEFFEELGIKDKLVYYPTVTREPFHTQGRVTTAIETGALFEKIGLPRFNRETDRAM
LCGSPAFLKDVAALLDQHGLVESPRMGEMGDYVIERAFVEK" /product="Ferredoxin--NADP(+)
reductase (EC 1.18.1.2) @ Flavodoxin--NADP(+) reductase (EC 1.19.1.1)"
/EC_number="1.18.1.2"
/transl_table=11
CDS 3277363..3278058
/locus_tag="AUO97b_03165" /db_xref="SEED:fig|400667.82.peg.3030"
/translation="MCSPFREKFGIPRQPNLVNIESYIEMVEPYNDLLAFEGIEQFSH
LWLIWQFHDNKNQETATKFRPQVRPPRLGGNEKIGVFATRSMYRPAPIGLSVVRLNKV
EKVGKSLRVYVTGSDLLNGTPILDIKPYIHYSDAIVDAQSGYAQAEPERKSVLWSENA
LSTQQSLFKNKEINAQYLDELEQVLSLDPRPAYQEDPERVYKMKFSNFDIHFKVNGNV ITVVDMVKDTSLV"
/product="tRNA (adenine(37)-N6)-methyltransferase" /transl_table=11
CDS complement(3278068..3279336)
/locus_tag="AUO97b_03166" /db_xref="SEED:fig|400667.82.peg.3031"

/translation="MNLPMTMSLQTVSFEEFITTIAAALGCGLLVGLERERSKLKHEY
KTFAGFRSFAISSLLGAICFLFGTAIGIVGALLIGAISIVSLKNQPNDPGVTTELAFI
MTYFIGALCIWNISLAAGLAVIMTIILLAKQSMHGIASQWITESELRDGIFLLALLLI
ALPLVPNKPFWGPVLNPHVILKLLTLILFVQALAHIAKRLLSSKNALLLSSLASGFVS
STATIASLGLEVRSGRANAKTNAGAALMSCVSTLVQTLIIVVGISLAWFKLIIFPTLI
ALAFLAVWAFILLRKAEPSTTSPELDSRMFSLKEAVIIAGTLTLIQAGVYGLSLYLGN
AGLIAGTLLASLFEIHAAIAAVIVQGEPNNSQTSLLIAFMGGFAVHAIAKSINSAISG
GLHYALAFIPAQILHMTIFIGLLWMNIHWF"
/product="MgtC family"
/transl_table=11
CDS 3279624..3281381
/locus_tag="AUO97b_03167" /db_xref="SEED:fig|400667.82.peg.3032"
/db_xref="GO:0005524"
/db_xref="GO:0005622" /db_xref="GO:0006810"
/translation="MNIHVEIDTHWCLEQLLQEGRITERDKLLVQTTNRQKDQLKWHP
LQWIAHFNLKDQHHVQAHLSLNRLCQWFADRAQLPLFVIDPLKADVSALTQVMSQEFA
IRNHILAVEVHADRIVIGTDQPFQTDWLNNLEKSLAPKKIERVFLNPEQLQRYLREYY
QVSRAVNSAQKDVAHDRDNKGVEALLQLGDSQNPDANDQHIVKLVDWILQFAFEQSAS
DIHMESRKDNGKVRFRIDGVLHTIYNMPSNTLTAVISRIKILGRLNVAEKRKPQDGRL
KTRTPKGQETELRLSTLPTAFGEKLVMRIFDPDVLVRSFQQLGFDQSLLQQWQRITQN
SHGIILVTGPTGSGKTTTLYSSLKQLATEQVNVCTIEDPIEMLEPSFNQMQVNHAIEL
GFADGVRALMRQDPDIIMIGEIRDQDTANMAIQAALTGHLVLSTLHTNDAPSSLTRLH
DLGVQPFLTAATILGVLAQRLVRKLCPNCKQQTHINEDEWQHLTFDYIMEMPEKVYRA
VGCEECRHTGYKGRIGIYEFMPVSLELKHLISSHATLNELRCQTKKESVELLRIAGAR
KVIEGLTTLEEVLRVVPLN"
/product="Type II secretory pathway, ATPase PulE/Tfp pilus assembly pathway, ATPase PilB" /transl_table=11
CDS 3281463..3281822
/locus_tag="AUO97b_03168" /db_xref="SEED:fig|400667.82.peg.3033"
/translation="MKMFKVILMTAGMATAGVVVANTPVNQAAIAPATVTTVKQALAS
KDNTPVKLHGQVVKSLGDEKYQFRDKSGSITIDVDDELWQGRPVSANTNVTLIGEVDI DYKPLKRVEIDVDQVQF"
/product="Protein co-occuring with molybdenum cofactor biosynthesis protein B" /transl_table=11
CDS 3281930..3282592
/locus_tag="AUO97b_03169" /db_xref="SEED:fig|400667.82.peg.3034"
/translation="MRILLAEDDRSQAESIQSWLELDGYQVDWVERGDYALTAIEQHD
YDCILLDRGLPQLTGEKVLTTIRHKQKNVPVIFITARDSIHDRVEGLDLGANDYLVKP
FSLEELSARIRAQLRKQTLSQQSILTWGDLQLDTQAKVVLCAGKTIDLTAKEFQILRK
LMVHPEHIITRDQLEEALYAWGEEIESNAIEVFIYQLRKKIGSSSIKTIRGLGYRMGD LK" /product="Two-component system response regulator QseB" /transl_table=11
CDS 3282589..3283947
/locus_tag="AUO97b_03170" /db_xref="SEED:fig|400667.82.peg.3035"
/translation="MRSNRSSLQSQLVKTTMWSSIVVGLLALSLLTIFSIYHNMSVQD
EIMDEISDTLLVSDLSKHSMKQFDELSDEFDIQYELLDTGQVLTHSHTYQHELFEKGN
LSEGFSYFWFDGQLWRSLAAQQEDSELQVEVFQPISTRIEEVLKALAGYSGLMVLFWL
LQWVIVSWRTEQQLAALNLLSKRIAQKTASNLEPIQEPDVITEIQPVIDALNQLLARL
QRALVAEQRFTADASHELRSPLSAIQMRLQVLQRKYQHIPELDHDFERIQEDVSRSTK
ILENLLLLARLEPNETEQLELSKSVVDLNHVLARVIDTVNIDAQAKHMVIETNILSNE
TKTFANEELMFIAFRNLFDNAIRYSPALGAIHVELSQYQQKLKISIEDTGNGVDDEVL
RRLGQRFFRVLGTKQQGSGLGLSITKKIIQLHGGELHFMHASQGGLKVEVILPFNREI QK"
/product="Sensor protein qseC (EC 2.7.3.-)" /transl_table=11
CDS complement(3284013..3284183)
/locus_tag="AUO97b_03171" /db_xref="SEED:fig|400667.82.peg.3036"
/translation="MNAPFSLFTRNTDSAHALPMLHSNNLFALGREIRIMHAGEEYRL RLTRNNRLILTK"

/product="Hemin uptake protein HemP/HmuP"
/transl_table=11
CDS 3284320..3285219
/locus_tag="AUO97b_03172" /db_xref="SEED:fig|400667.82.peg.3037"
/translation="MPKPIVDVAIAILIHRGKILVGWRGEQQHQGGKHEFPGGKVEQG
ETPEEACRREIYEEVGIGLKDWHQFDYIHHEYDDIIVNLHLFHSYVPDELLNLIHQPW
TWYTREQLLHLNFPKANKDIIKRLYWPHFIKISHTLTSVENSDALLYWRIEDEFGPRE
VEQLTALDEGQRSNLIINVDIWQQLSSELKKQIKTVHLKQSQLMSLHKGDLEVGIRFI
AACHDAVSLQHAQQIGCDAVFVSPVKVTATHPDVSALGWDRFADLIEKCQIPVFALGG
MSPDDLATAQQHGAYGLAGIRNF" /product="Mutator MutT protein (7,8-dihydro-8-oxoguanine-triphosphatase) (EC 3.6.1.-) / Thiazole tautomerase TenI-like domain" /transl_table=11
CDS complement(3285241..3285792)
/locus_tag="AUO97b_03173" /db_xref="SEED:fig|400667.82.peg.3038"
/translation="MLKKGVFYIGAVLMVGCTTLPDHSAPKEKTPPVSEKKKSESSSG
VKITPYDHPEIQRKSLQVIVPQQKKPQRFNDDGSQLPAFKTLMQKTEQAYKNQQWSEA
ERYALQAQRLAPQAAETYLFLALTANHKQQYSNAESLARRGLSFAQSQAMKKQLWLVI
LKAGQQRNNPKTVQEAQQALKAF"
/product="hypothetical protein"
/transl_table=11
CDS complement(3285813..3288209)
/locus_tag="AUO97b_03174" /db_xref="SEED:fig|400667.82.peg.3039"
/db_xref="GO:0008955"
/translation="MKFERGIGFFALIFSILVIGAFIALSIYLIRLDNIIREKFEGQR
WDIPAKVFARPLEIYNNAPITQANFTQELKLLGYKTSSNYDKSGTYVAQGSNMYVHTR
GFDYGDSVEPEQVLELSFANDQVVEVRSTKPSSTGVARLEPLLIGGIYPQHNEDRVLI
KLNSVPKPLIEALISTEDRNFYHHHGISIRGTARALVSNVTGGRRQGGSTLTQQLVKN
FYLTPERTLKRKVNEALMALLIELHYSKDEILEAYLNEVNLGQNGNYSINGYGLASQF
YFGLPLRELNVAQQAYLVGLVQGPSLYNPWKNPEGAKNRRDTVLNNMRVMGYLTQAEY
ETEIARPLNVLSKPSLGPAKFPDFLDIVRRQLRTEYQESDLTNQGLRIFTTLDPIAQT
QVQNAFKASVERLANSNPARLKNLQGAVLIAHPENGELIAAVGSTQDFTGFNRALDAK
RQVGSLLKPVIYLSAIESGRYNWASQIEDAPISVPVDGGKSWTPKNYSGGGHGIVSLS
EALANSYNLSAVRLGQEFGLSTFTNNLRKFGVESTIPAYPSIFLGAVNMSPMEVLGIY
ENFATGGFKYPTRAIRSVVDANGRLLDRYGLNVQQTIDPSVGYIMNYGLQQVMSSGTG
RAAYNSLSPALKLAGKSGTTNDTRDSWFAGYSGNHVAVVWLGLDDNKVTGLTGSSGAL
PVWINVMKQLRQTPVNLRQPDSVQWQWIDRASGDLSAQACDGAMYIPMLAHTVPHRAT
PCGAPYYQVDPTYTPQSDNTIPEPQDDNTDSYIRESENQMEQDLSNNTRIISSGSYNN "
/product="Multimodular transpeptidase-transglycosylase (EC 2.4.1.129) (EC 3.4.-.-)" /EC_number="2.4.1.129"
/transl_table=11
CDS 3288395..3289303
/locus_tag="AUO97b_03175" /db_xref="SEED:fig|400667.82.peg.3040"
/db_xref="GO:0003951"
/translation="MQISHKSFRNVGLIGRPDKSSVVETLCLIHDHLLSLGLNPIFDQ
ETAELVPYDHAQVVSRHLLGEVADLVIVVGGDGSLLHAARALVRYNTPVIGINRGRLG
FLTDIKPSEAIFKLDQVLQGHFQLDRRFLLEMEVRTNGEVIYDAIALNDVVLHSGKSV
HMIDFELNIDGQYVVRQHSDGLIVSTPTGSTAYALSGGGPILHPSMDAIALVPMHPHT
LSSRPIVVGGQSEIKIVIRENRVLPMVSADGQHSVSLNVGDSLHIRKHPFKLSLLHPP
GYDFYMACRTKLGWNQDFESFQRDES"
/product="NAD kinase (EC 2.7.1.23)" /EC_number="2.7.1.23"
/transl_table=11
CDS 3289300..3289572
/locus_tag="AUO97b_03176" /db_xref="SEED:fig|400667.82.peg.3041"
/translation="MNIEQMLSVLNPEIVERLKTAVEIGKWPNGVALTKEQRETCMQA

VIAWELKNLPEEQRSGYIDRGTKEEGEVCEDDHHKHEPEFKPIRFV" /product="hypothetical protein"
/transl_table=11
CDS complement(3289774..3290787)
/locus_tag="AUO97b_03177" /db_xref="SEED:fig|400667.82.peg.3042"
/translation="MNTKRNIYKDAEHPFAQYVRILGKGKTGSRSLSYEEAYQAFTMI
LKGEVLDVQLGAFLMLLRVKEESIDELAGFVQATKDQLHFAPLEVDLDWSSYAGKRKH
YPWFLLAALTLAHHGYKIVMHGASGHTINRVYTEQVLEYLDYKICHSEQEVRTQLAEQ
NFAYLPLDMISPVLSELISLRNVMGLRSPIHTLARLINPFNAKATLQAIFHPAYRTSH
QHSALRLGYQNSAVIKGEGGEFERNPDAKTLICGIKNGELYEHELPKLTPERSPIEEE
LDLATFKAVWLGQQHHEYGEMAVTETMGIALYAMGVVSNFEEAMQKAKVLWETRNLSN LNS"
/product="Anthranilate phosphoribosyltransferase-like protein"
/EC_number="2.4.2.18"
/transl_table=11
CDS 3291286..3292644
/locus_tag="AUO97b_03178" /db_xref="SEED:fig|400667.82.peg.3043"
/translation="MAKKPIYKSLYFQVIIAIIAGVLVGHFSPSTTQIVNGVEQHIPG
LGEQLKPLGDAFIRLIKMIIAPVIFCTVVSGIAGMESMKSVGKTGGVALLYFEIVSTI
ALLIGLVVINIAKPGVGMNVDPATLDTSGIQKYVTSGESQSTIDFLMHIIPDTVVGAF
ANGEILQVLLFAILFGFALHKLGDAGRPVLKLIDQIAHVFFNIVNMIMKLAPIGAFGA
MAFTIGKYGVGSLVQLGQLIICFYITCLLFIFLILGTISRISGFSILKMIRLIREELL
IVLGTSSSESVLPRMLRKLEIAGCEKSVVGLVIPTGYSFNLDGTSIYLTMAAIFIAQA
TNTQLDIQHQITLLLVLLISSKGAAGVTGSGFIVMAATLSAVGHIPVAGLALILGIDR
FMSEARALTNLVGNSLATIVVAKWVGALDKDKLNDALNNPDEVDRKMLAPKNPQVAEG LD"
/product="Na+/H+-dicarboxylate symporter"
/transl_table=11
CDS 3292808..3293953
/locus_tag="AUO97b_03179" /db_xref="SEED:fig|400667.82.peg.3044"
/db_xref="GO:0004085"
/translation="MLAYDADLELFRDNFKRFMNEHIAPHYDHWEREGIMPRSVWSQL
GENGFLCVDVPEEYGGYGVPTYYSLMLVEESARAGFCALSTAISCHSEIAAPYILHIG
TEEQKQYWLPKMVTGEVVGAIGMTEPGAGSDLQSMRTSAILQDDHYLLNGSKTFISNG
QHADLVVLAVKTDPQARAKGVSLLLVDTHLEGFKKGTNLDKIGLHSQDTSELFFDNVK
VPKNQLLGQAGQGFAYLMQELPRERTAIASTAVGAIRGAIDLATAYVKERHAFGQPIS
QFQNTRFVLAQAKIDELATTAFYERNVALYQEGKLDVETAAALKSFSTDMQMKVADDL
LQLFGGYGYMTEYPISRFFVDARIQRIYGGTNEIMKEIVARGLIGKA" /product="Acyl-CoA dehydrogenase" /EC_number="1.3.99.2"
/transl_table=11
CDS complement(3293997..3294875)
/locus_tag="AUO97b_03180" /db_xref="SEED:fig|400667.82.peg.3045"
/translation="MAQAPVKNQSKIDFHNNQAQFQIKSKNTGHEYLIQIYKPPVAPP
QHGYPVLYILDGNATFPSAMNIAQSIGAGSAKLGLDPLIIVAVGYPQQKTFDVQKRAY
DYTPKPSAEFQAQGKYKYGGADQFIAFLNNELKPEIAKQFPINSQQQSLYGHSFGGLF
VLYHFFQKPTAFQRYFAASPSLWFDQGLLFRELEQWQSQKQTVFPMLMTTVGTHEQGG
PHTQFNQRINKDDFFKVLENKRSDQFSYWHFNNPAEQHITNLYASLPKALMFASCQNL ESCKSLFDEPSQKTAK"
/product="IroE protein"
/transl_table=11
CDS 3295127..3296236
/locus_tag="AUO97b_03181" /db_xref="SEED:fig|400667.82.peg.3046"
/db_xref="GO:0004106"
/db_xref="GO:0004664"
/translation="MINDDQNKTTSLSLEQIREDIDSVDQQIQELLNRRASLAEAVAK
AKFASEENPLFYRPEREAQVLRKVMERNQGPLSDVTMARLFREIMSACLALEAPQSIA
FLGPEGTFTQSAVLKHFGKDAVVRPLPTIDEVFREVEAGSAHYGVVPVENSSEGIVNH

TLDCFKTSNLNVIGEVELRIHHQFLVSENTRKDSIKQIYAHQQTLAQCRQWLDAHYPG
VERVALNSNAEAARRIRNEWHSAAIASDIAAGMYNLEILHSNIEDNPENTTRFLVIGR
EKIPQSGNDKTSLLISAHDRAGALLEILAPFAKHNISLTSIETRPALPEKWAYVFFID
LEGHIDQENVAAAINEIRPMVKELRILGSYPAAVL"
/product="Chorismate mutase I (EC 5.4.99.5) / Prephenate dehydratase (EC 4.2.1.51)" /EC_number="5.4.99.5"
/EC_number="4.2.1.51"
/transl_table=11
CDS 3296321..3298591
/locus_tag="AUO97b_03182" /db_xref="SEED:fig|400667.82.peg.3047"
/translation="MSKSKVEVMSQPLFKKVAFIGLGLIGSSLARVIVAEKLATTIVA
STRSQKTLEDAKSLGLIQEGFSDPVEAVKGADLVVLALPVRATQKVLEQIKPYLSETT
IVTDVGSTKGNVVDAAKAVFGEDLPAGFVPGHPIAGSEHTGVHAGKVDLFANHKVILT
PLPTSAEWAVEKLIQLWSAAKAEVICMDVAKHDEVLAHTSHLPHLMAFNLVEQLANRE
DNLDIFRYAAGGFRDFSRIAASDPQMWHDIFFANKTAILNAVDGFEKQLTVLRKLIEN
EDSHALMGLLGHAQAARQHFNHMLAKKPLMEKNKVTQQFSILPGNKAFKGKFTVPGDK
SVSHRSIMFGAIAEGTTHVTGFLEGEDALATLQAFRDMGVSIEGPKNGEVTIHGVGMH
GLKAPASALYMGNSGTSMRLLSGMLSAQKFDSVMTGDASLSKRPMERIAKPLRLMGAQ
IQTTGEKGTPPVSITGGQQLKGIQYDLPMASAQVKSGILLAGLWAEGETSVTEPEPTR
DHTERMLRAFGYDVKTEGNKISLVGGGKLVGTNIQVPSDISSAAFFMVGAAITEGADV
VLEAVGINPTRTGVIEILKQMGADLTVENERIAGGEPIADIHIKGSRTLKGIHMPEDQ
VPLAIDEFPALFIAAACAEGQTVLTGAAELRVKESDRIQVMADGLKIMGIDCTPTEDG
IIIEGKGKSGDWSPIFAGGEIESHHDHRIAMSFSMAGLRTSGPITIHGTETVATSFPT FTELANRAGLTIEVSQ"
/product="Cyclohexadienyl dehydrogenase (EC 1.3.1.12)(EC 1.3.1.43) / 3-phosphoshikimate 1-carboxyvinyltransferase (EC 2.5.1.19)"
/EC_number="2.5.1.19"
/EC_number="1.3.1.12"
/transl_table=11
CDS 3298719..3299102
/locus_tag="AUO97b_03183" /db_xref="SEED:fig|400667.82.peg.3048"
/translation="MKKLQFIASCPQHGVLDHPPNECHITQEYVAALLFKQLGNYGFD
AQHIVHEHEKVEVSIDNHLLPLSITCQQTDHEGHLMCEISANPDEEQDWFEKIETQSI
IRQLAQAVENSLKADHSFSAFEWKS"
/product="hypothetical protein"
/transl_table=11
CDS 3299273..3300733
/locus_tag="AUO97b_03184" /db_xref="SEED:fig|400667.82.peg.3049"
/db_xref="GO:0008769"
/translation="MTQVDIAALSPQVVWQHFQTLCTIPRPSKHEQQLREFLRNWAES
RNLETYVDEVGNLIIRKNATLGKEHVSGVILQGHLDMVTQANTGTVHDFFKDPIRPVL
EDGWLIAKDTTLGADNGIGVALALAVLNSNDIAHGPIEVLLTVDEEAGMSGARLLETG
VLKGKWLFNIDTEEWGELYLGCAGSIDVEVEQPLNYEPIPENLNIVNIQVAGLKGGHS
GVDIHLGRGNANVILARFLNQYLAPLGGHLVEFTGGTARNALPREAVATIAISPNQLS
SLEKLLAEYQTAWKEQLKGIDDNLQLSIQSTGAEVTEVITQQQQNEWLQALATSSYGV
ASMSQVLPDVVETSNNIGVVRLNRESGKAVLMVRSMVNQEAQNFAEKIQAHFSQFNIG
STLTPLVSGWTPNPDSAALKCLQQAYQNAFNIEPNLKVIHAGLECGIIAEHYPHLQMV
SFGPDIQGAHAPCERVKVDTVEKCWKLLVTALASVE"
/product="Cytosol nonspecific dipeptidase (EC 3.4.13.18)"
/EC_number="3.4.13.3"
/transl_table=11
CDS complement(3300769..3301815)
/locus_tag="AUO97b_03185" /db_xref="SEED:fig|400667.82.peg.3050"
/db_xref="GO:0003963"
/translation="MNTVLANMPKAIQIDGSQGEGGGQILRTALTLSMITGQAFELIN

IRAGRKKPGLMRQHLVCVQASQQISQAYVEGAELHSQRLYFAPQHVQSGKYQFQIGSA
GSTTLVLQTLLPVLLLQNEATELTISGGTHNPLAPTADFIEHCFLPALTKIGINVDFR
LNKAGFFPIGAGEIQVTIQPWQHRNSLKLLDRGALQCNEAFAAVLNISEEAQIAQREL
ITLSKKLNLSSQQQFHLNGISQGNTVYVKVTHQYHTQLFTALGEMRKSAEQIADHLAK
DVNLYLSSQAVVDEYLADQLLLPLALGQGGEFTAQCISEHTRTQAAMIEKFIDCEIEF VELNPHHFHVKVKV"
/product="RNA 3'-terminal phosphate cyclase (EC 6.5.1.4)"
/EC_number="6.5.1.4"
/transl_table=11
CDS complement(3302075..3302533)
/locus_tag="AUO97b_03186" /db_xref="SEED:fig|400667.82.peg.3051"
/translation="MKLAEALLLRSNQQKKLALLKQRINANVLVQDGDEPSEDPNELI
KQVFALTQESNQLICRIHSTNAQAKLEDGKILLSLLSLRDSYAEQHKILIDAIANTHR
EPDRYSSREIKWKKVIPVSSLQKQADDISAKLRDLNVKIQAANWQIDLIN" /product="hypothetical
protein"
/transl_table=11
CDS complement(3302546..3302929)
/locus_tag="AUO97b_03187" /db_xref="SEED:fig|400667.82.peg.3052"
/translation="MEKDCSDIQSYRIRANEEKHLILPEQPYYIILVVESTQILPEWR
NWICEQIVYSKHCIQAMVTGYEGSIWDDVLDENYLVLYNYQPPVDHCFMTTWHEDETL
EDITECAKITMQLKDISNLVIVHIE"
/product="hypothetical protein"
/transl_table=11
CDS complement(3302932..3304200)
/locus_tag="AUO97b_03188" /db_xref="SEED:fig|400667.82.peg.3053"
/translation="MSVIEEINQQAQFEQKQQQRSYEVLQNEKSMPVKMWTKGVLVDD
NSKQQLLQTAQMPFIYKWMAVMPDVHFGLGATIGSVIPTKGAIIPAAVGVDIGCGMMA
TRTSLTASDLPDNLHALRSELERLIPHGMTKRGRDRGSWETPPEMVDQAWTDLVADFE
YICAKHPRLKNTNNRKHLGTLGTGNHFVEICLDEHDHVWIMLHSGSRGVGNAIGNHFI
ELARKDMQKHFINLPNKDLAYLVEGTEHFDDYWFAVGWAQRFAMKNREIMMQSAIKAL
ATIVPKPFQARLEAVNCHHNYVEKEEHYGEEVMVTRKGAVRARLGEYGIIPGSMGAKS
FIVRGLGNQEAFCSCSHGAGRVMSRTEAKRRFTVEDQIAQTEGVECRKDAAVIDEIPS
AYKPIEDVMKAQRDLVEVVYTLRQVVCVKG" /product="RNA-2',3'-PO4:RNA-5'-OH ligase"
/transl_table=11
CDS complement(3304456..3305430)
/locus_tag="AUO97b_03189" /db_xref="SEED:fig|400667.82.peg.3054"
/translation="MLHFTFLGTSSGVPTLTRNVSGLAVRNSKNKDWILIDAGEGTQH
RIQQAKLSLQNLIAICITHVHGDHCYGLVGLLASAGMNARSKPLIVIAPKEIQQWFEI
TAQLTDLYLPYSLQFIDVNEAMQPQQITDEFIVQAHPLSHRVPSFAFSIYIKSIQKKL
DTKALTQLGVPQGDMWGHLKRGYNIEFEGQTLKAQDFIKIQNHQVHAIIGGDNDQPEL
LVDACKDAQLLIHESTYLQTVLDKVGKGPMHSSAKMVAEFAEQQSLSNLILTHFSPRH
QDKAGQQAITEEVRQFYKGHFYLANDFDEFTLSEAGQLLKVDKNEKTH" /product="Ribonuclease Z
(EC 3.1.26.11)" /EC_number="3.1.26.11"
/transl_table=11
CDS 3305498..3307099
/locus_tag="AUO97b_03190" /db_xref="SEED:fig|400667.82.peg.3055"
/translation="MKDAKKTVVIGFVGSTLDQGRKPDRWQRWRPTISLLMHEDLLVD
ELVLLHDRQHHNLVKSIAADAKEISPQTQVSGHKVSIKDPWDFAEVYGALYDFVKTYP
FDVEKNNYLLHITTGTHVAQICWYLLVDAHYLPAQLIQSSPNQHKTSEGEYRIIDLDL
SRYDVLKQRFDDEQKQNWQQLKANISTHNKAFNQLIEEVELVATRSSAPILIMGATGV
GKSHLAKQIFQLKKDKFQLSGRFIDVNCATLRGDSAMSTLFGHIKGAFTGAAASRAGL
LKSADQGILFLDEIGELGLDEQAMLLKALEDKSFFPVGSDKEIQADFQLIAGTNRDLR
NEVQAGRFREDLWARLNTWTFFLPNLKDRVEDIAPNIDFELQRFAAIHQRQLRFQRDA
LEIYLKFAKSADASWQGNFRDLAASVTRLATLSQGELIRRDAVEKEIQRLKQLWLIQD
ESYNDKDADILLSYLSPEQLEQIDEFDQIQLRGVLKVCENSKSMAEAGRQLFSVSRQQ

RNTTNDSDRVKKYLARFGLSWNNFQ"
/product="Transcriptional regulatory protein RtcR" /transl_table=11
CDS complement(3307157..3307789)
/locus_tag="AUO97b_03191" /db_xref="SEED:fig|400667.82.peg.3056"
/db_xref="GO:0050129"
/translation="MSEQIFIQGPVGKIELFVDRPEGEIKGFAVVCHPHPLQGGTPQH
KVPALLTQIFNEYGCIVYRPSFRGLGGSEGVHDEGHGETEDILAVIEHVRKLHAGLPF
YAGGFSFGSHVLAKCHAQLSPELQPVQLILCGLPTATVVGLRHYKTPEIQGDILLIHG
EQDDITLLSDAIEWAKPQKHPITILPGANHFFTGYLKQLRQIITRFIIMK" /product="Hydrolase, alpha/beta fold family" /EC_number="3.5.1.68"
/transl_table=11
CDS 3307916..3308689 /locus_tag="AUO97b_03192" /db_xref="SEED:fig|400667.82.peg.3057"
/translation="MLALISPAKTLDYETALPTDEFTQPRLLENSAQLIDVCRKLSAS
EIANLMSVSEKIATLNADRFRDWKPEFDFSNARQAIYAFKGDVYTGLDAYHLKDKDID
FAQQHLRMLSGLYGLLRPLDLMMPYRLEMGTKLKNTRGHNLYEFWDDIITNQINEDLA
AIKSELLVNLASDEYYKSVNEKKIKAEIVKPVFLDQKNSKYKVISFYAKKARGLMARF
IIENQLNKAEDLKAFNTEGYYFDADNSSAKELVFKRDEQ"
/product="UPF0246 protein YaaA"
/transl_table=11
CDS 3308715..3309332
/locus_tag="AUO97b_03193" /db_xref="SEED:fig|400667.82.peg.3058"
/translation="MQLYLLKLELFWFELHQHYHFSEPQKILNEHVAAYSEKQRAYHT
VQHLYECLSLIETIQSELNDPYAVALALWFHDVVYEPQALDNELKSAELFEQLMAQDL
QLDTMQKIKRWILATQKHAPTDETDLQFLLDIDLAILAATPERFIQYEQQIQQEYSWV
DPEVYSIKRKEVLMNFYQSERLYQTAYFQKNFELNAKQNLKKILE" /product="COG4339 metal-dependent phosphohydrolase, HD superfamily" /transl_table=11
CDS complement(3309469..3310254)
/locus_tag="AUO97b_03194" /db_xref="SEED:fig|400667.82.peg.3059"
/db_xref="GO:0009228"
/translation="MQDTPLIIGSRSFQSRLLVGTGKYKDLNETDLAIQASGAEIVTV
AIRRVNIGQNPDQPNLLSVIPPEKYTILPNTAGCFDADSAVRTCMLARELLDGHNLVK
LEVLGDEKTLYPNVTETLKAARTLIDDGFEIMVYTSDDPIIAQELESMGCVAIMPLGS
LIGSGLGILNPHTISIIKENAKVPVLVDAGVGTASDAAIAMELGCDGVLMNTAIAAAQ
NPILMASAMKKAVEAGREAFLAGRMPRKRMANASSPETGYFFK" /product="Thiazole synthase (EC 2.8.1.10)"
/transl_table=11
CDS complement(3310268..3310465)
/locus_tag="AUO97b_03195" /db_xref="SEED:fig|400667.82.peg.3060"
/db_xref="GO:0006790"
/translation="MQIYLNGELTDTPSQNLLQLIQELALEGKRFAVEHNQQIVPKSK
LEQITIAQHDRIEIIHAVGGG"
/product="Sulfur carrier protein ThiS"
/transl_table=11
CDS complement(3310479..3310844)
/locus_tag="AUO97b_03196" /db_xref="SEED:fig|400667.82.peg.3061"
/translation="MWIAISALNLAFAVMLGAFGAHGLKAHASPEQLAWWQTATDYFF
YHALGLLALGILSKVLPHFPIKLSFLLIQIGILFFCGSLYIMALGLPRILGAITPIGG
ALMIAGWLILAWNAFKYAK"
/product="FIG00018398: hypothetical regulator" /transl_table=11
CDS complement(3310956..3311822)
/locus_tag="AUO97b_03197" /db_xref="SEED:fig|400667.82.peg.3062"
/db_xref="GO:0003677" /db_xref="GO:0003700"
/db_xref="GO:0006352"

/db_xref="GO:0016987"
/translation="MSDSSNQLMPLSLSAPGVNLGAYISTVNQIPILTAEQEKELAER
YYYDQDLDAAKMLVMSHLRFVVHIARSYAGYGLPQGDLIQEGNLGLMKAVKRFDPNMG
VRLVSFAVHWIKAEIHEYVIRNWRIVKIATTKAQRKLFFNLRSLKKSSKKLTLEEAQS
IANDLNVTPEQVLEMEGRLTAYDAAFEAQGDDDDDTPHTAPVLYLEDNRYDPARLVEN
EDWEEQSTSALHDAMNQLDDRSRNILQRRWLDDDKSTLHELAAEYNVSAERIRQLEKN AMEKIKVAMSAS"
/product="RNA polymerase sigma factor RpoH" /transl_table=11
CDS complement(3311956..3312138)
/locus_tag="AUO97b_03198" /db_xref="SEED:fig|400667.82.peg.3063"
/translation="MPLLMLKRELKKLSGKQLFLLKSSDPHSEIDVTRYCQLHHFTCQ TMQISEREFHYLIETQ"
/product="Uncharacterized UPF0033 protein" /transl_table=11
CDS 3312576..3312788
/locus_tag="AUO97b_03199" /db_xref="SEED:fig|400667.82.peg.3064"
/translation="MTAREQGVVKWFNDTKGFGFIQRNGGDDVFVHFRAIVGDGHRSL
RDGQRVEFSVVQGQKGFQAENVQPLD"
/product="Cold shock protein of CSP family" /transl_table=11
CDS 3312892..3314043
/locus_tag="AUO97b_03200" /db_xref="SEED:fig|400667.82.peg.3065"
/translation="MTSGFETLNLHPQLKKAIDALGFTQMTPIQQKVLKYTLAGHDAI
GRAQTGTGKTAAFLISVINDLLNNPVQEQRFRGEPRALILAPTRELALQIESDAKSLT
KFSNLHLVTLLGGVDFDKQKKQLDANFVDIMVATPGRLIDFVEQKEVWLDQIEFLVID
EADRLLDMGFIPSVKRIVRYSPRKEQRQTLMFSATFSYDVLNLARQWLFEPVTVEIEP
EQKTNNDVEQRVYVVAKQDKYRLLQDILREEPIDKVMIFANRRDQVRRLYDHLKKDGY
KVGMLSGEIAQDKRLKMLEQFKQGKHNIMIATDVAGRGIHVDGVSHVVNFTLPEQSDD
YVHRIGRTGRAGAQGVSISFLSEDDAFYLPEIEKAIGKKLPLTRLDGYC" /product="ATP-dependent
RNA helicase RhlB (EC 3.6.4.13)" /transl_table=11
CDS complement(3314080..3314460)
/locus_tag="AUO97b_03201" /db_xref="SEED:fig|400667.82.peg.3066"
/translation="MKKIILASAMSGVVLLLGACATTPKNTLAIQKENNQFEVTGIGK
TNLIAKNNAVTAAQKTCSKSTTPVIVDEKTSYHGVLKDVVSEKTGQMVEAAAGVIGTL
TGKNASLAKDDDYQTTLTFYCKIN"
/product="hypothetical protein; putative signal peptide" /transl_table=11
CDS 3314664..3315239
/locus_tag="AUO97b_03202" /db_xref="SEED:fig|400667.82.peg.3067"
/translation="MTDSAIQIIHQNIHQRQSIGQLVEPAPNTDQLELAFQAALTAPD
HHRLKPTRFVIVSGDQRAAFGEVLAKALVDLGESDPAQLERVKQHPFRAPLLILALTQ
LQDHPKVPHFEQILSTGAAVQNLLLSLQAQGFSTMWRSGAVVESNWLKQHLGLQPHDL
ISGIIYVGTAAKAIAPRANIDSKEFVKVWQA"
/product="Nitroreductase family protein"
/transl_table=11
CDS 3315275..3316348
/locus_tag="AUO97b_03203" /db_xref="SEED:fig|400667.82.peg.3068"
/db_xref="GO:0047952"
/translation="MAEFKFTDLVEPVAVDKKTALRITVLGGGSFGTAMANLAARNGC
DTMIWIRDAETAEEINKTHINKRYLPDFTLESSLRAVSDLEQAVCDRDIILVAIPSHS
FRDVLKQIAPYITAQAVVSLTKGVEAKTFSFMSDIIREELPEVPYGVLSGPNLAKEIM
AGMPSGTVIASDSELVRYAVQHALHSALFRVFGSDDVHGVELGGALKNIYAVAMGIGA
AYKIGENTKSMILTRALAEMSRFAVKQGANPLTFLGLSGVGDLFATCNSPLSRNYQIG
YALGSGKTLEQASKELGQTAEGINTIVQVRGKAQELDVYMPITNALYEVIFEGAPPLN
IALSLMKNGHRSDVEFVLPHHEV"
/product="Glycerol-3-phosphate dehydrogenase [NAD(P)+] (EC 1.1.1.94)"
/EC_number="1.1.1.94"
/transl_table=11
CDS 3316405..3316860

/locus_tag="AUO97b_03204" /db_xref="SEED:fig|400667.82.peg.3069"
/translation="MQLTLVRHGEAAPPVNGNDIKRPLTARGHAQAEQTATFLKDIVK
PDIFVVSPLLRAQETLAHIQTYFKDVPVLLCDKIKPDDDAKEAIEWLSQIPYESIVVV
CHMNVVGHIAELLTHENFNPFALAEARIYDQAVIANGLSTQKNSFIPTI" /product="Phosphohistidine phosphatase SixA" /transl_table=11
CDS 3316898..3318022
/locus_tag="AUO97b_03205" /db_xref="SEED:fig|400667.82.peg.3070"
/translation="MPETNGVWHWSNGENWLQAASLDQLIQDLQIHQGKEATVFFPSR
HAQMLQQTMAKSHYKQLGADGVKYLLEEFVTLPIDHMKVVHHFHADQLTVLGVAQGMV
ETWQHSLALLPTKLVALLPDFLVLPEPQAQQVILCNIGHQLLVRENKWLGNSIDDLGL
FLEFQSAETHYQYSGLTAEQLESLEAASSAEQRSEFVYQFQPLDKTKQHPFNVLPKSK
GQERTFSNYWKTCAAVVLAIIVVQFSYDLLRWVKLKKVADQTAEQAIEQYKYWFGPSS
RVTEQNIKGQFESHLRMSQQGDTQALSLLSRVGPILMQRQILAQQLNYDASILTMALK
AKSADDLQALTQQLNQQGFKAELGNVQADGSGAIGVVKIQ" /product="General secretion pathway protein L" /transl_table=11
CDS 3318022..3318501
/locus_tag="AUO97b_03206" /db_xref="SEED:fig|400667.82.peg.3071"
/db_xref="GO:0006858"
/translation="MKMLAQLQNRFDQWIEQIVQYLDRLTVRERIMVVFTTIFVVVVI
VGYSLWKMHSLAEQQQKRLNDLKDLMVWMQSNAVTMKPANELELDKSGKIQRVAQQQG
LTVSSQQNGEQLQIVVTHQNYAILANFLTQLAQMGLSIQKMEMVSSEGQIKLTATVQ" /product="General secretion pathway protein M" /transl_table=11 CDS 3318591..3319595
/locus_tag="AUO97b_03207" /db_xref="SEED:fig|400667.82.peg.3072"
/db_xref="GO:0004158"
/translation="MLYSLARPMLFSLAPERAHELTLSMLDKAHKLGMMRQTVEAKPT
TCMGIEFPNPVGLAAGLDKNGAHIDALAGLGFGFIEIGTITPRPQSGNPKPRLFRIPE
AKAIINRMGFNNDGVDKLIENVKASKFRGILGINIGKNADTPVEKAVDDYLICLEKVY
NYASYITVNISSPNTKNLRSLQSGDALTELLQTLKARQLELAEQYNHYVPLVLKVAPD
LTAEDVEFISAQLLDFKIDGLIVTNTTLSREGVENLPYGNESGGLSGAPVFEKSTECL
RLFAQTLKGQIPLIGVGGILSGEQAAAKQQAGATLVQIYSGLIYTGPTLVKQCVEAMT "
/product="Dihydroorotate dehydrogenase (quinone) (EC 1.3.5.2)"
/EC_number="1.3.3.1"
/transl_table=11
CDS 3319719..3320189
/locus_tag="AUO97b_03208" /db_xref="SEED:fig|400667.82.peg.3073"
/translation="MGAKYAVLLAPSMSGLSQDPVVQKIAAFAFIALLIIVLTWIVTA
FLNGLLKSLKLGPLNRLAGGAFGSLKGLLVVLITMQGVGPWVESSPHWKQSKFIQFLL
PYAPLATELSKDAASEAFHQITSGGGVTRTSPKPMDESEETELRPDHSTKNPFY" /product="Colicin V production protein"
/transl_table=11
CDS 3320215..3321756
/locus_tag="AUO97b_03209" /db_xref="SEED:fig|400667.82.peg.3074"
/db_xref="GO:0004044"
/translation="MCGVVGIAGKSPVNQMLFDALTMLQHRGQDAAGIVTCHEGRLFL
RKDNGMVRDVFHTRHMRALLGNYGIGHVRYPTAGSSSSAEAQPFYVNSPYGITLAHNG
NLTNAEEIHDDLFKTDLRHMNTDSDSEVLLNVFAHELQKIGTLNPTPEDIFHTVSRVH
ERCQGAYGVVAMITGHGLVGFRDPNGIRPLIYGSRVTEQGEMEYIIASESVAITALGF
KIERDIAPGEAIFINADGELFTKQCAADPKYRPCIFEYVYFARPDAIIDGISVYKARL
KMGEKLAHKILRDWGEDHDIDVVIPIPDTSRTSALELANILGVKFREGFMKNRYIGRT
FIMPGQQQRKKSVRQKLNPVELEFKGKNVLLVDDSIVRGTTCNEIIQMARDSGAKKVY
FASAAPKVMYPNVYGIDMPAKTELIASERSVEEIQEIIGADRLIFQDLEDLKNAVRTS
KVPTLTEFDCSVFDGIYVTGGIDADYLNNLEQKRNDSAKKKKDGYIDVNIDAASVDLT GIKEE"
/product="Amidophosphoribosyltransferase (EC 2.4.2.14)" /EC_number="2.4.2.14"
/transl_table=11

CDS 3321835..3323724
/locus_tag="AUO97b_03210" /db_xref="SEED:fig|400667.82.peg.3075"
/translation="MKEVSHYLIENKNMAWSITMCLVAVLLTIFILNLILGLLVHFFD
YSEPIWWHALSPYFIALLLFIMVWSVVTELYILRKGGHSLAKQLKARRLVKGESTPEE
HAALKITEHLAQTFSLNVPTLYVLPDEVGVNALTAGFHSNDIVIILTWGALQNLDELE
LYGLLGHEFNQILSGEAVENTKLKILYSGLTTFSQWGSKLAKQGFKRYSPGYKHKFET
VFVAVGGVIWLAGSLGVLITRFIKYLTLSGRTFRNDQKTMRLLKNSTNTQTLLRIYVH
HSGSQIHSAYSESIAHMCFANSLSPQSWMNIHPSIRERIYELNPTLLQDLQLENLKKL
RNRPLFSLFHVLEESETEIYTPWSSPQPLPLLRLSPISFALNDAIKPLSSDIRRNKKR
PELIQRALQTATGSREVMVAILMIRQYREFIPQDAPVSHAIVDALLNLDGRIHIQIFH
DACKNIGHMPASIARQFLTKLALIIQEDGEIGLLDALLLERVKYELNLMPLHLPTAFE
EVKPQIVRLIDALLHVQQINSPNQLEVRERILRSLLNPDEMHIYDEISDEPLDLAEIL
NDIAGLLLRDRLSILAIAEMCLWSDRIITQDELDVLELLYWRFGFESDEIVEQMQKKN SVMII"
/product="Zn-dependent protease with chaperone function" /transl_table=11
CDS 3323825..3324475
/locus_tag="AUO97b_03211" /db_xref="SEED:fig|400667.82.peg.3076"
/translation="MIGQQRNILATLGIDVWIPRTQVCQKNNAHTLWRDQVVEPHESI
TVPTIDVPAFEQKNTQPQVLEIPKVVEEPPIVVAEVSQPEILVEKPKVIEQETITPFE
LQAYCLEKCVIFVDVTALETEEKQLWANIQKAKVGQYSELRWPFPLAAYQDQRGVGSY
IQGFLDAVAAEKKILCLGKCTYIQHANIIHLASLKEMLDKPLLKKRLWQLMQDNNE"
/product="hypothetical protein"
/transl_table=11
CDS 3324487..3325452
/locus_tag="AUO97b_03212" /db_xref="SEED:fig|400667.82.peg.3077"
/translation="MKKVVVFSQIDEEILSRLQQDYHVVVLNPKLGDINEQIRQEVVD
ADGMIGAGRLLNESNLSPAQKLKIISSVTVGYDNYDVGYLNQKKIWLANTPHVLTETT
ADLAFTLLLSAARKVPLLDHWTKQGEWKRTVGPQQFGLDVFGKTLGIIGLGNIGAAIA
RRGFYGFNMNIVYHNRREKPELAEPLKAQYLGLEELLQQSDFVVTAVDLNAESKALMG
KAQFELMQKHAIFINIARGSVVDEQALIEALQNEVIFAAGLDVYEKEPLQESALFTLP
NVVTLPHVGSATAETRKKMANLAYKNLVEALEDKTPRYLVNPNFV" /product="Glyoxylate reductase
(EC 1.1.1.79) / Glyoxylate reductase (EC 1.1.1.26) / Hydroxypyruvate
reductase (EC 1.1.1.81); 2-ketoaldonate reductase, broad specificity (EC
1.1.1.215)" /EC_number="1.1.1.215"
/EC_number="1.1.1.79"
/EC_number="1.1.1.81"
/EC_number="1.1.1.26"
/transl_table=11
CDS 3325591..3327027
/locus_tag="AUO97b_03213" /db_xref="SEED:fig|400667.82.peg.3078"
/translation="MKFLPVILSLALITNASYANPFDPKPASGQVEIPNIGSGIGLLD
QQKEKFIGEKVFREVHKQMPVIQDVWLEDQFFQVFSNILSETQLGQPIALVVIKDPQI
NAFAVPGGLFALNTGLISSARNIDEIAGVMAHEIAHVSQRHFSRSEEAFKGQTLLSLA
GLLAGVALAAQAGGDAGAAVMLGTQAALLDKQLTYSRNQEREADRIGMQYMYAAGYNP
QSMADYFETMHRATSRVSFLPDFWLTHPLTSERMSEARLRANQMPKVKSRIYDVDFEI
LKWYTMVVAGEATENQLQSLASQKNLAGLLALSAFYLKQGDYTQAQATLEQAKSSGKP
LVALIQTDIYLGQNKLDQAYNSIAPLQMTMPENKAFSYKLAEVLLRQGKYAQVQTLVQ
RFINKNARDIQGWQLLQQAANLDKNSPLRAVNVLRYRAEAQYWSGSEEDAIKSMLHAQ
RLAKGNQAMSARIDSRLKQMQDERRMKI"
/product="Exported zinc metalloprotease YfgC precursor" /transl_table=11
CDS complement(3327118..3327564)
/locus_tag="AUO97b_03214" /db_xref="SEED:fig|400667.82.peg.3079"
/translation="MTTLKNQITDALKTSMRAKDMATVTVLRSLQAAIKQIEVDERIE
LDDAQVLAVIEKQIKQRKESIKAFEGAGRDDLASKEQAEAEVLSQFLPEAMTEEELDS
LIEQTIAAQEATSMKDMGKVMNSLRPIIAGRADPAQVSAKIKAKLA" /product="Transamidase GatB

domain protein" /transl_table=11
CDS complement(3327591..3327806)
/locus_tag="AUO97b_03215" /db_xref="SEED:fig|400667.82.peg.3080"
/db_xref="GO:0003735"
/db_xref="GO:0005622"
/db_xref="GO:0005840"
/db_xref="GO:0006412"
/translation="MPQVKLKEGEPVDVAIRRFKRSCEKAGVLADVRKREFYEKPTQE
RKRKKAAAVKRYQKKLARESVRTTRLY"
/product="SSU ribosomal protein S21p"
/transl_table=11
CDS 3327952..3328962
/locus_tag="AUO97b_03216" /db_xref="SEED:fig|400667.82.peg.3081"
/translation="MIVLGLETSCDETGLALYDSELGLRGQVLYSQIKLHAEYGGVVP
ELASRDHVRKLIPLMNQLLEQSGVKKQEIDAVAYTRGPGLMGALMTGALFGRTLAFSL
NKPAIGVHHMEGHMLAPLLSSQPPEFPFVALLVSGGHTQLMAAHGIGQYELLGESIDD
AAGEAFDKVAKMMNLPYPGGPNIAKLALSGDPLAFEFPRPMLHQGLDFSFSGLKTAVS
VQLKKLNGENRDADIAASFQEAIVDTLVKKSVKALKQTGLKRLVIAGGVSANLRLREQ
LETSLARIKAQVYYAEPALCTDNGAMIAFAGYQRLKAGQHDGLAVTTTPRWPMTELTI PE"
/product="N(6)-L-threonylcarbamoyladenine synthase (EC 2.3.1.234)"
/transl_table=11 CDS complement(3328965..3329849)
/locus_tag="AUO97b_03217" /db_xref="SEED:fig|400667.82.peg.3082"
/translation="MILIGTPGALAFDHDDIITGNPRKTATFFSSTAALEQALLKAYI
IPEDTKINKDGTLIELGQEISIQGKFPHIHINGHYDGFDFDFDIDITSHVSWFIKTPI
YDHFSLLAKFKGFLNYQAKHIETQGLCTYEYARAVGPHSITNKLISDAYKLPLDFFTY
QIINLNEATQLLLTKADIAGQTAAYTLHIRHLDQPAEIYTDVSFDIISHQVDDFVSPS
GQKMRLPKYFSWIARNDAKQIILNIQAEIDCPFRYGHGRGYASSYIFTGHYFGNEVQG
RGYIEYIDIENPQAFEDE"
/product="hypothetical protein"
/transl_table=11
CDS complement(3329812..3330069)
/locus_tag="AUO97b_03218" /db_xref="SEED:fig|400667.82.peg.3083"
/translation="MKKQLPKISTTGKALAKSLLLAQGVLDQAKKYSTLPFTQTHIIR
PRIDEKYYSWTHYGIFFPIFTRAASLLKYYDFNRYTRCTCF" /product="FIG00349962:
hypothetical protein" /transl_table=11
CDS complement(3330173..3331054)
/locus_tag="AUO97b_03219" /db_xref="SEED:fig|400667.82.peg.3084"
/translation="MATIDLPDNLVQTLSLVLNQLQQVLPEPKQETDFTAPAFRWENQ
QLKAIYTPKNIYLDDLKGIERQKEKIIQNTLQFLNGLPANDVLLTGSRGTGKSSIVRA
LLTEYAPQGLRLIEIERDDLADLPKIQKIIQNRPEKYIVYCDDLAFNAEDENYRSLKS
VLDGSLQSGSTNFIIYATSNRRHLLPEFMHENTPVTKVDVPQYTELHPQEAIEEKISL
SDRFGLWLSFYPMDQNLYLEIVEHYLDKANMPFNDEVRAESLRWCQMRGQRSGRAAYQ FSKHWIGLNALKDLSNN"
/product="ATP-DEPENDENT PROTEASE SUBUNIT"
/transl_table=11
CDS 3331233..3332741
/locus_tag="AUO97b_03220" /db_xref="SEED:fig|400667.82.peg.3085"
/translation="MFEKIRSDMNPHQAYRIKKLQRQLDMAESYEEWKSFALKLDEET
GAQEWKFDNSSPYFDAELISYRYTLLKRYRQQHRTLDLIYLLKEGLTYDIANIGHPML
FAATHVGTKKLIEDYIEEVSQSLAYIASSECITFQRKEKIEFFENCEKAYGQPALMFS
GGATLGLFHTGVCKALIEQDLMPKVLSGSSAGAIMTGMLGISASEDIQNLLNGEQFFS
DAFHFRKLRELIKGNGGIADVHYLKKFLIENLGDLTFEEAFKKSGLNINVAVAPYDAT
ENPRIMNAIMTPNVLVWSAVLASCAVPVLFPPVRLTSKRYDGEHTPYMANTKWVDGSV
RSDFPQERMARLYNLNYTIASQVNPHVVPFMQDDARRFRKDVLSWPERILRRQGKVLS
MGLMDFTRQRLGAISPVRRLLDHGYGVVGQRYYGDVNIIAKYSLKHYAYTLQNPRPHL

FKRLQREGERATWPKISSIETHARIGKTIQHCLEVLRFEEKKQQPESYYAEA" /product="hypothetical protein"
/transl_table=11
CDS 3332857..3333531
/locus_tag="AUO97b_03221" /db_xref="SEED:fig|400667.82.peg.3086"
/translation="MPNVHAQSEQDFLHELQFYEDITYKKANWLLPFKIIEGRTICQQ
TQFRGKVLVLPDTDCWFDDLDQKQNLKNINEKIYLALVKLAELHELGWIHGDIKKEHF
RKFKQELYLIDFEKTRLISSPDPITDATPRYMAPELFHGANKTVQSDLYALGIVLYEW
LTQTRLQANSYHEWAVLHCQKLNVELPSSFQIFLPLLSGLLQKQQQNRFSNVHEAINC LKALPT"
/product="Serine/threonine protein kinase"
/transl_table=11
CDS complement(3334160..3335077)
/locus_tag="AUO97b_03222" /db_xref="SEED:fig|400667.82.peg.3087"
/translation="MKPKKLNQVTDFDIKLLKIFKTVCDCHSFTSAESILGISRSAIS
LHMSDLENRLGIRLCQRGRAGFALTDEGREILEYIEVLTAAIEDFRSKVNQMHNRLKG
EFNIGIINNLVTMPRGYITNTLTQLAEEHAEVIINISMSTLSDIECRVLDNRLHAGVI
PLVMPLSGLDYFDLYSESSFLYCGKNHPLFNQCSSINLNELKKWQAILPNYAITSEAV
KLHQLLDCKATASDREGIAFLILTGKFLGFLPDHYAKKWVEDGVMQPVLKDKMHYSTP
MCLITHKGKSHNNILKTFMEMLEKRIANN"
/product="LysR family transcriptional regulator PA0133" /transl_table=11
CDS 3335181..3336557
/locus_tag="AUO97b_03223" /db_xref="SEED:fig|400667.82.peg.3088"
/db_xref="GO:0016223"
/translation="MFDTDKFSDSEHTLDAVQTNNNMHINYQAHWMPFSANRNFAKDP
RMIVGAKGSYLIDDSGREIFDSLSGLWTCGAGHTLPEIQQAVSAQLGQLDYSPAFQFG
HPLSFKLADKIVQHMPGKLQHVFFTNSGSESADTSIKMARAYWRIKGKPSKTKLIGRA
RGYHGVNVAGTSLGGIGGNRKMFGQLMDVDHLPHTLQPDLTFTKGCAETGGVELANEM
LKLIELHDASNIAAVIVEPISGSAGCIVPPTGYLQRLREICDEHDILLIFDEVITGFG
RLGTWTAAEYFGVTPDILNFAKQVTNGAIPLGGVVASHEIYSAFMQQDLPEHAIEFTH
GYTYSAHPVACAAALAALEILEKKNLLAQSAALAPSFEKMLHGLKGAPHILDIRNCGL
IGALQLAPRDGDATIRGFELGMKLWKEGFYVRFGGDTLQFGPMFNSTEADIDRLMNAV GDALYQVN"
/product="Omega-amino acid--pyruvate aminotransferase (EC 2.6.1.18)"
/EC_number="2.6.1.18"
/transl_table=11
CDS 3336668..3338236
/locus_tag="AUO97b_03224" /db_xref="SEED:fig|400667.82.peg.3089"
/db_xref="GO:0004491"
/translation="MTNGLEDFSYDEVSTENSTHSEQAKKVLGHFIQGRIVSKTARKQ
PVYNPATGEISKEVEIADAQTVNEAVQAAEQAFPAWRDTPVIKRARVMFKFKQLLEQN
AEKICALIGQEHGKISHDAQGELQRGIENVEYACGAPELLKGEFSKNVGPDIDSWSEF
QPLGVVAGITPFNFPAMVALWMFPMALVCGNCFILKPSEKAPSVVLFLAELLKQAGLP
DGVFNVVNGDKEAVDALLHHPRIQAVSFVGSTPIAEYIYRTATSTGKRCQALGGAKNH
AIIMPDADIDNVVTSLLGAAFGSSGERCMALSVAVAIGDEVADVVIDKLTQEMKKLKF
GNYADASNDFGPLITQAHKDKVQAYIQSAEQQGAKIVVDGRDAKPAGYEKGFFVGPTL
IDQVTPNMTSYQQEIFGPVLQVMRVNTMQEAMQLINDHEYGNGTCIYTRDGEAARYFS
SHIQVGMVGINVPLPVPVAYHSFGGWKRSLFGDLHAYGPDGVRFYTRRKTITQRWPSA QVREAKQFSMPTLN"
/product="Malonate-semialdehyde dehydrogenase [inositol] (EC 1.2.1.18)"
/EC_number="1.2.1.27"
/transl_table=11
CDS 3338339..3339736
/locus_tag="AUO97b_03225" /db_xref="SEED:fig|400667.82.peg.3090"
/translation="MNNDRTKKLGEGLSNRHITMISIGGVIGAGLFVGSSSAIAKAGP
AVILAYLITSIMVFLVMRMLGEMAVLEPDTGSFSTYARKAIGPWAGFTIGWLYWWFWV
LAIPVEAIAGAEILHSWFPSVSVSIYAFCFIVFLSLANFFSTKSFGEFEFWFSLVKVV

AIIGFIIIGILAISGIWPLAKNVSGVANLYNNAGFMPHGMGGILSAILITAFSFFGVE
IVSIAAAESSNPKQKIRRATNLVLYRIILFFVVSMFIAVSLVDWRSPDLQKYGTFQYV
LMSLNVPGTKLIIDTVVFIAVASCMNAAIYTSSRMLFRLGTRNEAPQAVTKVNYAGVP
AIAVFSSTFIGLVCCVINYVFPGKVFGLLLSSTGSIALLVYLVIAFSQLRLRHKLEAA
GAEIPFKMWLFPWLTWFVIIIILSVLAYMFFSPAYSYETTLSLGVTLGVVICGLFVTR KRKATRSVAINQSNL"
/product="gamma-aminobutyrate (GABA) permease" /transl_table=11
CDS complement(3339885..3340118)
/locus_tag="AUO97b_03226" /db_xref="SEED:fig|400667.82.peg.3091"
/translation="MNTLNINDIKKHADVIASCGTKVGTVDHLEGENQLKLTKDENDQ
HHLIPTSWIGEVKEDQVILNKNSEEVKENWQAI"
/product="Bsr8028 protein"
/transl_table=11
CDS 3340417..3341391
/locus_tag="AUO97b_03227" /db_xref="SEED:fig|400667.82.peg.3092"
/translation="MLNEFEHAQAENKSQLRQDLLYQLVSLSKSLPYPASGHTYQRWQ
LFAQIAGYDLSLAKLFESHHDALSILNELGYQDEIDDHTWAVWAAEGGPAPIQVQNNH
CNGIKTWCSGAEFIQKALMSYKDKEGQAQLCIVDLAHPSVHVDLSHWQAVGMQGTQTA
QVHFDNTPVIPIGLPNCYLERPGFWHGAAGVAACWYGAAVRLVSFLHKSCTLNPNAFK
KMYLGELAQQLSVTKQYFQYIAKLIDDEPALSHEREIRILRAQTEQCCQSVIQLVGKA
LGARPYCEEPTFSQLIADLPVFIRQSHAAFDYESIAELCLPEKSLWEL" /product="Acyl-CoA
dehydrogenase/oxidase domain protein" /transl_table=11
CDS 3341415..3342173
/locus_tag="AUO97b_03228" /db_xref="SEED:fig|400667.82.peg.3093"
/translation="MISGEGTPEFMWLEALKKNPLDRLNLKLFQSKRVVIVAPHPDDE
VLGCGGLMQQLVEQNCHIVILAVSNGTQSHPHSVKYTPDQLNDLRPQETLAALNTLGI
SAFSERIGLNLMDGQIHLQTDQLNQALSQIVQPEDILICSYALDGHPDHEAVGKTVQA
FAEARDLLCLHVLIWAWHWAEPFDTRINWSKAKAYALTENQLIKKHQAVMQFKTQLEA
DESTGNSAVLSSSAISRLLMPYEVYLSDSFSCVF"
/product="LmbE-like protein" /transl_table=11
CDS 3342148..3342747
/locus_tag="AUO97b_03229" /db_xref="SEED:fig|400667.82.peg.3094"
/translation="MIHSRVYFEELYRSNNDPWGYDYHWYEARKRQICLALLTRPHYE
KVLEIGCSNGHLSVYLAQRAAHLLCIDVSKQAVQLASQRLEAFEHVTVENRKIPEDFY
EQKFDLIVISEVAYYLTFDELGEFIEQLKQALNPEGEILCCHWRHDIQDFELNAQKVH
QSFQQSFPFHHYLSLNDPDFMVDVWTVNPSSIAQREQLR" /product="Methyltransferase type 12"
/transl_table=11
CDS 3342744..3343388
/locus_tag="AUO97b_03230" /db_xref="SEED:fig|400667.82.peg.3095"
/translation="MKIGVVVPAHNEEQHLPACLQSIQEAIEKVPDEQVEVMVVLDSC
TDQSRSIVQRYGVSWIECNYACVGKARDLGIRQLIQNGATWLACTDADSVVSPDWLRC
QILHQPTDAICGIVTLDDLSRLSVMKQKKYLSHYQDCMDHHHIHGANLSFSAAAYMQV
GGFEPVPCHEDVSLIQKLIRQCCNITWSNLVRVTTSSRLEGRAPEGLSKFLENL" /product="Glycosyl
transferase, group 2 family" /transl_table=11
CDS 3343675..3344037
/locus_tag="AUO97b_03231" /db_xref="SEED:fig|400667.82.peg.3096"
/translation="MKKILFTTLTGLVLLTSSAAFARTDPALLNQAAKNVVTVSKAKT
LADETGVTLTGTIVKHIAGDHYEFKDKTGSIVIDVDDDLANGWQLKVGDKVRIVGEVD
THRVKPTEIEVLQIERVK"
/product="hypothetical protein"
/transl_table=11
CDS 3344201..3344671
/locus_tag="AUO97b_03232" /db_xref="SEED:fig|400667.82.peg.3097"
/translation="MNVRLCYASQRNEKNEDLLQDLRDILTEARDFNDLNGICGVLYY
ADNAFFQCLEGEQEVVERLFEKIQKDQRHYNIKWLCTYSIDEHSFQRWSMKYVQRNTN

IETFFLNMGENTFNPLLLNQQNLKFFLNELLIAEQTKMNTVKKVGMVNRGVNPF"
/product="hypothetical protein"
/transl_table=11
CDS complement(3344668..3344994)
/locus_tag="AUO97b_03233" /db_xref="SEED:fig|400667.82.peg.3098"
/translation="MFIAKNIFVYLLSMLALCLLIILFNYLGMNQTLNLIISSALFGI
FITWYYKGSGLCIALFSFFYWAMFVISQSLEVIWMLSISVIVYLVMTQIVPKLKHIHI GITSNS"
/product="FIG00351612: hypothetical protein" /transl_table=11
CDS complement(3345561..3346166)
/locus_tag="AUO97b_03234" /db_xref="SEED:fig|400667.82.peg.3099"
/translation="MENLTAFSLFAIVASITPGPTNFIILSLSSHYKISKTLPVILGS
CIGAALLVLVVGIGLGSTILAYPAIQKIMTWSGLIWLTVLAWKLYNYNPVISLEKNEQ
YPPIGFKAAFLMQAINPKTWMMAFAVISVYTKQGQDILINVSILSCIFLLIAFPCLYL
WALVGRLSTRLLSKPKHINIFNKIMAIILLASVWWPMFPRF" /product="Transporter, LysE family"
/transl_table=11
CDS complement(3346217..3347053)
/locus_tag="AUO97b_03235" /db_xref="SEED:fig|400667.82.peg.3100"
/translation="MSSNWVDISKDTNTGIETIRAHFTGHAYDPHWHSSYLIGVTESG
IQQFHCRKKQINSHAGQVFMLEPEEIHDGHAPASDGFTYQMLYLHPEWLKQRLQGLFH
NSPDHYELVIDAPLKSDDQLAIITSQAFRHLHYKEMNIVKDNSLDVLIQYIAQGKWLK
KASQSSCIHVPQIAHQIRDILHDHLFEDIGLEDISRMVGLDRYHIHRIFKSTFHLAPH
QYLIQLRLTRARDLLARSISPAQVASDLCFSDQSHLSRWFKRCYGLTPAAYRKICTNL QD"
/product="Transcriptional regulator, AraC family" /transl_table=11
CDS complement(3347211..3348359)
/locus_tag="AUO97b_03236" /db_xref="SEED:fig|400667.82.peg.3101"
/db_xref="GO:0008415" /db_xref="GO:0042619"
/translation="MSLLLKVRQNVPQVQSIKTSLKKRAGQKIPHMLSNASDLFFRGS
EIALSGKTPFDVIYKHEIISLRHYRIETEASAKHRVPLVIVPPLAVNMLIYDLFPTRS
LIRYFLDQGFEVYLIDWGVPTRNQAKYNFGTYVKVFMPEMLKQVRIHSGQQQLSLHGW
SLGGALSLCYTALFKDKDIKNLMILASPIDTHKAGYMGKLYGSLTKPAQWVRKHTPFR
IRQHVPSEVFHIHGWQNTLGFKLTDPIGNLKTYWQLLKNLDNREFIVDHATSSSFIDN
MLAYPGGVMRDIILRFWIDNELSTDVIKFGELTAYLKDIDCSVLAVGGDTDIIVTADA
VKPLMDLISSQDKTFKTVPGGHMGVVSGSQAPTSVWPEMSNWLATRSD"
/product="Polyhydroxyalkanoic acid synthase" /transl_table=11
CDS 3348720..3349955
/locus_tag="AUO97b_03237" /db_xref="SEED:fig|400667.82.peg.3102"
/translation="MEFVFNGFYTLISAVIVLLLGRFLVNRIDFLKRYNIPEPVAGGL
VAAVVSLLVHTFWGYSIVFSSELQTSFMLVFFASIGLSANFMKLKEGGTALVIFLICV
ASFIVVQNAVGMSLATLLGLDPLIGLIAGSITLTGGHGTAGAWGEILESQHGIQGALA
LGMASATFGLIIGGVIGGPLAKLLINRYSLAQAKTNAEIQQRDTHVEQNSDDLAPFEN
PHQVRLITADNAITTLGMFAACLAFAEFMTGFSKGTWFELPTFVWALGGGVILRNILE
SVLKVDIFDRAIDVFGNASLSLYLAMALLSLKLWQLADLAGPLVVILGAQTLTMALYA
AFVTFRVMGKNYDAAVLAAGHCGFGMGATPTAVANMQAITNMYGPSHKAFLIVPLCGA
FFVDLINATVIQLMLKFIA"
/product="Sodium/glutamate symporter"
/transl_table=11
CDS complement(3350008..3350910)
/locus_tag="AUO97b_03238" /db_xref="SEED:fig|400667.82.peg.3103"
/translation="MELSQTISNLIFEIQKLTQAVEKISKTQEISVDVLDEAIAFRWE
RENNKGSLVAIRKPQLISFNHLCNIDSQLTKVKLNTEAFAKGLLANNVLMTGSRGTGK
SSIVKACLNEYHHLGLRVIELEKKYLEDLPKIINLLQDRTERFIIFCDDLAFEAGDSS
YATLKTVLDGSLASSSDNTLIYATSNRKHMVAEYNKDNTELNVGENGELRPGDSIEQK
ISLADRFGLQINFYGFSQQEYLKTVQYWLNEYKWQQRETWETIQLKAIQYATQQGNRS

GRIANQFAKMIVGQEMLSAADLTV"
/product="ATP/GTP-binding protein"
/transl_table=11
CDS 3351007..3351705
/locus_tag="AUO97b_03239" /db_xref="SEED:fig|400667.82.peg.3104"
/db_xref="GO:0003677"
/db_xref="GO:0003700"
/db_xref="GO:0005622"
/db_xref="GO:0006813"
/db_xref="GO:0008324"
/db_xref="GO:0009058"
/db_xref="GO:0030528"
/db_xref="GO:0045449"
/translation="MIEQIQKRSLVDEVIHVIRQNIKNDIWKVDEKIPTEPELVQGLG
VGRNTIREAIKILEYLGVLEVKQGLGTFVRTKNDFSIVINSIHHSDLYEHVEVRCLLE
IEIAKLAARHSTPEDMRDVIACLEHRAATNQQDVANFMLSDKKLHLAIAKATHNKALQ
ATYEYFLNSSYQYTLELVTNKNLPDPNQQIHSELVQAIQHKSESEAMRIAESMLAPIL RSLDSIKADFVQNH"
/product="Transcriptional regulator, GntR family" /transl_table=11
CDS 3351793..3351924
/locus_tag="AUO97b_03240" /db_xref="SEED:fig|400667.82.peg.3105"
/translation="MTAYYWATHQSQLALFDSYELAYRFYFPSRHILIRSEIKAFAQ"
/product="FIG00352060: hypothetical protein" /transl_table=11
CDS complement(3351993..3352631)
/locus_tag="AUO97b_03241" /db_xref="SEED:fig|400667.82.peg.3106"
/translation="MIKIDTFDTMQIKKSFTTLKGQERIKQILRNAEIVFLTKGYSGF
SIRGVATQSNISLSTLQHYFQNKDILLKALLNKLICDYIQRIEILINLNANEPPLIRF
MNIITNIIYEIEQPIITNAFKEFFSISDHLPYVYEALSIIQKYNLELIYKIILPIHNK
ISSEEYKERAIIIITQLNGYLVQHSNKNTDESYKEFLRSILLKNILRLVSEP"
/product="Transcriptional regulator, AcrR family" /transl_table=11
CDS 3353215..3353751
/locus_tag="AUO97b_03242" /db_xref="SEED:fig|400667.82.peg.3107"
/translation="MKNIQKSLLAALIVAGYAVNTQAAVTGQVDVKLNISTGCTVGGS
QTEGNMNKFGTLDFGKTSGTWNNVLTAEVASAATGGNISVTCDGTDPVDFTVAIDGGE
RTDRTLKNTASADVVAYNVYRDAARTNLYVVNQPQQFTTVSGQATAVPIFGAIAPNTG
TPKAQGDYKDTLLVTVNF"
/product="Sigma-fimbriae uncharacterized paralogous subunit" /transl_table=11
CDS 3353826..3354374
/locus_tag="AUO97b_03243" /db_xref="SEED:fig|400667.82.peg.3108"
/translation="MIFNRGSAFIISYFLISLVNAGEIGAKLTSQIELLPSCSVNNNV
VENNATNLNFGTIDFGEATTAFKGVLDASLVNNGNSGFQIECAGISTVKIIFGSGNND
SNIPASFSQNYYHALSNGRDFIAYNLLYGLNKQVIKANEAFILNDMNNKKNIDIFGQA
THDGSRISKGEYKDIVPITIEF"
/product="hypothetical protein"
/transl_table=11
CDS 3354380..3354898
/locus_tag="AUO97b_03244" /db_xref="SEED:fig|400667.82.peg.3109"
/translation="MKYLYFKAIFLLSVSQFIYAADPQLNSSFKVQAKIENGCSIDNI
EQNIDFGKYSALSKNKVVTNIINSKGSWNIRCTESLPVSISIDGGENLQNNTRRMKNS
SSTNYLSYKLYNSSSLSNEYIVGNKYLLPATTPTNRLANFEIYGVVDLENNNEPHAAG IYKDTVSIMITW"
/product="Sigma-fimbriae uncharacterized paralogous subunit" /transl_table=11
CDS 3354904..3355725
/locus_tag="AUO97b_03245" /db_xref="SEED:fig|400667.82.peg.3110"
/translation="MNNSAFIKNGILKSFLFASTLSLVTPVMAQATFLIWPIYPKIEA
NEKATAVWLQNTGKTDAMVQIRVFKWNQDGLKDNYSEQSEIIPSPPVAKIKAGEKHML

RLTKSANLPDGKEQSYRLIVDELPIRLSDGNEQDASKVSFQMRYSIPLFAYGKGIGSG
LTEESQKLNAKNALAKPVLQWSVRNNQQGQPELNLKNNGQKFARLSALKTSKTGNDIS
LGKAAFGYVLSNSTVKFAIDQSTAHELSKTSKIYGVDSSGIKQELIEITKMEDPS" /product="Sigma-fimbriae chaperone protein" /transl_table=11
CDS 3355722..3358220
/locus_tag="AUO97b_03246" /db_xref="SEED:fig|400667.82.peg.3111"
/translation="MILRHNNLCKSILLILLSGGCLNIPNTVFAGDLPPPPRDINEIN
QLFKLYLDLVVNQYSVQQVVPVIVKNDEYFIQKKKLIDELEINIPQELLTNTDTSLSN
QDILTLGFSGDASDWISLDKLKDVSYEYQSSNQYFKLNFPPAWMPTQVLGKDSWYKPE
VAQSGIGLLNNYDFYTYRPYQGGSTSSLFTEQRFFSPLGVIKNSGVYVKNQYKNEGNA
ESVDNDGYRRYDTSWQFDNQKNATSFLLGDIITGSKTTWGSSVRLGGFQVQRNYSTRP
DLITYPLPQFIGQAALPSTVDLIINGQKTSSTEVQSGPFILNNVPFINGKGEAVVVTT
DAVGRQVTTSVPFYISNTLLKPGLFDYSLSLGKIREDYGLKNFSYGKFASAADARYGV
NDWLTVEGRTELSSDLQLWGAGSVLKLANLGVLSASFTQSKADKSMSEDRTKDLEGNQ
YTVGYSYNRNRFGFSINHNQRDDEYTDLSRLQYSNLISVNSNKSLTANTYFATKNSGT
FGVGYINTKANDFKNRFLNLSWAPVLPTYMNGVTVSLSANRDFIEKEWSAAFQLSIPL
FQRNATVNSGYAFNKQGDTGYLNFNRSVPSEGGFGVDLTRRFNENSEDLNQARVNYRN
SYINTDFGLSGNHDYNYWFGLSGSLIYMAGDLFASNRLGESFALIDTNQVPDVLVRYE
NSLIGRSNKKGHIFVPSVTPYYSGKYSVDPIDLPSNFTITQVEQRIAAKRGSGVVIKF
PVHQSISANVYLTQADGKPMPVGSVVHRADQESSYVGMDGIVYLENLKPNNTVTVQRS
DQNICKADFSVDVEQAKQQIVVVKPVTCHEVSLP" /product="Sigma-fimbriae usher protein"
/transl_table=11
CDS 3358217..3359236 /locus_tag="AUO97b_03247" /db_xref="SEED:fig|400667.82.peg.3112"
/translation="MNIKTKKLLRHLCIFSGLMLTGNLAHAACSVSASGTSSISVPSI
YLMENGENSSQFNSGLSCTGFSLALANMTYLKYRVEQMSNSFTNAQTGEKLNAIILDS
NNEIISLGQEKDMSSFTLVNLFSGPDGNLPFYIRLPAGQSVSPGVYRADSPLKVKWFY
SVPAVAIVGIGAFFESPGFKRGVLGIGFNWGSGADSLGSLSITVLPDCRILAQDVNFG
TAAFASKLEPVQSSMGIRCSVNTPYYVSLNNGLSPQNGNQRAMKSQTGNTYLKYDIFK
NSSNDRWGSGNERWSSLNATINPGVHNGVTQQNYVFTTKIVDENADTIPAGTYQDTVT VQVEF"
/product="Sigma-fimbriae tip adhesin"
/transl_table=11
CDS complement(3359294..3359527)
/locus_tag="AUO97b_03248" /db_xref="SEED:fig|400667.82.peg.3113"
/translation="MEQQTLLQELNSLIEYYSKRTDCPPSRIRIGYRAYAKLMQCPPF
ADEVINSALDPNKRKYKKIKIKITKDDDQLEIE" /product="FIG00350954: hypothetical protein" /transl_table=11
CDS complement(3359953..3361440)
/locus_tag="AUO97b_03249" /db_xref="SEED:fig|400667.82.peg.3114"
/translation="MRTSEKQVKPIEIDDINIIENEKTKKAITAAALGNAIEWFDFGV
YGYVAYVLGKVFFPDASPSVQMIAALATFSVPFIFRPLGGLFFGHLGDKYGRQKVLAI
TVIIMSISTFGIGLIPSYETIGLWAPILLLIVKIVQGFSIGGEYSGAAIFVAEYSPDR
KRGFMGSWLDFGSIAGFVLGAATVALITHAVGEARFAEWGWRIPFFLALPLGIIGLYL
RNRLEETPVYQQHSEQQAQKSKPQKFSFKEIFVKHKRSLLVCIGLVISTNVTYYMLLT
YLPSYFSHNLGYSEAHGALIIIAVMVGMLFVQPVIGYLSDKFGRRPFIFIGSFSLIFL
SYPAFVLLNSGVNYQIFIGLLILALSLNMSIGVMASTLPALFPTEIRYSALGIAFNFS
VVIAGLTPTLTATLVETTHNLMVPAYYLMICGVVGIATAMYLKETANKPLIGGVPIAH
NIEEAQELLEEFHETIEQKIENIDQKIQELQQKRQKLADKHPDIA" /product="L-Proline/Glycine betaine transporter ProP" /transl_table=11
CDS complement(3361831..3362451)
/locus_tag="AUO97b_03250" /db_xref="SEED:fig|400667.82.peg.3115"
/translation="MNDSVKWLEILNKITGIAQTGLHYSKDVYDKERYEQLLGHIEHL
LELKEINTENFINNVLQDVGYATPKLDVRAVVFKENKLLLAKEIGDGRWSVPGGWADV
GYSASENAEKEVLEETGLRVKAIKLLALTDRTKHPHPPMFLHVYKAFFWCEIIDGELT

SSIETPEVGFFGRDELPPISTARVTEEQIQHFFDYLESIPEATKFD" /product="Putative ADP-ribose pyrophosphatase YjhB" /transl_table=11
CDS complement(3362587..3363345)
/locus_tag="AUO97b_03251" /db_xref="SEED:fig|400667.82.peg.3116"
/translation="MKNIRIGSYHWMQTTNGQLSFKEKIKLIQKIMVPNILSSIKINY
YRFQTGNDFDIGQIVIPDTQMIKIALEELESKASISIYNHSWRTYFWGAALGHLQNQQ
FDPELLLLASLFHDIGLTEQHMHSKGCQCFTYESAKQFEQKAKEYNFDDKKSEVIKDA
ICLHMNGYIEDSDPPEVVLLQQGASCDVISDNQYKLPLSFRNEILEKYPRNQFNKEFI
KLINLERKNVPSSRTGLLYDLGLPLMIKSNLYNE" /product="Metal-dependent phosphohydrolase, HD subdomain" /transl_table=11
CDS complement(3363458..3363967)
/locus_tag="AUO97b_03252" /db_xref="SEED:fig|400667.82.peg.3117"
/translation="MTLNTVTLSTQRLTLKPFTANDADETYNCITPTLTRYMAWDPQP
RHEFDQTWSNWLKNFALGTEIIFVIRETSSQVFIGLVGLHRMQTSNSELGIWIREDYH
YHGYGREAVTAVAKWAIDFVKPDFFVYPVAEENYPSRKIAESLGGKVVSKFSGPKYEV VTYEIPVSF"
/product="Ribosomal-protein-S5p-alanine acetyltransferase (EC 2.3.1.128)"
/transl_table=11
CDS 3364697..3365251
/locus_tag="AUO97b_03253" /db_xref="SEED:fig|400667.82.peg.3118"
/translation="MKNLGASFRALHVLHIASDLFKQYGFHKVGVDRIISETKISKAT
FYNYFHSKERLIEMCLLLQKDTLMEKVRVEIETTHYSTFAHKLRQIYLLHANLKSAYY
LLFKAIFEIKTSYPTAYQTAIRYRRWIKNEIFCLLMETKKAVSYAEAEIFIFMIDAAI
LGLLTSDRVEEQTKLLDYFLVRVN"
/product="Transcriptional regulator, AcrR family" /transl_table=11
CDS 3365486..3366376
/locus_tag="AUO97b_03254" /db_xref="SEED:fig|400667.82.peg.3119"
/db_xref="GO:0016020"
/translation="MDDHHLKRKVGTLEMVIAMLLSGSIGLFVIKSGQSPVNIVFFRC
LISALCLIPICYFYGHFRKVYFGKKELFLMVTSGLLIIFNWVLLFAAFPKTSISLATI
VYHVNPFVILFLGALVFHEKLNKNDVLWTIVAFIGLIVIIGLGSASVNSNELVGLGLV
LIATTLYSISVLITKKLSNTPPLLIVFIQTLSGAIAMTPFMSVFENPPIGQQWLFVVG
LGVLHTAFLYYLMYSAIKKIPLNNIAILSFIYPISTIVIDYFFFDHVLTSTQVLGAGL
ILLGVLGVKLHWNIFALKNA"
/product="Permease of the drug/metabolite transporter (DMT) superfamily"
/transl_table=11
CDS 3366609..3367226
/locus_tag="AUO97b_03255" /db_xref="SEED:fig|400667.82.peg.3120"
/translation="MKLKELAGCEECDTVYRRVPLAYGKRAYCVCCGAELYRHTKPFT
TLLALILTALIVFVIANSFPIVKIELQGNISETTLLGAVWVMFHYDRAFVGVLILITT
FIVPLTYLLLLGYVLGTVSILKKCPQFLVGALRTLYFMRVWGMVEVFLIGILVTLVKL
MGMVLVIPEIALWAFAVLSLLMVYITSVKVSDIWNEIDRSQPWQK" /product="Paraquat-inducible protein A"
/transl_table=11
CDS 3367214..3368020
/locus_tag="AUO97b_03256" /db_xref="SEED:fig|400667.82.peg.3121"
/translation="MAKIKNVTSEKITNTNMILKQYPRAKDLSLILCHCCGLLNSSKV
NKADAHKNQILRCVRCHSVLHERKPGSLNRTFALVVAATILYIPANVLPMTVTDSLLG
RQQDTIMSGVIYFWQSGDYLVSVVIFMASIFIPMLKLIILYFLLLVVYIQSSAAWKFA
PEQCIKLYRIVEFVGRWSMIDVFVVALLTALIQIQSLATILAGPGSIAFGAVVVLTMF
ASLSFDPRIIWDNFYASQNTNKPNRVSSEKNTIIQAEHSPLNNDSINPSQ" /product="Paraquat-inducible protein A"
/transl_table=11
CDS 3368028..3369695
/locus_tag="AUO97b_03257" /db_xref="SEED:fig|400667.82.peg.3122"

/translation="MSMSENKTTSETSDHHETDVELENMNDLPEPQEKKNRWKPLLIW
IIPLIALLIALSLAVKALLSNGPTIEVSFRTAEGLVAGKTTVRYKQVDIGVVRQIDLA
EDRSHIIARIDLRKDASNFAAQDSRFWVVRPRIGTSGVSGIDTLLSGSYIEVDGGKSP
EKKLEFTGLEIPPVITSDVPGKEFFLKADDLGSLDIGSPIYYRRINVGQITAYKLSDD
GKSVELQTFIRAPYDKFVTTDTRFWQASGIDVTLNASGFNLDTQSLASIVAGGIAFGF
PENSNATVAANKSRFNLWDSKAEALKEPDGLPRGVIMYFDHSLRGLAVGAPIDFMGIE
IGSIKSINAEFYNNYKQIRMRVEAVIYPSRVENGQALNPNSDIFKDFVEHGWRAQMRT
GNLLTGQNYIALDKFPKAKPATLQILNDGRVVIPTTATELSGLQAQVSQIADKLTKFP
LVEIGQDVRKTLKNMNTAIESTDKLVKQLDGKVAPGLQATLDDVRKTVRSSESILSSD
APLQQDVRKALQQMTRAAASLQLMADYIEQHPESIIRGKRPEANDEK" /product="Paraquat-inducible protein B"
/transl_table=11
CDS 3369757..3370395
/locus_tag="AUO97b_03258" /db_xref="SEED:fig|400667.82.peg.3123"
/translation="MTAIAMTACSSSPTPNYYTLTPKVTPVSNSTVQVIEVLPVGIPD
RLDRAPLVLQDSNGKSTVLDNERWTSTLGTQLRDTLSAGLQQKLGAIDSYSSGLSGNN
QPLYRVSTDFSHFDIVDKSYINIAVSWVVKRQIPPTQLESNNAKVITNAGSQLNCRIA
FKQPIDQKTKKMDAIVNASSEAMTQIINTVSSSIVALDSNKKMNINNGICSS" /product="putative lipoprotein"
/transl_table=11
CDS complement(3370475..3370969)
/locus_tag="AUO97b_03259" /db_xref="SEED:fig|400667.82.peg.3124"
/translation="MLETDRLILKPFVQENLSTVIEMLQDKDFMAFSPYGDLTTEAAK
TRFFEILEHYKNYNFGKMAIVLKDTHQIIGYCGFENCEVDGKKEAELGFRIIKSERNK
GYIVEASLKLIEDMKNRNFKHVIAFSEKDNLPAHNLLAKLGFIKTNSSHYLNMEVCFF KKDL"
/product="Acetyltransferase, GNAT family"
/transl_table=11
CDS 3371109..3371837
/locus_tag="AUO97b_03260" /db_xref="SEED:fig|400667.82.peg.3125"
/translation="MKTIGLLGGMSWESTSLYYQQINRMVQNKLGKLHSAKIILNSVD
FEEIAALQSKGLWQEAGAYLAEQAQNLEKAGADCILVCTNTMHKVAPQIEDAITVPFI
HIADATAKEILSQNIRKVALLGTAFTMEQDFYKARLQDLGIEVIIPNDANRKIVHSII
YDELCLGLINPDSQNKYMTIVEELIGQGAEGIILGCTEICMLIGDIKFSVPLFDTTTI
HAKEAVNFALEQKTAARSIAASSV"
/product="Aspartate racemase"
/EC_number="5.1.1.13"
/transl_table=11
CDS complement(3371852..3372763)
/locus_tag="AUO97b_03261" /db_xref="SEED:fig|400667.82.peg.3126"
/db_xref="GO:0016020"
/translation="MSKNLATLIGLSAILMWASMVGFVKHITTAIGPDVGITLIYSLS
ALLLLIIFRVPNFKLISKKYLILGAILFIAYELCFSFALAYSKTAQQAIEVSIVNYLW
PSLTVLAFVIFRELKFNVFIILGLLISISGIIFIQTGNGDFSLGRVVDNFHSNPLSYI
LAFIGAIIWAFYCVLTKKMGKGQNPISIFFLGVAITLWLKLLFSGQVSLPSLDLNTIL
SLIVASAAIGLGYAAWNIGIIHGNITMLVVASYFTPIISSLLAMLVLQTELSISFWQG
TAMVTAGSLICWLSTNWAVIRSYFRIN" /product="Permease of the drug/metabolite transporter (DMT) superfamily" /transl_table=11
CDS 3373114..3374481
/locus_tag="AUO97b_03262" /db_xref="SEED:fig|400667.82.peg.3127"
/translation="MLTRFLLPILGTTVLVTGCASSSQYPINESYGPEPKLPEPKTSL
FPTVNIAPAQGWPSGVMPTPAEGLKVKAFAKGLEHPRWLYVLPNGDVLVAETDAPPKP
EDSKGIKGKIMTFVMRRAGSSHPSANRISLLRDTNGDGIADQKTVFLKNLKSPFGMAL
VGNHLYVANTDSLMRFPYQEGETHITSTGTKVLDLPGGPLNHHWTKNVIANREGTKLY
ITVGSNSNVAENGLDQEKGRAQIMEFDIASGQSRPFATGLRNPNGMAWQPQSGKLWTV

VNERDEIGSDLVPDYITSVQDGAFYGWPYSYYGQHVDVRVKPQNPDMVARAIKPDYAL
GNHTASLGLAFYTAELMPQFRGGAFIGQHGSWNRKPHSGYKVIFVPFRSGQPSGPPQD
ILTGFLSEKGKAYGRPVGVAIDFSGAVLVADDVGNTIWRVSPVAETTYESPTQQSIRS SVTQP"
/product="L-sorbosone dehydrogenase" /transl_table=11
CDS 3374509..3374961
/locus_tag="AUO97b_03263" /db_xref="SEED:fig|400667.82.peg.3128"
/db_xref="GO:0008080"
/translation="MQILRLSDYPQYKEMAAQWFSEKWQIPVEAYLESIQISIDQRHA
IPQWYIVLNENKHLIAGAGVIDNDFHERKDLTPNLCALFVEENYRNQNIAKHILDFVR
EDLSNQGIQTLYLITDHTEFYEKCGWRFLTLVKDEEGEMVRMYVADTF"
/product="Acetyltransferase, GNAT family"
/transl_table=11
CDS complement(3375020..3376129)
/locus_tag="AUO97b_03264" /db_xref="SEED:fig|400667.82.peg.3129"
/translation="MAASSAPTPLYRLYQQLWQFSPVTLTLIFATYAFTLLGSLLIIG
SLSDYIGRRPVIIAAITLQIISMSFFLFASDVSMLFIARGLQGIATGLAVSAIGAAIL
DFSKLHGSLINSIAPMIGMAVGIFLTCSILQFSAHPLQLVFEFLCFLLICELILSFLT
PETAQRRSGALASLKPNMAIPPQTKSALLSISPINIALWMVSGFFLSLMPSLLAKIFH
TSSAWLNGIMFMALALSGAVGILTLRKSTNFRILLTGTLSIAIGAIVLFIAINLTNAV
VLFLGSIITGVGFGTAFMGAIRSVMPLALPEERAGLMAAFFVESYLAFSIPAILAGYF
VGKIGLMSTANSYIGFIILLSLVALLMIIKNFKNK" /product="Uncharacterized MFS-type
transporter" /transl_table=11
CDS 3376361..3376927
/locus_tag="AUO97b_03265" /db_xref="SEED:fig|400667.82.peg.3130"
/translation="MAKVVTGLRPGGRSERIQTAVHQAVKELQKDFEQSQITIPMIAA
QAGVTPSTIYRRWGDINQLFSDVALNELKPDMEPKDLGSFQQDITAWVEQYFEEYASE
VGQDLLRDVLYTSNSDSNARKCETLIIQQLDIIQNRAKLRNEFTIPNQVIIETVIAPM
LFRILFTNHDLSLEYVYDLLNRLFIKNK"
/product="Transcriptional regulator, AcrR family" /transl_table=11
CDS 3377356..3378654 /locus_tag="AUO97b_03266" /db_xref="SEED:fig|
400667.82.peg.3131"
/translation="MDFNTLDQETVQKKQKFHQILYVQVIFAIILGILIGHFYPELGE
SLKPLGDAFIKIVKMIIAPVIFLTVVTGIASMTNMSRVGRVTGKAMLYFLTFSSLALV
VGMIVANIIQPGKGLNIDPATLVNDKVNSYVEKAHATNITDFFMNIIPQTLVSPLAGG
EILQVLFVAVLFGISLAALGDRARPITDFLNNLTAPVFYLVGMLMKLAPIGAFGAMAF
TIGAYGIESIGNLLLLIMTFYITAVLFVLIILGAVARYNGFSIIDLIRYIKDELWLVL
GTSSSEAALPSLMDKMQKAGCEKSVVGLVIPTGYSFNLDGTNIYMTMAALFIAQACNV
DLSIGEQVALLLVAMVSSKGAAGVTGAGFITLAATLSVVPAVPVAGMALILGIDRFMS
ECRALTNLIGNACATIVVARWDKALDKDVLDKALRKSKTN" /product="Na+/H+-dicarboxylate
symporter"
/transl_table=11
CDS complement(3378890..3379093)
/locus_tag="AUO97b_03267" /db_xref="SEED:fig|400667.82.peg.3132"
/translation="MKKVVKAKNLIAFRIWLEKLGYSVKNLADGKGFTFSFKKEYGLV
TCDLAGNALAMQLGEEFEDHLKA"
/product="hypothetical protein"
/transl_table=11
CDS complement(3379450..3380181)
/locus_tag="AUO97b_03268" /db_xref="SEED:fig|400667.82.peg.3133"
/db_xref="GO:0004416"
/translation="MRYRIHYIDVQNALQNYIWILEDTETHEAVAVDPTEAELVIHFC
QEHQLTLKQIWLTHWHKDHIGGVADLTANTPMAVYGPRDELSKIPGITHPLQHDDHLK
FNDLKVEIIATPGHTLGHIVYFIEELNTLFCGDTLFAMGCGRLFEGTAEQMYHSLNRL
AALPTATKVYCTHEYTLSNAEFALSVEPENHALQERAEQVRMLRQEGKITLPSSIELE

LATNPFLRAESVDEFAHLRSLKDNF" /product="Hydroxyacylglutathione hydrolase (EC 3.1.2.6)" /EC_number="3.1.2.6"
/transl_table=11
CDS complement(3380339..3381034) /locus_tag="AUO97b_03269"
/db_xref="SEED:fig|400667.82.peg.3134" /db_xref="GO:0005215"
/db_xref="GO:0006810"
/db_xref="GO:0016020"
/translation="MRDLIVQWLTELTQPFWHSSLSIDQFVTALQETFHMVFFALLFG
GIWGFIQGIILLVTRPEGILPNKFIYHLLNPIVNALRSLPFIILLIAVIPLTKLLVGT
SIGTWAAIVPLTIYVGPYMGRLIETSLLEVNEGIIESAQAMGATPWQIISRFILPEAR
SSLILNLTTATISLIGATAMAGAVGAGGIGDLAISYGYQRFDTSVVILTVIVLLLLVQ IVQMLGNWLAKMR"
/product="Methionine ABC transporter permease protein" /transl_table=11
CDS complement(3381031..3382056)
/locus_tag="AUO97b_03270" /db_xref="SEED:fig|400667.82.peg.3135"
/db_xref="GO:0005524"
/db_xref="GO:0016887"
/translation="MIQFKNISKHYQLKGQTIRALDQINLEIPEGSIFGIIGYSGAGK
STLIRLINLLERPNEGQVIINQKDFTALDARSLRQERANIGMIFQHFNLLQTKTVAEN
IEMPMRLLNYSKAEREKRLNELLEFIDLKHKKDAYPDELSGGQKQRVGIARALANHPK
ILLCDEATSALDPQTTKSVLELLKKINQEQKITIVMVTHEMDVIETVCDHVAVMEAGK
VIEQGSTIDIFSNPQHPTTKNFIQTVLHQQLPVNILNQLENQHHHSIYCLQFLGRSAQ
EPVIQSLIKQFDISLNILFANMTEINGTVIGQMFVQLLGDPQLIRQAIEFLEKNEVSV VQSGDQV"
/product="Methionine ABC transporter ATP-binding protein" /transl_table=11
CDS complement(3382132..3382998)
/locus_tag="AUO97b_03271" /db_xref="SEED:fig|400667.82.peg.3136"
/translation="MKKLISLFLSVSVVLLAACGKQQNEPQNGKDSAQLQTVVIASTG
SDADIWRYIATLPETKAAGLKLEVKNFTDYVAMNTATANKEVDLNAFQSYAYLVAFNA
SNKDKIAPVATTYLEPMGIYSSKYKKVDEFPQGASIAIPNDAANEARALLLLQSAGLI
KLKADFDPAKGTPSDITDNSKKIDIKPIQMATAVRVKDEVDAIVLGNTLAMEGGLNVL
KDSIYYEPVDQSTKLNVNILATAESRKDDPVLQKVGQLYHTEAVKKYVEQHFGGTKVD VNQPISYLTQAK"
/product="Methionine ABC transporter substrate-binding protein"
/transl_table=11
CDS complement(3383247..3384533)
/locus_tag="AUO97b_03272" /db_xref="SEED:fig|400667.82.peg.3137"
/db_xref="GO:0004637"
/translation="MNILVLGSGGREHALAWKIAQDAKVTQVFVAPGNAGTATEPKCV
NVQLDILDNPAIIAFAKENNVELIVVGPEAPLVNGVVDAAREAGVKIWGPTQYAAQLE
GSKAFAKHFLKRHNIPTAFYEVFTEVDAAKAYVEKNGAPIVIKADGLAAGKGVIVAMT
NEEAFAAIDDMLAGNKFGDAGSRVVIEQFLAGEEASFICMIDGKNILPMATSQDHKRI
FEGDQGPNTGGMGAYSPAPVVTPDVFERVMNEIMRPTVDGMAADGHVYTGFLYAGLMI
DEQGQPRVIEFNCRFGDPETQPIMMRLKSSLVDLVEAGIAGNLPSEAEWDERKSIGIV
LAAEGYPDSVRKGDVISGIGQSPEDTKIFHAGTATREDGHIVTSGGRVLCVTALGDSV
LEAQINALEVCGQVTFTGMQYRSDIGYRAIAREKAE" /product="Phosphoribosylamine--glycine ligase (EC 6.3.4.13)" /EC_number="6.3.4.13"
/transl_table=11
CDS complement(3384659..3386233)
/locus_tag="AUO97b_03273" /db_xref="SEED:fig|400667.82.peg.3138"
/db_xref="GO:0003937"
/db_xref="GO:0004643"
/translation="MTIKRALISVSDKTGIVEFAQNLAALGVELLSTGGTYKLLKDNN
IAVVEVSEHTGFPEMMDGRVKTLHPKIHGGILARRGLDEAVMQEHNIDPIDLVVVNLY
PFAATVAKPNCSLANAIENIDIGGPTMVRAAAKNHASVGIVVNASDYDTVISELKASG
SLSYETRFDLAVKAFEHTAQYDGMIASYLGARVGKAEGEADLFPRTFNTQLNKAQDLR
YGENPHQSAAFYVEANAKEASVSTAKQLQGKELSYNNIADTDAALECVKSFAKPACVI

VKHANPCGVAVSLDGIKAAYDLAYATDPESAFGGIIAFNRELDVATAQAIVDRQFVEV
IIAPSIADGVLEVTGAKKNVRVLVCGELPAIDARAPQLDYKRVNGGLLVQDQDLGMIT
KDDLKVVTKRAPTEQEIDDLIFAWKVAKYVKSNAIVYAKNRQTIGVGAGQMSRVNSAR
IAAIKAEHAGLVVEGAVMASDAFFPFRDGIDNAAKAGIKCIIQPGGSMRDEEVIAAAD EAGIAMVFTGMRHFRH"
/product="IMP cyclohydrolase (EC 3.5.4.10) /
Phosphoribosylaminoimidazolecarboxamide formyltransferase (EC 2.1.2.3)"
/EC_number="3.5.4.10"
/EC_number="2.1.2.3"
/transl_table=11
CDS complement(3386323..3386592)
/locus_tag="AUO97b_03274" /db_xref="SEED:fig|400667.82.peg.3139"
/translation="MNSKSPIFTAQSDVALRIHVDRAVRHYFAQLQGEQPSQVYDMVL
AEMEKPLLSVVLEYTRGNQTRAAEILGLNRGTLRKKLKAHGLMSE" /product="DNA-binding protein Fis"
/transl_table=11 CDS complement(3386969..3387811)
/locus_tag="AUO97b_03275" /db_xref="SEED:fig|400667.82.peg.3140"
/translation="MNEKQTRCPKCLTVYKVSLTQLTVAQGMVCCPKCNLNFNALTNL
VNVEQPETVNSSALAHGSMQTRYMTHSHVLDIFDQKVENSNIDLLTYLNNLNYFHNEP
INALPNLNLAEQATSPEQDNGKKHSWLYYTLWSSANLILILVFTFQILWFNPKLMQQS
PAISHAFNYVCQILHCNKSAEQYQLLYIEKVKLRKIGTEHTQLSGVLINQNNKSIELP
NLKLSFGKETDQEEIIFTPQQYLVENLRGIQRIPTNTPFRFQVELNKSKKSLVNYRLE IVHP"
/product="hypothetical protein"
/transl_table=11
CDS complement(3387825..3388730)
/locus_tag="AUO97b_03276" /db_xref="SEED:fig|400667.82.peg.3141"
/db_xref="GO:0005737"
/db_xref="GO:0006479"
/db_xref="GO:0008276"
/translation="MKWLQIHITVDQEQVEFTETLLMSLGAVSVTLDDAEDQALLEPL
PGETPLWNKVIVTGIYQQDEQDPIDVDTLEAFLKAQLPDVPMRHEELEDQVWERAWMD
YYEPIQIGEKFWIVPEWLEPPEADATNIKLDPGLAFGTGNHASTFLCLQWLGKTDVKD
KIVIDYGCGSGILGVAALLLGAKKVYATDIDPQAVLATKQNAELNGVLDRLYVGLPEE
FDQEFKPQQADVLVANILAGPLMALAPEFAKLLKSDGDFALAGVIEEQVADVSGVYSE
FFDILDVEKREENWCRISGKRKTTN"
/product="Ribosomal protein L11 methyltransferase" /transl_table=11
CDS complement(3388887..3389117)
/locus_tag="AUO97b_03277" /db_xref="SEED:fig|400667.82.peg.3142"
/translation="MKTTMLKILTASLICFSGVTFTHAADTSKAETQKQIRVCAKKQQ
GEWVSYNHNGVTFNGTCQPNENGKLQFKAPAP"
/product="hypothetical protein"
/transl_table=11
CDS 3389350..3391230 /locus_tag="AUO97b_03278" /db_xref="SEED:fig|
400667.82.peg.3143"
/translation="MHYPKVYDVIVIGGGHAGTEAALAAARMGRQTLLLTHNIETLGQ
MSCNPAIGGIGKSHLVREIDALGGAMALAADKGGIQFRILNSRKGAAVRATRAQADRV
RYKAAIRETLENQANLDIFQQAADDLIVEGDTVKGVVTQMGIRFDAKTVVLTTGTFLG
GVIHVGLEKSSGGRAGDPPSIALAQRLRELKLPVGRLKTGTPPRIDARSVDFSVMTPQ
PGDFPSPVMSFMGDVSMHPEQVNCYITHTNEKTHDIIRGGLDRSPMYTGVIEGVGPRY
CPSIEDKIHRFSDKDSHQVFLEPEGLDTHELYPNGISTSLPFDVQFELVRSIRGMENA
HILRPGYAIEYDYFNPQALKFTLETKAINGLYFAGQINGTTGYEEAGAQGLLAGLNAA
RRAWEQEEWTPKRDQAYMGVLVDDLITLGTKEPYRMFTSRAEYRLMLREDNADQRLTT
IGRELGLVDDVRWAAYCEKMEAVERETSRLQHLWAAPNNPMGKKFVEMTGADLSKECS
AIDLLKRPNINFSQIAELTGSEVSQQVGEQIEIAVKYEGYINRQHEDVAQLKRLEETK
IPADFDYDVVSGLSREITQKLKTVRPETLAQASRIPGVTPAAVQLVMITIRKNNMTKK TA"

/product="tRNA-5-carboxymethylaminomethyl-2-thiouridine(34) synthesis protein MnmG" /transl_table=11
CDS complement(3391618..3392625)
/locus_tag="AUO97b_03279" /db_xref="SEED:fig|400667.82.peg.3144"
/translation="MSVTEKETSNSLYRQWQILSRLPTGKWIGTRELQEMLEREGIEI
SLRTIQRDLNQIAQRFPIESNKAVPQGWRWQSDAPIQSLPHMTSSQAVTFMMVEEHLK
HLLPPSLLDEMGPWFDLAKRSLSTQNNVRQWINRVRIVPASQPLIPPVVDRAAQQAIY
EGLLQDKQIECVYRARVNQGEDKTYILNPLALVQKGAVIYLICTRHDKTEVQTFALHR
FKSAKVLESRALHPVNFDIDHYIDSGALGFRVDYNQPTESIQLTLTMTEQTAKTFYES
QLSKDQTITPIEENIVEVTATVPFTSQLVWWLRSFGKKLLHIEPVQVHNAVREIEPDS K"
/product="hypothetical protein"
/transl_table=11
CDS 3392851..3394401
/locus_tag="AUO97b_03280" /db_xref="SEED:fig|400667.82.peg.3145"
/translation="MSEQEKDLQADIDYFLQEQATNALLNQTSFIENLSVQSNLFEQL
KSQFFNEMLDDATSNGVASKKIEQWLSIRTLDELKQLNAEAKQHFLYHGITFNVYGDK
EGVERTIPFDLIPRVIEKKQWEKIAAGCAQRVKALNYFLDDIYHGQHILKEQLIPESQ
IICHEAFQPHMLKHTLKGKIYSQISGIDIIRDSEGEFFVLEDNLRTPSGVSYMIECRN
ISQQLMPELCAANNLQGIEHYPSLLKEILQENSEVDQPFIVVLTPGRFNSAYYEHAFL
AREMDVPLVTNRDLFVENDQVFVKTIRGRQKVDVIYRRLDDDFLDPLAFRPDSALGVA
GLMSAYLQKNVVIANAPGTGVADDKSIYPYVDQMIQYYLGETPILKNVPTYQCREKEH
LDYVLAHLDQLVIKEAQGSGGYGMLIGPKASSCEIDEFRQKLIATPHEYIAQPTLALS
VAPTLTTAGVAERHIDLRPFVLSSPYRTEIVPGGLTRVAMQAGSLVVNSSQGGGIKDT WVVETLHS"
/product="Protein containing domains DUF404, DUF407" /transl_table=11
CDS 3394424..3395020
/locus_tag="AUO97b_03281" /db_xref="SEED:fig|400667.82.peg.3146"
/translation="MVLLNSSAHHIYWLGRYLMRIQFAVSHLPFTDDARAAQFAAAFG
LVIDQAELLNCYMLDTKQTYSLLNQFAIAKDNIQELRGILSSNAYAELNHAIKGVQAH
PDSLKQALAKCNQILDAEHEDIALFLHLGQKIELFDIQLRFQQDLTQLLQELEQLLQQ
LNDLGWNKLTQPWQLLKDHPNWEAYYNFTQQLEYMFEA" /product="hypothetical protein"
/transl_table=11
CDS 3395023..3395802
/locus_tag="AUO97b_03282" /db_xref="SEED:fig|400667.82.peg.3147"
/translation="MKLMVNHQTHYNYTETAKNSIQYIKMMPQTSLHQHVMNWAISVP
GDKTLKRDIFNNIWITASQRYEYQHLTFMAQGIVELLNTEIGGVDVSIPINLFLQSTE
ATKYDAEMLDFAQRIVTVKDRQHIALLSENILQKMPYQPDSTSVHTTATQAFQAGQGV
CQDHAHVLIAMCRALQLPARYVSGYLFDQNSPHLASHAWAEVFLDNQWYCFDVSNQLF
TPKHHIYLAVGRDYLDVAPIRGVREKGGTENMMSVVQVLAC" /product="Protein containing transglutaminase-like domain, putative cysteine protease" /transl_table=11
CDS 3395858..3396553
/locus_tag="AUO97b_03283" /db_xref="SEED:fig|400667.82.peg.3148"
/translation="MFISDTRTNAGVDHISVFRKLHTFGVTGERFIALQTAGNLATTQ
AVIGHLQNALILQQEPNLYSINTMFEVADLVGKTLKSVLEDISSDTQEQMNYACSILV
GGQIKGGDMQLYNVYPQGNFICATTDTPYFQIGESKYGKPILDRALYYAMPLDDALRC
SLISFDSTLRSNVSVGLPLDALVYSKDSFVIPMGKRITEDDPYFSQISRQWSDTLRRG LQEMPKPTDDYWR"
/product="hypothetical protein"
/transl_table=11
CDS complement(3396605..3397675)
/locus_tag="AUO97b_03284" /db_xref="SEED:fig|400667.82.peg.3149"
/db_xref="GO:0008784"
/translation="MRQATVYIDRQALQYNLNRVKQLAANSKIVSMVKANAYGHGVKD
CLAALNASDAFGVACLQEGLEIRELGFEQPVTLIEGVFSEDEMPIAIEQKFECVIHHQ
QQFEWLIKHKQAYITQGLKVWVKLNSGMNRLGFKNPEIIEVIKTLKSEGFTCVLAMHF
ANADVDHPLNEQQKQQFLHVKETCDPILASCCNSAAIFKWPELNFDYVRPGIMLYGAS

PFADKTVHDLDLKPVMTFTAEIIALNHIEAGEHVGYGSTFCAQQAMDIAIVSIGYGDG
YPRAYVKQNFVAIDSKLCPVVGRVSMDMIAIDVTNTQVKLGTQVELWGNHRLVDDVAE
ANGTIGYELLCRLSSRPVRQGT"
/product="Alanine racemase (EC 5.1.1.1)" /EC_number="5.1.1.1"
/transl_table=11
CDS complement(3397708..3399153)
/locus_tag="AUO97b_03285" /db_xref="SEED:fig|400667.82.peg.3150"
/db_xref="GO:0003678"
/db_xref="GO:0005524"
/db_xref="GO:0006260"
/translation="MSQANATSLTLSSPTENKTSESGQLKEFRTPPHNLAIEQAVLAA
LMTVAESFEQVSDLLKENDFYATRHKYIYRAIEKLAQENSPYDAVLVNDWLLKQNLLD
AIGGEEYLMQLMADSPSSFYNLETYATKIKEFSTLRSMIKVSTEILQNAYDTKGRGVS
EILDLAETSIFSLAEQHNNNKKDSGPKAISSVTADVISKLDELSKLDGNITGLTTGFL
ELDNKTSGMQPGDLIIVAARPSMGKTTFAMNLVESVLQYNKLPALVFSMEMPADSIAM
RLISAMGKVHQGHLRSGNLDADEWTKVTGTILQLQQMHLYIDDSSALPPTEVRARARR
IAKMHDGKIGCIMVDYLQLMKVPGMGDNRVGEISEISRSLKALAKEMNCPVIALSQLN
RSLENRPNKRPVMSDLRESGAIEQDADLIMFIYRDEVYNKESKEAGTAEIIIGKQRNG
PIGTVRLAFEGQYTRFSNLSPEYYSQYDDEE"
/product="Replicative DNA helicase (DnaB) (EC 3.6.4.12)" /transl_table=11
CDS complement(3399354..3399800)
/locus_tag="AUO97b_03286" /db_xref="SEED:fig|400667.82.peg.3151"
/db_xref="GO:0003735"
/db_xref="GO:0005622"
/db_xref="GO:0005840" /db_xref="GO:0006412"
/translation="MDVILLQRIKNLGKLGDKVSVKAGYGRNFLIPQGKAVAATEANT
AAFEARRAELEKQEAEVLAAAQARAEQLNEVNIVITAKAGDEGKLFGSIGTRDIADAL
TNAGLTVDRAEVRLPNGALRHTGEFNIAIQLHHDVVAEVLVTIVSE" /product="LSU ribosomal
protein L9p"
/transl_table=11
CDS complement(3399809..3400036)
/locus_tag="AUO97b_03287" /db_xref="SEED:fig|400667.82.peg.3152"
/db_xref="GO:0003735"
/db_xref="GO:0005622"
/db_xref="GO:0005840"
/db_xref="GO:0006412"
/translation="MARFYRRRKFCRFTAENVAYIDYKDIDTLKQYITENGKIVPSRI
TGTKARYQRQLALAIKQARYLSLIPYTDNHK"
/product="SSU ribosomal protein S18p @ SSU ribosomal protein S18p, zinc-
independent" /transl_table=11 CDS complement(3400048..3400431)
/locus_tag="AUO97b_03288" /db_xref="SEED:fig|400667.82.peg.3153"
/db_xref="GO:0003735"
/db_xref="GO:0005622"
/db_xref="GO:0005840"
/db_xref="GO:0006412"
/translation="MRHYEIVLLVHPDQSDQVVGMVERYISQIKEADGQIHRLEDWGR
RQLAYPINKIHKAHYILMNVECGQSTLDELEELFRYNDAIIRNLIIRREHAITEESLL
AKSAEEKRARKAQREEAQQVAQEAE"
/product="SSU ribosomal protein S6p"
/transl_table=11
CDS complement(3400616..3401494)
/locus_tag="AUO97b_03289" /db_xref="SEED:fig|400667.82.peg.3154"
/db_xref="GO:0004659"
/db_xref="GO:0016021"

/translation="MLKKYLFLTKPGILFGNFITTLGGFFLAAQGSIDILLLLLTLIG
TTLVVASGCVVNNVIDQDIDTKMQRTQNRALVKKTISPTVALVYAFVLGVMGFSILWF
GVNGYAFLFAMIGFIVYVGFYSLWTKRTSIHQTVIGSISGASPPVIGYTAVTHQFDVA
ALLLFLAYALWQMPHSWAIAIYRFDDYKNAGIPILPVARSIYRTKIECVIYILLFAAV
LNGLYCFGYTNVFFLITFNALTAYWFYLSIIGFKAENDQLWAKRFFLYSVILITLLSL SFSFTYQSPAPNLPLF"
/product="Heme O synthase, protoheme IX farnesyltransferase, COX10-CtaB"
/transl_table=11
CDS complement(3401505..3401834)
/locus_tag="AUO97b_03290" /db_xref="SEED:fig|400667.82.peg.3155"
/db_xref="GO:0006118"
/db_xref="GO:0016021"
/translation="MSSHEHNAAGASHGNFKQYTVGFILSVILTIIPFGMVMAGGFSR
GLLVTVIAITAVAQVLVQLVYFLHMNTSSEQRWNMIAFIYTILCIAVLLIGSVWIMNY LHYNMMI"
/product="Cytochrome O ubiquinol oxidase subunit IV (EC 1.10.3.-)"
/transl_table=11
CDS complement(3401834..3402454)
/locus_tag="AUO97b_03291" /db_xref="SEED:fig|400667.82.peg.3156"
/db_xref="GO:0004129"
/db_xref="GO:0006118"
/db_xref="GO:0016020"
/translation="MAEVLHHDNHGHDGHHEHDDTDLTVFGFWTYLMSDLILFGSLFI
AFAVLSSHVPPGTPSAYDLFHESLGYVLTETFALLISSVTFGFAVLASYKKNVNQVIT
WLFITFLFGATFIGMELYEFHHLVEEGHGPTHSAFLSSFFTLVGTHGIHVTSGLVWML
VLMYQIKTKGLTLPNTRRLACLSLFWHFLDIVWICVFSVVYLLGVL" /product="Cytochrome O
ubiquinol oxidase subunit III (EC 1.10.3.-)" /transl_table=11
CDS complement(3402459..3404450)
/locus_tag="AUO97b_03292" /db_xref="SEED:fig|400667.82.peg.3157"
/db_xref="GO:0004129"
/db_xref="GO:0006118" /db_xref="GO:0016020"
/translation="MDMIFGKLGWDSIPTEPIVLVTMVFMALGAIAVLGGITYFKKWG
YLWKEWFTTVDHKKIGIMYIIVSVVMLLRGFADAIMMRLQLFLAKGGGEGYLHPDHYD
QIFTAHGVIMIFFVAMGLVVGMMNISVPLQIGARDVAFPLLNSLSFWLFAGAAGLMML
SLVLGEFAATGWMAYPPLSGIQYSPGVGVDYYIWALQVSGLGTLLSGVNFFVTIIKMR
APGMKLMDMPIFTWTSLCTAVLIIASFPVLTGTLAMLTLDRYFGFHFFTNELGGSPML
YVNLIWTWGHPEVYILVLPAFGLYSEIVATFSRKALFGYKSMVYATIAITVLAFVVWL
HHFFTMGAGANVNAFFGIMTMVIAIPTGVKIFSWLFTMYKGRITFTTPMLWTLGFLVT
FGIGGLTGVLMAVPPADFLVHNSLFLIAHFHNVIIGGVVFGMFAGIIYYWPKMFGWKL
NEAWGKAAFWFWFFGFYFAFMPLYILGFMGMTRRLNTYDNPEWDPYLAIALFGAVLVA
IGIACFLMQIIVGFLQRHQNMDYTGDPWDARTLEWATSSPAPFYNFAHEPDASGIDRF
WTDKENGVAYARNTKYEDIHMPTDRAAGFVIAMFITLLGFALIWHIWWLVVVSFVAAV
VSLIVSSFTKNVDYYVPAAEVERIENERYALLEKHLKKD" /product="Cytochrome O ubiquinol
oxidase subunit I (EC 1.10.3.-)" /transl_table=11
CDS complement(3404454..3405506)
/locus_tag="AUO97b_03293" /db_xref="SEED:fig|400667.82.peg.3158"
/db_xref="GO:0004129"
/db_xref="GO:0005507"
/db_xref="GO:0006118"
/db_xref="GO:0008827"
/db_xref="GO:0016020"
/db_xref="GO:0016021"
/translation="MRQTILAVLSLSTLAALLTGCGGDMVLLNSKGPVGQGQSDLMMT
AIYLMLLVVIPSIIMALWFGWKYRASNKDADYKPTWAHSTAIEVVVWGIPVIIIGILA
WLTWWGSHKYDPYRPLESDKAPLTIQVIAEQFKWIFIYPEQNIATVNEVRFPEKTPLS
FKITSNFTMNSFFIPQLGGQIYAMAGMQTHLHLLANETGVYRGFSSNYSGYGFSQMRF

KAHSVTEQQFNEWVAAVKAGNGTTINPEAVQKTTLDQAELATLRDGDRSKHQIEHLVN
RAKAAGDQEALAKAEAMKPFPTKPHPVTYYSSVEPKLFETIINHYMSNYHGADHSAAH TAAETHVAAEHAAQGE"
/product="Cytochrome O ubiquinol oxidase subunit II (EC 1.10.3.-)"
/transl_table=11
CDS complement(3405822..3406577)
/locus_tag="AUO97b_03294" /db_xref="SEED:fig|400667.82.peg.3159"
/translation="MQIYLARNNQQAGPYTLEQLNQMLASQQVLLTDLAWHEGMTEWK
ALGELTQGKLVYQPTGYSAFSANTNTPYNETIQHIRVETKTHELASISSRALAKIIDL
LLWLPIAAIPSFFFNEAQYKQLFELQKQMQSAEVASTKAAELQQQLFTLIPIEAWHTM
LLYVVIMLAIQAFLLTKFGQSIGKKIVGIRIVDAETNGKVNLTRIFLLRSVVFIILNL
LFMPISTIIDYAFALGQKRQALHDKIARTKVIK"
/product="putative membrane protein"
/transl_table=11
CDS complement(3406642..3409020)
/locus_tag="AUO97b_03295" /db_xref="SEED:fig|400667.82.peg.3160"
/db_xref="GO:0008986"
/translation="MEARVIGLEKLGKHDVELVGGKNSSLGEMISHLSNAGVSVPGGF
ATTADAYREFLDQSGLNARIQAELAELNVDDVNALAETGAKIRQWIVETPLTPGLEQE
IREAFAALSNGNPDIAVAVRSSATAEDLPDASFAGQQETFLNIRGIDNVLIAVKEVFA
SLFNDRAIAYRVHQGFEHSLVALSAGVQRMVRSETGAAGVMFTLDTESGFRDVVFITA
SYGLGEMVVQGAVNPDEFYLSKPLLNAGKHAVLRRNLGSKHQKMIYGEEGSAGKSVVV
VDVEKQERQQFALNDHELQELAKQALIIEKHYGAPMDIEWAKDGDDGQIYIVQARPET
VKSRQNVGTMERYLLKQKGTVLCEGRSIGQRIGSGKVRIVNSIKEMDKVQDGDVLVSD
MTDPDWEPVMKRAAAIITNRGGRTCHAAIIARELGVPAIVGCGNATEVLTDGQEVTVS
CAEGDTGFIYEGALDFEVQRNSIESMPKLSFKIMMNVGNPDRAFDFAQIPNEGIGLAR
LEFIINRMIGVHPKALLNIESLPRETRAAVMTRTAGYASPVEFYVEKLVEGIATLAAA
FADKPVIVRMSDFKSNEYANLIGGKLYEPEEENPMLGFRGASRYVSDNFRDCFELECR
ALKKVRDEMGLTNIQIMIPFVRTVSEAKRVIELLAQNGLKRGENGLKVIMMCELPTNA
LLAEQFLEHFDGFSIGSNDLTQLTLGLDRDSGIVSHLFDERDAAVKALLSMAIHACRK
AGKYVGICGQGPSDHPDLAKWLMEQGIESVSLNPDSVLDTWFFLAEEKIKQI"
/product="Phosphoenolpyruvate synthase (EC 2.7.9.2)" /EC_number="2.7.9.2"
/transl_table=11
CDS 3409176..3410012
/locus_tag="AUO97b_03296" /db_xref="SEED:fig|400667.82.peg.3161"
/translation="MSESKQFKRSVFFISDGTAITAETLGHSLLAQFPNVDFDIHIMP
YITTEEAAMAVVVEINKCQTRDGCLPLVFDTLVDPHVREIINTAKAVNLDVFEGLISK
LEQELGTPPTTLVGQTHAVTDSEYYKARIDAVHFALDNDDGARTRHYDKADLILIGVS
RSGKTPTSIYLSLQFGIRVANYPLTEEDLDDNRLPAVLREHRSKLFGLMIDAERLVAI
RSERKANSRYASFSQCQMELRAIEGIYISEGIKYLNVTEMSIEEISTRILQMTGLKRR IG"
/product="Phosphoenolpyruvate synthase regulatory protein" /transl_table=11
CDS 3410434..3412836
/locus_tag="AUO97b_03297" /db_xref="SEED:fig|400667.82.peg.3162"
/translation="MPIHLYAAQVDVLREQAIQNYRAGQTQQAVSQLDQLLNKYPHDQ
KLLADYLFIMSSEKRDLTNFSRFLVNINYVDFPEYAKLPLIQNFRDFKHFKTAIDWSN
KFNIQKSVDGRILLSVLYAEAQDVANAKDQLSKIDIKGLTADQLVRVAYAYRLINLPV
DALATVEHAYQQQPKSLSVLQEYVYDLIAIGSYKKAQQLLQASEKTEQTVQMLKTLQV
SEFSQHINNAIARYKYLNREGLSDAESFAELDKVLEQGQKMHQQMNPSDPNYLRFYYD
YLYGLDFRGRSKAVIESFTQLNIPLEKLPAYVRHAIADSYLAEQKPKQAEFAYKTLLT
EKNYPDMTVYTGLYYSYIEQEKYKEAEQLLAEVDRLIPTYKYSQAKGVDKISHPDRDD
YIALQGMHLAYANHLDQAEKHFQKKVEQAPANESLINNLARVERWREKPLEAKKTISR
LNGIDPIAKDTRINEMQNAQALGDIPTWRKTTQNLVQYYPDDSGVIKSRKELEDRNRA
TISHSTTWGQSKADGRDTVSGQNGLKDREMETRLNSPWINDNYRLFAWHQDRYGEYRF
GDVHDQRYGVGAEWQANRKALSAIVSQSTDGGQAGVRLDWSQWLNDHWQYQLQYNSQA
DIPLQALDAGEDGQSYRAAVTWQKDESRQIGASYGLTDISDGNKQQEFSTFWRERLFD

APHHITYGTVRGFYGTNSQDQTAYFSPSSHYSAELNLSHDWVTWREYERSFKQHFEAG
VGLYKQADYSAKPTYSLQYQHQWQLSRTWQLNYGIGWQYHPYDGHDEQHTYGIFGFEG RF"
/product="Biofilm PGA outer membrane secretin PgaA" /transl_table=11
CDS 3412836..3414665
/locus_tag="AUO97b_03298" /db_xref="SEED:fig|400667.82.peg.3163"
/translation="MNNLLSKMLGCALVSTLLPLQFAYAQIEKDLPKNHTVSLTFHDV
RDDVLKEGDRDIYAIQTKNLAQFFDWLSQSDWKPIRLKDIEEARKQGKELPHNSILLT
FDDGALSSYSRVFPLLKQYQIPAVFALPTSWLNGNTQAGYEAYGQGNLVNWKQVREMQ
ASGLAEFASHSDDLHHGVLANPQGNEQPAATSYAYLKSQKRYETDVEYQQRILQDLKK
SYAVLKKEVGVEPKAIIWPYGAVNEQLEKLSQEAGFIFSFSLGRDGMNRVSDSTFKRS
LVTNNPTAEQLTEGMINILNFEELDLFKQPRHFVSMDLKQLTASTNTQSDEKLGLLLS
KLYSLKNNTLILKPLDDQDGDGQYDIAYFPTTQLSVQQDILNRTLWQAQTRAGQSVIL
ELPVYPQKNKPFLVADLAKDIARFNSNLSGIQLNAGTTLNCAMQSTTIKENACANQLK
QLTYVSQLTQKAVKPYLNMSNQAQFSLLLTPDFEHIENLPTLLKTLLSQHDLVNLKFN
IVGKQKQFNHVLAILNTLDSKYKQRIMLTLSLPENSQQNAWQEVKQGLFNIQRIGIQK
FGVDGYTNENSKNVHEYLYNPISLNSSSVMYQPFAGLATEGKK" /product="Biofilm PGA synthesis deacetylase PgaB (EC 3.-)" /transl_table=11
CDS 3414665..3415912
/locus_tag="AUO97b_03299" /db_xref="SEED:fig|400667.82.peg.3164"
/translation="MKVLLDILFAFAFLYPLLMAWTWMVGGLWFFFKREYHEQQLPEP
SSEGCSIIIPCFNEEAQVRQTIRYALQTKYPNFEVIAVNDGSSDSTAEILDELAAQDA
RLRVVHLAENQGKAVALRSGVLVSKYEYLVCIDGDALLHPHAVLWLMQPFLNFPRIGA
VTGNPRILNRSSILGKLQVGEFSSIIGLIKRAQRTYGRIFTVSGVIVAFRKTALVRVG
FWSDDKITEDIDISWKLQMDHWDIQYIPQALCYIYMPETFKGLWKQRLRWAQGGVEVL
LEYIPKMFKLRLRRMWPVMLEALVSIIWSYVMIMIFILFFVGLFVDLPQQFQINSLMP
QWYGVILGGTCLVQFLVSLWIDHRYDRGRLFRNYFWVIWYPLFFWLLTLFTSVVAVPK
TIFNTKKRARWVSPDRGFRGDHS"
/product="Biofilm PGA synthesis N-glycosyltransferase PgaC (EC 2.4.-.-)"
/transl_table=11
CDS 3415909..3416373
/locus_tag="AUO97b_03300" /db_xref="SEED:fig|400667.82.peg.3165"
/translation="MKNDANIDVEVLDIPEYIDQPEYVKNKTANYTLQTIGWFCLMWL
LFPLVSLFLWIFEGHLIYDYVWVDHVSEVKTLLHLLLLIGLSALILILWASYNWLRFH
GDDRRSKAPNSSVELLASQFMVSTESLSELQKSQRIILHYDEQGYLTRYDLK" /product="Biofilm PGA synthesis auxiliary protein PgaD" /transl_table=11 CDS complement (3416414..3416977)
/locus_tag="AUO97b_03301" /db_xref="SEED:fig|400667.82.peg.3166"
/translation="MSIDLKNNVAKSAIFQNTVEDFEAFTGKILPSNKLKEFDFSHLN
VAIIGTDQETVTHLEKICQQAKFVTVFQITPHFILPHSQIGIHRLITHPLIAKNRRLF
NNRVKSILALRFLETQVNETWLKRLLTPNTARANKTFFKSDSYYTALQRPNCKLQTWP
IVKVTDTAIYSMDGTQRPVDIIIRTTP"
/product="Cyclohexanone monooxygenase (EC 1.14.13.22)"
/EC_number="1.14.13.22"
/transl_table=11
CDS complement(3417124..3418128)
/locus_tag="AUO97b_03302" /db_xref="SEED:fig|400667.82.peg.3167"
/translation="MRSLQDTKFSILELAPIRDDKTIEFSLRHALELAQHAEKLGYDR
FWLAEHHNMDGIASSATAVLLGFIAAHTQRIRLGSGGIMLPNHAPLVVAEQFGTLATL
YPDRIELGLGRAPGTDQVTMRALRRGHQETEDQFPRDVVEILQYFKAPQVGQKIIATP
GQNTHVPVWLLGSSLFSAQLAAKLGLPYSFASHFAPRMLGQAIELYRENFEPSEYLDK
PYVSMGVPVVVANTDEEAQFLATSAYQRVLGLIRGESLKLKPPVTSMQGLWNTAEQLS
VENFYTMAQIGSVETVRSGLQKLLDTYEVDEFIFTCDVYDTVKRLDSFTQLMQVKSGN "
/product="Luciferase-like monooxygenase YhbW" /transl_table=11
CDS complement(3418263..3418766)

/locus_tag="AUO97b_03303" /db_xref="SEED:fig|400667.82.peg.3168"
/translation="MKMNVWLASALIATTSMVTVAHADNGTRVAATSALGSVVGTAIG
KSIGGTSGATIGAALGGAGGAAAASDRRNRTEAAIGGALGGGAGYTVGKNMGGTNGGY
IGAAVGAAGGSALGRKVAQDRNYDDRYDRRYDRDDRRYYDGDRRYYRGGNYRHDNGLH RGWYKHR"
/product="hypothetical protein"
/transl_table=11
CDS complement(3419001..3419423)
/locus_tag="AUO97b_03304" /db_xref="SEED:fig|400667.82.peg.3169"
/translation="MKLNALLCSAVLATSMVTVAHADNTTHVAAASALGSIAGTAIGK
SMGGTSGATIGAALGGAGGAAVASDRRHRTESAIGGGLGGGAGYTVGKSMGGTNGGYI
GAALGAAGGSALGNKISKDRDADHSSKYWKKKHRHHRH" /product="Glycine-rich cell wall
structural protein precursor" /transl_table=11
CDS complement(3419622..3420524)
/locus_tag="AUO97b_03305" /db_xref="SEED:fig|400667.82.peg.3170"
/translation="MSRQFDHLADVEVFLIVAEKLSISAAAIDLSTTPSVISRTITRL
EKKLGIQFFRRTTRHLSLTEAGQLYYEKMQHAFQLIDHAERVVQGEQLQISGKIKLSV
PTTYGHYRLPPLLEKFLLKYPDIQIEISISNRNVDLIAEGFDLAIRQGQLPNSTLIAR
PLEQAPLKLVASPAYLKRMGIPKNLEELKHHQCIAFELPSSGKITPWFLKNDNEEIQW
IPTNRILVKEDIVGVVTLAESGLGICQSYDFIVNEKIQQGKLQPILTTYAGHTRPFSL
LYAQHKHMSSATRALIDFLCSNDD" /product="Transcriptional regulator, LysR family"
/transl_table=11
CDS 3420665..3421483
/locus_tag="AUO97b_03306" /db_xref="SEED:fig|400667.82.peg.3171"
/translation="MFAMIAASAALSANAAHVLVVLSDEGQLQLKNKQVFKTGFYLNE
LMQPTKMLLDAGHTVTFATPKGKAPTLDESSNNAMYFNQDEKALKQYAGLLHDLKLTS
AQDSPVVSLARIEQIGIDQFDAIYIPGGHAPMQDLLKDKQLGKVLTAFHKAGKPTALV
CHGPIALMSALPHAAEAVKQLEQGKKVKTGEWIYKNYRMTVISNQEEEQAKGLLKGGE
MKFYPQTALESLGAKYQSNTKAWSPNVVVDRELITGQNPASAVLVGKSLLEKLK"
/product="Intracellular protease"
/transl_table=11
CDS 3421674..3422762
/locus_tag="AUO97b_03307" /db_xref="SEED:fig|400667.82.peg.3172"
/db_xref="GO:0003747"
/db_xref="GO:0006415"
/translation="MKASLRLRLDQLCDRHEELTALLADAEVISDNKRFRKLSREHSD
LTEITEVWGKYRQAEEDIETAEMMKSDPDFKDMAEEEIQANKALLEELESQLNILMIP
KDPNDSNAAYLEIRAGTGGDEAAIFSGDLFRMYSKYAESQGWRIEVLSENEGEHGGFK
EVICRVDGDGVYGRLKFESGAHRVQRVPATESQGRVHTSACTVAILPEIDVDTNVEIN
PADLRIDTYRASGAGGQHINKTDSAVRITHIPTGTVVECQEERSQHKNKAKAMALLVS
RLENAKRAAADAATSEMRRDLVGSGDRSERIRTYNYPQGRMTDHRINLTLYKLDAIME
GDLTELLDSLHREYQADQLAMLAQENGG"
/product="Peptide chain release factor 1"
/transl_table=11
CDS 3422762..3423583
/locus_tag="AUO97b_03308" /db_xref="SEED:fig|400667.82.peg.3173"
/translation="MNIAQALAIRGEPDSYERQENAWLLEHFLKINSLELKFRLEQEL
TAQQEQDYLAGLERIQNGEPLAYVTGSQPFWTLDLKVTSDTLVPRPDTEVLIETVLNL
NLPKNANIVDLGTGTGAIALALASERPNWFVTATDIYAPTLDVAKENAQTHGLHHVKF
ACGAWFEALEPQQFDLIVSNPPYIDPEDEHMQALATEPRRALVADHHGLADIEIIIAQ
GKNWLKPQGWIALEHGYDQGQAVRGIFAEHGFSEIKTIQDYGQNDRVTLASLLIK" /product="Peptide
chain release factor N(5)-glutamine methyltransferase (EC 2.1.1.297)"
/transl_table=11
CDS complement(3423591..3424619)
/locus_tag="AUO97b_03309" /db_xref="SEED:fig|400667.82.peg.3174"

/translation="MVVQSSLSKGTISIALVHEALSAAYAKGLNTQIILNKAGIPAEL
LMTPKARVPVATYAQLWIELANAMDDEFFGMDSHPMRRGSYKLLTKLVSTAETLEKAL
YDILKFFNFVLDDIRGELIREQEKAYLVIHDQKQPKRMFTYATFLMLVHGLMCWLVDQ
RIGLNRIAVRCPKPNDIQDYLVRFCEDIEFNAKTNQIEFDAHYLDIKIKKDKKALYDF
LEQTPQNLLVRFKNENALSVVIRRHLLKLHPAEWPELKDVAQQLNISEATVQRRLKHE
GVSYQQIKNEIRCDIAIERLSKSNDSIQDISEDLNFHDPSAFHRAFKKWTGVSPGAYR EATLNSTA"
/product="Transcriptional regulator, AraC family" /transl_table=11
CDS 3424785..3425555
/locus_tag="AUO97b_03310" /db_xref="SEED:fig|400667.82.peg.3175"
/db_xref="GO:0003857"
/translation="MKIQGKHFVITGGGSGLGAATAEYLVKQGASVTLVDMNVEAGEQ
QAKQLGPKADFVKLDVTDEAAAEQFFKDVLVKHGHLHGLVNCAGIGPSAKVVGREGVH
DLGLFAKTLNINVTGTFNMLRFAADAMSKNTVEEGEEDRGIIVNTASVAAFDGQIGQA
AYSASKGAIVAMTLPIARELARHAIRIMTIAPGIMETPMLKGMPQNVQDALGQMVPYP
SRLGKPEEFARLVAHIAENSYLNGEVIRLDGAIRMAAK" /product="3-hydroxyacyl-CoA
dehydrogenase" /EC_number="1.1.1.35"
/transl_table=11
CDS 3425574..3426710
/locus_tag="AUO97b_03311" /db_xref="SEED:fig|400667.82.peg.3176"
/translation="MILNAEQSMVQEMMRNYAQNQLKPTAAHRDKTHEFPAQELKDLG
ALGAMGMTVPDEWGGAGMDYVSLVLAIEEIAAGDGAISTIVSVQNSLICGITLAYGSE
QQKQTYLPKFASGEWLGCFCLTEPHVGSDASAILCKAERDGDHWILNGVKQFITSGKN
AQVALVFAVTDKQAGKKGISCFLVPTNTQGYLVTRIEDKMGQHASDTATITLEDCRIP
LENLVGQEGEGYKIALSNLAAGRIGIAAQSVGMARAAFDAAVQYANERKAFGVELVQH
QAVGFRLADMATQIEAAHQLVLHAATLKDAGLPCLKEASMAKLFASTMAERVCSDAIQ
IHGGYGYVSDFPVERIYRDVRVSQIYEGASDIQRLVIAREVAQV" /product="Acyl-CoA
dehydrogenase, short-chain specific (EC 1.3.8.1)" /EC_number="1.3.8.1"
/EC_number="1.3.8.4"
/transl_table=11
CDS complement(3426707..3426847)
/locus_tag="AUO97b_03312" /db_xref="SEED:fig|400667.82.peg.3177"
/translation="MKNISSTFPSISNKTNSKNASFQLQETAHGVRLLAVLSLKTQYF MI"
/product="hypothetical protein"
/transl_table=11
CDS 3426931..3428574
/locus_tag="AUO97b_03313" /db_xref="SEED:fig|400667.82.peg.3178"
/translation="MKTLAEAYQQFDLTRLIEEQLVGHPQAINAYVECCERYVGKNKT
ALIWEGKNGQAEHYTFEQLAELSGKLANFFKAQGIQAGDCIAGLLPRTPELLITILAA
WRIGAIYQPLFTAFEAKSIDHRITTAQTKLIVTNDEQRPKLNTLNVPVIVTVHQTGLL
SEHDFDFWQSLQRYSEQCEPVNRSFDDDFLMMFTSGTTGLAKSVPVPLKAILAFKGYM
THAVDLREEDMFWNLADPGWAYGLYYGITGPLSLGHSIIMDERSFNVDQAIELIKKYK
VSNLTGSPTAFRMFFGFKEKFDPSIKQHLRVVSSAGEPLTPEVVNWFKQDLEVNIFDQ
YGQTELGMVIANHHALEHPLKVGSAGFAIPGHRFAVLDQNYQELPTGGVGILAMDSEQ
SPLMWFKGYGGNNRKAFVGKYYLTGDTVTLNEHGGIDFVGRADDVITTSGYRVGPFDV
ESTLLECEAVLESAVIGKPDPERTEIVKAFVVLKPAYQACETLKDKLQQYVRNRLSRH
AYPKEIEFVDSLPKTTSGKIQRGLLKQQEIAKMQQAYAS" /product="Acyl-CoA synthetases (AMP-
forming)/AMP-acid ligases" /transl_table=11
CDS complement(3428619..3429197)
/locus_tag="AUO97b_03314" /db_xref="SEED:fig|400667.82.peg.3179"
/translation="MSQSYIAPVELEKAYRLLNHGPTVLVSAQHGDDRNVMAAAWACA
LEFKPAKVTVVLDKSTKTRQLVEQSGYFTLQVPCYAQLNMTKQLGTISKLDDPQKLEH
CGVELFYQKDLTSPLVSGCIAWLVCKLISEPHNQSAHDLFIGSVVGAWADSRVFRDGH
WHFQDAPKELRSLHYIAGGTFYLIGEEVKADL"
/product="NAD(P)H-flavin oxidoreductase"

/transl_table=11
CDS 3429367..3430143
/locus_tag="AUO97b_03315" /db_xref="SEED:fig|400667.82.peg.3180"
/translation="MTELQDIDFVELSDEEMHLYSRQILLDGWDIEAQEKLKLANVLI
VGAGGIGCSSAELLARAGVGKITLIDADTIEISNLQRQIAFGHEDIGRYKAEVLAKRL
QKINPYICVEYFNERLDEHNIDKLVEHQDVVLDGCDNFTTRYLVNAACKKHQVALISA
SAIGFQAQMFMVEGDSACYECLFPKEQHANEGLRCAESGVLATTPVMIASLQAHHTLL
YLGLNRTPLKQKLLLWDGLNMTQRIVSFDKDINCPLCQAS" /product="Molybdopterin-synthase adenylyltransferase (EC 2.7.7.80)" /transl_table=11
CDS 3430202..3431500
/locus_tag="AUO97b_03316" /db_xref="SEED:fig|400667.82.peg.3181"
/translation="MKKNILFDGLRSVARIGETAVVAAKAGIKYATDKPSNAKLMRET
FESLGSTYIKLGQFIASTPSLFPREYVEEFQGCLDQTPTLPFSYIQGVLASEFEGRDL
SQIFSYIDEKPLASASIAQVHAAKLTTGEDVVIKVQKPGVETILYTDLNVVHWAAKLL
ERAVPKIKFAALSEIVDEIKTRMVREVDFIEEAQNLDDFVEYLNISQNQAATAPKVYH
QFSTRRVLTMQRLYGVPLTDFSVVKQYAKDPSQVLITAMNTWFGSLMLCKSFHADLHA
GNLMLLEDGRVGFIDFGIVGQLKPEVWTACIAFMDALQKTDYQAMAENMLKMGMTHNK
IDVQVLAQDLERLFNGVLMADPQQILASNPADLNDIMMDMVAVGERHGIKFPRDFALL
FKQMLYFDRFMRVLAPYTDIYADQRLKMVQNMEPASLLKH" /product="Uncharacterized ABC1 family protein A1S_2145" /transl_table=11
CDS 3431516..3431896
/locus_tag="AUO97b_03317" /db_xref="SEED:fig|400667.82.peg.3182"
/db_xref="GO:0004150"
/translation="MDAIIIEGLKVETVIGCFNWERQIIQPLMLDLTIHNDLSRAAES
DKLEDTLNYAQICELSAQTIQQAKPELIEHAAHLVLQCLFETFPSIEKITITIRKPAI
IAEANAVGIRLERTRNNFCARPSE"
/product="Dihydroneopterin aldolase (EC 4.1.2.25)" /EC_number="4.1.2.25"
/transl_table=11
CDS 3431853..3432305
/locus_tag="AUO97b_03318" /db_xref="SEED:fig|400667.82.peg.3183"
/db_xref="GO:0003848"
/translation="MNAPETIFALALASNLDADQHFTFAYTQLATLGKVQFSSVYQIP
CRDGIGDDYWNSACLLKSTLSSEQIESFLKKLESDSGRVRPSHHISLDVDLIAWGADL
DHMQFNSKKLPLALDVKIPLYELWHCETLKADSTLYPVVNFKVECLSN" /product="2-amino-4-hydroxy-6-hydroxymethyldihydropteridine pyrophosphokinase (EC 2.7.6.3)"
/EC_number="2.7.6.3"
/transl_table=11
CDS 3432550..3434259
/locus_tag="AUO97b_03319" /db_xref="SEED:fig|400667.82.peg.3184"
/db_xref="GO:0008556"
/translation="MLELILVLFIAIFLAWCLSKYLSKVMANQPMWGDGLFRWIENPI
YRLLGVSPQQQMNWKQYSLAFVVSCIFLAVAILTIFMTQAWLPLNPNHAPNMSWDLAL
HTVISFLTNTNQQHYSGQAQLSYLSQMTGVVGLQVITPMMGLALVVATLRAFFYQRPS
HIAADIAEQPDQILIGNYWADVIRPTVRFLLPLCFVWSLLLNSQGVPSTFQSGPEVQL
IDKANSVETQKIPLGPVAPMVAIKQLGSNGGGWYGPNSSVPLENPTPFSNLLEMIAIL
LIPITVIFMVGHFTQRKKFAYFVFGSMLFMSIISGAAAVWSESMSSTASQFAVMEGKE
QRFGPAASAVWAALTTQVNNGSVNMMHDSSAPLTGLVELINMLINAIWGGVGCGLQQF
MIYLLLAVFIAGLMTGRTPELFGRKIEAAEIKLLAIIILIQPLVILAFTALSLSIPSI
SGISNPGPHGISQVFYEYVSAFANNGSGFEGLGDNTVWWNVTCSIALLLGRFPTLILP
LMIATRLAAKRKAPETSGSLQVETPTFALTLITIVVLLTLLQFMPVLVLGPIADQLLL VKG"
/product="Potassium-transporting ATPase A chain (EC 3.6.3.12) (TC 3.A.3.7.1)"
/EC_number="3.6.3.12"
/transl_table=11
CDS 3434274..3436307

/locus_tag="AUO97b_03320" /db_xref="SEED:fig|400667.82.peg.3185"
/db_xref="GO:0008556"
/translation="MNTQTHVNSHKQTMAVPNFEVWKNAFVKLLPQHAIKNPVMAIVW
LGTVVTGISTILGYTTLIFGLVVTAILFITILFANYAEAVAEARGRGQASSLRAAREN
LMARRLNSLTDRQATQVAATELHLNDFIEVHAGELVPADGEIVEGFATINESAVTGES
APVLREAGTDRSGVIGGTKVLTDRIVVQVTAESGQSFLDRMIALVEGSNRQKTPNEIA
LGFLLIVMTITFLIVVISLPFIADYLHIELDPVVLVALLVCLIPTTIGGLLPAIGIAG
MNRALKANVLAKSGKAVEVAGDIDVLLLDKTGTITYGDRQATSFYPLTSVTESELRAA
AWISSLADPTPEGKSIVKLAKEQGLKQQEPEQAEFISFSASTRISGVNLPNGDQIRKG
ALDAILKFVDEDYSQDLELKSRVEQVAKKGATPLVVANQHHVLGVIELSDVIKHGIKE
RFARLREMGIKTVMVTGDNPLTAAAIAAEAGVDDYIAEAKPEDKLACIRTEQQQGRLV
AMVGDGTNDAPALAQADIGLAMNSGTQAAKEAGNMVDLDSDPTKLLAVVEIGKQQLIT
RGALTTFSLANDVSKYFAILPALFAAAIPQMQVLNVMHLASPNSAILSALIFNAIIIP
LLIPIALRGVKFKPSMATQLLRRNMLIYGVGGVVLPFLAIKAIDVVIVQLFGL" /product="Potassium-transporting ATPase B chain (EC 3.6.3.12) (TC 3.A.3.7.1)"
/EC_number="3.6.3.12"
/transl_table=11
CDS 3436321..3436926 /locus_tag="AUO97b_03321" /db_xref="SEED:fig|400667.82.peg.3186" /db_xref="GO:0008556"
/translation="MKTYAQNSEANLGQTLRASFGLLIFTLVGCGAIYSAIATGAGQV
LFNNQANGSLIEIDHKVLGSKLVAQPFIGEAYFHPRPSAVAYDPMAMGATNLALTNPE
LQKQIDAQILEVKKQDHTGSTAIPSDLVTKSGSGIDPHISPESAQLQVARVAQARHLD
PKVVEELLQKHIEPVQFGVLGQARVNVLELNIALDQLQSIH" /product="Potassium-transporting ATPase C chain (EC 3.6.3.12) (TC 3.A.3.7.1)" /EC_number="3.6.3.12"
/transl_table=11
CDS 3436965..3439619
/locus_tag="AUO97b_03322" /db_xref="SEED:fig|400667.82.peg.3187"
/translation="MQSNRENQAEALLNHVNRYQAGRLTVFLGAAPGVGKTYAMLARA
KELYQQGTDVVVGIVETHGRIETLKILEGLPQIARKEMQYQGHTLEEMDLDAILLRHP
QIVLVDELAHRNVPNSRHERRWQDVNELLDAGIDVFTTINIQHLESLNDVVYQITGIR
VNETVPDRVFDRIRDIRLIDLPVSELIERLHQGKVYVPEQANLALQGFFSISNLTALR
ELAMQCVAEHVDSDLKESYASKGLKSISLQNELMIAIDGQGSSEYLVRAGCRLAERNG
ATWTVVNVAKSLDFGQSSVSSYKKEYIEIDRAFELARQLGGRTEVLYGPRVASVLMDA
AVDRGISNLVIGKSISPWWLKLFKKNLAQQLLNQENSIALTILHPEQGTKKINQLKKP
SFLSLKESVFVLAVTCGSIFIAHFAEVLFGIEDFSVIFIISVLIVATKTRMLAAVVAA
LICFLAYNFFFIAPRYTFQISAHQGVVTVVAFFAAALIAGRLASQLRQQVLSLKAANA
YTTVMQDLARKLSSAVNLEEVMQTGRMTLETQLQTKVWISIRDKIISSDIELNDKEKV
AAEWCLKHQQPCGRFTDTLSQSNWWFLPLLEQKNSLGIVGIYFKDEVVSLNFEQKKLT
ESVIEYIAQAALRTQLVNELEQAKVTSETERLRSALLSSVSHDLRSPLASIIGAADTL
ANFKAEMTEQDQQDLLETIHLEGERLDRYIQNLLDMTRLGHEGLTLKRDWIGVDELIG
SATRRLKRYKPDTQVVVQLPEQPISLYVHPALVEQAIFNVLENAANFSPPDESVMIRA
QLLSEDEVKIEIEDKGVGIPEDERHRIFDMFYTMERGDRGKFGTGLGLTIVKAIIGAH
MGTIEAFSGRQNKGTLIQIKLPLRPVKE" /product="Osmosensitive K+ channel histidine kinase KdpD" /transl_table=11
CDS 3439631..3440347
/locus_tag="AUO97b_03323" /db_xref="SEED:fig|400667.82.peg.3188"
/translation="MISSETSPVETRIVIIDDEPQIRKFLDIALRTQKYKTTLCETGY
KGLEALATQGADLVILDLGLPDLDGKEVLQELRSWSNVPVIVLSVRADEYEKVALLDA
GANDYMTKPFSVQELLARIRVILRNQPIQQEAHIYDDGYLKVDVTQRLVWIEQQPITL
TRKEFQLLALLMRYQGQLLTQPQLLKELWGPTHQEDTHYLRILVGKLRSKLGDNAIQP
RYIATEPGVGLRFLAKQKNH"
/product="DNA-binding response regulator KdpE" /transl_table=11
CDS complement(3440425..3441849)
/locus_tag="AUO97b_03324" /db_xref="SEED:fig|400667.82.peg.3189"

/translation="MSTLIYPSLNLKDSHFKAFKISDDFIPQTQIHTFNSDLFLKEVE
EYLSLYPHTQHIDICLHDLNGHIRGKRIDVKSLKNLSNGCYFPLSVYAMSLDGKVIEE
TGLGKYIGEPDRLCLPILGSLQPSALSPELNAQLYLSMQEEDGSDCRYEPRNILKKLL
NQLNANNYFPVMAAELEFYLFSPQHQSETCIENQCFDIDAPNNYQQVLDEVEKAALLQ
SIEITAIVAESSPGQYELNLQHSHDILKLCDQINALKRIVKQVARKHDLTACFMAKPN
LAKAGSGMHFHMSMLNQYHENIFSSKVQKDELSAKLLSAISGLIELMPASMAILAPNI
NSYRRFKIGHHVPLEANWDTNNRNVAIRIPCSDVQNQRLEYRVAGADCNPYLVTATIL
AGASYGLSHKLPLPKPAHLLKFPDEHILLANNQPEALKIFKGSLILKGYLGADFVEHW
YTVKQAEYQSIYSQMTEAEQHWDI"
/product="glutamine synthetase family protein" /transl_table=11
CDS 3442257..3442805
/locus_tag="AUO97b_03325" /db_xref="SEED:fig|400667.82.peg.3190"
/translation="MKNIGLAILAISLSGCAAMSVEECKTANWSLVGEKDGSKGSSPR
LDQYYKACGKANIVPDQKSYERGYKEGLGYYCQPANIFYNALEGNGNINVCPVEQRNR
LRPYYRAASDYYNAKAEYDRYDEKFKQYSDSAYNEKLKPEERERYRKLLRELQIDRDR
INRNYWNSIRDIERFKYDHGLK"
/product="ATPase involved in DNA repair"
/transl_table=11
CDS complement(3442853..3443488)
/locus_tag="AUO97b_03326" /db_xref="SEED:fig|400667.82.peg.3191"
/translation="MRHYTGIDQLINSFDQALRSLVPGATAAQRQNPAETVEAKLGVE
DARHVAGLMRVNHSGEVCAQALYHGQALTAKLPNVRREMQQAAIEEQDHLAWCEDRLK
ELNSHTSLLNPIWYGLSYGMGALAGIAGDKYSLGFVAETERQVSLHLQDHLNQLPAQD
ERSRKILEQMNEDELHHRHTALEAGGVELPYAVKITMTAISKLMTKTSYYL" /product="2-polyprenyl-3
-methyl-6-methoxy-1,4-benzoquinol hydroxylase, coq7 type" /transl_table=11
CDS complement(3443492..3444814)
/locus_tag="AUO97b_03327" /db_xref="SEED:fig|400667.82.peg.3192"
/translation="MMKQDESLMTSRRFVPMFFTQFFGALNDNVYKQALLLVITYGWI
QQQSAPVSTLNNLAALLFILPYFFFSATAGQIADKYERSQLVRGIKILEIIIMLIGSA
GFLLGHLWLLLLALFLMGTHSTFFGPIKYAILPEILKPHELMSGNALFQSGTSMAILF
GMILGGAVIASSDGNLLWISITVVAIACIGYLFSRFILPQKVAAPDLDIDWNFIRTSF
QTIKYAKSLPLVFTILLGNSWYWFYGATYLTQIPQLTQQNLHASENVVSLLLTFFSVG
IGVGSLLCRKIGGSEINIKMVPIGAVGLTVFALYLAASLAFVPERTGALLTLKDVFTQ
GWVYYHVMIAVTLLGISGGFYIVPLYAMMQAYSPRSHRARVVAANNILNAVFMVSSAI
FSILILSVLKIDIKILFIITAVLSAIFSIWLLARLKPMLATAHLSLED" /product="Lysophospholipid
transporter LplT" /transl_table=11
CDS 3444980..3447700
/locus_tag="AUO97b_03328" /db_xref="SEED:fig|400667.82.peg.3193"
/translation="MPSKIKFKQSTLSHSMHLILKMQSIPKLICSSLLLSLCVTPCYA
QSSAETVTPEANQTVTDSLVQQTNTNNPSDVPITDVATLVTQAQQQQDSLAILQQQEQ
FPNQIEEFKPITLDNLEDLPVMPVDQNMANEIYRVAEEAKNEAQNFQNGTQKQPEMVV
NDASQAELHEINQAPVNIDQLMHEIQSDSKIVVEANETGKTLPELTAAVEEPPEEKGF
FRRIFNKIRPPRVIPMEQIPRITAEVTGAPDDLAKNIKGKLSTFTQESFEDFNAALPQ
LRSLSNQAAQAVGYYNAEFRFEKLSASRVRVNVTPNEPVRINEQNIEFTGAGAKQPQF
QVIRLVPDQDVGDIFNHGLYETTKSRIVDAASDNGYFDAYWRLHDVKVSQPENKADIN
LKYETGERYKLGKVEFRMSDPSKPLPLNMNILESMAPWKEGDDYAFWRVNVLANNLTN
SRYFNYTLVDSIKPDPIEKPLELPPDLQALVDQQNVDIDESKLLPLEQQQLAKARQLA
SSSKEVTQNVVDEKQFAGTESVQAAPASLKAATVQHEEQESEQDRLQAQAREEKRIPV
IVTLNADKLNSLETGIGYGTDTGARLRSQYRRSIVNKYGHSFDANLELSQIRQSIDGR
YSIPYKHPLNDYFNIVGGYERETRDDIGPDVSLLTESAVLGGERVIKKPLGNWQHTIG
VRYRLDRLTQKGNVDISELPDAFKTAASEQEALLFSYETSKTSSNTRLNPTKAFKQTY
KLELGSESLLSDANMAIATAGWRFIYSLGENDDHQFVGRSDFSYIFTDEFDKVPYNLR
FFTGGDQTIRGFDYKSLSPEDNGYKIGGQALAVGSLEYNYQFKEGWRAAVFSDFGNAY
DKSFSNPTAYSVGVGIRWKSPIGPIRLDVASGISDDNHPIRLHFFIGPQL" /product="Outer membrane

component of TAM transport system" /transl_table=11
CDS 3447727..3452232
/locus_tag="AUO97b_03329" /db_xref="SEED:fig|400667.82.peg.3194"
/translation="MAEVEQQPTSAPSSPKKRRILRSFLLTILIILLLLVASIIIMMS
TDRGSRFLLDRVLQAQQVIKYEYEGGNLLRGIILKNIIVQLKEVDVTLDRADVRLGWR
SLILEKEVHLSNADVRNLVIINKAPPSDKPFEFKPIKLPFVLRVDVGDVDHLEIKNSG
SVVNFHDVHLNDALWSETKLKFENSSMDMGYLSVHNATGNMDFSGKYPLNATADLRIP
SLKSLNIQNIKVAARGSLDTLQAGVATTTPDLLTGWVVLHPVRHEVPMQGALLLKNYH
LPLLVEQKLFAKNGVIKFQGDIKQLNLALETDLKGENLPEGQYNALMNTDLVHQLNIT
DFNGQVMKGAVNLKGLVNWKDHVTWDIKGRLDHVNPKDKAIPQVVQDFLPPSLDAAVS
STGSLEKGTEVFANVDFDRYESWKLKLNQAPEKNKKPQPMFMNVAWANIDRAMPYIGW
LSSDSGQVDLTLRDGQQDIKVSTKVYQHEQTLLPAGQYLATLNVKDNILNVPSFSFAA
QKGSLTGQAKVLLPSEKRQLAWNALLNAKDFNPQSIHTAAPVNLLNGSIKASGFAKPN
QQIIQLEKIDLKGRLAQAGQEMVALAGKSTAALLFNDVKAGGGFKGFAVNYDGSLKAL
NQANGLLKFSIAGTPEFIRISQLQHDGVAGKIYATGALNLKDRIAWDINSSLVRFKPQ
YFASSVKGEISGNVKTQGIWSDKLKRIDIQQLNLAGYLNNKPVRGKGNLSILMDSNQK
GFLPQQFEANNLFLVYAQNQLQATGNAQNLKIKLNAPALYELYPGLSGRAYGDLSVQS
QPRLKATANIAVDNFAFNTLVSIKKLRIQGELPTSETTPTQLTAKLDNLRSGSRQIQS
AEVNLTGTRKAHLLKVQGNNNVSKFYVQLAGGFNAKNDWLGQIQKGSFDSRRIRLAQN
QNAPVVFSSARSELYVGQHCWQSTNSQLCLDQPVRVSKAQGNISFVTQNLDLGDFAAF
MPEGLAMTGQLNGYAKASWVNGGHPKLDARLVTRKGELGLAAEDPQDPPTTLAYDELS
VIAKSISEGLLFRVDVKTPDIGTGYANVIINPYQSSMPMHGEVAFNDVQLKVLKPFIQ
DVRSMSGTLALAGKVNGTLTQPQFTGEMRLKNGAISMISLPVNLTNVQVYSSIRQDMA
TIDGAFNSGQGVGLLKGSFDWKNAPRLQLNLKGDNLLVRQAPLITAIANPNLTLDMYP
FDKRLSLKGSVDVPRARISMPETTAPVVNTSSDVRIVRQGQDPLAILRAAKPWDIRAD
ISVNIGNQVIFQGFNSNIPLVGRLNLTQRGYETAMRAMGAIGVSQKVKIEAYGQSLDL
NRAIARFNGPLANPTLDIDANKNVQGSMVGVRVTGTASSPNIQVYNDAGLSEQEALNA
LVTGRINEGASGLSNAEGFKSDVNNTIAAAGISMGLGGTRALTNQIGRTFGLSGLALD
AQGTGDDTQVSLTGYITPDLFIRYGVGVFTPVNKLTLRYQMNRRLYLEASQSLERAID LFYNWRF"
/product="Putative exported protein precursor" /transl_table=11
CDS 3452378..3452737
/locus_tag="AUO97b_03330" /db_xref="SEED:fig|400667.82.peg.3195"
/translation="MKKVLFVLMGMLLVGCTEKKPLTPEEQWHGYCTSVGNAARSILF
DRQQAIEKSQAIEHANKIEDEITKKFIFNIIEKVYAIPQDELKTNPEALQEKIRKQMT DECLVTPHDKMPNYKKF"
/product="hypothetical protein"
/transl_table=11
CDS 3452996..3455635
/locus_tag="AUO97b_03331" /db_xref="SEED:fig|400667.82.peg.3196"
/db_xref="GO:0047456"
/db_xref="GO:0003994"
/translation="MLEAYRQHVAERAALGVPPKPLDDAQTAQLVELLKNPPAGEEAF
LVDLLENRVPAGVDQAAYVKAAFLAAIAKGEATSPLVSKERAVYLLGTMLGGYNVAPL
VELLDDAELGSLAAEALKKTLLVFDAFHDVADKAKAGNANAKAVLQSWADAEWFTSRK
DVPEEIKITVFKVTGETNTDDLSPAQDAWSRPDIPLHANAMLKNERDGINPEKPGEVG
PLSQIKELIAKGNQVAYVGDVVGTGSSRKSATNSVLWFFGDEIAHIPNKKDGGVCLGG
KIAPIFFNTMEDAGALPVEIDVSNMNMGDEVTLKIDHAAAKVTAFKNGEQIAESELKT
PVLLDEVRAGGRINLIIGRGLTAKAREALGLAPSTLFRTPVQPADTGKGFTLAQKMVG
RACGLPEGQGIRPGTYCEPKMTTVGSQDTTGPMTRDELKDLACLGFSADLVMQSFCHT
AAYPKPVDVQMQHTLPDFIMNRGGVSLRPGDGIIHSWLNRMLLPDTVGTGGDSHTRFP
IGISFPAGSGLVAFAAATGVMPLDMPESVLVKFKGKMQPGITLRDLVHAIPYYAIKEG
DLTVEKKGKKNIFSGRILEIDLTEMETDLTVEQAFELSDASAERSAAGCAITLSEEKV
AEYLRSNITMLKWMISQGYGDARTMARRVENMEKWLANPSLLKADADAEYTKVYEIDL
SEIKEPILCCPNDPDDAKLLSDVQGDKIDEVFIGSCMTNIGHFRAAGQLLEKVPSGSL
TTRLWLAPPTRMDEHQLMEEGFYNTYGRAGARTEMPGCSLCMGNQARVAPNTTVVSTS

TRNFPNRLGQGSNVYLASAELASVAAVLGKLPTPEEYQQYAAQIDSMSADIYKYLNFD
QMGEYTNAADKVDTKKIAAAQLT"
/product="Aconitate hydratase 2 (EC 4.2.1.3) @ 2-methylisocitrate dehydratase (EC 4.2.1.99)" /EC_number="4.2.1.3"
/EC_number="4.2.1.99"
/transl_table=11
CDS 3455863..3456297
/locus_tag="AUO97b_03332" /db_xref="SEED:fig|400667.82.peg.3197"
/translation="MLKTIVEFKFDDYQLYDQNLSAEDGNTLVQKTEDIAQYIYSILK
NRYHLSIELTAERWGWEIEVPLPEITMYIGISVYEEYSNGFAIFISPNTPILRRFLFR
KLDITHHIMLLQNYINVILKSHAGIYDVQWWEENEFRYVAAH" /product="FIG00349963: hypothetical protein" /transl_table=11
CDS 3456436..3457419
/locus_tag="AUO97b_03333" /db_xref="SEED:fig|400667.82.peg.3198"
/translation="MRILAWKTVRRFFYNNLHNHDYETTVSRCINYFLVFLIIGNVVA
VLLETVNDLYYSYRIWFDYFENVSIAIFSVEYLLRLWSIVERDLTQAAWKQRLAWMKS
GEALIDLMAILPAYLNFFVRIDLRMLRILRLLRLLKLTRYFISLQILLCVIKREKGSF
QAVIFILIIMIIMTASGIYVVENKAQPEAFSSIPKAMWWAVVTLTTVGYGDVTPVTSL
GKLLGALITILGVGIAALPAGILASGLANELNQRNQRLEQEFRELLQVRGIDIMHDEV
EIERIRQKVGLPKEQAHNLIIQIMREKVLEEKEAVREKKCYCPHCGEKLTD" /product="Potassium voltage-gated channel subfamily KQT; possible potassium channel, VIC family"
/transl_table=11
CDS complement(3457445..3458593)
/locus_tag="AUO97b_03334" /db_xref="SEED:fig|400667.82.peg.3199"
/translation="MAIFSLAVGGFCIGTTEFVAMGLIQEIAHNLKITVPEAGHFISA
YALGVVIGAPIIAILGAKVPRKTLLLCLMLFYGIANACTALAHTPETVLVSRFIAGLP
HGAYFGVGALVAAELAGPSRRASAVAQMMMGLTVATVIGVPLATWLGQHFGWRAGFEF
SATIAFFTLIAVACFVPNIPVQATASIKTELAGLKNINMWLTLAVGAIGFGGMFSVYS
YVSPILTEYTKVNIQIVPIALALWGIGMVIGGLAAGWLADKNLNKTIVGVLISSAIVF
VVASFLMSNIYSAIGSLFLIGLTVMGLGGALQTRLMDVAGDAQTLAASLNHSAFNLAN
ALGAFLGGWVLSHQMGWIAPIWVGFVLSLGGLIILLIAFAVEKATQKA" /product="Uncharacterized MFS-type transporter" /transl_table=11
CDS 3458912..3459301 /locus_tag="AUO97b_03335" /db_xref="SEED:fig|400667.82.peg.3200"
/translation="MLSMSGCAVFMAGNQPSKKNLNVLNPGSSRNYVIAEFGAPVLSE
YRDGTRVEIYTFQQGYSKWVKVGRAFGHGVADVASIGLWEVFGTPAEAYFNGKETTYE
VTYGEDDLVKSYKLIDFNQAFPKVKEQ"
/product="hypothetical protein"
/transl_table=11
CDS 3459459..3460253
/locus_tag="AUO97b_03336" /db_xref="SEED:fig|400667.82.peg.3201"
/translation="MNRLLVTSLAFVCSYSLAHEPYVAPLAYKTEQTQVPVVAGYAEE
ALNSEYALKDAKLTVITPKQDPKVINAEALHKSVTVFDVALPEDGTYILQTQASYPLK
YVYDQKEWHLFFDLPADKAPPRKERDYLIPADLKTKKIKTEEVTREWVLQSYLSKGKV
SDIQLPNTPVKVNFSVHPNQLKVTQAVQLTISEKGQNLPYAEINLREKGATDKQVLHF
KADDKGQVELKFPKAGEYLMEVTAPVDLKLKPKNQNYTIVSLQVTE" /product="hypothetical protein" /transl_table=11
CDS 3460479..3461399
/locus_tag="AUO97b_03337" /db_xref="SEED:fig|400667.82.peg.3202"
/translation="MLEIRHLKTLVALREHGSLVAAANDLCLTPSAISHQLKELDHWY
GVEVVNRRSRPVSFSNVGQRLLKLADDVLPQIQITQSDITRIVHGQTGRIIFSSECHS
CFDWLMPLLNQYRQQYPDVDLDFASGFEANPHELLQNAEFDLLITADPIALKGIEYFP
IFEYESRLVLSNTHRLVRAENITVQDLAEEVLITYPVDKHRLDIMSKLFIPANIQPKQ
IRTTDLTQMLIQLVASGRGIAALPDWVVNEYEQKGWVTSRRLDCVSPAGLRRTLYAGY

RTEEKEKSYFEGFLKQLEKFSLKRTSYYSG"
/product="Transcriptional activator MetR"
/transl_table=11
CDS complement(3461450..3461902)
/locus_tag="AUO97b_03338" /db_xref="SEED:fig|400667.82.peg.3203"
/translation="MLAQFDVNLVVLLVLLICGLLSQNAAVTIAAGILIVIKITPLNQ
FFPYIQAHGLNLGILILTIGVLTPIASGKLSGESILKSFISFKSLVAIAIGLLVAWLG
GRGVKLMSSQPDVVAGLLIGTVAGVALLRGVPVGPLIAAGLLSLFIGK" /product="UPF0756 membrane protein YeaL"
/transl_table=11
CDS 3462156..3462374
/locus_tag="AUO97b_03339" /db_xref="SEED:fig|400667.82.peg.3204"
/translation="MDIIDIKIKDQFDQIHDAKAQLKKNLVEHENEPLKLSQRIEHII
VDNEVILPTTELLFESEQNEKIYRVIEE"
/product="FIG00352022: hypothetical protein" /transl_table=11
CDS 3462475..3463179
/locus_tag="AUO97b_03340" /db_xref="SEED:fig|400667.82.peg.3205"
/db_xref="GO:0003723"
/translation="MLLEKILQSQGFGSRKYCQQLIKNGSVIIDGEVVSDLKKQFSPE
NFEFSVFGQNYQYREKIYIALRKPQGFECSHHPQHHQSVFSLLPETMIHRGVQAVGRL
DQDTTGLLLLTDDGKYLQALTHPRKHVPKVYHVTTYDPVTPEQIEMLTQGVNLHQEKG
GFAATDVALLGTHQLTMTIHQGVYHQVKRMIAAVGNKVEKLHRHQIGQLVLPEIEEGG WVYLSEQEKQLAQNII"
/product="SSU rRNA pseudouridine(516) synthase (EC 5.4.99.19)"
/EC_number="4.2.1.70"
/transl_table=11
CDS complement(3463172..3463615)
/locus_tag="AUO97b_03341" /db_xref="SEED:fig|400667.82.peg.3206"
/translation="MIVRRATYEDLSQLAVLFDEYRQFYGASSNLEESHHFLKQRFEN
KESVFFIHIKDEKITGFVLLYLGFSSVACSTYYILDDVYVTPPFRRQGSAKQLIDTAI
LFAKQENALRISLETQSNNHESHRLYEKMGFIRDSEFQTFHCFLK" /product="Acetyltransferase, GNAT family"
/transl_table=11
CDS complement(3463713..3464021)
[truncated: 527,104 more chars]
