# Supplementary material for: A novel antibiotic class targeting the lipopolysaccharide transporter
Source: Nature. 2024 Jan 3;625(7995):566–71. doi: 10.1038/s41586-023-06873-0 (PMC10794144; doi:10.1038/s41586-023-06873-0)
Supplement: Supplementary file 3 — Biochemical assay uncropped immunoblots. Uncropped gels and blots for data shown in Fig. 3. For each figure, the cropped regions are denoted by boxes. [file 41586_2023_6873_MOESM3_ESM.pdf]

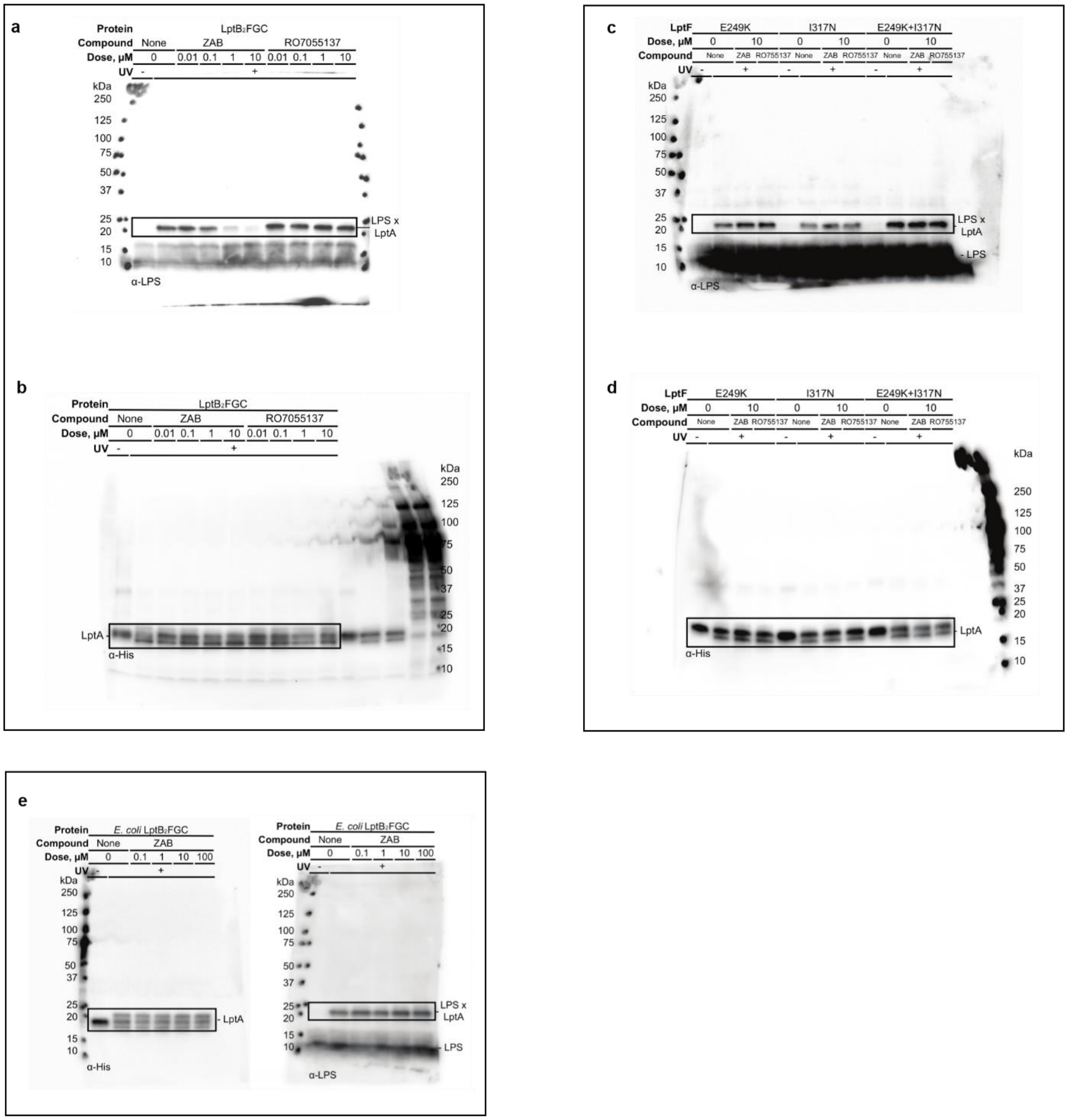

Supplementary Fig. 1. Biochemical assay uncropped immunoblots. Uncropped gels and blots for data shown in main-text (Fig. 3). For each figure, the cropped regions are denoted by boxes. **a**, **b**, **c and d**, source images of data shown in Fig. 3c, left top, left bottom, right top and right bottom respectively. Independent blots were probed for anti-LPS and anti-His. **e**, source images of data described in main text for the *E. coli* $LptB_2FGC$ control.
