# Supplementary material for: A novel antibiotic class targeting the lipopolysaccharide transporter
Source: Nature. 2024 Jan 3;625(7995):566–71. doi: 10.1038/s41586-023-06873-0 (PMC10794144; doi:10.1038/s41586-023-06873-0)
Supplement: Supplementary file 2 — Reporting Summary [file 41586_2023_6873_MOESM2_ESM.pdf]

nature portfolio

Corresponding author(s): Kenneth Bradley, Michael Lobritz

Last updated by author(s): Oct 24, 2023

Reporting Summary

Nature Portfolio wishes to improve the reproducibility of the work that we publish. This form provides structure for consistency and transparency in reporting. For further information on Nature Portfolio policies, see our Editorial Policies and the Editorial Policy Checklist.

Statistics

For all statistical analyses, confirm that the following items are present in the figure legend, table legend, main text, or Methods section.

n/a	Confirmed	
☐	☒	The exact sample size (*n*) for each experimental group/condition, given as a discrete number and unit of measurement
☐	☒	A statement on whether measurements were taken from distinct samples or whether the same sample was measured repeatedly
☐	☒	The statistical test(s) used AND whether they are one- or two-sided<br>*Only common tests should be described solely by name; describe more complex techniques in the Methods section.*
☒	☐	A description of all covariates tested
☒	☐	A description of any assumptions or corrections, such as tests of normality and adjustment for multiple comparisons
☐	☒	A full description of the statistical parameters including central tendency (e.g. means) or other basic estimates (e.g. regression coefficient) AND variation (e.g. standard deviation) or associated estimates of uncertainty (e.g. confidence intervals)
☐	☒	For null hypothesis testing, the test statistic (e.g. *F*, *t*, *r*) with confidence intervals, effect sizes, degrees of freedom and *P* value noted<br>*Give P values as exact values whenever suitable.*
☒	☐	For Bayesian analysis, information on the choice of priors and Markov chain Monte Carlo settings
☒	☐	For hierarchical and complex designs, identification of the appropriate level for tests and full reporting of outcomes
☒	☐	Estimates of effect sizes (e.g. Cohen's *d*, Pearson's *r*), indicating how they were calculated

Our web collection on statistics for biologists contains articles on many of the points above.

Software and code

Policy information about availability of computer code

Data collection	For the bacterial phenotypic fingerprint profiling, image analysis algorithm was developed for the Opera QEHS reader (Perkin-Elmer, Hamburg, Germany) instrument-integrated software Acapella.<br>Morbidostat data were collected using a custom made morbidostat device. The description of software and hardware implementation is deposited to GitHub page: https://github.com/sleyn/morbidostat_construction. The morbidostat interface was built based on MegunoLink software (v. 1.17.17239.0827).
Data analysis	Microsoft Excel 2016 and GraphPad Prism 8 were used to plot graphs.<br>Statistical calculations and Machine Learning are carried out using R, both for LOED detection (batch mode) and from within Spotfire v10.10 (TibcoSoftware Inc. Palo Alto, USA) by the user, i.e. interactively. Spotfire is attached to a relational database (Oracle Corporation, Redwood Shores, USA) which allows the user to dynamically add new data and annotations on the well level; Non compartmental PK analysis was performed using Phoenix v1.4; Morbidostat sequencing data was analyzed using custom software pipelines. Fastq files QC was analyzed with FastQC v. 0.11.8. Adapter and quality trimming was made by Trimmomatic v. 0.36. Reads from population sequencing data were processed with BWA v. 0.7.13, Picard v. 2.2.1, Samtools + bcftools v. 1.3, Genome analysis Toolkit (GATK) v. 3.5, LoFreq v. 2.1.3.1 and snpEff v. 4.3 to call SNPs and indels. IS elements reallocation in population data was predicted using custom iJump tool available on GitHub (https://github.com/sleyn/ijump). BAM files QC was done using Qualimap v. 2.2. Copy number variation was called using CNOGPro v. 1.1 package for R v. 3.6.0. Repeat regions were masked based on analysis produced by MUMmer v. 3.1.; AlogP was calculated using BIOVIA, Dassault Systèmes, Pipeline Pilot, 19.1, San Diego: Dassault Systèmes, 2018; For mutations identification in resistance studies: Hybrid assemblies were generated using a Roche-developed pipeline. Insertions were detected using a Roche-developed tool that makes use of the high-quality hybrid genome assemblies. Details are provided in methods and are available upon request from the corresponding author.

For manuscripts utilizing custom algorithms or software that are central to the research but not yet described in published literature, software must be made available to editors and reviewers. We strongly encourage code deposition in a community repository (e.g. GitHub). See the Nature Portfolio guidelines for submitting code & software for further information.

Data

Policy information about availability of data

All manuscripts must include a data availability statement. This statement should provide the following information, where applicable:

- Accession codes, unique identifiers, or web links for publicly available datasets
- A description of any restrictions on data availability
- For clinical datasets or third party data, please ensure that the statement adheres to our policy

All data supporting the finding of this study are available within the Article and its Supplementary Information (SI) or have been deposited to the indicated databases. Sequencing reads are deposited in the NCBI Sequence Read Archive (SRA) with the accession code PRJNA1026547 (spontaneous mutant profiling) and PRJNA1016345 (morbidostat). SI includes: i) Off-target activity screening of the MCP compounds (Supplementary table 1); ii) Phenotypic and genotypic characterization of the isolates used both in in vitro and in vivo studies (Supplementary table 2 and 3); iii) Line listing of all mutants obtained in spontaneous mutation studies (Supplementary table 4); iv) Line listing of the cumulative % MIC data shown in Fig. 4 (Supplementary table 7); v) Bacterial strains and plasmids used to construct A. baylyi mutants (Supplementary tables 8, 9 and plasmid sequences); vi) The uncropped gels and blots of the biochemical assay shown in Fig. 3 (Supplementary Fig. 1); vii) Synthesis and chemical characterization of all compounds described in this paper (analytical data of the target compounds and synthetic chemistry). Source data for Figs. 1 b, c; Fig 4 b; Extended Data (ED) Fig. 2; ED Fig. 3 b, c are provided with this paper. All other data are available from the corresponding authors.

Research involving human participants, their data, or biological material

Policy information about studies with human participants or human data. See also policy information about sex, gender (identity/presentation), and sexual orientation and race, ethnicity and racism.

Reporting on sex and gender	Human blood was provided by Roche medical services via an anonymous blood donation for research program.
Reporting on race, ethnicity, or other socially relevant groupings	All samples were fully anonymised prior to being provided for studies reported here. No demographic information is available.
Population characteristics	To be considered for the BDR program, all donors must be a Roche employee or contractor and be at least 18 years old. Employees and contractors are eligible for participation in the BDR program if ALL of the following criteria are met:<br>1. Potential donors will have blood drawn for a complete blood count as well as HBV surface antigen, HCV antibody, and HIV 1/2 antibody at their initial clinic visit, which may be at time of first donation<br>2. Potential donors must attend a once a year check-up by the Medical Services and provide a blood sample. Their blood is tested for the presence of the major viruses, i.e. HIV, hepatitis B and hepatitis C<br>3. Employees making donations >100 milliliters at one time must have a hemoglobin level within the normal range according to gender (males >14.0g/dL, females >12.0g/dL) unless approved by a physician<br>4. Potential donors must weigh at least 50 Kg
Recruitment	Donors are volunteers working for Roche that must:<br>1. Read and sign appropriate consent forms for the procedure(s) they voluntarily choose to take part in. The Informed Consent Form is signed once when registering in the tool. Subsequently for each donation a questionnaire needs to be completed & signed. A copy of this signed ICF should be kept by the donor.<br>2. Complete required screening forms and testing.<br>3. Keep scheduled appointment times and contact the BDR Coordinator or Program Manager as soon as possible if unable to make scheduled appointments.
Ethics oversight	Blood collection for research was approved by the Ethics Committee Northwestern Switzerland and Central Switzerland (EKNZ). All donors completed an informed consent form.

Note that full information on the approval of the study protocol must also be provided in the manuscript.

Field-specific reporting

Please select the one below that is the best fit for your research. If you are not sure, read the appropriate sections before making your selection.

☒ Life sciences ☐ Behavioural & social sciences ☐ Ecological, evolutionary & environmental sciences

For a reference copy of the document with all sections, see nature.com/documents/nr-reporting-summary-flat.pdf

Life sciences study design

All studies must disclose on these points even when the disclosure is negative.

Sample size	For animal studies sample size was chosen based on experience with the models used to enable identification of differences between treatment and control groups. Specifically, in Immunocompetent murine septicemia, n = 10/group, in Neutropenic murine thigh, n = 4/group and in Neutropenic murine lung, n = 6/group. For murine PK assessment, n = 3. For rat safety assessment, n = 4. For mouse PK,3 mice are considered the minimal for the purpose of the study, i.e. to provide descriptive statistics of the pharmacokinetic parameters with acceptable standard deviations. For rat safety, this study was considered the initial screen for major organ toxicity of the molecule and to guide the dose selection of subsequent toxicity studies in the species. For this, 4 animals per group was considered sufficient in order to get valid information on mortality, in-life observations, body weight and food consumption changes and any potential clinical and anatomical pathological changes.<br><br>Morbidostat (n=4 isolates) and spontaneous resistance (n=8 isolates) studies were designed to identify putative target(s) and resistance mechanisms for zosurabalpin. Multiple isolates were used to increase diversity, but experiments were not designed to provide complete (e.g. saturating) survey of mutational landscape.
Data exclusions	No data were excluded
Replication	Three independent experiments were conducted for the bacterial phenotypic fingerprint profiling with reproducible results.<br>In Morbidostat, 6 independent reactors were used for each experiment, corresponding to 6 runs with 6 single colonies, i.e. 1 colony per reactor, for each of the 4 tested isolates. Experiments were repeated with 2 media (with and w/o serum supplementation). Results from all replicates are reported.<br><br>Table 1: n = 3 except for RO7036668 (n=1) against all tested isolates and ATCC17978 (n = 1) against all tested compounds. Table 2: spontaneous resistance; n = 1. Figure 2: precipitation threshold n = 1. Figure 4: MIC90 (from SI table 7) n=1. Cell viability assay: n = 2. CEREP was performed with technical duplicates. ED Table 2: MICs versus efflux and porin deficient strains n = 1. ED Table 6: MIC fold increase in mutants n = 1. ED Table 7: MIC for constructed mutants n = 1
Randomization	Randomization was achieved where possible throughout the studies. Mice were randomly allocated to treatment groups on arrival. Rat were randomly assigned to group/cage based on body weight.
Blinding	Mouse PK: Blinding was not applied, as all animals received the same treatment without any randomization.<br>Rat safety: Blinding was not applied, as this is default practice for initial toxicology studies, where the toxicity of the molecule is not yet known and thus, dose levels can be adjusted for subsequent animals, in case severe adverse reactions are observed.<br>Mouse infection studies: blinding was not employed as objective measurements, i.e. bacterial burden, were used.

Reporting for specific materials, systems and methods

We require information from authors about some types of materials, experimental systems and methods used in many studies. Here, indicate whether each material, system or method listed is relevant to your study. If you are not sure if a list item applies to your research, read the appropriate section before selecting a response.

Materials & experimental systems

n/a	Involved in the study	
☐	☒	Antibodies
☐	☒	Eukaryotic cell lines
☒	☐	Palaeontology and archaeology
☐	☒	Animals and other organisms
☒	☐	Clinical data
☒	☐	Dual use research of concern
☒	☐	Plants

Methods

n/a	Involved in the study	
☒	☐	ChIP-seq
☒	☐	Flow cytometry
☒	☐	MRI-based neuroimaging

Antibodies

Antibodies used	Secondary antibodies used were: sheep-anti-mouse HRP conjugate (GE AMersham, LNA931V/AH, lot #14251045, 1:10,000 dilution). Commercially available primary antibodies used were : mouse anti-His HRP conjugate (Biolegend, 652504, 1:10,000 dilution), and anti-LPS core mouse monoclonal antibody (Hycult Biotechnology, HM6011, clone WN1 222-5, lot# 18419M0715-A, 1:5,000 dilution).
Validation	Antibodies were exclusively used for western blotting. Validation of the anti-His antibody can be found on the Biolegend website (https://www.biolegend.com/fr-lu/products/hrp-anti-his-tag-antibody-9873) Certificates of analysis for the anti-mouse antibodies made by GE-Amersham can be found by lot number at https://www.gelifesciences.com/en/us/support/quality/ certificates. Certificate of analysis for the anti-LPS antibody can be found at https://www.hycultbiotech.com/downloads/dl/file/id/1685/ product/814/hm6011.pdf.

Eukaryotic cell lines

Policy information about cell lines and Sex and Gender in Research

Cell line source(s)	HEK293 cells used in Cell viability assay were obtained from ATCC and stored as stocks in Roche repository
Authentication	Short Tandem Repeat PCR to cross-check against a database
Mycoplasma contamination	The cells were tested for mycoplasma after banking (creation of a cell bank) and are negative
Commonly misidentified lines (See ICLAC register)	No commonly misidentified lines were used in the study

Animals and other research organisms

Policy information about studies involving animals; ARRIVE guidelines recommended for reporting animal research, and Sex and Gender in Research

Laboratory animals	CD-1 mice, 6 weeks old at arrival (minimum acclimatisation 5 days). Rat/Wistar Han IGS Crl:WI(Han), 8 weeks old at start of dosing.<br><br>Mice were group housed in individually ventilated cages (IVCs) and maintained on a 12:12 hour light/dark cycle, with constant temperature (21–24°C) or (20-22°C) and humidity (40–80%) or (45-65%). Each cage was provided with unrestricted access to municipal water and sterilized food (Provimi Kliba 3436). The cage was supplied with autoclaved sawdust bedding and environmental enrichments, which were applied to best practice animals' welfare standards and rotated weekly. The mice were acclimated for at least 1 week before the start of the study.<br><br>Rats (4 per cage) was kept in air-conditioned animal room under periodic bacteriologic control, at 22°C ± 2°C (monitored), at 40%-80% (monitored), with fluorescent tubes at 12 hours light/dark cycle, and background music coordinated with light hours, in macrolon boxes (type IV, with autoclaved sawdust bedding). The rats received pelleted maintenance rodent diet Provimi Kliba 3436 ad libitum in food containers integrated in cage lid, tap water ad libitum (in water bottles) and were offered enriched environment.
Wild animals	No wild animals were used
Reporting on sex	All animals were male
Field-collected samples	No field-collected samples were used
Ethics oversight	Mouse pharmacokinetic studies and rat safety studies were conducted at Roche and all procedures were in accordance with the respective Swiss regulations and approved by the Cantonal Ethical Committee for Animal Research and conducted in a facility accredited by the Association for Assessment and Accreditation of Laboratory Animal Care International (AAALAC) (animal research permit #2395). The pharmacodynamics studies assessing the efficacy of the compounds were performed at Aptuit Verona, an Evotec company and subject to both the European directive 2010/63/UE governing animal welfare and protection, which is acknowledged by the Italian Legislative Decree no 26/2014 and the company policy on the care and use of laboratory animals. All animal studies were reviewed by the Animal Welfare Body and approved by Italian Ministry of Health (authorization n. 51/2014-PR) and conducted in a facility accredited by the Association for Assessment and Accreditation of Laboratory Animal Care International (AAALAC) (accredited unit #001090).

Note that full information on the approval of the study protocol must also be provided in the manuscript.

Plants

Seed stocks	*Report on the source of all seed stocks or other plant material used. If applicable, state the seed stock centre and catalogue number. If plant specimens were collected from the field, describe the collection location, date and sampling procedures.*
Novel plant genotypes	*Describe the methods by which all novel plant genotypes were produced. This includes those generated by transgenic approaches, gene editing, chemical/radiation-based mutagenesis and hybridization. For transgenic lines, describe the transformation method, the number of independent lines analyzed and the generation upon which experiments were performed. For gene-edited lines, describe the editor used, the endogenous sequence targeted for editing, the targeting guide RNA sequence (if applicable) and how the editor was applied.*
Authentication	*Describe any authentication procedures for each seed stock used or novel genotype generated. Describe any experiments used to assess the effect of a mutation and, where applicable, how potential secondary effects (e.g. second site T-DNA insertions, mosiacism, off-target gene editing) were examined.*
